# Characterization of dynamic patterns of human fetal to neonatal brain asymmetry with deformation-based morphometry

**DOI:** 10.3389/fnins.2023.1252850

**Published:** 2023-12-06

**Authors:** Céline Steger, Charles Moatti, Kelly Payette, Alexandra De Silvestro, Thi Dao Nguyen, Seline Coraj, Ninib Yakoub, Giancarlo Natalucci, Raimund Kottke, Ruth Tuura, Walter Knirsch, Andras Jakab

**Affiliations:** ^1^Center for MR Research, University Children’s Hospital Zurich, University of Zurich, Zürich, Switzerland; ^2^Children’s Research Center, University Children’s Hospital Zurich, University of Zurich, Zurich, Switzerland; ^3^Pediatric Heart Center, Division of Pediatric Cardiology, University Children’s Hospital Zurich, University of Zurich, Zurich, Switzerland; ^4^Neuroscience Center Zurich, University of Zurich and Swiss Federal Institute of Technology, Zurich, Switzerland; ^5^Department of Information Technology and Electrical Engineering, ETH Zurich, Zurich, Switzerland; ^6^Newborn Research, Department of Neonatology, University of Zurich and University Hospital Zurich, Zurich, Switzerland; ^7^Larsson-Rosenquist Foundation Center for Neurodevelopment, Growth and Nutrition of the Newborn, Department of Neonatology, University Hospital Zurich, University of Zurich, Zurich, Switzerland; ^8^Department of Diagnostic Imaging, University Children’s Hospital Zurich, University of Zurich, Zurich, Switzerland

**Keywords:** brain asymmetry, magnetic resonance imaging (MRI), fetal brain, neonatal brain, DBM = deformation-based morphometry, longitudinal

## Abstract

**Introduction:**

Despite established knowledge on the morphological and functional asymmetries in the human brain, the understanding of how brain asymmetry patterns change during late fetal to neonatal life remains incomplete. The goal of this study was to characterize the dynamic patterns of inter-hemispheric brain asymmetry over this critically important developmental stage using longitudinally acquired MRI scans.

**Methods:**

Super-resolution reconstructed T2-weighted MRI of 20 neurotypically developing participants were used, and for each participant fetal and neonatal MRI was acquired. To quantify brain morphological changes, deformation-based morphometry (DBM) on the longitudinal MRI scans was utilized. Two registration frameworks were evaluated and used in our study: (A) fetal to neonatal image registration and (B) registration through a mid-time template. Developmental changes of cerebral asymmetry were characterized as (A) the inter-hemispheric differences of the Jacobian determinant (JD) of fetal to neonatal morphometry change and the (B) time-dependent change of the JD capturing left-right differences at fetal or neonatal time points. Left-right and fetal-neonatal differences were statistically tested using multivariate linear models, corrected for participants’ age and sex and using threshold-free cluster enhancement.

**Results:**

Fetal to neonatal morphometry changes demonstrated asymmetry in the temporal pole, and left-right asymmetry differences between fetal and neonatal timepoints revealed temporal changes in the temporal pole, likely to go from right dominant in fetal to a bilateral morphology in neonatal timepoint. Furthermore, the analysis revealed right-dominant subcortical gray matter in neonates and three clusters of increased JD values in the left hemisphere from fetal to neonatal timepoints.

**Discussion:**

While these findings provide evidence that morphological asymmetry gradually emerges during development, discrepancies between registration frameworks require careful considerations when using DBM for longitudinal data of early brain development.

## 1 Introduction

Asymmetry of the brain manifests on various levels, including genetic, morphological, and functional dimensions (Wan et al., 2022). From the neuroscience perspective, studying anatomical asymmetries and their development can reveal insights into the emergence of brain functional specialization, using macro-morphological inter-hemispheric asymmetry as a proxy. The lateralization of sensory-motor functions, behavioral and cognitive processes in the brain is a well-described phenomenon, which is not exclusive to humans. It is widely believed that this lateralization provides evolutionary advantages at the behavioral level, including decreased reaction time and parallel processing in both hemispheres (Rogers et al., 2004; Dongen, 2006; Güntürkün et al., 2020). On a morphological level, post-mortem studies in humans provide evidence for cytoarchitectonic asymmetries (Galaburda et al., 1978) and macroscopic asymmetries such as the ‘Yakovlevian-Torque’ can be observed by pure visual inspection. From the clinical perspective, there is emerging evidence for the association of altered or disrupted hemispheric asymmetry in neurological disorders and neuropsychological conditions (Postema et al., 2019; Lubben et al., 2021; Sone et al., 2022).

Brain asymmetry is likely not a static or linearly emerging property during development; instead, it might involve a complex dynamic process where patterns of left-right asymmetry may appear or diminish due to variations in the expression of developmental genetic programs that control the formation of the characteristic cortical surface patters and brain specialization. Inter-hemispheric asymmetry already develops in the embryonic stage and is induced not only by genetic components but also dependent on environmental factors (Schmitz et al., 2019; Güntürkün et al., 2020). Post-mortem studies have indicated that right hemispheric structures and a leftward planum temporale (PT) asymmetry have an early origin in fetal brain development (Wada et al., 1975; Chi et al., 1977). While some works found no morphological asymmetries in the fetus using post-mortem analysis (Zhang et al., 2013), asymmetrical cortical folding patterns were described in-vivo as early as the 22nd gestational week (Habas et al., 2012). Several studies described volumetric changes throughout late fetal development for each hemisphere separately to assess their asymmetry (Cai et al., 2020; Vasung et al., 2020; Kienast et al., 2021; Machado-Rivas et al., 2022; Yun et al., 2022). For example, temporal lobe asymmetries are known to change dynamically during the second half of gestation (Kasprian et al., 2011). Asymmetries in the PT and superior temporal sulcus are believed to persist throughout infancy and remain relatively stable until adulthood in healthy human brains (Dubois et al., 2009; Hill et al., 2010).

In addition to the neuroscientific significance of inter-hemispheric asymmetries throughout cerebral development, the final stage of this development holds considerable biological and clinical importance, warranting further investigation in this domain. During the last 8 weeks of gestation and early newborn phase, the brain undergoes rapid growth and morphological features, such as secondary and tertiary gyri emerge. In addition, birth takes place, undoubtedly a crucial moment, where a lot of mechanical stress is experienced (Ami et al., 2022) and the entire organism needs to adapt to new conditions and is no longer supported by the mother. Furthermore, this timeframe is of interest, as this is a realistic timeframe to acquire longitudinal data in patients which show altered brain development and risk for brain injuries (Peyvandi et al., 2021) and alternatives to tissue specific volume measurements could provide new insights.

In recent years, advances in fetal and infant MRI have improved access to studying *in vivo* human brain development. However, limited literature exists on longitudinally acquired data and there is a clear limitation of these studies because of their cross-sectional design (that is, each developmental stage is represented by a different individual). Many studies report brain asymmetry, but quantitative MRI data on gestational time-dependent brain morphology changes primarily come from cross-sectional studies, which are constrained by significant inter-individual brain surface variability. Consequently, inferring developmental trends from cross-sectional data can be confounded by this variability and may not accurately represent a within-individual dynamic asymmetry evolution. Furthermore, technical challenges may have contributed to the lack of studies quantifying the dynamic patterns of brain asymmetry over the fetal to neonatal period. Popular neuroscience software and toolboxes are very limited for use with fetal or neonatal MRI data due to different MR image contrasts and not yet developed brain structures, highlighting the urgent need for specialized tools to examine early brain development. While brain volumetry is seen as the gold standard for analyzing brain growth, segmentation of the brain into consistent subregions throughout development remains challenging. Alternative techniques such as deformation-based morphometry (DBM) have been applied before (Jakab et al., 2019; Ng et al., 2020), relying on non-linear image registration. Using DBM, no consistent segmentation of subregions is required to detect local asymmetries of the brain.

The goal of our study was twofold: first, to improve our understanding of the neuroanatomical changes from late fetal to neonatal stage by describing the age-related changes of brain asymmetry based on real longitudinal MRI data acquired at two timepoints. Second, to address outstanding challenges in the deformation-based morphometry analysis of fetal-to-newborn longitudinal development. Specifically, by conducting our analysis using two plausible registration frameworks and evaluating the effects of image post processing options on the image registration accuracy.

## 2 Materials and methods

### 2.1 Participants

Data from twenty healthy participants recruited as participants of two ongoing, prospective studies (BrainDNIU study and BrainCHD study) between 2017 and 2022 were taken for this analysis. Based on patient history, ultrasound (US) and MR scans, all fetuses and newborns were classified as having normal brain development. The female/male ratio was 14/6. All participants were scanned twice: during fetal life and as a newborn. The gestational week (GW) at scan was calculated based on clinical US records. Mean ± standard deviation (SD) age at scan was 32.4 ± 1.1 GW for the fetal timepoint and 41.7 ± 1.4 GW for neonatal. All babies were born around term at a mean age of 39.7 ± 1.2 GW. The captured time interval between the two MRIs was 65 ± 12 days for each participant.

Parents gave written informed consent for the participation of the study they were enrolled in as well as consent for data sharing between the studies. BrainCHD and brainDNIU study were approved by the local ethics committee (BASEC IDs: 2019-01993, 2017-00885).

### 2.2 MRI acquisition

Fetal MRI was performed on a 1.5T clinical whole-body MRI scanner (GE Signa Discovery MR450) using a 32-channel cardiac coil or an 8-channel body array coil. The MRI scanner was upgraded to GE Signa Artist during the data collection. Post-upgrade fetal scans were performed with an AIR coil, with the anterior array (blanked coil) having 30 channels and table coil 40 channels. T2-weighted single shot Fast Spin Echo (ssFSE) sequences were acquired in axial, sagittal and coronal orientations, relative to the fetal brain. In-plane resolution was 0.5 mm × 0.5 mm and a slice thickness of 3–5 mm was applied, depending on the covered organ (placenta, whole fetus or brain only). The sequence parameters were the following: TR: 2,000–3,500 ms, TE: 120 ms (minimum), flip angle: 90. During the scan, the quality of the ssFSE scans was checked by the attending pediatric radiologist and were repeated in case excessive movement or other artifacts were detected. As a rule of thumb, at least two image sequences per orientation were acquired.

In the neonates, all MR scans were performed in natural sleep, without sedation. Neonates received protective earplugs and earmuffs. T2-weighted fast spin-echo (FSE) sequences in all three planes were acquired on a 3.0 T MRI scanner using an 8-channel head coil (GE Signa Discovery MR750). The sequence parameters were the following: TR: 5,900 ms, TE: 97 ms, flip angle: 90°. In-plane resolution was 0.35 × 0.35 mm, slice thickness: 2.5 mm with a 0.2 mm slice gap. For the neonatal MRI, only one image sequence per orientation was acquired. During data collection time, the MR scanner was upgraded to GE Signa Premier. Post-upgrade neonatal scans were performed with a 48-channel head coil.

### 2.3 Image pre-processing

The MR images were super-resolution (SR) reconstructed using the slice-to-volume reconstruction toolkit (Kuklisova-Murgasova et al., 2012). Fetal ssFSE images were first checked visually for quality, and at least one axial, sagittal and coronal image was selected for SR reconstruction. Similarly, for the neonatal data, one image from each of the three orientations were used. A reference image was chosen and a mask covering the area of the brain was manually drawn using 3D Slicer Software. Images were pre-processed with N4ITK filter in the 3D Slicer Software (Fedorov et al., 2012) and denoising BTK Toolkit (Rousseau et al., 2013). Both fetal and neonatal images were reconstructed to 0.75 mm isotropic voxel resolution. SR images were reoriented to an age-matched template using a 6 degrees-of-freedom transformation to ensure consistent anatomical orientation. In a final step, brains were skull-stripped using a binary mask based on brain segmentation labels (described below).

**FIGURE 1:**
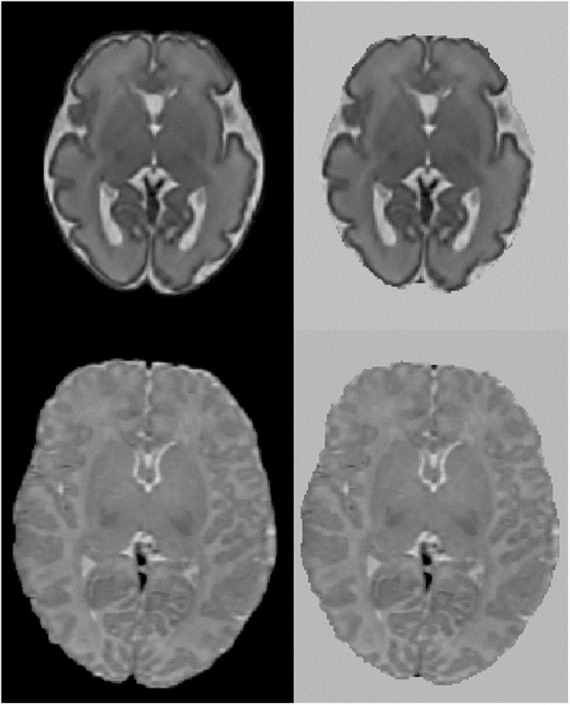
Axial view of fetal and neonatal image of a participant. **Top:** fetal, **bottom:** neonatal. Left original image, right image background replaced with bright values.

As part of the pre-processing for non-linear image registration, we explored whether replacing the black background voxels with bright voxels would affect the performance of the deformable image registration. The aim of this procedure was to reduce the confounding effect of the interface between dark voxels, such as the skull and background air, and the bright voxels of extracerebral fluid spaces, as the latter appear very different between the fetal and neonatal MR images. This step was performed by an in-house developed script with image processing steps that rely on FSL (Jenkinson et al., 2012) and AFNI (Cox, 1996). Brain and background classes were identified using 3dkmeans and the background was replaced by the 99th percentile value of the image. See Figure 1 for a fetal and neonatal image example with and without a “bleached” background.

### 2.4 Characterization of the temporal dynamics of morphological brain asymmetry

**FIGURE 2:**
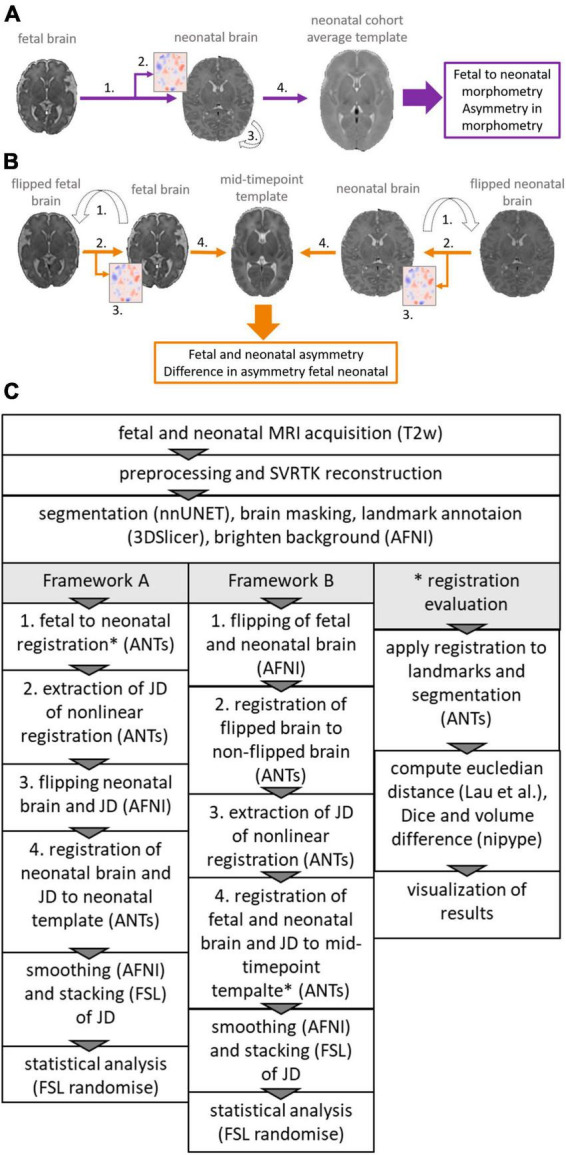
Overview of the two frameworks used to reveal asymmetry patterns and their temporal dynamics. **(A)** Framework A (violet), calculating the inter-hemispheric asymmetry of fetal to neonatal regional brain growth maps. **(B)** Framework B (orange), calculating fetal and neonatal inter-hemispheric asymmetry at the given timepoint, registering these to a mid-timepoint template and then calculating the differences between the inter-hemispheric asymmetry. **(C)** Flowchart showing steps required to extract JD maps and perform asymmetry analysis for framework A and B and steps required to extract metrics for the registration evaluation. *Indicates registration step was evaluated (see * registration evaluation).

To capture time-dependent changes of the inter-hemispheric asymmetry, multiple image registration frameworks are viable choices. Figure 2 gives an overview of the two frameworks used in our study to perform the asymmetry analysis.

In framework A (Figure 2A), we first defined brain growth based on image registrations that map the fetal to the neonatal image within the same participant, directly capturing dynamic morphometric changes from fetal to neonatal timepoints. Similar to Jakab et al. (2019), longitudinal MR image pairs were registered for each participant using subsequent linear and non-linear image registrations. A Jacobian determinant (JD) map was calculated from the nonlinear registration component of this step. To statistically test inter-hemispheric asymmetry, as a next step, the JD maps and neonatal MRI were then flipped along the midline axis. The flipped and non-flipped brain and JD pairs were registered to a neonatal template where the asymmetry analysis was performed. Inter-hemispheric difference in morphometric changes were tested as the differences between the flipped and non-flipped JD in the shared template space.

In framework B (Figure 2B), we first defined static inter-hemispheric asymmetries at the fetal and neonatal time points individually, and then captured their time-dependent changes after registering the asymmetry maps to a mid-age template. For the asymmetry analysis in framework B, fetal and neonatal images were first flipped along the midline axis for each participant. Each flipped image was then registered to the non-flipped image and the JD capturing left-right differences were extracted. Next, the images and JD maps corresponding to the fetal and neonatal timepoints were registered to a mid-age template. Asymmetry analysis was performed by statistically testing for differences between the fetal and neonatal JD maps.

All registration steps were done using the ANTs toolbox (Tustison et al., 2021). Figure 2C shows an overview of the steps, more details can be found in the Supplementary material.

#### 2.4.1 Evaluation of registration accuracy for large registration steps

Registration accuracy was tested using two volumetric (image segmentation-based) accuracy metrics and one anatomical landmark-based metric. The rationale for this was to obtain a set of quantitative measurements of accuracy. In the Supplementary section we provide an overview of the different registration parameters tried.

##### 2.4.1.1 Anatomical overlap and volume differences

**FIGURE 3:**
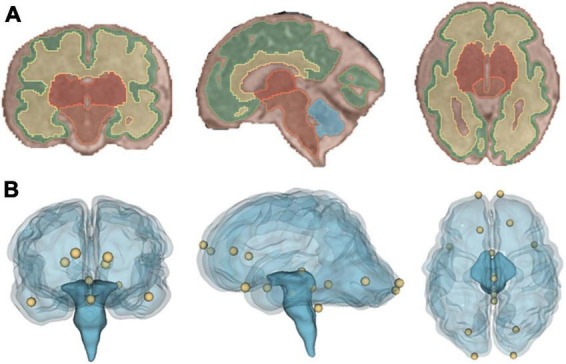
Visualization of the registration evaluation metrics used in our study. **(A)** Coronal, sagittal and axial view of segmentation overlaid on a T2-weighted SR image of a fetus. **(B)** Coronal, sagittal and axial view of a glass brain rendering showing all sixteen, manually placed landmarks that were used during the registration evaluation.

The fetal and neonatal SR images and the mid-age atlas MRI template were segmented into the following structures: cerebral spinal fluid (CSF), grey matter (GM), white matter (WM), deep grey matter (dGM), brain stem (BS), and cerebellum (Cer). To automate this step, two in-house trained nnUNets (Isensee et al., 2021) were used, corresponding to fetal and neonatal MRI segmentation tasks. The fetal brain segmentation network was trained using the FeTA dataset (Payette et al., 2021), which provides manually annotated ground truth data. For the neonatal data, the ground truth data was generated using the developing human connectome project (dHCP) structural pipeline (Makropoulos et al., 2018). To improve the consistency between the anatomical definition of the segmentations in the fetal and neonatal timepoints, several modifications of the tissue label maps were necessary. We combined the cerebral spinal fluid and ventricle labels. The hippocampus label generated by the neonatal network was assigned to gray matter. All segmentations were visually checked by the first author of this study. The segmented anatomical structures are illustrated in Figure 3A.

The two segmentation-based evaluation metrics, Dice overlap coefficient (Dice, 1945) and volume difference were evaluated using the co-registered segmentations (framework A: fetal, framework B: fetal and neonatal) and the target segmentation (framework A: participant specific neonatal segmentation, framework B: mid-age template). The Dice coefficient and volume difference were calculated using their implementation in nipype (Gorgolewski et al., 2011). Volume difference is described using the following definition:


$$\begin{array}{c} \mathrm{Volume}\mathrm{Difference}=\left({\mathrm{segment}}_{\mathrm{image1}}-{\mathrm{segment}}_{\mathrm{image2}}\right)/\\ {\mathrm{segment}}_{\mathrm{image1}} \end{array}$$

For the evaluation the absolute value of this metric was used.

##### 2.4.1.2 Anatomical landmark distances

The following landmarks were annotated in each image following Lau (Lau et al., 2019): posterior commissure, infracollicular sulcus, pontomesencephalic junction (PMJ). In addition, the most caudal point of the 4th ventricle and the optic chiasm were annotated and for left and right hemisphere, the rostral end of: hemisphere, temporal lobe and lateral ventricles; and the caudal end of: lateral ventricles and hemisphere were annotated. The landmarks were manually placed in the fetal and neonatal SR images as well as the mid-age template using the Markup module in 3D Slicer, and their coordinates were exported for the registration evaluation. After transforming each landmark with the given registration method, the distance between the ground truth and transformed points were calculated using the Euclidean distance. The anatomical landmarks are illustrated in Figure 3B.

### 2.5 Statistical analysis of inter-hemispheric brain asymmetry

As defined in Section “2.4 Characterization of the temporal dynamics of morphological brain asymmetry,” both frameworks used JD maps to capture brain morphometry changes or brain asymmetry. Following this logic, the statistical evaluations of brain asymmetry and the time-dependent change of brain asymmetry were performed as follows.

First, JD used for the given analysis were smoothed [using 3dBlurInMask (Cox, 1996)] and stacked into a 4D format using fslmerge (Jenkinson et al., 2012). Statistical brain asymmetry analysis was carried out using the randomize tool of FSL. Randomize is an implementation of randomized non-parametric permutation testing (Winkler et al., 2014). Paired sample designs were set up. Threshold-free cluster enhancement (TFCE) (Smith and Nichols, 2009) was used to correct for multiple comparisons, using TFCE with 5,000 permutations. In framework A the linear regression was performed contrasting two groups: flipped and non-flipped JD maps. Demeaned GW at fetal and neonatal scan were added as covariates. Similarly, in framework B the asymmetry analysis for the fetal and neonatal timepoint was performed. The two groups flipped and non-flipped were contrasted and the demeaned GW at scan and sex were added as covariate. To capture the temporal changes in asymmetry from fetal to neonatal in framework B the (non-flipped) fetal and (non-flipped) neonatal JD were tested for significant differences while including sex and the age variable of both timepoints as covariates.

### 2.6 Visualization and clusters

For visualizing the results of the statistical tests, the anatomical clusters were shown after thresholding them at a value of 0.975, corresponding to *p* < 0.025. FSL Cluster reported the cluster size and generated corresponding labels. Mean, minimum and maximum JD value for each cluster were extracted using the labels. All visualizations were done in fsleyes in the FSL tool, images in the axial view were mirrored to show then in neurological orientation (left hemisphere corresponding to left side in the image). Statistical images were overlaid on the corresponding T2-weighted template images.

## 3 Results

### 3.1 Registration accuracy

**FIGURE 4:**
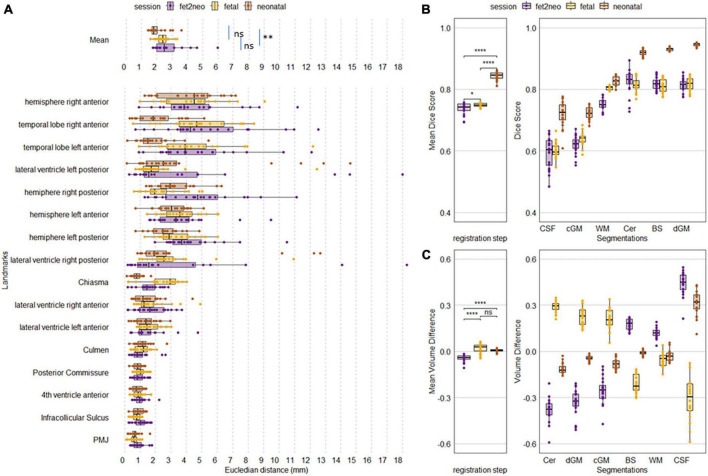
Achieved metric accuracy for each participant for each registration step. **(A)** Landmark accuracy as Euclidean distance in mm. Overall mean landmark distance per registration step (Mean, top row) and individual landmark distance. **(B)** Overall mean Dice score and Dice per segmentation label. **(C)** Overall mean volume difference and volume difference per segmentation label. fet2neo = fetal to neonatal registration, fetal = fetal to template registration, neonatal = neonatal to template registration. PMJ, pontomesencephalic junction. ns = *p*-value > 0.05, * = *p*-value < 0.05, ** = *p*-value < 0.01, **** = *p*-value < 0.0001.

In Figure 4 the achieved registration accuracy for the three registrations fetal to neonatal, fetal to mid-template and neonatal to mid-template are visualized. *Post-hoc* Wilcox test between registration showed significant difference between mean landmarks accuracy of fetal to neonatal and neonatal to template registration (*p* = 0.001). For the mean dice there was a significant difference for all comparisons (fet2neo vs. fetal: *p* = 0.03, fet2neo vs. neonatal: *p* < 0.001, fetal vs. neonatal: *p* < 0.001). Finally, mean volume difference was significantly different for fetal to neonatal registration compared to fetal (*p* < 0.001) and neonatal (*p* < 0.001) to template registration.

In addition, a pattern within the individual metrics seems to be visible. Landmarks located around the brainstem seem to have achieved consistently high alignment, whereas other landmarks had larger errors. For the segmentation-based metrics, the cerebellum, brainstem and deep grey matter achieved the highest dice score and there was lower accuracy for cortical grey matter and CSF. While the mean volume difference was close to zero, the individual labels had larger errors in both negative and positive directions.

Results of a complementary analysis, where we tested different settings for the registration can be found in the Supplementary material.

### 3.2 Dynamic patterns of brain morphometry and asymmetry

In all experiments, overall scaling, translation, and rotation from the linear registration component has been factored out since our analyses relied on the deformation component of the fetal to neonatal or mid-age template registration. Therefore, we refer to inter-hemispheric asymmetry as the asymmetry of morphometric change, meaning relative expansion or contraction of a given anatomical structure.

#### 3.2.1 Inter-hemispheric asymmetry of regional fetal to neonatal brain growth maps (framework A)

**FIGURE 5:**
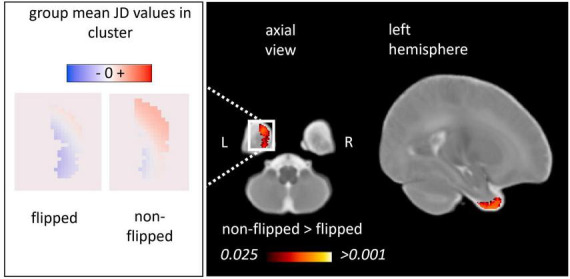
Inter-hemispheric asymmetry of the regional fetal to neonatal morphometry change maps (framework A). We illustrated the anatomical clusters in which flipped and non-flipped morphometry change maps were significantly different, referring to an inter-hemispheric difference in the regional fetal to neonatal morphometry change (TFCE-corrected *p* < 0.025). **Left panel:** population mean, voxel-level JD values in the left and right hemispheres characterizing regional brain growth (flipped to illustrate anatomical match). **Right panel:** anatomical clusters overlaid on a neonatal T2-weighted template.

We first quantified inter-hemispheric differences in regional fetal to neonatal morphometric change using the framework A. A repeated measures linear model (paired flipped vs. non-flipped measurements) revealed two anatomical clusters in which inter-hemispheric differences in the regional fetal to neonatal morphometry change maps were significant (Figure 5). The larger cluster (Table 1, FN 1) was localized to the left temporal pole region extending into cortical gray matter of the temporal lobe as well as the surrounding CSF. The mean values of the JD maps for the flipped and non-flipped hemispheres are visualized within the cluster in the axial view (Figure 5, left panel). This analysis revealed that the regional fetal to neonatal morphometry change map in the left hemisphere (non-flipped) is dominated by positive JD values, referring to regional expansion, however, due to the smoothing applied to the JD maps, it’s not possible to determine whether voxels in the CSF or GM are driving these results.

**TABLE 1:** Anatomical regions demonstrating inter-hemispheric morphometric asymmetry: fetal to neonatal asymmetry analysis (Framework A).

Cluster	Hemisphere	Anatomical location	Size (# voxels)	Lowest *p*-value	JD values ipsilateral	
					Min–max	Mean
FN1	Left	Temporal pole	1,462	0.006	−1.00 to 0.82	0.09

Clusters of a statistical threshold of TFCE-corrected *p* < 0.025 were included in this table. The clusters represent areas, where the JD values of the non-flipped images were significantly larger compared to the flipped images.

#### 3.2.2 Fetal to neonatal changes of inter-hemispheric asymmetry (framework B)

**FIGURE 6:**
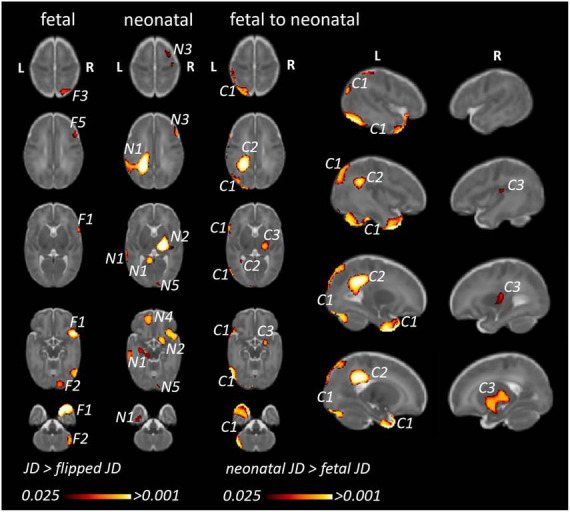
Fetal and neonatal inter-hemispheric asymmetry and dynamic, age-related changes of inter-hemispheric asymmetry (framework B). (Fetal/neonatal) Clusters corresponding to anatomical regions showing (static) inter-hemispheric asymmetry in either the fetal or neonatal time points. (Fetal to neonatal) Clusters corresponding to anatomical regions where inter-hemispheric asymmetry showed differences between the neonatal and fetal timepoints. We only illustrate clusters where neonatal JD was higher than fetal JD, since the opposite statistical test (fetal JD > neonatal) would reveal very similar findings in the homotopic ipsilateral anatomical regions. F1…N: clusters representing regional asymmetry in the fetal images (significant TFCE-corrected clusters where flipped JD > non-flipped JD). N1…N: clusters representing regional asymmetry in the neonatal images (significant TFCE-corrected clusters where flipped JD > non-flipped JD). C1…N: clusters where inter-hemispheric asymmetry changed from fetal to neonatal age.

In framework B, inter-hemispheric asymmetry at each timepoint (fetal or neonatal) was tested after transforming the JD maps to a mid-age template as shown in Figure 6. This approach enabled the quantification of inter-hemispheric asymmetry individually at the fetal and neonatal timepoint as well as the characterization of age-dependent changes.

Using a repeated measures linear model (fetal vs. neonatal measurements) regions with larger JD values in neonates were identified. This statistical analysis revealed three anatomical regions where inter-hemispheric asymmetry changed from fetal to neonatal timepoints. Table 2 summarizes the anatomical locations, cluster sizes, minimum *p*-values as well as JD (fetal and neonatal timepoints) of each cluster. Furthermore, we describe the localization of these clusters and link them to the previously described static asymmetries at the fetal and neonatal age to better characterize the dynamic changes from fetal to neonatal age. Cluster IDs are numbered in descending order of size.

**TABLE 2:** Anatomical regions demonstrating inter-hemispheric morphometric asymmetry: fetal and neonatal MRI to template analysis (Framework B).

Cluster	Hemisphere	Anatomical location	Most likely corresponding clusters	Size (# voxels)	Lowest *p*-value	JD values fetal		JD values neonatal	
						Min–max	Mean	Min–max	Mean
C1	Left	Temporal pole, temporo-occipital cortex, post-central gyrus, white matter of the parietal cortex	F1, F2, N2	27,513	<0.001	−1.33 to 0.67	−0.19	−0.71 to 0.46	−0.02
C2	Left	Centrum semiovale, white matter of the post-central and angular gyri, left ventricle frontal horn	N1	8,825	<0.001	−0.66 to 0.60	−0.07	−0.67 to 0.37	0.06
C3	Right	Thalamus, basal ganglia, claustrum, internal and external capsules, posterior most aspect of the lateral sulcus	N2	5,639	0.006	−0.32 to 0.24	−0.05	−0.15 to 0.29	0.05

The clusters represent areas, where the JD values of the neonatal group were significantly larger compared to the fetal group. Clusters were ordered according to descending size.

In the left temporal lobe, a large cluster (ID in Table 2 and Figure 6: C1) was localized to the temporal pole, extending superiorly into the temporo-occipital cortex as well as post-central gyrus in the parietal cortex. Within this cluster, a right-dominant temporal pole was shown at the fetal stage, while no right dominance was found in the neonatal age, therefore the neonatal JD > fetal JD was significant in the left hemisphere for the temporal pole region (Figure 6 C1). In the parietal cortex, extending into the deep white matter of the post-central gyrus and superior parietal lobule, more leftwards inter-hemispheric asymmetry was demonstrated in the neonates (Figure 6, N1/N4/N7). This marks a trend toward more bilateral morphology of the temporal lobes and temporo-occipital cortex in newborns compared to fetuses (or a left to right shift during fetal to neonatal development), and a trend toward leftwards asymmetry of the deep white matter of the post-central gyrus and superior temporal lobule (or a right to left shift during fetal to neonatal development).

In the parietal lobe, a cluster was found in the deep cerebral white matter, extending into to the body of the left lateral ventricle and extending superiorly into the parietal white matter of the post-central and angular gyri (ID in Table 2 and Figure 6: C2). Within the parietal aspect of this cluster, no fetal asymmetry was found (Figure 6), while strong, left dominant neonatal asymmetry was found in the static analysis (N1 in Figure 6). This refers to a trend toward more lateralized morphology in neonatal age in the left hemisphere.

In the deep white matter and subcortical gray matter, a cluster was revealed with its central aspect in the right thalamus, basal ganglia, claustrum, internal and external capsules, extending laterally to the posterior insula / posterior aspect of the central sulcus (ID in Table 2 and Figure 6: C3). In the fetal images, this cluster did not show inter-hemispheric asymmetry (Figure 6), referring to a trend toward more lateralized morphology in neonatal age in the right hemisphere.

While the static asymmetry pattern provided insights into what could have driven the observed dynamic patterns, they also revealed regions that did not correspond to the temporal analysis. In the analysis of the static neonatal asymmetry, cluster N3, located in the right frontal lobe, may correspond to parts of F1 of the fetal asymmetry analysis. The presence of these two clusters could indicate, that asymmetry is present in both timepoints to a similar degree. For F3 located in the occipital lobe and N6 (not visualized) located in the right cerebellum we could not find any correspondence between the time points. As these clusters were only identified in one either fetal or neonatal static asymmetry analysis, but not in the temporal dynamics, their temporal dynamics remain unclear.

#### 3.2.3 Common findings in framework A and framework B

Our working hypothesis was that the two methodological frameworks would yield comparable descriptions of the temporal dynamics of brain asymmetry, this way, cross-validating the results by using two different pipelines. The trend toward more bilateral morphology or a left to right shift of the pole of the left temporal lobe was the only consistent finding across the two image analysis frameworks in our study. However, in both approaches that investigated the temporal dynamics we found anatomical regions that showed different asymmetry patterns in late fetal than newborn age. In framework A the left temporal pole had higher JD values than the right, similarly, in framework B the left temporal pole revealed an asymmetry difference between fetal and neonatal timepoint. When looking at the static asymmetry results, this temporal dynamic seems to be driven by the smaller fetal left temporal pole, which is less asymmetric in the neonatal brains.

## 4 Discussion

Prenatal human brain development is a complex and precisely orchestrated process influenced by genetic programs and environmental factors. The appearance of macroscopic asymmetries between the brain’s hemispheres likely arises from a combination of various developmental events. To gain a deeper understanding of this phenomenon, our study examined the changing patterns of inter-hemispheric brain asymmetry from late fetal development to the neonatal stage. We employed an analytical approach with multiple tasks to optimize the image processing steps, all aimed at answering the fundamental neurobiological question: How does inter-hemispheric asymmetry change in the relatively short period from before birth to after birth? Out of the four areas showing significant differences in inter-hemispheric asymmetry between the two developmental stages, two were predominantly localized to the left temporal lobe and left parietal and post-central region, one in the right deep gray matter and white matter structures, and the smallest cluster found in the left inferior frontal gyrus. Contrary to our expectation of obtaining concordant results with the two frameworks, only one anatomical region (temporal lobe) survived the TFCE-based correction for multiplicity in two different frameworks of analysis.

### 4.1 The dynamic patterns of inter-hemispheric brain asymmetry over the fetal to neonatal developmental period

We have identified multiple brain regions that exhibit an increasing inter-hemispheric morphometric asymmetry from the fetal to neonatal timepoints. In our analysis, such a region could be detected either as a significant cluster found in both the late fetal and neonatal periods, which gradually became more lateralized as indicated by larger JD values in the ipsilateral hemisphere, or alternatively, a region that is not (significantly) detected in the fetal, but in the neonatal stage.

Specifically, the left-sided deep white matter of the post-central gyrus, superior parietal lobule, superior temporal gyrus, and planum temporale region present in the dynamic analysis (Figure 6, C2 cluster) was present in both the fetal (Figure 6, F4 cluster) and neonatal brain (Figure 6, N1 cluster). The most concordant finding in our study in terms of across-method reproducibility, cluster size or significance was the inter-hemispheric morphometric asymmetries of the temporal lobe. A cluster in the temporal pole in framework B (C1) was confluent with the post-central gyrus as well as deep white matter while the temporal pole was the only region to be consistently found in framework A and B.

The parietal lobe is known to display global leftwards dominance, which might be concordant with the leftwards localization of our clusters (C1, C2) in the deep white matter of the superior parietal lobule and angular gyrus (Lehtola et al., 2019). Several studies reported inter-hemispheric asymmetries in the temporal lobe during development, predominantly revealing an emerging left dominance for the planum temporale region and temporal lobe and Sylvian fissure length (Lyttelton et al., 2009; Mallela et al., 2020), but a rightwards dominance in terms of gyral and sulcal development (Chi et al., 1977; Kasprian et al., 2011; Habas et al., 2012; Rajagopalan et al., 2011, 2012). The results of multiple studies in infants are consistent with those found in adult brains (Kong et al., 2018), indicating that the structural differences responsible for hemispheric speech and language dominance may be formed during the final trimester of fetal development (Chi et al., 1977; Wada and Davis, 1977; Gilmore et al., 2007; Hill et al., 2010; Li et al., 2014). The literature regarding the temporal lobe and its development remains ambiguous since an overall rightwards asymmetry for the temporal lobe in newborns has been reported (Lehtola et al., 2019), and there is converging evidence for rightwards superior temporal sulcus asymmetry (Hill et al., 2010; Leroy et al., 2015; Specht and Wigglesworth, 2018). Our study, however, used longitudinal data, therefore removing some of the ambiguity of inter-individual variability that may hinder studies that concluded developmental changes of asymmetry based on cross-sectional data. A similar study using a longitudinal design and a versatile, surface-based analysis in preterm infants replicated well-known asymmetries in the Sylvian fissure and a more significant opercularization in the left hemisphere, in contrast to the previously documented early sulcation development in the right hemisphere (De Vareilles et al., 2023).

We identified a cluster with right sided (increasing) neonatal morphometric lateralization located in the subcortical gray matter, internal and external capsules, extending ventrally into the hippocampus or the temporal horn of the right lateral ventricle. Our finding is concordant with one of others, suggesting that morphological features of subcortical gray matter in neonates are right dominant (Ratnarajah et al., 2013; Dean et al., 2018). However, in the largest performed study of this kind using the data of the ENIGMA consortium, the thalamus and basal ganglia were overall left dominant, while the caudate nucleus, hippocampus and amygdala were right dominant (Guadalupe et al., 2017). While the cluster C3/N2 reached ventrally to the hippocampus, it is too early to conclude whether this is a finding concordant with the data from the ENIGMA study, since we also revealed a strong rightwards dominance in the neonatal brain (N2) in a region that encompasses the lateral aspects of the thalamus. It is highly likely that asymmetries in the thalamus reflect asymmetry of the white matter pathways they relay, and therefore might not be consistently left or right dominant for all parts of the thalamus and basal ganglia.

The smallest cluster showing dynamically changing inter-hemispheric asymmetry was localized to the left frontal lobe, around the region of the left inferior frontal gyrus. Data from adults in the ENIGMA consortium confirmed rightwards asymmetry of the surface area of the inferior frontal gyrus, however, there is a scarcity of developmental studies confirming this observation. While this is the least significant finding in our analysis, it is possible that it marks an emerging leftwards dominance of the inferior frontal gyrus.

There are several ambiguities in our findings. For instance, there are regions that were statistically significantly lateralized in the late fetal images but not in the neonatal images (such as F3, F5, F6, F7, see Table 2 and Figure 6) or regions that were only detected in the neonatal images (such as N4, N6 and N7, see Table 3 and Figure 6). There are several reasons for these changes. First, technical aspects such as the presence of clusters that are smaller than the registration inaccuracy or noise differed between fetal and neonatal images. Secondly, there is the possibility that very subtle changes did not survive the statistical correction for multiple comparisons.

**TABLE 3:** Anatomical regions demonstrating inter-hemispheric morphometric asymmetry, static asymmetry in the fetal and neonatal timepoints.

Cluster	Hemisphere with larger JD values	Anatomical location	Most likely corresponding clusters	Size (# voxels)	Lowest *p*-value	JD values ipsilateral	
						Min–max	Mean
**Fetal inter-hemispheric morphometric asymmetry**							
F1	Right	Superior frontal gyrus, temporal pole	N2, C1	13,191	<0.001	−0.31 to 1.03	0.15
F2	Right	Occipital pole, cerebellar hemisphere	N5	6,654	0.003	−0.52 to 0.74	0.13
F3	Right	Occipital cortex, cuneus	C1	1,995	0.013	−0.64 to 0.62	0.13
F4	Right	Superior frontal gyrus	N3	1,515	0.013	−0.27 to 0.57	0.11
F5	Right	Superior frontal gyrus	N3	273	0.02	−0.26 to 0.65	0.8
F6	Left	Occipital cortex, primary visual	N1	178	0.023	−0.13 to 0.28	0.09
**Neonatal inter-hemispheric morphometric asymmetry**							
N1	Left	White matter, post-central gyrus, superior parietal lobule, superior temporal gyrus / PT region	C2	22,230	<0.001	−0.61 to 0.40	0.05
N2	Right	Thalamus, basal ganglia, claustrum, internal and external capsules, posteriormost aspect of the lateral sulcus	F1, C1	18,602	<0.001	−0.54 to 0.49	0.06
N3	Right	Superior frontal gyrus	F5, F4	5,606	0.007	−0.22 to 0.60	0.04
N4	Left	Olfactory cortex, white matter of the superior frontal gyrus		4,230	0.005	−0.18 to 0.26	0.04
N5	Right	Occipital pole	F2	815	0.017	−0.66 to 0.49	0.12
N6	Right	Cerebellum		506	0.013	−0.07 to 0.29	0.07
N7	Left	Deep white matter of the frontal lobe		407	0.023	−0.13 to 0.22	0.05

The clusters represent areas, where the JD values on the ipsilateral side (non-flipped image) were larger than on the contralateral side (flipped image). Clusters were ordered according to descending size.

### 4.2 Selection of registration methods for DBM in longitudinal fetal to neonatal cohorts

For DBM, accurate registration is critical for obtaining reliable results. A lower degree of anatomical correspondence would require larger spatial smoothing, which is a crucial confounding factor in DBM, VBM and related studies (Scarpazza et al., 2015). Evaluation of registration accuracy was crucially important for our study as the size and morphology of the brain changes significantly from late fetal to newborn stage. Different frameworks may offer varying levels of precision in the registration process. In the next section, we provide a critical review and highlight the potential limitations of our methodology to select the best registration method for morphometry.

While visual inspection of the results is often advised, it still can be challenging to assess the best registration strategy for the given problem. Here, we used a set of landmarks and segmentation-based metrics to quantify the registration accuracy. Segmentation based metrics, similarly to volumetric studies, rely on consistent segmentation across the two timepoints. Therefore, we used tissue labels, which are easier to segment consistently across fetal and neonatal brains but allow for less local evaluation of the accuracy than finer segmentation labels. The distance between two landmarks on the other hand is a single point and therefore describes the accuracy on a very local level. However, it is challenging to establish a set of suitable landmarks, as they need to be visible in the images in both timepoints.

After nonlinear registration, the achieved accuracy was moderate (Mean Dice of 0.75, 0.85, and 0.74 and mean landmark distance of 2.4, 2.0, and 2.8 mm, respectively for fetal to template, neonatal to template and fetal to neonatal registration). These results were achieved by increasing the number of iterations, reducing the radius for the cross-correlation to two and replacing the background of the fetal images with a bright value (See Supplementary section Registration testing).

When using segmentation and landmark based metrics, certain errors have to be expected. Oishi et al. (2011) performed registration accuracy tests for their atlas based anatomical segmentation of neonatal brains. The average Dice measure was 0.82, which was similar to the achieved accuracy using manual segmentation. However, the segmented structures were anatomically detailed which makes a direct comparison to our metrics not possible. Similarly, for the landmark-based metric, Lau et al. (2019) developed a landmark based framework to evaluate the anatomical correspondence between to brain images in adults. They reported a landmark localization error of less than 1 mm for landmarks such as PMJ, culmen and infracollicular sulcus and a mean error after nonlinear registration of 0.68, 2.7, and 0.93. For our worst performing registration setting, fetal to neonatal, we could report registration for the PMJ of 1.07 after linear and 0.82 after nonlinear registration. While some of the landmarks were inspired by the paper of Lau et al. (2019), we also identified our own landmarks that were further away from the brain stem which might have suffered from greater inaccuracy during the annotation. The motivation behind this was to capture the registration accuracy in the entire brain and not just core structures.

Our results imply that static asymmetry in the neonatal stage may be more easily and reliably detected. This is likely due to the larger size, better image quality, increased development and maturity of the neonatal brain, which makes it easier to identify morphometric features even with less precise registration.

### 4.3 Methodological limitations

Our findings on inter-hemispheric brain asymmetry are limited by the following methodological factors. In framework A, the large registration from the fetal to neonatal timepoints is challenging, however the JD which describes morphometric changes within each participant already captures the longitudinal aspect of the data. It describes the change between the two timepoints and simply by flipping the JD maps it is possible to perform longitudinal asymmetry testing. In framework B, the asymmetry at each timepoint is captured by registering the flipped to the non-flipped brain. By registration to a shared template the asymmetry then can be tested for differences. The registration to a shared template could be seen as less challenging as the timeframe and therefore difference between participant and template might be smaller. However, it is important to consider that the accuracy of the neonatal to template registration is higher than that for the fetal to template registration. Another inherent aspect of this framework is that the generation of JD maps based on flipped fetal to non-flipped fetal and flipped and non-flipped neonatal brains might produce subtle systematic differences due to the difference in the images.

In our study, we utilized image registrations in DBM, to infer inter-hemispheric asymmetry. However, this approach has some limitations with regard to the interpretability of our findings. We referred to our statistically significant “clusters” as anatomical regions where the morphometric features of the brain differ between homotopic regions of the two hemispheres. This approach is somewhat restrictive, as it relies on information contained within the JD maps to infer morphology. It is worth noting that the JD maps are based on image-based registration (and not surface or landmark-based), which aims to map visual features between a moving and target image. Consequently, the expansion or contraction of a brain region is only detected if there are features that can be matched and which expand or contract. In many brain regions, this is not the case during development. For instance, for the cortex, we primarily detected shrinkage (negative JD values) as the general tendency from fetal to neonatal age, which may be attributed to the increasing convolutional pattern (gyrification) with age after factoring out the majority of global brain scaling that occurred during the linear registration step. Furthermore, our non-linear registration methods are not likely to capture emerging structures such as two gyri appearing instead of one, or structures rotating, etc. As a result, while we employ a robust statistical approach and a dual-framework analysis, we cannot definitively conclude whether the asymmetries are driven by (asymmetric) isotropic growth, folding, rotation, or other types of morphometric differences between the two hemispheres.

A possible solution to these problems would be to perform landmark, sulcal, surface-based analyses (Lefèvre et al., 2016; Bozek et al., 2018; Lebenberg et al., 2018; Ahmad et al., 2019; Oishi et al., 2019), however, there the fidelity and topological correctness of meshes and segmentation accuracy in fetal brain remains a critical challenge (de Dumast et al., 2022; Payette et al., 2023). A newer approach might be to learn registrations by deep learning (Wei et al., 2020), however, it is not yet clear how efficient these networks are for fetal and neonatal development. Alternatively, a future approach could be to re-visit each region individually in each participant and provide an observer based manual annotations [such as in Kasprian et al. (2011)] to reveal concrete morphometric changes.

The present study is subject to limitations due to the relatively small sample size (*n* = 20), limiting the statistical power of our analyses. Nevertheless, this challenge of achieving substantial sample sizes is present in many studies using fetal or neonatal MRI of healthy pregnancies. To substantiate our findings, we suggest that validation of our results using comparable datasets or a database comprising preterm newborns, where clinically indicated longitudinal MRI scans are available.

## 5 Conclusion

In conclusion, our study complements existing neuroscientific studies on inter-hemispheric neuroanatomical asymmetry by providing evidence that morphological asymmetry is not static during late fetal development, and dynamic processes take place during late fetal development. Importantly, the neuroscientific value of our work is to characterize the dynamic patterns of asymmetry within-subject, therefore reducing some of the confounding effects of inter-individual variability that is present in cross-sectional studies on human brain development. Our results underscore the importance of the perinatal period for brain development, encompassing significant milestones such as tertiary gyri emergence and maturation, myelination, and cortical adaptation in response to external stimuli. The study also implies that a suitable image registration is critical when using DBM and that results might vary when using different frameworks, which must be considered when interpreting results.

## Data availability statement

The datasets presented in this article are not readily available because of restrictions in consent. Non-participant specific data supporting the conclusions of this article will be made available by the authors, without undue reservation. Code is available on GitHub: https://github.com/CST333/fet2neo_asymmetry. Requests to access the datasets should be directed to andras.jakab@kispi.uzh.ch.

## Ethics statement

The studies involving humans were approved by the Ethics Committee Zurich (Kantonale Ethikkommission Zürich). The studies were conducted in accordance with the local legislation and institutional requirements. Written informed consent for participation in this study was provided by the participants’ legal guardians/next of kin.

## Author contributions

GN, WK, AJ, and RT contributed to the conception and design of the studies. GN, WK, and AJ contributed to the funding acquisition. CS, AS, TN, NY, and SC recruited participants and collected the dataset. KP implemented the reconstruction tools. CM implemented the segmentation network. CS processed the dataset and performed the entire analysis. CS and AJ wrote the first draft of the manuscript. All authors contributed to manuscript revision and read and approved the submitted version.
