# Supplementary material for: Characterization of dynamic patterns of human fetal to neonatal brain asymmetry with deformation-based morphometry
Source: Front Neurosci. 2023 Dec 6;17:1252850. doi: 10.3389/fnins.2023.1252850 (PMC10734644; doi:10.3389/fnins.2023.1252850)
Supplement: Supplementary file 1 [file Data_Sheet_1.ZIP › ChallengeR/Framework A fetal to neonatal/linear/ranking_Fetal2Neonatal_Linear_Dice.pdf]

Benchmarking report for Fetal to Neonatal Registration Linear

created by challengeR v1.0.4

28 März, 2023

This document presents a systematic report on the benchmark study "Fetal to Neonatal Registration Linear". Input data comprises raw metric values for all algorithms and cases. Generated plots are:

- Visualization of assessment data: Dot- and boxplots, podium plots and ranking heatmaps
- Visualization of ranking stability: Blob plots, violin plots and significance maps, line plots
- Visualization of cross-task insights: Blob plots, stacked frequency plots, dendrograms

Details can be found in Wiesenfarth et al. (2021).

1 Rankings

Algorithms within a task are ranked according to the following ranking scheme:

aggregate using function ("mean") then rank

Ranking for each task:

Dice Brainstem : The analysis is based on 17 algorithms and 20 cases. 0 missing cases have been found in the data set.

	value_mean	rank
TRSAA_erroded_masked	0.7899105	1
TRSAA_masked	0.7805105	2
TRSAA_nomask	0.7613389	3
flirt_NA	0.7541856	4
Affine_nomask	0.7541103	5
Affine_masked	0.7290414	6
Similarity_nomask	0.7178786	7
Affine_erroded_masked	0.6639471	8
Similarity_masked	0.6497113	9
terminal_CC_nomask	0.6420108	10
terminal_CC_masked	0.6352044	11
terminal_CC_erroded_masked	0.6343858	12
Similarity_erroded_masked	0.6324760	13
terminal_MI_nomask	0.5832403	14
terminal_MI_erroded_masked	0.5828046	15
terminal_MI_masked	0.5775537	16
antsAI_masked	0.5707625	17

Dice Cerebellum : The analysis is based on 17 algorithms and 20 cases. 0 missing cases have been found in the data set.

	value_mean	rank
TRSAA_erroded_masked	0.7172282	1
flirt_NA	0.6788023	2
TRSAA_nomask	0.6784406	3
Affine_nomask	0.6765522	4
TRSAA_masked	0.6648065	5
Similarity_nomask	0.6596847	6
Affine_masked	0.6427158	7
Affine_erroded_masked	0.6150631	8
Similarity_masked	0.6115194	9
Similarity_erroded_masked	0.6022289	10
terminal_CC_masked	0.4945895	11
terminal_CC_nomask	0.4941648	12
terminal_CC_erroded_masked	0.4842885	13
terminal_MI_masked	0.4691219	14
terminal_MI_erroded_masked	0.4523159	15
terminal_MI_nomask	0.4486554	16
antsAI_masked	0.4250379	17

Dice Cortical grey matter : The analysis is based on 17 algorithms and 20 cases. 0 missing cases have been found in the data set.

	value_mean	rank
TRSAA_erroded_masked	0.4461191	1
flirt_NA	0.4186681	2
TRSAA_nomask	0.4179982	3
Affine_nomask	0.4176298	4
TRSAA_masked	0.4116589	5
Affine_masked	0.4055611	6
Affine_erroded_masked	0.4033985	7
Similarity_nomask	0.4022807	8
Similarity_masked	0.3810117	9
Similarity_erroded_masked	0.3788675	10
terminal_MI_erroded_masked	0.2764788	11
terminal_CC_nomask	0.2721102	12
terminal_MI_nomask	0.2672210	13
terminal_CC_erroded_masked	0.2666965	14
terminal_MI_masked	0.2666390	15
terminal_CC_masked	0.2648276	16
antsAI_masked	0.2543837	17

Dice CSF and ventricles : The analysis is based on 17 algorithms and 20 cases. 0 missing cases have been found in the data set.

	value_mean	rank
flirt_NA	0.4860896	1
TRSAA_nomask	0.4835936	2
Affine_nomask	0.4816155	3
TRSAA_erroded_masked	0.4525796	4
TRSAA_masked	0.4204157	5
Similarity_nomask	0.4174858	6

	value_mean	rank
Affine_masked	0.3733163	7
Affine_erroded_masked	0.3470518	8
Similarity_masked	0.3071579	9
Similarity_erroded_masked	0.3017342	10
terminal_MI_erroded_masked	0.2002177	11
terminal_MI_masked	0.1907151	12
terminal_CC_nomask	0.1899362	13
terminal_CC_erroded_masked	0.1866303	14
terminal_MI_nomask	0.1854870	15
terminal_CC_masked	0.1833186	16
antsAI_masked	0.1655684	17

Dice Deep grey matter : The analysis is based on 17 algorithms and 20 cases. 0 missing cases have been found in the data set.

	value_mean	rank
TRSAA_erroded_masked	0.7835607	1
TRSAA_masked	0.7613307	2
TRSAA_nomask	0.7496447	3
Affine_nomask	0.7488511	4
flirt_NA	0.7457330	5
Affine_masked	0.7404453	6
Similarity_nomask	0.7250278	7
Affine_erroded_masked	0.7199502	8
Similarity_masked	0.7088347	9
Similarity_erroded_masked	0.6974690	10
terminal_CC_nomask	0.6201574	11
terminal_CC_erroded_masked	0.6126447	12
terminal_CC_masked	0.6123666	13
terminal_MI_erroded_masked	0.5972101	14
antsAI_masked	0.5945193	15
terminal_MI_nomask	0.5879106	16
terminal_MI_masked	0.5828615	17

Dice White matter : The analysis is based on 17 algorithms and 20 cases. 0 missing cases have been found in the data set.

	value_mean	rank
TRSAA_erroded_masked	0.6888511	1
TRSAA_masked	0.6735692	2
TRSAA_nomask	0.6690568	3
Affine_nomask	0.6689226	4
flirt_NA	0.6685577	5
Affine_masked	0.6638978	6
Similarity_nomask	0.6564830	7
Affine_erroded_masked	0.6524649	8
Similarity_masked	0.6437591	9
Similarity_erroded_masked	0.6370602	10
terminal_CC_nomask	0.5462038	11
terminal_CC_erroded_masked	0.5392586	12

	value_mean	rank
terminal_CC_masked	0.5368943	13
terminal_MI_erroded_masked	0.5342979	14
terminal_MI_nomask	0.5320050	15
antsAI_masked	0.5290228	16
terminal_MI_masked	0.5244325	17

Consensus ranking across tasks according to chosen method “euclidean”:

	value	rank
TRSAA_erroded_masked	1.500	1
TRSAA_nomask	2.833	2
flirt_NA	3.167	3
TRSAA_masked	3.500	4
Affine_nomask	4.000	5
Affine_masked	6.333	6
Similarity_nomask	6.833	7
Affine_erroded_masked	7.833	8
Similarity_masked	9.000	9
Similarity_erroded_masked	10.500	10
terminal_CC_nomask	11.500	11
terminal_CC_erroded_masked	12.833	12
terminal_CC_masked	13.333	13
terminal_MI_erroded_masked	13.333	13
terminal_MI_nomask	14.833	15
terminal_MI_masked	15.167	16
antsAI_masked	16.500	17

2 Visualization of raw assessment data

The algorithms are ordered according to the computed ranks for each task.

2.1 Dot- and boxplot

Dot- and boxplots for visualizing raw assessment data separately for each algorithm. Boxplots representing descriptive statistics over all cases (median, quartiles and outliers) are combined with horizontally jittered dots representing individual cases.

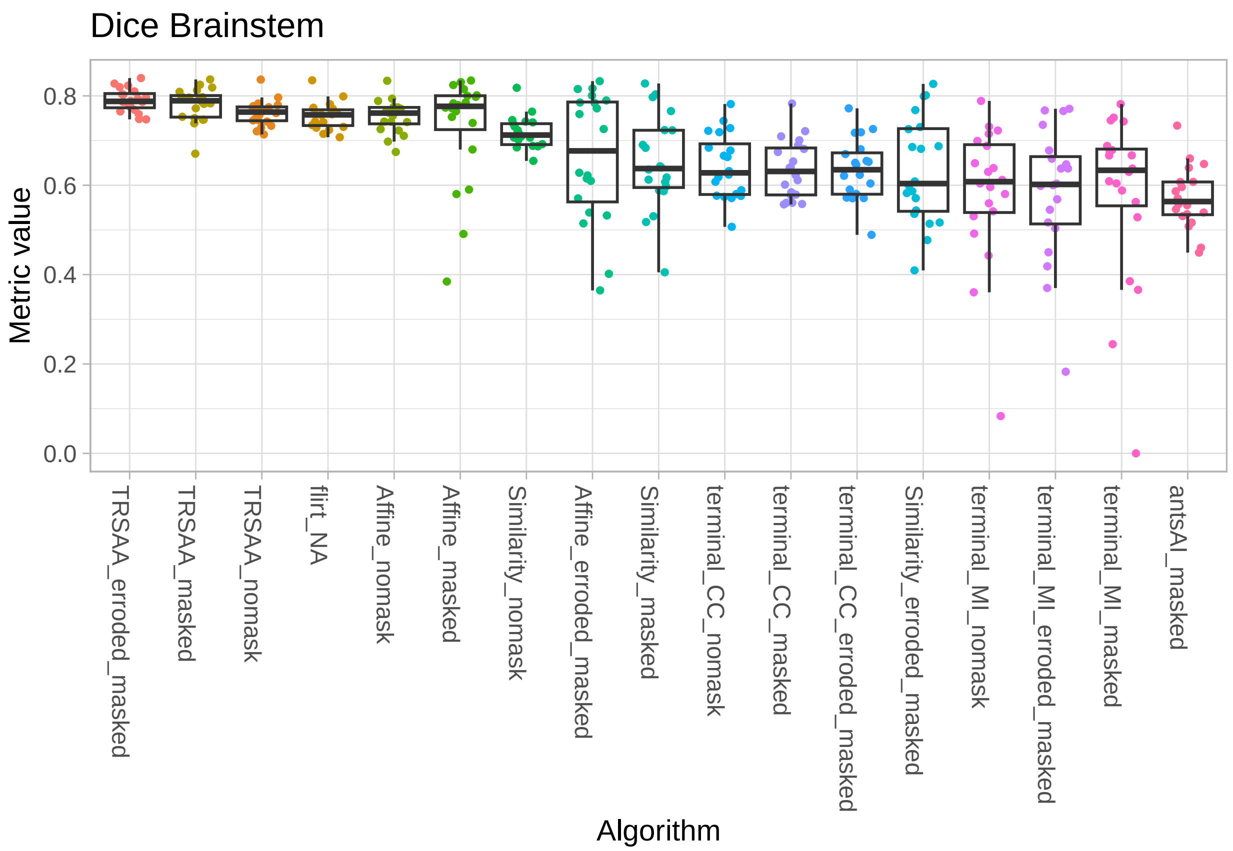

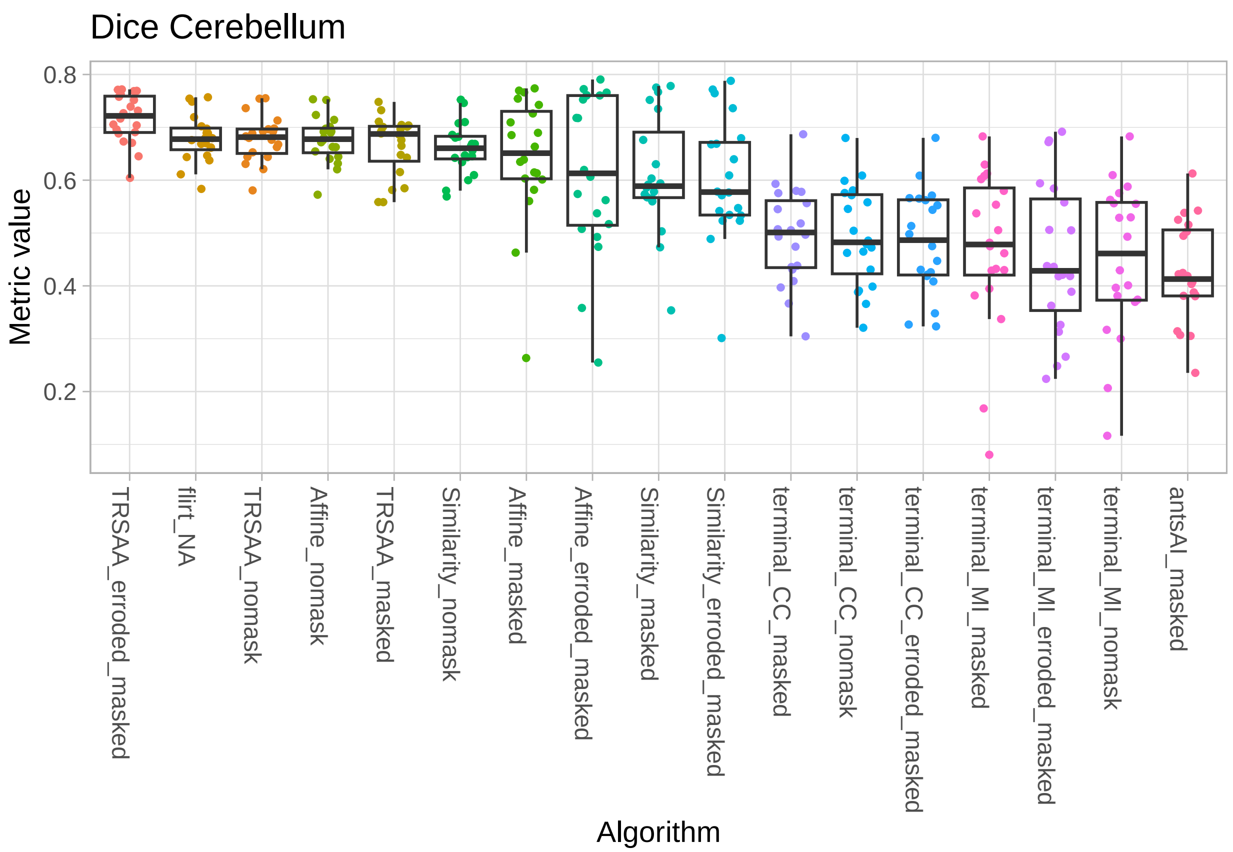
Dice Cerebellum
Metric value
0.8
0.6
0.4
0.2
TRSAA_erroded_masked
flirt_NA
TRSAA_nomask
Affine_nomask
TRSAA_masked
Similarity_nomask
Affine_masked
Affine_erroded_masked
Similarity_masked
Similarity_erroded_masked
terminal_CC_masked
terminal_CC_nomask
terminal_CC_erroded_masked
terminal_MI_masked
terminal_MI_erroded_masked
terminal_MI_nomask
antsAI_masked
Algorithm

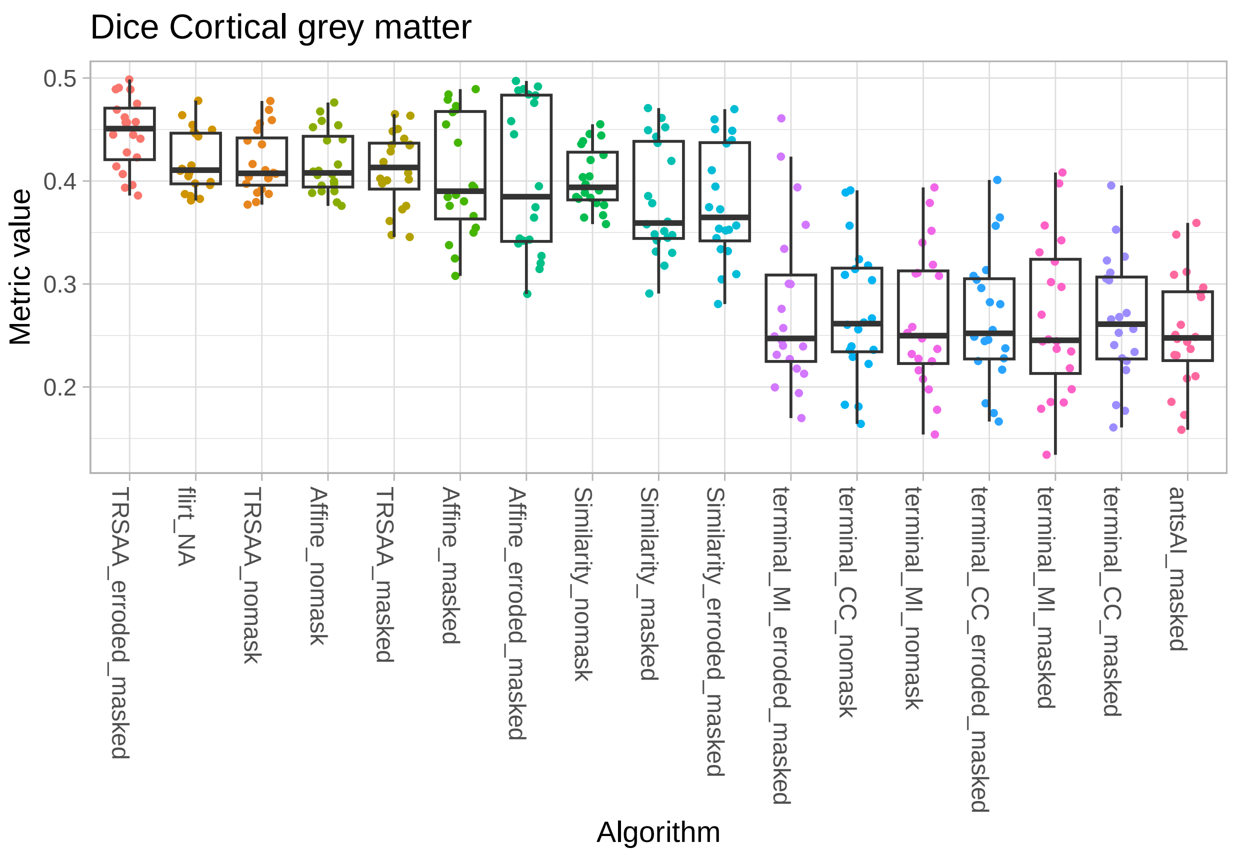
Dice Cortical grey matter
Metric value
0.5
0.4
0.3
0.2
TRSAA_erroded_masked
flirt_NA
TRSAA_nomask
Affine_nomask
TRSAA_masked
Affine_masked
Affine_erroded_masked
Similarity_nomask
Similarity_masked
Similarity_erroded_masked
terminal_MI_erroded_masked
terminal_CC_nomask
terminal_MI_nomask
terminal_CC_erroded_masked
terminal_MI_masked
terminal_CC_masked
antsAI_masked
Algorithm

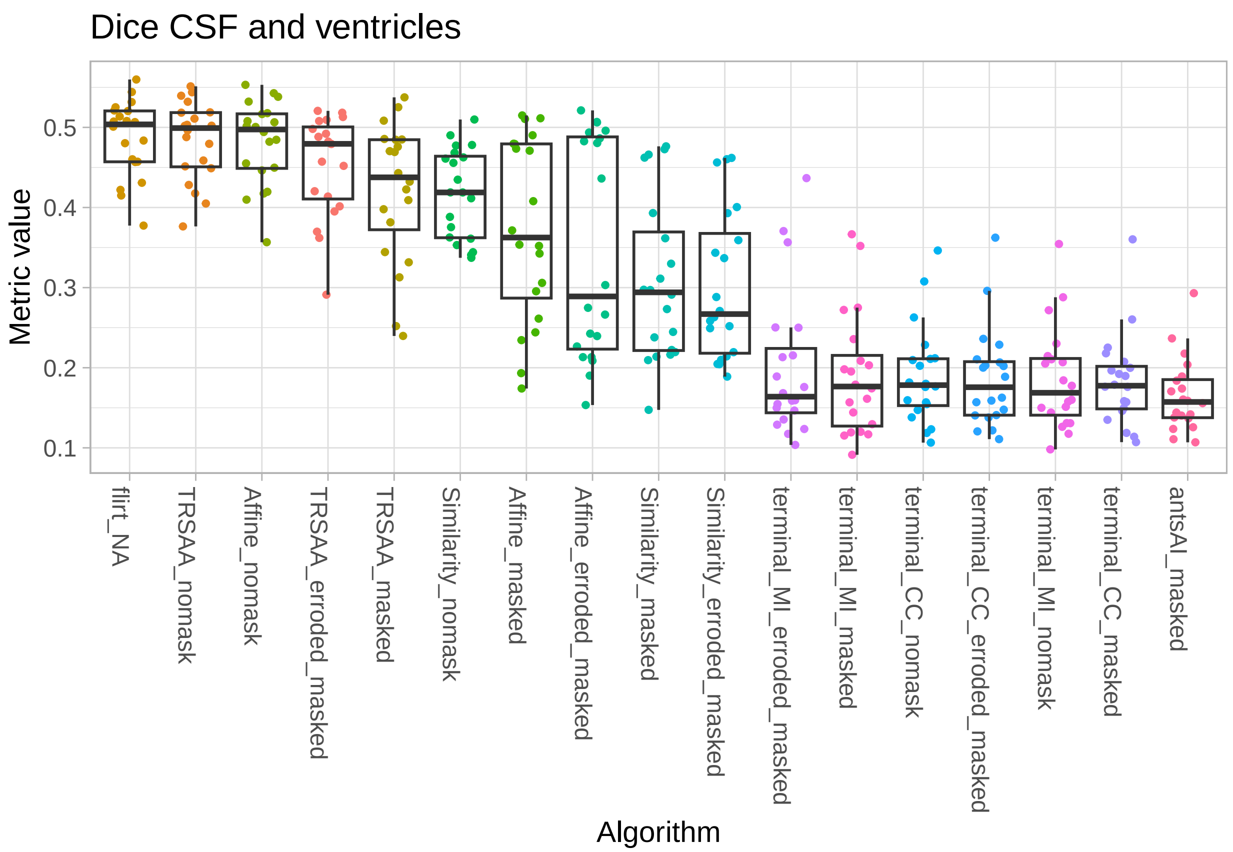
Dice CSF and ventricles
Metric value
0.5
0.4
0.3
0.2
0.1
flirt_NA
TRSAA_nomask
Affine_nomask
TRSAA_erroded_masked
TRSAA_masked
Similarity_nomask
Affine_masked
Affine_erroded_masked
Similarity_masked
Similarity_erroded_masked
terminal_MI_erroded_masked
terminal_MI_masked
terminal_CC_nomask
terminal_CC_erroded_masked
terminal_MI_nomask
terminal_CC_masked
antsAI_masked
Algorithm

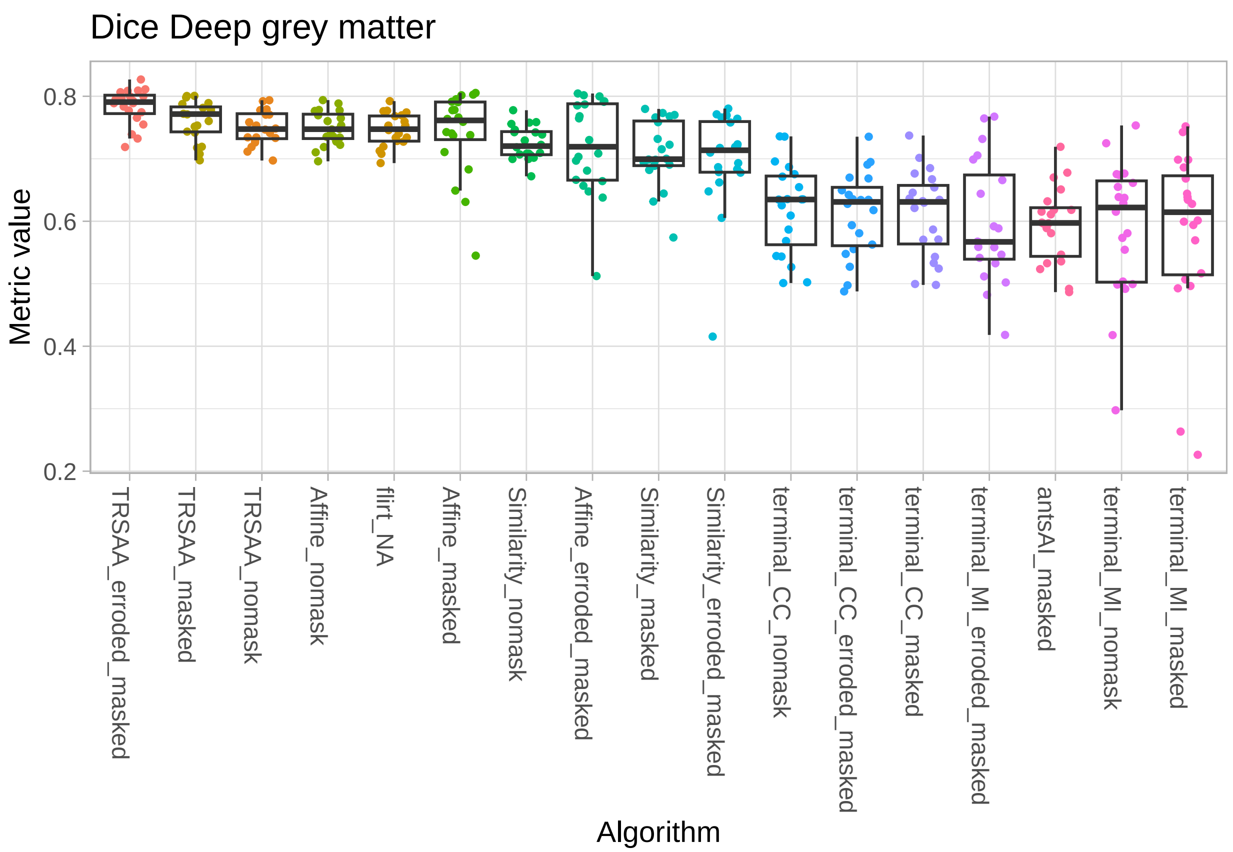
Dice Deep grey matter
Metric value
0.8
0.6
0.4
0.2
TRSAA_erroded_masked
TRSAA_masked
TRSAA_nomask
Affine_nomask
flirt_NA
Affine_masked
Similarity_nomask
Affine_erroded_masked
Similarity_masked
Similarity_erroded_masked
terminal_CC_nomask
terminal_CC_erroded_masked
terminal_CC_masked
terminal_MI_erroded_masked
antsAI_masked
terminal_MI_nomask
terminal_MI_masked
Algorithm

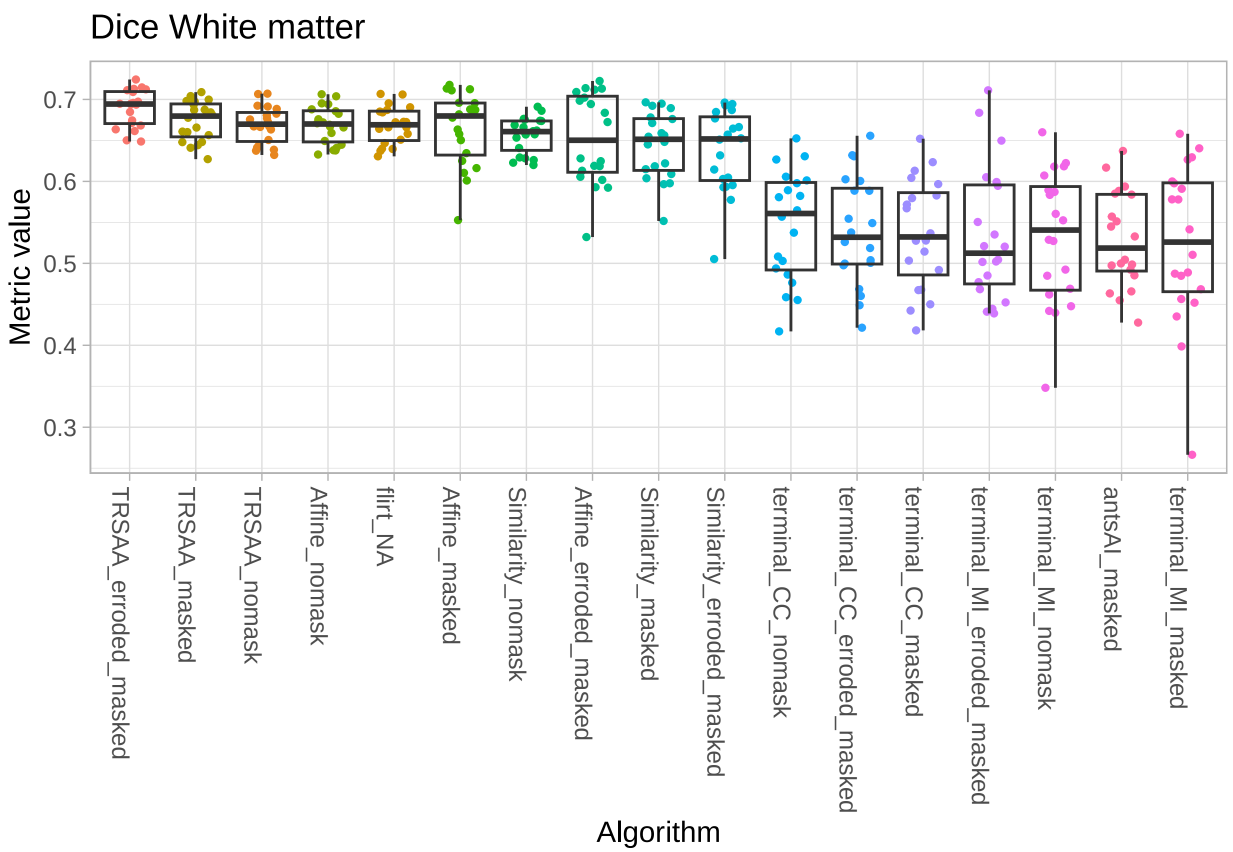
Dice White matter
Metric value
0.7
0.6
0.5
0.4
0.3
TRSAA_erroded_masked
TRSAA_masked
TRSAA_nomask
Affine_nomask
flirt_NA
Affine_masked
Similarity_nomask
Affine_erroded_masked
Similarity_masked
Similarity_erroded_masked
terminal_CC_nomask
terminal_CC_erroded_masked
terminal_CC_masked
terminal_MI_erroded_masked
terminal_MI_nomask
antsAI_masked
terminal_MI_masked
Algorithm

2.2 Podium plot

Podium plots (see also Eugster et al., 2008) for visualizing raw assessment data. Upper part (spaghetti plot): Participating algorithms are color-coded, and each colored dot in the plot represents a metric value achieved with the respective algorithm. The actual metric value is encoded by the y-axis. Each podium (here: p=17) represents one possible rank, ordered from best (1) to last (here: 17). The assignment of metric values (i.e. colored dots) to one of the podiums is based on the rank that the respective algorithm achieved on the corresponding case. Note that the plot part above each podium place is further subdivided into p "columns", where each column represents one participating algorithm (here: $p = 17$). Dots corresponding to identical cases are connected by a line, leading to the shown spaghetti structure. Lower part: Bar charts represent the relative frequency for each algorithm to achieve the rank encoded by the podium place.

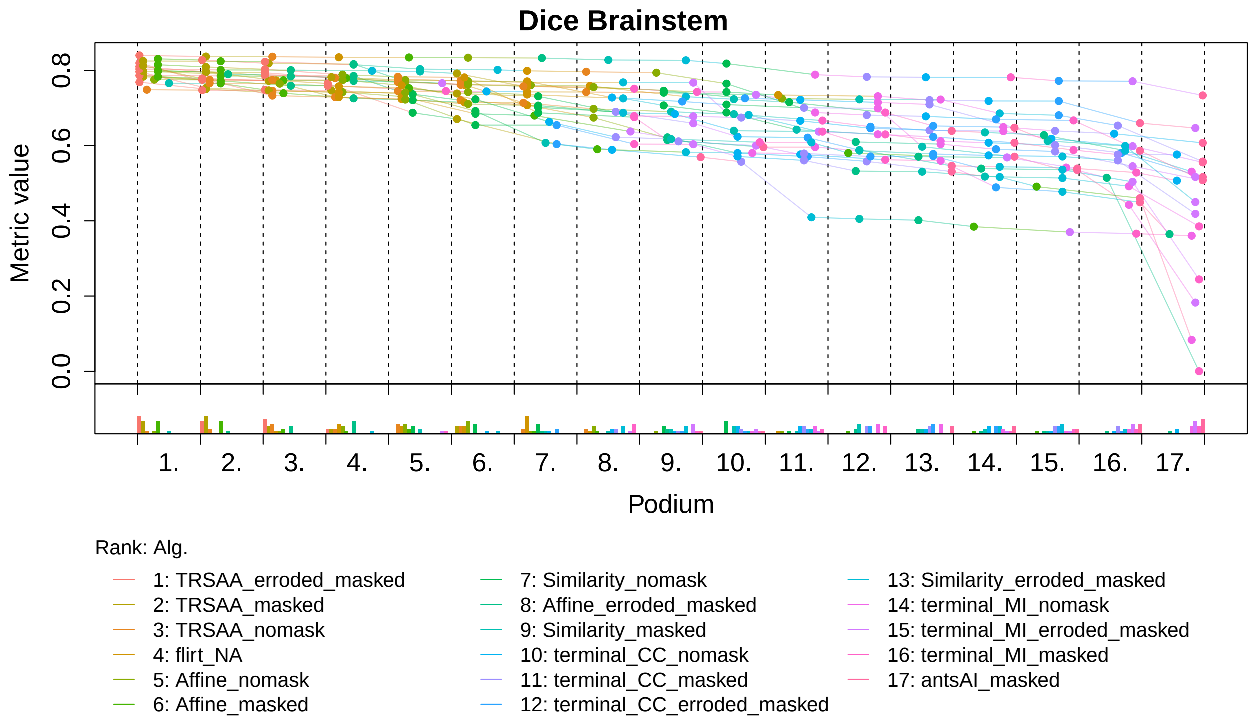

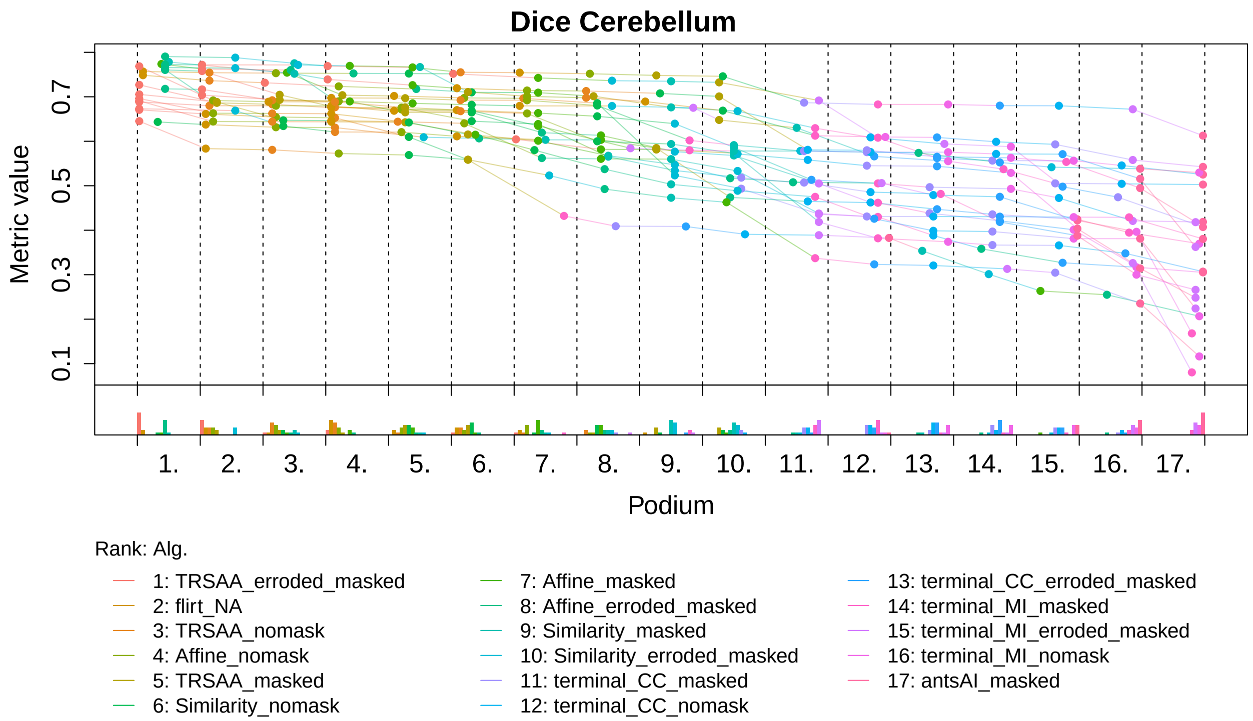
Dice Cerebellum
Metric value
0.1
0.3
0.5
0.7
1.
2.
3.
4.
5.
6.
7.
8.
9.
10.
11.
12.
13.
14.
15.
16.
17.
Podium
Rank: Alg.
1: TRSAA_erroded_masked
2: flirt_NA
3: TRSAA_nomask
4: Affine_nomask
5: TRSAA_masked
6: Similarity_nomask
7: Affine_masked
8: Affine_erroded_masked
9: Similarity_masked
10: Similarity_erroded_masked
11: terminal_CC_masked
12: terminal_CC_nomask
13: terminal_CC_erroded_masked
14: terminal_MI_masked
15: terminal_MI_erroded_masked
16: terminal_MI_nomask
17: antsAI_masked

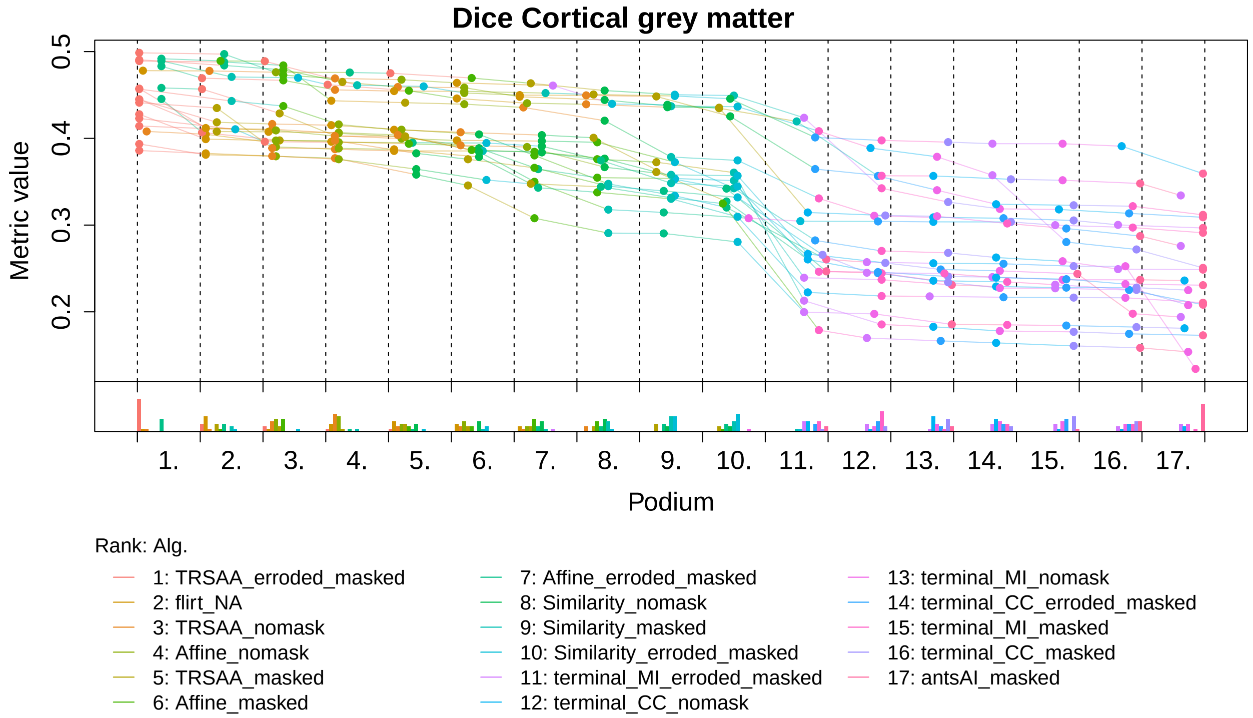
Dice Cortical grey matter
Metric value
0.2
0.3
0.4
0.5
1.
2.
3.
4.
5.
6.
7.
8.
9.
10.
11.
12.
13.
14.
15.
16.
17.
Podium
Rank: Alg.
1: TRSAA_erroded_masked
2: flirt_NA
3: TRSAA_nomask
4: Affine_nomask
5: TRSAA_masked
6: Affine_masked
7: Affine_erroded_masked
8: Similarity_nomask
9: Similarity_masked
10: Similarity_erroded_masked
11: terminal_MI_erroded_masked
12: terminal_CC_nomask
13: terminal_MI_nomask
14: terminal_CC_erroded_masked
15: terminal_MI_masked
16: terminal_CC_masked
17: antsAI_masked

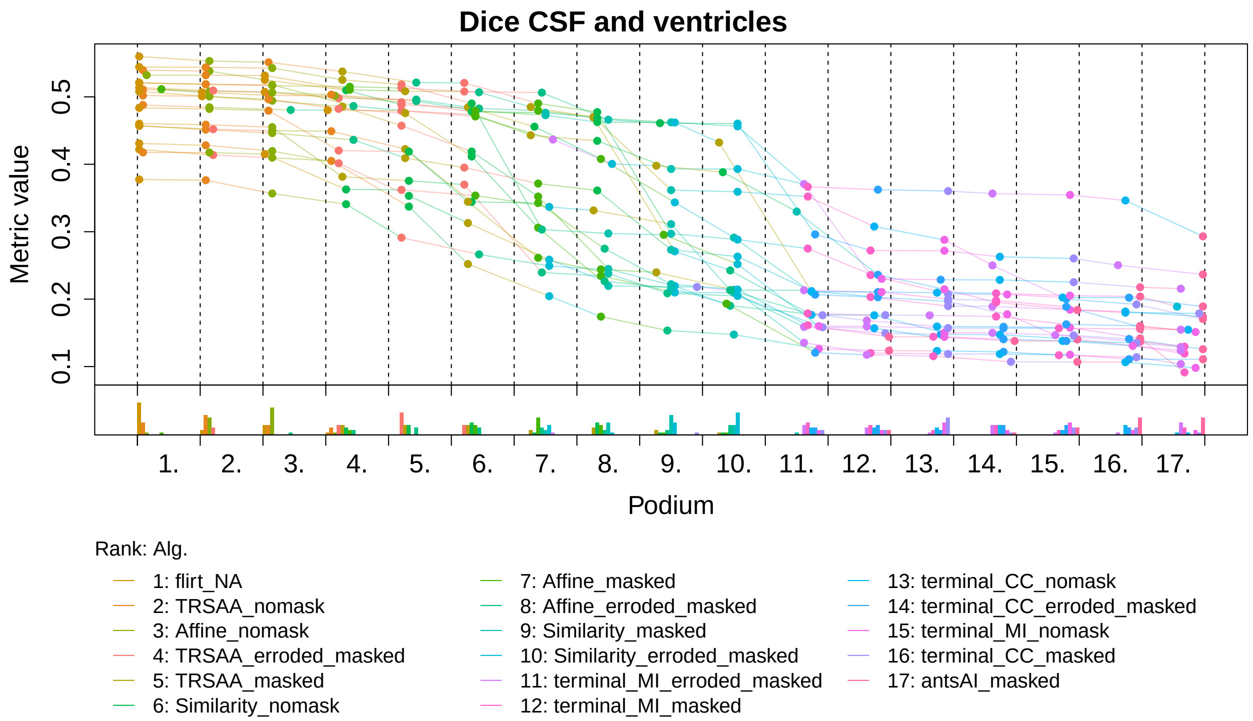
Dice CSF and ventricles
Metric value
0.1
0.2
0.3
0.4
0.5
1.
2.
3.
4.
5.
6.
7.
8.
9.
10.
11.
12.
13.
14.
15.
16.
17.
Podium
Rank: Alg.
1: flirt_NA
2: TRSAA_nomask
3: Affine_nomask
4: TRSAA_erroded_masked
5: TRSAA_masked
6: Similarity_nomask
7: Affine_masked
8: Affine_erroded_masked
9: Similarity_masked
10: Similarity_erroded_masked
11: terminal_MI_erroded_masked
12: terminal_MI_masked
13: terminal_CC_nomask
14: terminal_CC_erroded_masked
15: terminal_MI_nomask
16: terminal_CC_masked
17: antsAI_masked

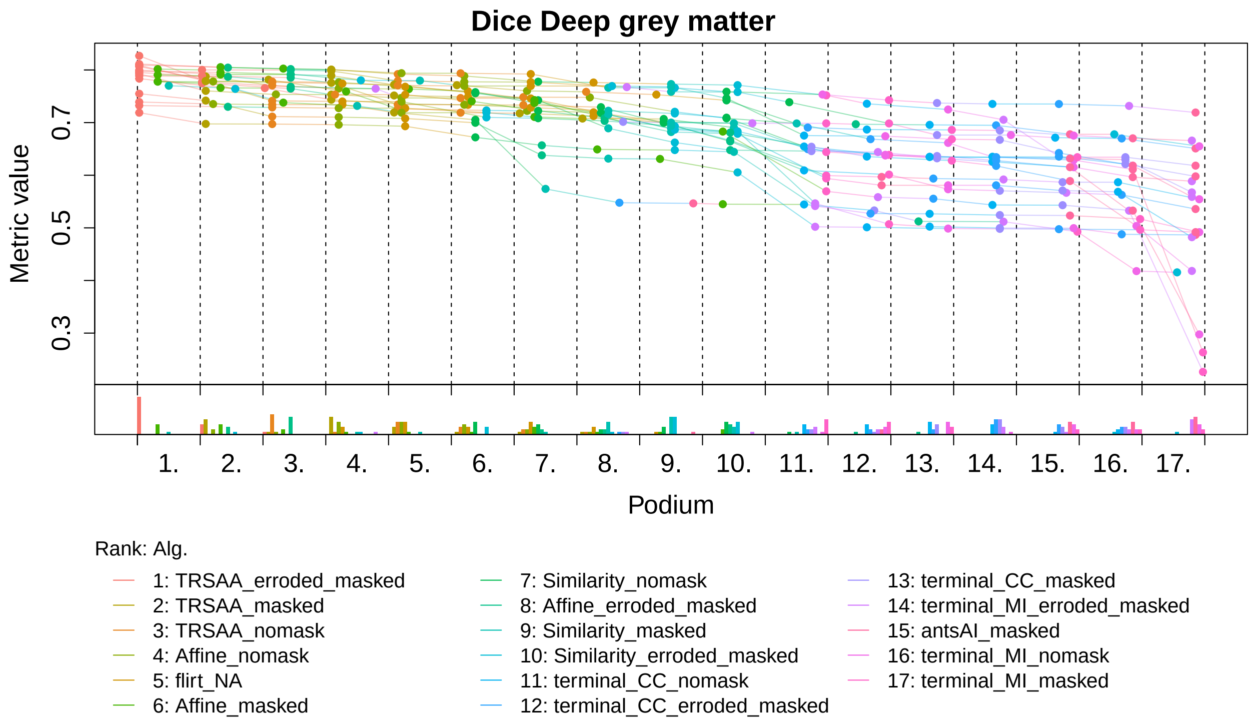
Dice Deep grey matter
Metric value
0.3
0.5
0.7
1.
2.
3.
4.
5.
6.
7.
8.
9.
10.
11.
12.
13.
14.
15.
16.
17.
Podium
Rank: Alg.
1: TRSAA_erroded_masked
2: TRSAA_masked
3: TRSAA_nomask
4: Affine_nomask
5: flirt_NA
6: Affine_masked
7: Similarity_nomask
8: Affine_erroded_masked
9: Similarity_masked
10: Similarity_erroded_masked
11: terminal_CC_nomask
12: terminal_CC_erroded_masked
13: terminal_CC_masked
14: terminal_MI_erroded_masked
15: antsAI_masked
16: terminal_MI_nomask
17: terminal_MI_masked

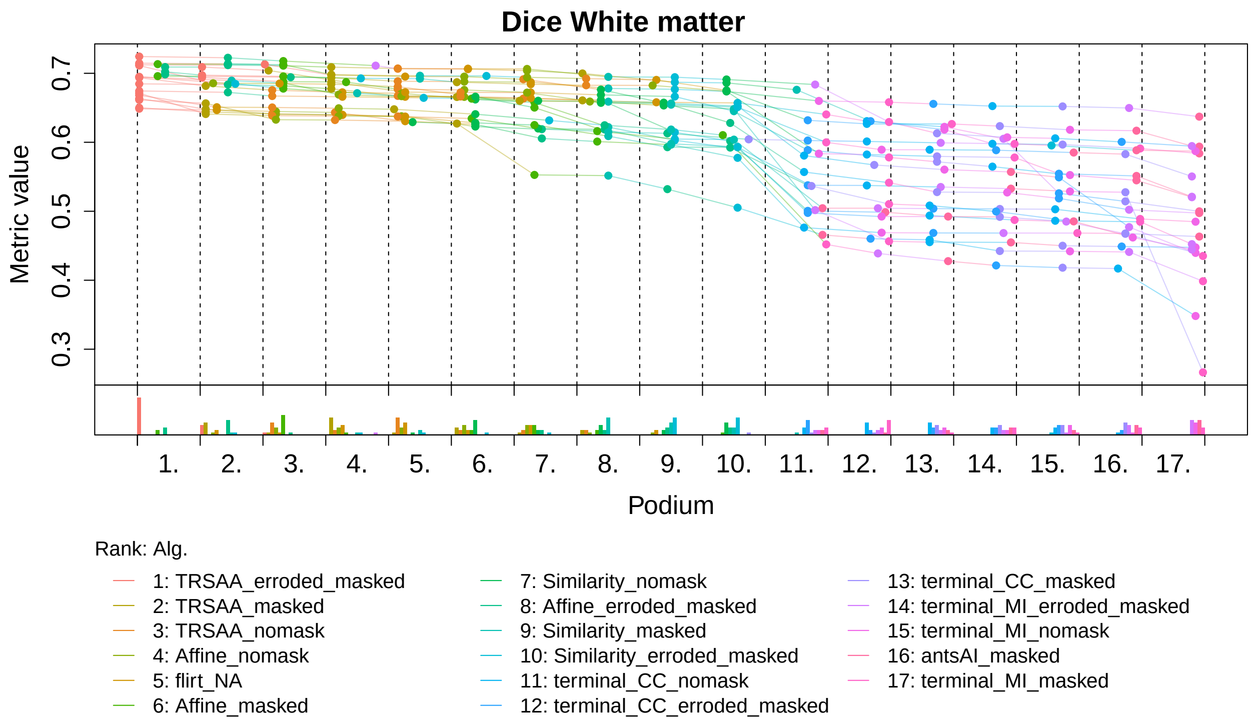
Dice White matter
Metric value
0.3
0.4
0.5
0.6
0.7
1.
2.
3.
4.
5.
6.
7.
8.
9.
10.
11.
12.
13.
14.
15.
16.
17.
Podium
Rank: Alg.
1: TRSAA_erroded_masked
2: TRSAA_masked
3: TRSAA_nomask
4: Affine_nomask
5: flirt_NA
6: Affine_masked
7: Similarity_nomask
8: Affine_erroded_masked
9: Similarity_masked
10: Similarity_erroded_masked
11: terminal_CC_nomask
12: terminal_CC_erroded_masked
13: terminal_CC_masked
14: terminal_MI_erroded_masked
15: terminal_MI_nomask
16: antsAI_masked
17: terminal_MI_masked

2.3 Ranking heatmap

Ranking heatmaps for visualizing raw assessment data. Each cell (i, A_j) shows the absolute frequency of cases in which algorithm A_j achieved rank i.

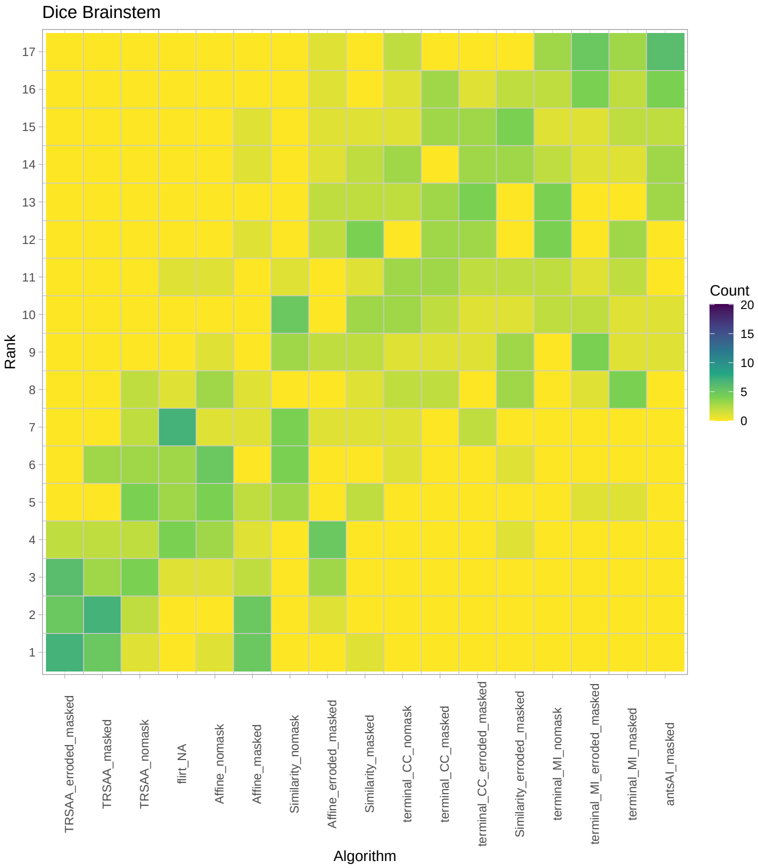

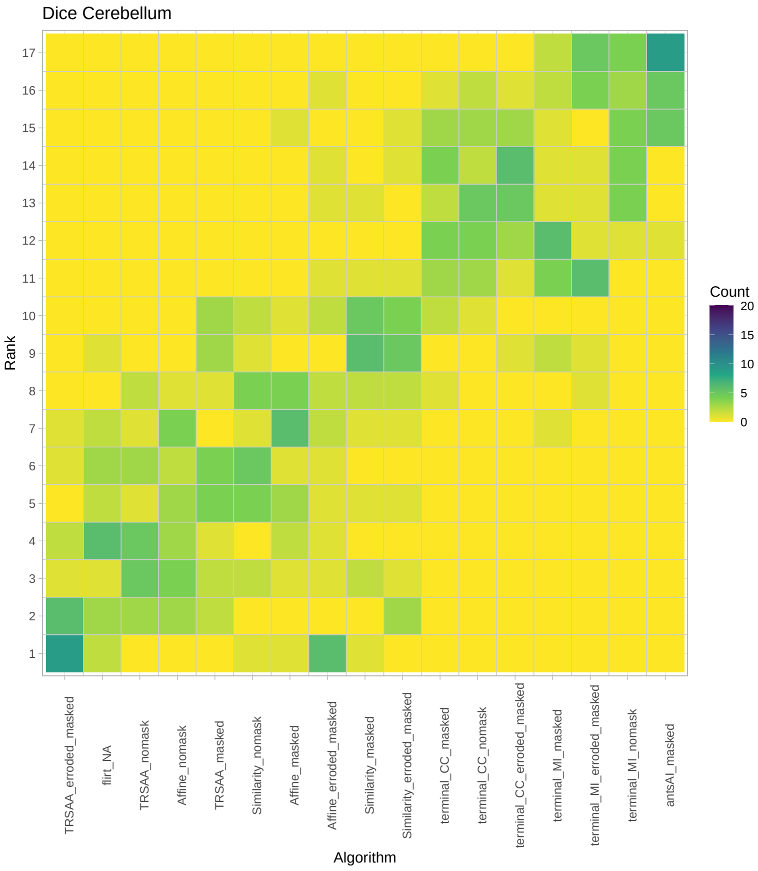

Dice Cerebellum
Rank
17
16
15
14
13
12
11
10
9
8
7
6
5
4
3
2
1
Count
20
15
10
5
0
TRSAA_erroded_masked
flirt_NA
TRSAA_nomask
Affine_nomask
TRSAA_masked
Similarity_nomask
Affine_masked
Affine_erroded_masked
Similarity_masked
Similarity_erroded_masked
terminal_CC_masked
terminal_CC_nomask
terminal_CC_erroded_masked
terminal_MI_masked
terminal_MI_erroded_masked
terminal_MI_nomask
antsAI_masked
Algorithm

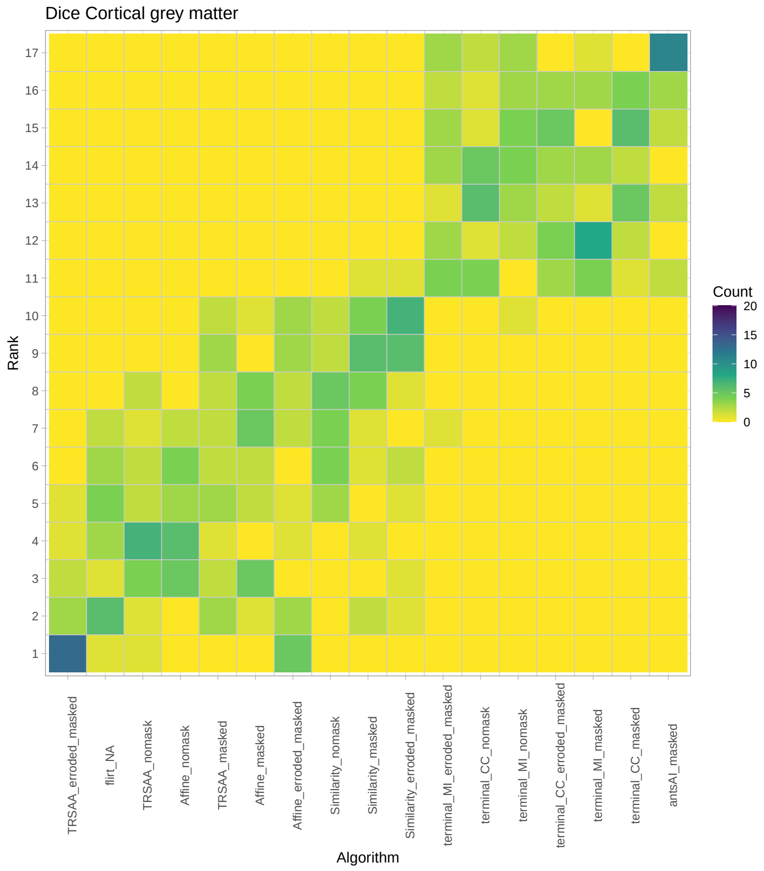
Dice Cortical grey matter
Rank
17
16
15
14
13
12
11
10
9
8
7
6
5
4
3
2
1
Count
20
15
10
5
0
TRSAA_erroded_masked
flirt_NA
TRSAA_nomask
Affine_nomask
TRSAA_masked
Affine_masked
Affine_erroded_masked
Similarity_nomask
Similarity_masked
Similarity_erroded_masked
terminal_MI_erroded_masked
terminal_CC_nomask
terminal_MI_nomask
terminal_CC_erroded_masked
terminal_MI_masked
terminal_CC_masked
antsAI_masked
Algorithm

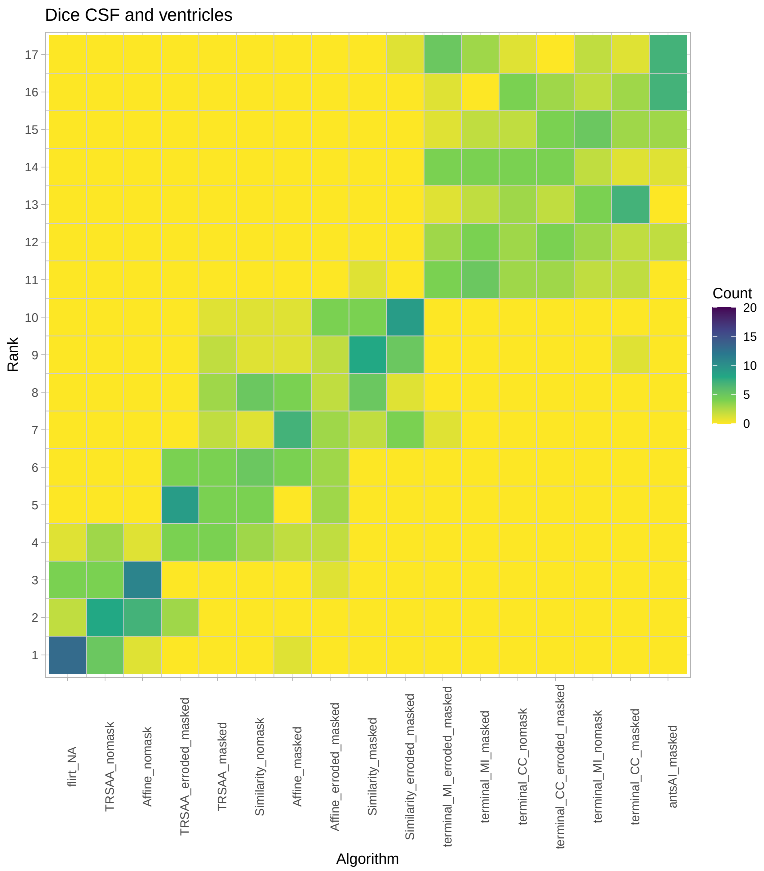

Dice CSF and ventricles
Rank
Algorithm
Count
20
15
10
5
0
17
16
15
14
13
12
11
10
9
8
7
6
5
4
3
2
1
flirt_NA
TRSAA_nomask
Affine_nomask
TRSAA_erroded_masked
TRSAA_masked
Similarity_nomask
Affine_masked
Affine_erroded_masked
Similarity_masked
Similarity_erroded_masked
terminal_MI_erroded_masked
terminal_MI_masked
terminal_CC_nomask
terminal_CC_erroded_masked
terminal_MI_nomask
terminal_CC_masked
antsAI_masked

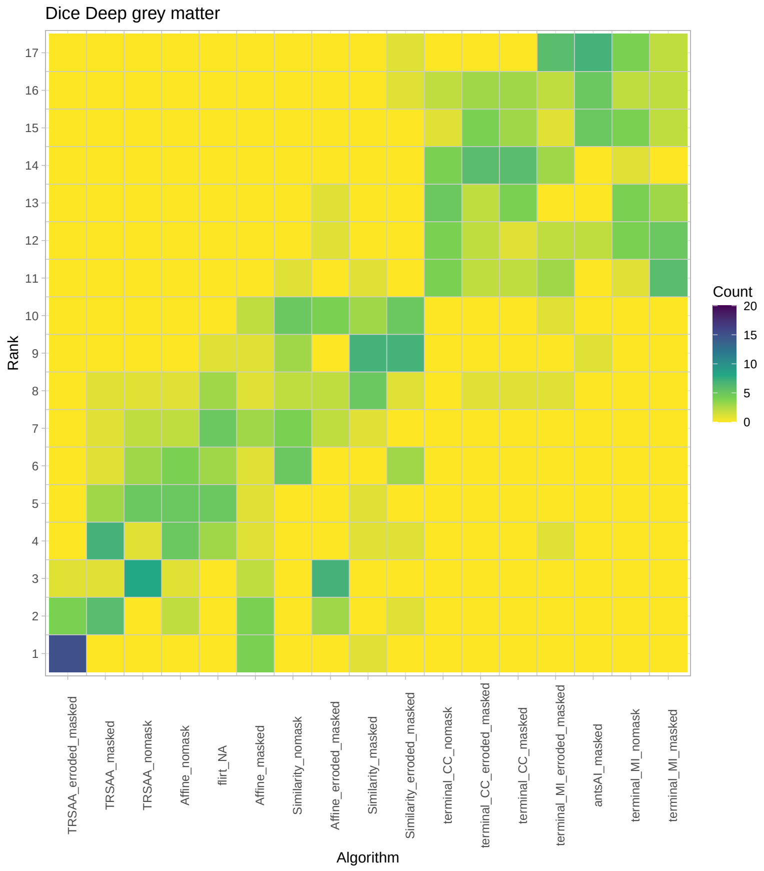

Dice Deep grey matter
Rank
17
16
15
14
13
12
11
10
9
8
7
6
5
4
3
2
1
Count
20
15
10
5
0
TRSAA_erroded_masked
TRSAA_masked
TRSAA_nomask
Affine_nomask
flirt_NA
Affine_masked
Similarity_nomask
Affine_erroded_masked
Similarity_masked
Similarity_erroded_masked
terminal_CC_nomask
terminal_CC_erroded_masked
terminal_CC_masked
terminal_MI_erroded_masked
antsAI_masked
terminal_MI_nomask
terminal_MI_masked
Algorithm

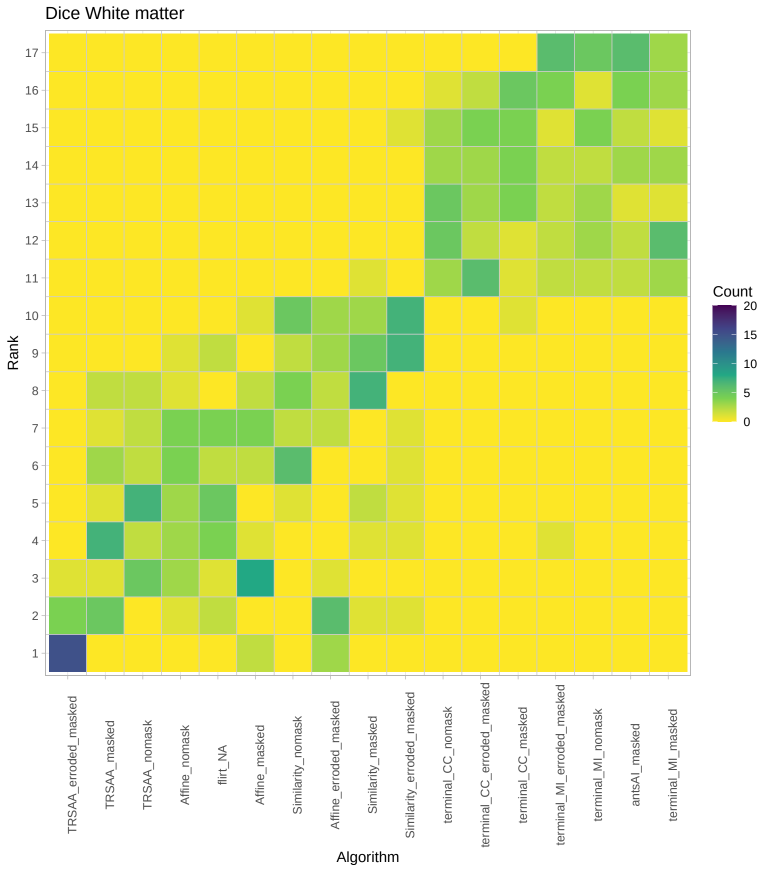

Dice White matter
Rank
17
16
15
14
13
12
11
10
9
8
7
6
5
4
3
2
1
TRSAA_erroded_masked
TRSAA_masked
TRSAA_nomask
Affine_nomask
flirt_NA
Affine_masked
Similarity_nomask
Affine_erroded_masked
Similarity_masked
Similarity_erroded_masked
terminal_CC_nomask
terminal_CC_erroded_masked
terminal_CC_masked
terminal_MI_erroded_masked
terminal_MI_nomask
antsAI_masked
terminal_MI_masked
Algorithm
Count
20
15
10
5
0

3 Visualization of ranking stability

3.1 *Blob plot* for visualizing ranking stability based on bootstrap sampling

Algorithms are color-coded, and the area of each blob at position $(A_i, \text{rank } j)$ is proportional to the relative frequency A_i achieved rank j across $b = 1000$ bootstrap samples. The median rank for each algorithm is indicated by a black cross. 95% bootstrap intervals across bootstrap samples are indicated by black lines.

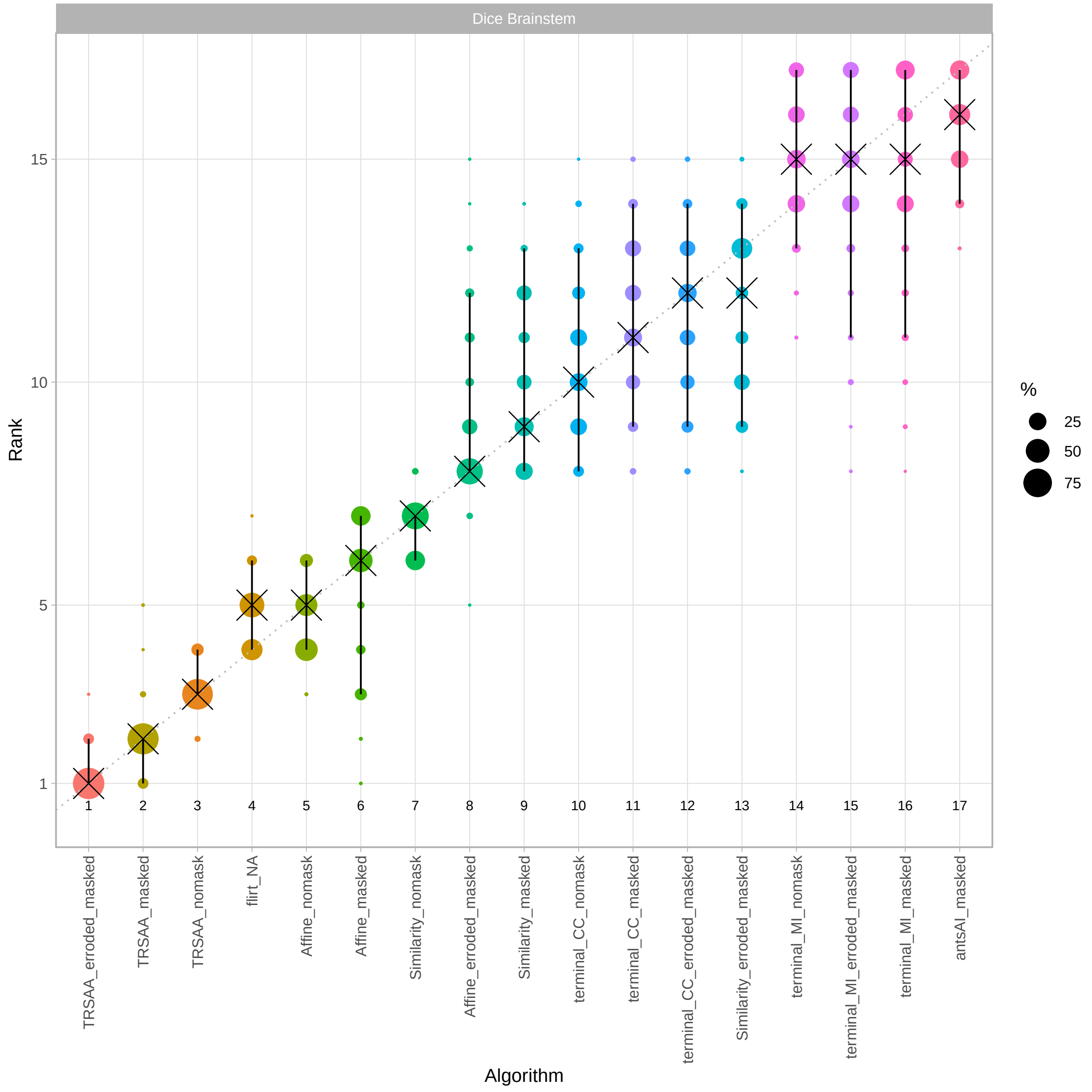

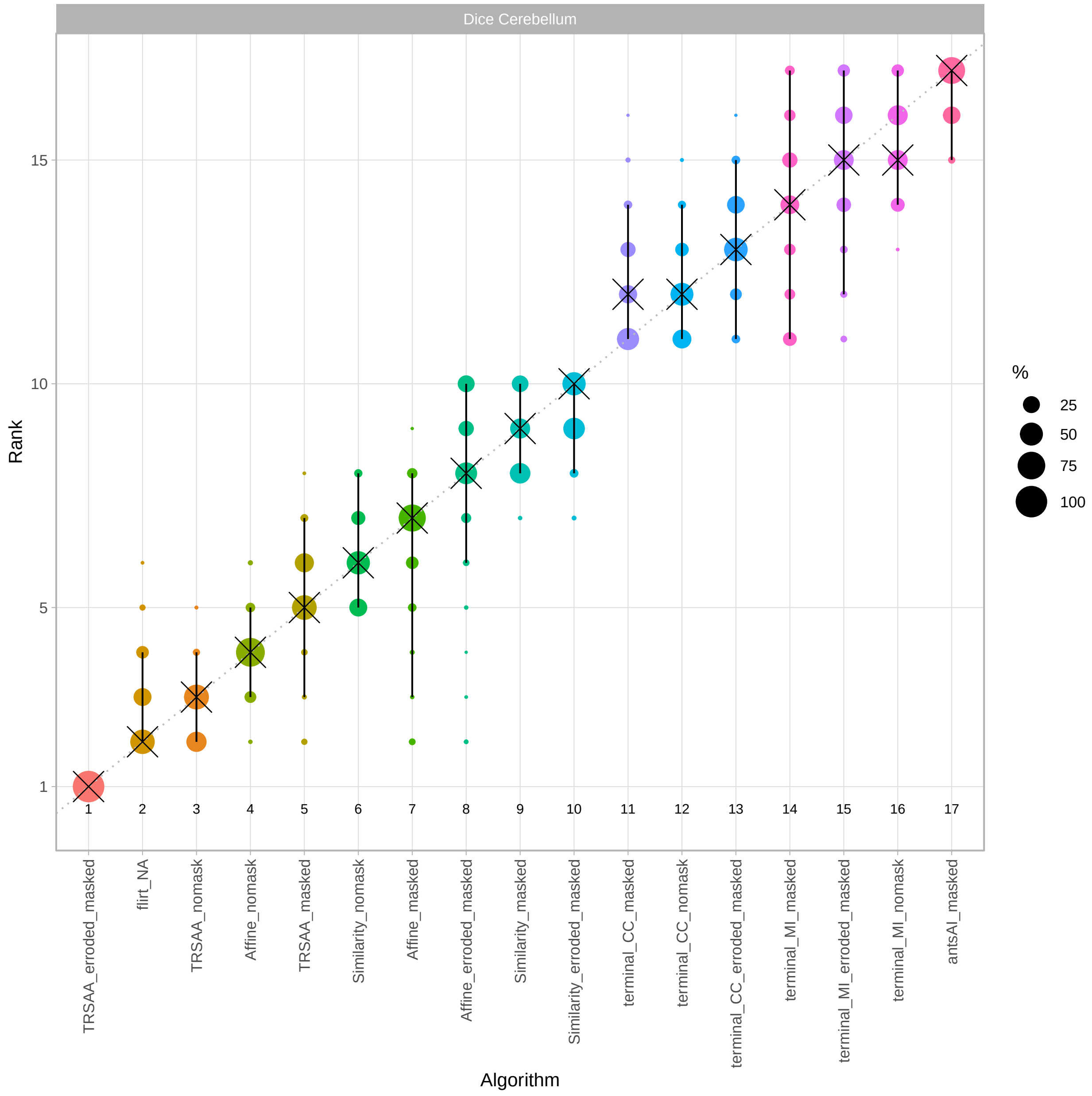

Dice Cerebellum
Rank
15
10
5
1
1
2
3
4
5
6
7
8
9
10
11
12
13
14
15
16
17
%
25
50
75
100
TRSAA_erroded_masked
flirt_NA
TRSAA_nomask
Affine_nomask
TRSAA_masked
Similarity_nomask
Affine_masked
Affine_erroded_masked
Similarity_masked
Similarity_erroded_masked
terminal_CC_masked
terminal_CC_nomask
terminal_CC_erroded_masked
terminal_MI_masked
terminal_MI_erroded_masked
terminal_MI_nomask
antsAI_masked
Algorithm

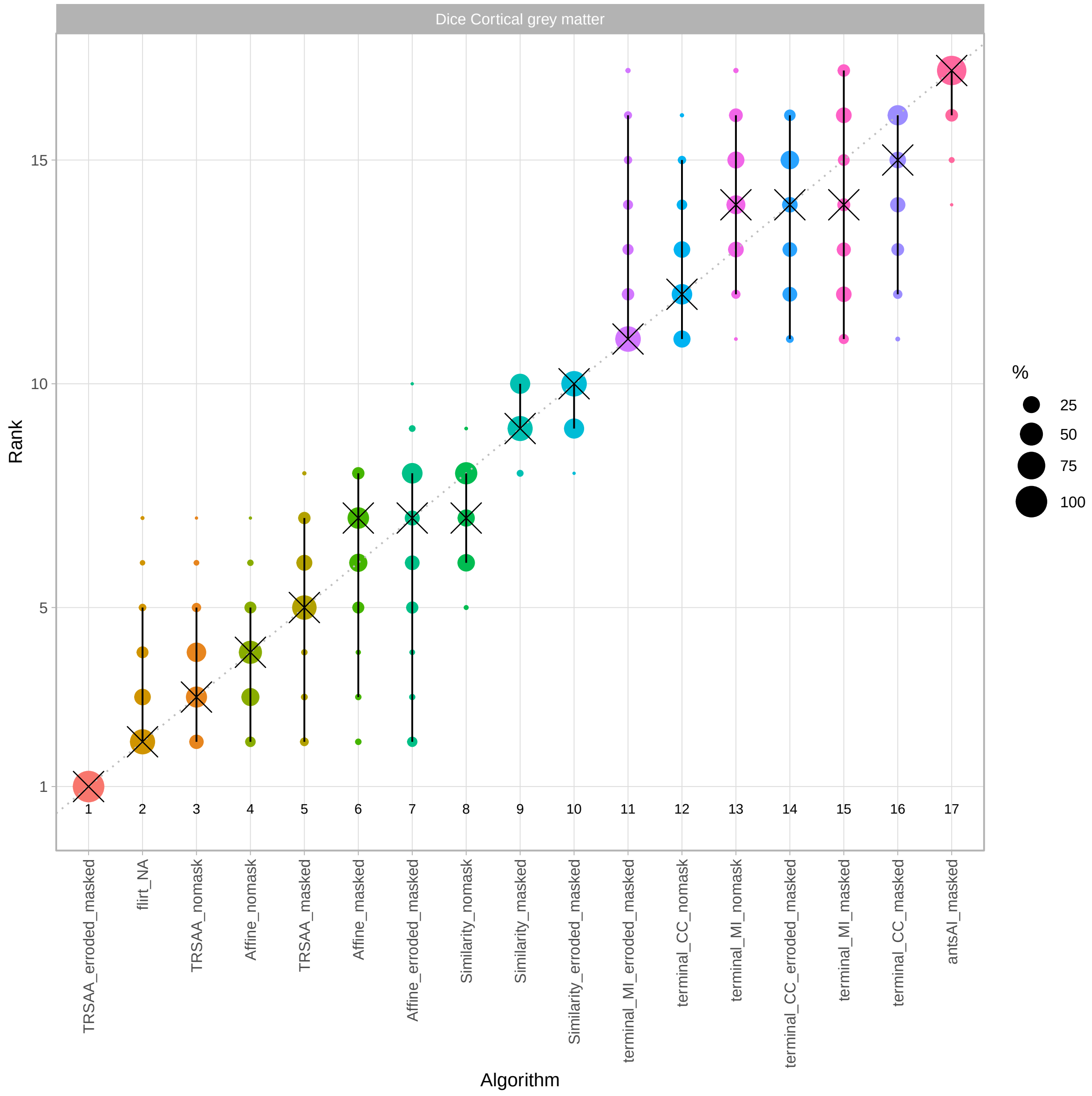

Dice Cortical grey matter
Rank
15
10
5
1
1
2
3
4
5
6
7
8
9
10
11
12
13
14
15
16
17
TRSAA_erroded_masked
flirt_NA
TRSAA_nomask
Affine_nomask
TRSAA_masked
Affine_masked
Affine_erroded_masked
Similarity_nomask
Similarity_masked
Similarity_erroded_masked
terminal_MI_erroded_masked
terminal_CC_nomask
terminal_MI_nomask
terminal_CC_erroded_masked
terminal_MI_masked
terminal_CC_masked
antsAI_masked
Algorithm
%
25
50
75
100

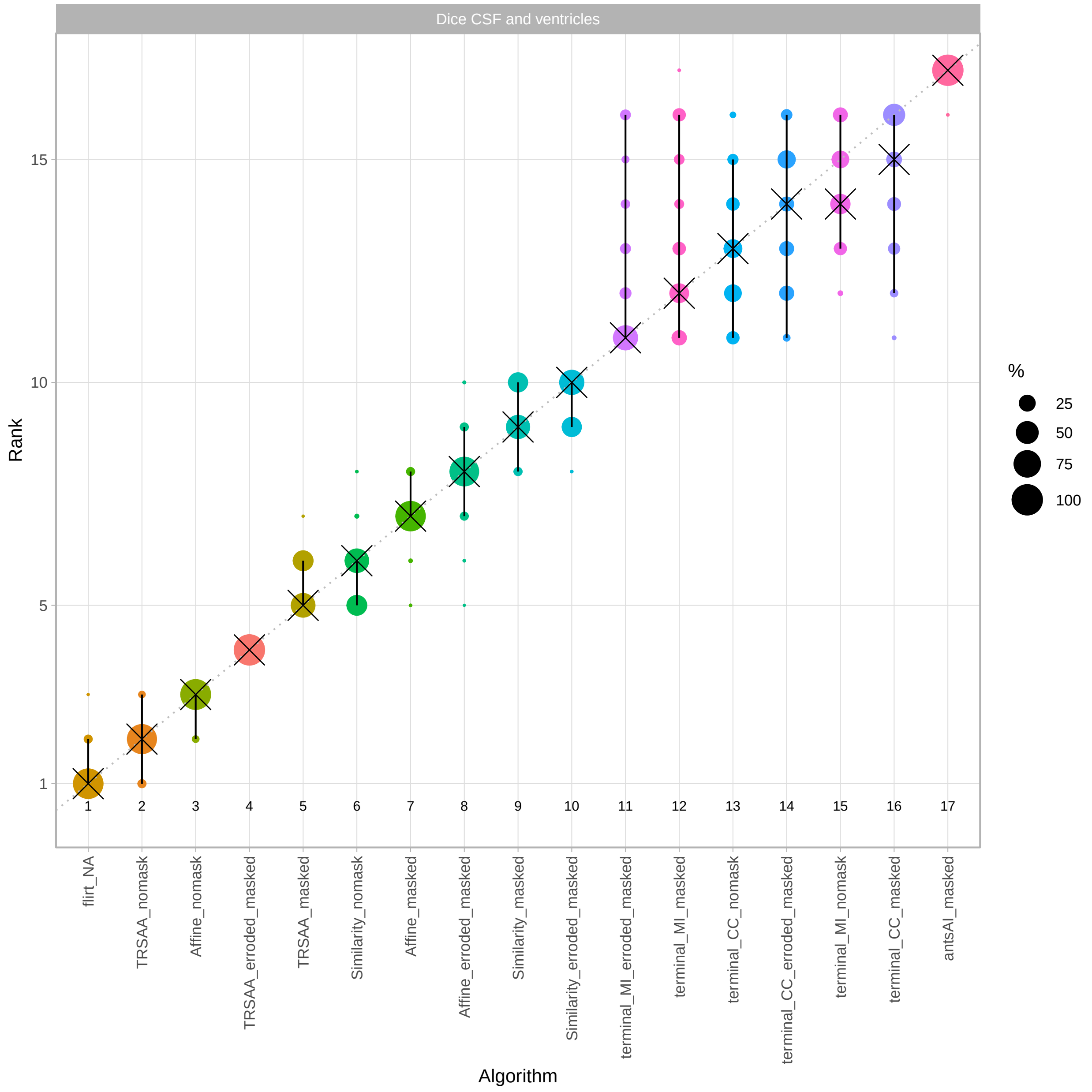
Dice CSF and ventricles
Rank
Algorithm
%
25
50
75
100
1
5
10
15
flirt_NA
TRSAA_nomask
Affine_nomask
TRSAA_erroded_masked
TRSAA_masked
Similarity_nomask
Affine_masked
Affine_erroded_masked
Similarity_masked
Similarity_erroded_masked
terminal_MI_erroded_masked
terminal_MI_masked
terminal_CC_nomask
terminal_CC_erroded_masked
terminal_MI_nomask
terminal_CC_masked
antsAI_masked

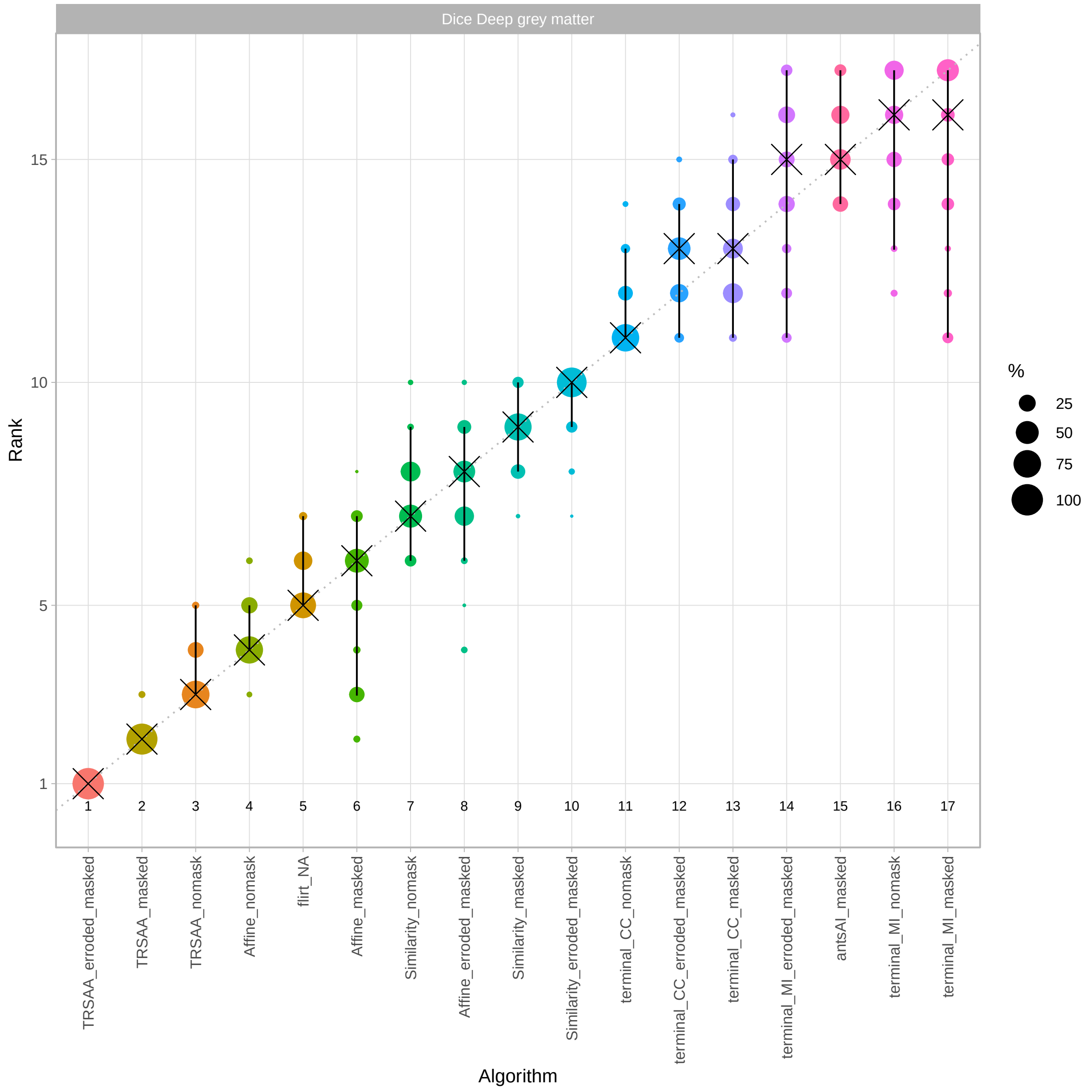
Dice Deep grey matter
Rank
15
10
5
1
1
2
3
4
5
6
7
8
9
10
11
12
13
14
15
16
17
%
25
50
75
100
TRSAA_erroded_masked
TRSAA_masked
TRSAA_nomask
Affine_nomask
flirt_NA
Affine_masked
Similarity_nomask
Affine_eroded_masked
Similarity_masked
Similarity_eroded_masked
terminal_CC_nomask
terminal_CC_eroded_masked
terminal_CC_masked
terminal_MI_eroded_masked
antsAI_masked
terminal_MI_nomask
terminal_MI_masked
Algorithm

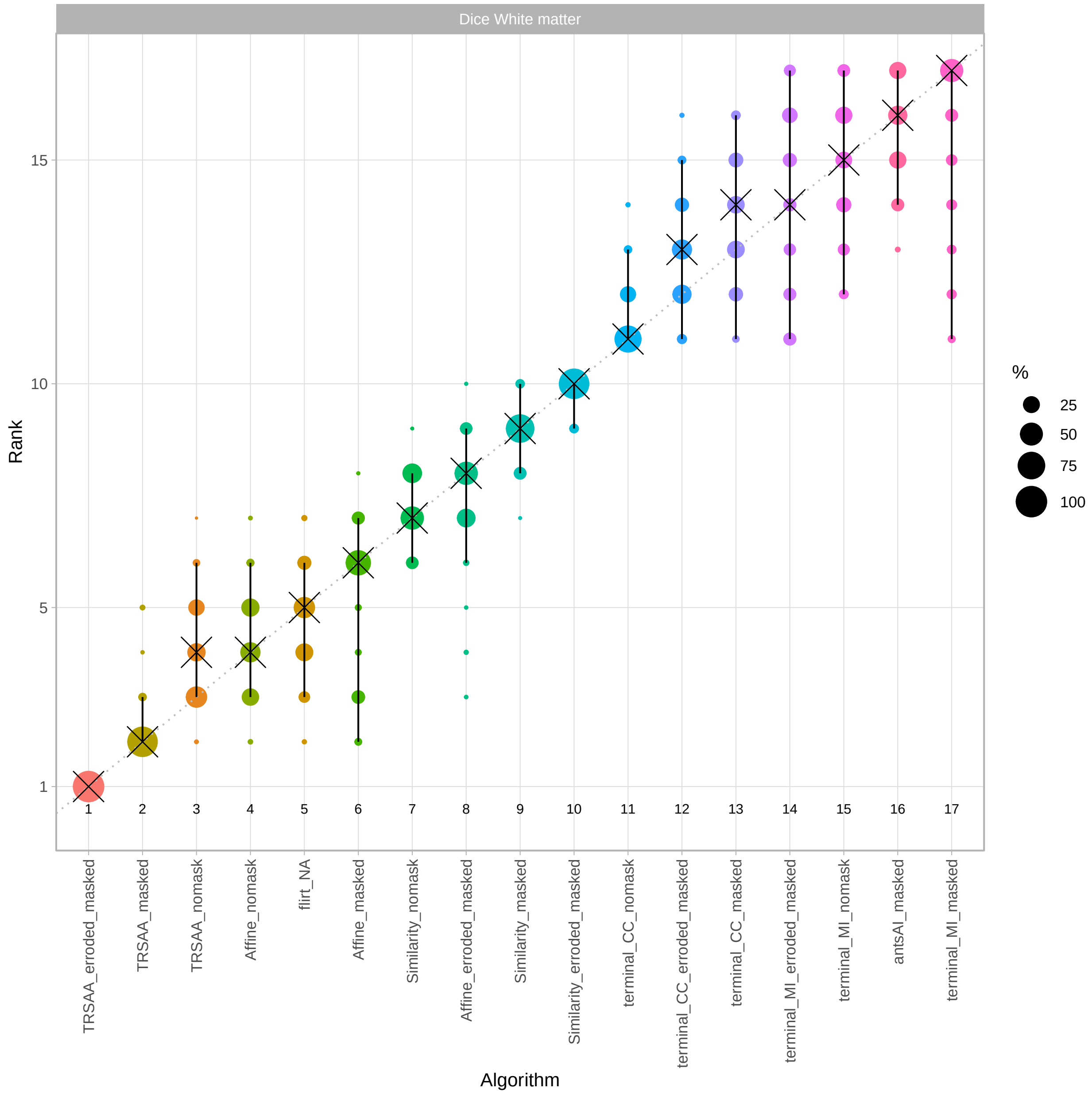

Dice White matter
Rank
1
5
10
15
1
2
3
4
5
6
7
8
9
10
11
12
13
14
15
16
17
TRSAA_erroded_masked
TRSAA_masked
TRSAA_nomask
Affine_nomask
flirt_NA
Affine_masked
Similarity_nomask
Affine_erroded_masked
Similarity_masked
Similarity_erroded_masked
terminal_CC_nomask
terminal_CC_erroded_masked
terminal_CC_masked
terminal_MI_erroded_masked
terminal_MI_nomask
antsAI_masked
terminal_MI_masked
Algorithm
%
25
50
75
100

3.2 *Violin plot* for visualizing ranking stability based on bootstrapping

The ranking list based on the full assessment data is pairwise compared with the ranking lists based on the individual bootstrap samples (here $b = 1000$ samples). For each pair of rankings, Kendall's τ correlation is computed. Kendall's τ is a scaled index determining the correlation between the lists. It is computed by evaluating the number of pairwise concordances and discordances between ranking lists and produces values between -1 (for inverted order) and 1 (for identical order). A violin plot, which simultaneously depicts a boxplot and a density plot, is generated from the results.

Summary Kendall's tau:

Task	mean	median	q25	q75
Dice CSF and ventricles	0.9292500	0.9411765	0.9117647	0.9558824
Dice Cerebellum	0.9202500	0.9264706	0.8970588	0.9558824
Dice Deep grey matter	0.9173529	0.9264706	0.8970588	0.9558824
Dice White matter	0.9000588	0.9117647	0.8676471	0.9411765
Dice Brainstem	0.8762500	0.8823529	0.8529412	0.9117647
Dice Cortical grey matter	0.8854412	0.8823529	0.8529412	0.9117647

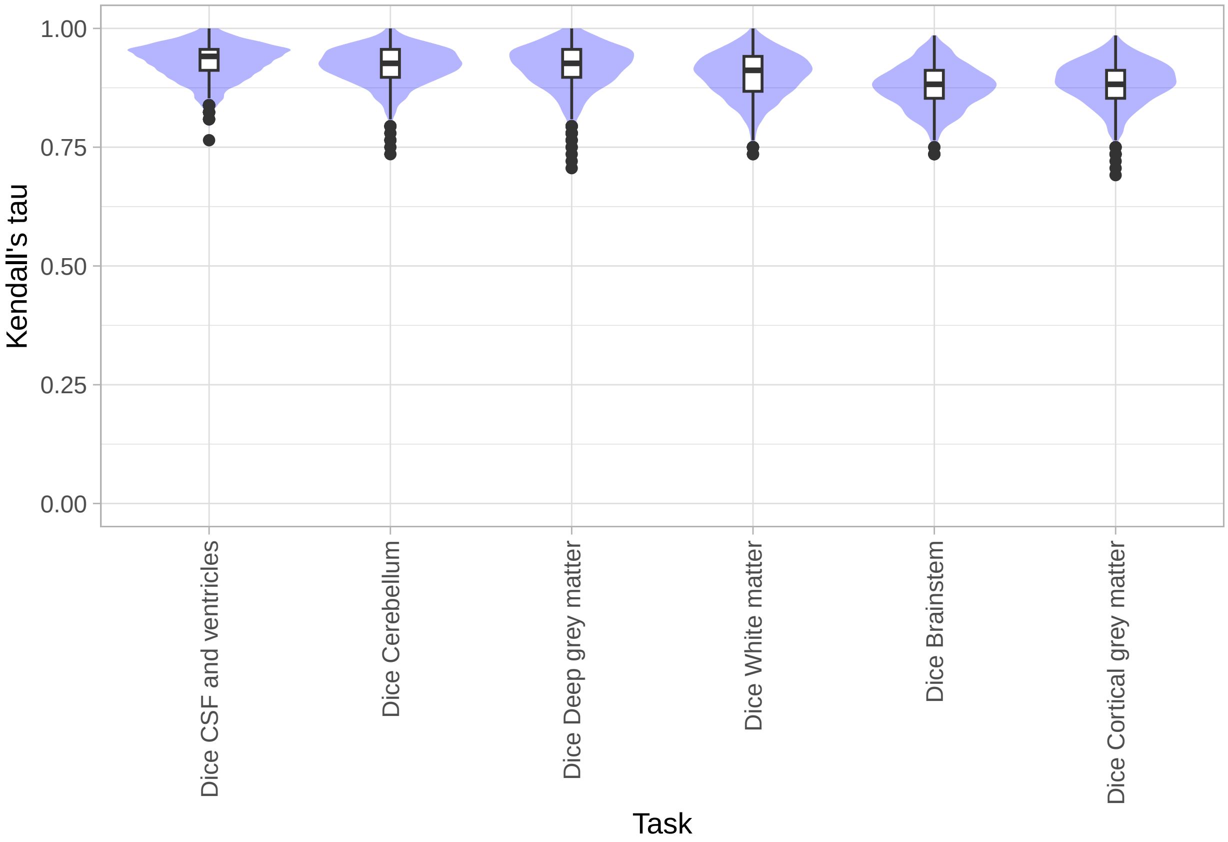

3.3 *Significance maps* for visualizing ranking stability based on statistical significance

Significance maps depict incidence matrices of pairwise significant test results for the one-sided Wilcoxon signed rank test at a 5% significance level with adjustment for multiple testing according to Holm. Yellow shading indicates that metric values from the algorithm on the x-axis were significantly superior to those from the algorithm on the y-axis, blue color indicates no significant difference.

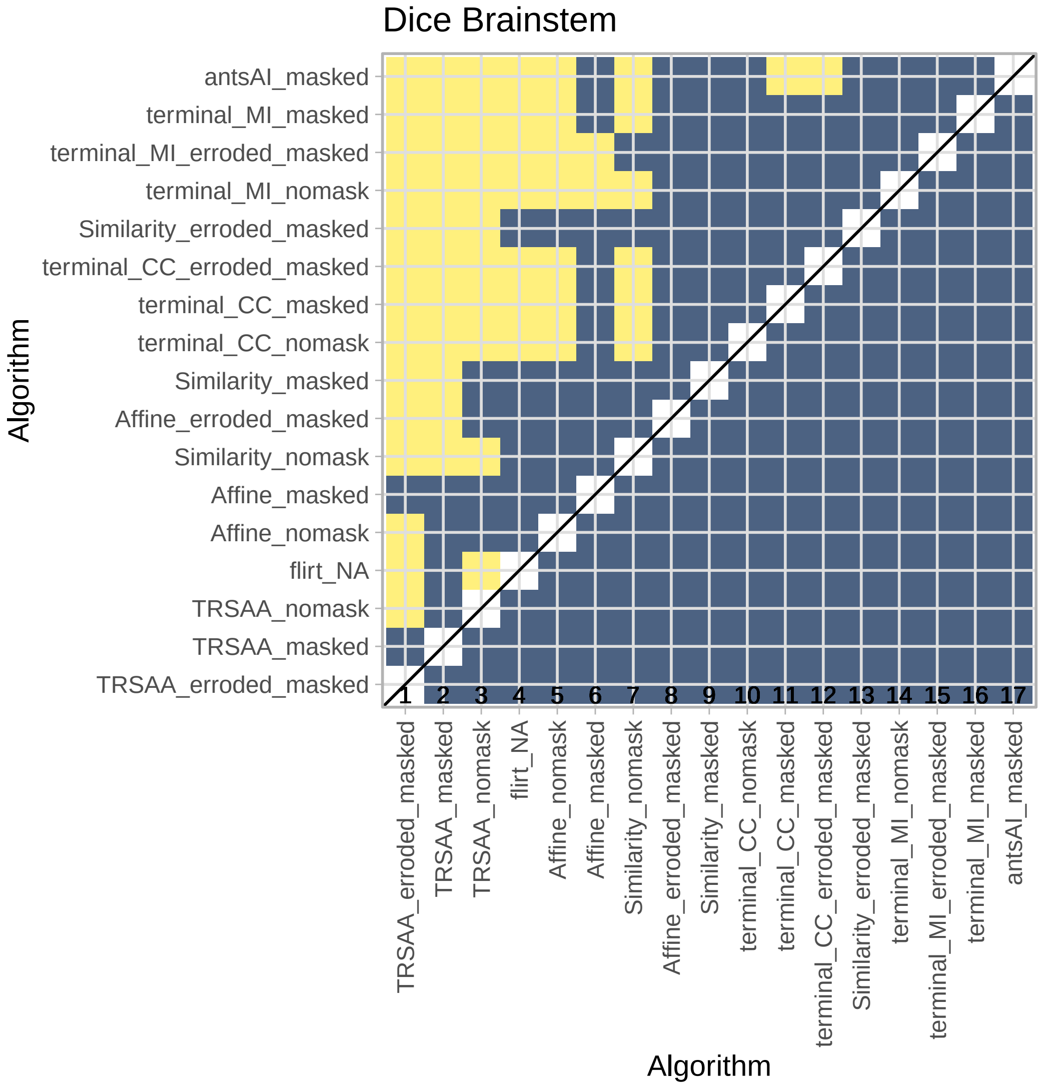

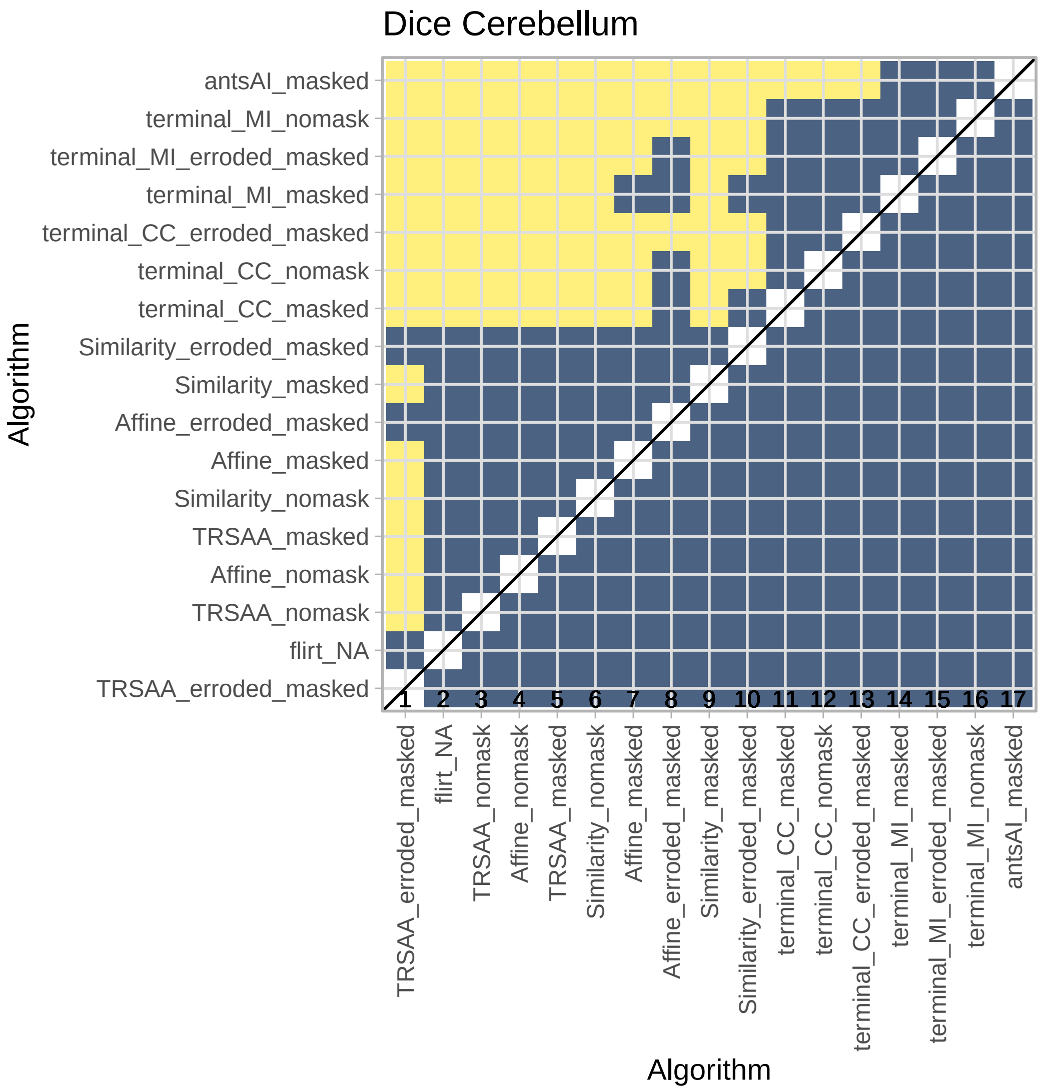
Dice Cerebellum
antsAI_masked
terminal_MI_nomask
terminal_MI_erroded_masked
terminal_MI_masked
terminal_CC_erroded_masked
terminal_CC_nomask
terminal_CC_masked
Similarity_erroded_masked
Similarity_masked
Affine_erroded_masked
Affine_masked
Similarity_nomask
TRSAA_masked
Affine_nomask
TRSAA_nomask
flirt_NA
TRSAA_erroded_masked
1 2 3 4 5 6 7 8 9 10 11 12 13 14 15 16 17
Algorithm
Algorithm

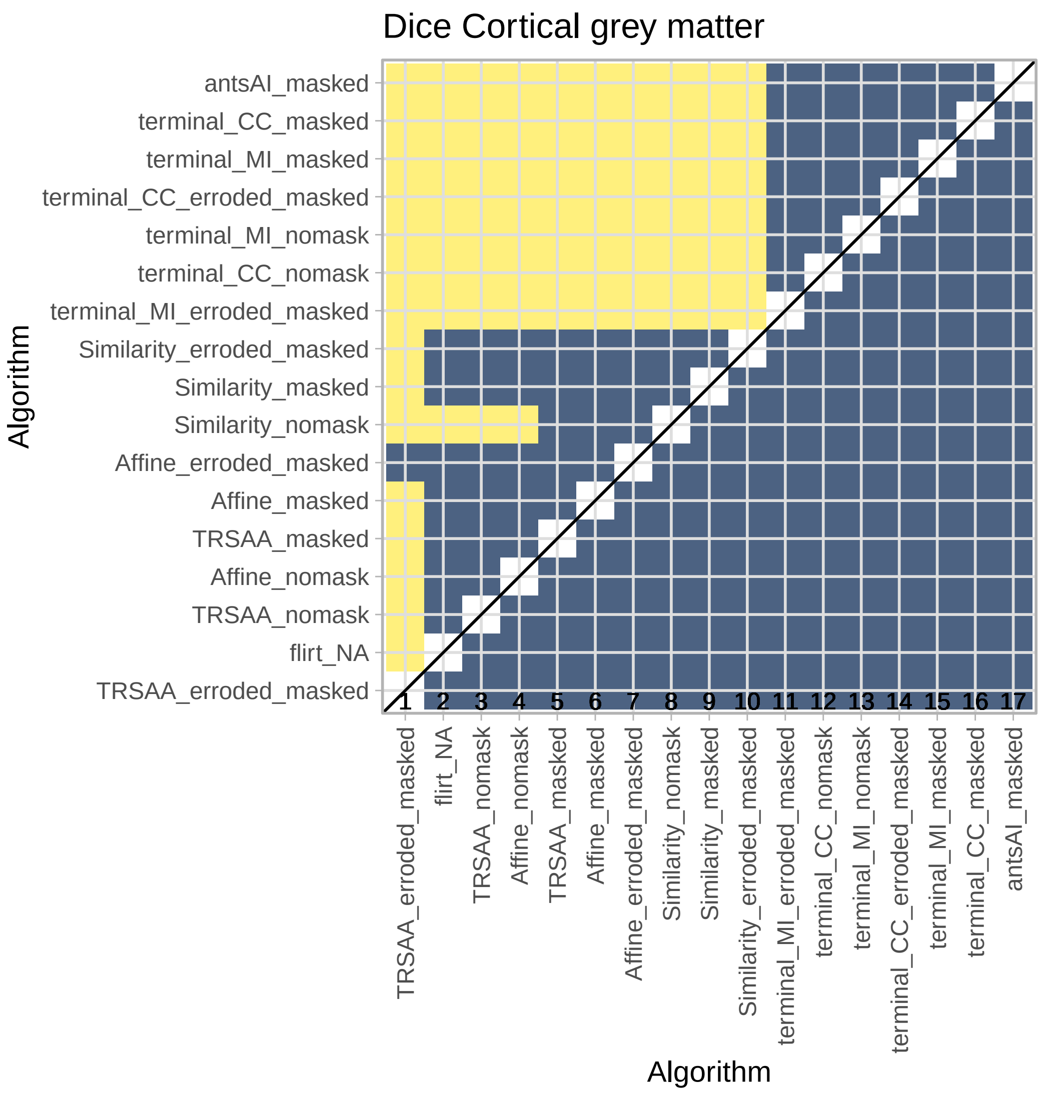

Dice Cortical grey matter
antsAI_masked
terminal_CC_masked
terminal_MI_masked
terminal_CC_erroded_masked
terminal_MI_nomask
terminal_CC_nomask
terminal_MI_erroded_masked
Similarity_erroded_masked
Similarity_masked
Similarity_nomask
Affine_erroded_masked
Affine_masked
TRSAA_masked
Affine_nomask
TRSAA_nomask
flirt_NA
TRSAA_erroded_masked
Algorithm
1 2 3 4 5 6 7 8 9 10 11 12 13 14 15 16 17
TRSAA_erroded_masked
flirt_NA
TRSAA_nomask
Affine_nomask
TRSAA_masked
Affine_masked
Affine_erroded_masked
Similarity_nomask
Similarity_masked
Similarity_erroded_masked
terminal_MI_erroded_masked
terminal_CC_nomask
terminal_MI_nomask
terminal_CC_erroded_masked
terminal_MI_masked
terminal_CC_masked
antsAI_masked
Algorithm

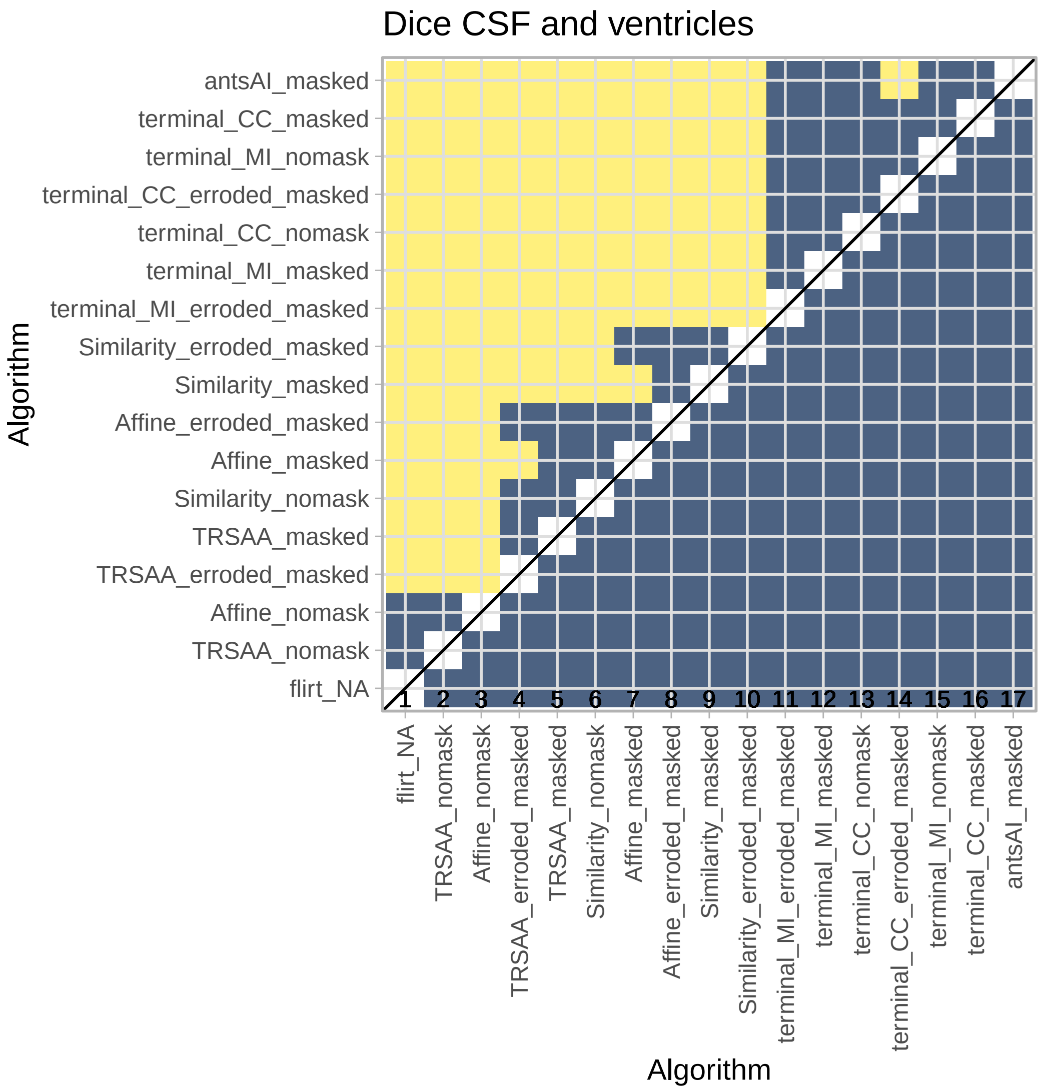
Dice CSF and ventricles
antsAI_masked
terminal_CC_masked
terminal_MI_nomask
terminal_CC_erroded_masked
terminal_CC_nomask
terminal_MI_masked
terminal_MI_erroded_masked
Similarity_erroded_masked
Similarity_masked
Affine_erroded_masked
Affine_masked
Similarity_nomask
TRSAA_masked
TRSAA_erroded_masked
Affine_nomask
TRSAA_nomask
flirt_NA
1 2 3 4 5 6 7 8 9 10 11 12 13 14 15 16 17
Algorithm
Algorithm

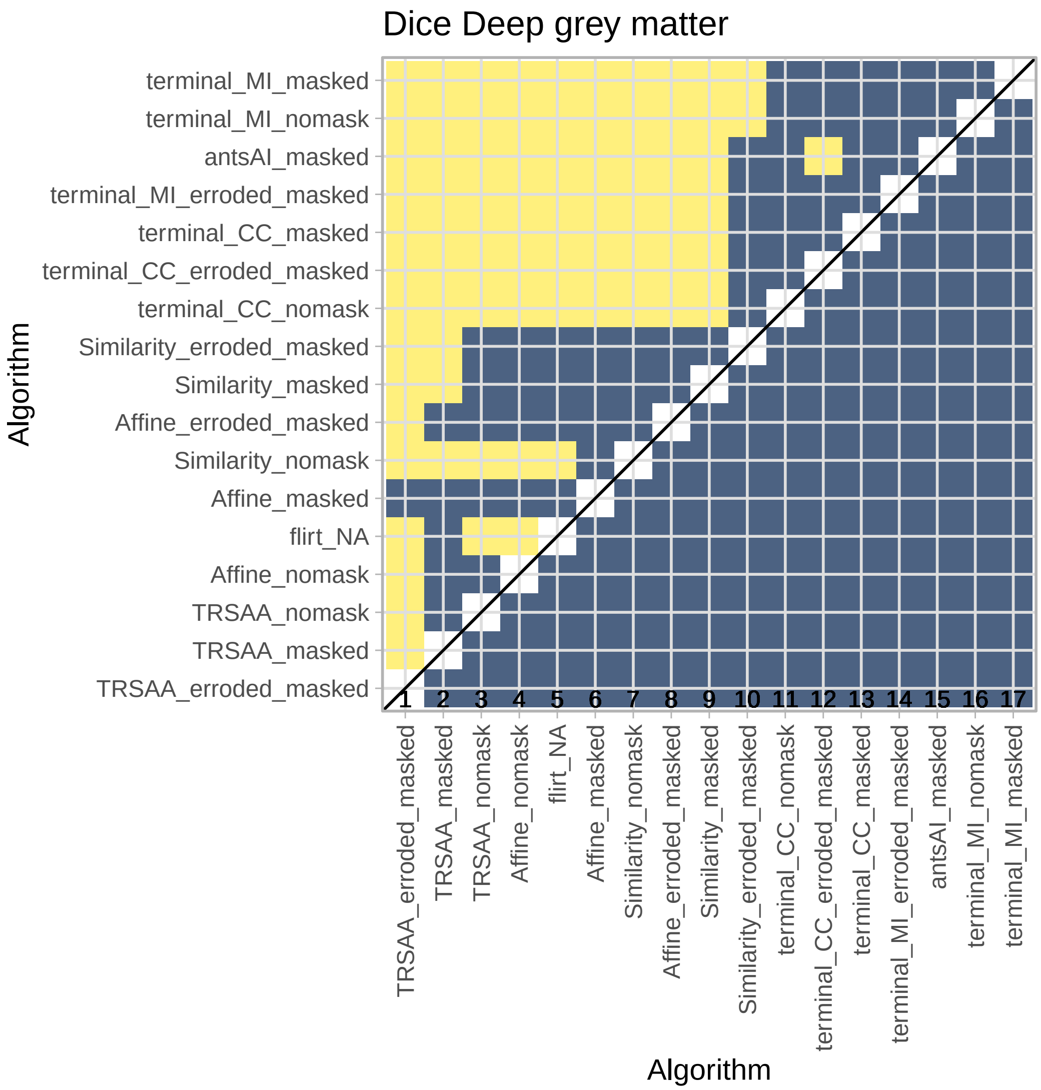
Dice Deep grey matter
terminal_MI_masked
terminal_MI_nomask
antsAI_masked
terminal_MI_erroded_masked
terminal_CC_masked
terminal_CC_erroded_masked
terminal_CC_nomask
Similarity_erroded_masked
Similarity_masked
Affine_erroded_masked
Similarity_nomask
Affine_masked
flirt_NA
Affine_nomask
TRSAA_nomask
TRSAA_masked
TRSAA_erroded_masked
Algorithm
1 2 3 4 5 6 7 8 9 10 11 12 13 14 15 16 17
TRSAA_erroded_masked
TRSAA_masked
TRSAA_nomask
Affine_nomask
flirt_NA
Affine_masked
Similarity_nomask
Affine_erroded_masked
Similarity_masked
Similarity_erroded_masked
terminal_CC_nomask
terminal_CC_erroded_masked
terminal_CC_masked
terminal_MI_erroded_masked
antsAI_masked
terminal_MI_nomask
terminal_MI_masked
Algorithm

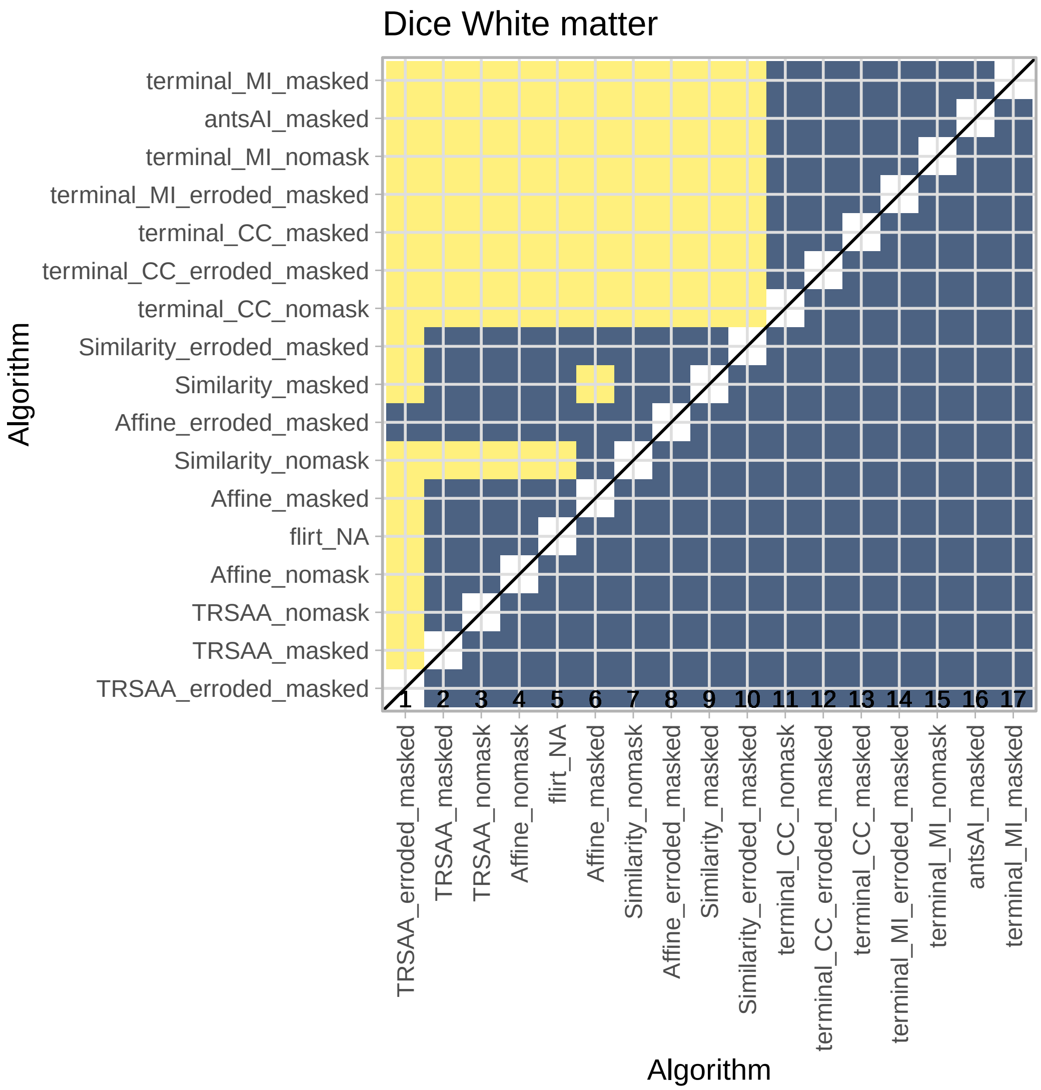

Dice White matter
terminal_MI_masked
antsAI_masked
terminal_MI_nomask
terminal_MI_erroded_masked
terminal_CC_masked
terminal_CC_erroded_masked
terminal_CC_nomask
Similarity_erroded_masked
Similarity_masked
Affine_erroded_masked
Similarity_nomask
Affine_masked
flirt_NA
Affine_nomask
TRSAA_nomask
TRSAA_masked
TRSAA_erroded_masked
Algorithm
1 2 3 4 5 6 7 8 9 10 11 12 13 14 15 16 17
TRSAA_erroded_masked
TRSAA_masked
TRSAA_nomask
Affine_nomask
flirt_NA
Affine_masked
Similarity_nomask
Affine_erroded_masked
Similarity_masked
Similarity_erroded_masked
terminal_CC_nomask
terminal_CC_erroded_masked
terminal_CC_masked
terminal_MI_erroded_masked
terminal_MI_nomask
antsAI_masked
terminal_MI_masked
Algorithm

3.4 Ranking robustness to ranking methods

Line plots for visualizing ranking robustness across different ranking methods. Each algorithm is represented by one colored line. For each ranking method encoded on the x-axis, the height of the line represents the corresponding rank. Horizontal lines indicate identical ranks for all methods.

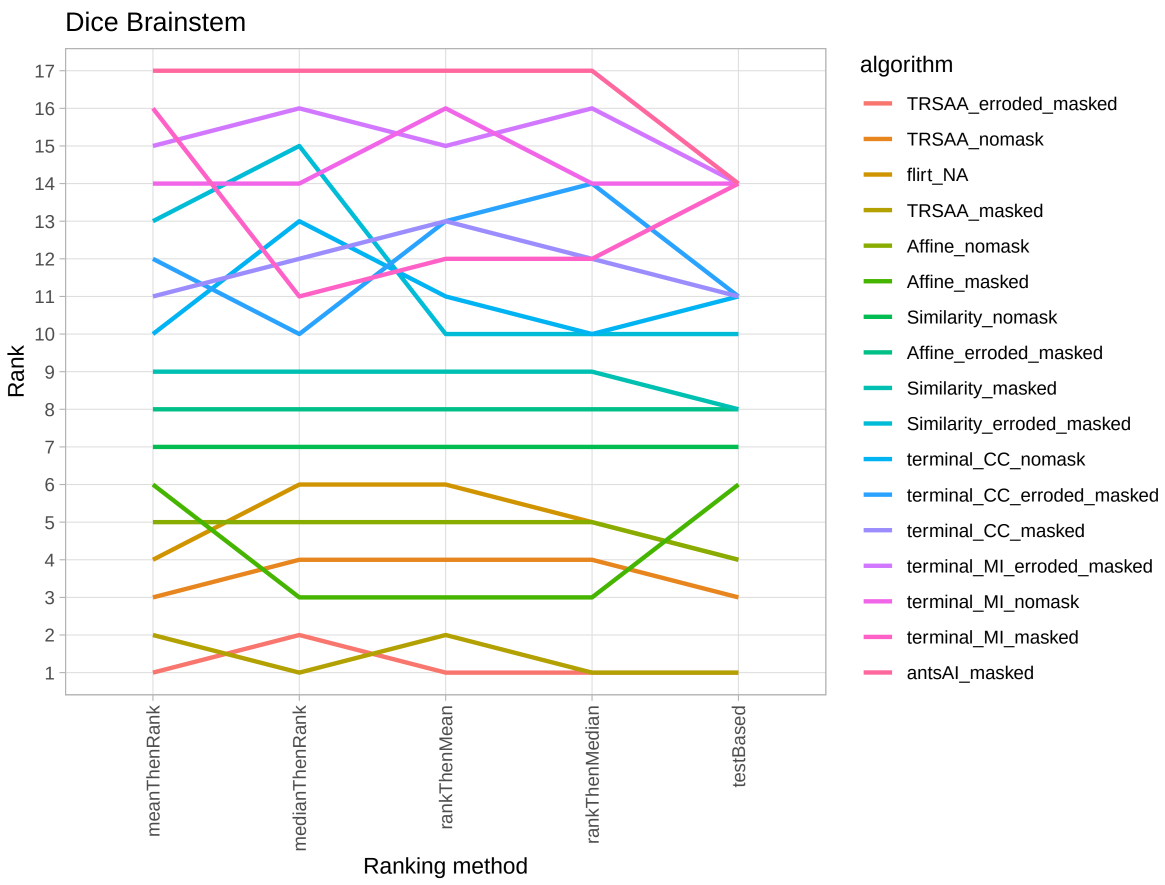

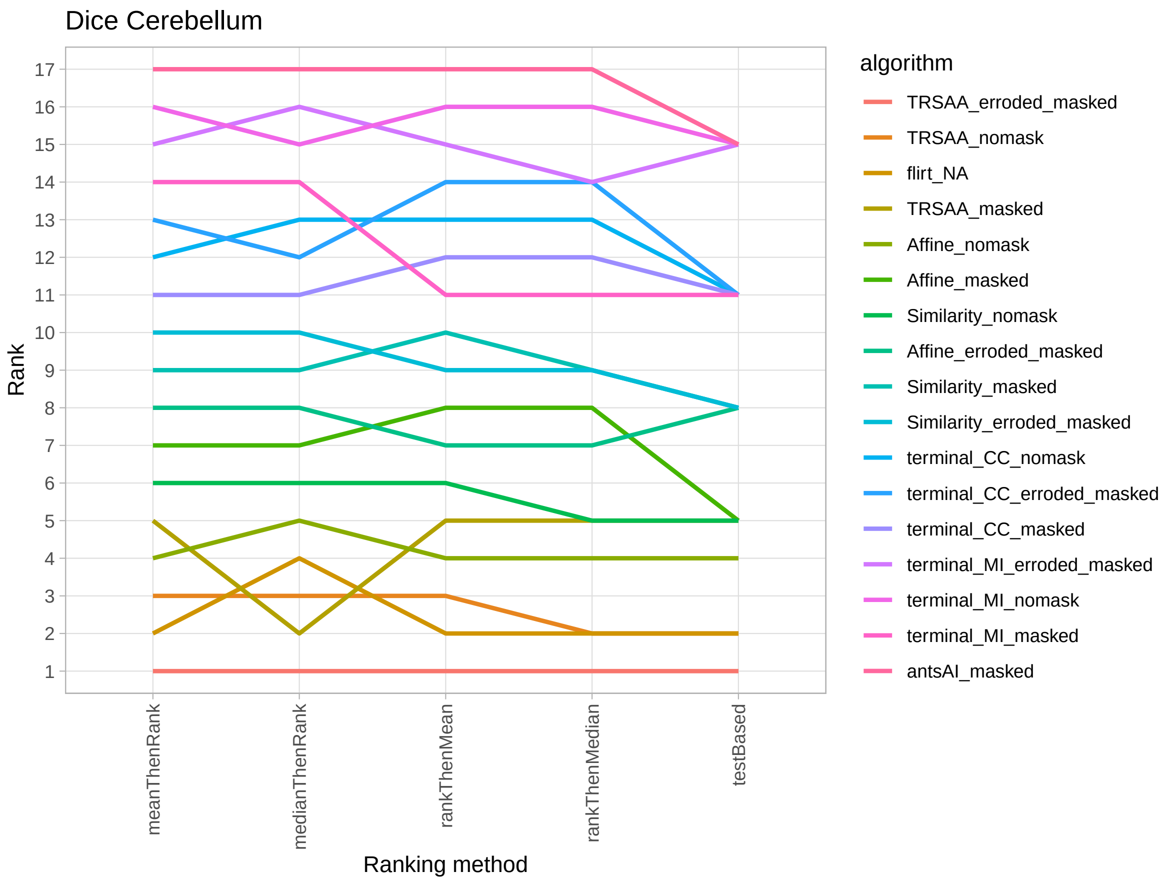
Dice Cerebellum
Rank
17
16
15
14
13
12
11
10
9
8
7
6
5
4
3
2
1
meanThenRank
medianThenRank
rankThenMean
rankThenMedian
testBased
Ranking method
algorithm
TRSAA_erroded_masked
TRSAA_nomask
flirt_NA
TRSAA_masked
Affine_nomask
Affine_masked
Similarity_nomask
Affine_erroded_masked
Similarity_masked
Similarity_erroded_masked
terminal_CC_nomask
terminal_CC_erroded_masked
terminal_CC_masked
terminal_MI_erroded_masked
terminal_MI_nomask
terminal_MI_masked
antsAI_masked

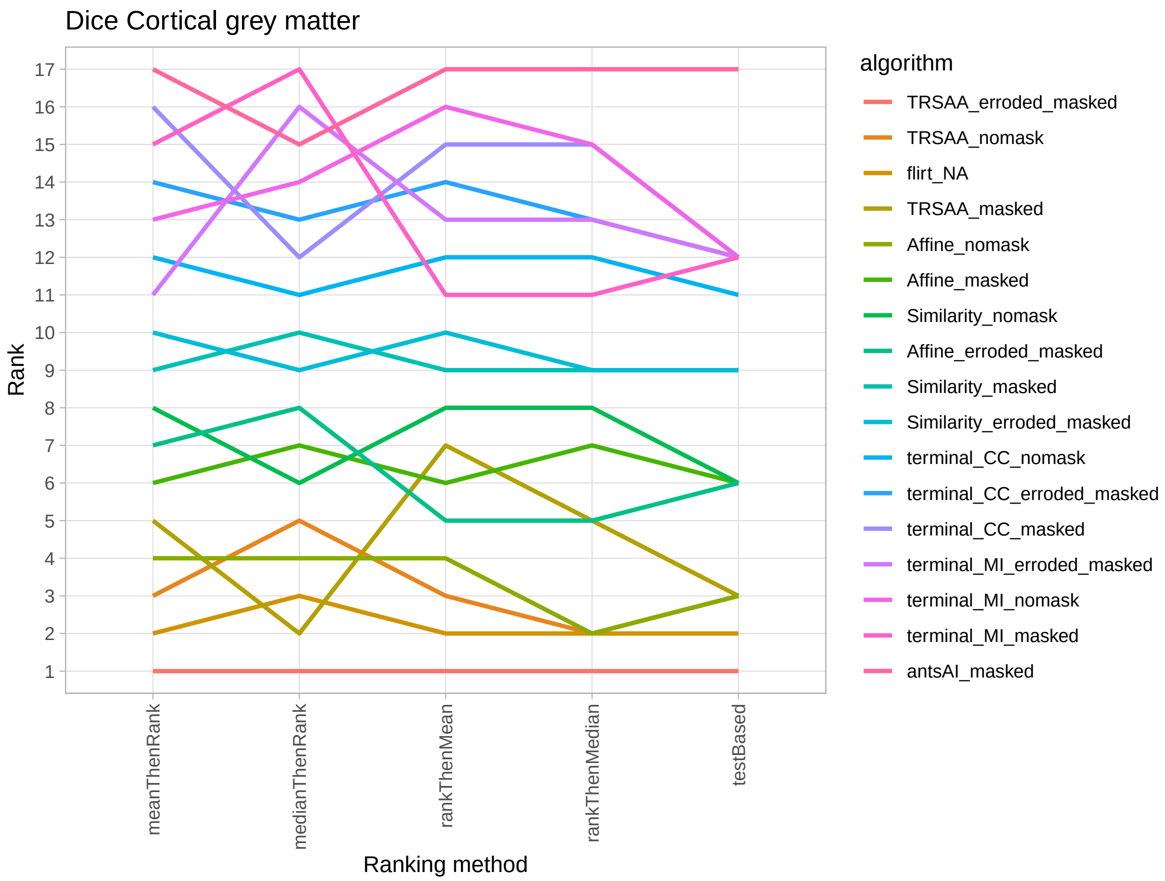
Dice Cortical grey matter
Rank
17
16
15
14
13
12
11
10
9
8
7
6
5
4
3
2
1
meanThenRank
medianThenRank
rankThenMean
rankThenMedian
testBased
Ranking method
algorithm
TRSAA_erroded_masked
TRSAA_nomask
flirt_NA
TRSAA_masked
Affine_nomask
Affine_masked
Similarity_nomask
Affine_erroded_masked
Similarity_masked
Similarity_erroded_masked
terminal_CC_nomask
terminal_CC_erroded_masked
terminal_CC_masked
terminal_MI_erroded_masked
terminal_MI_nomask
terminal_MI_masked
antsAI_masked

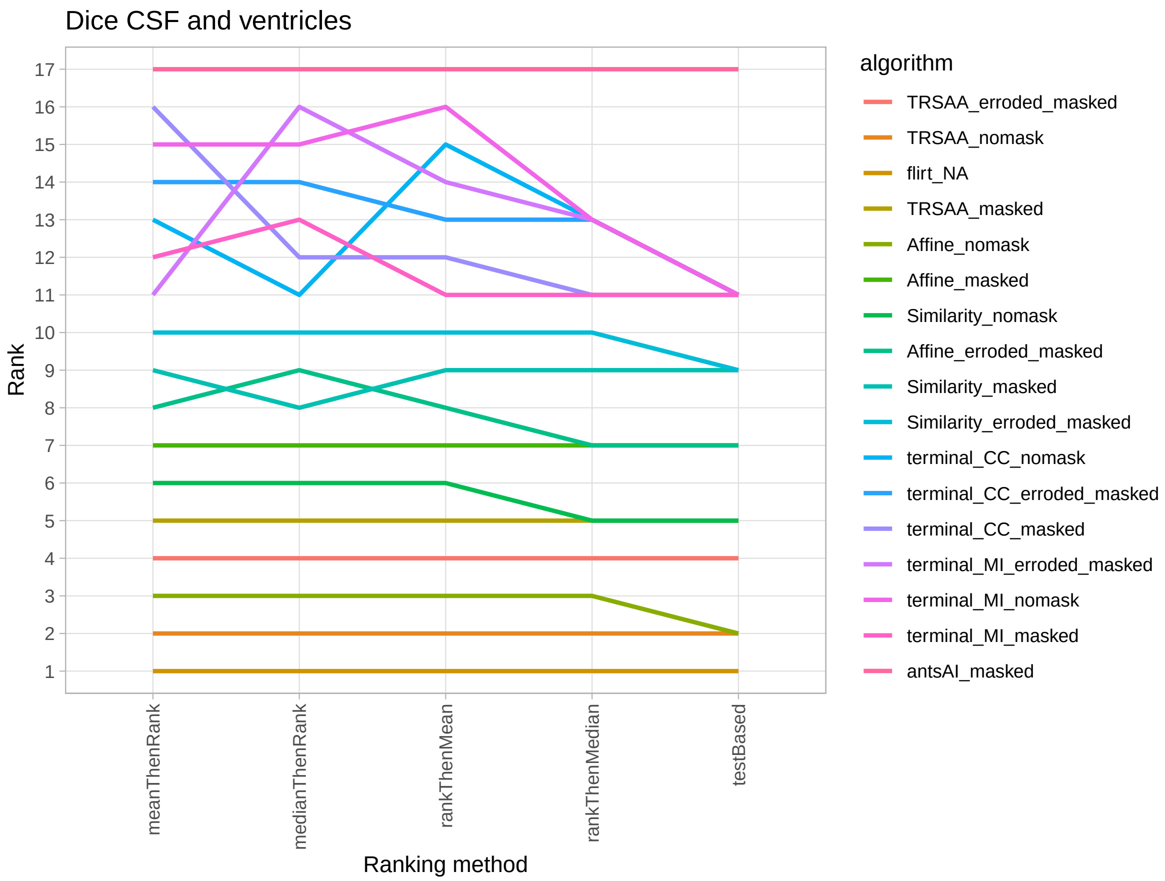
Dice CSF and ventricles
Rank
17
16
15
14
13
12
11
10
9
8
7
6
5
4
3
2
1
meanThenRank
medianThenRank
rankThenMean
rankThenMedian
testBased
Ranking method
algorithm
TRSAA_erroded_masked
TRSAA_nomask
flirt_NA
TRSAA_masked
Affine_nomask
Affine_masked
Similarity_nomask
Affine_erroded_masked
Similarity_masked
Similarity_erroded_masked
terminal_CC_nomask
terminal_CC_erroded_masked
terminal_CC_masked
terminal_MI_erroded_masked
terminal_MI_nomask
terminal_MI_masked
antsAI_masked

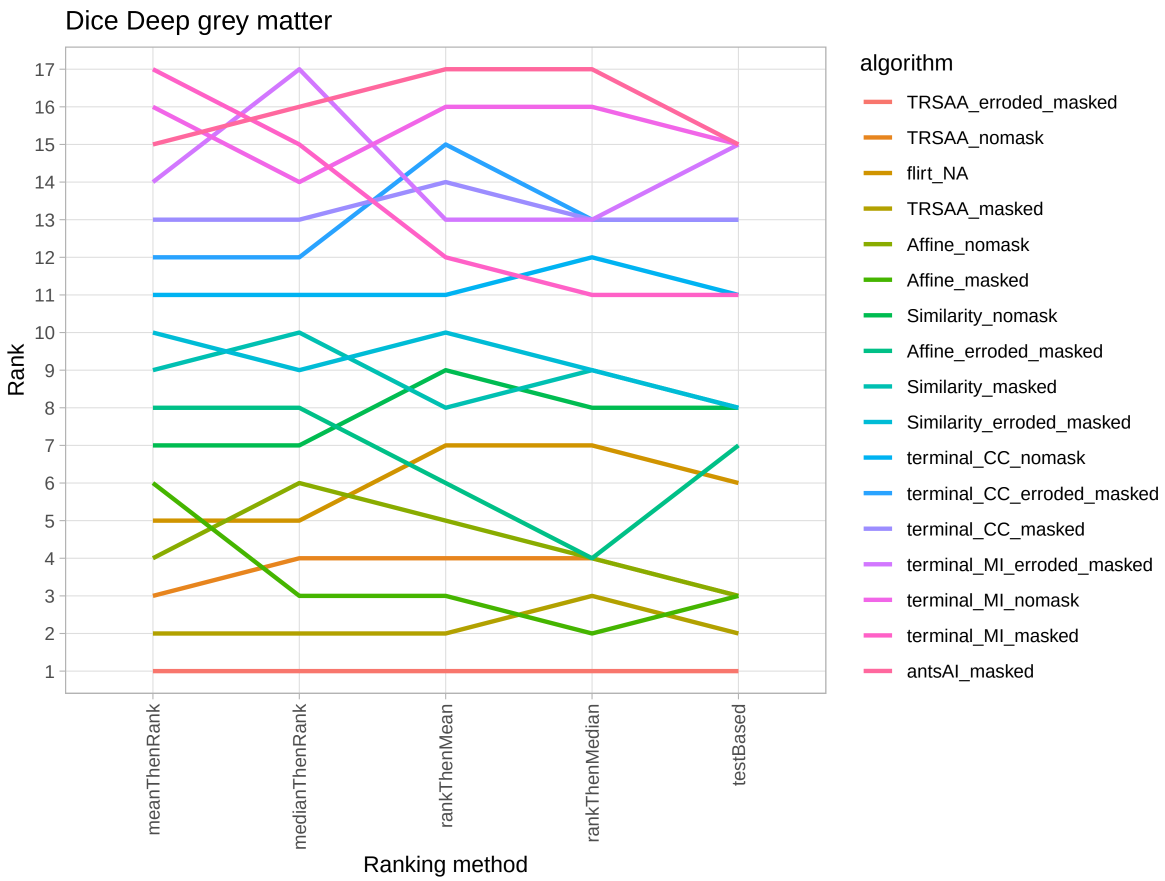

Dice Deep grey matter
Rank
17
16
15
14
13
12
11
10
9
8
7
6
5
4
3
2
1
meanThenRank
medianThenRank
rankThenMean
rankThenMedian
testBased
Ranking method
algorithm
TRSAA_erroded_masked
TRSAA_nomask
flirt_NA
TRSAA_masked
Affine_nomask
Affine_masked
Similarity_nomask
Affine_erroded_masked
Similarity_masked
Similarity_erroded_masked
terminal_CC_nomask
terminal_CC_erroded_masked
terminal_CC_masked
terminal_MI_erroded_masked
terminal_MI_nomask
terminal_MI_masked
antsAI_masked

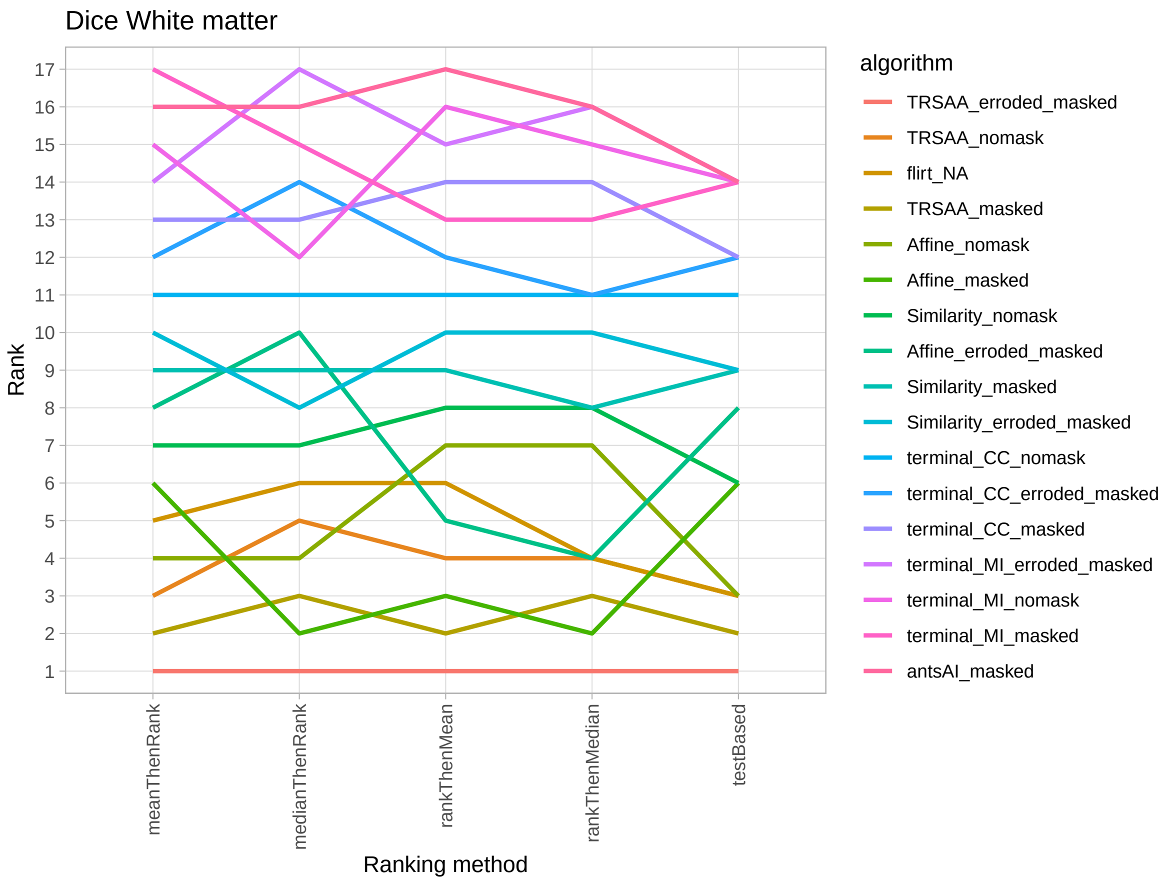

Dice White matter
Rank
17
16
15
14
13
12
11
10
9
8
7
6
5
4
3
2
1
meanThenRank
medianThenRank
rankThenMean
rankThenMedian
testBased
Ranking method
algorithm
TRSAA_erroded_masked
TRSAA_nomask
flirt_NA
TRSAA_masked
Affine_nomask
Affine_masked
Similarity_nomask
Affine_erroded_masked
Similarity_masked
Similarity_erroded_masked
terminal_CC_nomask
terminal_CC_erroded_masked
terminal_CC_masked
terminal_MI_erroded_masked
terminal_MI_nomask
terminal_MI_masked
antsAI_masked

4 Visualization of cross-task insights

The algorithms are ordered according to consensus ranking.

4.1 Characterization of algorithms

4.1.1 Ranking stability: Variability of achieved rankings across tasks

Algorithms are color-coded, and the area of each blob at position $(A_i, \text{rank } j)$ is proportional to the relative frequency A_i achieved rank j across multiple tasks. The median rank for each algorithm is indicated by a black cross. This way, the distribution of ranks across tasks can be intuitively visualized.

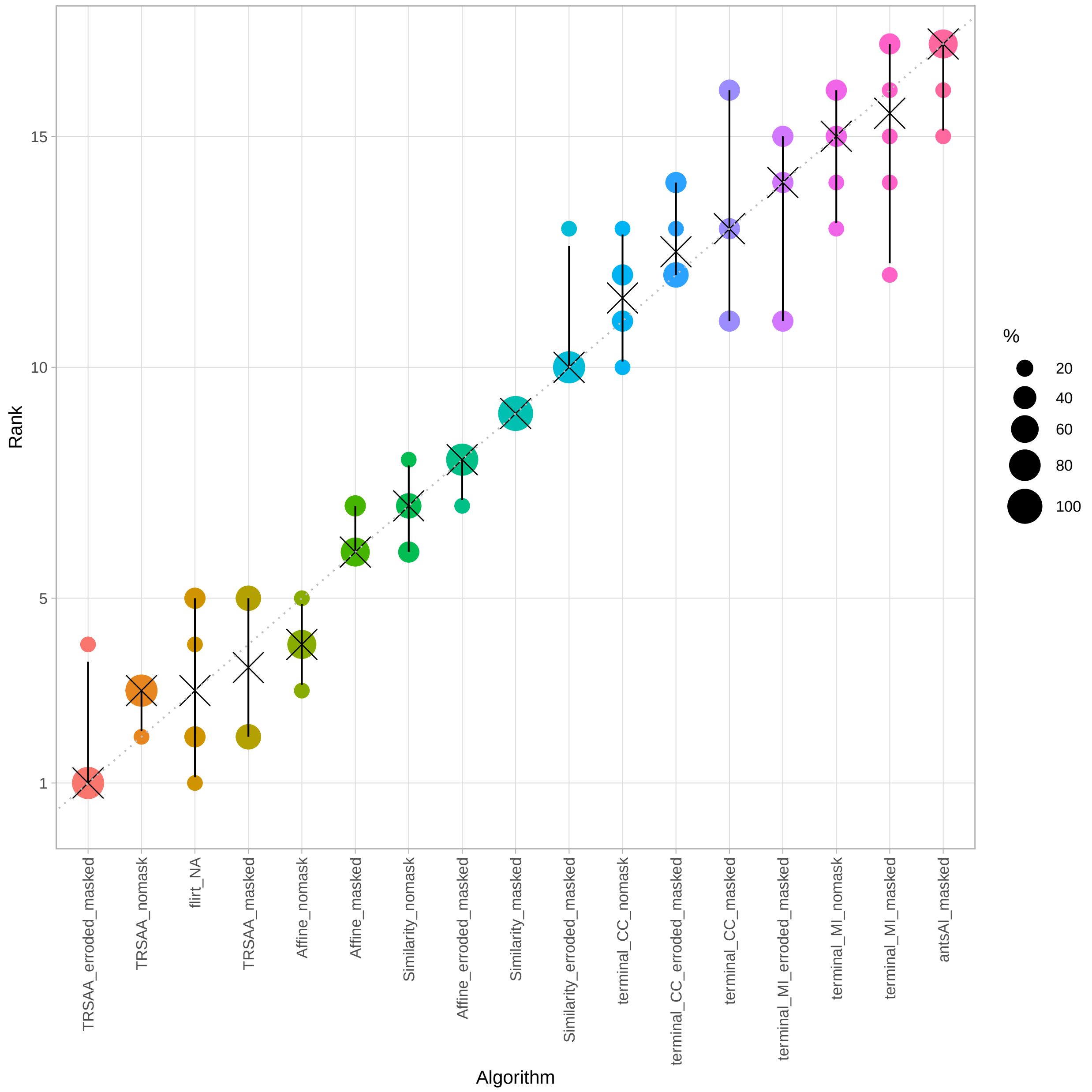

4.1.2 Ranking stability: Ranking variability via bootstrap approach

A blob plot of bootstrap results over the different tasks separated by algorithm allows another perspective on the assessment data. This gives deeper insights into the characteristics of tasks and the ranking uncertainty of the algorithms in each task.

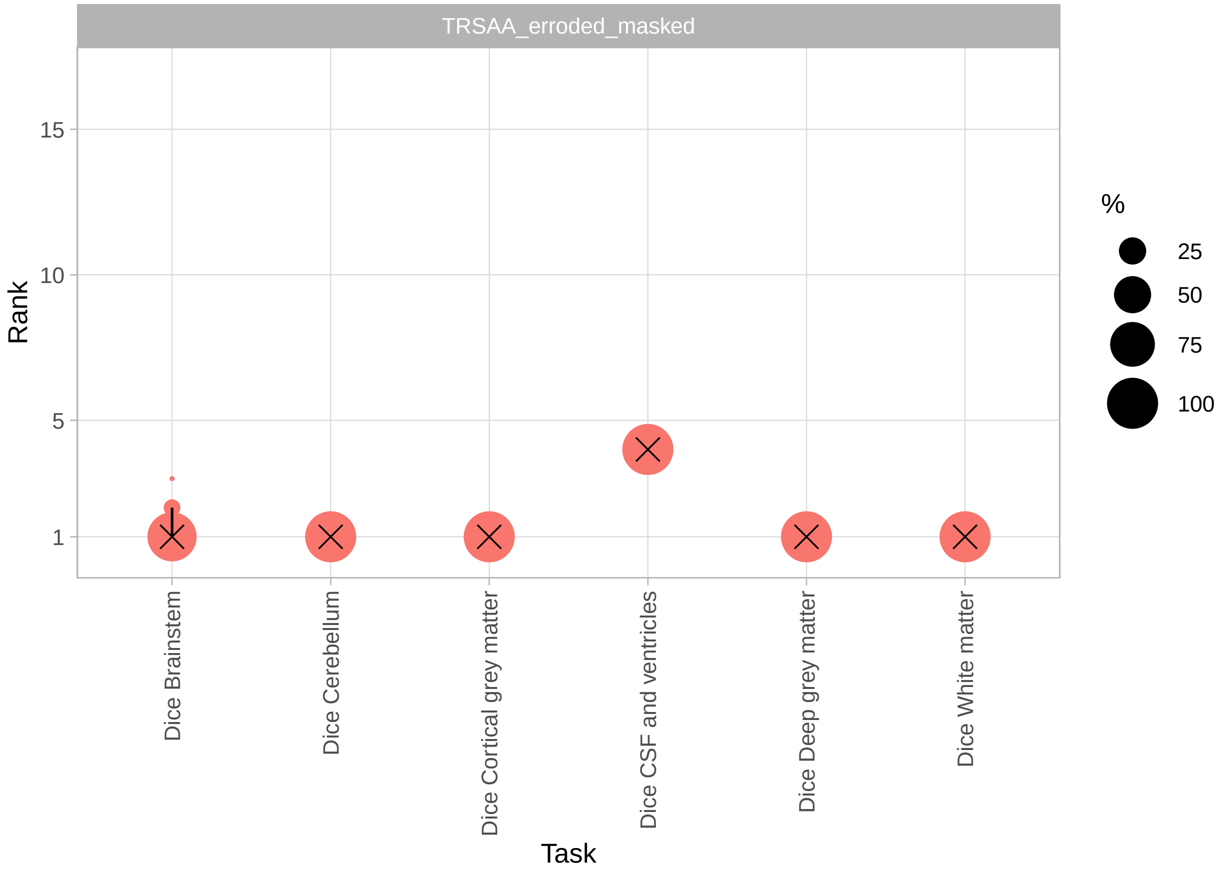

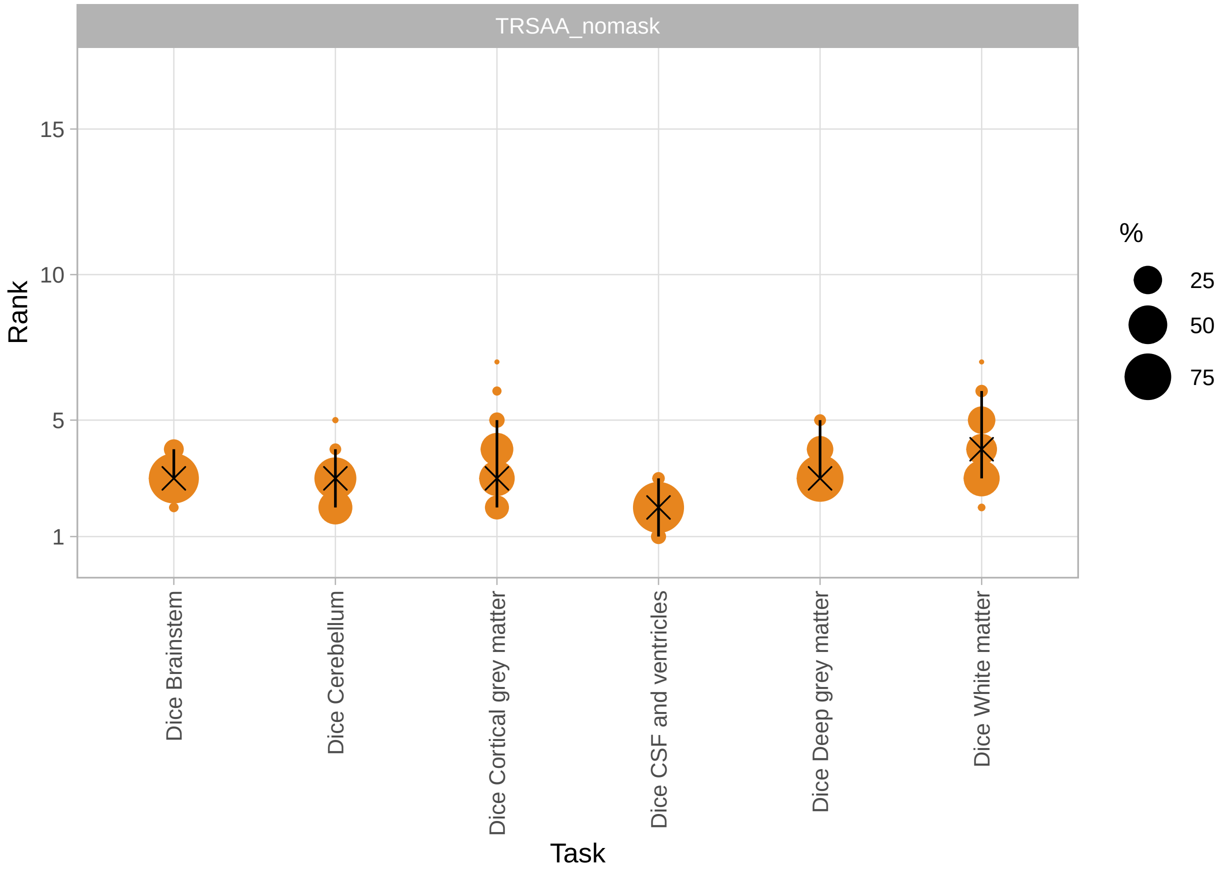

TRSAA_nomask
Rank
15
10
5
1
%
25
50
75
Dice Brainstem
Dice Cerebellum
Dice Cortical grey matter
Dice CSF and ventricles
Dice Deep grey matter
Dice White matter
Task

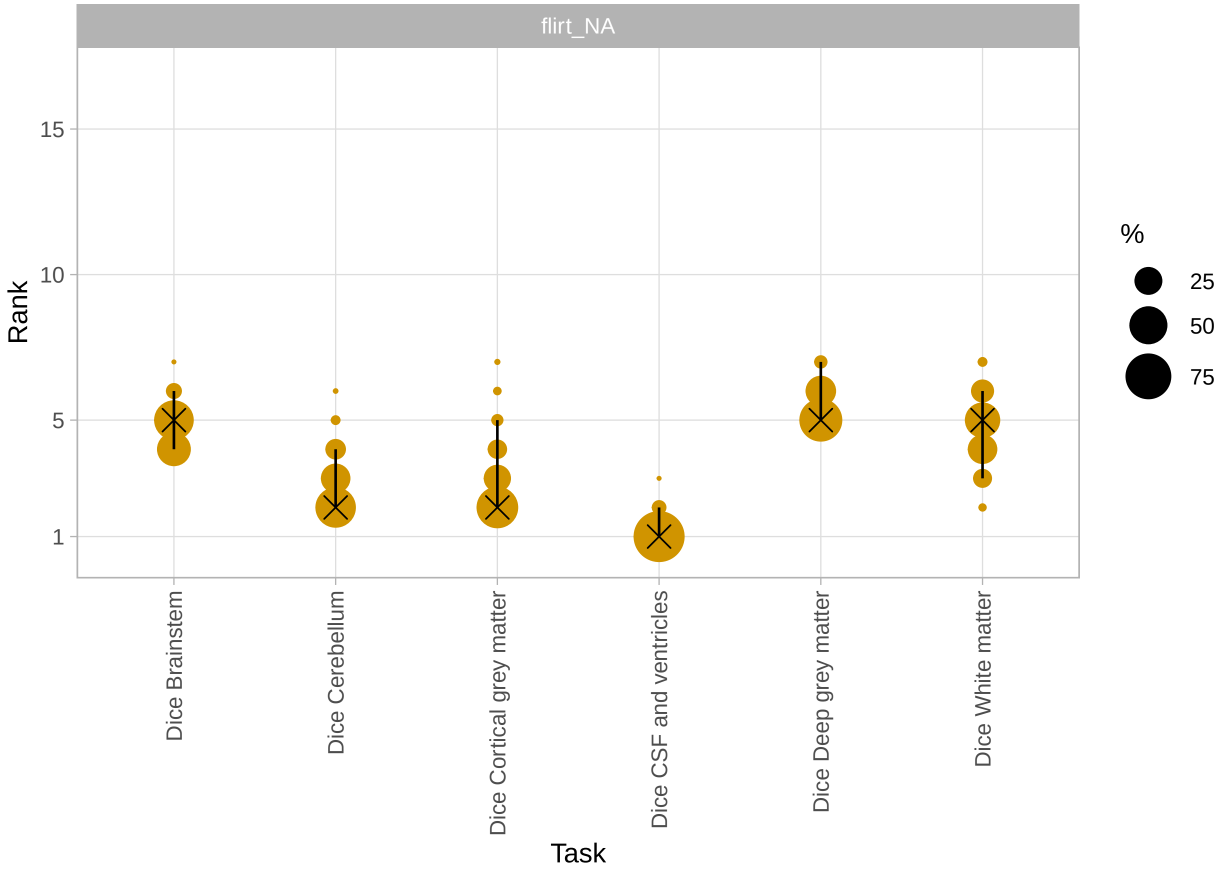
flirt_NA
Rank
15
10
5
1
%
25
50
75
Dice Brainstem
Dice Cerebellum
Dice Cortical grey matter
Dice CSF and ventricles
Dice Deep grey matter
Dice White matter
Task

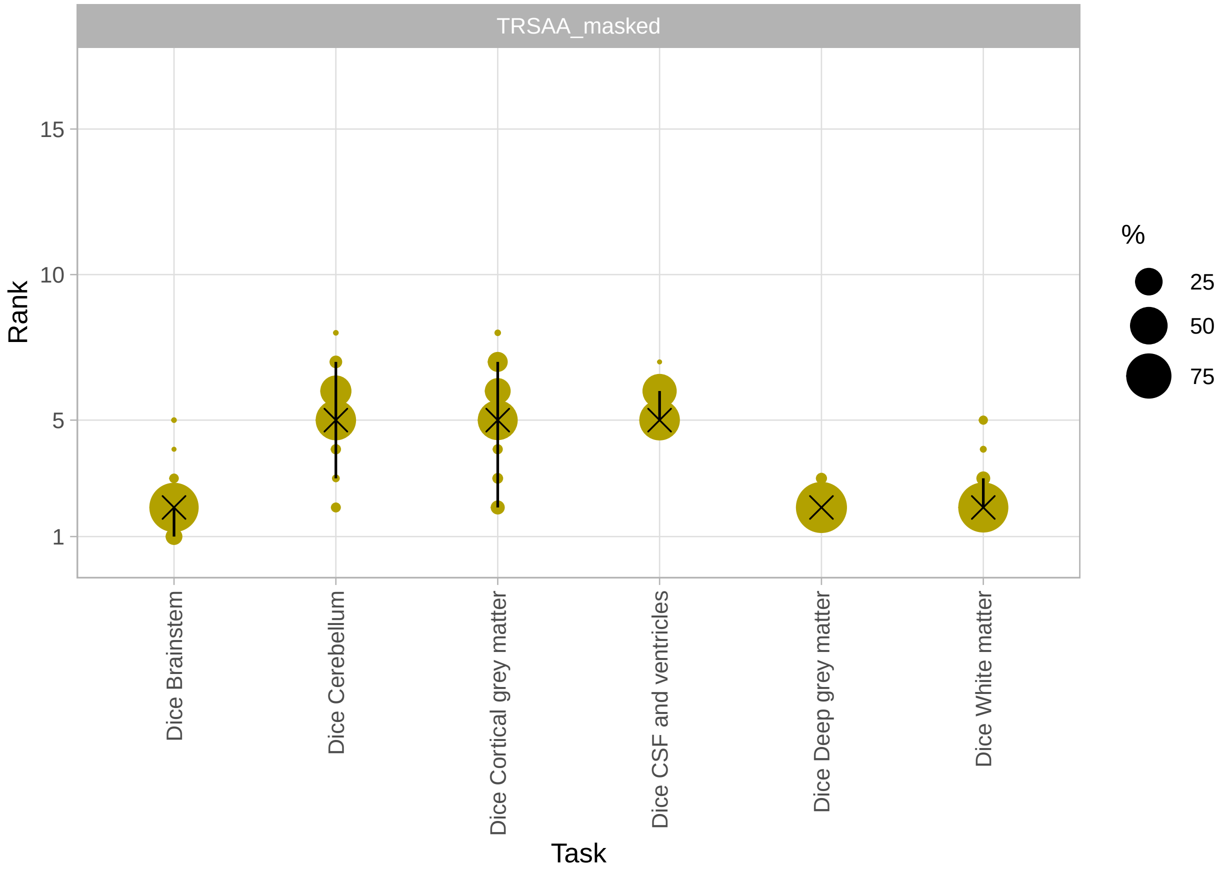
TRSAA_masked
Rank
15
10
5
1
%
25
50
75
Dice Brainstem
Dice Cerebellum
Dice Cortical grey matter
Dice CSF and ventricles
Dice Deep grey matter
Dice White matter
Task

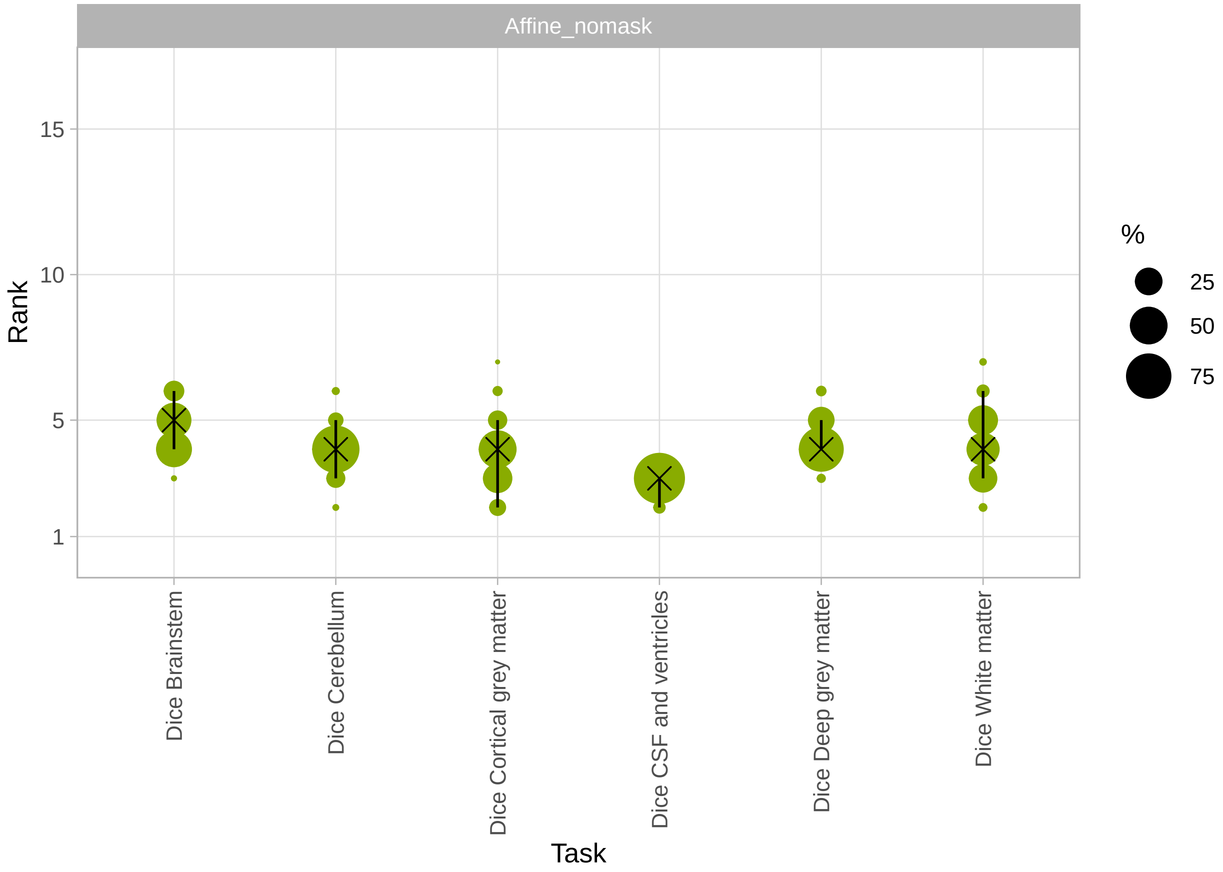
Affine_nomask
Rank
15
10
5
1
%
25
50
75
Dice Brainstem
Dice Cerebellum
Dice Cortical grey matter
Dice CSF and ventricles
Dice Deep grey matter
Dice White matter
Task

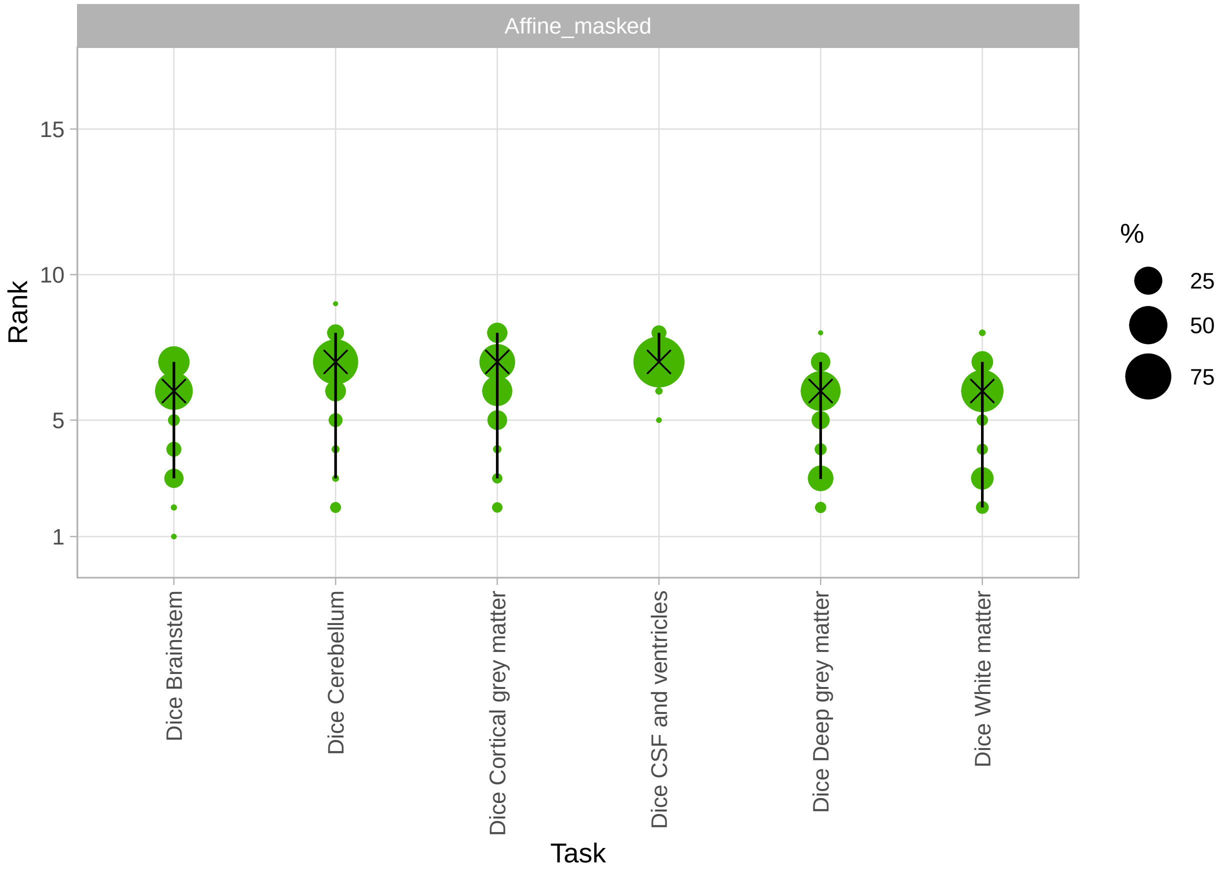
Affine_masked
Rank
15
10
5
1
%
25
50
75
Dice Brainstem
Dice Cerebellum
Dice Cortical grey matter
Dice CSF and ventricles
Dice Deep grey matter
Dice White matter
Task

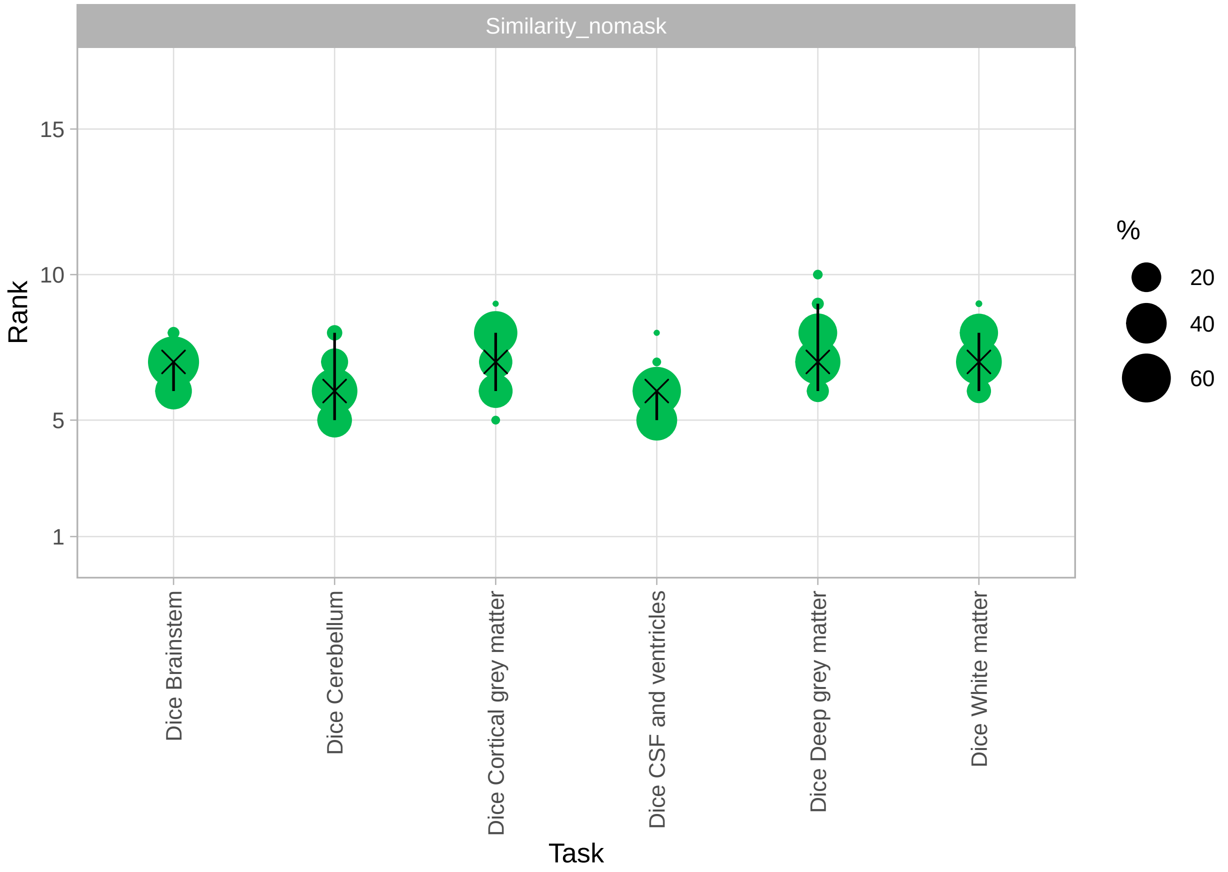
Similarity_nomask
Rank
15
10
5
1
%
20
40
60
Dice Brainstem
Dice Cerebellum
Dice Cortical grey matter
Dice CSF and ventricles
Dice Deep grey matter
Dice White matter
Task

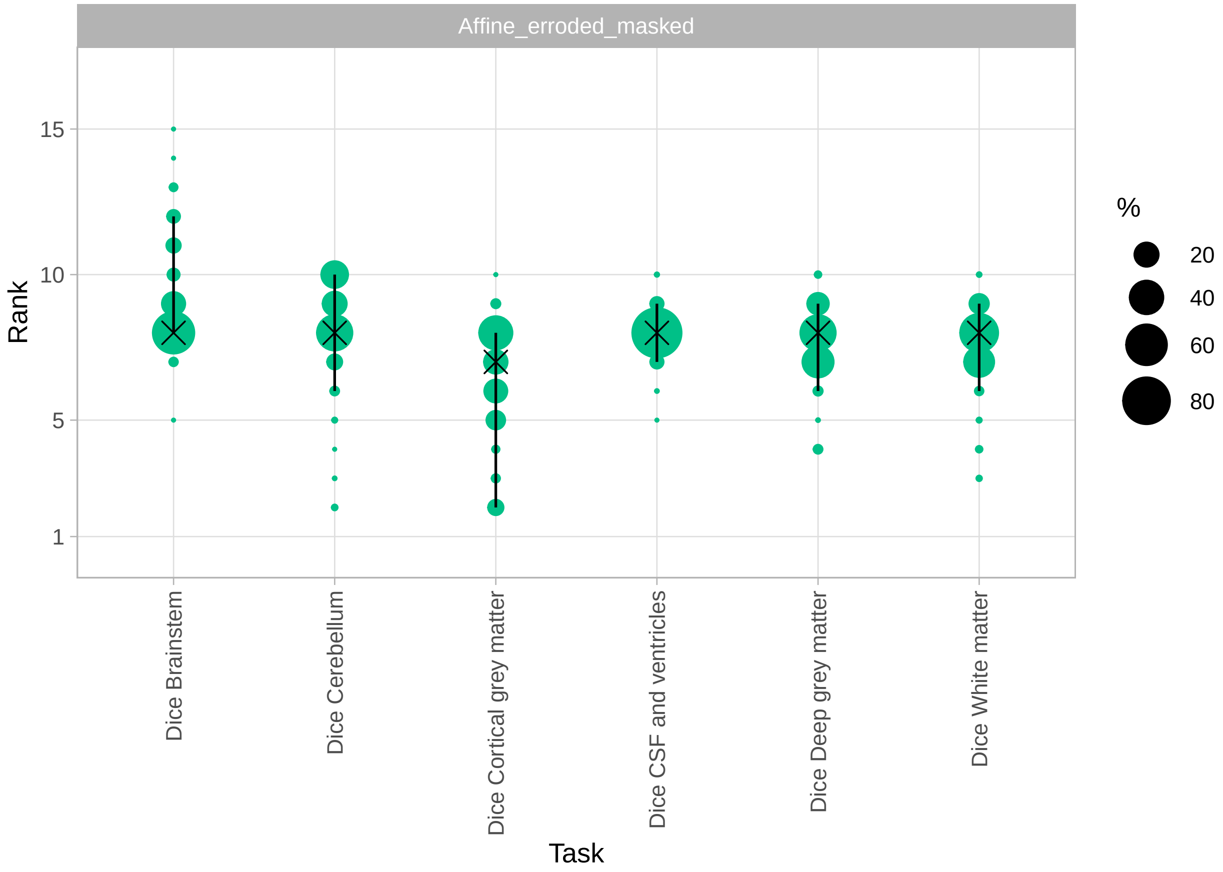
Affine_erroded_masked
Rank
15
10
5
1
Dice Brainstem
Dice Cerebellum
Dice Cortical grey matter
Dice CSF and ventricles
Dice Deep grey matter
Dice White matter
Task
%
20
40
60
80

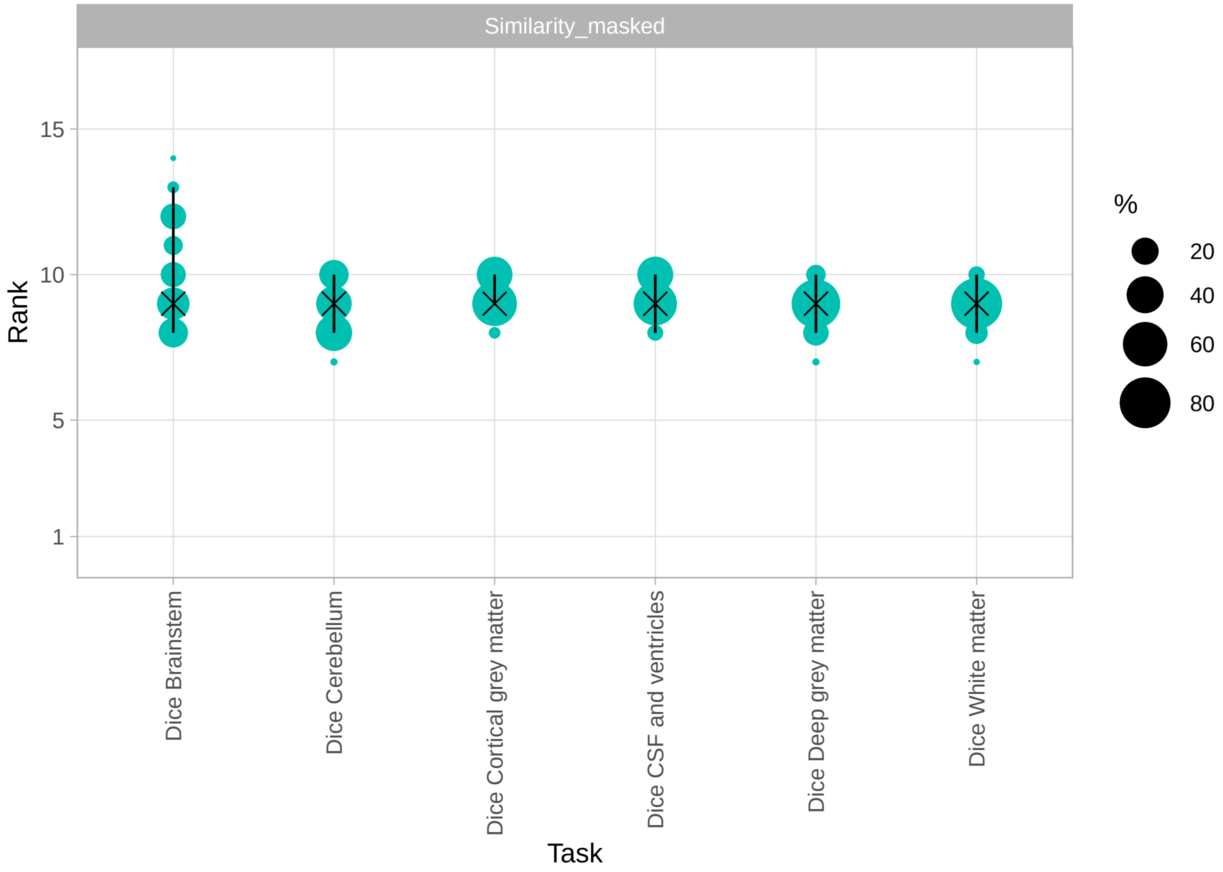

Similarity_masked
Rank
15
10
5
1
Dice Brainstem
Dice Cerebellum
Dice Cortical grey matter
Dice CSF and ventricles
Dice Deep grey matter
Dice White matter
Task
%
20
40
60
80

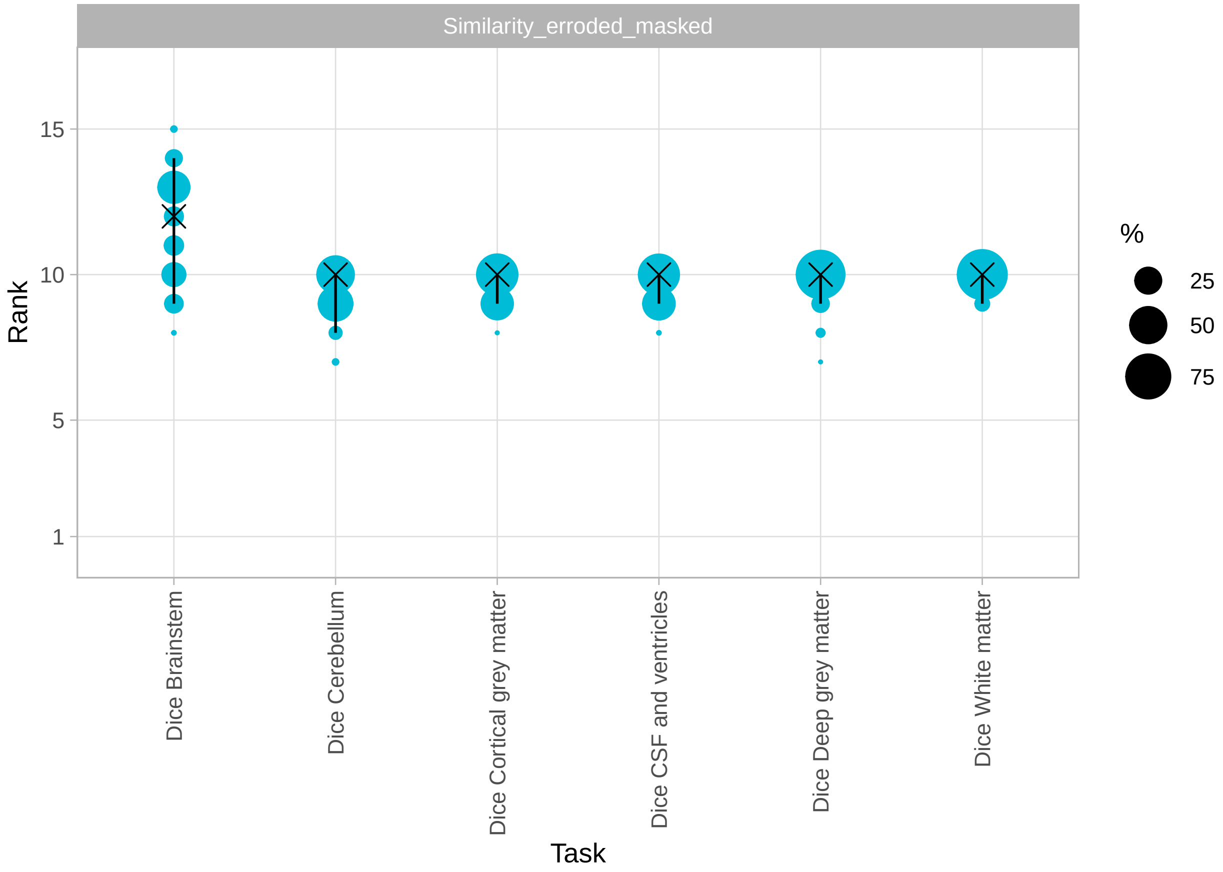

Similarity_erroded_masked
Rank
15
10
5
1
Dice Brainstem
Dice Cerebellum
Dice Cortical grey matter
Dice CSF and ventricles
Dice Deep grey matter
Dice White matter
Task
%
25
50
75

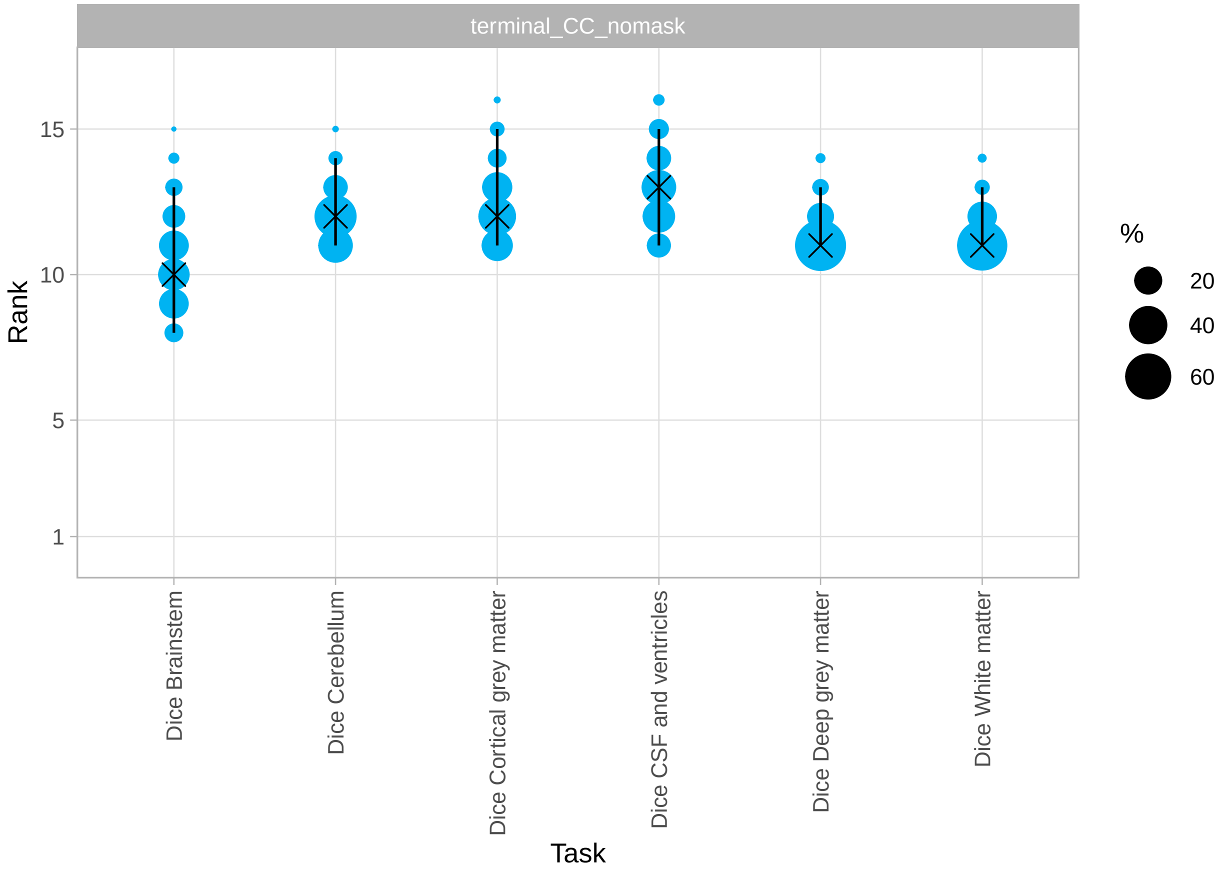
terminal_CC_nomask
Rank
15
10
5
1
Dice Brainstem
Dice Cerebellum
Dice Cortical grey matter
Dice CSF and ventricles
Dice Deep grey matter
Dice White matter
Task
%
20
40
60

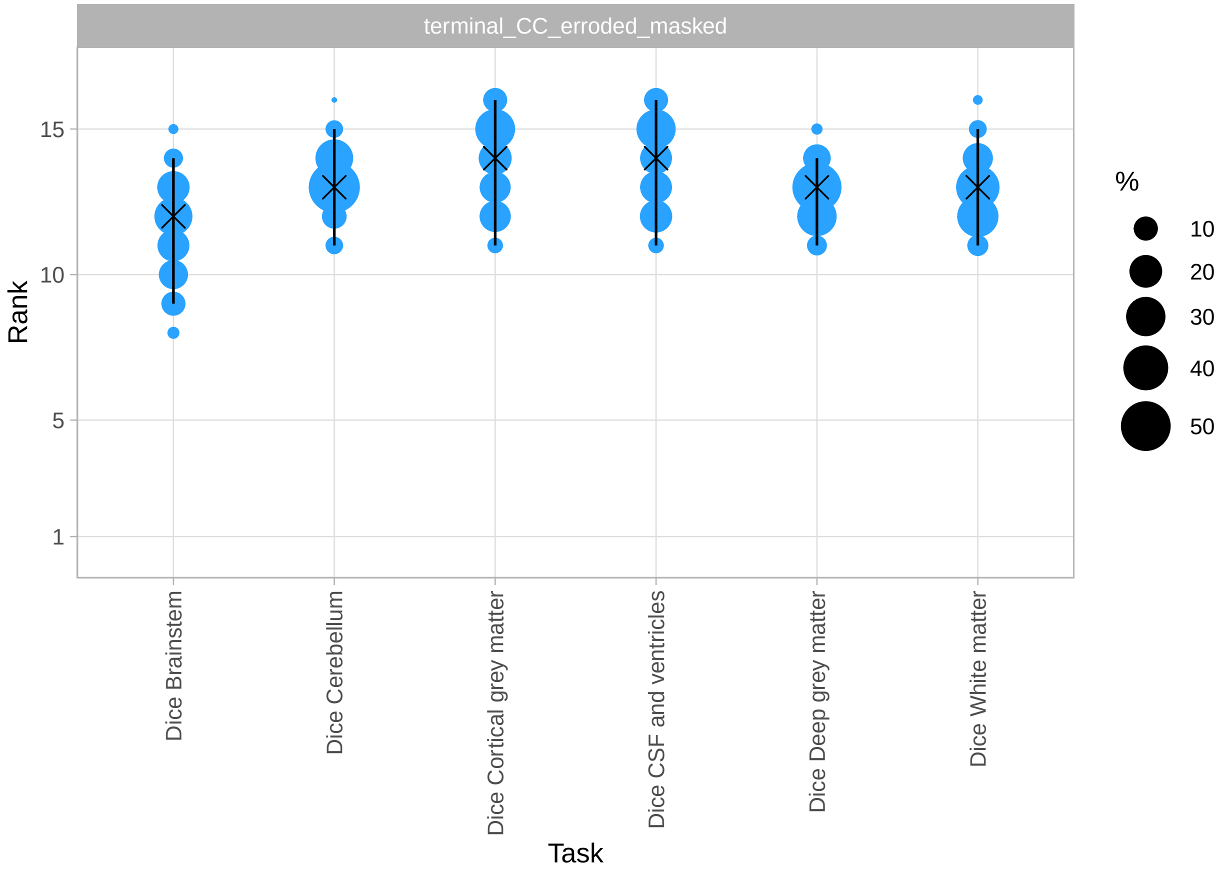
terminal_CC_erroded_masked
Rank
15
10
5
1
Dice Brainstem
Dice Cerebellum
Dice Cortical grey matter
Dice CSF and ventricles
Dice Deep grey matter
Dice White matter
Task
%
10
20
30
40
50

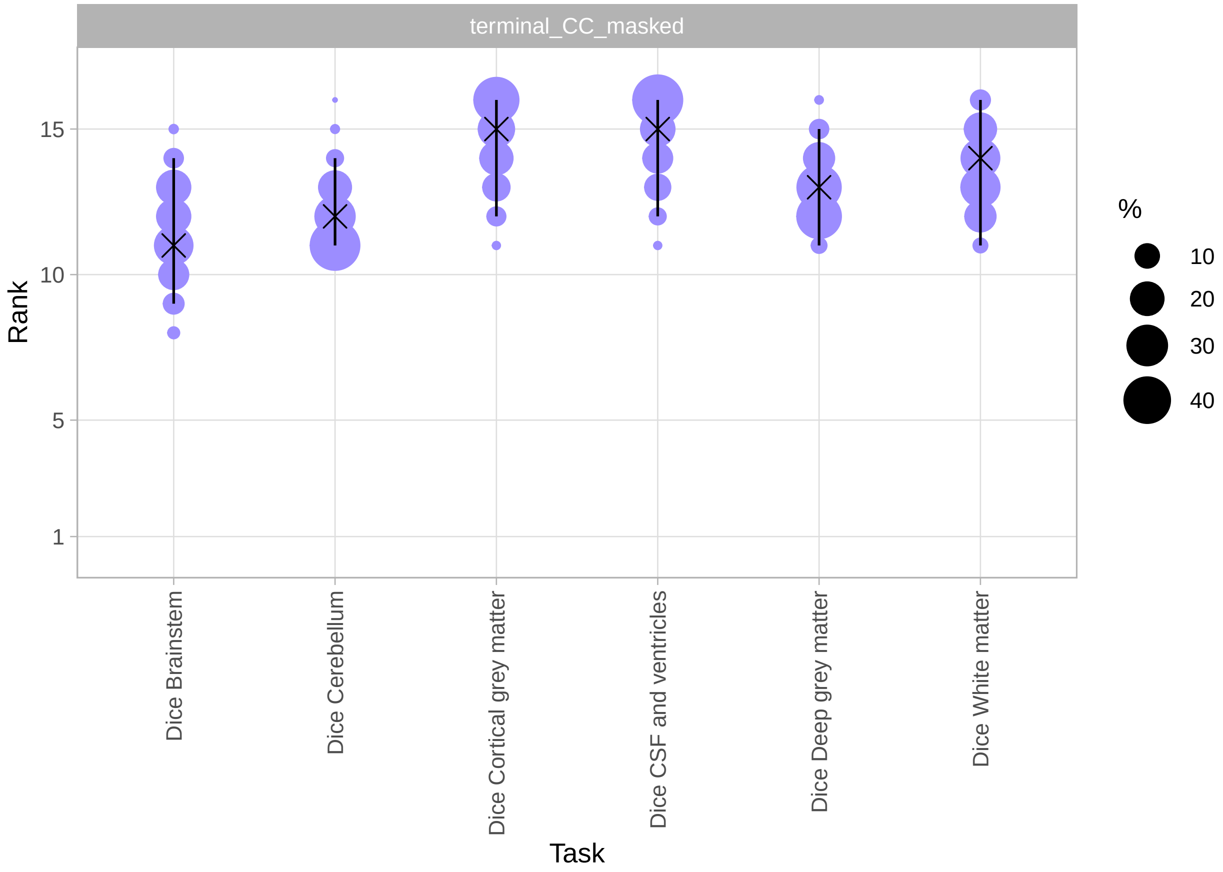
terminal_CC_masked
Rank
15
10
5
1
Dice Brainstem
Dice Cerebellum
Dice Cortical grey matter
Dice CSF and ventricles
Dice Deep grey matter
Dice White matter
Task
%
10
20
30
40

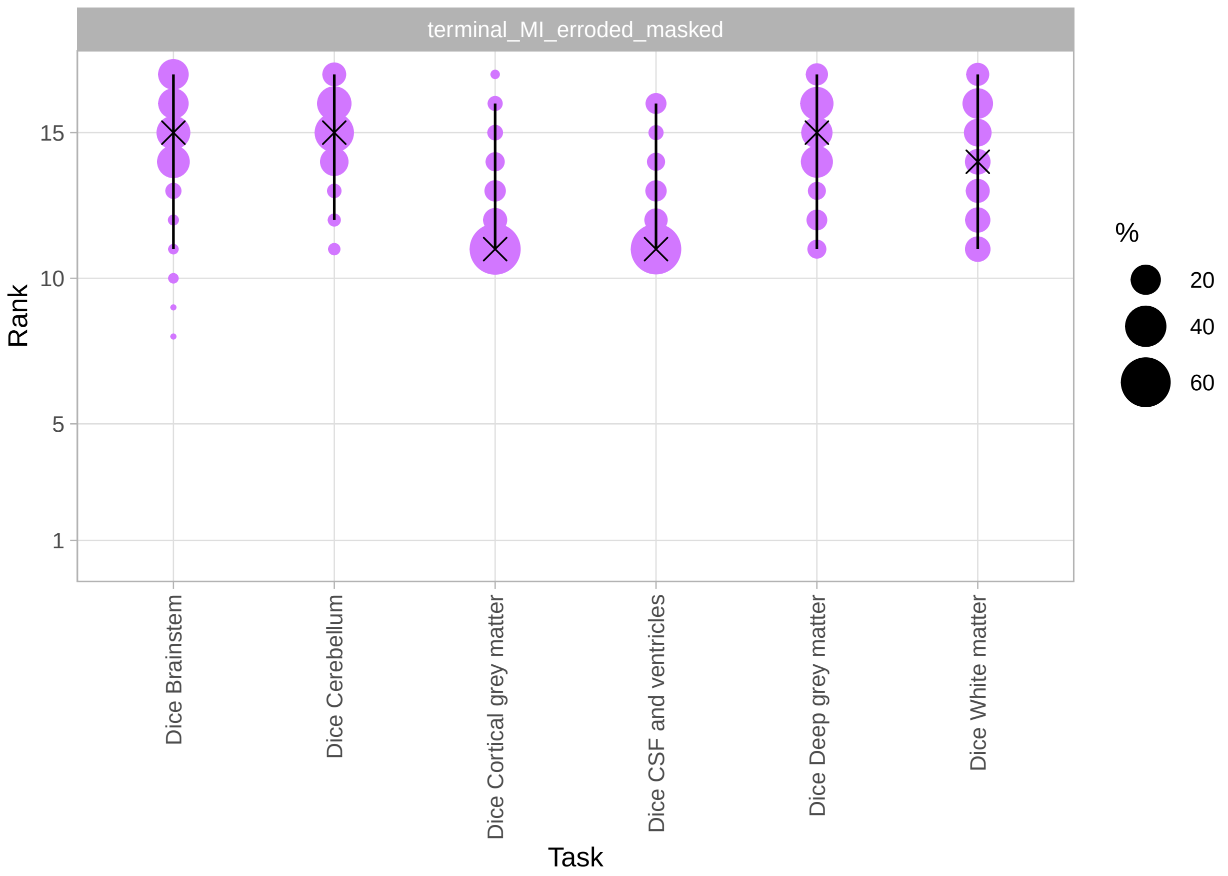
terminal_MI_erroded_masked
Rank
15
10
5
1
%
20
40
60
Dice Brainstem
Dice Cerebellum
Dice Cortical grey matter
Dice CSF and ventricles
Dice Deep grey matter
Dice White matter
Task

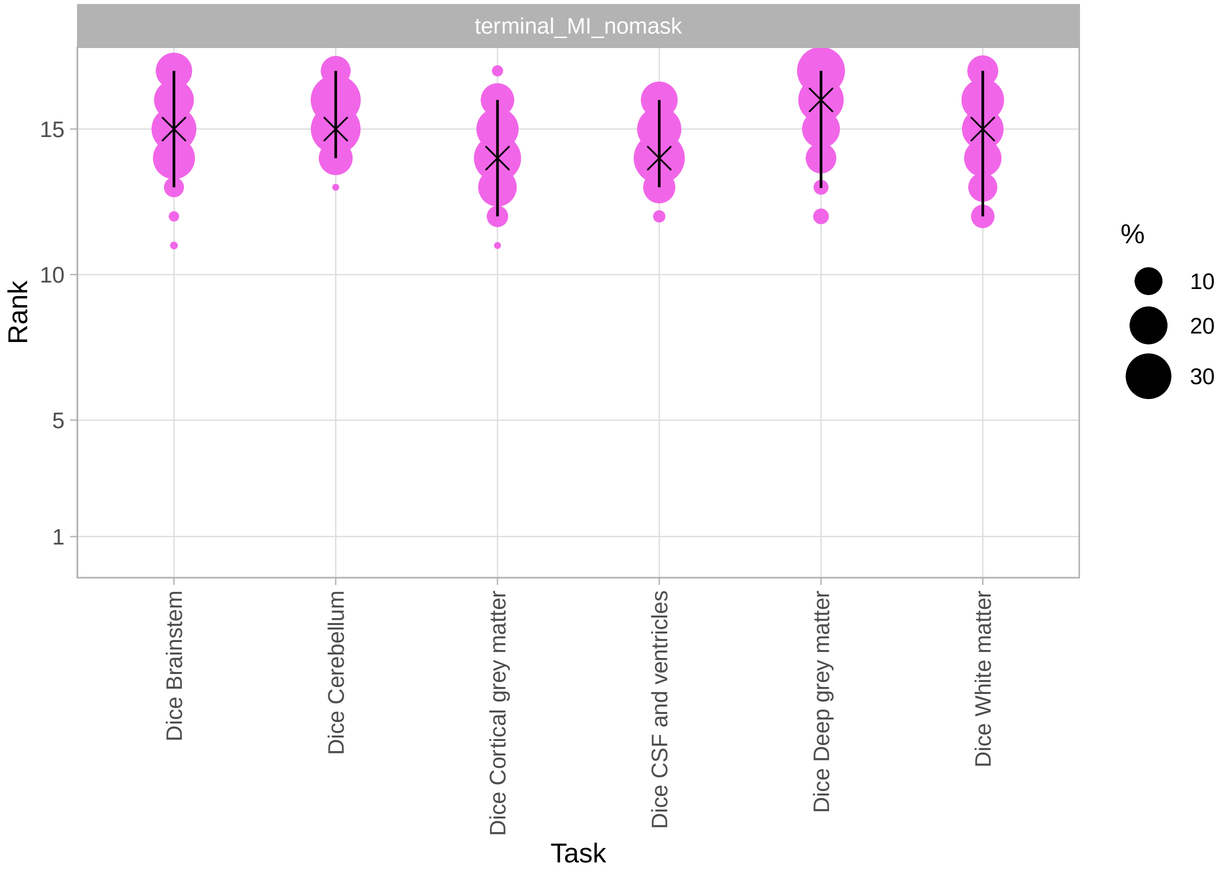
terminal_MI_nomask
Rank
15
10
5
1
Dice Brainstem
Dice Cerebellum
Dice Cortical grey matter
Dice CSF and ventricles
Dice Deep grey matter
Dice White matter
Task
%
10
20
30

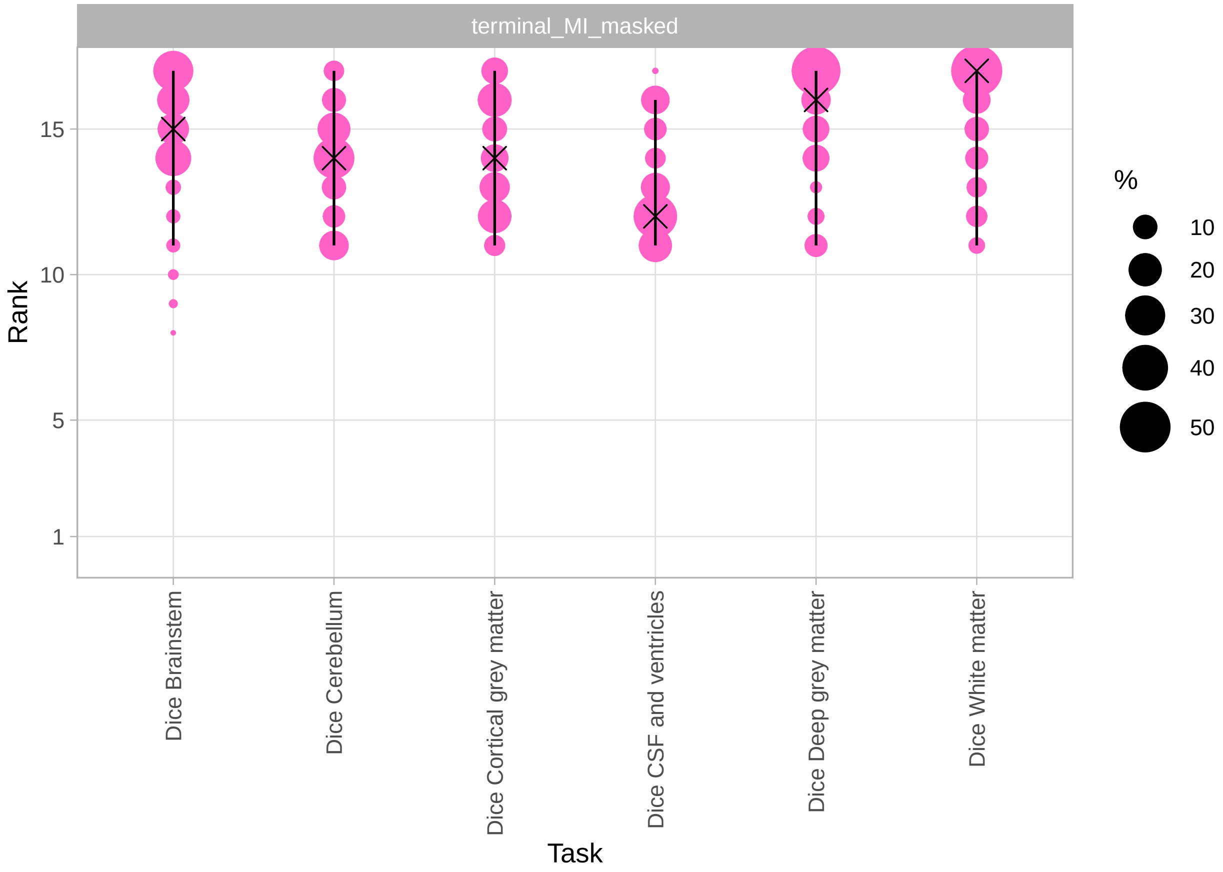
terminal_MI_masked
Rank
15
10
5
1
Dice Brainstem
Dice Cerebellum
Dice Cortical grey matter
Dice CSF and ventricles
Dice Deep grey matter
Dice White matter
Task
%
10
20
30
40
50

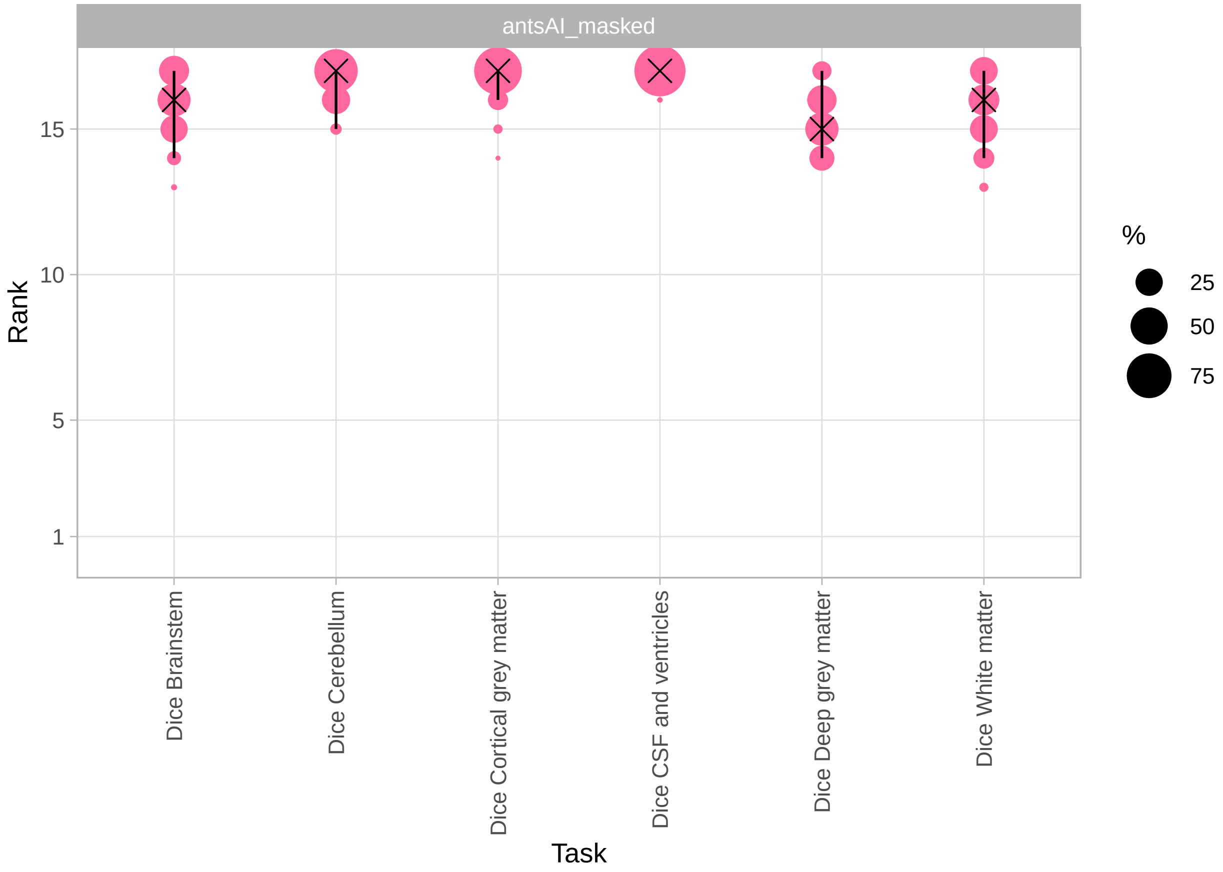
antsAI_masked
Rank
15
10
5
1
Dice Brainstem
Dice Cerebellum
Dice Cortical grey matter
Dice CSF and ventricles
Dice Deep grey matter
Dice White matter
Task
%
25
50
75

An alternative representation is provided by a stacked frequency plot of the observed ranks, separated by algorithm. Observed ranks across bootstrap samples are displayed with coloring according to the task. For algorithms that achieve the same rank in different tasks for the full assessment data set, vertical lines are on top of each other. Vertical lines allow to compare the achieved rank of each algorithm over different tasks.

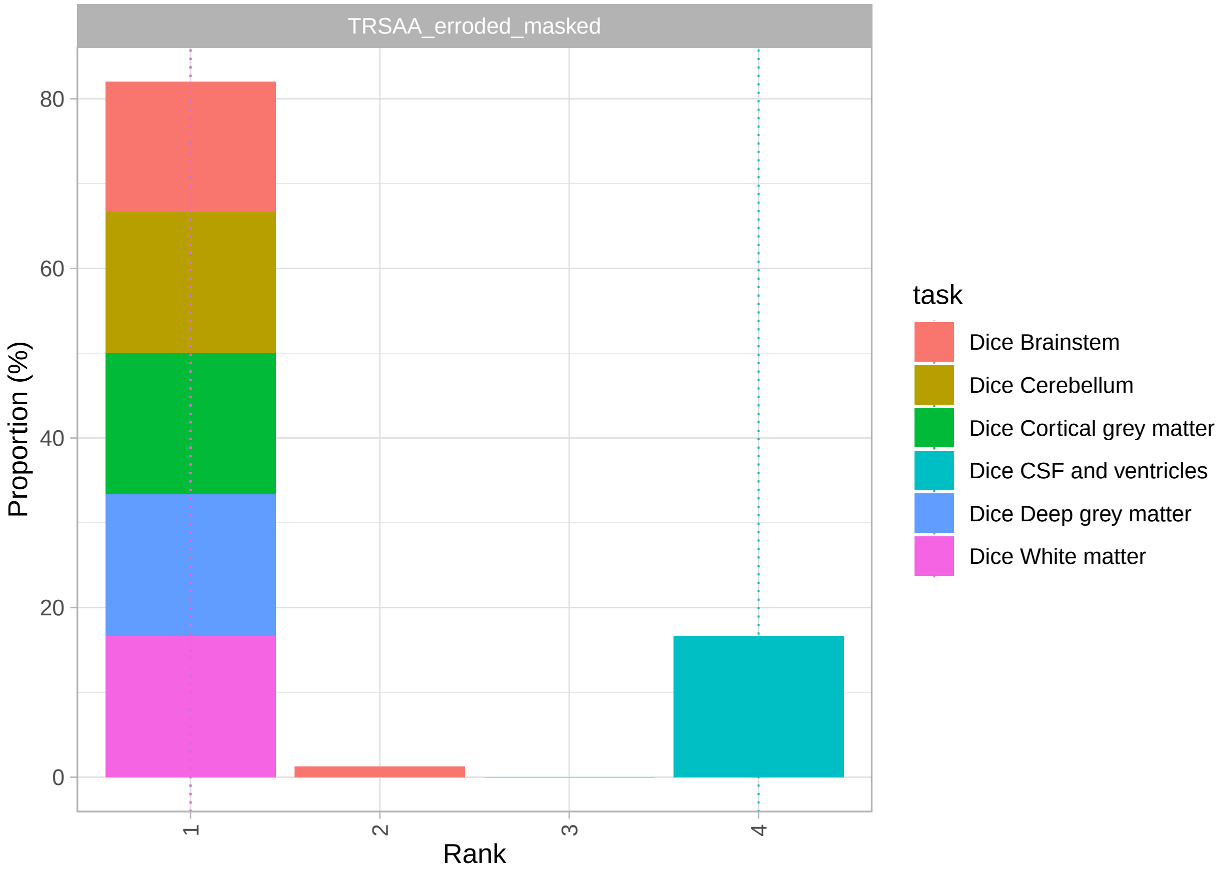

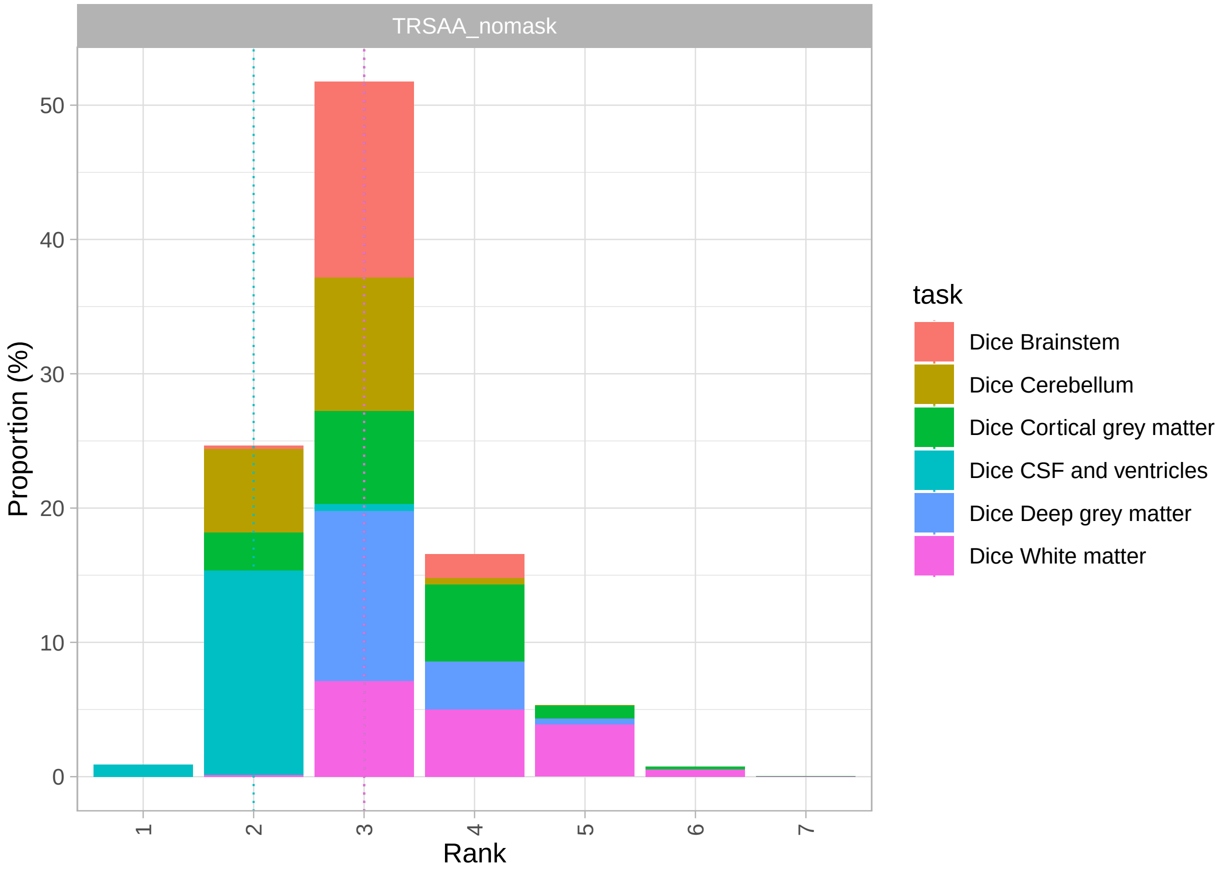
TRSAA_nomask
Proportion (%)
50
40
30
20
10
0
1
2
3
4
5
6
7
Rank
task
Dice Brainstem
Dice Cerebellum
Dice Cortical grey matter
Dice CSF and ventricles
Dice Deep grey matter
Dice White matter

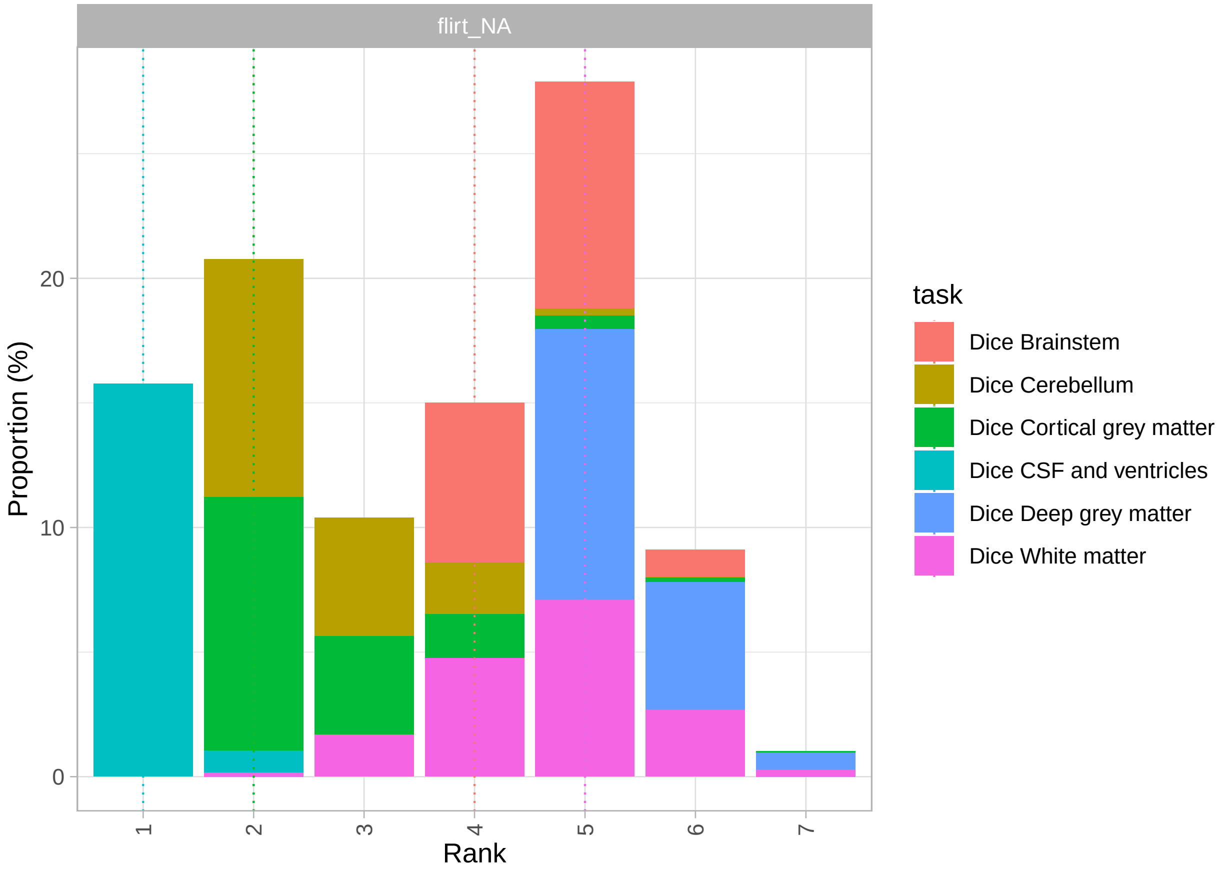
flirt_NA
Proportion (%)
20
10
0
1
2
3
4
5
6
7
Rank
task
Dice Brainstem
Dice Cerebellum
Dice Cortical grey matter
Dice CSF and ventricles
Dice Deep grey matter
Dice White matter

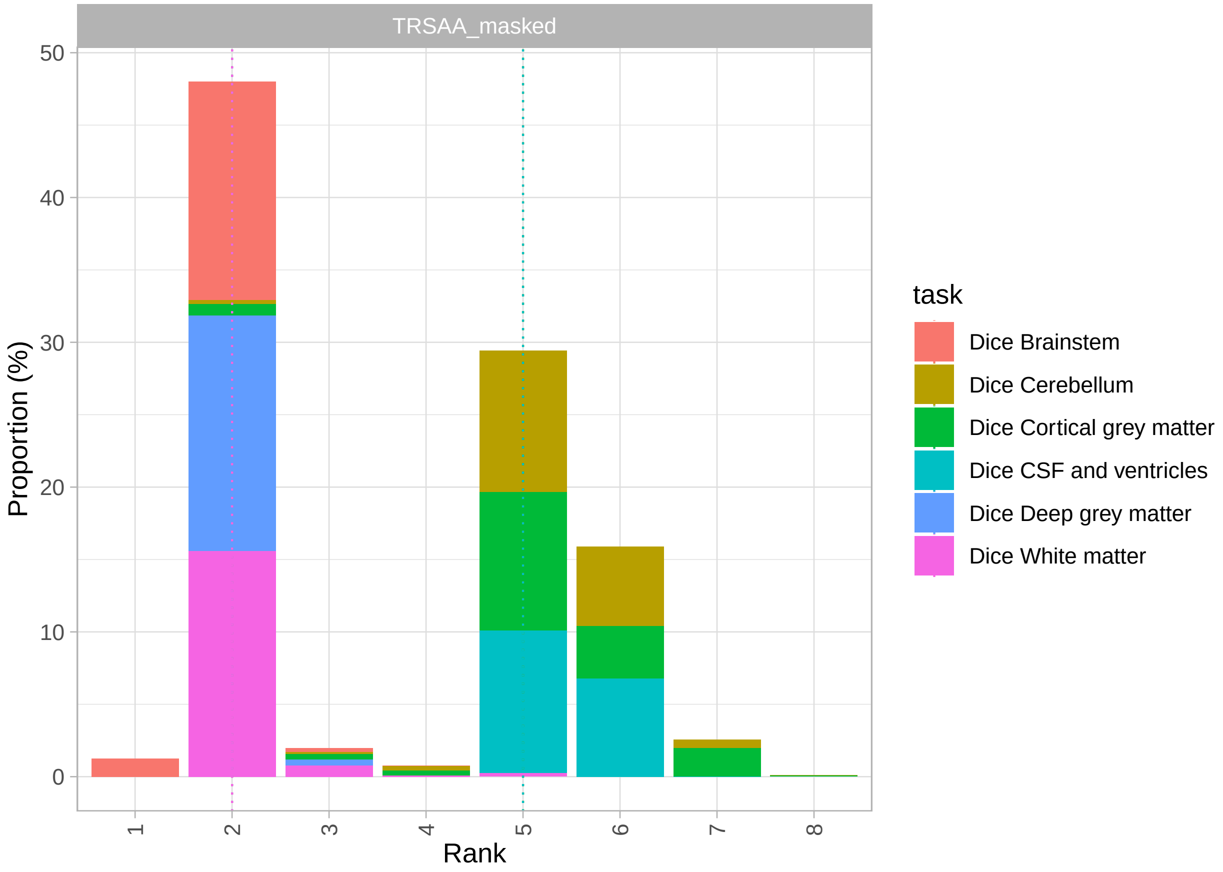
TRSAA_masked
Proportion (%)
50
40
30
20
10
0
1
2
3
4
5
6
7
8
Rank
task
Dice Brainstem
Dice Cerebellum
Dice Cortical grey matter
Dice CSF and ventricles
Dice Deep grey matter
Dice White matter

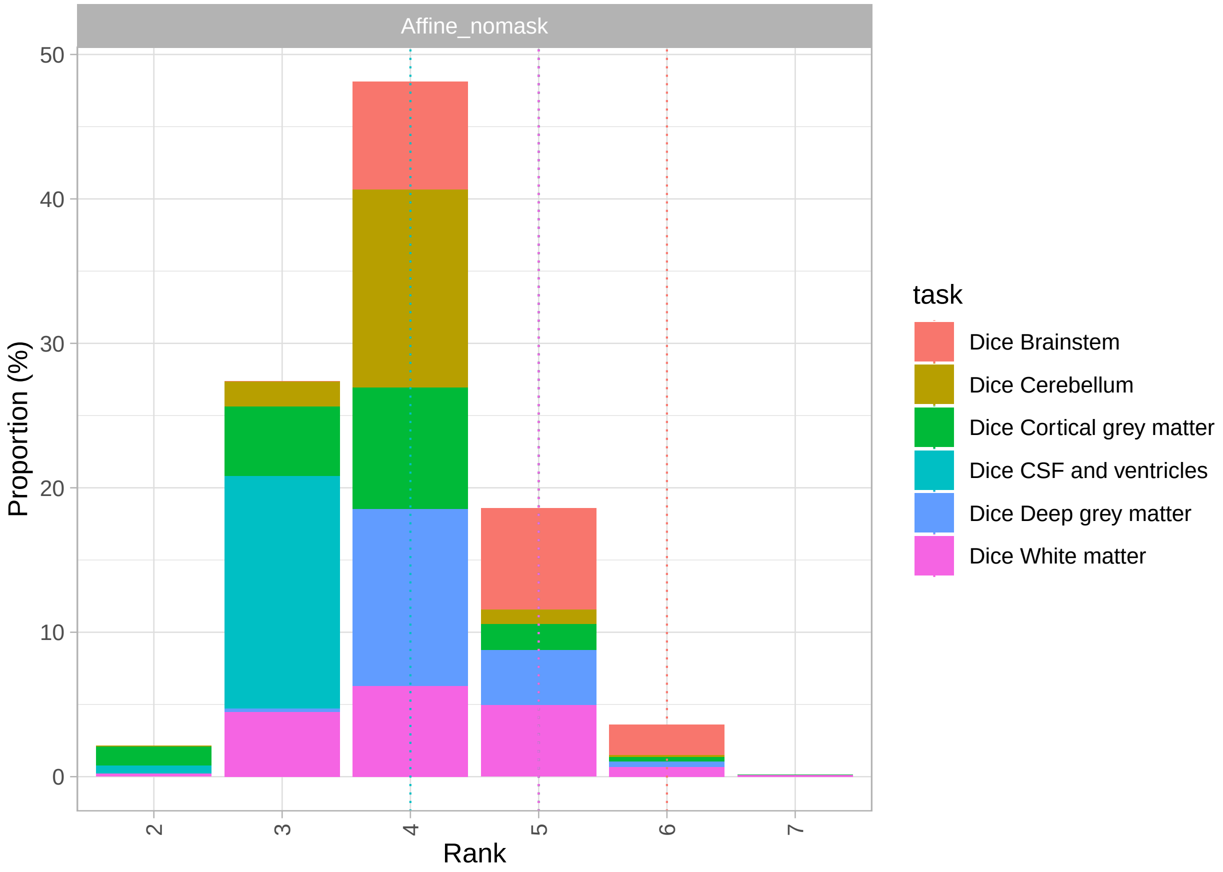
Affine_nomask
Proportion (%)
50
40
30
20
10
0
2
3
4
5
6
7
Rank
task
Dice Brainstem
Dice Cerebellum
Dice Cortical grey matter
Dice CSF and ventricles
Dice Deep grey matter
Dice White matter

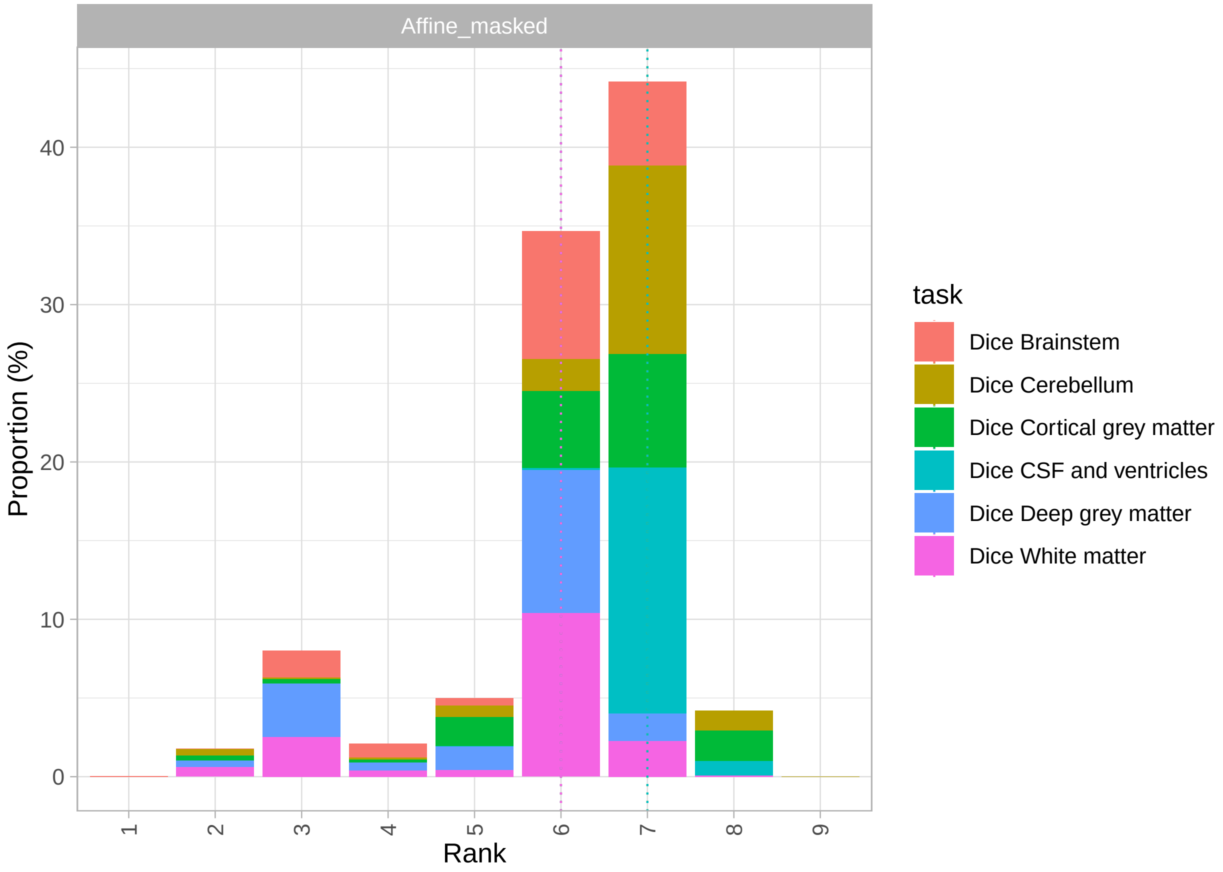
Affine_masked
Proportion (%)
40
30
20
10
0
1
2
3
4
5
6
7
8
9
Rank
task
Dice Brainstem
Dice Cerebellum
Dice Cortical grey matter
Dice CSF and ventricles
Dice Deep grey matter
Dice White matter

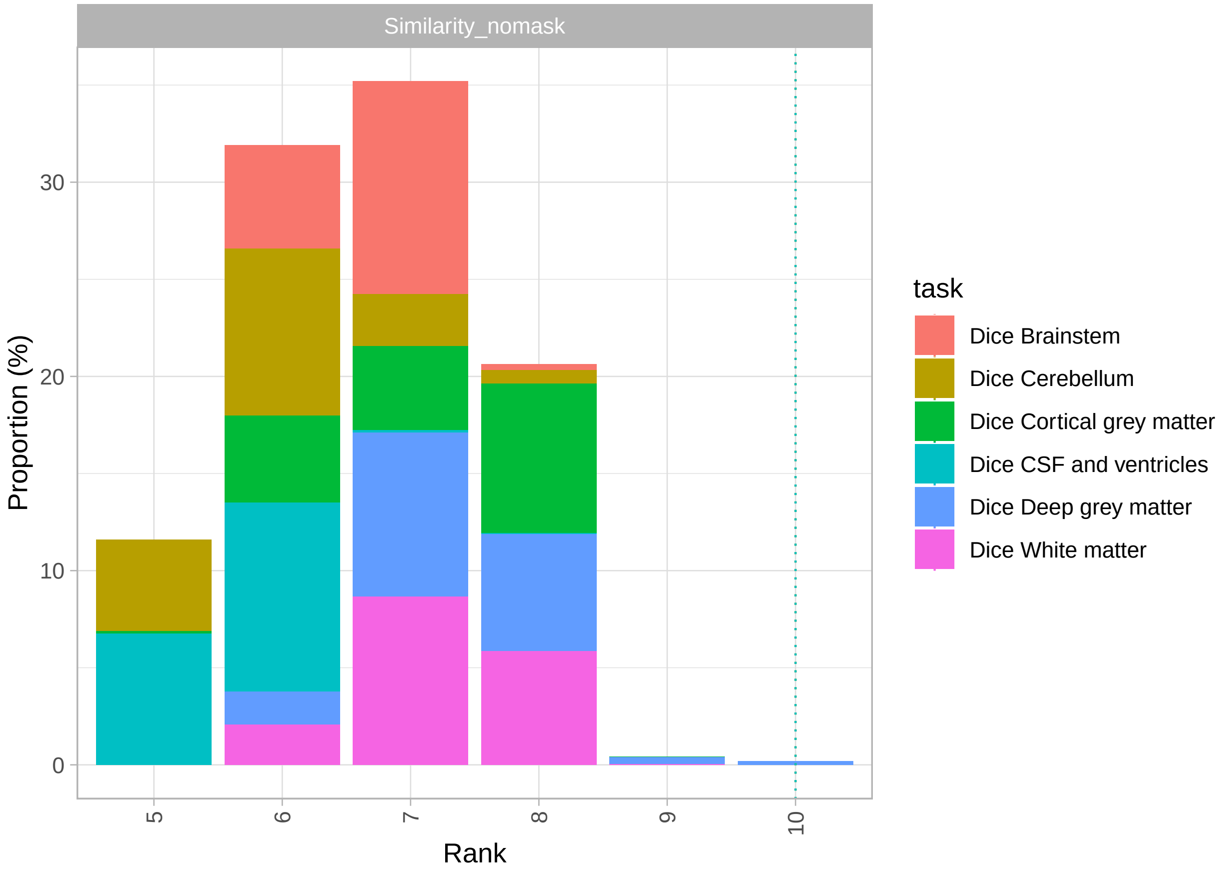
Similarity_nomask
Proportion (%)
30
20
10
0
5
6
7
8
9
10
Rank
task
Dice Brainstem
Dice Cerebellum
Dice Cortical grey matter
Dice CSF and ventricles
Dice Deep grey matter
Dice White matter

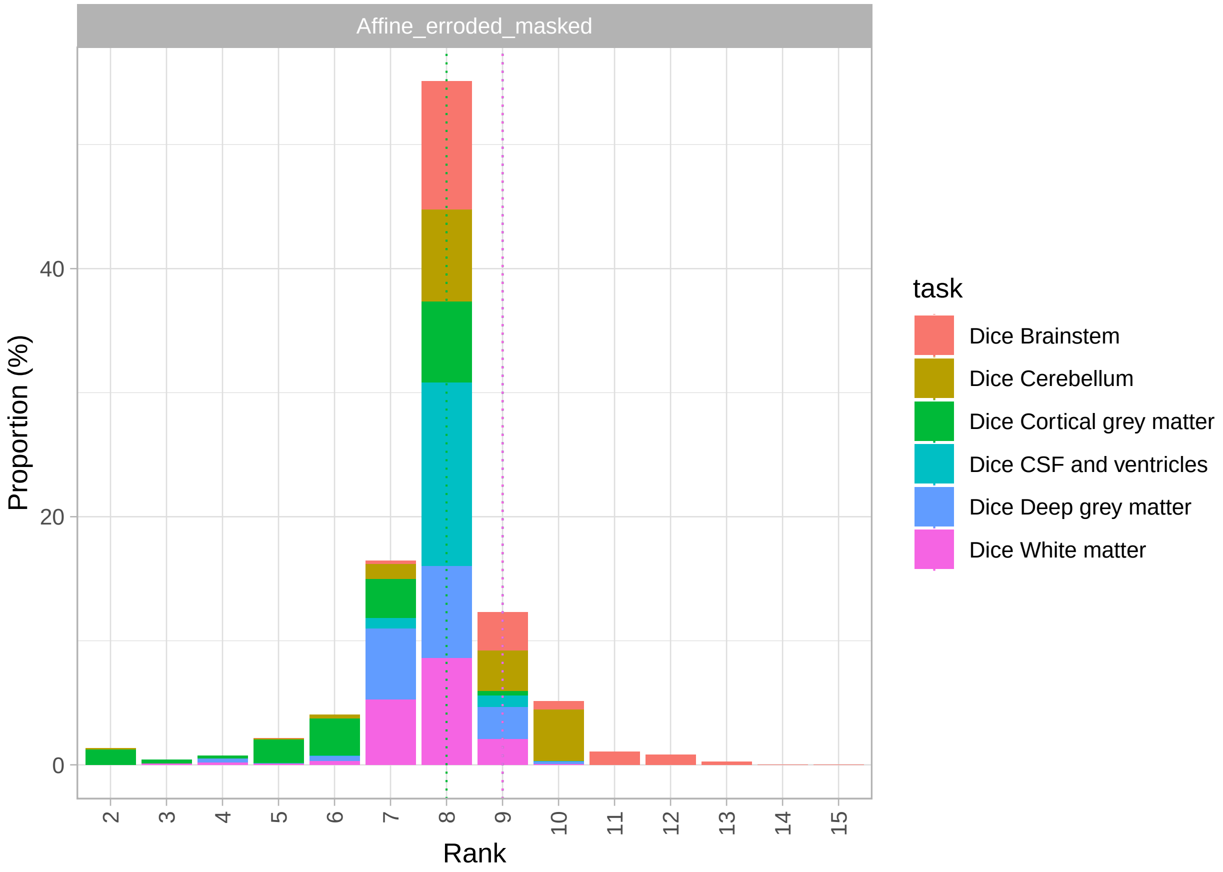
Affine_erroded_masked
Proportion (%)
40
20
0
2
3
4
5
6
7
8
9
10
11
12
13
14
15
Rank
task
Dice Brainstem
Dice Cerebellum
Dice Cortical grey matter
Dice CSF and ventricles
Dice Deep grey matter
Dice White matter

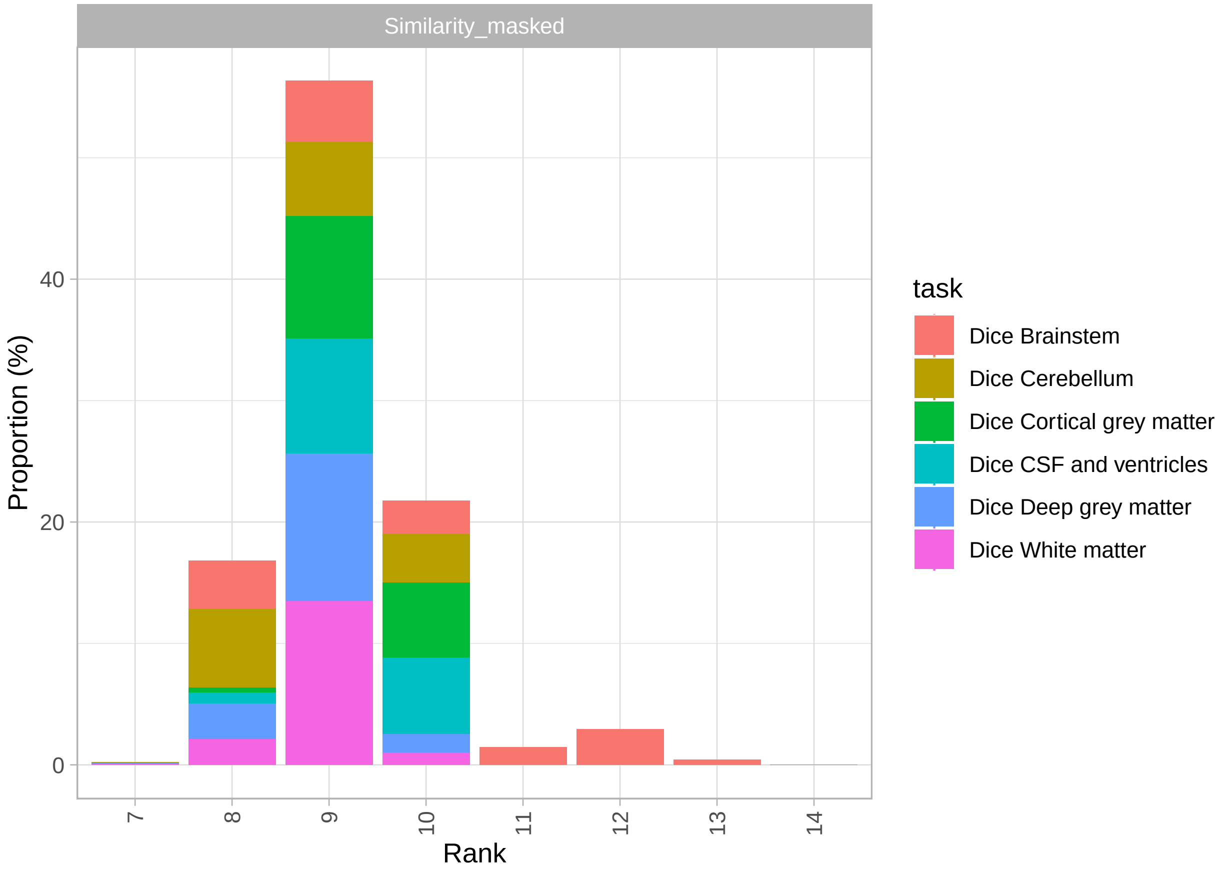
Similarity_masked
Proportion (%)
0
20
40
7
8
9
10
11
12
13
14
Rank
task
Dice Brainstem
Dice Cerebellum
Dice Cortical grey matter
Dice CSF and ventricles
Dice Deep grey matter
Dice White matter

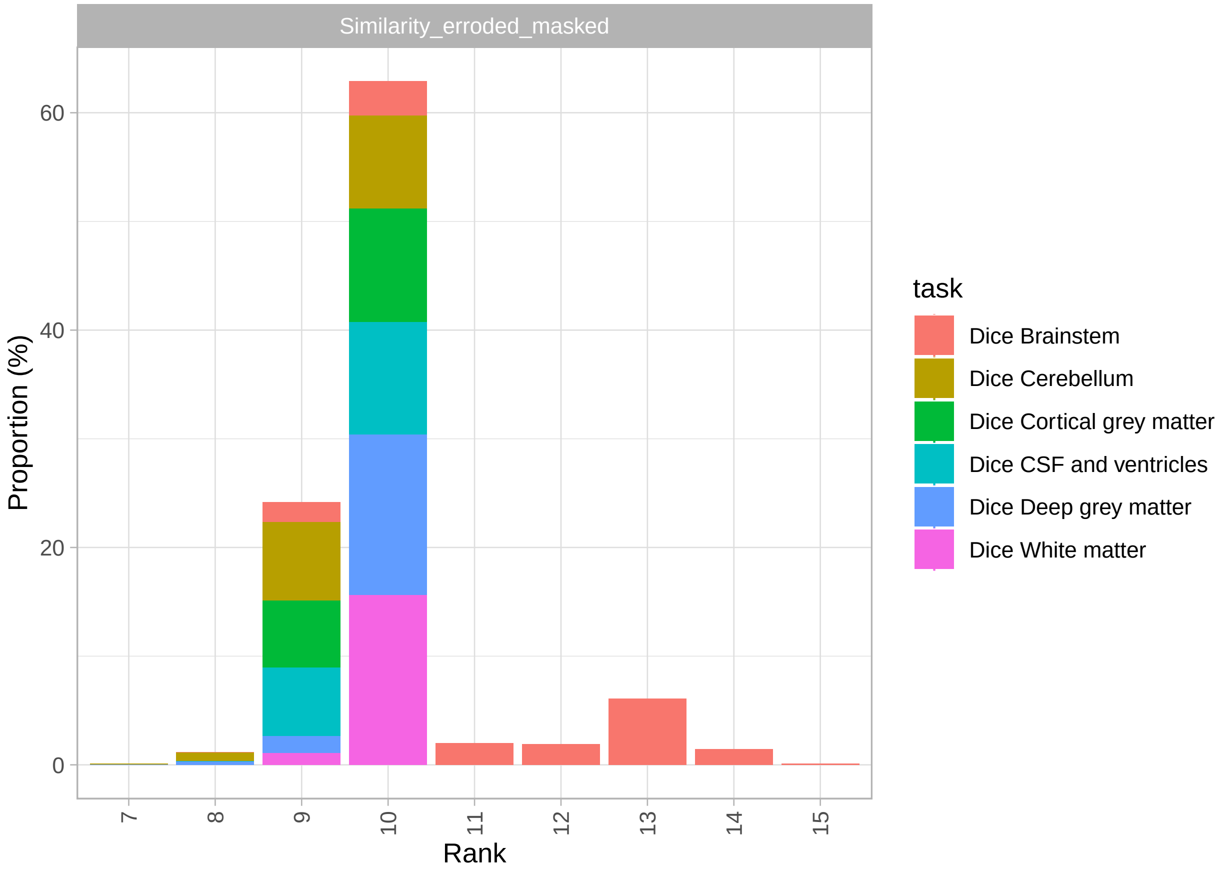

Similarity_erroded_masked
Proportion (%)
0
20
40
60
7
8
9
10
11
12
13
14
15
Rank
task
Dice Brainstem
Dice Cerebellum
Dice Cortical grey matter
Dice CSF and ventricles
Dice Deep grey matter
Dice White matter

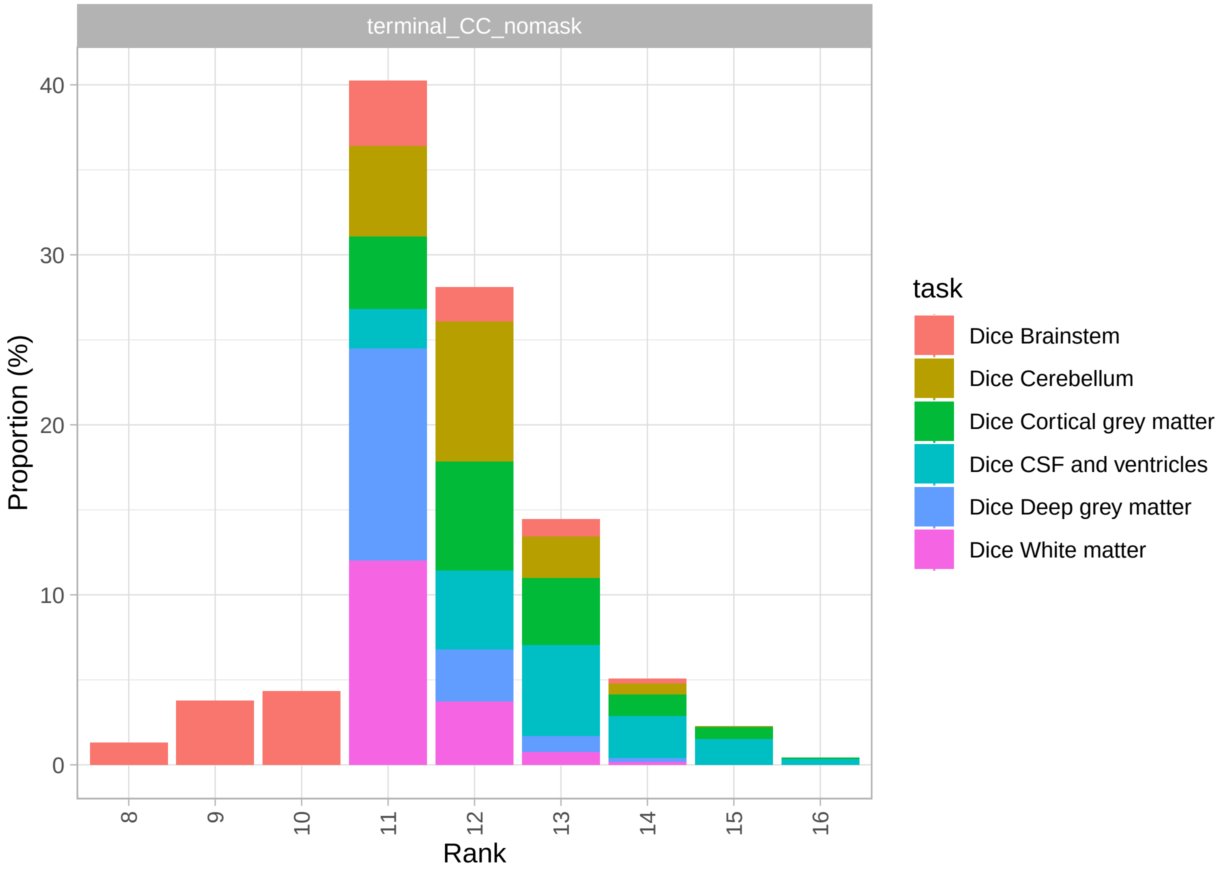
terminal_CC_nomask
Proportion (%)
Rank
task
Dice Brainstem
Dice Cerebellum
Dice Cortical grey matter
Dice CSF and ventricles
Dice Deep grey matter
Dice White matter
0
10
20
30
40
8
9
10
11
12
13
14
15
16

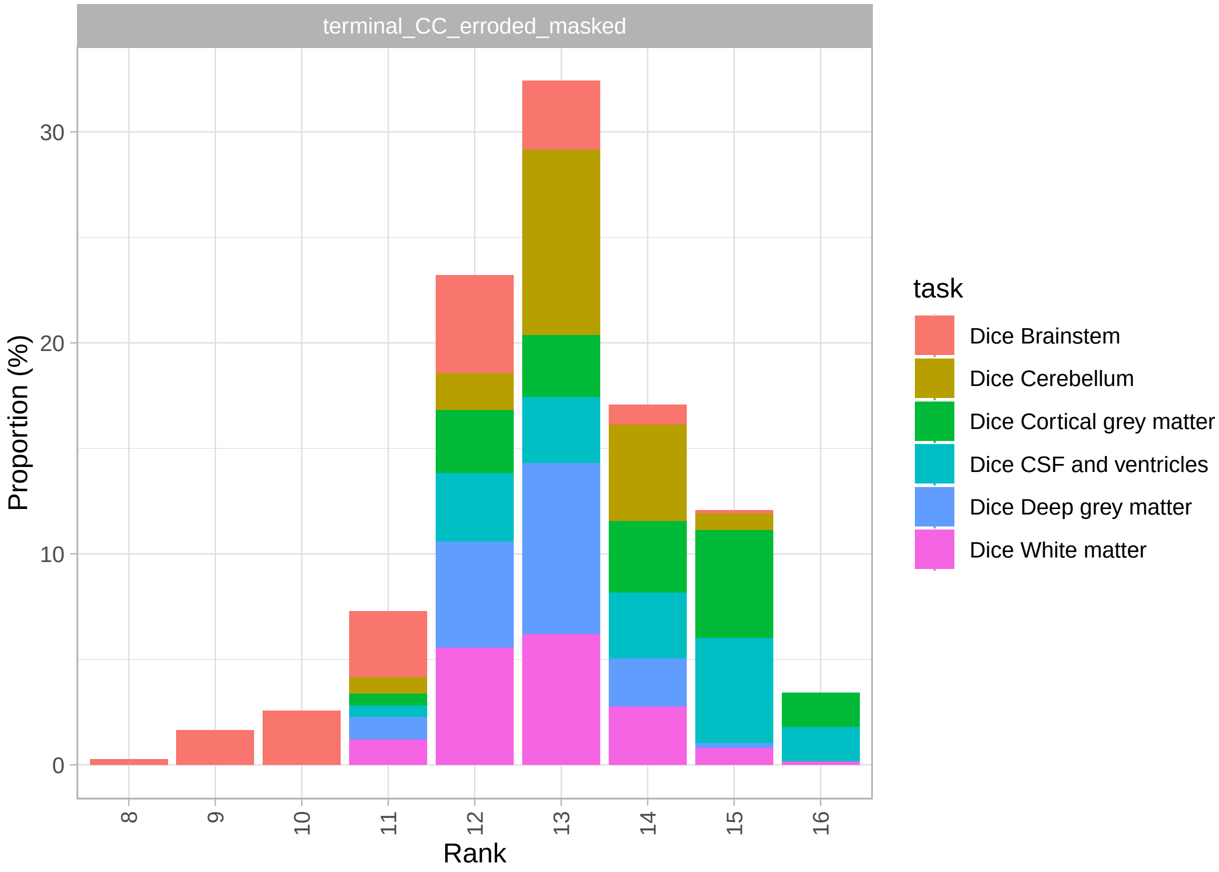
terminal_CC_erroded_masked
Proportion (%)
30
20
10
0
8
9
10
11
12
13
14
15
16
Rank
task
Dice Brainstem
Dice Cerebellum
Dice Cortical grey matter
Dice CSF and ventricles
Dice Deep grey matter
Dice White matter

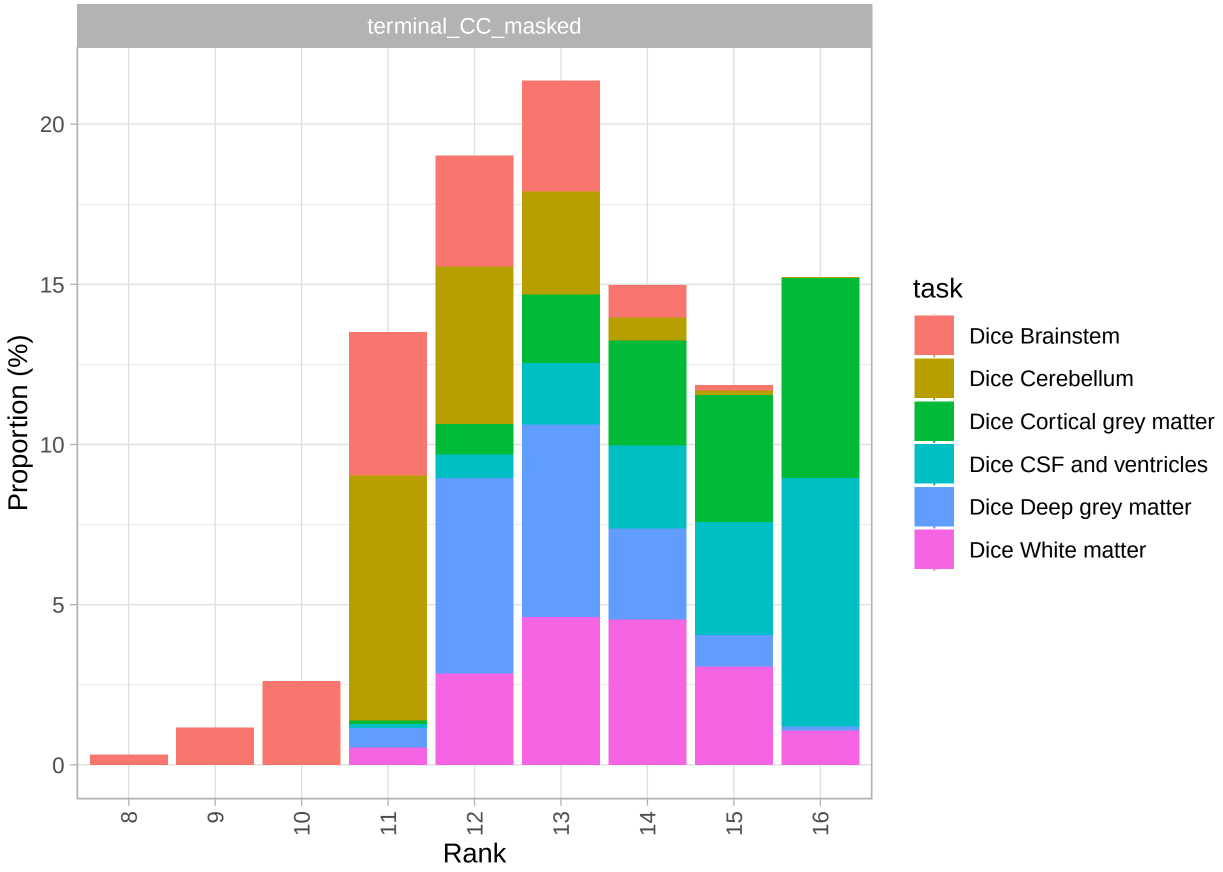
terminal_CC_masked
Proportion (%)
20
15
10
5
0
8
9
10
11
12
13
14
15
16
Rank
task
Dice Brainstem
Dice Cerebellum
Dice Cortical grey matter
Dice CSF and ventricles
Dice Deep grey matter
Dice White matter

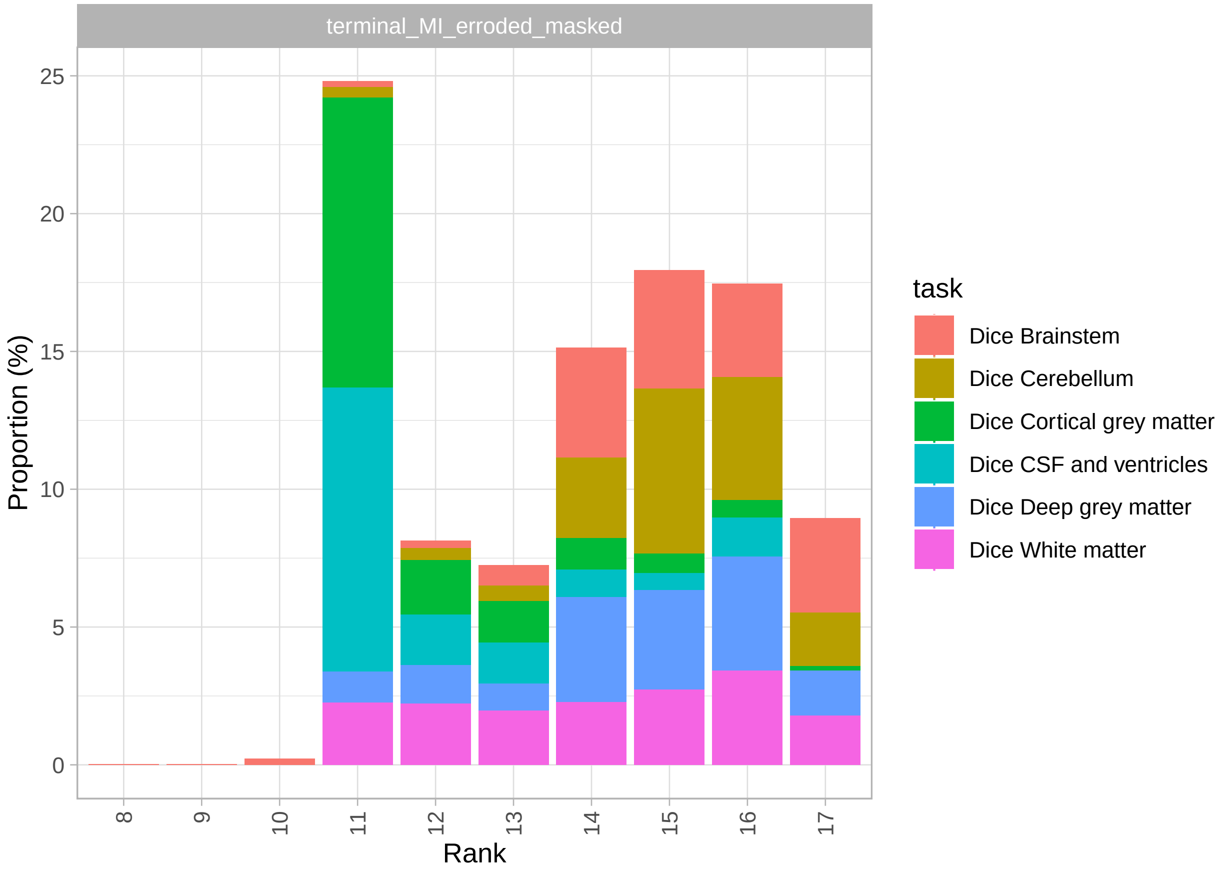
terminal_MI_erroded_masked
Proportion (%)
25
20
15
10
5
0
8
9
10
11
12
13
14
15
16
17
Rank
task
Dice Brainstem
Dice Cerebellum
Dice Cortical grey matter
Dice CSF and ventricles
Dice Deep grey matter
Dice White matter

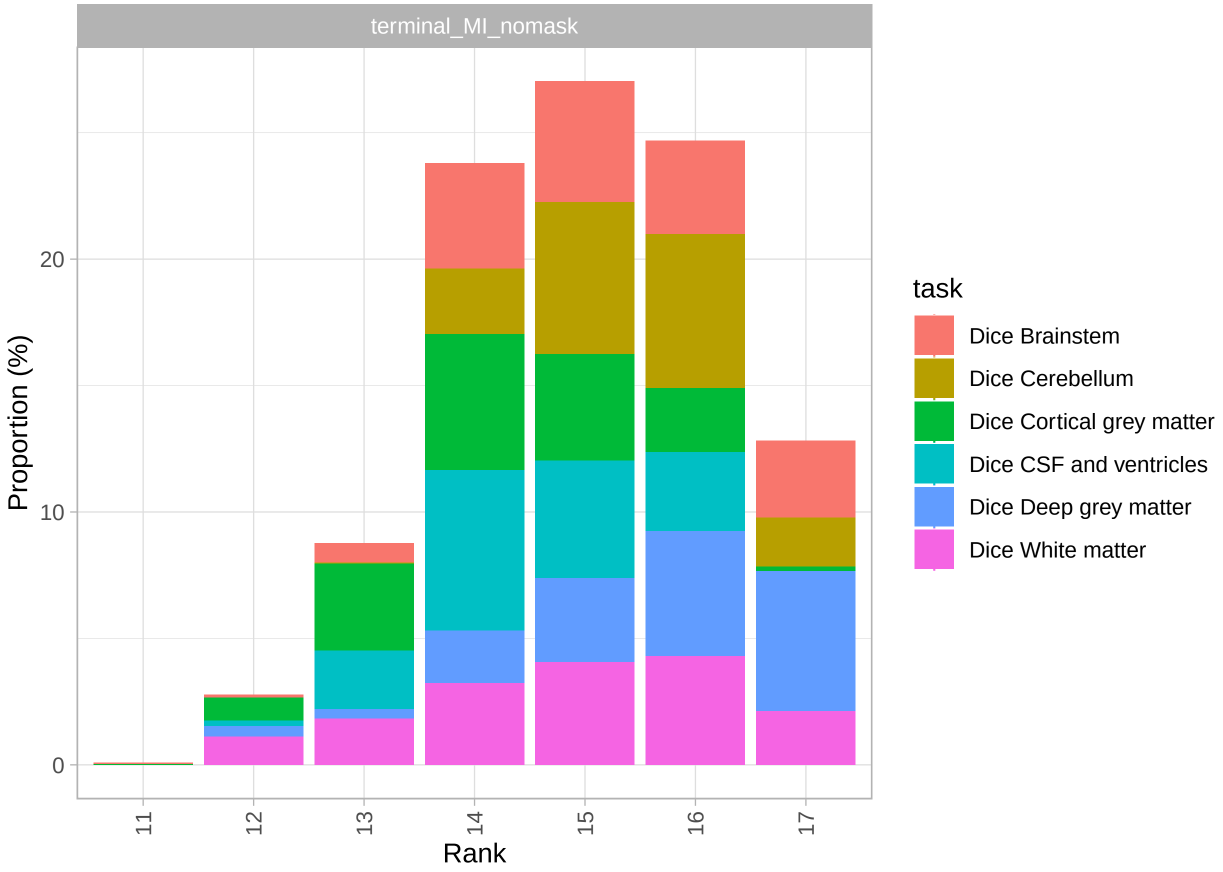
terminal_MI_nomask
Proportion (%)
0
10
20
11
12
13
14
15
16
17
Rank
task
Dice Brainstem
Dice Cerebellum
Dice Cortical grey matter
Dice CSF and ventricles
Dice Deep grey matter
Dice White matter

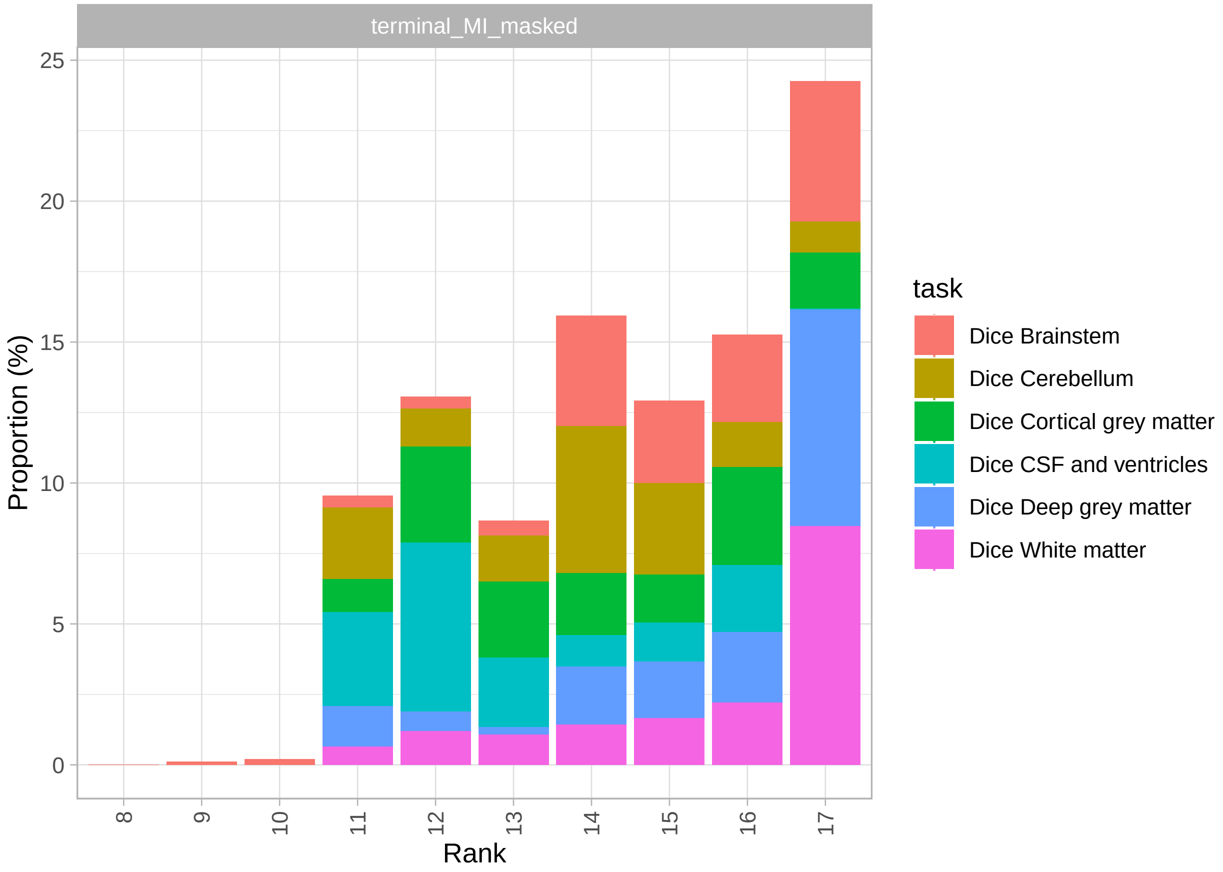
terminal_MI_masked
Proportion (%)
25
20
15
10
5
0
8
9
10
11
12
13
14
15
16
17
Rank
task
Dice Brainstem
Dice Cerebellum
Dice Cortical grey matter
Dice CSF and ventricles
Dice Deep grey matter
Dice White matter

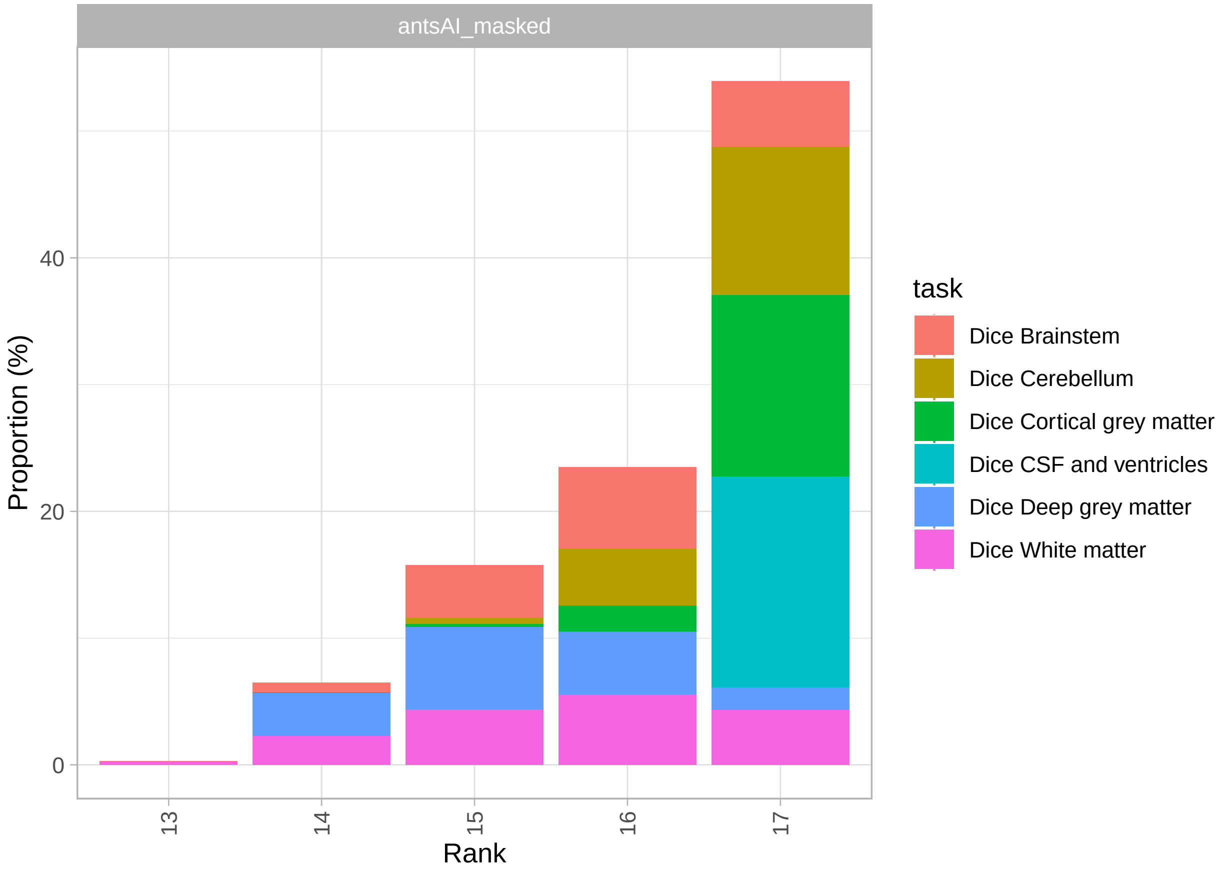
antsAI_masked
Proportion (%)
0
20
40
13
14
15
16
17
Rank
task
Dice Brainstem
Dice Cerebellum
Dice Cortical grey matter
Dice CSF and ventricles
Dice Deep grey matter
Dice White matter

4.2 Characterization of tasks

4.2.1 Visualizing bootstrap results

To investigate which tasks separate algorithms well (i.e., lead to a stable ranking), a blob plot is recommended.

Bootstrap results can be shown in a blob plot showing one plot for each task. In this view, the spread of the blobs for each algorithm can be compared across tasks. Deviations from the diagonal indicate deviations from the consensus ranking (over tasks). Specifically, if rank distribution of an algorithm is consistently below the diagonal, the algorithm performed better in this task than on average across tasks, while if the rank distribution of an algorithm is consistently above the diagonal, the algorithm performed worse in this task than on average across tasks. At the bottom of each panel, ranks for each algorithm in the tasks are provided.

Same as in Section 3.1 but now ordered according to consensus.

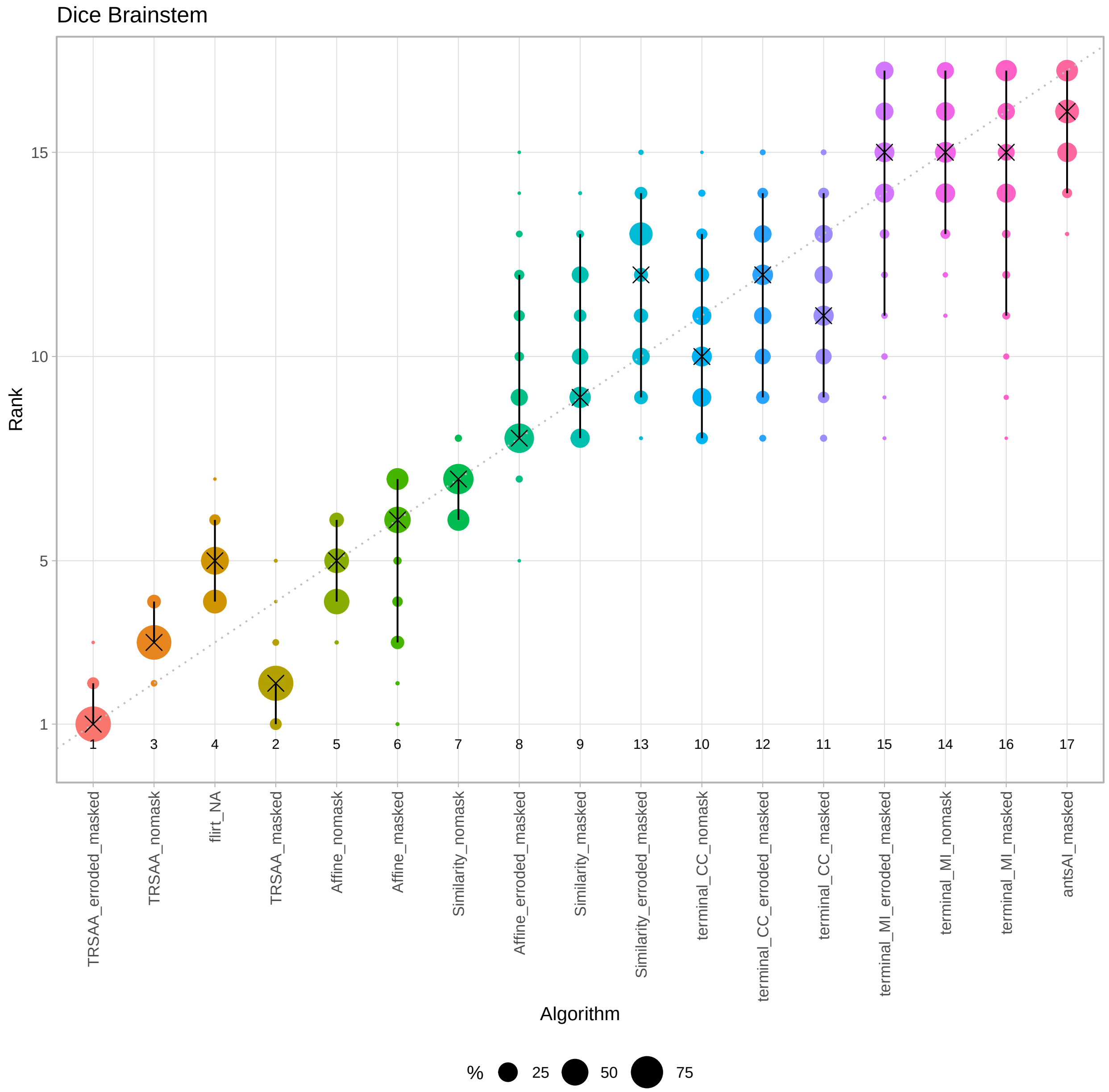

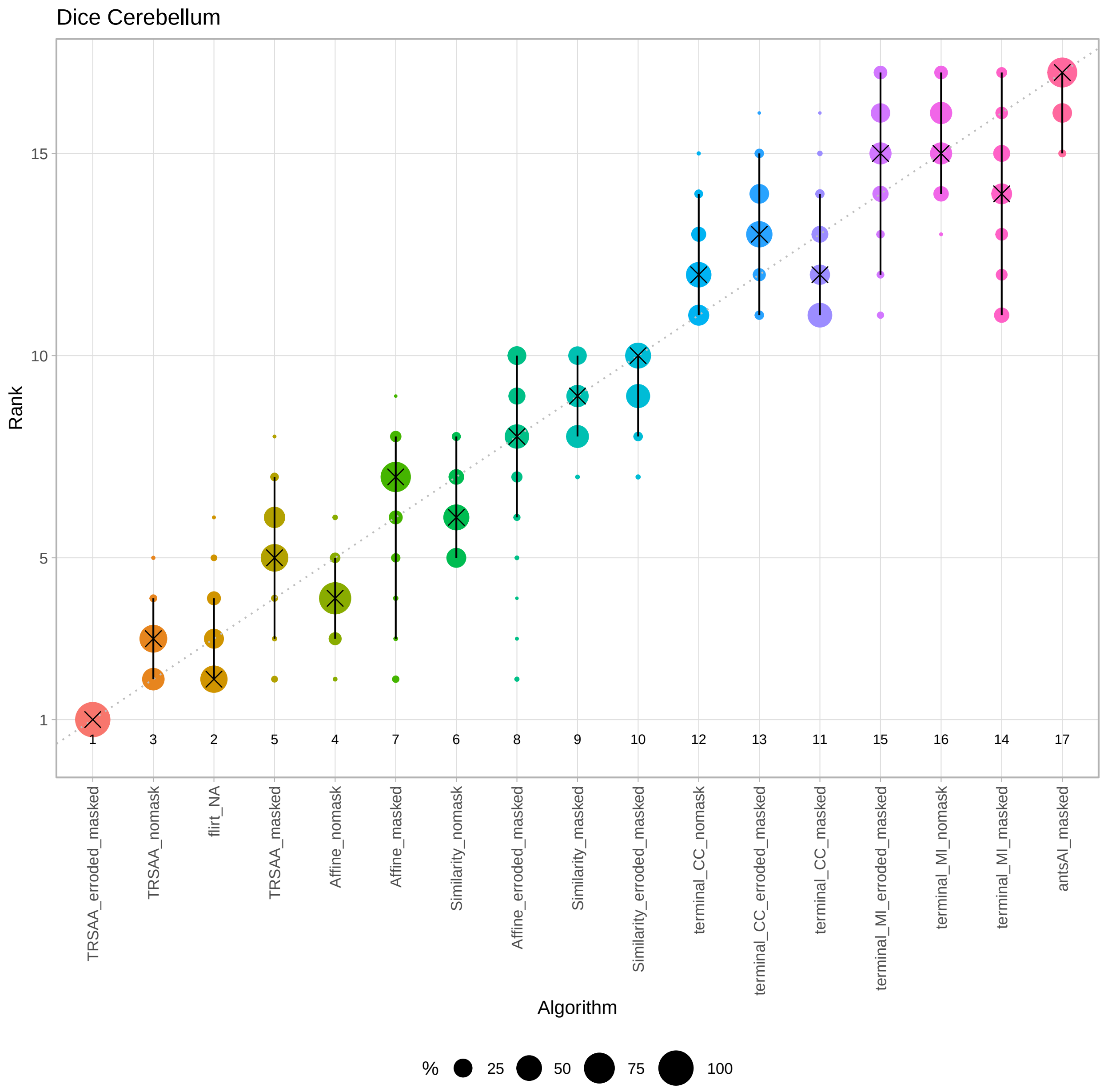
Dice Cerebellum
Rank
15
10
5
1
1
3
2
5
4
7
6
8
9
10
12
13
11
15
16
14
17
TRSAA_erroded_masked
TRSAA_nomask
flirt_NA
TRSAA_masked
Affine_nomask
Affine_masked
Similarity_nomask
Affine_erroded_masked
Similarity_masked
Similarity_erroded_masked
terminal_CC_nomask
terminal_CC_erroded_masked
terminal_CC_masked
terminal_MI_erroded_masked
terminal_MI_nomask
terminal_MI_masked
antsAI_masked
Algorithm
%
25
50
75
100

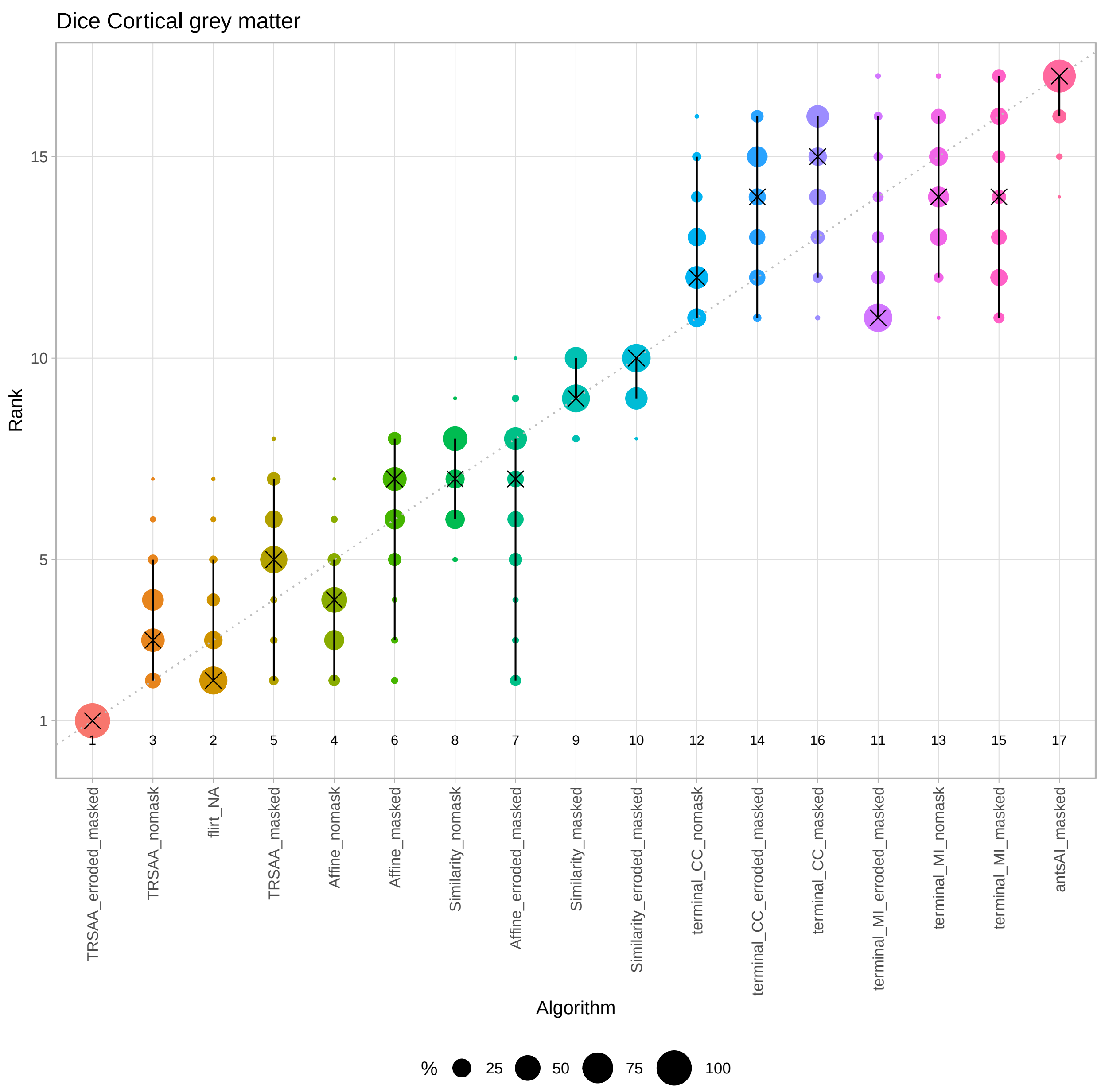
Dice Cortical grey matter
Rank
15
10
5
1
1
3
2
5
4
6
8
7
9
10
12
14
16
11
13
15
17
TRSAA_erroded_masked
TRSAA_nomask
flirt_NA
TRSAA_masked
Affine_nomask
Affine_masked
Similarity_nomask
Affine_erroded_masked
Similarity_masked
Similarity_erroded_masked
terminal_CC_nomask
terminal_CC_erroded_masked
terminal_CC_masked
terminal_MI_erroded_masked
terminal_MI_nomask
terminal_MI_masked
antsAI_masked
Algorithm
%
25
50
75
100

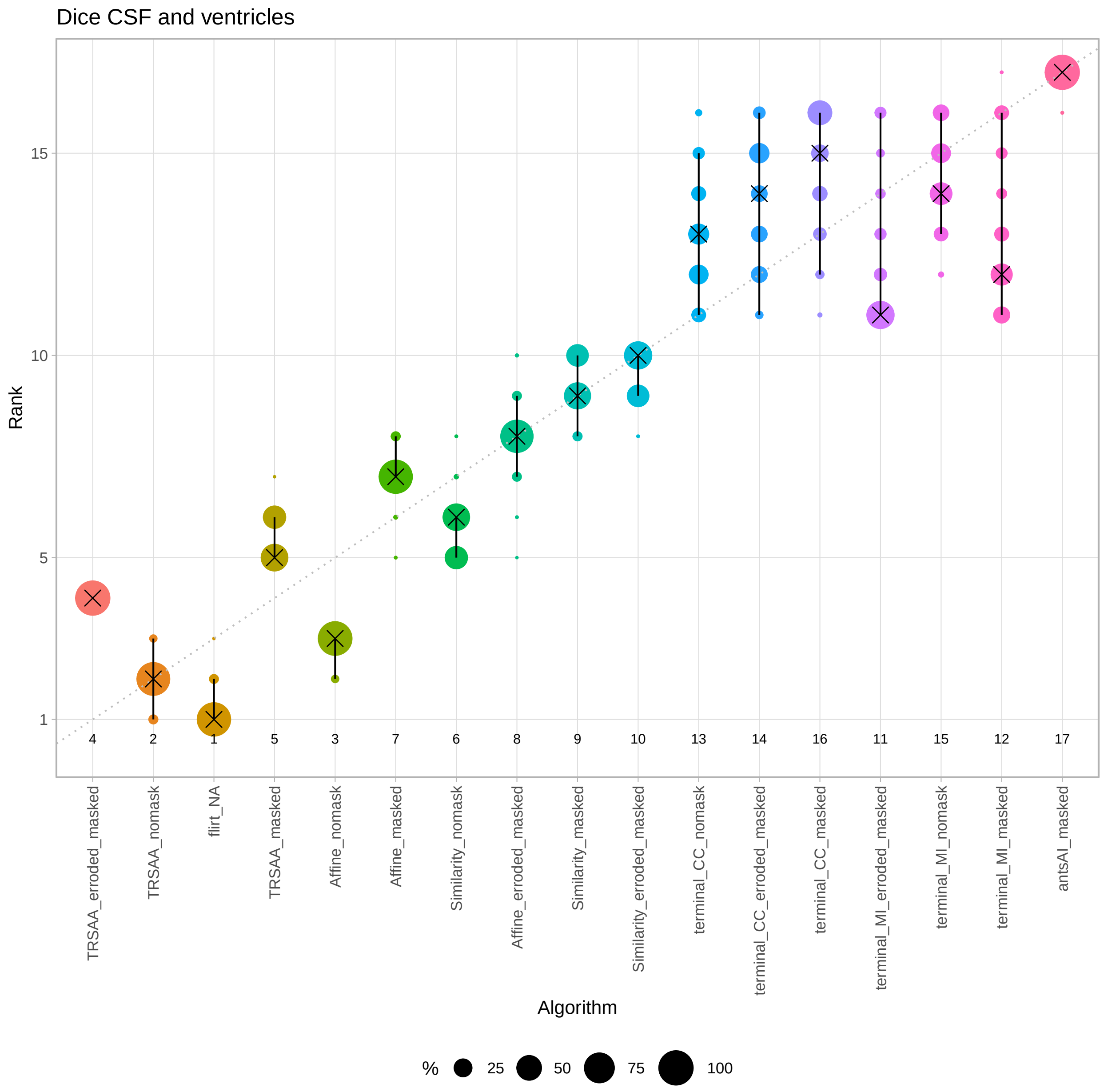

Dice CSF and ventricles
Rank
15
10
5
1
4
2
1
5
3
7
6
8
9
10
13
14
16
11
15
12
17
TRSAA_erroded_masked
TRSAA_nomask
flirt_NA
TRSAA_masked
Affine_nomask
Affine_masked
Similarity_nomask
Affine_erroded_masked
Similarity_masked
Similarity_erroded_masked
terminal_CC_nomask
terminal_CC_erroded_masked
terminal_CC_masked
terminal_MI_erroded_masked
terminal_MI_nomask
terminal_MI_masked
antsAI_masked
Algorithm
%
25
50
75
100

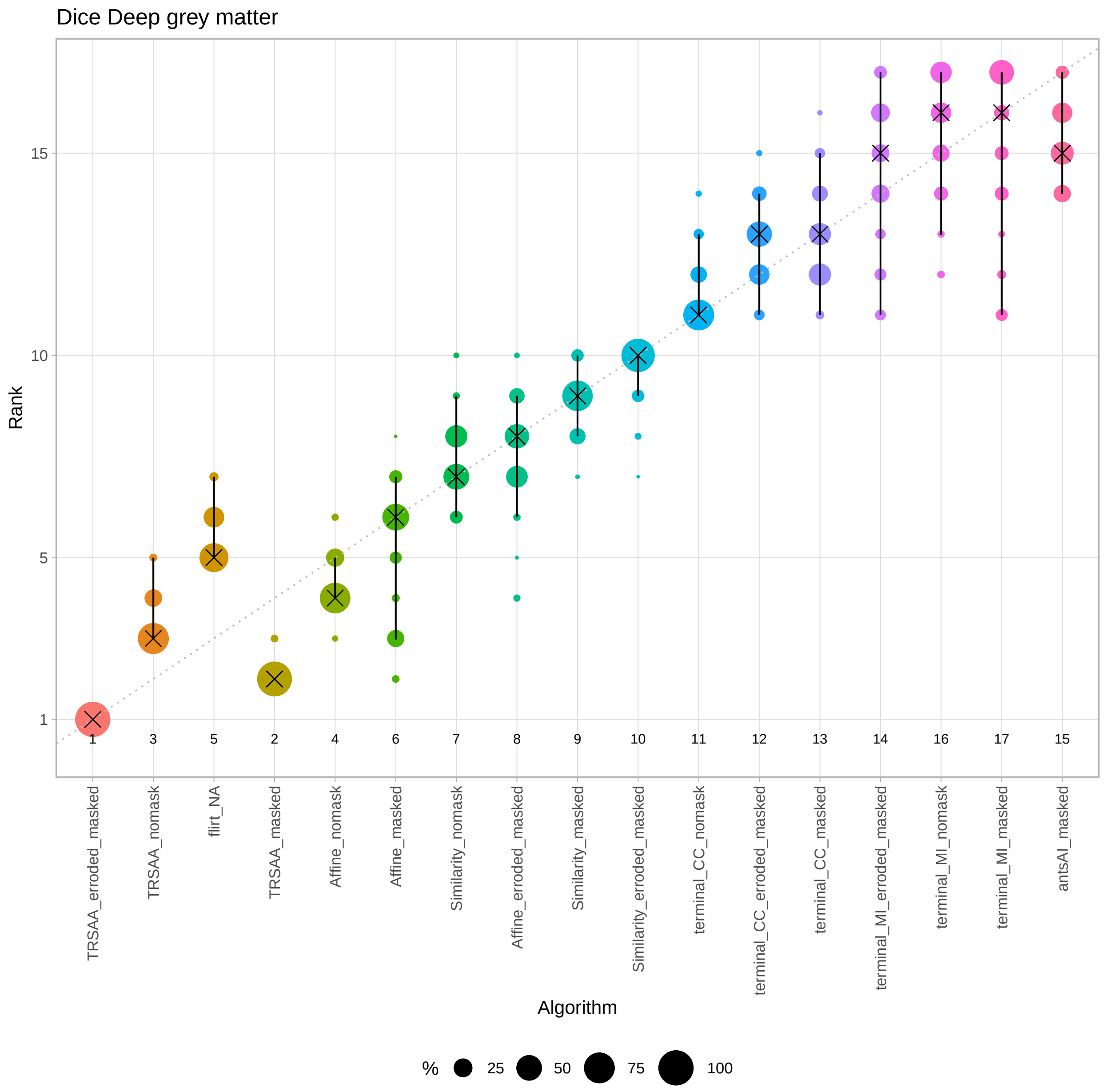

Dice Deep grey matter
Rank
15
10
5
1
1
3
5
2
4
6
7
8
9
10
11
12
13
14
16
17
15
TRSAA_erroded_masked
TRSAA_nomask
flirt_NA
TRSAA_masked
Affine_nomask
Affine_masked
Similarity_nomask
Affine_erroded_masked
Similarity_masked
Similarity_erroded_masked
terminal_CC_nomask
terminal_CC_erroded_masked
terminal_CC_masked
terminal_MI_erroded_masked
terminal_MI_nomask
terminal_MI_masked
antsAI_masked
Algorithm
%
25
50
75
100

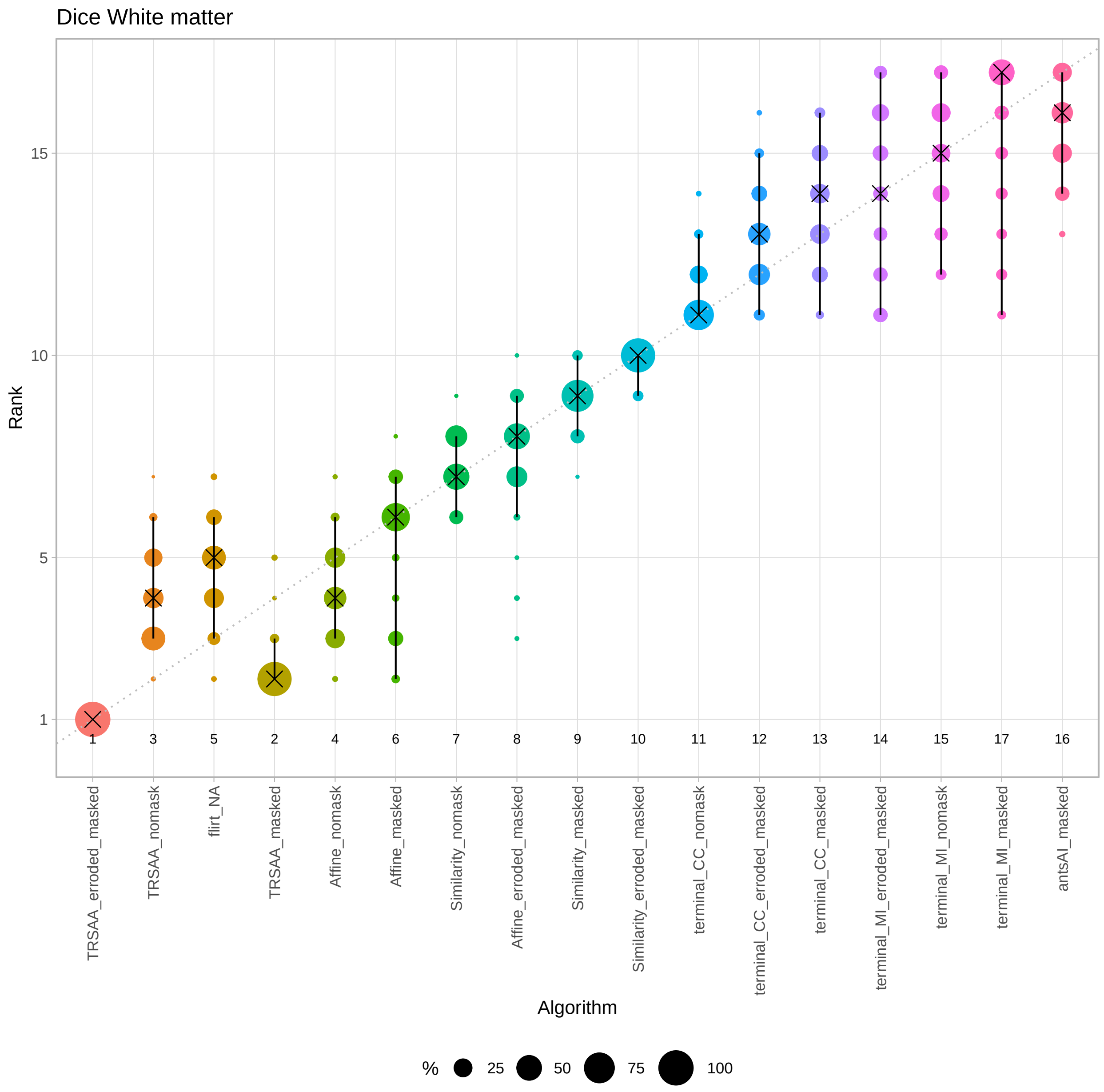

4.2.2 Cluster Analysis

Dendrogram from hierarchical cluster analysis and *network-type graphs* for assessing the similarity of tasks based on challenge rankings.

A dendrogram is a visualization approach based on hierarchical clustering. It depicts clusters according to a chosen distance measure (here: Spearman's footrule) as well as a chosen agglomeration method (here: complete and average agglomeration).

Cluster Dendrogram (complete agglomeration)

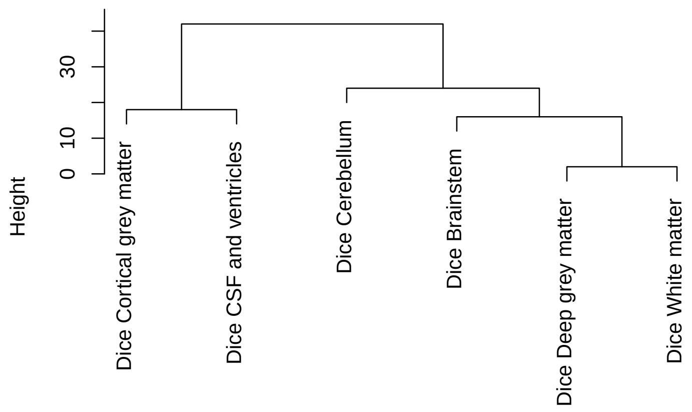

Cluster Dendrogram (average agglomeration)

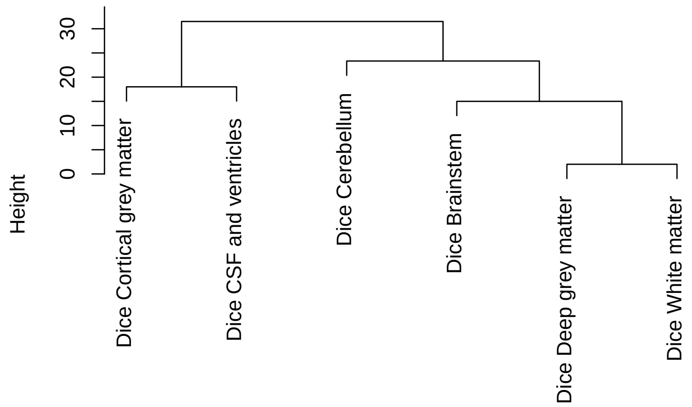

5 References

Wiesenfarth, M., Reinke, A., Landman, B.A., Eisenmann, M., Aguilera Saiz, L., Cardoso, M.J., Maier-Hein, L. and Kopp-Schneider, A. Methods and open-source toolkit for analyzing and visualizing challenge results. *Sci Rep* **11**, 2369 (2021). https://doi.org/10.1038/s41598-021-82017-6

M. J. A. Eugster, T. Hothorn, and F. Leisch, "Exploratory and inferential analysis of benchmark experiments," Institut fuer Statistik, Ludwig-Maximilians-Universitaet Muenchen, Germany, Technical Report 30, 2008. [Online]. Available: http://epub.ub.uni-muenchen.de/4134/.
