# Supplementary material for: Characterization of dynamic patterns of human fetal to neonatal brain asymmetry with deformation-based morphometry
Source: Front Neurosci. 2023 Dec 6;17:1252850. doi: 10.3389/fnins.2023.1252850 (PMC10734644; doi:10.3389/fnins.2023.1252850)
Supplement: Supplementary file 1 [file Data_Sheet_1.ZIP › ChallengeR/Framework A fetal to neonatal/linear/ranking_Fetal2Neonatal_Linear_Landmarks.pdf]

Benchmarking report for Fetal to Neonatal Registration Linear

created by challengeR v1.0.4

23 März, 2023

This document presents a systematic report on the benchmark study "Fetal to Neonatal Registration Linear". Input data comprises raw metric values for all algorithms and cases. Generated plots are:

- Visualization of assessment data: Dot- and boxplots, podium plots and ranking heatmaps
- Visualization of ranking stability: Blob plots, violin plots and significance maps, line plots
- Visualization of cross-task insights: Blob plots, stacked frequency plots, dendrograms

Details can be found in Wiesenfarth et al. (2021).

1 Rankings

Algorithms within a task are ranked according to the following ranking scheme:

aggregate using function ("mean") then rank

Ranking for each task:

4th Ventricle : The analysis is based on 17 algorithms and 20 cases. 0 missing cases have been found in the data set.

	value_mean	rank
TRSAA_eroded_masked	1.462086	1
TRSAA_masked	2.022630	2
TRSAA_nomask	2.282198	3
Affine_nomask	2.328755	4
flirt_na	2.475487	5
Affine_masked	2.481058	6
Similarity_nomask	2.555419	7
Similarity_masked	3.110864	8
Affine_eroded_masked	3.256383	9
Similarity_eroded_masked	3.280715	10
terminal_CC_masked	4.231688	11
terminal_CC_nomask	4.361980	12
terminal_CC_eroded_masked	4.530991	13
terminal_MI_eroded_masked	5.559728	14
terminal_MI_masked	5.618430	15
terminal_MI_nomask	5.685425	16
antsAI_masked	6.370732	17

Chiasma : The analysis is based on 17 algorithms and 20 cases. 0 missing cases have been found in the data set.

	value_mean	rank
TRSAA_eroded_masked	1.431613	1
TRSAA_masked	2.146924	2
TRSAA_nomask	2.208561	3
Affine_nomask	2.236711	4
flirt_na	2.289749	5
Affine_masked	3.043442	6
Similarity_nomask	3.307763	7
Affine_eroded_masked	4.138997	8
Similarity_masked	4.827657	9
Similarity_eroded_masked	5.114468	10
terminal_CC_nomask	5.655870	11
terminal_CC_eroded_masked	5.828189	12
terminal_CC_masked	5.979889	13
antsAI_masked	6.336980	14
terminal_MI_eroded_masked	6.430192	15
terminal_MI_nomask	6.787969	16
terminal_MI_masked	7.499810	17

Culmen : The analysis is based on 17 algorithms and 20 cases. 0 missing cases have been found in the data set.

	value_mean	rank
Similarity_nomask	1.490166	1
TRSAA_nomask	1.613594	2
Affine_nomask	1.619294	3
flirt_na	1.717534	4
TRSAA_masked	1.803020	5
TRSAA_eroded_masked	1.890118	6
Similarity_masked	1.900887	7
Affine_masked	2.173540	8
terminal_CC_masked	2.255597	9
terminal_CC_nomask	2.276977	10
Affine_eroded_masked	2.430624	11
terminal_CC_eroded_masked	2.434573	12
Similarity_eroded_masked	2.503945	13
terminal_MI_eroded_masked	3.447676	14
terminal_MI_nomask	3.470257	15
terminal_MI_masked	3.772614	16
antsAI_masked	4.013079	17

Hemisphere left caudal point : The analysis is based on 17 algorithms and 20 cases. 0 missing cases have been found in the data set.

	value_mean	rank
Similarity_nomask	4.498473	1
TRSAA_nomask	4.512604	2
flirt_na	4.520082	3
Affine_nomask	4.535073	4
TRSAA_masked	4.656060	5
Similarity_masked	4.956414	6

	value_mean	rank
Similarity_eroded_masked	4.958352	7
Affine_masked	5.095581	8
Affine_eroded_masked	5.286676	9
TRSAA_eroded_masked	5.303047	10
terminal_CC_masked	7.489344	11
terminal_CC_eroded_masked	7.835492	12
terminal_CC_nomask	7.844685	13
terminal_MI_masked	7.882690	14
terminal_MI_eroded_masked	7.977338	15
terminal_MI_nomask	8.614971	16
antsAI_masked	8.973703	17

Hemisphere left rostral point : The analysis is based on 17 algorithms and 20 cases. 0 missing cases have been found in the data set.

	value_mean	rank
TRSAA_eroded_masked	3.776227	1
TRSAA_nomask	4.182855	2
Similarity_nomask	4.227800	3
Affine_nomask	4.231908	4
flirt_na	4.236422	5
TRSAA_masked	5.139416	6
Affine_masked	5.574976	7
Affine_eroded_masked	5.707288	8
Similarity_masked	7.181503	9
Similarity_eroded_masked	7.198578	10
antsAI_masked	9.470861	11
terminal_CC_nomask	10.045326	12
terminal_CC_eroded_masked	10.107936	13
terminal_CC_masked	10.541001	14
terminal_MI_nomask	10.709258	15
terminal_MI_eroded_masked	10.933673	16
terminal_MI_masked	11.597765	17

Hemisphere right caudal point : The analysis is based on 17 algorithms and 20 cases. 0 missing cases have been found in the data set.

	value_mean	rank
TRSAA_masked	4.706767	1
Affine_nomask	4.872805	2
TRSAA_nomask	5.008515	3
flirt_na	5.068017	4
Affine_masked	5.085593	5
Similarity_nomask	5.147767	6
TRSAA_eroded_masked	5.230330	7
Similarity_eroded_masked	5.372054	8
Similarity_masked	5.406430	9
Affine_eroded_masked	5.413009	10
terminal_CC_masked	7.325028	11
terminal_CC_nomask	7.361004	12

	value_mean	rank
terminal_MI_masked	7.573255	13
terminal_CC_eroded_masked	7.627294	14
terminal_MI_eroded_masked	7.811233	15
terminal_MI_nomask	8.523361	16
antsAI_masked	8.837780	17

Hemisphere right rostral point : The analysis is based on 17 algorithms and 20 cases. 0 missing cases have been found in the data set.

	value_mean	rank
TRSAA_eroded_masked	4.476695	1
flirt_na	4.937498	2
TRSAA_nomask	4.941366	3
Affine_nomask	4.980555	4
Similarity_nomask	4.982439	5
TRSAA_masked	5.849687	6
Affine_masked	6.293895	7
Affine_eroded_masked	6.781789	8
Similarity_masked	7.674638	9
Similarity_eroded_masked	7.834415	10
antsAI_masked	10.131055	11
terminal_CC_nomask	10.504097	12
terminal_CC_eroded_masked	10.668481	13
terminal_CC_masked	11.100370	14
terminal_MI_nomask	11.202925	15
terminal_MI_eroded_masked	11.428025	16
terminal_MI_masked	12.560710	17

Infracollicular Sulcus : The analysis is based on 17 algorithms and 20 cases. 0 missing cases have been found in the data set.

	value_mean	rank
TRSAA_eroded_masked	1.081806	1
TRSAA_masked	1.412591	2
TRSAA_nomask	1.871164	3
Affine_nomask	1.931955	4
flirt_na	1.992209	5
Affine_masked	2.107945	6
Similarity_nomask	2.359560	7
Affine_eroded_masked	2.909913	8
Similarity_masked	3.020015	9
terminal_CC_masked	3.087593	10
terminal_CC_nomask	3.168838	11
terminal_CC_eroded_masked	3.271305	12
Similarity_eroded_masked	3.287117	13
terminal_MI_eroded_masked	4.270789	14
terminal_MI_nomask	4.516686	15
terminal_MI_masked	4.586168	16
antsAI_masked	4.917182	17

Lateral ventricle left caudal point : The analysis is based on 17 algorithms and 20 cases. 0 missing cases have been found in the data set.

	value_mean	rank
flirt_na	4.525309	1
TRSAA_masked	4.540669	2
Affine_nomask	4.548864	3
TRSAA_nomask	4.563954	4
Similarity_nomask	4.847784	5
terminal_CC_nomask	5.290458	6
terminal_CC_masked	5.379549	7
Similarity_masked	5.445659	8
Affine_masked	5.570655	9
terminal_CC_eroded_masked	5.646382	10
terminal_MI_eroded_masked	5.959601	11
Similarity_eroded_masked	5.998970	12
Affine_eroded_masked	6.170073	13
TRSAA_eroded_masked	6.236219	14
antsAI_masked	6.269476	15
terminal_MI_nomask	6.532767	16
terminal_MI_masked	6.840505	17

Lateral ventricle left rostral point : The analysis is based on 17 algorithms and 20 cases. 0 missing cases have been found in the data set.

	value_mean	rank
TRSAA_masked	1.832583	1
TRSAA_eroded_masked	1.888718	2
Affine_masked	2.339533	3
TRSAA_nomask	2.467345	4
Affine_nomask	2.538178	5
flirt_na	2.541671	6
Similarity_nomask	2.554349	7
Affine_eroded_masked	2.620085	8
Similarity_masked	2.832588	9
Similarity_eroded_masked	3.216436	10
antsAI_masked	4.480296	11
terminal_CC_eroded_masked	4.934913	12
terminal_CC_nomask	4.976401	13
terminal_CC_masked	5.362512	14
terminal_MI_nomask	5.593955	15
terminal_MI_eroded_masked	5.681074	16
terminal_MI_masked	6.683798	17

Lateral ventricle right caudal point : The analysis is based on 17 algorithms and 20 cases. 0 missing cases have been found in the data set.

	value_mean	rank
flirt_na	4.098268	1
TRSAA_nomask	4.155875	2
Affine_nomask	4.213826	3

	value_mean	rank
TRSAA_masked	4.319466	4
Similarity_nomask	4.461834	5
terminal_CC_nomask	4.666979	6
terminal_CC_masked	4.682279	7
antsAI_masked	4.885379	8
terminal_CC_eroded_masked	4.977990	9
Affine_masked	5.119985	10
Similarity_masked	5.207336	11
terminal_MI_nomask	5.347995	12
Affine_eroded_masked	5.369089	13
Similarity_eroded_masked	5.429287	14
terminal_MI_eroded_masked	5.558712	15
TRSAA_eroded_masked	5.651250	16
terminal_MI_masked	6.328314	17

Lateral ventricle right rostral point : The analysis is based on 17 algorithms and 20 cases. 0 missing cases have been found in the data set.

	value_mean	rank
TRSAA_masked	1.990263	1
TRSAA_eroded_masked	2.247290	2
Affine_masked	2.324098	3
Affine_eroded_masked	2.551377	4
Affine_nomask	2.635772	5
TRSAA_nomask	2.648071	6
Similarity_nomask	2.719781	7
flirt_na	2.848315	8
Similarity_masked	2.960952	9
Similarity_eroded_masked	3.225986	10
antsAI_masked	3.810097	11
terminal_CC_eroded_masked	4.060919	12
terminal_CC_nomask	4.088829	13
terminal_CC_masked	4.460678	14
terminal_MI_nomask	4.695193	15
terminal_MI_eroded_masked	5.235517	16
terminal_MI_masked	6.199445	17

PMJ : The analysis is based on 17 algorithms and 20 cases. 0 missing cases have been found in the data set.

	value_mean	rank
TRSAA_masked	1.090469	1
TRSAA_nomask	1.114945	2
Affine_nomask	1.195466	3
TRSAA_eroded_masked	1.229806	4
flirt_na	1.272703	5
Similarity_nomask	1.552765	6
Affine_masked	2.093527	7
Similarity_masked	2.919686	8
terminal_CC_nomask	2.928682	9
Affine_eroded_masked	2.939220	10

	value_mean	rank
terminal_CC_eroded_masked	3.019152	11
terminal_CC_masked	3.161590	12
Similarity_eroded_masked	3.296270	13
terminal_MI_eroded_masked	3.964441	14
antsAI_masked	4.139425	15
terminal_MI_nomask	4.200367	16
terminal_MI_masked	4.691568	17

Posterior Commissure : The analysis is based on 17 algorithms and 20 cases. 0 missing cases have been found in the data set.

	value_mean	rank
TRSAA_eroded_masked	1.483585	1
TRSAA_masked	1.490117	2
TRSAA_nomask	1.744955	3
Affine_nomask	1.781983	4
flirt_na	1.863989	5
Similarity_nomask	1.974144	6
Affine_masked	1.992381	7
terminal_CC_nomask	2.206002	8
terminal_CC_masked	2.238146	9
terminal_CC_eroded_masked	2.265304	10
Similarity_masked	2.614419	11
Affine_eroded_masked	2.838769	12
Similarity_eroded_masked	3.002541	13
terminal_MI_eroded_masked	3.017991	14
antsAI_masked	3.274046	15
terminal_MI_nomask	3.331993	16
terminal_MI_masked	3.843978	17

Temporal lobe left rostral point : The analysis is based on 17 algorithms and 20 cases. 0 missing cases have been found in the data set.

	value_mean	rank
TRSAA_eroded_masked	5.984417	1
flirt_na	6.663478	2
TRSAA_nomask	6.724031	3
TRSAA_masked	6.877779	4
Affine_nomask	6.913051	5
Affine_masked	7.678960	6
Similarity_nomask	8.305413	7
Affine_eroded_masked	8.831749	8
Similarity_masked	9.746314	9
Similarity_eroded_masked	10.027513	10
terminal_CC_nomask	11.453111	11
terminal_CC_eroded_masked	11.635633	12
terminal_CC_masked	11.778270	13
terminal_MI_eroded_masked	11.870308	14
antsAI_masked	12.003922	15
terminal_MI_nomask	12.422807	16

	value_mean	rank
terminal_MI_masked	13.098212	17

Temporal lobe ventricle right rostral point : The analysis is based on 17 algorithms and 20 cases. 0 missing cases have been found in the data set.

	value_mean	rank
TRSAA_eroded_masked	6.867329	1
Affine_nomask	7.395108	2
TRSAA_nomask	7.448144	3
flirt_na	7.455627	4
TRSAA_masked	7.686732	5
Affine_masked	8.613917	6
Similarity_nomask	8.803057	7
Affine_eroded_masked	9.790230	8
Similarity_masked	10.937325	9
Similarity_eroded_masked	11.117995	10
terminal_CC_nomask	11.522827	11
terminal_CC_eroded_masked	11.629302	12
antsAI_masked	11.757204	13
terminal_CC_masked	11.764739	14
terminal_MI_eroded_masked	12.153194	15
terminal_MI_nomask	12.198620	16
terminal_MI_masked	13.199817	17

Consensus ranking across tasks according to chosen method “euclidean”:

	value	rank
TRSAA_nomask	3.000	1
TRSAA_masked	3.062	2
Affine_nomask	3.688	3
flirt_na	4.062	4
TRSAA_eroded_masked	4.312	5
Similarity_nomask	5.438	6
Affine_masked	6.500	7
Similarity_masked	8.750	8
Affine_eroded_masked	9.188	9
terminal_CC_nomask	10.625	10
Similarity_eroded_masked	10.812	11
terminal_CC_masked	11.438	12
terminal_CC_eroded_masked	11.812	13
antsAI_masked	14.000	14
terminal_MI_eroded_masked	14.625	15
terminal_MI_nomask	15.375	16
terminal_MI_masked	16.312	17

2 Visualization of raw assessment data

The algorithms are ordered according to the computed ranks for each task.

2.1 Dot- and boxplot

Dot- and boxplots for visualizing raw assessment data separately for each algorithm. Boxplots representing descriptive statistics over all cases (median, quartiles and outliers) are combined with horizontally jittered dots representing individual cases.

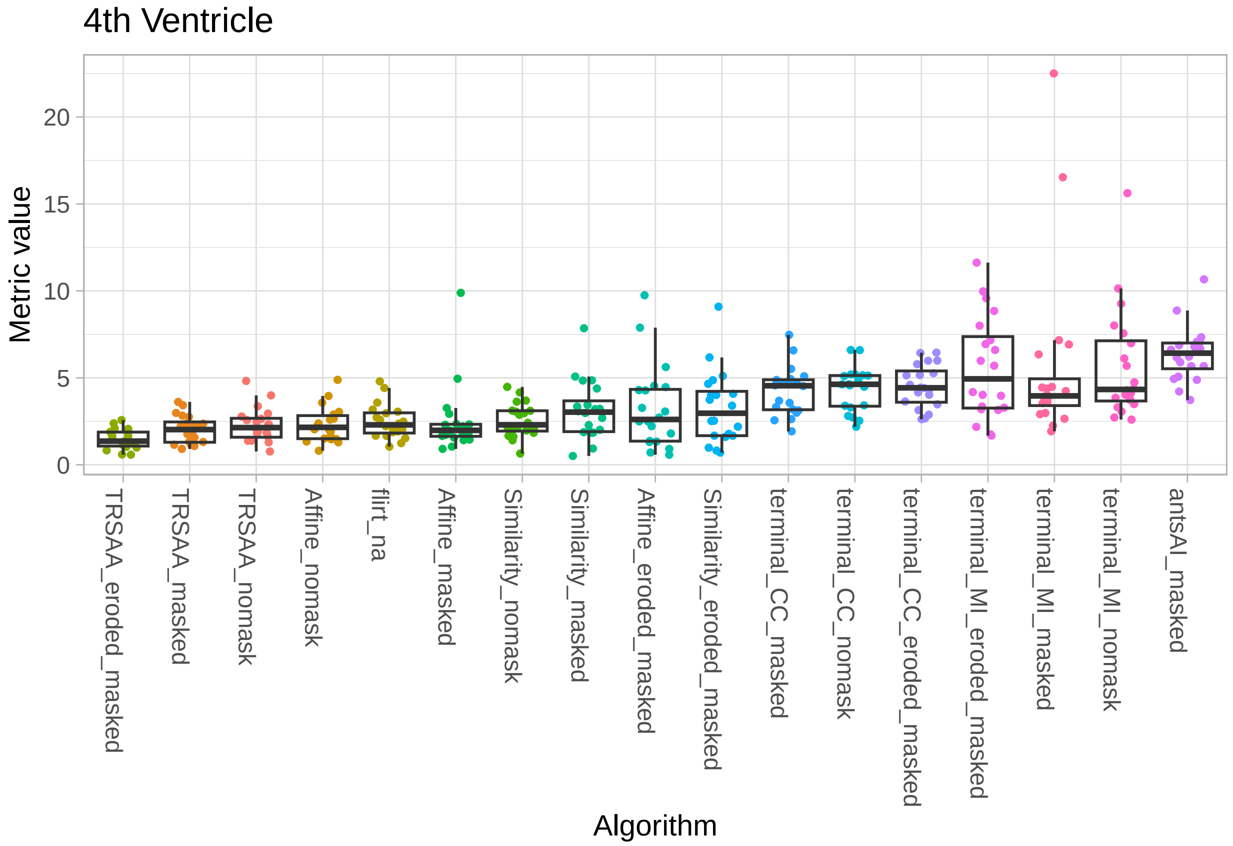

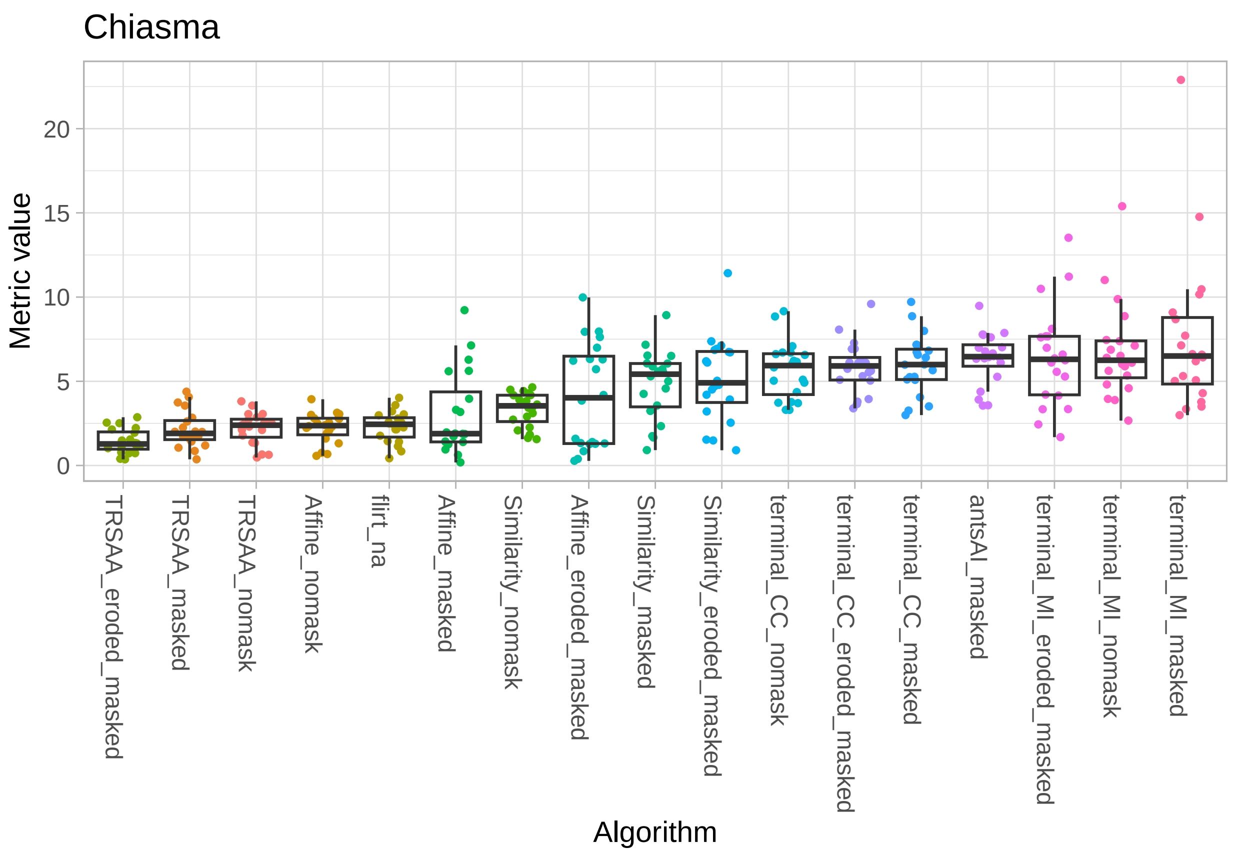
Chiasma
Metric value
0
5
10
15
20
TRSAA_eroded_masked
TRSAA_masked
TRSAA_nomask
Affine_nomask
flirt_na
Affine_masked
Similarity_nomask
Affine_eroded_masked
Similarity_masked
Similarity_eroded_masked
terminal_CC_nomask
terminal_CC_eroded_masked
terminal_CC_masked
antsAI_masked
terminal_MI_eroded_masked
terminal_MI_nomask
terminal_MI_masked
Algorithm

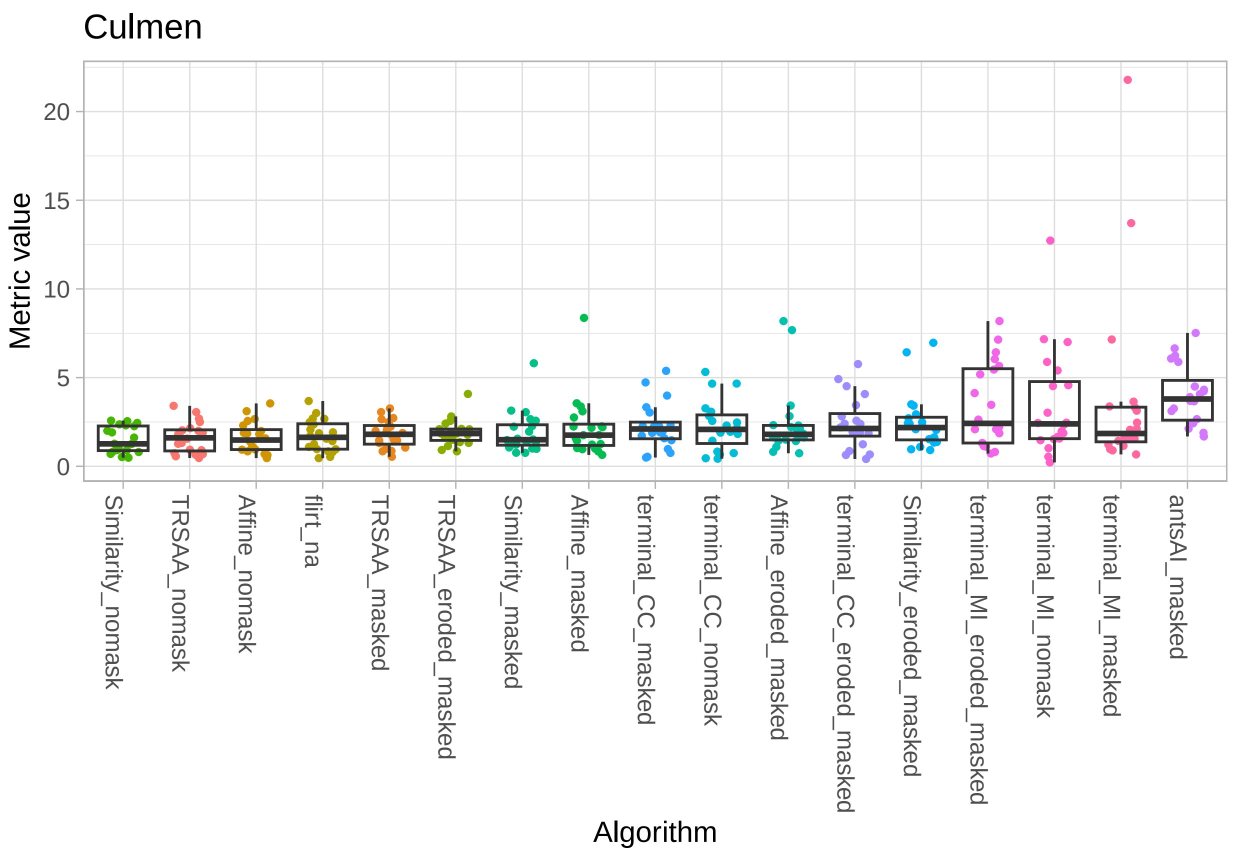

Culmen
Metric value
20
15
10
5
0
Similarity_nomask
TRSAA_nomask
Affine_nomask
flirt_na
TRSAA_masked
TRSAA_eroded_masked
Similarity_masked
Affine_masked
terminal_CC_masked
terminal_CC_nomask
Affine_eroded_masked
terminal_CC_eroded_masked
Similarity_eroded_masked
terminal_MI_eroded_masked
terminal_MI_nomask
terminal_MI_masked
antsAI_masked
Algorithm

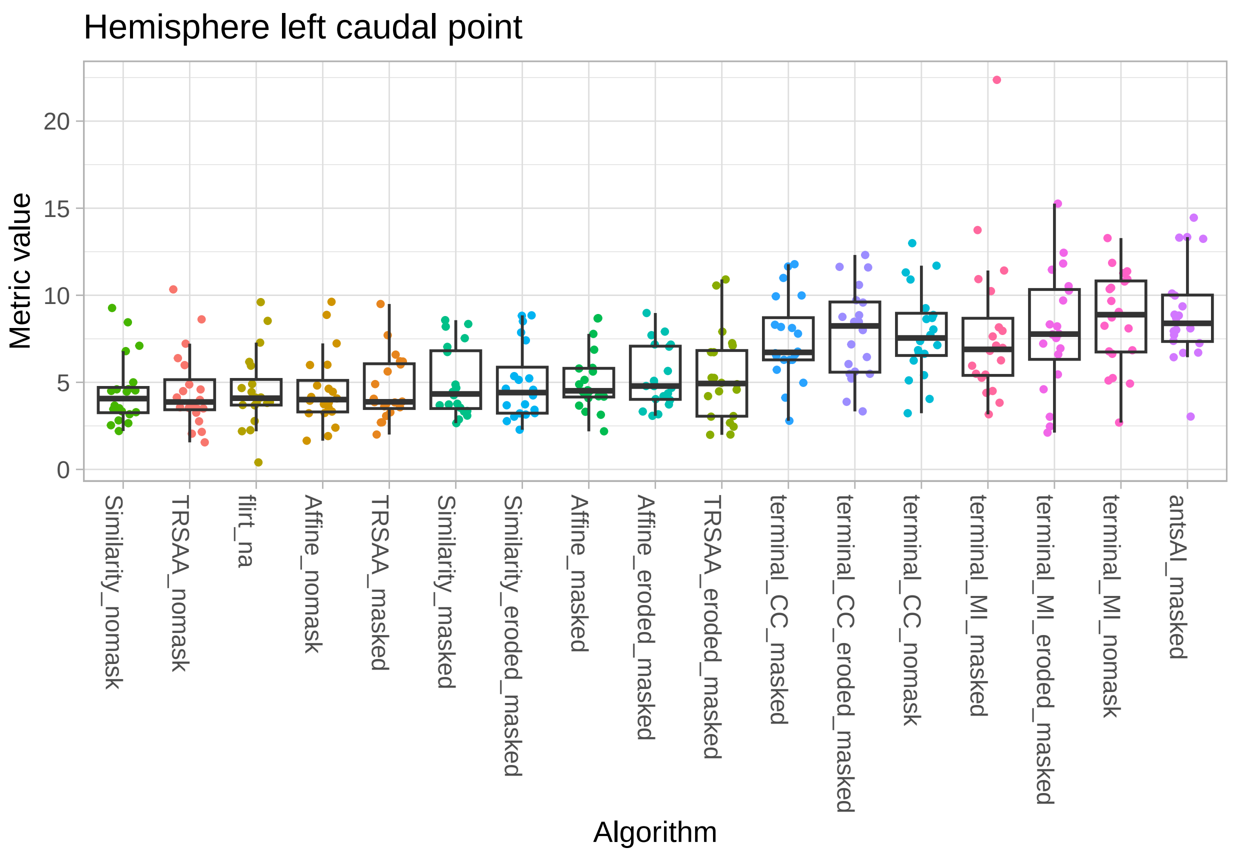
Hemisphere left caudal point
Metric value
0
5
10
15
20
Similarity_nomask
TRSAA_nomask
flirt_na
Affine_nomask
TRSAA_masked
Similarity_masked
Similarity_eroded_masked
Affine_masked
Affine_eroded_masked
TRSAA_eroded_masked
terminal_CC_masked
terminal_CC_eroded_masked
terminal_CC_nomask
terminal_MI_masked
terminal_MI_eroded_masked
terminal_MI_nomask
antsAI_masked
Algorithm

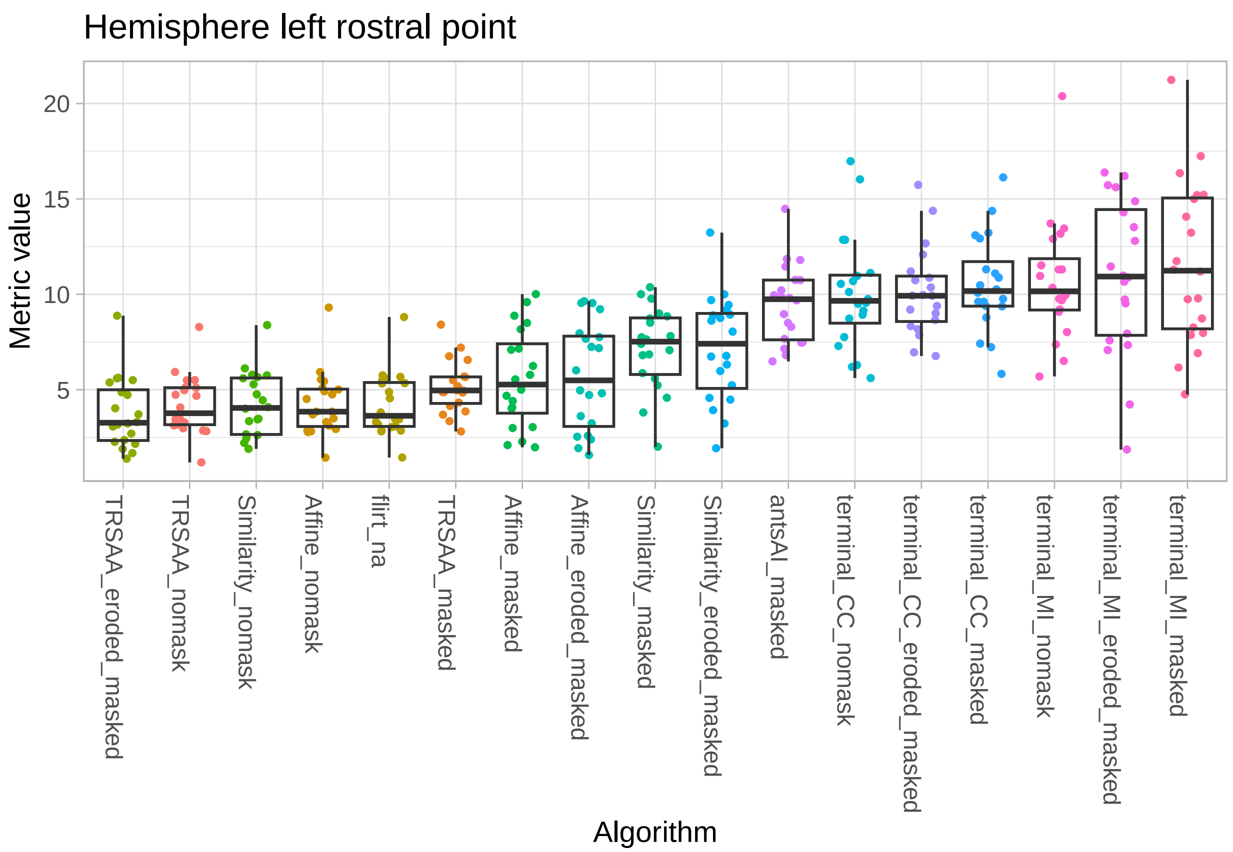

Hemisphere left rostral point
Metric value
20
15
10
5
TRSAA_eroded_masked
TRSAA_nomask
Similarity_nomask
Affine_nomask
flirt_na
TRSAA_masked
Affine_masked
Affine_eroded_masked
Similarity_masked
Similarity_eroded_masked
antsAI_masked
terminal_CC_nomask
terminal_CC_eroded_masked
terminal_CC_masked
terminal_MI_nomask
terminal_MI_eroded_masked
terminal_MI_masked
Algorithm

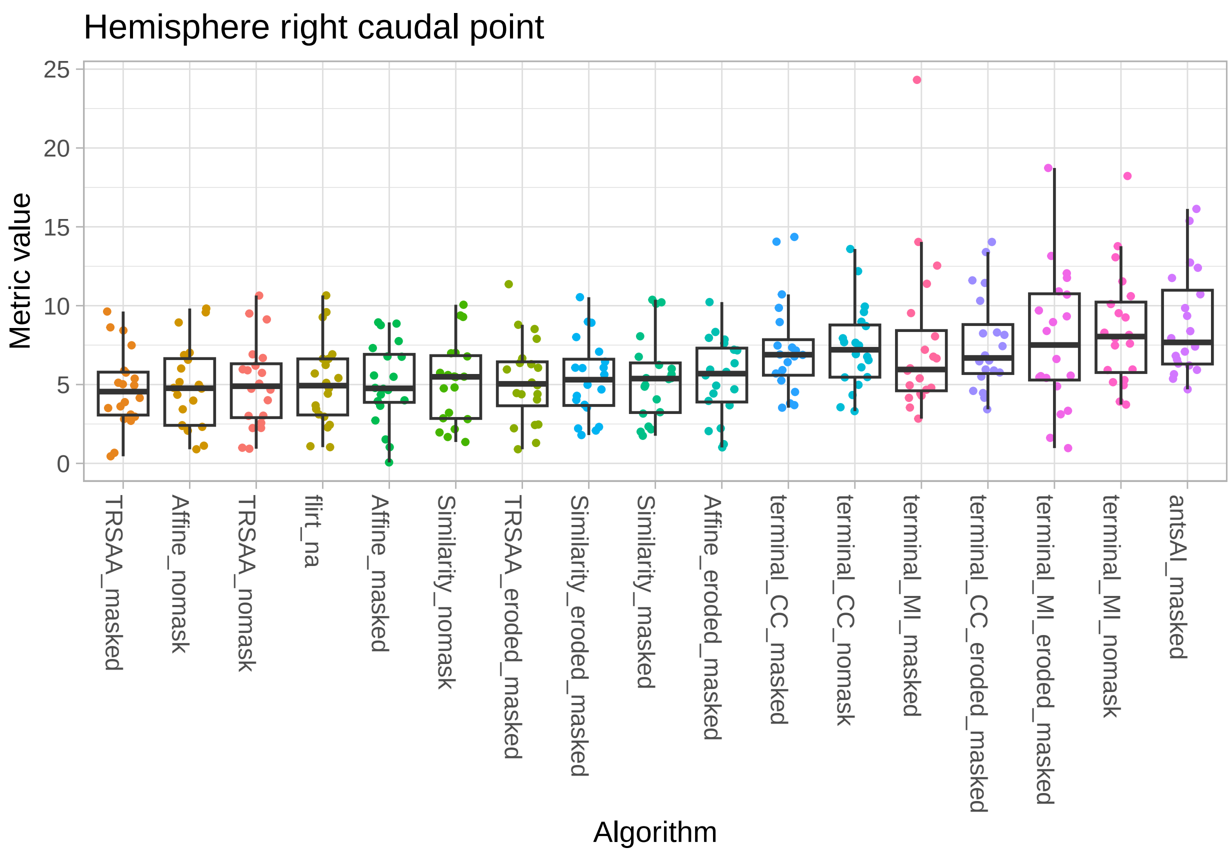
Hemisphere right caudal point
Metric value
0
5
10
15
20
25
TRSAA_masked
Affine_nomask
TRSAA_nomask
flirt_na
Affine_masked
Similarity_nomask
TRSAA_eroded_masked
Similarity_eroded_masked
Similarity_masked
Affine_eroded_masked
terminal_CC_masked
terminal_CC_nomask
terminal_MI_masked
terminal_CC_eroded_masked
terminal_MI_eroded_masked
terminal_MI_nomask
antsAI_masked
Algorithm

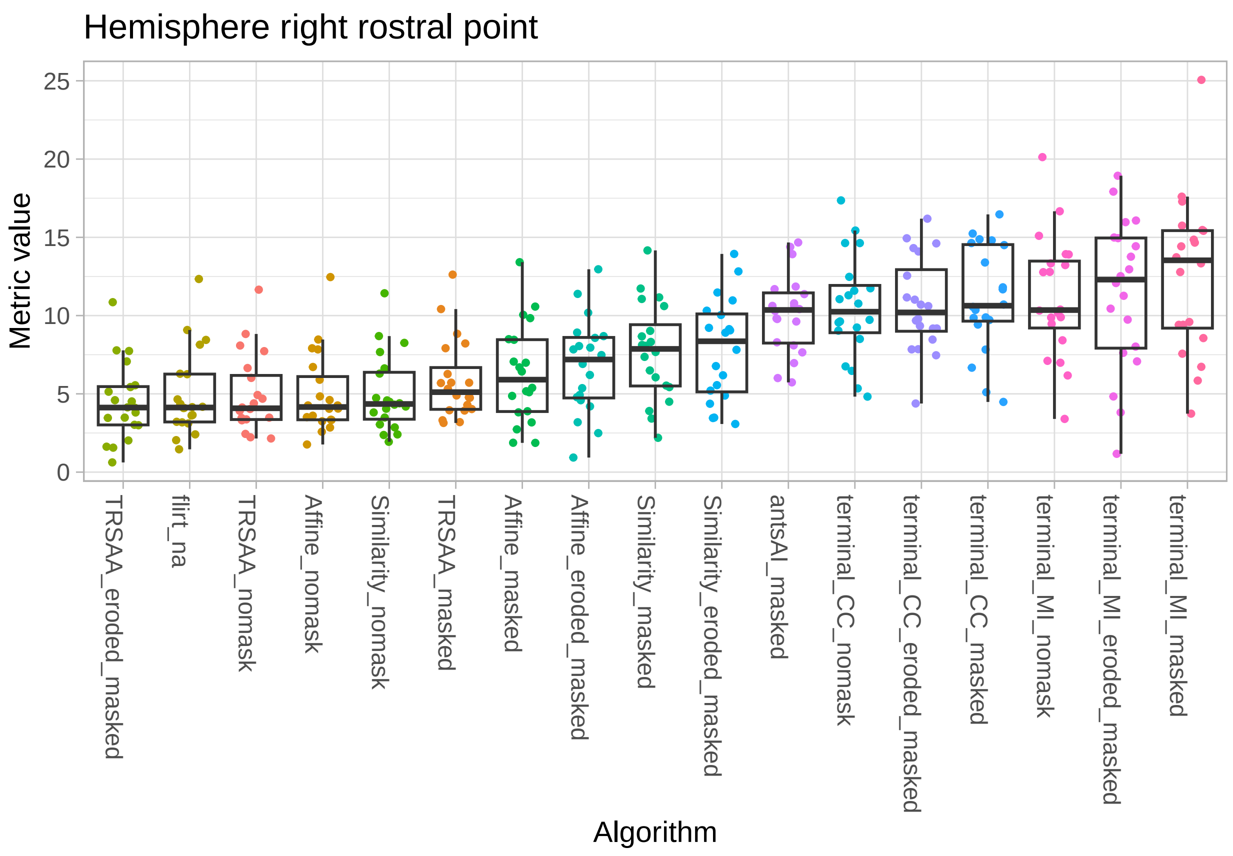
Hemisphere right rostral point
Metric value
0
5
10
15
20
25
TRSAA_eroded_masked
flirt_na
TRSAA_nomask
Affine_nomask
Similarity_nomask
TRSAA_masked
Affine_masked
Affine_eroded_masked
Similarity_masked
Similarity_eroded_masked
antsAI_masked
terminal_CC_nomask
terminal_CC_eroded_masked
terminal_CC_masked
terminal_MI_nomask
terminal_MI_eroded_masked
terminal_MI_masked
Algorithm

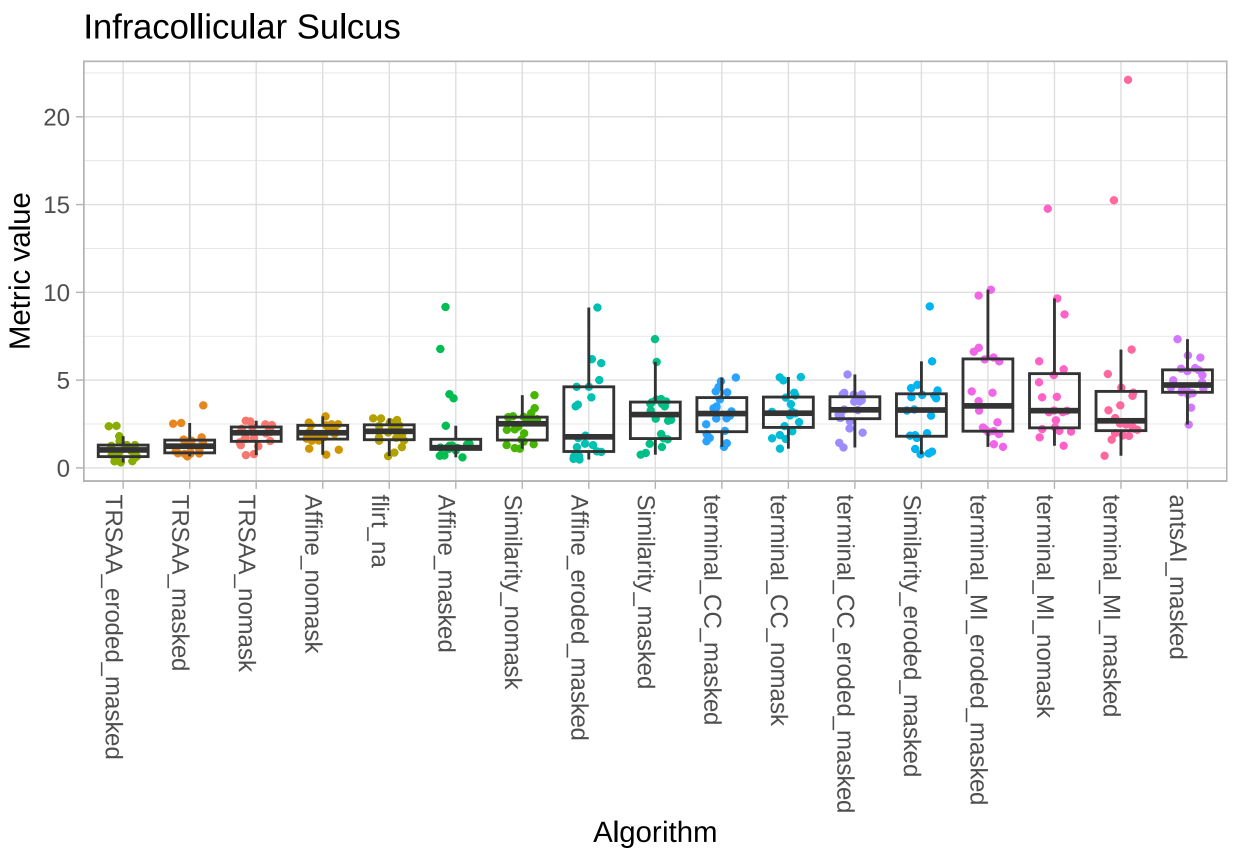
Infracollicular Sulcus
Metric value
20
15
10
5
0
TRSAA_eroded_masked
TRSAA_masked
TRSAA_nomask
Affine_nomask
flirt_na
Affine_masked
Similarity_nomask
Affine_eroded_masked
Similarity_masked
terminal_CC_masked
terminal_CC_nomask
terminal_CC_eroded_masked
Similarity_eroded_masked
terminal_MI_eroded_masked
terminal_MI_nomask
terminal_MI_masked
antsAI_masked
Algorithm

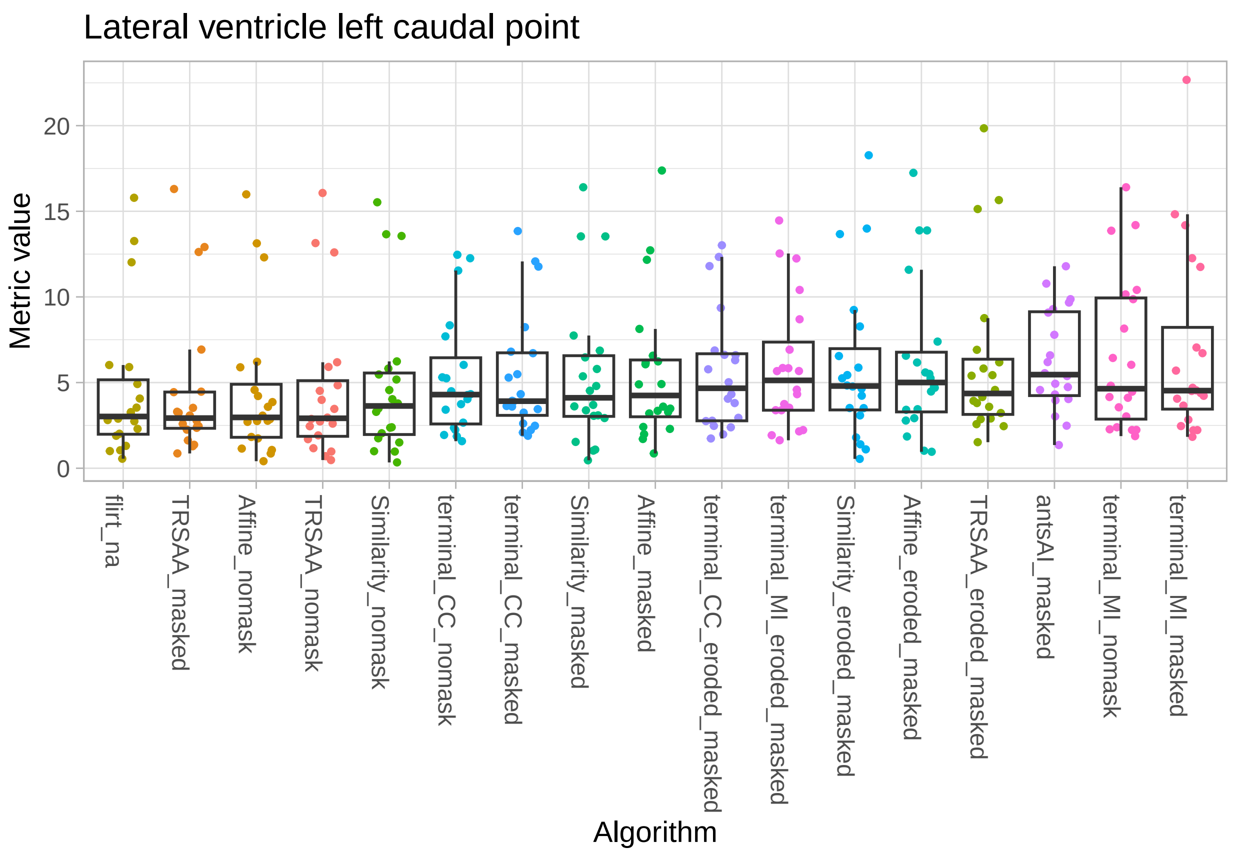

Lateral ventricle left caudal point
Metric value
20
15
10
5
0
flirt_na
TRSAA_masked
Affine_nomask
TRSAA_nomask
Similarity_nomask
terminal_CC_nomask
terminal_CC_masked
Similarity_masked
Affine_masked
terminal_CC_eroded_masked
terminal_MI_eroded_masked
Similarity_eroded_masked
Affine_eroded_masked
TRSAA_eroded_masked
antsAI_masked
terminal_MI_nomask
terminal_MI_masked
Algorithm

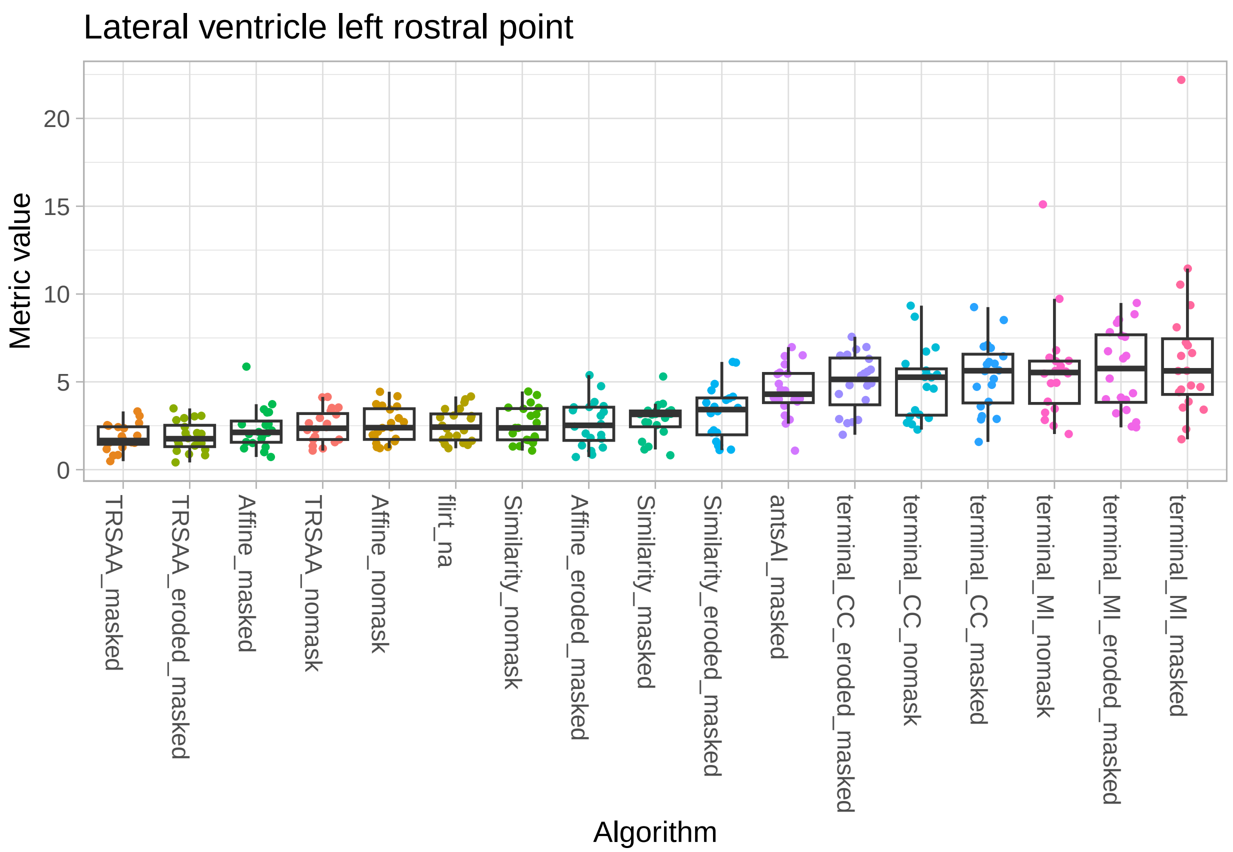
Lateral ventricle left rostral point
Metric value
0
5
10
15
20
TRSAA_masked
TRSAA_eroded_masked
Affine_masked
TRSAA_nomask
Affine_nomask
flirt_na
Similarity_nomask
Affine_eroded_masked
Similarity_masked
Similarity_eroded_masked
antsAI_masked
terminal_CC_eroded_masked
terminal_CC_nomask
terminal_CC_masked
terminal_MI_nomask
terminal_MI_eroded_masked
terminal_MI_masked
Algorithm

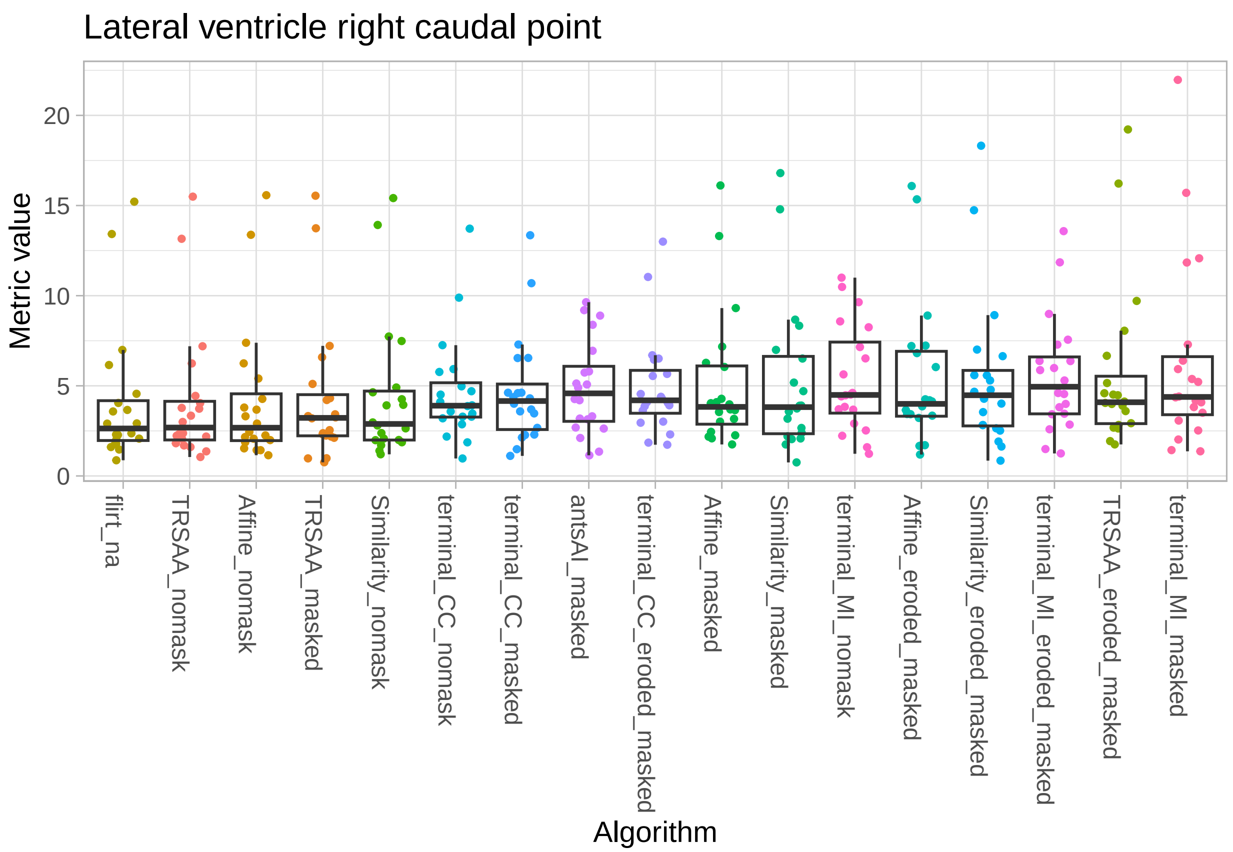

Lateral ventricle right caudal point
Metric value
0
5
10
15
20
flirt_na
TRSAA_nomask
Affine_nomask
TRSAA_masked
Similarity_nomask
terminal_CC_nomask
terminal_CC_masked
antsAI_masked
terminal_CC_eroded_masked
Affine_masked
Similarity_masked
terminal_MI_nomask
Affine_eroded_masked
Similarity_eroded_masked
terminal_MI_eroded_masked
TRSAA_eroded_masked
terminal_MI_masked
Algorithm

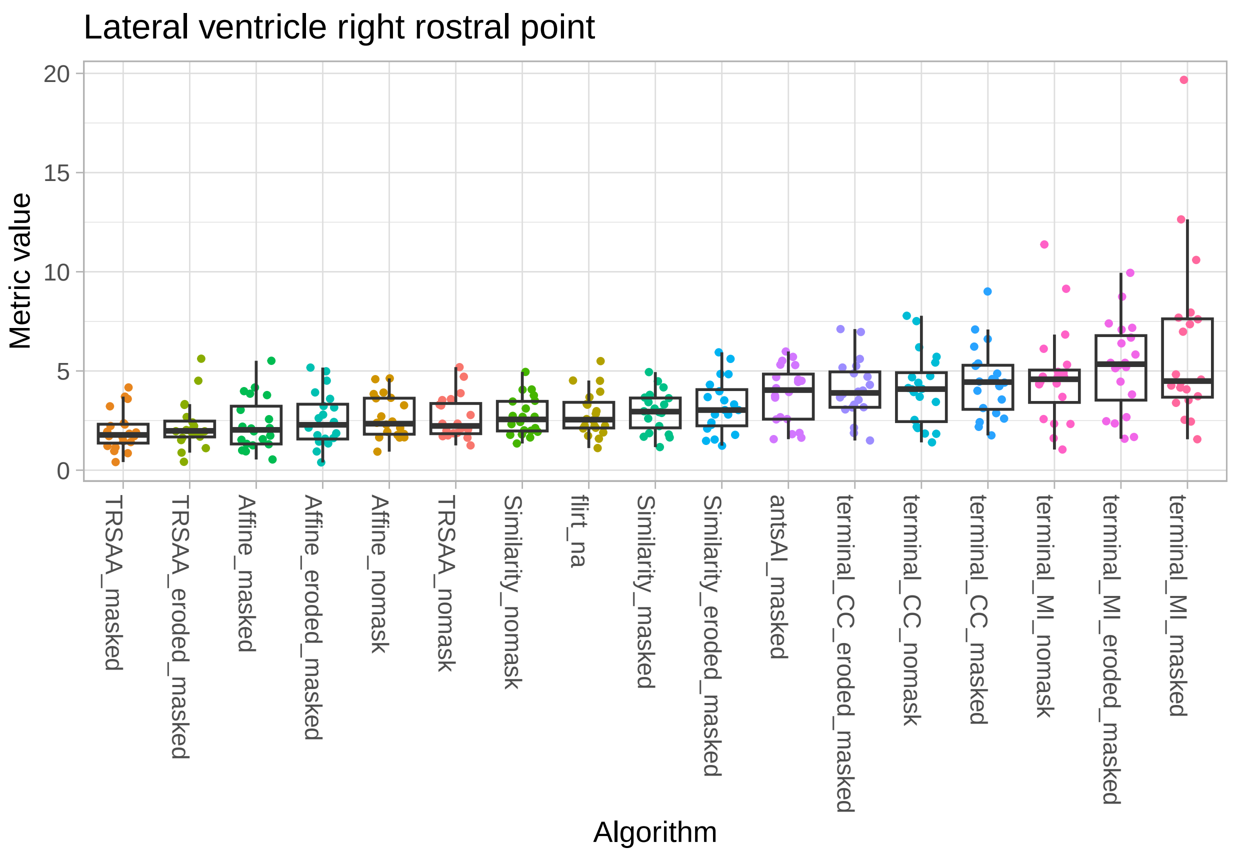

Lateral ventricle right rostral point
Metric value
20
15
10
5
0
TRSAA_masked
TRSAA_eroded_masked
Affine_masked
Affine_eroded_masked
Affine_nomask
TRSAA_nomask
Similarity_nomask
flirt_na
Similarity_masked
Similarity_eroded_masked
antsAI_masked
terminal_CC_eroded_masked
terminal_CC_nomask
terminal_CC_masked
terminal_MI_nomask
terminal_MI_eroded_masked
terminal_MI_masked
Algorithm

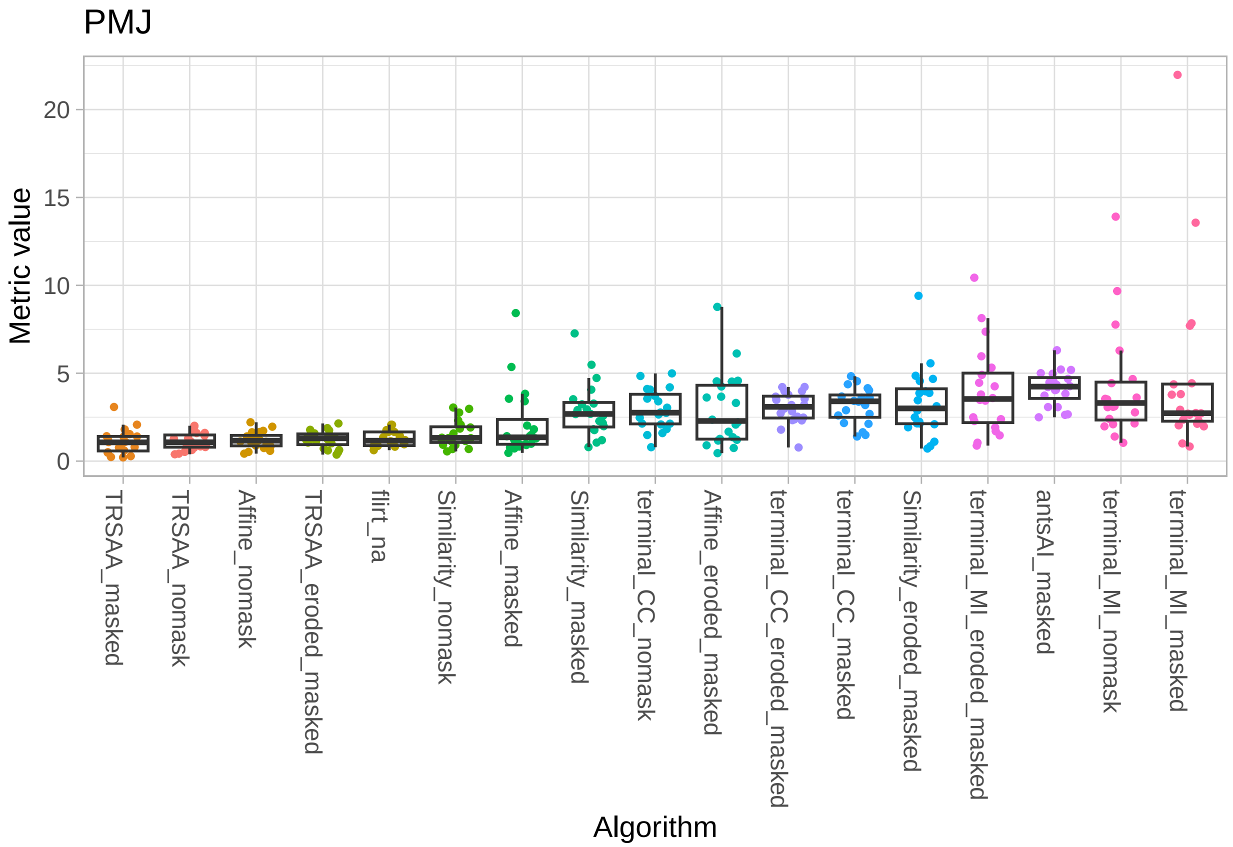
PMJ
Metric value
0
5
10
15
20
TRSAA_masked
TRSAA_nomask
Affine_nomask
TRSAA_eroded_masked
flirt_na
Similarity_nomask
Affine_masked
Similarity_masked
terminal_CC_nomask
Affine_eroded_masked
terminal_CC_eroded_masked
terminal_CC_masked
Similarity_eroded_masked
terminal_MI_eroded_masked
antsAI_masked
terminal_MI_nomask
terminal_MI_masked
Algorithm

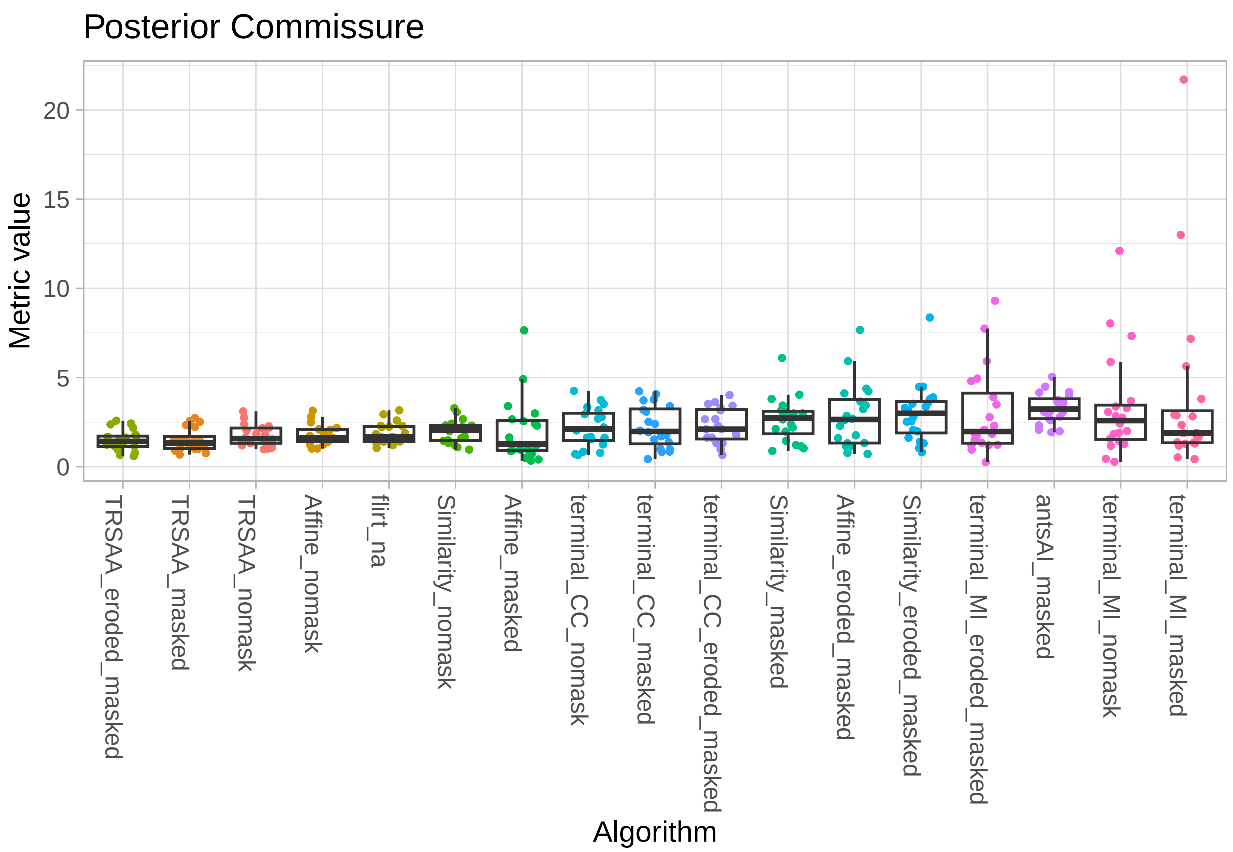

Posterior Commissure
Metric value
0
5
10
15
20
TRSAA_eroded_masked
TRSAA_masked
TRSAA_nomask
Affine_nomask
flirt_na
Similarity_nomask
Affine_masked
terminal_CC_nomask
terminal_CC_masked
terminal_CC_eroded_masked
Similarity_masked
Affine_eroded_masked
Similarity_eroded_masked
terminal_MI_eroded_masked
antsAI_masked
terminal_MI_nomask
terminal_MI_masked
Algorithm

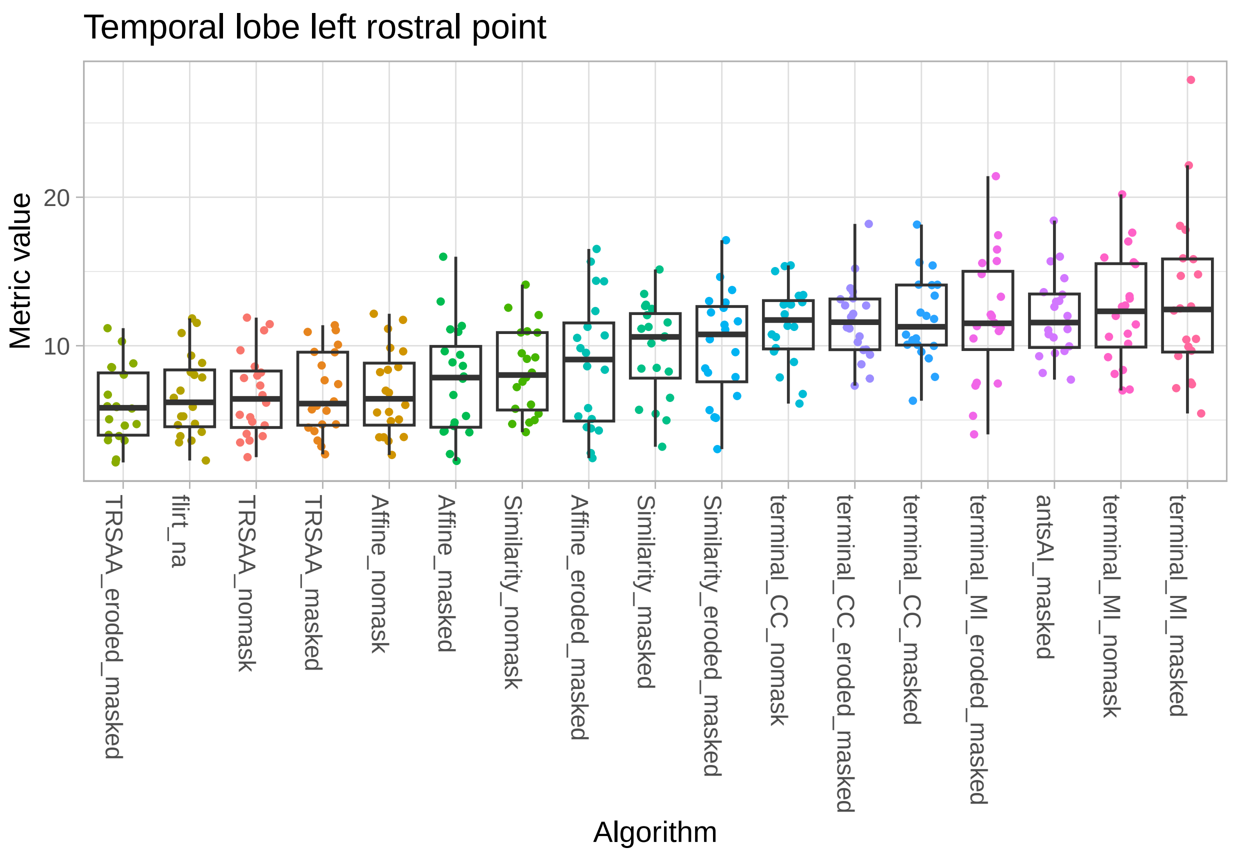

Temporal lobe left rostral point
Metric value
20
10
TRSAA_eroded_masked
flirt_na
TRSAA_nomask
TRSAA_masked
Affine_nomask
Affine_masked
Similarity_nomask
Affine_eroded_masked
Similarity_masked
Similarity_eroded_masked
terminal_CC_nomask
terminal_CC_eroded_masked
terminal_CC_masked
terminal_MI_eroded_masked
antsAI_masked
terminal_MI_nomask
terminal_MI_masked
Algorithm

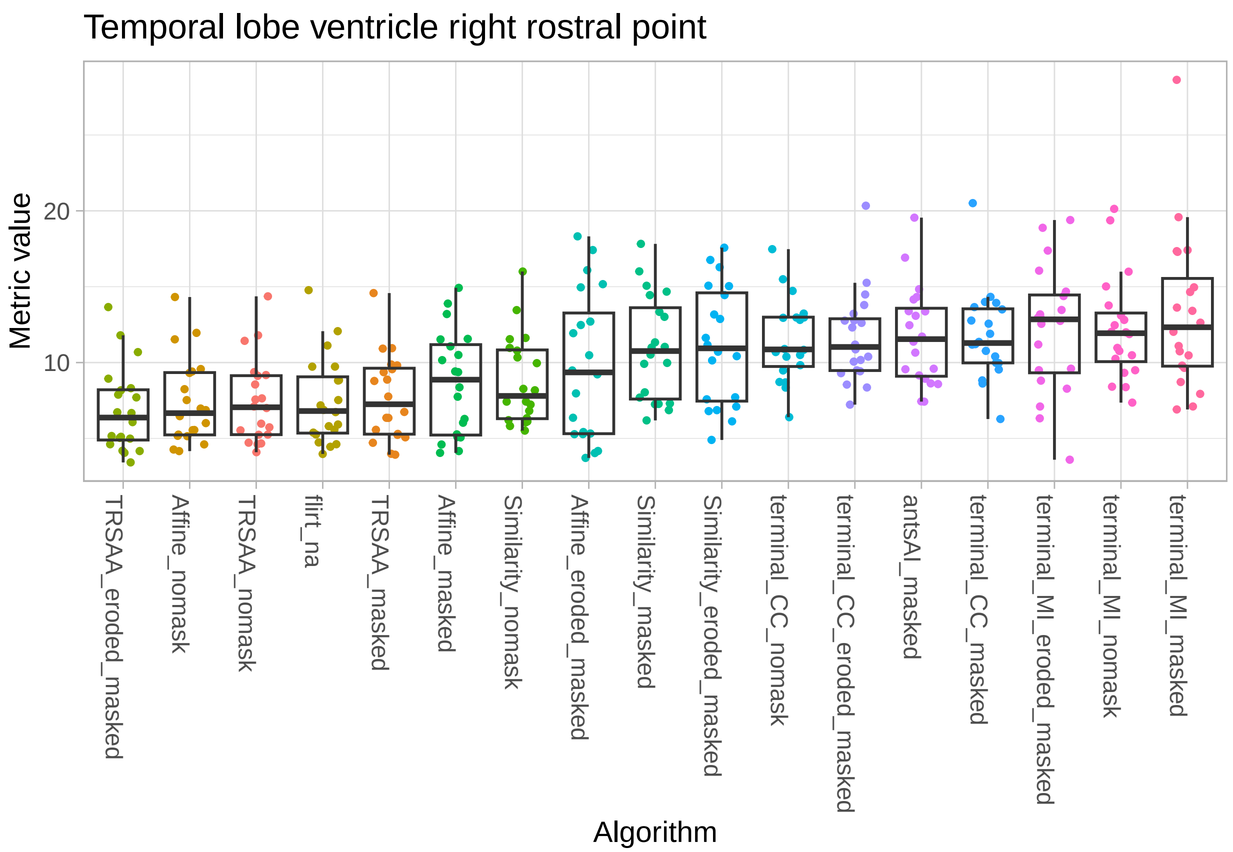
Temporal lobe ventricle right rostral point
Metric value
20
10
TRSAA_eroded_masked
Affine_nomask
TRSAA_nomask
flirt_na
TRSAA_masked
Affine_masked
Similarity_nomask
Affine_eroded_masked
Similarity_masked
Similarity_eroded_masked
terminal_CC_nomask
terminal_CC_eroded_masked
antsAI_masked
terminal_CC_masked
terminal_MI_eroded_masked
terminal_MI_nomask
terminal_MI_masked
Algorithm

2.2 Podium plot

Podium plots (see also Eugster et al., 2008) for visualizing raw assessment data. Upper part (spaghetti plot): Participating algorithms are color-coded, and each colored dot in the plot represents a metric value achieved with the respective algorithm. The actual metric value is encoded by the y-axis. Each podium (here: p=17) represents one possible rank, ordered from best (1) to last (here: 17). The assignment of metric values (i.e. colored dots) to one of the podiums is based on the rank that the respective algorithm achieved on the corresponding case. Note that the plot part above each podium place is further subdivided into p "columns", where each column represents one participating algorithm (here: $p = 17$). Dots corresponding to identical cases are connected by a line, leading to the shown spaghetti structure. Lower part: Bar charts represent the relative frequency for each algorithm to achieve the rank encoded by the podium place.

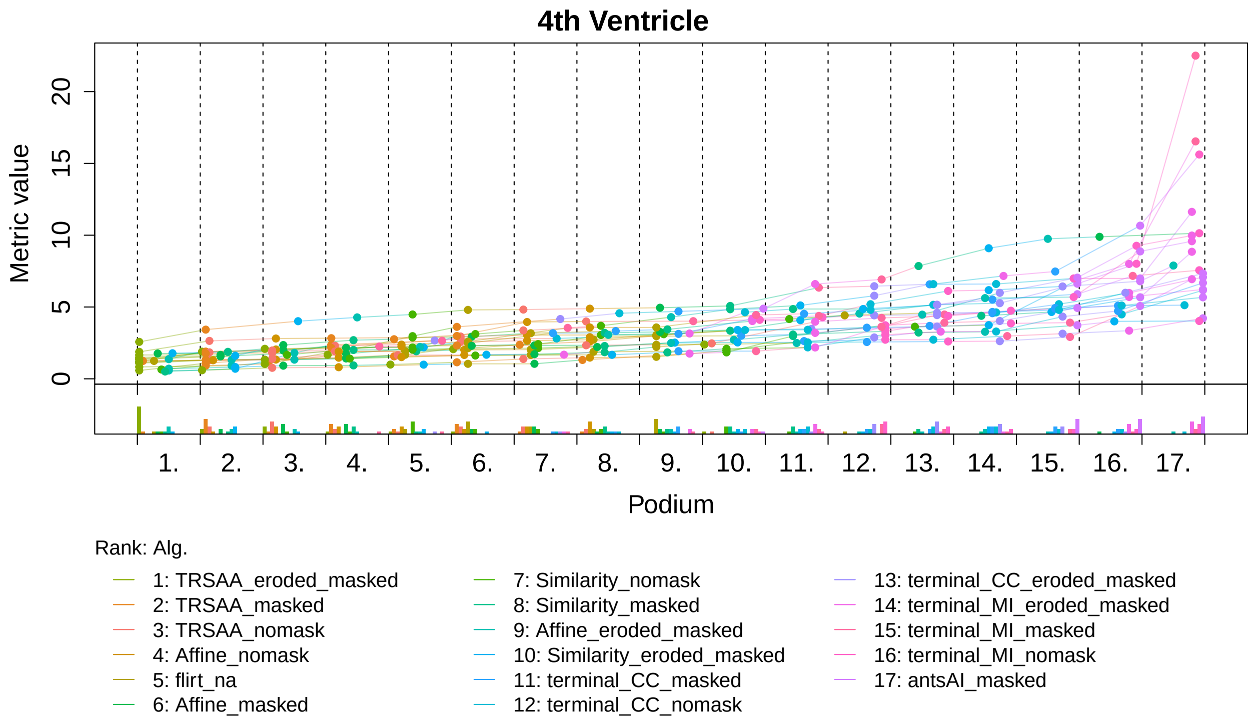

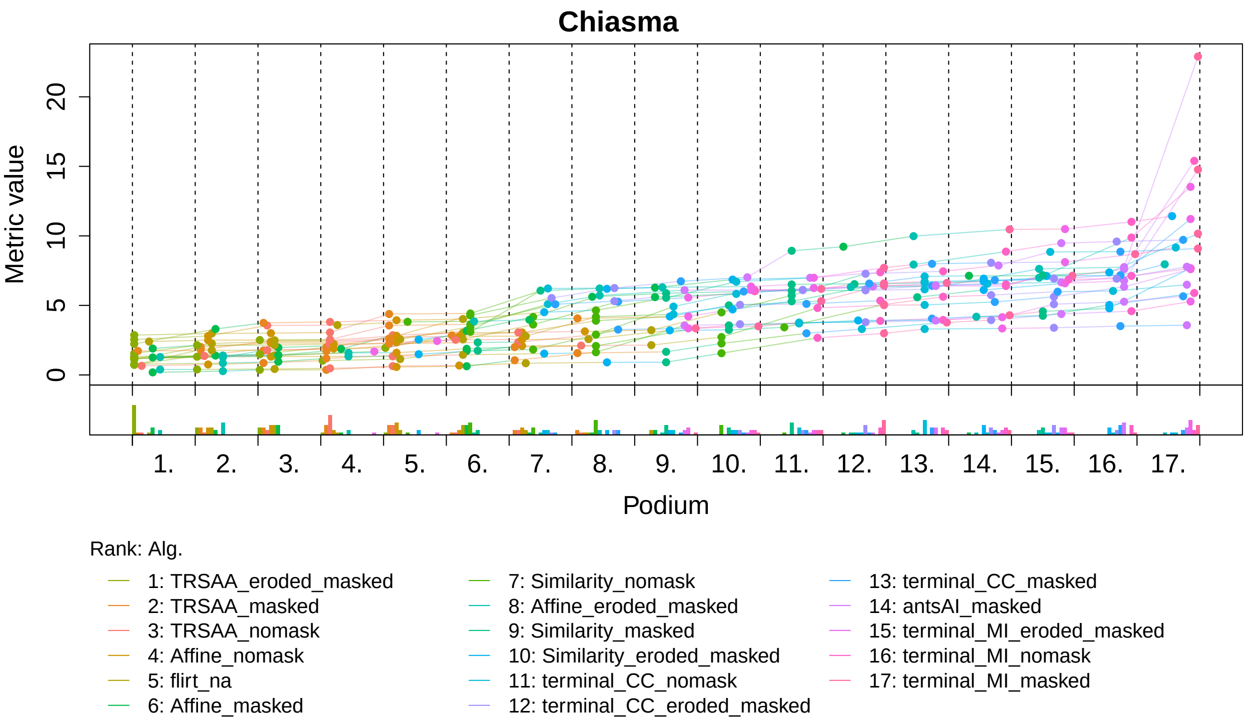
Chiasma
Metric value
0
5
10
15
20
1. 2. 3. 4. 5. 6. 7. 8. 9. 10. 11. 12. 13. 14. 15. 16. 17.
Podium
Rank: Alg.
1: TRSAA_eroded_masked
2: TRSAA_masked
3: TRSAA_nomask
4: Affine_nomask
5: flirt_na
6: Affine_masked
7: Similarity_nomask
8: Affine_eroded_masked
9: Similarity_masked
10: Similarity_eroded_masked
11: terminal_CC_nomask
12: terminal_CC_eroded_masked
13: terminal_CC_masked
14: antsAI_masked
15: terminal_MI_eroded_masked
16: terminal_MI_nomask
17: terminal_MI_masked

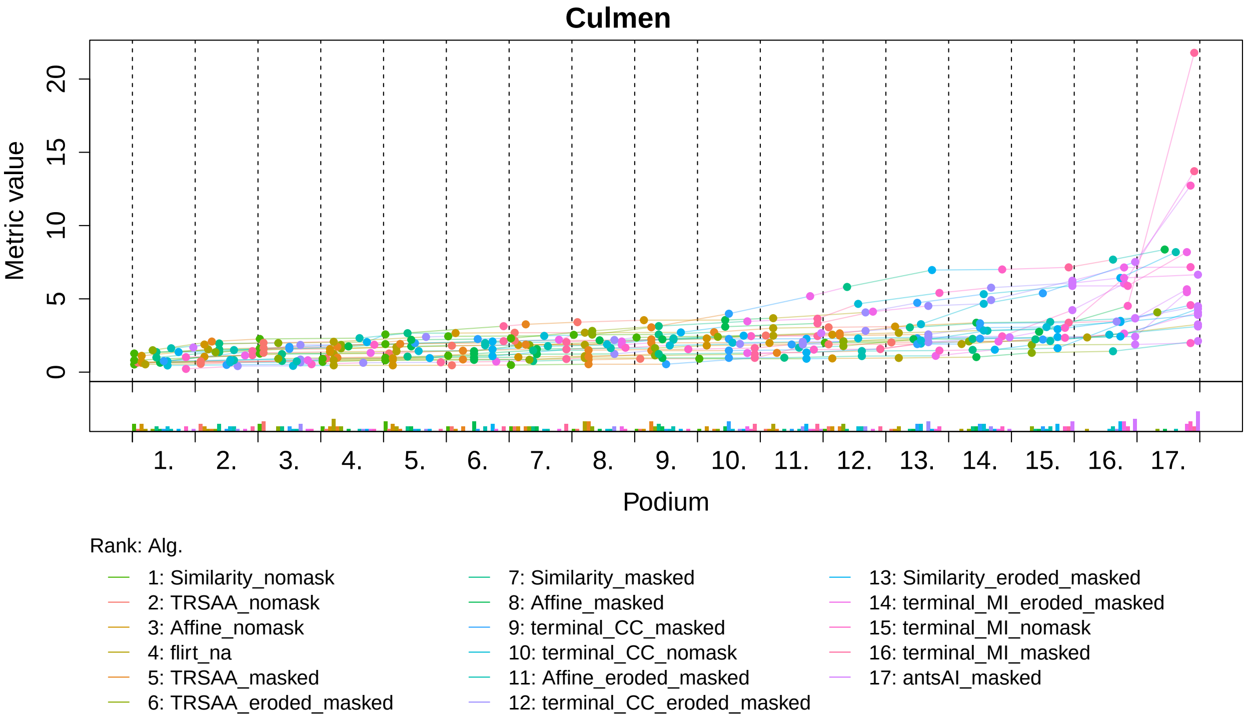
Culmen
Metric value
0
5
10
15
20
1. 2. 3. 4. 5. 6. 7. 8. 9. 10. 11. 12. 13. 14. 15. 16. 17.
Podium
Rank: Alg.
1: Similarity_nomask
2: TRSAA_nomask
3: Affine_nomask
4: flirt_na
5: TRSAA_masked
6: TRSAA_eroded_masked
7: Similarity_masked
8: Affine_masked
9: terminal_CC_masked
10: terminal_CC_nomask
11: Affine_eroded_masked
12: terminal_CC_eroded_masked
13: Similarity_eroded_masked
14: terminal_MI_eroded_masked
15: terminal_MI_nomask
16: terminal_MI_masked
17: antsAI_masked

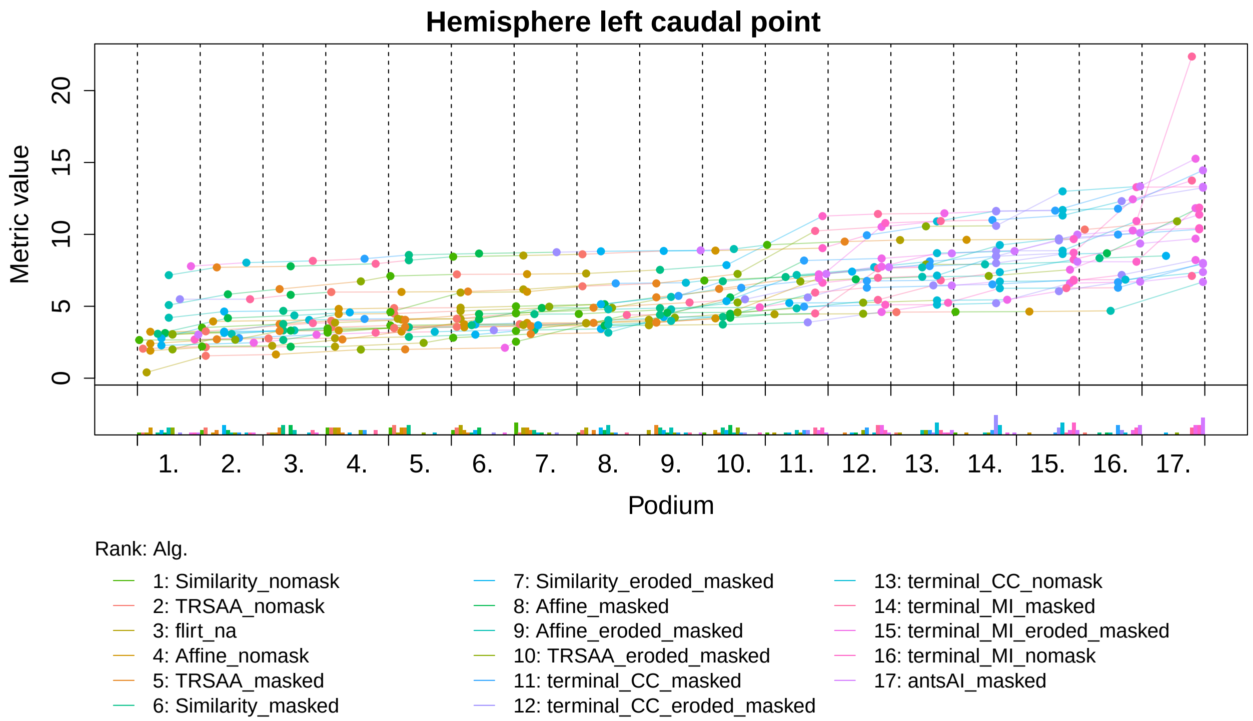
Hemisphere left caudal point
Metric value
0
5
10
15
20
1.
2.
3.
4.
5.
6.
7.
8.
9.
10.
11.
12.
13.
14.
15.
16.
17.
Podium
Rank: Alg.
1: Similarity_nomask
2: TRSAA_nomask
3: flirt_na
4: Affine_nomask
5: TRSAA_masked
6: Similarity_masked
7: Similarity_eroded_masked
8: Affine_masked
9: Affine_eroded_masked
10: TRSAA_eroded_masked
11: terminal_CC_masked
12: terminal_CC_eroded_masked
13: terminal_CC_nomask
14: terminal_MI_masked
15: terminal_MI_eroded_masked
16: terminal_MI_nomask
17: antsAI_masked

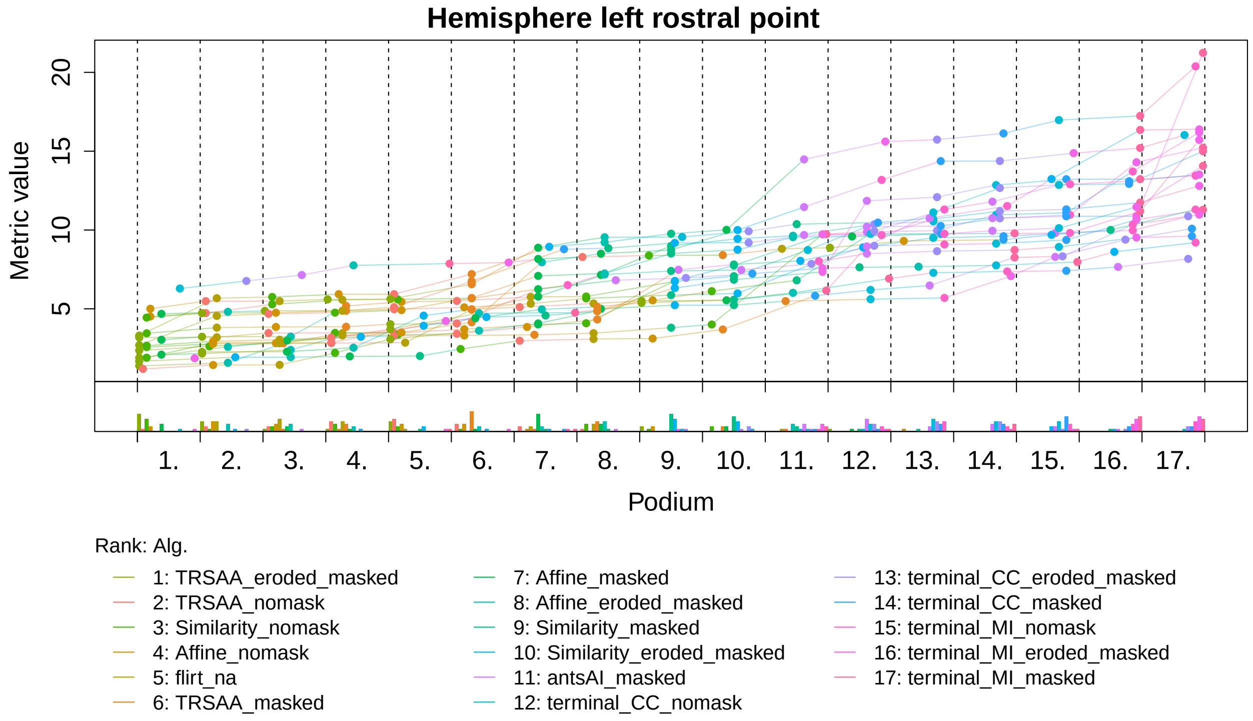
Hemisphere left rostral point
Metric value
5
10
15
20
1.
2.
3.
4.
5.
6.
7.
8.
9.
10.
11.
12.
13.
14.
15.
16.
17.
Podium
Rank: Alg.
1: TRSAA_eroded_masked
2: TRSAA_nomask
3: Similarity_nomask
4: Affine_nomask
5: flirt_na
6: TRSAA_masked
7: Affine_masked
8: Affine_eroded_masked
9: Similarity_masked
10: Similarity_eroded_masked
11: antsAI_masked
12: terminal_CC_nomask
13: terminal_CC_eroded_masked
14: terminal_CC_masked
15: terminal_MI_nomask
16: terminal_MI_eroded_masked
17: terminal_MI_masked

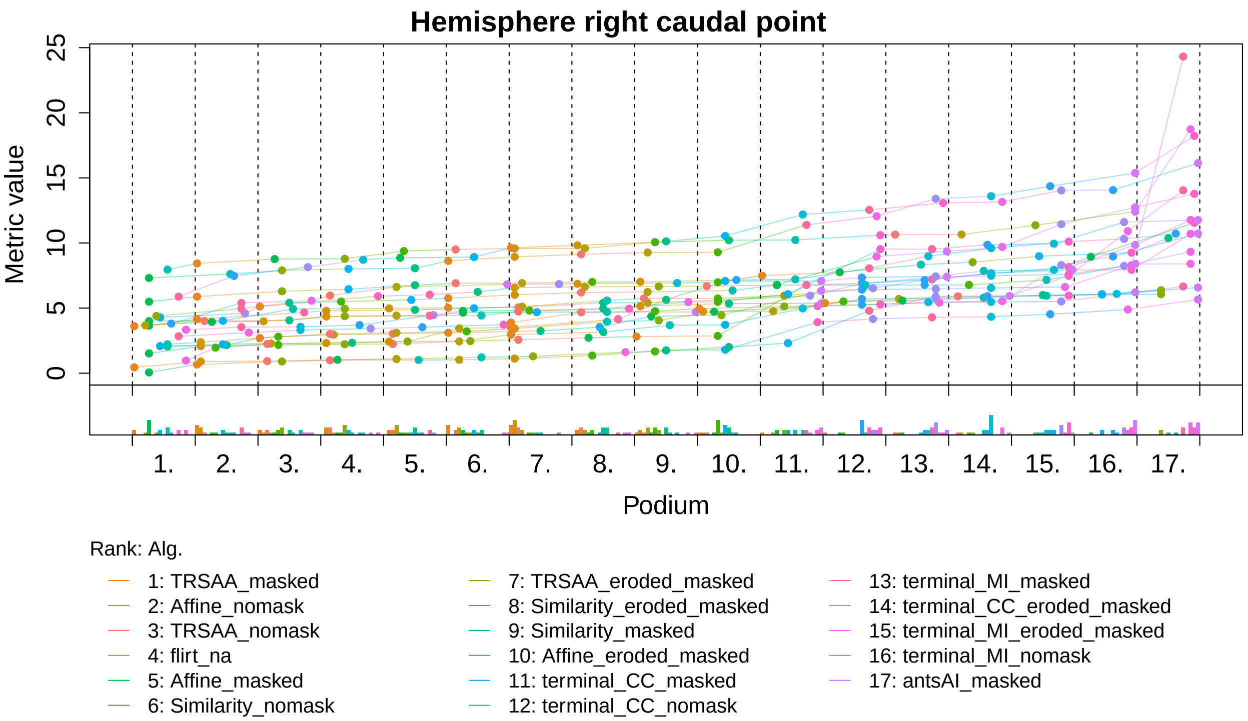
Hemisphere right caudal point
Metric value
0
5
10
15
20
25
1.
2.
3.
4.
5.
6.
7.
8.
9.
10.
11.
12.
13.
14.
15.
16.
17.
Podium
Rank: Alg.
1: TRSAA_masked
2: Affine_nomask
3: TRSAA_nomask
4: flirt_na
5: Affine_masked
6: Similarity_nomask
7: TRSAA_eroded_masked
8: Similarity_eroded_masked
9: Similarity_masked
10: Affine_eroded_masked
11: terminal_CC_masked
12: terminal_CC_nomask
13: terminal_MI_masked
14: terminal_CC_eroded_masked
15: terminal_MI_eroded_masked
16: terminal_MI_nomask
17: antsAI_masked

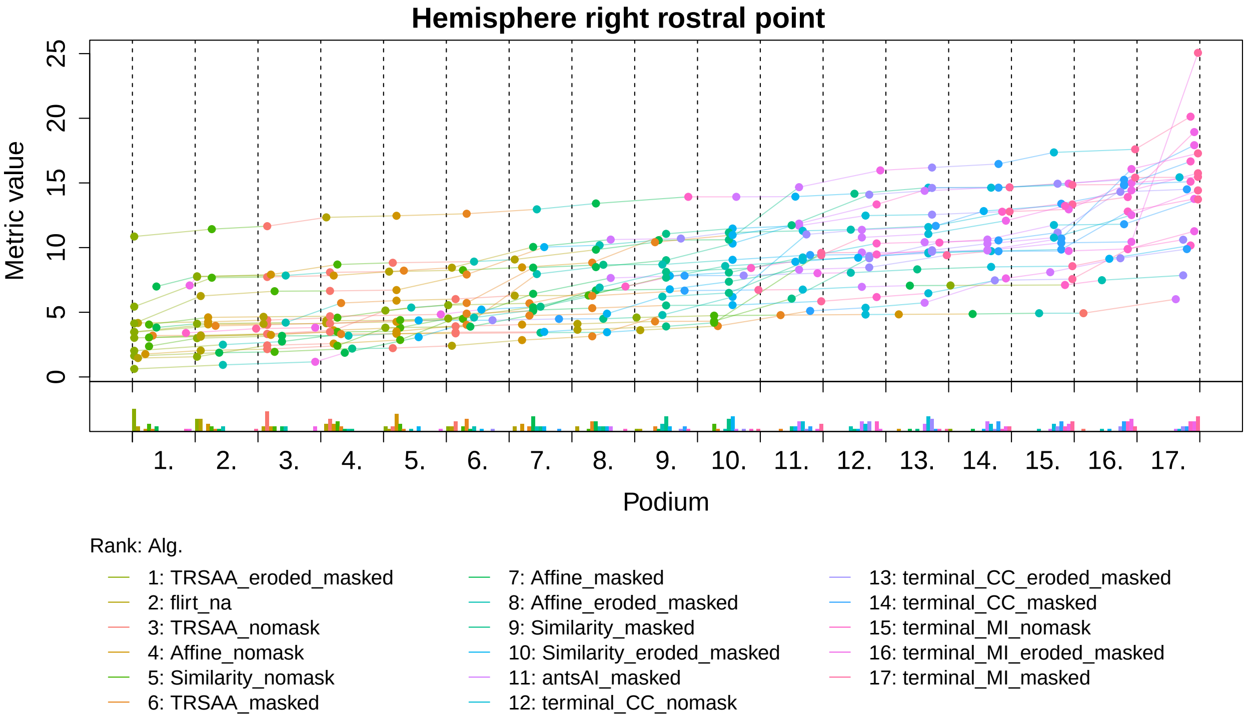
Hemisphere right rostral point
Metric value
0
5
10
15
20
25
1.
2.
3.
4.
5.
6.
7.
8.
9.
10.
11.
12.
13.
14.
15.
16.
17.
Podium
Rank: Alg.
1: TRSAA_eroded_masked
2: flirt_na
3: TRSAA_nomask
4: Affine_nomask
5: Similarity_nomask
6: TRSAA_masked
7: Affine_masked
8: Affine_eroded_masked
9: Similarity_masked
10: Similarity_eroded_masked
11: antsAI_masked
12: terminal_CC_nomask
13: terminal_CC_eroded_masked
14: terminal_CC_masked
15: terminal_MI_nomask
16: terminal_MI_eroded_masked
17: terminal_MI_masked

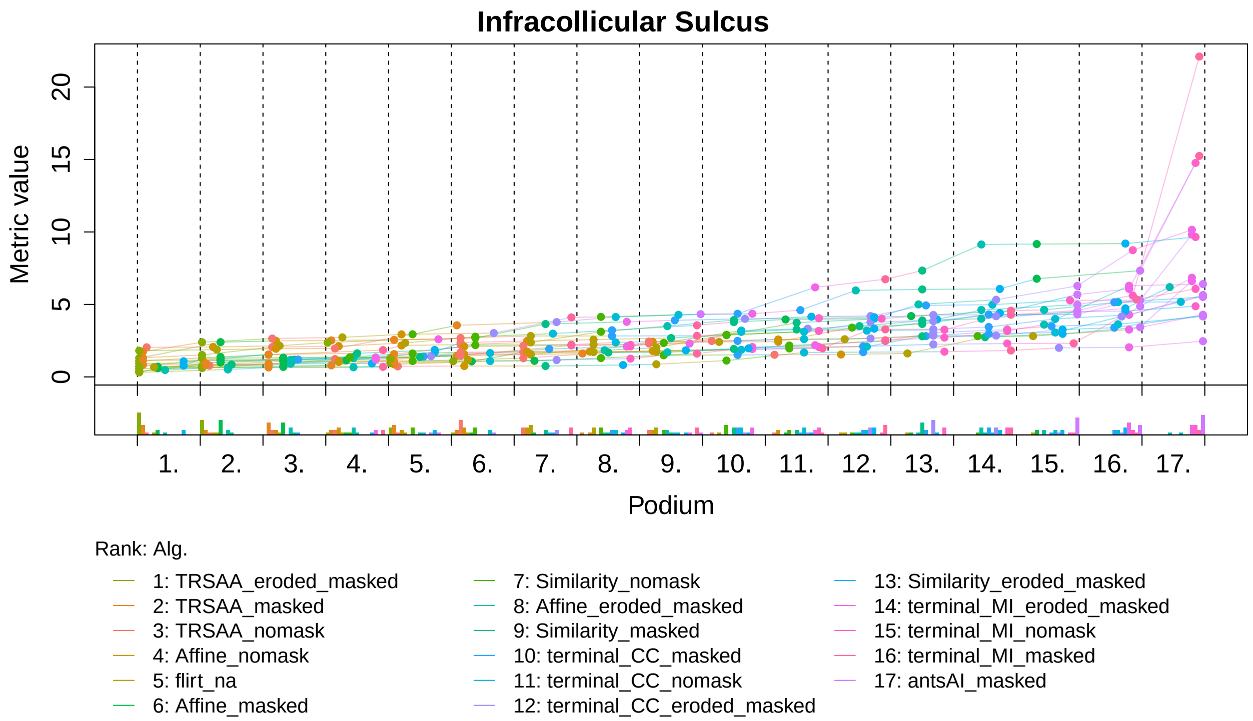
Infracollicular Sulcus
Metric value
0
5
10
15
20
1.
2.
3.
4.
5.
6.
7.
8.
9.
10.
11.
12.
13.
14.
15.
16.
17.
Podium
Rank: Alg.
1: TRSAA_eroded_masked
2: TRSAA_masked
3: TRSAA_nomask
4: Affine_nomask
5: flirt_na
6: Affine_masked
7: Similarity_nomask
8: Affine_eroded_masked
9: Similarity_masked
10: terminal_CC_masked
11: terminal_CC_nomask
12: terminal_CC_eroded_masked
13: Similarity_eroded_masked
14: terminal_MI_eroded_masked
15: terminal_MI_nomask
16: terminal_MI_masked
17: antsAI_masked

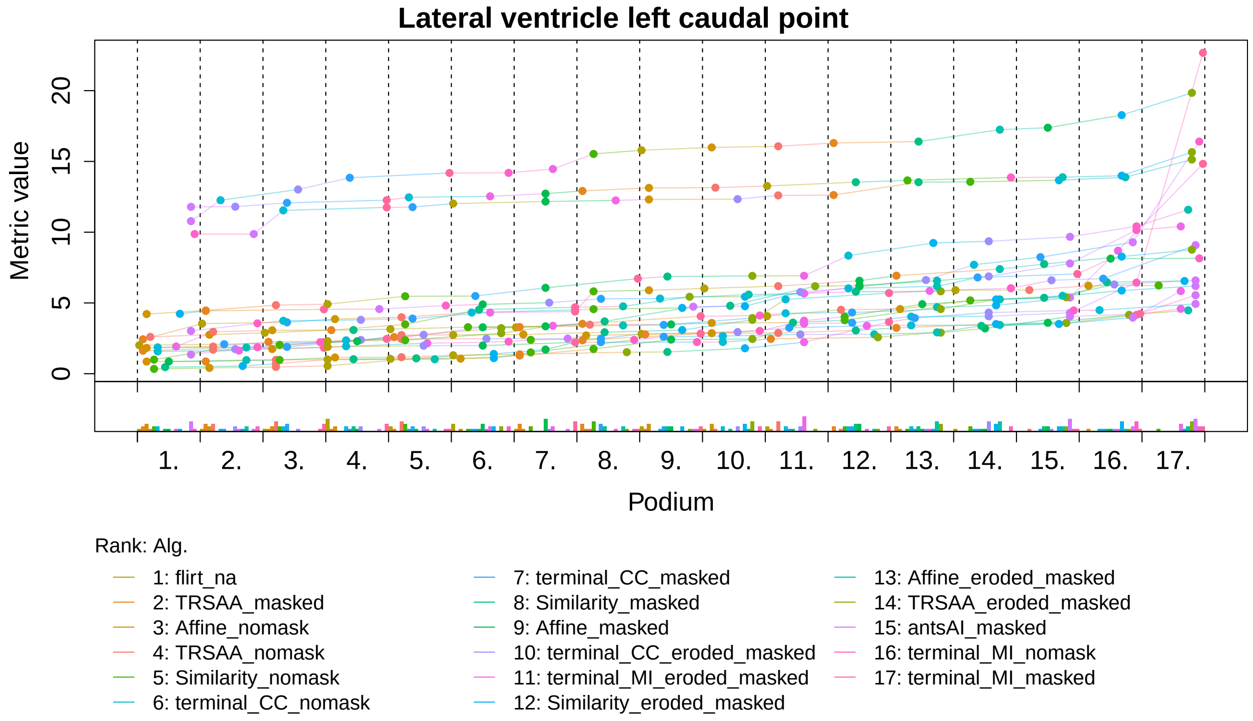
Lateral ventricle left caudal point
Metric value
0
5
10
15
20
1.
2.
3.
4.
5.
6.
7.
8.
9.
10.
11.
12.
13.
14.
15.
16.
17.
Podium
Rank: Alg.
1: flirt_na
2: TRSAA_masked
3: Affine_nomask
4: TRSAA_nomask
5: Similarity_nomask
6: terminal_CC_nomask
7: terminal_CC_masked
8: Similarity_masked
9: Affine_masked
10: terminal_CC_eroded_masked
11: terminal_MI_eroded_masked
12: Similarity_eroded_masked
13: Affine_eroded_masked
14: TRSAA_eroded_masked
15: antsAI_masked
16: terminal_MI_nomask
17: terminal_MI_masked

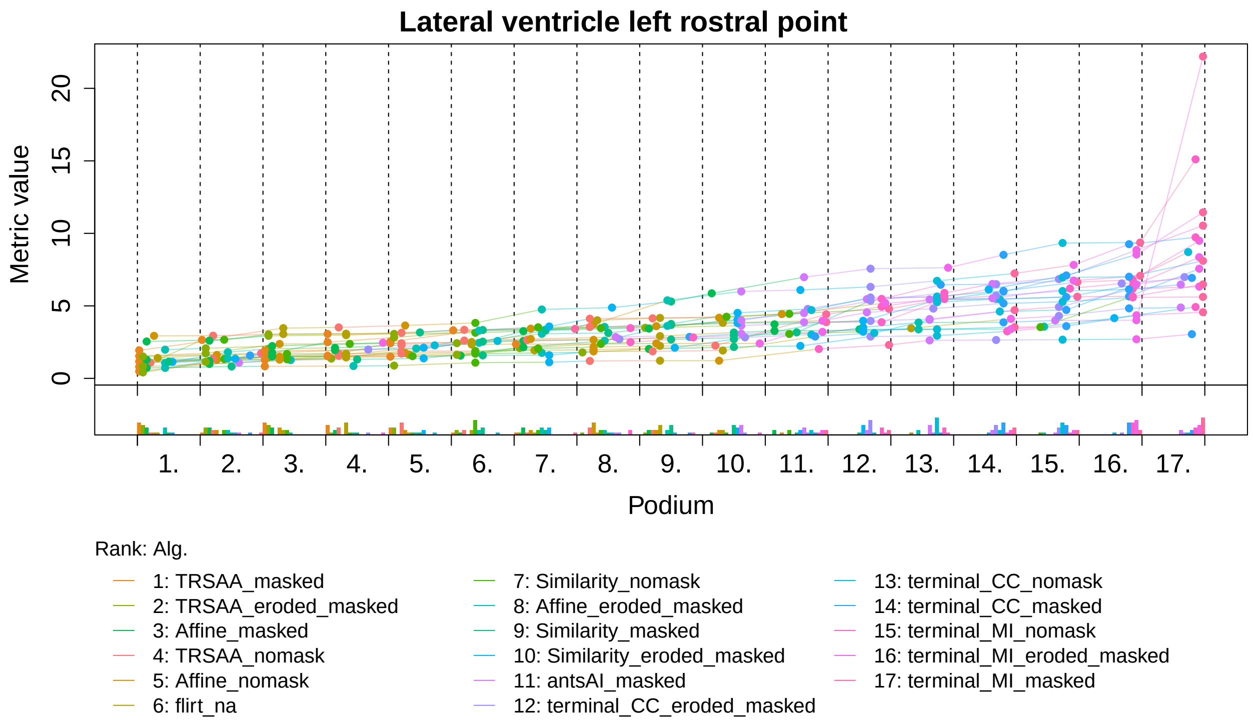
Lateral ventricle left rostral point
Metric value
0
5
10
15
20
Podium
1.
2.
3.
4.
5.
6.
7.
8.
9.
10.
11.
12.
13.
14.
15.
16.
17.
Rank: Alg.
1: TRSAA_masked
2: TRSAA_eroded_masked
3: Affine_masked
4: TRSAA_nomask
5: Affine_nomask
6: flirt_na
7: Similarity_nomask
8: Affine_eroded_masked
9: Similarity_masked
10: Similarity_eroded_masked
11: antsAI_masked
12: terminal_CC_eroded_masked
13: terminal_CC_nomask
14: terminal_CC_masked
15: terminal_MI_nomask
16: terminal_MI_eroded_masked
17: terminal_MI_masked

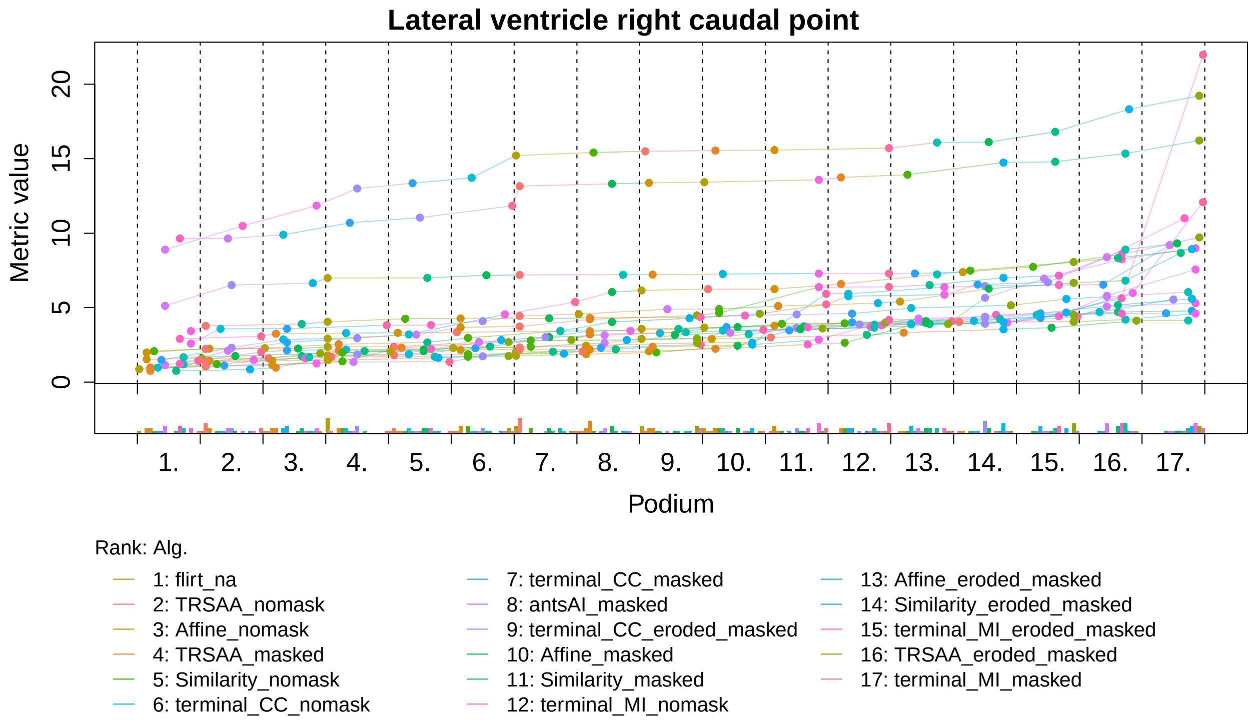
Lateral ventricle right caudal point
Metric value
0
5
10
15
20
Podium
1.
2.
3.
4.
5.
6.
7.
8.
9.
10.
11.
12.
13.
14.
15.
16.
17.
Rank: Alg.
1: flirt_na
2: TRSAA_nomask
3: Affine_nomask
4: TRSAA_masked
5: Similarity_nomask
6: terminal_CC_nomask
7: terminal_CC_masked
8: antsAI_masked
9: terminal_CC_eroded_masked
10: Affine_masked
11: Similarity_masked
12: terminal_MI_nomask
13: Affine_eroded_masked
14: Similarity_eroded_masked
15: terminal_MI_eroded_masked
16: TRSAA_eroded_masked
17: terminal_MI_masked

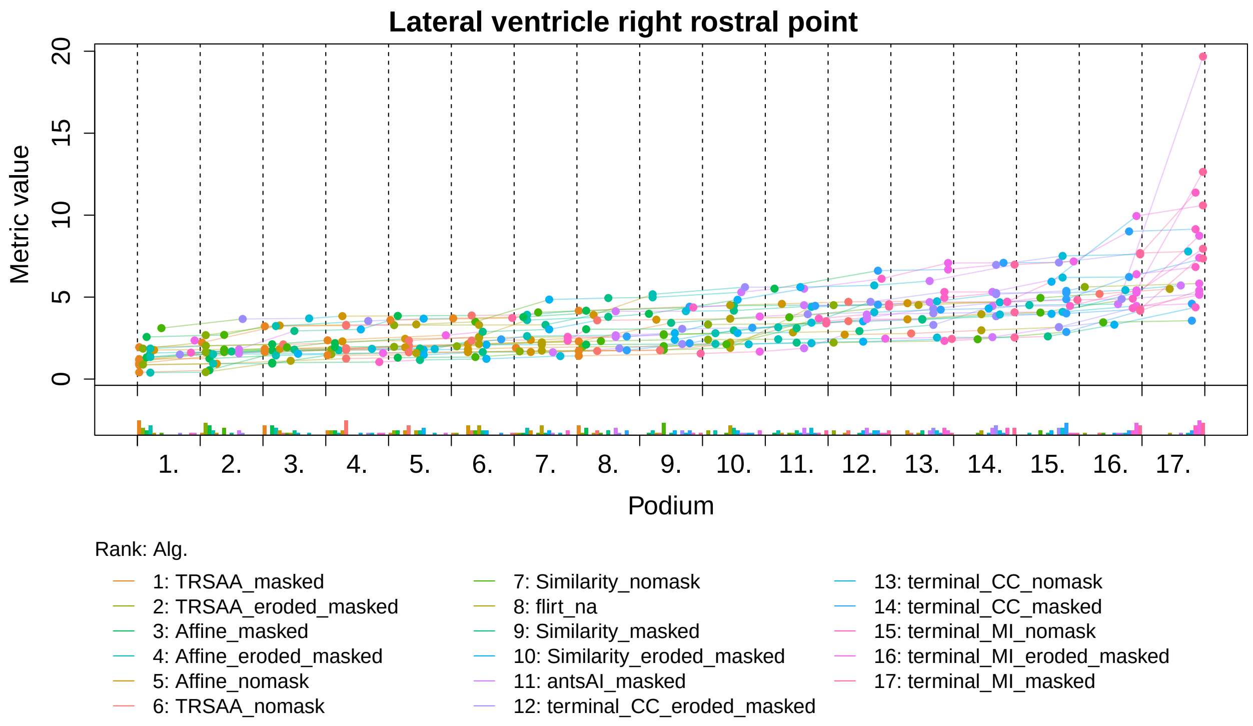
Lateral ventricle right rostral point
Metric value
0
5
10
15
20
1.
2.
3.
4.
5.
6.
7.
8.
9.
10.
11.
12.
13.
14.
15.
16.
17.
Podium
Rank: Alg.
1: TRSAA_masked
2: TRSAA_eroded_masked
3: Affine_masked
4: Affine_eroded_masked
5: Affine_nomask
6: TRSAA_nomask
7: Similarity_nomask
8: flirt_na
9: Similarity_masked
10: Similarity_eroded_masked
11: antsAI_masked
12: terminal_CC_eroded_masked
13: terminal_CC_nomask
14: terminal_CC_masked
15: terminal_MI_nomask
16: terminal_MI_eroded_masked
17: terminal_MI_masked

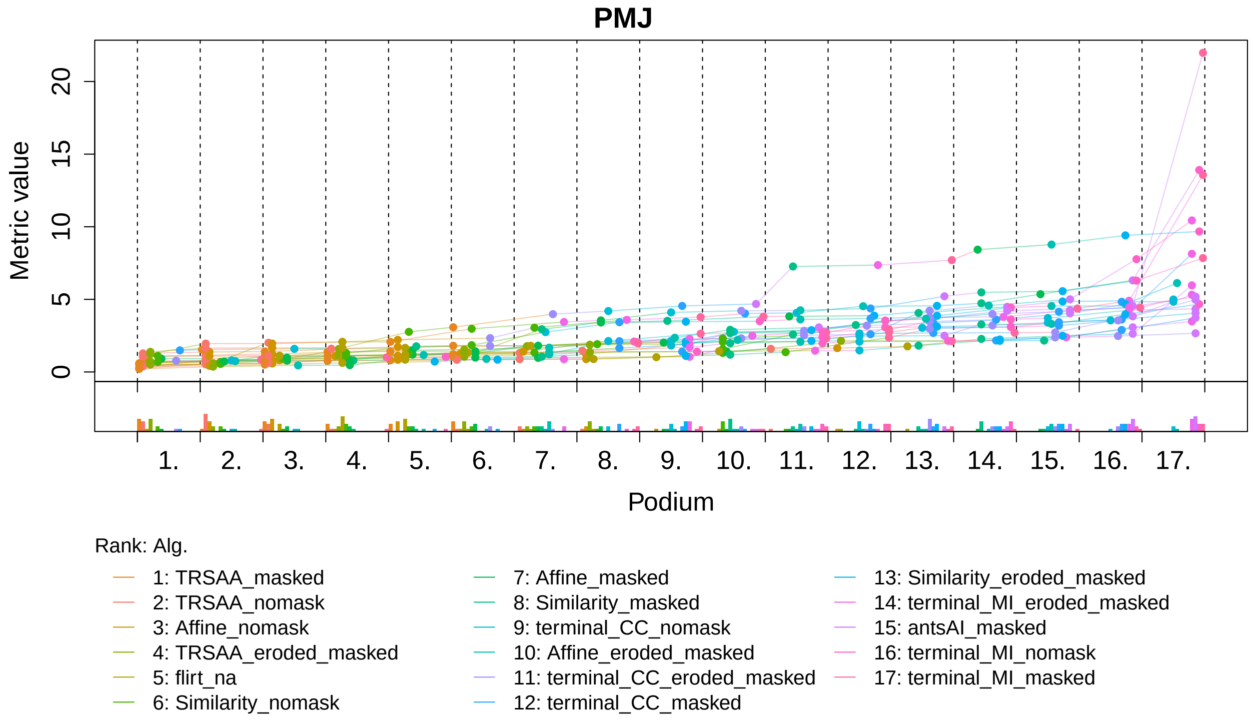
PMJ
Metric value
0
5
10
15
20
1.
2.
3.
4.
5.
6.
7.
8.
9.
10.
11.
12.
13.
14.
15.
16.
17.
Podium
Rank: Alg.
1: TRSAA_masked
2: TRSAA_nomask
3: Affine_nomask
4: TRSAA_eroded_masked
5: flirt_na
6: Similarity_nomask
7: Affine_masked
8: Similarity_masked
9: terminal_CC_nomask
10: Affine_eroded_masked
11: terminal_CC_eroded_masked
12: terminal_CC_masked
13: Similarity_eroded_masked
14: terminal_MI_eroded_masked
15: antsAI_masked
16: terminal_MI_nomask
17: terminal_MI_masked

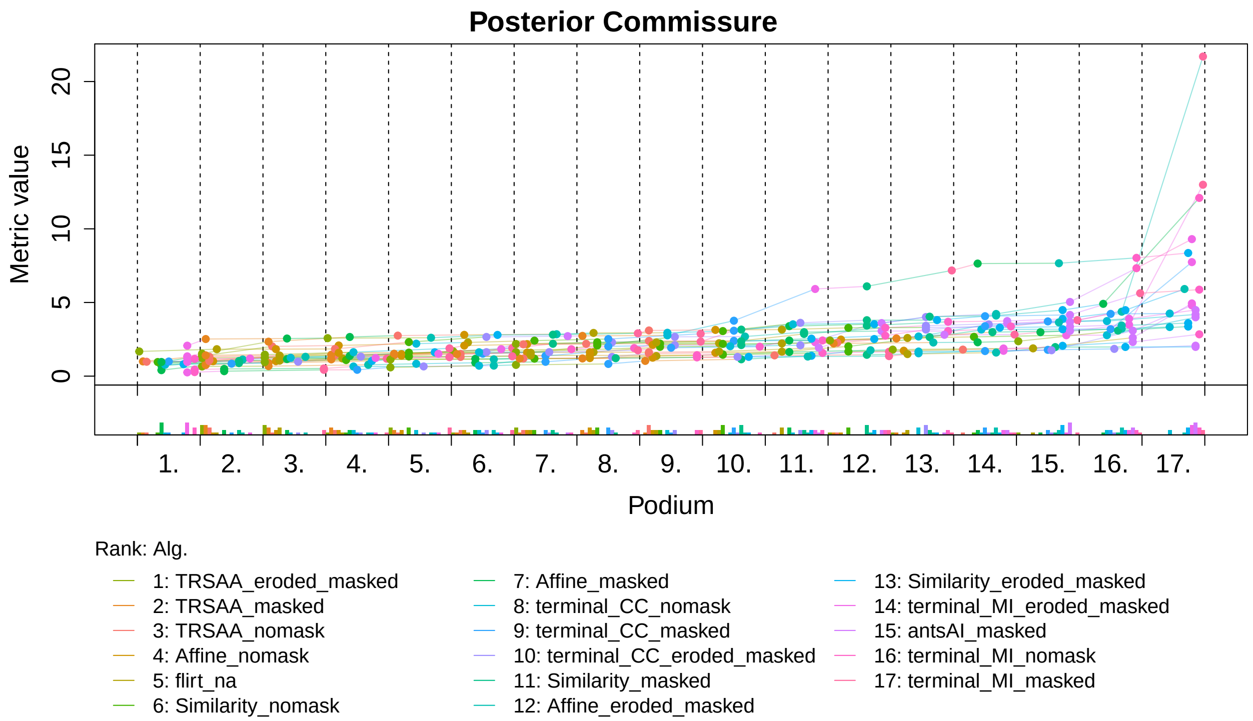

Posterior Commissure
Metric value
Podium
1. 2. 3. 4. 5. 6. 7. 8. 9. 10. 11. 12. 13. 14. 15. 16. 17.
Rank: Alg.
1: TRSAA_eroded_masked
2: TRSAA_masked
3: TRSAA_nomask
4: Affine_nomask
5: flirt_na
6: Similarity_nomask
7: Affine_masked
8: terminal_CC_nomask
9: terminal_CC_masked
10: terminal_CC_eroded_masked
11: Similarity_masked
12: Affine_eroded_masked
13: Similarity_eroded_masked
14: terminal_MI_eroded_masked
15: antsAI_masked
16: terminal_MI_nomask
17: terminal_MI_masked

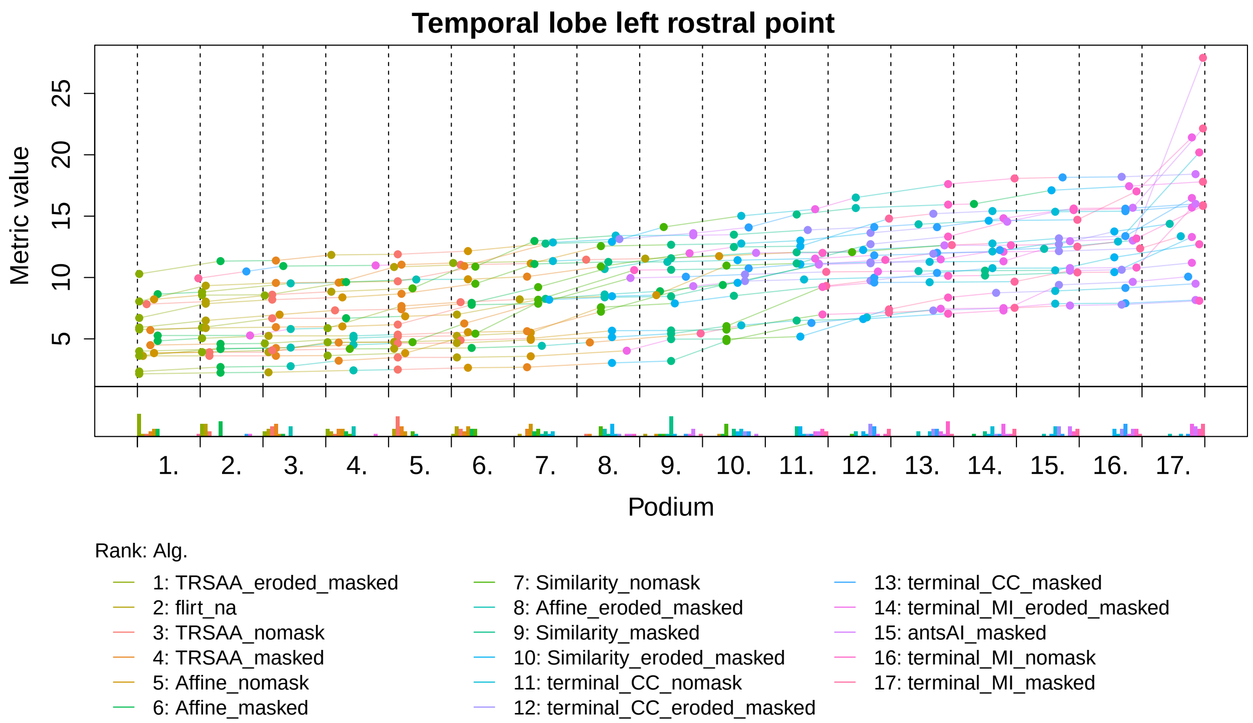

Temporal lobe left rostral point
Metric value
Podium
1. 2. 3. 4. 5. 6. 7. 8. 9. 10. 11. 12. 13. 14. 15. 16. 17.
Rank: Alg.
1: TRSAA_eroded_masked
2: flirt_na
3: TRSAA_nomask
4: TRSAA_masked
5: Affine_nomask
6: Affine_masked
7: Similarity_nomask
8: Affine_eroded_masked
9: Similarity_masked
10: Similarity_eroded_masked
11: terminal_CC_nomask
12: terminal_CC_eroded_masked
13: terminal_CC_masked
14: terminal_MI_eroded_masked
15: antsAI_masked
16: terminal_MI_nomask
17: terminal_MI_masked

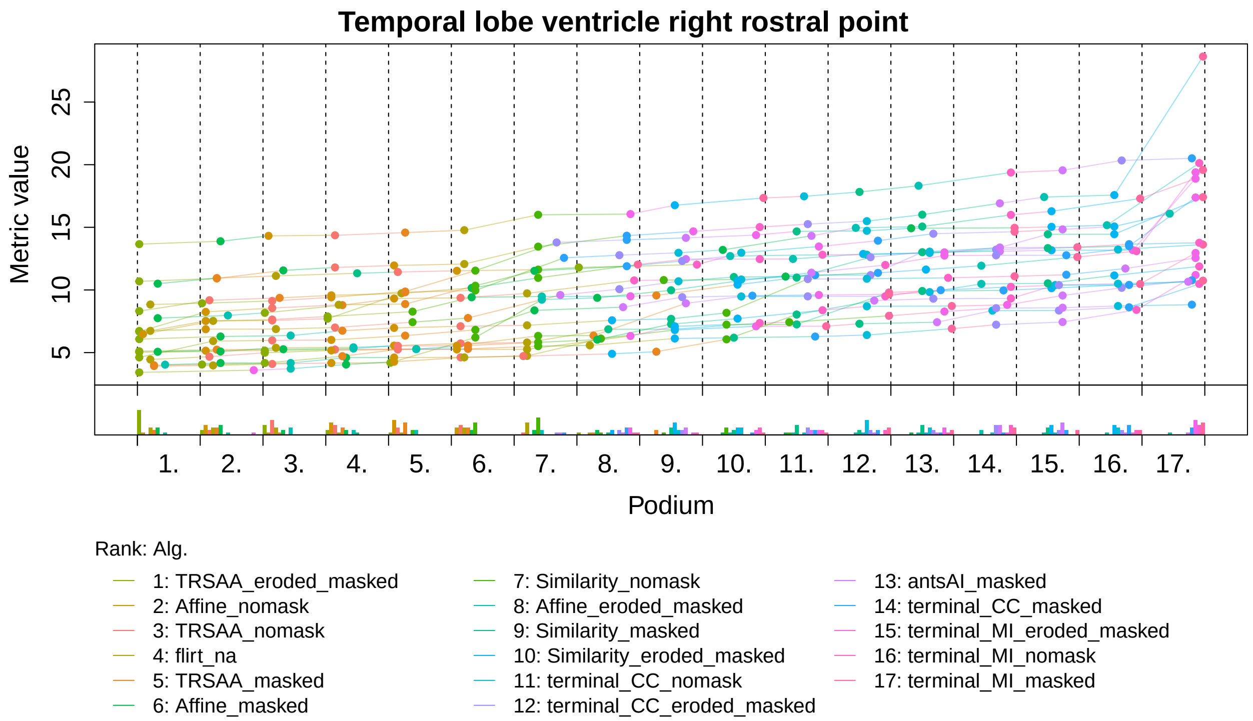
Temporal lobe ventricle right rostral point
Metric value
5
10
15
20
25
1.
2.
3.
4.
5.
6.
7.
8.
9.
10.
11.
12.
13.
14.
15.
16.
17.
Podium
Rank: Alg.
1: TRSAA_eroded_masked
2: Affine_nomask
3: TRSAA_nomask
4: flirt_na
5: TRSAA_masked
6: Affine_masked
7: Similarity_nomask
8: Affine_eroded_masked
9: Similarity_masked
10: Similarity_eroded_masked
11: terminal_CC_nomask
12: terminal_CC_eroded_masked
13: antsAI_masked
14: terminal_CC_masked
15: terminal_MI_eroded_masked
16: terminal_MI_nomask
17: terminal_MI_masked

2.3 Ranking heatmap

Ranking heatmaps for visualizing raw assessment data. Each cell (i, A_j) shows the absolute frequency of cases in which algorithm A_j achieved rank i.

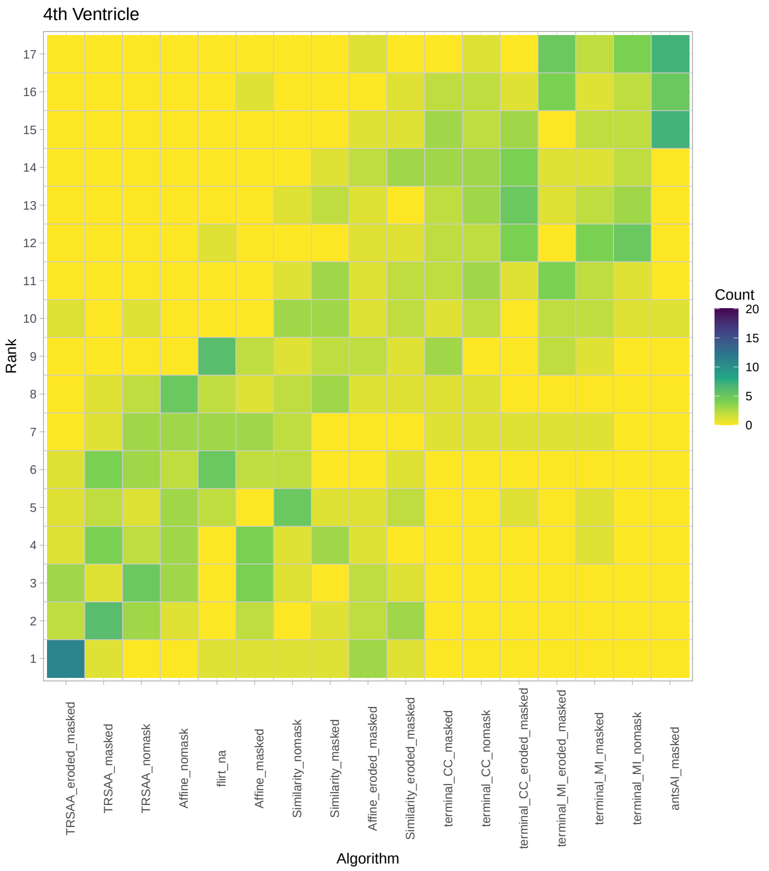

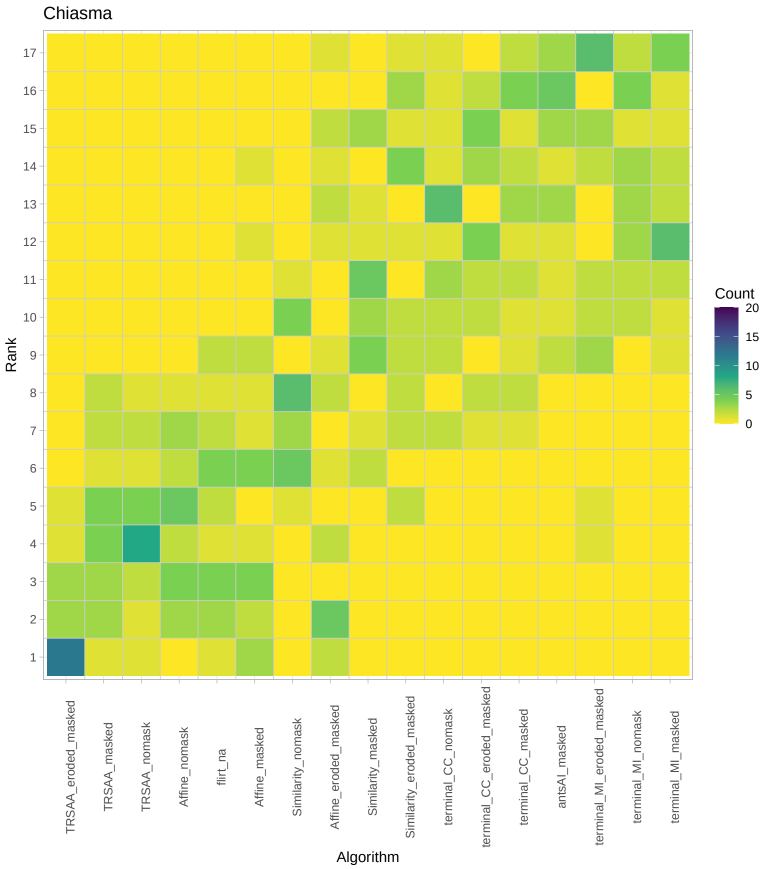

Chiasma
Rank
17
16
15
14
13
12
11
10
9
8
7
6
5
4
3
2
1
Count
20
15
10
5
0
TRSAA_eroded_masked
TRSAA_masked
TRSAA_nomask
Affine_nomask
flirt_na
Affine_masked
Similarity_nomask
Affine_eroded_masked
Similarity_masked
Similarity_eroded_masked
terminal_CC_nomask
terminal_CC_eroded_masked
terminal_CC_masked
antsAI_masked
terminal_MI_eroded_masked
terminal_MI_nomask
terminal_MI_masked
Algorithm

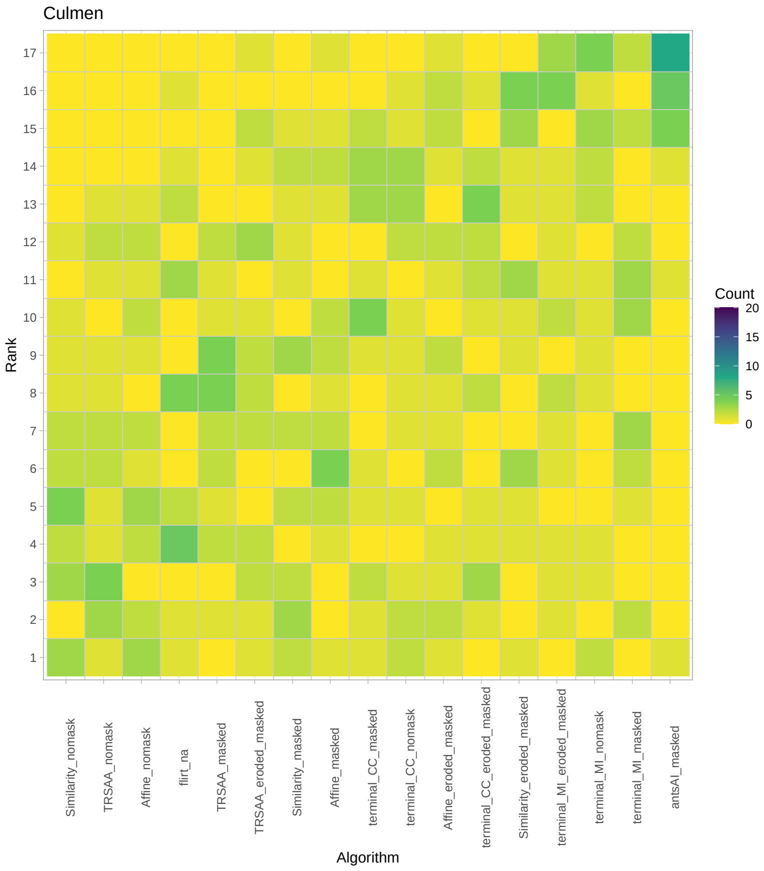

Culmen
Rank
17
16
15
14
13
12
11
10
9
8
7
6
5
4
3
2
1
Similarity_nomask
TRSAA_nomask
Affine_nomask
flirt_na
TRSAA_masked
TRSAA_eroded_masked
Similarity_masked
Affine_masked
terminal_CC_masked
terminal_CC_nomask
Affine_eroded_masked
terminal_CC_eroded_masked
Similarity_eroded_masked
terminal_MI_eroded_masked
terminal_MI_nomask
terminal_MI_masked
antsAI_masked
Algorithm
Count
20
15
10
5
0

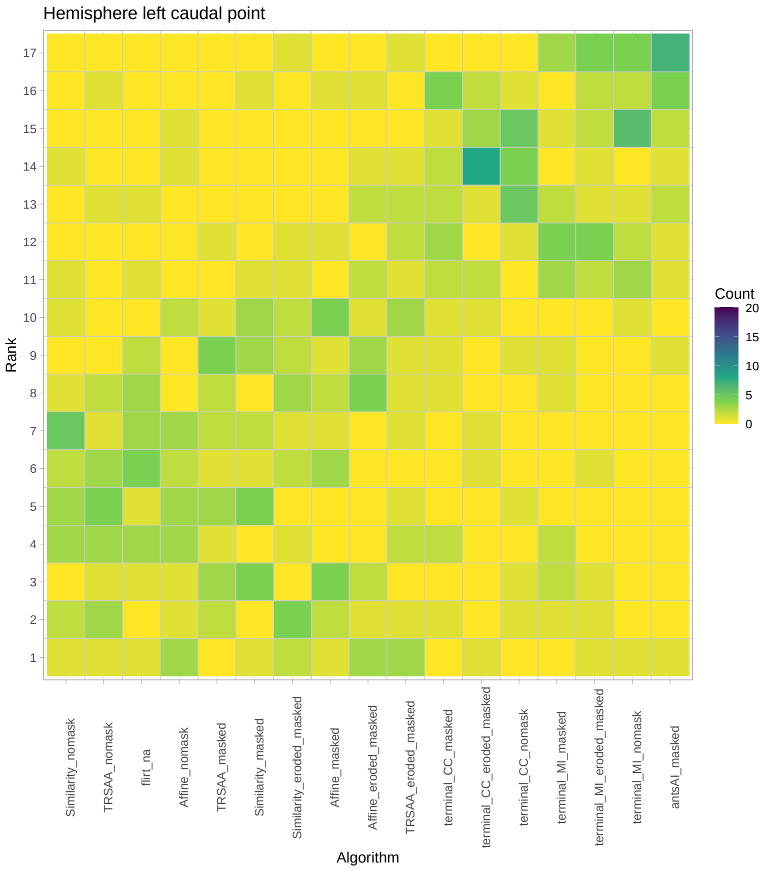
Hemisphere left caudal point
Rank
17
16
15
14
13
12
11
10
9
8
7
6
5
4
3
2
1
Count
20
15
10
5
0
Similarity_nomask
TRSAA_nomask
flirt_na
Affine_nomask
TRSAA_masked
Similarity_masked
Similarity_eroded_masked
Affine_masked
Affine_eroded_masked
TRSAA_eroded_masked
terminal_CC_masked
terminal_CC_eroded_masked
terminal_CC_nomask
terminal_MI_masked
terminal_MI_eroded_masked
terminal_MI_nomask
antsAI_masked
Algorithm

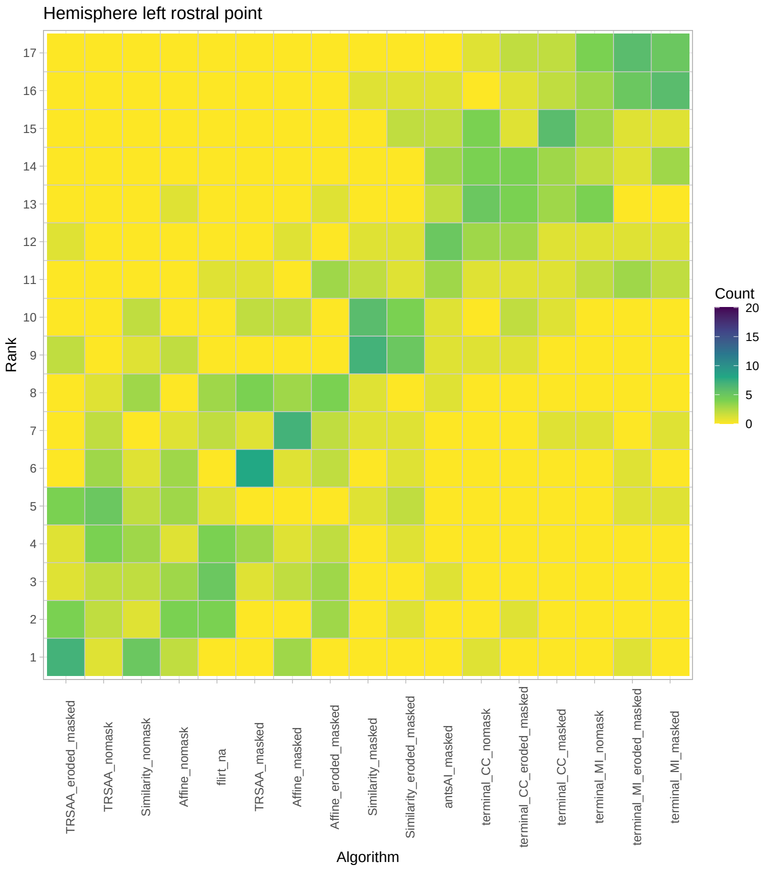
Hemisphere left rostral point
Rank
17
16
15
14
13
12
11
10
9
8
7
6
5
4
3
2
1
TRSAA_eroded_masked
TRSAA_nomask
Similarity_nomask
Affine_nomask
flirt_na
TRSAA_masked
Affine_masked
Affine_eroded_masked
Similarity_masked
Similarity_eroded_masked
antsAI_masked
terminal_CC_nomask
terminal_CC_eroded_masked
terminal_CC_masked
terminal_MI_nomask
terminal_MI_eroded_masked
terminal_MI_masked
Algorithm
Count
20
15
10
5
0

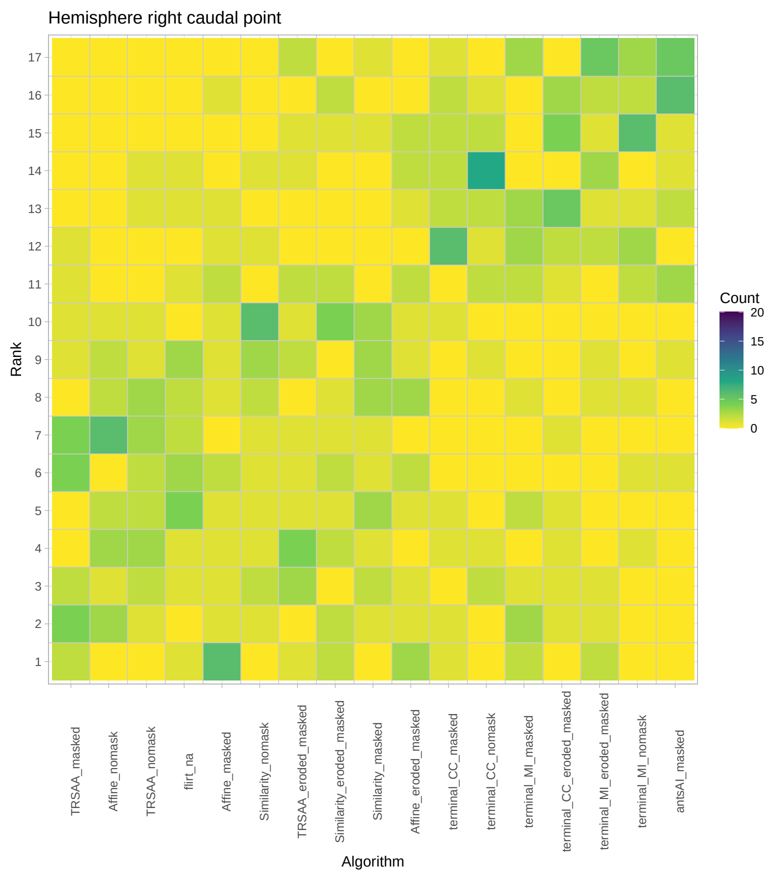

Hemisphere right caudal point
Rank
17
16
15
14
13
12
11
10
9
8
7
6
5
4
3
2
1
TRSAA_masked
Affine_nomask
TRSAA_nomask
flirt_na
Affine_masked
Similarity_nomask
TRSAA_eroded_masked
Similarity_eroded_masked
Similarity_masked
Affine_eroded_masked
terminal_CC_masked
terminal_CC_nomask
terminal_MI_masked
terminal_CC_eroded_masked
terminal_MI_eroded_masked
terminal_MI_nomask
antsAI_masked
Algorithm
Count
20
15
10
5
0

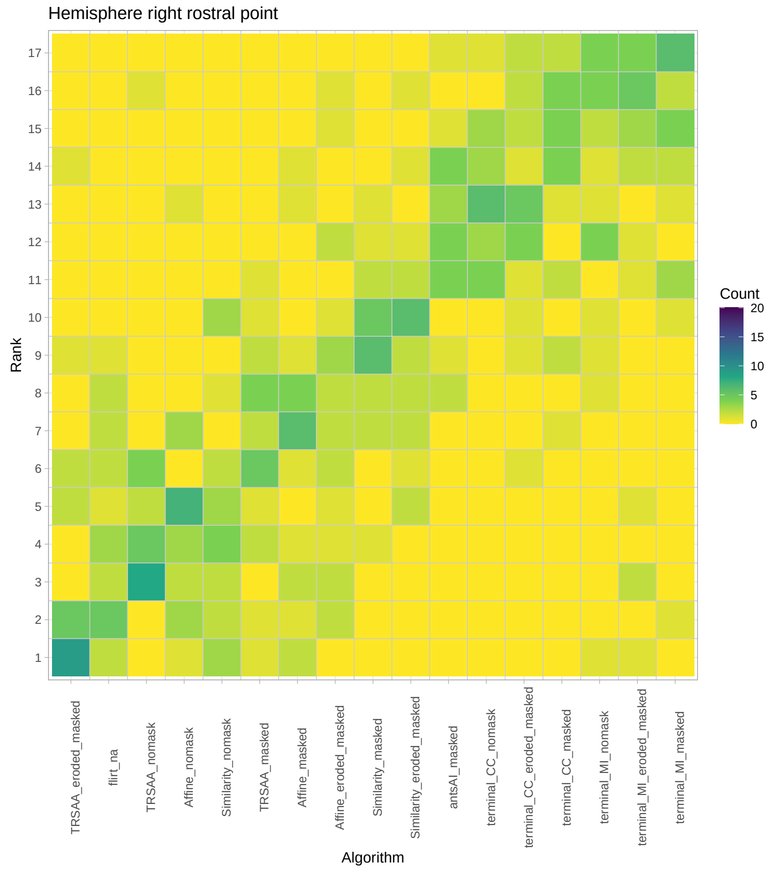
Hemisphere right rostral point
Rank
17
16
15
14
13
12
11
10
9
8
7
6
5
4
3
2
1
TRSAA_eroded_masked
flirt_na
TRSAA_nomask
Affine_nomask
Similarity_nomask
TRSAA_masked
Affine_masked
Affine_eroded_masked
Similarity_masked
Similarity_eroded_masked
antsAI_masked
terminal_CC_nomask
terminal_CC_eroded_masked
terminal_CC_masked
terminal_MI_nomask
terminal_MI_eroded_masked
terminal_MI_masked
Algorithm
Count
20
15
10
5
0

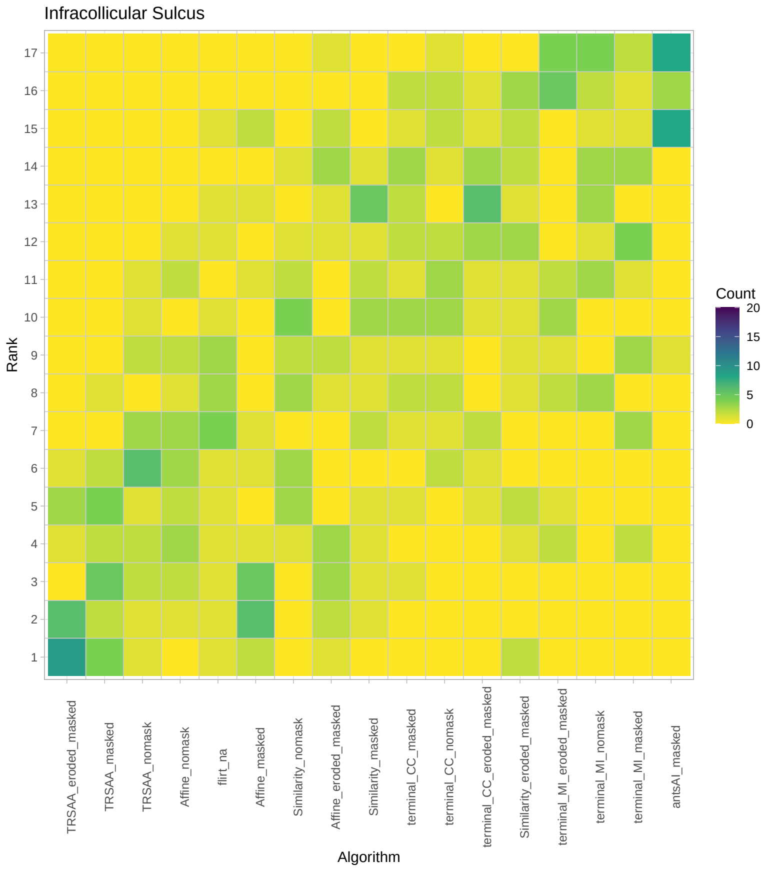
Infracollicular Sulcus
Rank
17
16
15
14
13
12
11
10
9
8
7
6
5
4
3
2
1
TRSAA_eroded_masked
TRSAA_masked
TRSAA_nomask
Affine_nomask
flirt_na
Affine_masked
Similarity_nomask
Affine_eroded_masked
Similarity_masked
terminal_CC_masked
terminal_CC_nomask
terminal_CC_eroded_masked
Similarity_eroded_masked
terminal_MI_eroded_masked
terminal_MI_nomask
terminal_MI_masked
antsAI_masked
Algorithm
Count
20
15
10
5
0

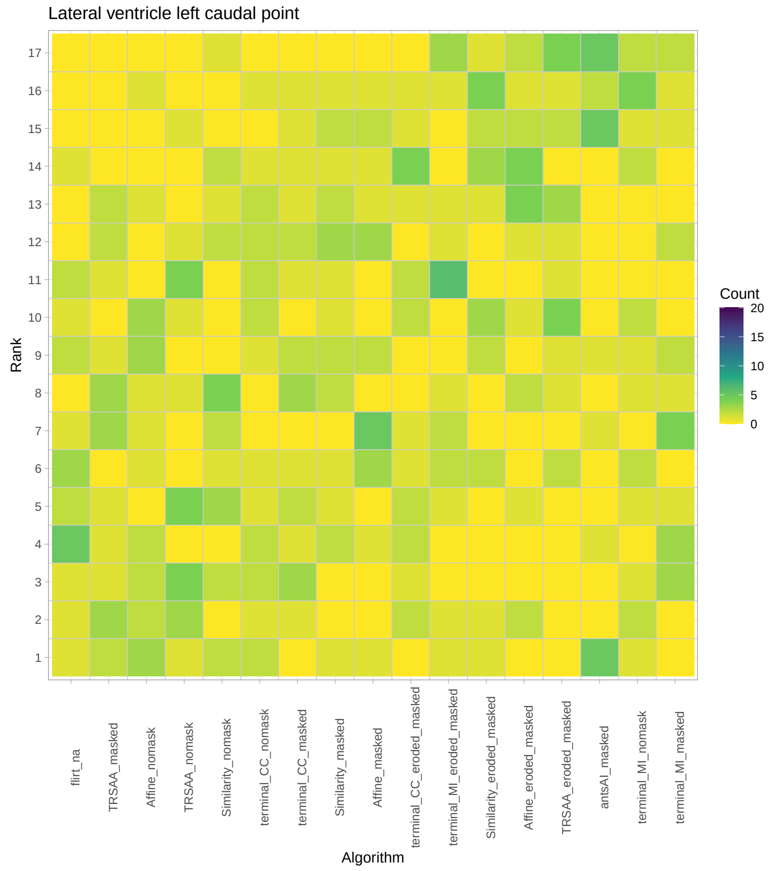
Lateral ventricle left caudal point
Rank
17
16
15
14
13
12
11
10
9
8
7
6
5
4
3
2
1
Count
20
15
10
5
0
flirt_na
TRSAA_masked
Affine_nomask
TRSAA_nomask
Similarity_nomask
terminal_CC_nomask
terminal_CC_masked
Similarity_masked
Affine_masked
terminal_CC_eroded_masked
terminal_MI_eroded_masked
Similarity_eroded_masked
Affine_eroded_masked
TRSAA_eroded_masked
antsAI_masked
terminal_MI_nomask
terminal_MI_masked
Algorithm

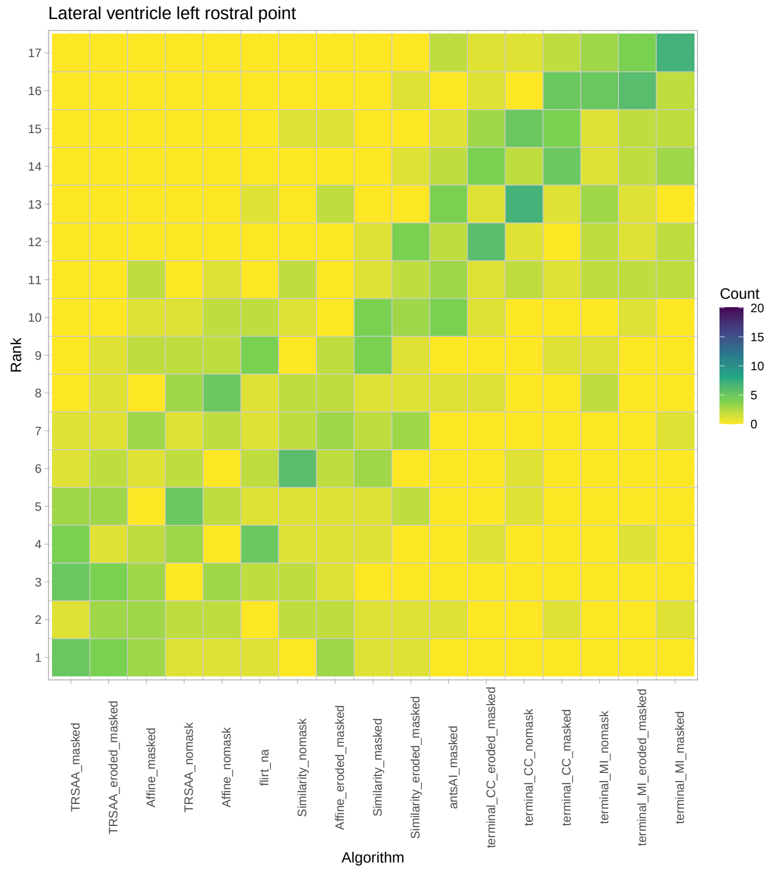

Lateral ventricle left rostral point
Rank
17
16
15
14
13
12
11
10
9
8
7
6
5
4
3
2
1
Count
20
15
10
5
0
TRSAA_masked
TRSAA_eroded_masked
Affine_masked
TRSAA_nomask
Affine_nomask
flirt_na
Similarity_nomask
Affine_eroded_masked
Similarity_masked
Similarity_eroded_masked
antsAI_masked
terminal_CC_eroded_masked
terminal_CC_nomask
terminal_CC_masked
terminal_MI_nomask
terminal_MI_eroded_masked
terminal_MI_masked
Algorithm

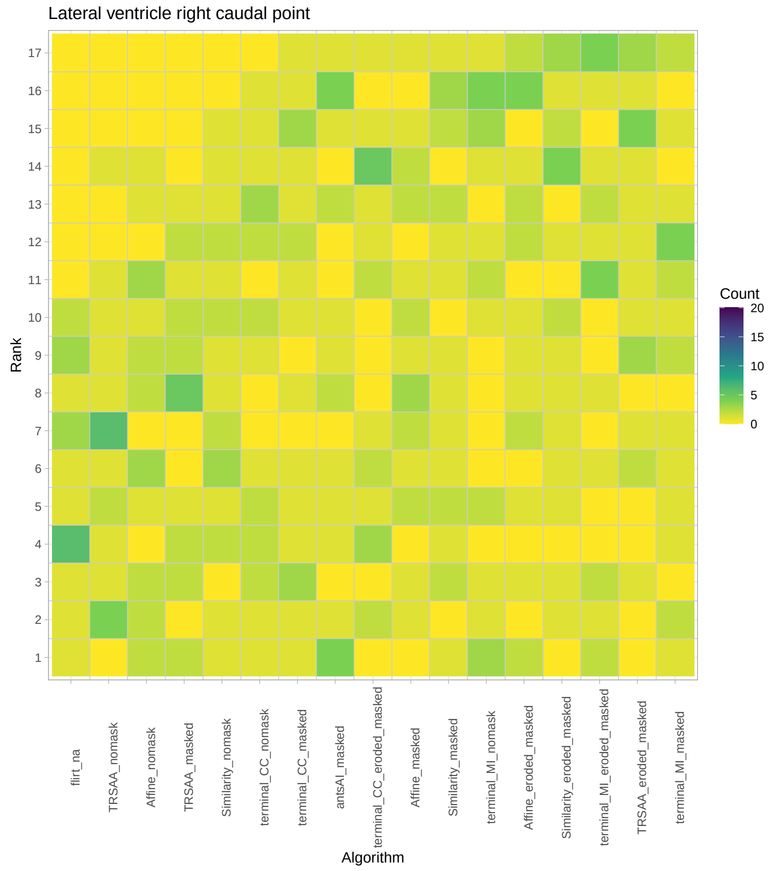
Lateral ventricle right caudal point
Rank
17
16
15
14
13
12
11
10
9
8
7
6
5
4
3
2
1
Count
20
15
10
5
0
flirt_na
TRSAA_nomask
Affine_nomask
TRSAA_masked
Similarity_nomask
terminal_CC_nomask
terminal_CC_masked
antsAI_masked
terminal_CC_eroded_masked
Affine_masked
Similarity_masked
terminal_MI_nomask
Affine_eroded_masked
Similarity_eroded_masked
terminal_MI_eroded_masked
TRSAA_eroded_masked
terminal_MI_masked
Algorithm

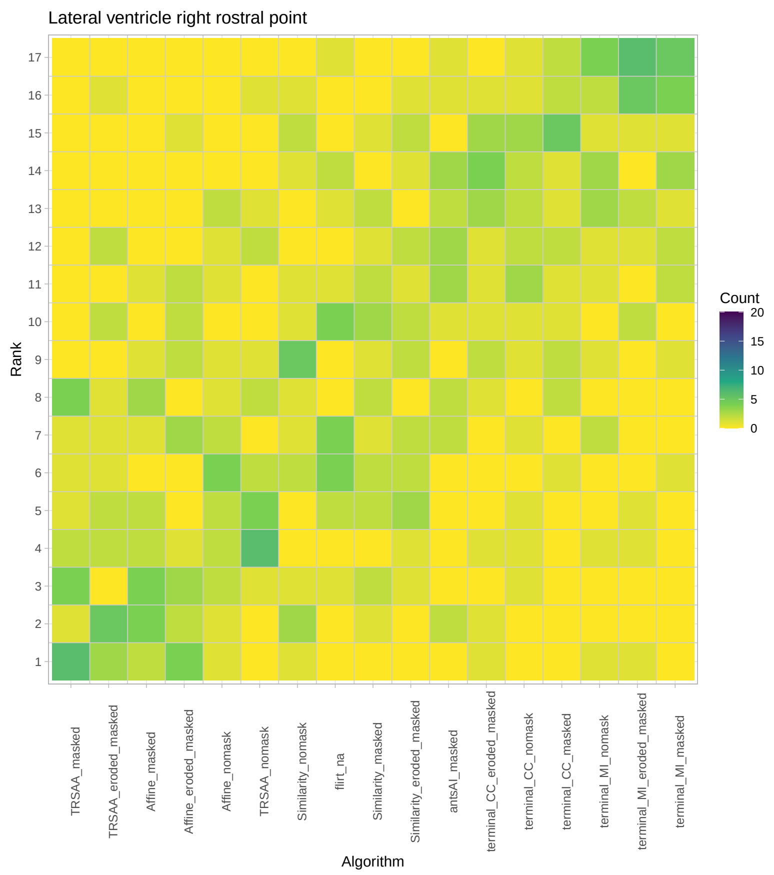

Lateral ventricle right rostral point
Rank
Algorithm
Count
20
15
10
5
0
17
16
15
14
13
12
11
10
9
8
7
6
5
4
3
2
1
TRSAA_masked
TRSAA_eroded_masked
Affine_masked
Affine_eroded_masked
Affine_nomask
TRSAA_nomask
Similarity_nomask
flirt_na
Similarity_masked
Similarity_eroded_masked
antsAI_masked
terminal_CC_eroded_masked
terminal_CC_nomask
terminal_CC_masked
terminal_MI_nomask
terminal_MI_eroded_masked
terminal_MI_masked

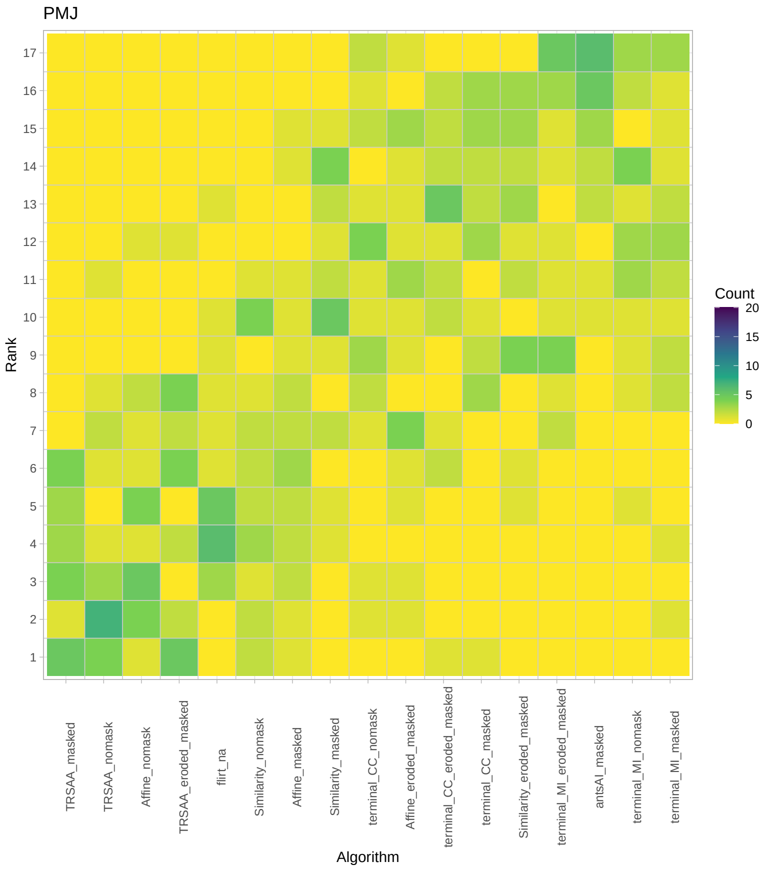
PMJ
Rank
Algorithm
Count
20
15
10
5
0
17
16
15
14
13
12
11
10
9
8
7
6
5
4
3
2
1
TRSAA_masked
TRSAA_nomask
Affine_nomask
TRSAA_eroded_masked
flirt_na
Similarity_nomask
Affine_masked
Similarity_masked
terminal_CC_nomask
Affine_eroded_masked
terminal_CC_eroded_masked
terminal_CC_masked
Similarity_eroded_masked
terminal_MI_eroded_masked
antsAI_masked
terminal_MI_nomask
terminal_MI_masked

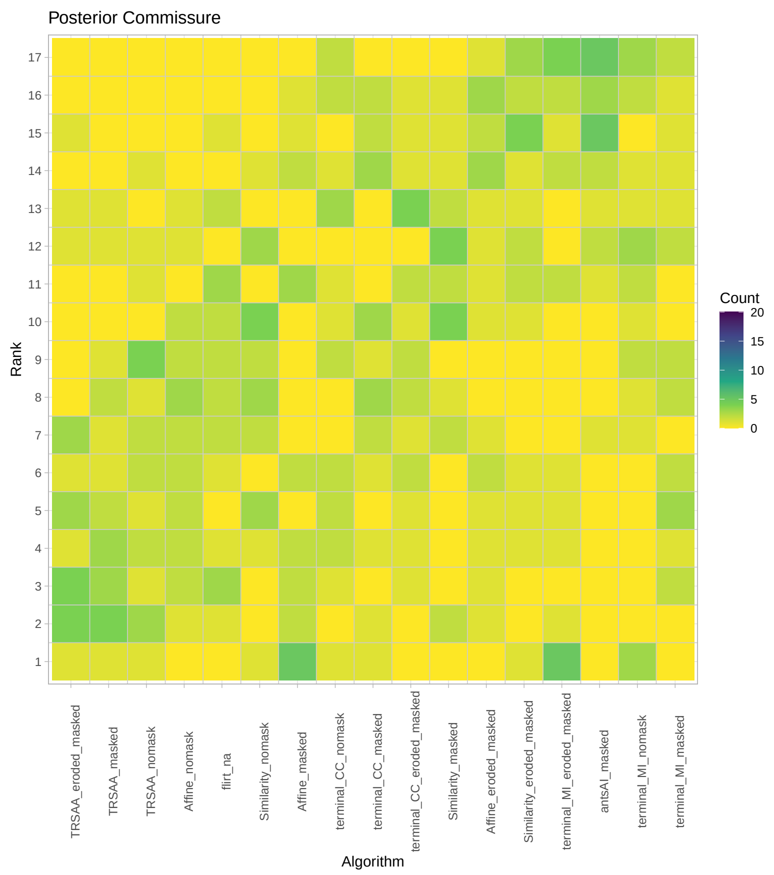
Posterior Commissure
Rank
Algorithm
Count
20
15
10
5
0
TRSAA_eroded_masked
TRSAA_masked
TRSAA_nomask
Affine_nomask
flirt_na
Similarity_nomask
Affine_masked
terminal_CC_nomask
terminal_CC_masked
terminal_CC_eroded_masked
Similarity_masked
Affine_eroded_masked
Similarity_eroded_masked
terminal_MI_eroded_masked
antsAI_masked
terminal_MI_nomask
terminal_MI_masked

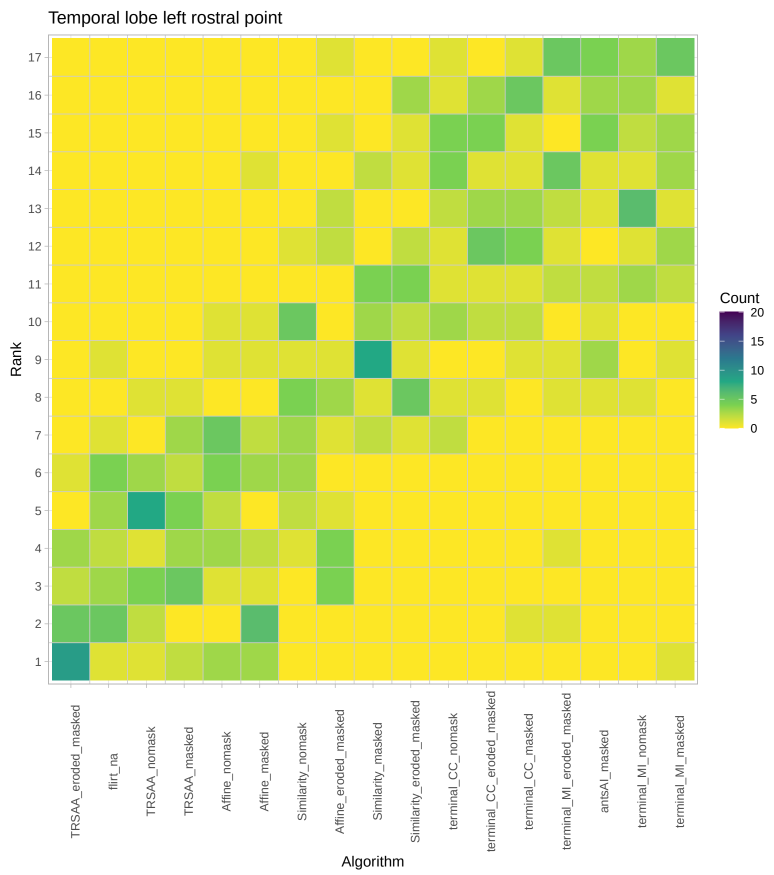
Temporal lobe left rostral point
Rank
17
16
15
14
13
12
11
10
9
8
7
6
5
4
3
2
1
Count
20
15
10
5
0
TRSAA_eroded_masked
flirt_na
TRSAA_nomask
TRSAA_masked
Affine_nomask
Affine_masked
Similarity_nomask
Affine_eroded_masked
Similarity_masked
Similarity_eroded_masked
terminal_CC_nomask
terminal_CC_eroded_masked
terminal_CC_masked
terminal_MI_eroded_masked
antsAI_masked
terminal_MI_nomask
terminal_MI_masked
Algorithm

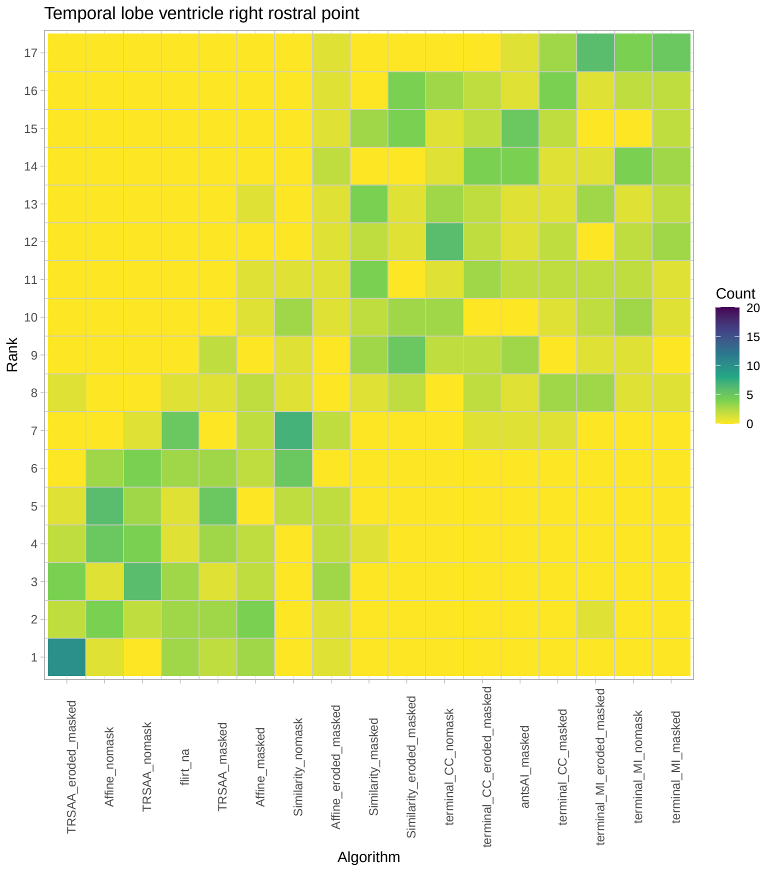
Temporal lobe ventricle right rostral point
Rank
17
16
15
14
13
12
11
10
9
8
7
6
5
4
3
2
1
Algorithm
TRSAA_eroded_masked
Affine_nomask
TRSAA_nomask
flirt_na
TRSAA_masked
Affine_masked
Similarity_nomask
Affine_eroded_masked
Similarity_masked
Similarity_eroded_masked
terminal_CC_nomask
terminal_CC_eroded_masked
antsAI_masked
terminal_CC_masked
terminal_MI_eroded_masked
terminal_MI_nomask
terminal_MI_masked
Count
20
15
10
5
0

3 Visualization of ranking stability

3.1 *Blob plot* for visualizing ranking stability based on bootstrap sampling

Algorithms are color-coded, and the area of each blob at position $(A_i, \text{rank } j)$ is proportional to the relative frequency A_i achieved rank j across $b = 1000$ bootstrap samples. The median rank for each algorithm is indicated by a black cross. 95% bootstrap intervals across bootstrap samples are indicated by black lines.

```
## Warning: `stat(prop * 100)` was deprecated in ggplot2 3.4.0.
## i Please use `after_stat(prop * 100)` instead.
## i The deprecated feature was likely used in the challengeR package.
##   Please report the issue to the authors.
```

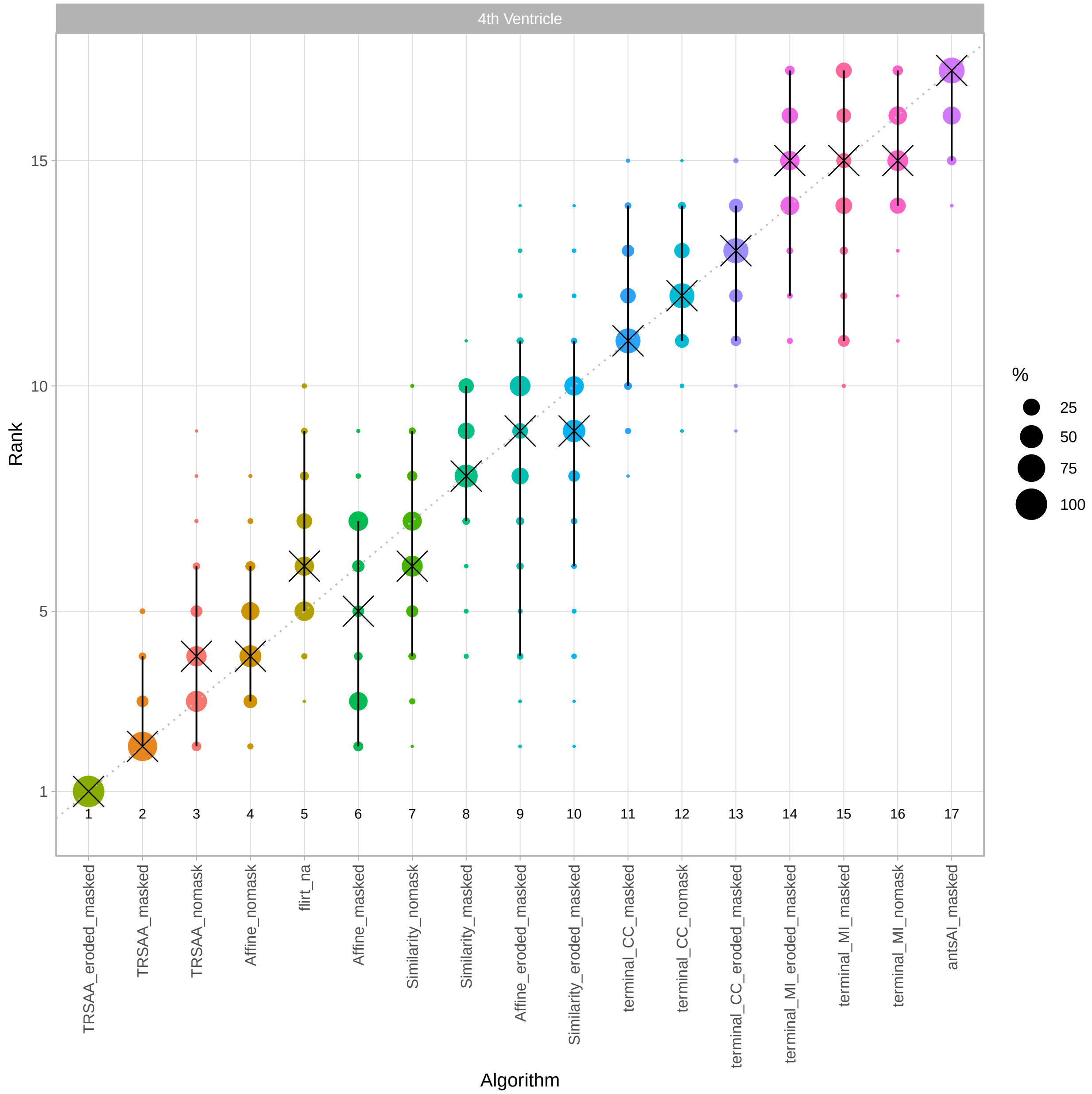

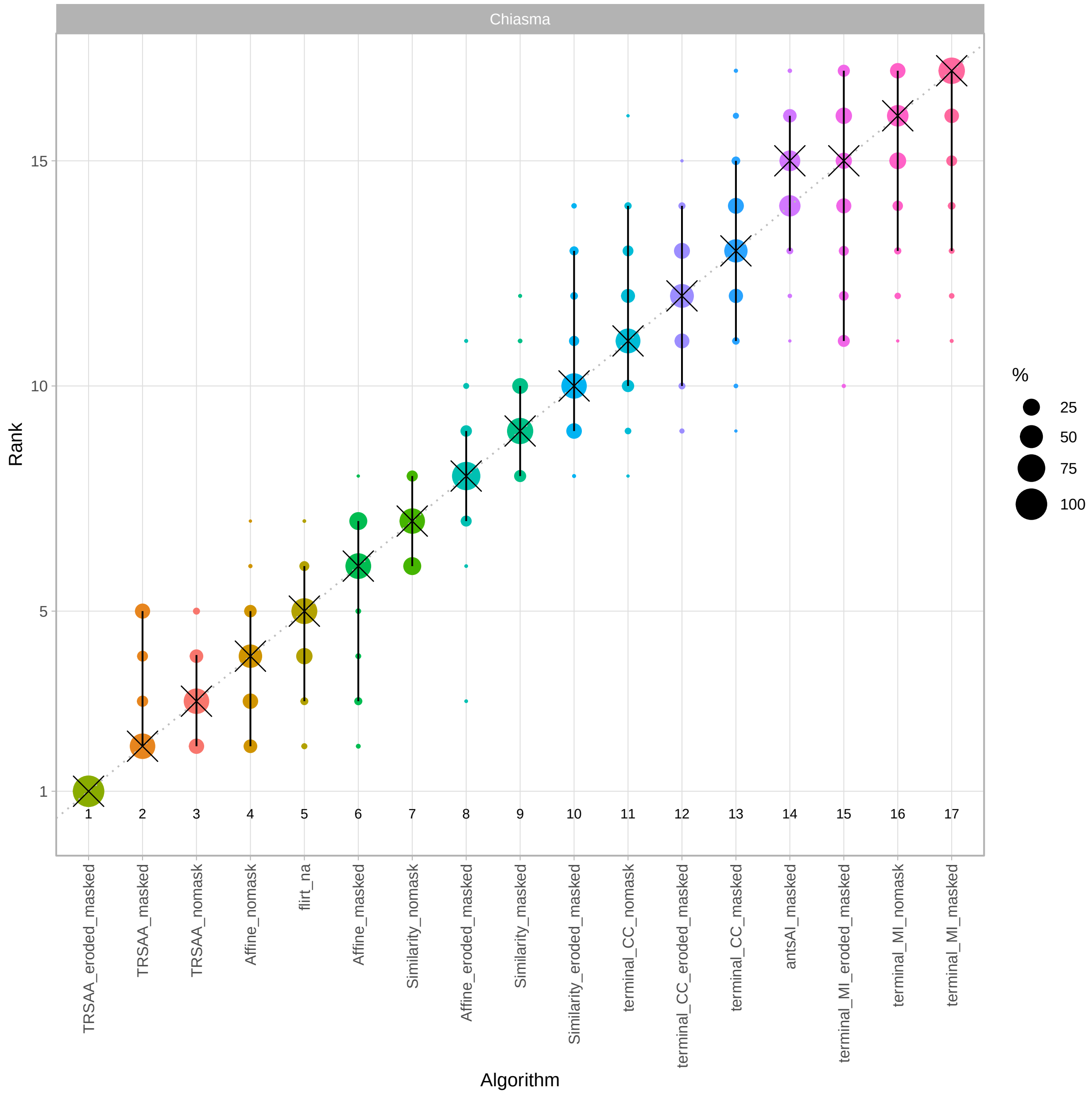
Chiasma
Rank
15
10
5
1
1
2
3
4
5
6
7
8
9
10
11
12
13
14
15
16
17
TRSAA_eroded_masked
TRSAA_masked
TRSAA_nomask
Affine_nomask
flirt_na
Affine_masked
Similarity_nomask
Affine_eroded_masked
Similarity_masked
Similarity_eroded_masked
terminal_CC_nomask
terminal_CC_eroded_masked
terminal_CC_masked
antsAI_masked
terminal_MI_eroded_masked
terminal_MI_nomask
terminal_MI_masked
Algorithm
%
25
50
75
100

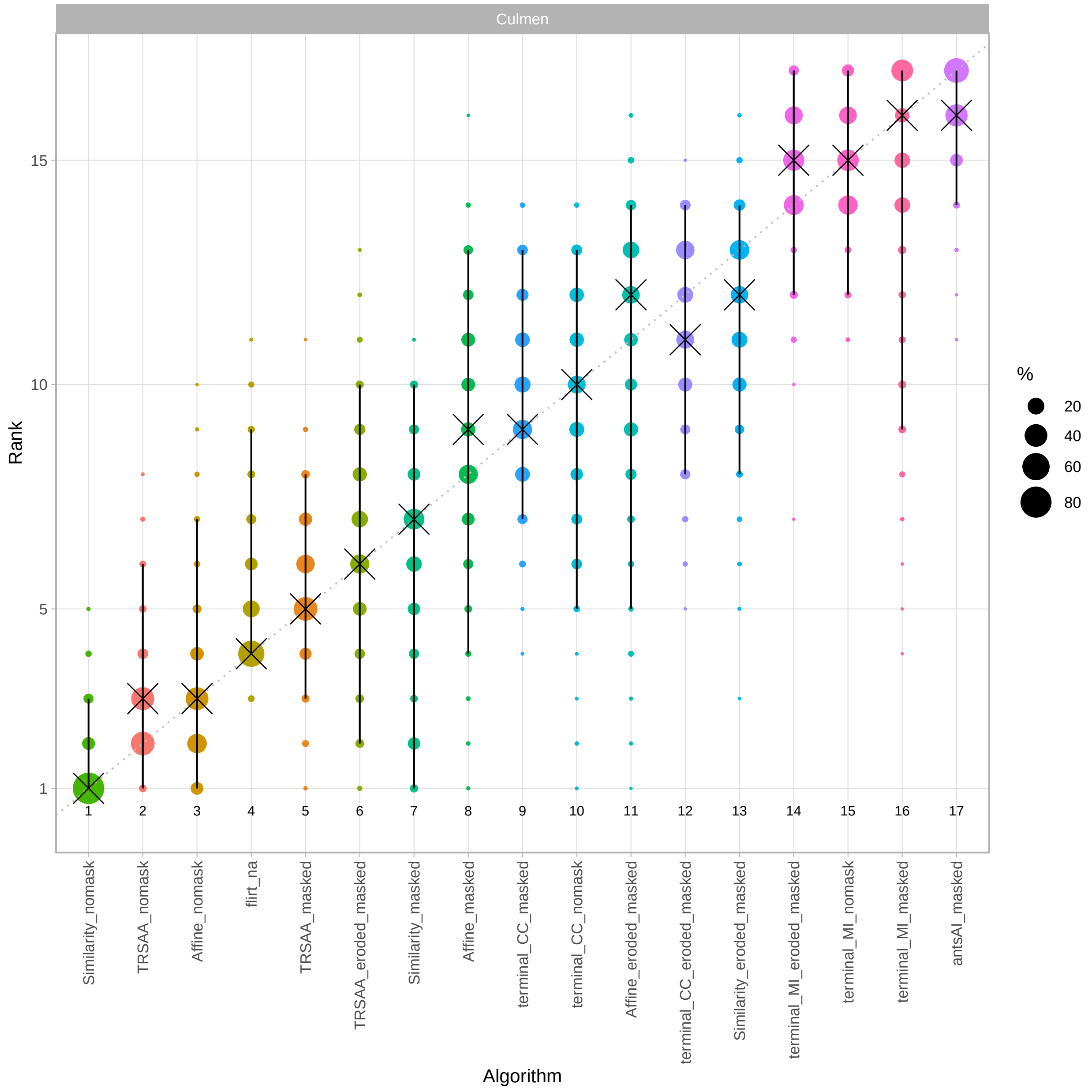

Culmen
Rank
Algorithm
%
20
40
60
80
1
5
10
15
1
2
3
4
5
6
7
8
9
10
11
12
13
14
15
16
17
Similarity_nomask
TRSAA_nomask
Affine_nomask
flirt_na
TRSAA_masked
TRSAA_eroded_masked
Similarity_masked
Affine_masked
terminal_CC_masked
terminal_CC_nomask
Affine_eroded_masked
terminal_CC_eroded_masked
Similarity_eroded_masked
terminal_MI_eroded_masked
terminal_MI_nomask
terminal_MI_masked
antsAI_masked

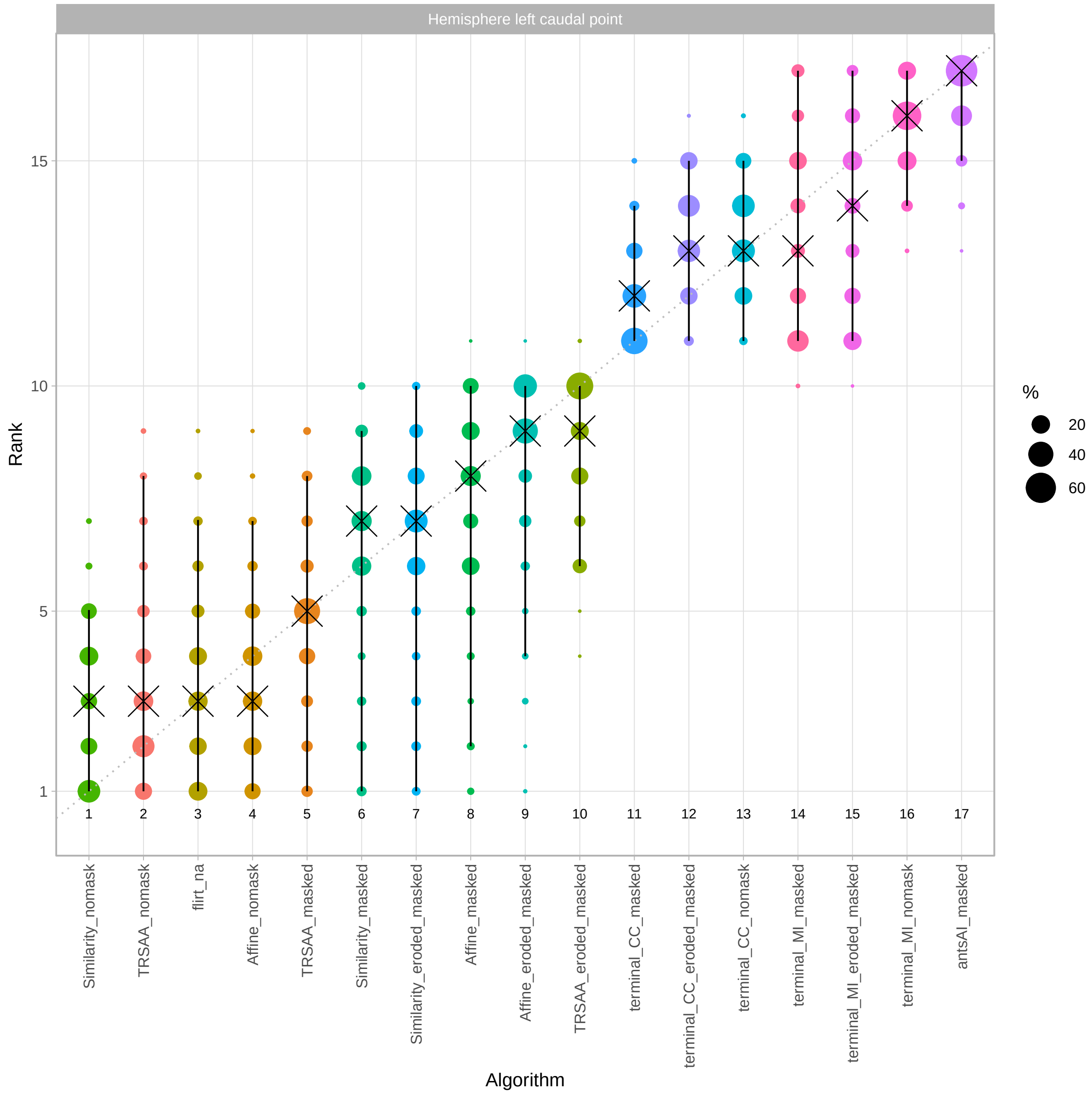

Hemisphere left caudal point
Rank
15
10
5
1
1
2
3
4
5
6
7
8
9
10
11
12
13
14
15
16
17
Similarity_nomask
TRSAA_nomask
flirt_na
Affine_nomask
TRSAA_masked
Similarity_masked
Similarity_eroded_masked
Affine_masked
Affine_eroded_masked
TRSAA_eroded_masked
terminal_CC_masked
terminal_CC_eroded_masked
terminal_CC_nomask
terminal_MI_masked
terminal_MI_eroded_masked
terminal_MI_nomask
antsAI_masked
Algorithm
%
20
40
60

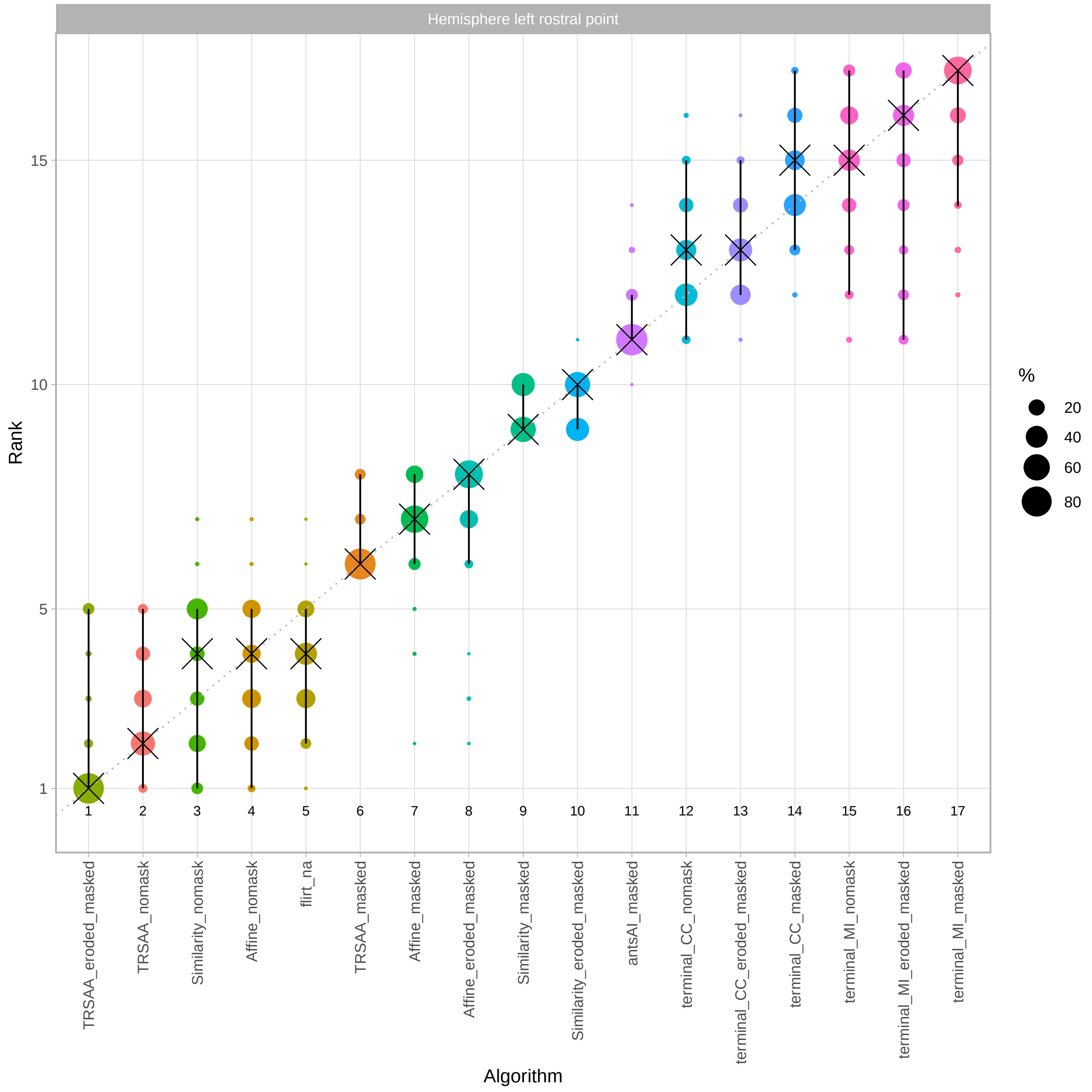
Hemisphere left rostral point
Rank
15
10
5
1
1
2
3
4
5
6
7
8
9
10
11
12
13
14
15
16
17
%
20
40
60
80
TRSAA_eroded_masked
TRSAA_nomask
Similarity_nomask
Affine_nomask
flirt_na
TRSAA_masked
Affine_masked
Affine_eroded_masked
Similarity_masked
Similarity_eroded_masked
antsAI_masked
terminal_CC_nomask
terminal_CC_eroded_masked
terminal_CC_masked
terminal_MI_nomask
terminal_MI_eroded_masked
terminal_MI_masked
Algorithm

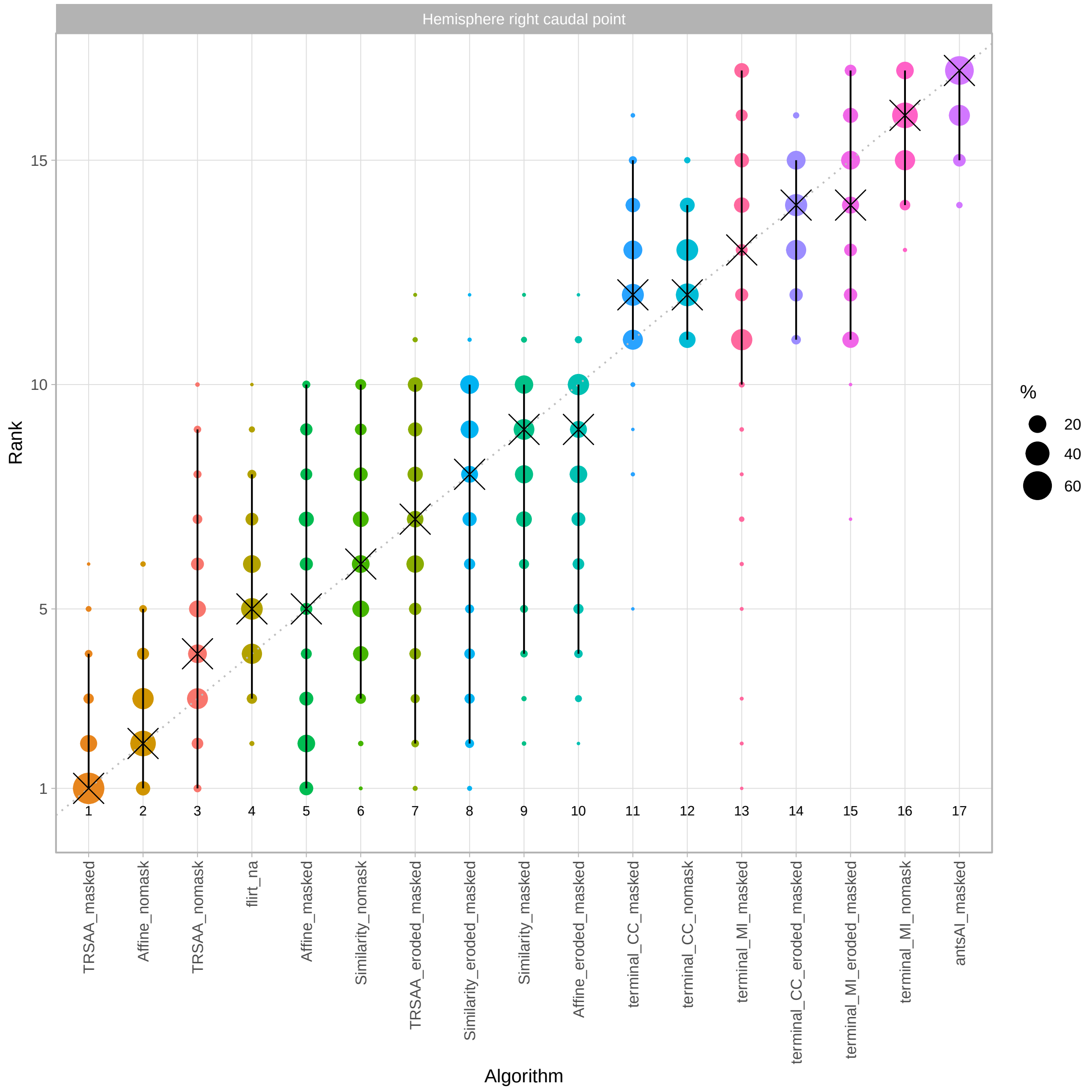

Hemisphere right caudal point
Rank
Algorithm
%
20
40
60
1
5
10
15
1
2
3
4
5
6
7
8
9
10
11
12
13
14
15
16
17
TRSAA_masked
Affine_nomask
TRSAA_nomask
flirt_na
Affine_masked
Similarity_nomask
TRSAA_eroded_masked
Similarity_eroded_masked
Similarity_masked
Affine_eroded_masked
terminal_CC_masked
terminal_CC_nomask
terminal_MI_masked
terminal_CC_eroded_masked
terminal_MI_eroded_masked
terminal_MI_nomask
antsAI_masked

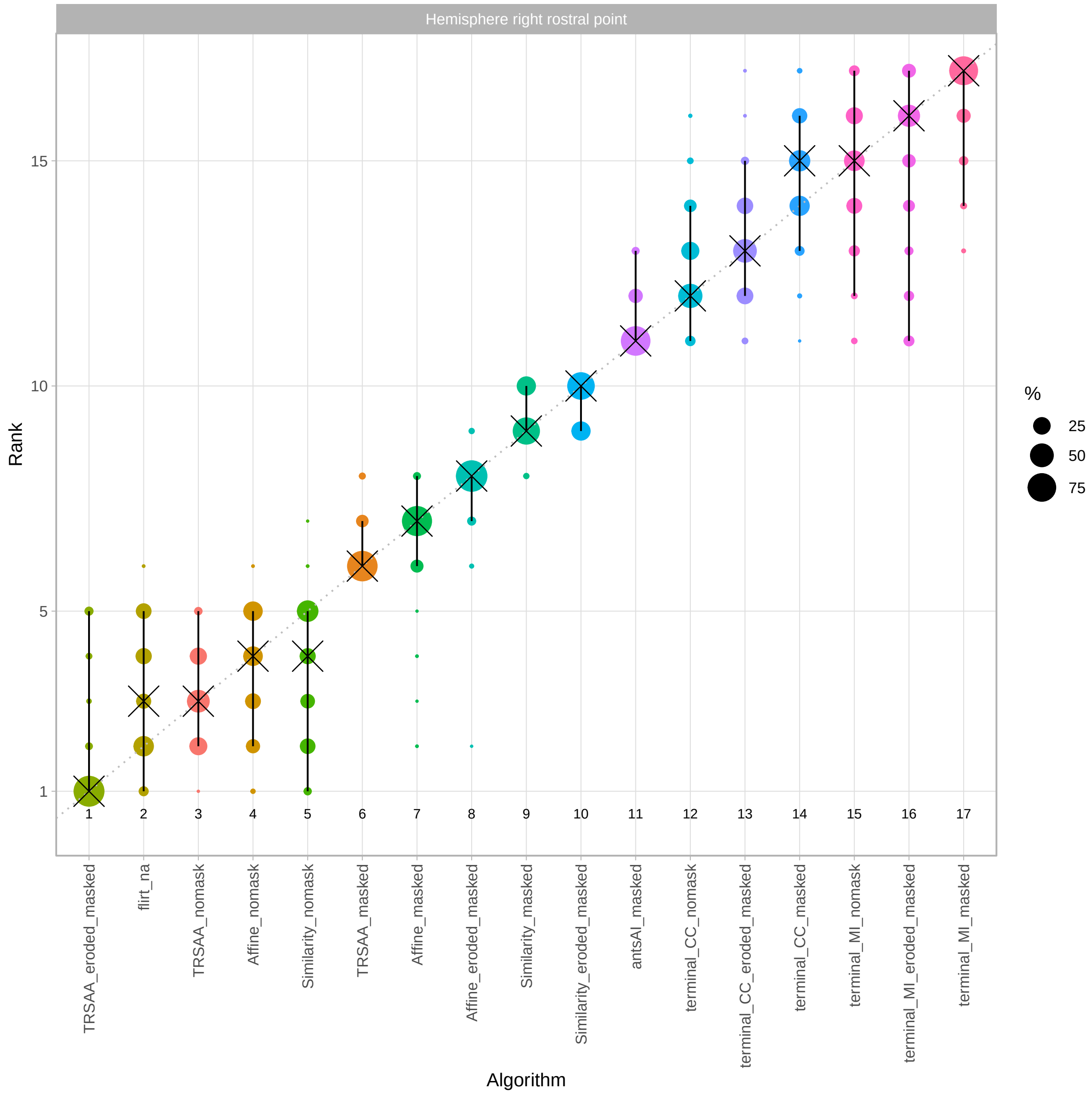

Hemisphere right rostral point
Rank
1
5
10
15
1
2
3
4
5
6
7
8
9
10
11
12
13
14
15
16
17
TRSAA_eroded_masked
flirt_na
TRSAA_nomask
Affine_nomask
Similarity_nomask
TRSAA_masked
Affine_masked
Affine_eroded_masked
Similarity_masked
Similarity_eroded_masked
antsAI_masked
terminal_CC_nomask
terminal_CC_eroded_masked
terminal_CC_masked
terminal_MI_nomask
terminal_MI_eroded_masked
terminal_MI_masked
Algorithm
%
25
50
75

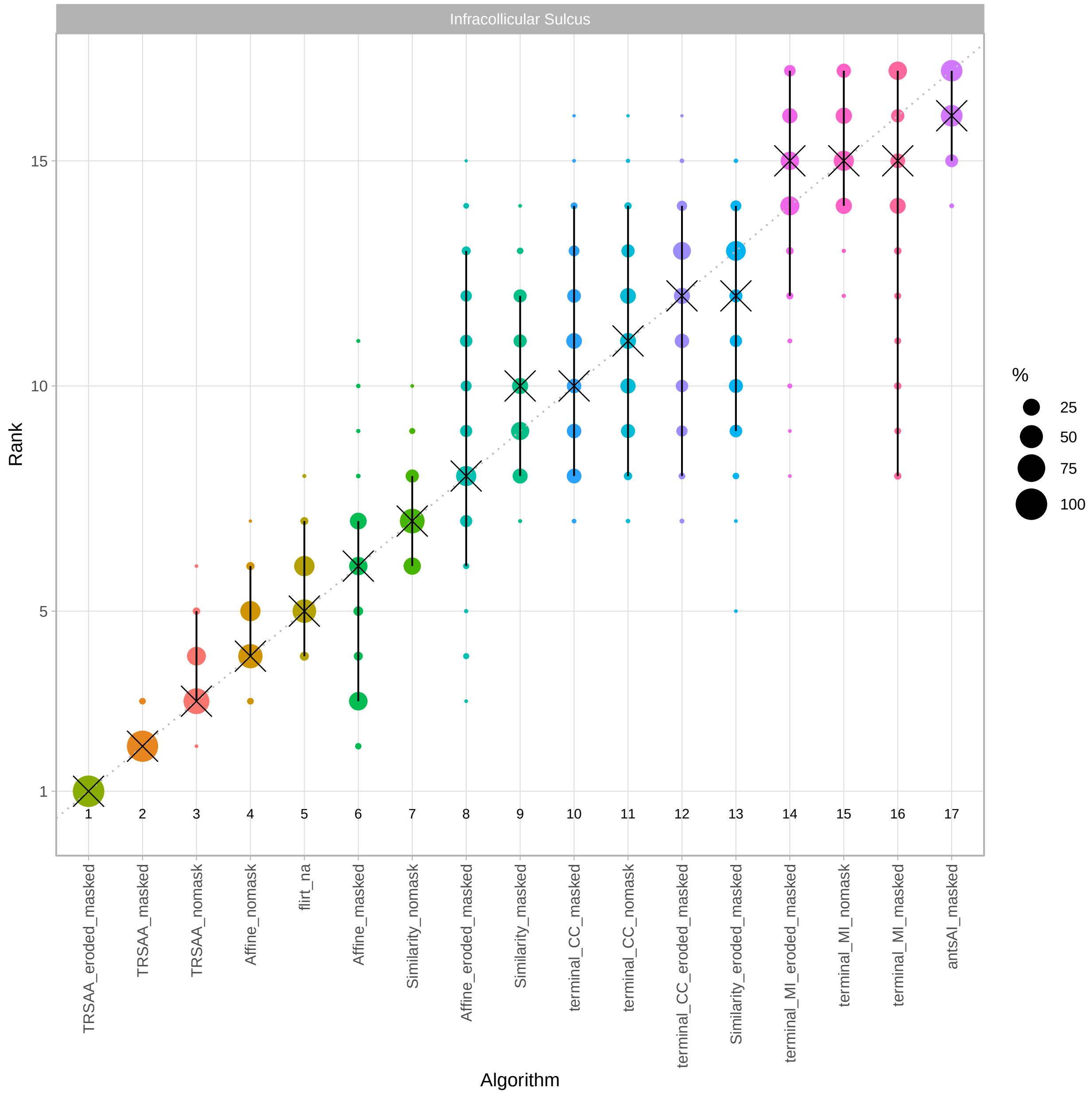

Infracollicular Sulcus
Rank
15
10
5
1
1
2
3
4
5
6
7
8
9
10
11
12
13
14
15
16
17
TRSAA_eroded_masked
TRSAA_masked
TRSAA_nomask
Affine_nomask
flirt_na
Affine_masked
Similarity_nomask
Affine_eroded_masked
Similarity_masked
terminal_CC_masked
terminal_CC_nomask
terminal_CC_eroded_masked
Similarity_eroded_masked
terminal_MI_eroded_masked
terminal_MI_nomask
terminal_MI_masked
antsAI_masked
Algorithm
%
25
50
75
100

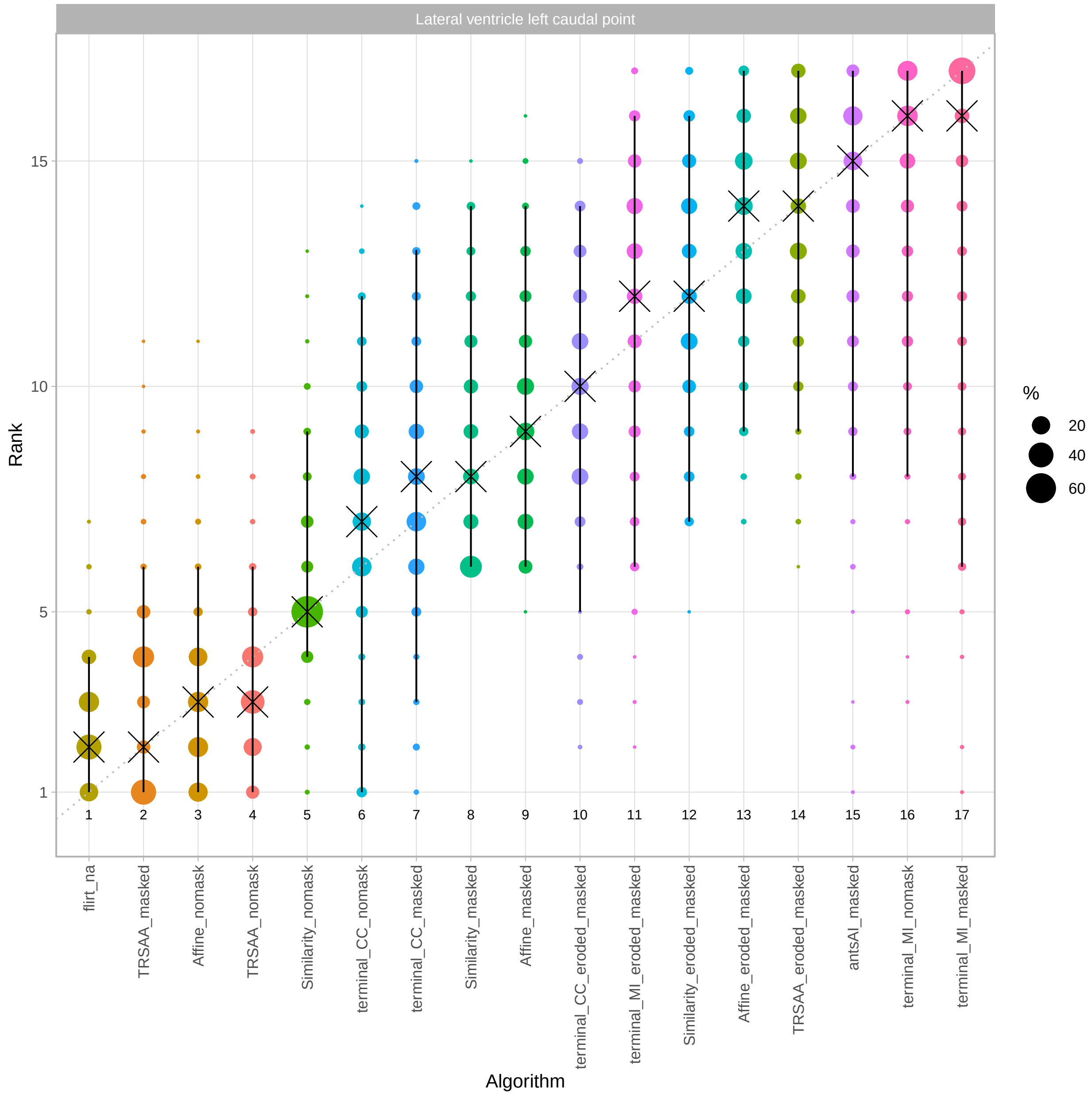
Lateral ventricle left caudal point
Rank
Algorithm
%
20
40
60
flirt_na
TRSAA_masked
Affine_nomask
TRSAA_nomask
Similarity_nomask
terminal_CC_nomask
terminal_CC_masked
Similarity_masked
Affine_masked
terminal_CC_eroded_masked
terminal_MI_eroded_masked
Similarity_eroded_masked
Affine_eroded_masked
TRSAA_eroded_masked
antsAI_masked
terminal_MI_nomask
terminal_MI_masked

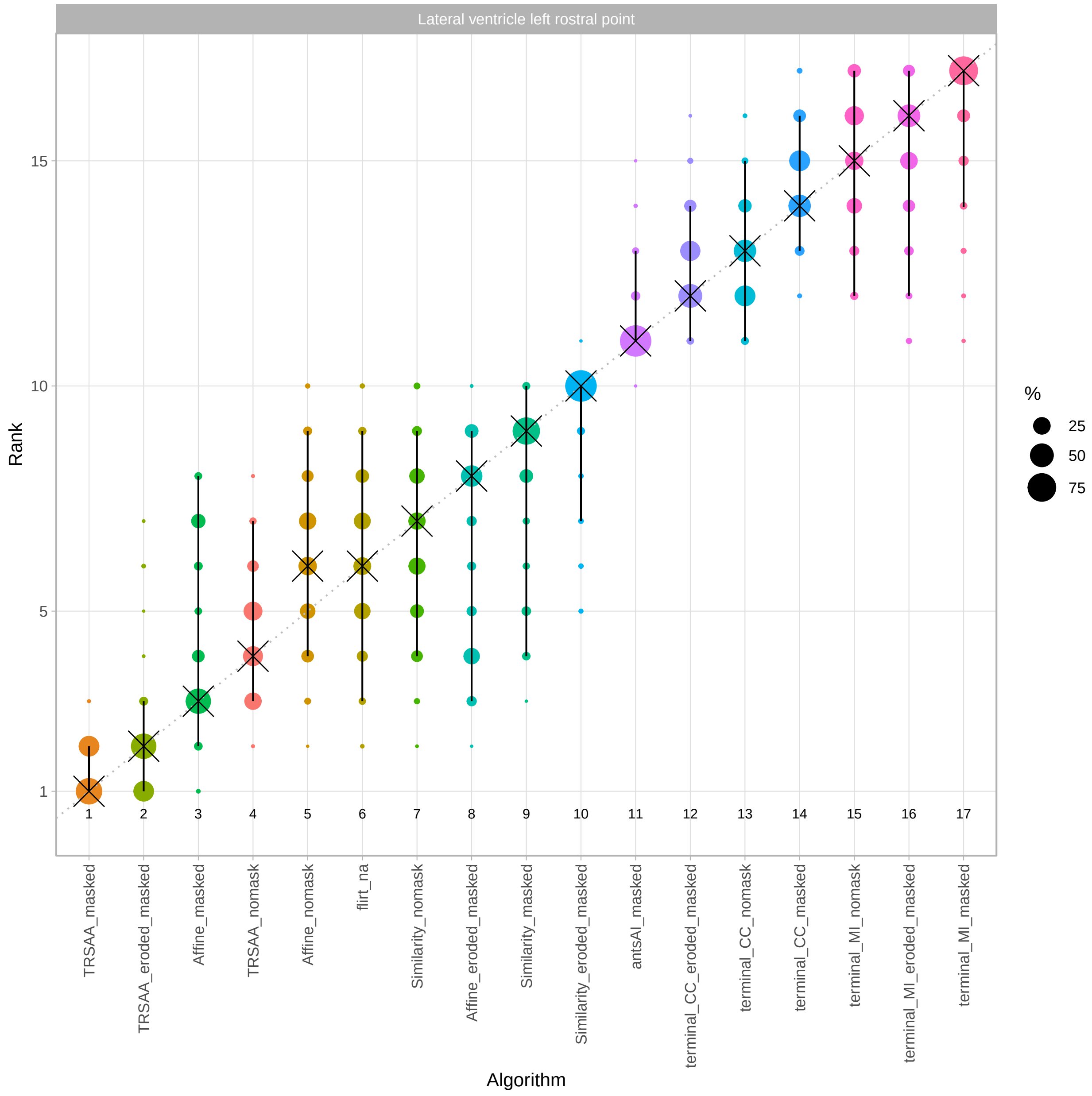
Lateral ventricle left rostral point
Rank
15
10
5
1
1
2
3
4
5
6
7
8
9
10
11
12
13
14
15
16
17
TRSAA_masked
TRSAA_eroded_masked
Affine_masked
TRSAA_nomask
Affine_nomask
flirt_na
Similarity_nomask
Affine_eroded_masked
Similarity_masked
Similarity_eroded_masked
antsAI_masked
terminal_CC_eroded_masked
terminal_CC_nomask
terminal_CC_masked
terminal_MI_nomask
terminal_MI_eroded_masked
terminal_MI_masked
Algorithm
%
25
50
75

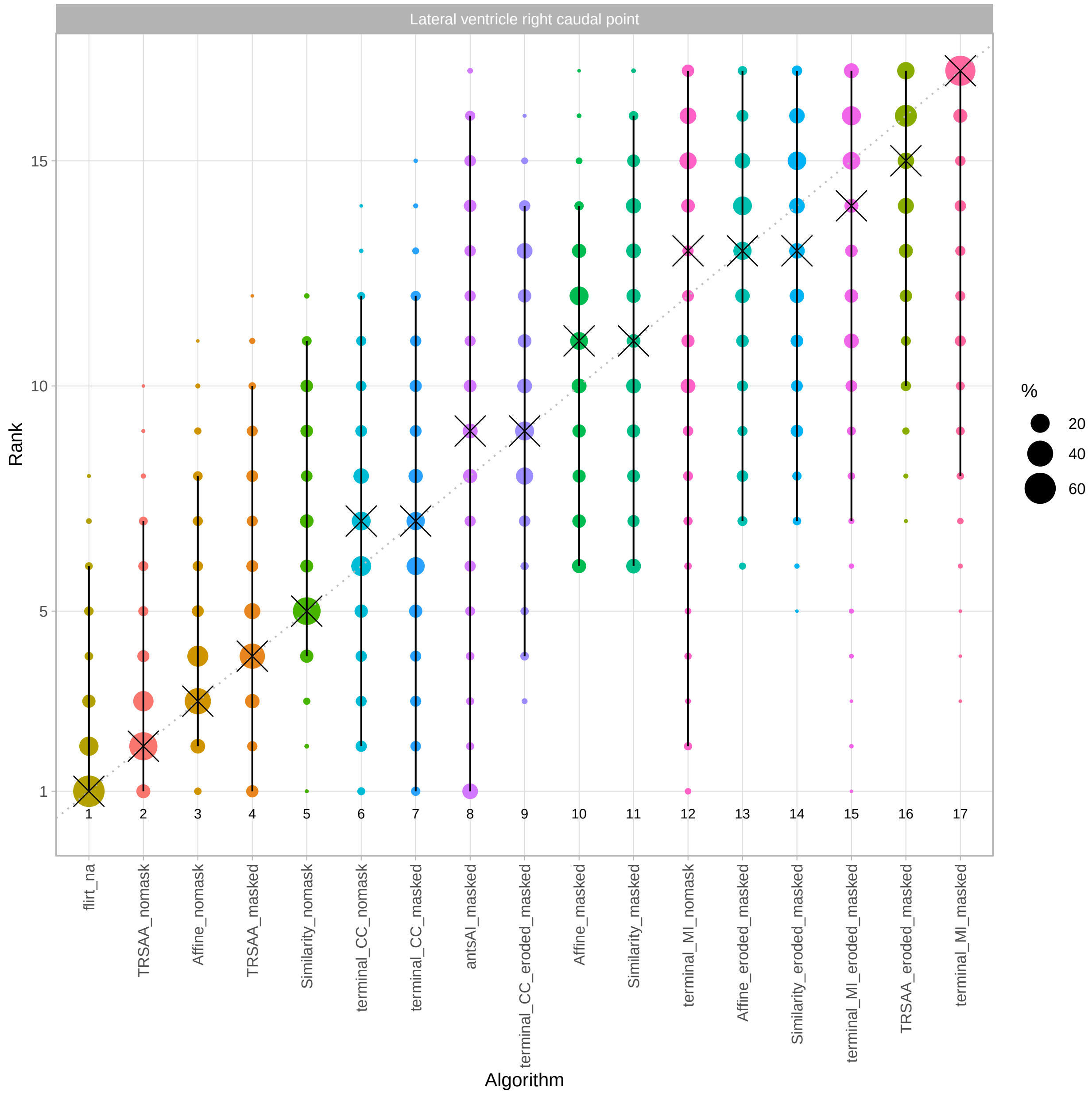
Lateral ventricle right caudal point
Rank
15
10
5
1
1
2
3
4
5
6
7
8
9
10
11
12
13
14
15
16
17
flirt_na
TRSAA_nomask
Affine_nomask
TRSAA_masked
Similarity_nomask
terminal_CC_nomask
terminal_CC_masked
antsAI_masked
terminal_CC_eroded_masked
Affine_masked
Similarity_masked
terminal_MI_nomask
Affine_eroded_masked
Similarity_eroded_masked
terminal_MI_eroded_masked
TRSAA_eroded_masked
terminal_MI_masked
Algorithm
%
20
40
60

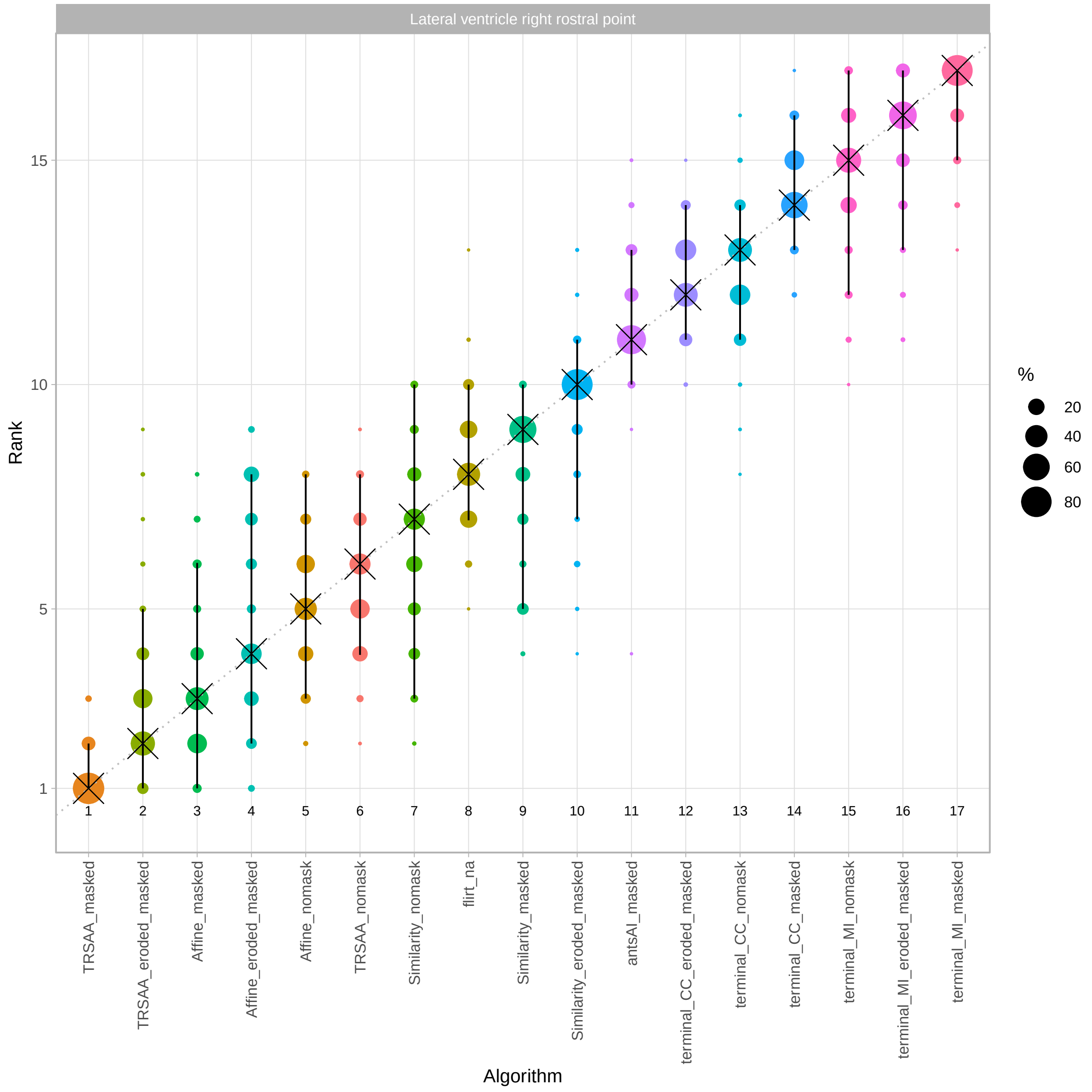
Lateral ventricle right rostral point
Rank
Algorithm
%
20
40
60
80
1
5
10
15
TRSAA_masked
TRSAA_eroded_masked
Affine_masked
Affine_eroded_masked
Affine_nomask
TRSAA_nomask
Similarity_nomask
flirt_na
Similarity_masked
Similarity_eroded_masked
antsAI_masked
terminal_CC_eroded_masked
terminal_CC_nomask
terminal_CC_masked
terminal_MI_nomask
terminal_MI_eroded_masked
terminal_MI_masked

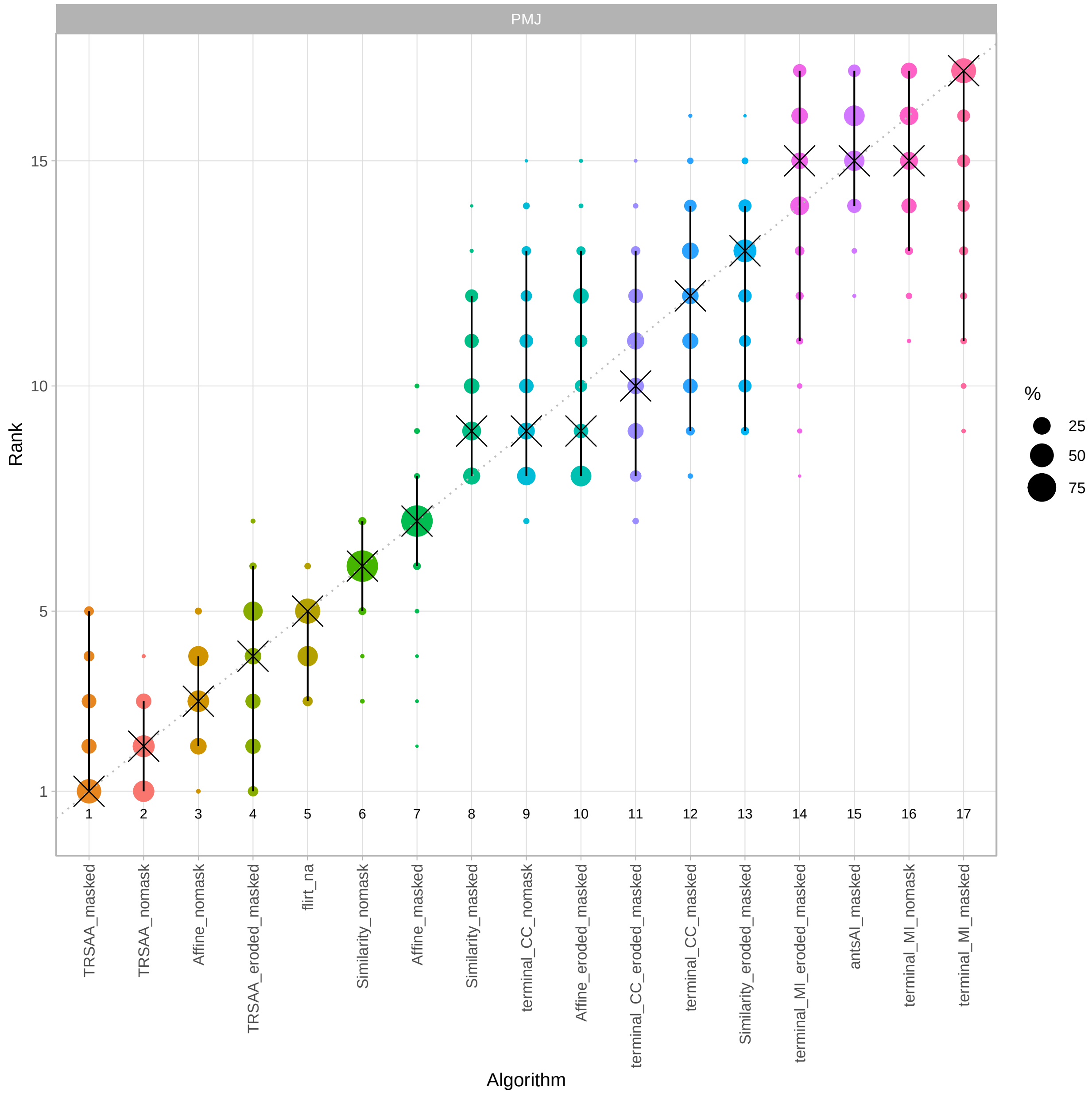

PMJ
Rank
15
10
5
1
1
2
3
4
5
6
7
8
9
10
11
12
13
14
15
16
17
TRSAA_masked
TRSAA_nomask
Affine_nomask
TRSAA_eroded_masked
flirt_na
Similarity_nomask
Affine_masked
Similarity_masked
terminal_CC_nomask
Affine_eroded_masked
terminal_CC_eroded_masked
terminal_CC_masked
Similarity_eroded_masked
terminal_MI_eroded_masked
antsAI_masked
terminal_MI_nomask
terminal_MI_masked
Algorithm
%
25
50
75

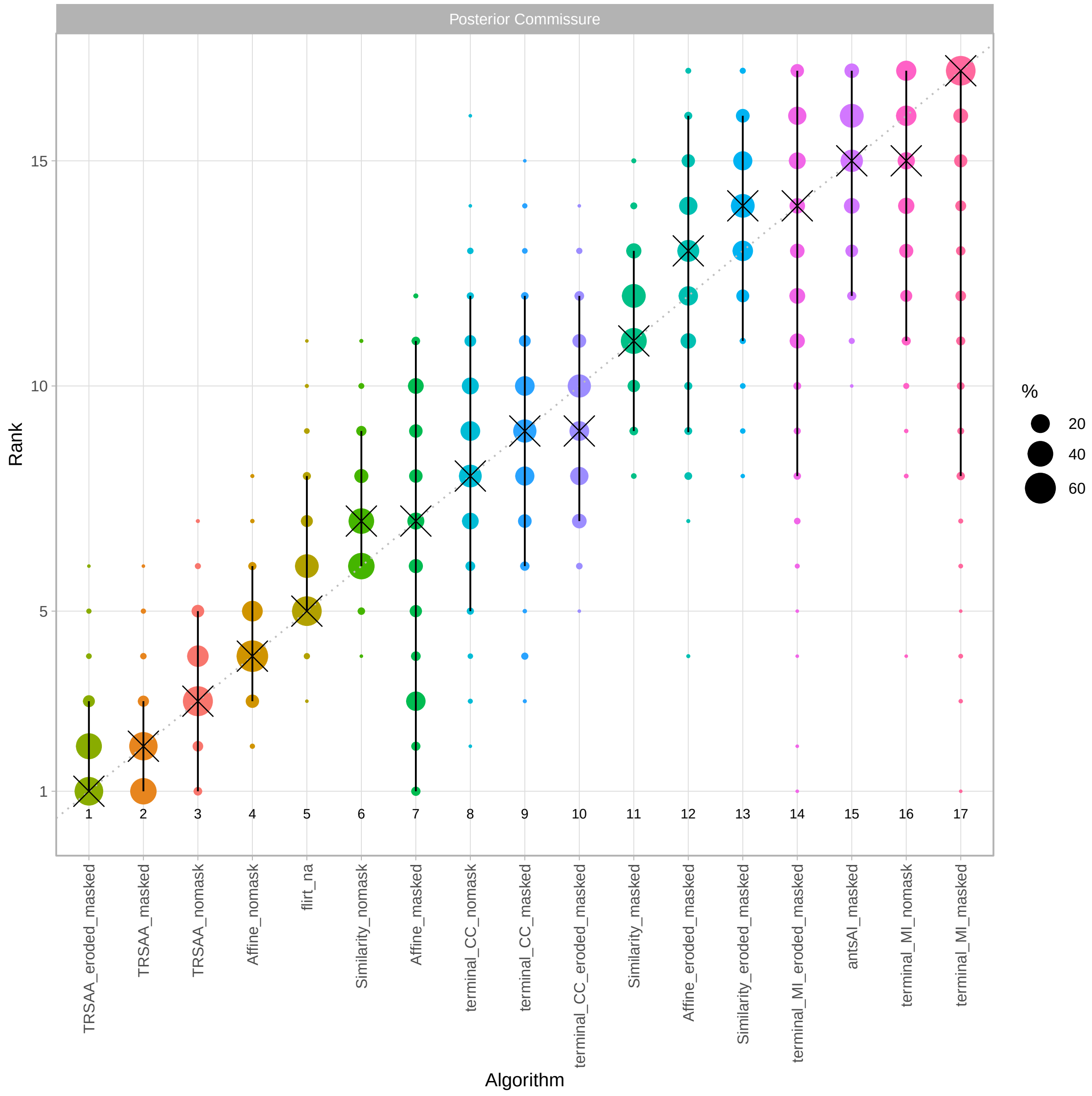

Posterior Commissure
Rank
Algorithm
%
20
40
60
1
5
10
15
1
2
3
4
5
6
7
8
9
10
11
12
13
14
15
16
17
TRSAA_eroded_masked
TRSAA_masked
TRSAA_nomask
Affine_nomask
flirt_na
Similarity_nomask
Affine_masked
terminal_CC_nomask
terminal_CC_masked
terminal_CC_eroded_masked
Similarity_masked
Affine_eroded_masked
Similarity_eroded_masked
terminal_MI_eroded_masked
antsAI_masked
terminal_MI_nomask
terminal_MI_masked

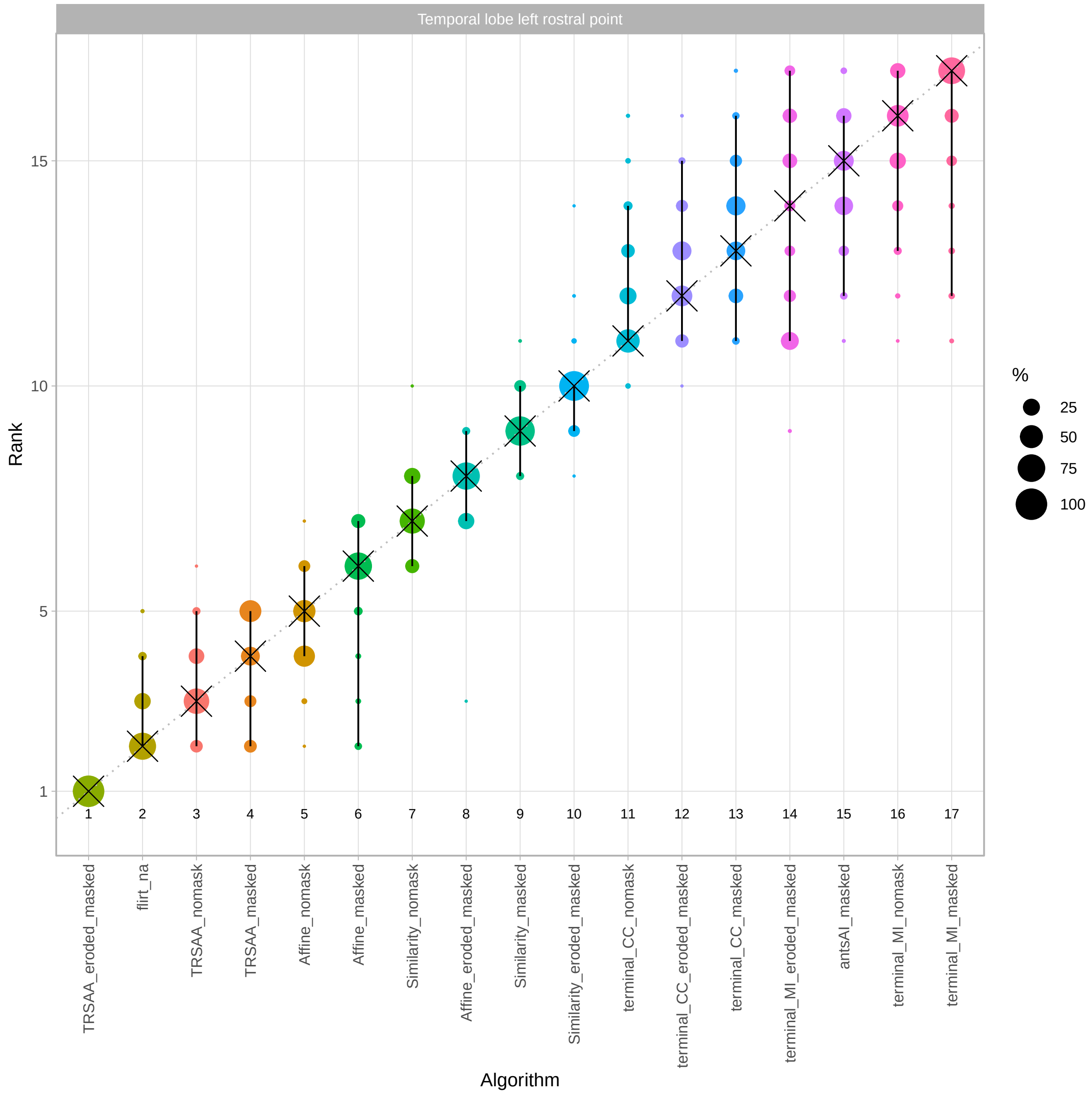

Temporal lobe left rostral point
Rank
Algorithm
%
25
50
75
100
TRSAA_eroded_masked
flirt_na
TRSAA_nomask
TRSAA_masked
Affine_nomask
Affine_masked
Similarity_nomask
Affine_eroded_masked
Similarity_masked
Similarity_eroded_masked
terminal_CC_nomask
terminal_CC_eroded_masked
terminal_CC_masked
terminal_MI_eroded_masked
antsAI_masked
terminal_MI_nomask
terminal_MI_masked

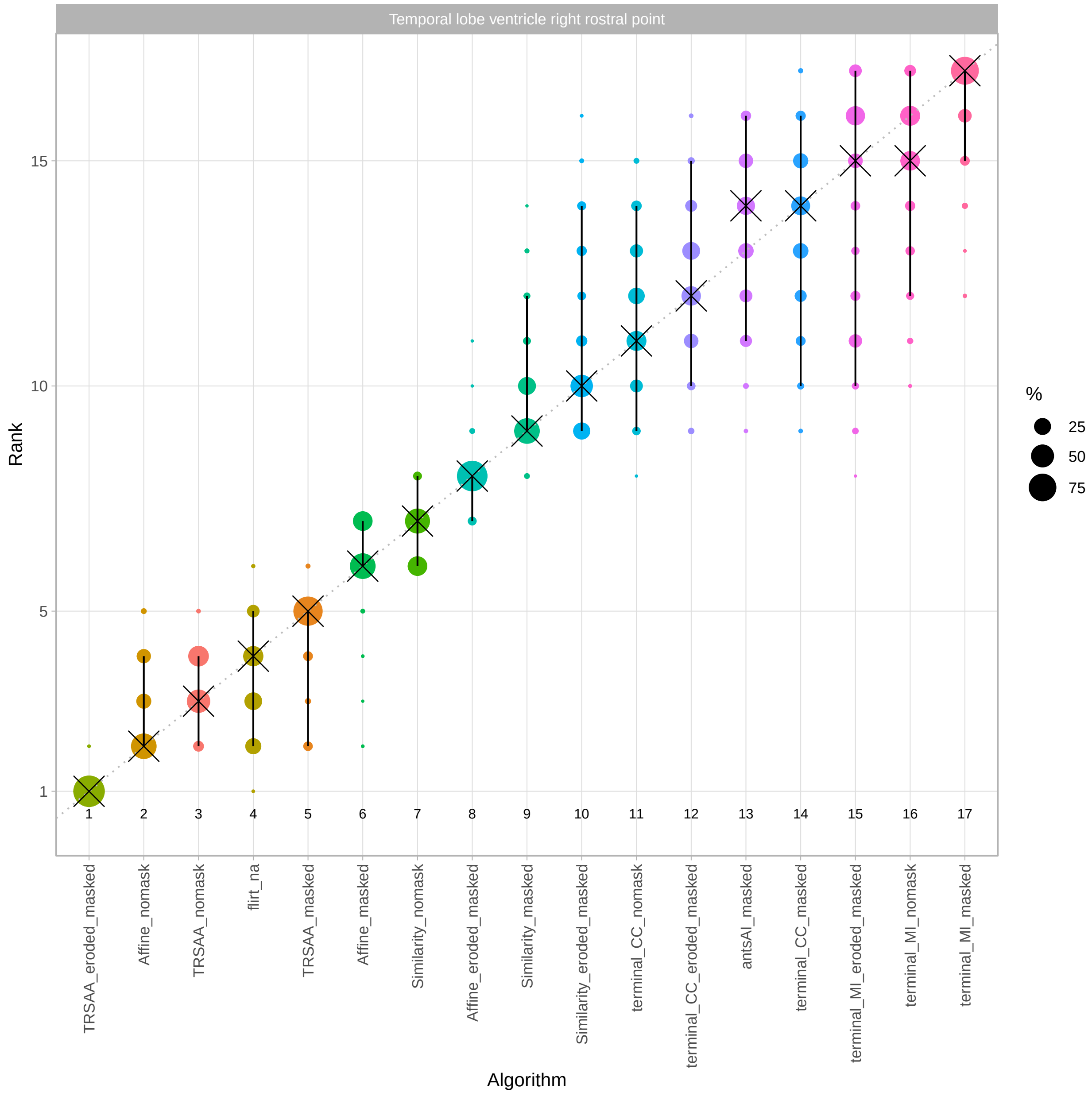
Temporal lobe ventricle right rostral point
Rank
15
10
5
1
1
2
3
4
5
6
7
8
9
10
11
12
13
14
15
16
17
TRSAA_eroded_masked
Affine_nomask
TRSAA_nomask
flirt_na
TRSAA_masked
Affine_masked
Similarity_nomask
Affine_eroded_masked
Similarity_masked
Similarity_eroded_masked
terminal_CC_nomask
terminal_CC_eroded_masked
antsAI_masked
terminal_CC_masked
terminal_MI_eroded_masked
terminal_MI_nomask
terminal_MI_masked
Algorithm
%
25
50
75

3.2 *Violin plot* for visualizing ranking stability based on bootstrapping

The ranking list based on the full assessment data is pairwise compared with the ranking lists based on the individual bootstrap samples (here $b = 1000$ samples). For each pair of rankings, Kendall's τ correlation is computed. Kendall's τ is a scaled index determining the correlation between the lists. It is computed by evaluating the number of pairwise concordances and discordances between ranking lists and produces values between -1 (for inverted order) and 1 (for identical order). A violin plot, which simultaneously depicts a boxplot and a density plot, is generated from the results.

Summary Kendall's tau:

Task	mean	median	q25	q75
Chiasma	0.9232647	0.9264706	0.8970588	0.9558824
Hemisphere right rostral point	0.9135735	0.9264706	0.8823529	0.9558824
Temporal lobe left rostral point	0.9197059	0.9264706	0.8970588	0.9411765
Hemisphere left rostral point	0.9029412	0.9117647	0.8676471	0.9411765
Lateral ventricle right rostral point	0.9045147	0.9117647	0.8676471	0.9411765
Temporal lobe ventricle right rostral point	0.8999118	0.9117647	0.8676471	0.9264706
4th Ventricle	0.8922500	0.8970588	0.8676471	0.9264706
Lateral ventricle left rostral point	0.8843235	0.8970588	0.8529412	0.9264706
Infracollicular Sulcus	0.8641029	0.8676471	0.8382353	0.8970588
PMJ	0.8614118	0.8676471	0.8382353	0.8970588
Posterior Commissure	0.8370147	0.8529412	0.7941176	0.8823529
Culmen	0.8215147	0.8382353	0.7794118	0.8823529
Hemisphere left caudal point	0.8077206	0.8235294	0.7647059	0.8529412
Hemisphere right caudal point	0.8086029	0.8088235	0.7647059	0.8529412
Lateral ventricle left caudal point	0.7383824	0.7500000	0.6764706	0.8088235
Lateral ventricle right caudal point	0.6972647	0.7205882	0.6029412	0.8088235

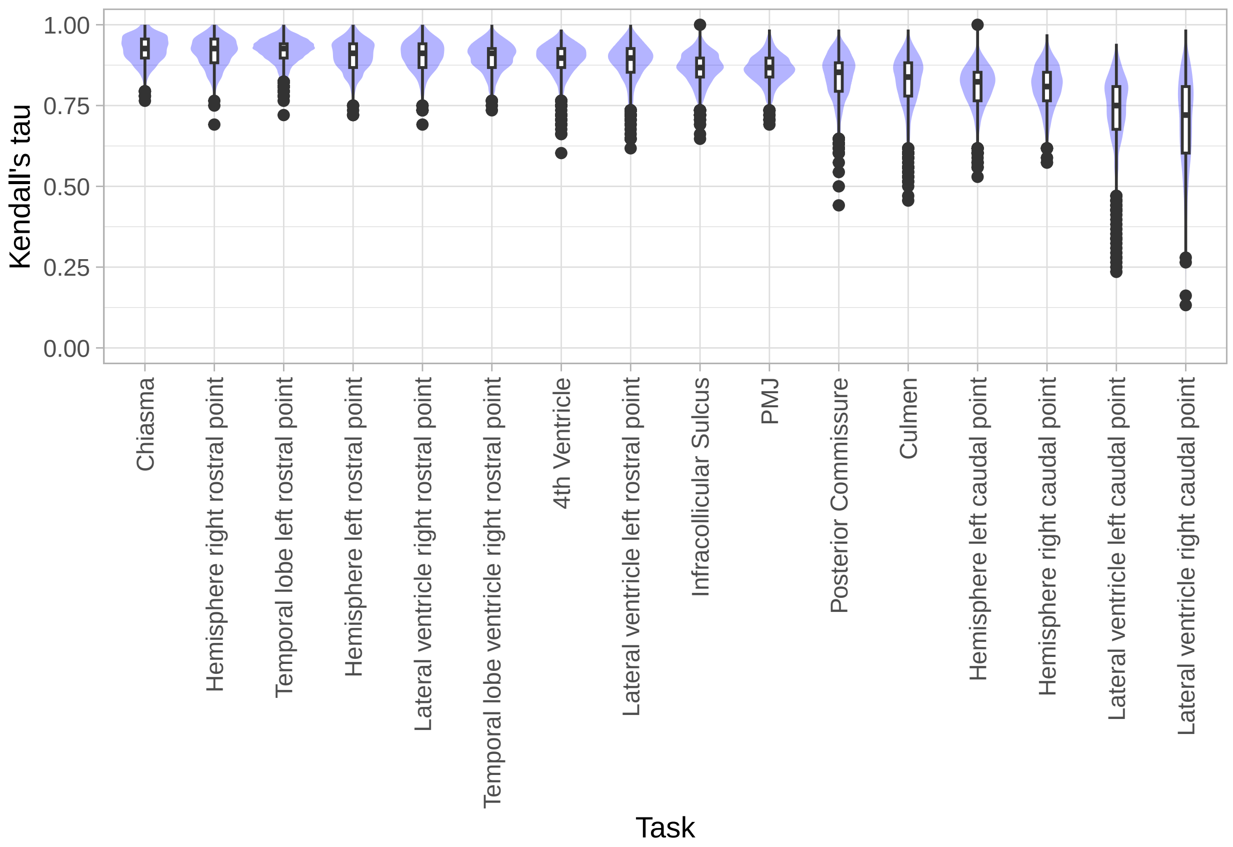
Kendall's tau
1.00
0.75
0.50
0.25
0.00
Chiasma
Hemisphere right rostral point
Temporal lobe left rostral point
Hemisphere left rostral point
Lateral ventricle right rostral point
Temporal lobe ventricle right rostral point
4th Ventricle
Lateral ventricle left rostral point
Infracollicular Sulcus
PMJ
Posterior Commissure
Culmen
Hemisphere left caudal point
Hemisphere right caudal point
Lateral ventricle left caudal point
Lateral ventricle right caudal point
Task

3.3 *Significance maps* for visualizing ranking stability based on statistical significance

Significance maps depict incidence matrices of pairwise significant test results for the one-sided Wilcoxon signed rank test at a 5% significance level with adjustment for multiple testing according to Holm. Yellow shading indicates that metric values from the algorithm on the x-axis were significantly superior to those from the algorithm on the y-axis, blue color indicates no significant difference.

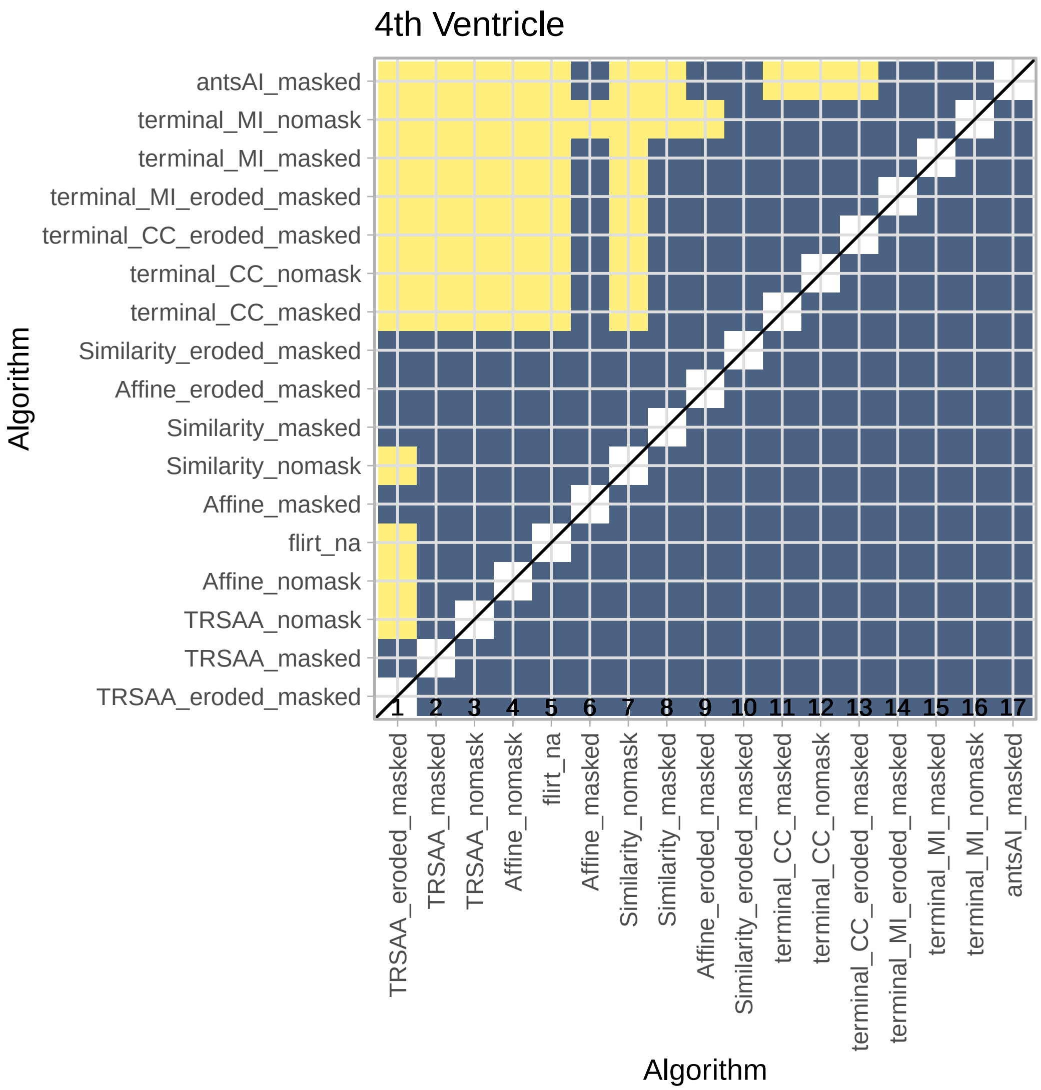

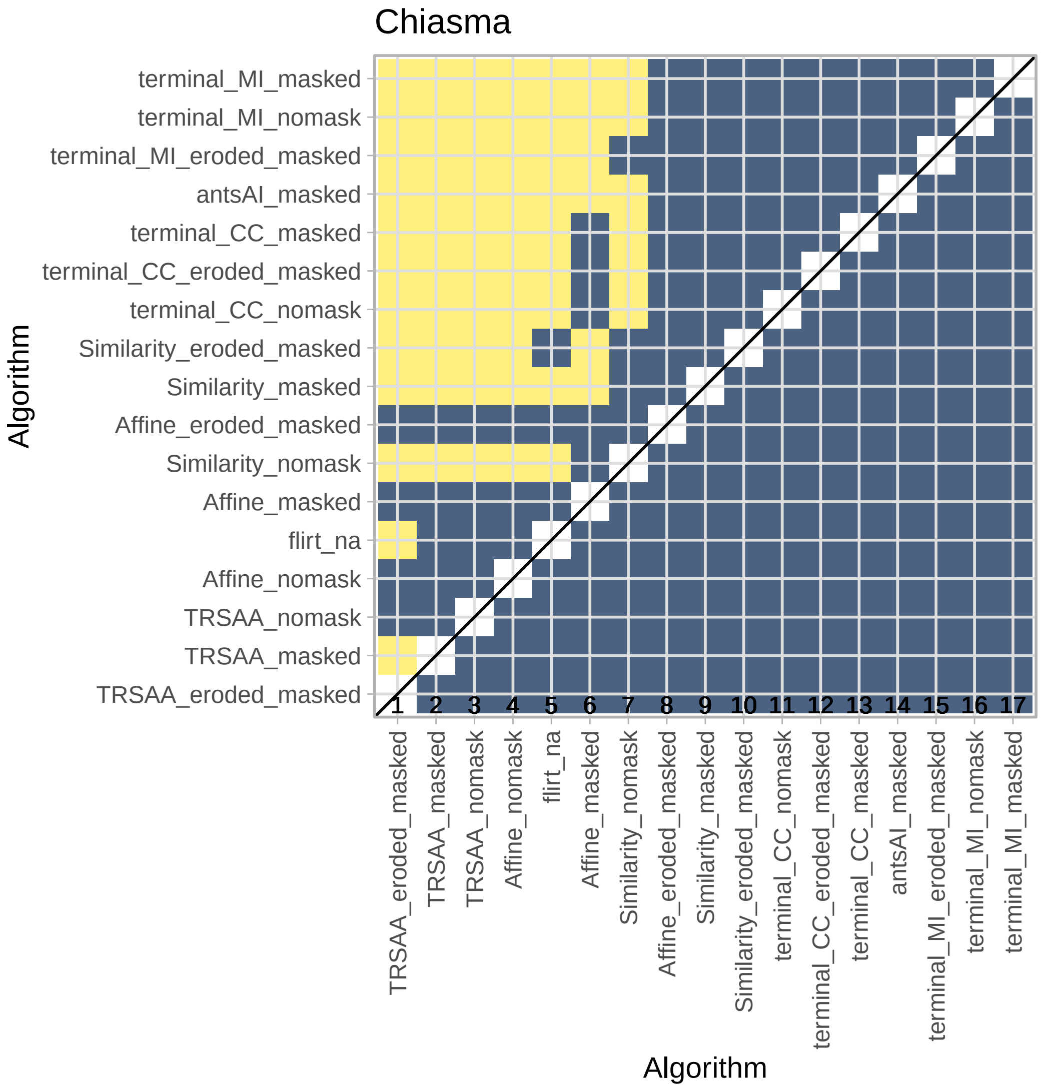

Chiasma
Algorithm
terminal_MI_masked
terminal_MI_nomask
terminal_MI_eroded_masked
antsAI_masked
terminal_CC_masked
terminal_CC_eroded_masked
terminal_CC_nomask
Similarity_eroded_masked
Similarity_masked
Affine_eroded_masked
Similarity_nomask
Affine_masked
flirt_na
Affine_nomask
TRSAA_nomask
TRSAA_masked
TRSAA_eroded_masked
1 2 3 4 5 6 7 8 9 10 11 12 13 14 15 16 17
TRSAA_eroded_masked
TRSAA_masked
TRSAA_nomask
Affine_nomask
flirt_na
Affine_masked
Similarity_nomask
Affine_eroded_masked
Similarity_masked
Similarity_eroded_masked
terminal_CC_nomask
terminal_CC_eroded_masked
terminal_CC_masked
antsAI_masked
terminal_MI_eroded_masked
terminal_MI_nomask
terminal_MI_masked
Algorithm

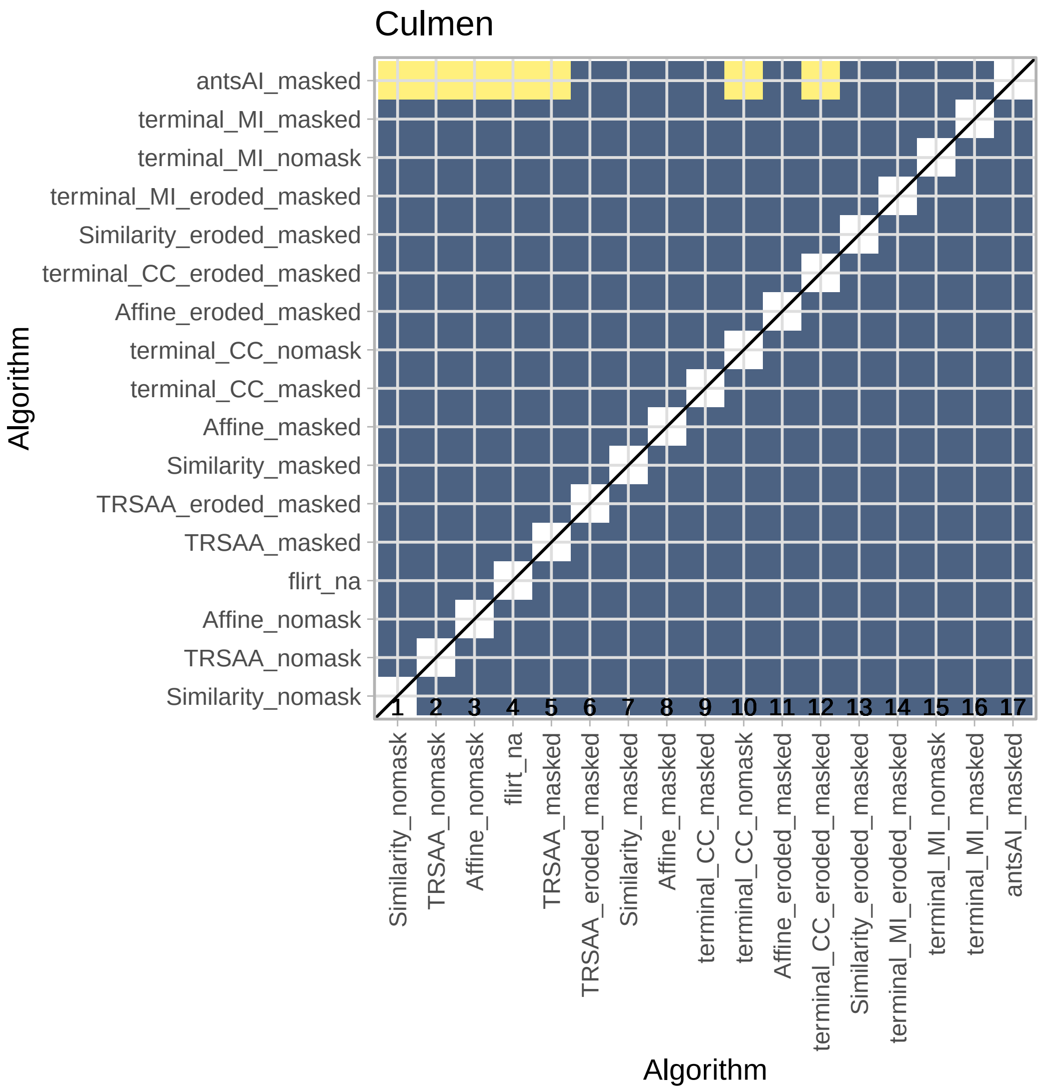
Culmen
Algorithm
antsAI_masked
terminal_MI_masked
terminal_MI_nomask
terminal_MI_eroded_masked
Similarity_eroded_masked
terminal_CC_eroded_masked
Affine_eroded_masked
terminal_CC_nomask
terminal_CC_masked
Affine_masked
Similarity_masked
TRSAA_eroded_masked
TRSAA_masked
flirt_na
Affine_nomask
TRSAA_nomask
Similarity_nomask
1 2 3 4 5 6 7 8 9 10 11 12 13 14 15 16 17
Similarity_nomask
TRSAA_nomask
Affine_nomask
flirt_na
TRSAA_masked
TRSAA_eroded_masked
Similarity_masked
Affine_masked
terminal_CC_masked
terminal_CC_nomask
Affine_eroded_masked
terminal_CC_eroded_masked
Similarity_eroded_masked
terminal_MI_eroded_masked
terminal_MI_nomask
terminal_MI_masked
antsAI_masked
Algorithm

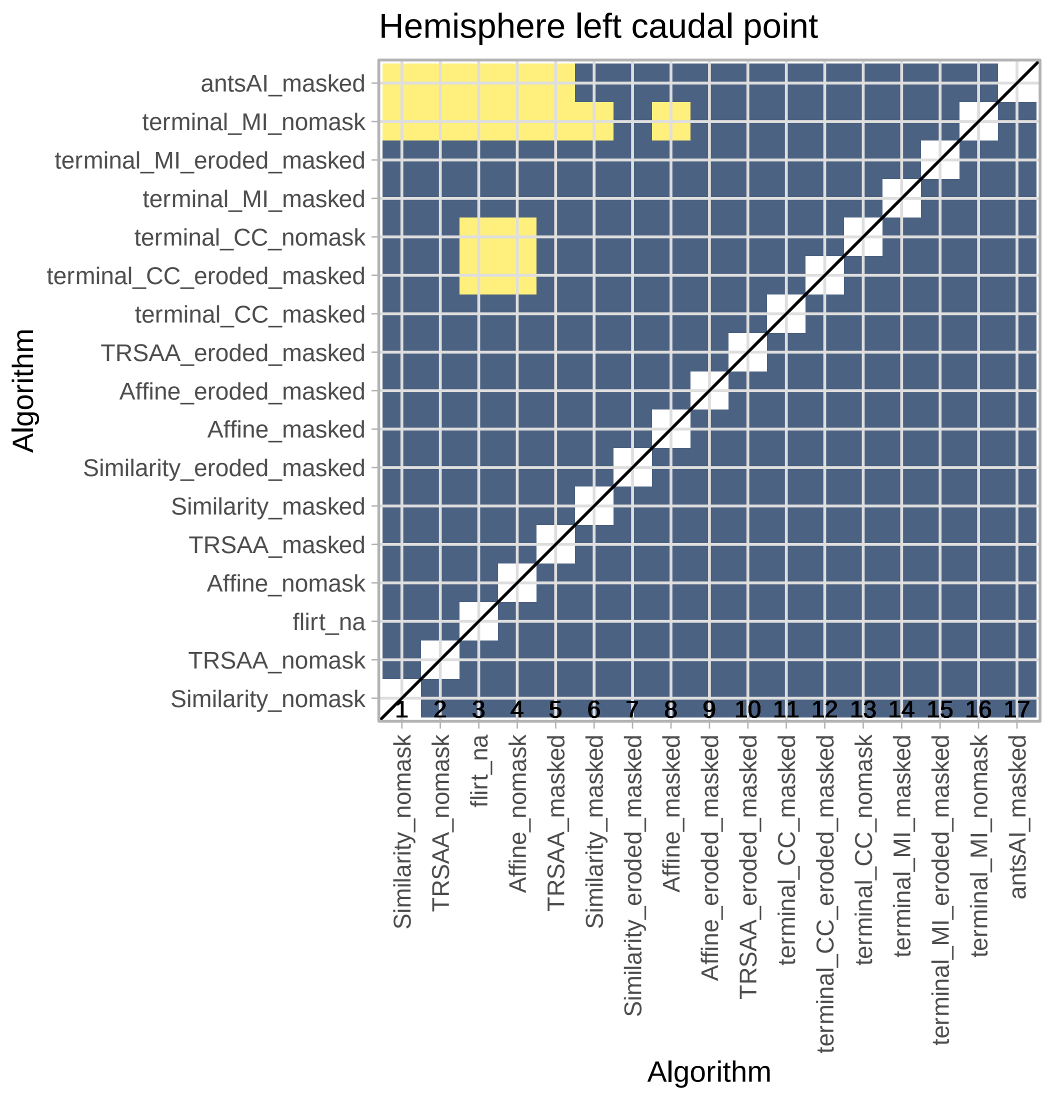

Hemisphere left caudal point
Algorithm
antsAI_masked
terminal_MI_nomask
terminal_MI_eroded_masked
terminal_MI_masked
terminal_CC_nomask
terminal_CC_eroded_masked
terminal_CC_masked
TRSAA_eroded_masked
Affine_eroded_masked
Affine_masked
Similarity_eroded_masked
Similarity_masked
TRSAA_masked
Affine_nomask
flirt_na
TRSAA_nomask
Similarity_nomask
1 2 3 4 5 6 7 8 9 10 11 12 13 14 15 16 17
Similarity_nomask
TRSAA_nomask
flirt_na
Affine_nomask
TRSAA_masked
Similarity_masked
Similarity_eroded_masked
Affine_masked
Affine_eroded_masked
TRSAA_eroded_masked
terminal_CC_masked
terminal_CC_eroded_masked
terminal_CC_nomask
terminal_MI_masked
terminal_MI_eroded_masked
terminal_MI_nomask
antsAI_masked
Algorithm

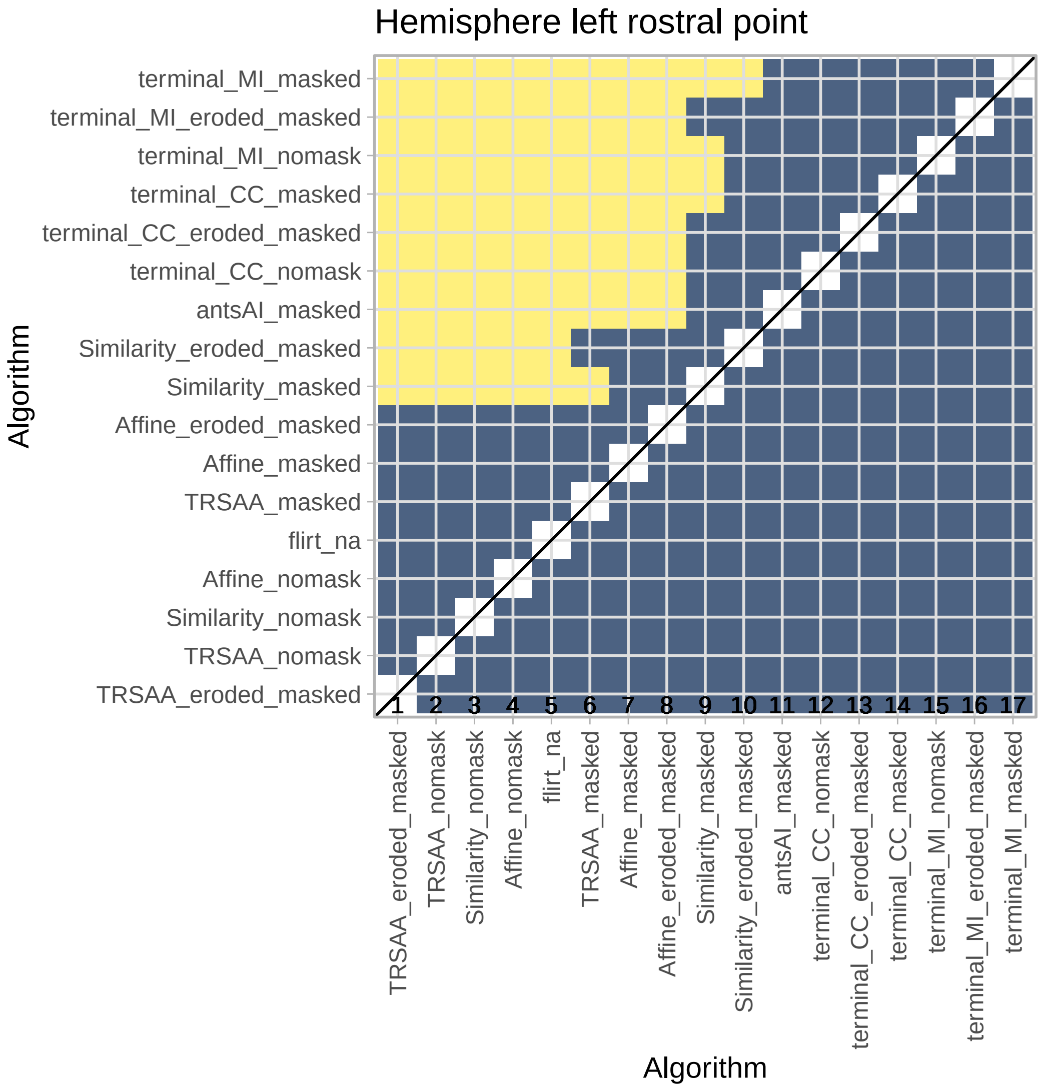
Hemisphere left rostral point
terminal_MI_masked
terminal_MI_eroded_masked
terminal_MI_nomask
terminal_CC_masked
terminal_CC_eroded_masked
terminal_CC_nomask
antsAI_masked
Similarity_eroded_masked
Similarity_masked
Affine_eroded_masked
Affine_masked
TRSAA_masked
flirt_na
Affine_nomask
Similarity_nomask
TRSAA_nomask
TRSAA_eroded_masked
1 2 3 4 5 6 7 8 9 10 11 12 13 14 15 16 17
Algorithm
Algorithm

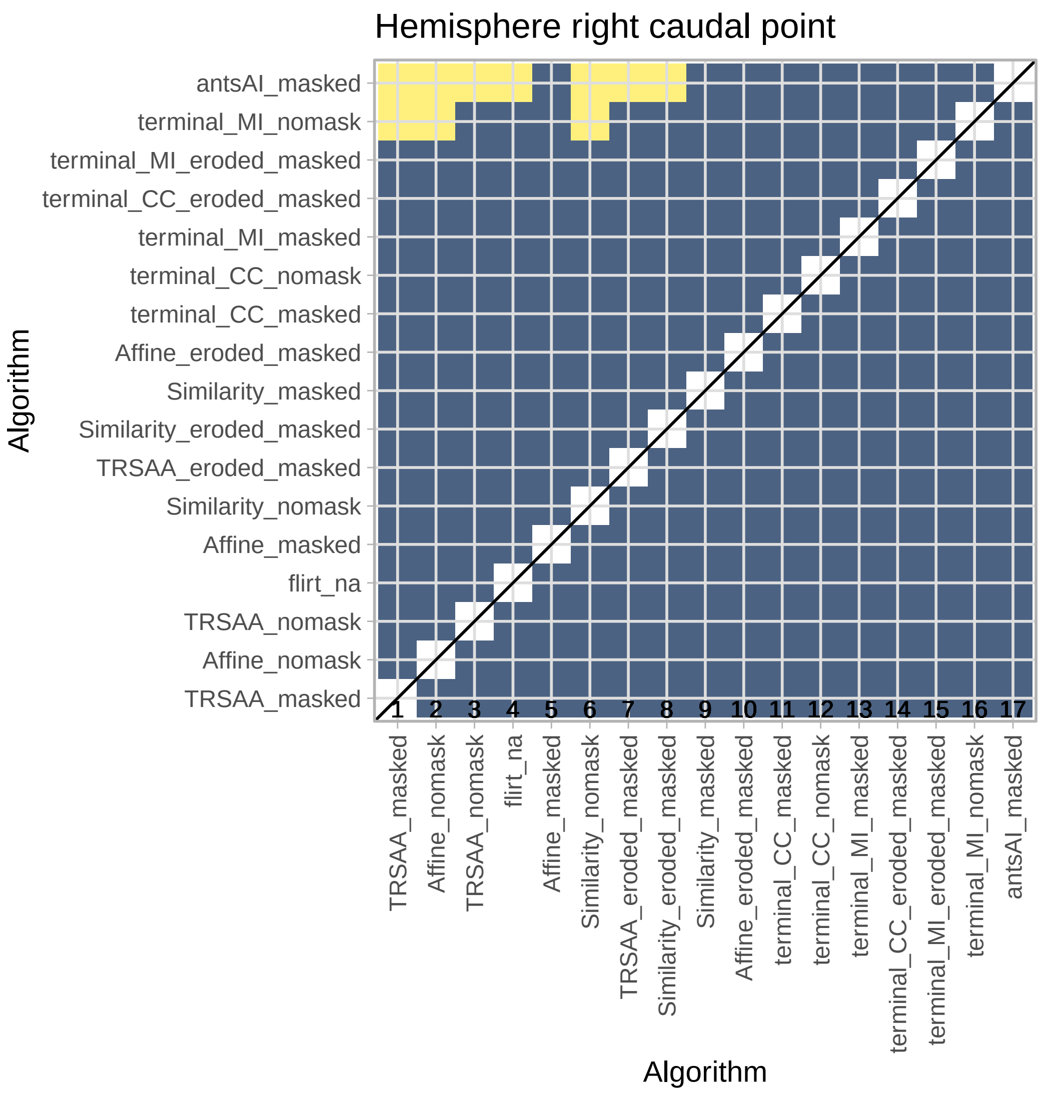
Hemisphere right caudal point
Algorithm
antsAI_masked
terminal_MI_nomask
terminal_MI_eroded_masked
terminal_CC_eroded_masked
terminal_MI_masked
terminal_CC_nomask
terminal_CC_masked
Affine_eroded_masked
Similarity_masked
Similarity_eroded_masked
TRSAA_eroded_masked
Similarity_nomask
Affine_masked
flirt_na
TRSAA_nomask
Affine_nomask
TRSAA_masked
1 2 3 4 5 6 7 8 9 10 11 12 13 14 15 16 17
TRSAA_masked
Affine_nomask
TRSAA_nomask
flirt_na
Affine_masked
Similarity_nomask
TRSAA_eroded_masked
Similarity_eroded_masked
Similarity_masked
Affine_eroded_masked
terminal_CC_masked
terminal_CC_nomask
terminal_MI_masked
terminal_CC_eroded_masked
terminal_MI_eroded_masked
terminal_MI_nomask
antsAI_masked
Algorithm

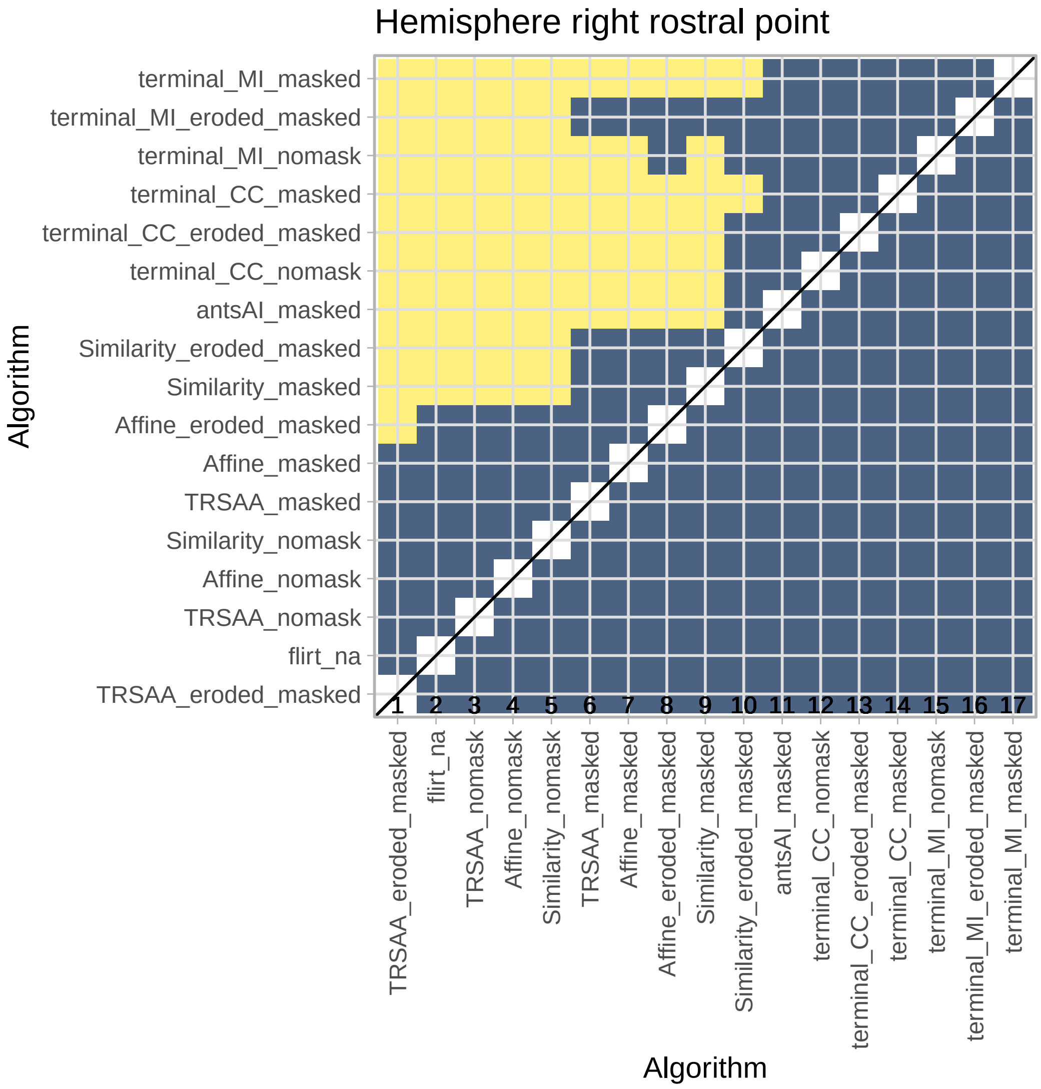

Hemisphere right rostral point
Algorithm
terminal_MI_masked
terminal_MI_eroded_masked
terminal_MI_nomask
terminal_CC_masked
terminal_CC_eroded_masked
terminal_CC_nomask
antsAI_masked
Similarity_eroded_masked
Similarity_masked
Affine_eroded_masked
Affine_masked
TRSAA_masked
Similarity_nomask
Affine_nomask
TRSAA_nomask
flirt_na
TRSAA_eroded_masked
1 2 3 4 5 6 7 8 9 10 11 12 13 14 15 16 17
TRSAA_eroded_masked
flirt_na
TRSAA_nomask
Affine_nomask
Similarity_nomask
TRSAA_masked
Affine_masked
Affine_eroded_masked
Similarity_masked
Similarity_eroded_masked
antsAI_masked
terminal_CC_nomask
terminal_CC_eroded_masked
terminal_CC_masked
terminal_MI_nomask
terminal_MI_eroded_masked
terminal_MI_masked
Algorithm

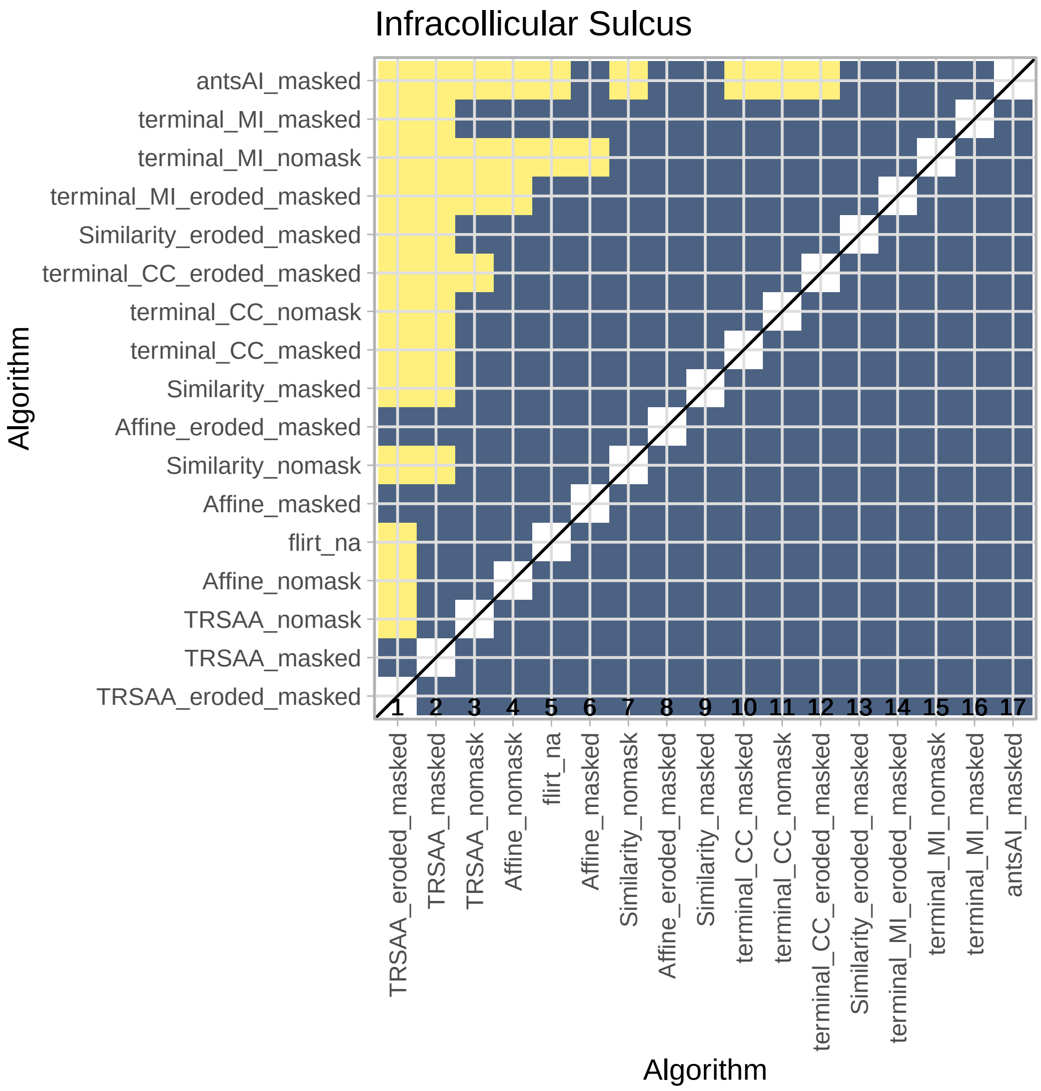
Infracollicular Sulcus
antsAI_masked
terminal_MI_masked
terminal_MI_nomask
terminal_MI_eroded_masked
Similarity_eroded_masked
terminal_CC_eroded_masked
terminal_CC_nomask
terminal_CC_masked
Similarity_masked
Affine_eroded_masked
Similarity_nomask
Affine_masked
flirt_na
Affine_nomask
TRSAA_nomask
TRSAA_masked
TRSAA_eroded_masked
Algorithm
1 2 3 4 5 6 7 8 9 10 11 12 13 14 15 16 17
TRSAA_eroded_masked
TRSAA_masked
TRSAA_nomask
Affine_nomask
flirt_na
Affine_masked
Similarity_nomask
Affine_eroded_masked
Similarity_masked
terminal_CC_masked
terminal_CC_nomask
terminal_CC_eroded_masked
Similarity_eroded_masked
terminal_MI_eroded_masked
terminal_MI_nomask
terminal_MI_masked
antsAI_masked
Algorithm

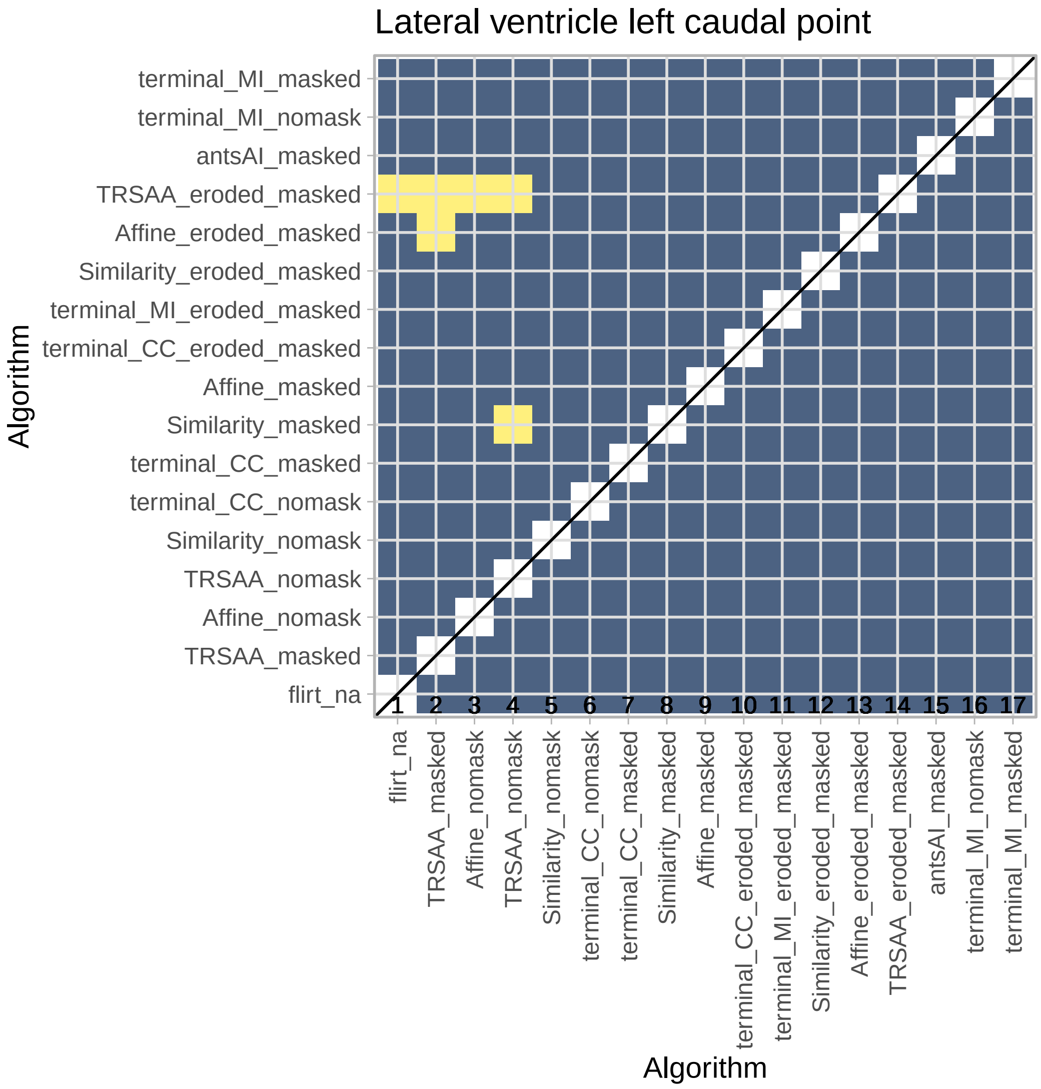
Lateral ventricle left caudal point
Algorithm
terminal_MI_masked
terminal_MI_nomask
antsAI_masked
TRSAA_eroded_masked
Affine_eroded_masked
Similarity_eroded_masked
terminal_MI_eroded_masked
terminal_CC_eroded_masked
Affine_masked
Similarity_masked
terminal_CC_masked
terminal_CC_nomask
Similarity_nomask
TRSAA_nomask
Affine_nomask
TRSAA_masked
flirt_na
1 2 3 4 5 6 7 8 9 10 11 12 13 14 15 16 17
flirt_na
TRSAA_masked
Affine_nomask
TRSAA_nomask
Similarity_nomask
terminal_CC_nomask
terminal_CC_masked
Similarity_masked
Affine_masked
terminal_CC_eroded_masked
terminal_MI_eroded_masked
Similarity_eroded_masked
Affine_eroded_masked
TRSAA_eroded_masked
antsAI_masked
terminal_MI_nomask
terminal_MI_masked
Algorithm

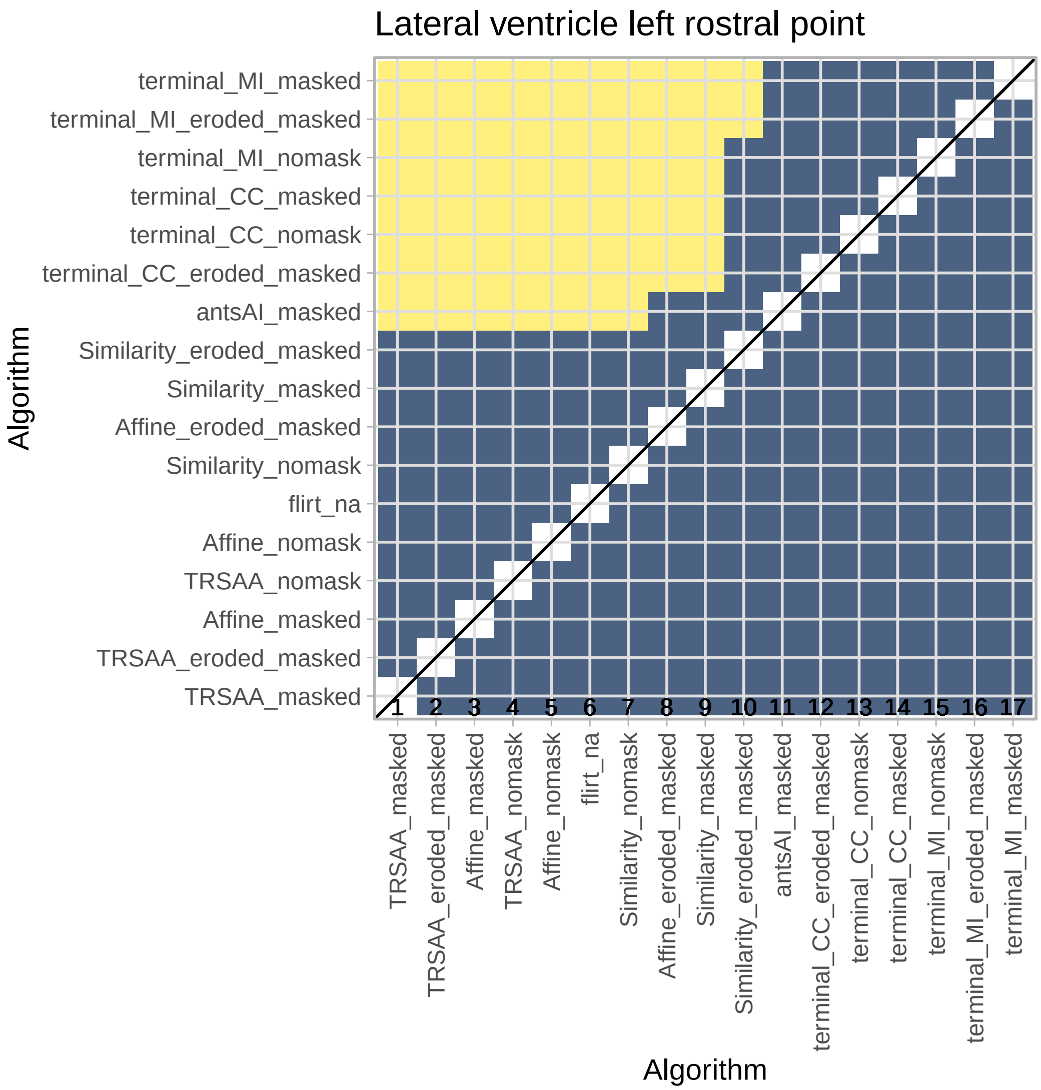

Lateral ventricle left rostral point
Algorithm
terminal_MI_masked
terminal_MI_eroded_masked
terminal_MI_nomask
terminal_CC_masked
terminal_CC_nomask
terminal_CC_eroded_masked
antsAI_masked
Similarity_eroded_masked
Similarity_masked
Affine_eroded_masked
Similarity_nomask
flirt_na
Affine_nomask
TRSAA_nomask
Affine_masked
TRSAA_eroded_masked
TRSAA_masked
1
2
3
4
5
6
7
8
9
10
11
12
13
14
15
16
17
TRSAA_masked
TRSAA_eroded_masked
Affine_masked
TRSAA_nomask
Affine_nomask
flirt_na
Similarity_nomask
Affine_eroded_masked
Similarity_masked
Similarity_eroded_masked
antsAI_masked
terminal_CC_eroded_masked
terminal_CC_nomask
terminal_CC_masked
terminal_MI_nomask
terminal_MI_eroded_masked
terminal_MI_masked
Algorithm

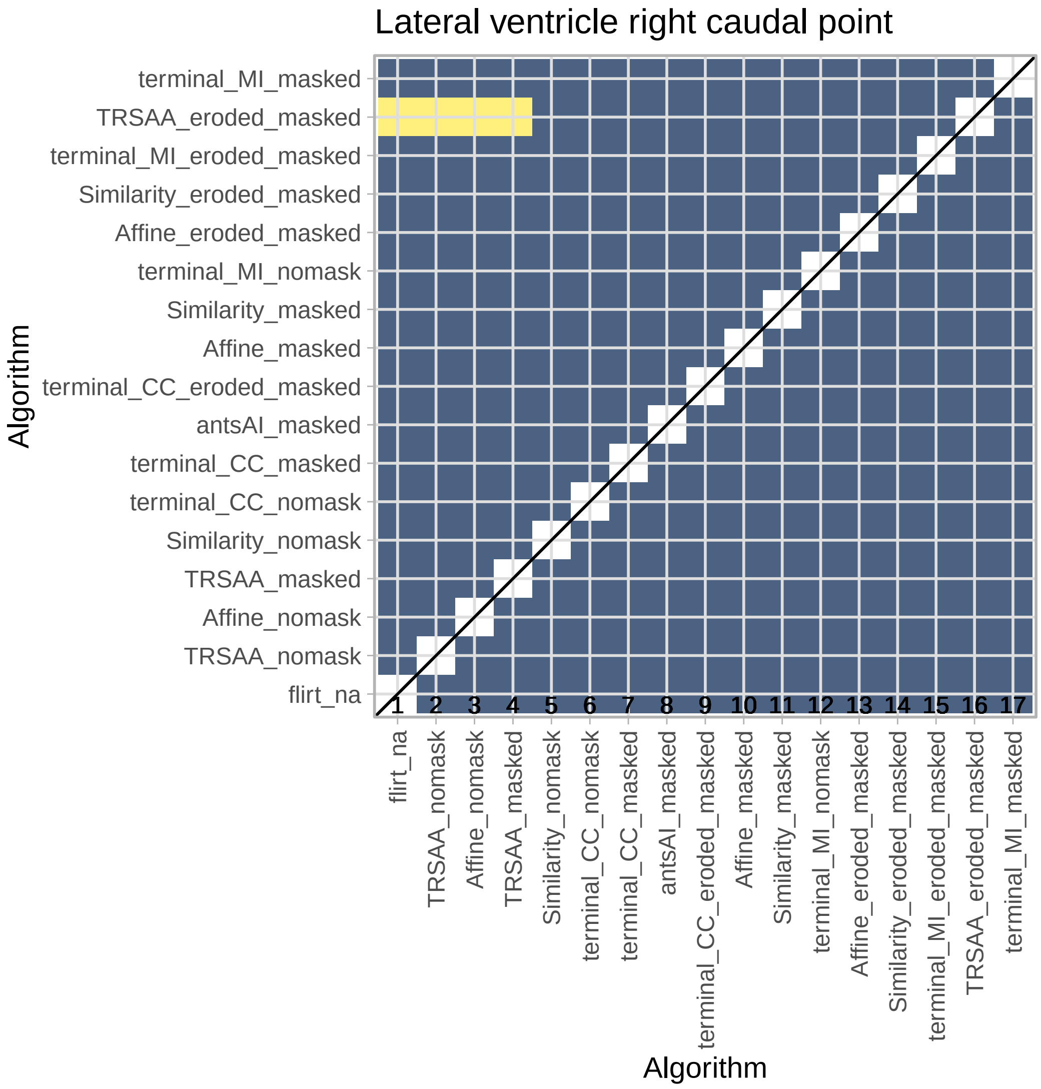
Lateral ventricle right caudal point
Algorithm
terminal_MI_masked
TRSAA_eroded_masked
terminal_MI_eroded_masked
Similarity_eroded_masked
Affine_eroded_masked
terminal_MI_nomask
Similarity_masked
Affine_masked
terminal_CC_eroded_masked
antsAI_masked
terminal_CC_masked
terminal_CC_nomask
Similarity_nomask
TRSAA_masked
Affine_nomask
TRSAA_nomask
flirt_na
1 2 3 4 5 6 7 8 9 10 11 12 13 14 15 16 17
flirt_na
TRSAA_nomask
Affine_nomask
TRSAA_masked
Similarity_nomask
terminal_CC_nomask
terminal_CC_masked
antsAI_masked
terminal_CC_eroded_masked
Affine_masked
Similarity_masked
terminal_MI_nomask
Affine_eroded_masked
Similarity_eroded_masked
terminal_MI_eroded_masked
TRSAA_eroded_masked
terminal_MI_masked
Algorithm

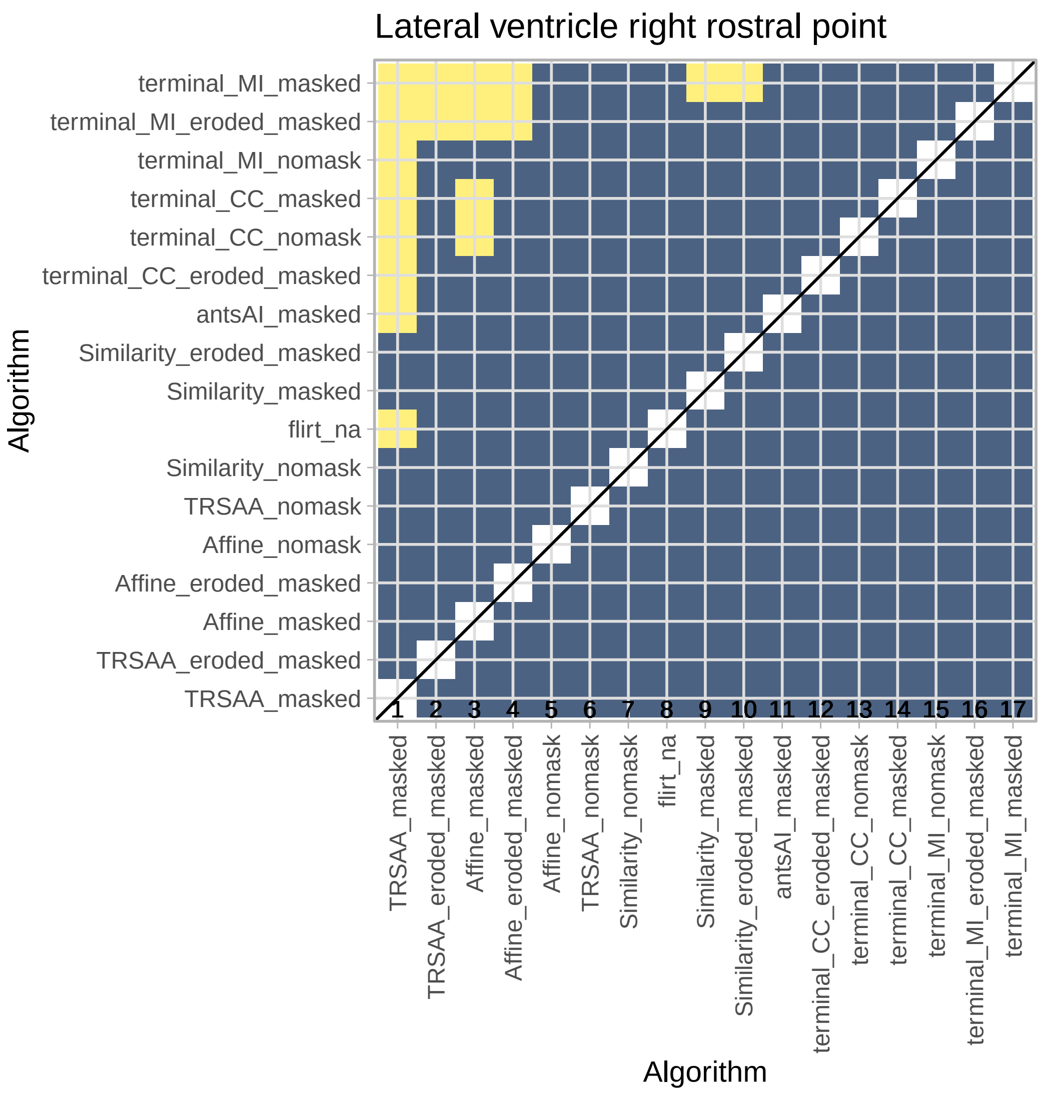
Lateral ventricle right rostral point
Algorithm
terminal_MI_masked
terminal_MI_eroded_masked
terminal_MI_nomask
terminal_CC_masked
terminal_CC_nomask
terminal_CC_eroded_masked
antsAI_masked
Similarity_eroded_masked
Similarity_masked
flirt_na
Similarity_nomask
TRSAA_nomask
Affine_nomask
Affine_eroded_masked
Affine_masked
TRSAA_eroded_masked
TRSAA_masked
1 2 3 4 5 6 7 8 9 10 11 12 13 14 15 16 17
TRSAA_masked
TRSAA_eroded_masked
Affine_masked
Affine_eroded_masked
Affine_nomask
TRSAA_nomask
Similarity_nomask
flirt_na
Similarity_masked
Similarity_eroded_masked
antsAI_masked
terminal_CC_eroded_masked
terminal_CC_nomask
terminal_CC_masked
terminal_MI_nomask
terminal_MI_eroded_masked
terminal_MI_masked
Algorithm

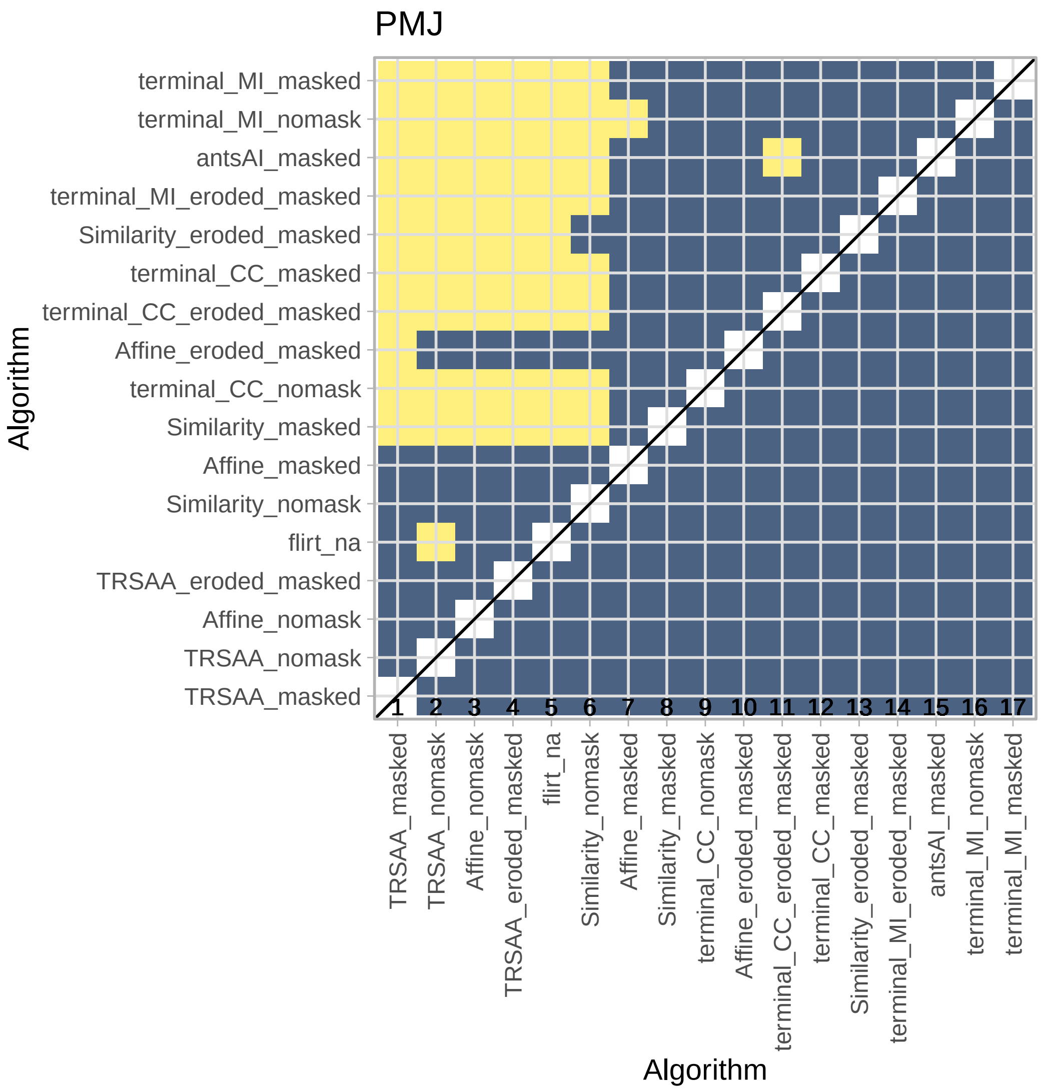
PMJ
Algorithm
terminal_MI_masked
terminal_MI_nomask
antsAI_masked
terminal_MI_eroded_masked
Similarity_eroded_masked
terminal_CC_masked
terminal_CC_eroded_masked
Affine_eroded_masked
terminal_CC_nomask
Similarity_masked
Affine_masked
Similarity_nomask
flirt_na
TRSAA_eroded_masked
Affine_nomask
TRSAA_nomask
TRSAA_masked
1 2 3 4 5 6 7 8 9 10 11 12 13 14 15 16 17
TRSAA_masked
TRSAA_nomask
Affine_nomask
TRSAA_eroded_masked
flirt_na
Similarity_nomask
Affine_masked
Similarity_masked
terminal_CC_nomask
Affine_eroded_masked
terminal_CC_eroded_masked
terminal_CC_masked
Similarity_eroded_masked
terminal_MI_eroded_masked
antsAI_masked
terminal_MI_nomask
terminal_MI_masked
Algorithm

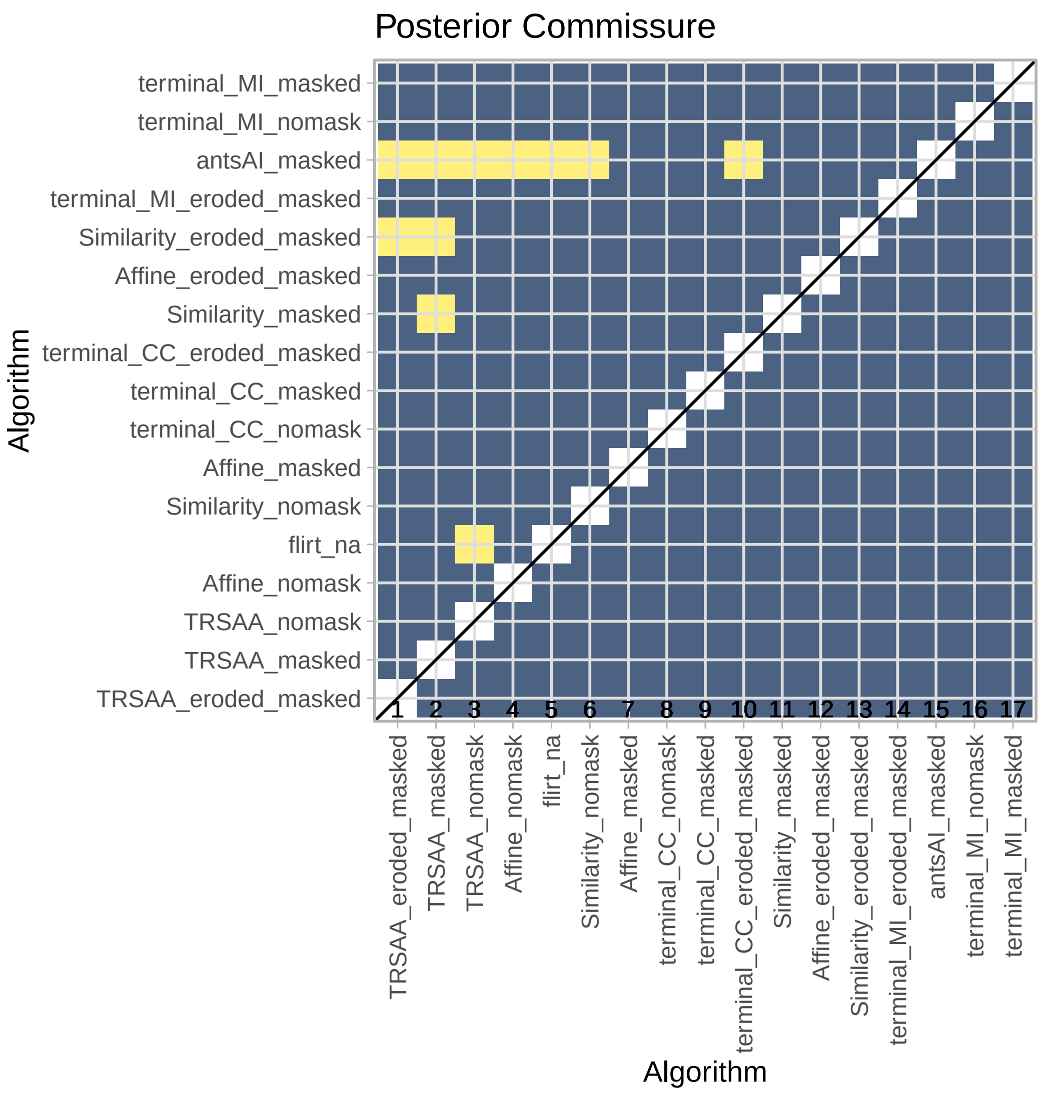

Posterior Commissure
terminal_MI_masked
terminal_MI_nomask
antsAI_masked
terminal_MI_eroded_masked
Similarity_eroded_masked
Affine_eroded_masked
Similarity_masked
terminal_CC_eroded_masked
terminal_CC_masked
terminal_CC_nomask
Affine_masked
Similarity_nomask
flirt_na
Affine_nomask
TRSAA_nomask
TRSAA_masked
TRSAA_eroded_masked
Algorithm
1 2 3 4 5 6 7 8 9 10 11 12 13 14 15 16 17
TRSAA_eroded_masked
TRSAA_masked
TRSAA_nomask
Affine_nomask
flirt_na
Similarity_nomask
Affine_masked
terminal_CC_nomask
terminal_CC_masked
terminal_CC_eroded_masked
Similarity_masked
Affine_eroded_masked
Similarity_eroded_masked
terminal_MI_eroded_masked
antsAI_masked
terminal_MI_nomask
terminal_MI_masked
Algorithm

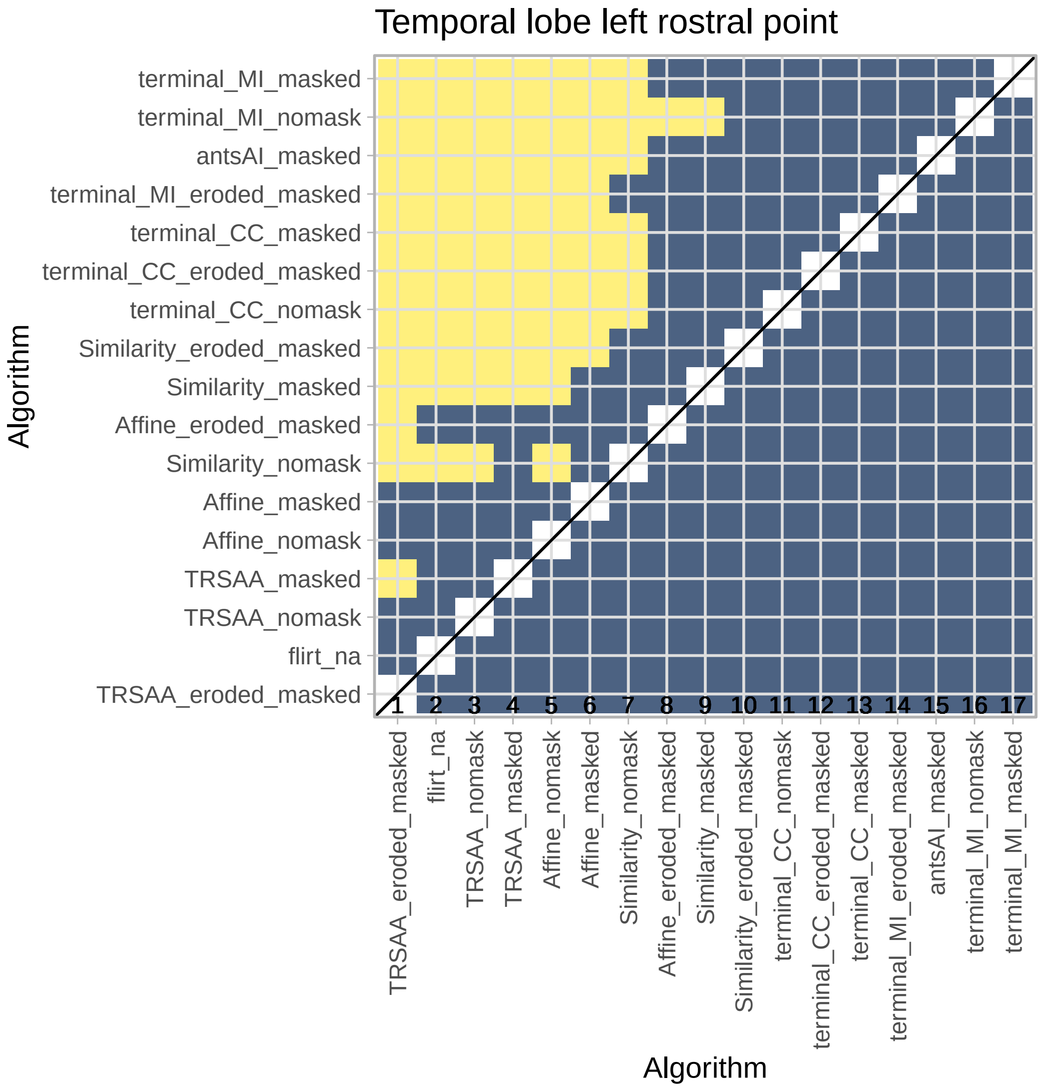
Temporal lobe left rostral point
Algorithm
terminal_MI_masked
terminal_MI_nomask
antsAI_masked
terminal_MI_eroded_masked
terminal_CC_masked
terminal_CC_eroded_masked
terminal_CC_nomask
Similarity_eroded_masked
Similarity_masked
Affine_eroded_masked
Similarity_nomask
Affine_masked
Affine_nomask
TRSAA_masked
TRSAA_nomask
flirt_na
TRSAA_eroded_masked
1 2 3 4 5 6 7 8 9 10 11 12 13 14 15 16 17
TRSAA_eroded_masked
flirt_na
TRSAA_nomask
TRSAA_masked
Affine_nomask
Affine_masked
Similarity_nomask
Affine_eroded_masked
Similarity_masked
Similarity_eroded_masked
terminal_CC_nomask
terminal_CC_eroded_masked
terminal_CC_masked
terminal_MI_eroded_masked
antsAI_masked
terminal_MI_nomask
terminal_MI_masked
Algorithm

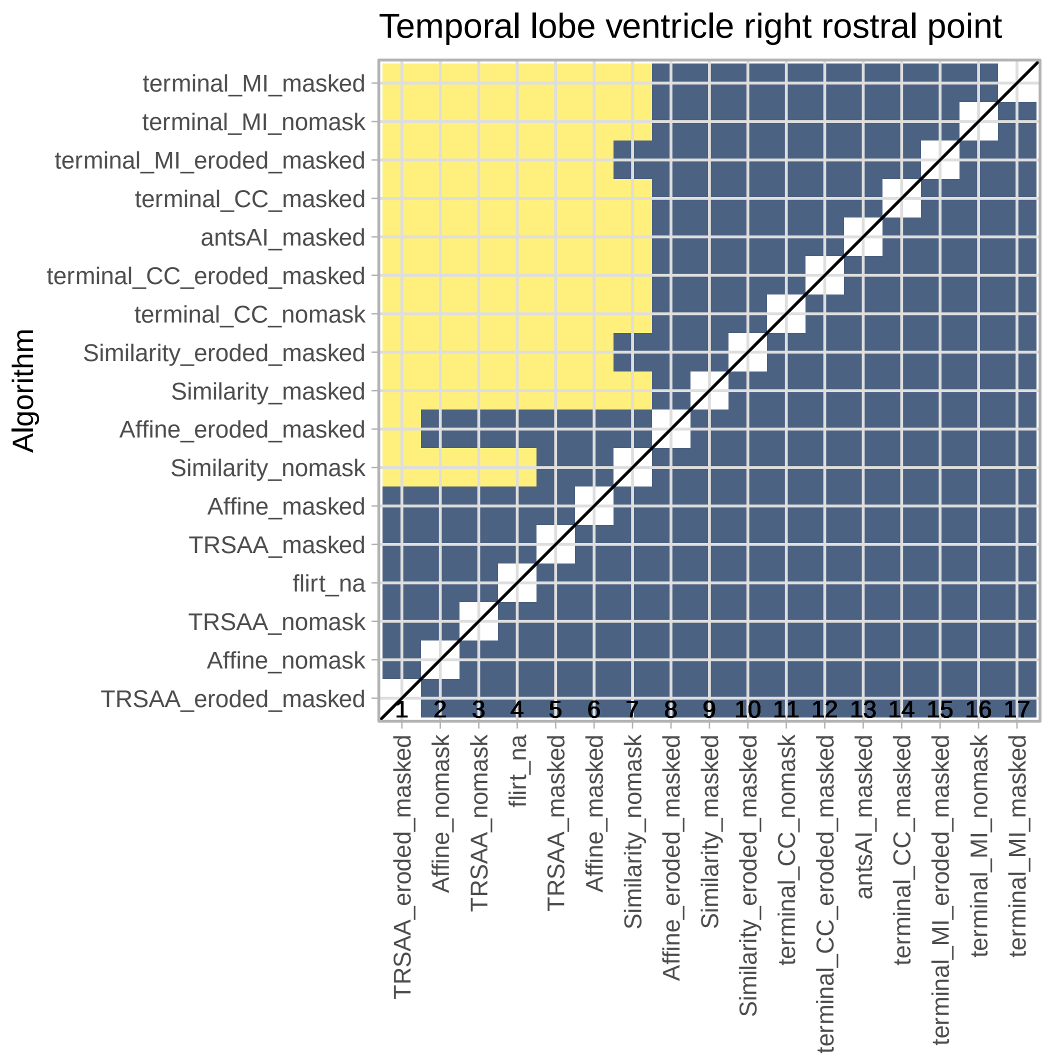
Temporal lobe ventricle right rostral point
terminal_MI_masked
terminal_MI_nomask
terminal_MI_eroded_masked
terminal_CC_masked
antsAI_masked
terminal_CC_eroded_masked
terminal_CC_nomask
Similarity_eroded_masked
Similarity_masked
Affine_eroded_masked
Similarity_nomask
Affine_masked
TRSAA_masked
flirt_na
TRSAA_nomask
Affine_nomask
TRSAA_eroded_masked
Algorithm
1 2 3 4 5 6 7 8 9 10 11 12 13 14 15 16 17
TRSAA_eroded_masked
Affine_nomask
TRSAA_nomask
flirt_na
TRSAA_masked
Affine_masked
Similarity_nomask
Affine_eroded_masked
Similarity_masked
Similarity_eroded_masked
terminal_CC_nomask
terminal_CC_eroded_masked
antsAI_masked
terminal_CC_masked
terminal_MI_eroded_masked
terminal_MI_nomask
terminal_MI_masked
Algorithm

3.4 Ranking robustness to ranking methods

Line plots for visualizing ranking robustness across different ranking methods. Each algorithm is represented by one colored line. For each ranking method encoded on the x-axis, the height of the line represents the corresponding rank. Horizontal lines indicate identical ranks for all methods.

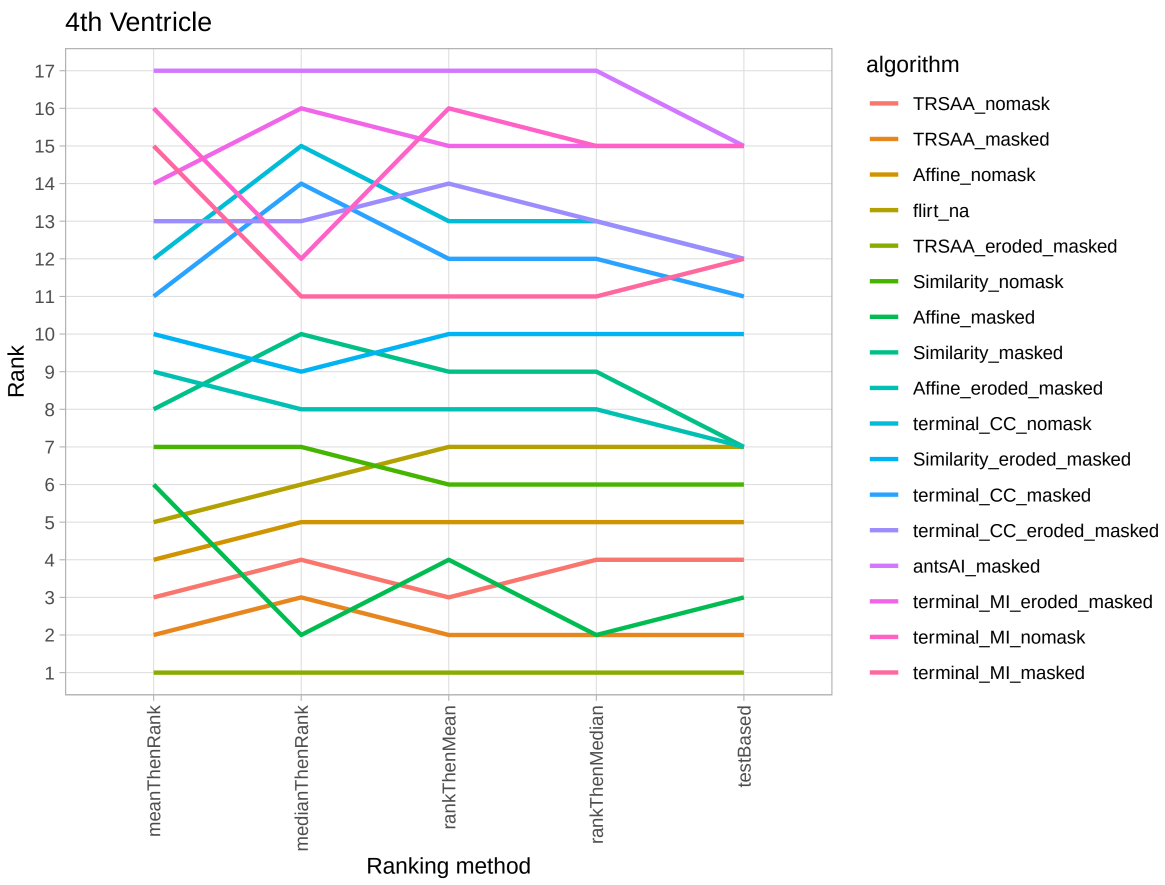

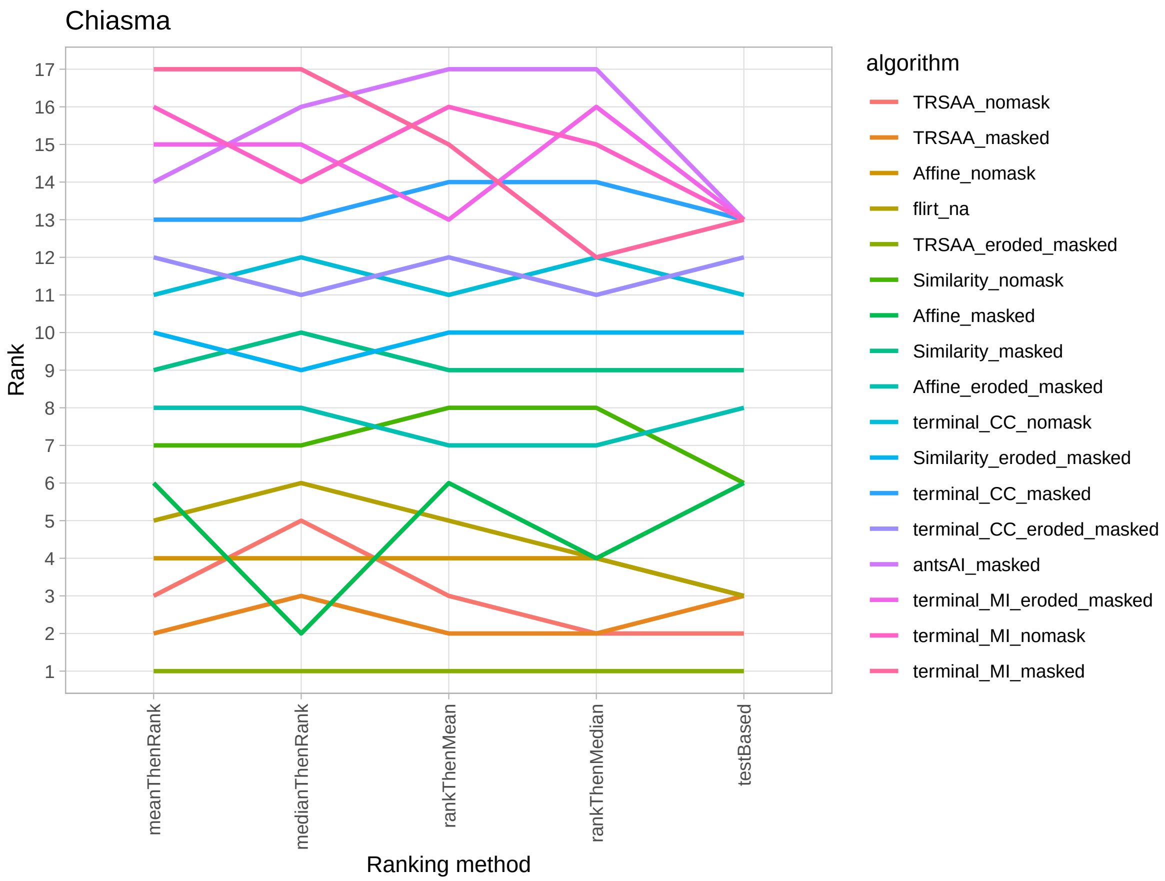

Chiasma
Rank
17
16
15
14
13
12
11
10
9
8
7
6
5
4
3
2
1
meanThenRank
medianThenRank
rankThenMean
rankThenMedian
testBased
Ranking method
algorithm
TRSAA_nomask
TRSAA_masked
Affine_nomask
flirt_na
TRSAA_eroded_masked
Similarity_nomask
Affine_masked
Similarity_masked
Affine_eroded_masked
terminal_CC_nomask
Similarity_eroded_masked
terminal_CC_masked
terminal_CC_eroded_masked
antsAI_masked
terminal_MI_eroded_masked
terminal_MI_nomask
terminal_MI_masked

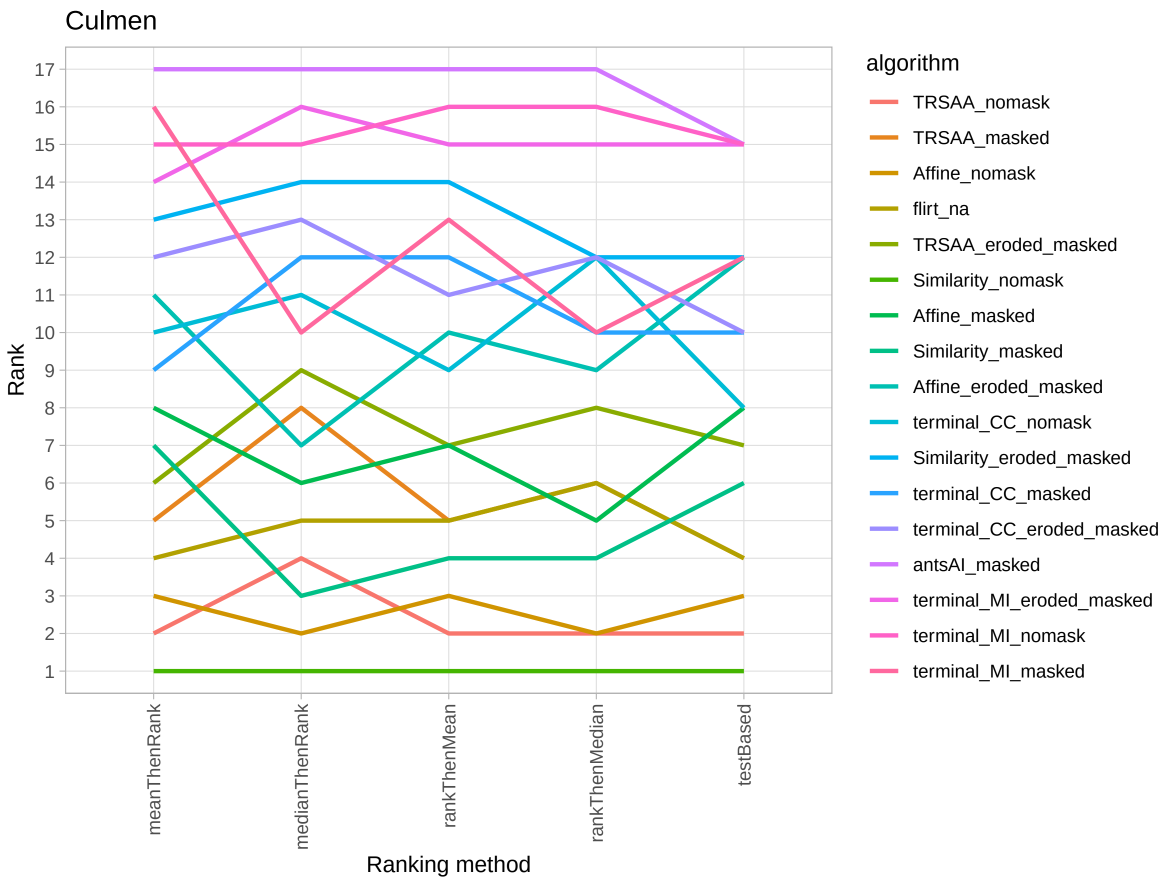
Culmen
Rank
17
16
15
14
13
12
11
10
9
8
7
6
5
4
3
2
1
meanThenRank
medianThenRank
rankThenMean
rankThenMedian
testBased
Ranking method
algorithm
TRSAA_nomask
TRSAA_masked
Affine_nomask
flirt_na
TRSAA_eroded_masked
Similarity_nomask
Affine_masked
Similarity_masked
Affine_eroded_masked
terminal_CC_nomask
Similarity_eroded_masked
terminal_CC_masked
terminal_CC_eroded_masked
antsAI_masked
terminal_MI_eroded_masked
terminal_MI_nomask
terminal_MI_masked

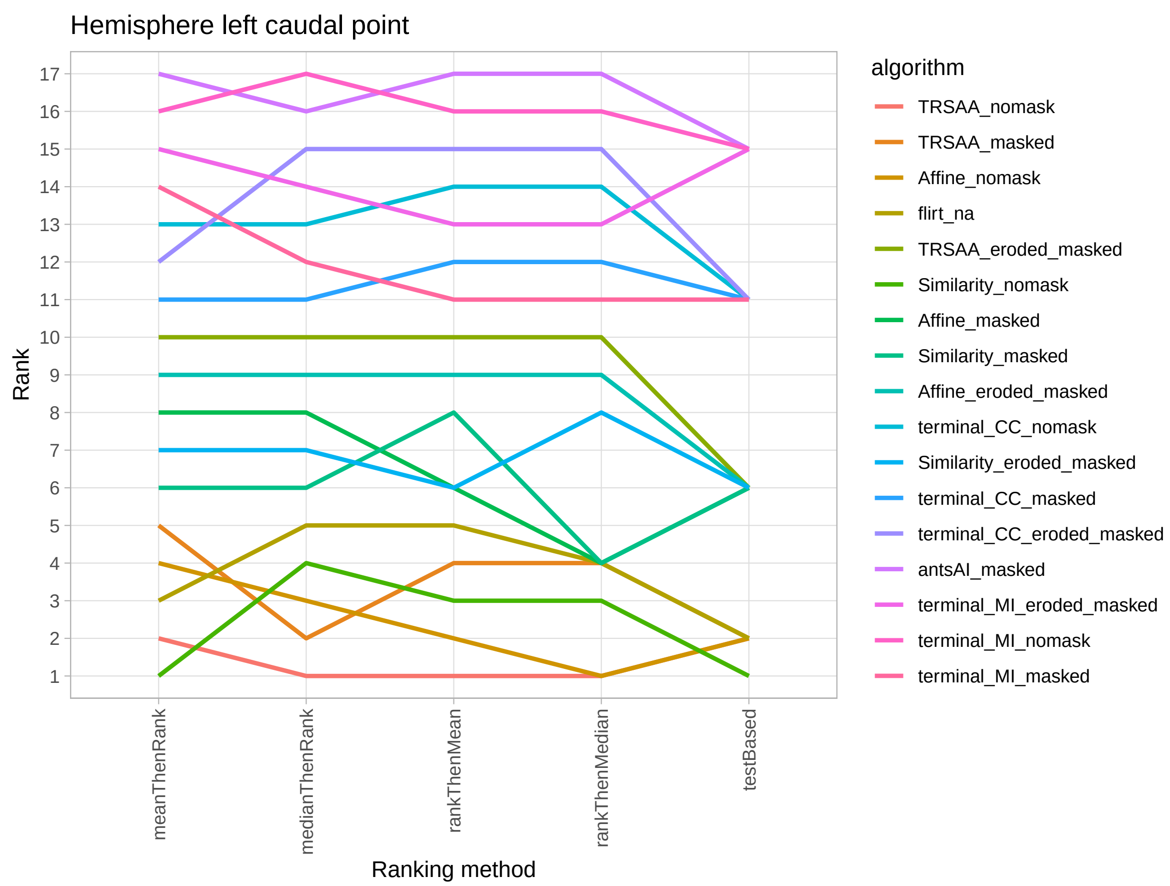
Hemisphere left caudal point
Rank
17
16
15
14
13
12
11
10
9
8
7
6
5
4
3
2
1
meanThenRank
medianThenRank
rankThenMean
rankThenMedian
testBased
Ranking method
algorithm
TRSAA_nomask
TRSAA_masked
Affine_nomask
flirt_na
TRSAA_eroded_masked
Similarity_nomask
Affine_masked
Similarity_masked
Affine_eroded_masked
terminal_CC_nomask
Similarity_eroded_masked
terminal_CC_masked
terminal_CC_eroded_masked
antsAI_masked
terminal_MI_eroded_masked
terminal_MI_nomask
terminal_MI_masked

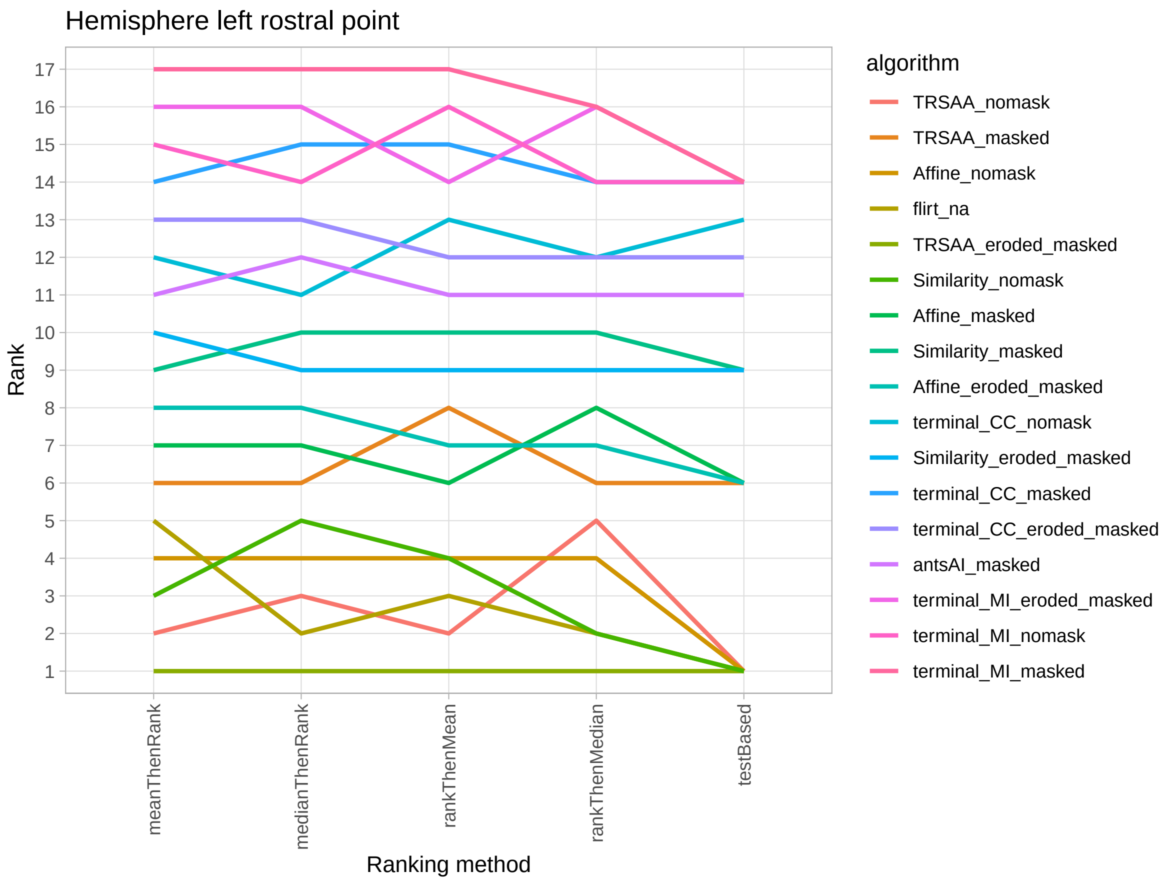
Hemisphere left rostral point
Rank
17
16
15
14
13
12
11
10
9
8
7
6
5
4
3
2
1
meanThenRank
medianThenRank
rankThenMean
rankThenMedian
testBased
Ranking method
algorithm
TRSAA_nomask
TRSAA_masked
Affine_nomask
flirt_na
TRSAA_eroded_masked
Similarity_nomask
Affine_masked
Similarity_masked
Affine_eroded_masked
terminal_CC_nomask
Similarity_eroded_masked
terminal_CC_masked
terminal_CC_eroded_masked
antsAI_masked
terminal_MI_eroded_masked
terminal_MI_nomask
terminal_MI_masked

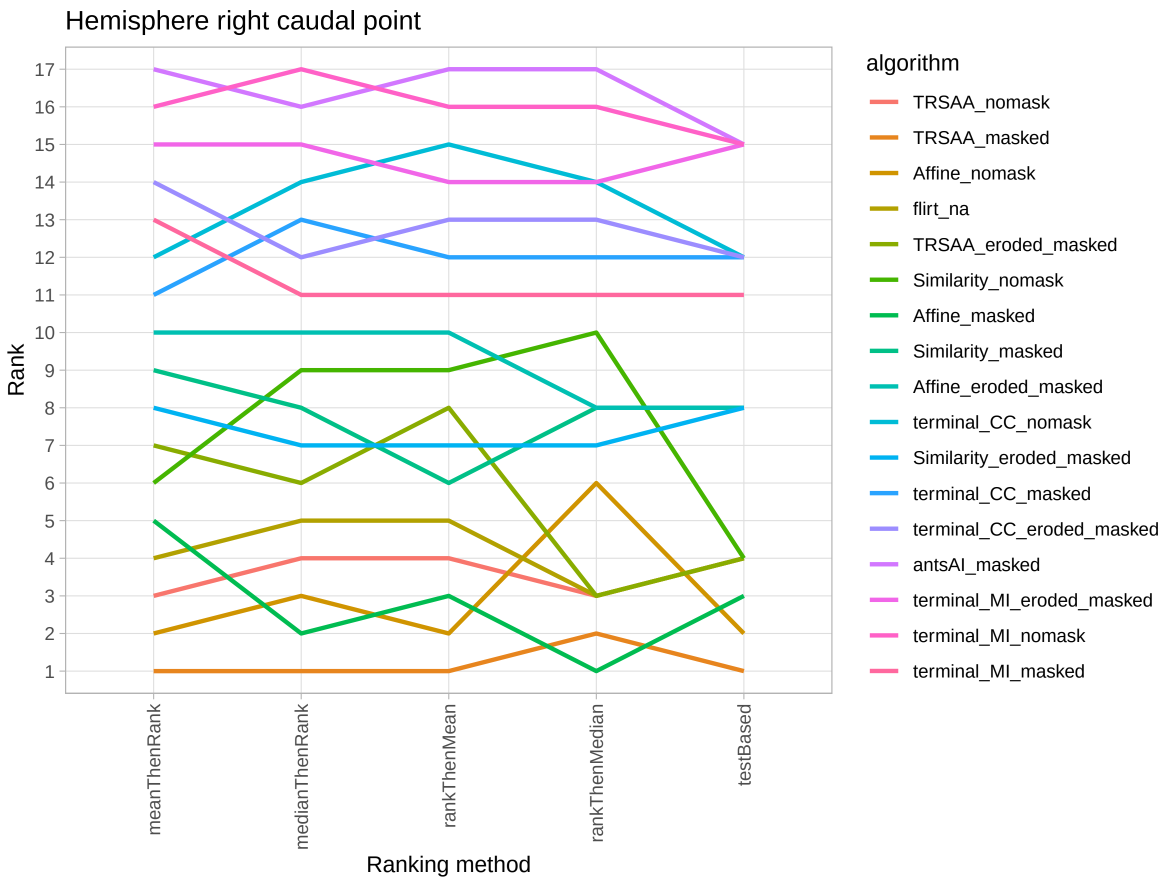

Hemisphere right caudal point
Rank
17
16
15
14
13
12
11
10
9
8
7
6
5
4
3
2
1
meanThenRank
medianThenRank
rankThenMean
rankThenMedian
testBased
Ranking method
algorithm
TRSAA_nomask
TRSAA_masked
Affine_nomask
flirt_na
TRSAA_eroded_masked
Similarity_nomask
Affine_masked
Similarity_masked
Affine_eroded_masked
terminal_CC_nomask
Similarity_eroded_masked
terminal_CC_masked
terminal_CC_eroded_masked
antsAI_masked
terminal_MI_eroded_masked
terminal_MI_nomask
terminal_MI_masked

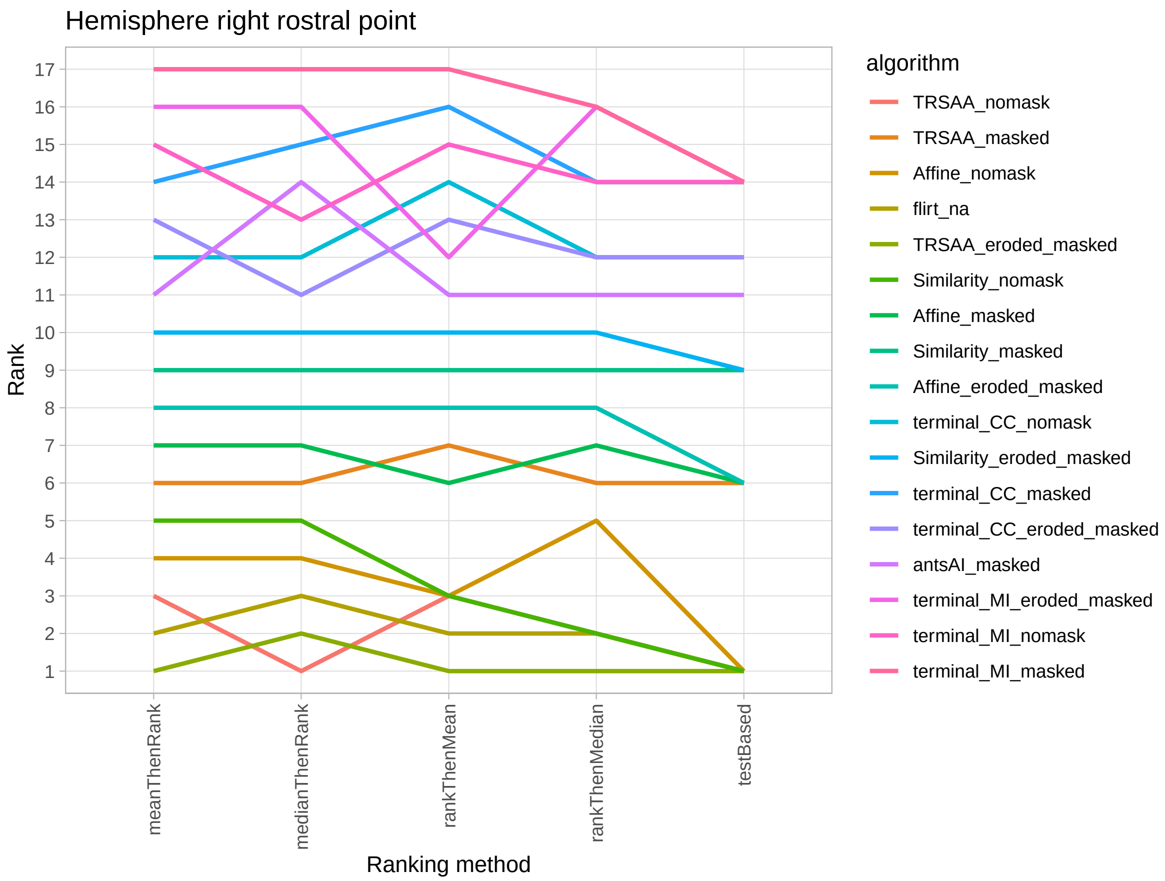
Hemisphere right rostral point
Rank
17
16
15
14
13
12
11
10
9
8
7
6
5
4
3
2
1
meanThenRank
medianThenRank
rankThenMean
rankThenMedian
testBased
Ranking method
algorithm
TRSAA_nomask
TRSAA_masked
Affine_nomask
flirt_na
TRSAA_eroded_masked
Similarity_nomask
Affine_masked
Similarity_masked
Affine_eroded_masked
terminal_CC_nomask
Similarity_eroded_masked
terminal_CC_masked
terminal_CC_eroded_masked
antsAI_masked
terminal_MI_eroded_masked
terminal_MI_nomask
terminal_MI_masked

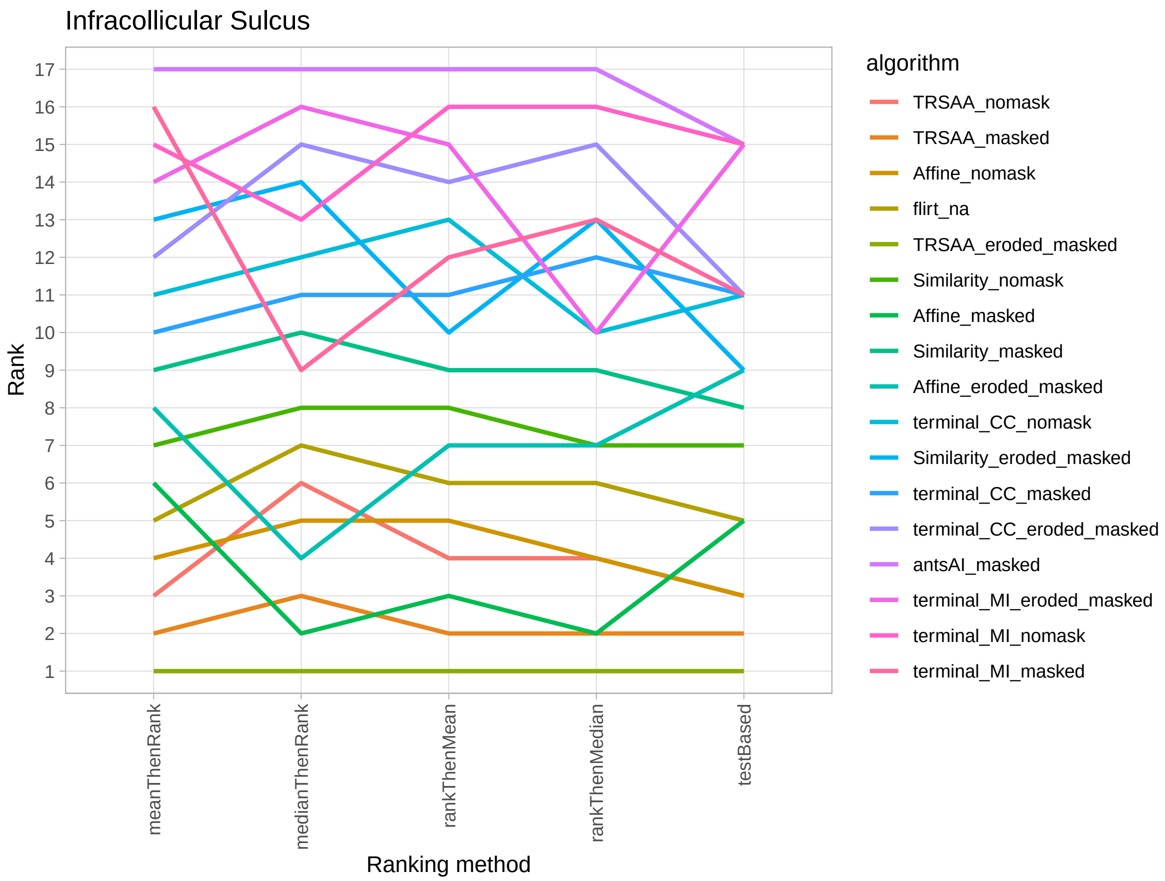

Infracollicular Sulcus
Rank
17
16
15
14
13
12
11
10
9
8
7
6
5
4
3
2
1
meanThenRank
medianThenRank
rankThenMean
rankThenMedian
testBased
Ranking method
algorithm
TRSAA_nomask
TRSAA_masked
Affine_nomask
flirt_na
TRSAA_eroded_masked
Similarity_nomask
Affine_masked
Similarity_masked
Affine_eroded_masked
terminal_CC_nomask
Similarity_eroded_masked
terminal_CC_masked
terminal_CC_eroded_masked
antsAI_masked
terminal_MI_eroded_masked
terminal_MI_nomask
terminal_MI_masked

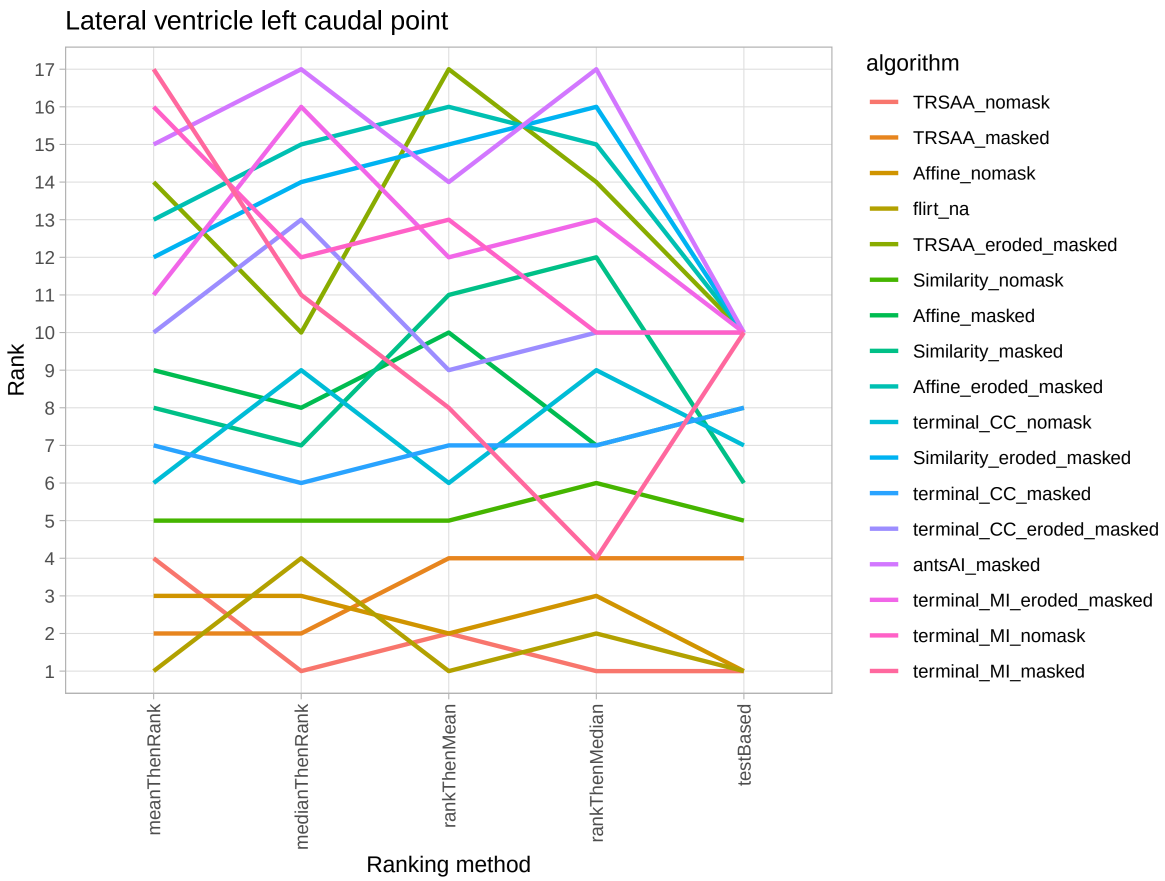
Lateral ventricle left caudal point
Rank
17
16
15
14
13
12
11
10
9
8
7
6
5
4
3
2
1
meanThenRank
medianThenRank
rankThenMean
rankThenMedian
testBased
Ranking method
algorithm
TRSAA_nomask
TRSAA_masked
Affine_nomask
flirt_na
TRSAA_eroded_masked
Similarity_nomask
Affine_masked
Similarity_masked
Affine_eroded_masked
terminal_CC_nomask
Similarity_eroded_masked
terminal_CC_masked
terminal_CC_eroded_masked
antsAI_masked
terminal_MI_eroded_masked
terminal_MI_nomask
terminal_MI_masked

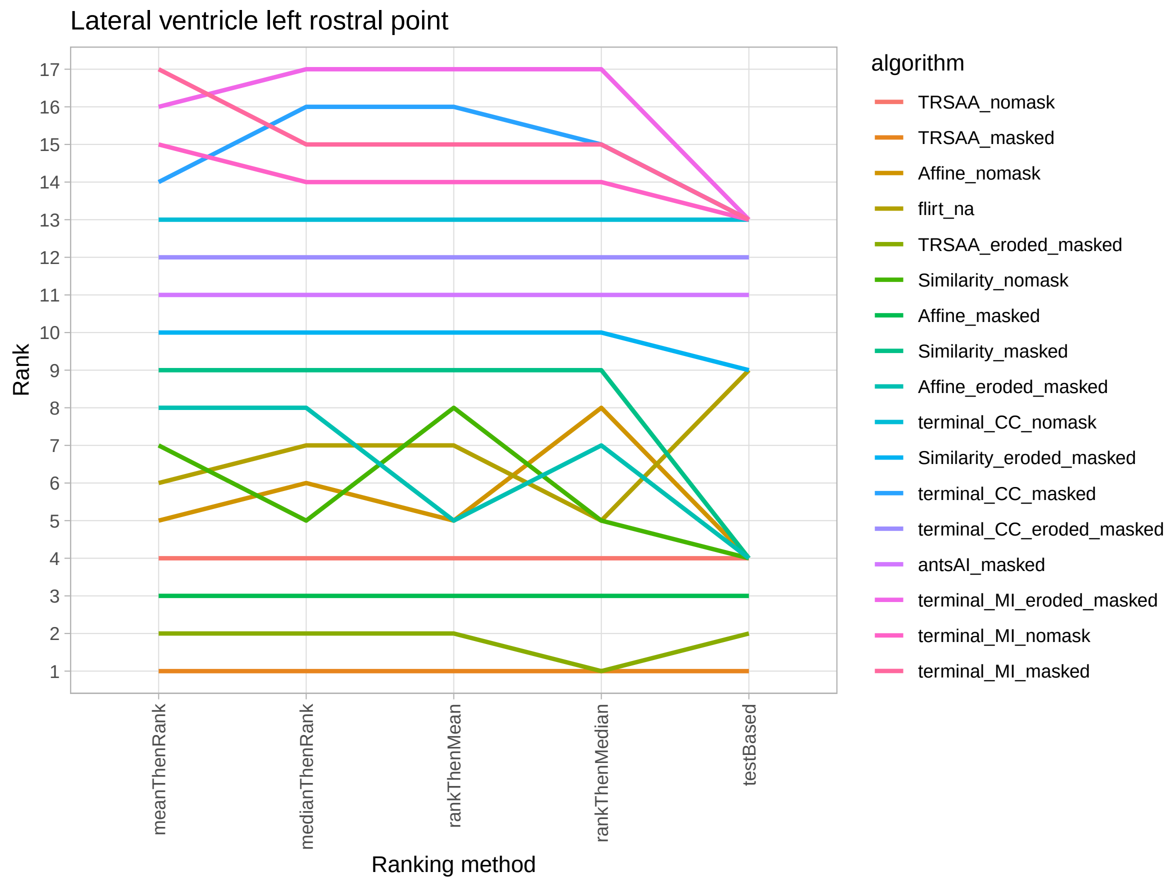

Lateral ventricle left rostral point
Rank
17
16
15
14
13
12
11
10
9
8
7
6
5
4
3
2
1
meanThenRank
medianThenRank
rankThenMean
rankThenMedian
testBased
Ranking method
algorithm
TRSAA_nomask
TRSAA_masked
Affine_nomask
flirt_na
TRSAA_eroded_masked
Similarity_nomask
Affine_masked
Similarity_masked
Affine_eroded_masked
terminal_CC_nomask
Similarity_eroded_masked
terminal_CC_masked
terminal_CC_eroded_masked
antsAI_masked
terminal_MI_eroded_masked
terminal_MI_nomask
terminal_MI_masked

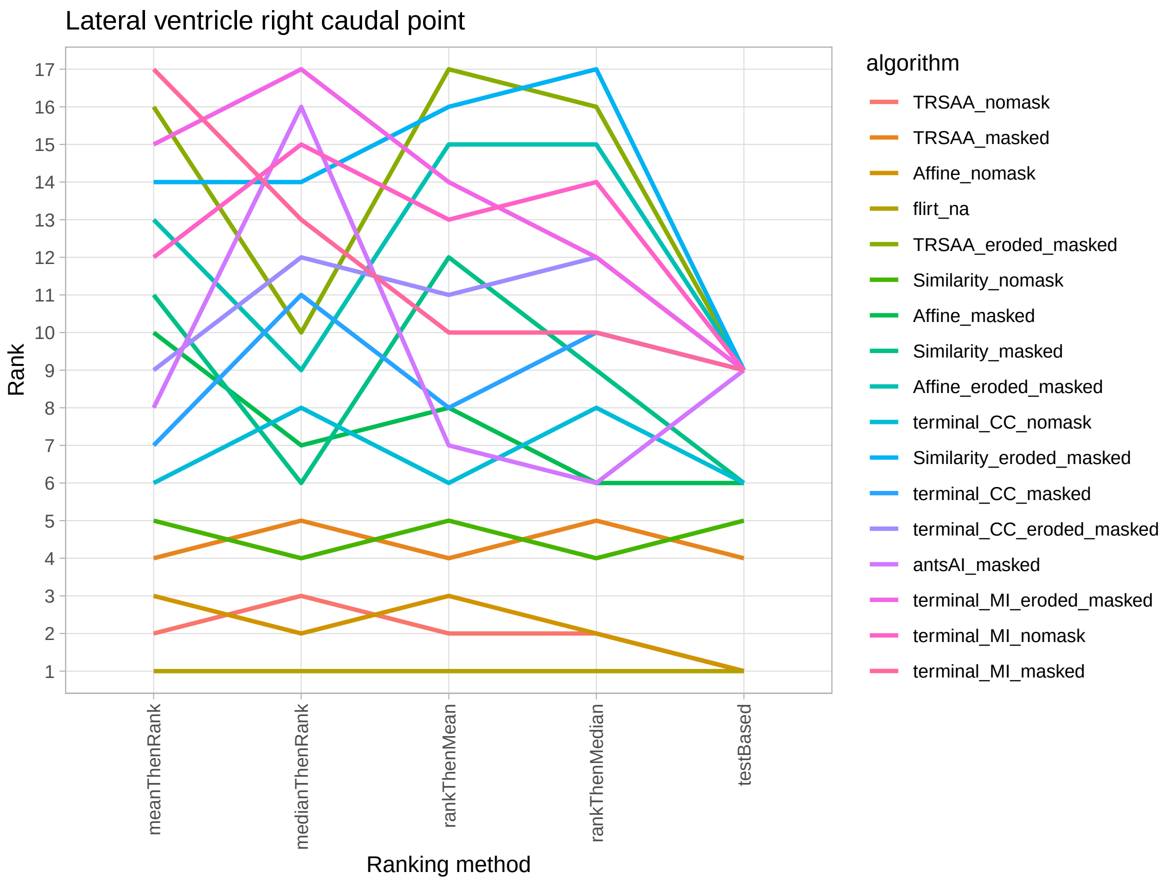
Lateral ventricle right caudal point
Rank
17
16
15
14
13
12
11
10
9
8
7
6
5
4
3
2
1
meanThenRank
medianThenRank
rankThenMean
rankThenMedian
testBased
Ranking method
algorithm
TRSAA_nomask
TRSAA_masked
Affine_nomask
flirt_na
TRSAA_eroded_masked
Similarity_nomask
Affine_masked
Similarity_masked
Affine_eroded_masked
terminal_CC_nomask
Similarity_eroded_masked
terminal_CC_masked
terminal_CC_eroded_masked
antsAI_masked
terminal_MI_eroded_masked
terminal_MI_nomask
terminal_MI_masked

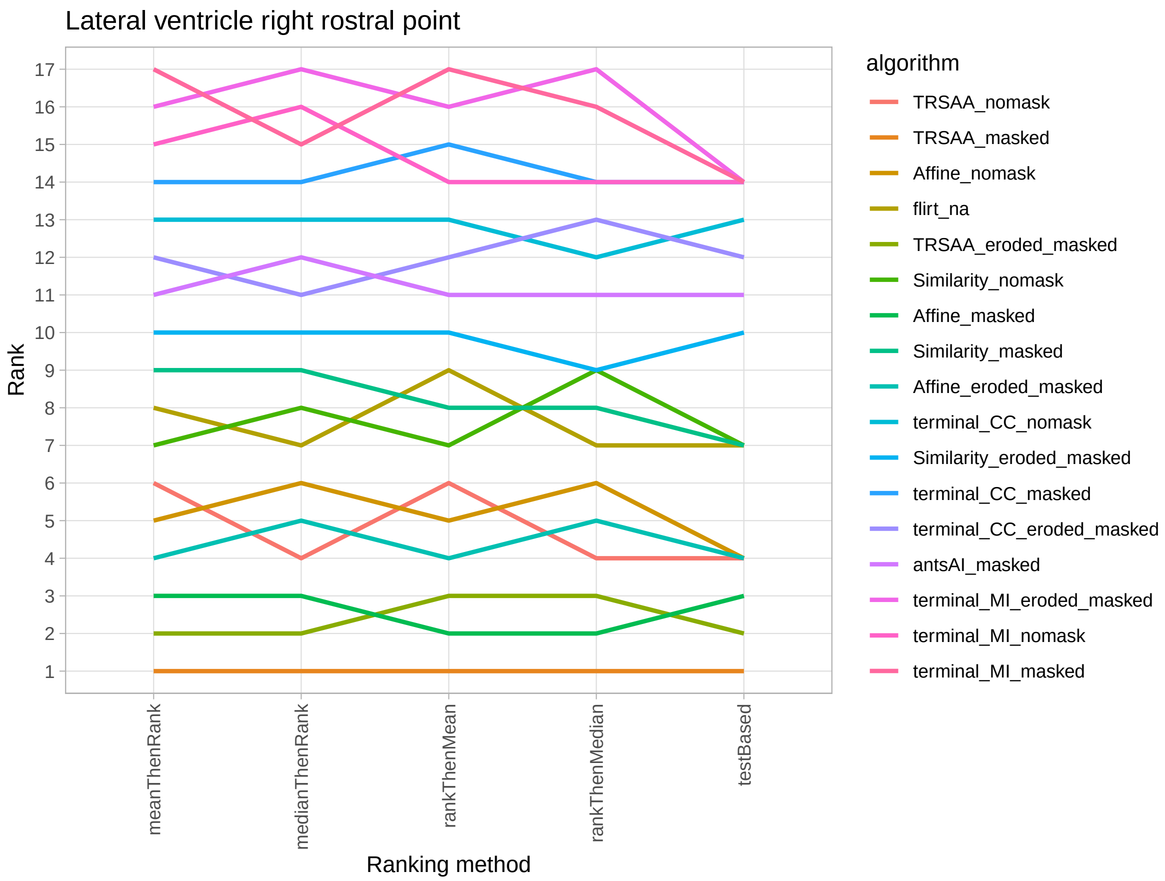

Lateral ventricle right rostral point
Rank
17
16
15
14
13
12
11
10
9
8
7
6
5
4
3
2
1
meanThenRank
medianThenRank
rankThenMean
rankThenMedian
testBased
Ranking method
algorithm
TRSAA_nomask
TRSAA_masked
Affine_nomask
flirt_na
TRSAA_eroded_masked
Similarity_nomask
Affine_masked
Similarity_masked
Affine_eroded_masked
terminal_CC_nomask
Similarity_eroded_masked
terminal_CC_masked
terminal_CC_eroded_masked
antsAI_masked
terminal_MI_eroded_masked
terminal_MI_nomask
terminal_MI_masked

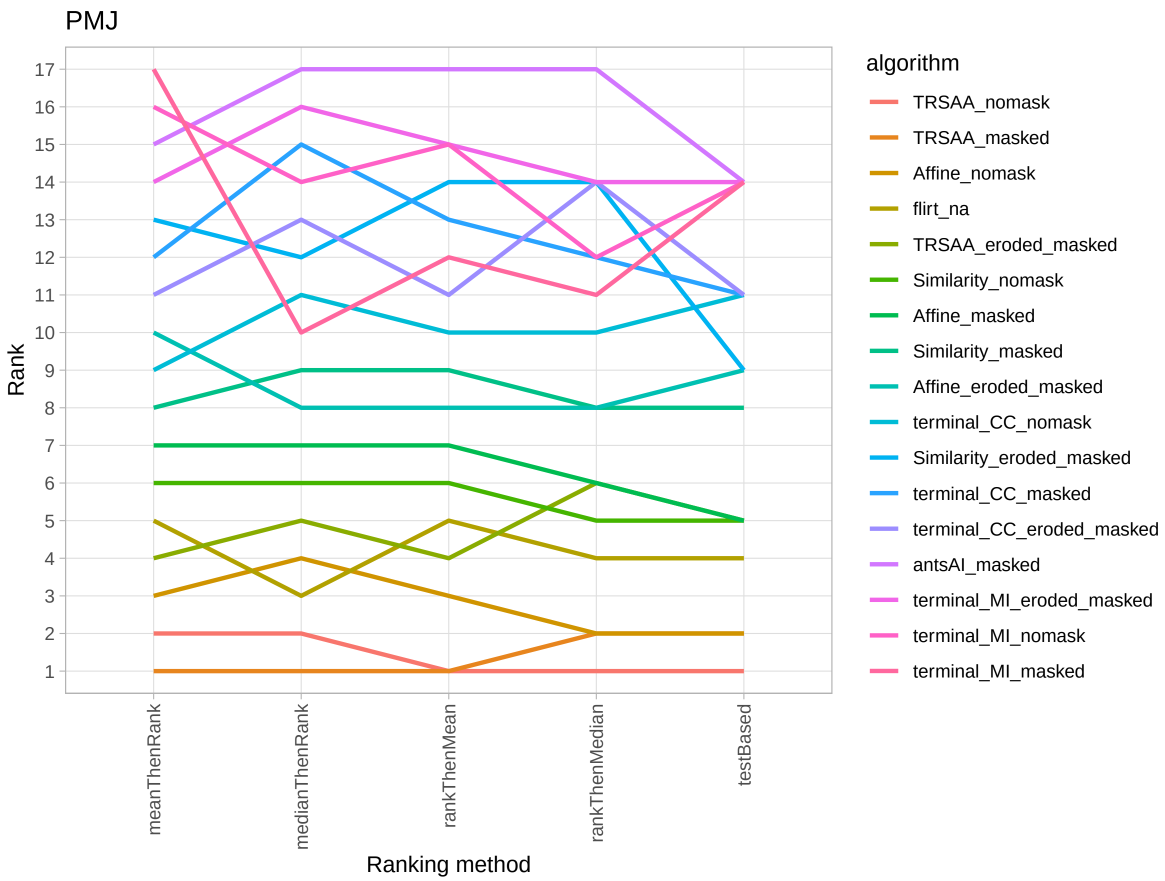
PMJ
Rank
17
16
15
14
13
12
11
10
9
8
7
6
5
4
3
2
1
meanThenRank
medianThenRank
rankThenMean
rankThenMedian
testBased
Ranking method
algorithm
TRSAA_nomask
TRSAA_masked
Affine_nomask
flirt_na
TRSAA_eroded_masked
Similarity_nomask
Affine_masked
Similarity_masked
Affine_eroded_masked
terminal_CC_nomask
Similarity_eroded_masked
terminal_CC_masked
terminal_CC_eroded_masked
antsAI_masked
terminal_MI_eroded_masked
terminal_MI_nomask
terminal_MI_masked

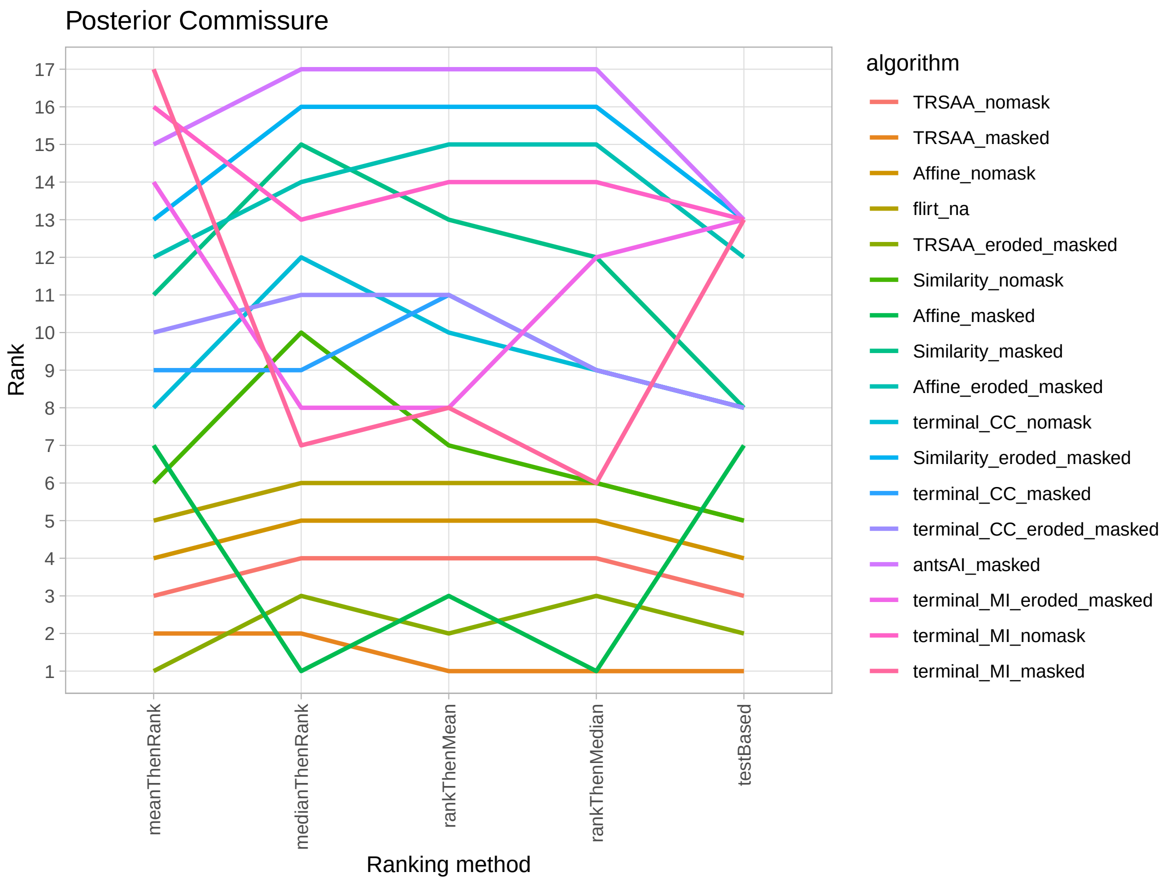

Posterior Commissure
Rank
17
16
15
14
13
12
11
10
9
8
7
6
5
4
3
2
1
meanThenRank
medianThenRank
rankThenMean
rankThenMedian
testBased
Ranking method
algorithm
TRSAA_nomask
TRSAA_masked
Affine_nomask
flirt_na
TRSAA_eroded_masked
Similarity_nomask
Affine_masked
Similarity_masked
Affine_eroded_masked
terminal_CC_nomask
Similarity_eroded_masked
terminal_CC_masked
terminal_CC_eroded_masked
antsAI_masked
terminal_MI_eroded_masked
terminal_MI_nomask
terminal_MI_masked

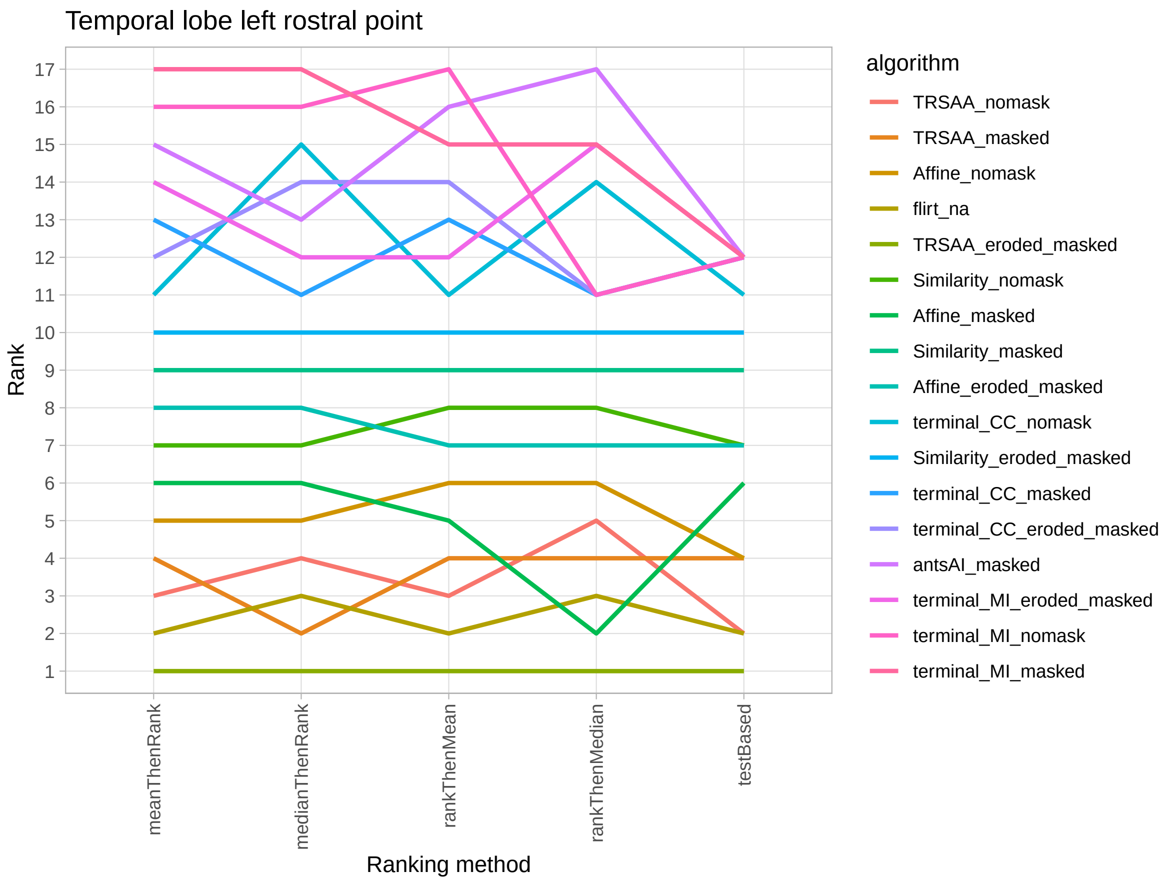
Temporal lobe left rostral point
Rank
17
16
15
14
13
12
11
10
9
8
7
6
5
4
3
2
1
meanThenRank
medianThenRank
rankThenMean
rankThenMedian
testBased
Ranking method
algorithm
TRSAA_nomask
TRSAA_masked
Affine_nomask
flirt_na
TRSAA_eroded_masked
Similarity_nomask
Affine_masked
Similarity_masked
Affine_eroded_masked
terminal_CC_nomask
Similarity_eroded_masked
terminal_CC_masked
terminal_CC_eroded_masked
antsAI_masked
terminal_MI_eroded_masked
terminal_MI_nomask
terminal_MI_masked

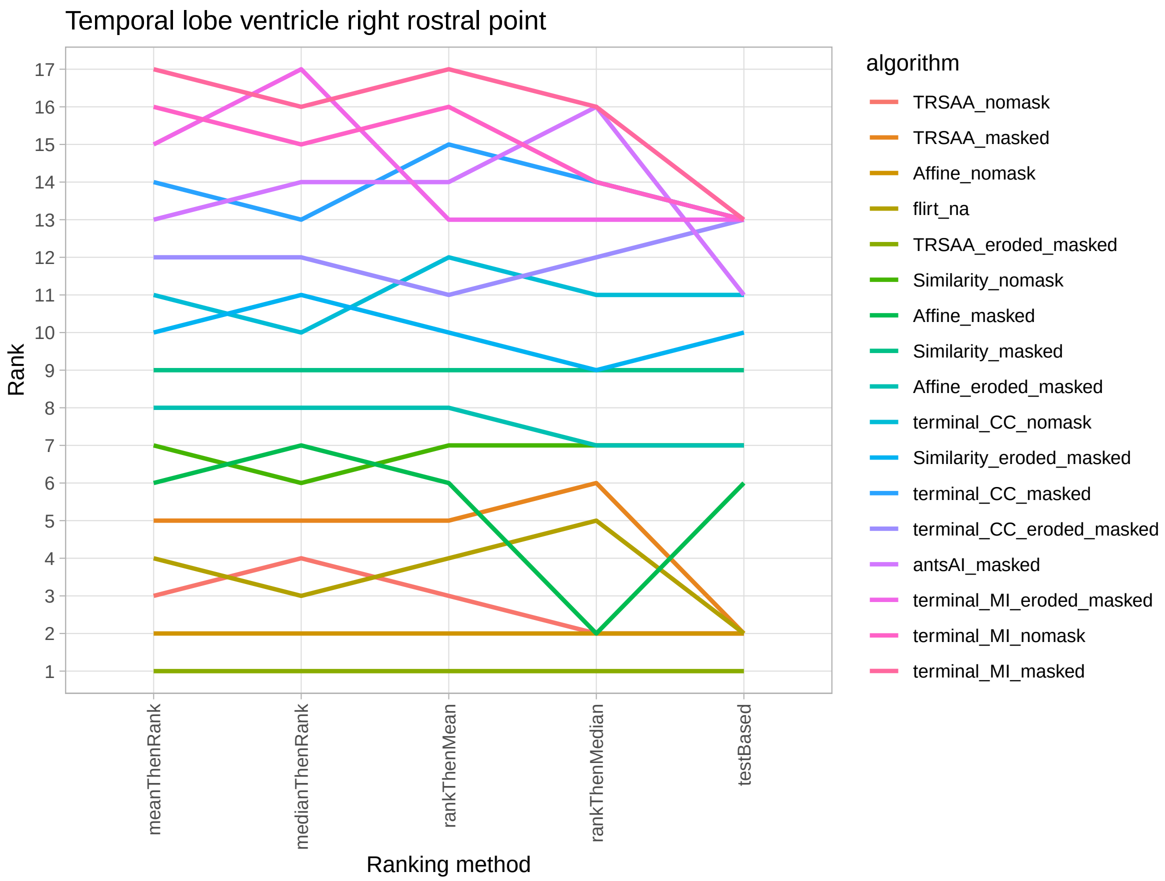
Temporal lobe ventricle right rostral point
Rank
Ranking method
meanThenRank
medianThenRank
rankThenMean
rankThenMedian
testBased
algorithm
TRSAA_nomask
TRSAA_masked
Affine_nomask
flirt_na
TRSAA_eroded_masked
Similarity_nomask
Affine_masked
Similarity_masked
Affine_eroded_masked
terminal_CC_nomask
Similarity_eroded_masked
terminal_CC_masked
terminal_CC_eroded_masked
antsAI_masked
terminal_MI_eroded_masked
terminal_MI_nomask
terminal_MI_masked

4 Visualization of cross-task insights

The algorithms are ordered according to consensus ranking.

4.1 Characterization of algorithms

4.1.1 Ranking stability: Variability of achieved rankings across tasks

Algorithms are color-coded, and the area of each blob at position $(A_i, \text{rank } j)$ is proportional to the relative frequency A_i achieved rank j across multiple tasks. The median rank for each algorithm is indicated by a black cross. This way, the distribution of ranks across tasks can be intuitively visualized.

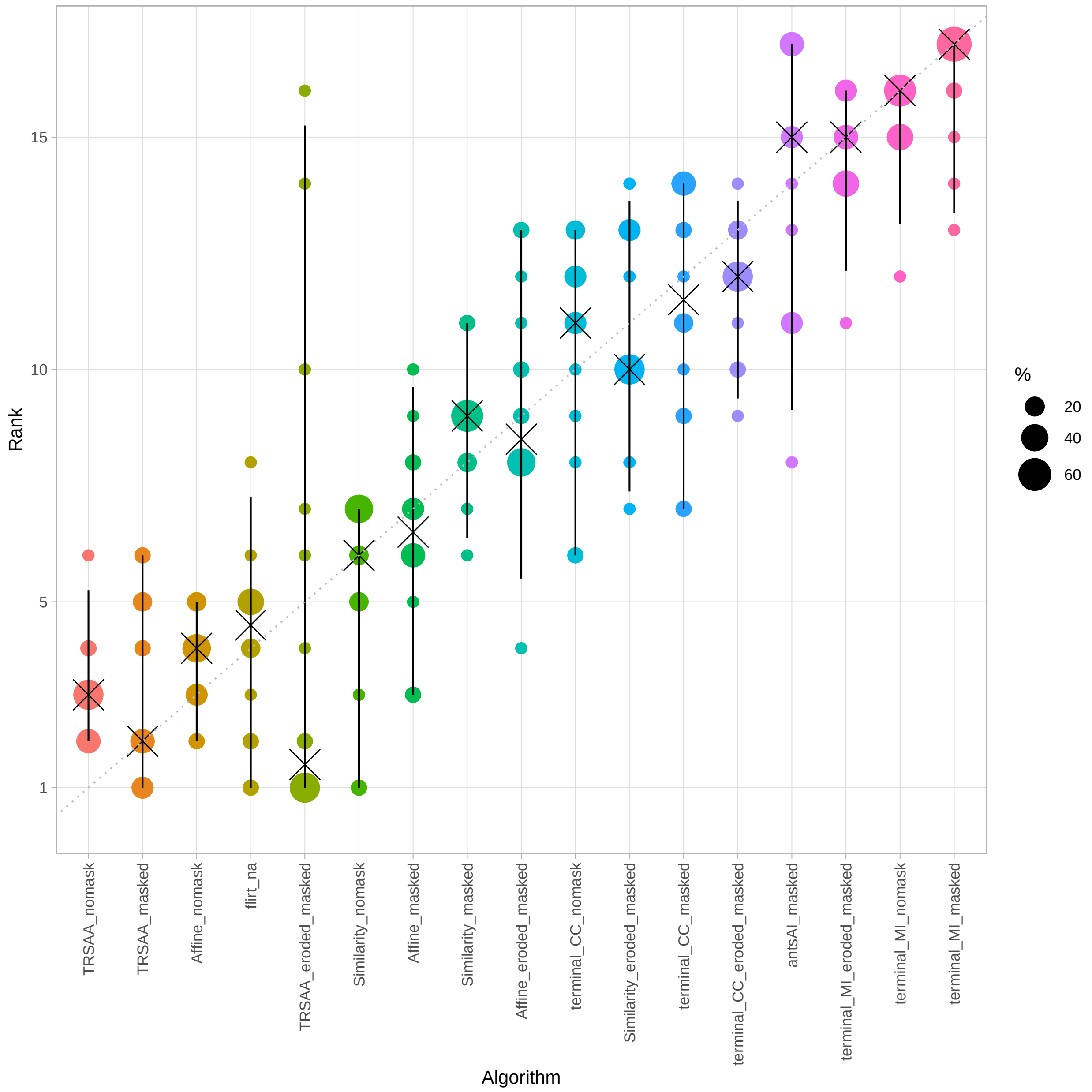

4.1.2 Ranking stability: Ranking variability via bootstrap approach

A blob plot of bootstrap results over the different tasks separated by algorithm allows another perspective on the assessment data. This gives deeper insights into the characteristics of tasks and the ranking uncertainty of the algorithms in each task.

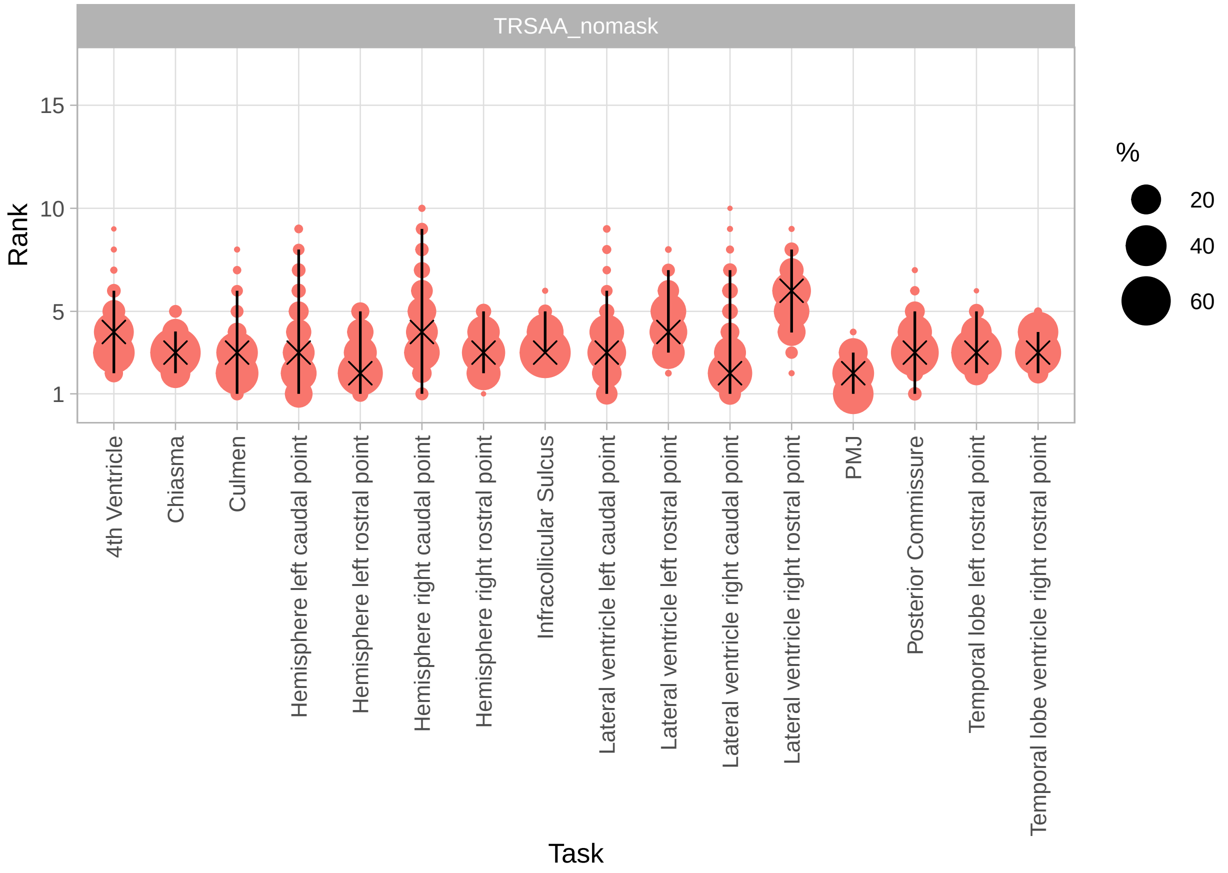

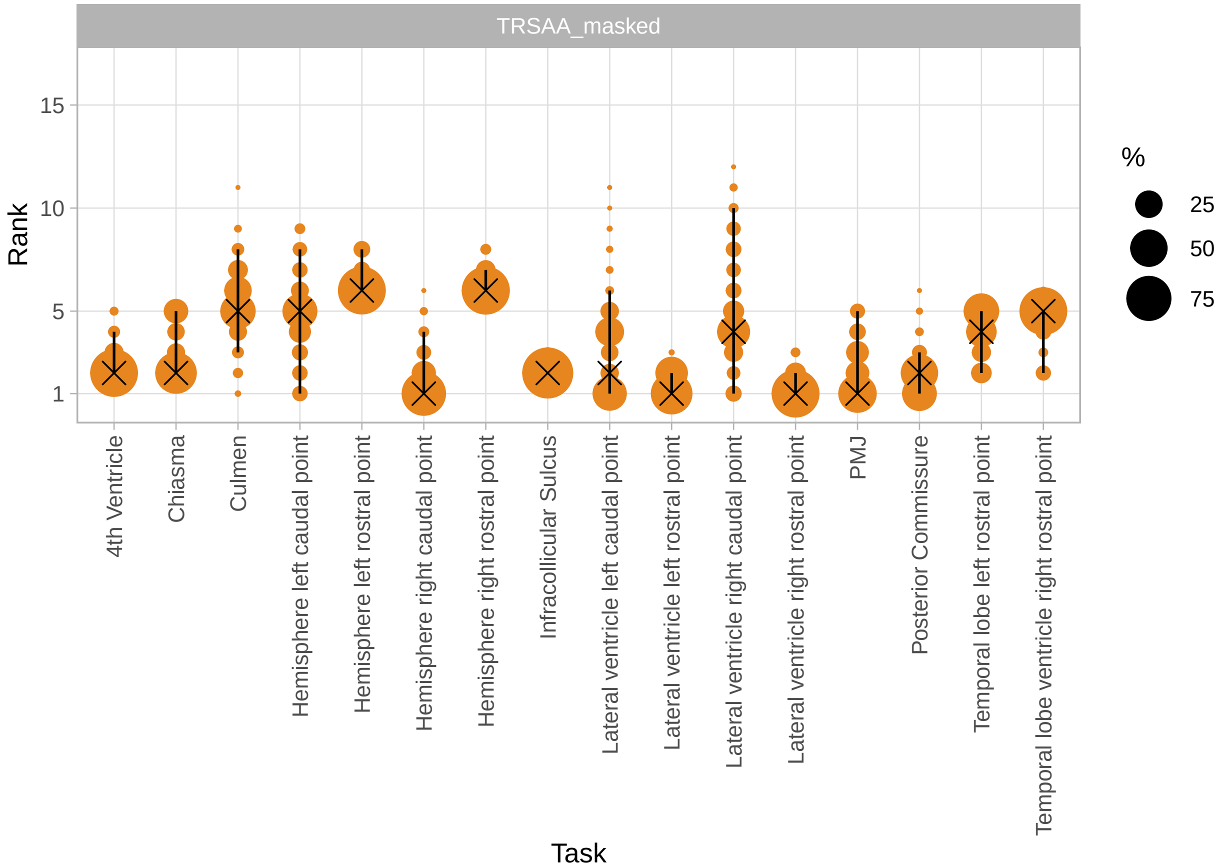
TRSAA_masked
Rank
15
10
5
1
%
25
50
75
4th Ventricle
Chiasma
Culmen
Hemisphere left caudal point
Hemisphere left rostral point
Hemisphere right caudal point
Hemisphere right rostral point
Infracollicular Sulcus
Lateral ventricle left caudal point
Lateral ventricle left rostral point
Lateral ventricle right caudal point
Lateral ventricle right rostral point
PMJ
Posterior Commissure
Temporal lobe left rostral point
Temporal lobe ventricle right rostral point
Task

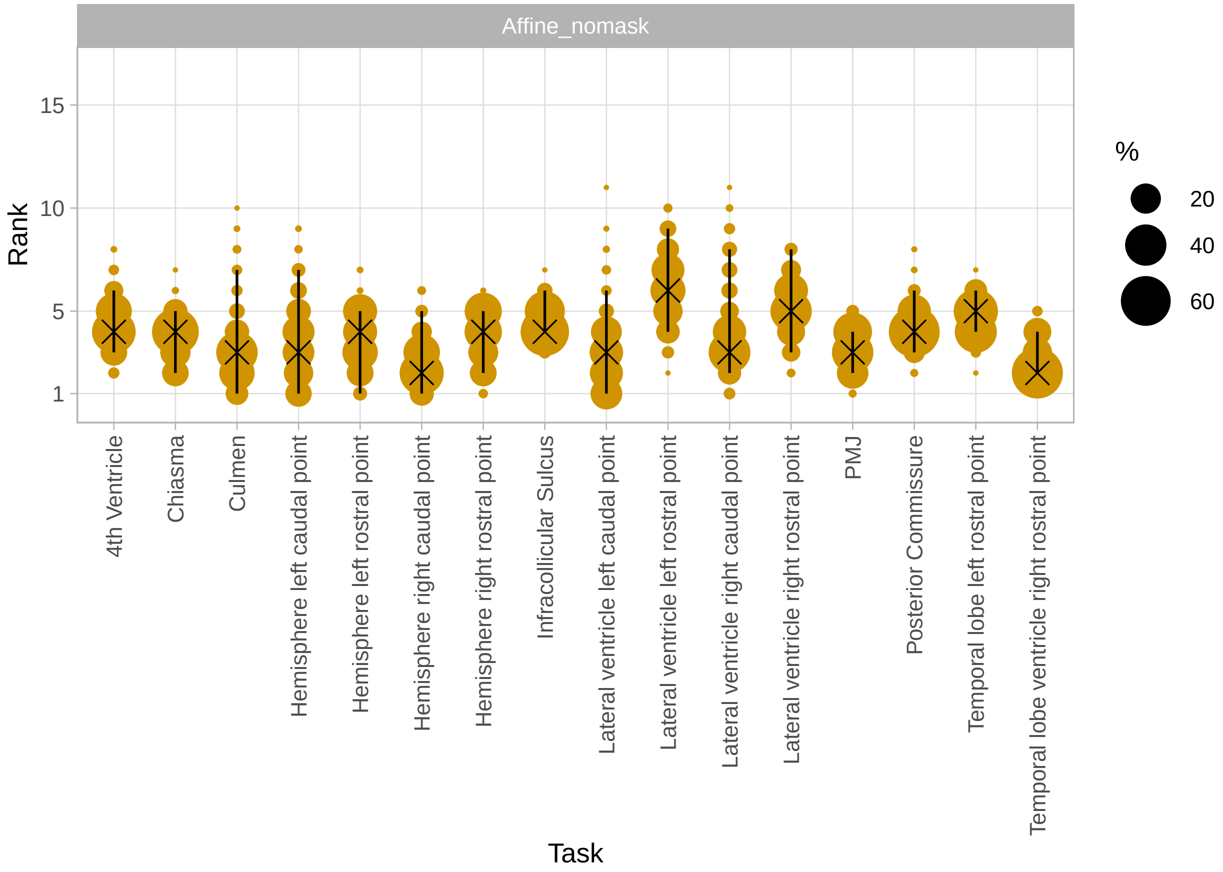
Affine_nomask
Rank
15
10
5
1
%
20
40
60
4th Ventricle
Chiasma
Culmen
Hemisphere left caudal point
Hemisphere left rostral point
Hemisphere right caudal point
Hemisphere right rostral point
Infracollicular Sulcus
Lateral ventricle left caudal point
Lateral ventricle left rostral point
Lateral ventricle right caudal point
Lateral ventricle right rostral point
PMJ
Posterior Commissure
Temporal lobe left rostral point
Temporal lobe ventricle right rostral point
Task

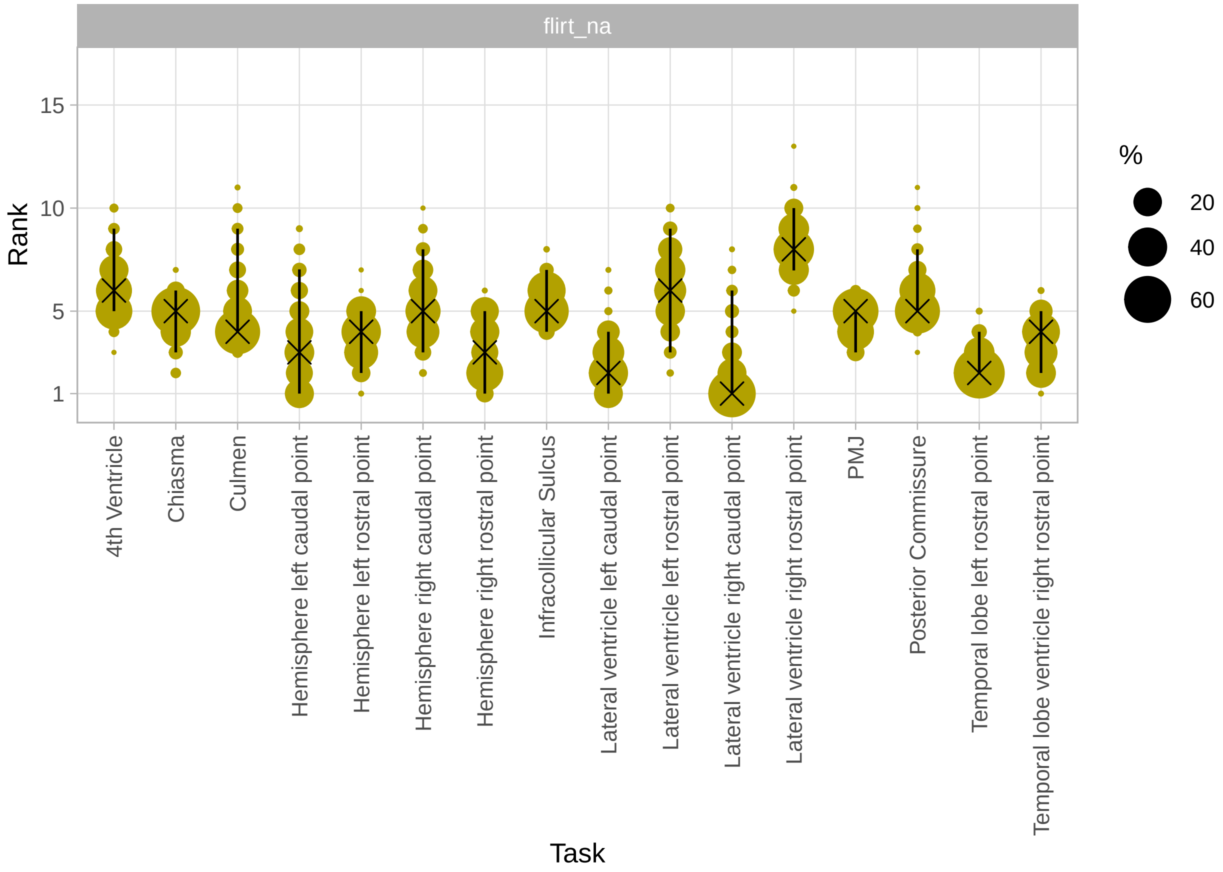
flirt_na
Rank
15
10
5
1
%
20
40
60
4th Ventricle
Chiasma
Culmen
Hemisphere left caudal point
Hemisphere left rostral point
Hemisphere right caudal point
Hemisphere right rostral point
Infracollicular Sulcus
Lateral ventricle left caudal point
Lateral ventricle left rostral point
Lateral ventricle right caudal point
Lateral ventricle right rostral point
PMJ
Posterior Commissure
Temporal lobe left rostral point
Temporal lobe ventricle right rostral point
Task

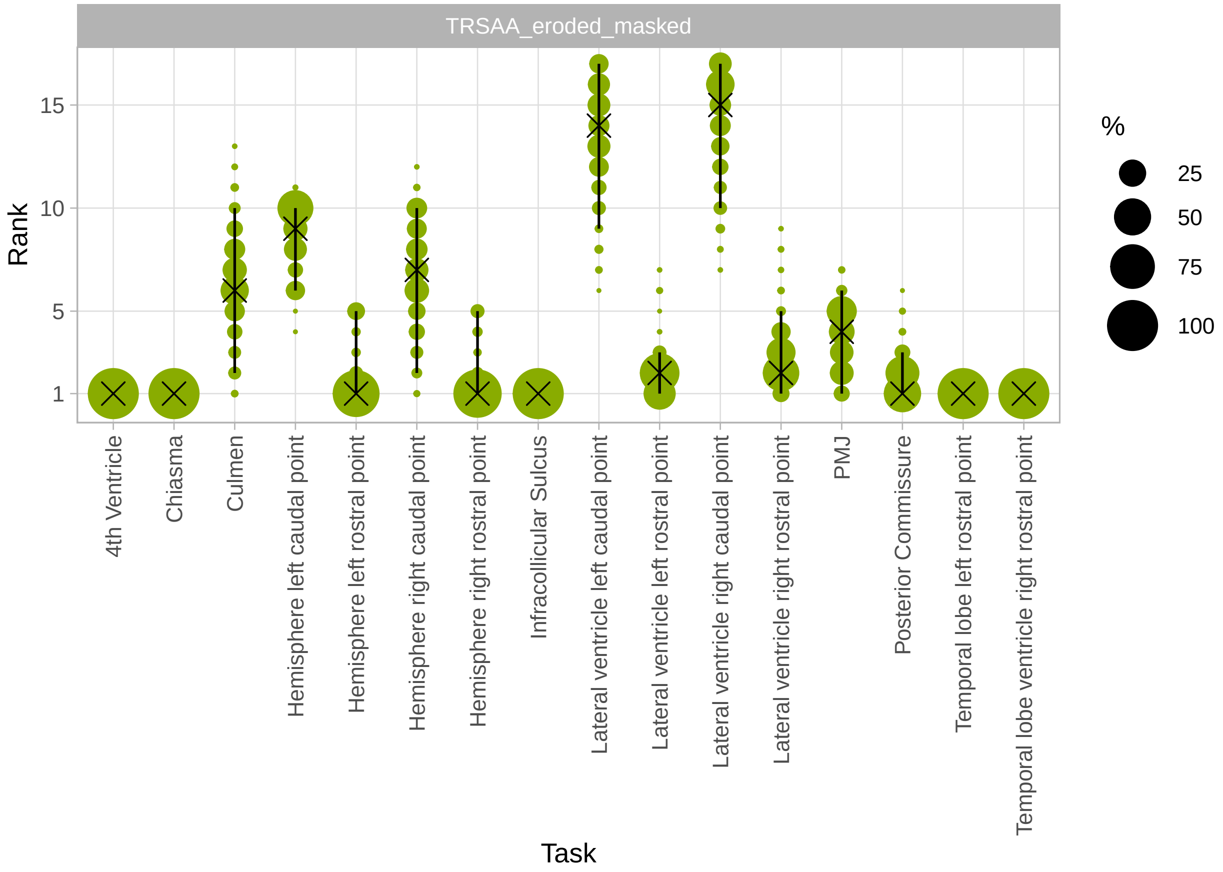

TRSAA_eroded_masked
Rank
15
10
5
1
%
25
50
75
100
4th Ventricle
Chiasma
Culmen
Hemisphere left caudal point
Hemisphere left rostral point
Hemisphere right caudal point
Hemisphere right rostral point
Infracollicular Sulcus
Lateral ventricle left caudal point
Lateral ventricle left rostral point
Lateral ventricle right caudal point
Lateral ventricle right rostral point
PMJ
Posterior Commissure
Temporal lobe left rostral point
Temporal lobe ventricle right rostral point
Task

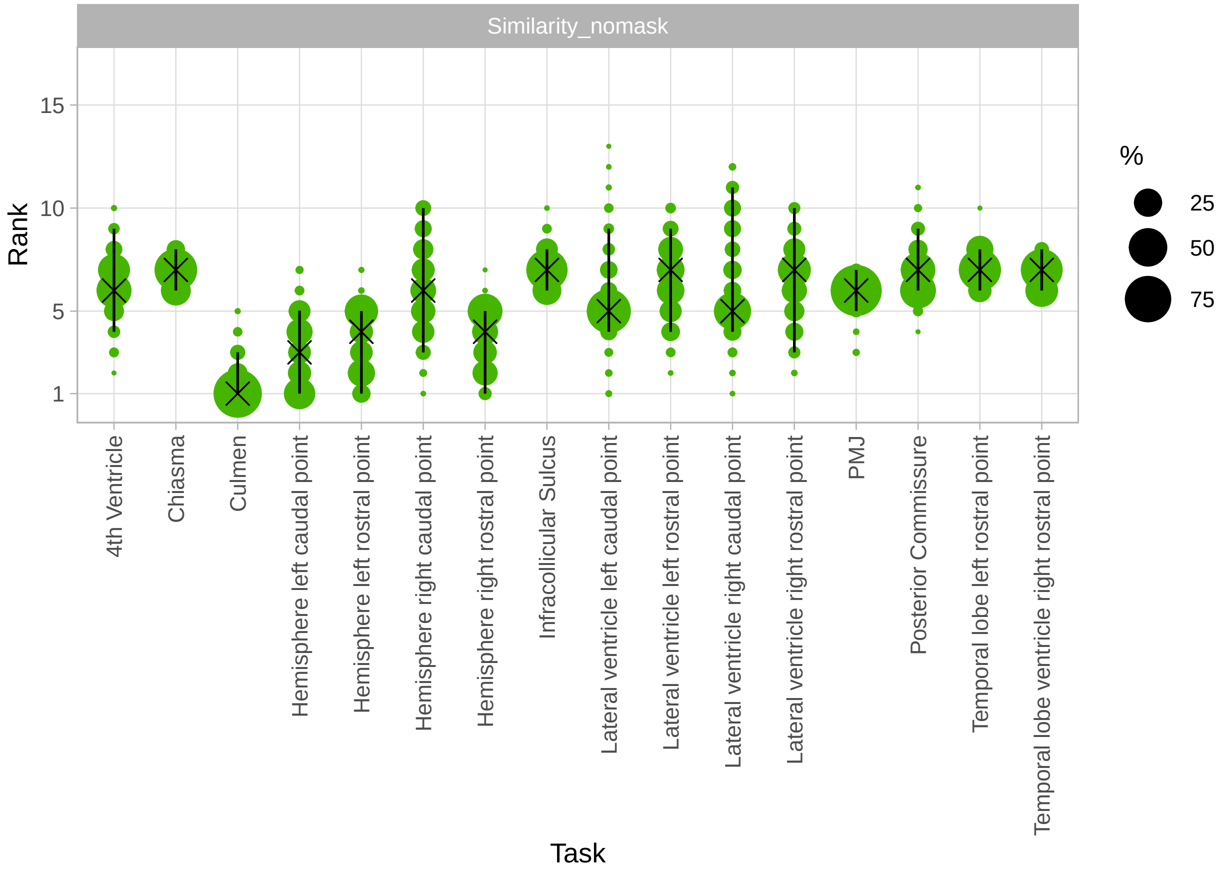
Similarity_nomask
Rank
15
10
5
1
4th Ventricle
Chiasma
Culmen
Hemisphere left caudal point
Hemisphere left rostral point
Hemisphere right caudal point
Hemisphere right rostral point
Infracollicular Sulcus
Lateral ventricle left caudal point
Lateral ventricle left rostral point
Lateral ventricle right caudal point
Lateral ventricle right rostral point
PMJ
Posterior Commissure
Temporal lobe left rostral point
Temporal lobe ventricle right rostral point
Task
%
25
50
75

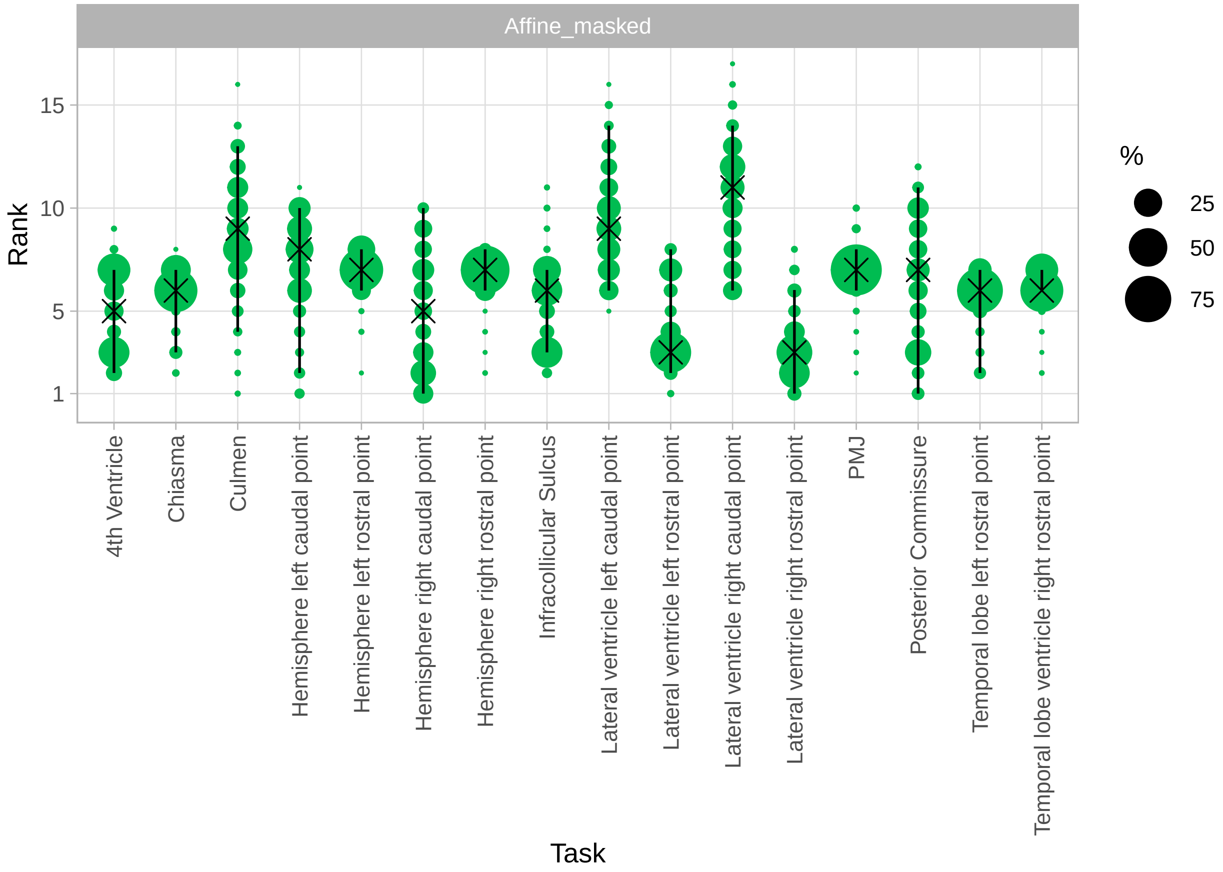
Affine_masked
Rank
15
10
5
1
4th Ventricle
Chiasma
Culmen
Hemisphere left caudal point
Hemisphere left rostral point
Hemisphere right caudal point
Hemisphere right rostral point
Infracollicular Sulcus
Lateral ventricle left caudal point
Lateral ventricle left rostral point
Lateral ventricle right caudal point
Lateral ventricle right rostral point
PMJ
Posterior Commissure
Temporal lobe left rostral point
Temporal lobe ventricle right rostral point
Task
%
25
50
75

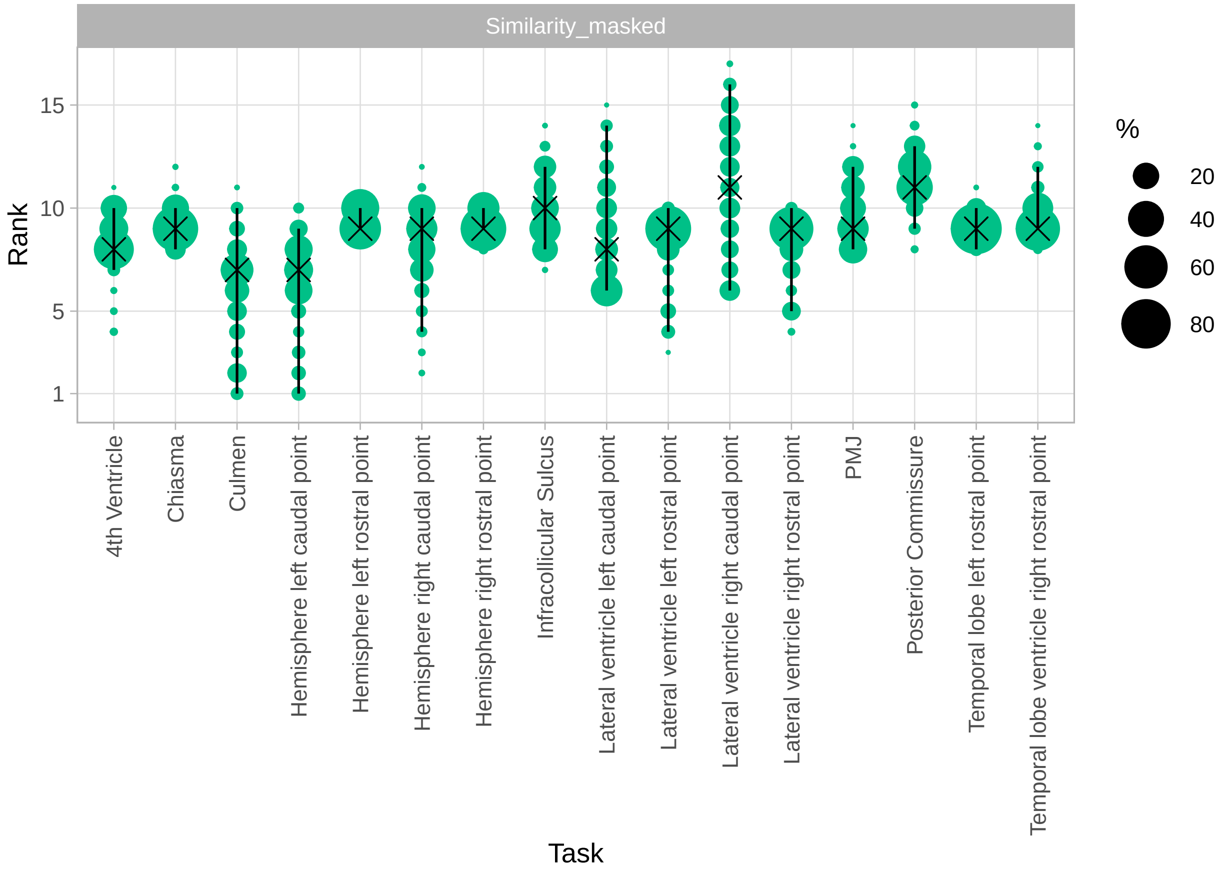
Similarity_masked
Rank
15
10
5
1
%
20
40
60
80
4th Ventricle
Chiasma
Culmen
Hemisphere left caudal point
Hemisphere left rostral point
Hemisphere right caudal point
Hemisphere right rostral point
Infracollicular Sulcus
Lateral ventricle left caudal point
Lateral ventricle left rostral point
Lateral ventricle right caudal point
Lateral ventricle right rostral point
PMJ
Posterior Commissure
Temporal lobe left rostral point
Temporal lobe ventricle right rostral point
Task

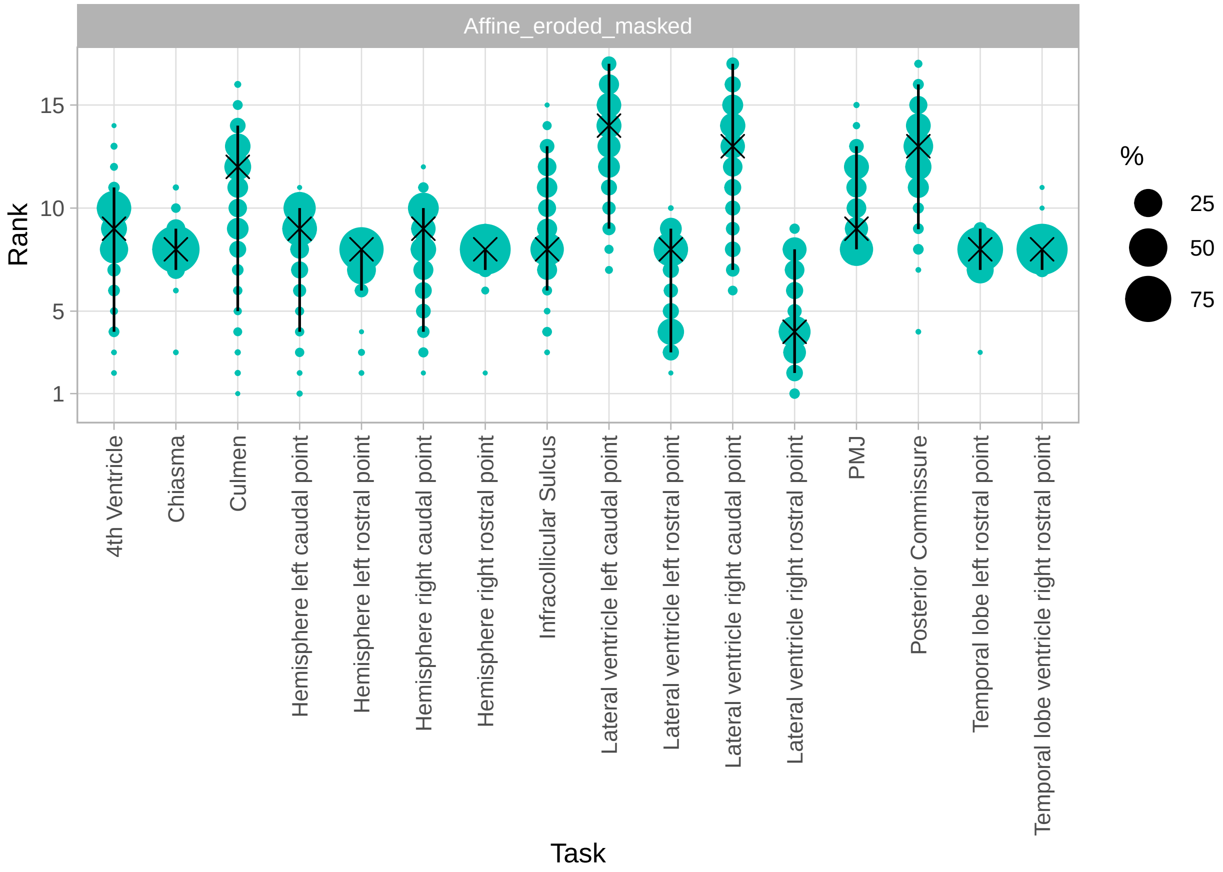

Affine_eroded_masked
Rank
15
10
5
1
4th Ventricle
Chiasma
Culmen
Hemisphere left caudal point
Hemisphere left rostral point
Hemisphere right caudal point
Hemisphere right rostral point
Infracollicular Sulcus
Lateral ventricle left caudal point
Lateral ventricle left rostral point
Lateral ventricle right caudal point
Lateral ventricle right rostral point
PMJ
Posterior Commissure
Temporal lobe left rostral point
Temporal lobe ventricle right rostral point
Task
%
25
50
75

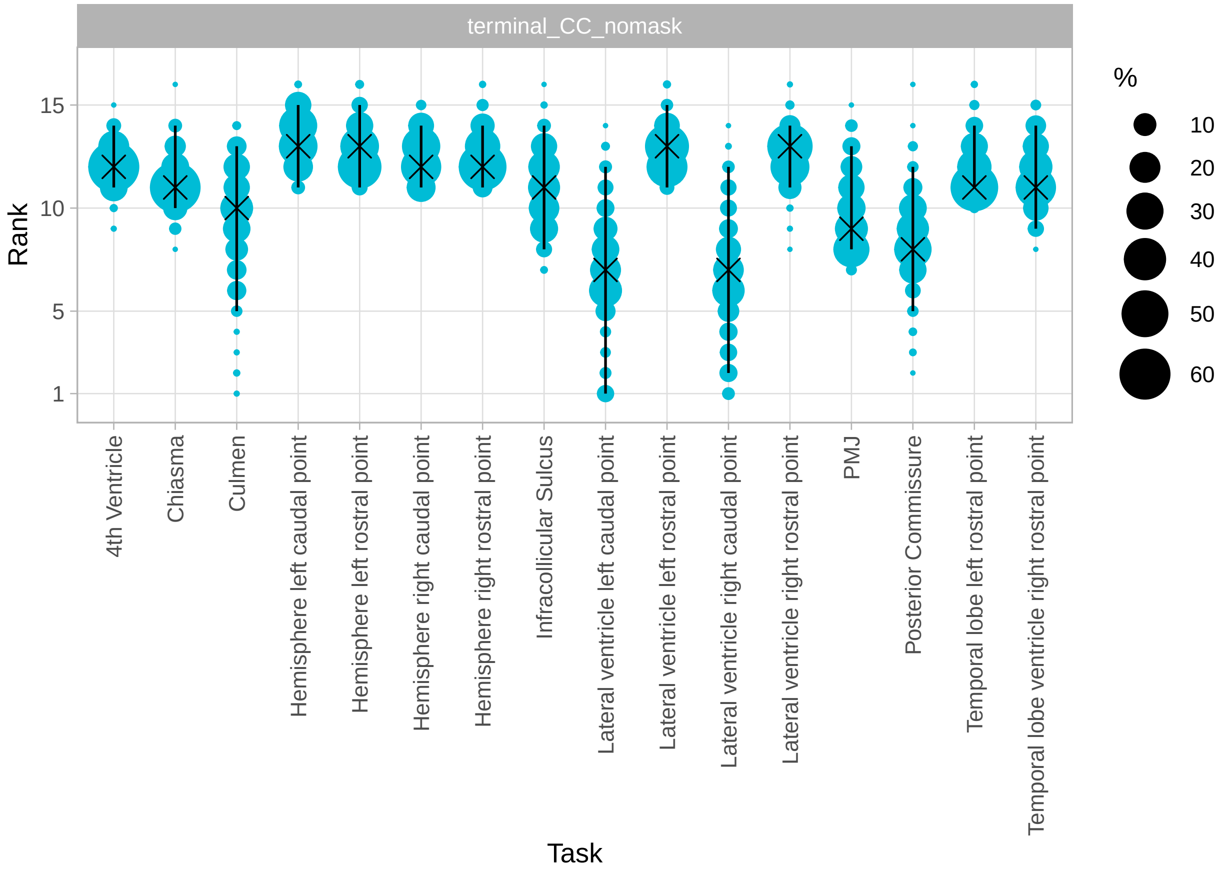
terminal_CC_nomask
Rank
15
10
5
1
4th Ventricle
Chiasma
Culmen
Hemisphere left caudal point
Hemisphere left rostral point
Hemisphere right caudal point
Hemisphere right rostral point
Infracollicular Sulcus
Lateral ventricle left caudal point
Lateral ventricle left rostral point
Lateral ventricle right caudal point
Lateral ventricle right rostral point
PMJ
Posterior Commissure
Temporal lobe left rostral point
Temporal lobe ventricle right rostral point
Task
%
10
20
30
40
50
60

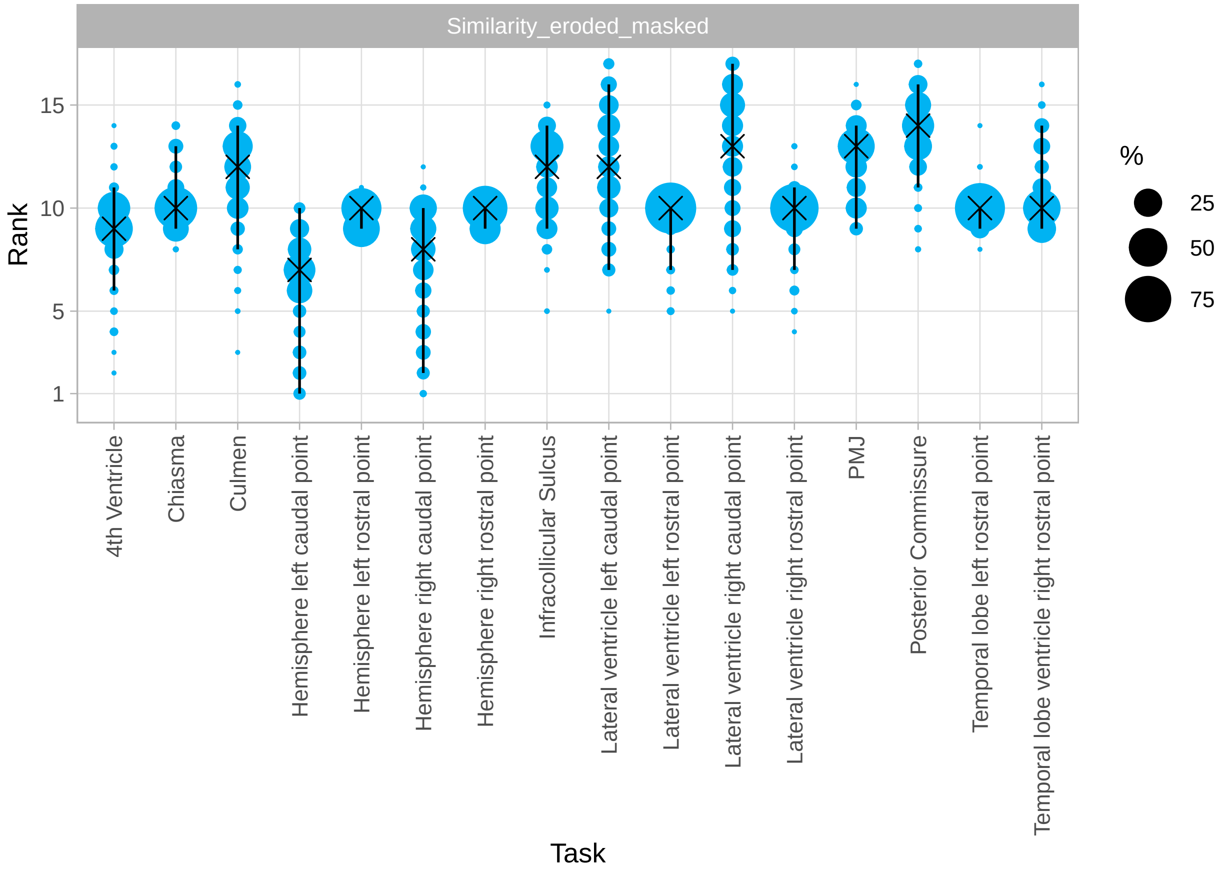

Similarity_eroded_masked
Rank
15
10
5
1
%
25
50
75
4th Ventricle
Chiasma
Culmen
Hemisphere left caudal point
Hemisphere left rostral point
Hemisphere right caudal point
Hemisphere right rostral point
Infracollicular Sulcus
Lateral ventricle left caudal point
Lateral ventricle left rostral point
Lateral ventricle right caudal point
Lateral ventricle right rostral point
PMJ
Posterior Commissure
Temporal lobe left rostral point
Temporal lobe ventricle right rostral point
Task

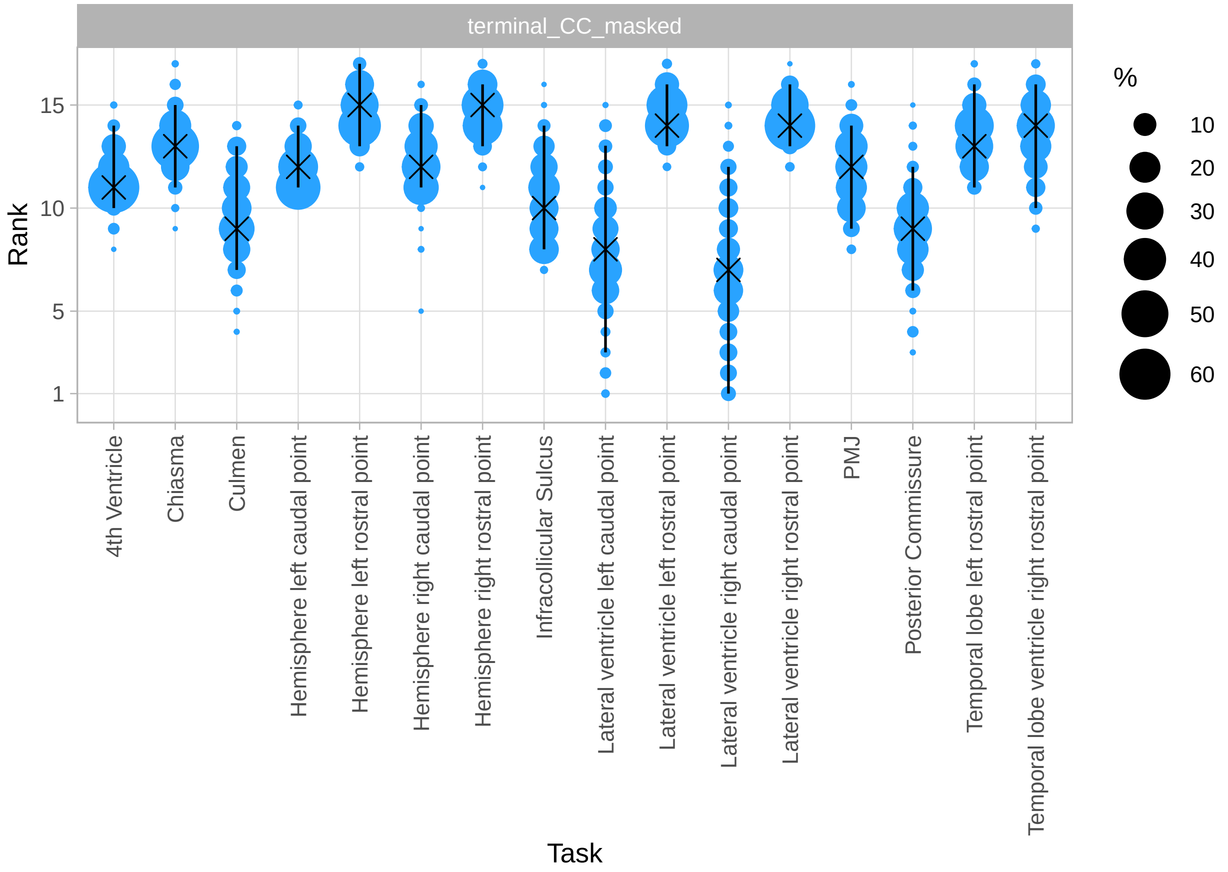
terminal_CC_masked
Rank
15
10
5
1
Task
4th Ventricle
Chiasma
Culmen
Hemisphere left caudal point
Hemisphere left rostral point
Hemisphere right caudal point
Hemisphere right rostral point
Infracollicular Sulcus
Lateral ventricle left caudal point
Lateral ventricle left rostral point
Lateral ventricle right caudal point
Lateral ventricle right rostral point
PMJ
Posterior Commissure
Temporal lobe left rostral point
Temporal lobe ventricle right rostral point
%
10
20
30
40
50
60

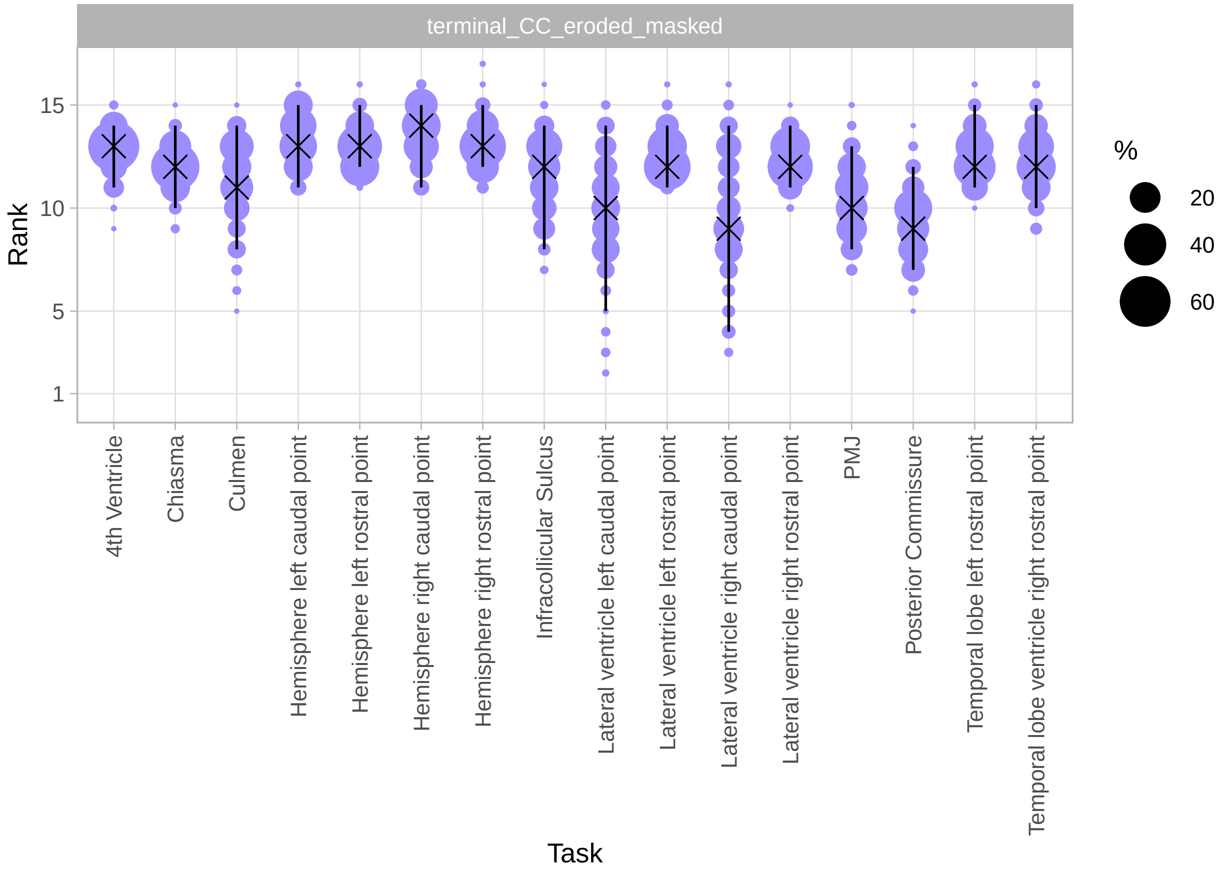

terminal_CC_eroded_masked
Rank
15
10
5
1
%
20
40
60
4th Ventricle
Chiasma
Culmen
Hemisphere left caudal point
Hemisphere left rostral point
Hemisphere right caudal point
Hemisphere right rostral point
Infracollicular Sulcus
Lateral ventricle left caudal point
Lateral ventricle left rostral point
Lateral ventricle right caudal point
Lateral ventricle right rostral point
PMJ
Posterior Commissure
Temporal lobe left rostral point
Temporal lobe ventricle right rostral point
Task

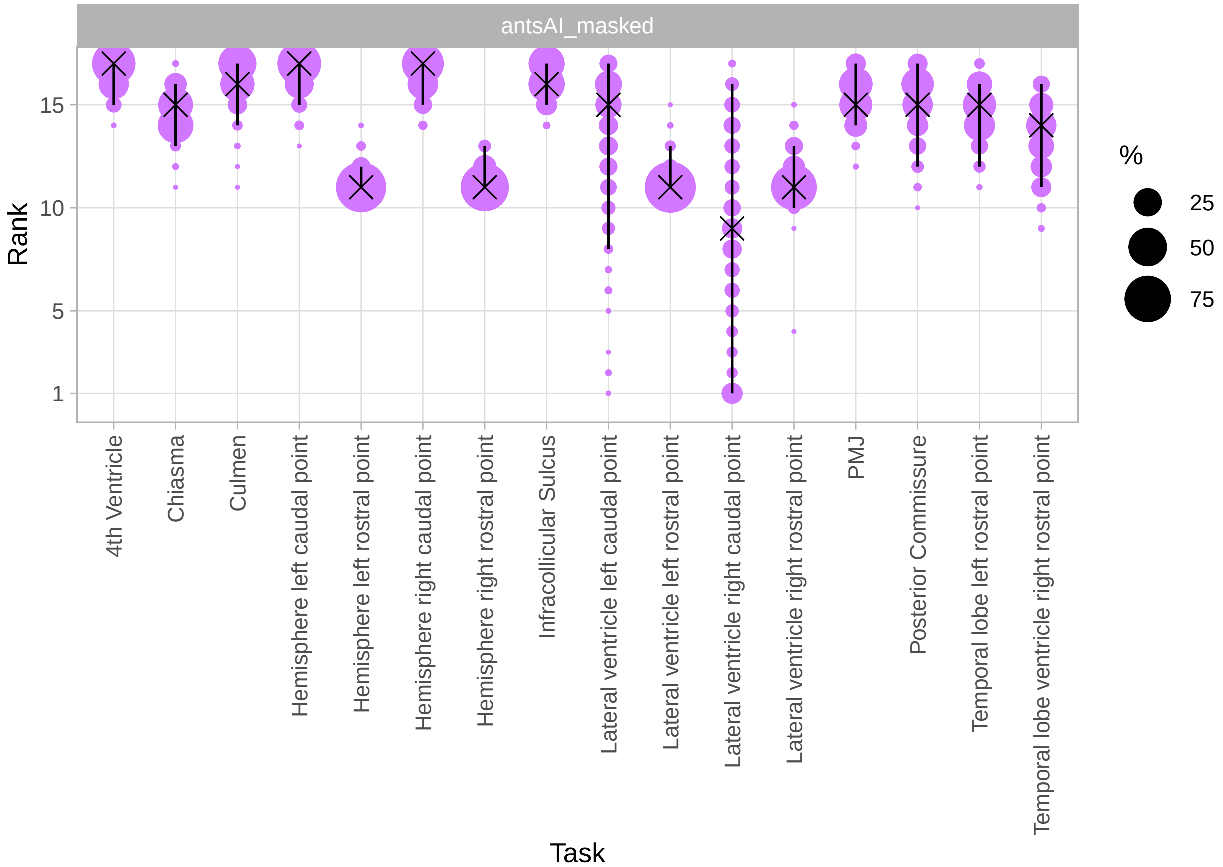
antsAI_masked
Rank
15
10
5
1
4th Ventricle
Chiasma
Culmen
Hemisphere left caudal point
Hemisphere left rostral point
Hemisphere right caudal point
Hemisphere right rostral point
Infracollicular Sulcus
Lateral ventricle left caudal point
Lateral ventricle left rostral point
Lateral ventricle right caudal point
Lateral ventricle right rostral point
PMJ
Posterior Commissure
Temporal lobe left rostral point
Temporal lobe ventricle right rostral point
Task
%
25
50
75

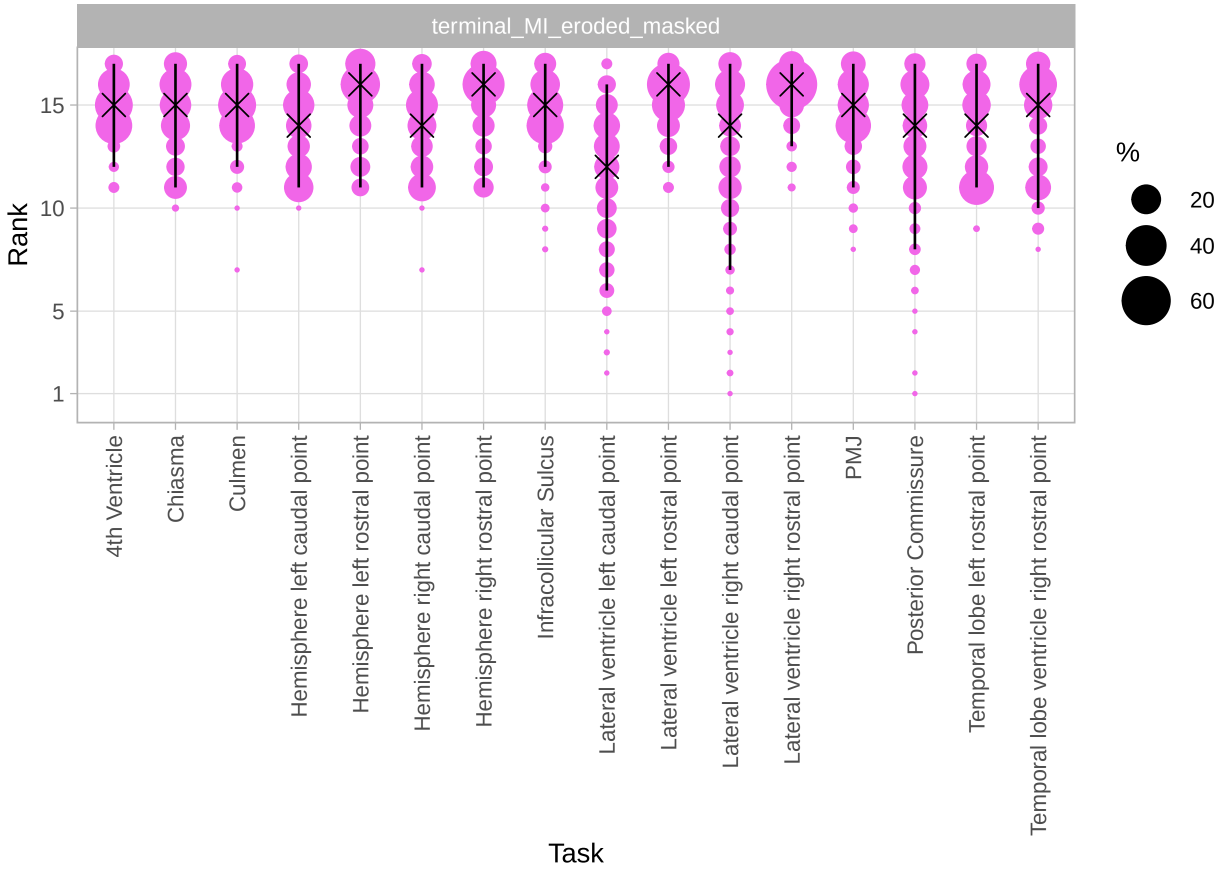
terminal_MI_eroded_masked
Rank
15
10
5
1
4th Ventricle
Chiasma
Culmen
Hemisphere left caudal point
Hemisphere left rostral point
Hemisphere right caudal point
Hemisphere right rostral point
Infracollicular Sulcus
Lateral ventricle left caudal point
Lateral ventricle left rostral point
Lateral ventricle right caudal point
Lateral ventricle right rostral point
PMJ
Posterior Commissure
Temporal lobe left rostral point
Temporal lobe ventricle right rostral point
Task
%
20
40
60

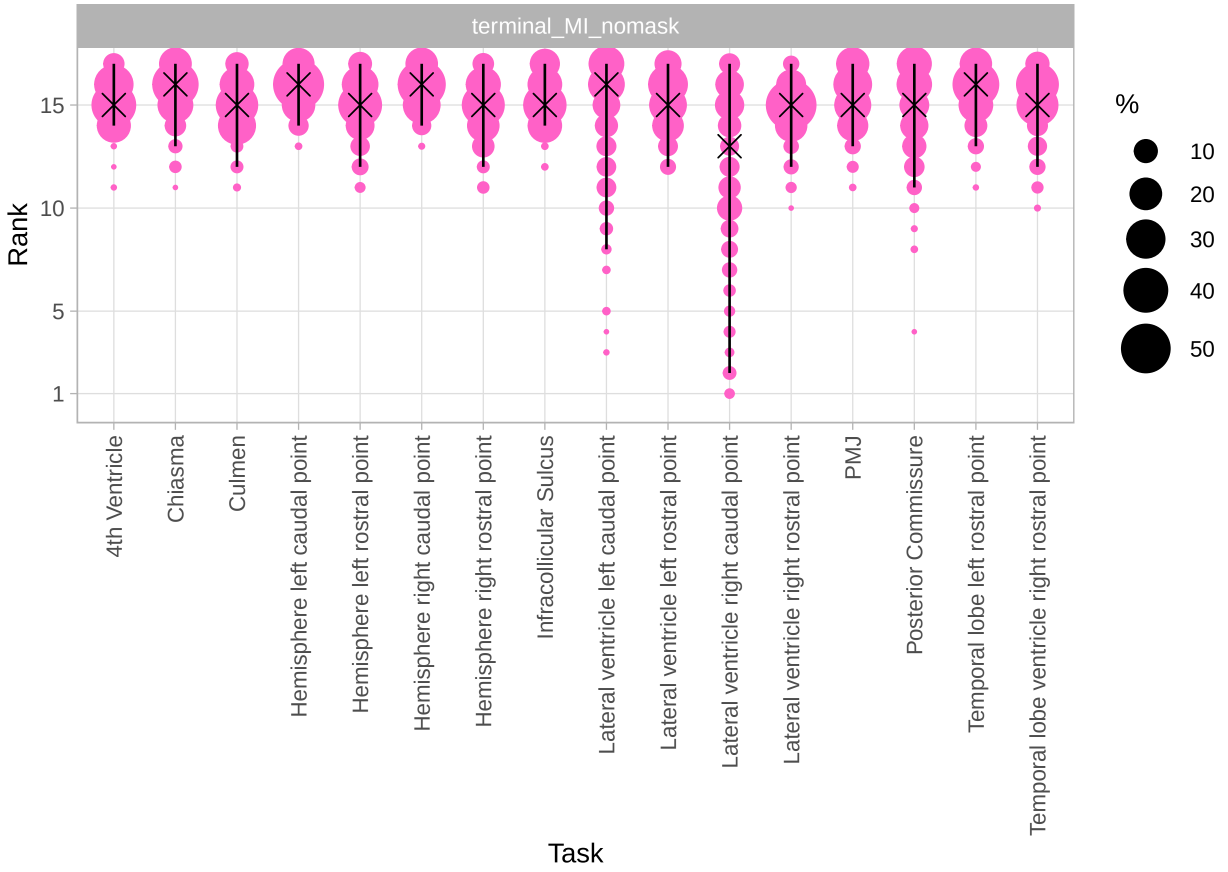
terminal_MI_nomask
Rank
15
10
5
1
4th Ventricle
Chiasma
Culmen
Hemisphere left caudal point
Hemisphere left rostral point
Hemisphere right caudal point
Hemisphere right rostral point
Infracollicular Sulcus
Lateral ventricle left caudal point
Lateral ventricle left rostral point
Lateral ventricle right caudal point
Lateral ventricle right rostral point
PMJ
Posterior Commissure
Temporal lobe left rostral point
Temporal lobe ventricle right rostral point
Task
%
10
20
30
40
50

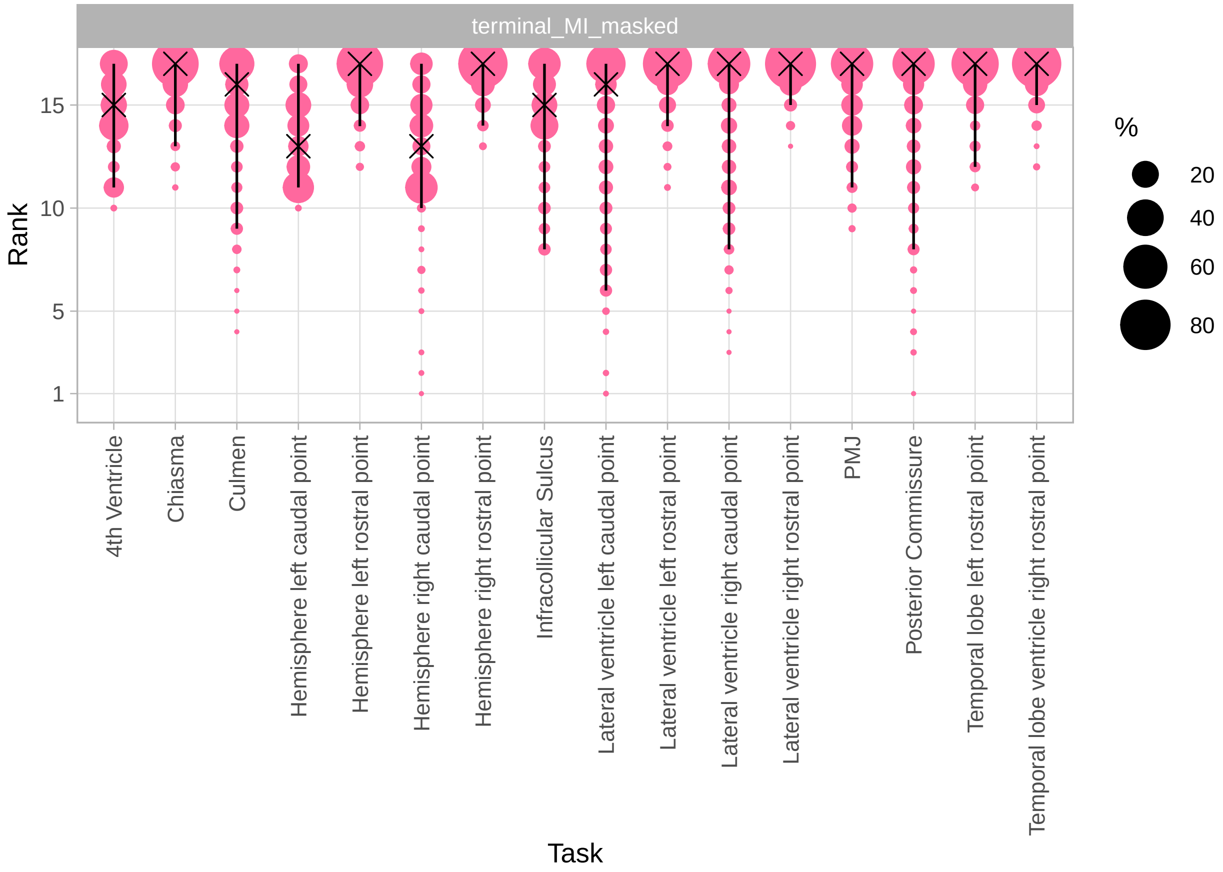
terminal_MI_masked
Rank
15
10
5
1
%
20
40
60
80
4th Ventricle
Chiasma
Culmen
Hemisphere left caudal point
Hemisphere left rostral point
Hemisphere right caudal point
Hemisphere right rostral point
Infracollicular Sulcus
Lateral ventricle left caudal point
Lateral ventricle left rostral point
Lateral ventricle right caudal point
Lateral ventricle right rostral point
PMJ
Posterior Commissure
Temporal lobe left rostral point
Temporal lobe ventricle right rostral point
Task

An alternative representation is provided by a stacked frequency plot of the observed ranks, separated by algorithm. Observed ranks across bootstrap samples are displayed with coloring according to the task. For algorithms that achieve the same rank in different tasks for the full assessment data set, vertical lines are on top of each other. Vertical lines allow to compare the achieved rank of each algorithm over different tasks.

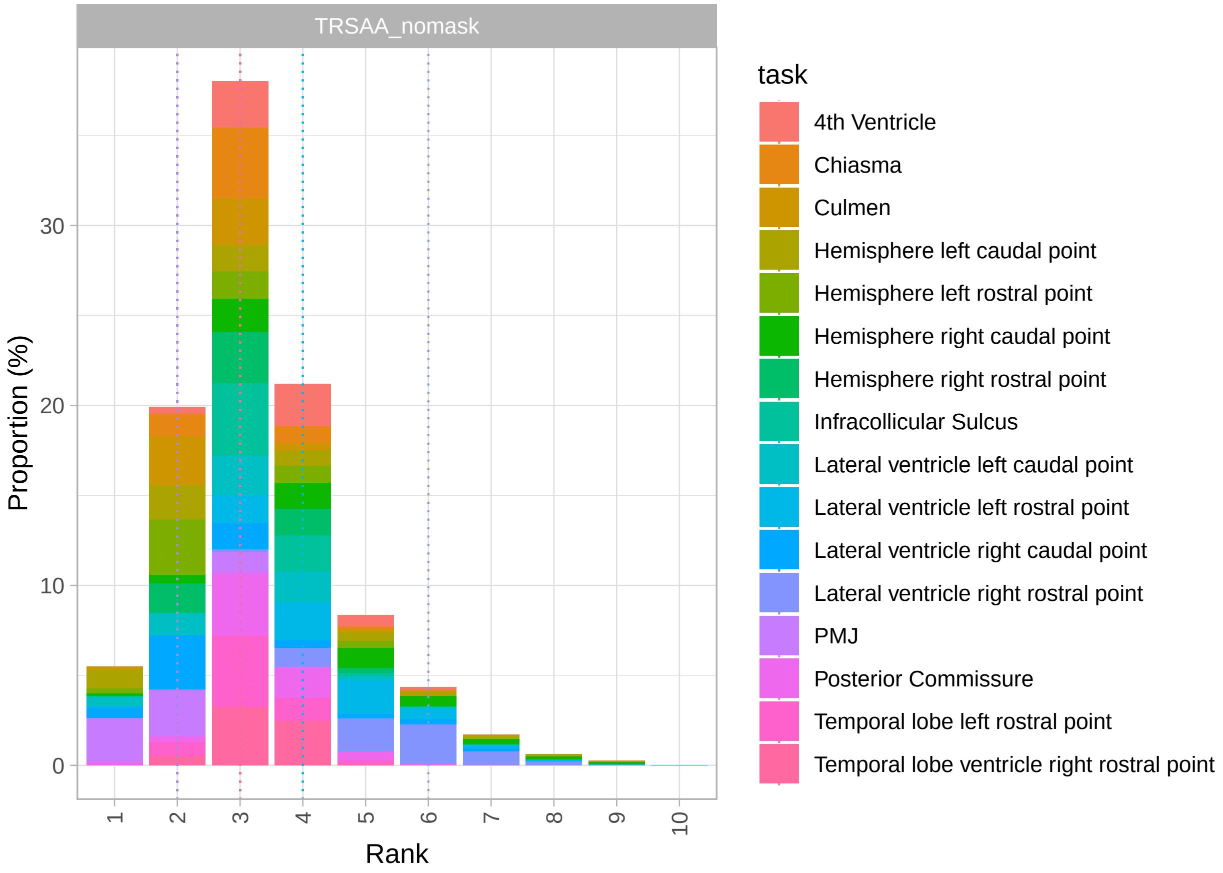

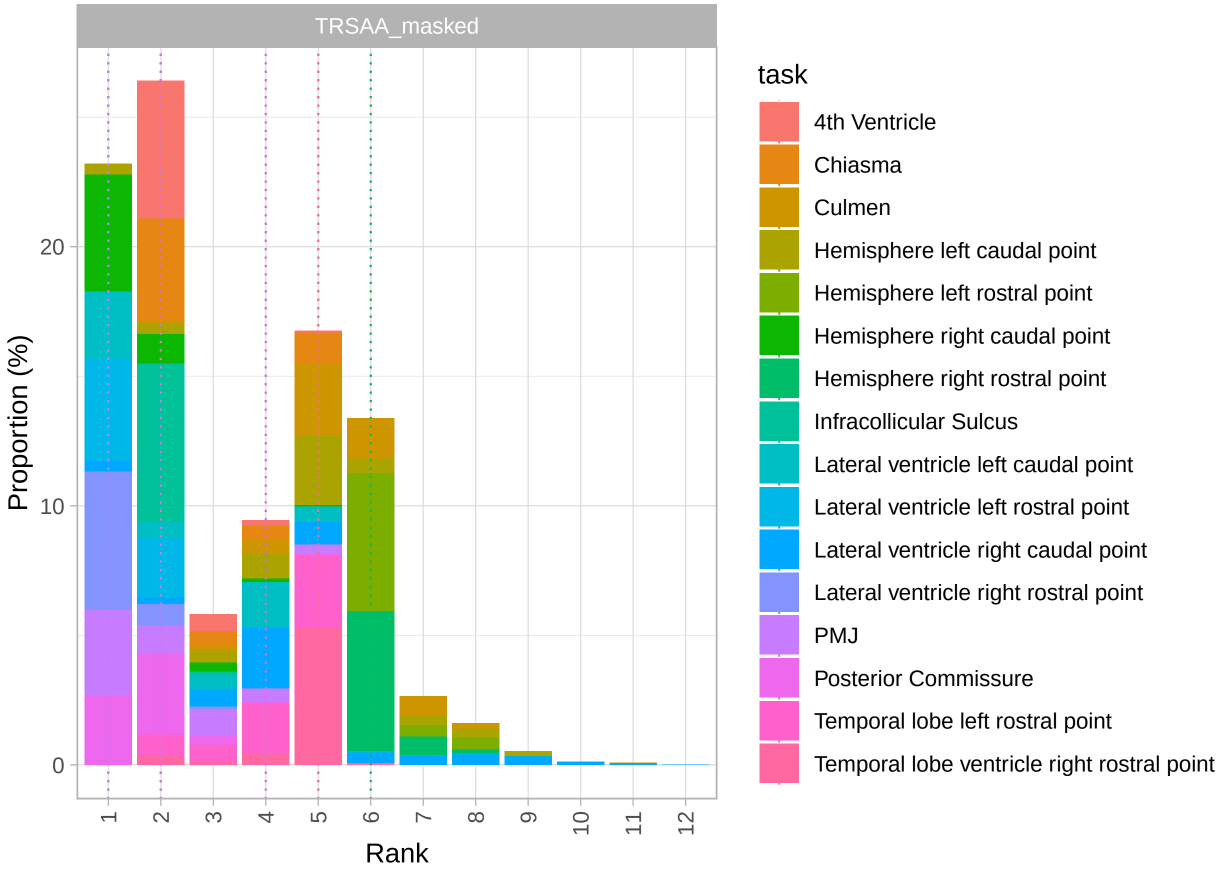
TRSAA_masked
Proportion (%)
0
10
20
1
2
3
4
5
6
7
8
9
10
11
12
Rank
task
4th Ventricle
Chiasma
Culmen
Hemisphere left caudal point
Hemisphere left rostral point
Hemisphere right caudal point
Hemisphere right rostral point
Infracollicular Sulcus
Lateral ventricle left caudal point
Lateral ventricle left rostral point
Lateral ventricle right caudal point
Lateral ventricle right rostral point
PMJ
Posterior Commissure
Temporal lobe left rostral point
Temporal lobe ventricle right rostral point

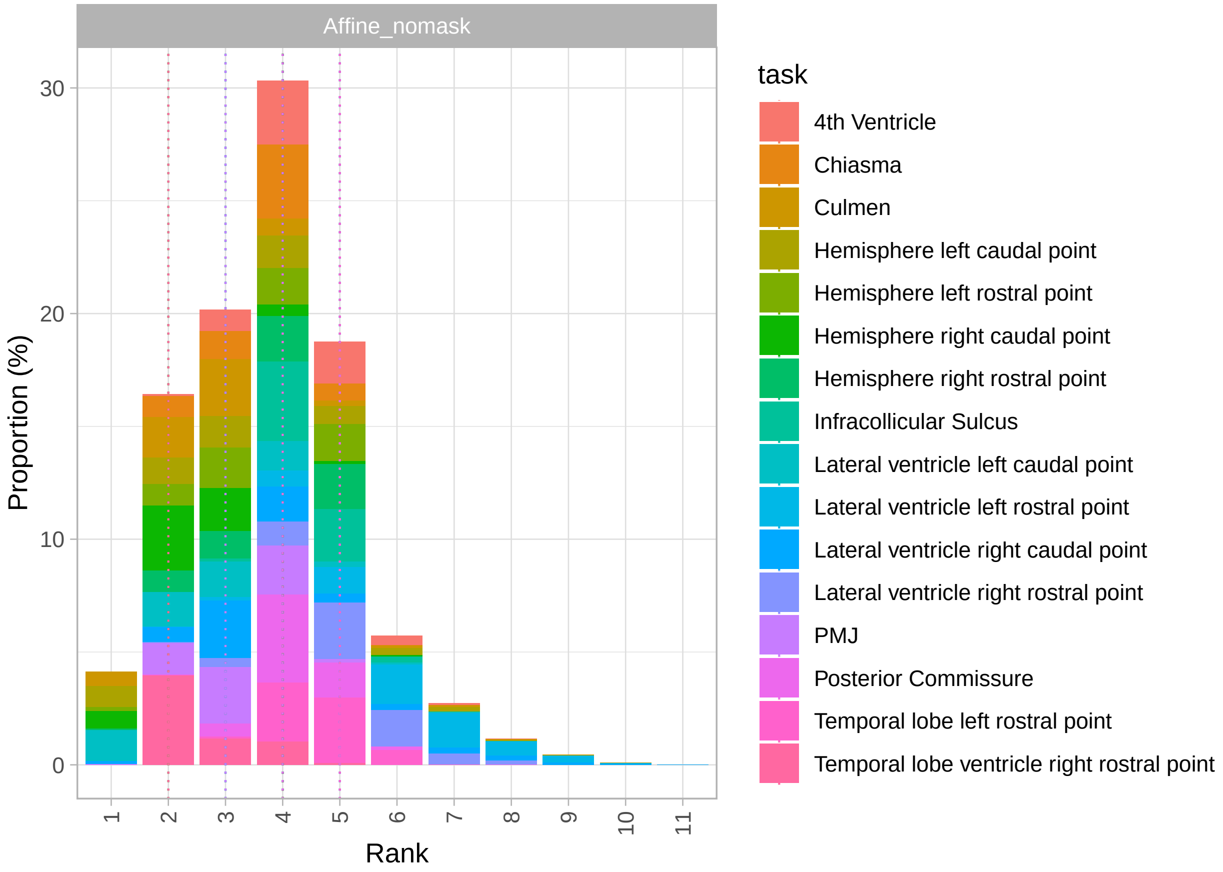
Affine_nomask
Proportion (%)
30
20
10
0
1
2
3
4
5
6
7
8
9
10
11
Rank
task
4th Ventricle
Chiasma
Culmen
Hemisphere left caudal point
Hemisphere left rostral point
Hemisphere right caudal point
Hemisphere right rostral point
Infracollicular Sulcus
Lateral ventricle left caudal point
Lateral ventricle left rostral point
Lateral ventricle right caudal point
Lateral ventricle right rostral point
PMJ
Posterior Commissure
Temporal lobe left rostral point
Temporal lobe ventricle right rostral point

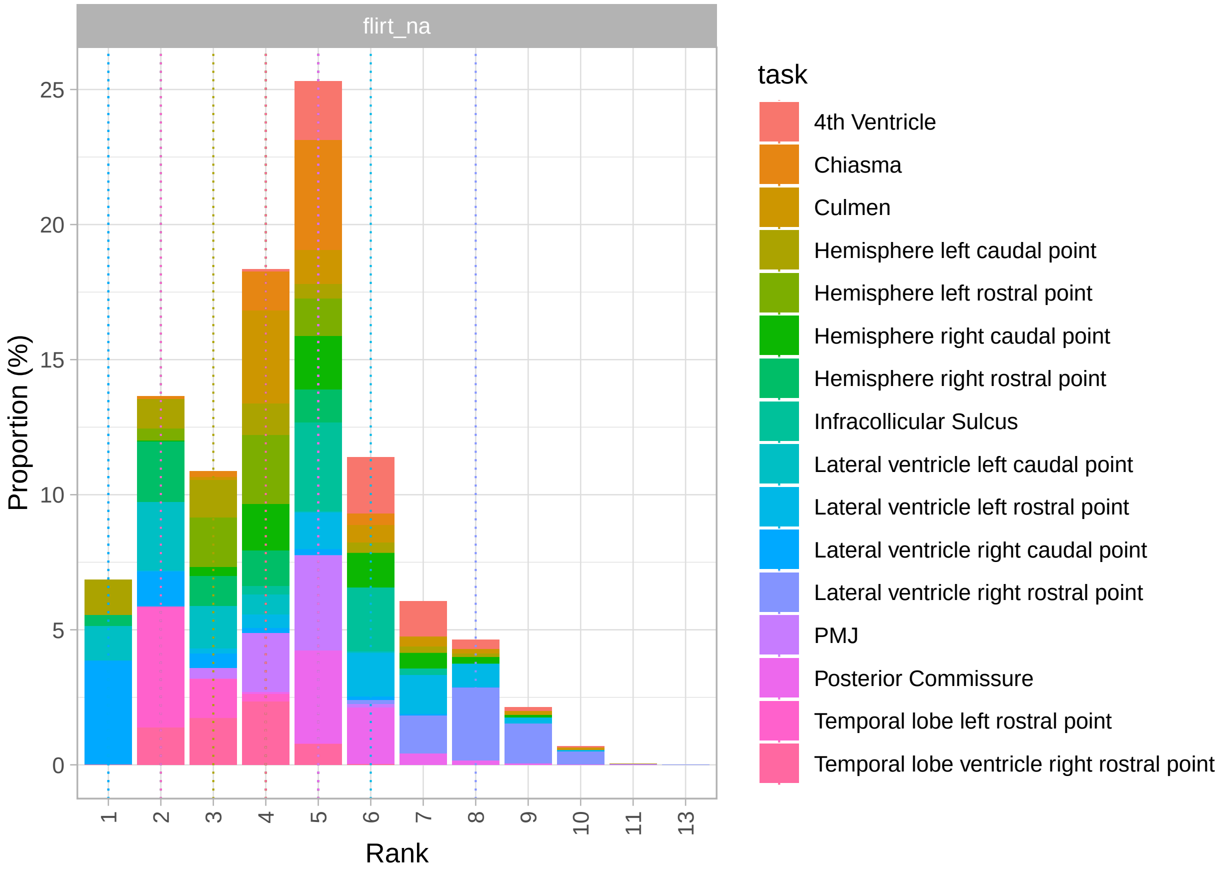
flirt_na
Proportion (%)
25
20
15
10
5
0
Rank
1
2
3
4
5
6
7
8
9
10
11
13
task
4th Ventricle
Chiasma
Culmen
Hemisphere left caudal point
Hemisphere left rostral point
Hemisphere right caudal point
Hemisphere right rostral point
Infracollicular Sulcus
Lateral ventricle left caudal point
Lateral ventricle left rostral point
Lateral ventricle right caudal point
Lateral ventricle right rostral point
PMJ
Posterior Commissure
Temporal lobe left rostral point
Temporal lobe ventricle right rostral point

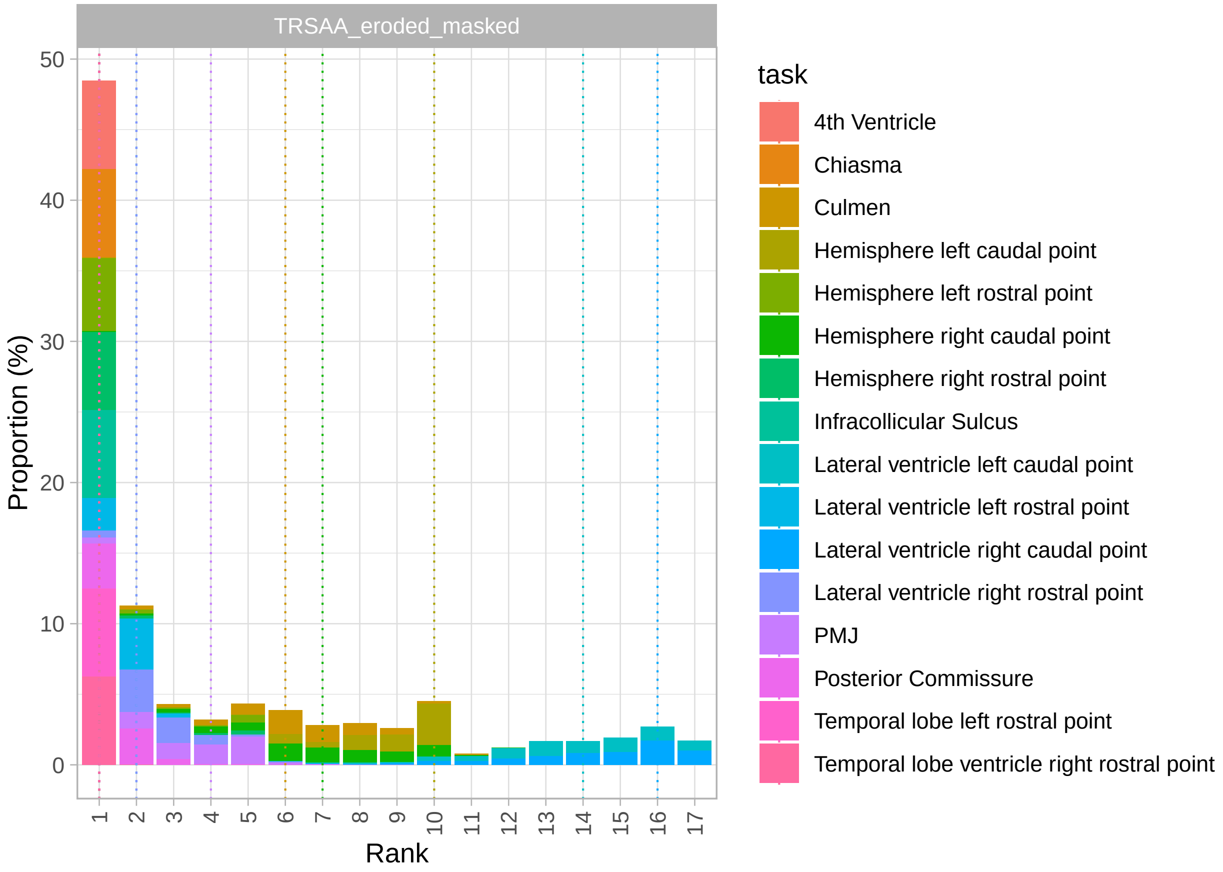

TRSAA_eroded_masked
Proportion (%)
50
40
30
20
10
0
1
2
3
4
5
6
7
8
9
10
11
12
13
14
15
16
17
Rank
task
4th Ventricle
Chiasma
Culmen
Hemisphere left caudal point
Hemisphere left rostral point
Hemisphere right caudal point
Hemisphere right rostral point
Infracollicular Sulcus
Lateral ventricle left caudal point
Lateral ventricle left rostral point
Lateral ventricle right caudal point
Lateral ventricle right rostral point
PMJ
Posterior Commissure
Temporal lobe left rostral point
Temporal lobe ventricle right rostral point

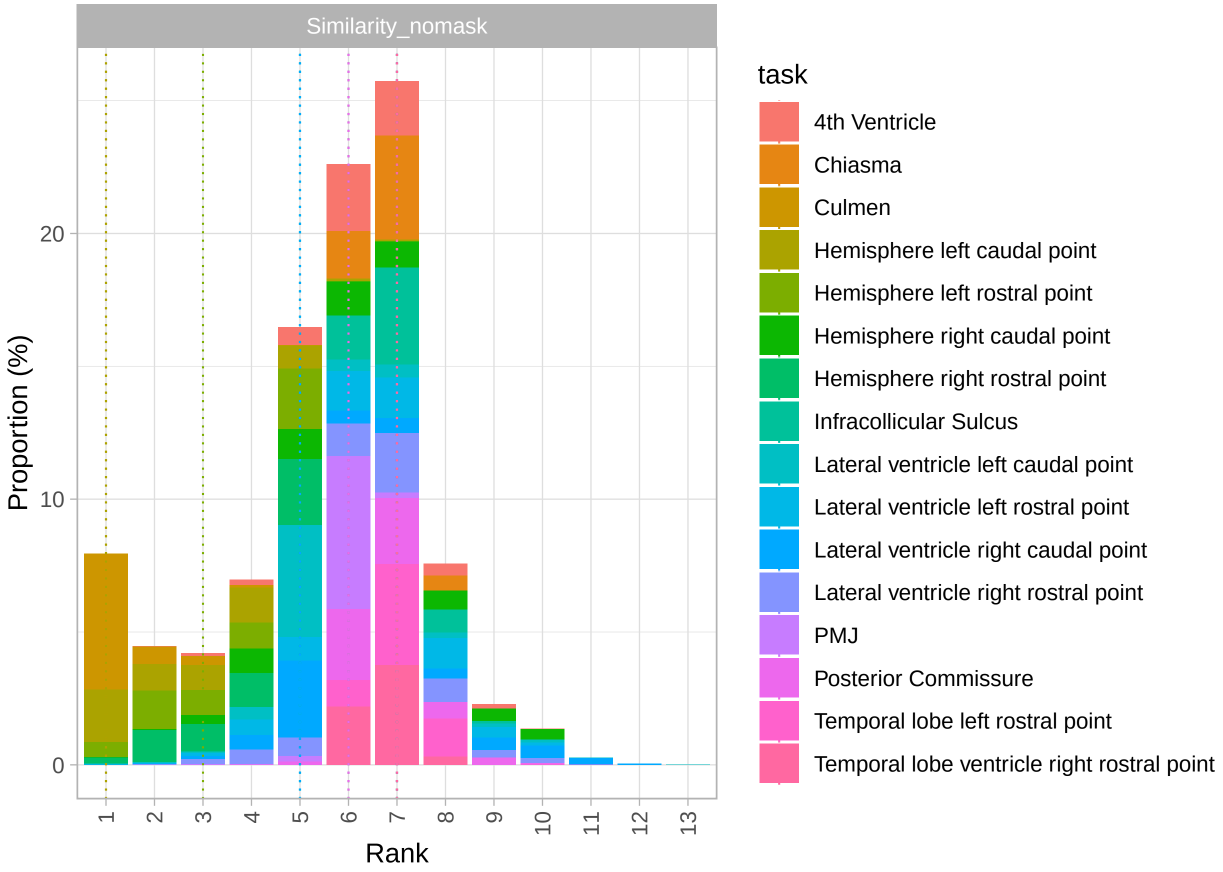

Similarity_nomask
Proportion (%)
0
10
20
1
2
3
4
5
6
7
8
9
10
11
12
13
Rank
task
4th Ventricle
Chiasma
Culmen
Hemisphere left caudal point
Hemisphere left rostral point
Hemisphere right caudal point
Hemisphere right rostral point
Infracollicular Sulcus
Lateral ventricle left caudal point
Lateral ventricle left rostral point
Lateral ventricle right caudal point
Lateral ventricle right rostral point
PMJ
Posterior Commissure
Temporal lobe left rostral point
Temporal lobe ventricle right rostral point

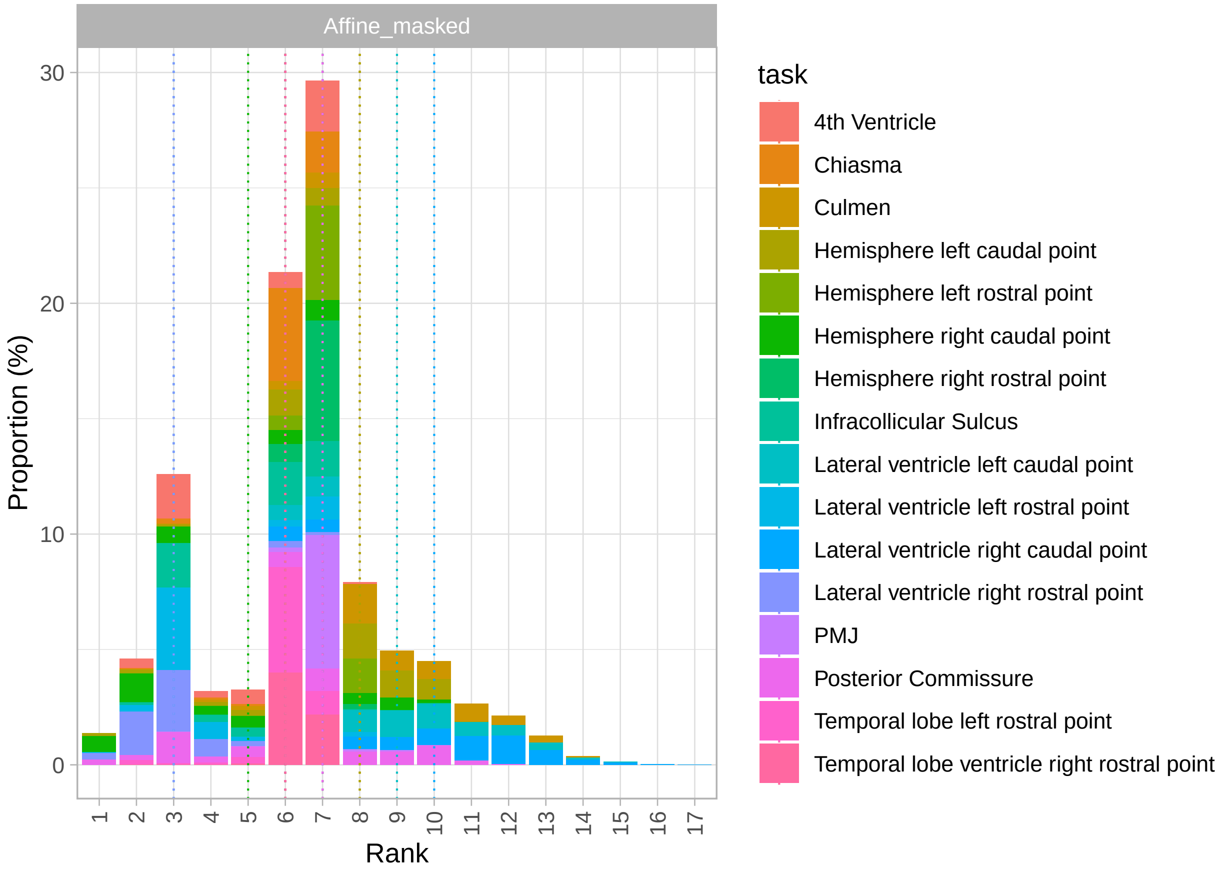
Affine_masked
Proportion (%)
30
20
10
0
1
2
3
4
5
6
7
8
9
10
11
12
13
14
15
16
17
Rank
task
4th Ventricle
Chiasma
Culmen
Hemisphere left caudal point
Hemisphere left rostral point
Hemisphere right caudal point
Hemisphere right rostral point
Infracollicular Sulcus
Lateral ventricle left caudal point
Lateral ventricle left rostral point
Lateral ventricle right caudal point
Lateral ventricle right rostral point
PMJ
Posterior Commissure
Temporal lobe left rostral point
Temporal lobe ventricle right rostral point

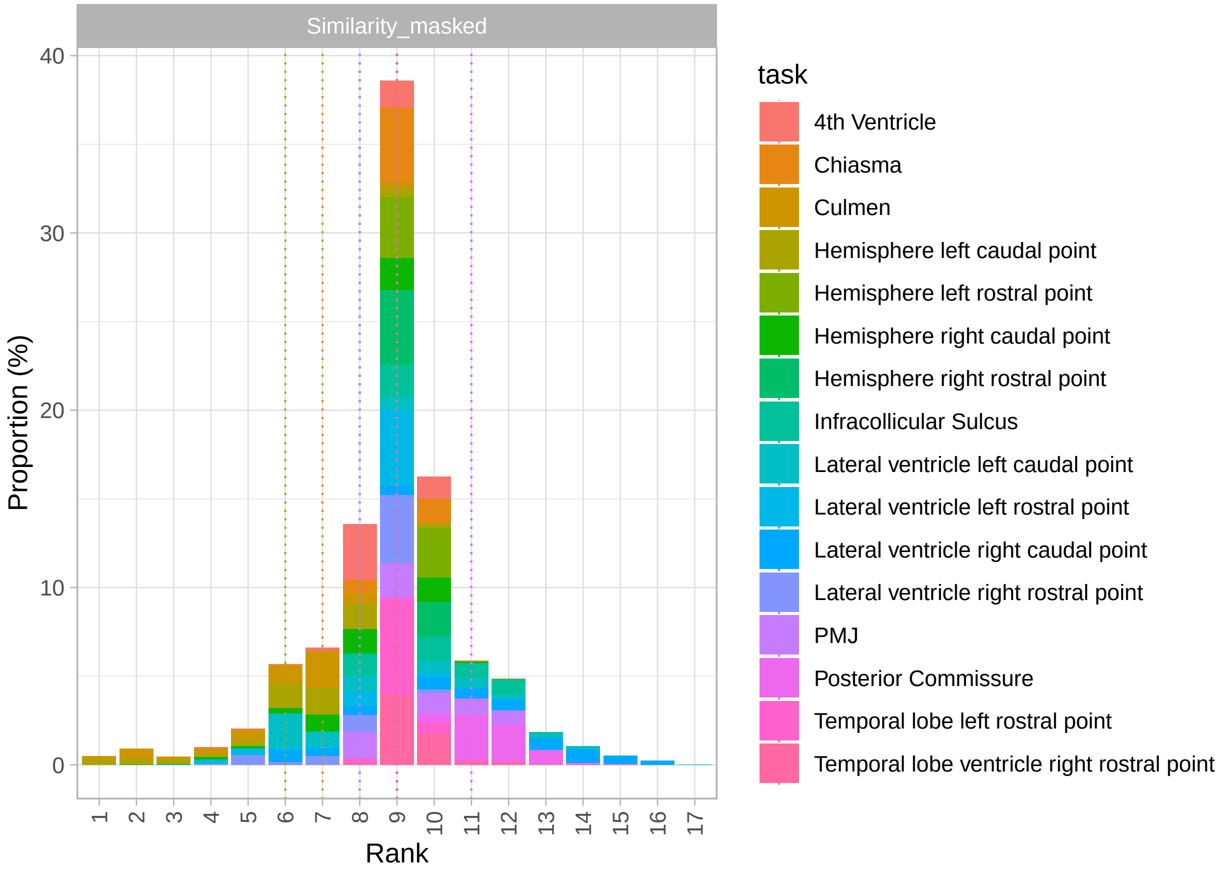
Similarity_masked
Proportion (%)
40
30
20
10
0
1
2
3
4
5
6
7
8
9
10
11
12
13
14
15
16
17
Rank
task
4th Ventricle
Chiasma
Culmen
Hemisphere left caudal point
Hemisphere left rostral point
Hemisphere right caudal point
Hemisphere right rostral point
Infracollicular Sulcus
Lateral ventricle left caudal point
Lateral ventricle left rostral point
Lateral ventricle right caudal point
Lateral ventricle right rostral point
PMJ
Posterior Commissure
Temporal lobe left rostral point
Temporal lobe ventricle right rostral point

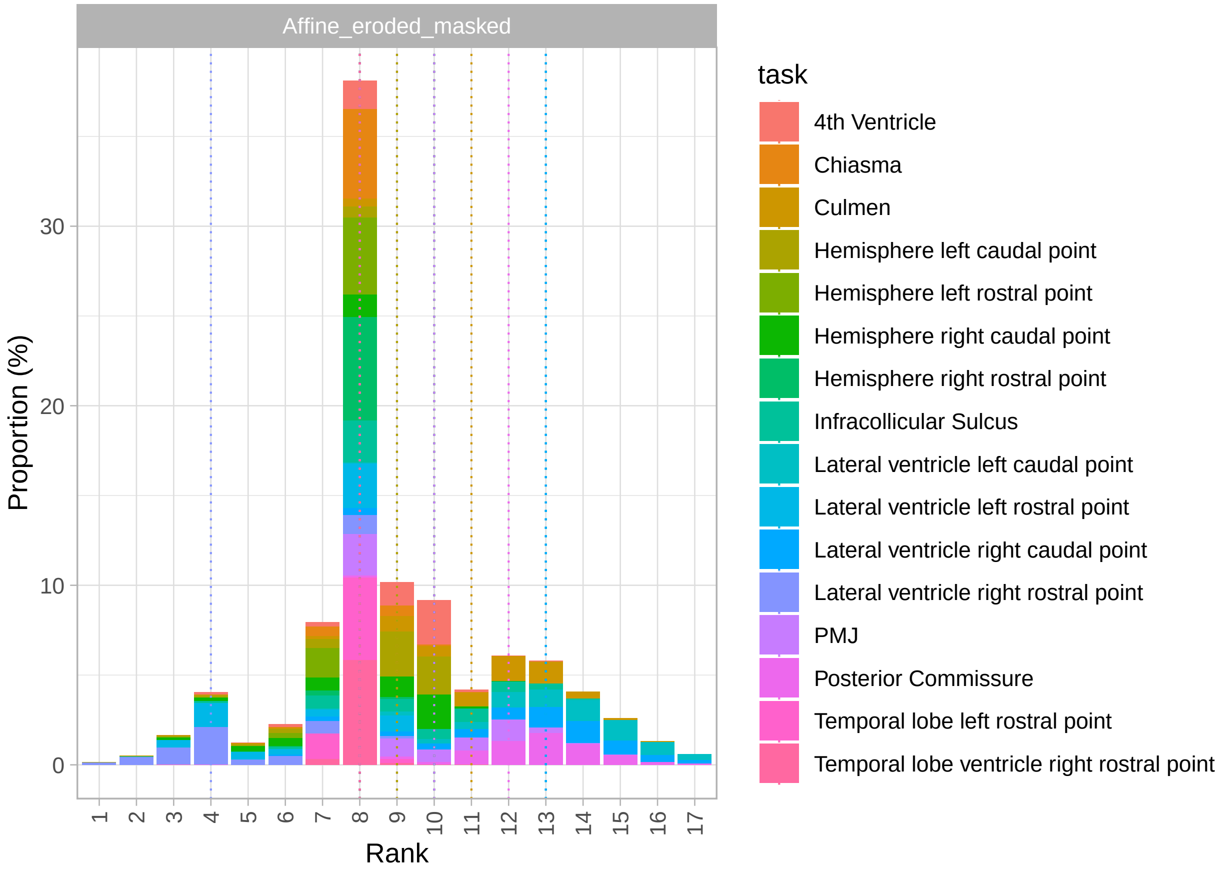
Affine_eroded_masked
Proportion (%)
Rank
0
10
20
30
1
2
3
4
5
6
7
8
9
10
11
12
13
14
15
16
17
task
4th Ventricle
Chiasma
Culmen
Hemisphere left caudal point
Hemisphere left rostral point
Hemisphere right caudal point
Hemisphere right rostral point
Infracollicular Sulcus
Lateral ventricle left caudal point
Lateral ventricle left rostral point
Lateral ventricle right caudal point
Lateral ventricle right rostral point
PMJ
Posterior Commissure
Temporal lobe left rostral point
Temporal lobe ventricle right rostral point

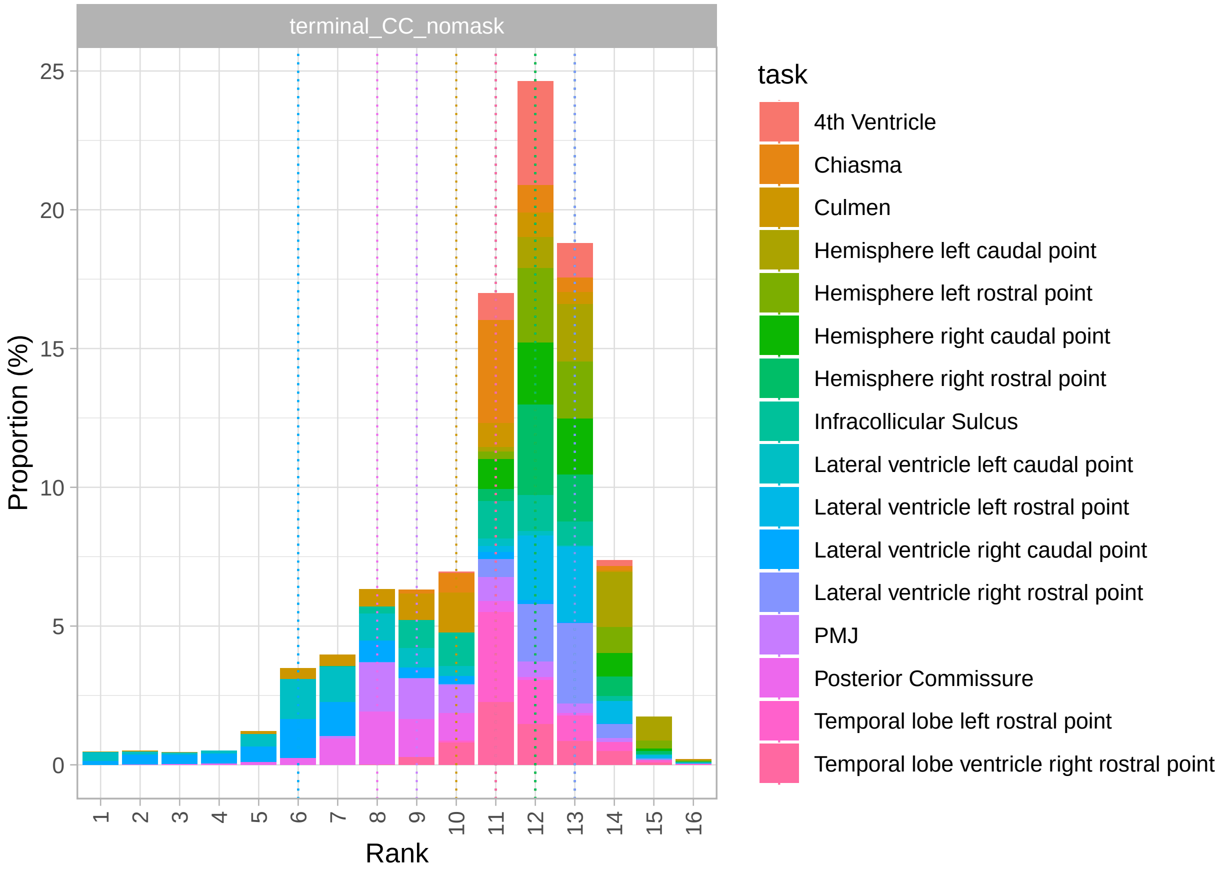
terminal_CC_nomask
Proportion (%)
25
20
15
10
5
0
1
2
3
4
5
6
7
8
9
10
11
12
13
14
15
16
Rank
task
4th Ventricle
Chiasma
Culmen
Hemisphere left caudal point
Hemisphere left rostral point
Hemisphere right caudal point
Hemisphere right rostral point
Infracollicular Sulcus
Lateral ventricle left caudal point
Lateral ventricle left rostral point
Lateral ventricle right caudal point
Lateral ventricle right rostral point
PMJ
Posterior Commissure
Temporal lobe left rostral point
Temporal lobe ventricle right rostral point

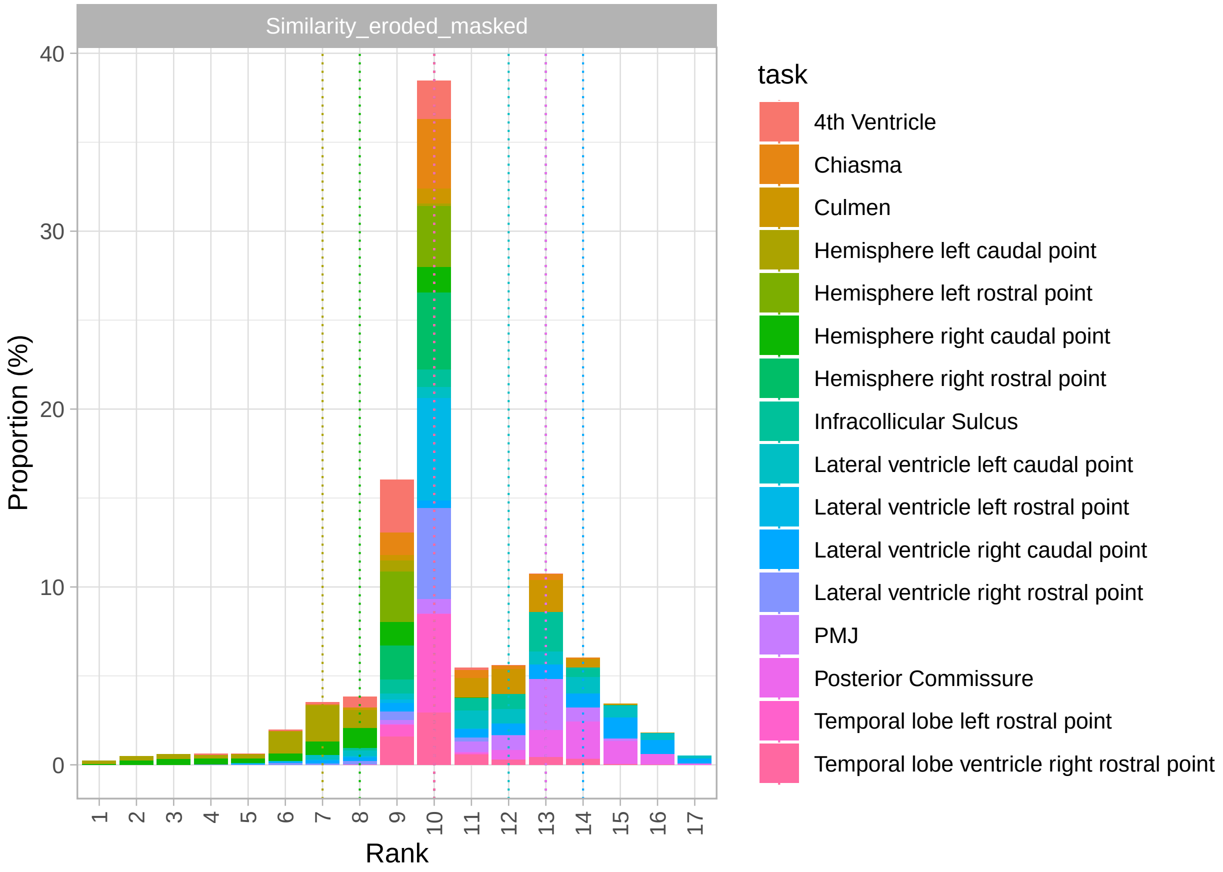

Similarity_eroded_masked
Proportion (%)
40
30
20
10
0
1
2
3
4
5
6
7
8
9
10
11
12
13
14
15
16
17
Rank
task
4th Ventricle
Chiasma
Culmen
Hemisphere left caudal point
Hemisphere left rostral point
Hemisphere right caudal point
Hemisphere right rostral point
Infracollicular Sulcus
Lateral ventricle left caudal point
Lateral ventricle left rostral point
Lateral ventricle right caudal point
Lateral ventricle right rostral point
PMJ
Posterior Commissure
Temporal lobe left rostral point
Temporal lobe ventricle right rostral point

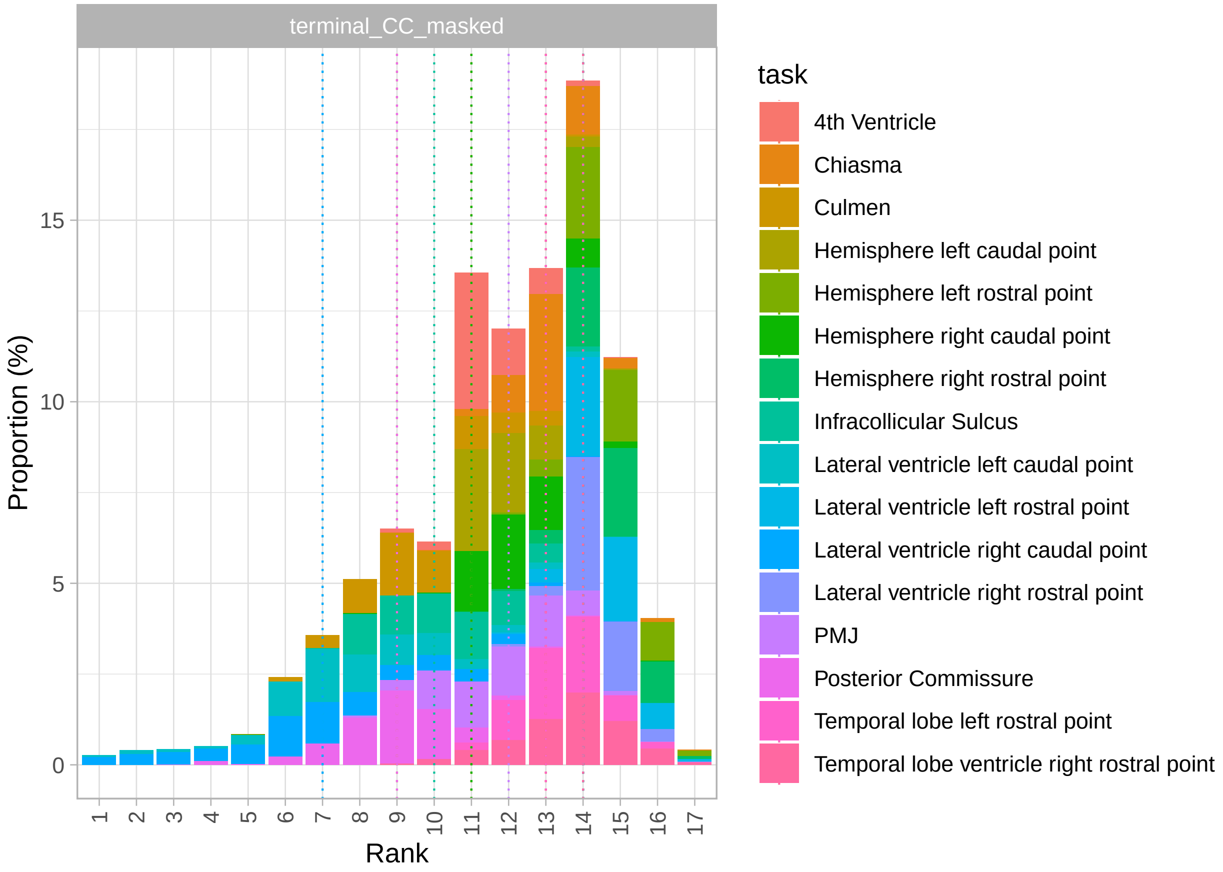

terminal_CC_masked
Proportion (%)
15
10
5
0
1
2
3
4
5
6
7
8
9
10
11
12
13
14
15
16
17
Rank
task
4th Ventricle
Chiasma
Culmen
Hemisphere left caudal point
Hemisphere left rostral point
Hemisphere right caudal point
Hemisphere right rostral point
Infracollicular Sulcus
Lateral ventricle left caudal point
Lateral ventricle left rostral point
Lateral ventricle right caudal point
Lateral ventricle right rostral point
PMJ
Posterior Commissure
Temporal lobe left rostral point
Temporal lobe ventricle right rostral point

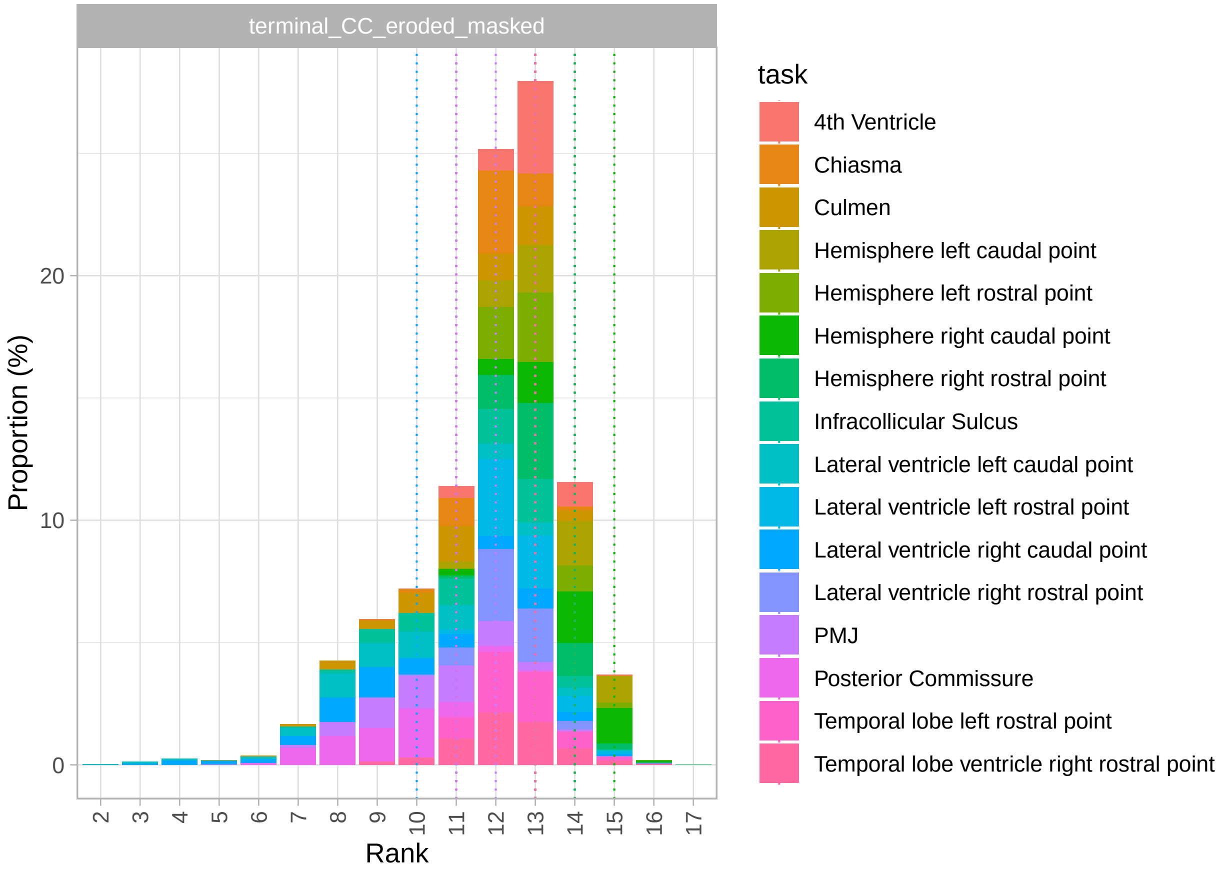
terminal_CC_eroded_masked
Proportion (%)
0
10
20
2
3
4
5
6
7
8
9
10
11
12
13
14
15
16
17
Rank
task
4th Ventricle
Chiasma
Culmen
Hemisphere left caudal point
Hemisphere left rostral point
Hemisphere right caudal point
Hemisphere right rostral point
Infracollicular Sulcus
Lateral ventricle left caudal point
Lateral ventricle left rostral point
Lateral ventricle right caudal point
Lateral ventricle right rostral point
PMJ
Posterior Commissure
Temporal lobe left rostral point
Temporal lobe ventricle right rostral point

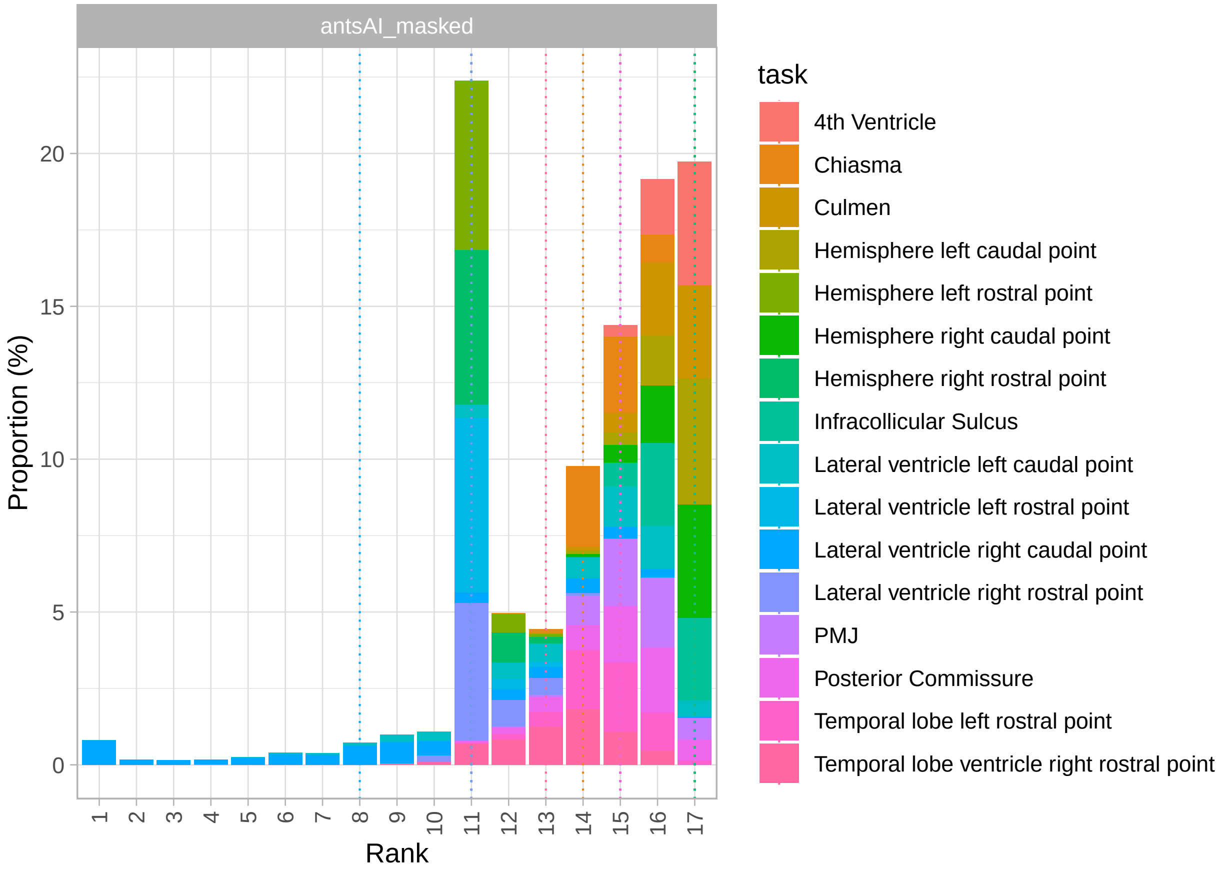
antsAI_masked
Proportion (%)
0
5
10
15
20
1
2
3
4
5
6
7
8
9
10
11
12
13
14
15
16
17
Rank
task
4th Ventricle
Chiasma
Culmen
Hemisphere left caudal point
Hemisphere left rostral point
Hemisphere right caudal point
Hemisphere right rostral point
Infracollicular Sulcus
Lateral ventricle left caudal point
Lateral ventricle left rostral point
Lateral ventricle right caudal point
Lateral ventricle right rostral point
PMJ
Posterior Commissure
Temporal lobe left rostral point
Temporal lobe ventricle right rostral point

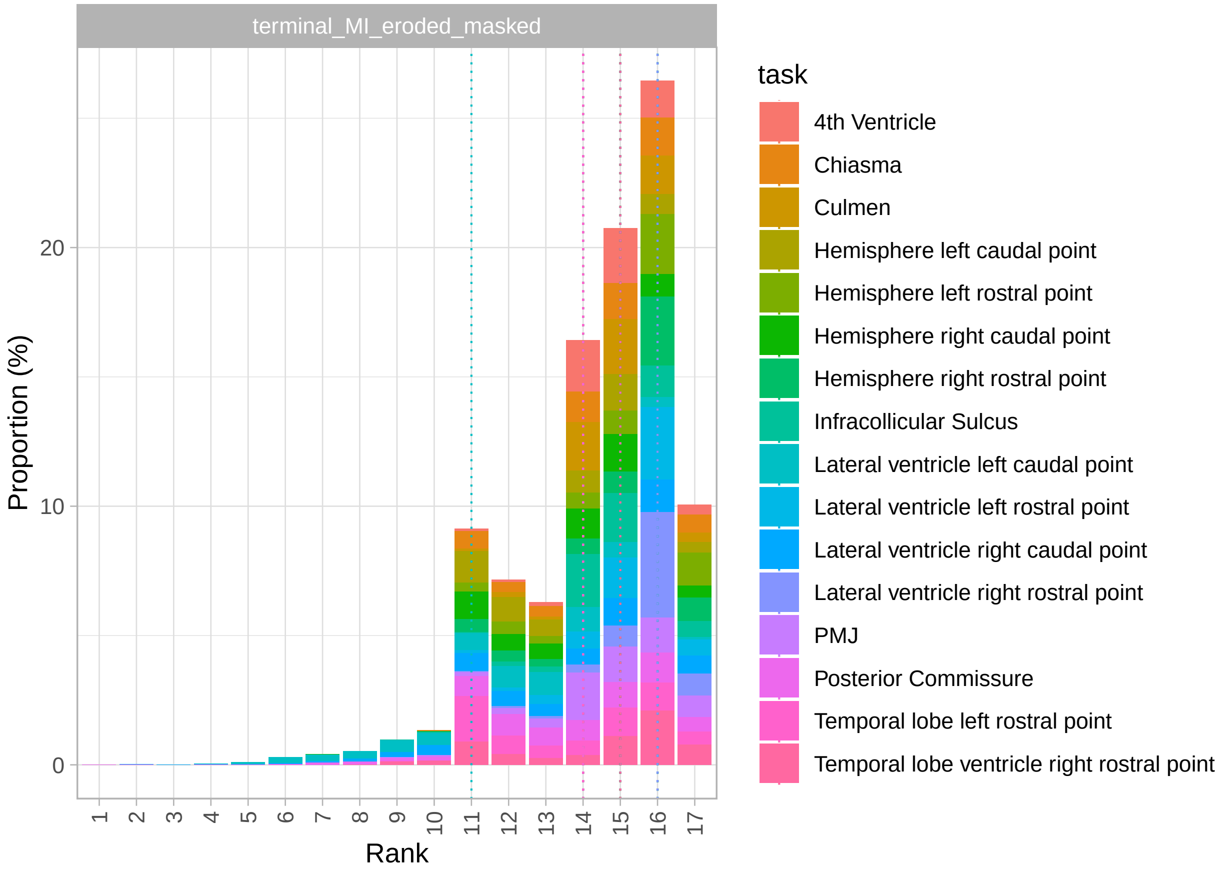
terminal_MI_eroded_masked
Proportion (%)
0
10
20
1
2
3
4
5
6
7
8
9
10
11
12
13
14
15
16
17
Rank
task
4th Ventricle
Chiasma
Culmen
Hemisphere left caudal point
Hemisphere left rostral point
Hemisphere right caudal point
Hemisphere right rostral point
Infracollicular Sulcus
Lateral ventricle left caudal point
Lateral ventricle left rostral point
Lateral ventricle right caudal point
Lateral ventricle right rostral point
PMJ
Posterior Commissure
Temporal lobe left rostral point
Temporal lobe ventricle right rostral point

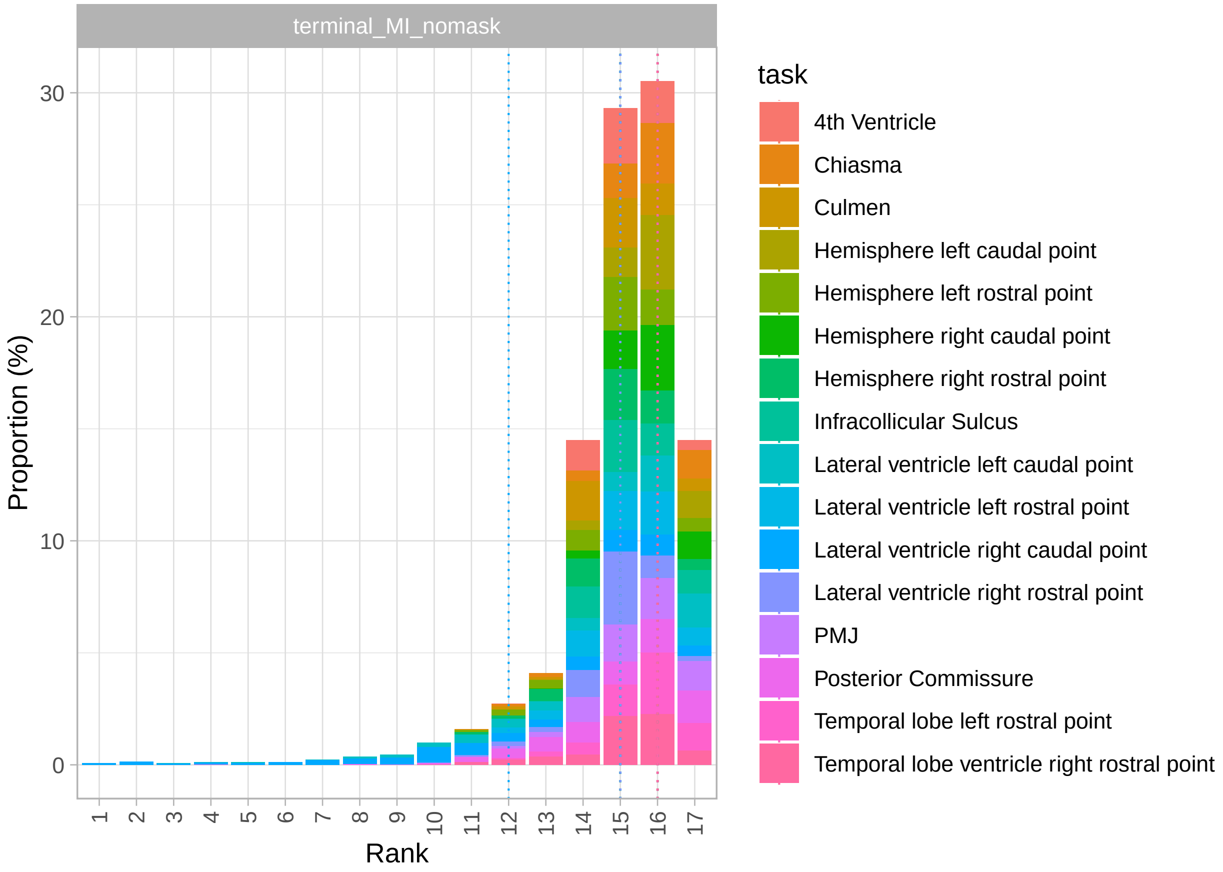

terminal_MI_nomask
Proportion (%)
30
20
10
0
1
2
3
4
5
6
7
8
9
10
11
12
13
14
15
16
17
Rank
task
4th Ventricle
Chiasma
Culmen
Hemisphere left caudal point
Hemisphere left rostral point
Hemisphere right caudal point
Hemisphere right rostral point
Infracollicular Sulcus
Lateral ventricle left caudal point
Lateral ventricle left rostral point
Lateral ventricle right caudal point
Lateral ventricle right rostral point
PMJ
Posterior Commissure
Temporal lobe left rostral point
Temporal lobe ventricle right rostral point

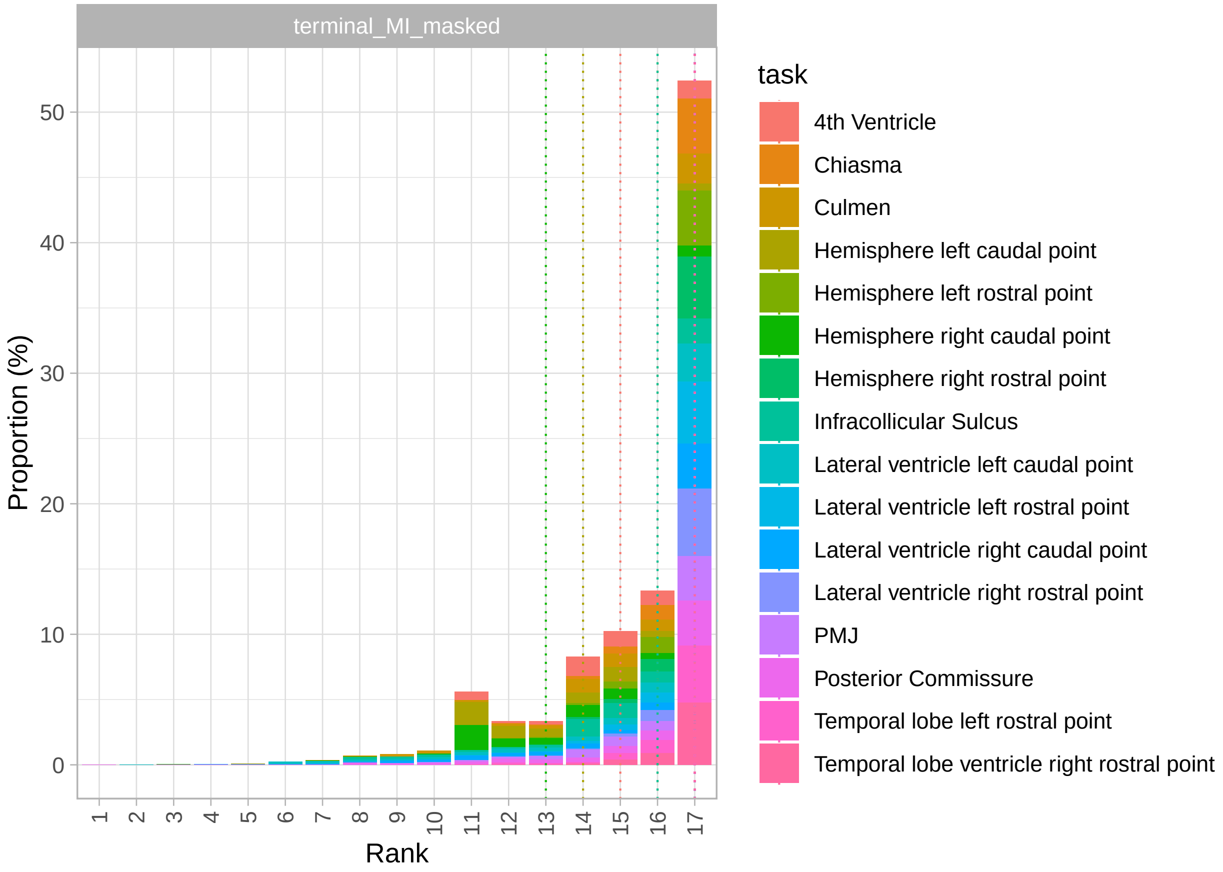
terminal_MI_masked
Proportion (%)
0
10
20
30
40
50
Rank
1
2
3
4
5
6
7
8
9
10
11
12
13
14
15
16
17
task
4th Ventricle
Chiasma
Culmen
Hemisphere left caudal point
Hemisphere left rostral point
Hemisphere right caudal point
Hemisphere right rostral point
Infracollicular Sulcus
Lateral ventricle left caudal point
Lateral ventricle left rostral point
Lateral ventricle right caudal point
Lateral ventricle right rostral point
PMJ
Posterior Commissure
Temporal lobe left rostral point
Temporal lobe ventricle right rostral point

4.2 Characterization of tasks

4.2.1 Visualizing bootstrap results

To investigate which tasks separate algorithms well (i.e., lead to a stable ranking), a blob plot is recommended.

Bootstrap results can be shown in a blob plot showing one plot for each task. In this view, the spread of the blobs for each algorithm can be compared across tasks. Deviations from the diagonal indicate deviations from the consensus ranking (over tasks). Specifically, if rank distribution of an algorithm is consistently below the diagonal, the algorithm performed better in this task than on average across tasks, while if the rank distribution of an algorithm is consistently above the diagonal, the algorithm performed worse in this task than on average across tasks. At the bottom of each panel, ranks for each algorithm in the tasks are provided.

Same as in Section 3.1 but now ordered according to consensus.

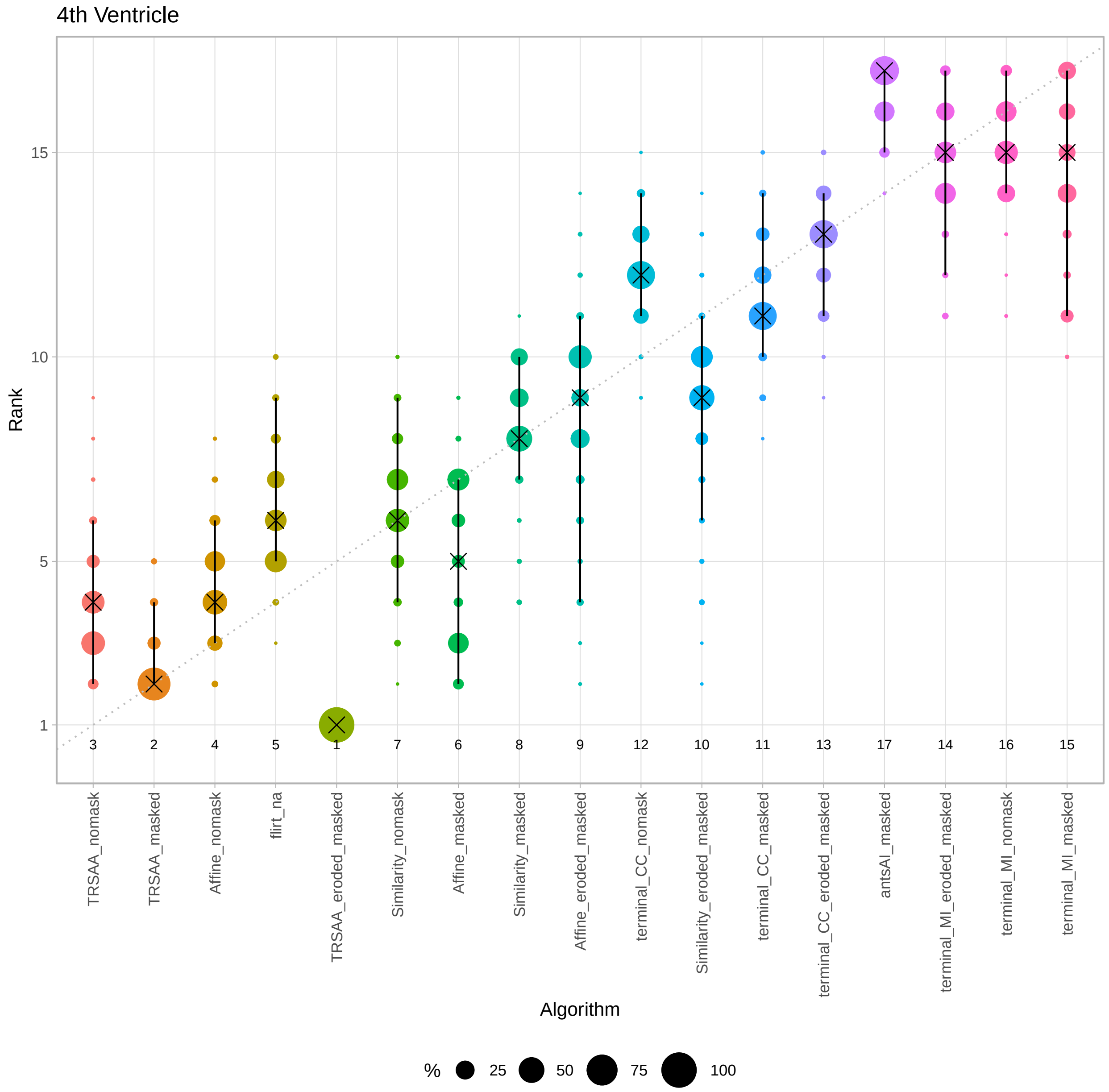

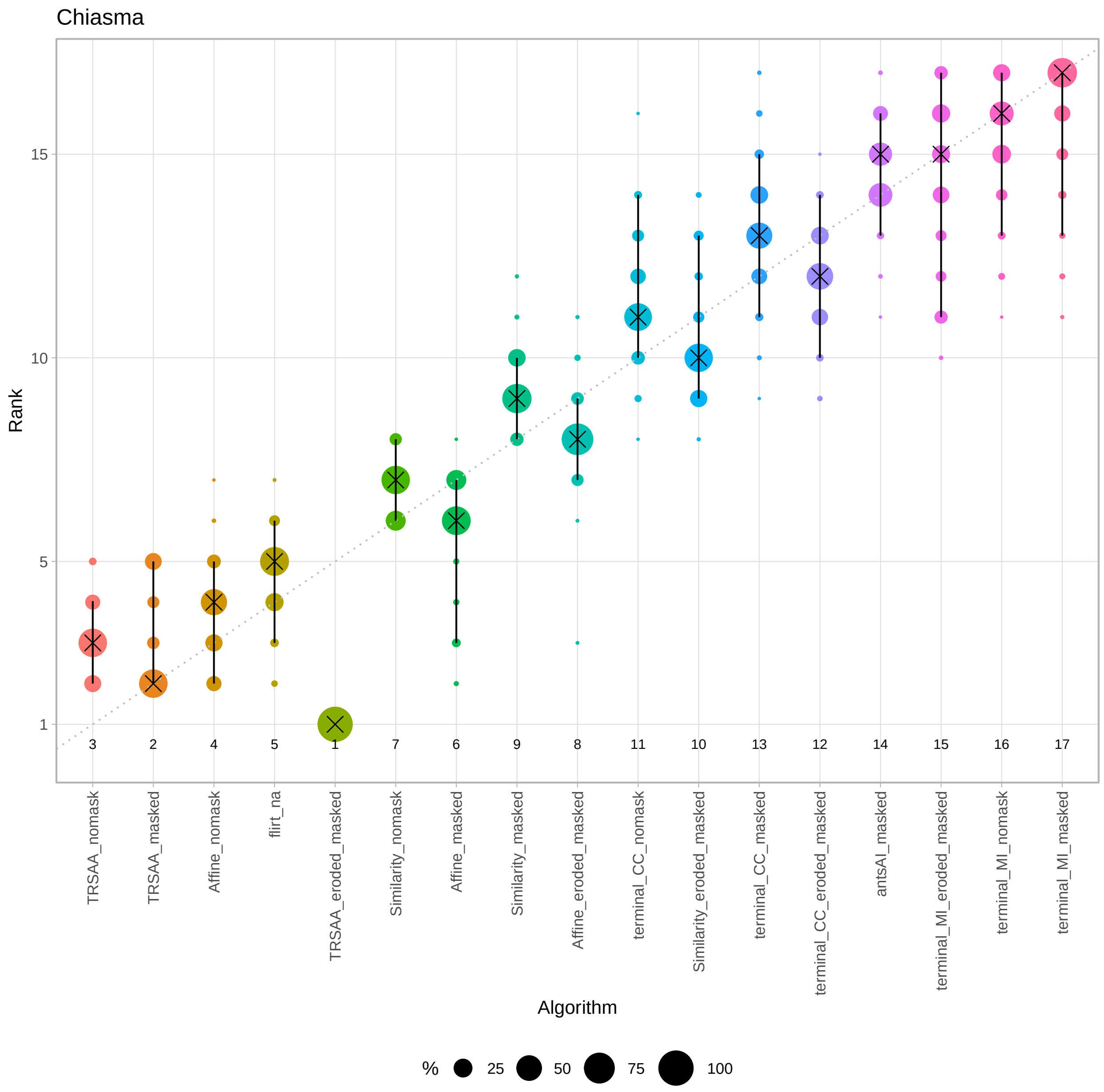

Chiasma
Rank
15
10
5
1
3
2
4
5
1
7
6
9
8
11
10
13
12
14
15
16
17
TRSAA_nomask
TRSAA_masked
Affine_nomask
flirt_na
TRSAA_eroded_masked
Similarity_nomask
Affine_masked
Similarity_masked
Affine_eroded_masked
terminal_CC_nomask
Similarity_eroded_masked
terminal_CC_masked
terminal_CC_eroded_masked
antsAI_masked
terminal_MI_eroded_masked
terminal_MI_nomask
terminal_MI_masked
Algorithm
%
25
50
75
100

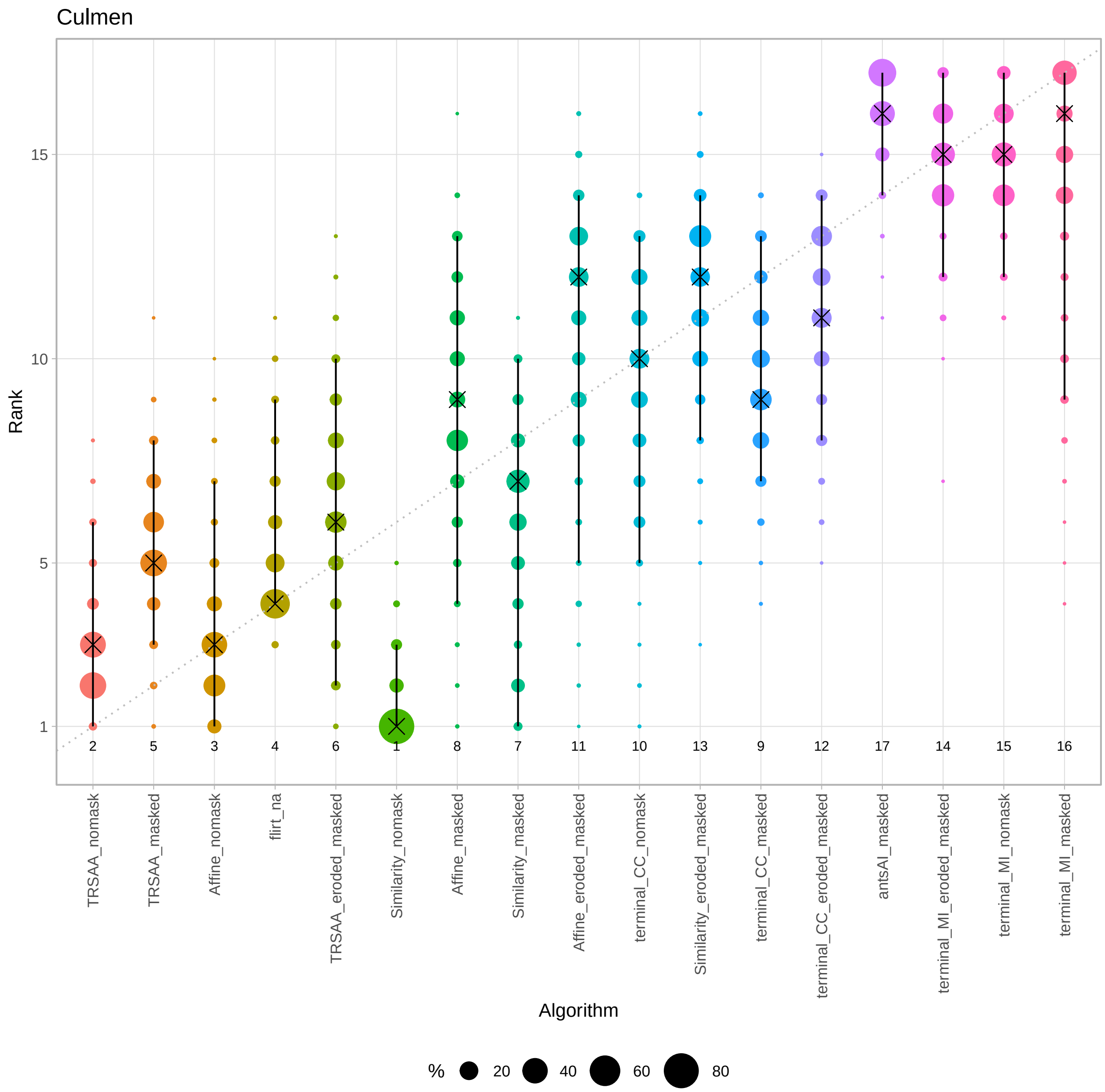
Culmen
Rank
15
10
5
1
2
5
3
4
6
1
8
7
11
10
13
9
12
17
14
15
16
TRSAA_nomask
TRSAA_masked
Affine_nomask
flirt_na
TRSAA_eroded_masked
Similarity_nomask
Affine_masked
Similarity_masked
Affine_eroded_masked
terminal_CC_nomask
Similarity_eroded_masked
terminal_CC_masked
terminal_CC_eroded_masked
antsAI_masked
terminal_MI_eroded_masked
terminal_MI_nomask
terminal_MI_masked
Algorithm
% 20 40 60 80

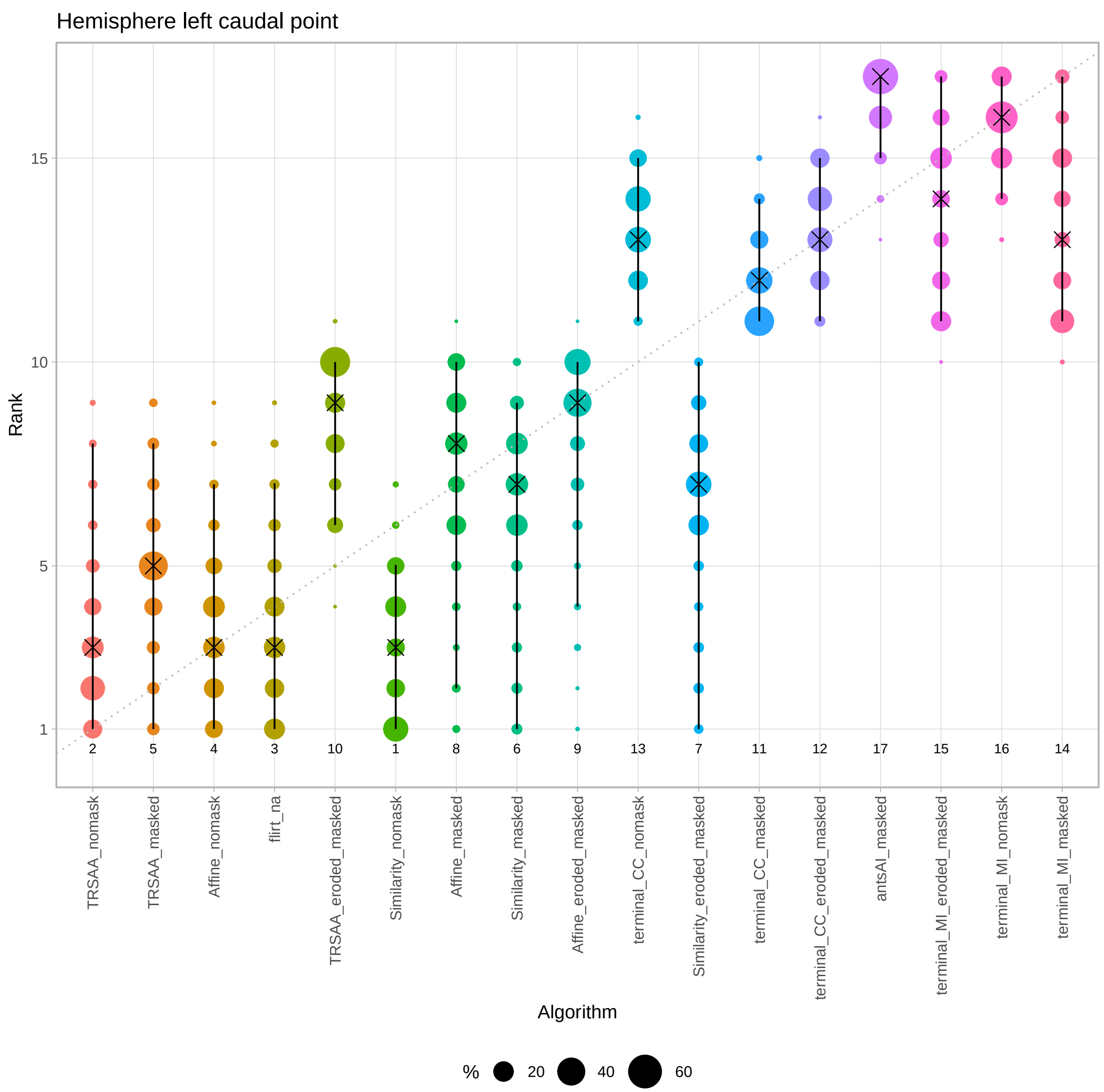

Hemisphere left caudal point
Rank
15
10
5
1
2
5
4
3
10
1
8
6
9
13
7
11
12
17
15
16
14
TRSAA_nomask
TRSAA_masked
Affine_nomask
flirt_na
TRSAA_eroded_masked
Similarity_nomask
Affine_masked
Similarity_masked
Affine_eroded_masked
terminal_CC_nomask
Similarity_eroded_masked
terminal_CC_masked
terminal_CC_eroded_masked
antsAI_masked
terminal_MI_eroded_masked
terminal_MI_nomask
terminal_MI_masked
Algorithm
%
20
40
60

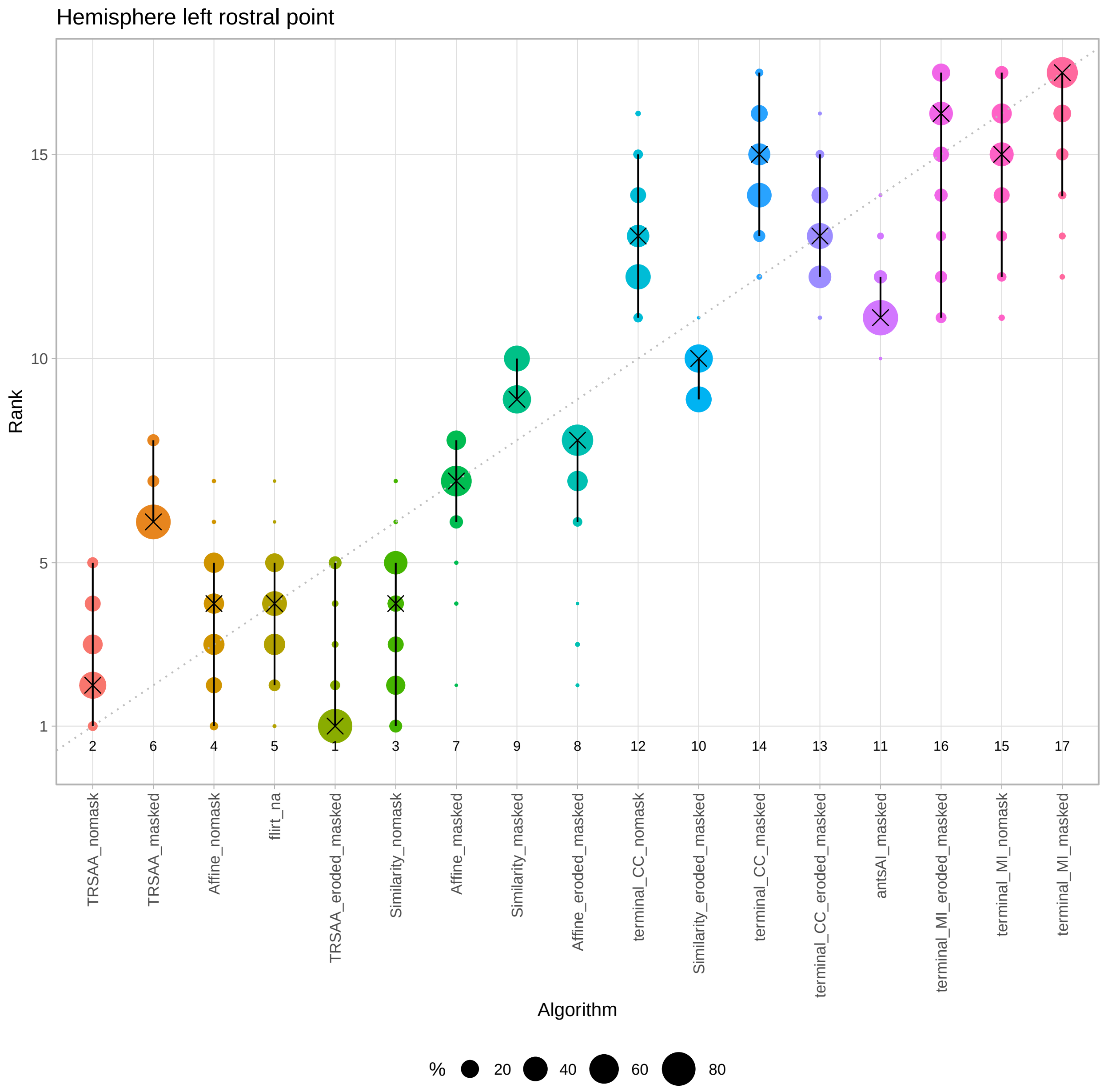

Hemisphere left rostral point
Rank
15
10
5
1
2
6
4
5
1
3
7
9
8
12
10
14
13
11
16
15
17
TRSAA_nomask
TRSAA_masked
Affine_nomask
flirt_na
TRSAA_eroded_masked
Similarity_nomask
Affine_masked
Similarity_masked
Affine_eroded_masked
terminal_CC_nomask
Similarity_eroded_masked
terminal_CC_masked
terminal_CC_eroded_masked
antsAI_masked
terminal_MI_eroded_masked
terminal_MI_nomask
terminal_MI_masked
Algorithm
%
20
40
60
80

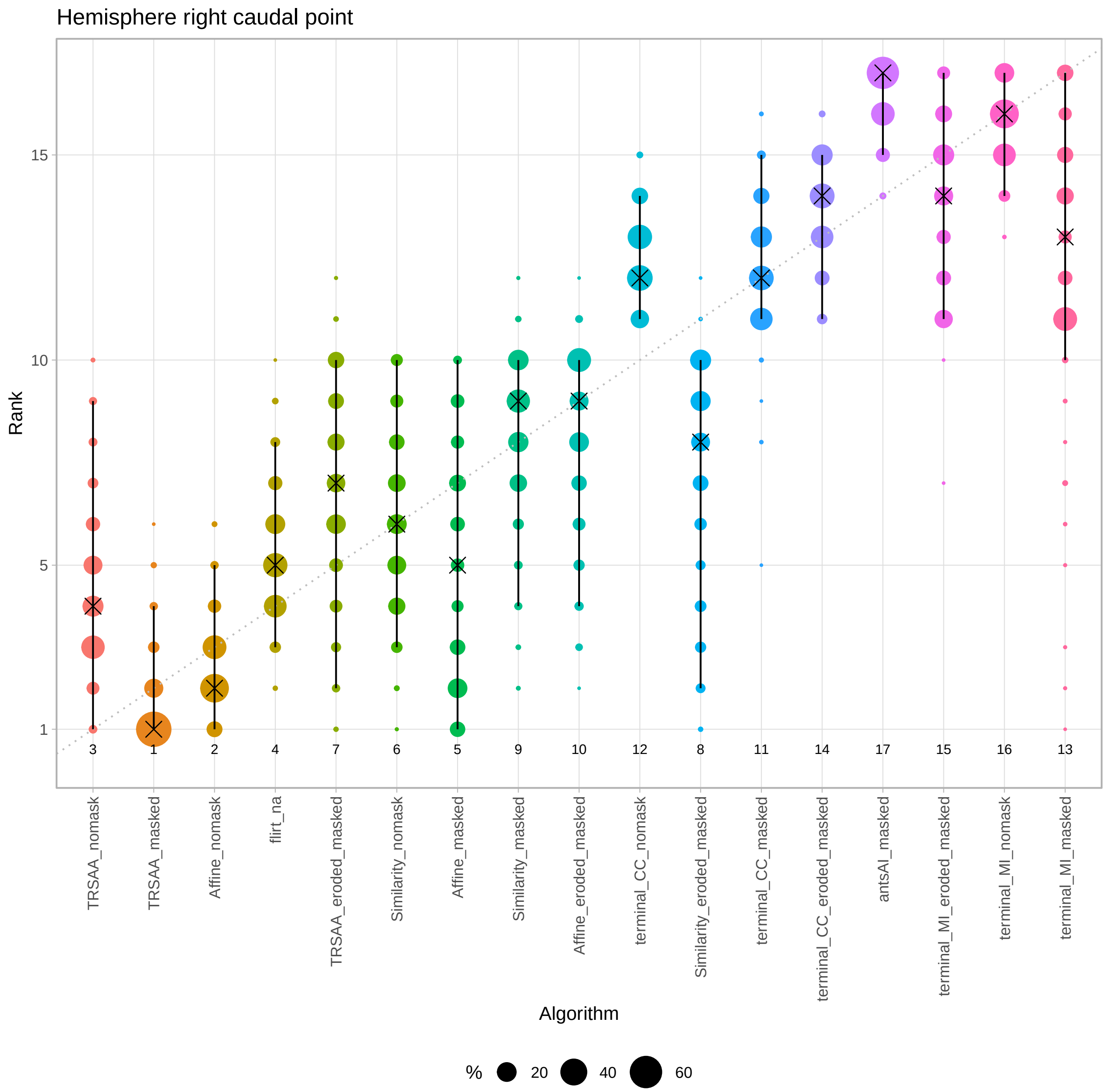
Hemisphere right caudal point
Rank
15
10
5
1
3
1
2
4
7
6
5
9
10
12
8
11
14
17
15
16
13
TRSAA_nomask
TRSAA_masked
Affine_nomask
flirt_na
TRSAA_eroded_masked
Similarity_nomask
Affine_masked
Similarity_masked
Affine_eroded_masked
terminal_CC_nomask
Similarity_eroded_masked
terminal_CC_masked
terminal_CC_eroded_masked
antsAI_masked
terminal_MI_eroded_masked
terminal_MI_nomask
terminal_MI_masked
Algorithm
%
20
40
60

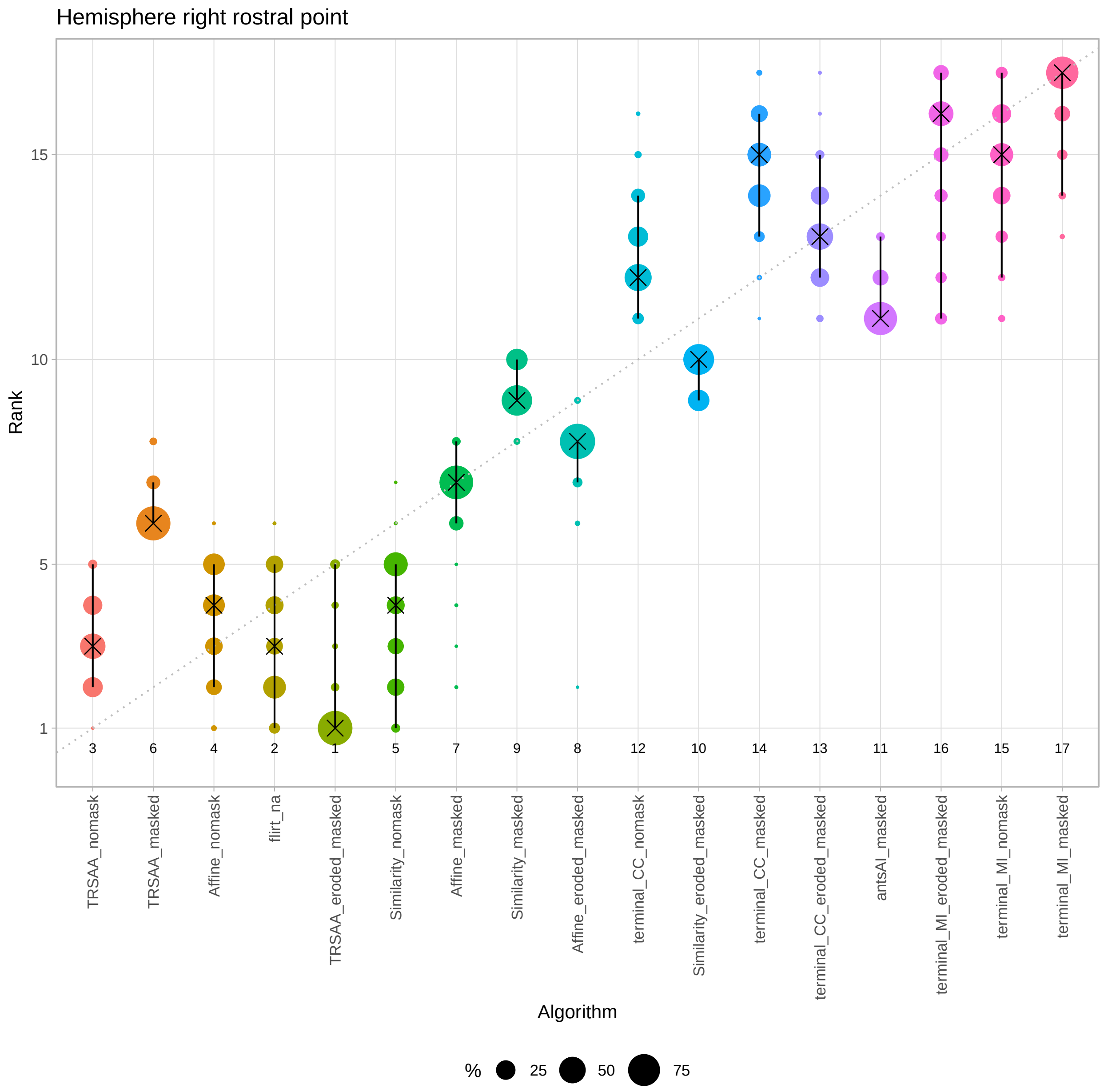
Hemisphere right rostral point
Rank
15
10
5
1
3
6
4
2
1
5
7
9
8
12
10
14
13
11
16
15
17
TRSAA_nomask
TRSAA_masked
Affine_nomask
flirt_na
TRSAA_eroded_masked
Similarity_nomask
Affine_masked
Similarity_masked
Affine_eroded_masked
terminal_CC_nomask
Similarity_eroded_masked
terminal_CC_masked
terminal_CC_eroded_masked
antsAI_masked
terminal_MI_eroded_masked
terminal_MI_nomask
terminal_MI_masked
Algorithm
%
25
50
75

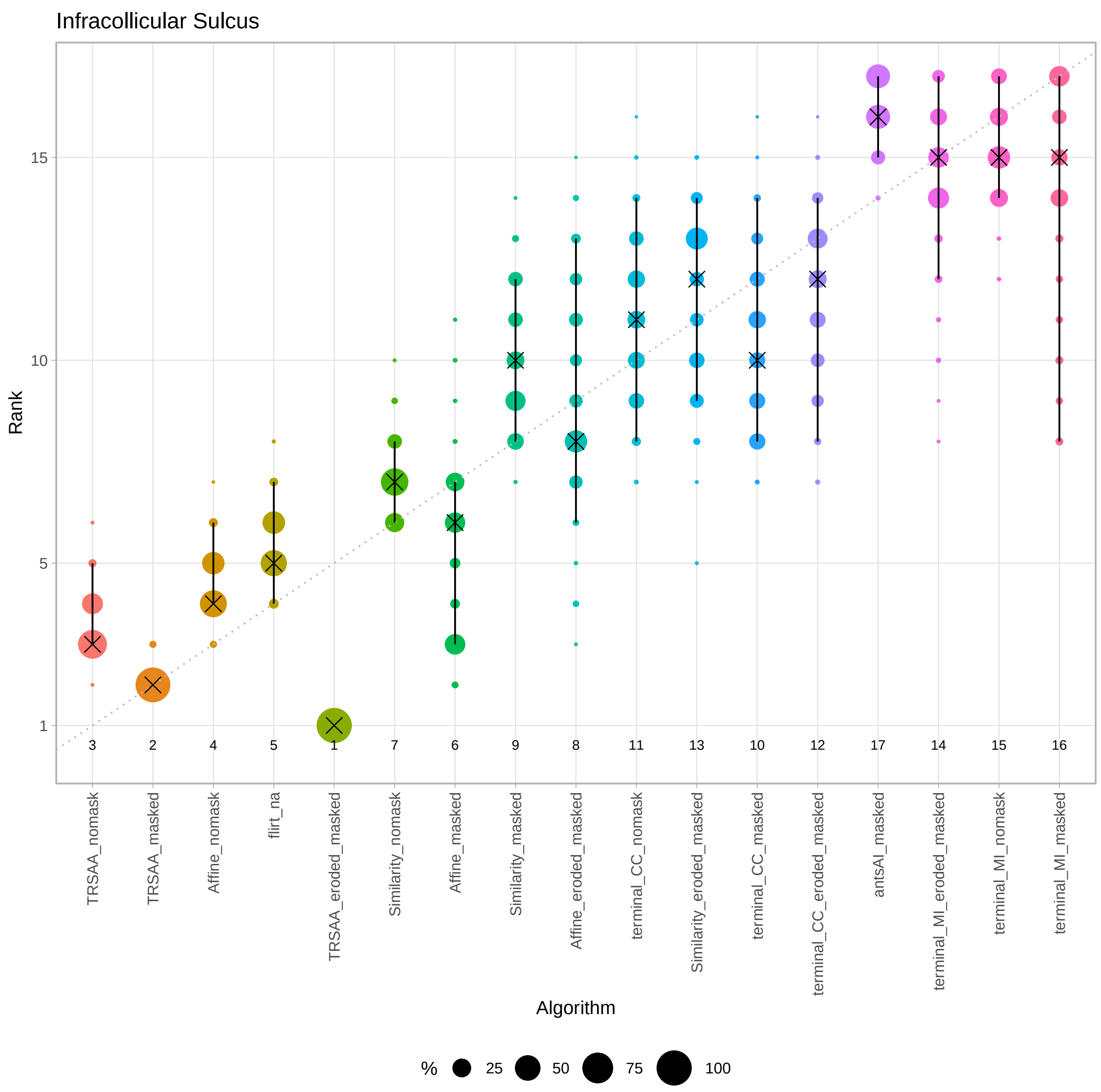

Infracollicular Sulcus
Rank
15
10
5
1
3
2
4
5
1
7
6
9
8
11
13
10
12
17
14
15
16
TRSAA_nomask
TRSAA_masked
Affine_nomask
flirt_na
TRSAA_eroded_masked
Similarity_nomask
Affine_masked
Similarity_masked
Affine_eroded_masked
terminal_CC_nomask
Similarity_eroded_masked
terminal_CC_masked
terminal_CC_eroded_masked
antsAI_masked
terminal_MI_eroded_masked
terminal_MI_nomask
terminal_MI_masked
Algorithm
%
25
50
75
100

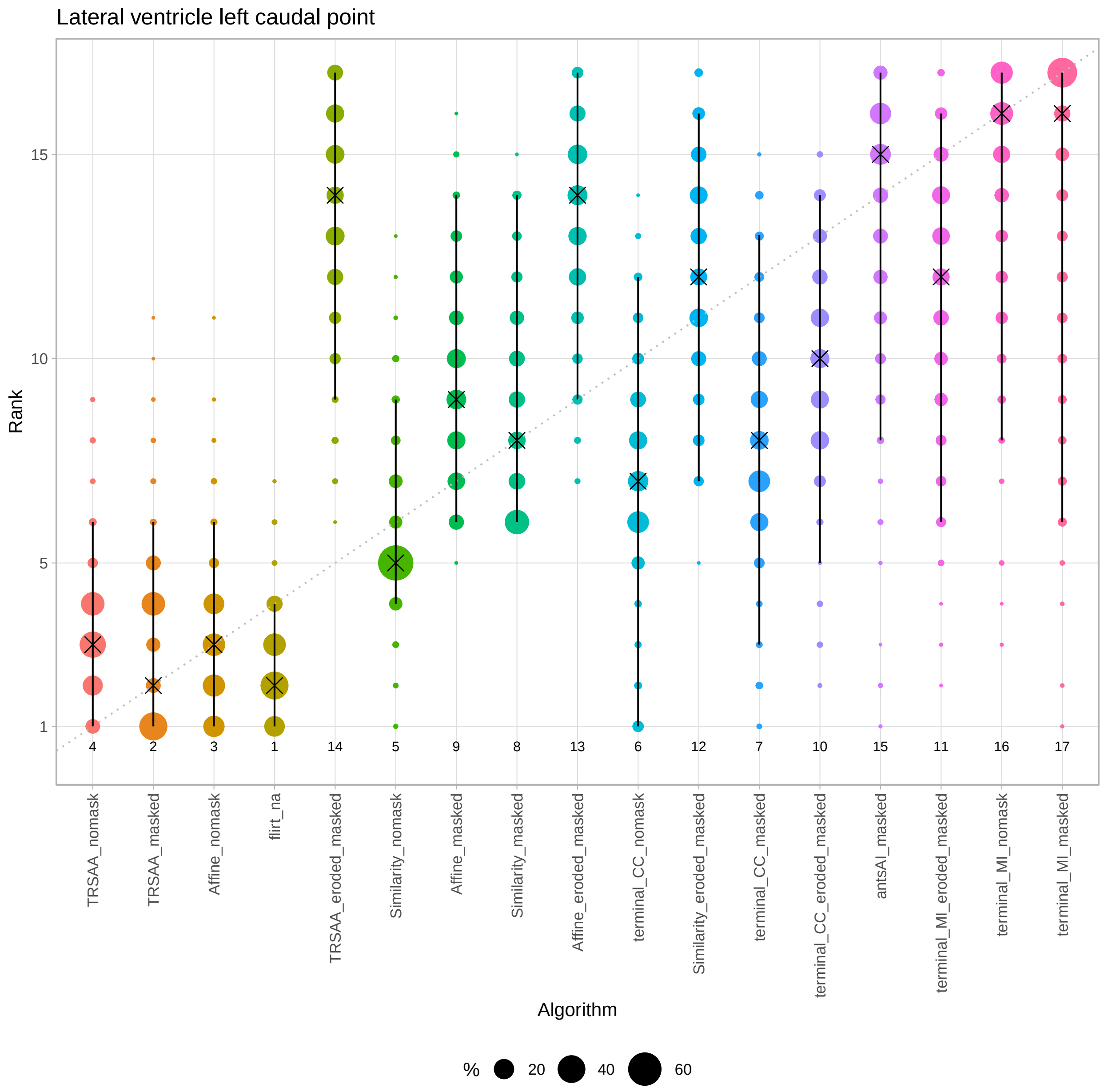
Lateral ventricle left caudal point
Rank
15
10
5
1
4
2
3
1
14
5
9
8
13
6
12
7
10
15
11
16
17
TRSAA_nomask
TRSAA_masked
Affine_nomask
flirt_na
TRSAA_eroded_masked
Similarity_nomask
Affine_masked
Similarity_masked
Affine_eroded_masked
terminal_CC_nomask
Similarity_eroded_masked
terminal_CC_masked
terminal_CC_eroded_masked
antsAI_masked
terminal_MI_eroded_masked
terminal_MI_nomask
terminal_MI_masked
Algorithm
%
20
40
60

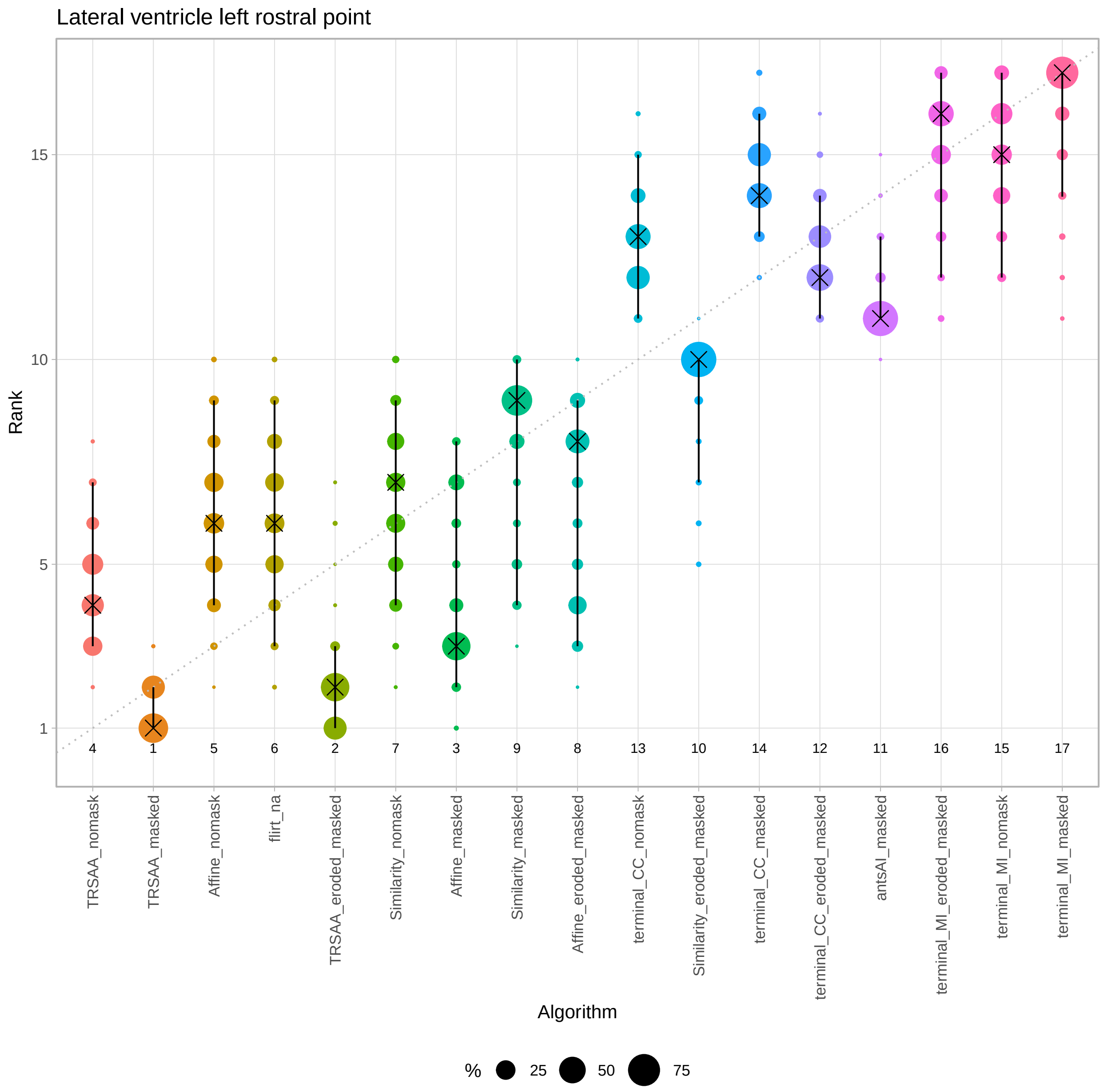

Lateral ventricle left rostral point
Rank
15
10
5
1
4
1
5
6
2
7
3
9
8
13
10
14
12
11
16
15
17
TRSAA_nomask
TRSAA_masked
Affine_nomask
flirt_na
TRSAA_eroded_masked
Similarity_nomask
Affine_masked
Similarity_masked
Affine_eroded_masked
terminal_CC_nomask
Similarity_eroded_masked
terminal_CC_masked
terminal_CC_eroded_masked
antsAI_masked
terminal_MI_eroded_masked
terminal_MI_nomask
terminal_MI_masked
Algorithm
%
25
50
75

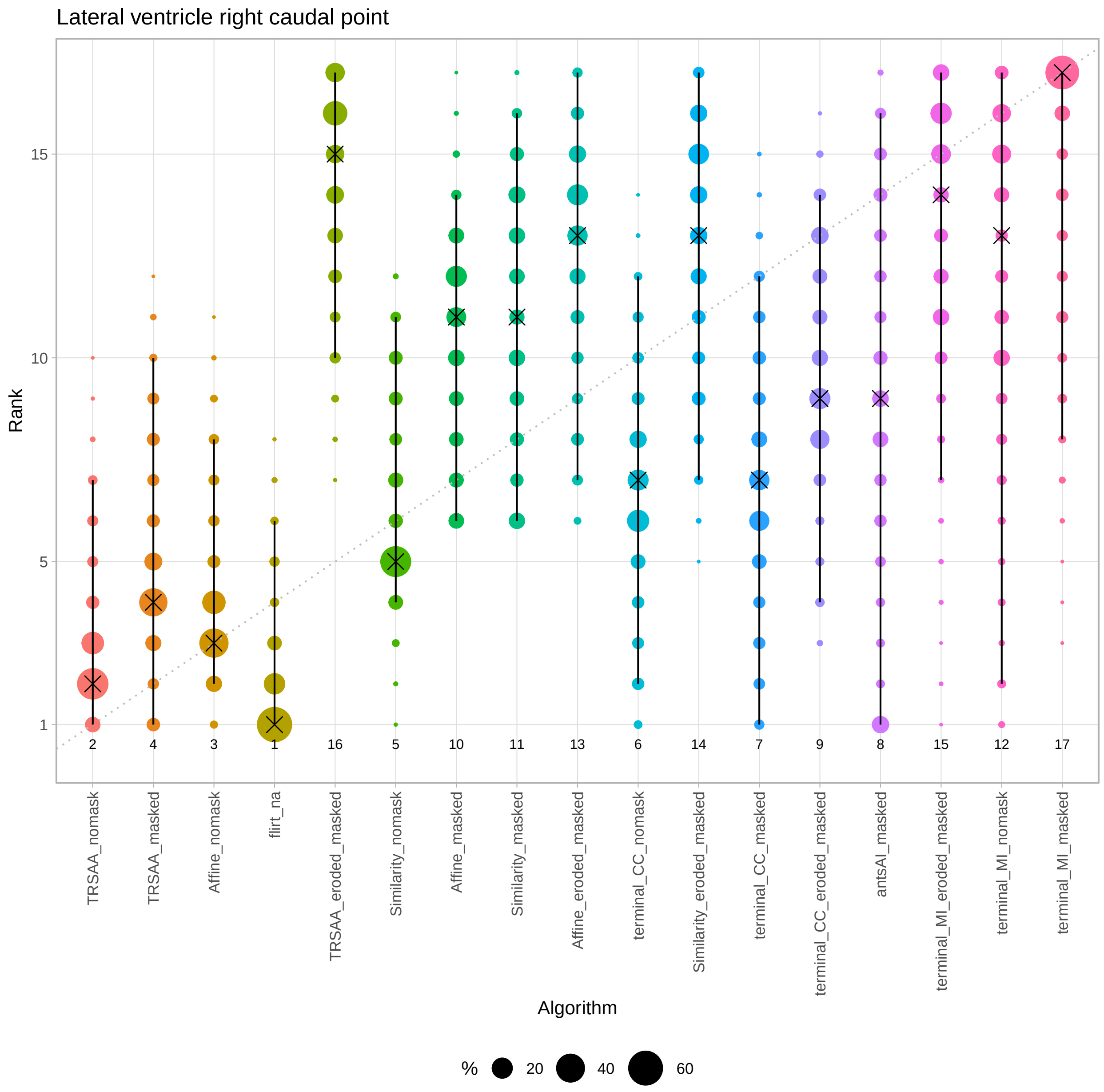
Lateral ventricle right caudal point
Rank
15
10
5
1
2
4
3
1
16
5
10
11
13
6
14
7
9
8
15
12
17
TRSAA_nomask
TRSAA_masked
Affine_nomask
flirt_na
TRSAA_eroded_masked
Similarity_nomask
Affine_masked
Similarity_masked
Affine_eroded_masked
terminal_CC_nomask
Similarity_eroded_masked
terminal_CC_masked
terminal_CC_eroded_masked
antsAI_masked
terminal_MI_eroded_masked
terminal_MI_nomask
terminal_MI_masked
Algorithm
%
20
40
60

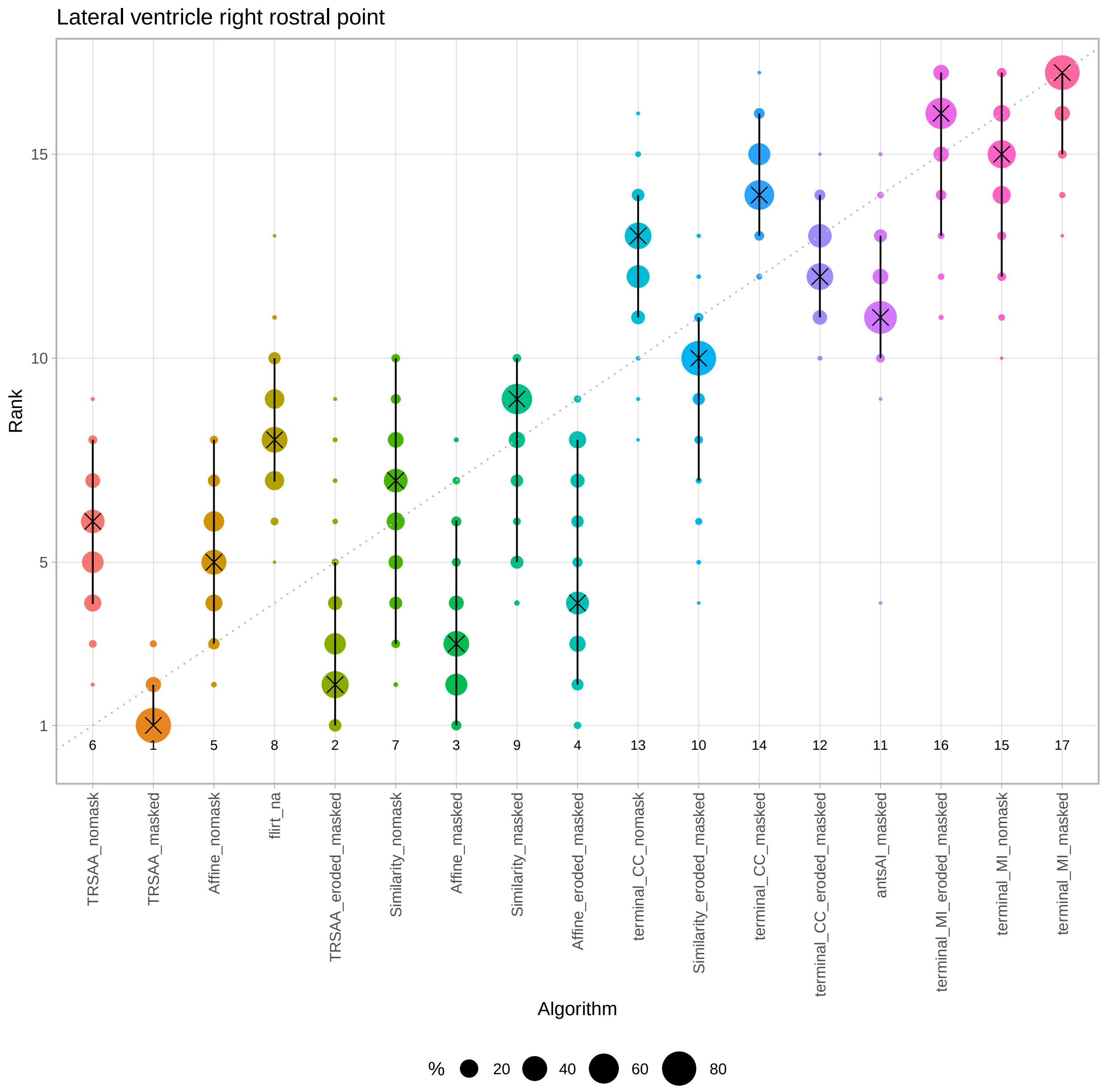
Lateral ventricle right rostral point
Rank
Algorithm
1
5
10
15
6
1
5
8
2
7
3
9
4
13
10
14
12
11
16
15
17
TRSAA_nomask
TRSAA_masked
Affine_nomask
flirt_na
TRSAA_eroded_masked
Similarity_nomask
Affine_masked
Similarity_masked
Affine_eroded_masked
terminal_CC_nomask
Similarity_eroded_masked
terminal_CC_masked
terminal_CC_eroded_masked
antsAI_masked
terminal_MI_eroded_masked
terminal_MI_nomask
terminal_MI_masked
%
20
40
60
80

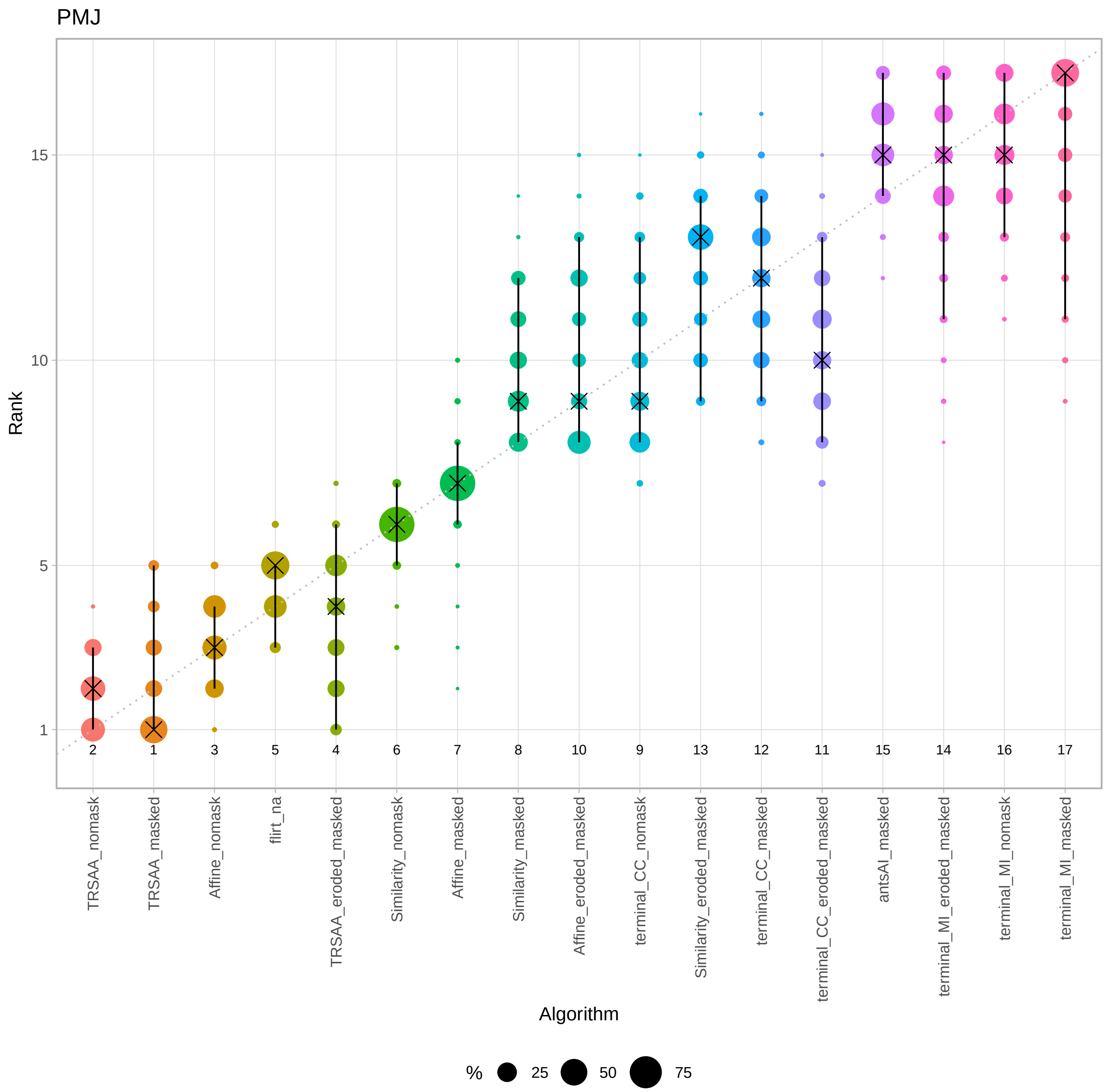
PMJ
Rank
15
10
5
1
2
1
3
5
4
6
7
8
10
9
13
12
11
15
14
16
17
TRSAA_nomask
TRSAA_masked
Affine_nomask
flirt_na
TRSAA_eroded_masked
Similarity_nomask
Affine_masked
Similarity_masked
Affine_eroded_masked
terminal_CC_nomask
Similarity_eroded_masked
terminal_CC_masked
terminal_CC_eroded_masked
antsAI_masked
terminal_MI_eroded_masked
terminal_MI_nomask
terminal_MI_masked
Algorithm
%
25
50
75

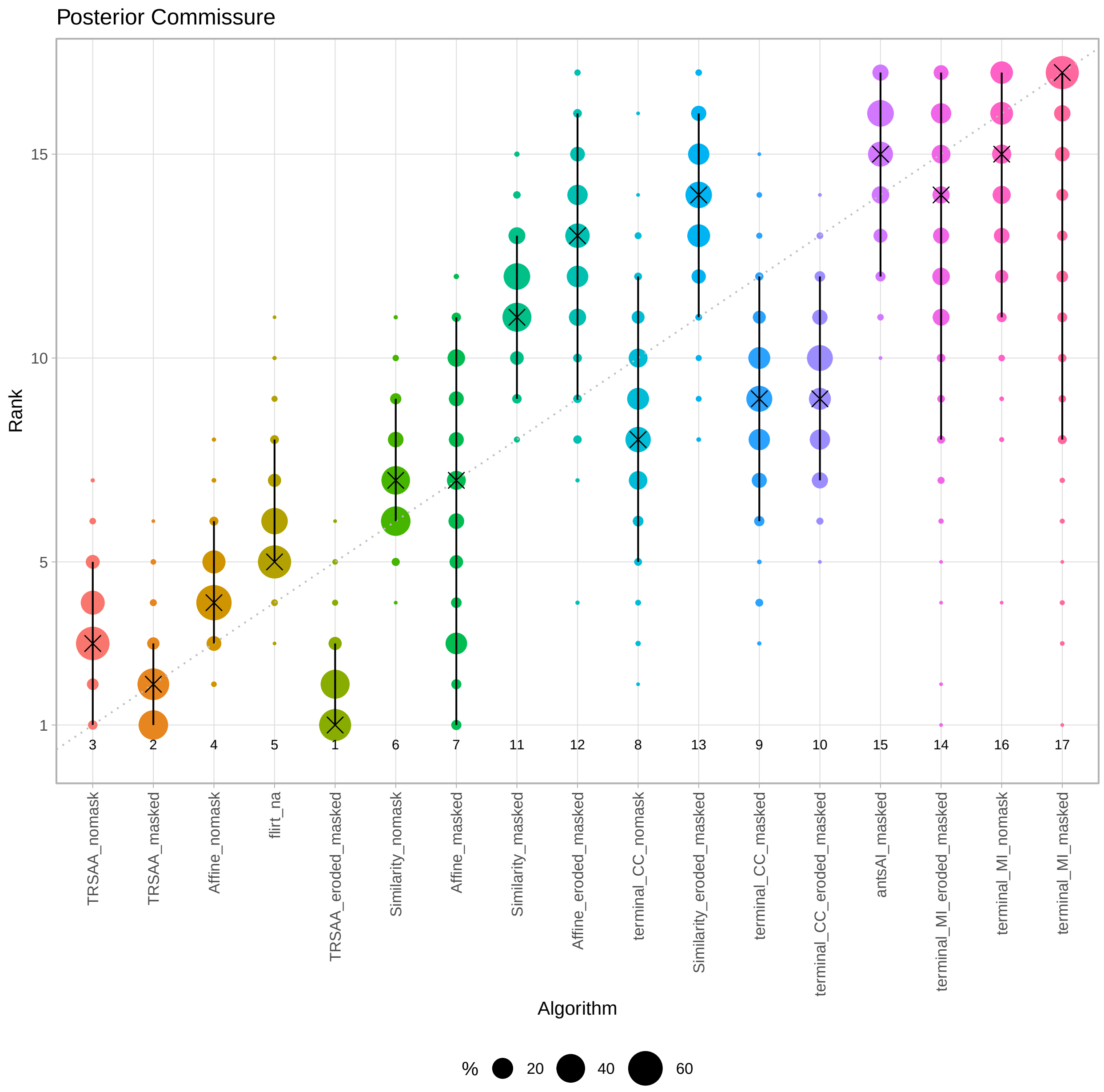

Posterior Commissure
Rank
15
10
5
1
3
2
4
5
1
6
7
11
12
8
13
9
10
15
14
16
17
TRSAA_nomask
TRSAA_masked
Affine_nomask
flirt_na
TRSAA_eroded_masked
Similarity_nomask
Affine_masked
Similarity_masked
Affine_eroded_masked
terminal_CC_nomask
Similarity_eroded_masked
terminal_CC_masked
terminal_CC_eroded_masked
antsAI_masked
terminal_MI_eroded_masked
terminal_MI_nomask
terminal_MI_masked
Algorithm
%
20
40
60

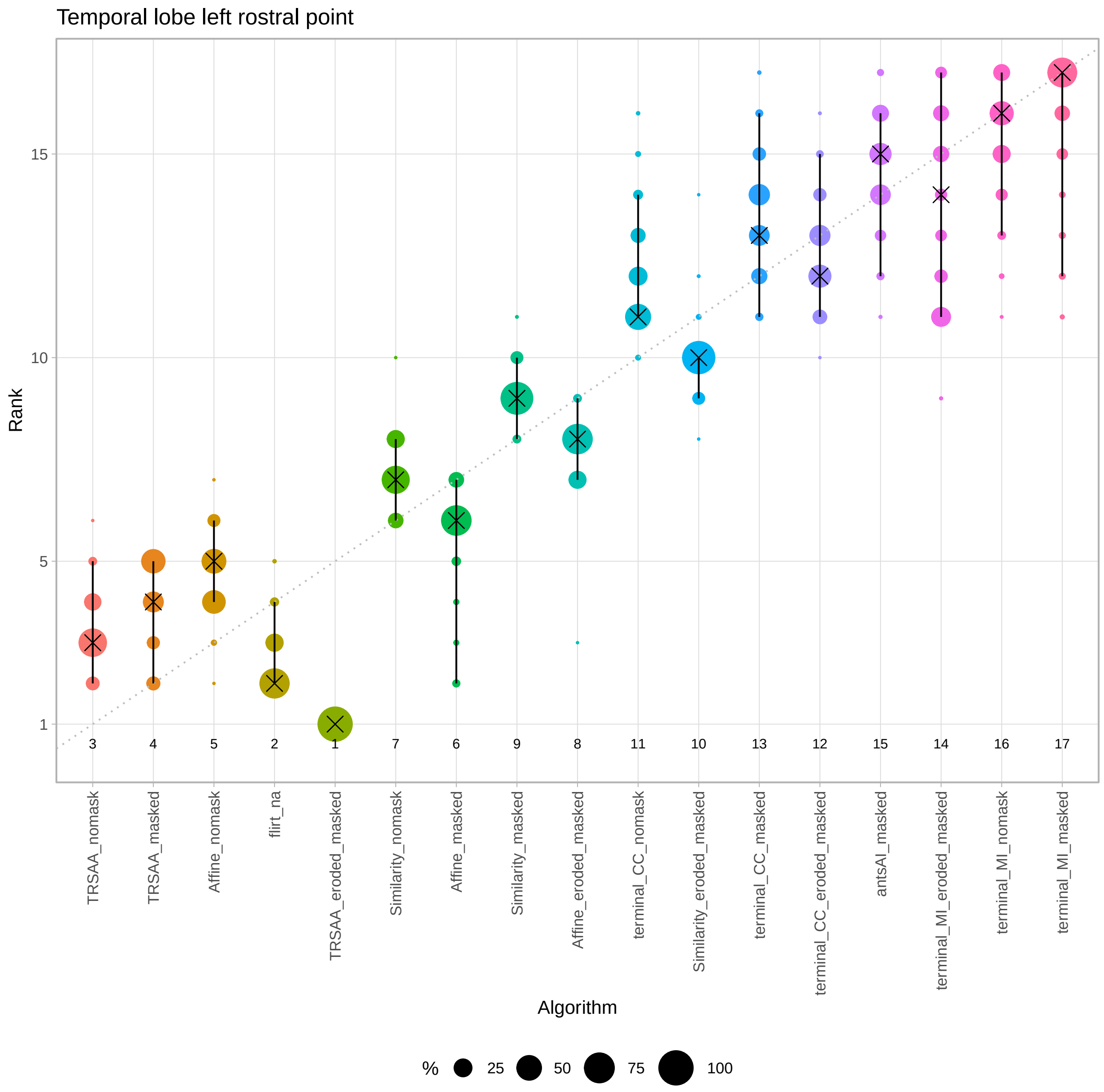
Temporal lobe left rostral point
Rank
15
10
5
1
3
4
5
2
1
7
6
9
8
11
10
13
12
15
14
16
17
TRSAA_nomask
TRSAA_masked
Affine_nomask
flirt_na
TRSAA_eroded_masked
Similarity_nomask
Affine_masked
Similarity_masked
Affine_eroded_masked
terminal_CC_nomask
Similarity_eroded_masked
terminal_CC_masked
terminal_CC_eroded_masked
antsAI_masked
terminal_MI_eroded_masked
terminal_MI_nomask
terminal_MI_masked
Algorithm
%
25
50
75
100

Temporal lobe ventricle right rostral point

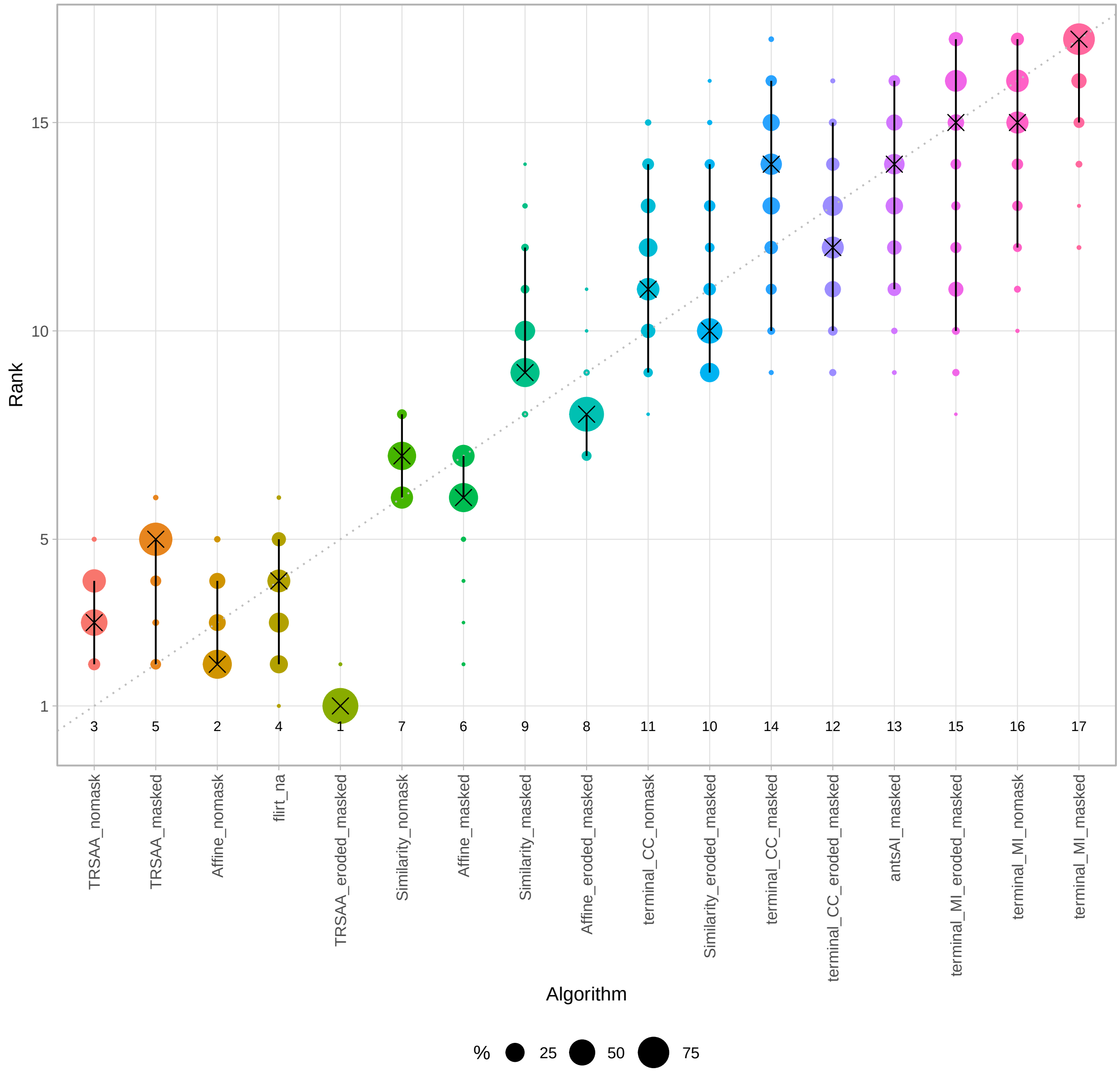

4.2.2 Cluster Analysis

Dendrogram from hierarchical cluster analysis and *network-type graphs* for assessing the similarity of tasks based on challenge rankings.

A dendrogram is a visualization approach based on hierarchical clustering. It depicts clusters according to a chosen distance measure (here: Spearman's footrule) as well as a chosen agglomeration method (here: complete and average agglomeration).

Cluster Dendrogram (complete agglomeration)

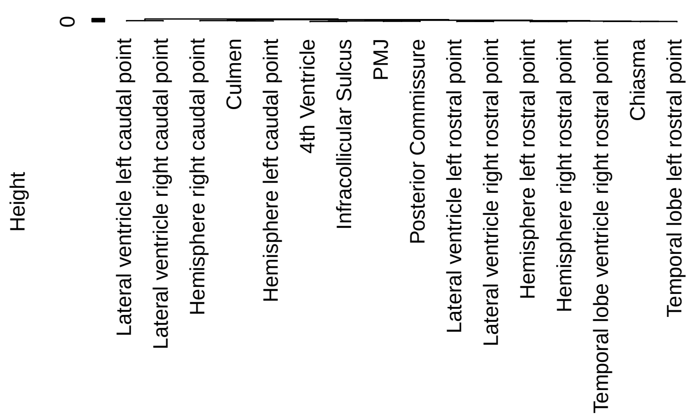

Cluster Dendrogram (average agglomeration)

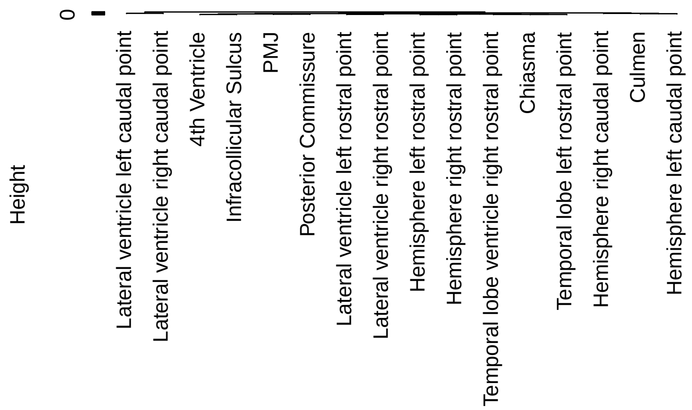

5 References

Wiesenfarth, M., Reinke, A., Landman, B.A., Eisenmann, M., Aguilera Saiz, L., Cardoso, M.J., Maier-Hein, L. and Kopp-Schneider, A. Methods and open-source toolkit for analyzing and visualizing challenge results. *Sci Rep* **11**, 2369 (2021). https://doi.org/10.1038/s41598-021-82017-6

M. J. A. Eugster, T. Hothorn, and F. Leisch, "Exploratory and inferential analysis of benchmark experiments," Institut fuer Statistik, Ludwig-Maximilians-Universitaet Muenchen, Germany, Technical Report 30, 2008. [Online]. Available: http://epub.ub.uni-muenchen.de/4134/.
