# Supplementary material for: Characterization of dynamic patterns of human fetal to neonatal brain asymmetry with deformation-based morphometry
Source: Front Neurosci. 2023 Dec 6;17:1252850. doi: 10.3389/fnins.2023.1252850 (PMC10734644; doi:10.3389/fnins.2023.1252850)
Supplement: Supplementary file 1 [file Data_Sheet_1.ZIP › ChallengeR/Framework A fetal to neonatal/nonlinear/ranking_Fetal2Neonatal_nonlinear_normal_and_bleached.pdf]

Benchmarking report for Fetal to neonatal registration nonlinear normal and bleached combined

created by challengeR v1.0.4

29 März, 2023

This document presents a systematic report on the benchmark study "Fetal to neonatal registration nonlinear normal and bleached combined". Input data comprises raw metric values for all algorithms and cases. Generated plots are:

- Visualization of assessment data: Dot- and boxplots, podium plots and ranking heatmaps
- Visualization of ranking stability: Blob plots, violin plots and significance maps, line plots
- Visualization of cross-task insights: Blob plots, stacked frequency plots, dendrograms

Details can be found in Wiesenfarth et al. (2021).

1 Rankings

Algorithms within a task are ranked according to the following ranking scheme:

aggregate using function ("mean") then rank

Ranking for each task:

4th Ventricle : The analysis is based on 20 algorithms and 20 cases. 0 missing cases have been found in the data set.

	value_mean	rank
SyNOnly__CC__2__reg__iter__1__bleached__masked	1.007558	1
SyNOnly__MI__32__reg__iter__1__bleached__masked	1.010491	2
SyNOnly__CC__4__reg__iter__1__bleached__masked	1.028764	3
SyNOnly__MI__32__reg__iter__1__normal__masked	1.040251	4
SyNOnly__CC__6__reg__iter__1__bleached__masked	1.044712	5
SyNOnly__CC__6__reg__iter__0__bleached__masked	1.067497	6
SyNOnly__CC__4__reg__iter__0__bleached__masked	1.090169	7
SyNOnly__CC__2__reg__iter__1__normal__masked	1.092836	8
SyNOnly__CC__6__reg__iter__1__normal__masked	1.095944	9
SyNOnly__CC__2__reg__iter__0__bleached__masked	1.102014	10
SyNOnly__MI__32__reg__iter__0__bleached__masked	1.113995	11
SyNOnly__CC__4__reg__iter__1__normal__masked	1.126186	12
SyNOnly__MI__32__reg__iter__0__normal__masked	1.167196	13
SyNOnly__CC__6__reg__iter__0__normal__masked	1.181540	14
SyNOnly__CC__4__reg__iter__0__normal__masked	1.183640	15
SyNOnly__CC__2__reg__iter__0__normal__masked	1.184196	16
TVMSQ__bleached__masked	2.126241	17
TVMSQ__normal__masked	2.216452	18
TVMSQC__normal__masked	2.470736	19
TVMSQC__bleached__masked	7.130142	20

Chiasma : The analysis is based on 20 algorithms and 20 cases. 0 missing cases have been found in the data set.

	value_mean	rank
SyNOnly_CC_6_reg_iter_0_normal_masked	1.365183	1
SyNOnly_CC_4_reg_iter_0_normal_masked	1.366260	2
SyNOnly_CC_6_reg_iter_1_normal_masked	1.388588	3
SyNOnly_CC_2_reg_iter_0_normal_masked	1.404164	4
SyNOnly_CC_4_reg_iter_1_normal_masked	1.429871	5
SyNOnly_CC_2_reg_iter_1_normal_masked	1.491635	6
SyNOnly_CC_2_reg_iter_1_bleached_masked	1.506134	7
SyNOnly_CC_4_reg_iter_1_bleached_masked	1.555175	8
SyNOnly_CC_2_reg_iter_0_bleached_masked	1.617652	9
SyNOnly_CC_6_reg_iter_1_bleached_masked	1.688652	10
TVMSQC_normal_masked	1.723710	11
SyNOnly_MI_32_reg_iter_1_normal_masked	1.778266	12
SyNOnly_CC_4_reg_iter_0_bleached_masked	1.881271	13
SyNOnly_MI_32_reg_iter_0_normal_masked	1.937091	14
SyNOnly_CC_6_reg_iter_0_bleached_masked	2.161225	15
TVMSQ_normal_masked	2.172587	16
TVMSQ_bleached_masked	2.318229	17
SyNOnly_MI_32_reg_iter_1_bleached_masked	2.785446	18
SyNOnly_MI_32_reg_iter_0_bleached_masked	3.520806	19
TVMSQC_bleached_masked	3.832329	20

Culmen : The analysis is based on 20 algorithms and 20 cases. 0 missing cases have been found in the data set.

	value_mean	rank
SyNOnly_CC_2_reg_iter_1_bleached_masked	0.9945523	1
SyNOnly_CC_4_reg_iter_1_bleached_masked	1.0597162	2
SyNOnly_CC_6_reg_iter_1_bleached_masked	1.1538463	3
SyNOnly_MI_32_reg_iter_1_bleached_masked	1.1580855	4
SyNOnly_CC_2_reg_iter_1_normal_masked	1.1900787	5
SyNOnly_CC_2_reg_iter_0_bleached_masked	1.3120627	6
SyNOnly_MI_32_reg_iter_0_bleached_masked	1.3500181	7
SyNOnly_CC_4_reg_iter_1_normal_masked	1.4235272	8
SyNOnly_CC_2_reg_iter_0_normal_masked	1.4307791	9
SyNOnly_MI_32_reg_iter_1_normal_masked	1.4723411	10
SyNOnly_MI_32_reg_iter_0_normal_masked	1.4986568	11
SyNOnly_CC_4_reg_iter_0_bleached_masked	1.5131989	12
SyNOnly_CC_6_reg_iter_0_bleached_masked	1.5139891	13
SyNOnly_CC_6_reg_iter_1_normal_masked	1.5228120	14
SyNOnly_CC_6_reg_iter_0_normal_masked	1.5665304	15
TVMSQ_bleached_masked	1.5679798	16
TVMSQ_normal_masked	1.5962558	17
SyNOnly_CC_4_reg_iter_0_normal_masked	1.6164512	18
TVMSQC_normal_masked	2.0810691	19
TVMSQC_bleached_masked	8.7870752	20

Hemisphere left caudal point : The analysis is based on 20 algorithms and 20 cases. 0 missing cases have

been found in the data set.

	value_mean	rank
SyNOnly_MI_32_reg_iter_1_bleached_masked	4.234404	1
SyNOnly_MI_32_reg_iter_0_bleached_masked	4.242198	2
SyNOnly_CC_2_reg_iter_0_bleached_masked	4.281430	3
SyNOnly_CC_2_reg_iter_1_bleached_masked	4.354779	4
SyNOnly_CC_2_reg_iter_1_normal_masked	4.373509	5
SyNOnly_CC_4_reg_iter_0_bleached_masked	4.383674	6
SyNOnly_CC_4_reg_iter_0_normal_masked	4.390875	7
SyNOnly_CC_2_reg_iter_0_normal_masked	4.403928	8
SyNOnly_CC_6_reg_iter_0_bleached_masked	4.415644	9
SyNOnly_CC_4_reg_iter_1_normal_masked	4.492616	10
SyNOnly_CC_6_reg_iter_0_normal_masked	4.493571	11
TVMSQ_bleached_masked	4.511581	12
TVMSQ_normal_masked	4.514428	13
SyNOnly_CC_4_reg_iter_1_bleached_masked	4.537767	14
SyNOnly_CC_6_reg_iter_1_bleached_masked	4.560100	15
SyNOnly_MI_32_reg_iter_0_normal_masked	4.582268	16
SyNOnly_CC_6_reg_iter_1_normal_masked	4.614341	17
SyNOnly_MI_32_reg_iter_1_normal_masked	4.616147	18
TVMSQC_normal_masked	4.768778	19
TVMSQC_bleached_masked	5.021359	20

Hemisphere left rostral point : The analysis is based on 20 algorithms and 20 cases. 0 missing cases have been found in the data set.

	value_mean	rank
SyNOnly_MI_32_reg_iter_1_bleached_masked	3.316835	1
SyNOnly_CC_6_reg_iter_1_bleached_masked	3.408787	2
SyNOnly_CC_4_reg_iter_1_bleached_masked	3.467160	3
SyNOnly_MI_32_reg_iter_0_bleached_masked	3.524174	4
SyNOnly_CC_2_reg_iter_1_bleached_masked	3.623052	5
SyNOnly_CC_6_reg_iter_0_bleached_masked	3.665575	6
SyNOnly_CC_4_reg_iter_0_bleached_masked	3.692736	7
SyNOnly_CC_2_reg_iter_0_bleached_masked	3.766302	8
SyNOnly_CC_2_reg_iter_1_normal_masked	4.008673	9
TVMSQC_bleached_masked	4.038123	10
SyNOnly_CC_2_reg_iter_0_normal_masked	4.128383	11
SyNOnly_CC_4_reg_iter_0_normal_masked	4.144351	12
SyNOnly_CC_4_reg_iter_1_normal_masked	4.160470	13
SyNOnly_CC_6_reg_iter_0_normal_masked	4.163450	14
TVMSQ_bleached_masked	4.169314	15
SyNOnly_MI_32_reg_iter_0_normal_masked	4.176474	16
TVMSQ_normal_masked	4.199419	17
SyNOnly_CC_6_reg_iter_1_normal_masked	4.217802	18
SyNOnly_MI_32_reg_iter_1_normal_masked	4.273882	19
TVMSQC_normal_masked	4.603021	20

Hemisphere right caudal point : The analysis is based on 20 algorithms and 20 cases. 0 missing cases have been found in the data set.

	value_mean	rank
SyNOnly_CC_2_reg_iter_0_bleached_masked	4.691161	1
SyNOnly_MI_32_reg_iter_0_bleached_masked	4.696726	2
SyNOnly_MI_32_reg_iter_1_bleached_masked	4.755507	3
SyNOnly_CC_2_reg_iter_1_normal_masked	4.775660	4
SyNOnly_CC_4_reg_iter_0_bleached_masked	4.782039	5
SyNOnly_CC_2_reg_iter_0_normal_masked	4.782146	6
SyNOnly_CC_4_reg_iter_0_normal_masked	4.782860	7
SyNOnly_CC_4_reg_iter_1_normal_masked	4.784695	8
SyNOnly_CC_6_reg_iter_0_bleached_masked	4.816800	9
SyNOnly_CC_6_reg_iter_0_normal_masked	4.851665	10
SyNOnly_CC_2_reg_iter_1_bleached_masked	4.867028	11
SyNOnly_MI_32_reg_iter_1_normal_masked	4.887755	12
SyNOnly_CC_6_reg_iter_1_normal_masked	4.895033	13
SyNOnly_MI_32_reg_iter_0_normal_masked	4.928101	14
TVMSQ_bleached_masked	5.011086	15
TVMSQ_normal_masked	5.011921	16
SyNOnly_CC_4_reg_iter_1_bleached_masked	5.028484	17
SyNOnly_CC_6_reg_iter_1_bleached_masked	5.035601	18
TVMSQC_normal_masked	5.069126	19
TVMSQC_bleached_masked	5.217433	20

Hemisphere right rostral point : The analysis is based on 20 algorithms and 20 cases. 0 missing cases have been found in the data set.

	value_mean	rank
SyNOnly_MI_32_reg_iter_1_bleached_masked	4.225013	1
SyNOnly_CC_6_reg_iter_1_bleached_masked	4.331213	2
SyNOnly_MI_32_reg_iter_0_bleached_masked	4.352432	3
SyNOnly_CC_4_reg_iter_1_bleached_masked	4.438871	4
SyNOnly_CC_6_reg_iter_0_bleached_masked	4.481628	5
SyNOnly_CC_4_reg_iter_0_bleached_masked	4.533790	6
SyNOnly_CC_2_reg_iter_1_bleached_masked	4.579931	7
TVMSQC_bleached_masked	4.649537	8
SyNOnly_CC_2_reg_iter_0_bleached_masked	4.715217	9
SyNOnly_CC_4_reg_iter_0_normal_masked	4.886802	10
SyNOnly_CC_6_reg_iter_0_normal_masked	4.897695	11
SyNOnly_CC_2_reg_iter_1_normal_masked	4.898434	12
TVMSQ_bleached_masked	4.926422	13
SyNOnly_MI_32_reg_iter_0_normal_masked	4.940364	14
TVMSQ_normal_masked	4.955426	15
SyNOnly_MI_32_reg_iter_1_normal_masked	4.958270	16
SyNOnly_CC_4_reg_iter_1_normal_masked	4.960446	17
SyNOnly_CC_6_reg_iter_1_normal_masked	4.986910	18
SyNOnly_CC_2_reg_iter_0_normal_masked	4.989217	19
TVMSQC_normal_masked	5.243124	20

Infracollicular Sulcus : The analysis is based on 20 algorithms and 20 cases. 0 missing cases have been found in the data set.

	value_mean	rank
SyNOnly_CC_2_reg_iter_1_bleached_masked	0.9973731	1
SyNOnly_CC_2_reg_iter_1_normal_masked	1.0104979	2
SyNOnly_CC_4_reg_iter_1_normal_masked	1.0768656	3
SyNOnly_CC_4_reg_iter_1_bleached_masked	1.0802887	4
SyNOnly_CC_6_reg_iter_1_bleached_masked	1.1352383	5
SyNOnly_CC_2_reg_iter_0_bleached_masked	1.1389133	6
SyNOnly_CC_6_reg_iter_1_normal_masked	1.1419708	7
SyNOnly_CC_2_reg_iter_0_normal_masked	1.1514515	8
SyNOnly_MI_32_reg_iter_1_normal_masked	1.1741775	9
SyNOnly_MI_32_reg_iter_1_bleached_masked	1.1834512	10
SyNOnly_MI_32_reg_iter_0_bleached_masked	1.2446247	11
SyNOnly_CC_4_reg_iter_0_bleached_masked	1.2564221	12
SyNOnly_CC_6_reg_iter_0_bleached_masked	1.2899647	13
SyNOnly_MI_32_reg_iter_0_normal_masked	1.3254515	14
SyNOnly_CC_4_reg_iter_0_normal_masked	1.3693778	15
SyNOnly_CC_6_reg_iter_0_normal_masked	1.4359053	16
TVMSQ_bleached_masked	1.7620939	17
TVMSQ_normal_masked	1.8241839	18
TVMSQC_normal_masked	2.2480910	19
TVMSQC_bleached_masked	8.4710750	20

Lateral ventricle left caudal point : The analysis is based on 20 algorithms and 20 cases. 0 missing cases have been found in the data set.

	value_mean	rank
SyNOnly_MI_32_reg_iter_1_normal_masked	3.875956	1
SyNOnly_CC_6_reg_iter_1_normal_masked	3.909332	2
SyNOnly_CC_4_reg_iter_1_normal_masked	3.930830	3
SyNOnly_CC_2_reg_iter_1_normal_masked	3.946177	4
TVMSQC_normal_masked	4.027188	5
SyNOnly_CC_2_reg_iter_0_normal_masked	4.172523	6
SyNOnly_CC_2_reg_iter_1_bleached_masked	4.174044	7
SyNOnly_CC_4_reg_iter_0_bleached_masked	4.191066	8
SyNOnly_CC_2_reg_iter_0_bleached_masked	4.191066	9
SyNOnly_CC_6_reg_iter_0_bleached_masked	4.226200	10
SyNOnly_CC_4_reg_iter_0_normal_masked	4.236284	11
SyNOnly_MI_32_reg_iter_0_bleached_masked	4.241299	12
SyNOnly_CC_4_reg_iter_1_bleached_masked	4.272787	13
SyNOnly_CC_6_reg_iter_0_normal_masked	4.310717	14
SyNOnly_MI_32_reg_iter_0_normal_masked	4.354068	15
SyNOnly_CC_6_reg_iter_1_bleached_masked	4.436546	16
SyNOnly_MI_32_reg_iter_1_bleached_masked	4.457931	17
TVMSQ_bleached_masked	4.536212	18
TVMSQ_normal_masked	4.542238	19
TVMSQC_bleached_masked	10.823763	20

Lateral ventricle left rostral point : The analysis is based on 20 algorithms and 20 cases. 0 missing cases have been found in the data set.

	value_mean	rank
SyNOnly_CC_2_reg_iter_1_bleached_masked	1.480944	1
SyNOnly_CC_2_reg_iter_0_bleached_masked	1.483362	2
SyNOnly_CC_2_reg_iter_1_normal_masked	1.497564	3
SyNOnly_CC_4_reg_iter_1_normal_masked	1.505121	4
SyNOnly_CC_2_reg_iter_0_normal_masked	1.522114	5
SyNOnly_CC_4_reg_iter_1_bleached_masked	1.541763	6
SyNOnly_CC_4_reg_iter_0_bleached_masked	1.558213	7
SyNOnly_CC_6_reg_iter_1_normal_masked	1.570783	8
SyNOnly_CC_6_reg_iter_0_bleached_masked	1.597059	9
SyNOnly_CC_6_reg_iter_1_bleached_masked	1.614885	10
SyNOnly_CC_4_reg_iter_0_normal_masked	1.624946	11
SyNOnly_MI_32_reg_iter_0_bleached_masked	1.720689	12
SyNOnly_MI_32_reg_iter_1_normal_masked	1.781213	13
SyNOnly_CC_6_reg_iter_0_normal_masked	1.827705	14
SyNOnly_MI_32_reg_iter_1_bleached_masked	1.909267	15
SyNOnly_MI_32_reg_iter_0_normal_masked	1.912949	16
TVMSQC_normal_masked	2.329211	17
TVMSQ_bleached_masked	2.408822	18
TVMSQ_normal_masked	2.446738	19
TVMSQC_bleached_masked	10.579151	20

Lateral ventricle right caudal point : The analysis is based on 20 algorithms and 20 cases. 0 missing cases have been found in the data set.

	value_mean	rank
SyNOnly_CC_2_reg_iter_1_normal_masked	3.505682	1
SyNOnly_CC_2_reg_iter_1_bleached_masked	3.535872	2
SyNOnly_CC_6_reg_iter_1_normal_masked	3.607703	3
SyNOnly_CC_4_reg_iter_1_normal_masked	3.633150	4
SyNOnly_MI_32_reg_iter_0_bleached_masked	3.648984	5
SyNOnly_CC_2_reg_iter_0_bleached_masked	3.661831	6
SyNOnly_CC_4_reg_iter_1_bleached_masked	3.663777	7
SyNOnly_MI_32_reg_iter_1_normal_masked	3.687797	8
SyNOnly_CC_4_reg_iter_0_bleached_masked	3.730181	9
SyNOnly_CC_2_reg_iter_0_normal_masked	3.730442	10
SyNOnly_CC_6_reg_iter_0_bleached_masked	3.736749	11
SyNOnly_CC_6_reg_iter_1_bleached_masked	3.788432	12
SyNOnly_MI_32_reg_iter_1_bleached_masked	3.811508	13
SyNOnly_CC_4_reg_iter_0_normal_masked	3.844389	14
SyNOnly_CC_6_reg_iter_0_normal_masked	3.924100	15
SyNOnly_MI_32_reg_iter_0_normal_masked	4.017111	16
TVMSQC_normal_masked	4.019909	17
TVMSQ_bleached_masked	4.203450	18
TVMSQ_normal_masked	4.221536	19
TVMSQC_bleached_masked	9.977902	20

Lateral ventricle right rostral point : The analysis is based on 20 algorithms and 20 cases. 0 missing cases have been found in the data set.

	value_mean	rank
SyNOnly_CC_4_reg_iter_1_bleached_masked	1.683668	1
SyNOnly_CC_2_reg_iter_0_normal_masked	1.702375	2
SyNOnly_CC_2_reg_iter_1_normal_masked	1.720208	3
SyNOnly_CC_2_reg_iter_1_bleached_masked	1.733207	4
SyNOnly_CC_6_reg_iter_0_bleached_masked	1.741007	5
SyNOnly_CC_6_reg_iter_1_bleached_masked	1.752490	6
SyNOnly_CC_4_reg_iter_0_bleached_masked	1.771707	7
SyNOnly_MI_32_reg_iter_0_bleached_masked	1.807803	8
SyNOnly_CC_4_reg_iter_1_normal_masked	1.822299	9
SyNOnly_CC_2_reg_iter_0_bleached_masked	1.829004	10
SyNOnly_MI_32_reg_iter_1_bleached_masked	1.833001	11
SyNOnly_CC_4_reg_iter_0_normal_masked	1.840944	12
SyNOnly_CC_6_reg_iter_1_normal_masked	1.950820	13
SyNOnly_MI_32_reg_iter_1_normal_masked	2.013549	14
SyNOnly_CC_6_reg_iter_0_normal_masked	2.017031	15
SyNOnly_MI_32_reg_iter_0_normal_masked	2.068242	16
TVMSQC_normal_masked	2.424929	17
TVMSQ_bleached_masked	2.552715	18
TVMSQ_normal_masked	2.588926	19
TVMSQC_bleached_masked	9.961393	20

PMJ : The analysis is based on 20 algorithms and 20 cases. 0 missing cases have been found in the data set.

	value_mean	rank
SyNOnly_CC_4_reg_iter_1_bleached_masked	0.8677087	1
SyNOnly_CC_2_reg_iter_1_bleached_masked	0.8704525	2
SyNOnly_CC_2_reg_iter_1_normal_masked	0.8791473	3
SyNOnly_CC_6_reg_iter_1_bleached_masked	0.8853157	4
SyNOnly_CC_4_reg_iter_0_bleached_masked	0.8995506	5
SyNOnly_CC_2_reg_iter_0_bleached_masked	0.9020969	6
SyNOnly_CC_6_reg_iter_0_normal_masked	0.9133852	7
SyNOnly_CC_4_reg_iter_0_normal_masked	0.9202404	8
SyNOnly_CC_4_reg_iter_1_normal_masked	0.9222701	9
SyNOnly_CC_2_reg_iter_0_normal_masked	0.9233732	10
SyNOnly_MI_32_reg_iter_0_normal_masked	0.9337110	11
SyNOnly_CC_6_reg_iter_0_bleached_masked	0.9350689	12
SyNOnly_MI_32_reg_iter_1_normal_masked	0.9604314	13
SyNOnly_CC_6_reg_iter_1_normal_masked	0.9761550	14
SyNOnly_MI_32_reg_iter_1_bleached_masked	1.0372510	15
TVMSQ_bleached_masked	1.0645140	16
SyNOnly_MI_32_reg_iter_0_bleached_masked	1.0816750	17
TVMSQC_normal_masked	1.1058681	18
TVMSQ_normal_masked	1.1283959	19
TVMSQC_bleached_masked	5.0399394	20

Posterior Commissure : The analysis is based on 20 algorithms and 20 cases. 0 missing cases have been found in the data set.

	value_mean	rank
SyNOnly_CC_2_reg_iter_1_bleached_masked	0.9179716	1
SyNOnly_CC_6_reg_iter_1_normal_masked	0.9408212	2
SyNOnly_CC_2_reg_iter_1_normal_masked	0.9440310	3
SyNOnly_CC_4_reg_iter_1_normal_masked	0.9441146	4
SyNOnly_CC_4_reg_iter_1_bleached_masked	0.9444601	5
SyNOnly_CC_2_reg_iter_0_normal_masked	0.9546083	6
SyNOnly_CC_4_reg_iter_0_normal_masked	0.9550994	7
SyNOnly_CC_6_reg_iter_1_bleached_masked	0.9599423	8
SyNOnly_CC_2_reg_iter_0_bleached_masked	0.9758154	9
SyNOnly_CC_4_reg_iter_0_bleached_masked	1.0155016	10
SyNOnly_CC_6_reg_iter_0_normal_masked	1.0312089	11
SyNOnly_CC_6_reg_iter_0_bleached_masked	1.0628642	12
SyNOnly_MI_32_reg_iter_1_normal_masked	1.1475701	13
SyNOnly_MI_32_reg_iter_1_bleached_masked	1.1574728	14
SyNOnly_MI_32_reg_iter_0_normal_masked	1.2240279	15
SyNOnly_MI_32_reg_iter_0_bleached_masked	1.2618942	16
TVMSQ_bleached_masked	1.6322494	17
TVMSQC_normal_masked	1.6895286	18
TVMSQ_normal_masked	1.6998685	19
TVMSQC_bleached_masked	5.0239015	20

Temporal lobe left rostral point : The analysis is based on 20 algorithms and 20 cases. 0 missing cases have been found in the data set.

	value_mean	rank
SyNOnly_CC_2_reg_iter_1_bleached_masked	4.727892	1
SyNOnly_CC_4_reg_iter_1_bleached_masked	4.803634	2
SyNOnly_CC_6_reg_iter_1_bleached_masked	4.881127	3
SyNOnly_MI_32_reg_iter_1_bleached_masked	4.981539	4
SyNOnly_CC_2_reg_iter_0_bleached_masked	5.018977	5
SyNOnly_CC_4_reg_iter_0_bleached_masked	5.090029	6
SyNOnly_CC_6_reg_iter_0_bleached_masked	5.116302	7
SyNOnly_CC_2_reg_iter_1_normal_masked	5.132434	8
SyNOnly_MI_32_reg_iter_0_bleached_masked	5.159744	9
SyNOnly_CC_4_reg_iter_1_normal_masked	5.500673	10
SyNOnly_CC_6_reg_iter_1_normal_masked	5.567054	11
SyNOnly_CC_2_reg_iter_0_normal_masked	5.598940	12
TVMSQC_bleached_masked	5.733779	13
SyNOnly_CC_4_reg_iter_0_normal_masked	5.895354	14
SyNOnly_CC_6_reg_iter_0_normal_masked	6.015260	15
SyNOnly_MI_32_reg_iter_1_normal_masked	6.126990	16
SyNOnly_MI_32_reg_iter_0_normal_masked	6.333040	17
TVMSQ_bleached_masked	6.713816	18
TVMSQ_normal_masked	6.724731	19
TVMSQC_normal_masked	7.152366	20

Temporal lobe ventricle right rostral point : The analysis is based on 20 algorithms and 20 cases. 0 missing cases have been found in the data set.

	value_mean	rank
SyNOnly_CC_2_reg_iter_1_bleached_masked	5.358119	1
SyNOnly_CC_4_reg_iter_1_bleached_masked	5.498523	2
SyNOnly_CC_6_reg_iter_1_bleached_masked	5.691225	3
SyNOnly_CC_2_reg_iter_0_bleached_masked	5.747832	4
TVMSQC_bleached_masked	5.853963	5
SyNOnly_CC_4_reg_iter_0_bleached_masked	5.855255	6
SyNOnly_CC_6_reg_iter_0_bleached_masked	5.971349	7
SyNOnly_CC_2_reg_iter_1_normal_masked	6.049703	8
SyNOnly_MI_32_reg_iter_1_bleached_masked	6.287636	9
SyNOnly_CC_2_reg_iter_0_normal_masked	6.364763	10
SyNOnly_CC_4_reg_iter_1_normal_masked	6.389161	11
SyNOnly_CC_6_reg_iter_1_normal_masked	6.439240	12
SyNOnly_MI_32_reg_iter_0_bleached_masked	6.621270	13
SyNOnly_CC_4_reg_iter_0_normal_masked	6.678810	14
SyNOnly_CC_6_reg_iter_0_normal_masked	6.793497	15
SyNOnly_MI_32_reg_iter_1_normal_masked	6.950680	16
SyNOnly_MI_32_reg_iter_0_normal_masked	7.093610	17
TVMSQ_normal_masked	7.449081	18
TVMSQ_bleached_masked	7.477404	19
TVMSQC_normal_masked	7.673168	20

Consensus ranking across tasks according to chosen method “euclidean”:

	value	rank
SyNOnly_CC_2_reg_iter_1_bleached_masked	3.500	1
SyNOnly_CC_2_reg_iter_1_normal_masked	5.250	2
SyNOnly_CC_4_reg_iter_1_bleached_masked	5.750	3
SyNOnly_CC_2_reg_iter_0_bleached_masked	6.438	4
SyNOnly_CC_6_reg_iter_1_bleached_masked	7.625	5
SyNOnly_CC_4_reg_iter_0_bleached_masked	7.875	6
SyNOnly_CC_4_reg_iter_1_normal_masked	8.125	7
SyNOnly_MI_32_reg_iter_1_bleached_masked	8.625	8
SyNOnly_CC_2_reg_iter_0_normal_masked	8.875	9
SyNOnly_CC_6_reg_iter_0_bleached_masked	9.312	10
SyNOnly_MI_32_reg_iter_0_bleached_masked	9.438	11
SyNOnly_CC_6_reg_iter_1_normal_masked	10.250	12
SyNOnly_CC_4_reg_iter_0_normal_masked	11.062	13
SyNOnly_MI_32_reg_iter_1_normal_masked	12.125	14
SyNOnly_CC_6_reg_iter_0_normal_masked	12.375	15
SyNOnly_MI_32_reg_iter_0_normal_masked	14.688	16
TVMSQ_bleached_masked	16.500	17
TVMSQC_bleached_masked	17.250	18
TVMSQC_normal_masked	17.375	19
TVMSQ_normal_masked	17.562	20

2 Visualization of raw assessment data

The algorithms are ordered according to the computed ranks for each task.

2.1 Dot- and boxplot

Dot- and boxplots for visualizing raw assessment data separately for each algorithm. Boxplots representing descriptive statistics over all cases (median, quartiles and outliers) are combined with horizontally jittered dots representing individual cases.

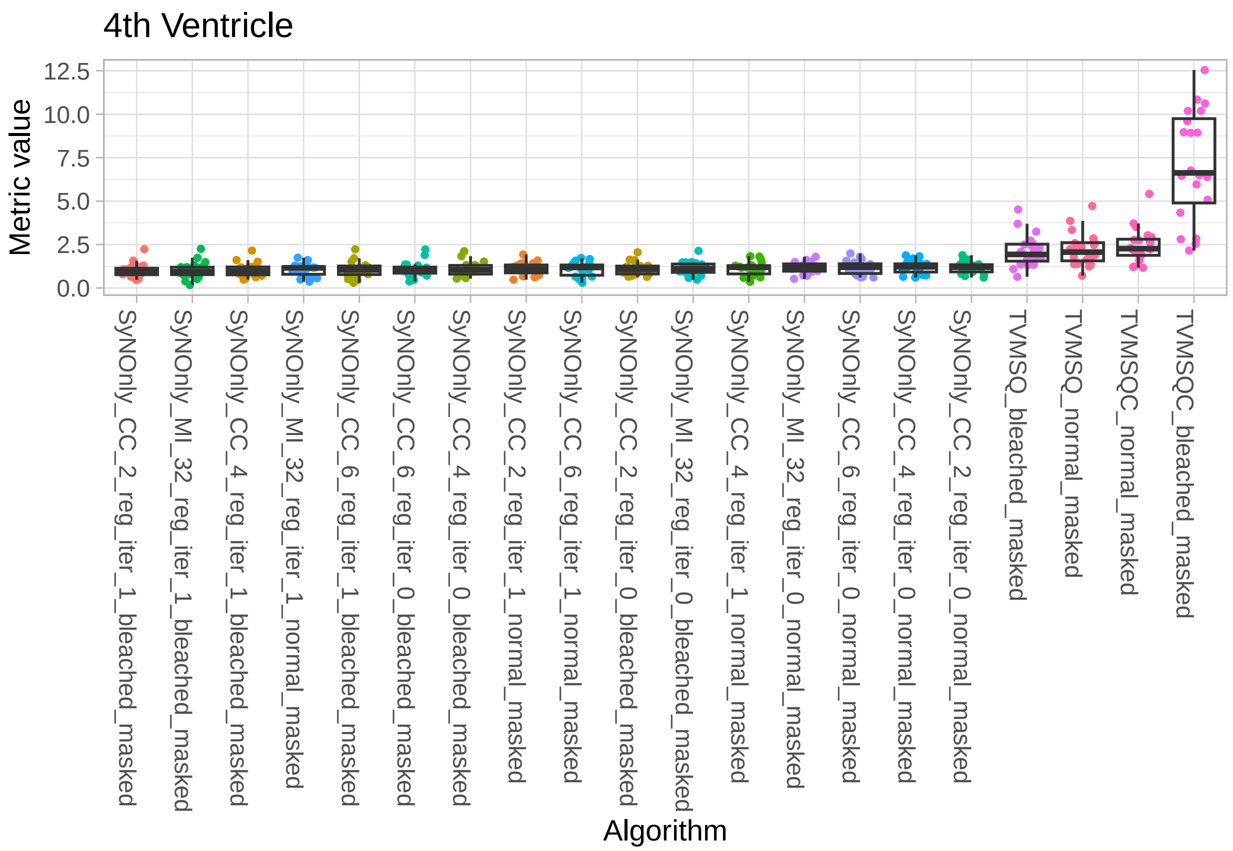

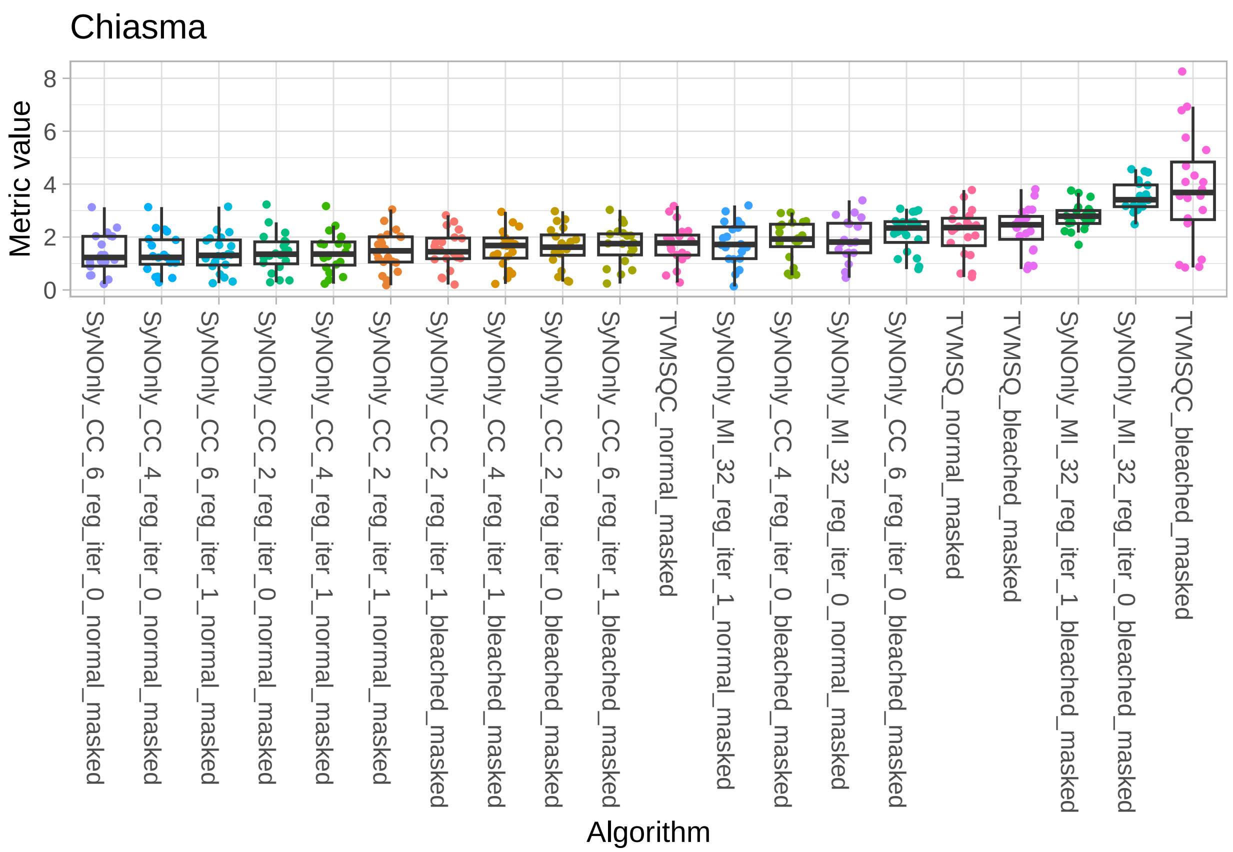
Chiasma
Metric value
0
2
4
6
8
SyNOnly_CC_6_reg_iter_0_normal_masked
SyNOnly_CC_4_reg_iter_0_normal_masked
SyNOnly_CC_6_reg_iter_1_normal_masked
SyNOnly_CC_2_reg_iter_0_normal_masked
SyNOnly_CC_4_reg_iter_1_normal_masked
SyNOnly_CC_2_reg_iter_1_normal_masked
SyNOnly_CC_2_reg_iter_1_bleached_masked
SyNOnly_CC_4_reg_iter_1_bleached_masked
SyNOnly_CC_2_reg_iter_0_bleached_masked
SyNOnly_CC_6_reg_iter_1_bleached_masked
TVMSQC_normal_masked
SyNOnly_MI_32_reg_iter_1_normal_masked
SyNOnly_CC_4_reg_iter_0_bleached_masked
SyNOnly_MI_32_reg_iter_0_normal_masked
SyNOnly_CC_6_reg_iter_0_bleached_masked
TVMSQ_normal_masked
TVMSQ_bleached_masked
SyNOnly_MI_32_reg_iter_1_bleached_masked
SyNOnly_MI_32_reg_iter_0_bleached_masked
TVMSQC_bleached_masked
Algorithm

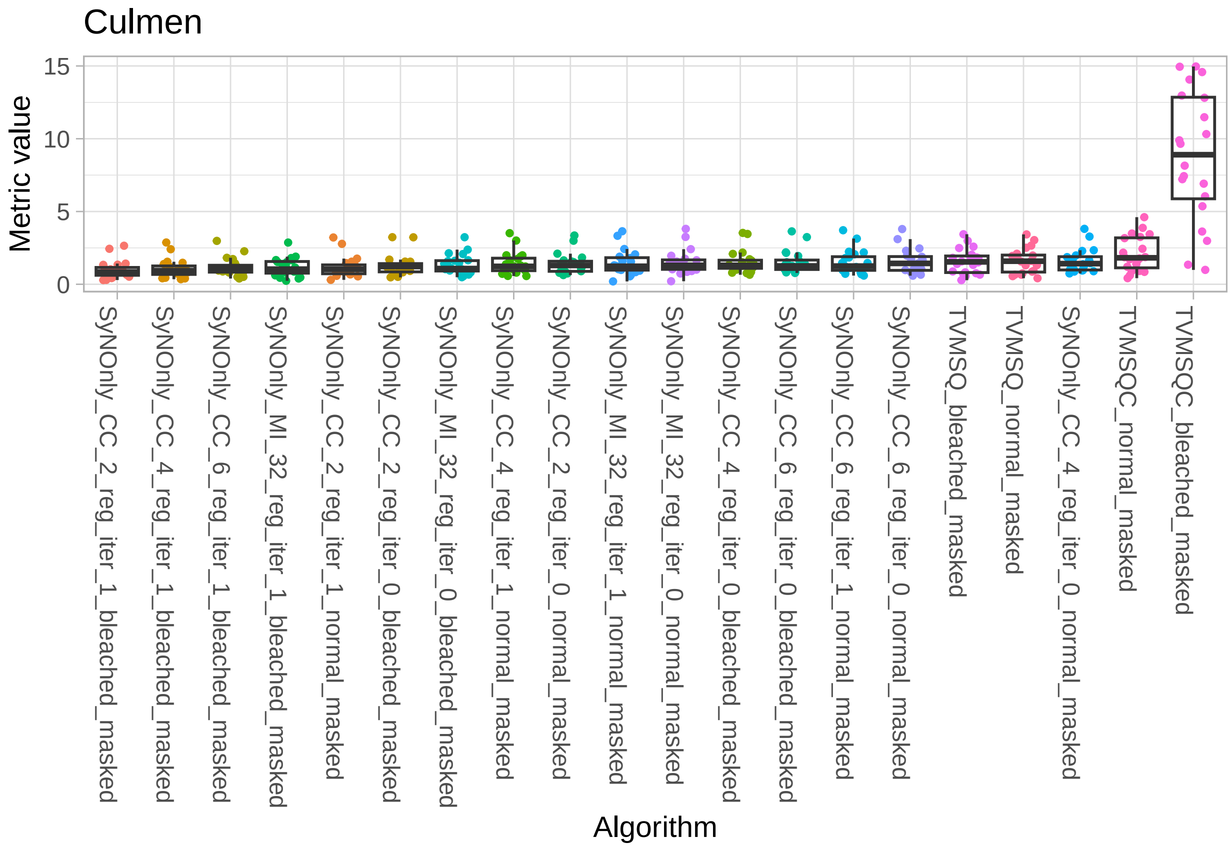
Culmen
Metric value
15
10
5
0
SyNOnly_CC_2_reg_iter_1_bleached_masked
SyNOnly_CC_4_reg_iter_1_bleached_masked
SyNOnly_CC_6_reg_iter_1_bleached_masked
SyNOnly_MI_32_reg_iter_1_bleached_masked
SyNOnly_CC_2_reg_iter_1_normal_masked
SyNOnly_CC_2_reg_iter_0_bleached_masked
SyNOnly_MI_32_reg_iter_0_bleached_masked
SyNOnly_CC_4_reg_iter_1_normal_masked
SyNOnly_CC_2_reg_iter_0_normal_masked
SyNOnly_MI_32_reg_iter_1_normal_masked
SyNOnly_MI_32_reg_iter_0_normal_masked
SyNOnly_CC_4_reg_iter_0_bleached_masked
SyNOnly_CC_6_reg_iter_0_bleached_masked
SyNOnly_CC_6_reg_iter_1_normal_masked
SyNOnly_CC_6_reg_iter_0_normal_masked
TVMSQ_bleached_masked
TVMSQ_normal_masked
SyNOnly_CC_4_reg_iter_0_normal_masked
TVMSQC_normal_masked
TVMSQC_bleached_masked
Algorithm

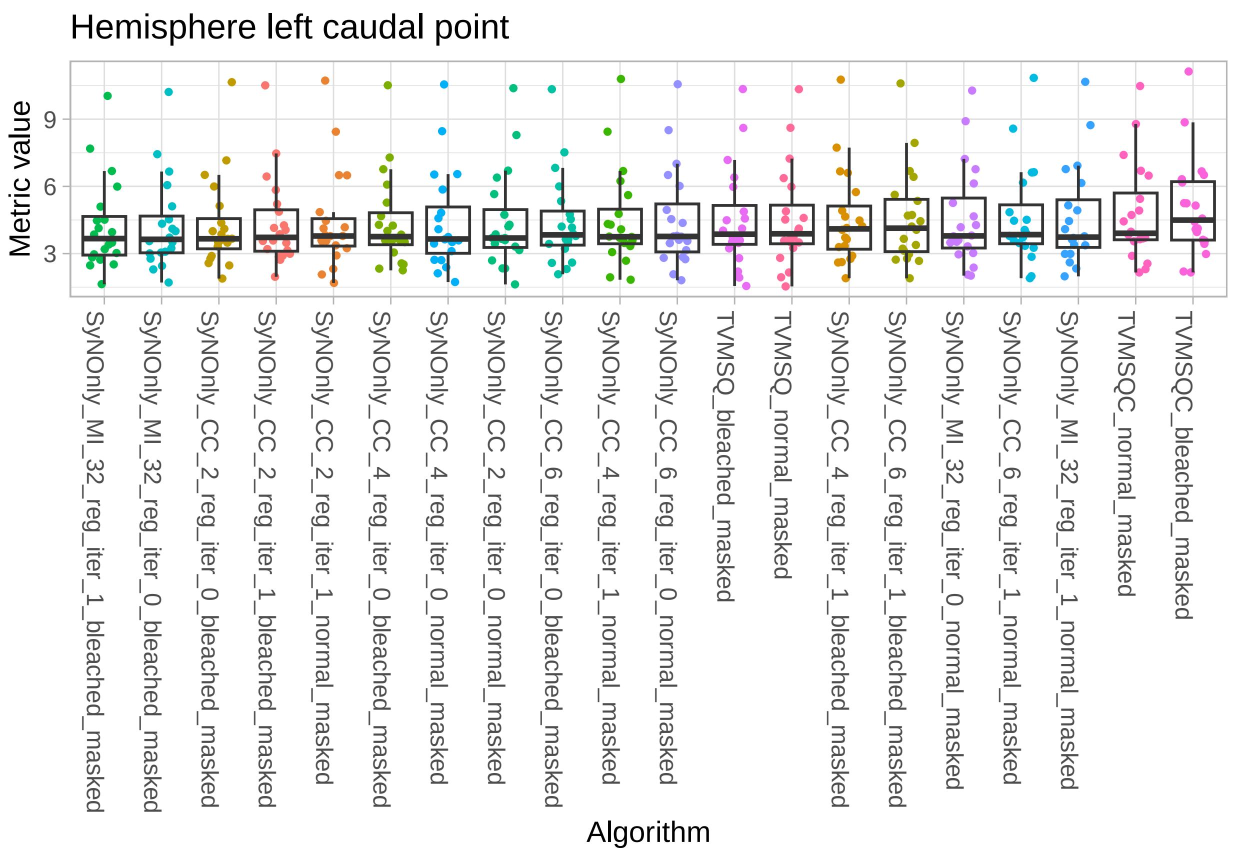

Hemisphere left caudal point
Metric value
9
6
3
SyNOnly_MI_32_reg_iter_1_bleached_masked
SyNOnly_MI_32_reg_iter_0_bleached_masked
SyNOnly_CC_2_reg_iter_0_bleached_masked
SyNOnly_CC_2_reg_iter_1_bleached_masked
SyNOnly_CC_2_reg_iter_1_normal_masked
SyNOnly_CC_4_reg_iter_0_bleached_masked
SyNOnly_CC_4_reg_iter_0_normal_masked
SyNOnly_CC_2_reg_iter_0_normal_masked
SyNOnly_CC_6_reg_iter_0_bleached_masked
SyNOnly_CC_4_reg_iter_1_normal_masked
SyNOnly_CC_6_reg_iter_0_normal_masked
TVMSQ_bleached_masked
TVMSQ_normal_masked
SyNOnly_CC_4_reg_iter_1_bleached_masked
SyNOnly_CC_6_reg_iter_1_bleached_masked
SyNOnly_MI_32_reg_iter_0_normal_masked
SyNOnly_CC_6_reg_iter_1_normal_masked
SyNOnly_MI_32_reg_iter_1_normal_masked
TVMSQC_normal_masked
TVMSQC_bleached_masked
Algorithm

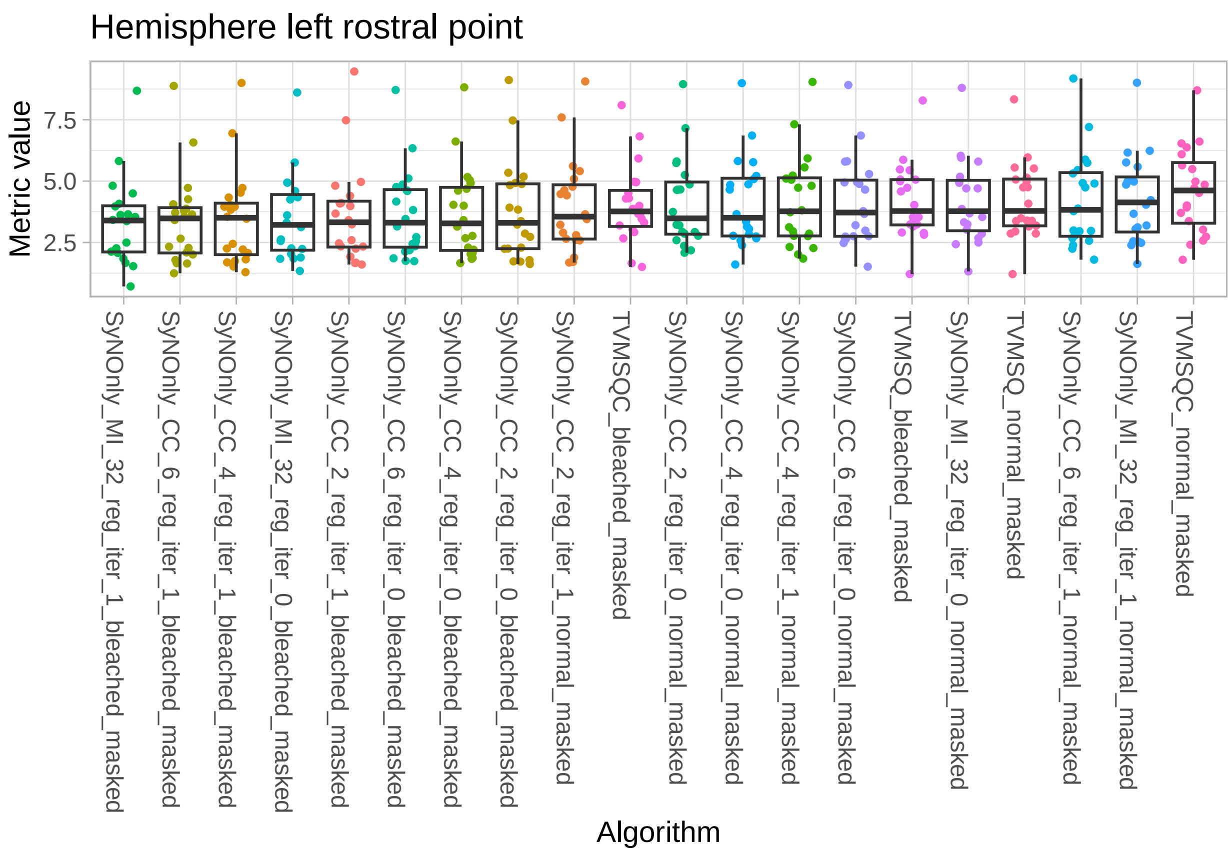
Hemisphere left rostral point
Metric value
7.5
5.0
2.5
SyNOnly_MI_32_reg_iter_1_bleached_masked
SyNOnly_CC_6_reg_iter_1_bleached_masked
SyNOnly_CC_4_reg_iter_1_bleached_masked
SyNOnly_MI_32_reg_iter_0_bleached_masked
SyNOnly_CC_2_reg_iter_1_bleached_masked
SyNOnly_CC_6_reg_iter_0_bleached_masked
SyNOnly_CC_4_reg_iter_0_bleached_masked
SyNOnly_CC_2_reg_iter_0_bleached_masked
SyNOnly_CC_2_reg_iter_1_normal_masked
TVMSQC_bleached_masked
SyNOnly_CC_2_reg_iter_0_normal_masked
SyNOnly_CC_4_reg_iter_0_normal_masked
SyNOnly_CC_4_reg_iter_1_normal_masked
SyNOnly_CC_6_reg_iter_0_normal_masked
TVMSQ_bleached_masked
SyNOnly_MI_32_reg_iter_0_normal_masked
TVMSQ_normal_masked
SyNOnly_CC_6_reg_iter_1_normal_masked
SyNOnly_MI_32_reg_iter_1_normal_masked
TVMSQC_normal_masked
Algorithm

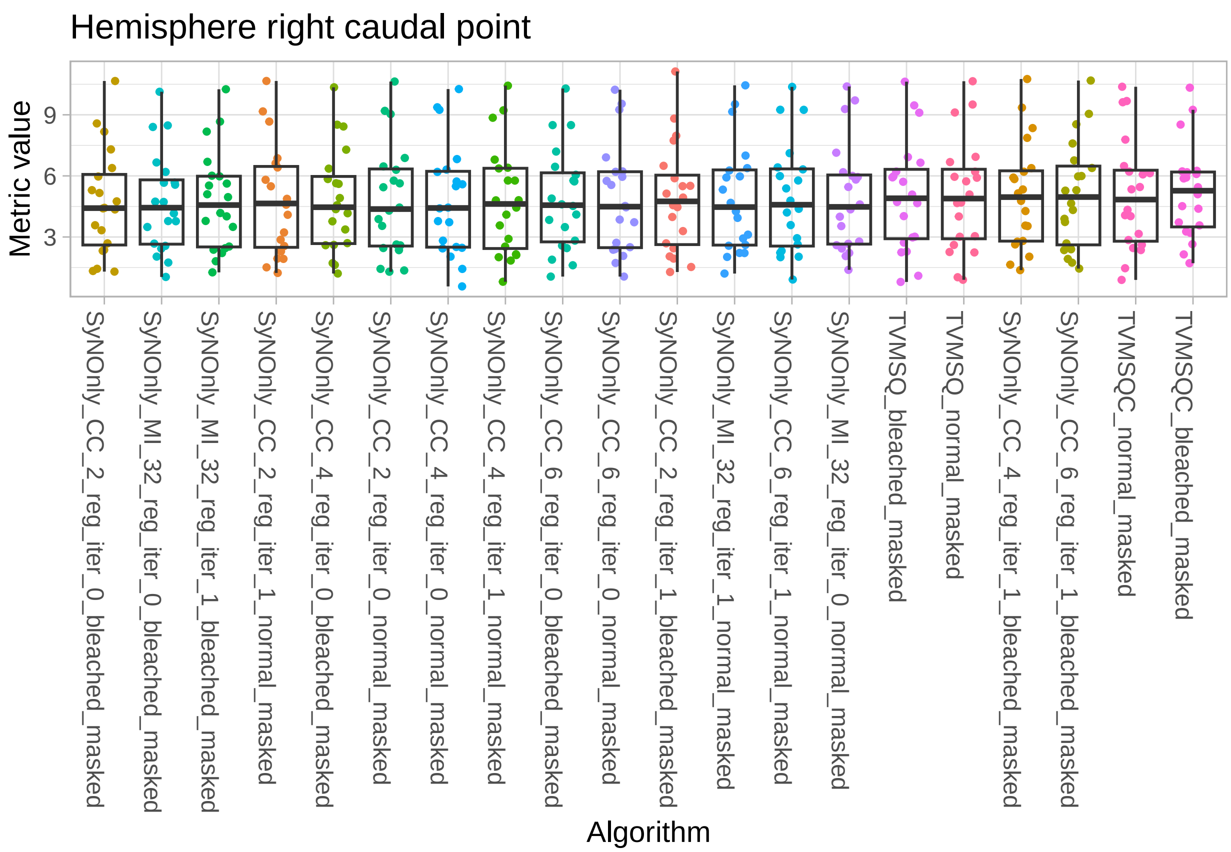
Hemisphere right caudal point
Metric value
9
6
3
SyNOnly_CC_2_reg_iter_0_bleached_masked
SyNOnly_MI_32_reg_iter_0_bleached_masked
SyNOnly_MI_32_reg_iter_1_bleached_masked
SyNOnly_CC_2_reg_iter_1_normal_masked
SyNOnly_CC_4_reg_iter_0_bleached_masked
SyNOnly_CC_2_reg_iter_0_normal_masked
SyNOnly_CC_4_reg_iter_0_normal_masked
SyNOnly_CC_4_reg_iter_1_normal_masked
SyNOnly_CC_6_reg_iter_0_bleached_masked
SyNOnly_CC_6_reg_iter_0_normal_masked
SyNOnly_CC_2_reg_iter_1_bleached_masked
SyNOnly_MI_32_reg_iter_1_normal_masked
SyNOnly_CC_6_reg_iter_1_normal_masked
SyNOnly_MI_32_reg_iter_0_normal_masked
TVMSQ_bleached_masked
TVMSQ_normal_masked
SyNOnly_CC_4_reg_iter_1_bleached_masked
SyNOnly_CC_6_reg_iter_1_bleached_masked
TVMSQC_normal_masked
TVMSQC_bleached_masked
Algorithm

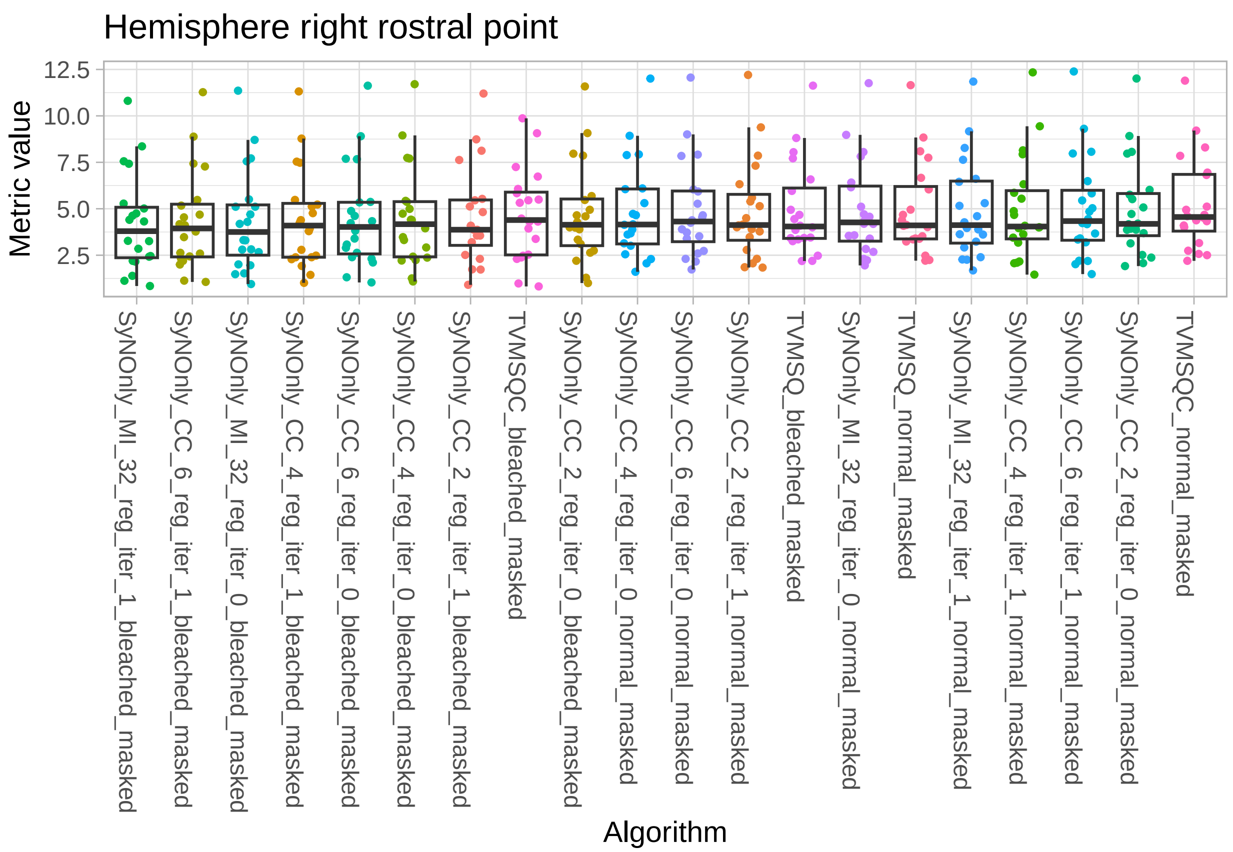
Hemisphere right rostral point
Metric value
12.5
10.0
7.5
5.0
2.5
SyNOnly_MI_32_reg_iter_1_bleached_masked
SyNOnly_CC_6_reg_iter_1_bleached_masked
SyNOnly_MI_32_reg_iter_0_bleached_masked
SyNOnly_CC_4_reg_iter_1_bleached_masked
SyNOnly_CC_6_reg_iter_0_bleached_masked
SyNOnly_CC_4_reg_iter_0_bleached_masked
SyNOnly_CC_2_reg_iter_1_bleached_masked
TVMSQC_bleached_masked
SyNOnly_CC_2_reg_iter_0_bleached_masked
SyNOnly_CC_4_reg_iter_0_normal_masked
SyNOnly_CC_6_reg_iter_0_normal_masked
SyNOnly_CC_2_reg_iter_1_normal_masked
TVMSQ_bleached_masked
SyNOnly_MI_32_reg_iter_0_normal_masked
TVMSQ_normal_masked
SyNOnly_MI_32_reg_iter_1_normal_masked
SyNOnly_CC_4_reg_iter_1_normal_masked
SyNOnly_CC_6_reg_iter_1_normal_masked
SyNOnly_CC_2_reg_iter_0_normal_masked
TVMSQC_normal_masked
Algorithm

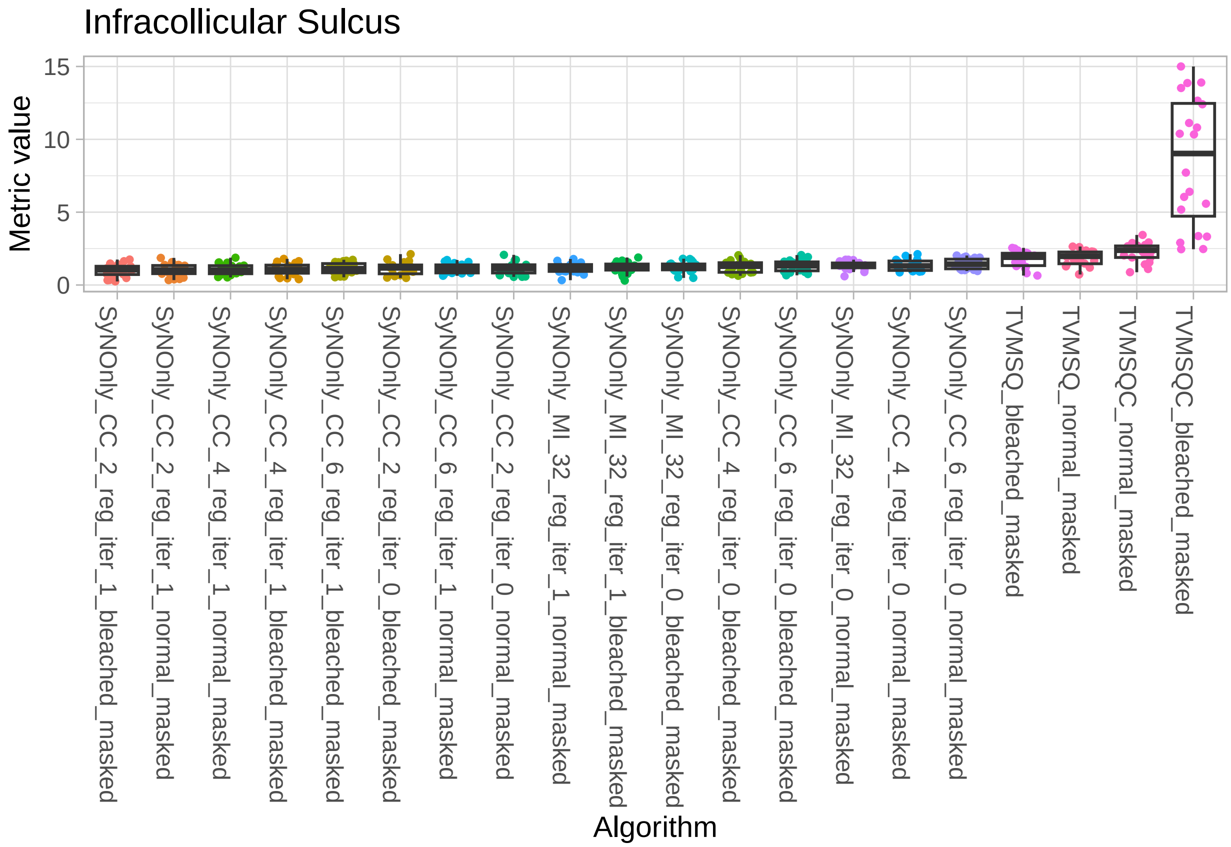

Infracollicular Sulcus
Metric value
15
10
5
0
SyNOnly_CC_2_reg_iter_1_bleached_masked
SyNOnly_CC_2_reg_iter_1_normal_masked
SyNOnly_CC_4_reg_iter_1_normal_masked
SyNOnly_CC_4_reg_iter_1_bleached_masked
SyNOnly_CC_6_reg_iter_1_bleached_masked
SyNOnly_CC_2_reg_iter_0_bleached_masked
SyNOnly_CC_6_reg_iter_1_normal_masked
SyNOnly_CC_2_reg_iter_0_normal_masked
SyNOnly_MI_32_reg_iter_1_normal_masked
SyNOnly_MI_32_reg_iter_1_bleached_masked
SyNOnly_MI_32_reg_iter_0_bleached_masked
SyNOnly_CC_4_reg_iter_0_bleached_masked
SyNOnly_CC_6_reg_iter_0_bleached_masked
SyNOnly_MI_32_reg_iter_0_normal_masked
SyNOnly_CC_4_reg_iter_0_normal_masked
SyNOnly_CC_6_reg_iter_0_normal_masked
TVMSQ_bleached_masked
TVMSQ_normal_masked
TVMSQC_normal_masked
TVMSQC_bleached_masked
Algorithm

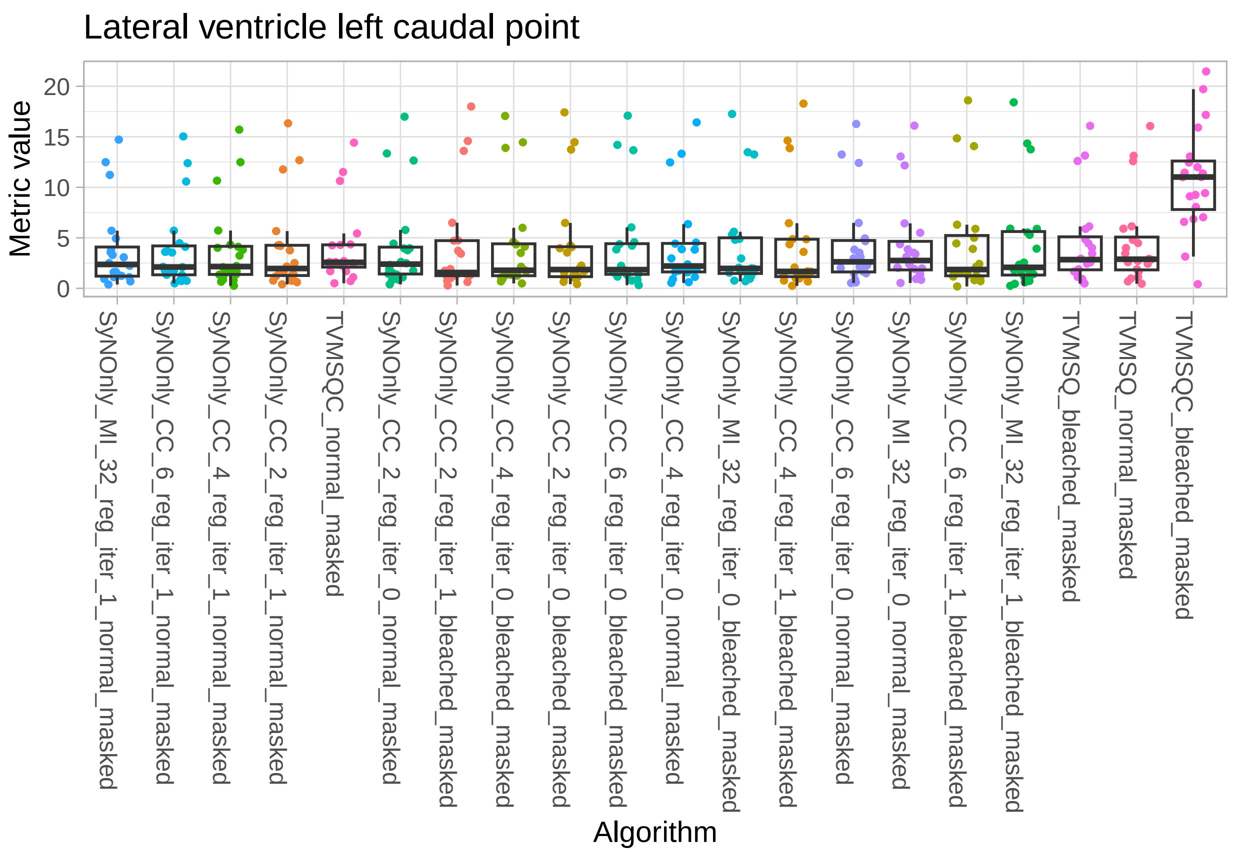

Lateral ventricle left caudal point
Metric value
20
15
10
5
0
SyNOnly_MI_32_reg_iter_1_normal_masked
SyNOnly_CC_6_reg_iter_1_normal_masked
SyNOnly_CC_4_reg_iter_1_normal_masked
SyNOnly_CC_2_reg_iter_1_normal_masked
TVMSQC_normal_masked
SyNOnly_CC_2_reg_iter_0_normal_masked
SyNOnly_CC_2_reg_iter_1_bleached_masked
SyNOnly_CC_4_reg_iter_0_bleached_masked
SyNOnly_CC_2_reg_iter_0_bleached_masked
SyNOnly_CC_6_reg_iter_0_bleached_masked
SyNOnly_CC_4_reg_iter_0_normal_masked
SyNOnly_MI_32_reg_iter_0_bleached_masked
SyNOnly_CC_4_reg_iter_1_bleached_masked
SyNOnly_CC_6_reg_iter_0_normal_masked
SyNOnly_MI_32_reg_iter_0_normal_masked
SyNOnly_CC_6_reg_iter_1_bleached_masked
SyNOnly_MI_32_reg_iter_1_bleached_masked
TVMSQ_bleached_masked
TVMSQ_normal_masked
TVMSQC_bleached_masked
Algorithm

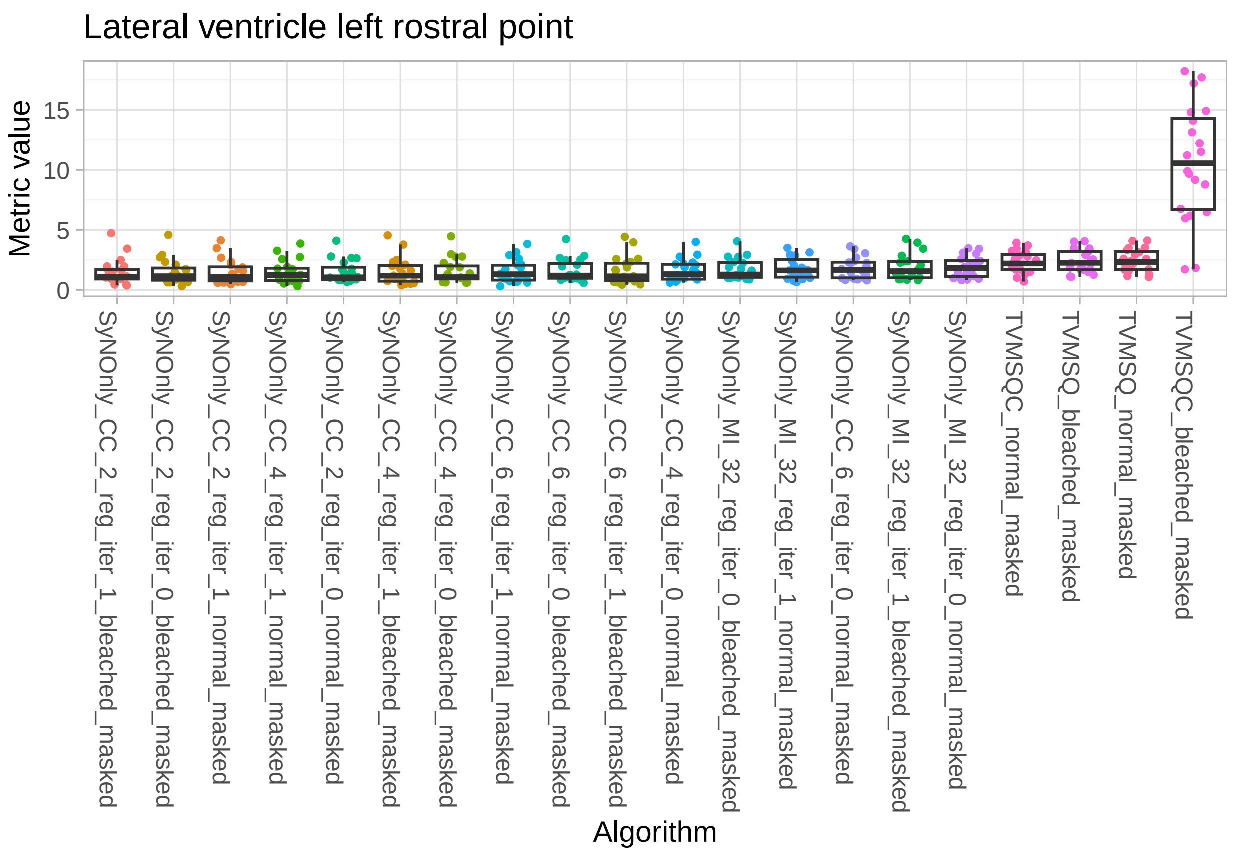
Lateral ventricle left rostral point
Metric value
0
5
10
15
SyNOnly_CC_2_reg_iter_1_bleached_masked
SyNOnly_CC_2_reg_iter_0_bleached_masked
SyNOnly_CC_2_reg_iter_1_normal_masked
SyNOnly_CC_4_reg_iter_1_normal_masked
SyNOnly_CC_2_reg_iter_0_normal_masked
SyNOnly_CC_4_reg_iter_1_bleached_masked
SyNOnly_CC_4_reg_iter_0_bleached_masked
SyNOnly_CC_6_reg_iter_1_normal_masked
SyNOnly_CC_6_reg_iter_0_bleached_masked
SyNOnly_CC_6_reg_iter_1_bleached_masked
SyNOnly_CC_4_reg_iter_0_normal_masked
SyNOnly_MI_32_reg_iter_0_bleached_masked
SyNOnly_MI_32_reg_iter_1_normal_masked
SyNOnly_CC_6_reg_iter_0_normal_masked
SyNOnly_MI_32_reg_iter_1_bleached_masked
SyNOnly_MI_32_reg_iter_0_normal_masked
TVMSQC_normal_masked
TVMSQ_bleached_masked
TVMSQ_normal_masked
TVMSQC_bleached_masked
Algorithm

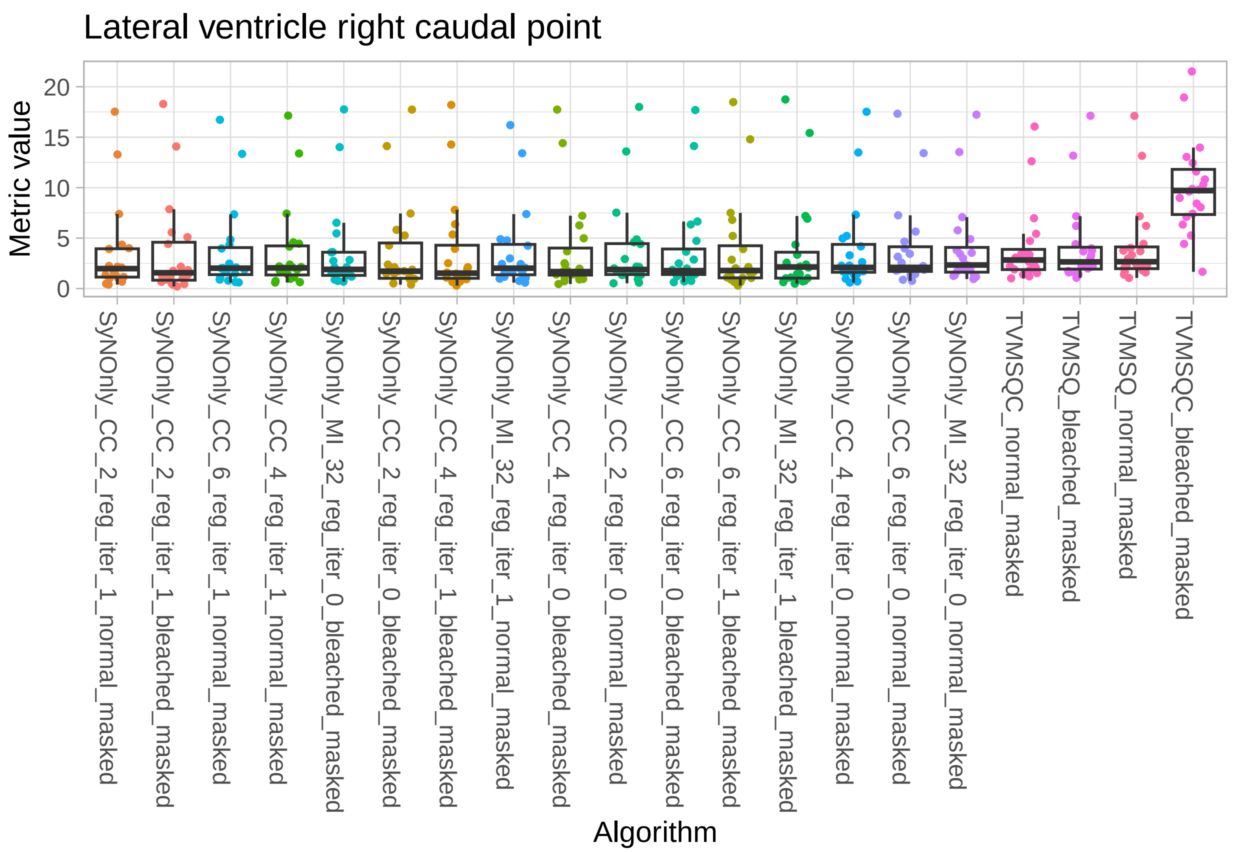

Lateral ventricle right caudal point
Metric value
0
5
10
15
20
SyNOnly_CC_2_reg_iter_1_normal_masked
SyNOnly_CC_2_reg_iter_1_bleached_masked
SyNOnly_CC_6_reg_iter_1_normal_masked
SyNOnly_CC_4_reg_iter_1_normal_masked
SyNOnly_MI_32_reg_iter_0_bleached_masked
SyNOnly_CC_2_reg_iter_0_bleached_masked
SyNOnly_CC_4_reg_iter_1_bleached_masked
SyNOnly_MI_32_reg_iter_1_normal_masked
SyNOnly_CC_4_reg_iter_0_bleached_masked
SyNOnly_CC_2_reg_iter_0_normal_masked
SyNOnly_CC_6_reg_iter_0_bleached_masked
SyNOnly_CC_6_reg_iter_1_bleached_masked
SyNOnly_MI_32_reg_iter_1_bleached_masked
SyNOnly_CC_4_reg_iter_0_normal_masked
SyNOnly_CC_6_reg_iter_0_normal_masked
SyNOnly_MI_32_reg_iter_0_normal_masked
TVMSQC_normal_masked
TVMSQ_bleached_masked
TVMSQ_normal_masked
TVMSQC_bleached_masked
Algorithm

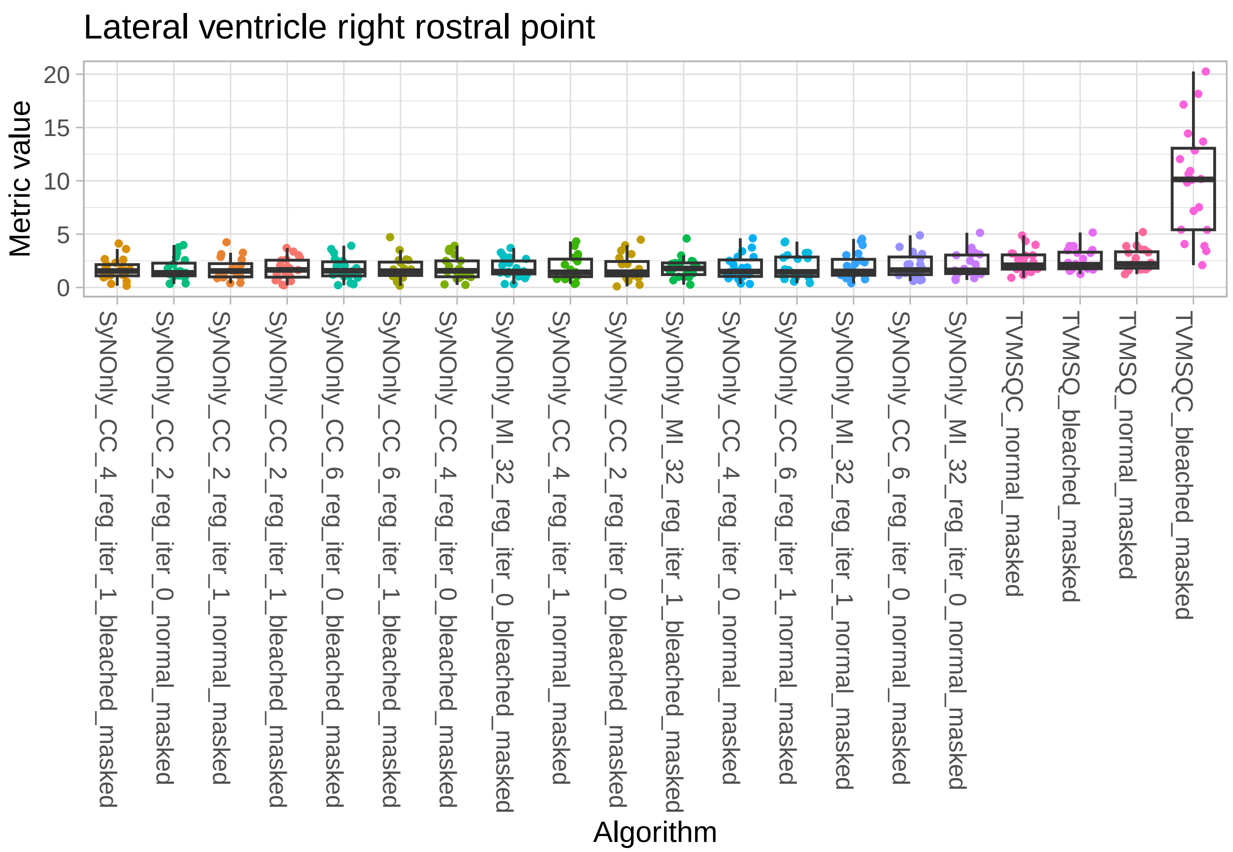
Lateral ventricle right rostral point
Metric value
20
15
10
5
0
SyNOnly_CC_4_reg_iter_1_bleached_masked
SyNOnly_CC_2_reg_iter_0_normal_masked
SyNOnly_CC_2_reg_iter_1_normal_masked
SyNOnly_CC_2_reg_iter_1_bleached_masked
SyNOnly_CC_6_reg_iter_0_bleached_masked
SyNOnly_CC_6_reg_iter_1_bleached_masked
SyNOnly_CC_4_reg_iter_0_bleached_masked
SyNOnly_MI_32_reg_iter_0_bleached_masked
SyNOnly_CC_4_reg_iter_1_normal_masked
SyNOnly_CC_2_reg_iter_0_bleached_masked
SyNOnly_MI_32_reg_iter_1_bleached_masked
SyNOnly_CC_4_reg_iter_0_normal_masked
SyNOnly_CC_6_reg_iter_1_normal_masked
SyNOnly_MI_32_reg_iter_1_normal_masked
SyNOnly_CC_6_reg_iter_0_normal_masked
SyNOnly_MI_32_reg_iter_0_normal_masked
TVMSQC_normal_masked
TVMSQ_bleached_masked
TVMSQ_normal_masked
TVMSQC_bleached_masked
Algorithm

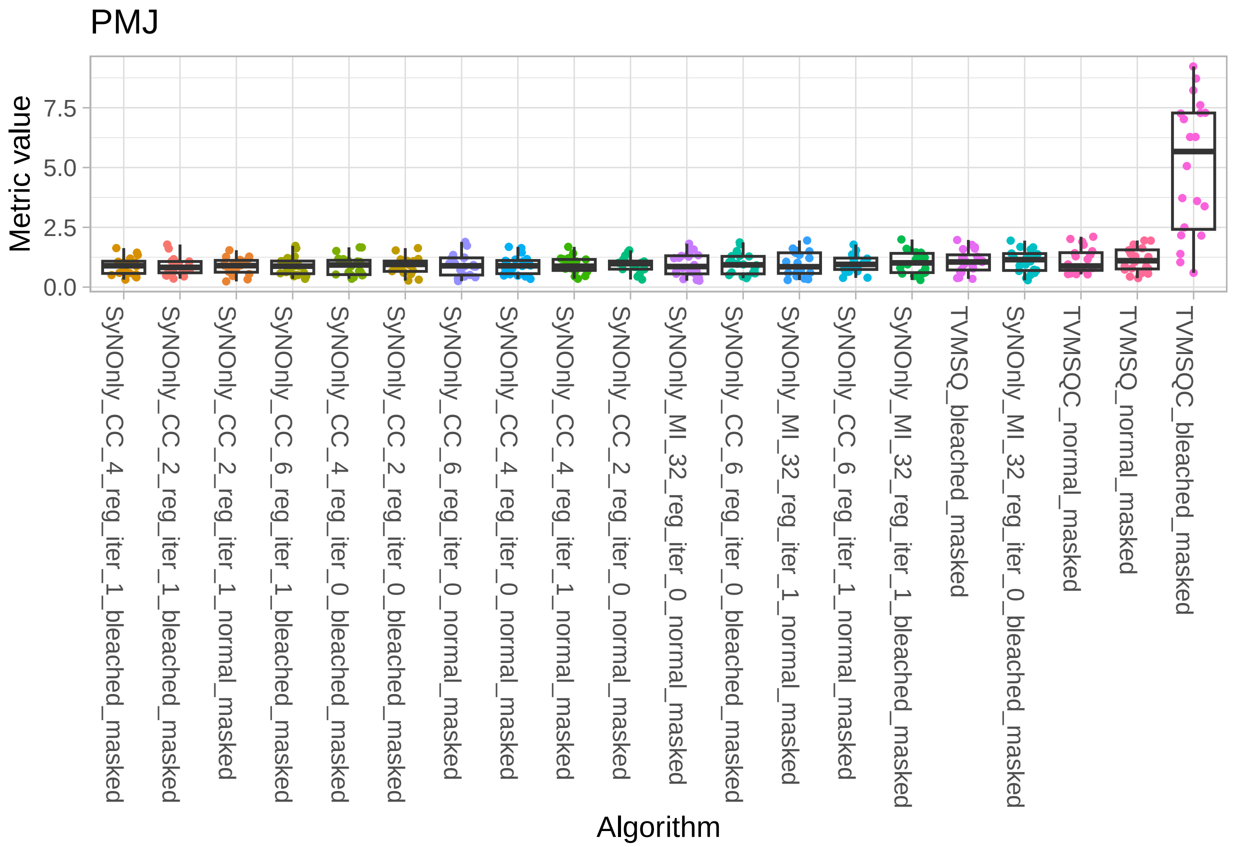
PMJ
Metric value
7.5
5.0
2.5
0.0
SyNOnly_CC_4_reg_iter_1_bleached_masked
SyNOnly_CC_2_reg_iter_1_bleached_masked
SyNOnly_CC_2_reg_iter_1_normal_masked
SyNOnly_CC_6_reg_iter_1_bleached_masked
SyNOnly_CC_4_reg_iter_0_bleached_masked
SyNOnly_CC_2_reg_iter_0_bleached_masked
SyNOnly_CC_6_reg_iter_0_normal_masked
SyNOnly_CC_4_reg_iter_0_normal_masked
SyNOnly_CC_4_reg_iter_1_normal_masked
SyNOnly_CC_2_reg_iter_0_normal_masked
SyNOnly_MI_32_reg_iter_0_normal_masked
SyNOnly_CC_6_reg_iter_0_bleached_masked
SyNOnly_MI_32_reg_iter_1_normal_masked
SyNOnly_CC_6_reg_iter_1_normal_masked
SyNOnly_MI_32_reg_iter_1_bleached_masked
TVMSQ_bleached_masked
SyNOnly_MI_32_reg_iter_0_bleached_masked
TVMSQC_normal_masked
TVMSQ_normal_masked
TVMSQC_bleached_masked
Algorithm

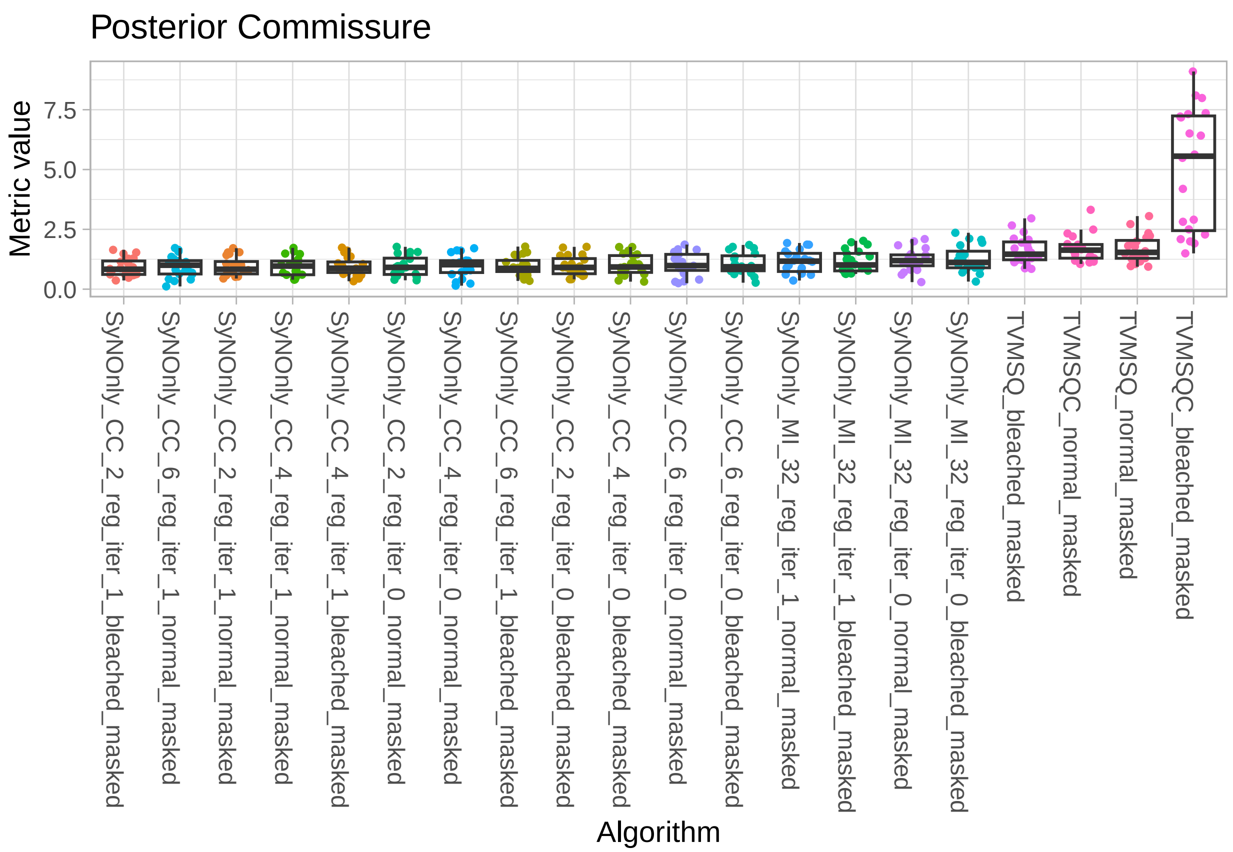
Posterior Commissure
Metric value
7.5
5.0
2.5
0.0
SyNOnly_CC_2_reg_iter_1_bleached_masked
SyNOnly_CC_6_reg_iter_1_normal_masked
SyNOnly_CC_2_reg_iter_1_normal_masked
SyNOnly_CC_4_reg_iter_1_normal_masked
SyNOnly_CC_4_reg_iter_1_bleached_masked
SyNOnly_CC_2_reg_iter_0_normal_masked
SyNOnly_CC_4_reg_iter_0_normal_masked
SyNOnly_CC_6_reg_iter_1_bleached_masked
SyNOnly_CC_2_reg_iter_0_bleached_masked
SyNOnly_CC_4_reg_iter_0_bleached_masked
SyNOnly_CC_6_reg_iter_0_normal_masked
SyNOnly_CC_6_reg_iter_0_bleached_masked
SyNOnly_MI_32_reg_iter_1_normal_masked
SyNOnly_MI_32_reg_iter_1_bleached_masked
SyNOnly_MI_32_reg_iter_0_normal_masked
SyNOnly_MI_32_reg_iter_0_bleached_masked
TVMSQ_bleached_masked
TVMSQC_normal_masked
TVMSQ_normal_masked
TVMSQC_bleached_masked
Algorithm

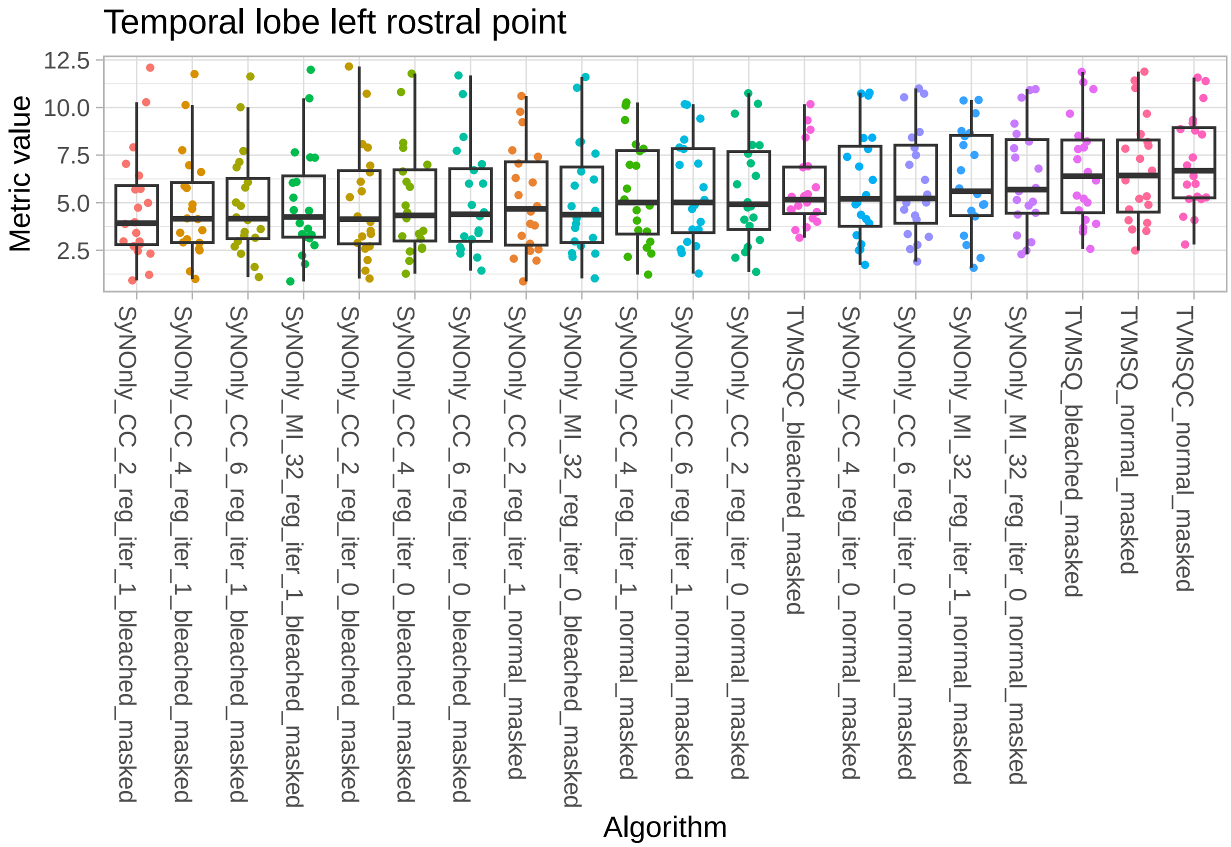

Temporal lobe left rostral point
Metric value
12.5
10.0
7.5
5.0
2.5
SyNOnly_CC_2_reg_iter_1_bleached_masked
SyNOnly_CC_4_reg_iter_1_bleached_masked
SyNOnly_CC_6_reg_iter_1_bleached_masked
SyNOnly_MI_32_reg_iter_1_bleached_masked
SyNOnly_CC_2_reg_iter_0_bleached_masked
SyNOnly_CC_4_reg_iter_0_bleached_masked
SyNOnly_CC_6_reg_iter_0_bleached_masked
SyNOnly_CC_2_reg_iter_1_normal_masked
SyNOnly_MI_32_reg_iter_0_bleached_masked
SyNOnly_CC_4_reg_iter_1_normal_masked
SyNOnly_CC_6_reg_iter_1_normal_masked
SyNOnly_CC_2_reg_iter_0_normal_masked
TVMSQC_bleached_masked
SyNOnly_CC_4_reg_iter_0_normal_masked
SyNOnly_CC_6_reg_iter_0_normal_masked
SyNOnly_MI_32_reg_iter_1_normal_masked
SyNOnly_MI_32_reg_iter_0_normal_masked
TVMSQ_bleached_masked
TVMSQ_normal_masked
TVMSQC_normal_masked
Algorithm

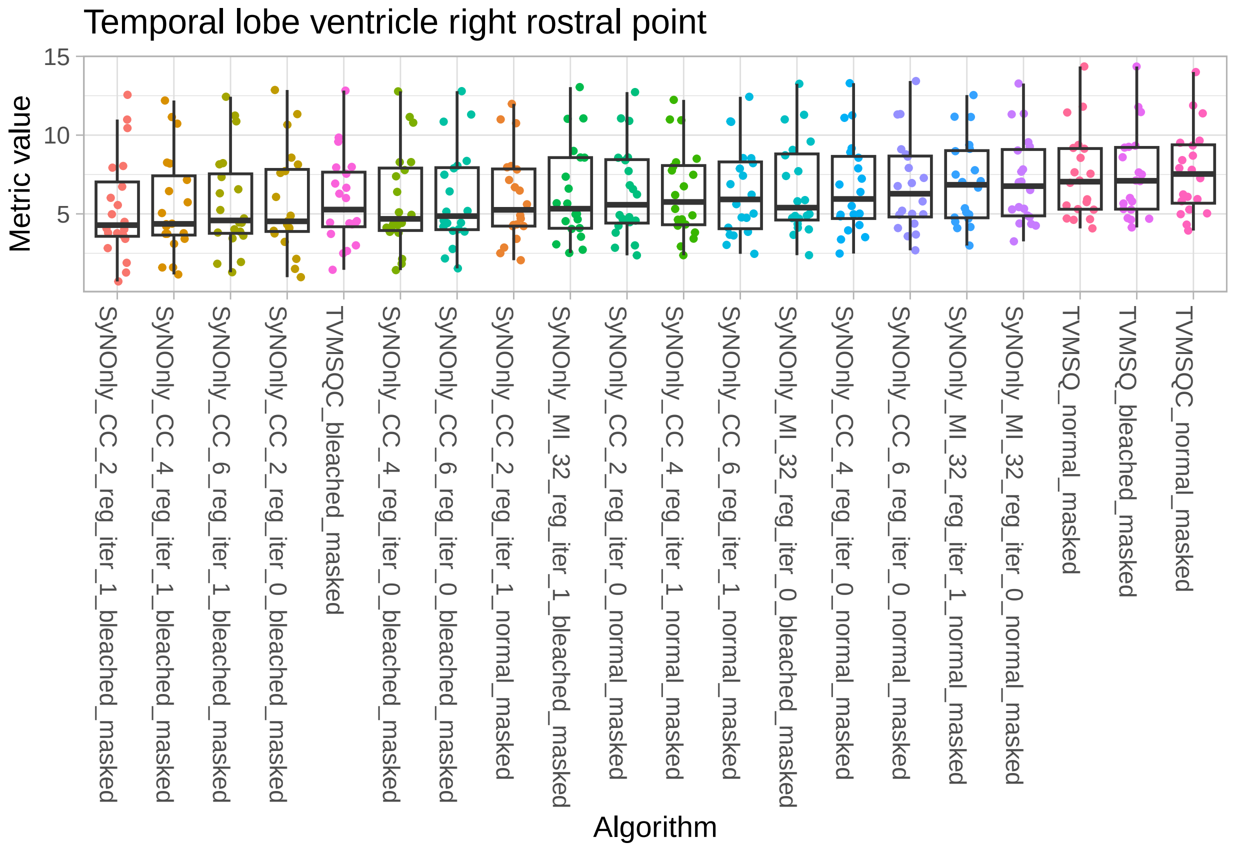
Temporal lobe ventricle right rostral point
Metric value
15
10
5
SyNOnly_CC_2_reg_iter_1_bleached_masked
SyNOnly_CC_4_reg_iter_1_bleached_masked
SyNOnly_CC_6_reg_iter_1_bleached_masked
SyNOnly_CC_2_reg_iter_0_bleached_masked
TVMSQC_bleached_masked
SyNOnly_CC_4_reg_iter_0_bleached_masked
SyNOnly_CC_6_reg_iter_0_bleached_masked
SyNOnly_CC_2_reg_iter_1_normal_masked
SyNOnly_MI_32_reg_iter_1_bleached_masked
SyNOnly_CC_2_reg_iter_0_normal_masked
SyNOnly_CC_4_reg_iter_1_normal_masked
SyNOnly_CC_6_reg_iter_1_normal_masked
SyNOnly_MI_32_reg_iter_0_bleached_masked
SyNOnly_CC_4_reg_iter_0_normal_masked
SyNOnly_CC_6_reg_iter_0_normal_masked
SyNOnly_MI_32_reg_iter_1_normal_masked
SyNOnly_MI_32_reg_iter_0_normal_masked
TVMSQ_normal_masked
TVMSQ_bleached_masked
TVMSQC_normal_masked
Algorithm

2.2 Podium plot

Podium plots (see also Eugster et al., 2008) for visualizing raw assessment data. Upper part (spaghetti plot): Participating algorithms are color-coded, and each colored dot in the plot represents a metric value achieved with the respective algorithm. The actual metric value is encoded by the y-axis. Each podium (here: p=20) represents one possible rank, ordered from best (1) to last (here: 20). The assignment of metric values (i.e. colored dots) to one of the podiums is based on the rank that the respective algorithm achieved on the corresponding case. Note that the plot part above each podium place is further subdivided into p "columns", where each column represents one participating algorithm (here: $p = 20$). Dots corresponding to identical cases are connected by a line, leading to the shown spaghetti structure. Lower part: Bar charts represent the relative frequency for each algorithm to achieve the rank encoded by the podium place.

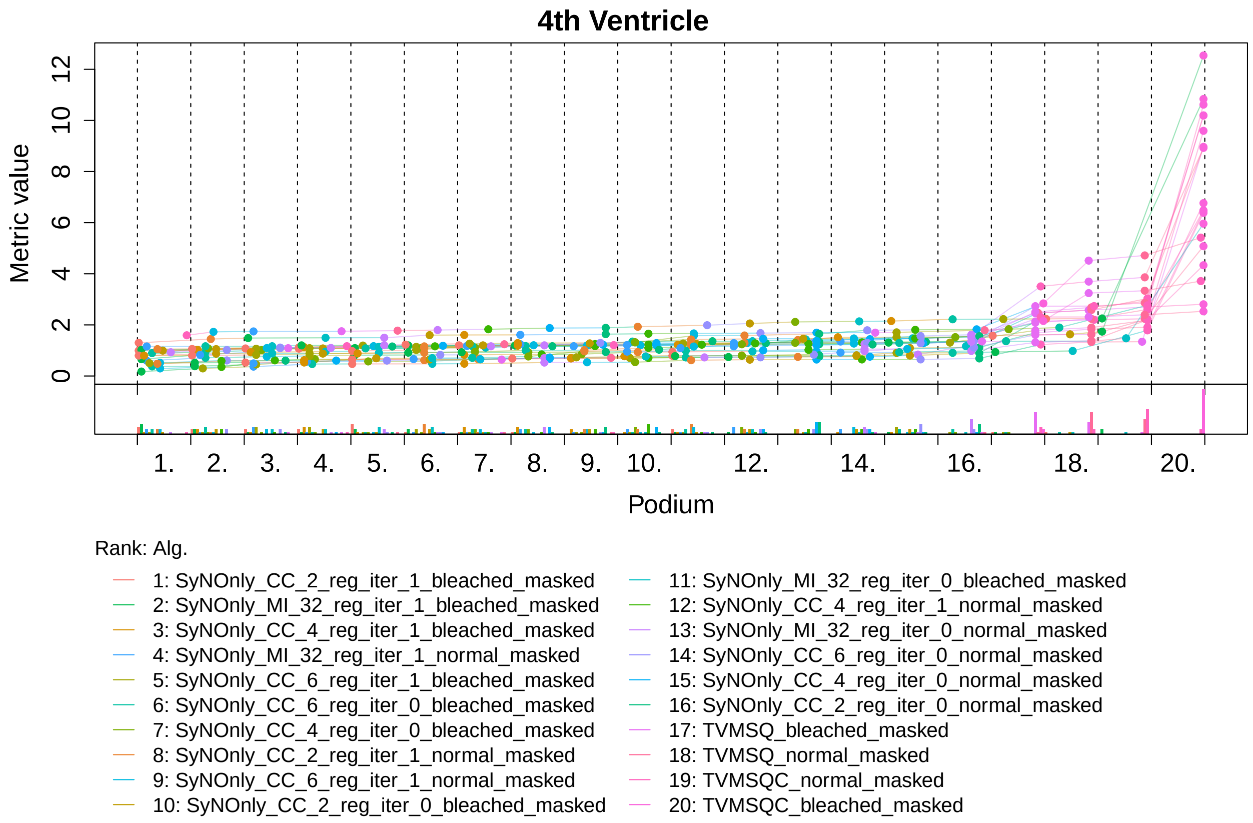

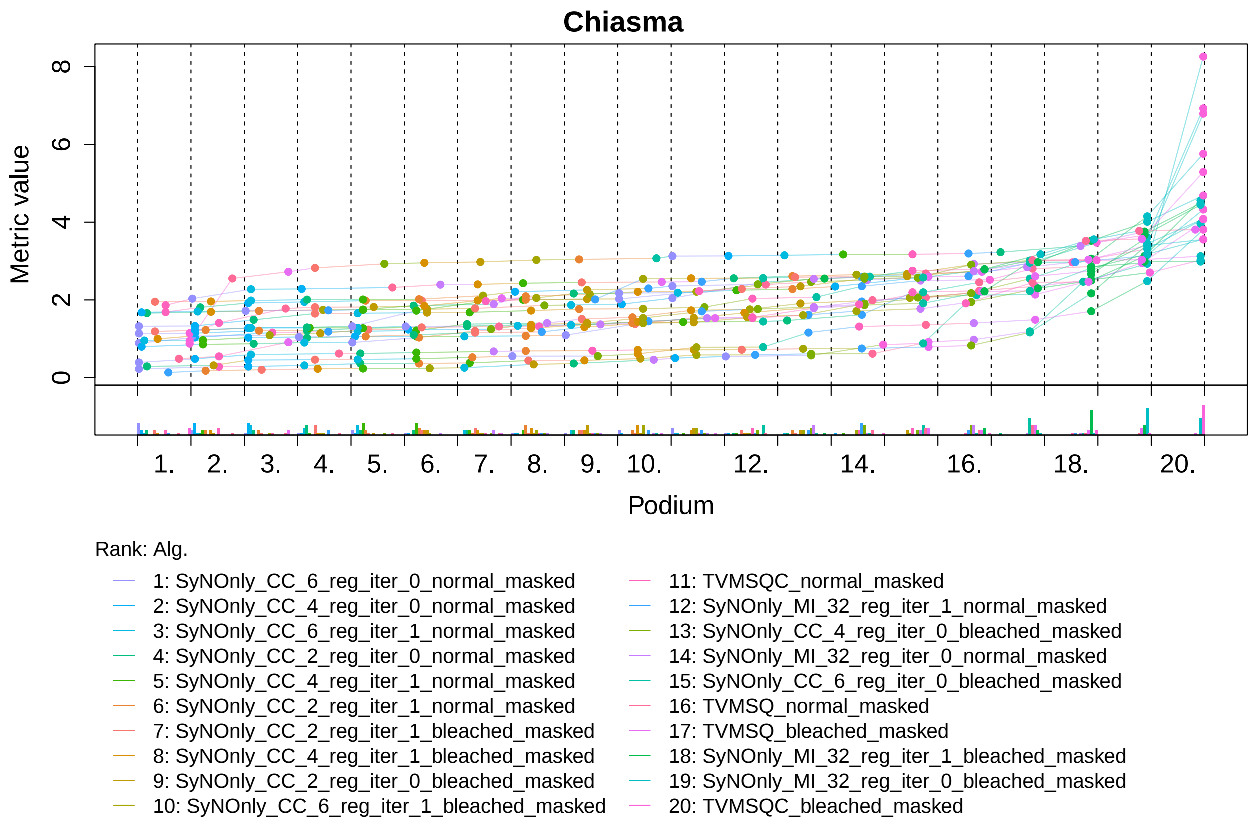
Chiasma
Metric value
0
2
4
6
8
1.
2.
3.
4.
5.
6.
7.
8.
9.
10.
12.
14.
16.
18.
20.
Podium
Rank: Alg.
1: SyNOnly_CC_6_reg_iter_0_normal_masked
2: SyNOnly_CC_4_reg_iter_0_normal_masked
3: SyNOnly_CC_6_reg_iter_1_normal_masked
4: SyNOnly_CC_2_reg_iter_0_normal_masked
5: SyNOnly_CC_4_reg_iter_1_normal_masked
6: SyNOnly_CC_2_reg_iter_1_normal_masked
7: SyNOnly_CC_2_reg_iter_1_bleached_masked
8: SyNOnly_CC_4_reg_iter_1_bleached_masked
9: SyNOnly_CC_2_reg_iter_0_bleached_masked
10: SyNOnly_CC_6_reg_iter_1_bleached_masked
11: TVMSQC_normal_masked
12: SyNOnly_MI_32_reg_iter_1_normal_masked
13: SyNOnly_CC_4_reg_iter_0_bleached_masked
14: SyNOnly_MI_32_reg_iter_0_normal_masked
15: SyNOnly_CC_6_reg_iter_0_bleached_masked
16: TVMSQ_normal_masked
17: TVMSQ_bleached_masked
18: SyNOnly_MI_32_reg_iter_1_bleached_masked
19: SyNOnly_MI_32_reg_iter_0_bleached_masked
20: TVMSQC_bleached_masked

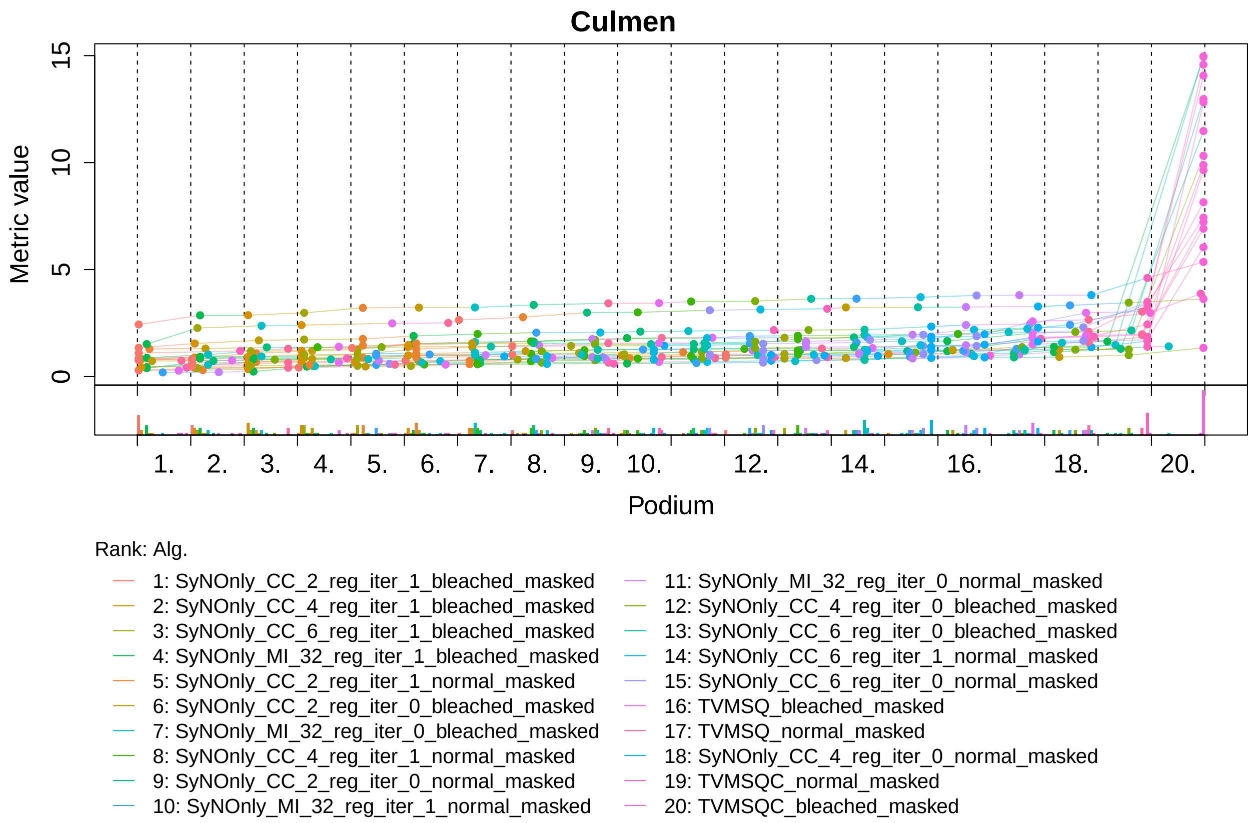
Culmen
Metric value
0
5
10
15
1.
2.
3.
4.
5.
6.
7.
8.
9.
10.
12.
14.
16.
18.
20.
Podium
Rank: Alg.
1: SyNOnly_CC_2_reg_iter_1_bleached_masked
2: SyNOnly_CC_4_reg_iter_1_bleached_masked
3: SyNOnly_CC_6_reg_iter_1_bleached_masked
4: SyNOnly_MI_32_reg_iter_1_bleached_masked
5: SyNOnly_CC_2_reg_iter_1_normal_masked
6: SyNOnly_CC_2_reg_iter_0_bleached_masked
7: SyNOnly_MI_32_reg_iter_0_bleached_masked
8: SyNOnly_CC_4_reg_iter_1_normal_masked
9: SyNOnly_CC_2_reg_iter_0_normal_masked
10: SyNOnly_MI_32_reg_iter_1_normal_masked
11: SyNOnly_MI_32_reg_iter_0_normal_masked
12: SyNOnly_CC_4_reg_iter_0_bleached_masked
13: SyNOnly_CC_6_reg_iter_0_bleached_masked
14: SyNOnly_CC_6_reg_iter_1_normal_masked
15: SyNOnly_CC_6_reg_iter_0_normal_masked
16: TVMSQ_bleached_masked
17: TVMSQ_normal_masked
18: SyNOnly_CC_4_reg_iter_0_normal_masked
19: TVMSQC_normal_masked
20: TVMSQC_bleached_masked

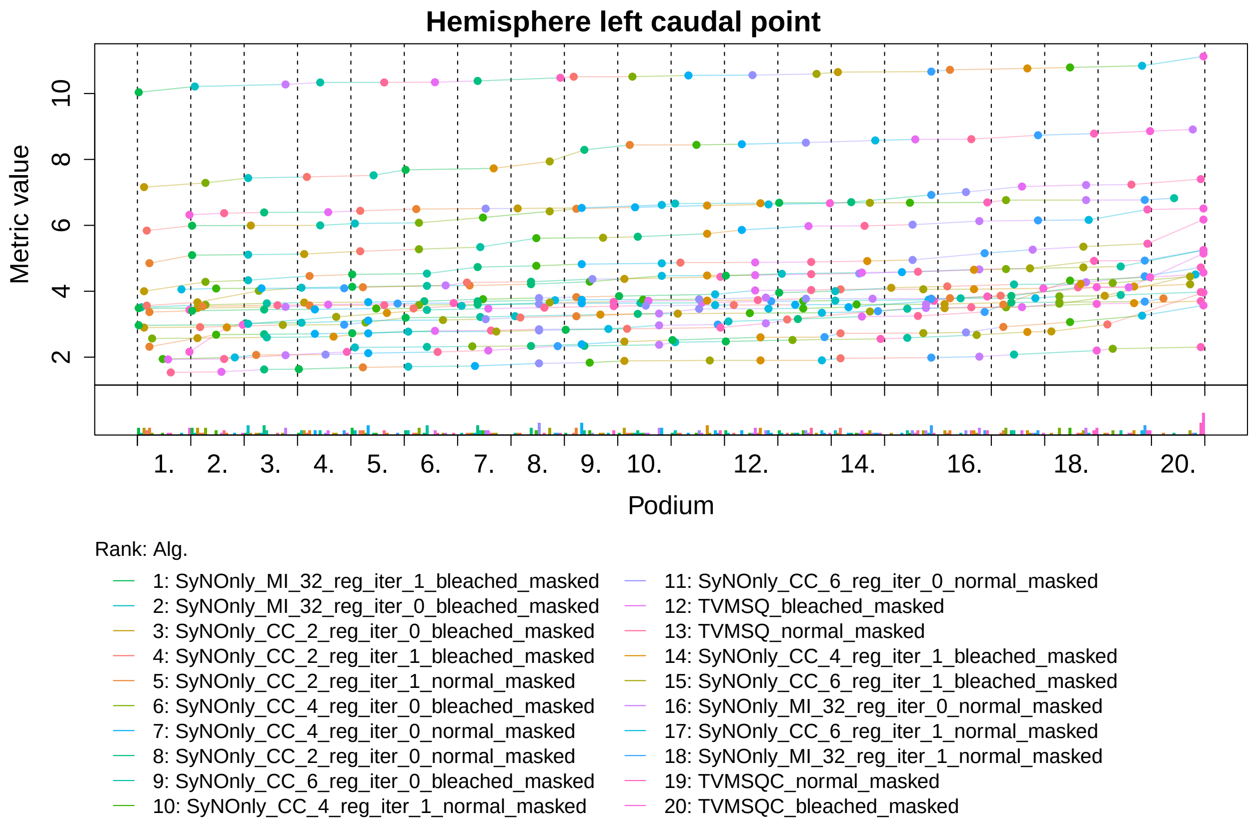

Hemisphere left caudal point
Metric value
Podium
Rank: Alg.
1: SyNOnly_MI_32_reg_iter_1_bleached_masked
2: SyNOnly_MI_32_reg_iter_0_bleached_masked
3: SyNOnly_CC_2_reg_iter_0_bleached_masked
4: SyNOnly_CC_2_reg_iter_1_bleached_masked
5: SyNOnly_CC_2_reg_iter_1_normal_masked
6: SyNOnly_CC_4_reg_iter_0_bleached_masked
7: SyNOnly_CC_4_reg_iter_0_normal_masked
8: SyNOnly_CC_2_reg_iter_0_normal_masked
9: SyNOnly_CC_6_reg_iter_0_bleached_masked
10: SyNOnly_CC_4_reg_iter_1_normal_masked
11: SyNOnly_CC_6_reg_iter_0_normal_masked
12: TVMSQ_bleached_masked
13: TVMSQ_normal_masked
14: SyNOnly_CC_4_reg_iter_1_bleached_masked
15: SyNOnly_CC_6_reg_iter_1_bleached_masked
16: SyNOnly_MI_32_reg_iter_0_normal_masked
17: SyNOnly_CC_6_reg_iter_1_normal_masked
18: SyNOnly_MI_32_reg_iter_1_normal_masked
19: TVMSQC_normal_masked
20: TVMSQC_bleached_masked

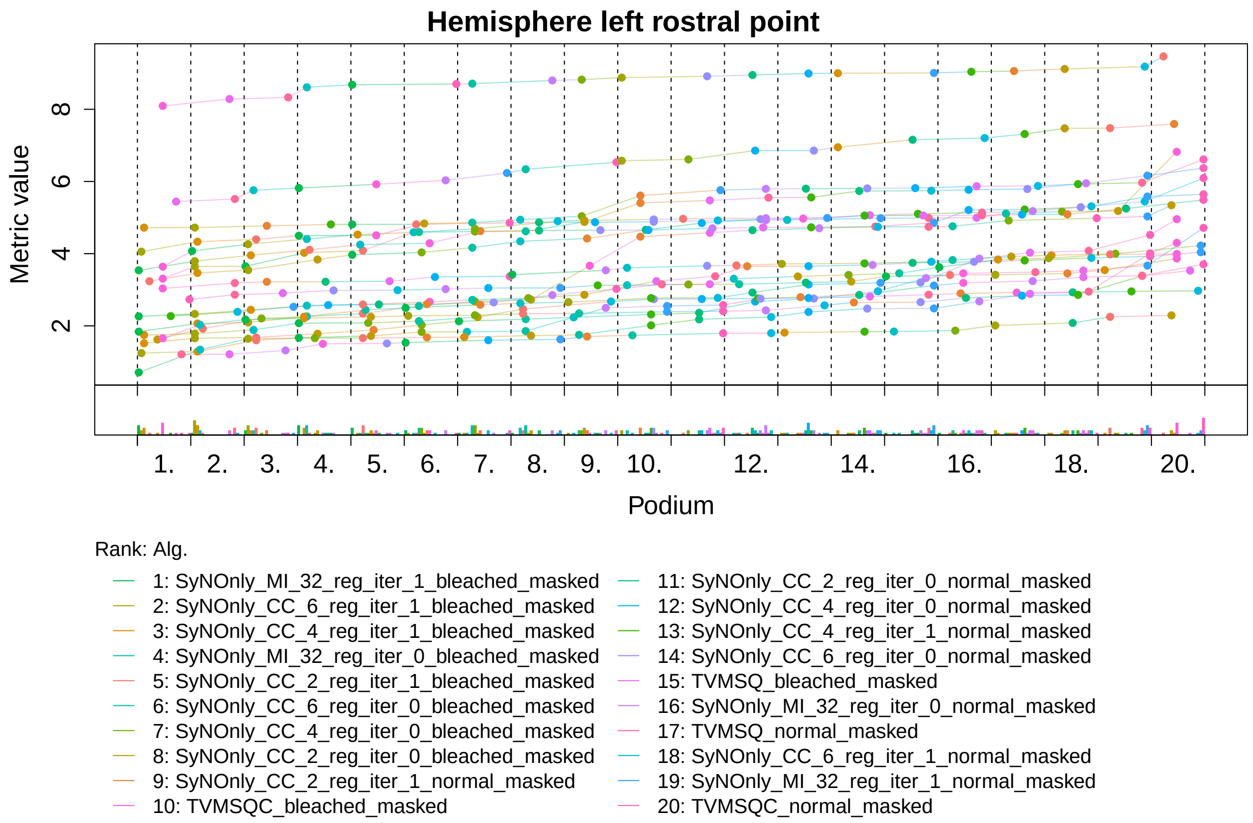
Hemisphere left rostral point
Metric value
2
4
6
8
1.
2.
3.
4.
5.
6.
7.
8.
9.
10.
12.
14.
16.
18.
20.
Podium
Rank: Alg.
1: SyNOnly_MI_32_reg_iter_1_bleached_masked
2: SyNOnly_CC_6_reg_iter_1_bleached_masked
3: SyNOnly_CC_4_reg_iter_1_bleached_masked
4: SyNOnly_MI_32_reg_iter_0_bleached_masked
5: SyNOnly_CC_2_reg_iter_1_bleached_masked
6: SyNOnly_CC_6_reg_iter_0_bleached_masked
7: SyNOnly_CC_4_reg_iter_0_bleached_masked
8: SyNOnly_CC_2_reg_iter_0_bleached_masked
9: SyNOnly_CC_2_reg_iter_1_normal_masked
10: TVMSQC_bleached_masked
11: SyNOnly_CC_2_reg_iter_0_normal_masked
12: SyNOnly_CC_4_reg_iter_0_normal_masked
13: SyNOnly_CC_4_reg_iter_1_normal_masked
14: SyNOnly_CC_6_reg_iter_0_normal_masked
15: TVMSQ_bleached_masked
16: SyNOnly_MI_32_reg_iter_0_normal_masked
17: TVMSQ_normal_masked
18: SyNOnly_CC_6_reg_iter_1_normal_masked
19: SyNOnly_MI_32_reg_iter_1_normal_masked
20: TVMSQC_normal_masked

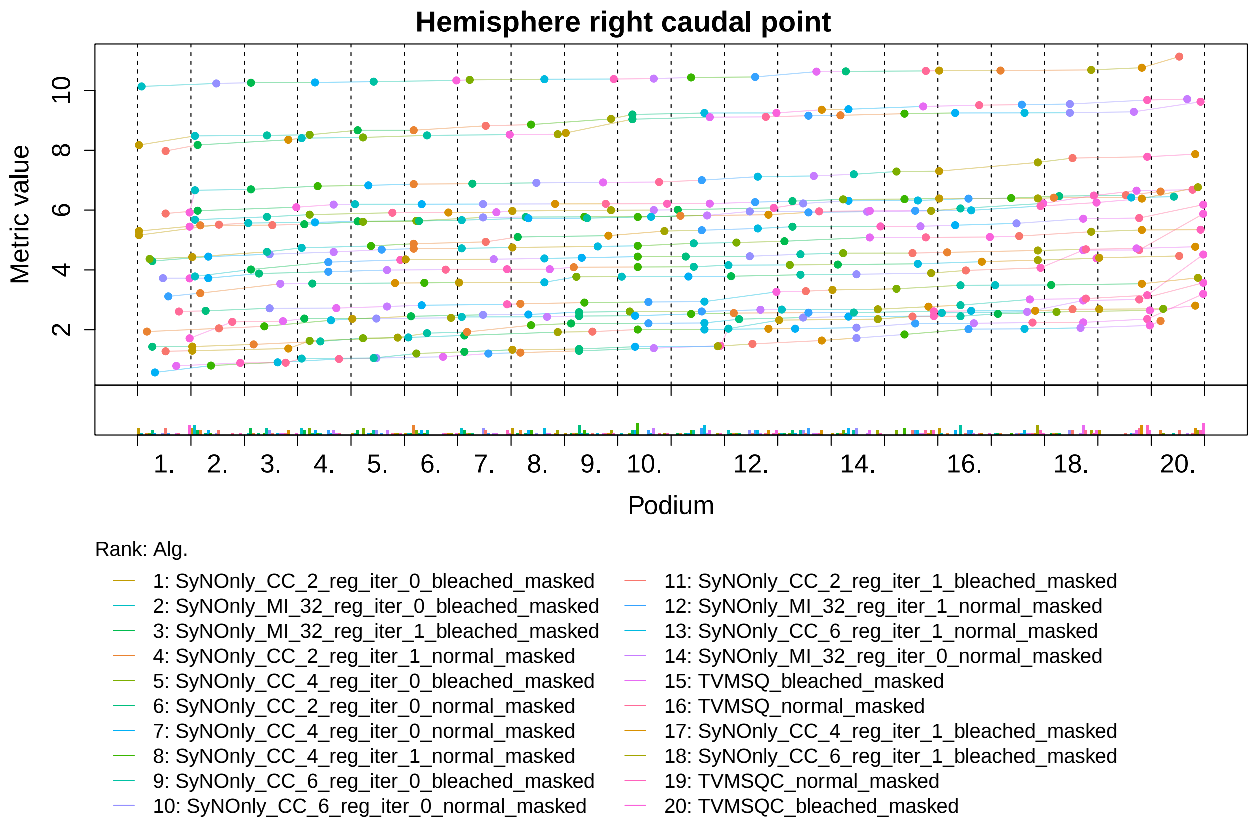
Hemisphere right caudal point
Metric value
2
4
6
8
10
Podium
1.
2.
3.
4.
5.
6.
7.
8.
9.
10.
12.
14.
16.
18.
20.
Rank: Alg.
1: SyNOnly_CC_2_reg_iter_0_bleached_masked
2: SyNOnly_MI_32_reg_iter_0_bleached_masked
3: SyNOnly_MI_32_reg_iter_1_bleached_masked
4: SyNOnly_CC_2_reg_iter_1_normal_masked
5: SyNOnly_CC_4_reg_iter_0_bleached_masked
6: SyNOnly_CC_2_reg_iter_0_normal_masked
7: SyNOnly_CC_4_reg_iter_0_normal_masked
8: SyNOnly_CC_4_reg_iter_1_normal_masked
9: SyNOnly_CC_6_reg_iter_0_bleached_masked
10: SyNOnly_CC_6_reg_iter_0_normal_masked
11: SyNOnly_CC_2_reg_iter_1_bleached_masked
12: SyNOnly_MI_32_reg_iter_1_normal_masked
13: SyNOnly_CC_6_reg_iter_1_normal_masked
14: SyNOnly_MI_32_reg_iter_0_normal_masked
15: TVMSQ_bleached_masked
16: TVMSQ_normal_masked
17: SyNOnly_CC_4_reg_iter_1_bleached_masked
18: SyNOnly_CC_6_reg_iter_1_bleached_masked
19: TVMSQC_normal_masked
20: TVMSQC_bleached_masked

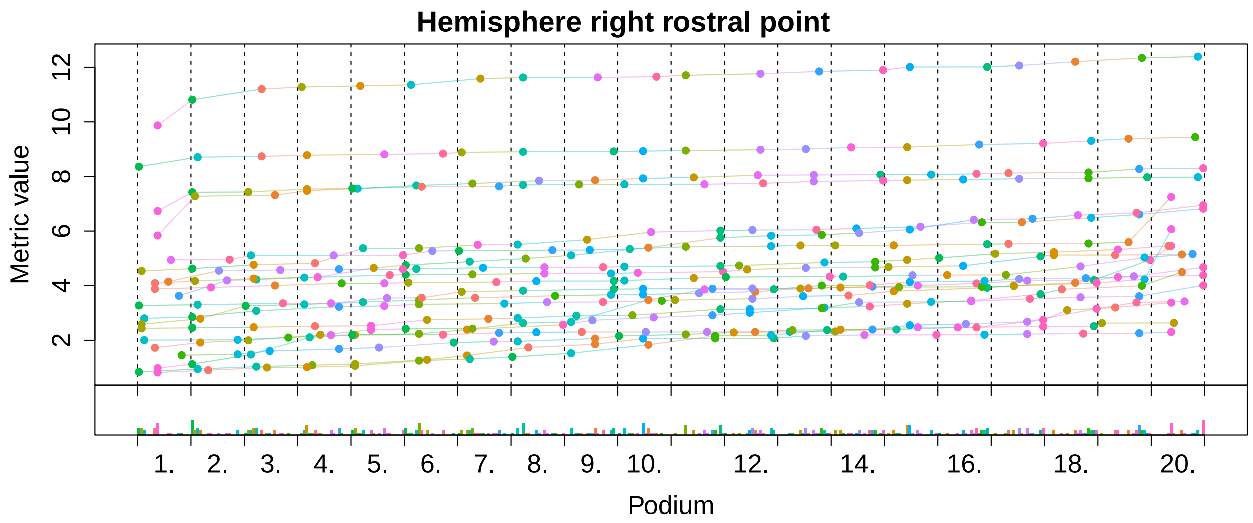

Rank: Alg.

- 1: SyNOnly_MI_32_reg_iter_1_bleached_masked
- 2: SyNOnly_CC_6_reg_iter_1_bleached_masked
- 3: SyNOnly_MI_32_reg_iter_0_bleached_masked
- 4: SyNOnly_CC_4_reg_iter_1_bleached_masked
- 5: SyNOnly_CC_6_reg_iter_0_bleached_masked
- 6: SyNOnly_CC_4_reg_iter_0_bleached_masked
- 7: SyNOnly_CC_2_reg_iter_1_bleached_masked
- 8: TVMSQC_bleached_masked
- 9: SyNOnly_CC_2_reg_iter_0_bleached_masked
- 10: SyNOnly_CC_4_reg_iter_0_normal_masked
- 11: SyNOnly_CC_6_reg_iter_0_normal_masked
- 12: SyNOnly_CC_2_reg_iter_1_normal_masked
- 13: TVMSQ_bleached_masked
- 14: SyNOnly_MI_32_reg_iter_0_normal_masked
- 15: TVMSQ_normal_masked
- 16: SyNOnly_MI_32_reg_iter_1_normal_masked
- 17: SyNOnly_CC_4_reg_iter_1_normal_masked
- 18: SyNOnly_CC_6_reg_iter_1_normal_masked
- 19: SyNOnly_CC_2_reg_iter_0_normal_masked
- 20: TVMSQC_normal_masked

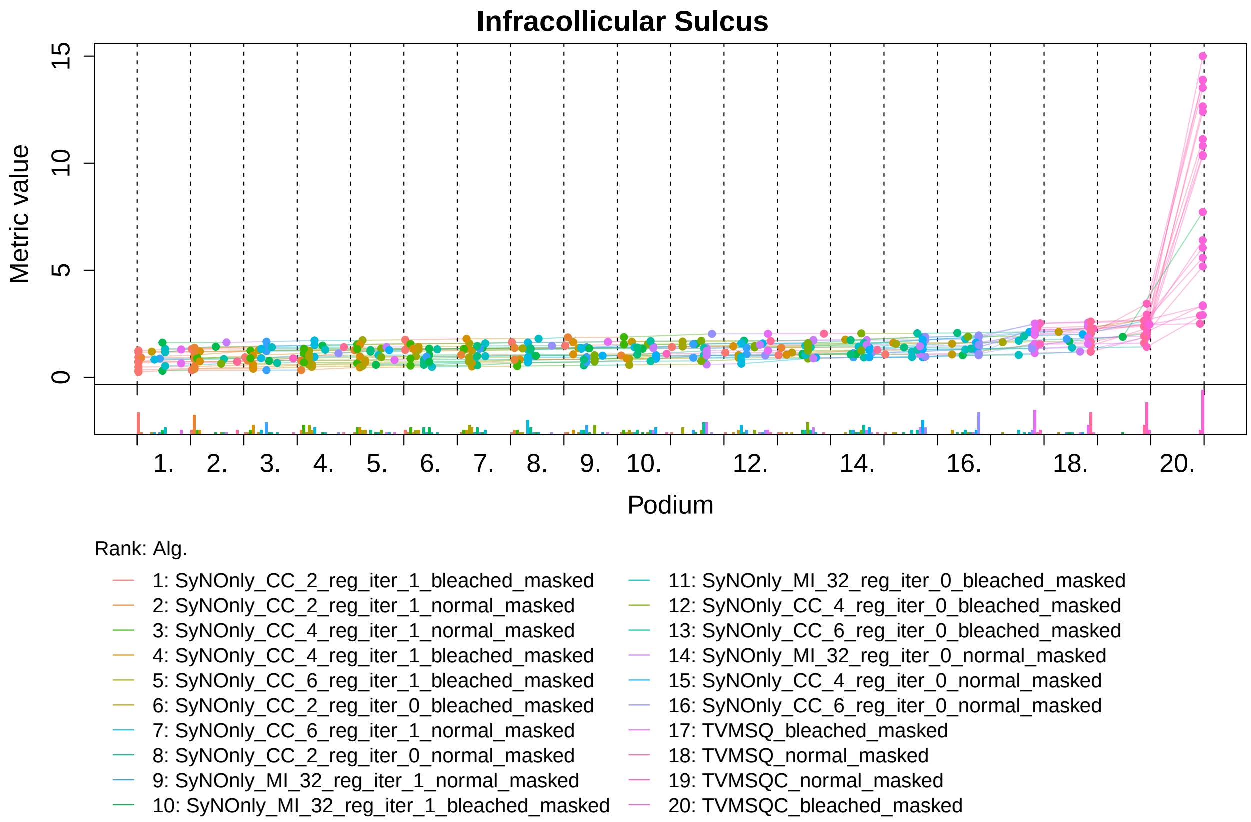
Infracollicular Sulcus
Metric value
0
5
10
15
1.
2.
3.
4.
5.
6.
7.
8.
9.
10.
12.
14.
16.
18.
20.
Podium
Rank: Alg.
1: SyNOnly_CC_2_reg_iter_1_bleached_masked
2: SyNOnly_CC_2_reg_iter_1_normal_masked
3: SyNOnly_CC_4_reg_iter_1_normal_masked
4: SyNOnly_CC_4_reg_iter_1_bleached_masked
5: SyNOnly_CC_6_reg_iter_1_bleached_masked
6: SyNOnly_CC_2_reg_iter_0_bleached_masked
7: SyNOnly_CC_6_reg_iter_1_normal_masked
8: SyNOnly_CC_2_reg_iter_0_normal_masked
9: SyNOnly_MI_32_reg_iter_1_normal_masked
10: SyNOnly_MI_32_reg_iter_1_bleached_masked
11: SyNOnly_MI_32_reg_iter_0_bleached_masked
12: SyNOnly_CC_4_reg_iter_0_bleached_masked
13: SyNOnly_CC_6_reg_iter_0_bleached_masked
14: SyNOnly_MI_32_reg_iter_0_normal_masked
15: SyNOnly_CC_4_reg_iter_0_normal_masked
16: SyNOnly_CC_6_reg_iter_0_normal_masked
17: TVMSQ_bleached_masked
18: TVMSQ_normal_masked
19: TVMSQC_normal_masked
20: TVMSQC_bleached_masked

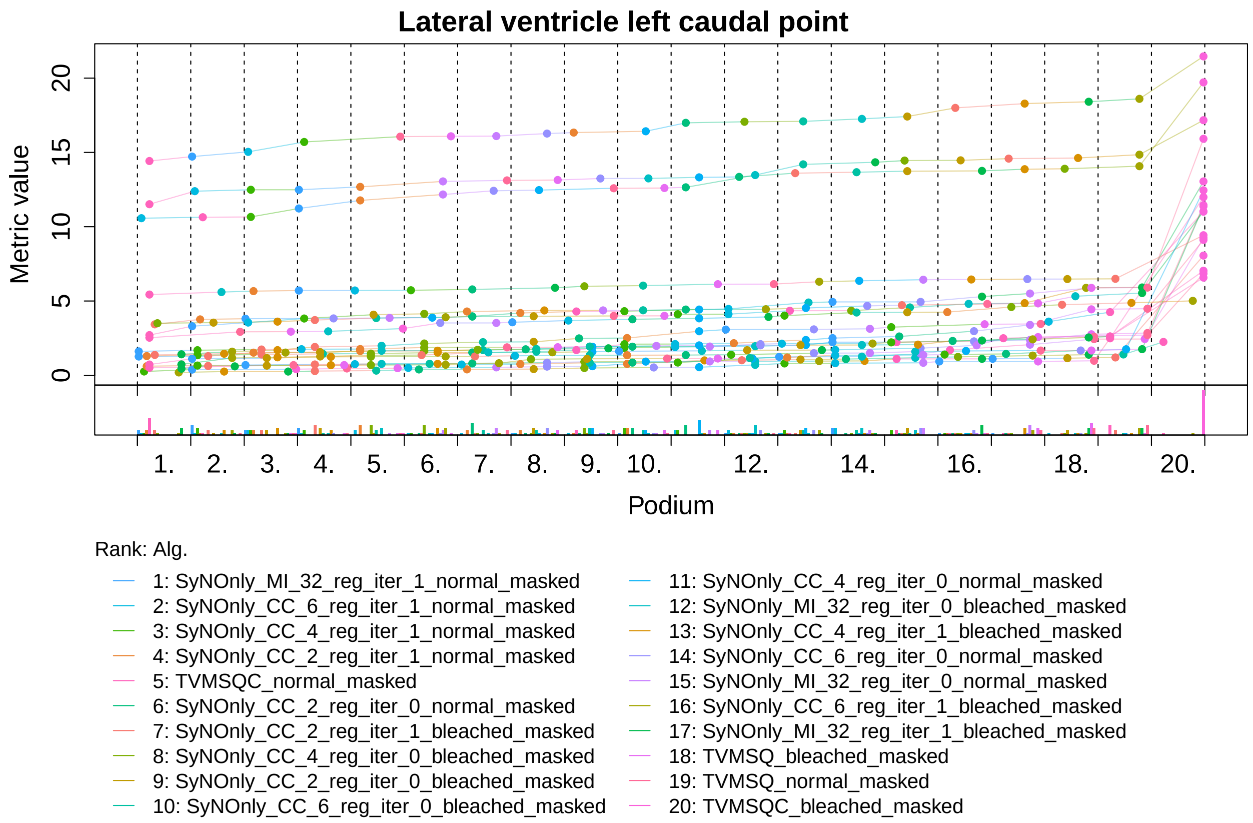

Lateral ventricle left caudal point
Metric value
0
5
10
15
20
1.
2.
3.
4.
5.
6.
7.
8.
9.
10.
12.
14.
16.
18.
20.
Podium
Rank: Alg.
1: SyNOnly_MI_32_reg_iter_1_normal_masked
2: SyNOnly_CC_6_reg_iter_1_normal_masked
3: SyNOnly_CC_4_reg_iter_1_normal_masked
4: SyNOnly_CC_2_reg_iter_1_normal_masked
5: TVMSQC_normal_masked
6: SyNOnly_CC_2_reg_iter_0_normal_masked
7: SyNOnly_CC_2_reg_iter_1_bleached_masked
8: SyNOnly_CC_4_reg_iter_0_bleached_masked
9: SyNOnly_CC_2_reg_iter_0_bleached_masked
10: SyNOnly_CC_6_reg_iter_0_bleached_masked
11: SyNOnly_CC_4_reg_iter_0_normal_masked
12: SyNOnly_MI_32_reg_iter_0_bleached_masked
13: SyNOnly_CC_4_reg_iter_1_bleached_masked
14: SyNOnly_CC_6_reg_iter_0_normal_masked
15: SyNOnly_MI_32_reg_iter_0_normal_masked
16: SyNOnly_CC_6_reg_iter_1_bleached_masked
17: SyNOnly_MI_32_reg_iter_1_bleached_masked
18: TVMSQ_bleached_masked
19: TVMSQ_normal_masked
20: TVMSQC_bleached_masked

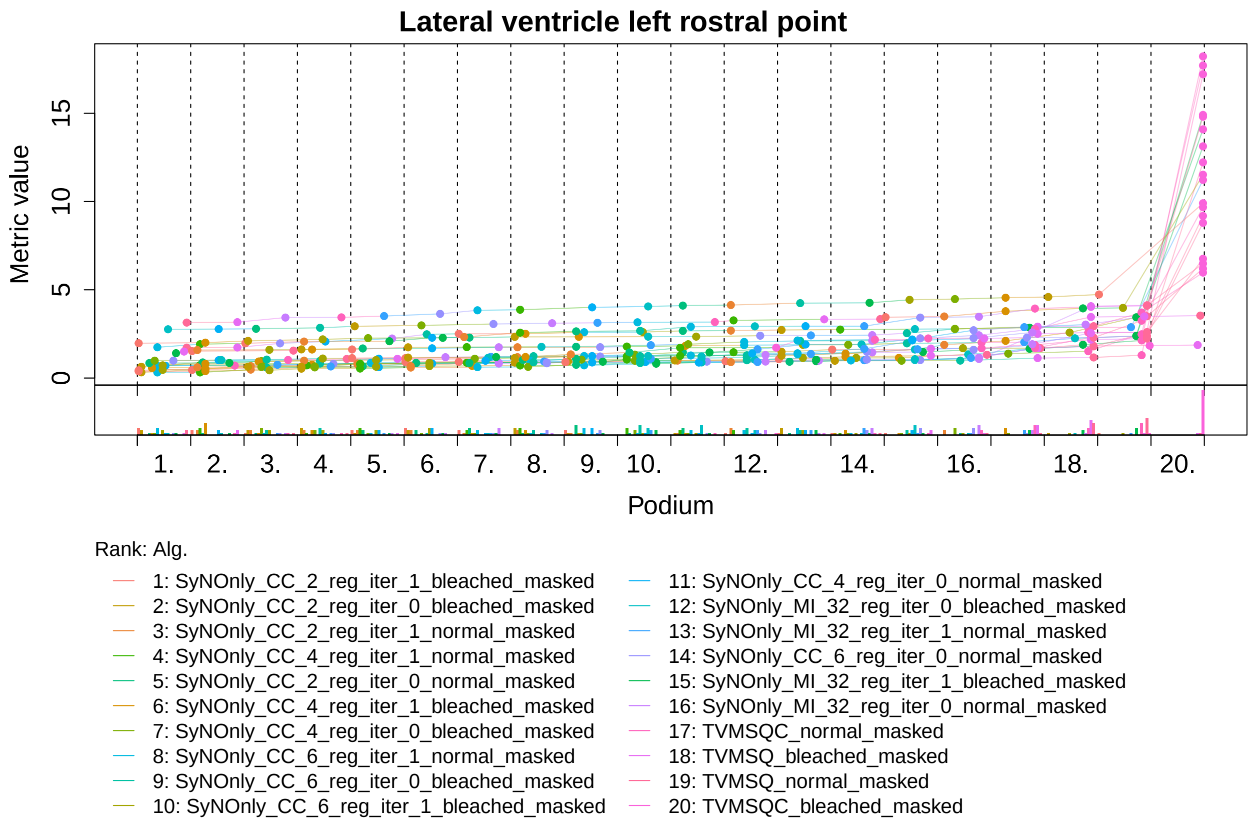

Lateral ventricle left rostral point
Metric value
0
5
10
15
1.
2.
3.
4.
5.
6.
7.
8.
9.
10.
12.
14.
16.
18.
20.
Podium
Rank: Alg.
1: SyNOnly_CC_2_reg_iter_1_bleached_masked
2: SyNOnly_CC_2_reg_iter_0_bleached_masked
3: SyNOnly_CC_2_reg_iter_1_normal_masked
4: SyNOnly_CC_4_reg_iter_1_normal_masked
5: SyNOnly_CC_2_reg_iter_0_normal_masked
6: SyNOnly_CC_4_reg_iter_1_bleached_masked
7: SyNOnly_CC_4_reg_iter_0_bleached_masked
8: SyNOnly_CC_6_reg_iter_1_normal_masked
9: SyNOnly_CC_6_reg_iter_0_bleached_masked
10: SyNOnly_CC_6_reg_iter_1_bleached_masked
11: SyNOnly_CC_4_reg_iter_0_normal_masked
12: SyNOnly_MI_32_reg_iter_0_bleached_masked
13: SyNOnly_MI_32_reg_iter_1_normal_masked
14: SyNOnly_CC_6_reg_iter_0_normal_masked
15: SyNOnly_MI_32_reg_iter_1_bleached_masked
16: SyNOnly_MI_32_reg_iter_0_normal_masked
17: TVMSQC_normal_masked
18: TVMSQ_bleached_masked
19: TVMSQ_normal_masked
20: TVMSQC_bleached_masked

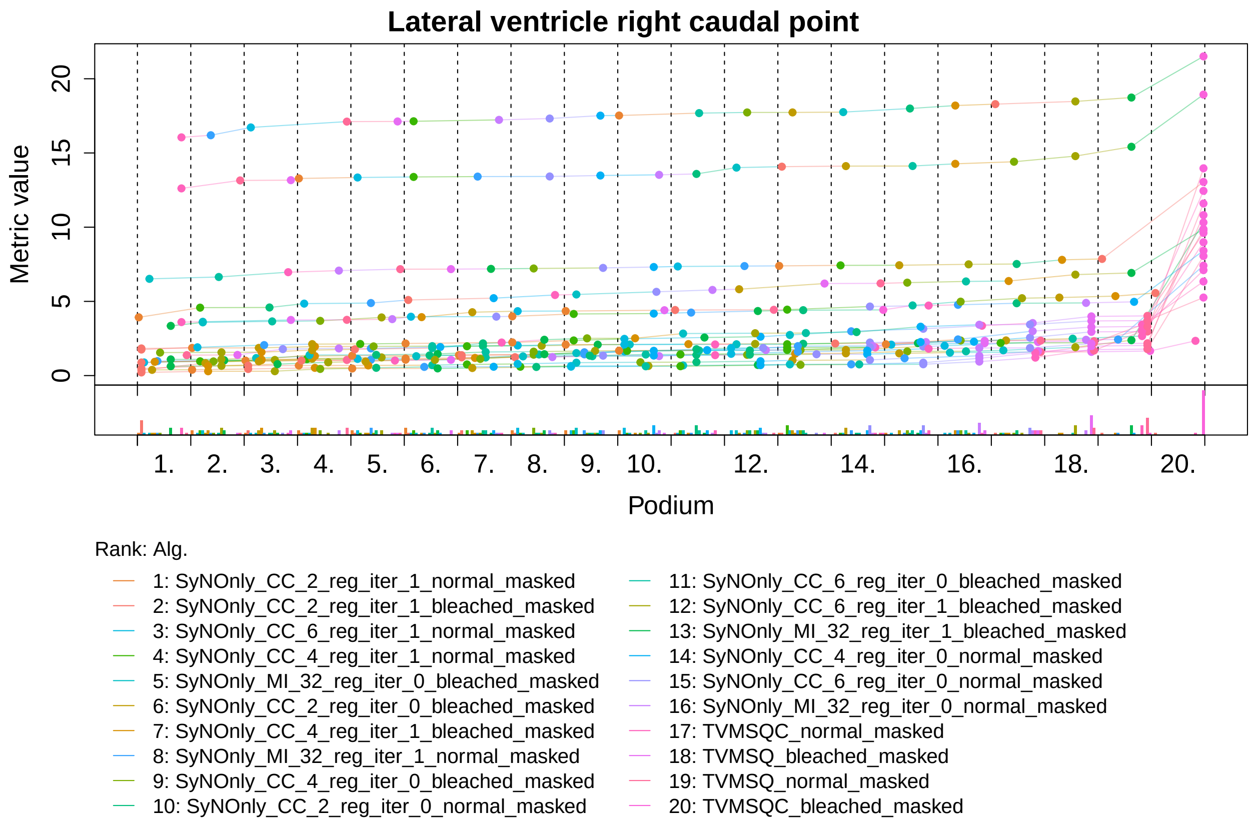
Lateral ventricle right caudal point
Metric value
0
5
10
15
20
Podium
1.
2.
3.
4.
5.
6.
7.
8.
9.
10.
12.
14.
16.
18.
20.
Rank: Alg.
1: SyNOnly_CC_2_reg_iter_1_normal_masked
2: SyNOnly_CC_2_reg_iter_1_bleached_masked
3: SyNOnly_CC_6_reg_iter_1_normal_masked
4: SyNOnly_CC_4_reg_iter_1_normal_masked
5: SyNOnly_MI_32_reg_iter_0_bleached_masked
6: SyNOnly_CC_2_reg_iter_0_bleached_masked
7: SyNOnly_CC_4_reg_iter_1_bleached_masked
8: SyNOnly_MI_32_reg_iter_1_normal_masked
9: SyNOnly_CC_4_reg_iter_0_bleached_masked
10: SyNOnly_CC_2_reg_iter_0_normal_masked
11: SyNOnly_CC_6_reg_iter_0_bleached_masked
12: SyNOnly_CC_6_reg_iter_1_bleached_masked
13: SyNOnly_MI_32_reg_iter_1_bleached_masked
14: SyNOnly_CC_4_reg_iter_0_normal_masked
15: SyNOnly_CC_6_reg_iter_0_normal_masked
16: SyNOnly_MI_32_reg_iter_0_normal_masked
17: TVMSQC_normal_masked
18: TVMSQ_bleached_masked
19: TVMSQ_normal_masked
20: TVMSQC_bleached_masked

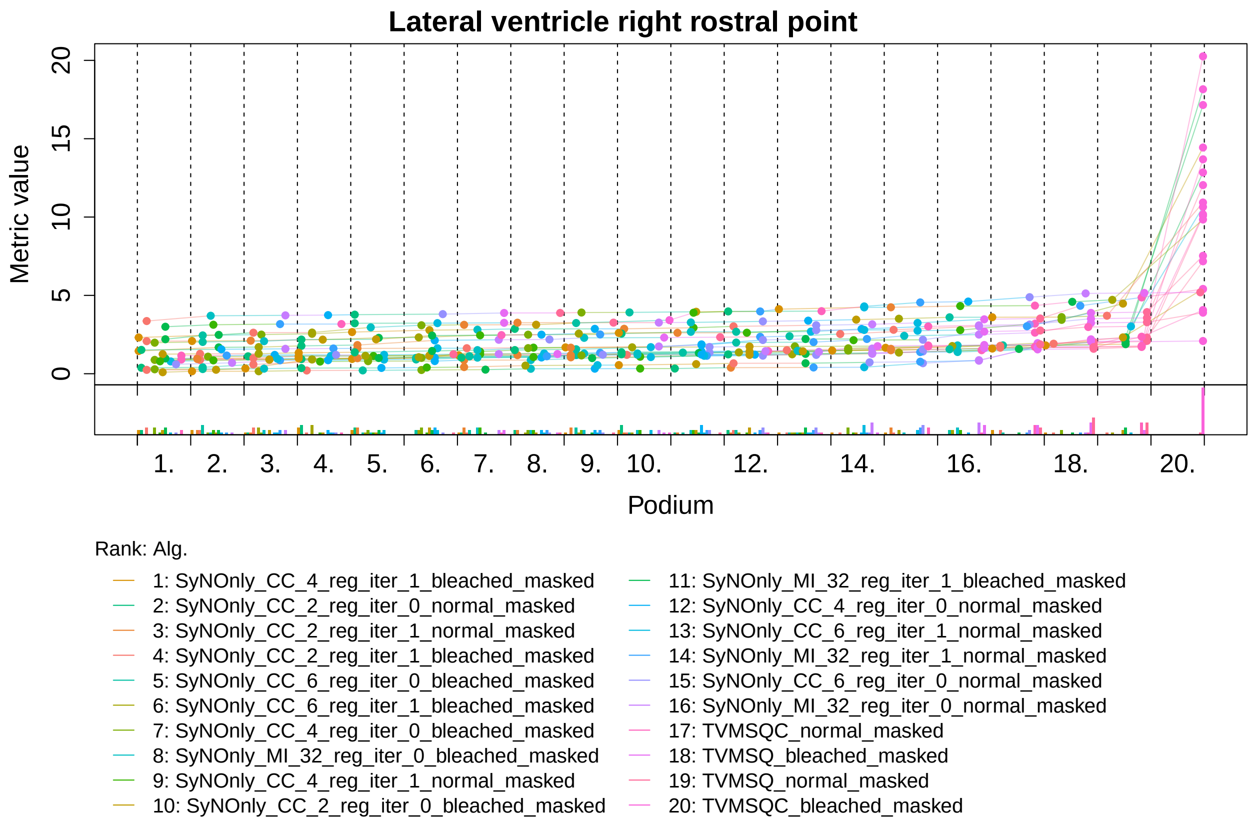

Lateral ventricle right rostral point
Metric value
0
5
10
15
20
1.
2.
3.
4.
5.
6.
7.
8.
9.
10.
12.
14.
16.
18.
20.
Podium
Rank: Alg.
1: SyNOnly_CC_4_reg_iter_1_bleached_masked
2: SyNOnly_CC_2_reg_iter_0_normal_masked
3: SyNOnly_CC_2_reg_iter_1_normal_masked
4: SyNOnly_CC_2_reg_iter_1_bleached_masked
5: SyNOnly_CC_6_reg_iter_0_bleached_masked
6: SyNOnly_CC_6_reg_iter_1_bleached_masked
7: SyNOnly_CC_4_reg_iter_0_bleached_masked
8: SyNOnly_MI_32_reg_iter_0_bleached_masked
9: SyNOnly_CC_4_reg_iter_1_normal_masked
10: SyNOnly_CC_2_reg_iter_0_bleached_masked
11: SyNOnly_MI_32_reg_iter_1_bleached_masked
12: SyNOnly_CC_4_reg_iter_0_normal_masked
13: SyNOnly_CC_6_reg_iter_1_normal_masked
14: SyNOnly_MI_32_reg_iter_1_normal_masked
15: SyNOnly_CC_6_reg_iter_0_normal_masked
16: SyNOnly_MI_32_reg_iter_0_normal_masked
17: TVMSQC_normal_masked
18: TVMSQ_bleached_masked
19: TVMSQ_normal_masked
20: TVMSQC_bleached_masked

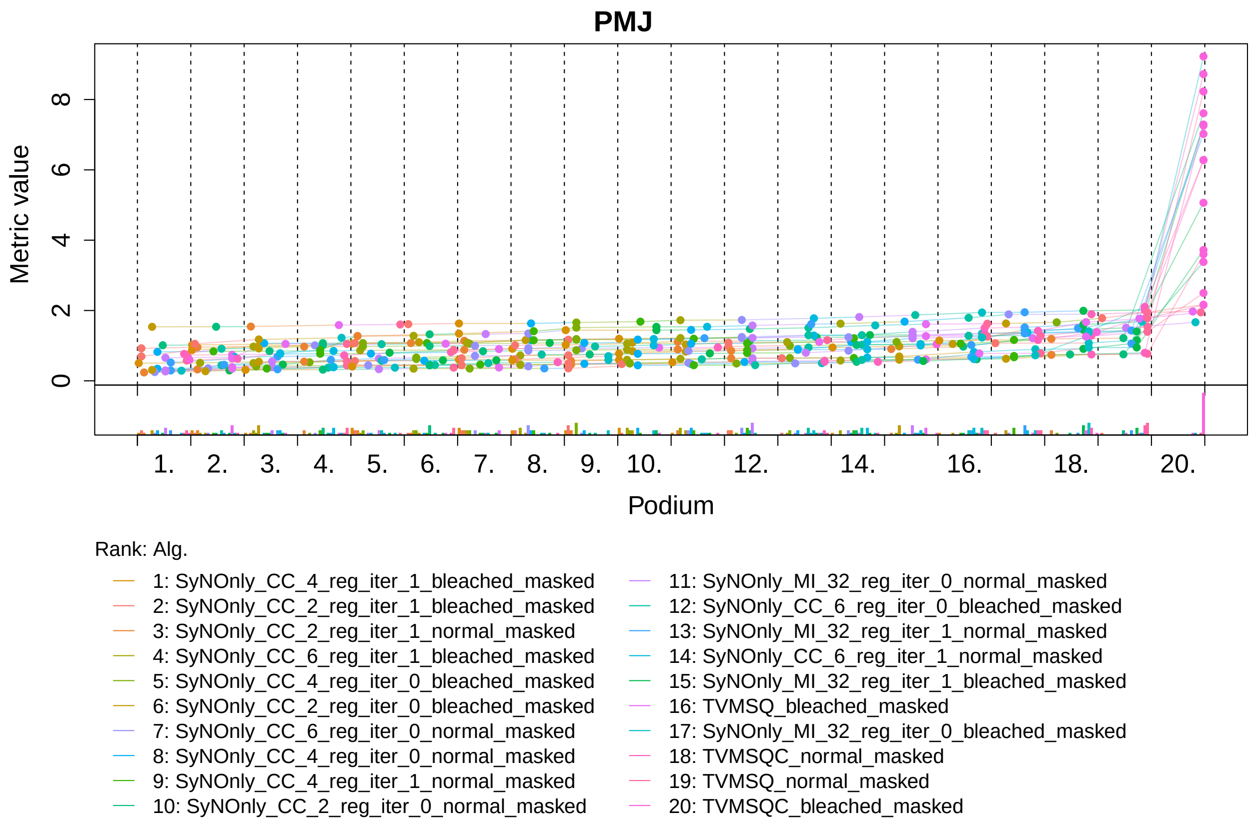
PMJ
Metric value
0
2
4
6
8
1.
2.
3.
4.
5.
6.
7.
8.
9.
10.
12.
14.
16.
18.
20.
Podium
Rank: Alg.
1: SyNOnly_CC_4_reg_iter_1_bleached_masked
2: SyNOnly_CC_2_reg_iter_1_bleached_masked
3: SyNOnly_CC_2_reg_iter_1_normal_masked
4: SyNOnly_CC_6_reg_iter_1_bleached_masked
5: SyNOnly_CC_4_reg_iter_0_bleached_masked
6: SyNOnly_CC_2_reg_iter_0_bleached_masked
7: SyNOnly_CC_6_reg_iter_0_normal_masked
8: SyNOnly_CC_4_reg_iter_0_normal_masked
9: SyNOnly_CC_4_reg_iter_1_normal_masked
10: SyNOnly_CC_2_reg_iter_0_normal_masked
11: SyNOnly_MI_32_reg_iter_0_normal_masked
12: SyNOnly_CC_6_reg_iter_0_bleached_masked
13: SyNOnly_MI_32_reg_iter_1_normal_masked
14: SyNOnly_CC_6_reg_iter_1_normal_masked
15: SyNOnly_MI_32_reg_iter_1_bleached_masked
16: TVMSQ_bleached_masked
17: SyNOnly_MI_32_reg_iter_0_bleached_masked
18: TVMSQC_normal_masked
19: TVMSQ_normal_masked
20: TVMSQC_bleached_masked

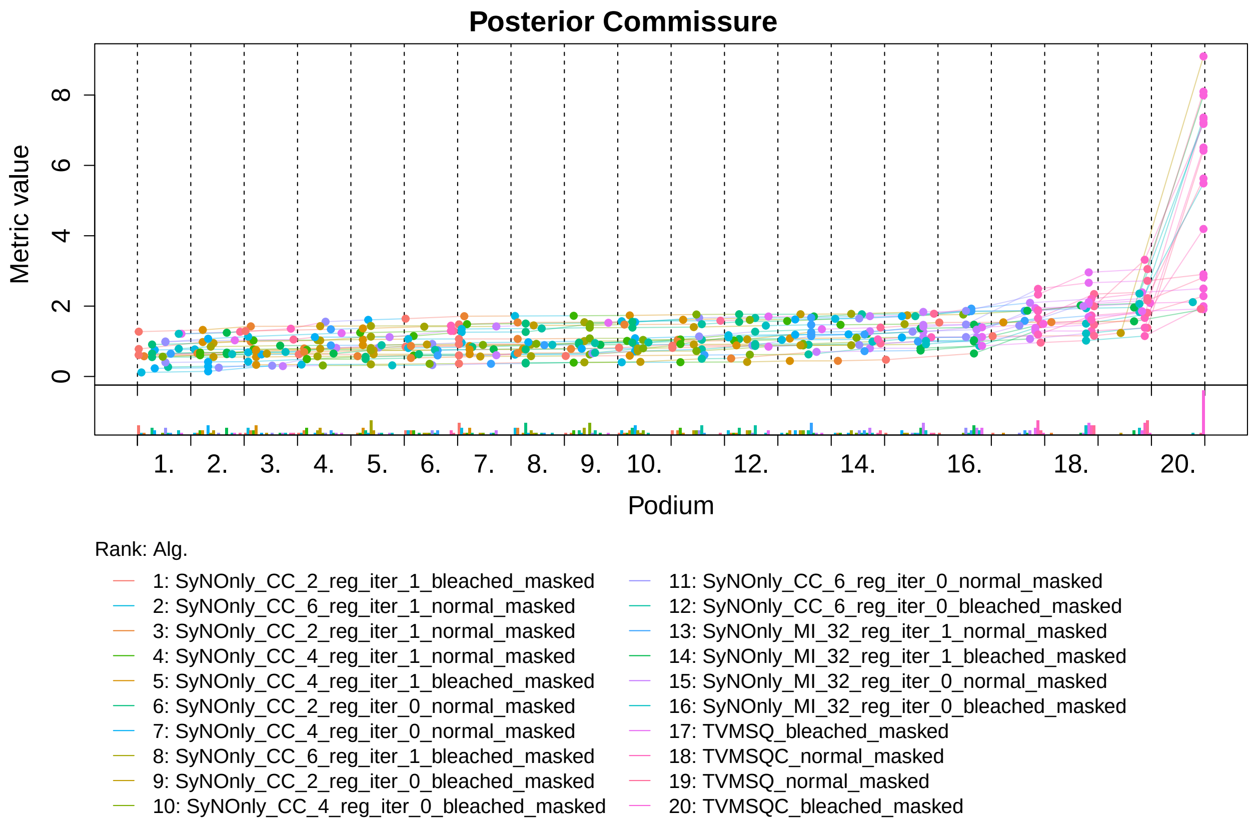

Posterior Commissure
Metric value
0
2
4
6
8
Podium
1.
2.
3.
4.
5.
6.
7.
8.
9.
10.
12.
14.
16.
18.
20.
Rank: Alg.
1: SyNOnly_CC_2_reg_iter_1_bleached_masked
2: SyNOnly_CC_6_reg_iter_1_normal_masked
3: SyNOnly_CC_2_reg_iter_1_normal_masked
4: SyNOnly_CC_4_reg_iter_1_normal_masked
5: SyNOnly_CC_4_reg_iter_1_bleached_masked
6: SyNOnly_CC_2_reg_iter_0_normal_masked
7: SyNOnly_CC_4_reg_iter_0_normal_masked
8: SyNOnly_CC_6_reg_iter_1_bleached_masked
9: SyNOnly_CC_2_reg_iter_0_bleached_masked
10: SyNOnly_CC_4_reg_iter_0_bleached_masked
11: SyNOnly_CC_6_reg_iter_0_normal_masked
12: SyNOnly_CC_6_reg_iter_0_bleached_masked
13: SyNOnly_MI_32_reg_iter_1_normal_masked
14: SyNOnly_MI_32_reg_iter_1_bleached_masked
15: SyNOnly_MI_32_reg_iter_0_normal_masked
16: SyNOnly_MI_32_reg_iter_0_bleached_masked
17: TVMSQ_bleached_masked
18: TVMSQC_normal_masked
19: TVMSQ_normal_masked
20: TVMSQC_bleached_masked

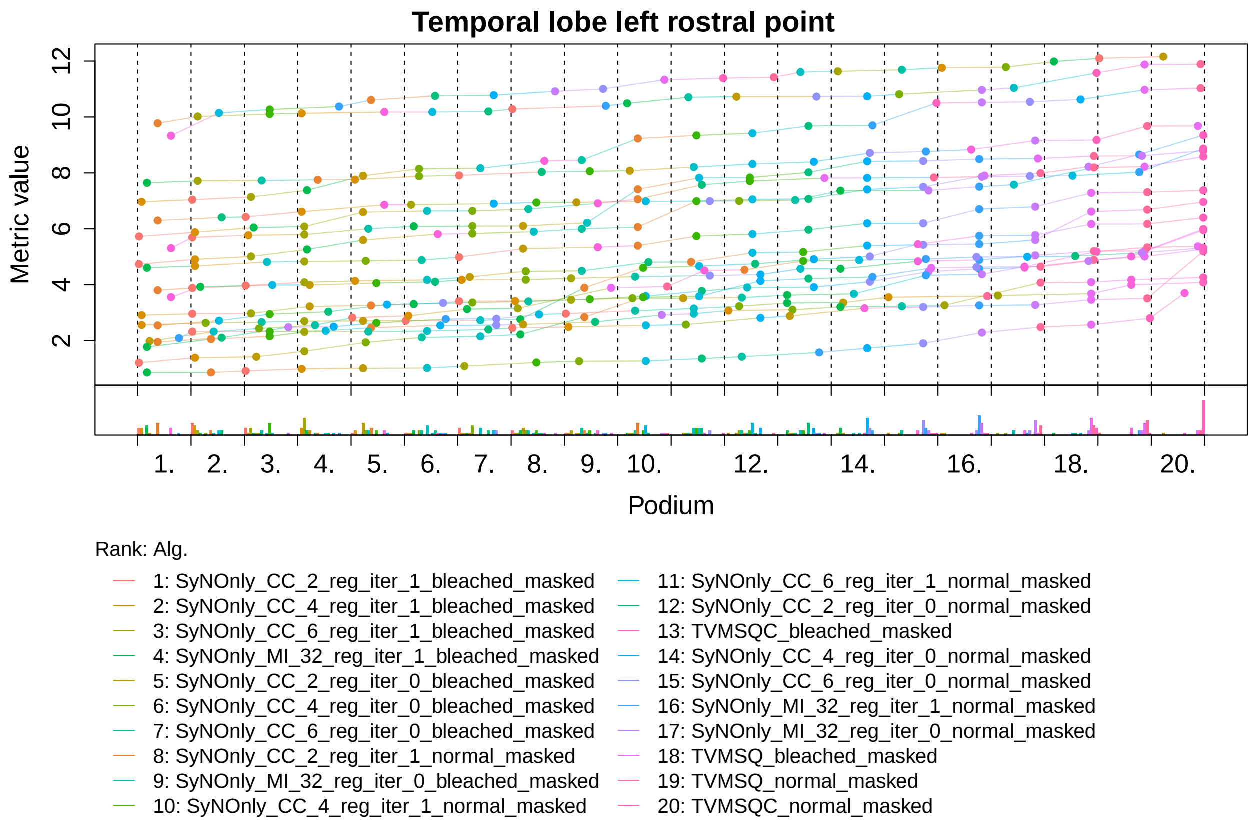

Temporal lobe left rostral point
Metric value
2
4
6
8
10
12
1.
2.
3.
4.
5.
6.
7.
8.
9.
10.
12.
14.
16.
18.
20.
Podium
Rank: Alg.
1: SyNOnly_CC_2_reg_iter_1_bleached_masked
2: SyNOnly_CC_4_reg_iter_1_bleached_masked
3: SyNOnly_CC_6_reg_iter_1_bleached_masked
4: SyNOnly_MI_32_reg_iter_1_bleached_masked
5: SyNOnly_CC_2_reg_iter_0_bleached_masked
6: SyNOnly_CC_4_reg_iter_0_bleached_masked
7: SyNOnly_CC_6_reg_iter_0_bleached_masked
8: SyNOnly_CC_2_reg_iter_1_normal_masked
9: SyNOnly_MI_32_reg_iter_0_bleached_masked
10: SyNOnly_CC_4_reg_iter_1_normal_masked
11: SyNOnly_CC_6_reg_iter_1_normal_masked
12: SyNOnly_CC_2_reg_iter_0_normal_masked
13: TVMSQC_bleached_masked
14: SyNOnly_CC_4_reg_iter_0_normal_masked
15: SyNOnly_CC_6_reg_iter_0_normal_masked
16: SyNOnly_MI_32_reg_iter_1_normal_masked
17: SyNOnly_MI_32_reg_iter_0_normal_masked
18: TVMSQ_bleached_masked
19: TVMSQ_normal_masked
20: TVMSQC_normal_masked

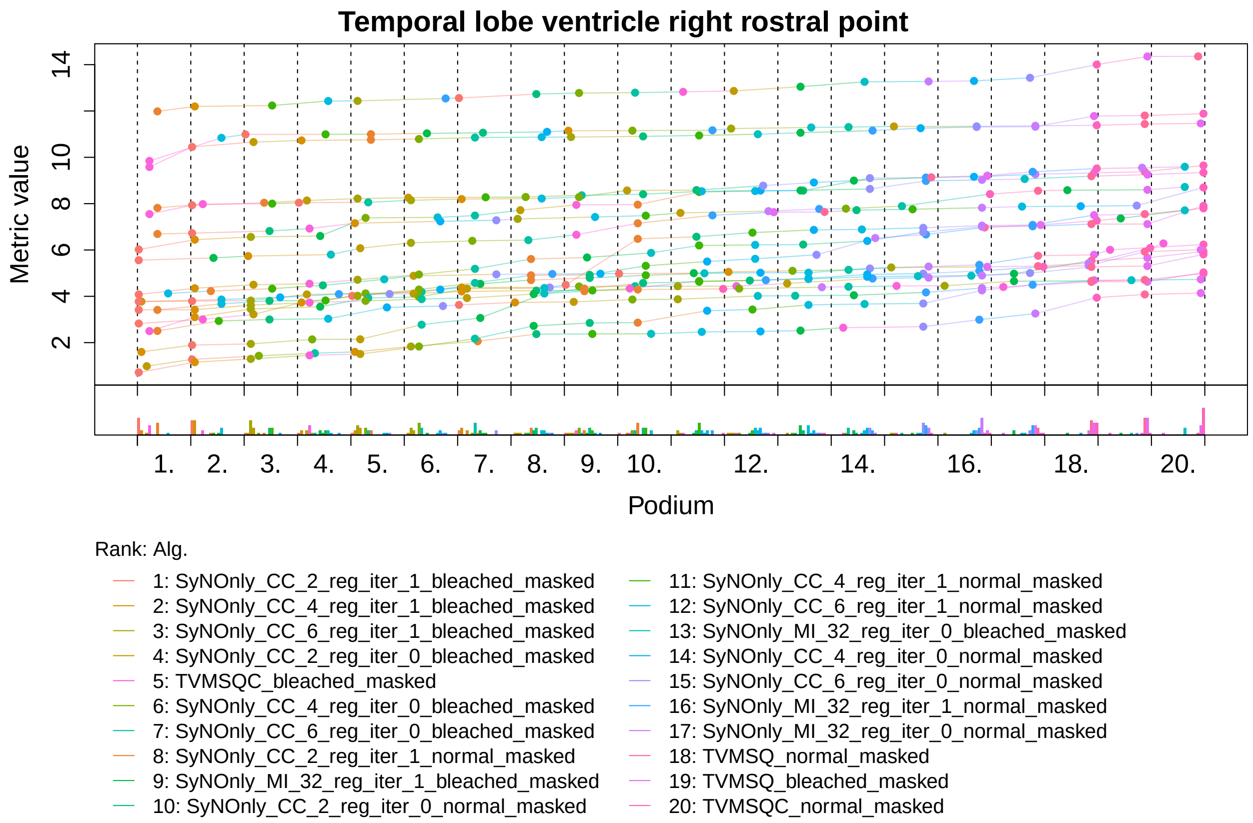
Temporal lobe ventricle right rostral point
Metric value
2
4
6
8
10
14
1.
2.
3.
4.
5.
6.
7.
8.
9.
10.
12.
14.
16.
18.
20.
Podium
Rank: Alg.
1: SyNOnly_CC_2_reg_iter_1_bleached_masked
2: SyNOnly_CC_4_reg_iter_1_bleached_masked
3: SyNOnly_CC_6_reg_iter_1_bleached_masked
4: SyNOnly_CC_2_reg_iter_0_bleached_masked
5: TVMSQC_bleached_masked
6: SyNOnly_CC_4_reg_iter_0_bleached_masked
7: SyNOnly_CC_6_reg_iter_0_bleached_masked
8: SyNOnly_CC_2_reg_iter_1_normal_masked
9: SyNOnly_MI_32_reg_iter_1_bleached_masked
10: SyNOnly_CC_2_reg_iter_0_normal_masked
11: SyNOnly_CC_4_reg_iter_1_normal_masked
12: SyNOnly_CC_6_reg_iter_1_normal_masked
13: SyNOnly_MI_32_reg_iter_0_bleached_masked
14: SyNOnly_CC_4_reg_iter_0_normal_masked
15: SyNOnly_CC_6_reg_iter_0_normal_masked
16: SyNOnly_MI_32_reg_iter_1_normal_masked
17: SyNOnly_MI_32_reg_iter_0_normal_masked
18: TVMSQ_normal_masked
19: TVMSQ_bleached_masked
20: TVMSQC_normal_masked

2.3 Ranking heatmap

Ranking heatmaps for visualizing raw assessment data. Each cell (i, A_j) shows the absolute frequency of cases in which algorithm A_j achieved rank i.

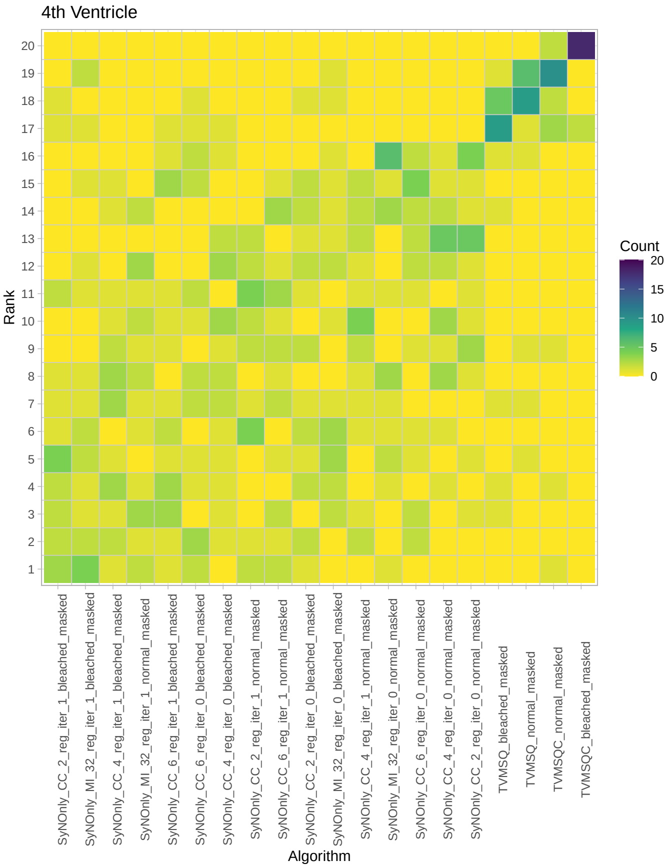

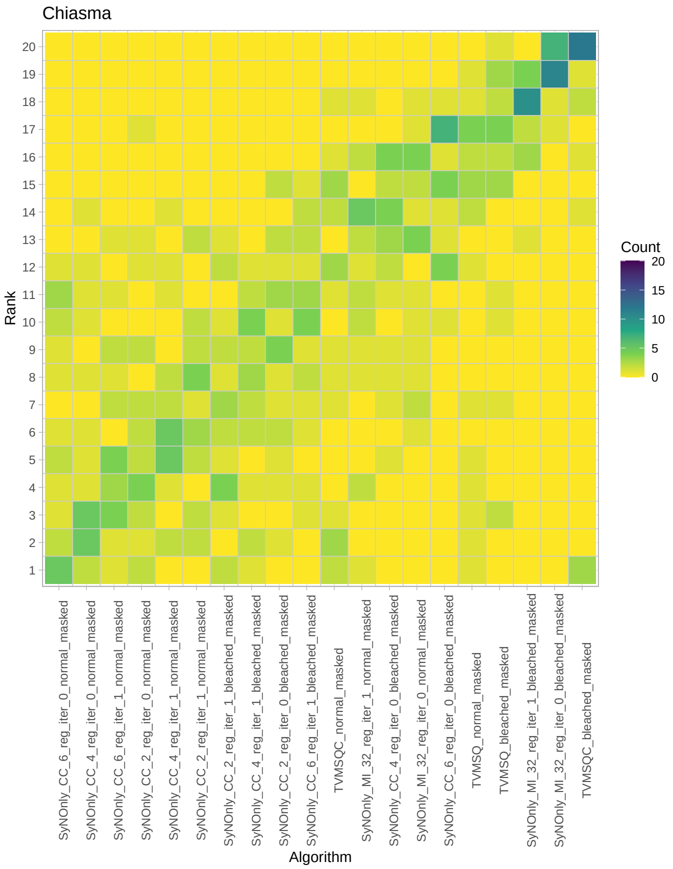
Chiasma
Rank
Algorithm
Count
20
15
10
5
0
SyNOnly_CC_6_reg_iter_0_normal_masked
SyNOnly_CC_4_reg_iter_0_normal_masked
SyNOnly_CC_6_reg_iter_1_normal_masked
SyNOnly_CC_2_reg_iter_0_normal_masked
SyNOnly_CC_4_reg_iter_1_normal_masked
SyNOnly_CC_2_reg_iter_1_normal_masked
SyNOnly_CC_2_reg_iter_1_bleached_masked
SyNOnly_CC_4_reg_iter_1_bleached_masked
SyNOnly_CC_2_reg_iter_0_bleached_masked
SyNOnly_CC_6_reg_iter_1_bleached_masked
TVMSQC_normal_masked
SyNOnly_MI_32_reg_iter_1_normal_masked
SyNOnly_CC_4_reg_iter_0_bleached_masked
SyNOnly_MI_32_reg_iter_0_normal_masked
SyNOnly_CC_6_reg_iter_0_bleached_masked
TVMSQ_normal_masked
TVMSQ_bleached_masked
SyNOnly_MI_32_reg_iter_1_bleached_masked
SyNOnly_MI_32_reg_iter_0_bleached_masked
TVMSQC_bleached_masked

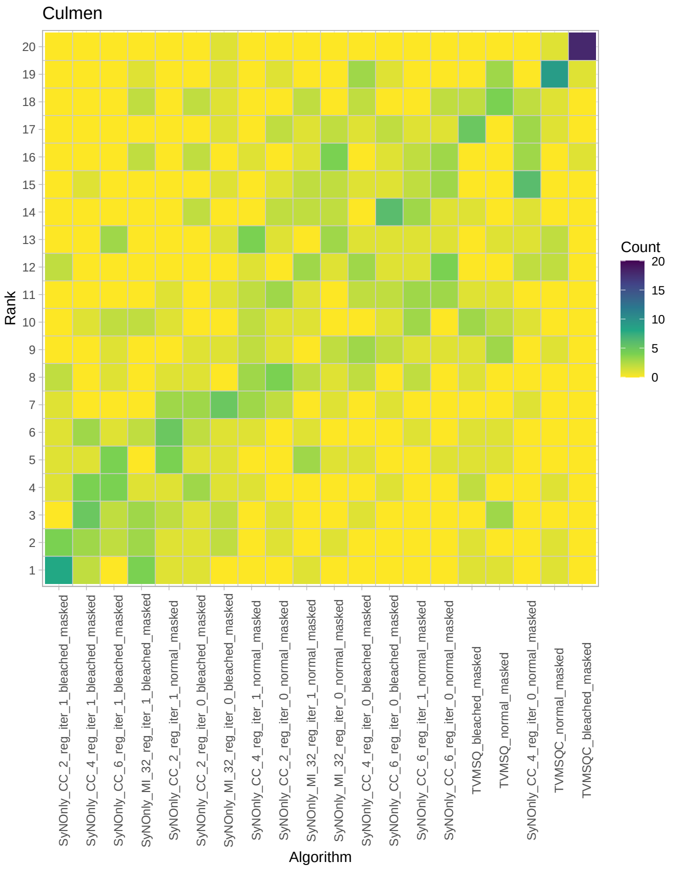
Culmen
Rank
Count
20
15
10
5
0
SyNOnly_CC_2_reg_iter_1_bleached_masked
SyNOnly_CC_4_reg_iter_1_bleached_masked
SyNOnly_CC_6_reg_iter_1_bleached_masked
SyNOnly_MI_32_reg_iter_1_bleached_masked
SyNOnly_CC_2_reg_iter_1_normal_masked
SyNOnly_CC_2_reg_iter_0_bleached_masked
SyNOnly_MI_32_reg_iter_0_bleached_masked
SyNOnly_CC_4_reg_iter_1_normal_masked
SyNOnly_CC_2_reg_iter_0_normal_masked
SyNOnly_MI_32_reg_iter_1_normal_masked
SyNOnly_MI_32_reg_iter_0_normal_masked
SyNOnly_CC_4_reg_iter_0_bleached_masked
SyNOnly_CC_6_reg_iter_0_bleached_masked
SyNOnly_CC_6_reg_iter_1_normal_masked
SyNOnly_CC_6_reg_iter_0_normal_masked
TVMSQ_bleached_masked
TVMSQ_normal_masked
SyNOnly_CC_4_reg_iter_0_normal_masked
TVMSQC_normal_masked
TVMSQC_bleached_masked
Algorithm

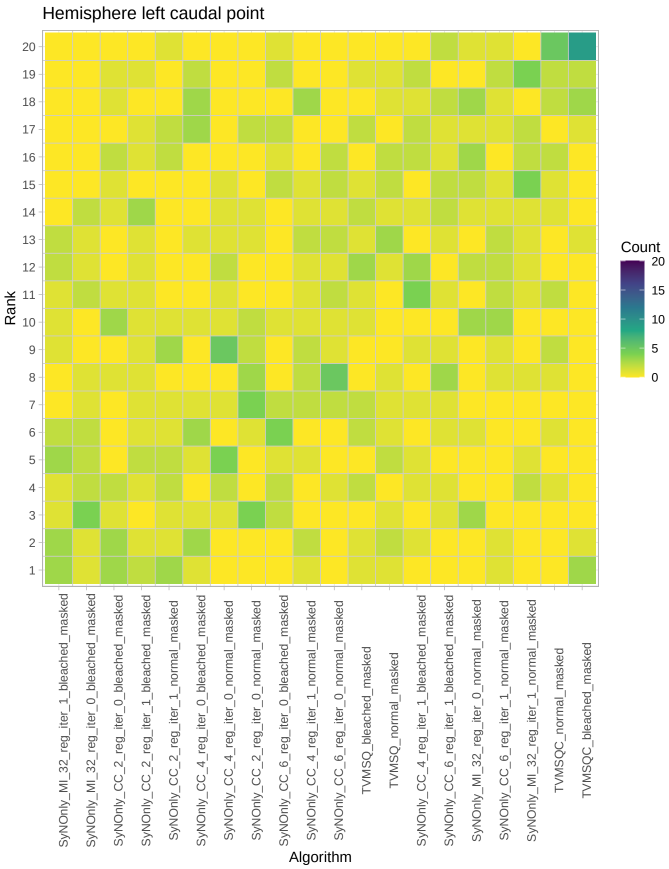
Hemisphere left caudal point
Rank
Algorithm
Count
20
15
10
5
0
SyNOnly_MI_32_reg_iter_1_bleached_masked
SyNOnly_MI_32_reg_iter_0_bleached_masked
SyNOnly_CC_2_reg_iter_0_bleached_masked
SyNOnly_CC_2_reg_iter_1_bleached_masked
SyNOnly_CC_2_reg_iter_1_normal_masked
SyNOnly_CC_4_reg_iter_0_bleached_masked
SyNOnly_CC_4_reg_iter_0_normal_masked
SyNOnly_CC_2_reg_iter_0_normal_masked
SyNOnly_CC_6_reg_iter_0_bleached_masked
SyNOnly_CC_4_reg_iter_1_normal_masked
SyNOnly_CC_6_reg_iter_0_normal_masked
TVMSQ_bleached_masked
TVMSQ_normal_masked
SyNOnly_CC_4_reg_iter_1_bleached_masked
SyNOnly_CC_6_reg_iter_1_bleached_masked
SyNOnly_MI_32_reg_iter_0_normal_masked
SyNOnly_CC_6_reg_iter_1_normal_masked
SyNOnly_MI_32_reg_iter_1_normal_masked
TVMSQC_normal_masked
TVMSQC_bleached_masked

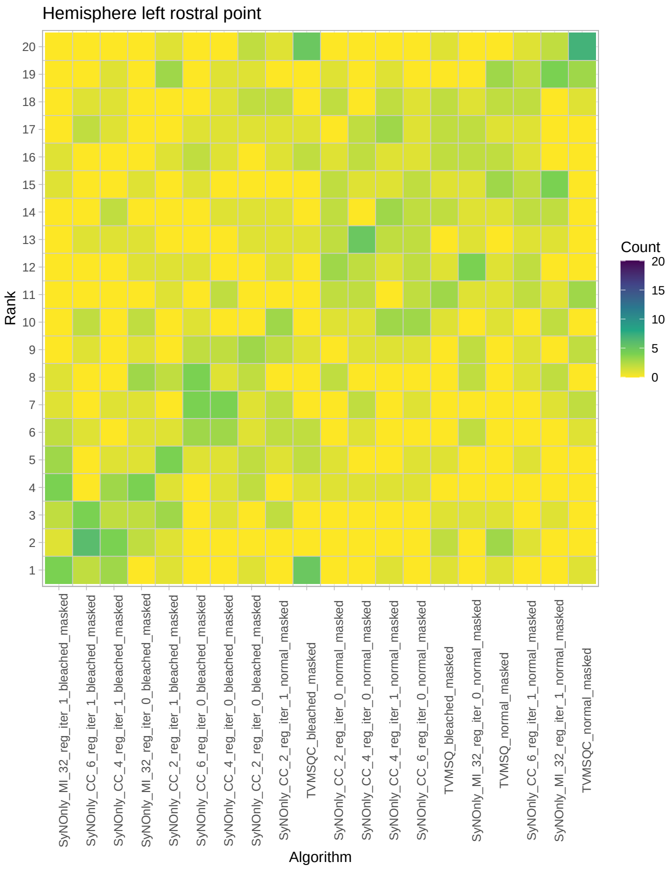
Hemisphere left rostral point
Rank
Algorithm
Count
20
15
10
5
0
SyNOnly_MI_32_reg_iter_1_bleached_masked
SyNOnly_CC_6_reg_iter_1_bleached_masked
SyNOnly_CC_4_reg_iter_1_bleached_masked
SyNOnly_MI_32_reg_iter_0_bleached_masked
SyNOnly_CC_2_reg_iter_1_bleached_masked
SyNOnly_CC_6_reg_iter_0_bleached_masked
SyNOnly_CC_4_reg_iter_0_bleached_masked
SyNOnly_CC_2_reg_iter_0_bleached_masked
SyNOnly_CC_2_reg_iter_1_normal_masked
TVMSQC_bleached_masked
SyNOnly_CC_2_reg_iter_0_normal_masked
SyNOnly_CC_4_reg_iter_0_normal_masked
SyNOnly_CC_4_reg_iter_1_normal_masked
SyNOnly_CC_6_reg_iter_0_normal_masked
TVMSQ_bleached_masked
SyNOnly_MI_32_reg_iter_0_normal_masked
TVMSQ_normal_masked
SyNOnly_CC_6_reg_iter_1_normal_masked
SyNOnly_MI_32_reg_iter_1_normal_masked
TVMSQC_normal_masked

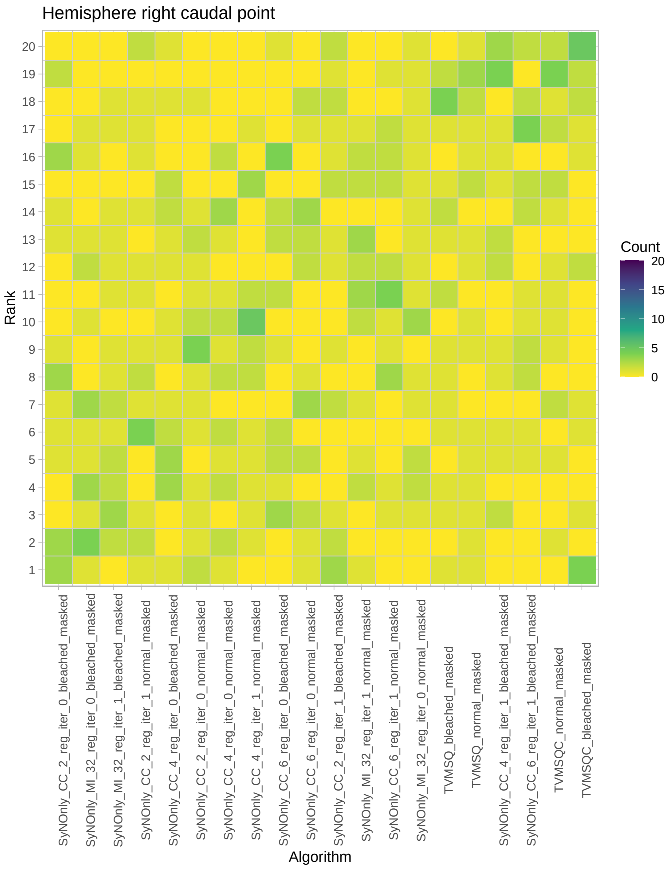
Hemisphere right caudal point
Rank
Algorithm
Count
20
15
10
5
0
SyNOnly_CC_2_reg_iter_0_bleached_masked
SyNOnly_MI_32_reg_iter_0_bleached_masked
SyNOnly_MI_32_reg_iter_1_bleached_masked
SyNOnly_CC_2_reg_iter_1_normal_masked
SyNOnly_CC_4_reg_iter_0_bleached_masked
SyNOnly_CC_2_reg_iter_0_normal_masked
SyNOnly_CC_4_reg_iter_0_normal_masked
SyNOnly_CC_4_reg_iter_1_normal_masked
SyNOnly_CC_6_reg_iter_0_bleached_masked
SyNOnly_CC_6_reg_iter_0_normal_masked
SyNOnly_CC_2_reg_iter_1_bleached_masked
SyNOnly_MI_32_reg_iter_1_normal_masked
SyNOnly_CC_6_reg_iter_1_normal_masked
SyNOnly_MI_32_reg_iter_0_normal_masked
TVMSQ_bleached_masked
TVMSQ_normal_masked
SyNOnly_CC_4_reg_iter_1_bleached_masked
SyNOnly_CC_6_reg_iter_1_bleached_masked
TVMSQC_normal_masked
TVMSQC_bleached_masked

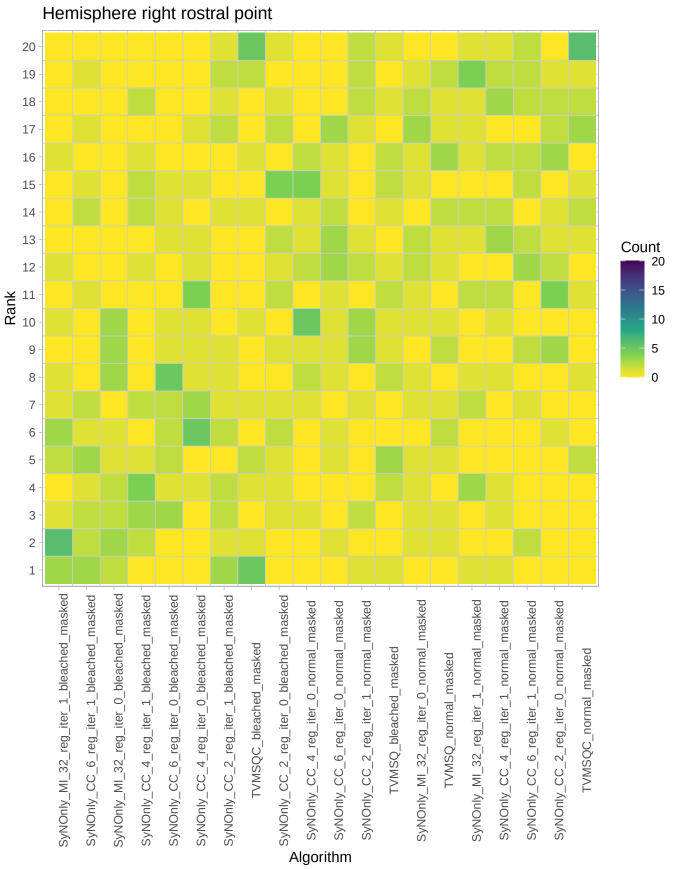
Hemisphere right rostral point
Rank
20
19
18
17
16
15
14
13
12
11
10
9
8
7
6
5
4
3
2
1
Count
20
15
10
5
0
SyNOnly_MI_32_reg_iter_1_bleached_masked
SyNOnly_CC_6_reg_iter_1_bleached_masked
SyNOnly_MI_32_reg_iter_0_bleached_masked
SyNOnly_CC_4_reg_iter_1_bleached_masked
SyNOnly_CC_6_reg_iter_0_bleached_masked
SyNOnly_CC_4_reg_iter_0_bleached_masked
SyNOnly_CC_2_reg_iter_1_bleached_masked
TVMSQC_bleached_masked
SyNOnly_CC_2_reg_iter_0_bleached_masked
SyNOnly_CC_4_reg_iter_0_normal_masked
SyNOnly_CC_6_reg_iter_0_normal_masked
SyNOnly_CC_2_reg_iter_1_normal_masked
TVMSQ_bleached_masked
SyNOnly_MI_32_reg_iter_0_normal_masked
TVMSQ_normal_masked
SyNOnly_MI_32_reg_iter_1_normal_masked
SyNOnly_CC_4_reg_iter_1_normal_masked
SyNOnly_CC_6_reg_iter_1_normal_masked
SyNOnly_CC_2_reg_iter_0_normal_masked
TVMSQC_normal_masked
Algorithm

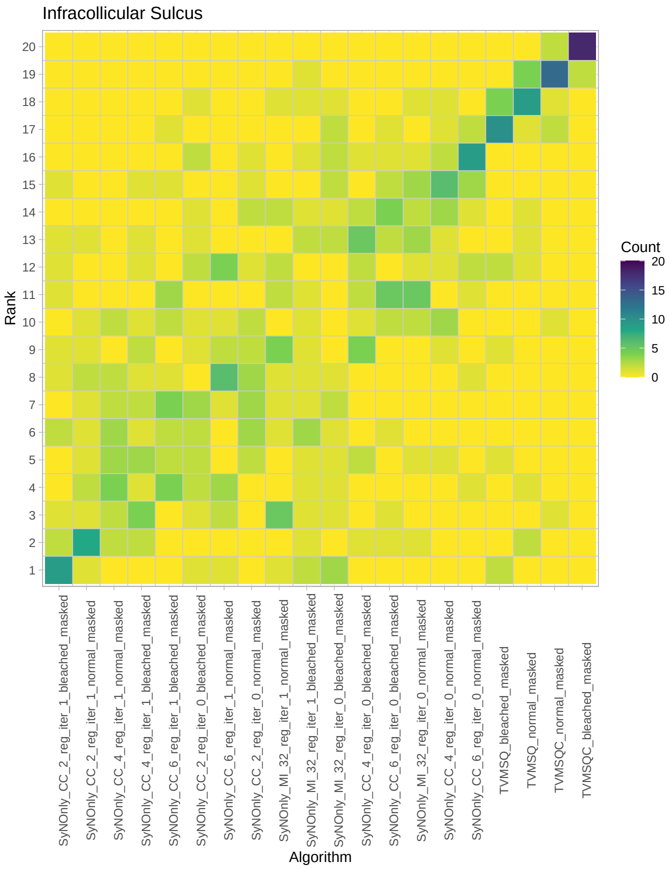
Infracollicular Sulcus
Rank
20
19
18
17
16
15
14
13
12
11
10
9
8
7
6
5
4
3
2
1
Count
20
15
10
5
0
SyNOnly_CC_2_reg_iter_1_bleached_masked
SyNOnly_CC_2_reg_iter_1_normal_masked
SyNOnly_CC_4_reg_iter_1_normal_masked
SyNOnly_CC_4_reg_iter_1_bleached_masked
SyNOnly_CC_6_reg_iter_1_bleached_masked
SyNOnly_CC_2_reg_iter_0_bleached_masked
SyNOnly_CC_6_reg_iter_1_normal_masked
SyNOnly_CC_2_reg_iter_0_normal_masked
SyNOnly_MI_32_reg_iter_1_normal_masked
SyNOnly_MI_32_reg_iter_1_bleached_masked
SyNOnly_MI_32_reg_iter_0_bleached_masked
SyNOnly_CC_4_reg_iter_0_bleached_masked
SyNOnly_CC_6_reg_iter_0_bleached_masked
SyNOnly_MI_32_reg_iter_0_normal_masked
SyNOnly_CC_4_reg_iter_0_normal_masked
SyNOnly_CC_6_reg_iter_0_normal_masked
TVMSQ_bleached_masked
TVMSQ_normal_masked
TVMSQC_normal_masked
TVMSQC_bleached_masked
Algorithm

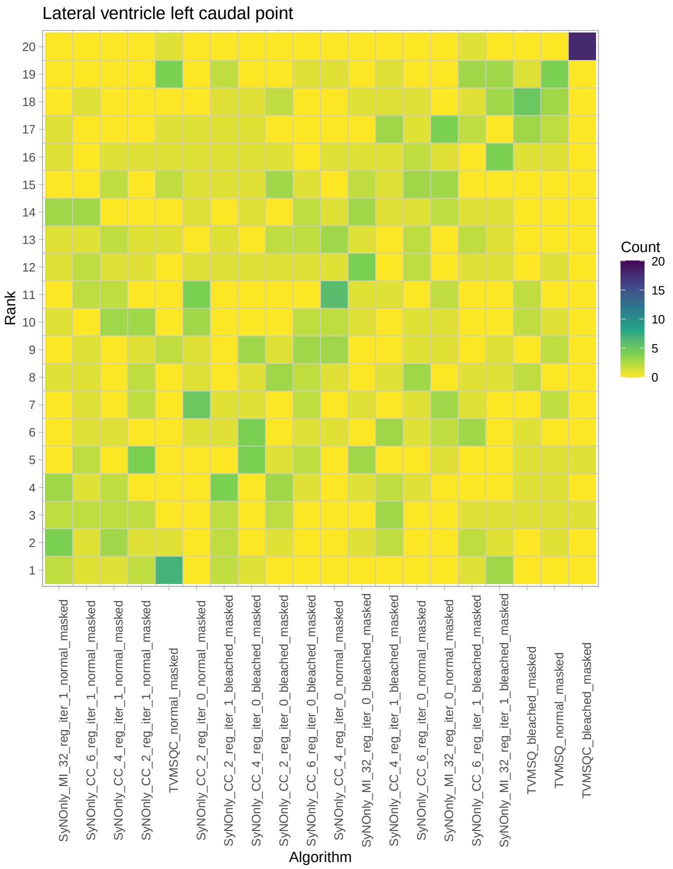

Lateral ventricle left caudal point
Rank
Algorithm
Count
20
15
10
5
0
SyNOnly_MI_32_reg_iter_1_normal_masked
SyNOnly_CC_6_reg_iter_1_normal_masked
SyNOnly_CC_4_reg_iter_1_normal_masked
SyNOnly_CC_2_reg_iter_1_normal_masked
TVMSQC_normal_masked
SyNOnly_CC_2_reg_iter_0_normal_masked
SyNOnly_CC_2_reg_iter_1_bleached_masked
SyNOnly_CC_4_reg_iter_0_bleached_masked
SyNOnly_CC_2_reg_iter_0_bleached_masked
SyNOnly_CC_6_reg_iter_0_bleached_masked
SyNOnly_CC_4_reg_iter_0_normal_masked
SyNOnly_MI_32_reg_iter_0_bleached_masked
SyNOnly_CC_4_reg_iter_1_bleached_masked
SyNOnly_CC_6_reg_iter_0_normal_masked
SyNOnly_MI_32_reg_iter_0_normal_masked
SyNOnly_CC_6_reg_iter_1_bleached_masked
SyNOnly_MI_32_reg_iter_1_bleached_masked
TVMSQ_bleached_masked
TVMSQ_normal_masked
TVMSQC_bleached_masked

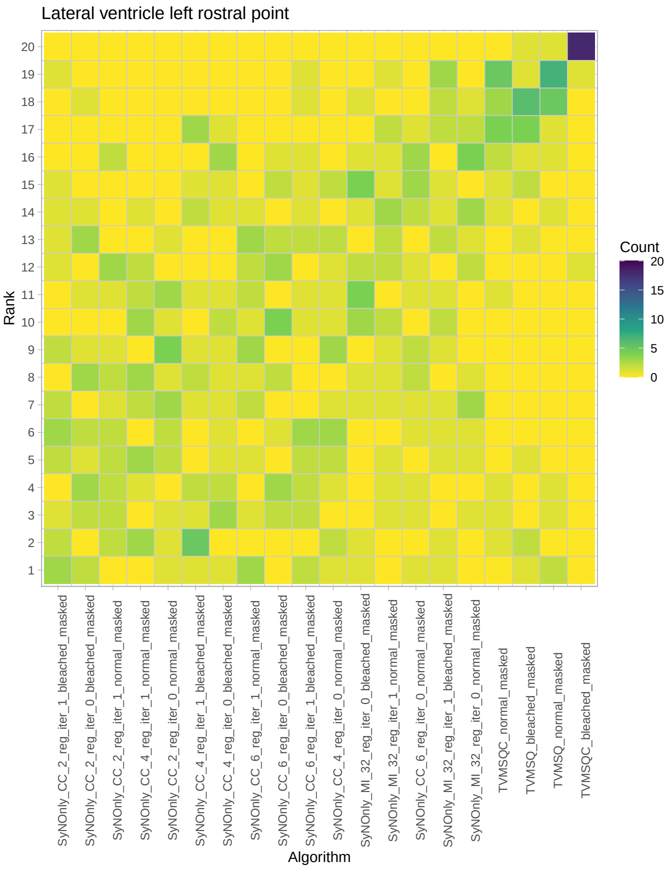

Lateral ventricle left rostral point
Rank
Algorithm
Count
SyNOnly_CC_2_reg_iter_1_bleached_masked
SyNOnly_CC_2_reg_iter_0_bleached_masked
SyNOnly_CC_2_reg_iter_1_normal_masked
SyNOnly_CC_4_reg_iter_1_normal_masked
SyNOnly_CC_2_reg_iter_0_normal_masked
SyNOnly_CC_4_reg_iter_1_bleached_masked
SyNOnly_CC_4_reg_iter_0_bleached_masked
SyNOnly_CC_6_reg_iter_1_normal_masked
SyNOnly_CC_6_reg_iter_0_bleached_masked
SyNOnly_CC_6_reg_iter_1_bleached_masked
SyNOnly_CC_4_reg_iter_0_normal_masked
SyNOnly_MI_32_reg_iter_0_bleached_masked
SyNOnly_MI_32_reg_iter_1_normal_masked
SyNOnly_CC_6_reg_iter_0_normal_masked
SyNOnly_MI_32_reg_iter_1_bleached_masked
SyNOnly_MI_32_reg_iter_0_normal_masked
TVMSQC_normal_masked
TVMSQ_bleached_masked
TVMSQ_normal_masked
TVMSQC_bleached_masked

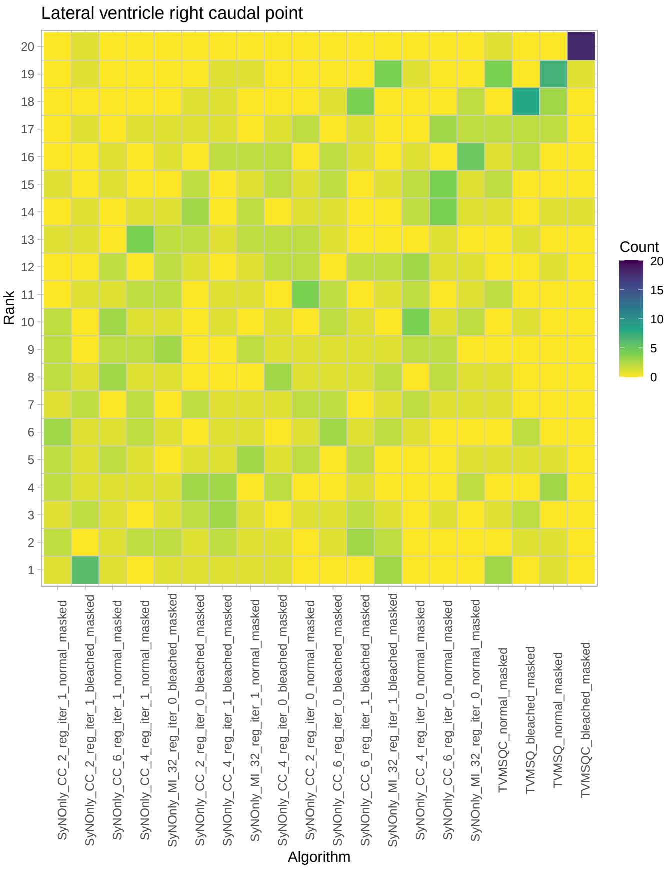
Lateral ventricle right caudal point
Rank
Algorithm
Count
20
15
10
5
0
SyNOnly_CC_2_reg_iter_1_normal_masked
SyNOnly_CC_2_reg_iter_1_bleached_masked
SyNOnly_CC_6_reg_iter_1_normal_masked
SyNOnly_CC_4_reg_iter_1_normal_masked
SyNOnly_MI_32_reg_iter_0_bleached_masked
SyNOnly_CC_2_reg_iter_0_bleached_masked
SyNOnly_CC_4_reg_iter_1_bleached_masked
SyNOnly_MI_32_reg_iter_1_normal_masked
SyNOnly_CC_4_reg_iter_0_bleached_masked
SyNOnly_CC_2_reg_iter_0_normal_masked
SyNOnly_CC_6_reg_iter_0_bleached_masked
SyNOnly_CC_6_reg_iter_1_bleached_masked
SyNOnly_MI_32_reg_iter_1_bleached_masked
SyNOnly_CC_4_reg_iter_0_normal_masked
SyNOnly_CC_6_reg_iter_0_normal_masked
SyNOnly_MI_32_reg_iter_0_normal_masked
TVMSQC_normal_masked
TVMSQ_bleached_masked
TVMSQ_normal_masked
TVMSQC_bleached_masked

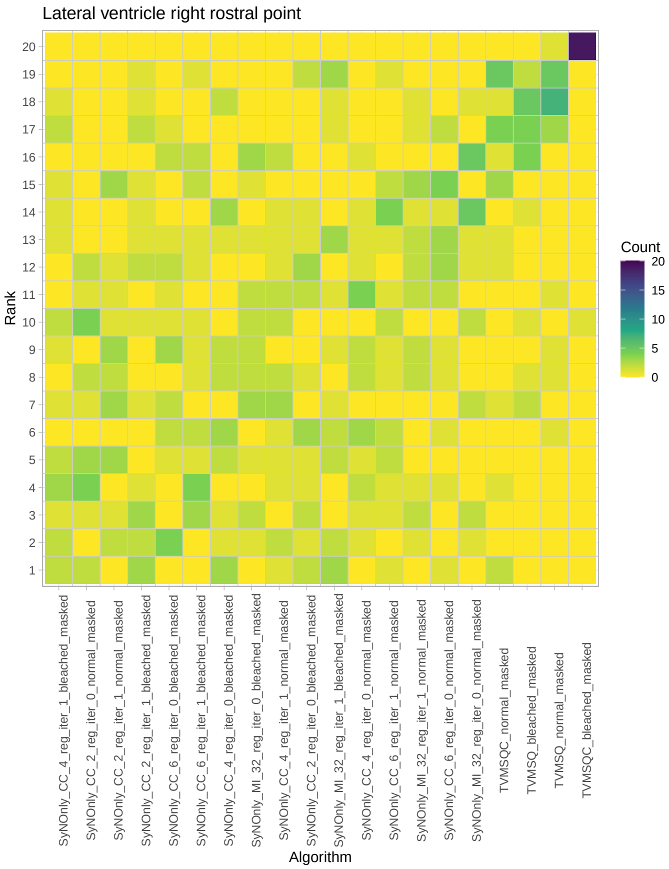
Lateral ventricle right rostral point
Rank
Algorithm
Count
20
15
10
5
0
SyNOnly_CC_4_reg_iter_1_bleached_masked
SyNOnly_CC_2_reg_iter_0_normal_masked
SyNOnly_CC_2_reg_iter_1_normal_masked
SyNOnly_CC_2_reg_iter_1_bleached_masked
SyNOnly_CC_6_reg_iter_0_bleached_masked
SyNOnly_CC_6_reg_iter_1_bleached_masked
SyNOnly_CC_4_reg_iter_0_bleached_masked
SyNOnly_MI_32_reg_iter_0_bleached_masked
SyNOnly_CC_4_reg_iter_1_normal_masked
SyNOnly_CC_2_reg_iter_0_bleached_masked
SyNOnly_MI_32_reg_iter_1_bleached_masked
SyNOnly_CC_4_reg_iter_0_normal_masked
SyNOnly_CC_6_reg_iter_1_normal_masked
SyNOnly_MI_32_reg_iter_1_normal_masked
SyNOnly_CC_6_reg_iter_0_normal_masked
SyNOnly_MI_32_reg_iter_0_normal_masked
TVMSQC_normal_masked
TVMSQ_bleached_masked
TVMSQ_normal_masked
TVMSQC_bleached_masked

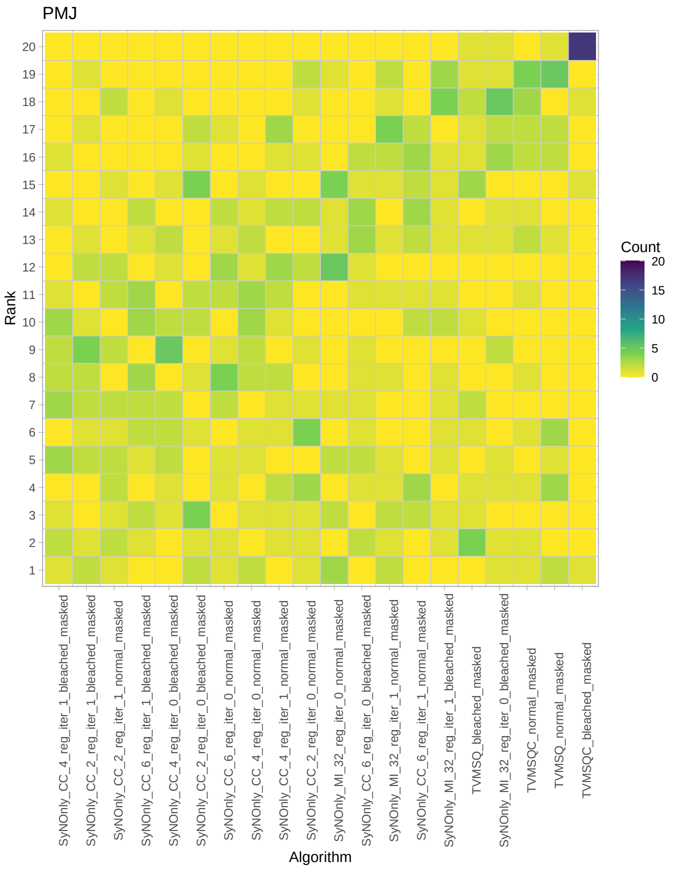
PMJ
Rank
Algorithm
Count
20
15
10
5
0
SyNOnly_CC_4_reg_iter_1_bleached_masked
SyNOnly_CC_2_reg_iter_1_bleached_masked
SyNOnly_CC_2_reg_iter_1_normal_masked
SyNOnly_CC_6_reg_iter_1_bleached_masked
SyNOnly_CC_4_reg_iter_0_bleached_masked
SyNOnly_CC_2_reg_iter_0_bleached_masked
SyNOnly_CC_6_reg_iter_0_normal_masked
SyNOnly_CC_4_reg_iter_0_normal_masked
SyNOnly_CC_4_reg_iter_1_normal_masked
SyNOnly_CC_2_reg_iter_0_normal_masked
SyNOnly_MI_32_reg_iter_0_normal_masked
SyNOnly_CC_6_reg_iter_0_bleached_masked
SyNOnly_MI_32_reg_iter_1_normal_masked
SyNOnly_CC_6_reg_iter_1_normal_masked
SyNOnly_MI_32_reg_iter_1_bleached_masked
TVMSQ_bleached_masked
SyNOnly_MI_32_reg_iter_0_bleached_masked
TVMSQC_normal_masked
TVMSQ_normal_masked
TVMSQC_bleached_masked

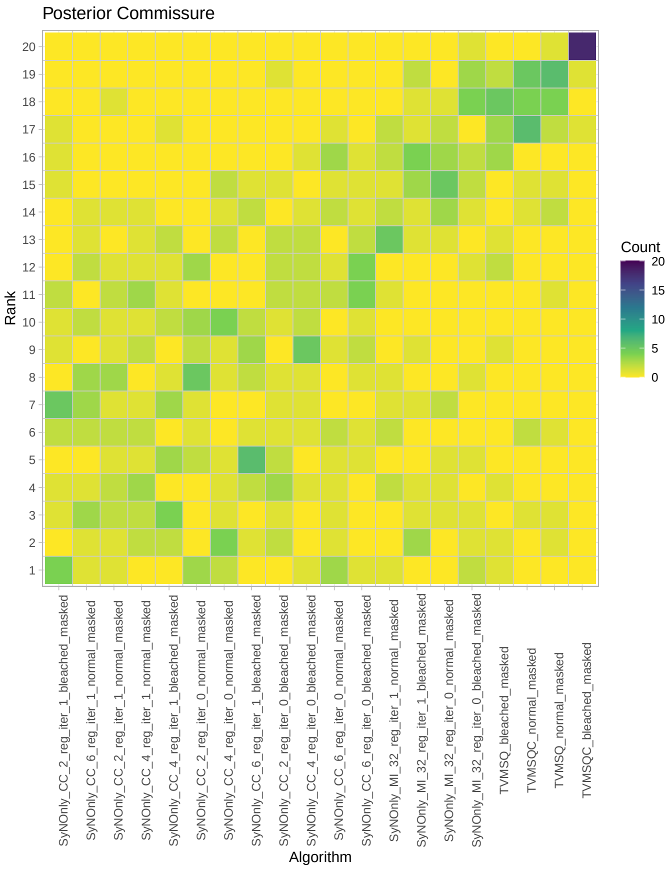
Posterior Commissure
Rank
Algorithm
Count
20
15
10
5
0
SyNOnly_CC_2_reg_iter_1_bleached_masked
SyNOnly_CC_6_reg_iter_1_normal_masked
SyNOnly_CC_2_reg_iter_1_normal_masked
SyNOnly_CC_4_reg_iter_1_normal_masked
SyNOnly_CC_4_reg_iter_1_bleached_masked
SyNOnly_CC_2_reg_iter_0_normal_masked
SyNOnly_CC_4_reg_iter_0_normal_masked
SyNOnly_CC_6_reg_iter_1_bleached_masked
SyNOnly_CC_2_reg_iter_0_bleached_masked
SyNOnly_CC_4_reg_iter_0_bleached_masked
SyNOnly_CC_6_reg_iter_0_normal_masked
SyNOnly_CC_6_reg_iter_0_bleached_masked
SyNOnly_MI_32_reg_iter_1_normal_masked
SyNOnly_MI_32_reg_iter_1_bleached_masked
SyNOnly_MI_32_reg_iter_0_normal_masked
SyNOnly_MI_32_reg_iter_0_bleached_masked
TVMSQ_bleached_masked
TVMSQC_normal_masked
TVMSQ_normal_masked
TVMSQC_bleached_masked

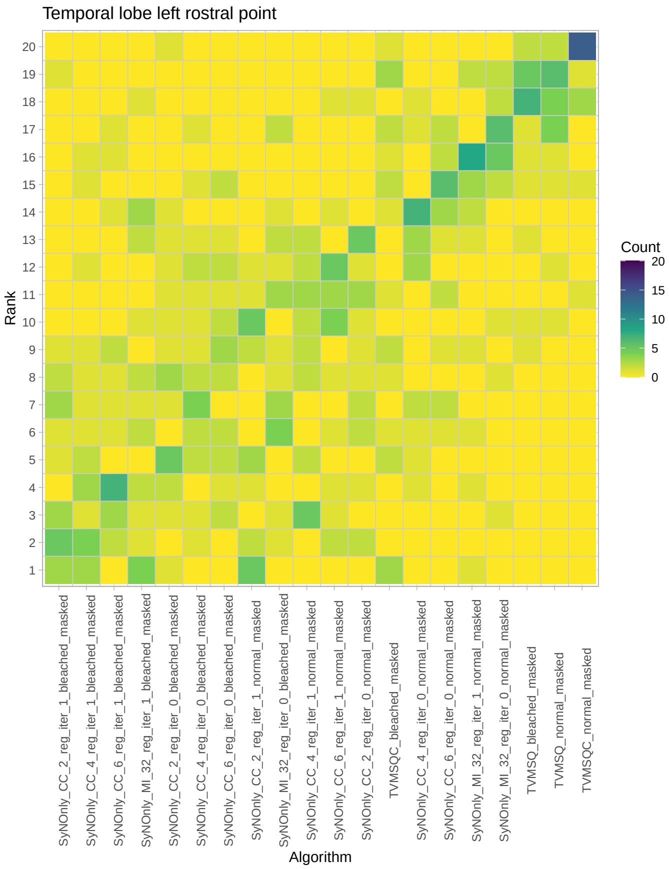

Temporal lobe left rostral point
Rank
Algorithm
Count
20
15
10
5
0
SyNOnly_CC_2_reg_iter_1_bleached_masked
SyNOnly_CC_4_reg_iter_1_bleached_masked
SyNOnly_CC_6_reg_iter_1_bleached_masked
SyNOnly_MI_32_reg_iter_1_bleached_masked
SyNOnly_CC_2_reg_iter_0_bleached_masked
SyNOnly_CC_4_reg_iter_0_bleached_masked
SyNOnly_CC_6_reg_iter_0_bleached_masked
SyNOnly_CC_2_reg_iter_1_normal_masked
SyNOnly_MI_32_reg_iter_0_bleached_masked
SyNOnly_CC_4_reg_iter_1_normal_masked
SyNOnly_CC_6_reg_iter_1_normal_masked
SyNOnly_CC_2_reg_iter_0_normal_masked
TVMSQC_bleached_masked
SyNOnly_CC_4_reg_iter_0_normal_masked
SyNOnly_CC_6_reg_iter_0_normal_masked
SyNOnly_MI_32_reg_iter_1_normal_masked
SyNOnly_MI_32_reg_iter_0_normal_masked
TVMSQ_bleached_masked
TVMSQ_normal_masked
TVMSQC_normal_masked

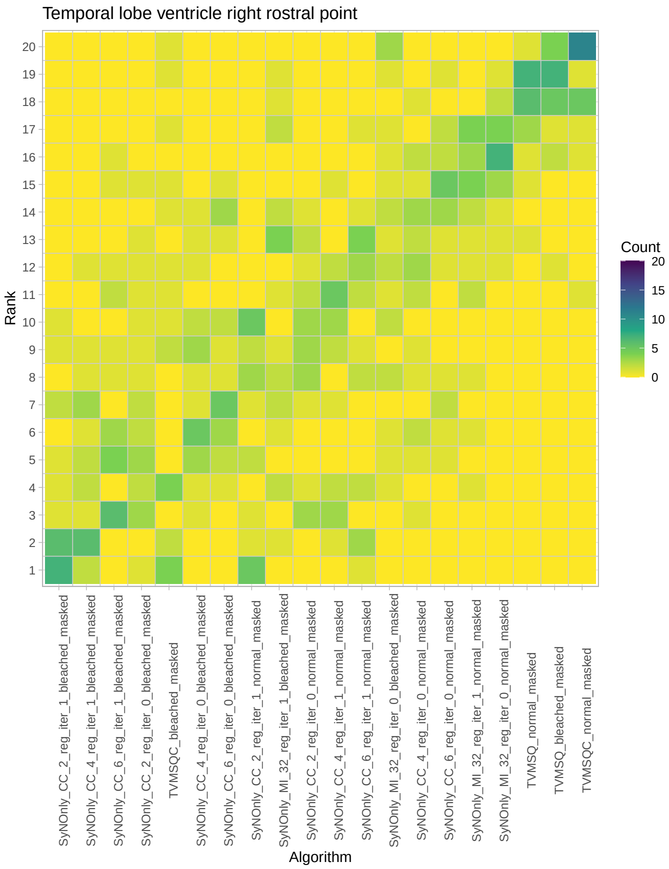
Temporal lobe ventricle right rostral point
Rank
Algorithm
Count
20
15
10
5
0
SyNOnly_CC_2_reg_iter_1_bleached_masked
SyNOnly_CC_4_reg_iter_1_bleached_masked
SyNOnly_CC_6_reg_iter_1_bleached_masked
SyNOnly_CC_2_reg_iter_0_bleached_masked
TVMSQC_bleached_masked
SyNOnly_CC_4_reg_iter_0_bleached_masked
SyNOnly_CC_6_reg_iter_0_bleached_masked
SyNOnly_CC_2_reg_iter_1_normal_masked
SyNOnly_MI_32_reg_iter_1_bleached_masked
SyNOnly_CC_2_reg_iter_0_normal_masked
SyNOnly_CC_4_reg_iter_1_normal_masked
SyNOnly_CC_6_reg_iter_1_normal_masked
SyNOnly_MI_32_reg_iter_0_bleached_masked
SyNOnly_CC_4_reg_iter_0_normal_masked
SyNOnly_CC_6_reg_iter_0_normal_masked
SyNOnly_MI_32_reg_iter_1_normal_masked
SyNOnly_MI_32_reg_iter_0_normal_masked
TVMSQ_normal_masked
TVMSQ_bleached_masked
TVMSQC_normal_masked

3 Visualization of ranking stability

3.1 *Blob plot* for visualizing ranking stability based on bootstrap sampling

Algorithms are color-coded, and the area of each blob at position $(A_i, \text{rank } j)$ is proportional to the relative frequency A_i achieved rank j across $b = 1000$ bootstrap samples. The median rank for each algorithm is indicated by a black cross. 95% bootstrap intervals across bootstrap samples are indicated by black lines.

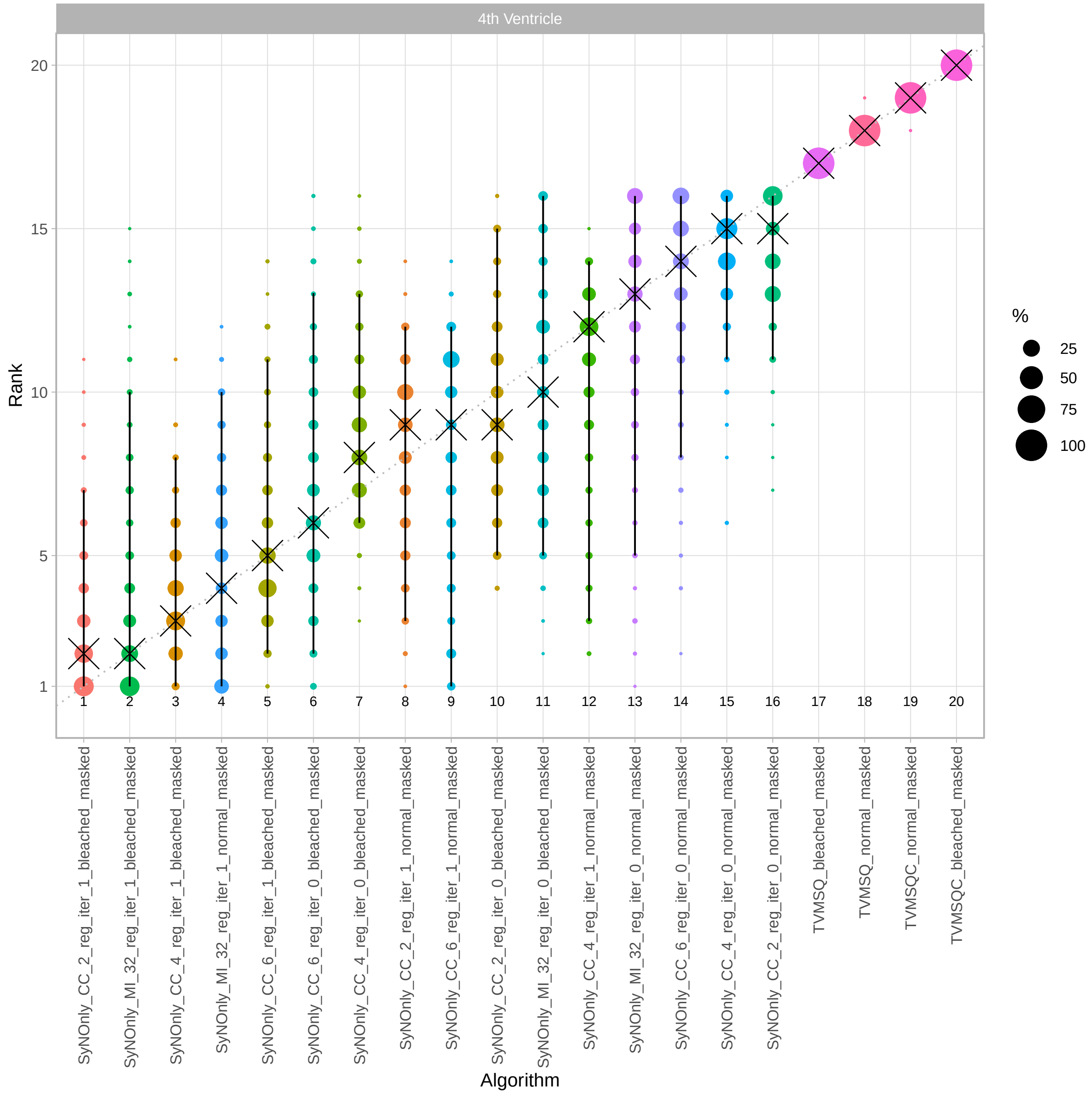

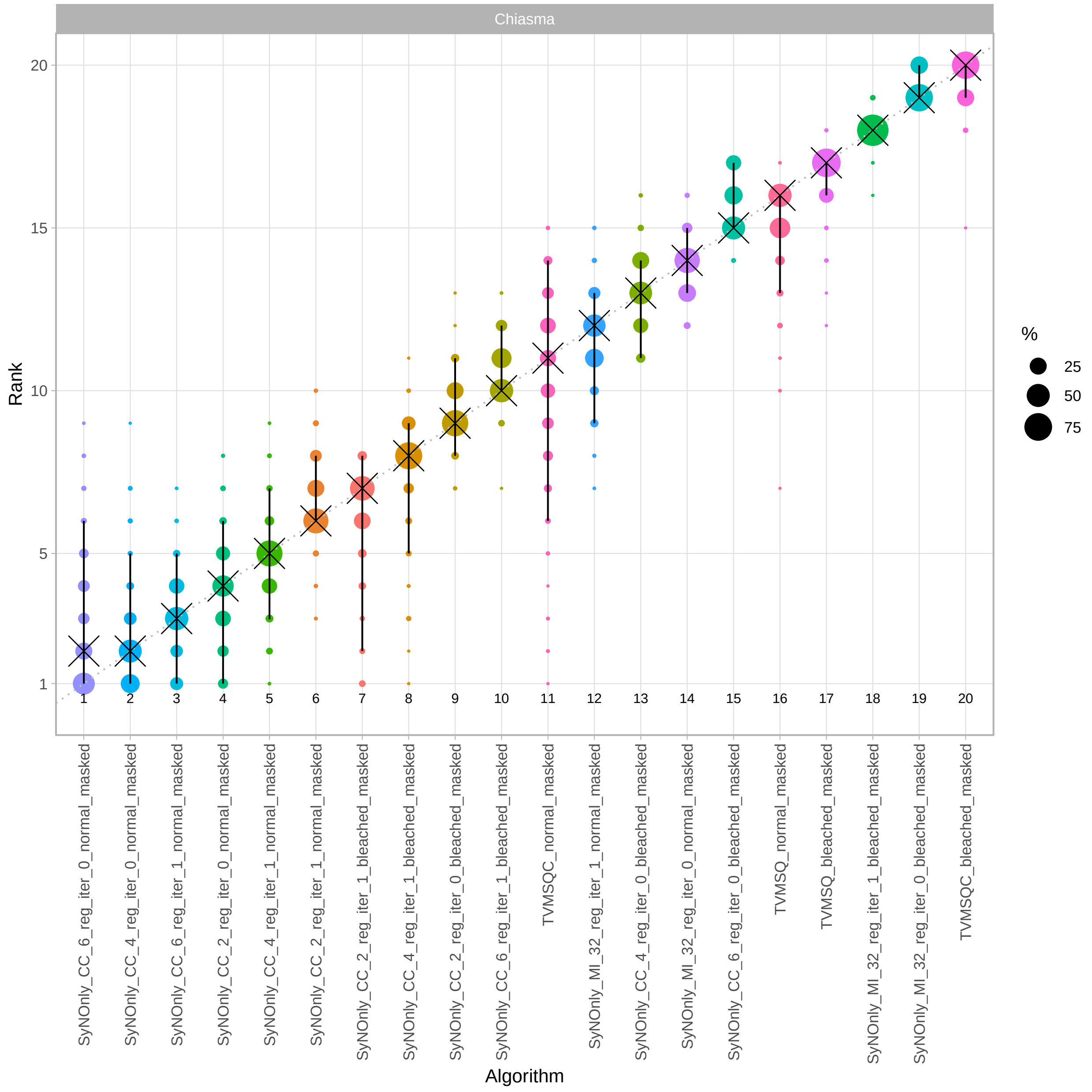

Chiasma
Rank
Algorithm
%
25
50
75
SyNOnly_CC_6_reg_iter_0_normal_masked
SyNOnly_CC_4_reg_iter_0_normal_masked
SyNOnly_CC_6_reg_iter_1_normal_masked
SyNOnly_CC_2_reg_iter_0_normal_masked
SyNOnly_CC_4_reg_iter_1_normal_masked
SyNOnly_CC_2_reg_iter_1_normal_masked
SyNOnly_CC_2_reg_iter_1_bleached_masked
SyNOnly_CC_4_reg_iter_1_bleached_masked
SyNOnly_CC_2_reg_iter_0_bleached_masked
SyNOnly_CC_6_reg_iter_1_bleached_masked
TVMSQC_normal_masked
SyNOnly_MI_32_reg_iter_1_normal_masked
SyNOnly_CC_4_reg_iter_0_bleached_masked
SyNOnly_MI_32_reg_iter_0_normal_masked
SyNOnly_CC_6_reg_iter_0_bleached_masked
TVMSQ_normal_masked
TVMSQ_bleached_masked
SyNOnly_MI_32_reg_iter_1_bleached_masked
SyNOnly_MI_32_reg_iter_0_bleached_masked
TVMSQC_bleached_masked

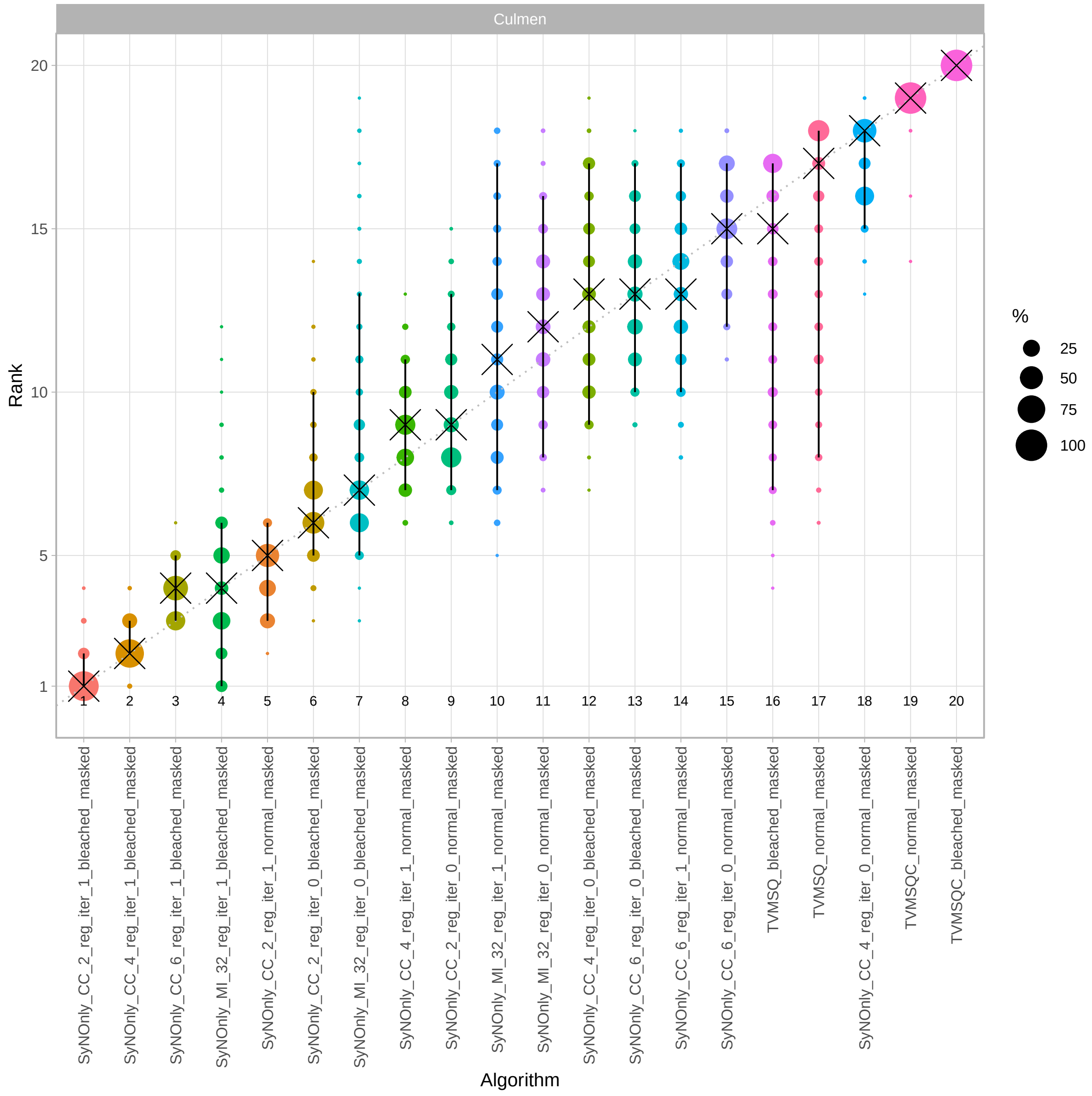
Culmen
Rank
Algorithm
%
25
50
75
100
SyNOnly_CC_2_reg_iter_1_bleached_masked
SyNOnly_CC_4_reg_iter_1_bleached_masked
SyNOnly_CC_6_reg_iter_1_bleached_masked
SyNOnly_MI_32_reg_iter_1_bleached_masked
SyNOnly_CC_2_reg_iter_1_normal_masked
SyNOnly_CC_2_reg_iter_0_bleached_masked
SyNOnly_MI_32_reg_iter_0_bleached_masked
SyNOnly_CC_4_reg_iter_1_normal_masked
SyNOnly_CC_2_reg_iter_0_normal_masked
SyNOnly_MI_32_reg_iter_1_normal_masked
SyNOnly_MI_32_reg_iter_0_normal_masked
SyNOnly_CC_4_reg_iter_0_bleached_masked
SyNOnly_CC_6_reg_iter_0_bleached_masked
SyNOnly_CC_6_reg_iter_1_normal_masked
SyNOnly_CC_6_reg_iter_0_normal_masked
TVMSQ_bleached_masked
TVMSQ_normal_masked
SyNOnly_CC_4_reg_iter_0_normal_masked
TVMSQC_normal_masked
TVMSQC_bleached_masked

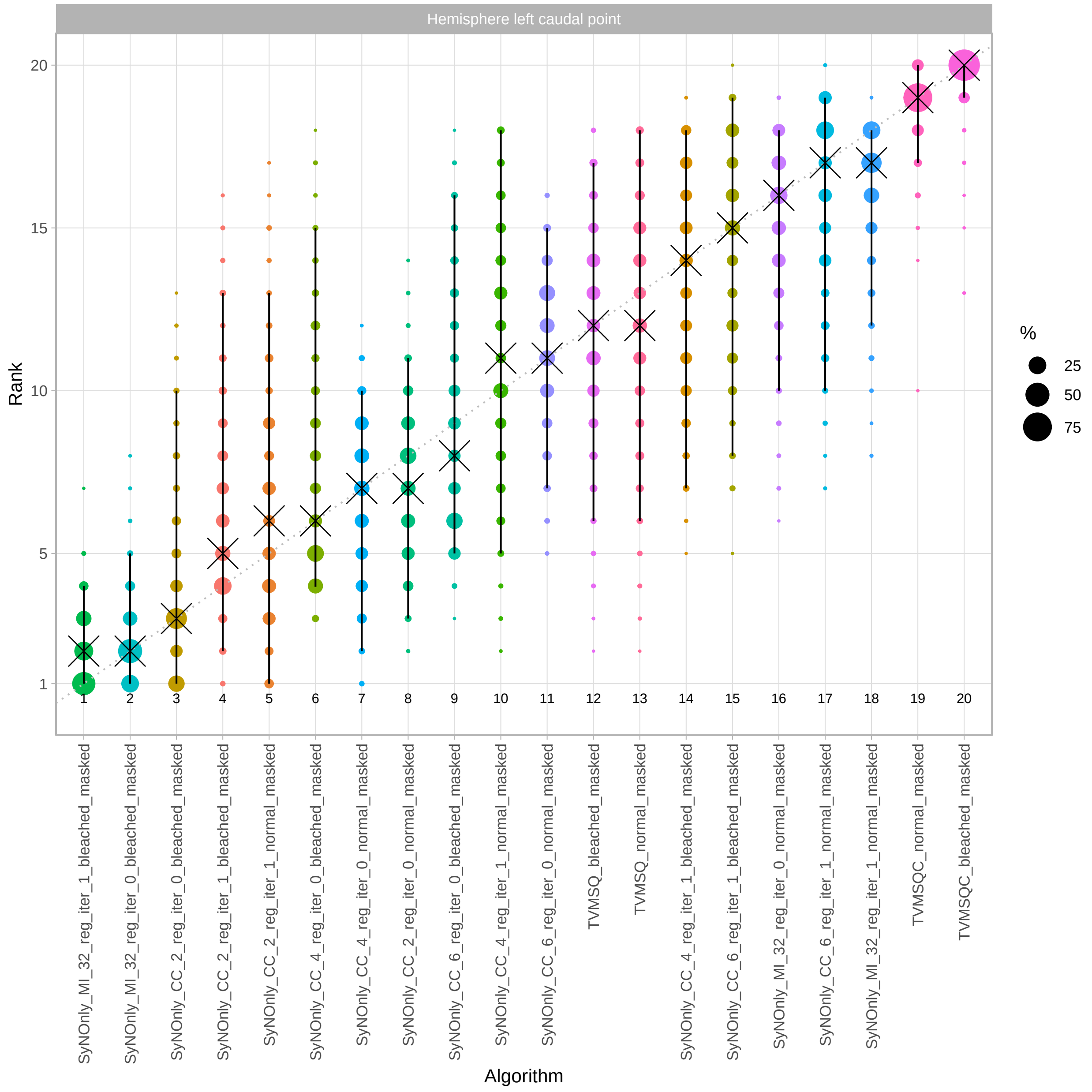
Hemisphere left caudal point
Rank
20
15
10
5
1
1 2 3 4 5 6 7 8 9 10 11 12 13 14 15 16 17 18 19 20
SyNOnly_MI_32_reg_iter_1_bleached_masked
SyNOnly_MI_32_reg_iter_0_bleached_masked
SyNOnly_CC_2_reg_iter_0_bleached_masked
SyNOnly_CC_2_reg_iter_1_bleached_masked
SyNOnly_CC_2_reg_iter_1_normal_masked
SyNOnly_CC_4_reg_iter_0_bleached_masked
SyNOnly_CC_4_reg_iter_0_normal_masked
SyNOnly_CC_2_reg_iter_0_normal_masked
SyNOnly_CC_6_reg_iter_0_bleached_masked
SyNOnly_CC_4_reg_iter_1_normal_masked
SyNOnly_CC_6_reg_iter_0_normal_masked
TVMSQ_bleached_masked
TVMSQ_normal_masked
SyNOnly_CC_4_reg_iter_1_bleached_masked
SyNOnly_CC_6_reg_iter_1_bleached_masked
SyNOnly_MI_32_reg_iter_0_normal_masked
SyNOnly_CC_6_reg_iter_1_normal_masked
SyNOnly_MI_32_reg_iter_1_normal_masked
TVMSQC_normal_masked
TVMSQC_bleached_masked
Algorithm
%
25
50
75

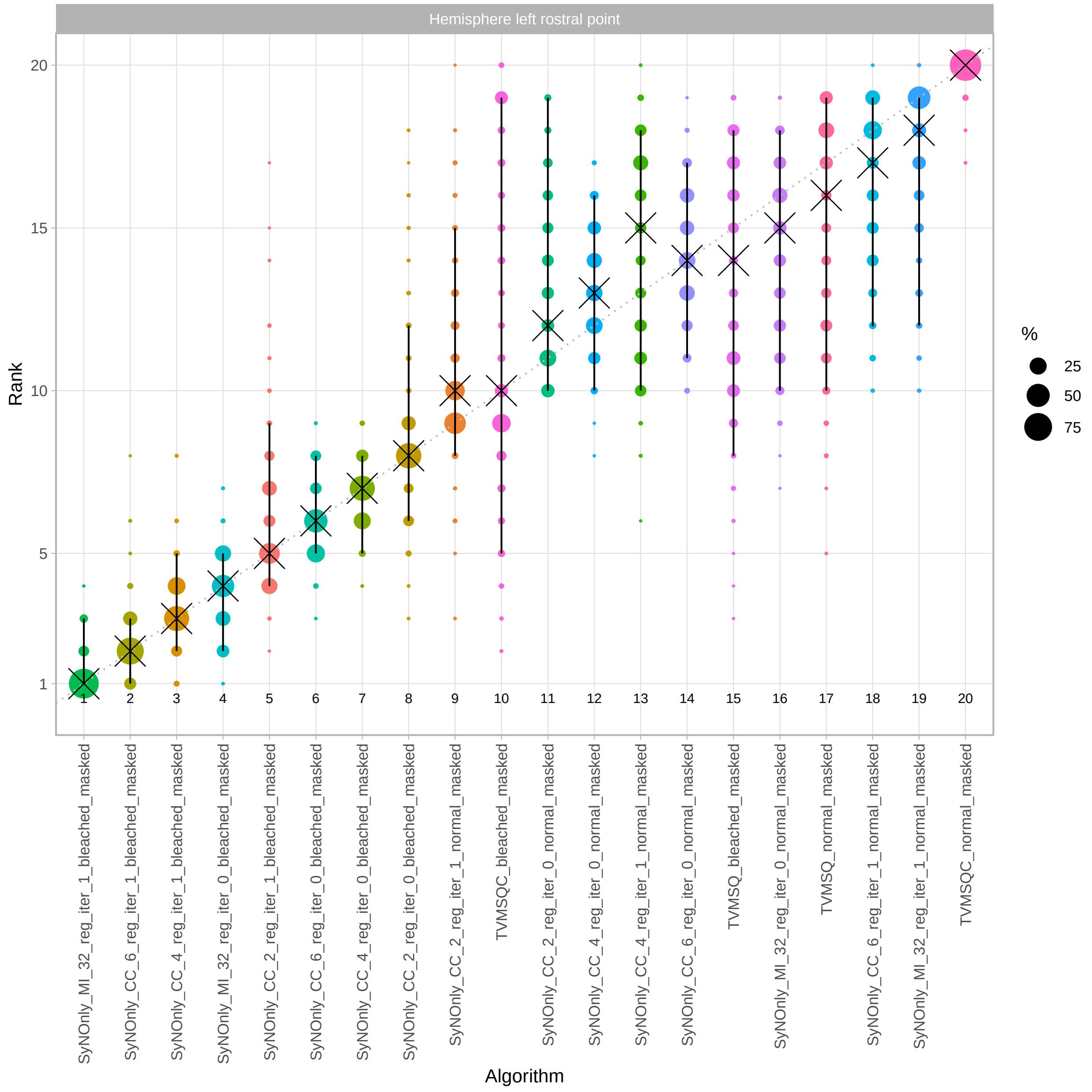

Hemisphere left rostral point
Rank
Algorithm
%
25
50
75
20
15
10
5
1
1 2 3 4 5 6 7 8 9 10 11 12 13 14 15 16 17 18 19 20
SyNOnly_MI_32_reg_iter_1_bleached_masked
SyNOnly_CC_6_reg_iter_1_bleached_masked
SyNOnly_CC_4_reg_iter_1_bleached_masked
SyNOnly_MI_32_reg_iter_0_bleached_masked
SyNOnly_CC_2_reg_iter_1_bleached_masked
SyNOnly_CC_6_reg_iter_0_bleached_masked
SyNOnly_CC_4_reg_iter_0_bleached_masked
SyNOnly_CC_2_reg_iter_0_bleached_masked
SyNOnly_CC_2_reg_iter_1_normal_masked
TVMSQC_bleached_masked
SyNOnly_CC_2_reg_iter_0_normal_masked
SyNOnly_CC_4_reg_iter_0_normal_masked
SyNOnly_CC_4_reg_iter_1_normal_masked
SyNOnly_CC_6_reg_iter_0_normal_masked
TVMSQ_bleached_masked
SyNOnly_MI_32_reg_iter_0_normal_masked
TVMSQ_normal_masked
SyNOnly_CC_6_reg_iter_1_normal_masked
SyNOnly_MI_32_reg_iter_1_normal_masked
TVMSQC_normal_masked

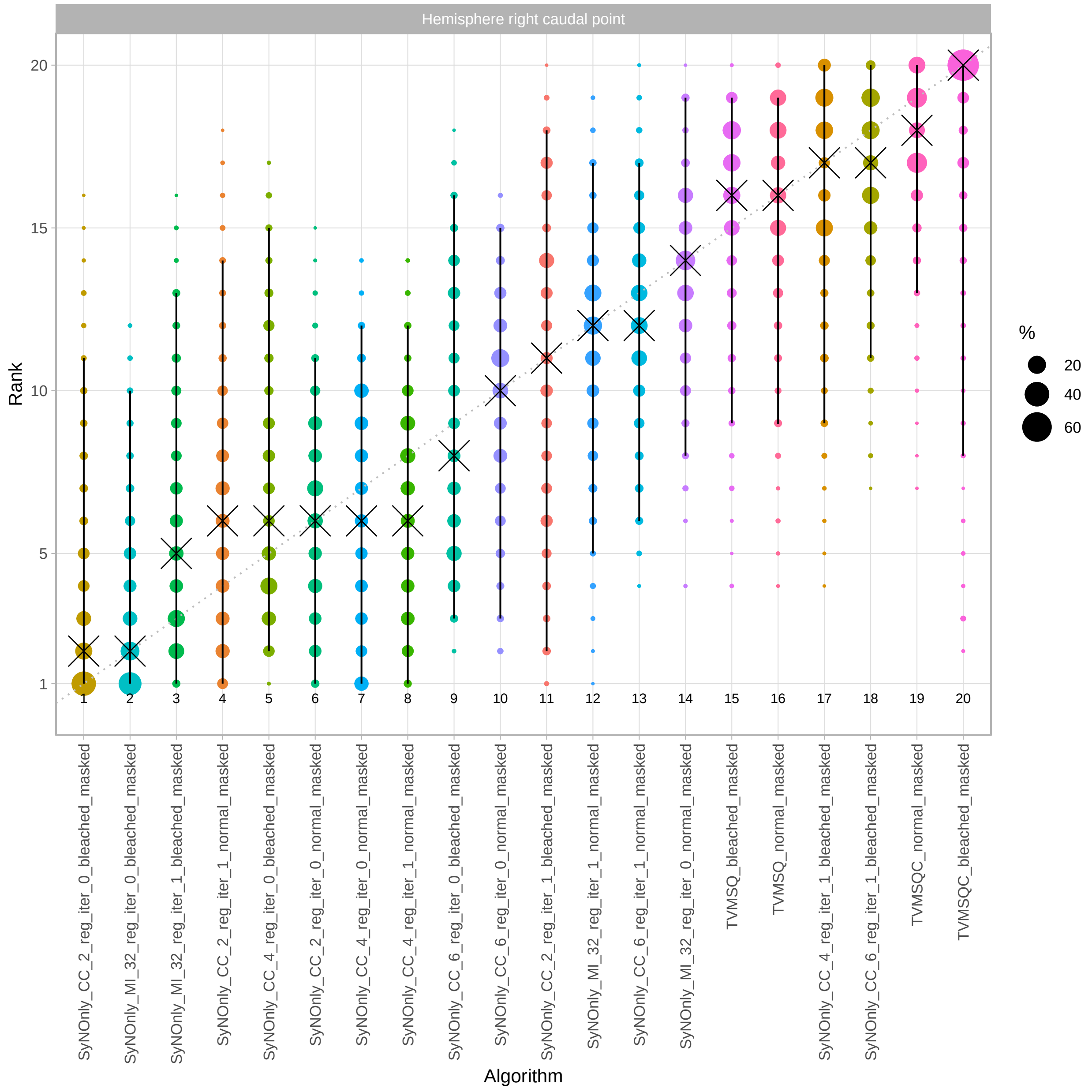
Hemisphere right caudal point
Rank
Algorithm
%
20
40
60
SyNOnly_CC_2_reg_iter_0_bleached_masked
SyNOnly_MI_32_reg_iter_0_bleached_masked
SyNOnly_MI_32_reg_iter_1_bleached_masked
SyNOnly_CC_2_reg_iter_1_normal_masked
SyNOnly_CC_4_reg_iter_0_bleached_masked
SyNOnly_CC_2_reg_iter_0_normal_masked
SyNOnly_CC_4_reg_iter_0_normal_masked
SyNOnly_CC_4_reg_iter_1_normal_masked
SyNOnly_CC_6_reg_iter_0_bleached_masked
SyNOnly_CC_6_reg_iter_0_normal_masked
SyNOnly_CC_2_reg_iter_1_bleached_masked
SyNOnly_MI_32_reg_iter_1_normal_masked
SyNOnly_CC_6_reg_iter_1_normal_masked
SyNOnly_MI_32_reg_iter_0_normal_masked
TVMSQ_bleached_masked
TVMSQ_normal_masked
SyNOnly_CC_4_reg_iter_1_bleached_masked
SyNOnly_CC_6_reg_iter_1_bleached_masked
TVMSQC_normal_masked
TVMSQC_bleached_masked

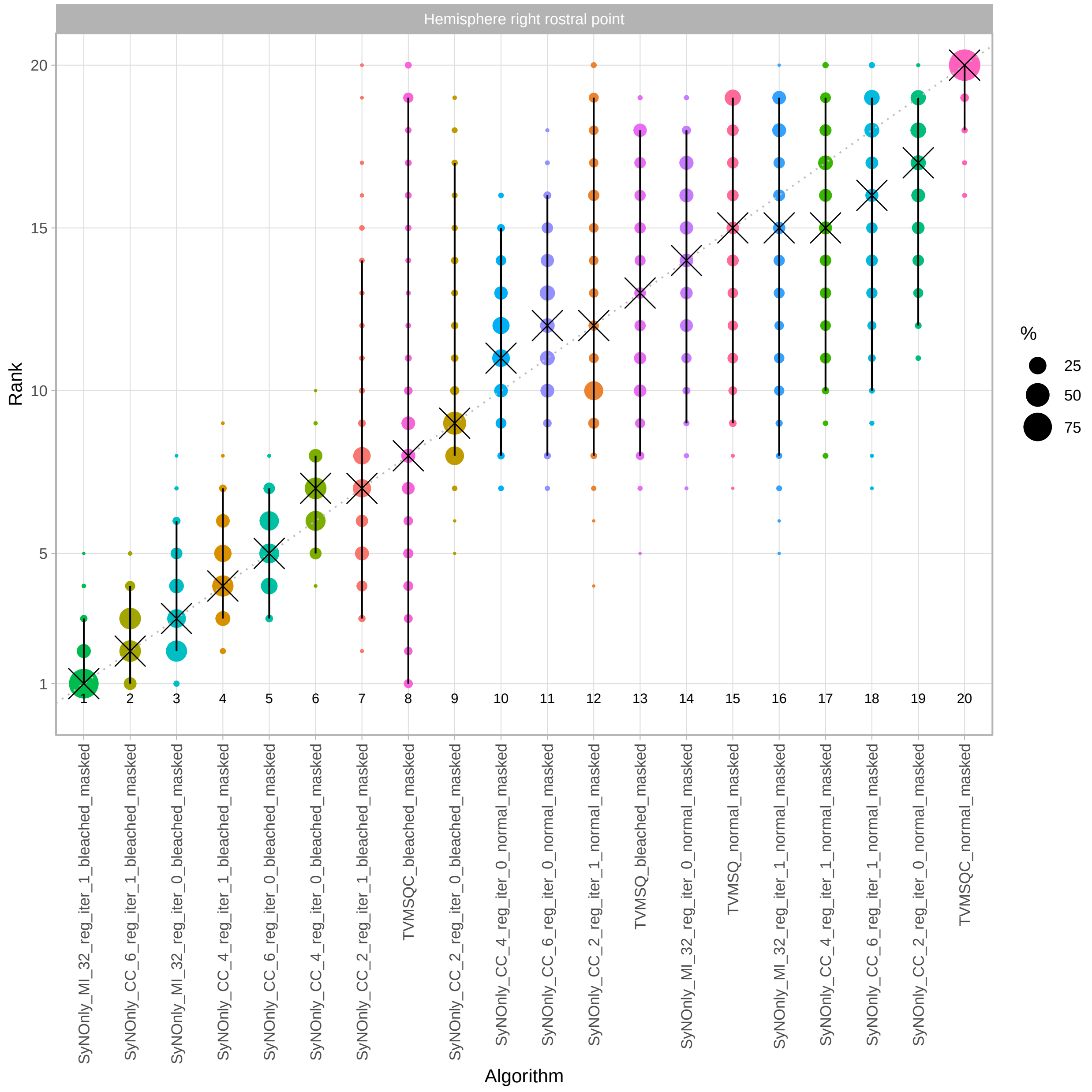

Hemisphere right rostral point
Rank
Algorithm
%
25
50
75
SyNOnly_MI_32_reg_iter_1_bleached_masked
SyNOnly_CC_6_reg_iter_1_bleached_masked
SyNOnly_MI_32_reg_iter_0_bleached_masked
SyNOnly_CC_4_reg_iter_1_bleached_masked
SyNOnly_CC_6_reg_iter_0_bleached_masked
SyNOnly_CC_4_reg_iter_0_bleached_masked
SyNOnly_CC_2_reg_iter_1_bleached_masked
TVMSQC_bleached_masked
SyNOnly_CC_2_reg_iter_0_bleached_masked
SyNOnly_CC_4_reg_iter_0_normal_masked
SyNOnly_CC_6_reg_iter_0_normal_masked
SyNOnly_CC_2_reg_iter_1_normal_masked
TVMSQ_bleached_masked
SyNOnly_MI_32_reg_iter_0_normal_masked
TVMSQ_normal_masked
SyNOnly_MI_32_reg_iter_1_normal_masked
SyNOnly_CC_4_reg_iter_1_normal_masked
SyNOnly_CC_6_reg_iter_1_normal_masked
SyNOnly_CC_2_reg_iter_0_normal_masked
TVMSQC_normal_masked

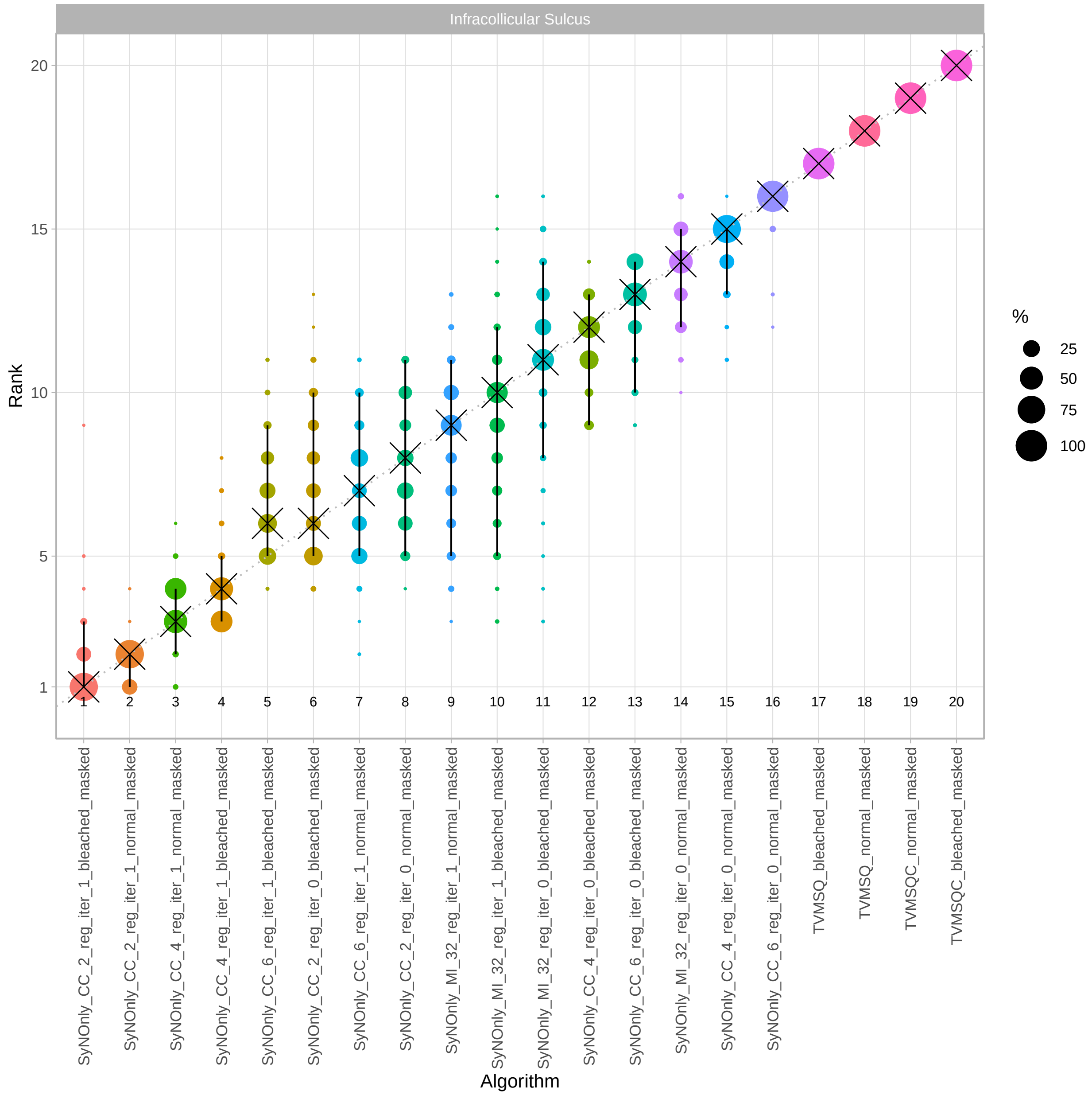

Infracollicular Sulcus
Rank
Algorithm
%
25
50
75
100
SyNOnly_CC_2_reg_iter_1_bleached_masked
SyNOnly_CC_2_reg_iter_1_normal_masked
SyNOnly_CC_4_reg_iter_1_normal_masked
SyNOnly_CC_4_reg_iter_1_bleached_masked
SyNOnly_CC_6_reg_iter_1_bleached_masked
SyNOnly_CC_2_reg_iter_0_bleached_masked
SyNOnly_CC_6_reg_iter_1_normal_masked
SyNOnly_CC_2_reg_iter_0_normal_masked
SyNOnly_MI_32_reg_iter_1_normal_masked
SyNOnly_MI_32_reg_iter_1_bleached_masked
SyNOnly_MI_32_reg_iter_0_bleached_masked
SyNOnly_CC_4_reg_iter_0_bleached_masked
SyNOnly_CC_6_reg_iter_0_bleached_masked
SyNOnly_MI_32_reg_iter_0_normal_masked
SyNOnly_CC_4_reg_iter_0_normal_masked
SyNOnly_CC_6_reg_iter_0_normal_masked
TVMSQ_bleached_masked
TVMSQ_normal_masked
TVMSQC_normal_masked
TVMSQC_bleached_masked

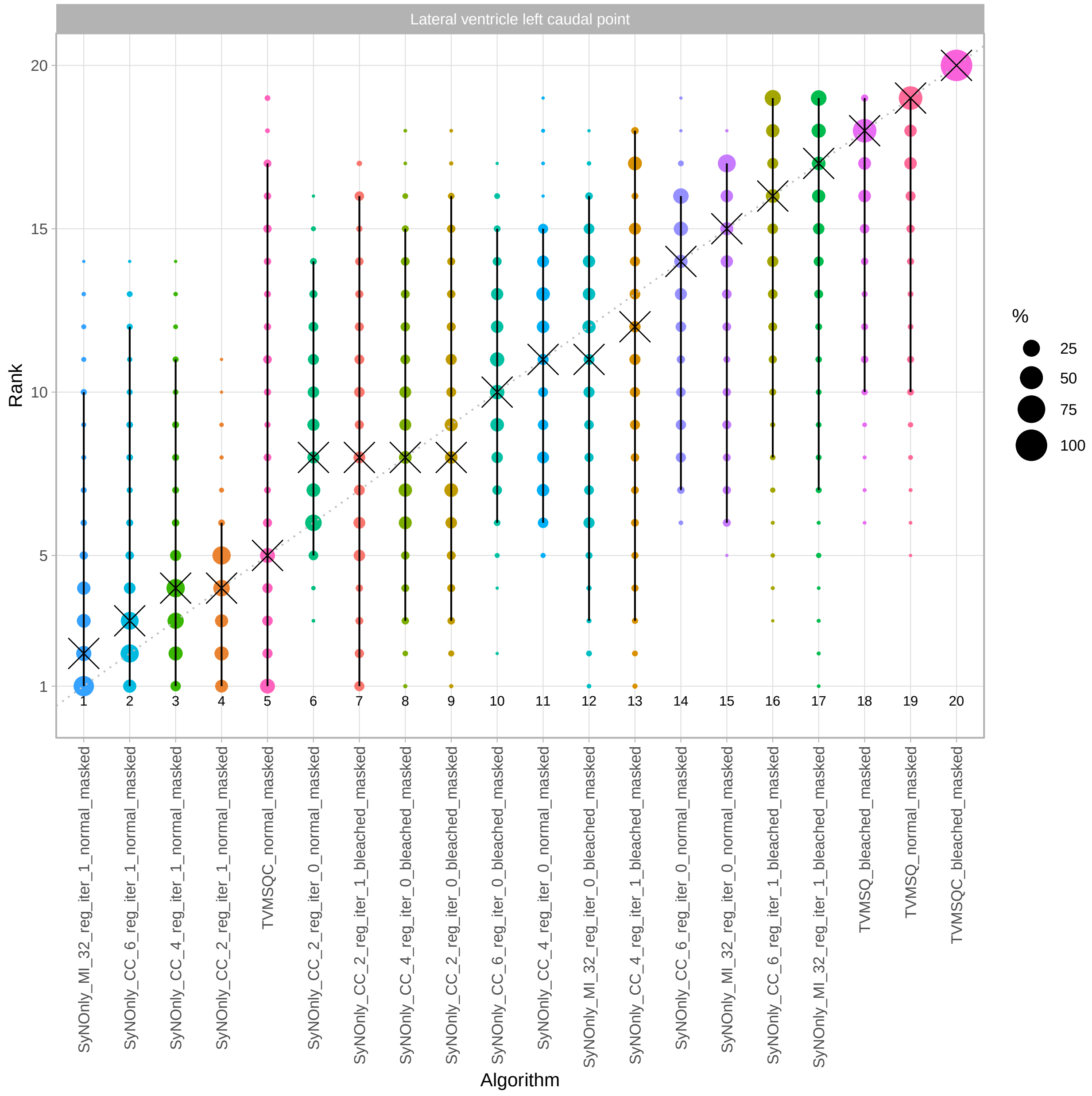

Lateral ventricle left caudal point
Rank
Algorithm
%
25
50
75
100
SyNOnly_MI_32_reg_iter_1_normal_masked
SyNOnly_CC_6_reg_iter_1_normal_masked
SyNOnly_CC_4_reg_iter_1_normal_masked
SyNOnly_CC_2_reg_iter_1_normal_masked
TVMSQC_normal_masked
SyNOnly_CC_2_reg_iter_0_normal_masked
SyNOnly_CC_2_reg_iter_1_bleached_masked
SyNOnly_CC_4_reg_iter_0_bleached_masked
SyNOnly_CC_2_reg_iter_0_bleached_masked
SyNOnly_CC_6_reg_iter_0_bleached_masked
SyNOnly_CC_4_reg_iter_0_normal_masked
SyNOnly_MI_32_reg_iter_0_bleached_masked
SyNOnly_CC_4_reg_iter_1_bleached_masked
SyNOnly_CC_6_reg_iter_0_normal_masked
SyNOnly_MI_32_reg_iter_0_normal_masked
SyNOnly_CC_6_reg_iter_1_bleached_masked
SyNOnly_MI_32_reg_iter_1_bleached_masked
TVMSQ_bleached_masked
TVMSQ_normal_masked
TVMSQC_bleached_masked

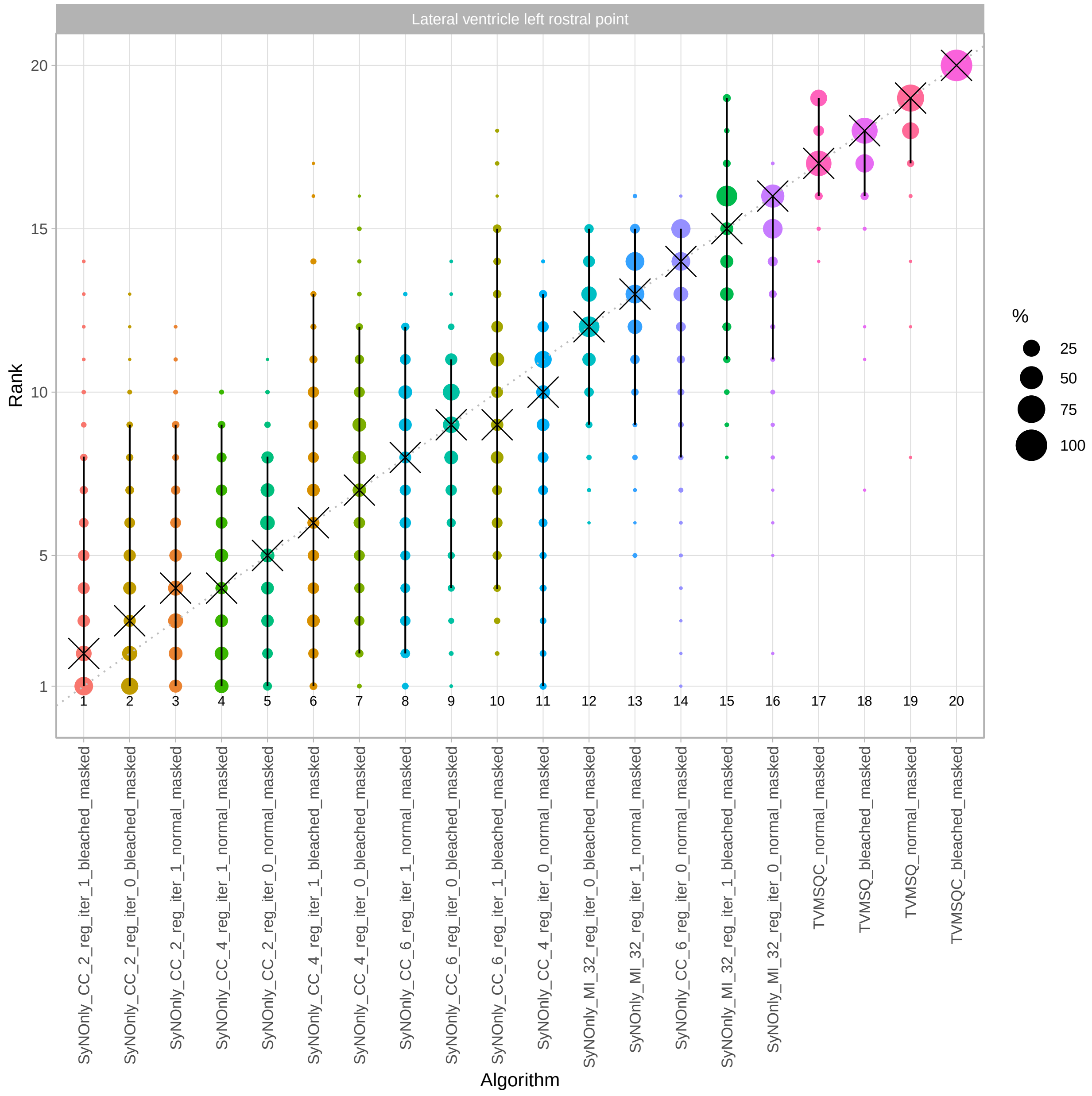
Lateral ventricle left rostral point
Rank
Algorithm
%
25
50
75
100
SyNOnly_CC_2_reg_iter_1_bleached_masked
SyNOnly_CC_2_reg_iter_0_bleached_masked
SyNOnly_CC_2_reg_iter_1_normal_masked
SyNOnly_CC_4_reg_iter_1_normal_masked
SyNOnly_CC_2_reg_iter_0_normal_masked
SyNOnly_CC_4_reg_iter_1_bleached_masked
SyNOnly_CC_4_reg_iter_0_bleached_masked
SyNOnly_CC_6_reg_iter_1_normal_masked
SyNOnly_CC_6_reg_iter_0_bleached_masked
SyNOnly_CC_6_reg_iter_1_bleached_masked
SyNOnly_CC_4_reg_iter_0_normal_masked
SyNOnly_MI_32_reg_iter_0_bleached_masked
SyNOnly_MI_32_reg_iter_1_normal_masked
SyNOnly_CC_6_reg_iter_0_normal_masked
SyNOnly_MI_32_reg_iter_1_bleached_masked
SyNOnly_MI_32_reg_iter_0_normal_masked
TVMSQC_normal_masked
TVMSQ_bleached_masked
TVMSQ_normal_masked
TVMSQC_bleached_masked

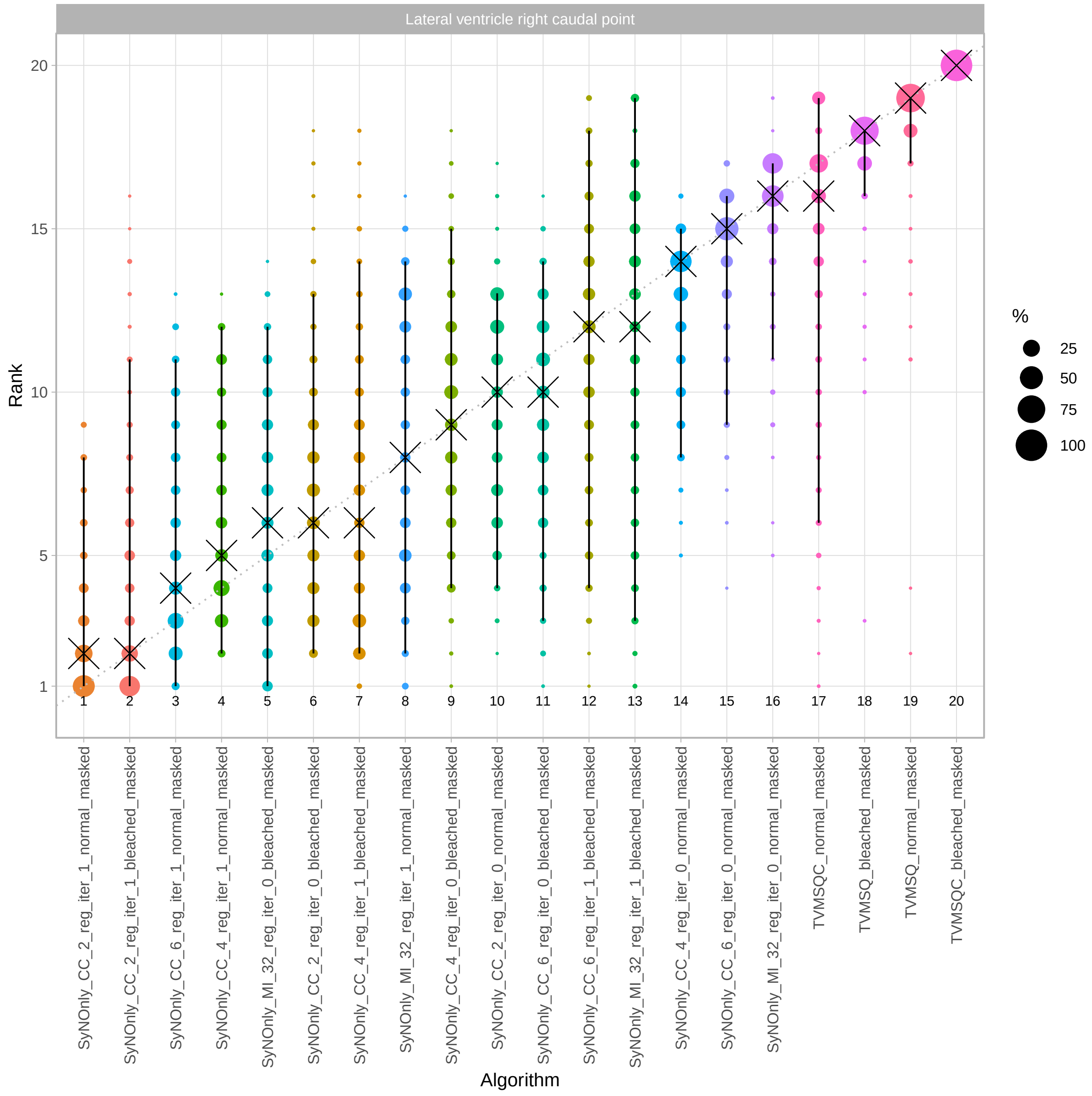

Lateral ventricle right caudal point
Rank
Algorithm
%
25
50
75
100
SyNOnly_CC_2_reg_iter_1_normal_masked
SyNOnly_CC_2_reg_iter_1_bleached_masked
SyNOnly_CC_6_reg_iter_1_normal_masked
SyNOnly_CC_4_reg_iter_1_normal_masked
SyNOnly_MI_32_reg_iter_0_bleached_masked
SyNOnly_CC_2_reg_iter_0_bleached_masked
SyNOnly_CC_4_reg_iter_1_bleached_masked
SyNOnly_MI_32_reg_iter_1_normal_masked
SyNOnly_CC_4_reg_iter_0_bleached_masked
SyNOnly_CC_2_reg_iter_0_normal_masked
SyNOnly_CC_6_reg_iter_0_bleached_masked
SyNOnly_CC_6_reg_iter_1_bleached_masked
SyNOnly_MI_32_reg_iter_1_bleached_masked
SyNOnly_CC_4_reg_iter_0_normal_masked
SyNOnly_CC_6_reg_iter_0_normal_masked
SyNOnly_MI_32_reg_iter_0_normal_masked
TVMSQC_normal_masked
TVMSQ_bleached_masked
TVMSQ_normal_masked
TVMSQC_bleached_masked

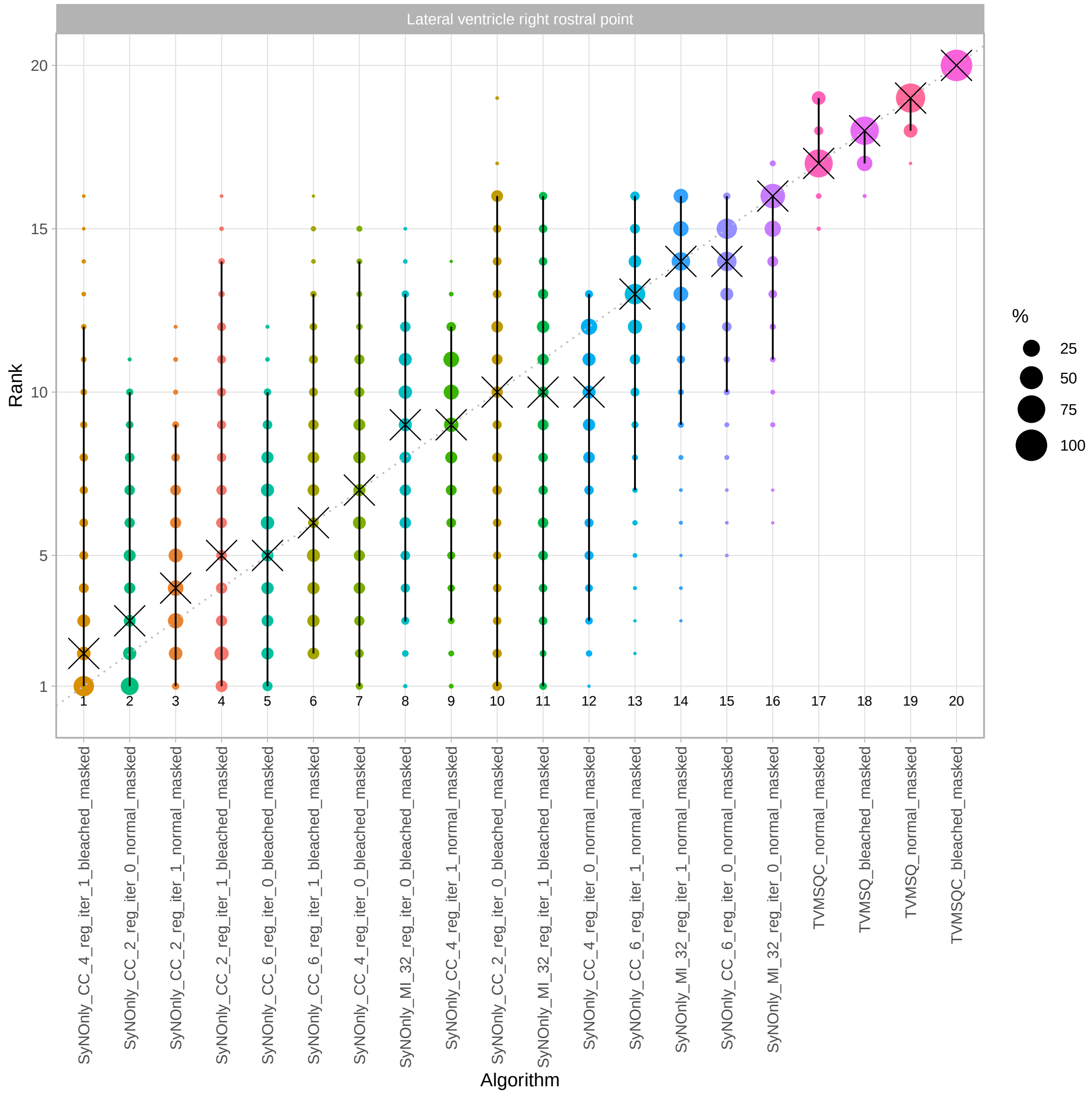
Lateral ventricle right rostral point
Rank
20
15
10
5
1
1 2 3 4 5 6 7 8 9 10 11 12 13 14 15 16 17 18 19 20
%
25
50
75
100
SyNOnly_CC_4_reg_iter_1_bleached_masked
SyNOnly_CC_2_reg_iter_0_normal_masked
SyNOnly_CC_2_reg_iter_1_normal_masked
SyNOnly_CC_2_reg_iter_1_bleached_masked
SyNOnly_CC_6_reg_iter_0_bleached_masked
SyNOnly_CC_6_reg_iter_1_bleached_masked
SyNOnly_CC_4_reg_iter_0_bleached_masked
SyNOnly_MI_32_reg_iter_0_bleached_masked
SyNOnly_CC_4_reg_iter_1_normal_masked
SyNOnly_CC_2_reg_iter_0_bleached_masked
SyNOnly_MI_32_reg_iter_1_bleached_masked
SyNOnly_CC_4_reg_iter_0_normal_masked
SyNOnly_CC_6_reg_iter_1_normal_masked
SyNOnly_MI_32_reg_iter_1_normal_masked
SyNOnly_CC_6_reg_iter_0_normal_masked
SyNOnly_MI_32_reg_iter_0_normal_masked
TVMSQC_normal_masked
TVMSQ_bleached_masked
TVMSQ_normal_masked
TVMSQC_bleached_masked
Algorithm

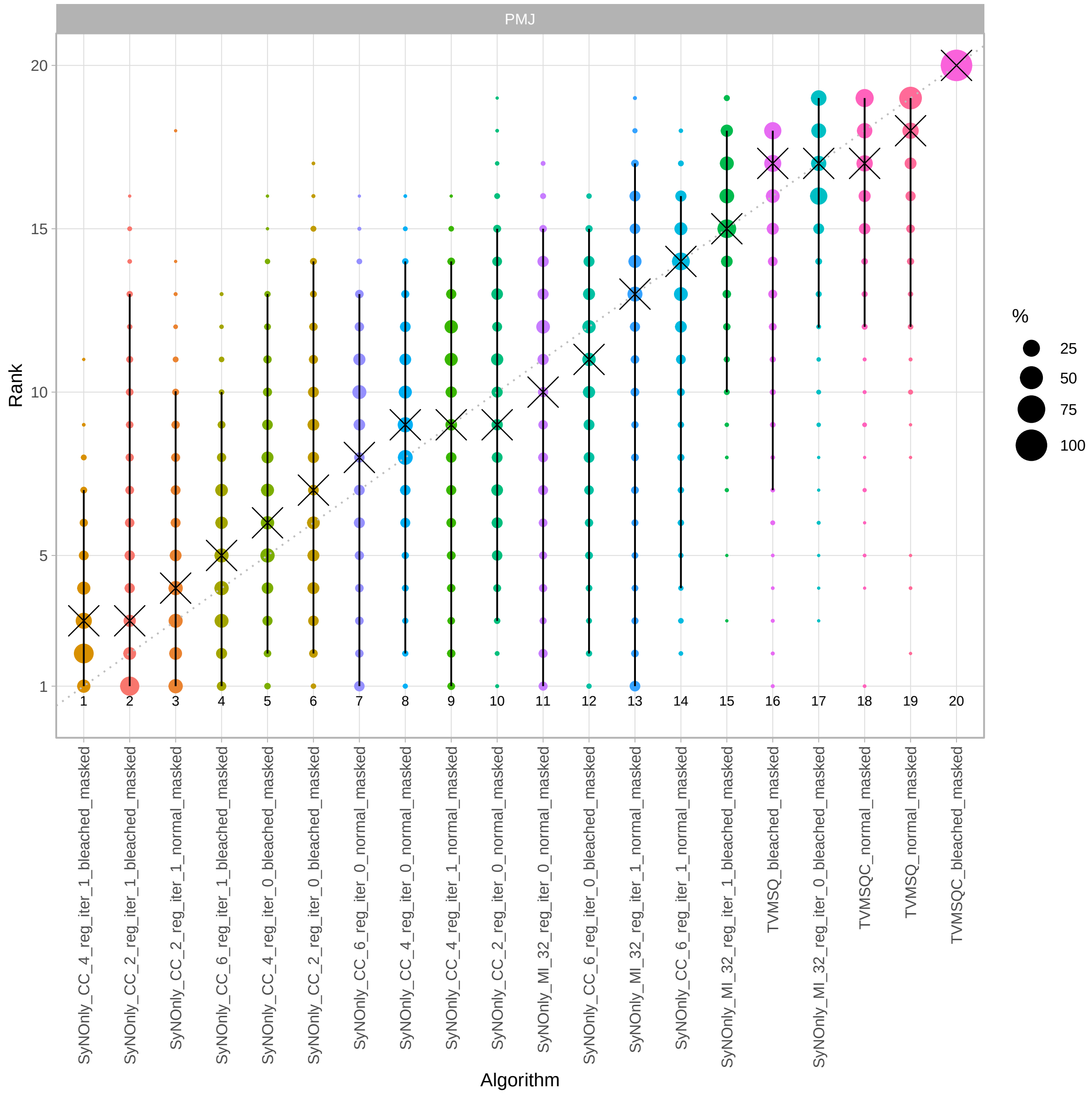
PMJ
Rank
Algorithm
%
25
50
75
100
SyNOnly_CC_4_reg_iter_1_bleached_masked
SyNOnly_CC_2_reg_iter_1_bleached_masked
SyNOnly_CC_2_reg_iter_1_normal_masked
SyNOnly_CC_6_reg_iter_1_bleached_masked
SyNOnly_CC_4_reg_iter_0_bleached_masked
SyNOnly_CC_2_reg_iter_0_bleached_masked
SyNOnly_CC_6_reg_iter_0_normal_masked
SyNOnly_CC_4_reg_iter_0_normal_masked
SyNOnly_CC_4_reg_iter_1_normal_masked
SyNOnly_CC_2_reg_iter_0_normal_masked
SyNOnly_MI_32_reg_iter_0_normal_masked
SyNOnly_CC_6_reg_iter_0_bleached_masked
SyNOnly_MI_32_reg_iter_1_normal_masked
SyNOnly_CC_6_reg_iter_1_normal_masked
SyNOnly_MI_32_reg_iter_1_bleached_masked
TVMSQ_bleached_masked
SyNOnly_MI_32_reg_iter_0_bleached_masked
TVMSQC_normal_masked
TVMSQ_normal_masked
TVMSQC_bleached_masked

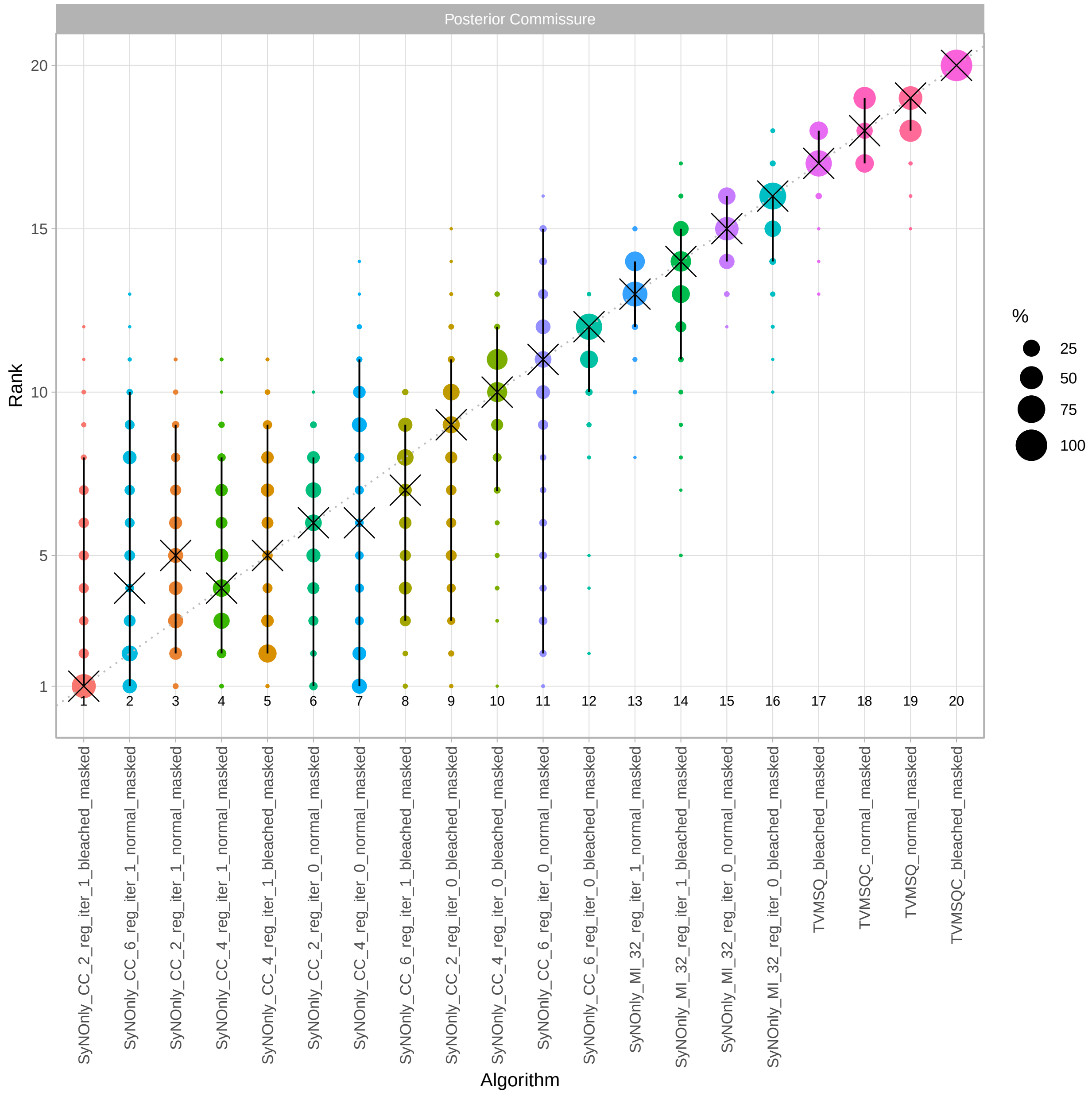
Posterior Commissure
Rank
Algorithm
%
25
50
75
100
SyNOnly_CC_2_reg_iter_1_bleached_masked
SyNOnly_CC_6_reg_iter_1_normal_masked
SyNOnly_CC_2_reg_iter_1_normal_masked
SyNOnly_CC_4_reg_iter_1_normal_masked
SyNOnly_CC_4_reg_iter_1_bleached_masked
SyNOnly_CC_2_reg_iter_0_normal_masked
SyNOnly_CC_4_reg_iter_0_normal_masked
SyNOnly_CC_6_reg_iter_1_bleached_masked
SyNOnly_CC_2_reg_iter_0_bleached_masked
SyNOnly_CC_4_reg_iter_0_bleached_masked
SyNOnly_CC_6_reg_iter_0_normal_masked
SyNOnly_CC_6_reg_iter_0_bleached_masked
SyNOnly_MI_32_reg_iter_1_normal_masked
SyNOnly_MI_32_reg_iter_1_bleached_masked
SyNOnly_MI_32_reg_iter_0_normal_masked
SyNOnly_MI_32_reg_iter_0_bleached_masked
TVMSQ_bleached_masked
TVMSQC_normal_masked
TVMSQ_normal_masked
TVMSQC_bleached_masked

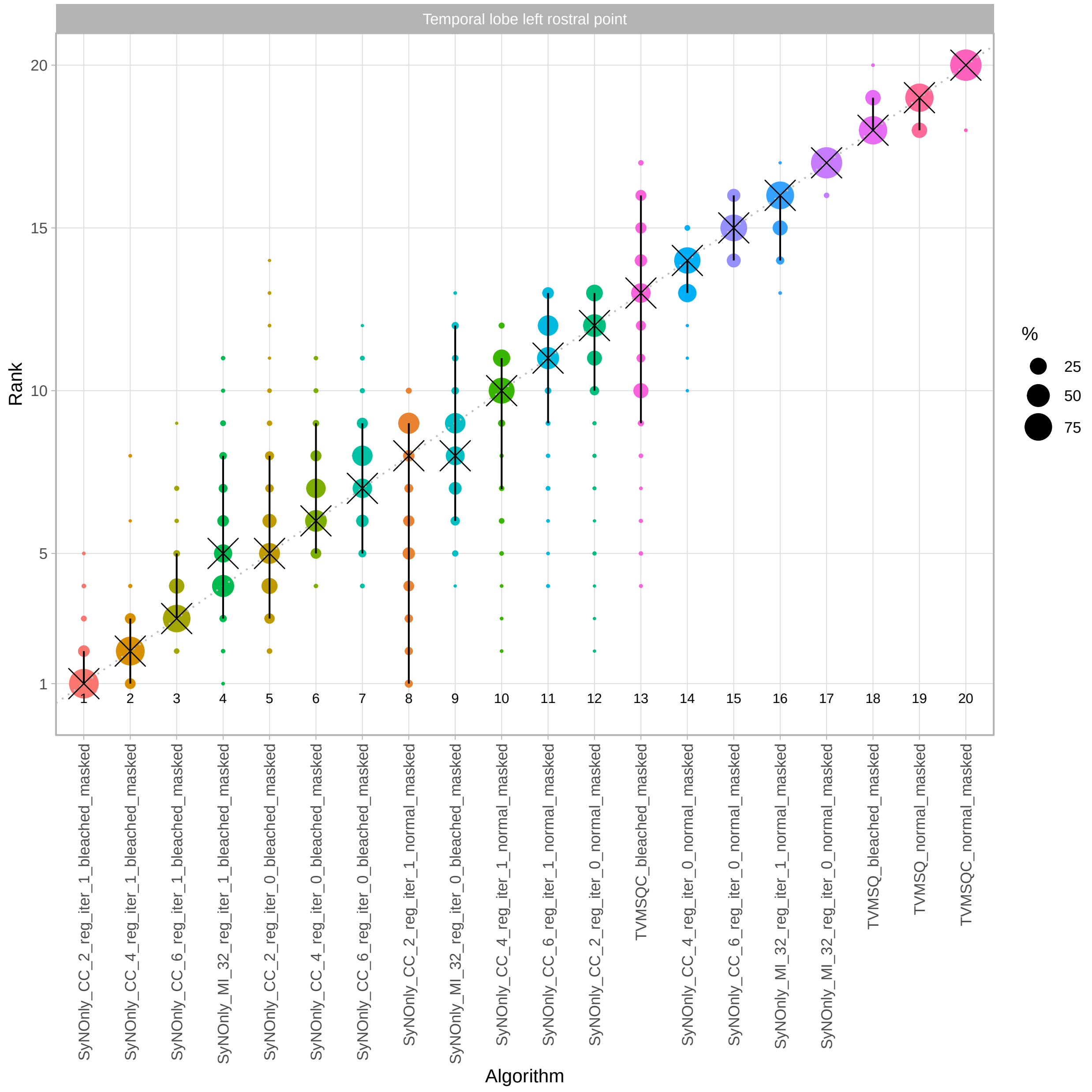

Temporal lobe left rostral point
Rank
Algorithm
%
25
50
75
SyNOnly_CC_2_reg_iter_1_bleached_masked
SyNOnly_CC_4_reg_iter_1_bleached_masked
SyNOnly_CC_6_reg_iter_1_bleached_masked
SyNOnly_MI_32_reg_iter_1_bleached_masked
SyNOnly_CC_2_reg_iter_0_bleached_masked
SyNOnly_CC_4_reg_iter_0_bleached_masked
SyNOnly_CC_6_reg_iter_0_bleached_masked
SyNOnly_CC_2_reg_iter_1_normal_masked
SyNOnly_MI_32_reg_iter_0_bleached_masked
SyNOnly_CC_4_reg_iter_1_normal_masked
SyNOnly_CC_6_reg_iter_1_normal_masked
SyNOnly_CC_2_reg_iter_0_normal_masked
TVMSQC_bleached_masked
SyNOnly_CC_4_reg_iter_0_normal_masked
SyNOnly_CC_6_reg_iter_0_normal_masked
SyNOnly_MI_32_reg_iter_1_normal_masked
SyNOnly_MI_32_reg_iter_0_normal_masked
TVMSQ_bleached_masked
TVMSQ_normal_masked
TVMSQC_normal_masked

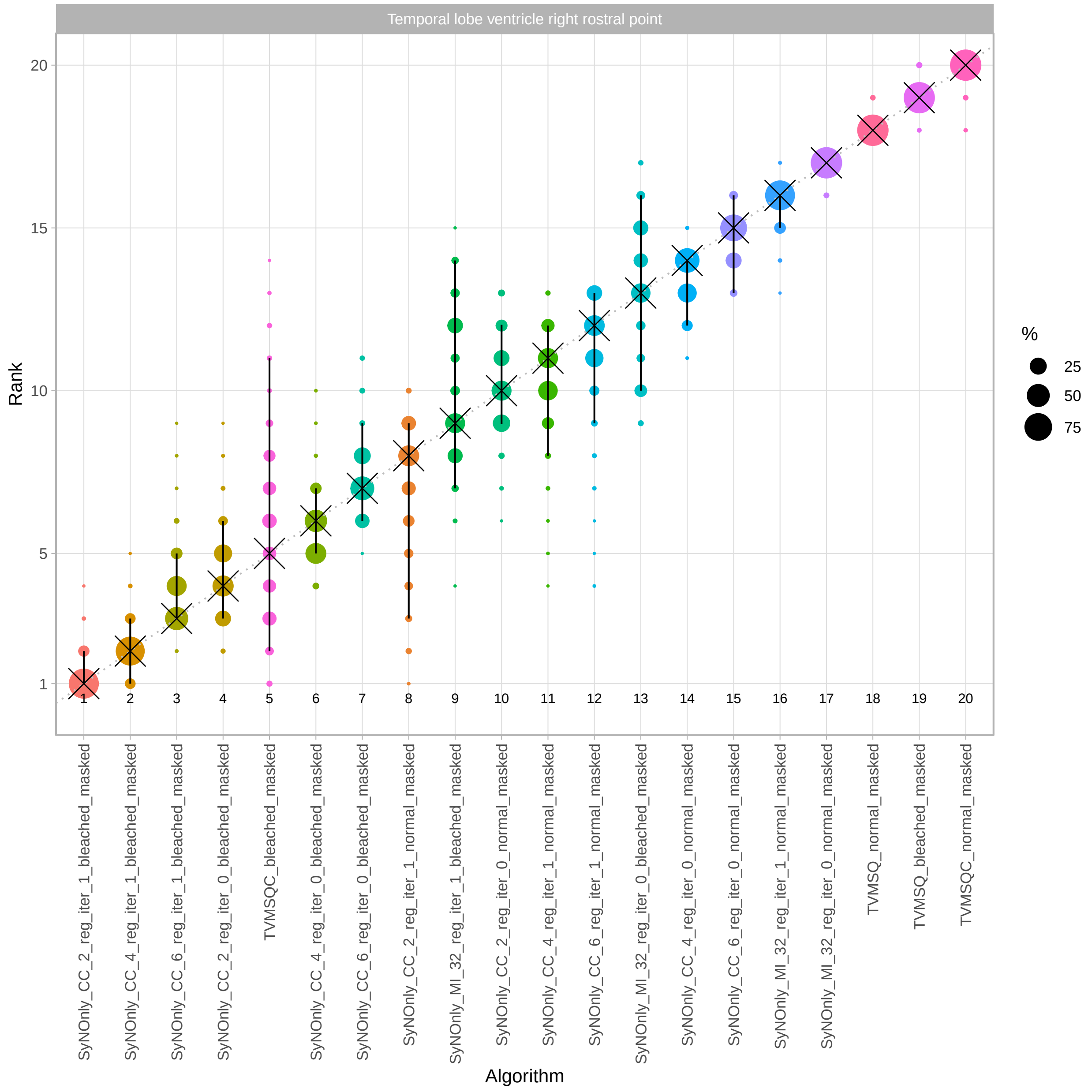

Temporal lobe ventricle right rostral point
Rank
Algorithm
%
25
50
75
SyNOnly_CC_2_reg_iter_1_bleached_masked
SyNOnly_CC_4_reg_iter_1_bleached_masked
SyNOnly_CC_6_reg_iter_1_bleached_masked
SyNOnly_CC_2_reg_iter_0_bleached_masked
TVMSQC_bleached_masked
SyNOnly_CC_4_reg_iter_0_bleached_masked
SyNOnly_CC_6_reg_iter_0_bleached_masked
SyNOnly_CC_2_reg_iter_1_normal_masked
SyNOnly_MI_32_reg_iter_1_bleached_masked
SyNOnly_CC_2_reg_iter_0_normal_masked
SyNOnly_CC_4_reg_iter_1_normal_masked
SyNOnly_CC_6_reg_iter_1_normal_masked
SyNOnly_MI_32_reg_iter_0_bleached_masked
SyNOnly_CC_4_reg_iter_0_normal_masked
SyNOnly_CC_6_reg_iter_0_normal_masked
SyNOnly_MI_32_reg_iter_1_normal_masked
SyNOnly_MI_32_reg_iter_0_normal_masked
TVMSQ_normal_masked
TVMSQ_bleached_masked
TVMSQC_normal_masked

3.2 *Violin plot* for visualizing ranking stability based on bootstrapping

The ranking list based on the full assessment data is pairwise compared with the ranking lists based on the individual bootstrap samples (here $b = 1000$ samples). For each pair of rankings, Kendall's τ correlation is computed. Kendall's τ is a scaled index determining the correlation between the lists. It is computed by evaluating the number of pairwise concordances and discordances between ranking lists and produces values between -1 (for inverted order) and 1 (for identical order). A violin plot, which simultaneously depicts a boxplot and a density plot, is generated from the results.

Summary Kendall's tau:

Task	mean	median	q25	q75
Temporal lobe left rostral point	0.9305263	0.9473684	0.9157895	0.9578947
Chiasma	0.9313895	0.9368421	0.9157895	0.9578947
Infracollicular Sulcus	0.9263263	0.9368421	0.9052632	0.9578947
Temporal lobe ventricle right rostral point	0.9310947	0.9368421	0.9052632	0.9684211
Culmen	0.8520211	0.8736842	0.8105263	0.9052632
4th Ventricle	0.8096737	0.8315789	0.7789474	0.8736842
Hemisphere left rostral point	0.8191263	0.8315789	0.7789474	0.8842105
Lateral ventricle left rostral point	0.8110737	0.8315789	0.7789474	0.8736842
Posterior Commissure	0.8302421	0.8315789	0.7789474	0.8842105
Hemisphere left caudal point	0.7706316	0.7894737	0.7157895	0.8421053
Hemisphere right rostral point	0.7735579	0.7894737	0.7368421	0.8315789
Lateral ventricle right rostral point	0.7560211	0.7684211	0.7052632	0.8210526
Lateral ventricle right caudal point	0.7440947	0.7578947	0.6736842	0.8315789
PMJ	0.7072737	0.7368421	0.6315789	0.8105263
Lateral ventricle left caudal point	0.7021263	0.7263158	0.6210526	0.8105263
Hemisphere right caudal point	0.6906526	0.7157895	0.6210526	0.7894737

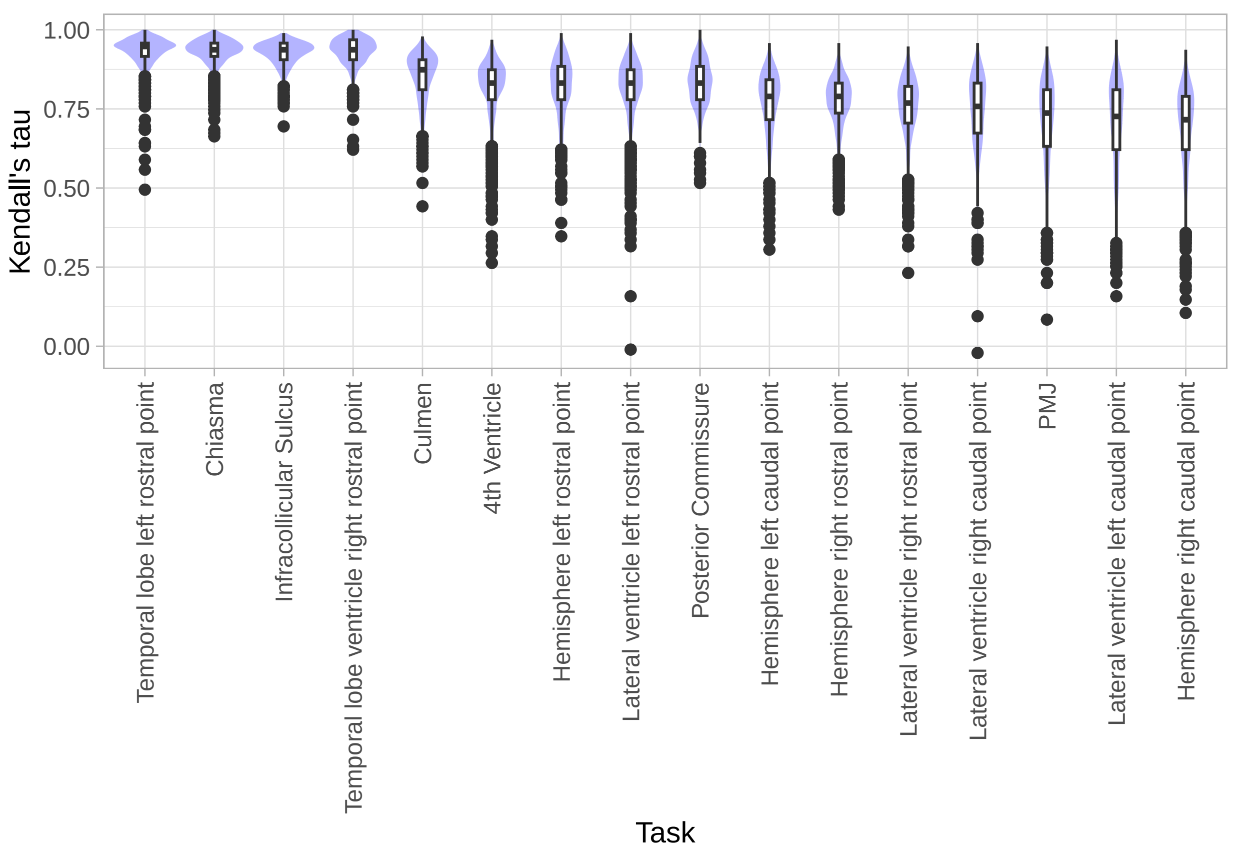

Kendall's tau
1.00
0.75
0.50
0.25
0.00
Temporal lobe left rostral point
Chiasma
Infracollicular Sulcus
Temporal lobe ventricle right rostral point
Culmen
4th Ventricle
Hemisphere left rostral point
Lateral ventricle left rostral point
Posterior Commissure
Hemisphere left caudal point
Hemisphere right rostral point
Lateral ventricle right rostral point
Lateral ventricle right caudal point
PMJ
Lateral ventricle left caudal point
Hemisphere right caudal point
Task

3.3 *Significance maps* for visualizing ranking stability based on statistical significance

Significance maps depict incidence matrices of pairwise significant test results for the one-sided Wilcoxon signed rank test at a 5% significance level with adjustment for multiple testing according to Holm. Yellow shading indicates that metric values from the algorithm on the x-axis were significantly superior to those from the algorithm on the y-axis, blue color indicates no significant difference.

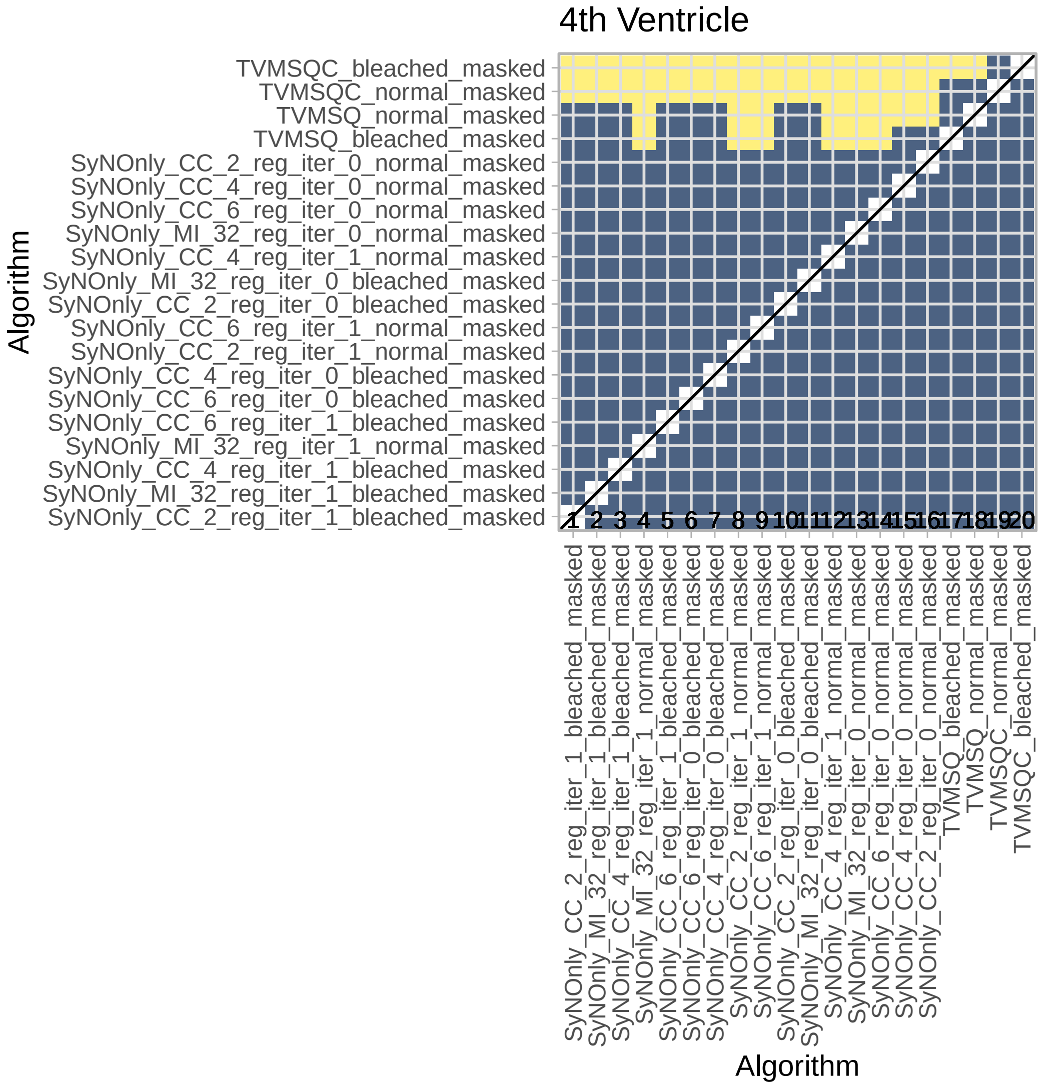

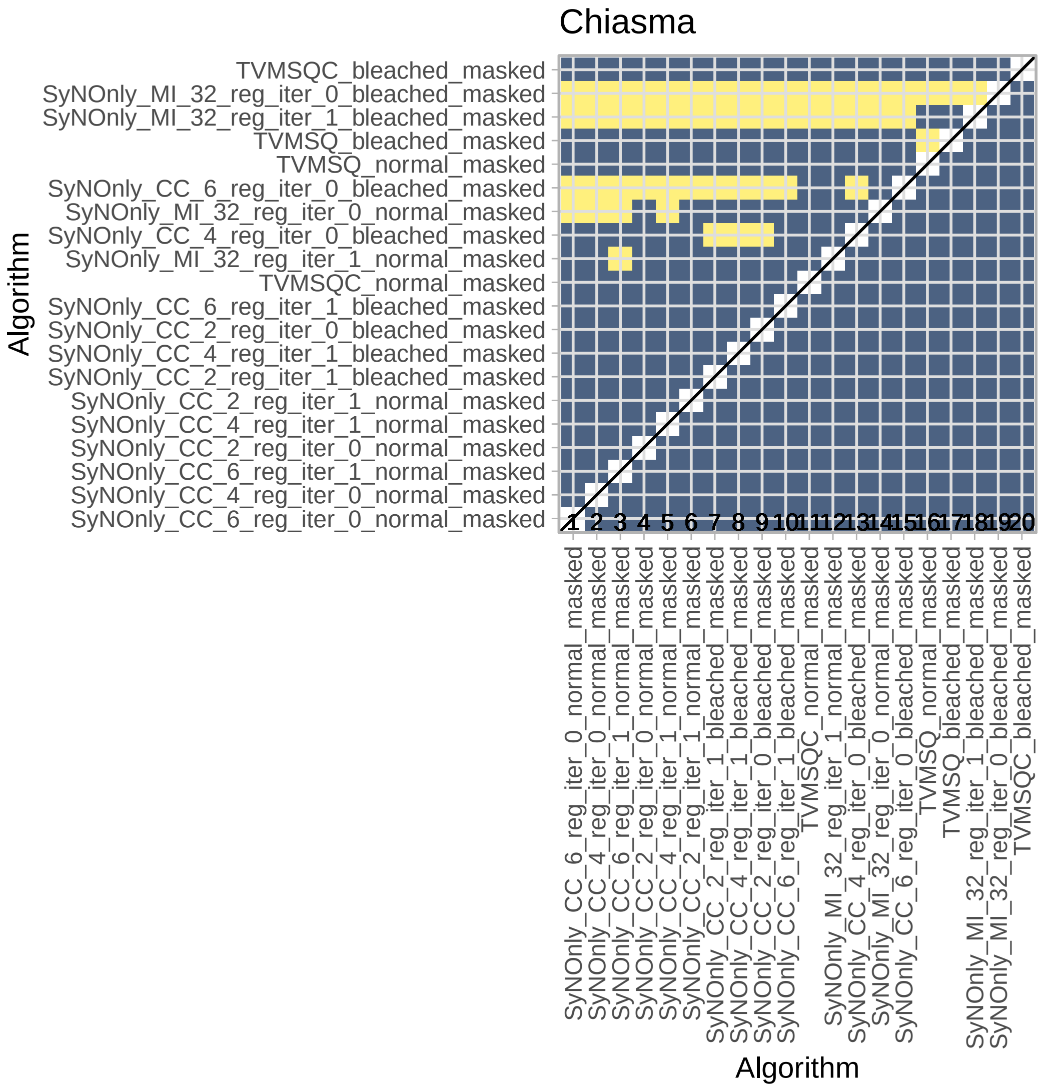
Chiasma
TVMSQC_bleached_masked
SyNOnly_MI_32_reg_iter_0_bleached_masked
SyNOnly_MI_32_reg_iter_1_bleached_masked
TVMSQ_bleached_masked
TVMSQ_normal_masked
SyNOnly_CC_6_reg_iter_0_bleached_masked
SyNOnly_MI_32_reg_iter_0_normal_masked
SyNOnly_CC_4_reg_iter_0_bleached_masked
SyNOnly_MI_32_reg_iter_1_normal_masked
TVMSQC_normal_masked
SyNOnly_CC_6_reg_iter_1_bleached_masked
SyNOnly_CC_2_reg_iter_0_bleached_masked
SyNOnly_CC_4_reg_iter_1_bleached_masked
SyNOnly_CC_2_reg_iter_1_bleached_masked
SyNOnly_CC_2_reg_iter_1_normal_masked
SyNOnly_CC_4_reg_iter_1_normal_masked
SyNOnly_CC_2_reg_iter_0_normal_masked
SyNOnly_CC_6_reg_iter_1_normal_masked
SyNOnly_CC_4_reg_iter_0_normal_masked
SyNOnly_CC_6_reg_iter_0_normal_masked
Algorithm
1 2 3 4 5 6 7 8 9 10 11 12 13 14 15 16 17 18 19 20
SyNOnly_CC_6_reg_iter_0_normal_masked
SyNOnly_CC_4_reg_iter_0_normal_masked
SyNOnly_CC_6_reg_iter_1_normal_masked
SyNOnly_CC_2_reg_iter_0_normal_masked
SyNOnly_CC_4_reg_iter_1_normal_masked
SyNOnly_CC_2_reg_iter_1_normal_masked
SyNOnly_CC_2_reg_iter_1_bleached_masked
SyNOnly_CC_4_reg_iter_1_bleached_masked
SyNOnly_CC_2_reg_iter_0_bleached_masked
SyNOnly_CC_6_reg_iter_1_bleached_masked
TVMSQC_normal_masked
SyNOnly_MI_32_reg_iter_1_normal_masked
SyNOnly_CC_4_reg_iter_0_bleached_masked
SyNOnly_MI_32_reg_iter_0_normal_masked
SyNOnly_CC_6_reg_iter_0_bleached_masked
TVMSQ_normal_masked
TVMSQ_bleached_masked
SyNOnly_MI_32_reg_iter_1_bleached_masked
SyNOnly_MI_32_reg_iter_0_bleached_masked
TVMSQC_bleached_masked
Algorithm

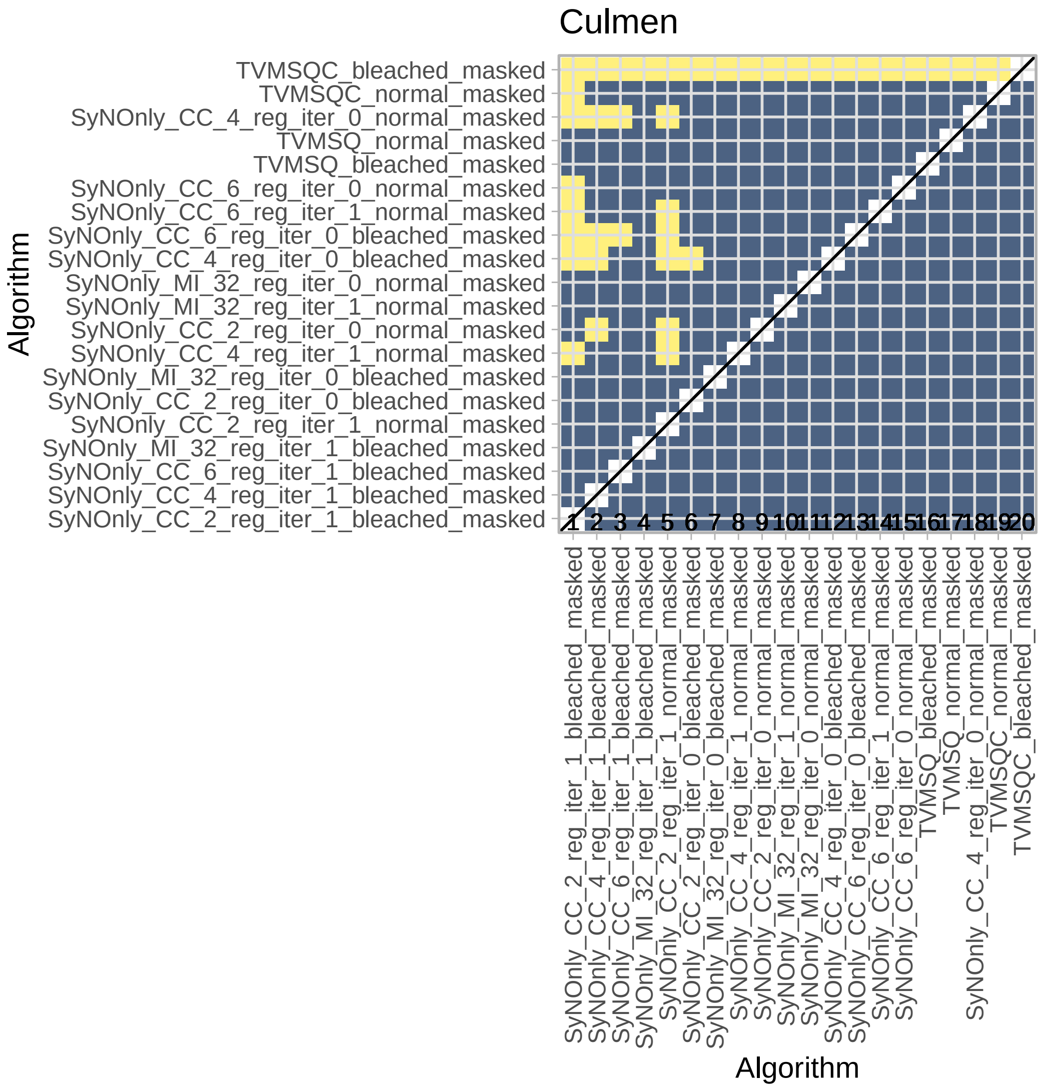

Culmen
Algorithm
TVMSQC_bleached_masked
TVMSQC_normal_masked
SyNOnly_CC_4_reg_iter_0_normal_masked
TVMSQ_normal_masked
TVMSQ_bleached_masked
SyNOnly_CC_6_reg_iter_0_normal_masked
SyNOnly_CC_6_reg_iter_1_normal_masked
SyNOnly_CC_6_reg_iter_0_bleached_masked
SyNOnly_CC_4_reg_iter_0_bleached_masked
SyNOnly_MI_32_reg_iter_0_normal_masked
SyNOnly_MI_32_reg_iter_1_normal_masked
SyNOnly_CC_2_reg_iter_0_normal_masked
SyNOnly_CC_4_reg_iter_1_normal_masked
SyNOnly_MI_32_reg_iter_0_bleached_masked
SyNOnly_CC_2_reg_iter_0_bleached_masked
SyNOnly_CC_2_reg_iter_1_normal_masked
SyNOnly_MI_32_reg_iter_1_bleached_masked
SyNOnly_CC_6_reg_iter_1_bleached_masked
SyNOnly_CC_4_reg_iter_1_bleached_masked
SyNOnly_CC_2_reg_iter_1_bleached_masked
1 2 3 4 5 6 7 8 9 10 11 12 13 14 15 16 17 18 19 20
SyNOnly_CC_2_reg_iter_1_bleached_masked
SyNOnly_CC_4_reg_iter_1_bleached_masked
SyNOnly_CC_6_reg_iter_1_bleached_masked
SyNOnly_MI_32_reg_iter_1_bleached_masked
SyNOnly_CC_2_reg_iter_1_normal_masked
SyNOnly_CC_2_reg_iter_0_bleached_masked
SyNOnly_MI_32_reg_iter_0_bleached_masked
SyNOnly_CC_4_reg_iter_1_normal_masked
SyNOnly_CC_2_reg_iter_0_normal_masked
SyNOnly_MI_32_reg_iter_1_normal_masked
SyNOnly_MI_32_reg_iter_0_normal_masked
SyNOnly_CC_4_reg_iter_0_bleached_masked
SyNOnly_CC_6_reg_iter_0_bleached_masked
SyNOnly_CC_6_reg_iter_1_normal_masked
SyNOnly_CC_6_reg_iter_0_normal_masked
TVMSQ_bleached_masked
TVMSQ_normal_masked
SyNOnly_CC_4_reg_iter_0_normal_masked
TVMSQC_normal_masked
TVMSQC_bleached_masked
Algorithm

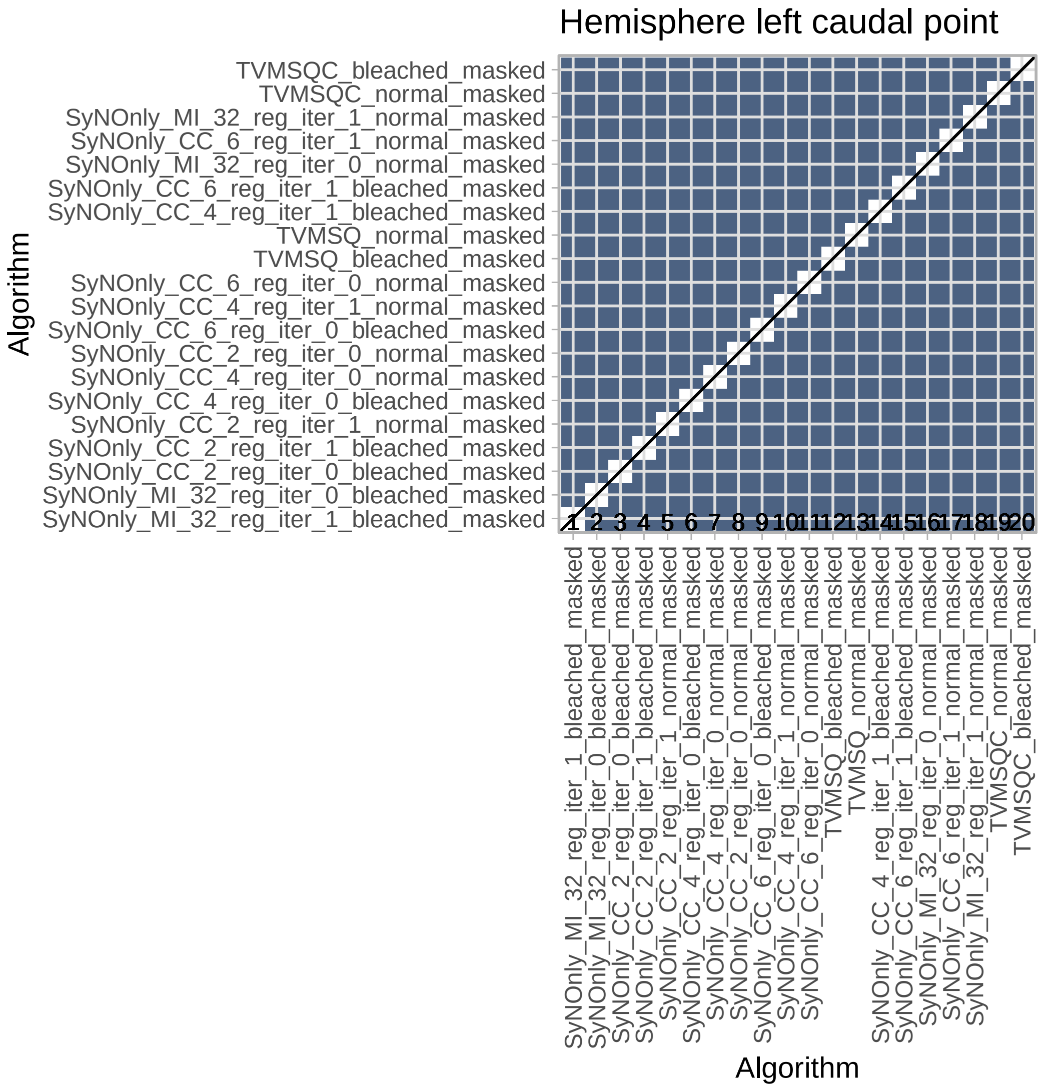
Hemisphere left caudal point
Algorithm
TVMSQC_bleached_masked
TVMSQC_normal_masked
SyNOnly_MI_32_reg_iter_1_normal_masked
SyNOnly_CC_6_reg_iter_1_normal_masked
SyNOnly_MI_32_reg_iter_0_normal_masked
SyNOnly_CC_6_reg_iter_1_bleached_masked
SyNOnly_CC_4_reg_iter_1_bleached_masked
TVMSQ_normal_masked
TVMSQ_bleached_masked
SyNOnly_CC_6_reg_iter_0_normal_masked
SyNOnly_CC_4_reg_iter_1_normal_masked
SyNOnly_CC_6_reg_iter_0_bleached_masked
SyNOnly_CC_2_reg_iter_0_normal_masked
SyNOnly_CC_4_reg_iter_0_normal_masked
SyNOnly_CC_4_reg_iter_0_bleached_masked
SyNOnly_CC_2_reg_iter_1_normal_masked
SyNOnly_CC_2_reg_iter_1_bleached_masked
SyNOnly_CC_2_reg_iter_0_bleached_masked
SyNOnly_MI_32_reg_iter_0_bleached_masked
SyNOnly_MI_32_reg_iter_1_bleached_masked
1 2 3 4 5 6 7 8 9 10 11 12 13 14 15 16 17 18 19 20
SyNOnly_MI_32_reg_iter_1_bleached_masked
SyNOnly_MI_32_reg_iter_0_bleached_masked
SyNOnly_CC_2_reg_iter_0_bleached_masked
SyNOnly_CC_2_reg_iter_1_bleached_masked
SyNOnly_CC_2_reg_iter_1_normal_masked
SyNOnly_CC_4_reg_iter_0_bleached_masked
SyNOnly_CC_4_reg_iter_0_normal_masked
SyNOnly_CC_2_reg_iter_0_normal_masked
SyNOnly_CC_6_reg_iter_0_bleached_masked
SyNOnly_CC_4_reg_iter_1_normal_masked
SyNOnly_CC_6_reg_iter_0_normal_masked
TVMSQ_bleached_masked
TVMSQ_normal_masked
SyNOnly_CC_4_reg_iter_1_bleached_masked
SyNOnly_CC_6_reg_iter_1_bleached_masked
SyNOnly_MI_32_reg_iter_0_normal_masked
SyNOnly_CC_6_reg_iter_1_normal_masked
SyNOnly_MI_32_reg_iter_1_normal_masked
TVMSQC_normal_masked
TVMSQC_bleached_masked
Algorithm

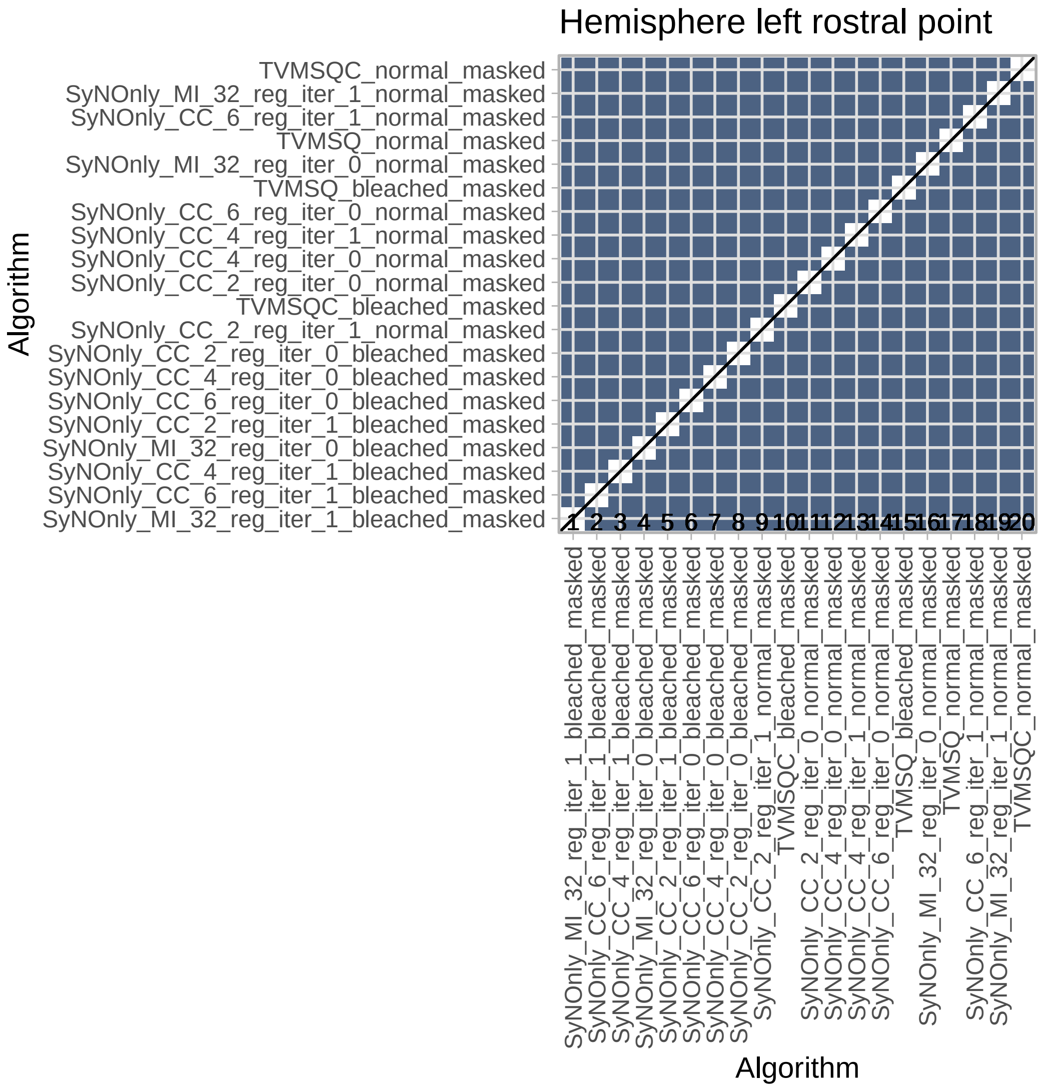
Hemisphere left rostral point
Algorithm
TVMSQC_normal_masked
SyNOnly_MI_32_reg_iter_1_normal_masked
SyNOnly_CC_6_reg_iter_1_normal_masked
TVMSQ_normal_masked
SyNOnly_MI_32_reg_iter_0_normal_masked
TVMSQ_bleached_masked
SyNOnly_CC_6_reg_iter_0_normal_masked
SyNOnly_CC_4_reg_iter_1_normal_masked
SyNOnly_CC_4_reg_iter_0_normal_masked
SyNOnly_CC_2_reg_iter_0_normal_masked
TVMSQC_bleached_masked
SyNOnly_CC_2_reg_iter_1_normal_masked
SyNOnly_CC_2_reg_iter_0_bleached_masked
SyNOnly_CC_4_reg_iter_0_bleached_masked
SyNOnly_CC_6_reg_iter_0_bleached_masked
SyNOnly_CC_2_reg_iter_1_bleached_masked
SyNOnly_MI_32_reg_iter_0_bleached_masked
SyNOnly_CC_4_reg_iter_1_bleached_masked
SyNOnly_CC_6_reg_iter_1_bleached_masked
SyNOnly_MI_32_reg_iter_1_bleached_masked
1 2 3 4 5 6 7 8 9 10 11 12 13 14 15 16 17 18 19 20
SyNOnly_MI_32_reg_iter_1_bleached_masked
SyNOnly_CC_6_reg_iter_1_bleached_masked
SyNOnly_CC_4_reg_iter_1_bleached_masked
SyNOnly_MI_32_reg_iter_0_bleached_masked
SyNOnly_CC_2_reg_iter_1_bleached_masked
SyNOnly_CC_6_reg_iter_0_bleached_masked
SyNOnly_CC_4_reg_iter_0_bleached_masked
SyNOnly_CC_2_reg_iter_0_bleached_masked
SyNOnly_CC_2_reg_iter_1_normal_masked
TVMSQC_bleached_masked
SyNOnly_CC_2_reg_iter_0_normal_masked
SyNOnly_CC_4_reg_iter_0_normal_masked
SyNOnly_CC_4_reg_iter_1_normal_masked
SyNOnly_CC_6_reg_iter_0_normal_masked
TVMSQ_bleached_masked
SyNOnly_MI_32_reg_iter_0_normal_masked
TVMSQ_normal_masked
SyNOnly_CC_6_reg_iter_1_normal_masked
SyNOnly_MI_32_reg_iter_1_normal_masked
TVMSQC_normal_masked
Algorithm

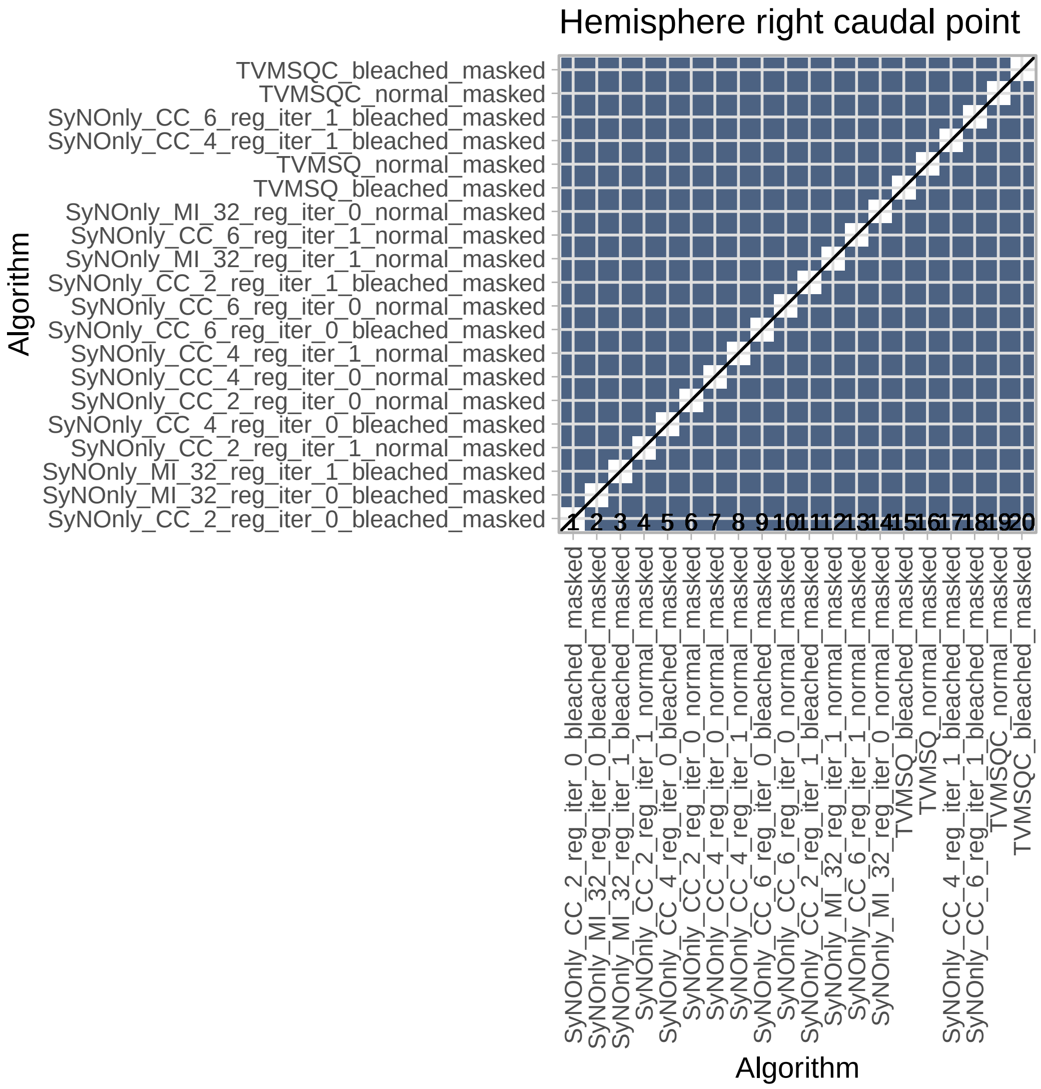
Hemisphere right caudal point
Algorithm
TVMSQC_bleached_masked
TVMSQC_normal_masked
SyNOnly_CC_6_reg_iter_1_bleached_masked
SyNOnly_CC_4_reg_iter_1_bleached_masked
TVMSQ_normal_masked
TVMSQ_bleached_masked
SyNOnly_MI_32_reg_iter_0_normal_masked
SyNOnly_CC_6_reg_iter_1_normal_masked
SyNOnly_MI_32_reg_iter_1_normal_masked
SyNOnly_CC_2_reg_iter_1_bleached_masked
SyNOnly_CC_6_reg_iter_0_normal_masked
SyNOnly_CC_6_reg_iter_0_bleached_masked
SyNOnly_CC_4_reg_iter_1_normal_masked
SyNOnly_CC_4_reg_iter_0_normal_masked
SyNOnly_CC_2_reg_iter_0_normal_masked
SyNOnly_CC_4_reg_iter_0_bleached_masked
SyNOnly_CC_2_reg_iter_1_normal_masked
SyNOnly_MI_32_reg_iter_1_bleached_masked
SyNOnly_MI_32_reg_iter_0_bleached_masked
SyNOnly_CC_2_reg_iter_0_bleached_masked
1 2 3 4 5 6 7 8 9 10 11 12 13 14 15 16 17 18 19 20
Algorithm

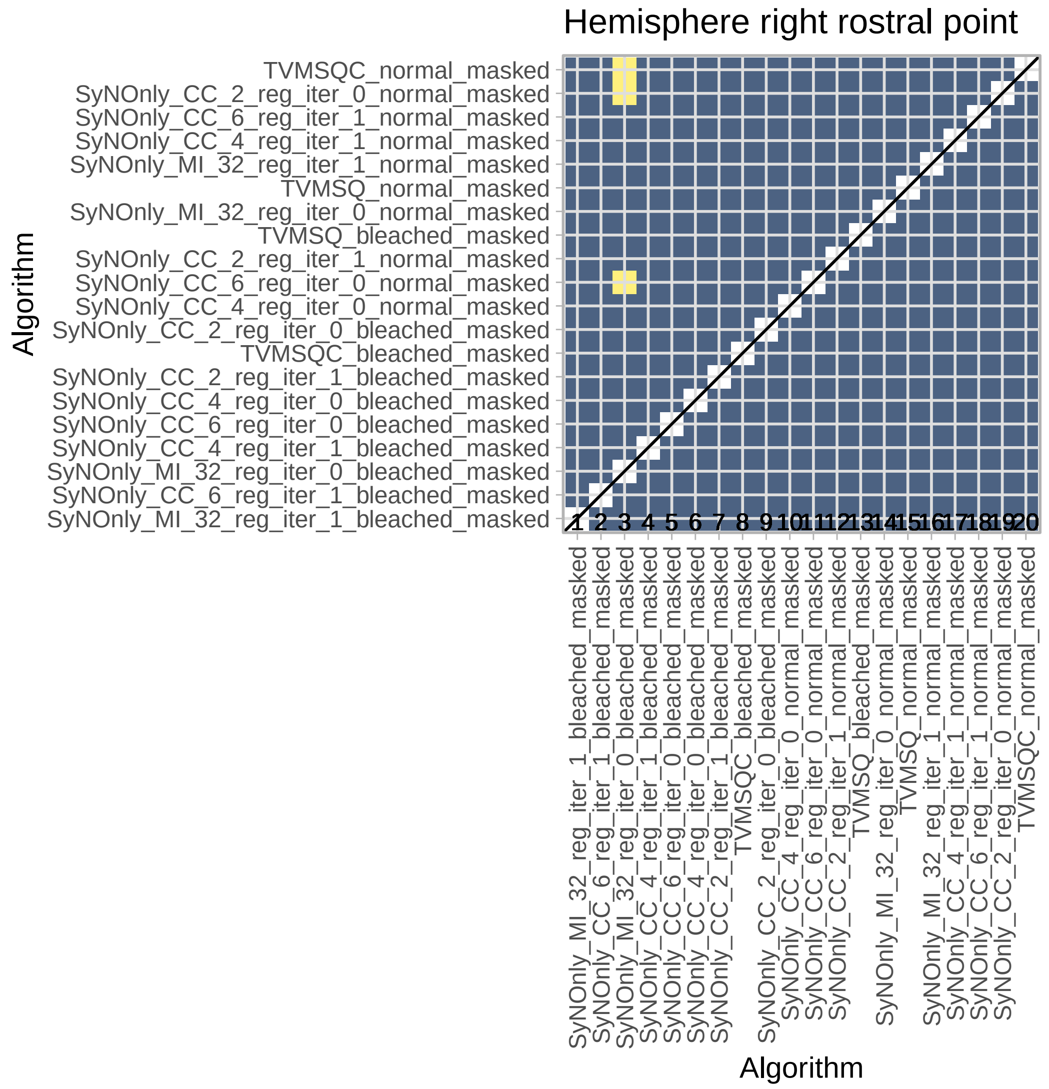
Hemisphere right rostral point
Algorithm
TVMSQC_normal_masked
SyNOnly_CC_2_reg_iter_0_normal_masked
SyNOnly_CC_6_reg_iter_1_normal_masked
SyNOnly_CC_4_reg_iter_1_normal_masked
SyNOnly_MI_32_reg_iter_1_normal_masked
TVMSQ_normal_masked
SyNOnly_MI_32_reg_iter_0_normal_masked
TVMSQ_bleached_masked
SyNOnly_CC_2_reg_iter_1_normal_masked
SyNOnly_CC_6_reg_iter_0_normal_masked
SyNOnly_CC_4_reg_iter_0_normal_masked
SyNOnly_CC_2_reg_iter_0_bleached_masked
TVMSQC_bleached_masked
SyNOnly_CC_2_reg_iter_1_bleached_masked
SyNOnly_CC_4_reg_iter_0_bleached_masked
SyNOnly_CC_6_reg_iter_0_bleached_masked
SyNOnly_CC_4_reg_iter_1_bleached_masked
SyNOnly_MI_32_reg_iter_0_bleached_masked
SyNOnly_CC_6_reg_iter_1_bleached_masked
SyNOnly_MI_32_reg_iter_1_bleached_masked
1 2 3 4 5 6 7 8 9 10 11 12 13 14 15 16 17 18 19 20
SyNOnly_MI_32_reg_iter_1_bleached_masked
SyNOnly_CC_6_reg_iter_1_bleached_masked
SyNOnly_MI_32_reg_iter_0_bleached_masked
SyNOnly_CC_4_reg_iter_1_bleached_masked
SyNOnly_CC_6_reg_iter_0_bleached_masked
SyNOnly_CC_4_reg_iter_0_bleached_masked
SyNOnly_CC_2_reg_iter_1_bleached_masked
TVMSQC_bleached_masked
SyNOnly_CC_2_reg_iter_0_bleached_masked
SyNOnly_CC_4_reg_iter_0_normal_masked
SyNOnly_CC_6_reg_iter_0_normal_masked
SyNOnly_CC_2_reg_iter_1_normal_masked
TVMSQ_bleached_masked
SyNOnly_MI_32_reg_iter_0_normal_masked
TVMSQ_normal_masked
SyNOnly_MI_32_reg_iter_1_normal_masked
SyNOnly_CC_4_reg_iter_1_normal_masked
SyNOnly_CC_6_reg_iter_1_normal_masked
SyNOnly_CC_2_reg_iter_0_normal_masked
TVMSQC_normal_masked
Algorithm

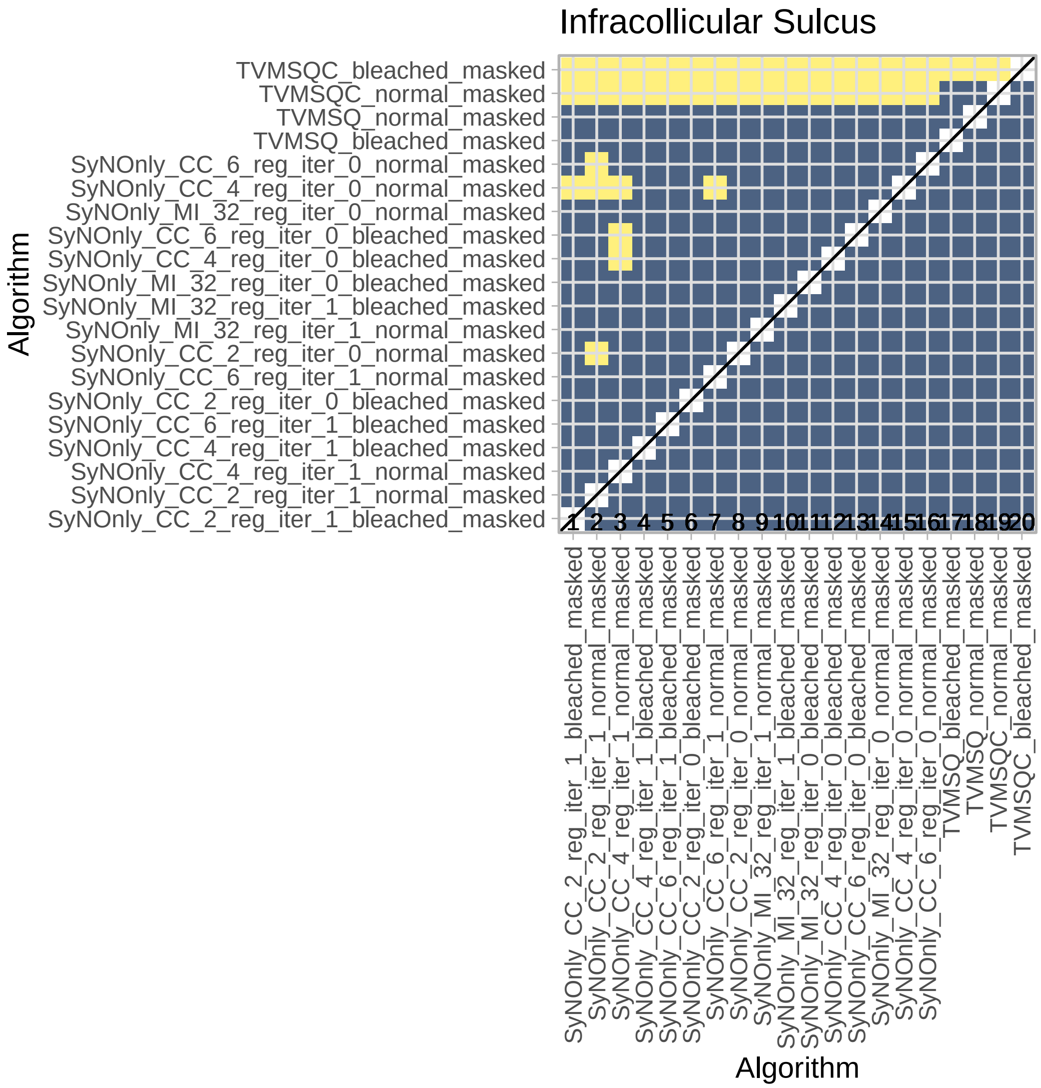
Infracollicular Sulcus
Algorithm
TVMSQC_bleached_masked
TVMSQC_normal_masked
TVMSQ_normal_masked
TVMSQ_bleached_masked
SyNOnly_CC_6_reg_iter_0_normal_masked
SyNOnly_CC_4_reg_iter_0_normal_masked
SyNOnly_MI_32_reg_iter_0_normal_masked
SyNOnly_CC_6_reg_iter_0_bleached_masked
SyNOnly_CC_4_reg_iter_0_bleached_masked
SyNOnly_MI_32_reg_iter_0_bleached_masked
SyNOnly_MI_32_reg_iter_1_bleached_masked
SyNOnly_MI_32_reg_iter_1_normal_masked
SyNOnly_CC_2_reg_iter_0_normal_masked
SyNOnly_CC_6_reg_iter_1_normal_masked
SyNOnly_CC_2_reg_iter_0_bleached_masked
SyNOnly_CC_6_reg_iter_1_bleached_masked
SyNOnly_CC_4_reg_iter_1_bleached_masked
SyNOnly_CC_4_reg_iter_1_normal_masked
SyNOnly_CC_2_reg_iter_1_normal_masked
SyNOnly_CC_2_reg_iter_1_bleached_masked
1 2 3 4 5 6 7 8 9 10 11 12 13 14 15 16 17 18 19 20
Algorithm

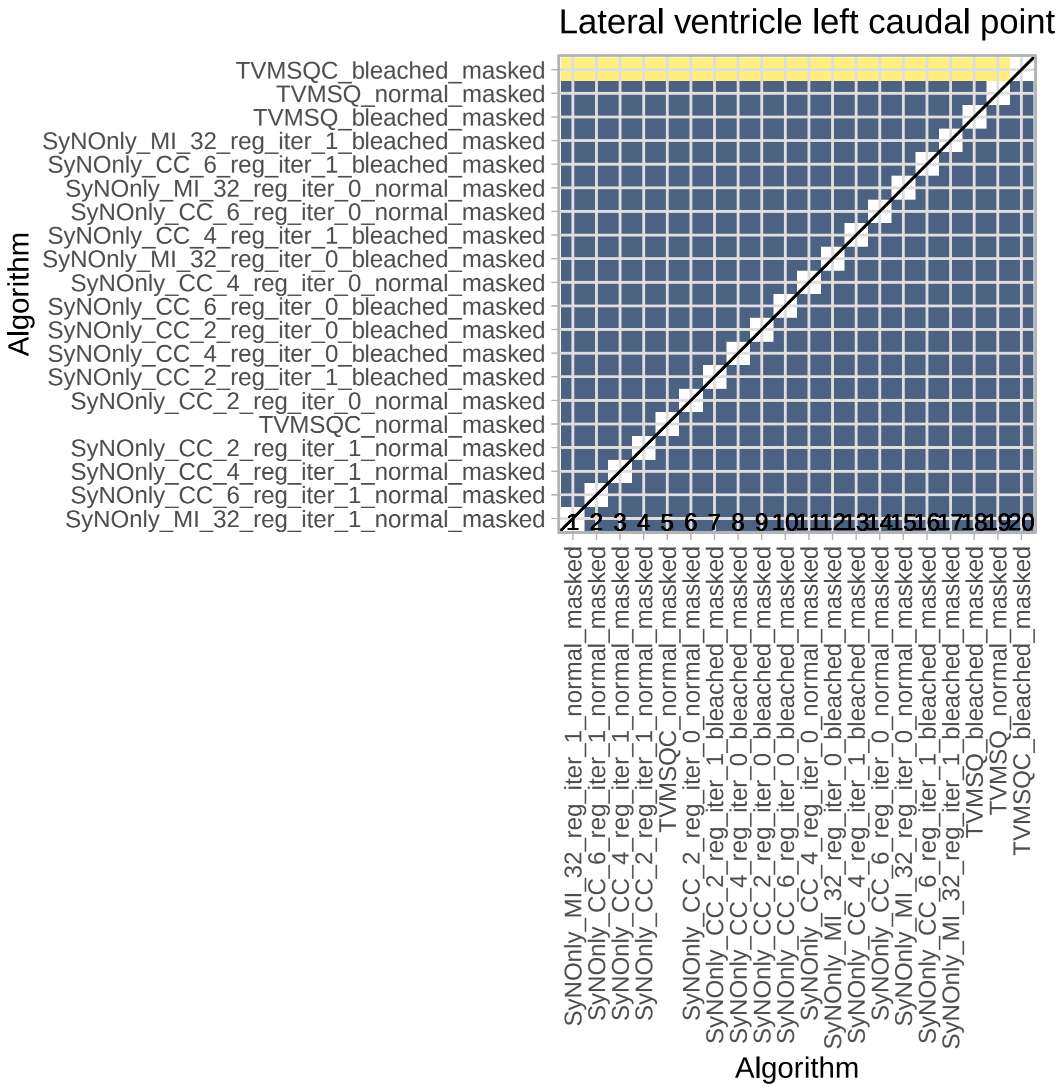
Lateral ventricle left caudal point
Algorithm
TVMSQC_bleached_masked
TVMSQ_normal_masked
TVMSQ_bleached_masked
SyNOnly_MI_32_reg_iter_1_bleached_masked
SyNOnly_CC_6_reg_iter_1_bleached_masked
SyNOnly_MI_32_reg_iter_0_normal_masked
SyNOnly_CC_6_reg_iter_0_normal_masked
SyNOnly_CC_4_reg_iter_1_bleached_masked
SyNOnly_MI_32_reg_iter_0_bleached_masked
SyNOnly_CC_4_reg_iter_0_normal_masked
SyNOnly_CC_6_reg_iter_0_bleached_masked
SyNOnly_CC_2_reg_iter_0_bleached_masked
SyNOnly_CC_4_reg_iter_0_bleached_masked
SyNOnly_CC_2_reg_iter_1_bleached_masked
SyNOnly_CC_2_reg_iter_0_normal_masked
TVMSQC_normal_masked
SyNOnly_CC_2_reg_iter_1_normal_masked
SyNOnly_CC_4_reg_iter_1_normal_masked
SyNOnly_CC_6_reg_iter_1_normal_masked
SyNOnly_MI_32_reg_iter_1_normal_masked
1 2 3 4 5 6 7 8 9 10 11 12 13 14 15 16 17 18 19 20
Algorithm

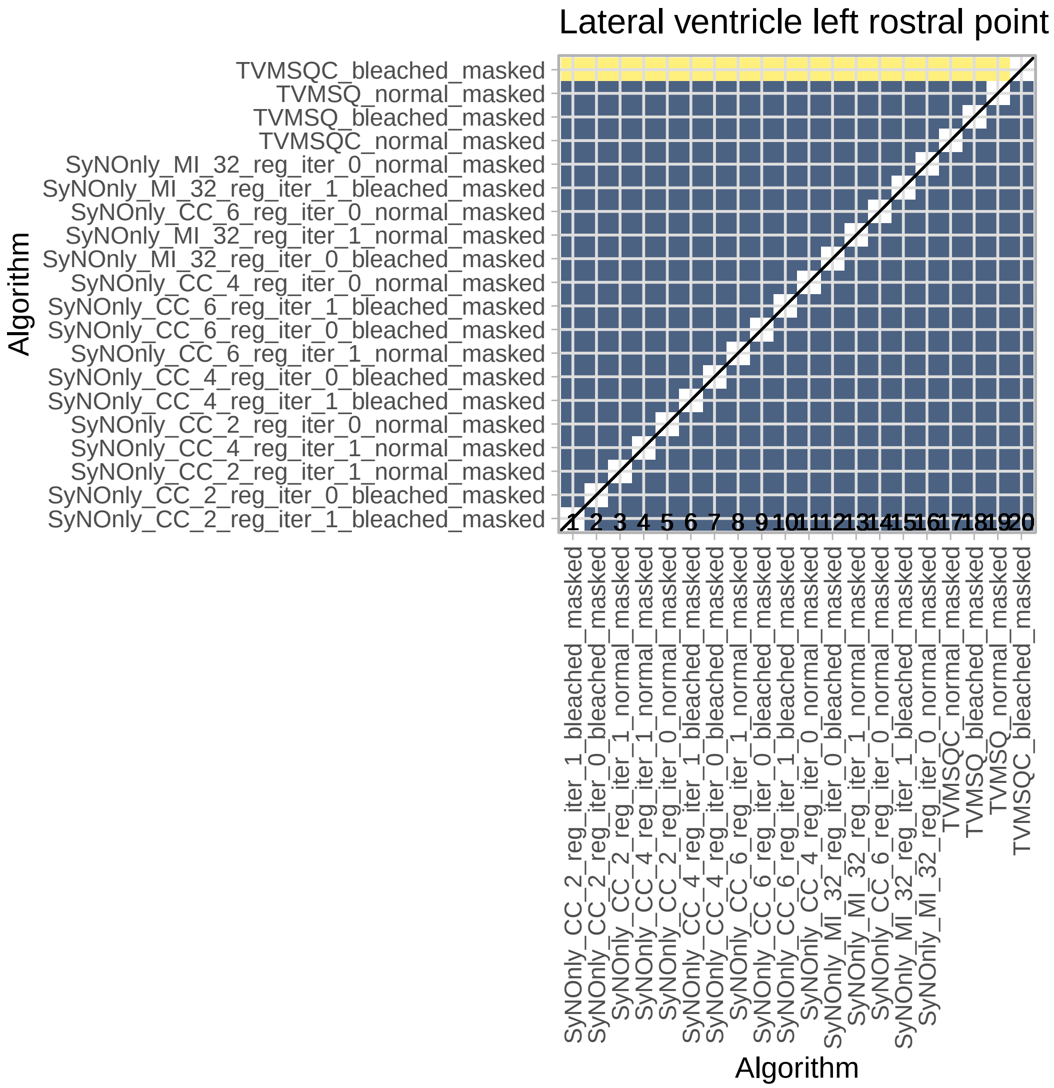
Lateral ventricle left rostral point
Algorithm
TVMSQC_bleached_masked
TVMSQ_normal_masked
TVMSQ_bleached_masked
TVMSQC_normal_masked
SyNOnly_MI_32_reg_iter_0_normal_masked
SyNOnly_MI_32_reg_iter_1_bleached_masked
SyNOnly_CC_6_reg_iter_0_normal_masked
SyNOnly_MI_32_reg_iter_1_normal_masked
SyNOnly_MI_32_reg_iter_0_bleached_masked
SyNOnly_CC_4_reg_iter_0_normal_masked
SyNOnly_CC_6_reg_iter_1_bleached_masked
SyNOnly_CC_6_reg_iter_0_bleached_masked
SyNOnly_CC_6_reg_iter_1_normal_masked
SyNOnly_CC_4_reg_iter_0_bleached_masked
SyNOnly_CC_4_reg_iter_1_bleached_masked
SyNOnly_CC_2_reg_iter_0_normal_masked
SyNOnly_CC_4_reg_iter_1_normal_masked
SyNOnly_CC_2_reg_iter_1_normal_masked
SyNOnly_CC_2_reg_iter_0_bleached_masked
SyNOnly_CC_2_reg_iter_1_bleached_masked
1 2 3 4 5 6 7 8 9 10 11 12 13 14 15 16 17 18 19 20
SyNOnly_CC_2_reg_iter_1_bleached_masked
SyNOnly_CC_2_reg_iter_0_bleached_masked
SyNOnly_CC_2_reg_iter_1_normal_masked
SyNOnly_CC_4_reg_iter_1_normal_masked
SyNOnly_CC_2_reg_iter_0_normal_masked
SyNOnly_CC_4_reg_iter_1_bleached_masked
SyNOnly_CC_4_reg_iter_0_bleached_masked
SyNOnly_CC_6_reg_iter_1_normal_masked
SyNOnly_CC_6_reg_iter_0_bleached_masked
SyNOnly_CC_6_reg_iter_1_bleached_masked
SyNOnly_CC_4_reg_iter_0_normal_masked
SyNOnly_MI_32_reg_iter_0_bleached_masked
SyNOnly_MI_32_reg_iter_1_normal_masked
SyNOnly_CC_6_reg_iter_0_normal_masked
SyNOnly_MI_32_reg_iter_1_bleached_masked
SyNOnly_MI_32_reg_iter_0_normal_masked
TVMSQC_normal_masked
TVMSQ_bleached_masked
TVMSQ_normal_masked
TVMSQC_bleached_masked
Algorithm

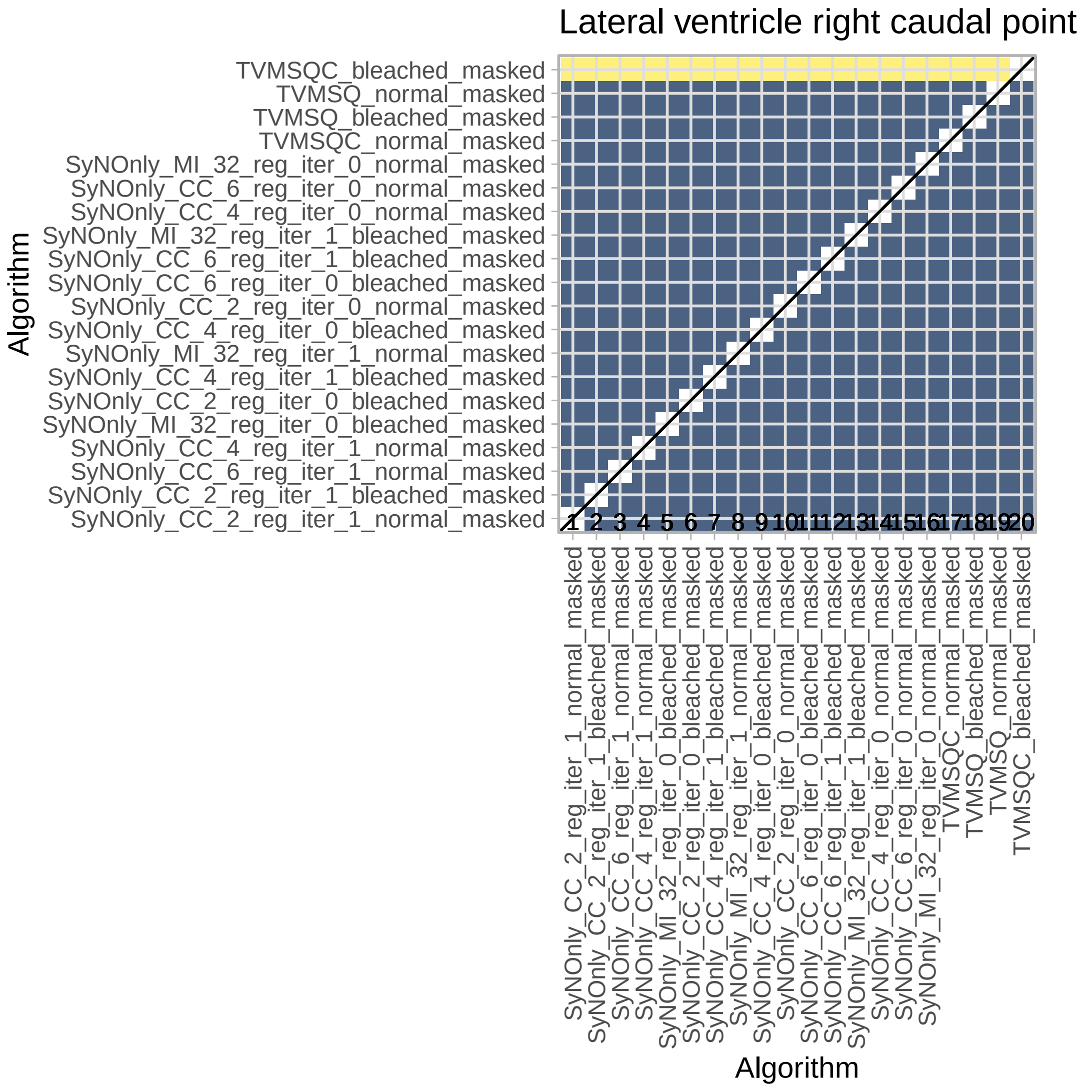

Lateral ventricle right caudal point
Algorithm
TVMSQC_bleached_masked
TVMSQ_normal_masked
TVMSQ_bleached_masked
TVMSQC_normal_masked
SyNOnly_MI_32_reg_iter_0_normal_masked
SyNOnly_CC_6_reg_iter_0_normal_masked
SyNOnly_CC_4_reg_iter_0_normal_masked
SyNOnly_MI_32_reg_iter_1_bleached_masked
SyNOnly_CC_6_reg_iter_1_bleached_masked
SyNOnly_CC_6_reg_iter_0_bleached_masked
SyNOnly_CC_2_reg_iter_0_normal_masked
SyNOnly_CC_4_reg_iter_0_bleached_masked
SyNOnly_MI_32_reg_iter_1_normal_masked
SyNOnly_CC_4_reg_iter_1_bleached_masked
SyNOnly_CC_2_reg_iter_0_bleached_masked
SyNOnly_MI_32_reg_iter_0_bleached_masked
SyNOnly_CC_4_reg_iter_1_normal_masked
SyNOnly_CC_6_reg_iter_1_normal_masked
SyNOnly_CC_2_reg_iter_1_bleached_masked
SyNOnly_CC_2_reg_iter_1_normal_masked
1 2 3 4 5 6 7 8 9 10 11 12 13 14 15 16 17 18 19 20
SyNOnly_CC_2_reg_iter_1_normal_masked
SyNOnly_CC_2_reg_iter_1_bleached_masked
SyNOnly_CC_6_reg_iter_1_normal_masked
SyNOnly_CC_4_reg_iter_1_normal_masked
SyNOnly_MI_32_reg_iter_0_bleached_masked
SyNOnly_CC_2_reg_iter_0_bleached_masked
SyNOnly_CC_4_reg_iter_1_bleached_masked
SyNOnly_MI_32_reg_iter_1_normal_masked
SyNOnly_CC_4_reg_iter_0_bleached_masked
SyNOnly_CC_2_reg_iter_0_normal_masked
SyNOnly_CC_6_reg_iter_0_bleached_masked
SyNOnly_CC_6_reg_iter_1_bleached_masked
SyNOnly_MI_32_reg_iter_1_bleached_masked
SyNOnly_CC_4_reg_iter_0_normal_masked
SyNOnly_CC_6_reg_iter_0_normal_masked
SyNOnly_MI_32_reg_iter_0_normal_masked
TVMSQC_normal_masked
TVMSQ_bleached_masked
TVMSQ_normal_masked
TVMSQC_bleached_masked
Algorithm

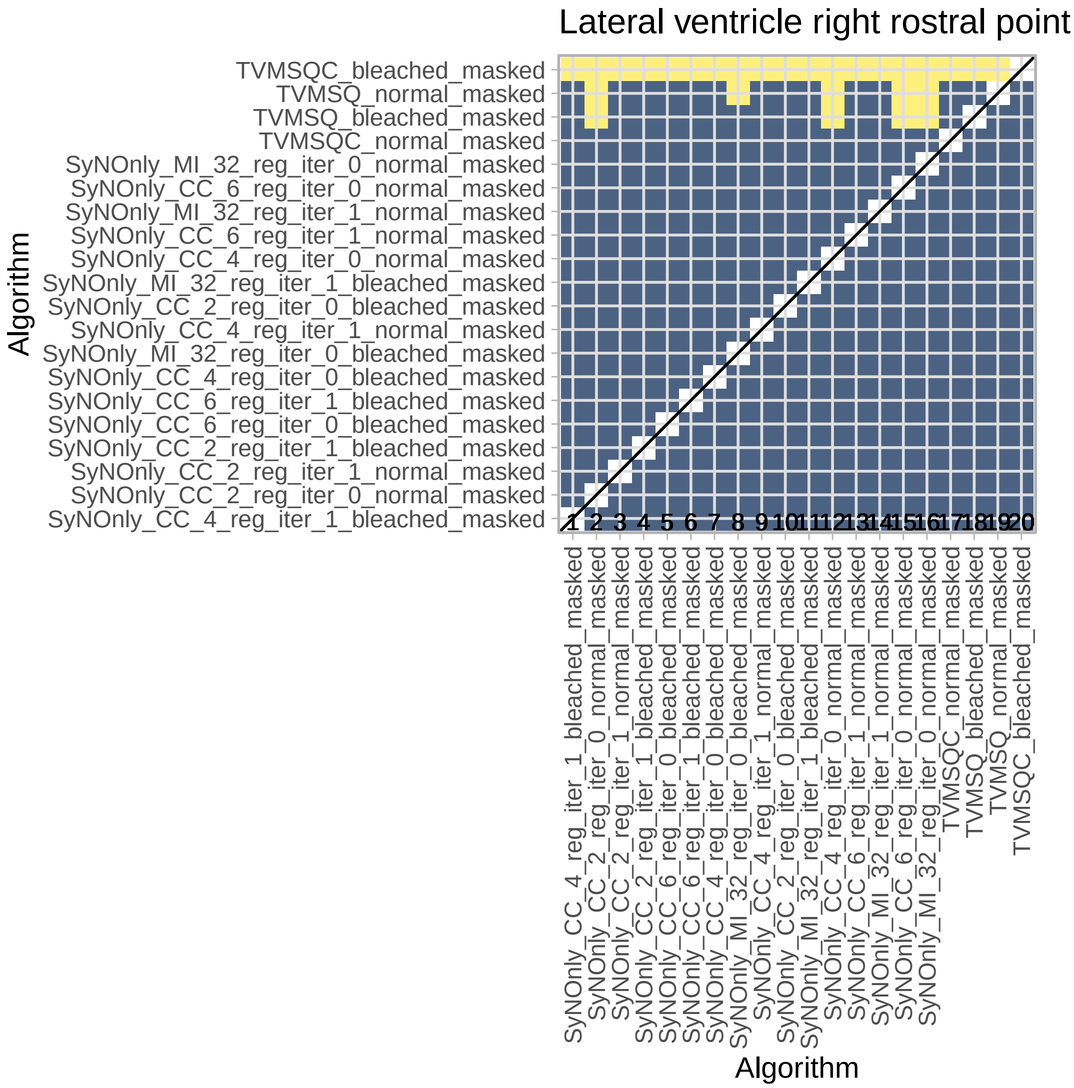

Lateral ventricle right rostral point
Algorithm
TVMSQC_bleached_masked
TVMSQ_normal_masked
TVMSQ_bleached_masked
TVMSQC_normal_masked
SyNOnly_MI_32_reg_iter_0_normal_masked
SyNOnly_CC_6_reg_iter_0_normal_masked
SyNOnly_MI_32_reg_iter_1_normal_masked
SyNOnly_CC_6_reg_iter_1_normal_masked
SyNOnly_CC_4_reg_iter_0_normal_masked
SyNOnly_MI_32_reg_iter_1_bleached_masked
SyNOnly_CC_2_reg_iter_0_bleached_masked
SyNOnly_CC_4_reg_iter_1_normal_masked
SyNOnly_MI_32_reg_iter_0_bleached_masked
SyNOnly_CC_4_reg_iter_0_bleached_masked
SyNOnly_CC_6_reg_iter_1_bleached_masked
SyNOnly_CC_6_reg_iter_0_bleached_masked
SyNOnly_CC_2_reg_iter_1_bleached_masked
SyNOnly_CC_2_reg_iter_1_normal_masked
SyNOnly_CC_2_reg_iter_0_normal_masked
SyNOnly_CC_4_reg_iter_1_bleached_masked
1 2 3 4 5 6 7 8 9 10 11 12 13 14 15 16 17 18 19 20
SyNOnly_CC_4_reg_iter_1_bleached_masked
SyNOnly_CC_2_reg_iter_0_normal_masked
SyNOnly_CC_2_reg_iter_1_normal_masked
SyNOnly_CC_2_reg_iter_1_bleached_masked
SyNOnly_CC_6_reg_iter_0_bleached_masked
SyNOnly_CC_6_reg_iter_1_bleached_masked
SyNOnly_CC_4_reg_iter_0_bleached_masked
SyNOnly_MI_32_reg_iter_0_bleached_masked
SyNOnly_CC_4_reg_iter_1_normal_masked
SyNOnly_CC_2_reg_iter_0_bleached_masked
SyNOnly_MI_32_reg_iter_1_bleached_masked
SyNOnly_CC_4_reg_iter_0_normal_masked
SyNOnly_CC_6_reg_iter_1_normal_masked
SyNOnly_MI_32_reg_iter_1_normal_masked
SyNOnly_CC_6_reg_iter_0_normal_masked
SyNOnly_MI_32_reg_iter_0_normal_masked
TVMSQC_normal_masked
TVMSQ_bleached_masked
TVMSQ_normal_masked
TVMSQC_bleached_masked
Algorithm

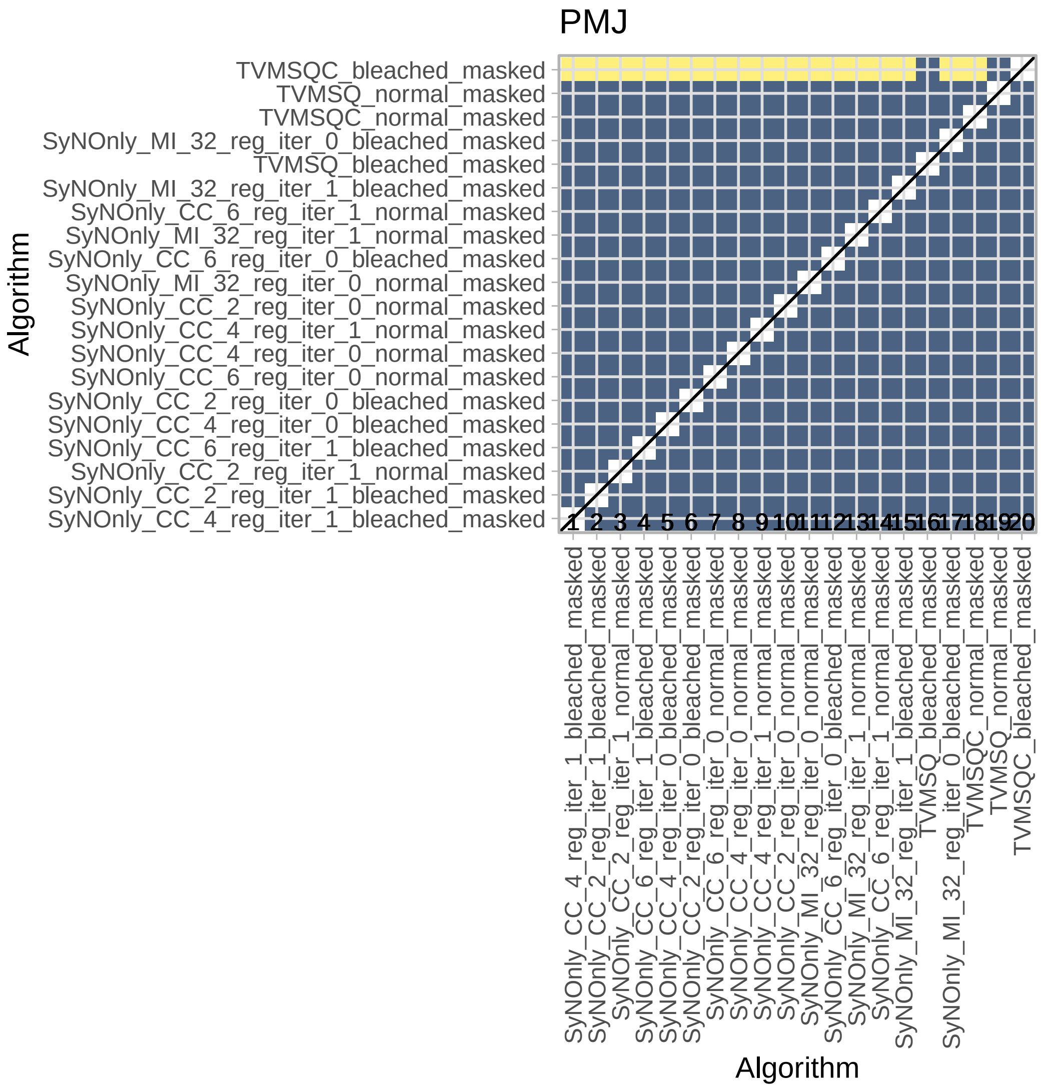

PMJ
Algorithm
TVMSQC_bleached_masked
TVMSQ_normal_masked
TVMSQC_normal_masked
SyNOnly_MI_32_reg_iter_0_bleached_masked
TVMSQ_bleached_masked
SyNOnly_MI_32_reg_iter_1_bleached_masked
SyNOnly_CC_6_reg_iter_1_normal_masked
SyNOnly_MI_32_reg_iter_1_normal_masked
SyNOnly_CC_6_reg_iter_0_bleached_masked
SyNOnly_MI_32_reg_iter_0_normal_masked
SyNOnly_CC_2_reg_iter_0_normal_masked
SyNOnly_CC_4_reg_iter_1_normal_masked
SyNOnly_CC_4_reg_iter_0_normal_masked
SyNOnly_CC_6_reg_iter_0_normal_masked
SyNOnly_CC_2_reg_iter_0_bleached_masked
SyNOnly_CC_4_reg_iter_0_bleached_masked
SyNOnly_CC_6_reg_iter_1_bleached_masked
SyNOnly_CC_2_reg_iter_1_normal_masked
SyNOnly_CC_2_reg_iter_1_bleached_masked
SyNOnly_CC_4_reg_iter_1_bleached_masked
1 2 3 4 5 6 7 8 9 10 11 12 13 14 15 16 17 18 19 20
SyNOnly_CC_4_reg_iter_1_bleached_masked
SyNOnly_CC_2_reg_iter_1_bleached_masked
SyNOnly_CC_2_reg_iter_1_normal_masked
SyNOnly_CC_6_reg_iter_1_bleached_masked
SyNOnly_CC_4_reg_iter_0_bleached_masked
SyNOnly_CC_2_reg_iter_0_bleached_masked
SyNOnly_CC_6_reg_iter_0_normal_masked
SyNOnly_CC_4_reg_iter_0_normal_masked
SyNOnly_CC_4_reg_iter_1_normal_masked
SyNOnly_CC_2_reg_iter_0_normal_masked
SyNOnly_MI_32_reg_iter_0_normal_masked
SyNOnly_CC_6_reg_iter_0_bleached_masked
SyNOnly_MI_32_reg_iter_1_normal_masked
SyNOnly_CC_6_reg_iter_1_normal_masked
SyNOnly_MI_32_reg_iter_1_bleached_masked
TVMSQ_bleached_masked
SyNOnly_MI_32_reg_iter_0_bleached_masked
TVMSQC_normal_masked
TVMSQ_normal_masked
TVMSQC_bleached_masked
Algorithm

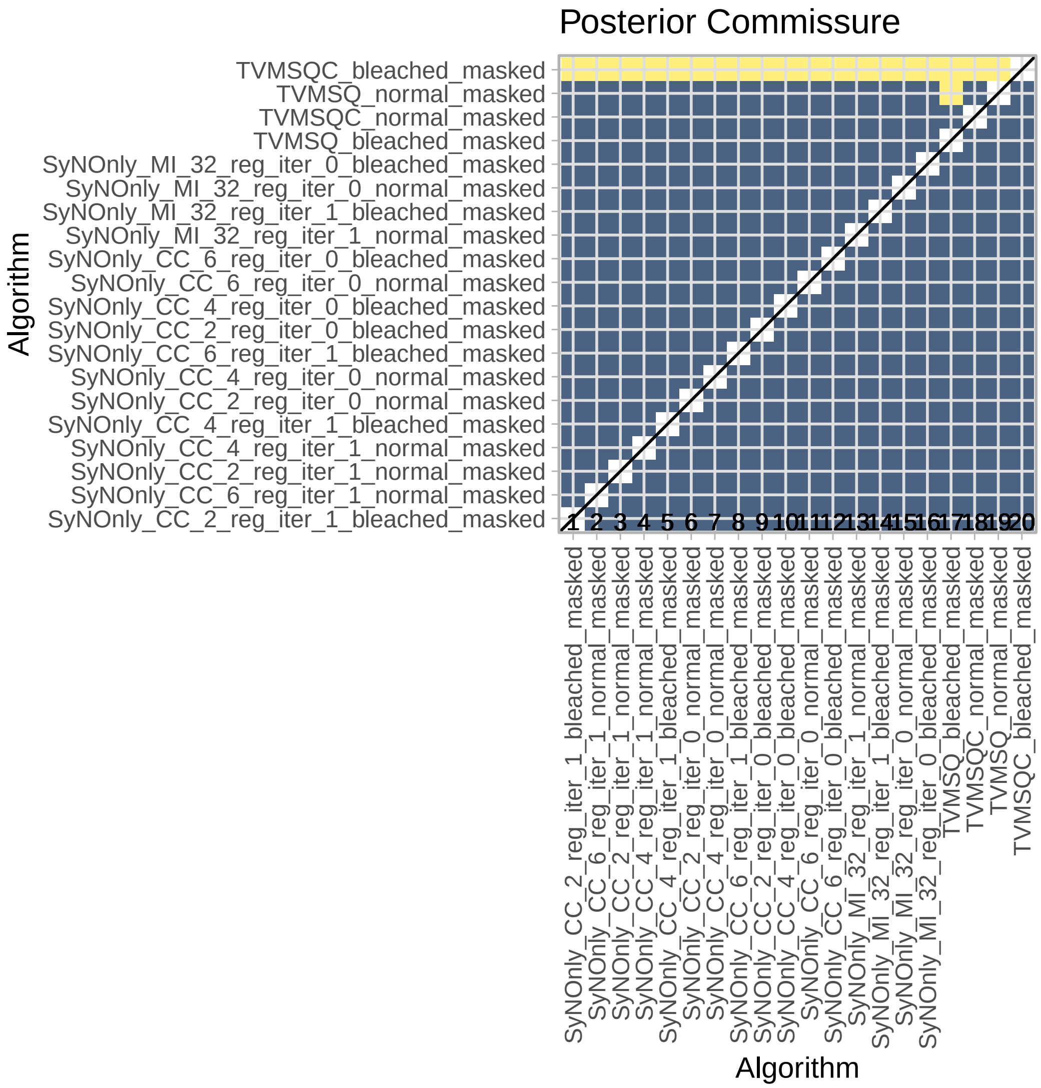
Posterior Commissure
Algorithm
TVMSQC_bleached_masked
TVMSQ_normal_masked
TVMSQC_normal_masked
TVMSQ_bleached_masked
SyNOnly_MI_32_reg_iter_0_bleached_masked
SyNOnly_MI_32_reg_iter_0_normal_masked
SyNOnly_MI_32_reg_iter_1_bleached_masked
SyNOnly_MI_32_reg_iter_1_normal_masked
SyNOnly_CC_6_reg_iter_0_bleached_masked
SyNOnly_CC_6_reg_iter_0_normal_masked
SyNOnly_CC_4_reg_iter_0_bleached_masked
SyNOnly_CC_2_reg_iter_0_bleached_masked
SyNOnly_CC_6_reg_iter_1_bleached_masked
SyNOnly_CC_4_reg_iter_0_normal_masked
SyNOnly_CC_2_reg_iter_0_normal_masked
SyNOnly_CC_4_reg_iter_1_bleached_masked
SyNOnly_CC_4_reg_iter_1_normal_masked
SyNOnly_CC_2_reg_iter_1_normal_masked
SyNOnly_CC_6_reg_iter_1_normal_masked
SyNOnly_CC_2_reg_iter_1_bleached_masked
1 2 3 4 5 6 7 8 9 10 11 12 13 14 15 16 17 18 19 20
Algorithm

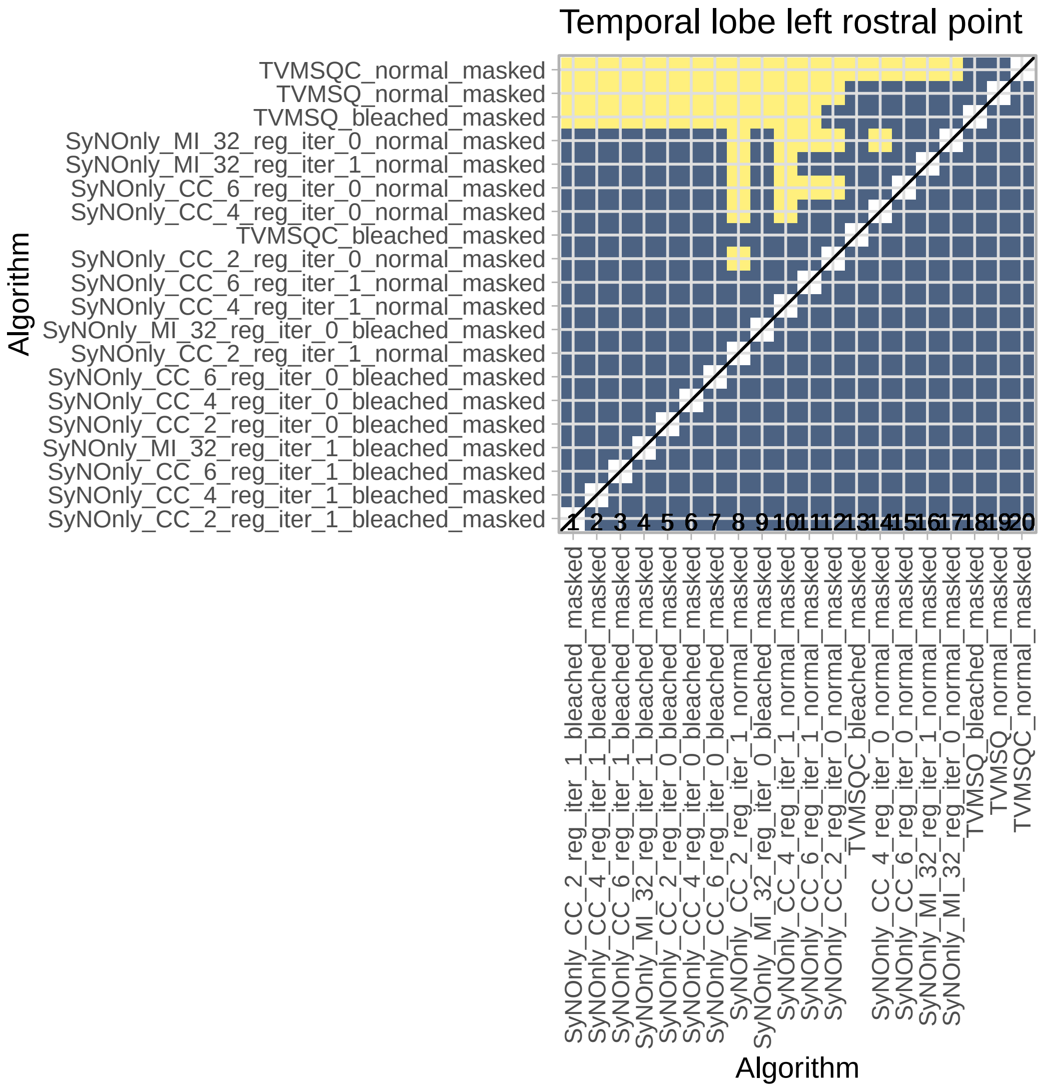

Temporal lobe left rostral point
Algorithm
TVMSQC_normal_masked
TVMSQ_normal_masked
TVMSQ_bleached_masked
SyNOnly_MI_32_reg_iter_0_normal_masked
SyNOnly_MI_32_reg_iter_1_normal_masked
SyNOnly_CC_6_reg_iter_0_normal_masked
SyNOnly_CC_4_reg_iter_0_normal_masked
TVMSQC_bleached_masked
SyNOnly_CC_2_reg_iter_0_normal_masked
SyNOnly_CC_6_reg_iter_1_normal_masked
SyNOnly_CC_4_reg_iter_1_normal_masked
SyNOnly_MI_32_reg_iter_0_bleached_masked
SyNOnly_CC_2_reg_iter_1_normal_masked
SyNOnly_CC_6_reg_iter_0_bleached_masked
SyNOnly_CC_4_reg_iter_0_bleached_masked
SyNOnly_CC_2_reg_iter_0_bleached_masked
SyNOnly_MI_32_reg_iter_1_bleached_masked
SyNOnly_CC_6_reg_iter_1_bleached_masked
SyNOnly_CC_4_reg_iter_1_bleached_masked
SyNOnly_CC_2_reg_iter_1_bleached_masked
1 2 3 4 5 6 7 8 9 10 11 12 13 14 15 16 17 18 19 20
SyNOnly_CC_2_reg_iter_1_bleached_masked
SyNOnly_CC_4_reg_iter_1_bleached_masked
SyNOnly_CC_6_reg_iter_1_bleached_masked
SyNOnly_MI_32_reg_iter_1_bleached_masked
SyNOnly_CC_2_reg_iter_0_bleached_masked
SyNOnly_CC_4_reg_iter_0_bleached_masked
SyNOnly_CC_6_reg_iter_0_bleached_masked
SyNOnly_CC_2_reg_iter_1_normal_masked
SyNOnly_MI_32_reg_iter_0_bleached_masked
SyNOnly_CC_4_reg_iter_1_normal_masked
SyNOnly_CC_6_reg_iter_1_normal_masked
SyNOnly_CC_2_reg_iter_0_normal_masked
TVMSQC_bleached_masked
SyNOnly_CC_4_reg_iter_0_normal_masked
SyNOnly_CC_6_reg_iter_0_normal_masked
SyNOnly_MI_32_reg_iter_1_normal_masked
SyNOnly_MI_32_reg_iter_0_normal_masked
TVMSQ_bleached_masked
TVMSQ_normal_masked
TVMSQC_normal_masked
Algorithm

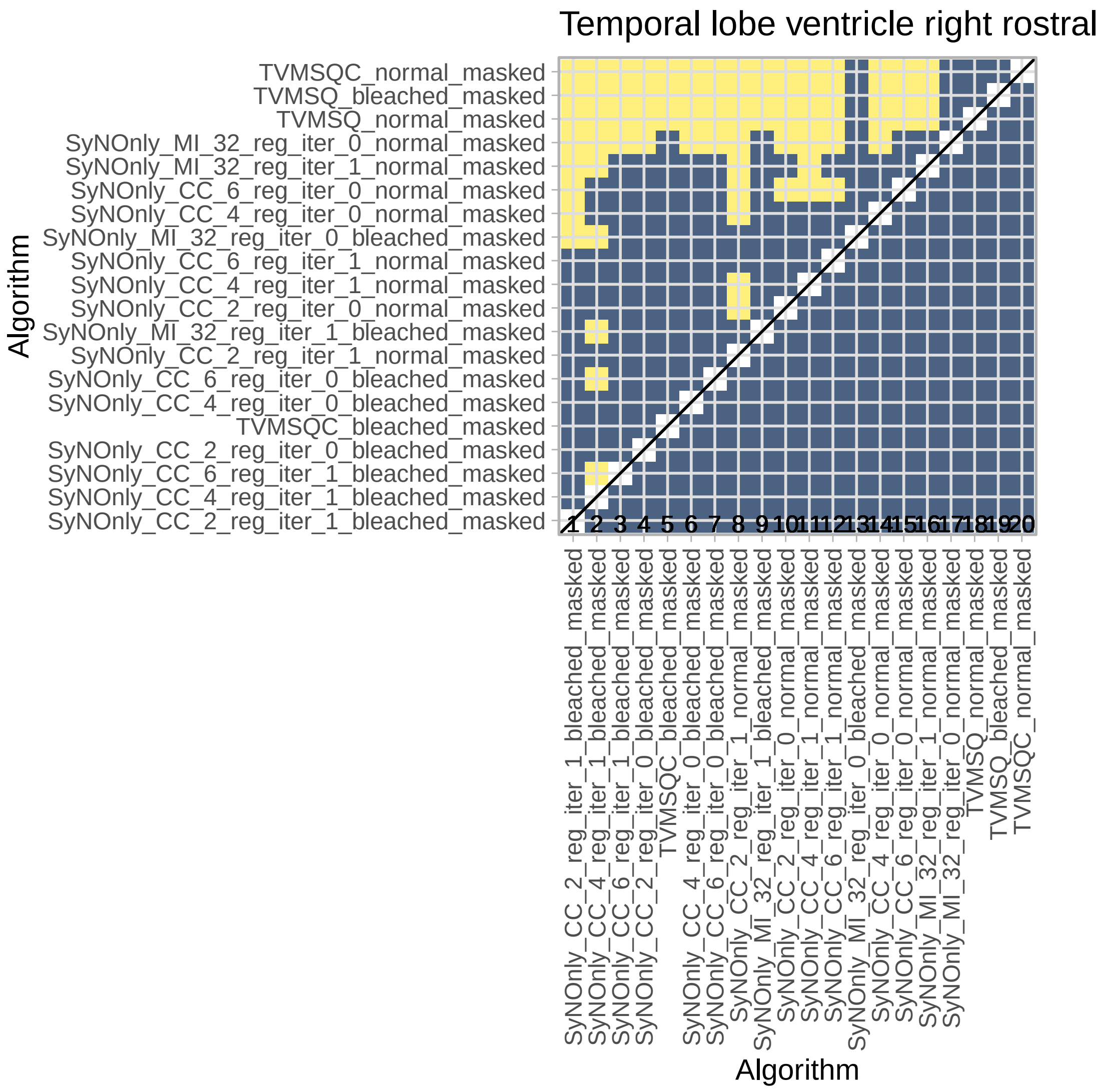
Temporal lobe ventricle right rostral
TVMSQC_normal_masked
TVMSQ_bleached_masked
TVMSQ_normal_masked
SyNOnly_MI_32_reg_iter_0_normal_masked
SyNOnly_MI_32_reg_iter_1_normal_masked
SyNOnly_CC_6_reg_iter_0_normal_masked
SyNOnly_CC_4_reg_iter_0_normal_masked
SyNOnly_MI_32_reg_iter_0_bleached_masked
SyNOnly_CC_6_reg_iter_1_normal_masked
SyNOnly_CC_4_reg_iter_1_normal_masked
SyNOnly_CC_2_reg_iter_0_normal_masked
SyNOnly_MI_32_reg_iter_1_bleached_masked
SyNOnly_CC_2_reg_iter_1_normal_masked
SyNOnly_CC_6_reg_iter_0_bleached_masked
SyNOnly_CC_4_reg_iter_0_bleached_masked
TVMSQC_bleached_masked
SyNOnly_CC_2_reg_iter_0_bleached_masked
SyNOnly_CC_6_reg_iter_1_bleached_masked
SyNOnly_CC_4_reg_iter_1_bleached_masked
SyNOnly_CC_2_reg_iter_1_bleached_masked
Algorithm
1 2 3 4 5 6 7 8 9 10 11 12 13 14 15 16 17 18 19 20
SyNOnly_CC_2_reg_iter_1_bleached_masked
SyNOnly_CC_4_reg_iter_1_bleached_masked
SyNOnly_CC_6_reg_iter_1_bleached_masked
SyNOnly_CC_2_reg_iter_0_bleached_masked
TVMSQC_bleached_masked
SyNOnly_CC_4_reg_iter_0_bleached_masked
SyNOnly_CC_6_reg_iter_0_bleached_masked
SyNOnly_CC_2_reg_iter_1_normal_masked
SyNOnly_MI_32_reg_iter_1_bleached_masked
SyNOnly_CC_2_reg_iter_0_normal_masked
SyNOnly_CC_4_reg_iter_1_normal_masked
SyNOnly_CC_6_reg_iter_1_normal_masked
SyNOnly_MI_32_reg_iter_0_bleached_masked
SyNOnly_CC_4_reg_iter_0_normal_masked
SyNOnly_CC_6_reg_iter_0_normal_masked
SyNOnly_MI_32_reg_iter_1_normal_masked
SyNOnly_MI_32_reg_iter_0_normal_masked
TVMSQ_normal_masked
TVMSQ_bleached_masked
TVMSQC_normal_masked
Algorithm

3.4 Ranking robustness to ranking methods

Line plots for visualizing ranking robustness across different ranking methods. Each algorithm is represented by one colored line. For each ranking method encoded on the x-axis, the height of the line represents the corresponding rank. Horizontal lines indicate identical ranks for all methods.

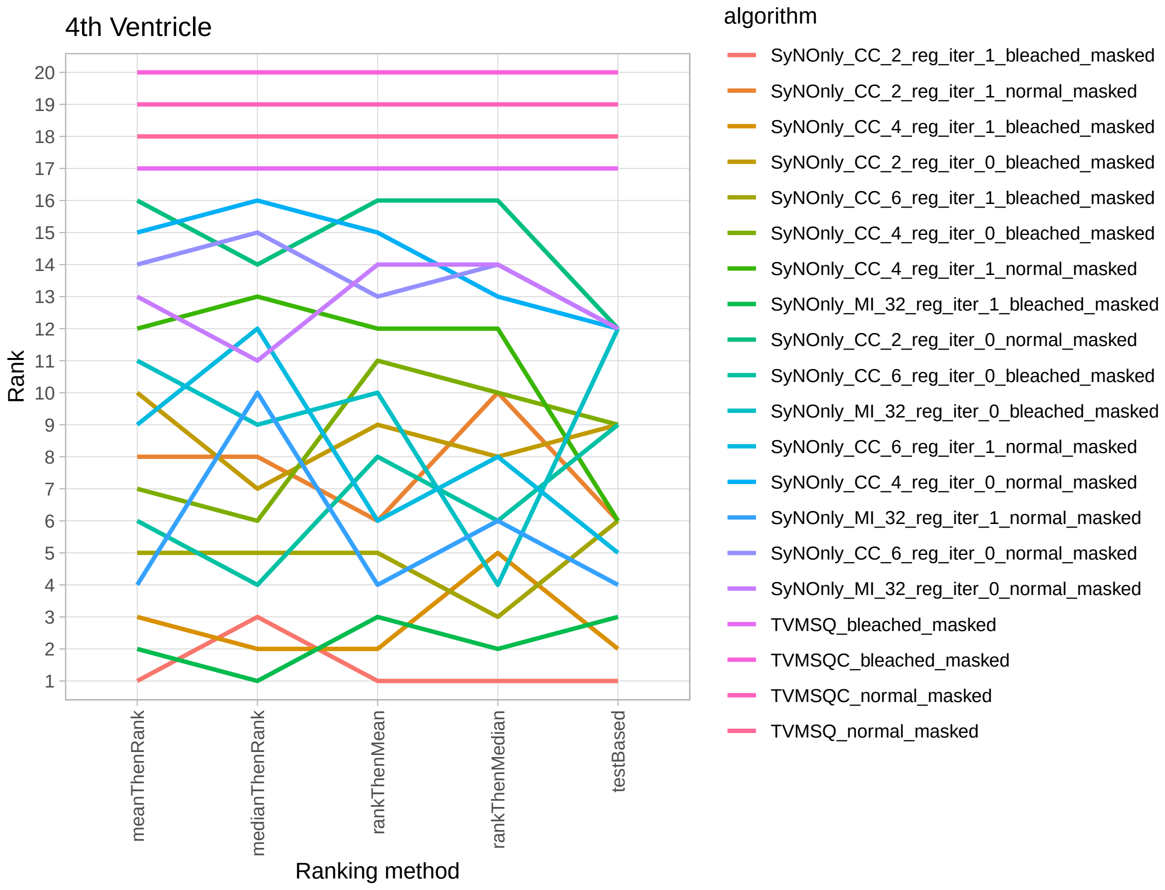

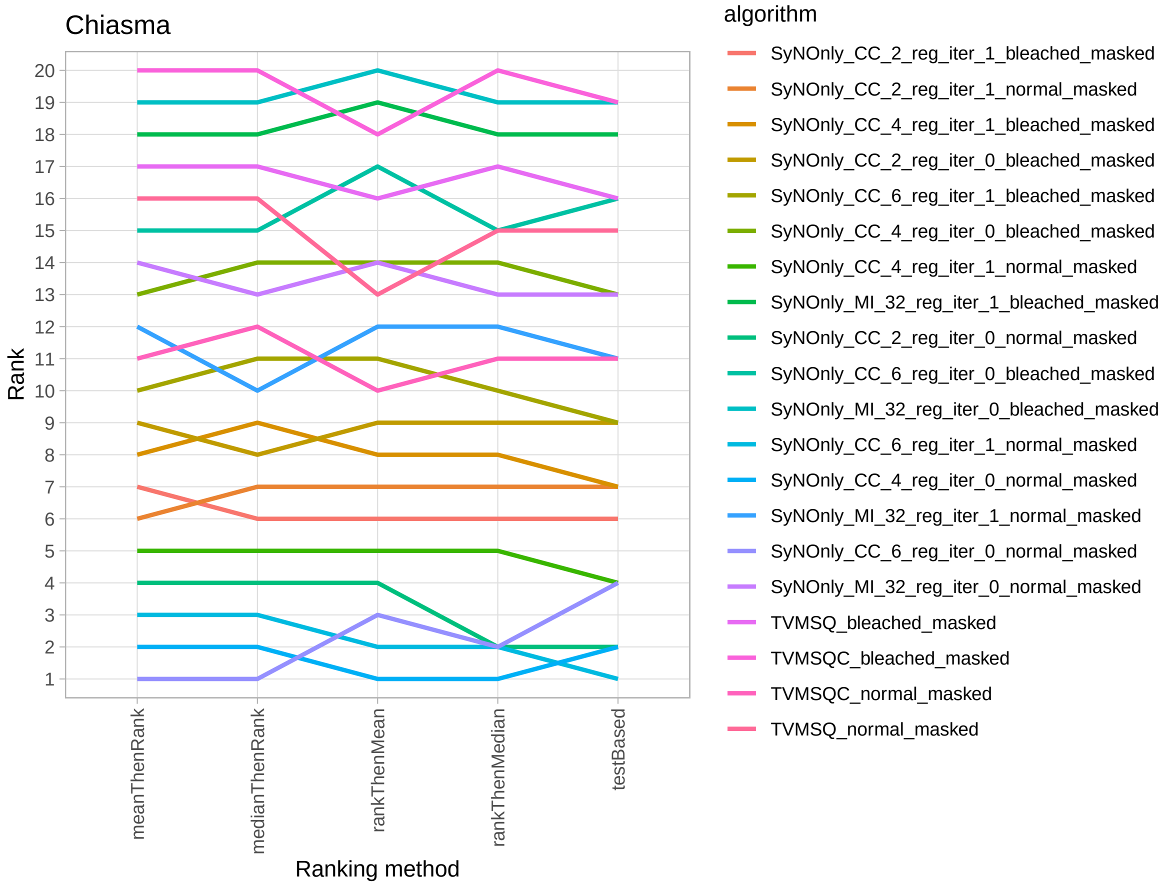
Chiasma
Rank
20
19
18
17
16
15
14
13
12
11
10
9
8
7
6
5
4
3
2
1
meanThenRank
medianThenRank
rankThenMean
rankThenMedian
testBased
Ranking method
algorithm
SyNOnly_CC_2_reg_iter_1_bleached_masked
SyNOnly_CC_2_reg_iter_1_normal_masked
SyNOnly_CC_4_reg_iter_1_bleached_masked
SyNOnly_CC_2_reg_iter_0_bleached_masked
SyNOnly_CC_6_reg_iter_1_bleached_masked
SyNOnly_CC_4_reg_iter_0_bleached_masked
SyNOnly_CC_4_reg_iter_1_normal_masked
SyNOnly_MI_32_reg_iter_1_bleached_masked
SyNOnly_CC_2_reg_iter_0_normal_masked
SyNOnly_CC_6_reg_iter_0_bleached_masked
SyNOnly_MI_32_reg_iter_0_bleached_masked
SyNOnly_CC_6_reg_iter_1_normal_masked
SyNOnly_CC_4_reg_iter_0_normal_masked
SyNOnly_MI_32_reg_iter_1_normal_masked
SyNOnly_CC_6_reg_iter_0_normal_masked
SyNOnly_MI_32_reg_iter_0_normal_masked
TVMSQ_bleached_masked
TVMSQC_bleached_masked
TVMSQC_normal_masked
TVMSQ_normal_masked

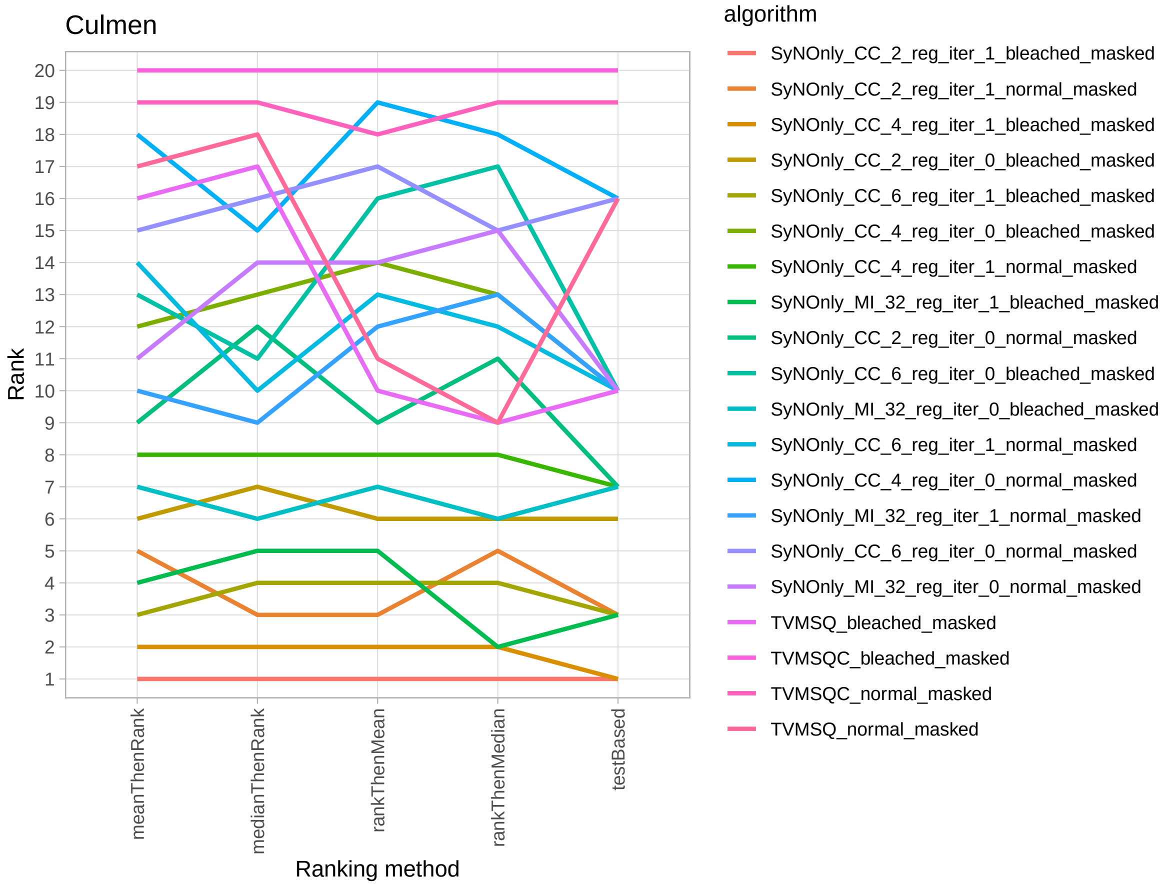
Culmen
Rank
20
19
18
17
16
15
14
13
12
11
10
9
8
7
6
5
4
3
2
1
meanThenRank
medianThenRank
rankThenMean
rankThenMedian
testBased
Ranking method
algorithm
SyNOnly_CC_2_reg_iter_1_bleached_masked
SyNOnly_CC_2_reg_iter_1_normal_masked
SyNOnly_CC_4_reg_iter_1_bleached_masked
SyNOnly_CC_2_reg_iter_0_bleached_masked
SyNOnly_CC_6_reg_iter_1_bleached_masked
SyNOnly_CC_4_reg_iter_0_bleached_masked
SyNOnly_CC_4_reg_iter_1_normal_masked
SyNOnly_MI_32_reg_iter_1_bleached_masked
SyNOnly_CC_2_reg_iter_0_normal_masked
SyNOnly_CC_6_reg_iter_0_bleached_masked
SyNOnly_MI_32_reg_iter_0_bleached_masked
SyNOnly_CC_6_reg_iter_1_normal_masked
SyNOnly_CC_4_reg_iter_0_normal_masked
SyNOnly_MI_32_reg_iter_1_normal_masked
SyNOnly_CC_6_reg_iter_0_normal_masked
SyNOnly_MI_32_reg_iter_0_normal_masked
TVMSQ_bleached_masked
TVMSQC_bleached_masked
TVMSQC_normal_masked
TVMSQ_normal_masked

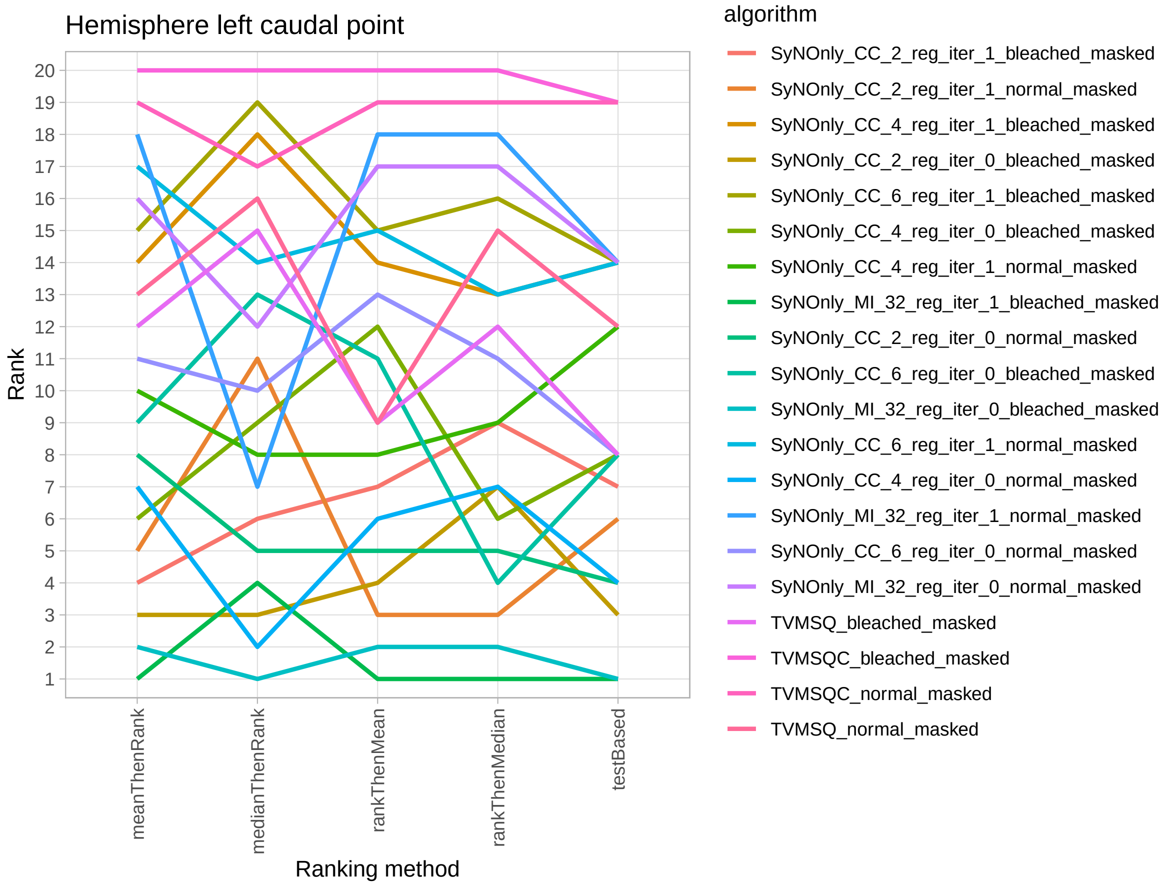
Hemisphere left caudal point
Rank
Ranking method
meanThenRank
medianThenRank
rankThenMean
rankThenMedian
testBased
algorithm
SyNOnly_CC_2_reg_iter_1_bleached_masked
SyNOnly_CC_2_reg_iter_1_normal_masked
SyNOnly_CC_4_reg_iter_1_bleached_masked
SyNOnly_CC_2_reg_iter_0_bleached_masked
SyNOnly_CC_6_reg_iter_1_bleached_masked
SyNOnly_CC_4_reg_iter_0_bleached_masked
SyNOnly_CC_4_reg_iter_1_normal_masked
SyNOnly_MI_32_reg_iter_1_bleached_masked
SyNOnly_CC_2_reg_iter_0_normal_masked
SyNOnly_CC_6_reg_iter_0_bleached_masked
SyNOnly_MI_32_reg_iter_0_bleached_masked
SyNOnly_CC_6_reg_iter_1_normal_masked
SyNOnly_CC_4_reg_iter_0_normal_masked
SyNOnly_MI_32_reg_iter_1_normal_masked
SyNOnly_CC_6_reg_iter_0_normal_masked
SyNOnly_MI_32_reg_iter_0_normal_masked
TVMSQ_bleached_masked
TVMSQC_bleached_masked
TVMSQC_normal_masked
TVMSQ_normal_masked

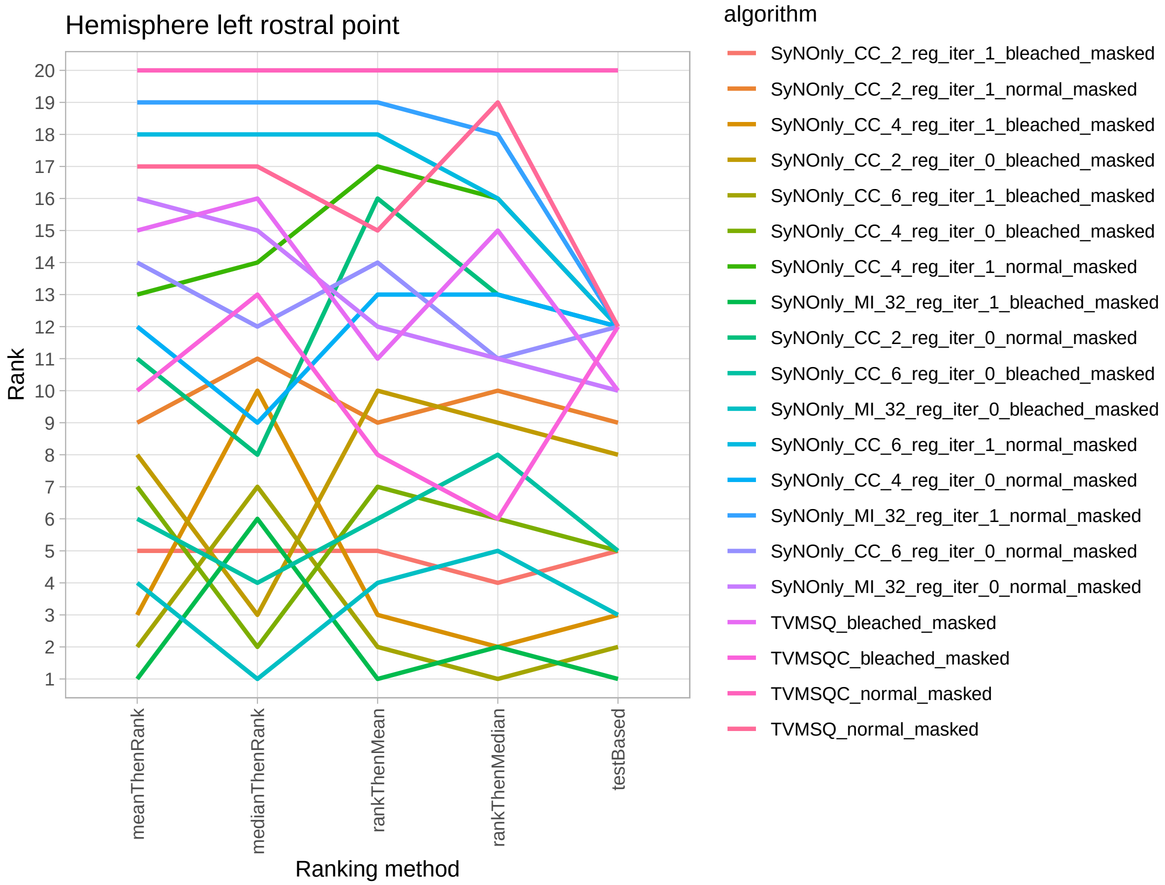
Hemisphere left rostral point
Rank
20
19
18
17
16
15
14
13
12
11
10
9
8
7
6
5
4
3
2
1
meanThenRank
medianThenRank
rankThenMean
rankThenMedian
testBased
Ranking method
algorithm
SyNOnly_CC_2_reg_iter_1_bleached_masked
SyNOnly_CC_2_reg_iter_1_normal_masked
SyNOnly_CC_4_reg_iter_1_bleached_masked
SyNOnly_CC_2_reg_iter_0_bleached_masked
SyNOnly_CC_6_reg_iter_1_bleached_masked
SyNOnly_CC_4_reg_iter_0_bleached_masked
SyNOnly_CC_4_reg_iter_1_normal_masked
SyNOnly_MI_32_reg_iter_1_bleached_masked
SyNOnly_CC_2_reg_iter_0_normal_masked
SyNOnly_CC_6_reg_iter_0_bleached_masked
SyNOnly_MI_32_reg_iter_0_bleached_masked
SyNOnly_CC_6_reg_iter_1_normal_masked
SyNOnly_CC_4_reg_iter_0_normal_masked
SyNOnly_MI_32_reg_iter_1_normal_masked
SyNOnly_CC_6_reg_iter_0_normal_masked
SyNOnly_MI_32_reg_iter_0_normal_masked
TVMSQ_bleached_masked
TVMSQC_bleached_masked
TVMSQC_normal_masked
TVMSQ_normal_masked

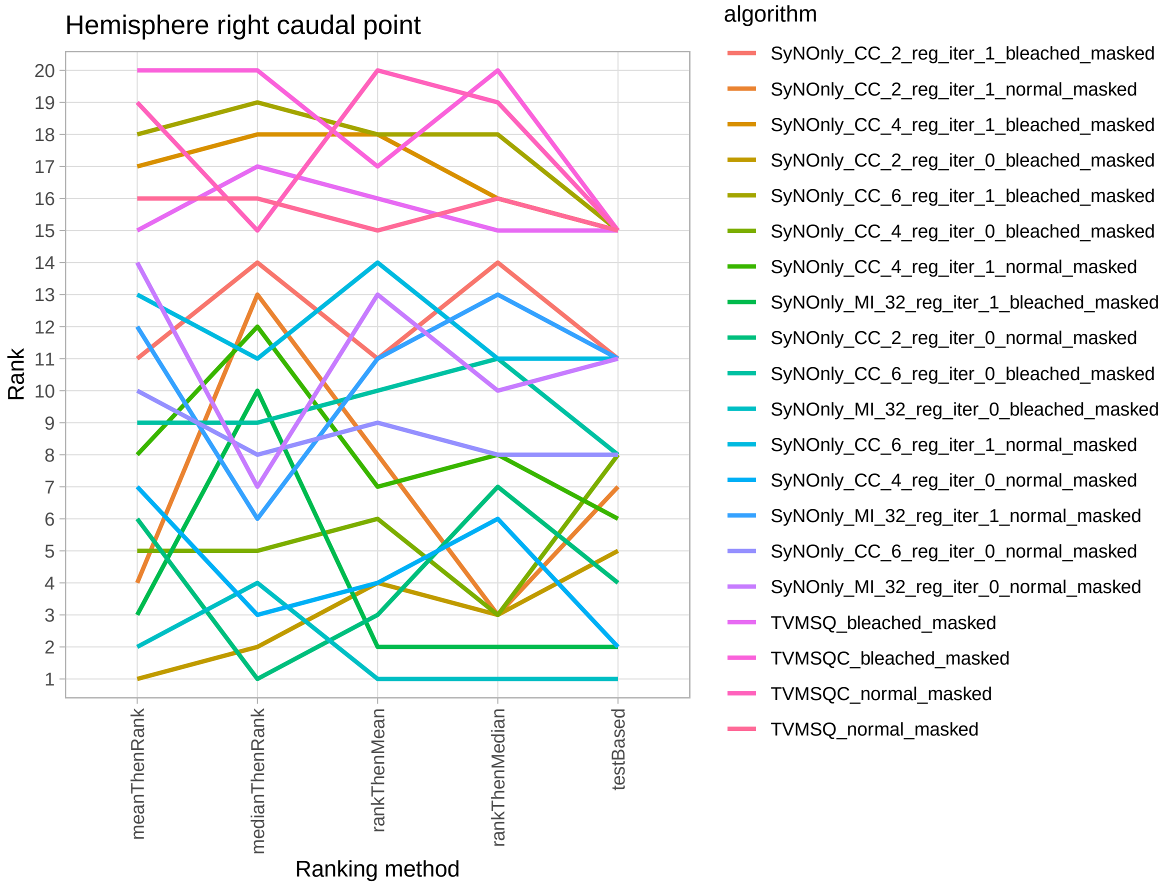
Hemisphere right caudal point
Rank
20
19
18
17
16
15
14
13
12
11
10
9
8
7
6
5
4
3
2
1
meanThenRank
medianThenRank
rankThenMean
rankThenMedian
testBased
Ranking method
algorithm
SyNOnly_CC_2_reg_iter_1_bleached_masked
SyNOnly_CC_2_reg_iter_1_normal_masked
SyNOnly_CC_4_reg_iter_1_bleached_masked
SyNOnly_CC_2_reg_iter_0_bleached_masked
SyNOnly_CC_6_reg_iter_1_bleached_masked
SyNOnly_CC_4_reg_iter_0_bleached_masked
SyNOnly_CC_4_reg_iter_1_normal_masked
SyNOnly_MI_32_reg_iter_1_bleached_masked
SyNOnly_CC_2_reg_iter_0_normal_masked
SyNOnly_CC_6_reg_iter_0_bleached_masked
SyNOnly_MI_32_reg_iter_0_bleached_masked
SyNOnly_CC_6_reg_iter_1_normal_masked
SyNOnly_CC_4_reg_iter_0_normal_masked
SyNOnly_MI_32_reg_iter_1_normal_masked
SyNOnly_CC_6_reg_iter_0_normal_masked
SyNOnly_MI_32_reg_iter_0_normal_masked
TVMSQ_bleached_masked
TVMSQC_bleached_masked
TVMSQC_normal_masked
TVMSQ_normal_masked

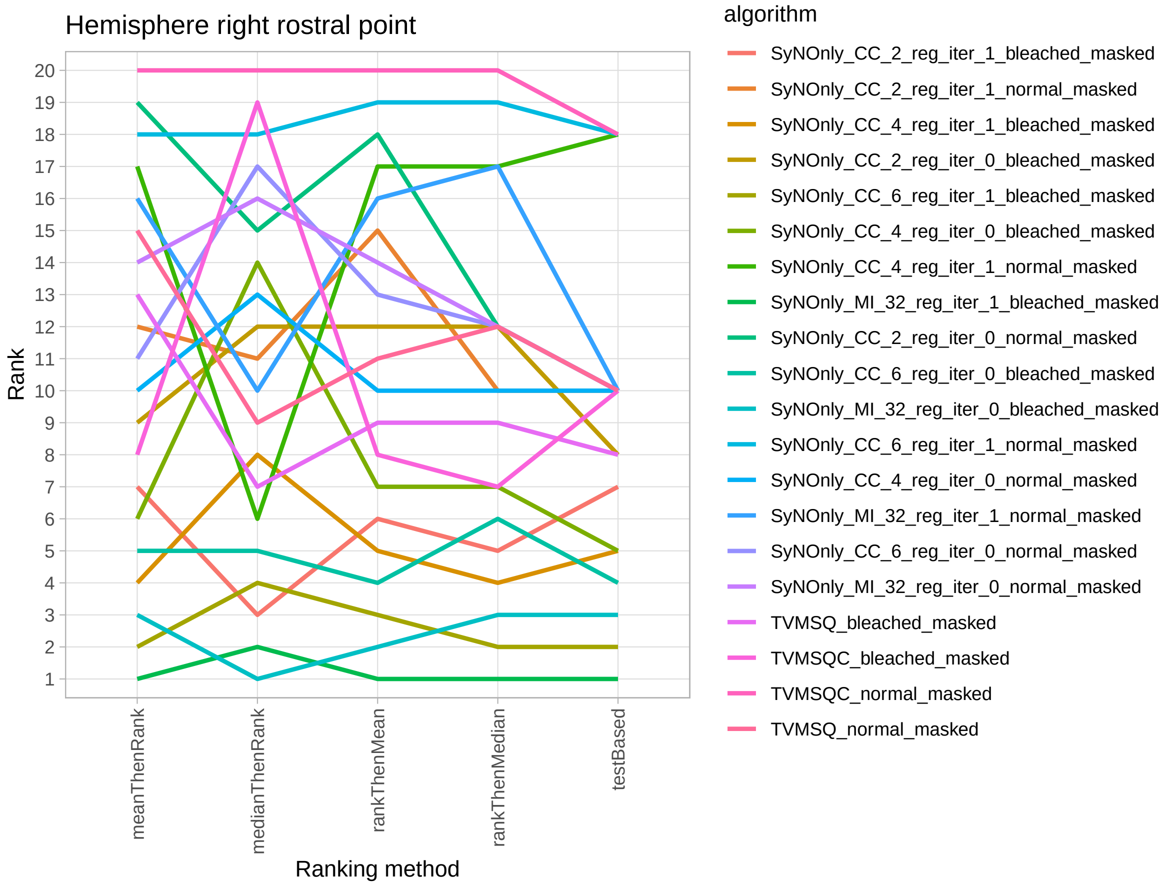
Hemisphere right rostral point
Rank
20
19
18
17
16
15
14
13
12
11
10
9
8
7
6
5
4
3
2
1
meanThenRank
medianThenRank
rankThenMean
rankThenMedian
testBased
Ranking method
algorithm
SyNOnly_CC_2_reg_iter_1_bleached_masked
SyNOnly_CC_2_reg_iter_1_normal_masked
SyNOnly_CC_4_reg_iter_1_bleached_masked
SyNOnly_CC_2_reg_iter_0_bleached_masked
SyNOnly_CC_6_reg_iter_1_bleached_masked
SyNOnly_CC_4_reg_iter_0_bleached_masked
SyNOnly_CC_4_reg_iter_1_normal_masked
SyNOnly_MI_32_reg_iter_1_bleached_masked
SyNOnly_CC_2_reg_iter_0_normal_masked
SyNOnly_CC_6_reg_iter_0_bleached_masked
SyNOnly_MI_32_reg_iter_0_bleached_masked
SyNOnly_CC_6_reg_iter_1_normal_masked
SyNOnly_CC_4_reg_iter_0_normal_masked
SyNOnly_MI_32_reg_iter_1_normal_masked
SyNOnly_CC_6_reg_iter_0_normal_masked
SyNOnly_MI_32_reg_iter_0_normal_masked
TVMSQ_bleached_masked
TVMSQC_bleached_masked
TVMSQC_normal_masked
TVMSQ_normal_masked

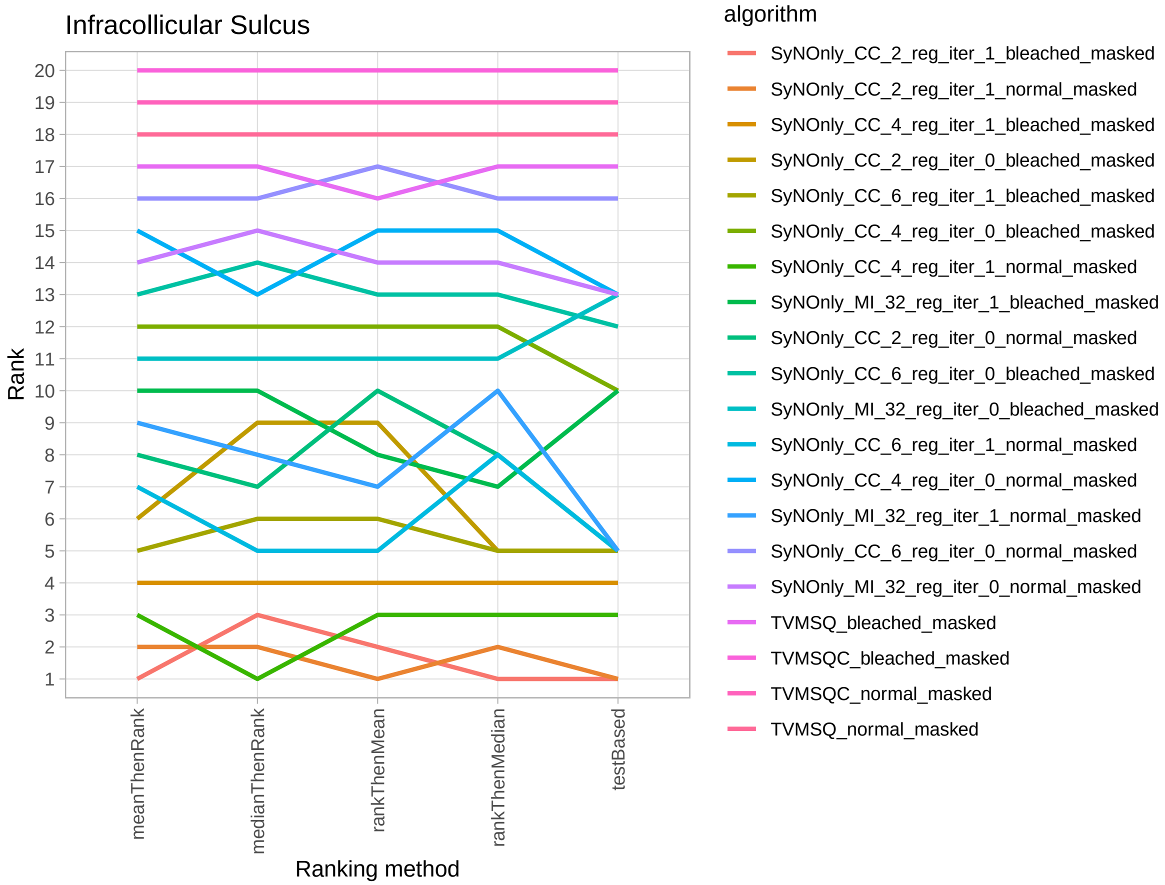
Infracollicular Sulcus
Rank
20
19
18
17
16
15
14
13
12
11
10
9
8
7
6
5
4
3
2
1
meanThenRank
medianThenRank
rankThenMean
rankThenMedian
testBased
Ranking method
algorithm
SyNOnly_CC_2_reg_iter_1_bleached_masked
SyNOnly_CC_2_reg_iter_1_normal_masked
SyNOnly_CC_4_reg_iter_1_bleached_masked
SyNOnly_CC_2_reg_iter_0_bleached_masked
SyNOnly_CC_6_reg_iter_1_bleached_masked
SyNOnly_CC_4_reg_iter_0_bleached_masked
SyNOnly_CC_4_reg_iter_1_normal_masked
SyNOnly_MI_32_reg_iter_1_bleached_masked
SyNOnly_CC_2_reg_iter_0_normal_masked
SyNOnly_CC_6_reg_iter_0_bleached_masked
SyNOnly_MI_32_reg_iter_0_bleached_masked
SyNOnly_CC_6_reg_iter_1_normal_masked
SyNOnly_CC_4_reg_iter_0_normal_masked
SyNOnly_MI_32_reg_iter_1_normal_masked
SyNOnly_CC_6_reg_iter_0_normal_masked
SyNOnly_MI_32_reg_iter_0_normal_masked
TVMSQ_bleached_masked
TVMSQC_bleached_masked
TVMSQC_normal_masked
TVMSQ_normal_masked

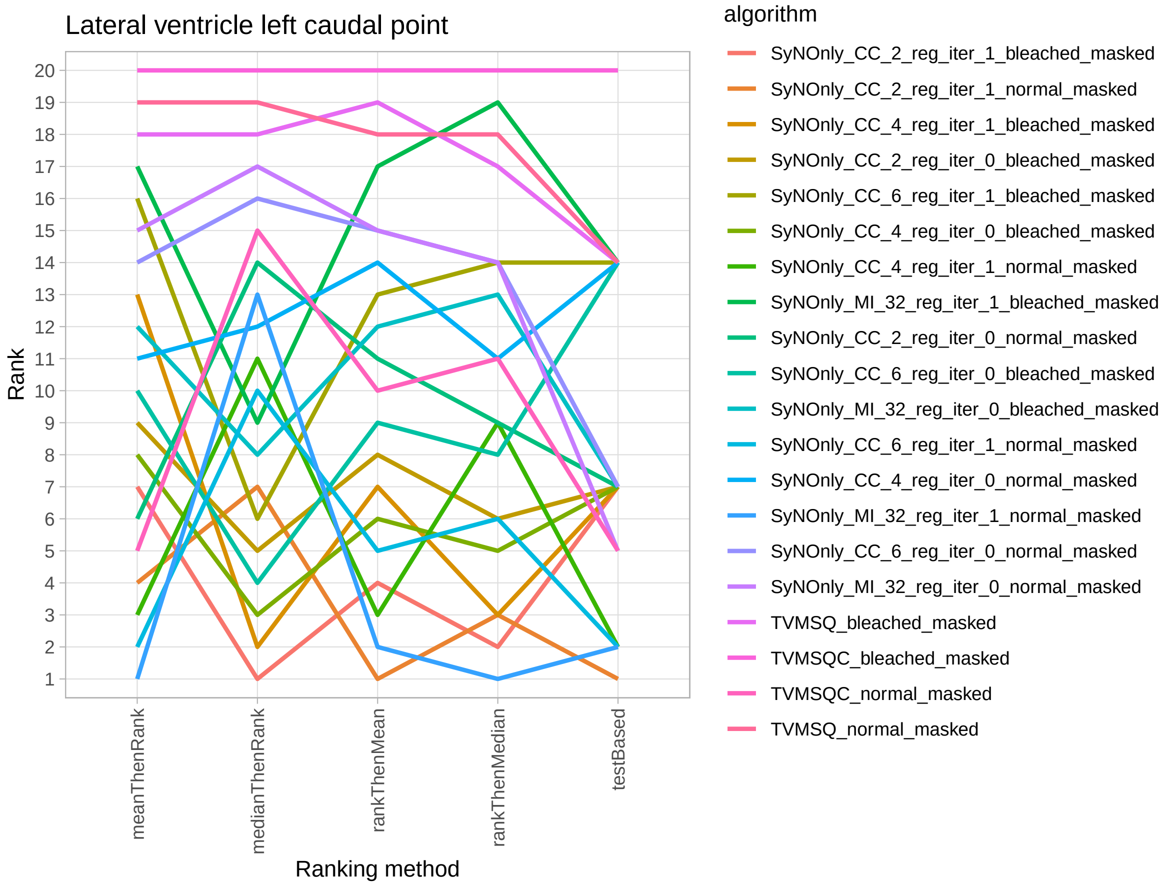

Lateral ventricle left caudal point
Rank
20
19
18
17
16
15
14
13
12
11
10
9
8
7
6
5
4
3
2
1
meanThenRank
medianThenRank
rankThenMean
rankThenMedian
testBased
Ranking method
algorithm
SyNOnly_CC_2_reg_iter_1_bleached_masked
SyNOnly_CC_2_reg_iter_1_normal_masked
SyNOnly_CC_4_reg_iter_1_bleached_masked
SyNOnly_CC_2_reg_iter_0_bleached_masked
SyNOnly_CC_6_reg_iter_1_bleached_masked
SyNOnly_CC_4_reg_iter_0_bleached_masked
SyNOnly_CC_4_reg_iter_1_normal_masked
SyNOnly_MI_32_reg_iter_1_bleached_masked
SyNOnly_CC_2_reg_iter_0_normal_masked
SyNOnly_CC_6_reg_iter_0_bleached_masked
SyNOnly_MI_32_reg_iter_0_bleached_masked
SyNOnly_CC_6_reg_iter_1_normal_masked
SyNOnly_CC_4_reg_iter_0_normal_masked
SyNOnly_MI_32_reg_iter_1_normal_masked
SyNOnly_CC_6_reg_iter_0_normal_masked
SyNOnly_MI_32_reg_iter_0_normal_masked
TVMSQ_bleached_masked
TVMSQC_bleached_masked
TVMSQC_normal_masked
TVMSQ_normal_masked

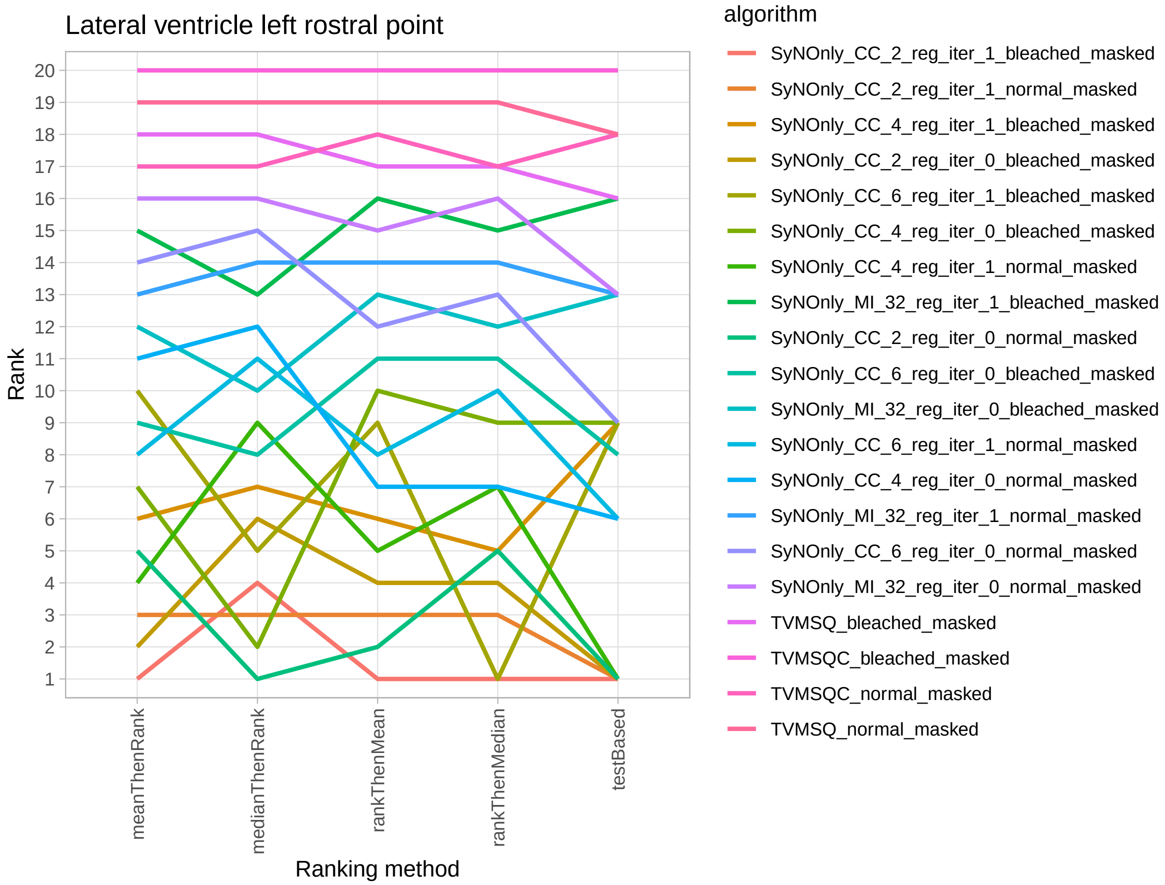
Lateral ventricle left rostral point
Rank
Ranking method
meanThenRank
medianThenRank
rankThenMean
rankThenMedian
testBased
algorithm
SyNOnly_CC_2_reg_iter_1_bleached_masked
SyNOnly_CC_2_reg_iter_1_normal_masked
SyNOnly_CC_4_reg_iter_1_bleached_masked
SyNOnly_CC_2_reg_iter_0_bleached_masked
SyNOnly_CC_6_reg_iter_1_bleached_masked
SyNOnly_CC_4_reg_iter_0_bleached_masked
SyNOnly_CC_4_reg_iter_1_normal_masked
SyNOnly_MI_32_reg_iter_1_bleached_masked
SyNOnly_CC_2_reg_iter_0_normal_masked
SyNOnly_CC_6_reg_iter_0_bleached_masked
SyNOnly_MI_32_reg_iter_0_bleached_masked
SyNOnly_CC_6_reg_iter_1_normal_masked
SyNOnly_CC_4_reg_iter_0_normal_masked
SyNOnly_MI_32_reg_iter_1_normal_masked
SyNOnly_CC_6_reg_iter_0_normal_masked
SyNOnly_MI_32_reg_iter_0_normal_masked
TVMSQ_bleached_masked
TVMSQC_bleached_masked
TVMSQC_normal_masked
TVMSQ_normal_masked

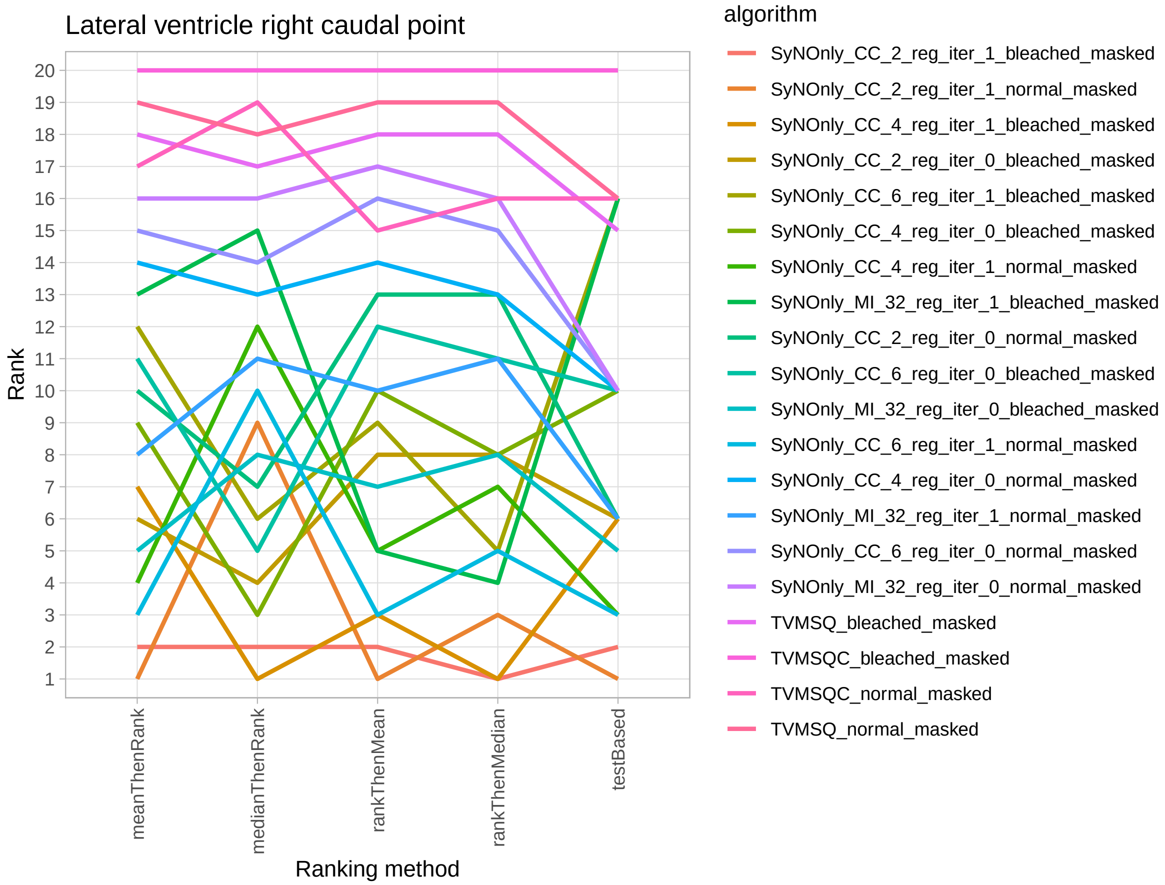
Lateral ventricle right caudal point
Rank
20
19
18
17
16
15
14
13
12
11
10
9
8
7
6
5
4
3
2
1
meanThenRank
medianThenRank
rankThenMean
rankThenMedian
testBased
Ranking method
algorithm
SyNOnly_CC_2_reg_iter_1_bleached_masked
SyNOnly_CC_2_reg_iter_1_normal_masked
SyNOnly_CC_4_reg_iter_1_bleached_masked
SyNOnly_CC_2_reg_iter_0_bleached_masked
SyNOnly_CC_6_reg_iter_1_bleached_masked
SyNOnly_CC_4_reg_iter_0_bleached_masked
SyNOnly_CC_4_reg_iter_1_normal_masked
SyNOnly_MI_32_reg_iter_1_bleached_masked
SyNOnly_CC_2_reg_iter_0_normal_masked
SyNOnly_CC_6_reg_iter_0_bleached_masked
SyNOnly_MI_32_reg_iter_0_bleached_masked
SyNOnly_CC_6_reg_iter_1_normal_masked
SyNOnly_CC_4_reg_iter_0_normal_masked
SyNOnly_MI_32_reg_iter_1_normal_masked
SyNOnly_CC_6_reg_iter_0_normal_masked
SyNOnly_MI_32_reg_iter_0_normal_masked
TVMSQ_bleached_masked
TVMSQC_bleached_masked
TVMSQC_normal_masked
TVMSQ_normal_masked

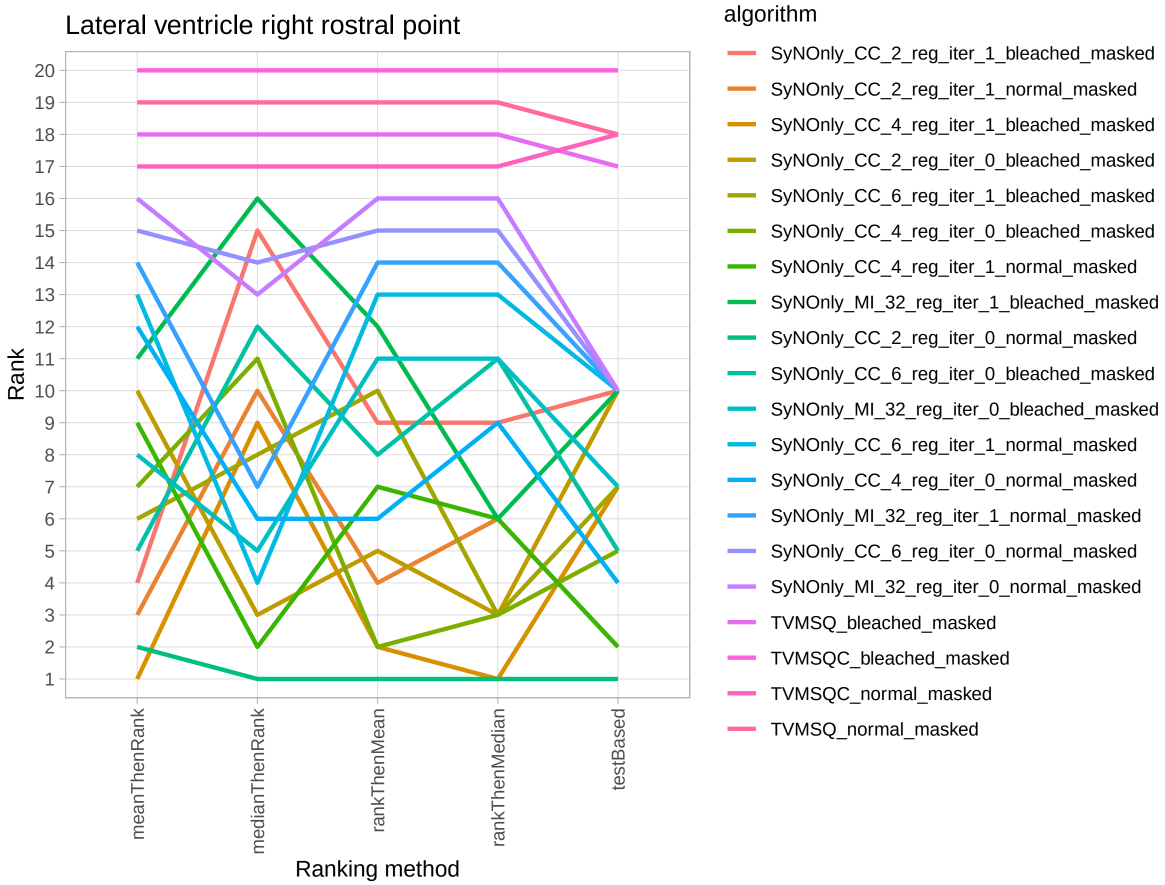

Lateral ventricle right rostral point
Rank
20
19
18
17
16
15
14
13
12
11
10
9
8
7
6
5
4
3
2
1
meanThenRank
medianThenRank
rankThenMean
rankThenMedian
testBased
Ranking method
algorithm
SyNOnly_CC_2_reg_iter_1_bleached_masked
SyNOnly_CC_2_reg_iter_1_normal_masked
SyNOnly_CC_4_reg_iter_1_bleached_masked
SyNOnly_CC_2_reg_iter_0_bleached_masked
SyNOnly_CC_6_reg_iter_1_bleached_masked
SyNOnly_CC_4_reg_iter_0_bleached_masked
SyNOnly_CC_4_reg_iter_1_normal_masked
SyNOnly_MI_32_reg_iter_1_bleached_masked
SyNOnly_CC_2_reg_iter_0_normal_masked
SyNOnly_CC_6_reg_iter_0_bleached_masked
SyNOnly_MI_32_reg_iter_0_bleached_masked
SyNOnly_CC_6_reg_iter_1_normal_masked
SyNOnly_CC_4_reg_iter_0_normal_masked
SyNOnly_MI_32_reg_iter_1_normal_masked
SyNOnly_CC_6_reg_iter_0_normal_masked
SyNOnly_MI_32_reg_iter_0_normal_masked
TVMSQ_bleached_masked
TVMSQC_bleached_masked
TVMSQC_normal_masked
TVMSQ_normal_masked

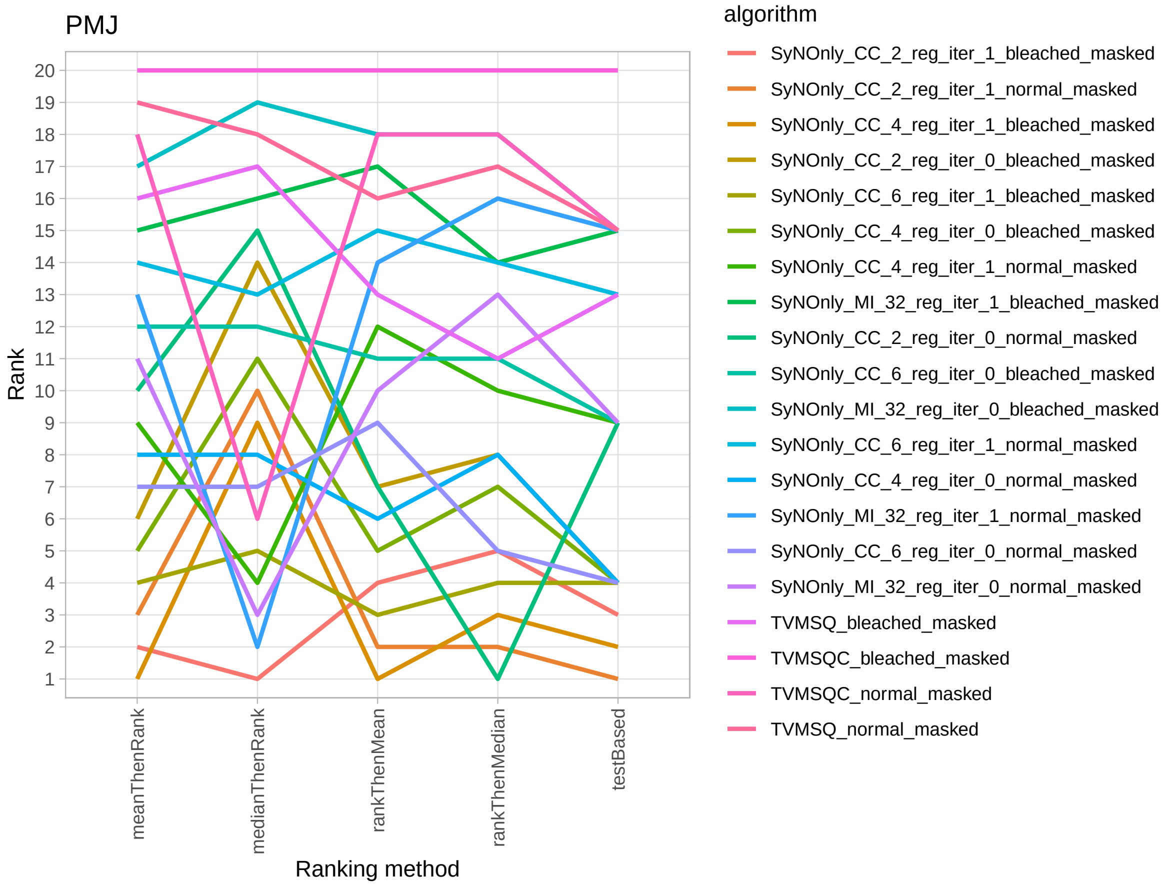
PMJ
Rank
Ranking method
meanThenRank
medianThenRank
rankThenMean
rankThenMedian
testBased
algorithm
SyNOnly_CC_2_reg_iter_1_bleached_masked
SyNOnly_CC_2_reg_iter_1_normal_masked
SyNOnly_CC_4_reg_iter_1_bleached_masked
SyNOnly_CC_2_reg_iter_0_bleached_masked
SyNOnly_CC_6_reg_iter_1_bleached_masked
SyNOnly_CC_4_reg_iter_0_bleached_masked
SyNOnly_CC_4_reg_iter_1_normal_masked
SyNOnly_MI_32_reg_iter_1_bleached_masked
SyNOnly_CC_2_reg_iter_0_normal_masked
SyNOnly_CC_6_reg_iter_0_bleached_masked
SyNOnly_MI_32_reg_iter_0_bleached_masked
SyNOnly_CC_6_reg_iter_1_normal_masked
SyNOnly_CC_4_reg_iter_0_normal_masked
SyNOnly_MI_32_reg_iter_1_normal_masked
SyNOnly_CC_6_reg_iter_0_normal_masked
SyNOnly_MI_32_reg_iter_0_normal_masked
TVMSQ_bleached_masked
TVMSQC_bleached_masked
TVMSQC_normal_masked
TVMSQ_normal_masked

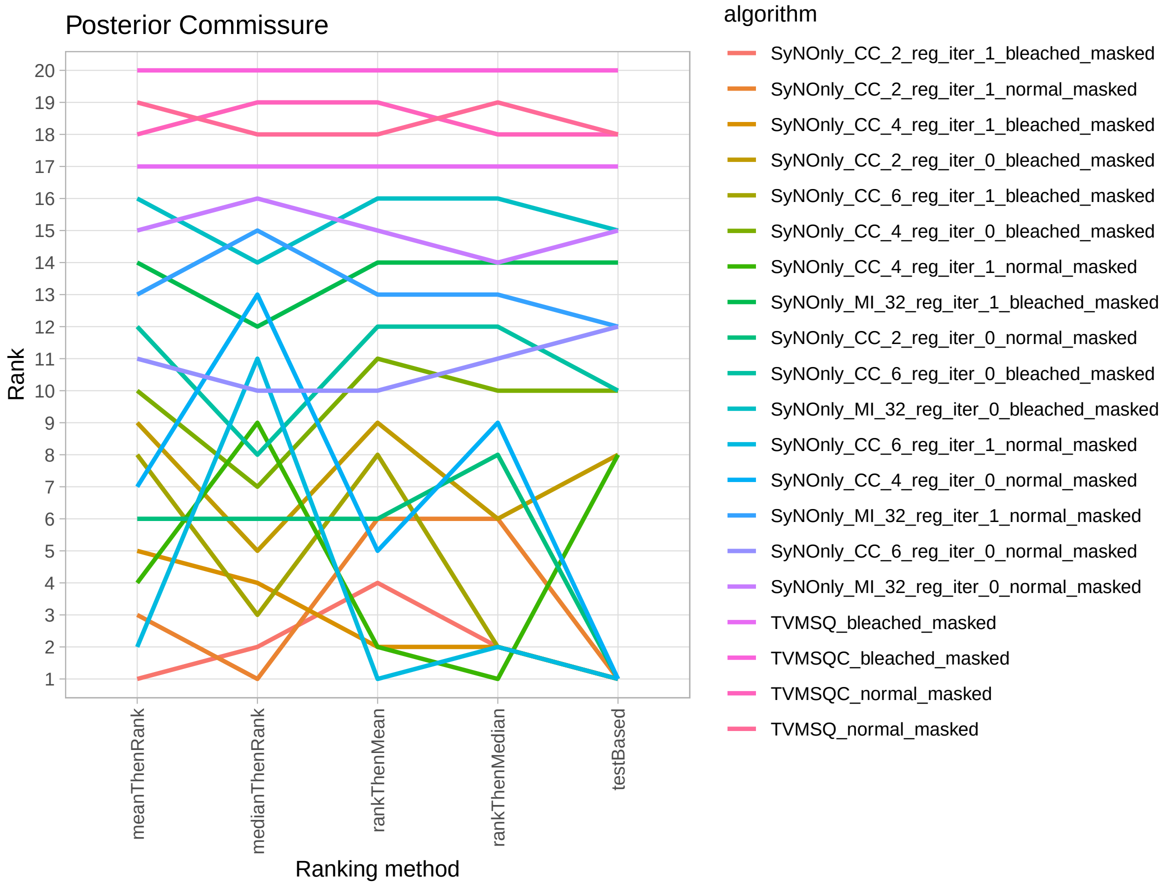
Posterior Commissure
algorithm
SyNOnly_CC_2_reg_iter_1_bleached_masked
SyNOnly_CC_2_reg_iter_1_normal_masked
SyNOnly_CC_4_reg_iter_1_bleached_masked
SyNOnly_CC_2_reg_iter_0_bleached_masked
SyNOnly_CC_6_reg_iter_1_bleached_masked
SyNOnly_CC_4_reg_iter_0_bleached_masked
SyNOnly_CC_4_reg_iter_1_normal_masked
SyNOnly_MI_32_reg_iter_1_bleached_masked
SyNOnly_CC_2_reg_iter_0_normal_masked
SyNOnly_CC_6_reg_iter_0_bleached_masked
SyNOnly_MI_32_reg_iter_0_bleached_masked
SyNOnly_CC_6_reg_iter_1_normal_masked
SyNOnly_CC_4_reg_iter_0_normal_masked
SyNOnly_MI_32_reg_iter_1_normal_masked
SyNOnly_CC_6_reg_iter_0_normal_masked
SyNOnly_MI_32_reg_iter_0_normal_masked
TVMSQ_bleached_masked
TVMSQC_bleached_masked
TVMSQC_normal_masked
TVMSQ_normal_masked
Rank
20
19
18
17
16
15
14
13
12
11
10
9
8
7
6
5
4
3
2
1
meanThenRank
medianThenRank
rankThenMean
rankThenMedian
testBased
Ranking method

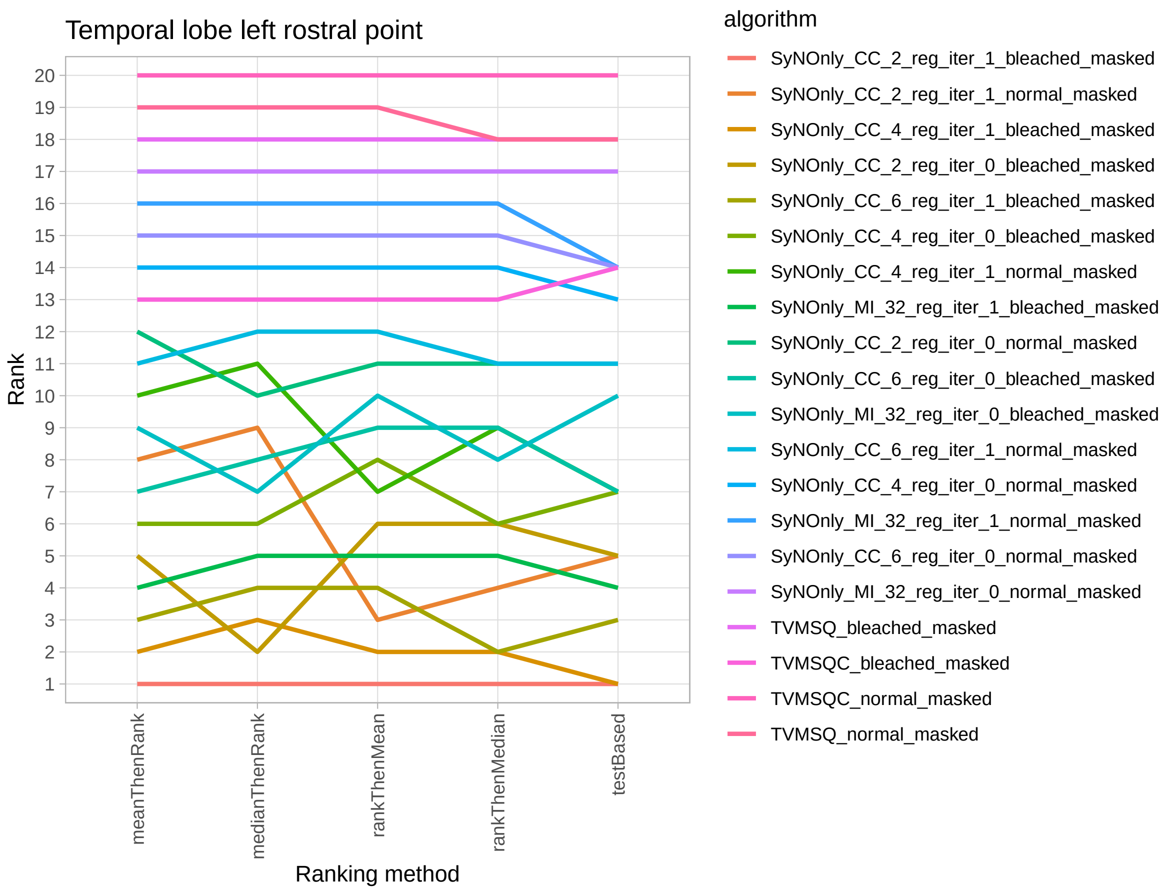
Temporal lobe left rostral point
Rank
20
19
18
17
16
15
14
13
12
11
10
9
8
7
6
5
4
3
2
1
meanThenRank
medianThenRank
rankThenMean
rankThenMedian
testBased
Ranking method
algorithm
SyNOnly_CC_2_reg_iter_1_bleached_masked
SyNOnly_CC_2_reg_iter_1_normal_masked
SyNOnly_CC_4_reg_iter_1_bleached_masked
SyNOnly_CC_2_reg_iter_0_bleached_masked
SyNOnly_CC_6_reg_iter_1_bleached_masked
SyNOnly_CC_4_reg_iter_0_bleached_masked
SyNOnly_CC_4_reg_iter_1_normal_masked
SyNOnly_MI_32_reg_iter_1_bleached_masked
SyNOnly_CC_2_reg_iter_0_normal_masked
SyNOnly_CC_6_reg_iter_0_bleached_masked
SyNOnly_MI_32_reg_iter_0_bleached_masked
SyNOnly_CC_6_reg_iter_1_normal_masked
SyNOnly_CC_4_reg_iter_0_normal_masked
SyNOnly_MI_32_reg_iter_1_normal_masked
SyNOnly_CC_6_reg_iter_0_normal_masked
SyNOnly_MI_32_reg_iter_0_normal_masked
TVMSQ_bleached_masked
TVMSQC_bleached_masked
TVMSQC_normal_masked
TVMSQ_normal_masked

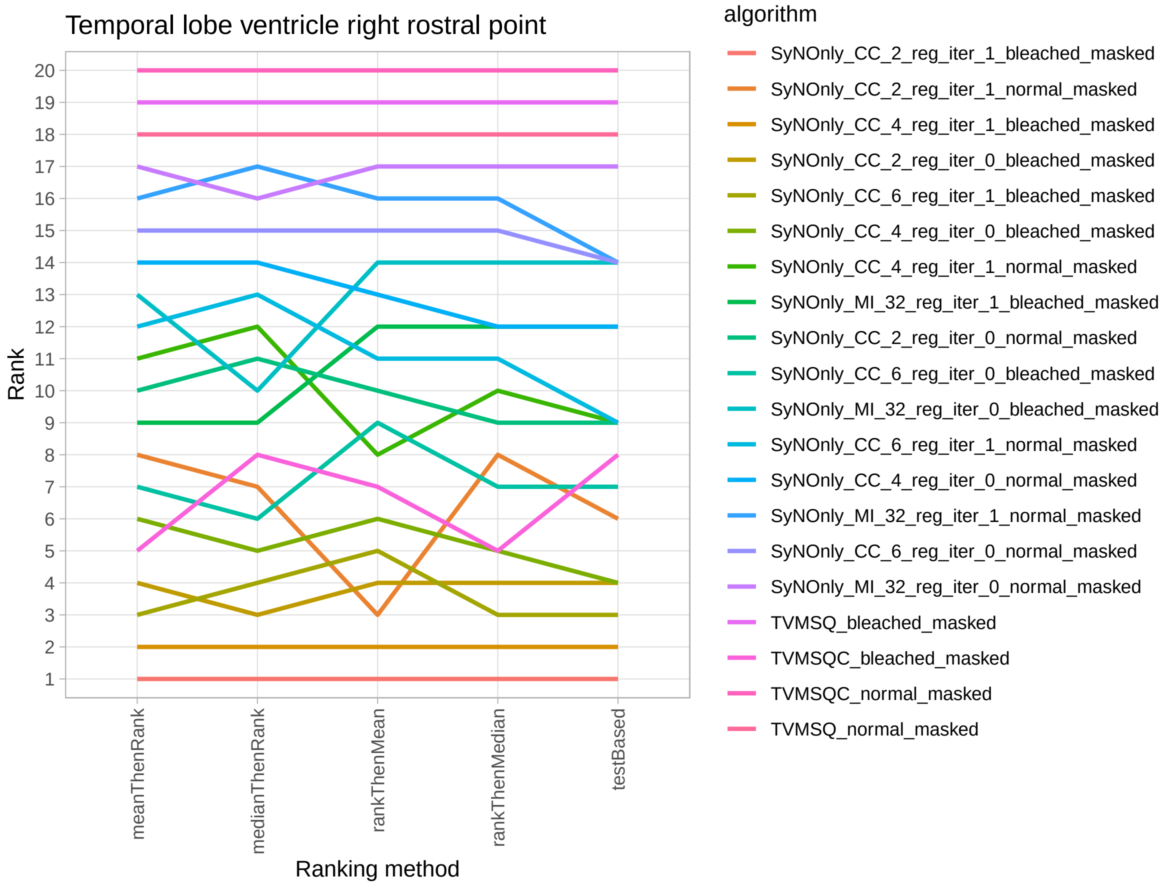
Temporal lobe ventricle right rostral point
Rank
20
19
18
17
16
15
14
13
12
11
10
9
8
7
6
5
4
3
2
1
meanThenRank
medianThenRank
rankThenMean
rankThenMedian
testBased
Ranking method
algorithm
SyNOnly_CC_2_reg_iter_1_bleached_masked
SyNOnly_CC_2_reg_iter_1_normal_masked
SyNOnly_CC_4_reg_iter_1_bleached_masked
SyNOnly_CC_2_reg_iter_0_bleached_masked
SyNOnly_CC_6_reg_iter_1_bleached_masked
SyNOnly_CC_4_reg_iter_0_bleached_masked
SyNOnly_CC_4_reg_iter_1_normal_masked
SyNOnly_MI_32_reg_iter_1_bleached_masked
SyNOnly_CC_2_reg_iter_0_normal_masked
SyNOnly_CC_6_reg_iter_0_bleached_masked
SyNOnly_MI_32_reg_iter_0_bleached_masked
SyNOnly_CC_6_reg_iter_1_normal_masked
SyNOnly_CC_4_reg_iter_0_normal_masked
SyNOnly_MI_32_reg_iter_1_normal_masked
SyNOnly_CC_6_reg_iter_0_normal_masked
SyNOnly_MI_32_reg_iter_0_normal_masked
TVMSQ_bleached_masked
TVMSQC_bleached_masked
TVMSQC_normal_masked
TVMSQ_normal_masked

4 Visualization of cross-task insights

The algorithms are ordered according to consensus ranking.

4.1 Characterization of algorithms

4.1.1 Ranking stability: Variability of achieved rankings across tasks

Algorithms are color-coded, and the area of each blob at position $(A_i, \text{rank } j)$ is proportional to the relative frequency A_i achieved rank j across multiple tasks. The median rank for each algorithm is indicated by a black cross. This way, the distribution of ranks across tasks can be intuitively visualized.

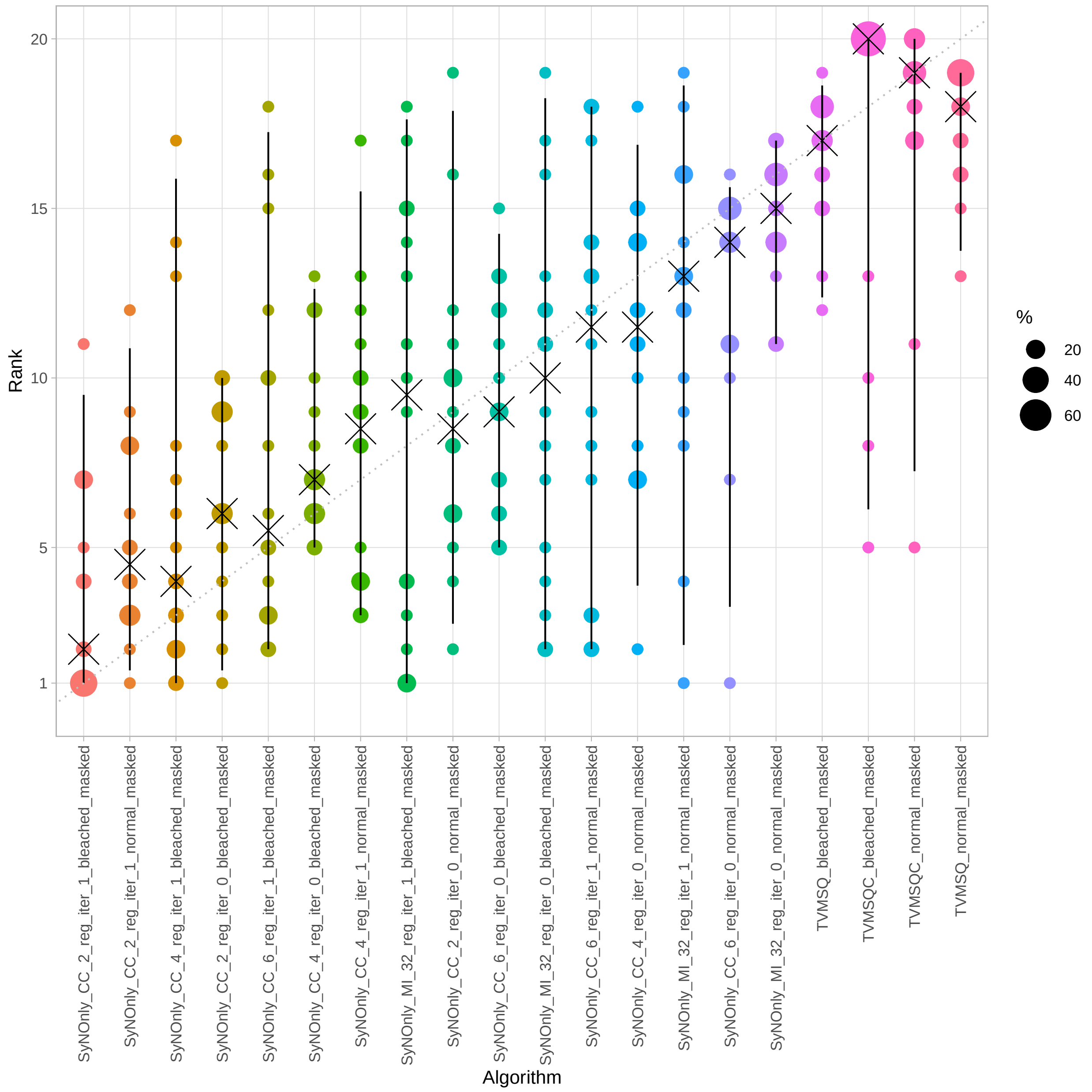

4.1.2 Ranking stability: Ranking variability via bootstrap approach

A blob plot of bootstrap results over the different tasks separated by algorithm allows another perspective on the assessment data. This gives deeper insights into the characteristics of tasks and the ranking uncertainty of the algorithms in each task.

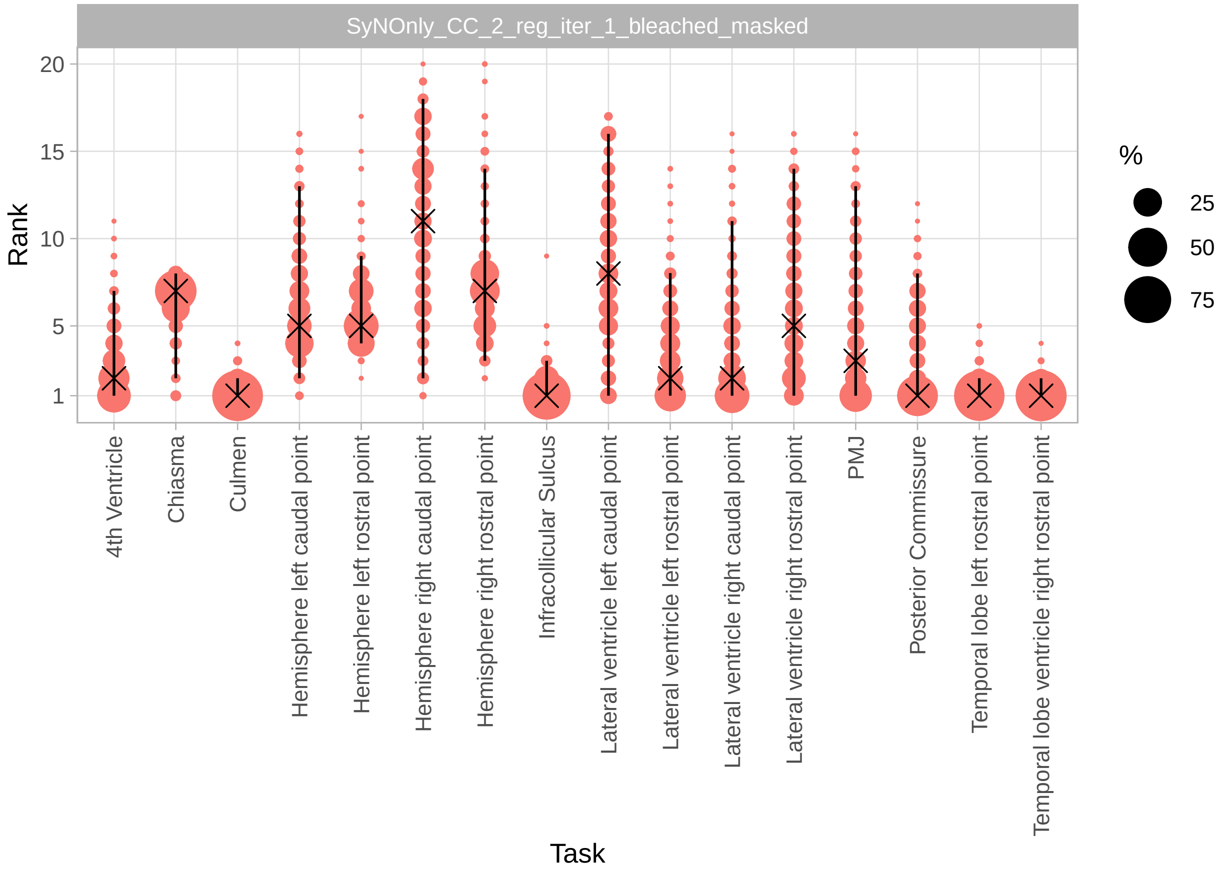

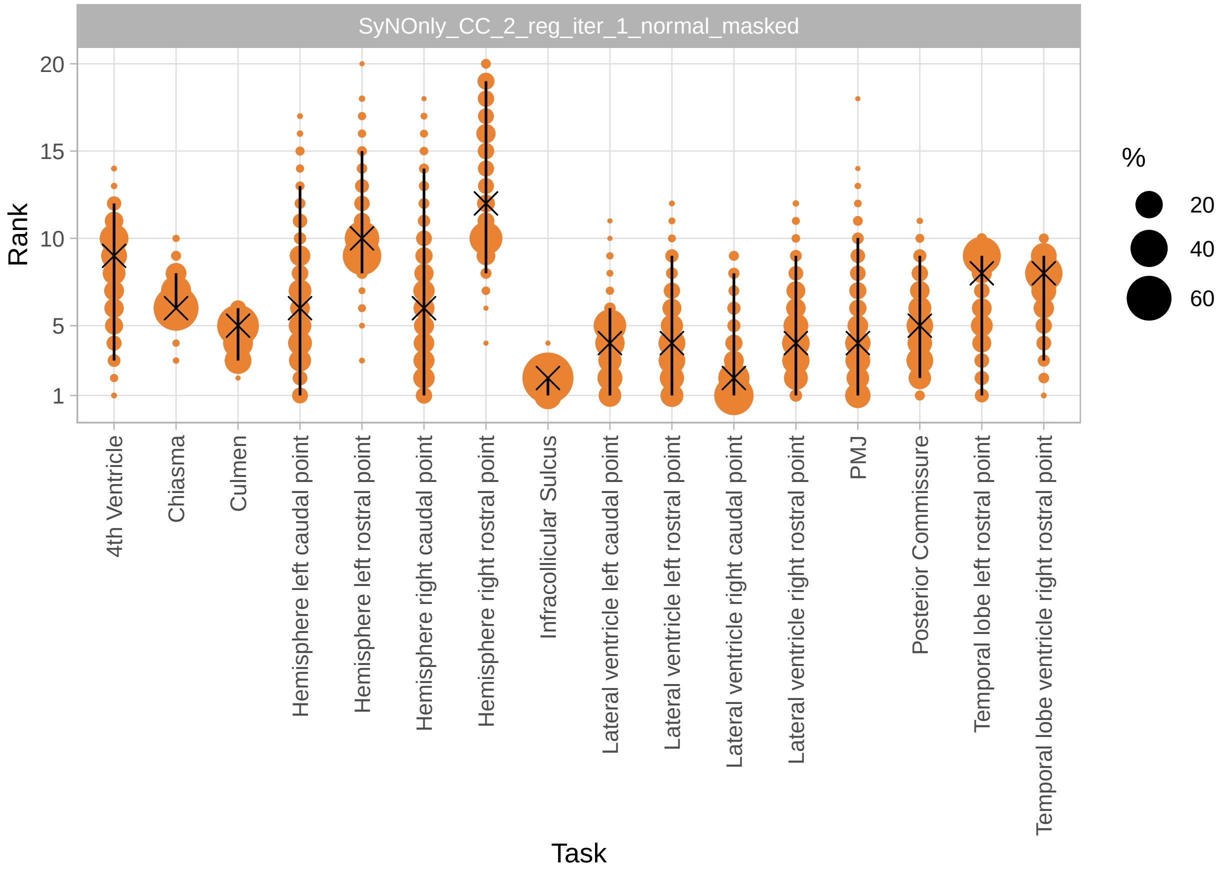
SyNOnly_CC_2_reg_iter_1_normal_masked
Rank
20
15
10
5
1
4th Ventricle
Chiasma
Culmen
Hemisphere left caudal point
Hemisphere left rostral point
Hemisphere right caudal point
Hemisphere right rostral point
Infracollicular Sulcus
Lateral ventricle left caudal point
Lateral ventricle left rostral point
Lateral ventricle right caudal point
Lateral ventricle right rostral point
PMJ
Posterior Commissure
Temporal lobe left rostral point
Temporal lobe ventricle right rostral point
Task
%
20
40
60

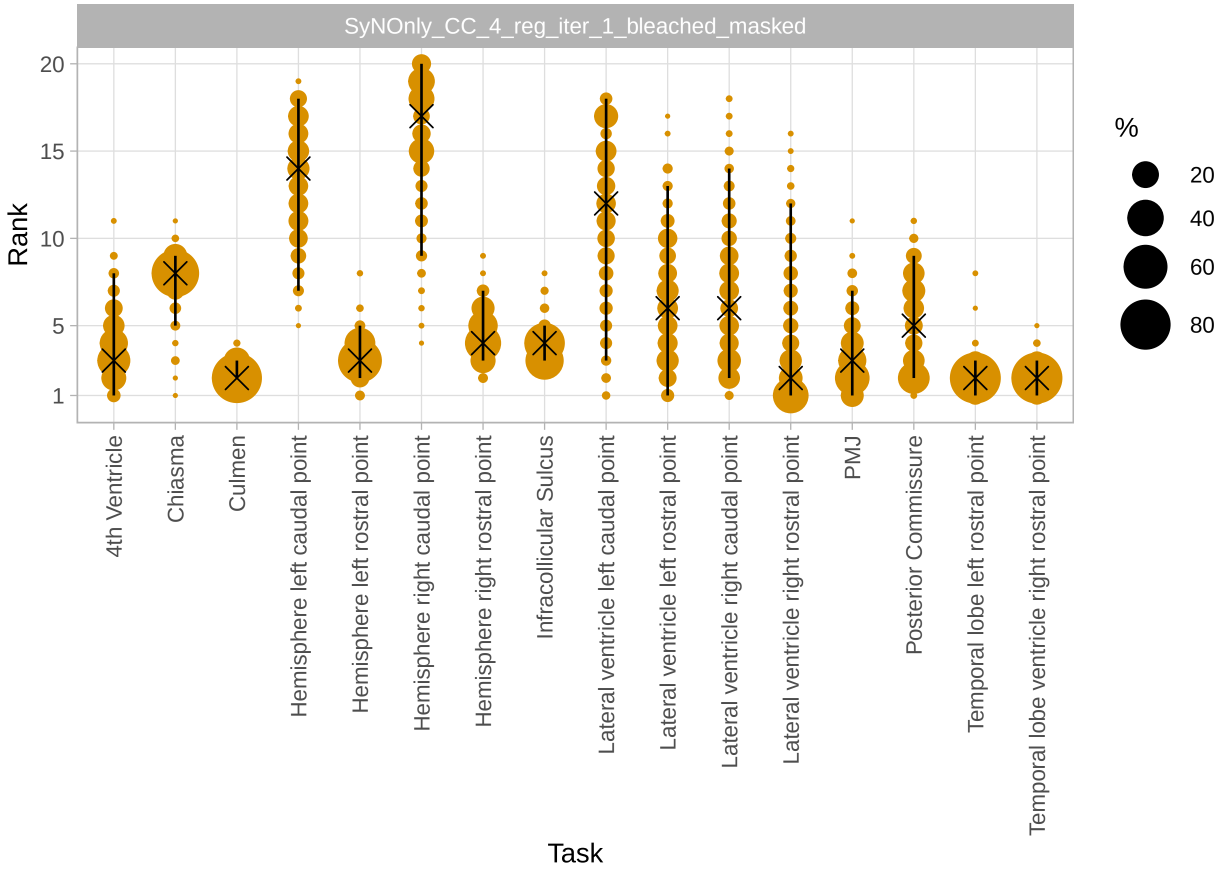
SyNOnly_CC_4_reg_iter_1_bleached_masked
Rank
20
15
10
5
1
%
20
40
60
80
4th Ventricle
Chiasma
Culmen
Hemisphere left caudal point
Hemisphere left rostral point
Hemisphere right caudal point
Hemisphere right rostral point
Infracollicular Sulcus
Lateral ventricle left caudal point
Lateral ventricle left rostral point
Lateral ventricle right caudal point
Lateral ventricle right rostral point
PMJ
Posterior Commissure
Temporal lobe left rostral point
Temporal lobe ventricle right rostral point
Task

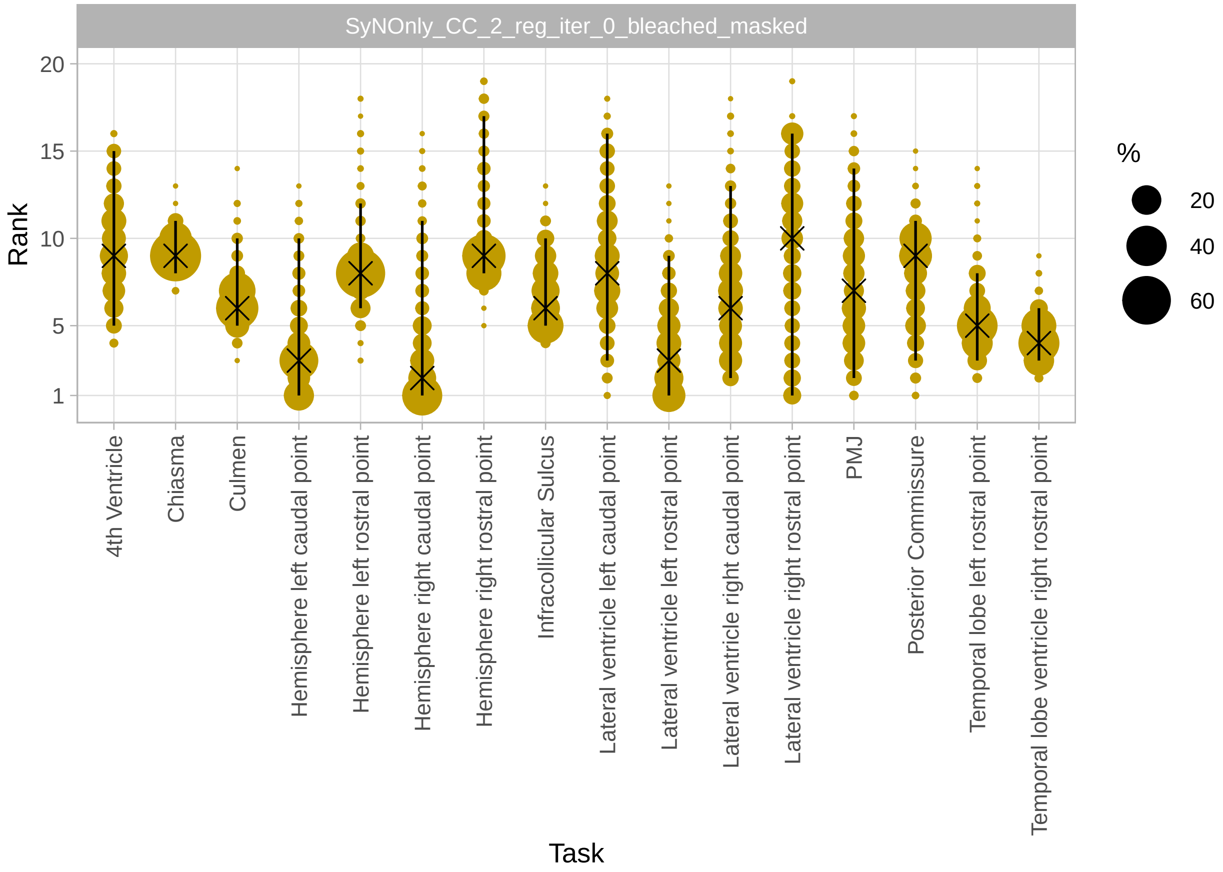
SyNOnly_CC_2_reg_iter_0_bleached_masked
Rank
20
15
10
5
1
Task
4th Ventricle
Chiasma
Culmen
Hemisphere left caudal point
Hemisphere left rostral point
Hemisphere right caudal point
Hemisphere right rostral point
Infracollicular Sulcus
Lateral ventricle left caudal point
Lateral ventricle left rostral point
Lateral ventricle right caudal point
Lateral ventricle right rostral point
PMJ
Posterior Commissure
Temporal lobe left rostral point
Temporal lobe ventricle right rostral point
%
20
40
60

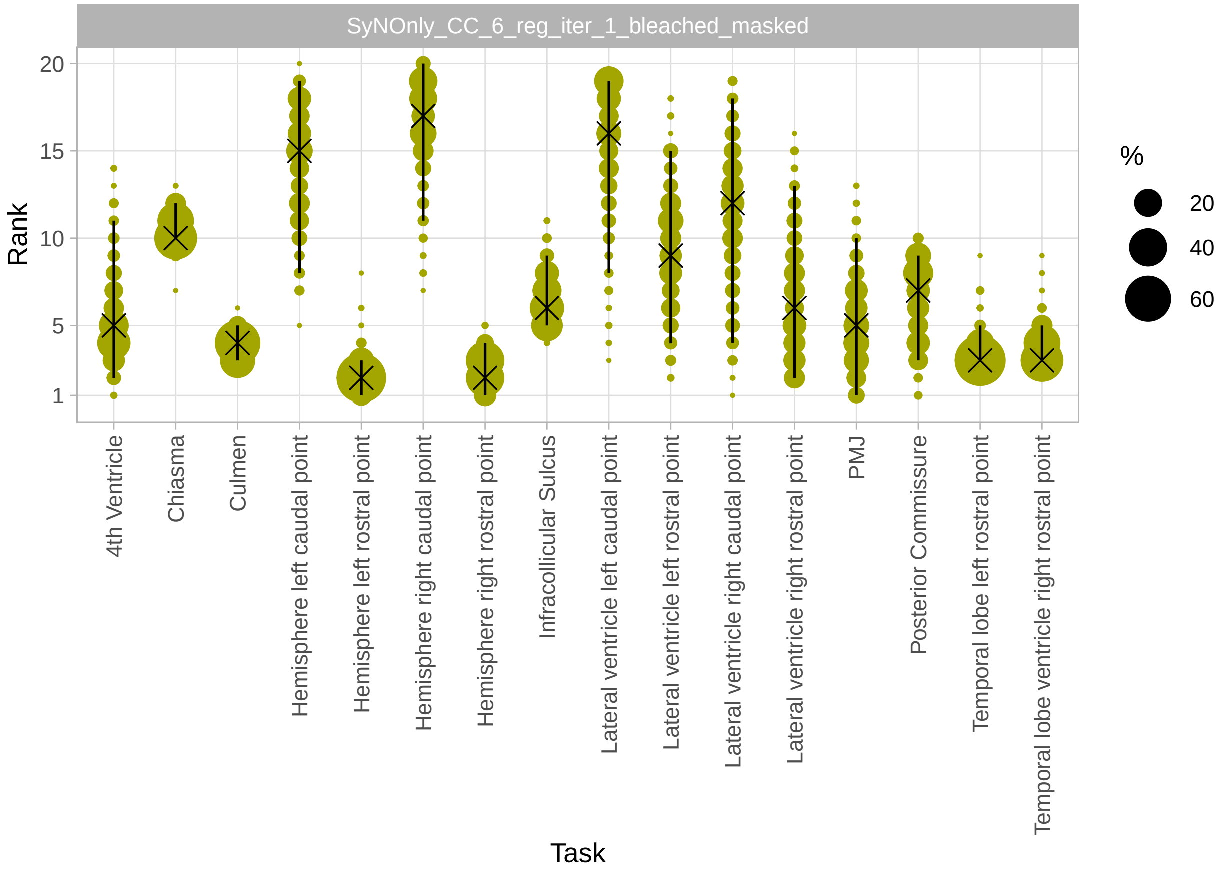
SyNOnly_CC_6_reg_iter_1_bleached_masked
Rank
20
15
10
5
1
%
20
40
60
4th Ventricle
Chiasma
Culmen
Hemisphere left caudal point
Hemisphere left rostral point
Hemisphere right caudal point
Hemisphere right rostral point
Infracollicular Sulcus
Lateral ventricle left caudal point
Lateral ventricle left rostral point
Lateral ventricle right caudal point
Lateral ventricle right rostral point
PMJ
Posterior Commissure
Temporal lobe left rostral point
Temporal lobe ventricle right rostral point
Task

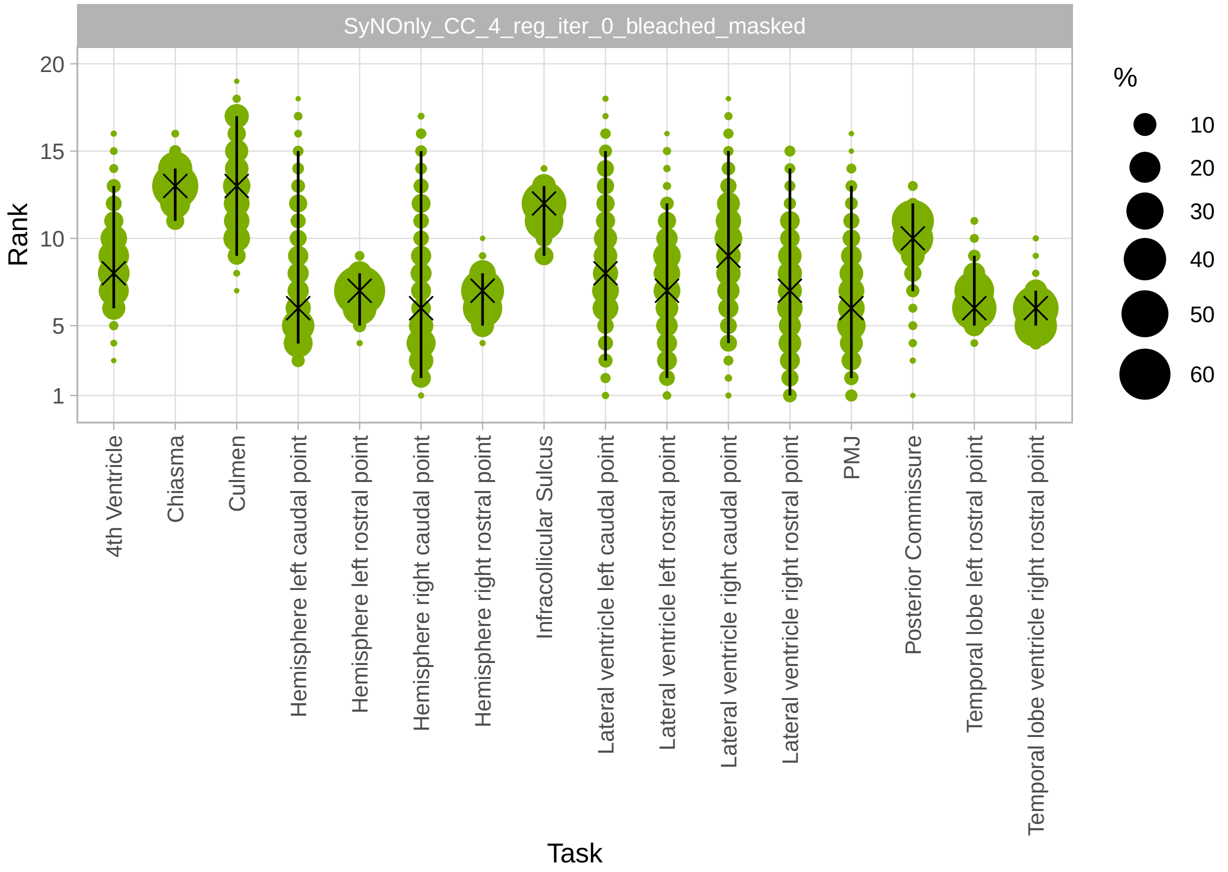

SyNOnly_CC_4_reg_iter_0_bleached_masked
Rank
20
15
10
5
1
Task
4th Ventricle
Chiasma
Culmen
Hemisphere left caudal point
Hemisphere left rostral point
Hemisphere right caudal point
Hemisphere right rostral point
Infracollicular Sulcus
Lateral ventricle left caudal point
Lateral ventricle left rostral point
Lateral ventricle right caudal point
Lateral ventricle right rostral point
PMJ
Posterior Commissure
Temporal lobe left rostral point
Temporal lobe ventricle right rostral point
%
10
20
30
40
50
60

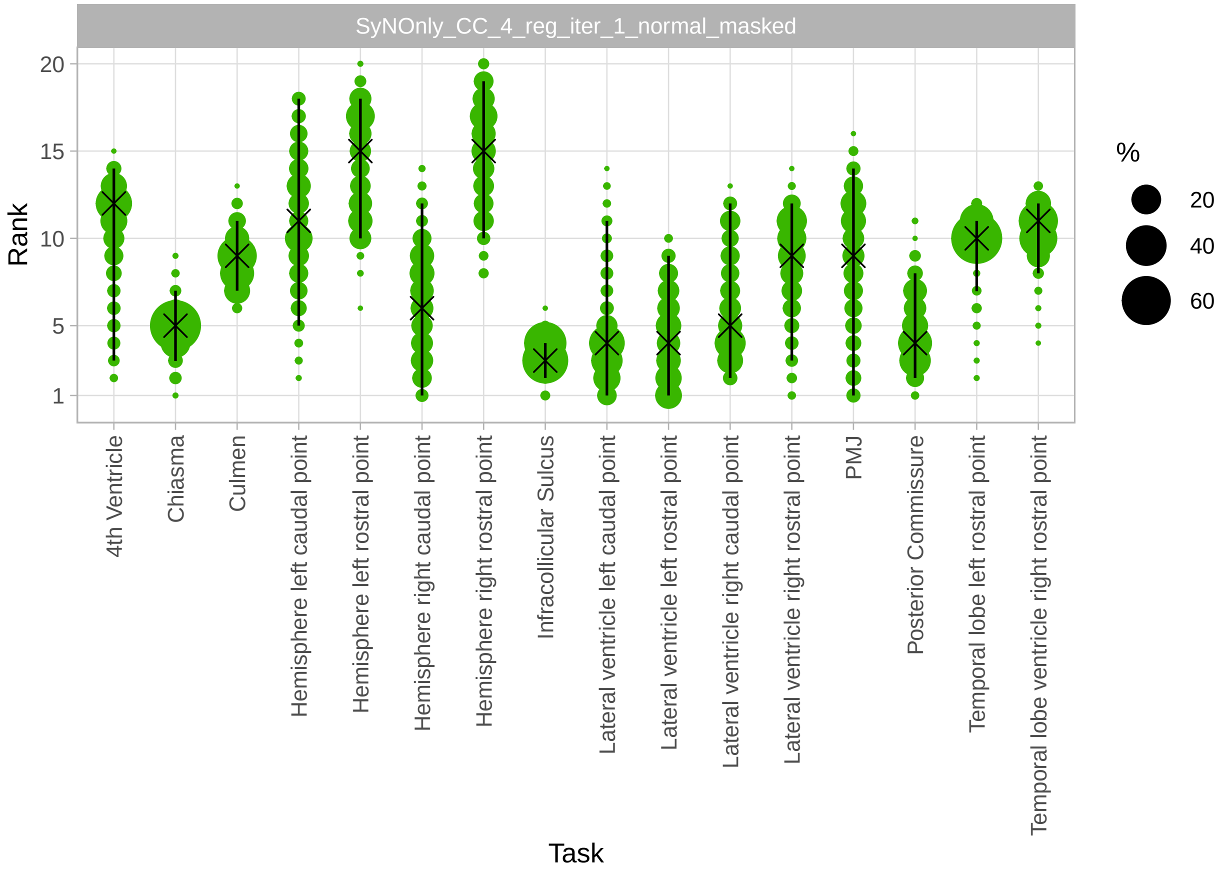
SyNOnly_CC_4_reg_iter_1_normal_masked
Rank
20
15
10
5
1
%
20
40
60
4th Ventricle
Chiasma
Culmen
Hemisphere left caudal point
Hemisphere left rostral point
Hemisphere right caudal point
Hemisphere right rostral point
Infracollicular Sulcus
Lateral ventricle left caudal point
Lateral ventricle left rostral point
Lateral ventricle right caudal point
Lateral ventricle right rostral point
PMJ
Posterior Commissure
Temporal lobe left rostral point
Temporal lobe ventricle right rostral point
Task

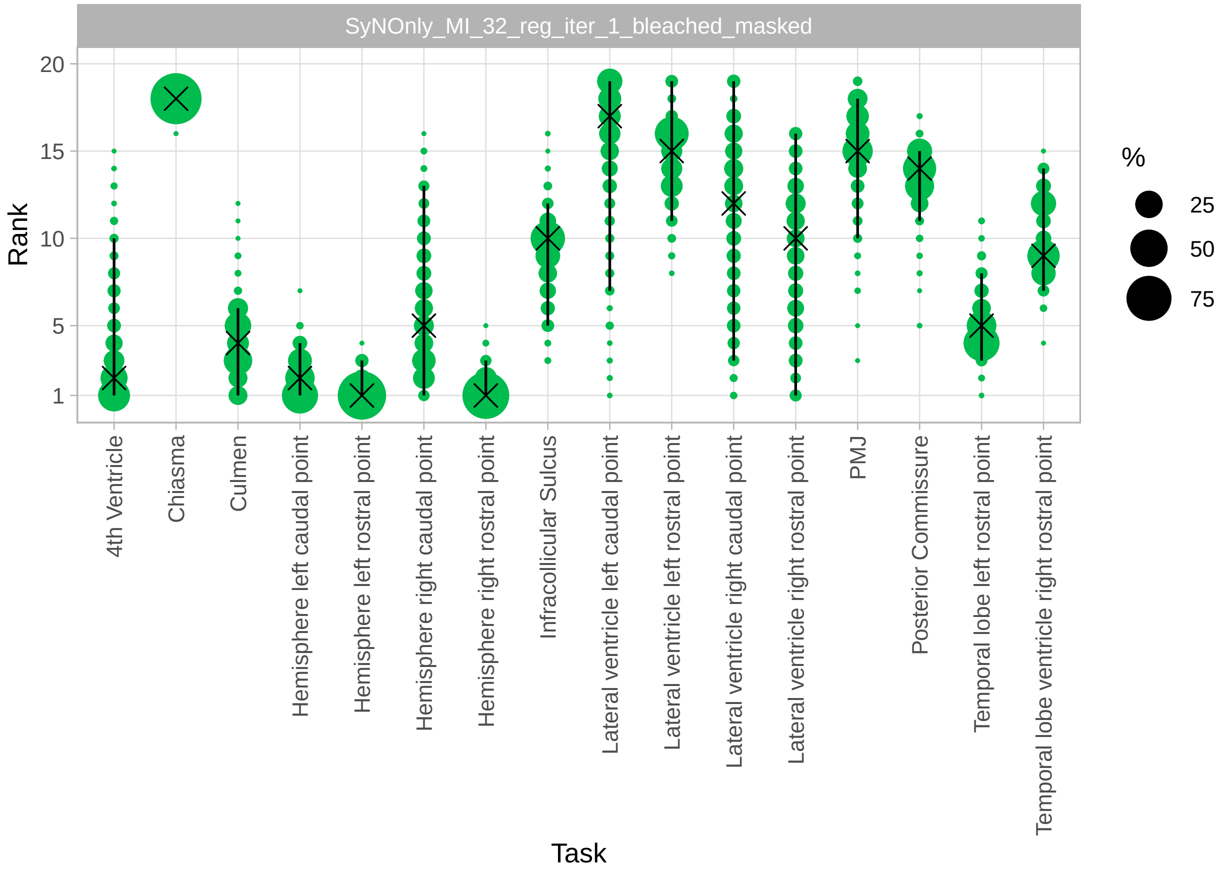

SyNOnly_MI_32_reg_iter_1_bleached_masked
Rank
20
15
10
5
1
4th Ventricle
Chiasma
Culmen
Hemisphere left caudal point
Hemisphere left rostral point
Hemisphere right caudal point
Hemisphere right rostral point
Infracollicular Sulcus
Lateral ventricle left caudal point
Lateral ventricle left rostral point
Lateral ventricle right caudal point
Lateral ventricle right rostral point
PMJ
Posterior Commissure
Temporal lobe left rostral point
Temporal lobe ventricle right rostral point
Task
%
25
50
75

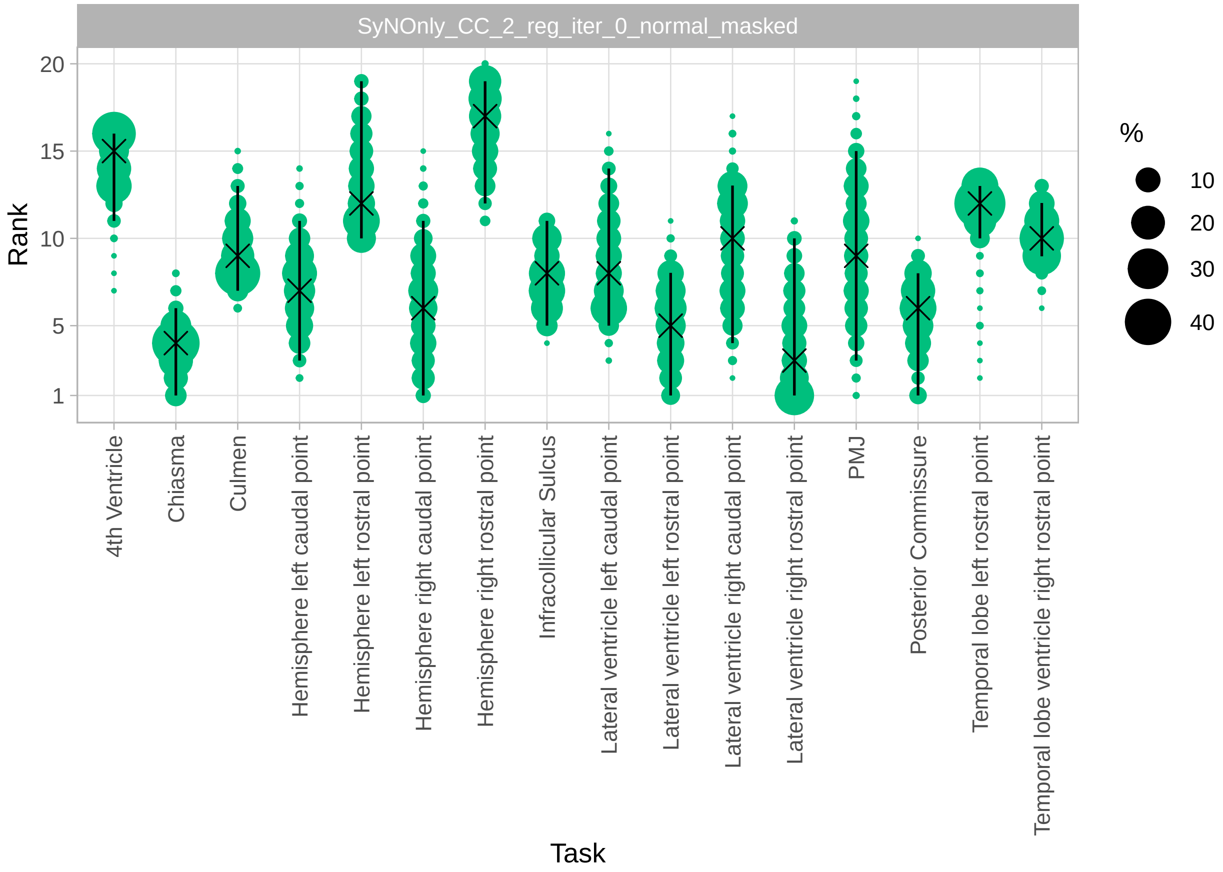
SyNOnly_CC_2_reg_iter_0_normal_masked
Rank
20
15
10
5
1
4th Ventricle
Chiasma
Culmen
Hemisphere left caudal point
Hemisphere left rostral point
Hemisphere right caudal point
Hemisphere right rostral point
Infracollicular Sulcus
Lateral ventricle left caudal point
Lateral ventricle left rostral point
Lateral ventricle right caudal point
Lateral ventricle right rostral point
PMJ
Posterior Commissure
Temporal lobe left rostral point
Temporal lobe ventricle right rostral point
Task
%
10
20
30
40

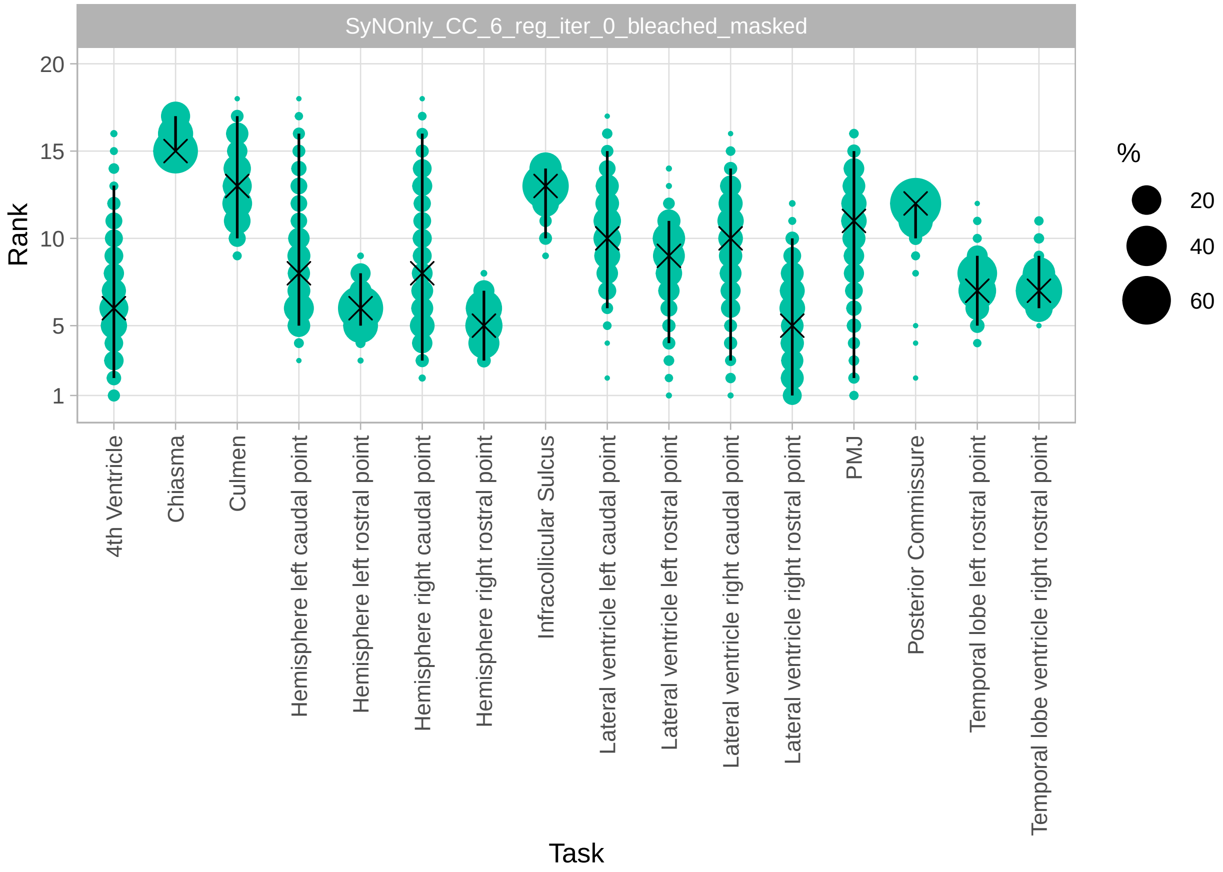
SyNOnly_CC_6_reg_iter_0_bleached_masked
Rank
20
15
10
5
1
4th Ventricle
Chiasma
Culmen
Hemisphere left caudal point
Hemisphere left rostral point
Hemisphere right caudal point
Hemisphere right rostral point
Infracollicular Sulcus
Lateral ventricle left caudal point
Lateral ventricle left rostral point
Lateral ventricle right caudal point
Lateral ventricle right rostral point
PMJ
Posterior Commissure
Temporal lobe left rostral point
Temporal lobe ventricle right rostral point
Task
%
20
40
60

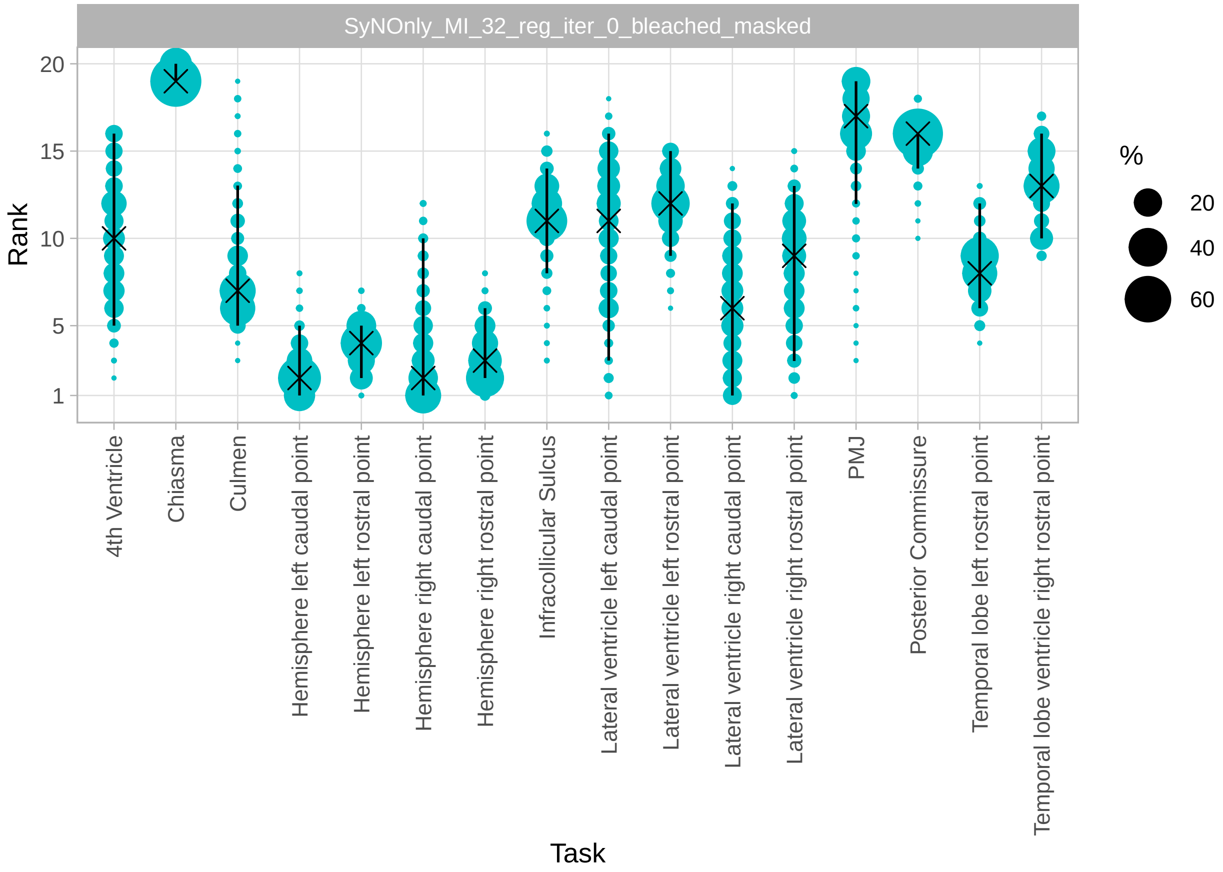
SyNOnly_MI_32_reg_iter_0_bleached_masked
Rank
20
15
10
5
1
4th Ventricle
Chiasma
Culmen
Hemisphere left caudal point
Hemisphere left rostral point
Hemisphere right caudal point
Hemisphere right rostral point
Infracollicular Sulcus
Lateral ventricle left caudal point
Lateral ventricle left rostral point
Lateral ventricle right caudal point
Lateral ventricle right rostral point
PMJ
Posterior Commissure
Temporal lobe left rostral point
Temporal lobe ventricle right rostral point
Task
%
20
40
60

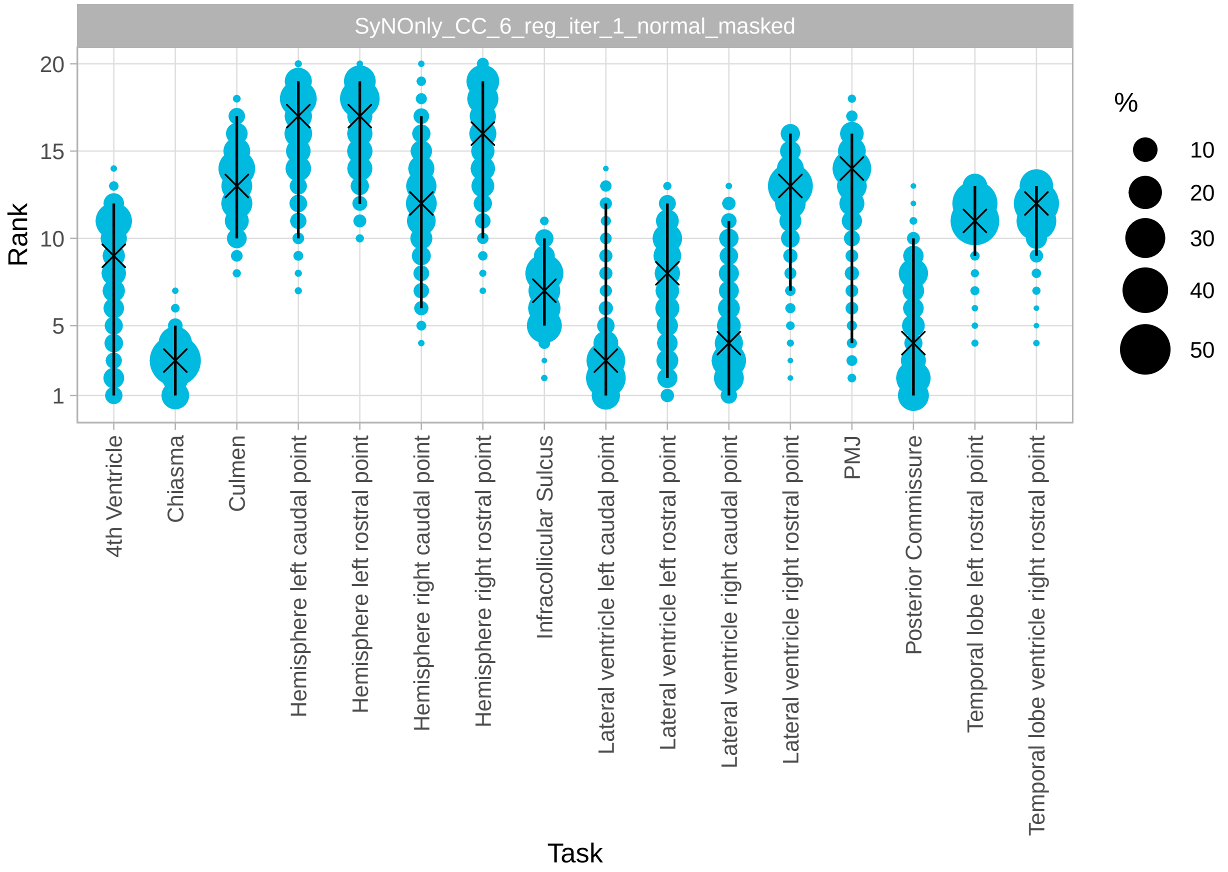
SyNOnly_CC_6_reg_iter_1_normal_masked
Rank
20
15
10
5
1
Task
4th Ventricle
Chiasma
Culmen
Hemisphere left caudal point
Hemisphere left rostral point
Hemisphere right caudal point
Hemisphere right rostral point
Infracollicular Sulcus
Lateral ventricle left caudal point
Lateral ventricle left rostral point
Lateral ventricle right caudal point
Lateral ventricle right rostral point
PMJ
Posterior Commissure
Temporal lobe left rostral point
Temporal lobe ventricle right rostral point
%
10
20
30
40
50

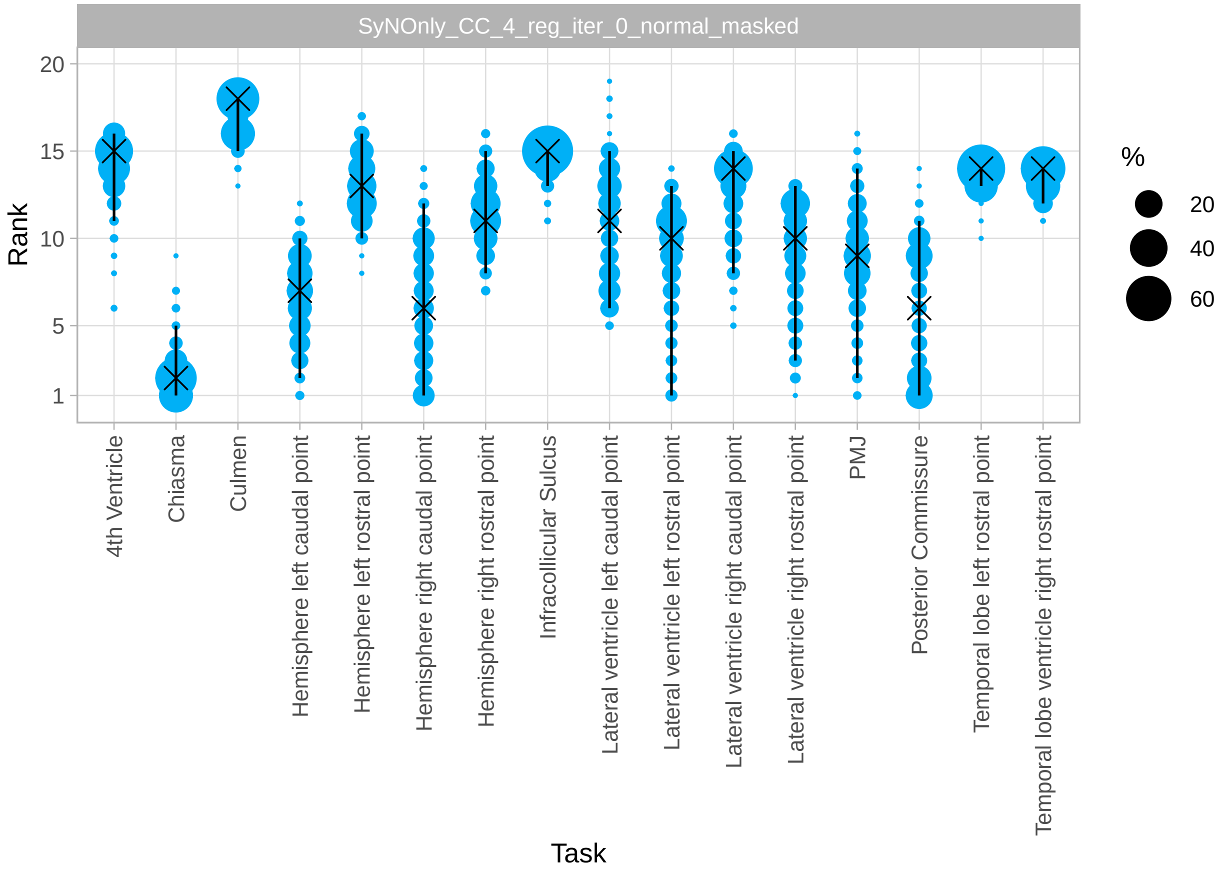
SyNOnly_CC_4_reg_iter_0_normal_masked
Rank
20
15
10
5
1
4th Ventricle
Chiasma
Culmen
Hemisphere left caudal point
Hemisphere left rostral point
Hemisphere right caudal point
Hemisphere right rostral point
Infracollicular Sulcus
Lateral ventricle left caudal point
Lateral ventricle left rostral point
Lateral ventricle right caudal point
Lateral ventricle right rostral point
PMJ
Posterior Commissure
Temporal lobe left rostral point
Temporal lobe ventricle right rostral point
Task
%
20
40
60

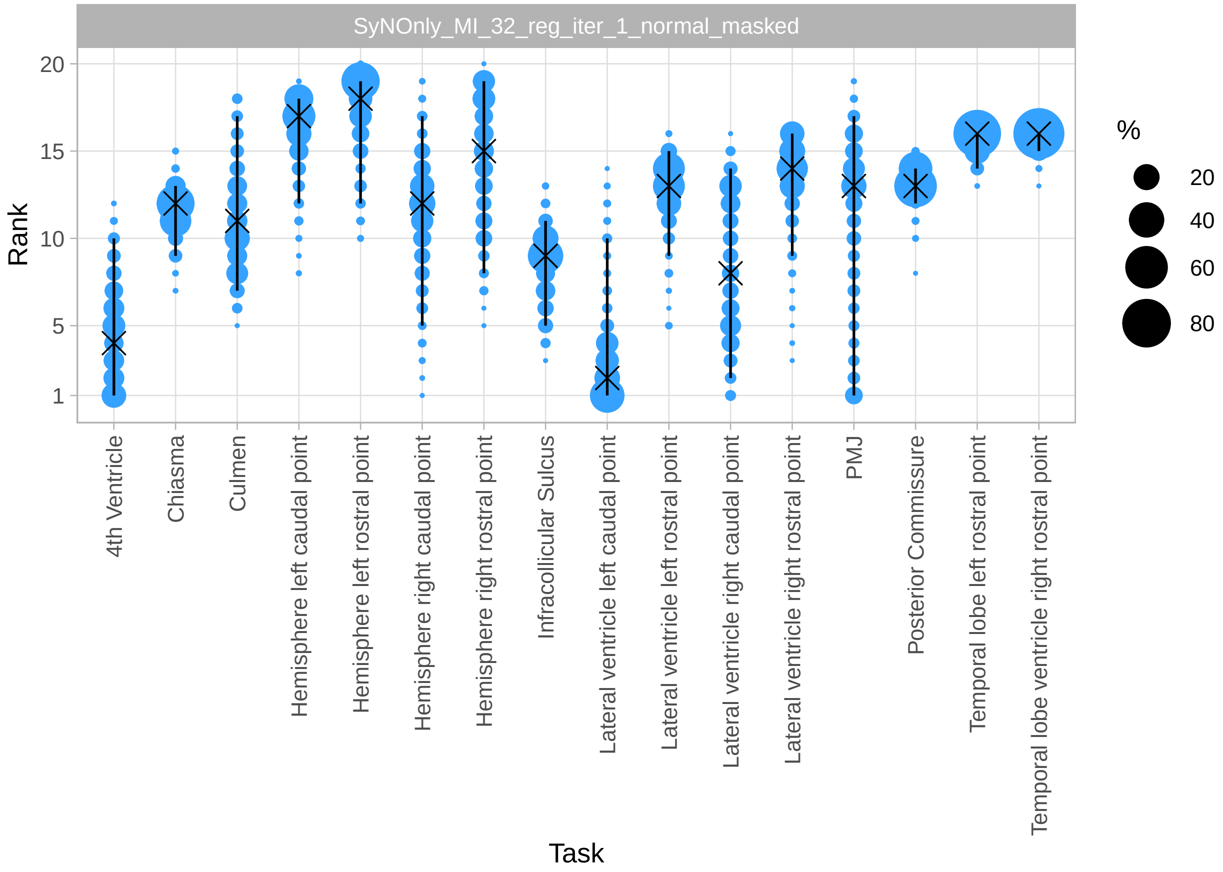

SyNOnly_MI_32_reg_iter_1_normal_masked
Rank
20
15
10
5
1
4th Ventricle
Chiasma
Culmen
Hemisphere left caudal point
Hemisphere left rostral point
Hemisphere right caudal point
Hemisphere right rostral point
Infracollicular Sulcus
Lateral ventricle left caudal point
Lateral ventricle left rostral point
Lateral ventricle right caudal point
Lateral ventricle right rostral point
PMJ
Posterior Commissure
Temporal lobe left rostral point
Temporal lobe ventricle right rostral point
Task
%
20
40
60
80

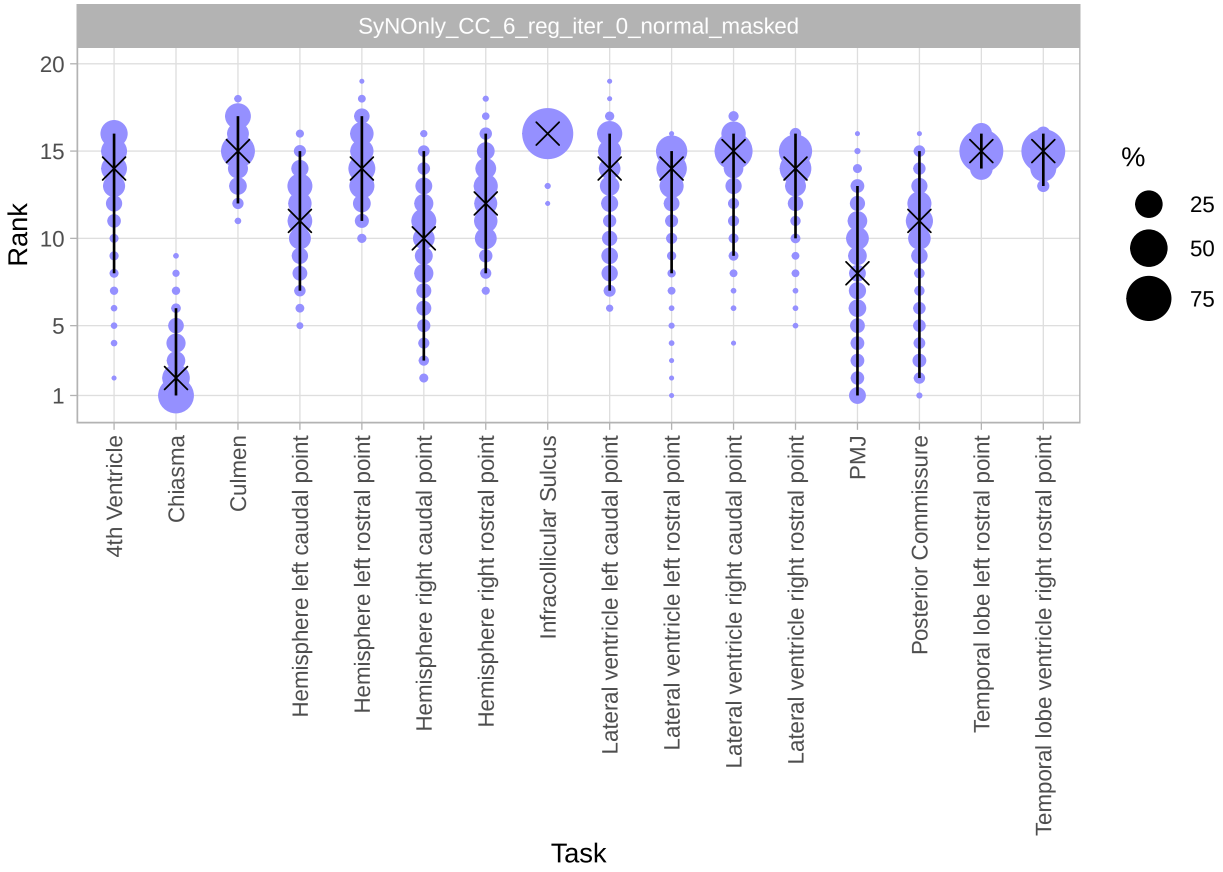
SyNOnly_CC_6_reg_iter_0_normal_masked
Rank
20
15
10
5
1
4th Ventricle
Chiasma
Culmen
Hemisphere left caudal point
Hemisphere left rostral point
Hemisphere right caudal point
Hemisphere right rostral point
Infracollicular Sulcus
Lateral ventricle left caudal point
Lateral ventricle left rostral point
Lateral ventricle right caudal point
Lateral ventricle right rostral point
PMJ
Posterior Commissure
Temporal lobe left rostral point
Temporal lobe ventricle right rostral point
Task
%
25
50
75

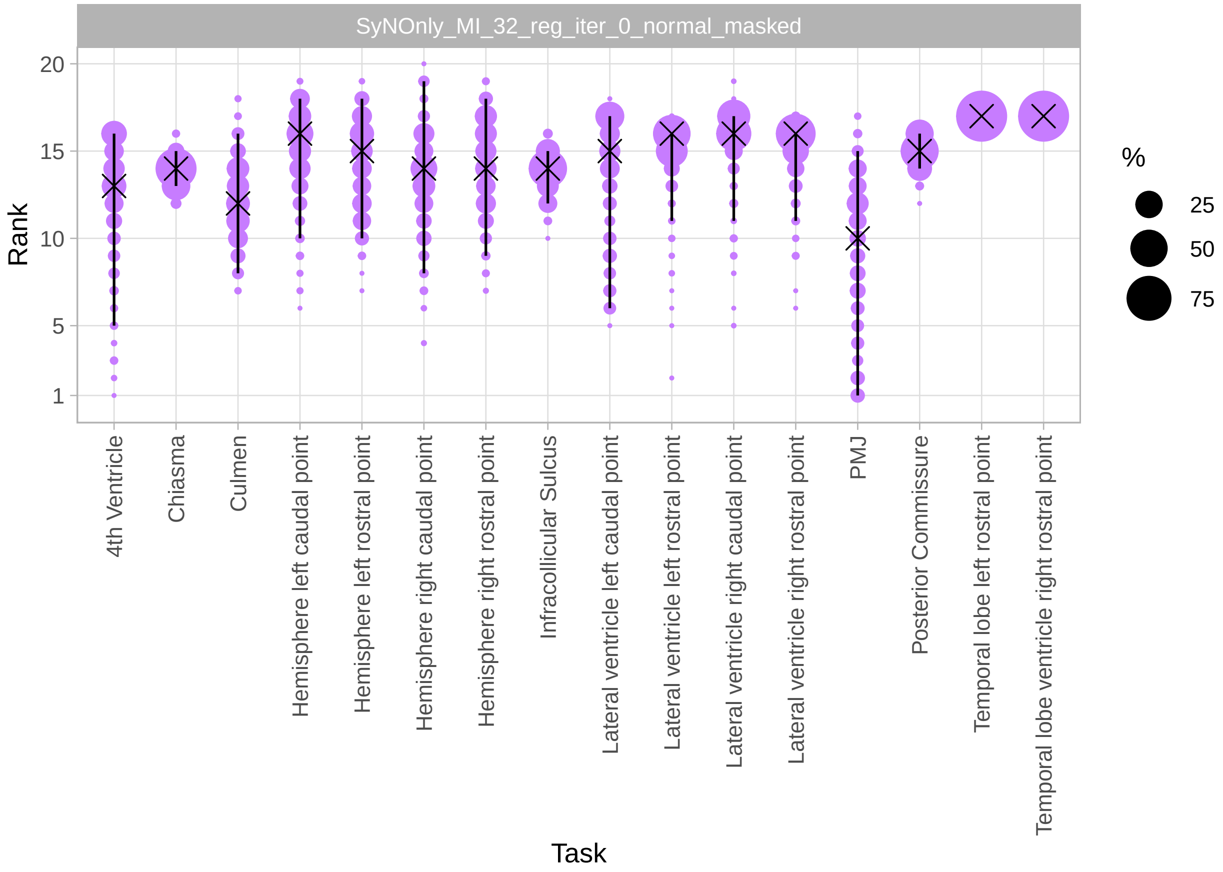

SyNOnly_MI_32_reg_iter_0_normal_masked
Rank
20
15
10
5
1
4th Ventricle
Chiasma
Culmen
Hemisphere left caudal point
Hemisphere left rostral point
Hemisphere right caudal point
Hemisphere right rostral point
Infracollicular Sulcus
Lateral ventricle left caudal point
Lateral ventricle left rostral point
Lateral ventricle right caudal point
Lateral ventricle right rostral point
PMJ
Posterior Commissure
Temporal lobe left rostral point
Temporal lobe ventricle right rostral point
Task
%
25
50
75

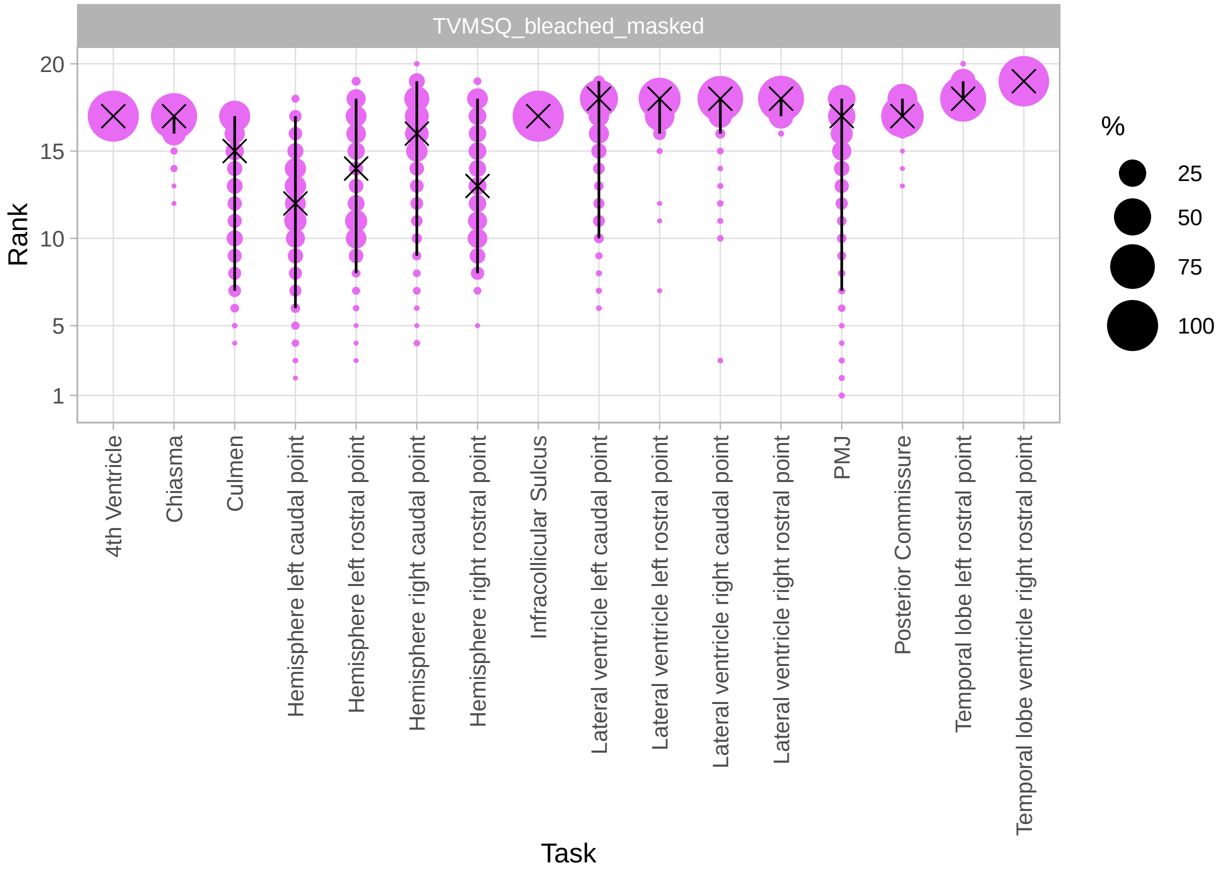
TVMSQ_bleached_masked
Rank
20
15
10
5
1
%
25
50
75
100
4th Ventricle
Chiasma
Culmen
Hemisphere left caudal point
Hemisphere left rostral point
Hemisphere right caudal point
Hemisphere right rostral point
Infracollicular Sulcus
Lateral ventricle left caudal point
Lateral ventricle left rostral point
Lateral ventricle right caudal point
Lateral ventricle right rostral point
PMJ
Posterior Commissure
Temporal lobe left rostral point
Temporal lobe ventricle right rostral point
Task

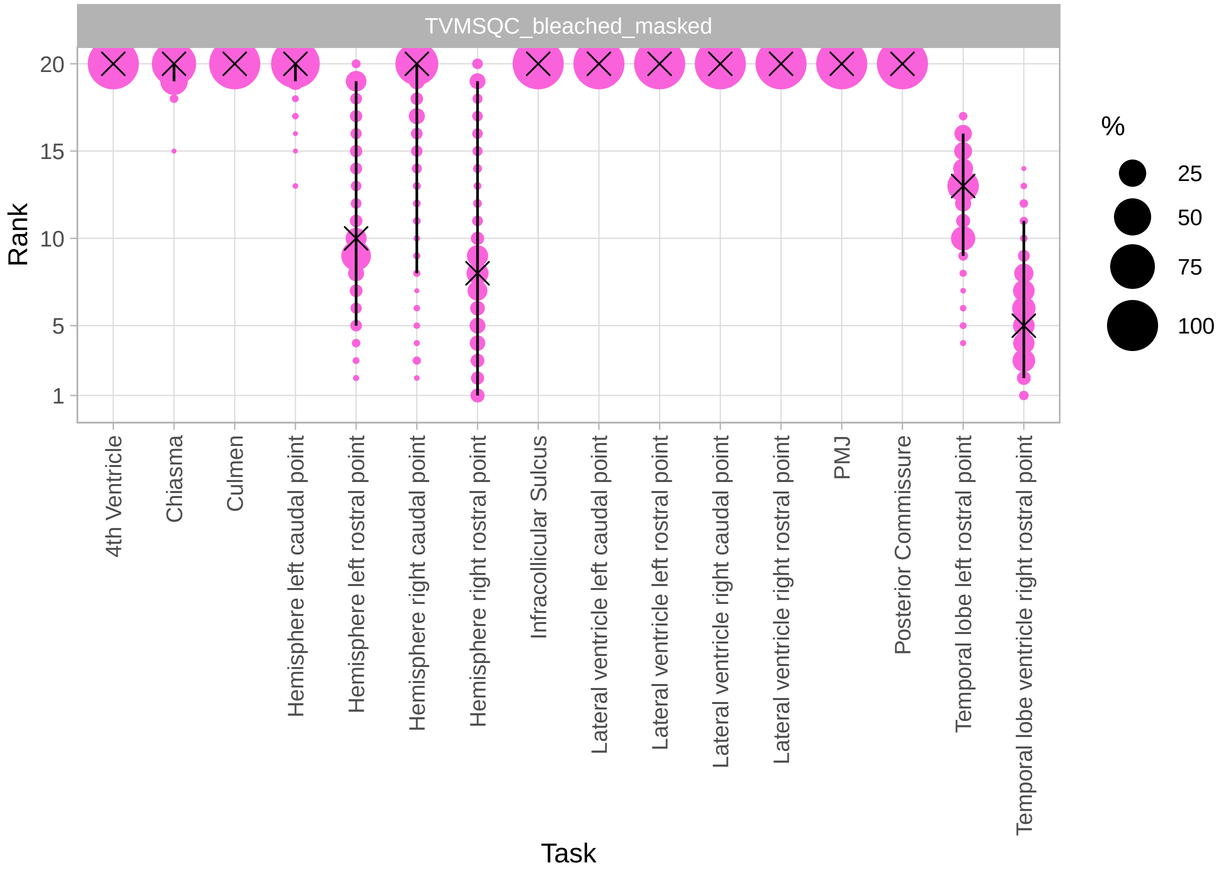
TVMSQC_bleached_masked
Rank
20
15
10
5
1
4th Ventricle
Chiasma
Culmen
Hemisphere left caudal point
Hemisphere left rostral point
Hemisphere right caudal point
Hemisphere right rostral point
Infracollicular Sulcus
Lateral ventricle left caudal point
Lateral ventricle left rostral point
Lateral ventricle right caudal point
Lateral ventricle right rostral point
PMJ
Posterior Commissure
Temporal lobe left rostral point
Temporal lobe ventricle right rostral point
Task
%
25
50
75
100

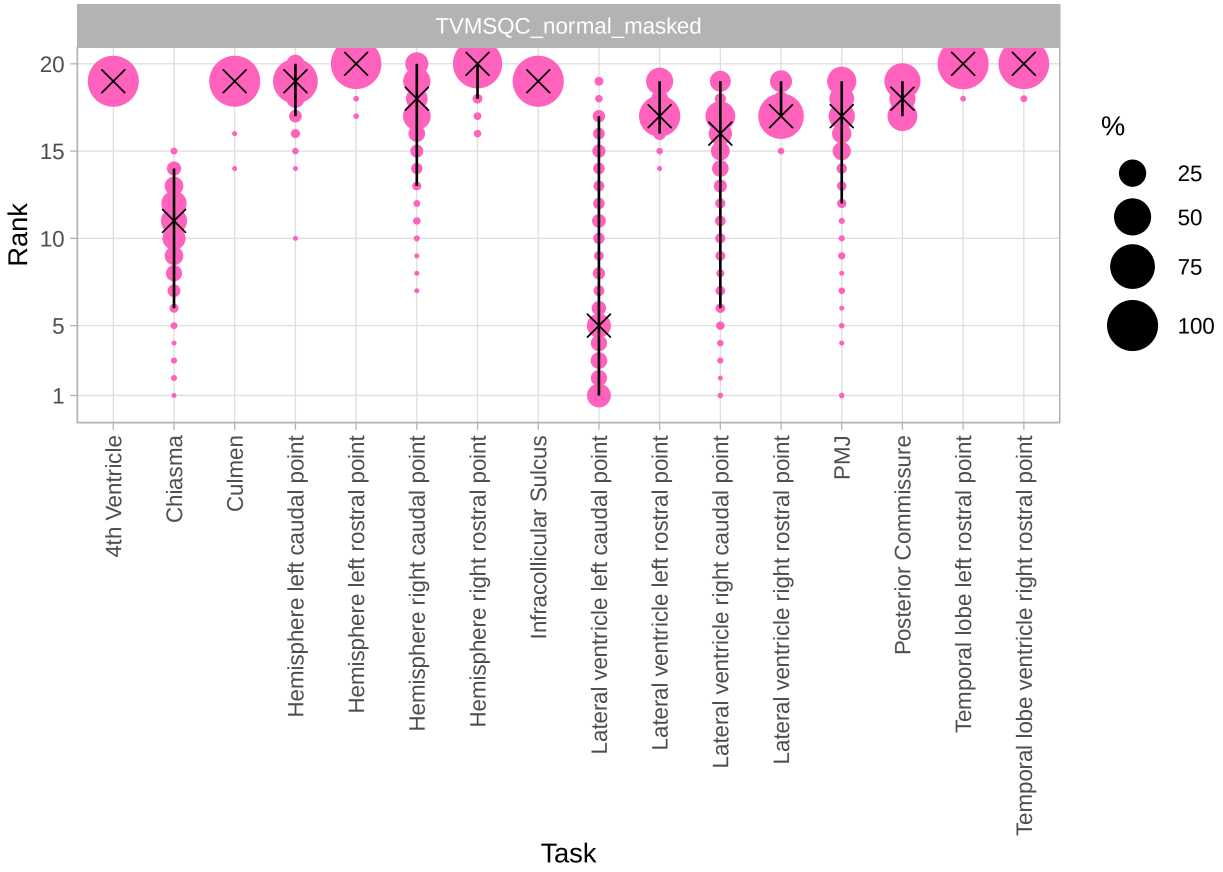

TVMSQC_normal_masked
Rank
20
15
10
5
1
4th Ventricle
Chiasma
Culmen
Hemisphere left caudal point
Hemisphere left rostral point
Hemisphere right caudal point
Hemisphere right rostral point
Infracollicular Sulcus
Lateral ventricle left caudal point
Lateral ventricle left rostral point
Lateral ventricle right caudal point
Lateral ventricle right rostral point
PMJ
Posterior Commissure
Temporal lobe left rostral point
Temporal lobe ventricle right rostral point
Task
%
25
50
75
100

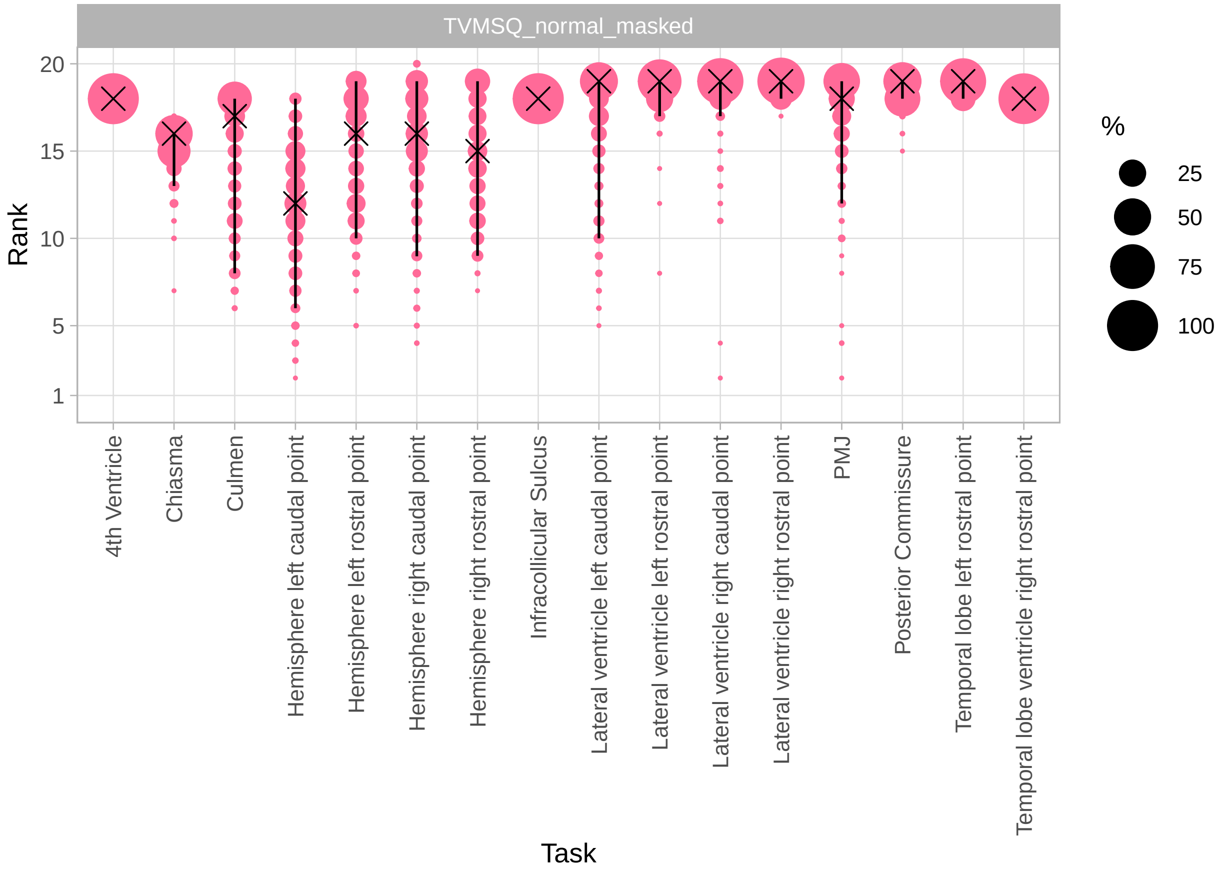
TVMSQ_normal_masked
Rank
20
15
10
5
1
Task
4th Ventricle
Chiasma
Culmen
Hemisphere left caudal point
Hemisphere left rostral point
Hemisphere right caudal point
Hemisphere right rostral point
Infracollicular Sulcus
Lateral ventricle left caudal point
Lateral ventricle left rostral point
Lateral ventricle right caudal point
Lateral ventricle right rostral point
PMJ
Posterior Commissure
Temporal lobe left rostral point
Temporal lobe ventricle right rostral point
%
25
50
75
100

An alternative representation is provided by a stacked frequency plot of the observed ranks, separated by algorithm. Observed ranks across bootstrap samples are displayed with coloring according to the task. For algorithms that achieve the same rank in different tasks for the full assessment data set, vertical lines are on top of each other. Vertical lines allow to compare the achieved rank of each algorithm over different tasks.

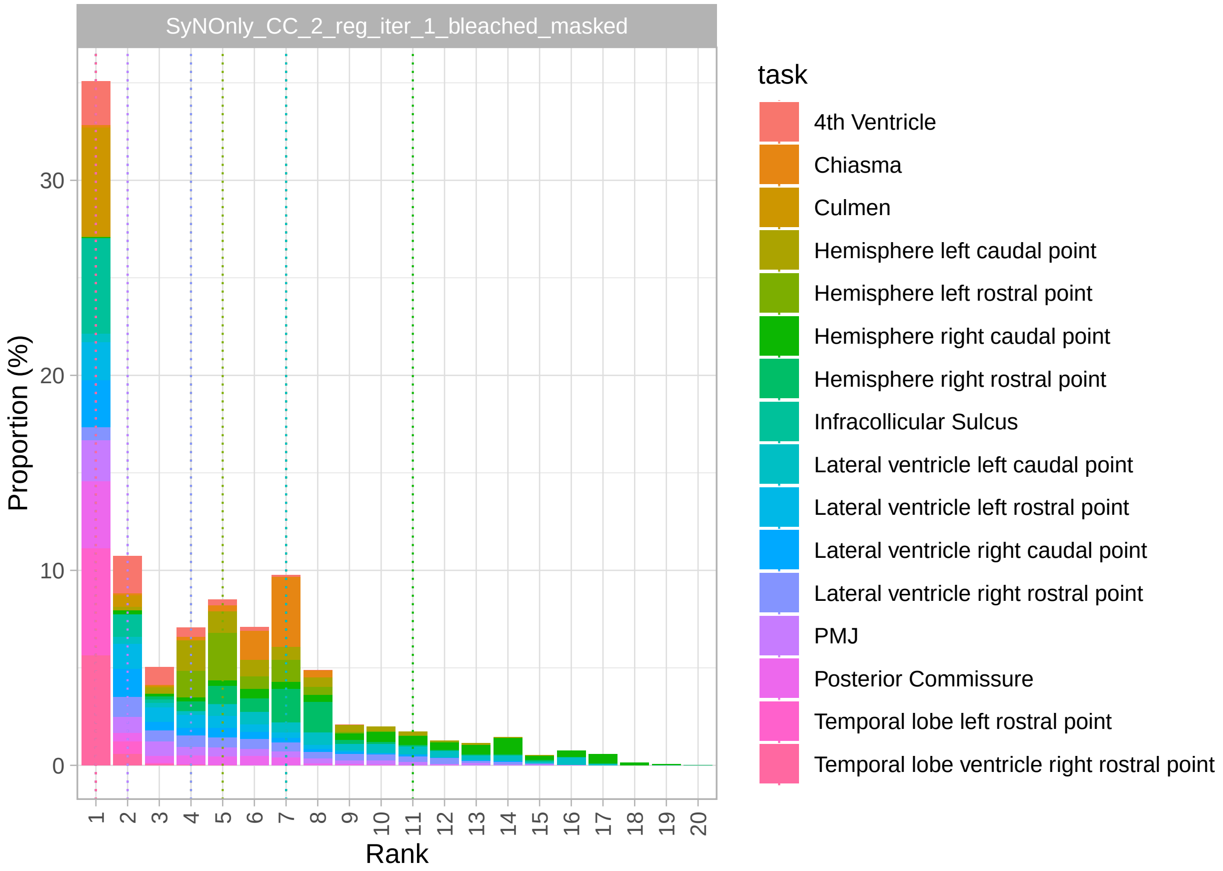

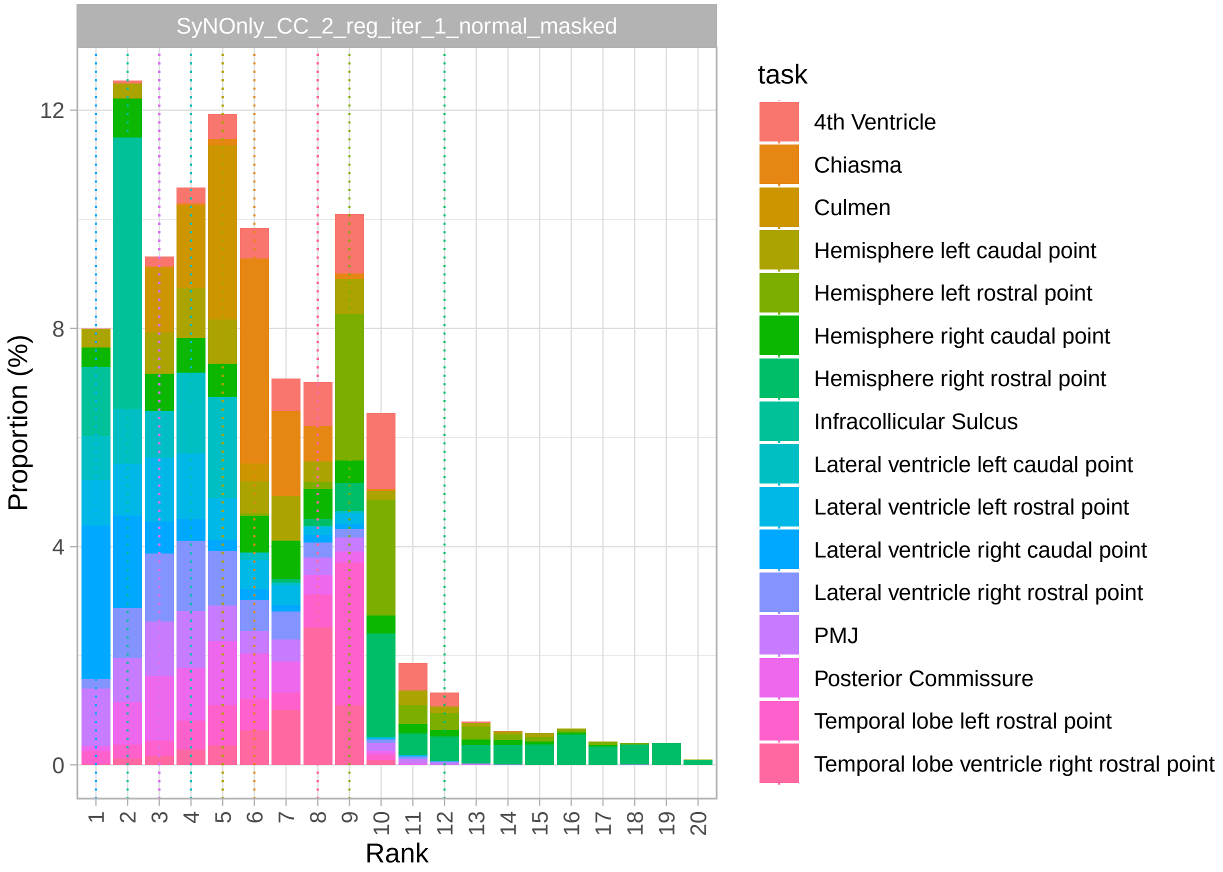
SyNOnly_CC_2_reg_iter_1_normal_masked
Proportion (%)
Rank
0
4
8
12
1 2 3 4 5 6 7 8 9 10 11 12 13 14 15 16 17 18 19 20
task
4th Ventricle
Chiasma
Culmen
Hemisphere left caudal point
Hemisphere left rostral point
Hemisphere right caudal point
Hemisphere right rostral point
Infracollicular Sulcus
Lateral ventricle left caudal point
Lateral ventricle left rostral point
Lateral ventricle right caudal point
Lateral ventricle right rostral point
PMJ
Posterior Commissure
Temporal lobe left rostral point
Temporal lobe ventricle right rostral point

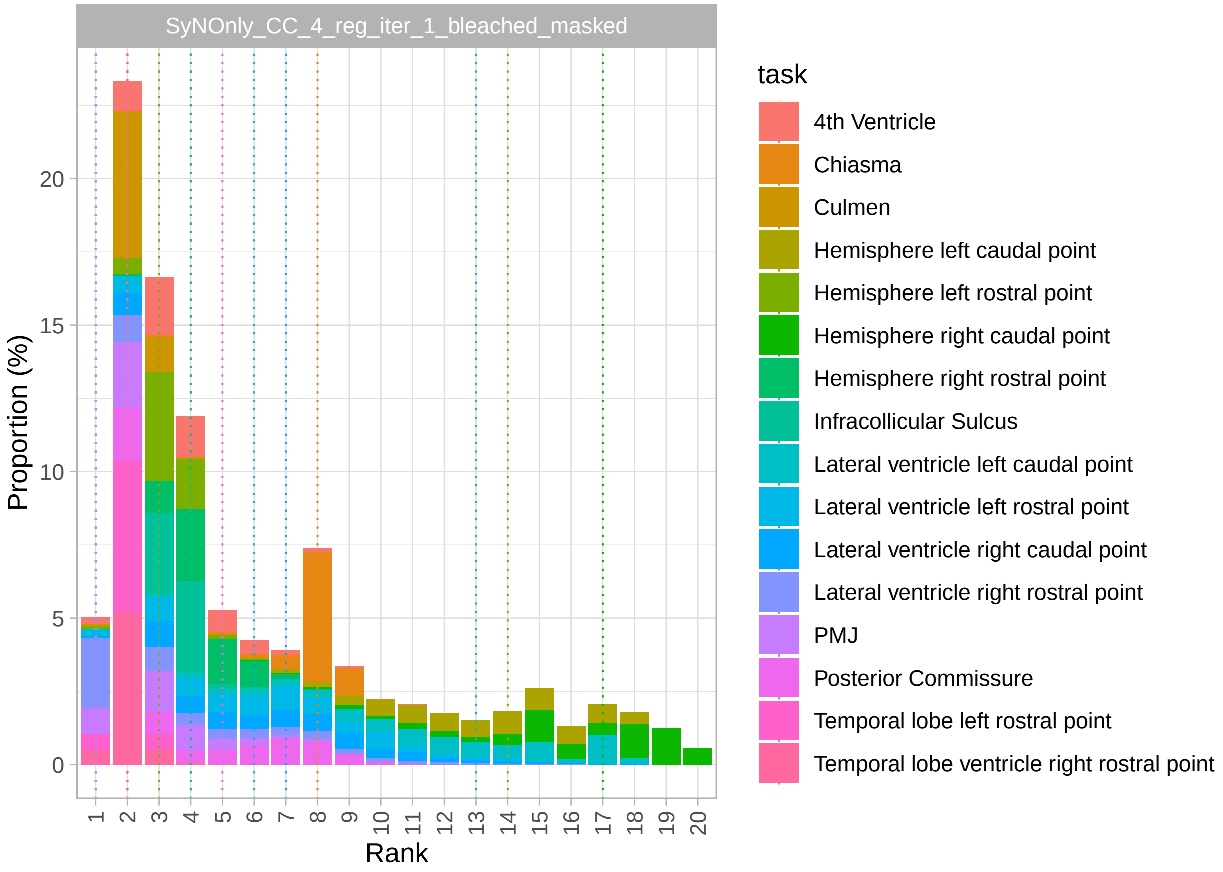
SyNOnly_CC_4_reg_iter_1_bleached_masked
Proportion (%)
0
5
10
15
20
1
2
3
4
5
6
7
8
9
10
11
12
13
14
15
16
17
18
19
20
Rank
task
4th Ventricle
Chiasma
Culmen
Hemisphere left caudal point
Hemisphere left rostral point
Hemisphere right caudal point
Hemisphere right rostral point
Infracollicular Sulcus
Lateral ventricle left caudal point
Lateral ventricle left rostral point
Lateral ventricle right caudal point
Lateral ventricle right rostral point
PMJ
Posterior Commissure
Temporal lobe left rostral point
Temporal lobe ventricle right rostral point

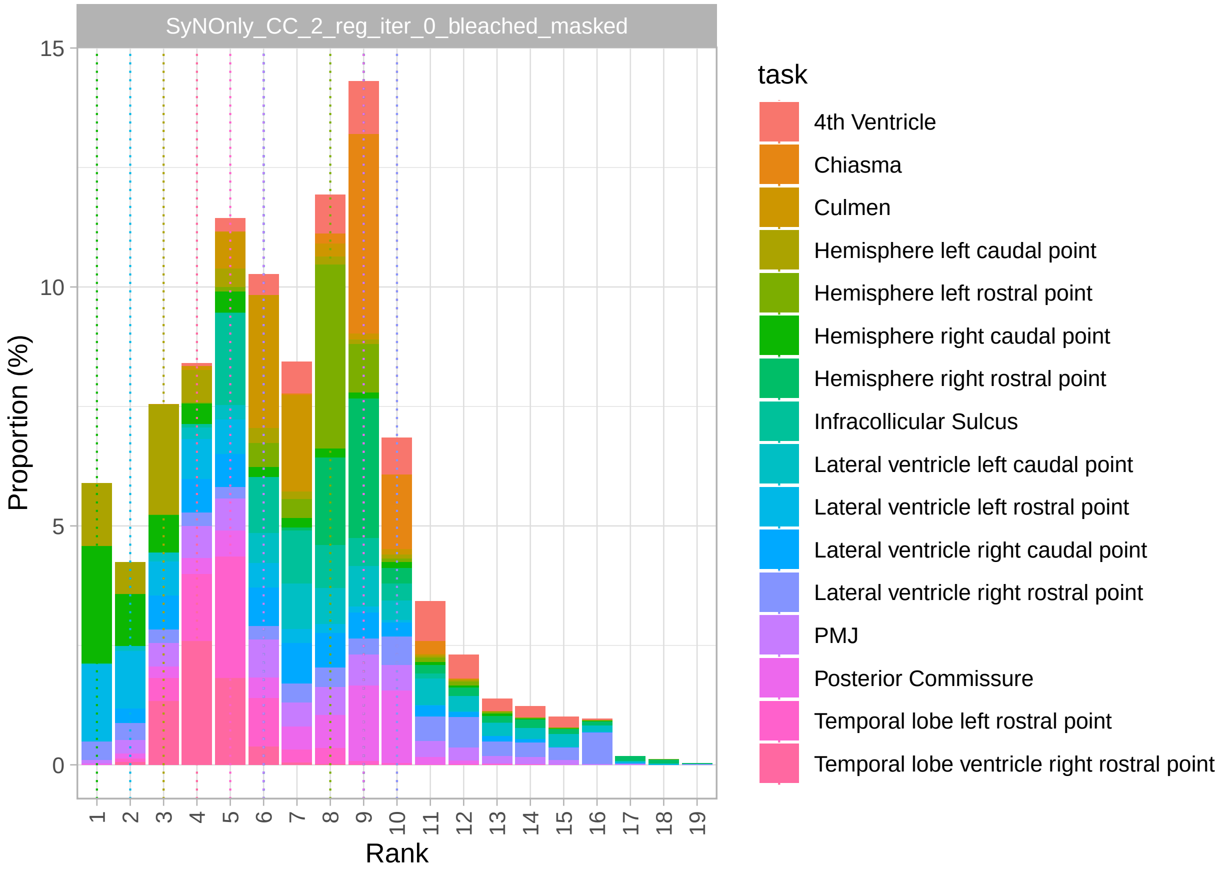
SyNOnly_CC_2_reg_iter_0_bleached_masked
Proportion (%)
Rank
task
4th Ventricle
Chiasma
Culmen
Hemisphere left caudal point
Hemisphere left rostral point
Hemisphere right caudal point
Hemisphere right rostral point
Infracollicular Sulcus
Lateral ventricle left caudal point
Lateral ventricle left rostral point
Lateral ventricle right caudal point
Lateral ventricle right rostral point
PMJ
Posterior Commissure
Temporal lobe left rostral point
Temporal lobe ventricle right rostral point

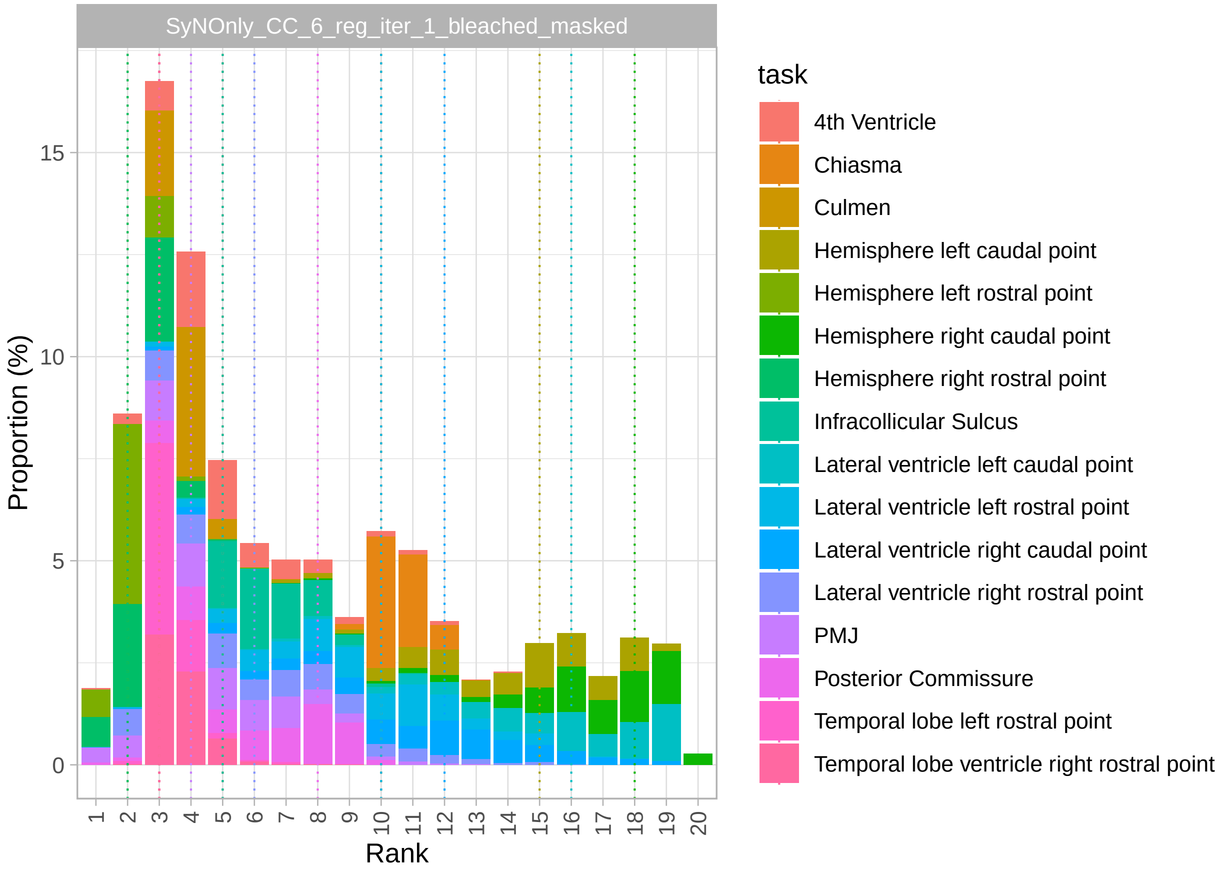
SyNOnly_CC_6_reg_iter_1_bleached_masked
Proportion (%)
0
5
10
15
1
2
3
4
5
6
7
8
9
10
11
12
13
14
15
16
17
18
19
20
Rank
task
4th Ventricle
Chiasma
Culmen
Hemisphere left caudal point
Hemisphere left rostral point
Hemisphere right caudal point
Hemisphere right rostral point
Infracollicular Sulcus
Lateral ventricle left caudal point
Lateral ventricle left rostral point
Lateral ventricle right caudal point
Lateral ventricle right rostral point
PMJ
Posterior Commissure
Temporal lobe left rostral point
Temporal lobe ventricle right rostral point

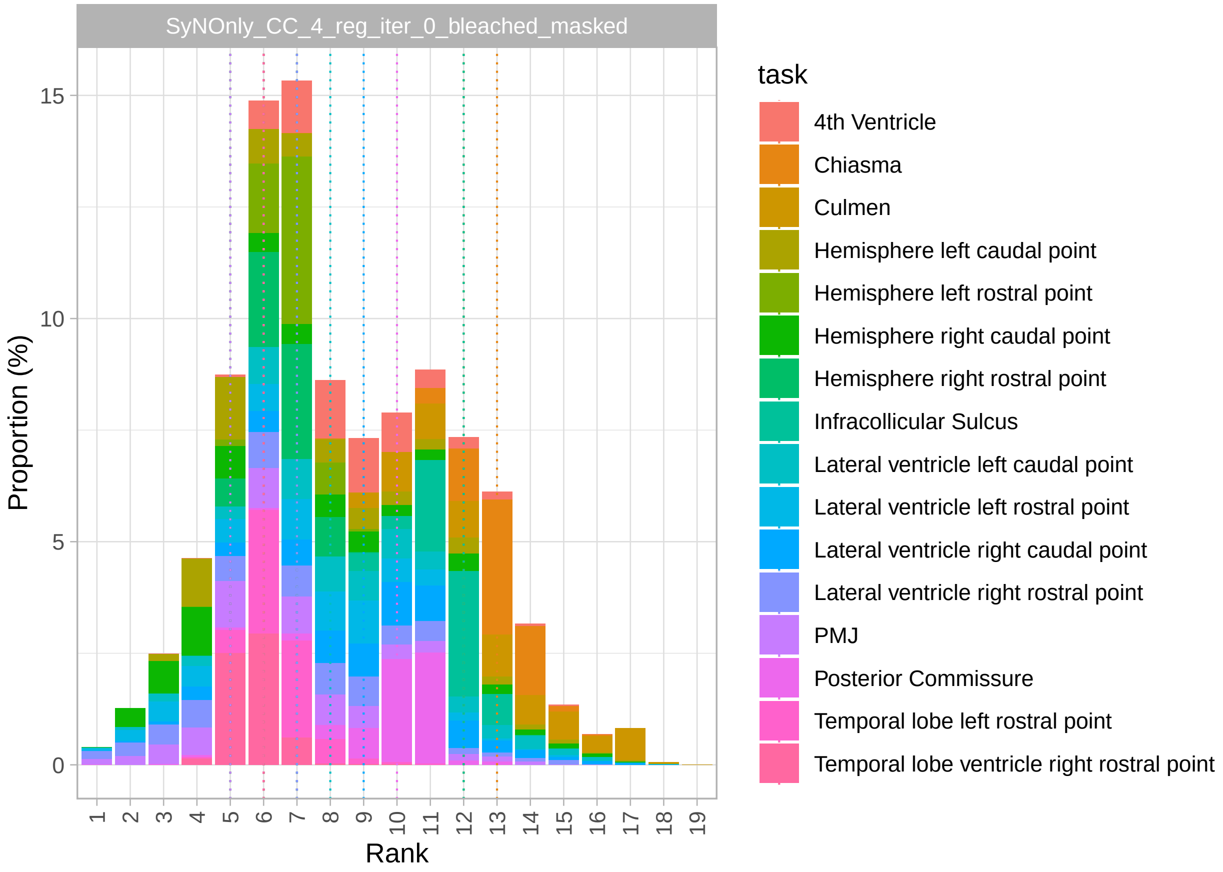
SyNOnly_CC_4_reg_iter_0_bleached_masked
Proportion (%)
15
10
5
0
1
2
3
4
5
6
7
8
9
10
11
12
13
14
15
16
17
18
19
Rank
task
4th Ventricle
Chiasma
Culmen
Hemisphere left caudal point
Hemisphere left rostral point
Hemisphere right caudal point
Hemisphere right rostral point
Infracollicular Sulcus
Lateral ventricle left caudal point
Lateral ventricle left rostral point
Lateral ventricle right caudal point
Lateral ventricle right rostral point
PMJ
Posterior Commissure
Temporal lobe left rostral point
Temporal lobe ventricle right rostral point

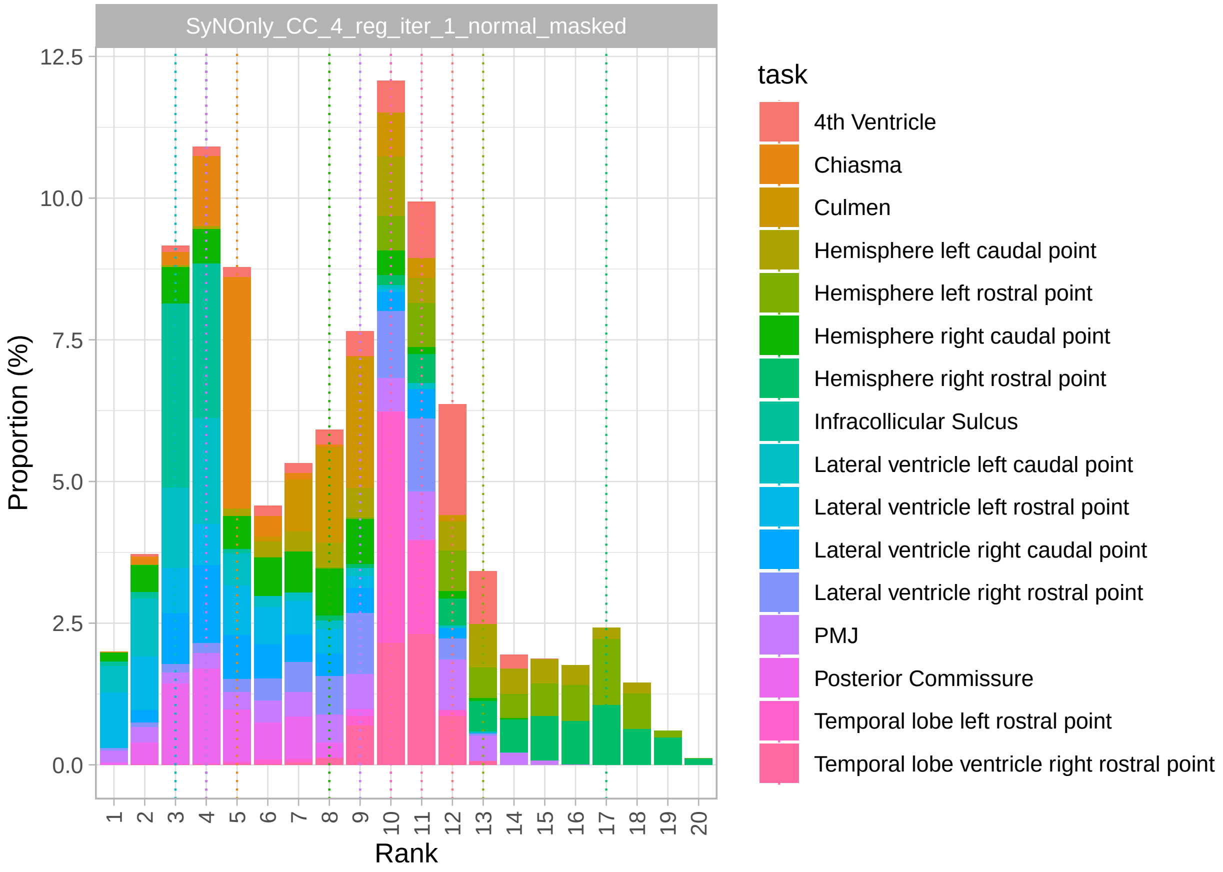
SyNOnly_CC_4_reg_iter_1_normal_masked
Proportion (%)
Rank
0.0
2.5
5.0
7.5
10.0
12.5
1 2 3 4 5 6 7 8 9 10 11 12 13 14 15 16 17 18 19 20
task
4th Ventricle
Chiasma
Culmen
Hemisphere left caudal point
Hemisphere left rostral point
Hemisphere right caudal point
Hemisphere right rostral point
Infracollicular Sulcus
Lateral ventricle left caudal point
Lateral ventricle left rostral point
Lateral ventricle right caudal point
Lateral ventricle right rostral point
PMJ
Posterior Commissure
Temporal lobe left rostral point
Temporal lobe ventricle right rostral point

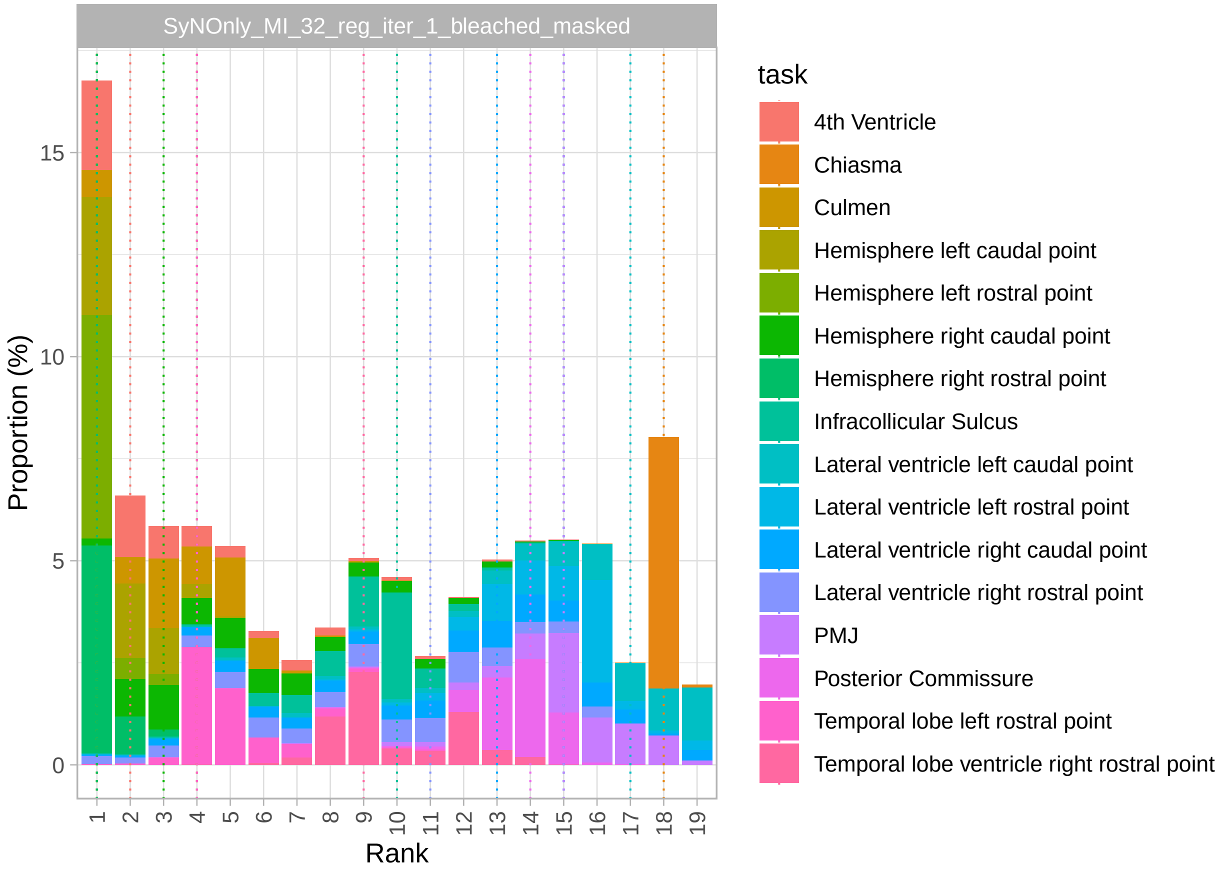
SyNOnly_MI_32_reg_iter_1_bleached_masked
Proportion (%)
0
5
10
15
Rank
1
2
3
4
5
6
7
8
9
10
11
12
13
14
15
16
17
18
19
task
4th Ventricle
Chiasma
Culmen
Hemisphere left caudal point
Hemisphere left rostral point
Hemisphere right caudal point
Hemisphere right rostral point
Infracollicular Sulcus
Lateral ventricle left caudal point
Lateral ventricle left rostral point
Lateral ventricle right caudal point
Lateral ventricle right rostral point
PMJ
Posterior Commissure
Temporal lobe left rostral point
Temporal lobe ventricle right rostral point

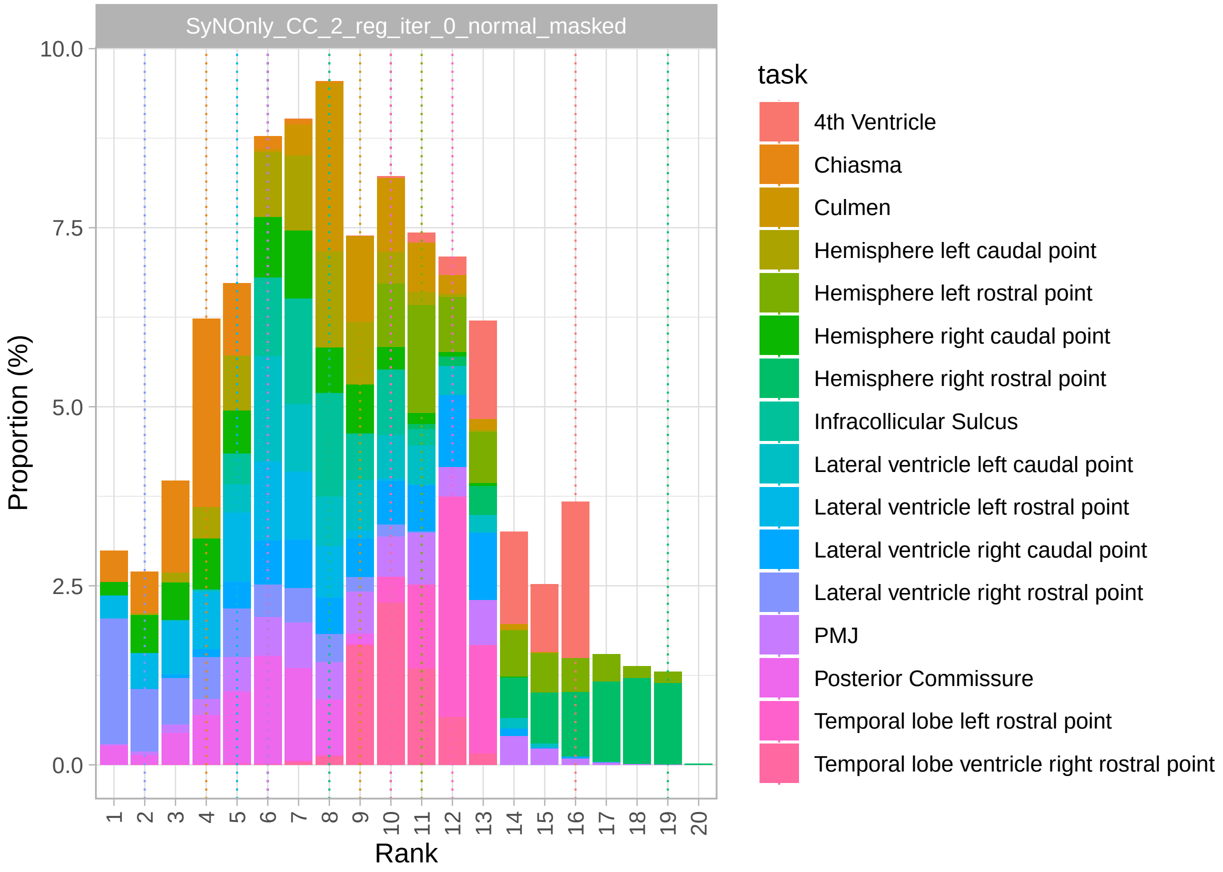
SyNOnly_CC_2_reg_iter_0_normal_masked
Proportion (%)
10.0
7.5
5.0
2.5
0.0
1
2
3
4
5
6
7
8
9
10
11
12
13
14
15
16
17
18
19
20
Rank
task
4th Ventricle
Chiasma
Culmen
Hemisphere left caudal point
Hemisphere left rostral point
Hemisphere right caudal point
Hemisphere right rostral point
Infracollicular Sulcus
Lateral ventricle left caudal point
Lateral ventricle left rostral point
Lateral ventricle right caudal point
Lateral ventricle right rostral point
PMJ
Posterior Commissure
Temporal lobe left rostral point
Temporal lobe ventricle right rostral point

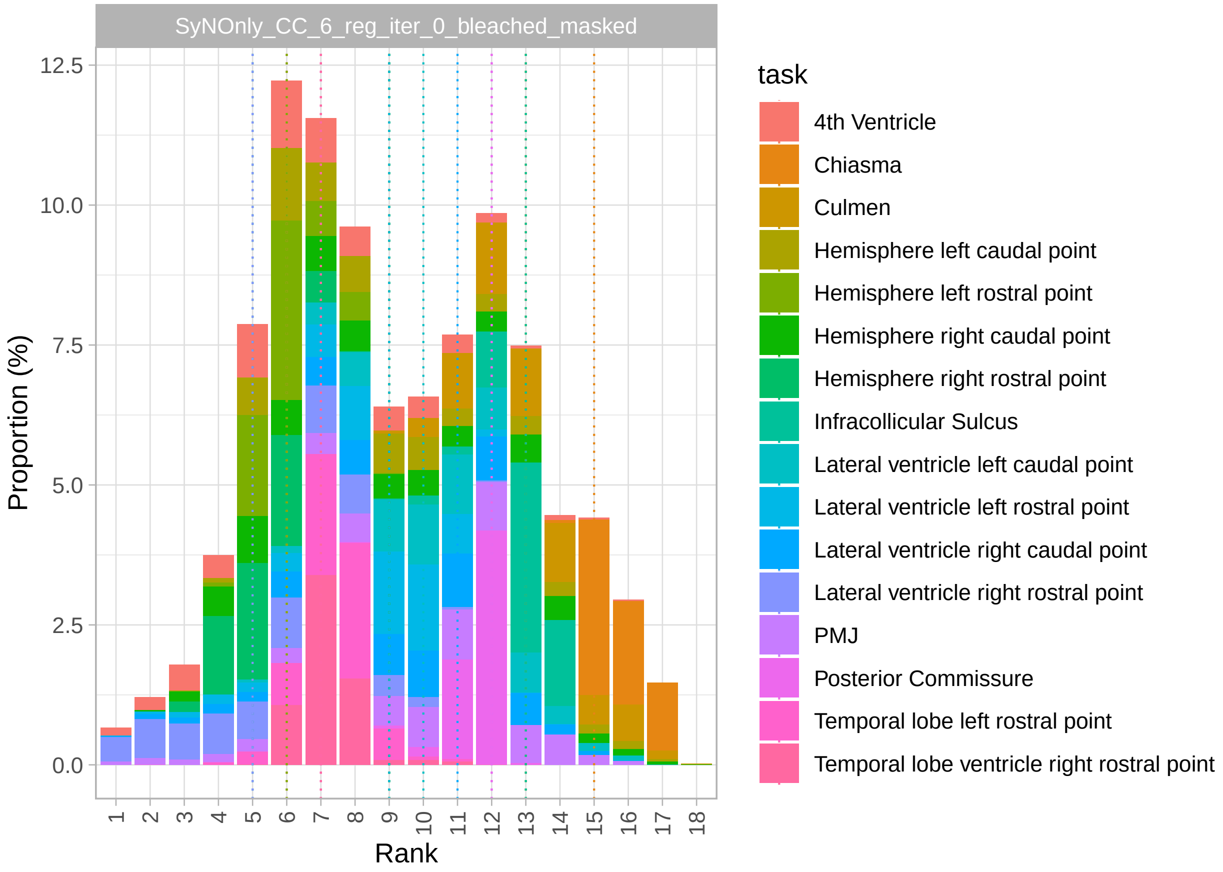
SyNOnly_CC_6_reg_iter_0_bleached_masked
Proportion (%)
12.5
10.0
7.5
5.0
2.5
0.0
1 2 3 4 5 6 7 8 9 10 11 12 13 14 15 16 17 18
Rank
task
4th Ventricle
Chiasma
Culmen
Hemisphere left caudal point
Hemisphere left rostral point
Hemisphere right caudal point
Hemisphere right rostral point
Infracollicular Sulcus
Lateral ventricle left caudal point
Lateral ventricle left rostral point
Lateral ventricle right caudal point
Lateral ventricle right rostral point
PMJ
Posterior Commissure
Temporal lobe left rostral point
Temporal lobe ventricle right rostral point

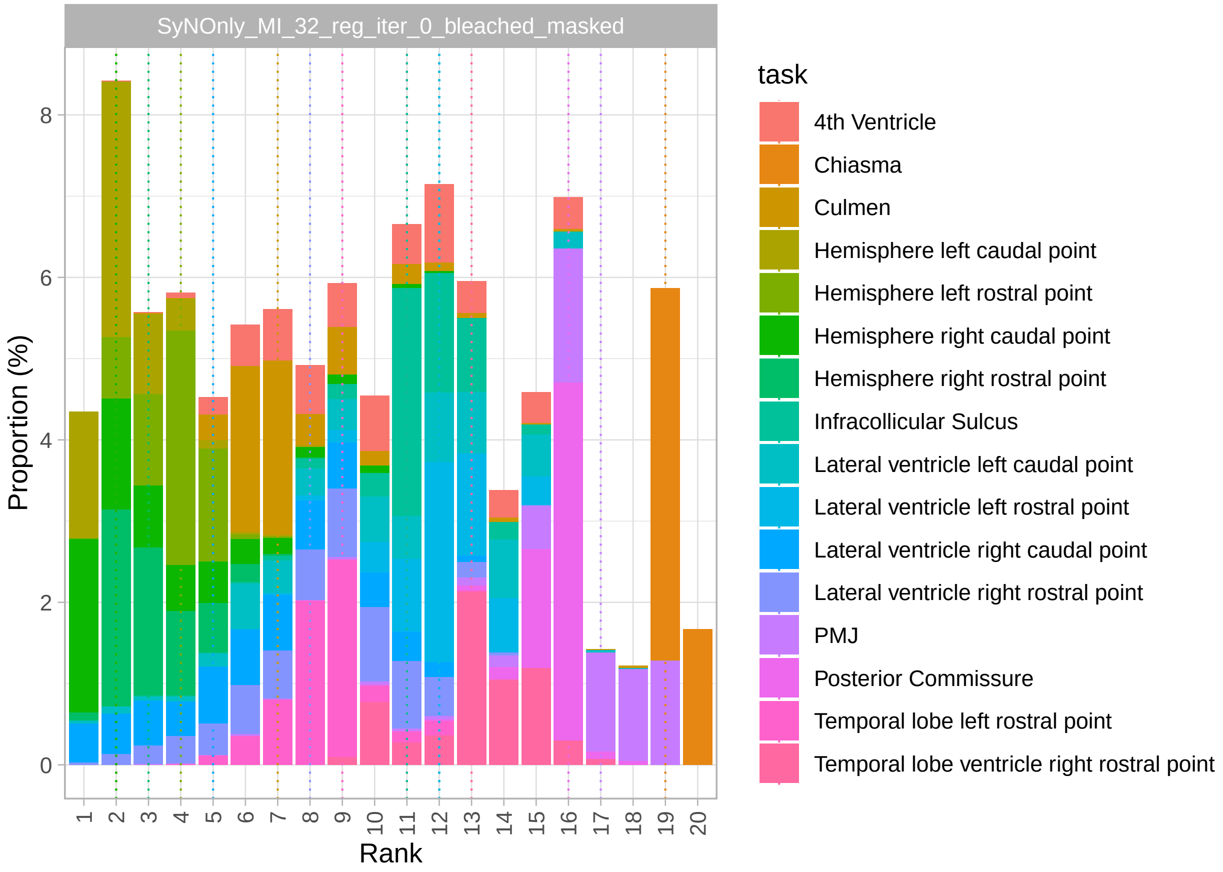
SyNOnly_MI_32_reg_iter_0_bleached_masked
Proportion (%)
0
2
4
6
8
Rank
1
2
3
4
5
6
7
8
9
10
11
12
13
14
15
16
17
18
19
20
task
4th Ventricle
Chiasma
Culmen
Hemisphere left caudal point
Hemisphere left rostral point
Hemisphere right caudal point
Hemisphere right rostral point
Infracollicular Sulcus
Lateral ventricle left caudal point
Lateral ventricle left rostral point
Lateral ventricle right caudal point
Lateral ventricle right rostral point
PMJ
Posterior Commissure
Temporal lobe left rostral point
Temporal lobe ventricle right rostral point

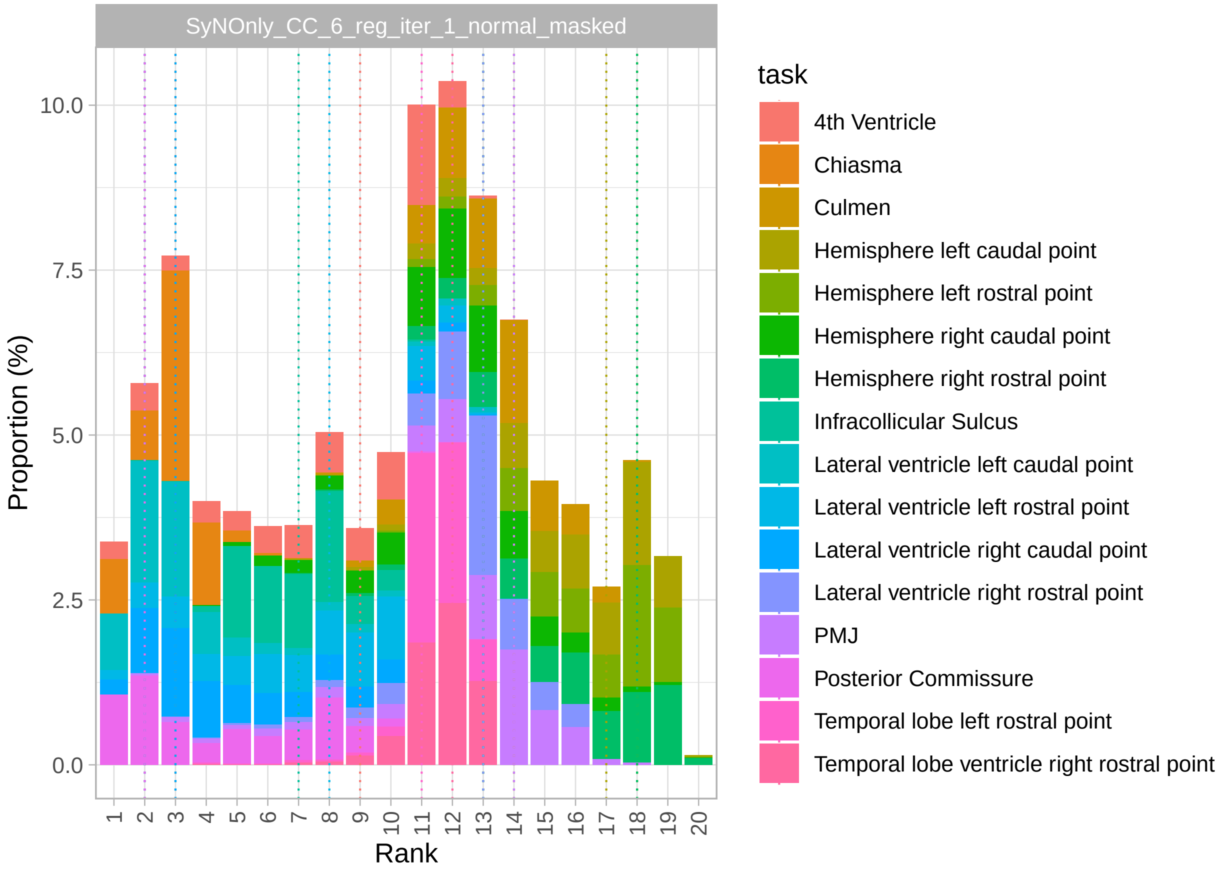
SyNOnly_CC_6_reg_iter_1_normal_masked
Proportion (%)
10.0
7.5
5.0
2.5
0.0
1
2
3
4
5
6
7
8
9
10
11
12
13
14
15
16
17
18
19
20
Rank
task
4th Ventricle
Chiasma
Culmen
Hemisphere left caudal point
Hemisphere left rostral point
Hemisphere right caudal point
Hemisphere right rostral point
Infracollicular Sulcus
Lateral ventricle left caudal point
Lateral ventricle left rostral point
Lateral ventricle right caudal point
Lateral ventricle right rostral point
PMJ
Posterior Commissure
Temporal lobe left rostral point
Temporal lobe ventricle right rostral point

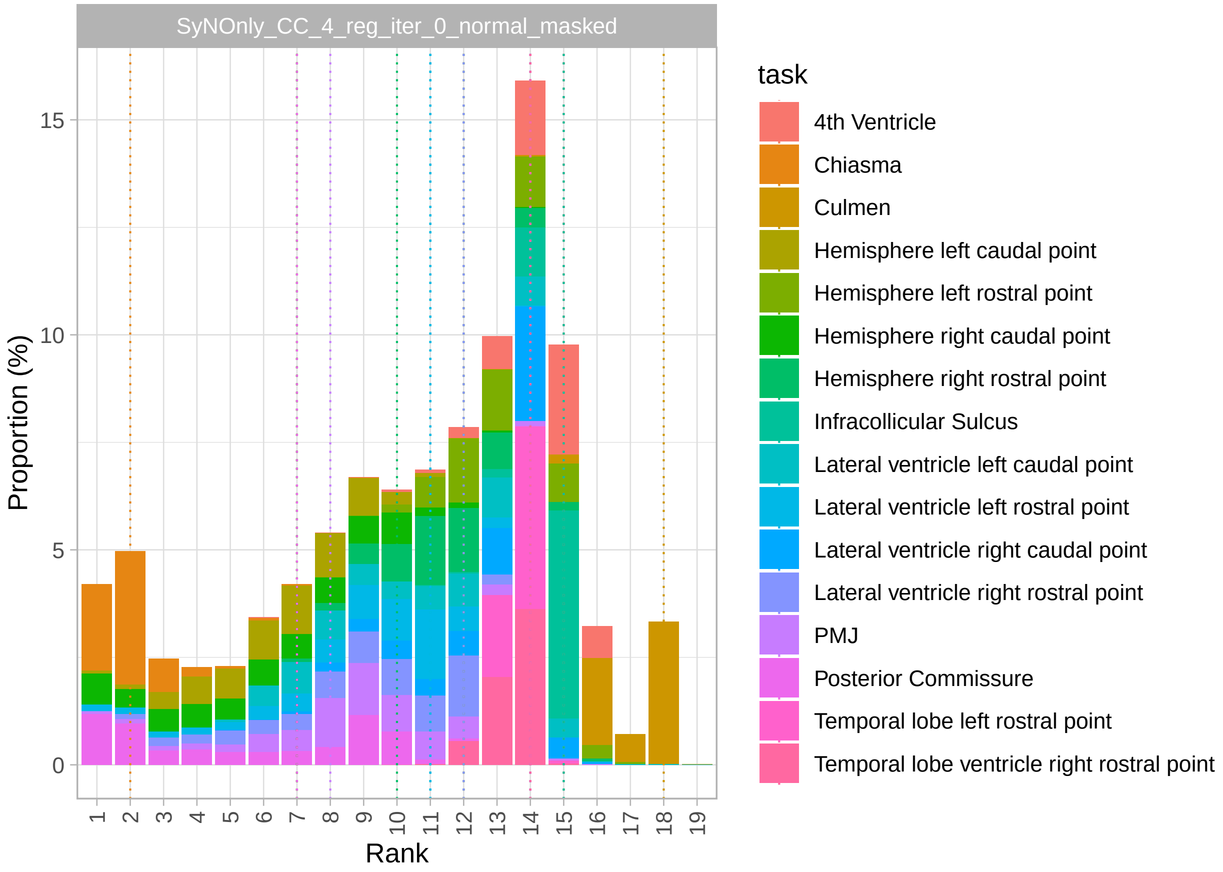
SyNOnly_CC_4_reg_iter_0_normal_masked
Proportion (%)
Rank
task
4th Ventricle
Chiasma
Culmen
Hemisphere left caudal point
Hemisphere left rostral point
Hemisphere right caudal point
Hemisphere right rostral point
Infracollicular Sulcus
Lateral ventricle left caudal point
Lateral ventricle left rostral point
Lateral ventricle right caudal point
Lateral ventricle right rostral point
PMJ
Posterior Commissure
Temporal lobe left rostral point
Temporal lobe ventricle right rostral point

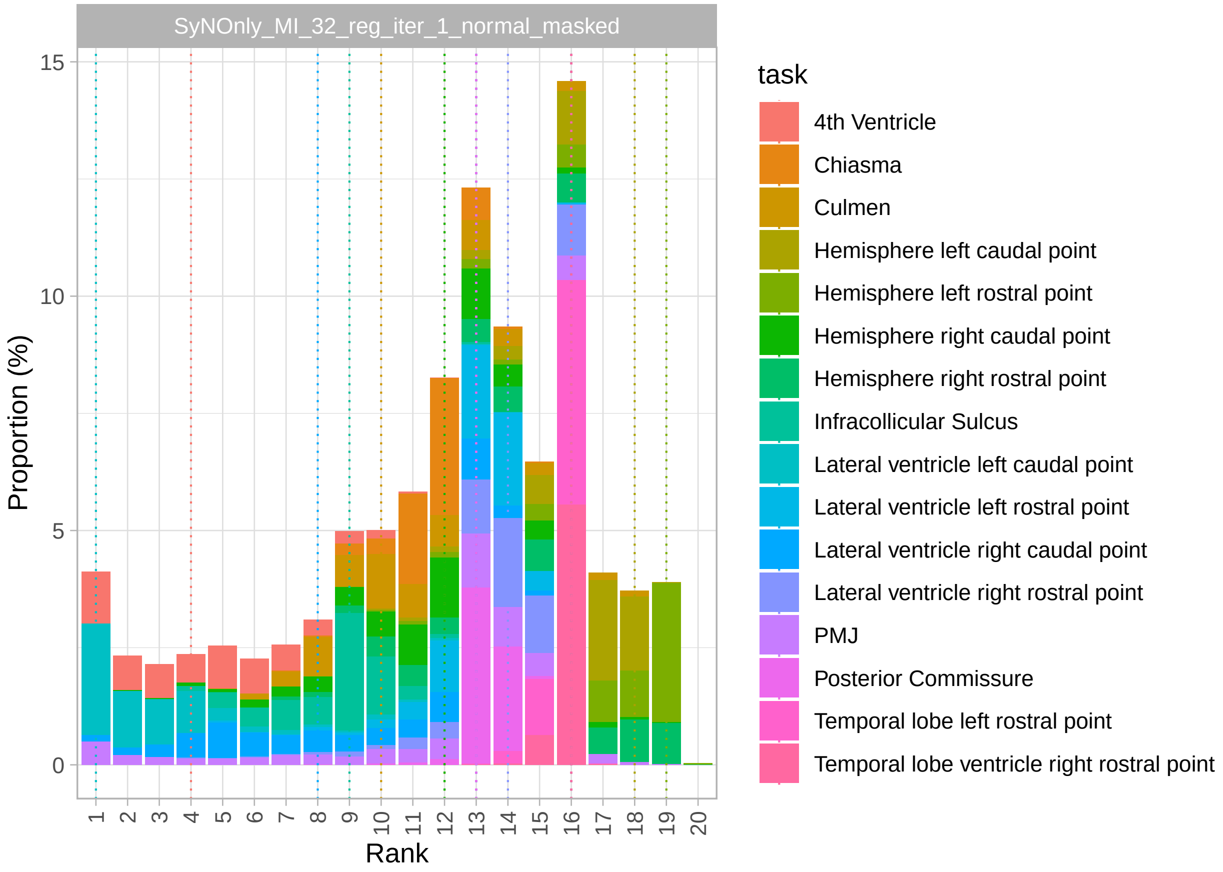
SyNOnly_MI_32_reg_iter_1_normal_masked
Proportion (%)
Rank
task
4th Ventricle
Chiasma
Culmen
Hemisphere left caudal point
Hemisphere left rostral point
Hemisphere right caudal point
Hemisphere right rostral point
Infracollicular Sulcus
Lateral ventricle left caudal point
Lateral ventricle left rostral point
Lateral ventricle right caudal point
Lateral ventricle right rostral point
PMJ
Posterior Commissure
Temporal lobe left rostral point
Temporal lobe ventricle right rostral point

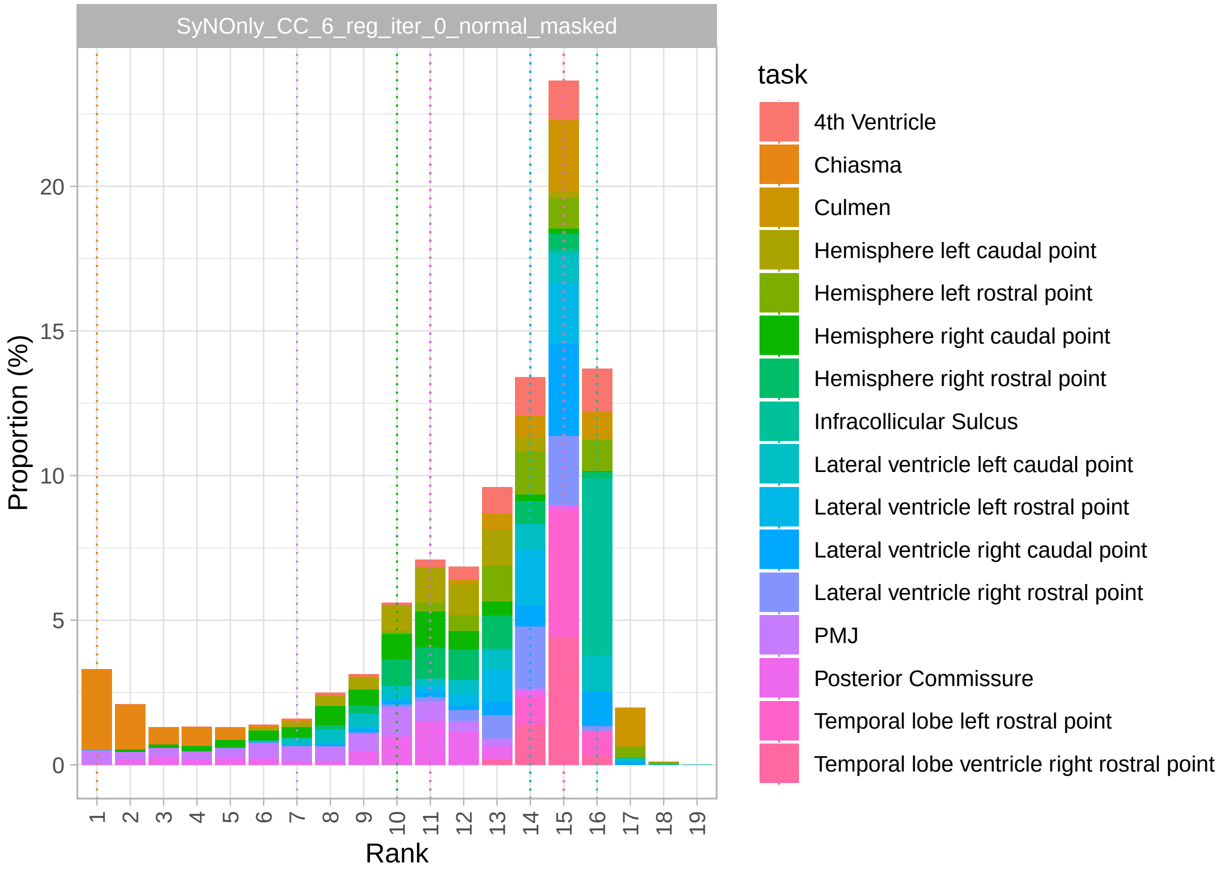

SyNOnly_CC_6_reg_iter_0_normal_masked
Proportion (%)
0
5
10
15
20
Rank
1
2
3
4
5
6
7
8
9
10
11
12
13
14
15
16
17
18
19
task
4th Ventricle
Chiasma
Culmen
Hemisphere left caudal point
Hemisphere left rostral point
Hemisphere right caudal point
Hemisphere right rostral point
Infracollicular Sulcus
Lateral ventricle left caudal point
Lateral ventricle left rostral point
Lateral ventricle right caudal point
Lateral ventricle right rostral point
PMJ
Posterior Commissure
Temporal lobe left rostral point
Temporal lobe ventricle right rostral point

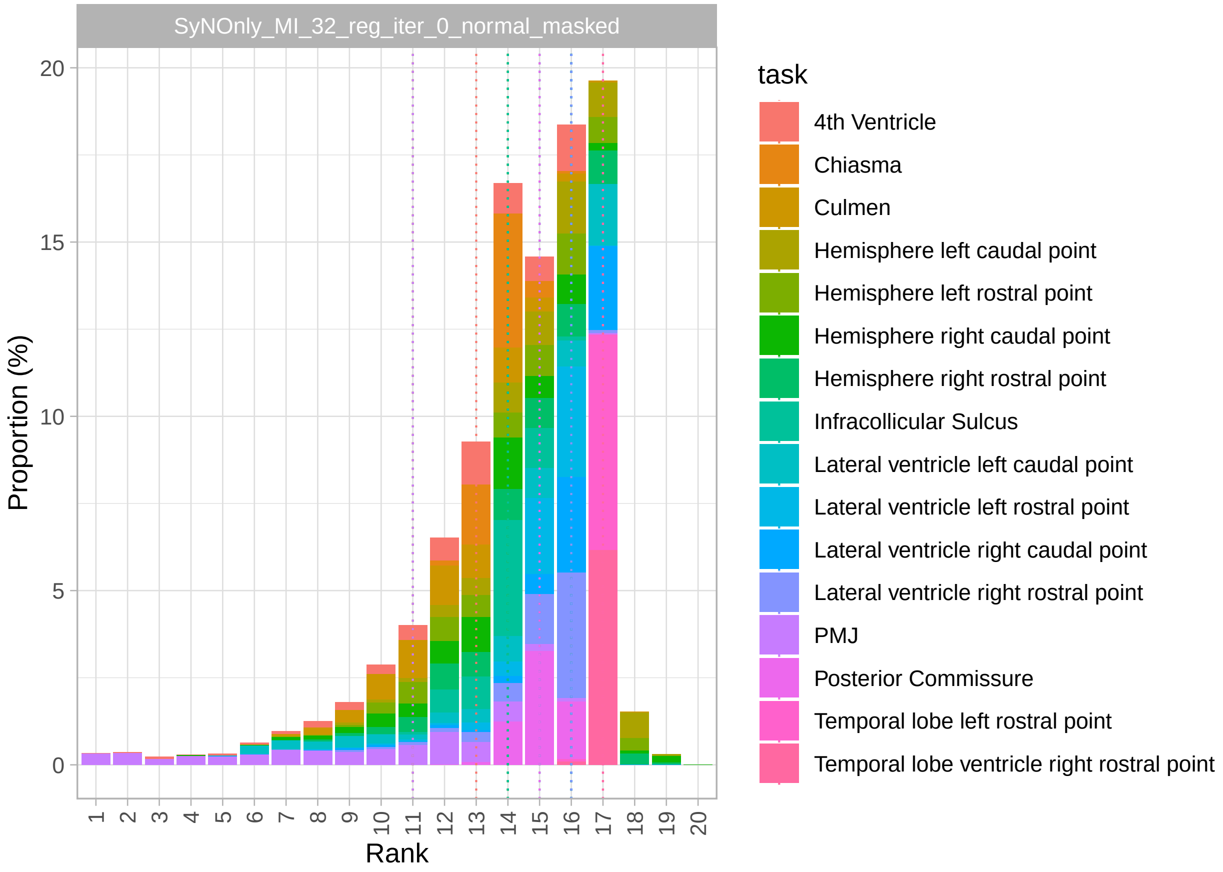
SyNOnly_MI_32_reg_iter_0_normal_masked
Proportion (%)
Rank
task
4th Ventricle
Chiasma
Culmen
Hemisphere left caudal point
Hemisphere left rostral point
Hemisphere right caudal point
Hemisphere right rostral point
Infracollicular Sulcus
Lateral ventricle left caudal point
Lateral ventricle left rostral point
Lateral ventricle right caudal point
Lateral ventricle right rostral point
PMJ
Posterior Commissure
Temporal lobe left rostral point
Temporal lobe ventricle right rostral point

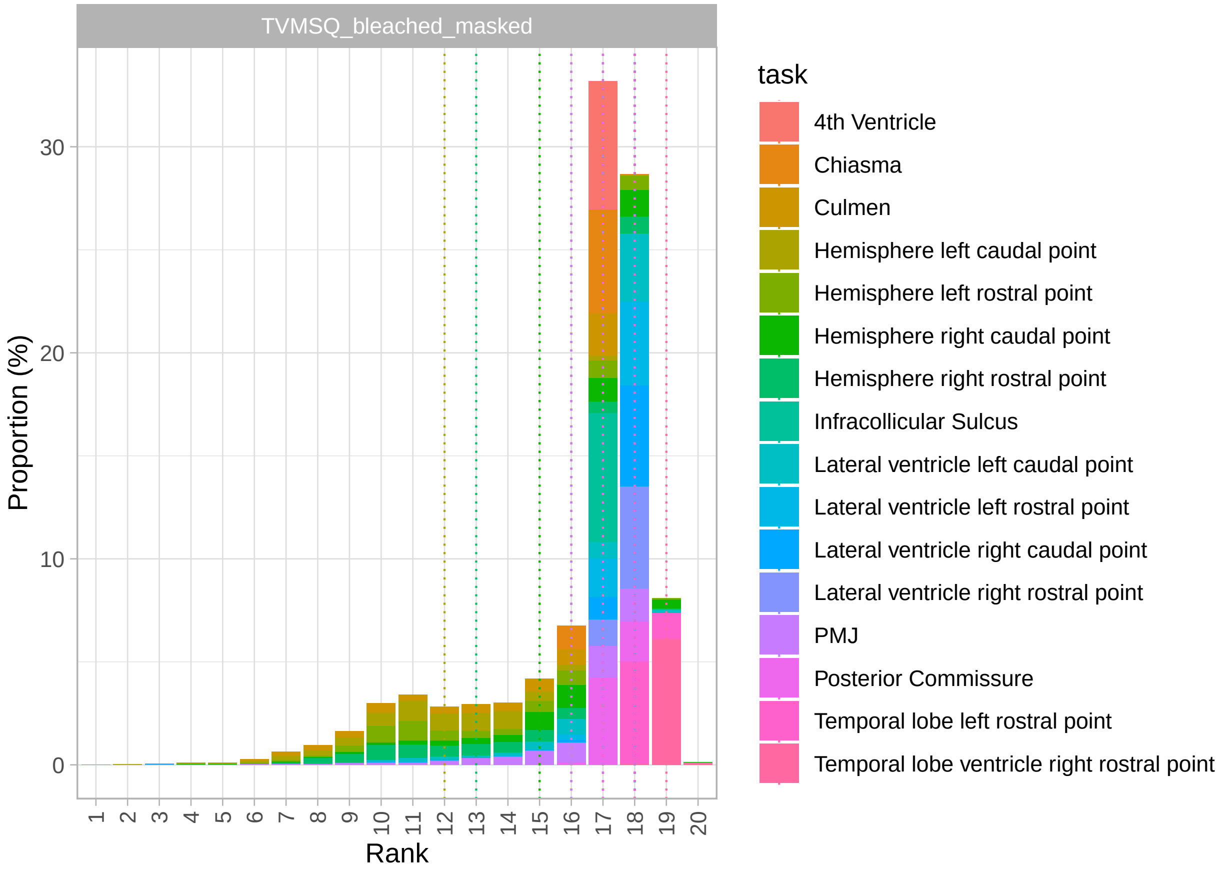
TVMSQ_bleached_masked
Proportion (%)
0
10
20
30
Rank
1 2 3 4 5 6 7 8 9 10 11 12 13 14 15 16 17 18 19 20
task
4th Ventricle
Chiasma
Culmen
Hemisphere left caudal point
Hemisphere left rostral point
Hemisphere right caudal point
Hemisphere right rostral point
Infracollicular Sulcus
Lateral ventricle left caudal point
Lateral ventricle left rostral point
Lateral ventricle right caudal point
Lateral ventricle right rostral point
PMJ
Posterior Commissure
Temporal lobe left rostral point
Temporal lobe ventricle right rostral point

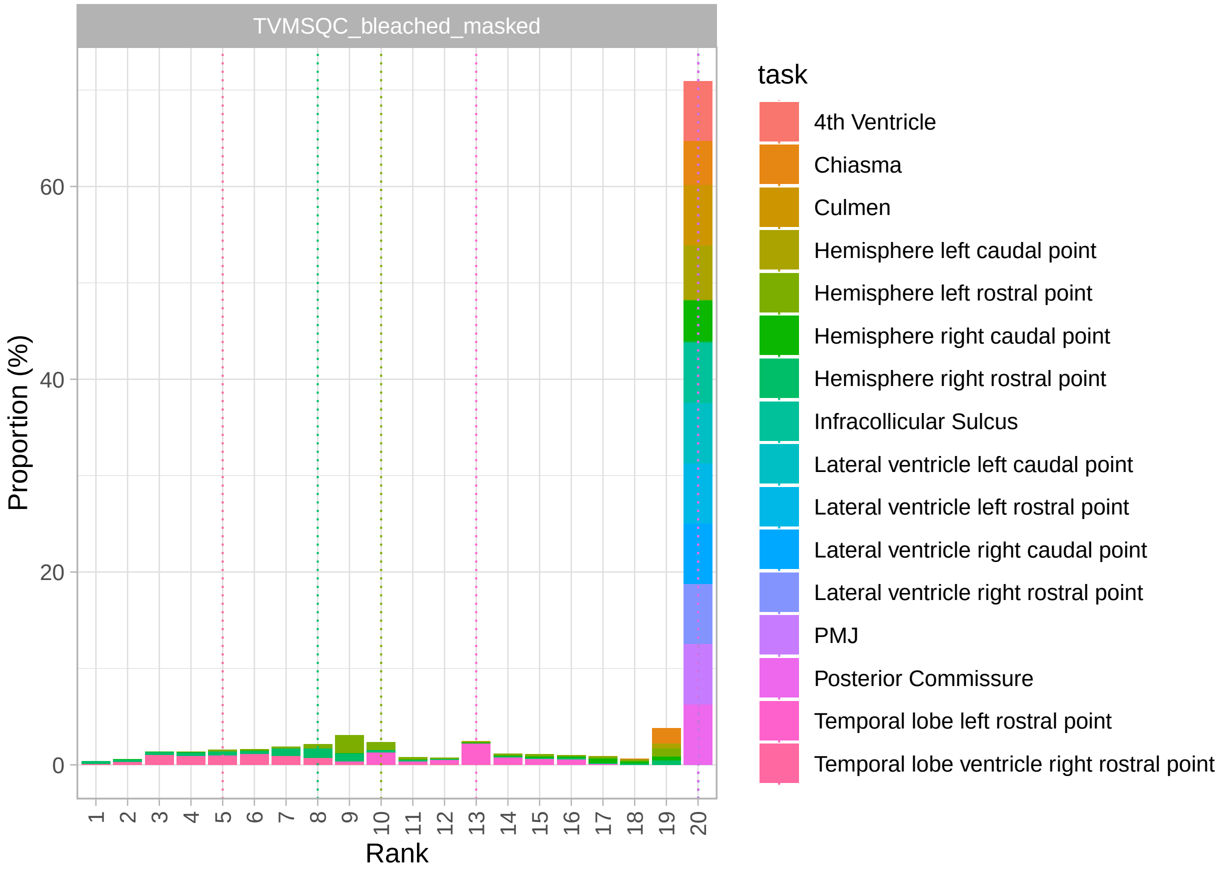
TVMSQC_bleached_masked
Proportion (%)
0
20
40
60
Rank
1 2 3 4 5 6 7 8 9 10 11 12 13 14 15 16 17 18 19 20
task
4th Ventricle
Chiasma
Culmen
Hemisphere left caudal point
Hemisphere left rostral point
Hemisphere right caudal point
Hemisphere right rostral point
Infracollicular Sulcus
Lateral ventricle left caudal point
Lateral ventricle left rostral point
Lateral ventricle right caudal point
Lateral ventricle right rostral point
PMJ
Posterior Commissure
Temporal lobe left rostral point
Temporal lobe ventricle right rostral point

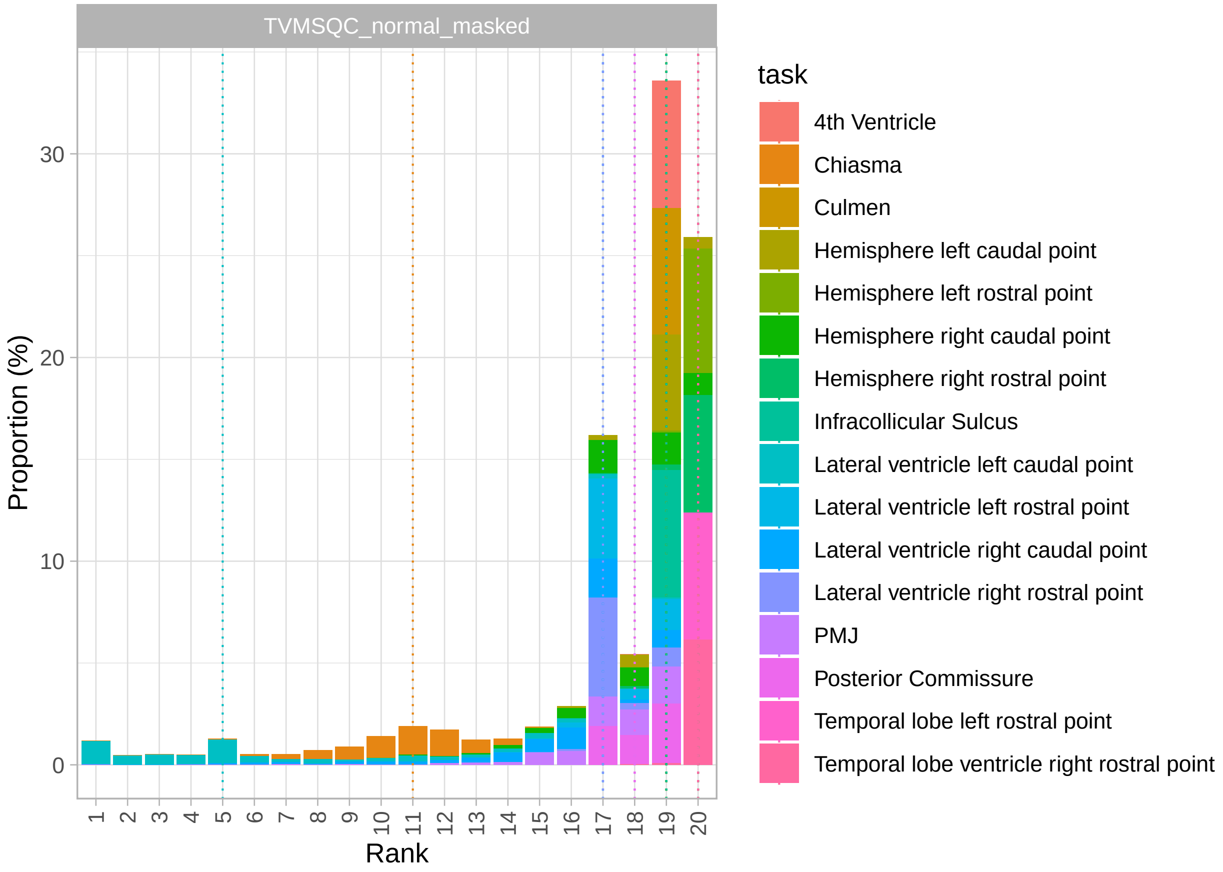
TVMSQC_normal_masked
Proportion (%)
Rank
task
4th Ventricle
Chiasma
Culmen
Hemisphere left caudal point
Hemisphere left rostral point
Hemisphere right caudal point
Hemisphere right rostral point
Infracollicular Sulcus
Lateral ventricle left caudal point
Lateral ventricle left rostral point
Lateral ventricle right caudal point
Lateral ventricle right rostral point
PMJ
Posterior Commissure
Temporal lobe left rostral point
Temporal lobe ventricle right rostral point

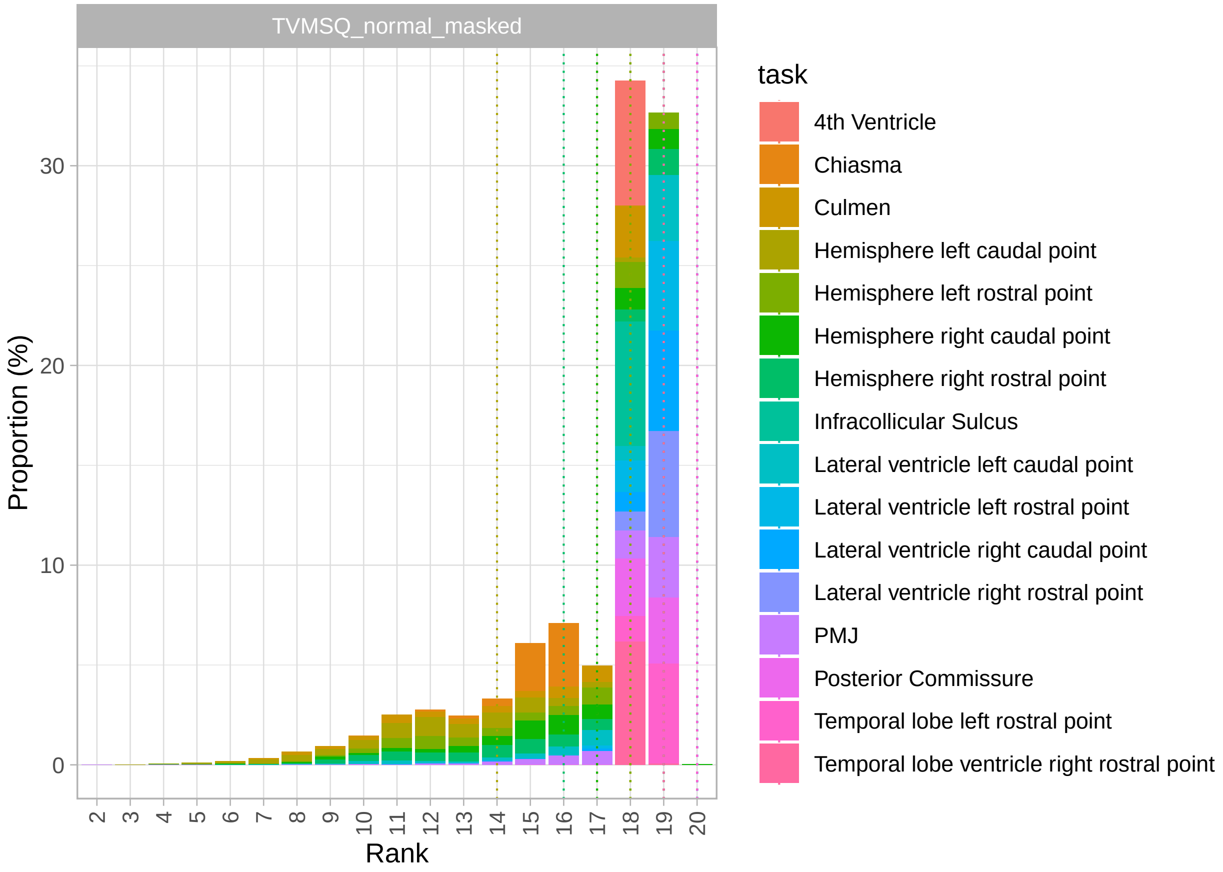

TVMSQ_normal_masked
Proportion (%)
Rank
task
4th Ventricle
Chiasma
Culmen
Hemisphere left caudal point
Hemisphere left rostral point
Hemisphere right caudal point
Hemisphere right rostral point
Infracollicular Sulcus
Lateral ventricle left caudal point
Lateral ventricle left rostral point
Lateral ventricle right caudal point
Lateral ventricle right rostral point
PMJ
Posterior Commissure
Temporal lobe left rostral point
Temporal lobe ventricle right rostral point

4.2 Characterization of tasks

4.2.1 Visualizing bootstrap results

To investigate which tasks separate algorithms well (i.e., lead to a stable ranking), a blob plot is recommended.

Bootstrap results can be shown in a blob plot showing one plot for each task. In this view, the spread of the blobs for each algorithm can be compared across tasks. Deviations from the diagonal indicate deviations from the consensus ranking (over tasks). Specifically, if rank distribution of an algorithm is consistently below the diagonal, the algorithm performed better in this task than on average across tasks, while if the rank distribution of an algorithm is consistently above the diagonal, the algorithm performed worse in this task than on average across tasks. At the bottom of each panel, ranks for each algorithm in the tasks are provided.

Same as in Section 3.1 but now ordered according to consensus.

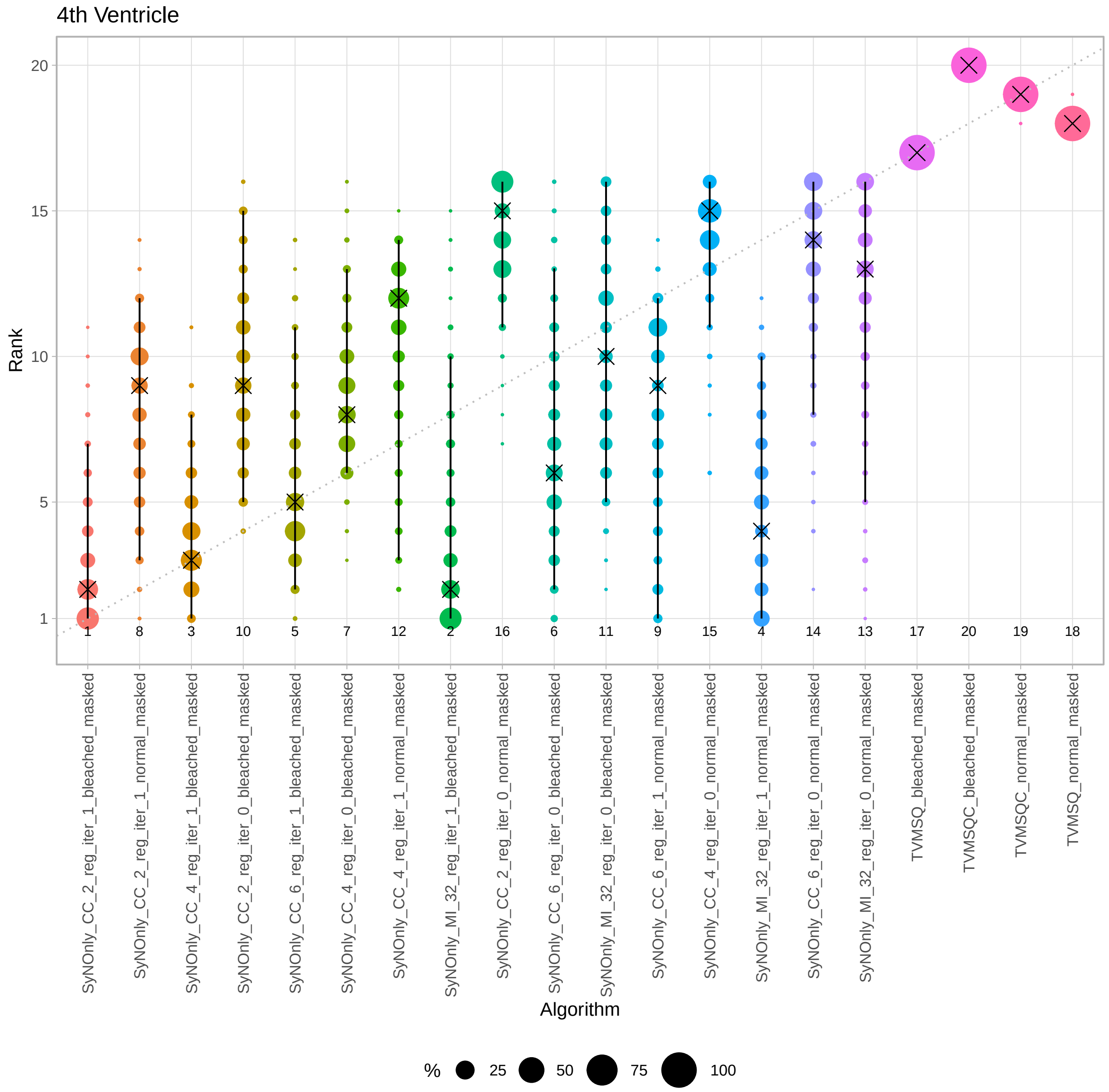

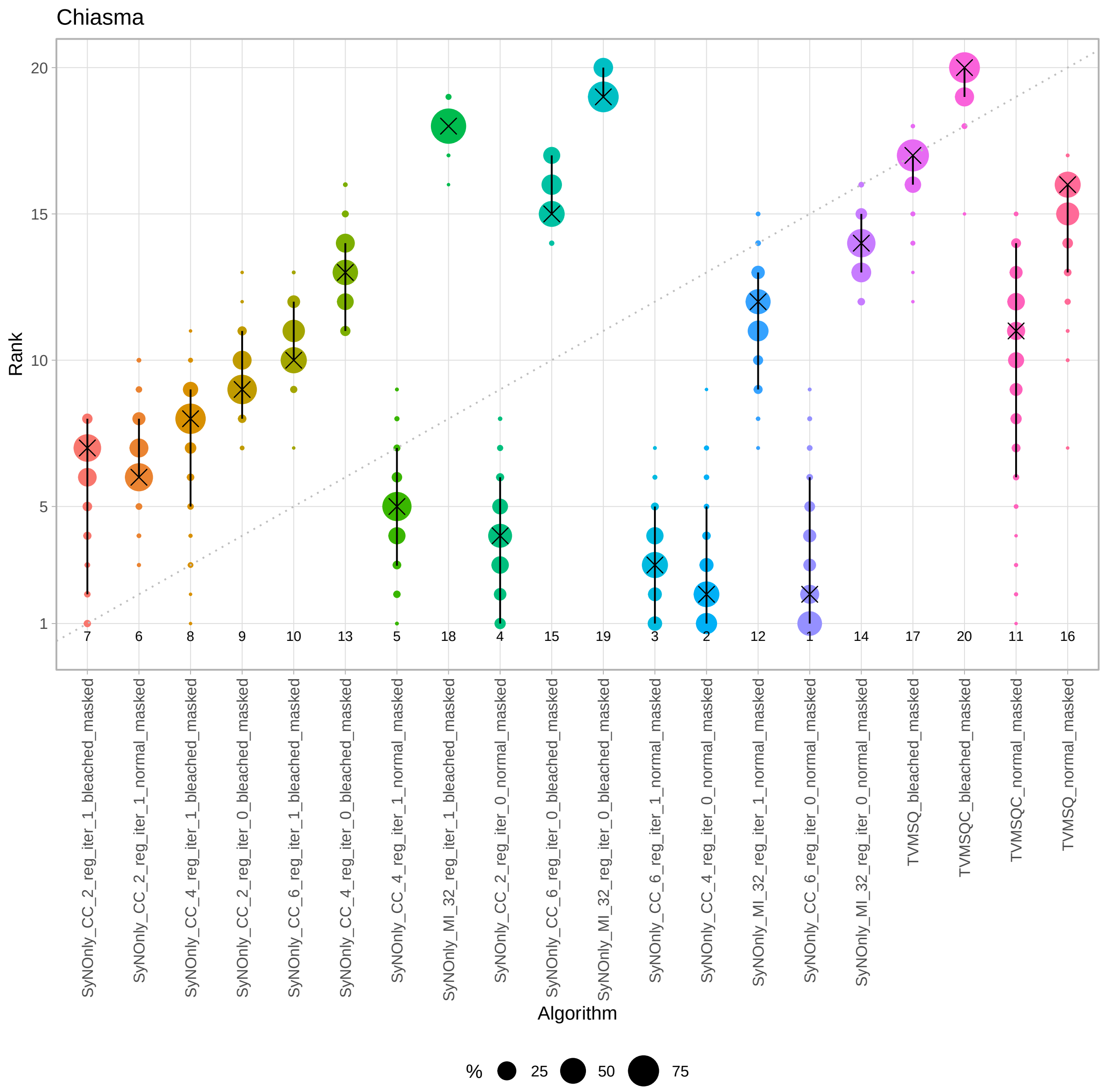
Chiasma
Rank
Algorithm
%
25
50
75
SyNOnly_CC_2_reg_iter_1_bleached_masked
SyNOnly_CC_2_reg_iter_1_normal_masked
SyNOnly_CC_4_reg_iter_1_bleached_masked
SyNOnly_CC_2_reg_iter_0_bleached_masked
SyNOnly_CC_6_reg_iter_1_bleached_masked
SyNOnly_CC_4_reg_iter_0_bleached_masked
SyNOnly_CC_4_reg_iter_1_normal_masked
SyNOnly_MI_32_reg_iter_1_bleached_masked
SyNOnly_CC_2_reg_iter_0_normal_masked
SyNOnly_CC_6_reg_iter_0_bleached_masked
SyNOnly_MI_32_reg_iter_0_bleached_masked
SyNOnly_CC_6_reg_iter_1_normal_masked
SyNOnly_CC_4_reg_iter_0_normal_masked
SyNOnly_MI_32_reg_iter_1_normal_masked
SyNOnly_CC_6_reg_iter_0_normal_masked
SyNOnly_MI_32_reg_iter_0_normal_masked
TVMSQ_bleached_masked
TVMSQC_bleached_masked
TVMSQC_normal_masked
TVMSQ_normal_masked
7 6 8 9 10 13 5 18 4 15 19 3 2 12 1 14 17 20 11 16

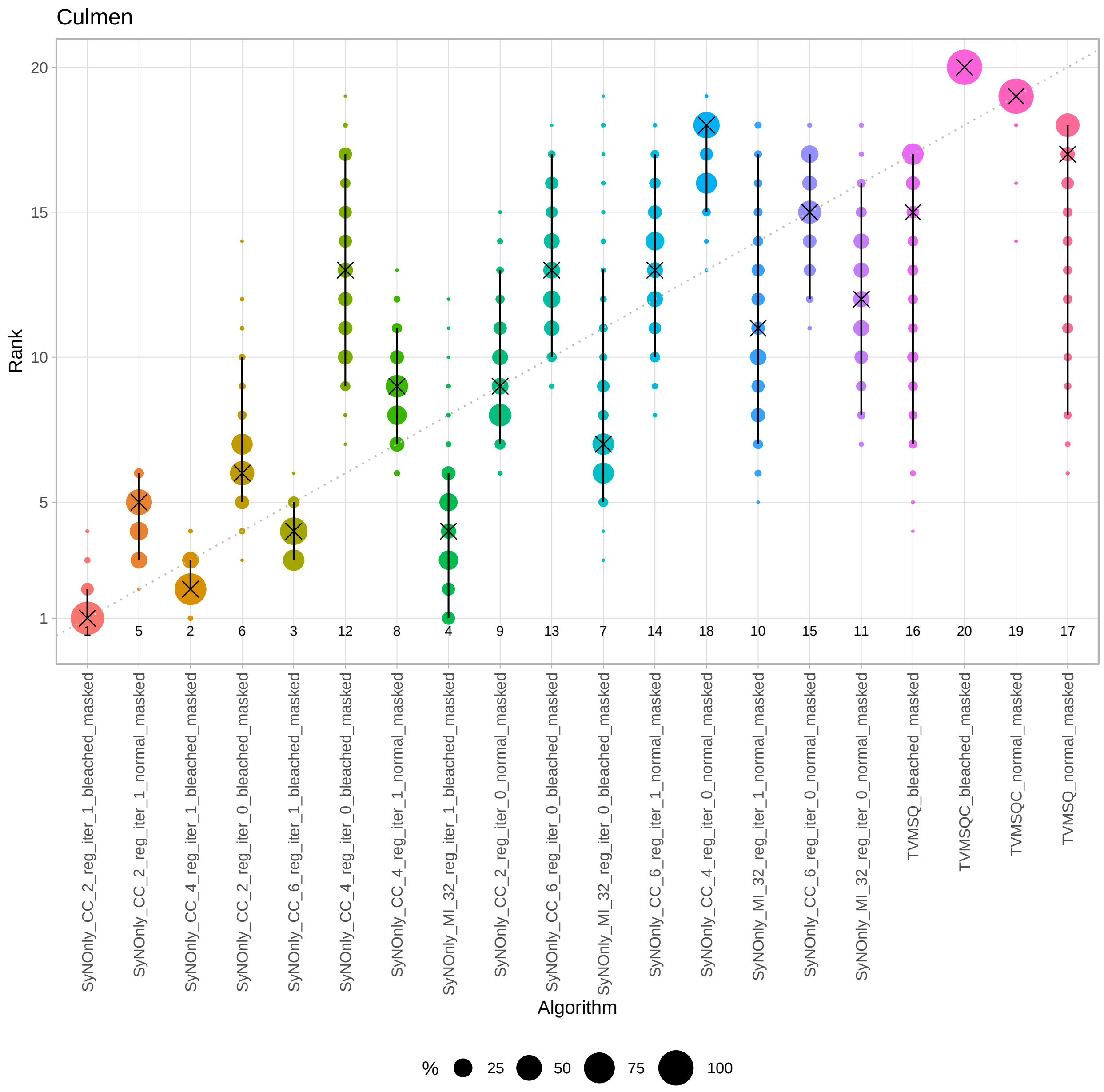
Culmen
Rank
Algorithm
20
15
10
5
1
1
5
2
6
3
12
8
4
9
13
7
14
18
10
15
11
16
20
19
17
SyNOnly_CC_2_reg_iter_1_bleached_masked
SyNOnly_CC_2_reg_iter_1_normal_masked
SyNOnly_CC_4_reg_iter_1_bleached_masked
SyNOnly_CC_2_reg_iter_0_bleached_masked
SyNOnly_CC_6_reg_iter_1_bleached_masked
SyNOnly_CC_4_reg_iter_0_bleached_masked
SyNOnly_CC_4_reg_iter_1_normal_masked
SyNOnly_MI_32_reg_iter_1_bleached_masked
SyNOnly_CC_2_reg_iter_0_normal_masked
SyNOnly_CC_6_reg_iter_0_bleached_masked
SyNOnly_MI_32_reg_iter_0_bleached_masked
SyNOnly_CC_6_reg_iter_1_normal_masked
SyNOnly_CC_4_reg_iter_0_normal_masked
SyNOnly_MI_32_reg_iter_1_normal_masked
SyNOnly_CC_6_reg_iter_0_normal_masked
SyNOnly_MI_32_reg_iter_0_normal_masked
TVMSQ_bleached_masked
TVMSQC_bleached_masked
TVMSQC_normal_masked
TVMSQ_normal_masked
%
25
50
75
100

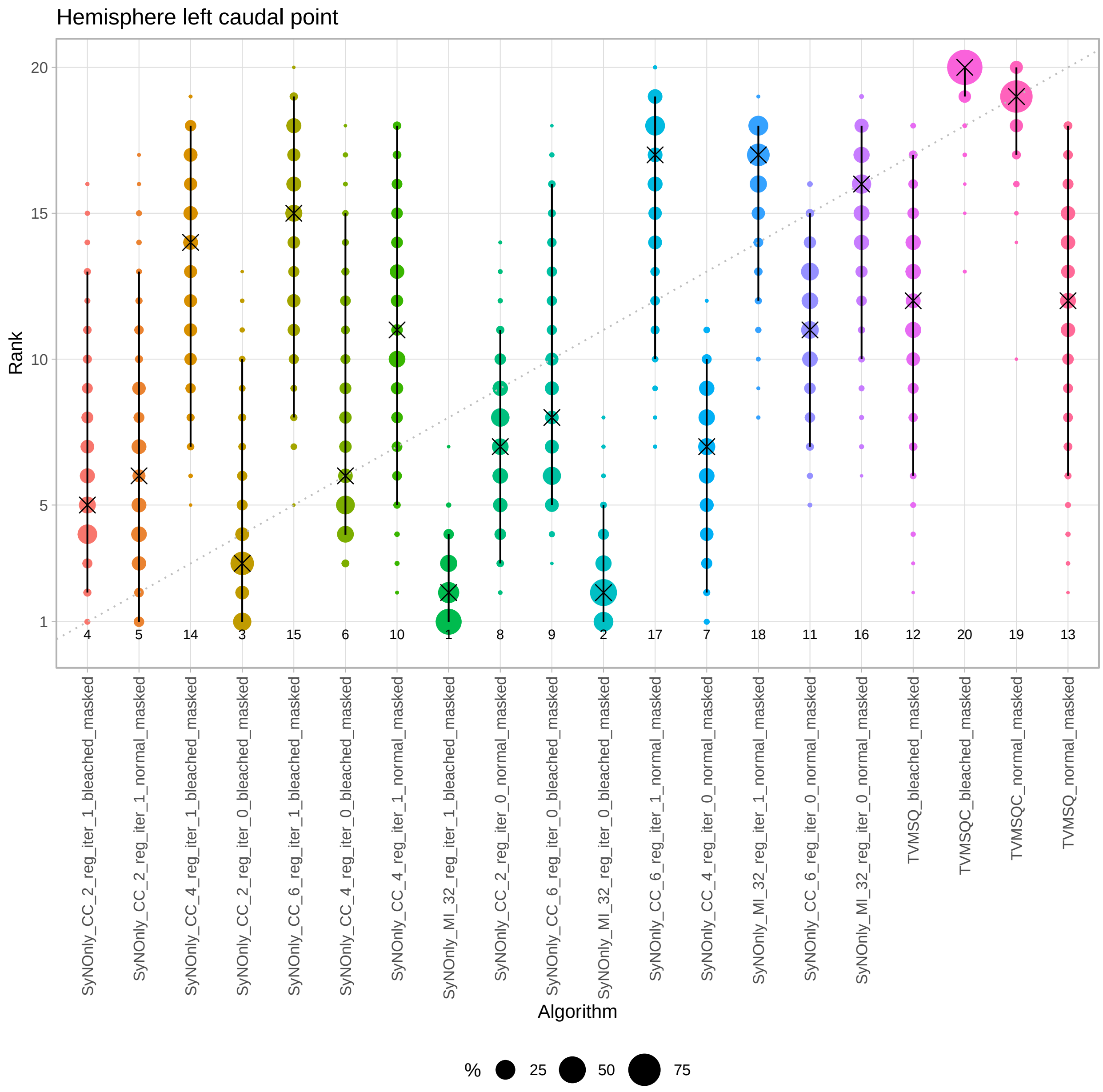
Hemisphere left caudal point
Rank
Algorithm
20
15
10
5
1
4
5
14
3
15
6
10
1
8
9
2
17
7
18
11
16
12
20
19
13
SyNOnly_CC_2_reg_iter_1_bleached_masked
SyNOnly_CC_2_reg_iter_1_normal_masked
SyNOnly_CC_4_reg_iter_1_bleached_masked
SyNOnly_CC_2_reg_iter_0_bleached_masked
SyNOnly_CC_6_reg_iter_1_bleached_masked
SyNOnly_CC_4_reg_iter_0_bleached_masked
SyNOnly_CC_4_reg_iter_1_normal_masked
SyNOnly_MI_32_reg_iter_1_bleached_masked
SyNOnly_CC_2_reg_iter_0_normal_masked
SyNOnly_CC_6_reg_iter_0_bleached_masked
SyNOnly_MI_32_reg_iter_0_bleached_masked
SyNOnly_CC_6_reg_iter_1_normal_masked
SyNOnly_CC_4_reg_iter_0_normal_masked
SyNOnly_MI_32_reg_iter_1_normal_masked
SyNOnly_CC_6_reg_iter_0_normal_masked
SyNOnly_MI_32_reg_iter_0_normal_masked
TVMSQ_bleached_masked
TVMSQC_bleached_masked
TVMSQC_normal_masked
TVMSQ_normal_masked
%
25
50
75

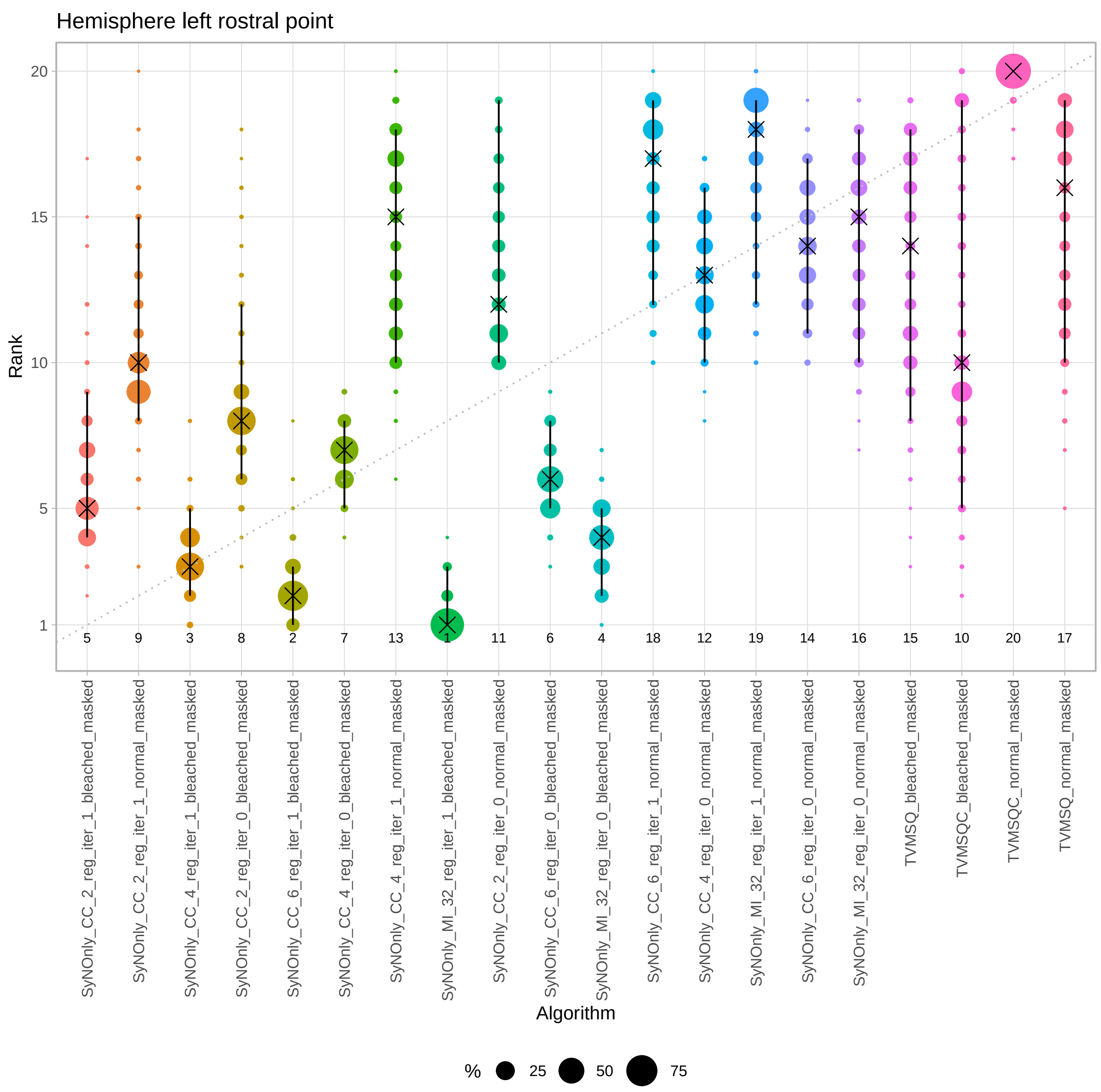
Hemisphere left rostral point
Rank
20
15
10
5
1
5
9
3
8
2
7
13
1
11
6
4
18
12
19
14
16
15
10
20
17
SyNOnly_CC_2_reg_iter_1_bleached_masked
SyNOnly_CC_2_reg_iter_1_normal_masked
SyNOnly_CC_4_reg_iter_1_bleached_masked
SyNOnly_CC_2_reg_iter_0_bleached_masked
SyNOnly_CC_6_reg_iter_1_bleached_masked
SyNOnly_CC_4_reg_iter_0_bleached_masked
SyNOnly_CC_4_reg_iter_1_normal_masked
SyNOnly_MI_32_reg_iter_1_bleached_masked
SyNOnly_CC_2_reg_iter_0_normal_masked
SyNOnly_CC_6_reg_iter_0_bleached_masked
SyNOnly_MI_32_reg_iter_0_bleached_masked
SyNOnly_CC_6_reg_iter_1_normal_masked
SyNOnly_CC_4_reg_iter_0_normal_masked
SyNOnly_MI_32_reg_iter_1_normal_masked
SyNOnly_CC_6_reg_iter_0_normal_masked
SyNOnly_MI_32_reg_iter_0_normal_masked
TVMSQ_bleached_masked
TVMSQC_bleached_masked
TVMSQC_normal_masked
TVMSQ_normal_masked
Algorithm
%
25
50
75

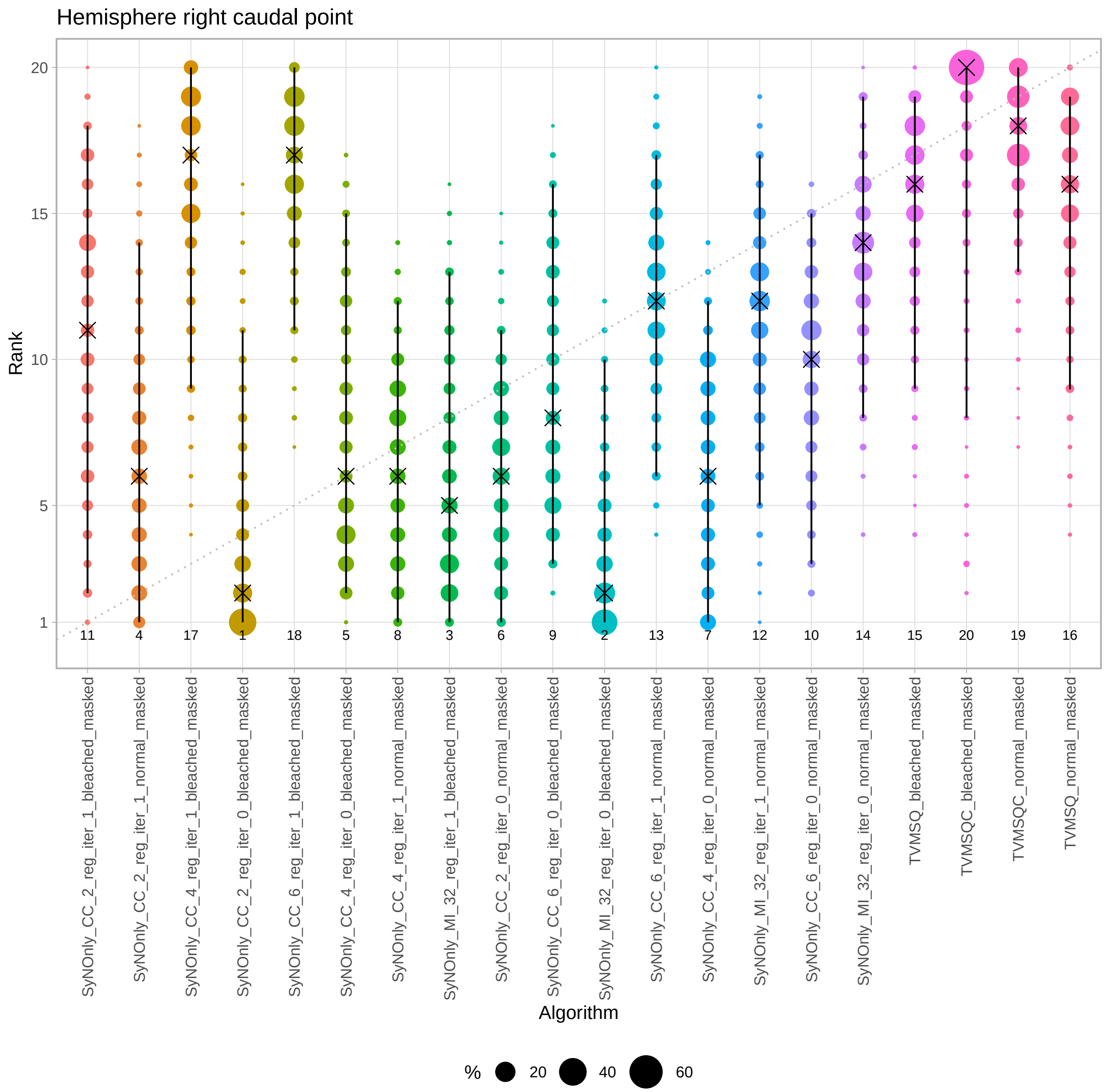

Hemisphere right caudal point
Rank
Algorithm
11 4 17 1 18 5 8 3 6 9 2 13 7 12 10 14 15 20 19 16
SyNOnly_CC_2_reg_iter_1_bleached_masked
SyNOnly_CC_2_reg_iter_1_normal_masked
SyNOnly_CC_4_reg_iter_1_bleached_masked
SyNOnly_CC_2_reg_iter_0_bleached_masked
SyNOnly_CC_6_reg_iter_1_bleached_masked
SyNOnly_CC_4_reg_iter_0_bleached_masked
SyNOnly_CC_4_reg_iter_1_normal_masked
SyNOnly_MI_32_reg_iter_1_bleached_masked
SyNOnly_CC_2_reg_iter_0_normal_masked
SyNOnly_CC_6_reg_iter_0_bleached_masked
SyNOnly_MI_32_reg_iter_0_bleached_masked
SyNOnly_CC_6_reg_iter_1_normal_masked
SyNOnly_CC_4_reg_iter_0_normal_masked
SyNOnly_MI_32_reg_iter_1_normal_masked
SyNOnly_CC_6_reg_iter_0_normal_masked
SyNOnly_MI_32_reg_iter_0_normal_masked
TVMSQ_bleached_masked
TVMSQC_bleached_masked
TVMSQC_normal_masked
TVMSQ_normal_masked
% 20 40 60

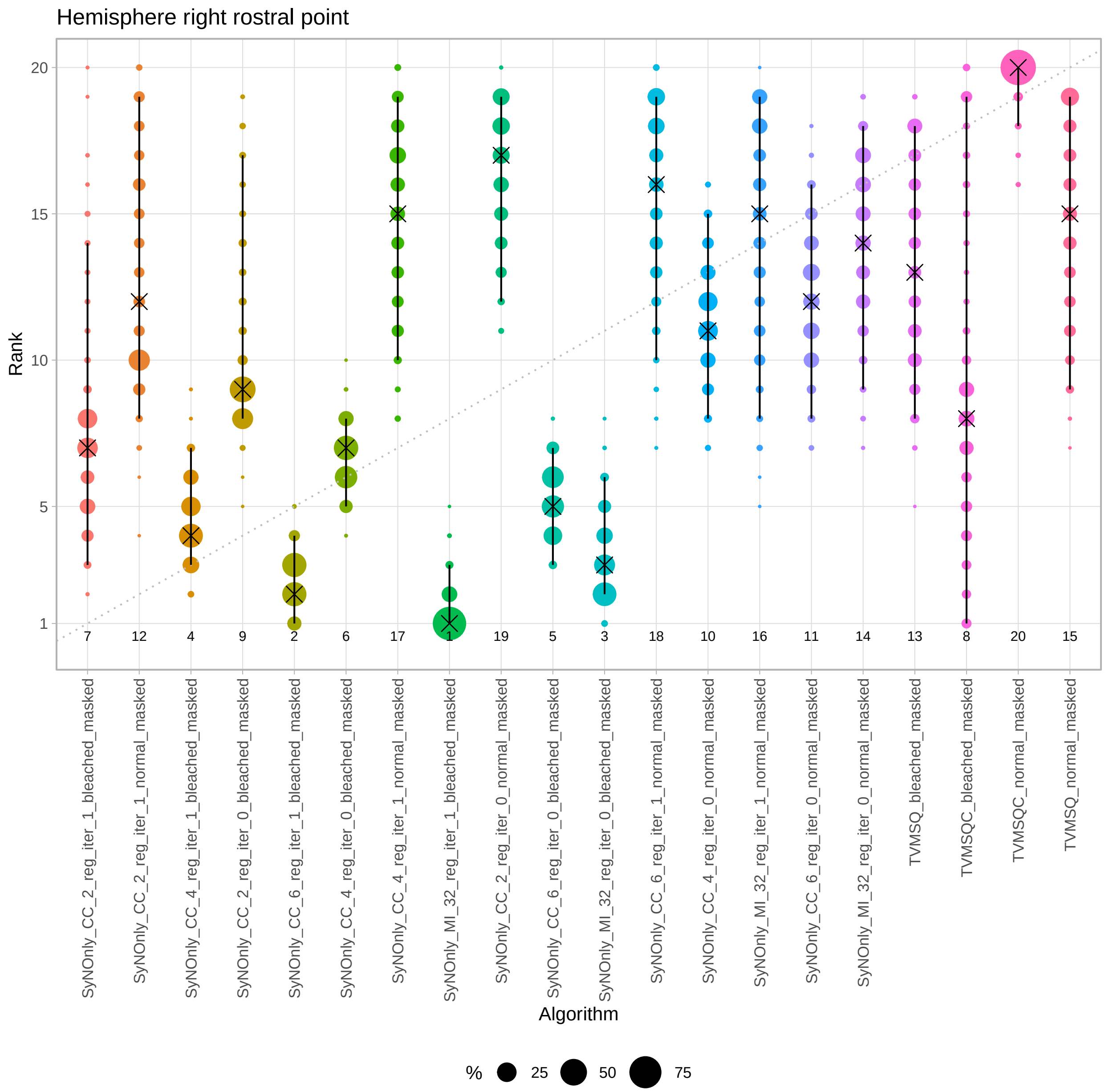
Hemisphere right rostral point
Rank
Algorithm
20
15
10
5
1
7
12
4
9
2
6
17
1
19
5
3
18
10
16
11
14
13
8
20
15
SyNOnly_CC_2_reg_iter_1_bleached_masked
SyNOnly_CC_2_reg_iter_1_normal_masked
SyNOnly_CC_4_reg_iter_1_bleached_masked
SyNOnly_CC_2_reg_iter_0_bleached_masked
SyNOnly_CC_6_reg_iter_1_bleached_masked
SyNOnly_CC_4_reg_iter_0_bleached_masked
SyNOnly_CC_4_reg_iter_1_normal_masked
SyNOnly_MI_32_reg_iter_1_bleached_masked
SyNOnly_CC_2_reg_iter_0_normal_masked
SyNOnly_CC_6_reg_iter_0_bleached_masked
SyNOnly_MI_32_reg_iter_0_bleached_masked
SyNOnly_CC_6_reg_iter_1_normal_masked
SyNOnly_CC_4_reg_iter_0_normal_masked
SyNOnly_MI_32_reg_iter_1_normal_masked
SyNOnly_CC_6_reg_iter_0_normal_masked
SyNOnly_MI_32_reg_iter_0_normal_masked
TVMSQ_bleached_masked
TVMSQC_bleached_masked
TVMSQC_normal_masked
TVMSQ_normal_masked
% 25 50 75

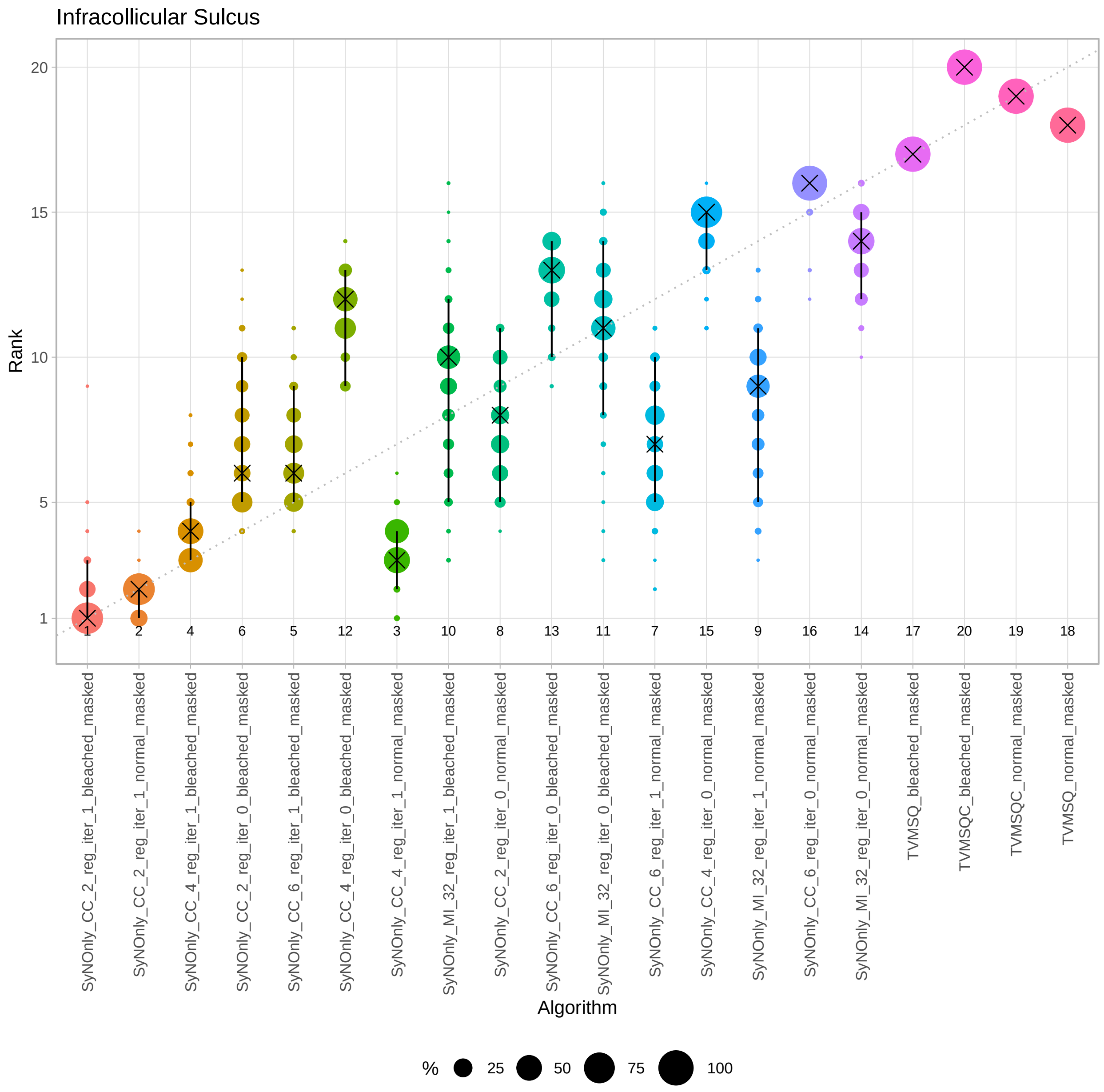

Infracollicular Sulcus
Rank
20
15
10
5
1
1
2
4
6
5
12
3
10
8
13
11
7
15
9
16
14
17
20
19
18
SyNOnly_CC_2_reg_iter_1_bleached_masked
SyNOnly_CC_2_reg_iter_1_normal_masked
SyNOnly_CC_4_reg_iter_1_bleached_masked
SyNOnly_CC_2_reg_iter_0_bleached_masked
SyNOnly_CC_6_reg_iter_1_bleached_masked
SyNOnly_CC_4_reg_iter_0_bleached_masked
SyNOnly_CC_4_reg_iter_1_normal_masked
SyNOnly_MI_32_reg_iter_1_bleached_masked
SyNOnly_CC_2_reg_iter_0_normal_masked
SyNOnly_CC_6_reg_iter_0_bleached_masked
SyNOnly_MI_32_reg_iter_0_bleached_masked
SyNOnly_CC_6_reg_iter_1_normal_masked
SyNOnly_CC_4_reg_iter_0_normal_masked
SyNOnly_MI_32_reg_iter_1_normal_masked
SyNOnly_CC_6_reg_iter_0_normal_masked
SyNOnly_MI_32_reg_iter_0_normal_masked
TVMSQ_bleached_masked
TVMSQC_bleached_masked
TVMSQC_normal_masked
TVMSQ_normal_masked
Algorithm
%
25
50
75
100

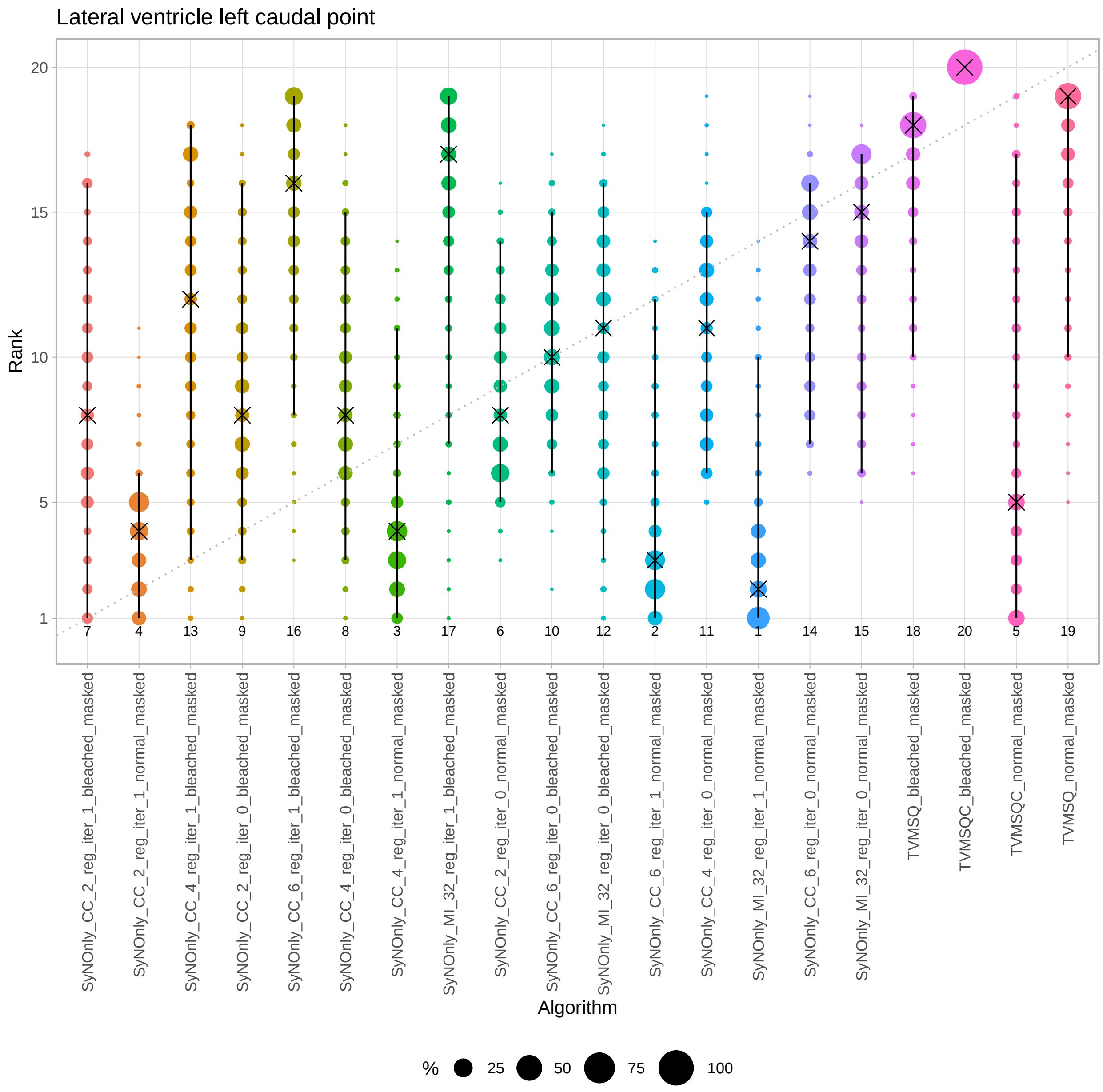
Lateral ventricle left caudal point
Rank
Algorithm
7 4 13 9 16 8 3 17 6 10 12 2 11 1 14 15 18 20 5 19
SyNOnly_CC_2_reg_iter_1_bleached_masked
SyNOnly_CC_2_reg_iter_1_normal_masked
SyNOnly_CC_4_reg_iter_1_bleached_masked
SyNOnly_CC_2_reg_iter_0_bleached_masked
SyNOnly_CC_6_reg_iter_1_bleached_masked
SyNOnly_CC_4_reg_iter_0_bleached_masked
SyNOnly_CC_4_reg_iter_1_normal_masked
SyNOnly_MI_32_reg_iter_1_bleached_masked
SyNOnly_CC_2_reg_iter_0_normal_masked
SyNOnly_CC_6_reg_iter_0_bleached_masked
SyNOnly_MI_32_reg_iter_0_bleached_masked
SyNOnly_CC_6_reg_iter_1_normal_masked
SyNOnly_CC_4_reg_iter_0_normal_masked
SyNOnly_MI_32_reg_iter_1_normal_masked
SyNOnly_CC_6_reg_iter_0_normal_masked
SyNOnly_MI_32_reg_iter_0_normal_masked
TVMSQ_bleached_masked
TVMSQC_bleached_masked
TVMSQC_normal_masked
TVMSQ_normal_masked
% 25 50 75 100

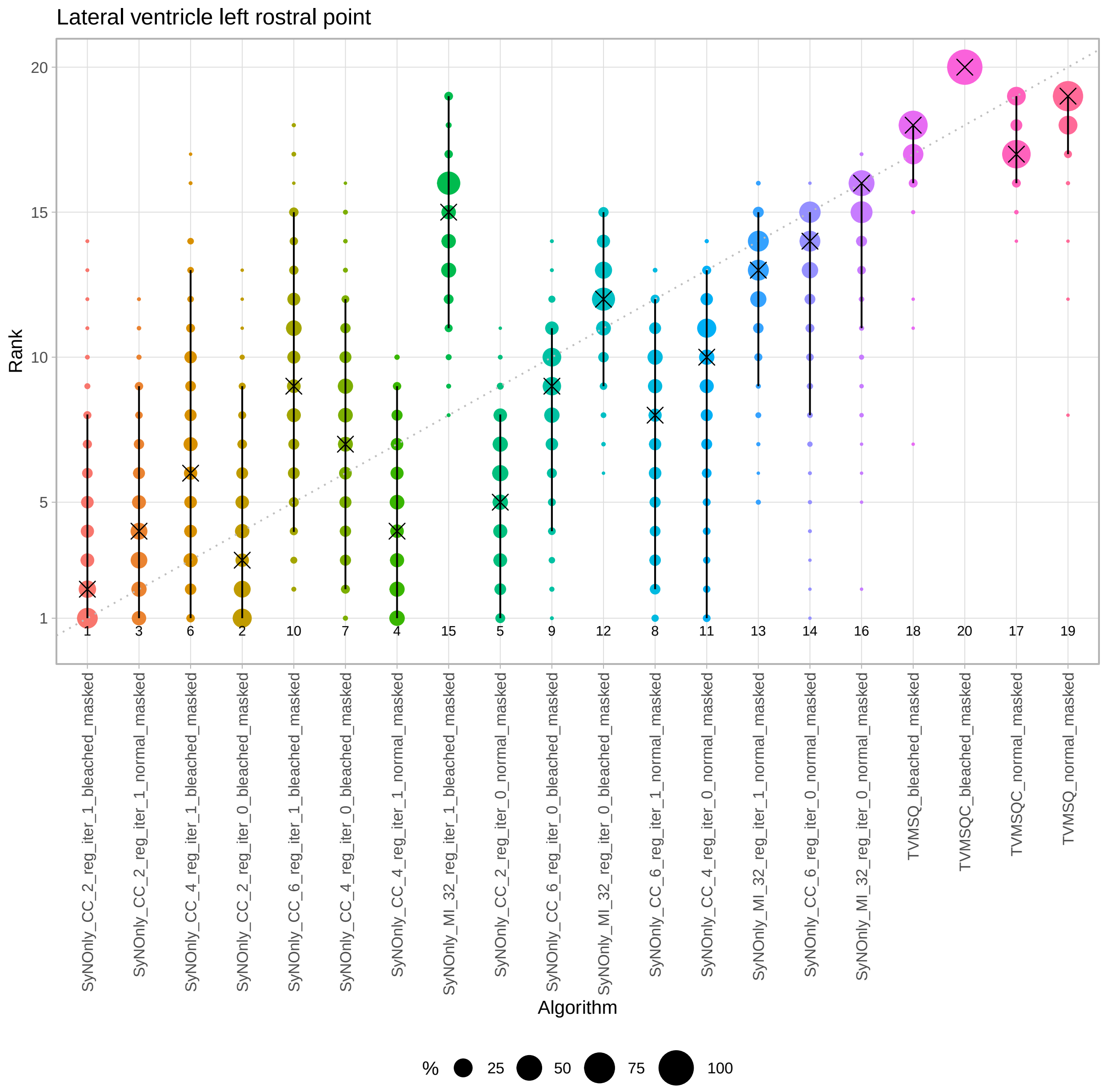

Lateral ventricle left rostral point
Rank
20
15
10
5
1
1 3 6 2 10 7 4 15 5 9 12 8 11 13 14 16 18 20 17 19
SyNOnly_CC_2_reg_iter_1_bleached_masked
SyNOnly_CC_2_reg_iter_1_normal_masked
SyNOnly_CC_4_reg_iter_1_bleached_masked
SyNOnly_CC_2_reg_iter_0_bleached_masked
SyNOnly_CC_6_reg_iter_1_bleached_masked
SyNOnly_CC_4_reg_iter_0_bleached_masked
SyNOnly_CC_4_reg_iter_1_normal_masked
SyNOnly_MI_32_reg_iter_1_bleached_masked
SyNOnly_CC_2_reg_iter_0_normal_masked
SyNOnly_CC_6_reg_iter_0_bleached_masked
SyNOnly_MI_32_reg_iter_0_bleached_masked
SyNOnly_CC_6_reg_iter_1_normal_masked
SyNOnly_CC_4_reg_iter_0_normal_masked
SyNOnly_MI_32_reg_iter_1_normal_masked
SyNOnly_CC_6_reg_iter_0_normal_masked
SyNOnly_MI_32_reg_iter_0_normal_masked
TVMSQ_bleached_masked
TVMSQC_bleached_masked
TVMSQC_normal_masked
TVMSQ_normal_masked
Algorithm
% 25 50 75 100

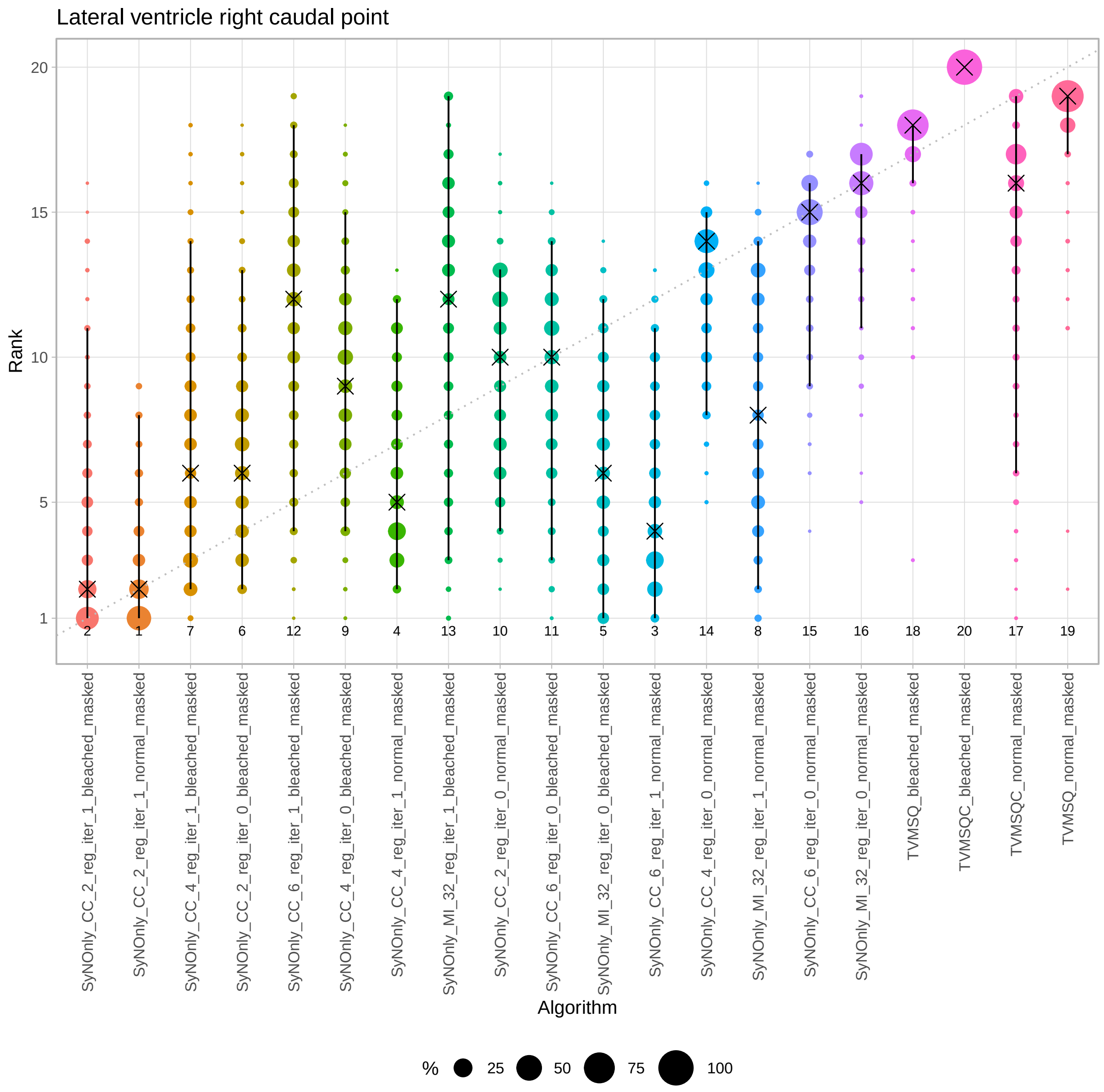
Lateral ventricle right caudal point
Rank
20
15
10
5
1
2
1
7
6
12
9
4
13
10
11
5
3
14
8
15
16
18
20
17
19
SyNOnly_CC_2_reg_iter_1_bleached_masked
SyNOnly_CC_2_reg_iter_1_normal_masked
SyNOnly_CC_4_reg_iter_1_bleached_masked
SyNOnly_CC_2_reg_iter_0_bleached_masked
SyNOnly_CC_6_reg_iter_1_bleached_masked
SyNOnly_CC_4_reg_iter_0_bleached_masked
SyNOnly_CC_4_reg_iter_1_normal_masked
SyNOnly_MI_32_reg_iter_1_bleached_masked
SyNOnly_CC_2_reg_iter_0_normal_masked
SyNOnly_CC_6_reg_iter_0_bleached_masked
SyNOnly_MI_32_reg_iter_0_bleached_masked
SyNOnly_CC_6_reg_iter_1_normal_masked
SyNOnly_CC_4_reg_iter_0_normal_masked
SyNOnly_MI_32_reg_iter_1_normal_masked
SyNOnly_CC_6_reg_iter_0_normal_masked
SyNOnly_MI_32_reg_iter_0_normal_masked
TVMSQ_bleached_masked
TVMSQC_bleached_masked
TVMSQC_normal_masked
TVMSQ_normal_masked
Algorithm
%
25
50
75
100

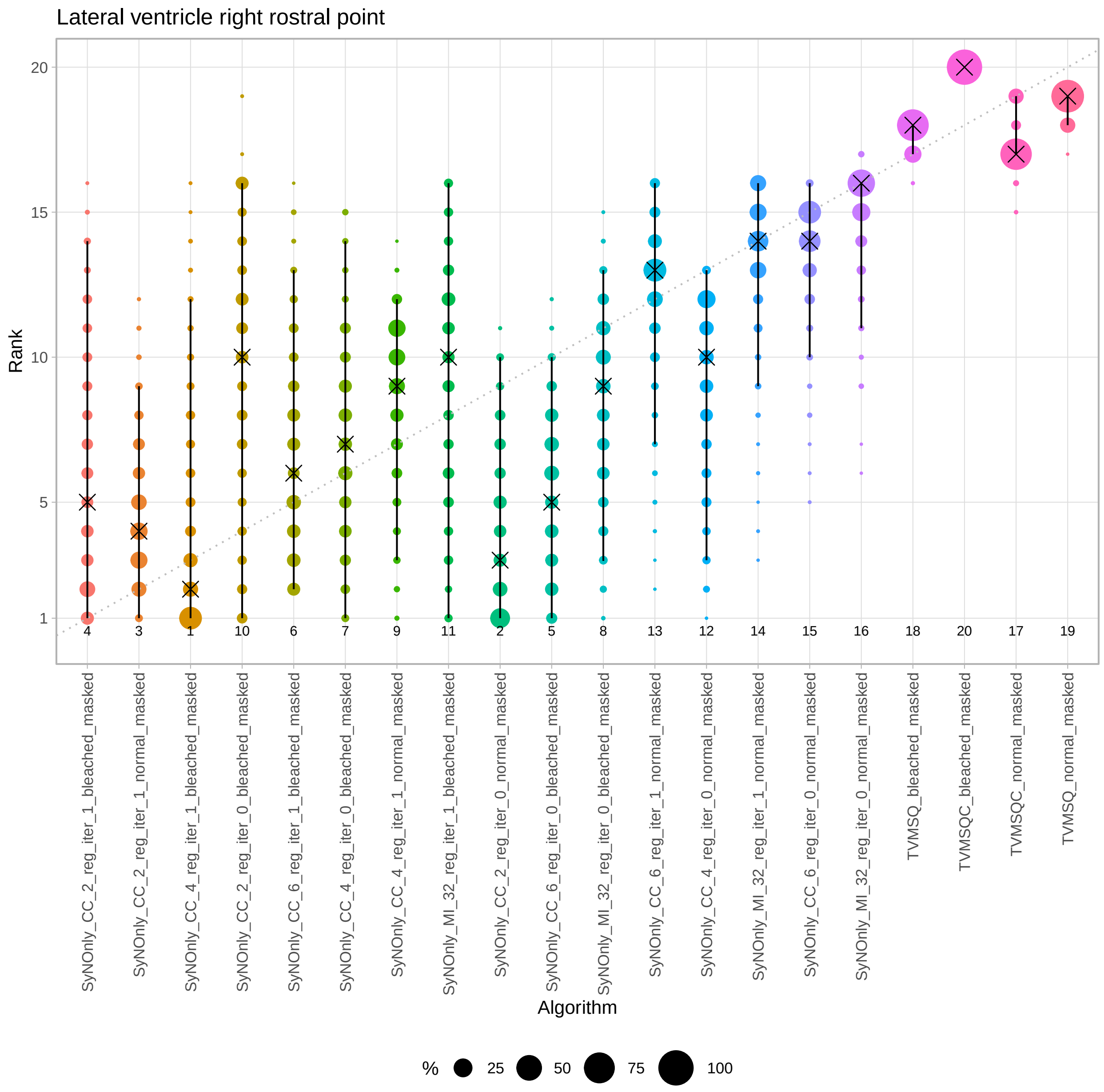
Lateral ventricle right rostral point
Rank
20
15
10
5
1
4
3
1
10
6
7
9
11
2
5
8
13
12
14
15
16
18
20
17
19
SyNOnly_CC_2_reg_iter_1_bleached_masked
SyNOnly_CC_2_reg_iter_1_normal_masked
SyNOnly_CC_4_reg_iter_1_bleached_masked
SyNOnly_CC_2_reg_iter_0_bleached_masked
SyNOnly_CC_6_reg_iter_1_bleached_masked
SyNOnly_CC_4_reg_iter_0_bleached_masked
SyNOnly_CC_4_reg_iter_1_normal_masked
SyNOnly_MI_32_reg_iter_1_bleached_masked
SyNOnly_CC_2_reg_iter_0_normal_masked
SyNOnly_CC_6_reg_iter_0_bleached_masked
SyNOnly_MI_32_reg_iter_0_bleached_masked
SyNOnly_CC_6_reg_iter_1_normal_masked
SyNOnly_CC_4_reg_iter_0_normal_masked
SyNOnly_MI_32_reg_iter_1_normal_masked
SyNOnly_CC_6_reg_iter_0_normal_masked
SyNOnly_MI_32_reg_iter_0_normal_masked
TVMSQ_bleached_masked
TVMSQC_bleached_masked
TVMSQC_normal_masked
TVMSQ_normal_masked
Algorithm
%
25
50
75
100

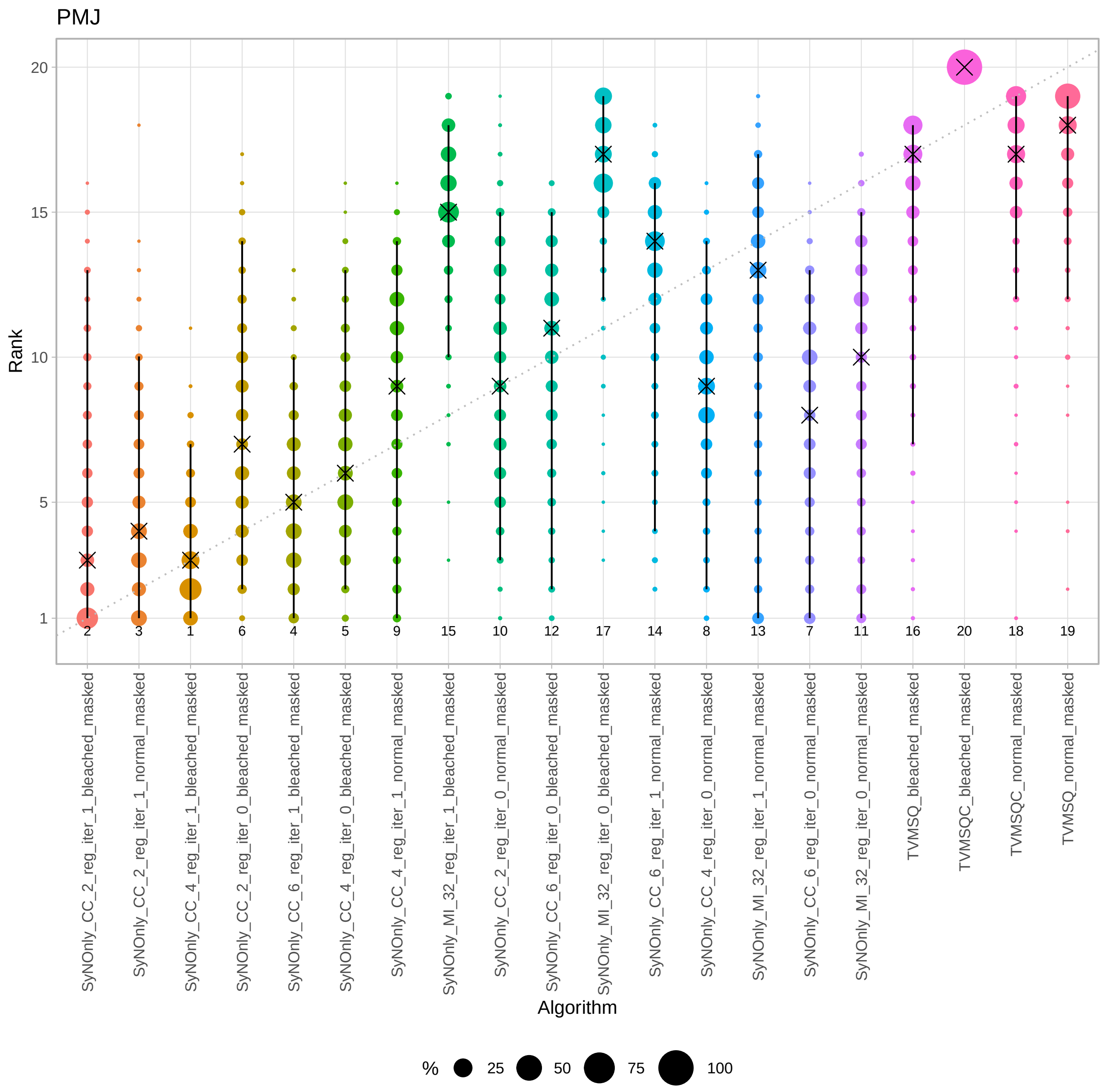
PMJ
Rank
Algorithm
SyNOnly_CC_2_reg_iter_1_bleached_masked
SyNOnly_CC_2_reg_iter_1_normal_masked
SyNOnly_CC_4_reg_iter_1_bleached_masked
SyNOnly_CC_2_reg_iter_0_bleached_masked
SyNOnly_CC_6_reg_iter_1_bleached_masked
SyNOnly_CC_4_reg_iter_0_bleached_masked
SyNOnly_CC_4_reg_iter_1_normal_masked
SyNOnly_MI_32_reg_iter_1_bleached_masked
SyNOnly_CC_2_reg_iter_0_normal_masked
SyNOnly_CC_6_reg_iter_0_bleached_masked
SyNOnly_MI_32_reg_iter_0_bleached_masked
SyNOnly_CC_6_reg_iter_1_normal_masked
SyNOnly_CC_4_reg_iter_0_normal_masked
SyNOnly_MI_32_reg_iter_1_normal_masked
SyNOnly_CC_6_reg_iter_0_normal_masked
SyNOnly_MI_32_reg_iter_0_normal_masked
TVMSQ_bleached_masked
TVMSQC_bleached_masked
TVMSQC_normal_masked
TVMSQ_normal_masked
2 3 1 6 4 5 9 15 10 12 17 14 8 13 7 11 16 20 18 19
% 25 50 75 100

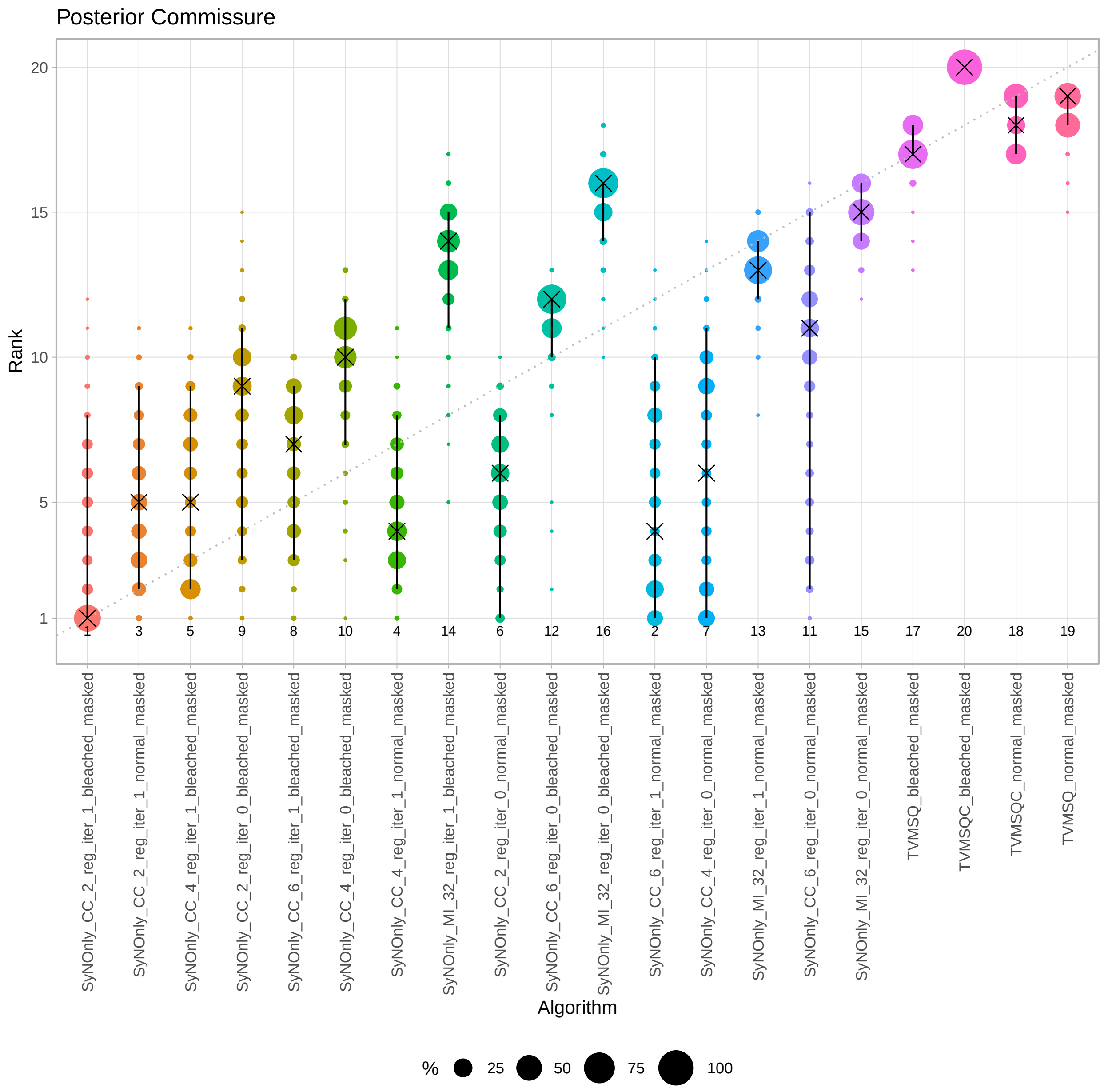

Posterior Commissure
Rank
Algorithm
%
25
50
75
100
1
3
5
9
8
10
4
14
6
12
16
2
7
13
11
15
17
20
18
19
SyNOnly_CC_2_reg_iter_1_bleached_masked
SyNOnly_CC_2_reg_iter_1_normal_masked
SyNOnly_CC_4_reg_iter_1_bleached_masked
SyNOnly_CC_2_reg_iter_0_bleached_masked
SyNOnly_CC_6_reg_iter_1_bleached_masked
SyNOnly_CC_4_reg_iter_0_bleached_masked
SyNOnly_CC_4_reg_iter_1_normal_masked
SyNOnly_MI_32_reg_iter_1_bleached_masked
SyNOnly_CC_2_reg_iter_0_normal_masked
SyNOnly_CC_6_reg_iter_0_bleached_masked
SyNOnly_MI_32_reg_iter_0_bleached_masked
SyNOnly_CC_6_reg_iter_1_normal_masked
SyNOnly_CC_4_reg_iter_0_normal_masked
SyNOnly_MI_32_reg_iter_1_normal_masked
SyNOnly_CC_6_reg_iter_0_normal_masked
SyNOnly_MI_32_reg_iter_0_normal_masked
TVMSQ_bleached_masked
TVMSQC_bleached_masked
TVMSQC_normal_masked
TVMSQ_normal_masked

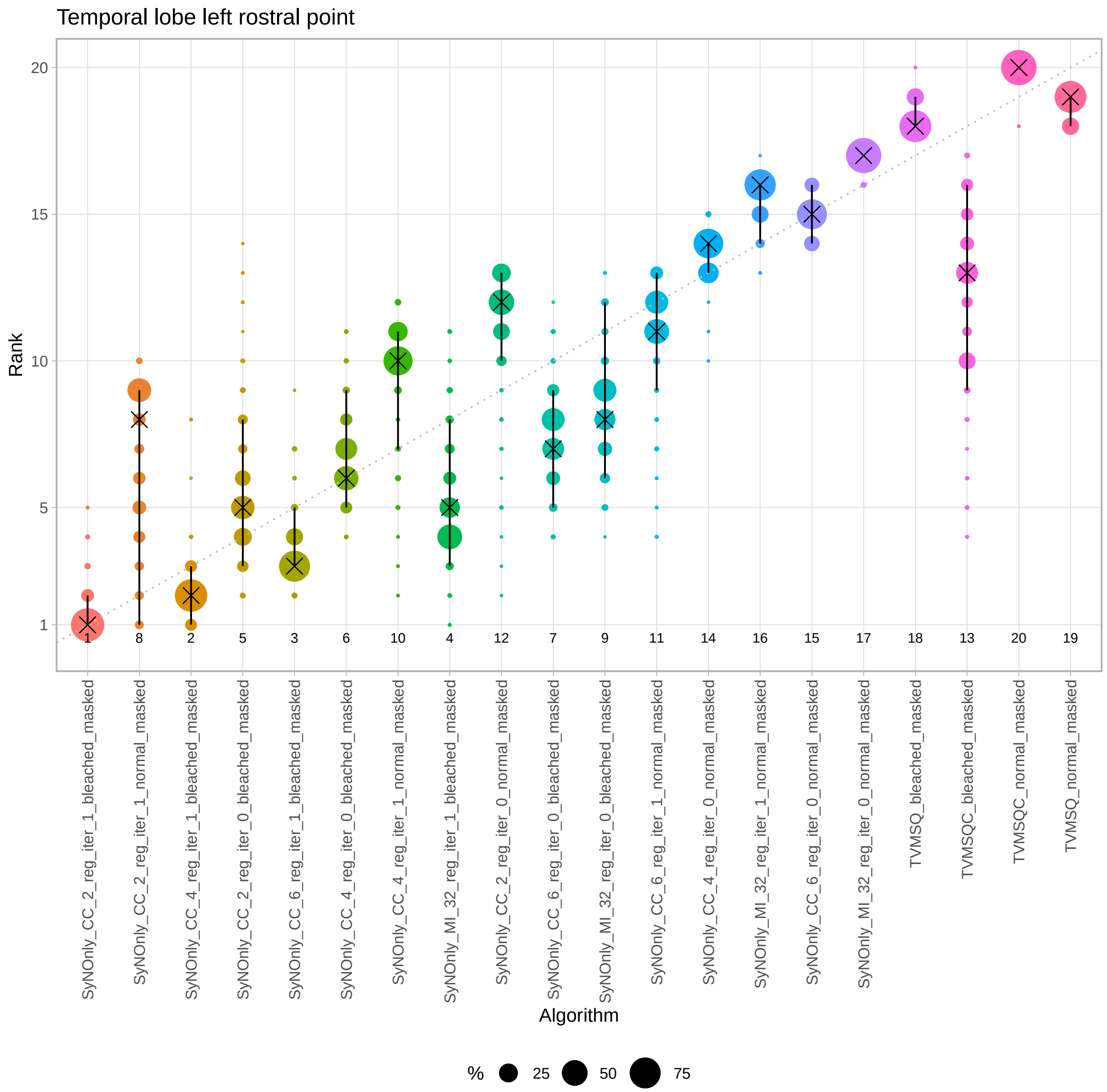
Temporal lobe left rostral point
Rank
20
15
10
5
1
1 8 2 5 3 6 10 4 12 7 9 11 14 16 15 17 18 13 20 19
SyNOnly_CC_2_reg_iter_1_bleached_masked
SyNOnly_CC_2_reg_iter_1_normal_masked
SyNOnly_CC_4_reg_iter_1_bleached_masked
SyNOnly_CC_2_reg_iter_0_bleached_masked
SyNOnly_CC_6_reg_iter_1_bleached_masked
SyNOnly_CC_4_reg_iter_0_bleached_masked
SyNOnly_CC_4_reg_iter_1_normal_masked
SyNOnly_MI_32_reg_iter_1_bleached_masked
SyNOnly_CC_2_reg_iter_0_normal_masked
SyNOnly_CC_6_reg_iter_0_bleached_masked
SyNOnly_MI_32_reg_iter_0_bleached_masked
SyNOnly_CC_6_reg_iter_1_normal_masked
SyNOnly_CC_4_reg_iter_0_normal_masked
SyNOnly_MI_32_reg_iter_1_normal_masked
SyNOnly_CC_6_reg_iter_0_normal_masked
SyNOnly_MI_32_reg_iter_0_normal_masked
TVMSQ_bleached_masked
TVMSQC_bleached_masked
TVMSQC_normal_masked
TVMSQ_normal_masked
Algorithm
% 25 50 75

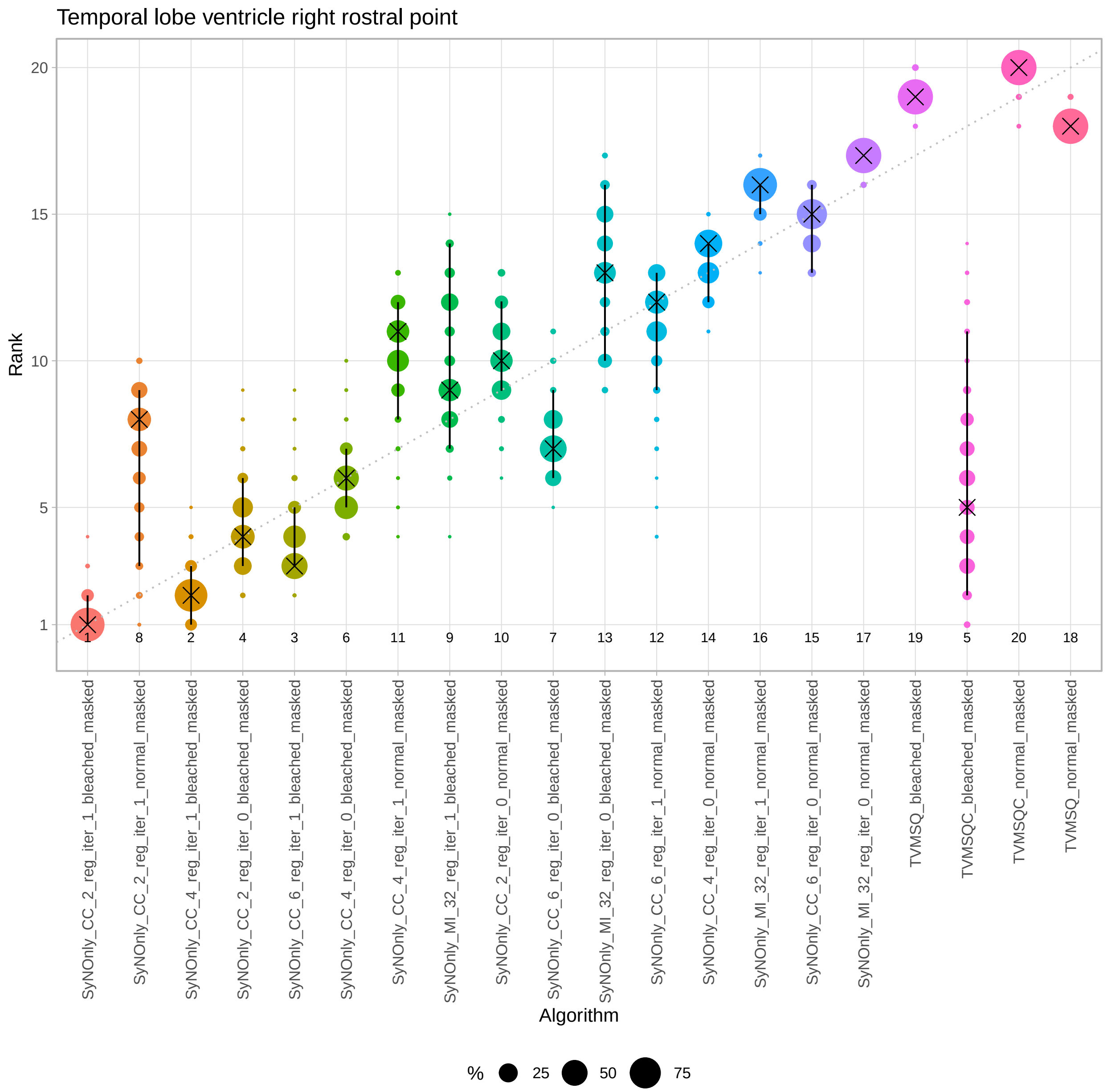

4.2.2 Cluster Analysis

Dendrogram from hierarchical cluster analysis and *network-type graphs* for assessing the similarity of tasks based on challenge rankings.

A dendrogram is a visualization approach based on hierarchical clustering. It depicts clusters according to a chosen distance measure (here: Spearman's footrule) as well as a chosen agglomeration method (here: complete and average agglomeration).

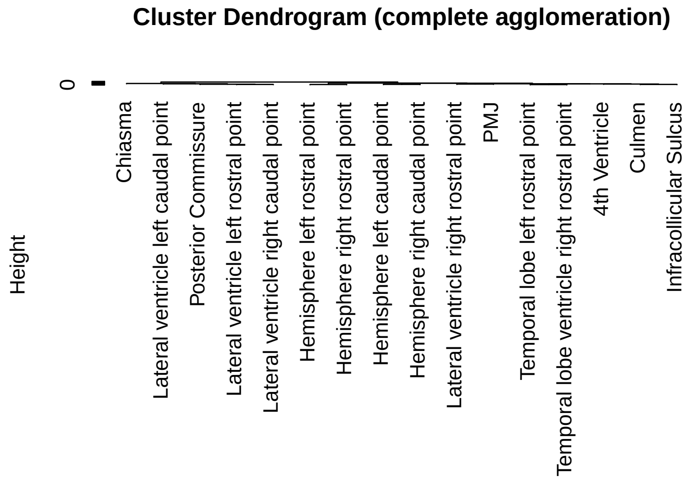
Cluster Dendrogram (complete agglomeration)
Height
0
Chiasma
Lateral ventricle left caudal point
Posterior Commissure
Lateral ventricle left rostral point
Lateral ventricle right caudal point
Hemisphere left rostral point
Hemisphere right rostral point
Hemisphere left caudal point
Hemisphere right caudal point
Lateral ventricle right rostral point
PMJ
Temporal lobe left rostral point
Temporal lobe ventricle right rostral point
4th Ventricle
Culmen
Infracollicular Sulcus

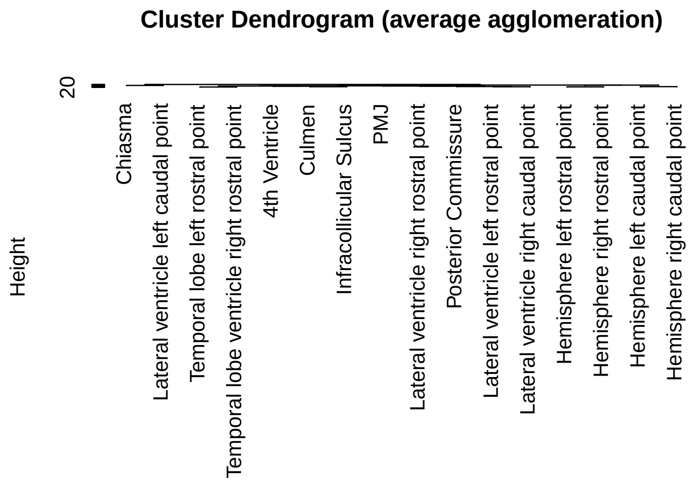
Cluster Dendrogram (average agglomeration)
Height
20
Chiasma
Lateral ventricle left caudal point
Temporal lobe left rostral point
Temporal lobe ventricle right rostral point
4th Ventricle
Culmen
Infracollicular Sulcus
PMJ
Lateral ventricle right rostral point
Posterior Commissure
Lateral ventricle left rostral point
Lateral ventricle right caudal point
Hemisphere left rostral point
Hemisphere right rostral point
Hemisphere left caudal point
Hemisphere right caudal point

5 References

Wiesenfarth, M., Reinke, A., Landman, B.A., Eisenmann, M., Aguilera Saiz, L., Cardoso, M.J., Maier-Hein, L. and Kopp-Schneider, A. Methods and open-source toolkit for analyzing and visualizing challenge results. *Sci Rep* **11**, 2369 (2021). https://doi.org/10.1038/s41598-021-82017-6

M. J. A. Eugster, T. Hothorn, and F. Leisch, “Exploratory and inferential analysis of benchmark experiments,” Institut fuer Statistik, Ludwig-Maximilians-Universitaet Muenchen, Germany, Technical Report 30, 2008. [Online]. Available: http://epub.ub.uni-muenchen.de/4134/.
