# Supplementary material for: Characterization of dynamic patterns of human fetal to neonatal brain asymmetry with deformation-based morphometry
Source: Front Neurosci. 2023 Dec 6;17:1252850. doi: 10.3389/fnins.2023.1252850 (PMC10734644; doi:10.3389/fnins.2023.1252850)
Supplement: Supplementary file 1 [file Data_Sheet_1.ZIP › ChallengeR/Framework A fetal to neonatal/nonlinear/ranking_Fetal2Neonatal_nonlinear_normal_and_bleached_dice.pdf]

Benchmarking report for Fetal to neonatal registration nonlinear normal and bleached combined

created by challengeR v1.0.4

29 März, 2023

This document presents a systematic report on the benchmark study "Fetal to neonatal registration nonlinear normal and bleached combined". Input data comprises raw metric values for all algorithms and cases. Generated plots are:

- Visualization of assessment data: Dot- and boxplots, podium plots and ranking heatmaps
- Visualization of ranking stability: Blob plots, violin plots and significance maps, line plots
- Visualization of cross-task insights: Blob plots, stacked frequency plots, dendrograms

Details can be found in Wiesenfarth et al. (2021).

1 Rankings

Algorithms within a task are ranked according to the following ranking scheme:

aggregate using function ("mean") then rank

Ranking for each task:

Dice Brainstem : The analysis is based on 20 algorithms and 20 cases. 0 missing cases have been found in the data set.

	value_mean	rank
SyNOnly_CC_2_reg_iter_1_bleached_masked	0.8184626	1
SyNOnly_CC_2_reg_iter_1_normal_masked	0.8170396	2
SyNOnly_CC_4_reg_iter_1_bleached_masked	0.8153900	3
SyNOnly_CC_6_reg_iter_1_bleached_masked	0.8109816	4
SyNOnly_CC_4_reg_iter_1_normal_masked	0.8103635	5
SyNOnly_CC_2_reg_iter_0_bleached_masked	0.8078925	6
SyNOnly_MI_32_reg_iter_1_bleached_masked	0.8071255	7
SyNOnly_MI_32_reg_iter_1_normal_masked	0.8065281	8
SyNOnly_CC_2_reg_iter_0_normal_masked	0.8050249	9
SyNOnly_CC_6_reg_iter_1_normal_masked	0.8049912	10
SyNOnly_CC_4_reg_iter_0_bleached_masked	0.8012812	11
SyNOnly_CC_6_reg_iter_0_bleached_masked	0.7954607	12
SyNOnly_CC_4_reg_iter_0_normal_masked	0.7933762	13
SyNOnly_MI_32_reg_iter_0_normal_masked	0.7888138	14
SyNOnly_CC_6_reg_iter_0_normal_masked	0.7868495	15
SyNOnly_MI_32_reg_iter_0_bleached_masked	0.7865253	16
TVMSQC_normal_masked	0.7678513	17
TVMSQ_bleached_masked	0.7678262	18
TVMSQ_normal_masked	0.7648188	19
TVMSQC_bleached_masked	0.5369389	20

Dice Cerebellum : The analysis is based on 20 algorithms and 20 cases. 0 missing cases have been found in the data set.

	value_mean	rank
SyNOnly_MI_32_reg_iter_1_bleached_masked	0.8614524	1
SyNOnly_MI_32_reg_iter_0_bleached_masked	0.8511791	2
SyNOnly_CC_6_reg_iter_1_bleached_masked	0.8314004	3
SyNOnly_CC_4_reg_iter_1_bleached_masked	0.8282637	4
SyNOnly_CC_6_reg_iter_0_bleached_masked	0.8274174	5
SyNOnly_CC_4_reg_iter_0_bleached_masked	0.8244772	6
SyNOnly_CC_2_reg_iter_1_bleached_masked	0.8233359	7
SyNOnly_CC_2_reg_iter_0_bleached_masked	0.8195777	8
SyNOnly_CC_2_reg_iter_1_normal_masked	0.7717719	9
SyNOnly_CC_2_reg_iter_0_normal_masked	0.7690770	10
SyNOnly_CC_4_reg_iter_1_normal_masked	0.7682311	11
SyNOnly_CC_6_reg_iter_1_normal_masked	0.7661851	12
SyNOnly_CC_4_reg_iter_0_normal_masked	0.7636917	13
SyNOnly_CC_6_reg_iter_0_normal_masked	0.7604740	14
SyNOnly_MI_32_reg_iter_1_normal_masked	0.7604491	15
SyNOnly_MI_32_reg_iter_0_normal_masked	0.7512749	16
TVMSQC_bleached_masked	0.7156338	17
TVMSQC_normal_masked	0.6937219	18
TVMSQ_bleached_masked	0.6834743	19
TVMSQ_normal_masked	0.6808001	20

Dice Cortical grey matter : The analysis is based on 20 algorithms and 20 cases. 0 missing cases have been found in the data set.

	value_mean	rank
SyNOnly_CC_2_reg_iter_1_bleached_masked	0.6195213	1
SyNOnly_CC_4_reg_iter_1_bleached_masked	0.5882170	2
SyNOnly_CC_2_reg_iter_0_bleached_masked	0.5708354	3
SyNOnly_CC_6_reg_iter_1_bleached_masked	0.5611788	4
SyNOnly_MI_32_reg_iter_1_bleached_masked	0.5497030	5
SyNOnly_CC_4_reg_iter_0_bleached_masked	0.5369098	6
SyNOnly_CC_6_reg_iter_0_bleached_masked	0.5189395	7
SyNOnly_MI_32_reg_iter_0_bleached_masked	0.5178763	8
SyNOnly_CC_2_reg_iter_1_normal_masked	0.4985320	9
SyNOnly_CC_2_reg_iter_0_normal_masked	0.4756768	10
SyNOnly_CC_4_reg_iter_1_normal_masked	0.4674907	11
SyNOnly_CC_6_reg_iter_1_normal_masked	0.4552828	12
SyNOnly_MI_32_reg_iter_1_normal_masked	0.4539971	13
SyNOnly_CC_4_reg_iter_0_normal_masked	0.4485545	14
SyNOnly_CC_6_reg_iter_0_normal_masked	0.4380188	15
SyNOnly_MI_32_reg_iter_0_normal_masked	0.4334599	16
TVMSQ_bleached_masked	0.4203272	17
TVMSQ_normal_masked	0.4179152	18
TVMSQC_normal_masked	0.3977607	19
TVMSQC_bleached_masked	0.3697429	20

Dice CSF and ventricles : The analysis is based on 20 algorithms and 20 cases. 0 missing cases have been

found in the data set.

	value_mean	rank
SyNOnly_CC_4_reg_iter_1_bleached_masked	0.5943140	1
SyNOnly_CC_6_reg_iter_1_bleached_masked	0.5935548	2
SyNOnly_CC_2_reg_iter_1_bleached_masked	0.5933386	3
SyNOnly_CC_2_reg_iter_0_bleached_masked	0.5925669	4
SyNOnly_CC_4_reg_iter_0_bleached_masked	0.5901970	5
SyNOnly_CC_6_reg_iter_0_bleached_masked	0.5854738	6
SyNOnly_MI_32_reg_iter_1_bleached_masked	0.5741598	7
SyNOnly_MI_32_reg_iter_0_bleached_masked	0.5660108	8
SyNOnly_CC_2_reg_iter_1_normal_masked	0.5555994	9
SyNOnly_CC_2_reg_iter_0_normal_masked	0.5493600	10
SyNOnly_CC_4_reg_iter_1_normal_masked	0.5458005	11
SyNOnly_CC_6_reg_iter_1_normal_masked	0.5430176	12
SyNOnly_CC_4_reg_iter_0_normal_masked	0.5415253	13
SyNOnly_MI_32_reg_iter_1_normal_masked	0.5413670	14
SyNOnly_CC_6_reg_iter_0_normal_masked	0.5374920	15
SyNOnly_MI_32_reg_iter_0_normal_masked	0.5313179	16
TVMSQ_bleached_masked	0.4873602	17
TVMSQ_normal_masked	0.4852561	18
TVMSQC_normal_masked	0.4716064	19
TVMSQC_bleached_masked	0.3293625	20

Dice Deep grey matter : The analysis is based on 20 algorithms and 20 cases. 0 missing cases have been found in the data set.

	value_mean	rank
SyNOnly_CC_2_reg_iter_1_bleached_masked	0.8139777	1
SyNOnly_CC_4_reg_iter_1_bleached_masked	0.8109514	2
SyNOnly_CC_2_reg_iter_1_normal_masked	0.8066134	3
SyNOnly_CC_2_reg_iter_0_bleached_masked	0.8055632	4
SyNOnly_CC_6_reg_iter_1_bleached_masked	0.8049829	5
SyNOnly_CC_2_reg_iter_0_normal_masked	0.7992474	6
SyNOnly_CC_4_reg_iter_0_bleached_masked	0.7968417	7
SyNOnly_CC_4_reg_iter_1_normal_masked	0.7945641	8
SyNOnly_CC_6_reg_iter_0_bleached_masked	0.7864162	9
SyNOnly_CC_6_reg_iter_1_normal_masked	0.7831171	10
SyNOnly_CC_4_reg_iter_0_normal_masked	0.7825788	11
SyNOnly_MI_32_reg_iter_1_bleached_masked	0.7792210	12
SyNOnly_CC_6_reg_iter_0_normal_masked	0.7699215	13
SyNOnly_MI_32_reg_iter_1_normal_masked	0.7658557	14
SyNOnly_MI_32_reg_iter_0_bleached_masked	0.7653583	15
SyNOnly_MI_32_reg_iter_0_normal_masked	0.7574218	16
TVMSQ_bleached_masked	0.7511072	17
TVMSQ_normal_masked	0.7502480	18
TVMSQC_normal_masked	0.7184481	19
TVMSQC_bleached_masked	0.5795666	20

Dice White matter : The analysis is based on 20 algorithms and 20 cases. 0 missing cases have been found in the data set.

	value_mean	rank
SyNOnly_CC_2_reg_iter_1_bleached_masked	0.7524861	1
SyNOnly_CC_4_reg_iter_1_bleached_masked	0.7407415	2
SyNOnly_CC_2_reg_iter_0_bleached_masked	0.7335093	3
SyNOnly_CC_6_reg_iter_1_bleached_masked	0.7307722	4
SyNOnly_MI_32_reg_iter_1_bleached_masked	0.7248913	5
SyNOnly_CC_4_reg_iter_0_bleached_masked	0.7221245	6
SyNOnly_CC_6_reg_iter_0_bleached_masked	0.7154463	7
SyNOnly_MI_32_reg_iter_0_bleached_masked	0.7115154	8
SyNOnly_CC_2_reg_iter_1_normal_masked	0.7066710	9
SyNOnly_CC_2_reg_iter_0_normal_masked	0.7002831	10
SyNOnly_CC_4_reg_iter_1_normal_masked	0.6932958	11
SyNOnly_CC_4_reg_iter_0_normal_masked	0.6879025	12
SyNOnly_MI_32_reg_iter_1_normal_masked	0.6869695	13
SyNOnly_CC_6_reg_iter_1_normal_masked	0.6862188	14
SyNOnly_CC_6_reg_iter_0_normal_masked	0.6820759	15
SyNOnly_MI_32_reg_iter_0_normal_masked	0.6805321	16
TVMSQ_bleached_masked	0.6699193	17
TVMSQ_normal_masked	0.6691549	18
TVMSQC_normal_masked	0.6594460	19
TVMSQC_bleached_masked	0.5762944	20

Consensus ranking across tasks according to chosen method “euclidean”:

	value	rank
SyNOnly_CC_2_reg_iter_1_bleached_masked	2.333	1
SyNOnly_CC_4_reg_iter_1_bleached_masked	2.333	1
SyNOnly_CC_6_reg_iter_1_bleached_masked	3.667	3
SyNOnly_CC_2_reg_iter_0_bleached_masked	4.667	4
SyNOnly_MI_32_reg_iter_1_bleached_masked	6.167	5
SyNOnly_CC_2_reg_iter_1_normal_masked	6.833	6
SyNOnly_CC_4_reg_iter_0_bleached_masked	6.833	6
SyNOnly_CC_6_reg_iter_0_bleached_masked	7.667	8
SyNOnly_CC_2_reg_iter_0_normal_masked	9.167	9
SyNOnly_CC_4_reg_iter_1_normal_masked	9.500	10
SyNOnly_MI_32_reg_iter_0_bleached_masked	9.500	10
SyNOnly_CC_6_reg_iter_1_normal_masked	11.667	12
SyNOnly_CC_4_reg_iter_0_normal_masked	12.667	13
SyNOnly_MI_32_reg_iter_1_normal_masked	12.833	14
SyNOnly_CC_6_reg_iter_0_normal_masked	14.500	15
SyNOnly_MI_32_reg_iter_0_normal_masked	15.667	16
TVMSQ_bleached_masked	17.500	17
TVMSQC_normal_masked	18.500	18
TVMSQ_normal_masked	18.500	18
TVMSQC_bleached_masked	19.500	20

2 Visualization of raw assessment data

The algorithms are ordered according to the computed ranks for each task.

2.1 Dot- and boxplot

Dot- and boxplots for visualizing raw assessment data separately for each algorithm. Boxplots representing descriptive statistics over all cases (median, quartiles and outliers) are combined with horizontally jittered dots representing individual cases.

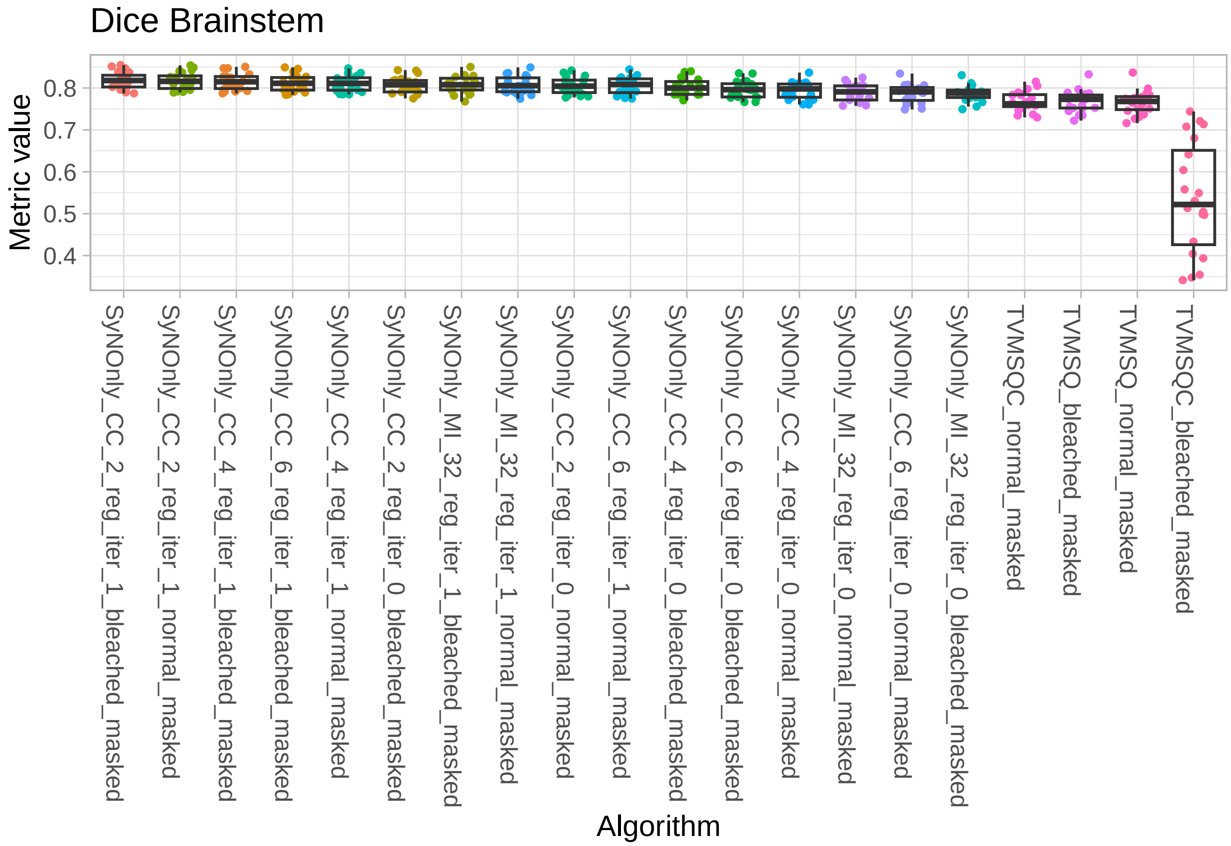

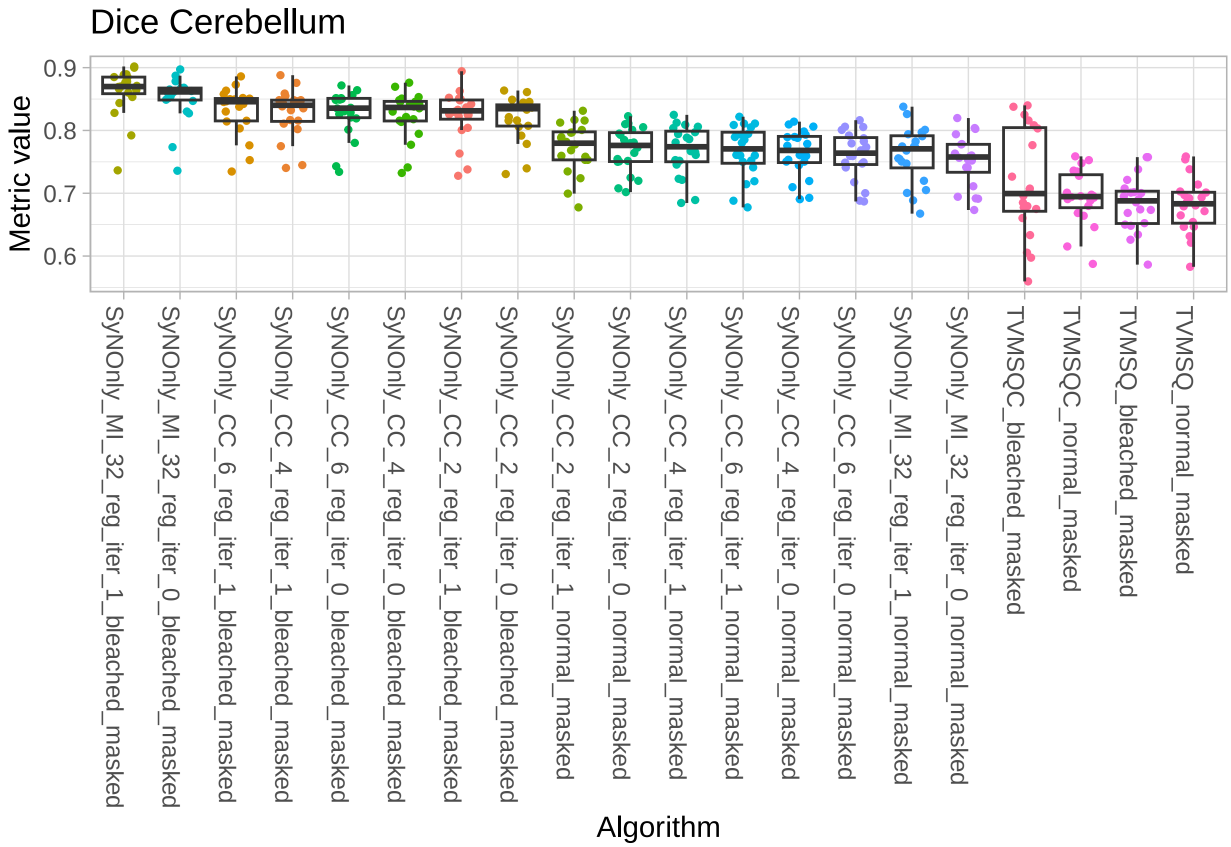

Dice Cerebellum
Metric value
0.9
0.8
0.7
0.6
SyNOnly_MI_32_reg_iter_1_bleached_masked
SyNOnly_MI_32_reg_iter_0_bleached_masked
SyNOnly_CC_6_reg_iter_1_bleached_masked
SyNOnly_CC_4_reg_iter_1_bleached_masked
SyNOnly_CC_6_reg_iter_0_bleached_masked
SyNOnly_CC_4_reg_iter_0_bleached_masked
SyNOnly_CC_2_reg_iter_1_bleached_masked
SyNOnly_CC_2_reg_iter_0_bleached_masked
SyNOnly_CC_2_reg_iter_1_normal_masked
SyNOnly_CC_2_reg_iter_0_normal_masked
SyNOnly_CC_4_reg_iter_1_normal_masked
SyNOnly_CC_6_reg_iter_1_normal_masked
SyNOnly_CC_4_reg_iter_0_normal_masked
SyNOnly_CC_6_reg_iter_0_normal_masked
SyNOnly_MI_32_reg_iter_1_normal_masked
SyNOnly_MI_32_reg_iter_0_normal_masked
TVMSQC_bleached_masked
TVMSQC_normal_masked
TVMSQ_bleached_masked
TVMSQ_normal_masked
Algorithm

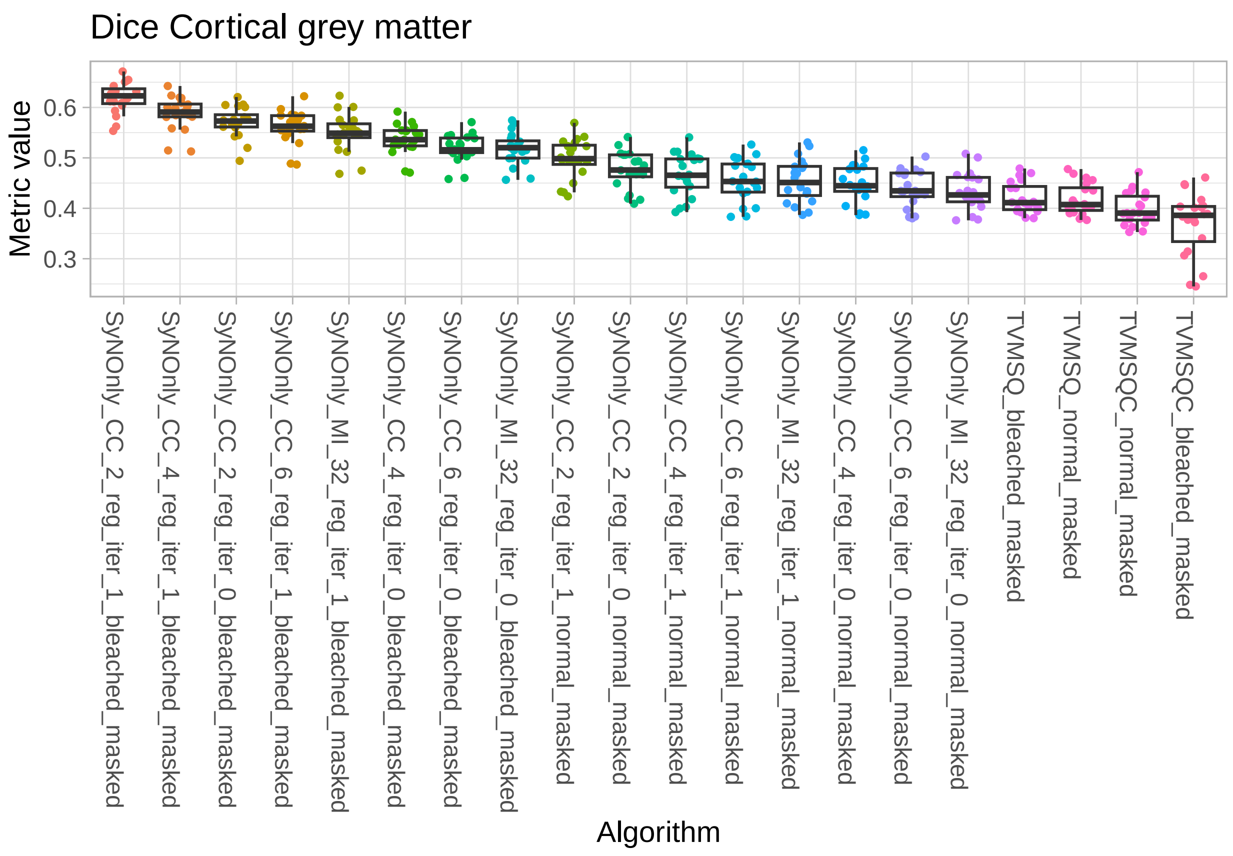

Dice Cortical grey matter
Metric value
0.6
0.5
0.4
0.3
SyNOnly_CC_2_reg_iter_1_bleached_masked
SyNOnly_CC_4_reg_iter_1_bleached_masked
SyNOnly_CC_2_reg_iter_0_bleached_masked
SyNOnly_CC_6_reg_iter_1_bleached_masked
SyNOnly_MI_32_reg_iter_1_bleached_masked
SyNOnly_CC_4_reg_iter_0_bleached_masked
SyNOnly_CC_6_reg_iter_0_bleached_masked
SyNOnly_MI_32_reg_iter_0_bleached_masked
SyNOnly_CC_2_reg_iter_1_normal_masked
SyNOnly_CC_2_reg_iter_0_normal_masked
SyNOnly_CC_4_reg_iter_1_normal_masked
SyNOnly_CC_6_reg_iter_1_normal_masked
SyNOnly_MI_32_reg_iter_1_normal_masked
SyNOnly_CC_4_reg_iter_0_normal_masked
SyNOnly_CC_6_reg_iter_0_normal_masked
SyNOnly_MI_32_reg_iter_0_normal_masked
TVMSQ_bleached_masked
TVMSQ_normal_masked
TVMSQC_normal_masked
TVMSQC_bleached_masked
Algorithm

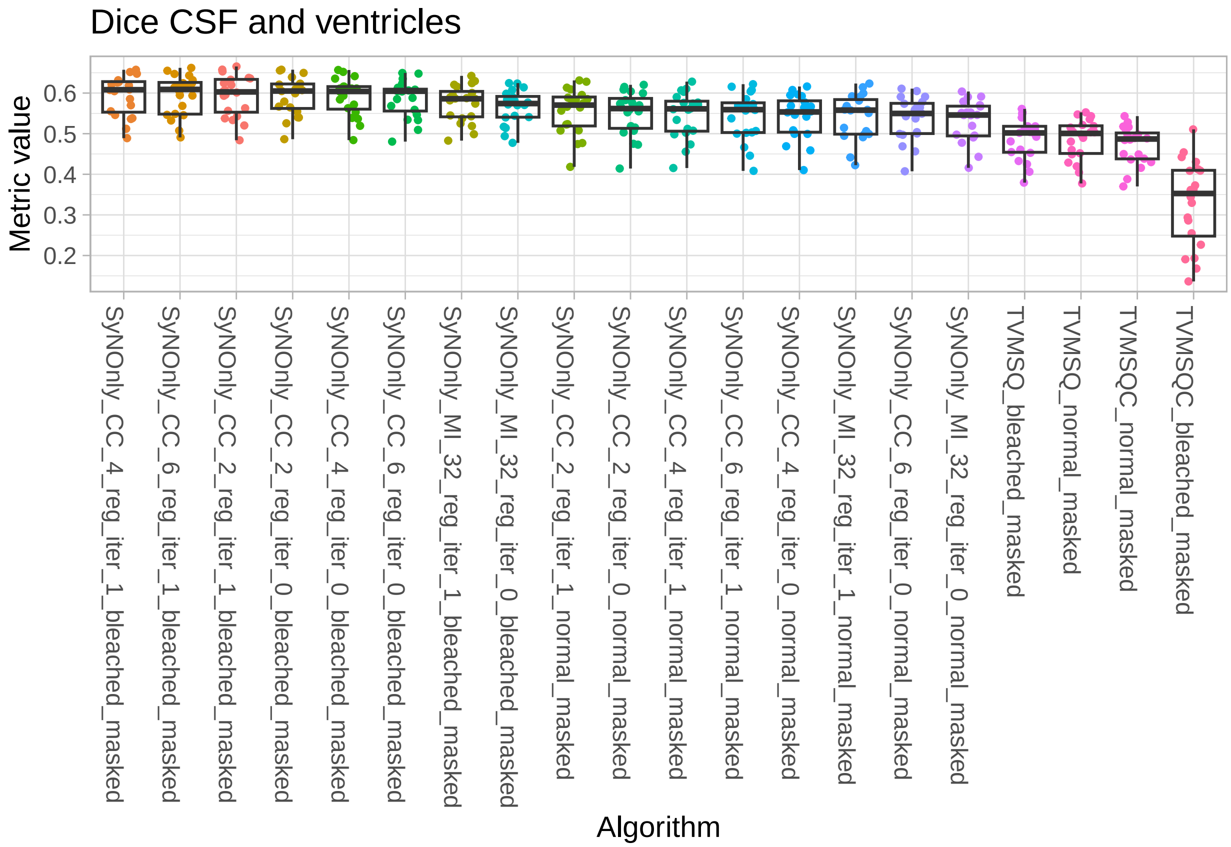
Dice CSF and ventricles
Metric value
0.6
0.5
0.4
0.3
0.2
SyNOnly_CC_4_reg_iter_1_bleached_masked
SyNOnly_CC_6_reg_iter_1_bleached_masked
SyNOnly_CC_2_reg_iter_1_bleached_masked
SyNOnly_CC_2_reg_iter_0_bleached_masked
SyNOnly_CC_4_reg_iter_0_bleached_masked
SyNOnly_CC_6_reg_iter_0_bleached_masked
SyNOnly_MI_32_reg_iter_1_bleached_masked
SyNOnly_MI_32_reg_iter_0_bleached_masked
SyNOnly_CC_2_reg_iter_1_normal_masked
SyNOnly_CC_2_reg_iter_0_normal_masked
SyNOnly_CC_4_reg_iter_1_normal_masked
SyNOnly_CC_6_reg_iter_1_normal_masked
SyNOnly_CC_4_reg_iter_0_normal_masked
SyNOnly_MI_32_reg_iter_1_normal_masked
SyNOnly_CC_6_reg_iter_0_normal_masked
SyNOnly_MI_32_reg_iter_0_normal_masked
TVMSQ_bleached_masked
TVMSQ_normal_masked
TVMSQC_normal_masked
TVMSQC_bleached_masked
Algorithm

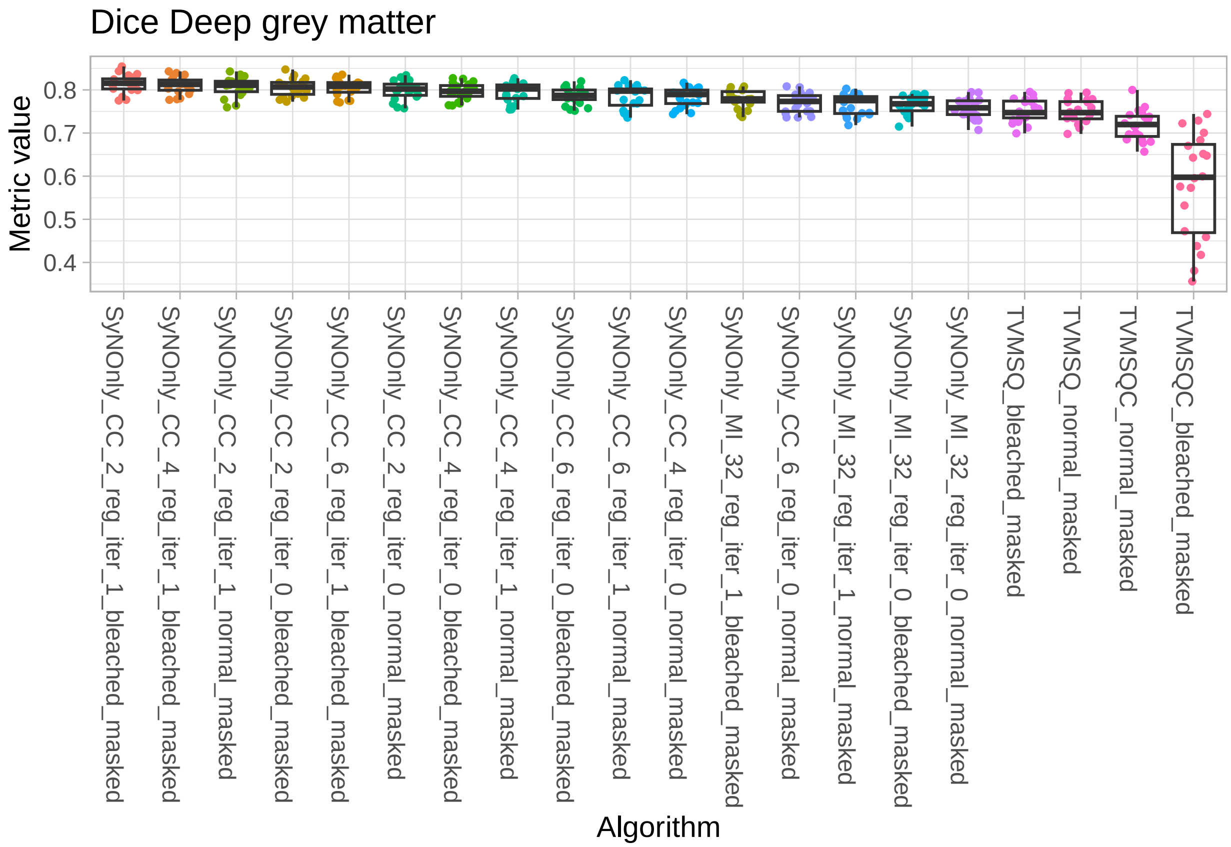
Dice Deep grey matter
Metric value
0.8
0.7
0.6
0.5
0.4
SyNOnly_CC_2_reg_iter_1_bleached_masked
SyNOnly_CC_4_reg_iter_1_bleached_masked
SyNOnly_CC_2_reg_iter_1_normal_masked
SyNOnly_CC_2_reg_iter_0_bleached_masked
SyNOnly_CC_6_reg_iter_1_bleached_masked
SyNOnly_CC_2_reg_iter_0_normal_masked
SyNOnly_CC_4_reg_iter_0_bleached_masked
SyNOnly_CC_4_reg_iter_1_normal_masked
SyNOnly_CC_6_reg_iter_0_bleached_masked
SyNOnly_CC_6_reg_iter_1_normal_masked
SyNOnly_CC_4_reg_iter_0_normal_masked
SyNOnly_MI_32_reg_iter_1_bleached_masked
SyNOnly_CC_6_reg_iter_0_normal_masked
SyNOnly_MI_32_reg_iter_1_normal_masked
SyNOnly_MI_32_reg_iter_0_bleached_masked
SyNOnly_MI_32_reg_iter_0_normal_masked
TVMSQ_bleached_masked
TVMSQ_normal_masked
TVMSQC_normal_masked
TVMSQC_bleached_masked
Algorithm

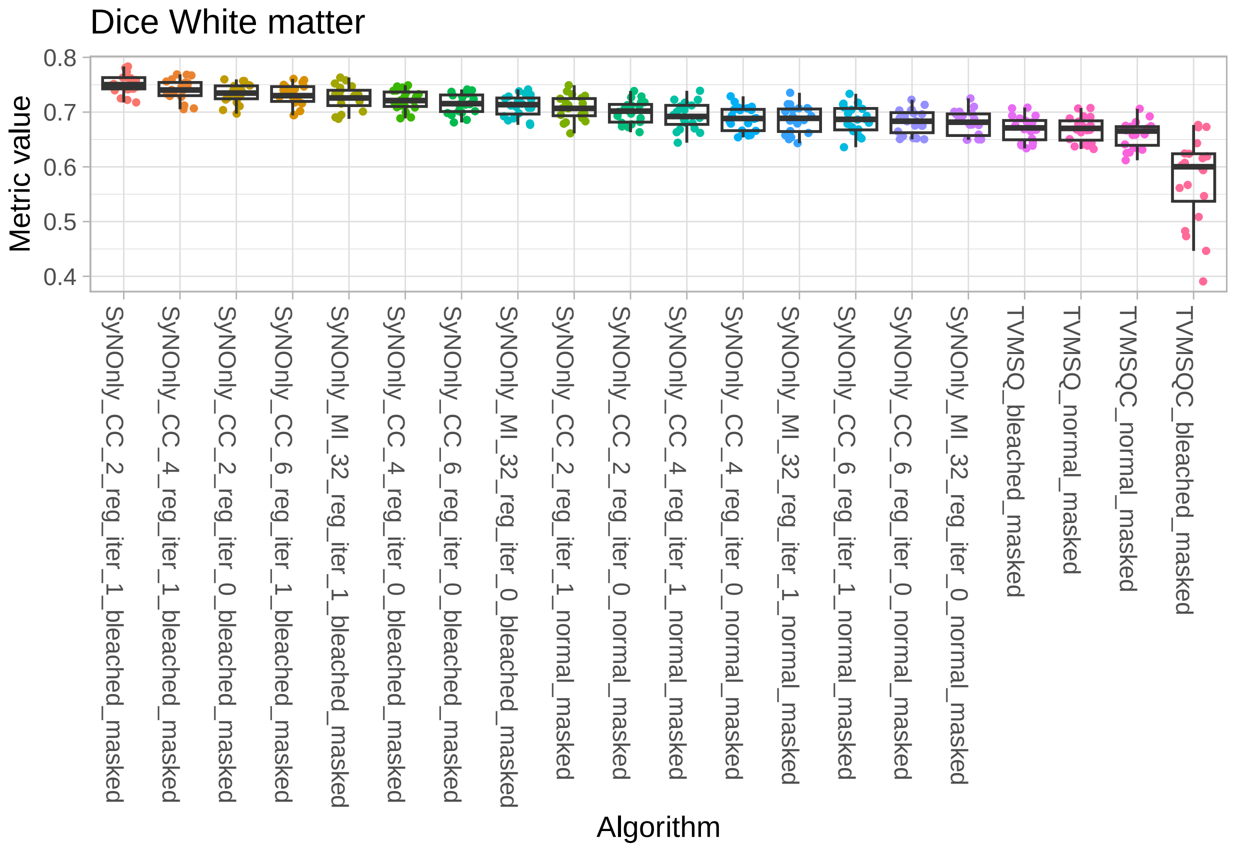

Dice White matter
Metric value
0.8
0.7
0.6
0.5
0.4
SyNOnly_CC_2_reg_iter_1_bleached_masked
SyNOnly_CC_4_reg_iter_1_bleached_masked
SyNOnly_CC_2_reg_iter_0_bleached_masked
SyNOnly_CC_6_reg_iter_1_bleached_masked
SyNOnly_MI_32_reg_iter_1_bleached_masked
SyNOnly_CC_4_reg_iter_0_bleached_masked
SyNOnly_CC_6_reg_iter_0_bleached_masked
SyNOnly_MI_32_reg_iter_0_bleached_masked
SyNOnly_CC_2_reg_iter_1_normal_masked
SyNOnly_CC_2_reg_iter_0_normal_masked
SyNOnly_CC_4_reg_iter_1_normal_masked
SyNOnly_CC_4_reg_iter_0_normal_masked
SyNOnly_MI_32_reg_iter_1_normal_masked
SyNOnly_CC_6_reg_iter_1_normal_masked
SyNOnly_CC_6_reg_iter_0_normal_masked
SyNOnly_MI_32_reg_iter_0_normal_masked
TVMSQ_bleached_masked
TVMSQ_normal_masked
TVMSQC_normal_masked
TVMSQC_bleached_masked
Algorithm

2.2 Podium plot

Podium plots (see also Eugster et al., 2008) for visualizing raw assessment data. Upper part (spaghetti plot): Participating algorithms are color-coded, and each colored dot in the plot represents a metric value achieved with the respective algorithm. The actual metric value is encoded by the y-axis. Each podium (here: p=20) represents one possible rank, ordered from best (1) to last (here: 20). The assignment of metric values (i.e. colored dots) to one of the podiums is based on the rank that the respective algorithm achieved on the corresponding case. Note that the plot part above each podium place is further subdivided into p "columns", where each column represents one participating algorithm (here: $p = 20$). Dots corresponding to identical cases are connected by a line, leading to the shown spaghetti structure. Lower part: Bar charts represent the relative frequency for each algorithm to achieve the rank encoded by the podium place.

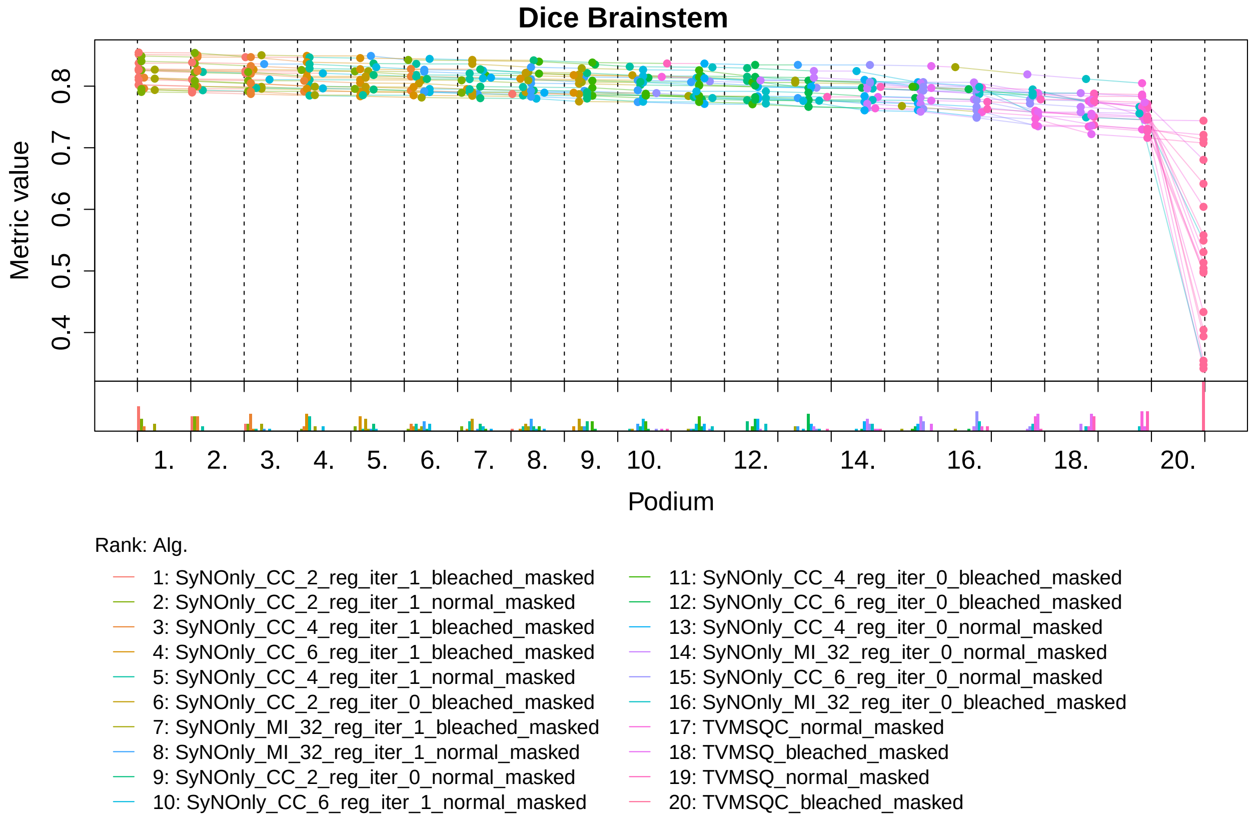

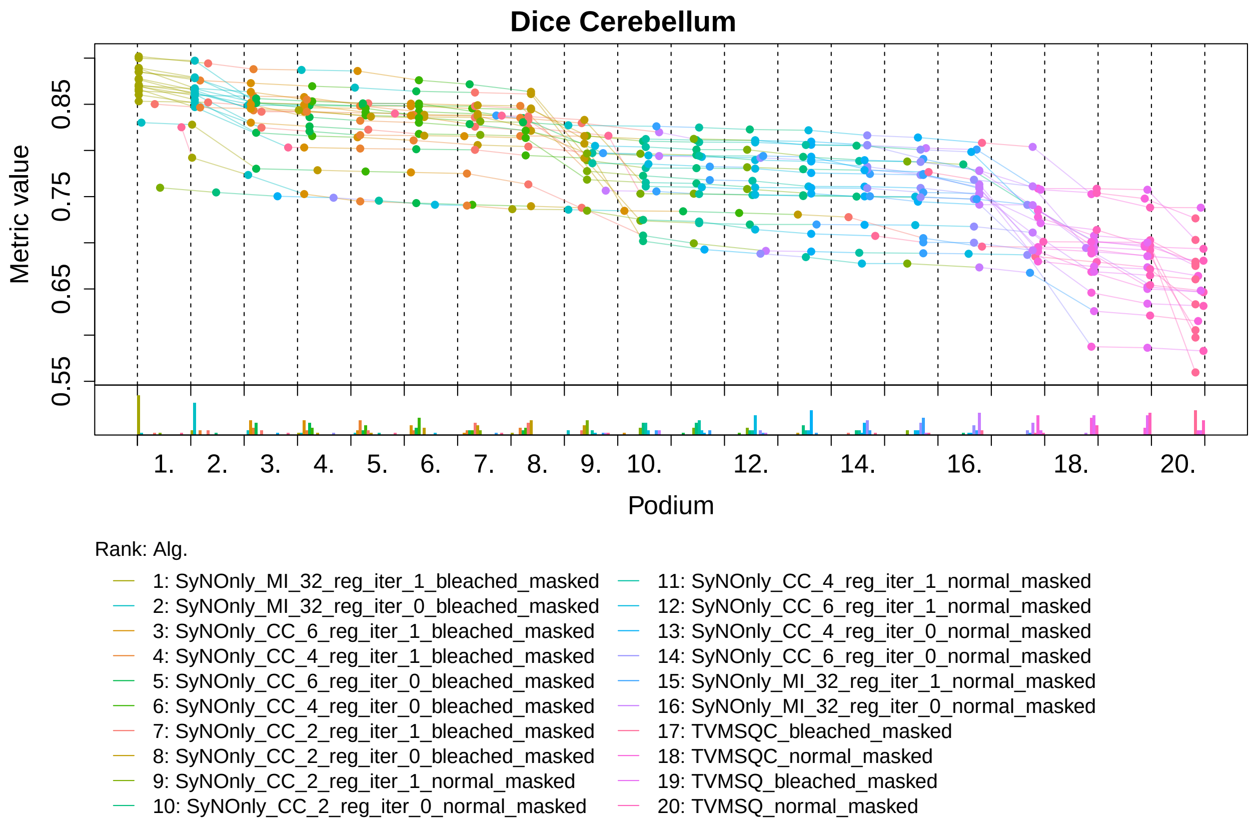
Dice Cerebellum
Metric value
0.55
0.65
0.75
0.85
1.
2.
3.
4.
5.
6.
7.
8.
9.
10.
12.
14.
16.
18.
20.
Podium
Rank: Alg.
1: SyNOnly_MI_32_reg_iter_1_bleached_masked
2: SyNOnly_MI_32_reg_iter_0_bleached_masked
3: SyNOnly_CC_6_reg_iter_1_bleached_masked
4: SyNOnly_CC_4_reg_iter_1_bleached_masked
5: SyNOnly_CC_6_reg_iter_0_bleached_masked
6: SyNOnly_CC_4_reg_iter_0_bleached_masked
7: SyNOnly_CC_2_reg_iter_1_bleached_masked
8: SyNOnly_CC_2_reg_iter_0_bleached_masked
9: SyNOnly_CC_2_reg_iter_1_normal_masked
10: SyNOnly_CC_2_reg_iter_0_normal_masked
11: SyNOnly_CC_4_reg_iter_1_normal_masked
12: SyNOnly_CC_6_reg_iter_1_normal_masked
13: SyNOnly_CC_4_reg_iter_0_normal_masked
14: SyNOnly_CC_6_reg_iter_0_normal_masked
15: SyNOnly_MI_32_reg_iter_1_normal_masked
16: SyNOnly_MI_32_reg_iter_0_normal_masked
17: TVMSQC_bleached_masked
18: TVMSQC_normal_masked
19: TVMSQ_bleached_masked
20: TVMSQ_normal_masked

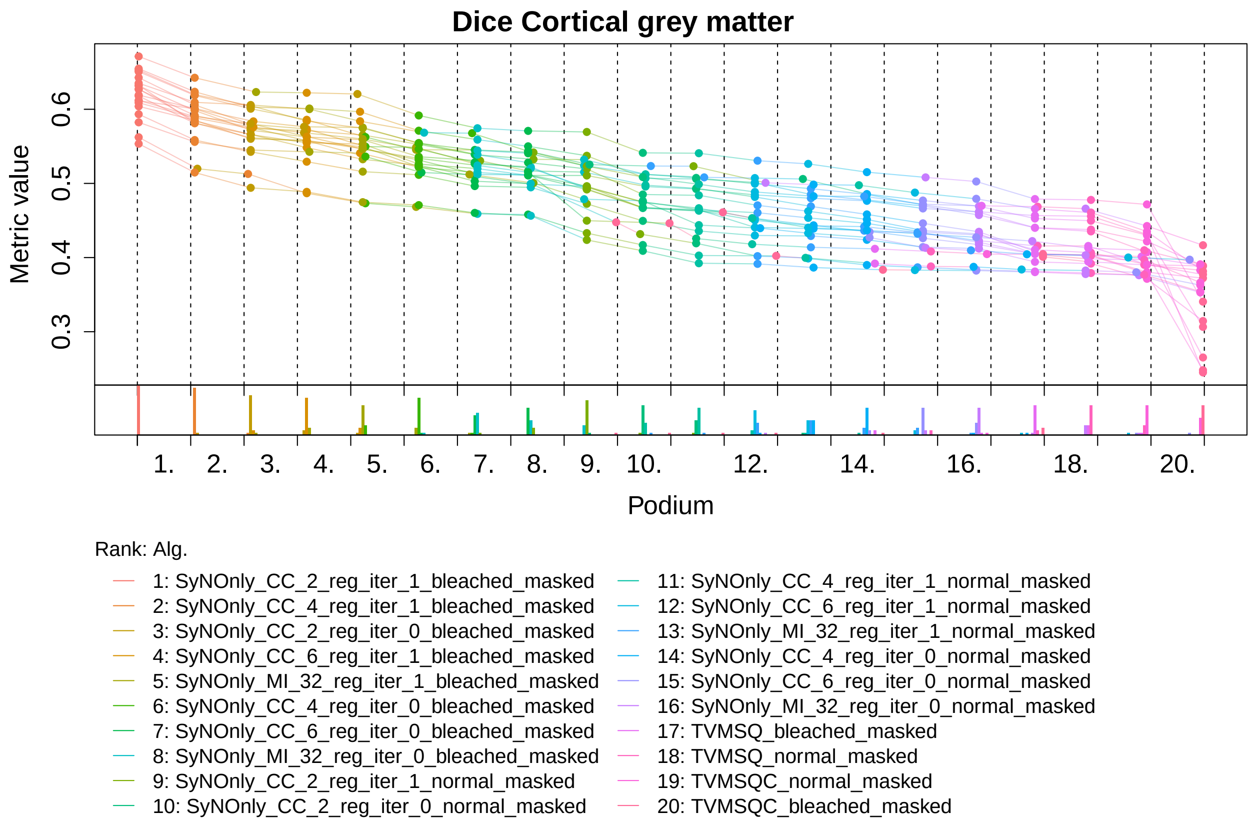
Dice Cortical grey matter
Metric value
0.3
0.4
0.5
0.6
Podium
1.
2.
3.
4.
5.
6.
7.
8.
9.
10.
12.
14.
16.
18.
20.
Rank: Alg.
1: SyNOnly_CC_2_reg_iter_1_bleached_masked
2: SyNOnly_CC_4_reg_iter_1_bleached_masked
3: SyNOnly_CC_2_reg_iter_0_bleached_masked
4: SyNOnly_CC_6_reg_iter_1_bleached_masked
5: SyNOnly_MI_32_reg_iter_1_bleached_masked
6: SyNOnly_CC_4_reg_iter_0_bleached_masked
7: SyNOnly_CC_6_reg_iter_0_bleached_masked
8: SyNOnly_MI_32_reg_iter_0_bleached_masked
9: SyNOnly_CC_2_reg_iter_1_normal_masked
10: SyNOnly_CC_2_reg_iter_0_normal_masked
11: SyNOnly_CC_4_reg_iter_1_normal_masked
12: SyNOnly_CC_6_reg_iter_1_normal_masked
13: SyNOnly_MI_32_reg_iter_1_normal_masked
14: SyNOnly_CC_4_reg_iter_0_normal_masked
15: SyNOnly_CC_6_reg_iter_0_normal_masked
16: SyNOnly_MI_32_reg_iter_0_normal_masked
17: TVMSQ_bleached_masked
18: TVMSQ_normal_masked
19: TVMSQC_normal_masked
20: TVMSQC_bleached_masked

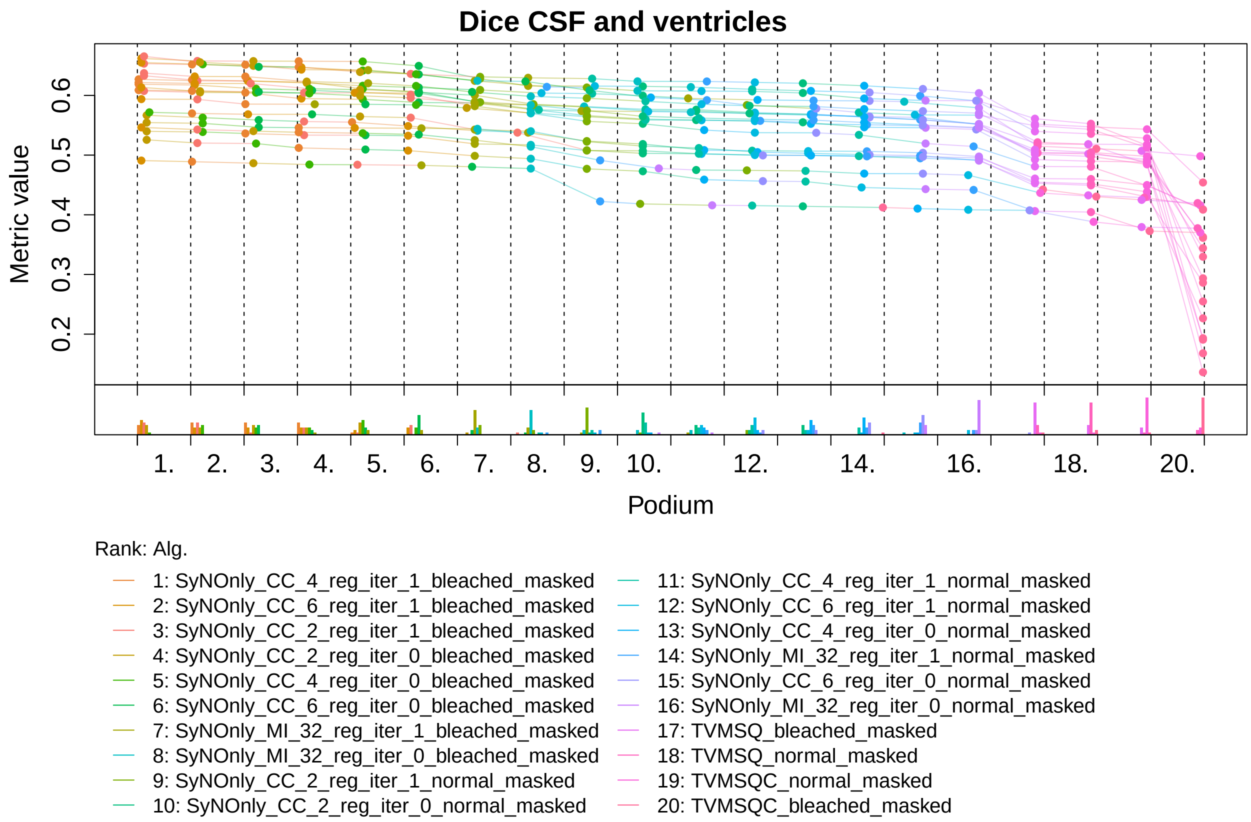

Dice CSF and ventricles
Metric value
0.2
0.3
0.4
0.5
0.6
1.
2.
3.
4.
5.
6.
7.
8.
9.
10.
12.
14.
16.
18.
20.
Podium
Rank: Alg.
1: SyNOnly_CC_4_reg_iter_1_bleached_masked
2: SyNOnly_CC_6_reg_iter_1_bleached_masked
3: SyNOnly_CC_2_reg_iter_1_bleached_masked
4: SyNOnly_CC_2_reg_iter_0_bleached_masked
5: SyNOnly_CC_4_reg_iter_0_bleached_masked
6: SyNOnly_CC_6_reg_iter_0_bleached_masked
7: SyNOnly_MI_32_reg_iter_1_bleached_masked
8: SyNOnly_MI_32_reg_iter_0_bleached_masked
9: SyNOnly_CC_2_reg_iter_1_normal_masked
10: SyNOnly_CC_2_reg_iter_0_normal_masked
11: SyNOnly_CC_4_reg_iter_1_normal_masked
12: SyNOnly_CC_6_reg_iter_1_normal_masked
13: SyNOnly_CC_4_reg_iter_0_normal_masked
14: SyNOnly_MI_32_reg_iter_1_normal_masked
15: SyNOnly_CC_6_reg_iter_0_normal_masked
16: SyNOnly_MI_32_reg_iter_0_normal_masked
17: TVMSQ_bleached_masked
18: TVMSQ_normal_masked
19: TVMSQC_normal_masked
20: TVMSQC_bleached_masked

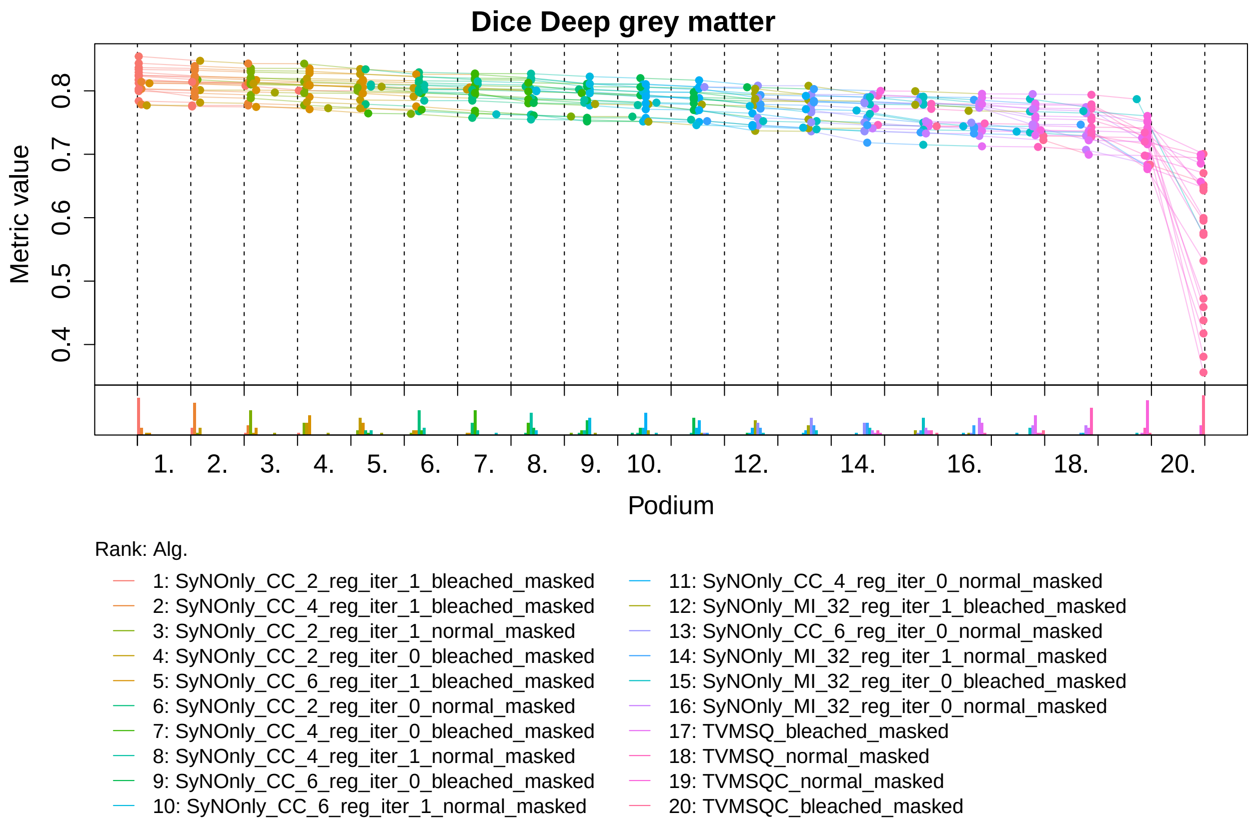
Dice Deep grey matter
Metric value
0.4
0.5
0.6
0.7
0.8
1.
2.
3.
4.
5.
6.
7.
8.
9.
10.
12.
14.
16.
18.
20.
Podium
Rank: Alg.
1: SyNOnly_CC_2_reg_iter_1_bleached_masked
2: SyNOnly_CC_4_reg_iter_1_bleached_masked
3: SyNOnly_CC_2_reg_iter_1_normal_masked
4: SyNOnly_CC_2_reg_iter_0_bleached_masked
5: SyNOnly_CC_6_reg_iter_1_bleached_masked
6: SyNOnly_CC_2_reg_iter_0_normal_masked
7: SyNOnly_CC_4_reg_iter_0_bleached_masked
8: SyNOnly_CC_4_reg_iter_1_normal_masked
9: SyNOnly_CC_6_reg_iter_0_bleached_masked
10: SyNOnly_CC_6_reg_iter_1_normal_masked
11: SyNOnly_CC_4_reg_iter_0_normal_masked
12: SyNOnly_MI_32_reg_iter_1_bleached_masked
13: SyNOnly_CC_6_reg_iter_0_normal_masked
14: SyNOnly_MI_32_reg_iter_1_normal_masked
15: SyNOnly_MI_32_reg_iter_0_bleached_masked
16: SyNOnly_MI_32_reg_iter_0_normal_masked
17: TVMSQ_bleached_masked
18: TVMSQ_normal_masked
19: TVMSQC_normal_masked
20: TVMSQC_bleached_masked

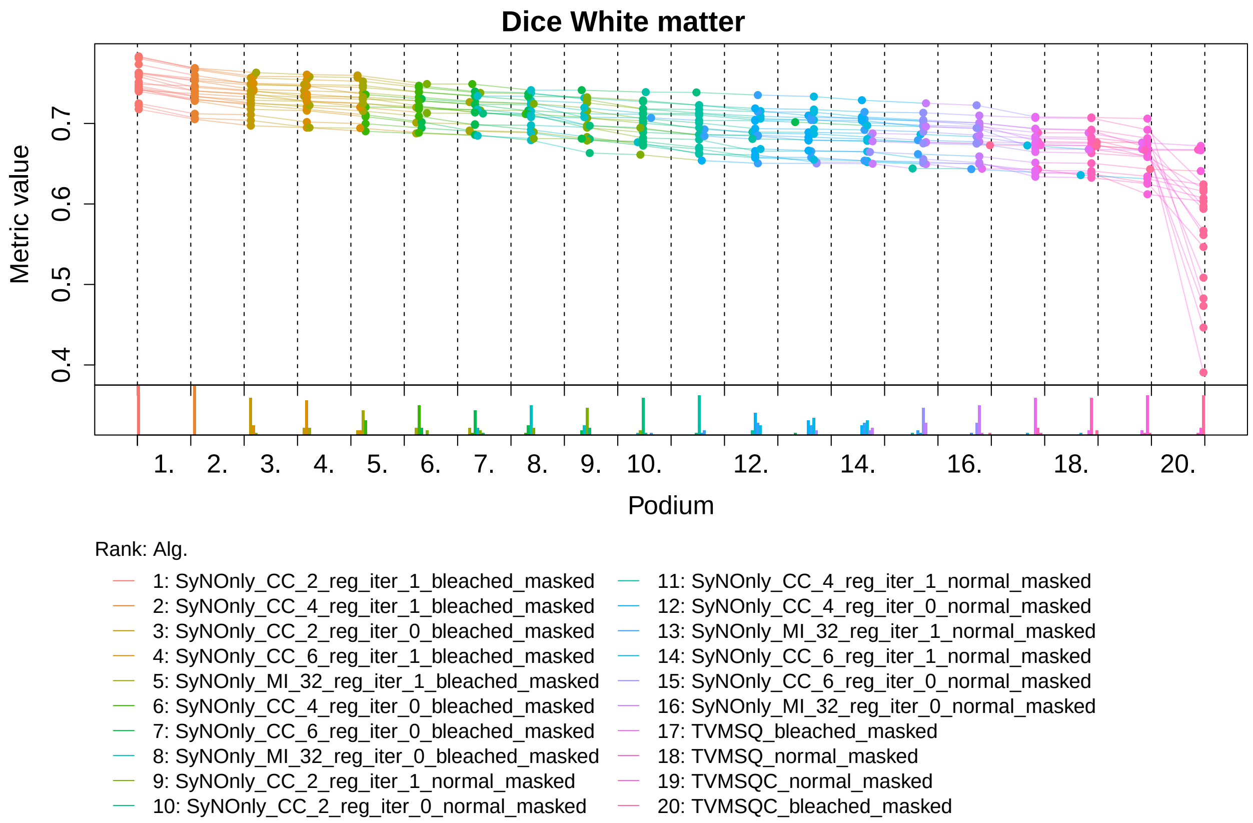
Dice White matter
Metric value
0.4
0.5
0.6
0.7
1.
2.
3.
4.
5.
6.
7.
8.
9.
10.
12.
14.
16.
18.
20.
Podium
Rank: Alg.
1: SyNOnly_CC_2_reg_iter_1_bleached_masked
2: SyNOnly_CC_4_reg_iter_1_bleached_masked
3: SyNOnly_CC_2_reg_iter_0_bleached_masked
4: SyNOnly_CC_6_reg_iter_1_bleached_masked
5: SyNOnly_MI_32_reg_iter_1_bleached_masked
6: SyNOnly_CC_4_reg_iter_0_bleached_masked
7: SyNOnly_CC_6_reg_iter_0_bleached_masked
8: SyNOnly_MI_32_reg_iter_0_bleached_masked
9: SyNOnly_CC_2_reg_iter_1_normal_masked
10: SyNOnly_CC_2_reg_iter_0_normal_masked
11: SyNOnly_CC_4_reg_iter_1_normal_masked
12: SyNOnly_CC_4_reg_iter_0_normal_masked
13: SyNOnly_MI_32_reg_iter_1_normal_masked
14: SyNOnly_CC_6_reg_iter_1_normal_masked
15: SyNOnly_CC_6_reg_iter_0_normal_masked
16: SyNOnly_MI_32_reg_iter_0_normal_masked
17: TVMSQ_bleached_masked
18: TVMSQ_normal_masked
19: TVMSQC_normal_masked
20: TVMSQC_bleached_masked

2.3 Ranking heatmap

Ranking heatmaps for visualizing raw assessment data. Each cell (i, A_j) shows the absolute frequency of cases in which algorithm A_j achieved rank i.

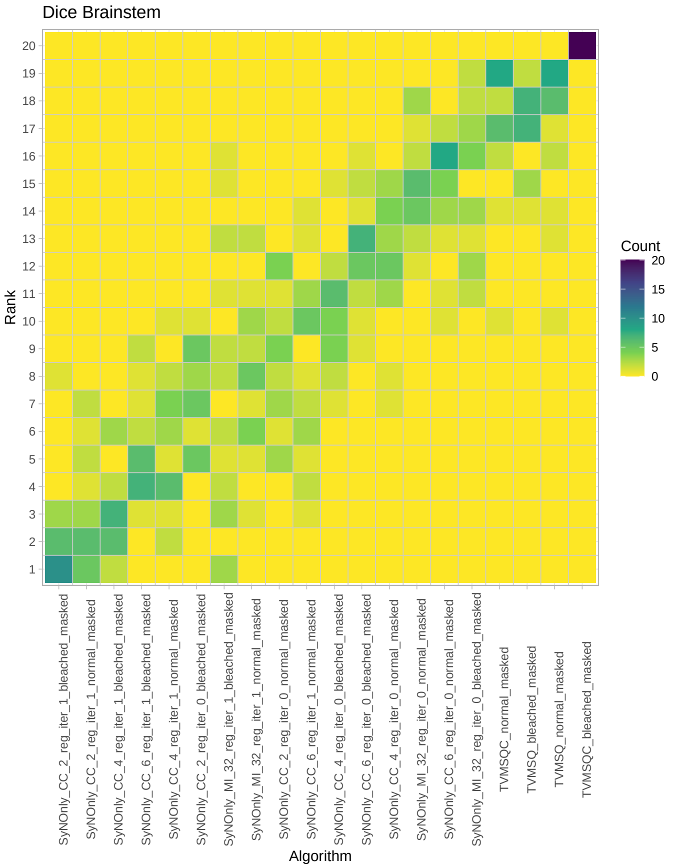

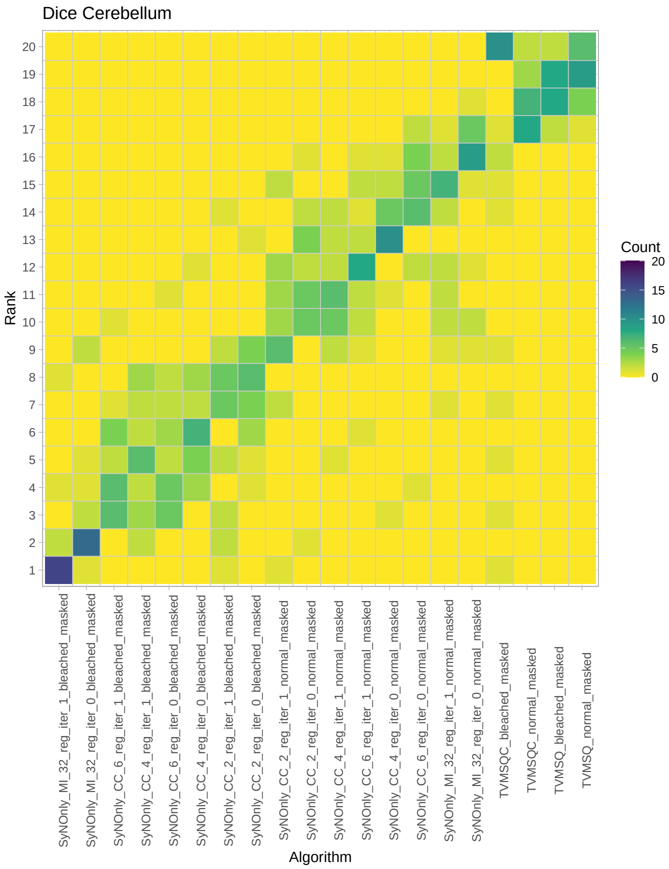
Dice Cerebellum
Rank
20
19
18
17
16
15
14
13
12
11
10
9
8
7
6
5
4
3
2
1
Count
20
15
10
5
0
SyNOnly_MI_32_reg_iter_1_bleached_masked
SyNOnly_MI_32_reg_iter_0_bleached_masked
SyNOnly_CC_6_reg_iter_1_bleached_masked
SyNOnly_CC_4_reg_iter_1_bleached_masked
SyNOnly_CC_6_reg_iter_0_bleached_masked
SyNOnly_CC_4_reg_iter_0_bleached_masked
SyNOnly_CC_2_reg_iter_1_bleached_masked
SyNOnly_CC_2_reg_iter_0_bleached_masked
SyNOnly_CC_2_reg_iter_1_normal_masked
SyNOnly_CC_2_reg_iter_0_normal_masked
SyNOnly_CC_4_reg_iter_1_normal_masked
SyNOnly_CC_6_reg_iter_1_normal_masked
SyNOnly_CC_4_reg_iter_0_normal_masked
SyNOnly_CC_6_reg_iter_0_normal_masked
SyNOnly_MI_32_reg_iter_1_normal_masked
SyNOnly_MI_32_reg_iter_0_normal_masked
TVMSQC_bleached_masked
TVMSQC_normal_masked
TVMSQ_bleached_masked
TVMSQ_normal_masked
Algorithm

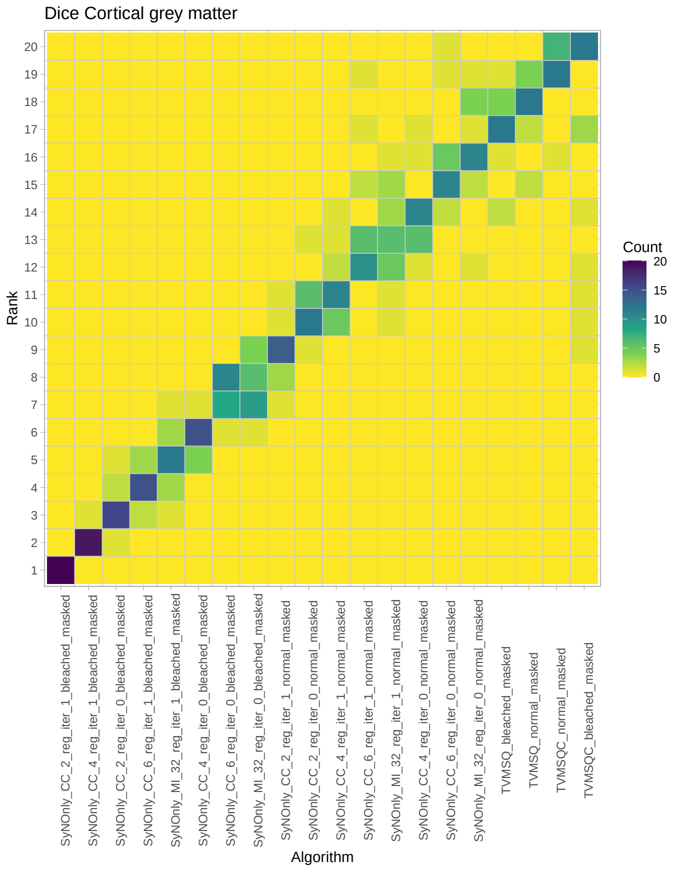
Dice Cortical grey matter
Rank
20
19
18
17
16
15
14
13
12
11
10
9
8
7
6
5
4
3
2
1
Count
20
15
10
5
0
SyNOnly_CC_2_reg_iter_1_bleached_masked
SyNOnly_CC_4_reg_iter_1_bleached_masked
SyNOnly_CC_2_reg_iter_0_bleached_masked
SyNOnly_CC_6_reg_iter_1_bleached_masked
SyNOnly_MI_32_reg_iter_1_bleached_masked
SyNOnly_CC_4_reg_iter_0_bleached_masked
SyNOnly_CC_6_reg_iter_0_bleached_masked
SyNOnly_MI_32_reg_iter_0_bleached_masked
SyNOnly_CC_2_reg_iter_1_normal_masked
SyNOnly_CC_2_reg_iter_0_normal_masked
SyNOnly_CC_4_reg_iter_1_normal_masked
SyNOnly_CC_6_reg_iter_1_normal_masked
SyNOnly_MI_32_reg_iter_1_normal_masked
SyNOnly_CC_4_reg_iter_0_normal_masked
SyNOnly_CC_6_reg_iter_0_normal_masked
SyNOnly_MI_32_reg_iter_0_normal_masked
TVMSQ_bleached_masked
TVMSQ_normal_masked
TVMSQC_normal_masked
TVMSQC_bleached_masked
Algorithm

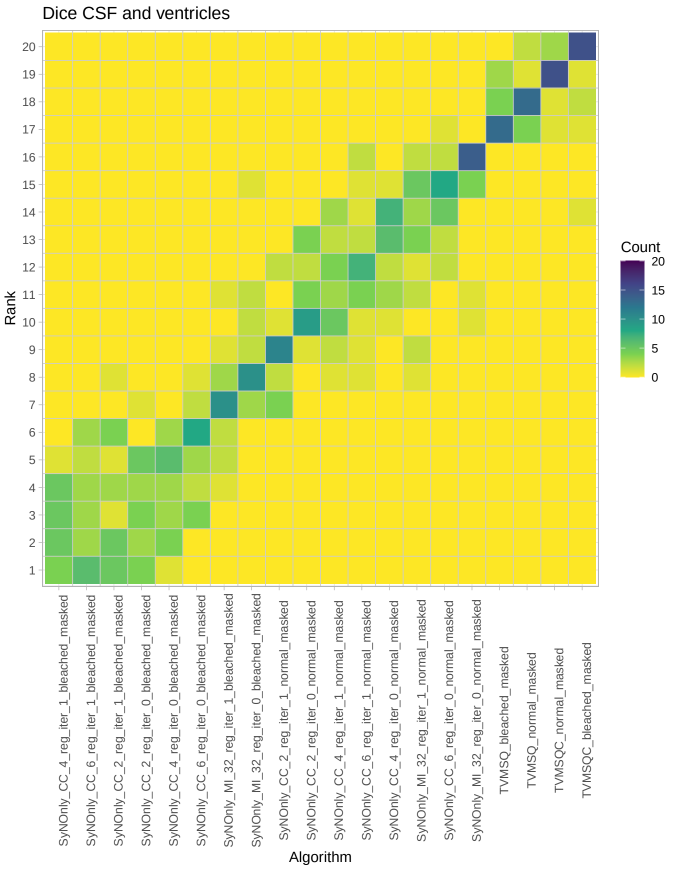
Dice CSF and ventricles
Rank
Algorithm
Count
20
15
10
5
0
SyNOnly_CC_4_reg_iter_1_bleached_masked
SyNOnly_CC_6_reg_iter_1_bleached_masked
SyNOnly_CC_2_reg_iter_1_bleached_masked
SyNOnly_CC_2_reg_iter_0_bleached_masked
SyNOnly_CC_4_reg_iter_0_bleached_masked
SyNOnly_CC_6_reg_iter_0_bleached_masked
SyNOnly_MI_32_reg_iter_1_bleached_masked
SyNOnly_MI_32_reg_iter_0_bleached_masked
SyNOnly_CC_2_reg_iter_1_normal_masked
SyNOnly_CC_2_reg_iter_0_normal_masked
SyNOnly_CC_4_reg_iter_1_normal_masked
SyNOnly_CC_6_reg_iter_1_normal_masked
SyNOnly_CC_4_reg_iter_0_normal_masked
SyNOnly_MI_32_reg_iter_1_normal_masked
SyNOnly_CC_6_reg_iter_0_normal_masked
SyNOnly_MI_32_reg_iter_0_normal_masked
TVMSQ_bleached_masked
TVMSQ_normal_masked
TVMSQC_normal_masked
TVMSQC_bleached_masked

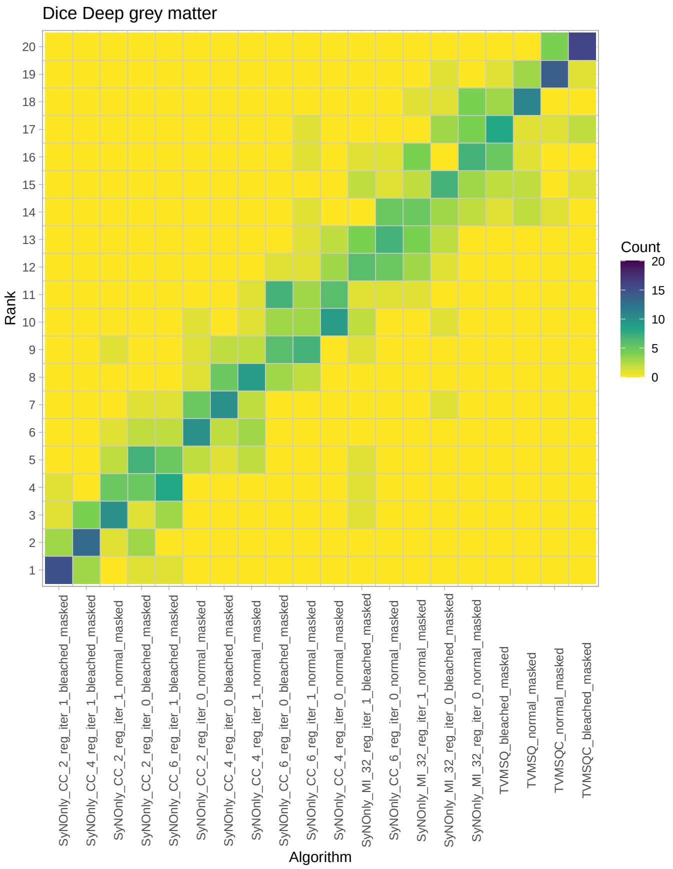
Dice Deep grey matter
Rank
Algorithm
Count
20
15
10
5
0
SyNOnly_CC_2_reg_iter_1_bleached_masked
SyNOnly_CC_4_reg_iter_1_bleached_masked
SyNOnly_CC_2_reg_iter_1_normal_masked
SyNOnly_CC_2_reg_iter_0_bleached_masked
SyNOnly_CC_6_reg_iter_1_bleached_masked
SyNOnly_CC_2_reg_iter_0_normal_masked
SyNOnly_CC_4_reg_iter_0_bleached_masked
SyNOnly_CC_4_reg_iter_1_normal_masked
SyNOnly_CC_6_reg_iter_0_bleached_masked
SyNOnly_CC_6_reg_iter_1_normal_masked
SyNOnly_CC_4_reg_iter_0_normal_masked
SyNOnly_MI_32_reg_iter_1_bleached_masked
SyNOnly_CC_6_reg_iter_0_normal_masked
SyNOnly_MI_32_reg_iter_1_normal_masked
SyNOnly_MI_32_reg_iter_0_bleached_masked
SyNOnly_MI_32_reg_iter_0_normal_masked
TVMSQ_bleached_masked
TVMSQ_normal_masked
TVMSQC_normal_masked
TVMSQC_bleached_masked

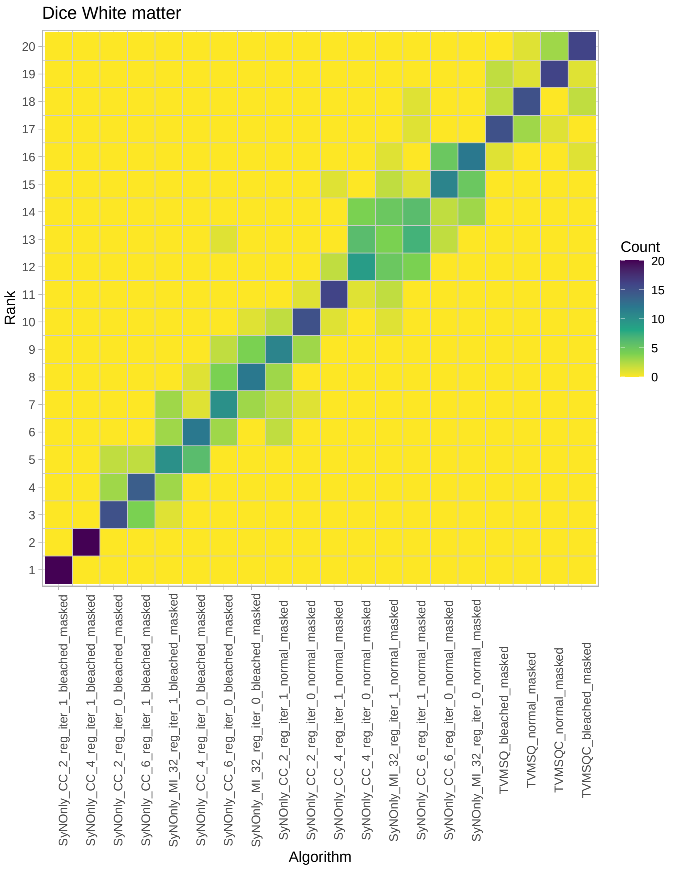

Dice White matter
Rank
Algorithm
Count
20
15
10
5
0
SyNOnly_CC_2_reg_iter_1_bleached_masked
SyNOnly_CC_4_reg_iter_1_bleached_masked
SyNOnly_CC_2_reg_iter_0_bleached_masked
SyNOnly_CC_6_reg_iter_1_bleached_masked
SyNOnly_MI_32_reg_iter_1_bleached_masked
SyNOnly_CC_4_reg_iter_0_bleached_masked
SyNOnly_CC_6_reg_iter_0_bleached_masked
SyNOnly_MI_32_reg_iter_0_bleached_masked
SyNOnly_CC_2_reg_iter_1_normal_masked
SyNOnly_CC_2_reg_iter_0_normal_masked
SyNOnly_CC_4_reg_iter_1_normal_masked
SyNOnly_CC_4_reg_iter_0_normal_masked
SyNOnly_MI_32_reg_iter_1_normal_masked
SyNOnly_CC_6_reg_iter_1_normal_masked
SyNOnly_CC_6_reg_iter_0_normal_masked
SyNOnly_MI_32_reg_iter_0_normal_masked
TVMSQ_bleached_masked
TVMSQ_normal_masked
TVMSQC_normal_masked
TVMSQC_bleached_masked

3 Visualization of ranking stability

3.1 *Blob plot* for visualizing ranking stability based on bootstrap sampling

Algorithms are color-coded, and the area of each blob at position $(A_i, \text{rank } j)$ is proportional to the relative frequency A_i achieved rank j across $b = 1000$ bootstrap samples. The median rank for each algorithm is indicated by a black cross. 95% bootstrap intervals across bootstrap samples are indicated by black lines.

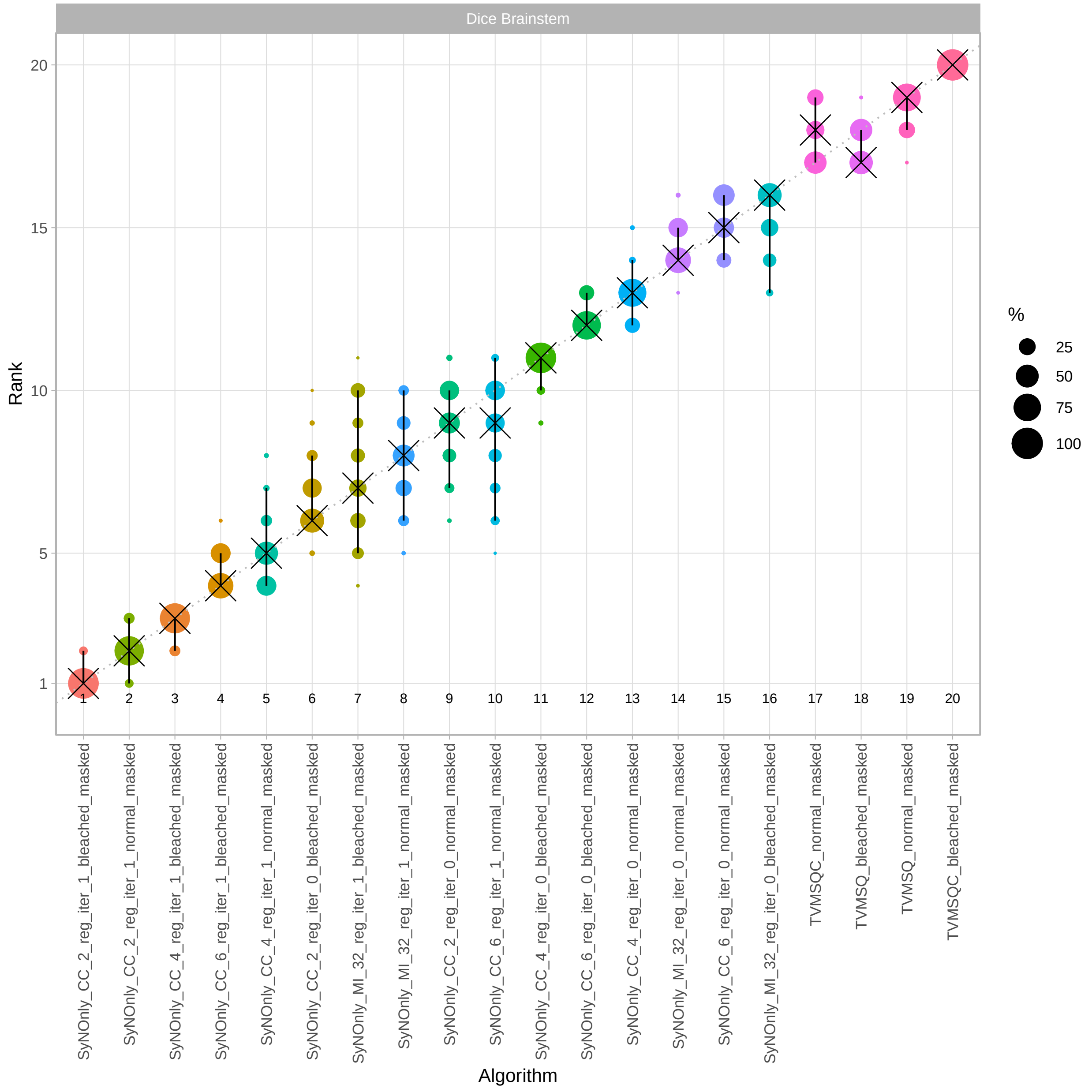

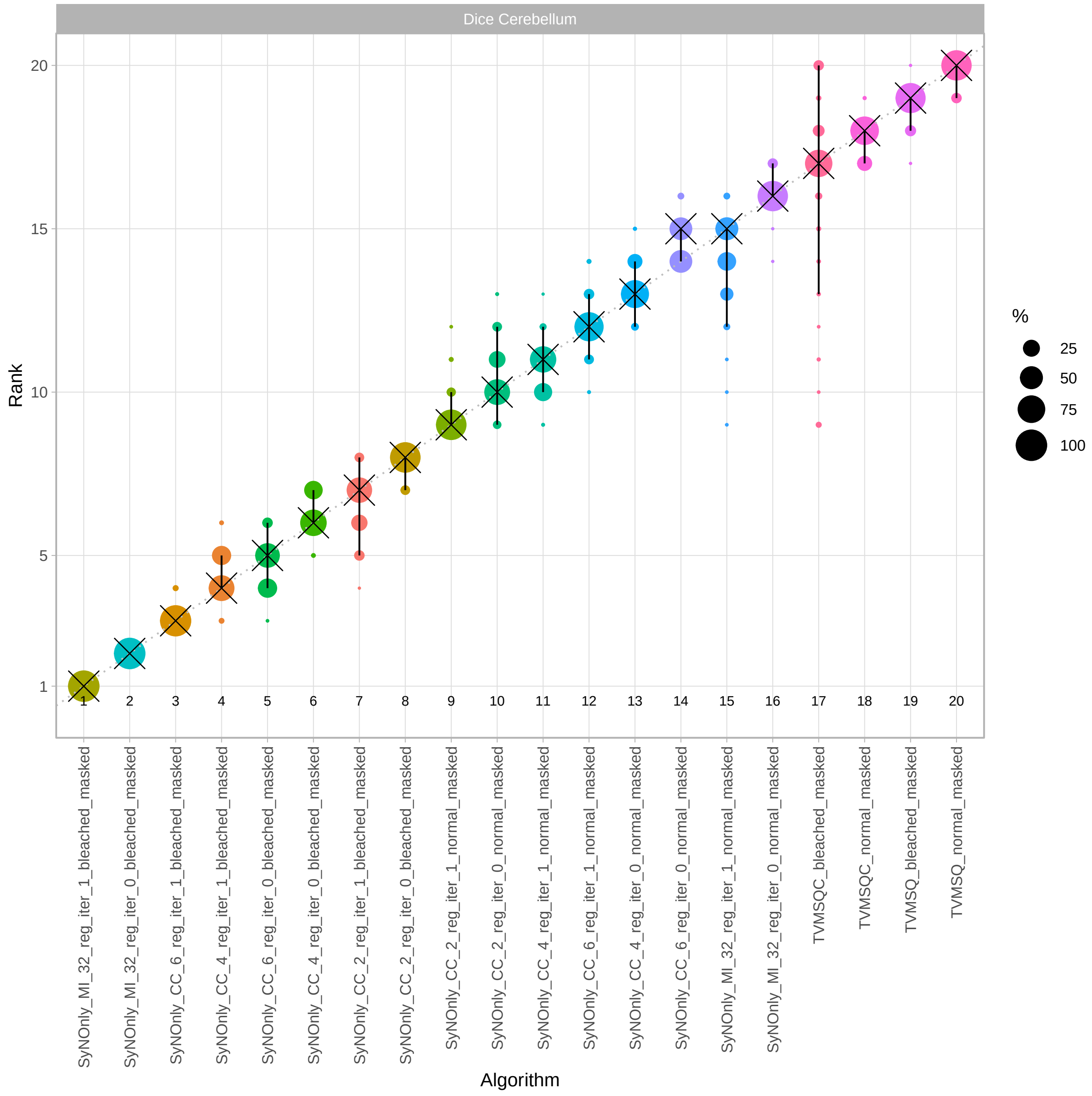
Dice Cerebellum
Rank
Algorithm
%
25
50
75
100
SyNOnly_MI_32_reg_iter_1_bleached_masked
SyNOnly_MI_32_reg_iter_0_bleached_masked
SyNOnly_CC_6_reg_iter_1_bleached_masked
SyNOnly_CC_4_reg_iter_1_bleached_masked
SyNOnly_CC_6_reg_iter_0_bleached_masked
SyNOnly_CC_4_reg_iter_0_bleached_masked
SyNOnly_CC_2_reg_iter_1_bleached_masked
SyNOnly_CC_2_reg_iter_0_bleached_masked
SyNOnly_CC_2_reg_iter_1_normal_masked
SyNOnly_CC_2_reg_iter_0_normal_masked
SyNOnly_CC_4_reg_iter_1_normal_masked
SyNOnly_CC_6_reg_iter_1_normal_masked
SyNOnly_CC_4_reg_iter_0_normal_masked
SyNOnly_CC_6_reg_iter_0_normal_masked
SyNOnly_MI_32_reg_iter_1_normal_masked
SyNOnly_MI_32_reg_iter_0_normal_masked
TVMSQC_bleached_masked
TVMSQC_normal_masked
TVMSQ_bleached_masked
TVMSQ_normal_masked

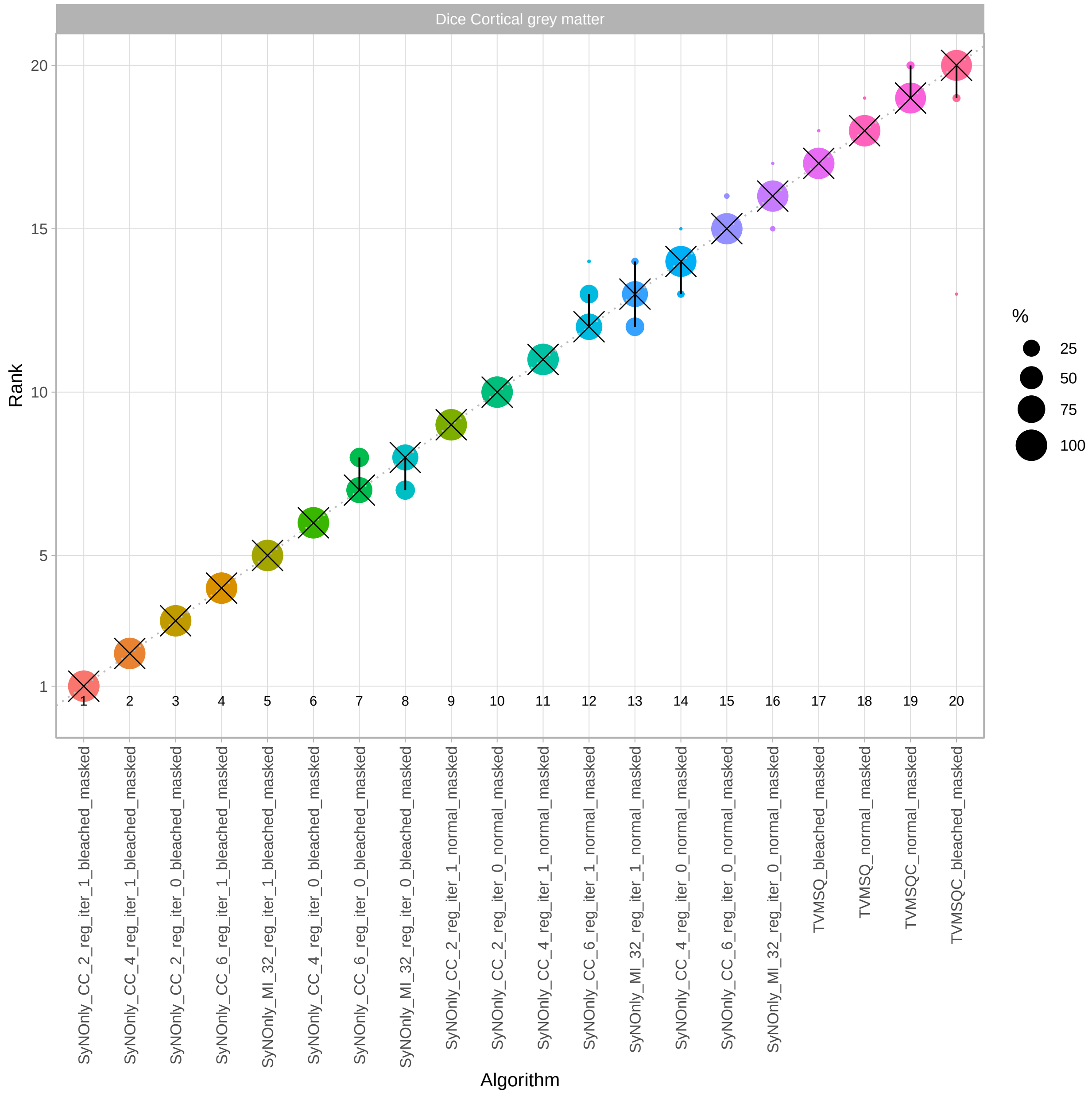
Dice Cortical grey matter
Rank
20
15
10
5
1
1
2
3
4
5
6
7
8
9
10
11
12
13
14
15
16
17
18
19
20
%
25
50
75
100
SyNOnly_CC_2_reg_iter_1_bleached_masked
SyNOnly_CC_4_reg_iter_1_bleached_masked
SyNOnly_CC_2_reg_iter_0_bleached_masked
SyNOnly_CC_6_reg_iter_1_bleached_masked
SyNOnly_MI_32_reg_iter_1_bleached_masked
SyNOnly_CC_4_reg_iter_0_bleached_masked
SyNOnly_CC_6_reg_iter_0_bleached_masked
SyNOnly_MI_32_reg_iter_0_bleached_masked
SyNOnly_CC_2_reg_iter_1_normal_masked
SyNOnly_CC_2_reg_iter_0_normal_masked
SyNOnly_CC_4_reg_iter_1_normal_masked
SyNOnly_CC_6_reg_iter_1_normal_masked
SyNOnly_MI_32_reg_iter_1_normal_masked
SyNOnly_CC_4_reg_iter_0_normal_masked
SyNOnly_CC_6_reg_iter_0_normal_masked
SyNOnly_MI_32_reg_iter_0_normal_masked
TVMSQ_bleached_masked
TVMSQ_normal_masked
TVMSQC_normal_masked
TVMSQC_bleached_masked
Algorithm

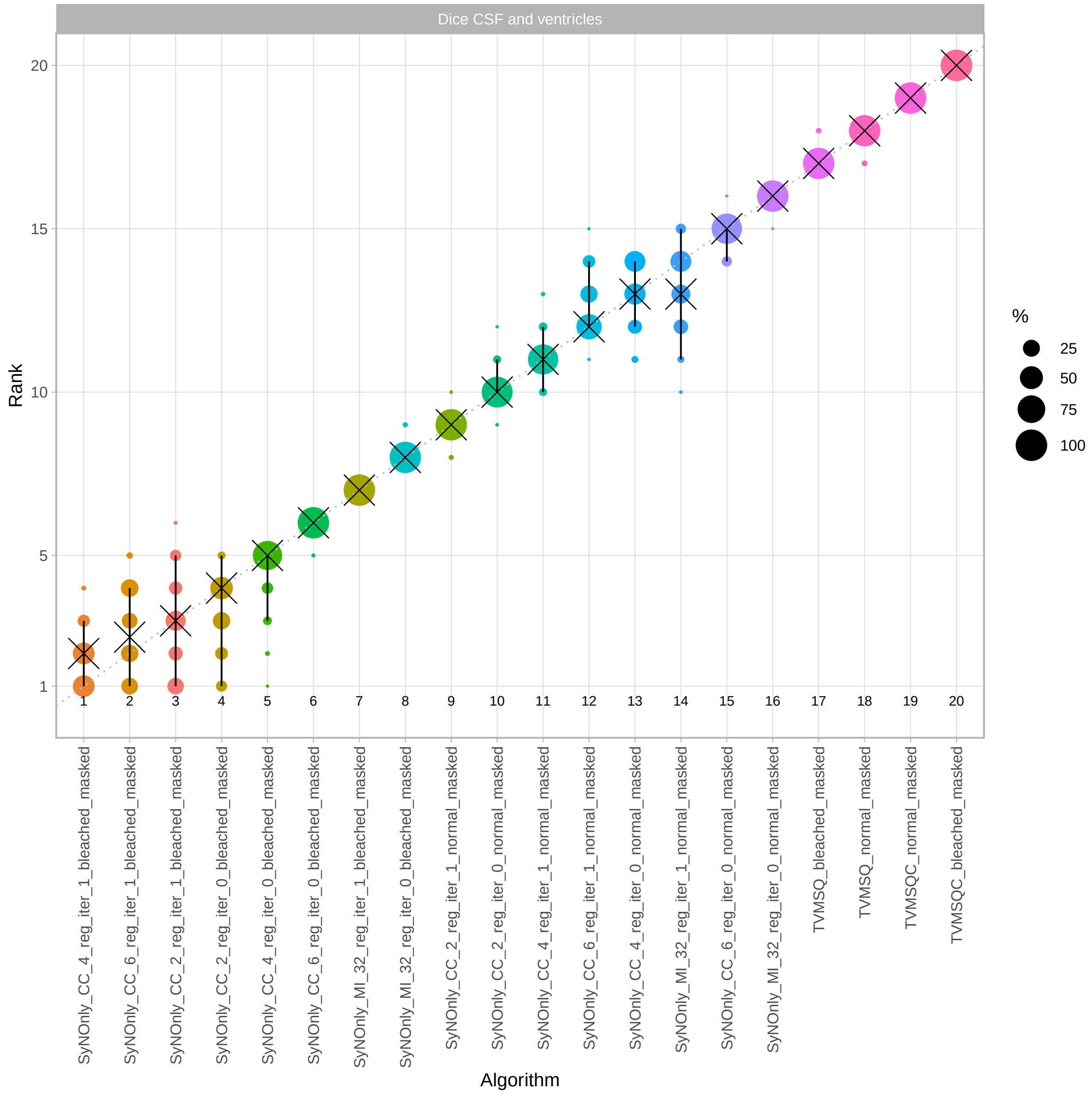

Dice CSF and ventricles
Rank
20
15
10
5
1
1
2
3
4
5
6
7
8
9
10
11
12
13
14
15
16
17
18
19
20
%
25
50
75
100
SyNOnly_CC_4_reg_iter_1_bleached_masked
SyNOnly_CC_6_reg_iter_1_bleached_masked
SyNOnly_CC_2_reg_iter_1_bleached_masked
SyNOnly_CC_2_reg_iter_0_bleached_masked
SyNOnly_CC_4_reg_iter_0_bleached_masked
SyNOnly_CC_6_reg_iter_0_bleached_masked
SyNOnly_MI_32_reg_iter_1_bleached_masked
SyNOnly_MI_32_reg_iter_0_bleached_masked
SyNOnly_CC_2_reg_iter_1_normal_masked
SyNOnly_CC_2_reg_iter_0_normal_masked
SyNOnly_CC_4_reg_iter_1_normal_masked
SyNOnly_CC_6_reg_iter_1_normal_masked
SyNOnly_CC_4_reg_iter_0_normal_masked
SyNOnly_MI_32_reg_iter_1_normal_masked
SyNOnly_CC_6_reg_iter_0_normal_masked
SyNOnly_MI_32_reg_iter_0_normal_masked
TVMSQ_bleached_masked
TVMSQ_normal_masked
TVMSQC_normal_masked
TVMSQC_bleached_masked
Algorithm

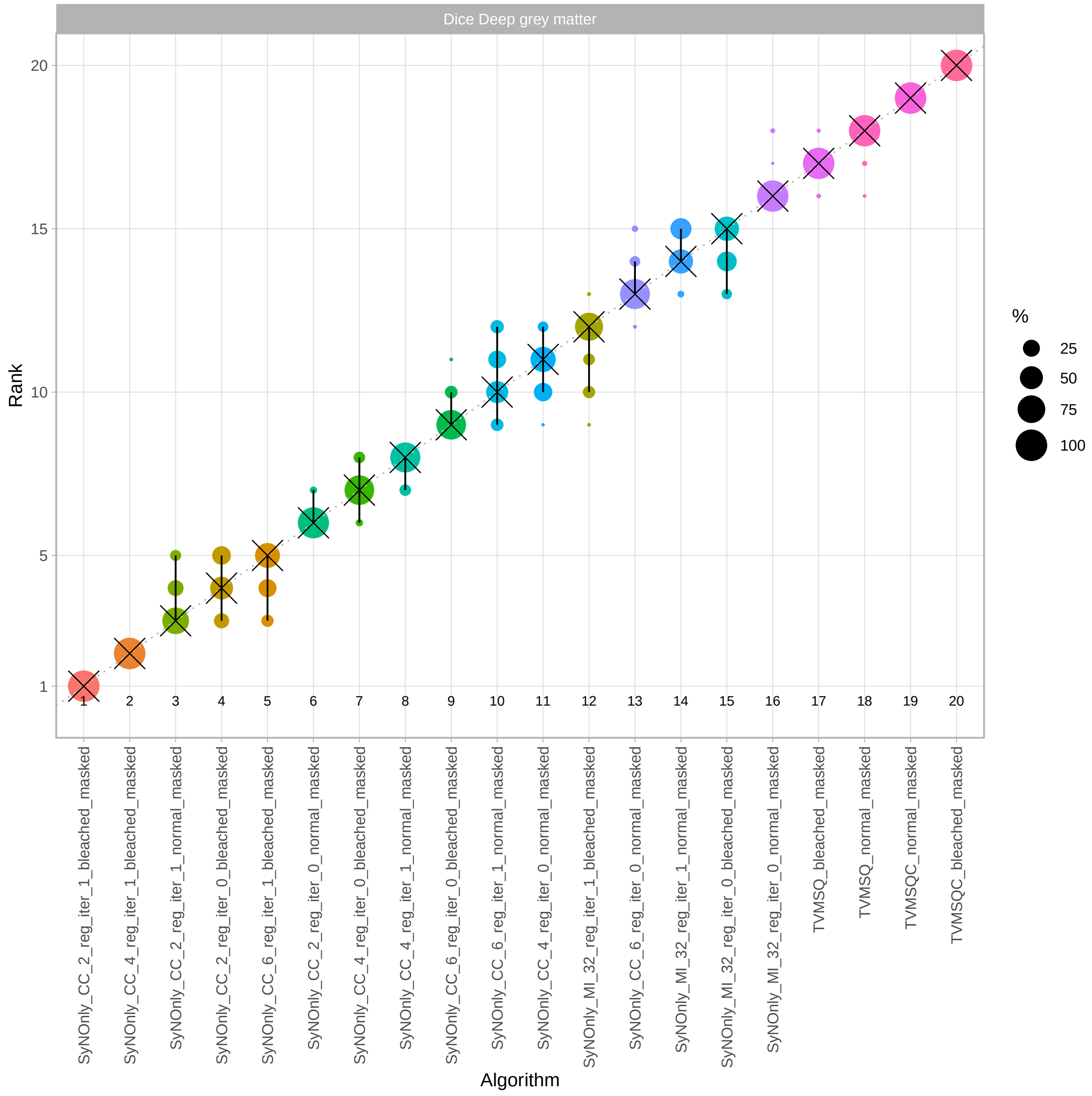
Dice Deep grey matter
Rank
20
15
10
5
1
1 2 3 4 5 6 7 8 9 10 11 12 13 14 15 16 17 18 19 20
%
25
50
75
100
SyNOnly_CC_2_reg_iter_1_bleached_masked
SyNOnly_CC_4_reg_iter_1_bleached_masked
SyNOnly_CC_2_reg_iter_1_normal_masked
SyNOnly_CC_2_reg_iter_0_bleached_masked
SyNOnly_CC_6_reg_iter_1_bleached_masked
SyNOnly_CC_2_reg_iter_0_normal_masked
SyNOnly_CC_4_reg_iter_0_bleached_masked
SyNOnly_CC_4_reg_iter_1_normal_masked
SyNOnly_CC_6_reg_iter_0_bleached_masked
SyNOnly_CC_6_reg_iter_1_normal_masked
SyNOnly_CC_4_reg_iter_0_normal_masked
SyNOnly_MI_32_reg_iter_1_bleached_masked
SyNOnly_CC_6_reg_iter_0_normal_masked
SyNOnly_MI_32_reg_iter_1_normal_masked
SyNOnly_MI_32_reg_iter_0_bleached_masked
SyNOnly_MI_32_reg_iter_0_normal_masked
TVMSQ_bleached_masked
TVMSQ_normal_masked
TVMSQC_normal_masked
TVMSQC_bleached_masked
Algorithm

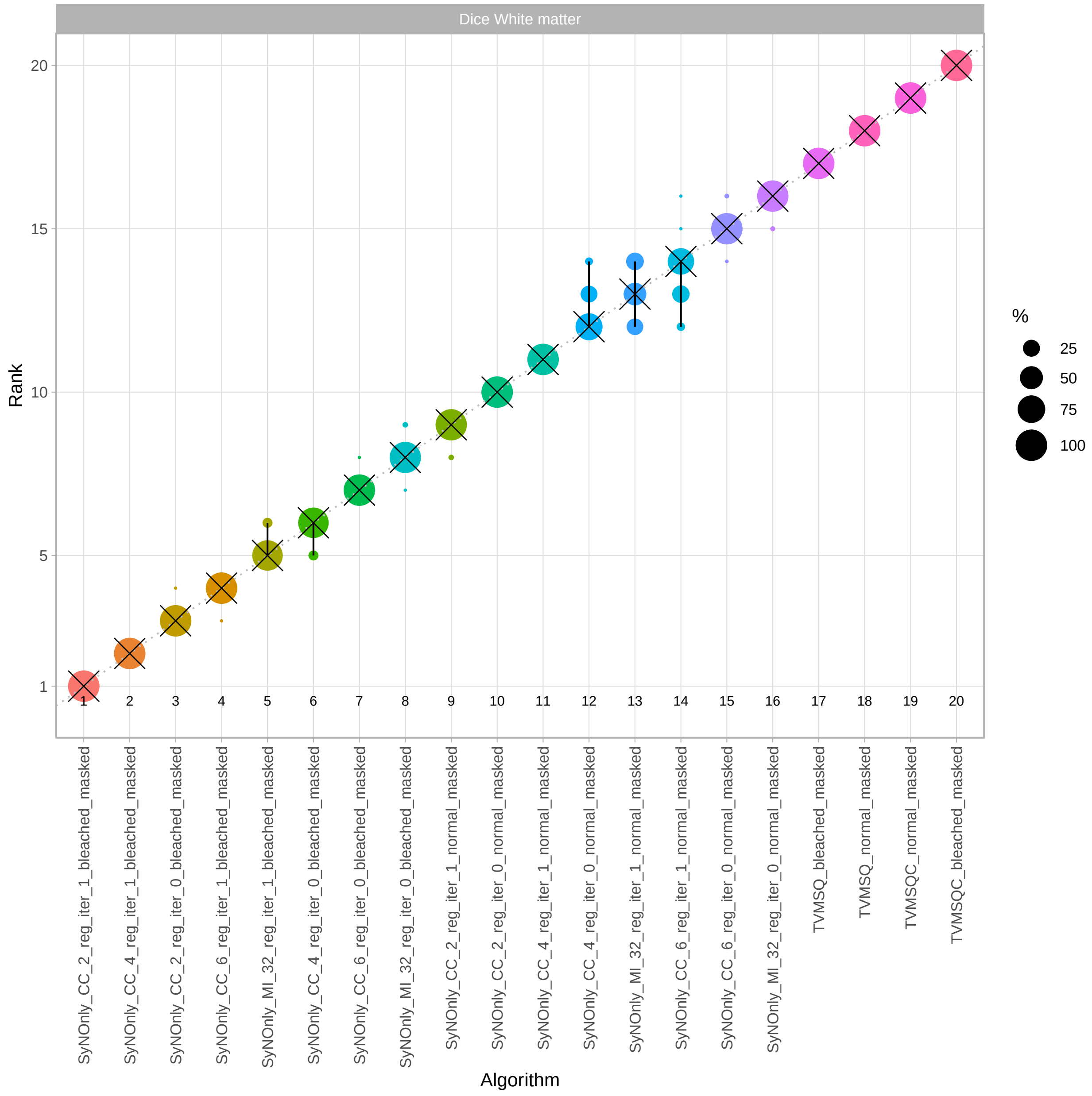
Dice White matter
Rank
Algorithm
%
25
50
75
100
SyNOnly_CC_2_reg_iter_1_bleached_masked
SyNOnly_CC_4_reg_iter_1_bleached_masked
SyNOnly_CC_2_reg_iter_0_bleached_masked
SyNOnly_CC_6_reg_iter_1_bleached_masked
SyNOnly_MI_32_reg_iter_1_bleached_masked
SyNOnly_CC_4_reg_iter_0_bleached_masked
SyNOnly_CC_6_reg_iter_0_bleached_masked
SyNOnly_MI_32_reg_iter_0_bleached_masked
SyNOnly_CC_2_reg_iter_1_normal_masked
SyNOnly_CC_2_reg_iter_0_normal_masked
SyNOnly_CC_4_reg_iter_1_normal_masked
SyNOnly_CC_4_reg_iter_0_normal_masked
SyNOnly_MI_32_reg_iter_1_normal_masked
SyNOnly_CC_6_reg_iter_1_normal_masked
SyNOnly_CC_6_reg_iter_0_normal_masked
SyNOnly_MI_32_reg_iter_0_normal_masked
TVMSQ_bleached_masked
TVMSQ_normal_masked
TVMSQC_normal_masked
TVMSQC_bleached_masked

3.2 *Violin plot* for visualizing ranking stability based on bootstrapping

The ranking list based on the full assessment data is pairwise compared with the ranking lists based on the individual bootstrap samples (here $b = 1000$ samples). For each pair of rankings, Kendall's τ correlation is computed. Kendall's τ is a scaled index determining the correlation between the lists. It is computed by evaluating the number of pairwise concordances and discordances between ranking lists and produces values between -1 (for inverted order) and 1 (for identical order). A violin plot, which simultaneously depicts a boxplot and a density plot, is generated from the results.

Summary Kendall's tau:

Task	mean	median	q25	q75
Dice Cortical grey matter	0.9922316	0.9894737	0.9894737	1.0000000
Dice White matter	0.9926842	0.9894737	0.9894737	1.0000000
Dice Cerebellum	0.9724947	0.9789474	0.9578947	0.9894737
Dice Deep grey matter	0.9758211	0.9789474	0.9684211	0.9894737
Dice CSF and ventricles	0.9661789	0.9684211	0.9578947	0.9789474
Dice Brainstem	0.9479895	0.9578947	0.9263158	0.9684211

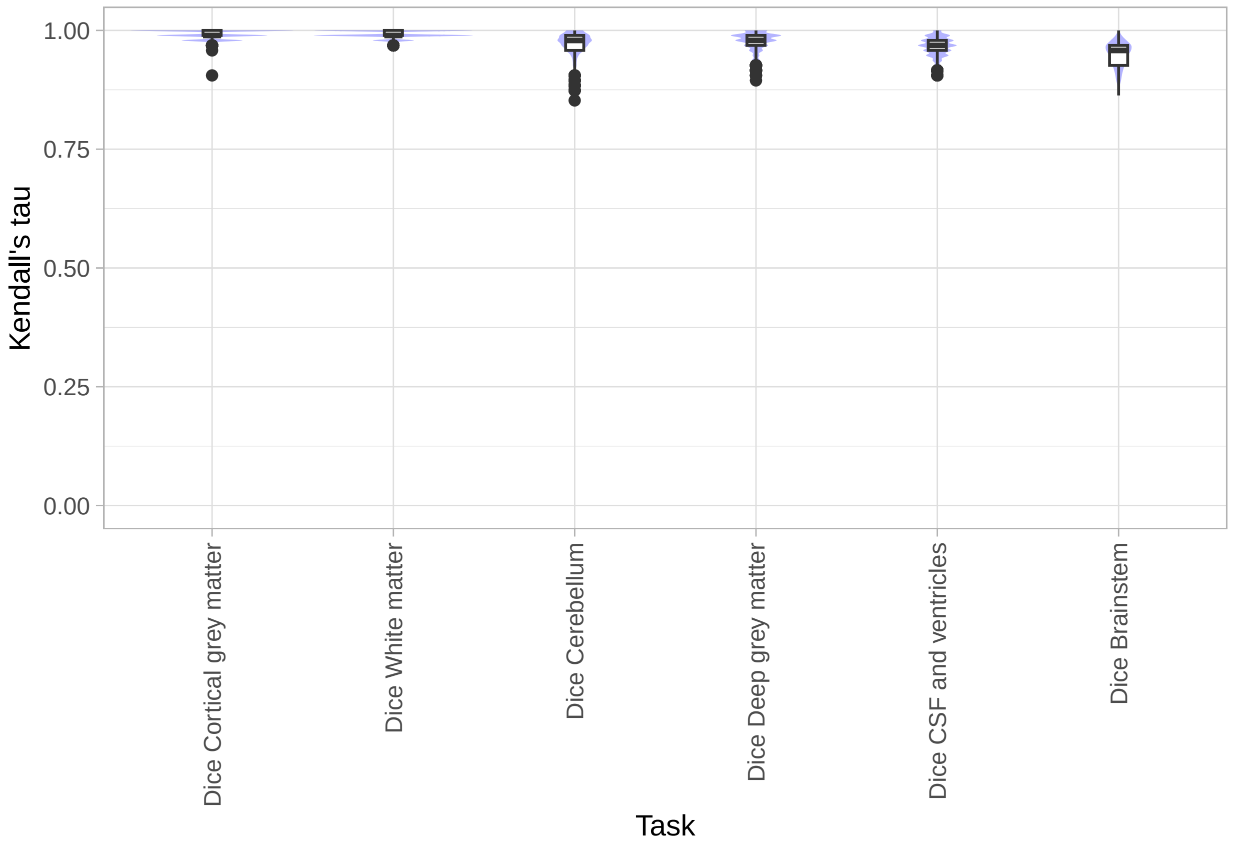

3.3 *Significance maps* for visualizing ranking stability based on statistical significance

Significance maps depict incidence matrices of pairwise significant test results for the one-sided Wilcoxon signed rank test at a 5% significance level with adjustment for multiple testing according to Holm. Yellow shading indicates that metric values from the algorithm on the x-axis were significantly superior to those from the algorithm on the y-axis, blue color indicates no significant difference.

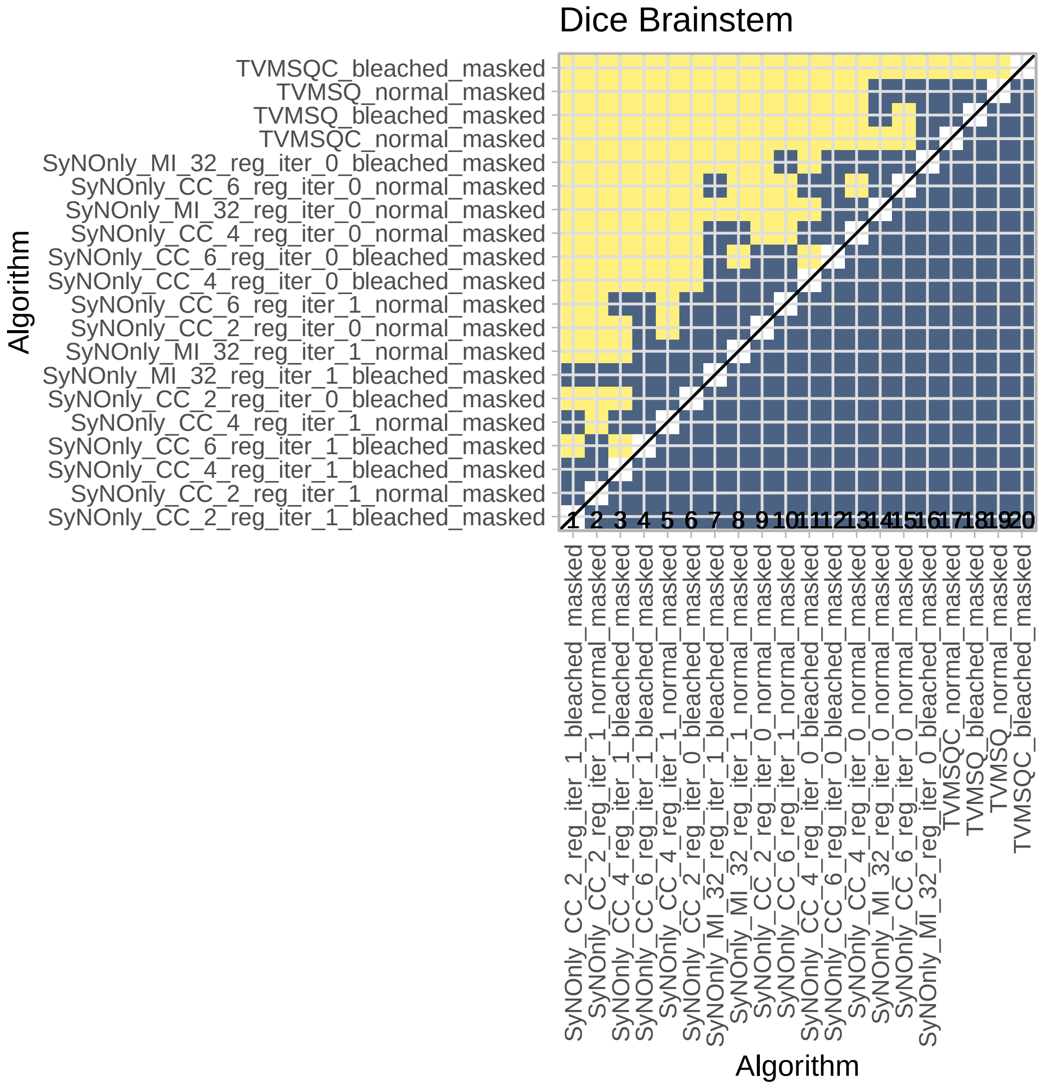

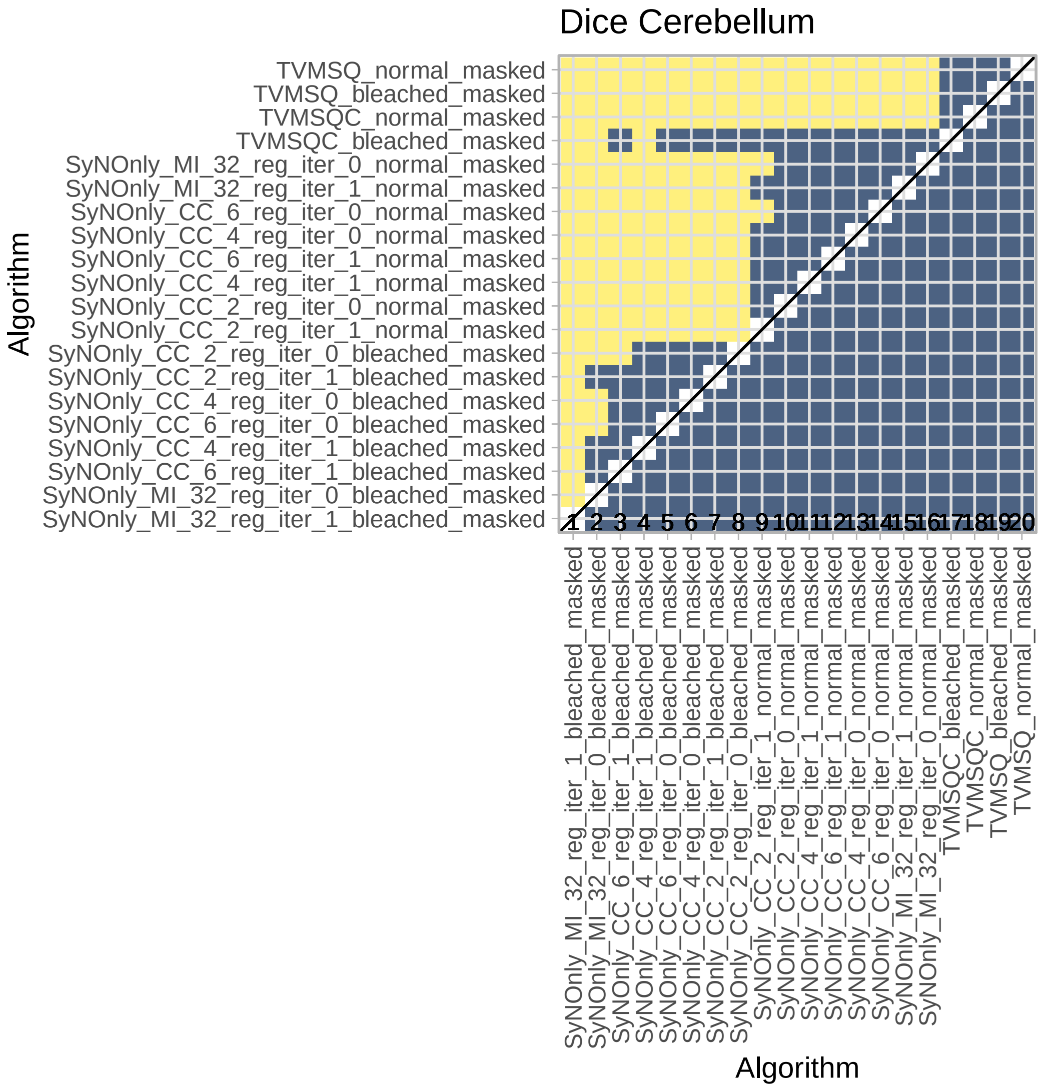
Dice Cerebellum
Algorithm
TVMSQ_normal_masked
TVMSQ_bleached_masked
TVMSQC_normal_masked
TVMSQC_bleached_masked
SyNOnly_MI_32_reg_iter_0_normal_masked
SyNOnly_MI_32_reg_iter_1_normal_masked
SyNOnly_CC_6_reg_iter_0_normal_masked
SyNOnly_CC_4_reg_iter_0_normal_masked
SyNOnly_CC_6_reg_iter_1_normal_masked
SyNOnly_CC_4_reg_iter_1_normal_masked
SyNOnly_CC_2_reg_iter_0_normal_masked
SyNOnly_CC_2_reg_iter_1_normal_masked
SyNOnly_CC_2_reg_iter_0_bleached_masked
SyNOnly_CC_2_reg_iter_1_bleached_masked
SyNOnly_CC_4_reg_iter_0_bleached_masked
SyNOnly_CC_6_reg_iter_0_bleached_masked
SyNOnly_CC_4_reg_iter_1_bleached_masked
SyNOnly_CC_6_reg_iter_1_bleached_masked
SyNOnly_MI_32_reg_iter_0_bleached_masked
SyNOnly_MI_32_reg_iter_1_bleached_masked
1 2 3 4 5 6 7 8 9 10 11 12 13 14 15 16 17 18 19 20
Algorithm

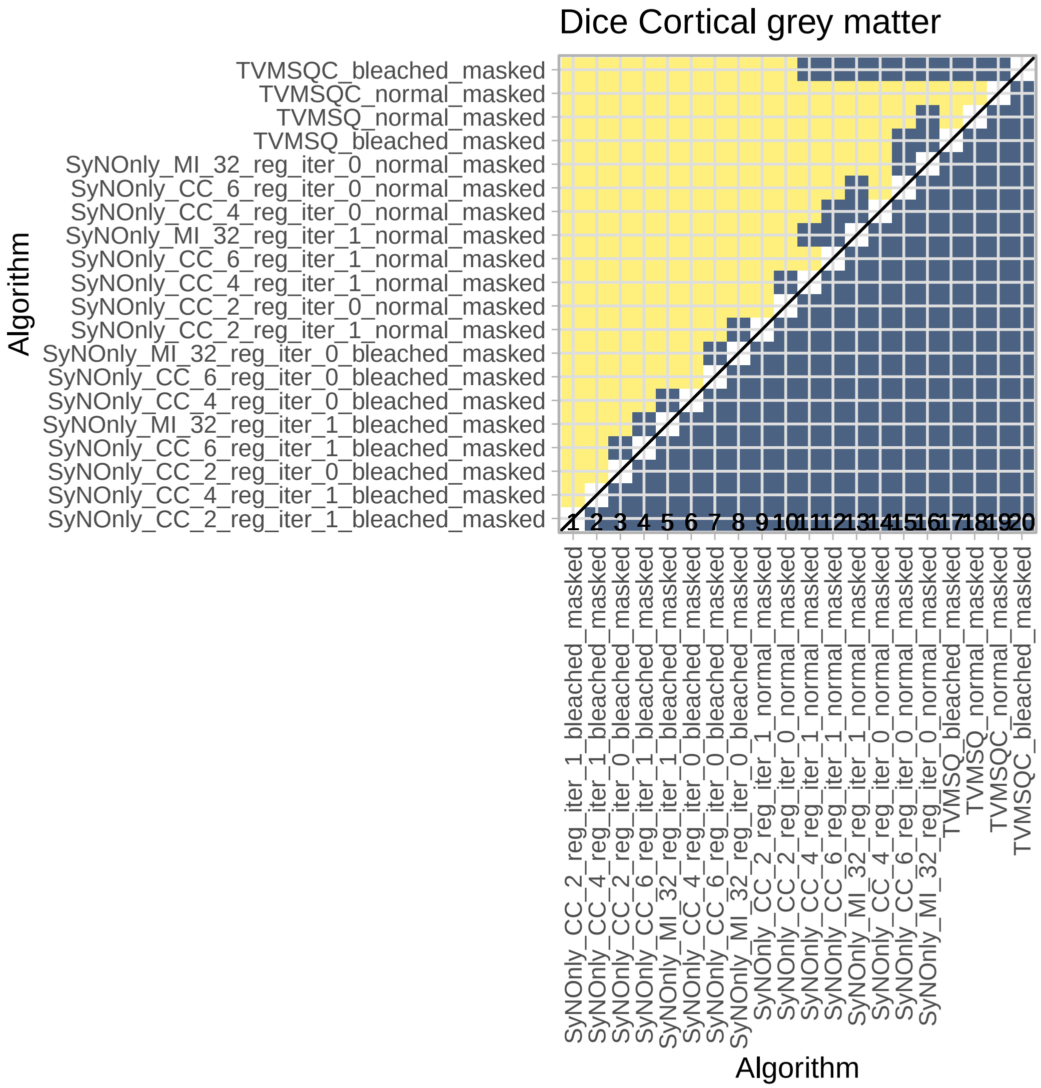
Dice Cortical grey matter
Algorithm
TVMSQC_bleached_masked
TVMSQC_normal_masked
TVMSQ_normal_masked
TVMSQ_bleached_masked
SyNOnly_MI_32_reg_iter_0_normal_masked
SyNOnly_CC_6_reg_iter_0_normal_masked
SyNOnly_CC_4_reg_iter_0_normal_masked
SyNOnly_MI_32_reg_iter_1_normal_masked
SyNOnly_CC_6_reg_iter_1_normal_masked
SyNOnly_CC_4_reg_iter_1_normal_masked
SyNOnly_CC_2_reg_iter_0_normal_masked
SyNOnly_CC_2_reg_iter_1_normal_masked
SyNOnly_MI_32_reg_iter_0_bleached_masked
SyNOnly_CC_6_reg_iter_0_bleached_masked
SyNOnly_CC_4_reg_iter_0_bleached_masked
SyNOnly_MI_32_reg_iter_1_bleached_masked
SyNOnly_CC_6_reg_iter_1_bleached_masked
SyNOnly_CC_2_reg_iter_0_bleached_masked
SyNOnly_CC_4_reg_iter_1_bleached_masked
SyNOnly_CC_2_reg_iter_1_bleached_masked
1 2 3 4 5 6 7 8 9 10 11 12 13 14 15 16 17 18 19 20
SyNOnly_CC_2_reg_iter_1_bleached_masked
SyNOnly_CC_4_reg_iter_1_bleached_masked
SyNOnly_CC_2_reg_iter_0_bleached_masked
SyNOnly_CC_6_reg_iter_1_bleached_masked
SyNOnly_MI_32_reg_iter_1_bleached_masked
SyNOnly_CC_4_reg_iter_0_bleached_masked
SyNOnly_CC_6_reg_iter_0_bleached_masked
SyNOnly_MI_32_reg_iter_0_bleached_masked
SyNOnly_CC_2_reg_iter_1_normal_masked
SyNOnly_CC_2_reg_iter_0_normal_masked
SyNOnly_CC_4_reg_iter_1_normal_masked
SyNOnly_CC_6_reg_iter_1_normal_masked
SyNOnly_MI_32_reg_iter_1_normal_masked
SyNOnly_CC_4_reg_iter_0_normal_masked
SyNOnly_CC_6_reg_iter_0_normal_masked
SyNOnly_MI_32_reg_iter_0_normal_masked
TVMSQ_bleached_masked
TVMSQ_normal_masked
TVMSQC_normal_masked
TVMSQC_bleached_masked
Algorithm

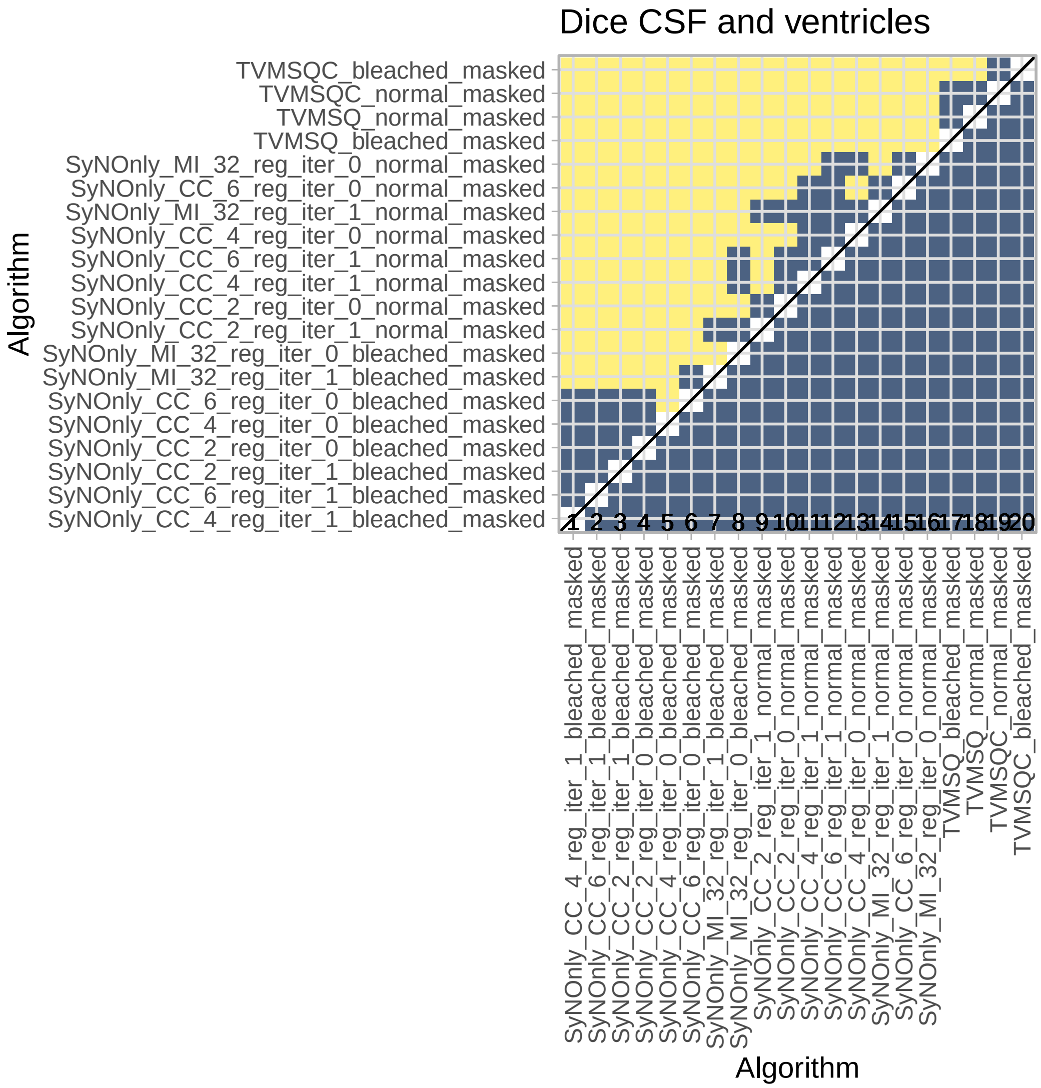
Dice CSF and ventricles
TVMSQC_bleached_masked
TVMSQC_normal_masked
TVMSQ_normal_masked
TVMSQ_bleached_masked
SyNOnly_MI_32_reg_iter_0_normal_masked
SyNOnly_CC_6_reg_iter_0_normal_masked
SyNOnly_MI_32_reg_iter_1_normal_masked
SyNOnly_CC_4_reg_iter_0_normal_masked
SyNOnly_CC_6_reg_iter_1_normal_masked
SyNOnly_CC_4_reg_iter_1_normal_masked
SyNOnly_CC_2_reg_iter_0_normal_masked
SyNOnly_CC_2_reg_iter_1_normal_masked
SyNOnly_MI_32_reg_iter_0_bleached_masked
SyNOnly_MI_32_reg_iter_1_bleached_masked
SyNOnly_CC_6_reg_iter_0_bleached_masked
SyNOnly_CC_4_reg_iter_0_bleached_masked
SyNOnly_CC_2_reg_iter_0_bleached_masked
SyNOnly_CC_2_reg_iter_1_bleached_masked
SyNOnly_CC_6_reg_iter_1_bleached_masked
SyNOnly_CC_4_reg_iter_1_bleached_masked
Algorithm
1 2 3 4 5 6 7 8 9 10 11 12 13 14 15 16 17 18 19 20
SyNOnly_CC_4_reg_iter_1_bleached_masked
SyNOnly_CC_6_reg_iter_1_bleached_masked
SyNOnly_CC_2_reg_iter_1_bleached_masked
SyNOnly_CC_2_reg_iter_0_bleached_masked
SyNOnly_CC_4_reg_iter_0_bleached_masked
SyNOnly_CC_6_reg_iter_0_bleached_masked
SyNOnly_MI_32_reg_iter_1_bleached_masked
SyNOnly_MI_32_reg_iter_0_bleached_masked
SyNOnly_CC_2_reg_iter_1_normal_masked
SyNOnly_CC_2_reg_iter_0_normal_masked
SyNOnly_CC_4_reg_iter_1_normal_masked
SyNOnly_CC_6_reg_iter_1_normal_masked
SyNOnly_CC_4_reg_iter_0_normal_masked
SyNOnly_MI_32_reg_iter_1_normal_masked
SyNOnly_CC_6_reg_iter_0_normal_masked
SyNOnly_MI_32_reg_iter_0_normal_masked
TVMSQ_bleached_masked
TVMSQ_normal_masked
TVMSQC_normal_masked
TVMSQC_bleached_masked
Algorithm

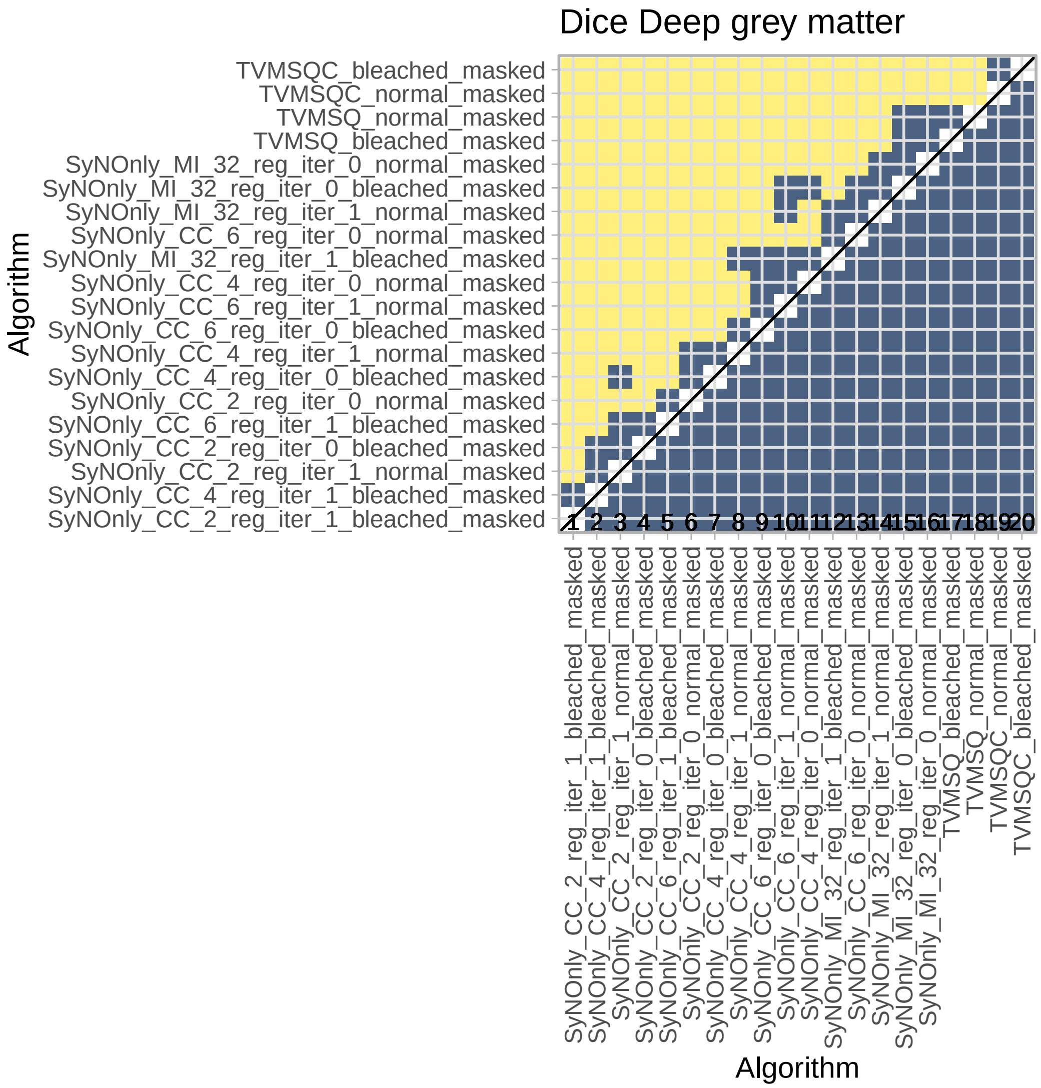

Dice Deep grey matter
Algorithm
TVMSQC_bleached_masked
TVMSQC_normal_masked
TVMSQ_normal_masked
TVMSQ_bleached_masked
SyNOnly_MI_32_reg_iter_0_normal_masked
SyNOnly_MI_32_reg_iter_0_bleached_masked
SyNOnly_MI_32_reg_iter_1_normal_masked
SyNOnly_CC_6_reg_iter_0_normal_masked
SyNOnly_MI_32_reg_iter_1_bleached_masked
SyNOnly_CC_4_reg_iter_0_normal_masked
SyNOnly_CC_6_reg_iter_1_normal_masked
SyNOnly_CC_6_reg_iter_0_bleached_masked
SyNOnly_CC_4_reg_iter_1_normal_masked
SyNOnly_CC_4_reg_iter_0_bleached_masked
SyNOnly_CC_2_reg_iter_0_normal_masked
SyNOnly_CC_6_reg_iter_1_bleached_masked
SyNOnly_CC_2_reg_iter_0_bleached_masked
SyNOnly_CC_2_reg_iter_1_normal_masked
SyNOnly_CC_4_reg_iter_1_bleached_masked
SyNOnly_CC_2_reg_iter_1_bleached_masked
1 2 3 4 5 6 7 8 9 10 11 12 13 14 15 16 17 18 19 20
SyNOnly_CC_2_reg_iter_1_bleached_masked
SyNOnly_CC_4_reg_iter_1_bleached_masked
SyNOnly_CC_2_reg_iter_1_normal_masked
SyNOnly_CC_2_reg_iter_0_bleached_masked
SyNOnly_CC_6_reg_iter_1_bleached_masked
SyNOnly_CC_2_reg_iter_0_normal_masked
SyNOnly_CC_4_reg_iter_0_bleached_masked
SyNOnly_CC_4_reg_iter_1_normal_masked
SyNOnly_CC_6_reg_iter_0_bleached_masked
SyNOnly_CC_6_reg_iter_1_normal_masked
SyNOnly_CC_4_reg_iter_0_normal_masked
SyNOnly_MI_32_reg_iter_1_bleached_masked
SyNOnly_CC_6_reg_iter_0_normal_masked
SyNOnly_MI_32_reg_iter_1_normal_masked
SyNOnly_MI_32_reg_iter_0_bleached_masked
SyNOnly_MI_32_reg_iter_0_normal_masked
TVMSQ_bleached_masked
TVMSQ_normal_masked
TVMSQC_normal_masked
TVMSQC_bleached_masked
Algorithm

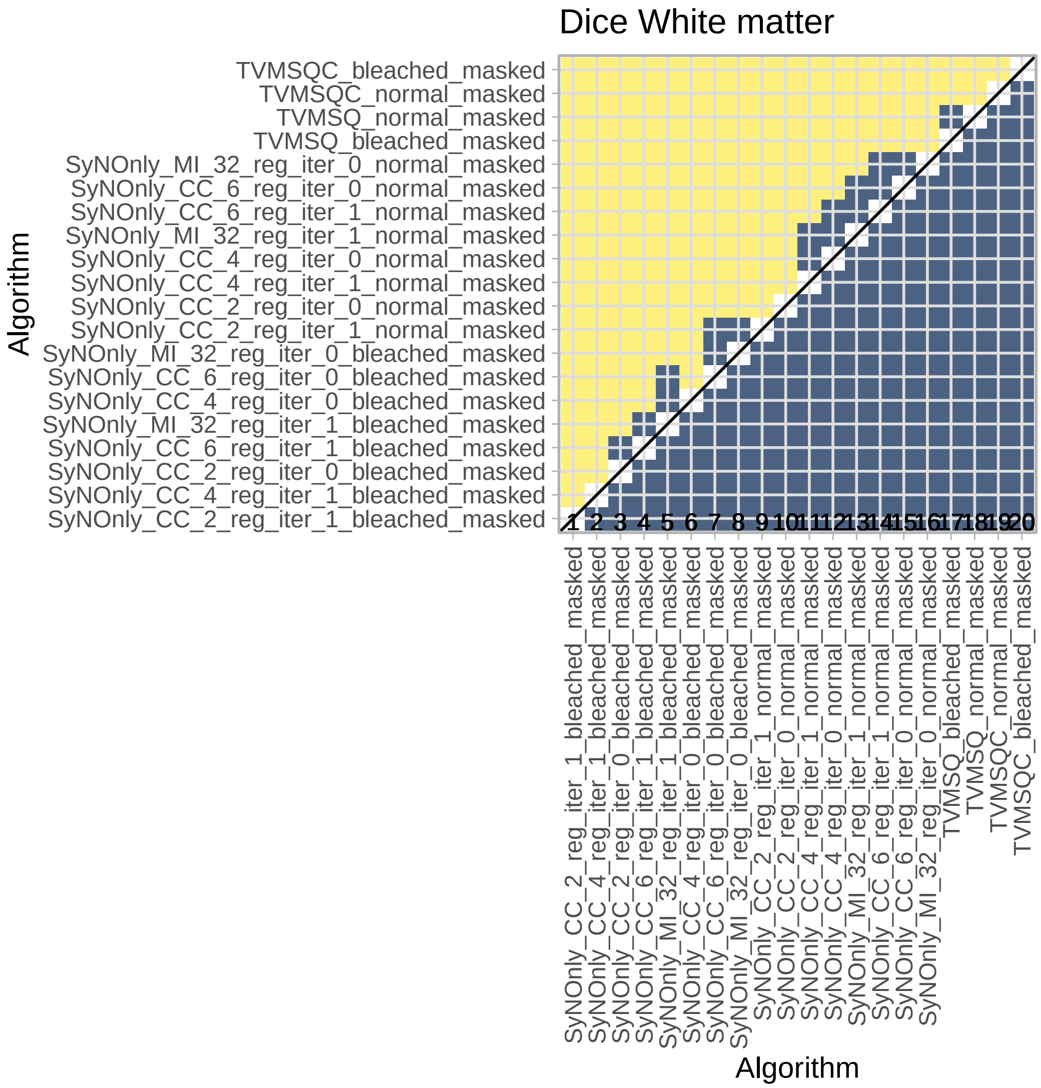
Dice White matter
Algorithm
TVMSQC_bleached_masked
TVMSQC_normal_masked
TVMSQ_normal_masked
TVMSQ_bleached_masked
SyNOnly_MI_32_reg_iter_0_normal_masked
SyNOnly_CC_6_reg_iter_0_normal_masked
SyNOnly_CC_6_reg_iter_1_normal_masked
SyNOnly_MI_32_reg_iter_1_normal_masked
SyNOnly_CC_4_reg_iter_0_normal_masked
SyNOnly_CC_4_reg_iter_1_normal_masked
SyNOnly_CC_2_reg_iter_0_normal_masked
SyNOnly_CC_2_reg_iter_1_normal_masked
SyNOnly_MI_32_reg_iter_0_bleached_masked
SyNOnly_CC_6_reg_iter_0_bleached_masked
SyNOnly_CC_4_reg_iter_0_bleached_masked
SyNOnly_MI_32_reg_iter_1_bleached_masked
SyNOnly_CC_6_reg_iter_1_bleached_masked
SyNOnly_CC_2_reg_iter_0_bleached_masked
SyNOnly_CC_4_reg_iter_1_bleached_masked
SyNOnly_CC_2_reg_iter_1_bleached_masked
1 2 3 4 5 6 7 8 9 10 11 12 13 14 15 16 17 18 19 20
SyNOnly_CC_2_reg_iter_1_bleached_masked
SyNOnly_CC_4_reg_iter_1_bleached_masked
SyNOnly_CC_2_reg_iter_0_bleached_masked
SyNOnly_CC_6_reg_iter_1_bleached_masked
SyNOnly_MI_32_reg_iter_1_bleached_masked
SyNOnly_CC_4_reg_iter_0_bleached_masked
SyNOnly_CC_6_reg_iter_0_bleached_masked
SyNOnly_MI_32_reg_iter_0_bleached_masked
SyNOnly_CC_2_reg_iter_1_normal_masked
SyNOnly_CC_2_reg_iter_0_normal_masked
SyNOnly_CC_4_reg_iter_1_normal_masked
SyNOnly_CC_4_reg_iter_0_normal_masked
SyNOnly_MI_32_reg_iter_1_normal_masked
SyNOnly_CC_6_reg_iter_1_normal_masked
SyNOnly_CC_6_reg_iter_0_normal_masked
SyNOnly_MI_32_reg_iter_0_normal_masked
TVMSQ_bleached_masked
TVMSQ_normal_masked
TVMSQC_normal_masked
TVMSQC_bleached_masked
Algorithm

3.4 Ranking robustness to ranking methods

Line plots for visualizing ranking robustness across different ranking methods. Each algorithm is represented by one colored line. For each ranking method encoded on the x-axis, the height of the line represents the corresponding rank. Horizontal lines indicate identical ranks for all methods.

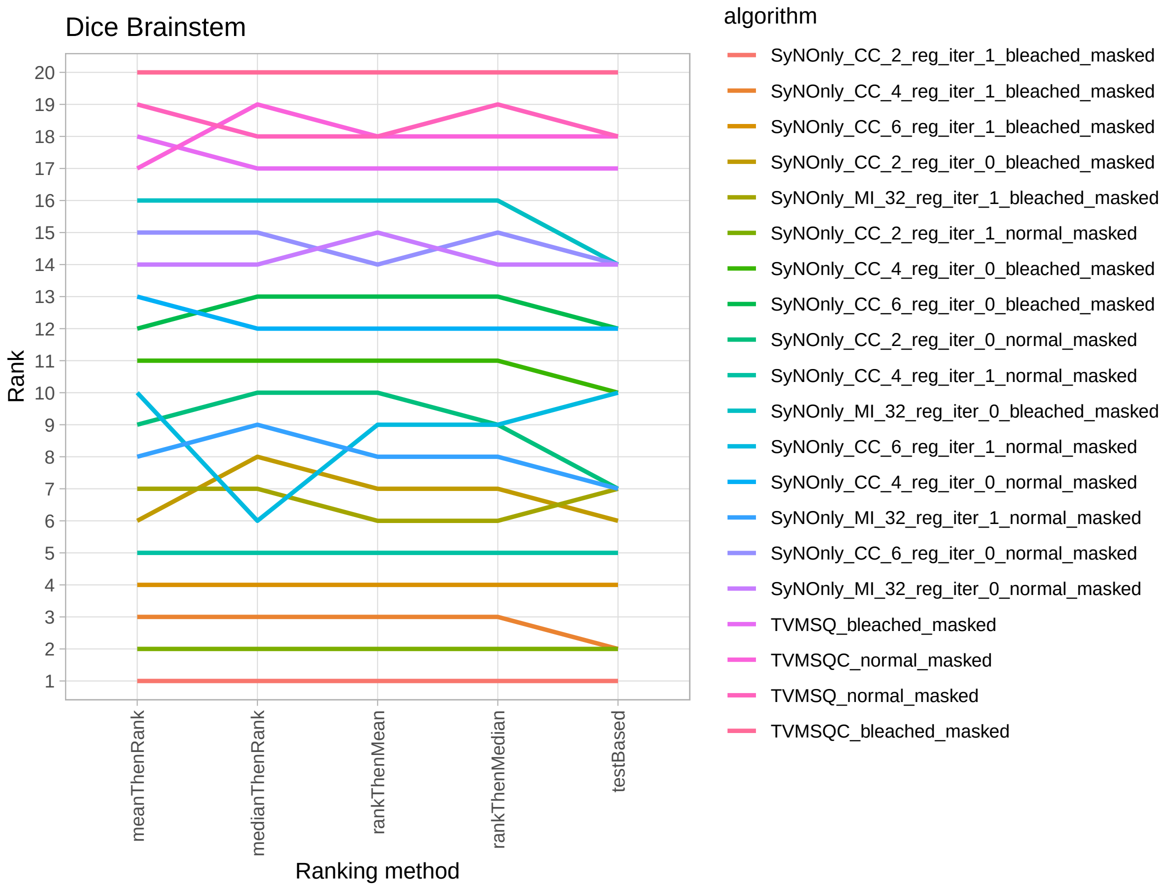

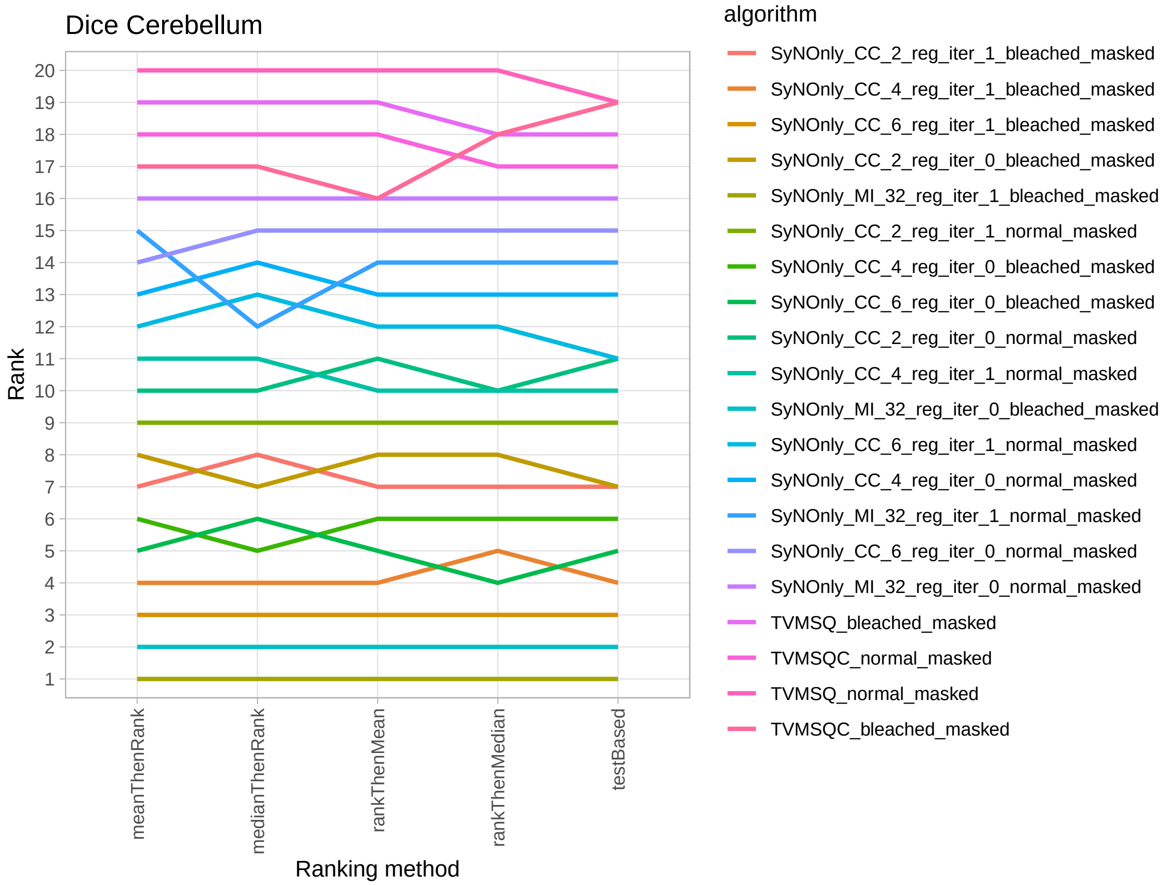
Dice Cerebellum
Rank
Ranking method
meanThenRank
medianThenRank
rankThenMean
rankThenMedian
testBased
algorithm
SyNOnly_CC_2_reg_iter_1_bleached_masked
SyNOnly_CC_4_reg_iter_1_bleached_masked
SyNOnly_CC_6_reg_iter_1_bleached_masked
SyNOnly_CC_2_reg_iter_0_bleached_masked
SyNOnly_MI_32_reg_iter_1_bleached_masked
SyNOnly_CC_2_reg_iter_1_normal_masked
SyNOnly_CC_4_reg_iter_0_bleached_masked
SyNOnly_CC_6_reg_iter_0_bleached_masked
SyNOnly_CC_2_reg_iter_0_normal_masked
SyNOnly_CC_4_reg_iter_1_normal_masked
SyNOnly_MI_32_reg_iter_0_bleached_masked
SyNOnly_CC_6_reg_iter_1_normal_masked
SyNOnly_CC_4_reg_iter_0_normal_masked
SyNOnly_MI_32_reg_iter_1_normal_masked
SyNOnly_CC_6_reg_iter_0_normal_masked
SyNOnly_MI_32_reg_iter_0_normal_masked
TVMSQ_bleached_masked
TVMSQC_normal_masked
TVMSQ_normal_masked
TVMSQC_bleached_masked

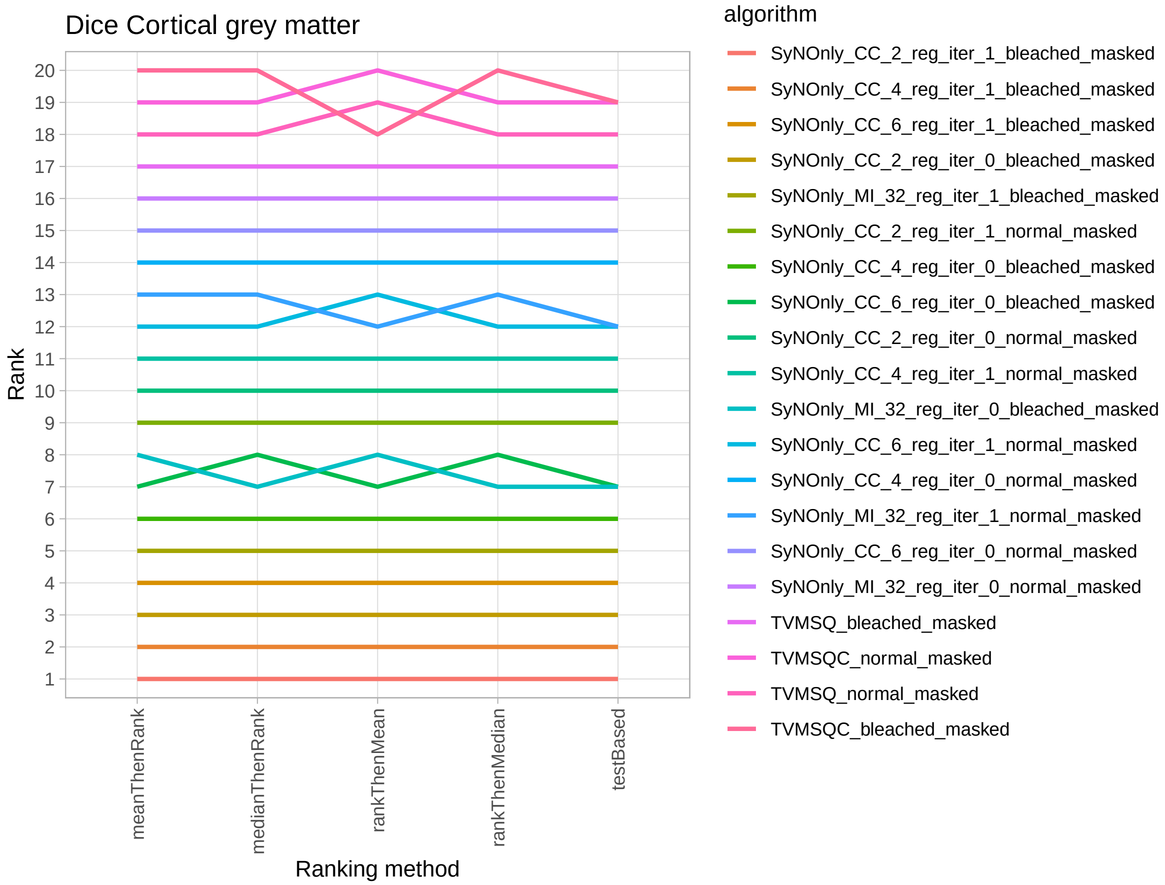
Dice Cortical grey matter
Rank
20
19
18
17
16
15
14
13
12
11
10
9
8
7
6
5
4
3
2
1
meanThenRank
medianThenRank
rankThenMean
rankThenMedian
testBased
Ranking method
algorithm
SyNOnly_CC_2_reg_iter_1_bleached_masked
SyNOnly_CC_4_reg_iter_1_bleached_masked
SyNOnly_CC_6_reg_iter_1_bleached_masked
SyNOnly_CC_2_reg_iter_0_bleached_masked
SyNOnly_MI_32_reg_iter_1_bleached_masked
SyNOnly_CC_2_reg_iter_1_normal_masked
SyNOnly_CC_4_reg_iter_0_bleached_masked
SyNOnly_CC_6_reg_iter_0_bleached_masked
SyNOnly_CC_2_reg_iter_0_normal_masked
SyNOnly_CC_4_reg_iter_1_normal_masked
SyNOnly_MI_32_reg_iter_0_bleached_masked
SyNOnly_CC_6_reg_iter_1_normal_masked
SyNOnly_CC_4_reg_iter_0_normal_masked
SyNOnly_MI_32_reg_iter_1_normal_masked
SyNOnly_CC_6_reg_iter_0_normal_masked
SyNOnly_MI_32_reg_iter_0_normal_masked
TVMSQ_bleached_masked
TVMSQC_normal_masked
TVMSQ_normal_masked
TVMSQC_bleached_masked

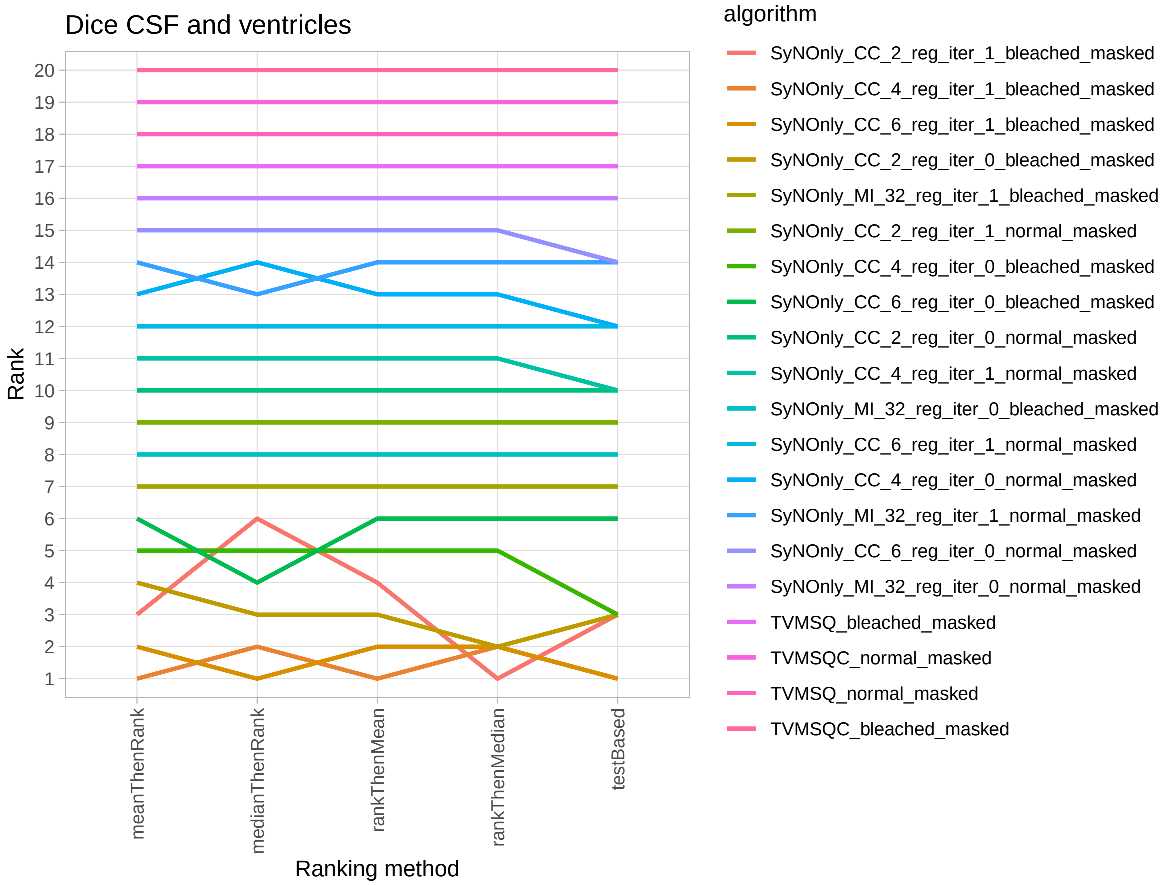
Dice CSF and ventricles
Rank
20
19
18
17
16
15
14
13
12
11
10
9
8
7
6
5
4
3
2
1
meanThenRank
medianThenRank
rankThenMean
rankThenMedian
testBased
Ranking method
algorithm
SyNOnly_CC_2_reg_iter_1_bleached_masked
SyNOnly_CC_4_reg_iter_1_bleached_masked
SyNOnly_CC_6_reg_iter_1_bleached_masked
SyNOnly_CC_2_reg_iter_0_bleached_masked
SyNOnly_MI_32_reg_iter_1_bleached_masked
SyNOnly_CC_2_reg_iter_1_normal_masked
SyNOnly_CC_4_reg_iter_0_bleached_masked
SyNOnly_CC_6_reg_iter_0_bleached_masked
SyNOnly_CC_2_reg_iter_0_normal_masked
SyNOnly_CC_4_reg_iter_1_normal_masked
SyNOnly_MI_32_reg_iter_0_bleached_masked
SyNOnly_CC_6_reg_iter_1_normal_masked
SyNOnly_CC_4_reg_iter_0_normal_masked
SyNOnly_MI_32_reg_iter_1_normal_masked
SyNOnly_CC_6_reg_iter_0_normal_masked
SyNOnly_MI_32_reg_iter_0_normal_masked
TVMSQ_bleached_masked
TVMSQC_normal_masked
TVMSQ_normal_masked
TVMSQC_bleached_masked

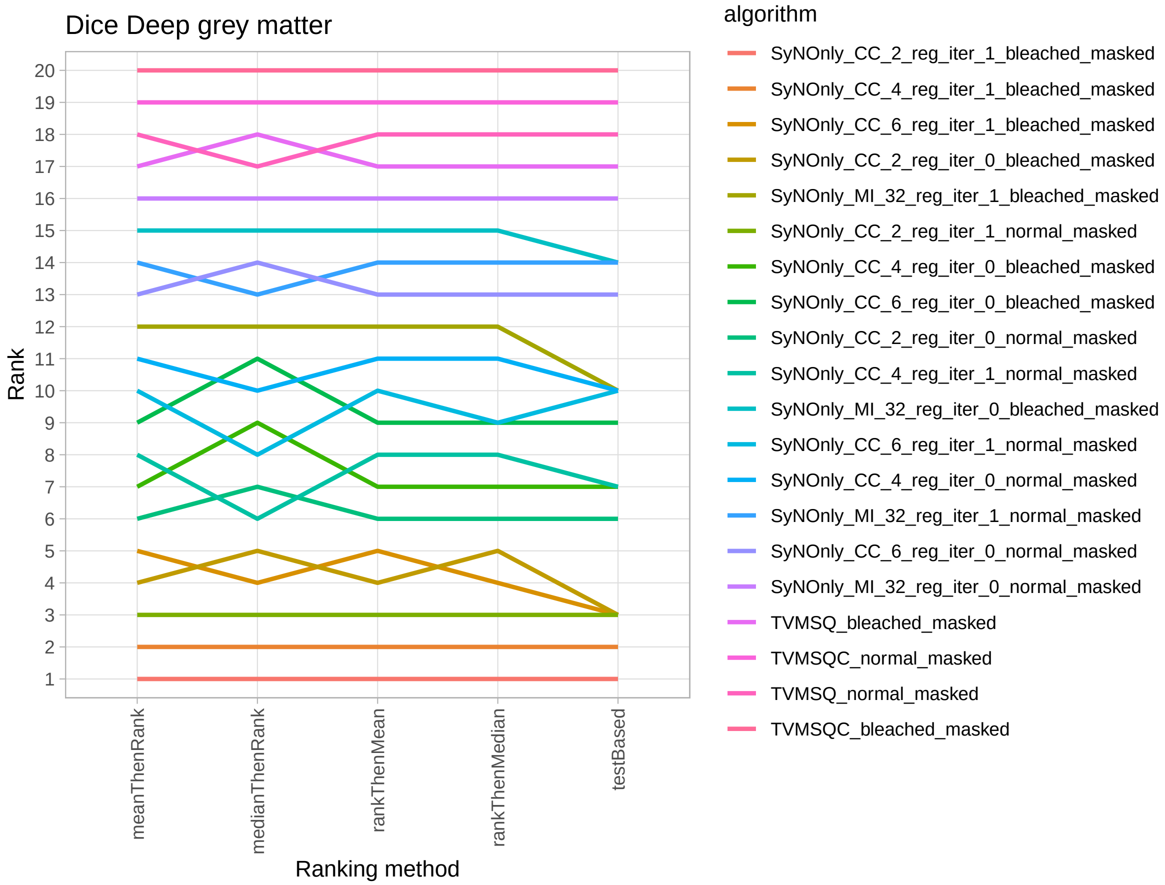
Dice Deep grey matter
algorithm
SyNOnly_CC_2_reg_iter_1_bleached_masked
SyNOnly_CC_4_reg_iter_1_bleached_masked
SyNOnly_CC_6_reg_iter_1_bleached_masked
SyNOnly_CC_2_reg_iter_0_bleached_masked
SyNOnly_MI_32_reg_iter_1_bleached_masked
SyNOnly_CC_2_reg_iter_1_normal_masked
SyNOnly_CC_4_reg_iter_0_bleached_masked
SyNOnly_CC_6_reg_iter_0_bleached_masked
SyNOnly_CC_2_reg_iter_0_normal_masked
SyNOnly_CC_4_reg_iter_1_normal_masked
SyNOnly_MI_32_reg_iter_0_bleached_masked
SyNOnly_CC_6_reg_iter_1_normal_masked
SyNOnly_CC_4_reg_iter_0_normal_masked
SyNOnly_MI_32_reg_iter_1_normal_masked
SyNOnly_CC_6_reg_iter_0_normal_masked
SyNOnly_MI_32_reg_iter_0_normal_masked
TVMSQ_bleached_masked
TVMSQC_normal_masked
TVMSQ_normal_masked
TVMSQC_bleached_masked
Rank
20
19
18
17
16
15
14
13
12
11
10
9
8
7
6
5
4
3
2
1
meanThenRank
medianThenRank
rankThenMean
rankThenMedian
testBased
Ranking method

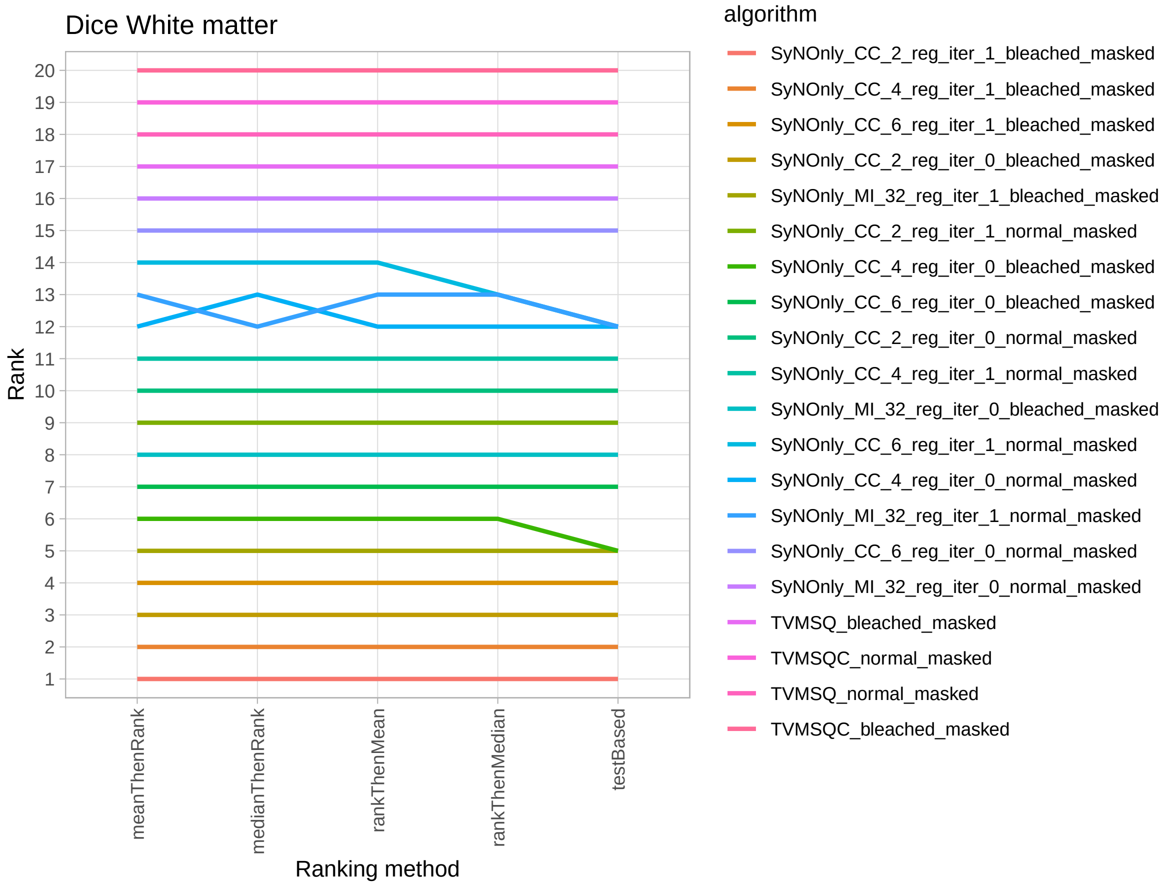
Dice White matter
Rank
20
19
18
17
16
15
14
13
12
11
10
9
8
7
6
5
4
3
2
1
meanThenRank
medianThenRank
rankThenMean
rankThenMedian
testBased
Ranking method
algorithm
SyNOnly_CC_2_reg_iter_1_bleached_masked
SyNOnly_CC_4_reg_iter_1_bleached_masked
SyNOnly_CC_6_reg_iter_1_bleached_masked
SyNOnly_CC_2_reg_iter_0_bleached_masked
SyNOnly_MI_32_reg_iter_1_bleached_masked
SyNOnly_CC_2_reg_iter_1_normal_masked
SyNOnly_CC_4_reg_iter_0_bleached_masked
SyNOnly_CC_6_reg_iter_0_bleached_masked
SyNOnly_CC_2_reg_iter_0_normal_masked
SyNOnly_CC_4_reg_iter_1_normal_masked
SyNOnly_MI_32_reg_iter_0_bleached_masked
SyNOnly_CC_6_reg_iter_1_normal_masked
SyNOnly_CC_4_reg_iter_0_normal_masked
SyNOnly_MI_32_reg_iter_1_normal_masked
SyNOnly_CC_6_reg_iter_0_normal_masked
SyNOnly_MI_32_reg_iter_0_normal_masked
TVMSQ_bleached_masked
TVMSQC_normal_masked
TVMSQ_normal_masked
TVMSQC_bleached_masked

4 Visualization of cross-task insights

The algorithms are ordered according to consensus ranking.

4.1 Characterization of algorithms

4.1.1 Ranking stability: Variability of achieved rankings across tasks

Algorithms are color-coded, and the area of each blob at position $(A_i, \text{rank } j)$ is proportional to the relative frequency A_i achieved rank j across multiple tasks. The median rank for each algorithm is indicated by a black cross. This way, the distribution of ranks across tasks can be intuitively visualized.

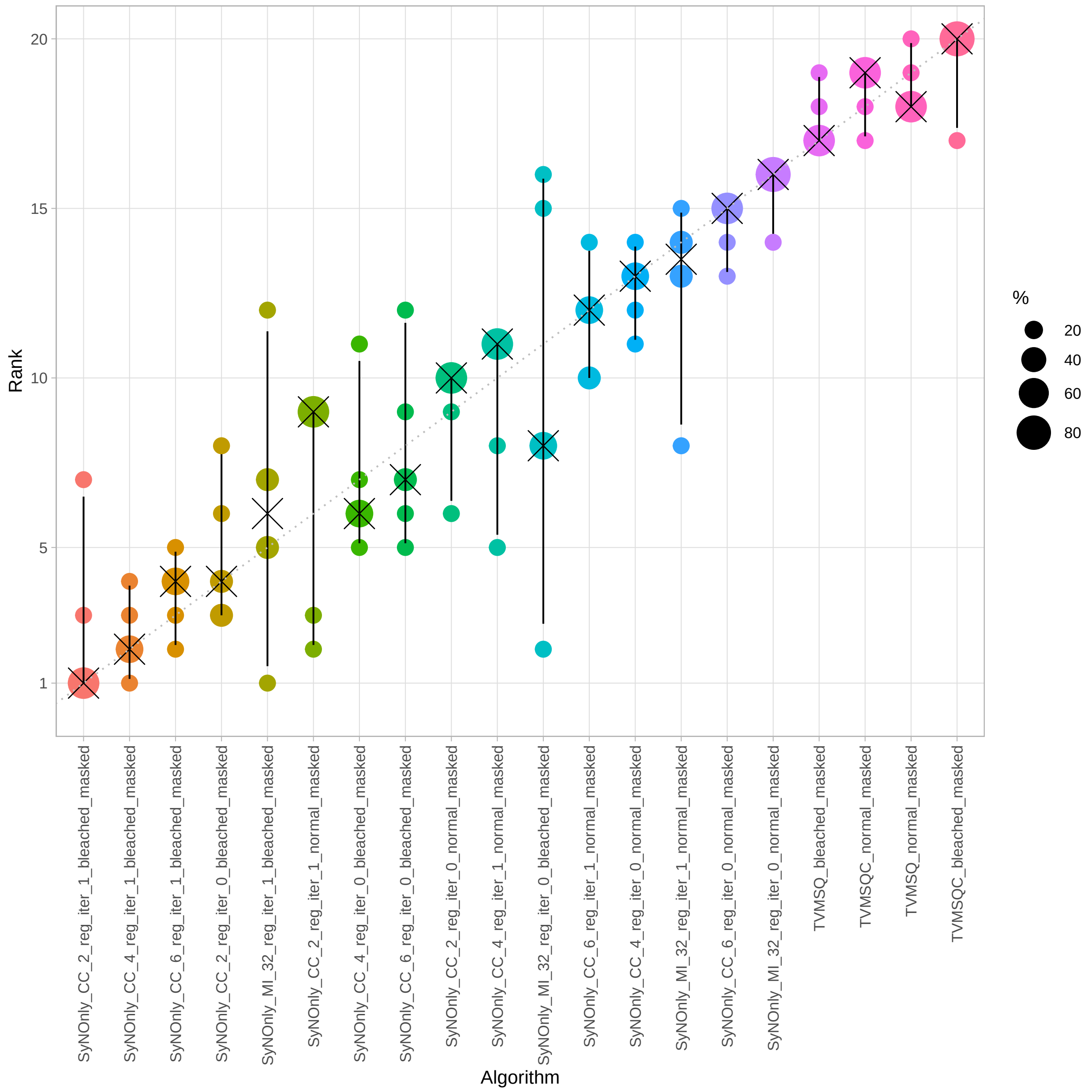

4.1.2 Ranking stability: Ranking variability via bootstrap approach

A blob plot of bootstrap results over the different tasks separated by algorithm allows another perspective on the assessment data. This gives deeper insights into the characteristics of tasks and the ranking uncertainty of the algorithms in each task.

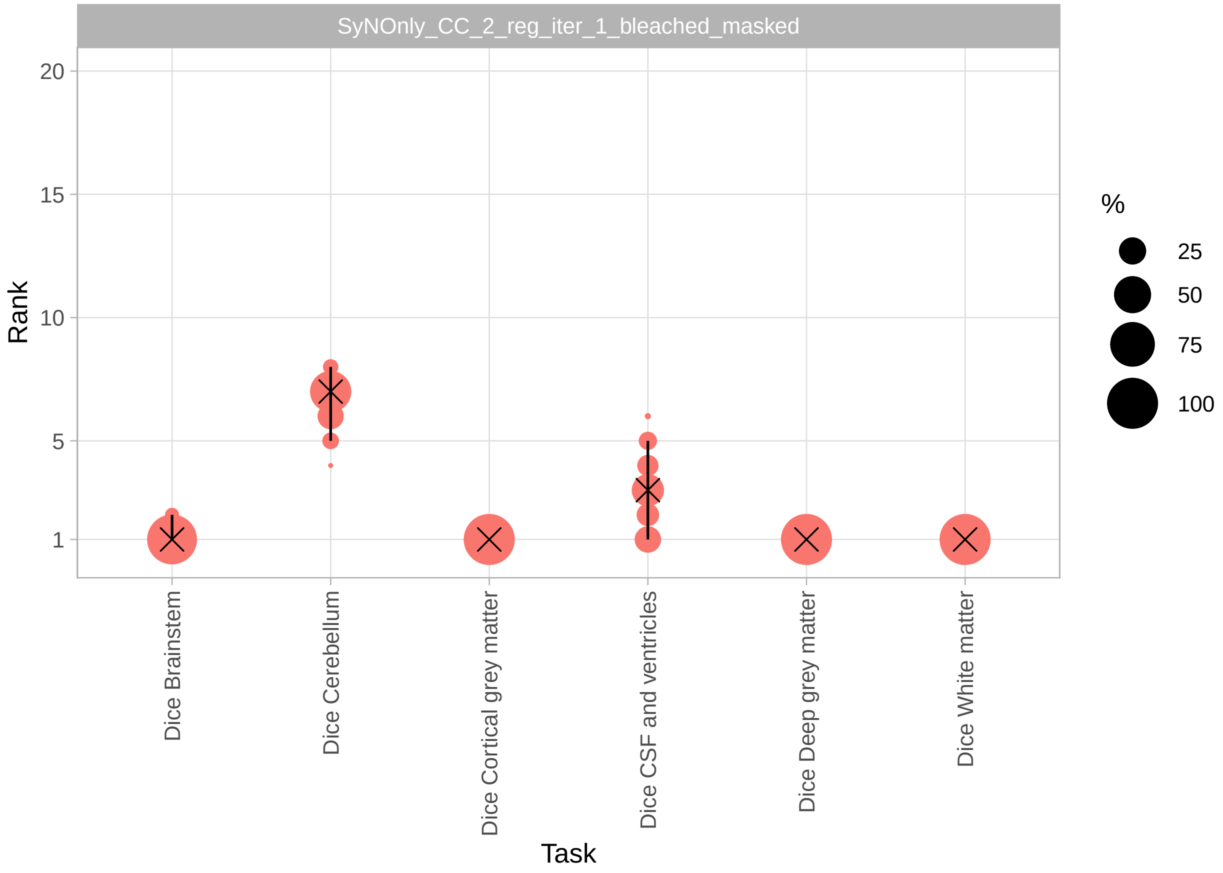

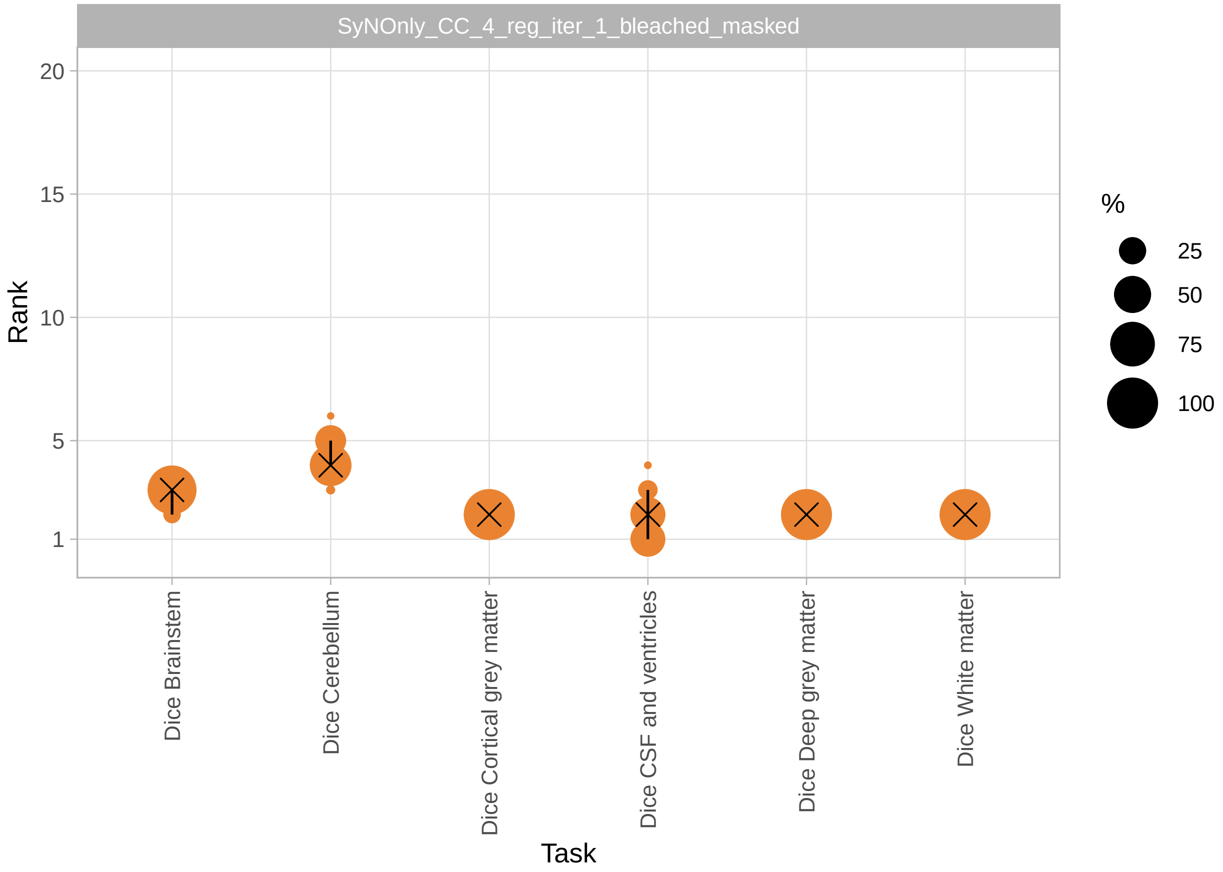
SyNOnly_CC_4_reg_iter_1_bleached_masked
Rank
20
15
10
5
1
Dice Brainstem
Dice Cerebellum
Dice Cortical grey matter
Dice CSF and ventricles
Dice Deep grey matter
Dice White matter
Task
%
25
50
75
100

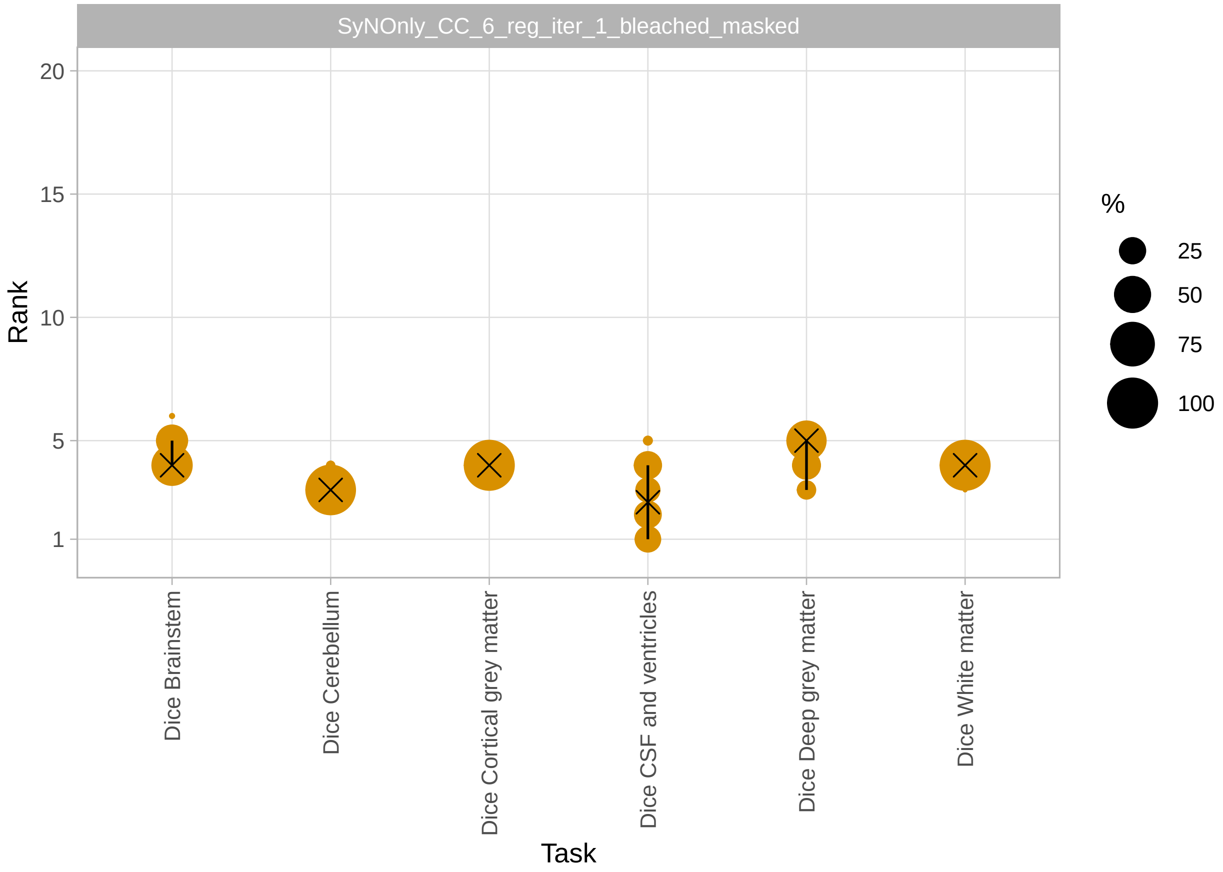
SyNOnly_CC_6_reg_iter_1_bleached_masked
Rank
20
15
10
5
1
Dice Brainstem
Dice Cerebellum
Dice Cortical grey matter
Dice CSF and ventricles
Dice Deep grey matter
Dice White matter
Task
%
25
50
75
100

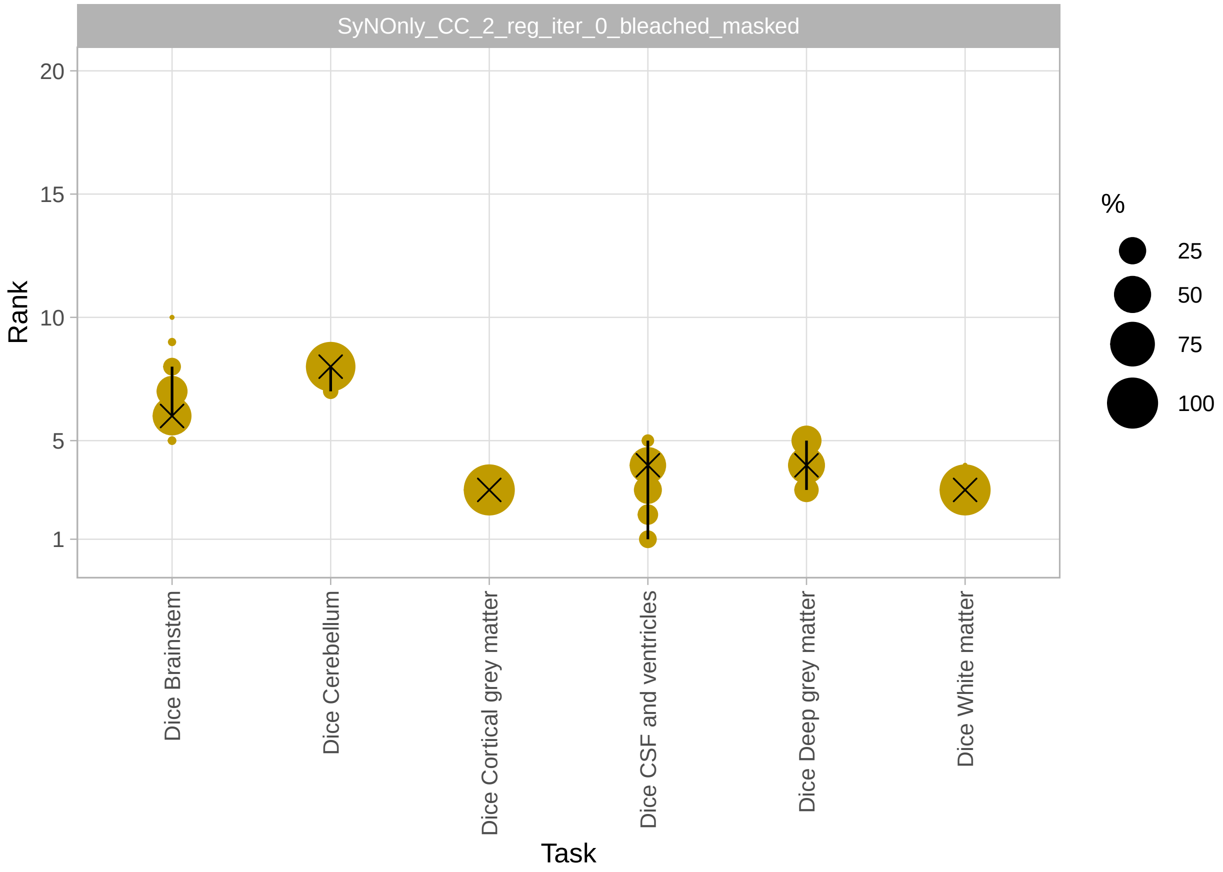

SyNOnly_CC_2_reg_iter_0_bleached_masked
Rank
20
15
10
5
1
Dice Brainstem
Dice Cerebellum
Dice Cortical grey matter
Dice CSF and ventricles
Dice Deep grey matter
Dice White matter
Task
%
25
50
75
100

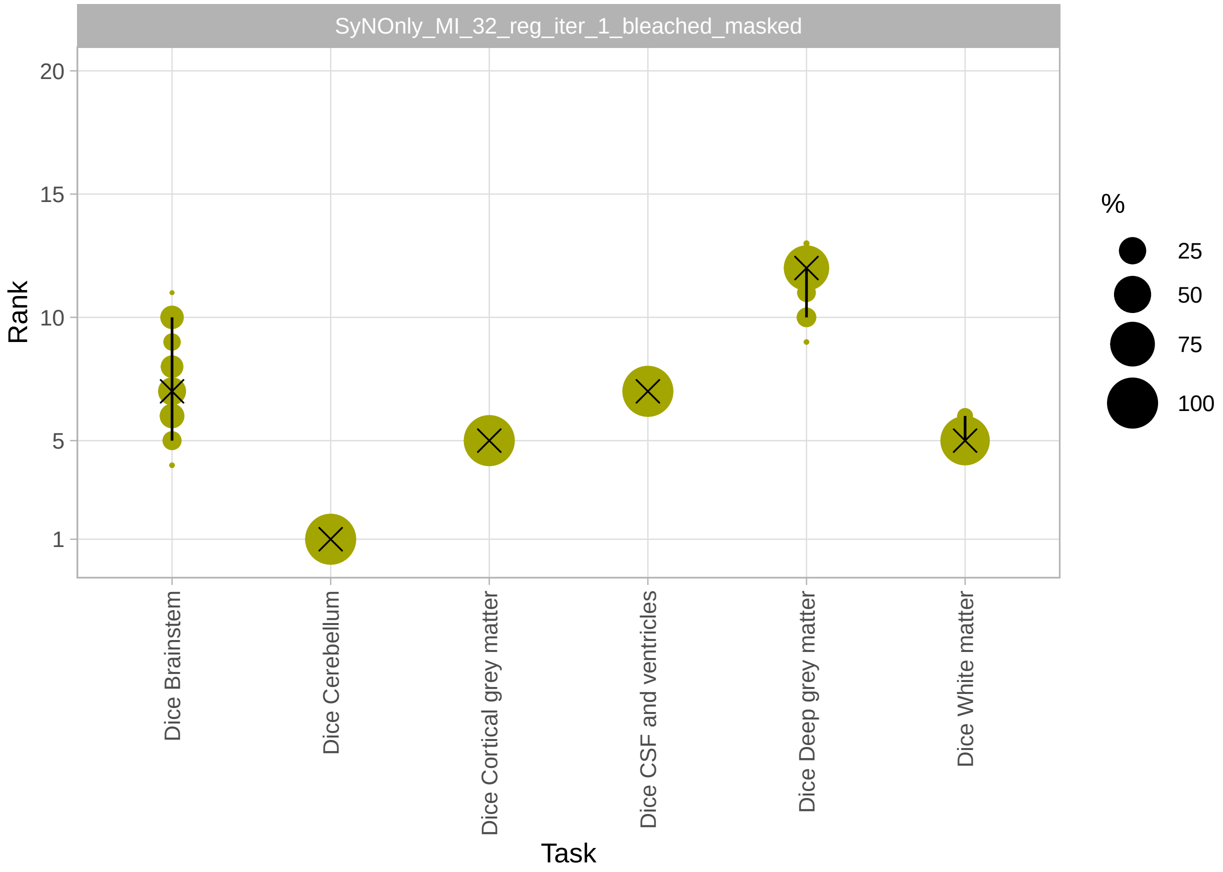

SyNOnly_MI_32_reg_iter_1_bleached_masked
Rank
20
15
10
5
1
Dice Brainstem
Dice Cerebellum
Dice Cortical grey matter
Dice CSF and ventricles
Dice Deep grey matter
Dice White matter
Task
%
25
50
75
100

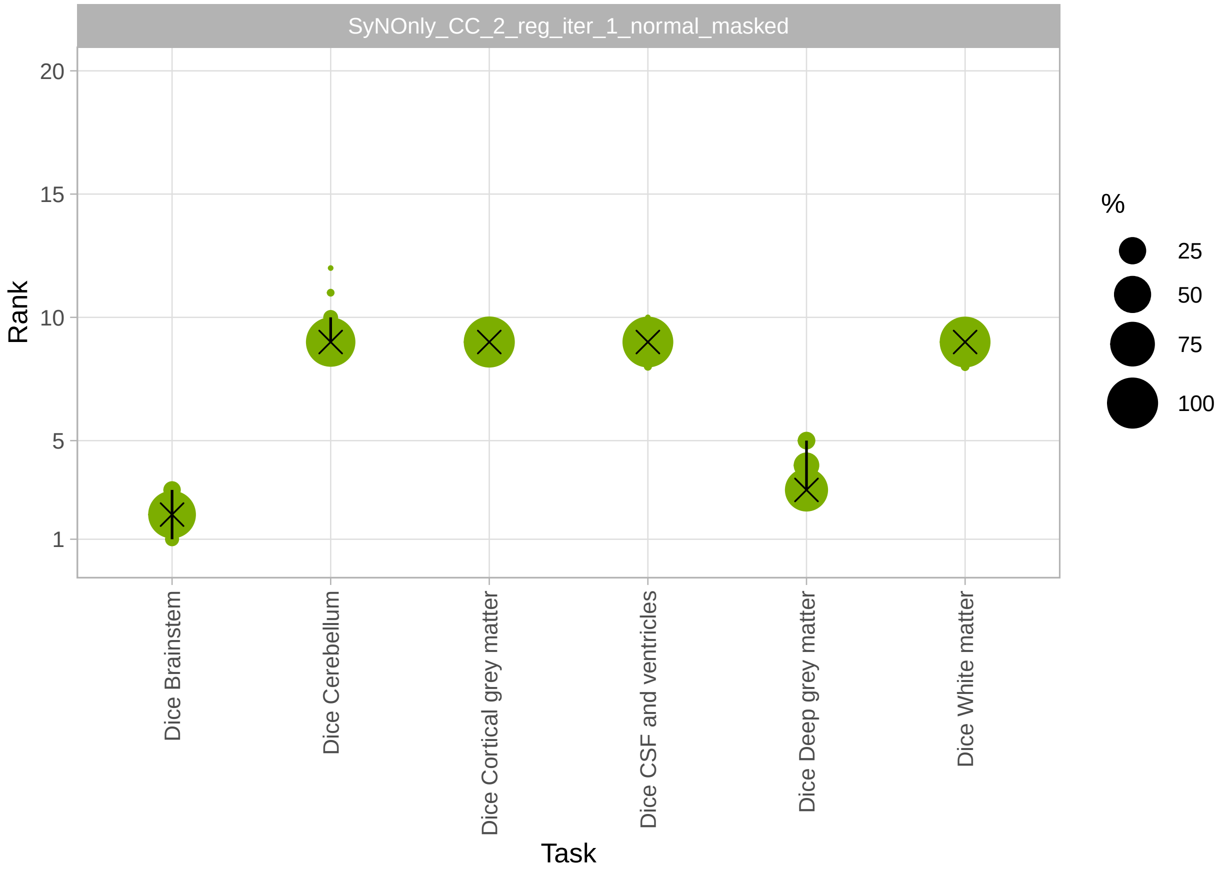
SyNOnly_CC_2_reg_iter_1_normal_masked
Rank
20
15
10
5
1
Dice Brainstem
Dice Cerebellum
Dice Cortical grey matter
Dice CSF and ventricles
Dice Deep grey matter
Dice White matter
Task
%
25
50
75
100

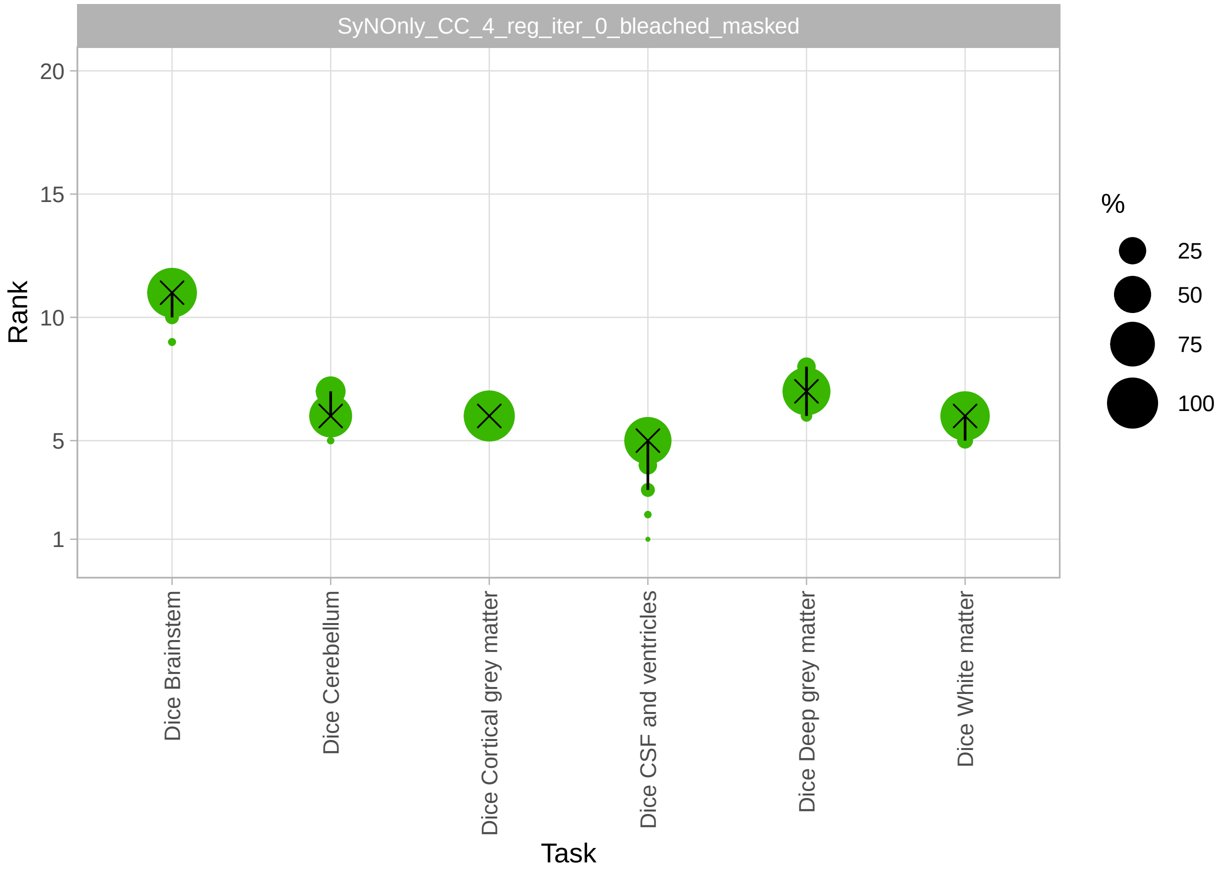
SyNOnly_CC_4_reg_iter_0_bleached_masked
Rank
20
15
10
5
1
Dice Brainstem
Dice Cerebellum
Dice Cortical grey matter
Dice CSF and ventricles
Dice Deep grey matter
Dice White matter
Task
%
25
50
75
100

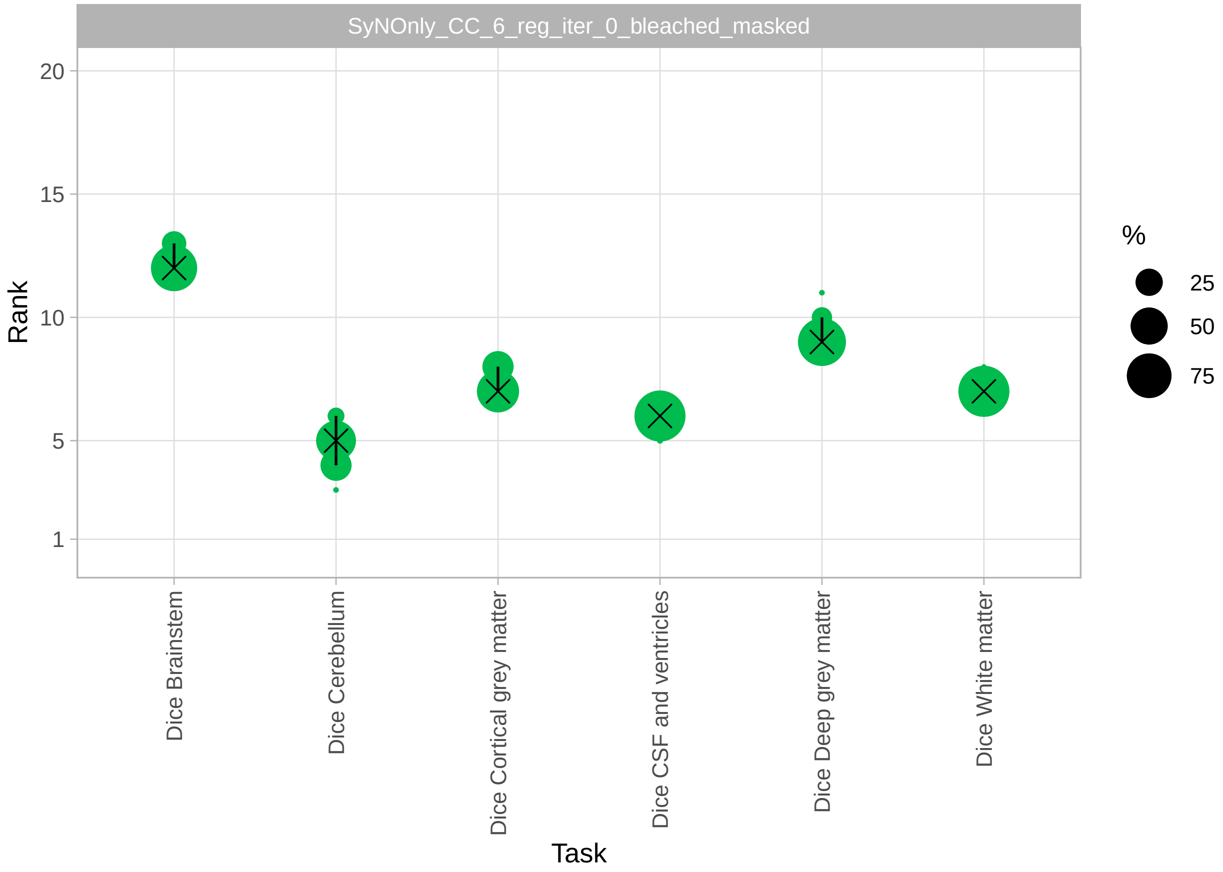
SyNOnly_CC_6_reg_iter_0_bleached_masked
Rank
20
15
10
5
1
%
25
50
75
Dice Brainstem
Dice Cerebellum
Dice Cortical grey matter
Dice CSF and ventricles
Dice Deep grey matter
Dice White matter
Task

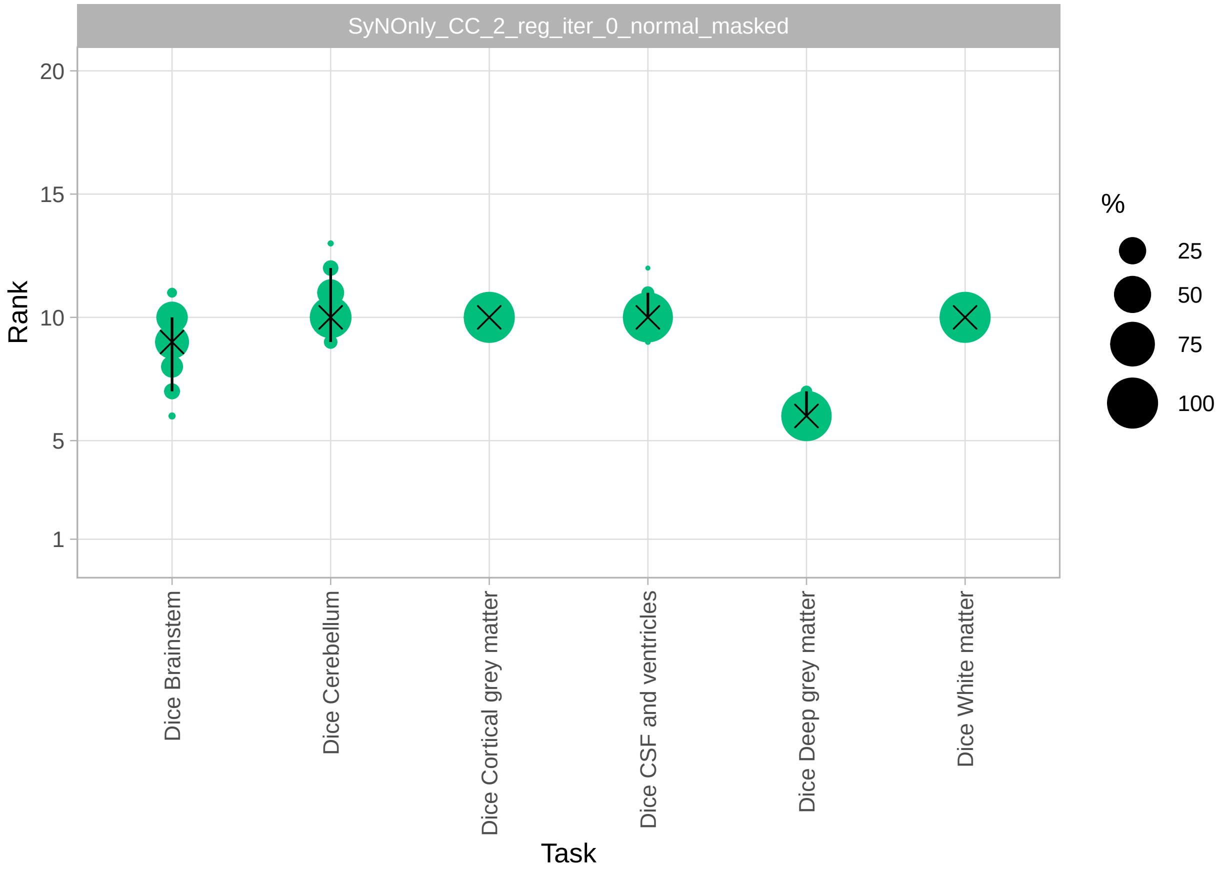
SyNOnly_CC_2_reg_iter_0_normal_masked
Rank
20
15
10
5
1
Dice Brainstem
Dice Cerebellum
Dice Cortical grey matter
Dice CSF and ventricles
Dice Deep grey matter
Dice White matter
Task
%
25
50
75
100

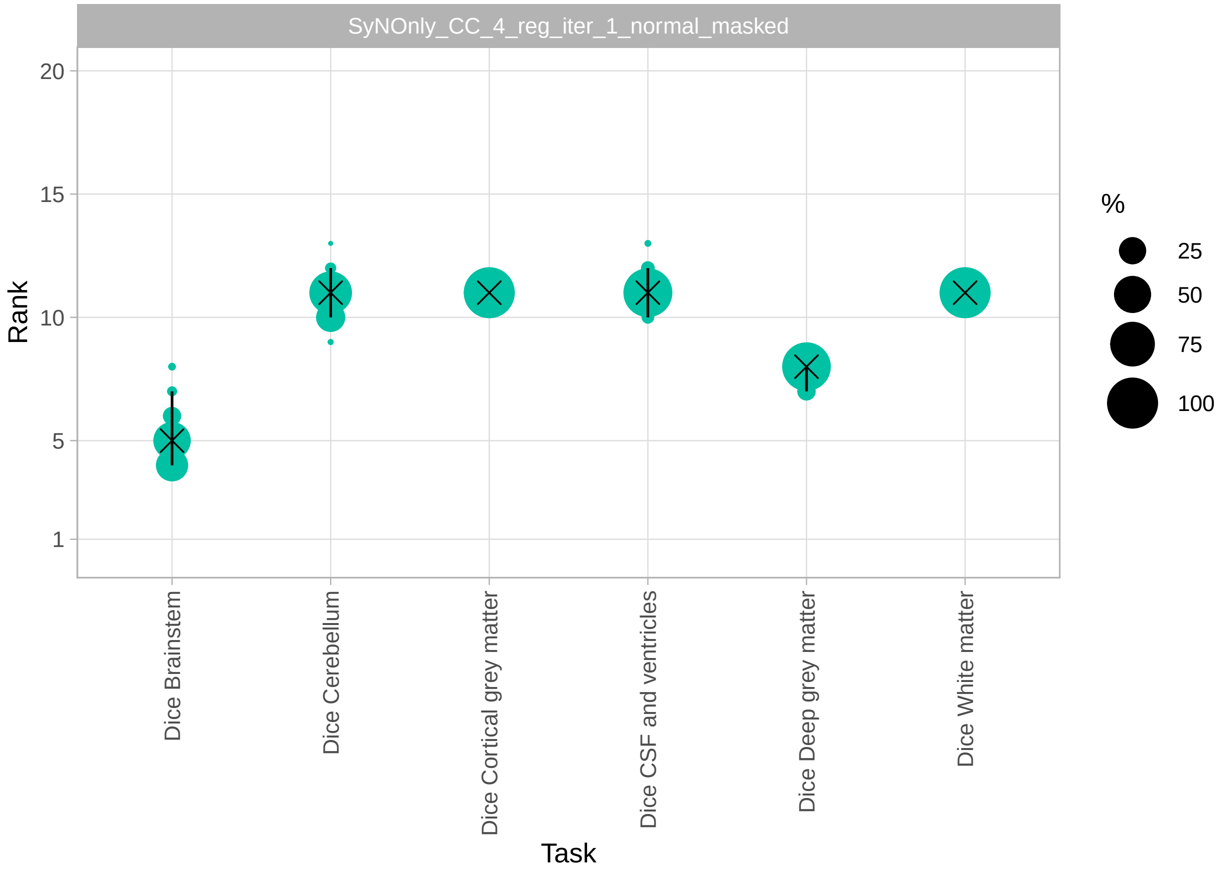

SyNOnly_CC_4_reg_iter_1_normal_masked
Rank
20
15
10
5
1
Dice Brainstem
Dice Cerebellum
Dice Cortical grey matter
Dice CSF and ventricles
Dice Deep grey matter
Dice White matter
Task
%
25
50
75
100

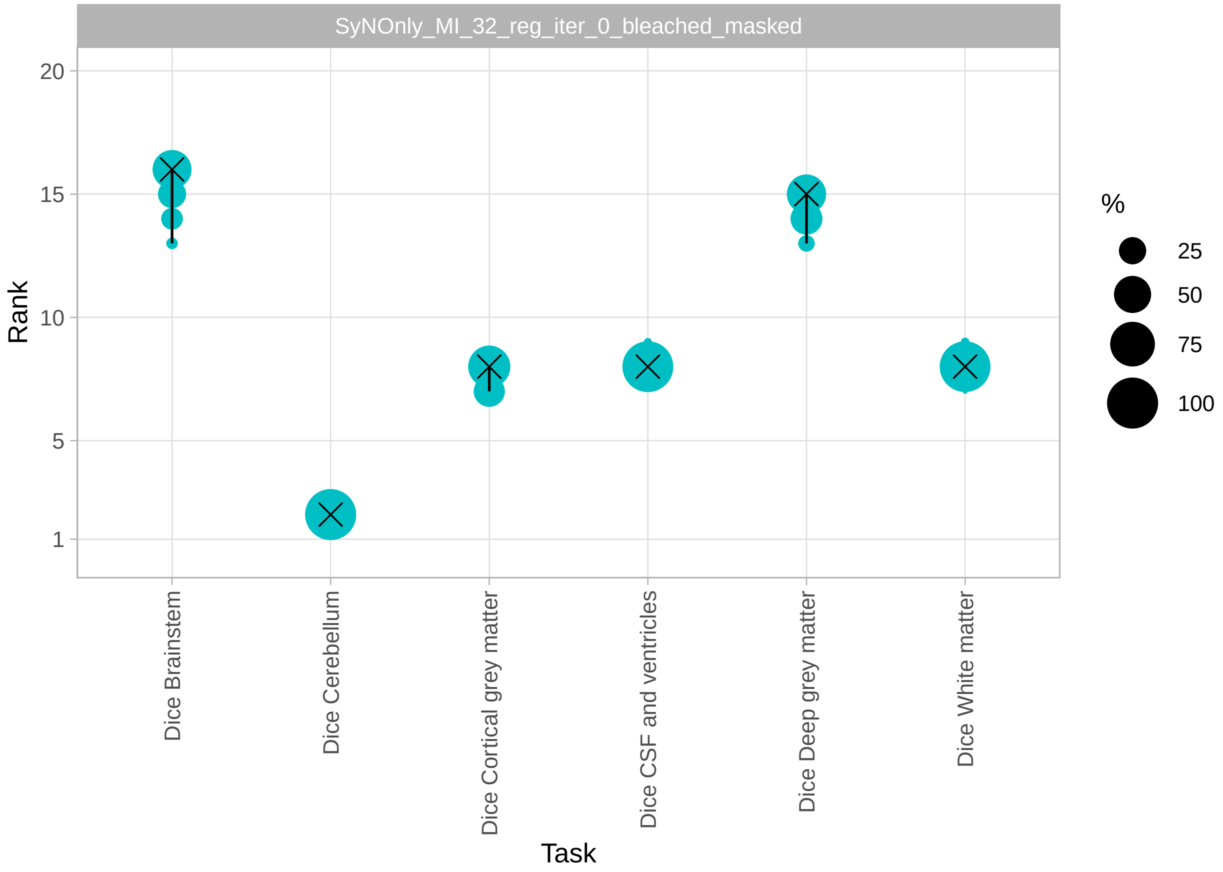
SyNOnly_MI_32_reg_iter_0_bleached_masked
Rank
20
15
10
5
1
%
25
50
75
100
Dice Brainstem
Dice Cerebellum
Dice Cortical grey matter
Dice CSF and ventricles
Dice Deep grey matter
Dice White matter
Task

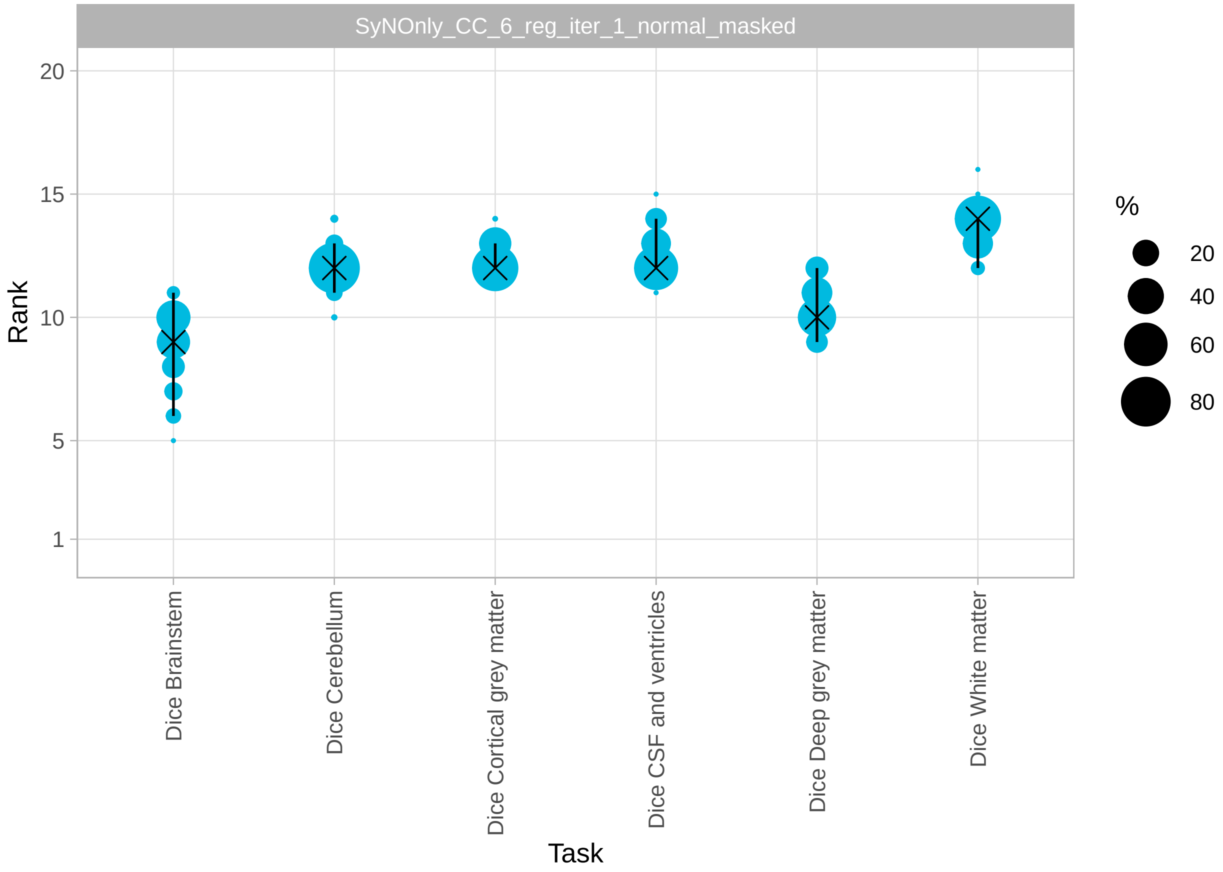
SyNOnly_CC_6_reg_iter_1_normal_masked
Rank
20
15
10
5
1
%
20
40
60
80
Dice Brainstem
Dice Cerebellum
Dice Cortical grey matter
Dice CSF and ventricles
Dice Deep grey matter
Dice White matter
Task

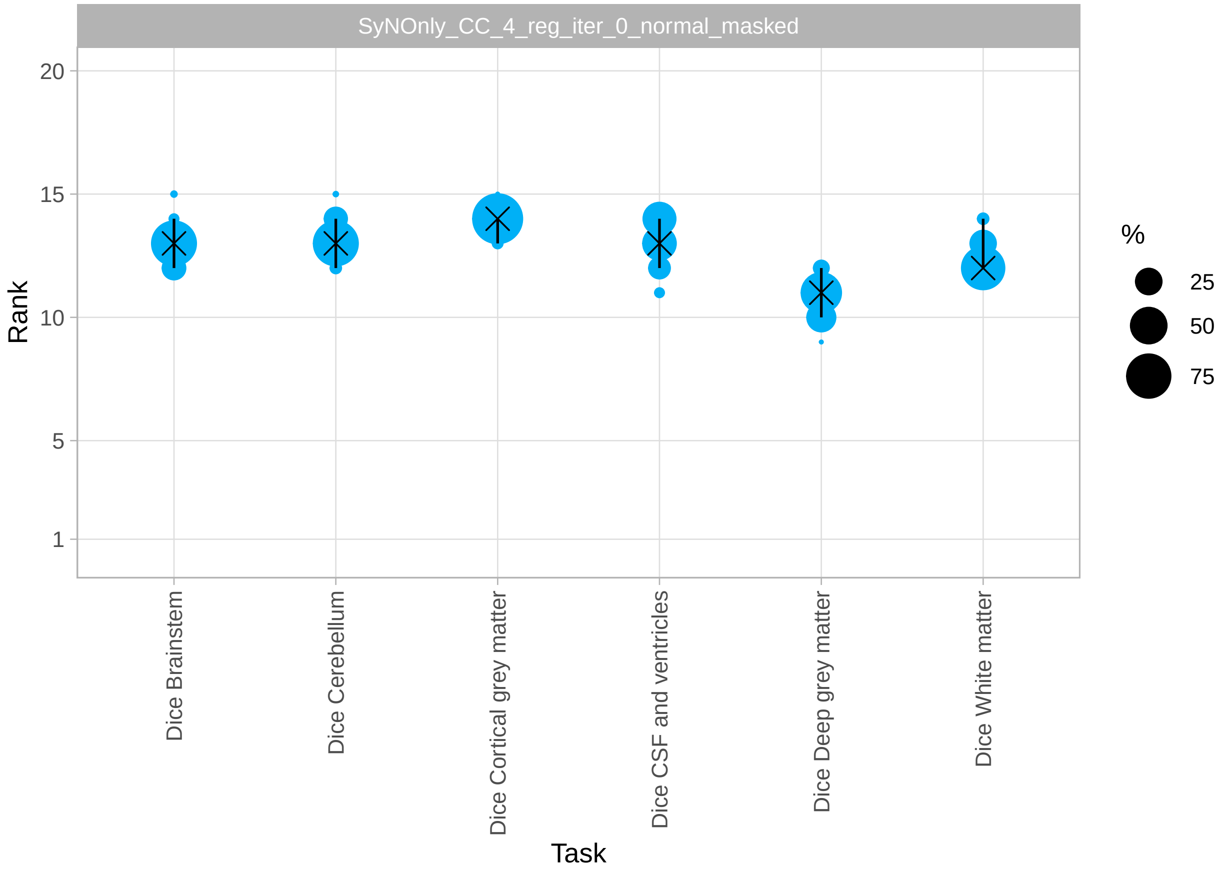
SyNOnly_CC_4_reg_iter_0_normal_masked
Rank
20
15
10
5
1
Dice Brainstem
Dice Cerebellum
Dice Cortical grey matter
Dice CSF and ventricles
Dice Deep grey matter
Dice White matter
Task
%
25
50
75

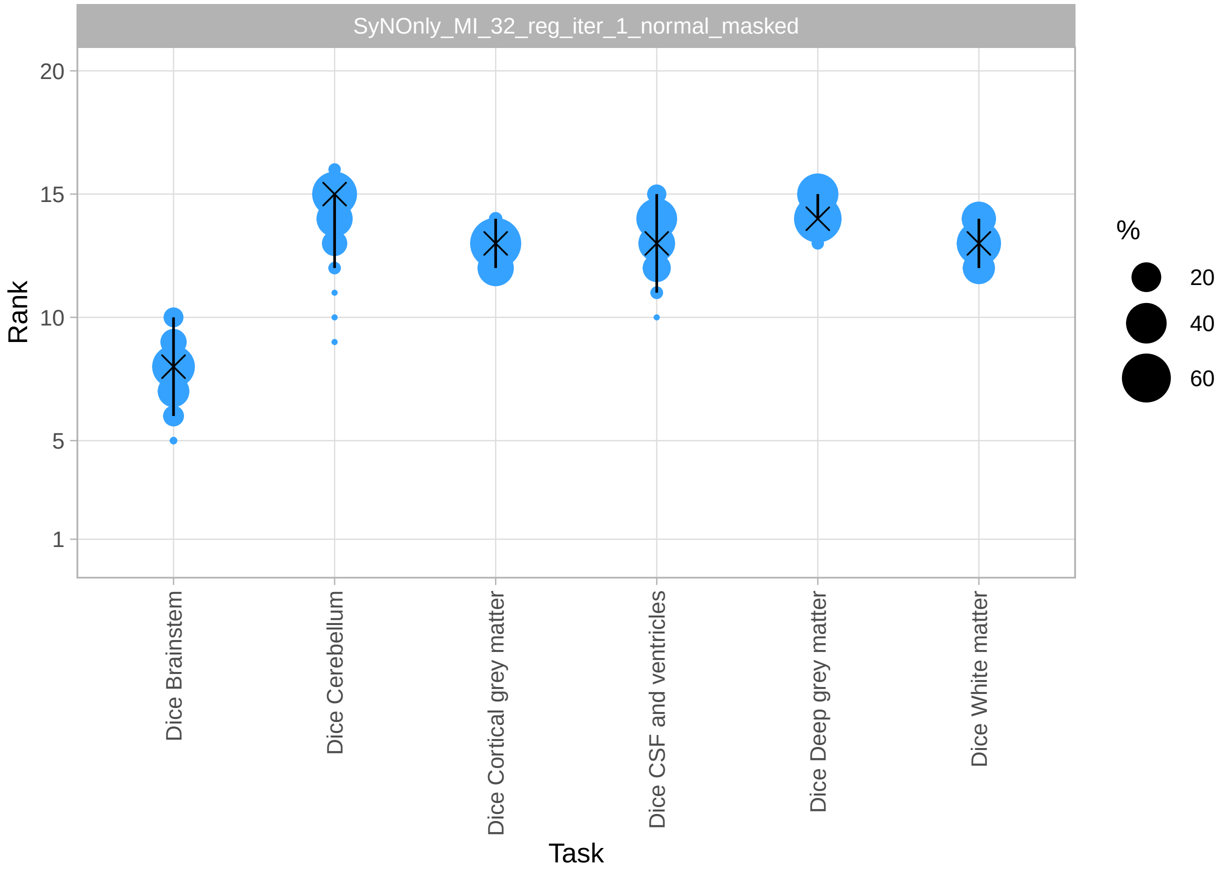
SyNOnly_MI_32_reg_iter_1_normal_masked
Rank
20
15
10
5
1
Dice Brainstem
Dice Cerebellum
Dice Cortical grey matter
Dice CSF and ventricles
Dice Deep grey matter
Dice White matter
Task
%
20
40
60

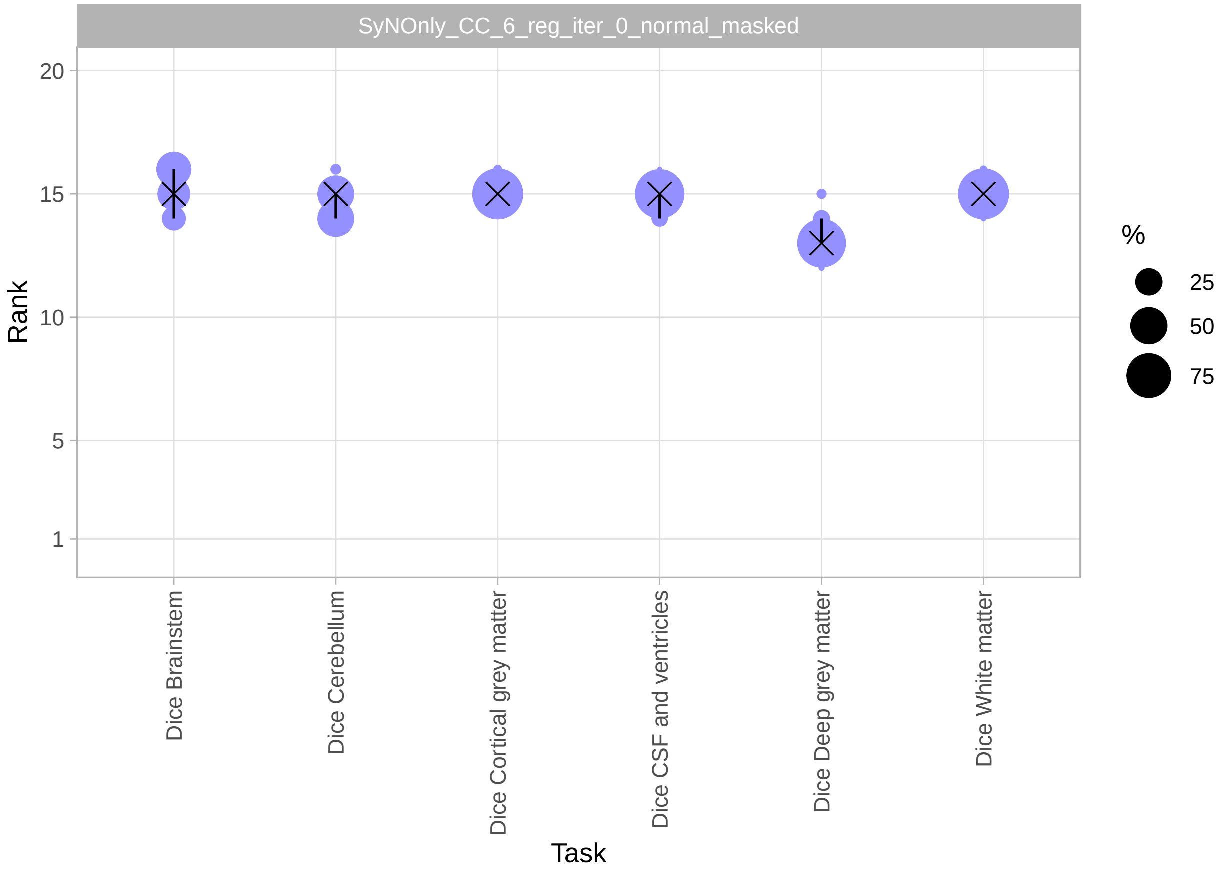

SyNOnly_CC_6_reg_iter_0_normal_masked
Rank
20
15
10
5
1
Dice Brainstem
Dice Cerebellum
Dice Cortical grey matter
Dice CSF and ventricles
Dice Deep grey matter
Dice White matter
Task
%
25
50
75

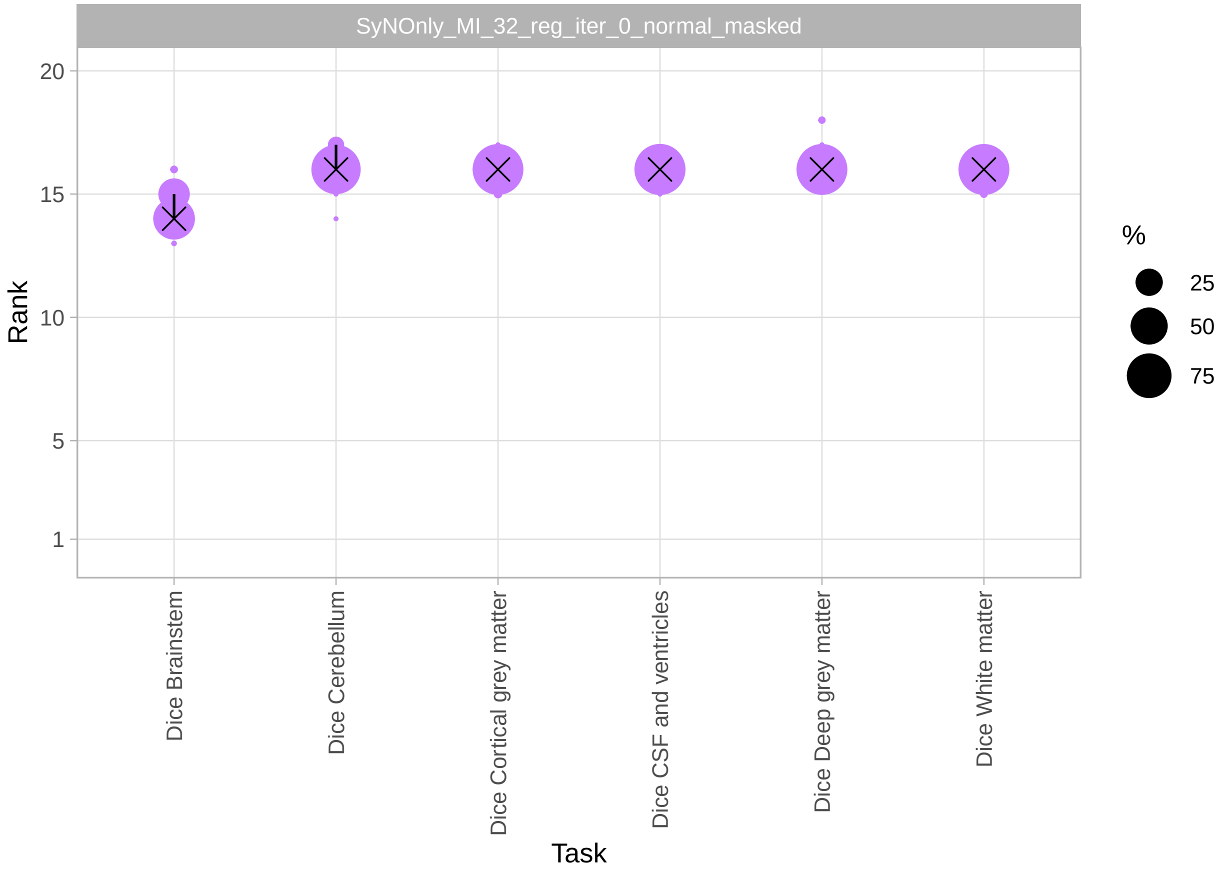
SyNOnly_MI_32_reg_iter_0_normal_masked
Rank
20
15
10
5
1
Dice Brainstem
Dice Cerebellum
Dice Cortical grey matter
Dice CSF and ventricles
Dice Deep grey matter
Dice White matter
Task
%
25
50
75

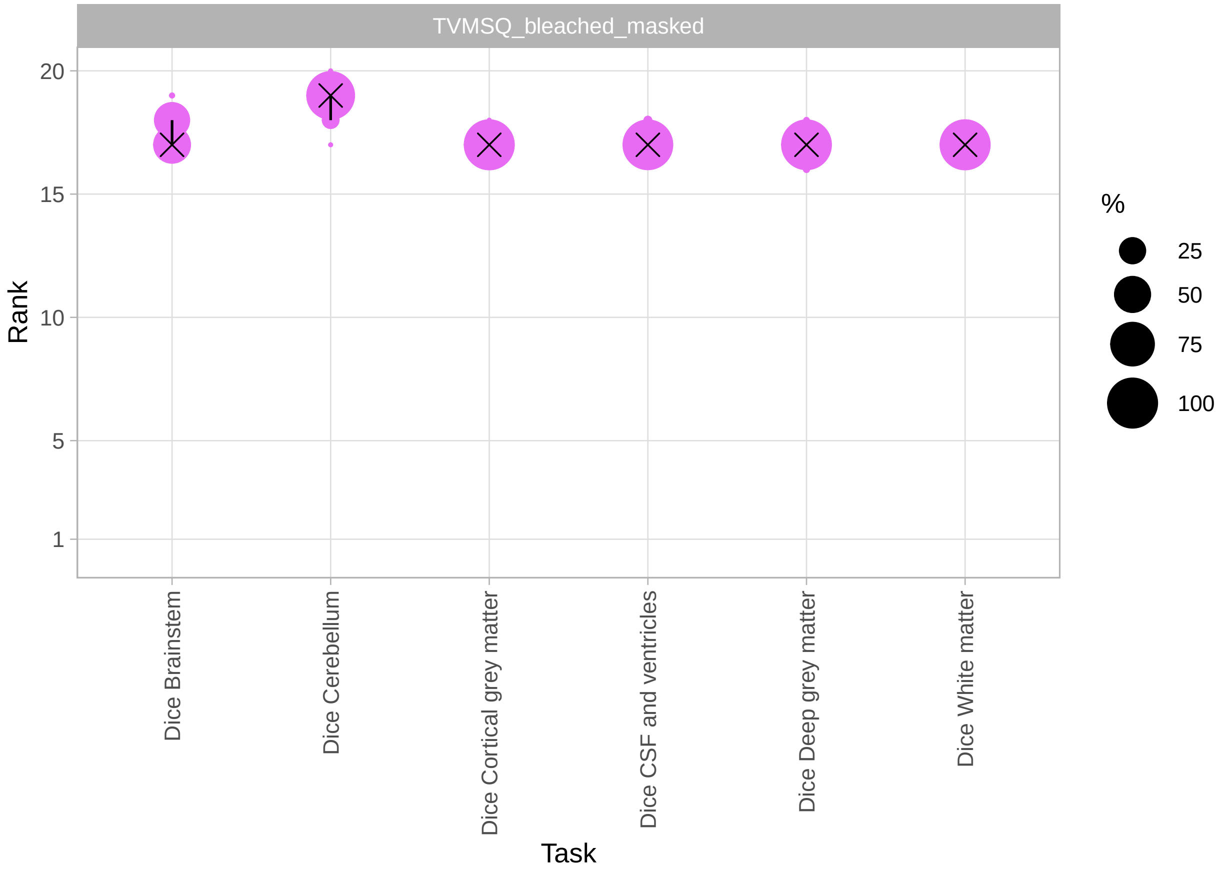

TVMSQ_bleached_masked
Rank
20
15
10
5
1
%
25
50
75
100
Dice Brainstem
Dice Cerebellum
Dice Cortical grey matter
Dice CSF and ventricles
Dice Deep grey matter
Dice White matter
Task

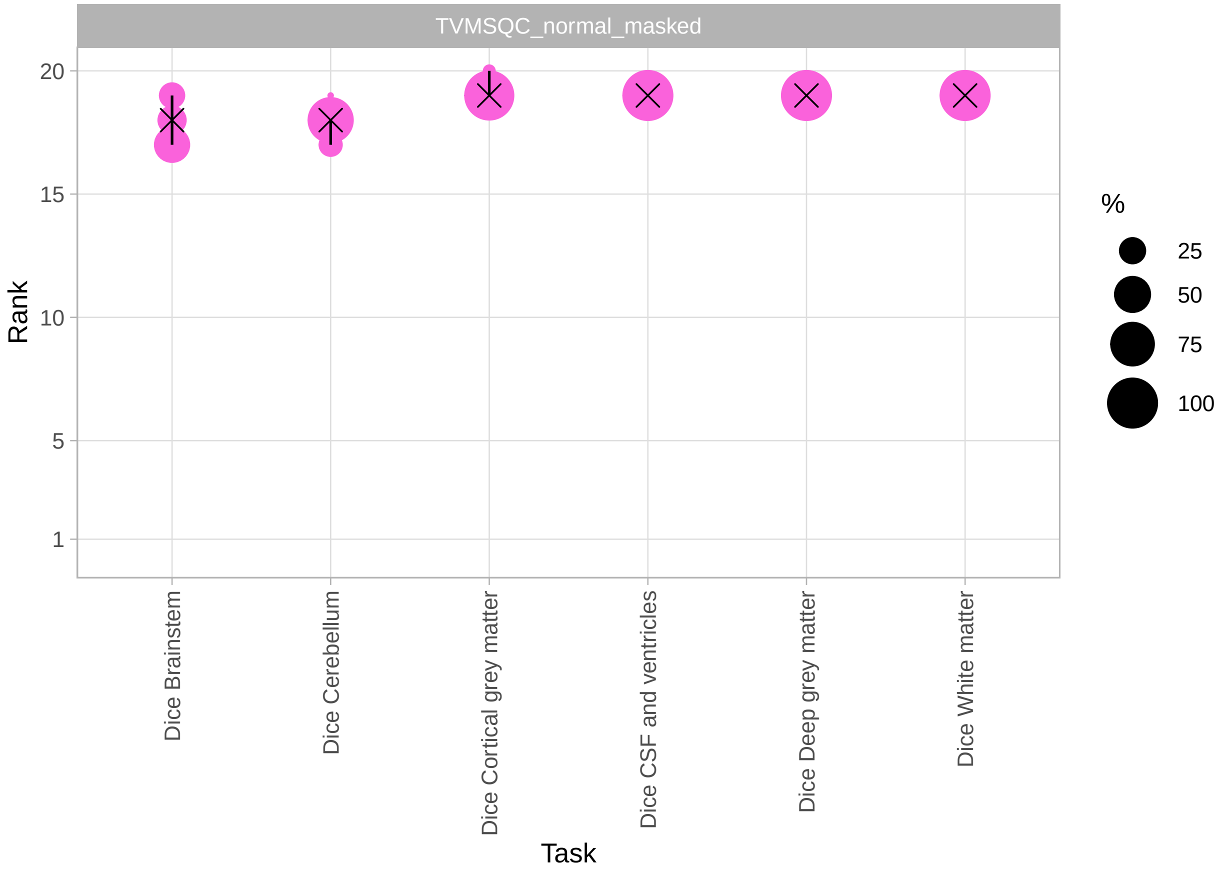

TVMSQC_normal_masked
Rank
20
15
10
5
1
Dice Brainstem
Dice Cerebellum
Dice Cortical grey matter
Dice CSF and ventricles
Dice Deep grey matter
Dice White matter
Task
%
25
50
75
100

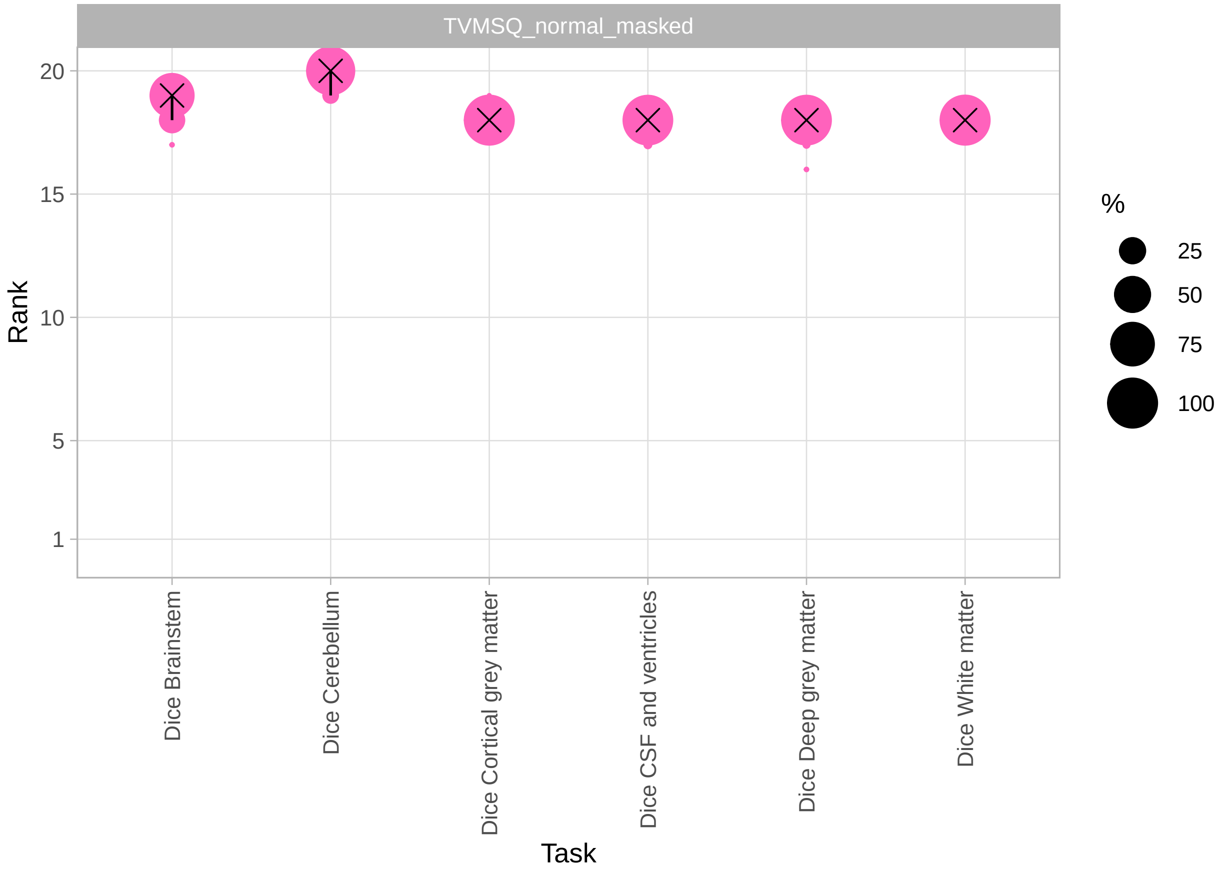
TVMSQ_normal_masked
Rank
20
15
10
5
1
Dice Brainstem
Dice Cerebellum
Dice Cortical grey matter
Dice CSF and ventricles
Dice Deep grey matter
Dice White matter
Task
%
25
50
75
100

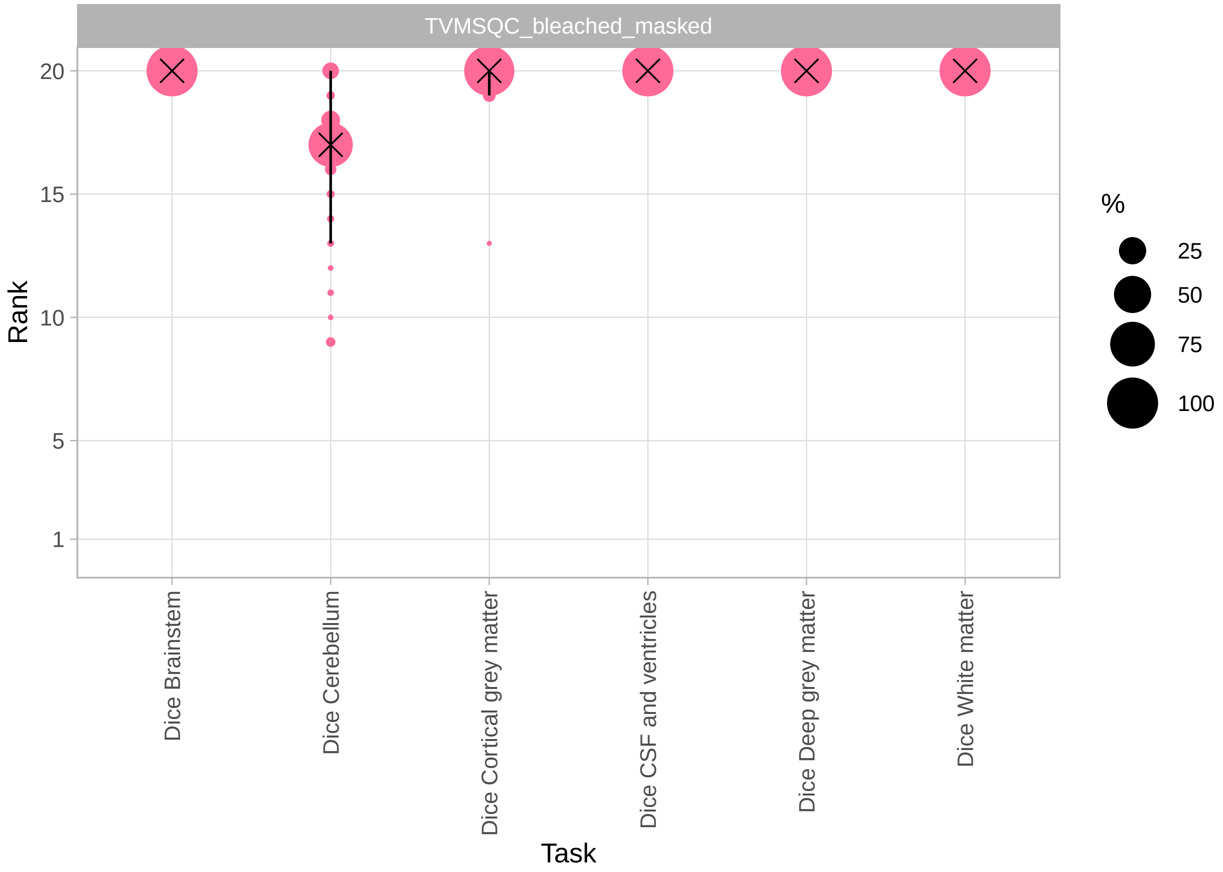

TVMSQC_bleached_masked
Rank
20
15
10
5
1
Dice Brainstem
Dice Cerebellum
Dice Cortical grey matter
Dice CSF and ventricles
Dice Deep grey matter
Dice White matter
Task
%
25
50
75
100

An alternative representation is provided by a stacked frequency plot of the observed ranks, separated by algorithm. Observed ranks across bootstrap samples are displayed with coloring according to the task. For algorithms that achieve the same rank in different tasks for the full assessment data set, vertical lines are on top of each other. Vertical lines allow to compare the achieved rank of each algorithm over different tasks.

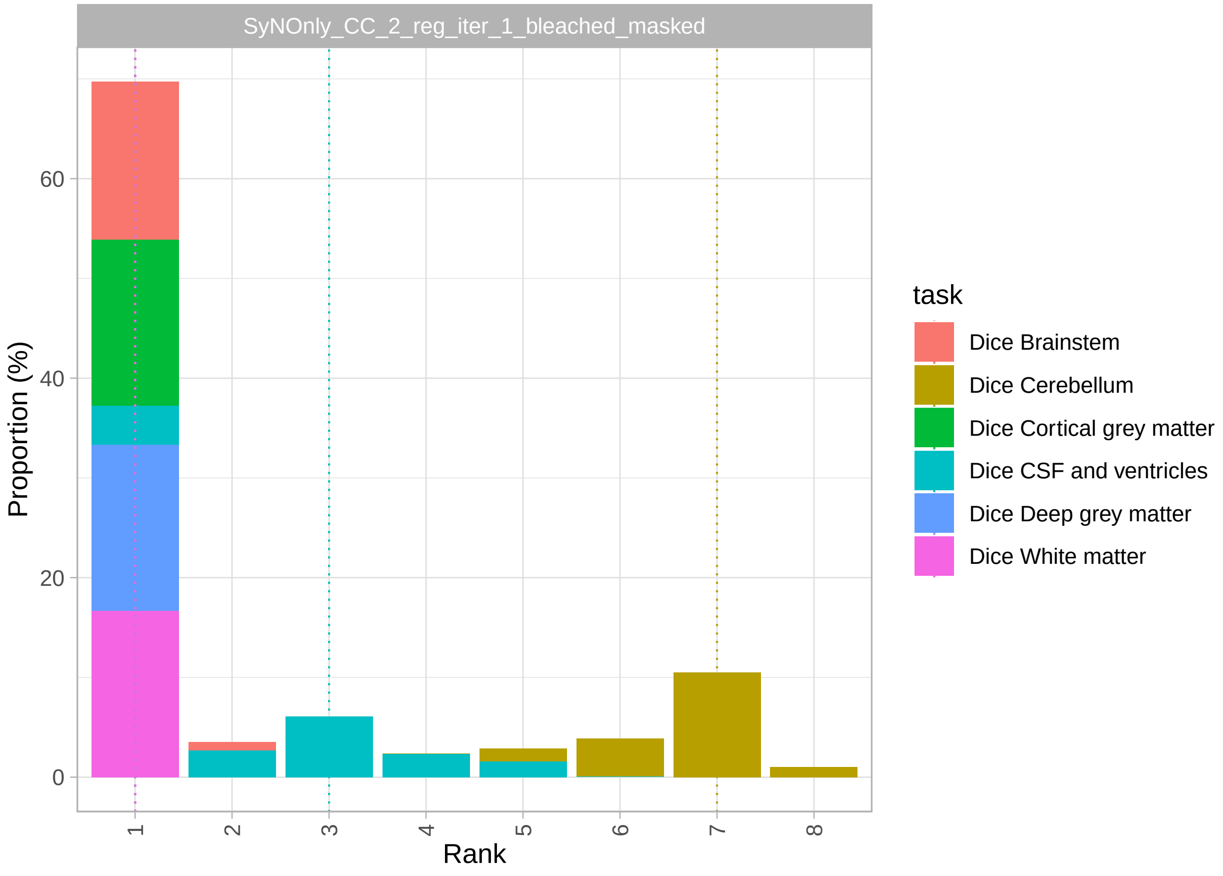

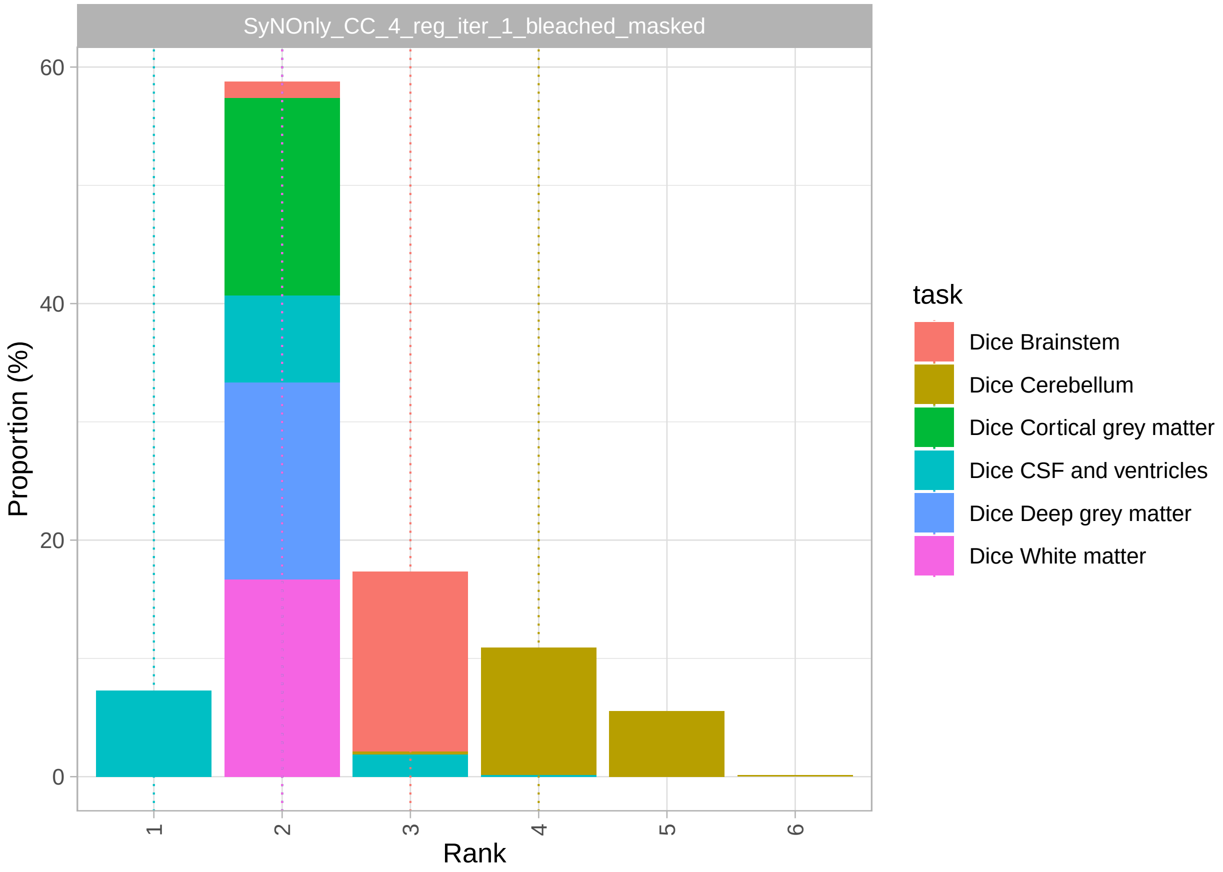

SyNOnly_CC_4_reg_iter_1_bleached_masked
Proportion (%)
60
40
20
0
1
2
3
4
5
6
Rank
task
Dice Brainstem
Dice Cerebellum
Dice Cortical grey matter
Dice CSF and ventricles
Dice Deep grey matter
Dice White matter

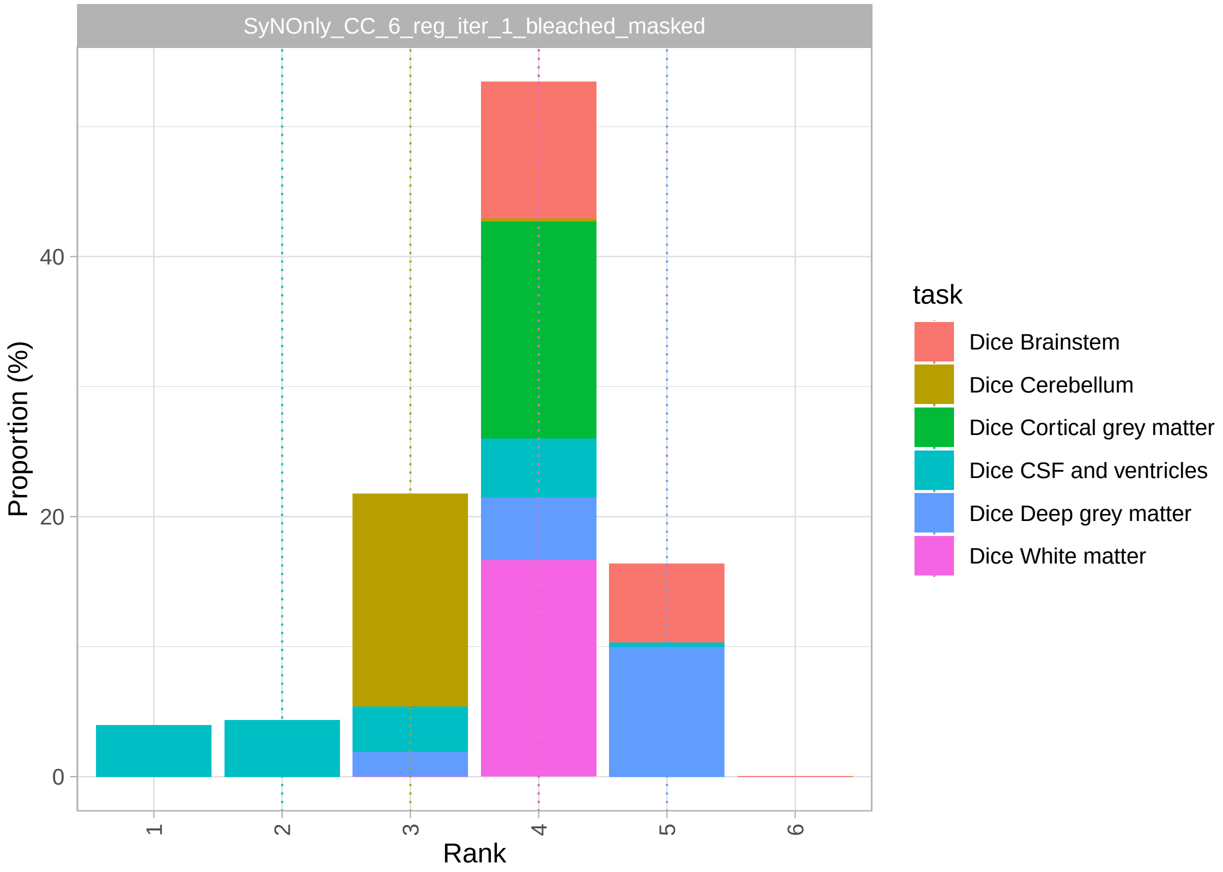
SyNOnly_CC_6_reg_iter_1_bleached_masked
Proportion (%)
0
20
40
Rank
1
2
3
4
5
6
task
Dice Brainstem
Dice Cerebellum
Dice Cortical grey matter
Dice CSF and ventricles
Dice Deep grey matter
Dice White matter

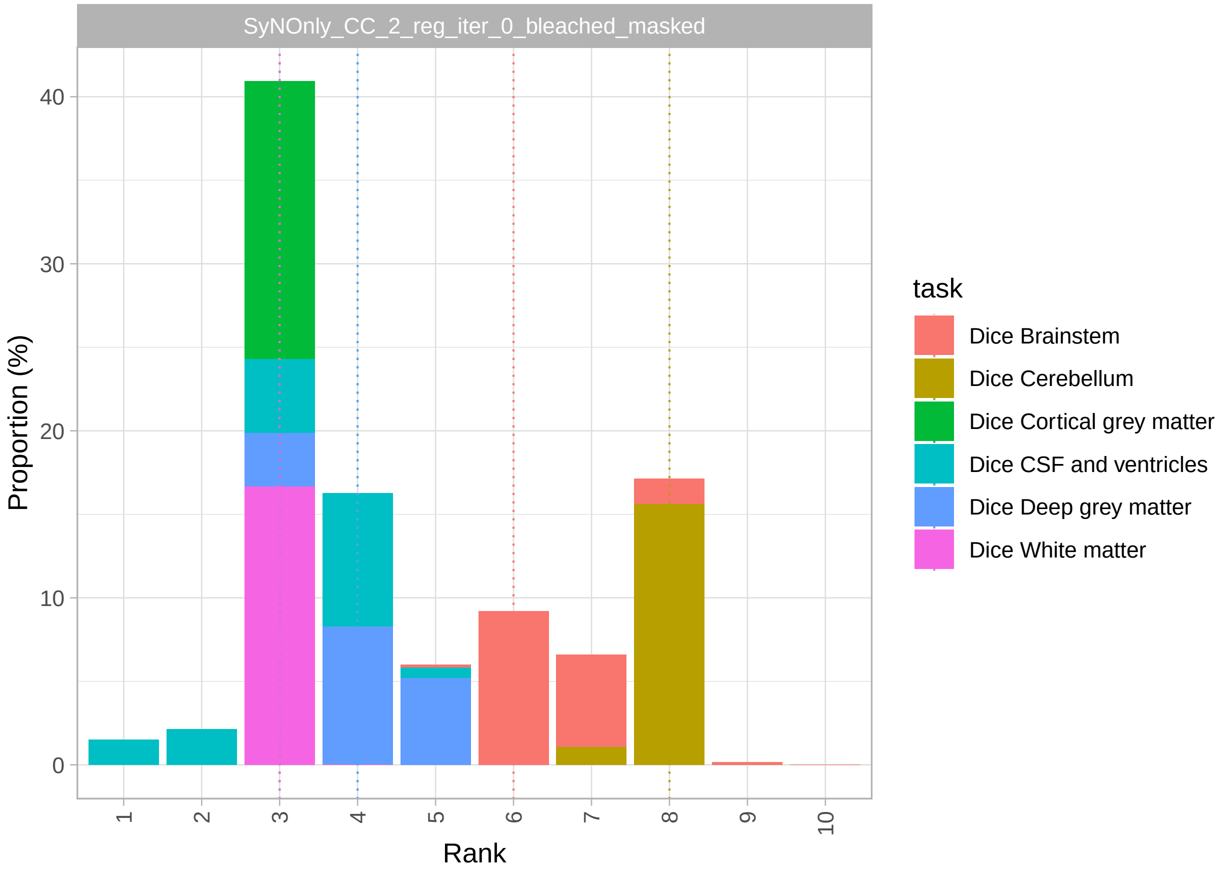
SyNOnly_CC_2_reg_iter_0_bleached_masked
Proportion (%)
0
10
20
30
40
1
2
3
4
5
6
7
8
9
10
Rank
task
Dice Brainstem
Dice Cerebellum
Dice Cortical grey matter
Dice CSF and ventricles
Dice Deep grey matter
Dice White matter

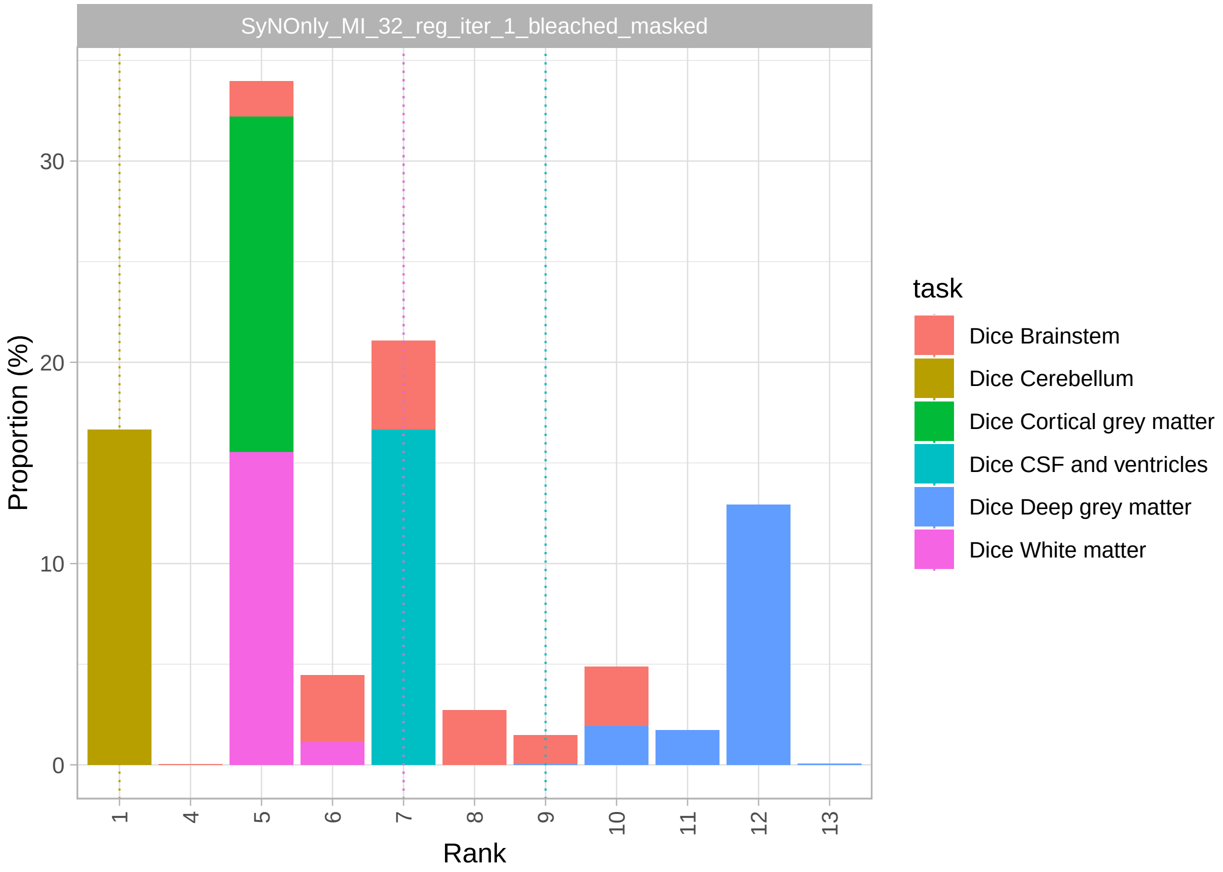
SyNOnly_MI_32_reg_iter_1_bleached_masked
Proportion (%)
0
10
20
30
Rank
1
4
5
6
7
8
9
10
11
12
13
task
Dice Brainstem
Dice Cerebellum
Dice Cortical grey matter
Dice CSF and ventricles
Dice Deep grey matter
Dice White matter

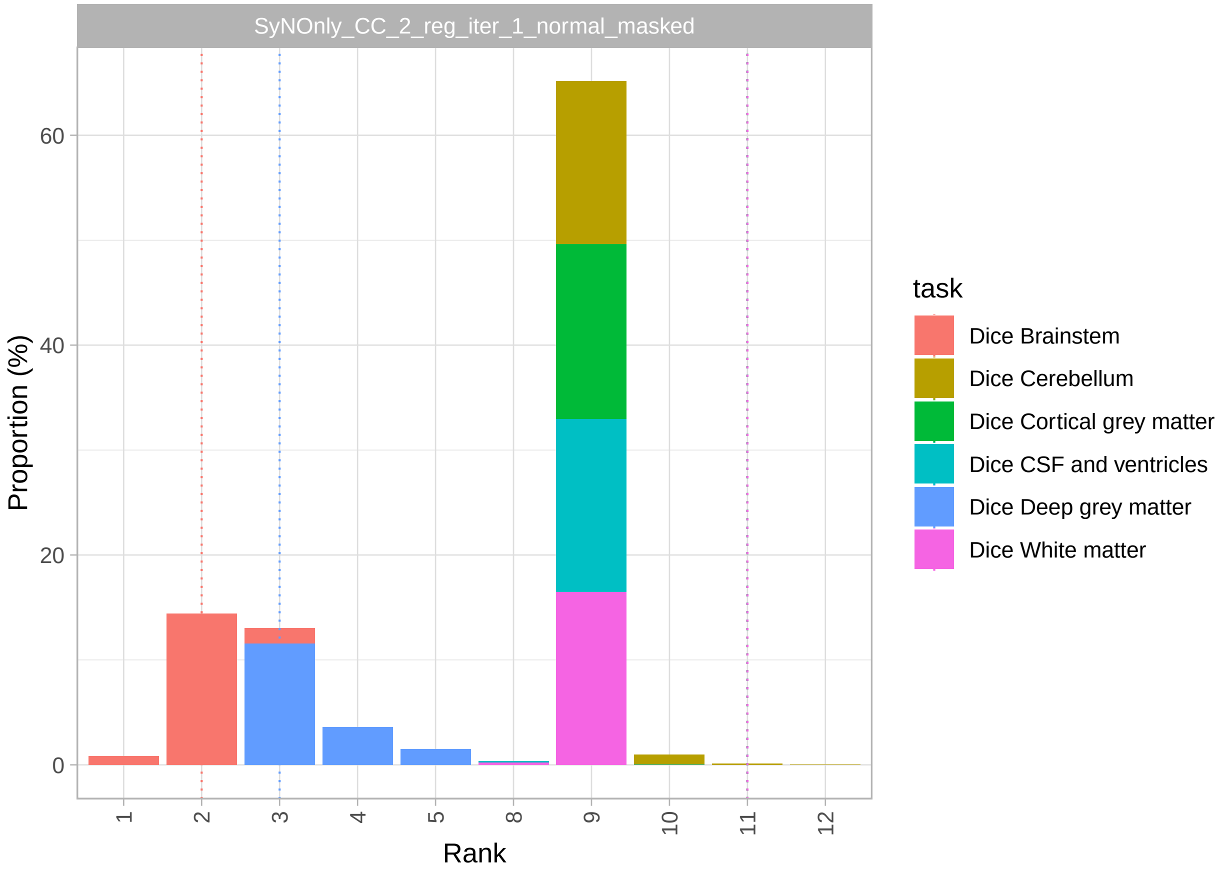

SyNOnly_CC_2_reg_iter_1_normal_masked
Proportion (%)
60
40
20
0
1
2
3
4
5
8
9
10
11
12
Rank
task
Dice Brainstem
Dice Cerebellum
Dice Cortical grey matter
Dice CSF and ventricles
Dice Deep grey matter
Dice White matter

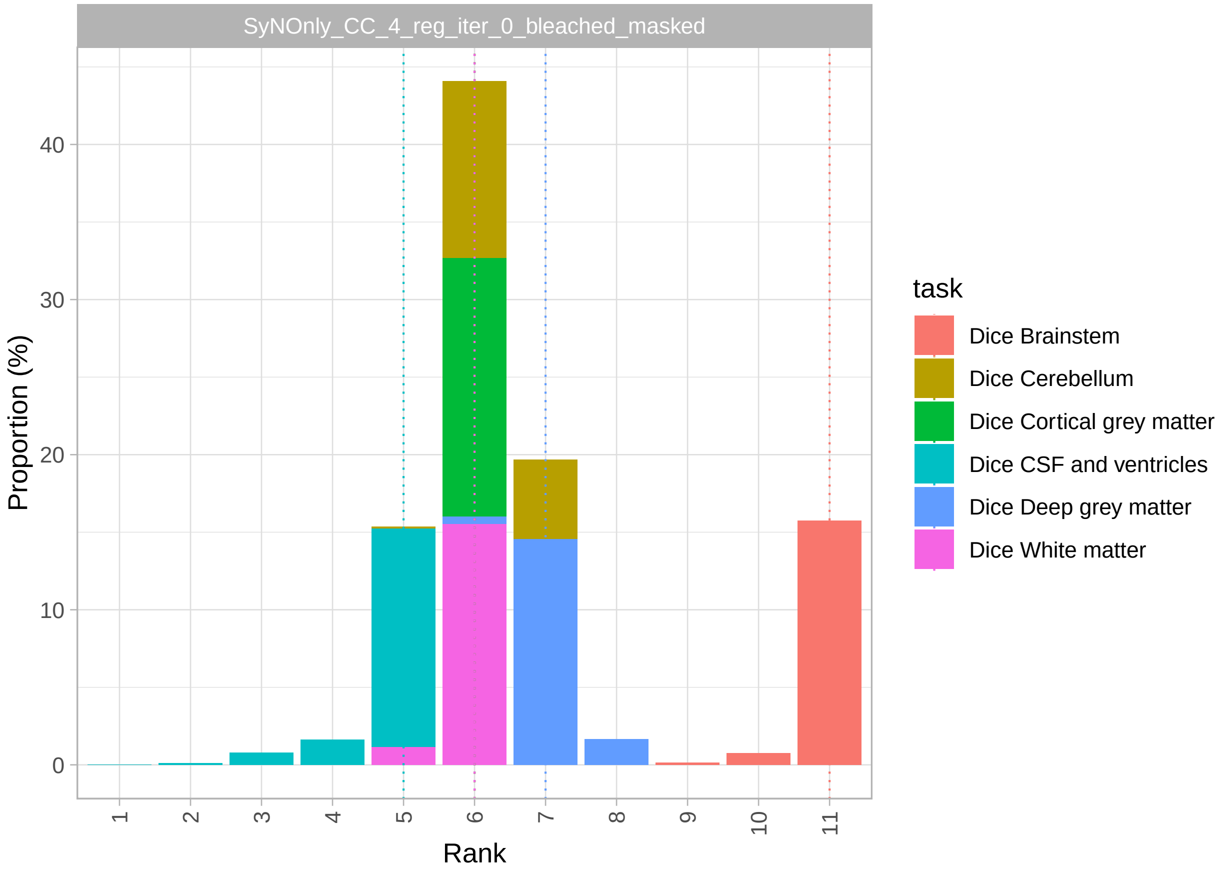
SyNOnly_CC_4_reg_iter_0_bleached_masked
Proportion (%)
40
30
20
10
0
1
2
3
4
5
6
7
8
9
10
11
Rank
task
Dice Brainstem
Dice Cerebellum
Dice Cortical grey matter
Dice CSF and ventricles
Dice Deep grey matter
Dice White matter

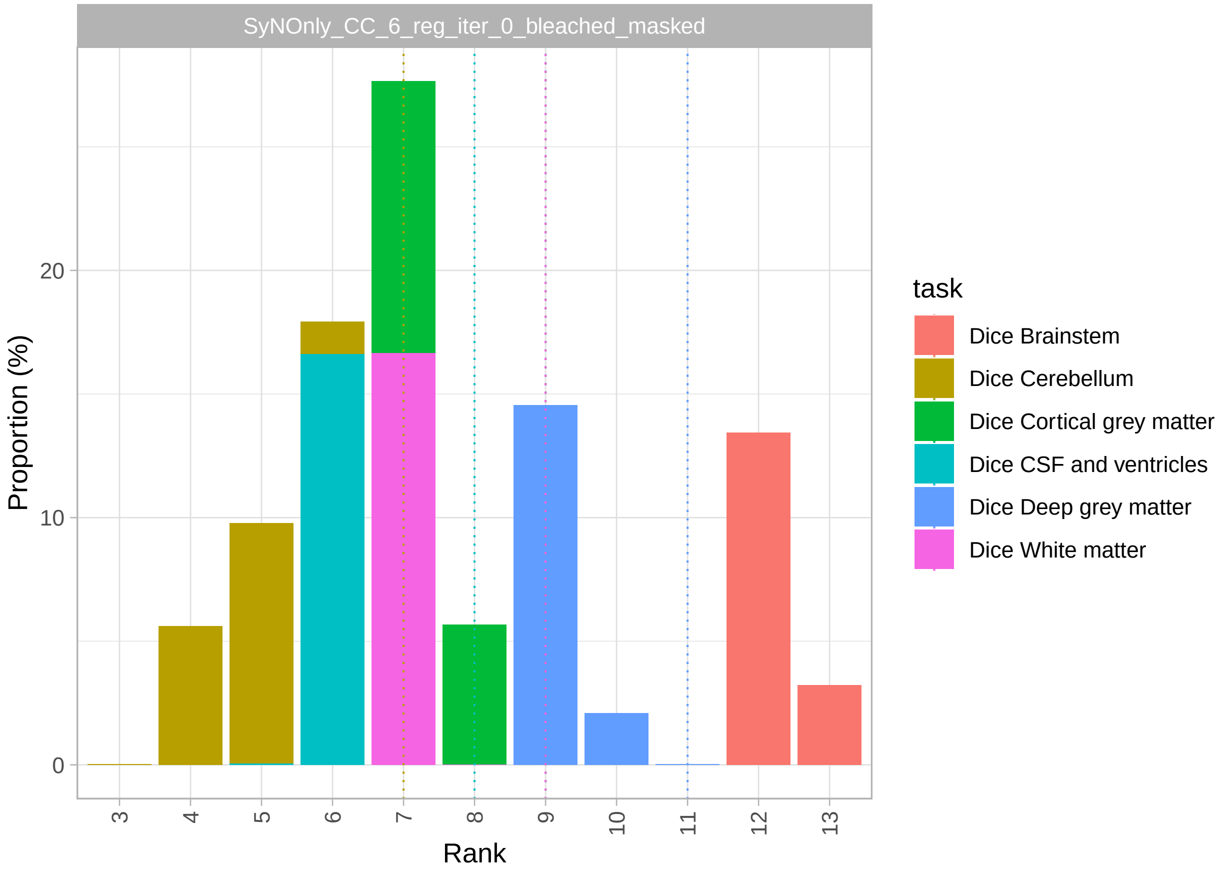
SyNOnly_CC_6_reg_iter_0_bleached_masked
Proportion (%)
20
10
0
3
4
5
6
7
8
9
10
11
12
13
Rank
task
Dice Brainstem
Dice Cerebellum
Dice Cortical grey matter
Dice CSF and ventricles
Dice Deep grey matter
Dice White matter

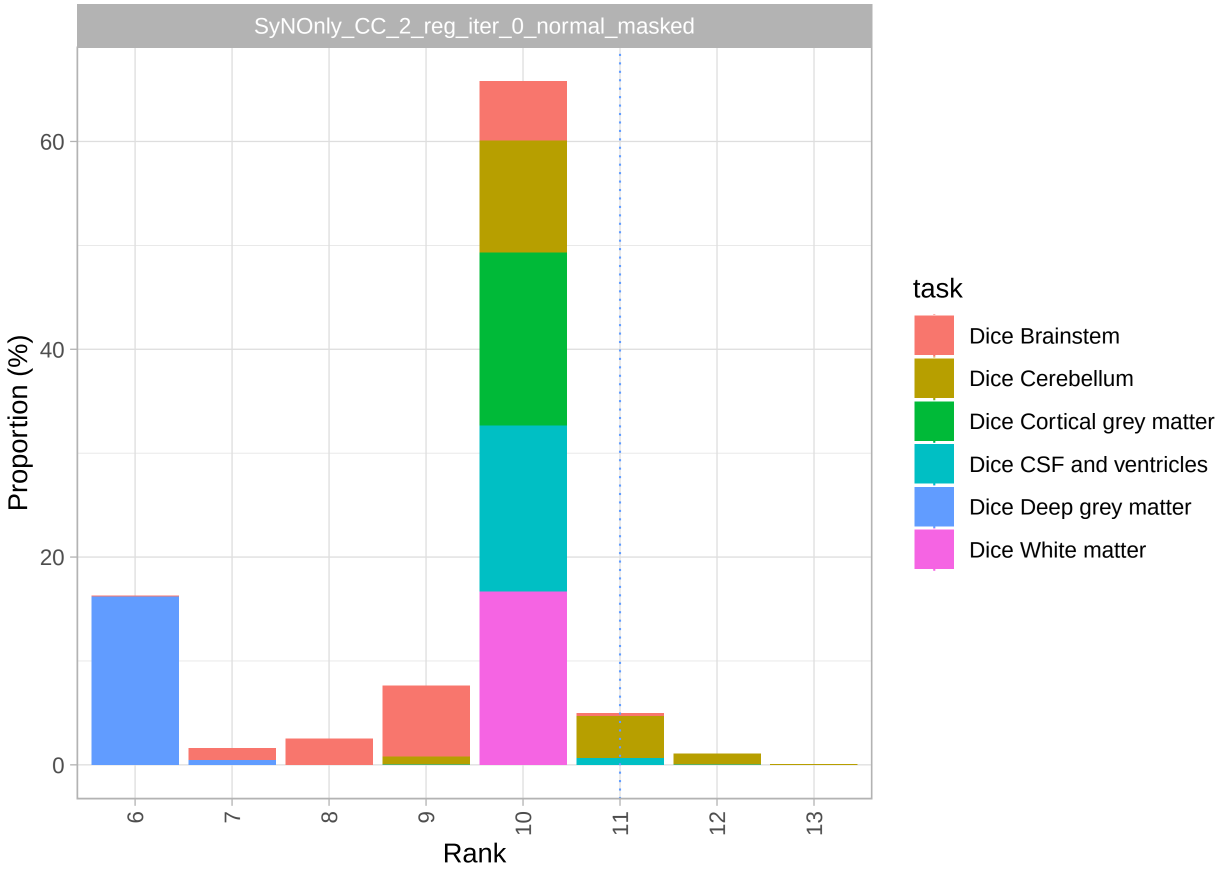

SyNOnly_CC_2_reg_iter_0_normal_masked
Proportion (%)
60
40
20
0
6
7
8
9
10
11
12
13
Rank
task
Dice Brainstem
Dice Cerebellum
Dice Cortical grey matter
Dice CSF and ventricles
Dice Deep grey matter
Dice White matter

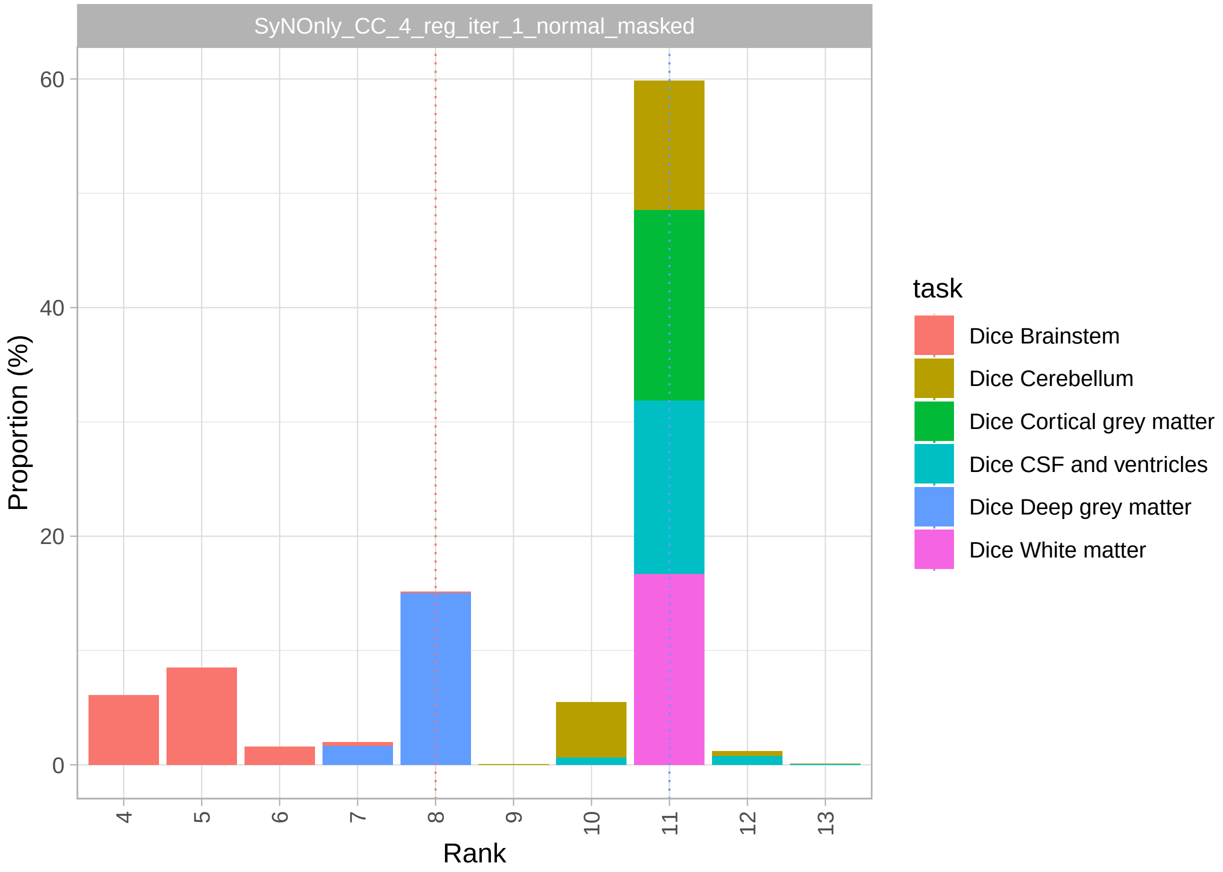
SyNOnly_CC_4_reg_iter_1_normal_masked
Proportion (%)
60
40
20
0
4
5
6
7
8
9
10
11
12
13
Rank
task
Dice Brainstem
Dice Cerebellum
Dice Cortical grey matter
Dice CSF and ventricles
Dice Deep grey matter
Dice White matter

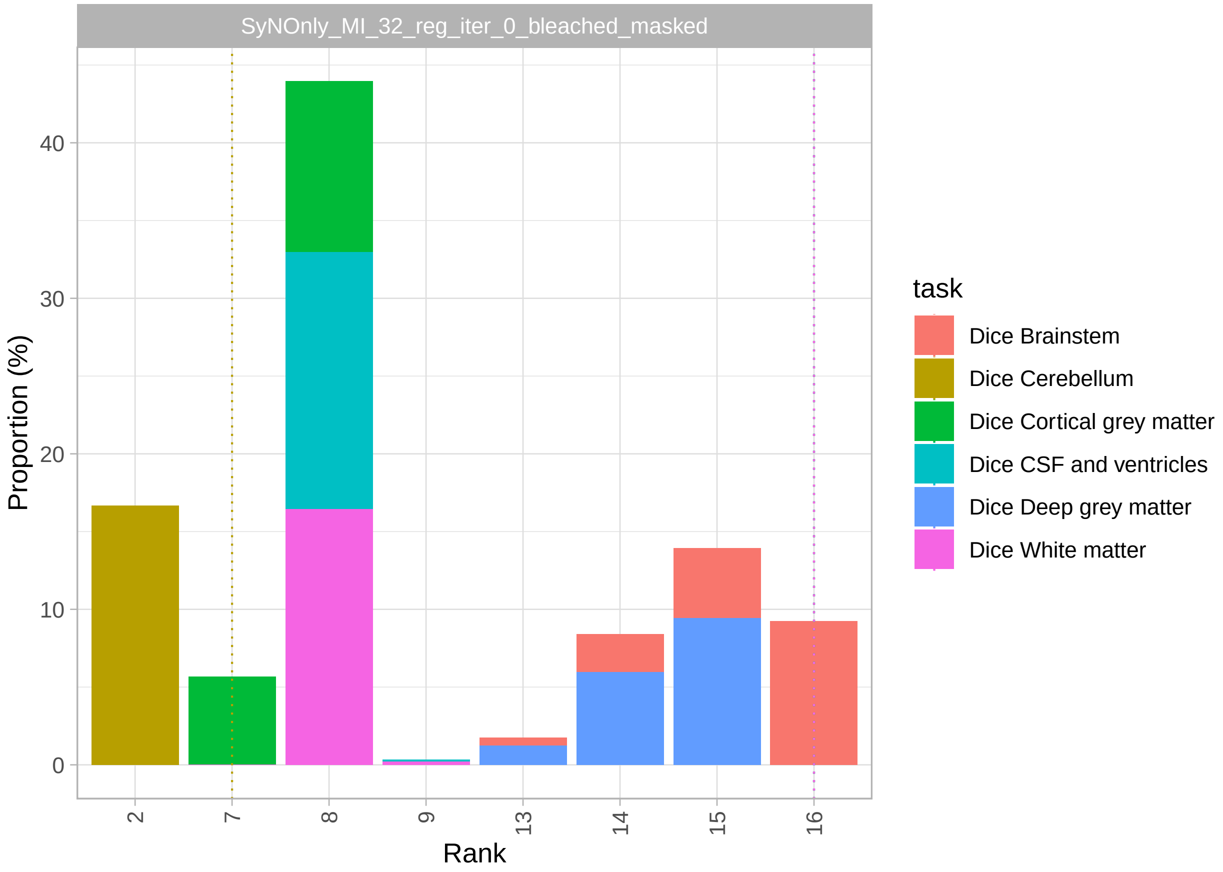

SyNOnly_MI_32_reg_iter_0_bleached_masked
Proportion (%)
0
10
20
30
40
2
7
8
9
13
14
15
16
Rank
task
Dice Brainstem
Dice Cerebellum
Dice Cortical grey matter
Dice CSF and ventricles
Dice Deep grey matter
Dice White matter

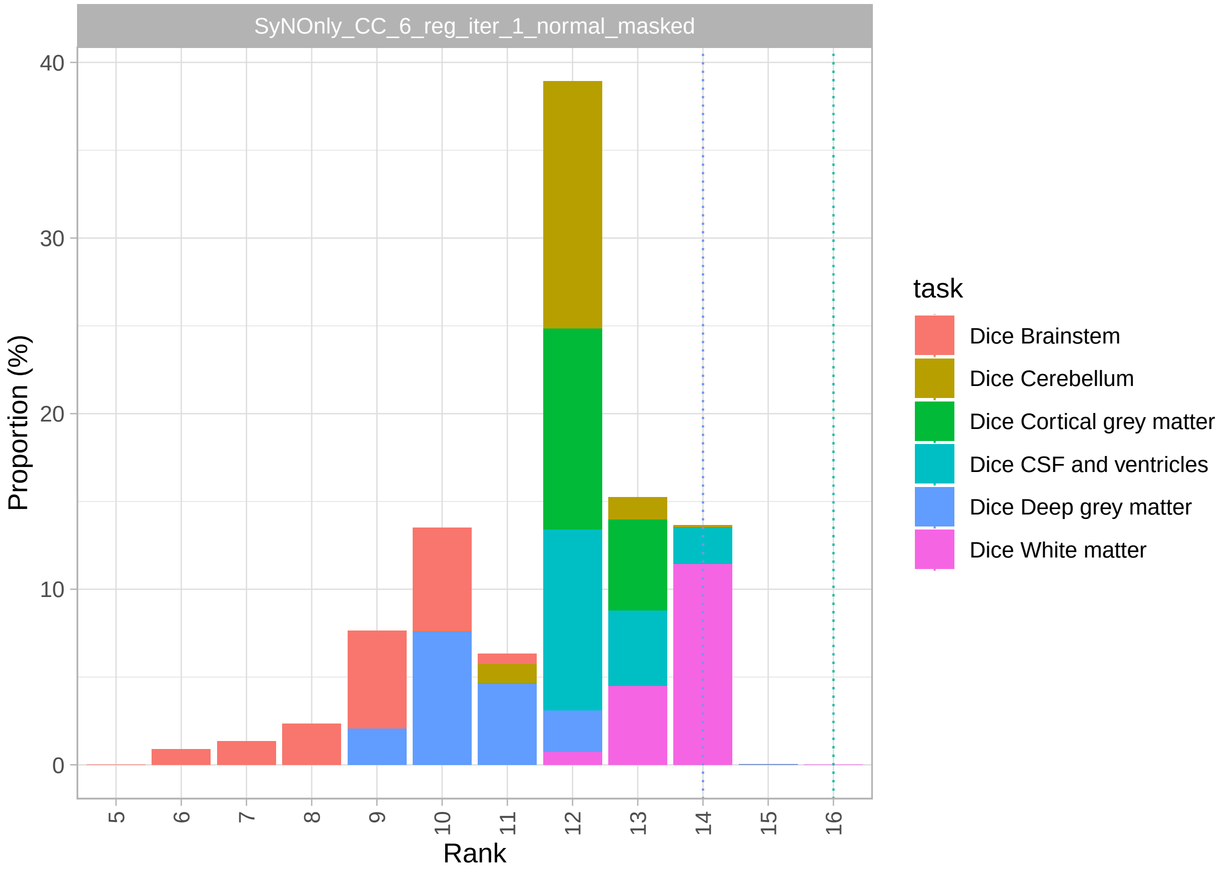
SyNOnly_CC_6_reg_iter_1_normal_masked
Proportion (%)
40
30
20
10
0
5
6
7
8
9
10
11
12
13
14
15
16
Rank
task
Dice Brainstem
Dice Cerebellum
Dice Cortical grey matter
Dice CSF and ventricles
Dice Deep grey matter
Dice White matter

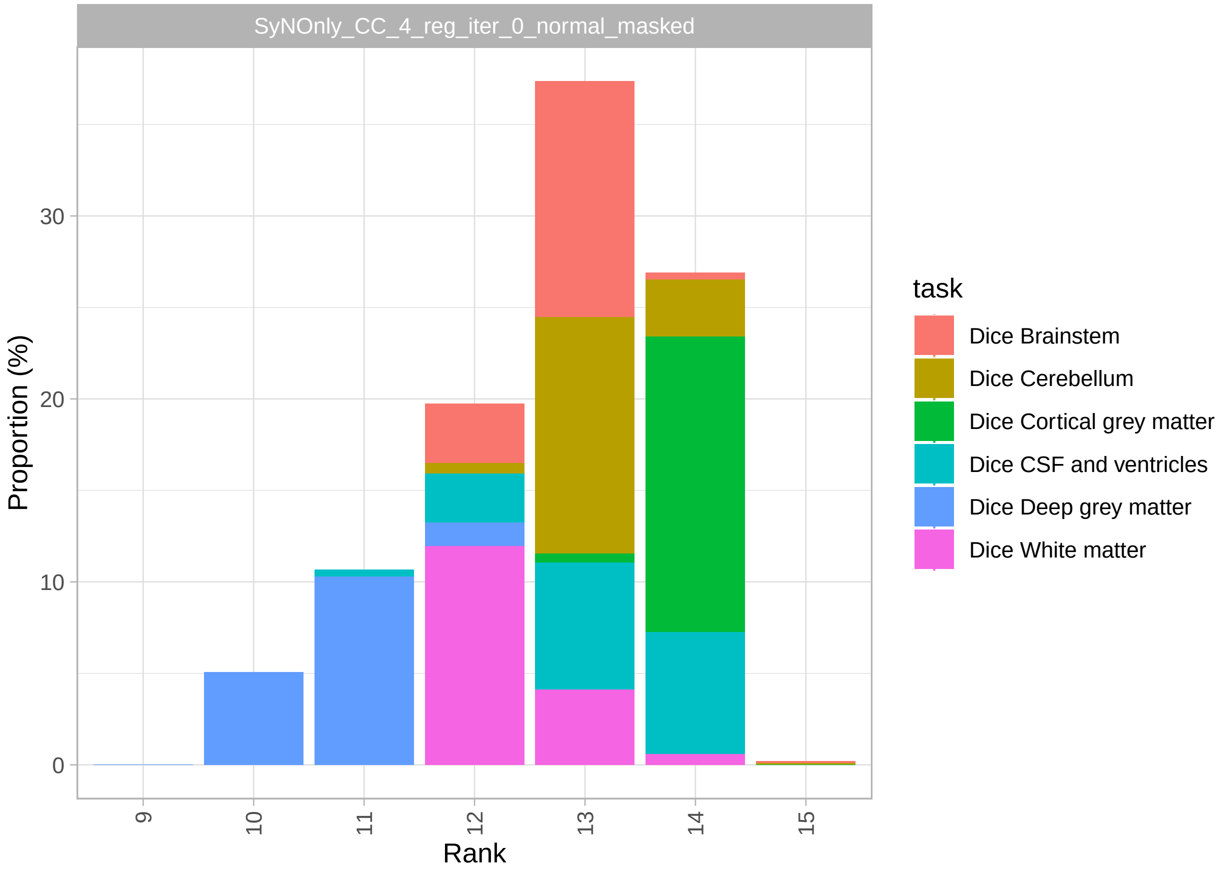
SyNOnly_CC_4_reg_iter_0_normal_masked
Proportion (%)
0
10
20
30
9
10
11
12
13
14
15
Rank
task
Dice Brainstem
Dice Cerebellum
Dice Cortical grey matter
Dice CSF and ventricles
Dice Deep grey matter
Dice White matter

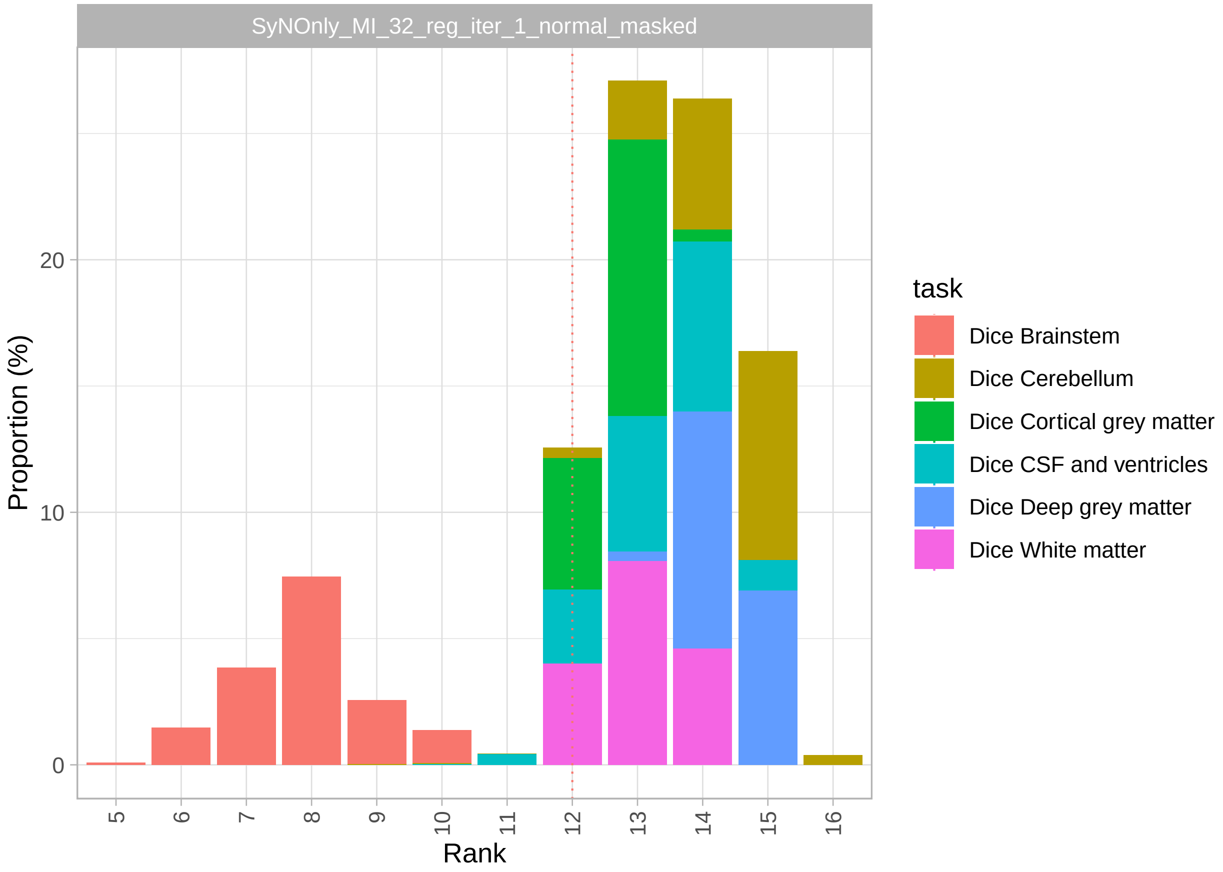
SyNOnly_MI_32_reg_iter_1_normal_masked
Proportion (%)
20
10
0
5
6
7
8
9
10
11
12
13
14
15
16
Rank
task
Dice Brainstem
Dice Cerebellum
Dice Cortical grey matter
Dice CSF and ventricles
Dice Deep grey matter
Dice White matter

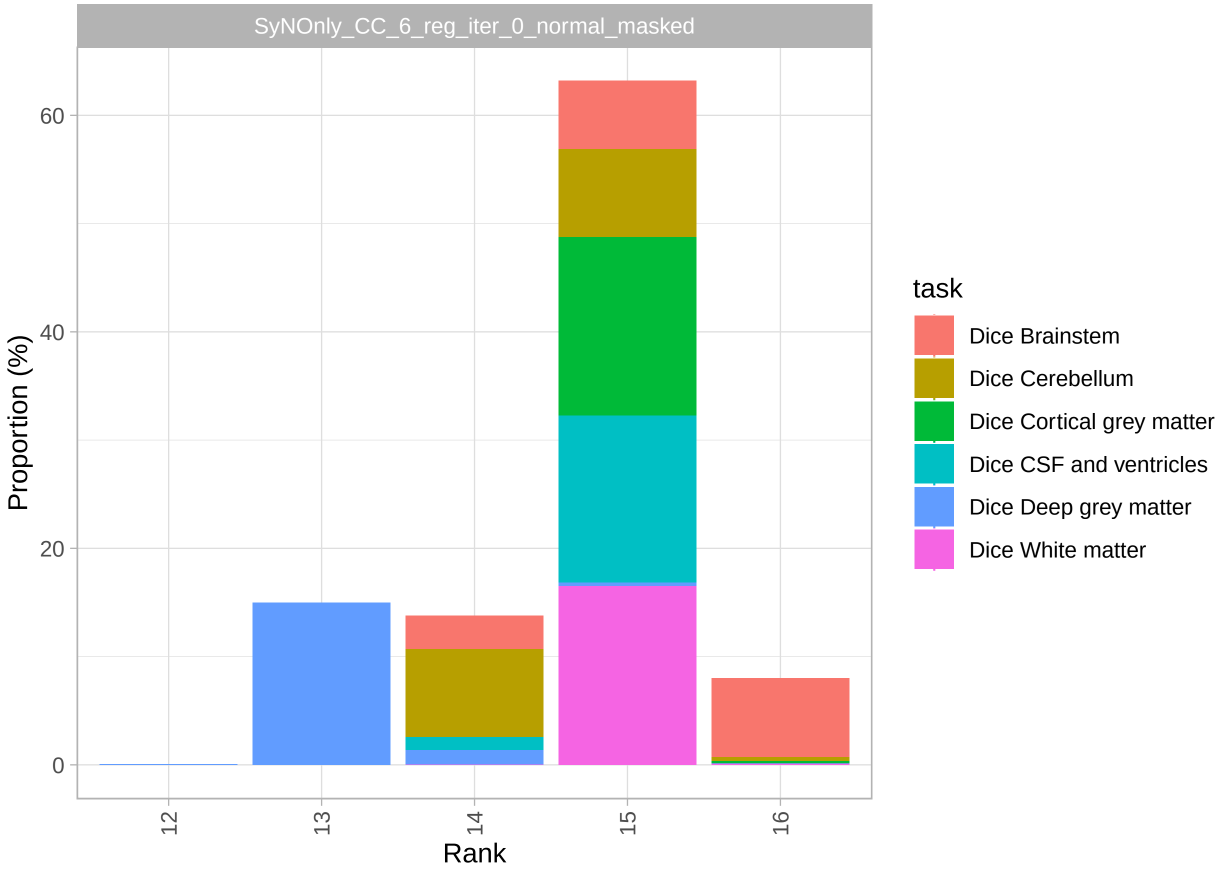
SyNOnly_CC_6_reg_iter_0_normal_masked
Proportion (%)
0
20
40
60
12
13
14
15
16
Rank
task
Dice Brainstem
Dice Cerebellum
Dice Cortical grey matter
Dice CSF and ventricles
Dice Deep grey matter
Dice White matter

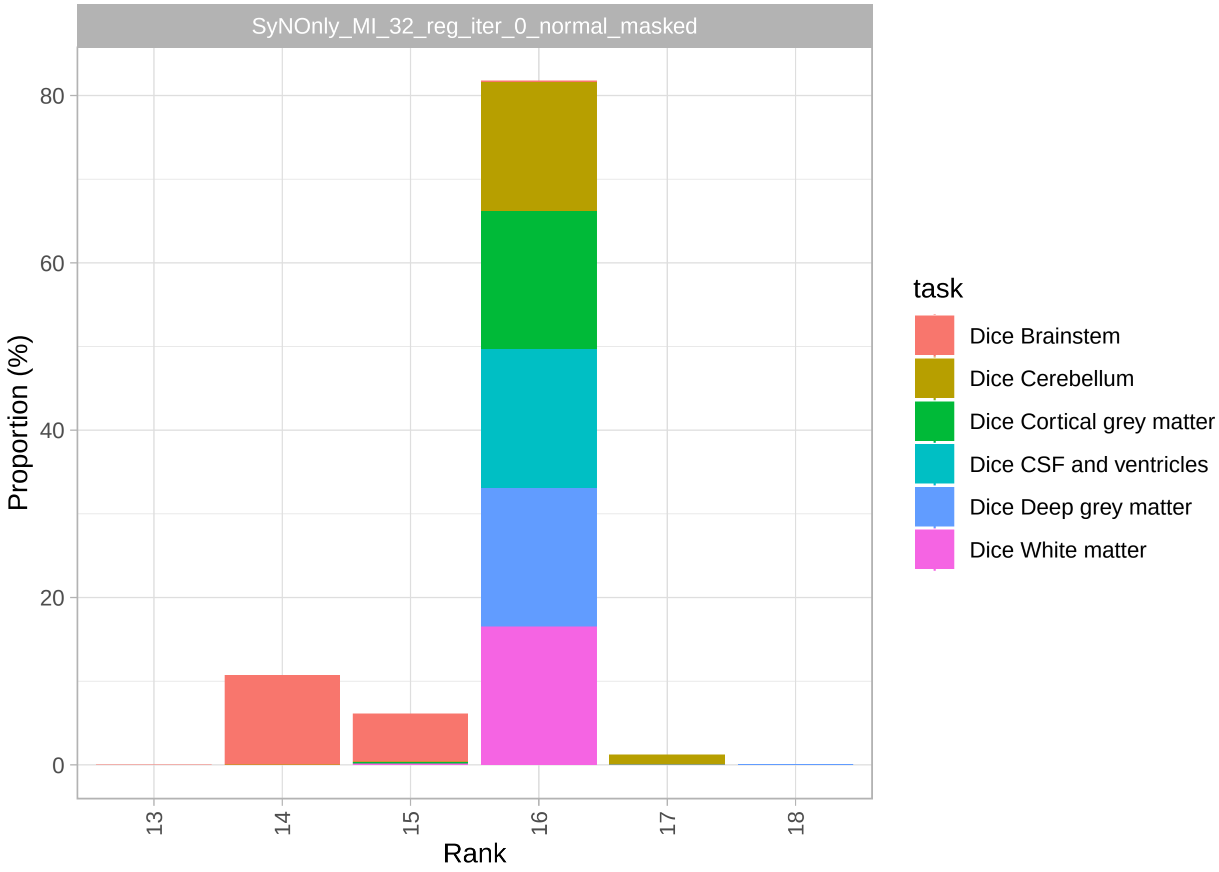
SyNOnly_MI_32_reg_iter_0_normal_masked
Proportion (%)
80
60
40
20
0
13
14
15
16
17
18
Rank
task
Dice Brainstem
Dice Cerebellum
Dice Cortical grey matter
Dice CSF and ventricles
Dice Deep grey matter
Dice White matter

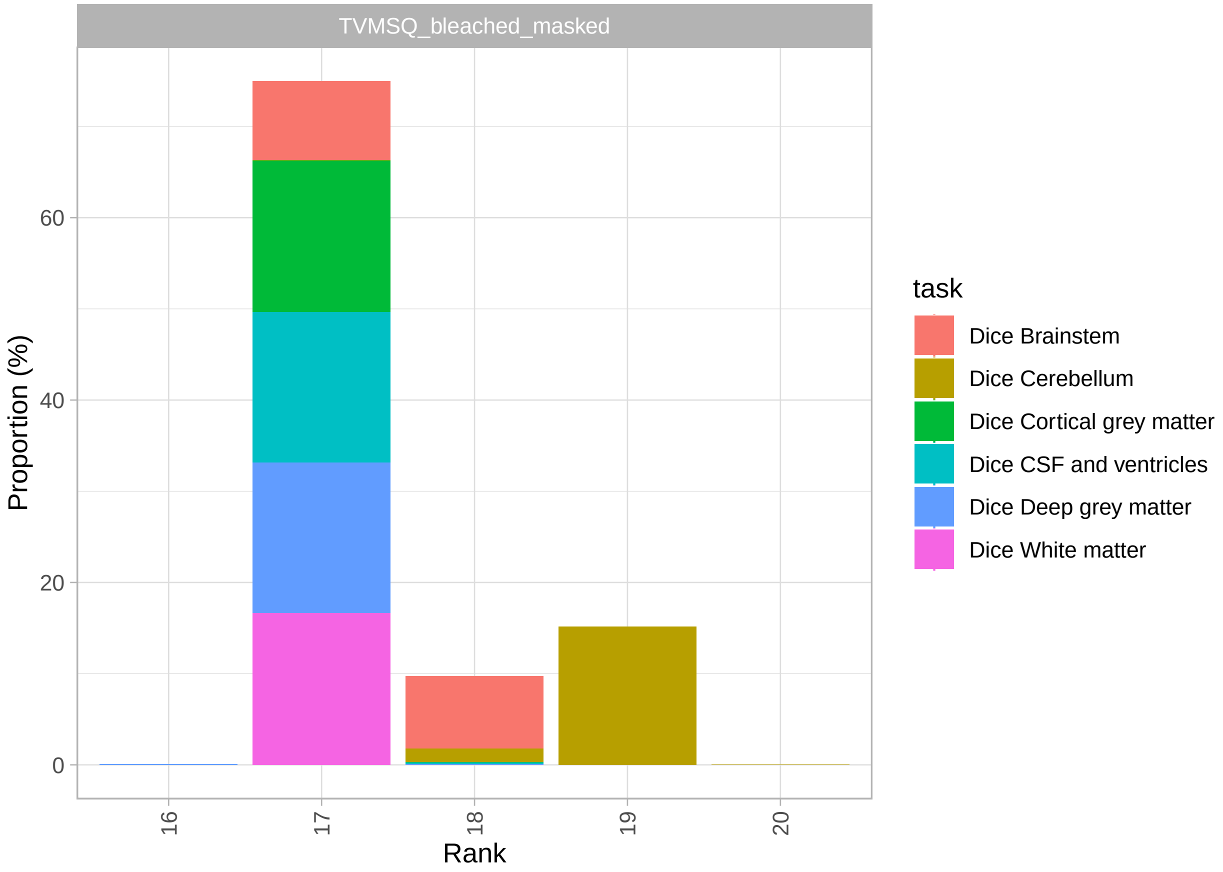
TVMSQ_bleached_masked
Proportion (%)
60
40
20
0
16
17
18
19
20
Rank
task
Dice Brainstem
Dice Cerebellum
Dice Cortical grey matter
Dice CSF and ventricles
Dice Deep grey matter
Dice White matter

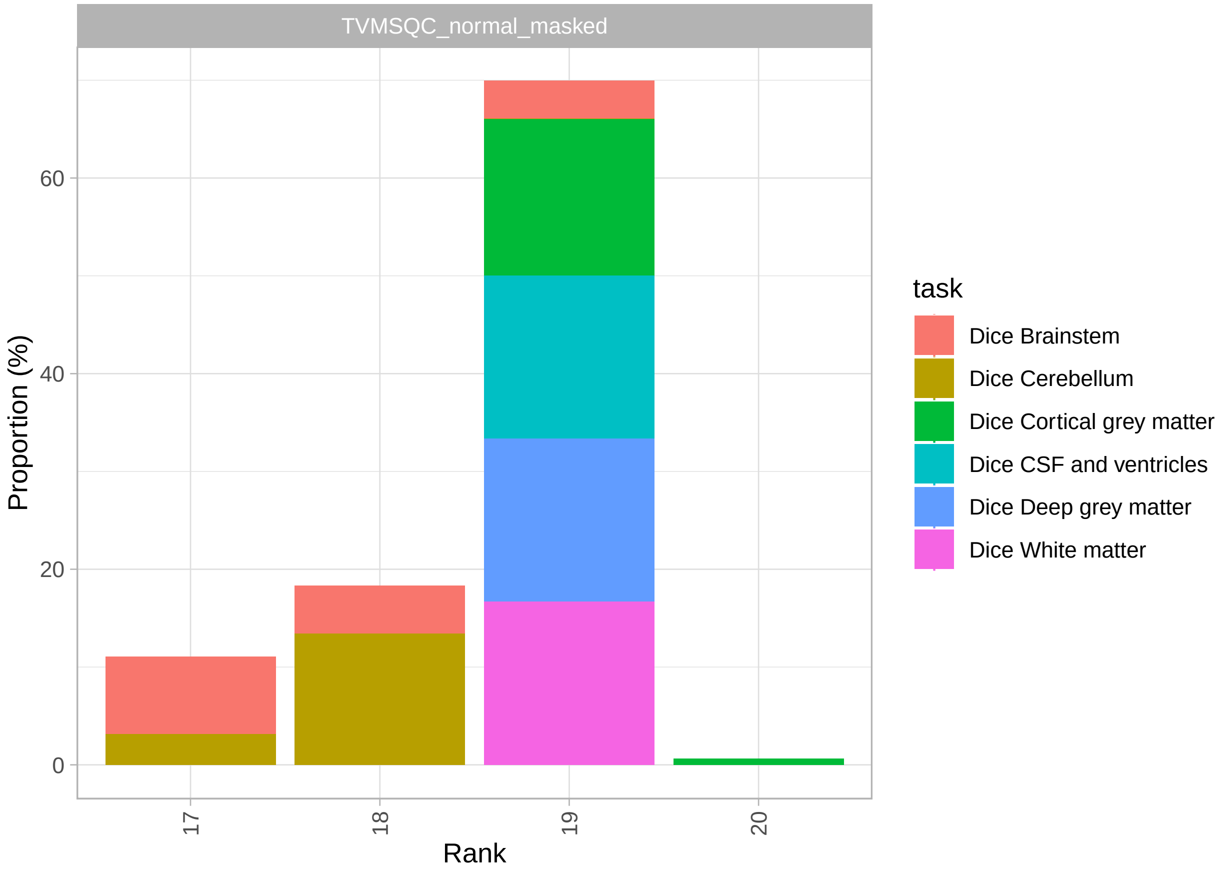
TVMSQC_normal_masked
Proportion (%)
60
40
20
0
17
18
19
20
Rank
task
Dice Brainstem
Dice Cerebellum
Dice Cortical grey matter
Dice CSF and ventricles
Dice Deep grey matter
Dice White matter

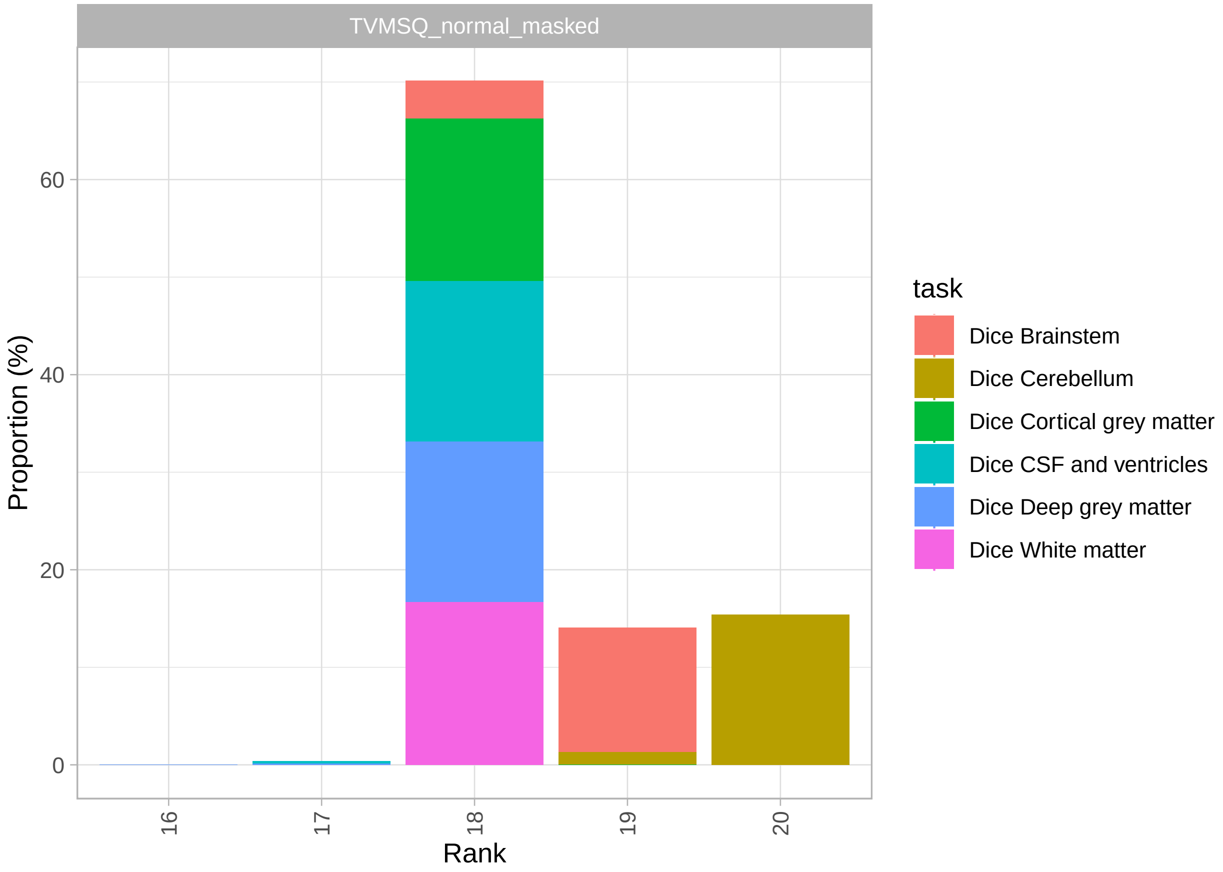

TVMSQ_normal_masked
Proportion (%)
60
40
20
0
16
17
18
19
20
Rank
task
Dice Brainstem
Dice Cerebellum
Dice Cortical grey matter
Dice CSF and ventricles
Dice Deep grey matter
Dice White matter

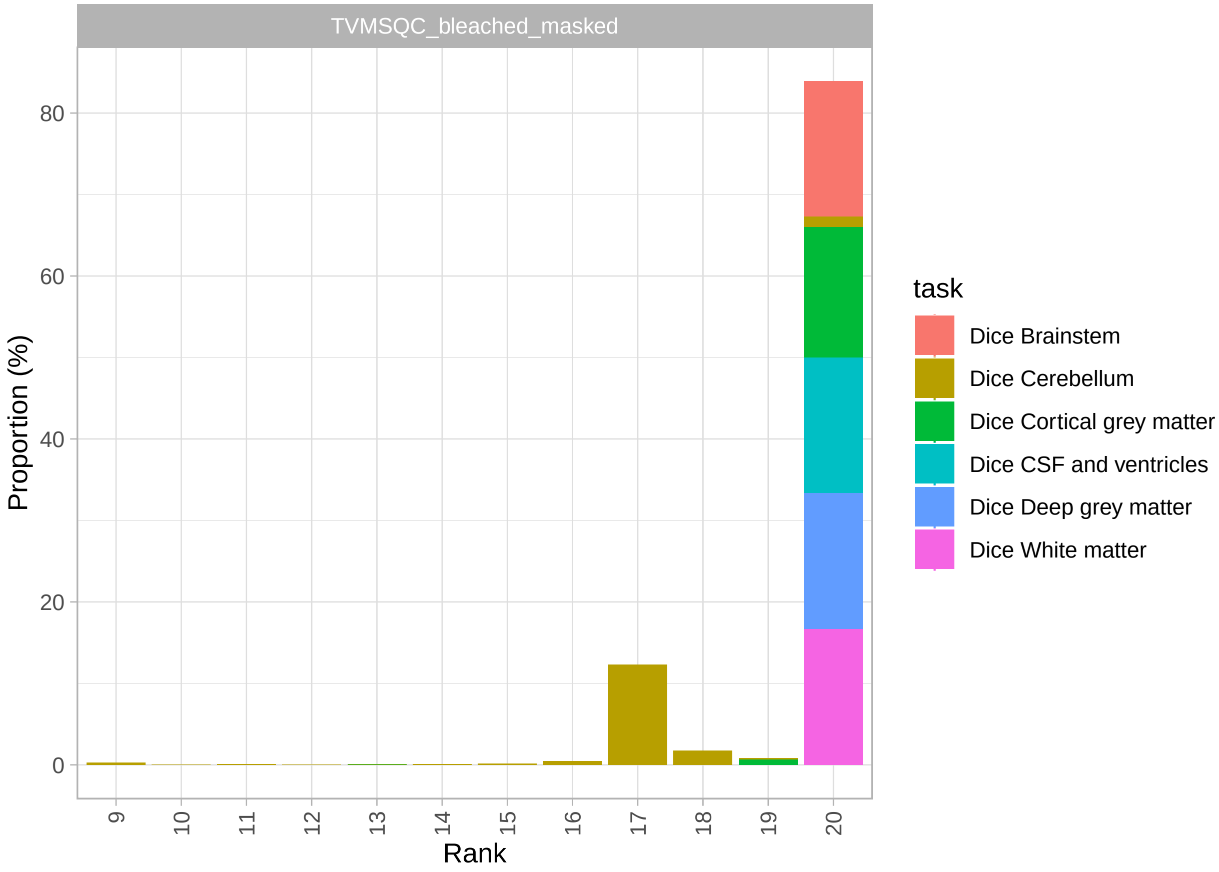

TVMSQC_bleached_masked
Proportion (%)
80
60
40
20
0
9
10
11
12
13
14
15
16
17
18
19
20
Rank
task
Dice Brainstem
Dice Cerebellum
Dice Cortical grey matter
Dice CSF and ventricles
Dice Deep grey matter
Dice White matter

4.2 Characterization of tasks

4.2.1 Visualizing bootstrap results

To investigate which tasks separate algorithms well (i.e., lead to a stable ranking), a blob plot is recommended.

Bootstrap results can be shown in a blob plot showing one plot for each task. In this view, the spread of the blobs for each algorithm can be compared across tasks. Deviations from the diagonal indicate deviations from the consensus ranking (over tasks). Specifically, if rank distribution of an algorithm is consistently below the diagonal, the algorithm performed better in this task than on average across tasks, while if the rank distribution of an algorithm is consistently above the diagonal, the algorithm performed worse in this task than on average across tasks. At the bottom of each panel, ranks for each algorithm in the tasks are provided.

Same as in Section 3.1 but now ordered according to consensus.

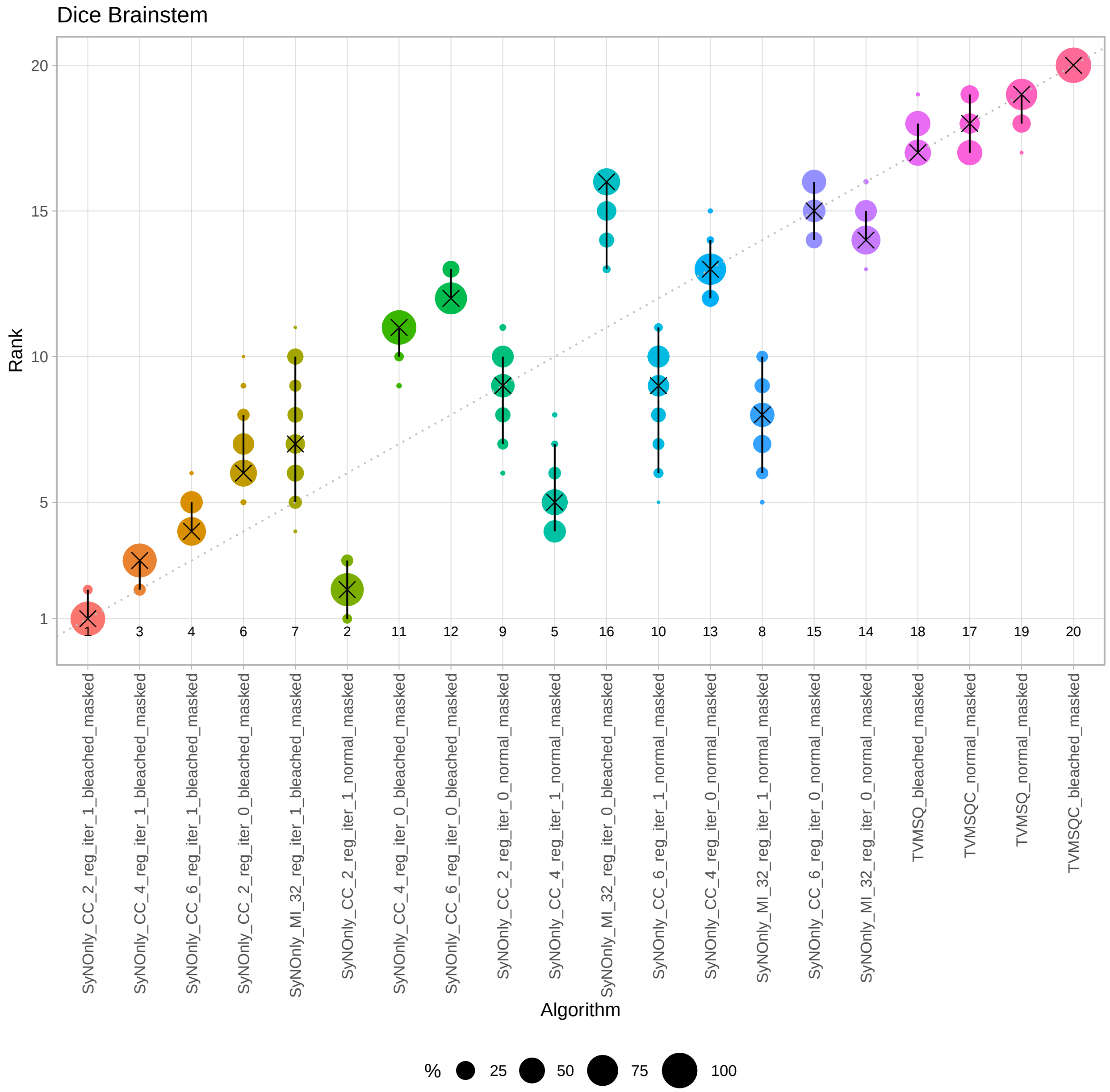

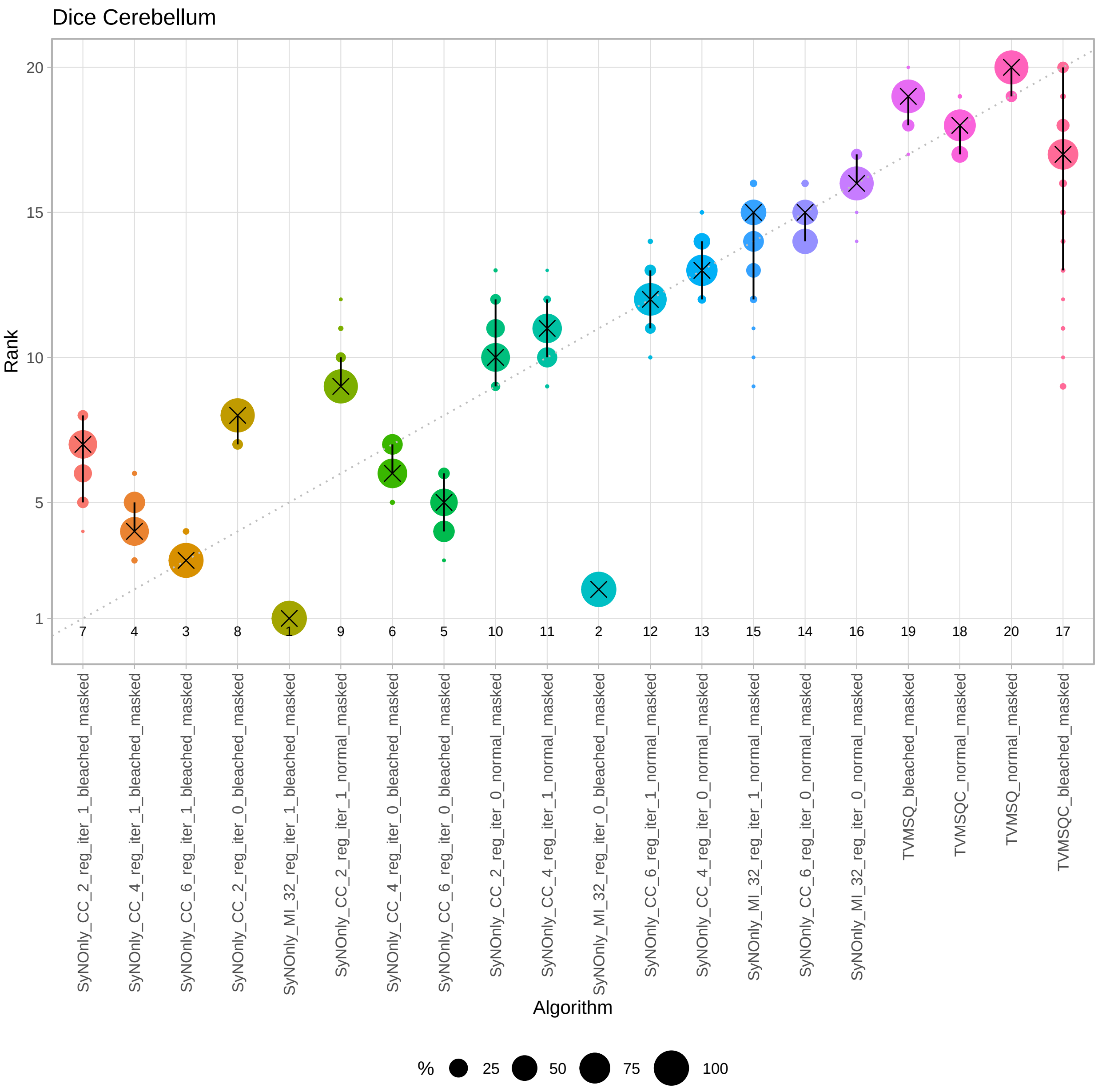
Dice Cerebellum
Rank
20
15
10
5
1
7
4
3
8
1
9
6
5
10
11
2
12
13
15
14
16
19
18
20
17
SyNOnly_CC_2_reg_iter_1_bleached_masked
SyNOnly_CC_4_reg_iter_1_bleached_masked
SyNOnly_CC_6_reg_iter_1_bleached_masked
SyNOnly_CC_2_reg_iter_0_bleached_masked
SyNOnly_MI_32_reg_iter_1_bleached_masked
SyNOnly_CC_2_reg_iter_1_normal_masked
SyNOnly_CC_4_reg_iter_0_bleached_masked
SyNOnly_CC_6_reg_iter_0_bleached_masked
SyNOnly_CC_2_reg_iter_0_normal_masked
SyNOnly_CC_4_reg_iter_1_normal_masked
SyNOnly_MI_32_reg_iter_0_bleached_masked
SyNOnly_CC_6_reg_iter_1_normal_masked
SyNOnly_CC_4_reg_iter_0_normal_masked
SyNOnly_MI_32_reg_iter_1_normal_masked
SyNOnly_CC_6_reg_iter_0_normal_masked
SyNOnly_MI_32_reg_iter_0_normal_masked
TVMSQ_bleached_masked
TVMSQC_normal_masked
TVMSQ_normal_masked
TVMSQC_bleached_masked
Algorithm
%
25
50
75
100

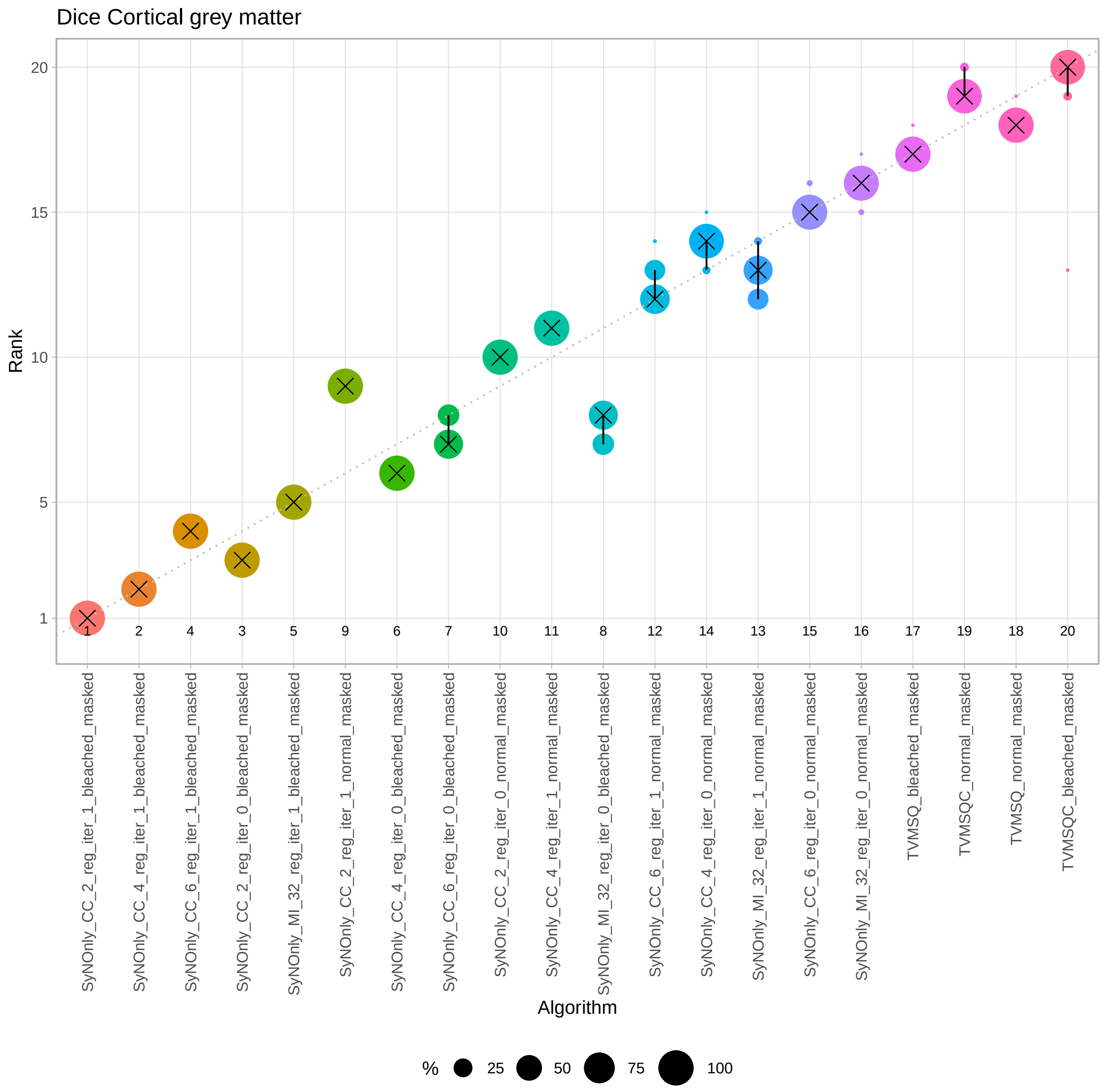
Dice Cortical grey matter
Rank
20
15
10
5
1
1
2
4
3
5
9
6
7
10
11
8
12
14
13
15
16
17
19
18
20
SyNOnly_CC_2_reg_iter_1_bleached_masked
SyNOnly_CC_4_reg_iter_1_bleached_masked
SyNOnly_CC_6_reg_iter_1_bleached_masked
SyNOnly_CC_2_reg_iter_0_bleached_masked
SyNOnly_MI_32_reg_iter_1_bleached_masked
SyNOnly_CC_2_reg_iter_1_normal_masked
SyNOnly_CC_4_reg_iter_0_bleached_masked
SyNOnly_CC_6_reg_iter_0_bleached_masked
SyNOnly_CC_2_reg_iter_0_normal_masked
SyNOnly_CC_4_reg_iter_1_normal_masked
SyNOnly_MI_32_reg_iter_0_bleached_masked
SyNOnly_CC_6_reg_iter_1_normal_masked
SyNOnly_CC_4_reg_iter_0_normal_masked
SyNOnly_MI_32_reg_iter_1_normal_masked
SyNOnly_CC_6_reg_iter_0_normal_masked
SyNOnly_MI_32_reg_iter_0_normal_masked
TVMSQ_bleached_masked
TVMSQC_normal_masked
TVMSQ_normal_masked
TVMSQC_bleached_masked
Algorithm
%
25
50
75
100

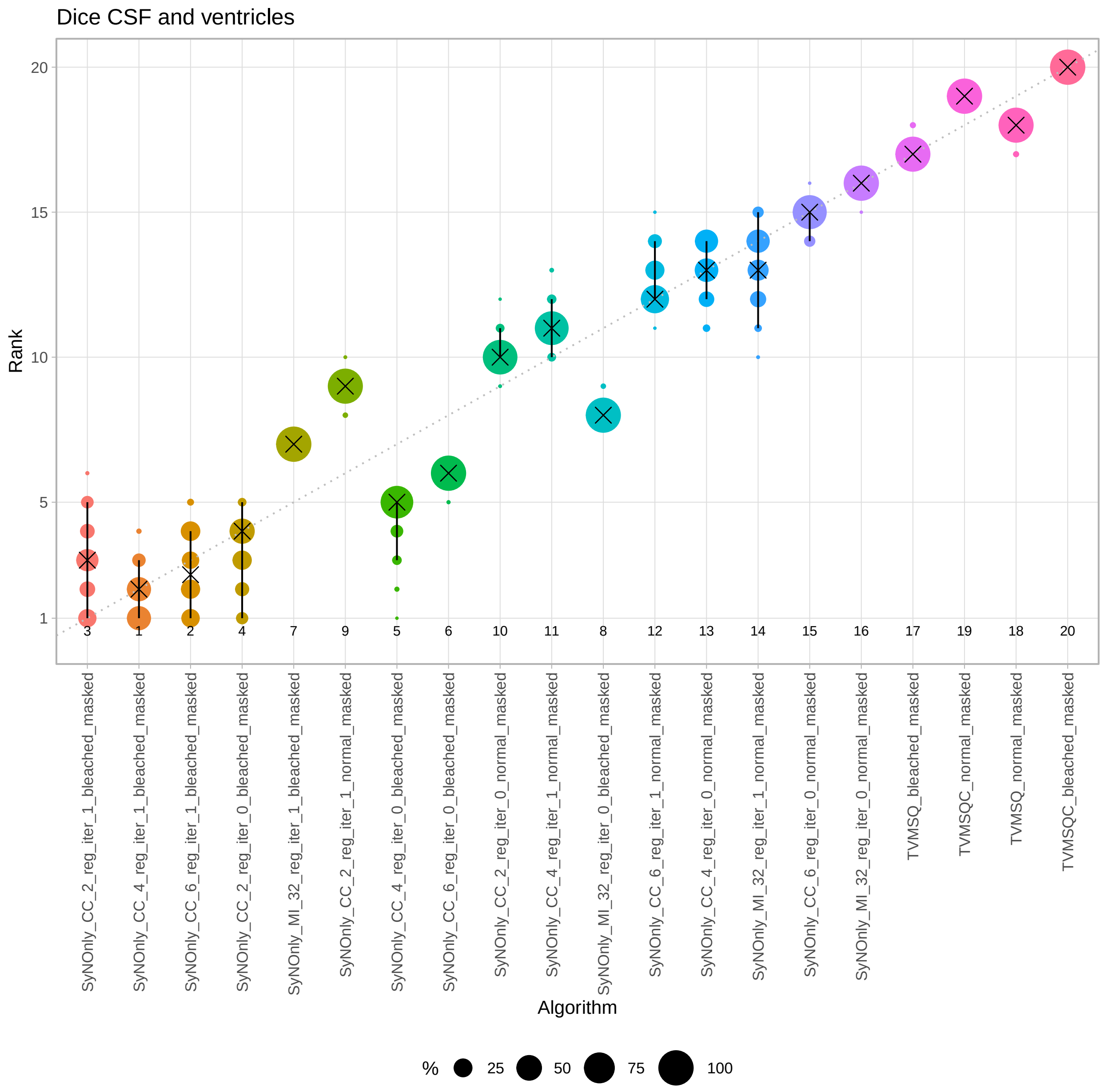
Dice CSF and ventricles
Rank
20
15
10
5
1
3 1 2 4 7 9 5 6 10 11 8 12 13 14 15 16 17 19 18 20
SyNOnly_CC_2_reg_iter_1_bleached_masked
SyNOnly_CC_4_reg_iter_1_bleached_masked
SyNOnly_CC_6_reg_iter_1_bleached_masked
SyNOnly_CC_2_reg_iter_0_bleached_masked
SyNOnly_MI_32_reg_iter_1_bleached_masked
SyNOnly_CC_2_reg_iter_1_normal_masked
SyNOnly_CC_4_reg_iter_0_bleached_masked
SyNOnly_CC_6_reg_iter_0_bleached_masked
SyNOnly_CC_2_reg_iter_0_normal_masked
SyNOnly_CC_4_reg_iter_1_normal_masked
SyNOnly_MI_32_reg_iter_0_bleached_masked
SyNOnly_CC_6_reg_iter_1_normal_masked
SyNOnly_CC_4_reg_iter_0_normal_masked
SyNOnly_MI_32_reg_iter_1_normal_masked
SyNOnly_CC_6_reg_iter_0_normal_masked
SyNOnly_MI_32_reg_iter_0_normal_masked
TVMSQ_bleached_masked
TVMSQC_normal_masked
TVMSQ_normal_masked
TVMSQC_bleached_masked
Algorithm
% 25 50 75 100

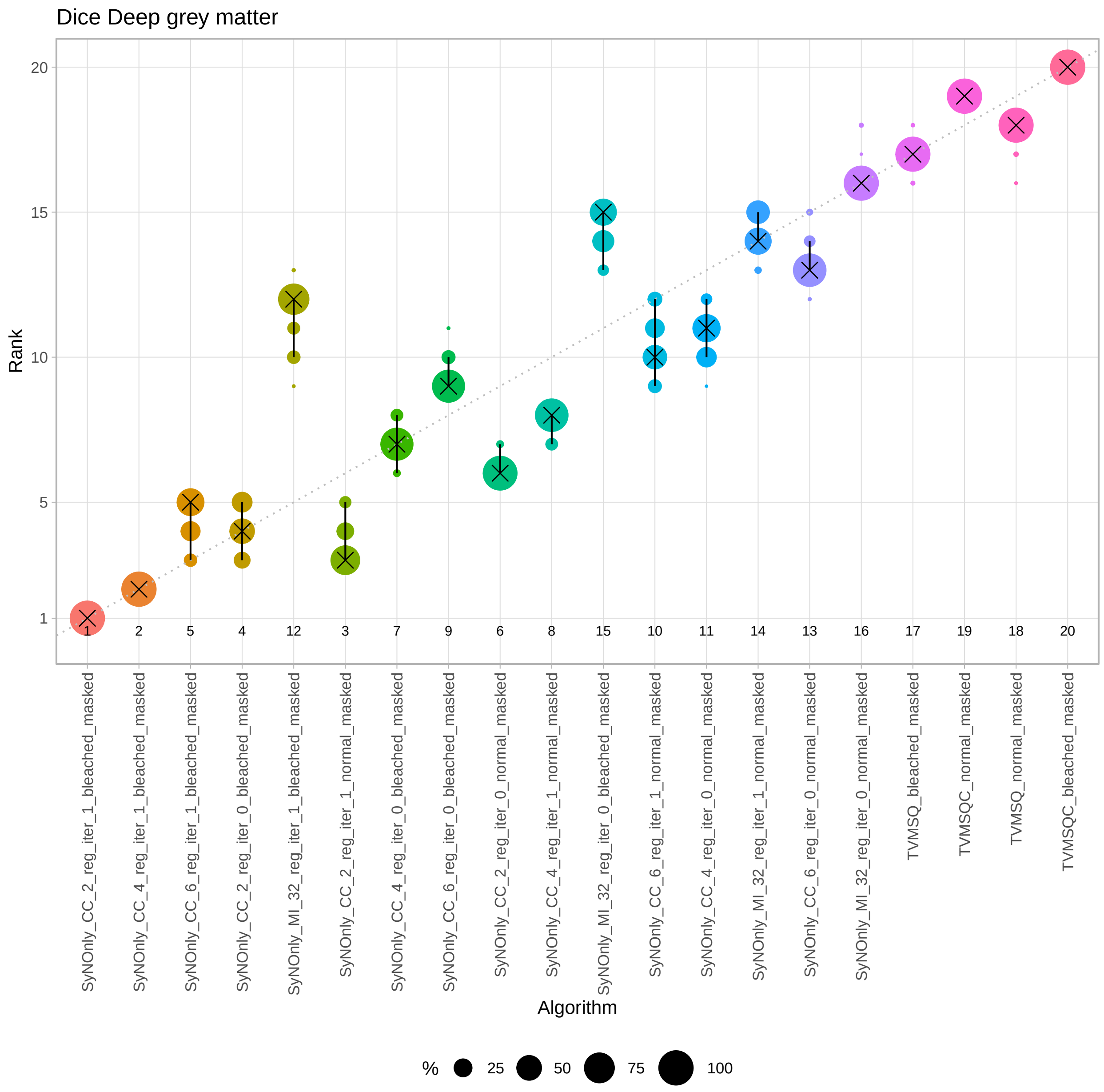
Dice Deep grey matter
Rank
20
15
10
5
1
1
2
5
4
12
3
7
9
6
8
15
10
11
14
13
16
17
19
18
20
SyNOnly_CC_2_reg_iter_1_bleached_masked
SyNOnly_CC_4_reg_iter_1_bleached_masked
SyNOnly_CC_6_reg_iter_1_bleached_masked
SyNOnly_CC_2_reg_iter_0_bleached_masked
SyNOnly_MI_32_reg_iter_1_bleached_masked
SyNOnly_CC_2_reg_iter_1_normal_masked
SyNOnly_CC_4_reg_iter_0_bleached_masked
SyNOnly_CC_6_reg_iter_0_bleached_masked
SyNOnly_CC_2_reg_iter_0_normal_masked
SyNOnly_CC_4_reg_iter_1_normal_masked
SyNOnly_MI_32_reg_iter_0_bleached_masked
SyNOnly_CC_6_reg_iter_1_normal_masked
SyNOnly_CC_4_reg_iter_0_normal_masked
SyNOnly_MI_32_reg_iter_1_normal_masked
SyNOnly_CC_6_reg_iter_0_normal_masked
SyNOnly_MI_32_reg_iter_0_normal_masked
TVMSQ_bleached_masked
TVMSQC_normal_masked
TVMSQ_normal_masked
TVMSQC_bleached_masked
Algorithm
%
25
50
75
100

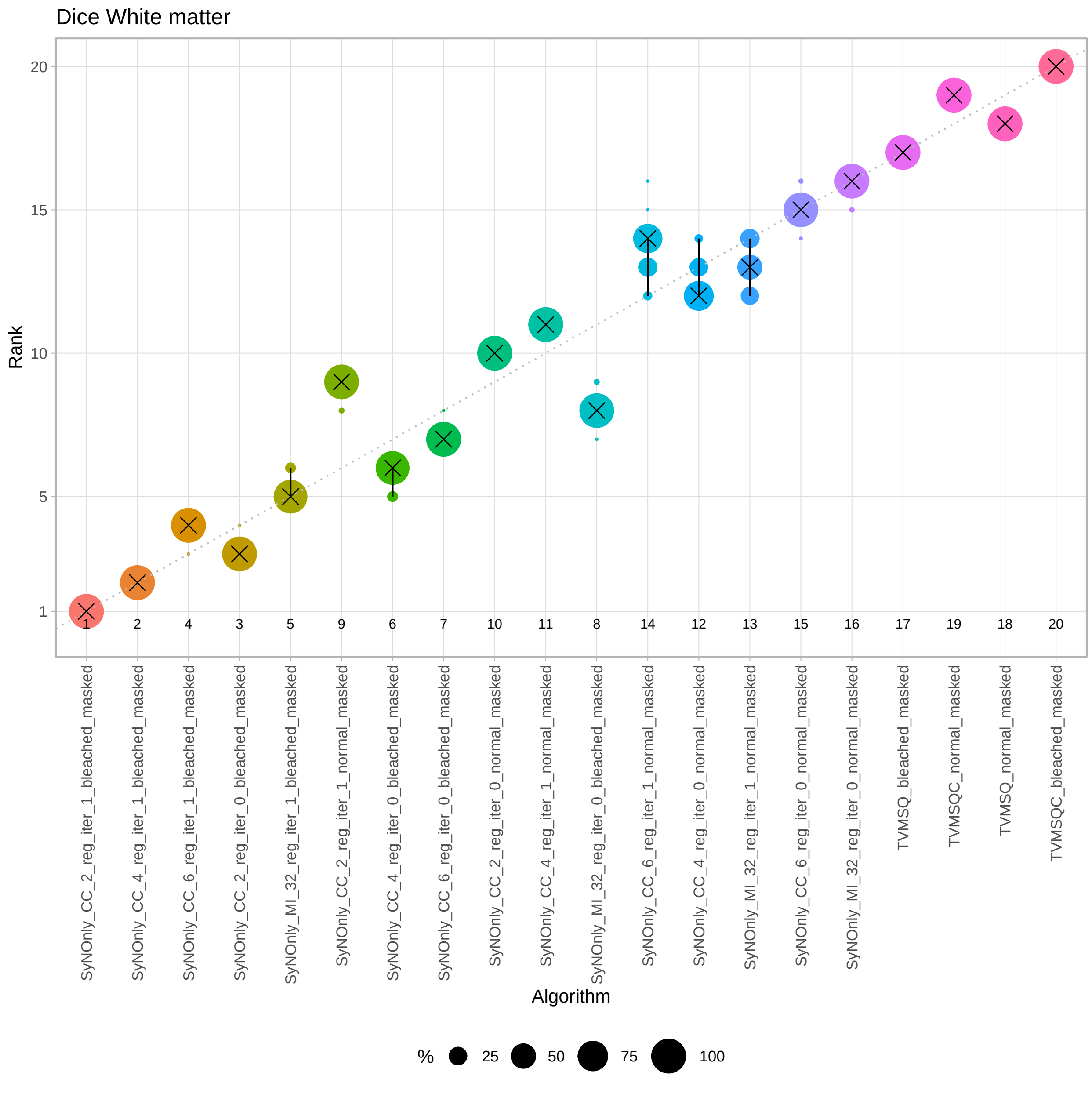

4.2.2 Cluster Analysis

Dendrogram from hierarchical cluster analysis and *network-type graphs* for assessing the similarity of tasks based on challenge rankings.

A dendrogram is a visualization approach based on hierarchical clustering. It depicts clusters according to a chosen distance measure (here: Spearman's footrule) as well as a chosen agglomeration method (here: complete and average agglomeration).

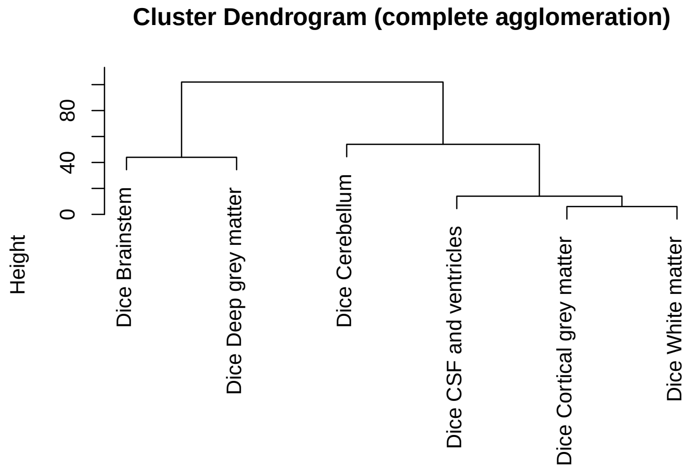
Cluster Dendrogram (complete agglomeration)
Height
0
40
80
Dice Brainstem
Dice Deep grey matter
Dice Cerebellum
Dice CSF and ventricles
Dice Cortical grey matter
Dice White matter

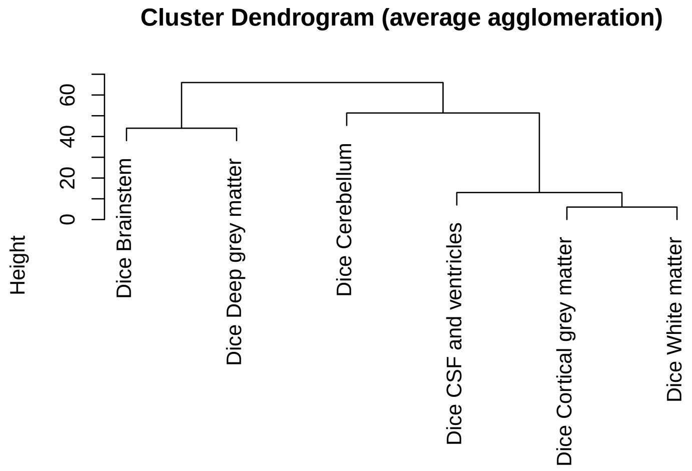
Cluster Dendrogram (average agglomeration)
Height
0
20
40
60
Dice Brainstem
Dice Deep grey matter
Dice Cerebellum
Dice CSF and ventricles
Dice Cortical grey matter
Dice White matter

5 References

Wiesenfarth, M., Reinke, A., Landman, B.A., Eisenmann, M., Aguilera Saiz, L., Cardoso, M.J., Maier-Hein, L. and Kopp-Schneider, A. Methods and open-source toolkit for analyzing and visualizing challenge results. *Sci Rep* **11**, 2369 (2021). https://doi.org/10.1038/s41598-021-82017-6

M. J. A. Eugster, T. Hothorn, and F. Leisch, "Exploratory and inferential analysis of benchmark experiments," Institut fuer Statistik, Ludwig-Maximilians-Universitaet Muenchen, Germany, Technical Report 30, 2008. [Online]. Available: http://epub.ub.uni-muenchen.de/4134/.
