# Supplementary material for: Characterization of dynamic patterns of human fetal to neonatal brain asymmetry with deformation-based morphometry
Source: Front Neurosci. 2023 Dec 6;17:1252850. doi: 10.3389/fnins.2023.1252850 (PMC10734644; doi:10.3389/fnins.2023.1252850)
Supplement: Supplementary file 1 [file Data_Sheet_1.ZIP › ChallengeR/Framework B to mid-age template/fetal2template_linear/Fetal2MidTemplate_linear_dice.pdf]

Benchmarking report for Fetal to Midtempalte Registration Linear

created by challengeR v1.0.4

29 März, 2023

This document presents a systematic report on the benchmark study "Fetal to Midtempalte Registration Linear". Input data comprises raw metric values for all algorithms and cases. Generated plots are:

- Visualization of assessment data: Dot- and boxplots, podium plots and ranking heatmaps
- Visualization of ranking stability: Blob plots, violin plots and significance maps, line plots
- Visualization of cross-task insights: Blob plots, stacked frequency plots, dendrograms

Details can be found in Wiesenfarth et al. (2021).

1 Rankings

Algorithms within a task are ranked according to the following ranking scheme:

aggregate using function ("mean") then rank

Ranking for each task:

Brainstem : The analysis is based on 17 algorithms and 20 cases. 0 missing cases have been found in the data set.

	value_mean	rank
Affine_eroded_masked	0.7908571	1
TRSAA_eroded_masked	0.7815757	2
Affine_masked	0.7751681	3
TRSAA_masked	0.7710142	4
TRSAA_nomask	0.7339533	5
Similarity_eroded_masked	0.7331557	6
Affine_nomask	0.7328631	7
Similarity_masked	0.7269161	8
Similarity_nomask	0.6717961	9
terminal_CC_masked	0.6369182	10
terminal_CC_nomask	0.6357372	11
flirt_NA	0.6134193	12
terminal_CC_eroded_masked	0.5997222	13
terminal_MI_masked	0.5535096	14
terminal_MI_nomask	0.5509198	15
antsAI_masked	0.5465163	16
terminal_MI_eroded_masked	0.5137839	17

Cerebellum : The analysis is based on 17 algorithms and 20 cases. 0 missing cases have been found in the data set.

	value_mean	rank
TRSAA_eroded_masked	0.7951723	1
TRSAA_nomask	0.7682495	2
Similarity_eroded_masked	0.7668211	3
Affine_nomask	0.7656431	4
Affine_eroded_masked	0.7614345	5
TRSAA_masked	0.7422855	6
Similarity_masked	0.7355196	7
Similarity_nomask	0.7342747	8
Affine_masked	0.7188360	9
flirt_NA	0.6350198	10
terminal_CC_nomask	0.5782561	11
terminal_CC_masked	0.5606140	12
terminal_CC_eroded_masked	0.5279431	13
terminal_MI_masked	0.4732053	14
terminal_MI_nomask	0.4583521	15
terminal_MI_eroded_masked	0.4359605	16
antsAI_masked	0.4309712	17

Cortical grey matter : The analysis is based on 17 algorithms and 20 cases. 0 missing cases have been found in the data set.

	value_mean	rank
TRSAA_eroded_masked	0.4663763	1
TRSAA_masked	0.4377515	2
Affine_eroded_masked	0.4353597	3
TRSAA_nomask	0.4332699	4
Affine_nomask	0.4325515	5
Affine_masked	0.4052818	6
Similarity_eroded_masked	0.3984923	7
Similarity_masked	0.3933406	8
flirt_NA	0.3928076	9
Similarity_nomask	0.3643936	10
terminal_CC_masked	0.3211563	11
terminal_CC_nomask	0.3209585	12
terminal_CC_eroded_masked	0.3037807	13
terminal_MI_nomask	0.2965788	14
terminal_MI_masked	0.2960202	15
terminal_MI_eroded_masked	0.2929226	16
antsAI_masked	0.2923243	17

CSF and ventricles : The analysis is based on 17 algorithms and 20 cases. 0 missing cases have been found in the data set.

	value_mean	rank
Affine_nomask	0.5294593	1
TRSAA_nomask	0.5292491	2
flirt_NA	0.4896304	3
TRSAA_masked	0.4660576	4
TRSAA_eroded_masked	0.4445984	5
Similarity_nomask	0.3990931	6

	value_mean	rank
Affine_eroded_masked	0.3989416	7
Affine_masked	0.3936700	8
Similarity_eroded_masked	0.3356319	9
Similarity_masked	0.3312821	10
terminal_CC_nomask	0.2796990	11
terminal_CC_masked	0.2780243	12
terminal_MI_nomask	0.2581208	13
antsAI_masked	0.2533153	14
terminal_CC_eroded_masked	0.2529898	15
terminal_MI_eroded_masked	0.2493639	16
terminal_MI_masked	0.2450127	17

Deep grey matter : The analysis is based on 17 algorithms and 20 cases. 0 missing cases have been found in the data set.

	value_mean	rank
TRSAA_eroded_masked	0.7930948	1
Affine_eroded_masked	0.7765327	2
TRSAA_masked	0.7632527	3
Affine_masked	0.7493515	4
Similarity_eroded_masked	0.7345197	5
Similarity_masked	0.7242240	6
TRSAA_nomask	0.7130442	7
Affine_nomask	0.7117499	8
Similarity_nomask	0.6701524	9
terminal_CC_nomask	0.6568040	10
terminal_CC_masked	0.6511121	11
flirt_NA	0.6358760	12
terminal_CC_eroded_masked	0.6270243	13
terminal_MI_masked	0.5894733	14
terminal_MI_nomask	0.5724374	15
antsAI_masked	0.5673390	16
terminal_MI_eroded_masked	0.5413141	17

White matter : The analysis is based on 17 algorithms and 20 cases. 0 missing cases have been found in the data set.

	value_mean	rank
TRSAA_eroded_masked	0.7366292	1
TRSAA_masked	0.7218997	2
Affine_eroded_masked	0.7213944	3
TRSAA_nomask	0.7131027	4
Affine_nomask	0.7125896	5
Affine_masked	0.7072164	6
Similarity_eroded_masked	0.6907332	7
Similarity_masked	0.6885260	8
flirt_NA	0.6702183	9
Similarity_nomask	0.6700596	10
terminal_CC_nomask	0.6200043	11
terminal_CC_masked	0.6176143	12

	value_mean	rank
terminal_CC_eroded_masked	0.6017118	13
terminal_MI_masked	0.5873799	14
antsAI_masked	0.5837887	15
terminal_MI_nomask	0.5816084	16
terminal_MI_eroded_masked	0.5673386	17

Consensus ranking across tasks according to chosen method "euclidean":

	value	rank
TRSAA_eroded_masked	1.833	1
Affine_eroded_masked	3.500	2
TRSAA_masked	3.500	2
TRSAA_nomask	4.000	4
Affine_nomask	5.000	5
Affine_masked	6.000	6
Similarity_eroded_masked	6.167	7
Similarity_masked	7.833	8
Similarity_nomask	8.667	9
flirt_NA	9.167	10
terminal_CC_nomask	11.000	11
terminal_CC_masked	11.333	12
terminal_CC_eroded_masked	13.333	13
terminal_MI_masked	14.667	14
terminal_MI_nomask	14.667	14
antsAI_masked	15.833	16
terminal_MI_eroded_masked	16.500	17

2 Visualization of raw assessment data

The algorithms are ordered according to the computed ranks for each task.

2.1 Dot- and boxplot

Dot- and boxplots for visualizing raw assessment data separately for each algorithm. Boxplots representing descriptive statistics over all cases (median, quartiles and outliers) are combined with horizontally jittered dots representing individual cases.

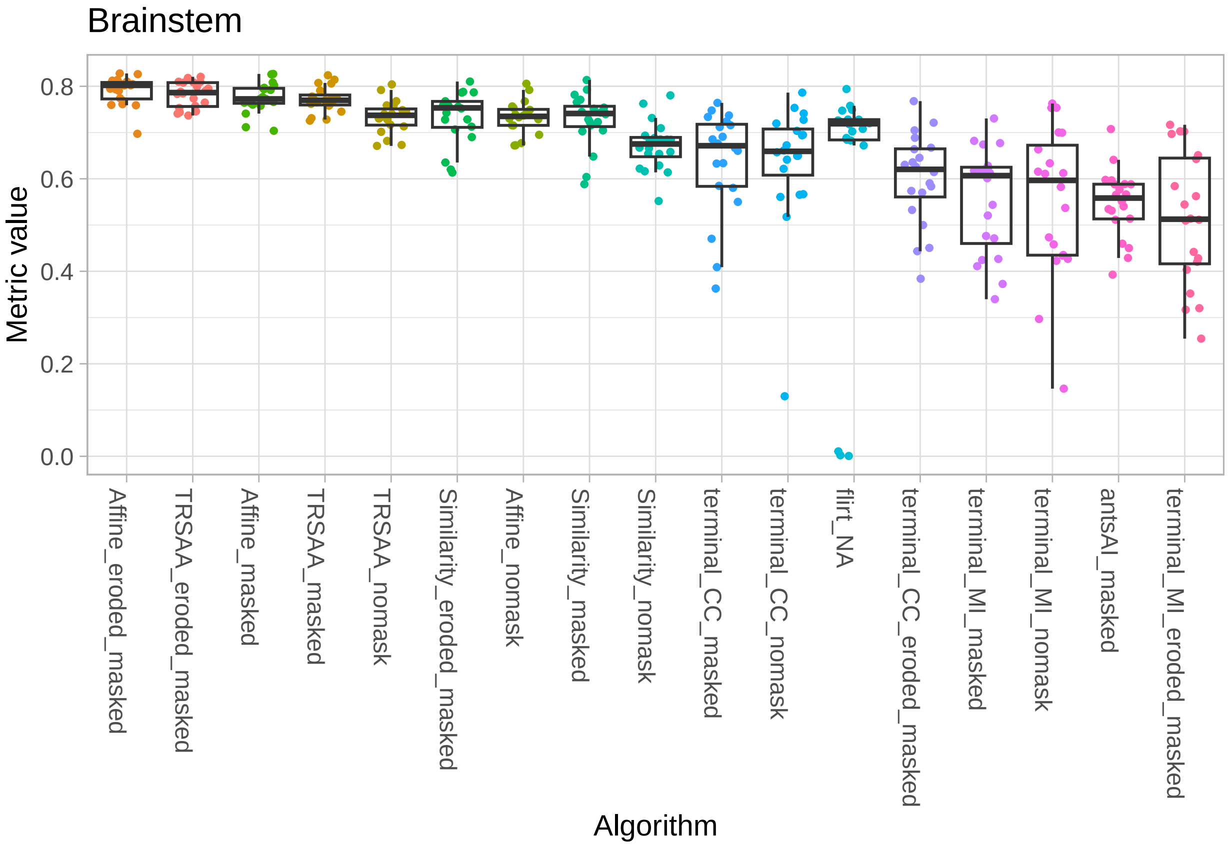

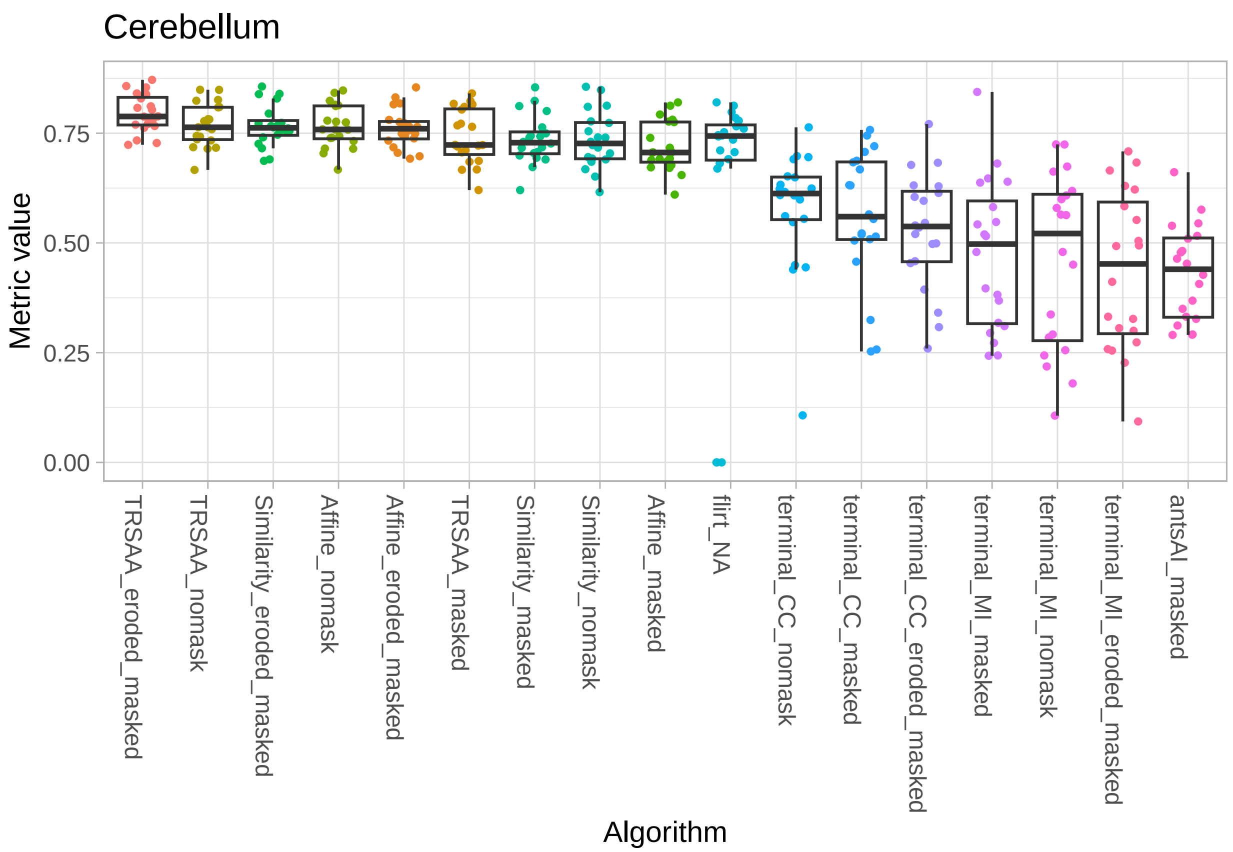
Cerebellum
Metric value
0.75
0.50
0.25
0.00
TRSAA_eroded_masked
TRSAA_nomask
Similarity_eroded_masked
Affine_nomask
Affine_eroded_masked
TRSAA_masked
Similarity_masked
Similarity_nomask
Affine_masked
flirt_NA
terminal_CC_nomask
terminal_CC_masked
terminal_CC_eroded_masked
terminal_MI_masked
terminal_MI_nomask
terminal_MI_eroded_masked
antsAI_masked
Algorithm

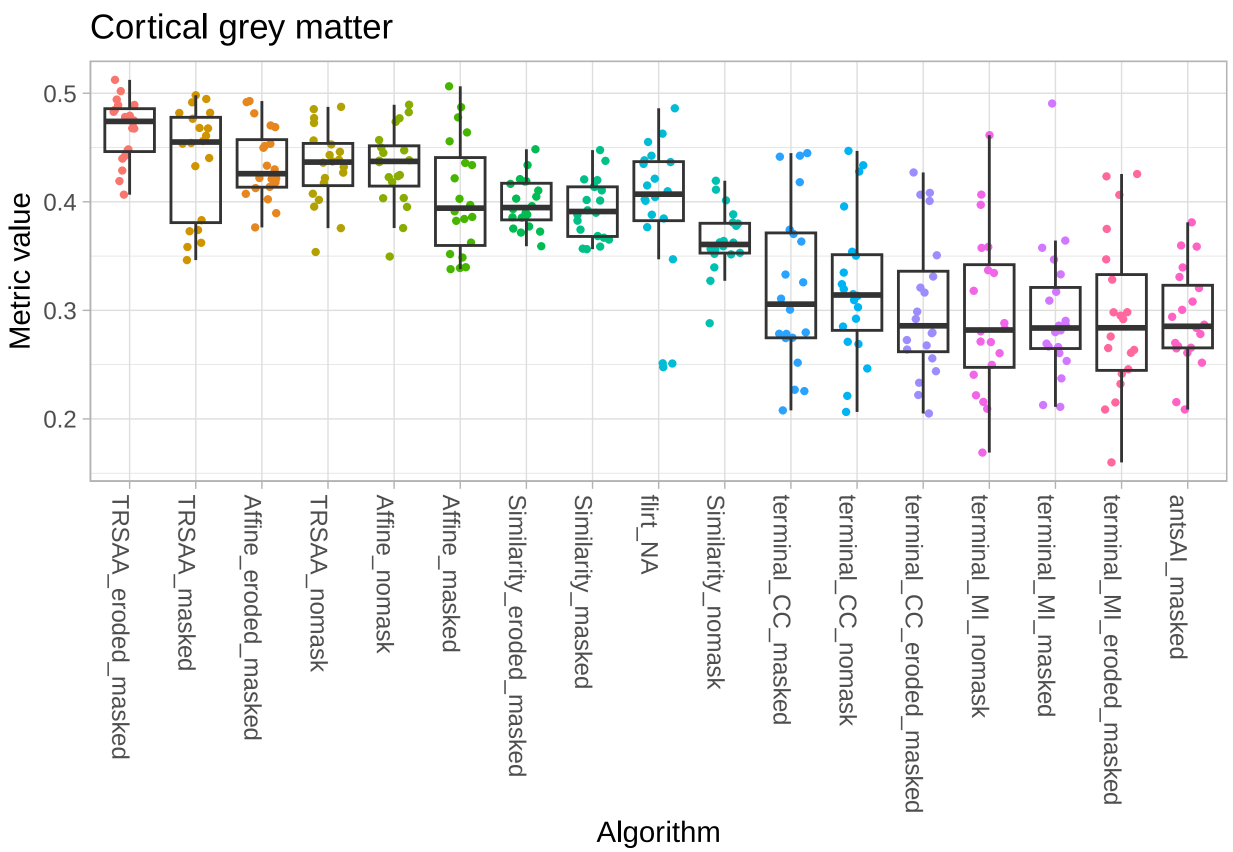
Cortical grey matter
Metric value
0.5
0.4
0.3
0.2
TRSAA_eroded_masked
TRSAA_masked
Affine_eroded_masked
TRSAA_nomask
Affine_nomask
Affine_masked
Similarity_eroded_masked
Similarity_masked
flirt_NA
Similarity_nomask
terminal_CC_masked
terminal_CC_nomask
terminal_CC_eroded_masked
terminal_MI_nomask
terminal_MI_masked
terminal_MI_eroded_masked
antsAI_masked
Algorithm

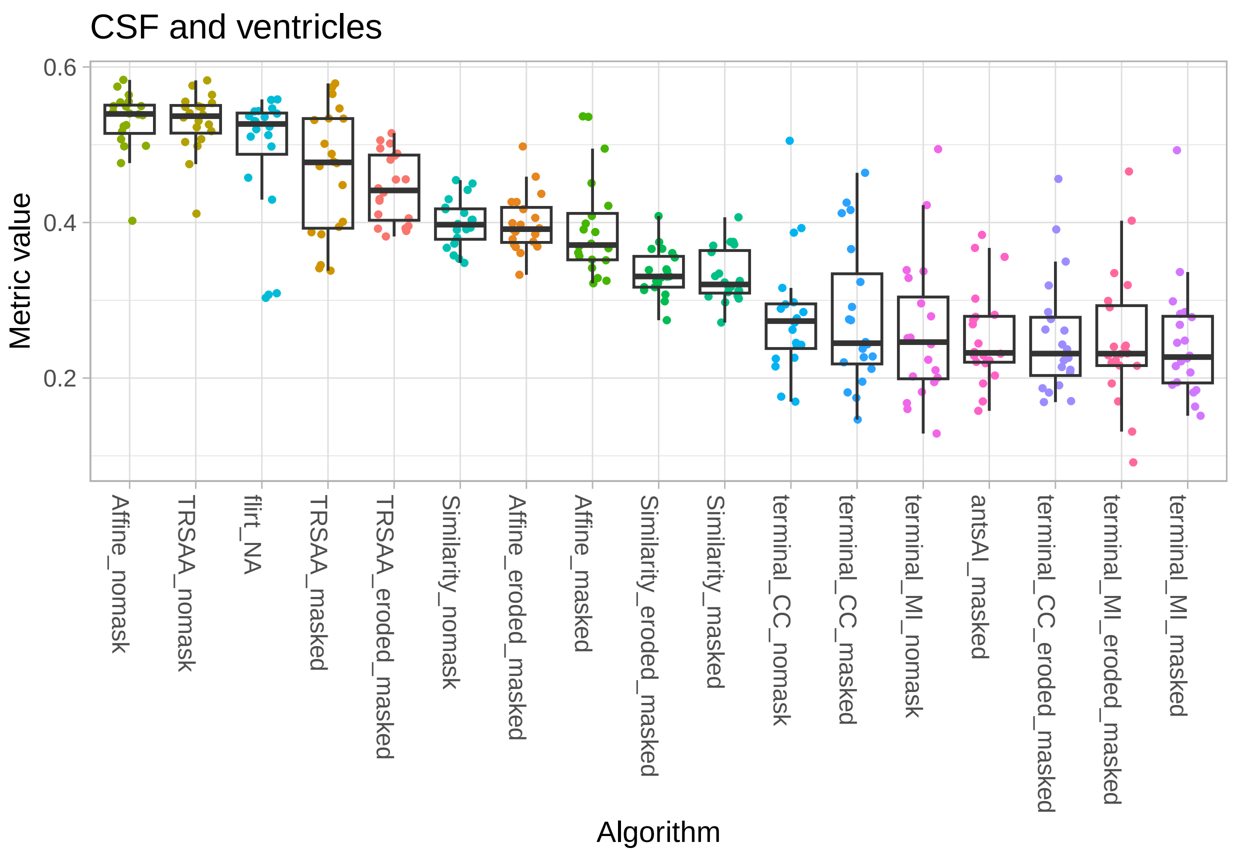

CSF and ventricles
Metric value
0.6
0.4
0.2
Affine_nomask
TRSAA_nomask
flirt_NA
TRSAA_masked
TRSAA_eroded_masked
Similarity_nomask
Affine_eroded_masked
Affine_masked
Similarity_eroded_masked
Similarity_masked
terminal_CC_nomask
terminal_CC_masked
terminal_MI_nomask
antsAI_masked
terminal_CC_eroded_masked
terminal_MI_eroded_masked
terminal_MI_masked
Algorithm

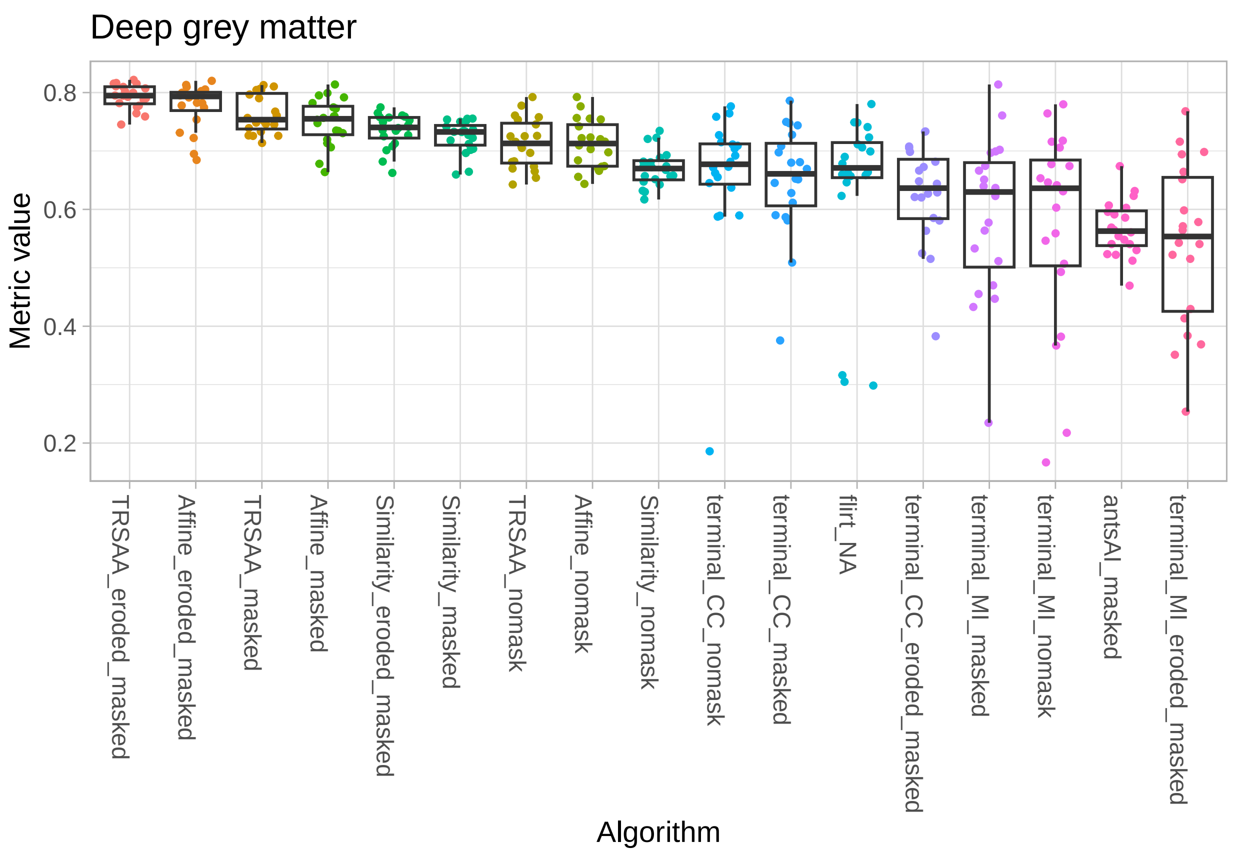
Deep grey matter
Metric value
0.8
0.6
0.4
0.2
TRSAA_eroded_masked
Affine_eroded_masked
TRSAA_masked
Affine_masked
Similarity_eroded_masked
Similarity_masked
TRSAA_nomask
Affine_nomask
Similarity_nomask
terminal_CC_nomask
terminal_CC_masked
flirt_NA
terminal_CC_eroded_masked
terminal_MI_masked
terminal_MI_nomask
antsAI_masked
terminal_MI_eroded_masked
Algorithm

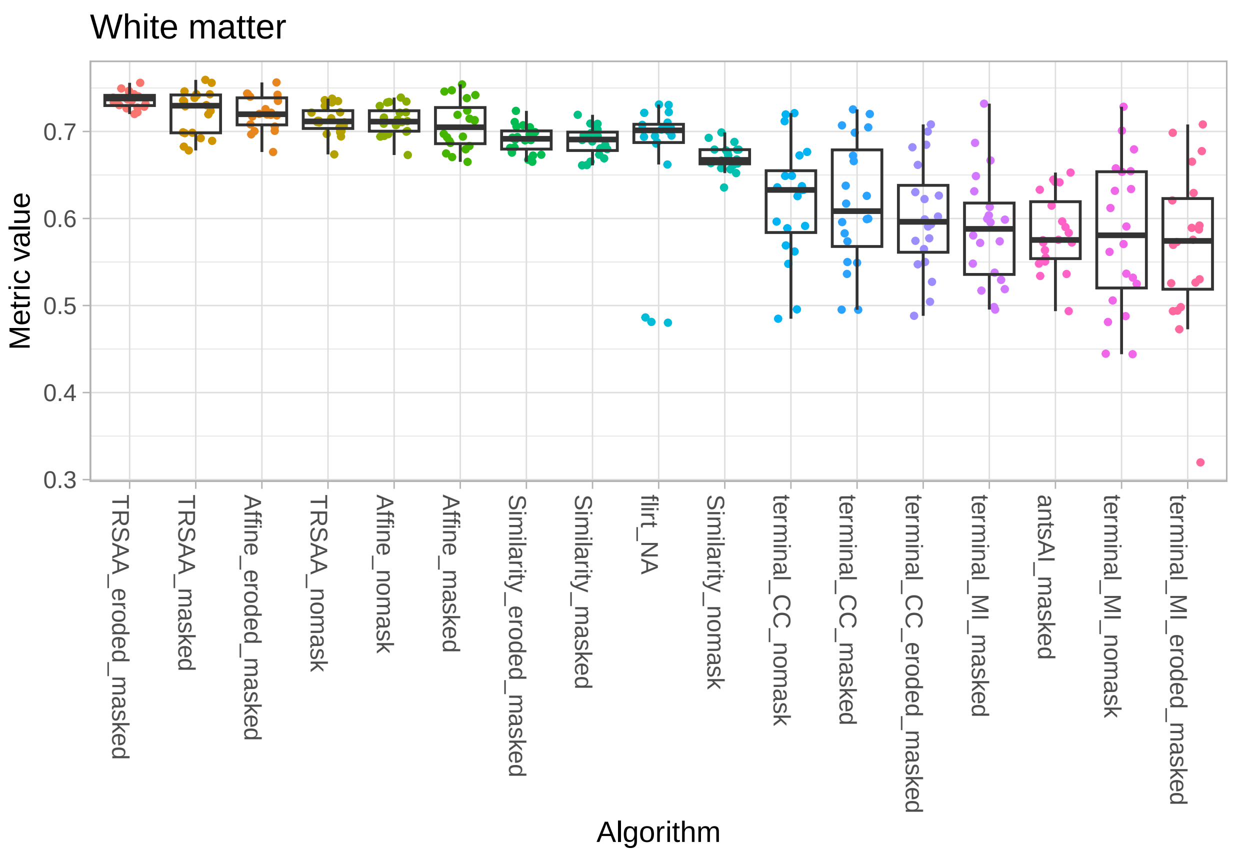

White matter
Metric value
0.7
0.6
0.5
0.4
0.3
TRSAA_eroded_masked
TRSAA_masked
Affine_eroded_masked
TRSAA_nomask
Affine_nomask
Affine_masked
Similarity_eroded_masked
Similarity_masked
flirt_NA
Similarity_nomask
terminal_CC_nomask
terminal_CC_masked
terminal_CC_eroded_masked
terminal_MI_masked
antsAI_masked
terminal_MI_nomask
terminal_MI_eroded_masked
Algorithm

2.2 Podium plot

Podium plots (see also Eugster et al., 2008) for visualizing raw assessment data. Upper part (spaghetti plot): Participating algorithms are color-coded, and each colored dot in the plot represents a metric value achieved with the respective algorithm. The actual metric value is encoded by the y-axis. Each podium (here: p=17) represents one possible rank, ordered from best (1) to last (here: 17). The assignment of metric values (i.e. colored dots) to one of the podiums is based on the rank that the respective algorithm achieved on the corresponding case. Note that the plot part above each podium place is further subdivided into p "columns", where each column represents one participating algorithm (here: p = 17). Dots corresponding to identical cases are connected by a line, leading to the shown spaghetti structure. Lower part: Bar charts represent the relative frequency for each algorithm to achieve the rank encoded by the podium place.

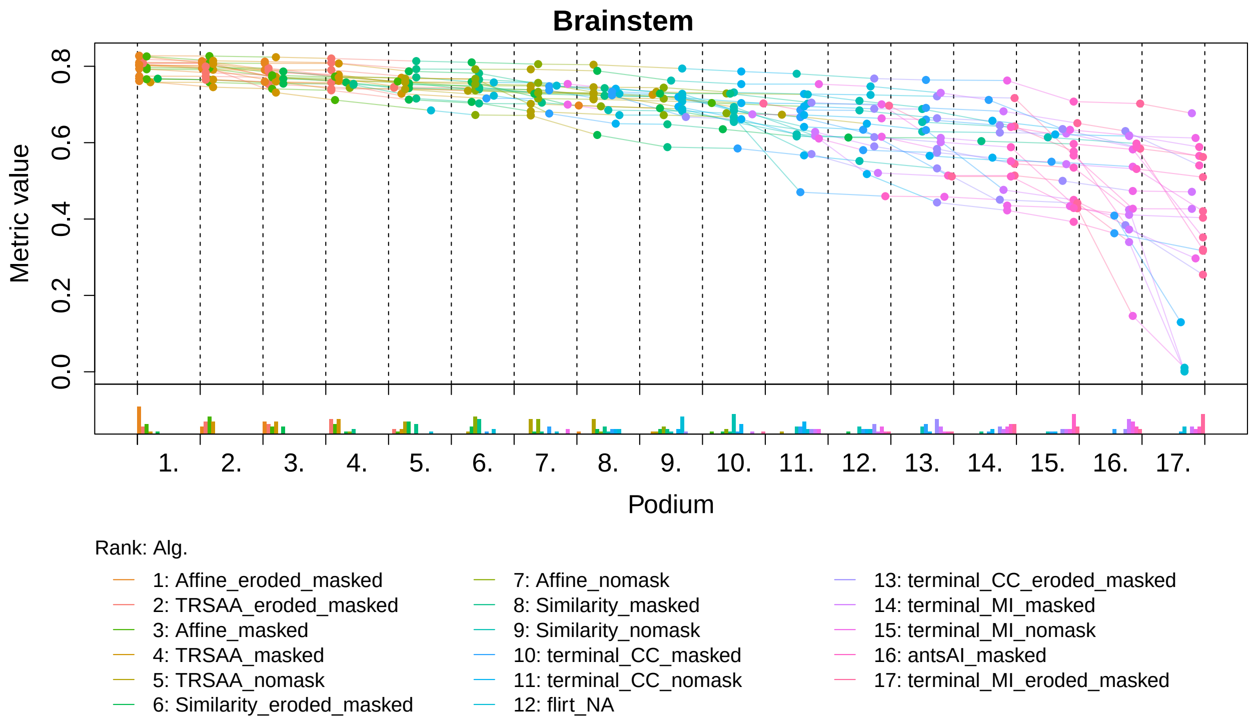

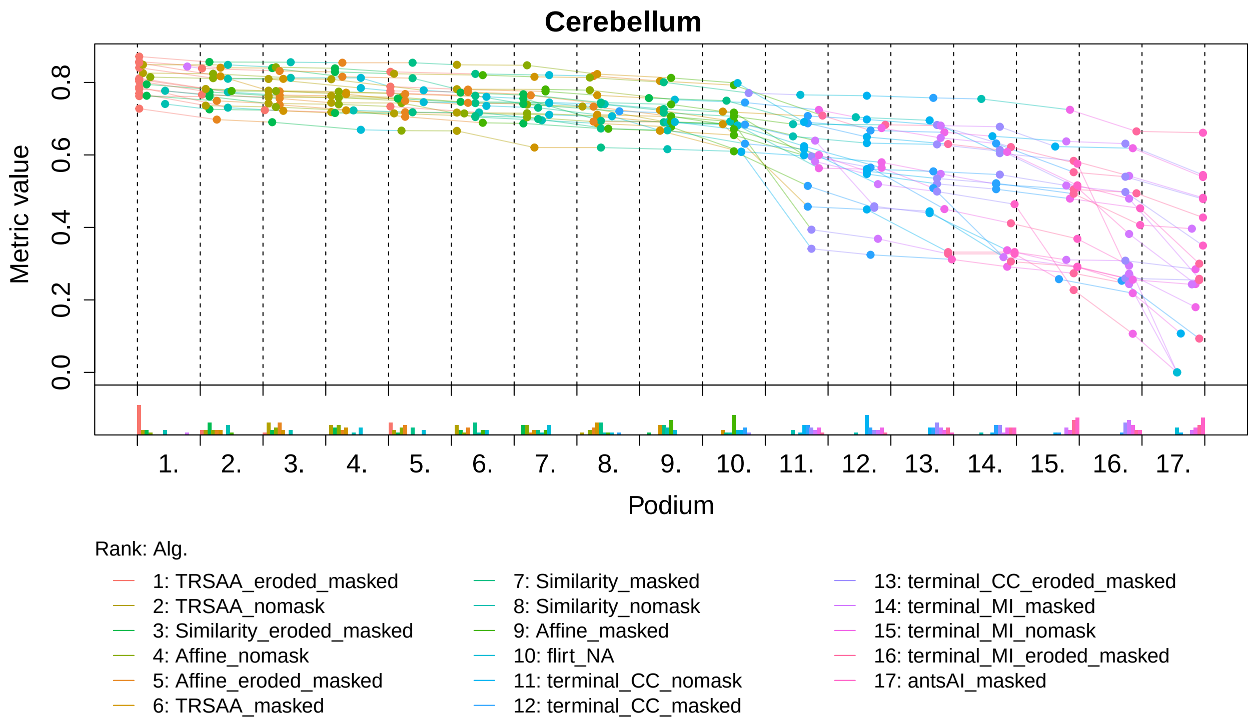
Cerebellum
Metric value
0.0
0.2
0.4
0.6
0.8
1.
2.
3.
4.
5.
6.
7.
8.
9.
10.
11.
12.
13.
14.
15.
16.
17.
Podium
Rank: Alg.
1: TRSAA_eroded_masked
2: TRSAA_nomask
3: Similarity_eroded_masked
4: Affine_nomask
5: Affine_eroded_masked
6: TRSAA_masked
7: Similarity_masked
8: Similarity_nomask
9: Affine_masked
10: flirt_NA
11: terminal_CC_nomask
12: terminal_CC_masked
13: terminal_CC_eroded_masked
14: terminal_MI_masked
15: terminal_MI_nomask
16: terminal_MI_eroded_masked
17: antsAI_masked

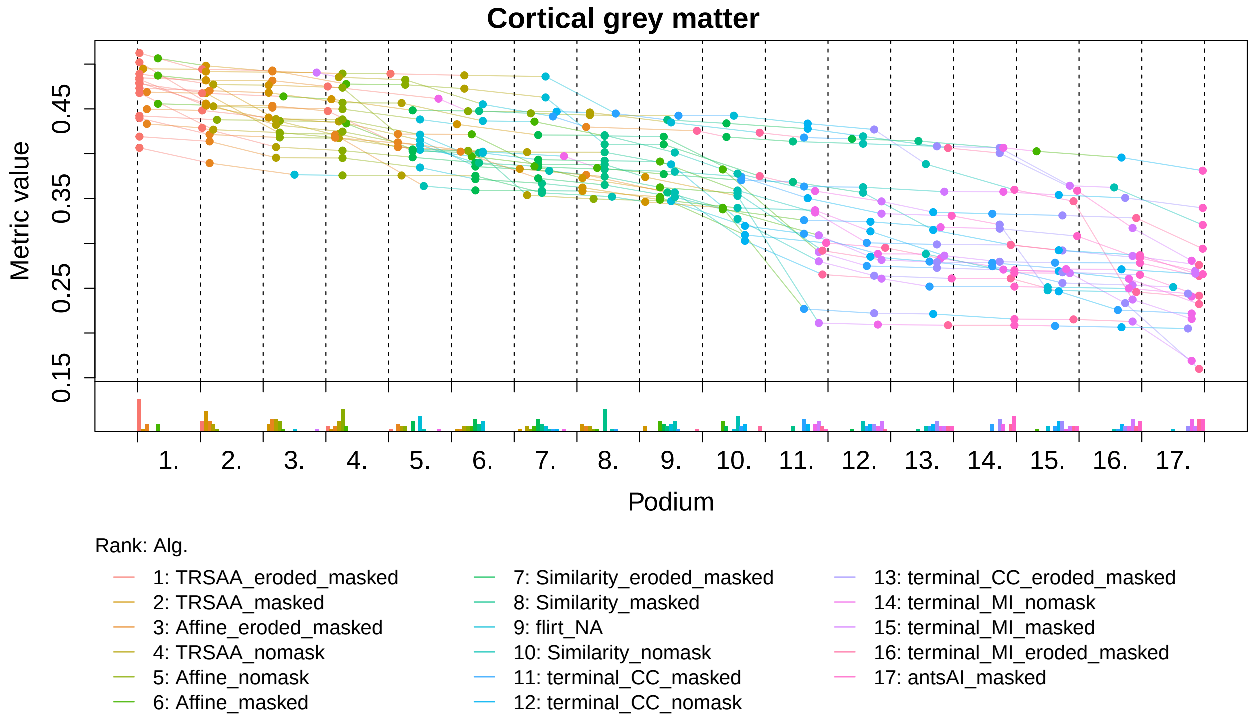
Cortical grey matter
Metric value
0.15
0.25
0.35
0.45
1.
2.
3.
4.
5.
6.
7.
8.
9.
10.
11.
12.
13.
14.
15.
16.
17.
Podium
Rank: Alg.
1: TRSAA_eroded_masked
2: TRSAA_masked
3: Affine_eroded_masked
4: TRSAA_nomask
5: Affine_nomask
6: Affine_masked
7: Similarity_eroded_masked
8: Similarity_masked
9: flirt_NA
10: Similarity_nomask
11: terminal_CC_masked
12: terminal_CC_nomask
13: terminal_CC_eroded_masked
14: terminal_MI_nomask
15: terminal_MI_masked
16: terminal_MI_eroded_masked
17: antsAI_masked

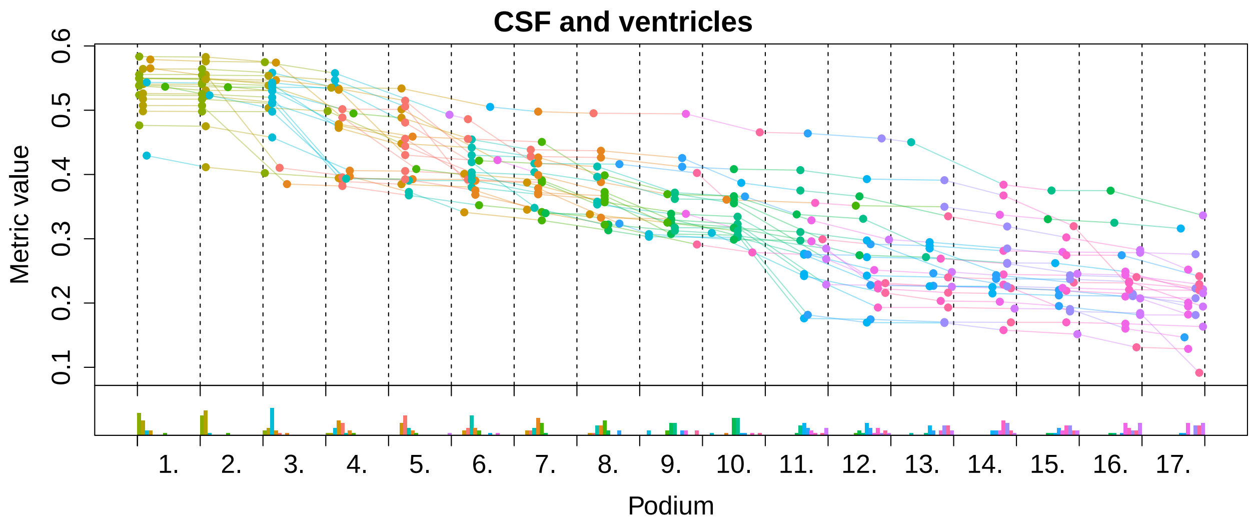

Rank: Alg.

1: Affine_nomask
2: TRSAA_nomask
3: flirt_NA
4: TRSAA_masked
5: TRSAA_eroded_masked
6: Similarity_nomask
7: Affine_eroded_masked
8: Affine_masked
9: Similarity_eroded_masked
10: Similarity_masked
11: terminal_CC_nomask
12: terminal_CC_masked
13: terminal_MI_nomask
14: antsAI_masked
15: terminal_CC_eroded_masked
16: terminal_MI_eroded_masked
17: terminal_MI_masked

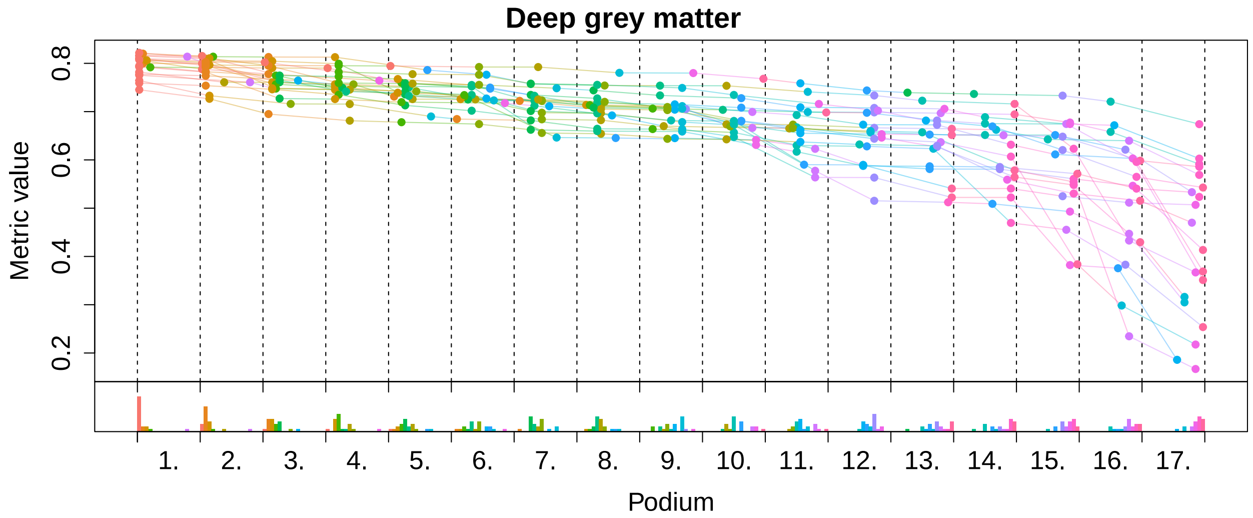

Rank: Alg.

1: TRSAA_eroded_masked
2: Affine_eroded_masked
3: TRSAA_masked
4: Affine_masked
5: Similarity_eroded_masked
6: Similarity_masked
7: TRSAA_nomask
8: Affine_nomask
9: Similarity_nomask
10: terminal_CC_nomask
11: terminal_CC_masked
12: flirt_NA
13: terminal_CC_eroded_masked
14: terminal_MI_masked
15: terminal_MI_nomask
16: antsAI_masked
17: terminal_MI_eroded_masked

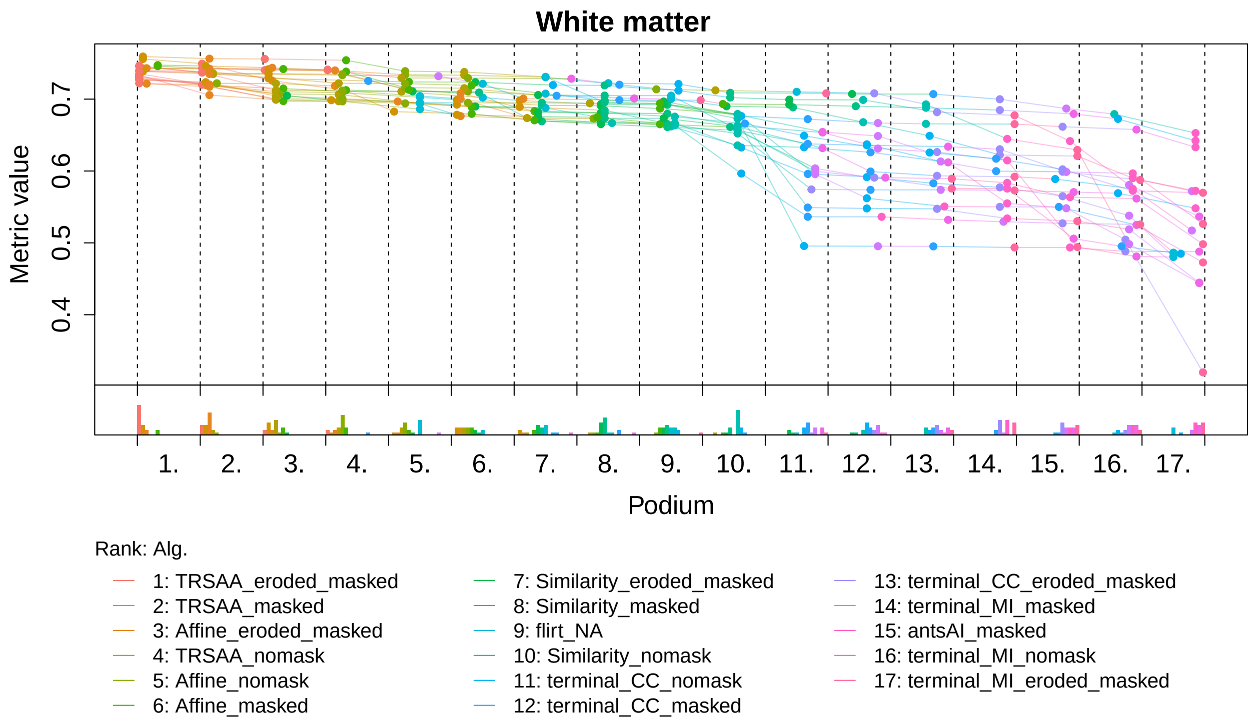
White matter
Metric value
0.4
0.5
0.6
0.7
1.
2.
3.
4.
5.
6.
7.
8.
9.
10.
11.
12.
13.
14.
15.
16.
17.
Podium
Rank: Alg.
1: TRSAA_eroded_masked
2: TRSAA_masked
3: Affine_eroded_masked
4: TRSAA_nomask
5: Affine_nomask
6: Affine_masked
7: Similarity_eroded_masked
8: Similarity_masked
9: flirt_NA
10: Similarity_nomask
11: terminal_CC_nomask
12: terminal_CC_masked
13: terminal_CC_eroded_masked
14: terminal_MI_masked
15: antsAI_masked
16: terminal_MI_nomask
17: terminal_MI_eroded_masked

2.3 Ranking heatmap

Ranking heatmaps for visualizing raw assessment data. Each cell (i, A_j) shows the absolute frequency of cases in which algorithm A_j achieved rank i.

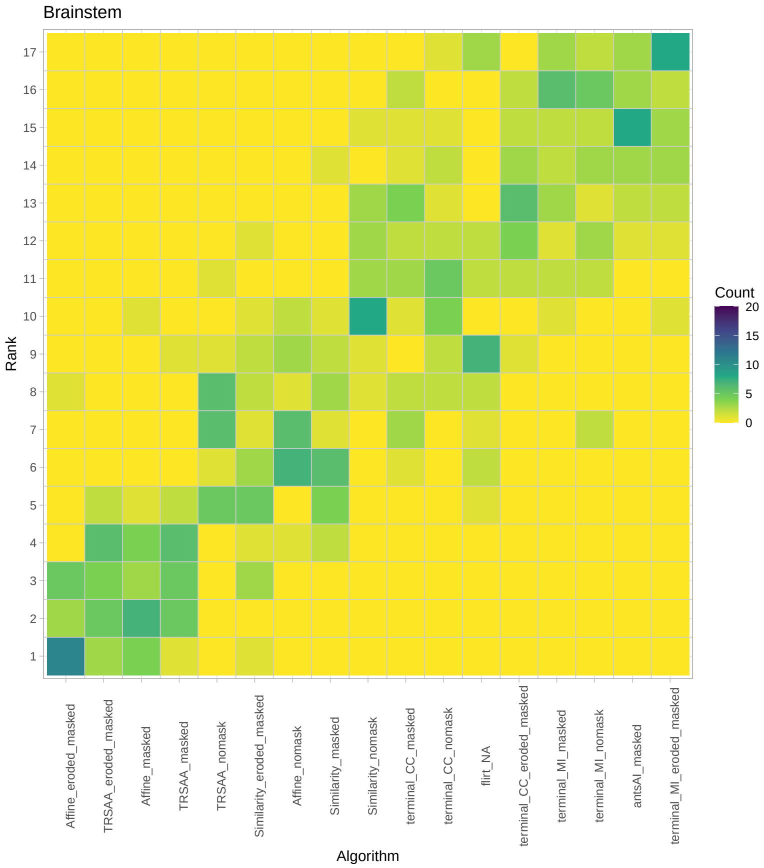

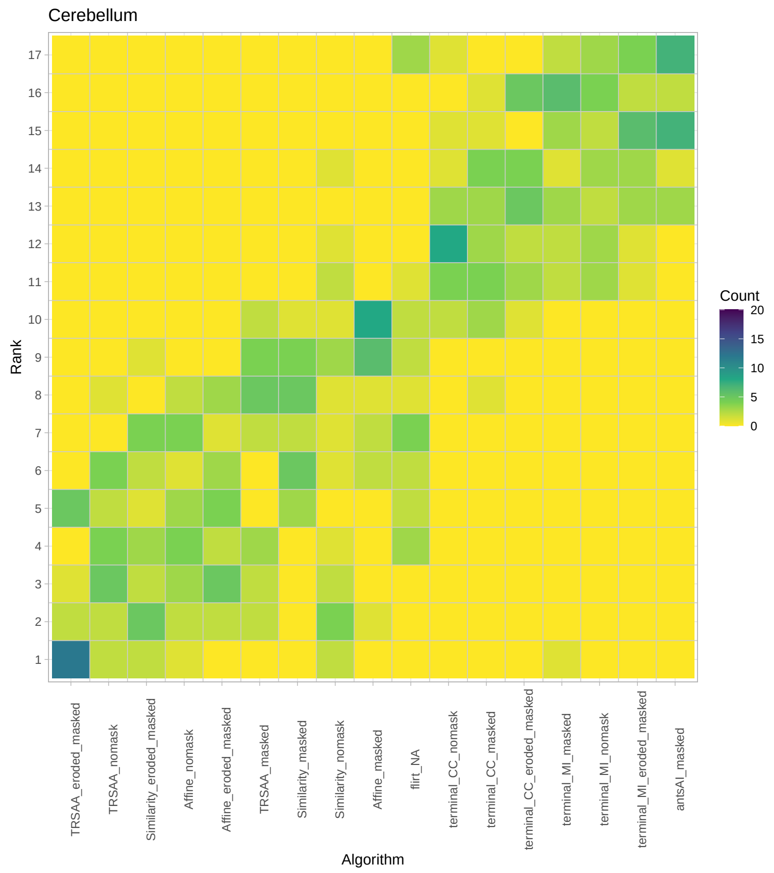

Cerebellum
Rank
17
16
15
14
13
12
11
10
9
8
7
6
5
4
3
2
1
Count
20
15
10
5
0
TRSAA_eroded_masked
TRSAA_nomask
Similarity_eroded_masked
Affine_nomask
Affine_eroded_masked
TRSAA_masked
Similarity_masked
Similarity_nomask
Affine_masked
flirt_NA
terminal_CC_nomask
terminal_CC_masked
terminal_CC_eroded_masked
terminal_MI_masked
terminal_MI_nomask
terminal_MI_eroded_masked
antsAI_masked
Algorithm

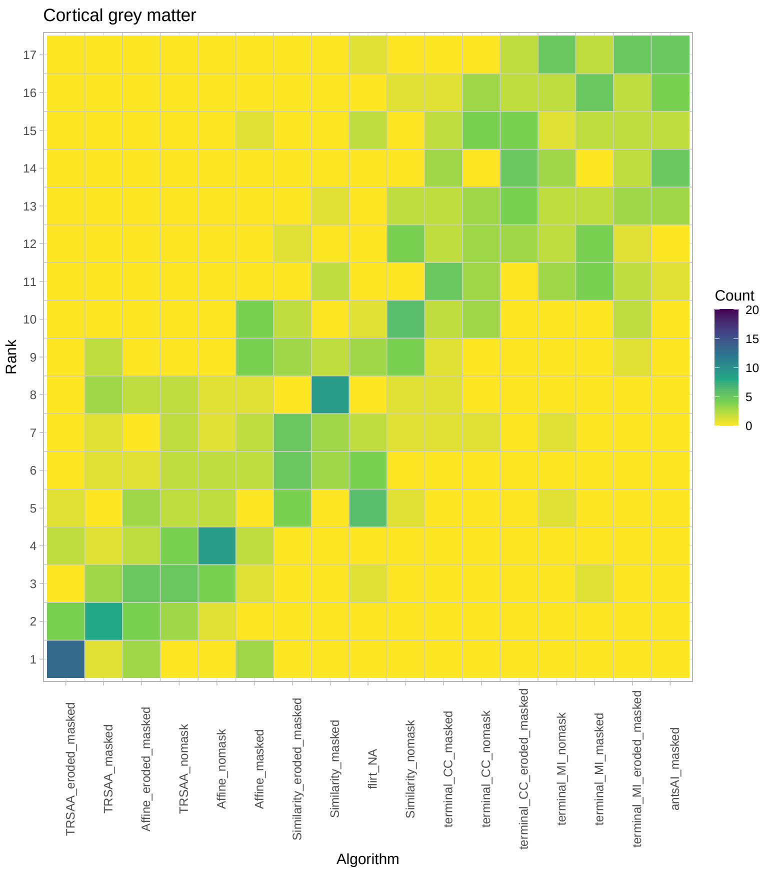

Cortical grey matter
Rank
17
16
15
14
13
12
11
10
9
8
7
6
5
4
3
2
1
Count
20
15
10
5
0
TRSAA_eroded_masked
TRSAA_masked
Affine_eroded_masked
TRSAA_nomask
Affine_nomask
Affine_masked
Similarity_eroded_masked
Similarity_masked
flirt_NA
Similarity_nomask
terminal_CC_masked
terminal_CC_nomask
terminal_CC_eroded_masked
terminal_MI_nomask
terminal_MI_masked
terminal_MI_eroded_masked
antsAI_masked
Algorithm

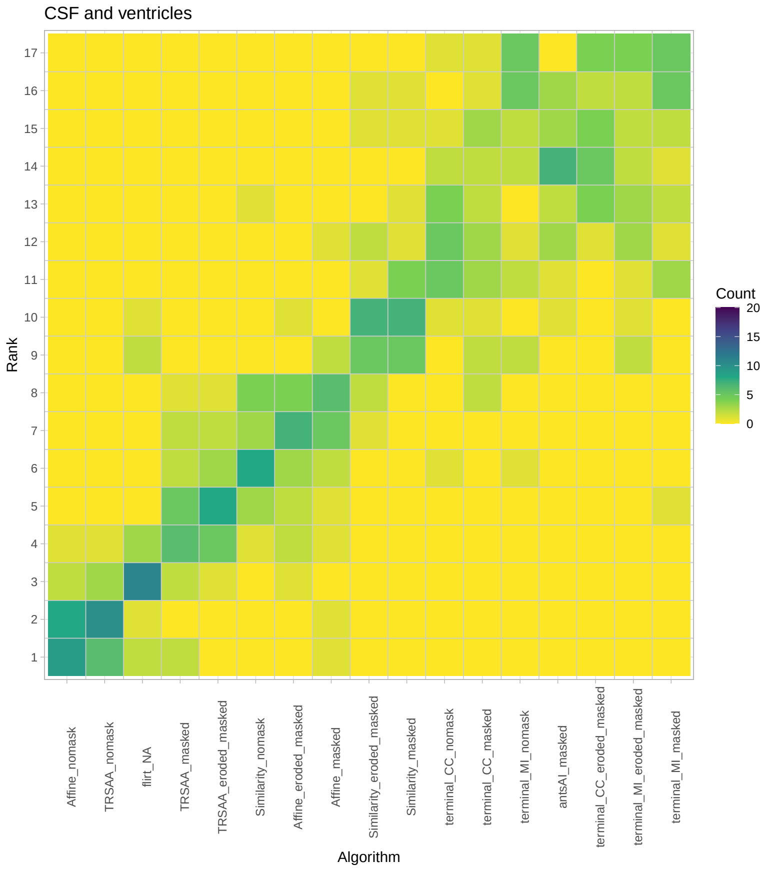
CSF and ventricles
Rank
17
16
15
14
13
12
11
10
9
8
7
6
5
4
3
2
1
Count
20
15
10
5
0
Affine_nomask
TRSAA_nomask
flirt_NA
TRSAA_masked
TRSAA_eroded_masked
Similarity_nomask
Affine_eroded_masked
Affine_masked
Similarity_eroded_masked
Similarity_masked
terminal_CC_nomask
terminal_CC_masked
terminal_MI_nomask
antsAI_masked
terminal_CC_eroded_masked
terminal_MI_eroded_masked
terminal_MI_masked
Algorithm

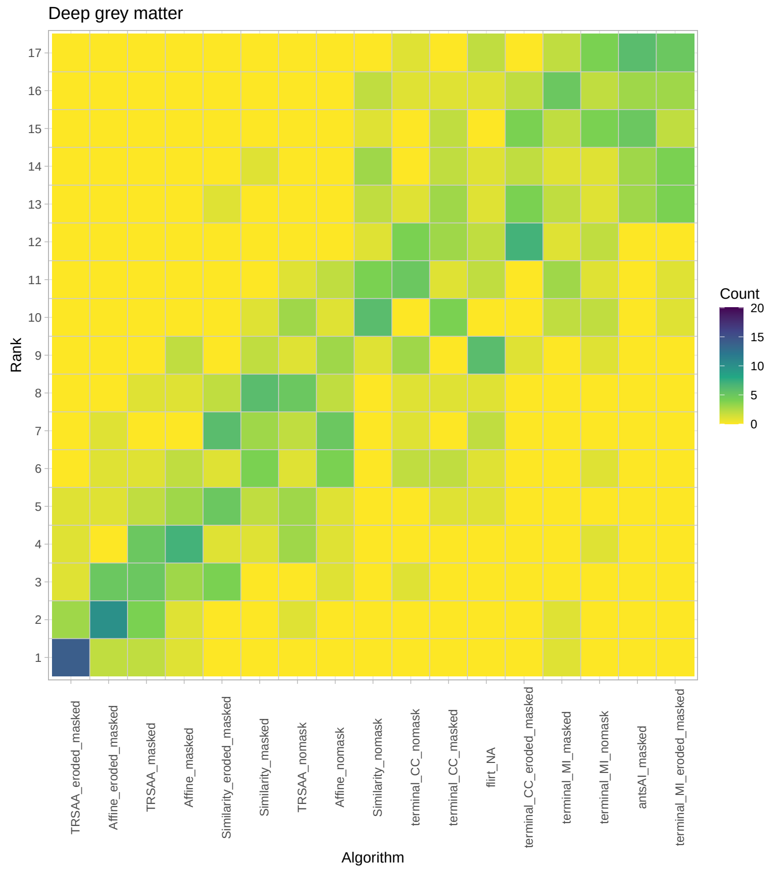

Deep grey matter
Rank
17
16
15
14
13
12
11
10
9
8
7
6
5
4
3
2
1
Count
20
15
10
5
0
TRSAA_eroded_masked
Affine_eroded_masked
TRSAA_masked
Affine_masked
Similarity_eroded_masked
Similarity_masked
TRSAA_nomask
Affine_nomask
Similarity_nomask
terminal_CC_nomask
terminal_CC_masked
flirt_NA
terminal_CC_eroded_masked
terminal_MI_masked
terminal_MI_nomask
antsAI_masked
terminal_MI_eroded_masked
Algorithm

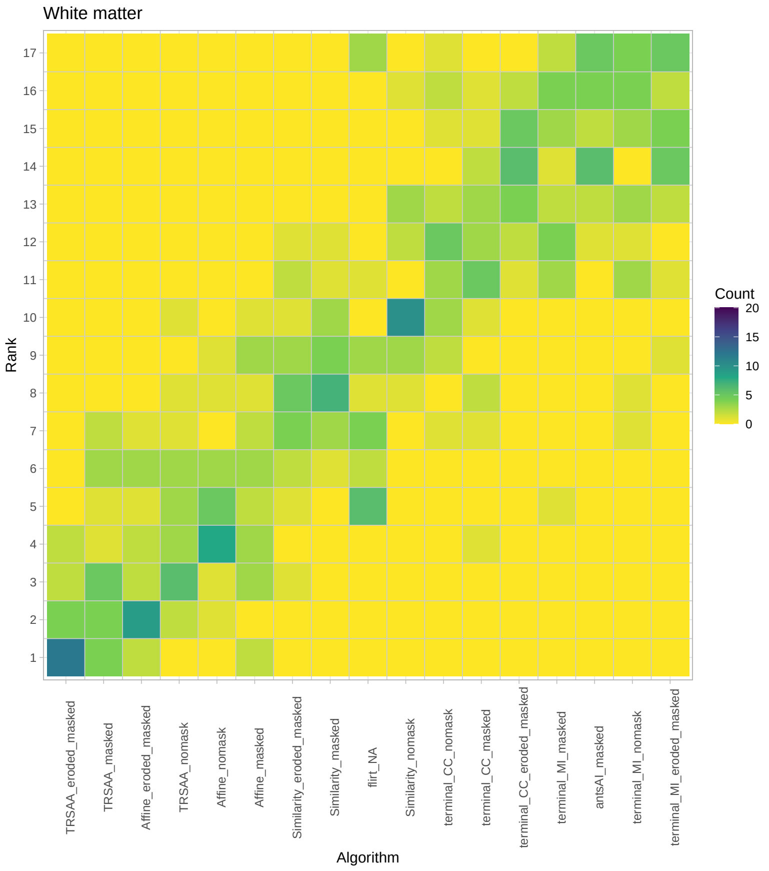
White matter
Count
20
15
10
5
0
Rank
17
16
15
14
13
12
11
10
9
8
7
6
5
4
3
2
1
TRSAA_eroded_masked
TRSAA_masked
Affine_eroded_masked
TRSAA_nomask
Affine_nomask
Affine_masked
Similarity_eroded_masked
Similarity_masked
flirt_NA
Similarity_nomask
terminal_CC_nomask
terminal_CC_masked
terminal_CC_eroded_masked
terminal_MI_masked
antsAI_masked
terminal_MI_nomask
terminal_MI_eroded_masked
Algorithm

3 Visualization of ranking stability

3.1 *Blob plot* for visualizing ranking stability based on bootstrap sampling

Algorithms are color-coded, and the area of each blob at position (A_i, rank j) is proportional to the relative frequency A_i achieved rank j across $b = 1000$ bootstrap samples. The median rank for each algorithm is indicated by a black cross. 95% bootstrap intervals across bootstrap samples are indicated by black lines.

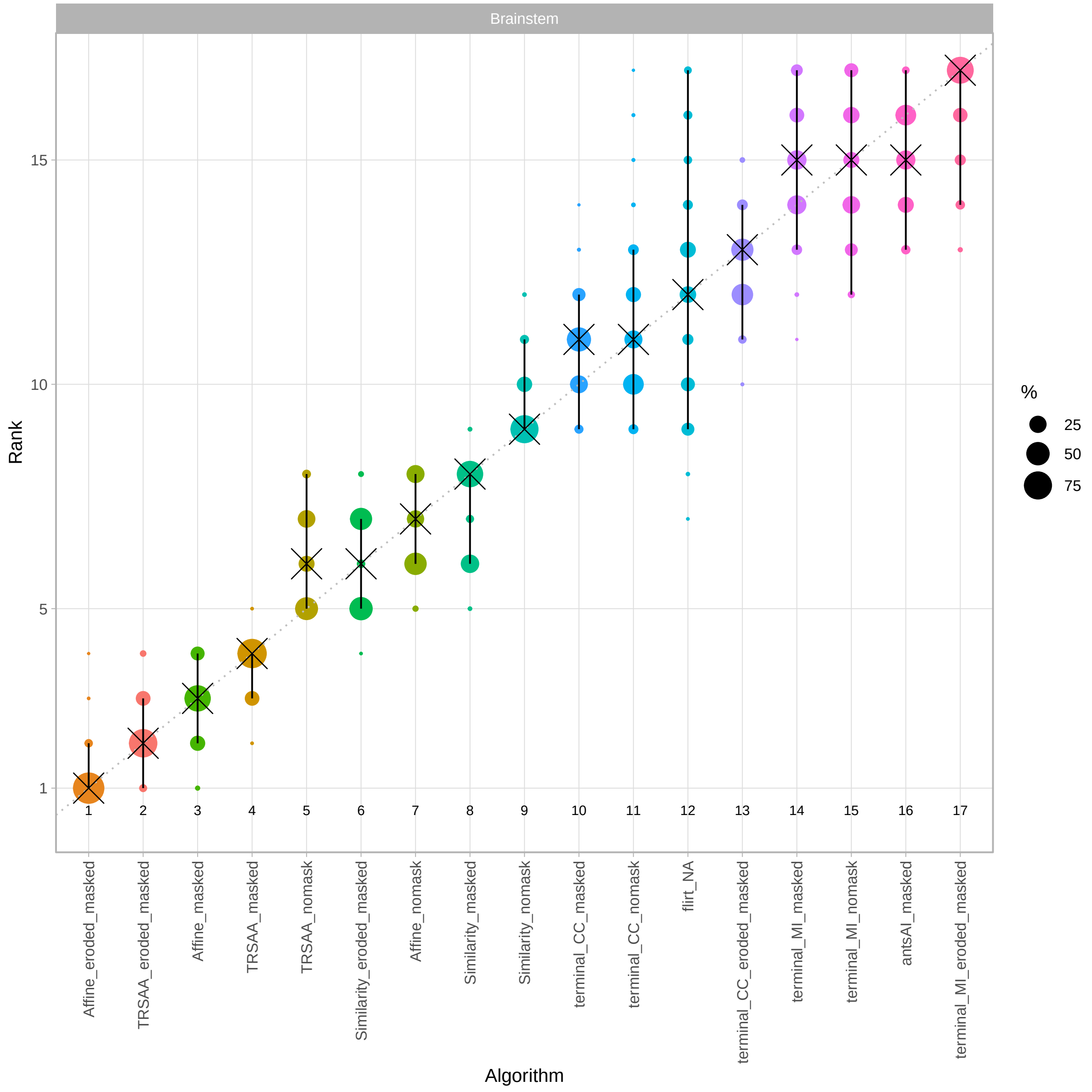

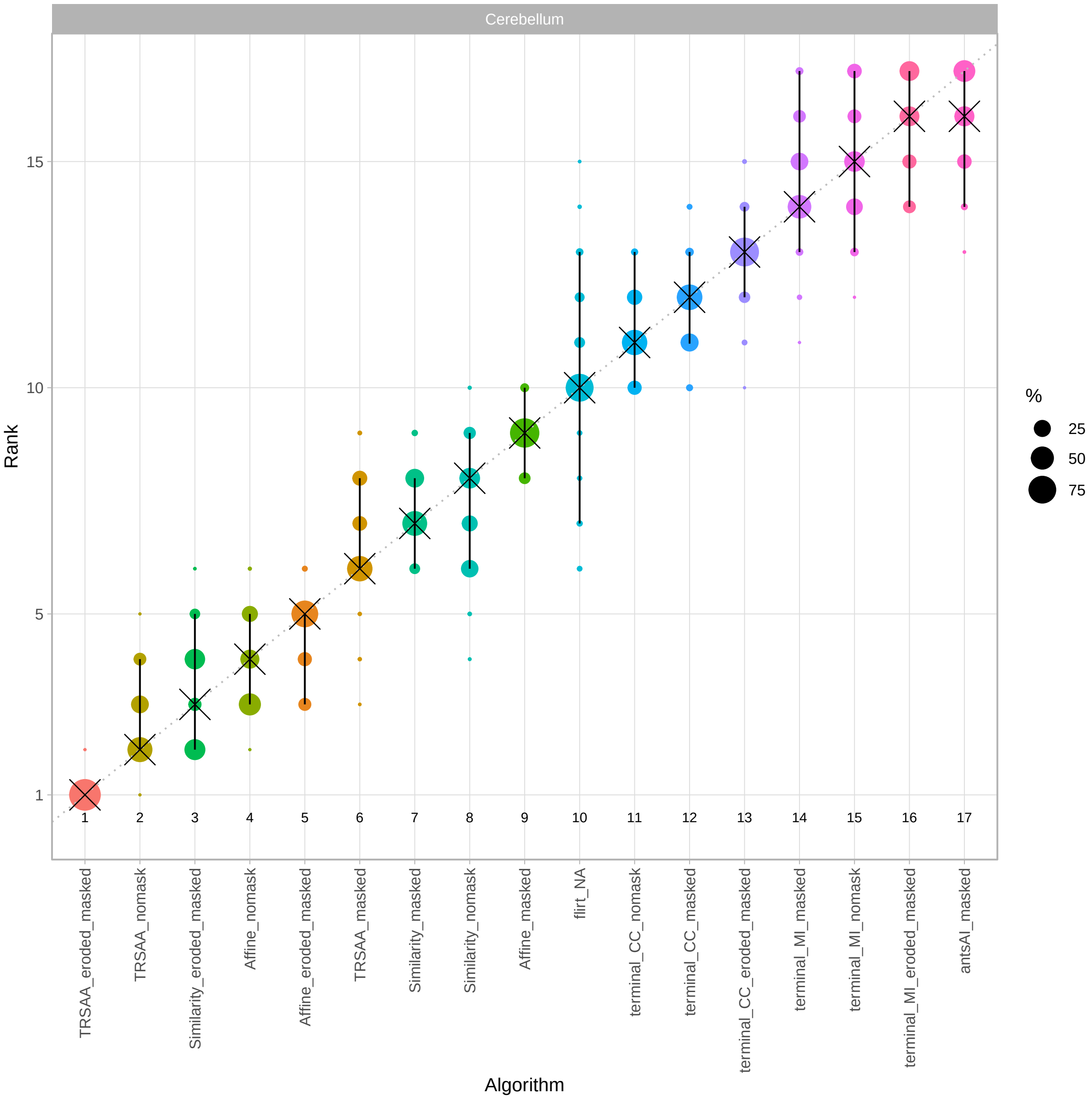
Cerebellum
Rank
Algorithm
%
25
50
75
TRSAA_eroded_masked
TRSAA_nomask
Similarity_eroded_masked
Affine_nomask
Affine_eroded_masked
TRSAA_masked
Similarity_masked
Similarity_nomask
Affine_masked
flirt_NA
terminal_CC_nomask
terminal_CC_masked
terminal_CC_eroded_masked
terminal_MI_masked
terminal_MI_nomask
terminal_MI_eroded_masked
antsAI_masked

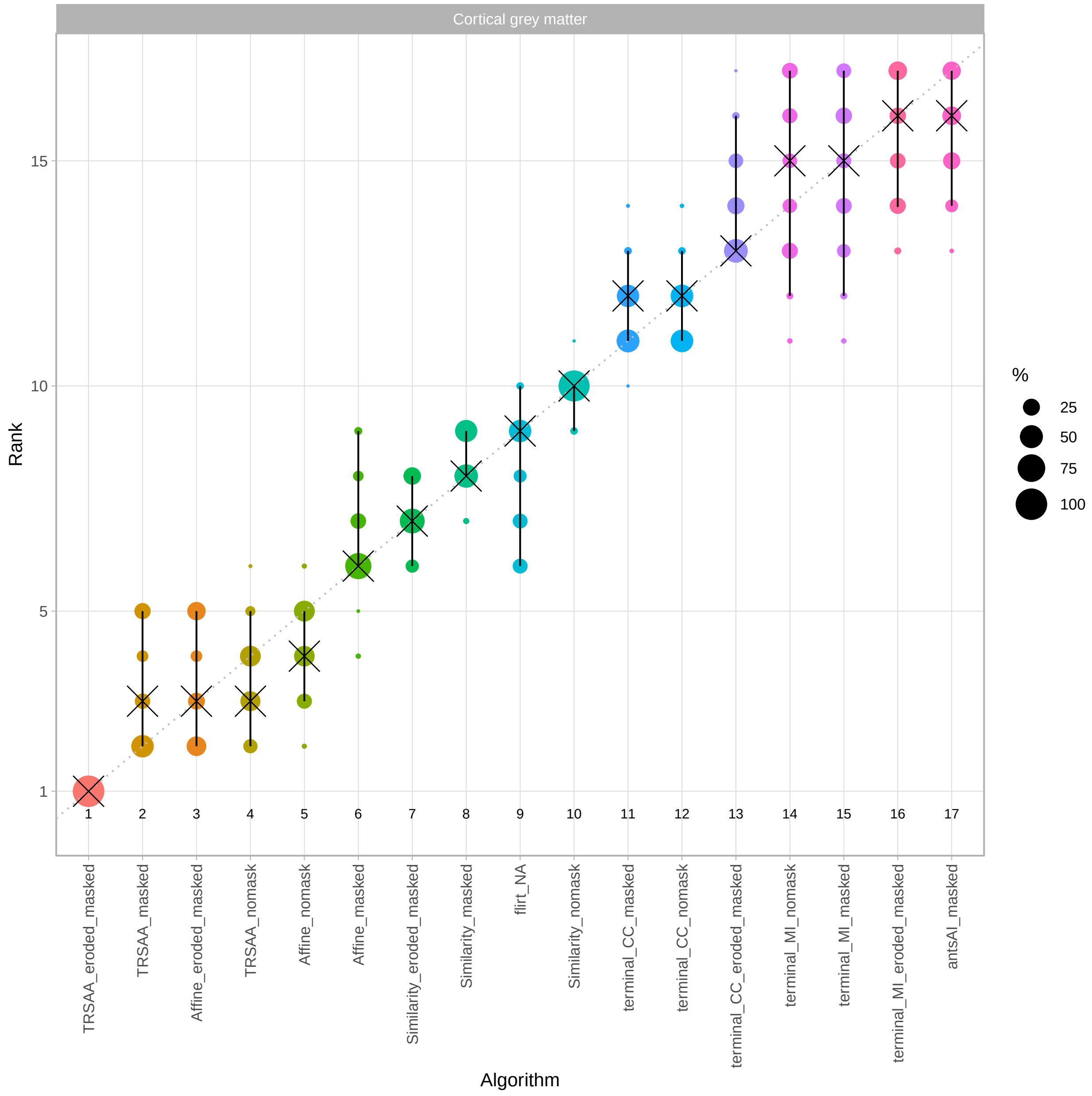

Cortical grey matter
Rank
Algorithm
%
25
50
75
100
TRSAA_eroded_masked
TRSAA_masked
Affine_eroded_masked
TRSAA_nomask
Affine_nomask
Affine_masked
Similarity_eroded_masked
Similarity_masked
flirt_NA
Similarity_nomask
terminal_CC_masked
terminal_CC_nomask
terminal_CC_eroded_masked
terminal_MI_nomask
terminal_MI_masked
terminal_MI_eroded_masked
antsAI_masked

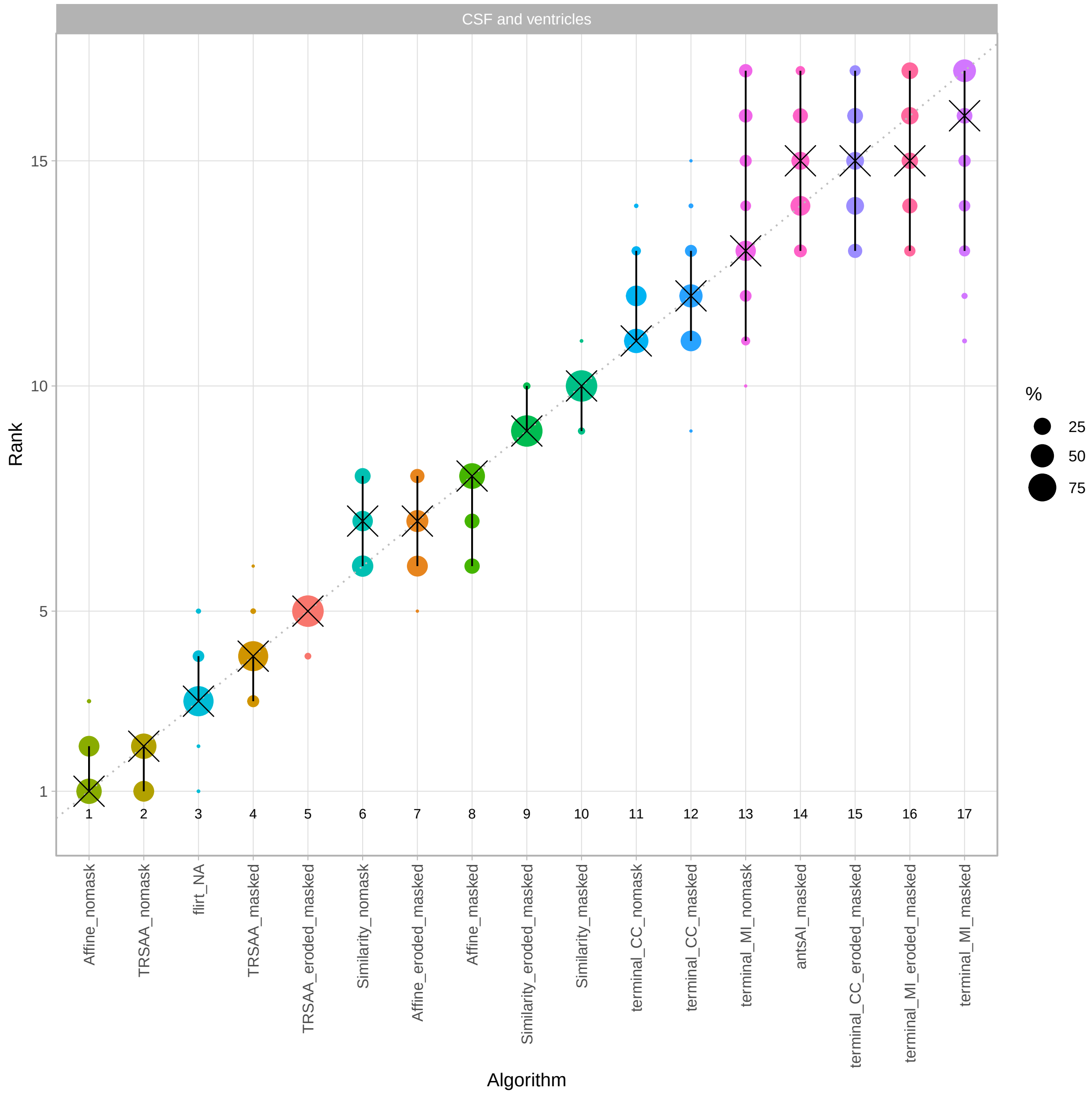

CSF and ventricles
Rank
Algorithm
%
25
50
75
1
5
10
15
1 2 3 4 5 6 7 8 9 10 11 12 13 14 15 16 17
Affine_nomask
TRSAA_nomask
flirt_NA
TRSAA_masked
TRSAA_eroded_masked
Similarity_nomask
Affine_eroded_masked
Affine_masked
Similarity_eroded_masked
Similarity_masked
terminal_CC_nomask
terminal_CC_masked
terminal_MI_nomask
antsAI_masked
terminal_CC_eroded_masked
terminal_MI_eroded_masked
terminal_MI_masked

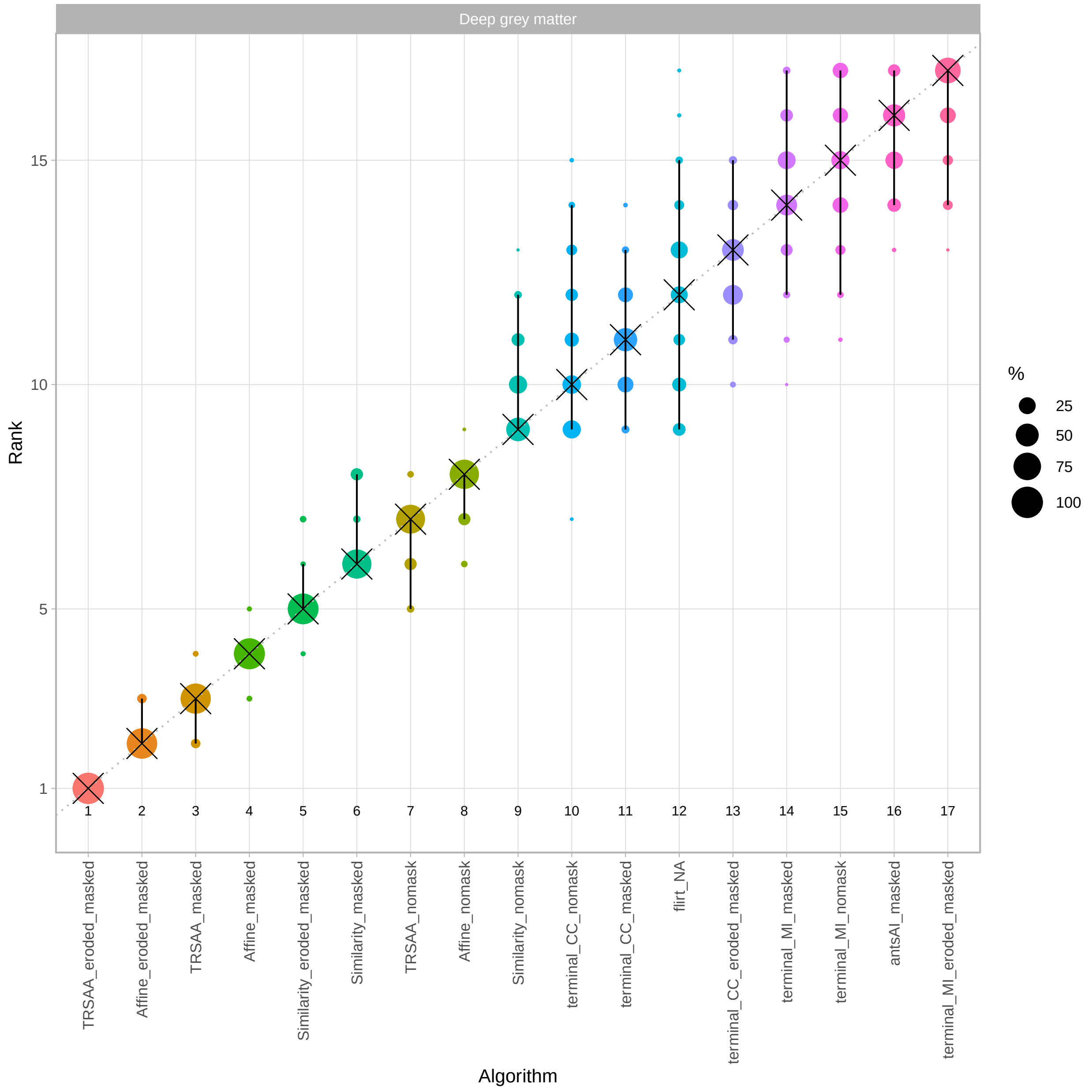

Deep grey matter
Rank
1
5
10
15
%
25
50
75
100
1
2
3
4
5
6
7
8
9
10
11
12
13
14
15
16
17
TRSAA_eroded_masked
Affine_eroded_masked
TRSAA_masked
Affine_masked
Similarity_eroded_masked
Similarity_masked
TRSAA_nomask
Affine_nomask
Similarity_nomask
terminal_CC_nomask
terminal_CC_masked
flirt_NA
terminal_CC_eroded_masked
terminal_MI_masked
terminal_MI_nomask
antsAI_masked
terminal_MI_eroded_masked
Algorithm

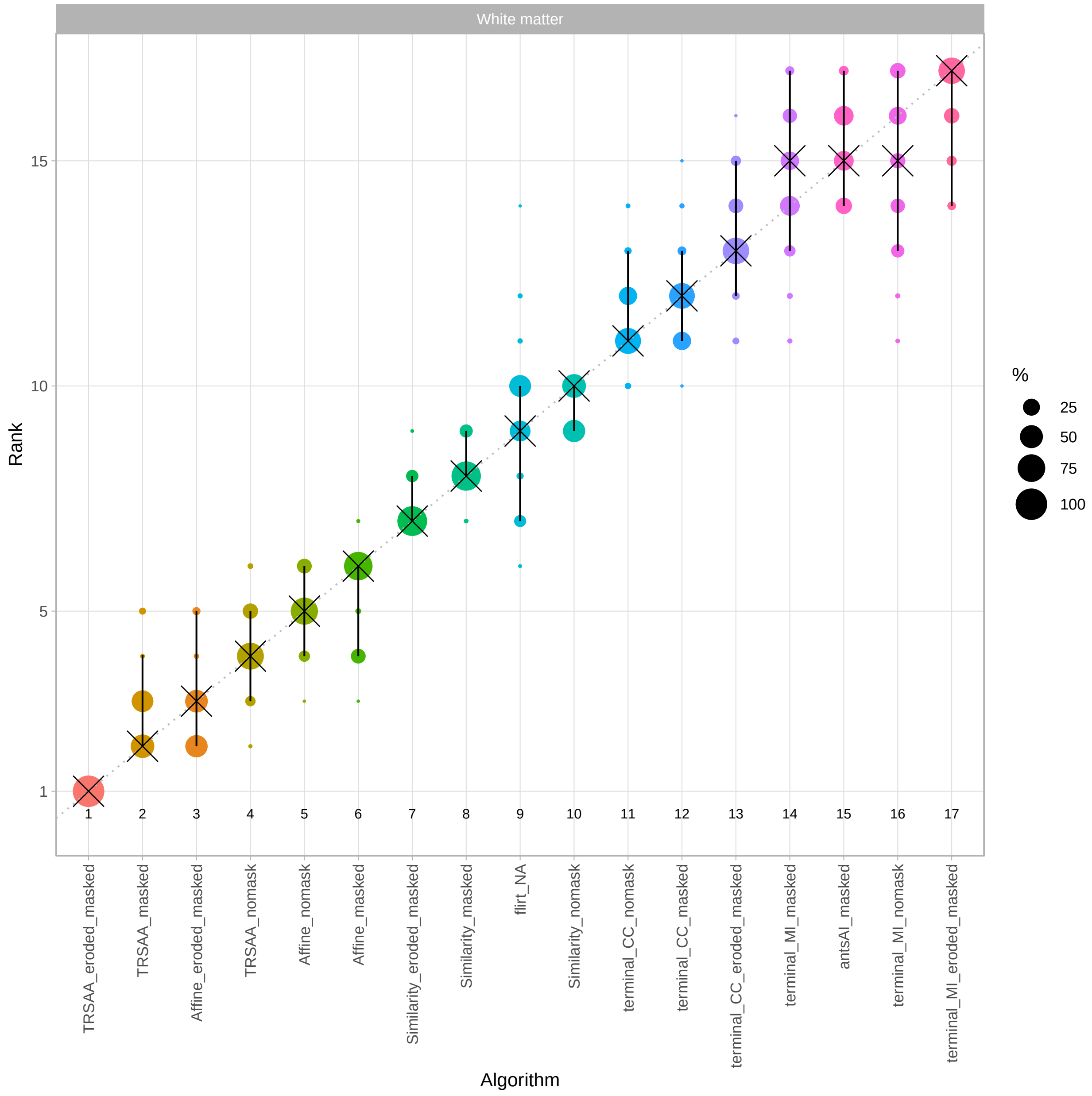
White matter
Rank
15
10
5
1
1
2
3
4
5
6
7
8
9
10
11
12
13
14
15
16
17
TRSAA_eroded_masked
TRSAA_masked
Affine_eroded_masked
TRSAA_nomask
Affine_nomask
Affine_masked
Similarity_eroded_masked
Similarity_masked
flirt_NA
Similarity_nomask
terminal_CC_nomask
terminal_CC_masked
terminal_CC_eroded_masked
terminal_MI_masked
antsAI_masked
terminal_MI_nomask
terminal_MI_eroded_masked
Algorithm
%
25
50
75
100

3.2 *Violin plot* for visualizing ranking stability based on bootstrapping

The ranking list based on the full assessment data is pairwise compared with the ranking lists based on the individual bootstrap samples (here $b = 1000$ samples). For each pair of rankings, Kendall's τ correlation is computed. Kendall's τ is a scaled index determining the correlation between the lists. It is computed by evaluating the number of pairwise concordances and discordances between ranking lists and produces values between -1 (for inverted order) and 1 (for identical order). A violin plot, which simultaneously depicts a boxplot and a density plot, is generated from the results.

Summary Kendall's tau:

Task	mean	median	q25	q75
Deep grey matter	0.9326176	0.9411765	0.9117647	0.9558824
White matter	0.9370441	0.9411765	0.9117647	0.9558824
Cerebellum	0.9266176	0.9264706	0.9117647	0.9558824
CSF and ventricles	0.9167353	0.9264706	0.8970588	0.9411765
Brainstem	0.9033971	0.9117647	0.8823529	0.9264706
Cortical grey matter	0.8951471	0.8970588	0.8676471	0.9264706

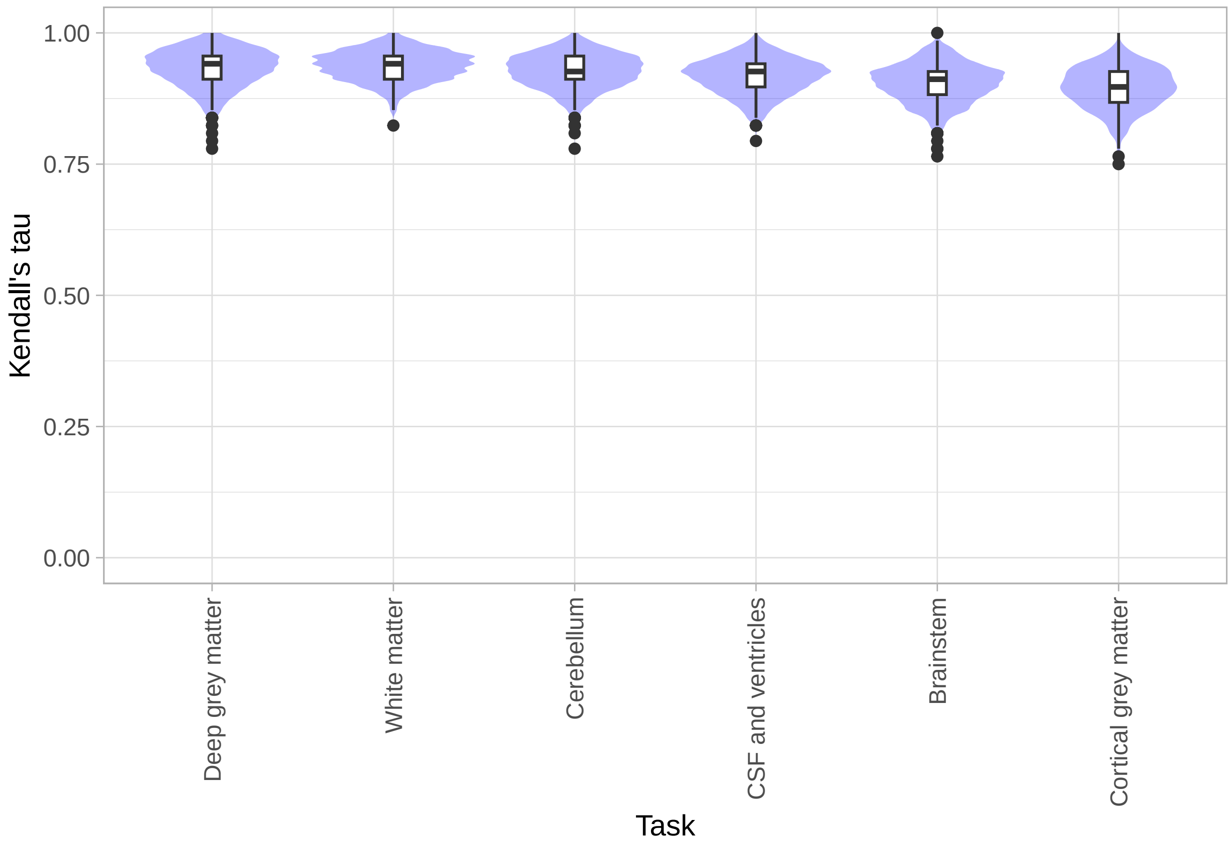

3.3 *Significance maps* for visualizing ranking stability based on statistical significance

Significance maps depict incidence matrices of pairwise significant test results for the one-sided Wilcoxon signed rank test at a 5% significance level with adjustment for multiple testing according to Holm. Yellow shading indicates that metric values from the algorithm on the x-axis were significantly superior to those from the algorithm on the y-axis, blue color indicates no significant difference.

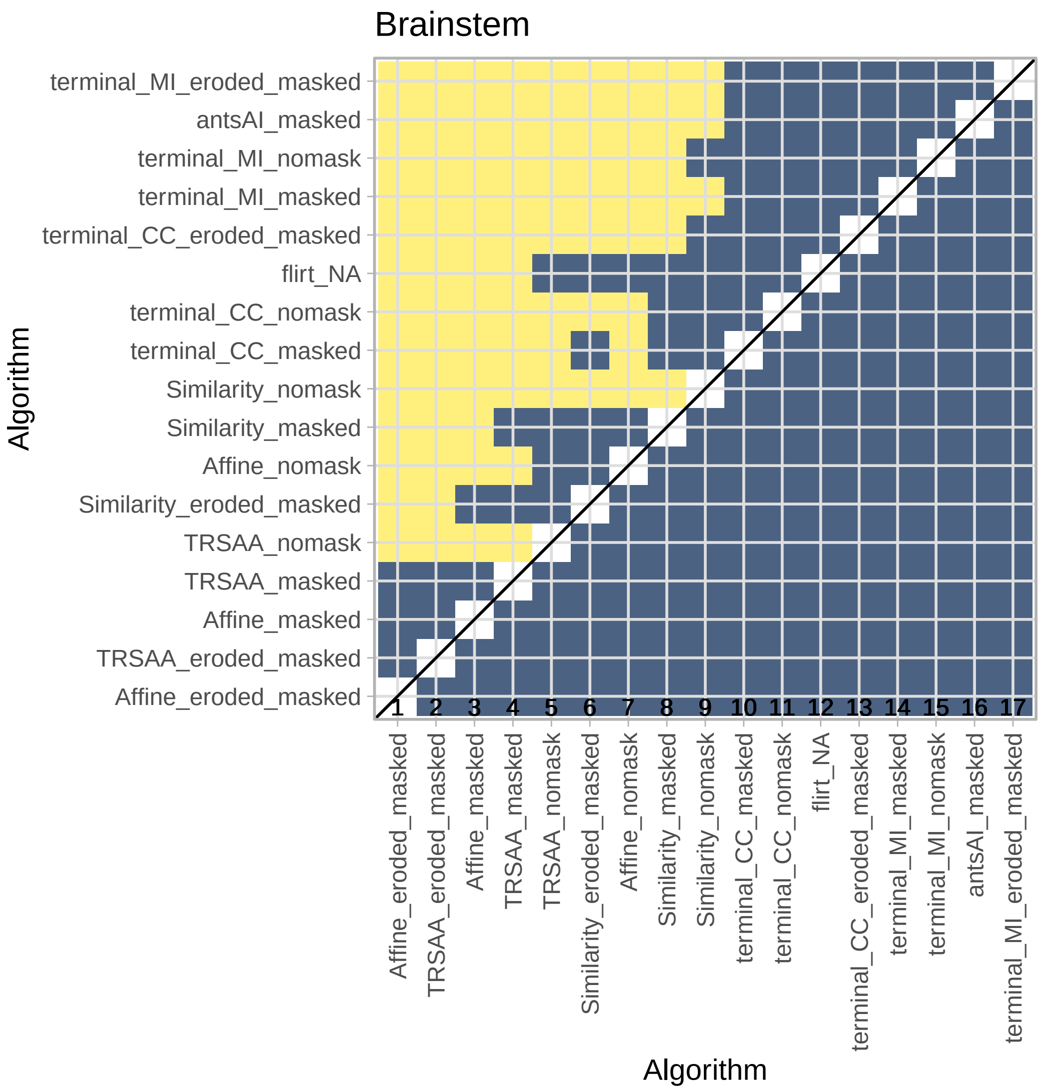

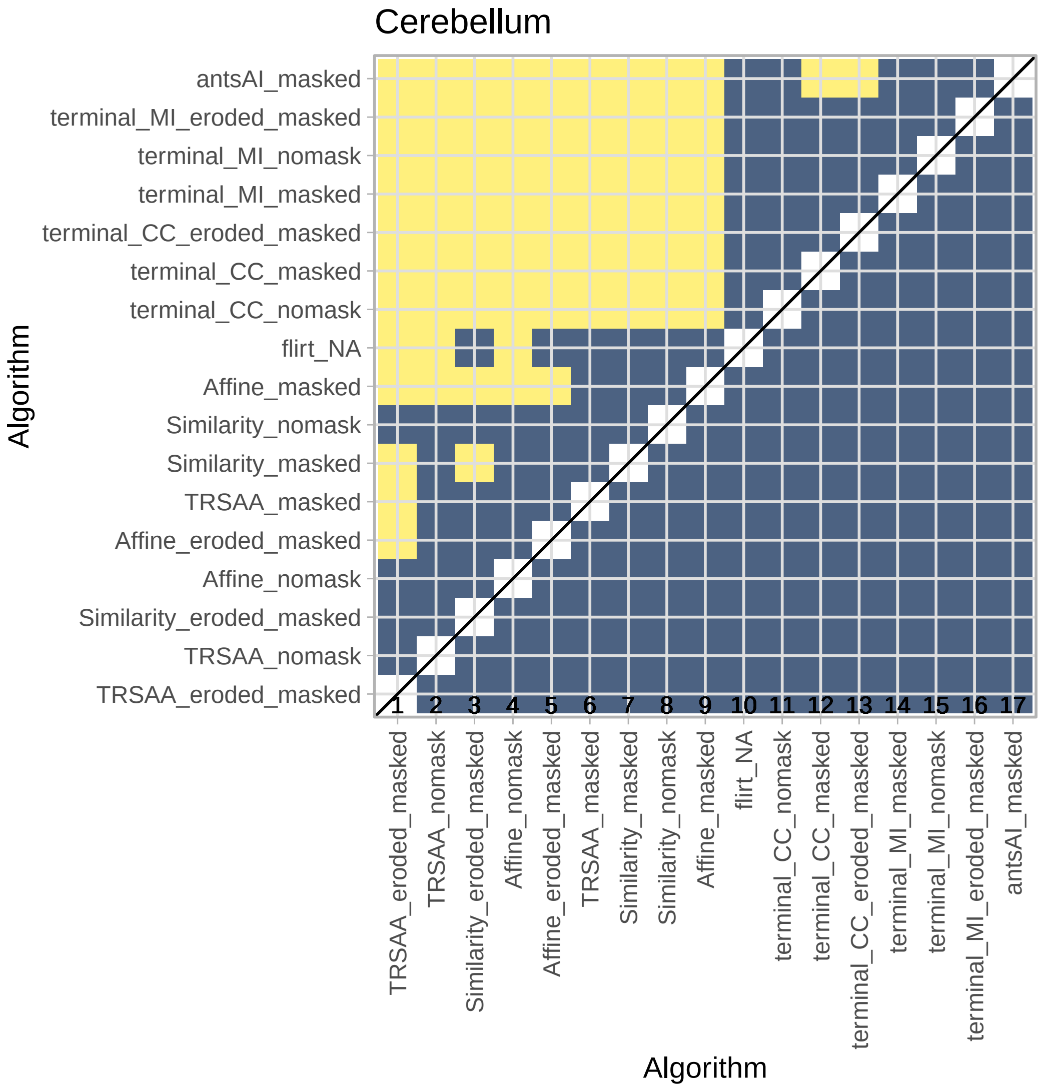
Cerebellum
antsAI_masked
terminal_MI_eroded_masked
terminal_MI_nomask
terminal_MI_masked
terminal_CC_eroded_masked
terminal_CC_masked
terminal_CC_nomask
flirt_NA
Affine_masked
Similarity_nomask
Similarity_masked
TRSAA_masked
Affine_eroded_masked
Affine_nomask
Similarity_eroded_masked
TRSAA_nomask
TRSAA_eroded_masked
Algorithm
1 2 3 4 5 6 7 8 9 10 11 12 13 14 15 16 17
TRSAA_eroded_masked
TRSAA_nomask
Similarity_eroded_masked
Affine_nomask
Affine_eroded_masked
TRSAA_masked
Similarity_masked
Similarity_nomask
Affine_masked
flirt_NA
terminal_CC_nomask
terminal_CC_masked
terminal_CC_eroded_masked
terminal_MI_masked
terminal_MI_nomask
terminal_MI_eroded_masked
antsAI_masked
Algorithm

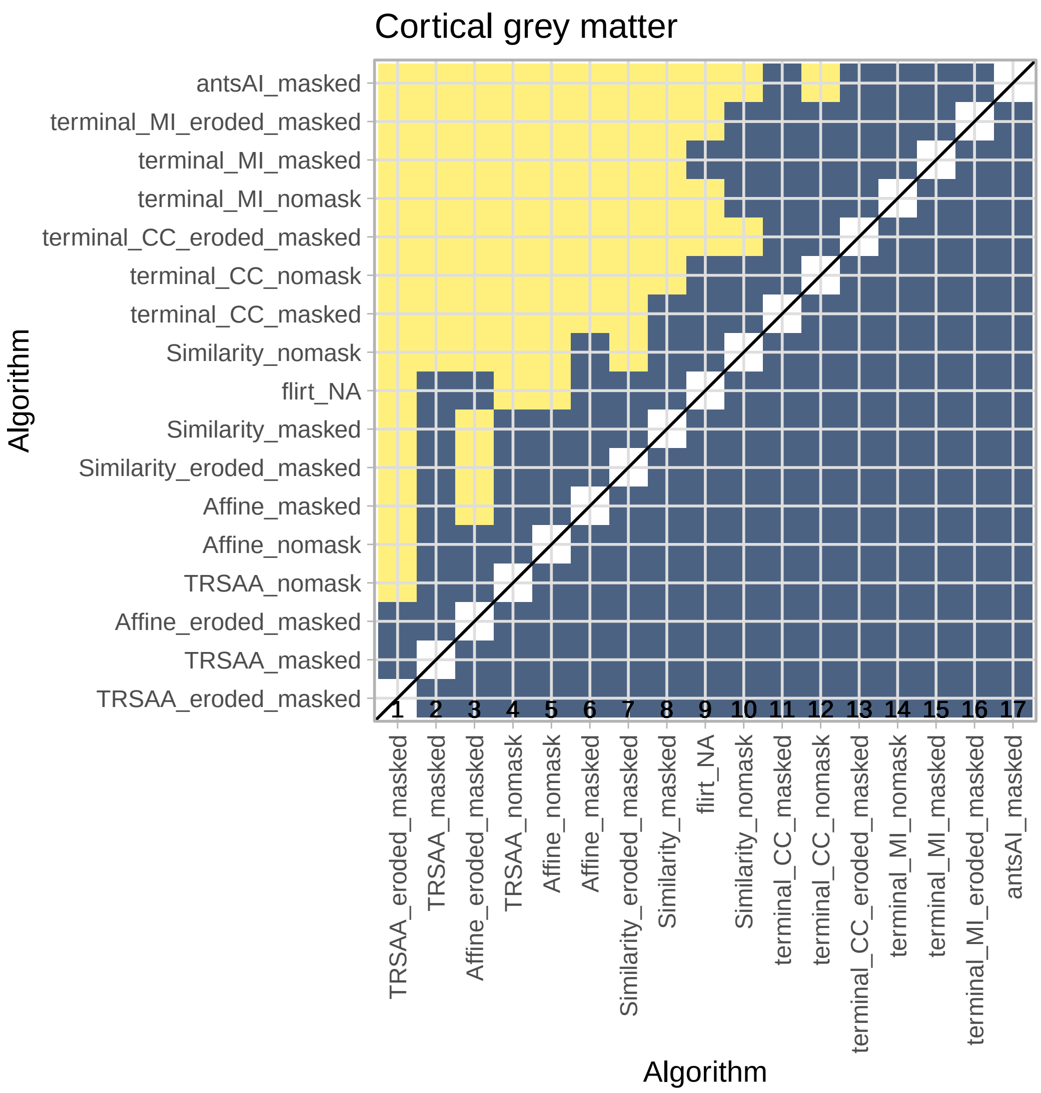
Cortical grey matter
antsAI_masked
terminal_MI_eroded_masked
terminal_MI_masked
terminal_MI_nomask
terminal_CC_eroded_masked
terminal_CC_nomask
terminal_CC_masked
Similarity_nomask
flirt_NA
Similarity_masked
Similarity_eroded_masked
Affine_masked
Affine_nomask
TRSAA_nomask
Affine_eroded_masked
TRSAA_masked
TRSAA_eroded_masked
Algorithm
1 2 3 4 5 6 7 8 9 10 11 12 13 14 15 16 17
TRSAA_eroded_masked
TRSAA_masked
Affine_eroded_masked
TRSAA_nomask
Affine_nomask
Affine_masked
Similarity_eroded_masked
Similarity_masked
flirt_NA
Similarity_nomask
terminal_CC_masked
terminal_CC_nomask
terminal_CC_eroded_masked
terminal_MI_nomask
terminal_MI_masked
terminal_MI_eroded_masked
antsAI_masked
Algorithm

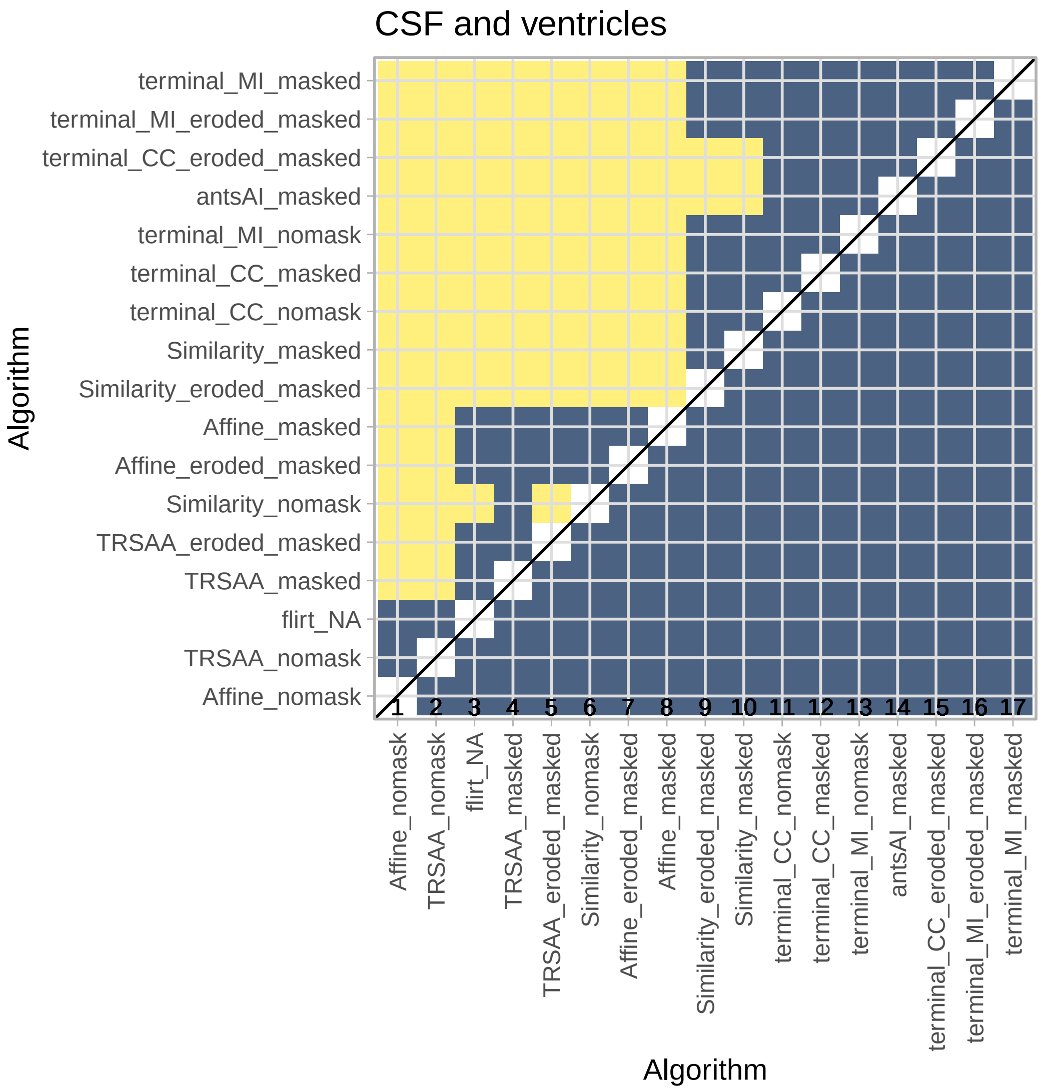
CSF and ventricles
Algorithm
terminal_MI_masked
terminal_MI_eroded_masked
terminal_CC_eroded_masked
antsAI_masked
terminal_MI_nomask
terminal_CC_masked
terminal_CC_nomask
Similarity_masked
Similarity_eroded_masked
Affine_masked
Affine_eroded_masked
Similarity_nomask
TRSAA_eroded_masked
TRSAA_masked
flirt_NA
TRSAA_nomask
Affine_nomask
1 2 3 4 5 6 7 8 9 10 11 12 13 14 15 16 17
Affine_nomask
TRSAA_nomask
flirt_NA
TRSAA_masked
TRSAA_eroded_masked
Similarity_nomask
Affine_eroded_masked
Affine_masked
Similarity_eroded_masked
Similarity_masked
terminal_CC_nomask
terminal_CC_masked
terminal_MI_nomask
antsAI_masked
terminal_CC_eroded_masked
terminal_MI_eroded_masked
terminal_MI_masked
Algorithm

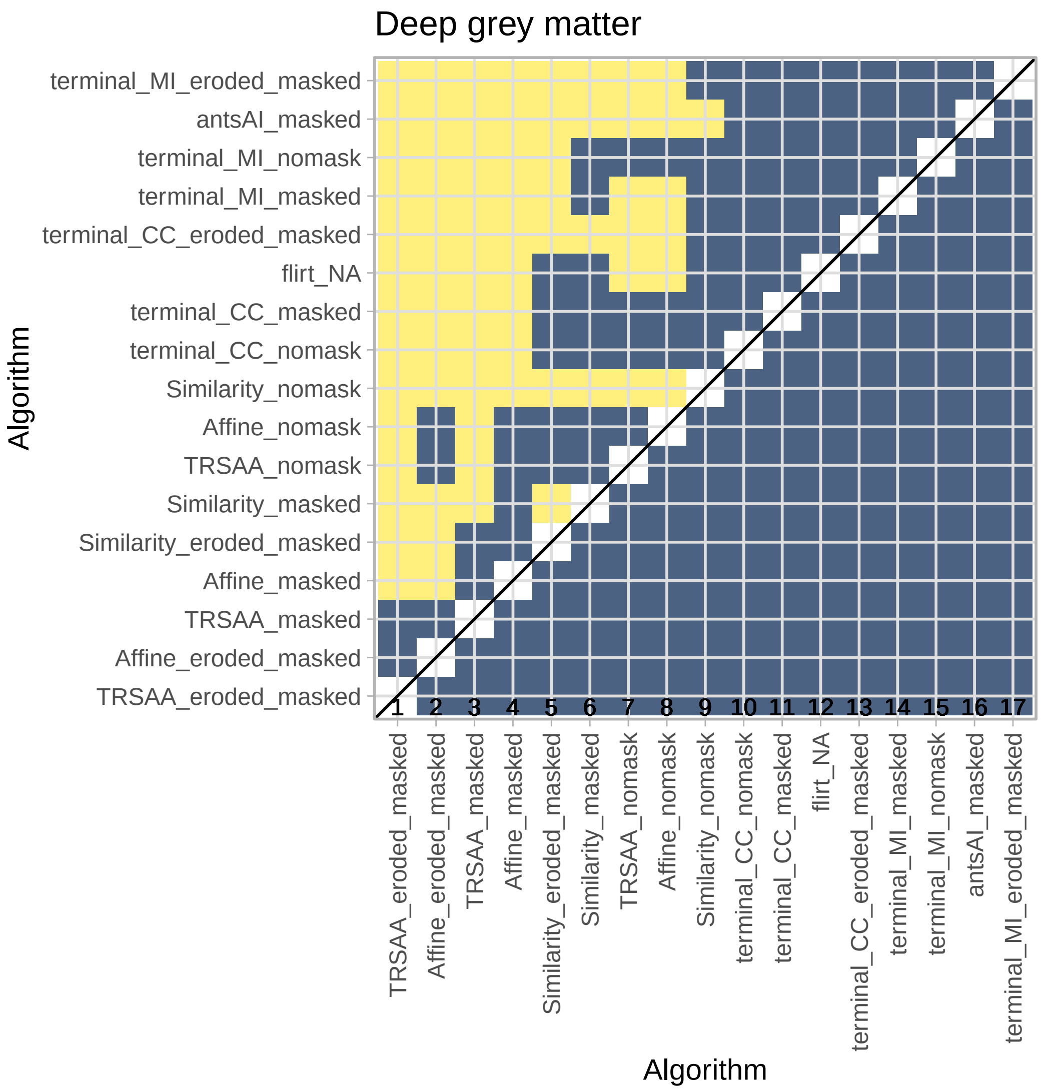
Deep grey matter
Algorithm
terminal_MI_eroded_masked
antsAI_masked
terminal_MI_nomask
terminal_MI_masked
terminal_CC_eroded_masked
flirt_NA
terminal_CC_masked
terminal_CC_nomask
Similarity_nomask
Affine_nomask
TRSAA_nomask
Similarity_masked
Similarity_eroded_masked
Affine_masked
TRSAA_masked
Affine_eroded_masked
TRSAA_eroded_masked
1 2 3 4 5 6 7 8 9 10 11 12 13 14 15 16 17
TRSAA_eroded_masked
Affine_eroded_masked
TRSAA_masked
Affine_masked
Similarity_eroded_masked
Similarity_masked
TRSAA_nomask
Affine_nomask
Similarity_nomask
terminal_CC_nomask
terminal_CC_masked
flirt_NA
terminal_CC_eroded_masked
terminal_MI_masked
terminal_MI_nomask
antsAI_masked
terminal_MI_eroded_masked
Algorithm

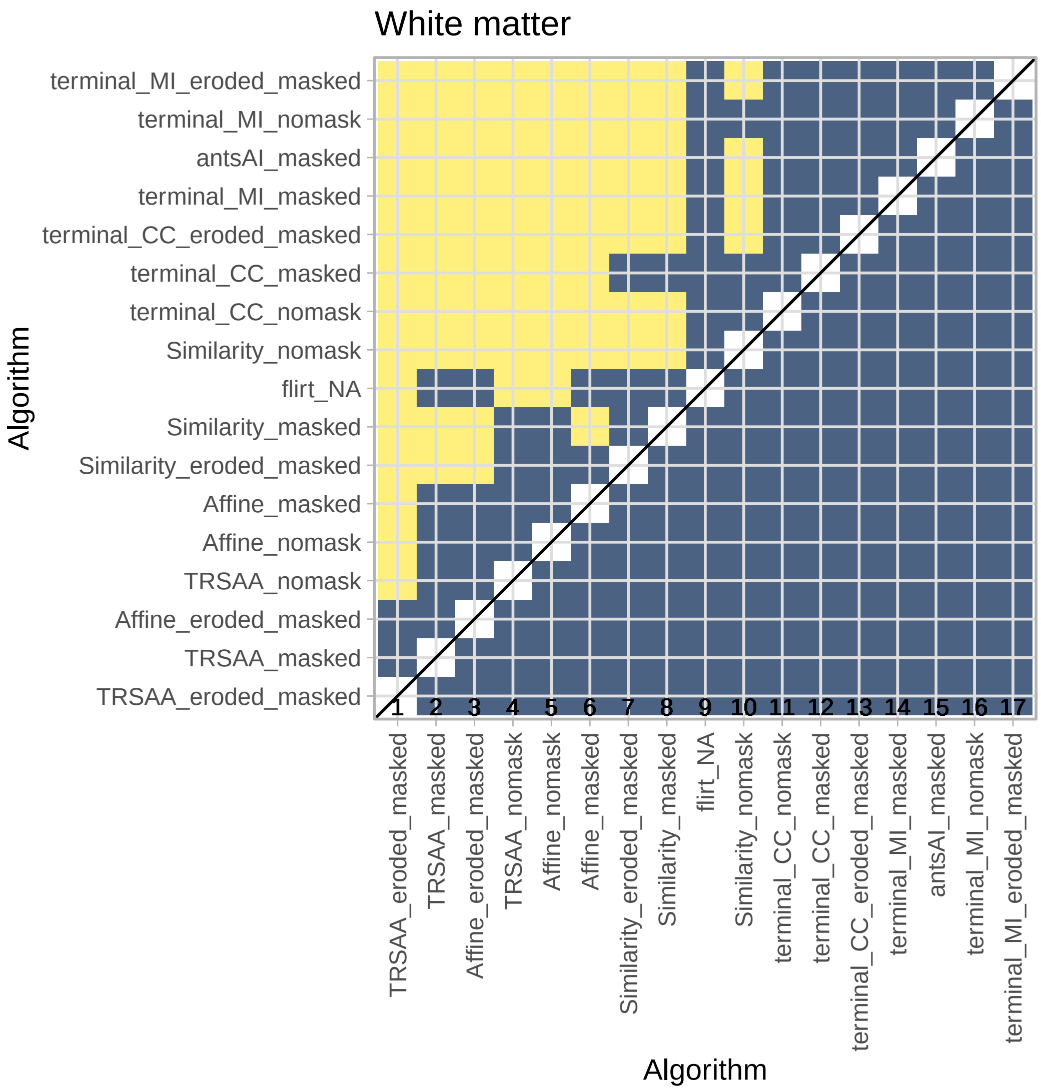
White matter
terminal_MI_eroded_masked
terminal_MI_nomask
antsAI_masked
terminal_MI_masked
terminal_CC_eroded_masked
terminal_CC_masked
terminal_CC_nomask
Similarity_nomask
flirt_NA
Similarity_masked
Similarity_eroded_masked
Affine_masked
Affine_nomask
TRSAA_nomask
Affine_eroded_masked
TRSAA_masked
TRSAA_eroded_masked
Algorithm
1 2 3 4 5 6 7 8 9 10 11 12 13 14 15 16 17
TRSAA_eroded_masked
TRSAA_masked
Affine_eroded_masked
TRSAA_nomask
Affine_nomask
Affine_masked
Similarity_eroded_masked
Similarity_masked
flirt_NA
Similarity_nomask
terminal_CC_nomask
terminal_CC_masked
terminal_CC_eroded_masked
terminal_MI_masked
antsAI_masked
terminal_MI_nomask
terminal_MI_eroded_masked
Algorithm

3.4 Ranking robustness to ranking methods

Line plots for visualizing ranking robustness across different ranking methods. Each algorithm is represented by one colored line. For each ranking method encoded on the x-axis, the height of the line represents the corresponding rank. Horizontal lines indicate identical ranks for all methods.

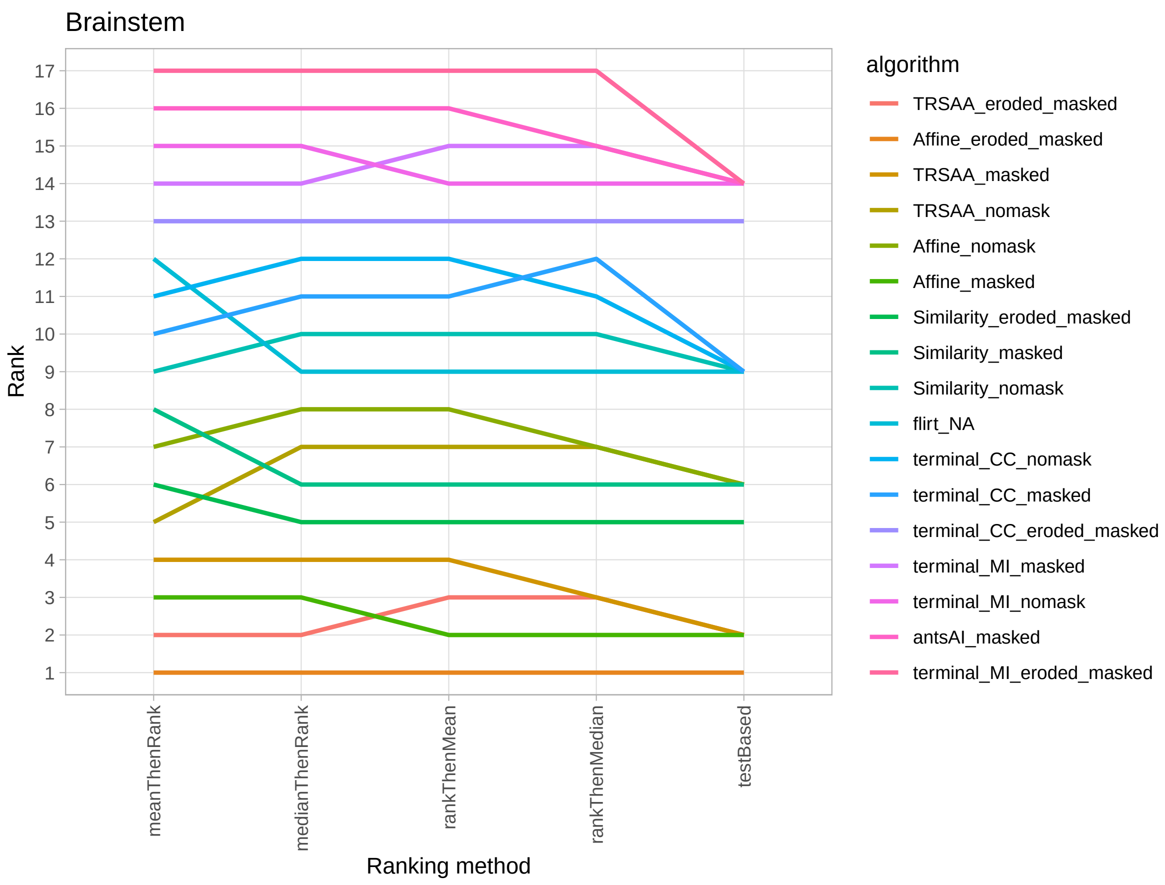

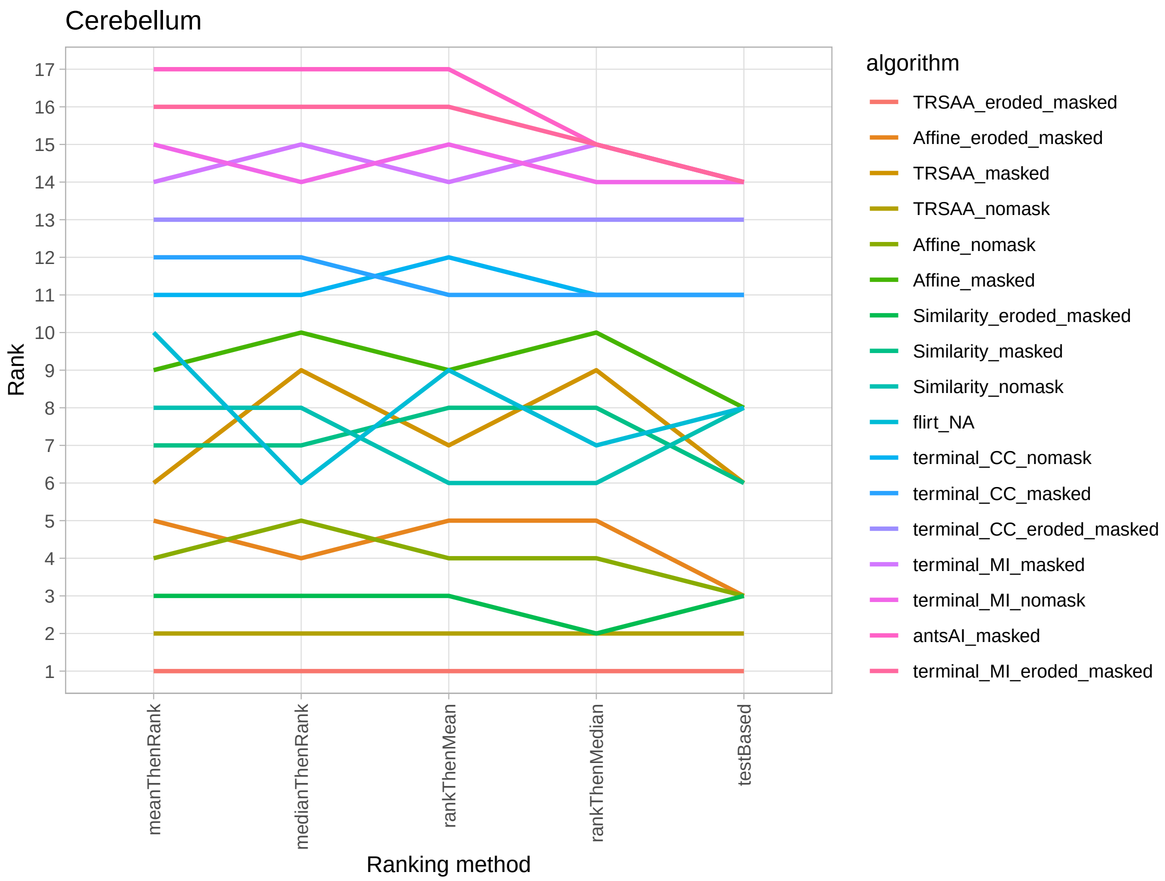
Cerebellum
Rank
17
16
15
14
13
12
11
10
9
8
7
6
5
4
3
2
1
meanThenRank
medianThenRank
rankThenMean
rankThenMedian
testBased
Ranking method
algorithm
TRSAA_eroded_masked
Affine_eroded_masked
TRSAA_masked
TRSAA_nomask
Affine_nomask
Affine_masked
Similarity_eroded_masked
Similarity_masked
Similarity_nomask
flirt_NA
terminal_CC_nomask
terminal_CC_masked
terminal_CC_eroded_masked
terminal_MI_masked
terminal_MI_nomask
antsAI_masked
terminal_MI_eroded_masked

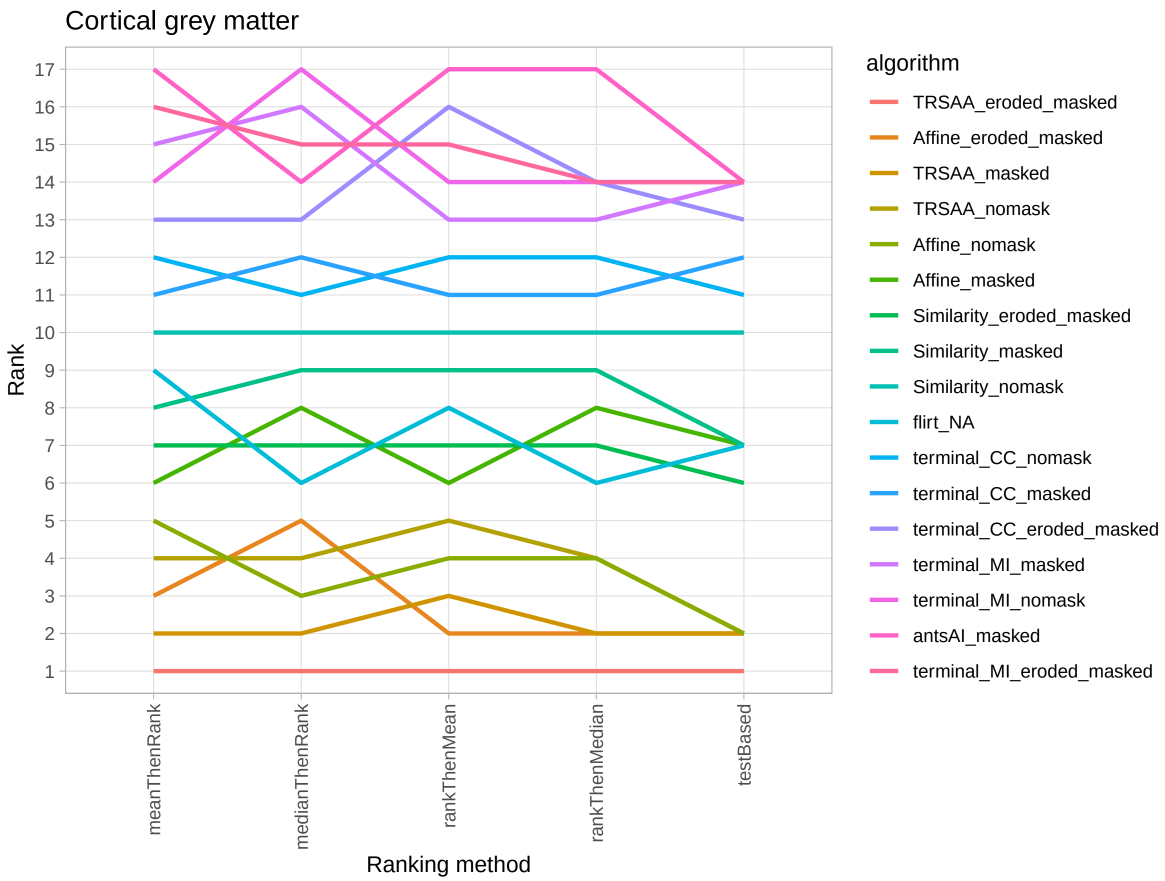
Cortical grey matter
Rank
17
16
15
14
13
12
11
10
9
8
7
6
5
4
3
2
1
meanThenRank
medianThenRank
rankThenMean
rankThenMedian
testBased
Ranking method
algorithm
TRSAA_eroded_masked
Affine_eroded_masked
TRSAA_masked
TRSAA_nomask
Affine_nomask
Affine_masked
Similarity_eroded_masked
Similarity_masked
Similarity_nomask
flirt_NA
terminal_CC_nomask
terminal_CC_masked
terminal_CC_eroded_masked
terminal_MI_masked
terminal_MI_nomask
antsAI_masked
terminal_MI_eroded_masked

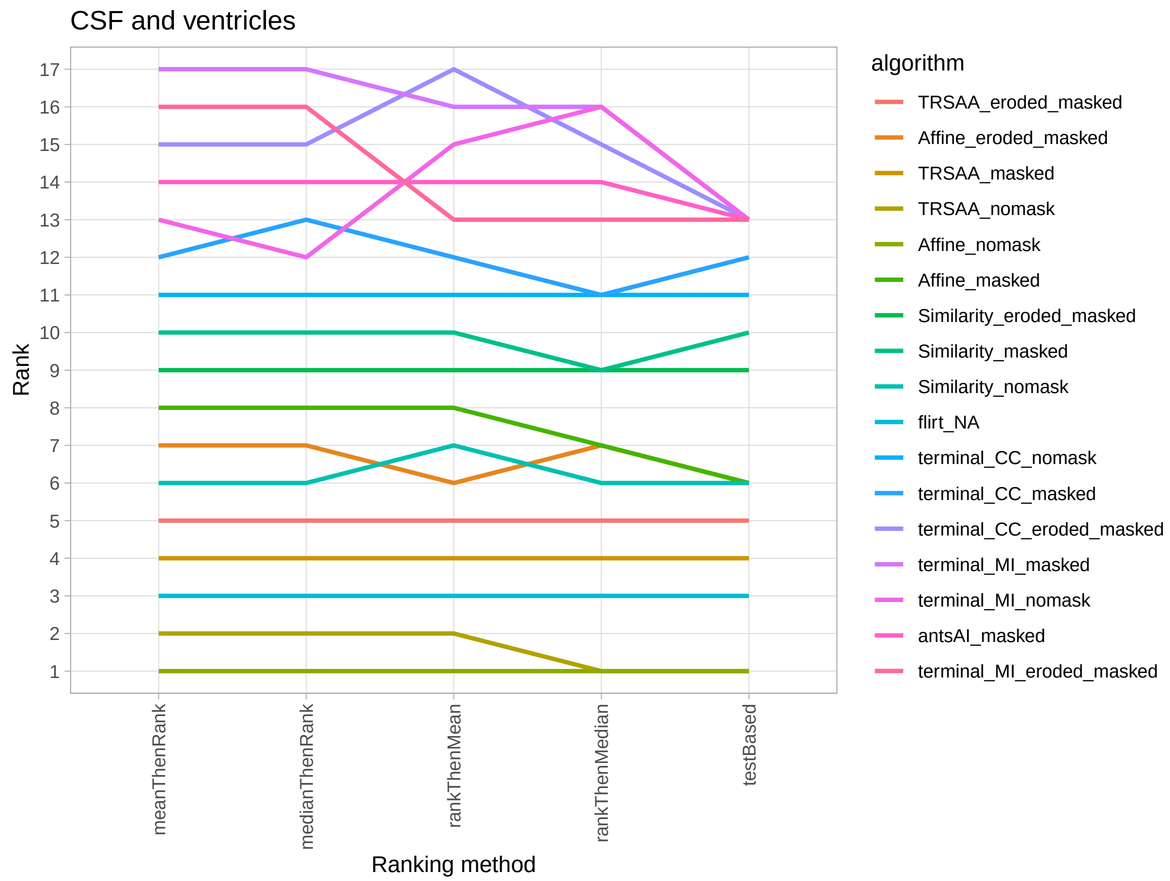

CSF and ventricles
Rank
17
16
15
14
13
12
11
10
9
8
7
6
5
4
3
2
1
meanThenRank
medianThenRank
rankThenMean
rankThenMedian
testBased
Ranking method
algorithm
TRSAA_eroded_masked
Affine_eroded_masked
TRSAA_masked
TRSAA_nomask
Affine_nomask
Affine_masked
Similarity_eroded_masked
Similarity_masked
Similarity_nomask
flirt_NA
terminal_CC_nomask
terminal_CC_masked
terminal_CC_eroded_masked
terminal_MI_masked
terminal_MI_nomask
antsAI_masked
terminal_MI_eroded_masked

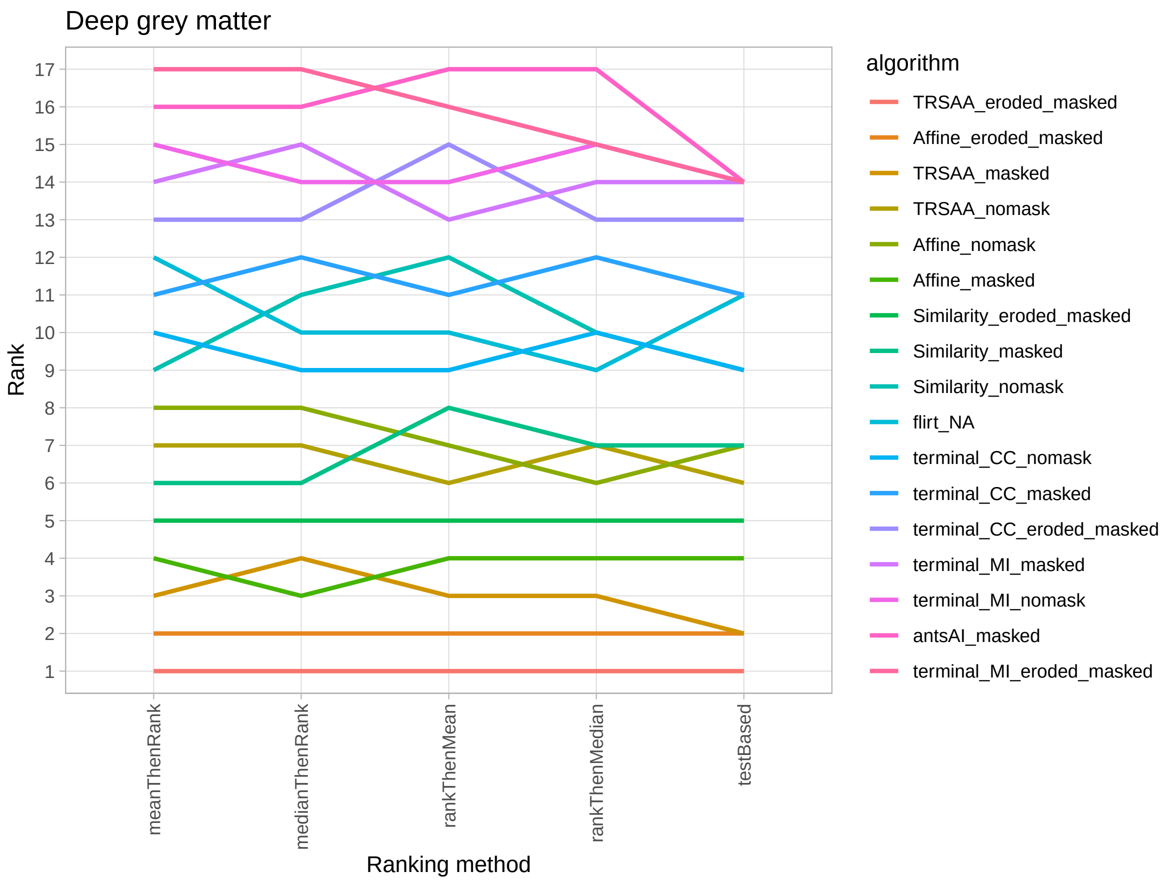
Deep grey matter
Rank
17
16
15
14
13
12
11
10
9
8
7
6
5
4
3
2
1
meanThenRank
medianThenRank
rankThenMean
rankThenMedian
testBased
Ranking method
algorithm
TRSAA_eroded_masked
Affine_eroded_masked
TRSAA_masked
TRSAA_nomask
Affine_nomask
Affine_masked
Similarity_eroded_masked
Similarity_masked
Similarity_nomask
flirt_NA
terminal_CC_nomask
terminal_CC_masked
terminal_CC_eroded_masked
terminal_MI_masked
terminal_MI_nomask
antsAI_masked
terminal_MI_eroded_masked

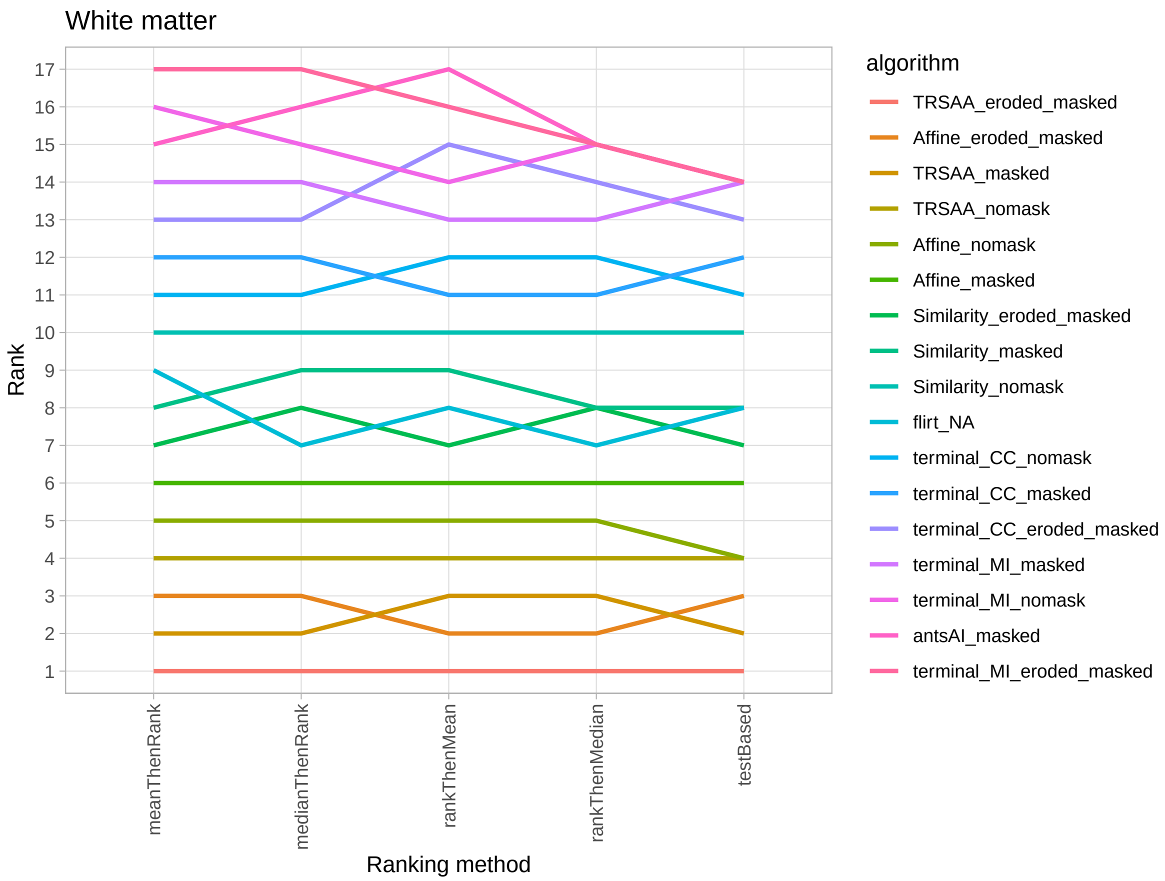
White matter
Rank
17
16
15
14
13
12
11
10
9
8
7
6
5
4
3
2
1
meanThenRank
medianThenRank
rankThenMean
rankThenMedian
testBased
Ranking method
algorithm
TRSAA_eroded_masked
Affine_eroded_masked
TRSAA_masked
TRSAA_nomask
Affine_nomask
Affine_masked
Similarity_eroded_masked
Similarity_masked
Similarity_nomask
flirt_NA
terminal_CC_nomask
terminal_CC_masked
terminal_CC_eroded_masked
terminal_MI_masked
terminal_MI_nomask
antsAI_masked
terminal_MI_eroded_masked

4 Visualization of cross-task insights

The algorithms are ordered according to consensus ranking.

4.1 Characterization of algorithms

4.1.1 Ranking stability: Variability of achieved rankings across tasks

Algorithms are color-coded, and the area of each blob at position $(A_i, \text{rank } j)$ is proportional to the relative frequency A_i achieved rank j across multiple tasks. The median rank for each algorithm is indicated by a black cross. This way, the distribution of ranks across tasks can be intuitively visualized.

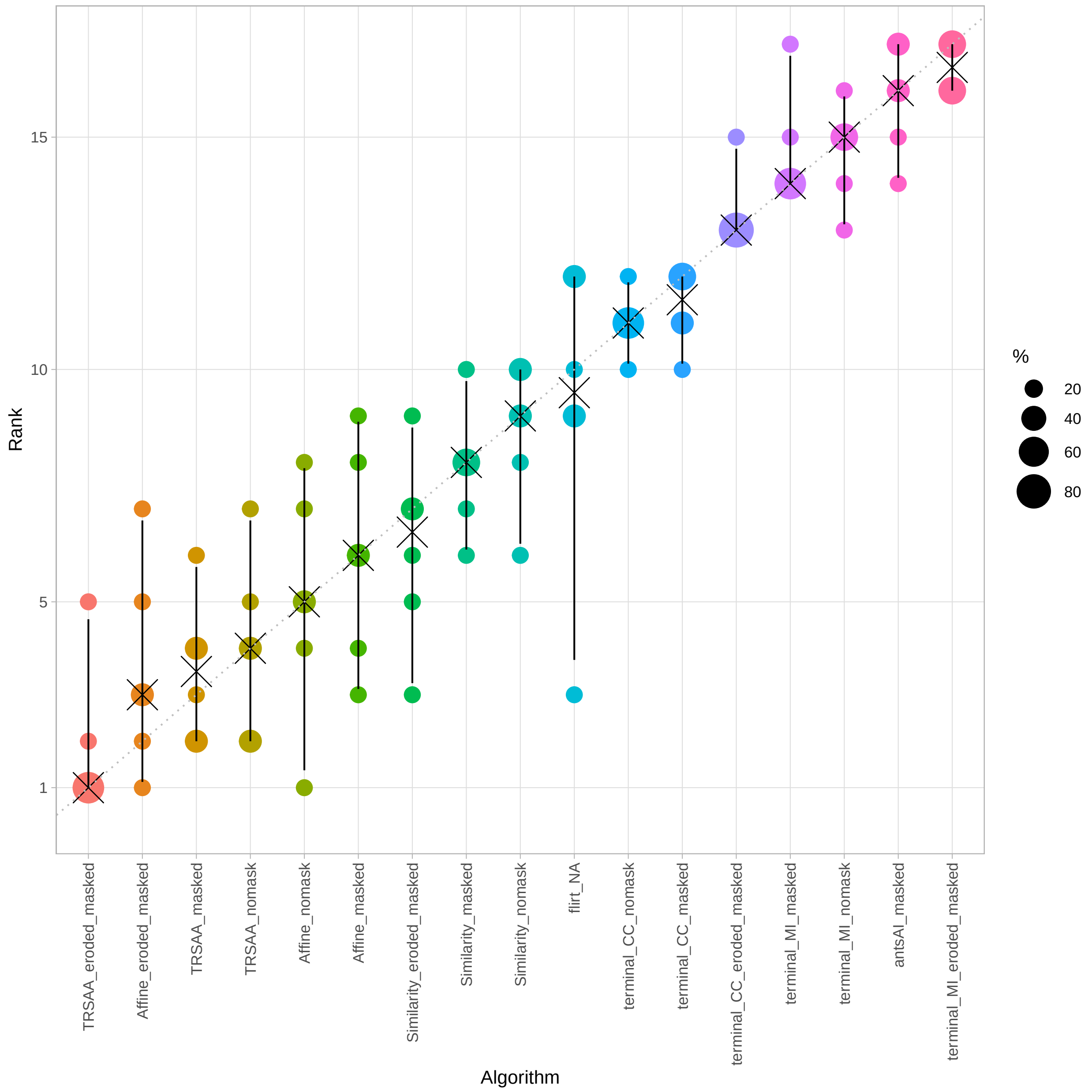

4.1.2 Ranking stability: Ranking variability via bootstrap approach

A blob plot of bootstrap results over the different tasks separated by algorithm allows another perspective on the assessment data. This gives deeper insights into the characteristics of tasks and the ranking uncertainty of the algorithms in each task.

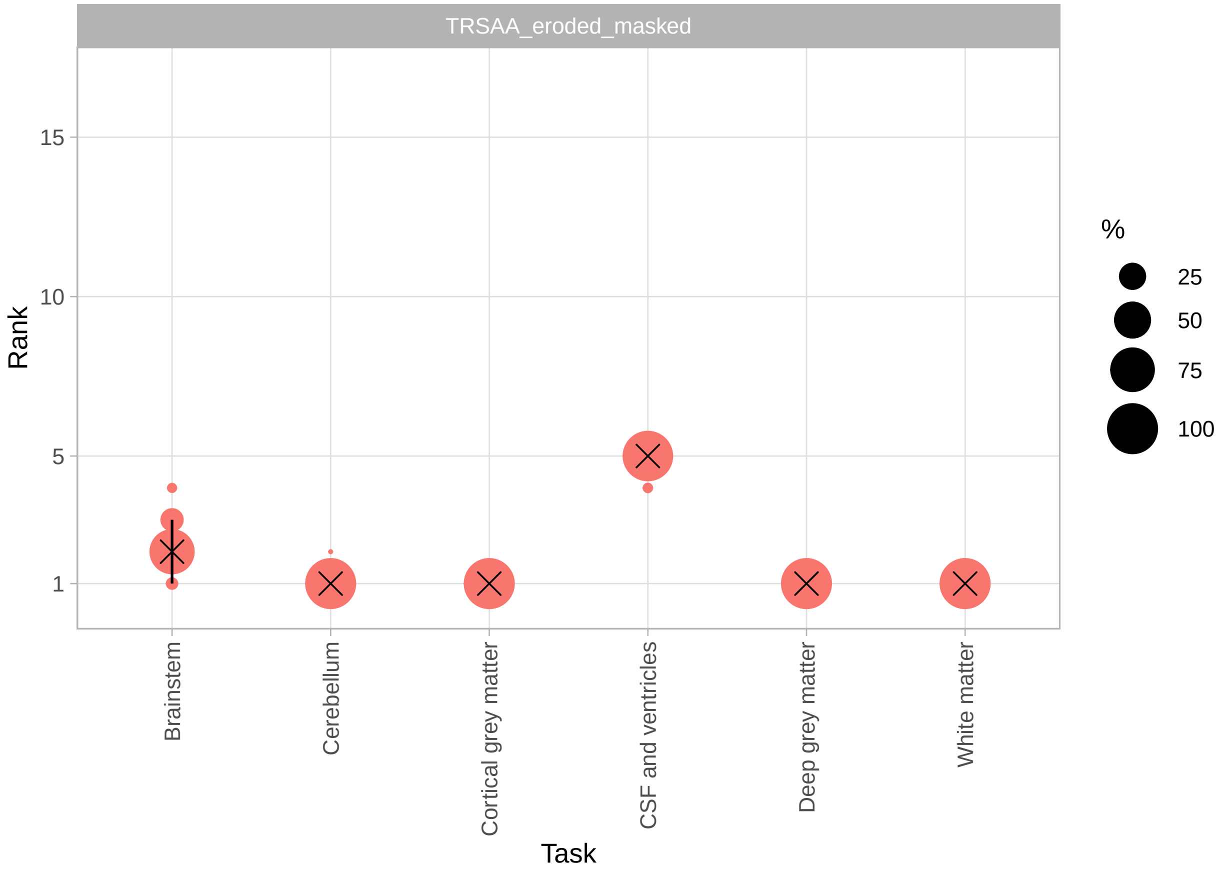

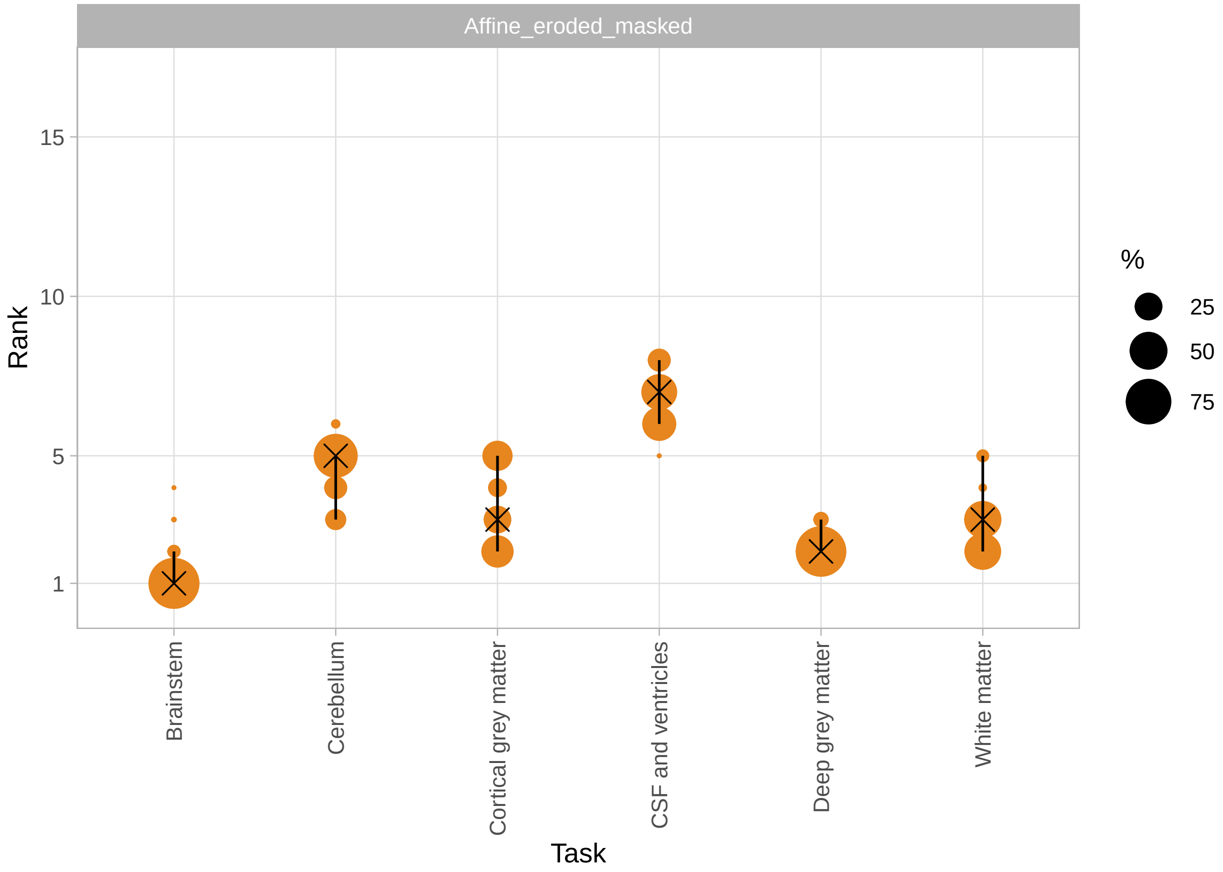

Affine_eroded_masked
Rank
15
10
5
1
%
25
50
75
Brainstem
Cerebellum
Cortical grey matter
CSF and ventricles
Deep grey matter
White matter
Task

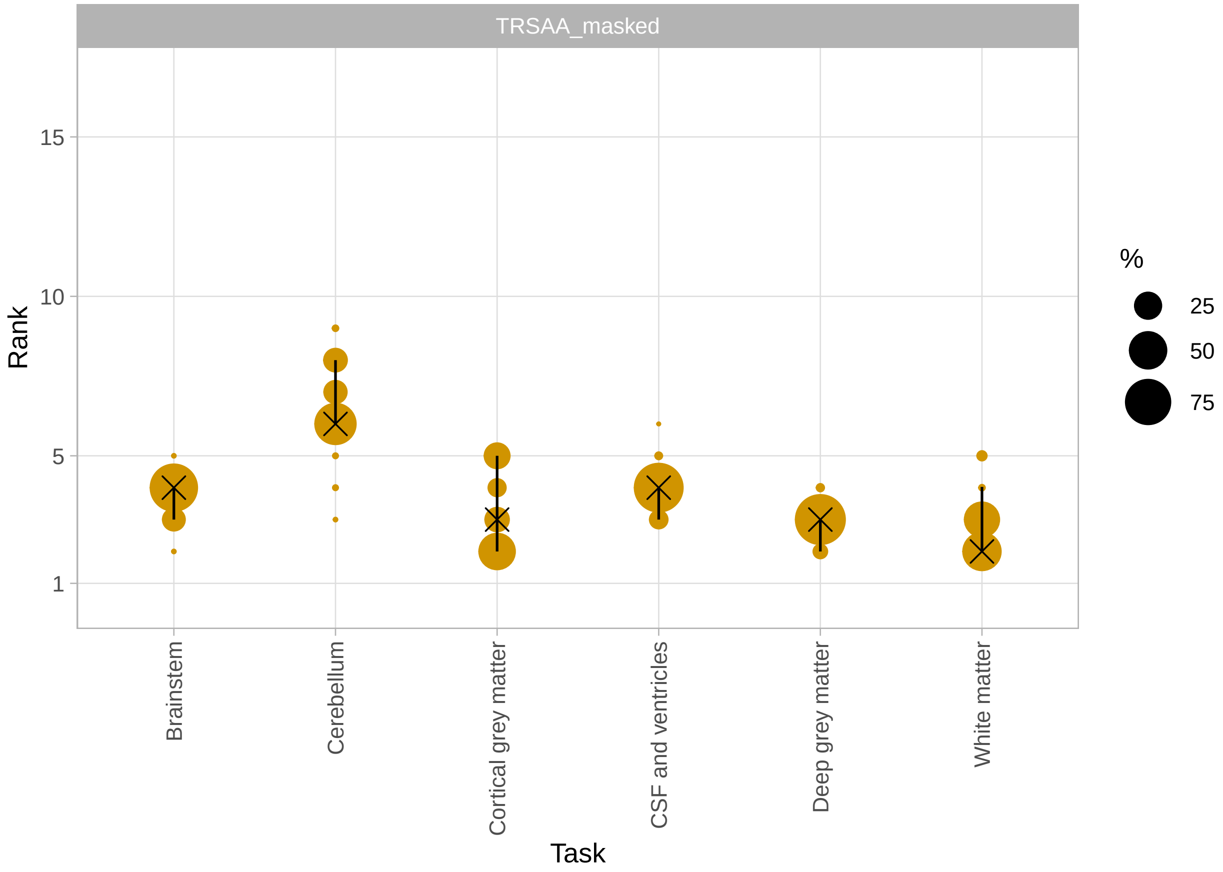

TRSAA_masked
Rank
15
10
5
1
Brainstem
Cerebellum
Cortical grey matter
CSF and ventricles
Deep grey matter
White matter
Task
%
25
50
75

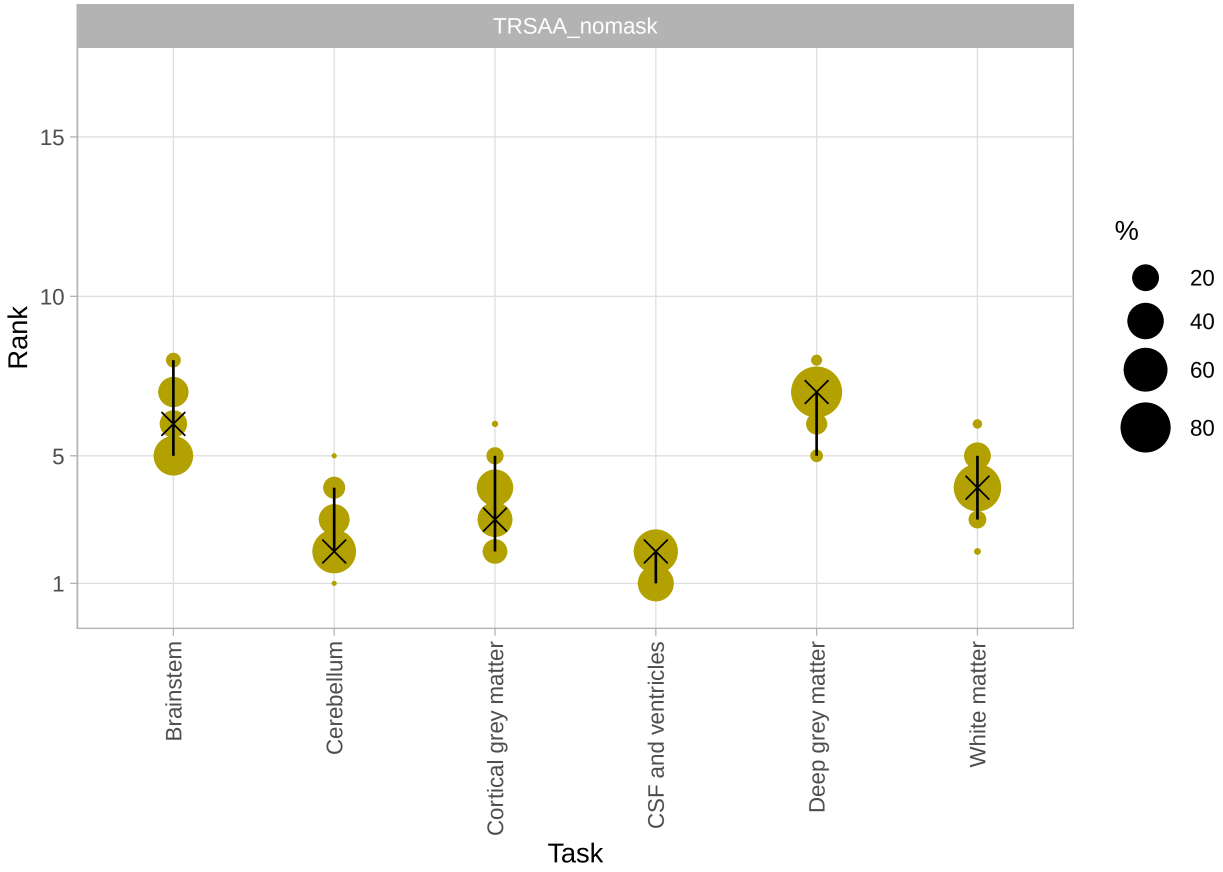
TRSAA_nomask
Rank
15
10
5
1
Brainstem
Cerebellum
Cortical grey matter
CSF and ventricles
Deep grey matter
White matter
Task
%
20
40
60
80

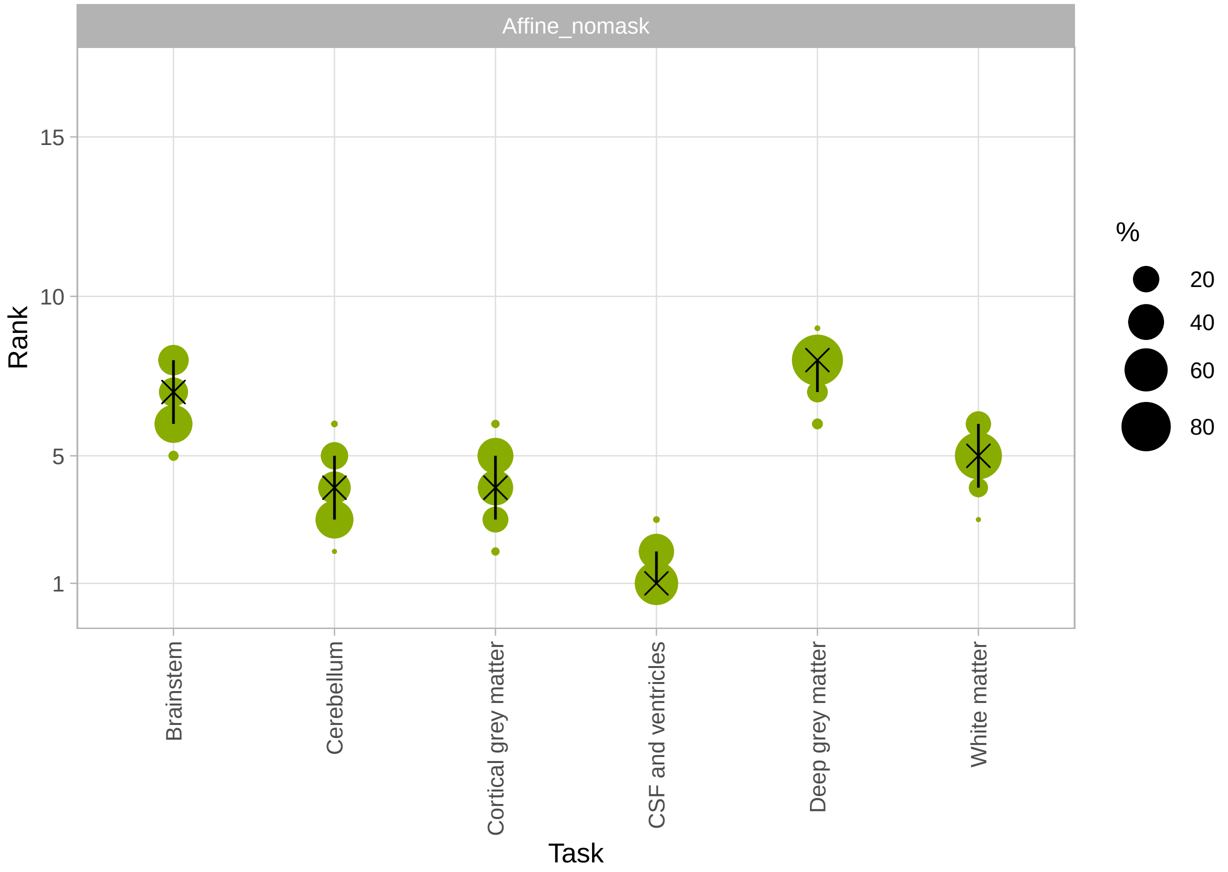

Affine_nomask
Rank
15
10
5
1
Brainstem
Cerebellum
Cortical grey matter
CSF and ventricles
Deep grey matter
White matter
Task
%
20
40
60
80

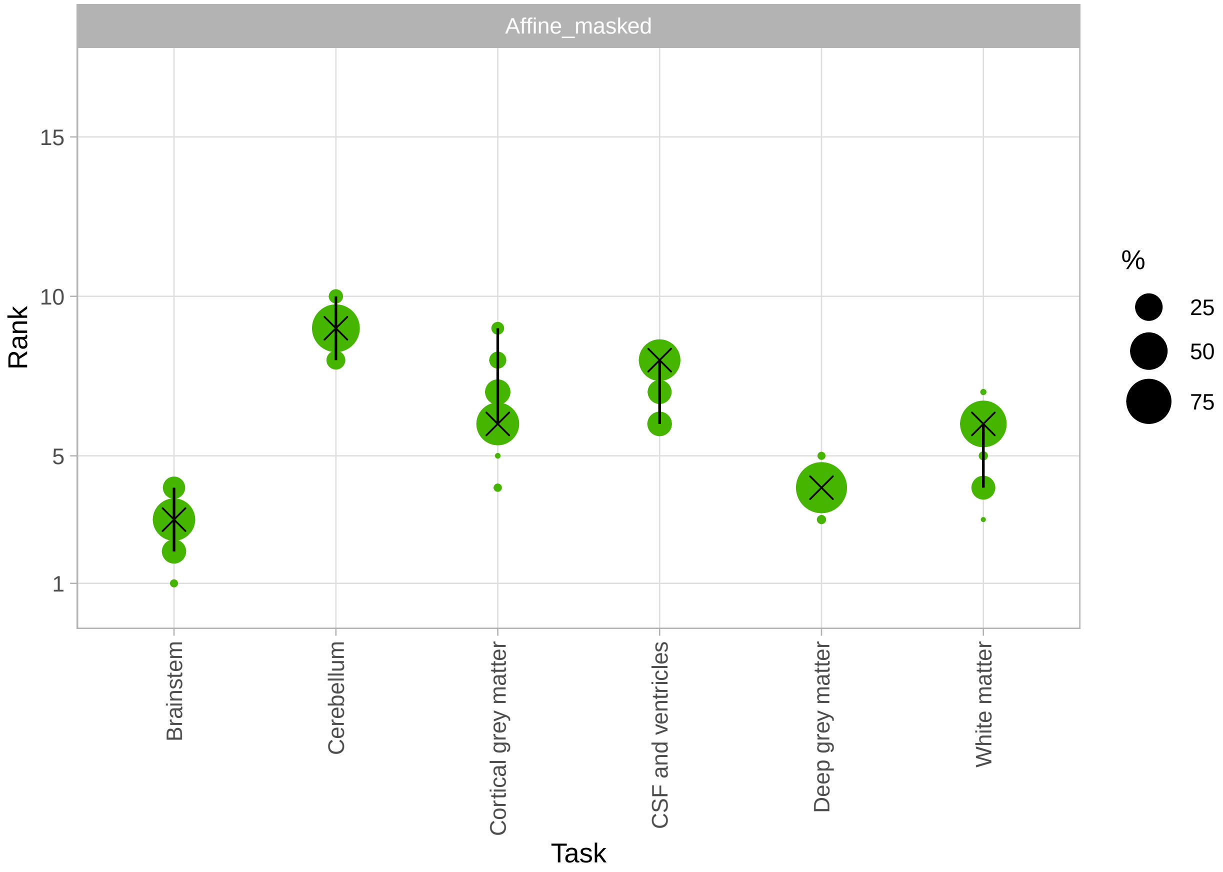

Affine_masked
Rank
15
10
5
1
Brainstem
Cerebellum
Cortical grey matter
CSF and ventricles
Deep grey matter
White matter
Task
%
25
50
75

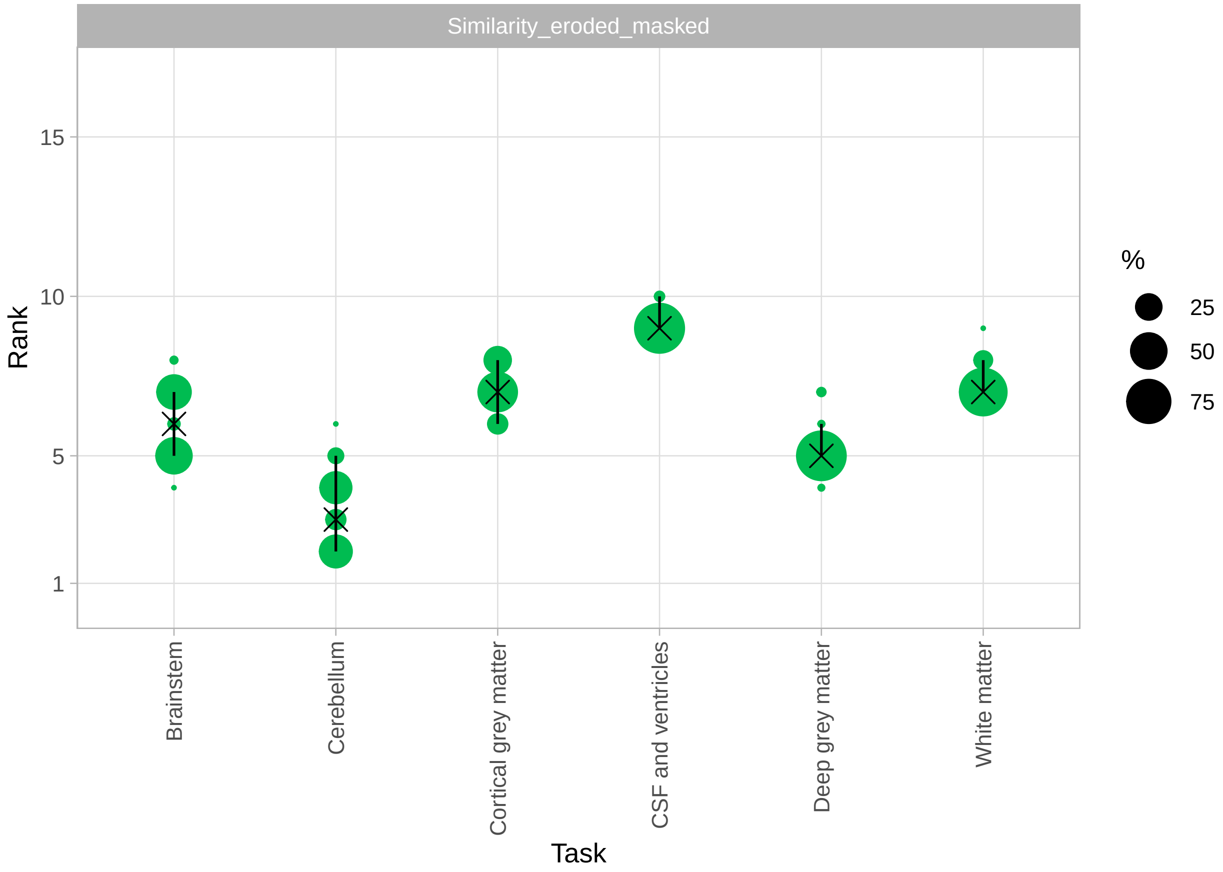
Similarity_eroded_masked
Rank
15
10
5
1
Brainstem
Cerebellum
Cortical grey matter
CSF and ventricles
Deep grey matter
White matter
Task
%
25
50
75

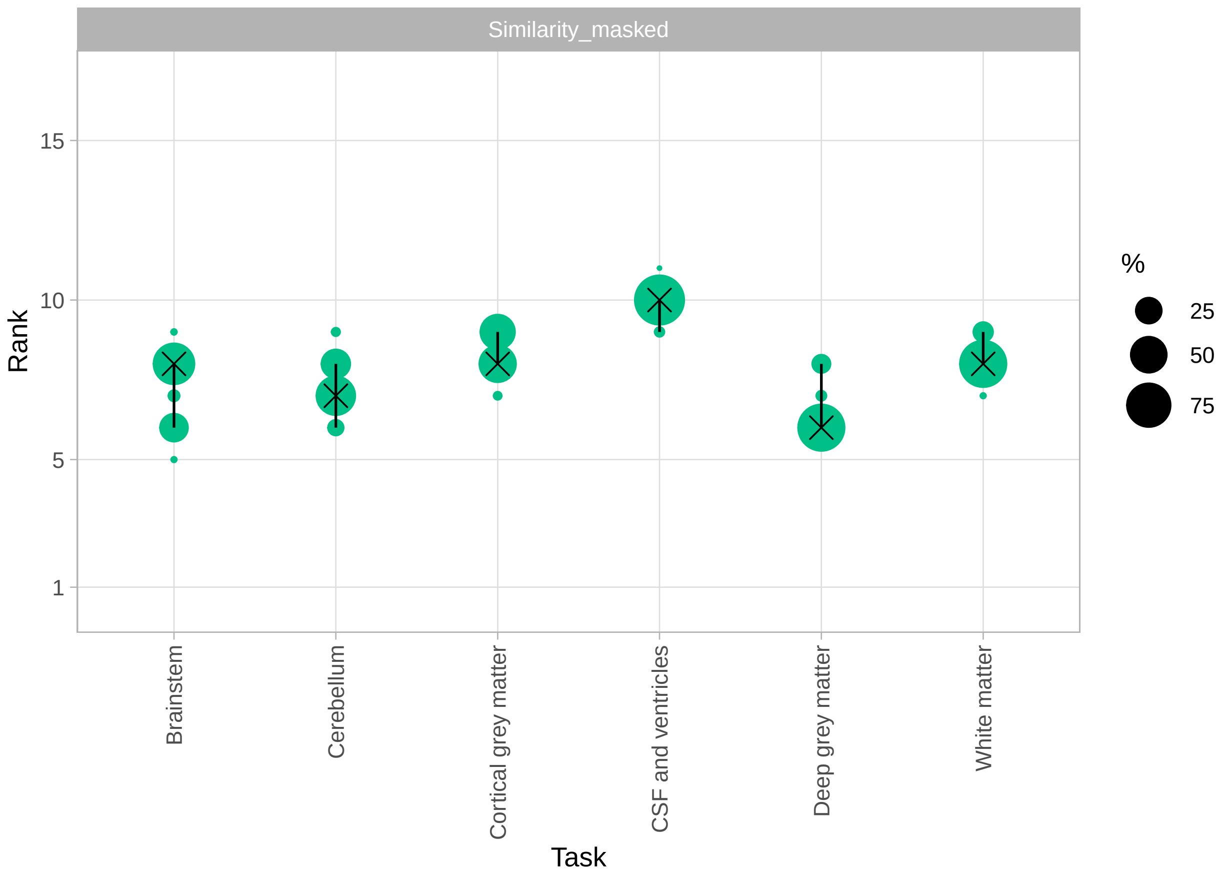
Similarity_masked
Rank
15
10
5
1
Brainstem
Cerebellum
Cortical grey matter
CSF and ventricles
Deep grey matter
White matter
Task
%
25
50
75

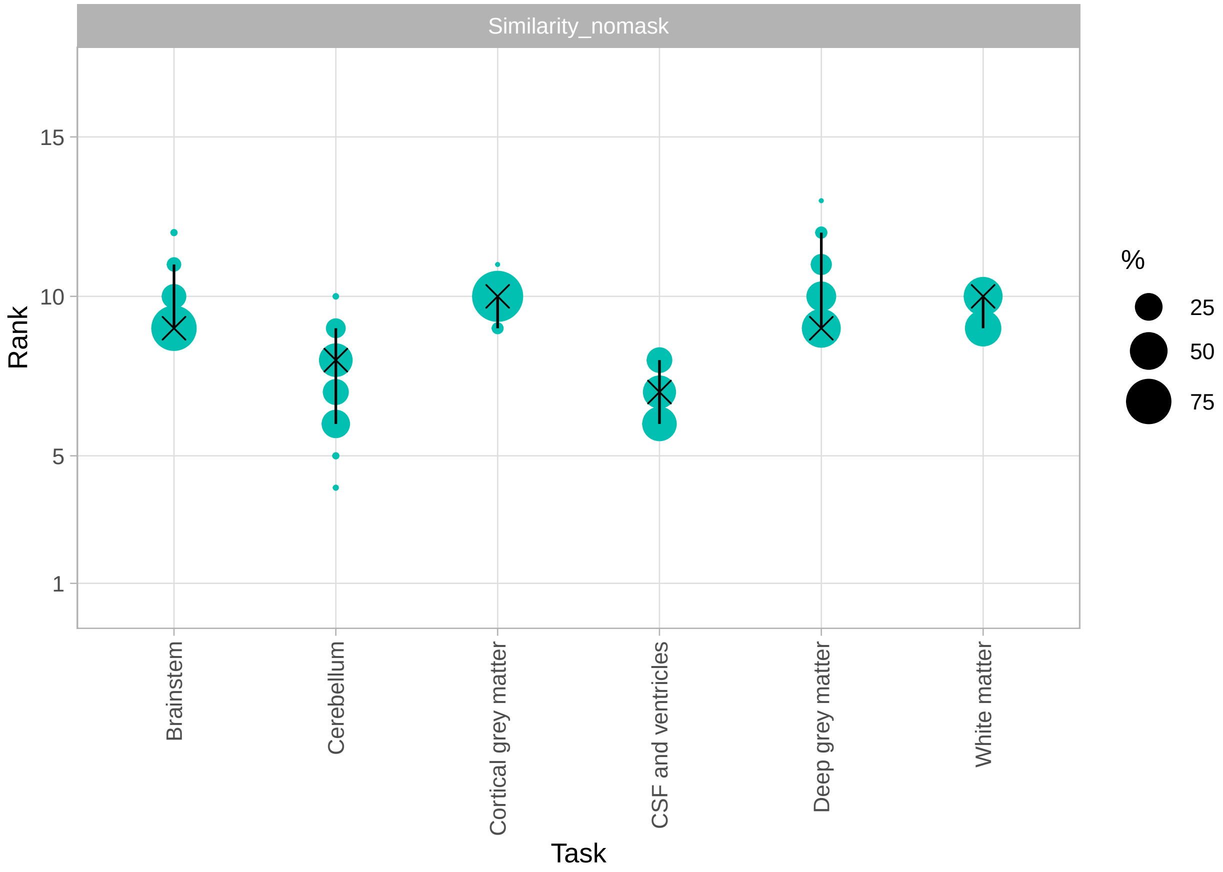
Similarity_nomask
Rank
15
10
5
1
Brainstem
Cerebellum
Cortical grey matter
CSF and ventricles
Deep grey matter
White matter
Task
%
25
50
75

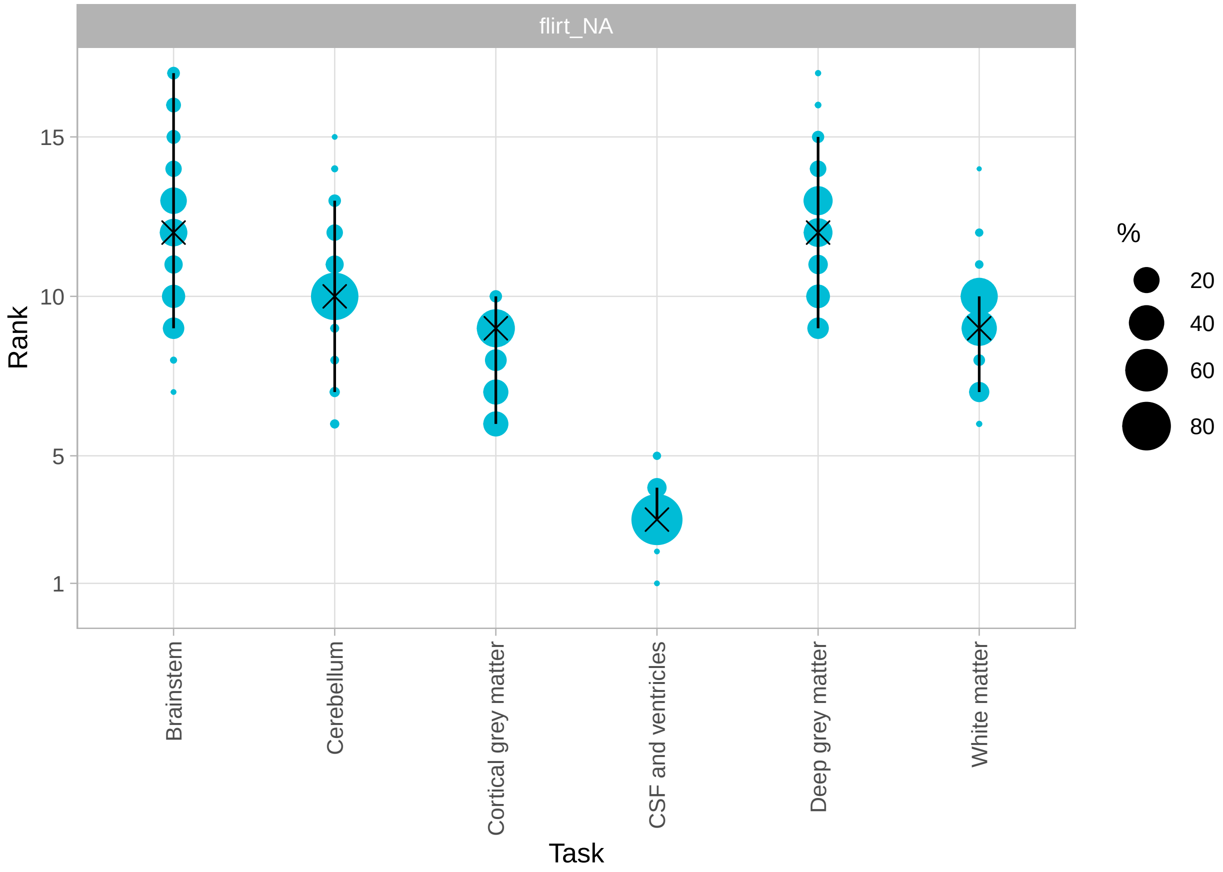
flirt_NA
Rank
15
10
5
1
Brainstem
Cerebellum
Cortical grey matter
CSF and ventricles
Deep grey matter
White matter
Task
%
20
40
60
80

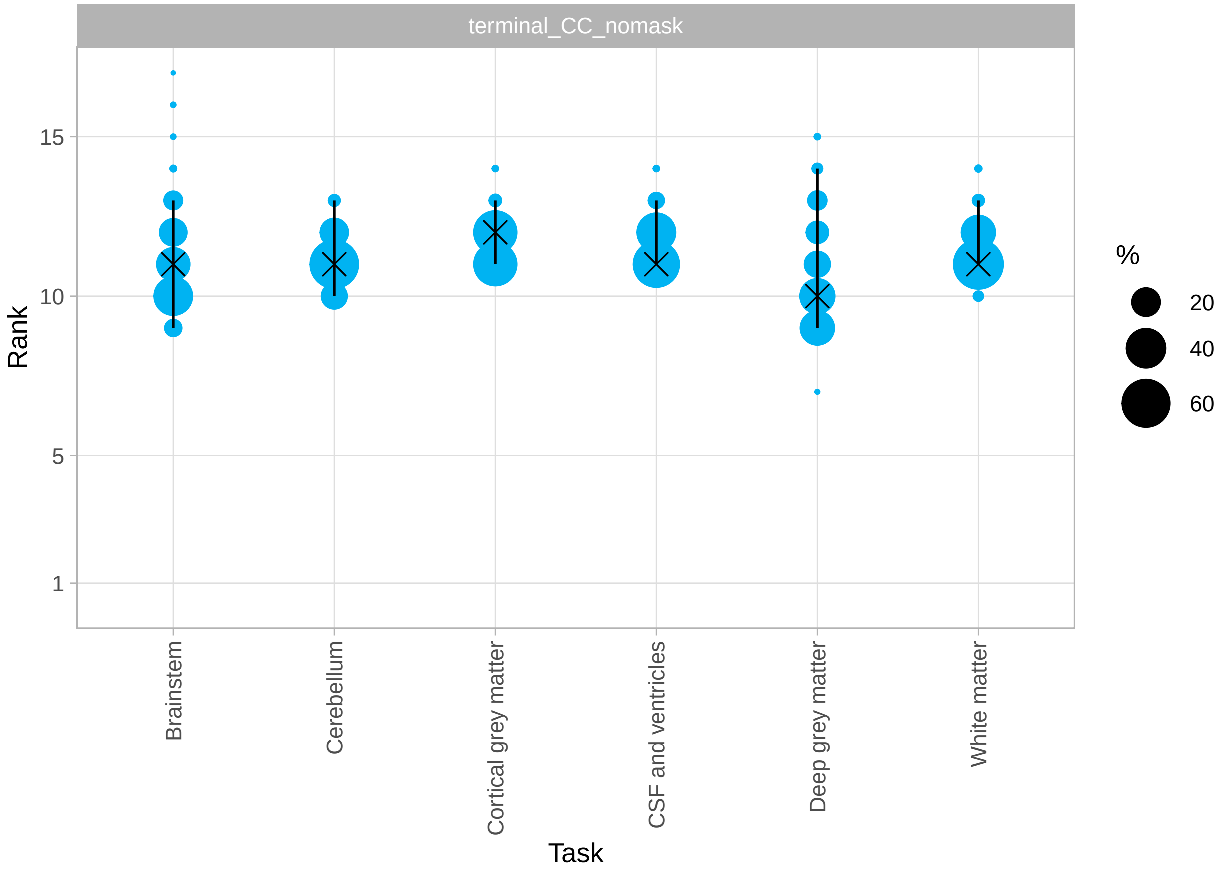
terminal_CC_nomask
Rank
15
10
5
1
Brainstem
Cerebellum
Cortical grey matter
CSF and ventricles
Deep grey matter
White matter
Task
%
20
40
60

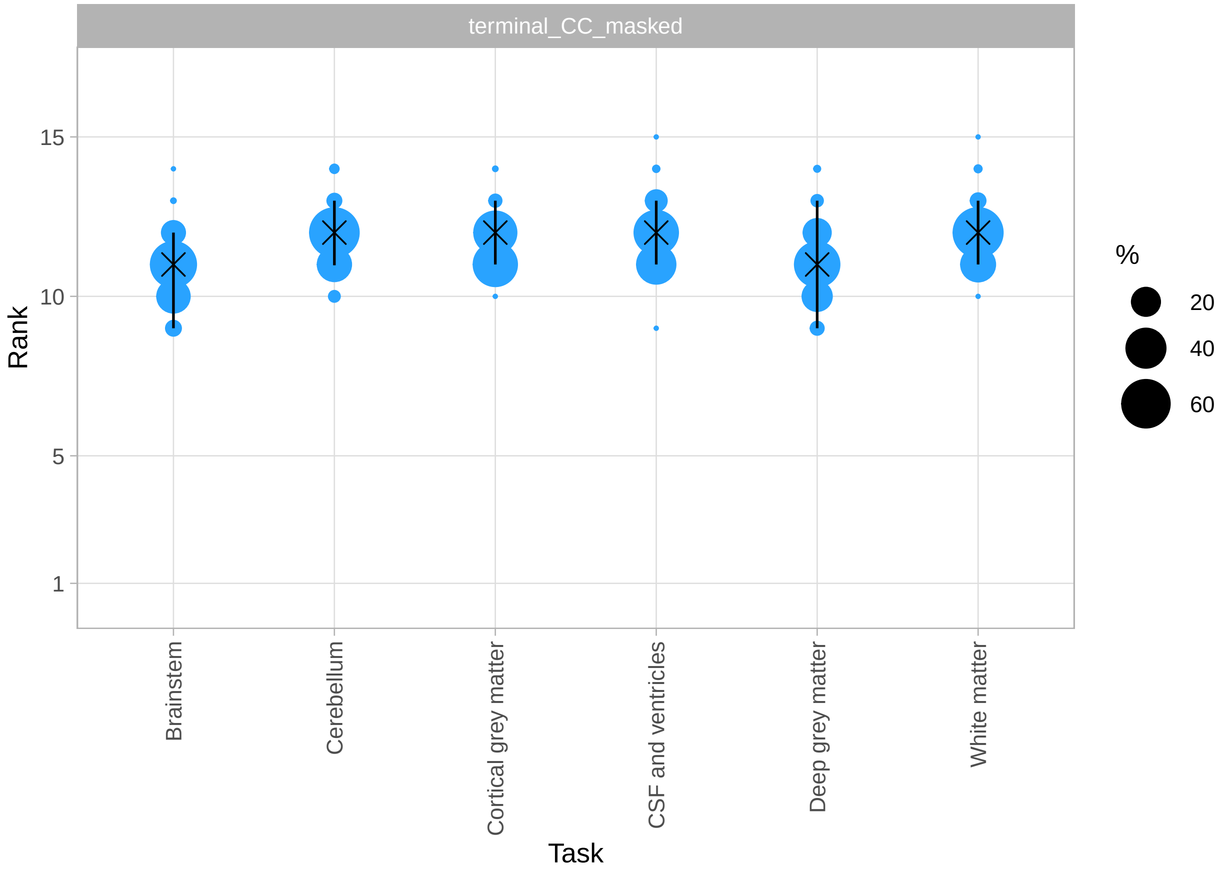

terminal_CC_masked
Rank
15
10
5
1
Brainstem
Cerebellum
Cortical grey matter
CSF and ventricles
Deep grey matter
White matter
Task
%
20
40
60

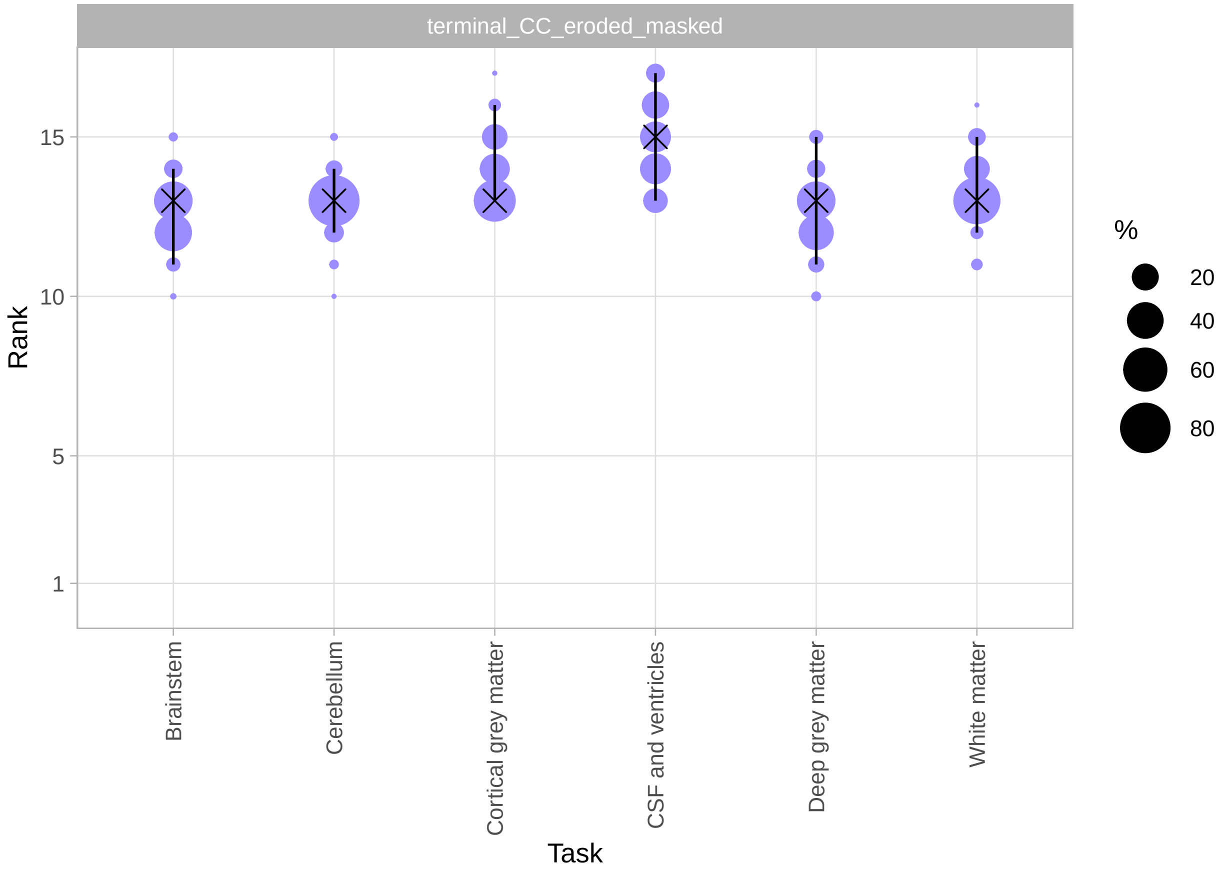
terminal_CC_eroded_masked
Rank
15
10
5
1
Brainstem
Cerebellum
Cortical grey matter
CSF and ventricles
Deep grey matter
White matter
Task
%
20
40
60
80

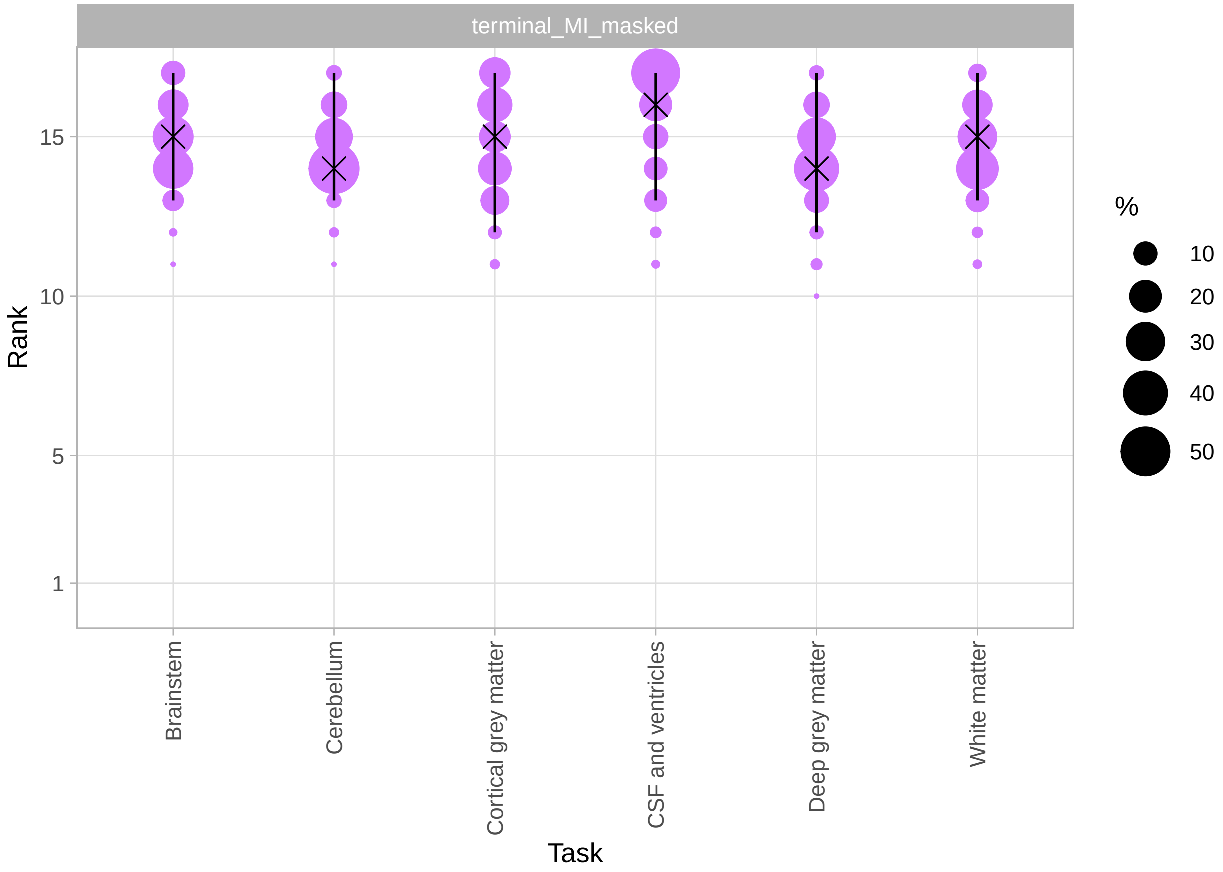
terminal_MI_masked
Rank
15
10
5
1
Brainstem
Cerebellum
Cortical grey matter
CSF and ventricles
Deep grey matter
White matter
Task
%
10
20
30
40
50

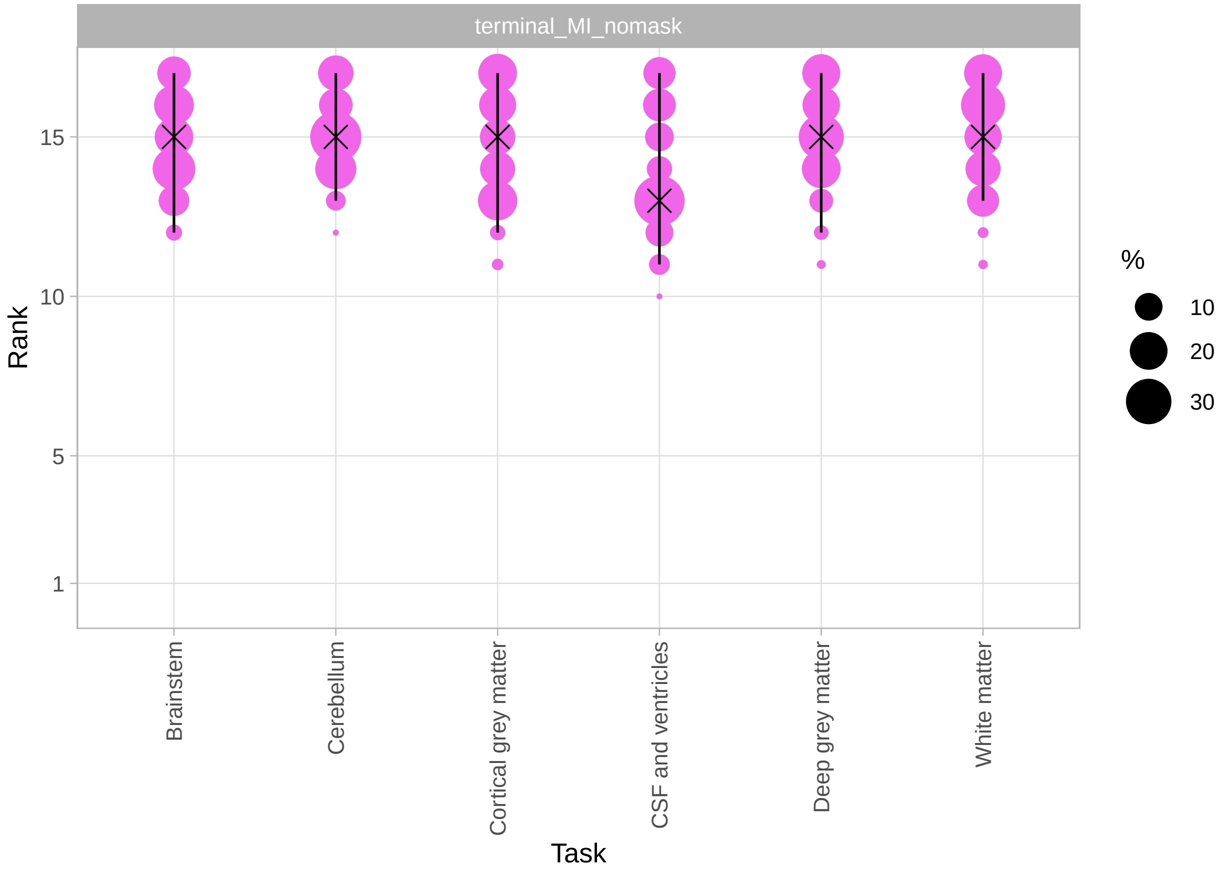

terminal_MI_nomask
Rank
15
10
5
1
Brainstem
Cerebellum
Cortical grey matter
CSF and ventricles
Deep grey matter
White matter
Task
%
10
20
30

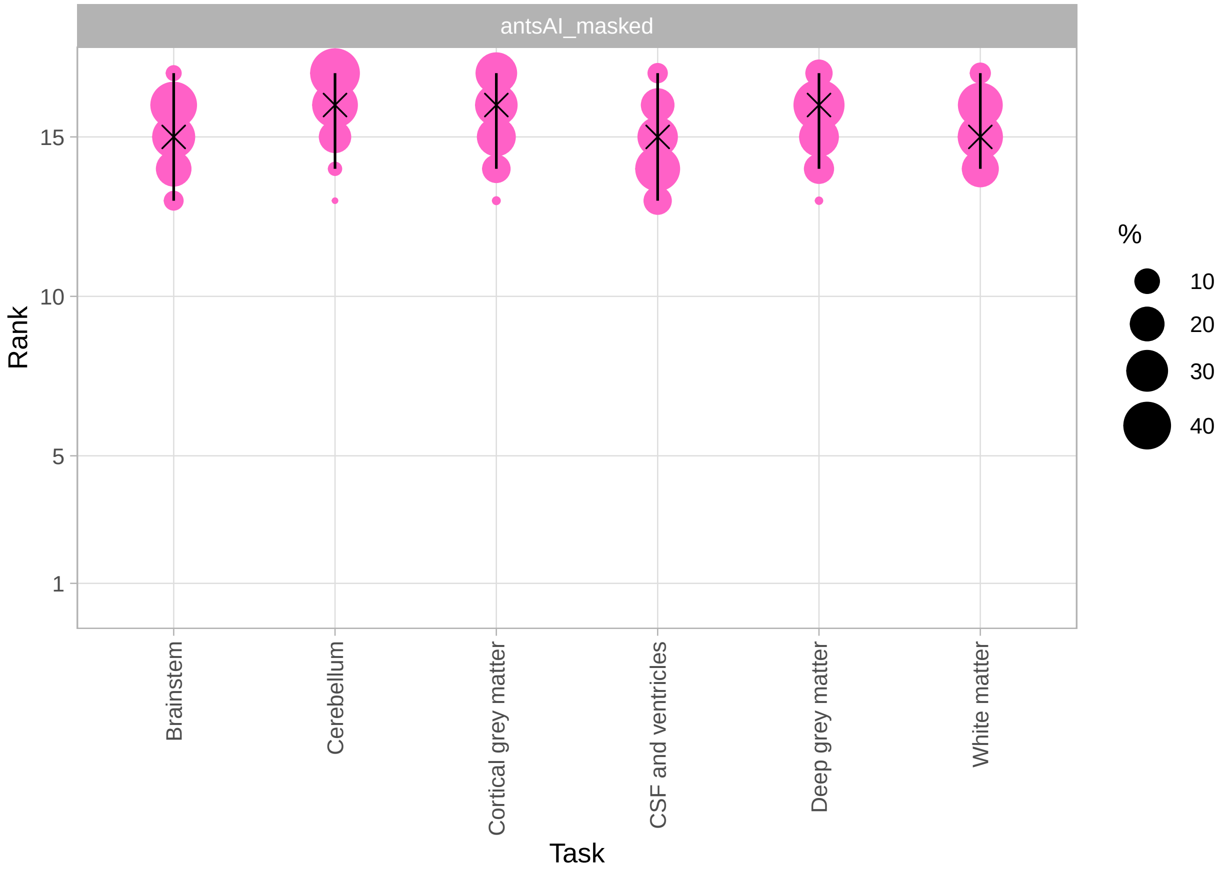
antsAI_masked
Rank
15
10
5
1
Brainstem
Cerebellum
Cortical grey matter
CSF and ventricles
Deep grey matter
White matter
Task
%
10
20
30
40

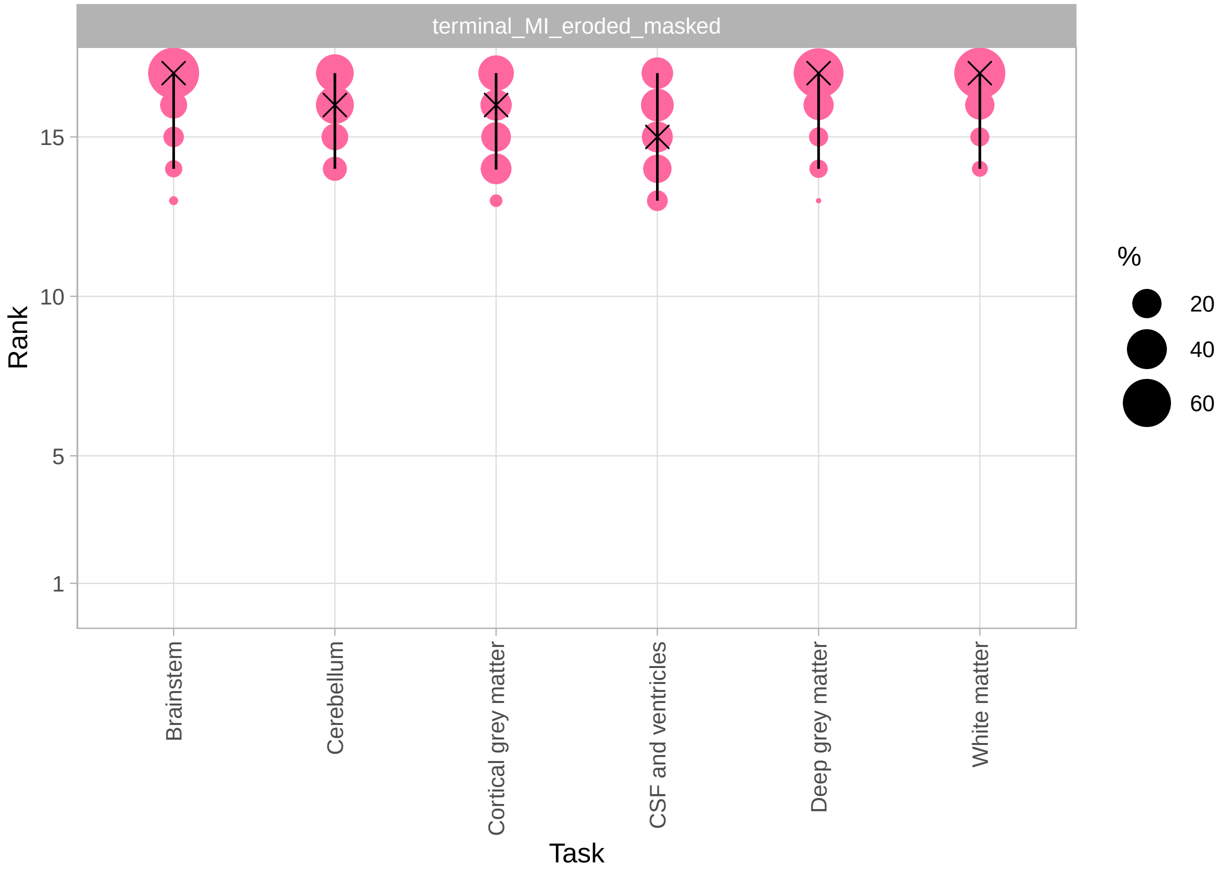

terminal_MI_eroded_masked
Rank
15
10
5
1
Brainstem
Cerebellum
Cortical grey matter
CSF and ventricles
Deep grey matter
White matter
Task
%
20
40
60

An alternative representation is provided by a stacked frequency plot of the observed ranks, separated by algorithm. Observed ranks across bootstrap samples are displayed with coloring according to the task. For algorithms that achieve the same rank in different tasks for the full assessment data set, vertical lines are on top of each other. Vertical lines allow to compare the achieved rank of each algorithm over different tasks.

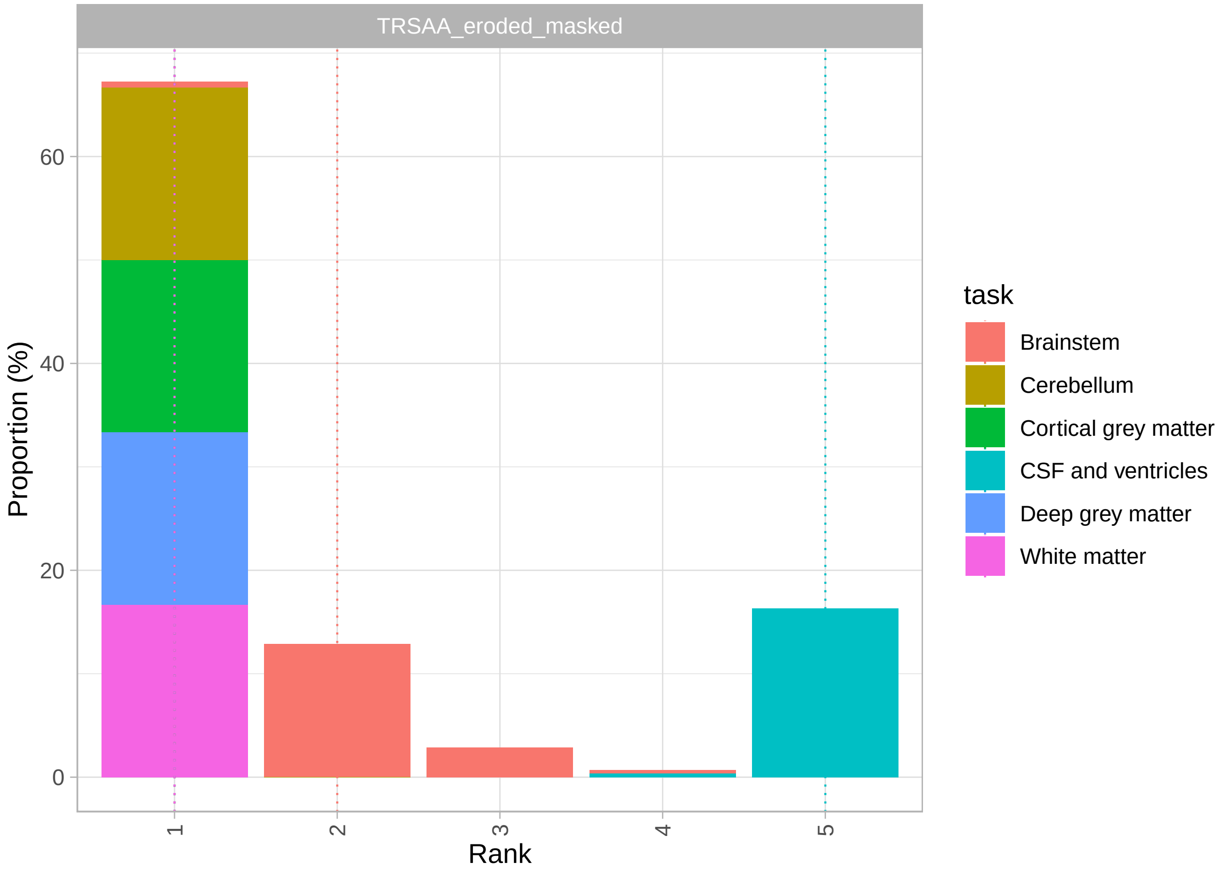

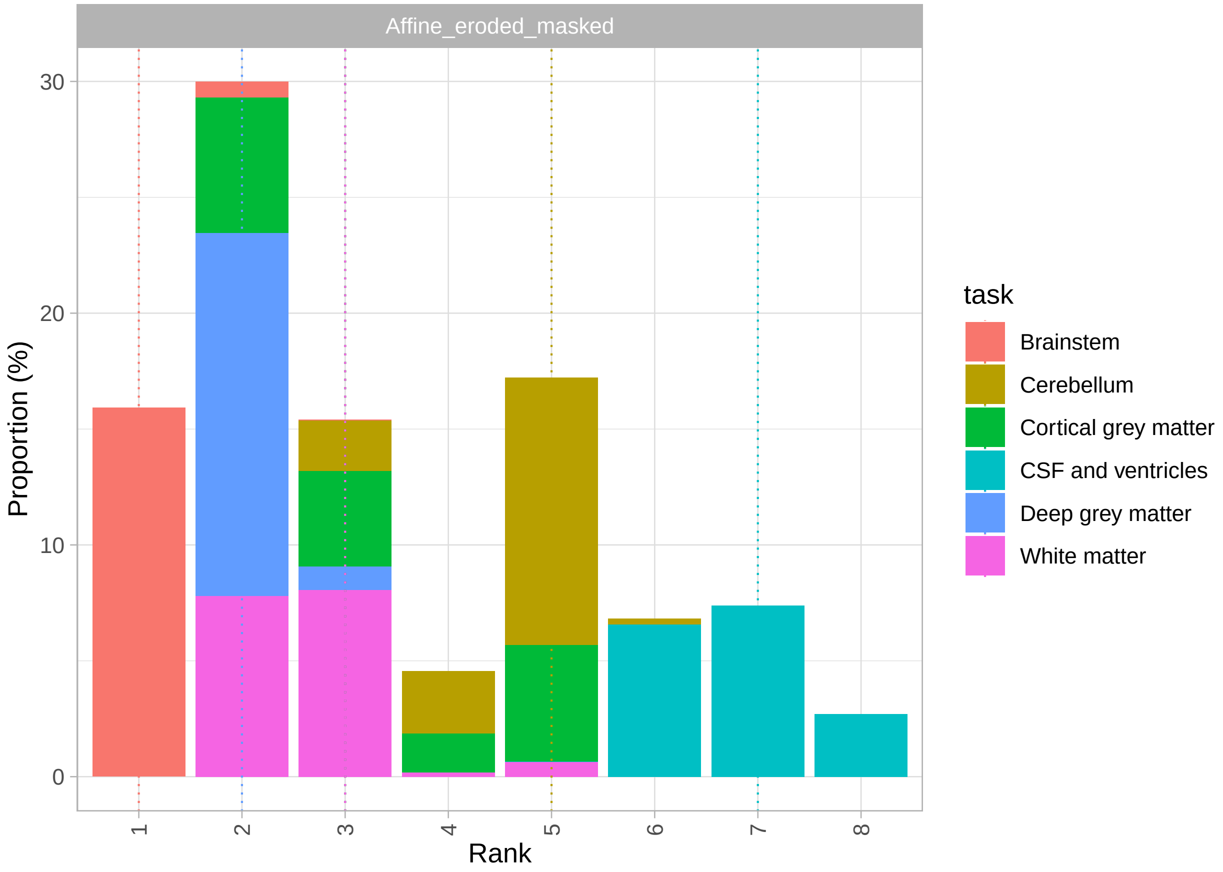

Affine_eroded_masked
Proportion (%)
30
20
10
0
1
2
3
4
5
6
7
8
Rank
task
Brainstem
Cerebellum
Cortical grey matter
CSF and ventricles
Deep grey matter
White matter

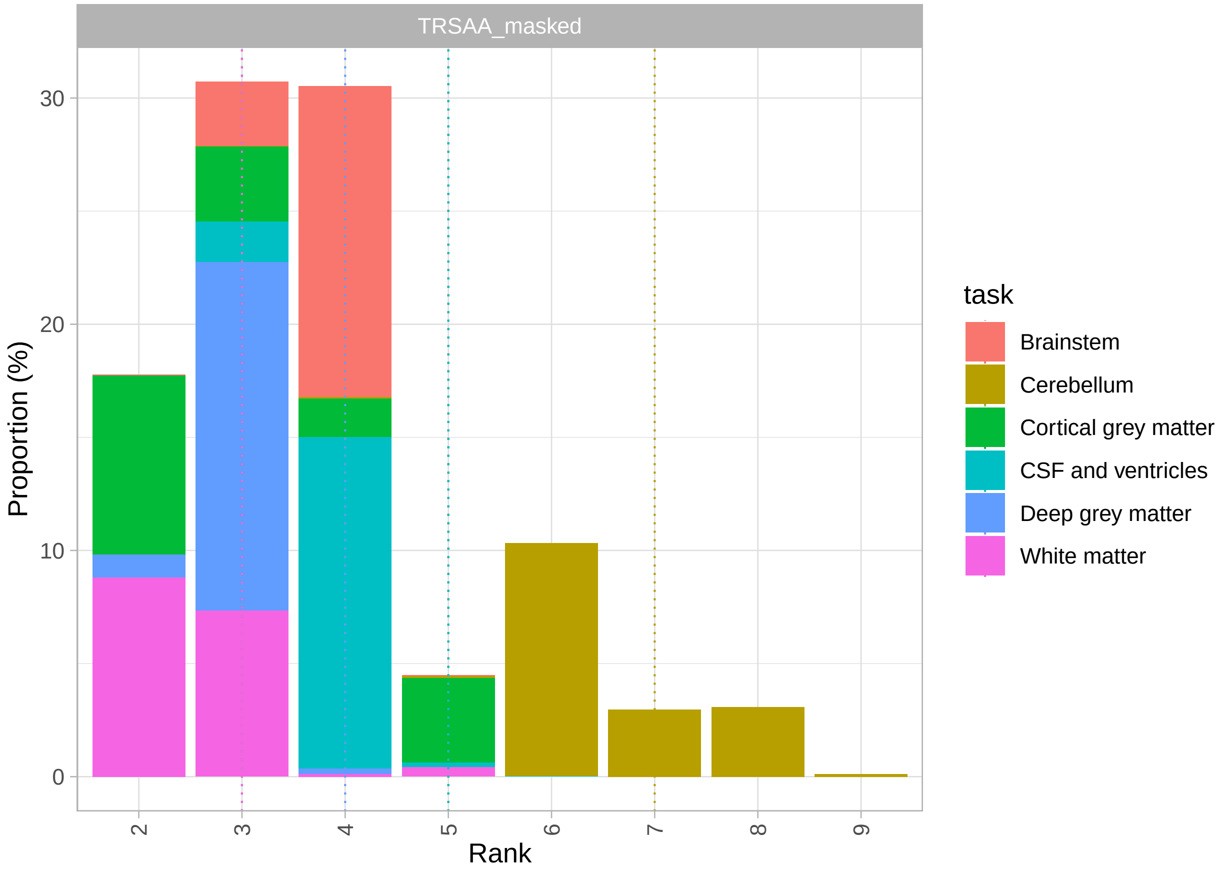
TRSAA_masked
Proportion (%)
30
20
10
0
2
3
4
5
6
7
8
9
Rank
task
Brainstem
Cerebellum
Cortical grey matter
CSF and ventricles
Deep grey matter
White matter

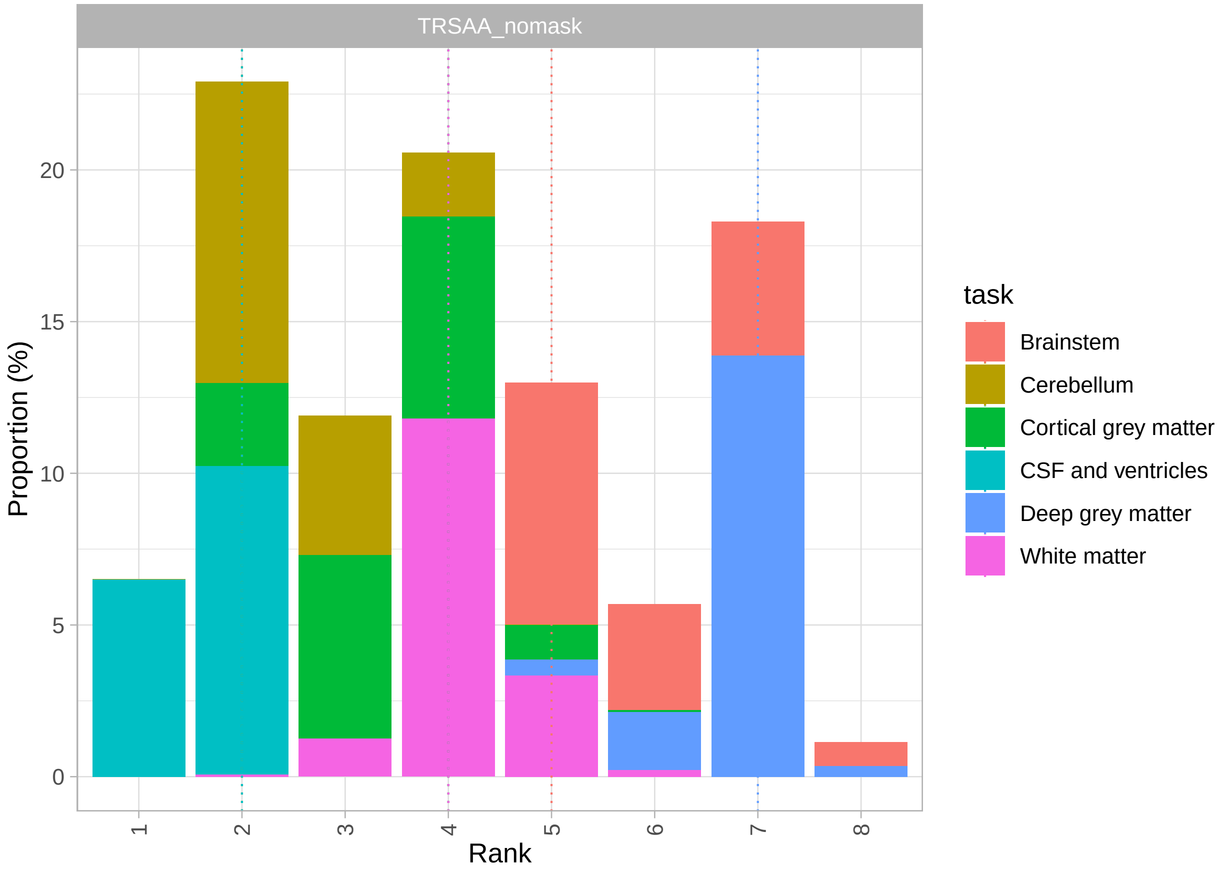

TRSAA_nomask
task
Brainstem
Cerebellum
Cortical grey matter
CSF and ventricles
Deep grey matter
White matter
Proportion (%)
Rank
0
5
10
15
20
1
2
3
4
5
6
7
8

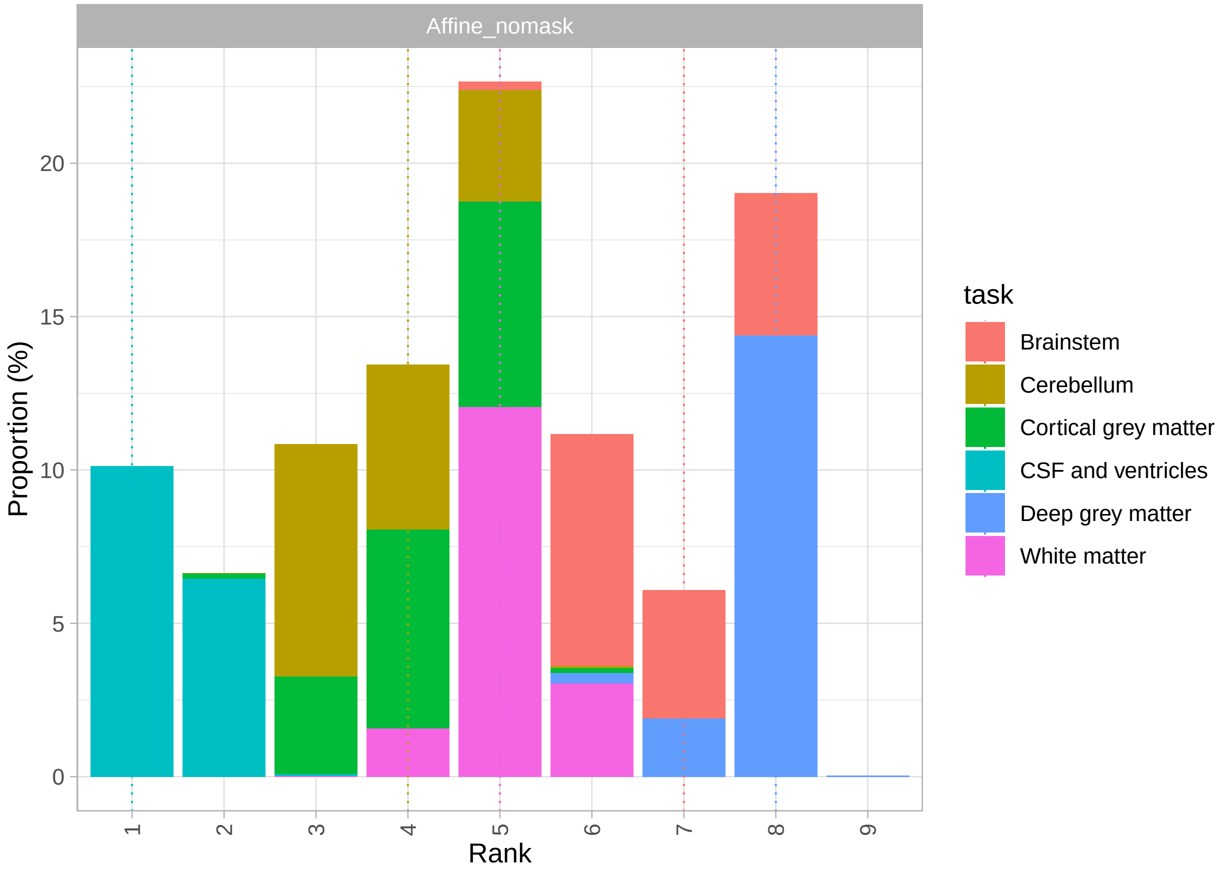
Affine_nomask
Proportion (%)
0
5
10
15
20
1
2
3
4
5
6
7
8
9
Rank
task
Brainstem
Cerebellum
Cortical grey matter
CSF and ventricles
Deep grey matter
White matter

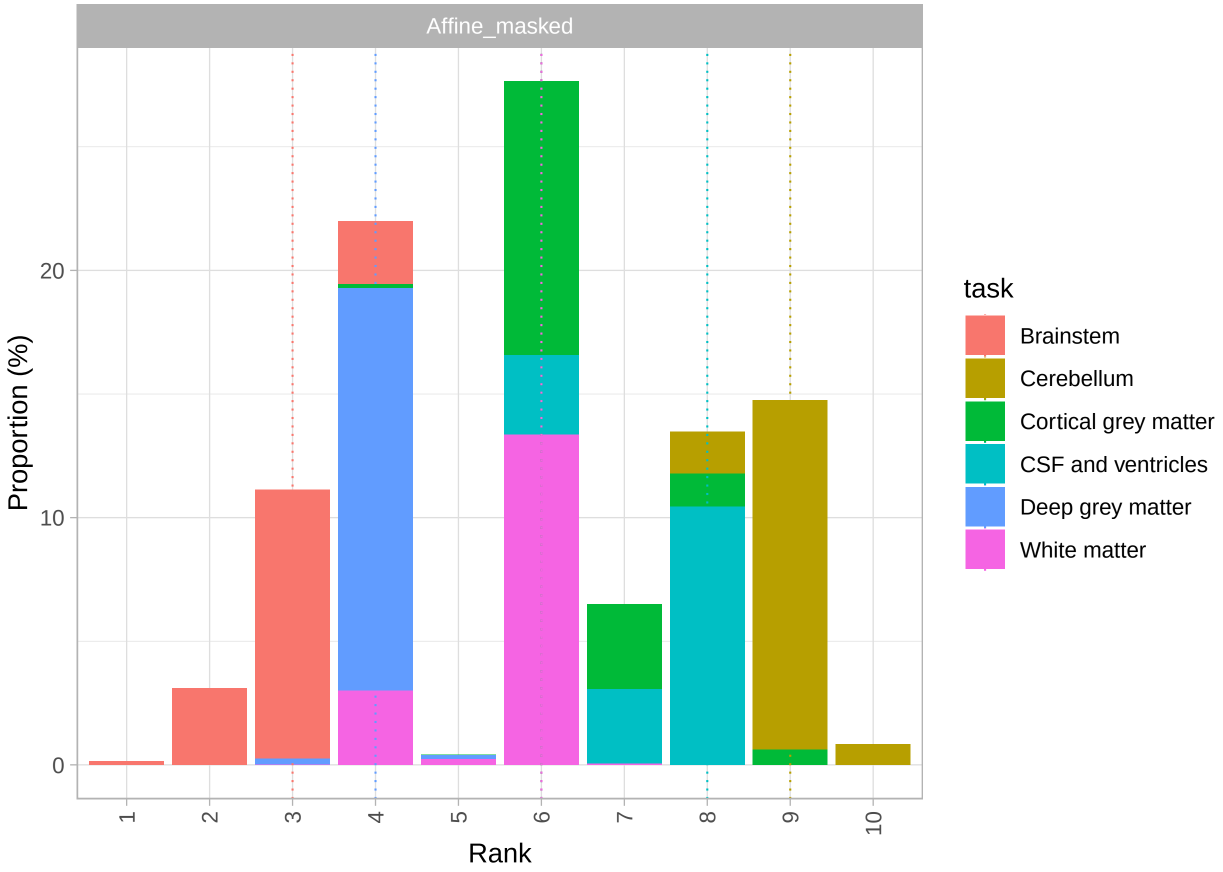
Affine_masked
Proportion (%)
0
10
20
1
2
3
4
5
6
7
8
9
10
Rank
task
Brainstem
Cerebellum
Cortical grey matter
CSF and ventricles
Deep grey matter
White matter

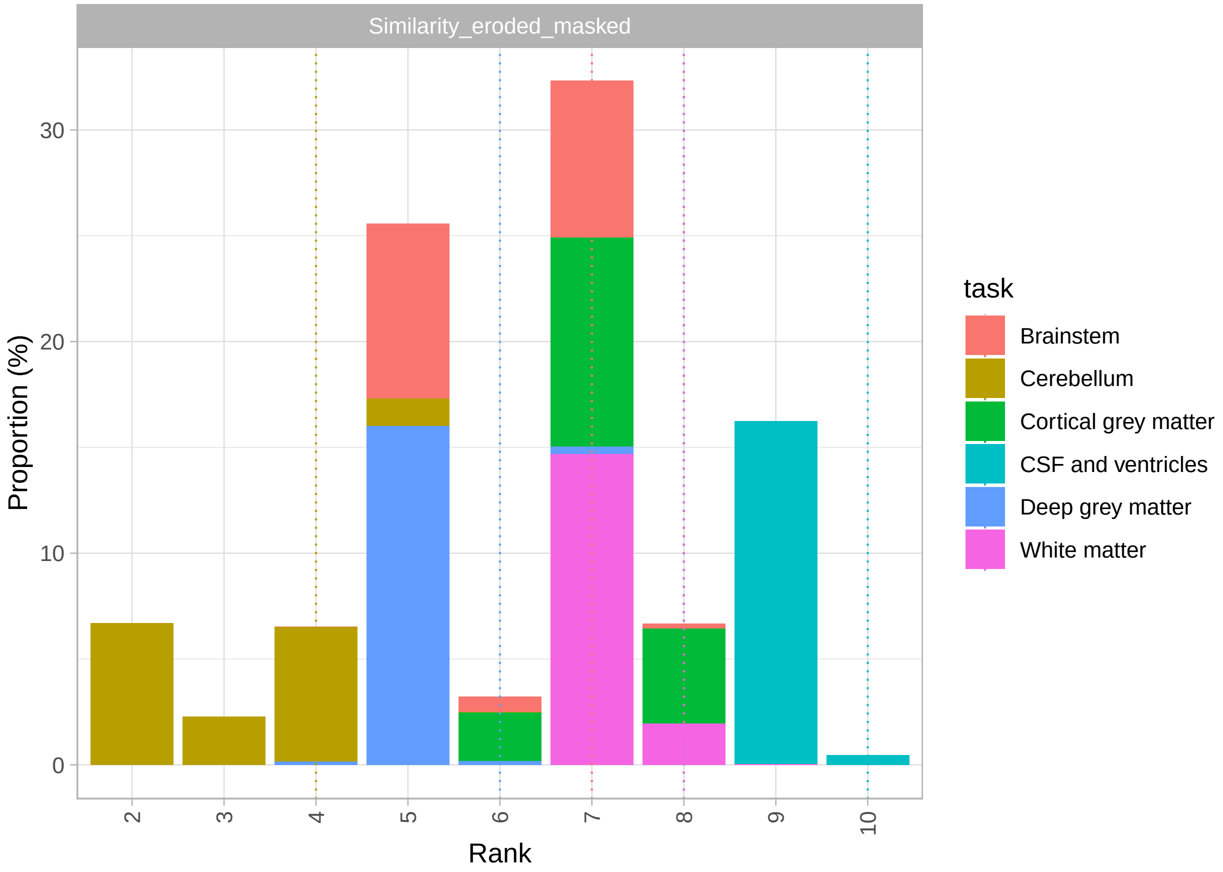
Similarity_eroded_masked
Proportion (%)
30
20
10
0
2
3
4
5
6
7
8
9
10
Rank
task
Brainstem
Cerebellum
Cortical grey matter
CSF and ventricles
Deep grey matter
White matter

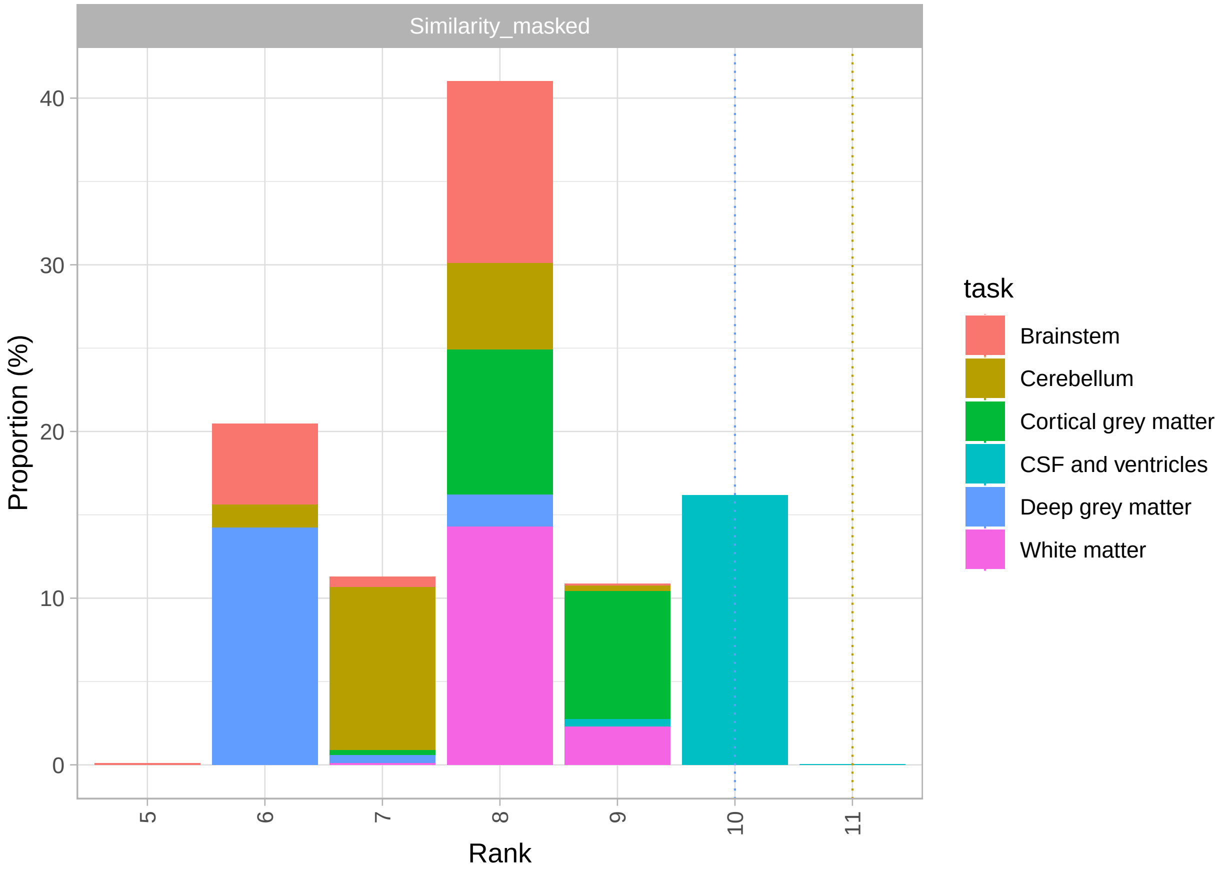
Similarity_masked
Proportion (%)
40
30
20
10
0
5
6
7
8
9
10
11
Rank
task
Brainstem
Cerebellum
Cortical grey matter
CSF and ventricles
Deep grey matter
White matter

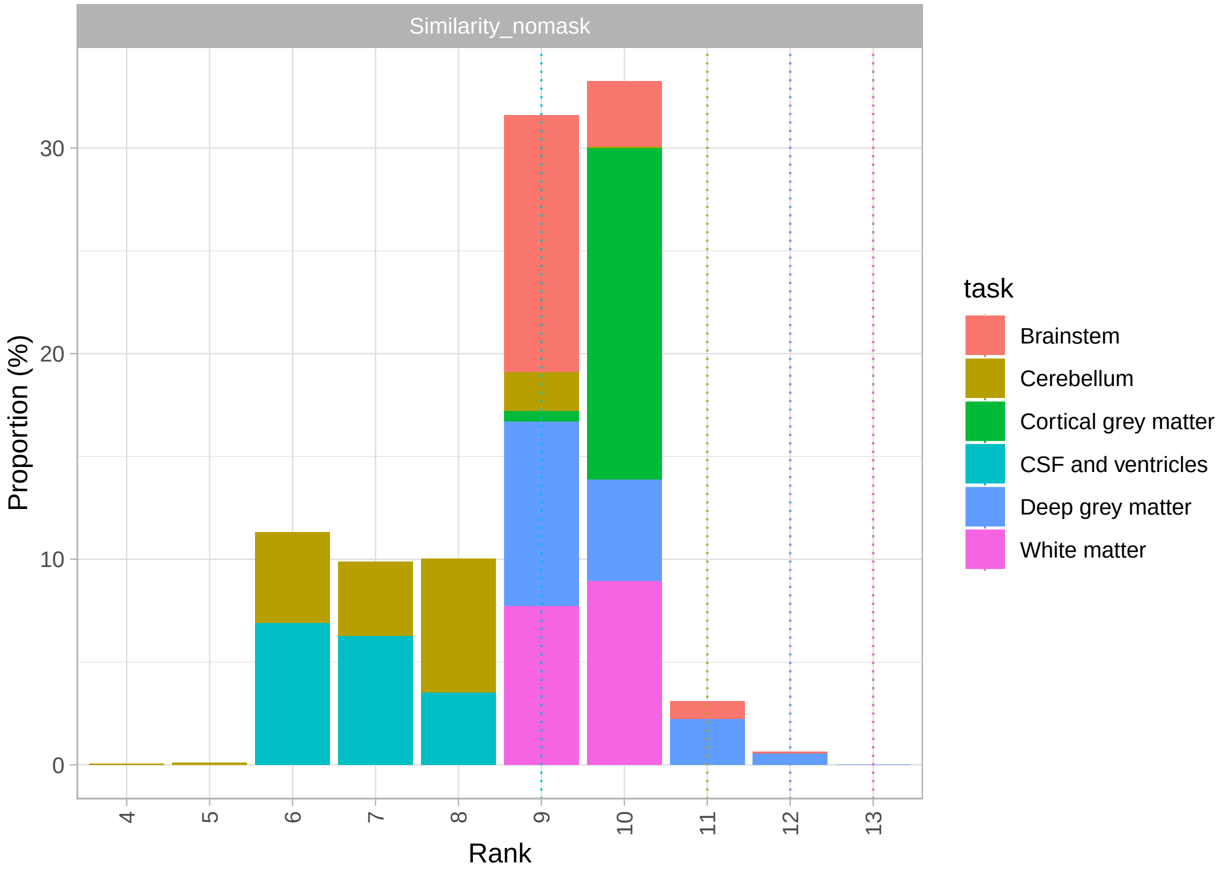
Similarity_nomask
Proportion (%)
30
20
10
0
4
5
6
7
8
9
10
11
12
13
Rank
task
Brainstem
Cerebellum
Cortical grey matter
CSF and ventricles
Deep grey matter
White matter

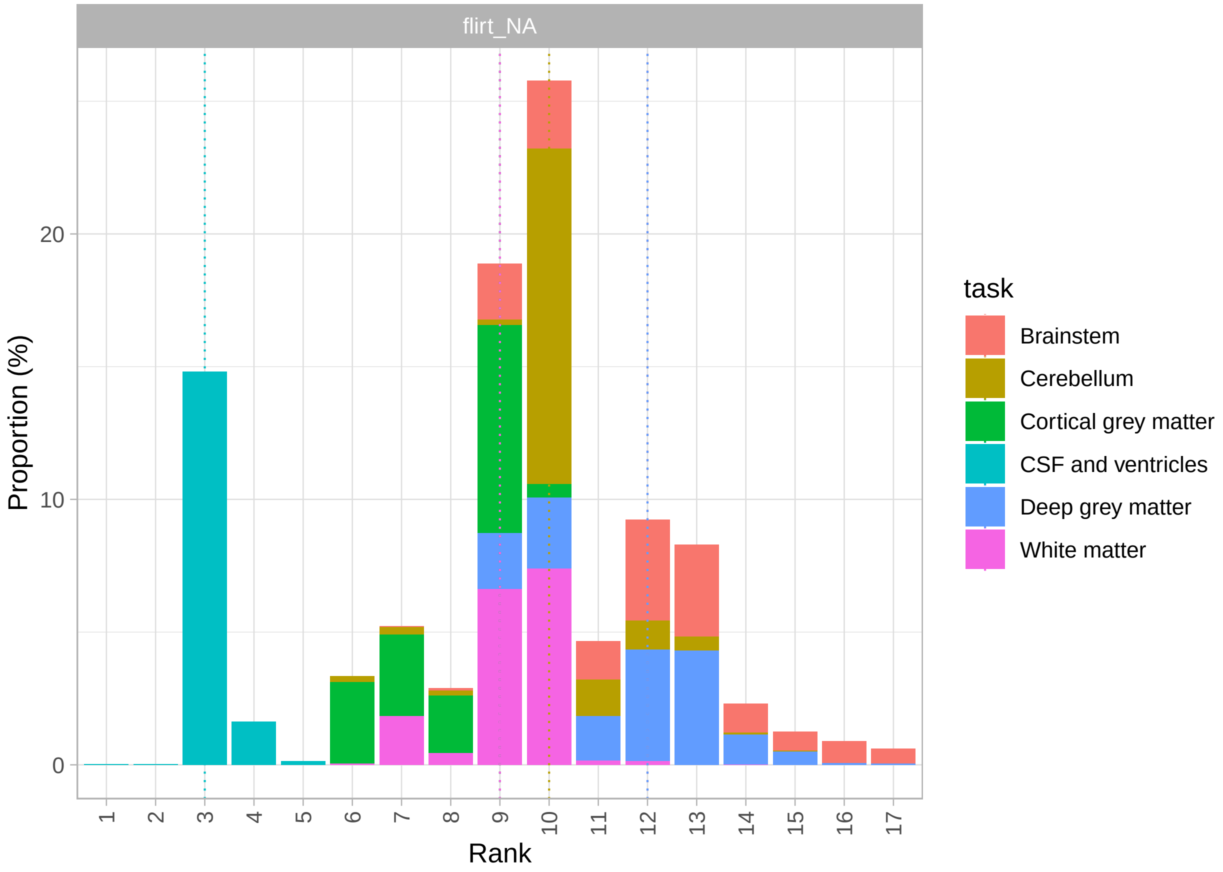
flirt_NA
Proportion (%)
20
10
0
1
2
3
4
5
6
7
8
9
10
11
12
13
14
15
16
17
Rank
task
Brainstem
Cerebellum
Cortical grey matter
CSF and ventricles
Deep grey matter
White matter

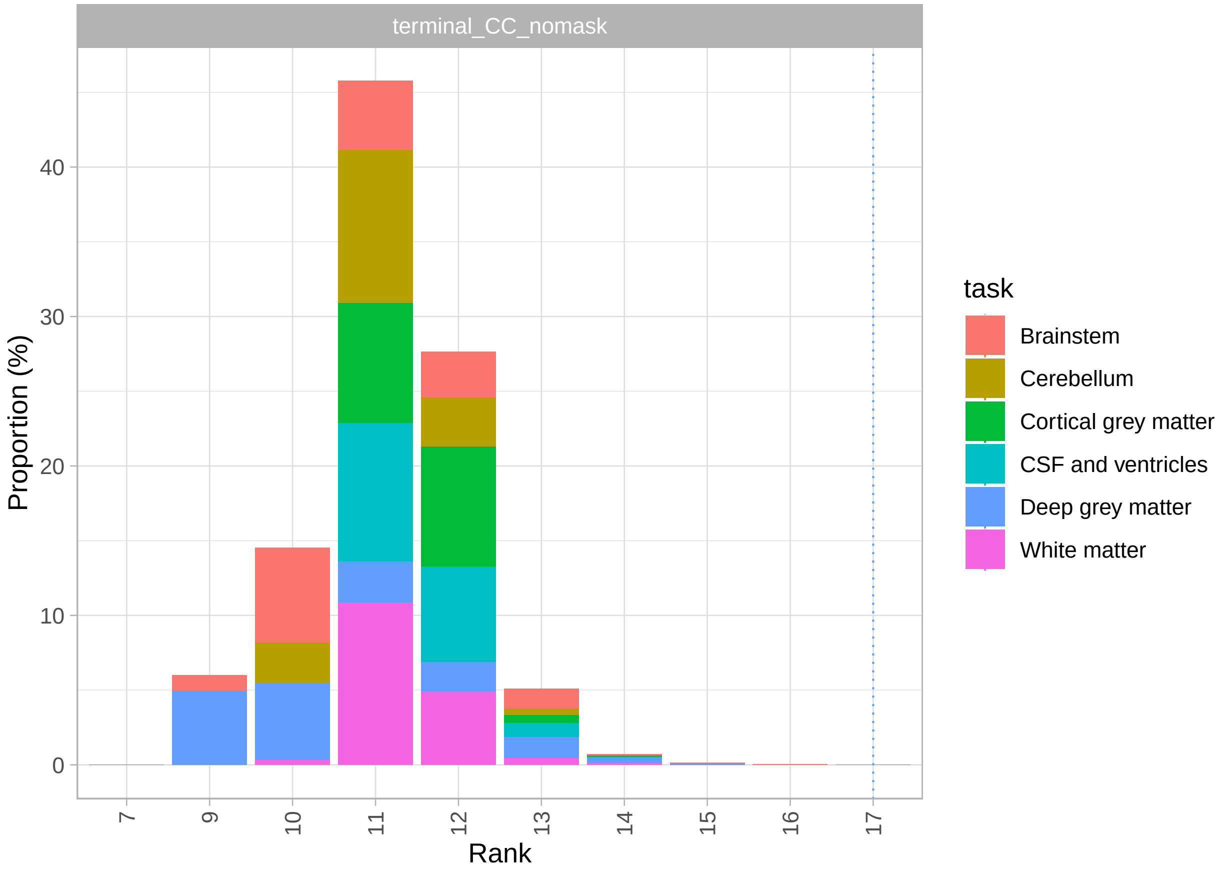
terminal_CC_nomask
Proportion (%)
40
30
20
10
0
7
9
10
11
12
13
14
15
16
17
Rank
task
Brainstem
Cerebellum
Cortical grey matter
CSF and ventricles
Deep grey matter
White matter

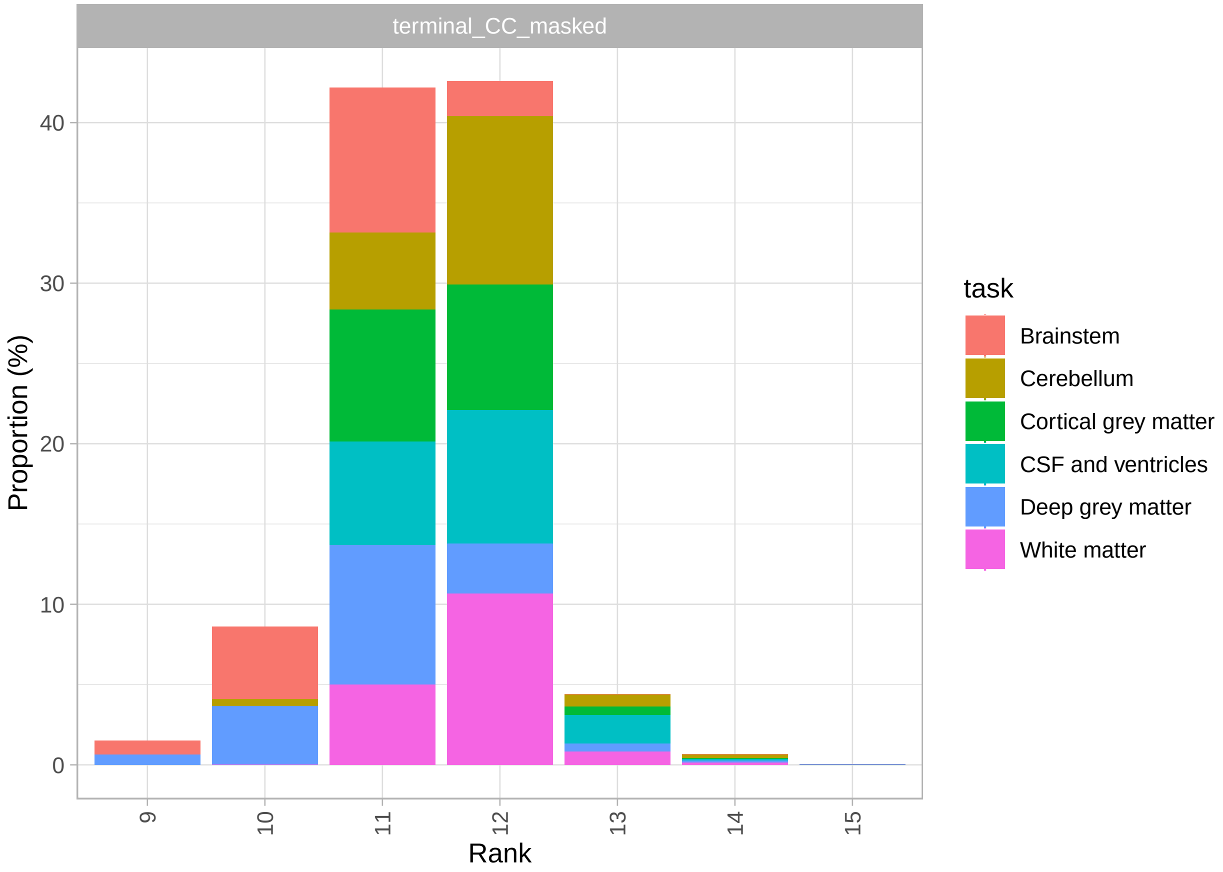
terminal_CC_masked
Proportion (%)
40
30
20
10
0
9
10
11
12
13
14
15
Rank
task
Brainstem
Cerebellum
Cortical grey matter
CSF and ventricles
Deep grey matter
White matter

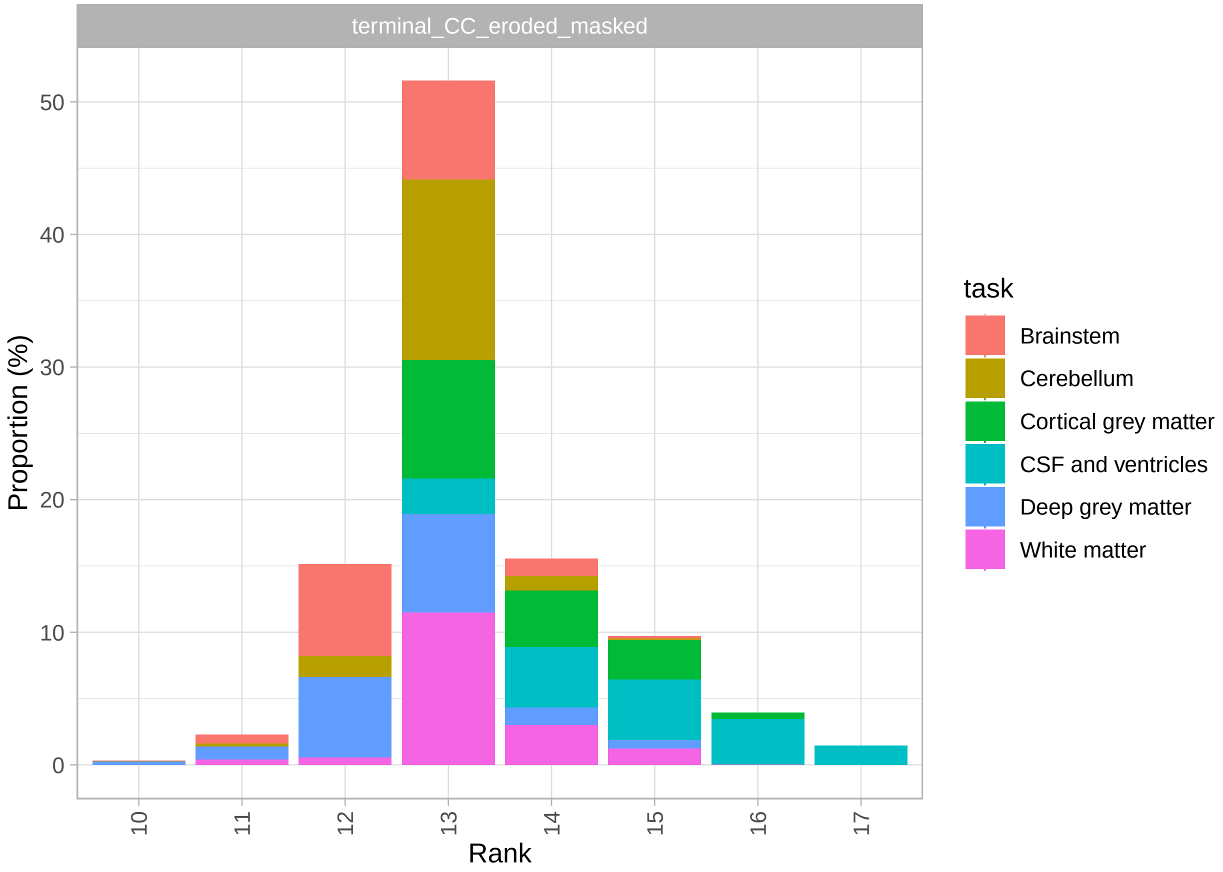
terminal_CC_eroded_masked
Proportion (%)
50
40
30
20
10
0
10
11
12
13
14
15
16
17
Rank
task
Brainstem
Cerebellum
Cortical grey matter
CSF and ventricles
Deep grey matter
White matter

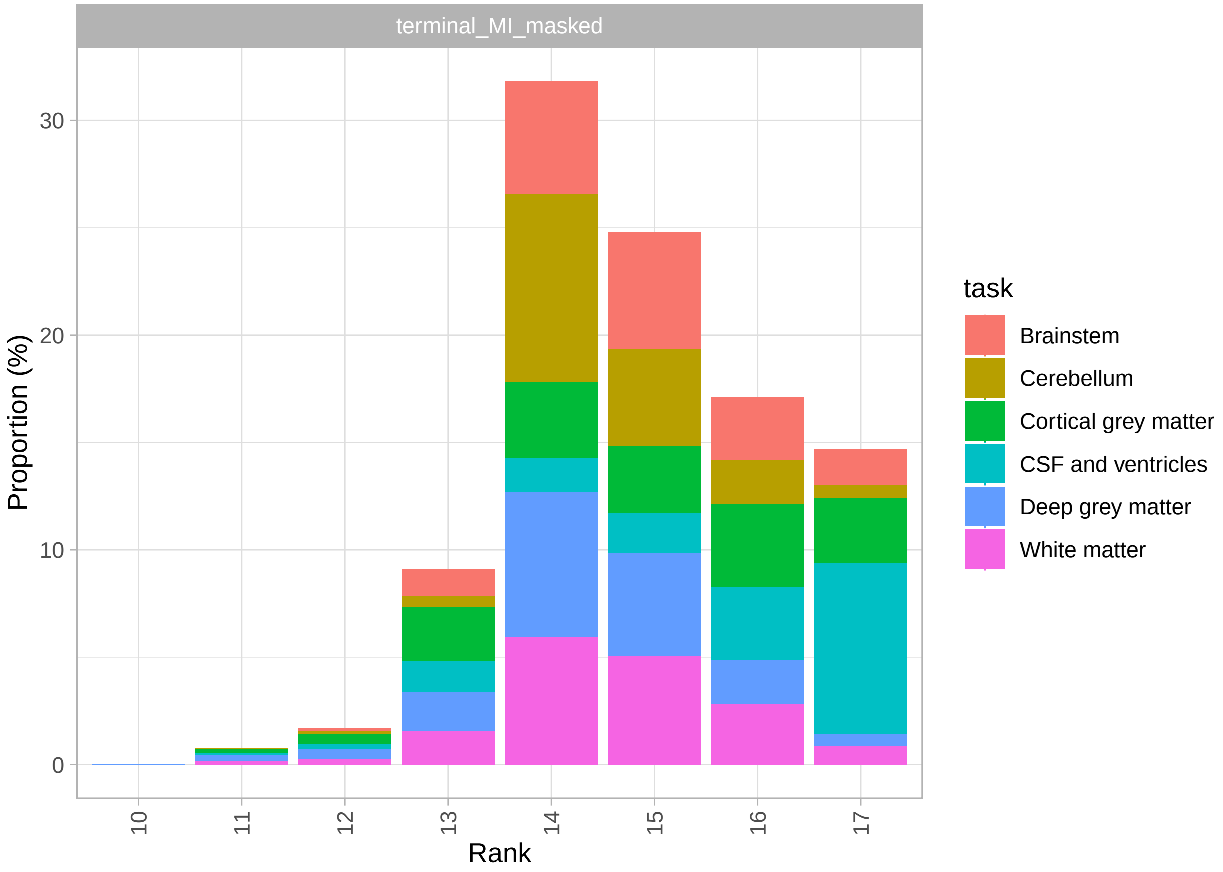
terminal_MI_masked
Proportion (%)
0
10
20
30
10
11
12
13
14
15
16
17
Rank
task
Brainstem
Cerebellum
Cortical grey matter
CSF and ventricles
Deep grey matter
White matter

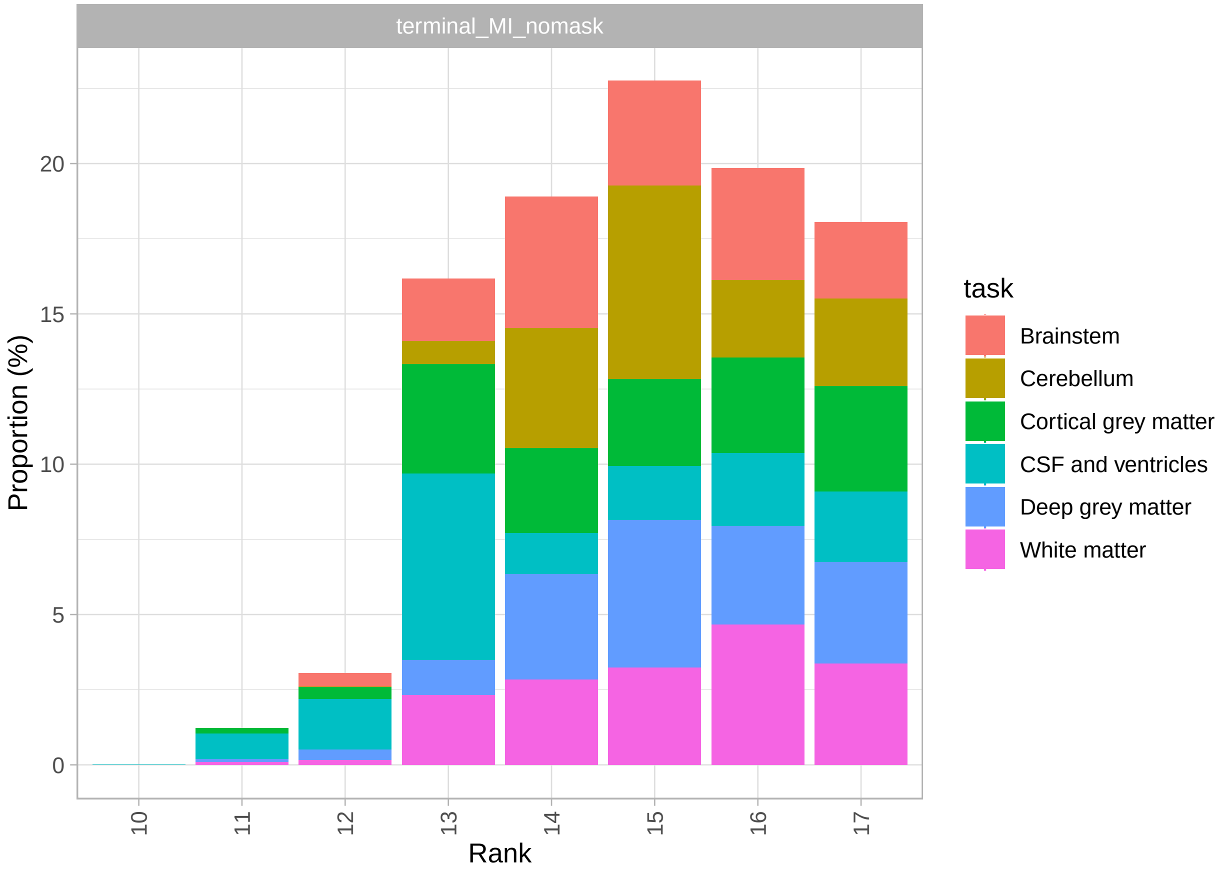
terminal_MI_nomask
Proportion (%)
0
5
10
15
20
10
11
12
13
14
15
16
17
Rank
task
Brainstem
Cerebellum
Cortical grey matter
CSF and ventricles
Deep grey matter
White matter

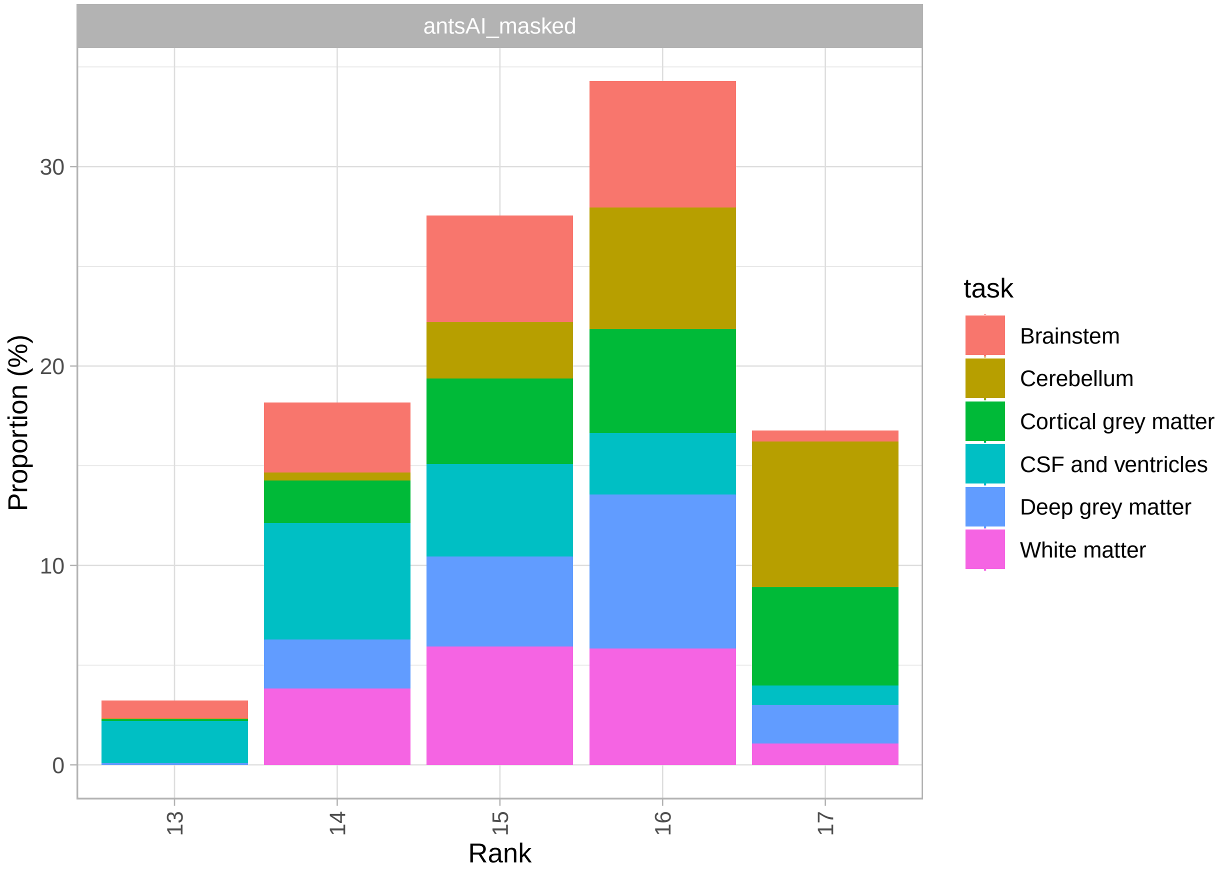
antsAI_masked
Proportion (%)
0
10
20
30
13
14
15
16
17
Rank
task
Brainstem
Cerebellum
Cortical grey matter
CSF and ventricles
Deep grey matter
White matter

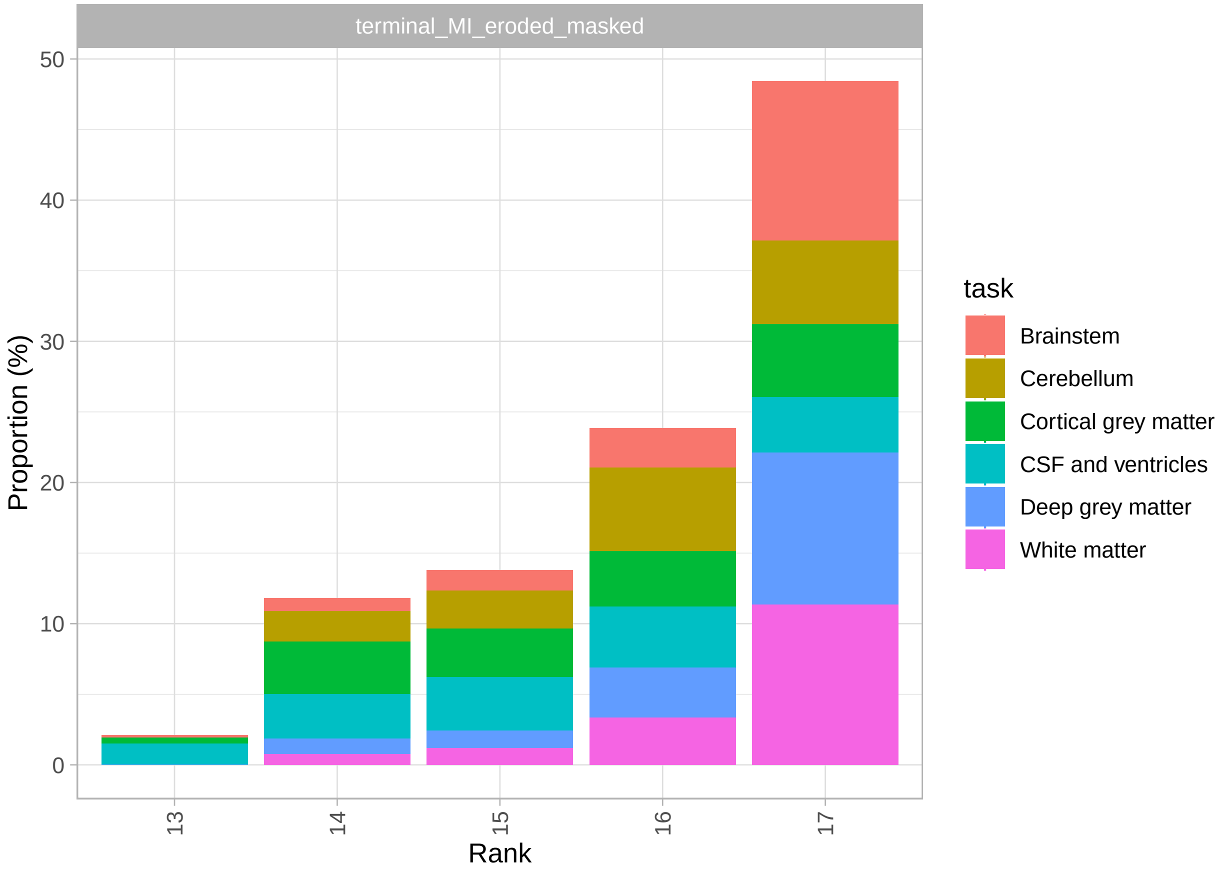
terminal_MI_eroded_masked
Proportion (%)
0
10
20
30
40
50
13
14
15
16
17
Rank
task
Brainstem
Cerebellum
Cortical grey matter
CSF and ventricles
Deep grey matter
White matter

4.2 Characterization of tasks

4.2.1 Visualizing bootstrap results

To investigate which tasks separate algorithms well (i.e., lead to a stable ranking), a blob plot is recommended.

Bootstrap results can be shown in a blob plot showing one plot for each task. In this view, the spread of the blobs for each algorithm can be compared across tasks. Deviations from the diagonal indicate deviations from the consensus ranking (over tasks). Specifically, if rank distribution of an algorithm is consistently below the diagonal, the algorithm performed better in this task than on average across tasks, while if the rank distribution of an algorithm is consistently above the diagonal, the algorithm performed worse in this task than on average across tasks. At the bottom of each panel, ranks for each algorithm in the tasks are provided.

Same as in Section 3.1 but now ordered according to consensus.

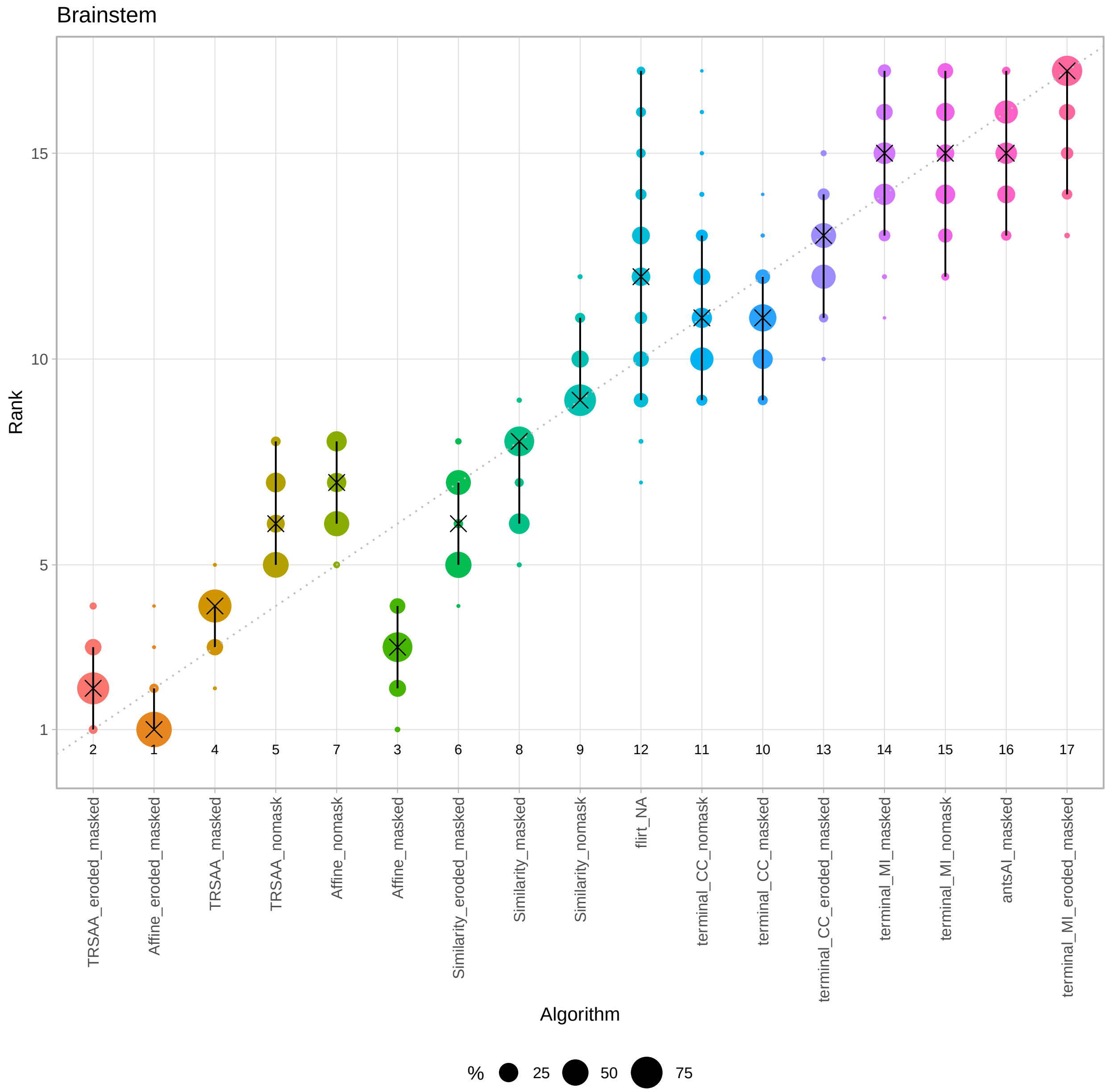

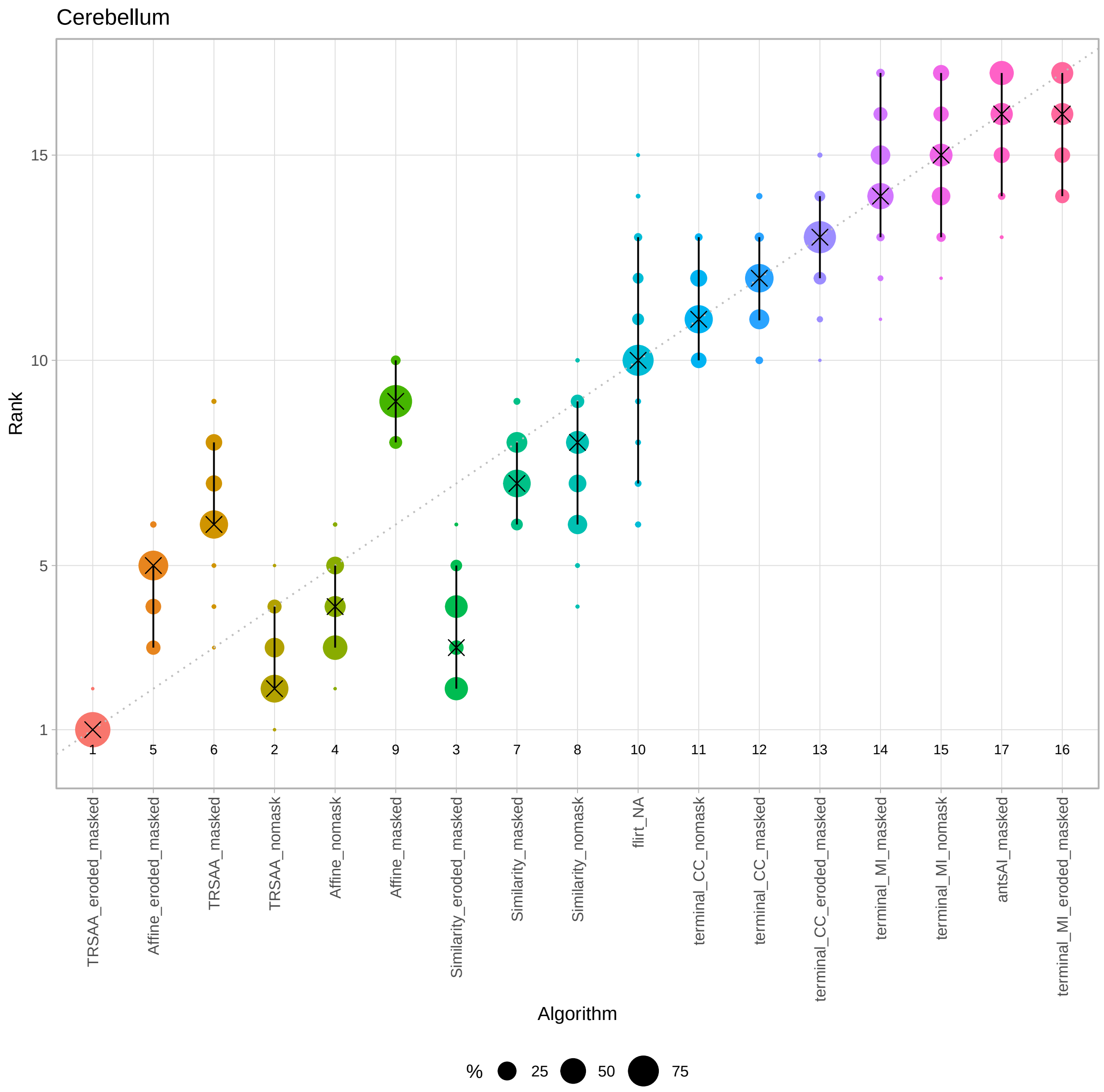
Cerebellum
Rank
15
10
5
1
1
5
6
2
4
9
3
7
8
10
11
12
13
14
15
17
16
TRSAA_eroded_masked
Affine_eroded_masked
TRSAA_masked
TRSAA_nomask
Affine_nomask
Affine_masked
Similarity_eroded_masked
Similarity_masked
Similarity_nomask
flirt_NA
terminal_CC_nomask
terminal_CC_masked
terminal_CC_eroded_masked
terminal_MI_masked
terminal_MI_nomask
antsAI_masked
terminal_MI_eroded_masked
Algorithm
%
25
50
75

Cortical grey matter

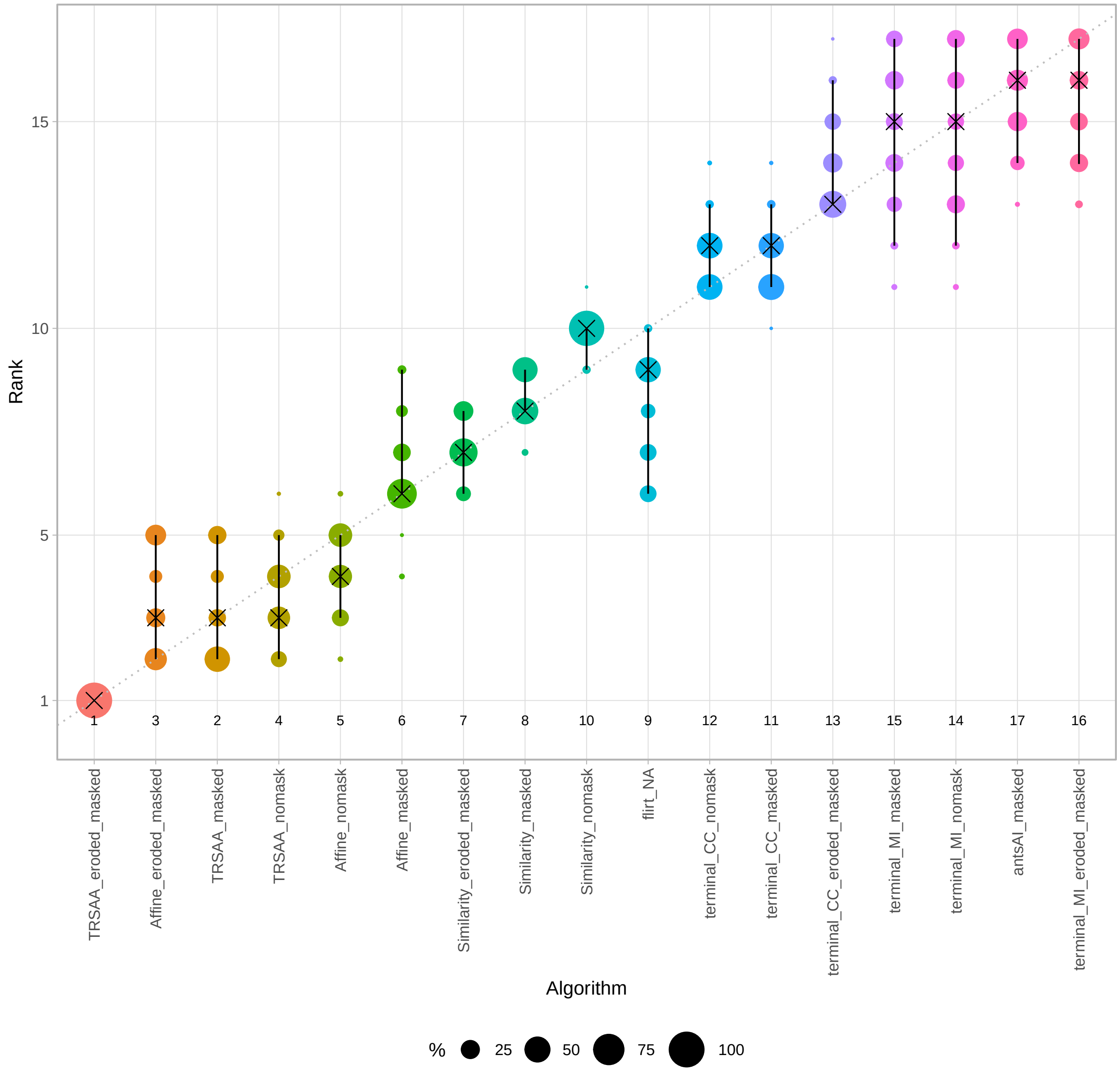

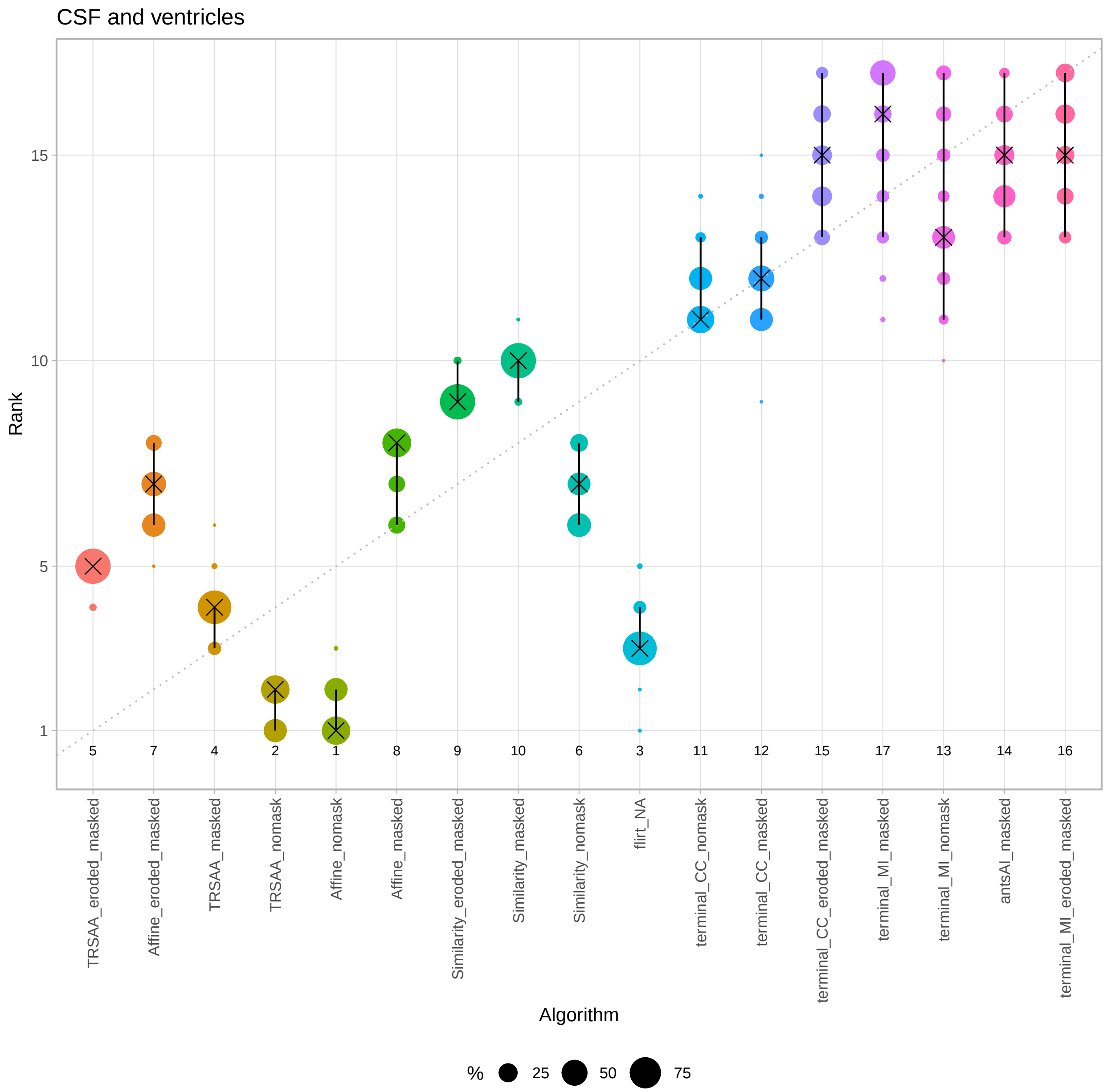

CSF and ventricles
Rank
15
10
5
1
5
7
4
2
1
8
9
10
6
3
11
12
15
17
13
14
16
TRSAA_eroded_masked
Affine_eroded_masked
TRSAA_masked
TRSAA_nomask
Affine_nomask
Affine_masked
Similarity_eroded_masked
Similarity_masked
Similarity_nomask
flirt_NA
terminal_CC_nomask
terminal_CC_masked
terminal_CC_eroded_masked
terminal_MI_masked
terminal_MI_nomask
antsAI_masked
terminal_MI_eroded_masked
Algorithm
%
25
50
75

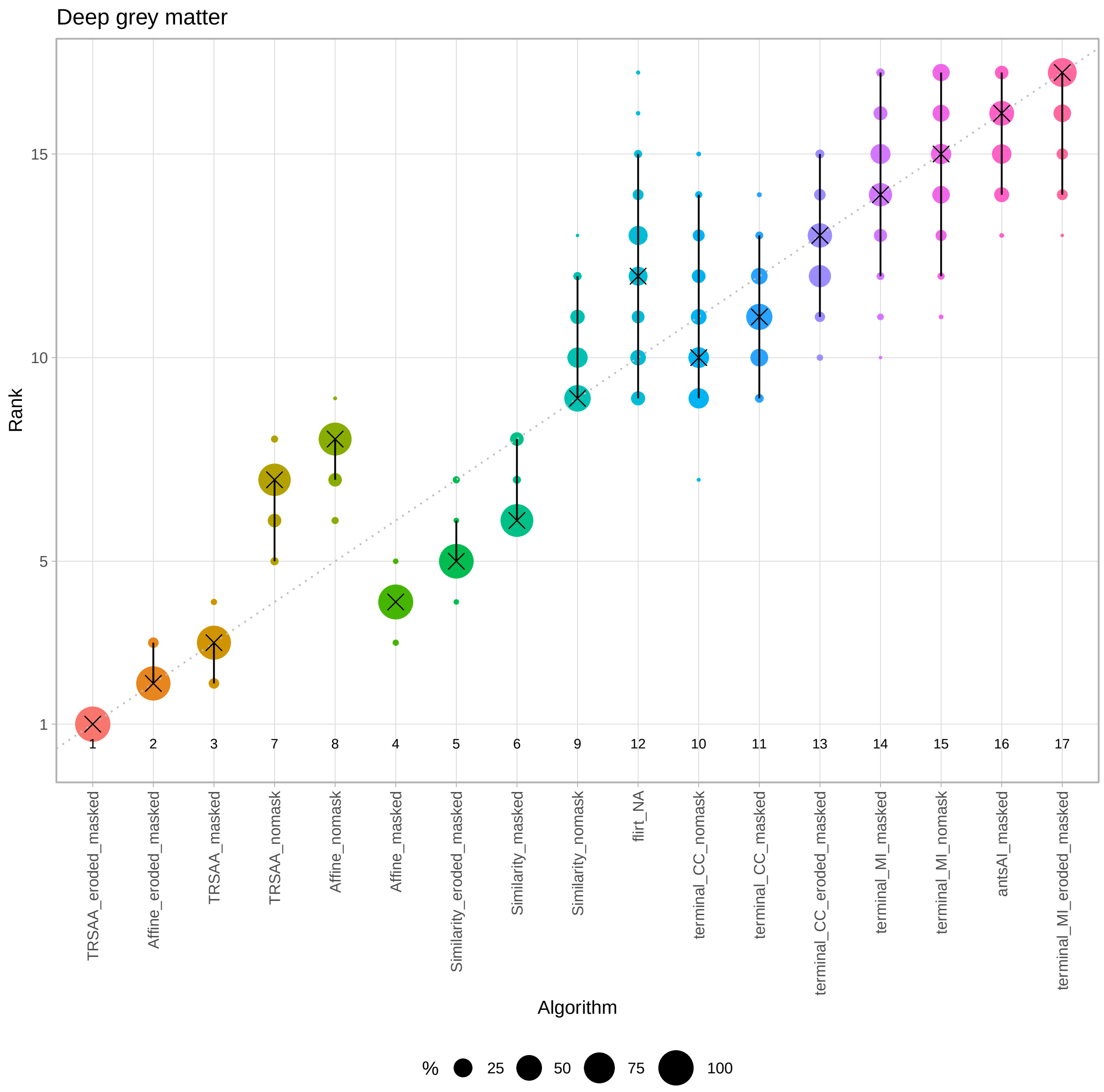
Deep grey matter
Rank
15
10
5
1
1
2
3
7
8
4
5
6
9
12
10
11
13
14
15
16
17
TRSAA_eroded_masked
Affine_eroded_masked
TRSAA_masked
TRSAA_nomask
Affine_nomask
Affine_masked
Similarity_eroded_masked
Similarity_masked
Similarity_nomask
flirt_NA
terminal_CC_nomask
terminal_CC_masked
terminal_CC_eroded_masked
terminal_MI_masked
terminal_MI_nomask
antsAI_masked
terminal_MI_eroded_masked
Algorithm
%
25
50
75
100

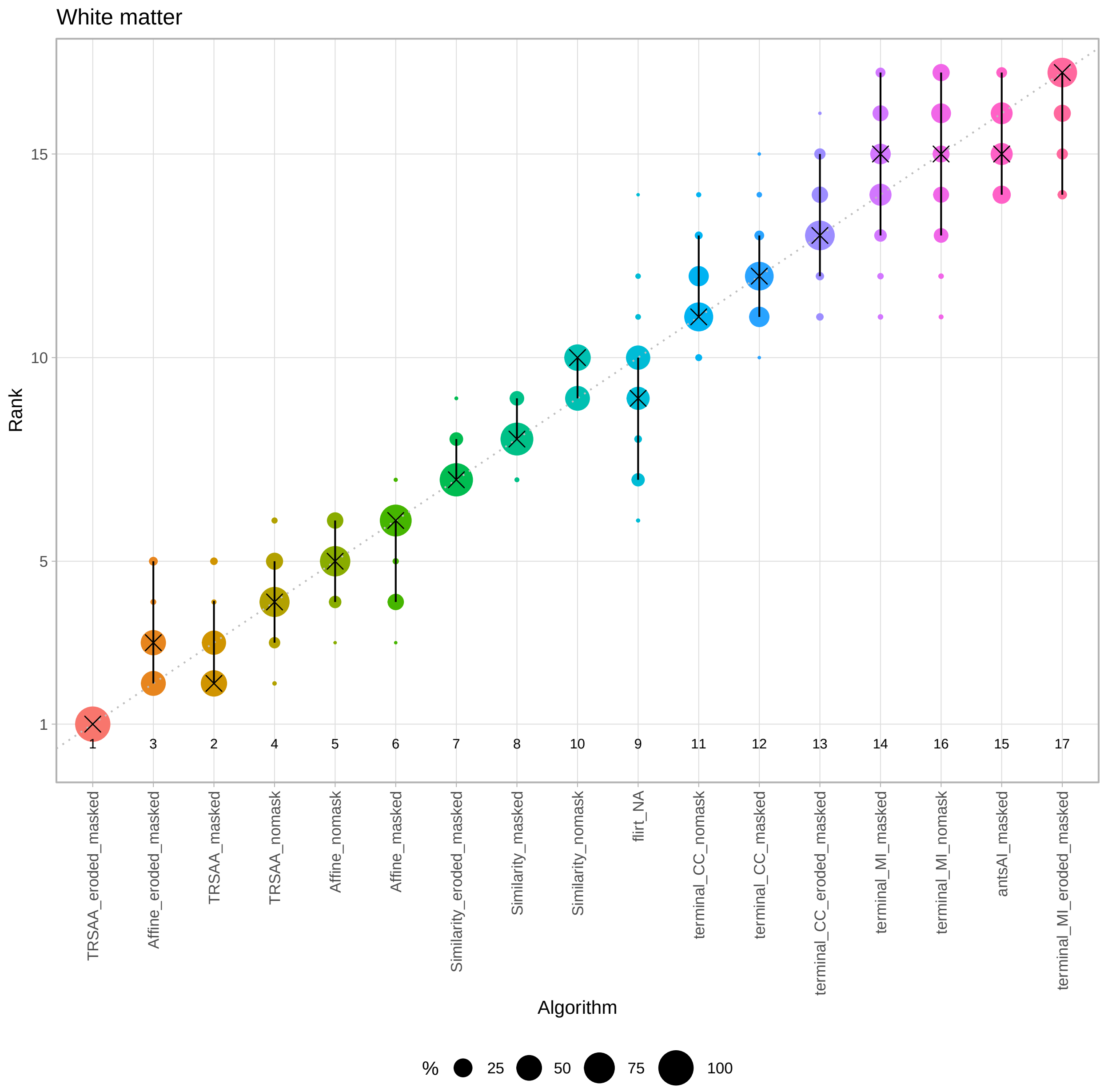

4.2.2 Cluster Analysis

Dendrogram from hierarchical cluster analysis and *network-type graphs* for assessing the similarity of tasks based on challenge rankings.

A dendrogram is a visualization approach based on hierarchical clustering. It depicts clusters according to a chosen distance measure (here: Spearman's footrule) as well as a chosen agglomeration method (here: complete and average agglomeration).

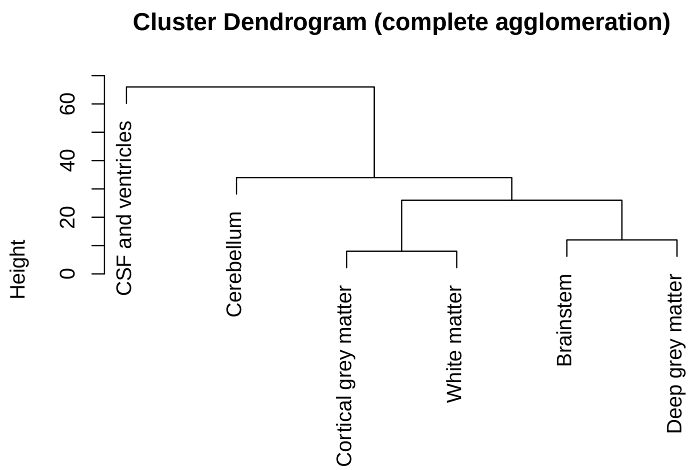

Cluster Dendrogram (complete agglomeration)
Height
0
20
40
60
CSF and ventricles
Cerebellum
Cortical grey matter
White matter
Brainstem
Deep grey matter

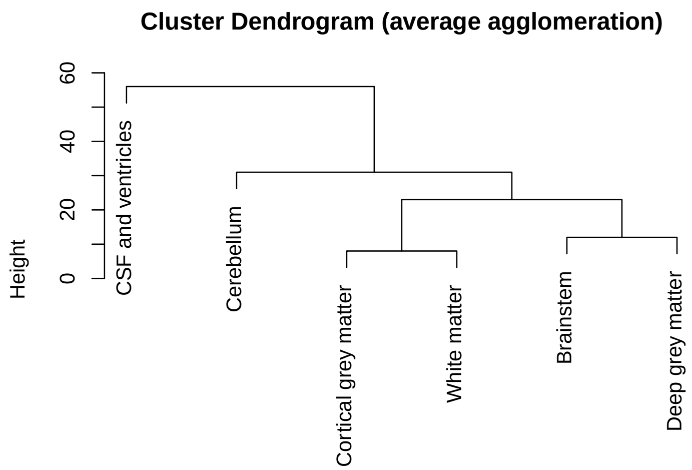

Cluster Dendrogram (average agglomeration)
Height
0
20
40
60
CSF and ventricles
Cerebellum
Cortical grey matter
White matter
Brainstem
Deep grey matter

5 References

Wiesenfarth, M., Reinke, A., Landman, B.A., Eisenmann, M., Aguilera Saiz, L., Cardoso, M.J., Maier-Hein, L. and Kopp-Schneider, A. Methods and open-source toolkit for analyzing and visualizing challenge results. *Sci Rep* **11**, 2369 (2021). https://doi.org/10.1038/s41598-021-82017-6

M. J. A. Eugster, T. Hothorn, and F. Leisch, “Exploratory and inferential analysis of benchmark experiments,” Institut fuer Statistik, Ludwig-Maximilians-Universitaet Muenchen, Germany, Technical Report 30, 2008. [Online]. Available: http://epub.ub.uni-muenchen.de/4134/.
