# Supplementary material for: Characterization of dynamic patterns of human fetal to neonatal brain asymmetry with deformation-based morphometry
Source: Front Neurosci. 2023 Dec 6;17:1252850. doi: 10.3389/fnins.2023.1252850 (PMC10734644; doi:10.3389/fnins.2023.1252850)
Supplement: Supplementary file 1 [file Data_Sheet_1.ZIP › ChallengeR/Framework B to mid-age template/fetal2template_linear/Fetal2MidTemplate_linear_landmarks.pdf]

Benchmarking report for Fetal to Midtempalte Registration Linear

created by challengeR v1.0.4

29 März, 2023

This document presents a systematic report on the benchmark study "Fetal to Midtempalte Registration Linear". Input data comprises raw metric values for all algorithms and cases. Generated plots are:

- Visualization of assessment data: Dot- and boxplots, podium plots and ranking heatmaps
- Visualization of ranking stability: Blob plots, violin plots and significance maps, line plots
- Visualization of cross-task insights: Blob plots, stacked frequency plots, dendrograms

Details can be found in Wiesenfarth et al. (2021).

1 Rankings

Algorithms within a task are ranked according to the following ranking scheme:

aggregate using function ("mean") then rank

Ranking for each task:

4th Ventricle : The analysis is based on 17 algorithms and 20 cases. 0 missing cases have been found in the data set.

	value_mean	rank
TRSAA_eroded_masked	1.463741	1
TRSAA_nomask	1.516579	2
Affine_eroded_masked	1.547894	3
Affine_nomask	1.574196	4
Affine_masked	1.638156	5
TRSAA_masked	1.670548	6
Similarity_eroded_masked	1.828205	7
Similarity_masked	1.934999	8
Similarity_nomask	2.314697	9
terminal_CC_nomask	3.677062	10
terminal_CC_masked	4.021930	11
terminal_CC_eroded_masked	4.763411	12
terminal_MI_masked	5.889456	13
terminal_MI_nomask	6.237009	14
flirt_na	6.259420	15
terminal_MI_eroded_masked	6.727437	16
antsAI_masked	6.908804	17

Chiasma : The analysis is based on 17 algorithms and 20 cases. 0 missing cases have been found in the data set.

	value_mean	rank
TRSAA_eroded_masked	1.664044	1
Affine_eroded_masked	2.166063	2
TRSAA_masked	2.222983	3
Affine_masked	2.652740	4
TRSAA_nomask	2.839755	5
Affine_nomask	2.869186	6
Similarity_eroded_masked	3.486184	7
Similarity_masked	3.758025	8
Similarity_nomask	3.791110	9
terminal_CC_nomask	6.028067	10
terminal_CC_masked	6.061036	11
terminal_CC_eroded_masked	6.818304	12
flirt_na	6.852910	13
terminal_MI_nomask	7.605479	14
antsAI_masked	7.758917	15
terminal_MI_masked	7.778375	16
terminal_MI_eroded_masked	8.727475	17

Culmen : The analysis is based on 17 algorithms and 20 cases. 0 missing cases have been found in the data set.

	value_mean	rank
TRSAA_eroded_masked	1.601476	1
Affine_masked	1.749783	2
Affine_eroded_masked	1.770352	3
TRSAA_masked	1.780677	4
TRSAA_nomask	1.999880	5
Affine_nomask	2.030687	6
Similarity_eroded_masked	2.050157	7
Similarity_masked	2.162640	8
Similarity_nomask	2.512621	9
terminal_CC_nomask	3.300016	10
terminal_CC_masked	3.625551	11
flirt_na	4.098273	12
terminal_CC_eroded_masked	4.131205	13
terminal_MI_masked	4.999536	14
terminal_MI_nomask	5.451284	15
terminal_MI_eroded_masked	6.071690	16
antsAI_masked	6.271638	17

Hemisphere left caudal point : The analysis is based on 17 algorithms and 20 cases. 0 missing cases have been found in the data set.

	value_mean	rank
Affine_masked	3.540560	1
Affine_nomask	3.682898	2
TRSAA_nomask	3.688081	3
TRSAA_masked	3.862523	4
Affine_eroded_masked	4.131498	5
Similarity_eroded_masked	4.372121	6

	value_mean	rank
Similarity_nomask	4.417288	7
Similarity_masked	4.433711	8
TRSAA_eroded_masked	4.992763	9
flirt_na	5.934364	10
terminal_CC_nomask	6.453737	11
terminal_CC_masked	6.621664	12
terminal_CC_eroded_masked	7.122716	13
terminal_MI_masked	8.386347	14
terminal_MI_nomask	8.590670	15
antsAI_masked	9.236920	16
terminal_MI_eroded_masked	9.361024	17

Hemisphere left rostral point : The analysis is based on 17 algorithms and 20 cases. 0 missing cases have been found in the data set.

	value_mean	rank
Affine_nomask	3.294530	1
TRSAA_nomask	3.381201	2
TRSAA_masked	4.439933	3
Similarity_nomask	4.443116	4
flirt_na	4.649800	5
Affine_masked	4.981491	6
TRSAA_eroded_masked	5.068986	7
Affine_eroded_masked	5.191343	8
Similarity_eroded_masked	6.391407	9
Similarity_masked	6.530372	10
antsAI_masked	7.856579	11
terminal_CC_masked	8.463927	12
terminal_CC_eroded_masked	8.840019	13
terminal_CC_nomask	9.192164	14
terminal_MI_nomask	9.509645	15
terminal_MI_masked	10.149612	16
terminal_MI_eroded_masked	10.480118	17

Hemisphere right caudal point : The analysis is based on 17 algorithms and 20 cases. 0 missing cases have been found in the data set.

	value_mean	rank
Affine_masked	3.093553	1
TRSAA_masked	3.263968	2
TRSAA_nomask	3.500018	3
Affine_nomask	3.510843	4
Affine_eroded_masked	3.712268	5
Similarity_masked	4.069543	6
Similarity_nomask	4.090952	7
Similarity_eroded_masked	4.222375	8
TRSAA_eroded_masked	4.633670	9
flirt_na	5.413703	10
terminal_CC_nomask	6.047592	11
terminal_CC_masked	6.397755	12

	value_mean	rank
terminal_CC_eroded_masked	6.666920	13
terminal_MI_nomask	7.713653	14
terminal_MI_masked	7.741223	15
antsAI_masked	8.182022	16
terminal_MI_eroded_masked	8.506129	17

Hemisphere right rostral point : The analysis is based on 17 algorithms and 20 cases. 0 missing cases have been found in the data set.

	value_mean	rank
TRSAA_nomask	4.014985	1
Affine_nomask	4.032955	2
flirt_na	4.718826	3
TRSAA_masked	4.806487	4
Similarity_nomask	4.860839	5
Affine_masked	4.991682	6
TRSAA_eroded_masked	5.188380	7
Affine_eroded_masked	5.395530	8
Similarity_eroded_masked	6.425635	9
Similarity_masked	6.507237	10
antsAI_masked	8.160550	11
terminal_CC_masked	8.379555	12
terminal_CC_eroded_masked	8.647354	13
terminal_CC_nomask	8.759089	14
terminal_MI_nomask	9.766782	15
terminal_MI_masked	10.423619	16
terminal_MI_eroded_masked	11.202312	17

Infracollicular Sulcus : The analysis is based on 17 algorithms and 20 cases. 0 missing cases have been found in the data set.

	value_mean	rank
Affine_eroded_masked	0.8213514	1
TRSAA_eroded_masked	0.9967417	2
Similarity_eroded_masked	1.1574466	3
Affine_masked	1.4629990	4
TRSAA_masked	1.4878571	5
Similarity_masked	1.5132613	6
TRSAA_nomask	2.1913330	7
Affine_nomask	2.2451717	8
Similarity_nomask	2.8639291	9
terminal_CC_nomask	3.4063459	10
terminal_CC_masked	3.7433969	11
terminal_CC_eroded_masked	4.3554632	12
flirt_na	5.3216318	13
terminal_MI_masked	5.4523729	14
terminal_MI_nomask	5.8007488	15
antsAI_masked	6.4838286	16
terminal_MI_eroded_masked	6.6694354	17

Lateral ventricle left caudal point : The analysis is based on 17 algorithms and 20 cases. 0 missing cases have been found in the data set.

	value_mean	rank
Similarity_masked	3.051791	1
Similarity_eroded_masked	3.064058	2
Affine_masked	3.178982	3
Affine_eroded_masked	3.206849	4
TRSAA_masked	3.652201	5
TRSAA_nomask	4.050691	6
Affine_nomask	4.080930	7
TRSAA_eroded_masked	4.101309	8
Similarity_nomask	4.803982	9
terminal_CC_nomask	5.229766	10
terminal_CC_masked	5.674564	11
terminal_CC_eroded_masked	6.147523	12
flirt_na	7.262446	13
terminal_MI_nomask	7.724030	14
terminal_MI_masked	7.811917	15
antsAI_masked	8.345593	16
terminal_MI_eroded_masked	8.509425	17

Lateral ventricle left rostral point : The analysis is based on 17 algorithms and 20 cases. 0 missing cases have been found in the data set.

	value_mean	rank
Affine_masked	1.783943	1
TRSAA_masked	1.915462	2
TRSAA_eroded_masked	2.019542	3
Similarity_nomask	2.033262	4
Affine_eroded_masked	2.034103	5
Similarity_masked	2.421159	6
Similarity_eroded_masked	2.475488	7
TRSAA_nomask	2.590469	8
Affine_nomask	2.605189	9
flirt_na	4.107677	10
terminal_CC_masked	4.648533	11
terminal_CC_eroded_masked	4.974062	12
antsAI_masked	4.986405	13
terminal_CC_nomask	5.031013	14
terminal_MI_nomask	6.123360	15
terminal_MI_masked	6.198359	16
terminal_MI_eroded_masked	7.132299	17

Lateral ventricle right caudal point : The analysis is based on 17 algorithms and 20 cases. 0 missing cases have been found in the data set.

	value_mean	rank
Affine_masked	3.348866	1
TRSAA_nomask	3.526999	2
Similarity_masked	3.530241	3

	value_mean	rank
Affine_nomask	3.572429	4
TRSAA_masked	3.789861	5
Similarity_nomask	3.875033	6
Affine_eroded_masked	4.024396	7
Similarity_eroded_masked	4.051023	8
terminal_CC_masked	4.646661	9
TRSAA_eroded_masked	4.727820	10
terminal_CC_nomask	4.770167	11
terminal_CC_eroded_masked	4.892936	12
terminal_MI_masked	5.625262	13
antsAI_masked	5.895231	14
terminal_MI_nomask	6.063486	15
flirt_na	6.101537	16
terminal_MI_eroded_masked	6.597639	17

Lateral ventricle right rostral point : The analysis is based on 17 algorithms and 20 cases. 0 missing cases have been found in the data set.

	value_mean	rank
Affine_masked	1.442829	1
Affine_eroded_masked	1.609315	2
TRSAA_eroded_masked	1.843696	3
TRSAA_masked	2.004762	4
Similarity_masked	2.202886	5
Similarity_eroded_masked	2.228984	6
Similarity_nomask	3.079011	7
TRSAA_nomask	3.247372	8
Affine_nomask	3.260408	9
terminal_CC_nomask	4.178371	10
terminal_CC_masked	4.258703	11
terminal_CC_eroded_masked	4.616309	12
flirt_na	4.765787	13
antsAI_masked	4.976142	14
terminal_MI_masked	5.641902	15
terminal_MI_nomask	5.824077	16
terminal_MI_eroded_masked	6.995799	17

PMJ : The analysis is based on 17 algorithms and 20 cases. 0 missing cases have been found in the data set.

	value_mean	rank
Affine_eroded_masked	0.9184347	1
TRSAA_masked	0.9319396	2
Affine_masked	0.9479540	3
TRSAA_eroded_masked	0.9578315	4
TRSAA_nomask	1.3385701	5
Affine_nomask	1.3629316	6
Similarity_masked	1.4533099	7
Similarity_eroded_masked	1.5016861	8
Similarity_nomask	2.0524832	9
terminal_CC_nomask	3.2897806	10

	value_mean	rank
terminal_CC_masked	3.4397985	11
terminal_CC_eroded_masked	4.2089960	12
flirt_na	5.2565464	13
terminal_MI_masked	5.2626248	14
terminal_MI_nomask	5.3059247	15
antsAI_masked	5.6577739	16
terminal_MI_eroded_masked	6.4079928	17

Posterior Commissure : The analysis is based on 17 algorithms and 20 cases. 0 missing cases have been found in the data set.

	value_mean	rank
Affine_masked	1.591438	1
Affine_eroded_masked	1.654941	2
TRSAA_masked	1.670174	3
Similarity_masked	1.690036	4
Similarity_eroded_masked	1.876353	5
TRSAA_eroded_masked	1.925154	6
TRSAA_nomask	2.279641	7
Affine_nomask	2.315064	8
Similarity_nomask	2.520747	9
terminal_CC_nomask	2.621366	10
terminal_CC_masked	2.820706	11
terminal_CC_eroded_masked	3.281547	12
flirt_na	3.793560	13
terminal_MI_masked	4.323721	14
terminal_MI_nomask	4.595936	15
antsAI_masked	4.614347	16
terminal_MI_eroded_masked	5.890102	17

Temporal lobe left rostral point : The analysis is based on 17 algorithms and 20 cases. 0 missing cases have been found in the data set.

	value_mean	rank
Affine_nomask	4.784696	1
TRSAA_masked	4.846894	2
TRSAA_nomask	4.850813	3
TRSAA_eroded_masked	5.024115	4
Affine_eroded_masked	5.318536	5
Affine_masked	5.360979	6
Similarity_eroded_masked	8.166506	7
Similarity_masked	8.224800	8
Similarity_nomask	8.434490	9
terminal_CC_masked	9.650747	10
terminal_CC_nomask	9.831001	11
terminal_CC_eroded_masked	10.344645	12
terminal_MI_nomask	10.532419	13
terminal_MI_masked	10.867226	14
antsAI_masked	11.013680	15
flirt_na	11.529623	16

	value_mean	rank
terminal_MI_eroded_masked	12.247091	17

Temporal lobe ventricle right rostral point : The analysis is based on 17 algorithms and 20 cases. 0 missing cases have been found in the data set.

	value_mean	rank
TRSAA_eroded_masked	5.610175	1
TRSAA_masked	5.704064	2
TRSAA_nomask	5.930372	3
Affine_nomask	6.010579	4
Affine_masked	6.141573	5
Affine_eroded_masked	6.199619	6
Similarity_eroded_masked	8.660394	7
Similarity_masked	8.670057	8
Similarity_nomask	9.288096	9
terminal_CC_nomask	9.488758	10
terminal_CC_masked	9.503098	11
terminal_CC_eroded_masked	10.117332	12
antsAI_masked	10.748665	13
flirt_na	11.177150	14
terminal_MI_nomask	11.399978	15
terminal_MI_masked	11.424257	16
terminal_MI_eroded_masked	12.028059	17

Consensus ranking across tasks according to chosen method “euclidean”:

	value	rank
Affine_masked	3.125	1
TRSAA_masked	3.500	2
Affine_eroded_masked	4.188	3
TRSAA_nomask	4.375	4
TRSAA_eroded_masked	4.750	5
Affine_nomask	5.062	6
Similarity_eroded_masked	6.625	7
Similarity_masked	6.625	7
Similarity_nomask	7.562	9
terminal_CC_nomask	11.000	10
terminal_CC_masked	11.062	11
flirt_na	11.812	12
terminal_CC_eroded_masked	12.312	13
terminal_MI_masked	14.688	14
terminal_MI_nomask	14.688	14
antsAI_masked	14.750	16
terminal_MI_eroded_masked	16.875	17

2 Visualization of raw assessment data

The algorithms are ordered according to the computed ranks for each task.

2.1 Dot- and boxplot

Dot- and boxplots for visualizing raw assessment data separately for each algorithm. Boxplots representing descriptive statistics over all cases (median, quartiles and outliers) are combined with horizontally jittered dots representing individual cases.

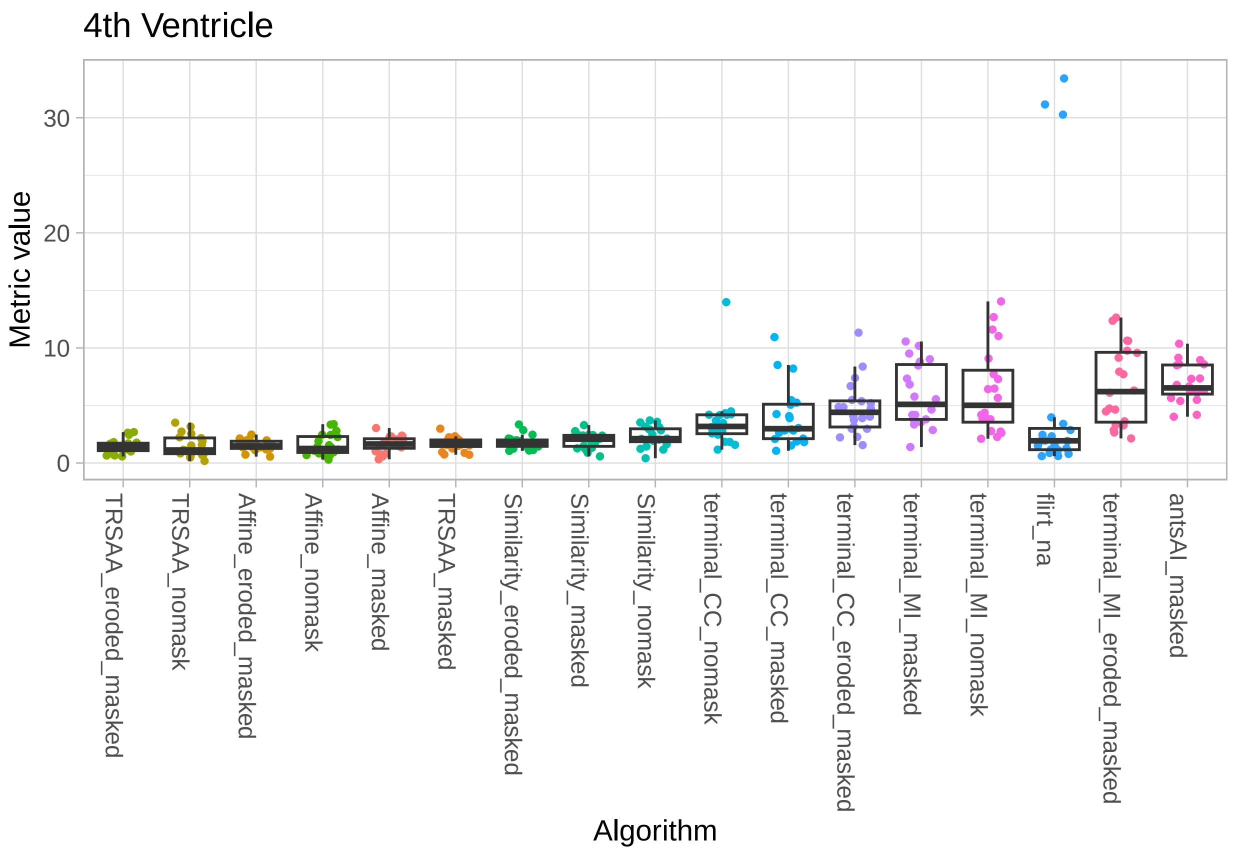

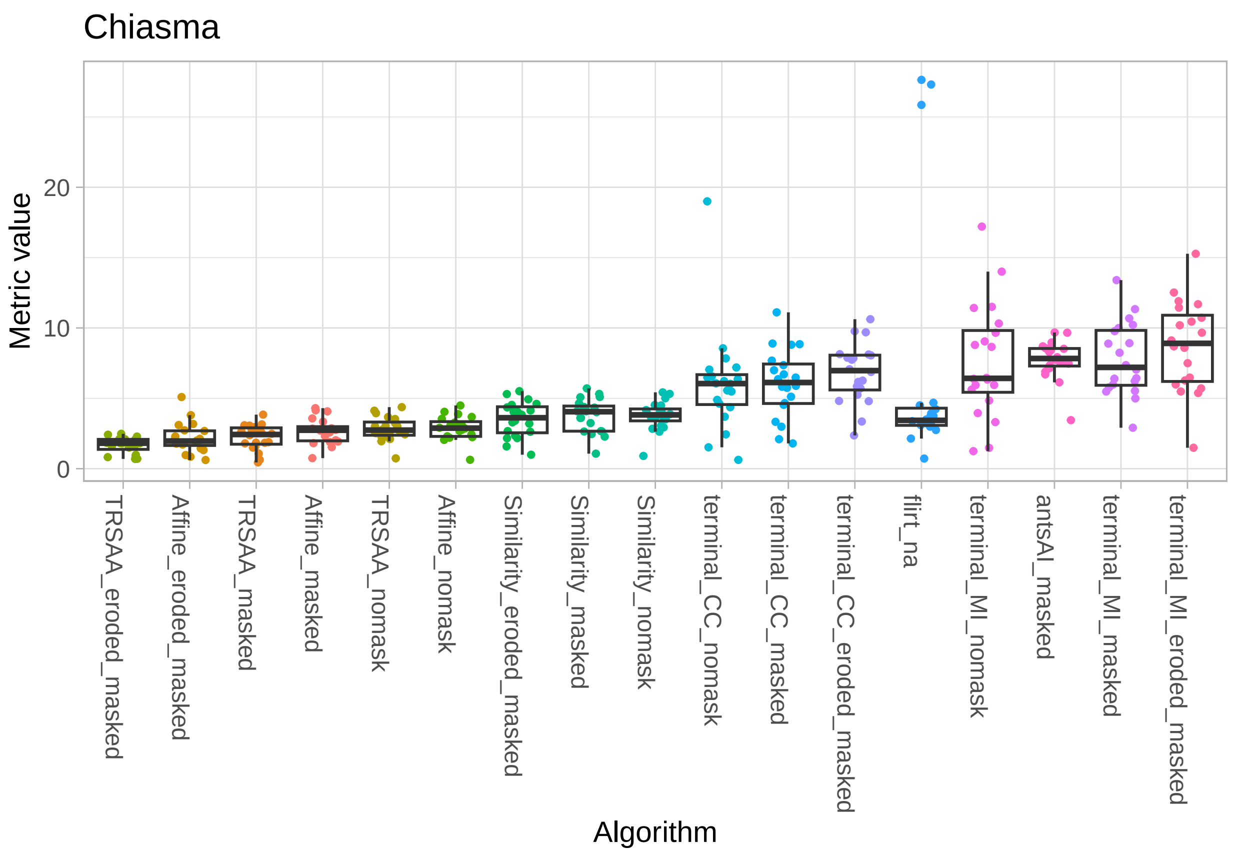
Chiasma
Metric value
0
10
20
TRSAA_eroded_masked
Affine_eroded_masked
TRSAA_masked
Affine_masked
TRSAA_nomask
Affine_nomask
Similarity_eroded_masked
Similarity_masked
Similarity_nomask
terminal_CC_nomask
terminal_CC_masked
terminal_CC_eroded_masked
flirt_na
terminal_MI_nomask
antsAI_masked
terminal_MI_masked
terminal_MI_eroded_masked
Algorithm

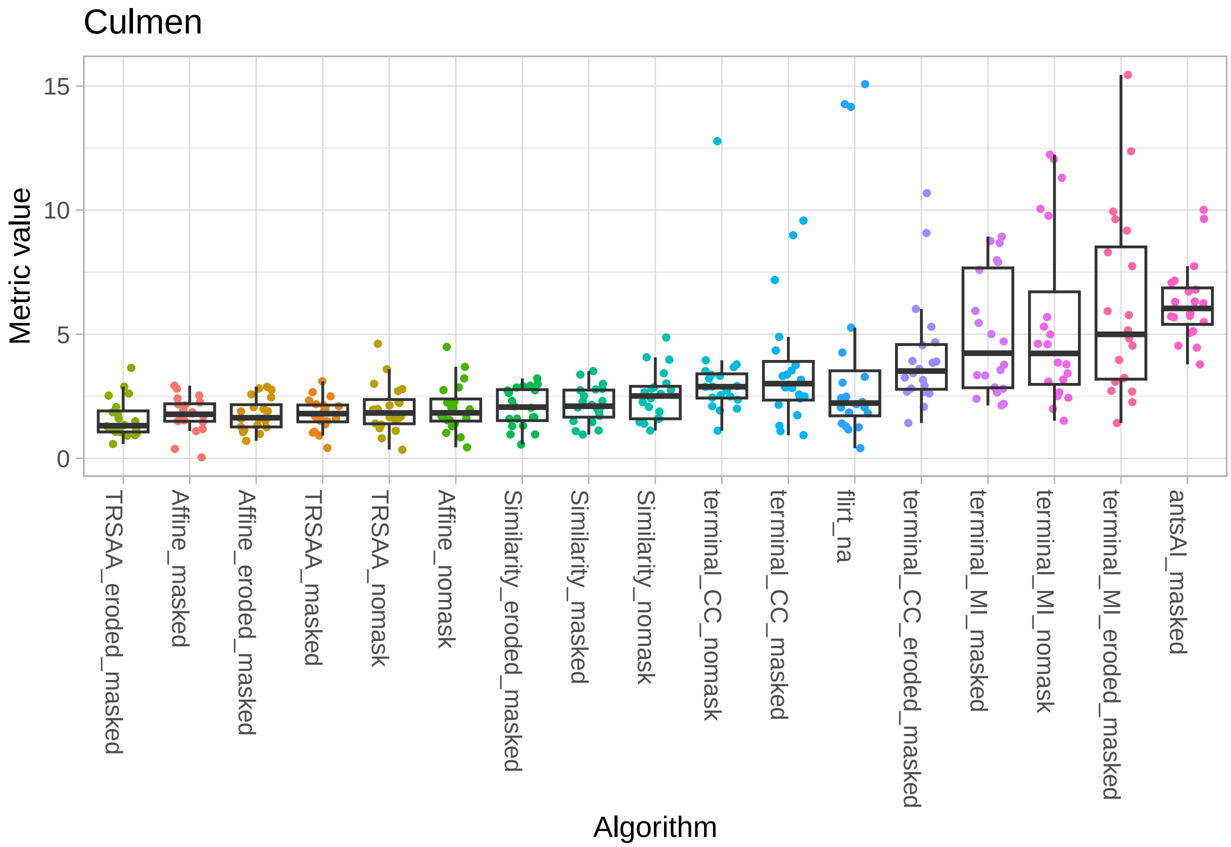

Culmen
Metric value
15
10
5
0
TRSAA_eroded_masked
Affine_masked
Affine_eroded_masked
TRSAA_masked
TRSAA_nomask
Affine_nomask
Similarity_eroded_masked
Similarity_masked
Similarity_nomask
terminal_CC_nomask
terminal_CC_masked
flirt_na
terminal_CC_eroded_masked
terminal_MI_masked
terminal_MI_nomask
terminal_MI_eroded_masked
antsAI_masked
Algorithm

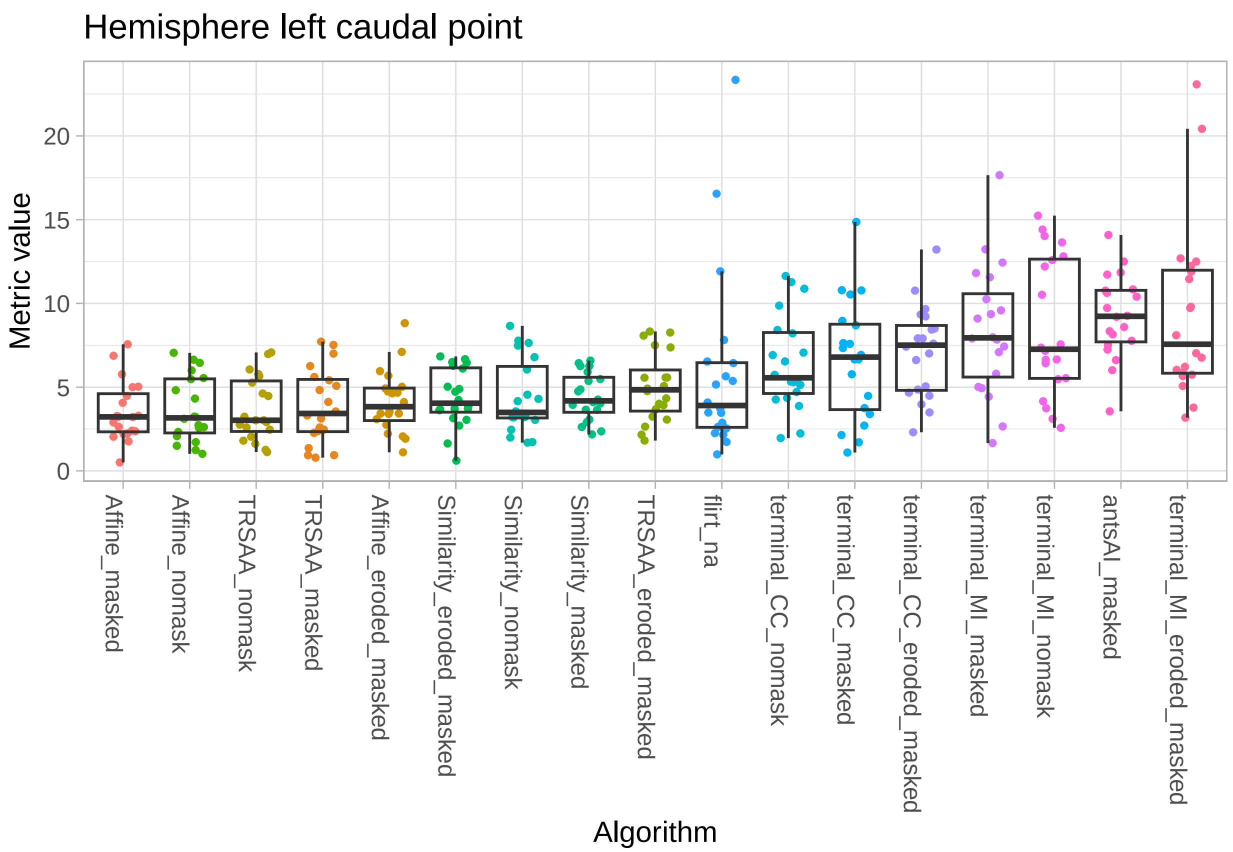

Hemisphere left caudal point
Metric value
0
5
10
15
20
Affine_masked
Affine_nomask
TRSAA_nomask
TRSAA_masked
Affine_eroded_masked
Similarity_eroded_masked
Similarity_nomask
Similarity_masked
TRSAA_eroded_masked
flirt_na
terminal_CC_nomask
terminal_CC_masked
terminal_CC_eroded_masked
terminal_MI_masked
terminal_MI_nomask
antsAI_masked
terminal_MI_eroded_masked
Algorithm

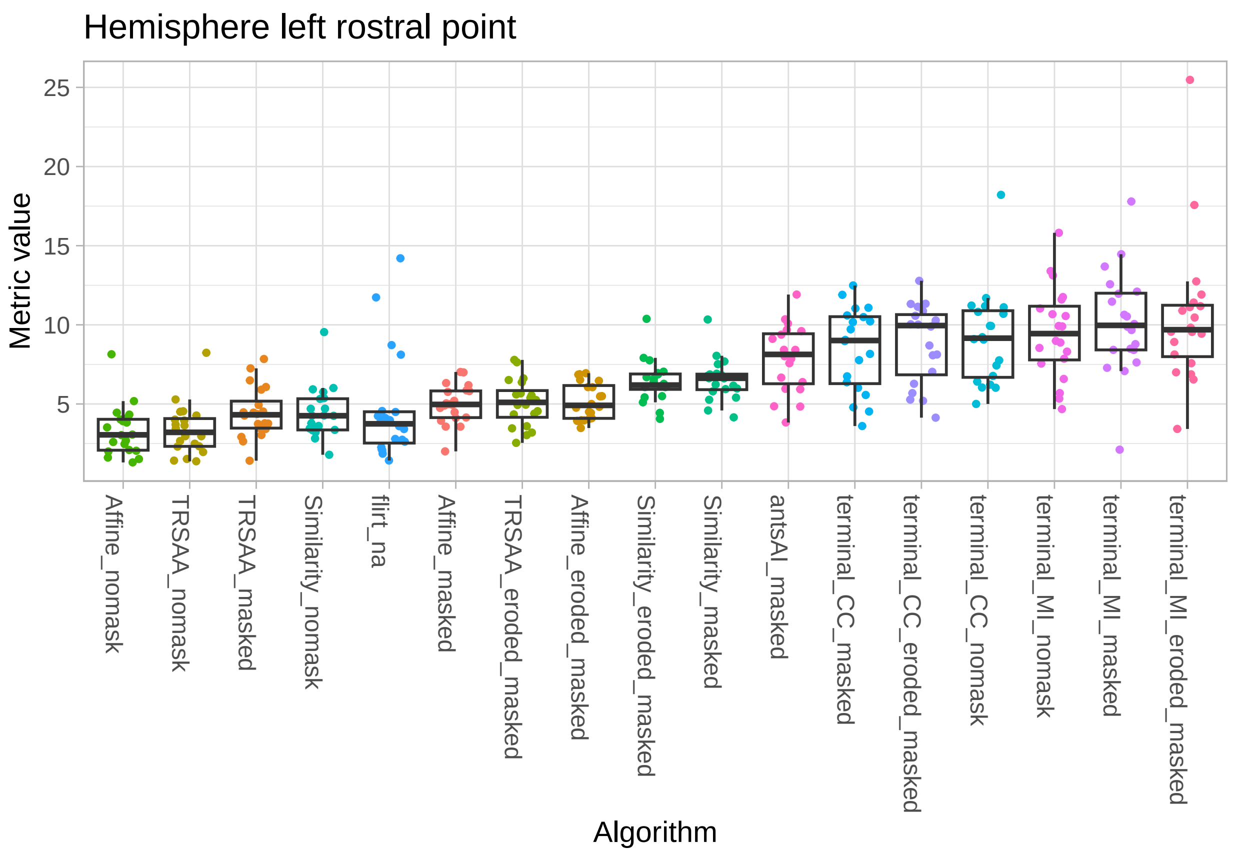

Hemisphere left rostral point
Metric value
25
20
15
10
5
Affine_nomask
TRSAA_nomask
TRSAA_masked
Similarity_nomask
flirt_na
Affine_masked
TRSAA_eroded_masked
Affine_eroded_masked
Similarity_eroded_masked
Similarity_masked
antsAI_masked
terminal_CC_masked
terminal_CC_eroded_masked
terminal_CC_nomask
terminal_MI_nomask
terminal_MI_masked
terminal_MI_eroded_masked
Algorithm

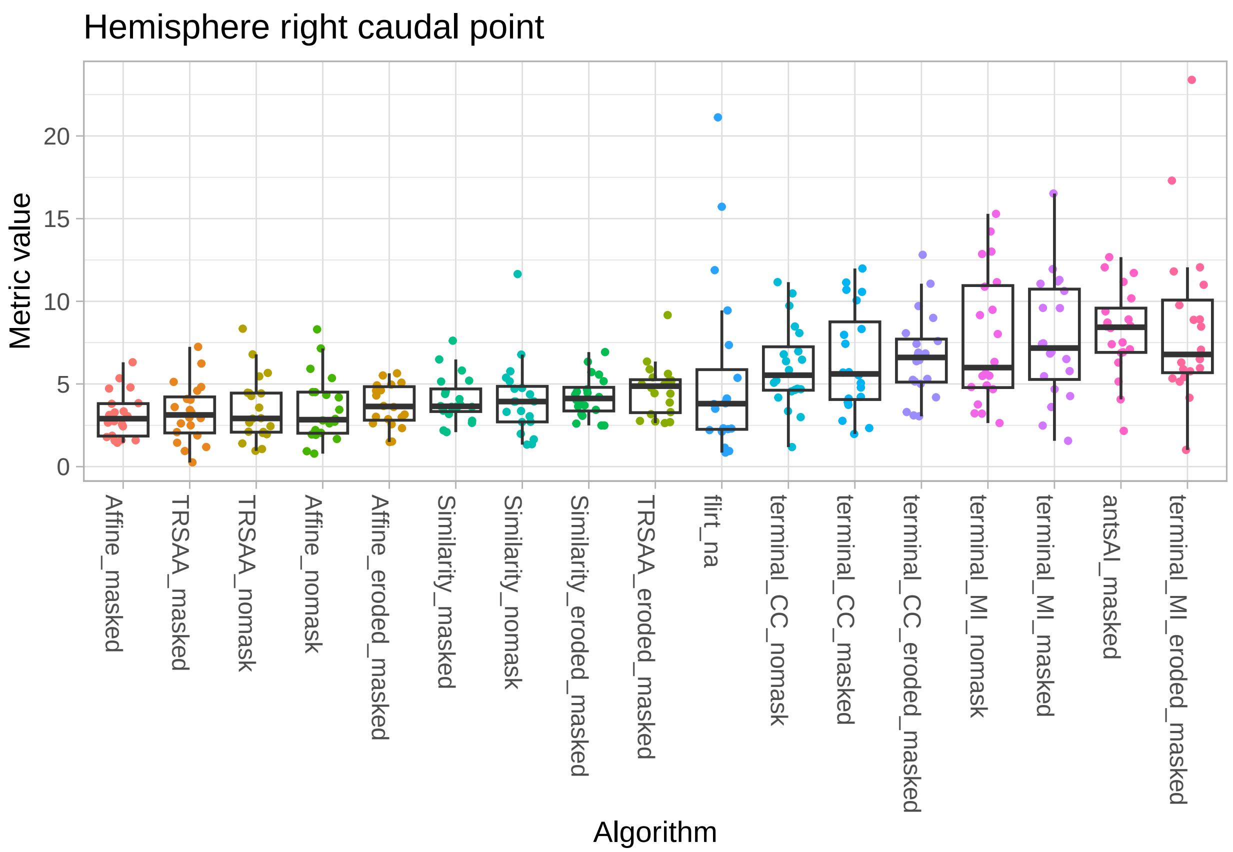
Hemisphere right caudal point
Metric value
20
15
10
5
0
Affine_masked
TRSAA_masked
TRSAA_nomask
Affine_nomask
Affine_eroded_masked
Similarity_masked
Similarity_nomask
Similarity_eroded_masked
TRSAA_eroded_masked
flirt_na
terminal_CC_nomask
terminal_CC_masked
terminal_CC_eroded_masked
terminal_MI_nomask
terminal_MI_masked
antsAI_masked
terminal_MI_eroded_masked
Algorithm

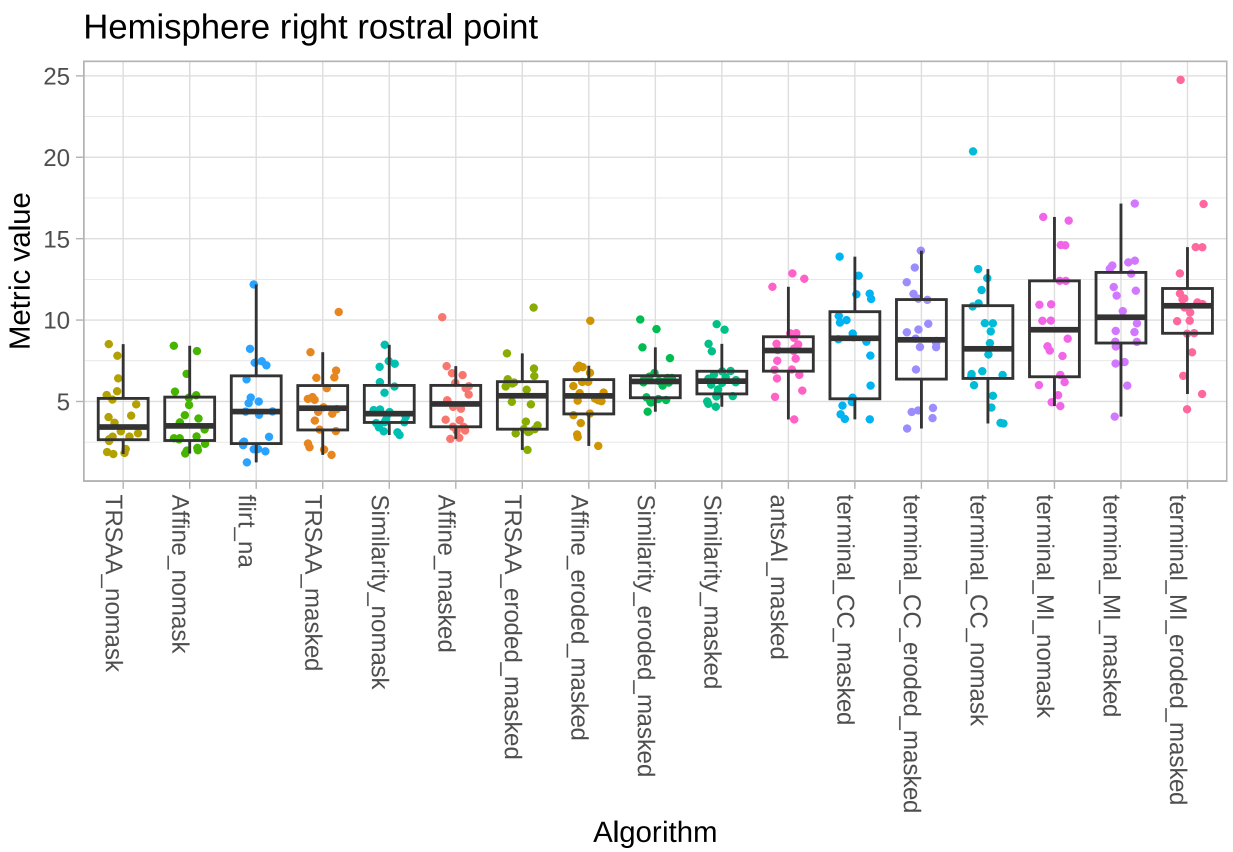

Hemisphere right rostral point
Metric value
25
20
15
10
5
TRSAA_nomask
Affine_nomask
flirt_na
TRSAA_masked
Similarity_nomask
Affine_masked
TRSAA_eroded_masked
Affine_eroded_masked
Similarity_eroded_masked
Similarity_masked
antsAI_masked
terminal_CC_masked
terminal_CC_eroded_masked
terminal_CC_nomask
terminal_MI_nomask
terminal_MI_masked
terminal_MI_eroded_masked
Algorithm

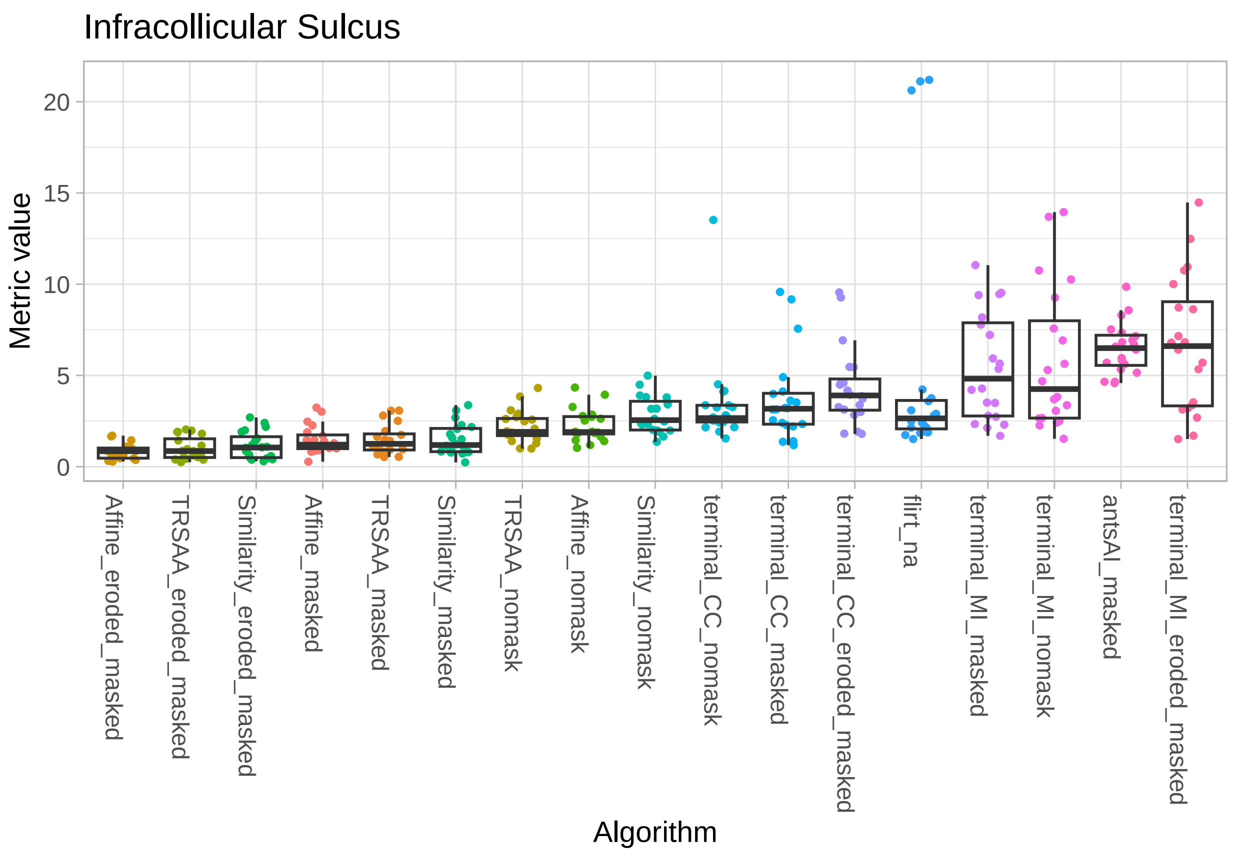

Infracollicular Sulcus
Metric value
0
5
10
15
20
Affine_eroded_masked
TRSAA_eroded_masked
Similarity_eroded_masked
Affine_masked
TRSAA_masked
Similarity_masked
TRSAA_nomask
Affine_nomask
Similarity_nomask
terminal_CC_nomask
terminal_CC_masked
terminal_CC_eroded_masked
flirt_na
terminal_MI_masked
terminal_MI_nomask
antsAI_masked
terminal_MI_eroded_masked
Algorithm

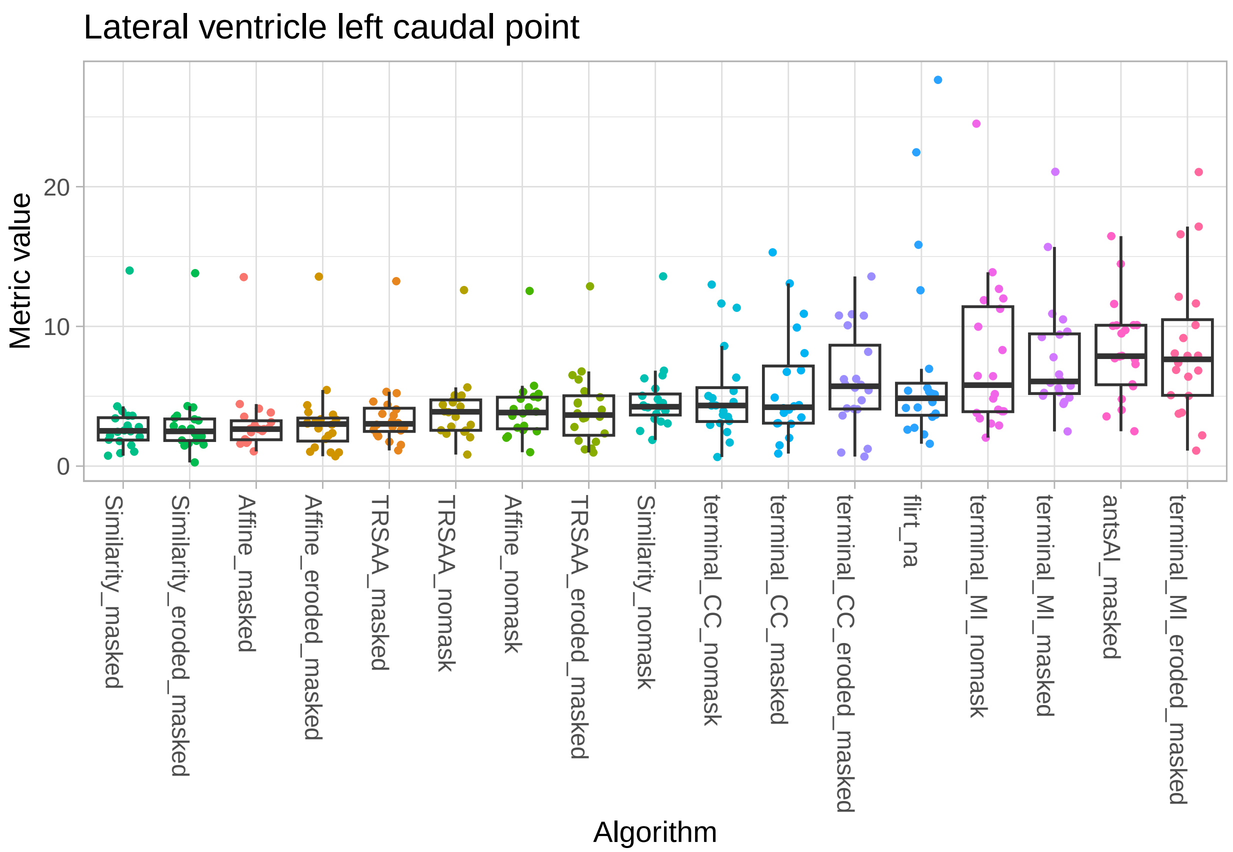
Lateral ventricle left caudal point
Metric value
0
10
20
Similarity_masked
Similarity_eroded_masked
Affine_masked
Affine_eroded_masked
TRSAA_masked
TRSAA_nomask
Affine_nomask
TRSAA_eroded_masked
Similarity_nomask
terminal_CC_nomask
terminal_CC_masked
terminal_CC_eroded_masked
flirt_na
terminal_MI_nomask
terminal_MI_masked
antsAI_masked
terminal_MI_eroded_masked
Algorithm

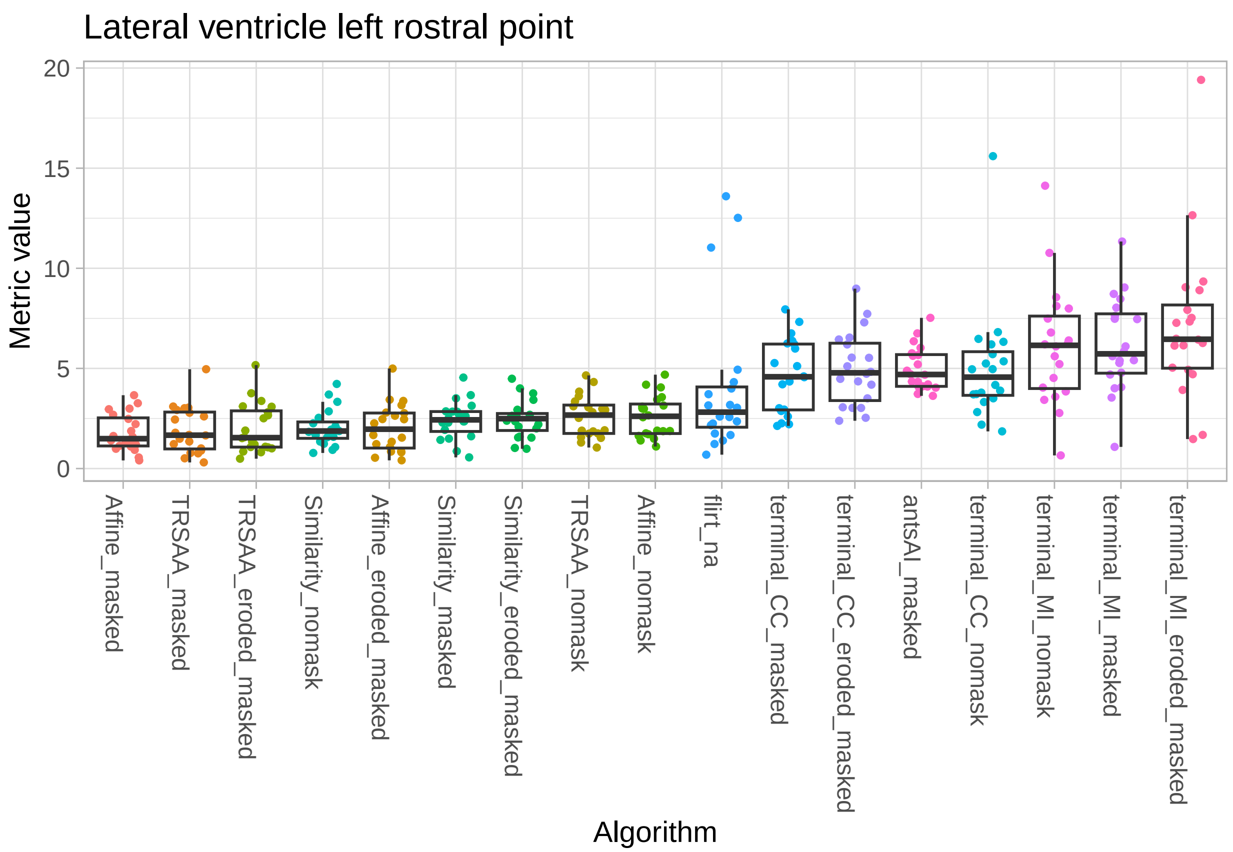
Lateral ventricle left rostral point
Metric value
20
15
10
5
0
Affine_masked
TRSAA_masked
TRSAA_eroded_masked
Similarity_nomask
Affine_eroded_masked
Similarity_masked
Similarity_eroded_masked
TRSAA_nomask
Affine_nomask
flirt_na
terminal_CC_masked
terminal_CC_eroded_masked
antsAI_masked
terminal_CC_nomask
terminal_MI_nomask
terminal_MI_masked
terminal_MI_eroded_masked
Algorithm

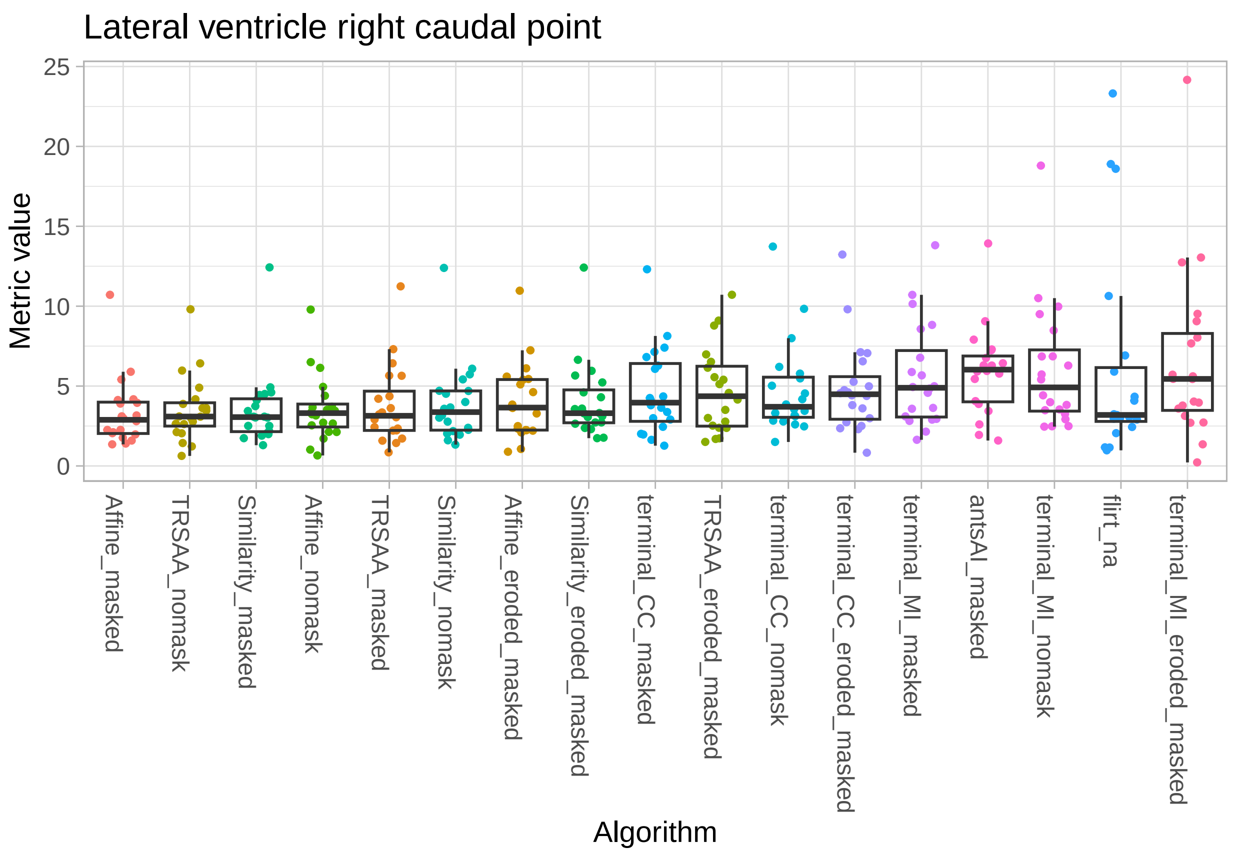

Lateral ventricle right caudal point
Metric value
0
5
10
15
20
25
Affine_masked
TRSAA_nomask
Similarity_masked
Affine_nomask
TRSAA_masked
Similarity_nomask
Affine_eroded_masked
Similarity_eroded_masked
terminal_CC_masked
TRSAA_eroded_masked
terminal_CC_nomask
terminal_CC_eroded_masked
terminal_MI_masked
antsAI_masked
terminal_MI_nomask
flirt_na
terminal_MI_eroded_masked
Algorithm

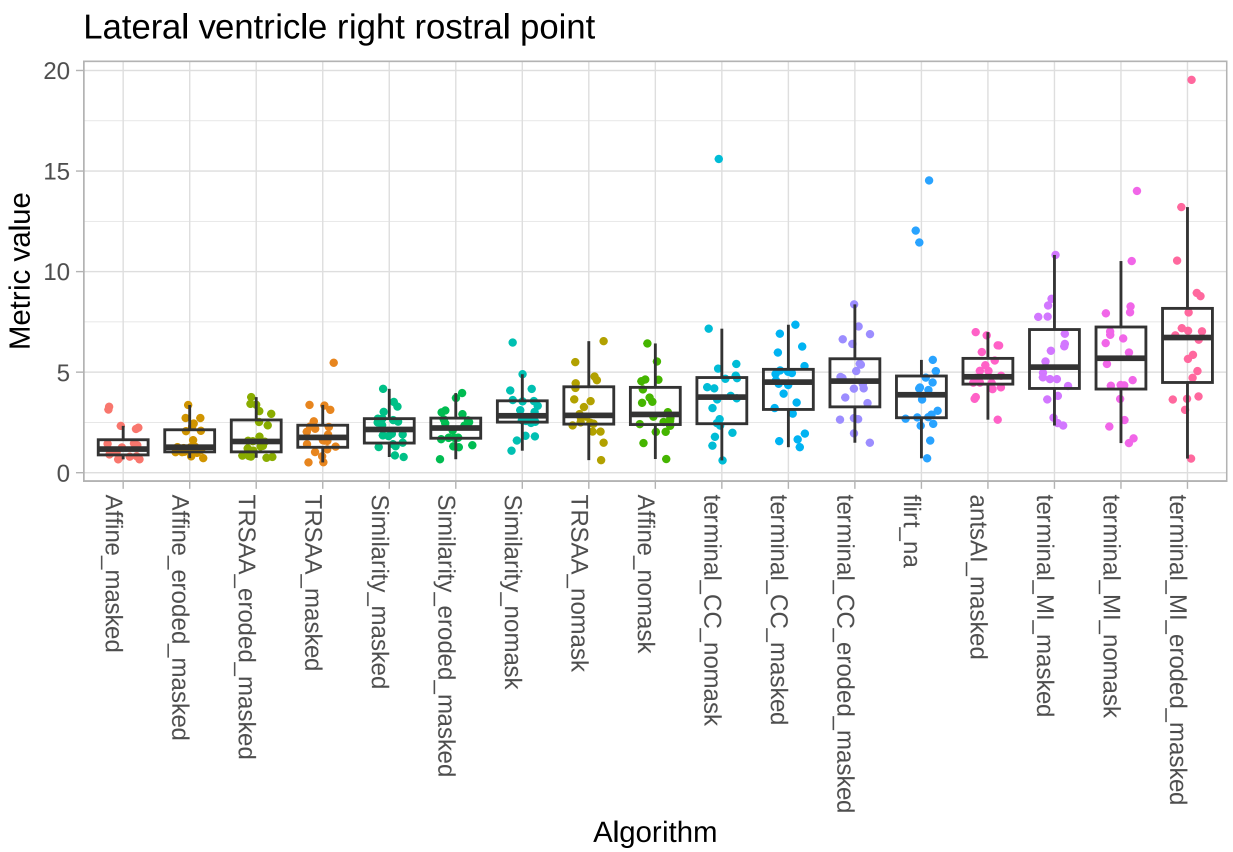
Lateral ventricle right rostral point
Metric value
0
5
10
15
20
Affine_masked
Affine_eroded_masked
TRSAA_eroded_masked
TRSAA_masked
Similarity_masked
Similarity_eroded_masked
Similarity_nomask
TRSAA_nomask
Affine_nomask
terminal_CC_nomask
terminal_CC_masked
terminal_CC_eroded_masked
flirt_na
antsAI_masked
terminal_MI_masked
terminal_MI_nomask
terminal_MI_eroded_masked
Algorithm

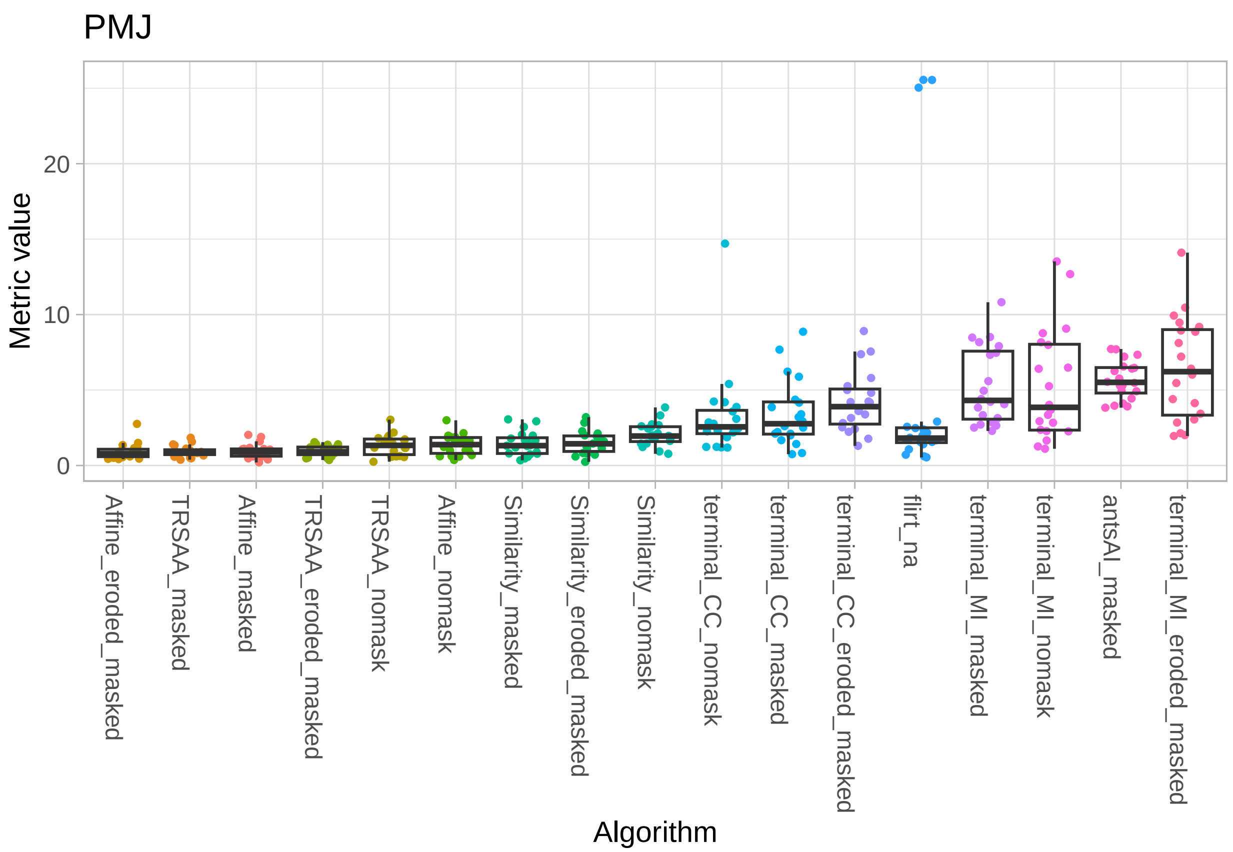
PMJ
Metric value
0
10
20
Affine_eroded_masked
TRSAA_masked
Affine_masked
TRSAA_eroded_masked
TRSAA_nomask
Affine_nomask
Similarity_masked
Similarity_eroded_masked
Similarity_nomask
terminal_CC_nomask
terminal_CC_masked
terminal_CC_eroded_masked
flirt_na
terminal_MI_masked
terminal_MI_nomask
antsAI_masked
terminal_MI_eroded_masked
Algorithm

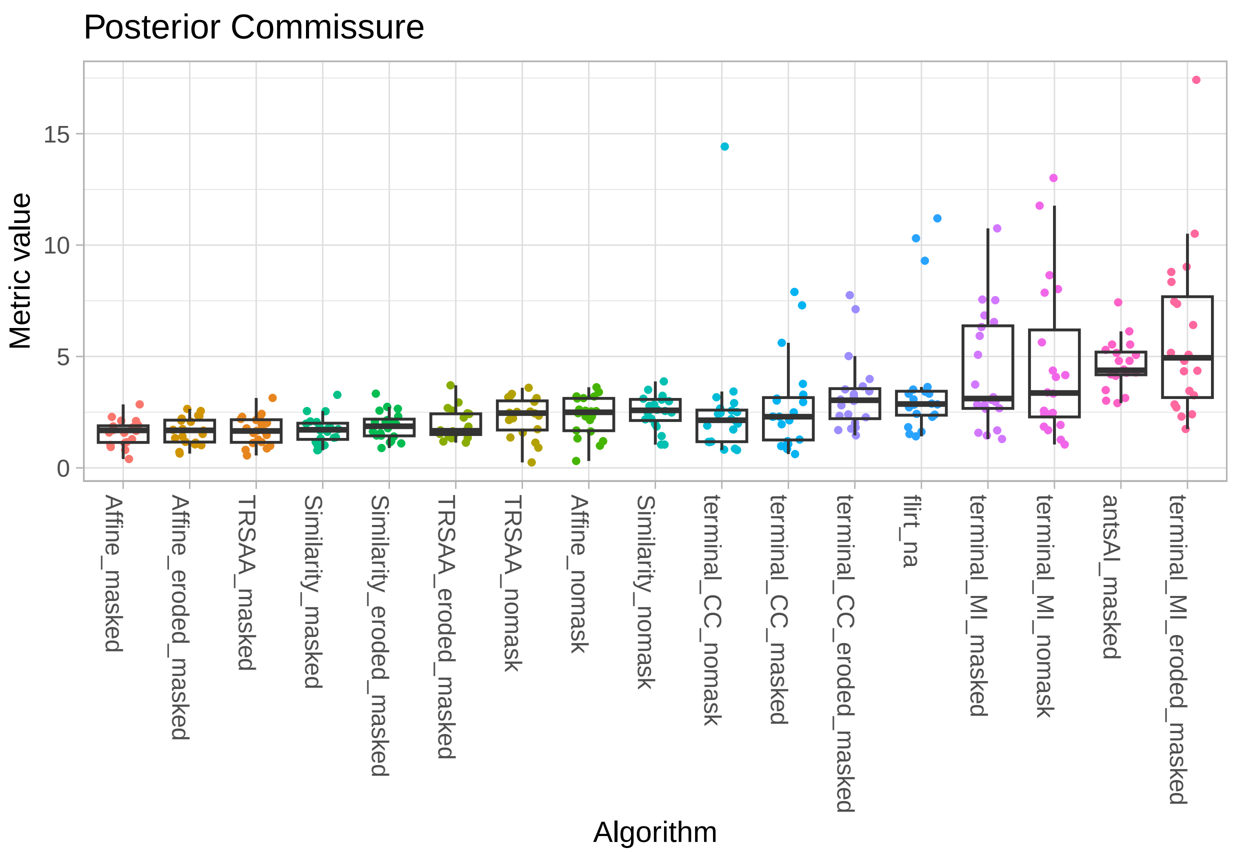
Posterior Commissure
Metric value
0
5
10
15
Affine_masked
Affine_eroded_masked
TRSAA_masked
Similarity_masked
Similarity_eroded_masked
TRSAA_eroded_masked
TRSAA_nomask
Affine_nomask
Similarity_nomask
terminal_CC_nomask
terminal_CC_masked
terminal_CC_eroded_masked
flirt_na
terminal_MI_masked
terminal_MI_nomask
antsAI_masked
terminal_MI_eroded_masked
Algorithm

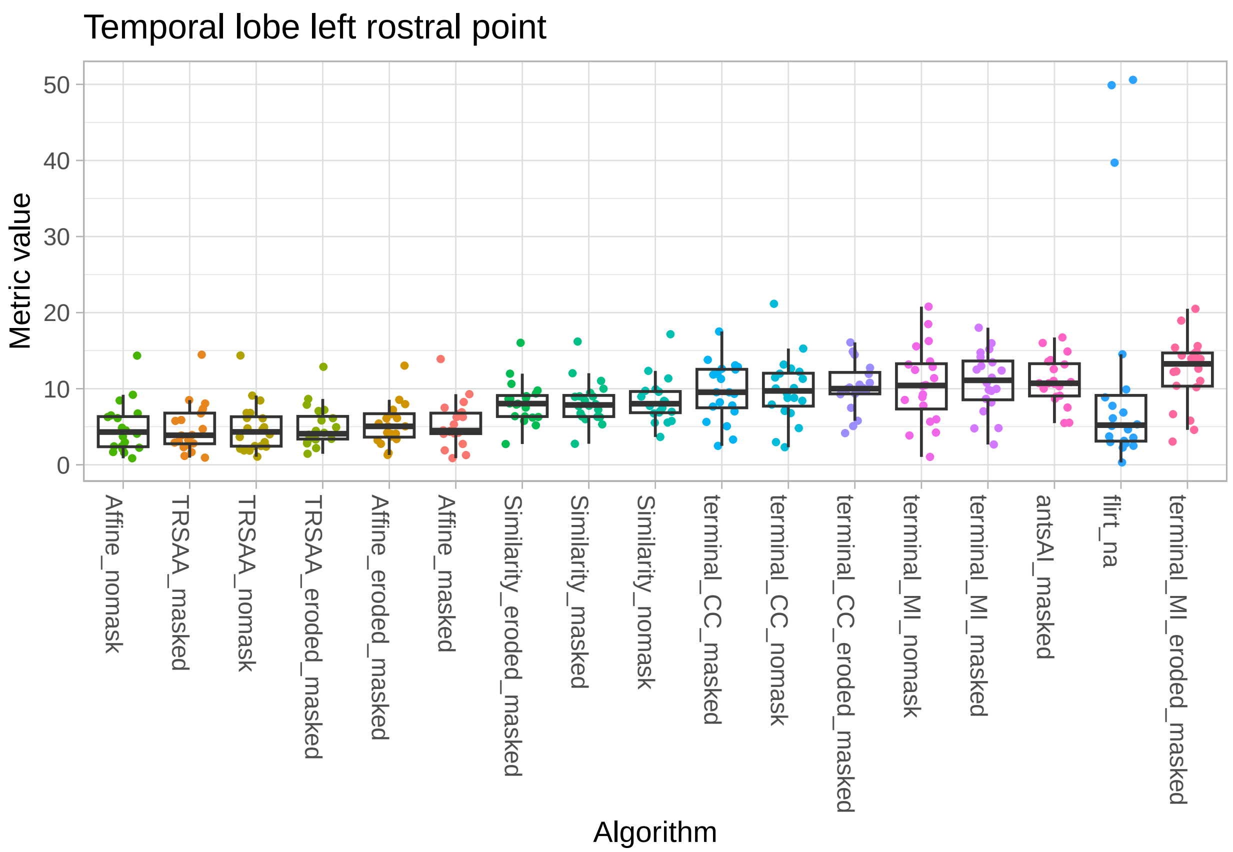

Temporal lobe left rostral point
Metric value
0
10
20
30
40
50
Affine_nomask
TRSAA_masked
TRSAA_nomask
TRSAA_eroded_masked
Affine_eroded_masked
Affine_masked
Similarity_eroded_masked
Similarity_masked
Similarity_nomask
terminal_CC_masked
terminal_CC_nomask
terminal_CC_eroded_masked
terminal_MI_nomask
terminal_MI_masked
antsAI_masked
flirt_na
terminal_MI_eroded_masked
Algorithm

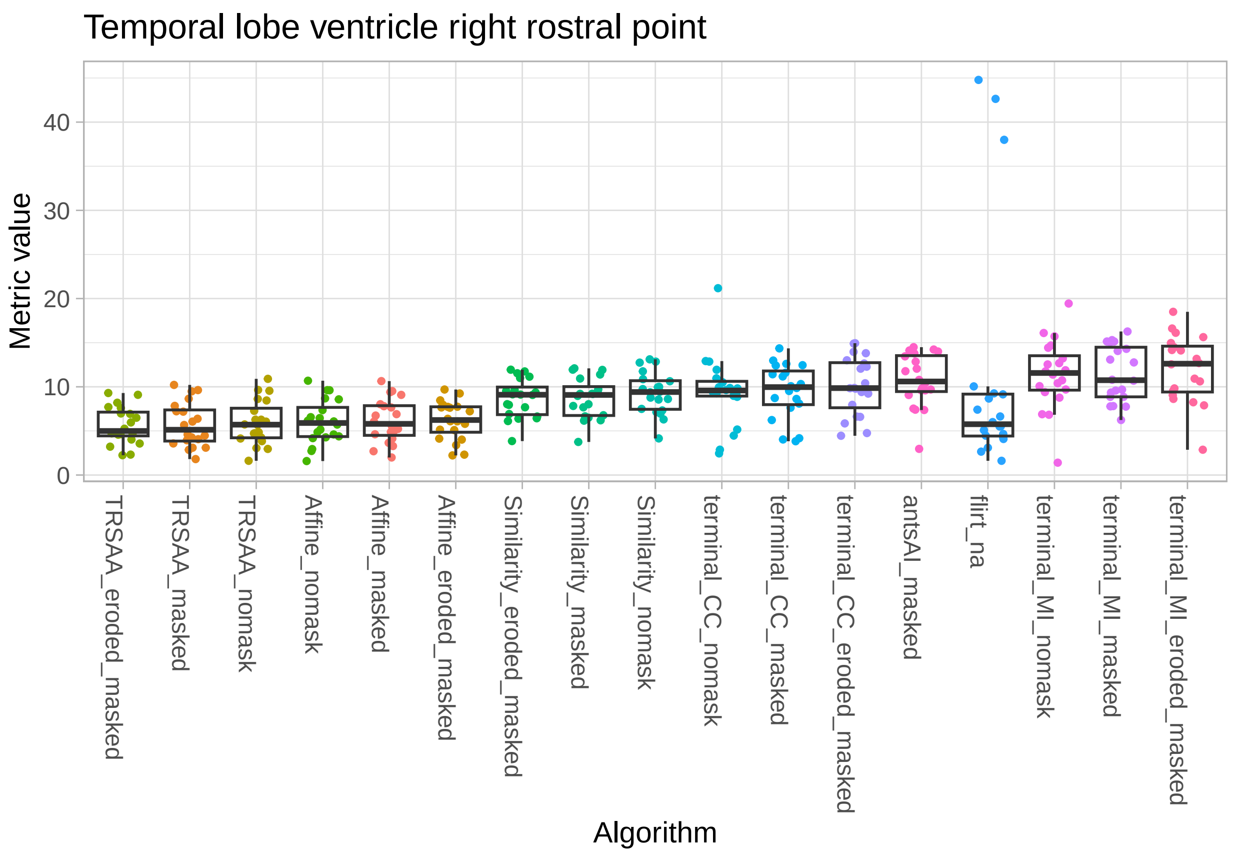

Temporal lobe ventricle right rostral point
Metric value
0
10
20
30
40
TRSAA_eroded_masked
TRSAA_masked
TRSAA_nomask
Affine_nomask
Affine_masked
Affine_eroded_masked
Similarity_eroded_masked
Similarity_masked
Similarity_nomask
terminal_CC_nomask
terminal_CC_masked
terminal_CC_eroded_masked
antsAI_masked
flirt_na
terminal_MI_nomask
terminal_MI_masked
terminal_MI_eroded_masked
Algorithm

2.2 Podium plot

Podium plots (see also Eugster et al., 2008) for visualizing raw assessment data. Upper part (spaghetti plot): Participating algorithms are color-coded, and each colored dot in the plot represents a metric value achieved with the respective algorithm. The actual metric value is encoded by the y-axis. Each podium (here: p=17) represents one possible rank, ordered from best (1) to last (here: 17). The assignment of metric values (i.e. colored dots) to one of the podiums is based on the rank that the respective algorithm achieved on the corresponding case. Note that the plot part above each podium place is further subdivided into p "columns", where each column represents one participating algorithm (here: $p = 17$). Dots corresponding to identical cases are connected by a line, leading to the shown spaghetti structure. Lower part: Bar charts represent the relative frequency for each algorithm to achieve the rank encoded by the podium place.

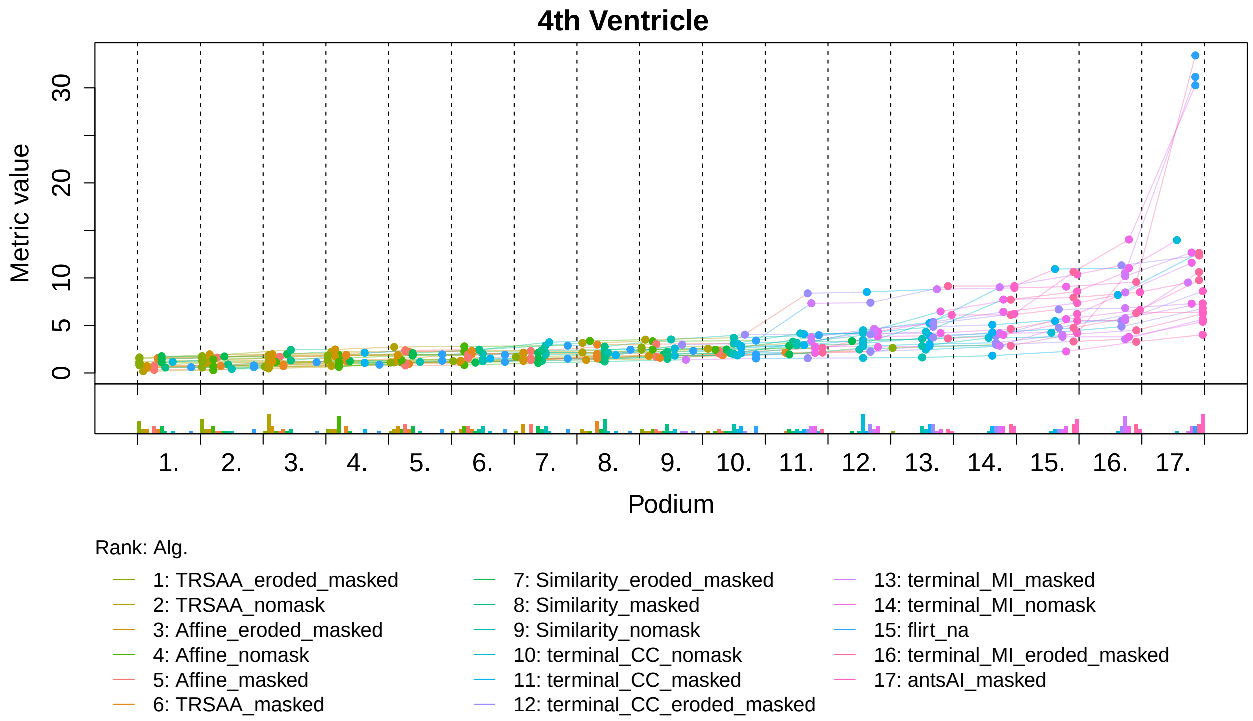

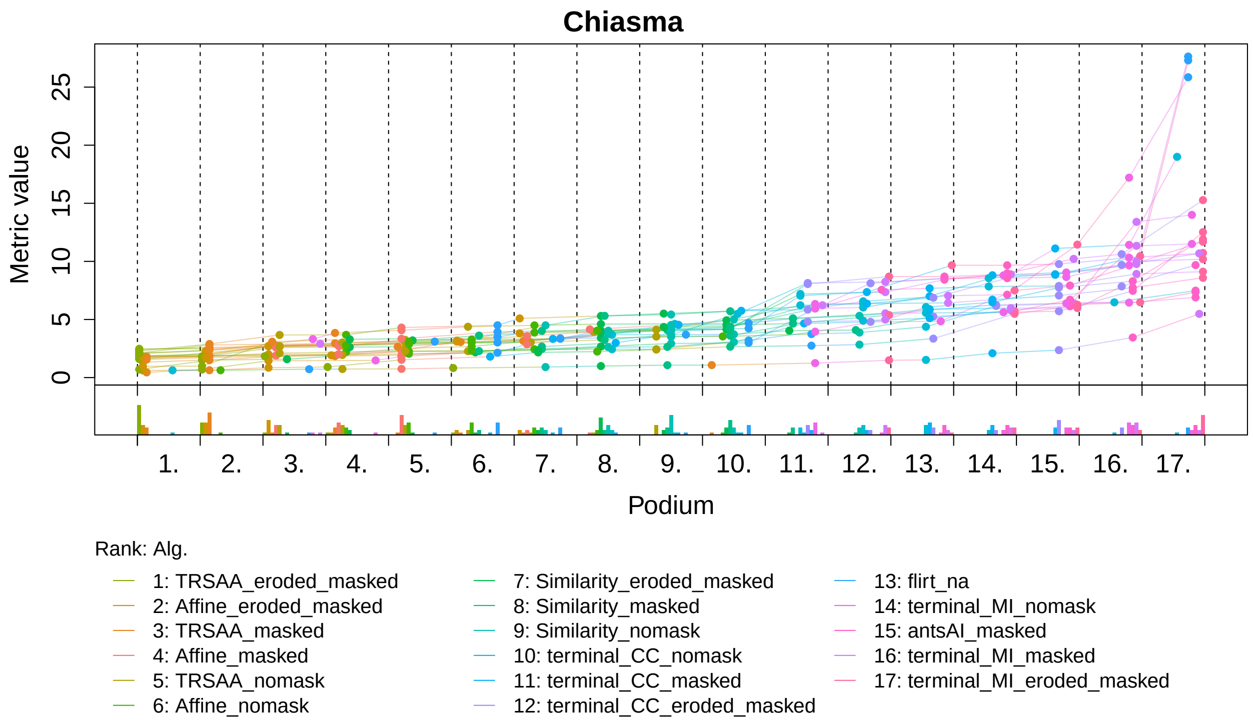
Chiasma
Metric value
Podium
1. 2. 3. 4. 5. 6. 7. 8. 9. 10. 11. 12. 13. 14. 15. 16. 17.
0 5 10 15 20 25
Rank: Alg.
1: TRSAA_eroded_masked
2: Affine_eroded_masked
3: TRSAA_masked
4: Affine_masked
5: TRSAA_nomask
6: Affine_nomask
7: Similarity_eroded_masked
8: Similarity_masked
9: Similarity_nomask
10: terminal_CC_nomask
11: terminal_CC_masked
12: terminal_CC_eroded_masked
13: flirt_na
14: terminal_MI_nomask
15: antsAI_masked
16: terminal_MI_masked
17: terminal_MI_eroded_masked

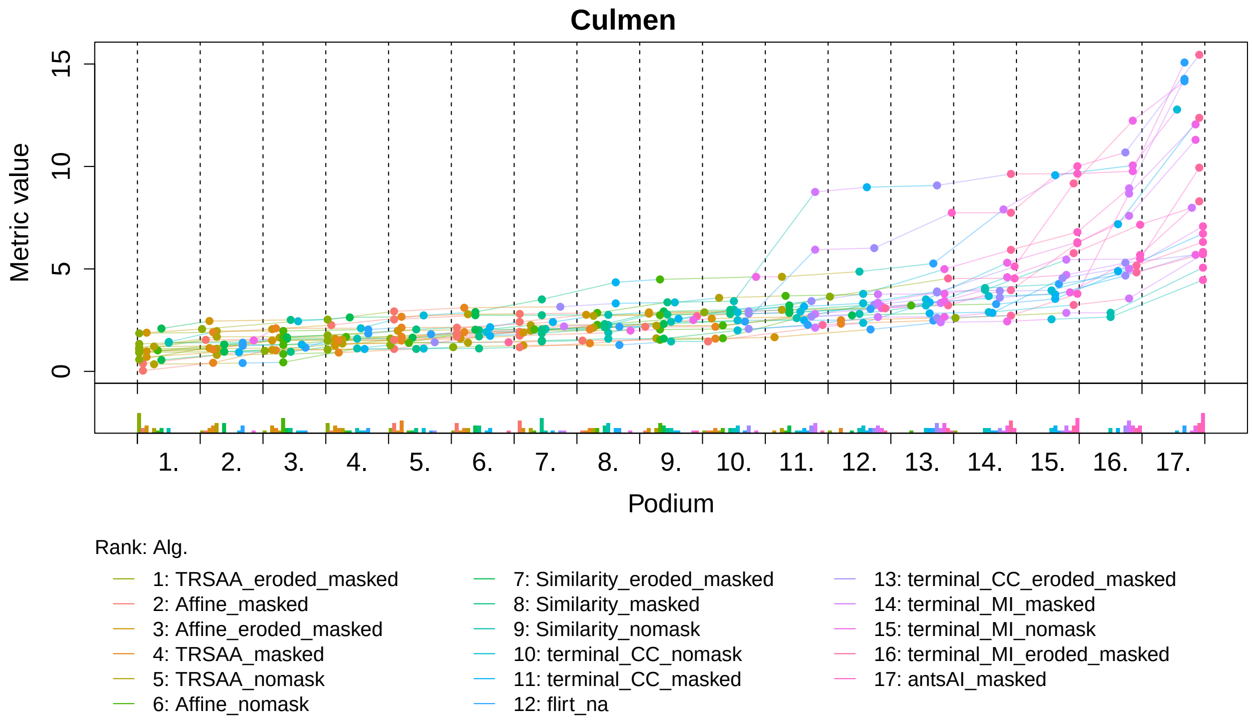
Culmen
Metric value
Podium
1. 2. 3. 4. 5. 6. 7. 8. 9. 10. 11. 12. 13. 14. 15. 16. 17.
0 5 10 15
Rank: Alg.
1: TRSAA_eroded_masked
2: Affine_masked
3: Affine_eroded_masked
4: TRSAA_masked
5: TRSAA_nomask
6: Affine_nomask
7: Similarity_eroded_masked
8: Similarity_masked
9: Similarity_nomask
10: terminal_CC_nomask
11: terminal_CC_masked
12: flirt_na
13: terminal_CC_eroded_masked
14: terminal_MI_masked
15: terminal_MI_nomask
16: terminal_MI_eroded_masked
17: antsAI_masked

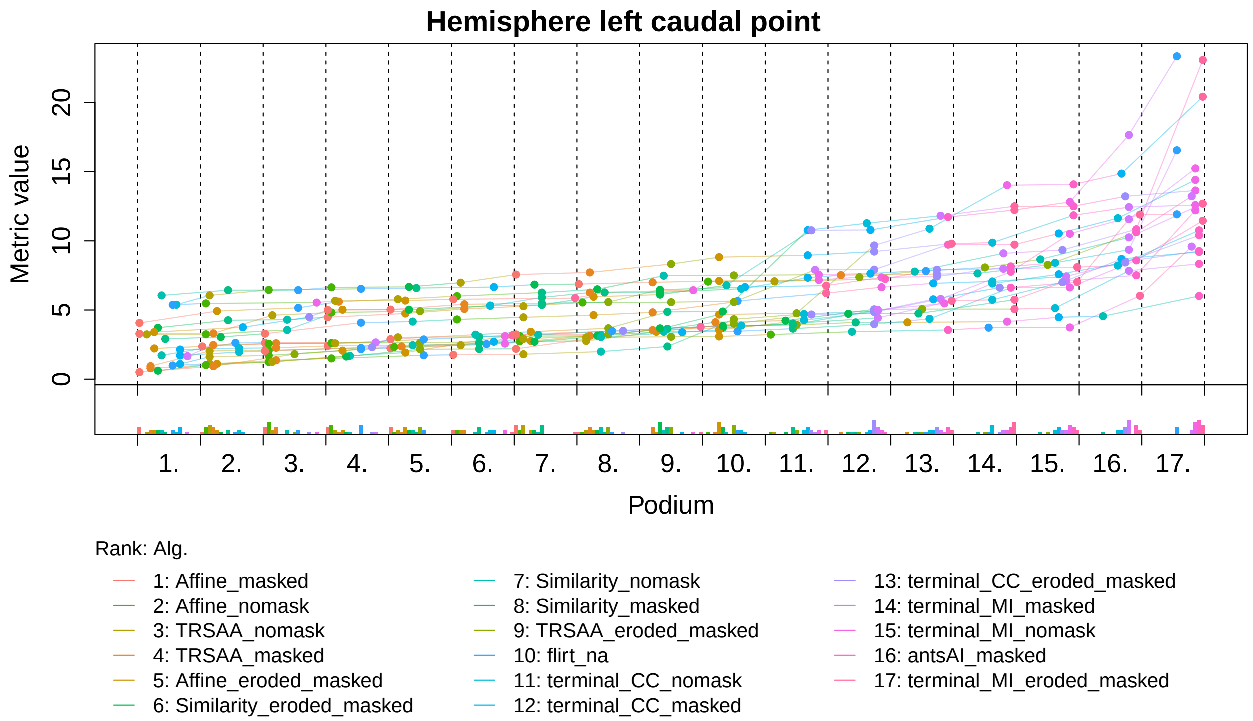
Hemisphere left caudal point
Metric value
0
5
10
15
20
Podium
1.
2.
3.
4.
5.
6.
7.
8.
9.
10.
11.
12.
13.
14.
15.
16.
17.
Rank: Alg.
1: Affine_masked
2: Affine_nomask
3: TRSAA_nomask
4: TRSAA_masked
5: Affine_eroded_masked
6: Similarity_eroded_masked
7: Similarity_nomask
8: Similarity_masked
9: TRSAA_eroded_masked
10: flirt_na
11: terminal_CC_nomask
12: terminal_CC_masked
13: terminal_CC_eroded_masked
14: terminal_MI_masked
15: terminal_MI_nomask
16: antsAI_masked
17: terminal_MI_eroded_masked

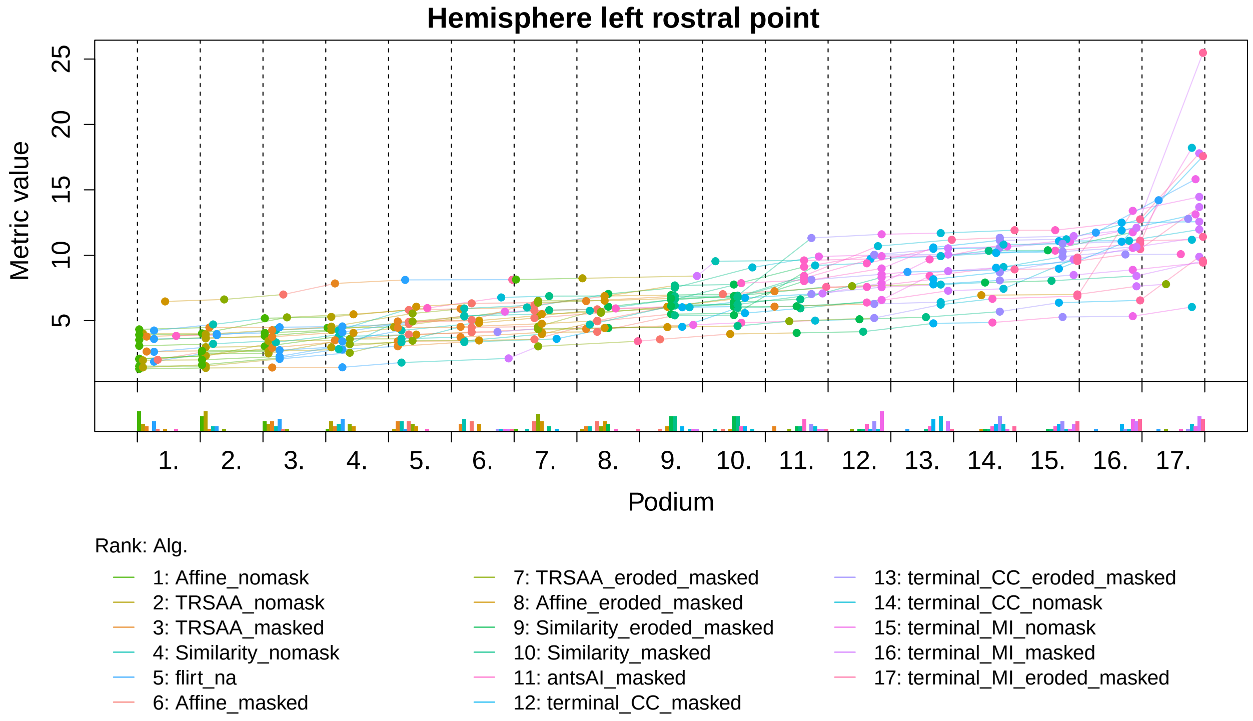
Hemisphere left rostral point
Metric value
5
10
15
20
25
Podium
1.
2.
3.
4.
5.
6.
7.
8.
9.
10.
11.
12.
13.
14.
15.
16.
17.
Rank: Alg.
1: Affine_nomask
2: TRSAA_nomask
3: TRSAA_masked
4: Similarity_nomask
5: flirt_na
6: Affine_masked
7: TRSAA_eroded_masked
8: Affine_eroded_masked
9: Similarity_eroded_masked
10: Similarity_masked
11: antsAI_masked
12: terminal_CC_masked
13: terminal_CC_eroded_masked
14: terminal_CC_nomask
15: terminal_MI_nomask
16: terminal_MI_masked
17: terminal_MI_eroded_masked

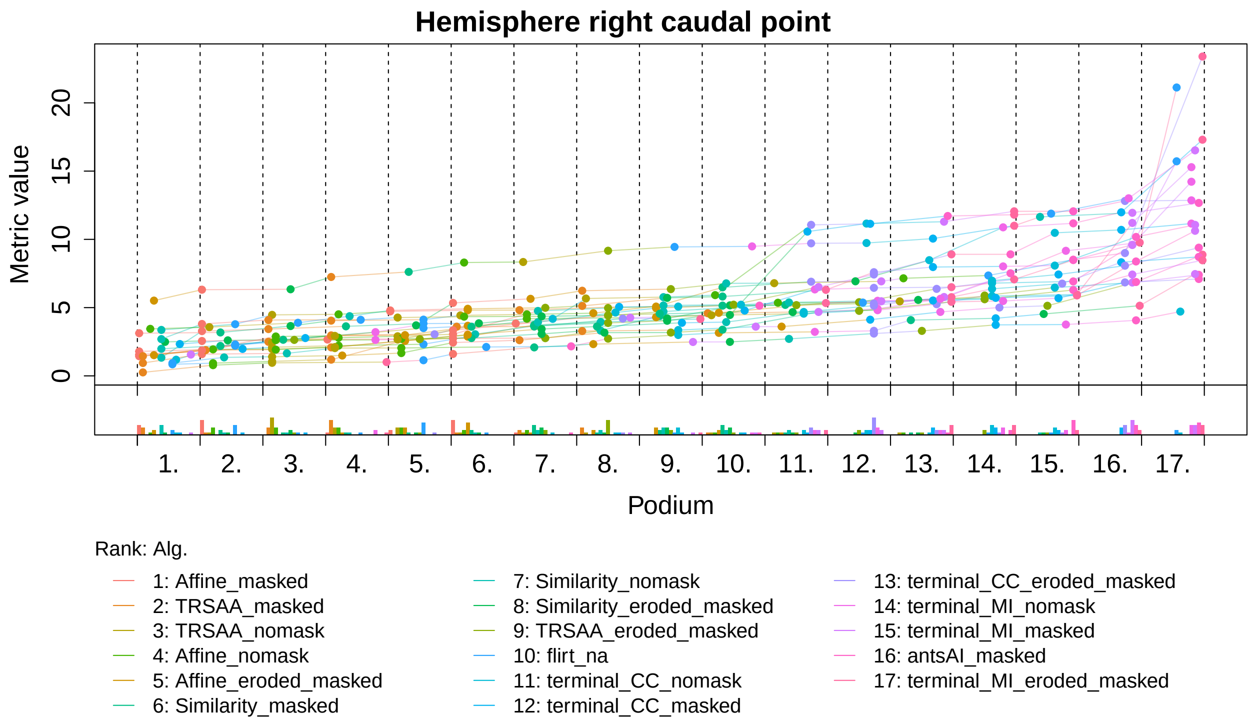
Hemisphere right caudal point
Metric value
Podium
1. 2. 3. 4. 5. 6. 7. 8. 9. 10. 11. 12. 13. 14. 15. 16. 17.
Rank: Alg.
1: Affine_masked
2: TRSAA_masked
3: TRSAA_nomask
4: Affine_nomask
5: Affine_eroded_masked
6: Similarity_masked
7: Similarity_nomask
8: Similarity_eroded_masked
9: TRSAA_eroded_masked
10: flirt_na
11: terminal_CC_nomask
12: terminal_CC_masked
13: terminal_CC_eroded_masked
14: terminal_MI_nomask
15: terminal_MI_masked
16: antsAI_masked
17: terminal_MI_eroded_masked

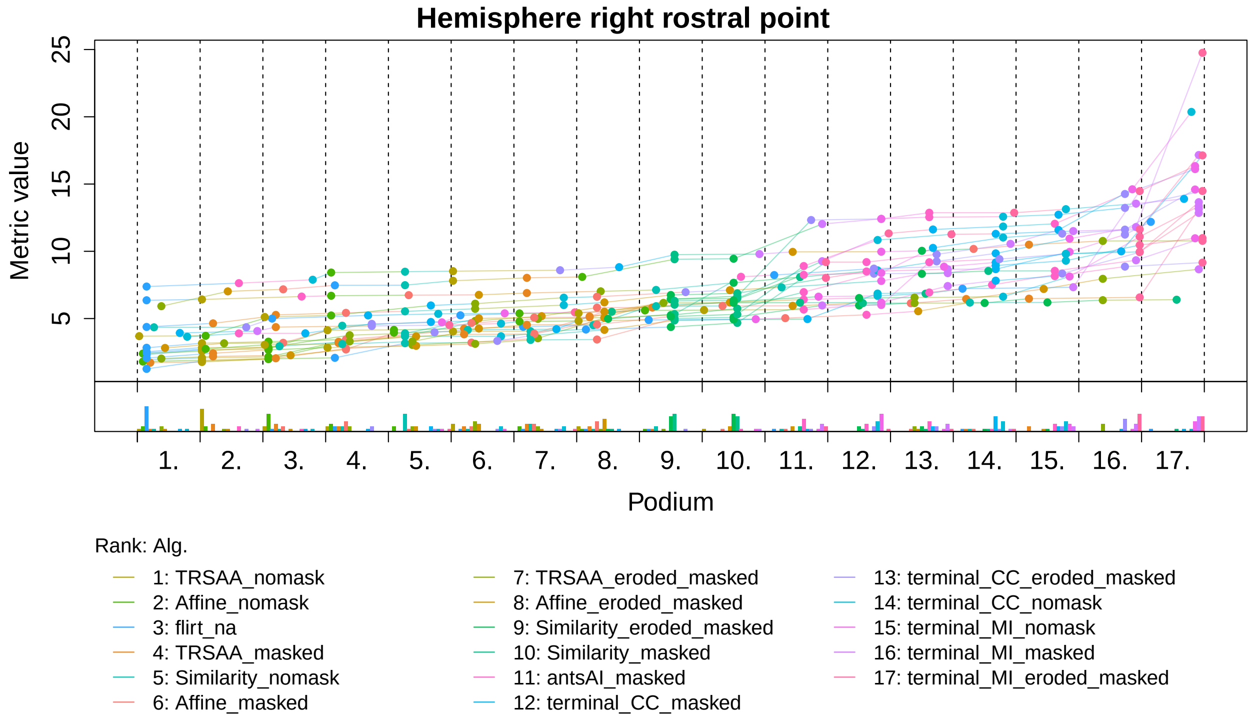
Hemisphere right rostral point
Metric value
Podium
1. 2. 3. 4. 5. 6. 7. 8. 9. 10. 11. 12. 13. 14. 15. 16. 17.
Rank: Alg.
1: TRSAA_nomask
2: Affine_nomask
3: flirt_na
4: TRSAA_masked
5: Similarity_nomask
6: Affine_masked
7: TRSAA_eroded_masked
8: Affine_eroded_masked
9: Similarity_eroded_masked
10: Similarity_masked
11: antsAI_masked
12: terminal_CC_masked
13: terminal_CC_eroded_masked
14: terminal_CC_nomask
15: terminal_MI_nomask
16: terminal_MI_masked
17: terminal_MI_eroded_masked

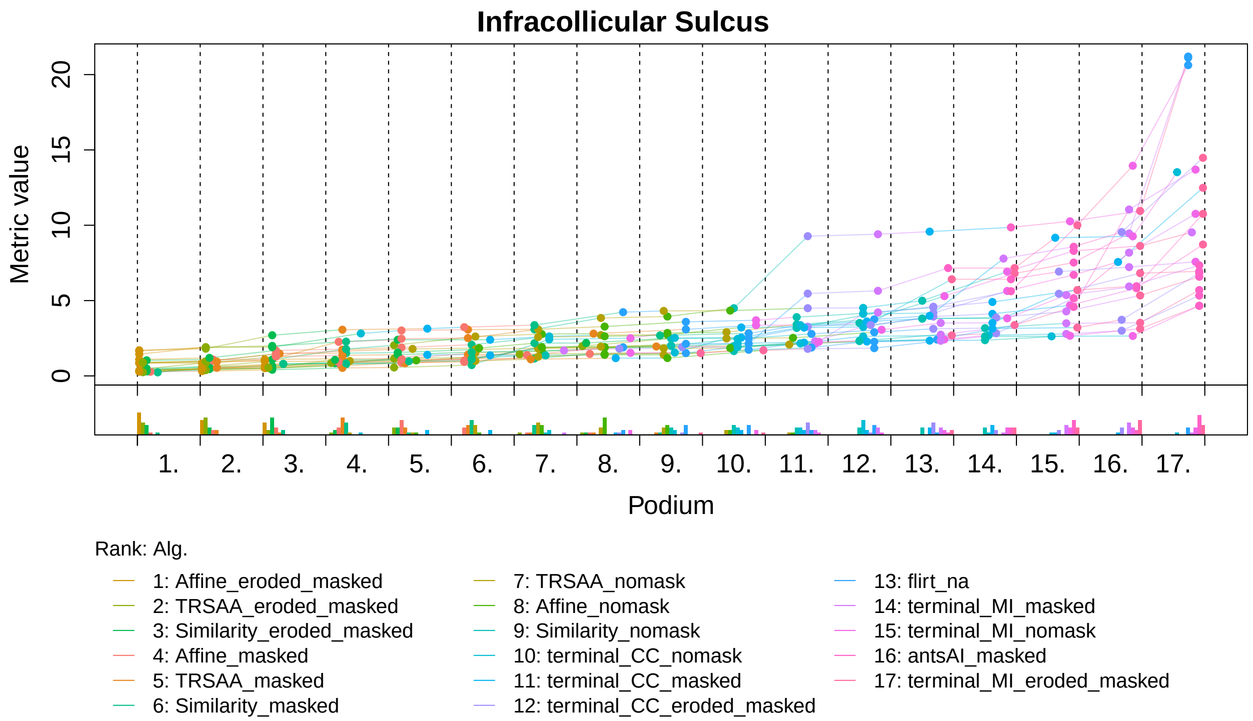
Infracollicular Sulcus
Metric value
0
5
10
15
20
1. 2. 3. 4. 5. 6. 7. 8. 9. 10. 11. 12. 13. 14. 15. 16. 17.
Podium
Rank: Alg.
1: Affine_eroded_masked
2: TRSAA_eroded_masked
3: Similarity_eroded_masked
4: Affine_masked
5: TRSAA_masked
6: Similarity_masked
7: TRSAA_nomask
8: Affine_nomask
9: Similarity_nomask
10: terminal_CC_nomask
11: terminal_CC_masked
12: terminal_CC_eroded_masked
13: flirt_na
14: terminal_MI_masked
15: terminal_MI_nomask
16: antsAI_masked
17: terminal_MI_eroded_masked

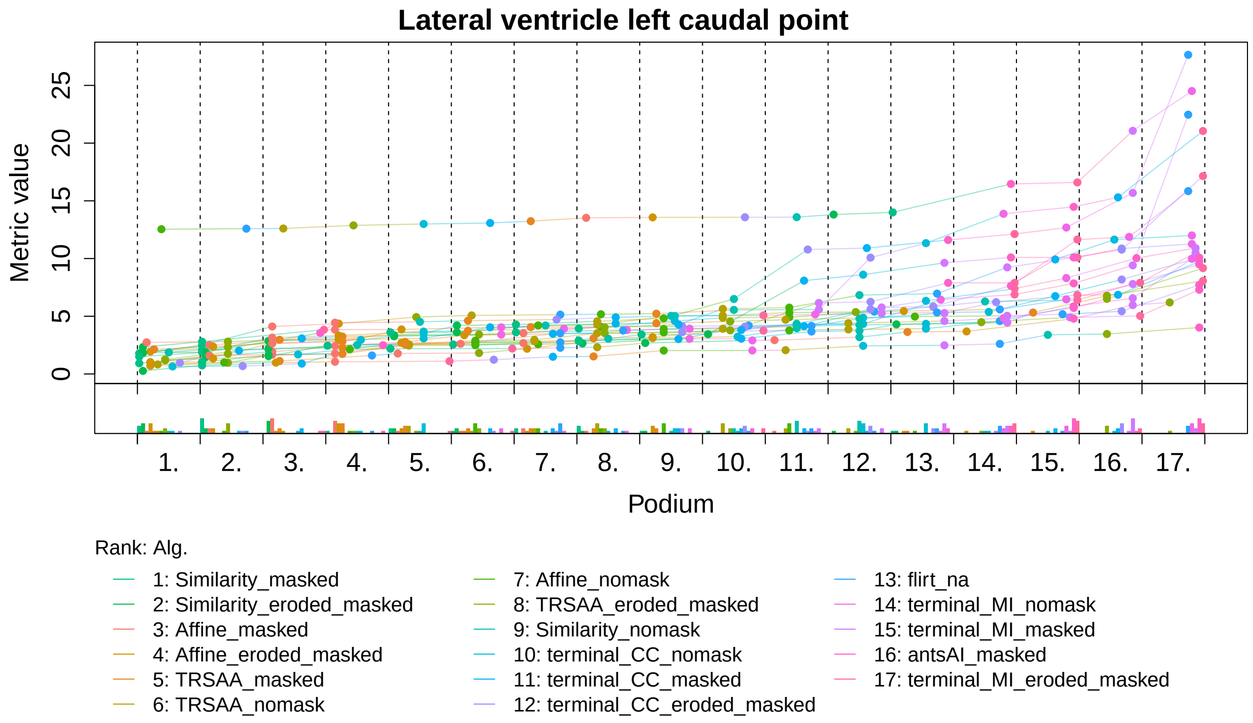
Lateral ventricle left caudal point
Metric value
0
5
10
15
20
25
1. 2. 3. 4. 5. 6. 7. 8. 9. 10. 11. 12. 13. 14. 15. 16. 17.
Podium
Rank: Alg.
1: Similarity_masked
2: Similarity_eroded_masked
3: Affine_masked
4: Affine_eroded_masked
5: TRSAA_masked
6: TRSAA_nomask
7: Affine_nomask
8: TRSAA_eroded_masked
9: Similarity_nomask
10: terminal_CC_nomask
11: terminal_CC_masked
12: terminal_CC_eroded_masked
13: flirt_na
14: terminal_MI_nomask
15: terminal_MI_masked
16: antsAI_masked
17: terminal_MI_eroded_masked

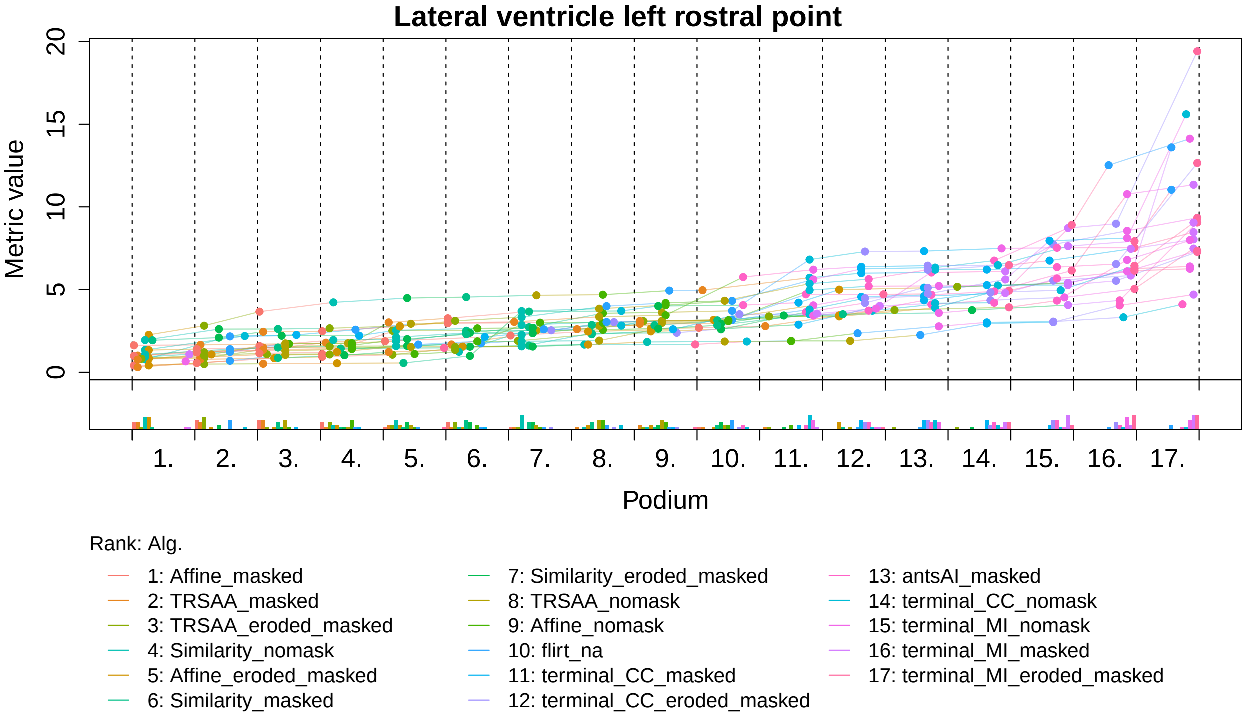
Lateral ventricle left rostral point
Metric value
0
5
10
15
20
1.
2.
3.
4.
5.
6.
7.
8.
9.
10.
11.
12.
13.
14.
15.
16.
17.
Podium
Rank: Alg.
1: Affine_masked
2: TRSAA_masked
3: TRSAA_eroded_masked
4: Similarity_nomask
5: Affine_eroded_masked
6: Similarity_masked
7: Similarity_eroded_masked
8: TRSAA_nomask
9: Affine_nomask
10: flirt_na
11: terminal_CC_masked
12: terminal_CC_eroded_masked
13: antsAI_masked
14: terminal_CC_nomask
15: terminal_MI_nomask
16: terminal_MI_masked
17: terminal_MI_eroded_masked

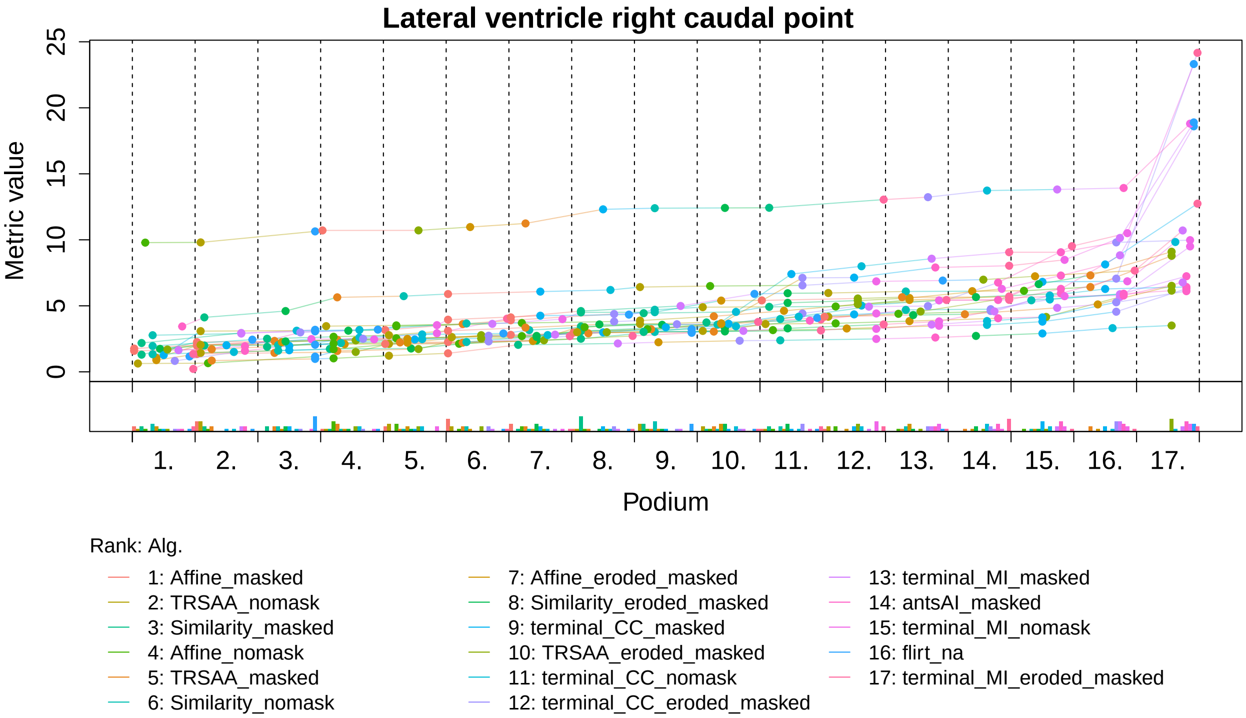
Lateral ventricle right caudal point
Metric value
0
5
10
15
20
25
1.
2.
3.
4.
5.
6.
7.
8.
9.
10.
11.
12.
13.
14.
15.
16.
17.
Podium
Rank: Alg.
1: Affine_masked
2: TRSAA_nomask
3: Similarity_masked
4: Affine_nomask
5: TRSAA_masked
6: Similarity_nomask
7: Affine_eroded_masked
8: Similarity_eroded_masked
9: terminal_CC_masked
10: TRSAA_eroded_masked
11: terminal_CC_nomask
12: terminal_CC_eroded_masked
13: terminal_MI_masked
14: antsAI_masked
15: terminal_MI_nomask
16: flirt_na
17: terminal_MI_eroded_masked

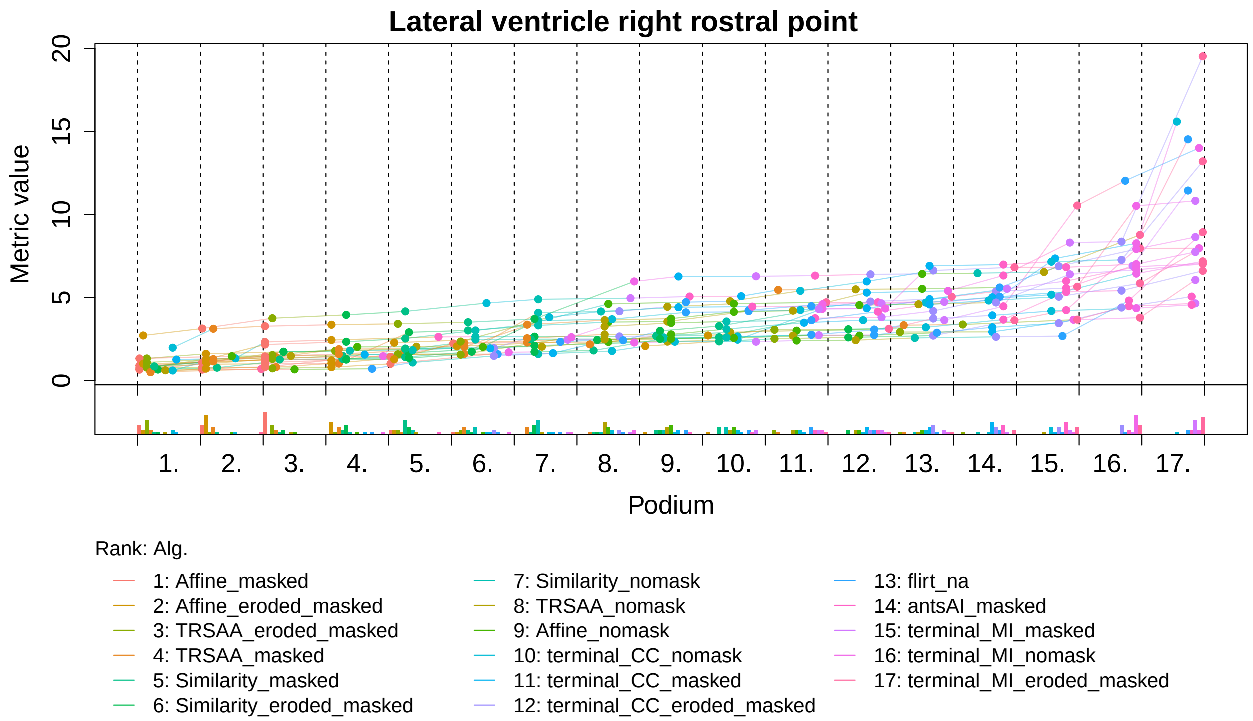
Lateral ventricle right rostral point
Metric value
0
5
10
15
20
1. 2. 3. 4. 5. 6. 7. 8. 9. 10. 11. 12. 13. 14. 15. 16. 17.
Podium
Rank: Alg.
1: Affine_masked
2: Affine_eroded_masked
3: TRSAA_eroded_masked
4: TRSAA_masked
5: Similarity_masked
6: Similarity_eroded_masked
7: Similarity_nomask
8: TRSAA_nomask
9: Affine_nomask
10: terminal_CC_nomask
11: terminal_CC_masked
12: terminal_CC_eroded_masked
13: flirt_na
14: antsAI_masked
15: terminal_MI_masked
16: terminal_MI_nomask
17: terminal_MI_eroded_masked

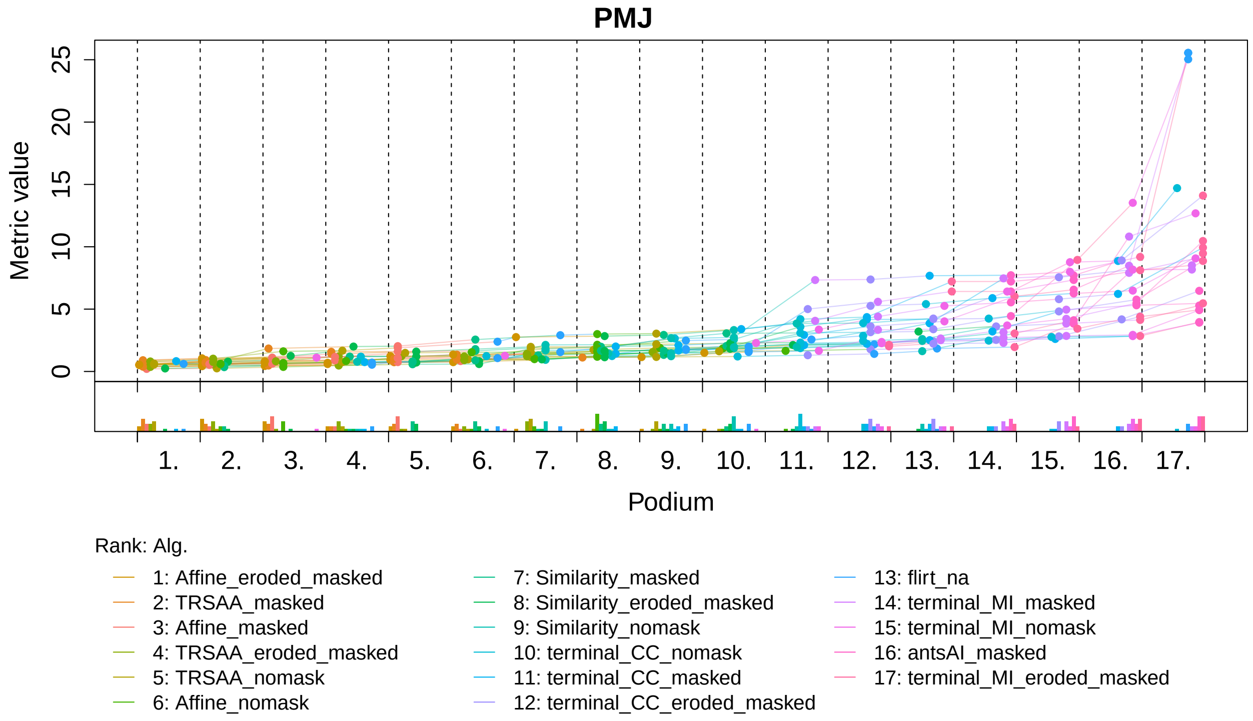
PMJ
Metric value
0
5
10
15
20
25
1. 2. 3. 4. 5. 6. 7. 8. 9. 10. 11. 12. 13. 14. 15. 16. 17.
Podium
Rank: Alg.
1: Affine_eroded_masked
2: TRSAA_masked
3: Affine_masked
4: TRSAA_eroded_masked
5: TRSAA_nomask
6: Affine_nomask
7: Similarity_masked
8: Similarity_eroded_masked
9: Similarity_nomask
10: terminal_CC_nomask
11: terminal_CC_masked
12: terminal_CC_eroded_masked
13: flirt_na
14: terminal_MI_masked
15: terminal_MI_nomask
16: antsAI_masked
17: terminal_MI_eroded_masked

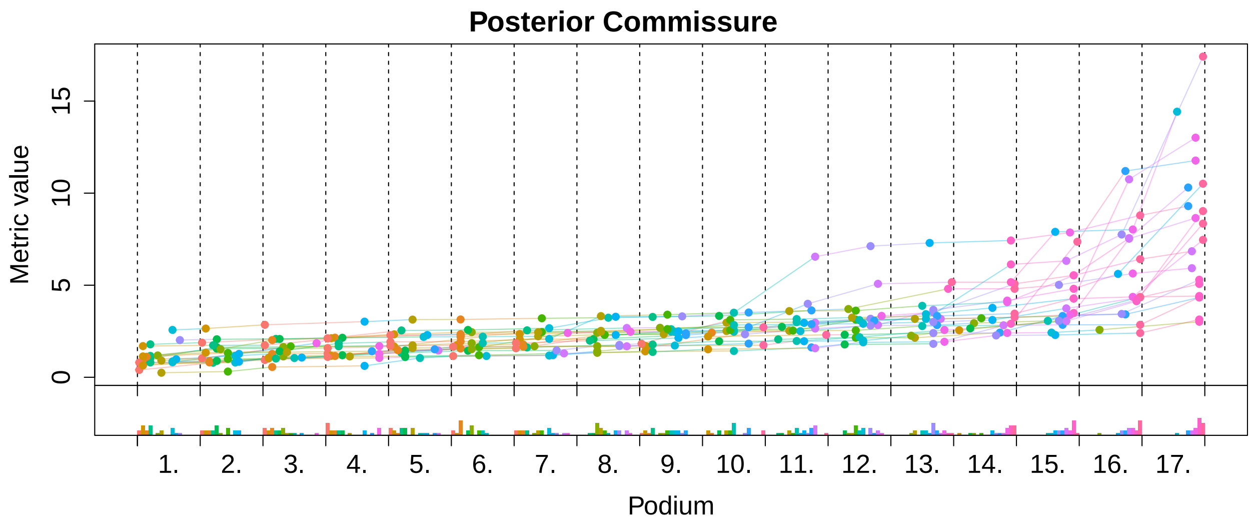

Rank: Alg.

- 1: Affine_masked
- 2: Affine_eroded_masked
- 3: TRSAA_masked
- 4: Similarity_masked
- 5: Similarity_eroded_masked
- 6: TRSAA_eroded_masked
- 7: TRSAA_nomask
- 8: Affine_nomask
- 9: Similarity_nomask
- 10: terminal_CC_nomask
- 11: terminal_CC_masked
- 12: terminal_CC_eroded_masked
- 13: flirt_na
- 14: terminal_MI_masked
- 15: terminal_MI_nomask
- 16: antsAI_masked
- 17: terminal_MI_eroded_masked

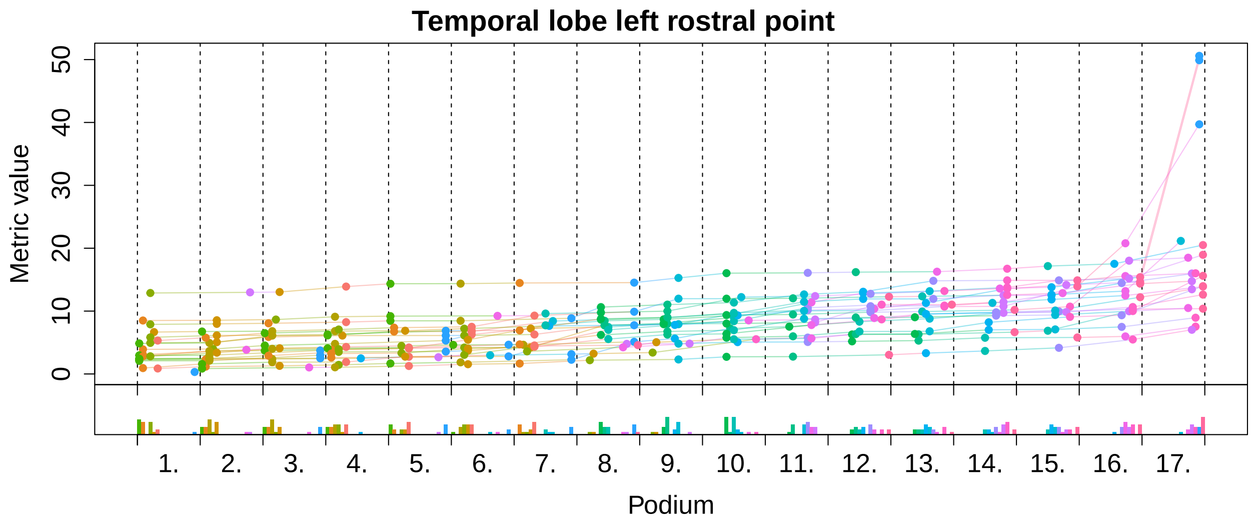

Rank: Alg.

- 1: Affine_nomask
- 2: TRSAA_masked
- 3: TRSAA_nomask
- 4: TRSAA_eroded_masked
- 5: Affine_eroded_masked
- 6: Affine_masked
- 7: Similarity_eroded_masked
- 8: Similarity_masked
- 9: Similarity_nomask
- 10: terminal_CC_masked
- 11: terminal_CC_nomask
- 12: terminal_CC_eroded_masked
- 13: terminal_MI_nomask
- 14: terminal_MI_masked
- 15: antsAI_masked
- 16: flirt_na
- 17: terminal_MI_eroded_masked

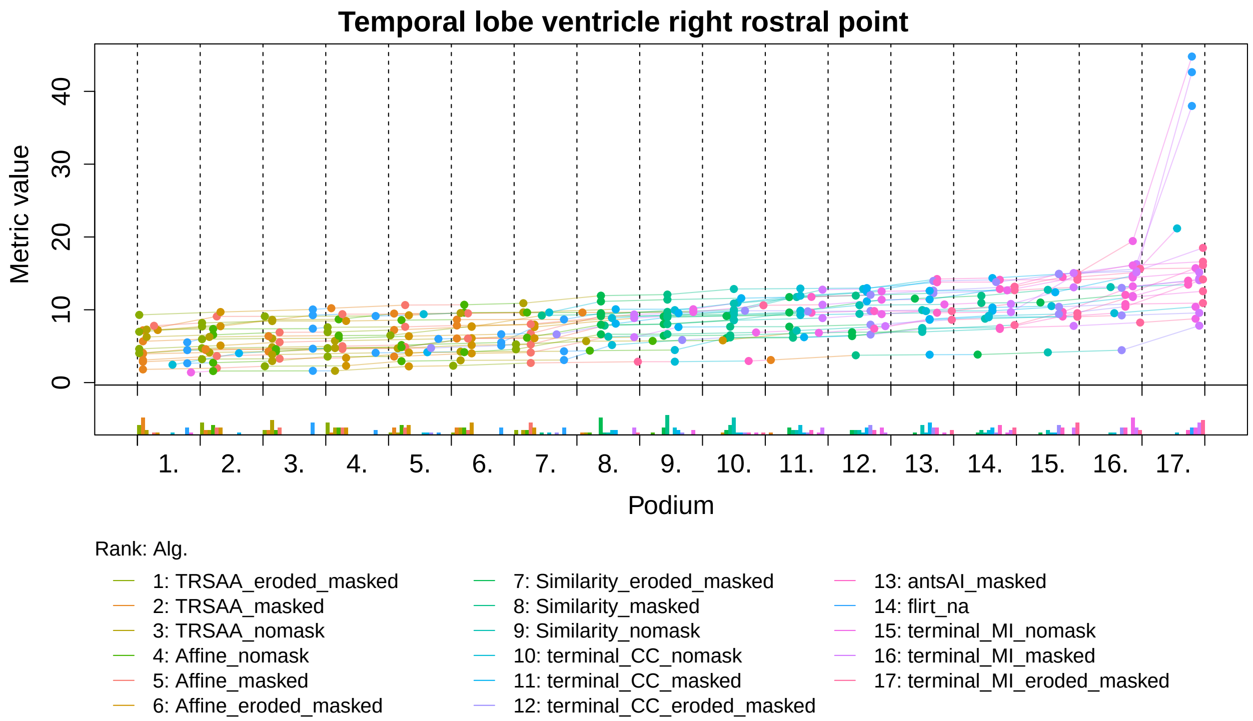
Temporal lobe ventricle right rostral point
Metric value
0
10
20
30
40
1.
2.
3.
4.
5.
6.
7.
8.
9.
10.
11.
12.
13.
14.
15.
16.
17.
Podium
Rank: Alg.
1: TRSAA_eroded_masked
2: TRSAA_masked
3: TRSAA_nomask
4: Affine_nomask
5: Affine_masked
6: Affine_eroded_masked
7: Similarity_eroded_masked
8: Similarity_masked
9: Similarity_nomask
10: terminal_CC_nomask
11: terminal_CC_masked
12: terminal_CC_eroded_masked
13: antsAI_masked
14: flirt_na
15: terminal_MI_nomask
16: terminal_MI_masked
17: terminal_MI_eroded_masked

2.3 Ranking heatmap

Ranking heatmaps for visualizing raw assessment data. Each cell (i, A_j) shows the absolute frequency of cases in which algorithm A_j achieved rank i.

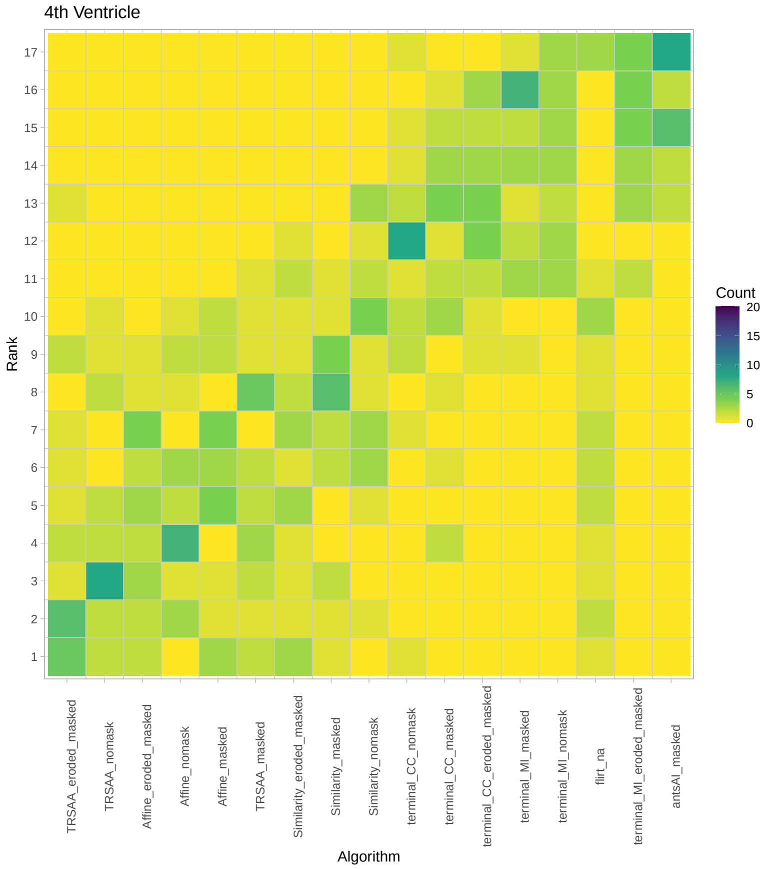

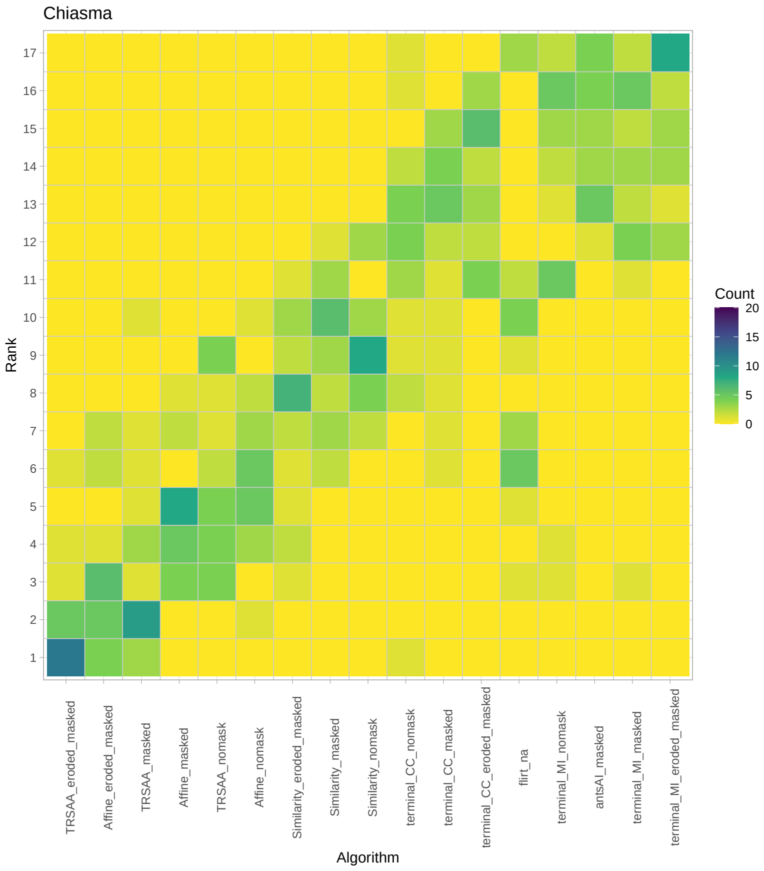

Chiasma
Rank
17
16
15
14
13
12
11
10
9
8
7
6
5
4
3
2
1
Count
20
15
10
5
0
TRSAA_eroded_masked
Affine_eroded_masked
TRSAA_masked
Affine_masked
TRSAA_nomask
Affine_nomask
Similarity_eroded_masked
Similarity_masked
Similarity_nomask
terminal_CC_nomask
terminal_CC_masked
terminal_CC_eroded_masked
flirt_na
terminal_MI_nomask
antsAI_masked
terminal_MI_masked
terminal_MI_eroded_masked
Algorithm

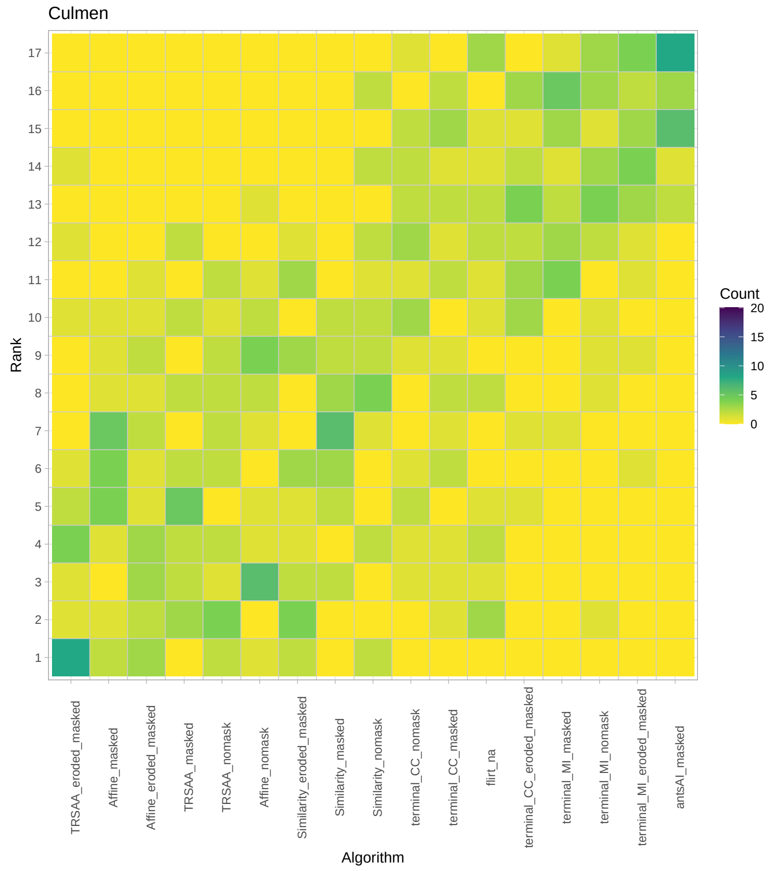
Culmen
Rank
Algorithm
Count
20
15
10
5
0
17
16
15
14
13
12
11
10
9
8
7
6
5
4
3
2
1
TRSAA_eroded_masked
Affine_masked
Affine_eroded_masked
TRSAA_masked
TRSAA_nomask
Affine_nomask
Similarity_eroded_masked
Similarity_masked
Similarity_nomask
terminal_CC_nomask
terminal_CC_masked
flirt_na
terminal_CC_eroded_masked
terminal_MI_masked
terminal_MI_nomask
terminal_MI_eroded_masked
antsAI_masked

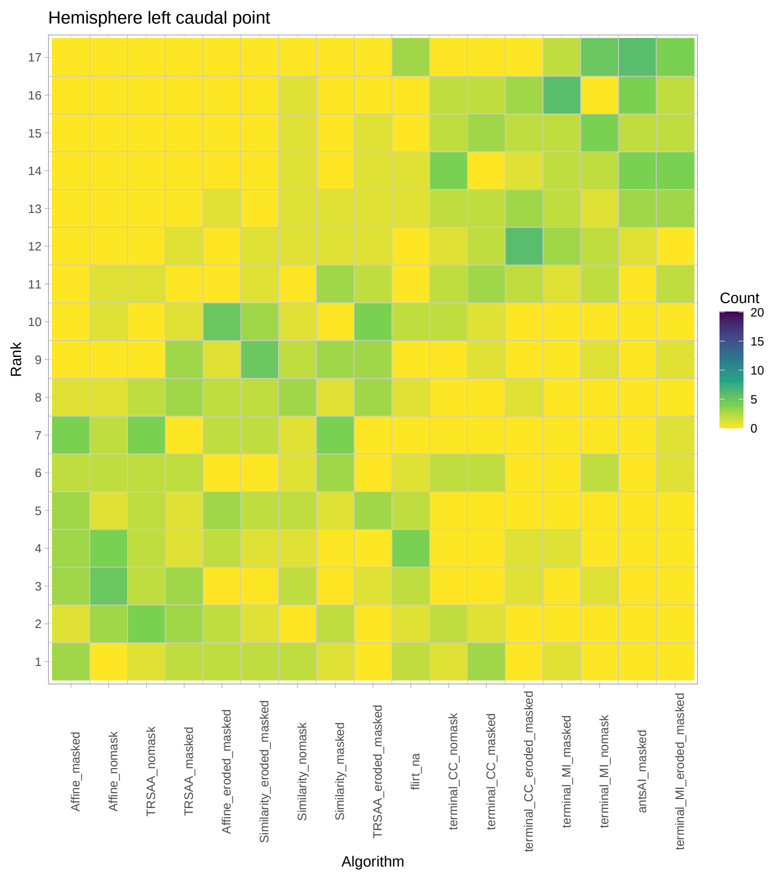

Hemisphere left caudal point
Rank
17
16
15
14
13
12
11
10
9
8
7
6
5
4
3
2
1
Count
20
15
10
5
0
Affine_masked
Affine_nomask
TRSAA_nomask
TRSAA_masked
Affine_eroded_masked
Similarity_eroded_masked
Similarity_nomask
Similarity_masked
TRSAA_eroded_masked
flirt_na
terminal_CC_nomask
terminal_CC_masked
terminal_CC_eroded_masked
terminal_MI_masked
terminal_MI_nomask
antsAI_masked
terminal_MI_eroded_masked
Algorithm

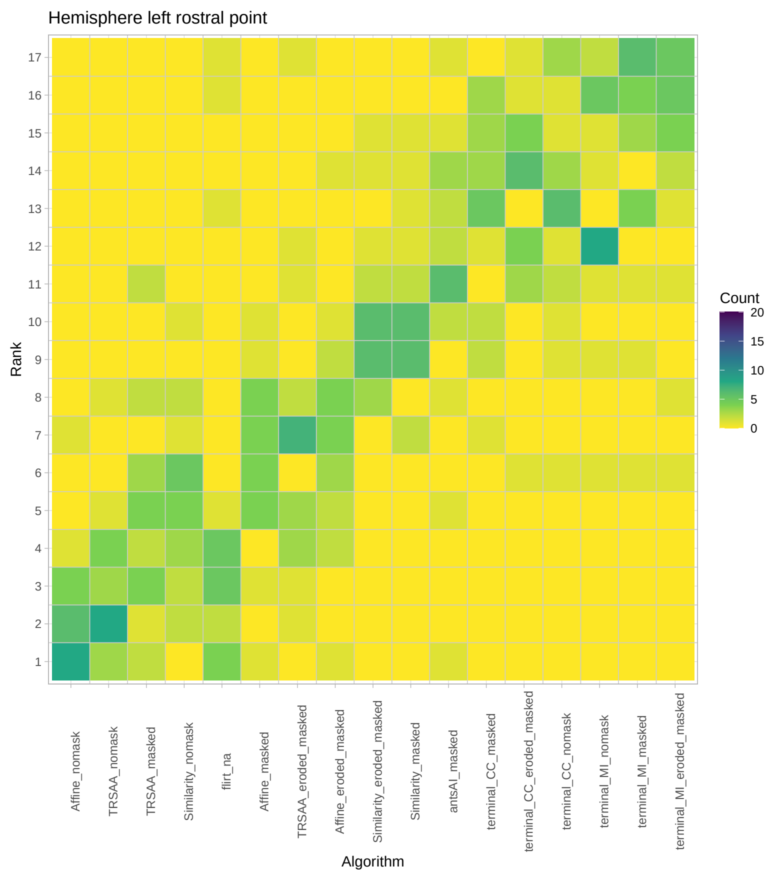

Hemisphere left rostral point
Rank
Algorithm
Count
20
15
10
5
0
17
16
15
14
13
12
11
10
9
8
7
6
5
4
3
2
1
Affine_nomask
TRSAA_nomask
TRSAA_masked
Similarity_nomask
flirt_na
Affine_masked
TRSAA_eroded_masked
Affine_eroded_masked
Similarity_eroded_masked
Similarity_masked
antsAI_masked
terminal_CC_masked
terminal_CC_eroded_masked
terminal_CC_nomask
terminal_MI_nomask
terminal_MI_masked
terminal_MI_eroded_masked

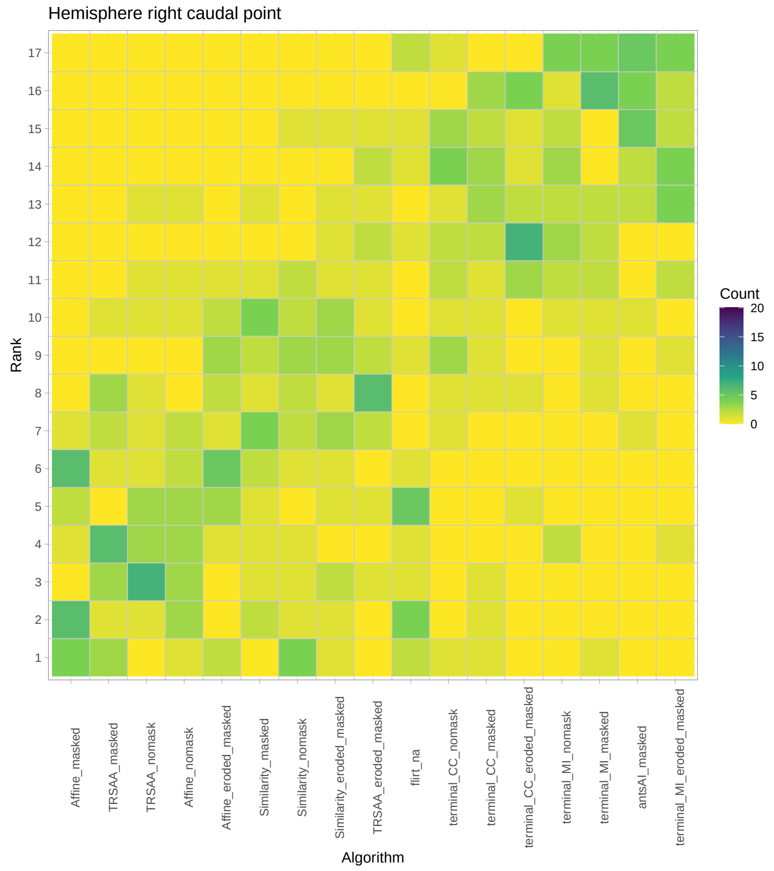
Hemisphere right caudal point
Rank
Algorithm
Count
20
15
10
5
0
17
16
15
14
13
12
11
10
9
8
7
6
5
4
3
2
1
Affine_masked
TRSAA_masked
TRSAA_nomask
Affine_nomask
Affine_eroded_masked
Similarity_masked
Similarity_nomask
Similarity_eroded_masked
TRSAA_eroded_masked
flirt_na
terminal_CC_nomask
terminal_CC_masked
terminal_CC_eroded_masked
terminal_MI_nomask
terminal_MI_masked
antsAI_masked
terminal_MI_eroded_masked

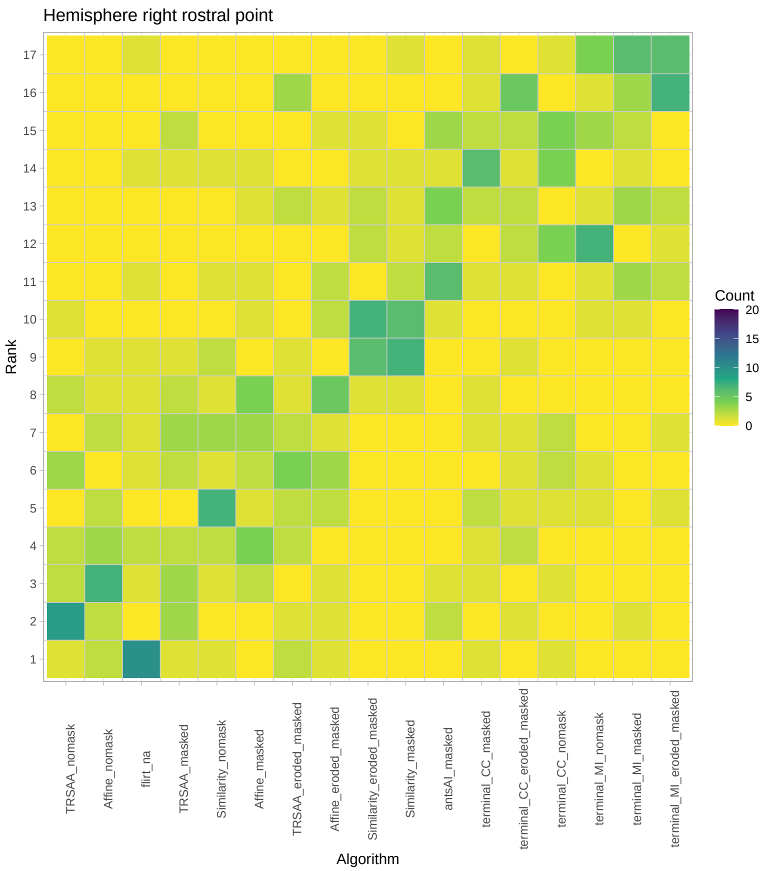
Hemisphere right rostral point
Rank
17
16
15
14
13
12
11
10
9
8
7
6
5
4
3
2
1
Count
20
15
10
5
0
TRSAA_nomask
Affine_nomask
flirt_na
TRSAA_masked
Similarity_nomask
Affine_masked
TRSAA_eroded_masked
Affine_eroded_masked
Similarity_eroded_masked
Similarity_masked
antsAI_masked
terminal_CC_masked
terminal_CC_eroded_masked
terminal_CC_nomask
terminal_MI_nomask
terminal_MI_masked
terminal_MI_eroded_masked
Algorithm

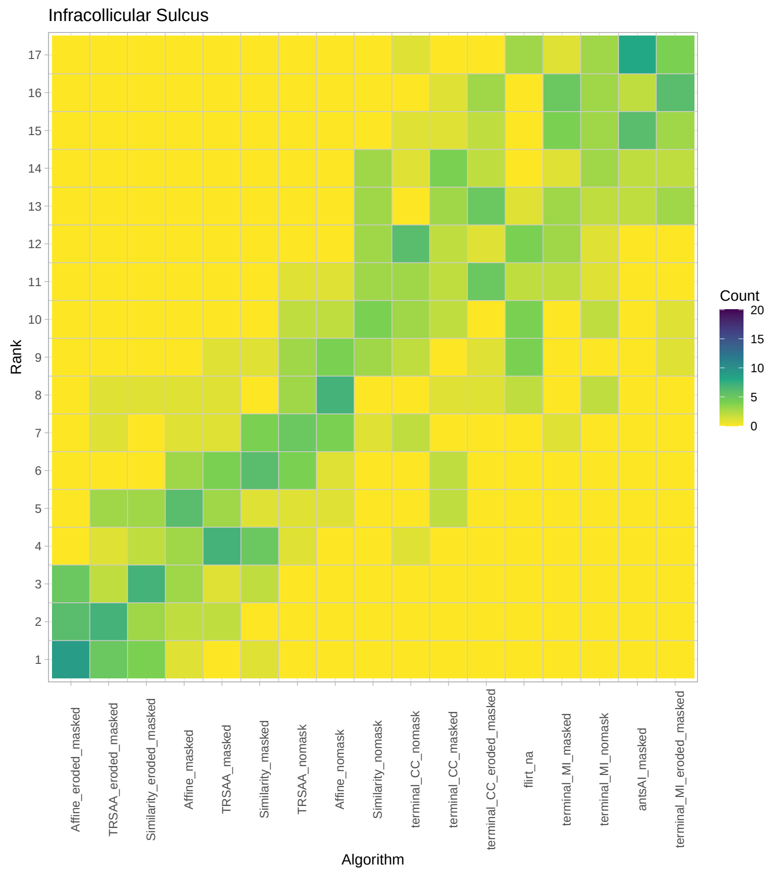

Infracollicular Sulcus
Rank
17
16
15
14
13
12
11
10
9
8
7
6
5
4
3
2
1
Affine_eroded_masked
TRSAA_eroded_masked
Similarity_eroded_masked
Affine_masked
TRSAA_masked
Similarity_masked
TRSAA_nomask
Affine_nomask
Similarity_nomask
terminal_CC_nomask
terminal_CC_masked
terminal_CC_eroded_masked
flirt_na
terminal_MI_masked
terminal_MI_nomask
antsAI_masked
terminal_MI_eroded_masked
Algorithm
Count
20
15
10
5
0

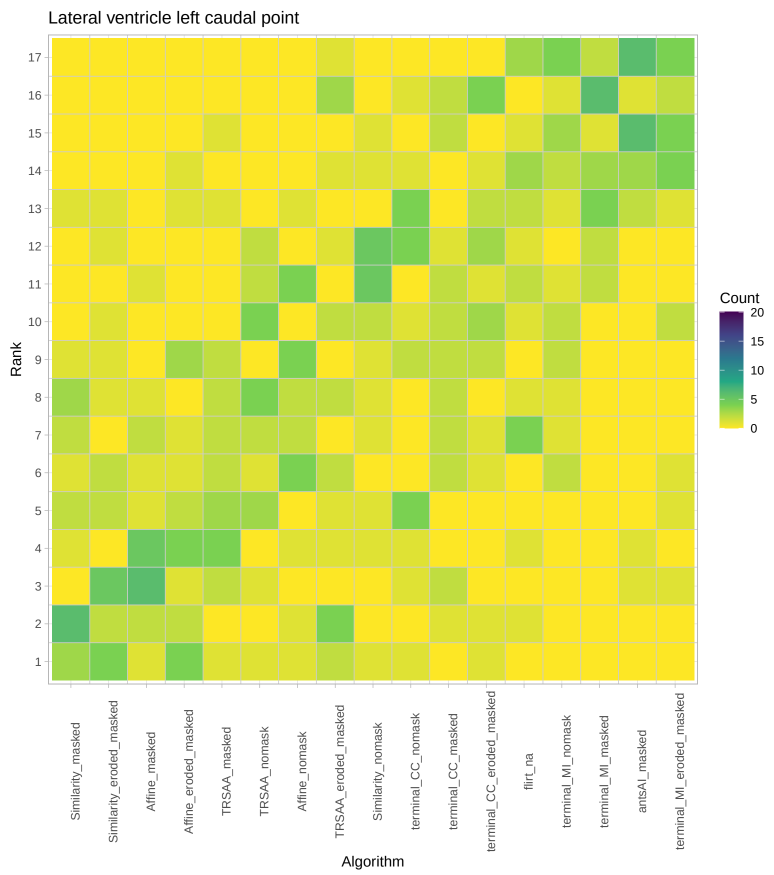

Lateral ventricle left caudal point
Rank
Count
20
15
10
5
0
17
16
15
14
13
12
11
10
9
8
7
6
5
4
3
2
1
Similarity_masked
Similarity_eroded_masked
Affine_masked
Affine_eroded_masked
TRSAA_masked
TRSAA_nomask
Affine_nomask
TRSAA_eroded_masked
Similarity_nomask
terminal_CC_nomask
terminal_CC_masked
terminal_CC_eroded_masked
flirt_na
terminal_MI_nomask
terminal_MI_masked
antsAI_masked
terminal_MI_eroded_masked
Algorithm

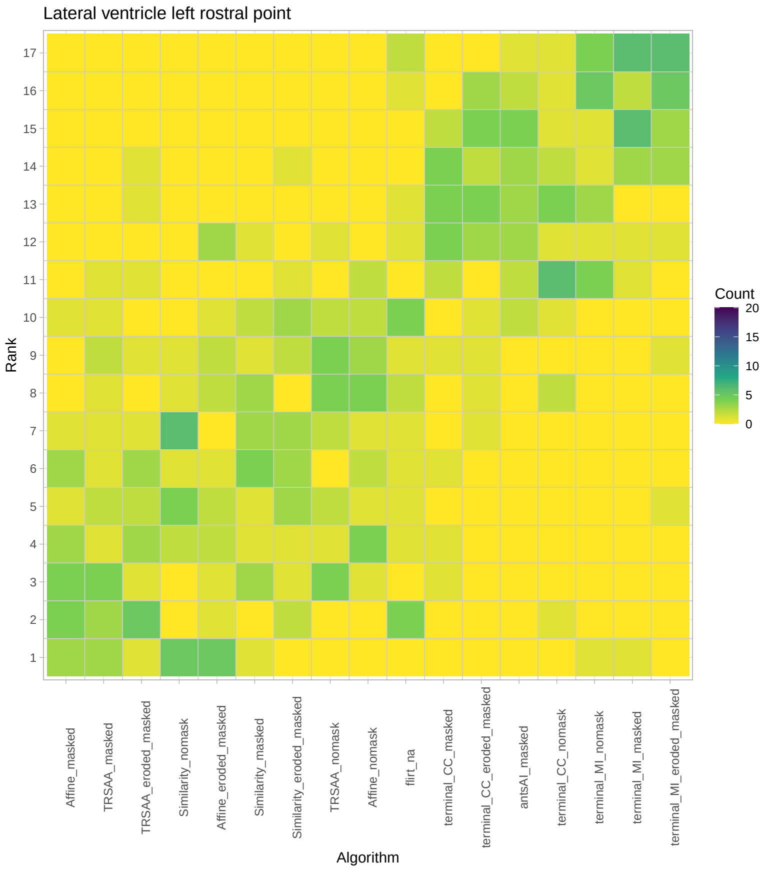

Lateral ventricle left rostral point
Rank
17
16
15
14
13
12
11
10
9
8
7
6
5
4
3
2
1
Algorithm
Affine_masked
TRSAA_masked
TRSAA_eroded_masked
Similarity_nomask
Affine_eroded_masked
Similarity_masked
Similarity_eroded_masked
TRSAA_nomask
Affine_nomask
flirt_na
terminal_CC_masked
terminal_CC_eroded_masked
antsAI_masked
terminal_CC_nomask
terminal_MI_nomask
terminal_MI_masked
terminal_MI_eroded_masked
Count
20
15
10
5
0

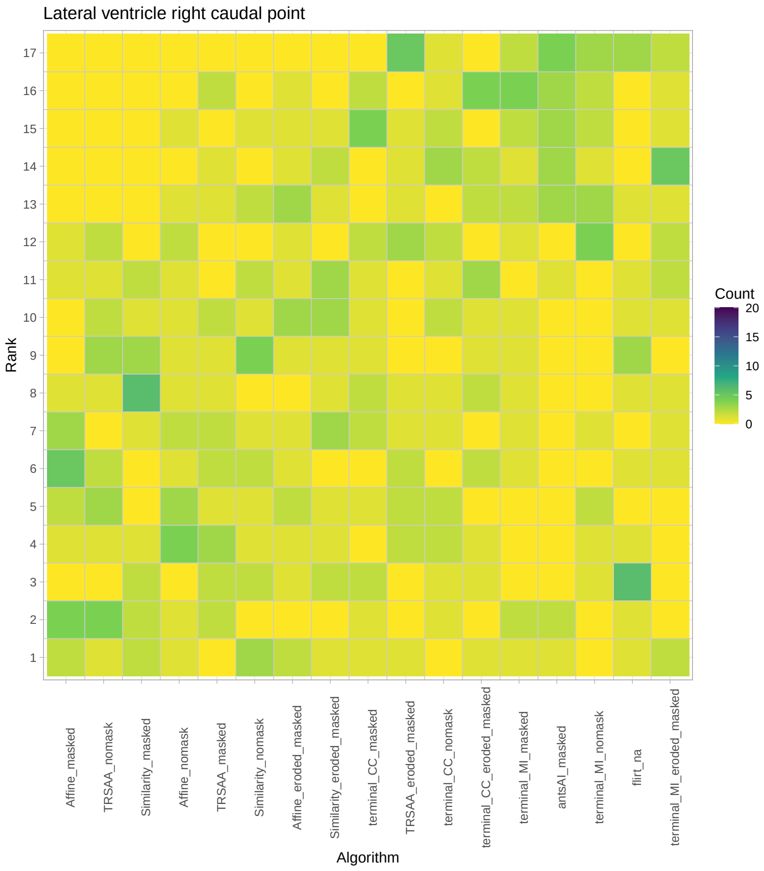

Lateral ventricle right caudal point
Rank
Algorithm
Count
20
15
10
5
0
17
16
15
14
13
12
11
10
9
8
7
6
5
4
3
2
1
Affine_masked
TRSAA_nomask
Similarity_masked
Affine_nomask
TRSAA_masked
Similarity_nomask
Affine_eroded_masked
Similarity_eroded_masked
terminal_CC_masked
TRSAA_eroded_masked
terminal_CC_nomask
terminal_CC_eroded_masked
terminal_MI_masked
antsAI_masked
terminal_MI_nomask
flirt_na
terminal_MI_eroded_masked

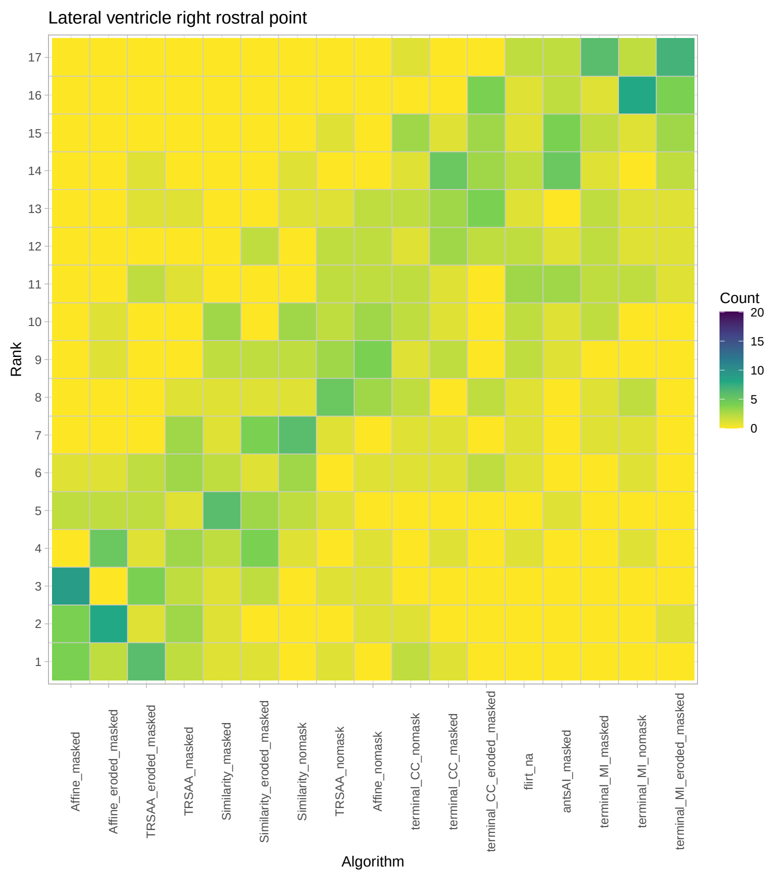
Lateral ventricle right rostral point
Rank
17
16
15
14
13
12
11
10
9
8
7
6
5
4
3
2
1
Count
20
15
10
5
0
Affine_masked
Affine_eroded_masked
TRSAA_eroded_masked
TRSAA_masked
Similarity_masked
Similarity_eroded_masked
Similarity_nomask
TRSAA_nomask
Affine_nomask
terminal_CC_nomask
terminal_CC_masked
terminal_CC_eroded_masked
flirt_na
antsAI_masked
terminal_MI_masked
terminal_MI_nomask
terminal_MI_eroded_masked
Algorithm

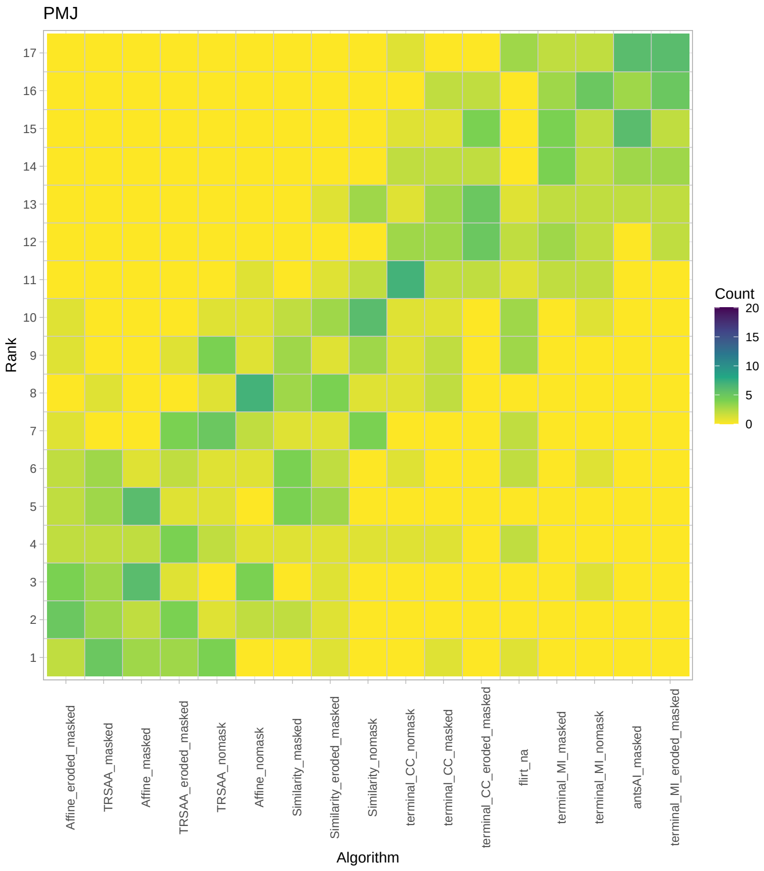
PMJ
Rank
17
16
15
14
13
12
11
10
9
8
7
6
5
4
3
2
1
Affine_eroded_masked
TRSAA_masked
Affine_masked
TRSAA_eroded_masked
TRSAA_nomask
Affine_nomask
Similarity_masked
Similarity_eroded_masked
Similarity_nomask
terminal_CC_nomask
terminal_CC_masked
terminal_CC_eroded_masked
flirt_na
terminal_MI_masked
terminal_MI_nomask
antsAI_masked
terminal_MI_eroded_masked
Algorithm
Count
20
15
10
5
0

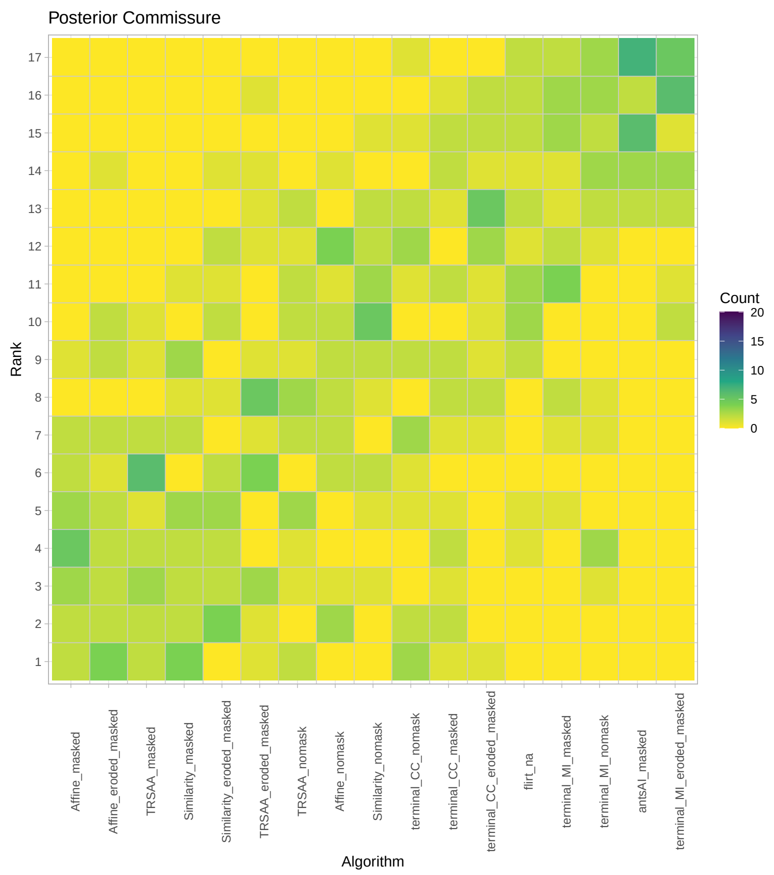
Posterior Commissure
Rank
17
16
15
14
13
12
11
10
9
8
7
6
5
4
3
2
1
Affine_masked
Affine_eroded_masked
TRSAA_masked
Similarity_masked
Similarity_eroded_masked
TRSAA_eroded_masked
TRSAA_nomask
Affine_nomask
Similarity_nomask
terminal_CC_nomask
terminal_CC_masked
terminal_CC_eroded_masked
flirt_na
terminal_MI_masked
terminal_MI_nomask
antsAI_masked
terminal_MI_eroded_masked
Algorithm
Count
20
15
10
5
0

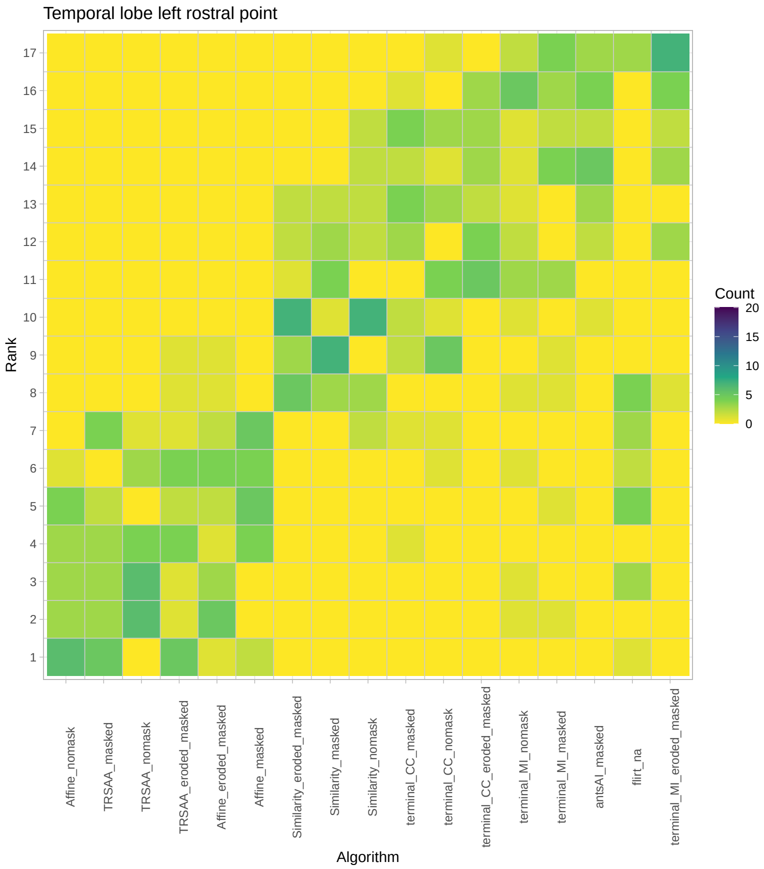

Temporal lobe left rostral point
Rank
Algorithm
Count
20
15
10
5
0
17
16
15
14
13
12
11
10
9
8
7
6
5
4
3
2
1
Affine_nomask
TRSAA_masked
TRSAA_nomask
TRSAA_eroded_masked
Affine_eroded_masked
Affine_masked
Similarity_eroded_masked
Similarity_masked
Similarity_nomask
terminal_CC_masked
terminal_CC_nomask
terminal_CC_eroded_masked
terminal_MI_nomask
terminal_MI_masked
antsAI_masked
flirt_na
terminal_MI_eroded_masked

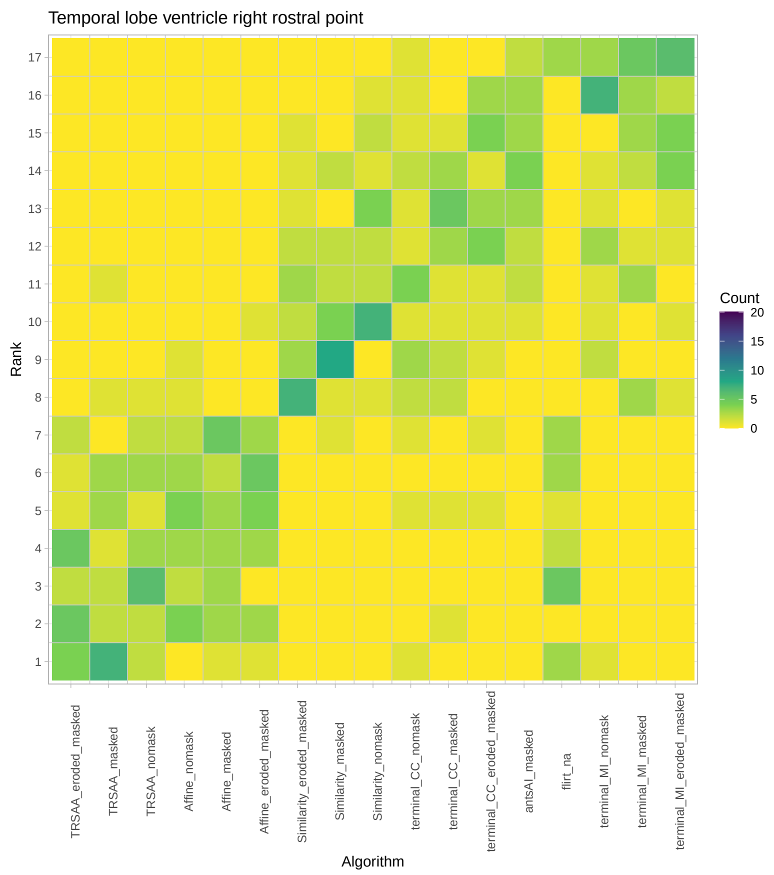

Temporal lobe ventricle right rostral point
Rank
Algorithm
Count
20
15
10
5
0
17
16
15
14
13
12
11
10
9
8
7
6
5
4
3
2
1
TRSAA_eroded_masked
TRSAA_masked
TRSAA_nomask
Affine_nomask
Affine_masked
Affine_eroded_masked
Similarity_eroded_masked
Similarity_masked
Similarity_nomask
terminal_CC_nomask
terminal_CC_masked
terminal_CC_eroded_masked
antsAI_masked
flirt_na
terminal_MI_nomask
terminal_MI_masked
terminal_MI_eroded_masked

3 Visualization of ranking stability

3.1 *Blob plot* for visualizing ranking stability based on bootstrap sampling

Algorithms are color-coded, and the area of each blob at position $(A_i, \text{rank } j)$ is proportional to the relative frequency A_i achieved rank j across $b = 1000$ bootstrap samples. The median rank for each algorithm is indicated by a black cross. 95% bootstrap intervals across bootstrap samples are indicated by black lines.

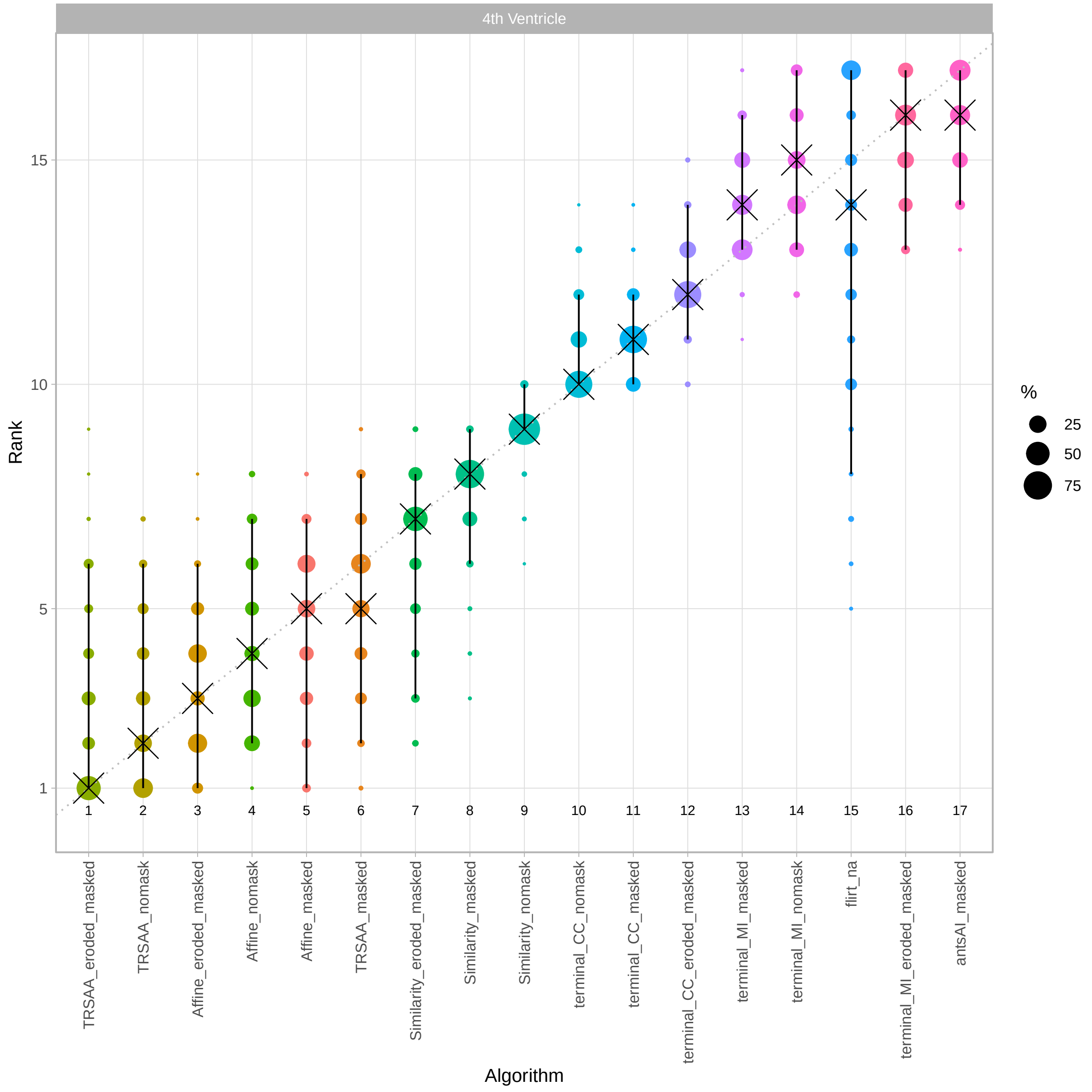

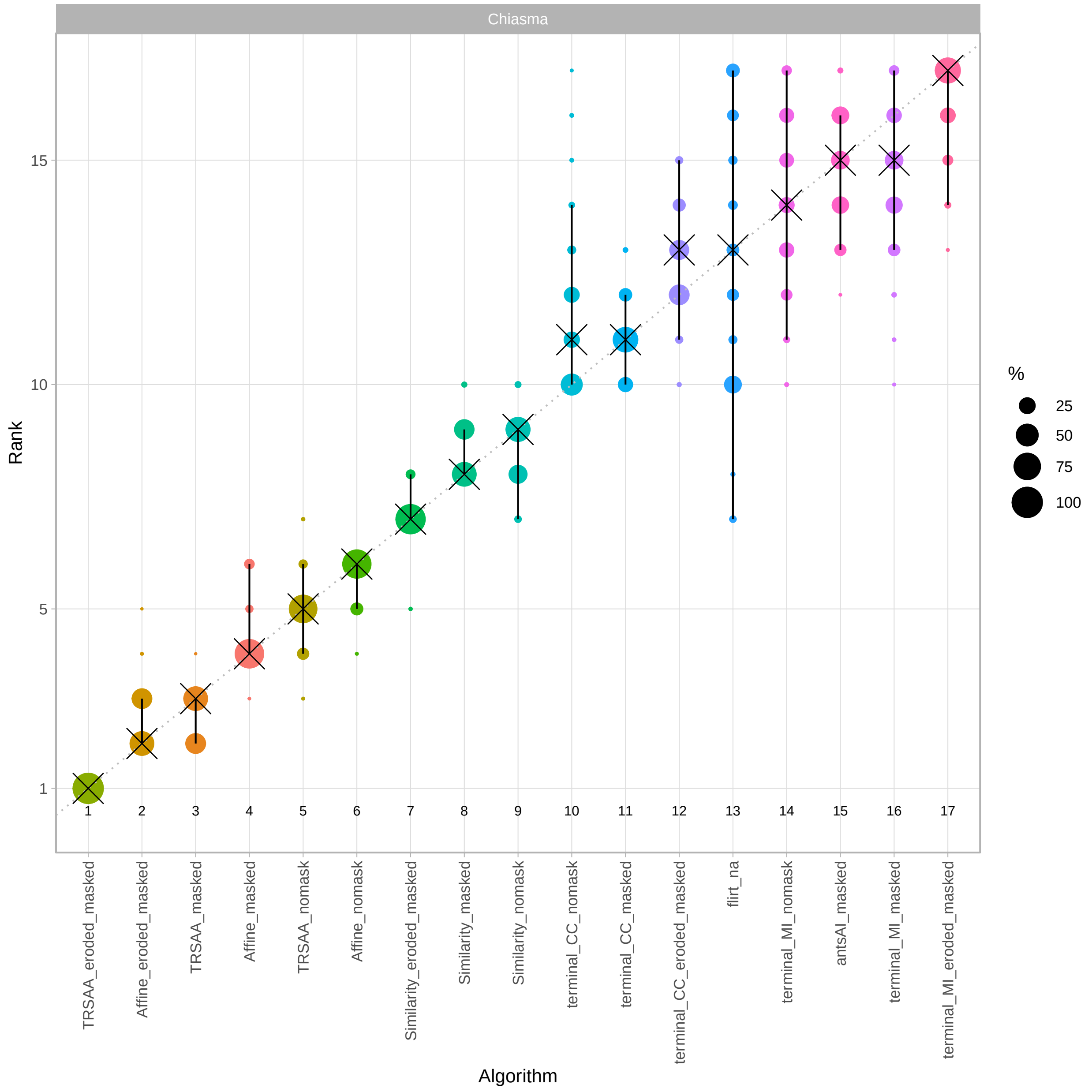

Chiasma
Rank
15
10
5
1
1
2
3
4
5
6
7
8
9
10
11
12
13
14
15
16
17
TRSAA_eroded_masked
Affine_eroded_masked
TRSAA_masked
Affine_masked
TRSAA_nomask
Affine_nomask
Similarity_eroded_masked
Similarity_masked
Similarity_nomask
terminal_CC_nomask
terminal_CC_masked
terminal_CC_eroded_masked
flirt_na
terminal_MI_nomask
antsAI_masked
terminal_MI_masked
terminal_MI_eroded_masked
Algorithm
%
25
50
75
100

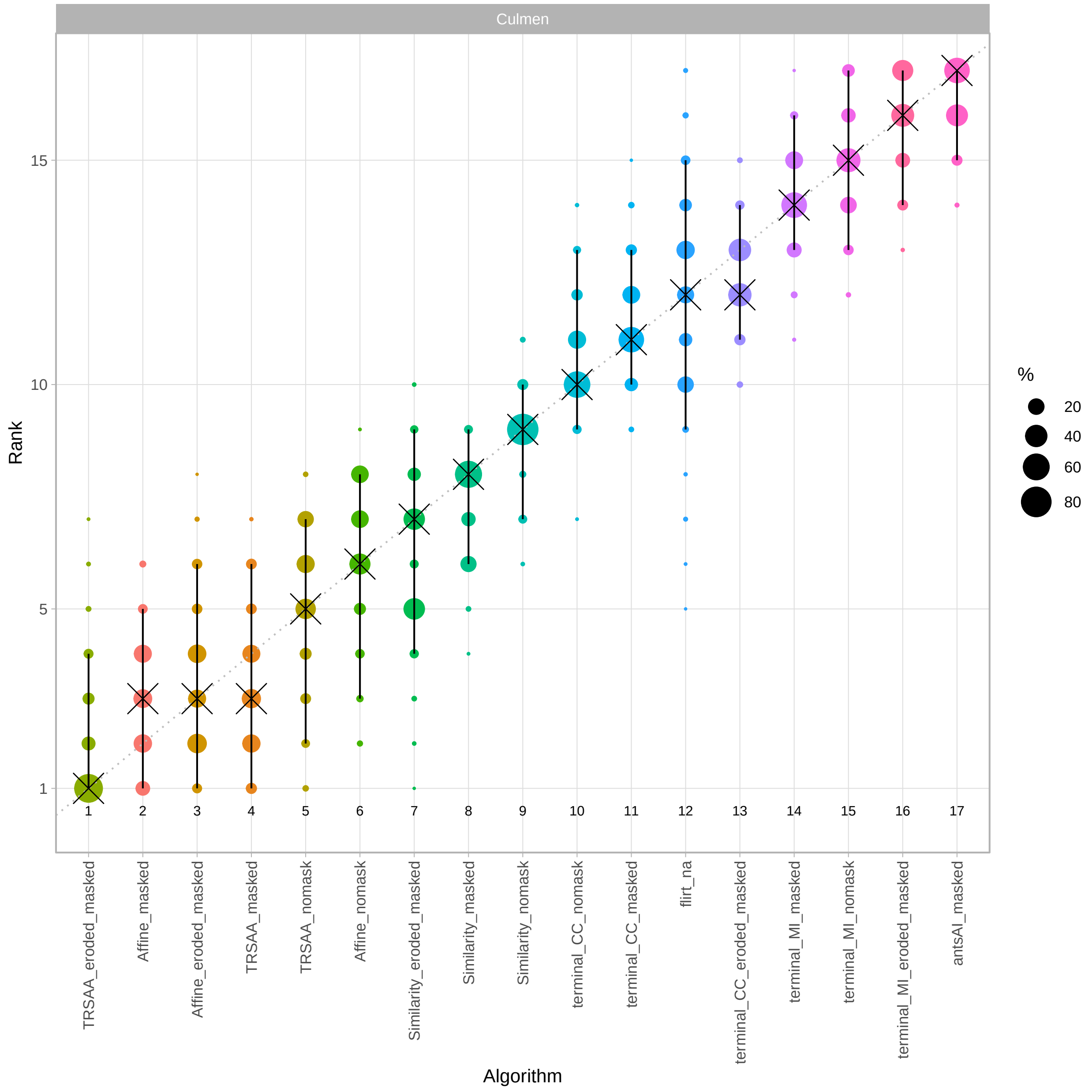
Culmen
Rank
15
10
5
1
1
2
3
4
5
6
7
8
9
10
11
12
13
14
15
16
17
%
20
40
60
80
TRSAA_eroded_masked
Affine_masked
Affine_eroded_masked
TRSAA_masked
TRSAA_nomask
Affine_nomask
Similarity_eroded_masked
Similarity_masked
Similarity_nomask
terminal_CC_nomask
terminal_CC_masked
flirt_na
terminal_CC_eroded_masked
terminal_MI_masked
terminal_MI_nomask
terminal_MI_eroded_masked
antsAI_masked
Algorithm

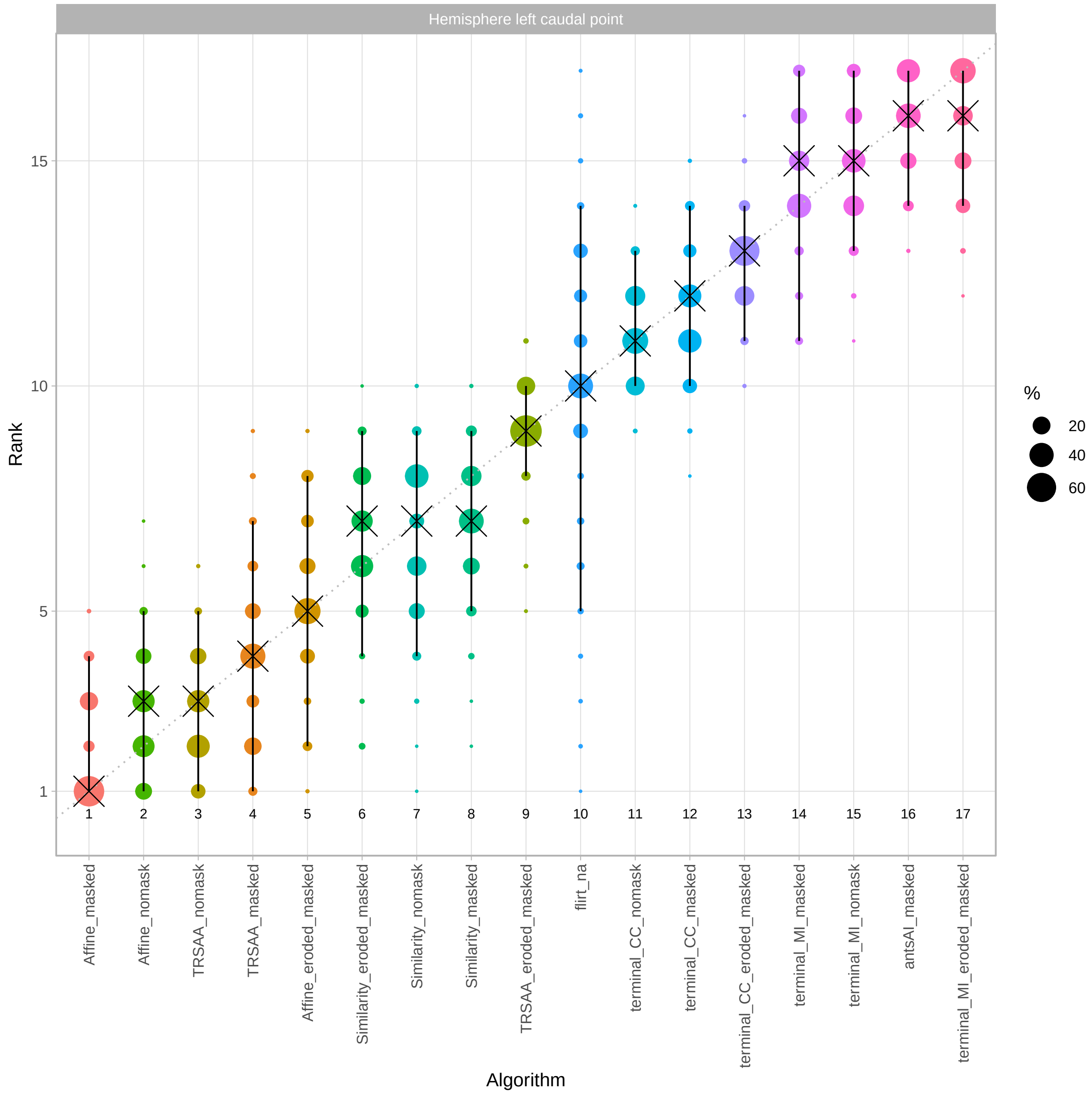
Hemisphere left caudal point
Rank
15
10
5
1
1 2 3 4 5 6 7 8 9 10 11 12 13 14 15 16 17
Affine_masked
Affine_nomask
TRSAA_nomask
TRSAA_masked
Affine_eroded_masked
Similarity_eroded_masked
Similarity_nomask
Similarity_masked
TRSAA_eroded_masked
flirt_na
terminal_CC_nomask
terminal_CC_masked
terminal_CC_eroded_masked
terminal_MI_masked
terminal_MI_nomask
antsAI_masked
terminal_MI_eroded_masked
Algorithm
%
20
40
60

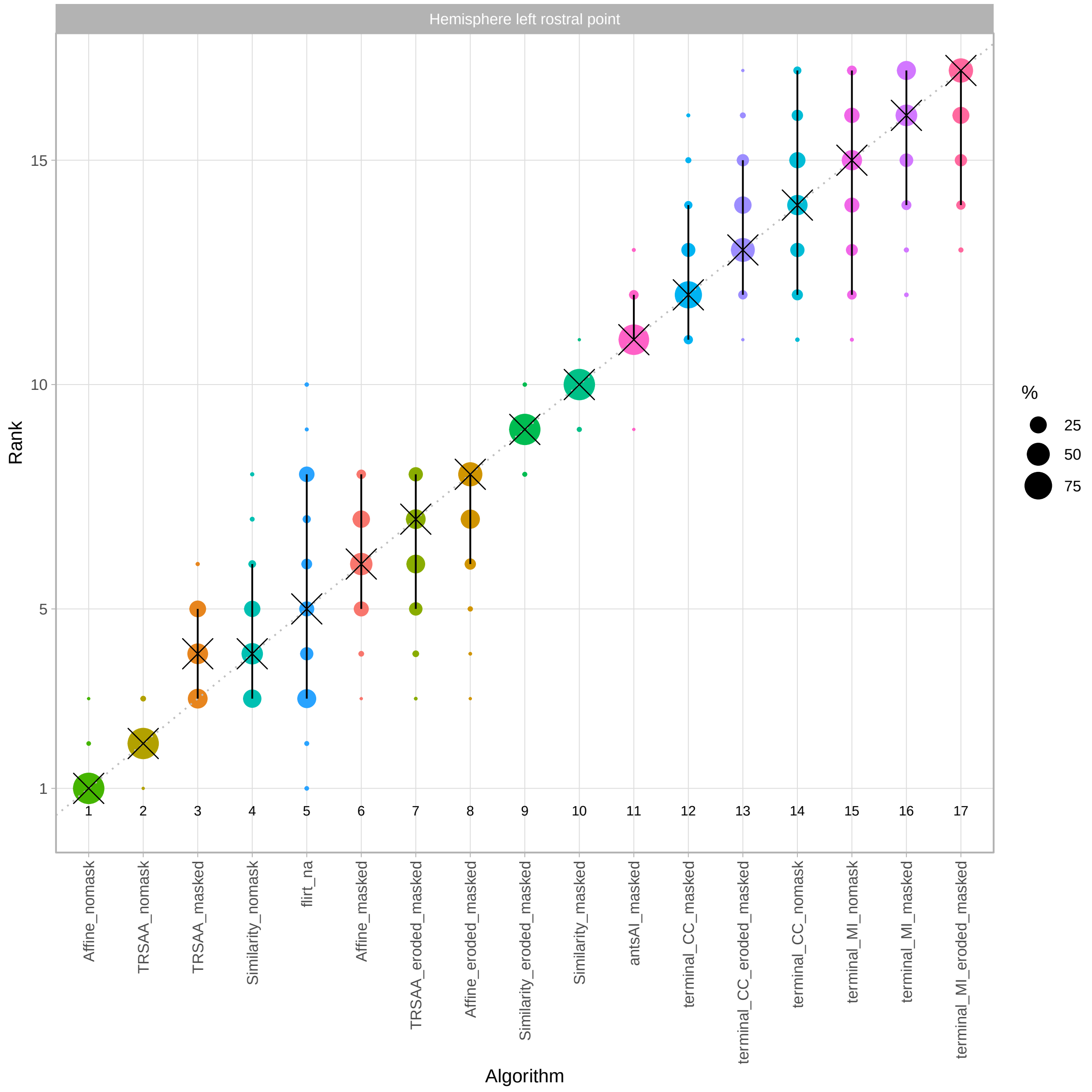
Hemisphere left rostral point
Rank
Algorithm
%
25
50
75
1
5
10
15
1 2 3 4 5 6 7 8 9 10 11 12 13 14 15 16 17
Affine_nomask
TRSAA_nomask
TRSAA_masked
Similarity_nomask
flirt_na
Affine_masked
TRSAA_eroded_masked
Affine_eroded_masked
Similarity_eroded_masked
Similarity_masked
antsAI_masked
terminal_CC_masked
terminal_CC_eroded_masked
terminal_CC_nomask
terminal_MI_nomask
terminal_MI_masked
terminal_MI_eroded_masked

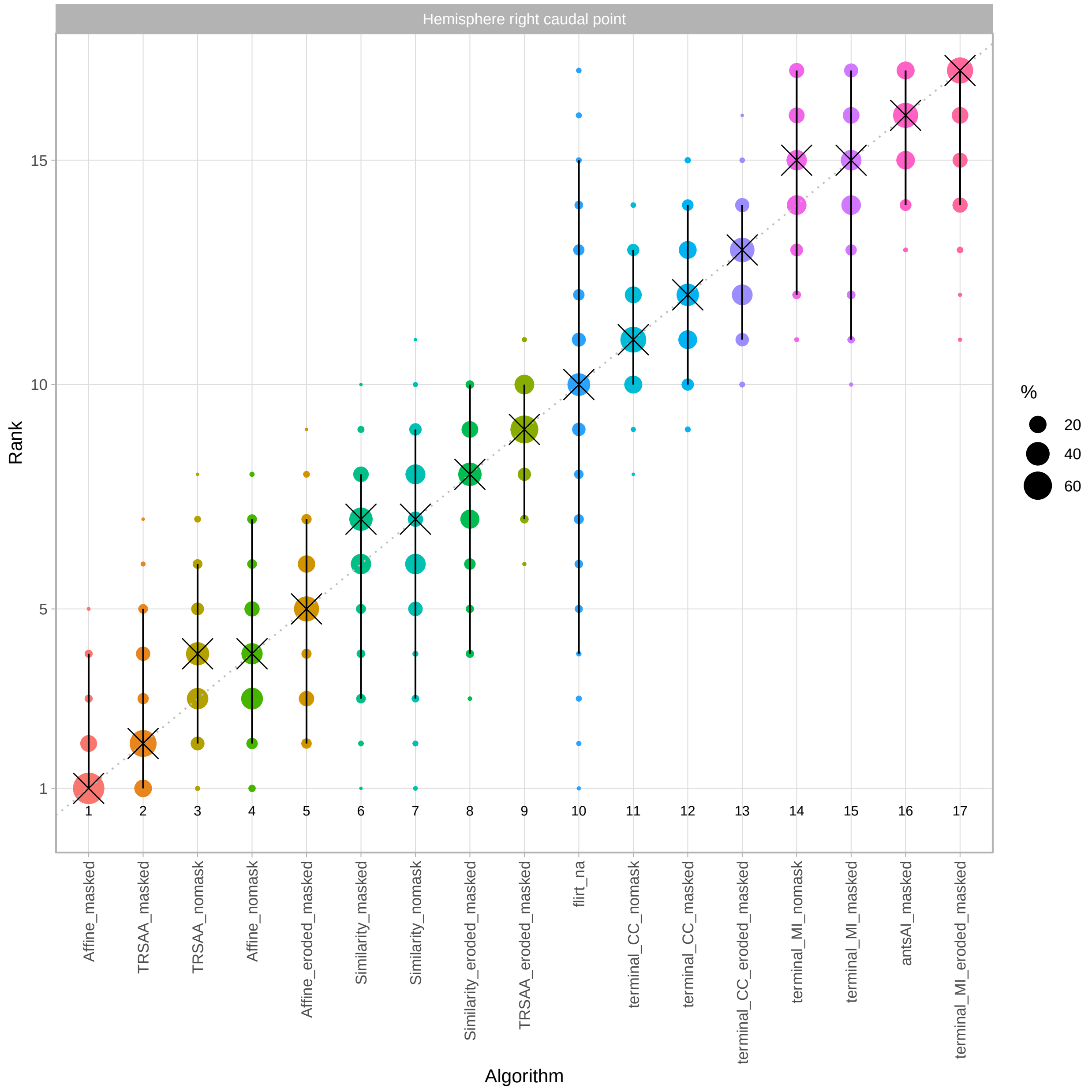

Hemisphere right caudal point
Rank
Algorithm
%
20
40
60
1
5
10
15
1
2
3
4
5
6
7
8
9
10
11
12
13
14
15
16
17
Affine_masked
TRSAA_masked
TRSAA_nomask
Affine_nomask
Affine_eroded_masked
Similarity_masked
Similarity_nomask
Similarity_eroded_masked
TRSAA_eroded_masked
flirt_na
terminal_CC_nomask
terminal_CC_masked
terminal_CC_eroded_masked
terminal_MI_nomask
terminal_MI_masked
antsAI_masked
terminal_MI_eroded_masked

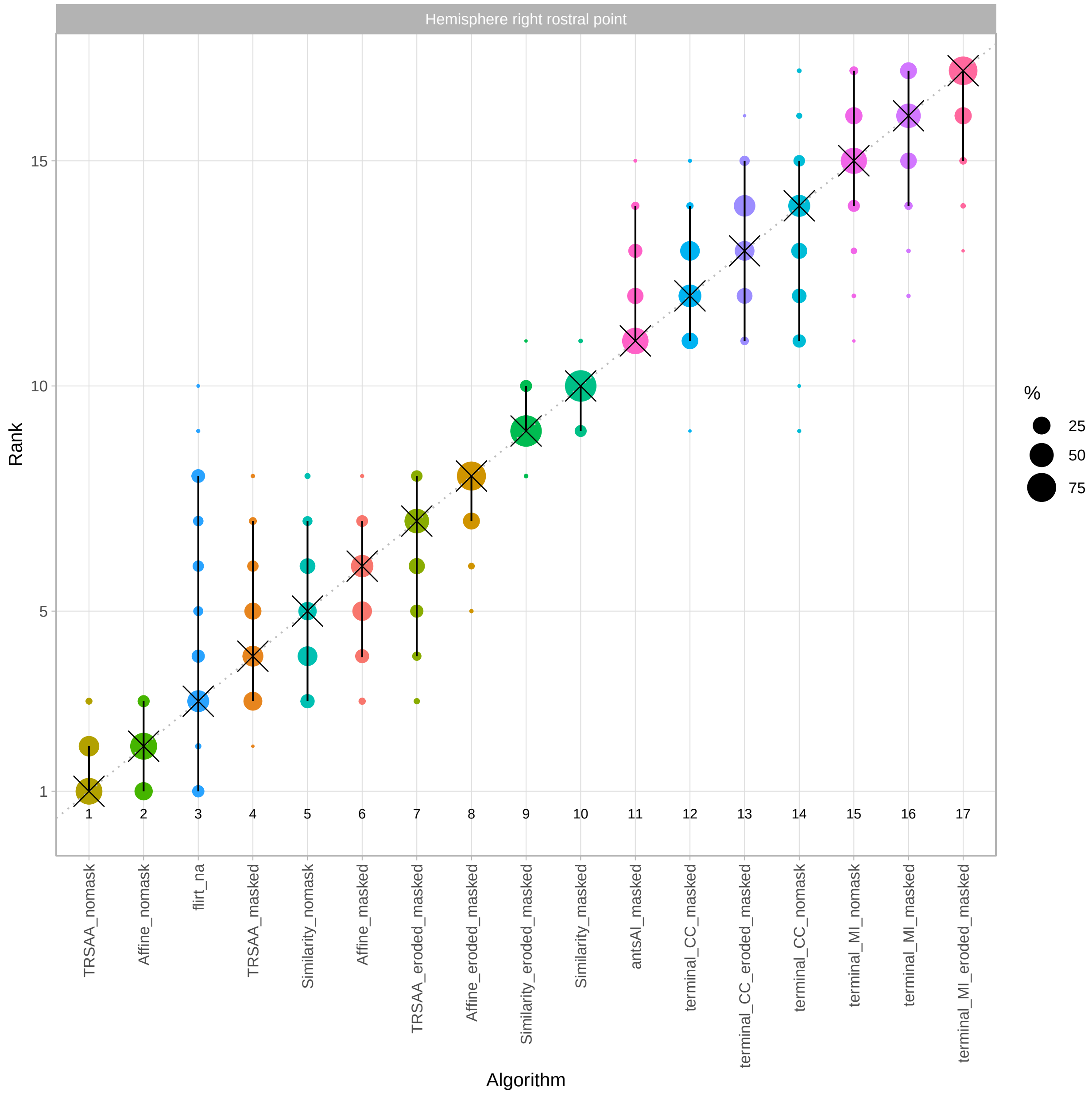

Hemisphere right rostral point
Rank
1
5
10
15
1
2
3
4
5
6
7
8
9
10
11
12
13
14
15
16
17
TRSAA_nomask
Affine_nomask
flirt_na
TRSAA_masked
Similarity_nomask
Affine_masked
TRSAA_eroded_masked
Affine_eroded_masked
Similarity_eroded_masked
Similarity_masked
antsAI_masked
terminal_CC_masked
terminal_CC_eroded_masked
terminal_CC_nomask
terminal_MI_nomask
terminal_MI_masked
terminal_MI_eroded_masked
Algorithm
%
25
50
75

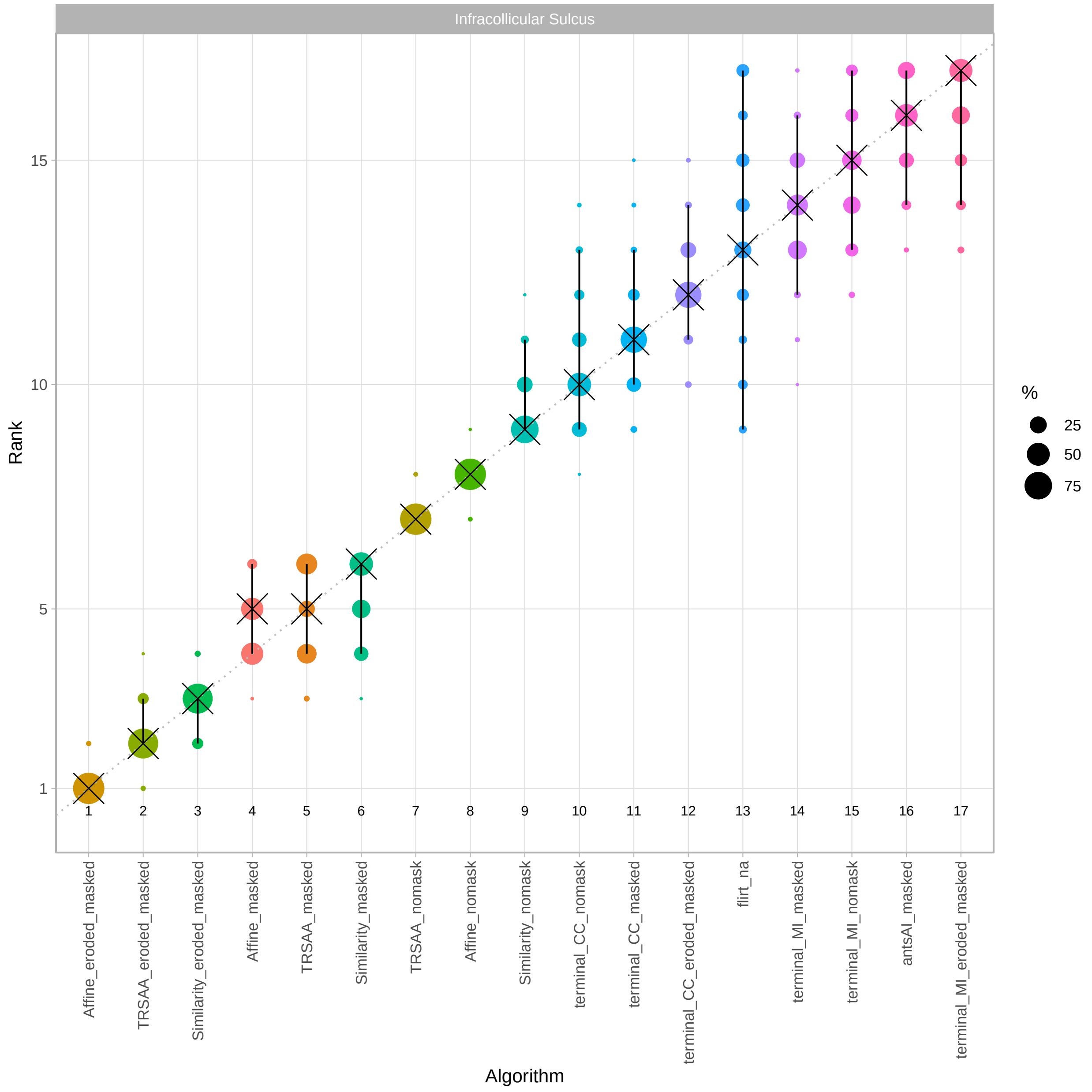

Infracollicular Sulcus
Rank
Algorithm
%
25
50
75
Affine_eroded_masked
TRSAA_eroded_masked
Similarity_eroded_masked
Affine_masked
TRSAA_masked
Similarity_masked
TRSAA_nomask
Affine_nomask
Similarity_nomask
terminal_CC_nomask
terminal_CC_masked
terminal_CC_eroded_masked
flirt_na
terminal_MI_masked
terminal_MI_nomask
antsAI_masked
terminal_MI_eroded_masked

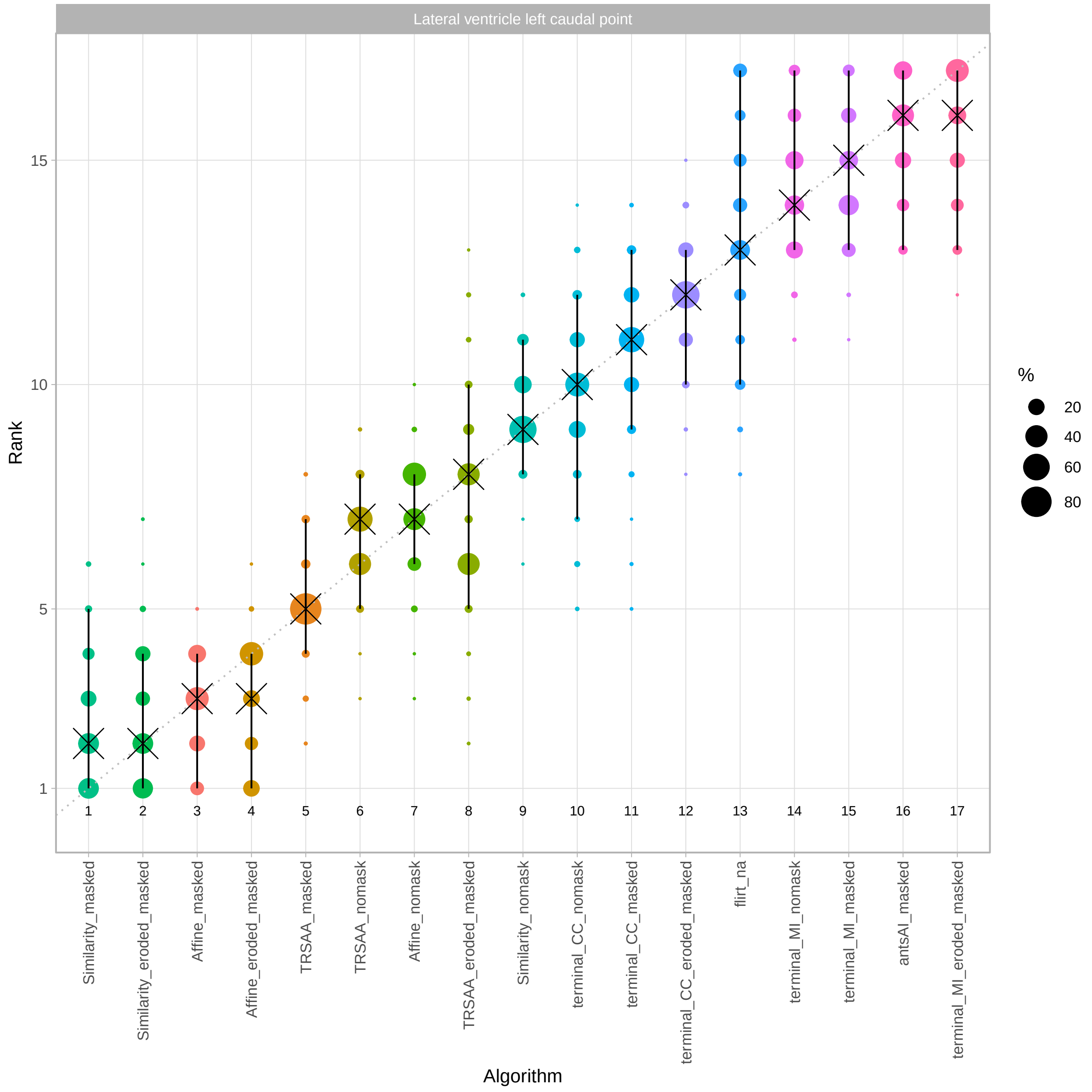

Lateral ventricle left caudal point
Rank
1
5
10
15
1 2 3 4 5 6 7 8 9 10 11 12 13 14 15 16 17
Similarity_masked
Similarity_eroded_masked
Affine_masked
Affine_eroded_masked
TRSAA_masked
TRSAA_nomask
Affine_nomask
TRSAA_eroded_masked
Similarity_nomask
terminal_CC_nomask
terminal_CC_masked
terminal_CC_eroded_masked
flirt_na
terminal_MI_nomask
terminal_MI_masked
antsAI_masked
terminal_MI_eroded_masked
Algorithm
%
20
40
60
80

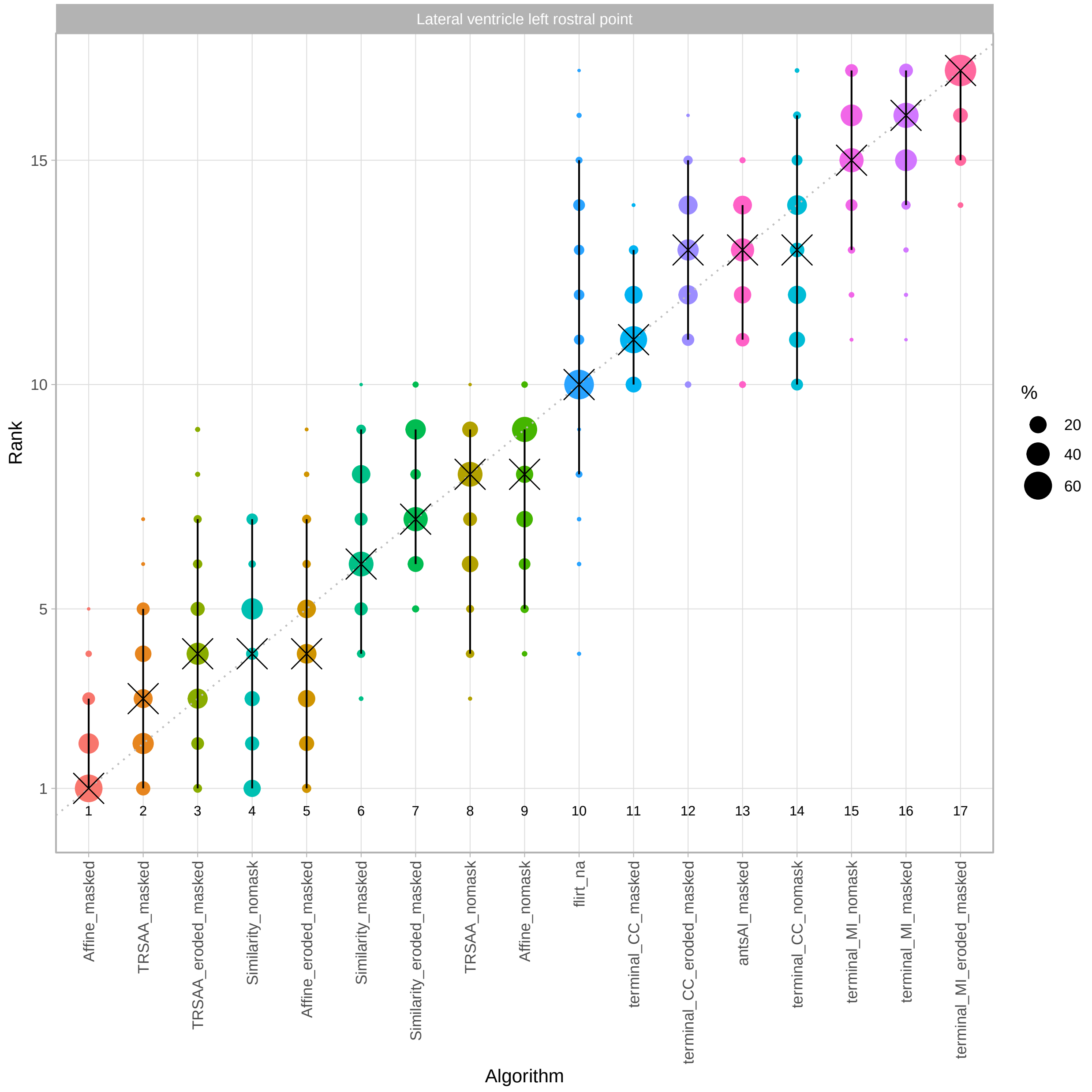

Lateral ventricle left rostral point
Rank
Algorithm
%
20
40
60
1
5
10
15
1 2 3 4 5 6 7 8 9 10 11 12 13 14 15 16 17
Affine_masked
TRSAA_masked
TRSAA_eroded_masked
Similarity_nomask
Affine_eroded_masked
Similarity_masked
Similarity_eroded_masked
TRSAA_nomask
Affine_nomask
flirt_na
terminal_CC_masked
terminal_CC_eroded_masked
antsAI_masked
terminal_CC_nomask
terminal_MI_nomask
terminal_MI_masked
terminal_MI_eroded_masked

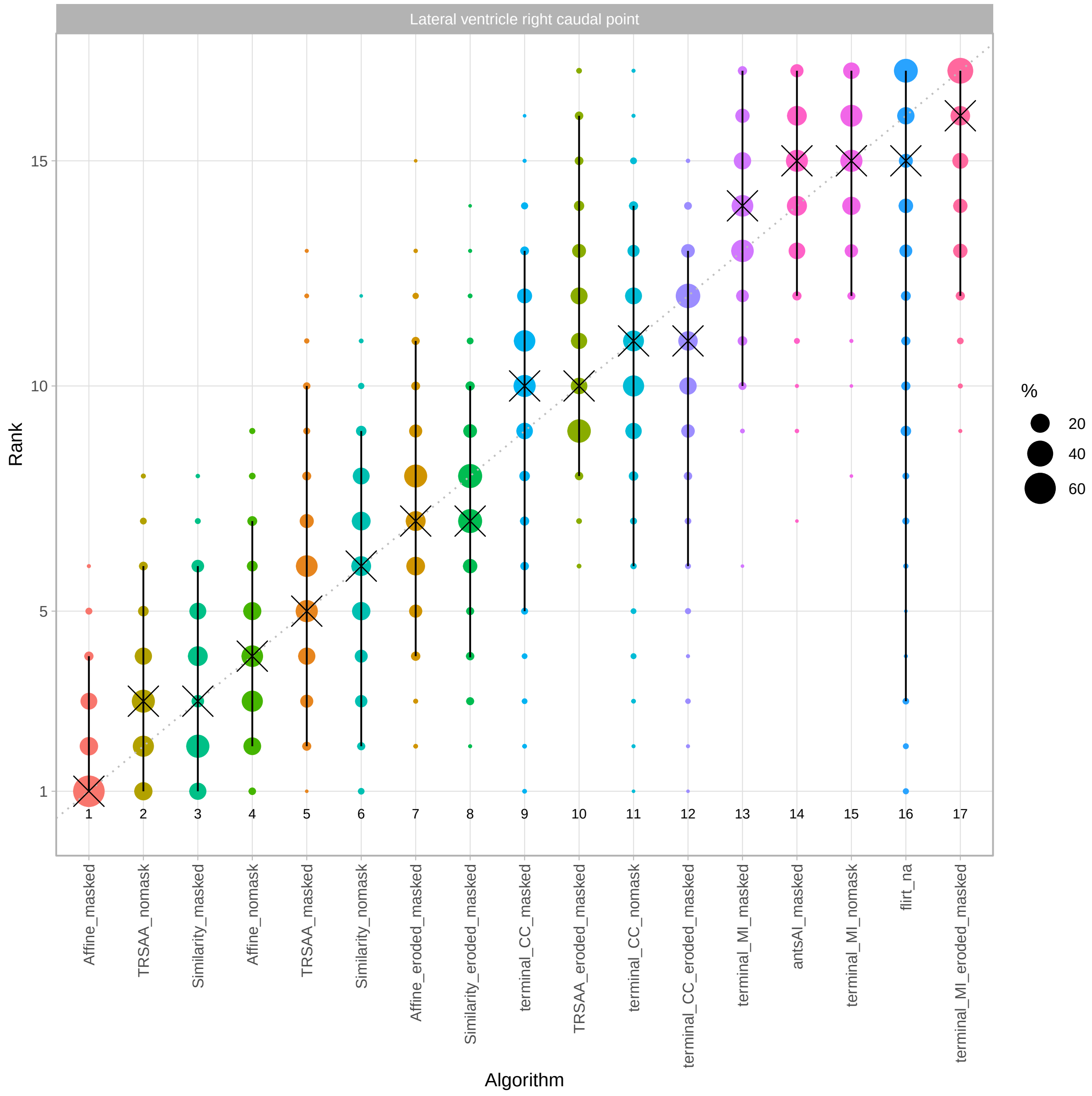
Lateral ventricle right caudal point
Rank
Algorithm
%
20
40
60
1
5
10
15
Affine_masked
TRSAA_nomask
Similarity_masked
Affine_nomask
TRSAA_masked
Similarity_nomask
Affine_eroded_masked
Similarity_eroded_masked
terminal_CC_masked
TRSAA_eroded_masked
terminal_CC_nomask
terminal_CC_eroded_masked
terminal_MI_masked
antsAI_masked
terminal_MI_nomask
flirt_na
terminal_MI_eroded_masked

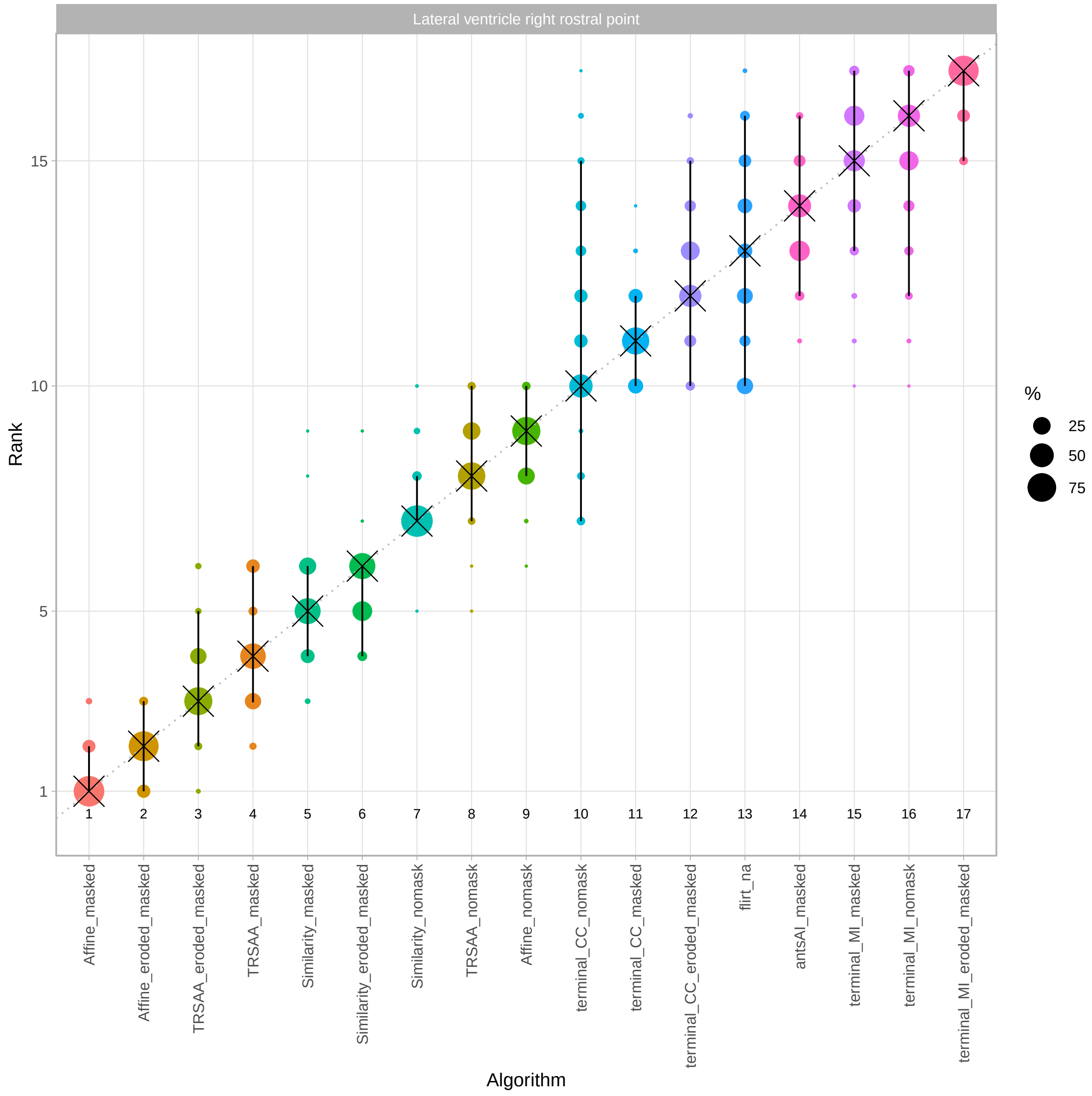

Lateral ventricle right rostral point
Rank
15
10
5
1
1
2
3
4
5
6
7
8
9
10
11
12
13
14
15
16
17
Affine_masked
Affine_eroded_masked
TRSAA_eroded_masked
TRSAA_masked
Similarity_masked
Similarity_eroded_masked
Similarity_nomask
TRSAA_nomask
Affine_nomask
terminal_CC_nomask
terminal_CC_masked
terminal_CC_eroded_masked
flirt_na
antsAI_masked
terminal_MI_masked
terminal_MI_nomask
terminal_MI_eroded_masked
Algorithm
%
25
50
75

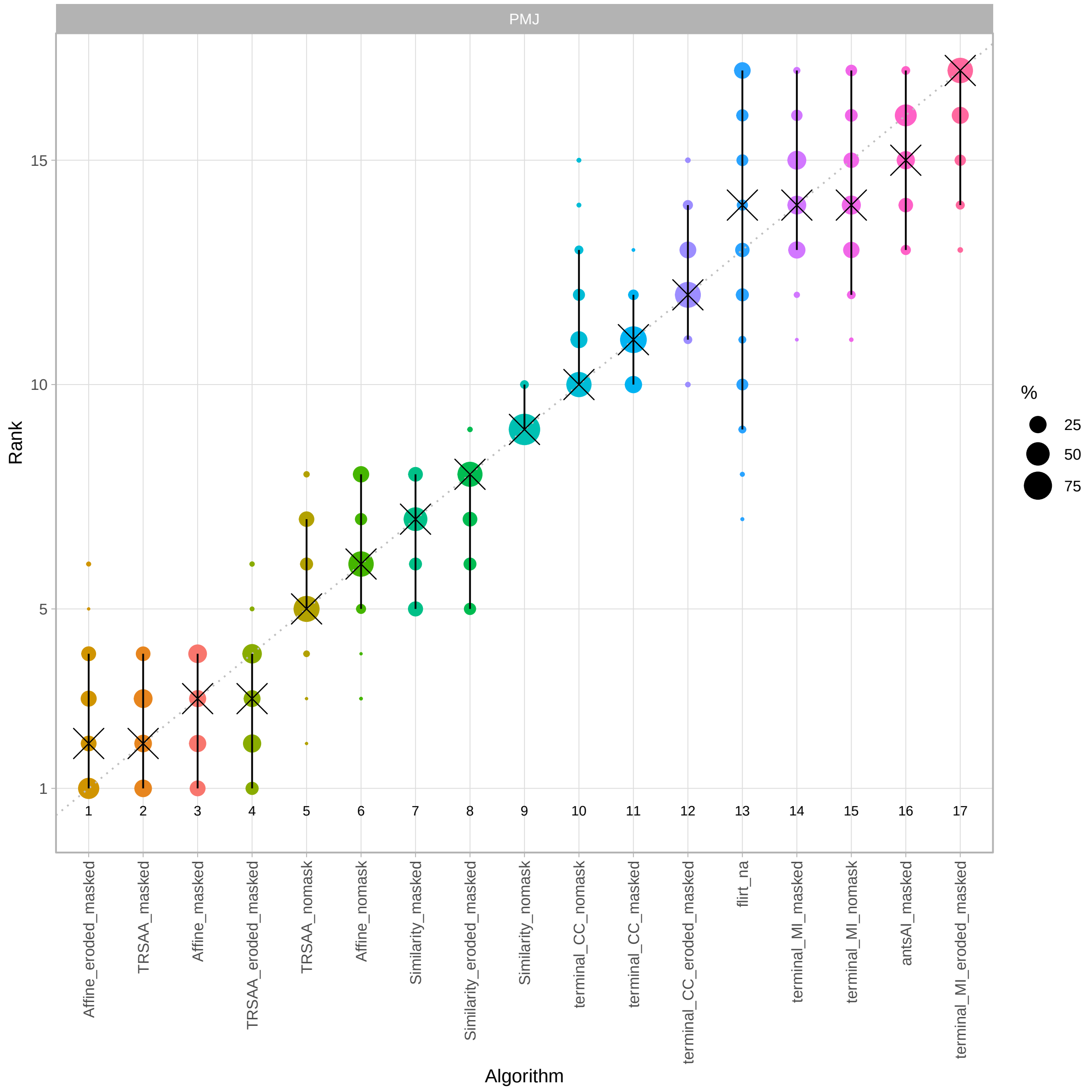

PMJ
Rank
Algorithm
%
25
50
75
1
5
10
15
1
2
3
4
5
6
7
8
9
10
11
12
13
14
15
16
17
Affine_eroded_masked
TRSAA_masked
Affine_masked
TRSAA_eroded_masked
TRSAA_nomask
Affine_nomask
Similarity_masked
Similarity_eroded_masked
Similarity_nomask
terminal_CC_nomask
terminal_CC_masked
terminal_CC_eroded_masked
flirt_na
terminal_MI_masked
terminal_MI_nomask
antsAI_masked
terminal_MI_eroded_masked

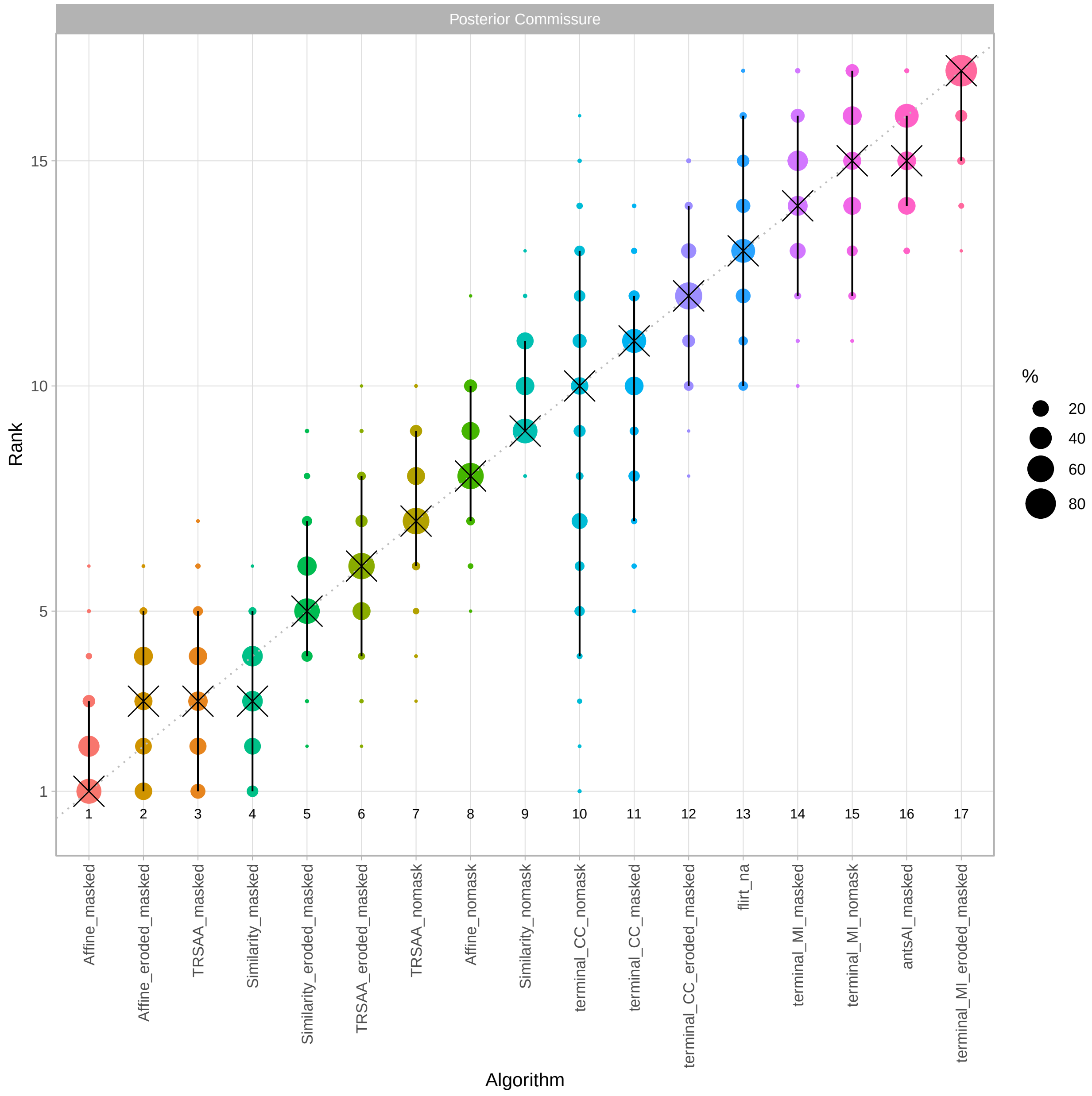
Posterior Commissure
Rank
15
10
5
1
1
2
3
4
5
6
7
8
9
10
11
12
13
14
15
16
17
%
20
40
60
80
Affine_masked
Affine_eroded_masked
TRSAA_masked
Similarity_masked
Similarity_eroded_masked
TRSAA_eroded_masked
TRSAA_nomask
Affine_nomask
Similarity_nomask
terminal_CC_nomask
terminal_CC_masked
terminal_CC_eroded_masked
flirt_na
terminal_MI_masked
terminal_MI_nomask
antsAI_masked
terminal_MI_eroded_masked
Algorithm

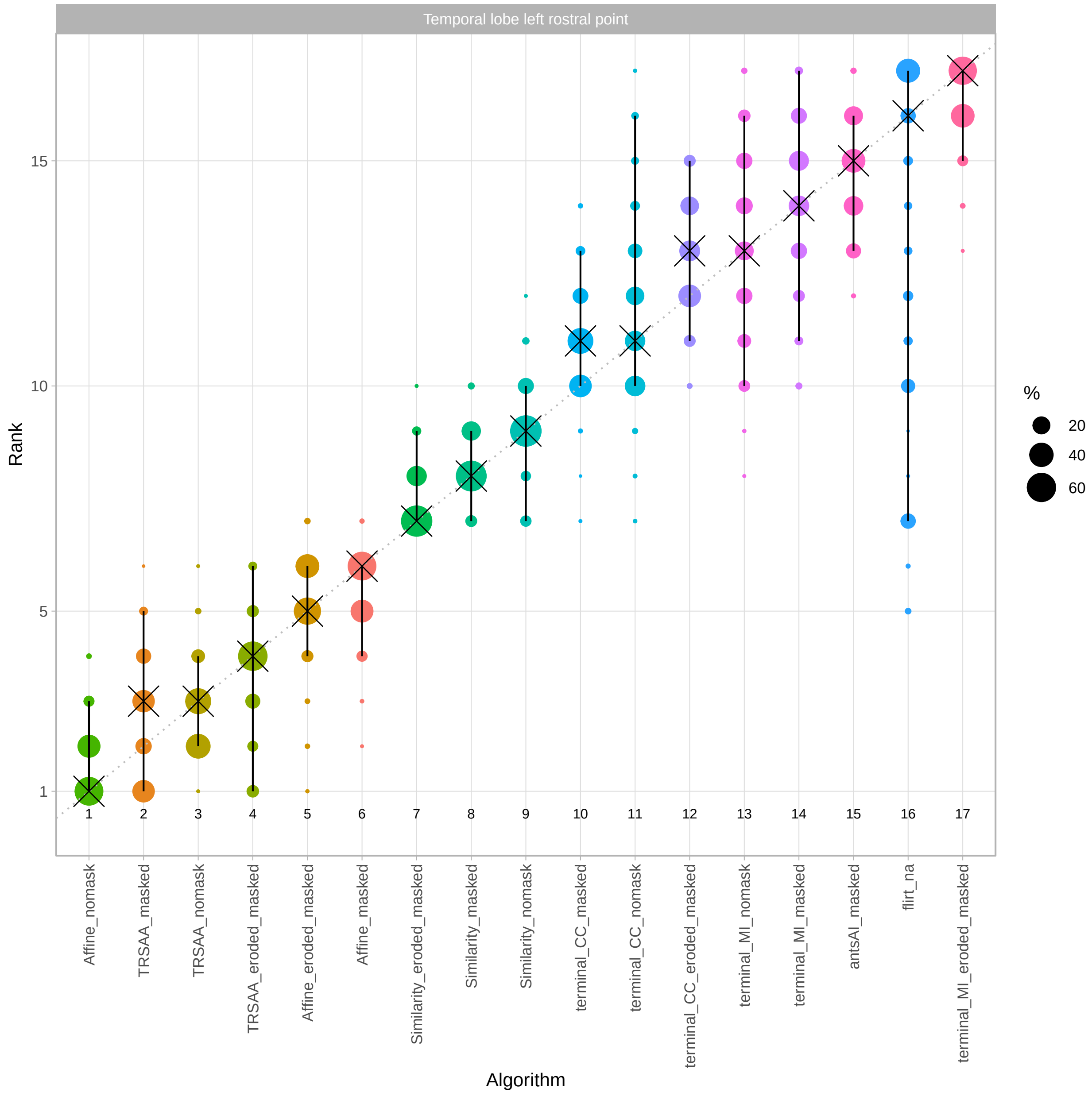

Temporal lobe left rostral point
Rank
Algorithm
%
20
40
60
1
5
10
15
1
2
3
4
5
6
7
8
9
10
11
12
13
14
15
16
17
Affine_nomask
TRSAA_masked
TRSAA_nomask
TRSAA_eroded_masked
Affine_eroded_masked
Affine_masked
Similarity_eroded_masked
Similarity_masked
Similarity_nomask
terminal_CC_masked
terminal_CC_nomask
terminal_CC_eroded_masked
terminal_MI_nomask
terminal_MI_masked
antsAI_masked
flirt_na
terminal_MI_eroded_masked

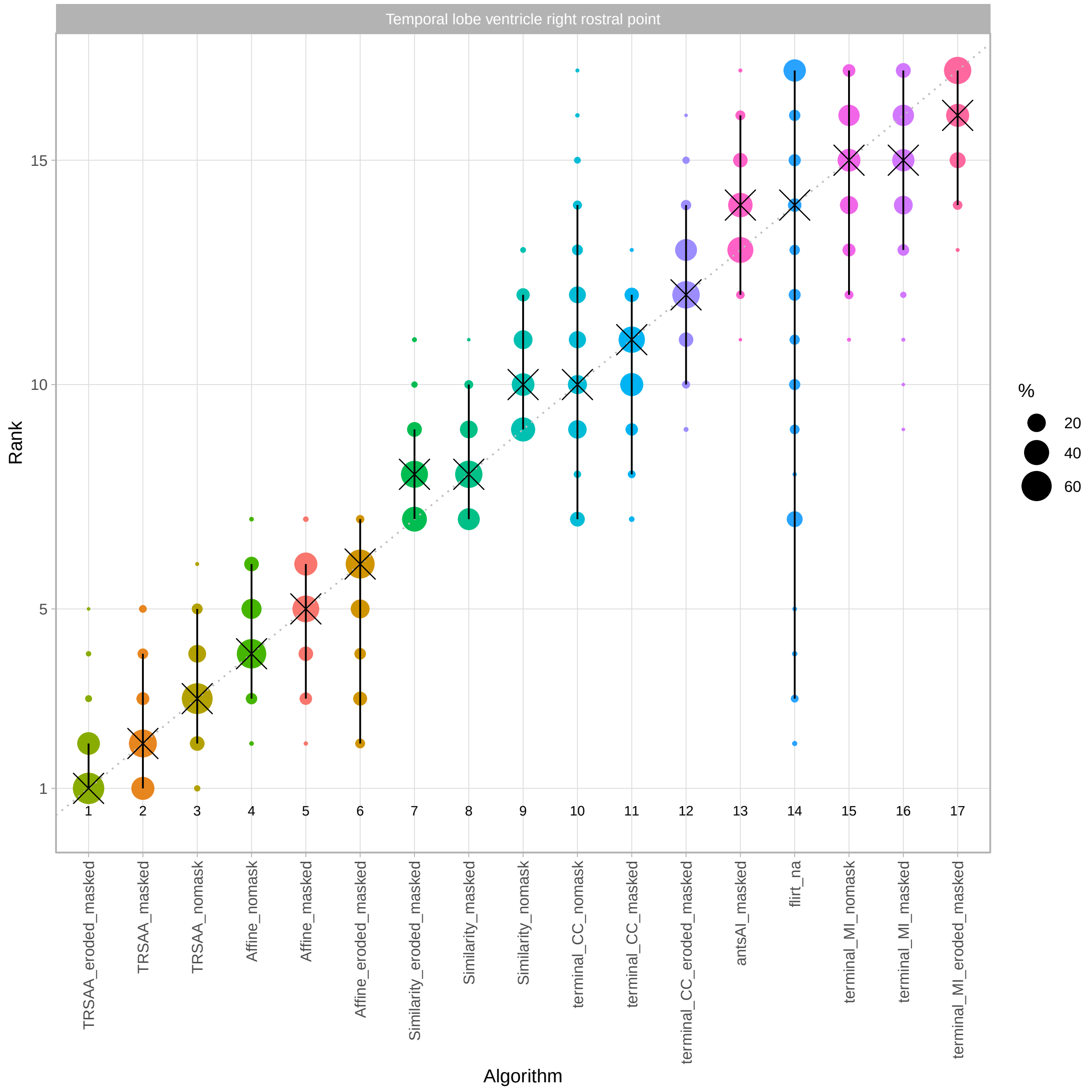

Temporal lobe ventricle right rostral point
Rank
15
10
5
1
1
2
3
4
5
6
7
8
9
10
11
12
13
14
15
16
17
%
20
40
60
TRSAA_eroded_masked
TRSAA_masked
TRSAA_nomask
Affine_nomask
Affine_masked
Affine_eroded_masked
Similarity_eroded_masked
Similarity_masked
Similarity_nomask
terminal_CC_nomask
terminal_CC_masked
terminal_CC_eroded_masked
antsAI_masked
flirt_na
terminal_MI_nomask
terminal_MI_masked
terminal_MI_eroded_masked
Algorithm

3.2 *Violin plot* for visualizing ranking stability based on bootstrapping

The ranking list based on the full assessment data is pairwise compared with the ranking lists based on the individual bootstrap samples (here $b = 1000$ samples). For each pair of rankings, Kendall's τ correlation is computed. Kendall's τ is a scaled index determining the correlation between the lists. It is computed by evaluating the number of pairwise concordances and discordances between ranking lists and produces values between -1 (for inverted order) and 1 (for identical order). A violin plot, which simultaneously depicts a boxplot and a density plot, is generated from the results.

Summary Kendall's tau:

Task	mean	median	q25	q75
Hemisphere left rostral point	0.9223676	0.9264706	0.8970588	0.9411765
Infracollicular Sulcus	0.9293971	0.9264706	0.9117647	0.9558824
Lateral ventricle right rostral point	0.9216912	0.9264706	0.8970588	0.9558824
Chiasma	0.9079265	0.9117647	0.8823529	0.9411765
Hemisphere right rostral point	0.9135000	0.9117647	0.8823529	0.9411765
Culmen	0.8894559	0.8970588	0.8529412	0.9264706
Posterior Commissure	0.8911324	0.8970588	0.8676471	0.9264706
Hemisphere left caudal point	0.8790882	0.8823529	0.8529412	0.9117647
Hemisphere right caudal point	0.8731618	0.8823529	0.8382353	0.9117647
Lateral ventricle left caudal point	0.8801765	0.8823529	0.8529412	0.9264706
PMJ	0.8789853	0.8823529	0.8529412	0.9117647
Temporal lobe left rostral point	0.8728824	0.8823529	0.8382353	0.9117647
Temporal lobe ventricle right rostral point	0.8697794	0.8823529	0.8382353	0.9117647
4th Ventricle	0.8683382	0.8676471	0.8382353	0.9117647
Lateral ventricle left rostral point	0.8659412	0.8676471	0.8382353	0.8970588
Lateral ventricle right caudal point	0.8014265	0.8235294	0.7647059	0.8676471

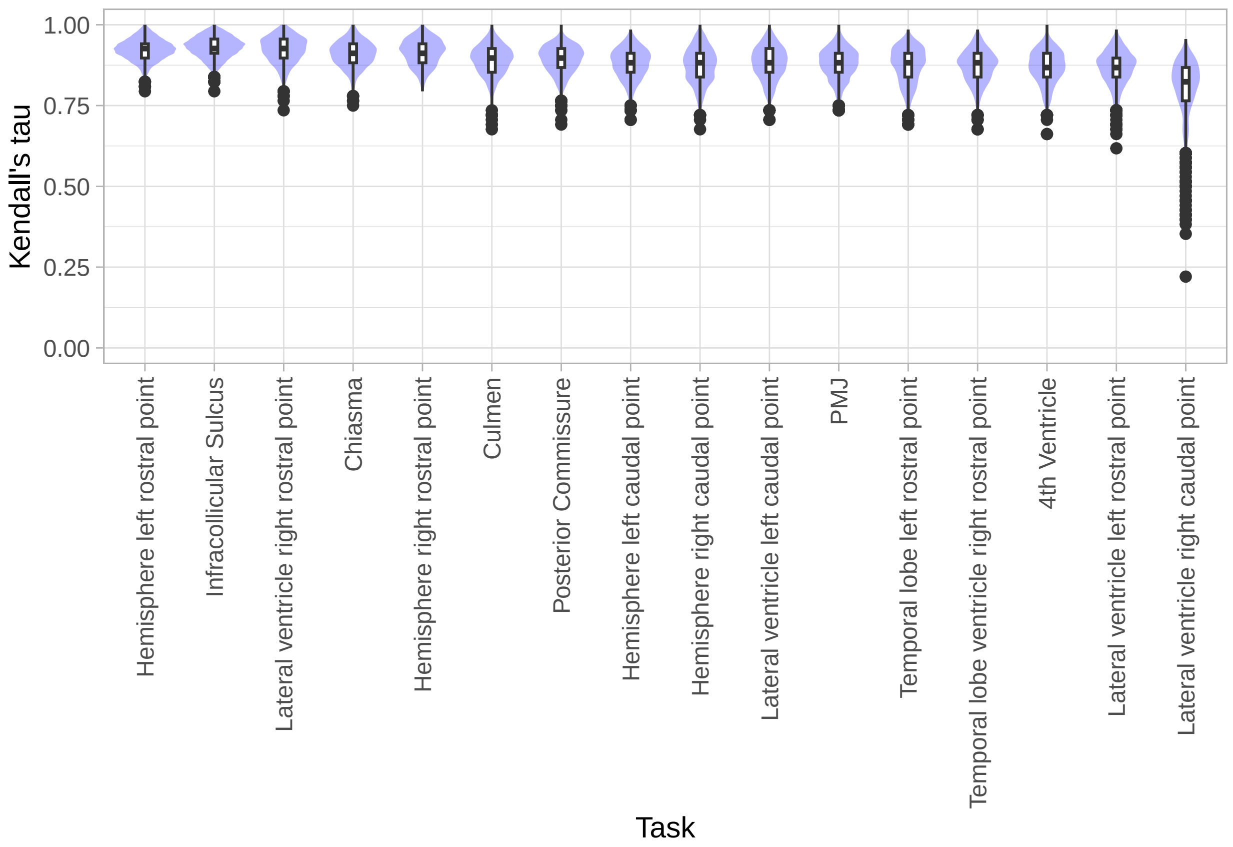
Kendall's tau
1.00
0.75
0.50
0.25
0.00
Hemisphere left rostral point
Infracollicular Sulcus
Lateral ventricle right rostral point
Chiasma
Hemisphere right rostral point
Culmen
Posterior Commissure
Hemisphere left caudal point
Hemisphere right caudal point
Lateral ventricle left caudal point
PMJ
Temporal lobe left rostral point
Temporal lobe ventricle right rostral point
4th Ventricle
Lateral ventricle left rostral point
Lateral ventricle right caudal point
Task

3.3 *Significance maps* for visualizing ranking stability based on statistical significance

Significance maps depict incidence matrices of pairwise significant test results for the one-sided Wilcoxon signed rank test at a 5% significance level with adjustment for multiple testing according to Holm. Yellow shading indicates that metric values from the algorithm on the x-axis were significantly superior to those from the algorithm on the y-axis, blue color indicates no significant difference.

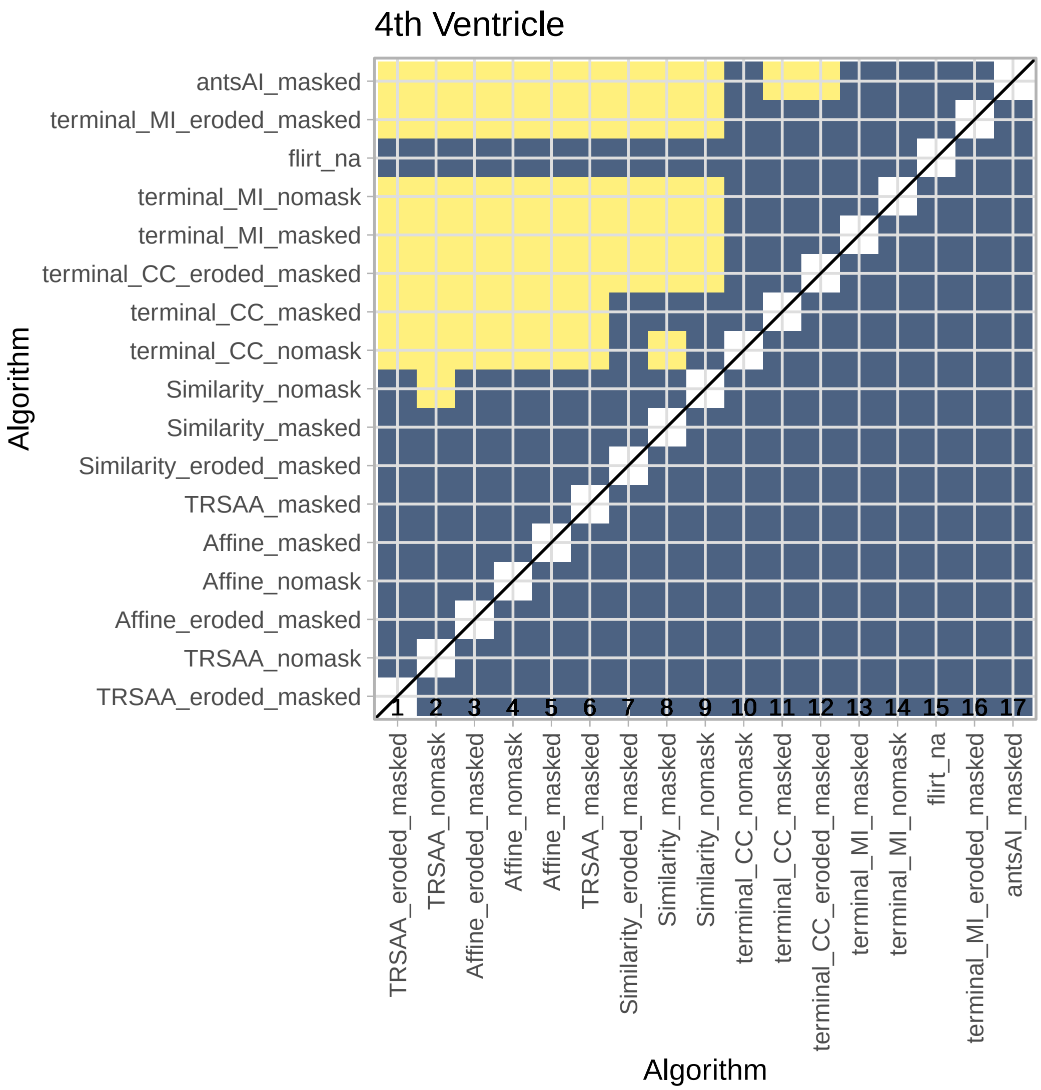

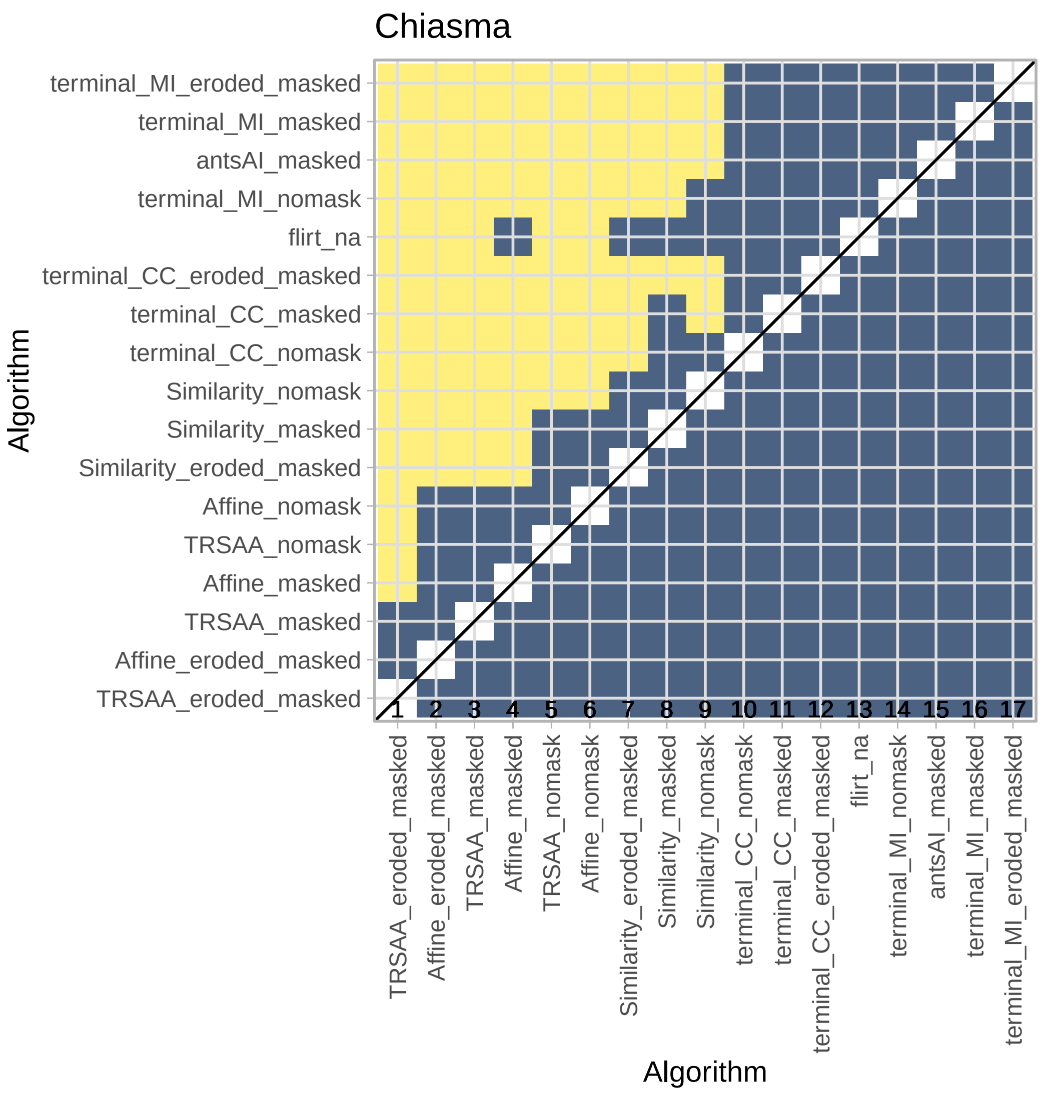

Chiasma
Algorithm
terminal_MI_eroded_masked
terminal_MI_masked
antsAI_masked
terminal_MI_nomask
flirt_na
terminal_CC_eroded_masked
terminal_CC_masked
terminal_CC_nomask
Similarity_nomask
Similarity_masked
Similarity_eroded_masked
Affine_nomask
TRSAA_nomask
Affine_masked
TRSAA_masked
Affine_eroded_masked
TRSAA_eroded_masked
1 2 3 4 5 6 7 8 9 10 11 12 13 14 15 16 17
Algorithm

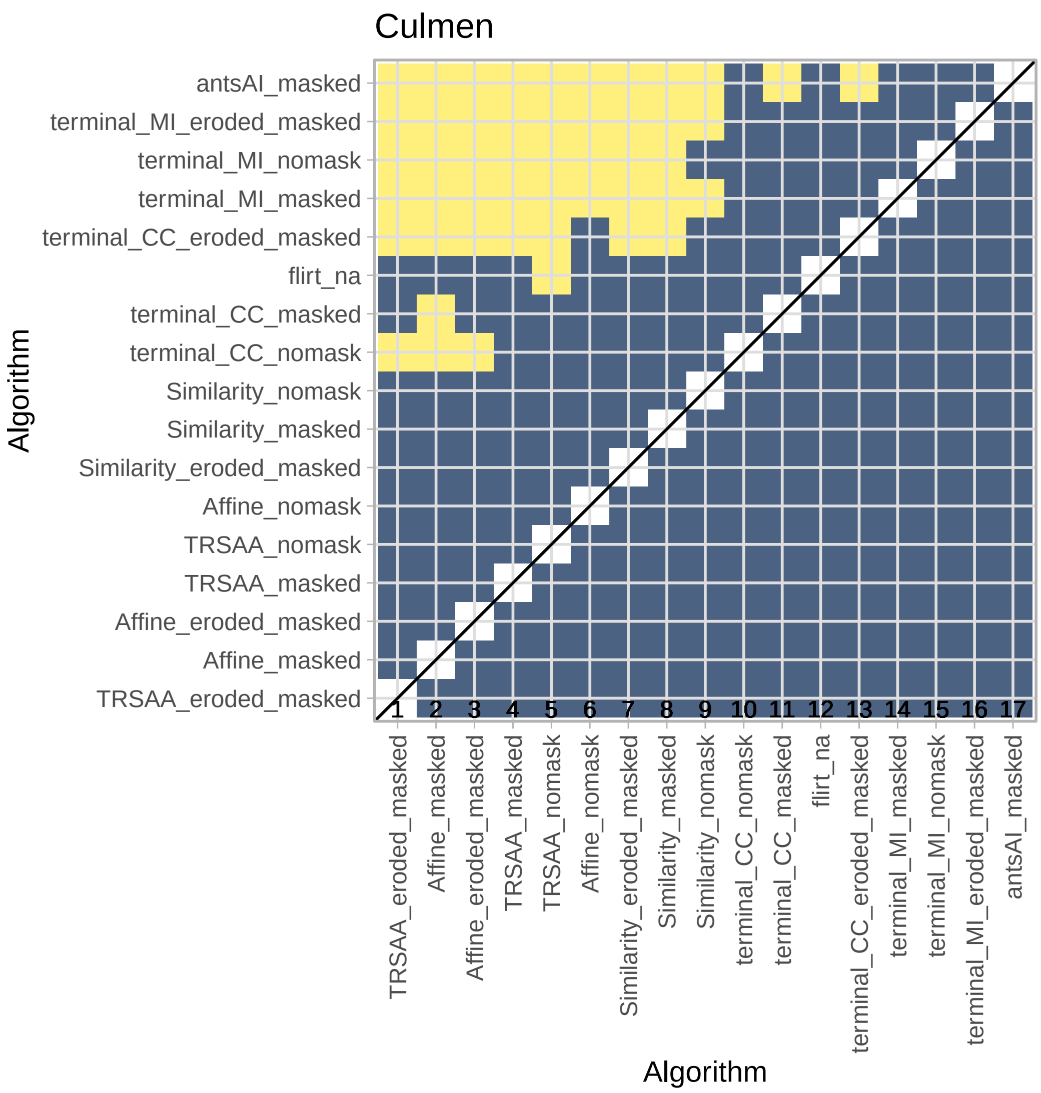

Culmen
Algorithm
antsAI_masked
terminal_MI_eroded_masked
terminal_MI_nomask
terminal_MI_masked
terminal_CC_eroded_masked
flirt_na
terminal_CC_masked
terminal_CC_nomask
Similarity_nomask
Similarity_masked
Similarity_eroded_masked
Affine_nomask
TRSAA_nomask
TRSAA_masked
Affine_eroded_masked
Affine_masked
TRSAA_eroded_masked
1 2 3 4 5 6 7 8 9 10 11 12 13 14 15 16 17
TRSAA_eroded_masked
Affine_masked
Affine_eroded_masked
TRSAA_masked
TRSAA_nomask
Affine_nomask
Similarity_eroded_masked
Similarity_masked
Similarity_nomask
terminal_CC_nomask
terminal_CC_masked
flirt_na
terminal_CC_eroded_masked
terminal_MI_masked
terminal_MI_nomask
terminal_MI_eroded_masked
antsAI_masked
Algorithm

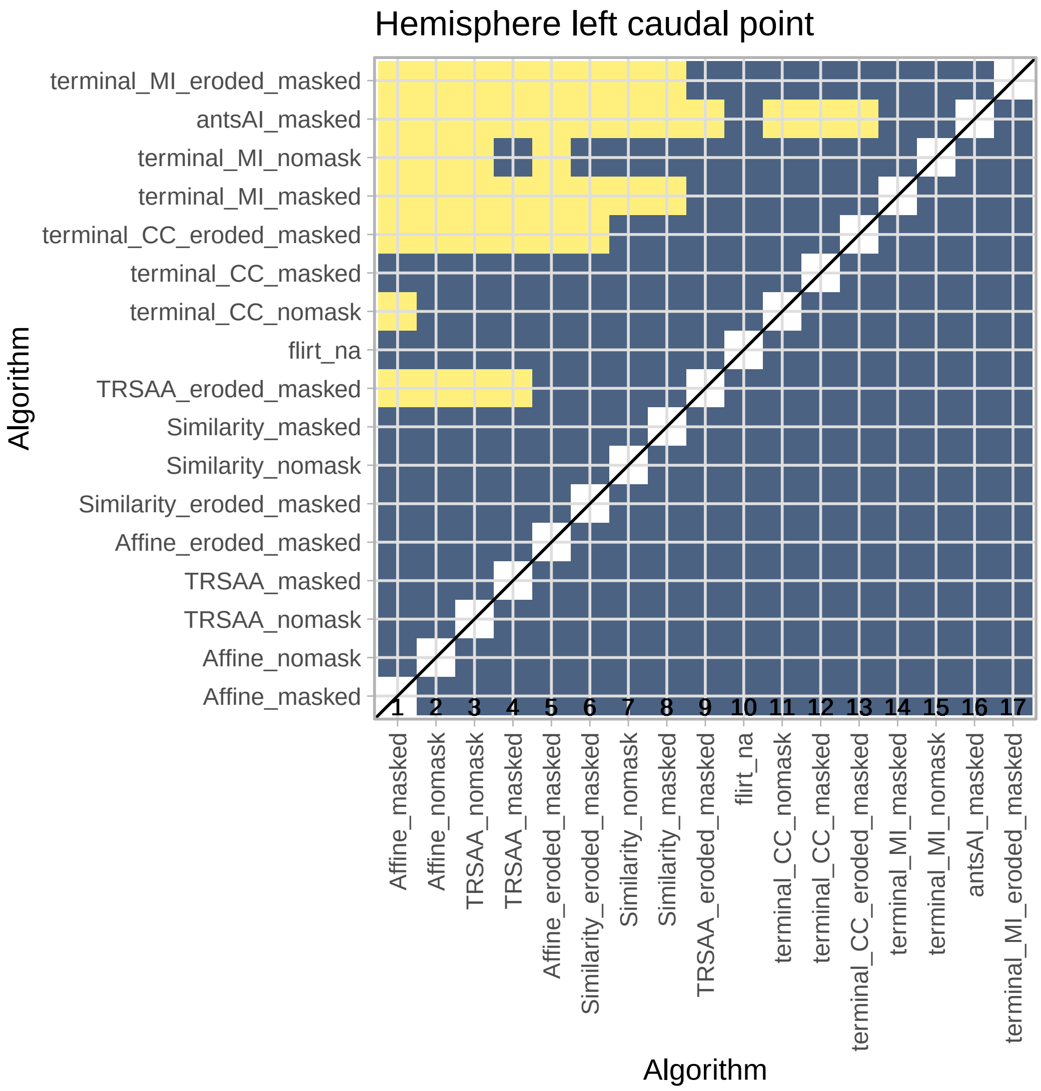
Hemisphere left caudal point
Algorithm
terminal_MI_eroded_masked
antsAI_masked
terminal_MI_nomask
terminal_MI_masked
terminal_CC_eroded_masked
terminal_CC_masked
terminal_CC_nomask
flirt_na
TRSAA_eroded_masked
Similarity_masked
Similarity_nomask
Similarity_eroded_masked
Affine_eroded_masked
TRSAA_masked
TRSAA_nomask
Affine_nomask
Affine_masked
1 2 3 4 5 6 7 8 9 10 11 12 13 14 15 16 17
Affine_masked
Affine_nomask
TRSAA_nomask
TRSAA_masked
Affine_eroded_masked
Similarity_eroded_masked
Similarity_nomask
Similarity_masked
TRSAA_eroded_masked
flirt_na
terminal_CC_nomask
terminal_CC_masked
terminal_CC_eroded_masked
terminal_MI_masked
terminal_MI_nomask
antsAI_masked
terminal_MI_eroded_masked
Algorithm

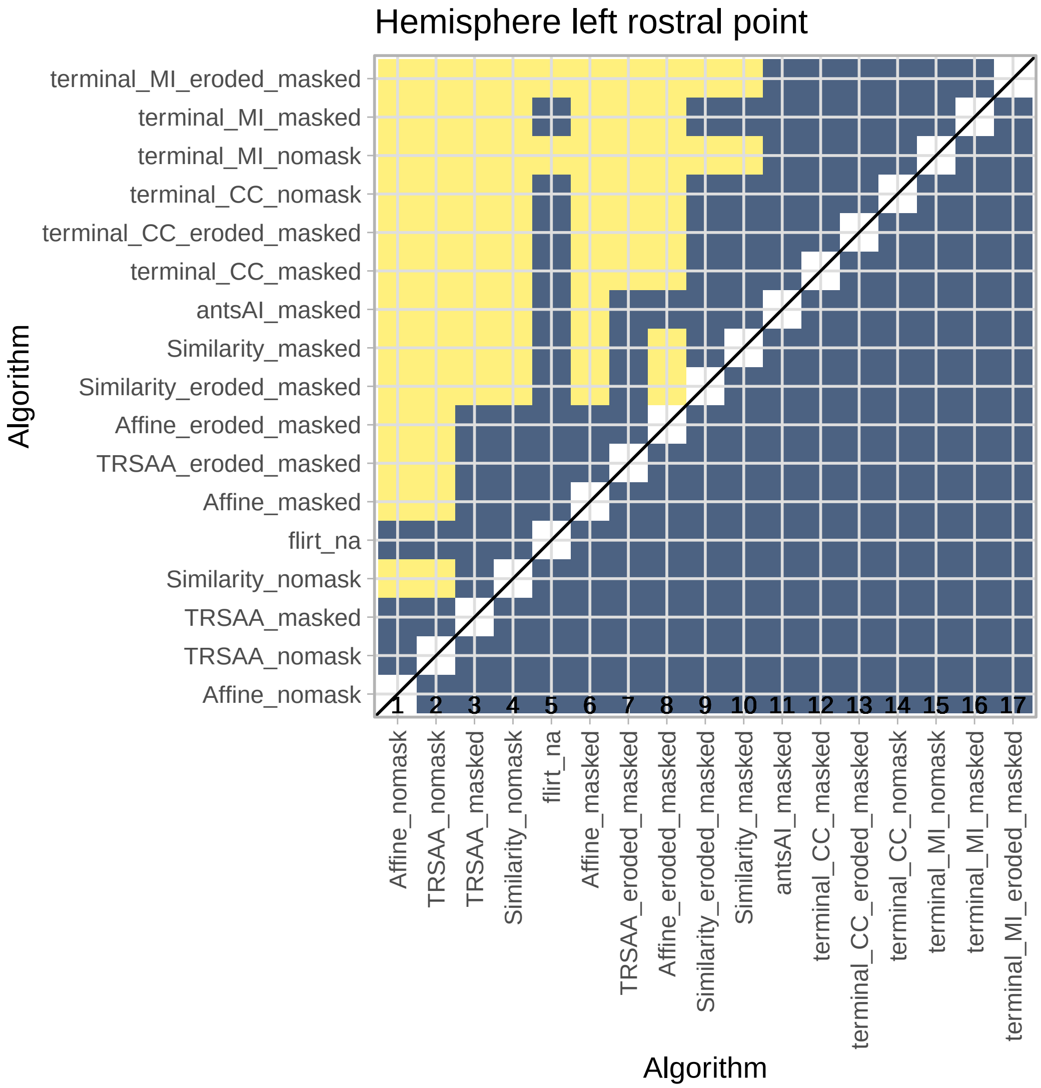
Hemisphere left rostral point
terminal_MI_eroded_masked
terminal_MI_masked
terminal_MI_nomask
terminal_CC_nomask
terminal_CC_eroded_masked
terminal_CC_masked
antsAI_masked
Similarity_masked
Similarity_eroded_masked
Affine_eroded_masked
TRSAA_eroded_masked
Affine_masked
flirt_na
Similarity_nomask
TRSAA_masked
TRSAA_nomask
Affine_nomask
Algorithm
1 2 3 4 5 6 7 8 9 10 11 12 13 14 15 16 17
Affine_nomask
TRSAA_nomask
TRSAA_masked
Similarity_nomask
flirt_na
Affine_masked
TRSAA_eroded_masked
Affine_eroded_masked
Similarity_eroded_masked
Similarity_masked
antsAI_masked
terminal_CC_masked
terminal_CC_eroded_masked
terminal_CC_nomask
terminal_MI_nomask
terminal_MI_masked
terminal_MI_eroded_masked
Algorithm

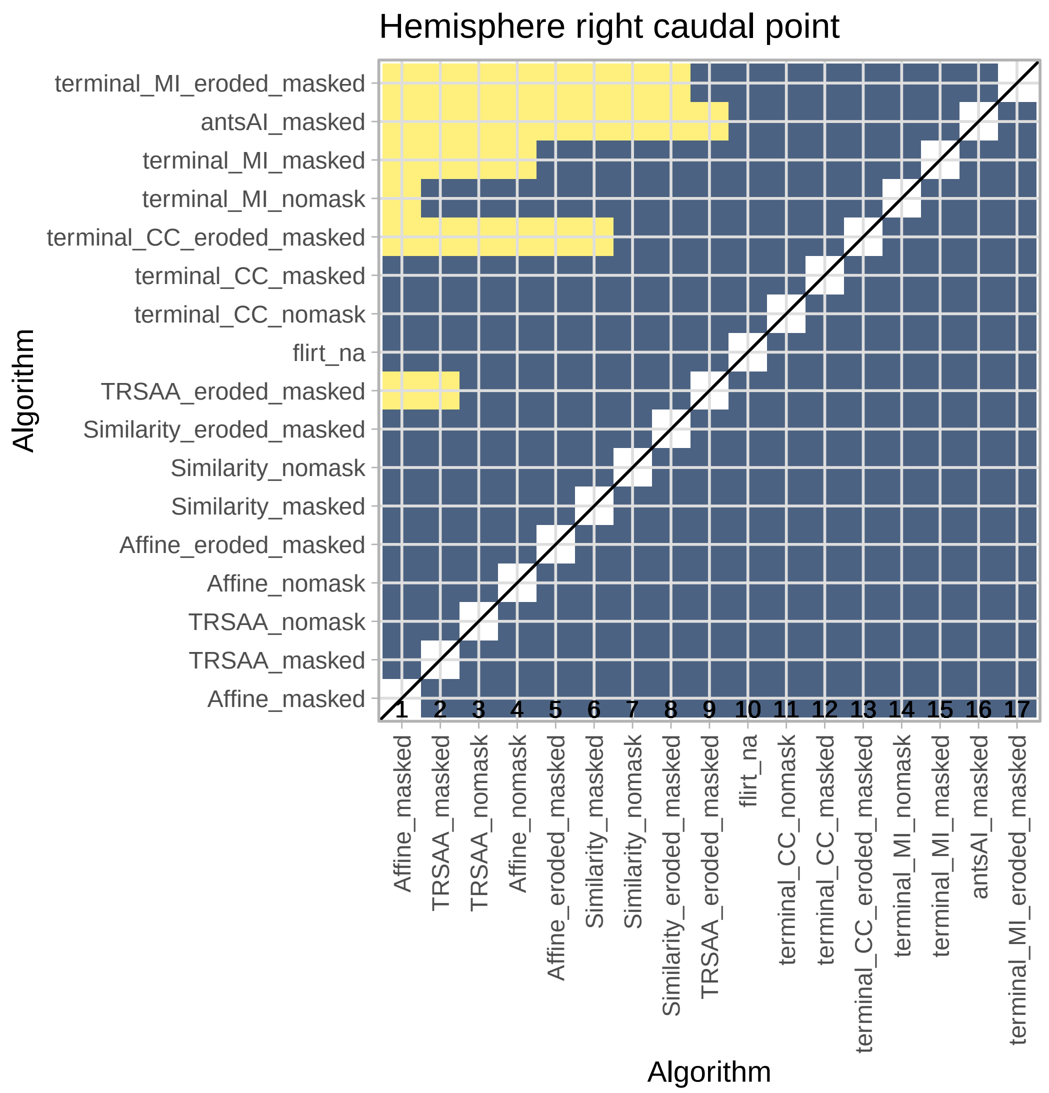
Hemisphere right caudal point
Algorithm
terminal_MI_eroded_masked
antsAI_masked
terminal_MI_masked
terminal_MI_nomask
terminal_CC_eroded_masked
terminal_CC_masked
terminal_CC_nomask
flirt_na
TRSAA_eroded_masked
Similarity_eroded_masked
Similarity_nomask
Similarity_masked
Affine_eroded_masked
Affine_nomask
TRSAA_nomask
TRSAA_masked
Affine_masked
1 2 3 4 5 6 7 8 9 10 11 12 13 14 15 16 17
Affine_masked
TRSAA_masked
TRSAA_nomask
Affine_nomask
Affine_eroded_masked
Similarity_masked
Similarity_nomask
Similarity_eroded_masked
TRSAA_eroded_masked
flirt_na
terminal_CC_nomask
terminal_CC_masked
terminal_CC_eroded_masked
terminal_MI_nomask
terminal_MI_masked
antsAI_masked
terminal_MI_eroded_masked
Algorithm

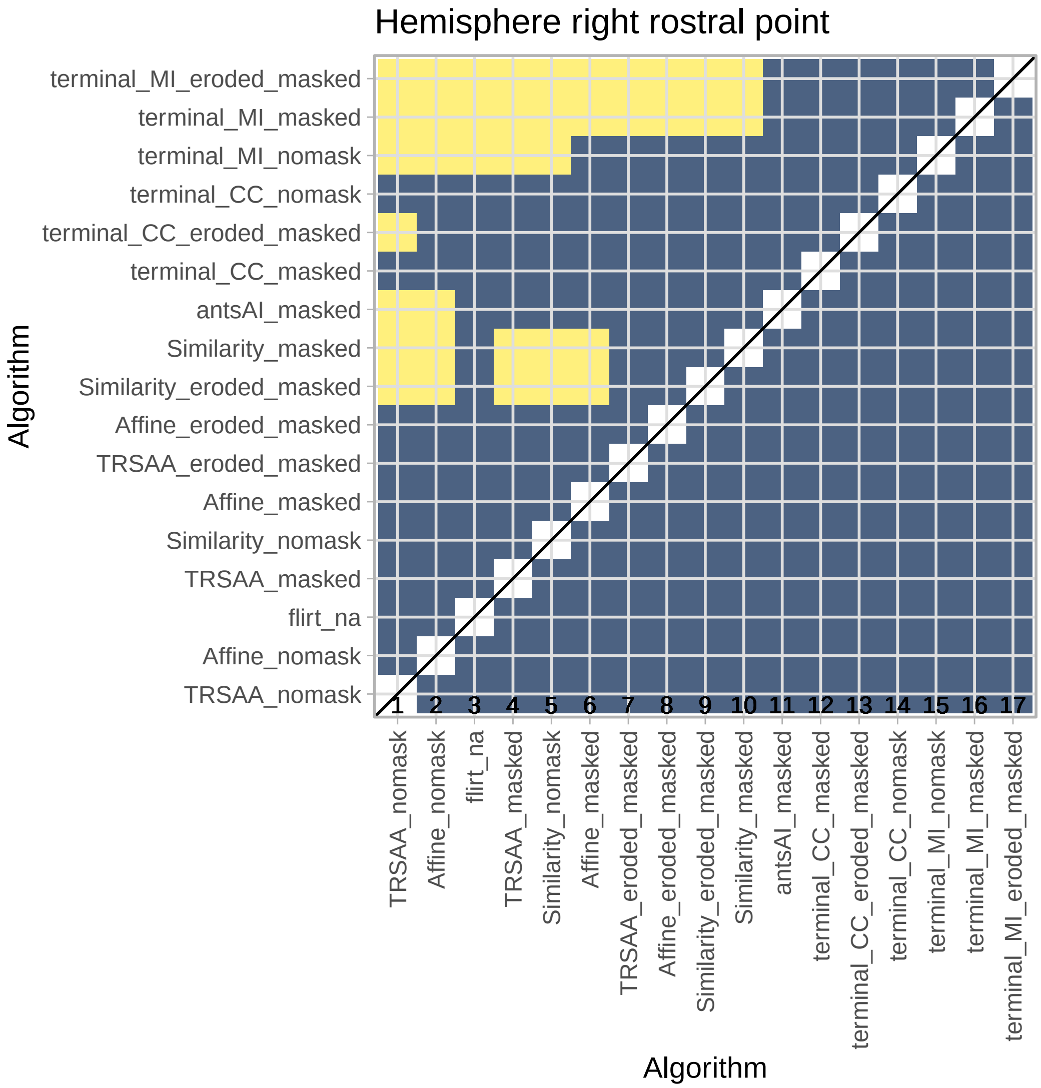
Hemisphere right rostral point
terminal_MI_eroded_masked
terminal_MI_masked
terminal_MI_nomask
terminal_CC_nomask
terminal_CC_eroded_masked
terminal_CC_masked
antsAI_masked
Similarity_masked
Similarity_eroded_masked
Affine_eroded_masked
TRSAA_eroded_masked
Affine_masked
Similarity_nomask
TRSAA_masked
flirt_na
Affine_nomask
TRSAA_nomask
Algorithm
1 2 3 4 5 6 7 8 9 10 11 12 13 14 15 16 17
TRSAA_nomask
Affine_nomask
flirt_na
TRSAA_masked
Similarity_nomask
Affine_masked
TRSAA_eroded_masked
Affine_eroded_masked
Similarity_eroded_masked
Similarity_masked
antsAI_masked
terminal_CC_masked
terminal_CC_eroded_masked
terminal_CC_nomask
terminal_MI_nomask
terminal_MI_masked
terminal_MI_eroded_masked
Algorithm

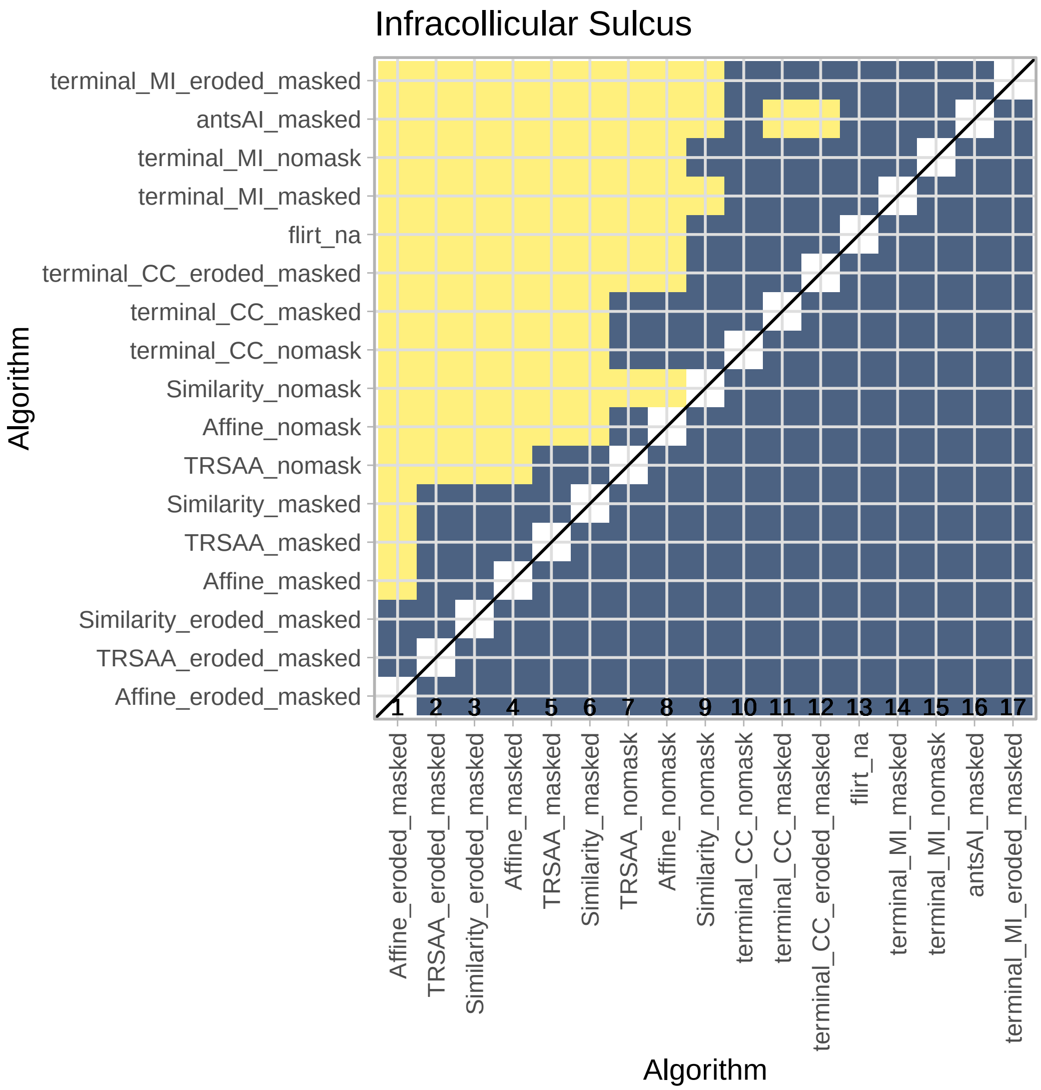
Infracollicular Sulcus
Algorithm
terminal_MI_eroded_masked
antsAI_masked
terminal_MI_nomask
terminal_MI_masked
flirt_na
terminal_CC_eroded_masked
terminal_CC_masked
terminal_CC_nomask
Similarity_nomask
Affine_nomask
TRSAA_nomask
Similarity_masked
TRSAA_masked
Affine_masked
Similarity_eroded_masked
TRSAA_eroded_masked
Affine_eroded_masked
1 2 3 4 5 6 7 8 9 10 11 12 13 14 15 16 17
Affine_eroded_masked
TRSAA_eroded_masked
Similarity_eroded_masked
Affine_masked
TRSAA_masked
Similarity_masked
TRSAA_nomask
Affine_nomask
Similarity_nomask
terminal_CC_nomask
terminal_CC_masked
terminal_CC_eroded_masked
flirt_na
terminal_MI_masked
terminal_MI_nomask
antsAI_masked
terminal_MI_eroded_masked
Algorithm

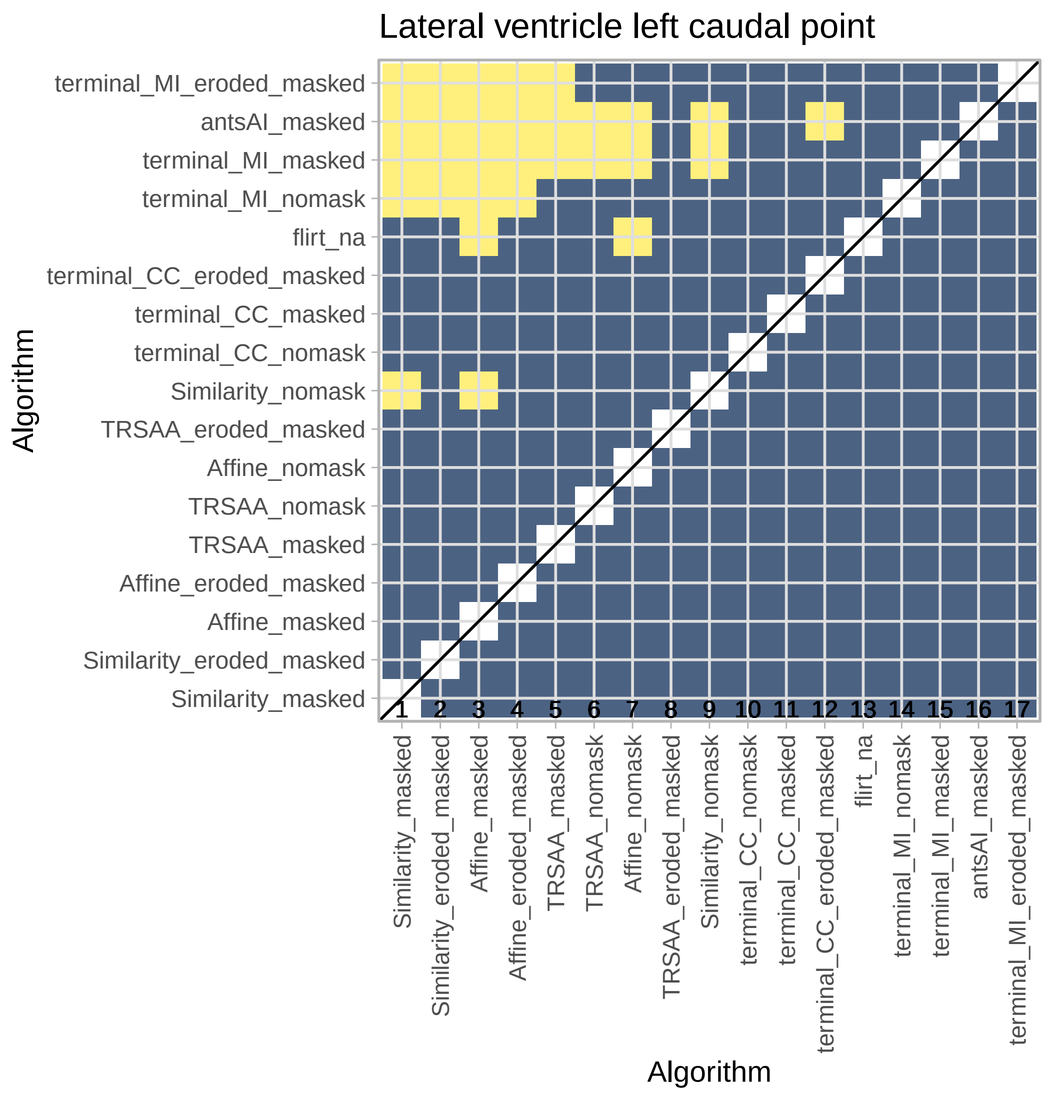

Lateral ventricle left caudal point
Algorithm
terminal_MI_eroded_masked
antsAI_masked
terminal_MI_masked
terminal_MI_nomask
flirt_na
terminal_CC_eroded_masked
terminal_CC_masked
terminal_CC_nomask
Similarity_nomask
TRSAA_eroded_masked
Affine_nomask
TRSAA_nomask
TRSAA_masked
Affine_eroded_masked
Affine_masked
Similarity_eroded_masked
Similarity_masked
1 2 3 4 5 6 7 8 9 10 11 12 13 14 15 16 17
Similarity_masked
Similarity_eroded_masked
Affine_masked
Affine_eroded_masked
TRSAA_masked
TRSAA_nomask
Affine_nomask
TRSAA_eroded_masked
Similarity_nomask
terminal_CC_nomask
terminal_CC_masked
terminal_CC_eroded_masked
flirt_na
terminal_MI_nomask
terminal_MI_masked
antsAI_masked
terminal_MI_eroded_masked
Algorithm

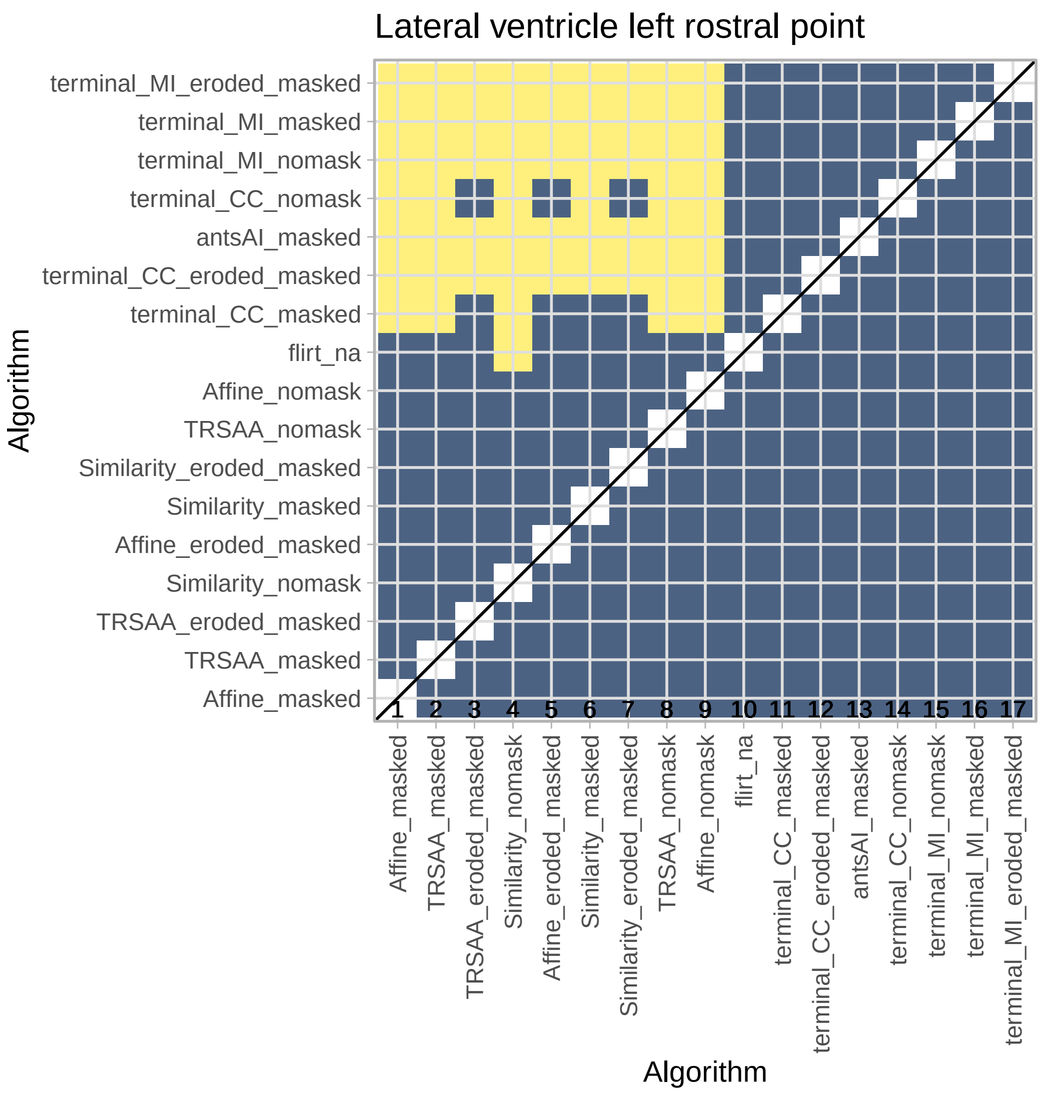
Lateral ventricle left rostral point
terminal_MI_eroded_masked
terminal_MI_masked
terminal_MI_nomask
terminal_CC_nomask
antsAI_masked
terminal_CC_eroded_masked
terminal_CC_masked
flirt_na
Affine_nomask
TRSAA_nomask
Similarity_eroded_masked
Similarity_masked
Affine_eroded_masked
Similarity_nomask
TRSAA_eroded_masked
TRSAA_masked
Affine_masked
Algorithm
1 2 3 4 5 6 7 8 9 10 11 12 13 14 15 16 17
Affine_masked
TRSAA_masked
TRSAA_eroded_masked
Similarity_nomask
Affine_eroded_masked
Similarity_masked
Similarity_eroded_masked
TRSAA_nomask
Affine_nomask
flirt_na
terminal_CC_masked
terminal_CC_eroded_masked
antsAI_masked
terminal_CC_nomask
terminal_MI_nomask
terminal_MI_masked
terminal_MI_eroded_masked
Algorithm

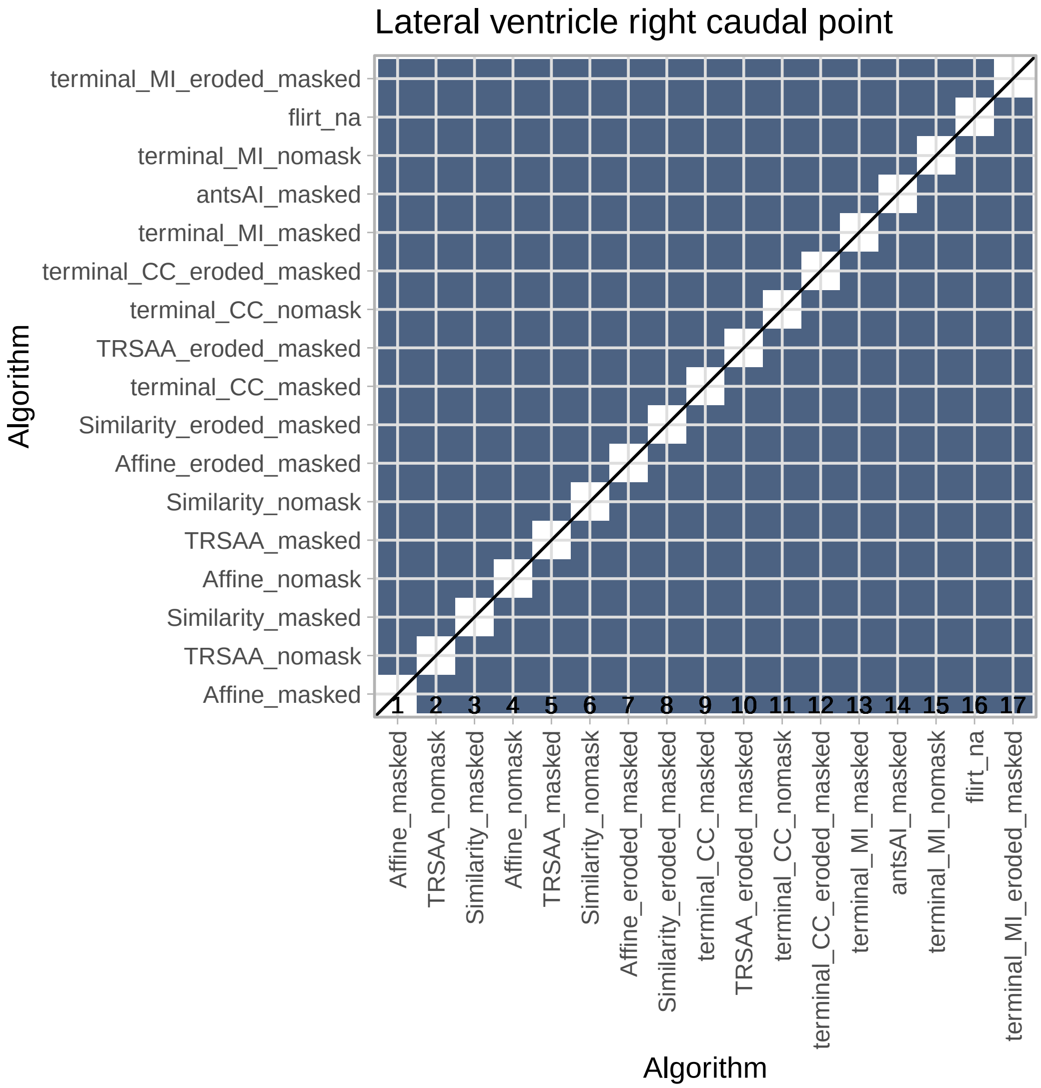
Lateral ventricle right caudal point
Algorithm
terminal_MI_eroded_masked
flirt_na
terminal_MI_nomask
antsAI_masked
terminal_MI_masked
terminal_CC_eroded_masked
terminal_CC_nomask
TRSAA_eroded_masked
terminal_CC_masked
Similarity_eroded_masked
Affine_eroded_masked
Similarity_nomask
TRSAA_masked
Affine_nomask
Similarity_masked
TRSAA_nomask
Affine_masked
1 2 3 4 5 6 7 8 9 10 11 12 13 14 15 16 17
Affine_masked
TRSAA_nomask
Similarity_masked
Affine_nomask
TRSAA_masked
Similarity_nomask
Affine_eroded_masked
Similarity_eroded_masked
terminal_CC_masked
TRSAA_eroded_masked
terminal_CC_nomask
terminal_CC_eroded_masked
terminal_MI_masked
antsAI_masked
terminal_MI_nomask
flirt_na
terminal_MI_eroded_masked
Algorithm

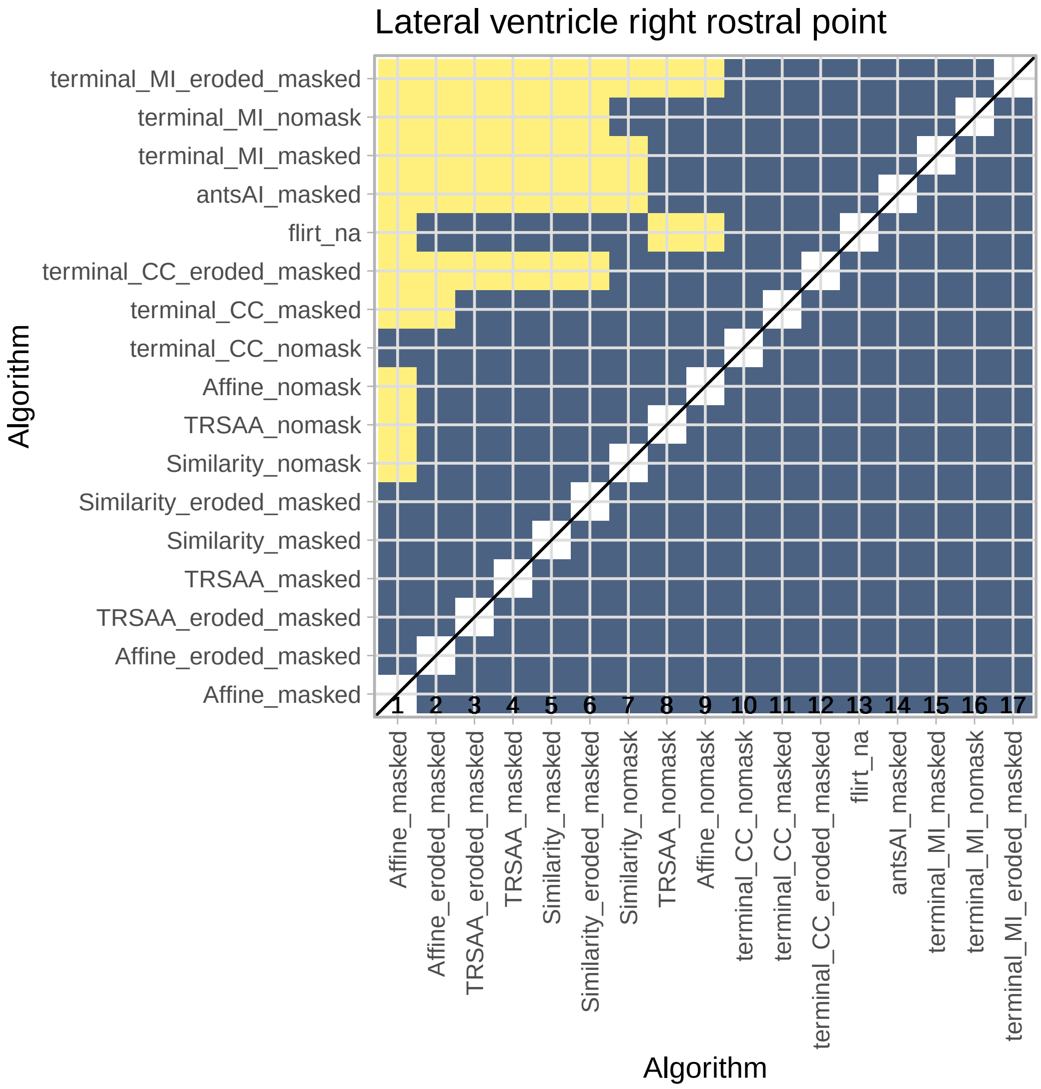
Lateral ventricle right rostral point
Algorithm
terminal_MI_eroded_masked
terminal_MI_nomask
terminal_MI_masked
antsAI_masked
flirt_na
terminal_CC_eroded_masked
terminal_CC_masked
terminal_CC_nomask
Affine_nomask
TRSAA_nomask
Similarity_nomask
Similarity_eroded_masked
Similarity_masked
TRSAA_masked
TRSAA_eroded_masked
Affine_eroded_masked
Affine_masked
1 2 3 4 5 6 7 8 9 10 11 12 13 14 15 16 17
Affine_masked
Affine_eroded_masked
TRSAA_eroded_masked
TRSAA_masked
Similarity_masked
Similarity_eroded_masked
Similarity_nomask
TRSAA_nomask
Affine_nomask
terminal_CC_nomask
terminal_CC_masked
terminal_CC_eroded_masked
flirt_na
antsAI_masked
terminal_MI_masked
terminal_MI_nomask
terminal_MI_eroded_masked
Algorithm

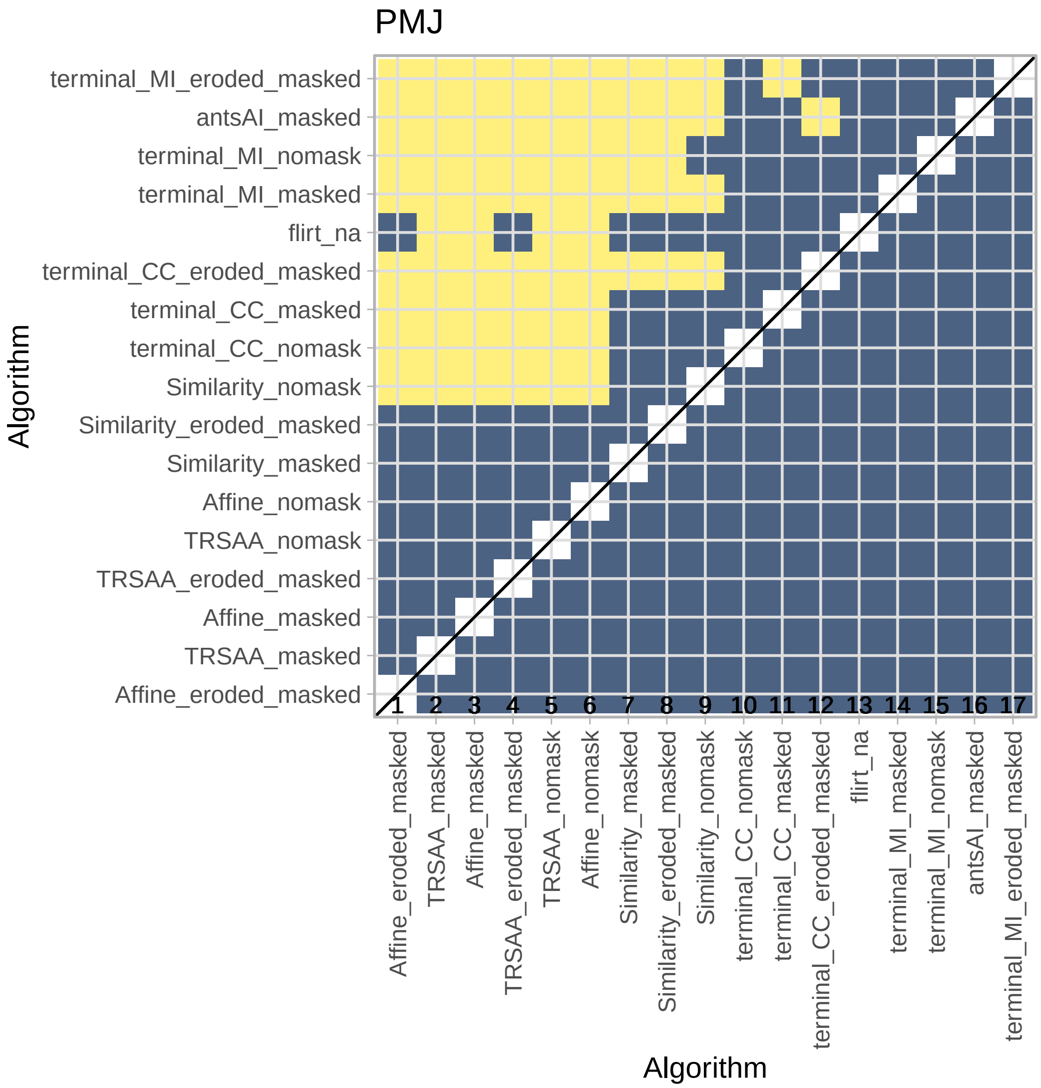
PMJ
Algorithm
terminal_MI_eroded_masked
antsAI_masked
terminal_MI_nomask
terminal_MI_masked
flirt_na
terminal_CC_eroded_masked
terminal_CC_masked
terminal_CC_nomask
Similarity_nomask
Similarity_eroded_masked
Similarity_masked
Affine_nomask
TRSAA_nomask
TRSAA_eroded_masked
Affine_masked
TRSAA_masked
Affine_eroded_masked
1 2 3 4 5 6 7 8 9 10 11 12 13 14 15 16 17
Affine_eroded_masked
TRSAA_masked
Affine_masked
TRSAA_eroded_masked
TRSAA_nomask
Affine_nomask
Similarity_masked
Similarity_eroded_masked
Similarity_nomask
terminal_CC_nomask
terminal_CC_masked
terminal_CC_eroded_masked
flirt_na
terminal_MI_masked
terminal_MI_nomask
antsAI_masked
terminal_MI_eroded_masked
Algorithm

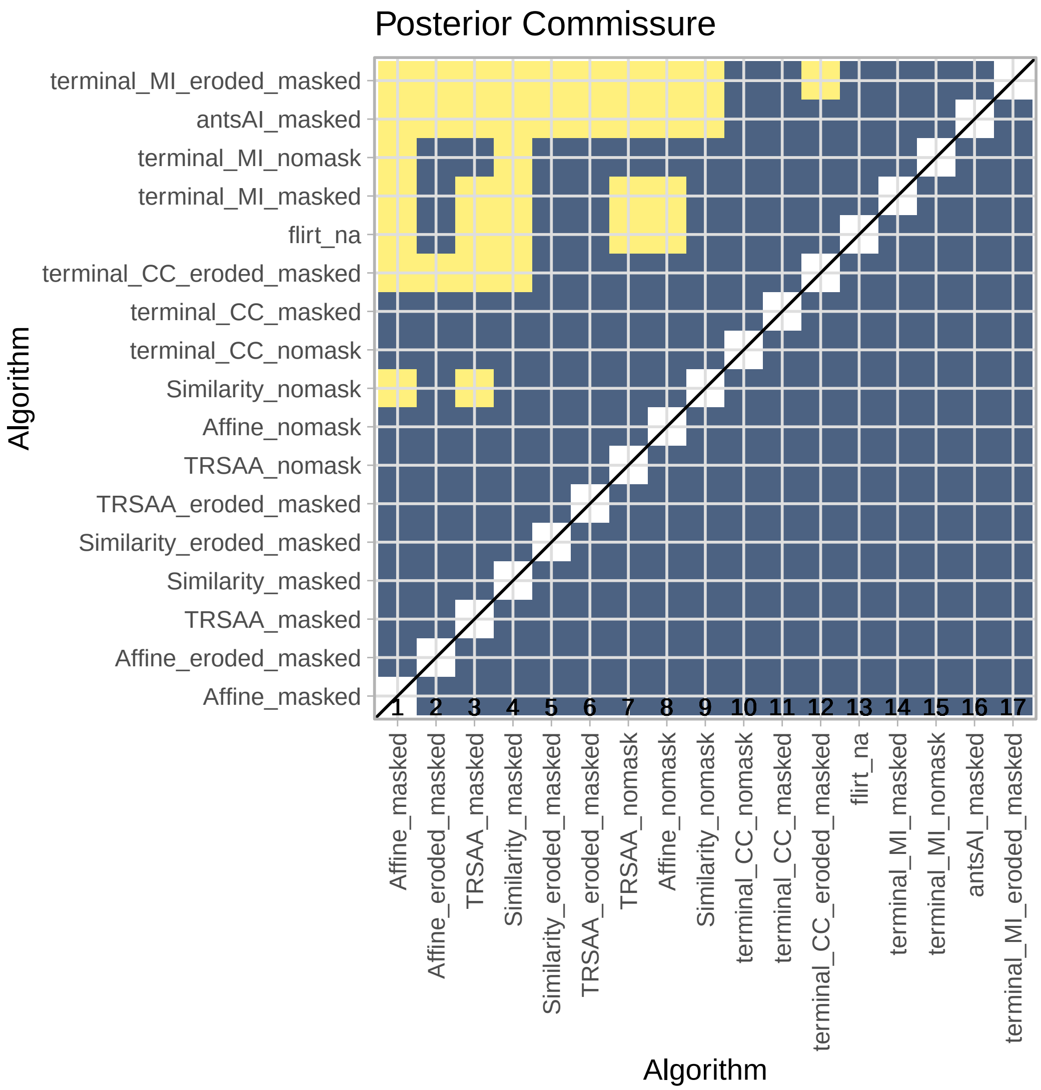
Posterior Commissure
Algorithm
terminal_MI_eroded_masked
antsAI_masked
terminal_MI_nomask
terminal_MI_masked
flirt_na
terminal_CC_eroded_masked
terminal_CC_masked
terminal_CC_nomask
Similarity_nomask
Affine_nomask
TRSAA_nomask
TRSAA_eroded_masked
Similarity_eroded_masked
Similarity_masked
TRSAA_masked
Affine_eroded_masked
Affine_masked
1 2 3 4 5 6 7 8 9 10 11 12 13 14 15 16 17
Affine_masked
Affine_eroded_masked
TRSAA_masked
Similarity_masked
Similarity_eroded_masked
TRSAA_eroded_masked
TRSAA_nomask
Affine_nomask
Similarity_nomask
terminal_CC_nomask
terminal_CC_masked
terminal_CC_eroded_masked
flirt_na
terminal_MI_masked
terminal_MI_nomask
antsAI_masked
terminal_MI_eroded_masked
Algorithm

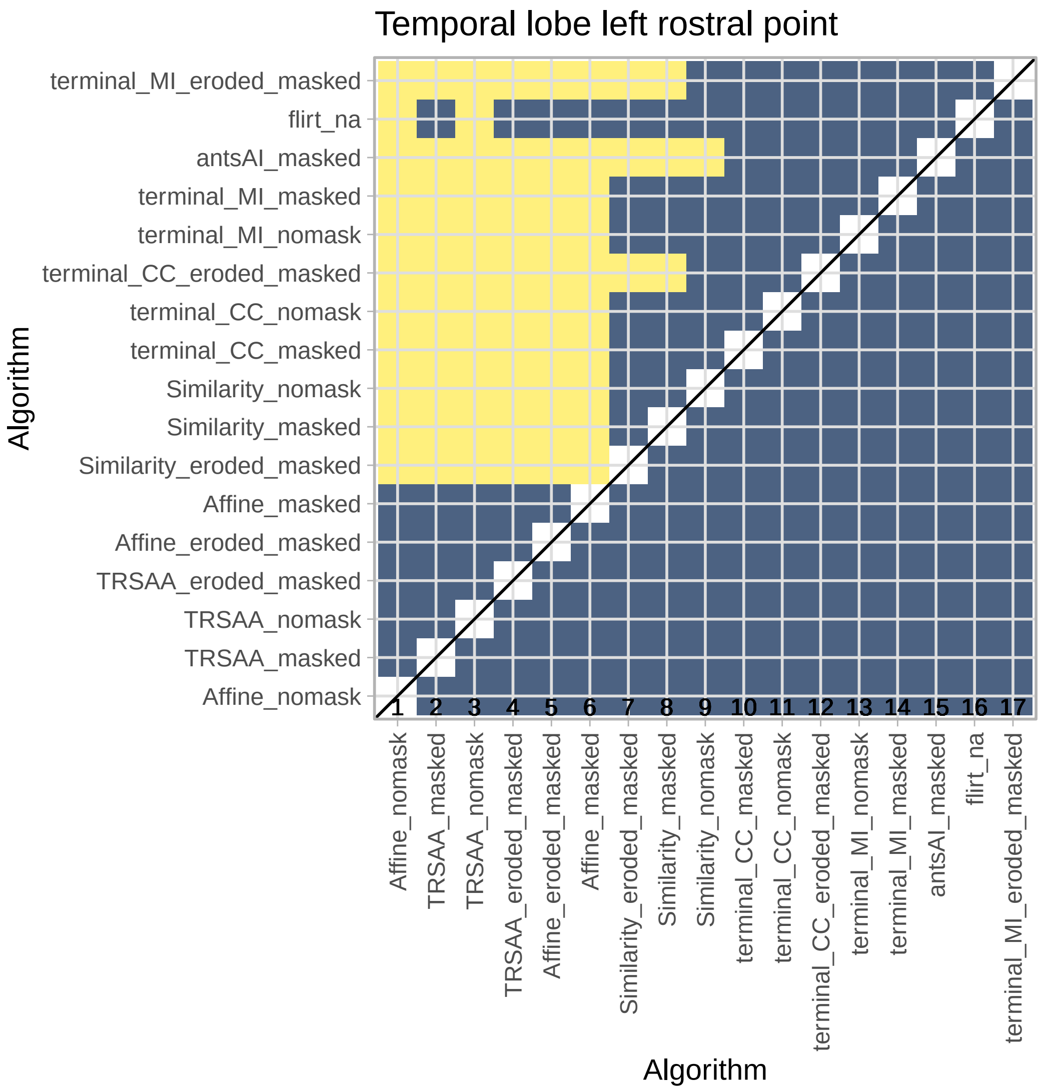
Temporal lobe left rostral point
Algorithm
terminal_MI_eroded_masked
flirt_na
antsAI_masked
terminal_MI_masked
terminal_MI_nomask
terminal_CC_eroded_masked
terminal_CC_nomask
terminal_CC_masked
Similarity_nomask
Similarity_masked
Similarity_eroded_masked
Affine_masked
Affine_eroded_masked
TRSAA_eroded_masked
TRSAA_nomask
TRSAA_masked
Affine_nomask
1 2 3 4 5 6 7 8 9 10 11 12 13 14 15 16 17
Affine_nomask
TRSAA_masked
TRSAA_nomask
TRSAA_eroded_masked
Affine_eroded_masked
Affine_masked
Similarity_eroded_masked
Similarity_masked
Similarity_nomask
terminal_CC_masked
terminal_CC_nomask
terminal_CC_eroded_masked
terminal_MI_nomask
terminal_MI_masked
antsAI_masked
flirt_na
terminal_MI_eroded_masked
Algorithm

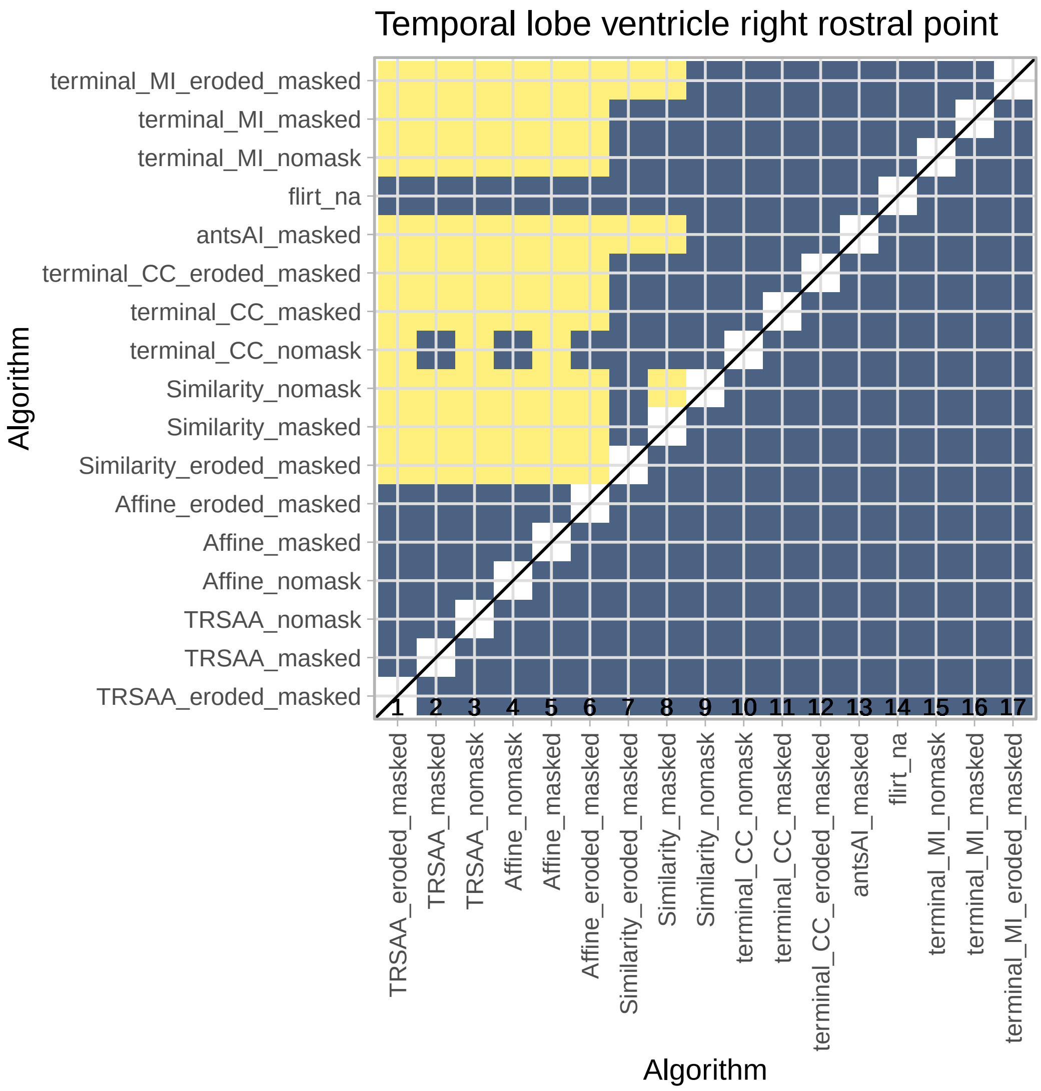
Temporal lobe ventricle right rostral point
Algorithm
terminal_MI_eroded_masked
terminal_MI_masked
terminal_MI_nomask
flirt_na
antsAI_masked
terminal_CC_eroded_masked
terminal_CC_masked
terminal_CC_nomask
Similarity_nomask
Similarity_masked
Similarity_eroded_masked
Affine_eroded_masked
Affine_masked
Affine_nomask
TRSAA_nomask
TRSAA_masked
TRSAA_eroded_masked
1 2 3 4 5 6 7 8 9 10 11 12 13 14 15 16 17
TRSAA_eroded_masked
TRSAA_masked
TRSAA_nomask
Affine_nomask
Affine_masked
Affine_eroded_masked
Similarity_eroded_masked
Similarity_masked
Similarity_nomask
terminal_CC_nomask
terminal_CC_masked
terminal_CC_eroded_masked
antsAI_masked
flirt_na
terminal_MI_nomask
terminal_MI_masked
terminal_MI_eroded_masked
Algorithm

3.4 Ranking robustness to ranking methods

Line plots for visualizing ranking robustness across different ranking methods. Each algorithm is represented by one colored line. For each ranking method encoded on the x-axis, the height of the line represents the corresponding rank. Horizontal lines indicate identical ranks for all methods.

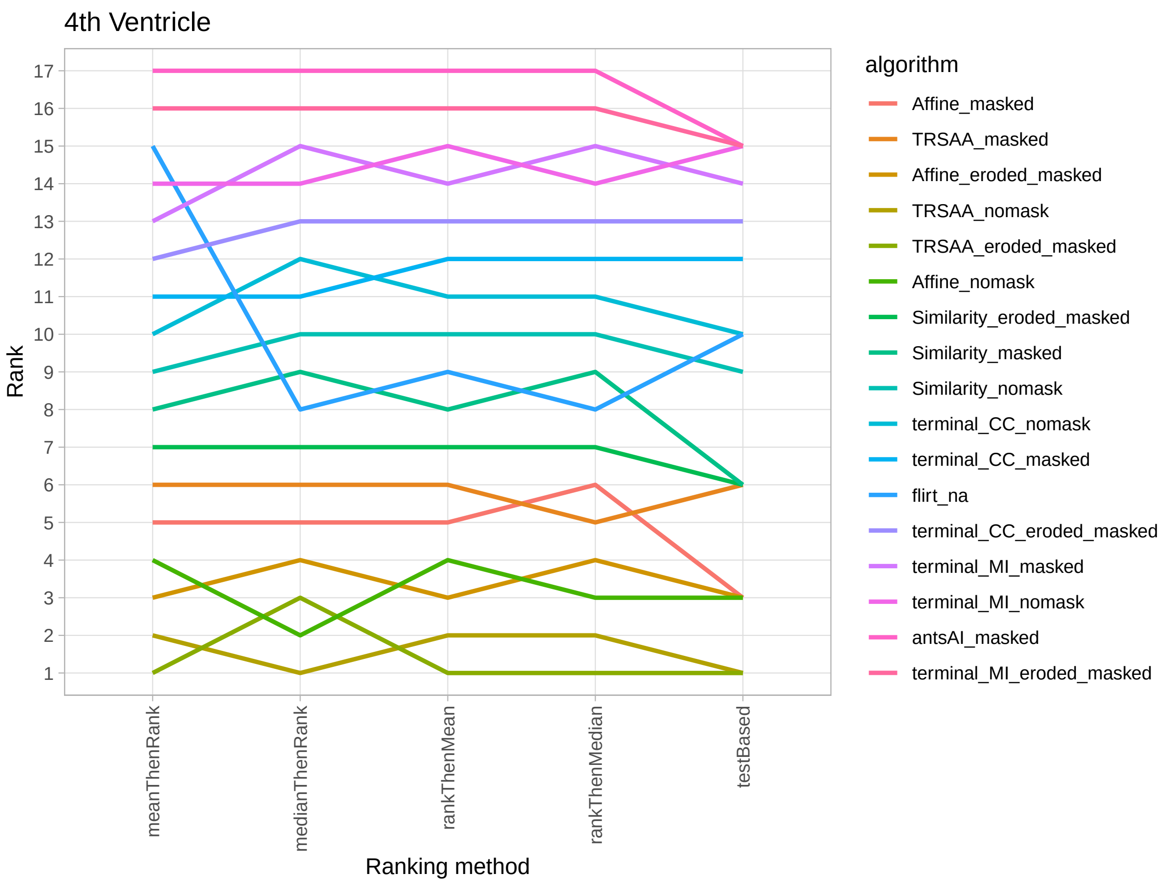

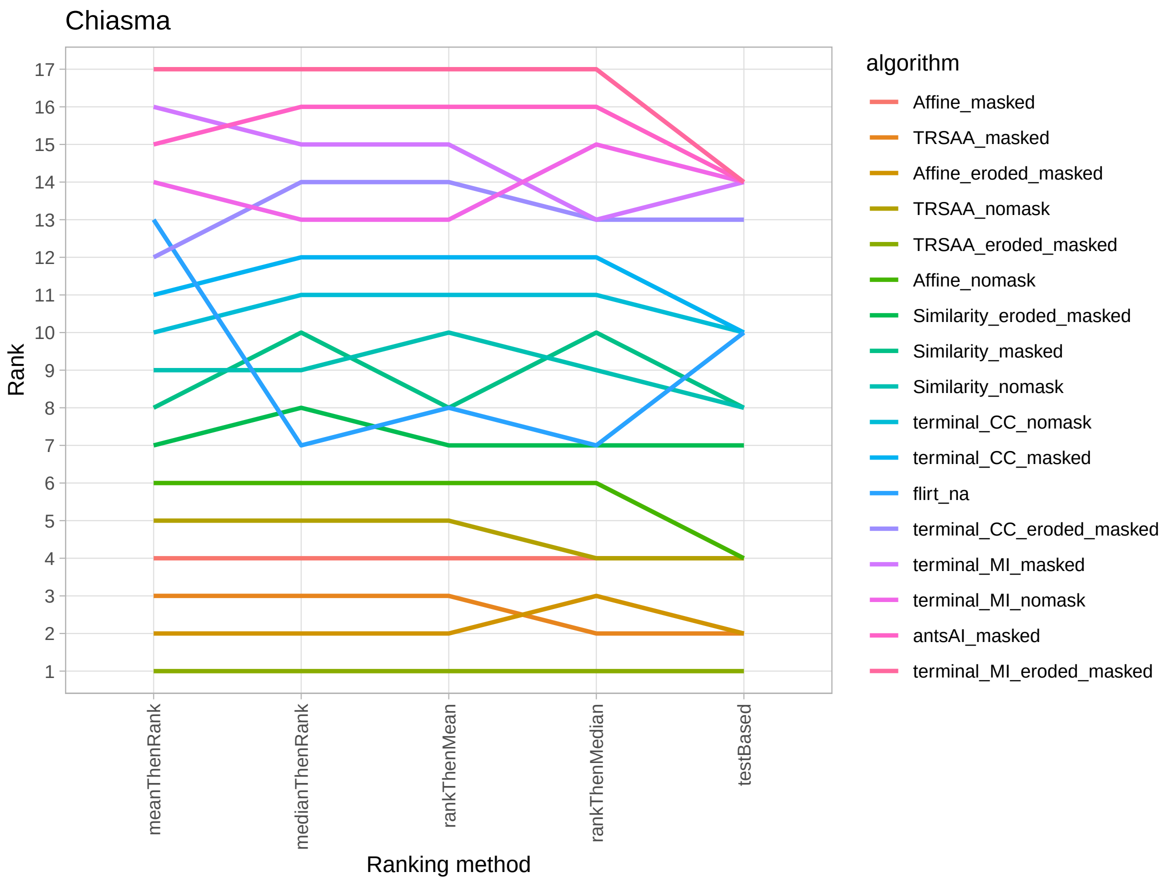
Chiasma
Rank
17
16
15
14
13
12
11
10
9
8
7
6
5
4
3
2
1
meanThenRank
medianThenRank
rankThenMean
rankThenMedian
testBased
Ranking method
algorithm
Affine_masked
TRSAA_masked
Affine_eroded_masked
TRSAA_nomask
TRSAA_eroded_masked
Affine_nomask
Similarity_eroded_masked
Similarity_masked
Similarity_nomask
terminal_CC_nomask
terminal_CC_masked
flirt_na
terminal_CC_eroded_masked
terminal_MI_masked
terminal_MI_nomask
antsAI_masked
terminal_MI_eroded_masked

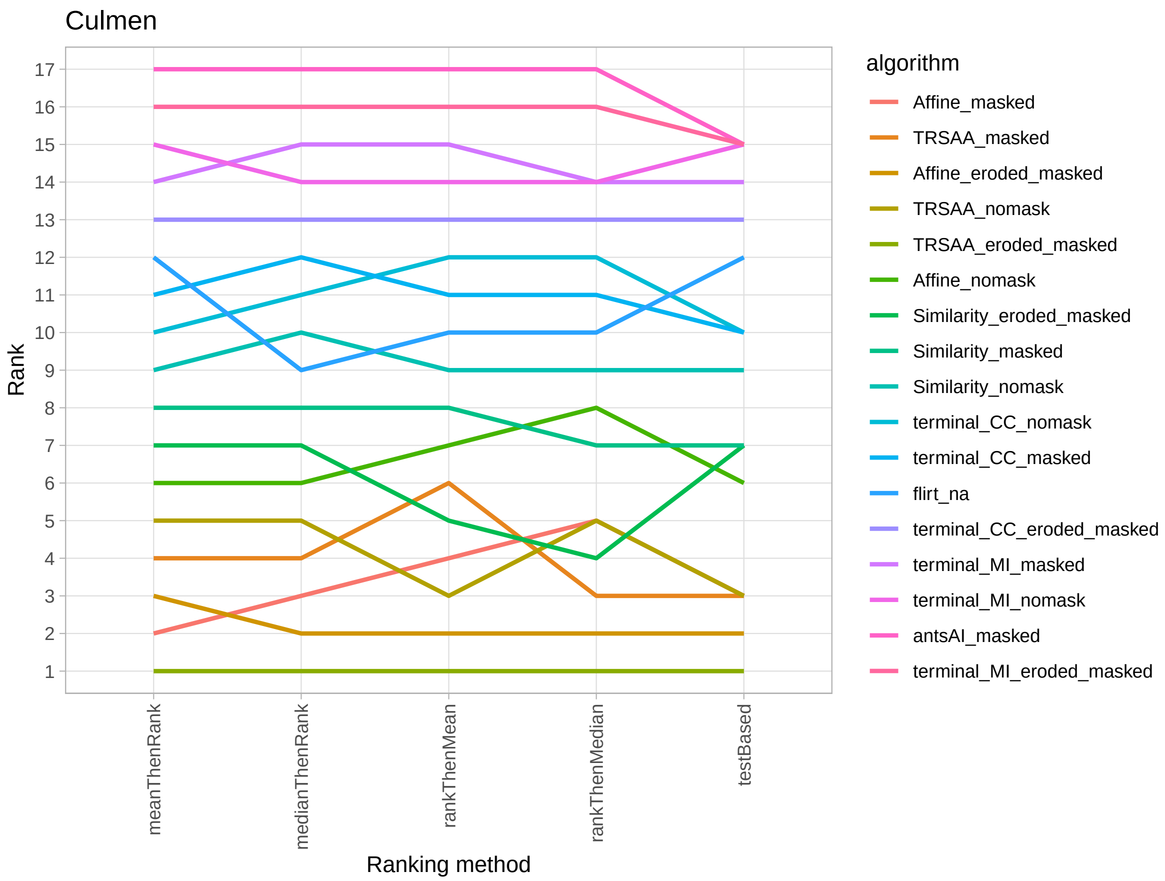
Culmen
Rank
Ranking method
meanThenRank
medianThenRank
rankThenMean
rankThenMedian
testBased
algorithm
Affine_masked
TRSAA_masked
Affine_eroded_masked
TRSAA_nomask
TRSAA_eroded_masked
Affine_nomask
Similarity_eroded_masked
Similarity_masked
Similarity_nomask
terminal_CC_nomask
terminal_CC_masked
flirt_na
terminal_CC_eroded_masked
terminal_MI_masked
terminal_MI_nomask
antsAI_masked
terminal_MI_eroded_masked

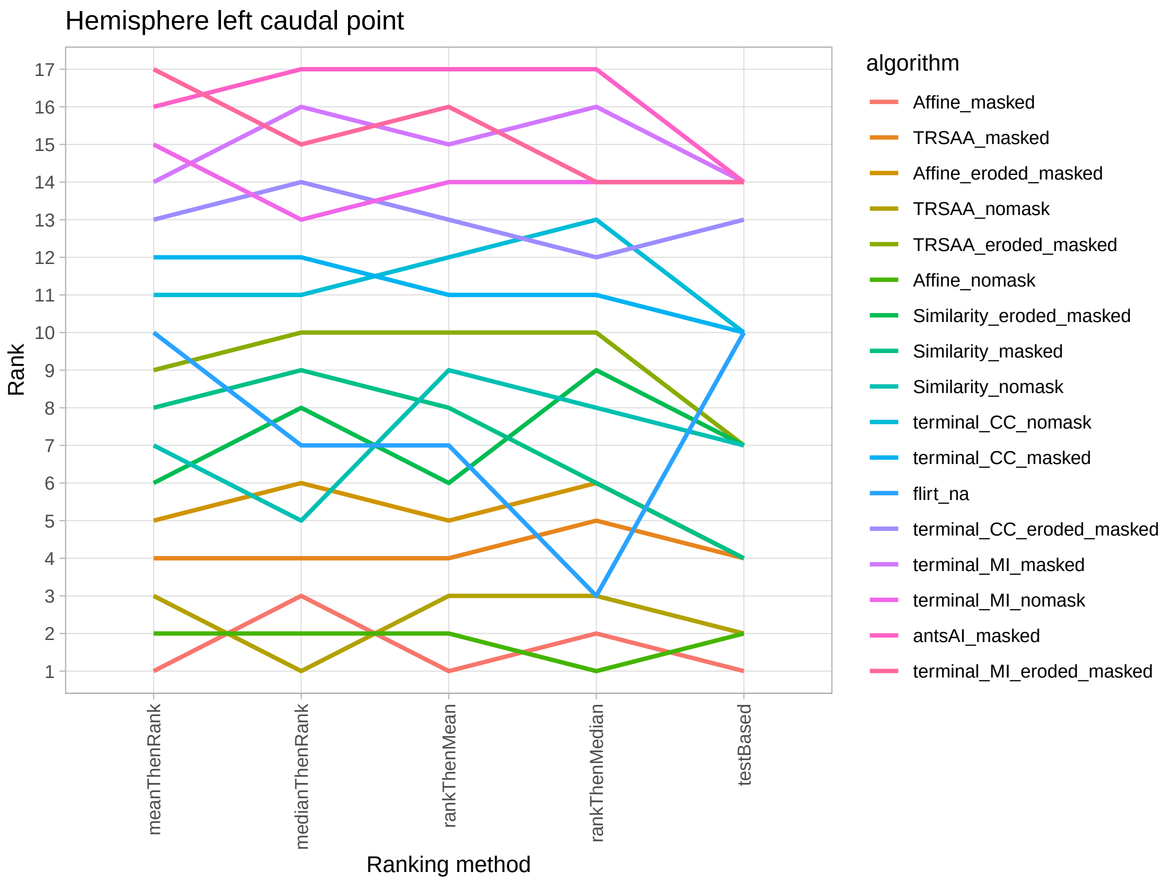
Hemisphere left caudal point
Rank
17
16
15
14
13
12
11
10
9
8
7
6
5
4
3
2
1
meanThenRank
medianThenRank
rankThenMean
rankThenMedian
testBased
Ranking method
algorithm
Affine_masked
TRSAA_masked
Affine_eroded_masked
TRSAA_nomask
TRSAA_eroded_masked
Affine_nomask
Similarity_eroded_masked
Similarity_masked
Similarity_nomask
terminal_CC_nomask
terminal_CC_masked
flirt_na
terminal_CC_eroded_masked
terminal_MI_masked
terminal_MI_nomask
antsAI_masked
terminal_MI_eroded_masked

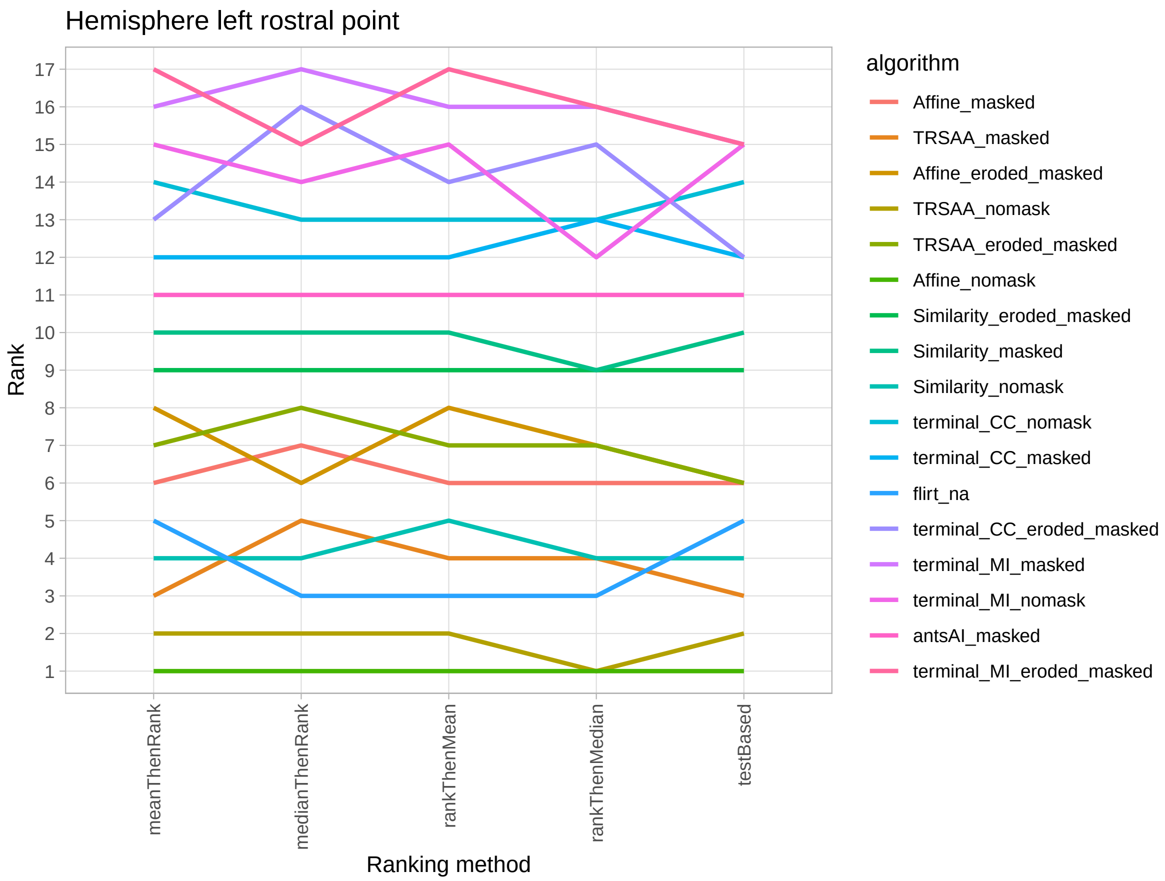
Hemisphere left rostral point
Rank
17
16
15
14
13
12
11
10
9
8
7
6
5
4
3
2
1
meanThenRank
medianThenRank
rankThenMean
rankThenMedian
testBased
Ranking method
algorithm
Affine_masked
TRSAA_masked
Affine_eroded_masked
TRSAA_nomask
TRSAA_eroded_masked
Affine_nomask
Similarity_eroded_masked
Similarity_masked
Similarity_nomask
terminal_CC_nomask
terminal_CC_masked
flirt_na
terminal_CC_eroded_masked
terminal_MI_masked
terminal_MI_nomask
antsAI_masked
terminal_MI_eroded_masked

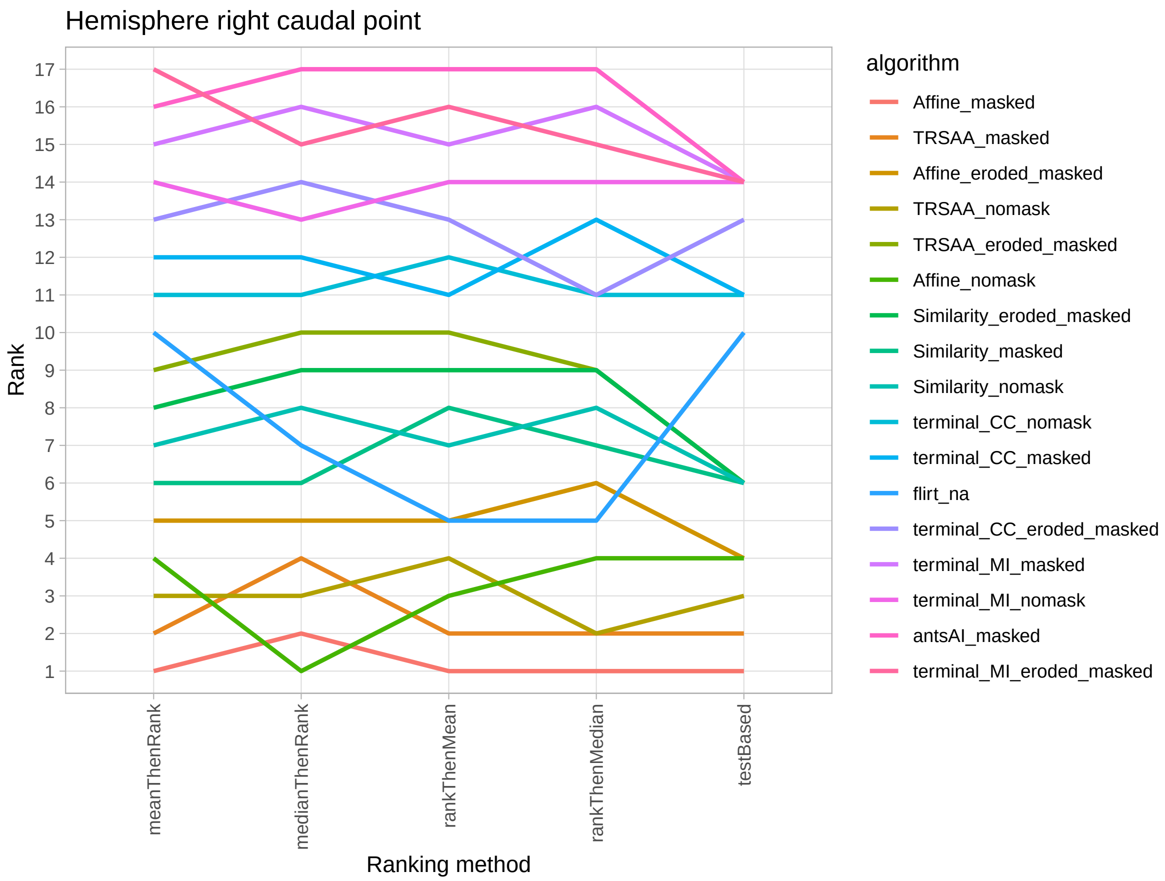
Hemisphere right caudal point
Rank
17
16
15
14
13
12
11
10
9
8
7
6
5
4
3
2
1
meanThenRank
medianThenRank
rankThenMean
rankThenMedian
testBased
Ranking method
algorithm
Affine_masked
TRSAA_masked
Affine_eroded_masked
TRSAA_nomask
TRSAA_eroded_masked
Affine_nomask
Similarity_eroded_masked
Similarity_masked
Similarity_nomask
terminal_CC_nomask
terminal_CC_masked
flirt_na
terminal_CC_eroded_masked
terminal_MI_masked
terminal_MI_nomask
antsAI_masked
terminal_MI_eroded_masked

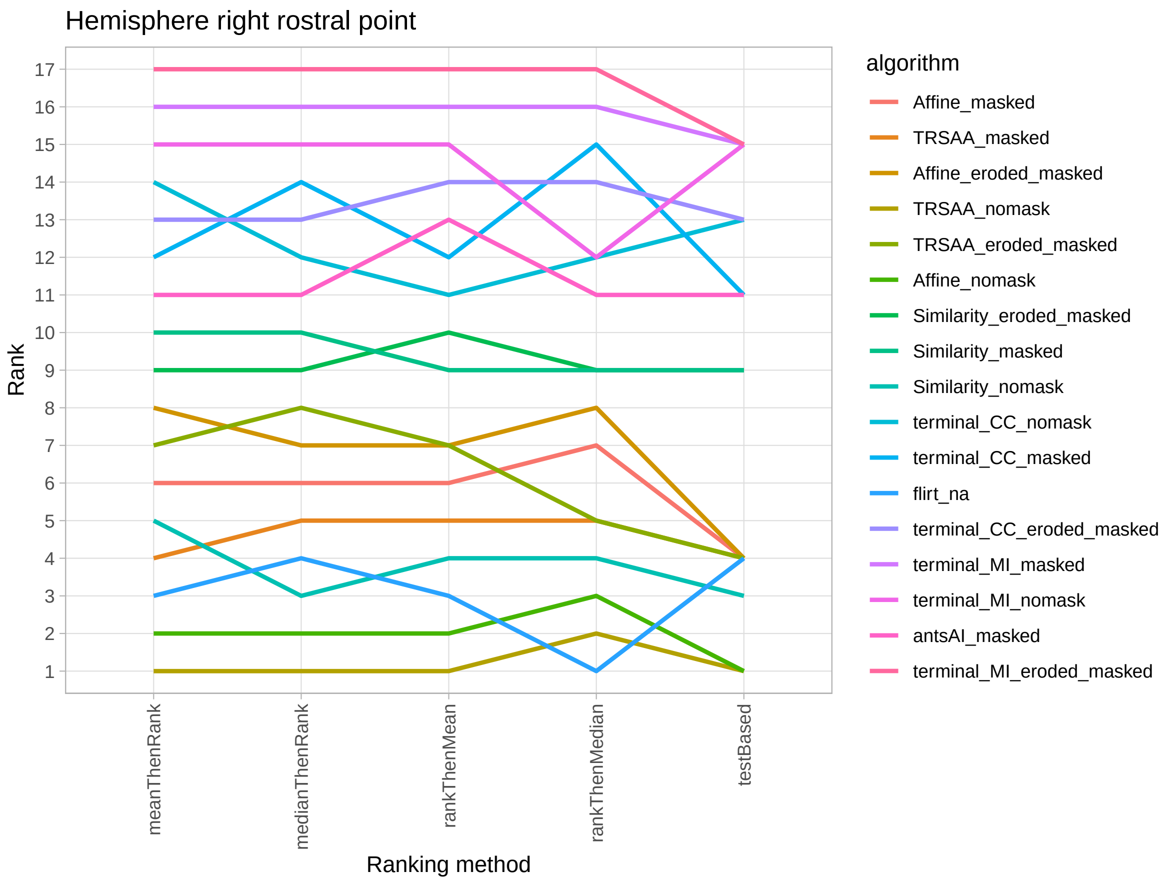
Hemisphere right rostral point
Rank
17
16
15
14
13
12
11
10
9
8
7
6
5
4
3
2
1
meanThenRank
medianThenRank
rankThenMean
rankThenMedian
testBased
Ranking method
algorithm
Affine_masked
TRSAA_masked
Affine_eroded_masked
TRSAA_nomask
TRSAA_eroded_masked
Affine_nomask
Similarity_eroded_masked
Similarity_masked
Similarity_nomask
terminal_CC_nomask
terminal_CC_masked
flirt_na
terminal_CC_eroded_masked
terminal_MI_masked
terminal_MI_nomask
antsAI_masked
terminal_MI_eroded_masked

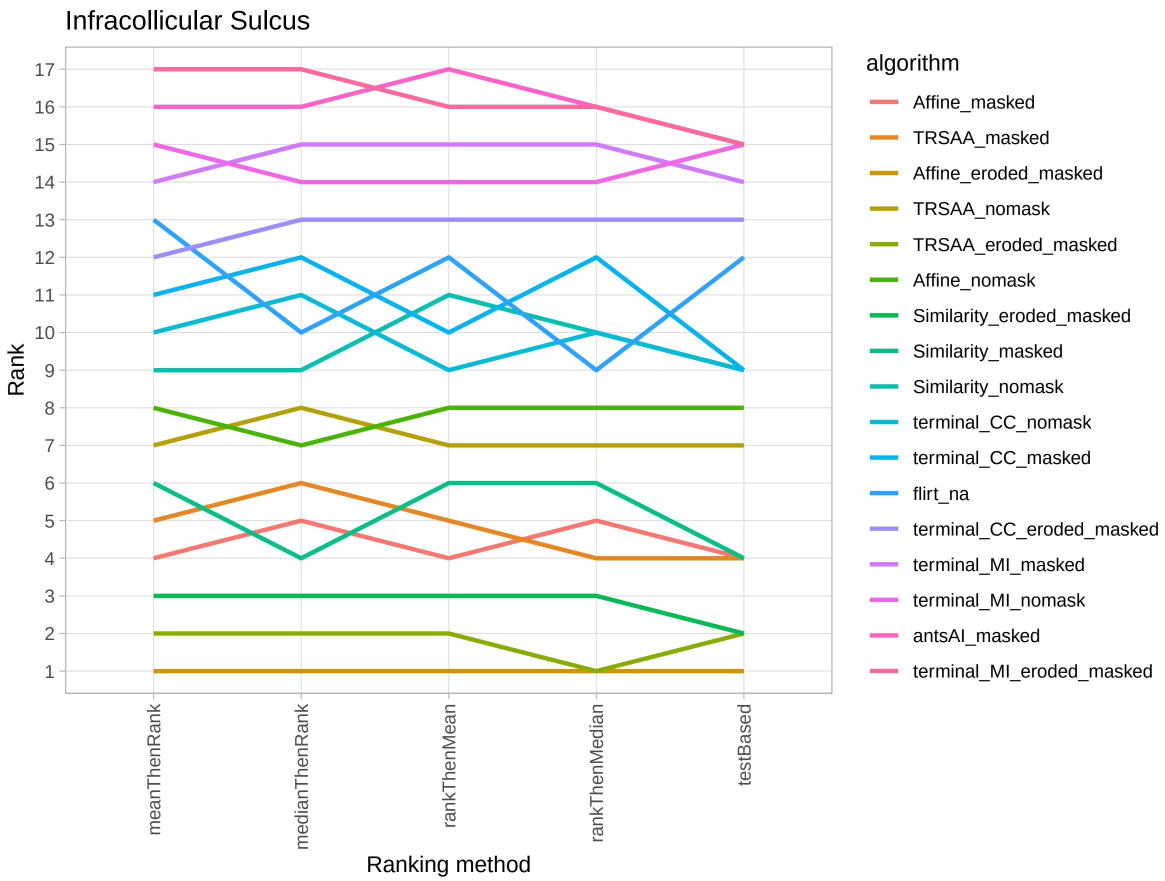
Infracollicular Sulcus
Rank
17
16
15
14
13
12
11
10
9
8
7
6
5
4
3
2
1
meanThenRank
medianThenRank
rankThenMean
rankThenMedian
testBased
Ranking method
algorithm
Affine_masked
TRSAA_masked
Affine_eroded_masked
TRSAA_nomask
TRSAA_eroded_masked
Affine_nomask
Similarity_eroded_masked
Similarity_masked
Similarity_nomask
terminal_CC_nomask
terminal_CC_masked
flirt_na
terminal_CC_eroded_masked
terminal_MI_masked
terminal_MI_nomask
antsAI_masked
terminal_MI_eroded_masked

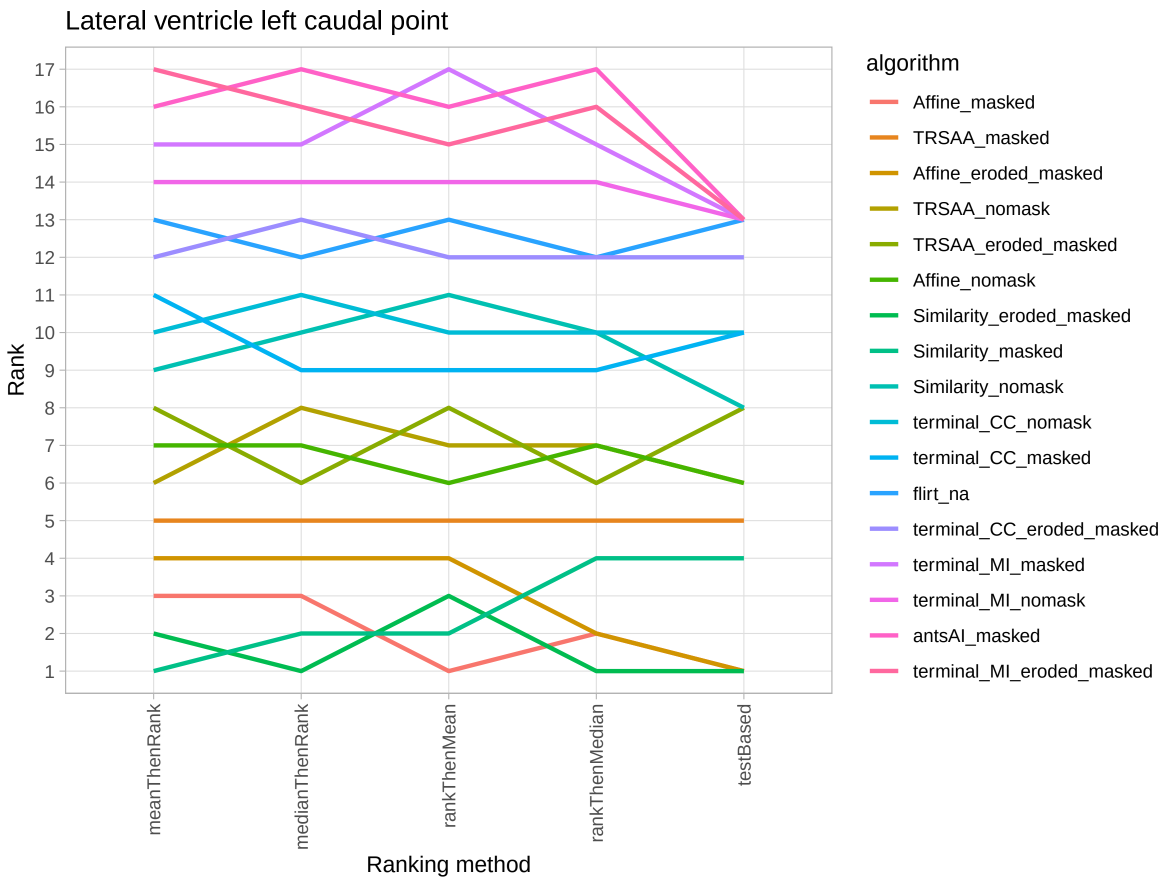

Lateral ventricle left caudal point
Rank
17
16
15
14
13
12
11
10
9
8
7
6
5
4
3
2
1
meanThenRank
medianThenRank
rankThenMean
rankThenMedian
testBased
Ranking method
algorithm
Affine_masked
TRSAA_masked
Affine_eroded_masked
TRSAA_nomask
TRSAA_eroded_masked
Affine_nomask
Similarity_eroded_masked
Similarity_masked
Similarity_nomask
terminal_CC_nomask
terminal_CC_masked
flirt_na
terminal_CC_eroded_masked
terminal_MI_masked
terminal_MI_nomask
antsAI_masked
terminal_MI_eroded_masked

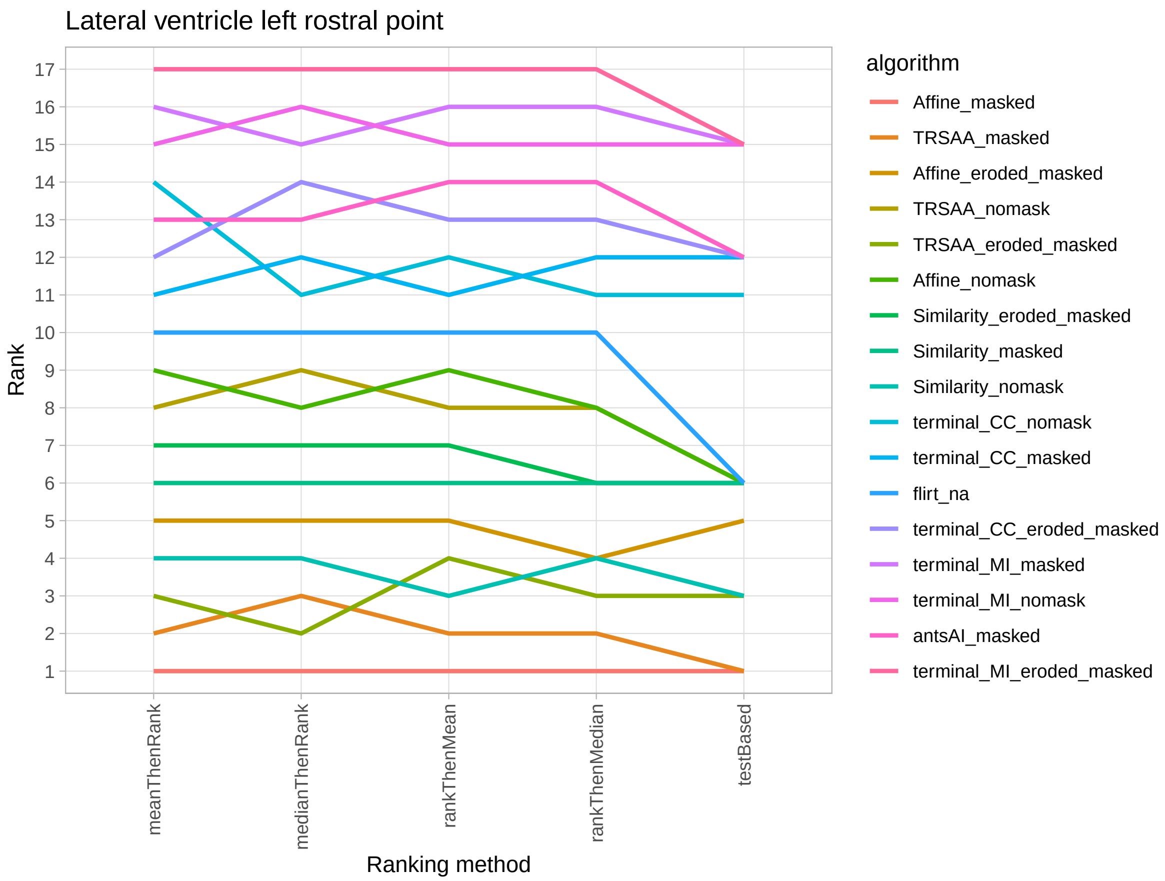

Lateral ventricle left rostral point
Rank
17
16
15
14
13
12
11
10
9
8
7
6
5
4
3
2
1
meanThenRank
medianThenRank
rankThenMean
rankThenMedian
testBased
Ranking method
algorithm
Affine_masked
TRSAA_masked
Affine_eroded_masked
TRSAA_nomask
TRSAA_eroded_masked
Affine_nomask
Similarity_eroded_masked
Similarity_masked
Similarity_nomask
terminal_CC_nomask
terminal_CC_masked
flirt_na
terminal_CC_eroded_masked
terminal_MI_masked
terminal_MI_nomask
antsAI_masked
terminal_MI_eroded_masked

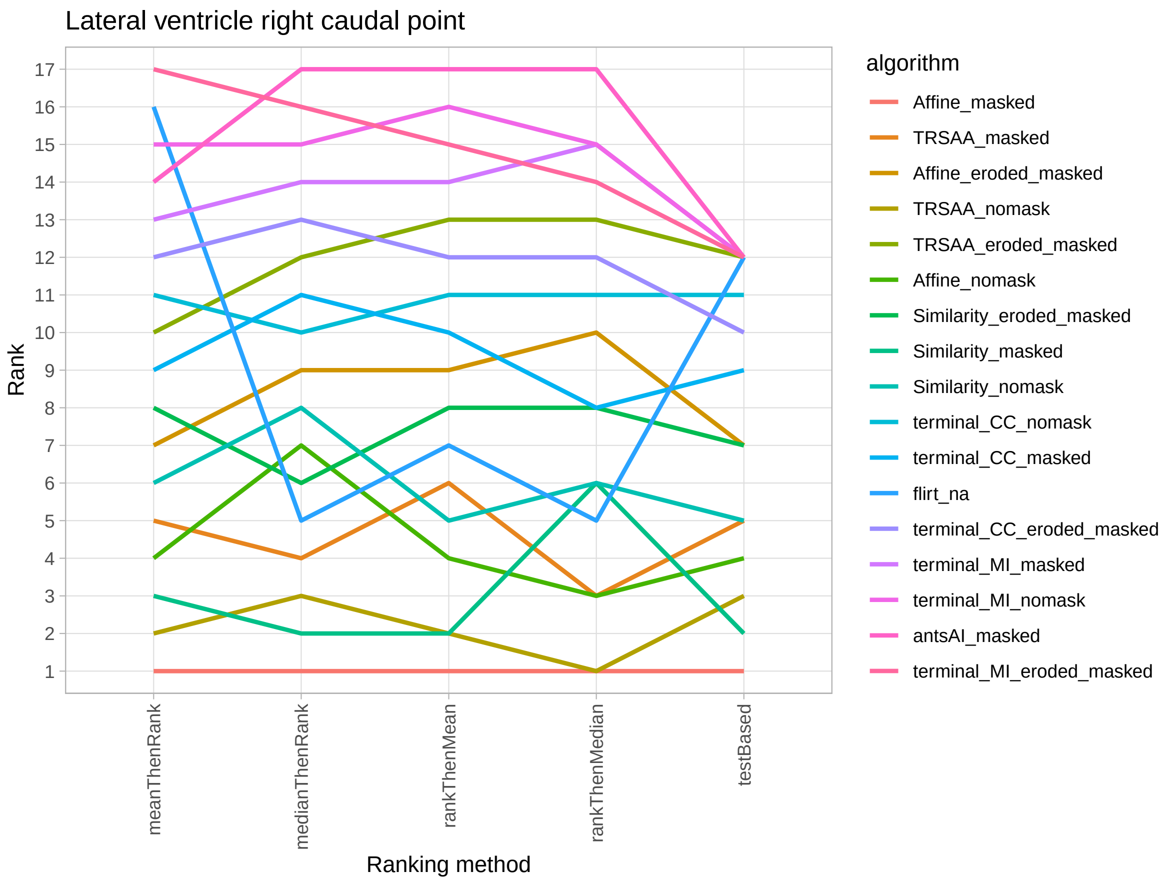

Lateral ventricle right caudal point
Rank
17
16
15
14
13
12
11
10
9
8
7
6
5
4
3
2
1
meanThenRank
medianThenRank
rankThenMean
rankThenMedian
testBased
Ranking method
algorithm
Affine_masked
TRSAA_masked
Affine_eroded_masked
TRSAA_nomask
TRSAA_eroded_masked
Affine_nomask
Similarity_eroded_masked
Similarity_masked
Similarity_nomask
terminal_CC_nomask
terminal_CC_masked
flirt_na
terminal_CC_eroded_masked
terminal_MI_masked
terminal_MI_nomask
antsAI_masked
terminal_MI_eroded_masked

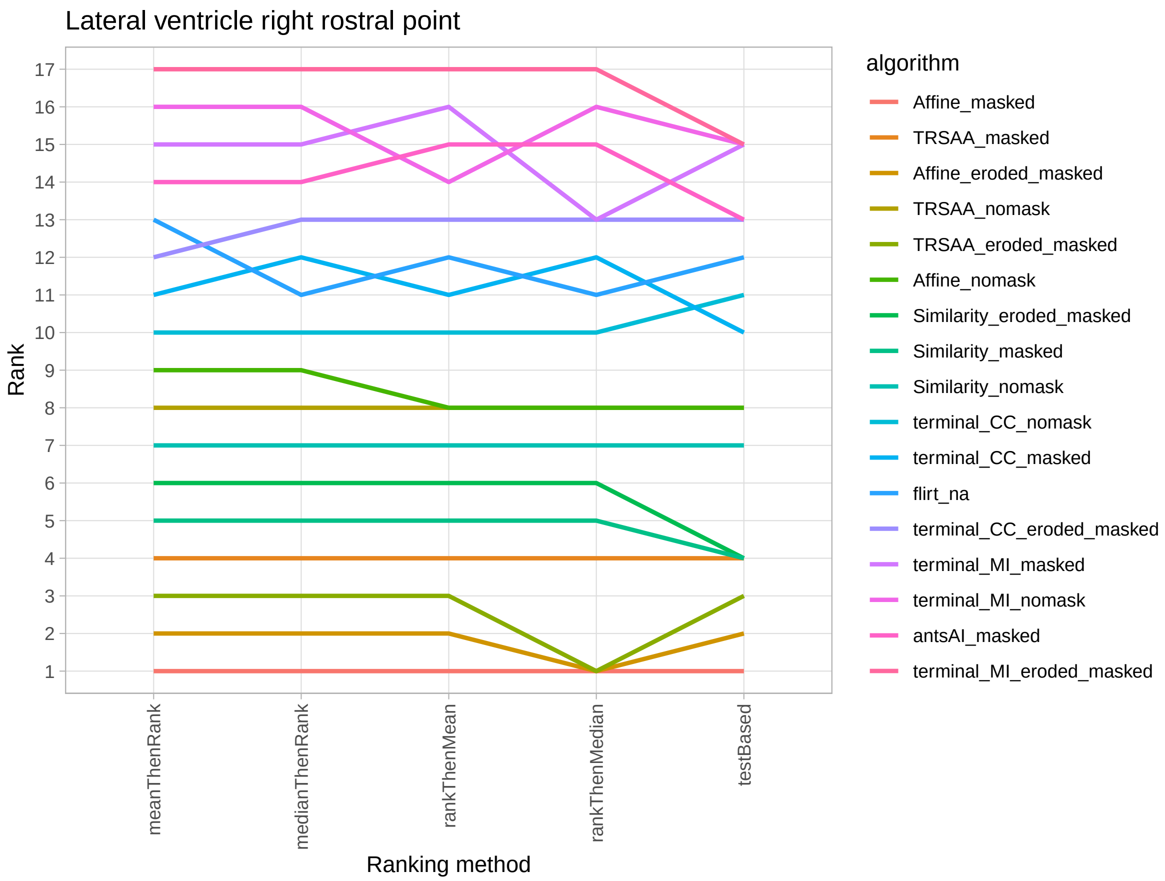

Lateral ventricle right rostral point
Rank
17
16
15
14
13
12
11
10
9
8
7
6
5
4
3
2
1
meanThenRank
medianThenRank
rankThenMean
rankThenMedian
testBased
Ranking method
algorithm
Affine_masked
TRSAA_masked
Affine_eroded_masked
TRSAA_nomask
TRSAA_eroded_masked
Affine_nomask
Similarity_eroded_masked
Similarity_masked
Similarity_nomask
terminal_CC_nomask
terminal_CC_masked
flirt_na
terminal_CC_eroded_masked
terminal_MI_masked
terminal_MI_nomask
antsAI_masked
terminal_MI_eroded_masked

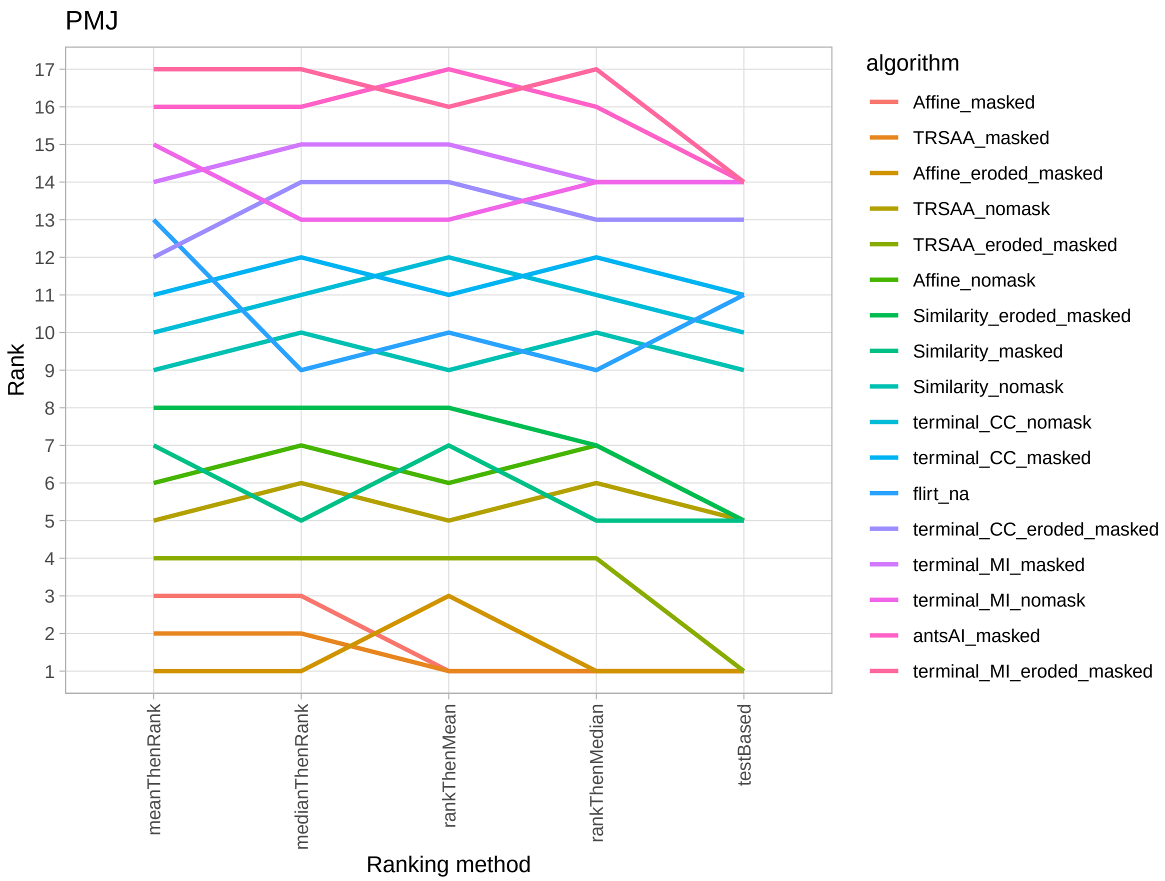
PMJ
Rank
Ranking method
meanThenRank
medianThenRank
rankThenMean
rankThenMedian
testBased
algorithm
Affine_masked
TRSAA_masked
Affine_eroded_masked
TRSAA_nomask
TRSAA_eroded_masked
Affine_nomask
Similarity_eroded_masked
Similarity_masked
Similarity_nomask
terminal_CC_nomask
terminal_CC_masked
flirt_na
terminal_CC_eroded_masked
terminal_MI_masked
terminal_MI_nomask
antsAI_masked
terminal_MI_eroded_masked

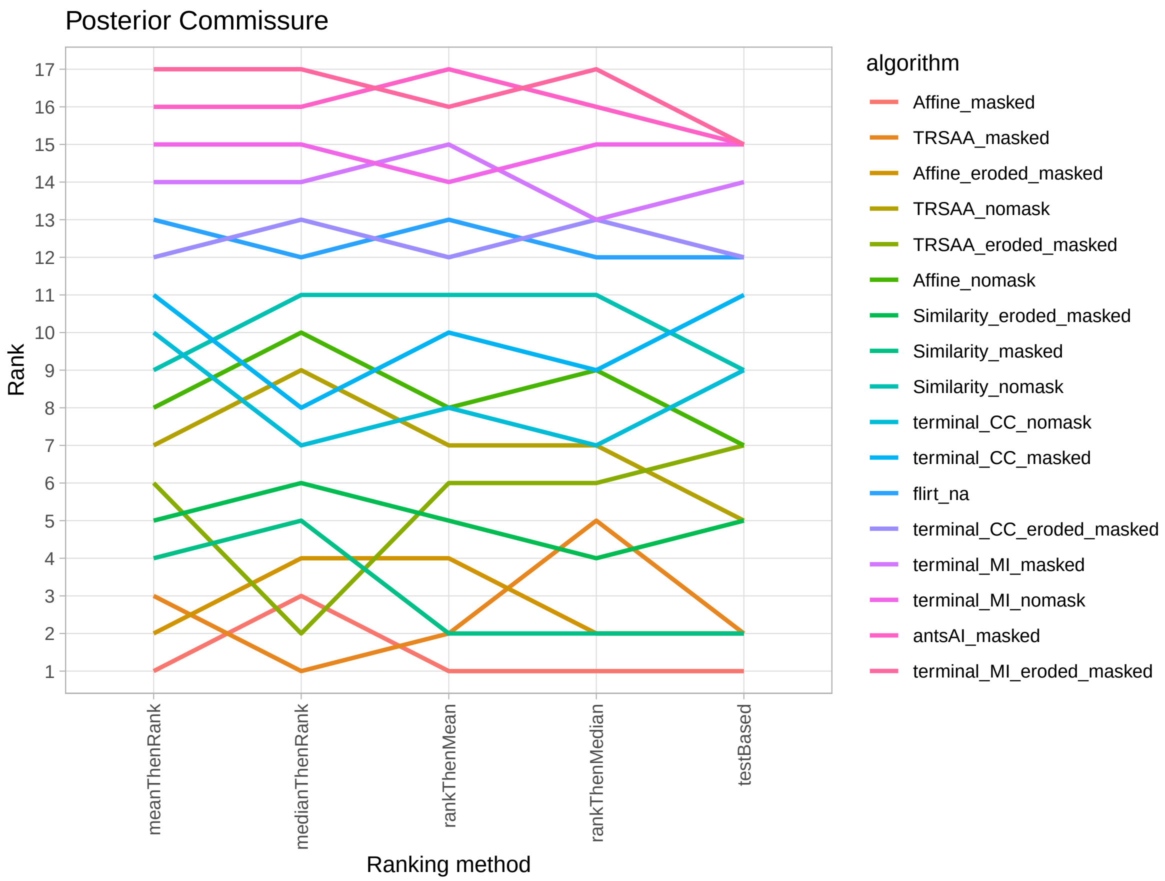

Posterior Commissure
Rank
17
16
15
14
13
12
11
10
9
8
7
6
5
4
3
2
1
meanThenRank
medianThenRank
rankThenMean
rankThenMedian
testBased
Ranking method
algorithm
Affine_masked
TRSAA_masked
Affine_eroded_masked
TRSAA_nomask
TRSAA_eroded_masked
Affine_nomask
Similarity_eroded_masked
Similarity_masked
Similarity_nomask
terminal_CC_nomask
terminal_CC_masked
flirt_na
terminal_CC_eroded_masked
terminal_MI_masked
terminal_MI_nomask
antsAI_masked
terminal_MI_eroded_masked

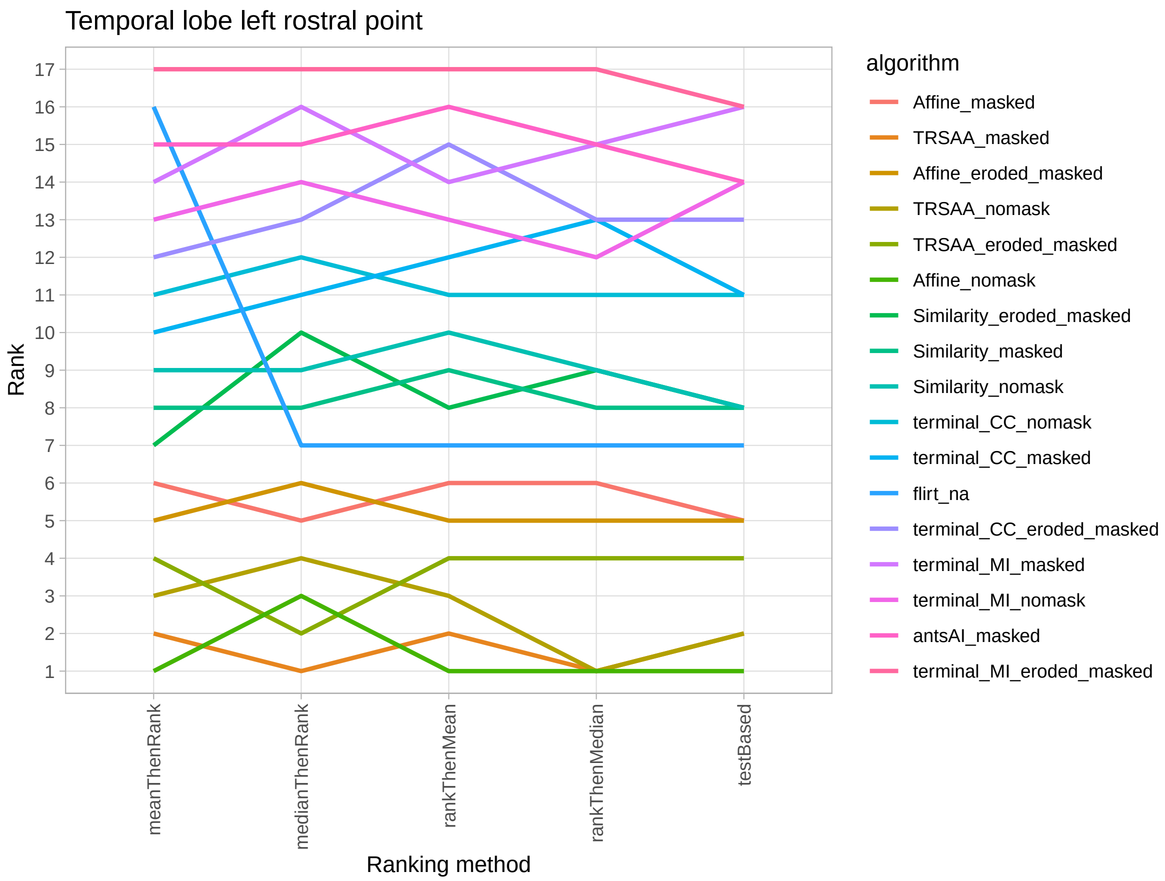
Temporal lobe left rostral point
Rank
17
16
15
14
13
12
11
10
9
8
7
6
5
4
3
2
1
meanThenRank
medianThenRank
rankThenMean
rankThenMedian
testBased
Ranking method
algorithm
Affine_masked
TRSAA_masked
Affine_eroded_masked
TRSAA_nomask
TRSAA_eroded_masked
Affine_nomask
Similarity_eroded_masked
Similarity_masked
Similarity_nomask
terminal_CC_nomask
terminal_CC_masked
flirt_na
terminal_CC_eroded_masked
terminal_MI_masked
terminal_MI_nomask
antsAI_masked
terminal_MI_eroded_masked

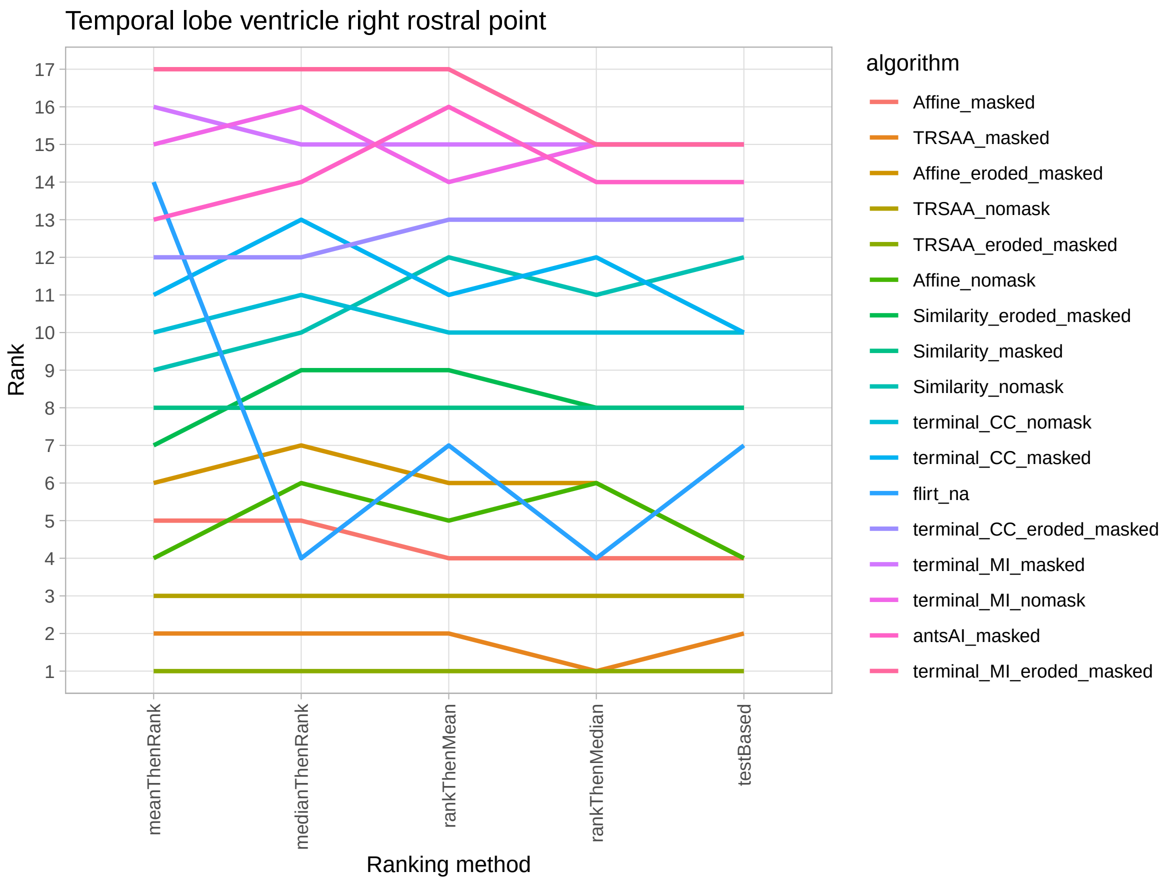

Temporal lobe ventricle right rostral point
Rank
17
16
15
14
13
12
11
10
9
8
7
6
5
4
3
2
1
meanThenRank
medianThenRank
rankThenMean
rankThenMedian
testBased
Ranking method
algorithm
Affine_masked
TRSAA_masked
Affine_eroded_masked
TRSAA_nomask
TRSAA_eroded_masked
Affine_nomask
Similarity_eroded_masked
Similarity_masked
Similarity_nomask
terminal_CC_nomask
terminal_CC_masked
flirt_na
terminal_CC_eroded_masked
terminal_MI_masked
terminal_MI_nomask
antsAI_masked
terminal_MI_eroded_masked

4 Visualization of cross-task insights

The algorithms are ordered according to consensus ranking.

4.1 Characterization of algorithms

4.1.1 Ranking stability: Variability of achieved rankings across tasks

Algorithms are color-coded, and the area of each blob at position $(A_i, \text{rank } j)$ is proportional to the relative frequency A_i achieved rank j across multiple tasks. The median rank for each algorithm is indicated by a black cross. This way, the distribution of ranks across tasks can be intuitively visualized.

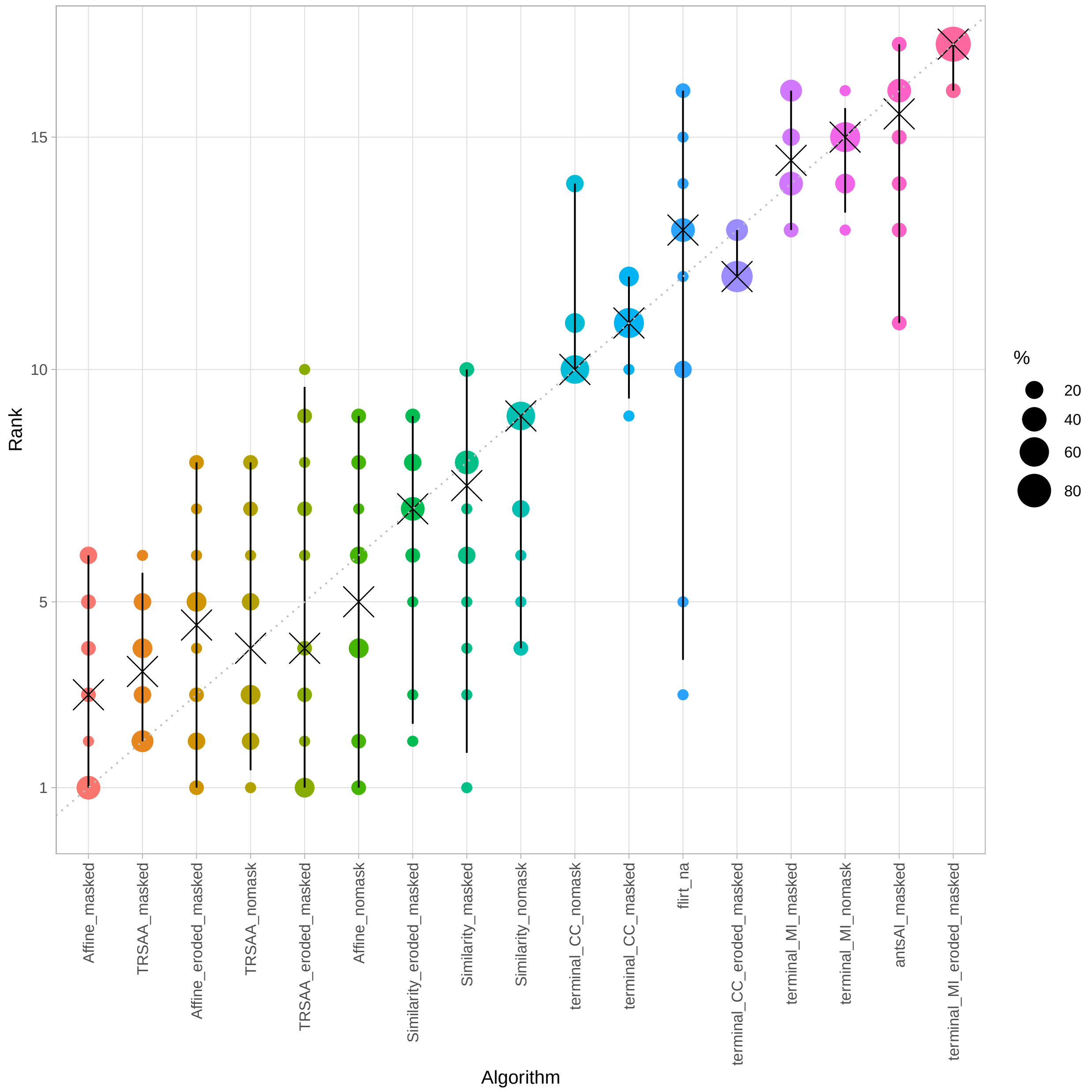

4.1.2 Ranking stability: Ranking variability via bootstrap approach

A blob plot of bootstrap results over the different tasks separated by algorithm allows another perspective on the assessment data. This gives deeper insights into the characteristics of tasks and the ranking uncertainty of the algorithms in each task.

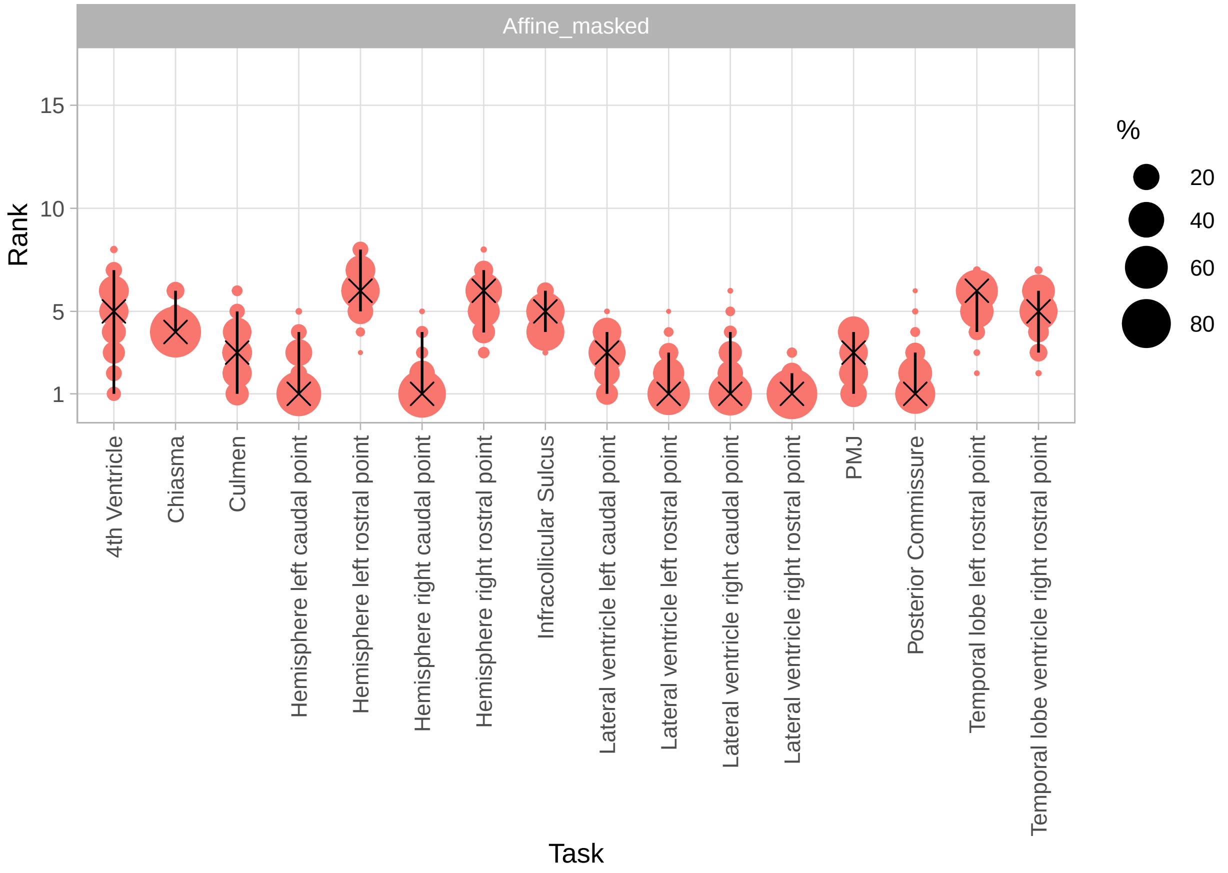

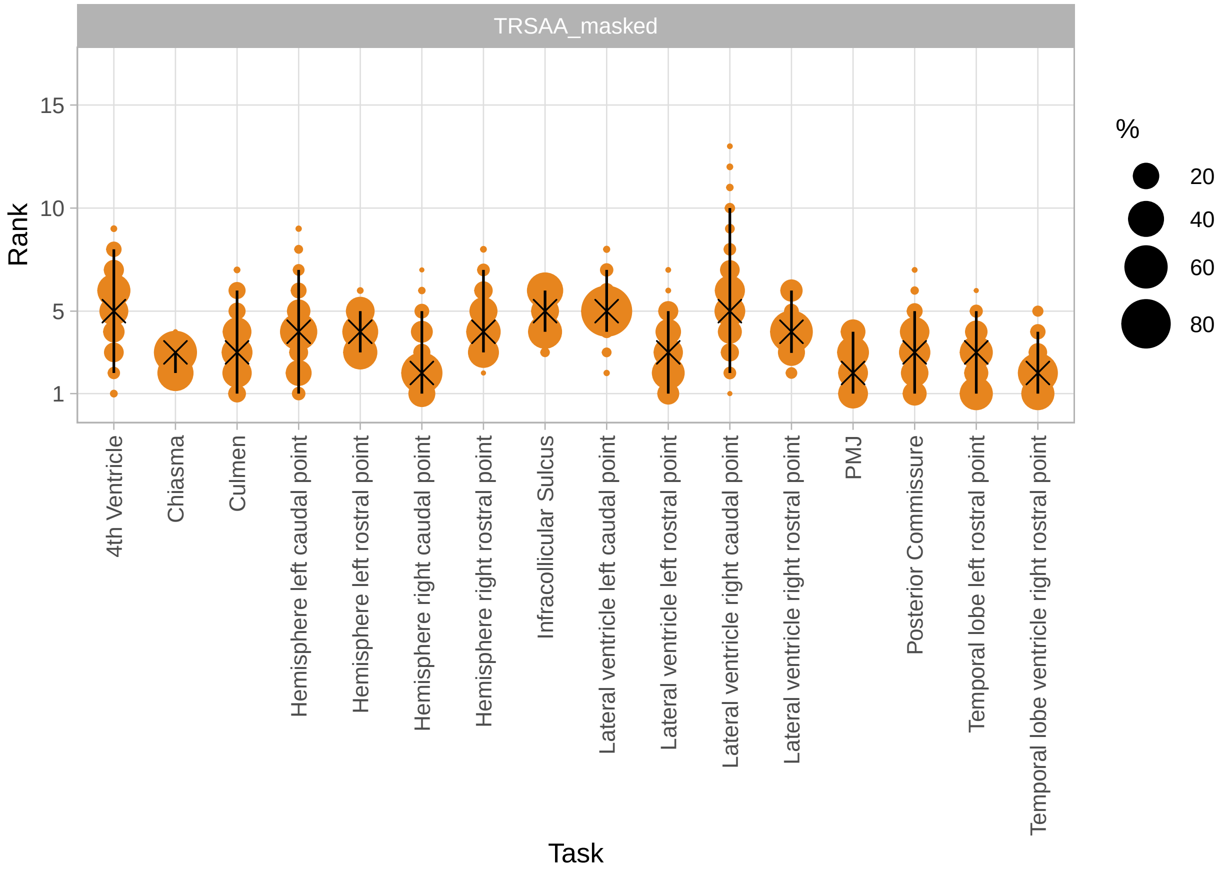
TRSAA_masked
Rank
15
10
5
1
%
20
40
60
80
4th Ventricle
Chiasma
Culmen
Hemisphere left caudal point
Hemisphere left rostral point
Hemisphere right caudal point
Hemisphere right rostral point
Infracollicular Sulcus
Lateral ventricle left caudal point
Lateral ventricle left rostral point
Lateral ventricle right caudal point
Lateral ventricle right rostral point
PMJ
Posterior Commissure
Temporal lobe left rostral point
Temporal lobe ventricle right rostral point
Task

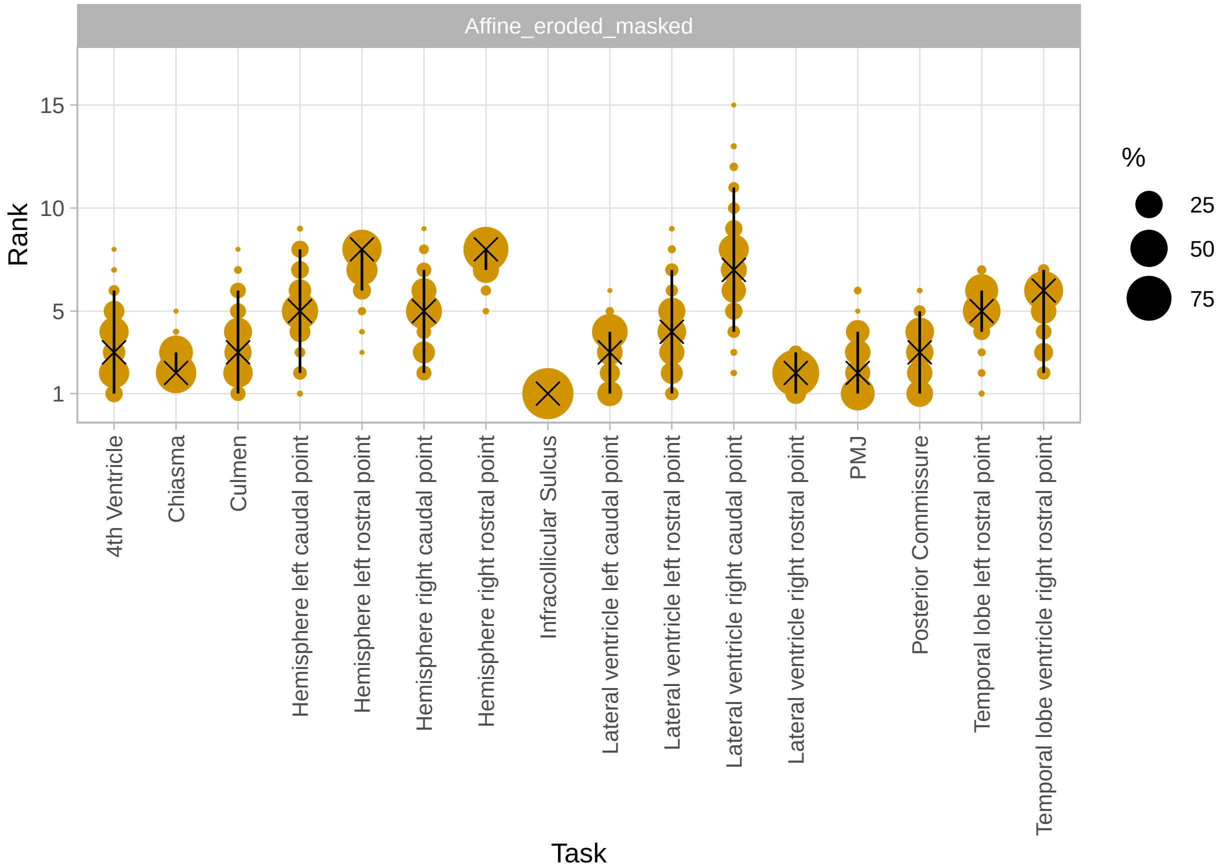
Affine_eroded_masked
Rank
15
10
5
1
Task
4th Ventricle
Chiasma
Culmen
Hemisphere left caudal point
Hemisphere left rostral point
Hemisphere right caudal point
Hemisphere right rostral point
Infracollicular Sulcus
Lateral ventricle left caudal point
Lateral ventricle left rostral point
Lateral ventricle right caudal point
Lateral ventricle right rostral point
PMJ
Posterior Commissure
Temporal lobe left rostral point
Temporal lobe ventricle right rostral point
%
25
50
75

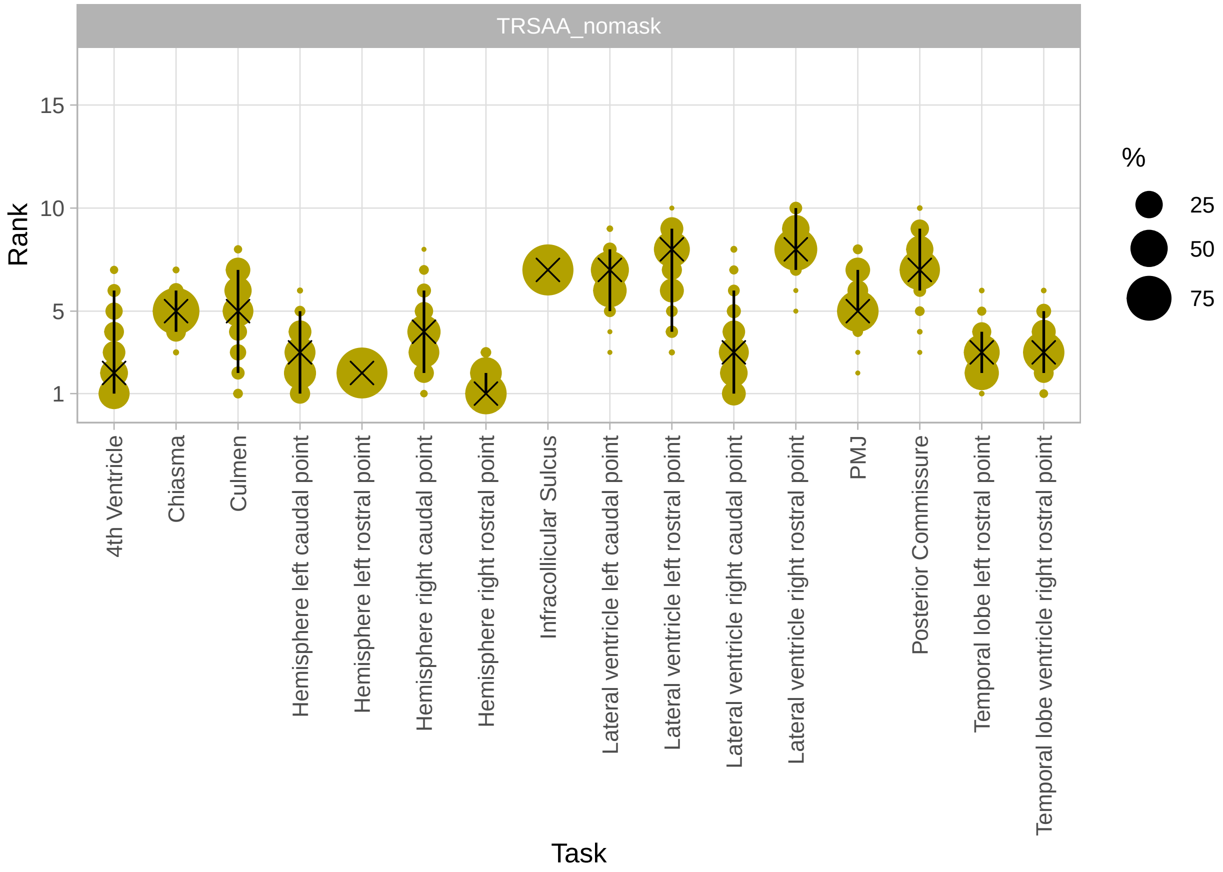
TRSAA_nomask
Rank
15
10
5
1
%
25
50
75
4th Ventricle
Chiasma
Culmen
Hemisphere left caudal point
Hemisphere left rostral point
Hemisphere right caudal point
Hemisphere right rostral point
Infracollicular Sulcus
Lateral ventricle left caudal point
Lateral ventricle left rostral point
Lateral ventricle right caudal point
Lateral ventricle right rostral point
PMJ
Posterior Commissure
Temporal lobe left rostral point
Temporal lobe ventricle right rostral point
Task

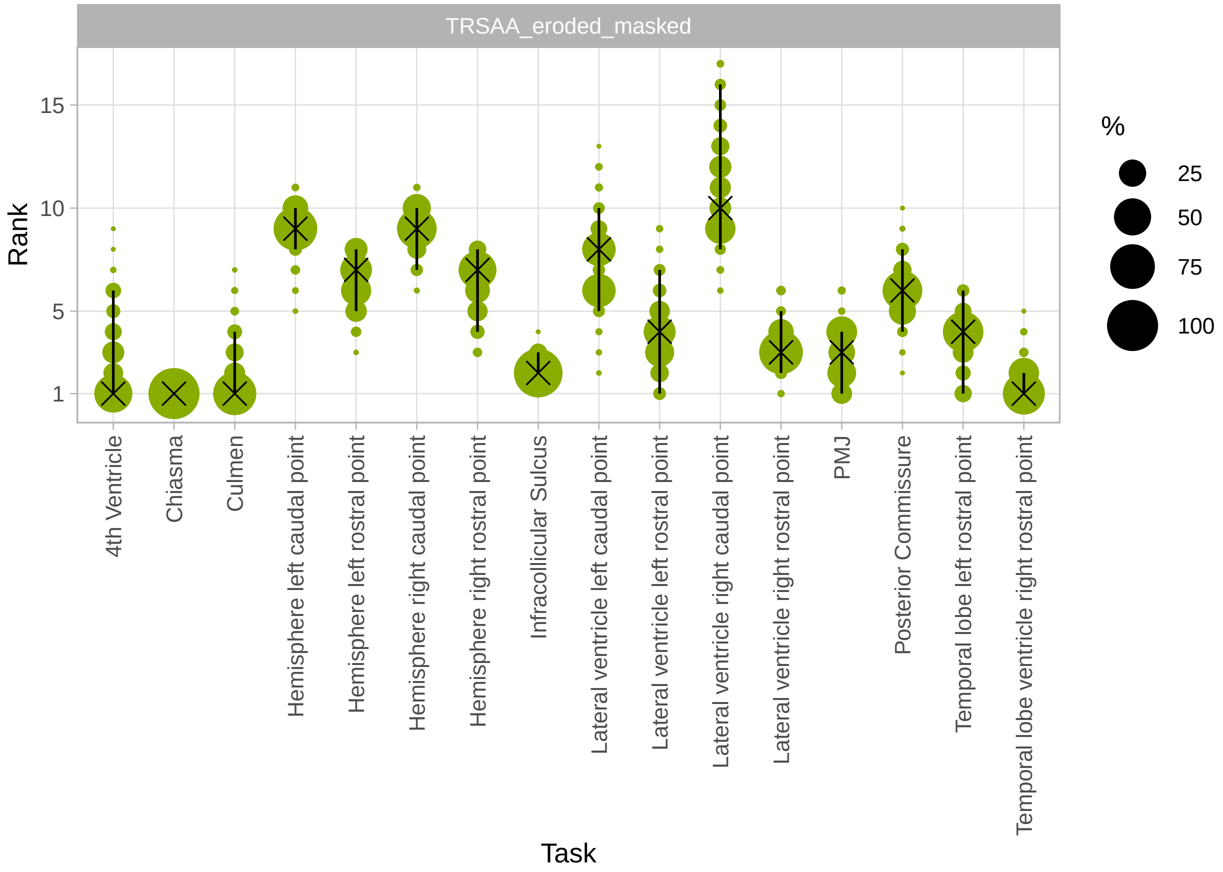

TRSAA_eroded_masked
Rank
15
10
5
1
Task
4th Ventricle
Chiasma
Culmen
Hemisphere left caudal point
Hemisphere left rostral point
Hemisphere right caudal point
Hemisphere right rostral point
Infracollicular Sulcus
Lateral ventricle left caudal point
Lateral ventricle left rostral point
Lateral ventricle right caudal point
Lateral ventricle right rostral point
PMJ
Posterior Commissure
Temporal lobe left rostral point
Temporal lobe ventricle right rostral point
%
25
50
75
100

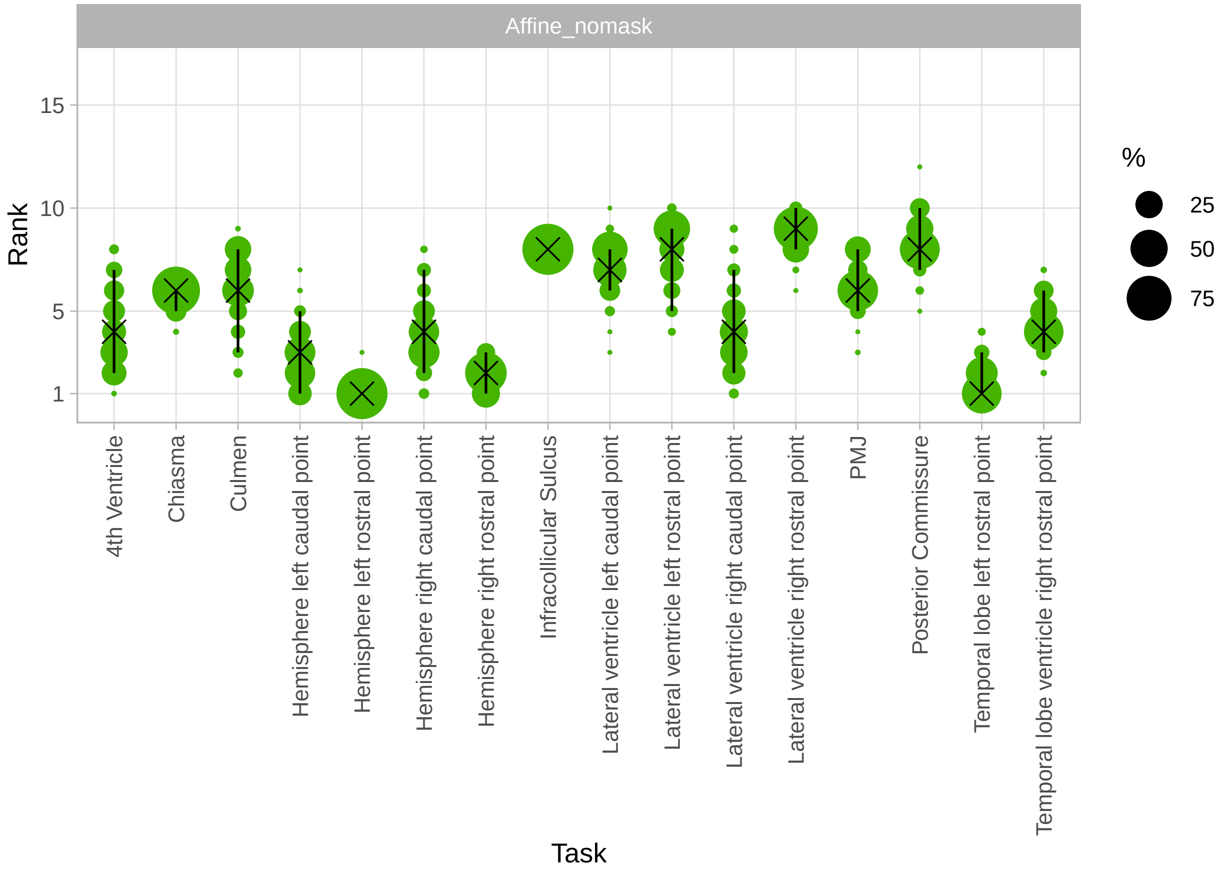
Affine_nomask
Rank
15
10
5
1
%
25
50
75
4th Ventricle
Chiasma
Culmen
Hemisphere left caudal point
Hemisphere left rostral point
Hemisphere right caudal point
Hemisphere right rostral point
Infracollicular Sulcus
Lateral ventricle left caudal point
Lateral ventricle left rostral point
Lateral ventricle right caudal point
Lateral ventricle right rostral point
PMJ
Posterior Commissure
Temporal lobe left rostral point
Temporal lobe ventricle right rostral point
Task

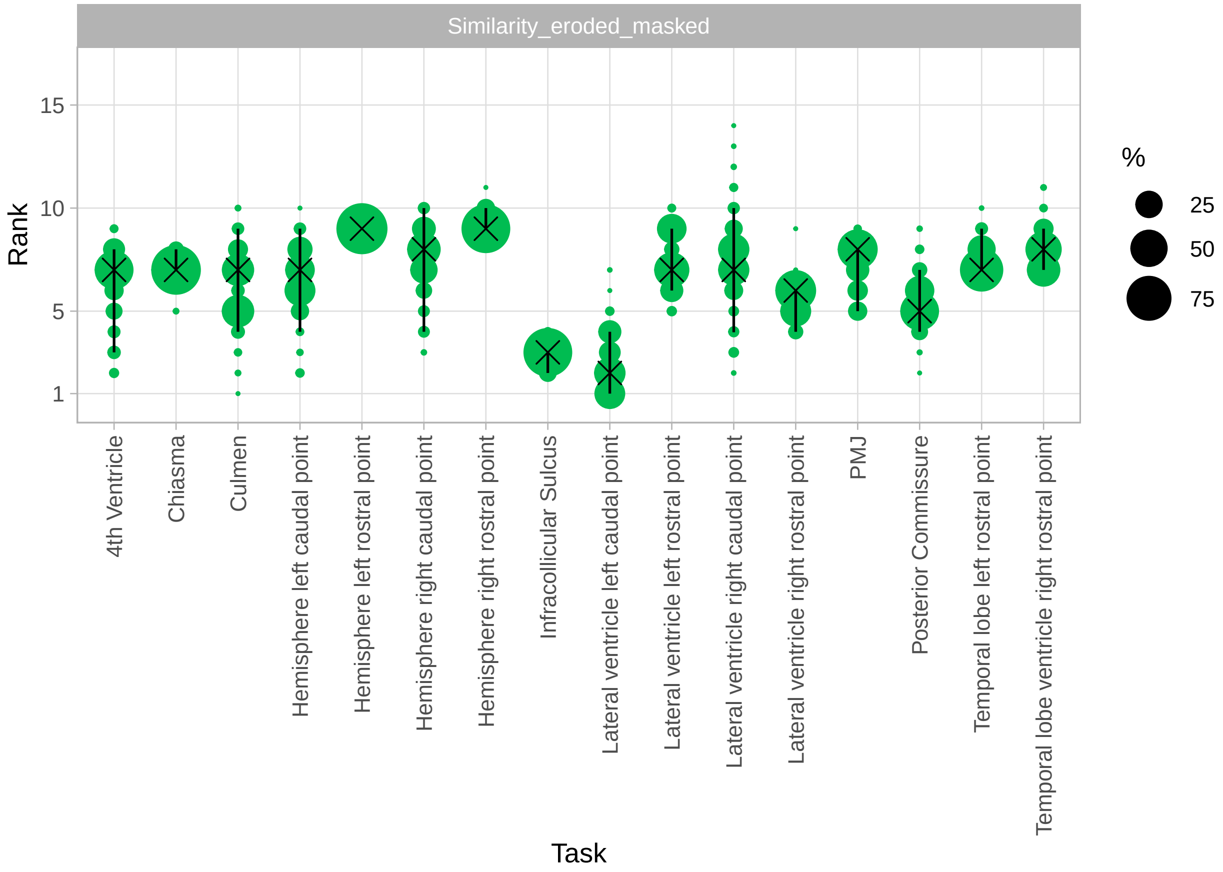
Similarity_eroded_masked
Rank
15
10
5
1
%
25
50
75
4th Ventricle
Chiasma
Culmen
Hemisphere left caudal point
Hemisphere left rostral point
Hemisphere right caudal point
Hemisphere right rostral point
Infracollicular Sulcus
Lateral ventricle left caudal point
Lateral ventricle left rostral point
Lateral ventricle right caudal point
Lateral ventricle right rostral point
PMJ
Posterior Commissure
Temporal lobe left rostral point
Temporal lobe ventricle right rostral point
Task

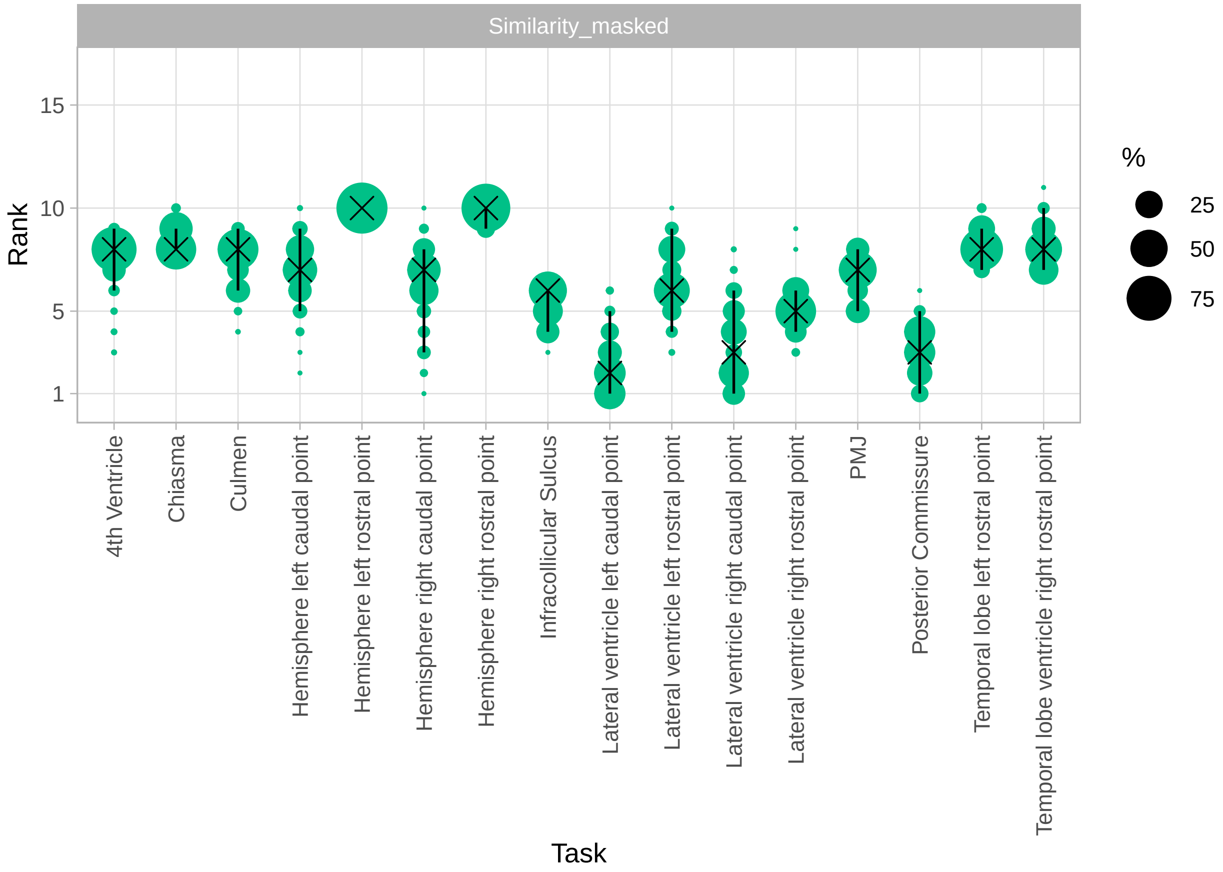

Similarity_masked
Rank
15
10
5
1
%
25
50
75
4th Ventricle
Chiasma
Culmen
Hemisphere left caudal point
Hemisphere left rostral point
Hemisphere right caudal point
Hemisphere right rostral point
Infracollicular Sulcus
Lateral ventricle left caudal point
Lateral ventricle left rostral point
Lateral ventricle right caudal point
Lateral ventricle right rostral point
PMJ
Posterior Commissure
Temporal lobe left rostral point
Temporal lobe ventricle right rostral point
Task

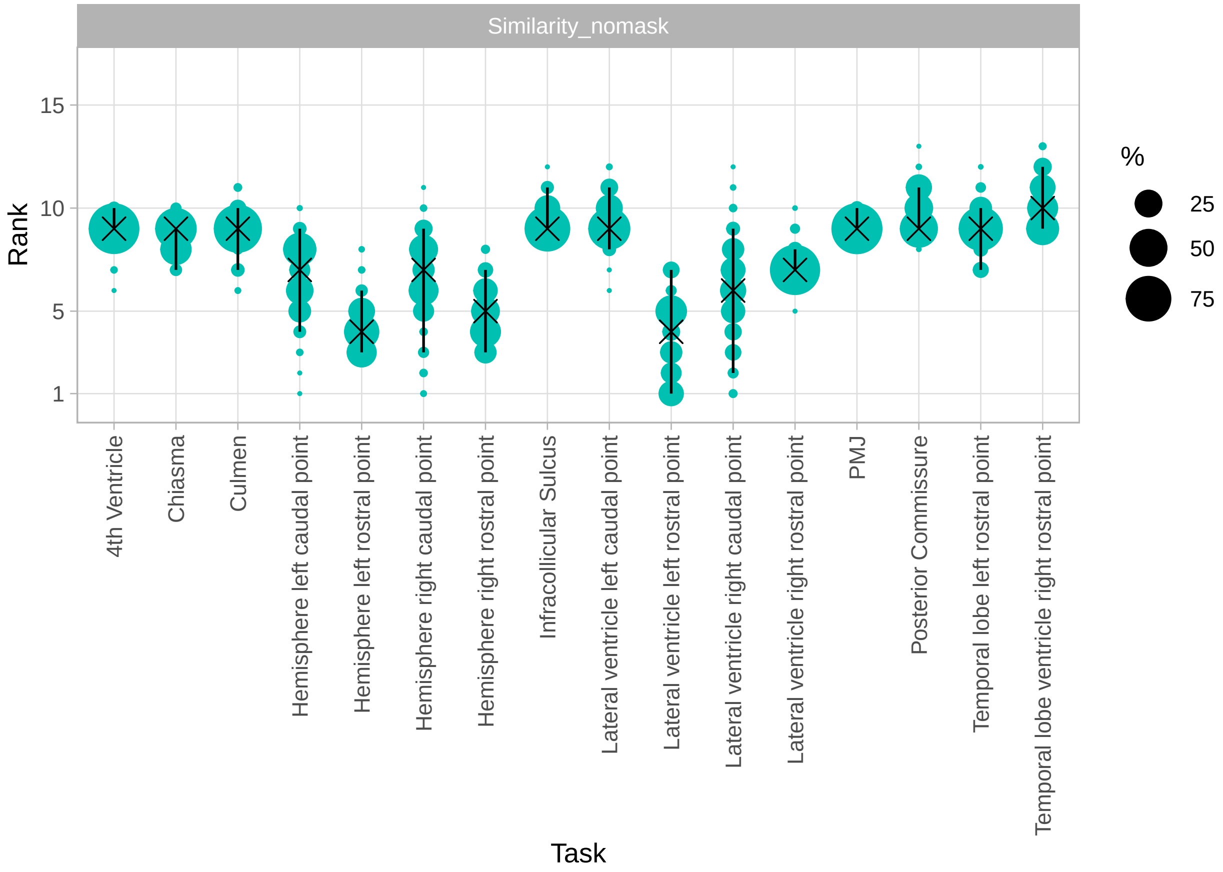

Similarity_nomask
Rank
15
10
5
1
4th Ventricle
Chiasma
Culmen
Hemisphere left caudal point
Hemisphere left rostral point
Hemisphere right caudal point
Hemisphere right rostral point
Infracollicular Sulcus
Lateral ventricle left caudal point
Lateral ventricle left rostral point
Lateral ventricle right caudal point
Lateral ventricle right rostral point
PMJ
Posterior Commissure
Temporal lobe left rostral point
Temporal lobe ventricle right rostral point
Task
%
25
50
75

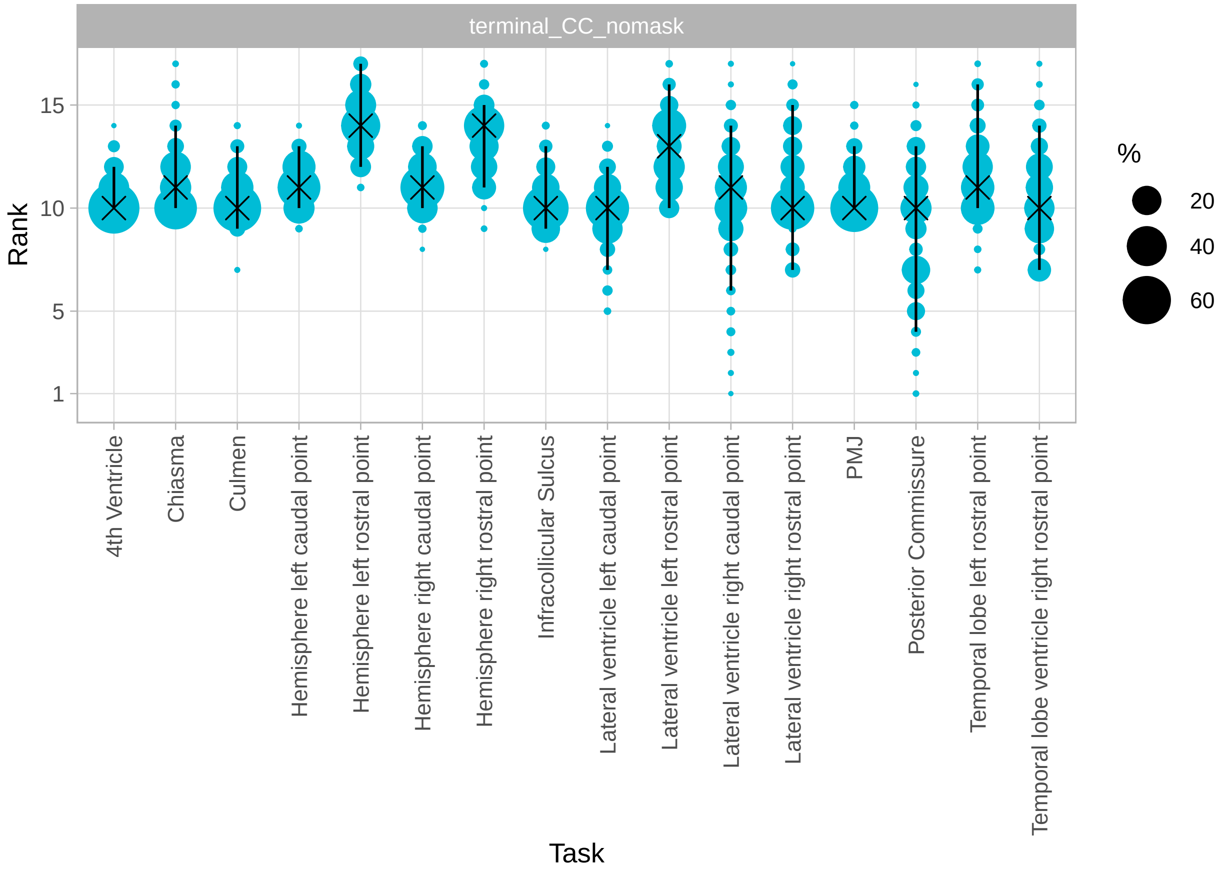
terminal_CC_nomask
Rank
15
10
5
1
%
20
40
60
4th Ventricle
Chiasma
Culmen
Hemisphere left caudal point
Hemisphere left rostral point
Hemisphere right caudal point
Hemisphere right rostral point
Infracollicular Sulcus
Lateral ventricle left caudal point
Lateral ventricle left rostral point
Lateral ventricle right caudal point
Lateral ventricle right rostral point
PMJ
Posterior Commissure
Temporal lobe left rostral point
Temporal lobe ventricle right rostral point
Task

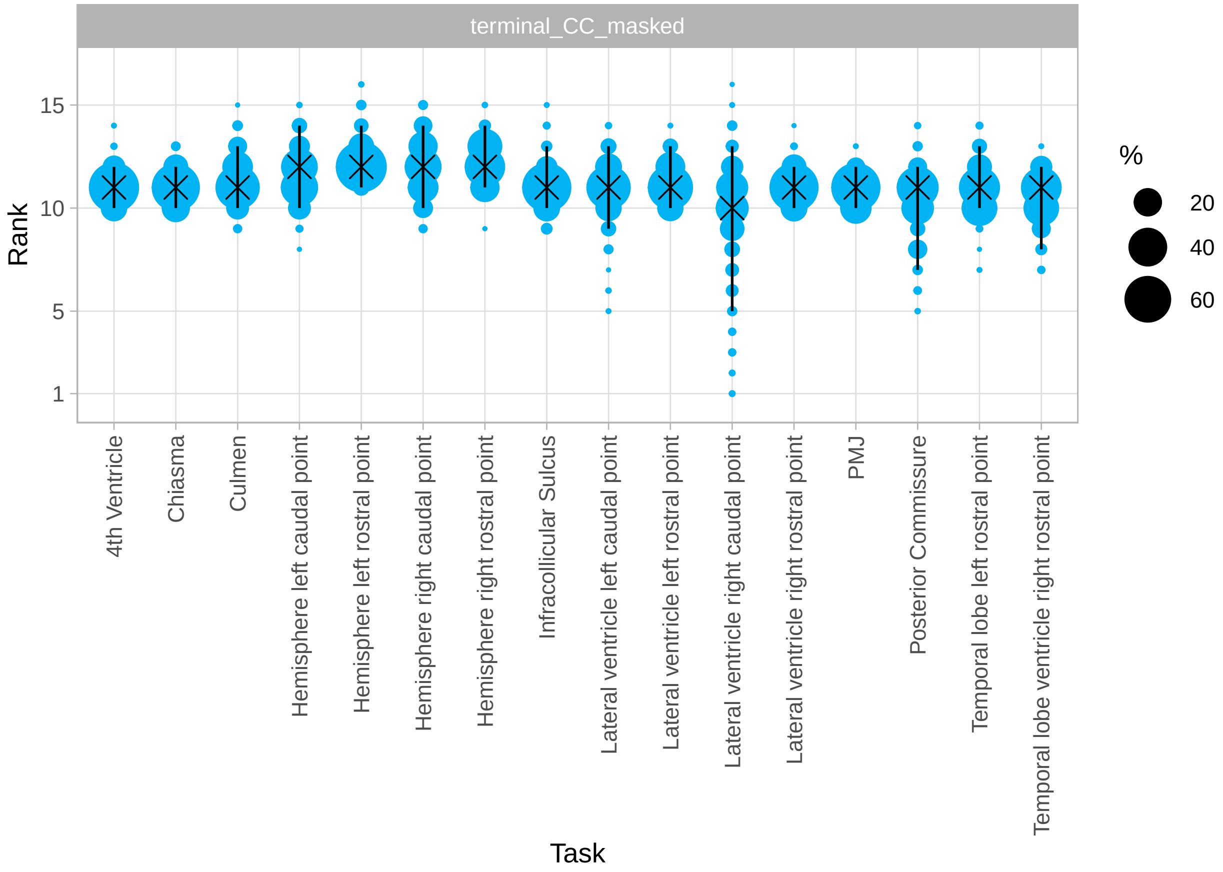

terminal_CC_masked
Rank
15
10
5
1
4th Ventricle
Chiasma
Culmen
Hemisphere left caudal point
Hemisphere left rostral point
Hemisphere right caudal point
Hemisphere right rostral point
Infracollicular Sulcus
Lateral ventricle left caudal point
Lateral ventricle left rostral point
Lateral ventricle right caudal point
Lateral ventricle right rostral point
PMJ
Posterior Commissure
Temporal lobe left rostral point
Temporal lobe ventricle right rostral point
Task
%
20
40
60

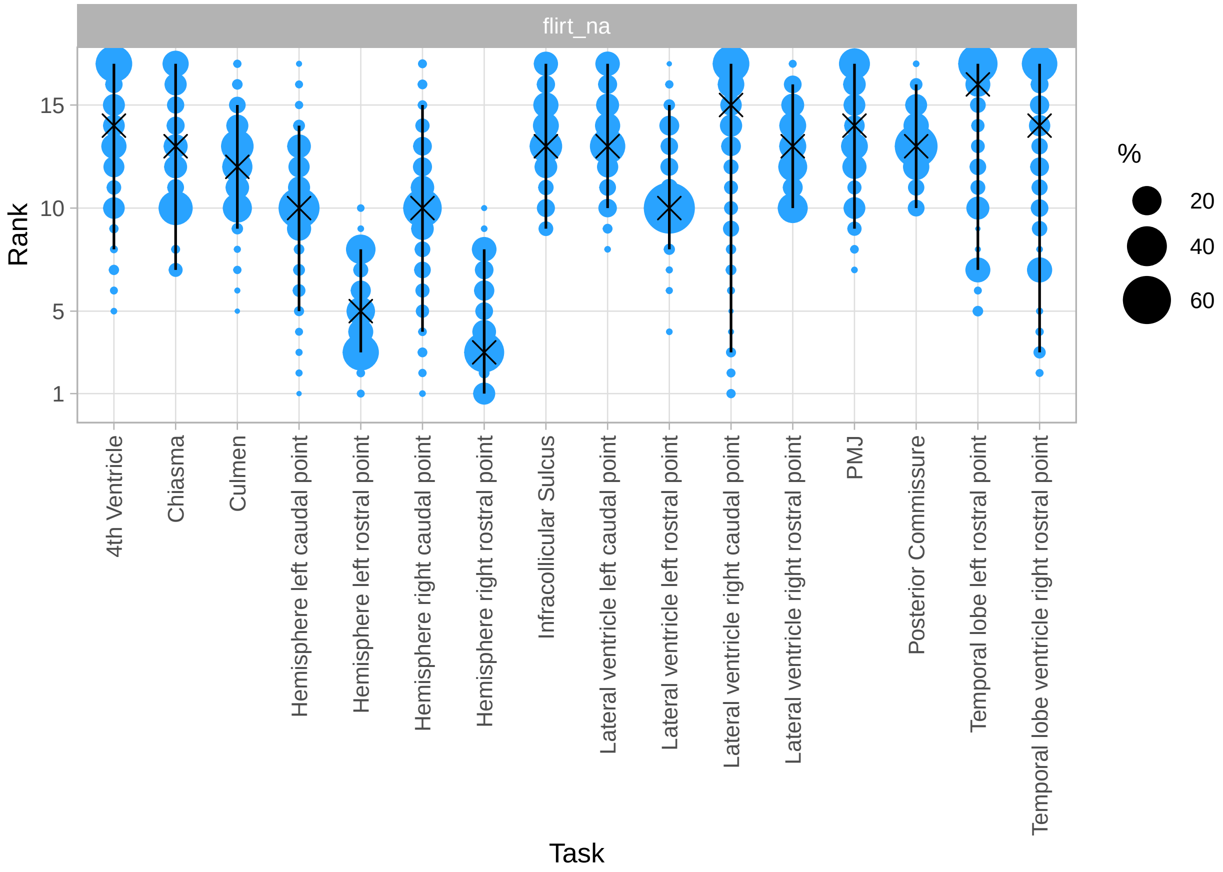
flirt_na
Rank
15
10
5
1
4th Ventricle
Chiasma
Culmen
Hemisphere left caudal point
Hemisphere left rostral point
Hemisphere right caudal point
Hemisphere right rostral point
Infracollicular Sulcus
Lateral ventricle left caudal point
Lateral ventricle left rostral point
Lateral ventricle right caudal point
Lateral ventricle right rostral point
PMJ
Posterior Commissure
Temporal lobe left rostral point
Temporal lobe ventricle right rostral point
Task
%
20
40
60

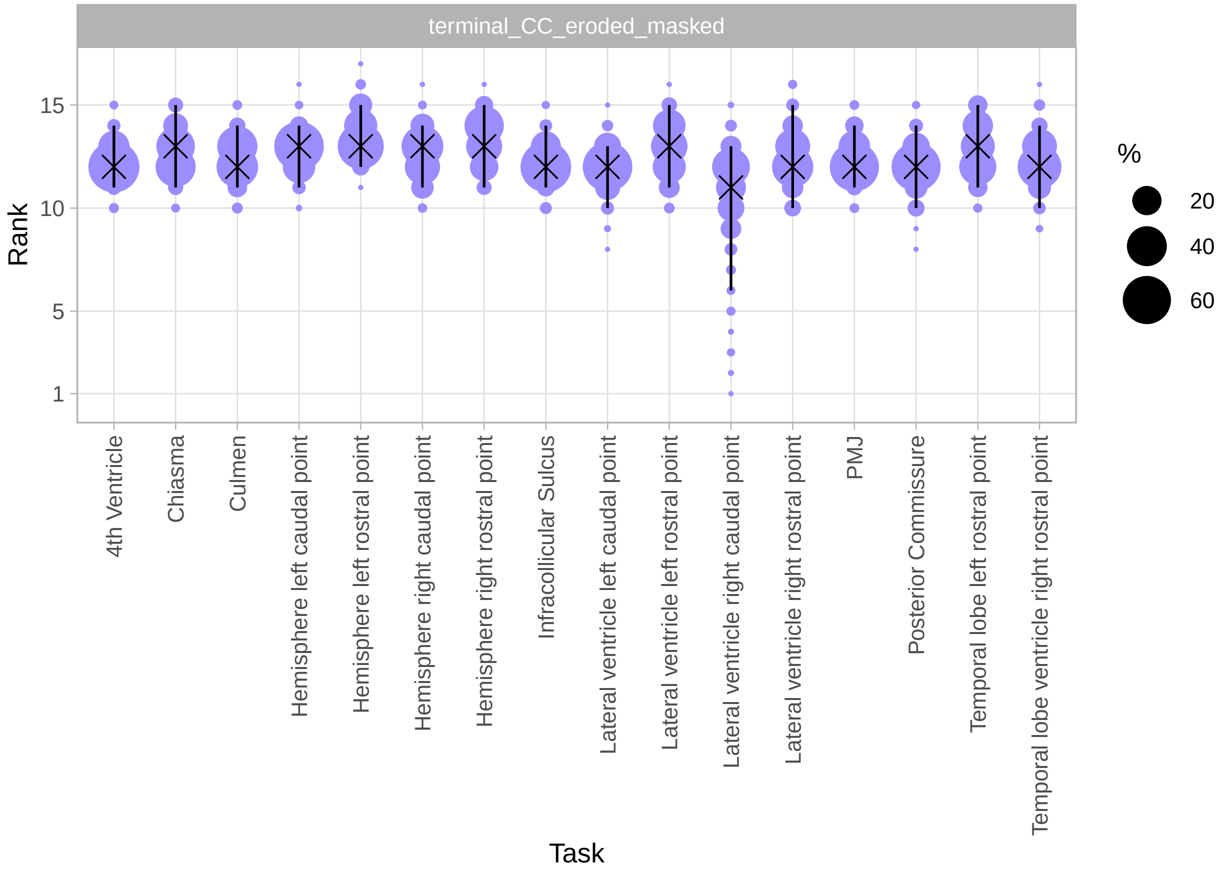

terminal_CC_eroded_masked
Rank
15
10
5
1
%
20
40
60
4th Ventricle
Chiasma
Culmen
Hemisphere left caudal point
Hemisphere left rostral point
Hemisphere right caudal point
Hemisphere right rostral point
Infracollicular Sulcus
Lateral ventricle left caudal point
Lateral ventricle left rostral point
Lateral ventricle right caudal point
Lateral ventricle right rostral point
PMJ
Posterior Commissure
Temporal lobe left rostral point
Temporal lobe ventricle right rostral point
Task

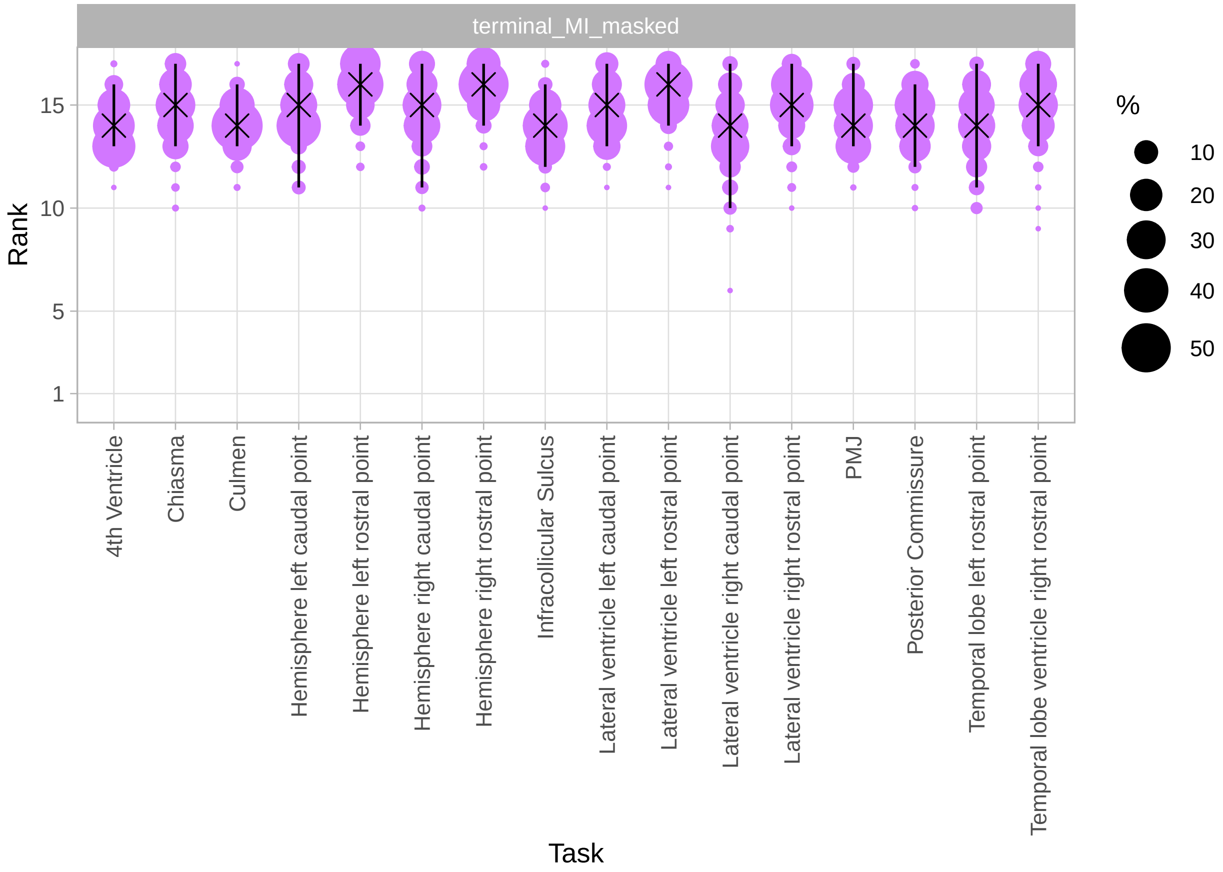
terminal_MI_masked
Rank
15
10
5
1
4th Ventricle
Chiasma
Culmen
Hemisphere left caudal point
Hemisphere left rostral point
Hemisphere right caudal point
Hemisphere right rostral point
Infracollicular Sulcus
Lateral ventricle left caudal point
Lateral ventricle left rostral point
Lateral ventricle right caudal point
Lateral ventricle right rostral point
PMJ
Posterior Commissure
Temporal lobe left rostral point
Temporal lobe ventricle right rostral point
Task
%
10
20
30
40
50

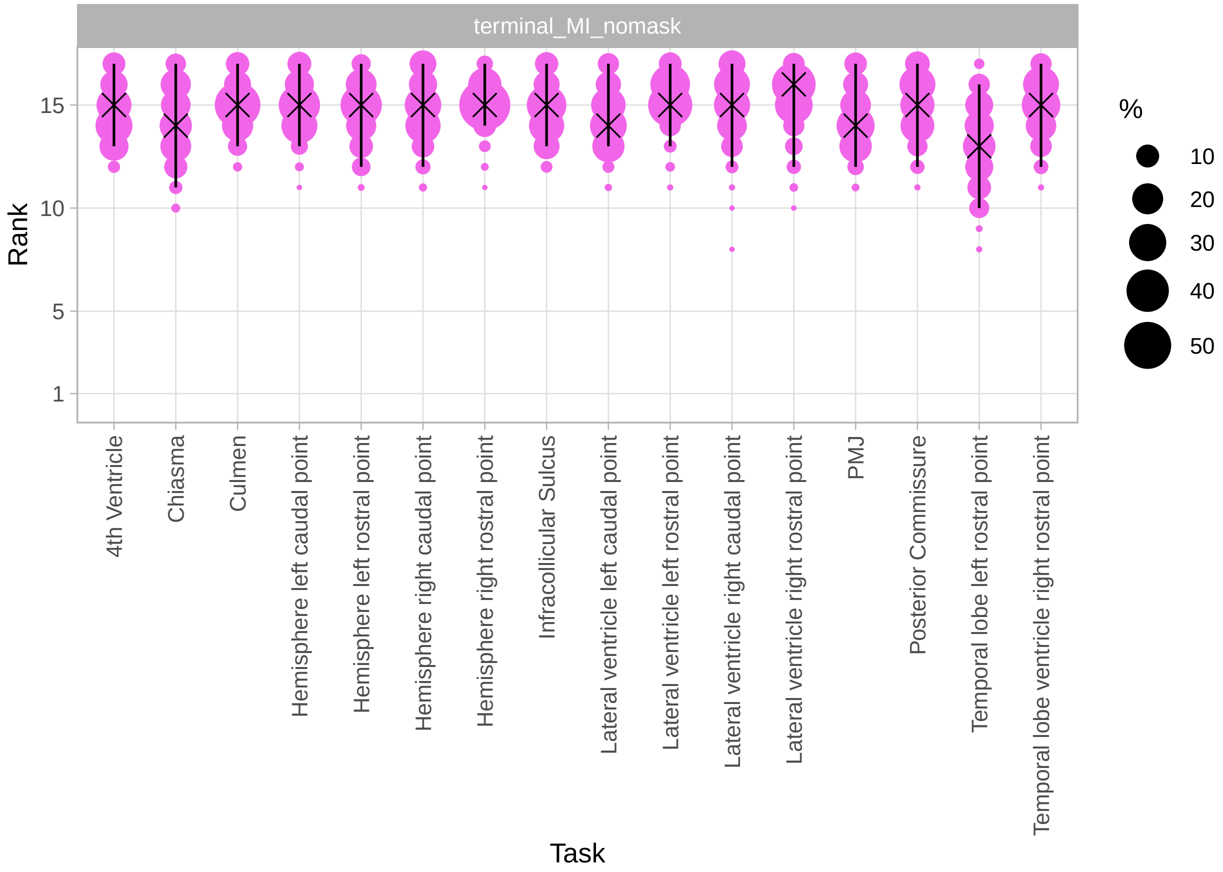
terminal_MI_nomask
Rank
15
10
5
1
%
10
20
30
40
50
4th Ventricle
Chiasma
Culmen
Hemisphere left caudal point
Hemisphere left rostral point
Hemisphere right caudal point
Hemisphere right rostral point
Infracollicular Sulcus
Lateral ventricle left caudal point
Lateral ventricle left rostral point
Lateral ventricle right caudal point
Lateral ventricle right rostral point
PMJ
Posterior Commissure
Temporal lobe left rostral point
Temporal lobe ventricle right rostral point
Task

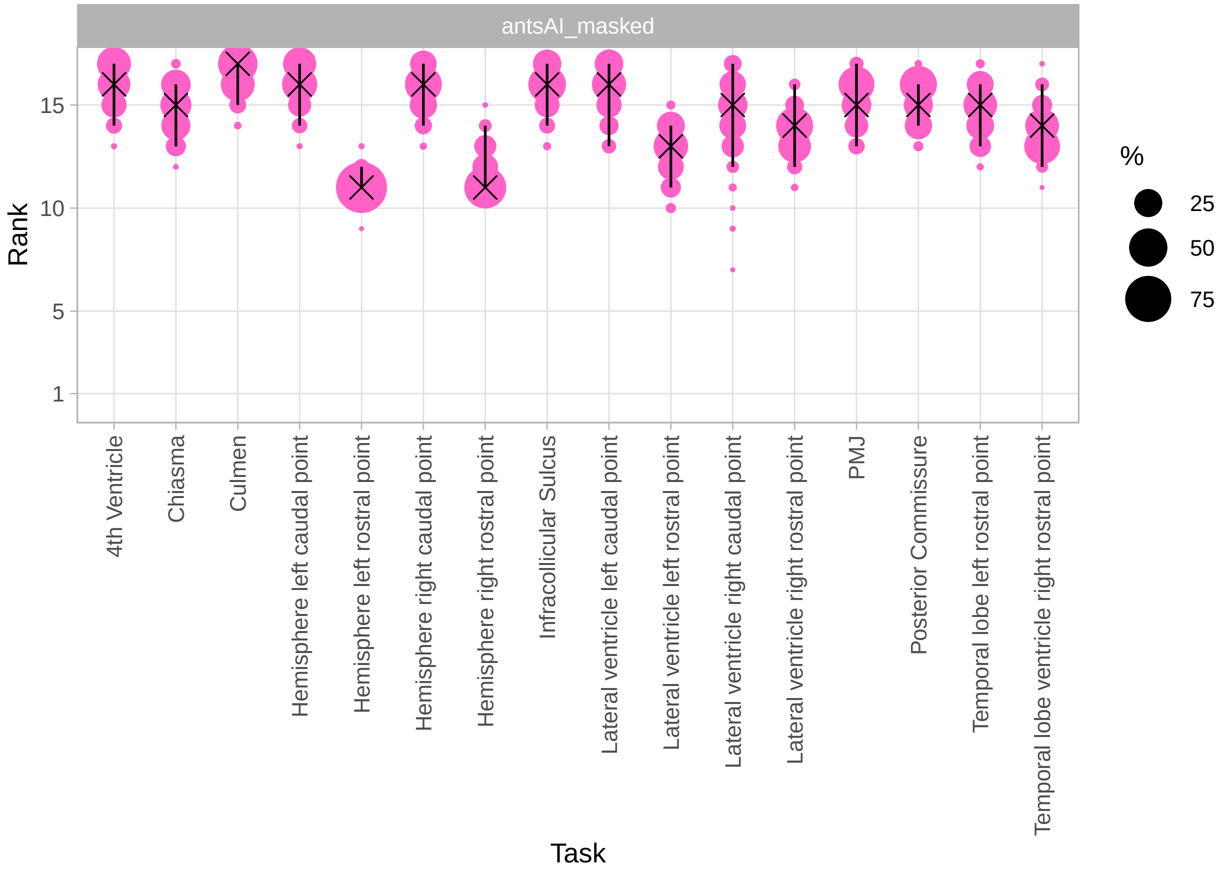
antsAI_masked
Rank
15
10
5
1
4th Ventricle
Chiasma
Culmen
Hemisphere left caudal point
Hemisphere left rostral point
Hemisphere right caudal point
Hemisphere right rostral point
Infracollicular Sulcus
Lateral ventricle left caudal point
Lateral ventricle left rostral point
Lateral ventricle right caudal point
Lateral ventricle right rostral point
PMJ
Posterior Commissure
Temporal lobe left rostral point
Temporal lobe ventricle right rostral point
Task
%
25
50
75

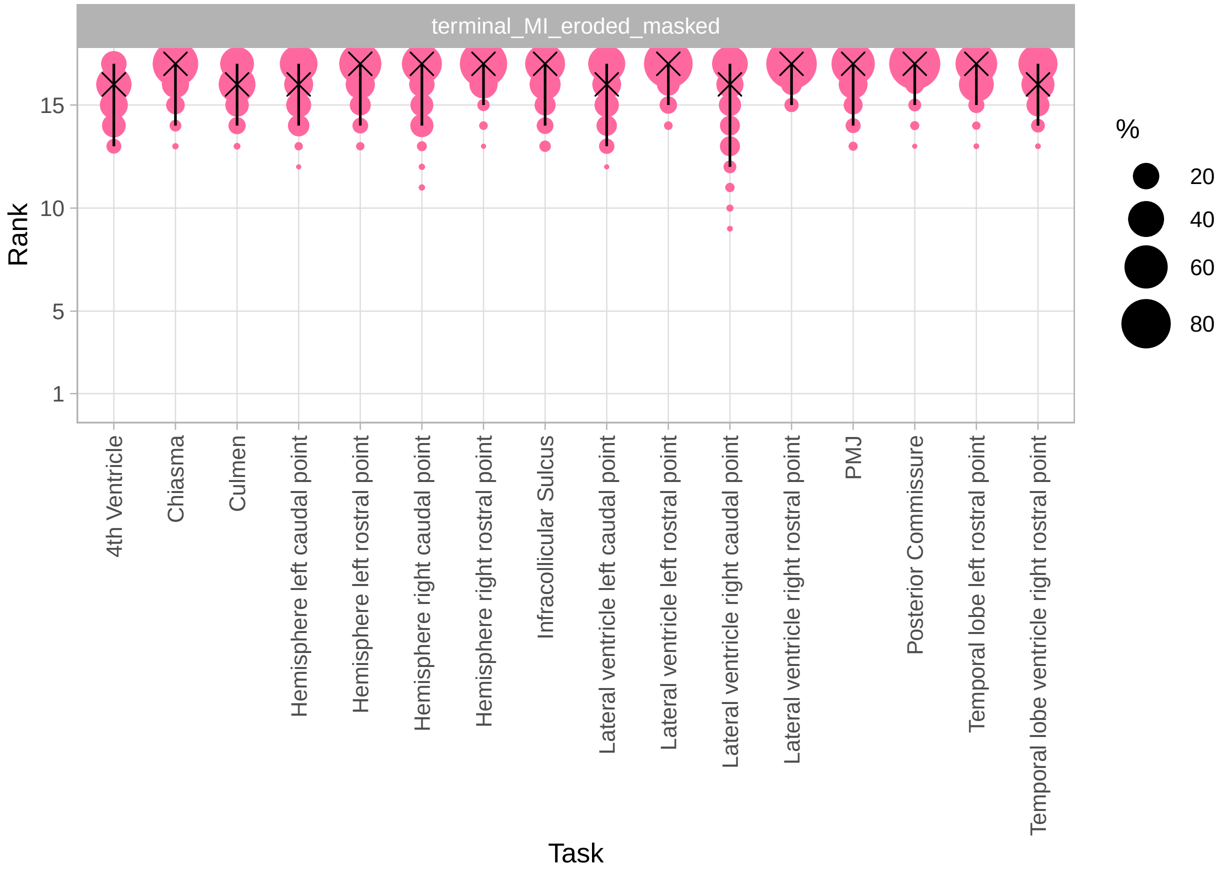
terminal_MI_eroded_masked
Rank
15
10
5
1
%
20
40
60
80
4th Ventricle
Chiasma
Culmen
Hemisphere left caudal point
Hemisphere left rostral point
Hemisphere right caudal point
Hemisphere right rostral point
Infracollicular Sulcus
Lateral ventricle left caudal point
Lateral ventricle left rostral point
Lateral ventricle right caudal point
Lateral ventricle right rostral point
PMJ
Posterior Commissure
Temporal lobe left rostral point
Temporal lobe ventricle right rostral point
Task

An alternative representation is provided by a stacked frequency plot of the observed ranks, separated by algorithm. Observed ranks across bootstrap samples are displayed with coloring according to the task. For algorithms that achieve the same rank in different tasks for the full assessment data set, vertical lines are on top of each other. Vertical lines allow to compare the achieved rank of each algorithm over different tasks.

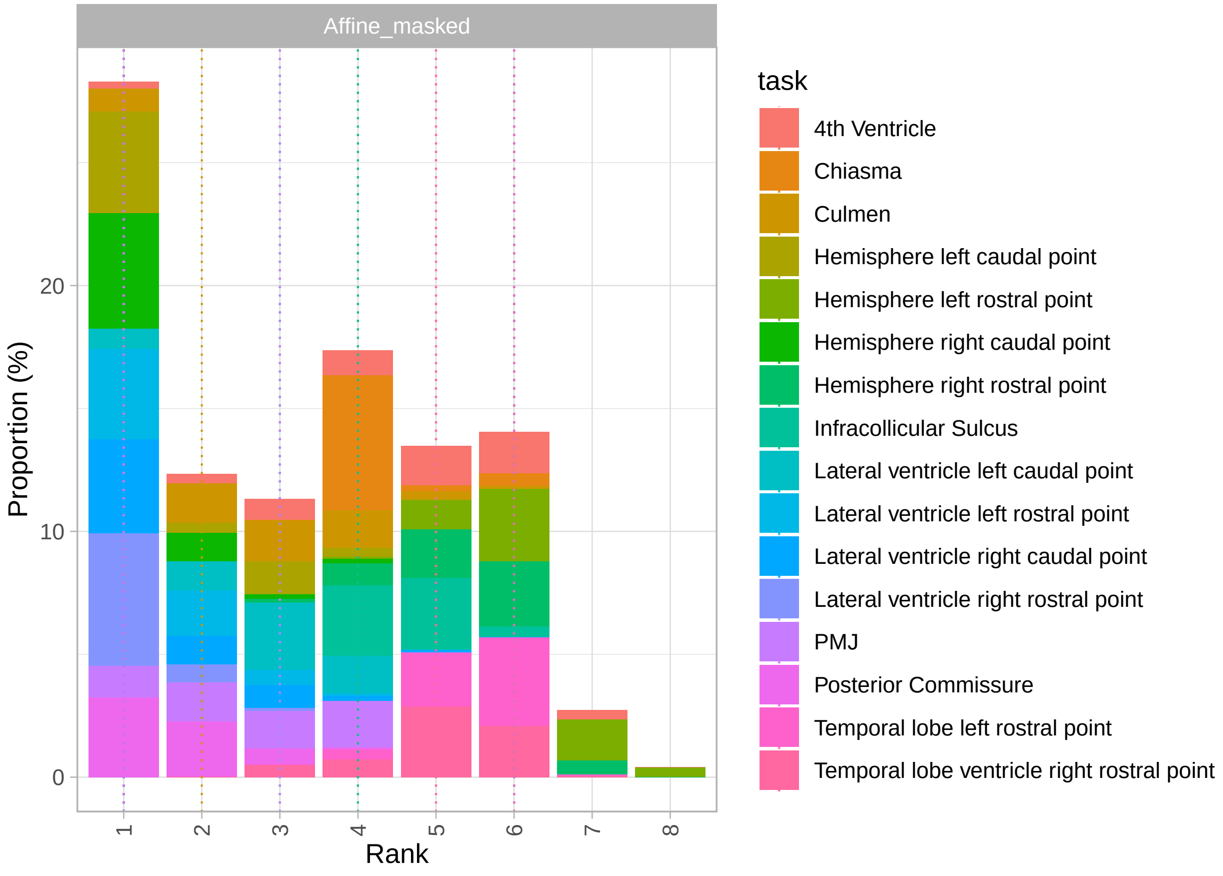

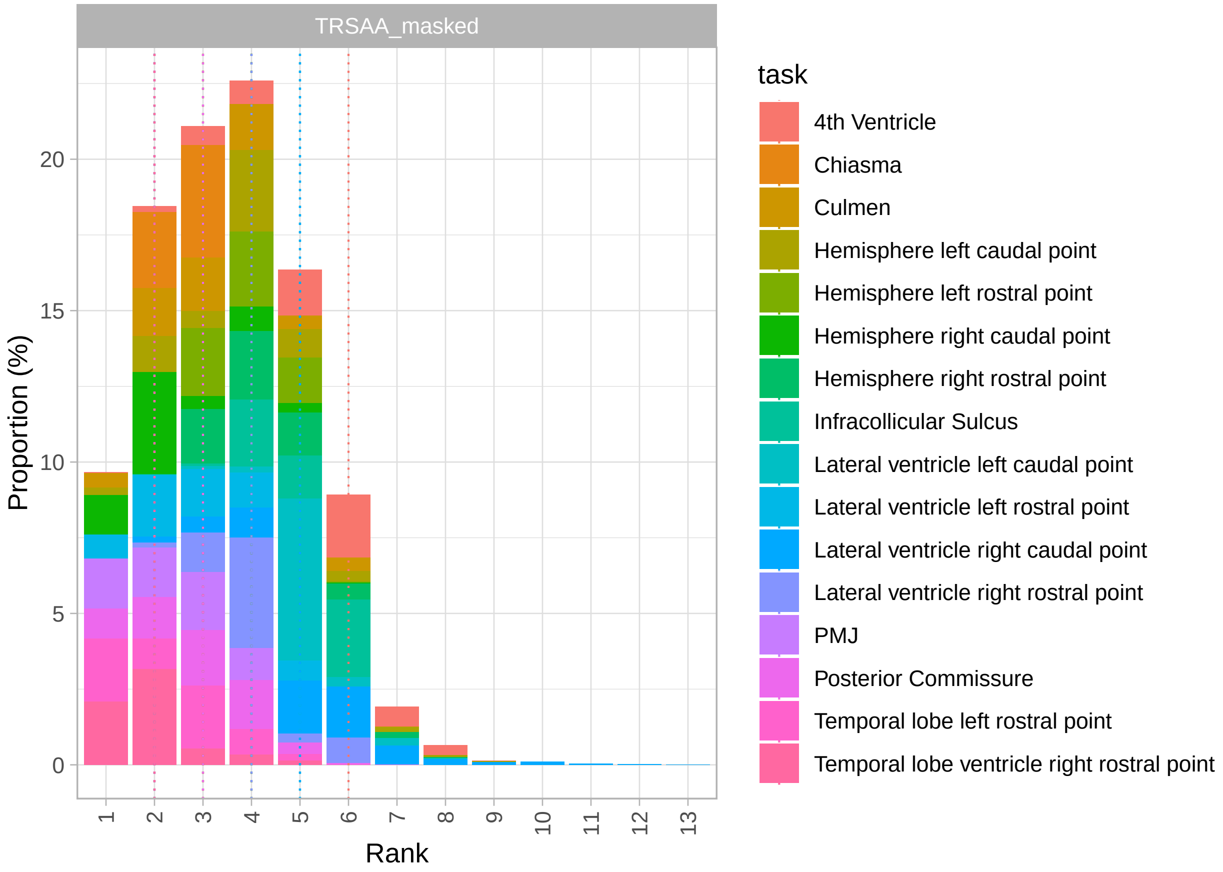
TRSAA_masked
Proportion (%)
Rank
0
5
10
15
20
1
2
3
4
5
6
7
8
9
10
11
12
13
task
4th Ventricle
Chiasma
Culmen
Hemisphere left caudal point
Hemisphere left rostral point
Hemisphere right caudal point
Hemisphere right rostral point
Infracollicular Sulcus
Lateral ventricle left caudal point
Lateral ventricle left rostral point
Lateral ventricle right caudal point
Lateral ventricle right rostral point
PMJ
Posterior Commissure
Temporal lobe left rostral point
Temporal lobe ventricle right rostral point

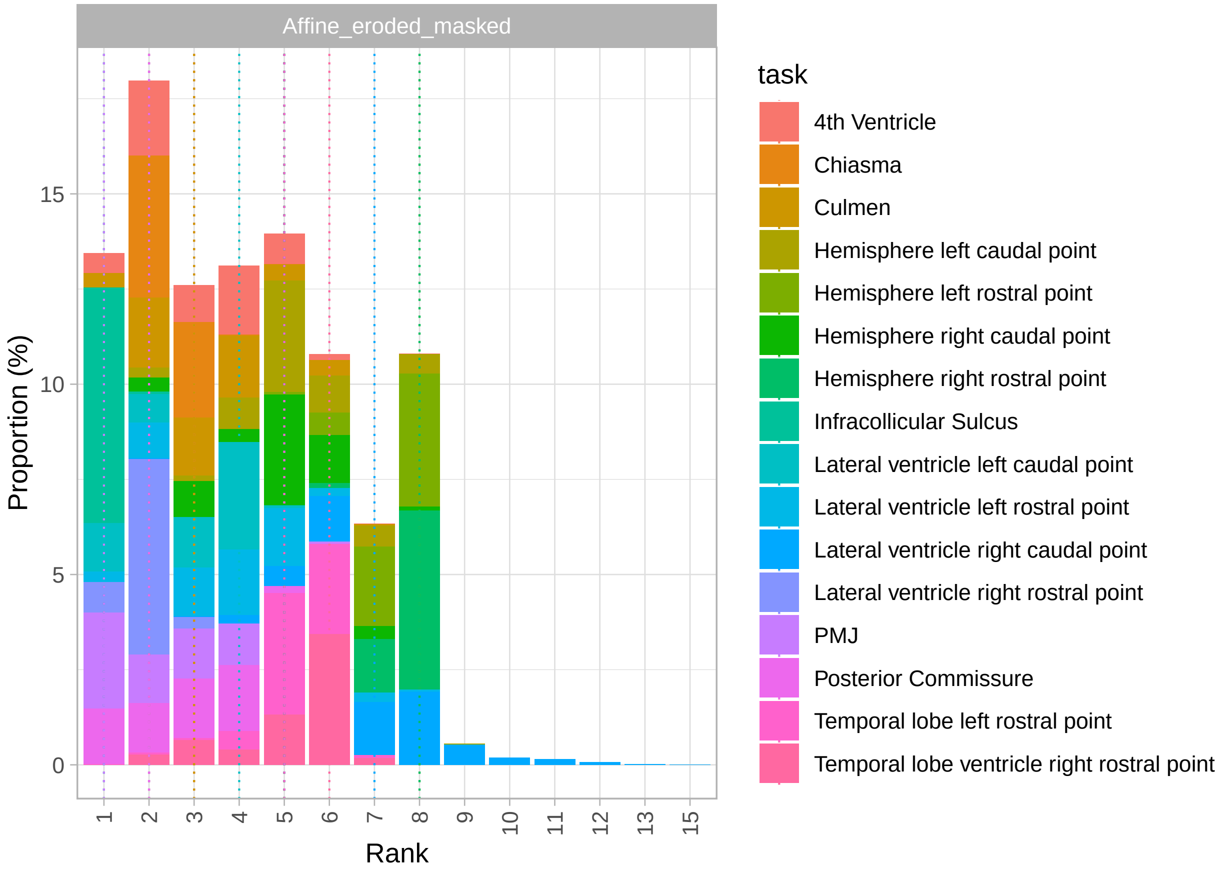

Affine_eroded_masked
Proportion (%)
0
5
10
15
Rank
1
2
3
4
5
6
7
8
9
10
11
12
13
15
task
4th Ventricle
Chiasma
Culmen
Hemisphere left caudal point
Hemisphere left rostral point
Hemisphere right caudal point
Hemisphere right rostral point
Infracollicular Sulcus
Lateral ventricle left caudal point
Lateral ventricle left rostral point
Lateral ventricle right caudal point
Lateral ventricle right rostral point
PMJ
Posterior Commissure
Temporal lobe left rostral point
Temporal lobe ventricle right rostral point

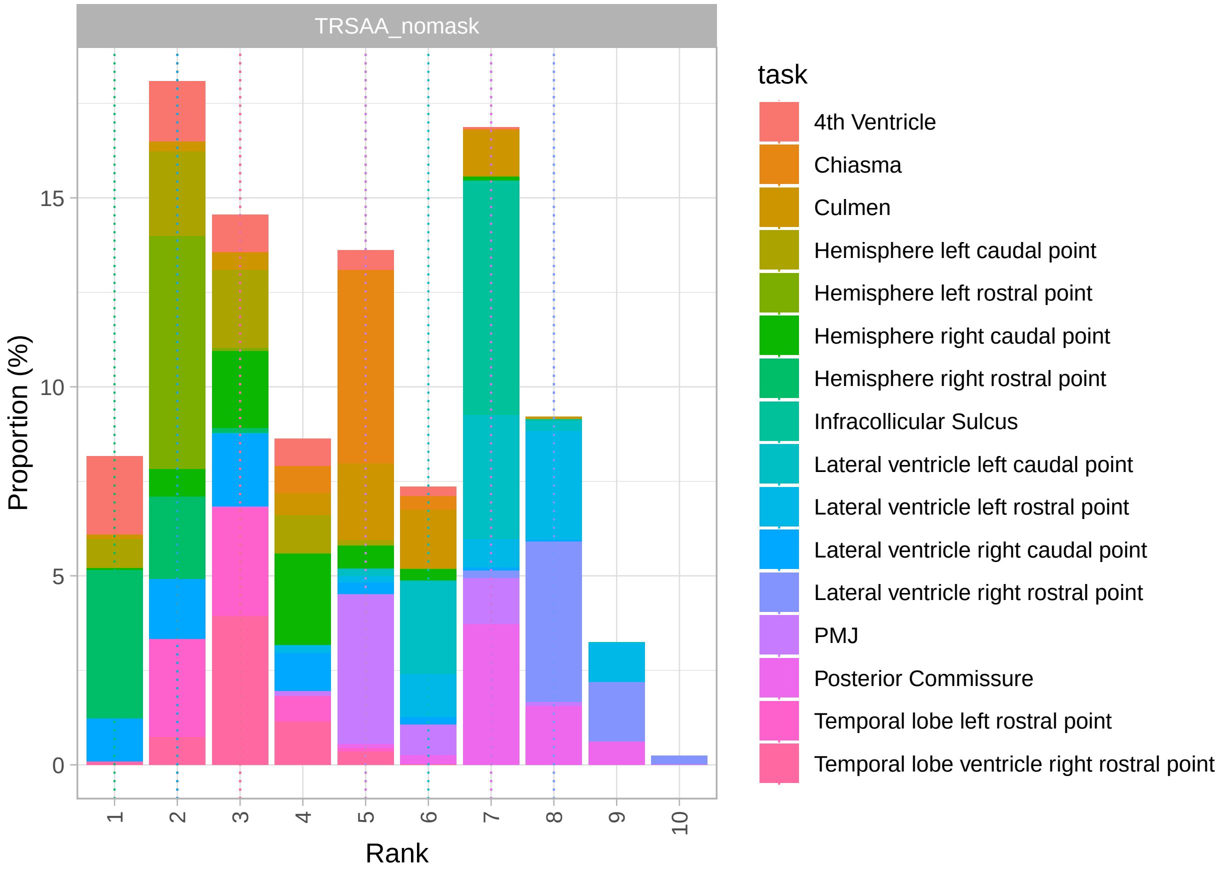

TRSAA_nomask
Proportion (%)
0
5
10
15
1
2
3
4
5
6
7
8
9
10
Rank
task
4th Ventricle
Chiasma
Culmen
Hemisphere left caudal point
Hemisphere left rostral point
Hemisphere right caudal point
Hemisphere right rostral point
Infracollicular Sulcus
Lateral ventricle left caudal point
Lateral ventricle left rostral point
Lateral ventricle right caudal point
Lateral ventricle right rostral point
PMJ
Posterior Commissure
Temporal lobe left rostral point
Temporal lobe ventricle right rostral point

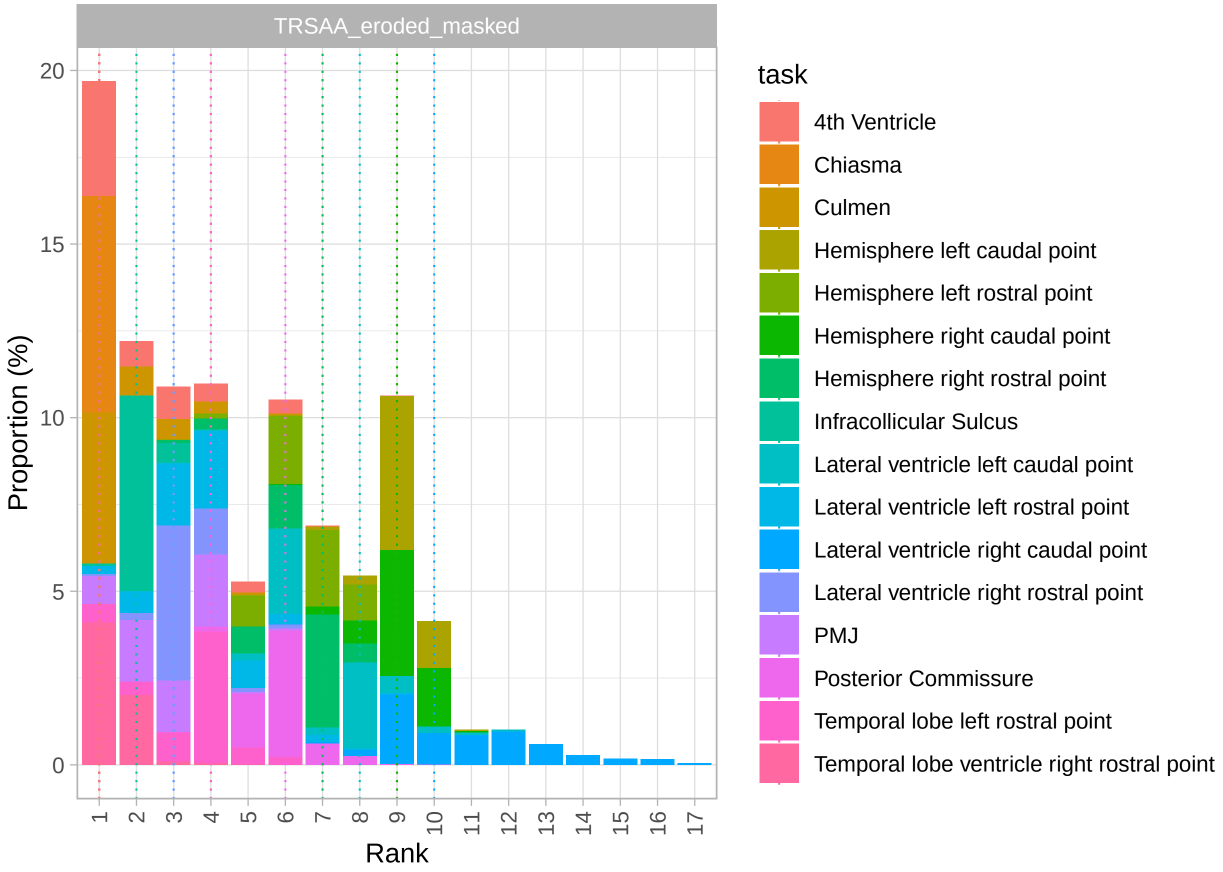
TRSAA_eroded_masked
Proportion (%)
20
15
10
5
0
1
2
3
4
5
6
7
8
9
10
11
12
13
14
15
16
17
Rank
task
4th Ventricle
Chiasma
Culmen
Hemisphere left caudal point
Hemisphere left rostral point
Hemisphere right caudal point
Hemisphere right rostral point
Infracollicular Sulcus
Lateral ventricle left caudal point
Lateral ventricle left rostral point
Lateral ventricle right caudal point
Lateral ventricle right rostral point
PMJ
Posterior Commissure
Temporal lobe left rostral point
Temporal lobe ventricle right rostral point

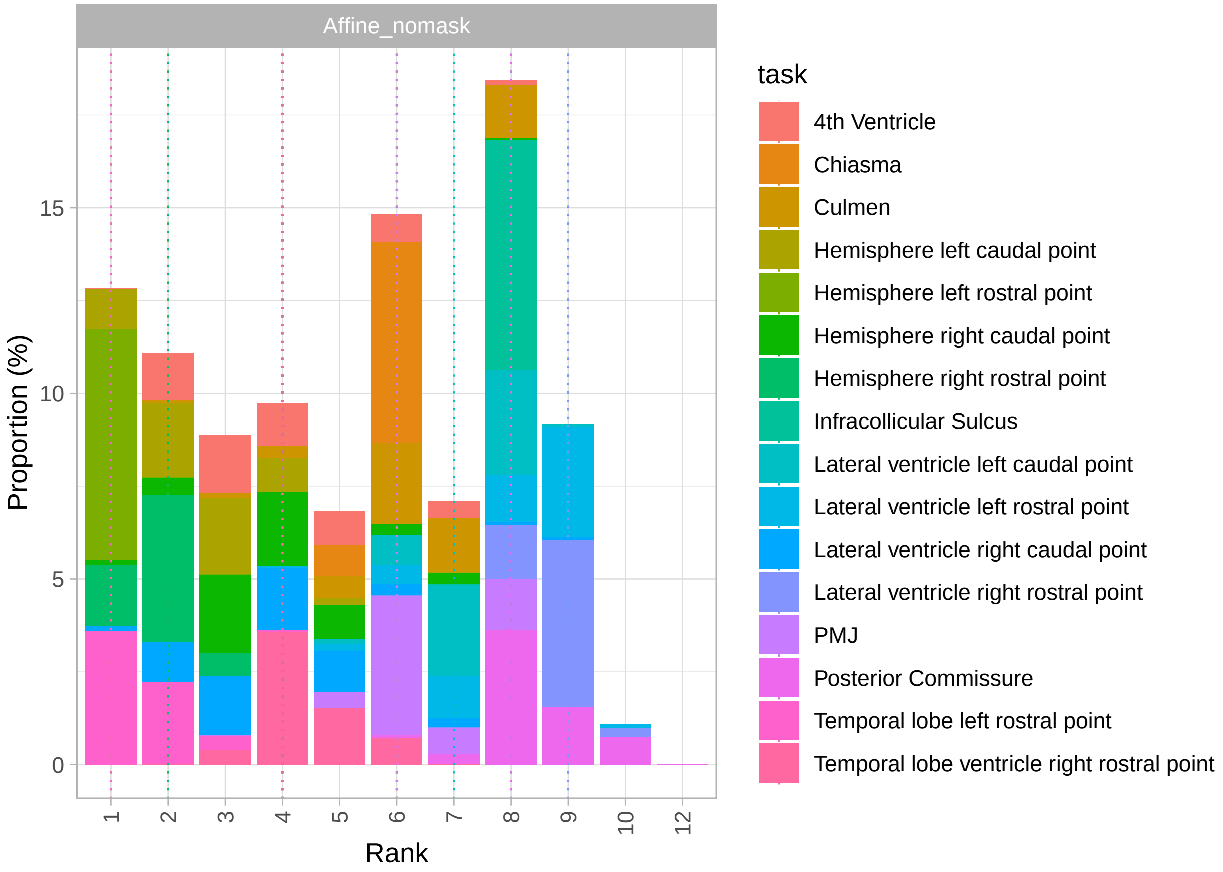

Affine_nomask
Proportion (%)
0
5
10
15
1
2
3
4
5
6
7
8
9
10
12
Rank
task
4th Ventricle
Chiasma
Culmen
Hemisphere left caudal point
Hemisphere left rostral point
Hemisphere right caudal point
Hemisphere right rostral point
Infracollicular Sulcus
Lateral ventricle left caudal point
Lateral ventricle left rostral point
Lateral ventricle right caudal point
Lateral ventricle right rostral point
PMJ
Posterior Commissure
Temporal lobe left rostral point
Temporal lobe ventricle right rostral point

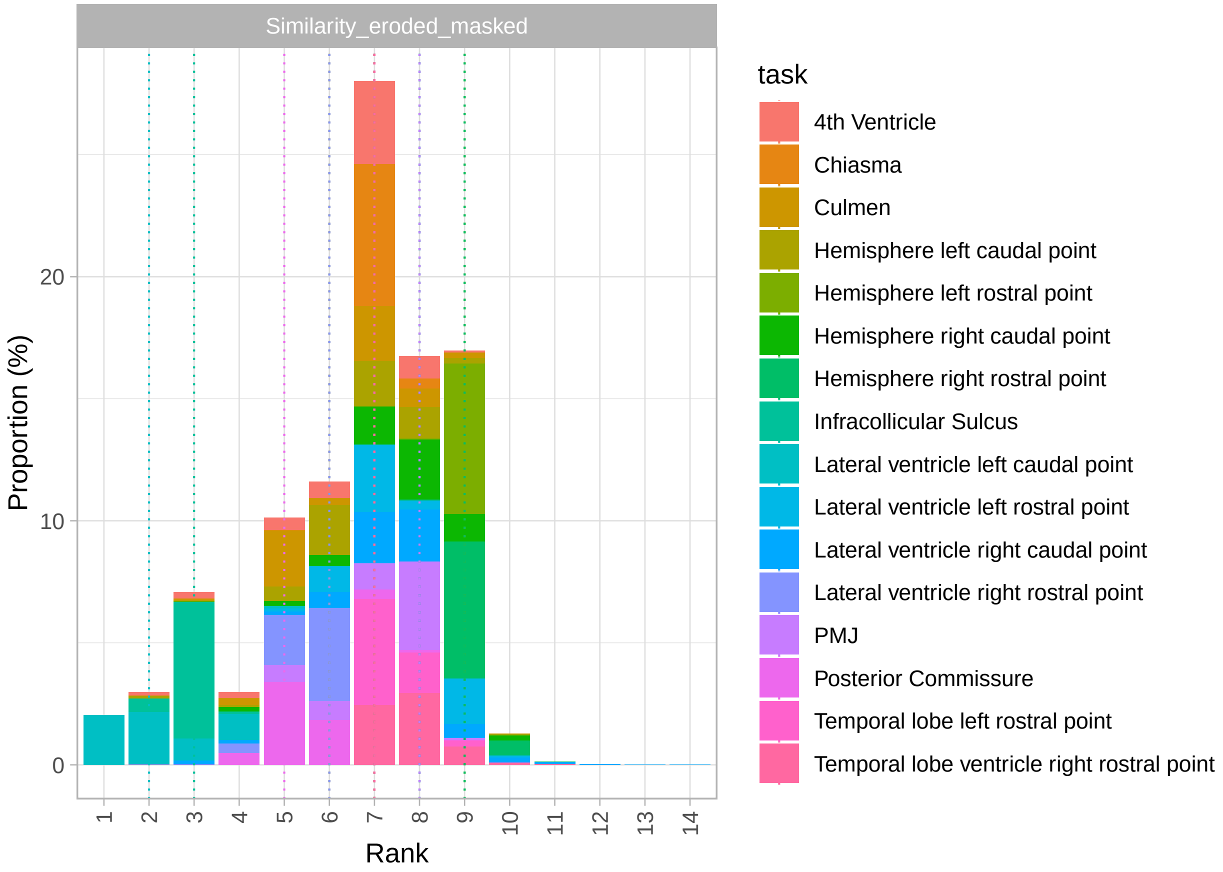
Similarity_eroded_masked
Proportion (%)
0
10
20
1
2
3
4
5
6
7
8
9
10
11
12
13
14
Rank
task
4th Ventricle
Chiasma
Culmen
Hemisphere left caudal point
Hemisphere left rostral point
Hemisphere right caudal point
Hemisphere right rostral point
Infracollicular Sulcus
Lateral ventricle left caudal point
Lateral ventricle left rostral point
Lateral ventricle right caudal point
Lateral ventricle right rostral point
PMJ
Posterior Commissure
Temporal lobe left rostral point
Temporal lobe ventricle right rostral point

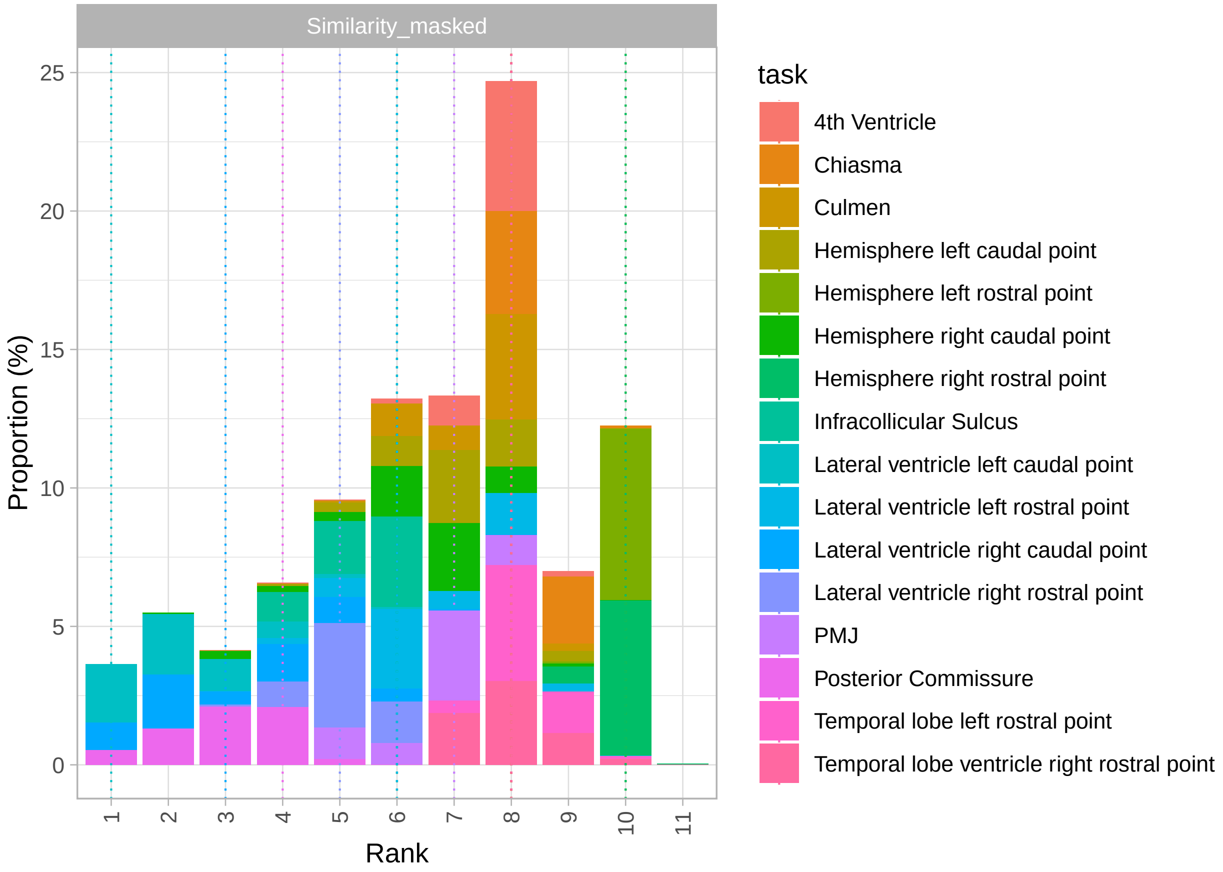
Similarity_masked
Proportion (%)
Rank
25
20
15
10
5
0
1
2
3
4
5
6
7
8
9
10
11
task
4th Ventricle
Chiasma
Culmen
Hemisphere left caudal point
Hemisphere left rostral point
Hemisphere right caudal point
Hemisphere right rostral point
Infracollicular Sulcus
Lateral ventricle left caudal point
Lateral ventricle left rostral point
Lateral ventricle right caudal point
Lateral ventricle right rostral point
PMJ
Posterior Commissure
Temporal lobe left rostral point
Temporal lobe ventricle right rostral point

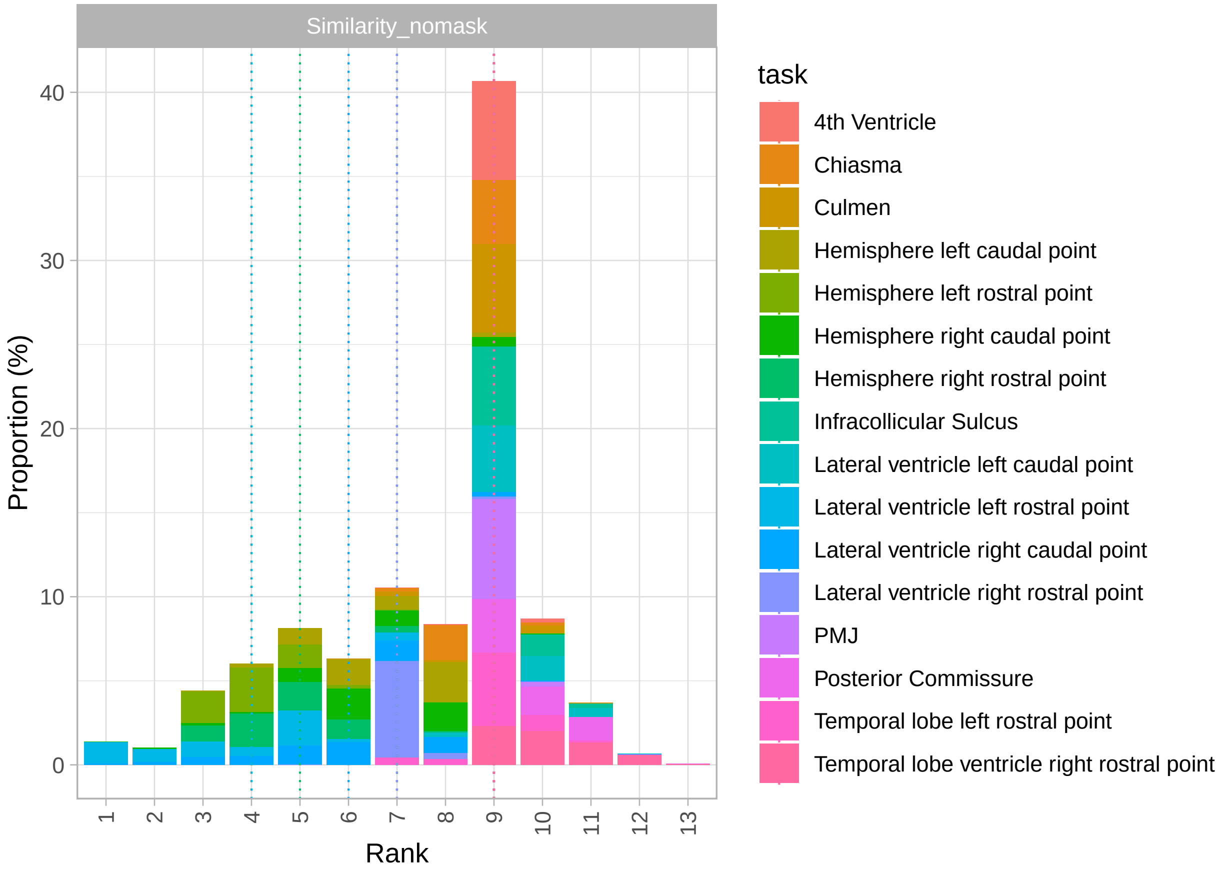
Similarity_nomask
Proportion (%)
0
10
20
30
40
Rank
1
2
3
4
5
6
7
8
9
10
11
12
13
task
4th Ventricle
Chiasma
Culmen
Hemisphere left caudal point
Hemisphere left rostral point
Hemisphere right caudal point
Hemisphere right rostral point
Infracollicular Sulcus
Lateral ventricle left caudal point
Lateral ventricle left rostral point
Lateral ventricle right caudal point
Lateral ventricle right rostral point
PMJ
Posterior Commissure
Temporal lobe left rostral point
Temporal lobe ventricle right rostral point

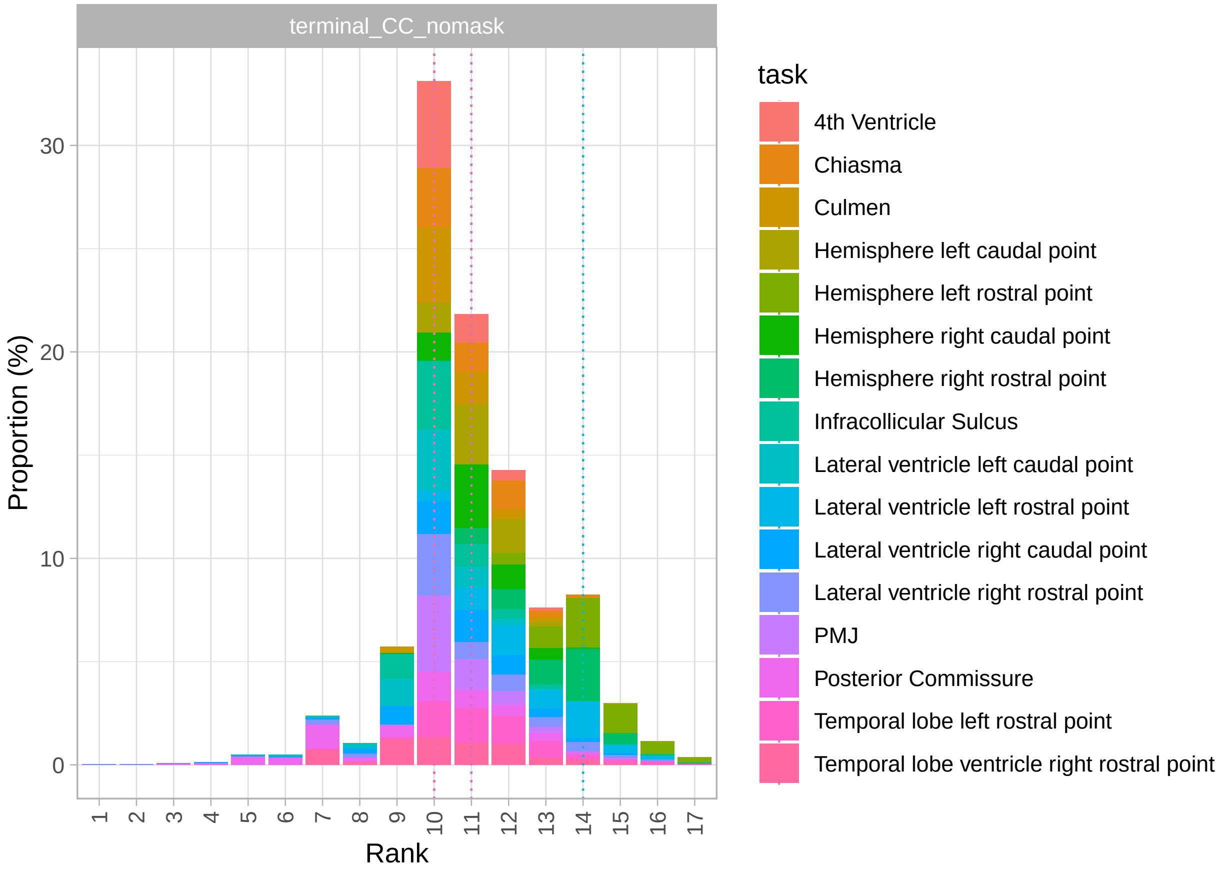

terminal_CC_nomask
Proportion (%)
Rank
task
4th Ventricle
Chiasma
Culmen
Hemisphere left caudal point
Hemisphere left rostral point
Hemisphere right caudal point
Hemisphere right rostral point
Infracollicular Sulcus
Lateral ventricle left caudal point
Lateral ventricle left rostral point
Lateral ventricle right caudal point
Lateral ventricle right rostral point
PMJ
Posterior Commissure
Temporal lobe left rostral point
Temporal lobe ventricle right rostral point

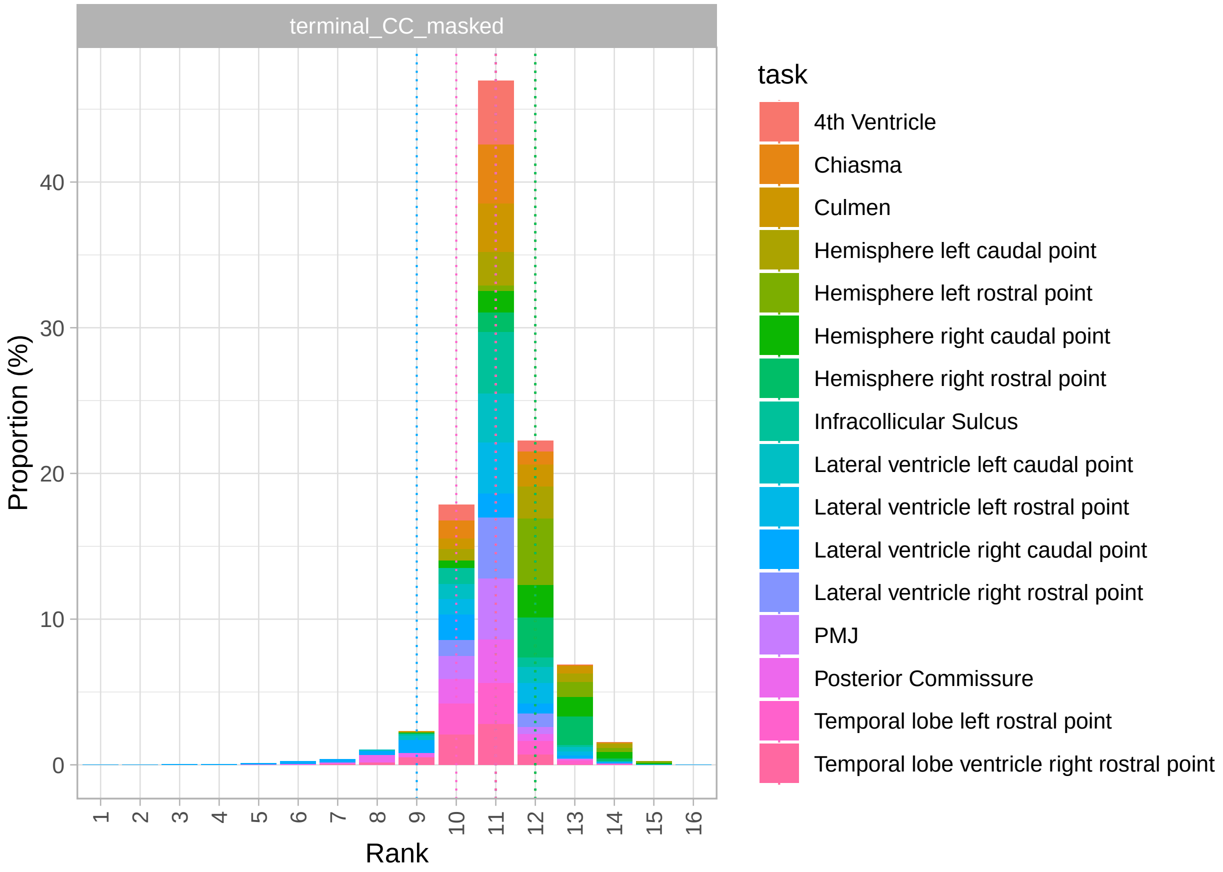
terminal_CC_masked
Proportion (%)
0
10
20
30
40
Rank
1
2
3
4
5
6
7
8
9
10
11
12
13
14
15
16
task
4th Ventricle
Chiasma
Culmen
Hemisphere left caudal point
Hemisphere left rostral point
Hemisphere right caudal point
Hemisphere right rostral point
Infracollicular Sulcus
Lateral ventricle left caudal point
Lateral ventricle left rostral point
Lateral ventricle right caudal point
Lateral ventricle right rostral point
PMJ
Posterior Commissure
Temporal lobe left rostral point
Temporal lobe ventricle right rostral point

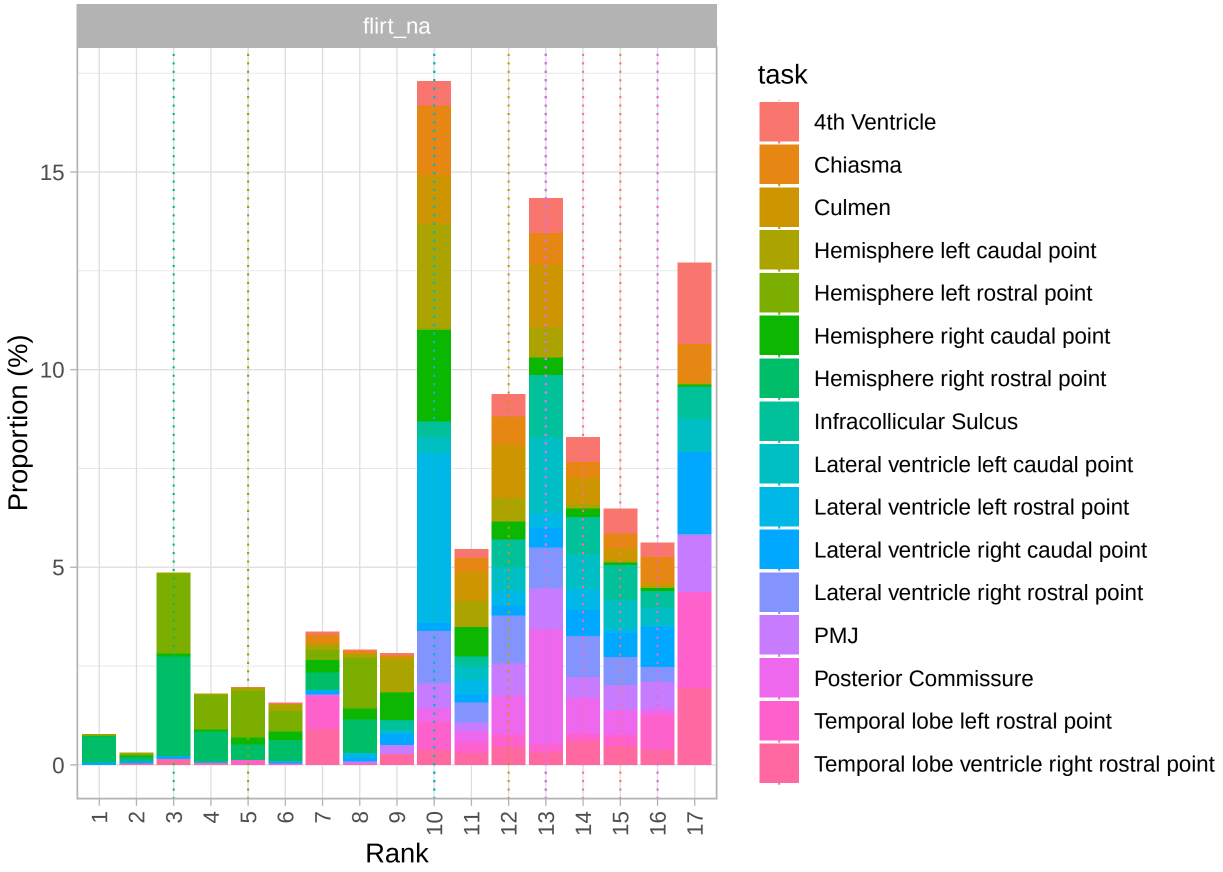
flirt_na
Proportion (%)
15
10
5
0
1
2
3
4
5
6
7
8
9
10
11
12
13
14
15
16
17
Rank
task
4th Ventricle
Chiasma
Culmen
Hemisphere left caudal point
Hemisphere left rostral point
Hemisphere right caudal point
Hemisphere right rostral point
Infracollicular Sulcus
Lateral ventricle left caudal point
Lateral ventricle left rostral point
Lateral ventricle right caudal point
Lateral ventricle right rostral point
PMJ
Posterior Commissure
Temporal lobe left rostral point
Temporal lobe ventricle right rostral point

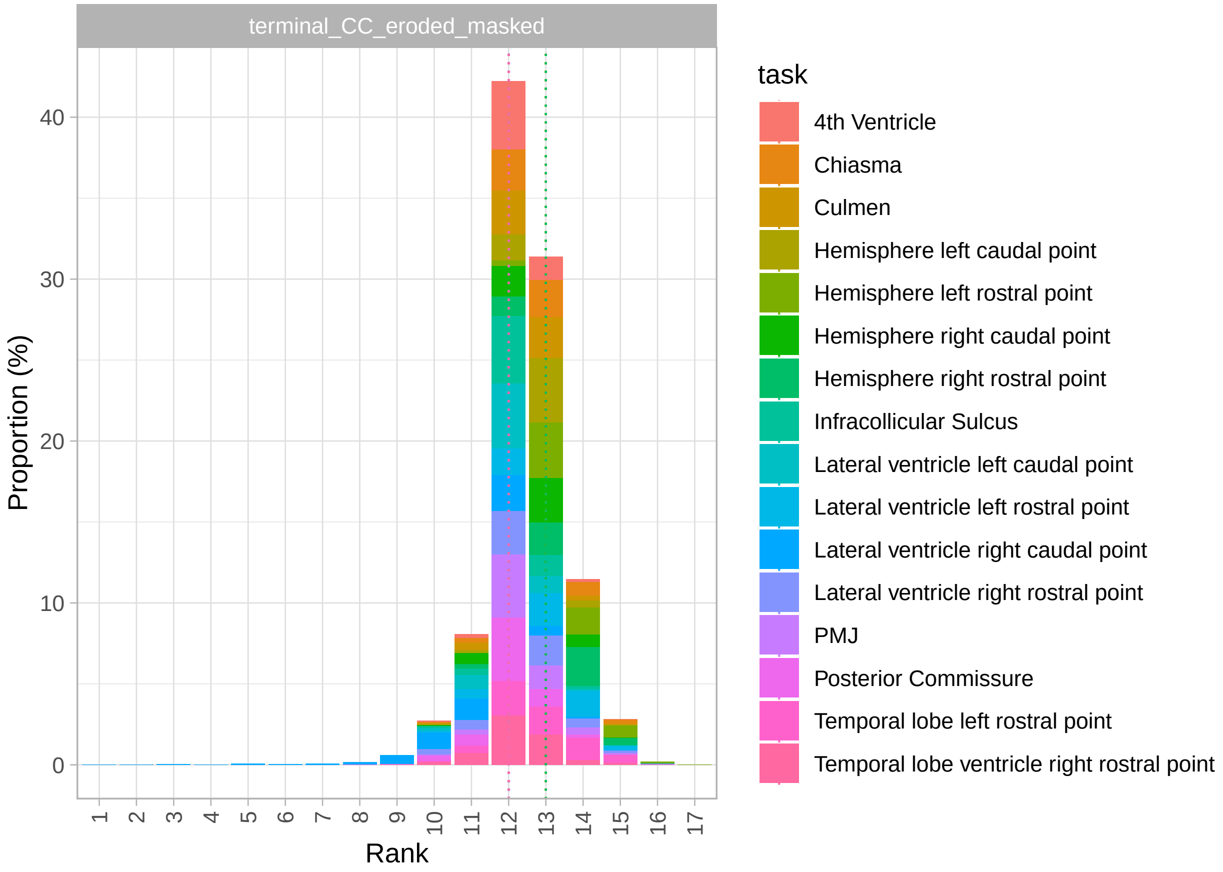

terminal_CC_eroded_masked
Proportion (%)
0
10
20
30
40
1
2
3
4
5
6
7
8
9
10
11
12
13
14
15
16
17
Rank
task
4th Ventricle
Chiasma
Culmen
Hemisphere left caudal point
Hemisphere left rostral point
Hemisphere right caudal point
Hemisphere right rostral point
Infracollicular Sulcus
Lateral ventricle left caudal point
Lateral ventricle left rostral point
Lateral ventricle right caudal point
Lateral ventricle right rostral point
PMJ
Posterior Commissure
Temporal lobe left rostral point
Temporal lobe ventricle right rostral point

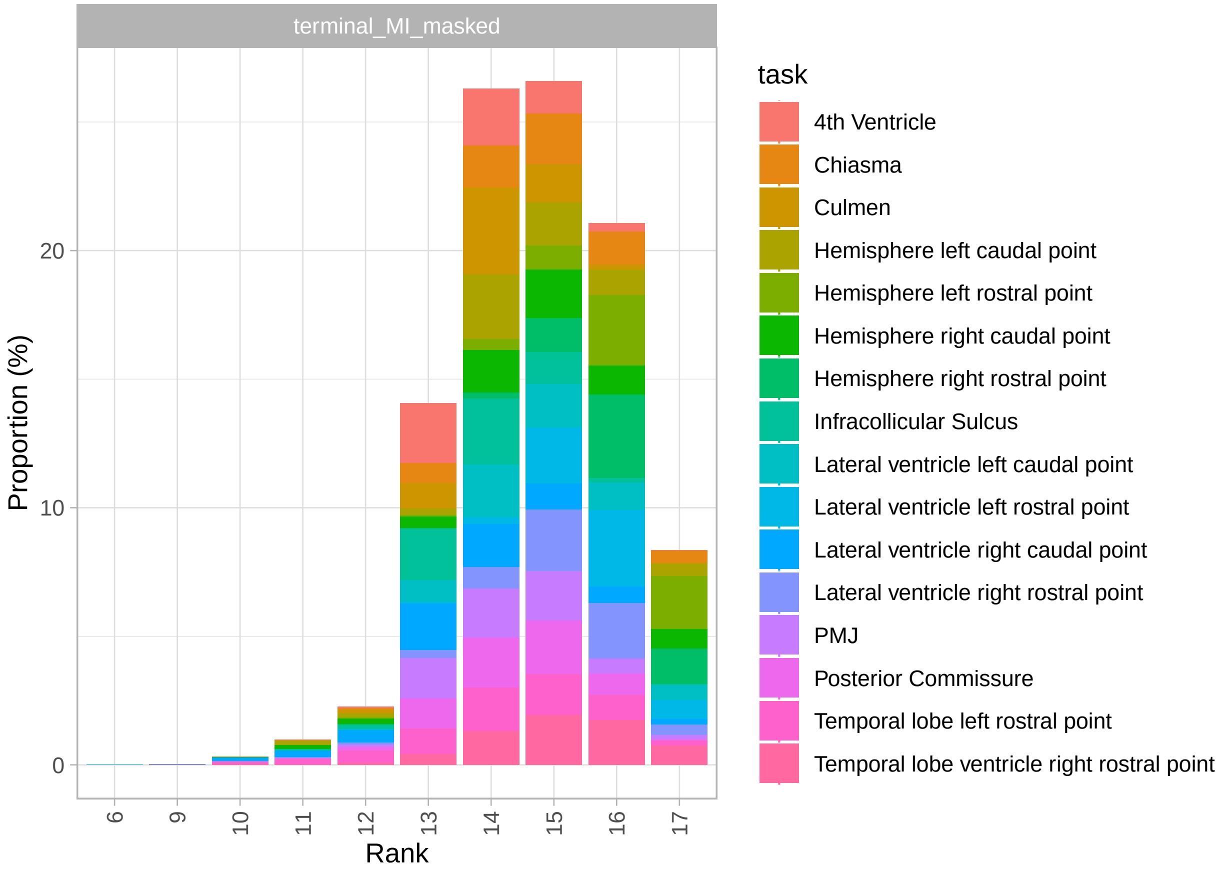

terminal_MI_masked
Proportion (%)
0
10
20
6
9
10
11
12
13
14
15
16
17
Rank
task
4th Ventricle
Chiasma
Culmen
Hemisphere left caudal point
Hemisphere left rostral point
Hemisphere right caudal point
Hemisphere right rostral point
Infracollicular Sulcus
Lateral ventricle left caudal point
Lateral ventricle left rostral point
Lateral ventricle right caudal point
Lateral ventricle right rostral point
PMJ
Posterior Commissure
Temporal lobe left rostral point
Temporal lobe ventricle right rostral point

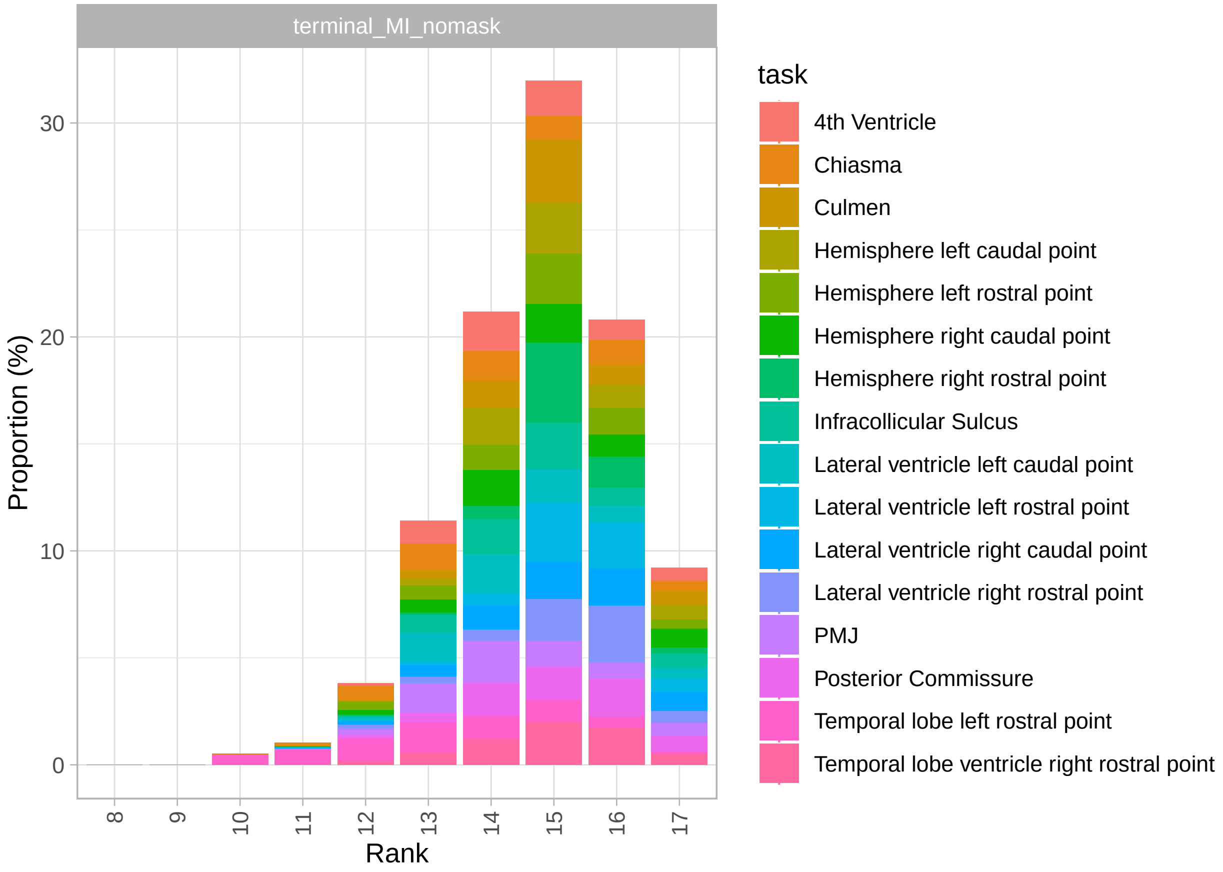
terminal_MI_nomask
Proportion (%)
30
20
10
0
8
9
10
11
12
13
14
15
16
17
Rank
task
4th Ventricle
Chiasma
Culmen
Hemisphere left caudal point
Hemisphere left rostral point
Hemisphere right caudal point
Hemisphere right rostral point
Infracollicular Sulcus
Lateral ventricle left caudal point
Lateral ventricle left rostral point
Lateral ventricle right caudal point
Lateral ventricle right rostral point
PMJ
Posterior Commissure
Temporal lobe left rostral point
Temporal lobe ventricle right rostral point

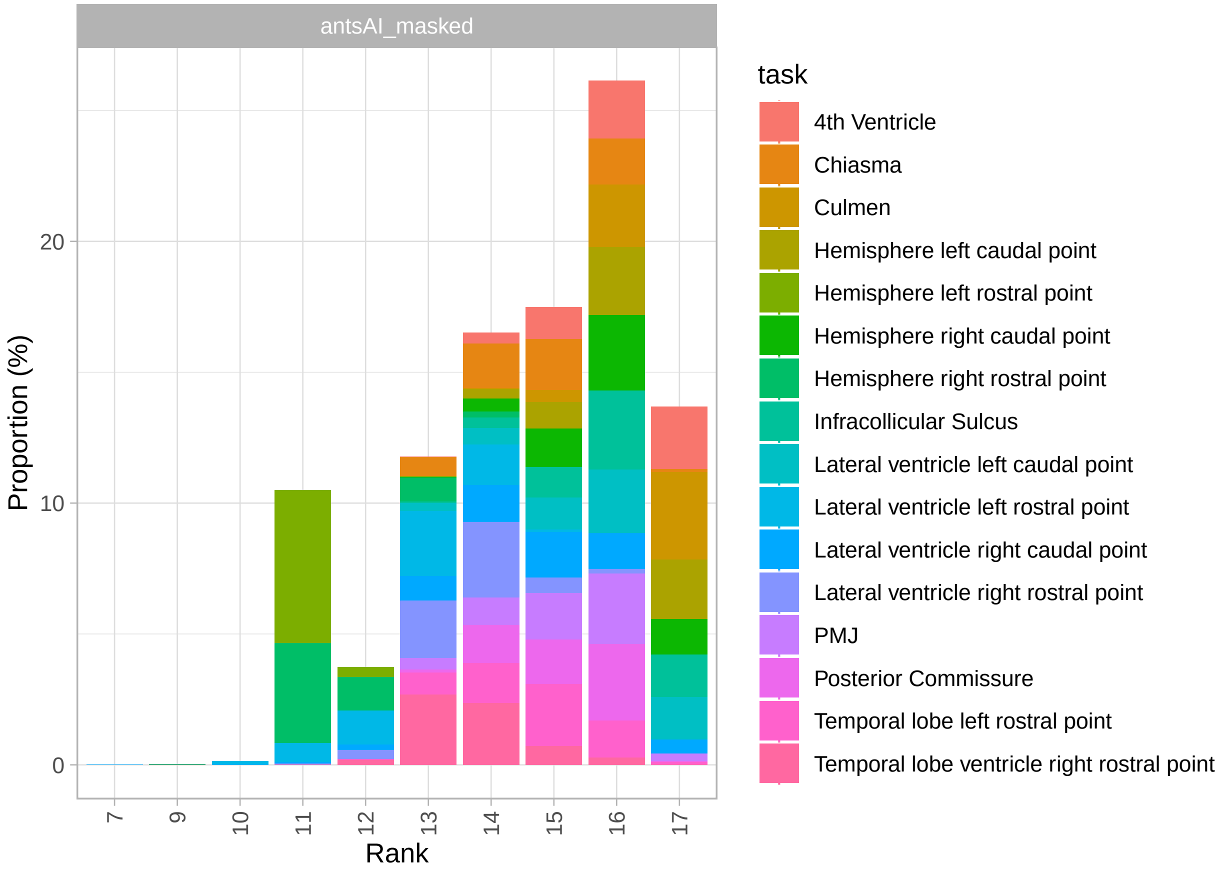
antsAI_masked
Proportion (%)
20
10
0
7
9
10
11
12
13
14
15
16
17
Rank
task
4th Ventricle
Chiasma
Culmen
Hemisphere left caudal point
Hemisphere left rostral point
Hemisphere right caudal point
Hemisphere right rostral point
Infracollicular Sulcus
Lateral ventricle left caudal point
Lateral ventricle left rostral point
Lateral ventricle right caudal point
Lateral ventricle right rostral point
PMJ
Posterior Commissure
Temporal lobe left rostral point
Temporal lobe ventricle right rostral point

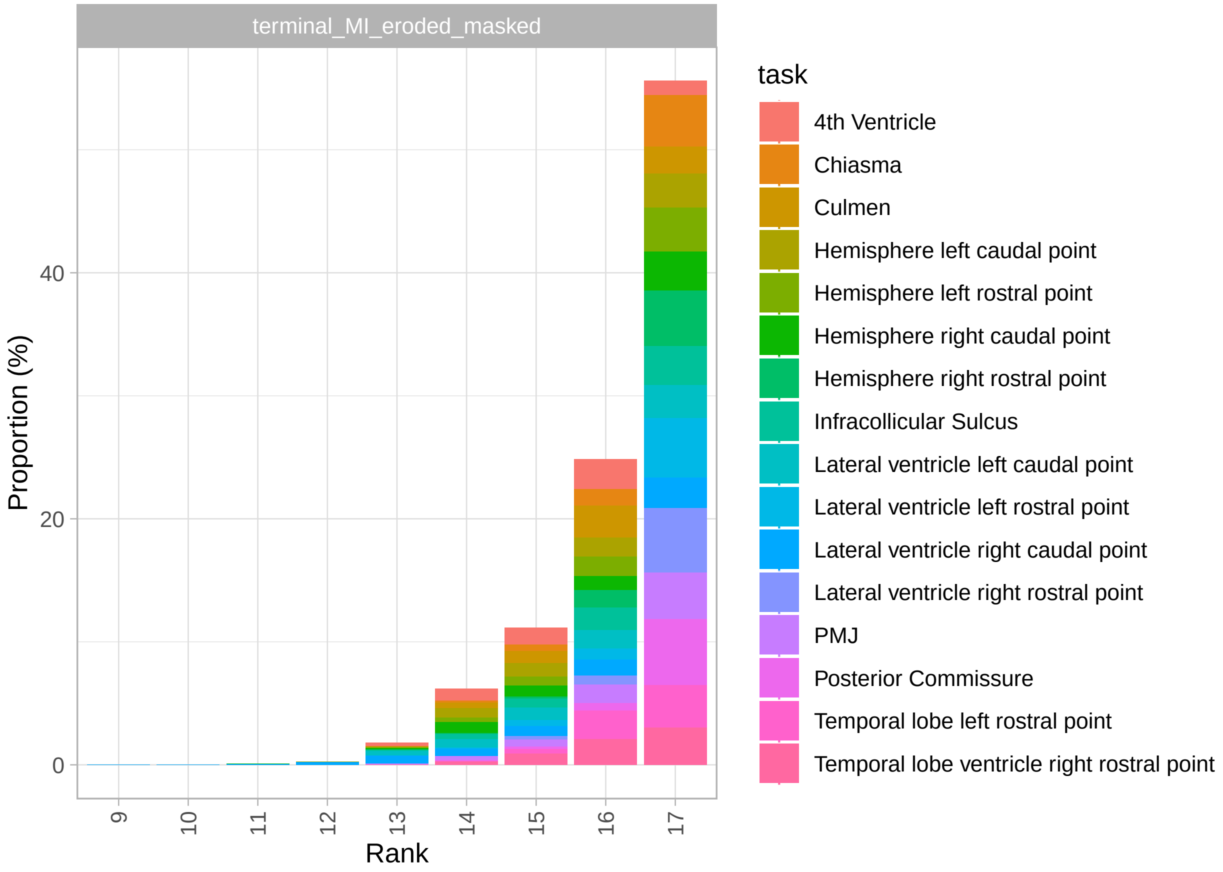
terminal_MI_eroded_masked
Proportion (%)
40
20
0
9
10
11
12
13
14
15
16
17
Rank
task
4th Ventricle
Chiasma
Culmen
Hemisphere left caudal point
Hemisphere left rostral point
Hemisphere right caudal point
Hemisphere right rostral point
Infracollicular Sulcus
Lateral ventricle left caudal point
Lateral ventricle left rostral point
Lateral ventricle right caudal point
Lateral ventricle right rostral point
PMJ
Posterior Commissure
Temporal lobe left rostral point
Temporal lobe ventricle right rostral point

4.2 Characterization of tasks

4.2.1 Visualizing bootstrap results

To investigate which tasks separate algorithms well (i.e., lead to a stable ranking), a blob plot is recommended.

Bootstrap results can be shown in a blob plot showing one plot for each task. In this view, the spread of the blobs for each algorithm can be compared across tasks. Deviations from the diagonal indicate deviations from the consensus ranking (over tasks). Specifically, if rank distribution of an algorithm is consistently below the diagonal, the algorithm performed better in this task than on average across tasks, while if the rank distribution of an algorithm is consistently above the diagonal, the algorithm performed worse in this task than on average across tasks. At the bottom of each panel, ranks for each algorithm in the tasks are provided.

Same as in Section 3.1 but now ordered according to consensus.

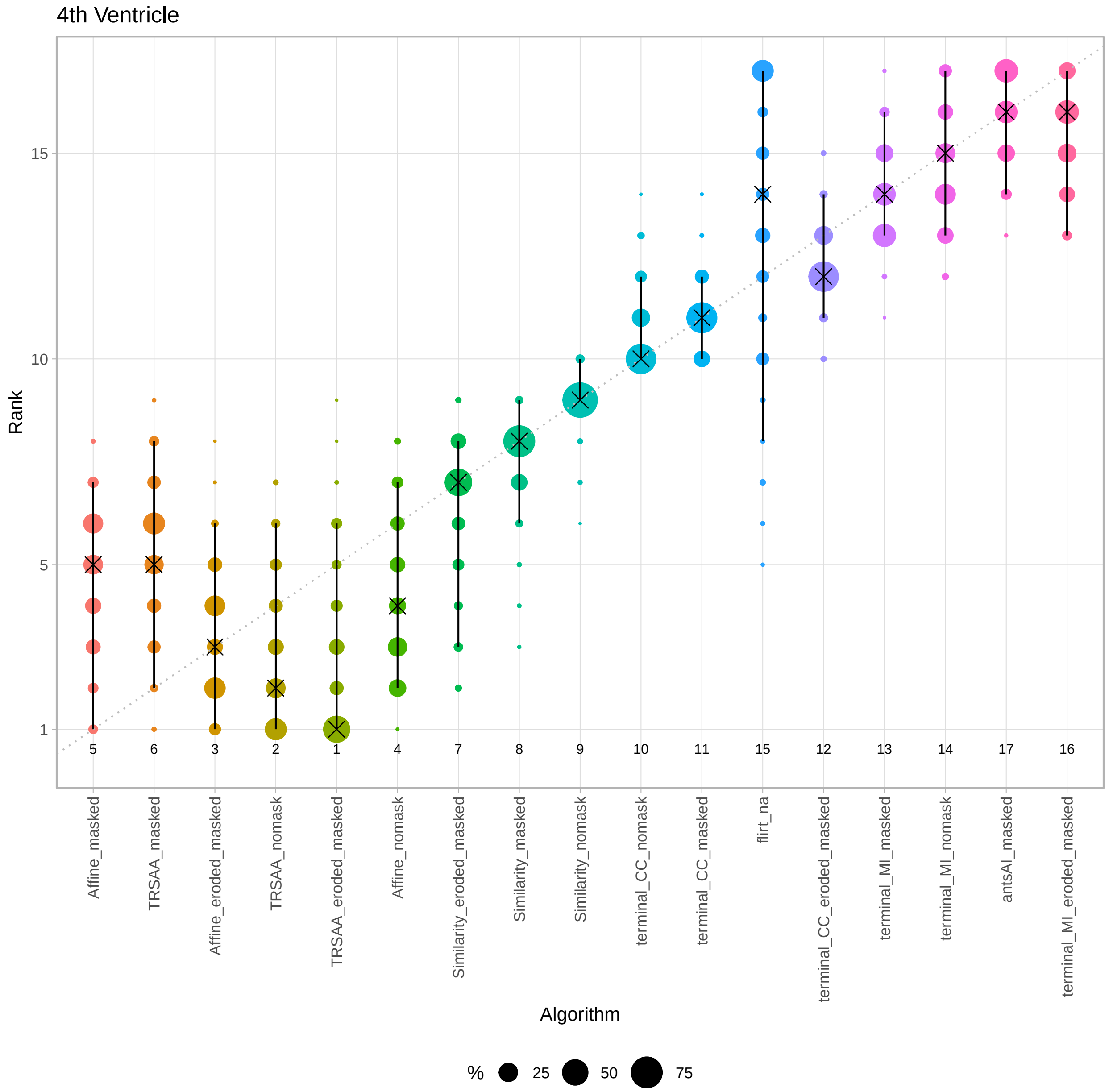

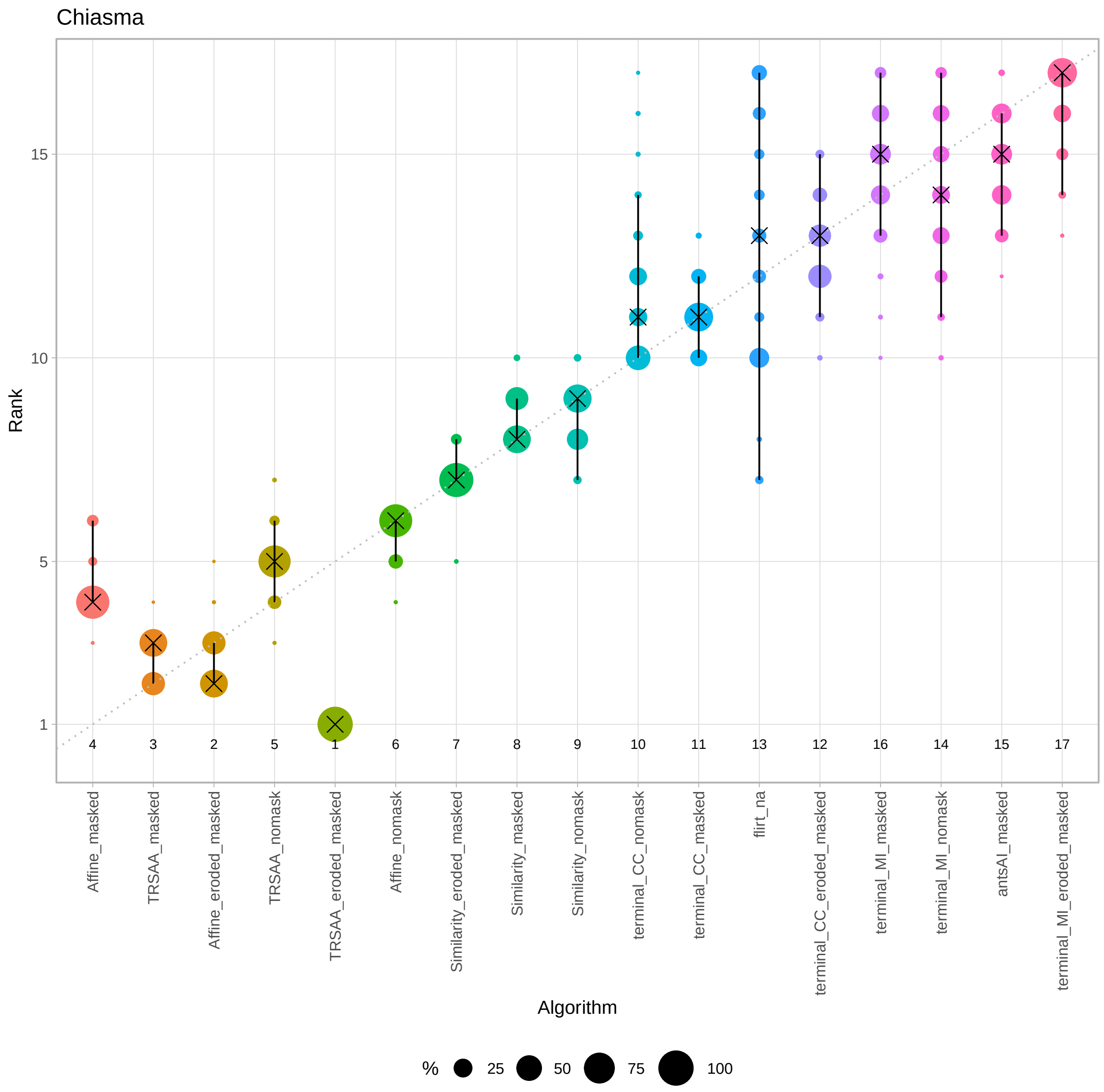

Chiasma
Rank
15
10
5
1
4
3
2
5
1
6
7
8
9
10
11
13
12
16
14
15
17
Affine_masked
TRSAA_masked
Affine_eroded_masked
TRSAA_nomask
TRSAA_eroded_masked
Affine_nomask
Similarity_eroded_masked
Similarity_masked
Similarity_nomask
terminal_CC_nomask
terminal_CC_masked
flirt_na
terminal_CC_eroded_masked
terminal_MI_masked
terminal_MI_nomask
antsAI_masked
terminal_MI_eroded_masked
Algorithm
%
25
50
75
100

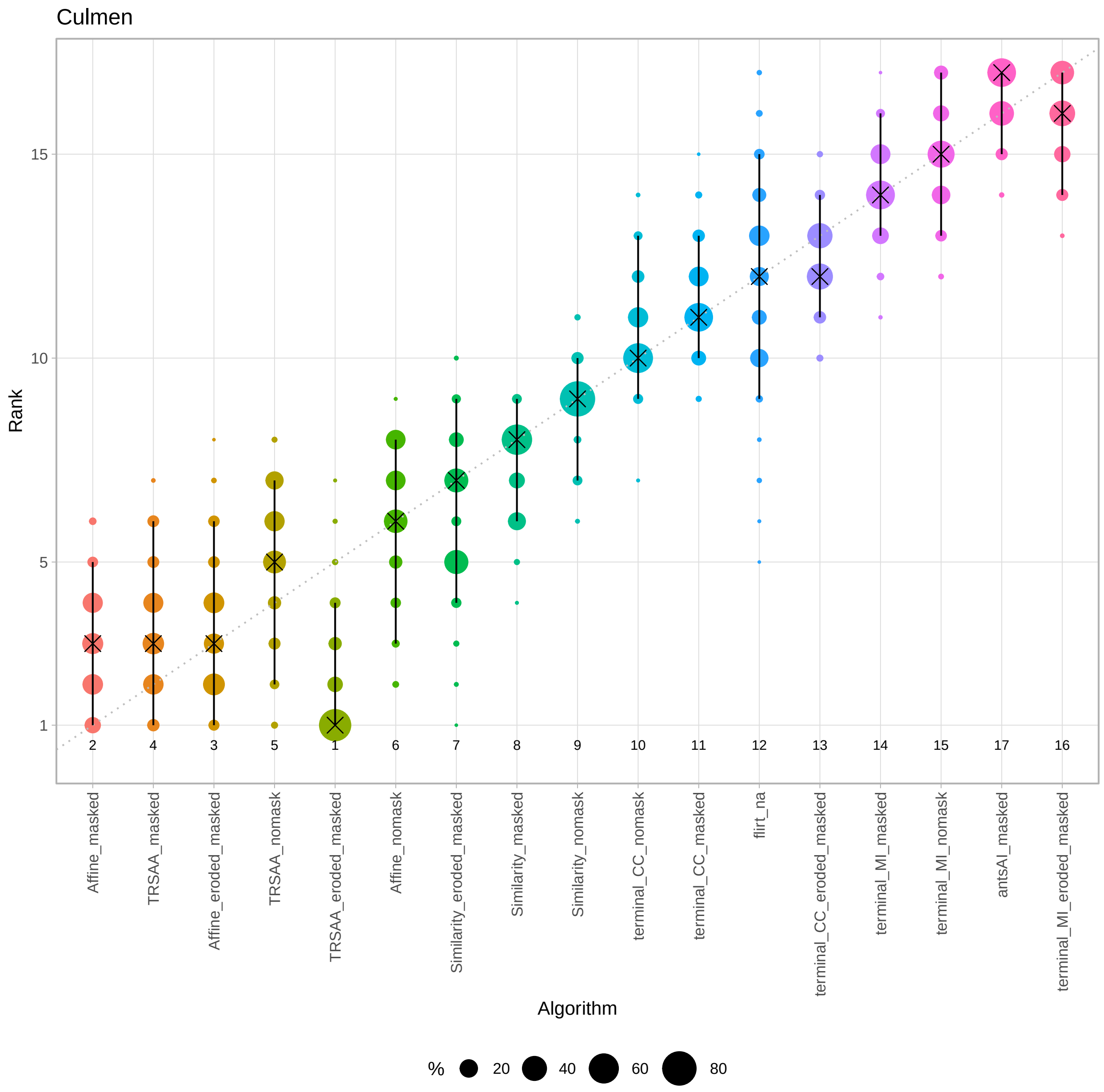
Culmen
Rank
15
10
5
1
2
4
3
5
1
6
7
8
9
10
11
12
13
14
15
17
16
Affine_masked
TRSAA_masked
Affine_eroded_masked
TRSAA_nomask
TRSAA_eroded_masked
Affine_nomask
Similarity_eroded_masked
Similarity_masked
Similarity_nomask
terminal_CC_nomask
terminal_CC_masked
flirt_na
terminal_CC_eroded_masked
terminal_MI_masked
terminal_MI_nomask
antsAI_masked
terminal_MI_eroded_masked
Algorithm
%
20
40
60
80

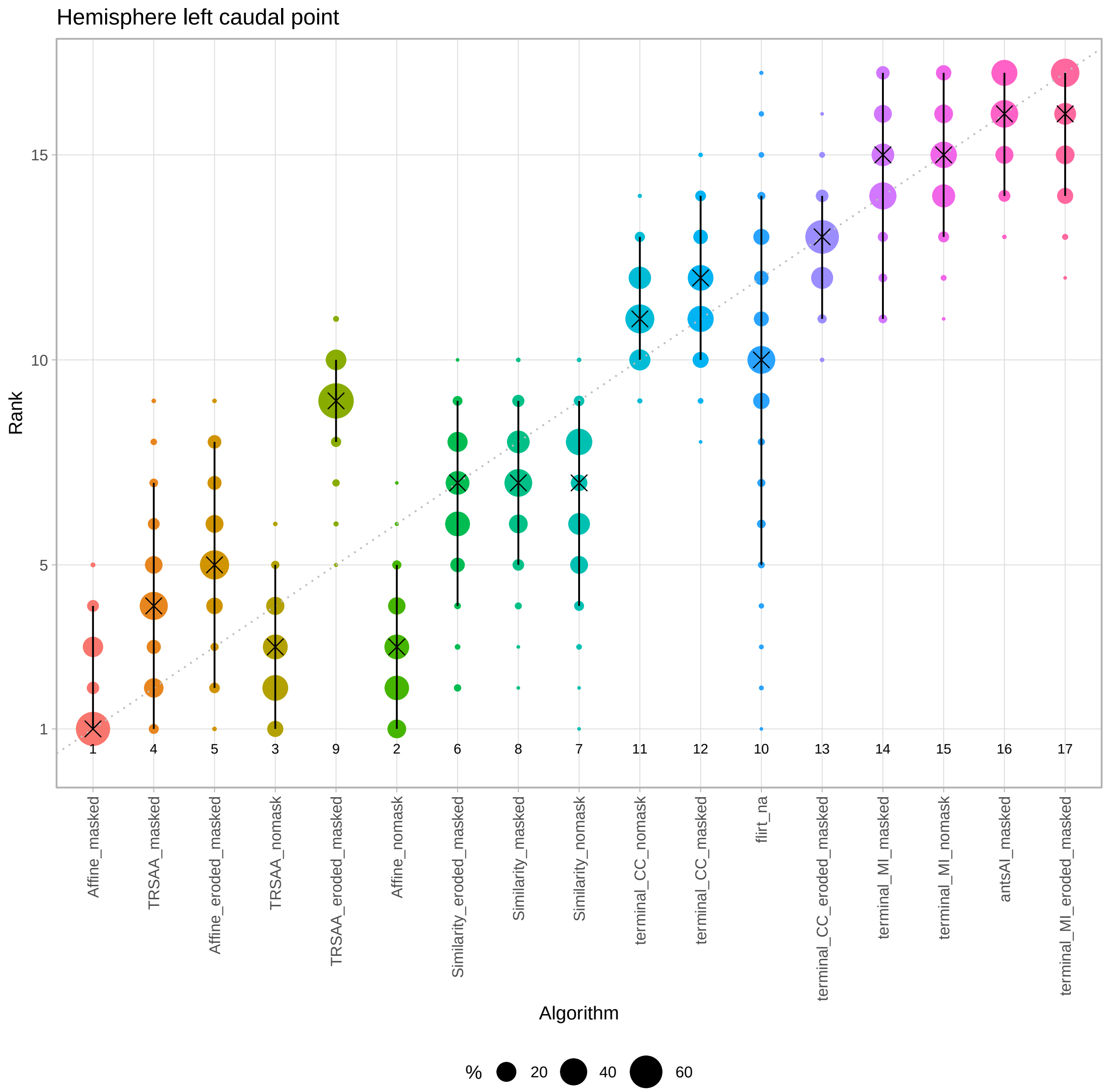
Hemisphere left caudal point
Rank
15
10
5
1
1
4
5
3
9
2
6
8
7
11
12
10
13
14
15
16
17
Affine_masked
TRSAA_masked
Affine_eroded_masked
TRSAA_nomask
TRSAA_eroded_masked
Affine_nomask
Similarity_eroded_masked
Similarity_masked
Similarity_nomask
terminal_CC_nomask
terminal_CC_masked
flirt_na
terminal_CC_eroded_masked
terminal_MI_masked
terminal_MI_nomask
antsAI_masked
terminal_MI_eroded_masked
Algorithm
%
20
40
60

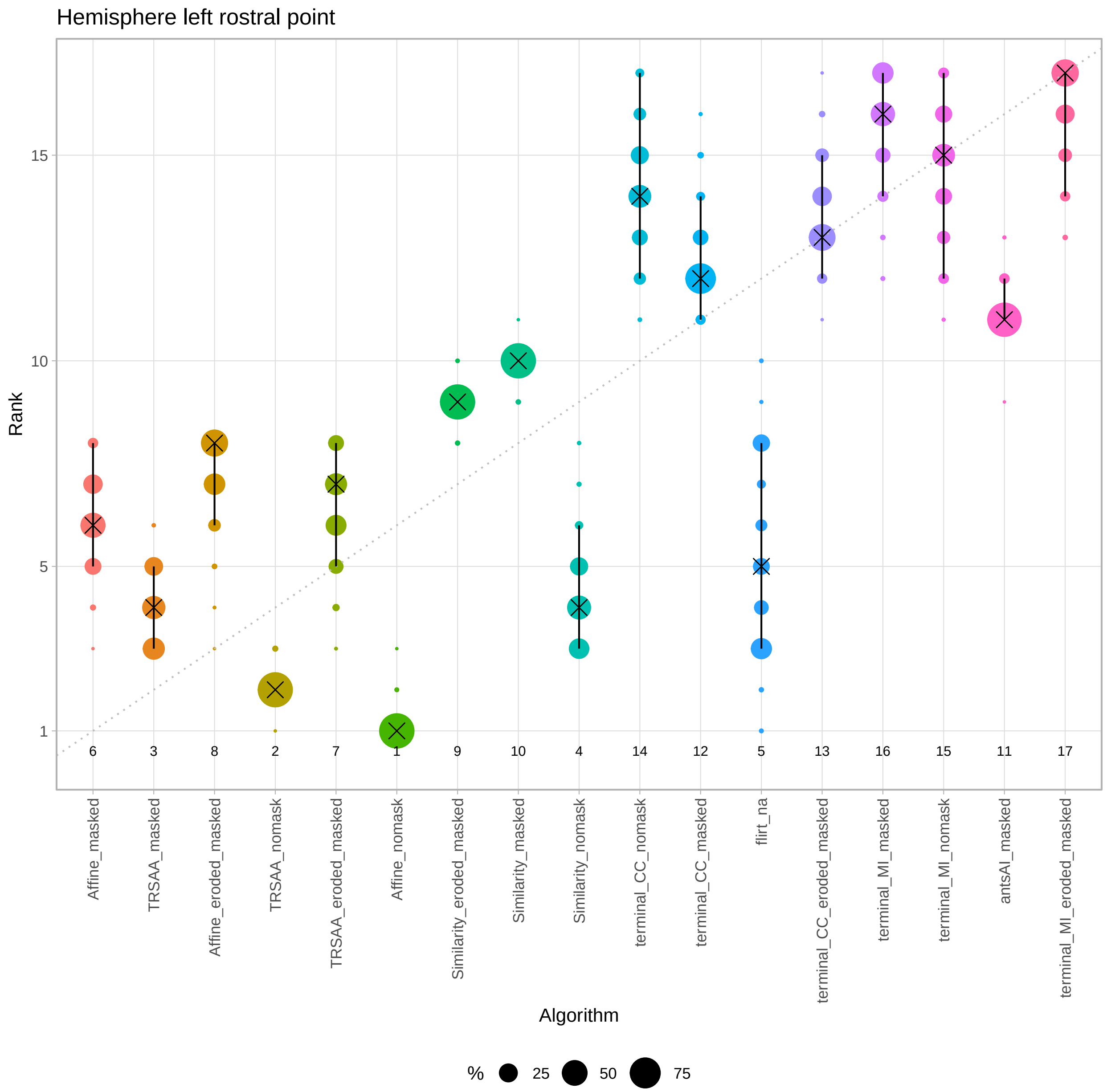
Hemisphere left rostral point
Rank
15
10
5
1
6
3
8
2
7
1
9
10
4
14
12
5
13
16
15
11
17
Affine_masked
TRSAA_masked
Affine_eroded_masked
TRSAA_nomask
TRSAA_eroded_masked
Affine_nomask
Similarity_eroded_masked
Similarity_masked
Similarity_nomask
terminal_CC_nomask
terminal_CC_masked
flirt_na
terminal_CC_eroded_masked
terminal_MI_masked
terminal_MI_nomask
antsAI_masked
terminal_MI_eroded_masked
Algorithm
%
25
50
75

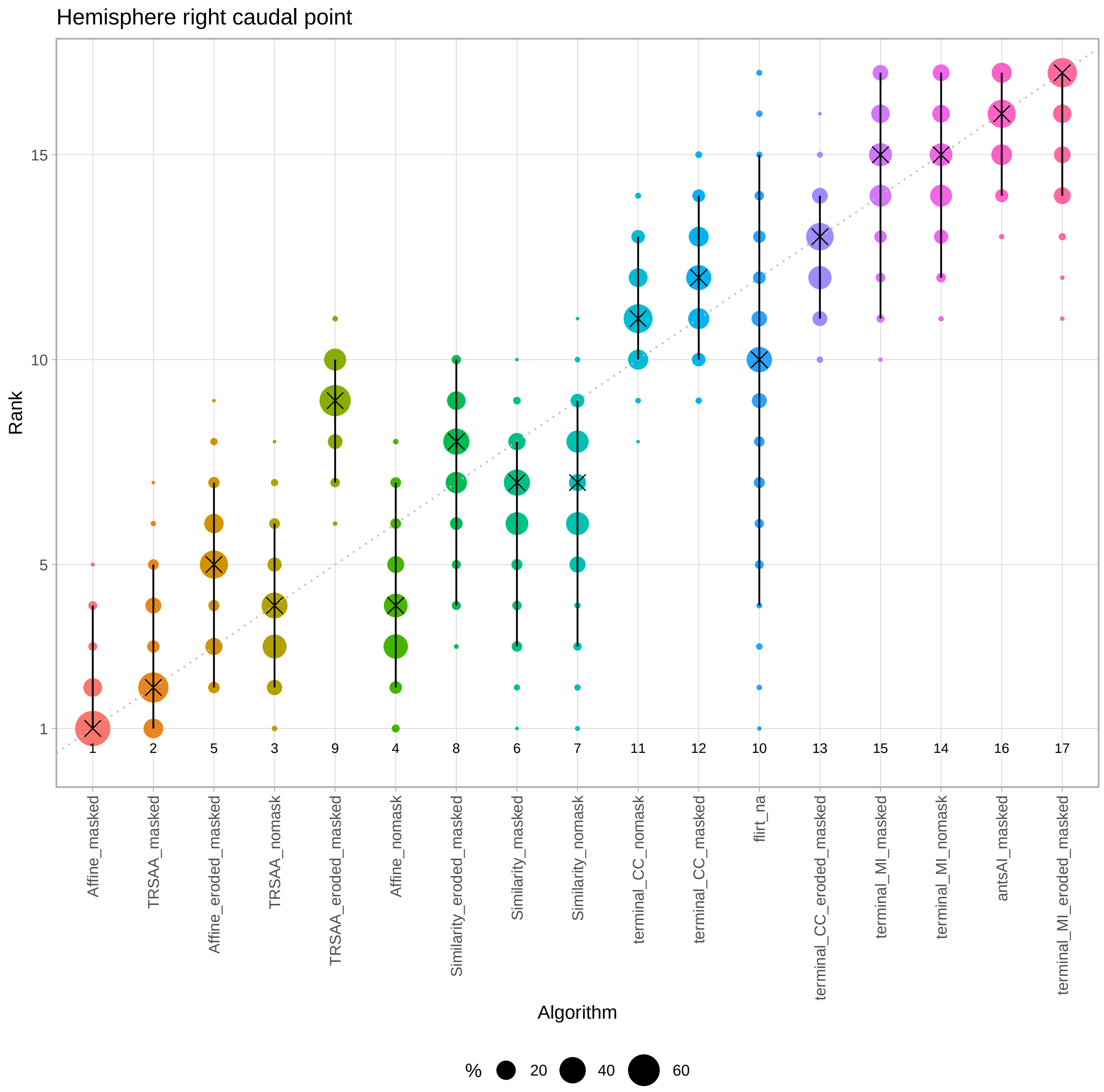
Hemisphere right caudal point
Rank
15
10
5
1
1
2
5
3
9
4
8
6
7
11
12
10
13
15
14
16
17
Affine_masked
TRSAA_masked
Affine_eroded_masked
TRSAA_nomask
TRSAA_eroded_masked
Affine_nomask
Similarity_eroded_masked
Similarity_masked
Similarity_nomask
terminal_CC_nomask
terminal_CC_masked
flirt_na
terminal_CC_eroded_masked
terminal_MI_masked
terminal_MI_nomask
antsAI_masked
terminal_MI_eroded_masked
Algorithm
%
20
40
60

Hemisphere right rostral point

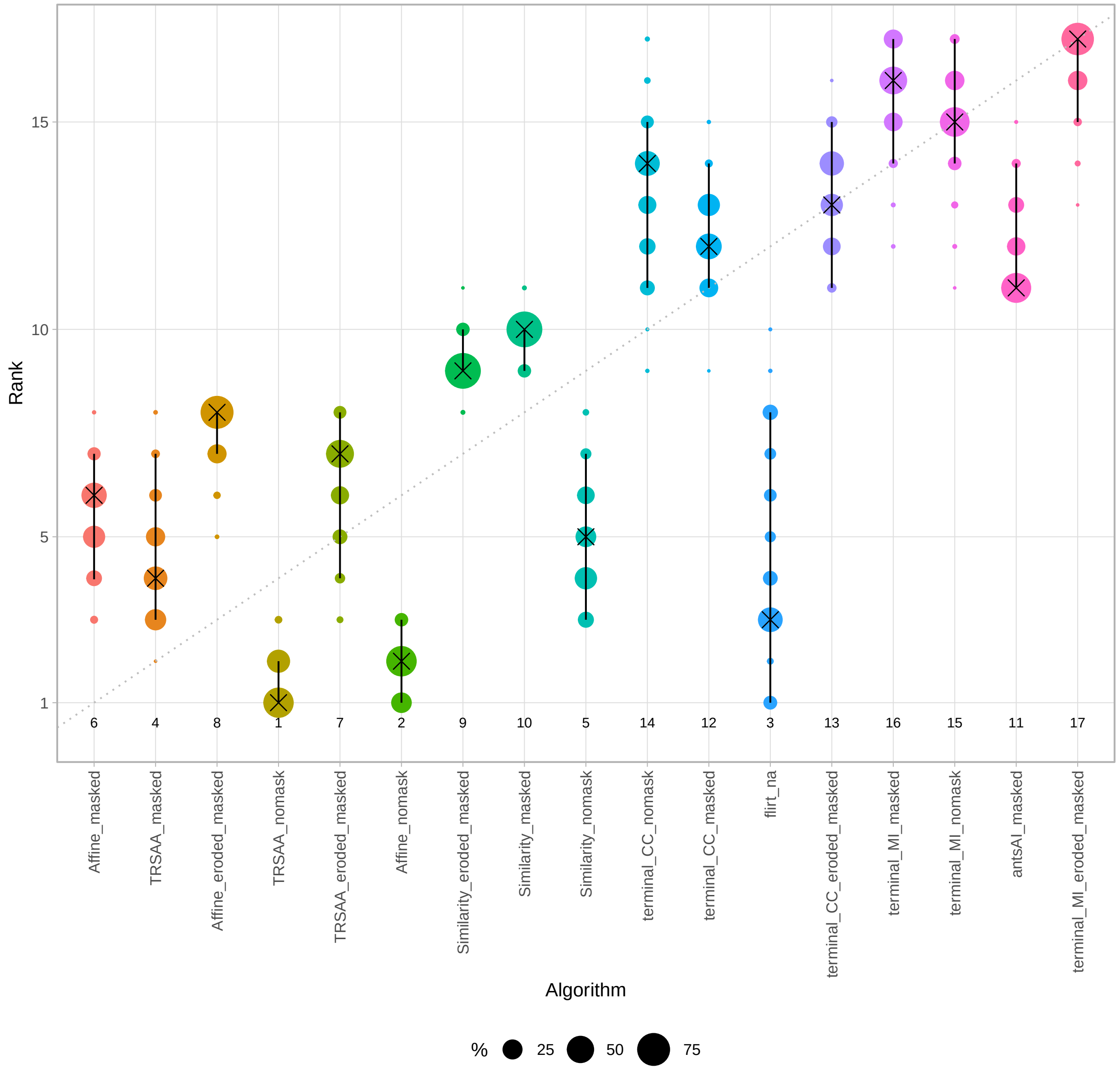

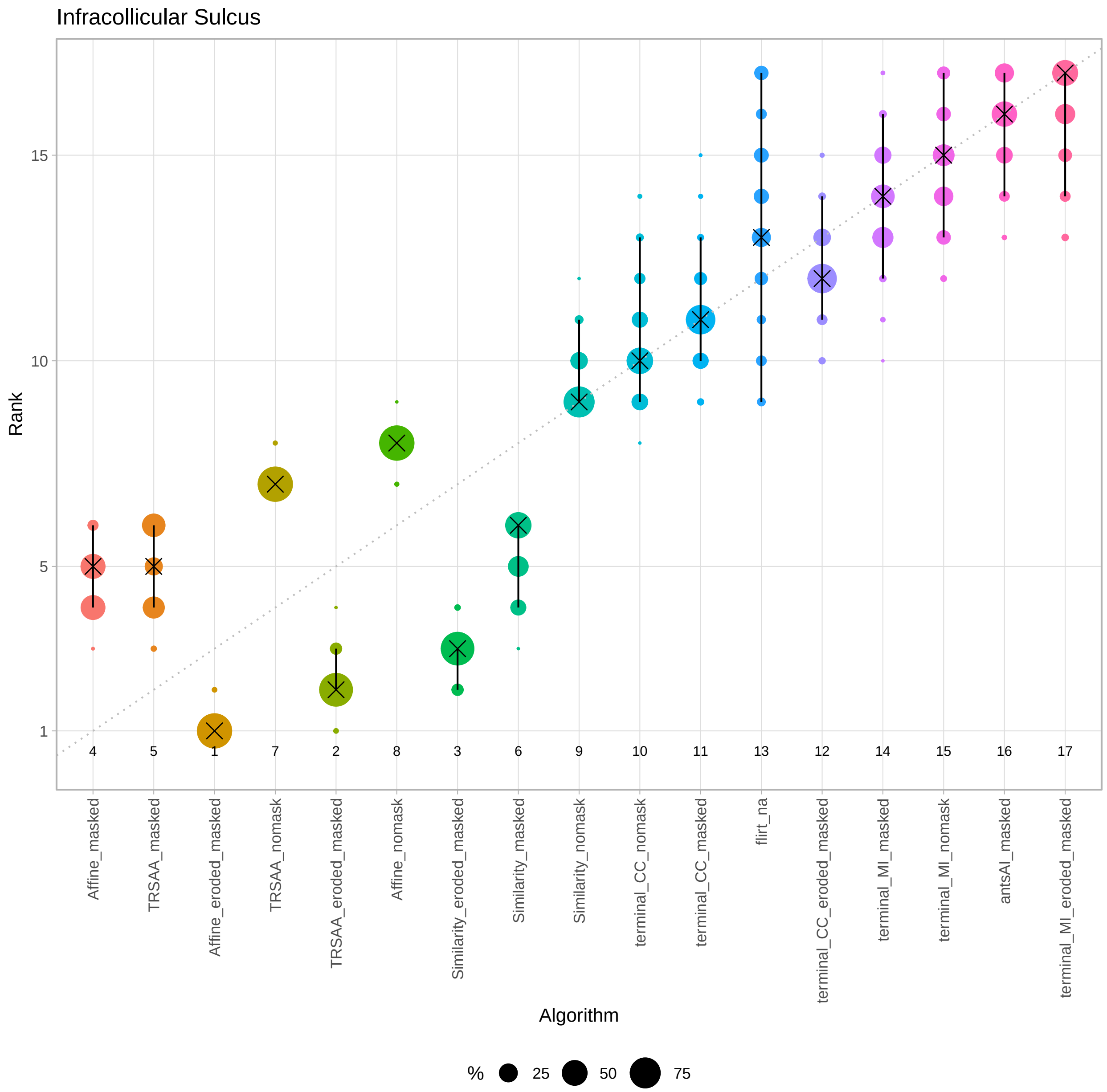

Infracollicular Sulcus
Rank
15
10
5
1
4
5
1
7
2
8
3
6
9
10
11
13
12
14
15
16
17
Affine_masked
TRSAA_masked
Affine_eroded_masked
TRSAA_nomask
TRSAA_eroded_masked
Affine_nomask
Similarity_eroded_masked
Similarity_masked
Similarity_nomask
terminal_CC_nomask
terminal_CC_masked
flirt_na
terminal_CC_eroded_masked
terminal_MI_masked
terminal_MI_nomask
antsAI_masked
terminal_MI_eroded_masked
Algorithm
%
25
50
75

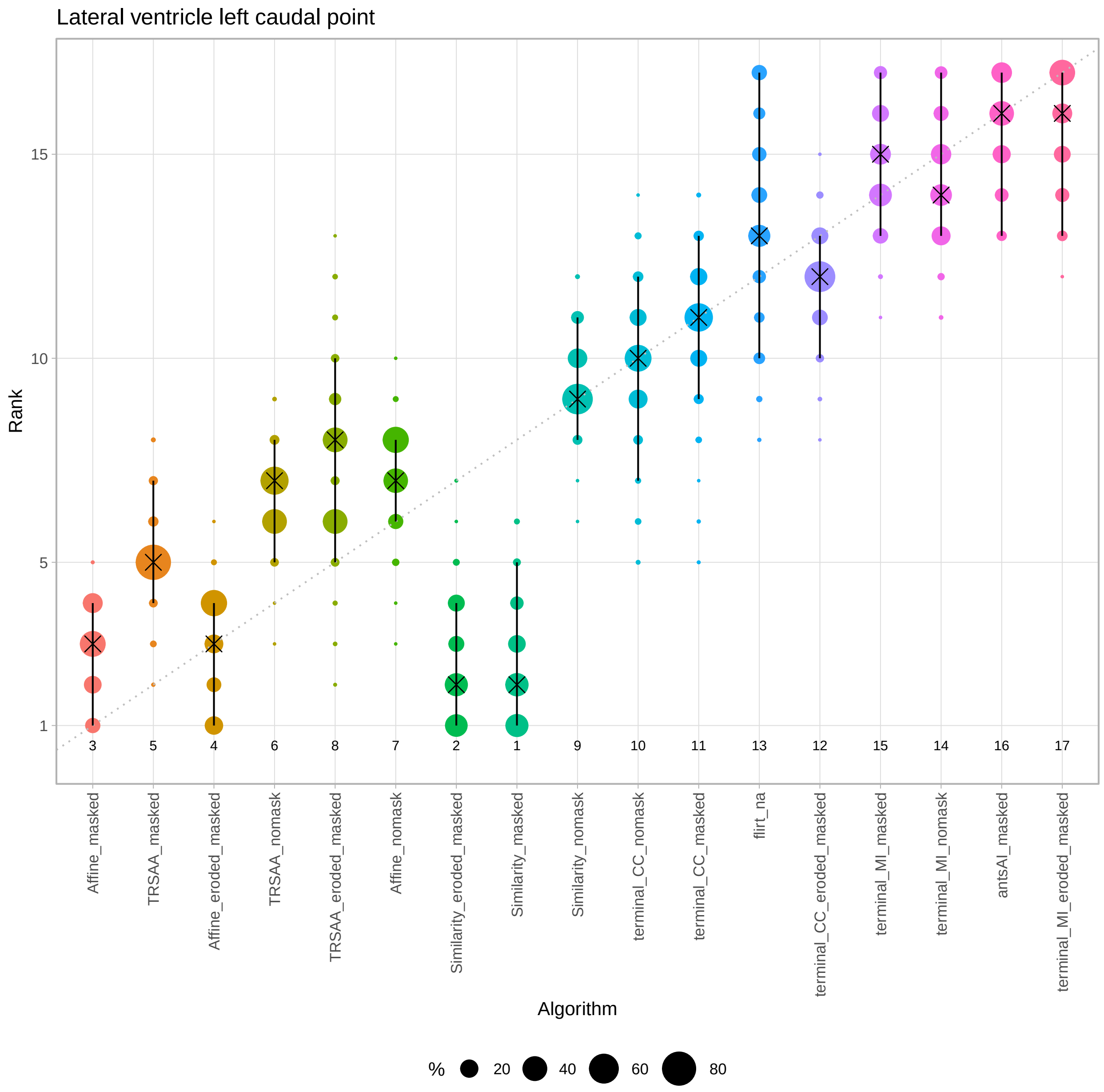

Lateral ventricle left caudal point
Rank
15
10
5
1
3
5
4
6
8
7
2
1
9
10
11
13
12
15
14
16
17
Affine_masked
TRSAA_masked
Affine_eroded_masked
TRSAA_nomask
TRSAA_eroded_masked
Affine_nomask
Similarity_eroded_masked
Similarity_masked
Similarity_nomask
terminal_CC_nomask
terminal_CC_masked
flirt_na
terminal_CC_eroded_masked
terminal_MI_masked
terminal_MI_nomask
antsAI_masked
terminal_MI_eroded_masked
Algorithm
%
20
40
60
80

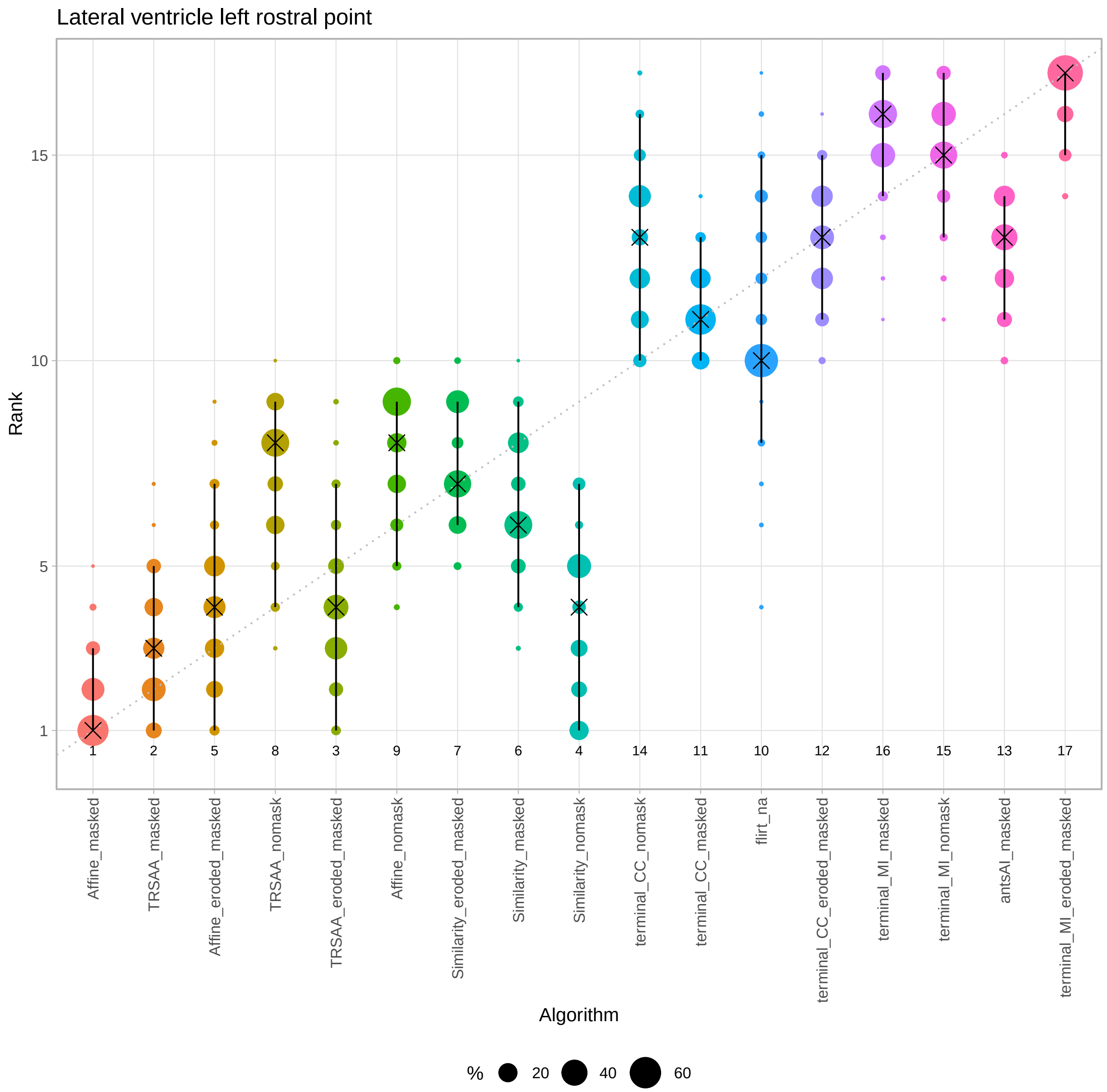
Lateral ventricle left rostral point
Rank
15
10
5
1
1
2
5
8
3
9
7
6
4
14
11
10
12
16
15
13
17
Affine_masked
TRSAA_masked
Affine_eroded_masked
TRSAA_nomask
TRSAA_eroded_masked
Affine_nomask
Similarity_eroded_masked
Similarity_masked
Similarity_nomask
terminal_CC_nomask
terminal_CC_masked
flirt_na
terminal_CC_eroded_masked
terminal_MI_masked
terminal_MI_nomask
antsAI_masked
terminal_MI_eroded_masked
Algorithm
%
20
40
60

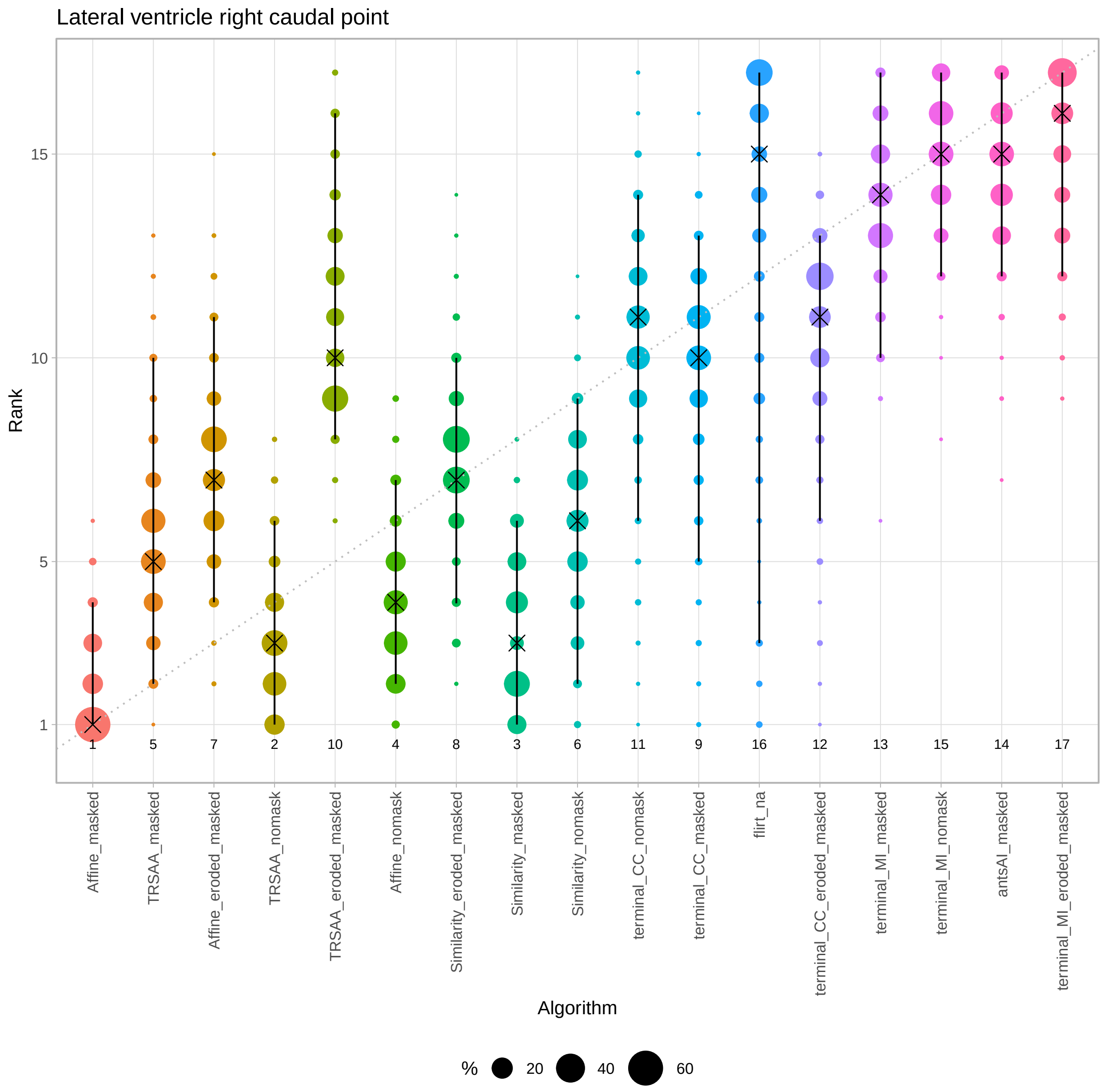

Lateral ventricle right caudal point
Rank
15
10
5
1
1
5
7
2
10
4
8
3
6
11
9
16
12
13
15
14
17
Affine_masked
TRSAA_masked
Affine_eroded_masked
TRSAA_nomask
TRSAA_eroded_masked
Affine_nomask
Similarity_eroded_masked
Similarity_masked
Similarity_nomask
terminal_CC_nomask
terminal_CC_masked
flirt_na
terminal_CC_eroded_masked
terminal_MI_masked
terminal_MI_nomask
antsAI_masked
terminal_MI_eroded_masked
Algorithm
%
20
40
60

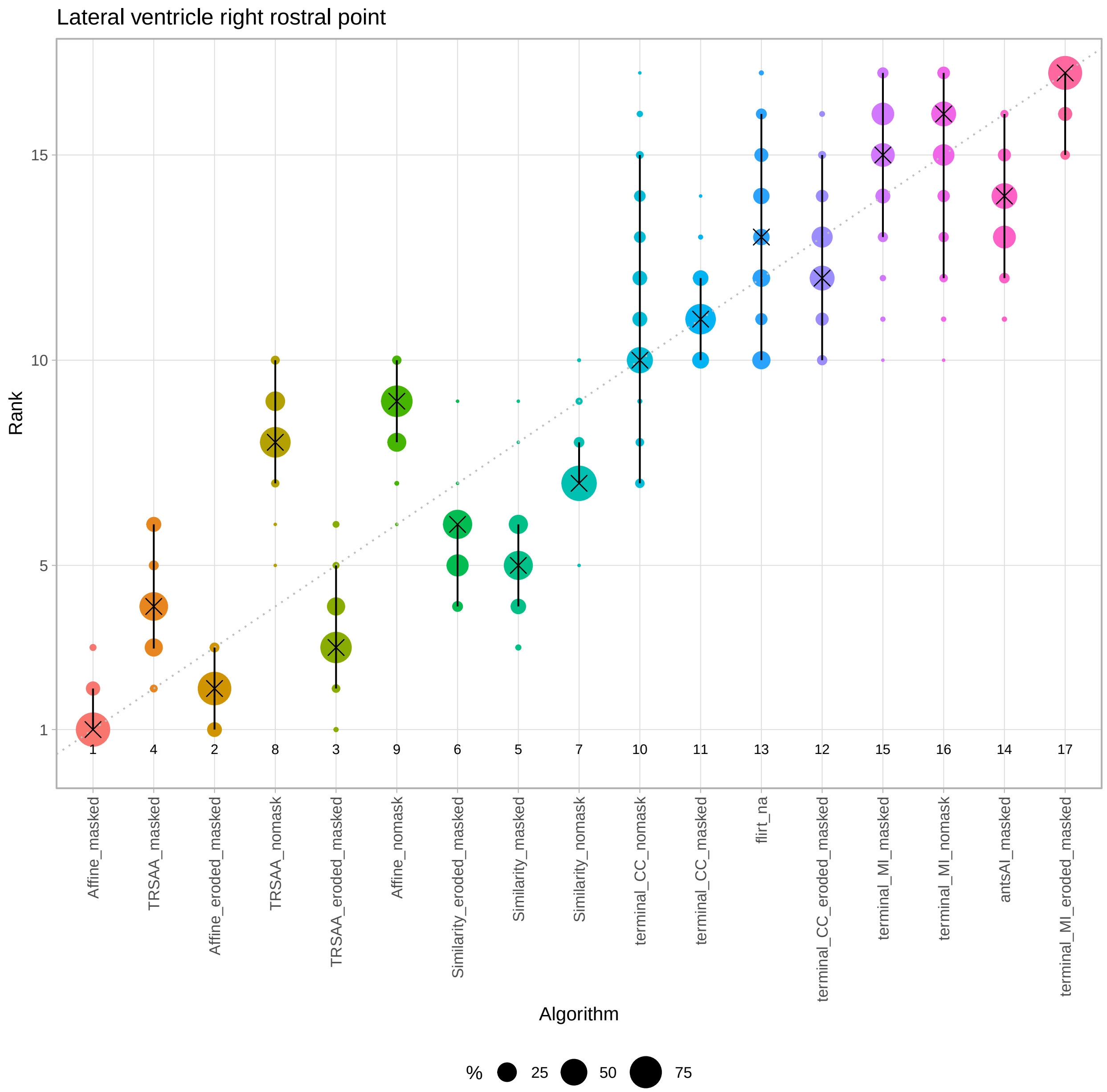
Lateral ventricle right rostral point
Rank
15
10
5
1
1
4
2
8
3
9
6
5
7
10
11
13
12
15
16
14
17
Affine_masked
TRSAA_masked
Affine_eroded_masked
TRSAA_nomask
TRSAA_eroded_masked
Affine_nomask
Similarity_eroded_masked
Similarity_masked
Similarity_nomask
terminal_CC_nomask
terminal_CC_masked
flirt_na
terminal_CC_eroded_masked
terminal_MI_masked
terminal_MI_nomask
antsAI_masked
terminal_MI_eroded_masked
Algorithm
%
25
50
75

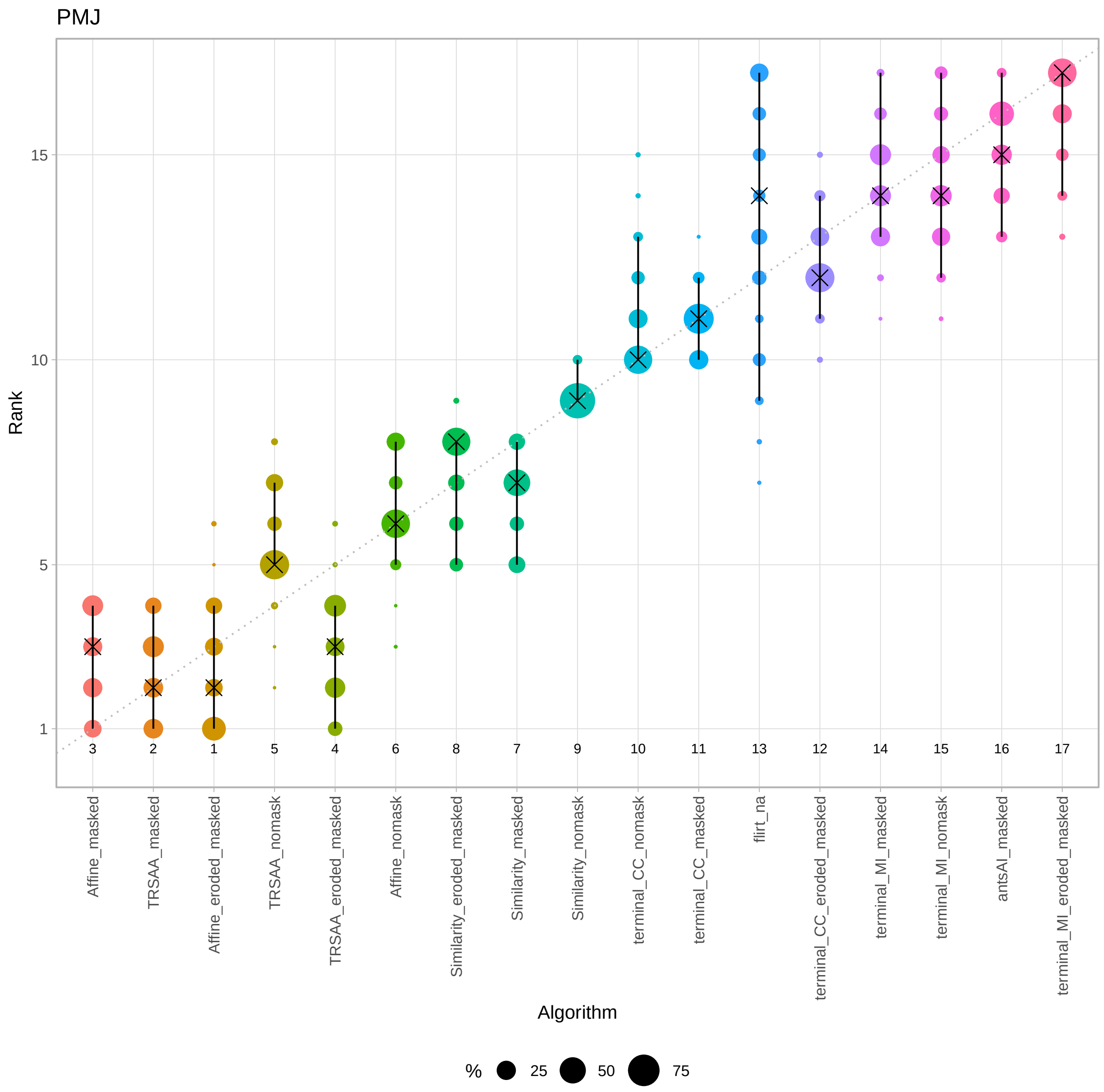
PMJ
Rank
15
10
5
1
3
2
1
5
4
6
8
7
9
10
11
13
12
14
15
16
17
Affine_masked
TRSAA_masked
Affine_eroded_masked
TRSAA_nomask
TRSAA_eroded_masked
Affine_nomask
Similarity_eroded_masked
Similarity_masked
Similarity_nomask
terminal_CC_nomask
terminal_CC_masked
flirt_na
terminal_CC_eroded_masked
terminal_MI_masked
terminal_MI_nomask
antsAI_masked
terminal_MI_eroded_masked
Algorithm
%
25
50
75

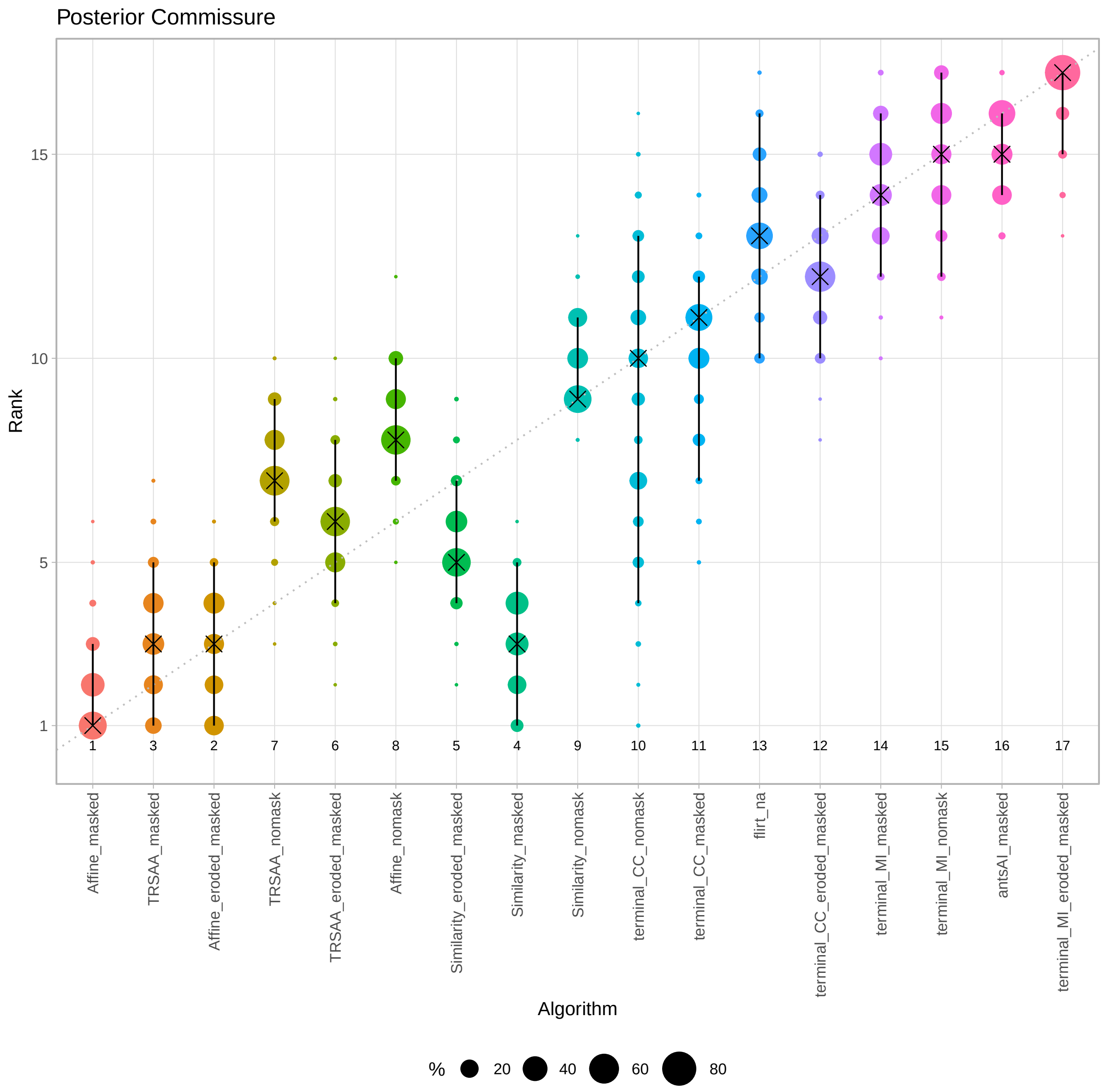
Posterior Commissure
Rank
15
10
5
1
1
3
2
7
6
8
5
4
9
10
11
13
12
14
15
16
17
Affine_masked
TRSAA_masked
Affine_eroded_masked
TRSAA_nomask
TRSAA_eroded_masked
Affine_nomask
Similarity_eroded_masked
Similarity_masked
Similarity_nomask
terminal_CC_nomask
terminal_CC_masked
flirt_na
terminal_CC_eroded_masked
terminal_MI_masked
terminal_MI_nomask
antsAI_masked
terminal_MI_eroded_masked
Algorithm
% 20 40 60 80

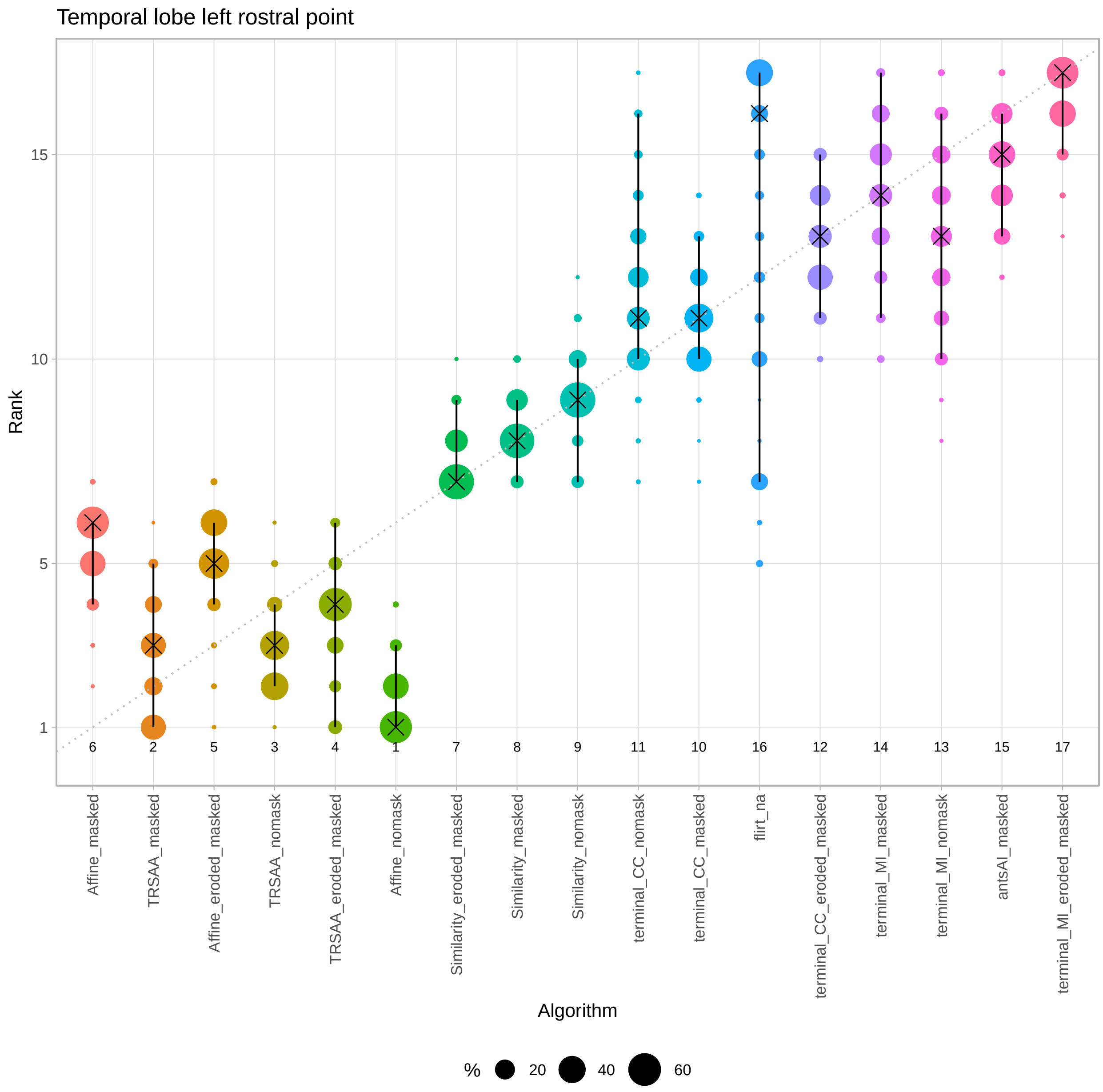

Temporal lobe left rostral point
Rank
15
10
5
1
6
2
5
3
4
1
7
8
9
11
10
16
12
14
13
15
17
Affine_masked
TRSAA_masked
Affine_eroded_masked
TRSAA_nomask
TRSAA_eroded_masked
Affine_nomask
Similarity_eroded_masked
Similarity_masked
Similarity_nomask
terminal_CC_nomask
terminal_CC_masked
flirt_na
terminal_CC_eroded_masked
terminal_MI_masked
terminal_MI_nomask
antsAI_masked
terminal_MI_eroded_masked
Algorithm
%
20
40
60

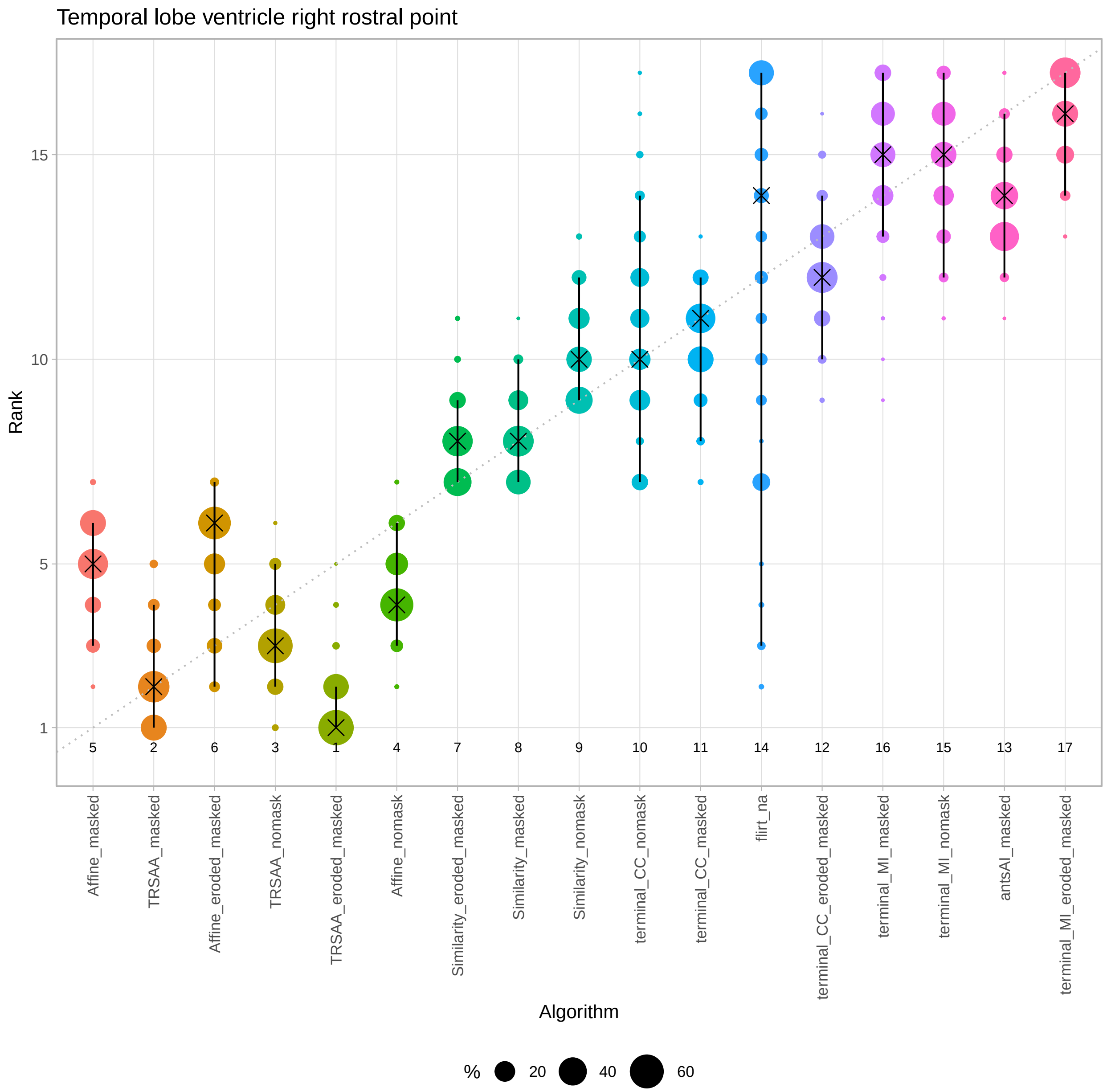

4.2.2 Cluster Analysis

Dendrogram from hierarchical cluster analysis and *network-type graphs* for assessing the similarity of tasks based on challenge rankings.

A dendrogram is a visualization approach based on hierarchical clustering. It depicts clusters according to a chosen distance measure (here: Spearman's footrule) as well as a chosen agglomeration method (here: complete and average agglomeration).

Cluster Dendrogram (complete agglomeration)

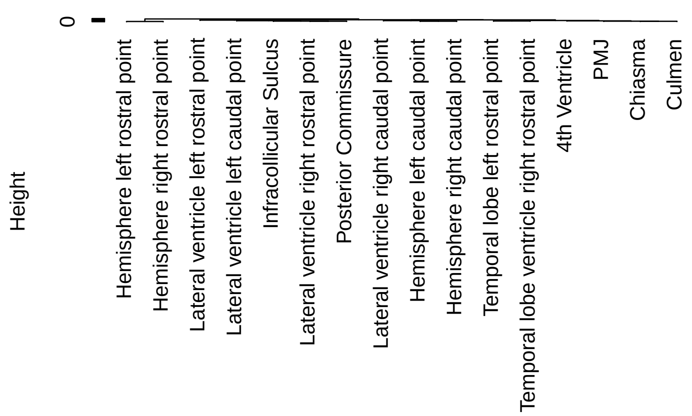

Cluster Dendrogram (average agglomeration)

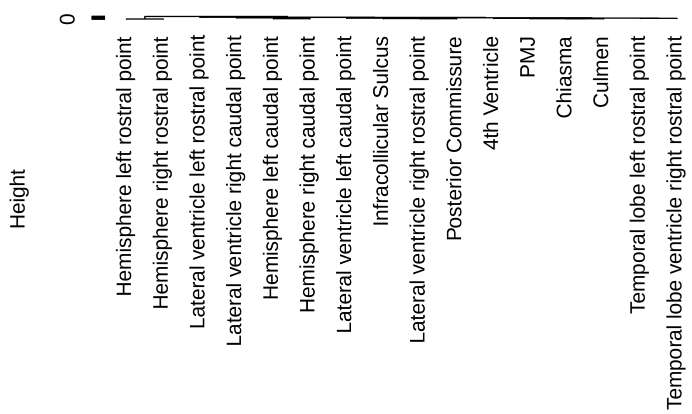

5 References

Wiesenfarth, M., Reinke, A., Landman, B.A., Eisenmann, M., Aguilera Saiz, L., Cardoso, M.J., Maier-Hein, L. and Kopp-Schneider, A. Methods and open-source toolkit for analyzing and visualizing challenge results. *Sci Rep* **11**, 2369 (2021). https://doi.org/10.1038/s41598-021-82017-6

M. J. A. Eugster, T. Hothorn, and F. Leisch, "Exploratory and inferential analysis of benchmark experiments," Institut fuer Statistik, Ludwig-Maximilians-Universitaet Muenchen, Germany, Technical Report 30, 2008. [Online]. Available: http://epub.ub.uni-muenchen.de/4134/.
