# Supplementary material for: Characterization of dynamic patterns of human fetal to neonatal brain asymmetry with deformation-based morphometry
Source: Front Neurosci. 2023 Dec 6;17:1252850. doi: 10.3389/fnins.2023.1252850 (PMC10734644; doi:10.3389/fnins.2023.1252850)
Supplement: Supplementary file 1 [file Data_Sheet_1.ZIP › ChallengeR/Framework B to mid-age template/fetal2template_linear/Fetal2MidTemplate_linear_voldiff.pdf]

Benchmarking report for Fetal to Midtempalte Registration Linear

created by challengeR v1.0.4

29 März, 2023

This document presents a systematic report on the benchmark study "Fetal to Midtempalte Registration Linear". Input data comprises raw metric values for all algorithms and cases. Generated plots are:

- Visualization of assessment data: Dot- and boxplots, podium plots and ranking heatmaps
- Visualization of ranking stability: Blob plots, violin plots and significance maps, line plots
- Visualization of cross-task insights: Blob plots, stacked frequency plots, dendrograms

Details can be found in Wiesenfarth et al. (2021).

1 Rankings

Algorithms within a task are ranked according to the following ranking scheme:

aggregate using function ("mean") then rank

Ranking for each task:

Brainstem : The analysis is based on 17 algorithms and 20 cases. 0 missing cases have been found in the data set.

	value_mean	rank
antsAI_masked	0.0808203	1
terminal_CC_eroded_masked	0.0809010	2
terminal_CC_nomask	0.0835164	3
terminal_MI_eroded_masked	0.0887933	4
terminal_CC_masked	0.0966856	5
terminal_MI_nomask	0.1002304	6
terminal_MI_masked	0.1129849	7
Affine_masked	0.1982602	8
Similarity_masked	0.2623243	9
TRSAA_masked	0.2663569	10
Affine_eroded_masked	0.2762309	11
Similarity_eroded_masked	0.3090982	12
Affine_nomask	0.3795760	13
TRSAA_nomask	0.3831976	14
TRSAA_eroded_masked	0.3894308	15
Similarity_nomask	0.4770912	16
flirt_NA	0.6134193	17

Cerebellum : The analysis is based on 17 algorithms and 20 cases. 0 missing cases have been found in the data set.

	value_mean	rank
Similarity__nomask	0.2561564	1
TRSAA__eroded__masked	0.2988744	2
TRSAA__nomask	0.3024273	3
Affine__nomask	0.3039857	4
flirt__NA	0.3209559	5
Similarity__eroded__masked	0.3398590	6
Affine__eroded__masked	0.3563756	7
TRSAA__masked	0.3596421	8
Similarity__masked	0.3628866	9
Affine__masked	0.3954879	10
terminal__MI__nomask	0.4715242	11
terminal__CC__nomask	0.4737181	12
terminal__MI__masked	0.4742566	13
terminal__CC__masked	0.4754262	14
terminal__MI__eroded__masked	0.4824273	15
antsAI__masked	0.4851949	16
terminal__CC__eroded__masked	0.4867654	17

Cortical grey matter : The analysis is based on 17 algorithms and 20 cases. 0 missing cases have been found in the data set.

	value_mean	rank
Similarity__nomask	0.2594327	1
TRSAA__eroded__masked	0.3035596	2
TRSAA__nomask	0.3060042	3
Affine__nomask	0.3077819	4
flirt__NA	0.3242267	5
Similarity__eroded__masked	0.3438563	6
Affine__eroded__masked	0.3596666	7
TRSAA__masked	0.3640548	8
Similarity__masked	0.3666987	9
Affine__masked	0.3983787	10
terminal__MI__nomask	0.4770530	11
terminal__CC__nomask	0.4777934	12
terminal__MI__masked	0.4785068	13
terminal__CC__masked	0.4796455	14
terminal__MI__eroded__masked	0.4858921	15
antsAI__masked	0.4893486	16
terminal__CC__eroded__masked	0.4905795	17

CSF and ventricles : The analysis is based on 17 algorithms and 20 cases. 0 missing cases have been found in the data set.

	value_mean	rank
terminal__CC__eroded__masked	0.2855554	1
antsAI__masked	0.2892832	2
terminal__MI__eroded__masked	0.2967445	3
terminal__MI__masked	0.3092797	4
terminal__CC__masked	0.3138804	5
terminal__CC__nomask	0.3185146	6

	value_mean	rank
terminal_MI_nomask	0.3363037	7
Affine_masked	0.5188484	8
Similarity_masked	0.6046632	9
TRSAA_masked	0.6136051	10
Affine_eroded_masked	0.6223773	11
Similarity_eroded_masked	0.6645975	12
flirt_NA	0.7103398	13
Affine_nomask	0.7521200	14
TRSAA_nomask	0.7568701	15
TRSAA_eroded_masked	0.7767097	16
Similarity_nomask	0.8742899	17

Deep grey matter : The analysis is based on 17 algorithms and 20 cases. 0 missing cases have been found in the data set.

	value_mean	rank
Similarity_nomask	0.2119638	1
TRSAA_eroded_masked	0.2585765	2
TRSAA_nomask	0.2612727	3
Affine_nomask	0.2629085	4
flirt_NA	0.2804026	5
Similarity_eroded_masked	0.2999487	6
Affine_eroded_masked	0.3172352	7
Similarity_masked	0.3250738	8
TRSAA_masked	0.3250985	9
Affine_masked	0.3606674	10
terminal_MI_nomask	0.4457034	11
terminal_CC_nomask	0.4463043	12
terminal_CC_masked	0.4485083	13
terminal_MI_masked	0.4486559	14
terminal_MI_eroded_masked	0.4546696	15
antsAI_masked	0.4586366	16
terminal_CC_eroded_masked	0.4600338	17

White matter : The analysis is based on 17 algorithms and 20 cases. 0 missing cases have been found in the data set.

	value_mean	rank
Similarity_masked	0.0593755	1
Similarity_eroded_masked	0.0603577	2
Affine_eroded_masked	0.0643653	3
TRSAA_masked	0.0654177	4
flirt_NA	0.0723768	5
Affine_nomask	0.0867266	6
TRSAA_nomask	0.0872023	7
TRSAA_eroded_masked	0.0875745	8
Affine_masked	0.0997921	9
Similarity_nomask	0.1530248	10
terminal_MI_nomask	0.1999037	11
terminal_CC_masked	0.2047076	12

	value_mean	rank
terminal_CC_nomask	0.2058858	13
terminal_MI_eroded_masked	0.2164074	14
antsAI_masked	0.2237717	15
terminal_CC_eroded_masked	0.2253450	16
terminal_MI_masked	0.2317049	17

Consensus ranking across tasks according to chosen method "euclidean":

	value	rank
Similarity_eroded_masked	7.333	1
Affine_nomask	7.500	2
Similarity_masked	7.500	2
TRSAA_eroded_masked	7.500	2
TRSAA_nomask	7.500	2
Affine_eroded_masked	7.667	6
Similarity_nomask	7.667	6
TRSAA_masked	8.167	8
flirt_NA	8.333	9
Affine_masked	9.167	10
terminal_MI_nomask	9.500	11
terminal_CC_nomask	9.667	12
terminal_CC_masked	10.500	13
antsAI_masked	11.000	14
terminal_MI_eroded_masked	11.000	14
terminal_MI_masked	11.333	16
terminal_CC_eroded_masked	11.667	17

2 Visualization of raw assessment data

The algorithms are ordered according to the computed ranks for each task.

2.1 Dot- and boxplot

Dot- and boxplots for visualizing raw assessment data separately for each algorithm. Boxplots representing descriptive statistics over all cases (median, quartiles and outliers) are combined with horizontally jittered dots representing individual cases.

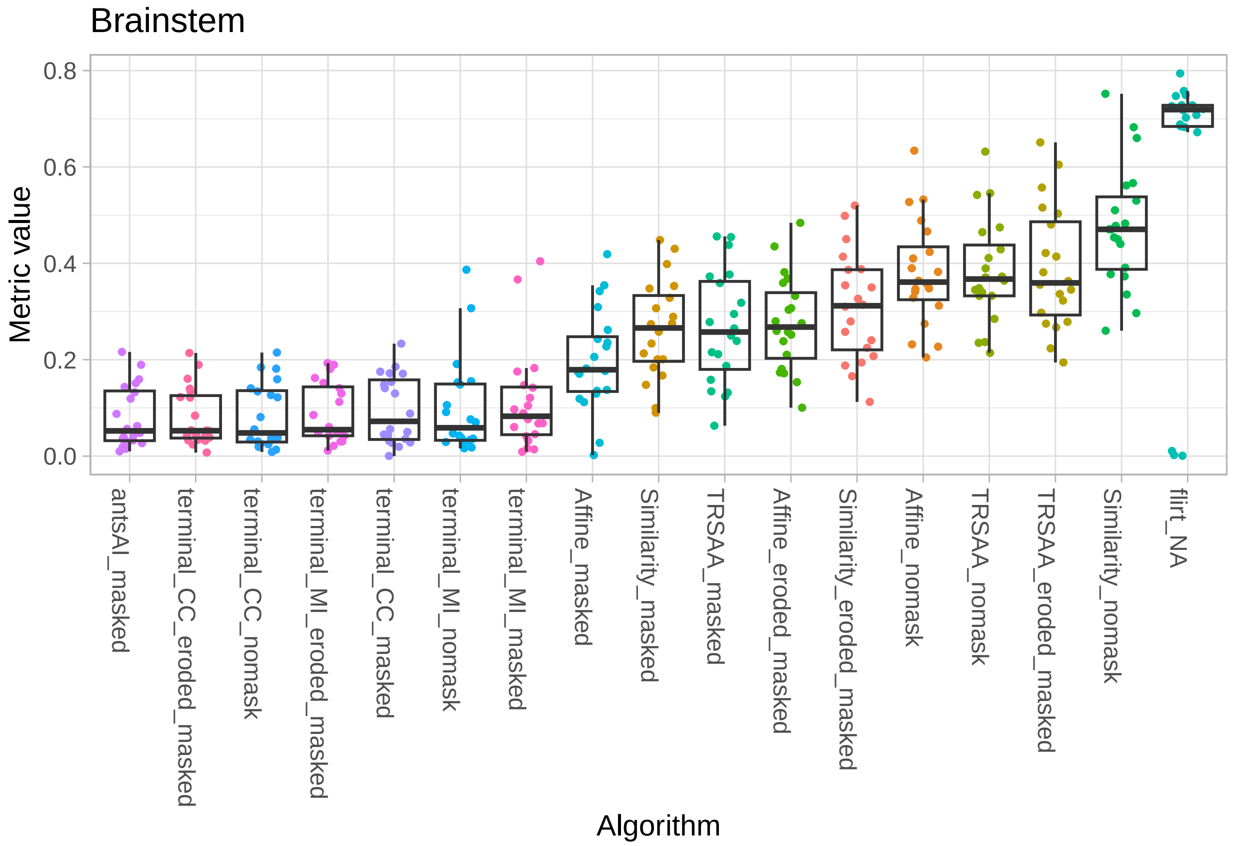

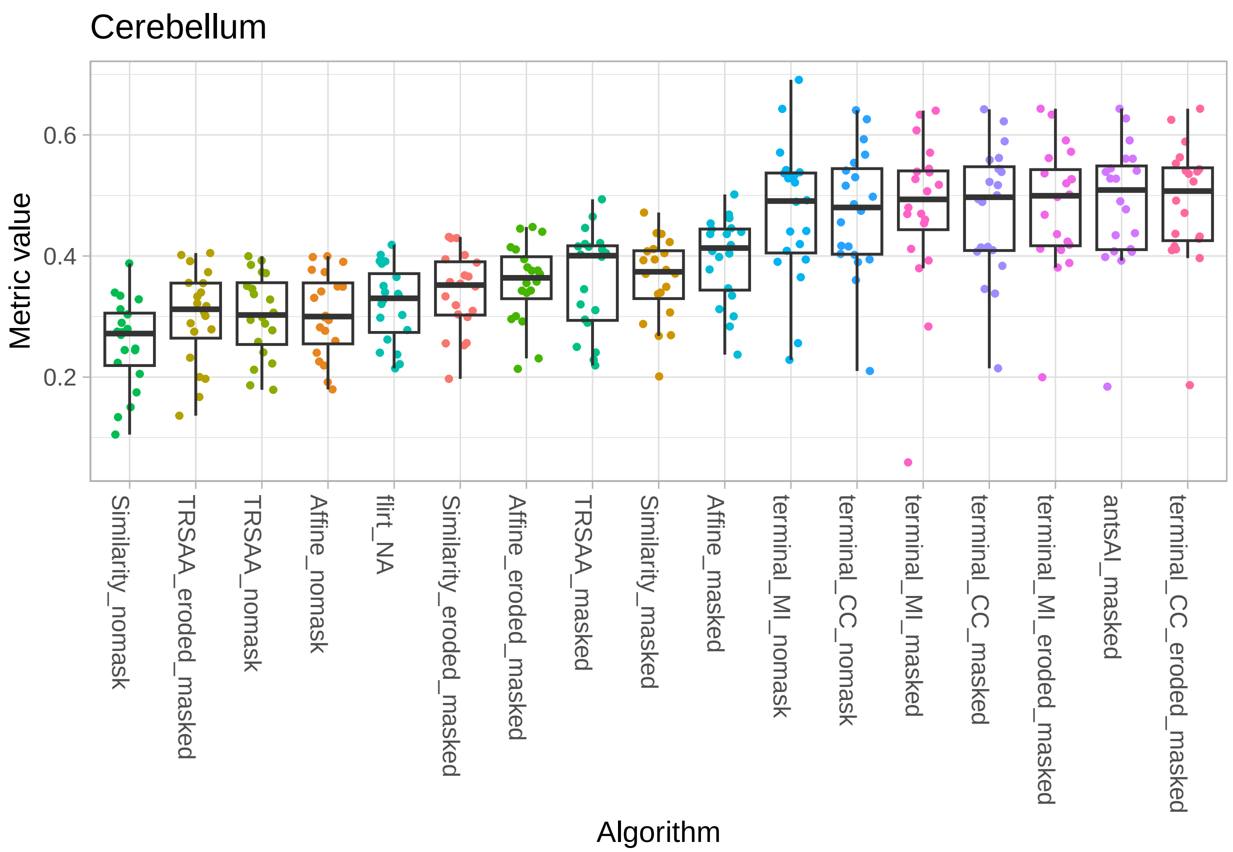
Cerebellum
Metric value
0.2
0.4
0.6
Algorithm
Similarity_nomask
TRSAA_eroded_masked
TRSAA_nomask
Affine_nomask
flirt_NA
Similarity_eroded_masked
Affine_eroded_masked
TRSAA_masked
Similarity_masked
Affine_masked
terminal_MI_nomask
terminal_CC_nomask
terminal_MI_masked
terminal_CC_masked
terminal_MI_eroded_masked
antsAI_masked
terminal_CC_eroded_masked

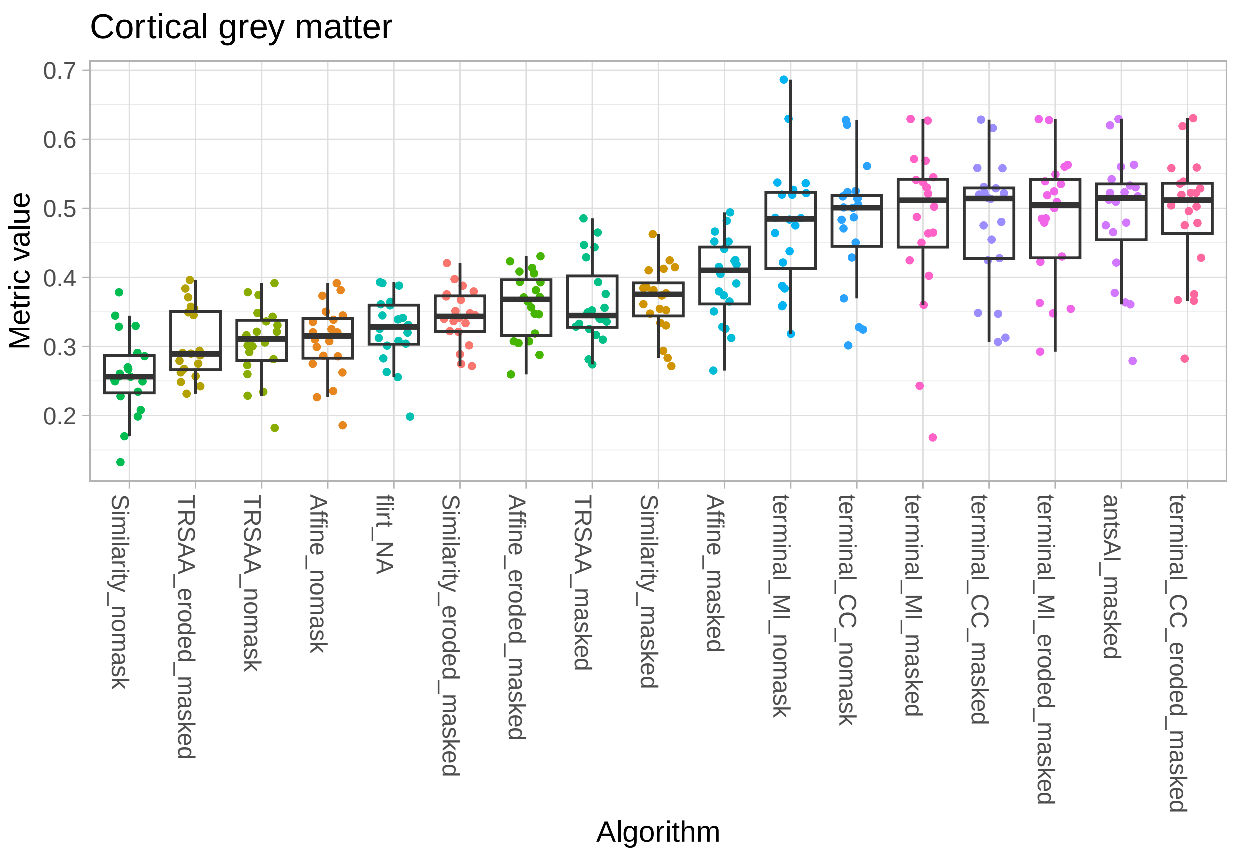
Cortical grey matter
Metric value
0.7
0.6
0.5
0.4
0.3
0.2
Similarity_nomask
TRSAA_eroded_masked
TRSAA_nomask
Affine_nomask
flirt_NA
Similarity_eroded_masked
Affine_eroded_masked
TRSAA_masked
Similarity_masked
Affine_masked
terminal_MI_nomask
terminal_CC_nomask
terminal_MI_masked
terminal_CC_masked
terminal_MI_eroded_masked
antsAI_masked
terminal_CC_eroded_masked
Algorithm

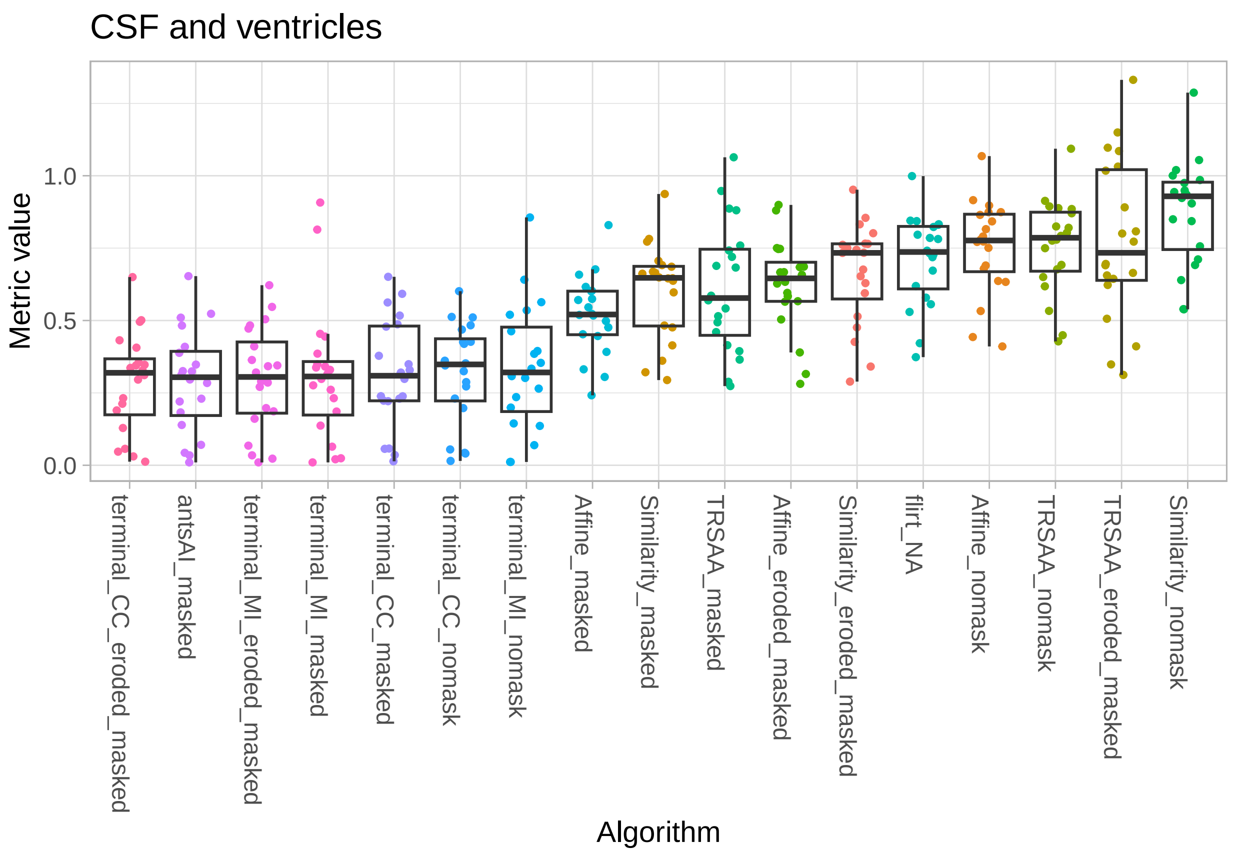
CSF and ventricles
Metric value
1.0
0.5
0.0
terminal_CC_eroded_masked
antsAI_masked
terminal_MI_eroded_masked
terminal_MI_masked
terminal_CC_masked
terminal_CC_nomask
terminal_MI_nomask
Affine_masked
Similarity_masked
TRSAA_masked
Affine_eroded_masked
Similarity_eroded_masked
flirt_NA
Affine_nomask
TRSAA_nomask
TRSAA_eroded_masked
Similarity_nomask
Algorithm

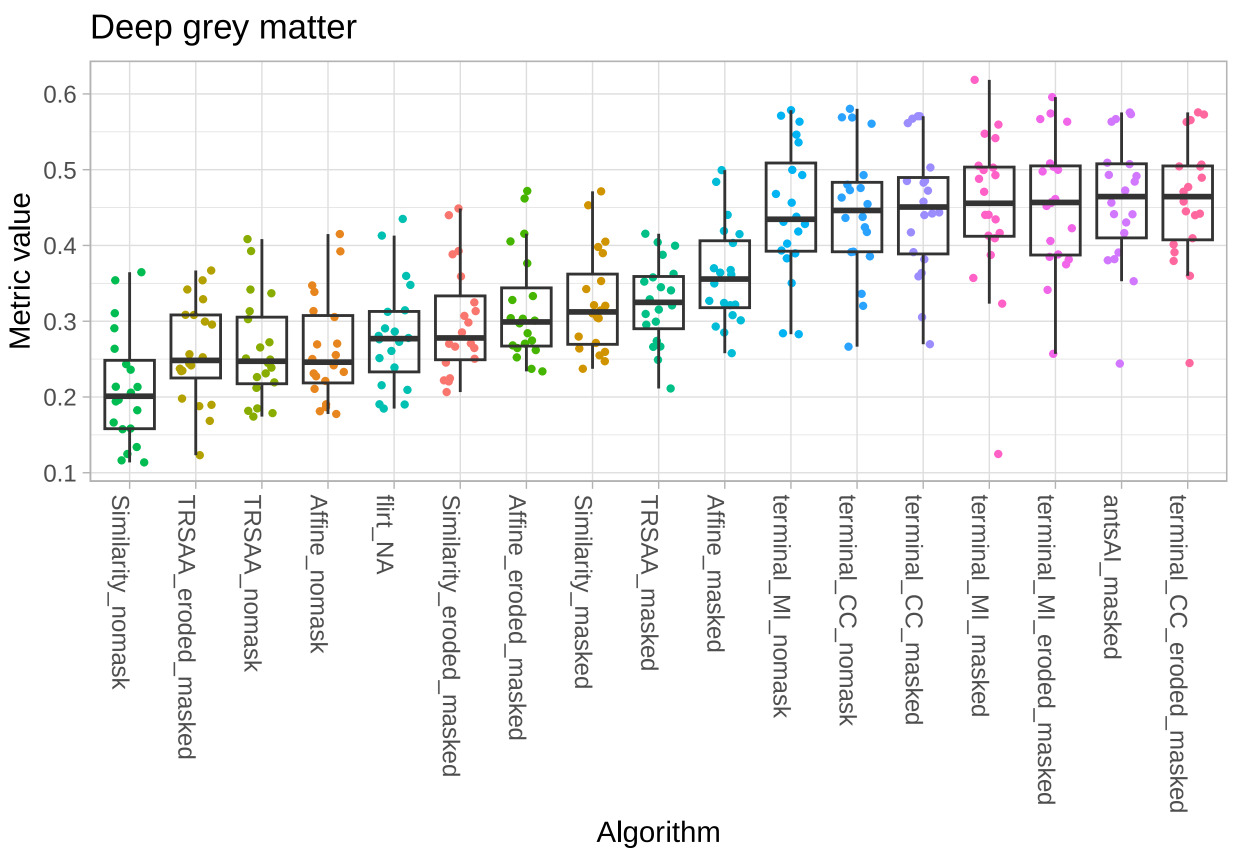

Deep grey matter
Metric value
0.6
0.5
0.4
0.3
0.2
0.1
Similarity_nomask
TRSAA_eroded_masked
TRSAA_nomask
Affine_nomask
flirt_NA
Similarity_eroded_masked
Affine_eroded_masked
Similarity_masked
TRSAA_masked
Affine_masked
terminal_MI_nomask
terminal_CC_nomask
terminal_CC_masked
terminal_MI_masked
terminal_MI_eroded_masked
antsAI_masked
terminal_CC_eroded_masked
Algorithm

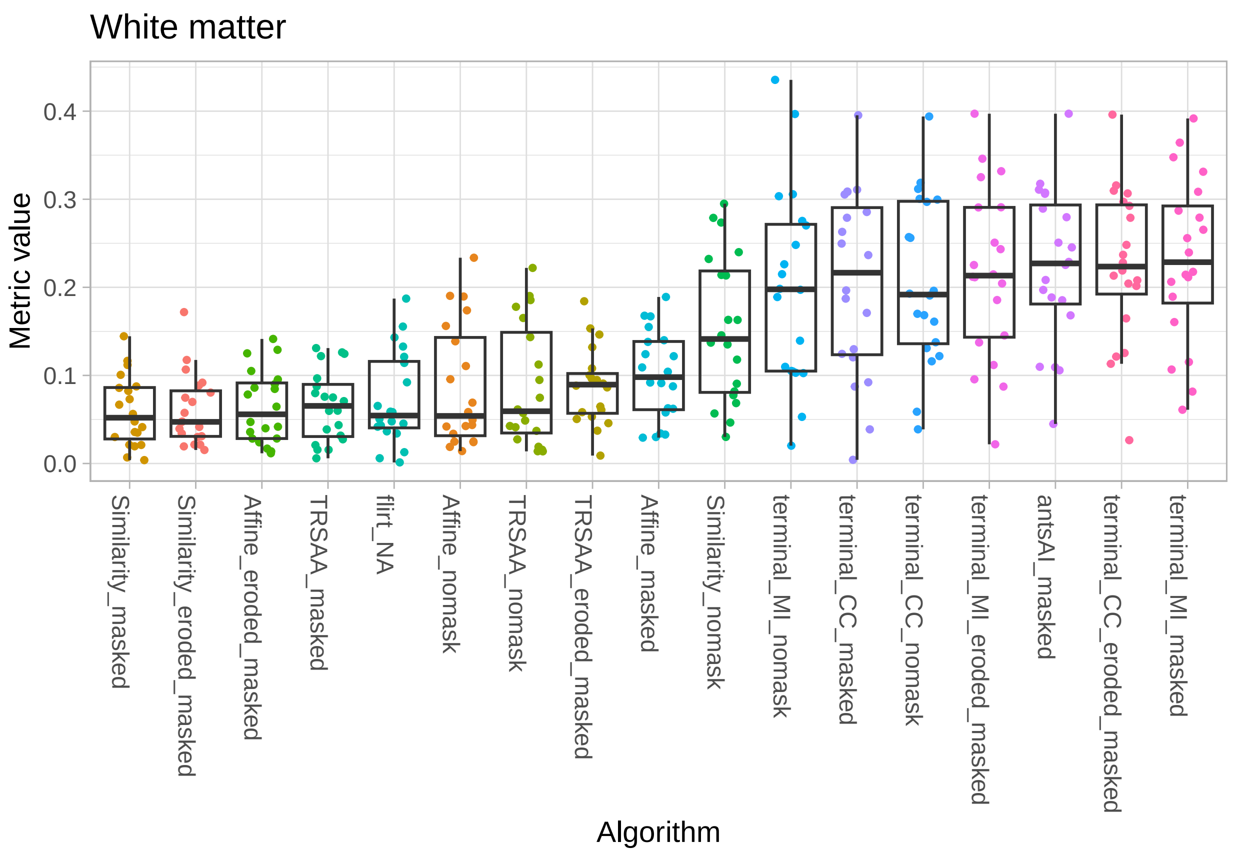
White matter
Metric value
0.0
0.1
0.2
0.3
0.4
Similarity_masked
Similarity_eroded_masked
Affine_eroded_masked
TRSAA_masked
flirt_NA
Affine_nomask
TRSAA_nomask
TRSAA_eroded_masked
Affine_masked
Similarity_nomask
terminal_MI_nomask
terminal_CC_masked
terminal_CC_nomask
terminal_MI_eroded_masked
antsAI_masked
terminal_CC_eroded_masked
terminal_MI_masked
Algorithm

2.2 Podium plot

Podium plots (see also Eugster et al., 2008) for visualizing raw assessment data. Upper part (spaghetti plot): Participating algorithms are color-coded, and each colored dot in the plot represents a metric value achieved with the respective algorithm. The actual metric value is encoded by the y-axis. Each podium (here: p=17) represents one possible rank, ordered from best (1) to last (here: 17). The assignment of metric values (i.e. colored dots) to one of the podiums is based on the rank that the respective algorithm achieved on the corresponding case. Note that the plot part above each podium place is further subdivided into p "columns", where each column represents one participating algorithm (here: $p = 17$). Dots corresponding to identical cases are connected by a line, leading to the shown spaghetti structure. Lower part: Bar charts represent the relative frequency for each algorithm to achieve the rank encoded by the podium place.

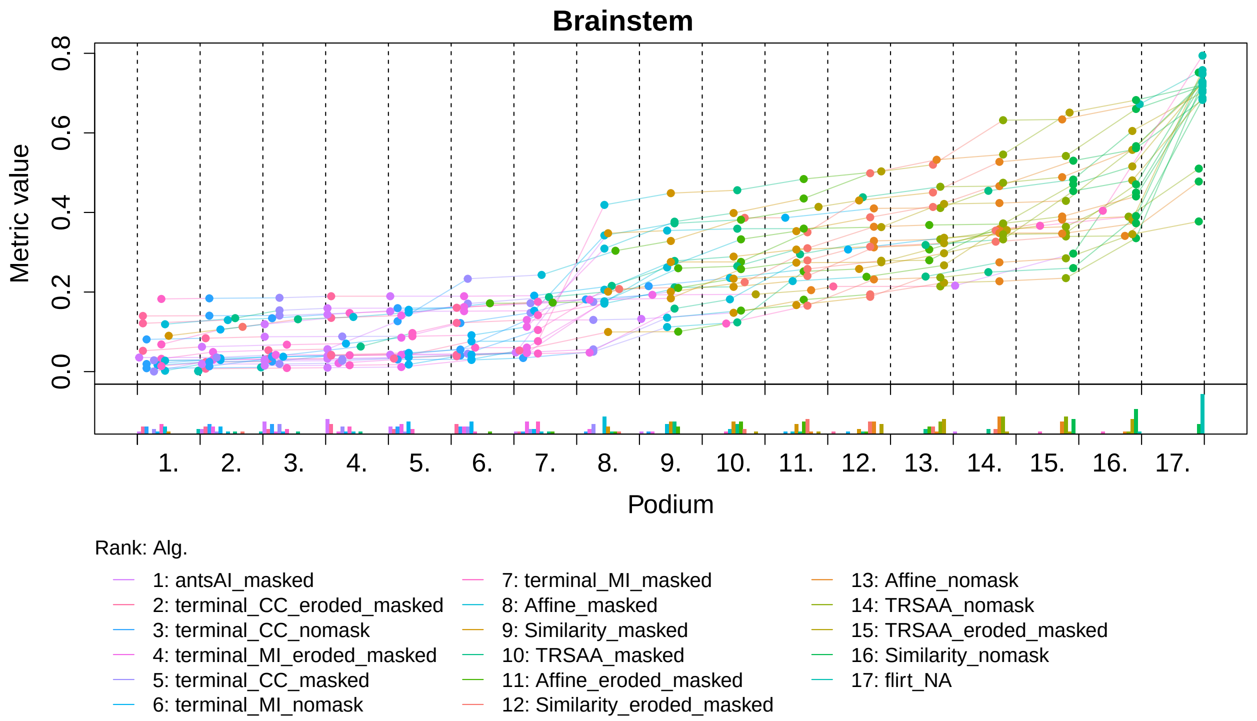

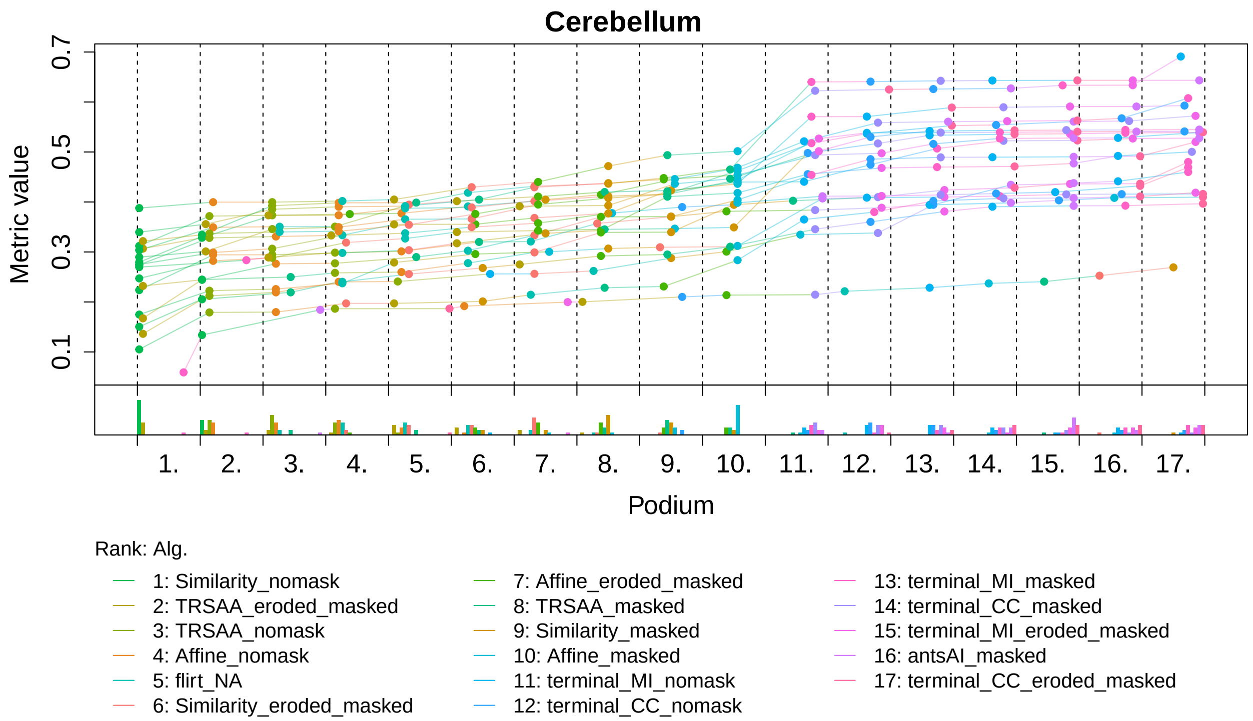
Cerebellum
Metric value
0.1
0.3
0.5
0.7
1.
2.
3.
4.
5.
6.
7.
8.
9.
10.
11.
12.
13.
14.
15.
16.
17.
Podium
Rank: Alg.
1: Similarity_nomask
2: TRSAA_eroded_masked
3: TRSAA_nomask
4: Affine_nomask
5: flirt_NA
6: Similarity_eroded_masked
7: Affine_eroded_masked
8: TRSAA_masked
9: Similarity_masked
10: Affine_masked
11: terminal_MI_nomask
12: terminal_CC_nomask
13: terminal_MI_masked
14: terminal_CC_masked
15: terminal_MI_eroded_masked
16: antsAI_masked
17: terminal_CC_eroded_masked

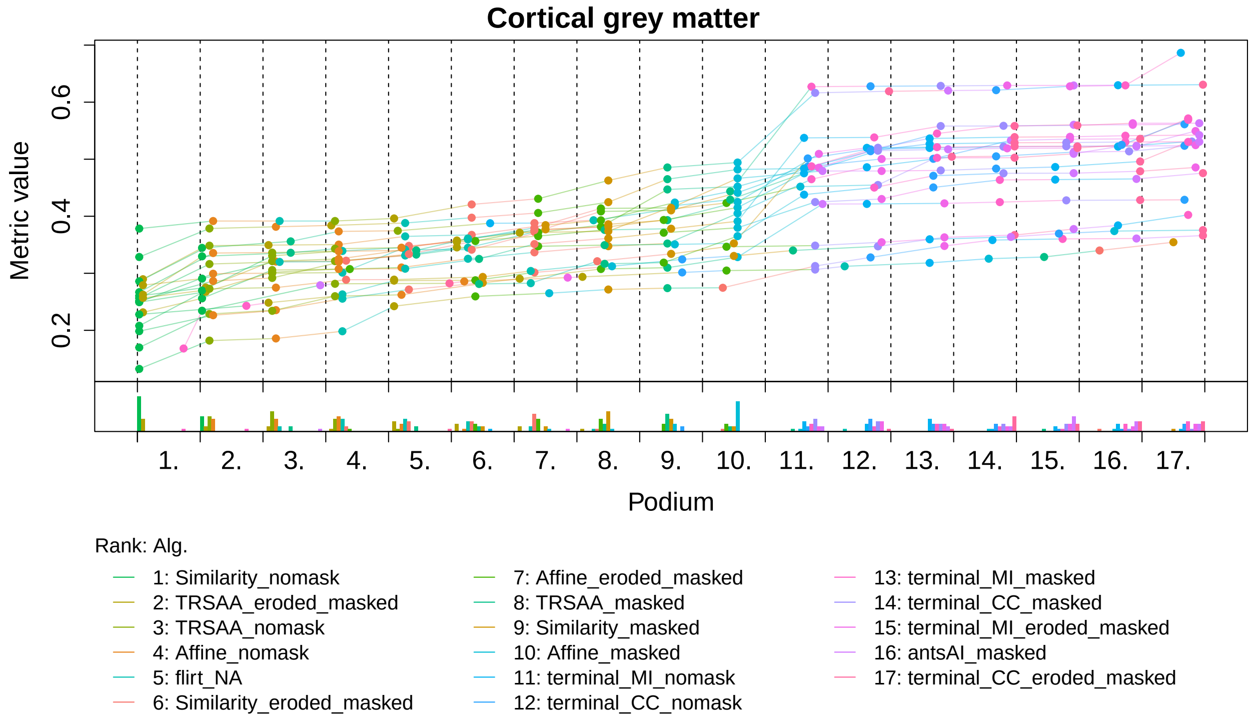
Cortical grey matter
Metric value
0.2
0.4
0.6
1.
2.
3.
4.
5.
6.
7.
8.
9.
10.
11.
12.
13.
14.
15.
16.
17.
Podium
Rank: Alg.
1: Similarity_nomask
2: TRSAA_eroded_masked
3: TRSAA_nomask
4: Affine_nomask
5: flirt_NA
6: Similarity_eroded_masked
7: Affine_eroded_masked
8: TRSAA_masked
9: Similarity_masked
10: Affine_masked
11: terminal_MI_nomask
12: terminal_CC_nomask
13: terminal_MI_masked
14: terminal_CC_masked
15: terminal_MI_eroded_masked
16: antsAI_masked
17: terminal_CC_eroded_masked

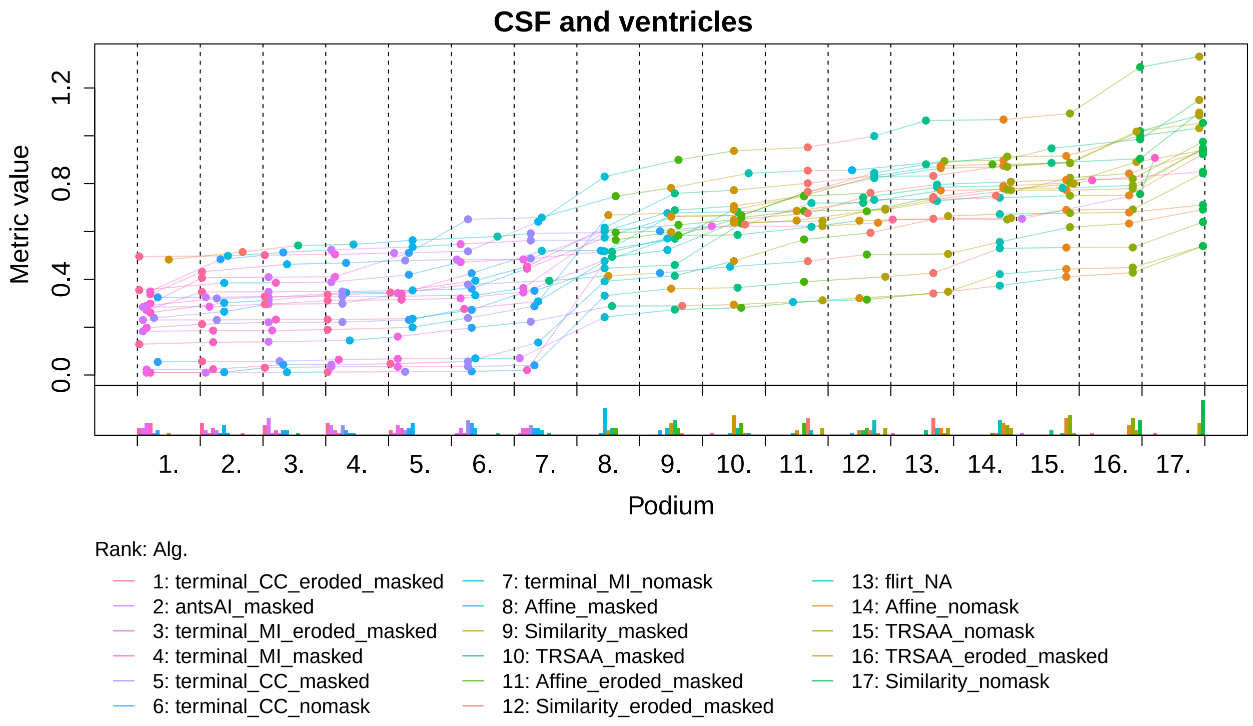
CSF and ventricles
Metric value
0.0
0.4
0.8
1.2
1. 2. 3. 4. 5. 6. 7. 8. 9. 10. 11. 12. 13. 14. 15. 16. 17.
Podium
Rank: Alg.
1: terminal_CC_eroded_masked
2: antsAI_masked
3: terminal_MI_eroded_masked
4: terminal_MI_masked
5: terminal_CC_masked
6: terminal_CC_nomask
7: terminal_MI_nomask
8: Affine_masked
9: Similarity_masked
10: TRSAA_masked
11: Affine_eroded_masked
12: Similarity_eroded_masked
13: flirt_NA
14: Affine_nomask
15: TRSAA_nomask
16: TRSAA_eroded_masked
17: Similarity_nomask

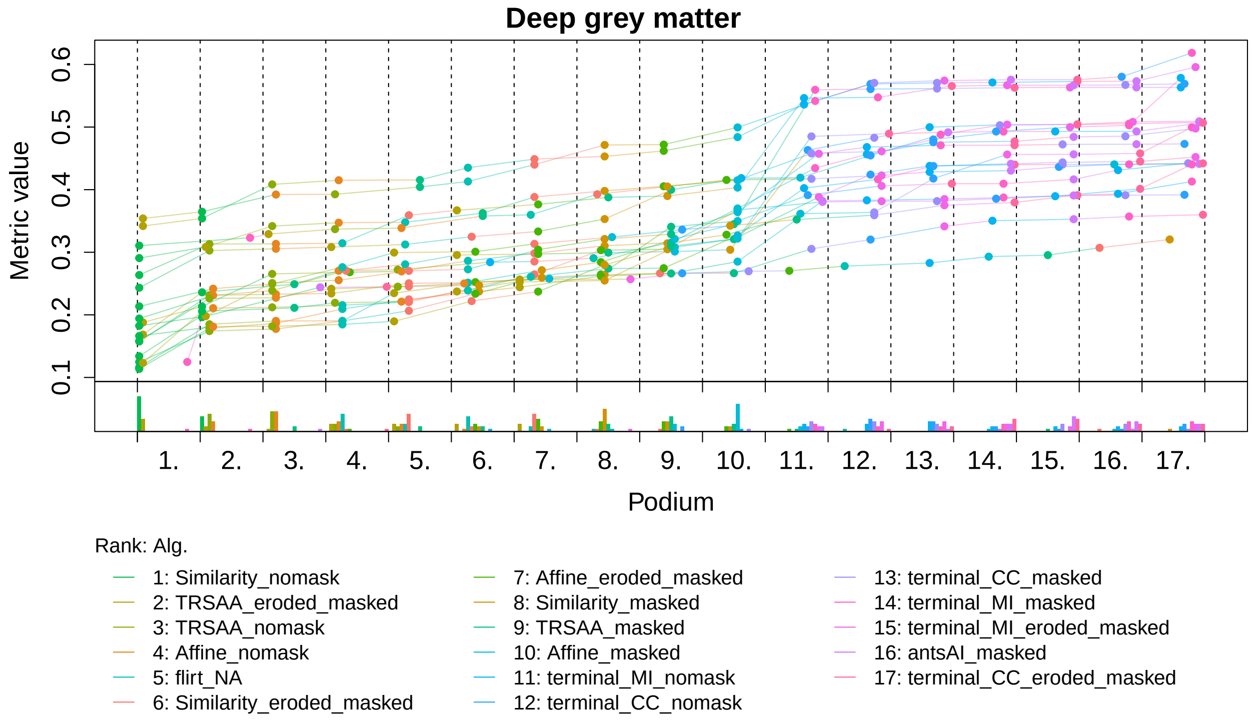
Deep grey matter
Metric value
0.1
0.2
0.3
0.4
0.5
0.6
1. 2. 3. 4. 5. 6. 7. 8. 9. 10. 11. 12. 13. 14. 15. 16. 17.
Podium
Rank: Alg.
1: Similarity_nomask
2: TRSAA_eroded_masked
3: TRSAA_nomask
4: Affine_nomask
5: flirt_NA
6: Similarity_eroded_masked
7: Affine_eroded_masked
8: Similarity_masked
9: TRSAA_masked
10: Affine_masked
11: terminal_MI_nomask
12: terminal_CC_nomask
13: terminal_CC_masked
14: terminal_MI_masked
15: terminal_MI_eroded_masked
16: antsAI_masked
17: terminal_CC_eroded_masked

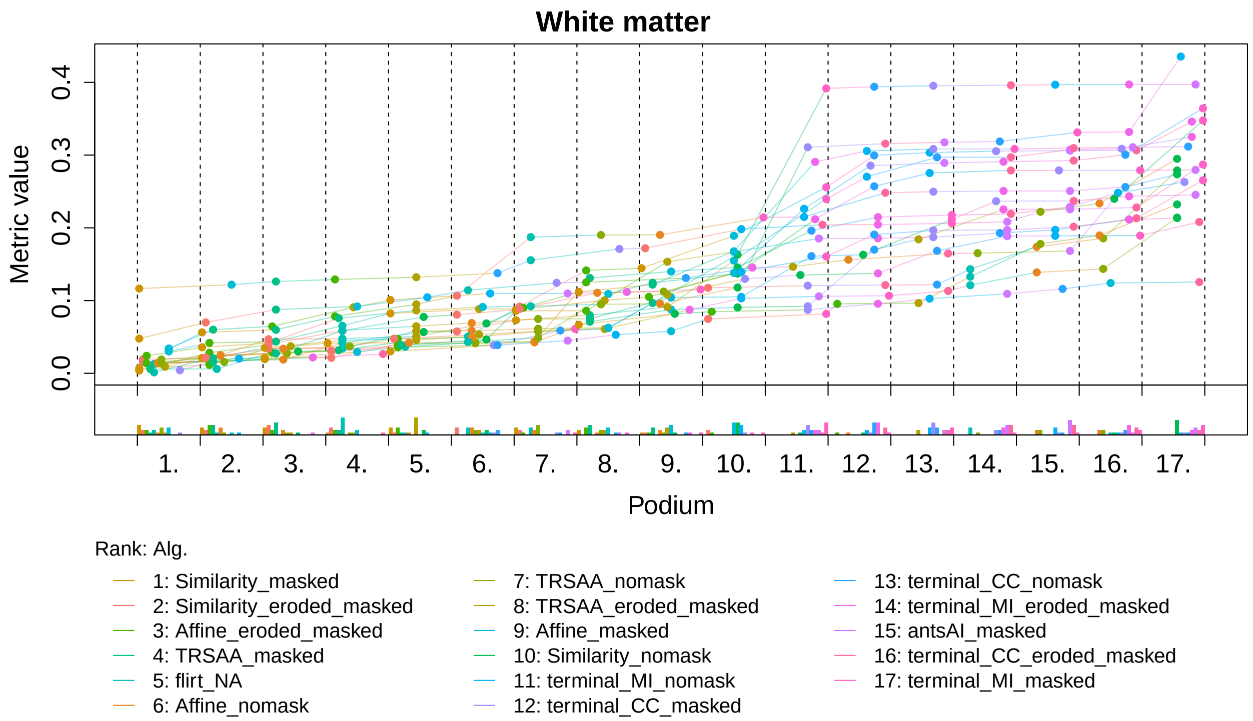
White matter
Metric value
0.0
0.1
0.2
0.3
0.4
1.
2.
3.
4.
5.
6.
7.
8.
9.
10.
11.
12.
13.
14.
15.
16.
17.
Podium
Rank: Alg.
1: Similarity_masked
2: Similarity_eroded_masked
3: Affine_eroded_masked
4: TRSAA_masked
5: flirt_NA
6: Affine_nomask
7: TRSAA_nomask
8: TRSAA_eroded_masked
9: Affine_masked
10: Similarity_nomask
11: terminal_MI_nomask
12: terminal_CC_masked
13: terminal_CC_nomask
14: terminal_MI_eroded_masked
15: antsAI_masked
16: terminal_CC_eroded_masked
17: terminal_MI_masked

2.3 Ranking heatmap

Ranking heatmaps for visualizing raw assessment data. Each cell (i, A_j) shows the absolute frequency of cases in which algorithm A_j achieved rank i.

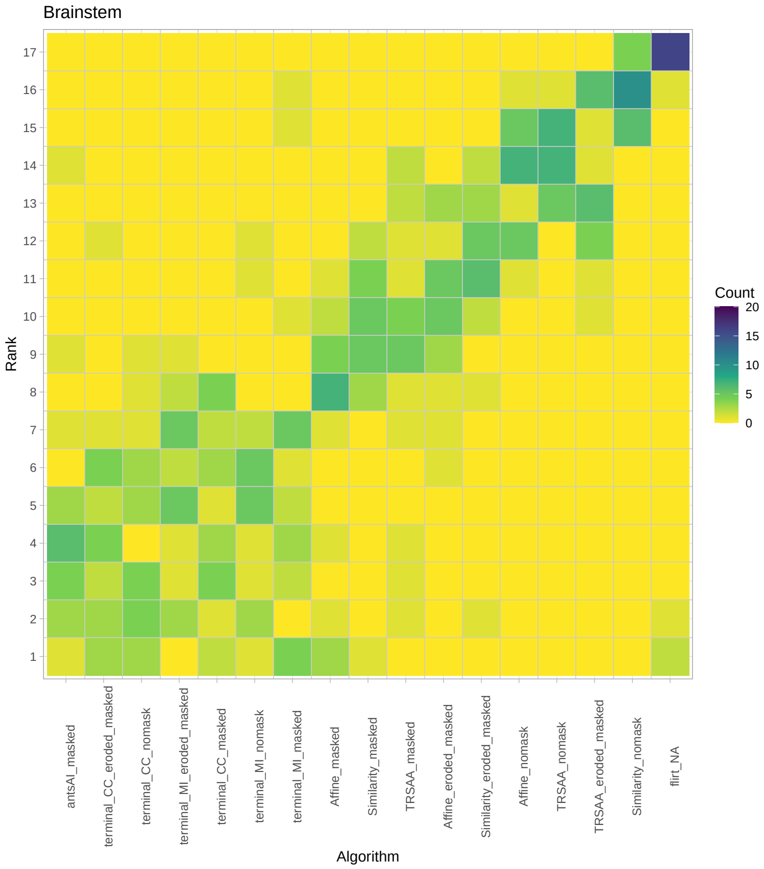

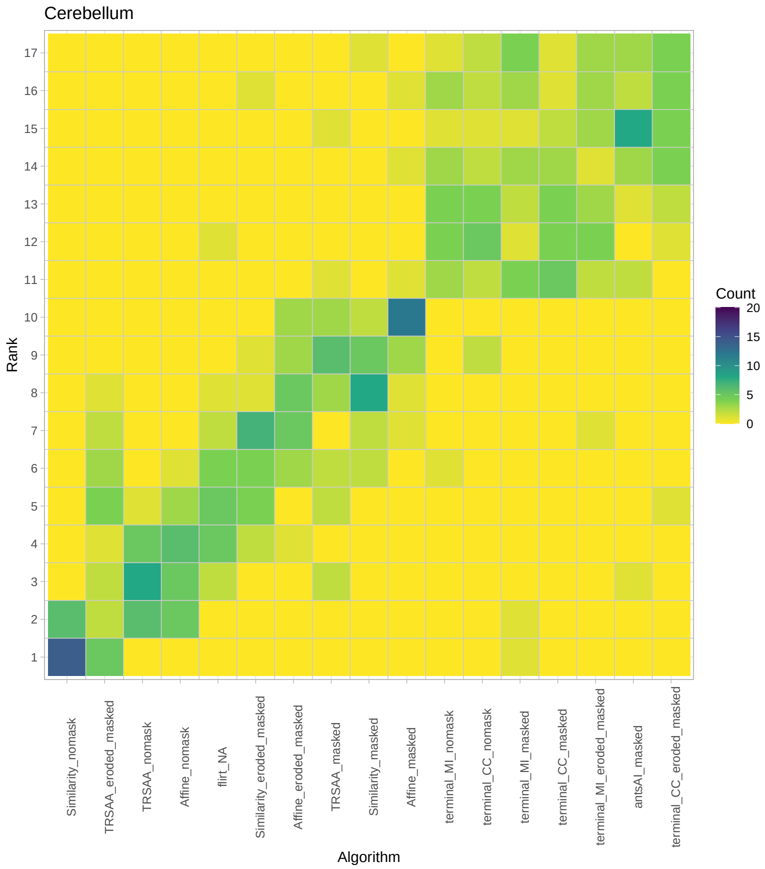

Cerebellum
Rank
17
16
15
14
13
12
11
10
9
8
7
6
5
4
3
2
1
Similarity_nomask
TRSAA_eroded_masked
TRSAA_nomask
Affine_nomask
flirt_NA
Similarity_eroded_masked
Affine_eroded_masked
TRSAA_masked
Similarity_masked
Affine_masked
terminal_MI_nomask
terminal_CC_nomask
terminal_MI_masked
terminal_CC_masked
terminal_MI_eroded_masked
antsAI_masked
terminal_CC_eroded_masked
Algorithm
Count
20
15
10
5
0

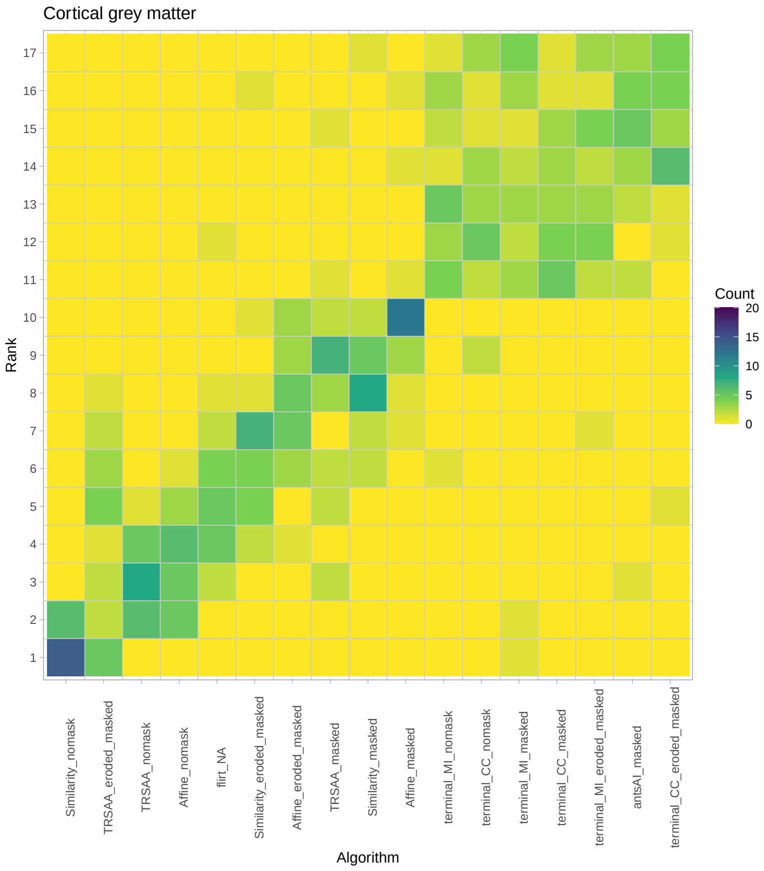

Cortical grey matter
Rank
17
16
15
14
13
12
11
10
9
8
7
6
5
4
3
2
1
Similarity_nomask
TRSAA_eroded_masked
TRSAA_nomask
Affine_nomask
flirt_NA
Similarity_eroded_masked
Affine_eroded_masked
TRSAA_masked
Similarity_masked
Affine_masked
terminal_MI_nomask
terminal_CC_nomask
terminal_MI_masked
terminal_CC_masked
terminal_MI_eroded_masked
antsAI_masked
terminal_CC_eroded_masked
Algorithm
Count
20
15
10
5
0

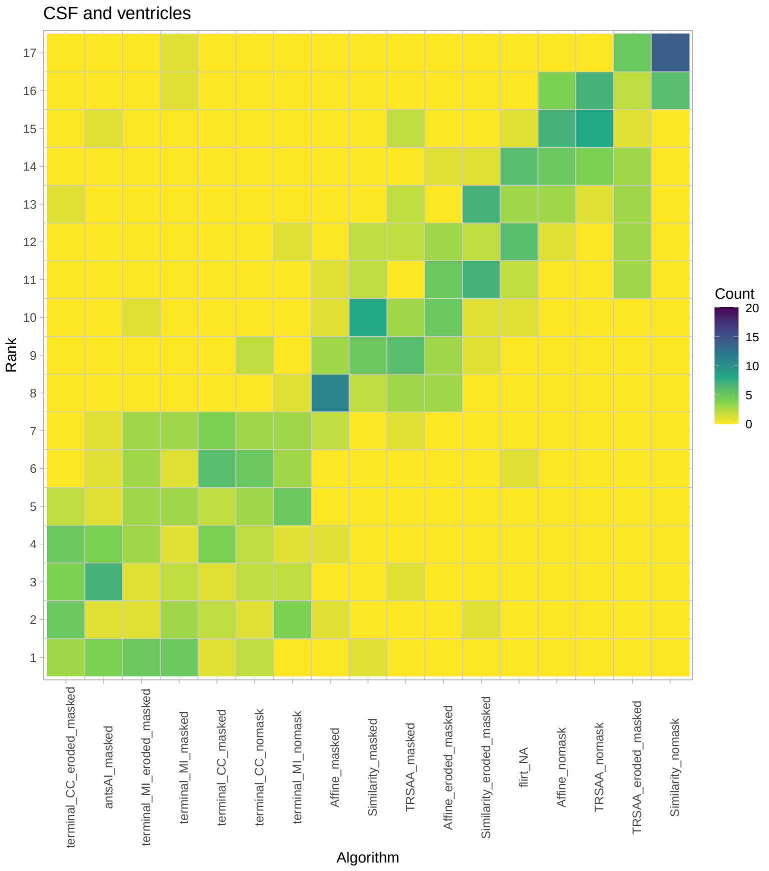
CSF and ventricles
Rank
17
16
15
14
13
12
11
10
9
8
7
6
5
4
3
2
1
Count
20
15
10
5
0
terminal_CC_eroded_masked
antsAI_masked
terminal_MI_eroded_masked
terminal_MI_masked
terminal_CC_masked
terminal_CC_nomask
terminal_MI_nomask
Affine_masked
Similarity_masked
TRSAA_masked
Affine_eroded_masked
Similarity_eroded_masked
flirt_NA
Affine_nomask
TRSAA_nomask
TRSAA_eroded_masked
Similarity_nomask
Algorithm

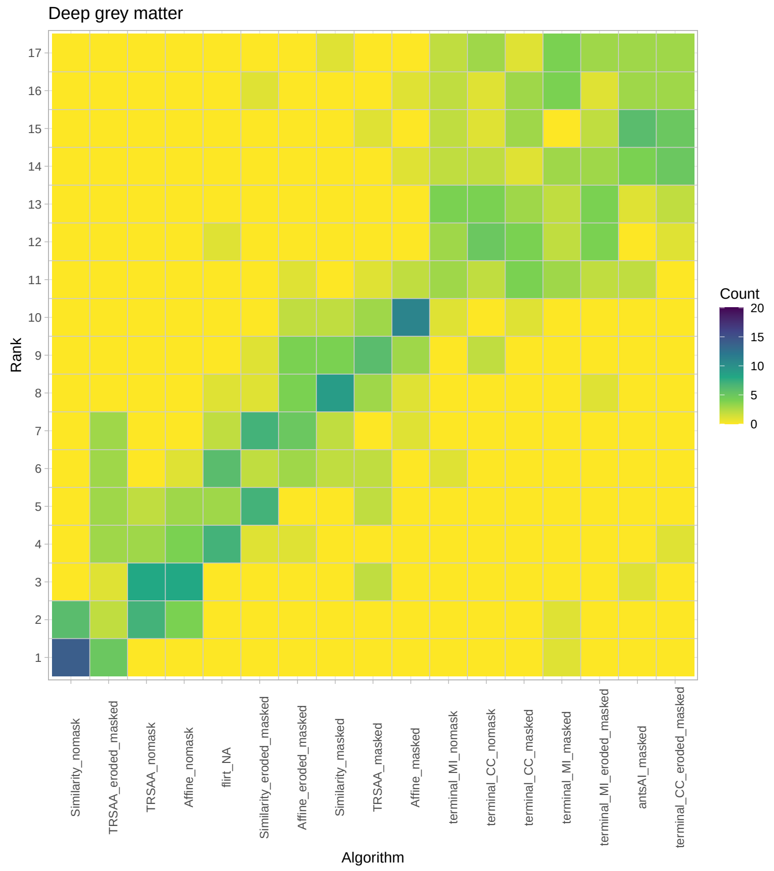

Deep grey matter
Rank
1
2
3
4
5
6
7
8
9
10
11
12
13
14
15
16
17
Algorithm
Similarity_nomask
TRSAA_eroded_masked
TRSAA_nomask
Affine_nomask
flirt_NA
Similarity_eroded_masked
Affine_eroded_masked
Similarity_masked
TRSAA_masked
Affine_masked
terminal_MI_nomask
terminal_CC_nomask
terminal_CC_masked
terminal_MI_masked
terminal_MI_eroded_masked
antsAI_masked
terminal_CC_eroded_masked
Count
20
15
10
5
0

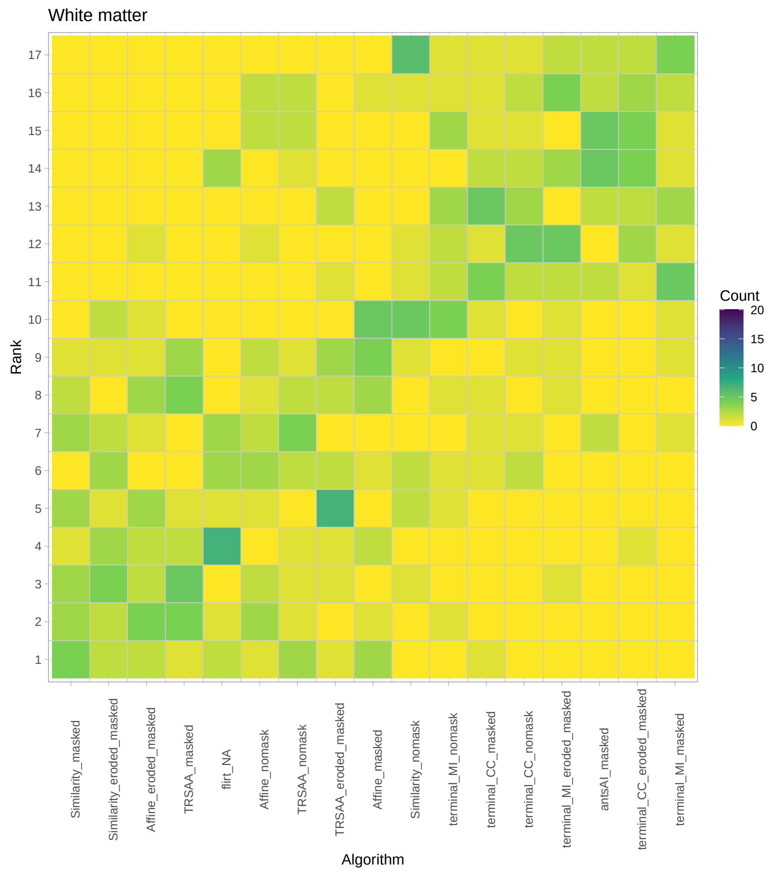
White matter
Rank
17
16
15
14
13
12
11
10
9
8
7
6
5
4
3
2
1
Similarity_masked
Similarity_eroded_masked
Affine_eroded_masked
TRSAA_masked
flirt_NA
Affine_nomask
TRSAA_nomask
TRSAA_eroded_masked
Affine_masked
Similarity_nomask
terminal_MI_nomask
terminal_CC_masked
terminal_CC_nomask
terminal_MI_eroded_masked
antsAI_masked
terminal_CC_eroded_masked
terminal_MI_masked
Algorithm
Count
20
15
10
5
0

3 Visualization of ranking stability

3.1 *Blob plot* for visualizing ranking stability based on bootstrap sampling

Algorithms are color-coded, and the area of each blob at position $(A_i, \text{rank } j)$ is proportional to the relative frequency A_i achieved rank j across $b = 1000$ bootstrap samples. The median rank for each algorithm is indicated by a black cross. 95% bootstrap intervals across bootstrap samples are indicated by black lines.

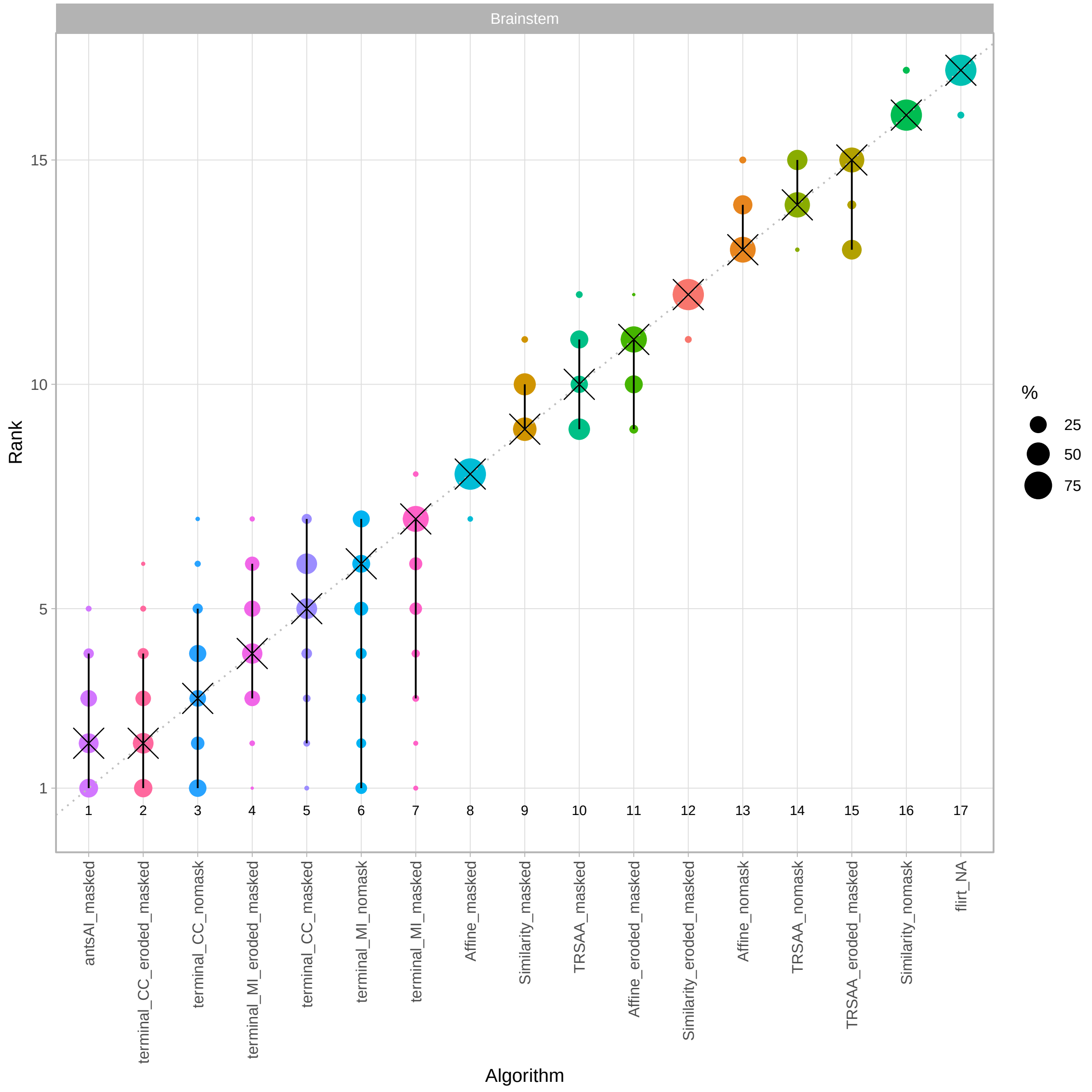

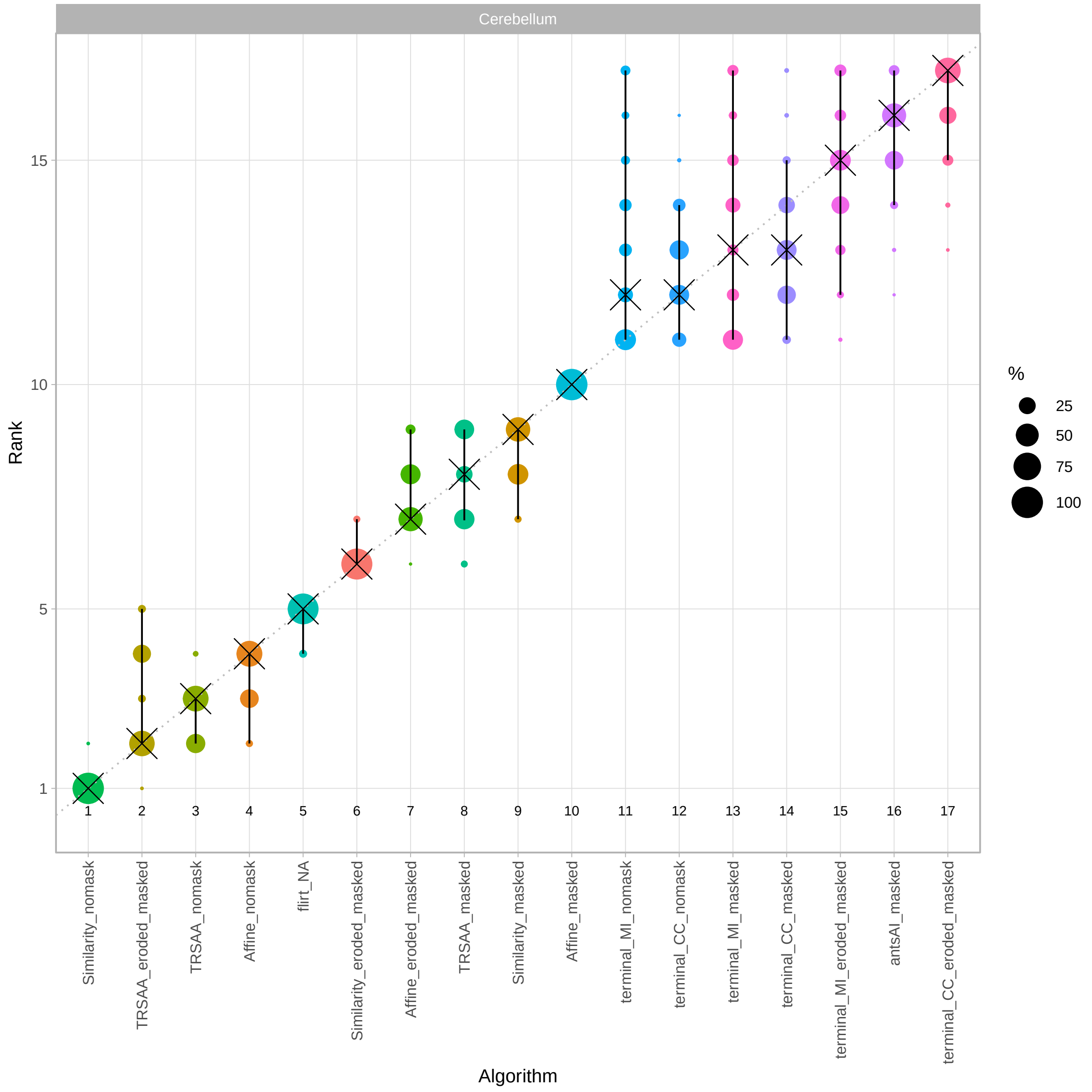

Cerebellum
Rank
1
5
10
15
1 2 3 4 5 6 7 8 9 10 11 12 13 14 15 16 17
Similarity_nomask
TRSAA_eroded_masked
TRSAA_nomask
Affine_nomask
flirt_NA
Similarity_eroded_masked
Affine_eroded_masked
TRSAA_masked
Similarity_masked
Affine_masked
terminal_MI_nomask
terminal_CC_nomask
terminal_MI_masked
terminal_CC_masked
terminal_MI_eroded_masked
antsAI_masked
terminal_CC_eroded_masked
Algorithm
%
25
50
75
100

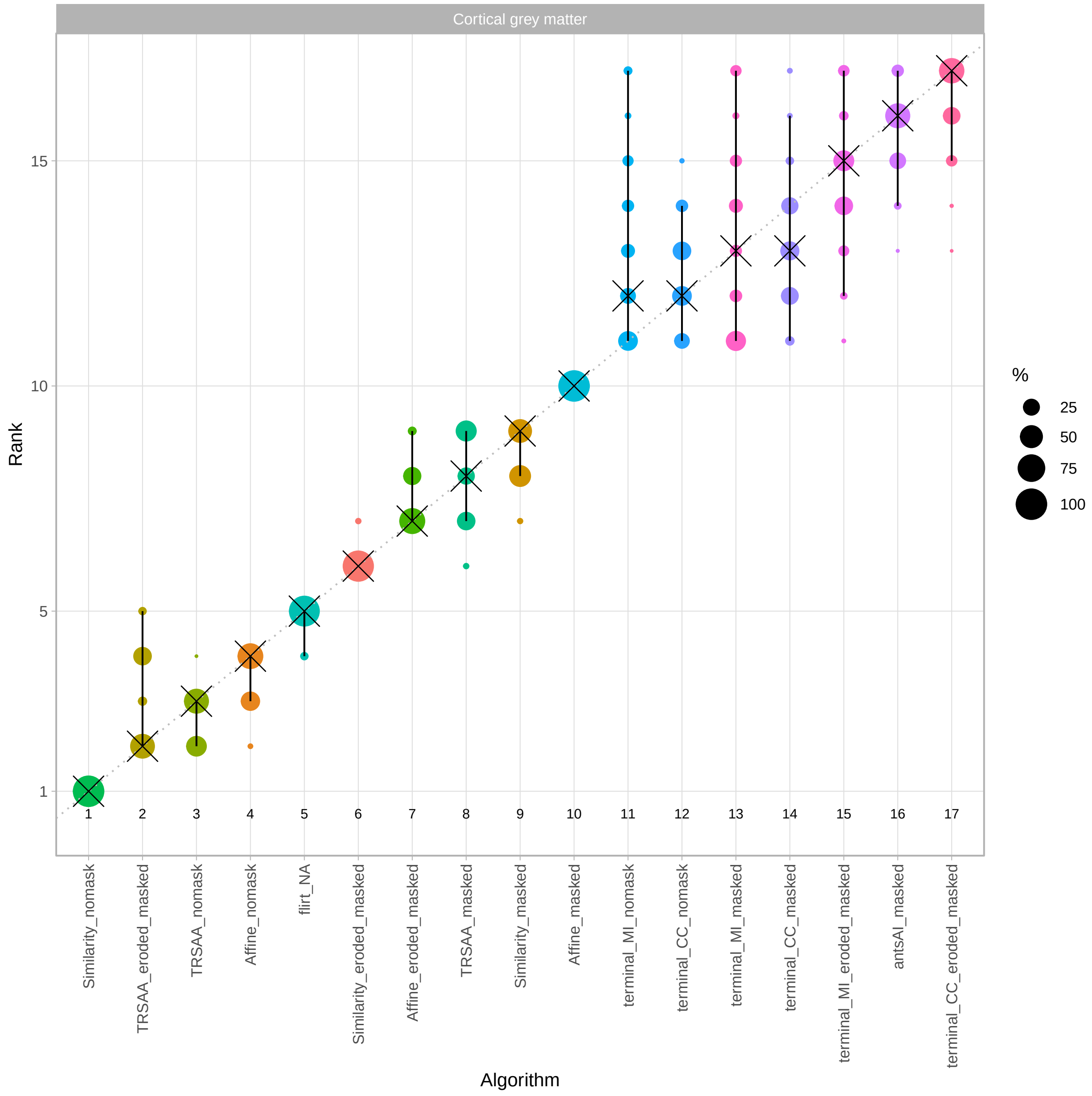
Cortical grey matter
Rank
15
10
5
1
1 2 3 4 5 6 7 8 9 10 11 12 13 14 15 16 17
Similarity_nomask
TRSAA_eroded_masked
TRSAA_nomask
Affine_nomask
flirt_NA
Similarity_eroded_masked
Affine_eroded_masked
TRSAA_masked
Similarity_masked
Affine_masked
terminal_MI_nomask
terminal_CC_nomask
terminal_MI_masked
terminal_CC_masked
terminal_MI_eroded_masked
antsAI_masked
terminal_CC_eroded_masked
Algorithm
%
25
50
75
100

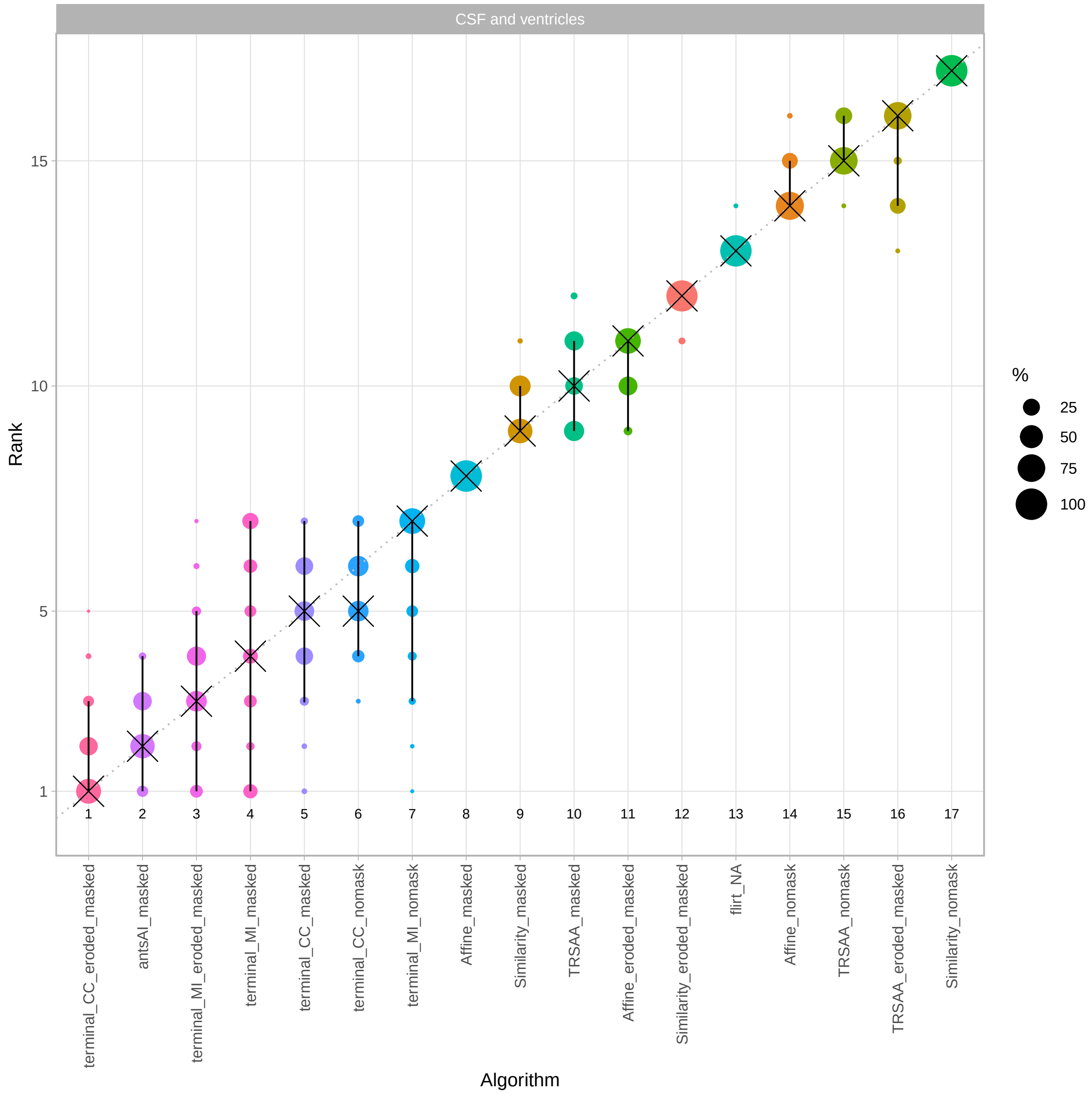
CSF and ventricles
Rank
15
10
5
1
1 2 3 4 5 6 7 8 9 10 11 12 13 14 15 16 17
terminal_CC_eroded_masked
antsAI_masked
terminal_MI_eroded_masked
terminal_MI_masked
terminal_CC_masked
terminal_CC_nomask
terminal_MI_nomask
Affine_masked
Similarity_masked
TRSAA_masked
Affine_eroded_masked
Similarity_eroded_masked
flirt_NA
Affine_nomask
TRSAA_nomask
TRSAA_eroded_masked
Similarity_nomask
Algorithm
%
25
50
75
100

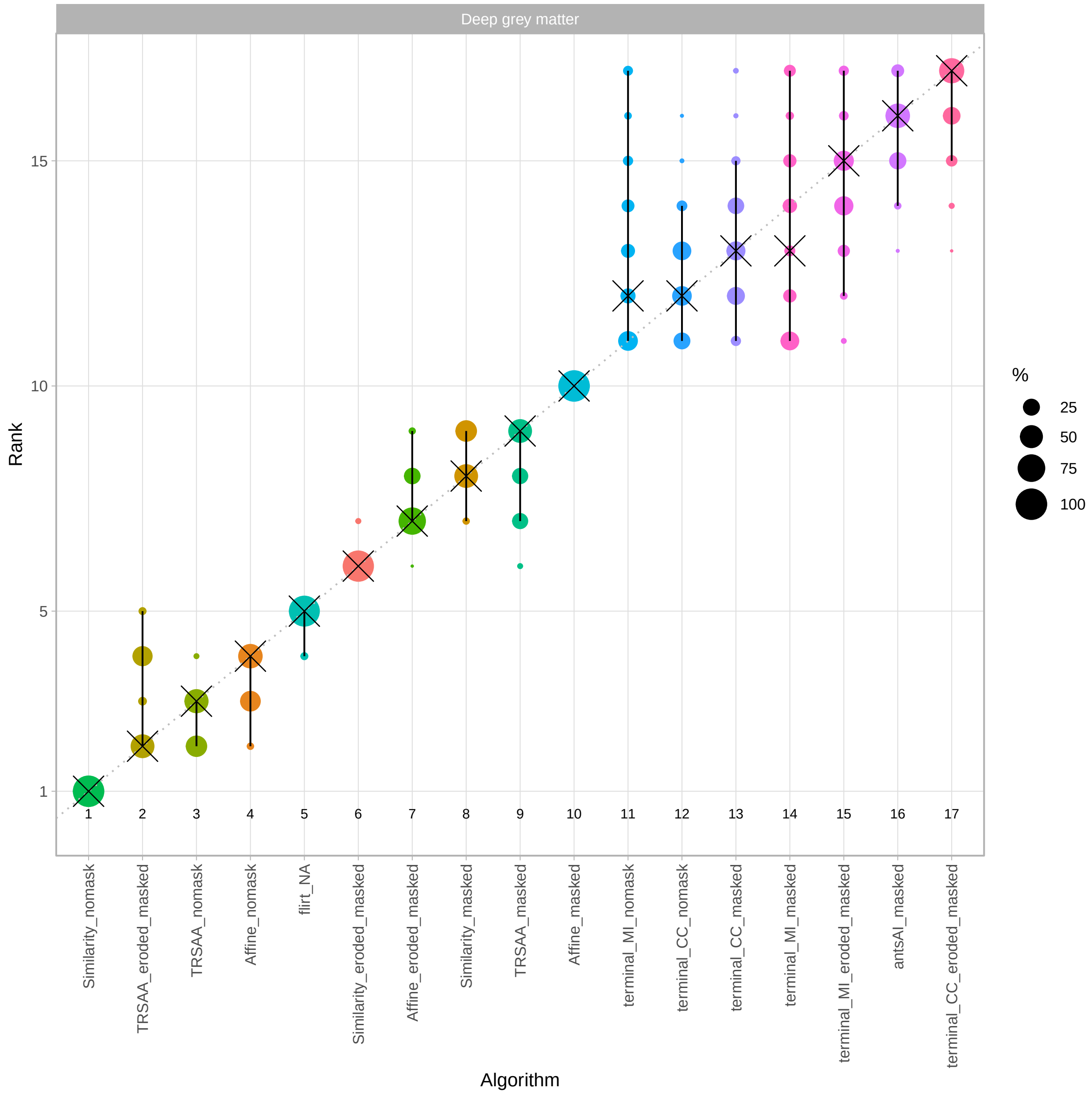
Deep grey matter
Rank
Algorithm
%
25
50
75
100
Similarity_nomask
TRSAA_eroded_masked
TRSAA_nomask
Affine_nomask
flirt_NA
Similarity_eroded_masked
Affine_eroded_masked
Similarity_masked
TRSAA_masked
Affine_masked
terminal_MI_nomask
terminal_CC_nomask
terminal_CC_masked
terminal_MI_masked
terminal_MI_eroded_masked
antsAI_masked
terminal_CC_eroded_masked

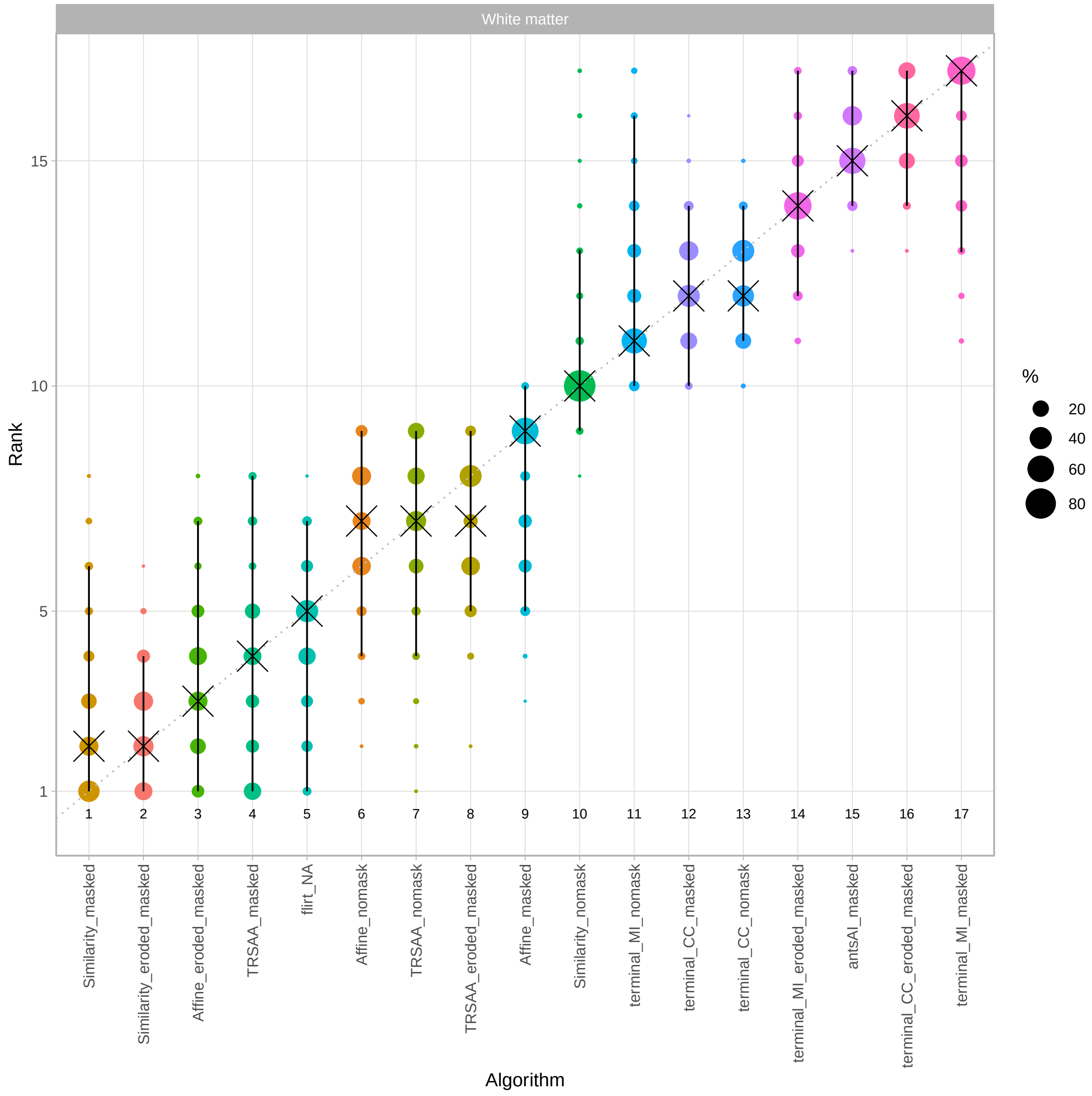
White matter
Rank
15
10
5
1
1
2
3
4
5
6
7
8
9
10
11
12
13
14
15
16
17
Similarity_masked
Similarity_eroded_masked
Affine_eroded_masked
TRSAA_masked
flirt_NA
Affine_nomask
TRSAA_nomask
TRSAA_eroded_masked
Affine_masked
Similarity_nomask
terminal_MI_nomask
terminal_CC_masked
terminal_CC_nomask
terminal_MI_eroded_masked
antsAI_masked
terminal_CC_eroded_masked
terminal_MI_masked
Algorithm
%
20
40
60
80

3.2 *Violin plot* for visualizing ranking stability based on bootstrapping

The ranking list based on the full assessment data is pairwise compared with the ranking lists based on the individual bootstrap samples (here $b = 1000$ samples). For each pair of rankings, Kendall's τ correlation is computed. Kendall's τ is a scaled index determining the correlation between the lists. It is computed by evaluating the number of pairwise concordances and discordances between ranking lists and produces values between -1 (for inverted order) and 1 (for identical order). A violin plot, which simultaneously depicts a boxplot and a density plot, is generated from the results.

Summary Kendall's tau:

Task	mean	median	q25	q75
CSF and ventricles	0.9323529	0.9411765	0.9117647	0.9558824
Brainstem	0.9177596	0.9264706	0.8970588	0.9558824
Cerebellum	0.9164664	0.9264706	0.8970588	0.9411765
Cortical grey matter	0.9157018	0.9117647	0.8970588	0.9411765
Deep grey matter	0.9124328	0.9117647	0.8823529	0.9411765
White matter	0.8684412	0.8823529	0.8235294	0.9117647

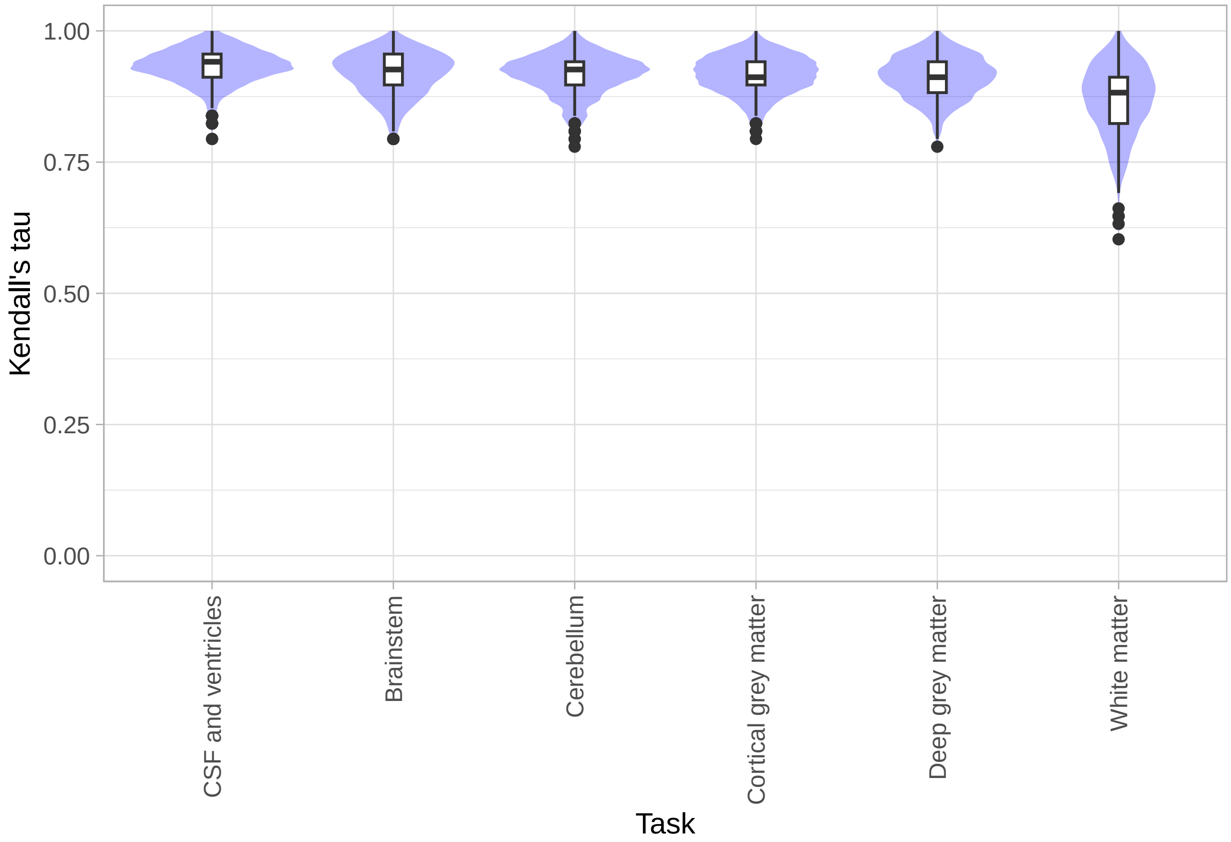

3.3 *Significance maps* for visualizing ranking stability based on statistical significance

Significance maps depict incidence matrices of pairwise significant test results for the one-sided Wilcoxon signed rank test at a 5% significance level with adjustment for multiple testing according to Holm. Yellow shading indicates that metric values from the algorithm on the x-axis were significantly superior to those from the algorithm on the y-axis, blue color indicates no significant difference.

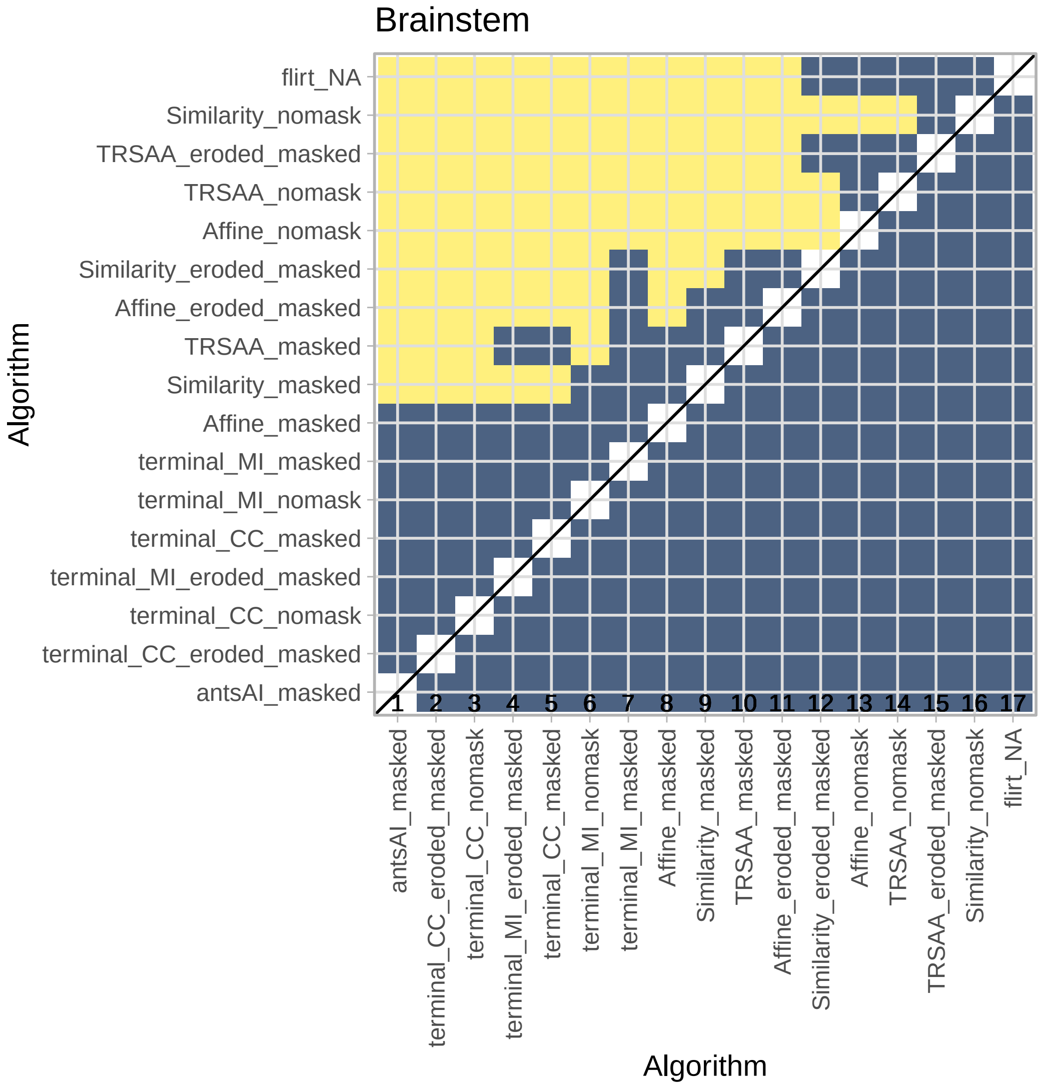

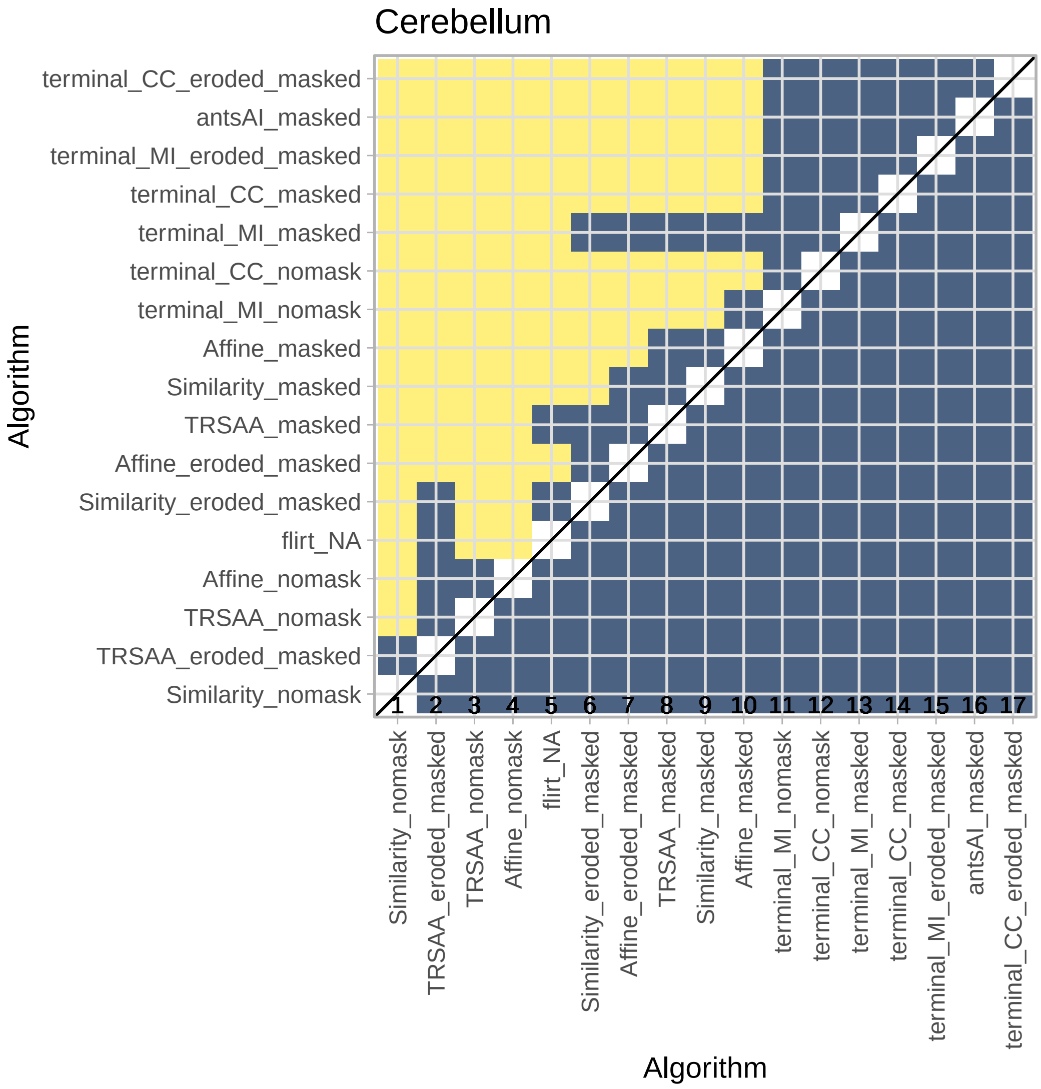
Cerebellum
terminal_CC_eroded_masked
antsAI_masked
terminal_MI_eroded_masked
terminal_CC_masked
terminal_MI_masked
terminal_CC_nomask
terminal_MI_nomask
Affine_masked
Similarity_masked
TRSAA_masked
Affine_eroded_masked
Similarity_eroded_masked
flirt_NA
Affine_nomask
TRSAA_nomask
TRSAA_eroded_masked
Similarity_nomask
Algorithm
1 2 3 4 5 6 7 8 9 10 11 12 13 14 15 16 17
Similarity_nomask
TRSAA_eroded_masked
TRSAA_nomask
Affine_nomask
flirt_NA
Similarity_eroded_masked
Affine_eroded_masked
TRSAA_masked
Similarity_masked
Affine_masked
terminal_MI_nomask
terminal_CC_nomask
terminal_MI_masked
terminal_CC_masked
terminal_MI_eroded_masked
antsAI_masked
terminal_CC_eroded_masked
Algorithm

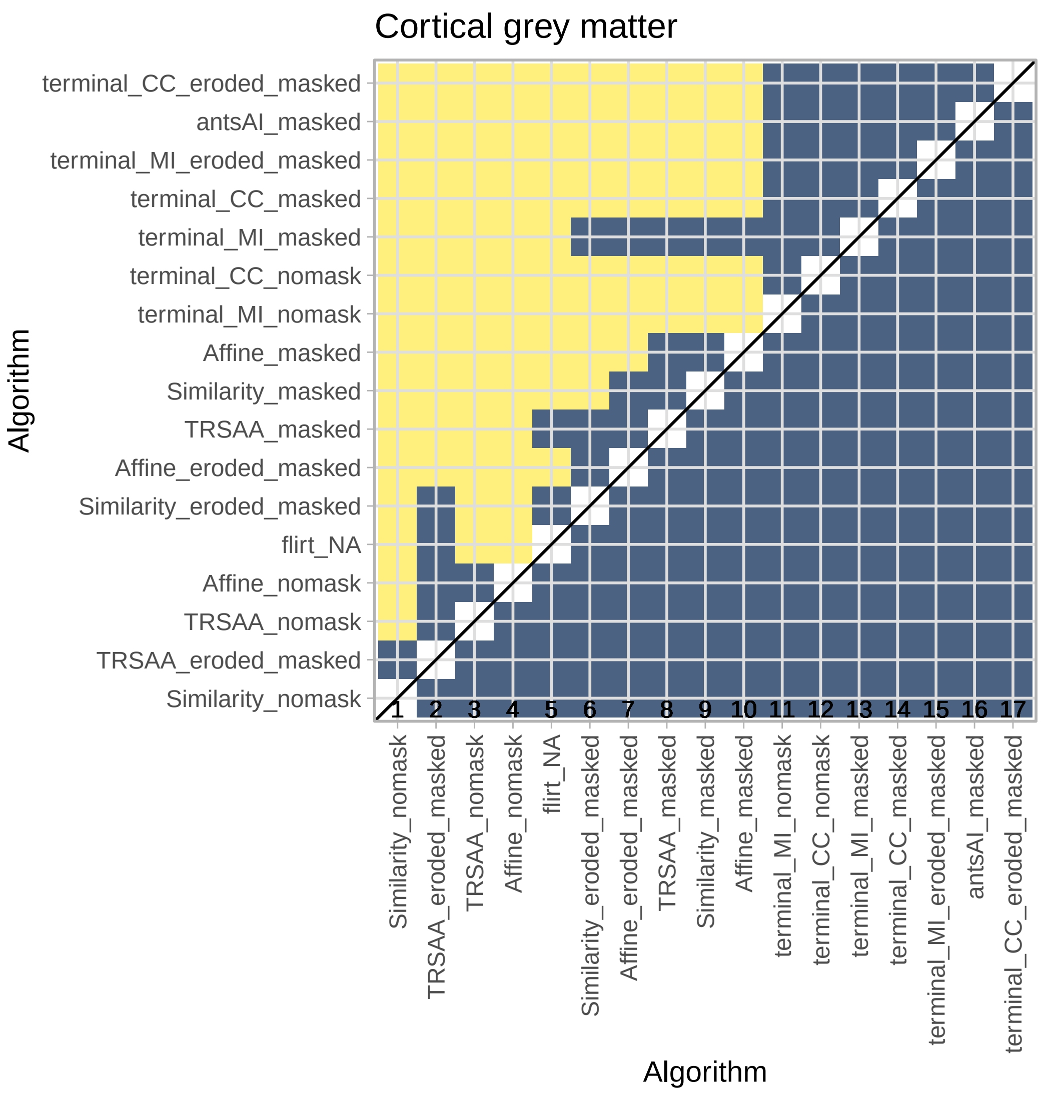
Cortical grey matter
Algorithm
terminal_CC_eroded_masked
antsAI_masked
terminal_MI_eroded_masked
terminal_CC_masked
terminal_MI_masked
terminal_CC_nomask
terminal_MI_nomask
Affine_masked
Similarity_masked
TRSAA_masked
Affine_eroded_masked
Similarity_eroded_masked
flirt_NA
Affine_nomask
TRSAA_nomask
TRSAA_eroded_masked
Similarity_nomask
1 2 3 4 5 6 7 8 9 10 11 12 13 14 15 16 17
Similarity_nomask
TRSAA_eroded_masked
TRSAA_nomask
Affine_nomask
flirt_NA
Similarity_eroded_masked
Affine_eroded_masked
TRSAA_masked
Similarity_masked
Affine_masked
terminal_MI_nomask
terminal_CC_nomask
terminal_MI_masked
terminal_CC_masked
terminal_MI_eroded_masked
antsAI_masked
terminal_CC_eroded_masked
Algorithm

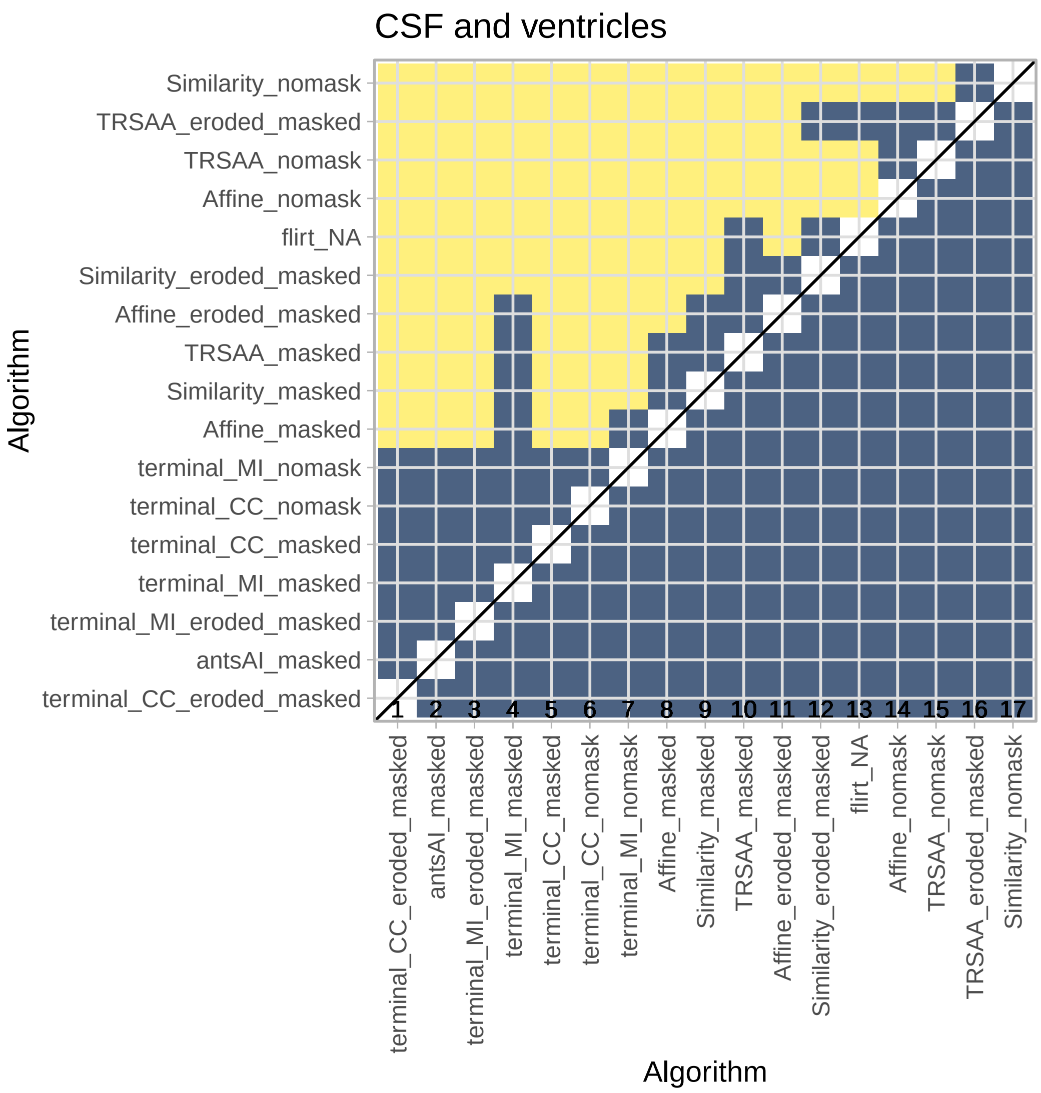

CSF and ventricles
Similarity_nomask
TRSAA_eroded_masked
TRSAA_nomask
Affine_nomask
flirt_NA
Similarity_eroded_masked
Affine_eroded_masked
TRSAA_masked
Similarity_masked
Affine_masked
terminal_MI_nomask
terminal_CC_nomask
terminal_CC_masked
terminal_MI_masked
terminal_MI_eroded_masked
antsAI_masked
terminal_CC_eroded_masked
Algorithm
1 2 3 4 5 6 7 8 9 10 11 12 13 14 15 16 17
terminal_CC_eroded_masked
antsAI_masked
terminal_MI_eroded_masked
terminal_MI_masked
terminal_CC_masked
terminal_CC_nomask
terminal_MI_nomask
Affine_masked
Similarity_masked
TRSAA_masked
Affine_eroded_masked
Similarity_eroded_masked
flirt_NA
Affine_nomask
TRSAA_nomask
TRSAA_eroded_masked
Similarity_nomask
Algorithm

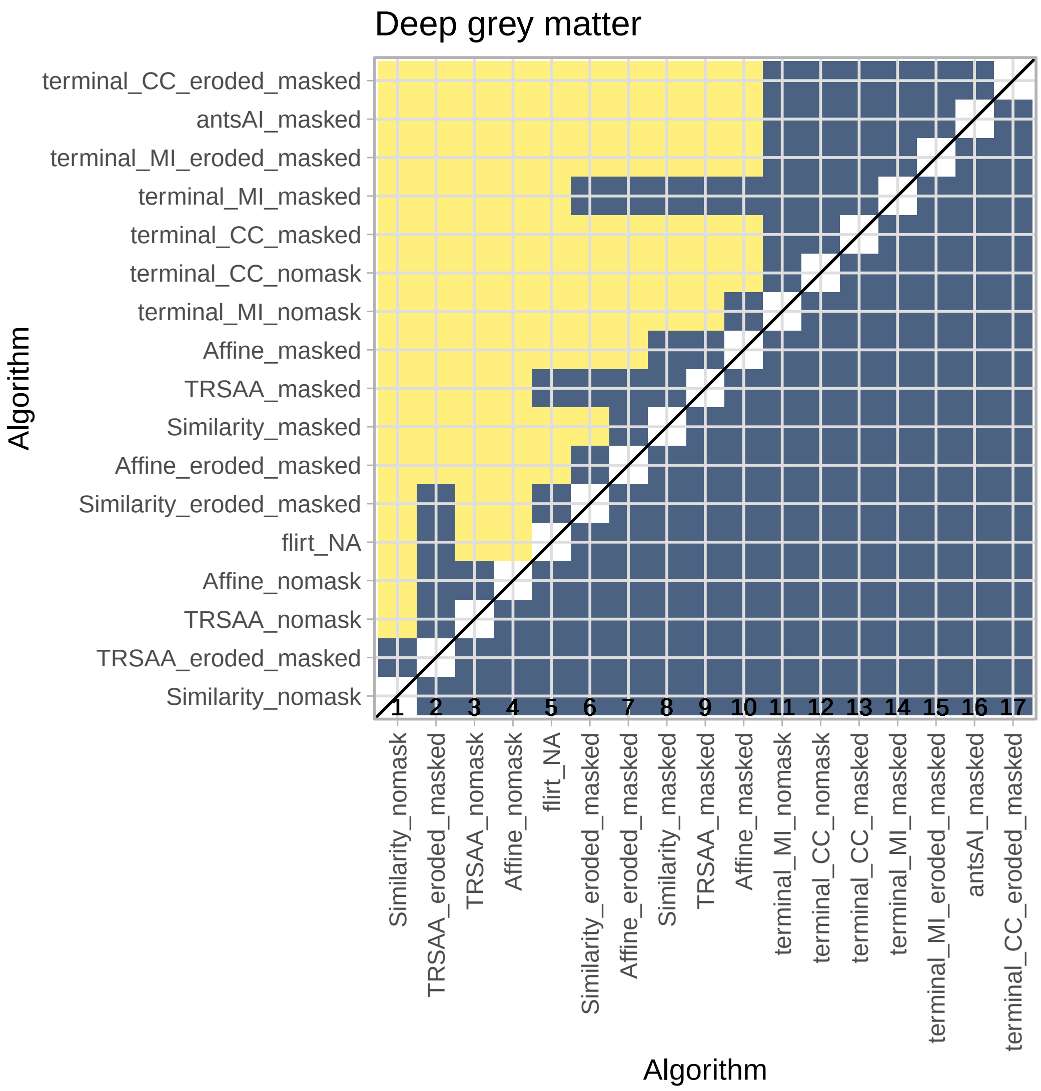

Deep grey matter
Algorithm
terminal_CC_eroded_masked
antsAI_masked
terminal_MI_eroded_masked
terminal_MI_masked
terminal_CC_masked
terminal_CC_nomask
terminal_MI_nomask
Affine_masked
TRSAA_masked
Similarity_masked
Affine_eroded_masked
Similarity_eroded_masked
flirt_NA
Affine_nomask
TRSAA_nomask
TRSAA_eroded_masked
Similarity_nomask
1 2 3 4 5 6 7 8 9 10 11 12 13 14 15 16 17
Similarity_nomask
TRSAA_eroded_masked
TRSAA_nomask
Affine_nomask
flirt_NA
Similarity_eroded_masked
Affine_eroded_masked
Similarity_masked
TRSAA_masked
Affine_masked
terminal_MI_nomask
terminal_CC_nomask
terminal_CC_masked
terminal_MI_masked
terminal_MI_eroded_masked
antsAI_masked
terminal_CC_eroded_masked
Algorithm

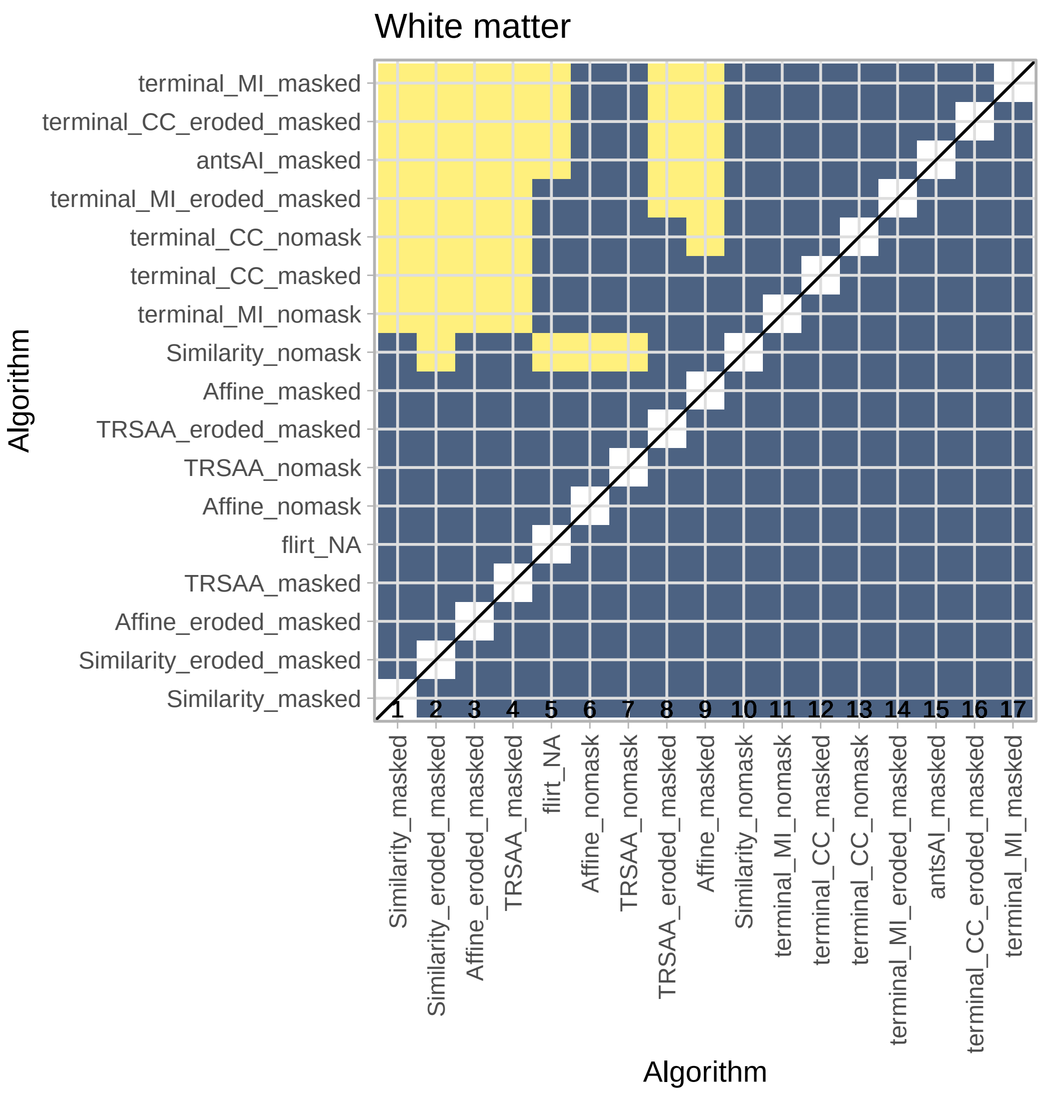
White matter
Algorithm
terminal_MI_masked
terminal_CC_eroded_masked
antsAI_masked
terminal_MI_eroded_masked
terminal_CC_nomask
terminal_CC_masked
terminal_MI_nomask
Similarity_nomask
Affine_masked
TRSAA_eroded_masked
TRSAA_nomask
Affine_nomask
flirt_NA
TRSAA_masked
Affine_eroded_masked
Similarity_eroded_masked
Similarity_masked
1 2 3 4 5 6 7 8 9 10 11 12 13 14 15 16 17
Similarity_masked
Similarity_eroded_masked
Affine_eroded_masked
TRSAA_masked
flirt_NA
Affine_nomask
TRSAA_nomask
TRSAA_eroded_masked
Affine_masked
Similarity_nomask
terminal_MI_nomask
terminal_CC_masked
terminal_CC_nomask
terminal_MI_eroded_masked
antsAI_masked
terminal_CC_eroded_masked
terminal_MI_masked
Algorithm

3.4 Ranking robustness to ranking methods

Line plots for visualizing ranking robustness across different ranking methods. Each algorithm is represented by one colored line. For each ranking method encoded on the x-axis, the height of the line represents the corresponding rank. Horizontal lines indicate identical ranks for all methods.

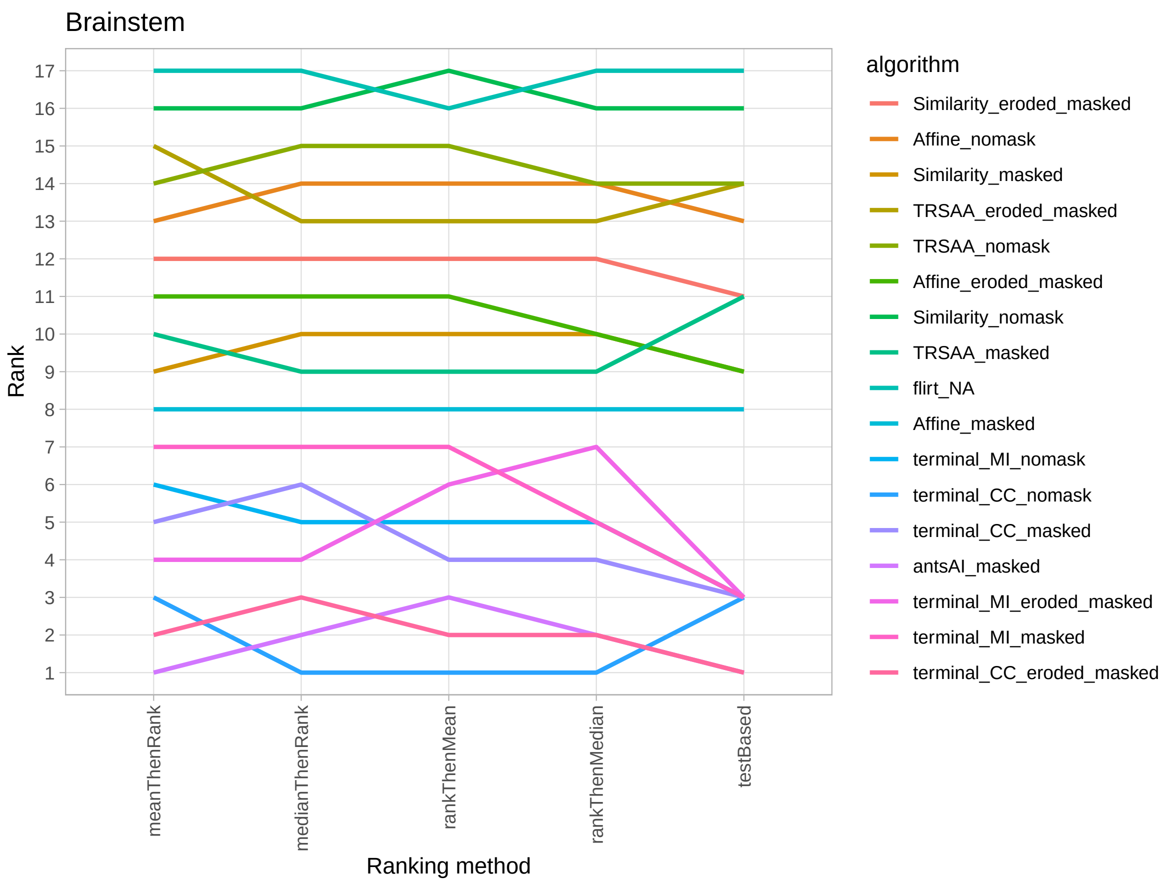

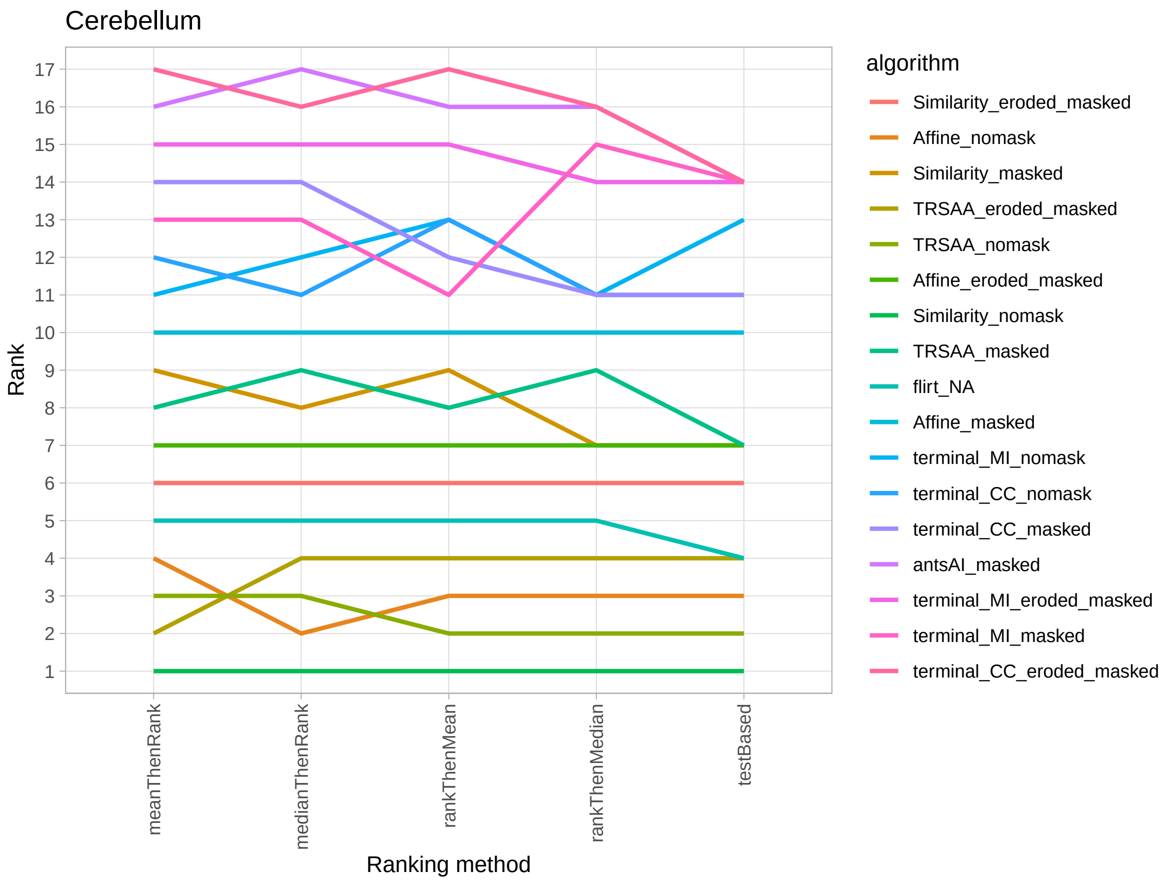
Cerebellum
Rank
17
16
15
14
13
12
11
10
9
8
7
6
5
4
3
2
1
meanThenRank
medianThenRank
rankThenMean
rankThenMedian
testBased
Ranking method
algorithm
Similarity_eroded_masked
Affine_nomask
Similarity_masked
TRSAA_eroded_masked
TRSAA_nomask
Affine_eroded_masked
Similarity_nomask
TRSAA_masked
flirt_NA
Affine_masked
terminal_MI_nomask
terminal_CC_nomask
terminal_CC_masked
antsAI_masked
terminal_MI_eroded_masked
terminal_MI_masked
terminal_CC_eroded_masked

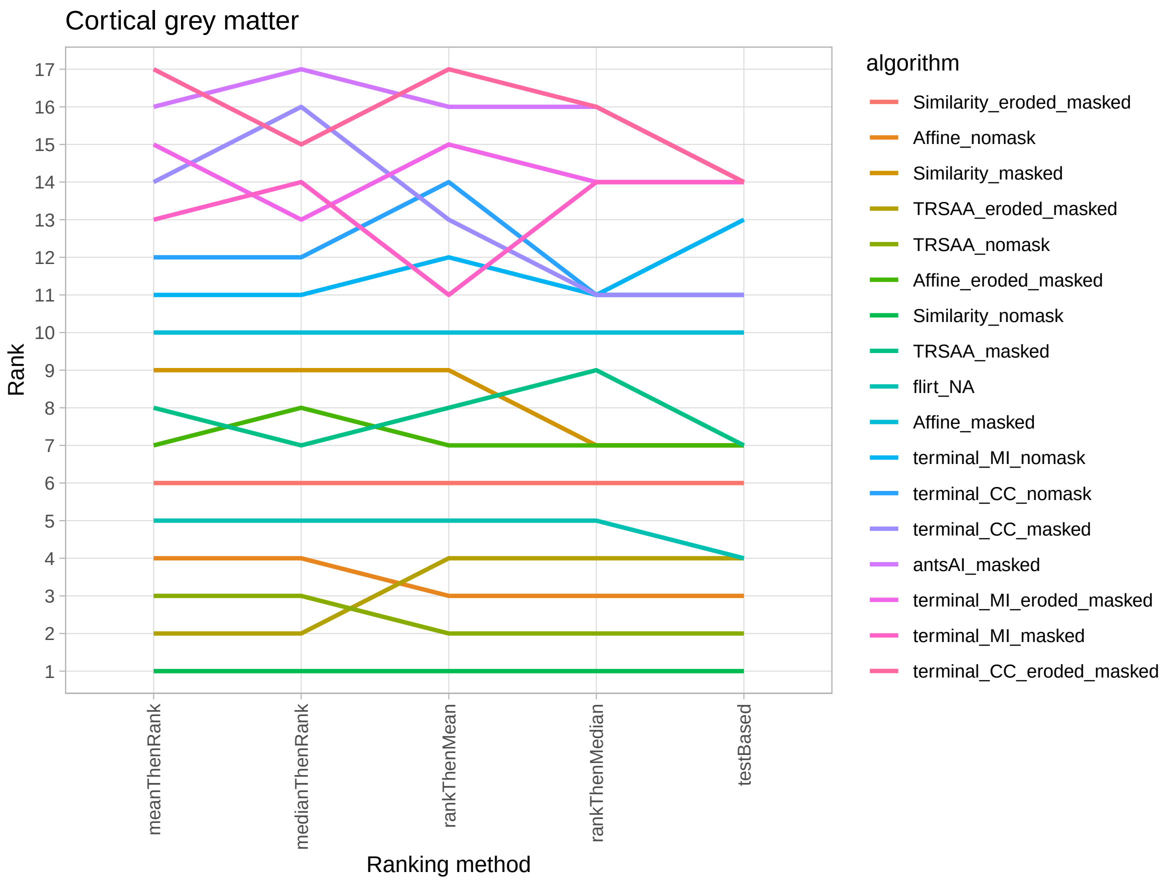

Cortical grey matter
Rank
Ranking method
meanThenRank
medianThenRank
rankThenMean
rankThenMedian
testBased
algorithm
Similarity_eroded_masked
Affine_nomask
Similarity_masked
TRSAA_eroded_masked
TRSAA_nomask
Affine_eroded_masked
Similarity_nomask
TRSAA_masked
flirt_NA
Affine_masked
terminal_MI_nomask
terminal_CC_nomask
terminal_CC_masked
antsAI_masked
terminal_MI_eroded_masked
terminal_MI_masked
terminal_CC_eroded_masked

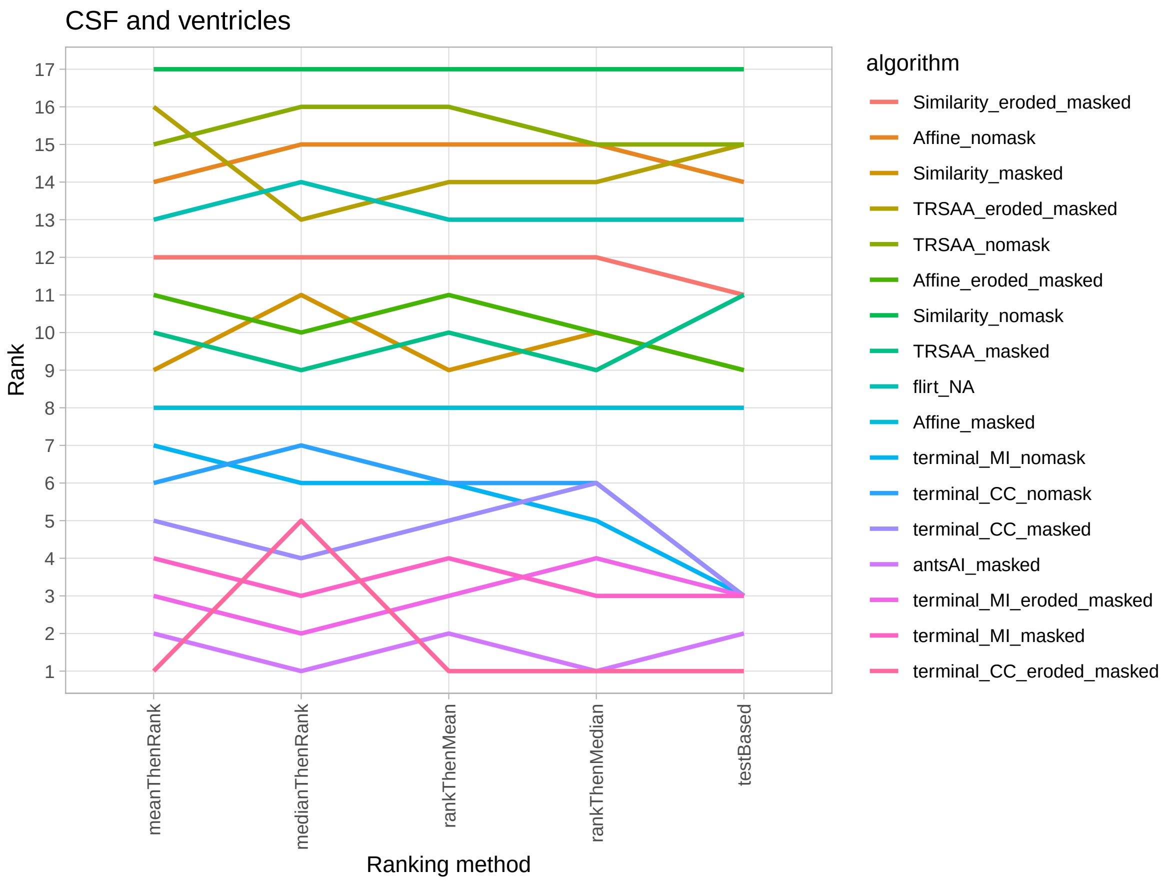
CSF and ventricles
Rank
17
16
15
14
13
12
11
10
9
8
7
6
5
4
3
2
1
meanThenRank
medianThenRank
rankThenMean
rankThenMedian
testBased
Ranking method
algorithm
Similarity_eroded_masked
Affine_nomask
Similarity_masked
TRSAA_eroded_masked
TRSAA_nomask
Affine_eroded_masked
Similarity_nomask
TRSAA_masked
flirt_NA
Affine_masked
terminal_MI_nomask
terminal_CC_nomask
terminal_CC_masked
antsAI_masked
terminal_MI_eroded_masked
terminal_MI_masked
terminal_CC_eroded_masked

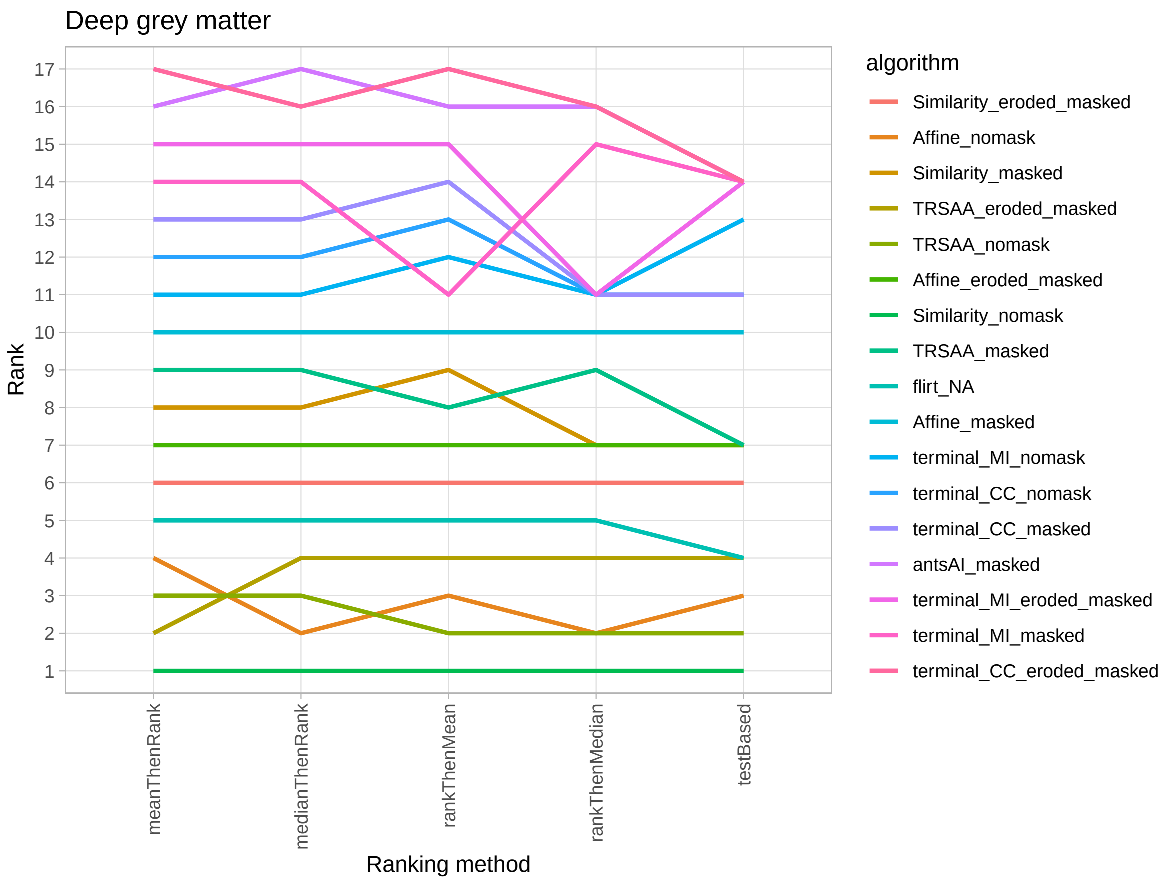
Deep grey matter
Rank
17
16
15
14
13
12
11
10
9
8
7
6
5
4
3
2
1
meanThenRank
medianThenRank
rankThenMean
rankThenMedian
testBased
Ranking method
algorithm
Similarity_eroded_masked
Affine_nomask
Similarity_masked
TRSAA_eroded_masked
TRSAA_nomask
Affine_eroded_masked
Similarity_nomask
TRSAA_masked
flirt_NA
Affine_masked
terminal_MI_nomask
terminal_CC_nomask
terminal_CC_masked
antsAI_masked
terminal_MI_eroded_masked
terminal_MI_masked
terminal_CC_eroded_masked

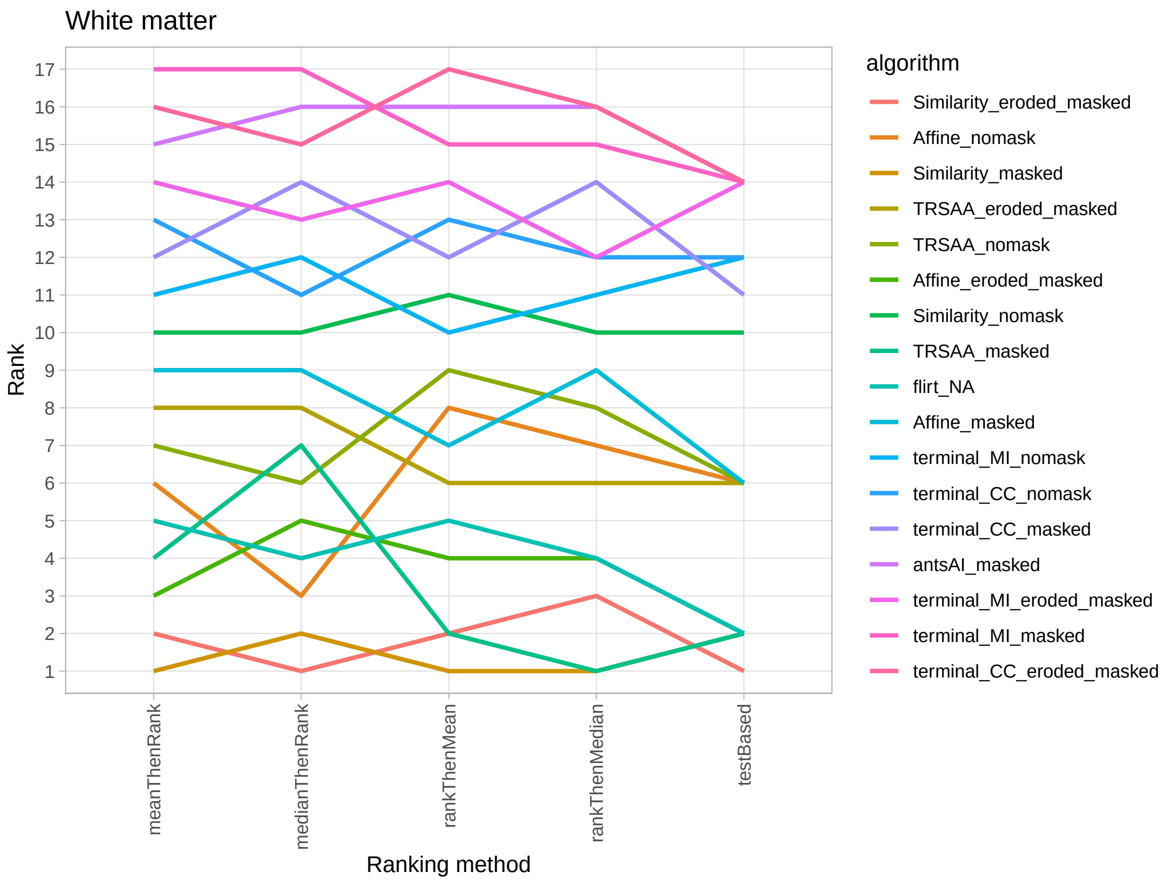
White matter
Rank
17
16
15
14
13
12
11
10
9
8
7
6
5
4
3
2
1
meanThenRank
medianThenRank
rankThenMean
rankThenMedian
testBased
Ranking method
algorithm
Similarity_eroded_masked
Affine_nomask
Similarity_masked
TRSAA_eroded_masked
TRSAA_nomask
Affine_eroded_masked
Similarity_nomask
TRSAA_masked
flirt_NA
Affine_masked
terminal_MI_nomask
terminal_CC_nomask
terminal_CC_masked
antsAI_masked
terminal_MI_eroded_masked
terminal_MI_masked
terminal_CC_eroded_masked

4 Visualization of cross-task insights

The algorithms are ordered according to consensus ranking.

4.1 Characterization of algorithms

4.1.1 Ranking stability: Variability of achieved rankings across tasks

Algorithms are color-coded, and the area of each blob at position $(A_i, \text{rank } j)$ is proportional to the relative frequency A_i achieved rank j across multiple tasks. The median rank for each algorithm is indicated by a black cross. This way, the distribution of ranks across tasks can be intuitively visualized.

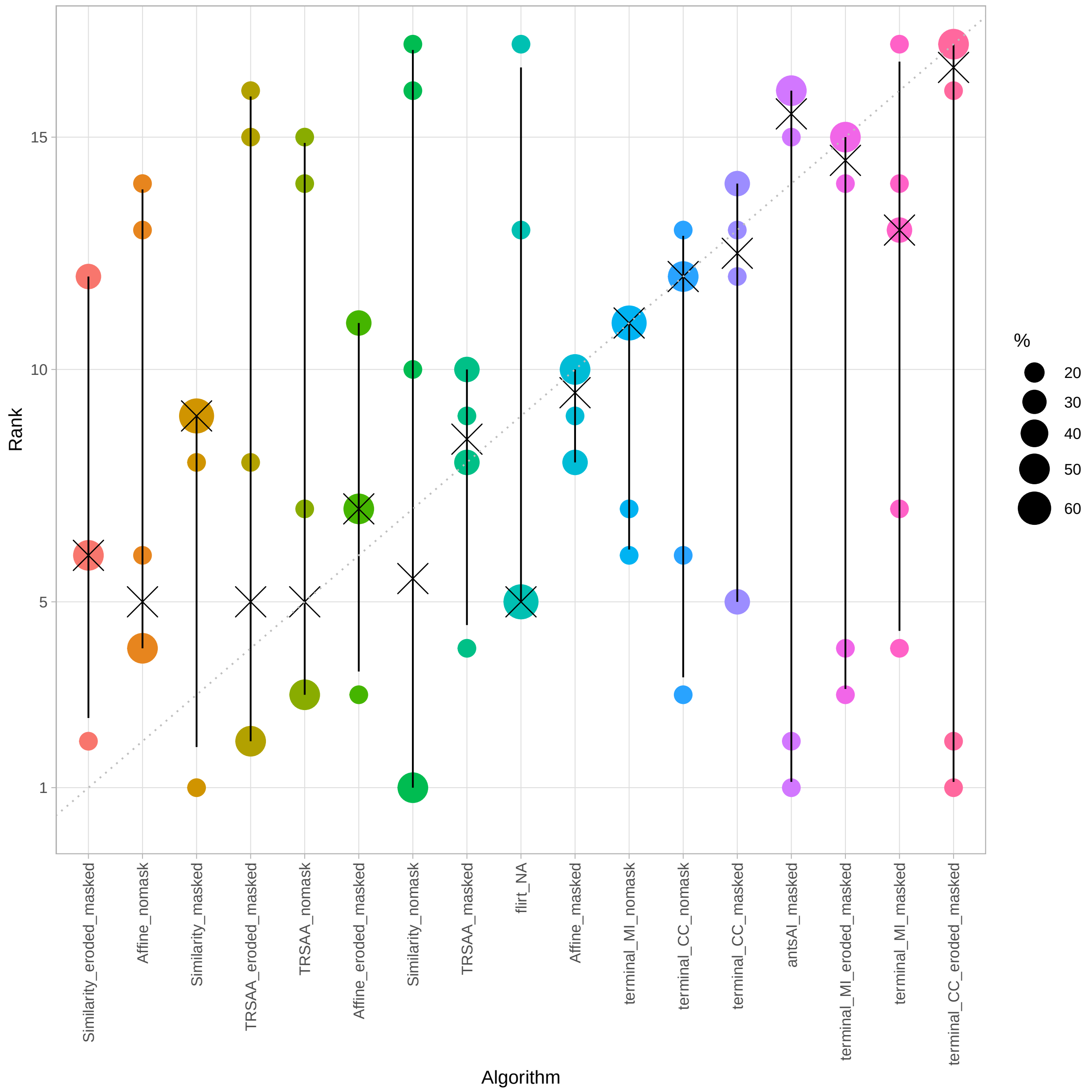

4.1.2 Ranking stability: Ranking variability via bootstrap approach

A blob plot of bootstrap results over the different tasks separated by algorithm allows another perspective on the assessment data. This gives deeper insights into the characteristics of tasks and the ranking uncertainty of the algorithms in each task.

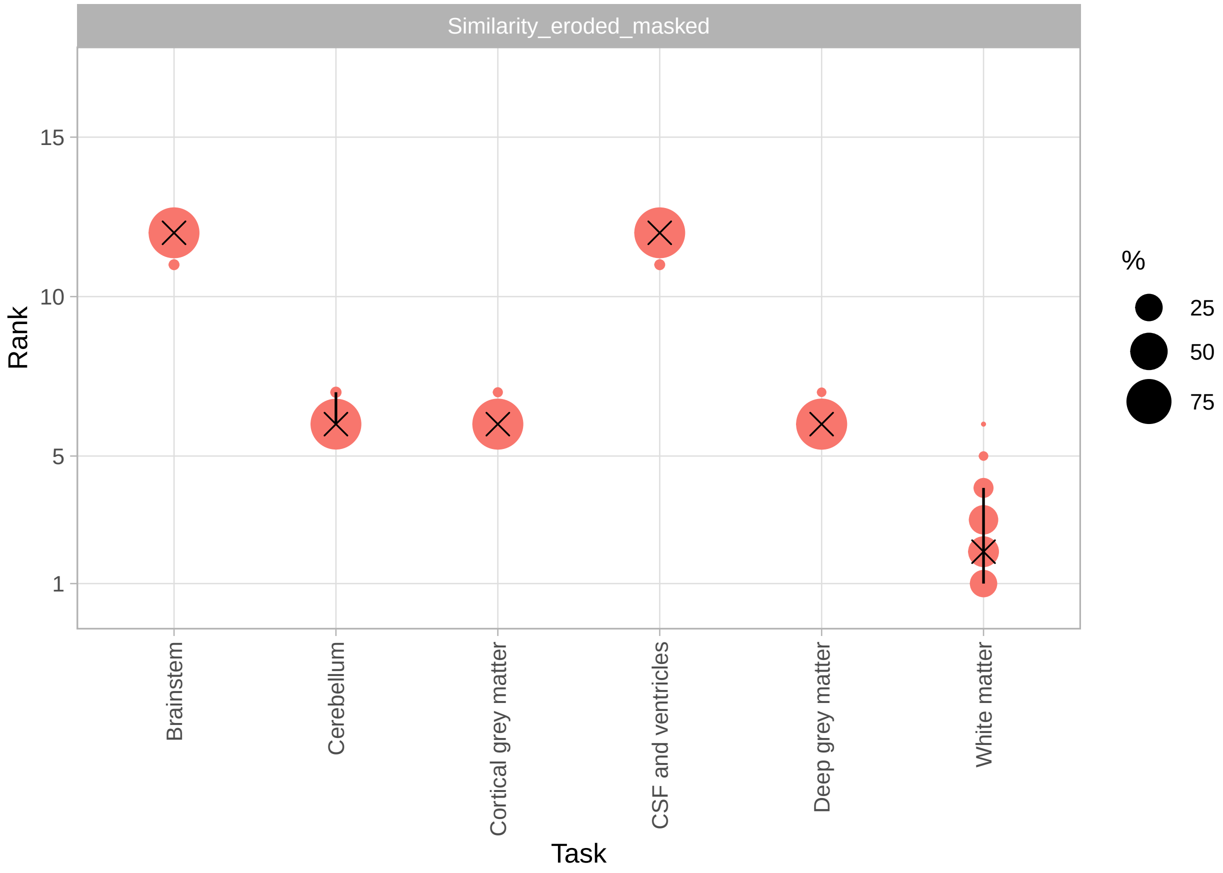

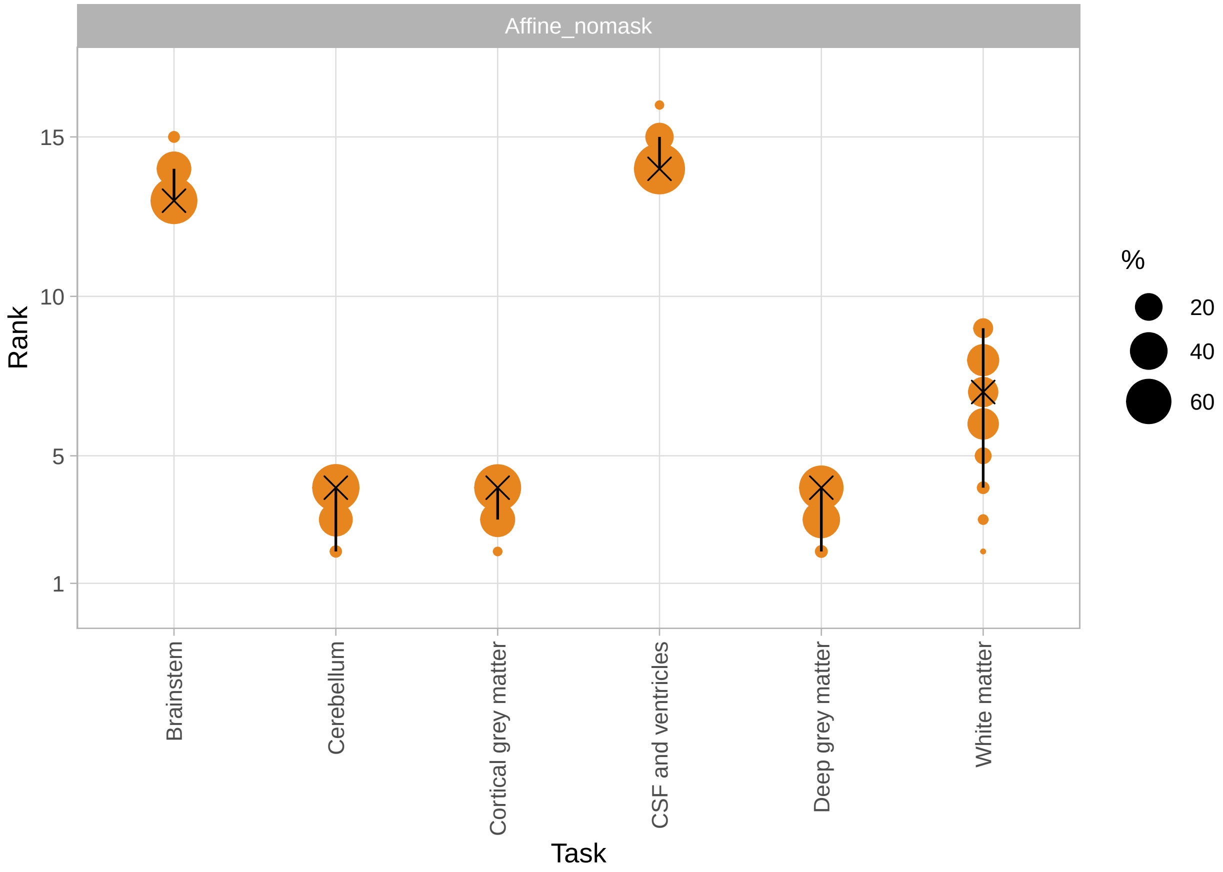
Affine_nomask
Rank
15
10
5
1
Brainstem
Cerebellum
Cortical grey matter
CSF and ventricles
Deep grey matter
White matter
Task
%
20
40
60

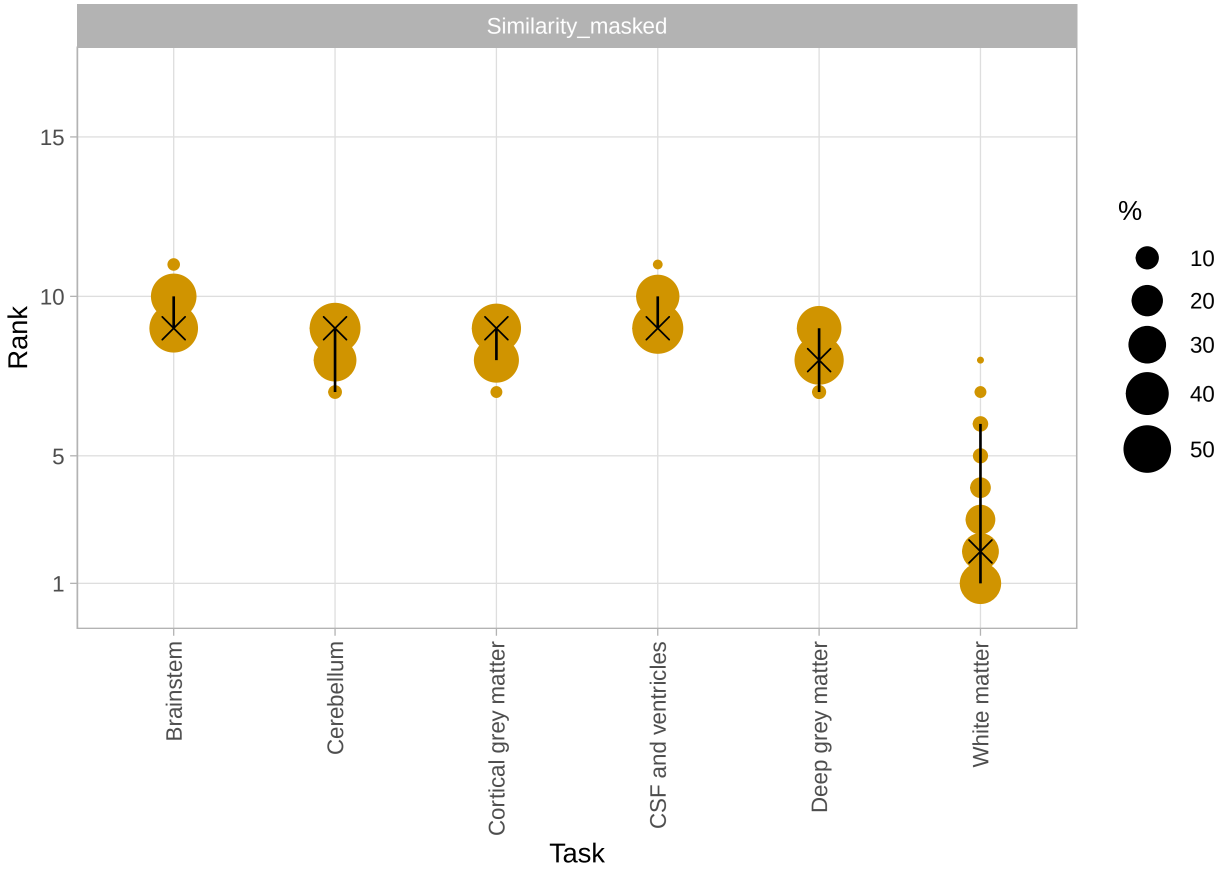

Similarity_masked
15
10
5
1
Rank
Brainstem
Cerebellum
Cortical grey matter
CSF and ventricles
Deep grey matter
White matter
Task
%
10
20
30
40
50

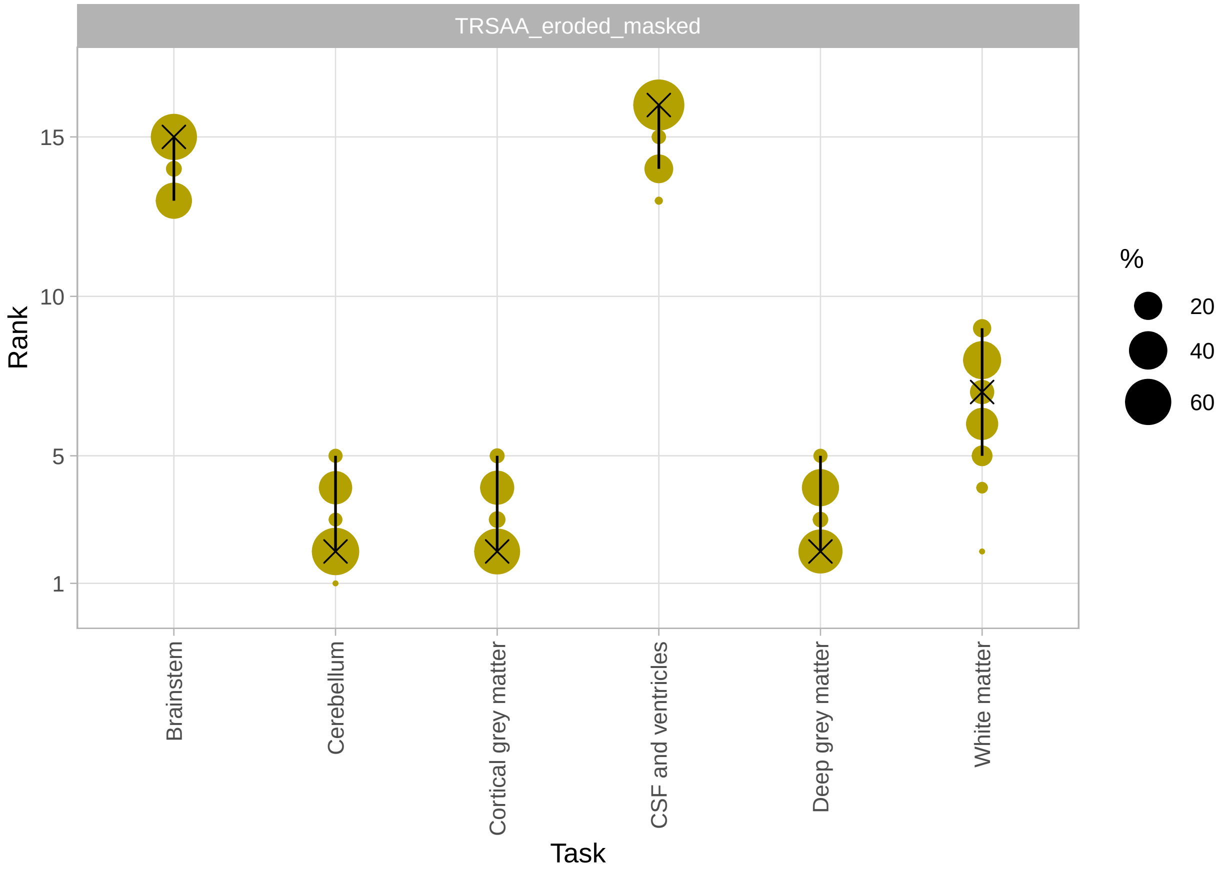
TRSAA_eroded_masked
Rank
15
10
5
1
Brainstem
Cerebellum
Cortical grey matter
CSF and ventricles
Deep grey matter
White matter
Task
%
20
40
60

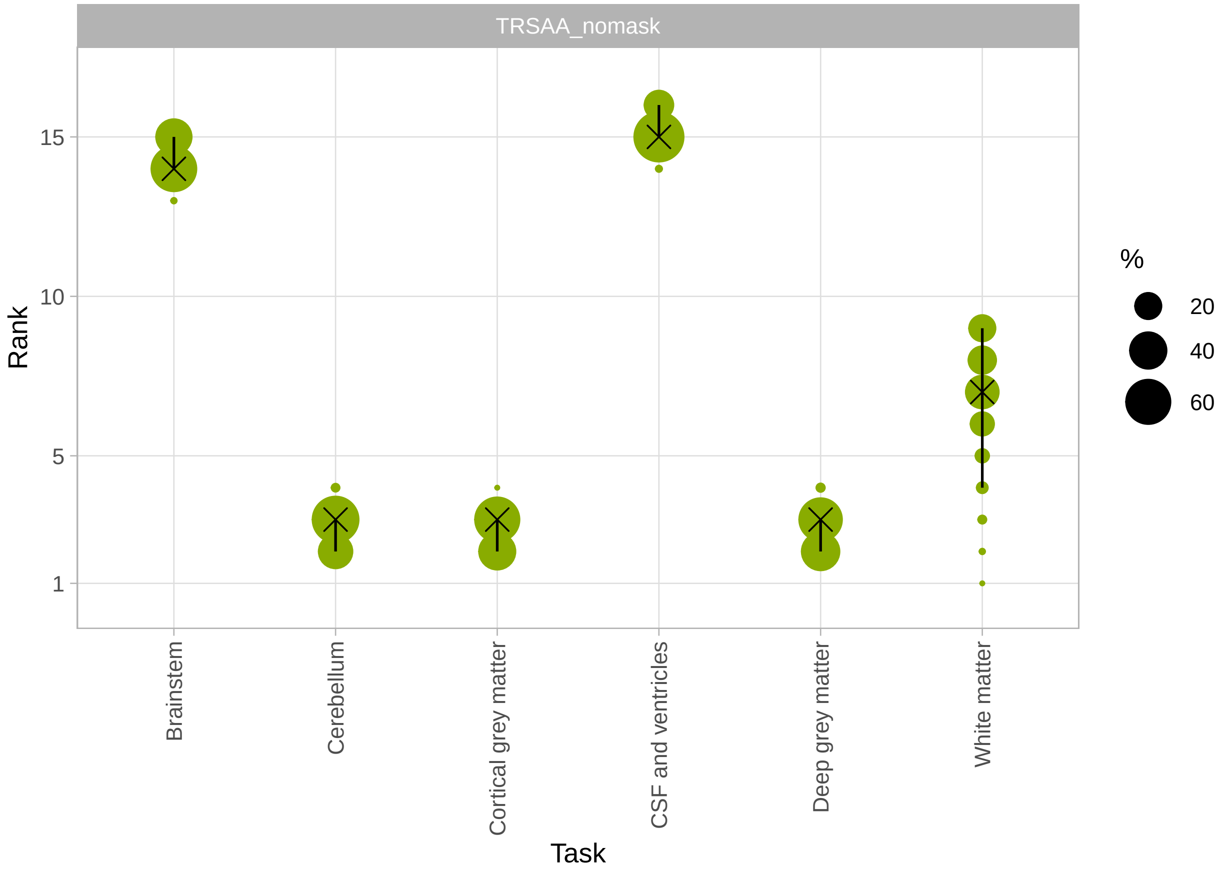

TRSAA_nomask
Rank
15
10
5
1
Brainstem
Cerebellum
Cortical grey matter
CSF and ventricles
Deep grey matter
White matter
Task
%
20
40
60

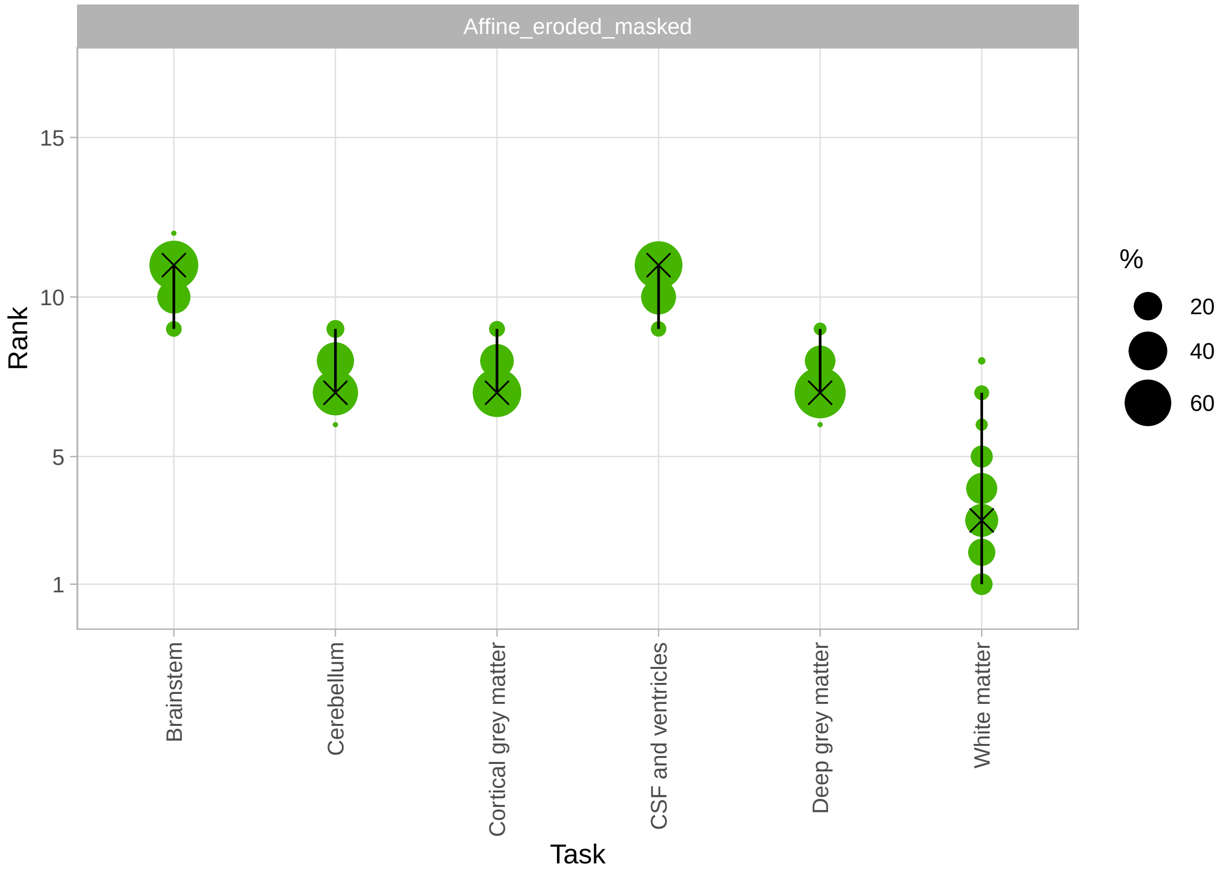
Affine_eroded_masked
Rank
15
10
5
1
Brainstem
Cerebellum
Cortical grey matter
CSF and ventricles
Deep grey matter
White matter
Task
%
20
40
60

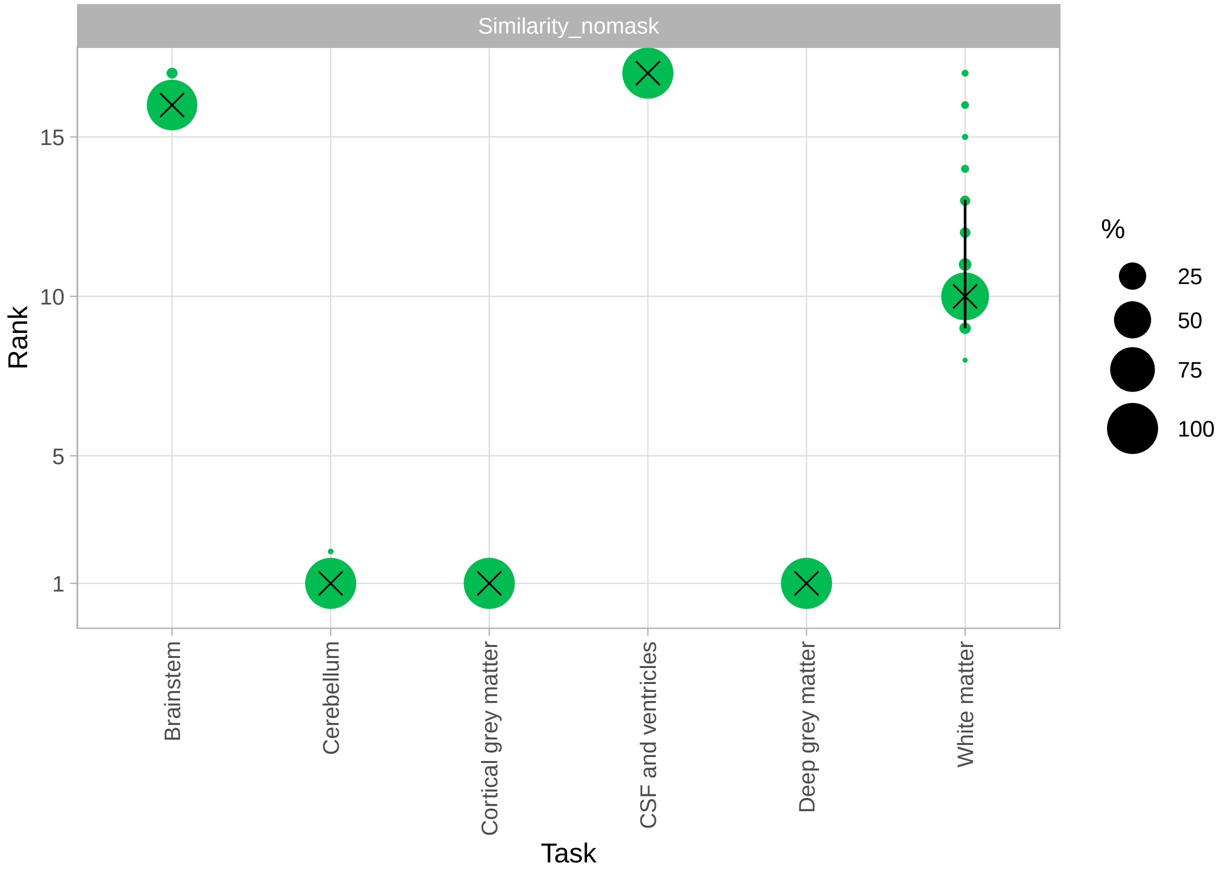

Similarity_nomask
Rank
15
10
5
1
Brainstem
Cerebellum
Cortical grey matter
CSF and ventricles
Deep grey matter
White matter
Task
%
25
50
75
100

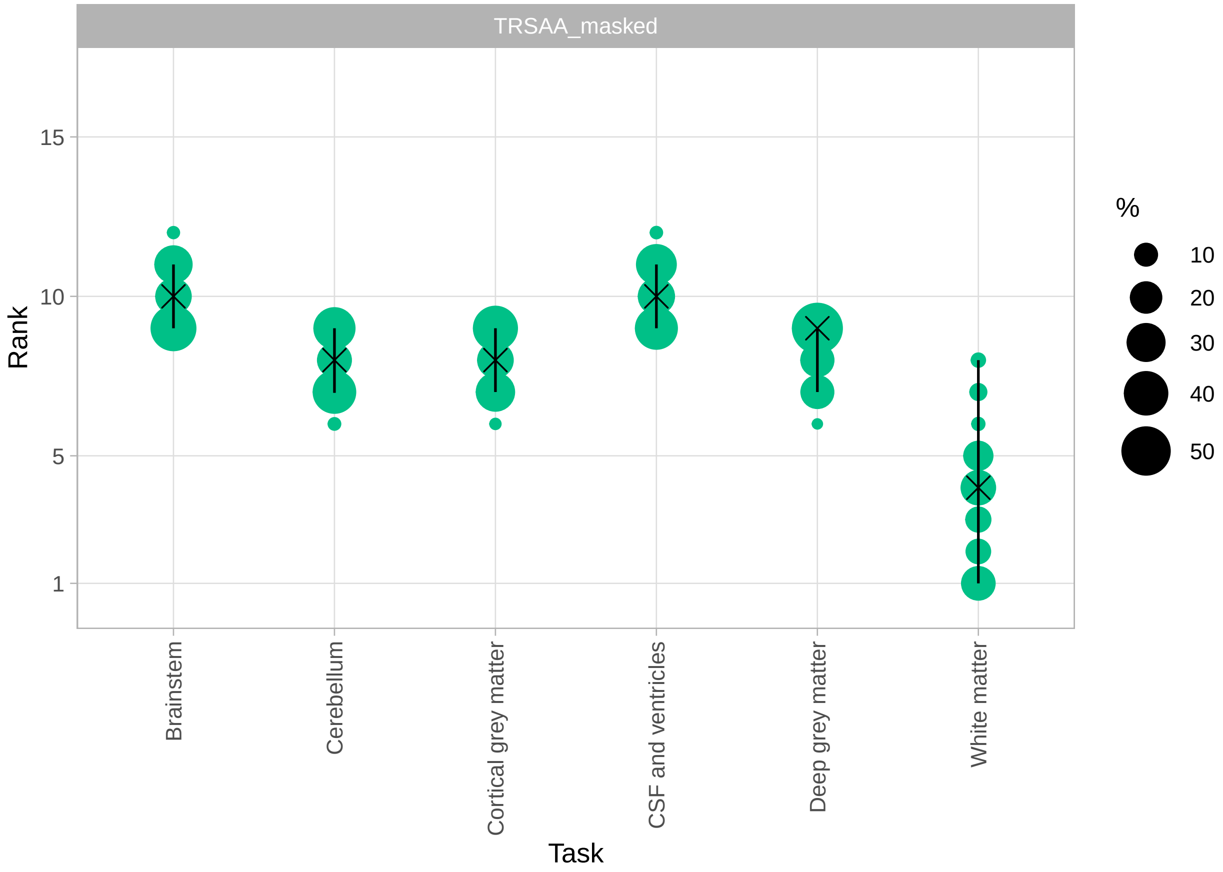

TRSAA_masked
Rank
15
10
5
1
Brainstem
Cerebellum
Cortical grey matter
CSF and ventricles
Deep grey matter
White matter
Task
%
10
20
30
40
50

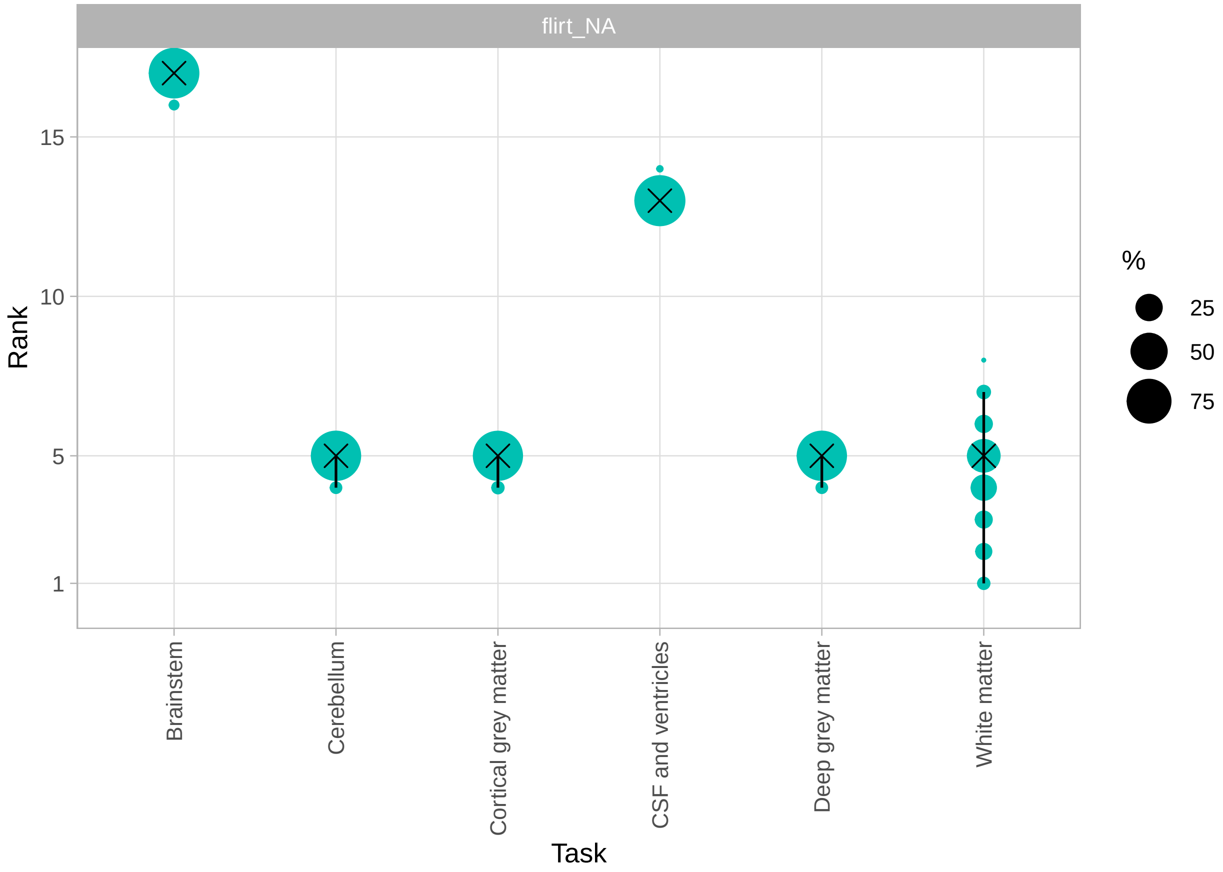

flirt_NA
Rank
15
10
5
1
Brainstem
Cerebellum
Cortical grey matter
CSF and ventricles
Deep grey matter
White matter
Task
%
25
50
75

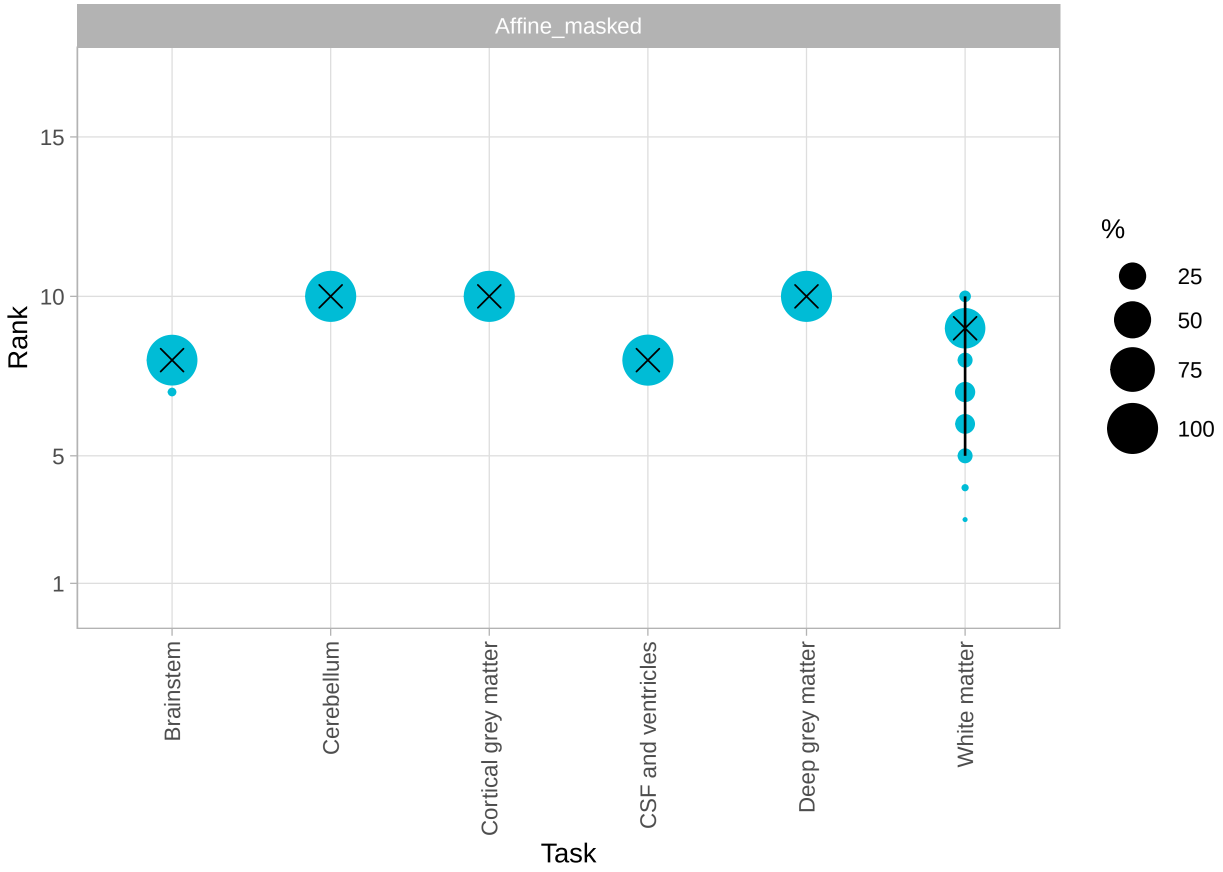
Affine_masked
Rank
15
10
5
1
Brainstem
Cerebellum
Cortical grey matter
CSF and ventricles
Deep grey matter
White matter
Task
%
25
50
75
100

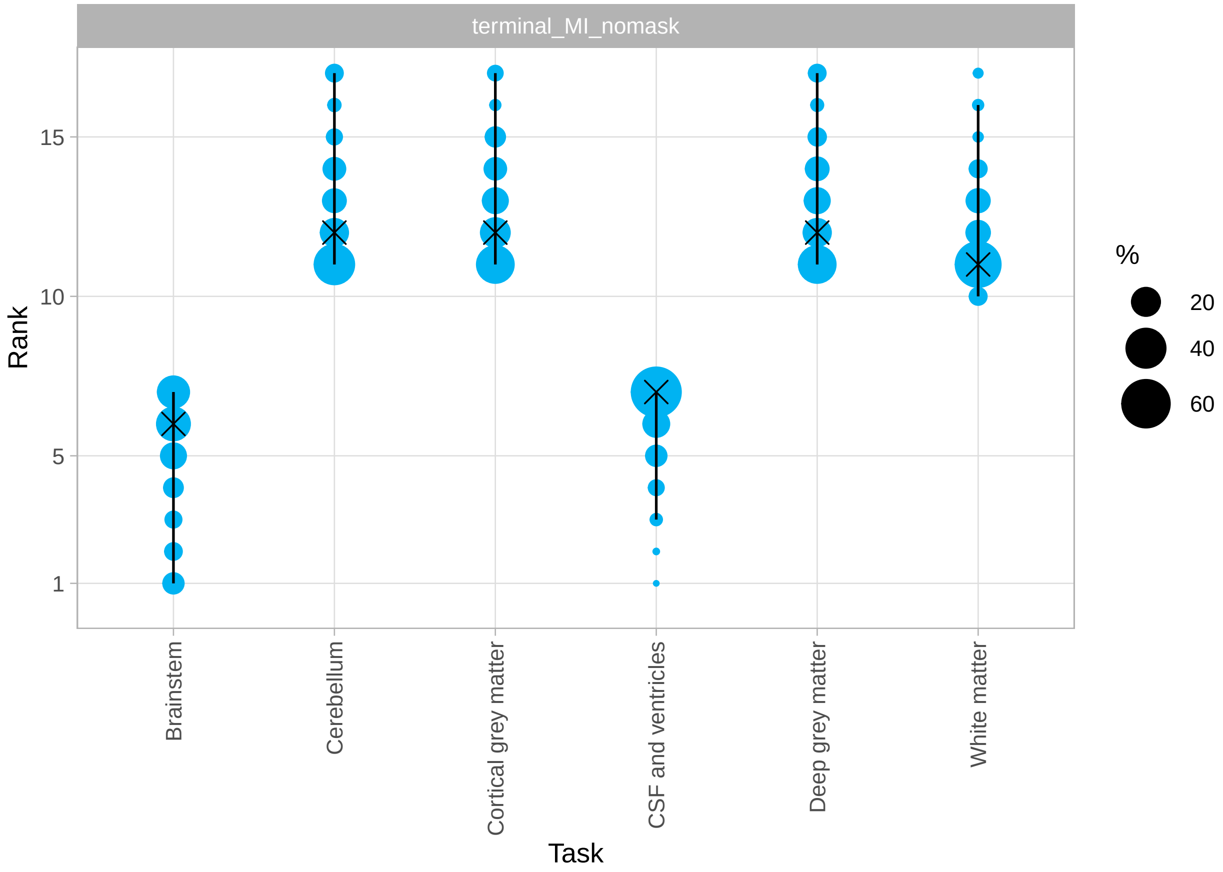
terminal_MI_nomask
Rank
15
10
5
1
Brainstem
Cerebellum
Cortical grey matter
CSF and ventricles
Deep grey matter
White matter
Task
%
20
40
60

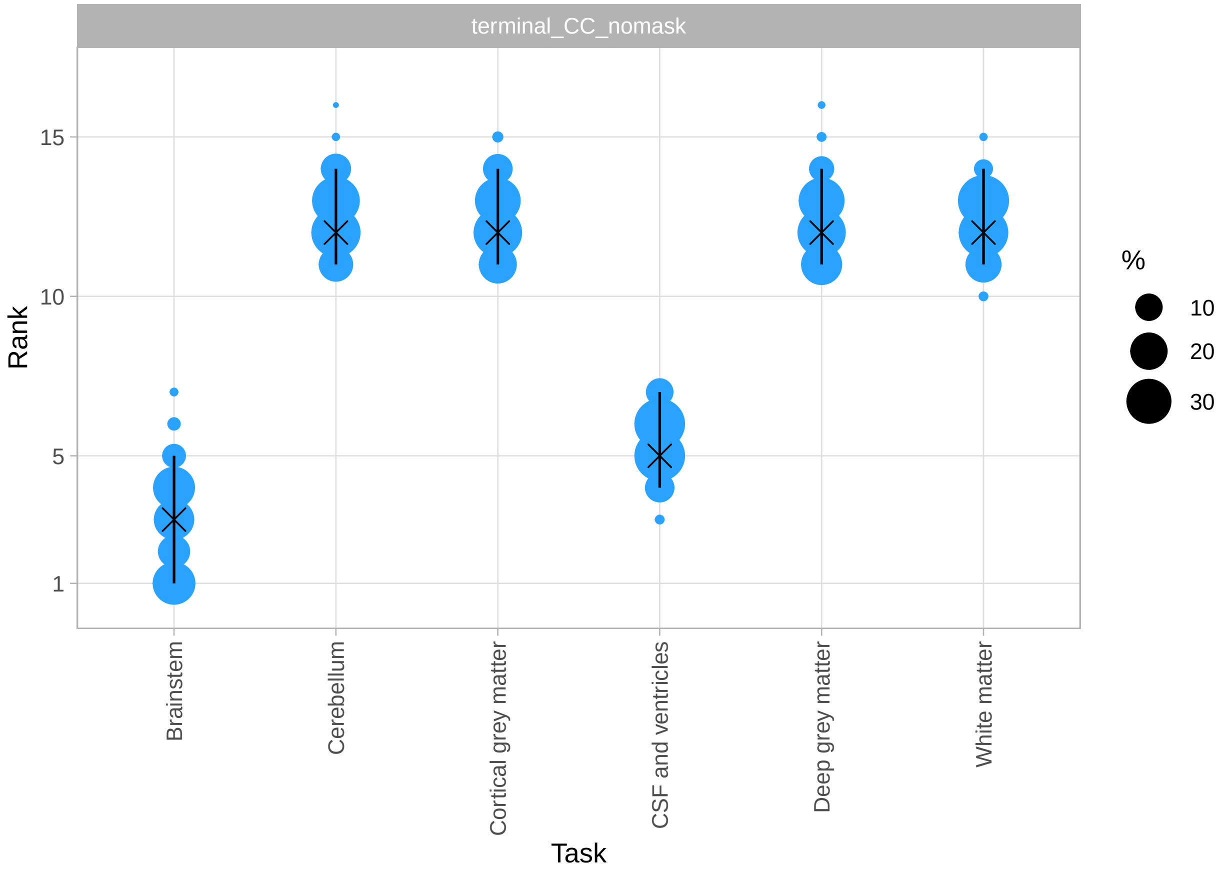

terminal_CC_nomask
Rank
15
10
5
1
Brainstem
Cerebellum
Cortical grey matter
CSF and ventricles
Deep grey matter
White matter
Task
%
10
20
30

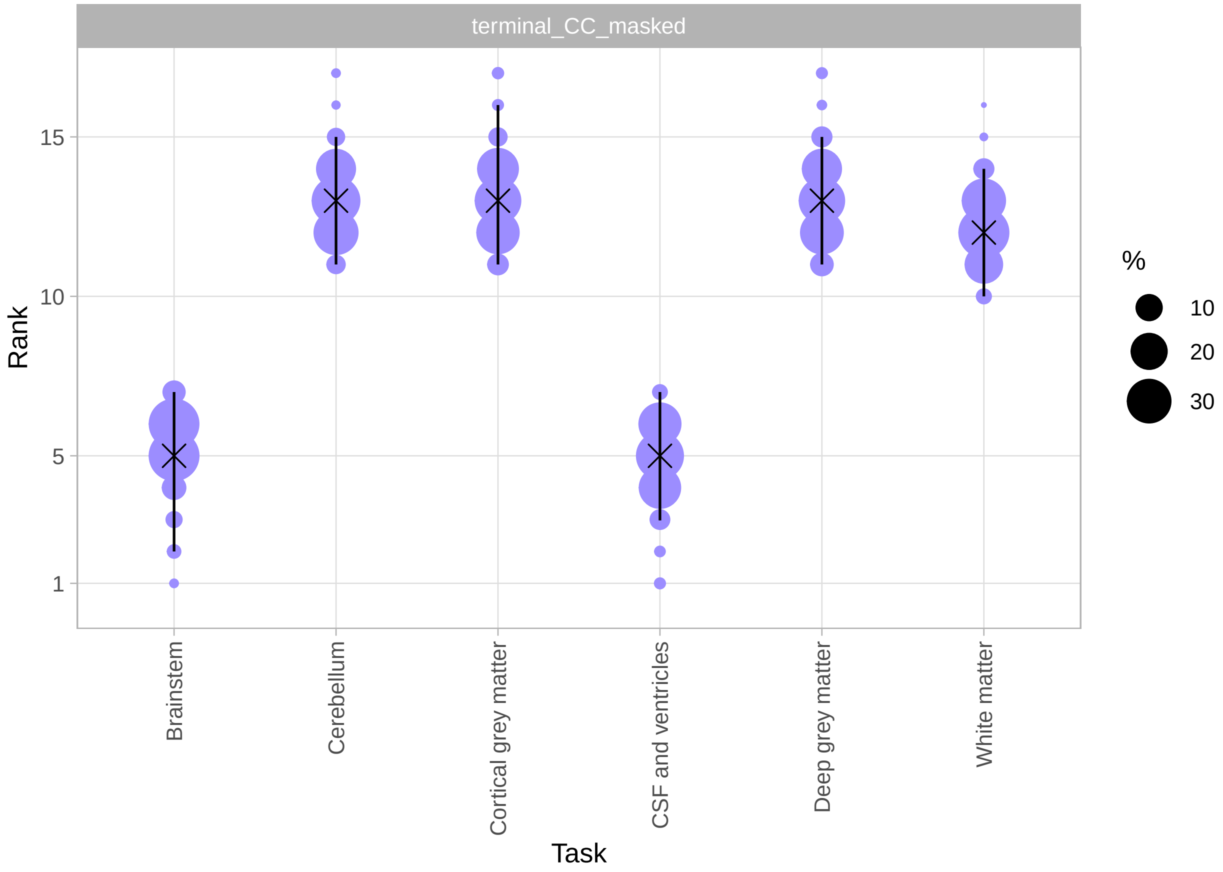

terminal_CC_masked
Rank
15
10
5
1
Brainstem
Cerebellum
Cortical grey matter
CSF and ventricles
Deep grey matter
White matter
Task
%
10
20
30

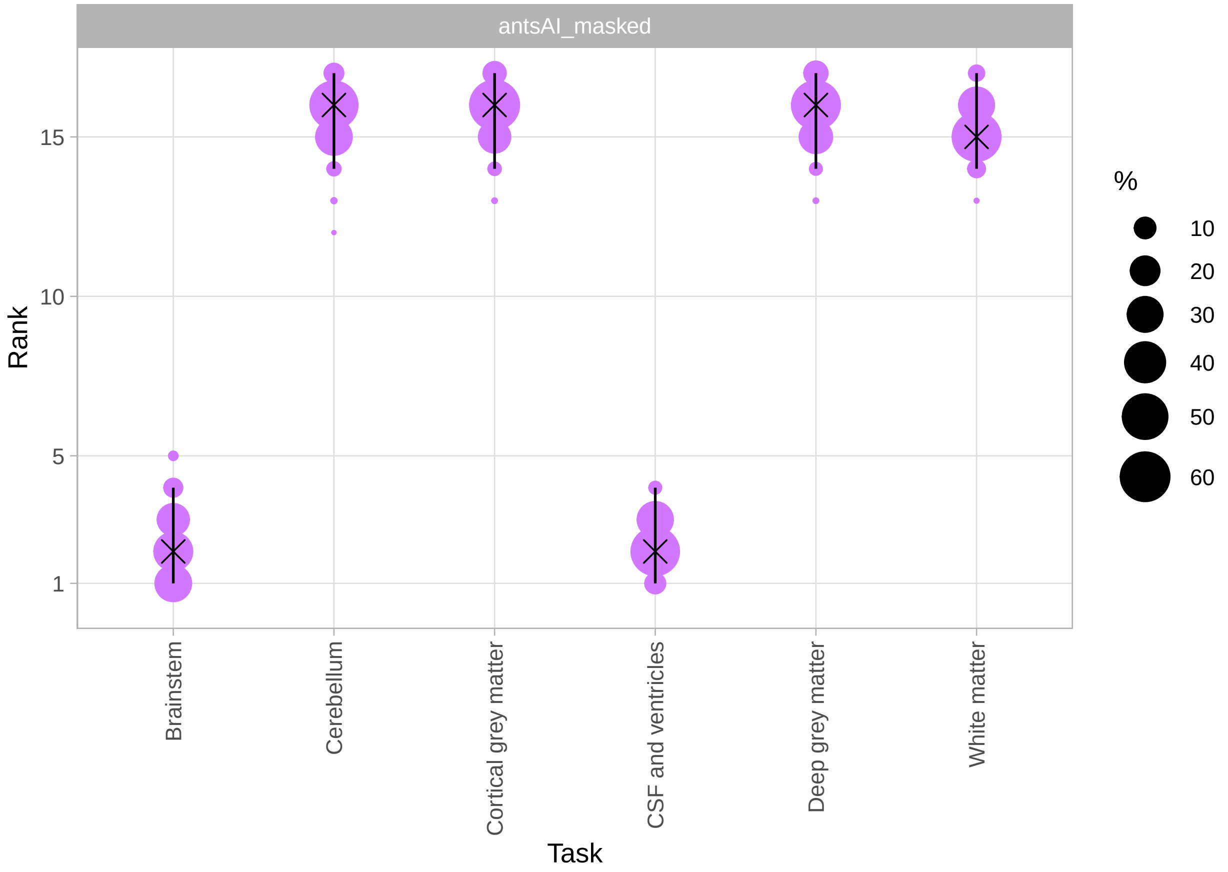
antsAI_masked
Rank
15
10
5
1
Brainstem
Cerebellum
Cortical grey matter
CSF and ventricles
Deep grey matter
White matter
Task
%
10
20
30
40
50
60

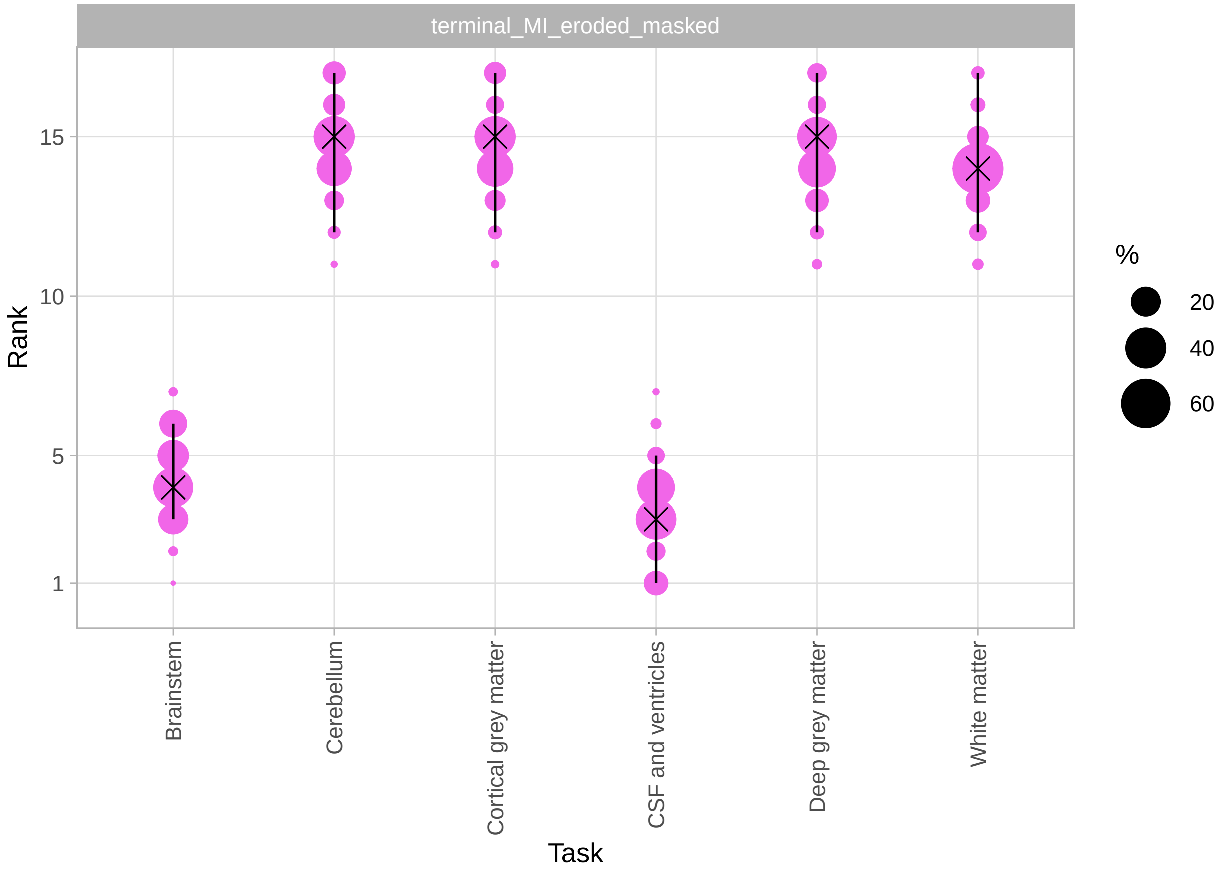

terminal_MI_eroded_masked
Rank
15
10
5
1
%
20
40
60
Brainstem
Cerebellum
Cortical grey matter
CSF and ventricles
Deep grey matter
White matter
Task

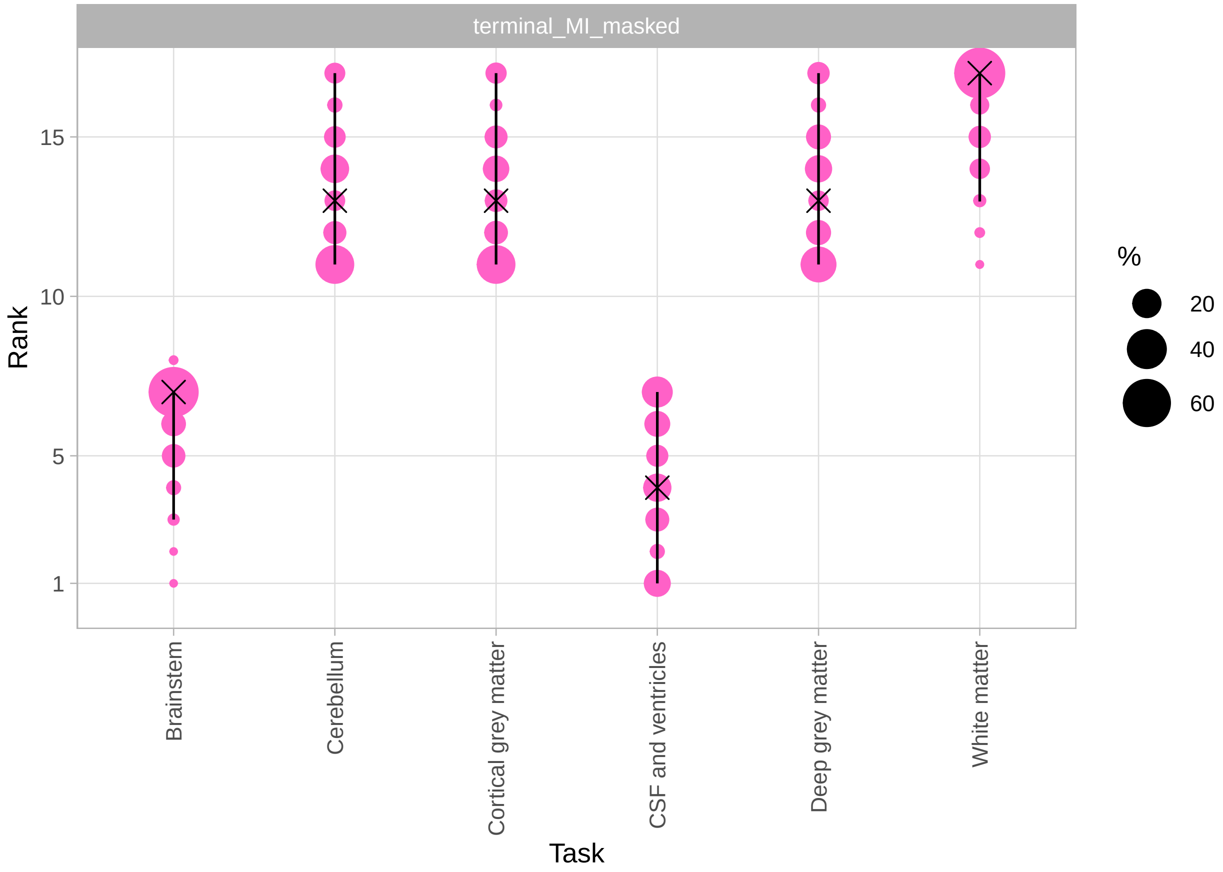
terminal_MI_masked
Rank
15
10
5
1
Brainstem
Cerebellum
Cortical grey matter
CSF and ventricles
Deep grey matter
White matter
Task
%
20
40
60

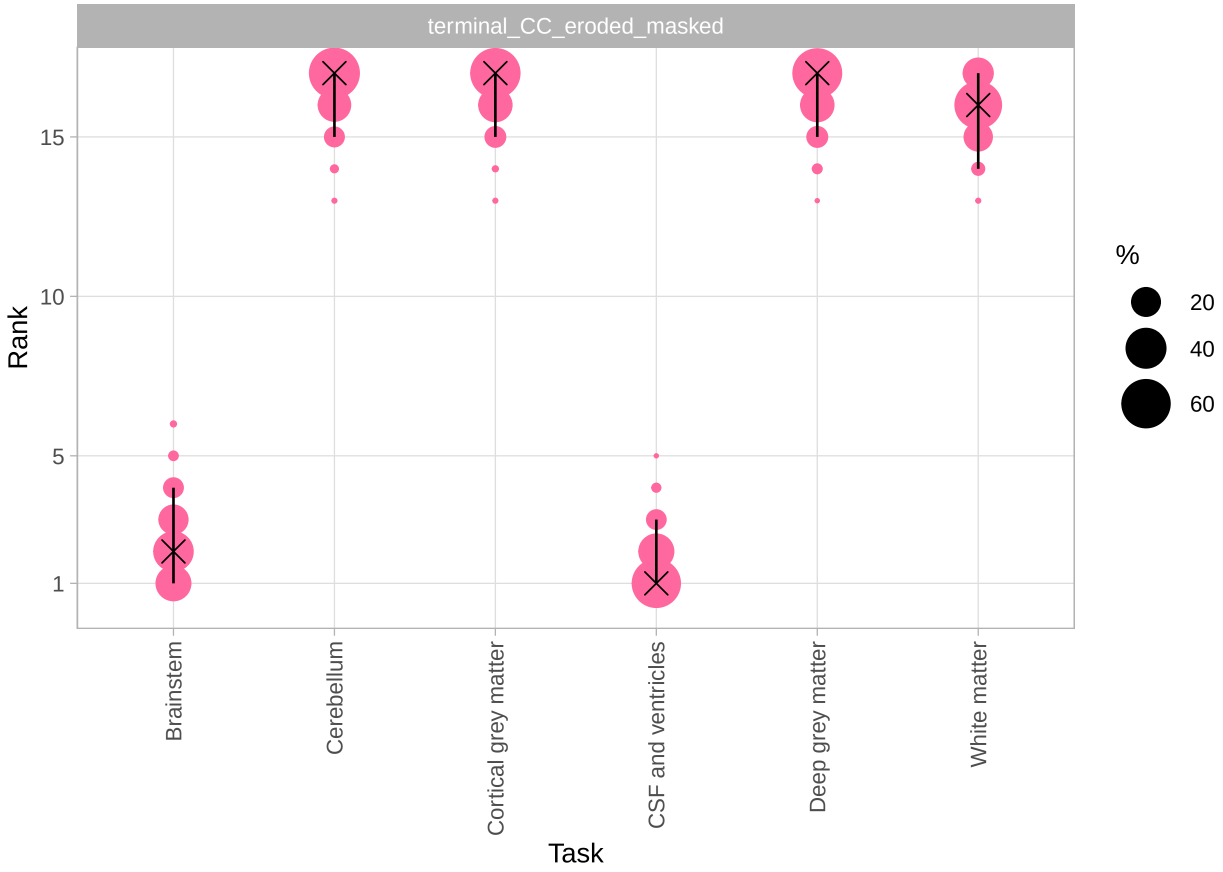
terminal_CC_eroded_masked
Rank
15
10
5
1
Brainstem
Cerebellum
Cortical grey matter
CSF and ventricles
Deep grey matter
White matter
Task
%
20
40
60

An alternative representation is provided by a stacked frequency plot of the observed ranks, separated by algorithm. Observed ranks across bootstrap samples are displayed with coloring according to the task. For algorithms that achieve the same rank in different tasks for the full assessment data set, vertical lines are on top of each other. Vertical lines allow to compare the achieved rank of each algorithm over different tasks.

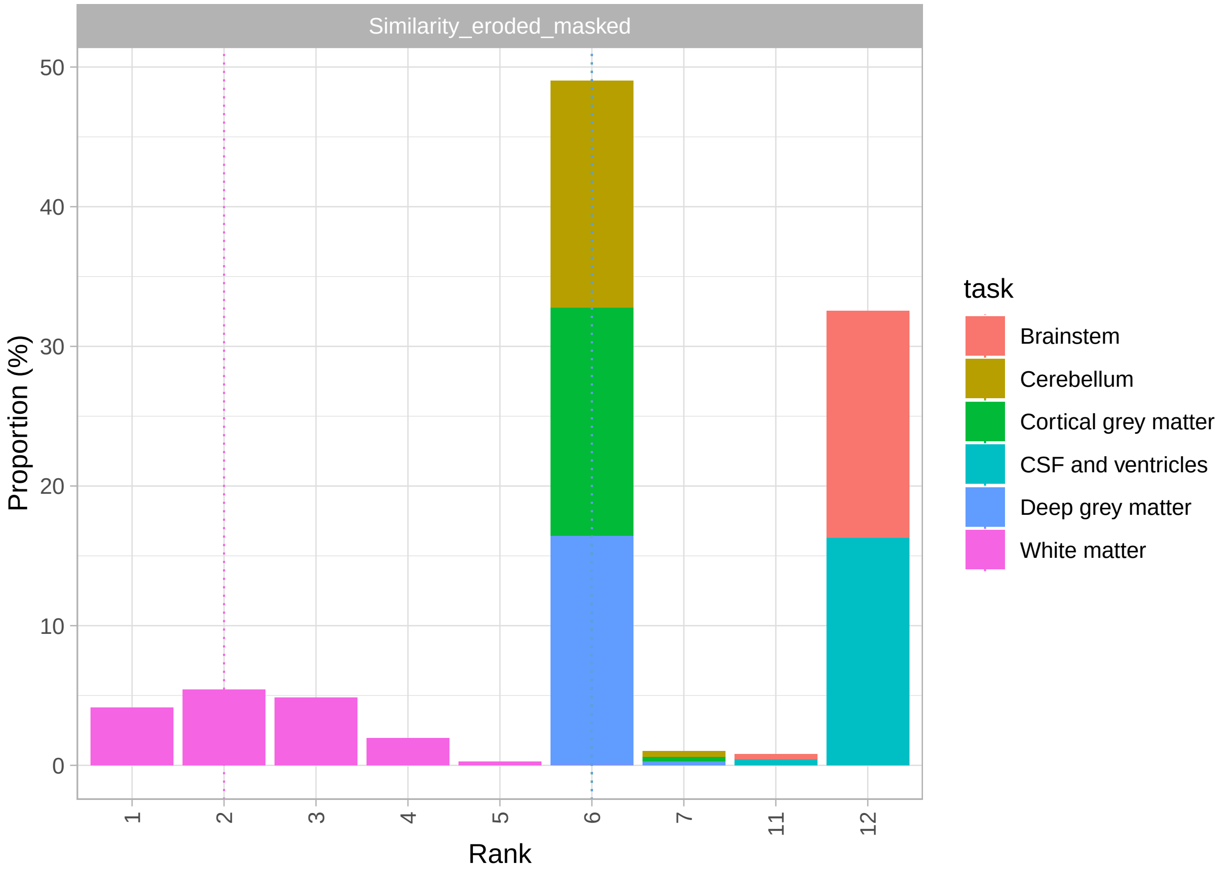

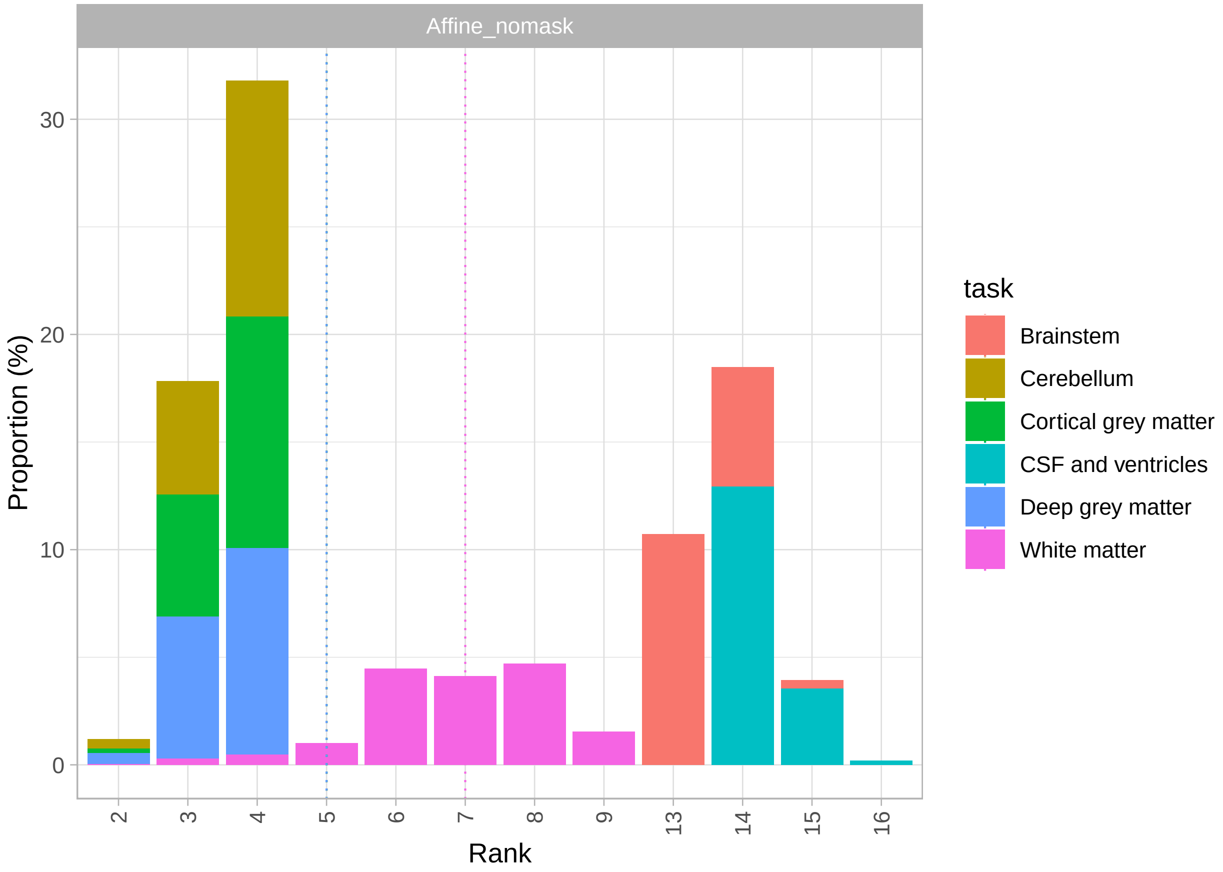
Affine_nomask
Proportion (%)
30
20
10
0
2
3
4
5
6
7
8
9
13
14
15
16
Rank
task
Brainstem
Cerebellum
Cortical grey matter
CSF and ventricles
Deep grey matter
White matter

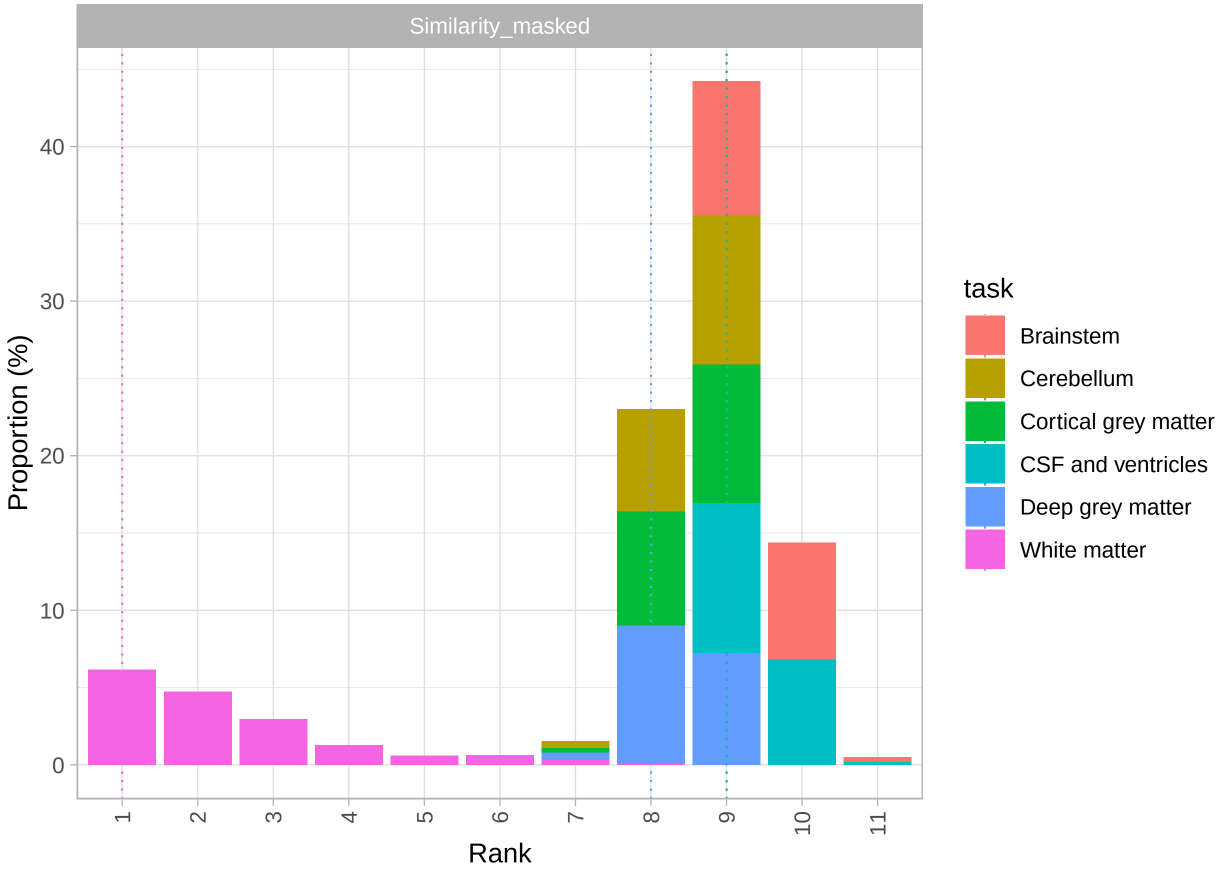

Similarity_masked
Proportion (%)
40
30
20
10
0
1
2
3
4
5
6
7
8
9
10
11
Rank
task
Brainstem
Cerebellum
Cortical grey matter
CSF and ventricles
Deep grey matter
White matter

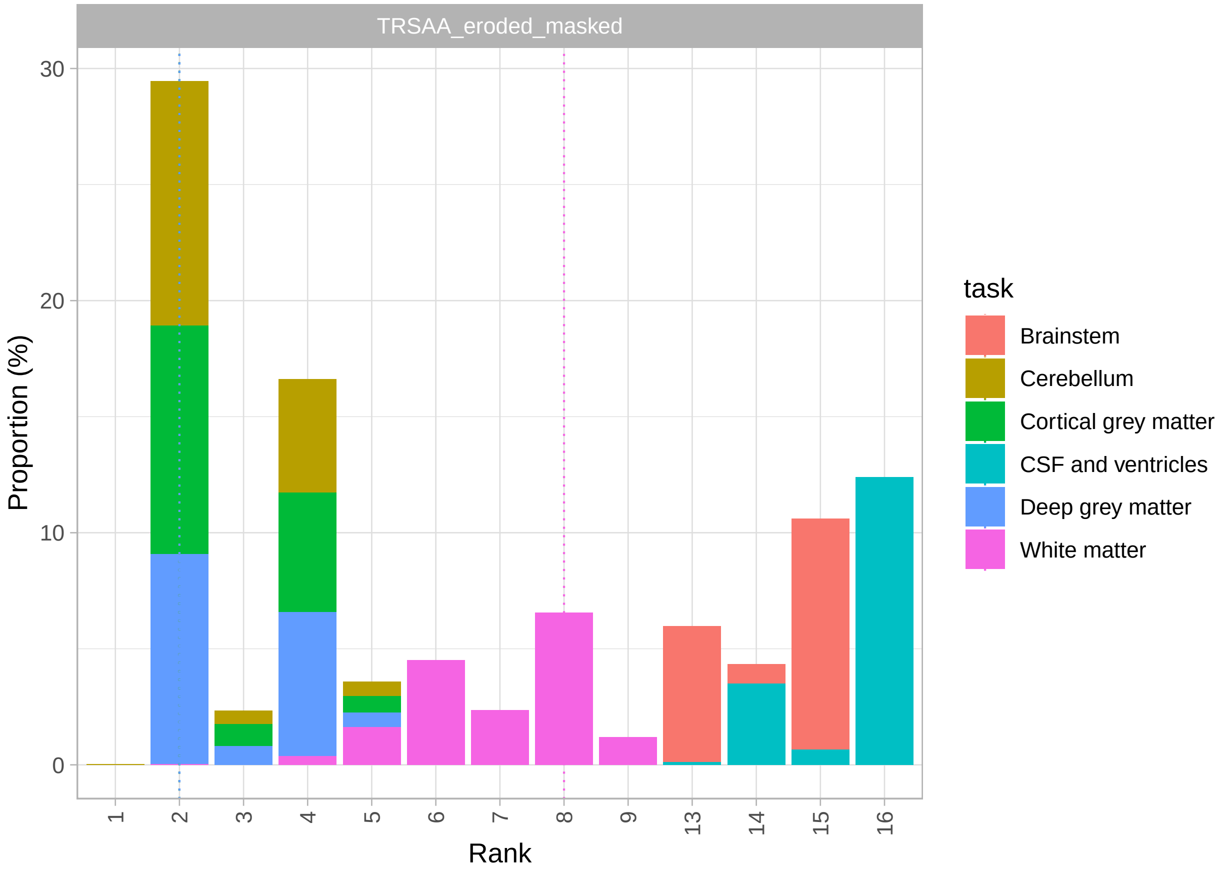

TRSAA_eroded_masked
Proportion (%)
30
20
10
0
1
2
3
4
5
6
7
8
9
13
14
15
16
Rank
task
Brainstem
Cerebellum
Cortical grey matter
CSF and ventricles
Deep grey matter
White matter

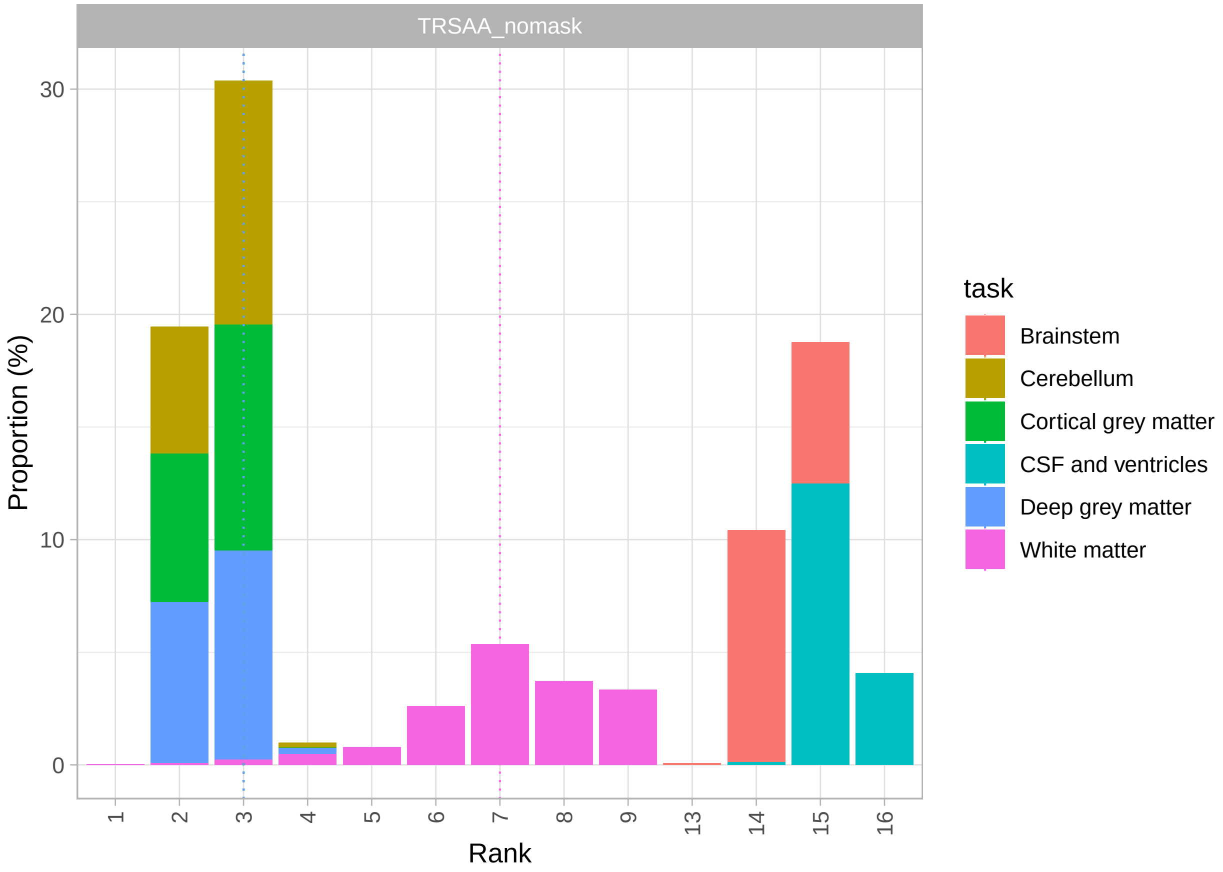

TRSAA_nomask
Proportion (%)
0
10
20
30
1
2
3
4
5
6
7
8
9
13
14
15
16
Rank
task
Brainstem
Cerebellum
Cortical grey matter
CSF and ventricles
Deep grey matter
White matter

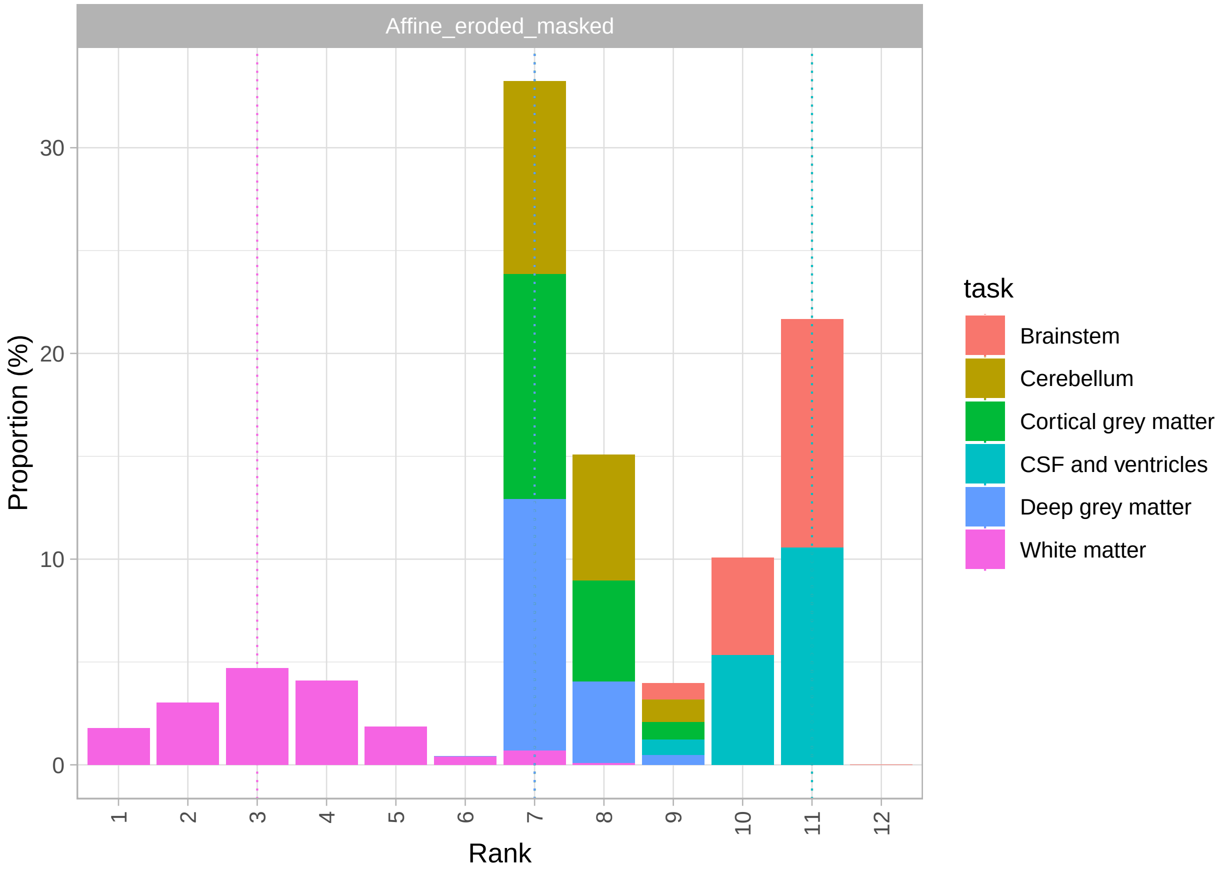
Affine_eroded_masked
Proportion (%)
0
10
20
30
1
2
3
4
5
6
7
8
9
10
11
12
Rank
task
Brainstem
Cerebellum
Cortical grey matter
CSF and ventricles
Deep grey matter
White matter

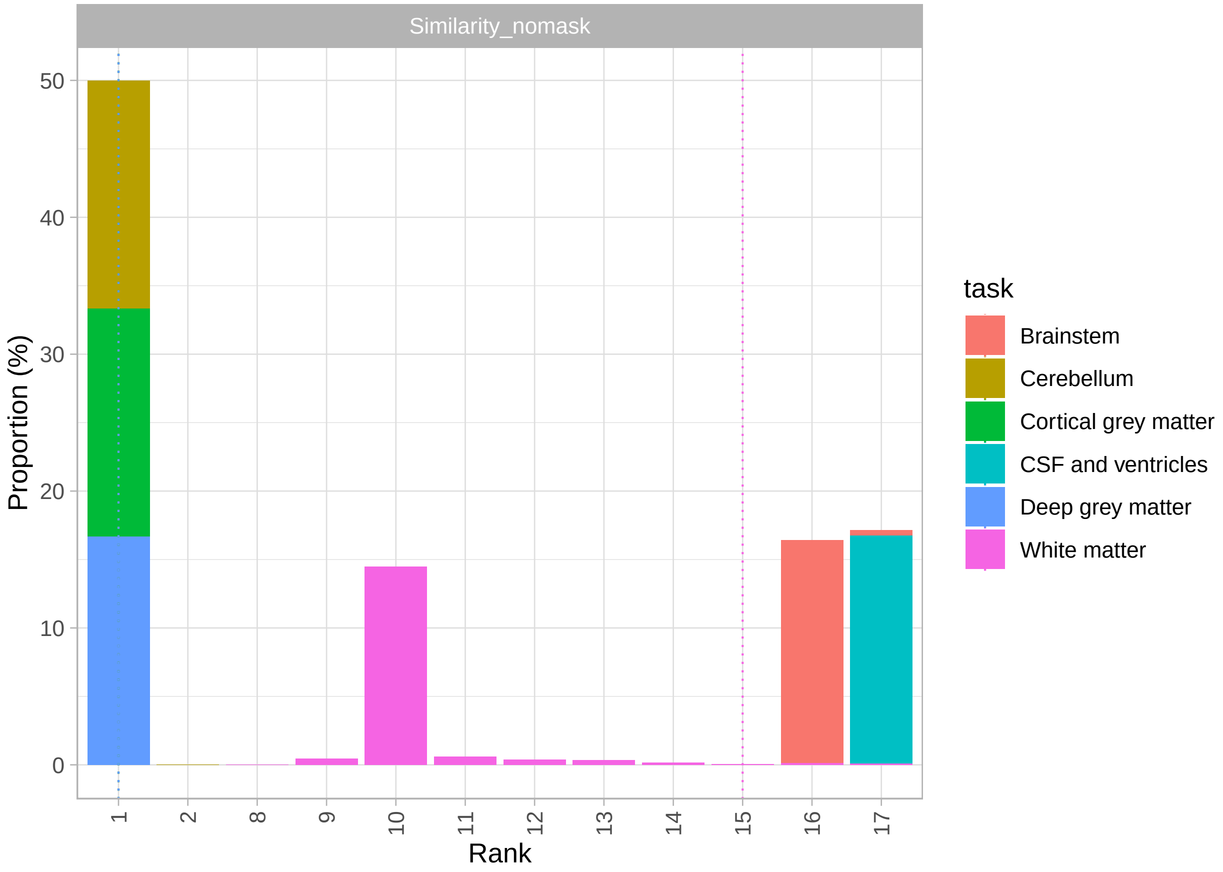
Similarity_nomask
Proportion (%)
50
40
30
20
10
0
1
2
8
9
10
11
12
13
14
15
16
17
Rank
task
Brainstem
Cerebellum
Cortical grey matter
CSF and ventricles
Deep grey matter
White matter

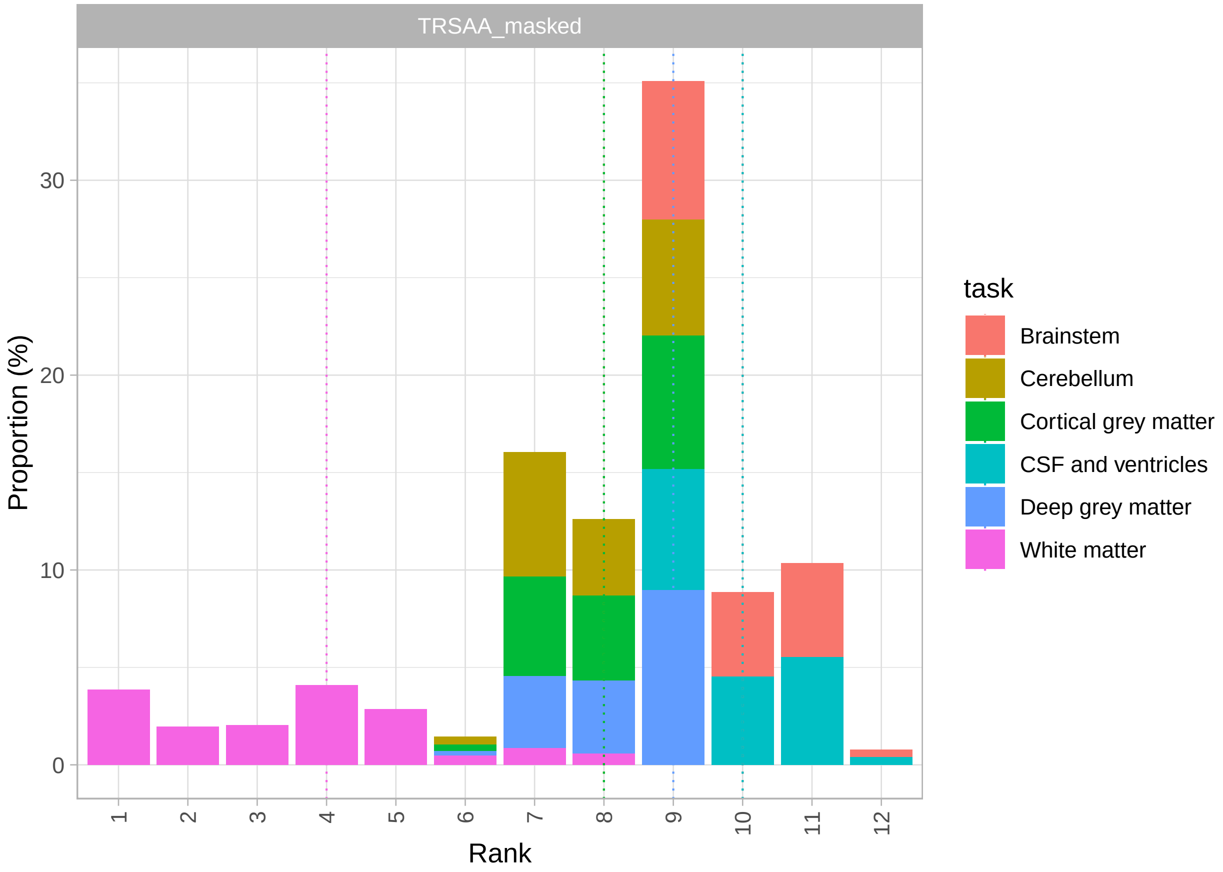
TRSAA_masked
Proportion (%)
0
10
20
30
1
2
3
4
5
6
7
8
9
10
11
12
Rank
task
Brainstem
Cerebellum
Cortical grey matter
CSF and ventricles
Deep grey matter
White matter

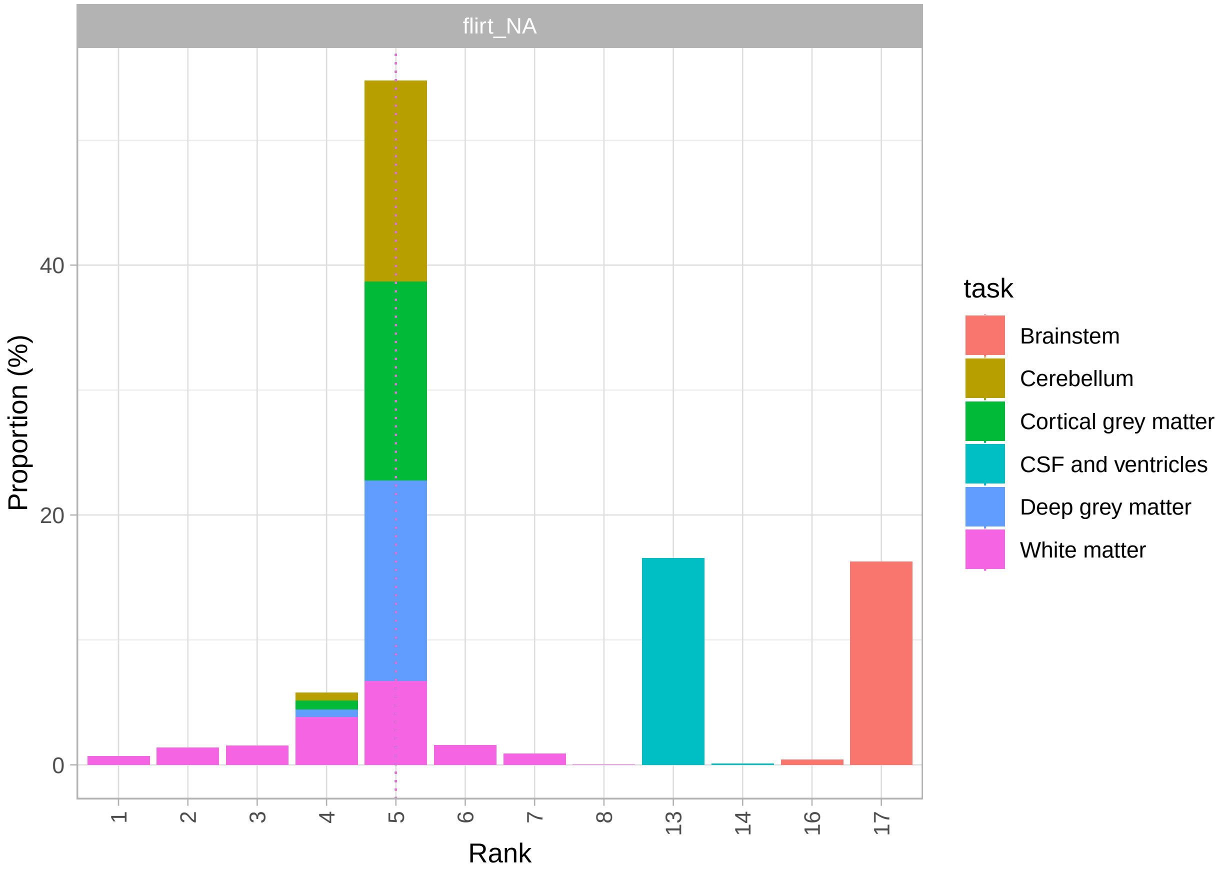
flirt_NA
Proportion (%)
0
20
40
1
2
3
4
5
6
7
8
13
14
16
17
Rank
task
Brainstem
Cerebellum
Cortical grey matter
CSF and ventricles
Deep grey matter
White matter

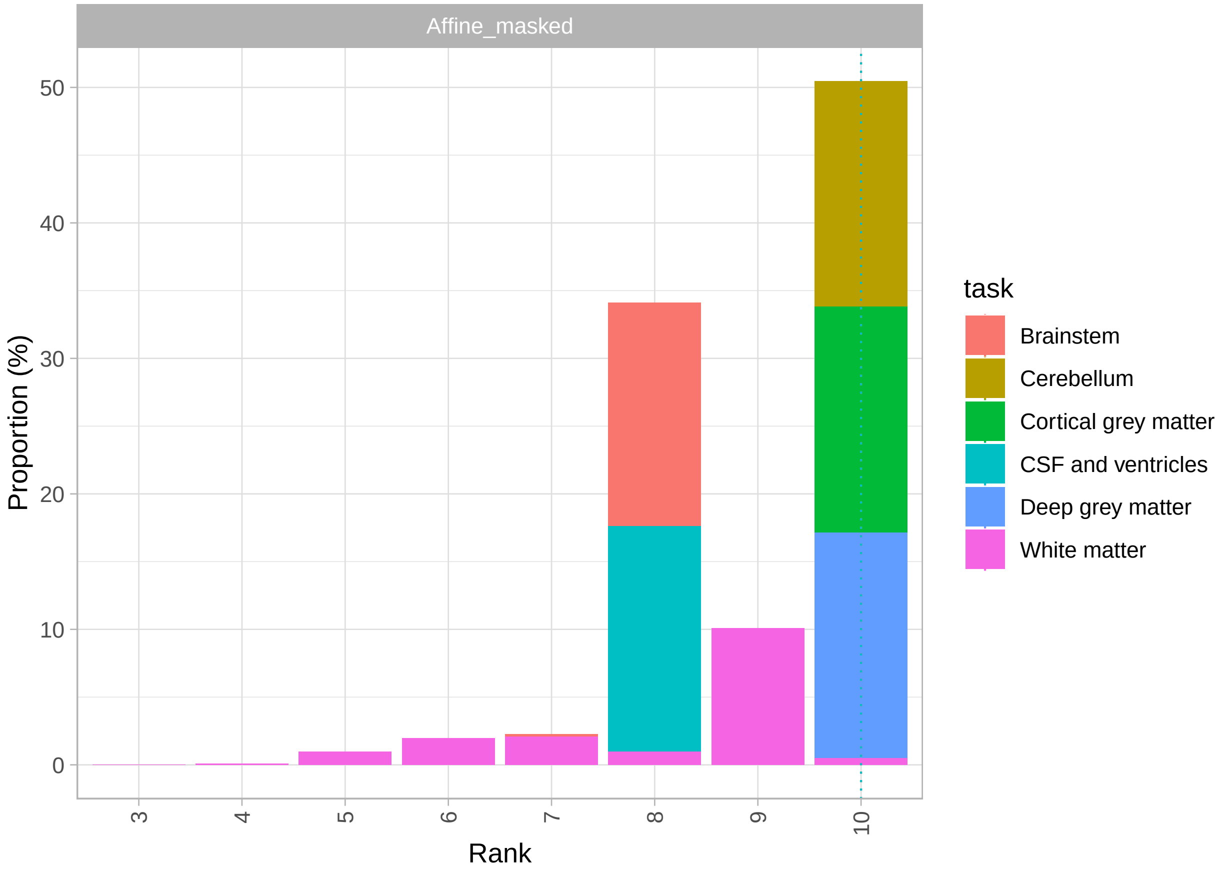
Affine_masked
Proportion (%)
0
10
20
30
40
50
3
4
5
6
7
8
9
10
Rank
task
Brainstem
Cerebellum
Cortical grey matter
CSF and ventricles
Deep grey matter
White matter

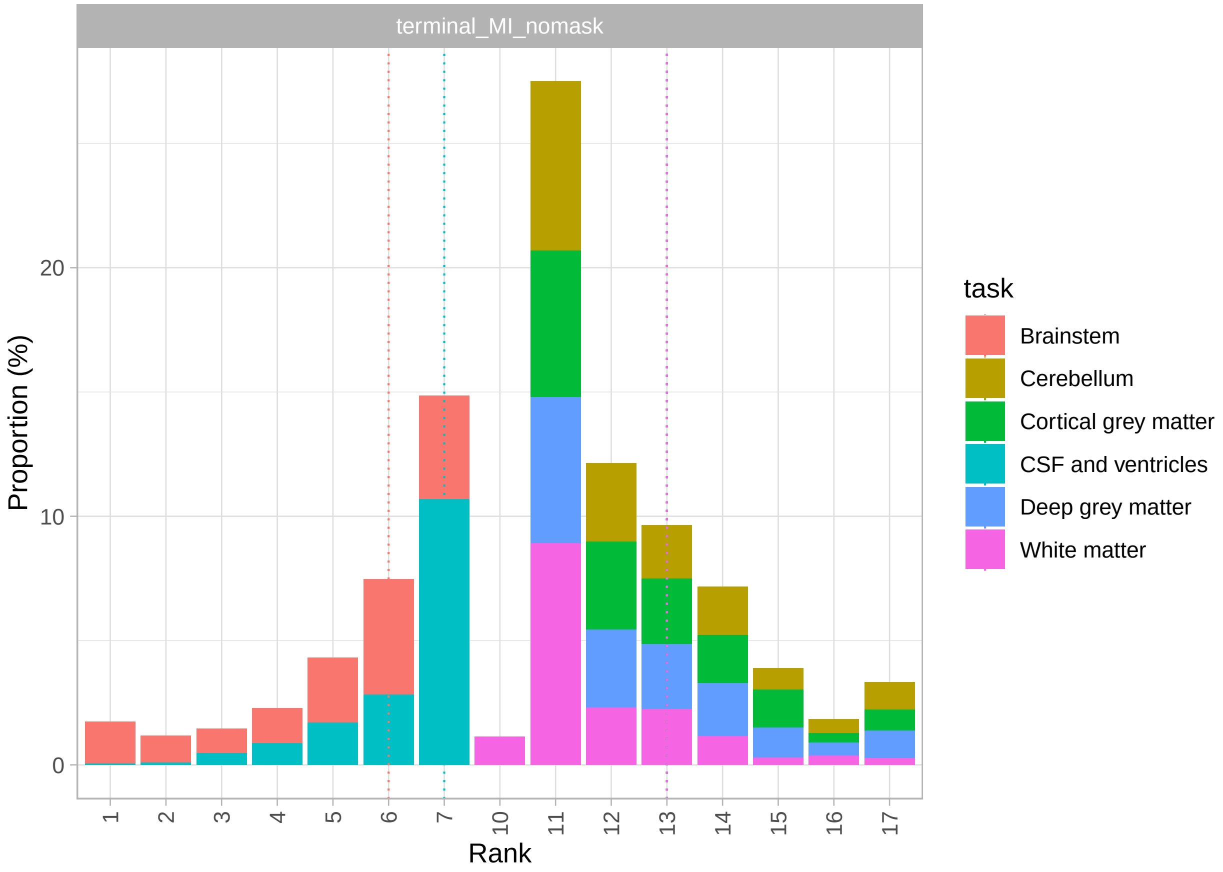
terminal_MI_nomask
Proportion (%)
0
10
20
1
2
3
4
5
6
7
10
11
12
13
14
15
16
17
Rank
task
Brainstem
Cerebellum
Cortical grey matter
CSF and ventricles
Deep grey matter
White matter

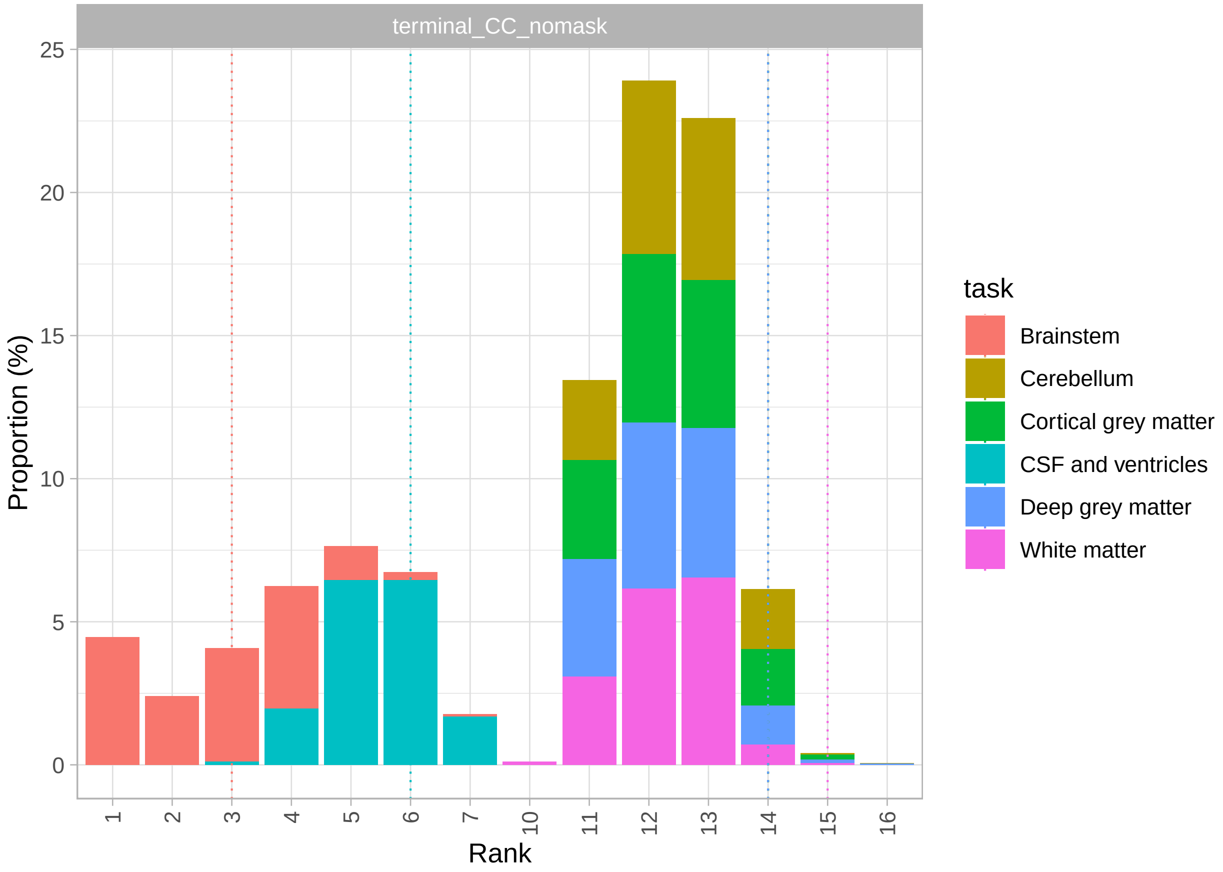
terminal_CC_nomask
Proportion (%)
25
20
15
10
5
0
1
2
3
4
5
6
7
10
11
12
13
14
15
16
Rank
task
Brainstem
Cerebellum
Cortical grey matter
CSF and ventricles
Deep grey matter
White matter

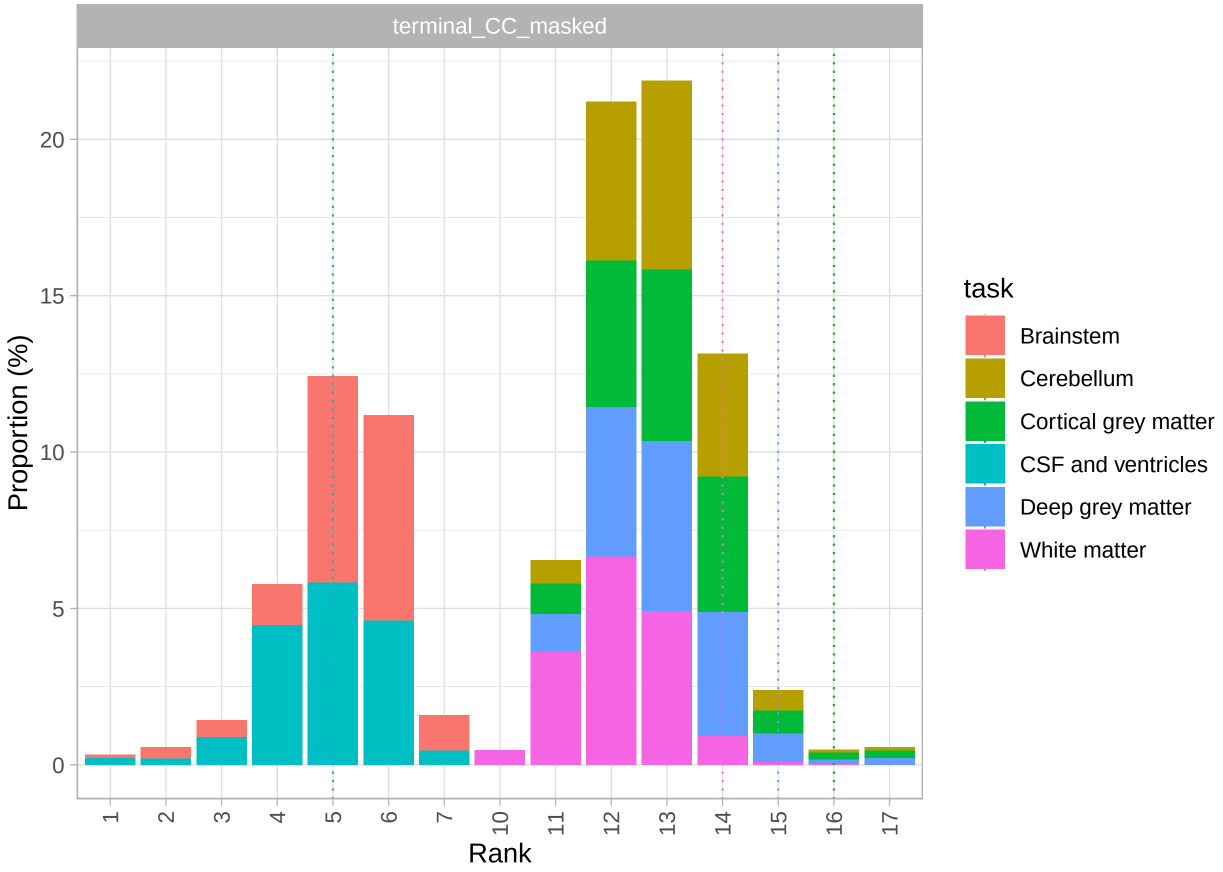
terminal_CC_masked
Proportion (%)
0
5
10
15
20
Rank
1
2
3
4
5
6
7
10
11
12
13
14
15
16
17
task
Brainstem
Cerebellum
Cortical grey matter
CSF and ventricles
Deep grey matter
White matter

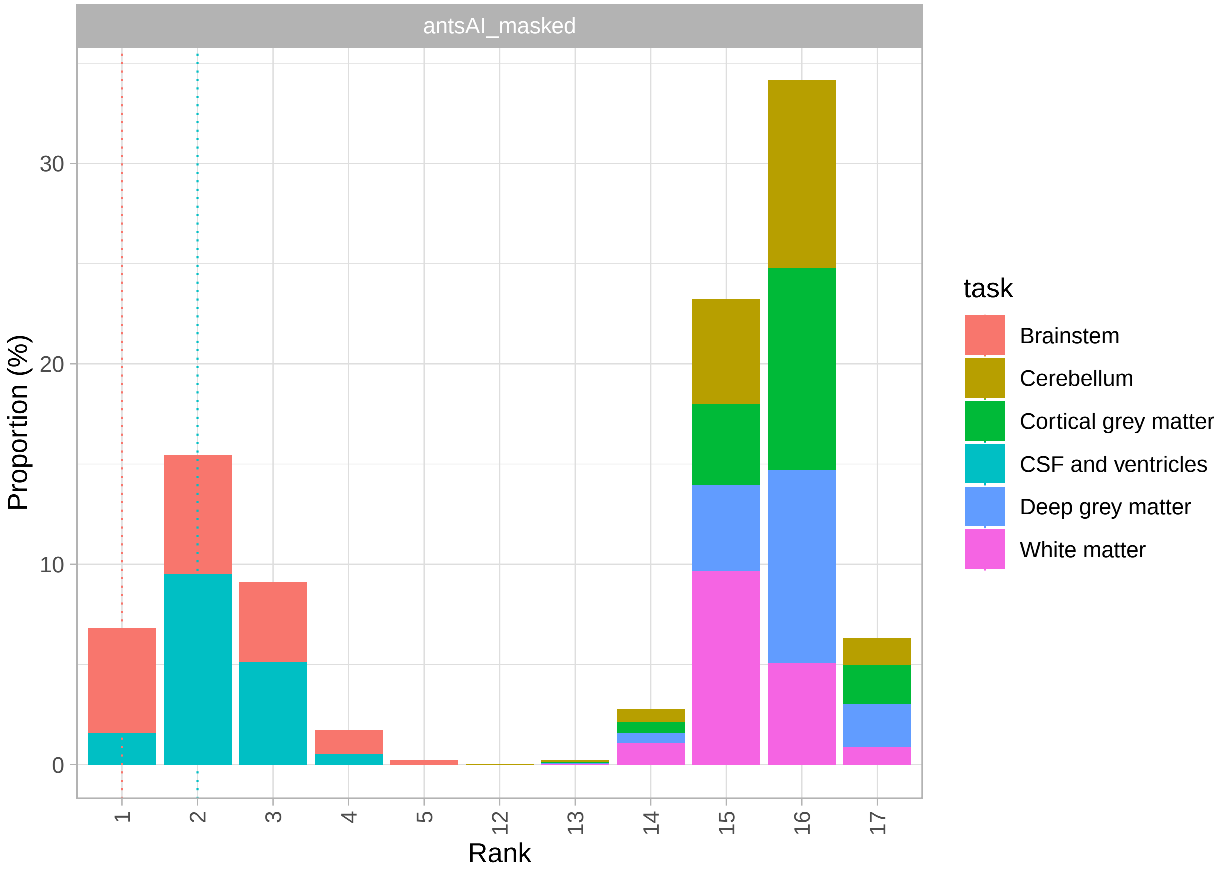
antsAI_masked
Proportion (%)
30
20
10
0
1
2
3
4
5
12
13
14
15
16
17
Rank
task
Brainstem
Cerebellum
Cortical grey matter
CSF and ventricles
Deep grey matter
White matter

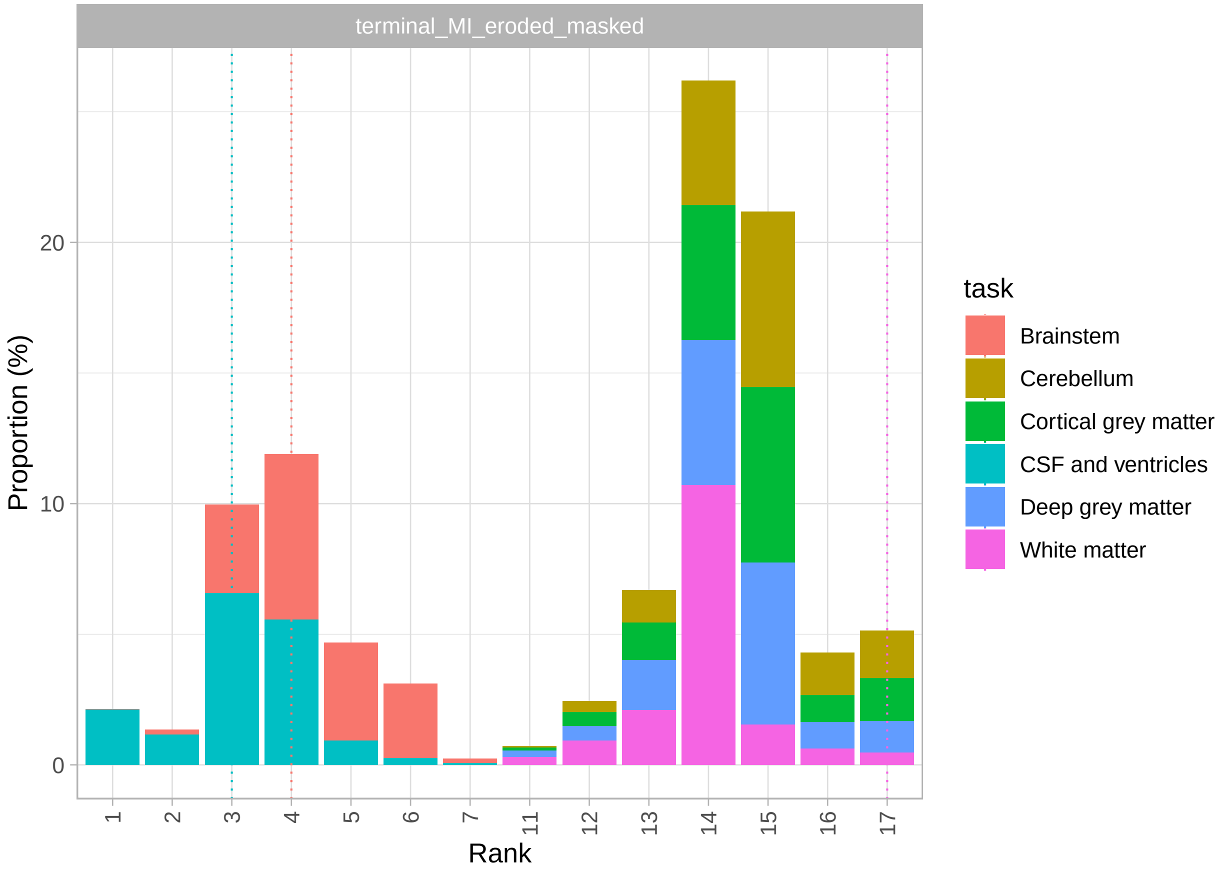
terminal_MI_eroded_masked
Proportion (%)
Rank
0
10
20
1
2
3
4
5
6
7
11
12
13
14
15
16
17
task
Brainstem
Cerebellum
Cortical grey matter
CSF and ventricles
Deep grey matter
White matter

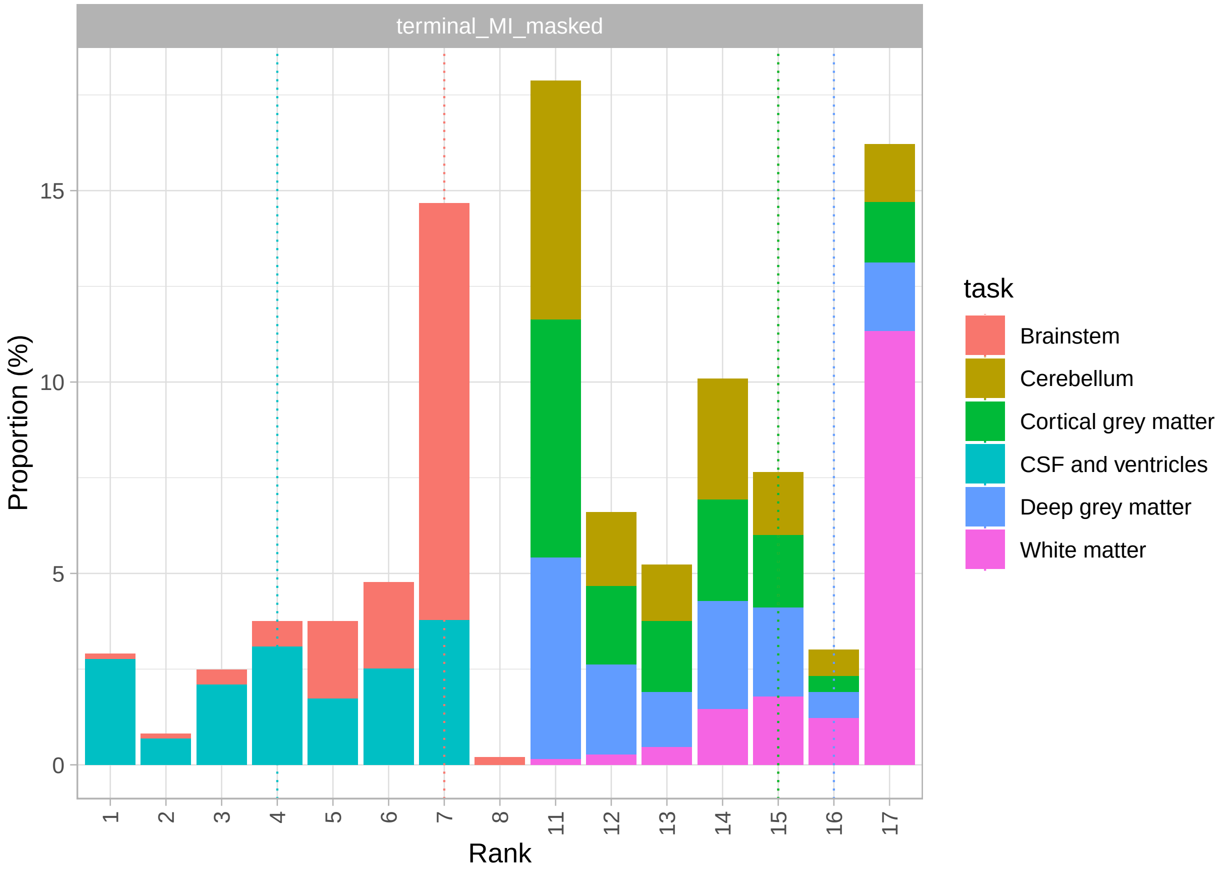
terminal_MI_masked
Proportion (%)
15
10
5
0
1
2
3
4
5
6
7
8
11
12
13
14
15
16
17
Rank
task
Brainstem
Cerebellum
Cortical grey matter
CSF and ventricles
Deep grey matter
White matter

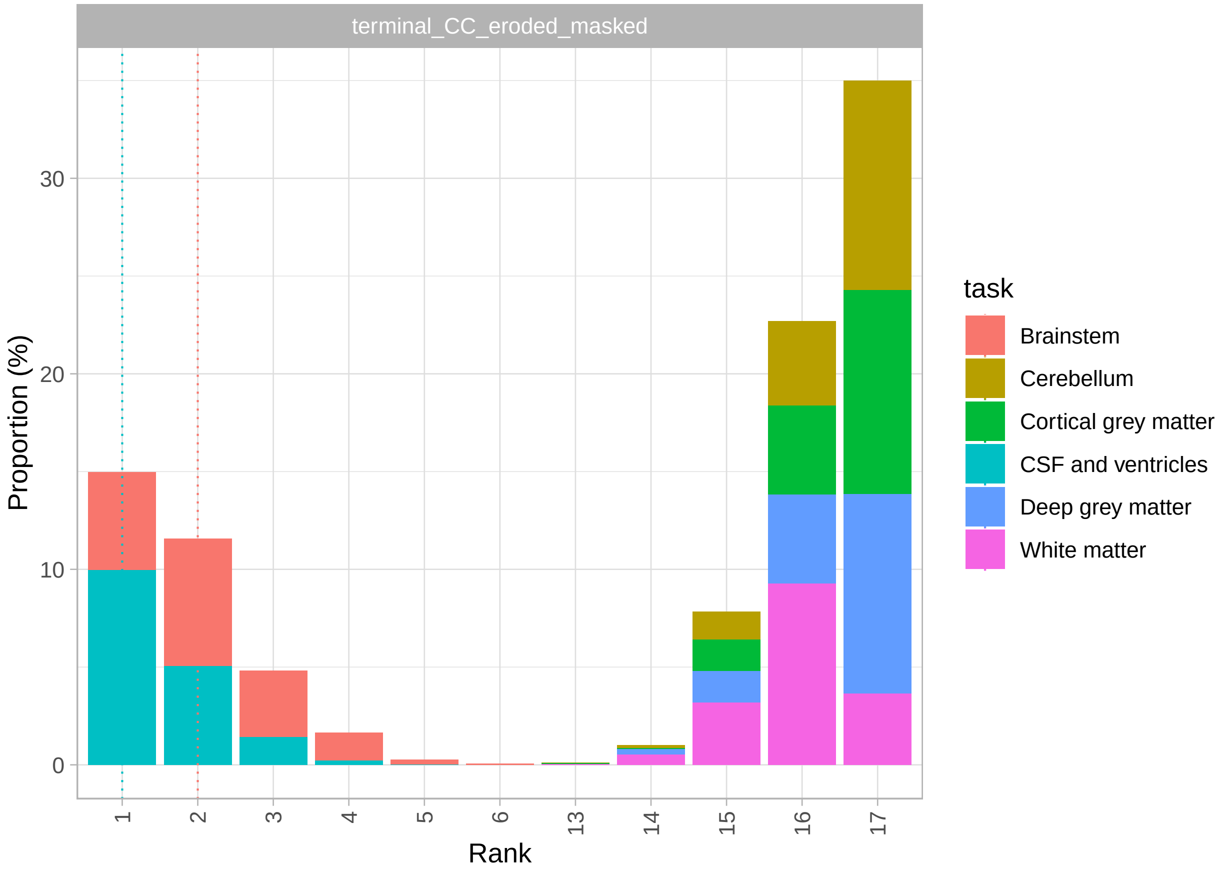

terminal_CC_eroded_masked
Proportion (%)
30
20
10
0
1
2
3
4
5
6
13
14
15
16
17
Rank
task
Brainstem
Cerebellum
Cortical grey matter
CSF and ventricles
Deep grey matter
White matter

4.2 Characterization of tasks

4.2.1 Visualizing bootstrap results

To investigate which tasks separate algorithms well (i.e., lead to a stable ranking), a blob plot is recommended.

Bootstrap results can be shown in a blob plot showing one plot for each task. In this view, the spread of the blobs for each algorithm can be compared across tasks. Deviations from the diagonal indicate deviations from the consensus ranking (over tasks). Specifically, if rank distribution of an algorithm is consistently below the diagonal, the algorithm performed better in this task than on average across tasks, while if the rank distribution of an algorithm is consistently above the diagonal, the algorithm performed worse in this task than on average across tasks. At the bottom of each panel, ranks for each algorithm in the tasks are provided.

Same as in Section 3.1 but now ordered according to consensus.

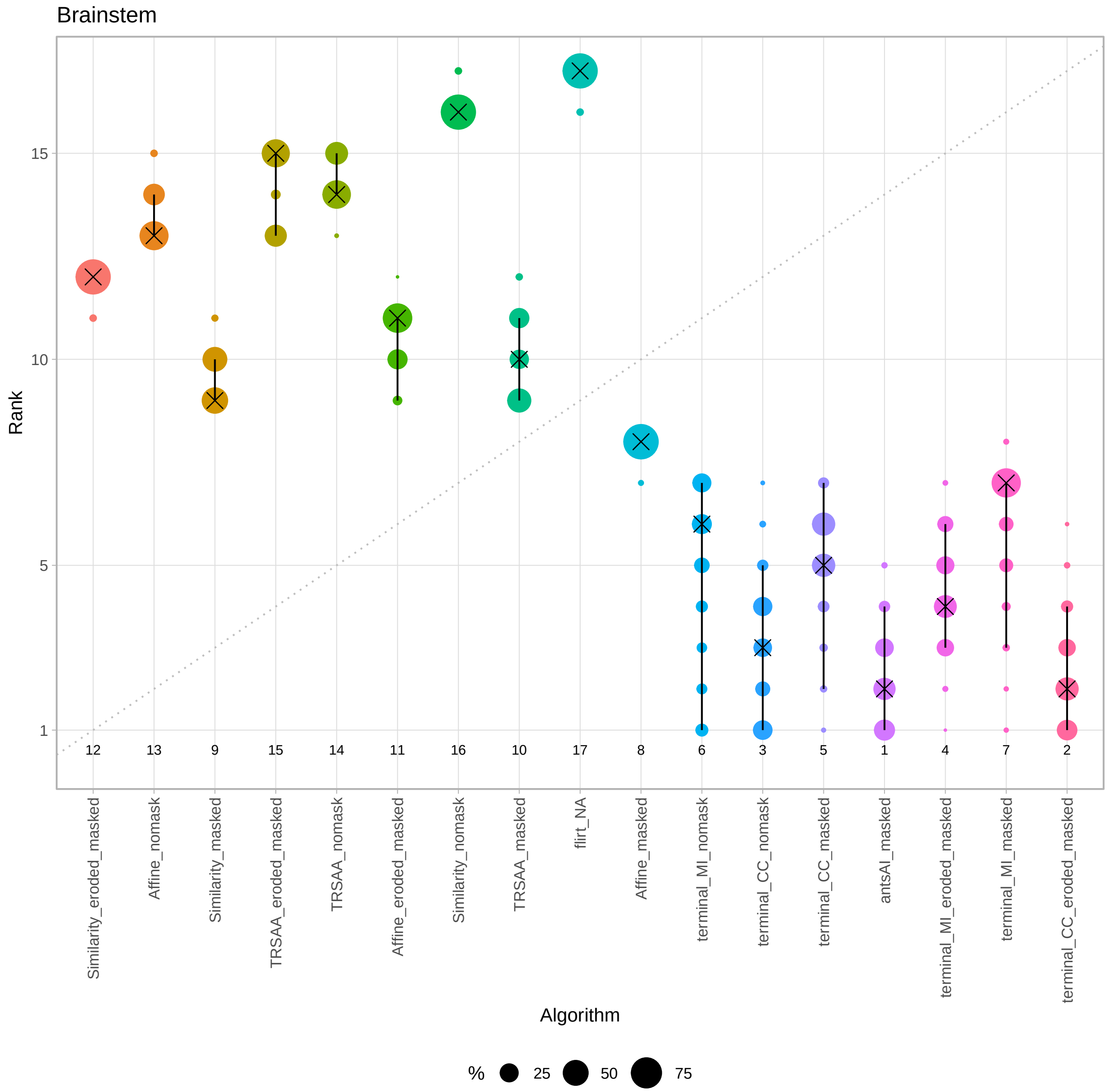

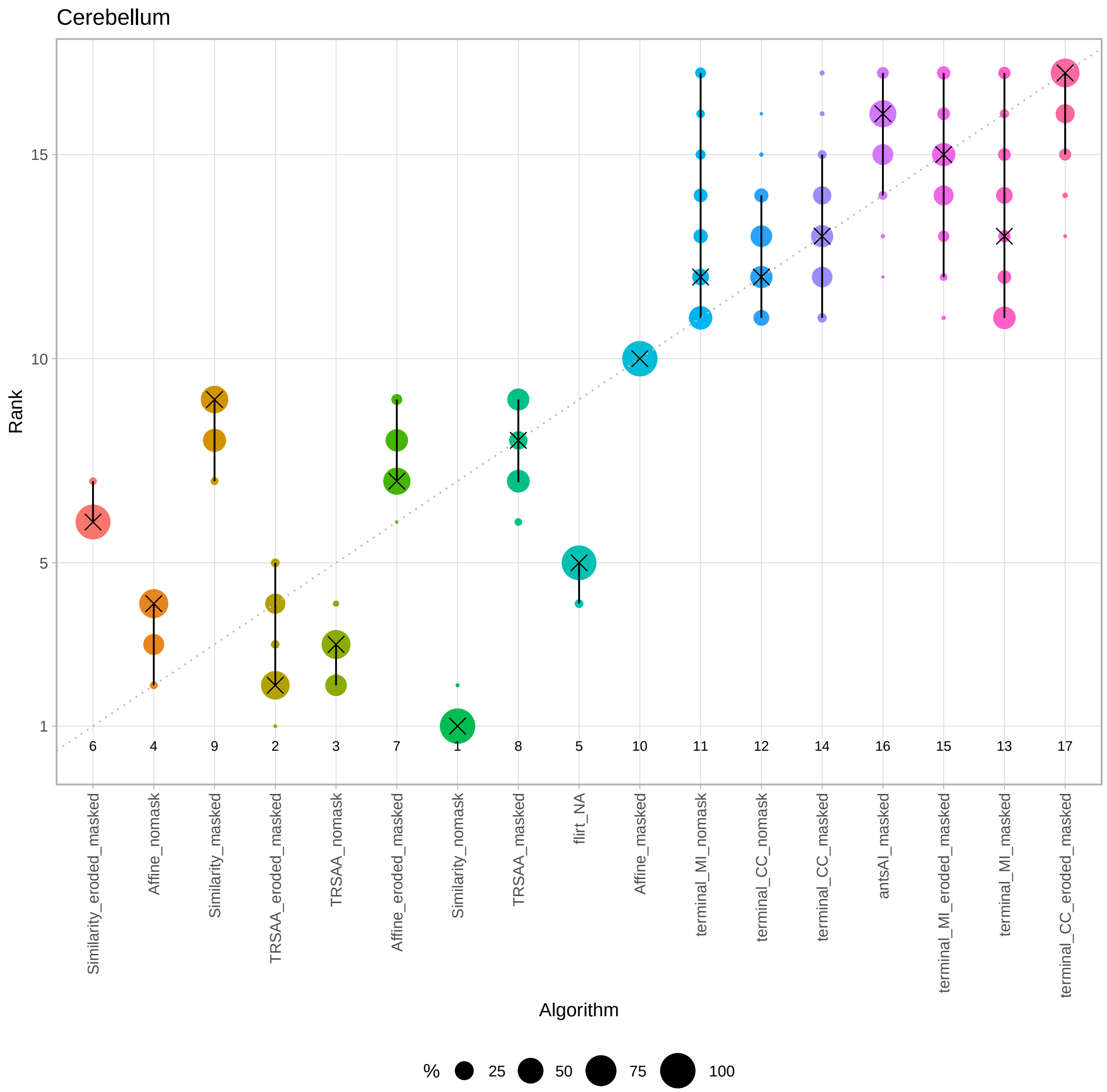

Cerebellum
Rank
15
10
5
1
6
4
9
2
3
7
1
8
5
10
11
12
14
16
15
13
17
Similarity_eroded_masked
Affine_nomask
Similarity_masked
TRSAA_eroded_masked
TRSAA_nomask
Affine_eroded_masked
Similarity_nomask
TRSAA_masked
flirt_NA
Affine_masked
terminal_MI_nomask
terminal_CC_nomask
terminal_CC_masked
antsAI_masked
terminal_MI_eroded_masked
terminal_MI_masked
terminal_CC_eroded_masked
Algorithm
%
25
50
75
100

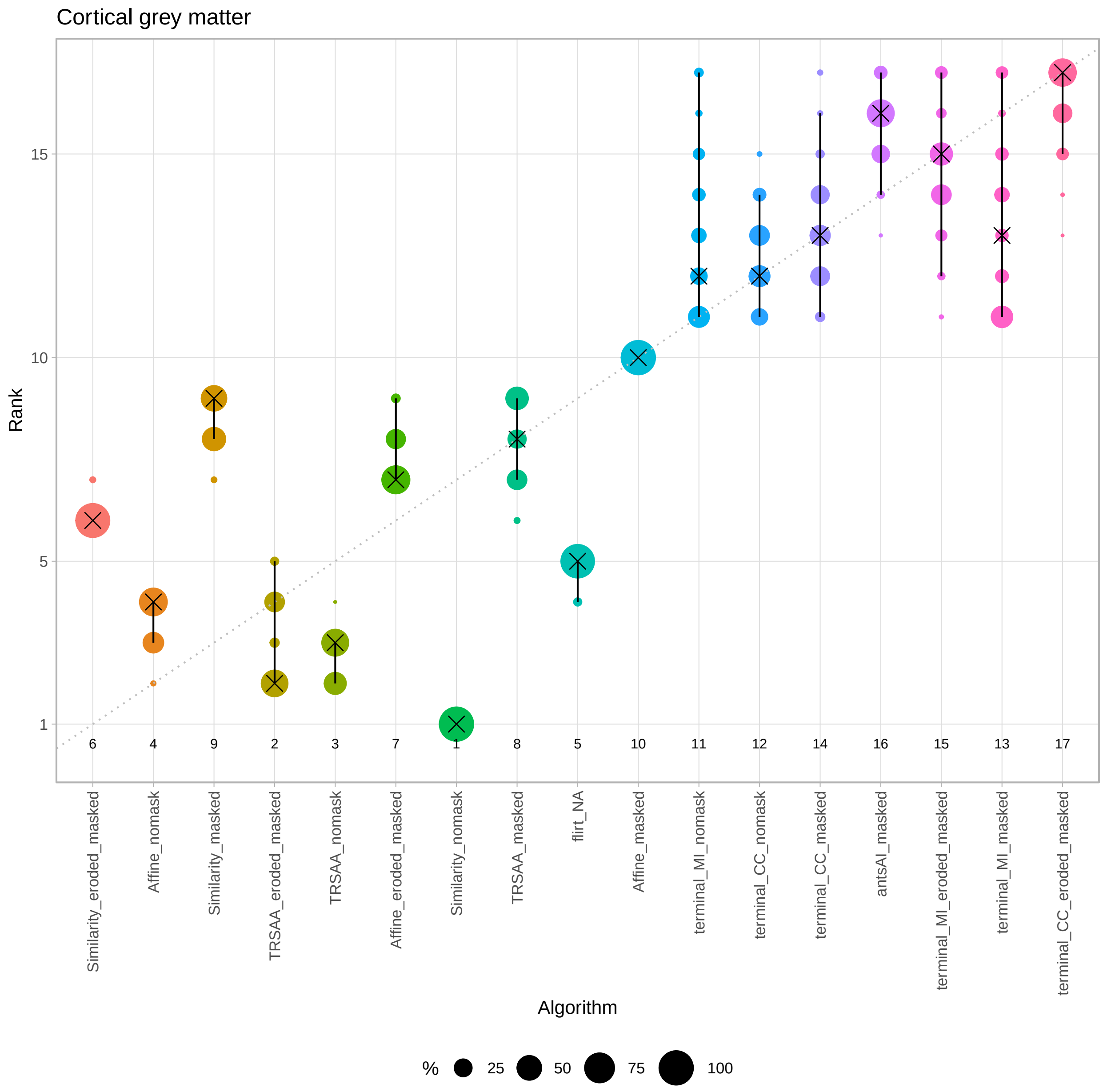
Cortical grey matter
Rank
15
10
5
1
6
4
9
2
3
7
1
8
5
10
11
12
14
16
15
13
17
Similarity_eroded_masked
Affine_nomask
Similarity_masked
TRSAA_eroded_masked
TRSAA_nomask
Affine_eroded_masked
Similarity_nomask
TRSAA_masked
flirt_NA
Affine_masked
terminal_MI_nomask
terminal_CC_nomask
terminal_CC_masked
antsAI_masked
terminal_MI_eroded_masked
terminal_MI_masked
terminal_CC_eroded_masked
Algorithm
%
25
50
75
100

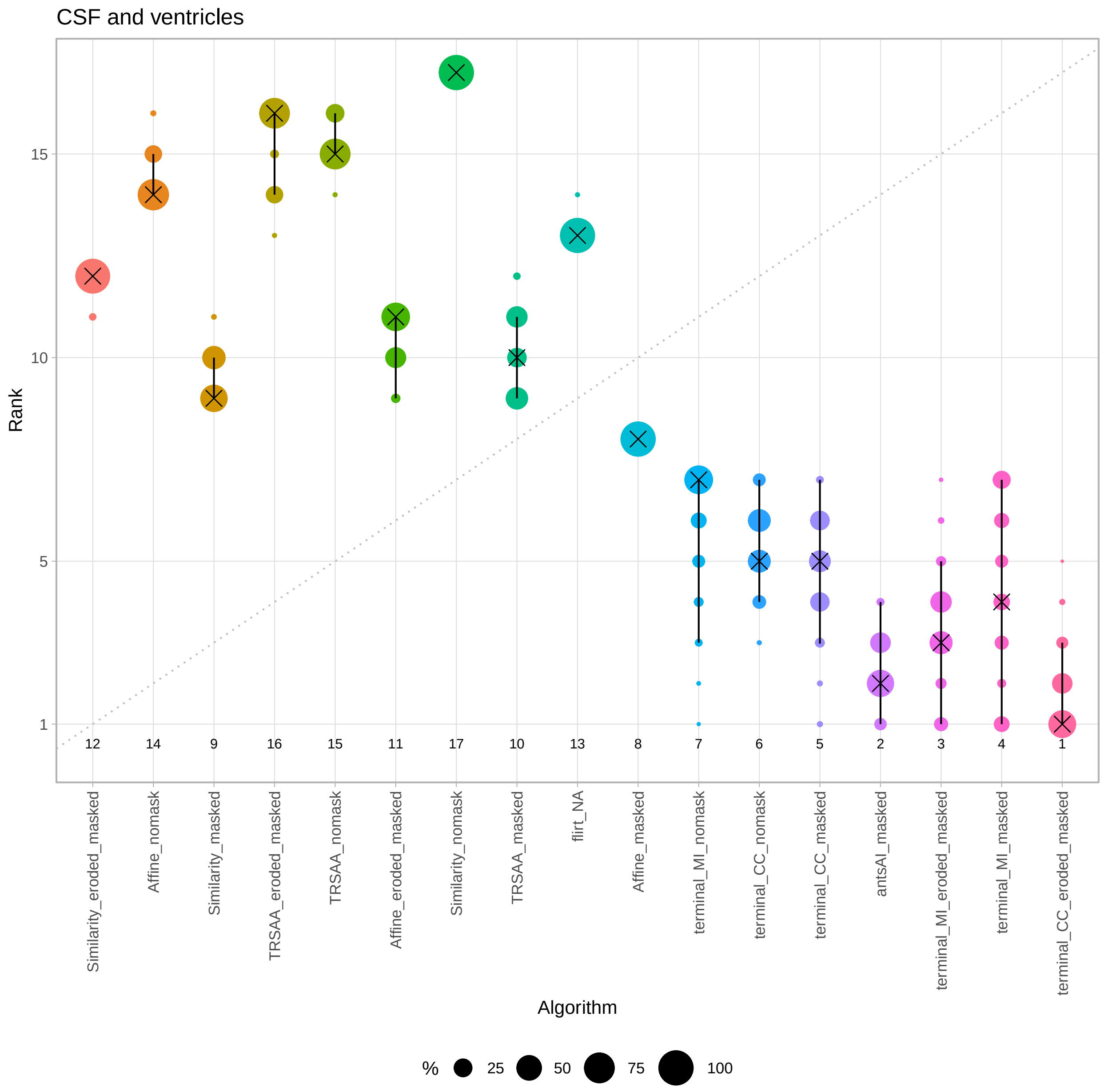

CSF and ventricles
Rank
15
10
5
1
12
14
9
16
15
11
17
10
13
8
7
6
5
2
3
4
1
Similarity_eroded_masked
Affine_nomask
Similarity_masked
TRSAA_eroded_masked
TRSAA_nomask
Affine_eroded_masked
Similarity_nomask
TRSAA_masked
flirt_NA
Affine_masked
terminal_MI_nomask
terminal_CC_nomask
terminal_CC_masked
antsAI_masked
terminal_MI_eroded_masked
terminal_MI_masked
terminal_CC_eroded_masked
Algorithm
%
25
50
75
100

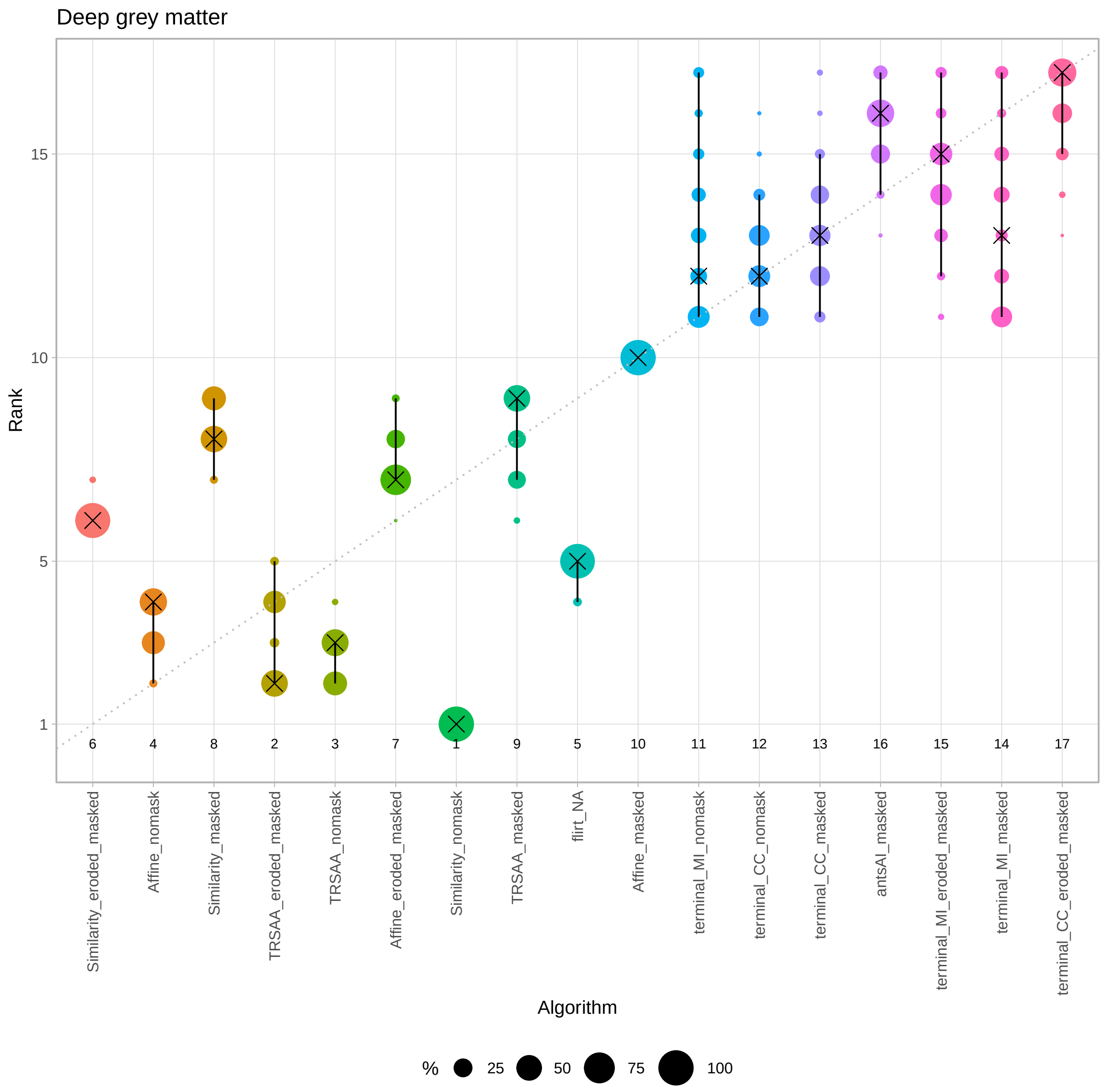
Deep grey matter
Rank
Algorithm
%
25
50
75
100
Similarity_eroded_masked
Affine_nomask
Similarity_masked
TRSAA_eroded_masked
TRSAA_nomask
Affine_eroded_masked
Similarity_nomask
TRSAA_masked
flirt_NA
Affine_masked
terminal_MI_nomask
terminal_CC_nomask
terminal_CC_masked
antsAI_masked
terminal_MI_eroded_masked
terminal_MI_masked
terminal_CC_eroded_masked

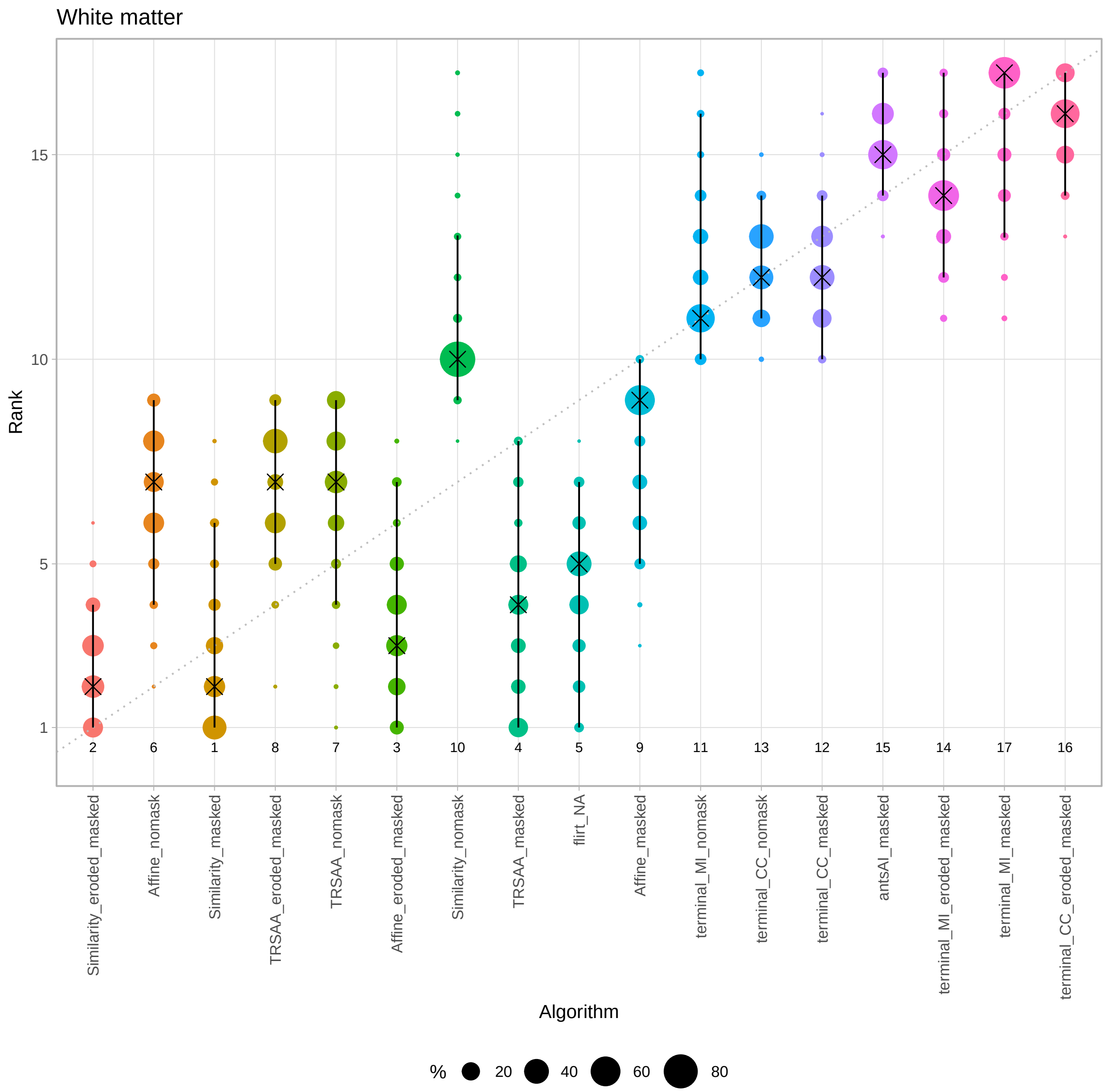

4.2.2 Cluster Analysis

Dendrogram from hierarchical cluster analysis and *network-type graphs* for assessing the similarity of tasks based on challenge rankings.

A dendrogram is a visualization approach based on hierarchical clustering. It depicts clusters according to a chosen distance measure (here: Spearman's footrule) as well as a chosen agglomeration method (here: complete and average agglomeration).

Cluster Dendrogram (complete agglomeration)

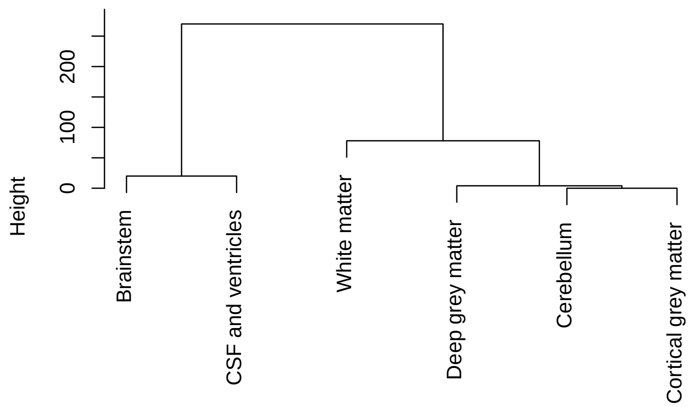

Cluster Dendrogram (average agglomeration)

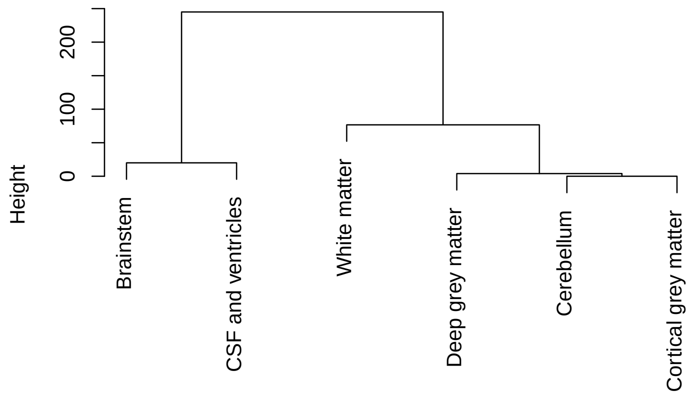

5 References

Wiesenfarth, M., Reinke, A., Landman, B.A., Eisenmann, M., Aguilera Saiz, L., Cardoso, M.J., Maier-Hein, L. and Kopp-Schneider, A. Methods and open-source toolkit for analyzing and visualizing challenge results. *Sci Rep* **11**, 2369 (2021). https://doi.org/10.1038/s41598-021-82017-6

M. J. A. Eugster, T. Hothorn, and F. Leisch, "Exploratory and inferential analysis of benchmark experiments," Institut fuer Statistik, Ludwig-Maximilians-Universitaet Muenchen, Germany, Technical Report 30, 2008. [Online]. Available: http://epub.ub.uni-muenchen.de/4134/.
