# Supplementary material for: Characterization of dynamic patterns of human fetal to neonatal brain asymmetry with deformation-based morphometry
Source: Front Neurosci. 2023 Dec 6;17:1252850. doi: 10.3389/fnins.2023.1252850 (PMC10734644; doi:10.3389/fnins.2023.1252850)
Supplement: Supplementary file 1 [file Data_Sheet_1.ZIP › ChallengeR/Framework B to mid-age template/fetal2template_nonlinear/ranking_Fetal2Template_nonlinear_normal_and_bleached_landmarks.pdf]

Benchmarking report for Fetal to Template registration nonlinear normal and bleached combined

created by challengeR v1.0.4

31 März, 2023

This document presents a systematic report on the benchmark study "Fetal to Template registration nonlinear normal and bleached combined". Input data comprises raw metric values for all algorithms and cases. Generated plots are:

- Visualization of assessment data: Dot- and boxplots, podium plots and ranking heatmaps
- Visualization of ranking stability: Blob plots, violin plots and significance maps, line plots
- Visualization of cross-task insights: Blob plots, stacked frequency plots, dendrograms

Details can be found in Wiesenfarth et al. (2021).

1 Rankings

Algorithms within a task are ranked according to the following ranking scheme:

aggregate using function ("mean") then rank

Ranking for each task:

4th Ventricle : The analysis is based on 20 algorithms and 20 cases. 0 missing cases have been found in the data set.

	value_mean	rank
SyNOnly_CC_4_reg_iter_1_normal_masked	0.7957943	1
SyNOnly_CC_2_reg_iter_1_normal_masked	0.8223177	2
SyNOnly_CC_6_reg_iter_1_normal_masked	0.8353085	3
SyNOnly_CC_2_reg_iter_1_bleached_masked	0.8739682	4
SyNOnly_MI_32_reg_iter_1_normal_masked	0.8874548	5
SyNOnly_CC_4_reg_iter_1_bleached_masked	0.9059319	6
SyNOnly_CC_2_reg_iter_0_normal_masked	0.9584546	7
SyNOnly_CC_4_reg_iter_0_normal_masked	0.9769451	8
SyNOnly_CC_6_reg_iter_1_bleached_masked	0.9891147	9
SyNOnly_MI_32_reg_iter_1_bleached_masked	0.9892344	10
SyNOnly_CC_2_reg_iter_0_bleached_masked	1.0080581	11
SyNOnly_CC_4_reg_iter_0_bleached_masked	1.0106075	12
SyNOnly_CC_6_reg_iter_0_normal_masked	1.0892518	13
SyNOnly_MI_32_reg_iter_0_normal_masked	1.0925781	14
SyNOnly_MI_32_reg_iter_0_bleached_masked	1.0926066	15
SyNOnly_CC_6_reg_iter_0_bleached_masked	1.1347836	16
TVMSQ_bleached_masked	1.3673374	17
TVMSQ_normal_masked	1.4180015	18
TVMSQC_bleached_masked	1.4522238	19
TVMSQC_normal_masked	1.5258220	20

Chiasma : The analysis is based on 20 algorithms and 20 cases. 0 missing cases have been found in the data set.

	value_mean	rank
SyNOnly_CC_4_reg_iter_1_normal_masked	1.196757	1
SyNOnly_CC_2_reg_iter_1_normal_masked	1.207379	2
SyNOnly_CC_2_reg_iter_0_normal_masked	1.226431	3
SyNOnly_CC_6_reg_iter_1_normal_masked	1.260916	4
SyNOnly_CC_4_reg_iter_0_normal_masked	1.326097	5
SyNOnly_CC_6_reg_iter_0_normal_masked	1.420710	6
TVMSQ_normal_masked	1.662932	7
TVMSQ_bleached_masked	1.711911	8
TVMSQC_normal_masked	1.748370	9
SyNOnly_MI_32_reg_iter_1_normal_masked	2.077942	10
SyNOnly_MI_32_reg_iter_0_normal_masked	2.104215	11
SyNOnly_CC_2_reg_iter_1_bleached_masked	2.572732	12
SyNOnly_MI_32_reg_iter_1_bleached_masked	3.252774	13
SyNOnly_CC_4_reg_iter_1_bleached_masked	3.311803	14
SyNOnly_CC_2_reg_iter_0_bleached_masked	3.513439	15
SyNOnly_MI_32_reg_iter_0_bleached_masked	3.531944	16
SyNOnly_CC_6_reg_iter_1_bleached_masked	3.602605	17
SyNOnly_CC_4_reg_iter_0_bleached_masked	3.838755	18
SyNOnly_CC_6_reg_iter_0_bleached_masked	4.069266	19
TVMSQC_bleached_masked	4.968119	20

Culmen : The analysis is based on 20 algorithms and 20 cases. 0 missing cases have been found in the data set.

	value_mean	rank
SyNOnly_CC_2_reg_iter_1_normal_masked	1.115010	1
SyNOnly_CC_2_reg_iter_1_bleached_masked	1.128035	2
SyNOnly_CC_4_reg_iter_1_bleached_masked	1.140895	3
SyNOnly_CC_4_reg_iter_1_normal_masked	1.186403	4
SyNOnly_CC_6_reg_iter_1_normal_masked	1.215328	5
SyNOnly_CC_2_reg_iter_0_normal_masked	1.259294	6
SyNOnly_CC_6_reg_iter_1_bleached_masked	1.292774	7
SyNOnly_CC_2_reg_iter_0_bleached_masked	1.321435	8
SyNOnly_MI_32_reg_iter_1_normal_masked	1.348676	9
SyNOnly_CC_4_reg_iter_0_normal_masked	1.366910	10
SyNOnly_CC_4_reg_iter_0_bleached_masked	1.377592	11
SyNOnly_CC_6_reg_iter_0_normal_masked	1.416646	12
SyNOnly_MI_32_reg_iter_0_normal_masked	1.444919	13
SyNOnly_CC_6_reg_iter_0_bleached_masked	1.489926	14
TVMSQ_bleached_masked	1.572527	15
TVMSQ_normal_masked	1.585932	16
SyNOnly_MI_32_reg_iter_1_bleached_masked	1.704181	17
SyNOnly_MI_32_reg_iter_0_bleached_masked	1.753997	18
TVMSQC_bleached_masked	1.960249	19
TVMSQC_normal_masked	2.112372	20

Hemisphere left caudal point : The analysis is based on 20 algorithms and 20 cases. 0 missing cases have

been found in the data set.

	value_mean	rank
SyNOnly_CC_2_reg_iter_0_bleached_masked	3.131941	1
SyNOnly_CC_4_reg_iter_0_bleached_masked	3.220206	2
SyNOnly_CC_4_reg_iter_1_bleached_masked	3.225684	3
SyNOnly_MI_32_reg_iter_0_bleached_masked	3.245508	4
SyNOnly_CC_6_reg_iter_0_bleached_masked	3.272409	5
SyNOnly_CC_6_reg_iter_1_bleached_masked	3.279972	6
SyNOnly_CC_2_reg_iter_1_bleached_masked	3.306759	7
SyNOnly_MI_32_reg_iter_1_bleached_masked	3.346412	8
SyNOnly_CC_4_reg_iter_1_normal_masked	3.447892	9
SyNOnly_CC_6_reg_iter_1_normal_masked	3.522198	10
SyNOnly_CC_2_reg_iter_1_normal_masked	3.529110	11
SyNOnly_CC_4_reg_iter_0_normal_masked	3.701503	12
SyNOnly_CC_6_reg_iter_0_normal_masked	3.704499	13
TVMSQC_bleached_masked	3.771841	14
SyNOnly_MI_32_reg_iter_1_normal_masked	3.787303	15
TVMSQC_normal_masked	3.806708	16
SyNOnly_CC_2_reg_iter_0_normal_masked	3.854163	17
SyNOnly_MI_32_reg_iter_0_normal_masked	3.860587	18
TVMSQ_bleached_masked	4.959269	19
TVMSQ_normal_masked	4.983527	20

Hemisphere left rostral point : The analysis is based on 20 algorithms and 20 cases. 0 missing cases have been found in the data set.

	value_mean	rank
SyNOnly_CC_4_reg_iter_1_normal_masked	3.464861	1
SyNOnly_CC_2_reg_iter_1_normal_masked	3.465381	2
SyNOnly_CC_6_reg_iter_1_normal_masked	3.490326	3
SyNOnly_CC_2_reg_iter_1_bleached_masked	3.629399	4
SyNOnly_CC_4_reg_iter_1_bleached_masked	3.738116	5
SyNOnly_CC_6_reg_iter_1_bleached_masked	3.746756	6
SyNOnly_CC_2_reg_iter_0_bleached_masked	3.912345	7
SyNOnly_CC_2_reg_iter_0_normal_masked	3.935691	8
SyNOnly_CC_4_reg_iter_0_bleached_masked	3.967075	9
SyNOnly_MI_32_reg_iter_1_normal_masked	3.975699	10
SyNOnly_CC_6_reg_iter_0_bleached_masked	4.003004	11
SyNOnly_CC_6_reg_iter_0_normal_masked	4.035681	12
SyNOnly_MI_32_reg_iter_1_bleached_masked	4.059370	13
SyNOnly_CC_4_reg_iter_0_normal_masked	4.156581	14
TVMSQC_normal_masked	4.184999	15
TVMSQC_bleached_masked	4.259301	16
SyNOnly_MI_32_reg_iter_0_bleached_masked	4.266297	17
SyNOnly_MI_32_reg_iter_0_normal_masked	4.528092	18
TVMSQ_bleached_masked	5.027786	19
TVMSQ_normal_masked	5.061474	20

Hemisphere right caudal point : The analysis is based on 20 algorithms and 20 cases. 0 missing cases have been found in the data set.

	value_mean	rank
SyNOnly_MI_32_reg_iter_0_bleached_masked	2.247982	1
SyNOnly_CC_2_reg_iter_0_bleached_masked	2.334993	2
SyNOnly_MI_32_reg_iter_1_bleached_masked	2.337691	3
SyNOnly_CC_2_reg_iter_1_bleached_masked	2.347456	4
SyNOnly_CC_4_reg_iter_0_bleached_masked	2.407023	5
SyNOnly_CC_6_reg_iter_0_bleached_masked	2.416593	6
SyNOnly_CC_4_reg_iter_1_bleached_masked	2.427720	7
SyNOnly_CC_4_reg_iter_1_normal_masked	2.438029	8
SyNOnly_CC_6_reg_iter_1_bleached_masked	2.438870	9
SyNOnly_CC_6_reg_iter_1_normal_masked	2.534483	10
SyNOnly_CC_2_reg_iter_1_normal_masked	2.598384	11
SyNOnly_MI_32_reg_iter_1_normal_masked	2.660077	12
SyNOnly_CC_6_reg_iter_0_normal_masked	2.884739	13
SyNOnly_CC_4_reg_iter_0_normal_masked	2.930442	14
SyNOnly_CC_2_reg_iter_0_normal_masked	3.170447	15
TVMSQC_bleached_masked	3.258923	16
SyNOnly_MI_32_reg_iter_0_normal_masked	3.321334	17
TVMSQC_normal_masked	3.421009	18
TVMSQ_bleached_masked	4.593679	19
TVMSQ_normal_masked	4.618701	20

Hemisphere right rostral point : The analysis is based on 20 algorithms and 20 cases. 0 missing cases have been found in the data set.

	value_mean	rank
SyNOnly_CC_4_reg_iter_1_normal_masked	4.051724	1
SyNOnly_CC_2_reg_iter_1_normal_masked	4.090282	2
SyNOnly_CC_6_reg_iter_1_normal_masked	4.094405	3
SyNOnly_CC_4_reg_iter_1_bleached_masked	4.288901	4
SyNOnly_CC_6_reg_iter_1_bleached_masked	4.288965	5
SyNOnly_CC_2_reg_iter_1_bleached_masked	4.289423	6
SyNOnly_CC_2_reg_iter_0_bleached_masked	4.424610	7
SyNOnly_MI_32_reg_iter_1_bleached_masked	4.445352	8
SyNOnly_CC_2_reg_iter_0_normal_masked	4.458859	9
SyNOnly_MI_32_reg_iter_1_normal_masked	4.459329	10
SyNOnly_CC_6_reg_iter_0_normal_masked	4.468517	11
SyNOnly_CC_6_reg_iter_0_bleached_masked	4.475288	12
SyNOnly_CC_4_reg_iter_0_bleached_masked	4.482522	13
TVMSQC_bleached_masked	4.529782	14
TVMSQC_normal_masked	4.576712	15
SyNOnly_MI_32_reg_iter_0_bleached_masked	4.601126	16
SyNOnly_CC_4_reg_iter_0_normal_masked	4.741102	17
SyNOnly_MI_32_reg_iter_0_normal_masked	4.826126	18
TVMSQ_bleached_masked	5.150019	19
TVMSQ_normal_masked	5.183502	20

Infracollicular Sulcus : The analysis is based on 20 algorithms and 20 cases. 0 missing cases have been found in the data set.

	value_mean	rank
SyNOnly_CC_2_reg_iter_1_normal_masked	0.7487551	1
SyNOnly_CC_2_reg_iter_1_bleached_masked	0.7543839	2
SyNOnly_CC_4_reg_iter_1_bleached_masked	0.8612488	3
SyNOnly_CC_4_reg_iter_1_normal_masked	0.8651878	4
SyNOnly_CC_2_reg_iter_0_normal_masked	0.9414249	5
SyNOnly_CC_2_reg_iter_0_bleached_masked	0.9489682	6
TVMSQ_bleached_masked	0.9630013	7
TVMSQ_normal_masked	0.9735582	8
SyNOnly_CC_6_reg_iter_1_normal_masked	0.9737970	9
SyNOnly_CC_6_reg_iter_1_bleached_masked	1.0103234	10
SyNOnly_CC_4_reg_iter_0_normal_masked	1.1680197	11
SyNOnly_MI_32_reg_iter_1_normal_masked	1.1690672	12
SyNOnly_CC_4_reg_iter_0_bleached_masked	1.2271247	13
SyNOnly_CC_6_reg_iter_0_normal_masked	1.2375169	14
SyNOnly_MI_32_reg_iter_0_normal_masked	1.2662968	15
SyNOnly_MI_32_reg_iter_1_bleached_masked	1.2730391	16
SyNOnly_CC_6_reg_iter_0_bleached_masked	1.3205901	17
SyNOnly_MI_32_reg_iter_0_bleached_masked	1.4232598	18
TVMSQC_bleached_masked	1.4502868	19
TVMSQC_normal_masked	1.9390991	20

Lateral ventricle left caudal point : The analysis is based on 20 algorithms and 20 cases. 0 missing cases have been found in the data set.

	value_mean	rank
SyNOnly_CC_2_reg_iter_1_bleached_masked	2.302002	1
SyNOnly_MI_32_reg_iter_1_bleached_masked	2.416980	2
SyNOnly_CC_4_reg_iter_1_bleached_masked	2.484091	3
SyNOnly_CC_6_reg_iter_1_bleached_masked	2.513174	4
SyNOnly_CC_2_reg_iter_0_bleached_masked	2.752619	5
SyNOnly_CC_4_reg_iter_0_bleached_masked	2.860892	6
SyNOnly_MI_32_reg_iter_0_bleached_masked	2.876394	7
SyNOnly_CC_6_reg_iter_0_bleached_masked	2.934678	8
SyNOnly_CC_2_reg_iter_1_normal_masked	2.964345	9
SyNOnly_CC_4_reg_iter_1_normal_masked	3.058119	10
SyNOnly_CC_2_reg_iter_0_normal_masked	3.246642	11
SyNOnly_CC_4_reg_iter_0_normal_masked	3.248608	12
SyNOnly_CC_6_reg_iter_1_normal_masked	3.248785	13
SyNOnly_MI_32_reg_iter_1_normal_masked	3.289722	14
SyNOnly_CC_6_reg_iter_0_normal_masked	3.321289	15
SyNOnly_MI_32_reg_iter_0_normal_masked	3.393105	16
TVMSQC_bleached_masked	3.564501	17
TVMSQC_normal_masked	4.032912	18
TVMSQ_bleached_masked	4.045538	19
TVMSQ_normal_masked	4.084950	20

Lateral ventricle left rostral point : The analysis is based on 20 algorithms and 20 cases. 0 missing cases have been found in the data set.

	value_mean	rank
SyNOnly_CC_6_reg_iter_1_normal_masked	1.327226	1
SyNOnly_CC_4_reg_iter_1_normal_masked	1.412075	2
SyNOnly_CC_2_reg_iter_1_normal_masked	1.548247	3
SyNOnly_CC_2_reg_iter_1_bleached_masked	1.615754	4
SyNOnly_CC_2_reg_iter_0_normal_masked	1.665444	5
SyNOnly_MI_32_reg_iter_1_normal_masked	1.707933	6
SyNOnly_CC_2_reg_iter_0_bleached_masked	1.728737	7
SyNOnly_CC_6_reg_iter_0_normal_masked	1.732438	8
SyNOnly_CC_4_reg_iter_0_normal_masked	1.763182	9
SyNOnly_CC_4_reg_iter_1_bleached_masked	1.786299	10
SyNOnly_CC_6_reg_iter_1_bleached_masked	1.787384	11
SyNOnly_CC_6_reg_iter_0_bleached_masked	1.921540	12
SyNOnly_CC_4_reg_iter_0_bleached_masked	1.966868	13
TVMSQ_bleached_masked	1.980951	14
TVMSQ_normal_masked	2.000422	15
SyNOnly_MI_32_reg_iter_0_normal_masked	2.022290	16
TVMSQC_bleached_masked	2.035755	17
SyNOnly_MI_32_reg_iter_0_bleached_masked	2.066161	18
SyNOnly_MI_32_reg_iter_1_bleached_masked	2.071106	19
TVMSQC_normal_masked	2.333909	20

Lateral ventricle right caudal point : The analysis is based on 20 algorithms and 20 cases. 0 missing cases have been found in the data set.

	value_mean	rank
SyNOnly_CC_6_reg_iter_1_normal_masked	2.696731	1
SyNOnly_CC_4_reg_iter_1_normal_masked	2.847044	2
SyNOnly_CC_2_reg_iter_1_bleached_masked	3.040732	3
SyNOnly_CC_2_reg_iter_1_normal_masked	3.179328	4
SyNOnly_CC_4_reg_iter_1_bleached_masked	3.248618	5
SyNOnly_CC_6_reg_iter_1_bleached_masked	3.419967	6
SyNOnly_MI_32_reg_iter_1_bleached_masked	3.480880	7
SyNOnly_CC_2_reg_iter_0_bleached_masked	3.519446	8
TVMSQC_normal_masked	3.598895	9
SyNOnly_CC_4_reg_iter_0_bleached_masked	3.643275	10
SyNOnly_MI_32_reg_iter_0_bleached_masked	3.667721	11
SyNOnly_CC_6_reg_iter_0_bleached_masked	3.689135	12
SyNOnly_CC_6_reg_iter_0_normal_masked	3.690688	13
SyNOnly_MI_32_reg_iter_1_normal_masked	3.705119	14
SyNOnly_CC_4_reg_iter_0_normal_masked	3.794002	15
SyNOnly_CC_2_reg_iter_0_normal_masked	4.019427	16
SyNOnly_MI_32_reg_iter_0_normal_masked	4.047228	17
TVMSQC_bleached_masked	4.269019	18
TVMSQ_bleached_masked	4.671093	19
TVMSQ_normal_masked	4.711181	20

Lateral ventricle right rostral point : The analysis is based on 20 algorithms and 20 cases. 0 missing cases have been found in the data set.

	value_mean	rank
SyNOnly_CC_4_reg_iter_1_normal_masked	1.470067	1
SyNOnly_CC_4_reg_iter_0_normal_masked	1.513319	2
SyNOnly_CC_6_reg_iter_1_normal_masked	1.527096	3
SyNOnly_CC_2_reg_iter_0_normal_masked	1.533887	4
SyNOnly_CC_2_reg_iter_1_normal_masked	1.549441	5
SyNOnly_CC_6_reg_iter_0_normal_masked	1.566477	6
SyNOnly_CC_6_reg_iter_0_bleached_masked	1.614470	7
SyNOnly_CC_6_reg_iter_1_bleached_masked	1.637388	8
SyNOnly_CC_2_reg_iter_1_bleached_masked	1.665046	9
SyNOnly_CC_2_reg_iter_0_bleached_masked	1.678942	10
SyNOnly_CC_4_reg_iter_0_bleached_masked	1.682500	11
SyNOnly_MI_32_reg_iter_1_normal_masked	1.688488	12
SyNOnly_CC_4_reg_iter_1_bleached_masked	1.714870	13
SyNOnly_MI_32_reg_iter_0_normal_masked	1.762403	14
SyNOnly_MI_32_reg_iter_0_bleached_masked	1.775603	15
TVMSQ_bleached_masked	1.835375	16
TVMSQ_normal_masked	1.836231	17
SyNOnly_MI_32_reg_iter_1_bleached_masked	1.861561	18
TVMSQC_bleached_masked	2.239570	19
TVMSQC_normal_masked	2.781268	20

PMJ : The analysis is based on 20 algorithms and 20 cases. 0 missing cases have been found in the data set.

	value_mean	rank
SyNOnly_CC_4_reg_iter_1_bleached_masked	0.6191343	1
SyNOnly_CC_2_reg_iter_1_bleached_masked	0.6236349	2
SyNOnly_CC_6_reg_iter_1_bleached_masked	0.6270491	3
SyNOnly_MI_32_reg_iter_1_bleached_masked	0.6551282	4
SyNOnly_CC_2_reg_iter_0_bleached_masked	0.6846222	5
SyNOnly_CC_4_reg_iter_0_bleached_masked	0.6907422	6
SyNOnly_CC_2_reg_iter_1_normal_masked	0.7038924	7
SyNOnly_MI_32_reg_iter_0_bleached_masked	0.7263729	8
SyNOnly_MI_32_reg_iter_1_normal_masked	0.7411542	9
SyNOnly_CC_6_reg_iter_0_bleached_masked	0.7508412	10
SyNOnly_CC_4_reg_iter_0_normal_masked	0.7780549	11
SyNOnly_CC_2_reg_iter_0_normal_masked	0.7799124	12
SyNOnly_MI_32_reg_iter_0_normal_masked	0.7930260	13
SyNOnly_CC_4_reg_iter_1_normal_masked	0.8010265	14
SyNOnly_CC_6_reg_iter_0_normal_masked	0.8188654	15
SyNOnly_CC_6_reg_iter_1_normal_masked	0.8443894	16
TVMSQC_bleached_masked	0.8896446	17
TVMSQ_bleached_masked	0.9035157	18
TVMSQ_normal_masked	0.9335764	19
TVMSQC_normal_masked	1.0938578	20

Posterior Commissure : The analysis is based on 20 algorithms and 20 cases. 0 missing cases have been found in the data set.

	value_mean	rank
SyNOnly_CC_6_reg_iter_1_bleached_masked	0.9433645	1
SyNOnly_CC_4_reg_iter_0_bleached_masked	0.9459734	2
SyNOnly_MI_32_reg_iter_0_normal_masked	0.9697749	3
SyNOnly_CC_6_reg_iter_0_normal_masked	0.9910999	4
SyNOnly_CC_4_reg_iter_0_normal_masked	0.9953122	5
SyNOnly_CC_6_reg_iter_0_bleached_masked	1.0013158	6
SyNOnly_CC_4_reg_iter_1_bleached_masked	1.0066580	7
SyNOnly_CC_2_reg_iter_0_bleached_masked	1.0124059	8
SyNOnly_MI_32_reg_iter_1_normal_masked	1.0223888	9
SyNOnly_CC_6_reg_iter_1_normal_masked	1.0266386	10
SyNOnly_CC_2_reg_iter_1_bleached_masked	1.0369388	11
SyNOnly_CC_4_reg_iter_1_normal_masked	1.0632894	12
SyNOnly_CC_2_reg_iter_0_normal_masked	1.0636793	13
SyNOnly_CC_2_reg_iter_1_normal_masked	1.0716374	14
TVMSQC_bleached_masked	1.0718714	15
SyNOnly_MI_32_reg_iter_0_bleached_masked	1.0779783	16
SyNOnly_MI_32_reg_iter_1_bleached_masked	1.1107028	17
TVMSQC_normal_masked	1.3931805	18
TVMSQ_bleached_masked	1.8260123	19
TVMSQ_normal_masked	1.8647205	20

Temporal lobe left rostral point : The analysis is based on 20 algorithms and 20 cases. 0 missing cases have been found in the data set.

	value_mean	rank
SyNOnly_CC_2_reg_iter_1_normal_masked	3.856193	1
SyNOnly_CC_6_reg_iter_1_normal_masked	4.004571	2
SyNOnly_CC_4_reg_iter_1_normal_masked	4.069971	3
SyNOnly_MI_32_reg_iter_1_normal_masked	4.153656	4
SyNOnly_CC_6_reg_iter_1_bleached_masked	4.155701	5
SyNOnly_MI_32_reg_iter_1_bleached_masked	4.170792	6
SyNOnly_CC_4_reg_iter_1_bleached_masked	4.235550	7
SyNOnly_MI_32_reg_iter_0_normal_masked	4.258428	8
SyNOnly_MI_32_reg_iter_0_bleached_masked	4.282950	9
SyNOnly_CC_2_reg_iter_0_normal_masked	4.349359	10
SyNOnly_CC_2_reg_iter_1_bleached_masked	4.392449	11
SyNOnly_CC_6_reg_iter_0_normal_masked	4.499253	12
SyNOnly_CC_6_reg_iter_0_bleached_masked	4.519164	13
SyNOnly_CC_4_reg_iter_0_normal_masked	4.521824	14
SyNOnly_CC_4_reg_iter_0_bleached_masked	4.604104	15
TVMSQC_bleached_masked	4.783301	16
SyNOnly_CC_2_reg_iter_0_bleached_masked	4.831120	17
TVMSQ_bleached_masked	4.987878	18
TVMSQ_normal_masked	5.012416	19
TVMSQC_normal_masked	5.048956	20

Temporal lobe ventricle right rostral point : The analysis is based on 20 algorithms and 20 cases. 0 missing cases have been found in the data set.

	value_mean	rank
SyNOnly_CC_2_reg_iter_1_normal_masked	4.479125	1
SyNOnly_CC_4_reg_iter_1_normal_masked	4.610618	2
SyNOnly_CC_6_reg_iter_1_normal_masked	4.611462	3
SyNOnly_CC_6_reg_iter_1_bleached_masked	4.634345	4
SyNOnly_CC_4_reg_iter_1_bleached_masked	4.655664	5
SyNOnly_CC_2_reg_iter_1_bleached_masked	4.744910	6
SyNOnly_MI_32_reg_iter_1_bleached_masked	4.805202	7
SyNOnly_CC_2_reg_iter_0_normal_masked	4.959021	8
SyNOnly_CC_6_reg_iter_0_bleached_masked	5.014281	9
SyNOnly_CC_6_reg_iter_0_normal_masked	5.030757	10
SyNOnly_CC_4_reg_iter_0_normal_masked	5.050531	11
SyNOnly_CC_4_reg_iter_0_bleached_masked	5.052545	12
SyNOnly_MI_32_reg_iter_0_bleached_masked	5.062948	13
SyNOnly_MI_32_reg_iter_0_normal_masked	5.149910	14
SyNOnly_CC_2_reg_iter_0_bleached_masked	5.162147	15
SyNOnly_MI_32_reg_iter_1_normal_masked	5.208728	16
TVMSQ_bleached_masked	5.595119	17
TVMSQ_normal_masked	5.604591	18
TVMSQC_bleached_masked	5.810255	19
TVMSQC_normal_masked	5.964472	20

Consensus ranking across tasks according to chosen method “euclidean”:

	value	rank
SyNOnly_CC_4_reg_iter_1_normal_masked	4.688	1
SyNOnly_CC_2_reg_iter_1_normal_masked	4.750	2
SyNOnly_CC_2_reg_iter_1_bleached_masked	5.500	3
SyNOnly_CC_4_reg_iter_1_bleached_masked	6.000	4
SyNOnly_CC_6_reg_iter_1_normal_masked	6.000	4
SyNOnly_CC_6_reg_iter_1_bleached_masked	6.938	6
SyNOnly_CC_2_reg_iter_0_bleached_masked	8.250	7
SyNOnly_CC_2_reg_iter_0_normal_masked	9.312	8
SyNOnly_CC_4_reg_iter_0_bleached_masked	9.875	9
SyNOnly_MI_32_reg_iter_1_normal_masked	10.438	10
SyNOnly_MI_32_reg_iter_1_bleached_masked	10.500	11
SyNOnly_CC_4_reg_iter_0_normal_masked	10.625	12
SyNOnly_CC_6_reg_iter_0_bleached_masked	11.062	13
SyNOnly_CC_6_reg_iter_0_normal_masked	11.062	13
SyNOnly_MI_32_reg_iter_0_bleached_masked	12.625	15
SyNOnly_MI_32_reg_iter_0_normal_masked	14.062	16
TVMSQ_bleached_masked	16.438	17
TVMSQC_bleached_masked	17.188	18
TVMSQ_normal_masked	17.312	19
TVMSQC_normal_masked	17.375	20

2 Visualization of raw assessment data

The algorithms are ordered according to the computed ranks for each task.

2.1 Dot- and boxplot

Dot- and boxplots for visualizing raw assessment data separately for each algorithm. Boxplots representing descriptive statistics over all cases (median, quartiles and outliers) are combined with horizontally jittered dots representing individual cases.

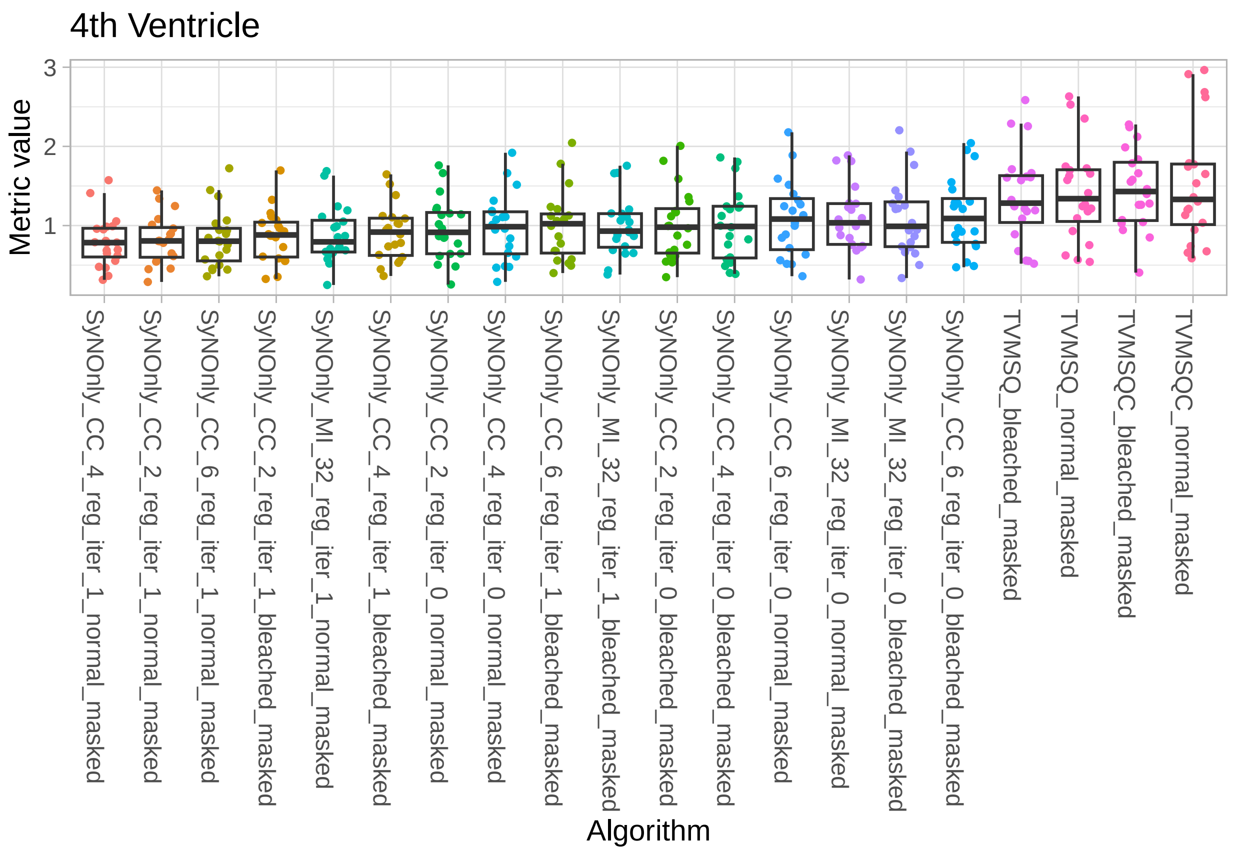

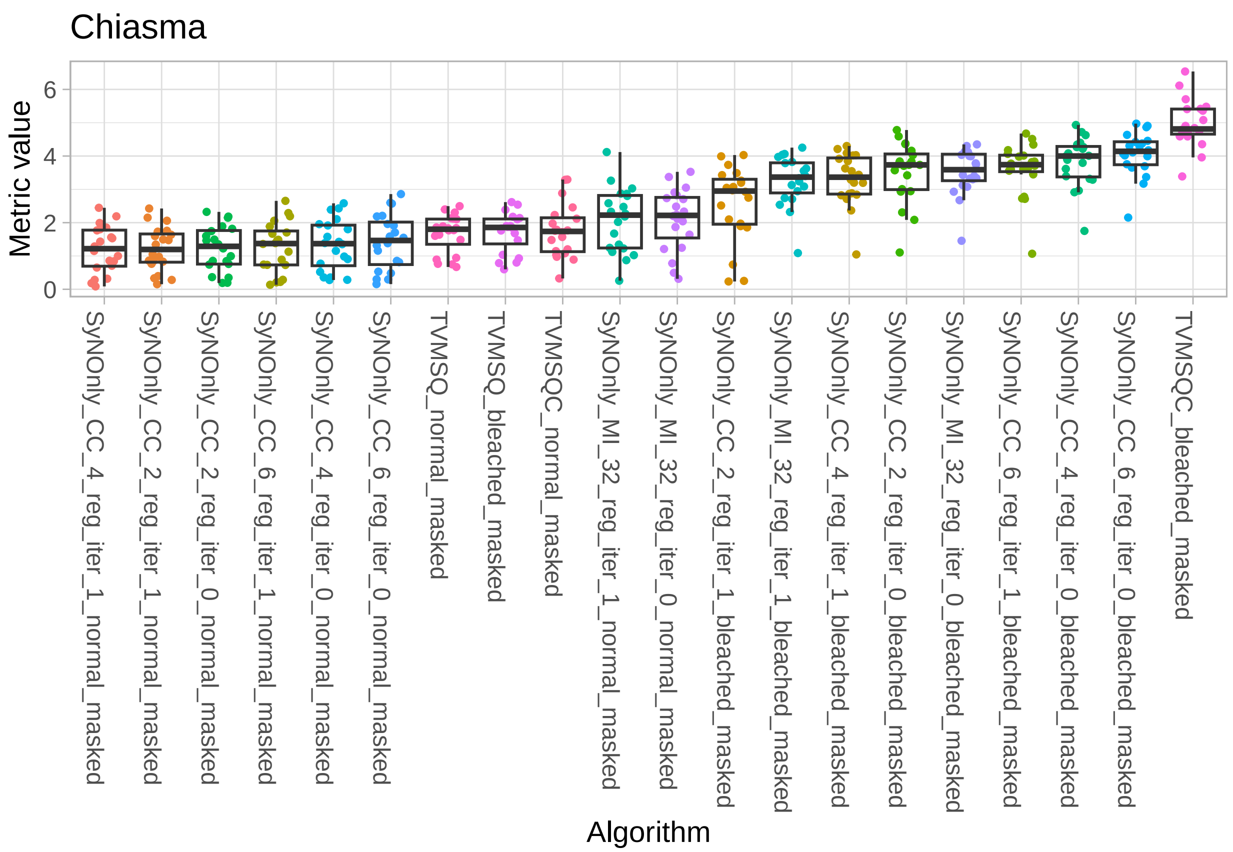
Chiasma
Metric value
0
2
4
6
SyNOnly_CC_4_reg_iter_1_normal_masked
SyNOnly_CC_2_reg_iter_1_normal_masked
SyNOnly_CC_2_reg_iter_0_normal_masked
SyNOnly_CC_6_reg_iter_1_normal_masked
SyNOnly_CC_4_reg_iter_0_normal_masked
SyNOnly_CC_6_reg_iter_0_normal_masked
TVMSQ_normal_masked
TVMSQ_bleached_masked
TVMSQC_normal_masked
SyNOnly_MI_32_reg_iter_1_normal_masked
SyNOnly_MI_32_reg_iter_0_normal_masked
SyNOnly_CC_2_reg_iter_1_bleached_masked
SyNOnly_MI_32_reg_iter_1_bleached_masked
SyNOnly_CC_4_reg_iter_1_bleached_masked
SyNOnly_CC_2_reg_iter_0_bleached_masked
SyNOnly_MI_32_reg_iter_0_bleached_masked
SyNOnly_CC_6_reg_iter_1_bleached_masked
SyNOnly_CC_4_reg_iter_0_bleached_masked
SyNOnly_CC_6_reg_iter_0_bleached_masked
TVMSQC_bleached_masked
Algorithm

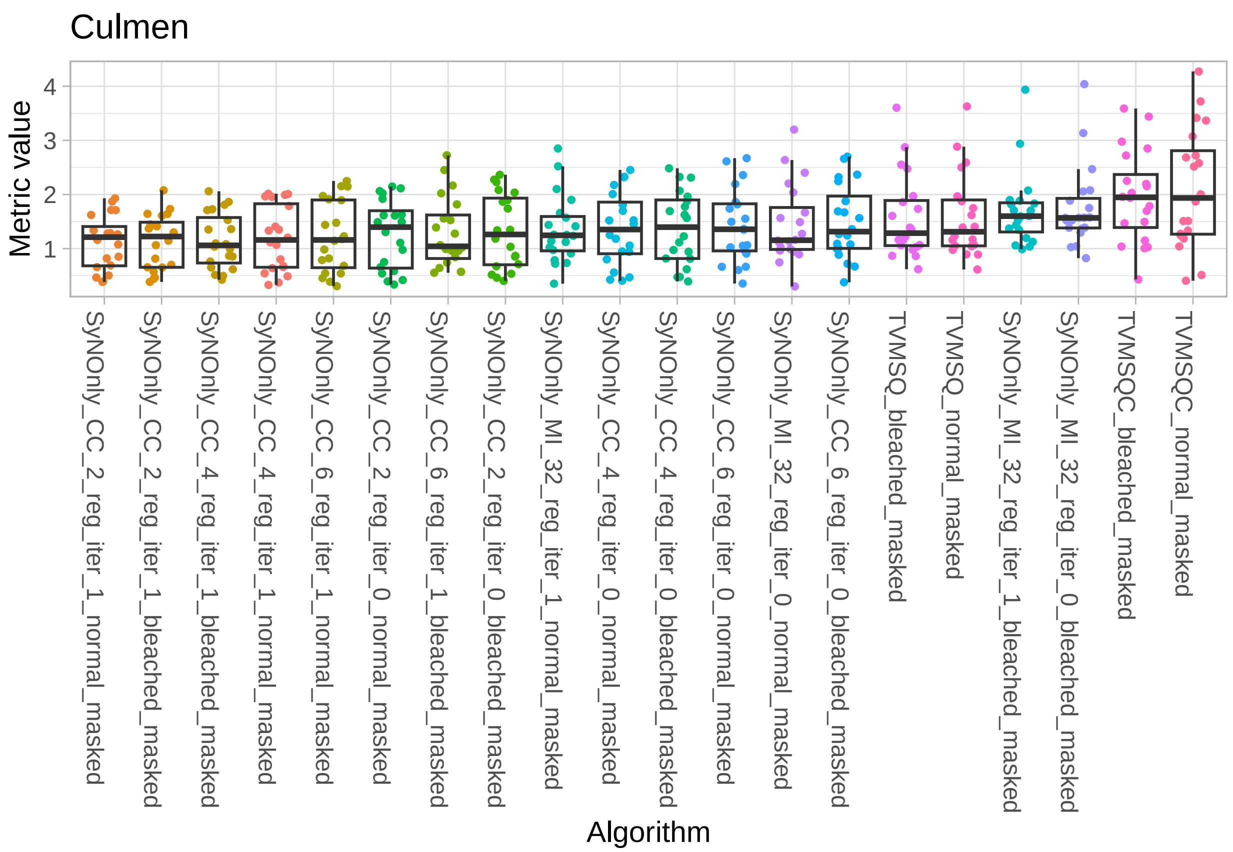

Culmen
Metric value
4
3
2
1
SyNOnly_CC_2_reg_iter_1_normal_masked
SyNOnly_CC_2_reg_iter_1_bleached_masked
SyNOnly_CC_4_reg_iter_1_bleached_masked
SyNOnly_CC_4_reg_iter_1_normal_masked
SyNOnly_CC_6_reg_iter_1_normal_masked
SyNOnly_CC_2_reg_iter_0_normal_masked
SyNOnly_CC_6_reg_iter_1_bleached_masked
SyNOnly_CC_2_reg_iter_0_bleached_masked
SyNOnly_MI_32_reg_iter_1_normal_masked
SyNOnly_CC_4_reg_iter_0_normal_masked
SyNOnly_CC_4_reg_iter_0_bleached_masked
SyNOnly_CC_6_reg_iter_0_normal_masked
SyNOnly_MI_32_reg_iter_0_normal_masked
SyNOnly_CC_6_reg_iter_0_bleached_masked
TVMSQ_bleached_masked
TVMSQ_normal_masked
SyNOnly_MI_32_reg_iter_1_bleached_masked
SyNOnly_MI_32_reg_iter_0_bleached_masked
TVMSQC_bleached_masked
TVMSQC_normal_masked
Algorithm

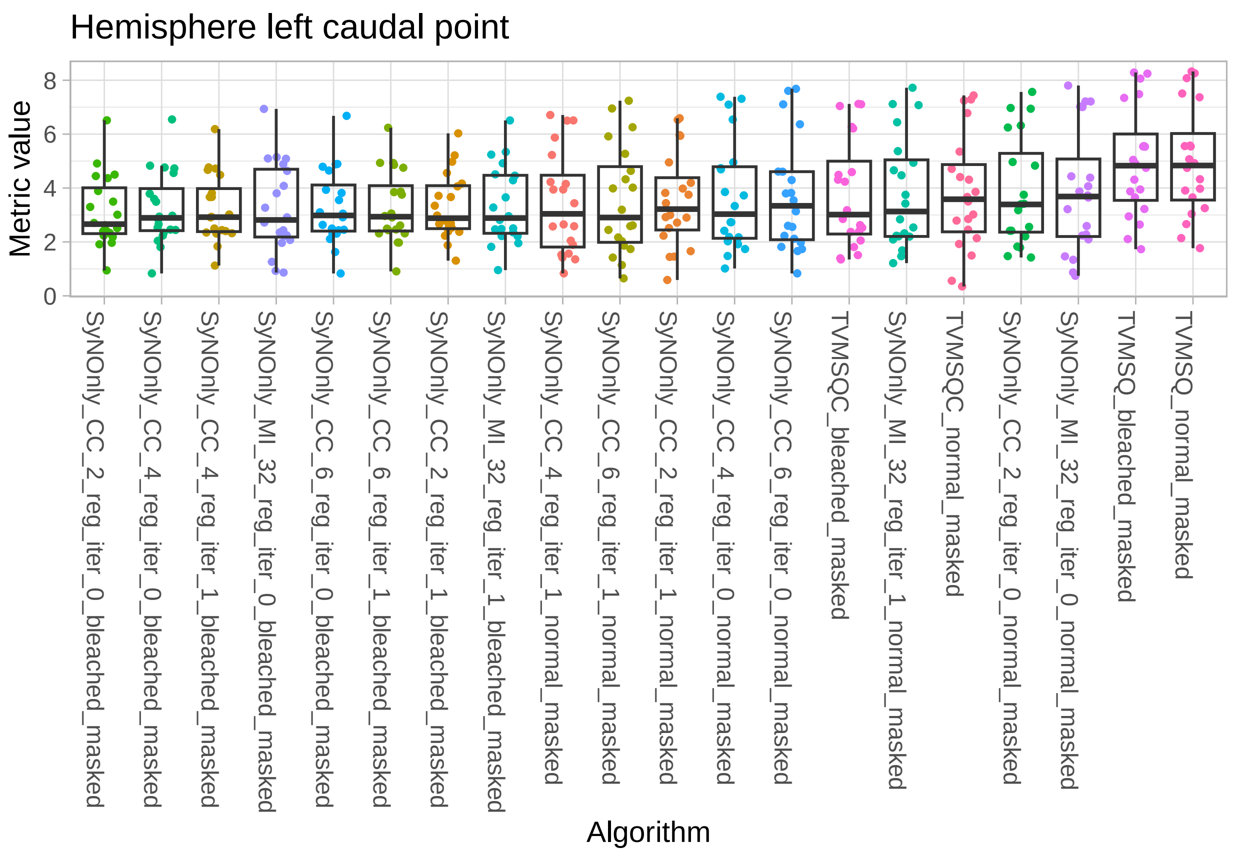

Hemisphere left caudal point
Metric value
0
2
4
6
8
SyNOnly_CC_2_reg_iter_0_bleached_masked
SyNOnly_CC_4_reg_iter_0_bleached_masked
SyNOnly_CC_4_reg_iter_1_bleached_masked
SyNOnly_MI_32_reg_iter_0_bleached_masked
SyNOnly_CC_6_reg_iter_0_bleached_masked
SyNOnly_CC_6_reg_iter_1_bleached_masked
SyNOnly_CC_2_reg_iter_1_bleached_masked
SyNOnly_MI_32_reg_iter_1_bleached_masked
SyNOnly_CC_4_reg_iter_1_normal_masked
SyNOnly_CC_6_reg_iter_1_normal_masked
SyNOnly_CC_2_reg_iter_1_normal_masked
SyNOnly_CC_4_reg_iter_0_normal_masked
SyNOnly_CC_6_reg_iter_0_normal_masked
TVMSQC_bleached_masked
SyNOnly_MI_32_reg_iter_1_normal_masked
TVMSQC_normal_masked
SyNOnly_CC_2_reg_iter_0_normal_masked
SyNOnly_MI_32_reg_iter_0_normal_masked
TVMSQ_bleached_masked
TVMSQ_normal_masked
Algorithm

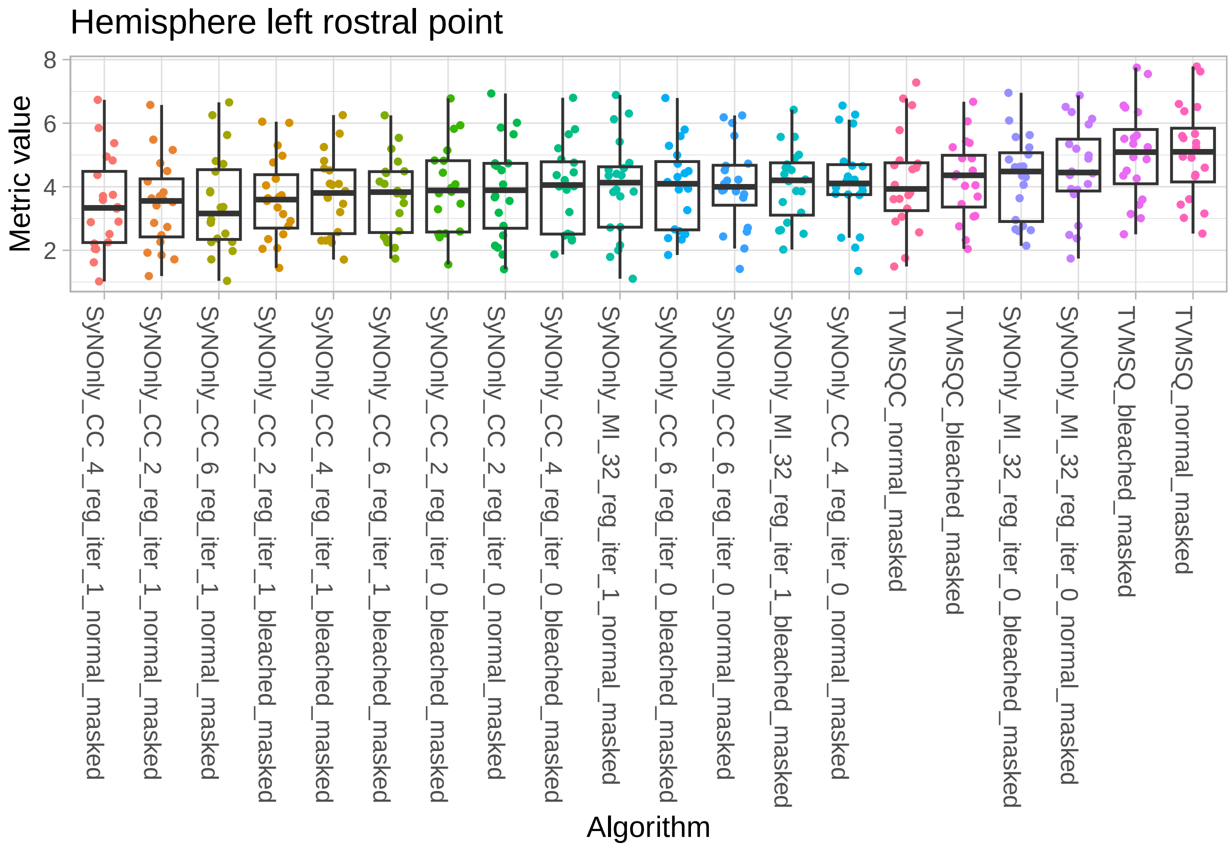
Hemisphere left rostral point
Metric value
8
6
4
2
SyNOnly_CC_4_reg_iter_1_normal_masked
SyNOnly_CC_2_reg_iter_1_normal_masked
SyNOnly_CC_6_reg_iter_1_normal_masked
SyNOnly_CC_2_reg_iter_1_bleached_masked
SyNOnly_CC_4_reg_iter_1_bleached_masked
SyNOnly_CC_6_reg_iter_1_bleached_masked
SyNOnly_CC_2_reg_iter_0_bleached_masked
SyNOnly_CC_2_reg_iter_0_normal_masked
SyNOnly_CC_4_reg_iter_0_bleached_masked
SyNOnly_MI_32_reg_iter_1_normal_masked
SyNOnly_CC_6_reg_iter_0_bleached_masked
SyNOnly_CC_6_reg_iter_0_normal_masked
SyNOnly_MI_32_reg_iter_1_bleached_masked
SyNOnly_CC_4_reg_iter_0_normal_masked
TVMSQC_normal_masked
TVMSQC_bleached_masked
SyNOnly_MI_32_reg_iter_0_bleached_masked
SyNOnly_MI_32_reg_iter_0_normal_masked
TVMSQ_bleached_masked
TVMSQ_normal_masked
Algorithm

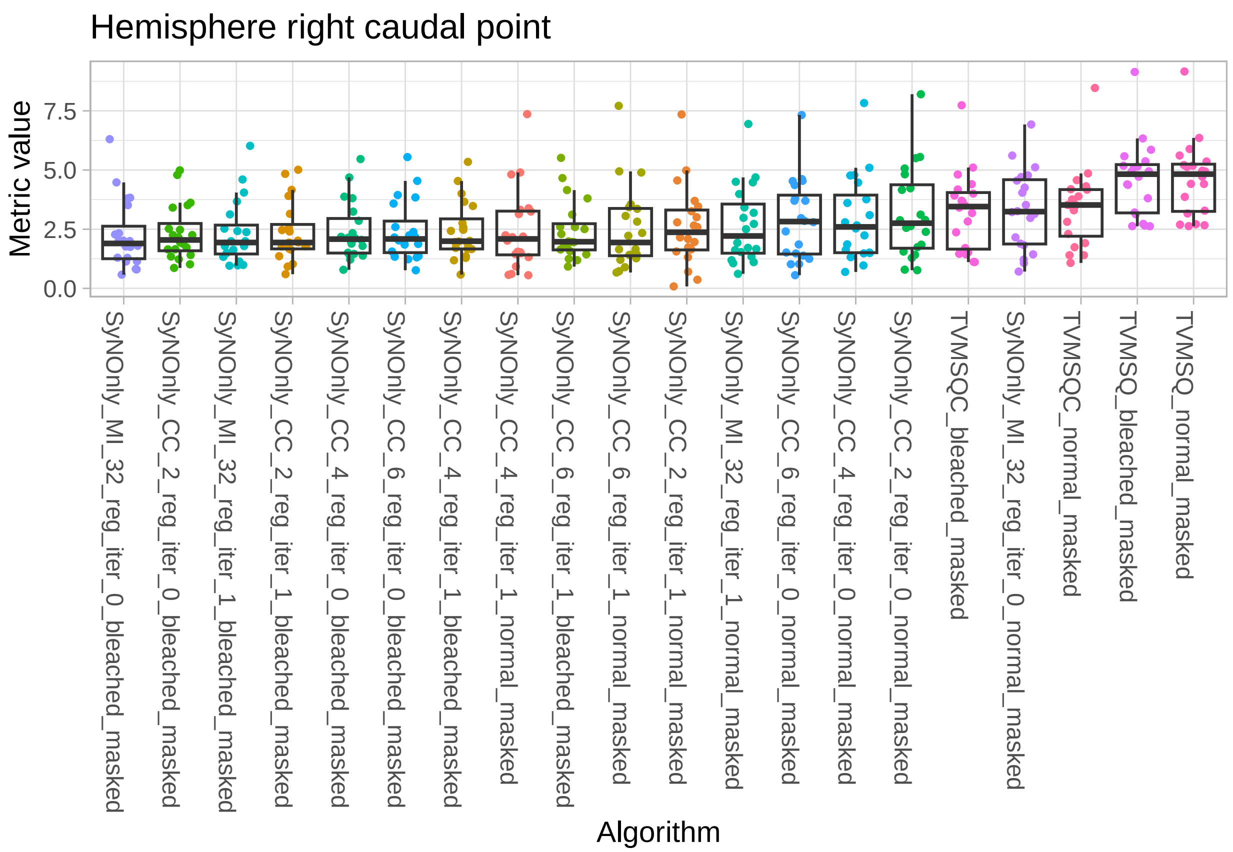

Hemisphere right caudal point
Metric value
0.0
2.5
5.0
7.5
Algorithm
SyNOnly_MI_32_reg_iter_0_bleached_masked
SyNOnly_CC_2_reg_iter_0_bleached_masked
SyNOnly_MI_32_reg_iter_1_bleached_masked
SyNOnly_CC_2_reg_iter_1_bleached_masked
SyNOnly_CC_4_reg_iter_0_bleached_masked
SyNOnly_CC_6_reg_iter_0_bleached_masked
SyNOnly_CC_4_reg_iter_1_bleached_masked
SyNOnly_CC_4_reg_iter_1_normal_masked
SyNOnly_CC_6_reg_iter_1_bleached_masked
SyNOnly_CC_6_reg_iter_1_normal_masked
SyNOnly_CC_2_reg_iter_1_normal_masked
SyNOnly_MI_32_reg_iter_1_normal_masked
SyNOnly_CC_6_reg_iter_0_normal_masked
SyNOnly_CC_4_reg_iter_0_normal_masked
SyNOnly_CC_2_reg_iter_0_normal_masked
TVMSQC_bleached_masked
SyNOnly_MI_32_reg_iter_0_normal_masked
TVMSQC_normal_masked
TVMSQ_bleached_masked
TVMSQ_normal_masked

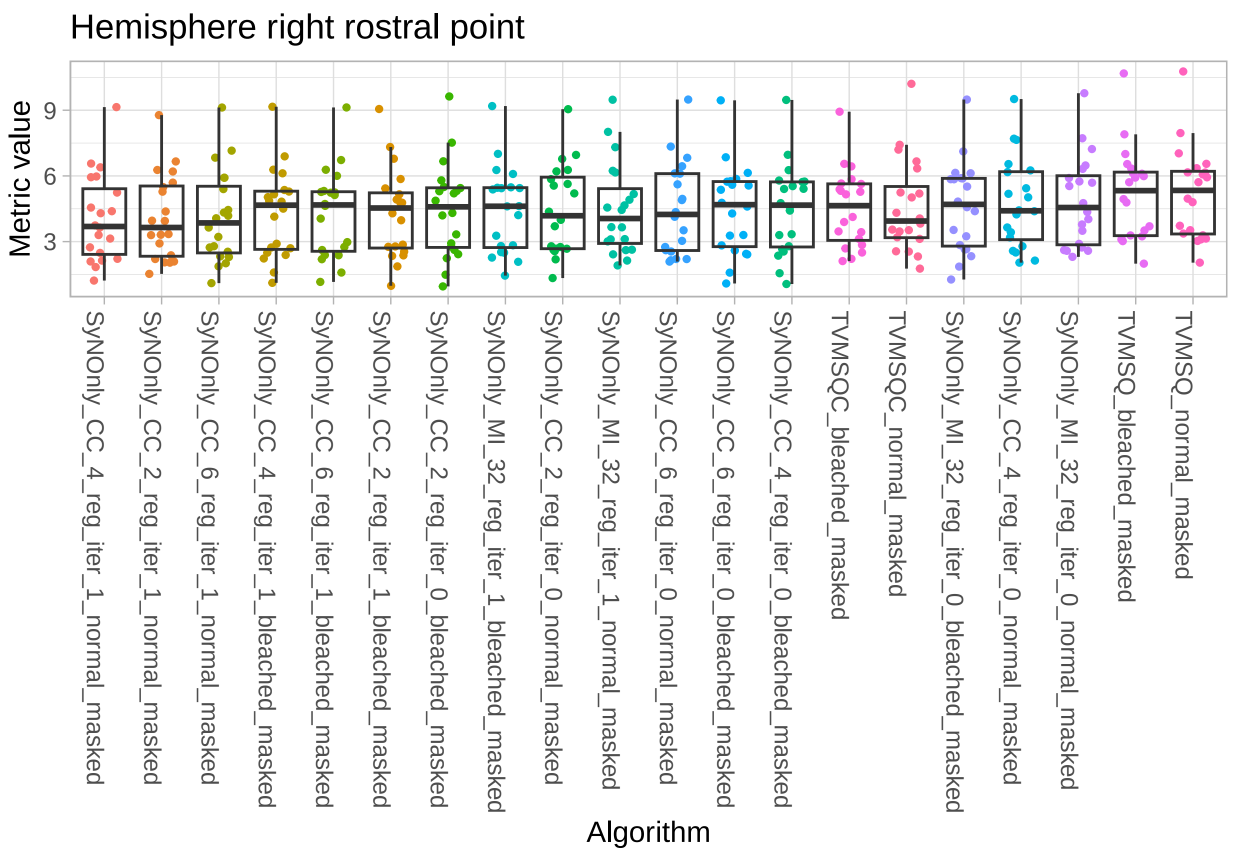
Hemisphere right rostral point
Metric value
9
6
3
SyNOnly_CC_4_reg_iter_1_normal_masked
SyNOnly_CC_2_reg_iter_1_normal_masked
SyNOnly_CC_6_reg_iter_1_normal_masked
SyNOnly_CC_4_reg_iter_1_bleached_masked
SyNOnly_CC_6_reg_iter_1_bleached_masked
SyNOnly_CC_2_reg_iter_1_bleached_masked
SyNOnly_CC_2_reg_iter_0_bleached_masked
SyNOnly_MI_32_reg_iter_1_bleached_masked
SyNOnly_CC_2_reg_iter_0_normal_masked
SyNOnly_MI_32_reg_iter_1_normal_masked
SyNOnly_CC_6_reg_iter_0_normal_masked
SyNOnly_CC_6_reg_iter_0_bleached_masked
SyNOnly_CC_4_reg_iter_0_bleached_masked
TVMSQC_bleached_masked
TVMSQC_normal_masked
SyNOnly_MI_32_reg_iter_0_bleached_masked
SyNOnly_CC_4_reg_iter_0_normal_masked
SyNOnly_MI_32_reg_iter_0_normal_masked
TVMSQ_bleached_masked
TVMSQ_normal_masked
Algorithm

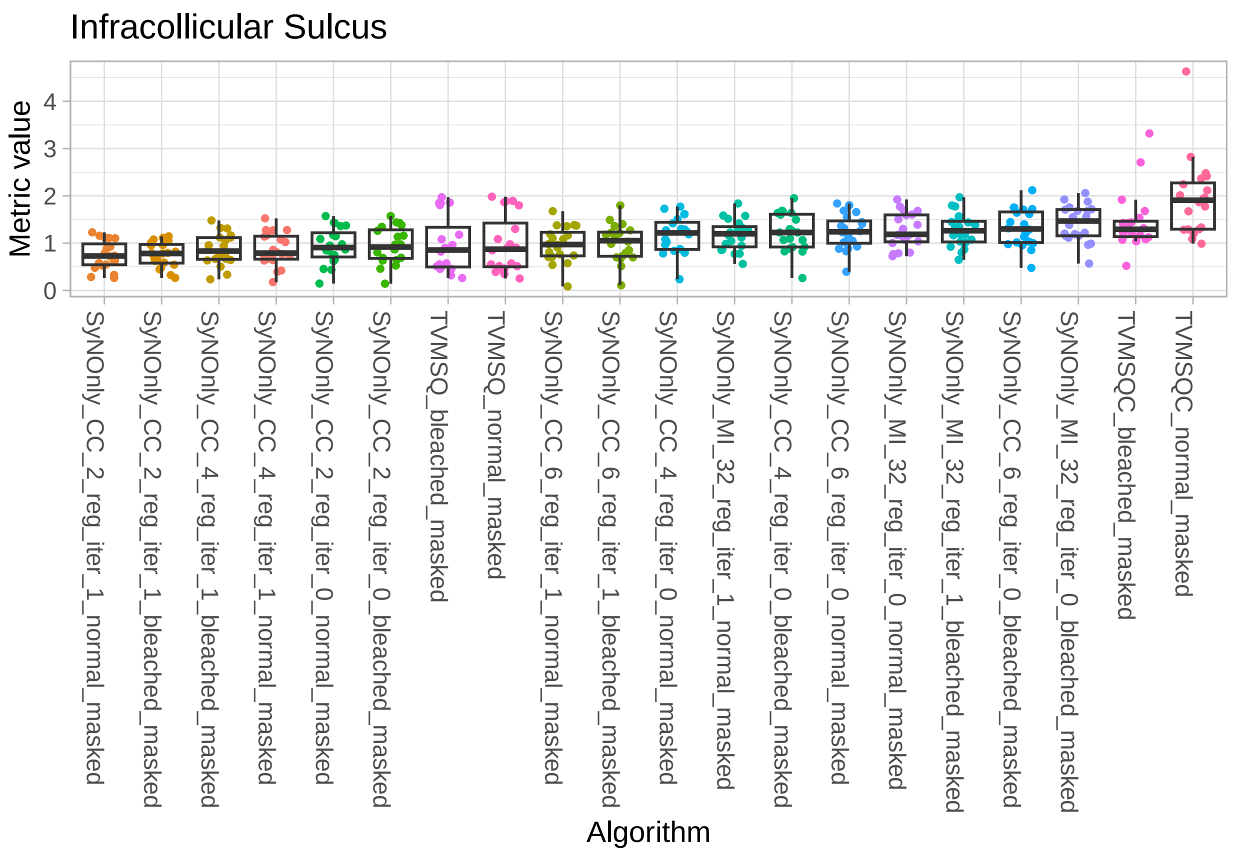

Infracollicular Sulcus
Metric value
0
1
2
3
4
SyNOnly_CC_2_reg_iter_1_normal_masked
SyNOnly_CC_2_reg_iter_1_bleached_masked
SyNOnly_CC_4_reg_iter_1_bleached_masked
SyNOnly_CC_4_reg_iter_1_normal_masked
SyNOnly_CC_2_reg_iter_0_normal_masked
SyNOnly_CC_2_reg_iter_0_bleached_masked
TVMSQ_bleached_masked
TVMSQ_normal_masked
SyNOnly_CC_6_reg_iter_1_normal_masked
SyNOnly_CC_6_reg_iter_1_bleached_masked
SyNOnly_CC_4_reg_iter_0_normal_masked
SyNOnly_MI_32_reg_iter_1_normal_masked
SyNOnly_CC_4_reg_iter_0_bleached_masked
SyNOnly_CC_6_reg_iter_0_normal_masked
SyNOnly_MI_32_reg_iter_0_normal_masked
SyNOnly_MI_32_reg_iter_1_bleached_masked
SyNOnly_CC_6_reg_iter_0_bleached_masked
SyNOnly_MI_32_reg_iter_0_bleached_masked
TVMSQC_bleached_masked
TVMSQC_normal_masked
Algorithm

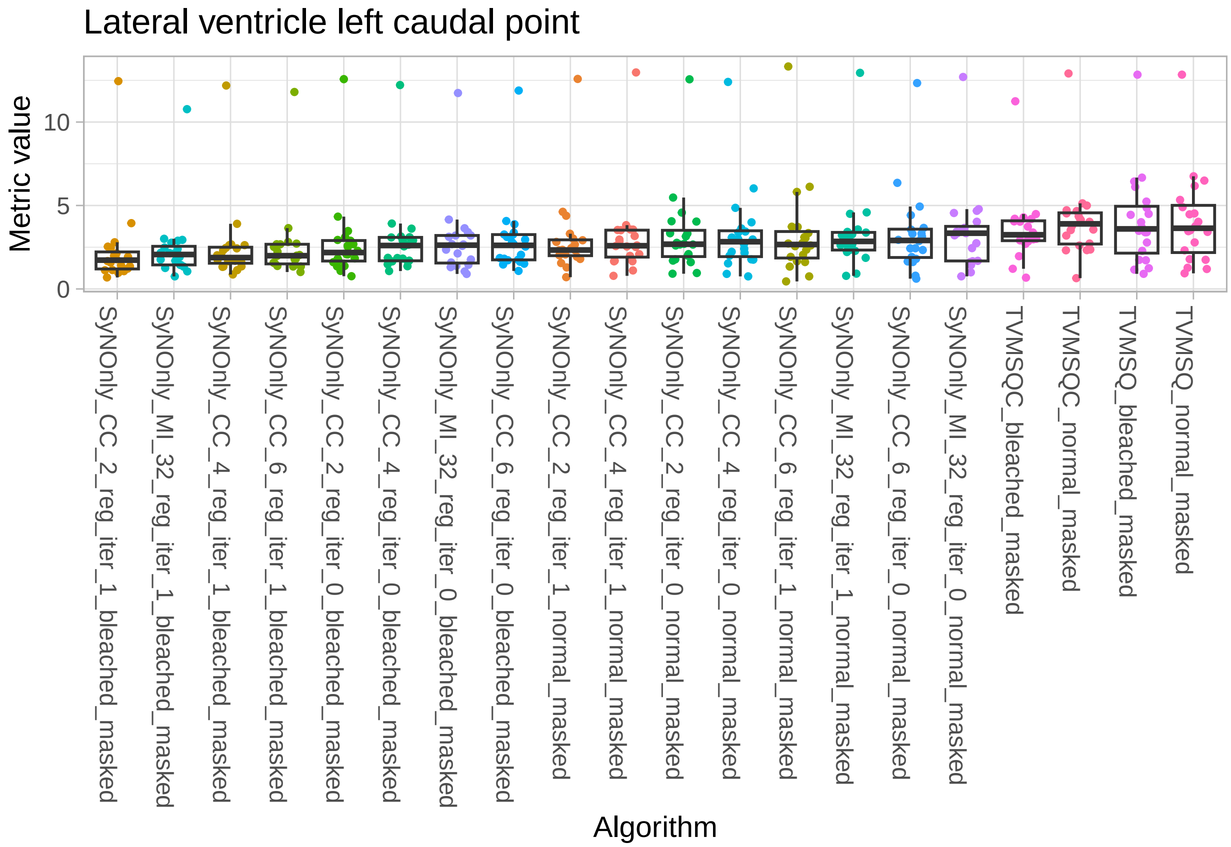

Lateral ventricle left caudal point
Metric value
10
5
0
SyNOnly_CC_2_reg_iter_1_bleached_masked
SyNOnly_MI_32_reg_iter_1_bleached_masked
SyNOnly_CC_4_reg_iter_1_bleached_masked
SyNOnly_CC_6_reg_iter_1_bleached_masked
SyNOnly_CC_2_reg_iter_0_bleached_masked
SyNOnly_CC_4_reg_iter_0_bleached_masked
SyNOnly_MI_32_reg_iter_0_bleached_masked
SyNOnly_CC_6_reg_iter_0_bleached_masked
SyNOnly_CC_2_reg_iter_1_normal_masked
SyNOnly_CC_4_reg_iter_1_normal_masked
SyNOnly_CC_2_reg_iter_0_normal_masked
SyNOnly_CC_4_reg_iter_0_normal_masked
SyNOnly_CC_6_reg_iter_1_normal_masked
SyNOnly_MI_32_reg_iter_1_normal_masked
SyNOnly_CC_6_reg_iter_0_normal_masked
SyNOnly_MI_32_reg_iter_0_normal_masked
TVMSQC_bleached_masked
TVMSQC_normal_masked
TVMSQ_bleached_masked
TVMSQ_normal_masked
Algorithm

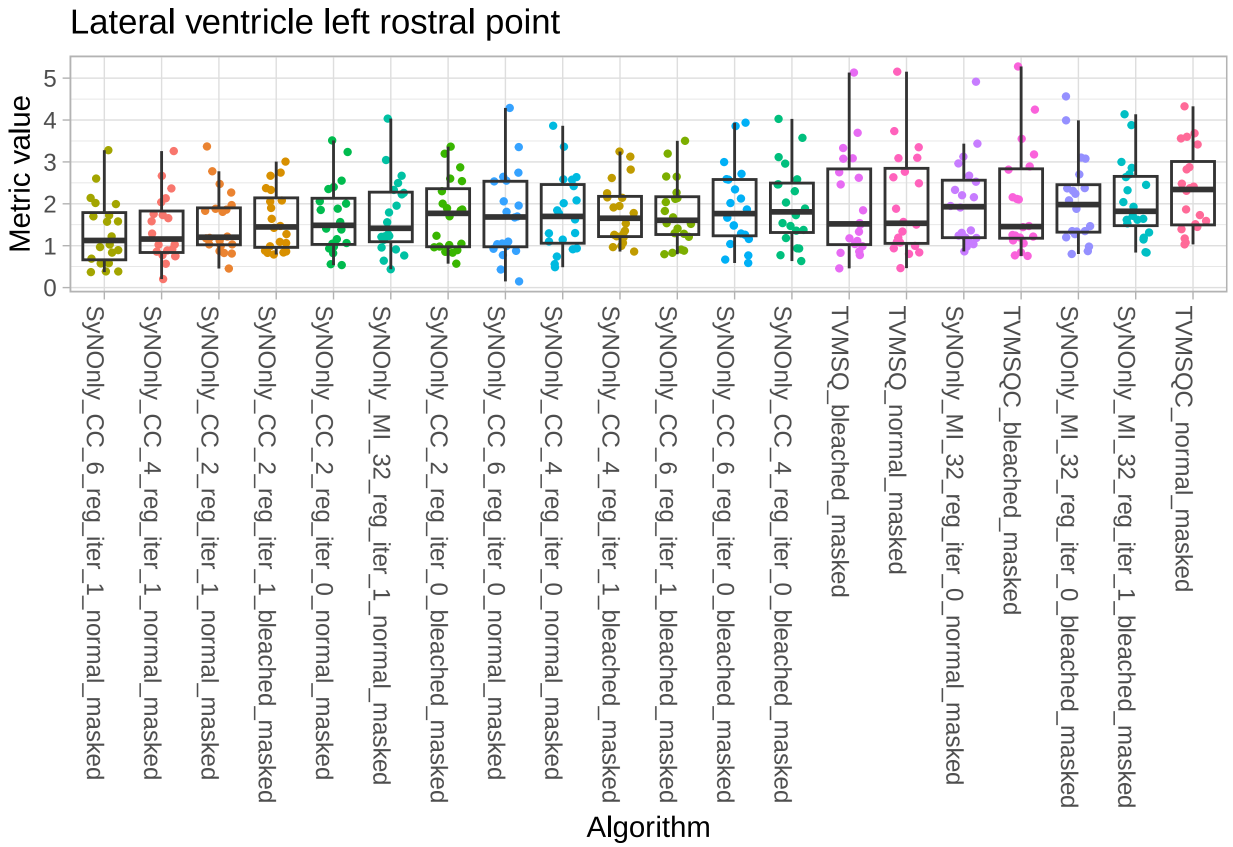
Lateral ventricle left rostral point
Metric value
0
1
2
3
4
5
SyNOnly_CC_6_reg_iter_1_normal_masked
SyNOnly_CC_4_reg_iter_1_normal_masked
SyNOnly_CC_2_reg_iter_1_normal_masked
SyNOnly_CC_2_reg_iter_1_bleached_masked
SyNOnly_CC_2_reg_iter_0_normal_masked
SyNOnly_MI_32_reg_iter_1_normal_masked
SyNOnly_CC_2_reg_iter_0_bleached_masked
SyNOnly_CC_6_reg_iter_0_normal_masked
SyNOnly_CC_4_reg_iter_0_normal_masked
SyNOnly_CC_4_reg_iter_1_bleached_masked
SyNOnly_CC_6_reg_iter_1_bleached_masked
SyNOnly_CC_6_reg_iter_0_bleached_masked
SyNOnly_CC_4_reg_iter_0_bleached_masked
TVMSQ_bleached_masked
TVMSQ_normal_masked
SyNOnly_MI_32_reg_iter_0_normal_masked
TVMSQC_bleached_masked
SyNOnly_MI_32_reg_iter_0_bleached_masked
SyNOnly_MI_32_reg_iter_1_bleached_masked
TVMSQC_normal_masked
Algorithm

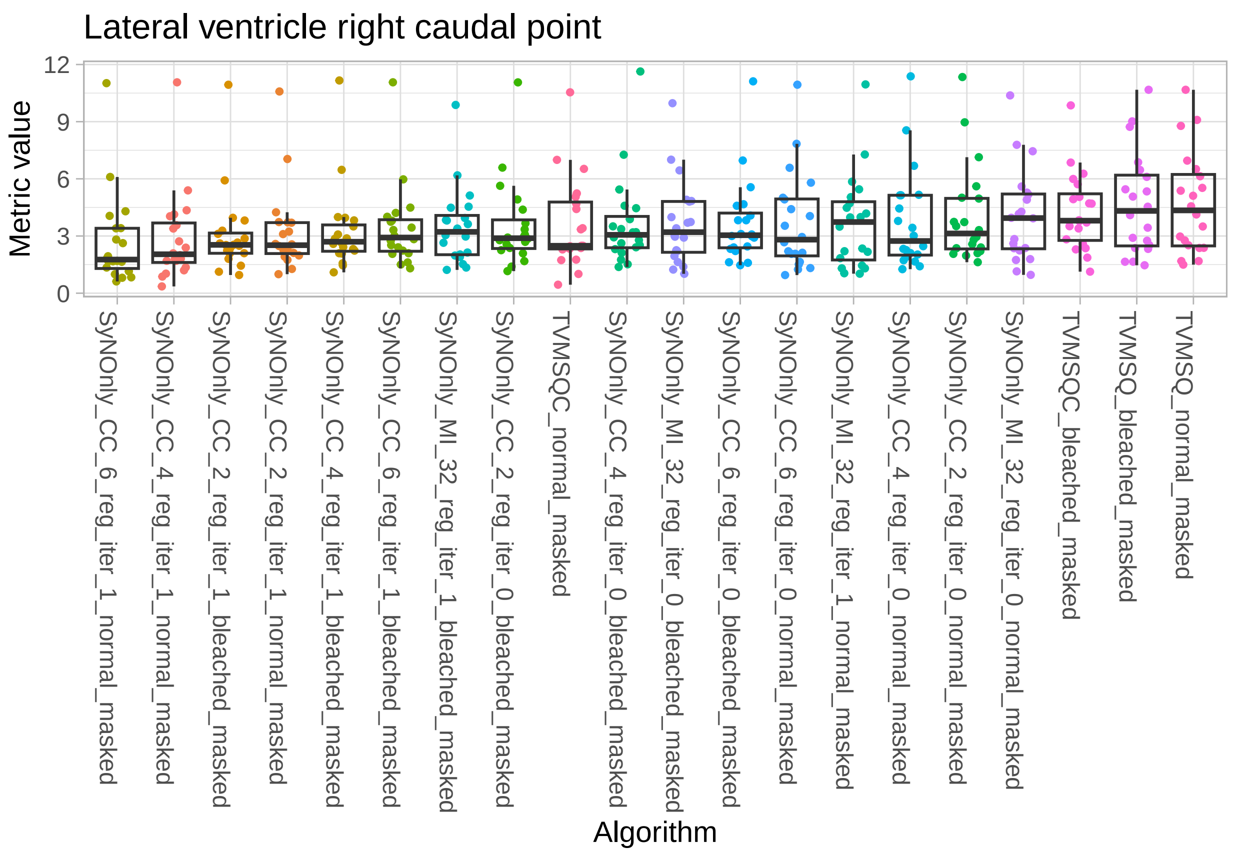
Lateral ventricle right caudal point
Metric value
12
9
6
3
0
SyNOnly_CC_6_reg_iter_1_normal_masked
SyNOnly_CC_4_reg_iter_1_normal_masked
SyNOnly_CC_2_reg_iter_1_bleached_masked
SyNOnly_CC_2_reg_iter_1_normal_masked
SyNOnly_CC_4_reg_iter_1_bleached_masked
SyNOnly_CC_6_reg_iter_1_bleached_masked
SyNOnly_MI_32_reg_iter_1_bleached_masked
SyNOnly_CC_2_reg_iter_0_bleached_masked
TVMSQC_normal_masked
SyNOnly_CC_4_reg_iter_0_bleached_masked
SyNOnly_MI_32_reg_iter_0_bleached_masked
SyNOnly_CC_6_reg_iter_0_bleached_masked
SyNOnly_CC_6_reg_iter_0_normal_masked
SyNOnly_MI_32_reg_iter_1_normal_masked
SyNOnly_CC_4_reg_iter_0_normal_masked
SyNOnly_CC_2_reg_iter_0_normal_masked
SyNOnly_MI_32_reg_iter_0_normal_masked
TVMSQC_bleached_masked
TVMSQ_bleached_masked
TVMSQ_normal_masked
Algorithm

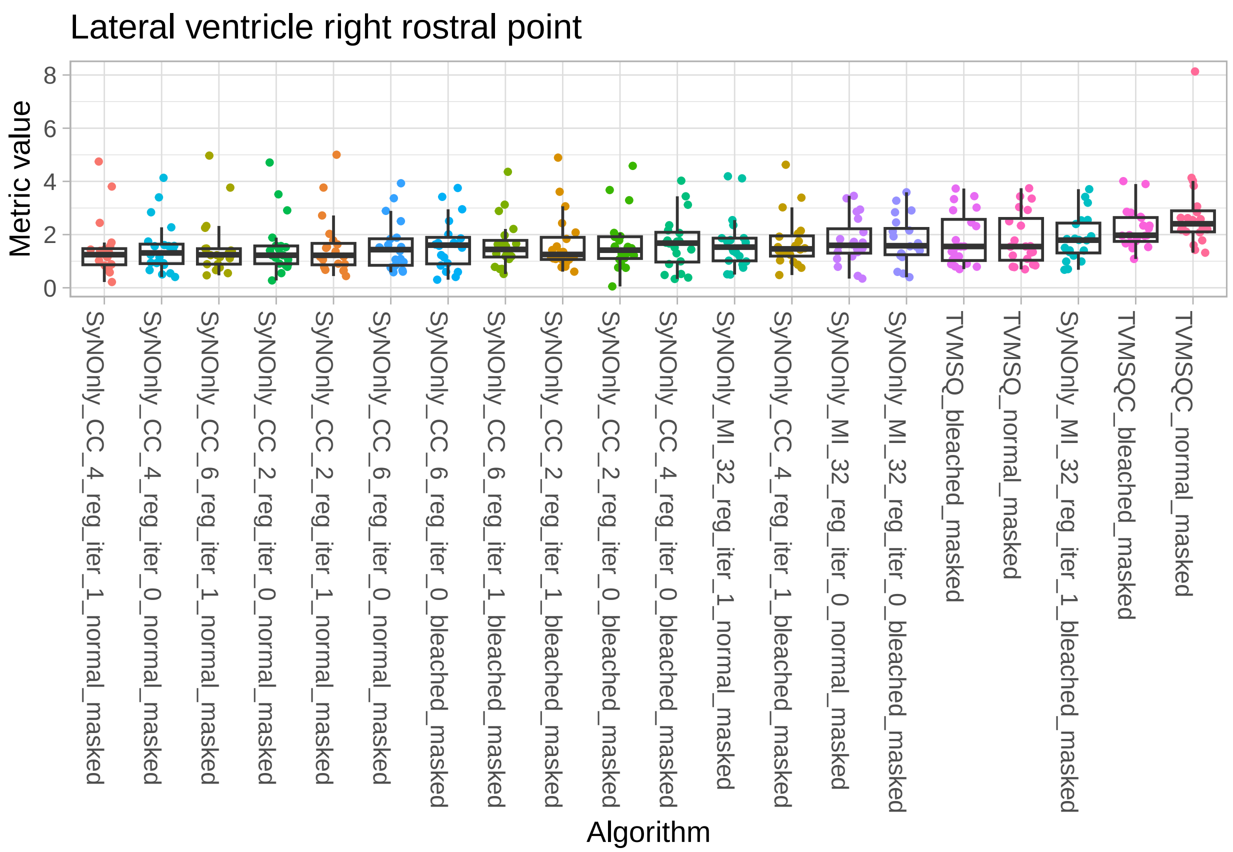
Lateral ventricle right rostral point
Metric value
0
2
4
6
8
SyNOnly_CC_4_reg_iter_1_normal_masked
SyNOnly_CC_4_reg_iter_0_normal_masked
SyNOnly_CC_6_reg_iter_1_normal_masked
SyNOnly_CC_2_reg_iter_0_normal_masked
SyNOnly_CC_2_reg_iter_1_normal_masked
SyNOnly_CC_6_reg_iter_0_normal_masked
SyNOnly_CC_6_reg_iter_0_bleached_masked
SyNOnly_CC_6_reg_iter_1_bleached_masked
SyNOnly_CC_2_reg_iter_1_bleached_masked
SyNOnly_CC_2_reg_iter_0_bleached_masked
SyNOnly_CC_4_reg_iter_0_bleached_masked
SyNOnly_MI_32_reg_iter_1_normal_masked
SyNOnly_CC_4_reg_iter_1_bleached_masked
SyNOnly_MI_32_reg_iter_0_normal_masked
SyNOnly_MI_32_reg_iter_0_bleached_masked
TVMSQ_bleached_masked
TVMSQ_normal_masked
SyNOnly_MI_32_reg_iter_1_bleached_masked
TVMSQC_bleached_masked
TVMSQC_normal_masked
Algorithm

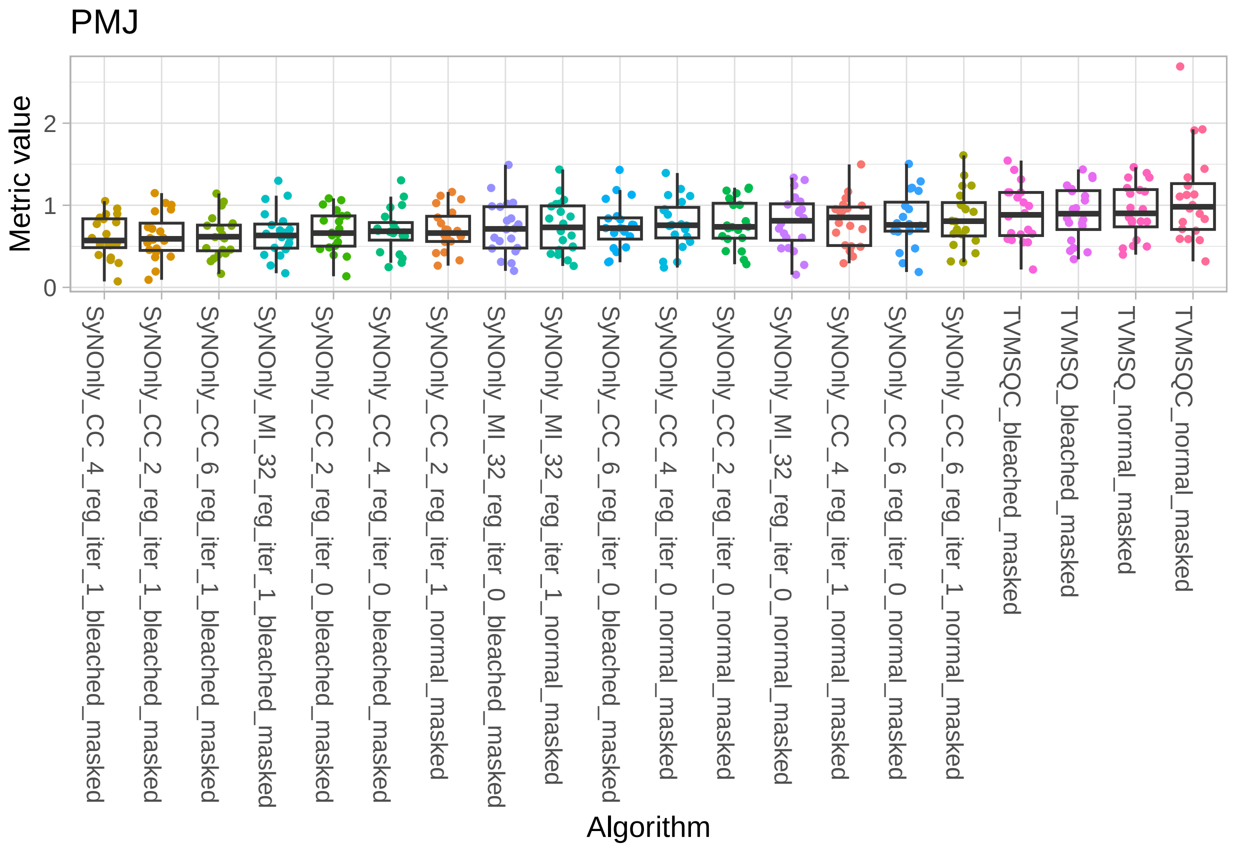
PMJ
Metric value
0
1
2
SyNOnly_CC_4_reg_iter_1_bleached_masked
SyNOnly_CC_2_reg_iter_1_bleached_masked
SyNOnly_CC_6_reg_iter_1_bleached_masked
SyNOnly_MI_32_reg_iter_1_bleached_masked
SyNOnly_CC_2_reg_iter_0_bleached_masked
SyNOnly_CC_4_reg_iter_0_bleached_masked
SyNOnly_CC_2_reg_iter_1_normal_masked
SyNOnly_MI_32_reg_iter_0_bleached_masked
SyNOnly_MI_32_reg_iter_1_normal_masked
SyNOnly_CC_6_reg_iter_0_bleached_masked
SyNOnly_CC_4_reg_iter_0_normal_masked
SyNOnly_CC_2_reg_iter_0_normal_masked
SyNOnly_MI_32_reg_iter_0_normal_masked
SyNOnly_CC_4_reg_iter_1_normal_masked
SyNOnly_CC_6_reg_iter_0_normal_masked
SyNOnly_CC_6_reg_iter_1_normal_masked
TVMSQC_bleached_masked
TVMSQ_bleached_masked
TVMSQ_normal_masked
TVMSQC_normal_masked
Algorithm

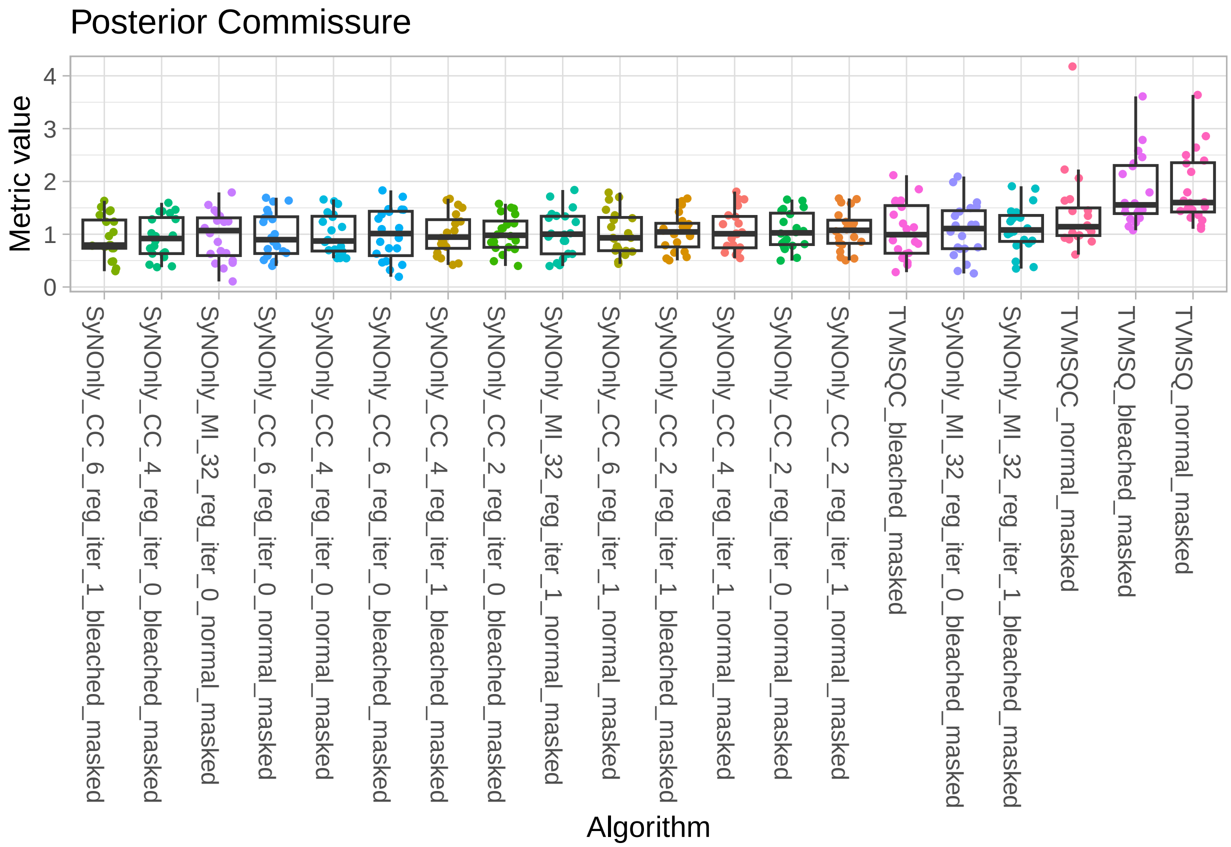

Posterior Commissure
Metric value
0
1
2
3
4
SyNOnly_CC_6_reg_iter_1_bleached_masked
SyNOnly_CC_4_reg_iter_0_bleached_masked
SyNOnly_MI_32_reg_iter_0_normal_masked
SyNOnly_CC_6_reg_iter_0_normal_masked
SyNOnly_CC_4_reg_iter_0_normal_masked
SyNOnly_CC_6_reg_iter_0_bleached_masked
SyNOnly_CC_4_reg_iter_1_bleached_masked
SyNOnly_CC_2_reg_iter_0_bleached_masked
SyNOnly_MI_32_reg_iter_1_normal_masked
SyNOnly_CC_6_reg_iter_1_normal_masked
SyNOnly_CC_2_reg_iter_1_bleached_masked
SyNOnly_CC_4_reg_iter_1_normal_masked
SyNOnly_CC_2_reg_iter_0_normal_masked
SyNOnly_CC_2_reg_iter_1_normal_masked
TVMSQC_bleached_masked
SyNOnly_MI_32_reg_iter_0_bleached_masked
SyNOnly_MI_32_reg_iter_1_bleached_masked
TVMSQC_normal_masked
TVMSQ_bleached_masked
TVMSQ_normal_masked
Algorithm

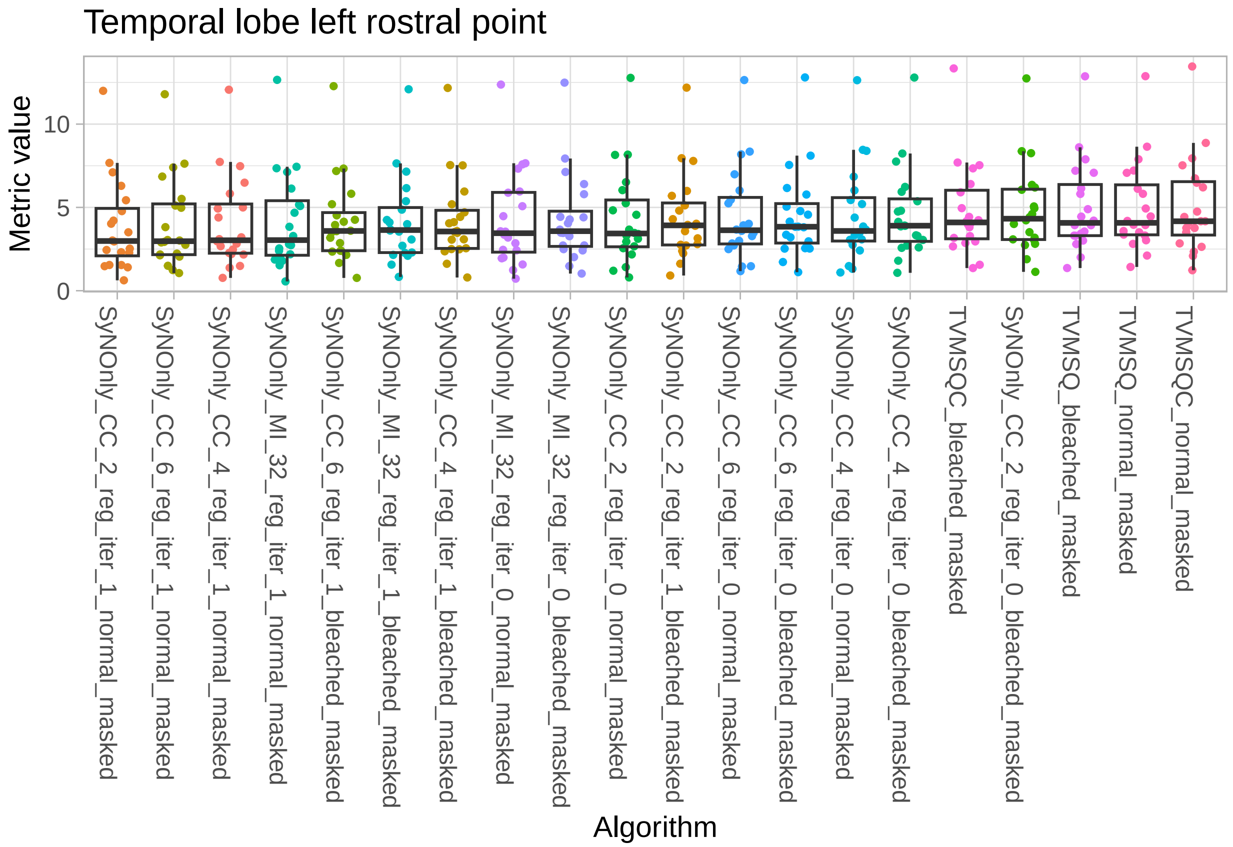

Temporal lobe left rostral point
Metric value
10
5
0
SyNOnly_CC_2_reg_iter_1_normal_masked
SyNOnly_CC_6_reg_iter_1_normal_masked
SyNOnly_CC_4_reg_iter_1_normal_masked
SyNOnly_MI_32_reg_iter_1_normal_masked
SyNOnly_CC_6_reg_iter_1_bleached_masked
SyNOnly_MI_32_reg_iter_1_bleached_masked
SyNOnly_CC_4_reg_iter_1_bleached_masked
SyNOnly_MI_32_reg_iter_0_normal_masked
SyNOnly_MI_32_reg_iter_0_bleached_masked
SyNOnly_CC_2_reg_iter_0_normal_masked
SyNOnly_CC_2_reg_iter_1_bleached_masked
SyNOnly_CC_6_reg_iter_0_normal_masked
SyNOnly_CC_6_reg_iter_0_bleached_masked
SyNOnly_CC_4_reg_iter_0_normal_masked
SyNOnly_CC_4_reg_iter_0_bleached_masked
TVMSQC_bleached_masked
SyNOnly_CC_2_reg_iter_0_bleached_masked
TVMSQ_bleached_masked
TVMSQ_normal_masked
TVMSQC_normal_masked
Algorithm

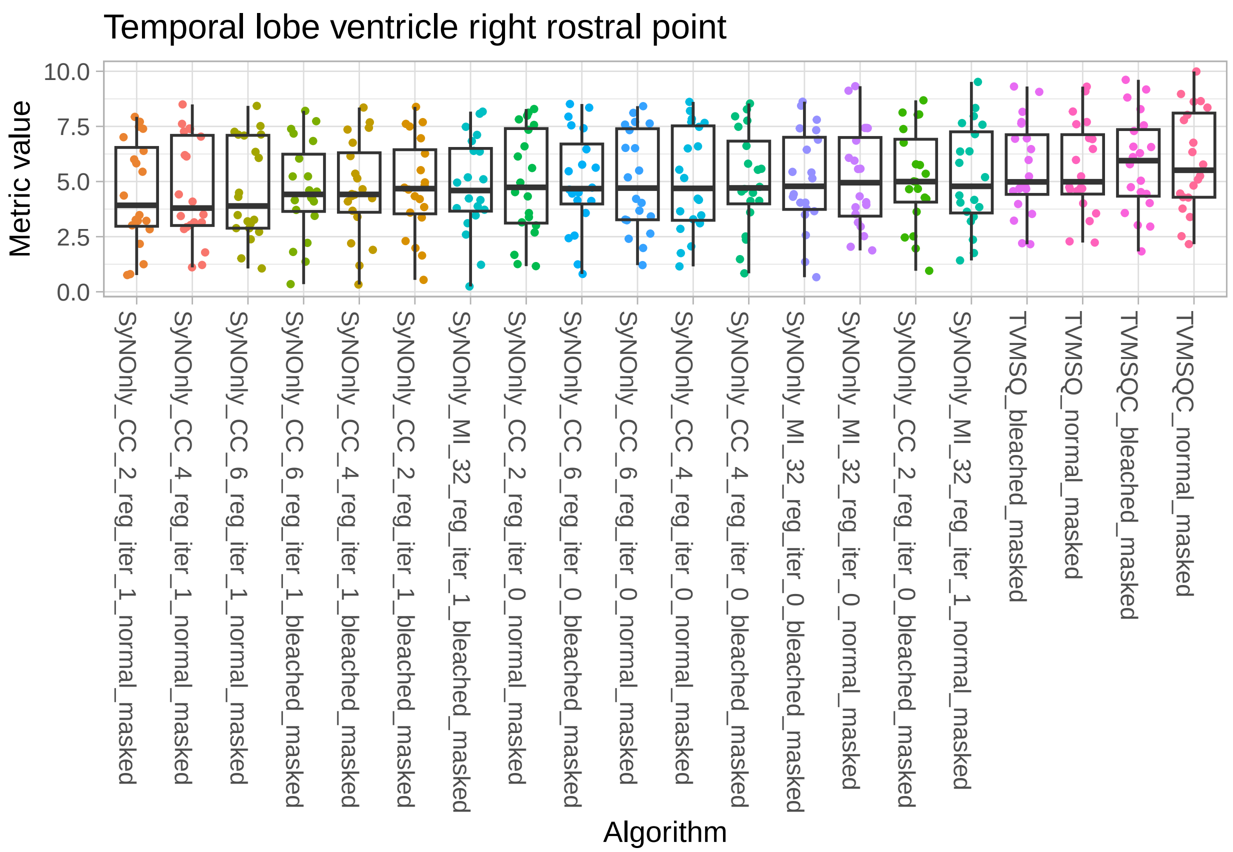

Temporal lobe ventricle right rostral point
Metric value
10.0
7.5
5.0
2.5
0.0
SyNOnly_CC_2_reg_iter_1_normal_masked
SyNOnly_CC_4_reg_iter_1_normal_masked
SyNOnly_CC_6_reg_iter_1_normal_masked
SyNOnly_CC_6_reg_iter_1_bleached_masked
SyNOnly_CC_4_reg_iter_1_bleached_masked
SyNOnly_CC_2_reg_iter_1_bleached_masked
SyNOnly_MI_32_reg_iter_1_bleached_masked
SyNOnly_CC_2_reg_iter_0_normal_masked
SyNOnly_CC_6_reg_iter_0_bleached_masked
SyNOnly_CC_6_reg_iter_0_normal_masked
SyNOnly_CC_4_reg_iter_0_normal_masked
SyNOnly_CC_4_reg_iter_0_bleached_masked
SyNOnly_MI_32_reg_iter_0_bleached_masked
SyNOnly_MI_32_reg_iter_0_normal_masked
SyNOnly_CC_2_reg_iter_0_bleached_masked
SyNOnly_MI_32_reg_iter_1_normal_masked
TVMSQ_bleached_masked
TVMSQ_normal_masked
TVMSQC_bleached_masked
TVMSQC_normal_masked
Algorithm

2.2 Podium plot

Podium plots (see also Eugster et al., 2008) for visualizing raw assessment data. Upper part (spaghetti plot): Participating algorithms are color-coded, and each colored dot in the plot represents a metric value achieved with the respective algorithm. The actual metric value is encoded by the y-axis. Each podium (here: p=20) represents one possible rank, ordered from best (1) to last (here: 20). The assignment of metric values (i.e. colored dots) to one of the podiums is based on the rank that the respective algorithm achieved on the corresponding case. Note that the plot part above each podium place is further subdivided into p “columns”, where each column represents one participating algorithm (here: p = 20). Dots corresponding to identical cases are connected by a line, leading to the shown spaghetti structure. Lower part: Bar charts represent the relative frequency for each algorithm to achieve the rank encoded by the podium place.

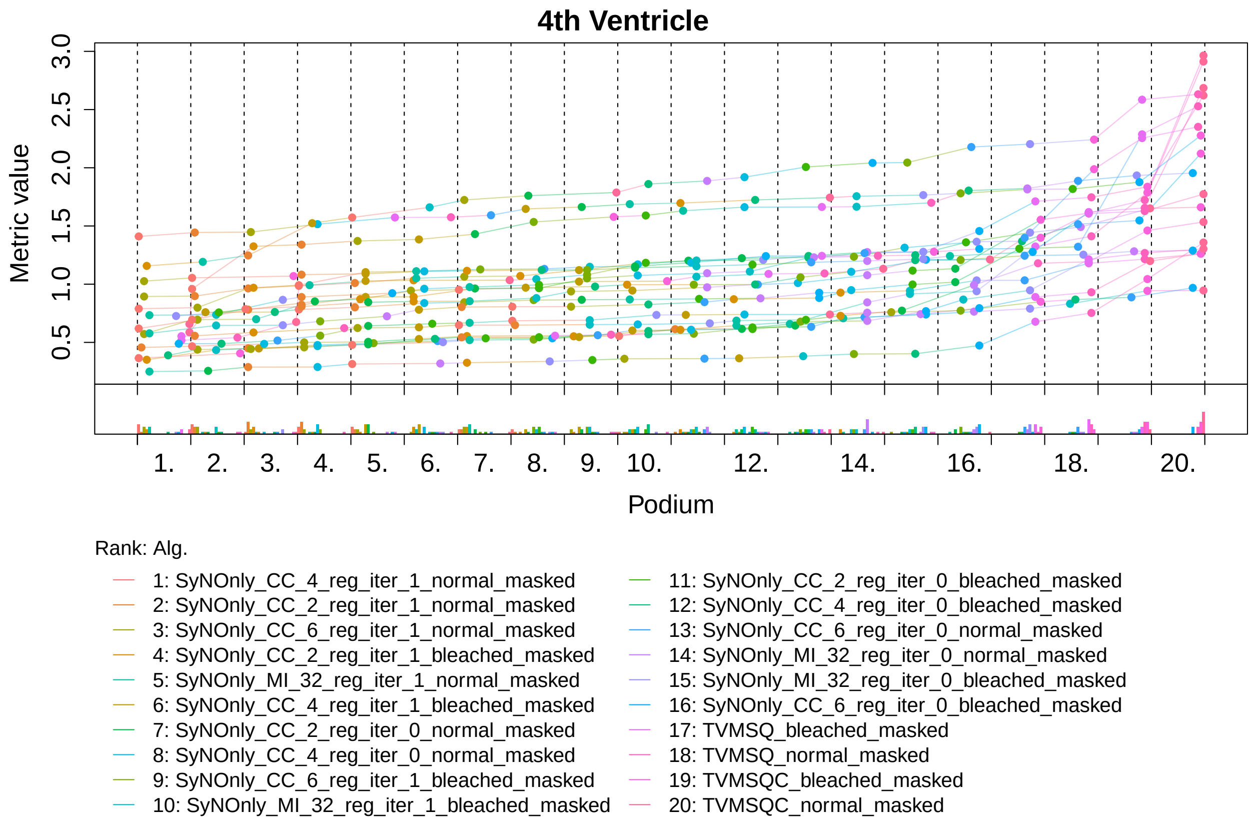

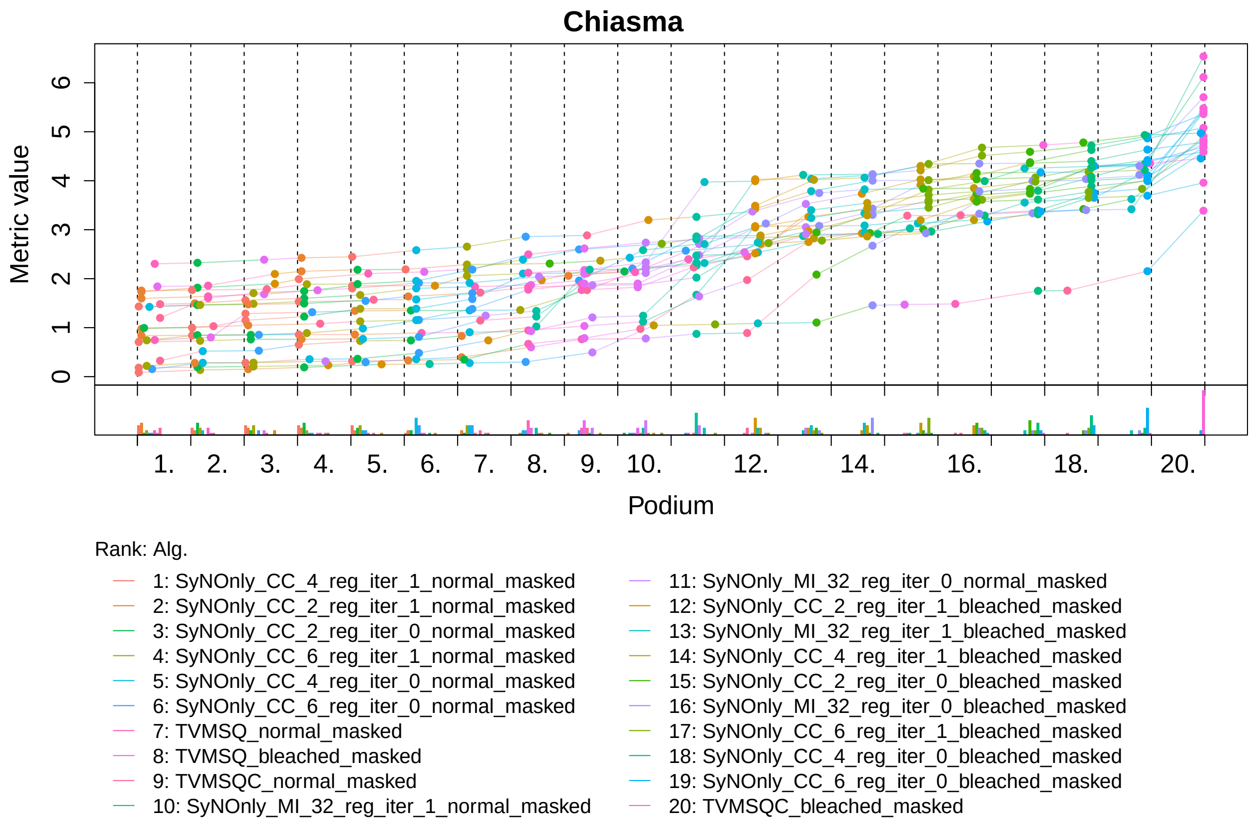
Chiasma
Metric value
0
1
2
3
4
5
6
1.
2.
3.
4.
5.
6.
7.
8.
9.
10.
12.
14.
16.
18.
20.
Podium
Rank: Alg.
1: SyNOnly_CC_4_reg_iter_1_normal_masked
2: SyNOnly_CC_2_reg_iter_1_normal_masked
3: SyNOnly_CC_2_reg_iter_0_normal_masked
4: SyNOnly_CC_6_reg_iter_1_normal_masked
5: SyNOnly_CC_4_reg_iter_0_normal_masked
6: SyNOnly_CC_6_reg_iter_0_normal_masked
7: TVMSQ_normal_masked
8: TVMSQ_bleached_masked
9: TVMSQC_normal_masked
10: SyNOnly_MI_32_reg_iter_1_normal_masked
11: SyNOnly_MI_32_reg_iter_0_normal_masked
12: SyNOnly_CC_2_reg_iter_1_bleached_masked
13: SyNOnly_MI_32_reg_iter_1_bleached_masked
14: SyNOnly_CC_4_reg_iter_1_bleached_masked
15: SyNOnly_CC_2_reg_iter_0_bleached_masked
16: SyNOnly_MI_32_reg_iter_0_bleached_masked
17: SyNOnly_CC_6_reg_iter_1_bleached_masked
18: SyNOnly_CC_4_reg_iter_0_bleached_masked
19: SyNOnly_CC_6_reg_iter_0_bleached_masked
20: TVMSQC_bleached_masked

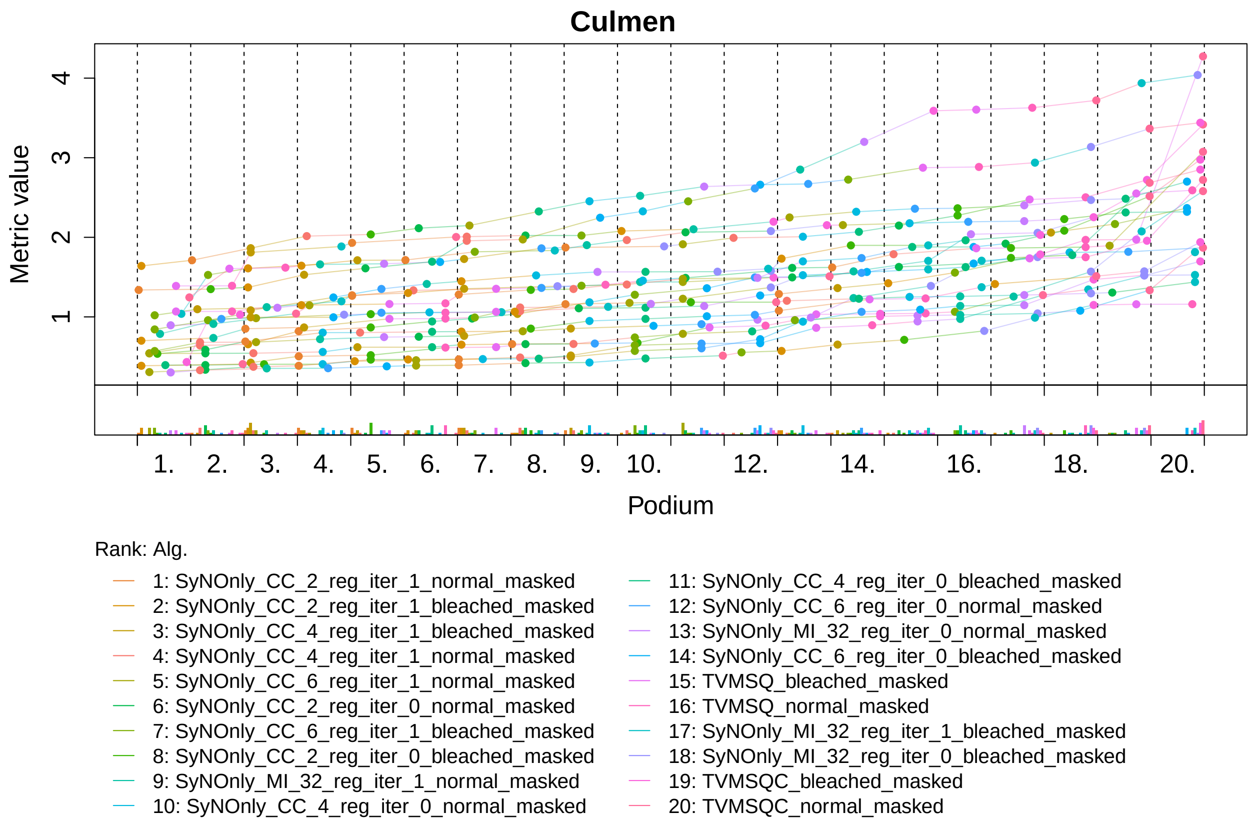

Culmen
Metric value
4
3
2
1
1.
2.
3.
4.
5.
6.
7.
8.
9.
10.
12.
14.
16.
18.
20.
Podium
Rank: Alg.
1: SyNOnly_CC_2_reg_iter_1_normal_masked
2: SyNOnly_CC_2_reg_iter_1_bleached_masked
3: SyNOnly_CC_4_reg_iter_1_bleached_masked
4: SyNOnly_CC_4_reg_iter_1_normal_masked
5: SyNOnly_CC_6_reg_iter_1_normal_masked
6: SyNOnly_CC_2_reg_iter_0_normal_masked
7: SyNOnly_CC_6_reg_iter_1_bleached_masked
8: SyNOnly_CC_2_reg_iter_0_bleached_masked
9: SyNOnly_MI_32_reg_iter_1_normal_masked
10: SyNOnly_CC_4_reg_iter_0_normal_masked
11: SyNOnly_CC_4_reg_iter_0_bleached_masked
12: SyNOnly_CC_6_reg_iter_0_normal_masked
13: SyNOnly_MI_32_reg_iter_0_normal_masked
14: SyNOnly_CC_6_reg_iter_0_bleached_masked
15: TVMSQ_bleached_masked
16: TVMSQ_normal_masked
17: SyNOnly_MI_32_reg_iter_1_bleached_masked
18: SyNOnly_MI_32_reg_iter_0_bleached_masked
19: TVMSQC_bleached_masked
20: TVMSQC_normal_masked

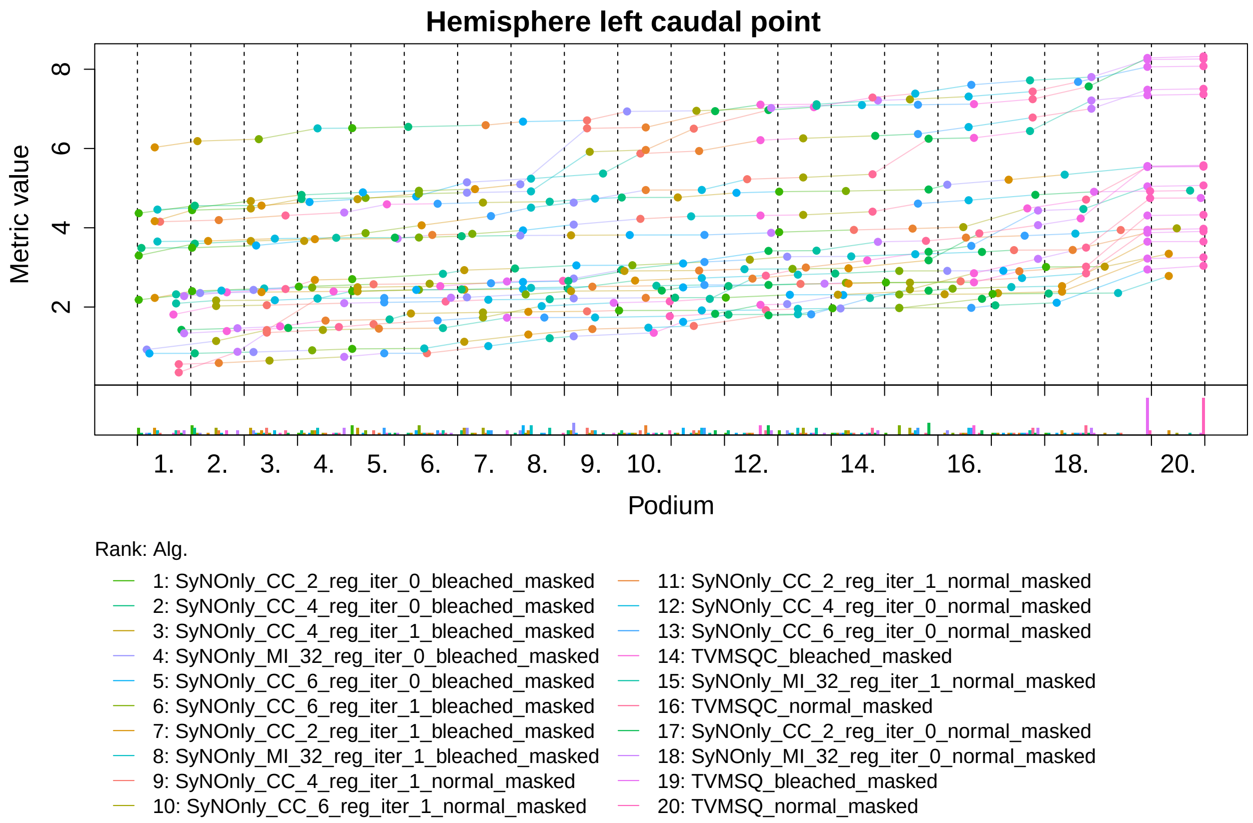
Hemisphere left caudal point
Metric value
Podium
Rank: Alg.
1: SyNOnly_CC_2_reg_iter_0_bleached_masked
2: SyNOnly_CC_4_reg_iter_0_bleached_masked
3: SyNOnly_CC_4_reg_iter_1_bleached_masked
4: SyNOnly_MI_32_reg_iter_0_bleached_masked
5: SyNOnly_CC_6_reg_iter_0_bleached_masked
6: SyNOnly_CC_6_reg_iter_1_bleached_masked
7: SyNOnly_CC_2_reg_iter_1_bleached_masked
8: SyNOnly_MI_32_reg_iter_1_bleached_masked
9: SyNOnly_CC_4_reg_iter_1_normal_masked
10: SyNOnly_CC_6_reg_iter_1_normal_masked
11: SyNOnly_CC_2_reg_iter_1_normal_masked
12: SyNOnly_CC_4_reg_iter_0_normal_masked
13: SyNOnly_CC_6_reg_iter_0_normal_masked
14: TVMSQC_bleached_masked
15: SyNOnly_MI_32_reg_iter_1_normal_masked
16: TVMSQC_normal_masked
17: SyNOnly_CC_2_reg_iter_0_normal_masked
18: SyNOnly_MI_32_reg_iter_0_normal_masked
19: TVMSQ_bleached_masked
20: TVMSQ_normal_masked

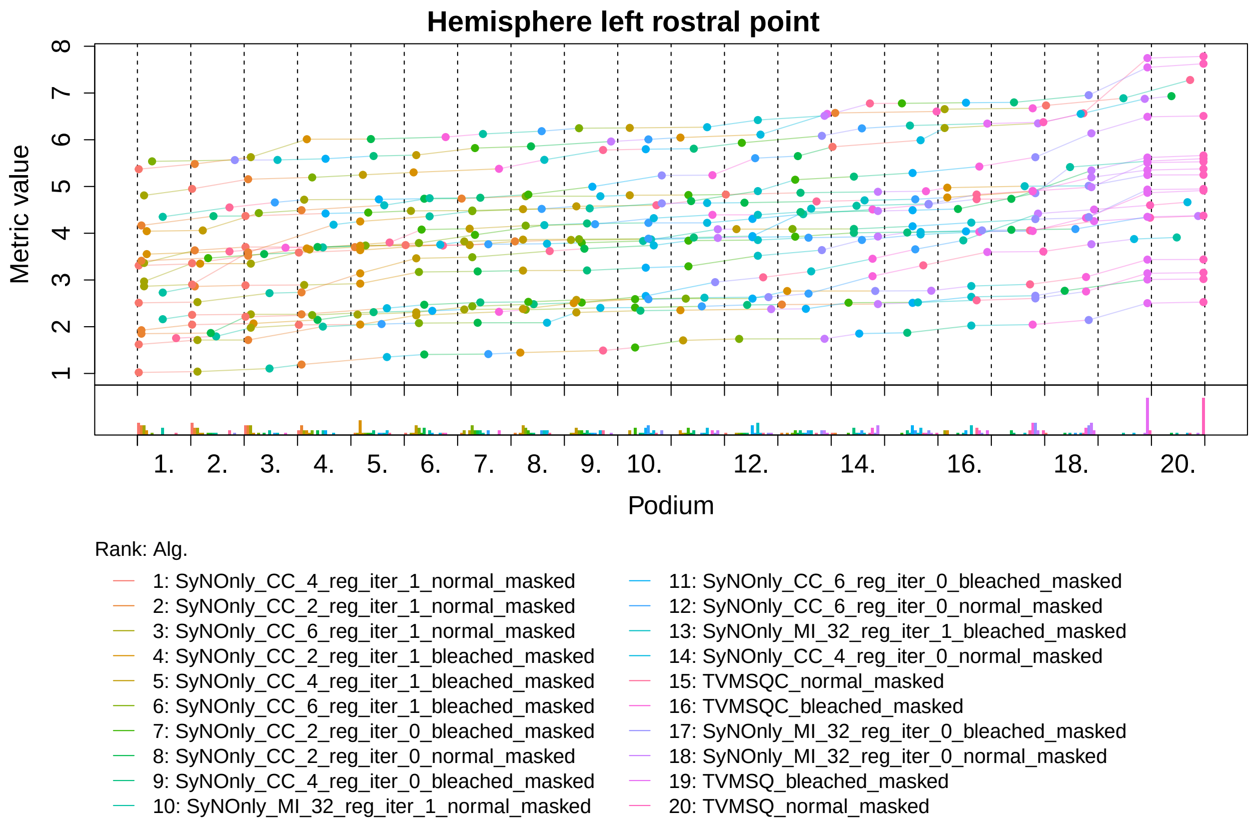

Hemisphere left rostral point
Metric value
1
2
3
4
5
6
7
8
1.
2.
3.
4.
5.
6.
7.
8.
9.
10.
12.
14.
16.
18.
20.
Podium
Rank: Alg.
1: SyNOnly_CC_4_reg_iter_1_normal_masked
2: SyNOnly_CC_2_reg_iter_1_normal_masked
3: SyNOnly_CC_6_reg_iter_1_normal_masked
4: SyNOnly_CC_2_reg_iter_1_bleached_masked
5: SyNOnly_CC_4_reg_iter_1_bleached_masked
6: SyNOnly_CC_6_reg_iter_1_bleached_masked
7: SyNOnly_CC_2_reg_iter_0_bleached_masked
8: SyNOnly_CC_2_reg_iter_0_normal_masked
9: SyNOnly_CC_4_reg_iter_0_bleached_masked
10: SyNOnly_MI_32_reg_iter_1_normal_masked
11: SyNOnly_CC_6_reg_iter_0_bleached_masked
12: SyNOnly_CC_6_reg_iter_0_normal_masked
13: SyNOnly_MI_32_reg_iter_1_bleached_masked
14: SyNOnly_CC_4_reg_iter_0_normal_masked
15: TVMSQC_normal_masked
16: TVMSQC_bleached_masked
17: SyNOnly_MI_32_reg_iter_0_bleached_masked
18: SyNOnly_MI_32_reg_iter_0_normal_masked
19: TVMSQ_bleached_masked
20: TVMSQ_normal_masked

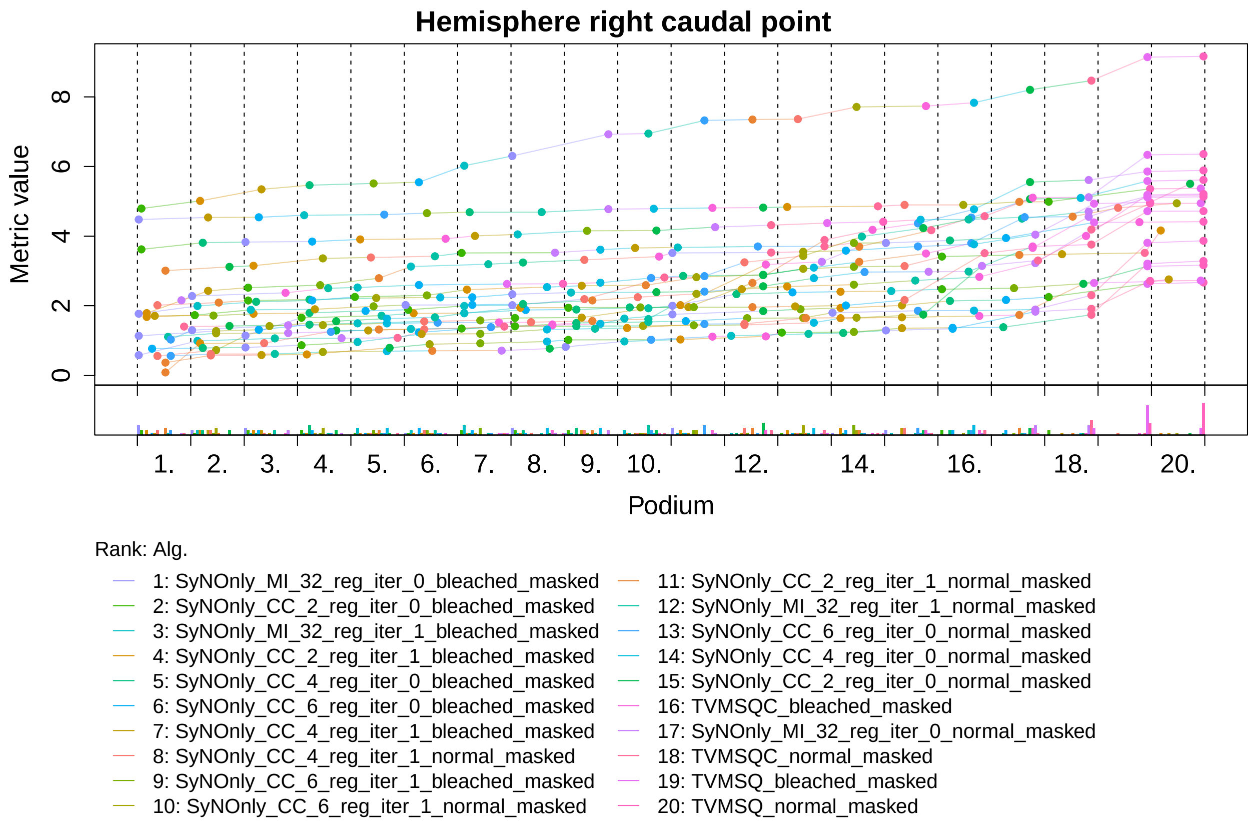

Hemisphere right caudal point
Metric value
0
2
4
6
8
1.
2.
3.
4.
5.
6.
7.
8.
9.
10.
12.
14.
16.
18.
20.
Podium
Rank: Alg.
1: SyNOnly_MI_32_reg_iter_0_bleached_masked
2: SyNOnly_CC_2_reg_iter_0_bleached_masked
3: SyNOnly_MI_32_reg_iter_1_bleached_masked
4: SyNOnly_CC_2_reg_iter_1_bleached_masked
5: SyNOnly_CC_4_reg_iter_0_bleached_masked
6: SyNOnly_CC_6_reg_iter_0_bleached_masked
7: SyNOnly_CC_4_reg_iter_1_bleached_masked
8: SyNOnly_CC_4_reg_iter_1_normal_masked
9: SyNOnly_CC_6_reg_iter_1_bleached_masked
10: SyNOnly_CC_6_reg_iter_1_normal_masked
11: SyNOnly_CC_2_reg_iter_1_normal_masked
12: SyNOnly_MI_32_reg_iter_1_normal_masked
13: SyNOnly_CC_6_reg_iter_0_normal_masked
14: SyNOnly_CC_4_reg_iter_0_normal_masked
15: SyNOnly_CC_2_reg_iter_0_normal_masked
16: TVMSQC_bleached_masked
17: SyNOnly_MI_32_reg_iter_0_normal_masked
18: TVMSQC_normal_masked
19: TVMSQ_bleached_masked
20: TVMSQ_normal_masked

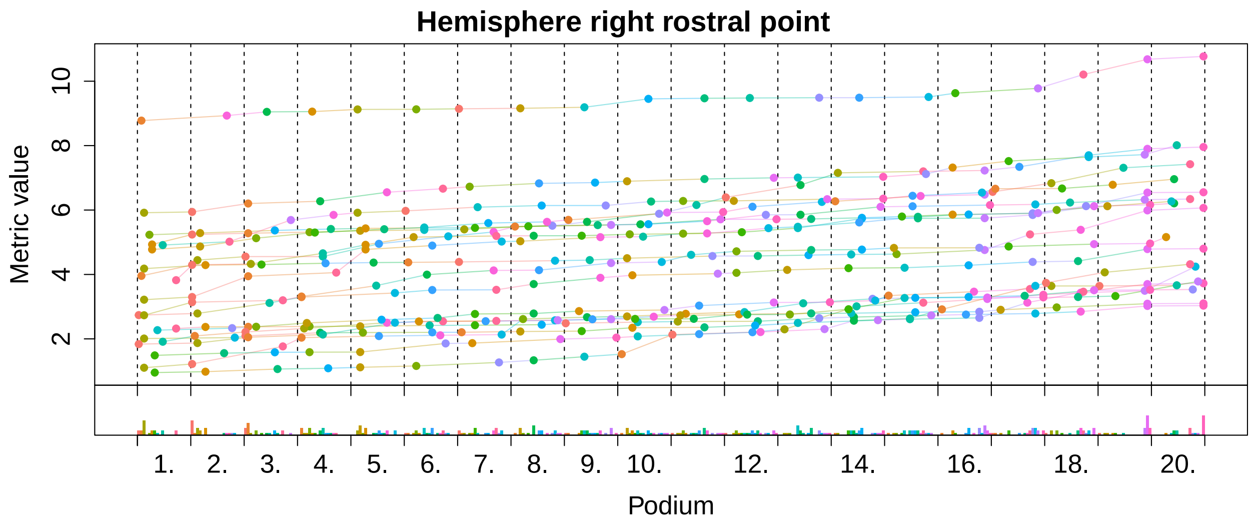

Rank: Alg.

- 1: SyNOnly_CC_4_reg_iter_1_normal_masked
- 2: SyNOnly_CC_2_reg_iter_1_normal_masked
- 3: SyNOnly_CC_6_reg_iter_1_normal_masked
- 4: SyNOnly_CC_4_reg_iter_1_bleached_masked
- 5: SyNOnly_CC_6_reg_iter_1_bleached_masked
- 6: SyNOnly_CC_2_reg_iter_1_bleached_masked
- 7: SyNOnly_CC_2_reg_iter_0_bleached_masked
- 8: SyNOnly_MI_32_reg_iter_1_bleached_masked
- 9: SyNOnly_CC_2_reg_iter_0_normal_masked
- 10: SyNOnly_MI_32_reg_iter_1_normal_masked
- 11: SyNOnly_CC_6_reg_iter_0_normal_masked
- 12: SyNOnly_CC_6_reg_iter_0_bleached_masked
- 13: SyNOnly_CC_4_reg_iter_0_bleached_masked
- 14: TVMSQC_bleached_masked
- 15: TVMSQC_normal_masked
- 16: SyNOnly_MI_32_reg_iter_0_bleached_masked
- 17: SyNOnly_CC_4_reg_iter_0_normal_masked
- 18: SyNOnly_MI_32_reg_iter_0_normal_masked
- 19: TVMSQ_bleached_masked
- 20: TVMSQ_normal_masked

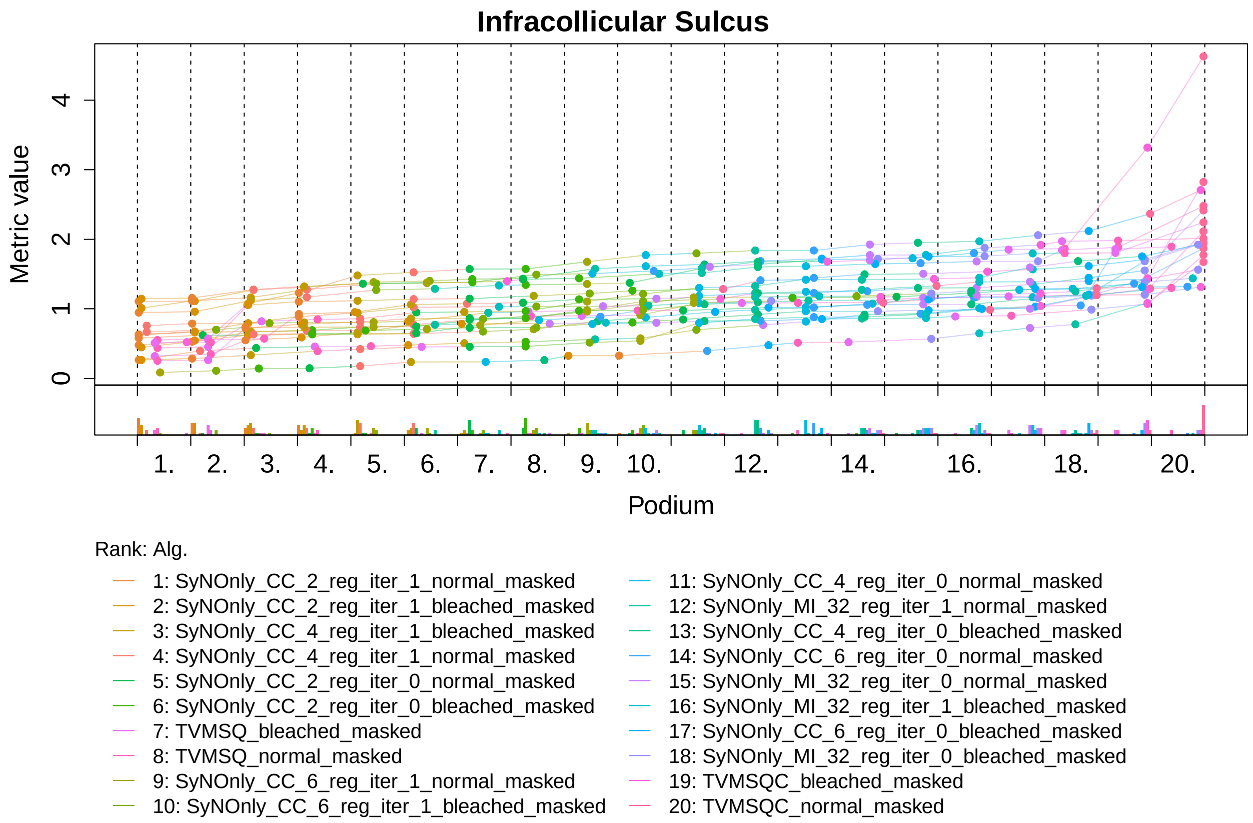

Infracollicular Sulcus
Metric value
0
1
2
3
4
1.
2.
3.
4.
5.
6.
7.
8.
9.
10.
12.
14.
16.
18.
20.
Podium
Rank: Alg.
1: SyNOnly_CC_2_reg_iter_1_normal_masked
2: SyNOnly_CC_2_reg_iter_1_bleached_masked
3: SyNOnly_CC_4_reg_iter_1_bleached_masked
4: SyNOnly_CC_4_reg_iter_1_normal_masked
5: SyNOnly_CC_2_reg_iter_0_normal_masked
6: SyNOnly_CC_2_reg_iter_0_bleached_masked
7: TVMSQ_bleached_masked
8: TVMSQ_normal_masked
9: SyNOnly_CC_6_reg_iter_1_normal_masked
10: SyNOnly_CC_6_reg_iter_1_bleached_masked
11: SyNOnly_CC_4_reg_iter_0_normal_masked
12: SyNOnly_MI_32_reg_iter_1_normal_masked
13: SyNOnly_CC_4_reg_iter_0_bleached_masked
14: SyNOnly_CC_6_reg_iter_0_normal_masked
15: SyNOnly_MI_32_reg_iter_0_normal_masked
16: SyNOnly_MI_32_reg_iter_1_bleached_masked
17: SyNOnly_CC_6_reg_iter_0_bleached_masked
18: SyNOnly_MI_32_reg_iter_0_bleached_masked
19: TVMSQC_bleached_masked
20: TVMSQC_normal_masked

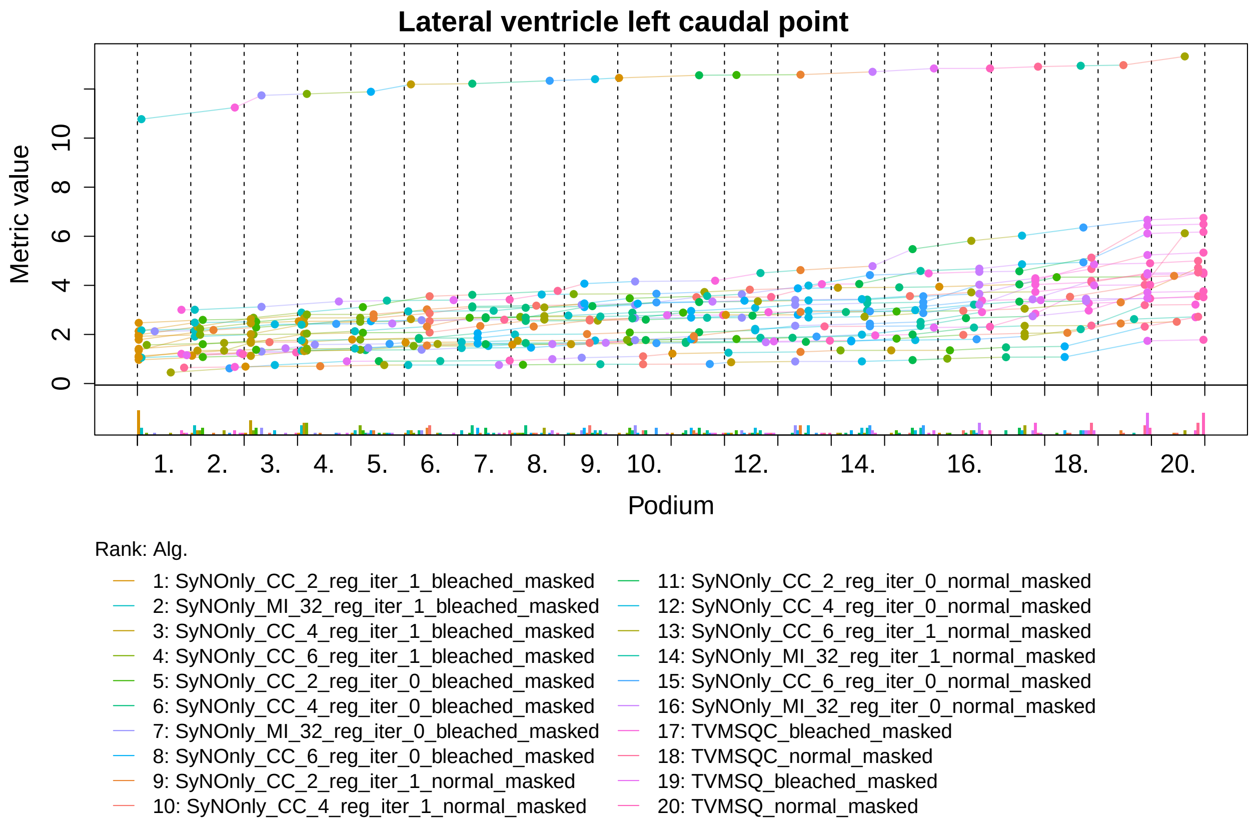

Lateral ventricle left caudal point
Metric value
0
2
4
6
8
10
Podium
1.
2.
3.
4.
5.
6.
7.
8.
9.
10.
12.
14.
16.
18.
20.
Rank: Alg.
1: SyNOnly_CC_2_reg_iter_1_bleached_masked
2: SyNOnly_MI_32_reg_iter_1_bleached_masked
3: SyNOnly_CC_4_reg_iter_1_bleached_masked
4: SyNOnly_CC_6_reg_iter_1_bleached_masked
5: SyNOnly_CC_2_reg_iter_0_bleached_masked
6: SyNOnly_CC_4_reg_iter_0_bleached_masked
7: SyNOnly_MI_32_reg_iter_0_bleached_masked
8: SyNOnly_CC_6_reg_iter_0_bleached_masked
9: SyNOnly_CC_2_reg_iter_1_normal_masked
10: SyNOnly_CC_4_reg_iter_1_normal_masked
11: SyNOnly_CC_2_reg_iter_0_normal_masked
12: SyNOnly_CC_4_reg_iter_0_normal_masked
13: SyNOnly_CC_6_reg_iter_1_normal_masked
14: SyNOnly_MI_32_reg_iter_1_normal_masked
15: SyNOnly_CC_6_reg_iter_0_normal_masked
16: SyNOnly_MI_32_reg_iter_0_normal_masked
17: TVMSQC_bleached_masked
18: TVMSQC_normal_masked
19: TVMSQ_bleached_masked
20: TVMSQ_normal_masked

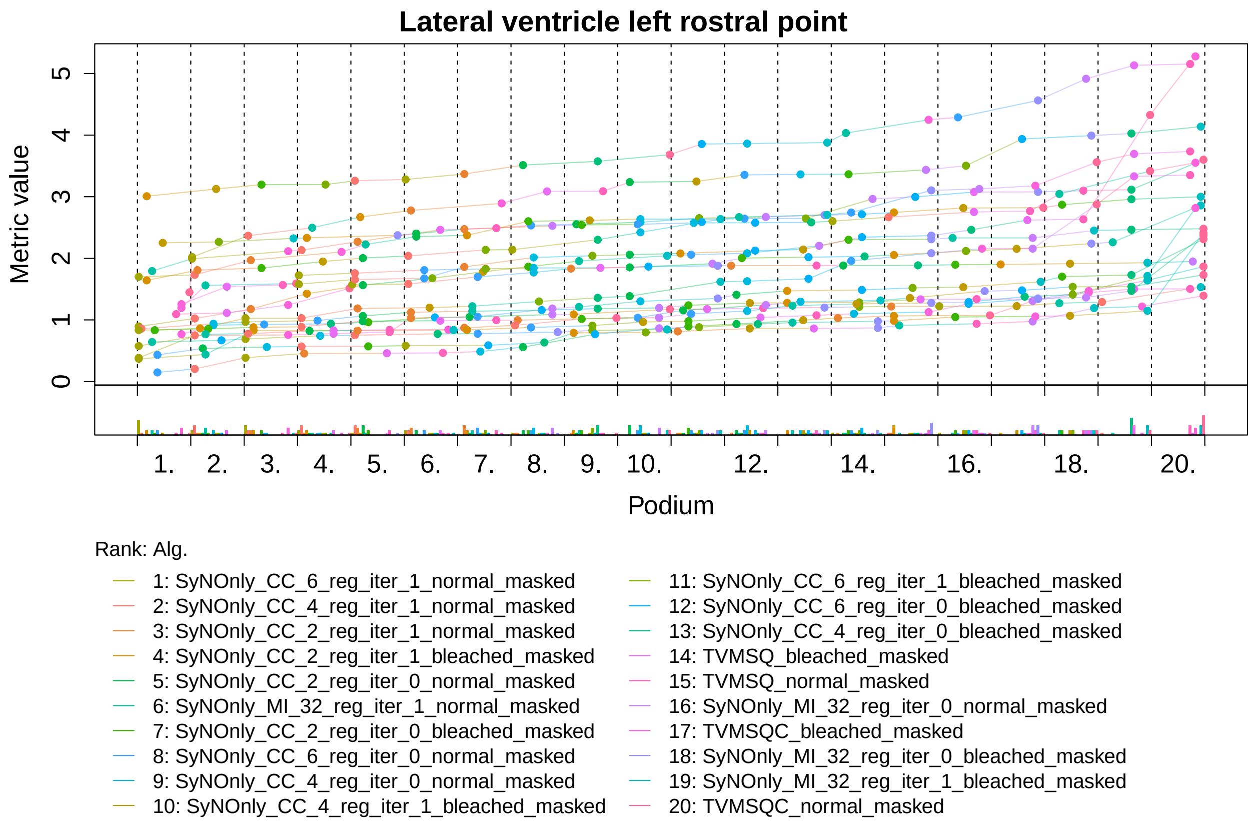
Lateral ventricle left rostral point
Metric value
0
1
2
3
4
5
1.
2.
3.
4.
5.
6.
7.
8.
9.
10.
12.
14.
16.
18.
20.
Podium
Rank: Alg.
1: SyNOnly_CC_6_reg_iter_1_normal_masked
2: SyNOnly_CC_4_reg_iter_1_normal_masked
3: SyNOnly_CC_2_reg_iter_1_normal_masked
4: SyNOnly_CC_2_reg_iter_1_bleached_masked
5: SyNOnly_CC_2_reg_iter_0_normal_masked
6: SyNOnly_MI_32_reg_iter_1_normal_masked
7: SyNOnly_CC_2_reg_iter_0_bleached_masked
8: SyNOnly_CC_6_reg_iter_0_normal_masked
9: SyNOnly_CC_4_reg_iter_0_normal_masked
10: SyNOnly_CC_4_reg_iter_1_bleached_masked
11: SyNOnly_CC_6_reg_iter_1_bleached_masked
12: SyNOnly_CC_6_reg_iter_0_bleached_masked
13: SyNOnly_CC_4_reg_iter_0_bleached_masked
14: TVMSQ_bleached_masked
15: TVMSQ_normal_masked
16: SyNOnly_MI_32_reg_iter_0_normal_masked
17: TVMSQC_bleached_masked
18: SyNOnly_MI_32_reg_iter_0_bleached_masked
19: SyNOnly_MI_32_reg_iter_1_bleached_masked
20: TVMSQC_normal_masked

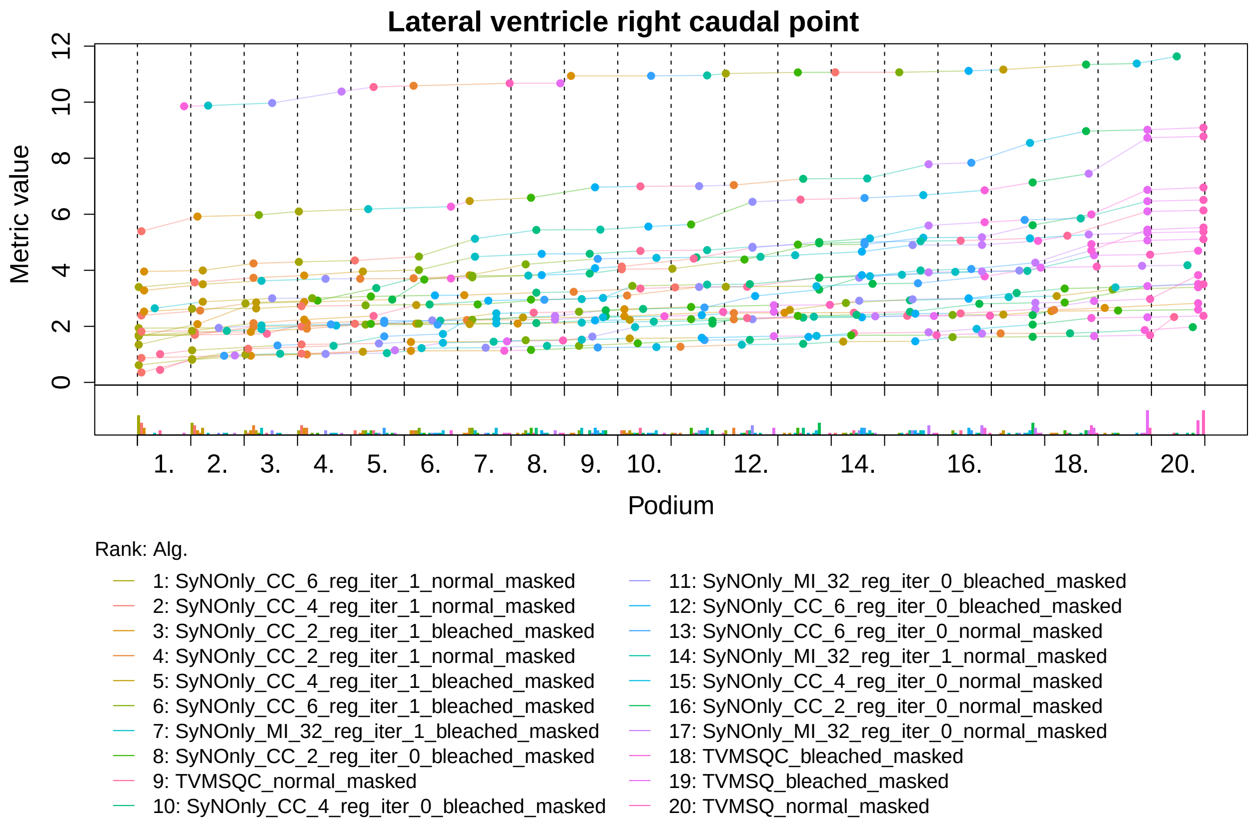

Lateral ventricle right caudal point
Metric value
0
2
4
6
8
10
12
1.
2.
3.
4.
5.
6.
7.
8.
9.
10.
12.
14.
16.
18.
20.
Podium
Rank: Alg.
1: SyNOnly_CC_6_reg_iter_1_normal_masked
2: SyNOnly_CC_4_reg_iter_1_normal_masked
3: SyNOnly_CC_2_reg_iter_1_bleached_masked
4: SyNOnly_CC_2_reg_iter_1_normal_masked
5: SyNOnly_CC_4_reg_iter_1_bleached_masked
6: SyNOnly_CC_6_reg_iter_1_bleached_masked
7: SyNOnly_MI_32_reg_iter_1_bleached_masked
8: SyNOnly_CC_2_reg_iter_0_bleached_masked
9: TVMSQC_normal_masked
10: SyNOnly_CC_4_reg_iter_0_bleached_masked
11: SyNOnly_MI_32_reg_iter_0_bleached_masked
12: SyNOnly_CC_6_reg_iter_0_bleached_masked
13: SyNOnly_CC_6_reg_iter_0_normal_masked
14: SyNOnly_MI_32_reg_iter_1_normal_masked
15: SyNOnly_CC_4_reg_iter_0_normal_masked
16: SyNOnly_CC_2_reg_iter_0_normal_masked
17: SyNOnly_MI_32_reg_iter_0_normal_masked
18: TVMSQC_bleached_masked
19: TVMSQ_bleached_masked
20: TVMSQ_normal_masked

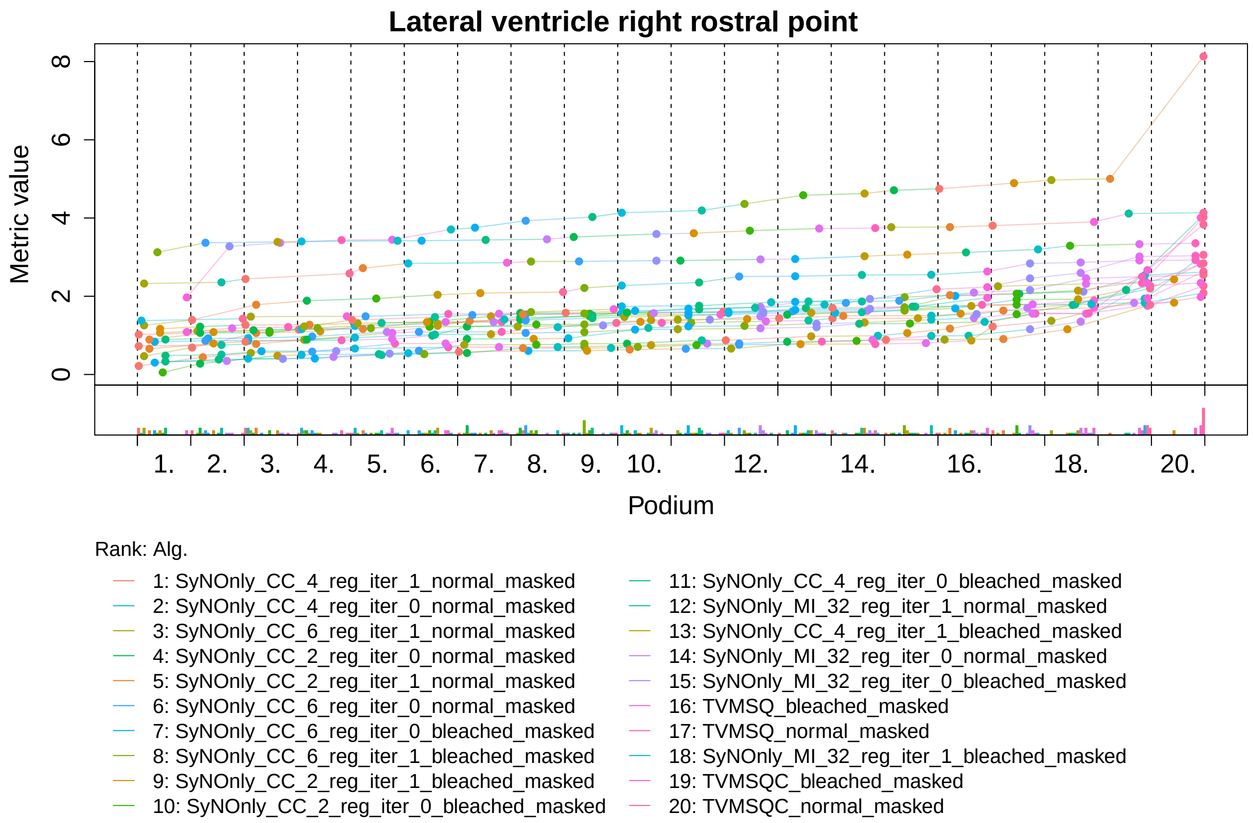
Lateral ventricle right rostral point
Metric value
0
2
4
6
8
1.
2.
3.
4.
5.
6.
7.
8.
9.
10.
12.
14.
16.
18.
20.
Podium
Rank: Alg.
1: SyNOnly_CC_4_reg_iter_1_normal_masked
2: SyNOnly_CC_4_reg_iter_0_normal_masked
3: SyNOnly_CC_6_reg_iter_1_normal_masked
4: SyNOnly_CC_2_reg_iter_0_normal_masked
5: SyNOnly_CC_2_reg_iter_1_normal_masked
6: SyNOnly_CC_6_reg_iter_0_normal_masked
7: SyNOnly_CC_6_reg_iter_0_bleached_masked
8: SyNOnly_CC_6_reg_iter_1_bleached_masked
9: SyNOnly_CC_2_reg_iter_1_bleached_masked
10: SyNOnly_CC_2_reg_iter_0_bleached_masked
11: SyNOnly_CC_4_reg_iter_0_bleached_masked
12: SyNOnly_MI_32_reg_iter_1_normal_masked
13: SyNOnly_CC_4_reg_iter_1_bleached_masked
14: SyNOnly_MI_32_reg_iter_0_normal_masked
15: SyNOnly_MI_32_reg_iter_0_bleached_masked
16: TVMSQ_bleached_masked
17: TVMSQ_normal_masked
18: SyNOnly_MI_32_reg_iter_1_bleached_masked
19: TVMSQC_bleached_masked
20: TVMSQC_normal_masked

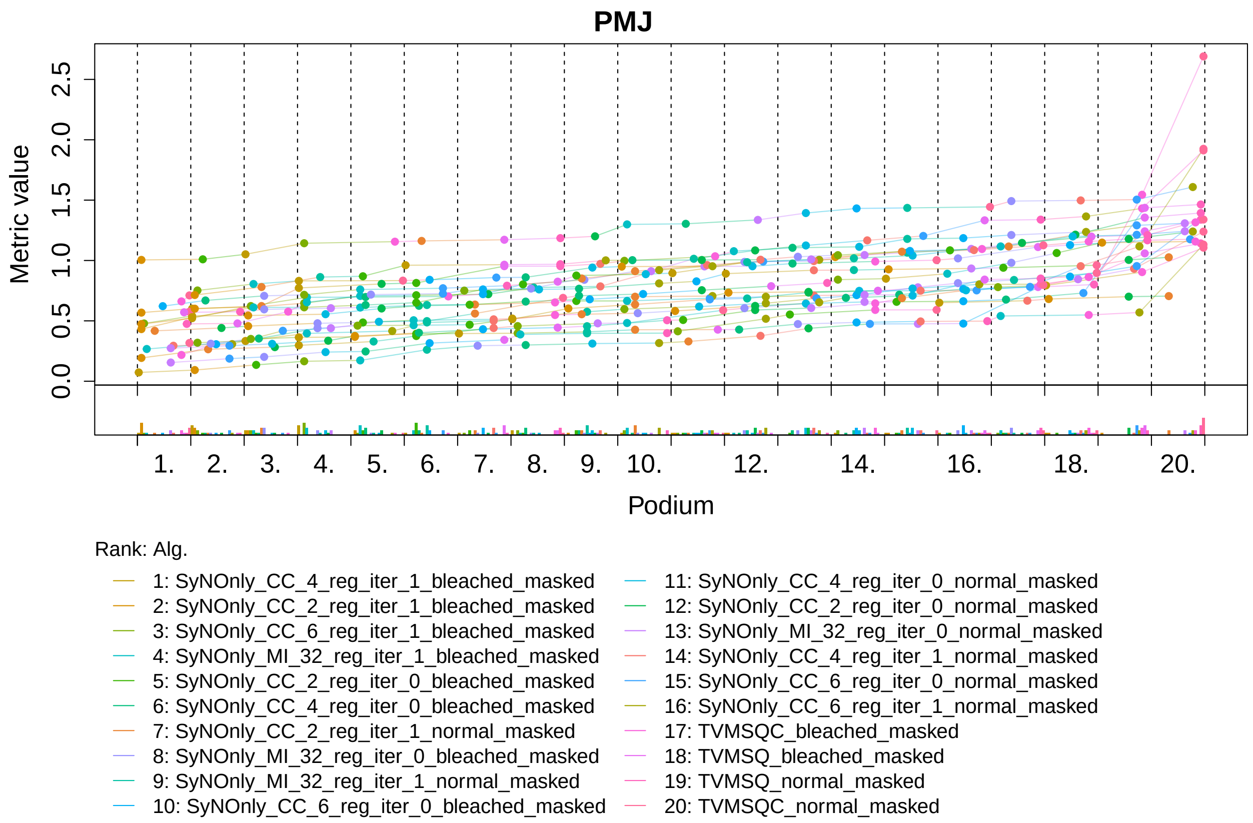
PMJ
Metric value
0.0
0.5
1.0
1.5
2.0
2.5
1.
2.
3.
4.
5.
6.
7.
8.
9.
10.
12.
14.
16.
18.
20.
Podium
Rank: Alg.
1: SyNOnly_CC_4_reg_iter_1_bleached_masked
2: SyNOnly_CC_2_reg_iter_1_bleached_masked
3: SyNOnly_CC_6_reg_iter_1_bleached_masked
4: SyNOnly_MI_32_reg_iter_1_bleached_masked
5: SyNOnly_CC_2_reg_iter_0_bleached_masked
6: SyNOnly_CC_4_reg_iter_0_bleached_masked
7: SyNOnly_CC_2_reg_iter_1_normal_masked
8: SyNOnly_MI_32_reg_iter_0_bleached_masked
9: SyNOnly_MI_32_reg_iter_1_normal_masked
10: SyNOnly_CC_6_reg_iter_0_bleached_masked
11: SyNOnly_CC_4_reg_iter_0_normal_masked
12: SyNOnly_CC_2_reg_iter_0_normal_masked
13: SyNOnly_MI_32_reg_iter_0_normal_masked
14: SyNOnly_CC_4_reg_iter_1_normal_masked
15: SyNOnly_CC_6_reg_iter_0_normal_masked
16: SyNOnly_CC_6_reg_iter_1_normal_masked
17: TVMSQC_bleached_masked
18: TVMSQ_bleached_masked
19: TVMSQ_normal_masked
20: TVMSQC_normal_masked

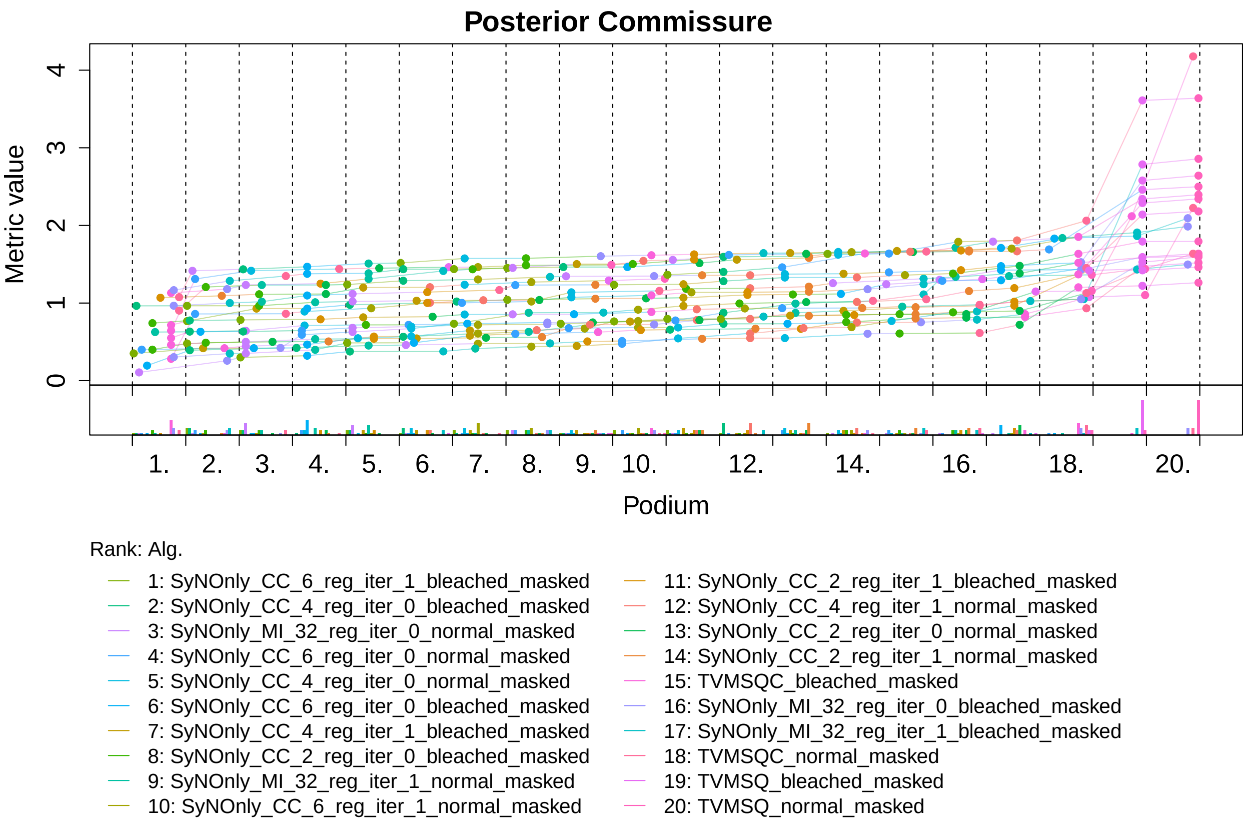
Posterior Commissure
Metric value
0
1
2
3
4
Podium
1.
2.
3.
4.
5.
6.
7.
8.
9.
10.
12.
14.
16.
18.
20.
Rank: Alg.
1: SyNOnly_CC_6_reg_iter_1_bleached_masked
2: SyNOnly_CC_4_reg_iter_0_bleached_masked
3: SyNOnly_MI_32_reg_iter_0_normal_masked
4: SyNOnly_CC_6_reg_iter_0_normal_masked
5: SyNOnly_CC_4_reg_iter_0_normal_masked
6: SyNOnly_CC_6_reg_iter_0_bleached_masked
7: SyNOnly_CC_4_reg_iter_1_bleached_masked
8: SyNOnly_CC_2_reg_iter_0_bleached_masked
9: SyNOnly_MI_32_reg_iter_1_normal_masked
10: SyNOnly_CC_6_reg_iter_1_normal_masked
11: SyNOnly_CC_2_reg_iter_1_bleached_masked
12: SyNOnly_CC_4_reg_iter_1_normal_masked
13: SyNOnly_CC_2_reg_iter_0_normal_masked
14: SyNOnly_CC_2_reg_iter_1_normal_masked
15: TVMSQC_bleached_masked
16: SyNOnly_MI_32_reg_iter_0_bleached_masked
17: SyNOnly_MI_32_reg_iter_1_bleached_masked
18: TVMSQC_normal_masked
19: TVMSQ_bleached_masked
20: TVMSQ_normal_masked

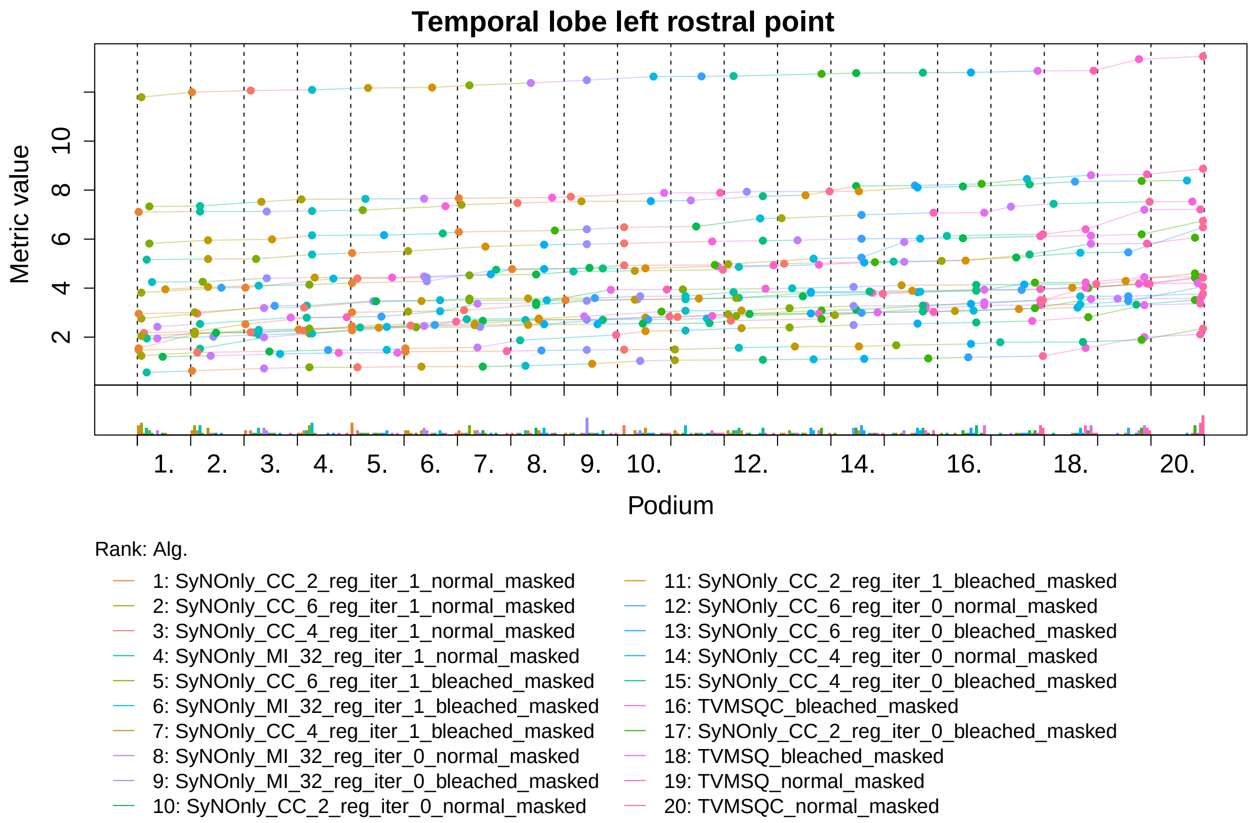
Temporal lobe left rostral point
Metric value
2
4
6
8
10
1.
2.
3.
4.
5.
6.
7.
8.
9.
10.
12.
14.
16.
18.
20.
Podium
Rank: Alg.
1: SyNOnly_CC_2_reg_iter_1_normal_masked
2: SyNOnly_CC_6_reg_iter_1_normal_masked
3: SyNOnly_CC_4_reg_iter_1_normal_masked
4: SyNOnly_MI_32_reg_iter_1_normal_masked
5: SyNOnly_CC_6_reg_iter_1_bleached_masked
6: SyNOnly_MI_32_reg_iter_1_bleached_masked
7: SyNOnly_CC_4_reg_iter_1_bleached_masked
8: SyNOnly_MI_32_reg_iter_0_normal_masked
9: SyNOnly_MI_32_reg_iter_0_bleached_masked
10: SyNOnly_CC_2_reg_iter_0_normal_masked
11: SyNOnly_CC_2_reg_iter_1_bleached_masked
12: SyNOnly_CC_6_reg_iter_0_normal_masked
13: SyNOnly_CC_6_reg_iter_0_bleached_masked
14: SyNOnly_CC_4_reg_iter_0_normal_masked
15: SyNOnly_CC_4_reg_iter_0_bleached_masked
16: TVMSQC_bleached_masked
17: SyNOnly_CC_2_reg_iter_0_bleached_masked
18: TVMSQ_bleached_masked
19: TVMSQ_normal_masked
20: TVMSQC_normal_masked

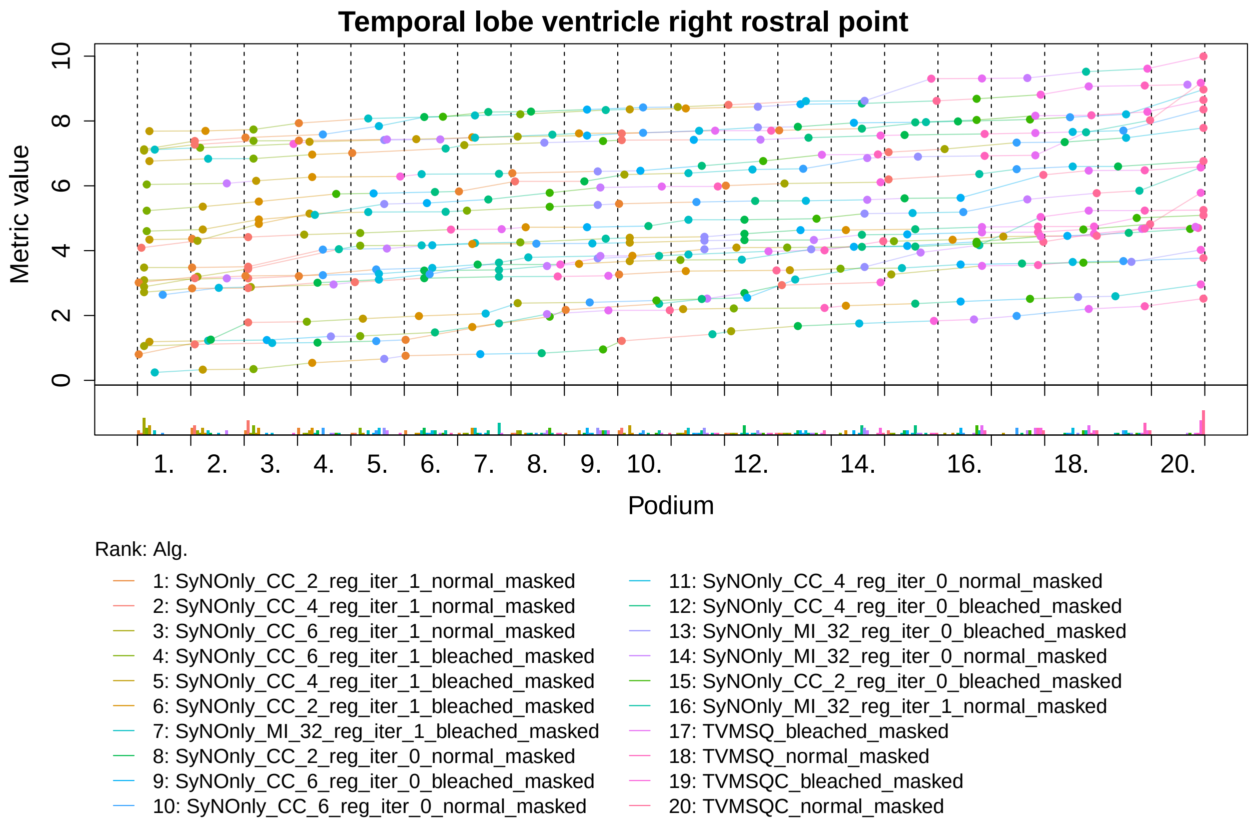
Temporal lobe ventricle right rostral point
Metric value
0
2
4
6
8
10
1.
2.
3.
4.
5.
6.
7.
8.
9.
10.
12.
14.
16.
18.
20.
Podium
Rank: Alg.
1: SyNOnly_CC_2_reg_iter_1_normal_masked
2: SyNOnly_CC_4_reg_iter_1_normal_masked
3: SyNOnly_CC_6_reg_iter_1_normal_masked
4: SyNOnly_CC_6_reg_iter_1_bleached_masked
5: SyNOnly_CC_4_reg_iter_1_bleached_masked
6: SyNOnly_CC_2_reg_iter_1_bleached_masked
7: SyNOnly_MI_32_reg_iter_1_bleached_masked
8: SyNOnly_CC_2_reg_iter_0_normal_masked
9: SyNOnly_CC_6_reg_iter_0_bleached_masked
10: SyNOnly_CC_6_reg_iter_0_normal_masked
11: SyNOnly_CC_4_reg_iter_0_normal_masked
12: SyNOnly_CC_4_reg_iter_0_bleached_masked
13: SyNOnly_MI_32_reg_iter_0_bleached_masked
14: SyNOnly_MI_32_reg_iter_0_normal_masked
15: SyNOnly_CC_2_reg_iter_0_bleached_masked
16: SyNOnly_MI_32_reg_iter_1_normal_masked
17: TVMSQ_bleached_masked
18: TVMSQ_normal_masked
19: TVMSQC_bleached_masked
20: TVMSQC_normal_masked

2.3 Ranking heatmap

Ranking heatmaps for visualizing raw assessment data. Each cell (i, A_j) shows the absolute frequency of cases in which algorithm A_j achieved rank i.

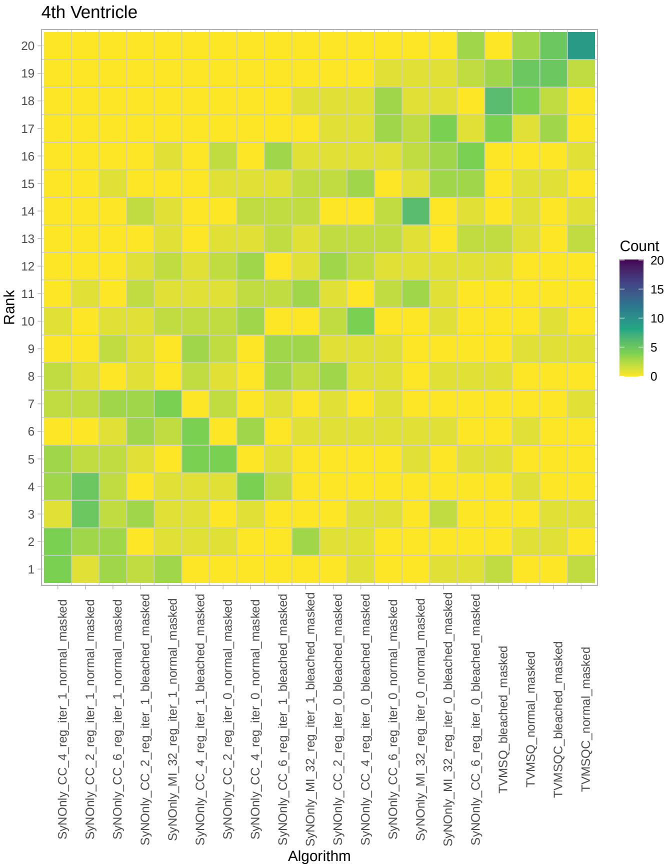

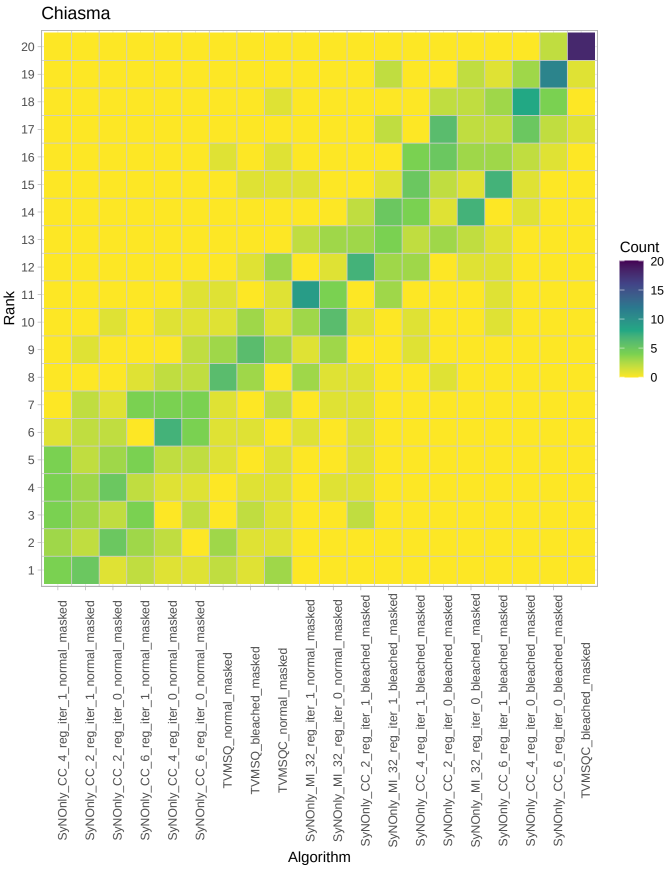
Chiasma
Rank
Algorithm
Count
20
15
10
5
0
SyNOnly_CC_4_reg_iter_1_normal_masked
SyNOnly_CC_2_reg_iter_1_normal_masked
SyNOnly_CC_2_reg_iter_0_normal_masked
SyNOnly_CC_6_reg_iter_1_normal_masked
SyNOnly_CC_4_reg_iter_0_normal_masked
SyNOnly_CC_6_reg_iter_0_normal_masked
TVMSQ_normal_masked
TVMSQ_bleached_masked
TVMSQC_normal_masked
SyNOnly_MI_32_reg_iter_1_normal_masked
SyNOnly_MI_32_reg_iter_0_normal_masked
SyNOnly_CC_2_reg_iter_1_bleached_masked
SyNOnly_MI_32_reg_iter_1_bleached_masked
SyNOnly_CC_4_reg_iter_1_bleached_masked
SyNOnly_CC_2_reg_iter_0_bleached_masked
SyNOnly_MI_32_reg_iter_0_bleached_masked
SyNOnly_CC_6_reg_iter_1_bleached_masked
SyNOnly_CC_4_reg_iter_0_bleached_masked
SyNOnly_CC_6_reg_iter_0_bleached_masked
TVMSQC_bleached_masked

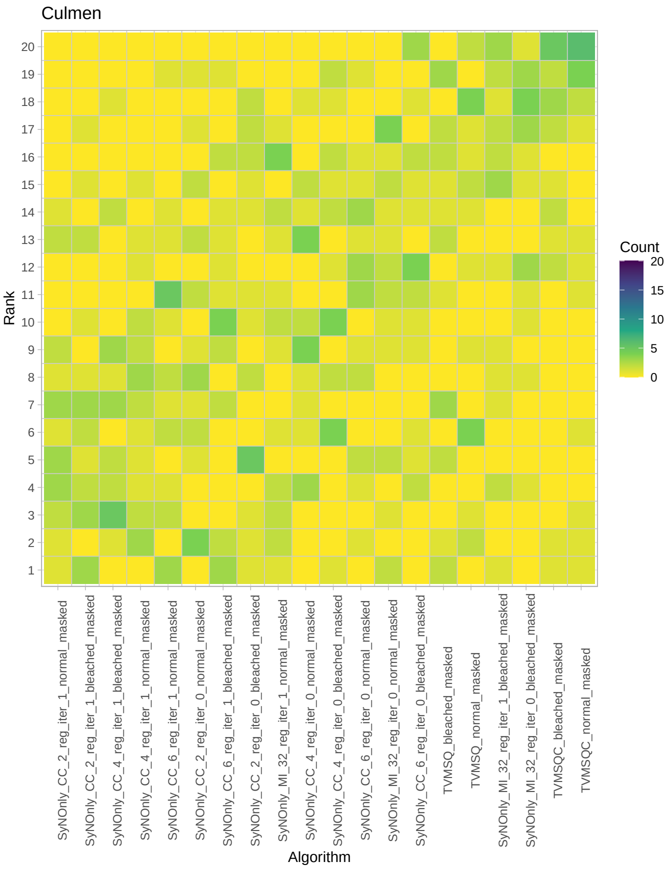
Culmen
Rank
Algorithm
Count
20
15
10
5
0
SyNOnly_CC_2_reg_iter_1_normal_masked
SyNOnly_CC_2_reg_iter_1_bleached_masked
SyNOnly_CC_4_reg_iter_1_bleached_masked
SyNOnly_CC_4_reg_iter_1_normal_masked
SyNOnly_CC_6_reg_iter_1_normal_masked
SyNOnly_CC_2_reg_iter_0_normal_masked
SyNOnly_CC_6_reg_iter_1_bleached_masked
SyNOnly_CC_2_reg_iter_0_bleached_masked
SyNOnly_MI_32_reg_iter_1_normal_masked
SyNOnly_CC_4_reg_iter_0_normal_masked
SyNOnly_CC_4_reg_iter_0_bleached_masked
SyNOnly_CC_6_reg_iter_0_normal_masked
SyNOnly_MI_32_reg_iter_0_normal_masked
SyNOnly_CC_6_reg_iter_0_bleached_masked
TVMSQ_bleached_masked
TVMSQ_normal_masked
SyNOnly_MI_32_reg_iter_1_bleached_masked
SyNOnly_MI_32_reg_iter_0_bleached_masked
TVMSQC_bleached_masked
TVMSQC_normal_masked

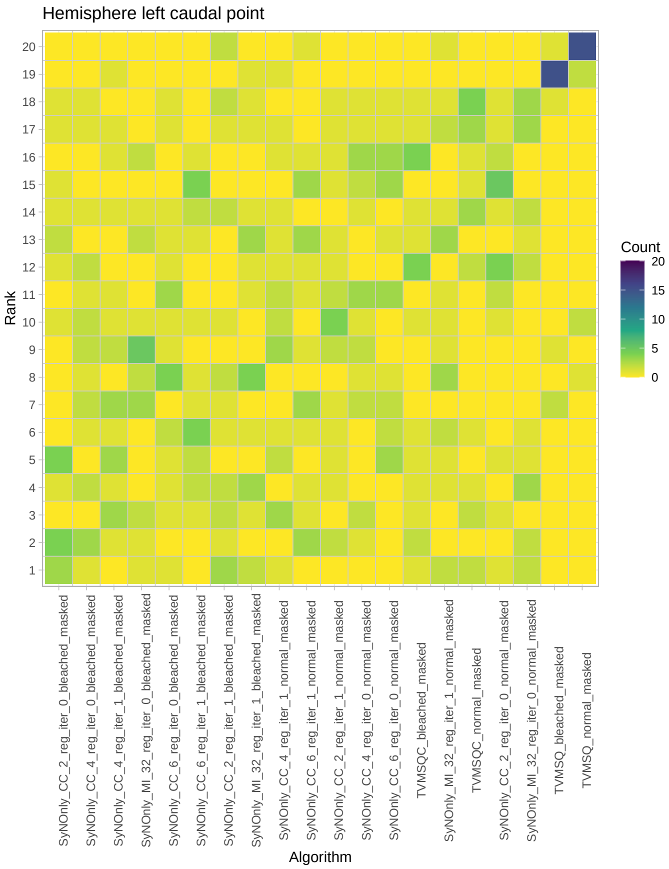
Hemisphere left caudal point
Rank
Algorithm
Count
20
15
10
5
0
SyNOnly_CC_2_reg_iter_0_bleached_masked
SyNOnly_CC_4_reg_iter_0_bleached_masked
SyNOnly_CC_4_reg_iter_1_bleached_masked
SyNOnly_MI_32_reg_iter_0_bleached_masked
SyNOnly_CC_6_reg_iter_0_bleached_masked
SyNOnly_CC_6_reg_iter_1_bleached_masked
SyNOnly_CC_2_reg_iter_1_bleached_masked
SyNOnly_MI_32_reg_iter_1_bleached_masked
SyNOnly_CC_4_reg_iter_1_normal_masked
SyNOnly_CC_6_reg_iter_1_normal_masked
SyNOnly_CC_2_reg_iter_1_normal_masked
SyNOnly_CC_4_reg_iter_0_normal_masked
SyNOnly_CC_6_reg_iter_0_normal_masked
TVMSQC_bleached_masked
SyNOnly_MI_32_reg_iter_1_normal_masked
TVMSQC_normal_masked
SyNOnly_CC_2_reg_iter_0_normal_masked
SyNOnly_MI_32_reg_iter_0_normal_masked
TVMSQ_bleached_masked
TVMSQ_normal_masked

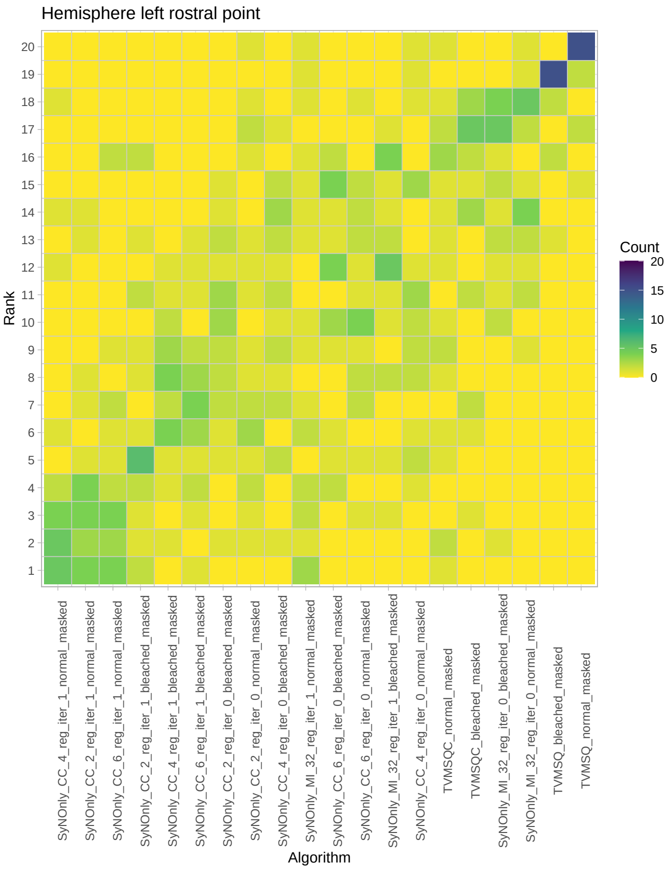

Hemisphere left rostral point
Rank
20
19
18
17
16
15
14
13
12
11
10
9
8
7
6
5
4
3
2
1
Count
20
15
10
5
0
SyNOnly_CC_4_reg_iter_1_normal_masked
SyNOnly_CC_2_reg_iter_1_normal_masked
SyNOnly_CC_6_reg_iter_1_normal_masked
SyNOnly_CC_2_reg_iter_1_bleached_masked
SyNOnly_CC_4_reg_iter_1_bleached_masked
SyNOnly_CC_6_reg_iter_1_bleached_masked
SyNOnly_CC_2_reg_iter_0_bleached_masked
SyNOnly_CC_2_reg_iter_0_normal_masked
SyNOnly_CC_4_reg_iter_0_bleached_masked
SyNOnly_MI_32_reg_iter_1_normal_masked
SyNOnly_CC_6_reg_iter_0_bleached_masked
SyNOnly_CC_6_reg_iter_0_normal_masked
SyNOnly_MI_32_reg_iter_1_bleached_masked
SyNOnly_CC_4_reg_iter_0_normal_masked
TVMSQC_normal_masked
TVMSQC_bleached_masked
SyNOnly_MI_32_reg_iter_0_bleached_masked
SyNOnly_MI_32_reg_iter_0_normal_masked
TVMSQ_bleached_masked
TVMSQ_normal_masked
Algorithm

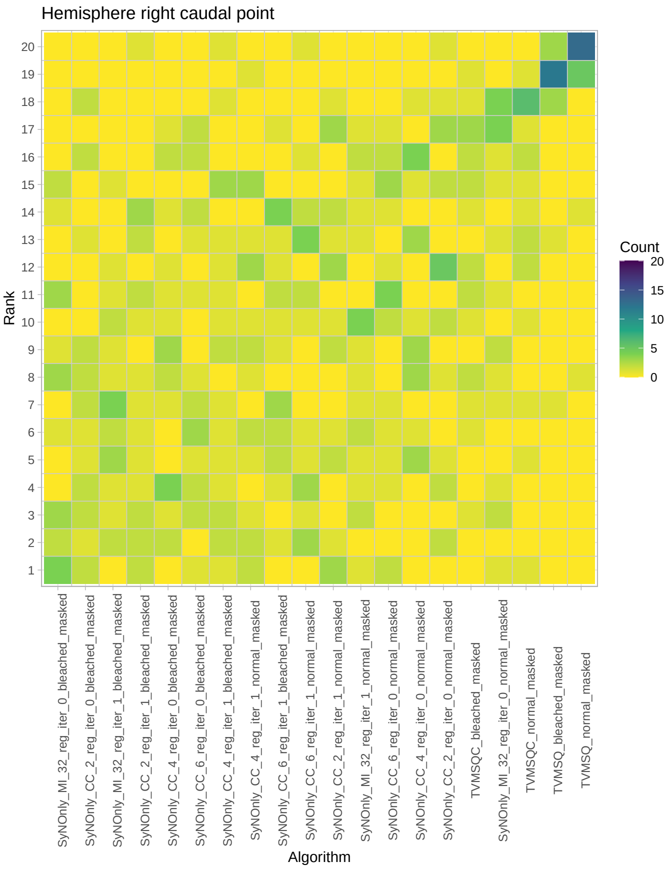
Hemisphere right caudal point
Rank
Algorithm
Count
20
15
10
5
0
SyNOnly_MI_32_reg_iter_0_bleached_masked
SyNOnly_CC_2_reg_iter_0_bleached_masked
SyNOnly_MI_32_reg_iter_1_bleached_masked
SyNOnly_CC_2_reg_iter_1_bleached_masked
SyNOnly_CC_4_reg_iter_0_bleached_masked
SyNOnly_CC_6_reg_iter_0_bleached_masked
SyNOnly_CC_4_reg_iter_1_bleached_masked
SyNOnly_CC_4_reg_iter_1_normal_masked
SyNOnly_CC_6_reg_iter_1_bleached_masked
SyNOnly_CC_6_reg_iter_1_normal_masked
SyNOnly_CC_2_reg_iter_1_normal_masked
SyNOnly_MI_32_reg_iter_1_normal_masked
SyNOnly_CC_6_reg_iter_0_normal_masked
SyNOnly_CC_4_reg_iter_0_normal_masked
SyNOnly_CC_2_reg_iter_0_normal_masked
TVMSQC_bleached_masked
SyNOnly_MI_32_reg_iter_0_normal_masked
TVMSQC_normal_masked
TVMSQ_bleached_masked
TVMSQ_normal_masked

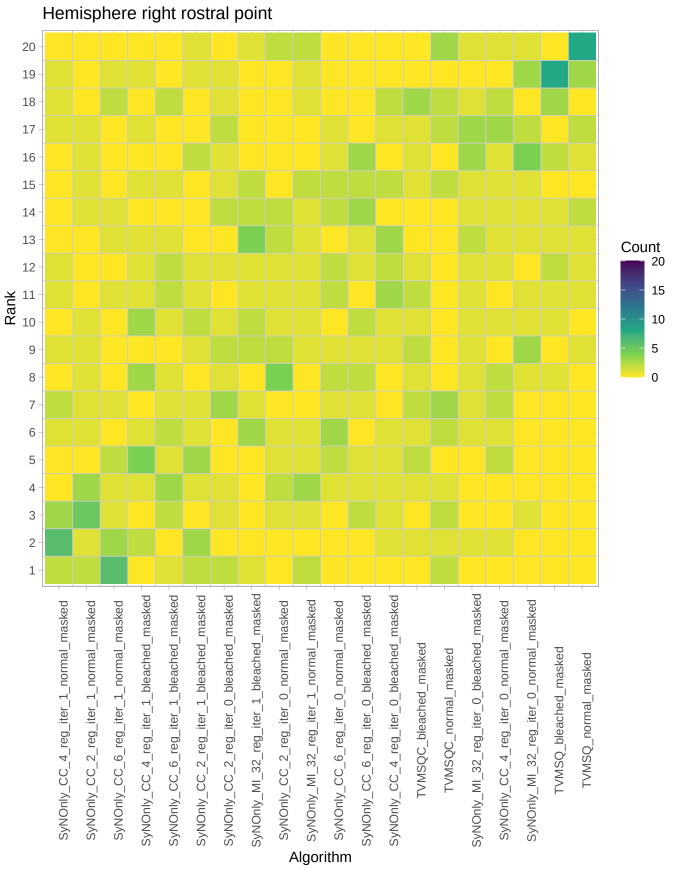

Hemisphere right rostral point
Rank
20
19
18
17
16
15
14
13
12
11
10
9
8
7
6
5
4
3
2
1
Count
20
15
10
5
0
SyNOnly_CC_4_reg_iter_1_normal_masked
SyNOnly_CC_2_reg_iter_1_normal_masked
SyNOnly_CC_6_reg_iter_1_normal_masked
SyNOnly_CC_4_reg_iter_1_bleached_masked
SyNOnly_CC_6_reg_iter_1_bleached_masked
SyNOnly_CC_2_reg_iter_1_bleached_masked
SyNOnly_CC_2_reg_iter_0_bleached_masked
SyNOnly_MI_32_reg_iter_1_bleached_masked
SyNOnly_CC_2_reg_iter_0_normal_masked
SyNOnly_MI_32_reg_iter_1_normal_masked
SyNOnly_CC_6_reg_iter_0_normal_masked
SyNOnly_CC_6_reg_iter_0_bleached_masked
SyNOnly_CC_4_reg_iter_0_bleached_masked
TVMSQC_bleached_masked
TVMSQC_normal_masked
SyNOnly_MI_32_reg_iter_0_bleached_masked
SyNOnly_CC_4_reg_iter_0_normal_masked
SyNOnly_MI_32_reg_iter_0_normal_masked
TVMSQ_bleached_masked
TVMSQ_normal_masked
Algorithm

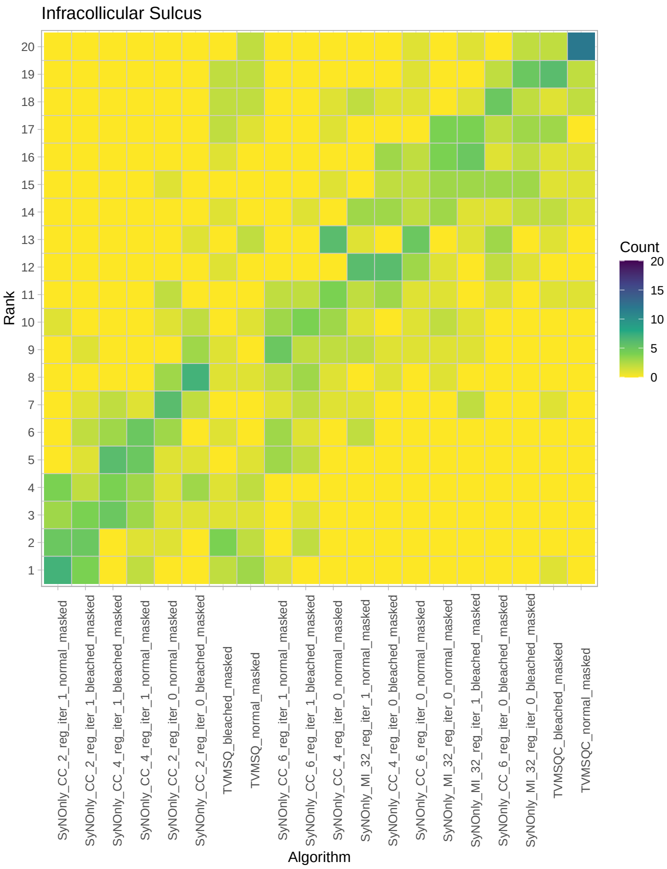
Infracollicular Sulcus
Rank
20
19
18
17
16
15
14
13
12
11
10
9
8
7
6
5
4
3
2
1
Count
20
15
10
5
0
SyNOnly_CC_2_reg_iter_1_normal_masked
SyNOnly_CC_2_reg_iter_1_bleached_masked
SyNOnly_CC_4_reg_iter_1_bleached_masked
SyNOnly_CC_4_reg_iter_1_normal_masked
SyNOnly_CC_2_reg_iter_0_normal_masked
SyNOnly_CC_2_reg_iter_0_bleached_masked
TVMSQ_bleached_masked
TVMSQ_normal_masked
SyNOnly_CC_6_reg_iter_1_normal_masked
SyNOnly_CC_6_reg_iter_1_bleached_masked
SyNOnly_CC_4_reg_iter_0_normal_masked
SyNOnly_MI_32_reg_iter_1_normal_masked
SyNOnly_CC_4_reg_iter_0_bleached_masked
SyNOnly_CC_6_reg_iter_0_normal_masked
SyNOnly_MI_32_reg_iter_0_normal_masked
SyNOnly_MI_32_reg_iter_1_bleached_masked
SyNOnly_CC_6_reg_iter_0_bleached_masked
SyNOnly_MI_32_reg_iter_0_bleached_masked
TVMSQC_bleached_masked
TVMSQC_normal_masked
Algorithm

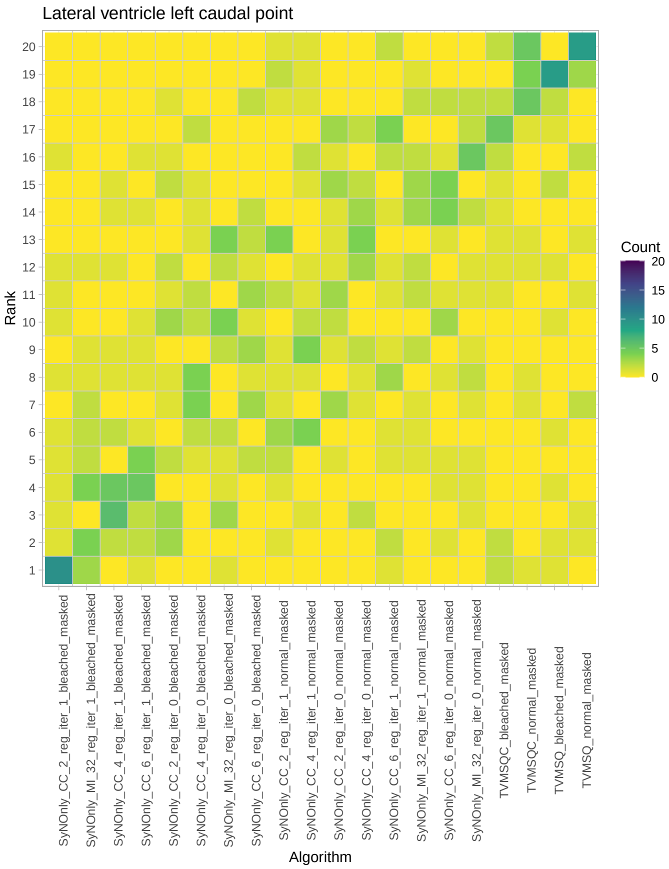
Lateral ventricle left caudal point
Rank
Algorithm
Count
SyNOnly_CC_2_reg_iter_1_bleached_masked
SyNOnly_MI_32_reg_iter_1_bleached_masked
SyNOnly_CC_4_reg_iter_1_bleached_masked
SyNOnly_CC_6_reg_iter_1_bleached_masked
SyNOnly_CC_2_reg_iter_0_bleached_masked
SyNOnly_CC_4_reg_iter_0_bleached_masked
SyNOnly_MI_32_reg_iter_0_bleached_masked
SyNOnly_CC_6_reg_iter_0_bleached_masked
SyNOnly_CC_2_reg_iter_1_normal_masked
SyNOnly_CC_4_reg_iter_1_normal_masked
SyNOnly_CC_2_reg_iter_0_normal_masked
SyNOnly_CC_4_reg_iter_0_normal_masked
SyNOnly_CC_6_reg_iter_1_normal_masked
SyNOnly_MI_32_reg_iter_1_normal_masked
SyNOnly_CC_6_reg_iter_0_normal_masked
SyNOnly_MI_32_reg_iter_0_normal_masked
TVMSQC_bleached_masked
TVMSQC_normal_masked
TVMSQ_bleached_masked
TVMSQ_normal_masked

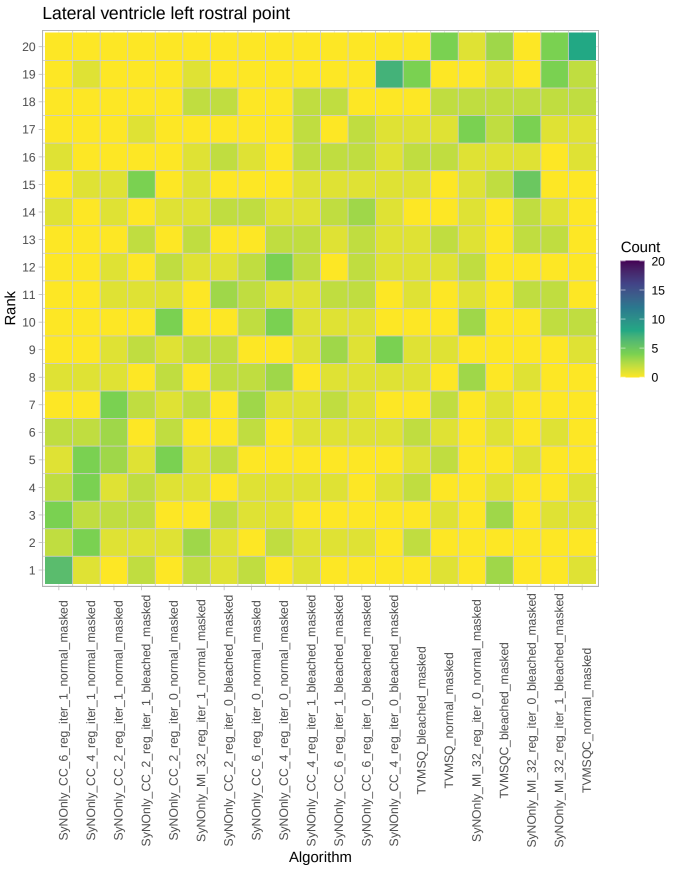

Lateral ventricle left rostral point
Rank
Algorithm
Count
20
15
10
5
0
SyNOnly_CC_6_reg_iter_1_normal_masked
SyNOnly_CC_4_reg_iter_1_normal_masked
SyNOnly_CC_2_reg_iter_1_normal_masked
SyNOnly_CC_2_reg_iter_1_bleached_masked
SyNOnly_CC_2_reg_iter_0_normal_masked
SyNOnly_MI_32_reg_iter_1_normal_masked
SyNOnly_CC_2_reg_iter_0_bleached_masked
SyNOnly_CC_6_reg_iter_0_normal_masked
SyNOnly_CC_4_reg_iter_0_normal_masked
SyNOnly_CC_4_reg_iter_1_bleached_masked
SyNOnly_CC_6_reg_iter_1_bleached_masked
SyNOnly_CC_6_reg_iter_0_bleached_masked
SyNOnly_CC_4_reg_iter_0_bleached_masked
TVMSQ_bleached_masked
TVMSQ_normal_masked
SyNOnly_MI_32_reg_iter_0_normal_masked
TVMSQC_bleached_masked
SyNOnly_MI_32_reg_iter_0_bleached_masked
SyNOnly_MI_32_reg_iter_1_bleached_masked
TVMSQC_normal_masked

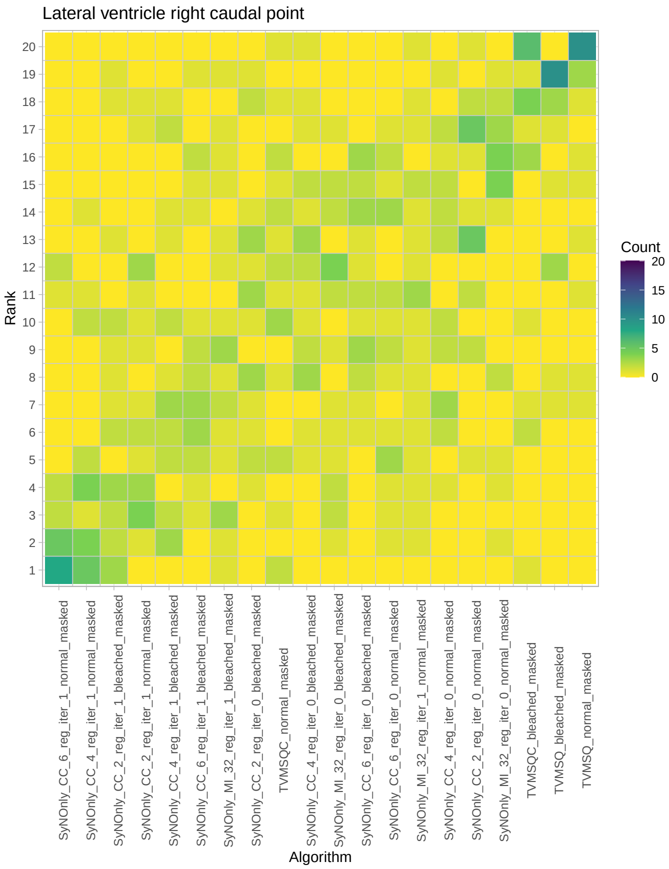

Lateral ventricle right caudal point
Rank
Algorithm
Count
SyNOnly_CC_6_reg_iter_1_normal_masked
SyNOnly_CC_4_reg_iter_1_normal_masked
SyNOnly_CC_2_reg_iter_1_bleached_masked
SyNOnly_CC_2_reg_iter_1_normal_masked
SyNOnly_CC_4_reg_iter_1_bleached_masked
SyNOnly_CC_6_reg_iter_1_bleached_masked
SyNOnly_MI_32_reg_iter_1_bleached_masked
SyNOnly_CC_2_reg_iter_0_bleached_masked
TVMSQC_normal_masked
SyNOnly_CC_4_reg_iter_0_bleached_masked
SyNOnly_MI_32_reg_iter_0_bleached_masked
SyNOnly_CC_6_reg_iter_0_bleached_masked
SyNOnly_CC_6_reg_iter_0_normal_masked
SyNOnly_MI_32_reg_iter_1_normal_masked
SyNOnly_CC_4_reg_iter_0_normal_masked
SyNOnly_CC_2_reg_iter_0_normal_masked
SyNOnly_MI_32_reg_iter_0_normal_masked
TVMSQC_bleached_masked
TVMSQ_bleached_masked
TVMSQ_normal_masked

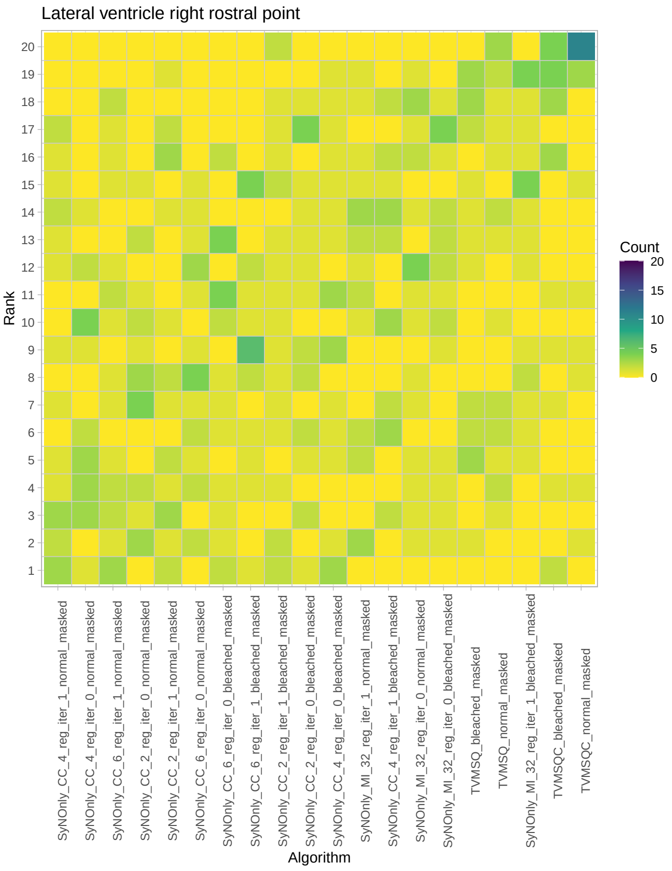
Lateral ventricle right rostral point
Rank
20
19
18
17
16
15
14
13
12
11
10
9
8
7
6
5
4
3
2
1
Count
20
15
10
5
0
SyNOnly_CC_4_reg_iter_1_normal_masked
SyNOnly_CC_4_reg_iter_0_normal_masked
SyNOnly_CC_6_reg_iter_1_normal_masked
SyNOnly_CC_2_reg_iter_0_normal_masked
SyNOnly_CC_2_reg_iter_1_normal_masked
SyNOnly_CC_6_reg_iter_0_normal_masked
SyNOnly_CC_6_reg_iter_0_bleached_masked
SyNOnly_CC_6_reg_iter_1_bleached_masked
SyNOnly_CC_2_reg_iter_1_bleached_masked
SyNOnly_CC_2_reg_iter_0_bleached_masked
SyNOnly_CC_4_reg_iter_0_bleached_masked
SyNOnly_MI_32_reg_iter_1_normal_masked
SyNOnly_CC_4_reg_iter_1_bleached_masked
SyNOnly_MI_32_reg_iter_0_normal_masked
SyNOnly_MI_32_reg_iter_0_bleached_masked
TVMSQ_bleached_masked
TVMSQ_normal_masked
SyNOnly_MI_32_reg_iter_1_bleached_masked
TVMSQC_bleached_masked
TVMSQC_normal_masked
Algorithm

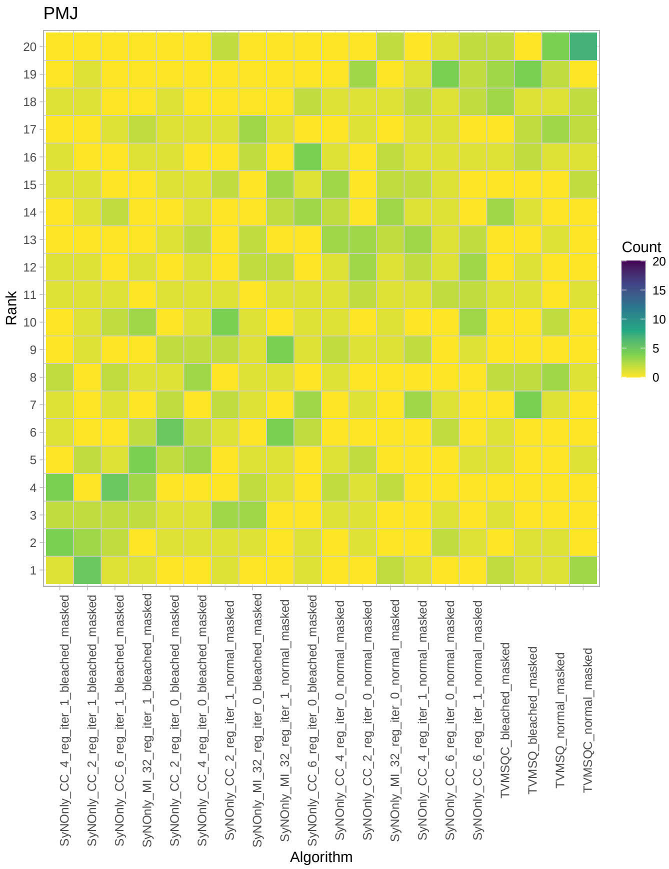
PMJ
Rank
Algorithm
Count
20
15
10
5
0
SyNOnly_CC_4_reg_iter_1_bleached_masked
SyNOnly_CC_2_reg_iter_1_bleached_masked
SyNOnly_CC_6_reg_iter_1_bleached_masked
SyNOnly_MI_32_reg_iter_1_bleached_masked
SyNOnly_CC_2_reg_iter_0_bleached_masked
SyNOnly_CC_4_reg_iter_0_bleached_masked
SyNOnly_CC_2_reg_iter_1_normal_masked
SyNOnly_MI_32_reg_iter_0_bleached_masked
SyNOnly_MI_32_reg_iter_1_normal_masked
SyNOnly_CC_6_reg_iter_0_bleached_masked
SyNOnly_CC_4_reg_iter_0_normal_masked
SyNOnly_CC_2_reg_iter_0_normal_masked
SyNOnly_MI_32_reg_iter_0_normal_masked
SyNOnly_CC_4_reg_iter_1_normal_masked
SyNOnly_CC_6_reg_iter_0_normal_masked
SyNOnly_CC_6_reg_iter_1_normal_masked
TVMSQC_bleached_masked
TVMSQ_bleached_masked
TVMSQ_normal_masked
TVMSQC_normal_masked

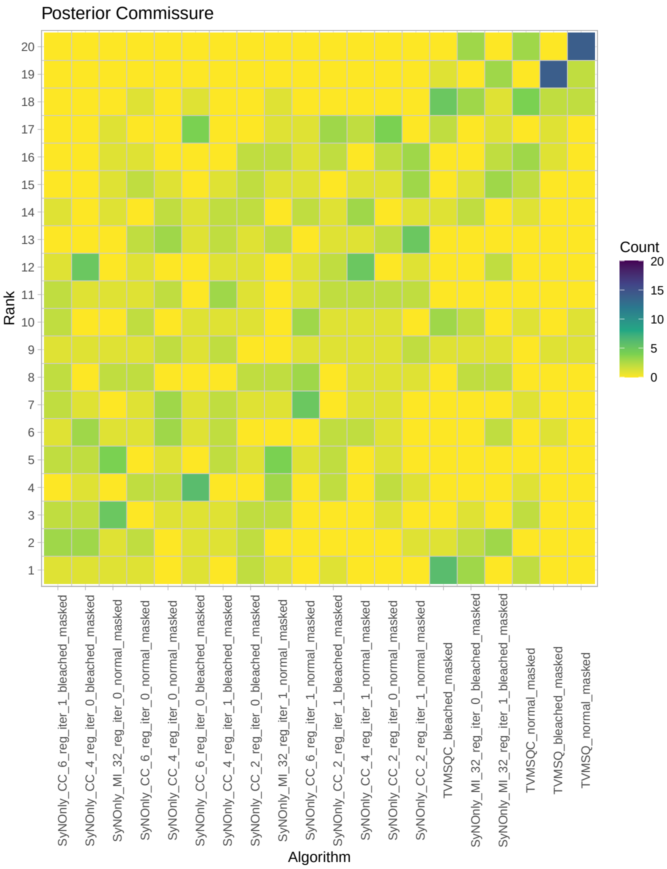

Posterior Commissure
Rank
20
19
18
17
16
15
14
13
12
11
10
9
8
7
6
5
4
3
2
1
Count
20
15
10
5
0
SyNOnly_CC_6_reg_iter_1_bleached_masked
SyNOnly_CC_4_reg_iter_0_bleached_masked
SyNOnly_MI_32_reg_iter_0_normal_masked
SyNOnly_CC_6_reg_iter_0_normal_masked
SyNOnly_CC_4_reg_iter_0_normal_masked
SyNOnly_CC_6_reg_iter_0_bleached_masked
SyNOnly_CC_4_reg_iter_1_bleached_masked
SyNOnly_CC_2_reg_iter_0_bleached_masked
SyNOnly_MI_32_reg_iter_1_normal_masked
SyNOnly_CC_6_reg_iter_1_normal_masked
SyNOnly_CC_2_reg_iter_1_bleached_masked
SyNOnly_CC_4_reg_iter_1_normal_masked
SyNOnly_CC_2_reg_iter_0_normal_masked
SyNOnly_CC_2_reg_iter_1_normal_masked
TVMSQC_bleached_masked
SyNOnly_MI_32_reg_iter_0_bleached_masked
SyNOnly_MI_32_reg_iter_1_bleached_masked
TVMSQC_normal_masked
TVMSQ_bleached_masked
TVMSQ_normal_masked
Algorithm

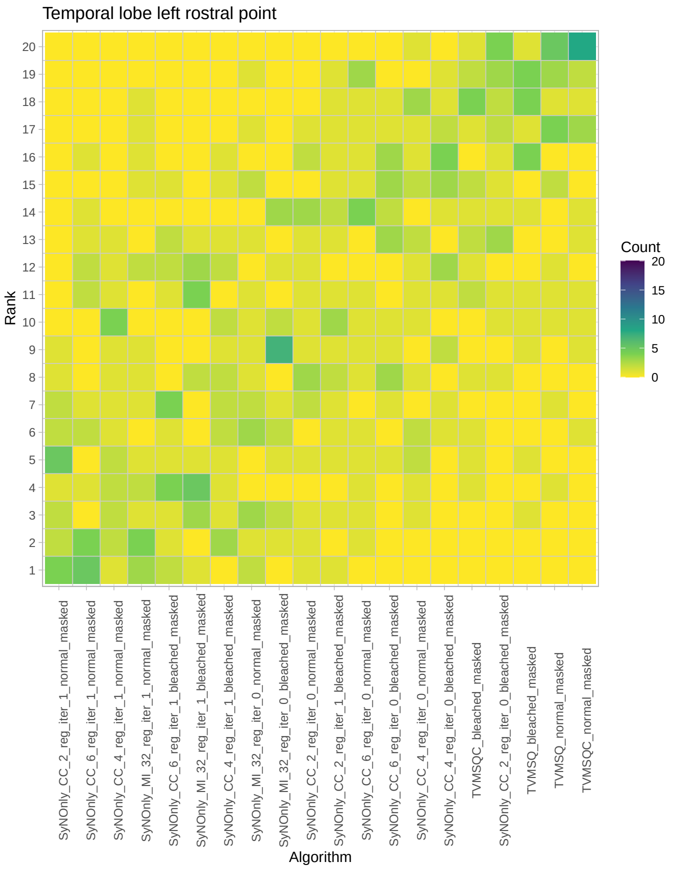

Temporal lobe left rostral point
Rank
Algorithm
Count
SyNOnly_CC_2_reg_iter_1_normal_masked
SyNOnly_CC_6_reg_iter_1_normal_masked
SyNOnly_CC_4_reg_iter_1_normal_masked
SyNOnly_MI_32_reg_iter_1_normal_masked
SyNOnly_CC_6_reg_iter_1_bleached_masked
SyNOnly_MI_32_reg_iter_1_bleached_masked
SyNOnly_CC_4_reg_iter_1_bleached_masked
SyNOnly_MI_32_reg_iter_0_normal_masked
SyNOnly_MI_32_reg_iter_0_bleached_masked
SyNOnly_CC_2_reg_iter_0_normal_masked
SyNOnly_CC_2_reg_iter_1_bleached_masked
SyNOnly_CC_6_reg_iter_0_normal_masked
SyNOnly_CC_6_reg_iter_0_bleached_masked
SyNOnly_CC_4_reg_iter_0_normal_masked
SyNOnly_CC_4_reg_iter_0_bleached_masked
TVMSQC_bleached_masked
SyNOnly_CC_2_reg_iter_0_bleached_masked
TVMSQ_bleached_masked
TVMSQ_normal_masked
TVMSQC_normal_masked

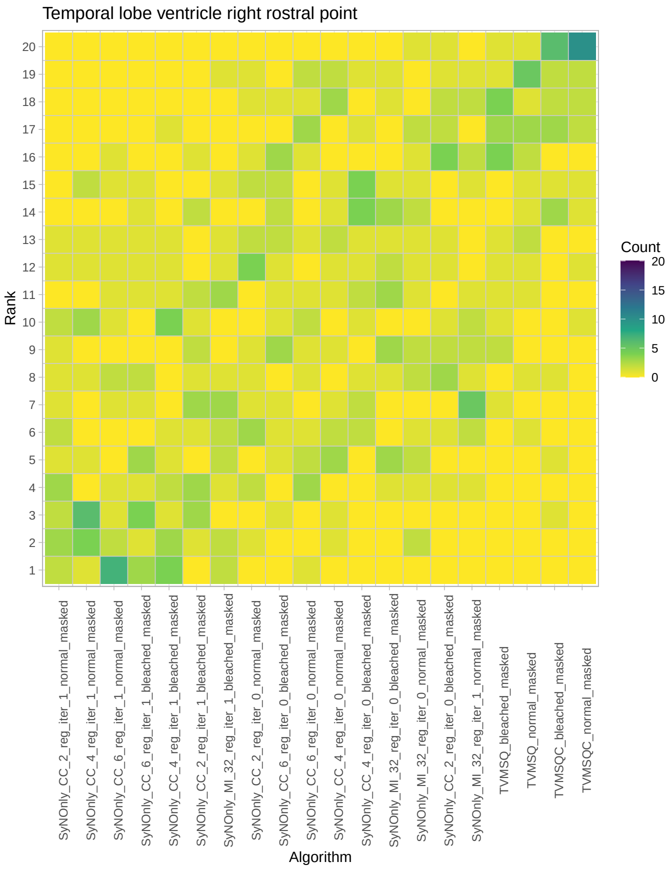
Temporal lobe ventricle right rostral point
Rank
20
19
18
17
16
15
14
13
12
11
10
9
8
7
6
5
4
3
2
1
Count
20
15
10
5
0
SyNOnly_CC_2_reg_iter_1_normal_masked
SyNOnly_CC_4_reg_iter_1_normal_masked
SyNOnly_CC_6_reg_iter_1_normal_masked
SyNOnly_CC_6_reg_iter_1_bleached_masked
SyNOnly_CC_4_reg_iter_1_bleached_masked
SyNOnly_CC_2_reg_iter_1_bleached_masked
SyNOnly_MI_32_reg_iter_1_bleached_masked
SyNOnly_CC_2_reg_iter_0_normal_masked
SyNOnly_CC_6_reg_iter_0_bleached_masked
SyNOnly_CC_6_reg_iter_0_normal_masked
SyNOnly_CC_4_reg_iter_0_normal_masked
SyNOnly_CC_4_reg_iter_0_bleached_masked
SyNOnly_MI_32_reg_iter_0_bleached_masked
SyNOnly_MI_32_reg_iter_0_normal_masked
SyNOnly_CC_2_reg_iter_0_bleached_masked
SyNOnly_MI_32_reg_iter_1_normal_masked
TVMSQ_bleached_masked
TVMSQ_normal_masked
TVMSQC_bleached_masked
TVMSQC_normal_masked
Algorithm

3 Visualization of ranking stability

3.1 *Blob plot* for visualizing ranking stability based on bootstrap sampling

Algorithms are color-coded, and the area of each blob at position $(A_i, \text{rank } j)$ is proportional to the relative frequency A_i achieved rank j across $b = 1000$ bootstrap samples. The median rank for each algorithm is indicated by a black cross. 95% bootstrap intervals across bootstrap samples are indicated by black lines.

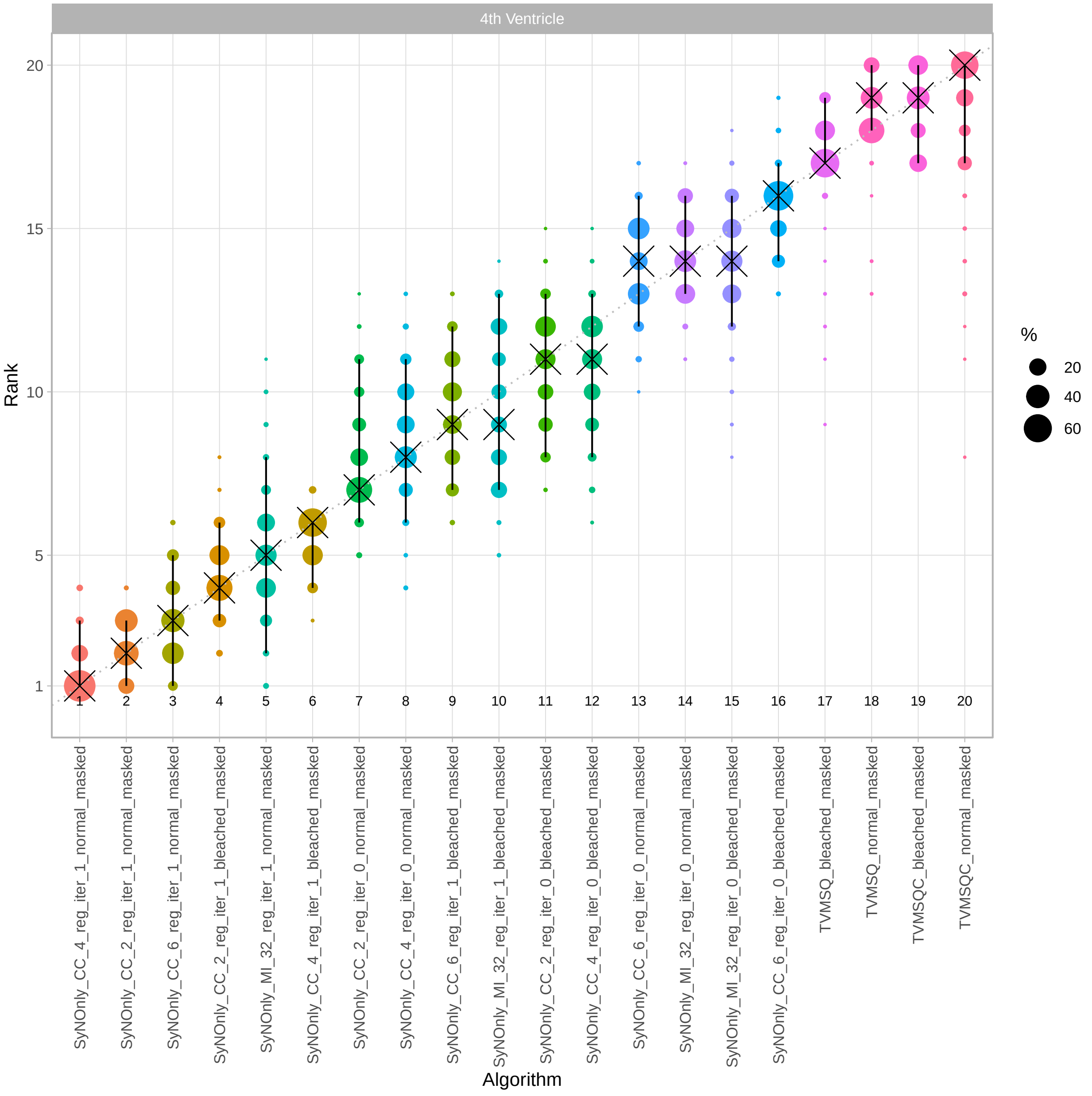

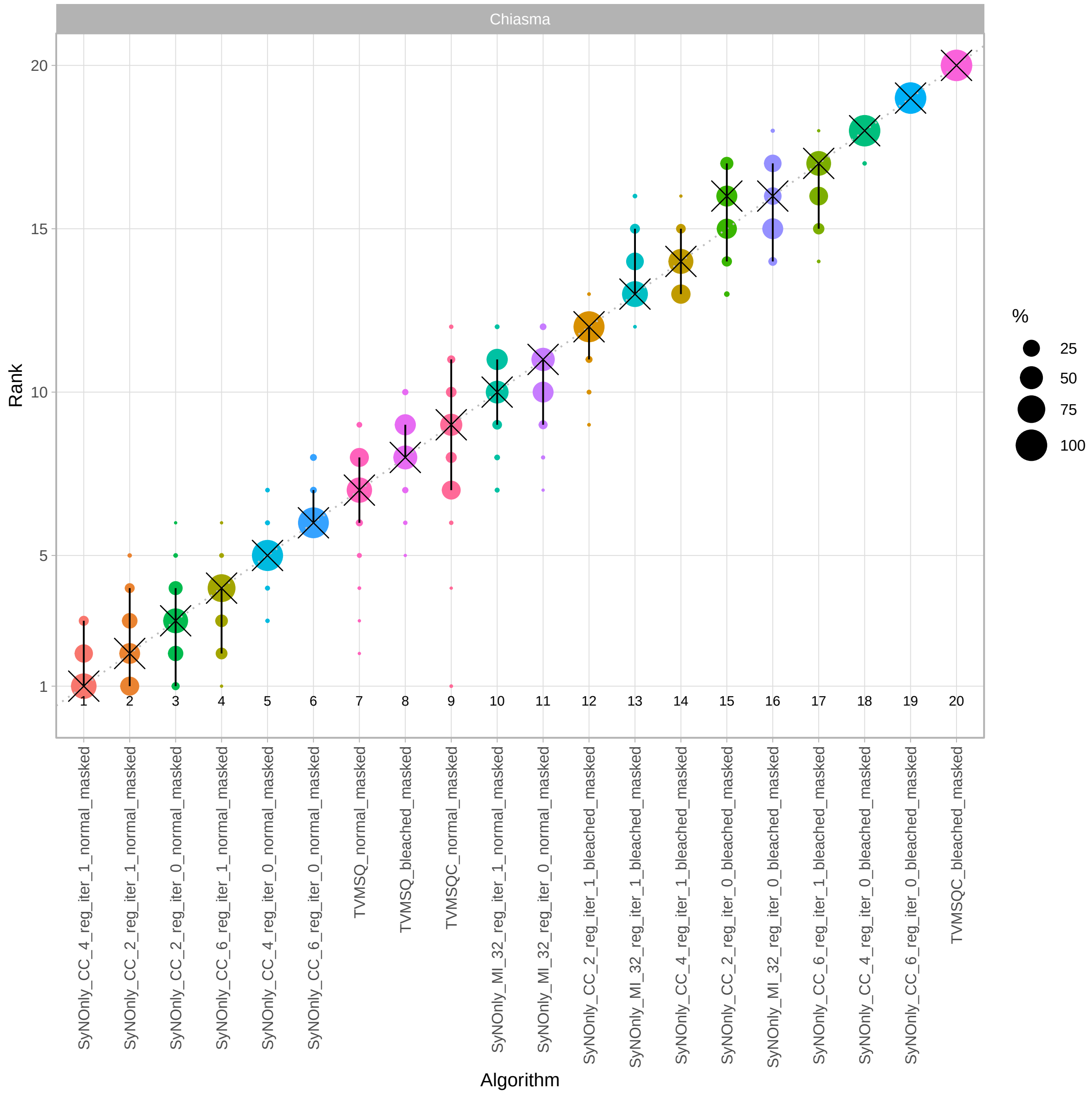

Chiasma
Rank
Algorithm
%
25
50
75
100
SyNOnly_CC_4_reg_iter_1_normal_masked
SyNOnly_CC_2_reg_iter_1_normal_masked
SyNOnly_CC_2_reg_iter_0_normal_masked
SyNOnly_CC_6_reg_iter_1_normal_masked
SyNOnly_CC_4_reg_iter_0_normal_masked
SyNOnly_CC_6_reg_iter_0_normal_masked
TVMSQ_normal_masked
TVMSQ_bleached_masked
TVMSQC_normal_masked
SyNOnly_MI_32_reg_iter_1_normal_masked
SyNOnly_MI_32_reg_iter_0_normal_masked
SyNOnly_CC_2_reg_iter_1_bleached_masked
SyNOnly_MI_32_reg_iter_1_bleached_masked
SyNOnly_CC_4_reg_iter_1_bleached_masked
SyNOnly_CC_2_reg_iter_0_bleached_masked
SyNOnly_MI_32_reg_iter_0_bleached_masked
SyNOnly_CC_6_reg_iter_1_bleached_masked
SyNOnly_CC_4_reg_iter_0_bleached_masked
SyNOnly_CC_6_reg_iter_0_bleached_masked
TVMSQC_bleached_masked

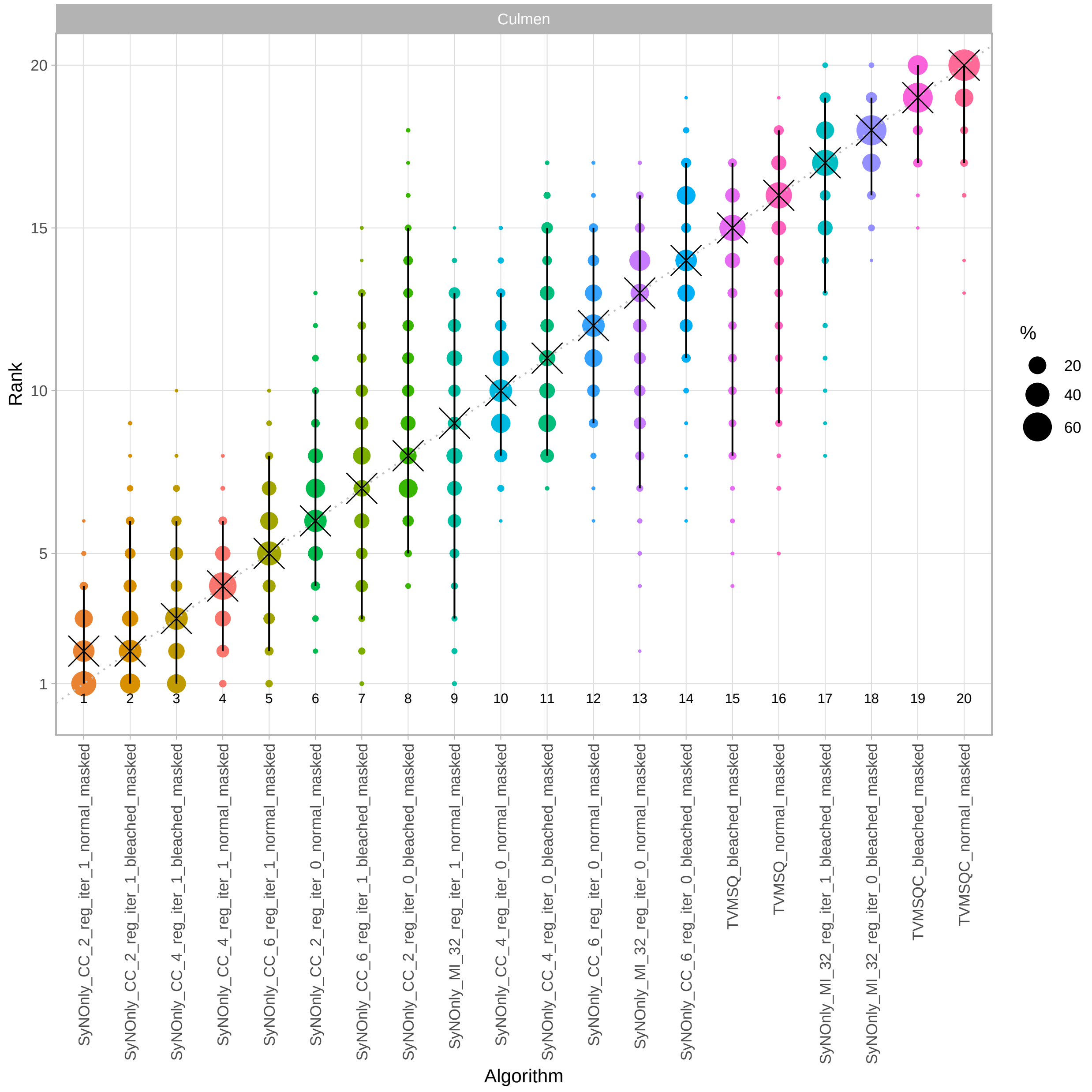
Culmen
Rank
20
15
10
5
1
1 2 3 4 5 6 7 8 9 10 11 12 13 14 15 16 17 18 19 20
%
20
40
60
SyNOnly_CC_2_reg_iter_1_normal_masked
SyNOnly_CC_2_reg_iter_1_bleached_masked
SyNOnly_CC_4_reg_iter_1_bleached_masked
SyNOnly_CC_4_reg_iter_1_normal_masked
SyNOnly_CC_6_reg_iter_1_normal_masked
SyNOnly_CC_2_reg_iter_0_normal_masked
SyNOnly_CC_6_reg_iter_1_bleached_masked
SyNOnly_CC_2_reg_iter_0_bleached_masked
SyNOnly_MI_32_reg_iter_1_normal_masked
SyNOnly_CC_4_reg_iter_0_normal_masked
SyNOnly_CC_4_reg_iter_0_bleached_masked
SyNOnly_CC_6_reg_iter_0_normal_masked
SyNOnly_MI_32_reg_iter_0_normal_masked
SyNOnly_CC_6_reg_iter_0_bleached_masked
TVMSQ_bleached_masked
TVMSQ_normal_masked
SyNOnly_MI_32_reg_iter_1_bleached_masked
SyNOnly_MI_32_reg_iter_0_bleached_masked
TVMSQC_bleached_masked
TVMSQC_normal_masked
Algorithm

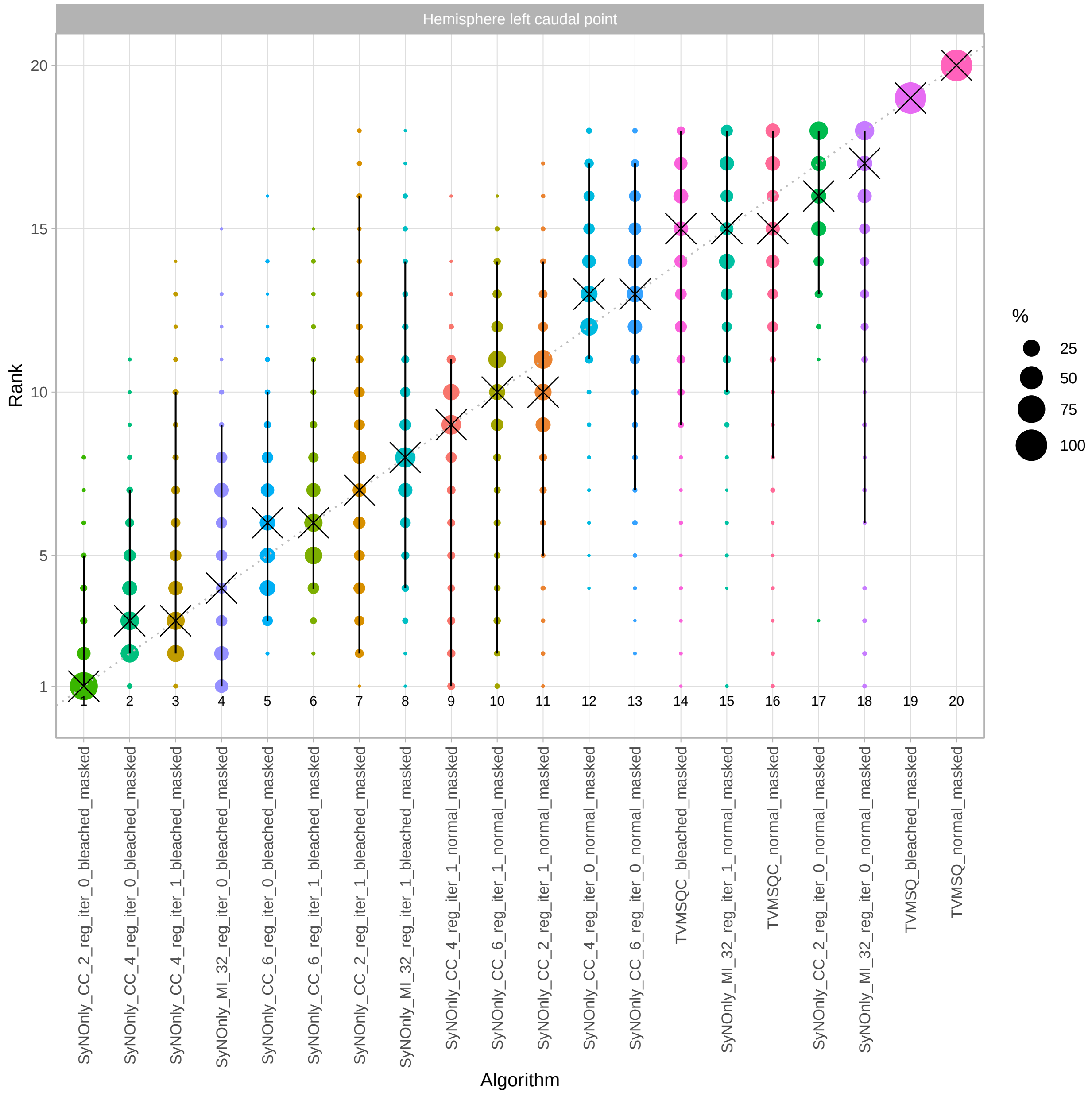
Hemisphere left caudal point
Rank
20
15
10
5
1
1
2
3
4
5
6
7
8
9
10
11
12
13
14
15
16
17
18
19
20
%
25
50
75
100
SyNOnly_CC_2_reg_iter_0_bleached_masked
SyNOnly_CC_4_reg_iter_0_bleached_masked
SyNOnly_CC_4_reg_iter_1_bleached_masked
SyNOnly_MI_32_reg_iter_0_bleached_masked
SyNOnly_CC_6_reg_iter_0_bleached_masked
SyNOnly_CC_6_reg_iter_1_bleached_masked
SyNOnly_CC_2_reg_iter_1_bleached_masked
SyNOnly_MI_32_reg_iter_1_bleached_masked
SyNOnly_CC_4_reg_iter_1_normal_masked
SyNOnly_CC_6_reg_iter_1_normal_masked
SyNOnly_CC_2_reg_iter_1_normal_masked
SyNOnly_CC_4_reg_iter_0_normal_masked
SyNOnly_CC_6_reg_iter_0_normal_masked
TVMSQC_bleached_masked
SyNOnly_MI_32_reg_iter_1_normal_masked
TVMSQC_normal_masked
SyNOnly_CC_2_reg_iter_0_normal_masked
SyNOnly_MI_32_reg_iter_0_normal_masked
TVMSQ_bleached_masked
TVMSQ_normal_masked
Algorithm

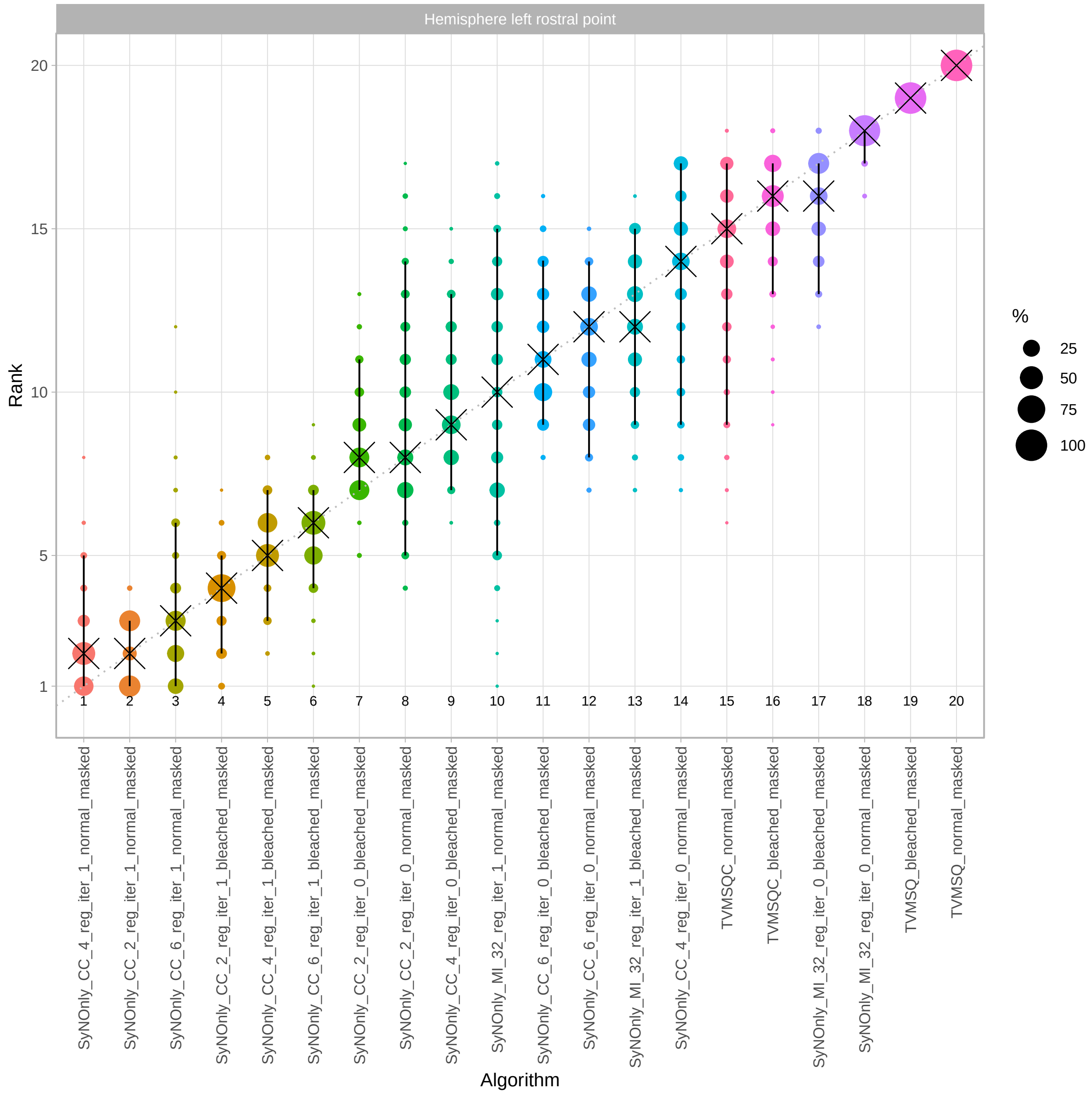

Hemisphere left rostral point
Rank
20
15
10
5
1
1 2 3 4 5 6 7 8 9 10 11 12 13 14 15 16 17 18 19 20
%
25
50
75
100
SyNOnly_CC_4_reg_iter_1_normal_masked
SyNOnly_CC_2_reg_iter_1_normal_masked
SyNOnly_CC_6_reg_iter_1_normal_masked
SyNOnly_CC_2_reg_iter_1_bleached_masked
SyNOnly_CC_4_reg_iter_1_bleached_masked
SyNOnly_CC_6_reg_iter_1_bleached_masked
SyNOnly_CC_2_reg_iter_0_bleached_masked
SyNOnly_CC_2_reg_iter_0_normal_masked
SyNOnly_CC_4_reg_iter_0_bleached_masked
SyNOnly_MI_32_reg_iter_1_normal_masked
SyNOnly_CC_6_reg_iter_0_bleached_masked
SyNOnly_CC_6_reg_iter_0_normal_masked
SyNOnly_MI_32_reg_iter_1_bleached_masked
SyNOnly_CC_4_reg_iter_0_normal_masked
TVMSQC_normal_masked
TVMSQC_bleached_masked
SyNOnly_MI_32_reg_iter_0_bleached_masked
SyNOnly_MI_32_reg_iter_0_normal_masked
TVMSQ_bleached_masked
TVMSQ_normal_masked
Algorithm

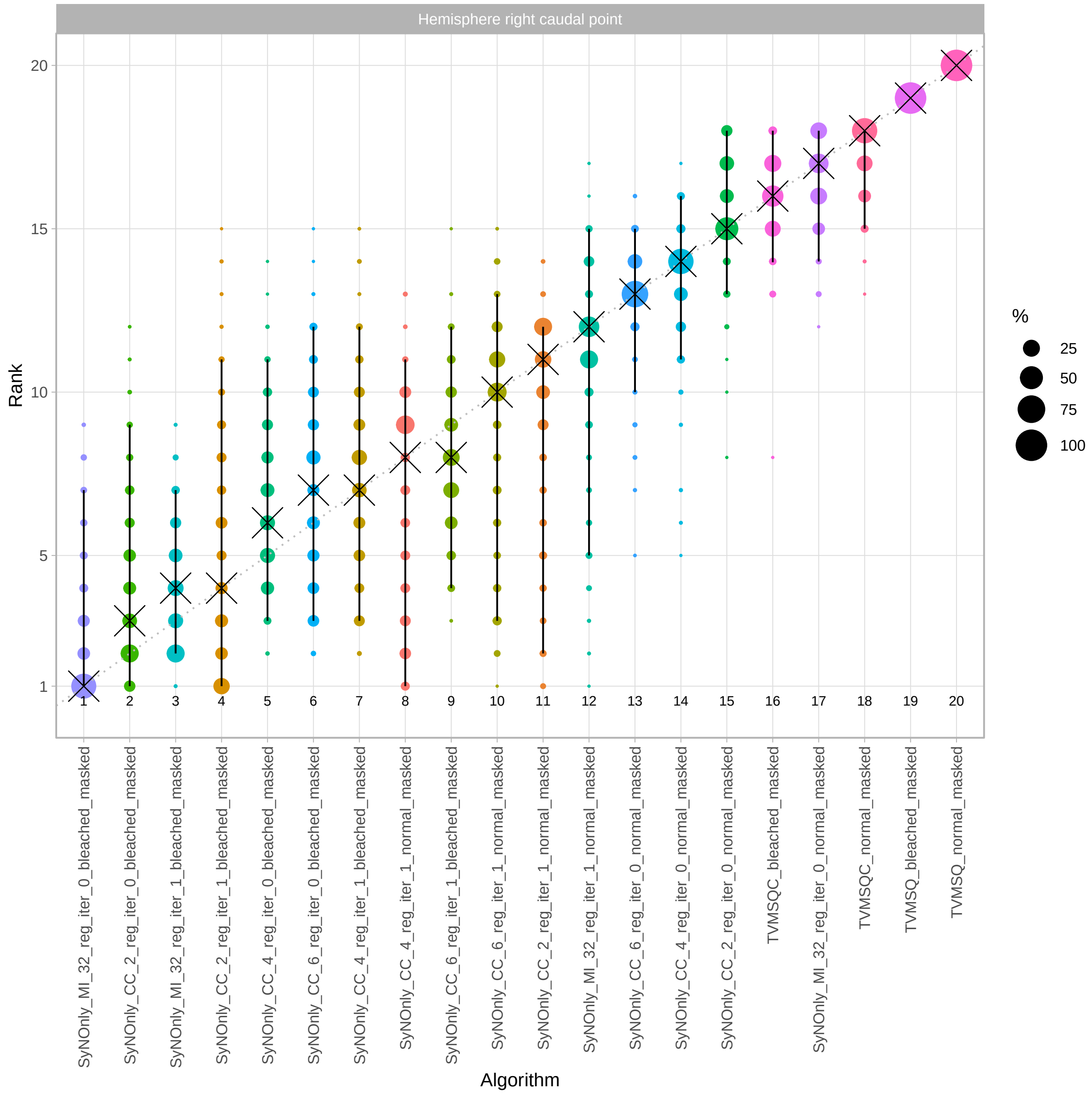
Hemisphere right caudal point
Rank
1
5
10
15
20
1
2
3
4
5
6
7
8
9
10
11
12
13
14
15
16
17
18
19
20
%
25
50
75
100
SyNOnly_MI_32_reg_iter_0_bleached_masked
SyNOnly_CC_2_reg_iter_0_bleached_masked
SyNOnly_MI_32_reg_iter_1_bleached_masked
SyNOnly_CC_2_reg_iter_1_bleached_masked
SyNOnly_CC_4_reg_iter_0_bleached_masked
SyNOnly_CC_6_reg_iter_0_bleached_masked
SyNOnly_CC_4_reg_iter_1_bleached_masked
SyNOnly_CC_4_reg_iter_1_normal_masked
SyNOnly_CC_6_reg_iter_1_bleached_masked
SyNOnly_CC_6_reg_iter_1_normal_masked
SyNOnly_CC_2_reg_iter_1_normal_masked
SyNOnly_MI_32_reg_iter_1_normal_masked
SyNOnly_CC_6_reg_iter_0_normal_masked
SyNOnly_CC_4_reg_iter_0_normal_masked
SyNOnly_CC_2_reg_iter_0_normal_masked
TVMSQC_bleached_masked
SyNOnly_MI_32_reg_iter_0_normal_masked
TVMSQC_normal_masked
TVMSQ_bleached_masked
TVMSQ_normal_masked
Algorithm

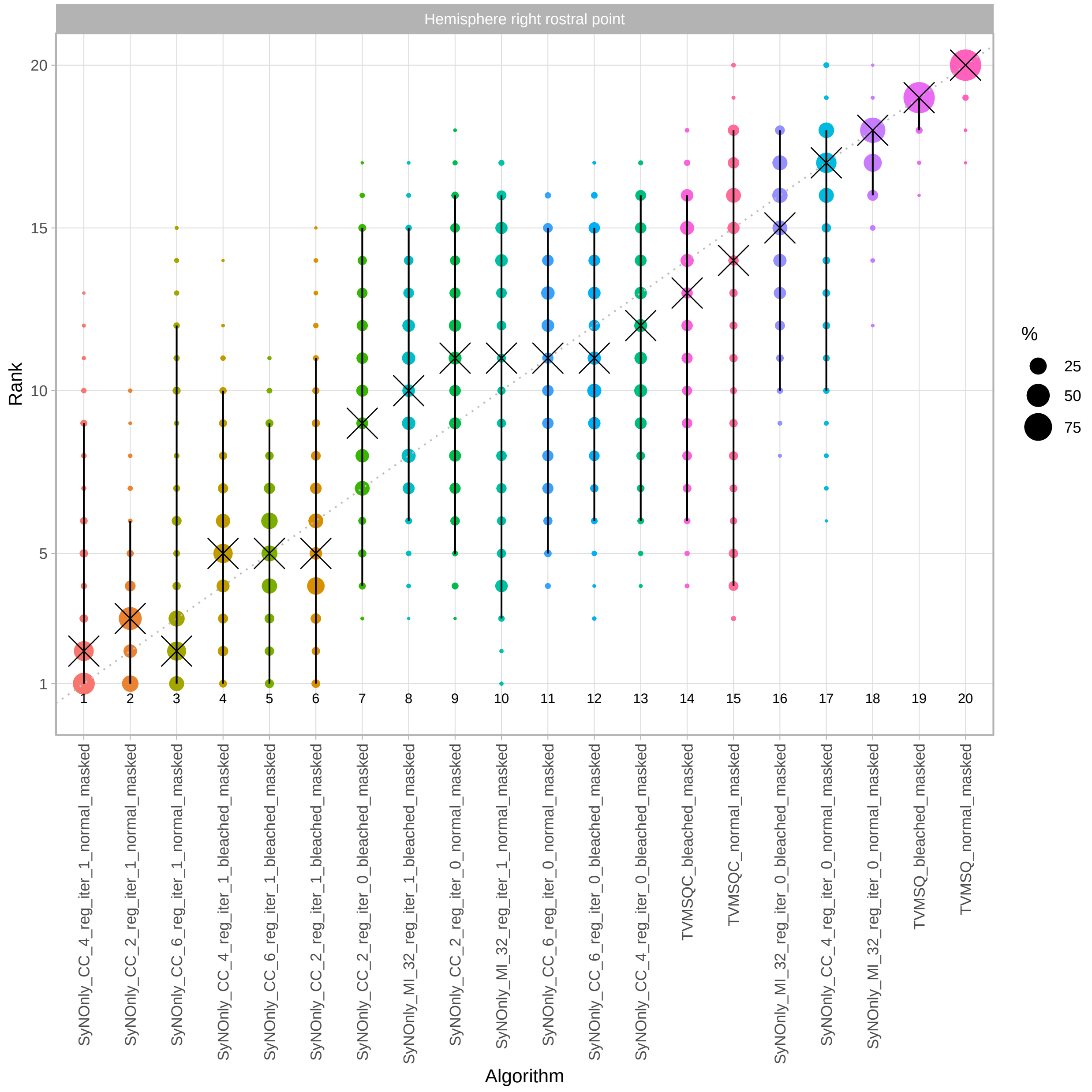
Hemisphere right rostral point
Rank
Algorithm
%
25
50
75
SyNOnly_CC_4_reg_iter_1_normal_masked
SyNOnly_CC_2_reg_iter_1_normal_masked
SyNOnly_CC_6_reg_iter_1_normal_masked
SyNOnly_CC_4_reg_iter_1_bleached_masked
SyNOnly_CC_6_reg_iter_1_bleached_masked
SyNOnly_CC_2_reg_iter_1_bleached_masked
SyNOnly_CC_2_reg_iter_0_bleached_masked
SyNOnly_MI_32_reg_iter_1_bleached_masked
SyNOnly_CC_2_reg_iter_0_normal_masked
SyNOnly_MI_32_reg_iter_1_normal_masked
SyNOnly_CC_6_reg_iter_0_normal_masked
SyNOnly_CC_6_reg_iter_0_bleached_masked
SyNOnly_CC_4_reg_iter_0_bleached_masked
TVMSQC_bleached_masked
TVMSQC_normal_masked
SyNOnly_MI_32_reg_iter_0_bleached_masked
SyNOnly_CC_4_reg_iter_0_normal_masked
SyNOnly_MI_32_reg_iter_0_normal_masked
TVMSQ_bleached_masked
TVMSQ_normal_masked

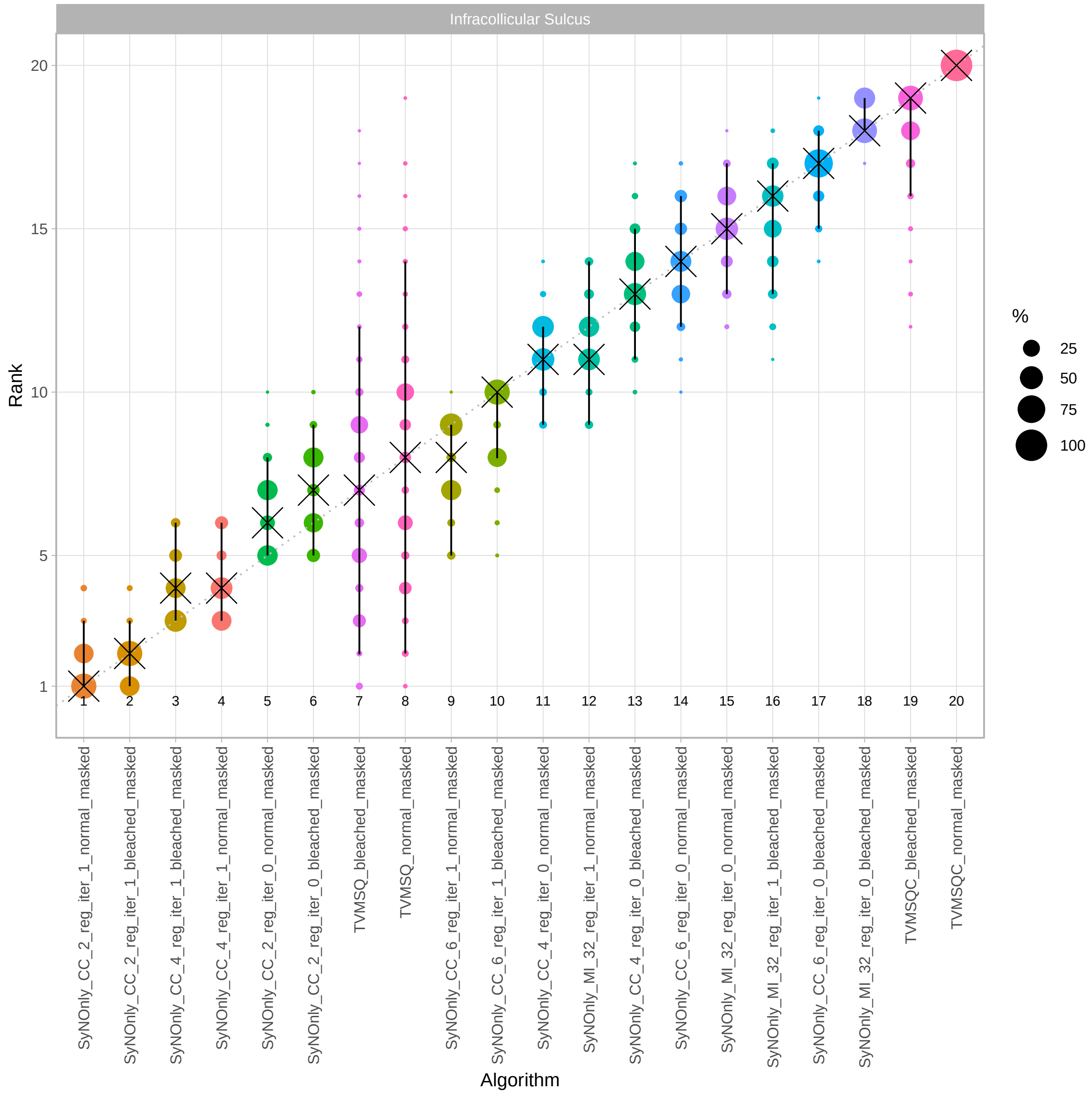
Infracollicular Sulcus
Rank
Algorithm
%
25
50
75
100
SyNOnly_CC_2_reg_iter_1_normal_masked
SyNOnly_CC_2_reg_iter_1_bleached_masked
SyNOnly_CC_4_reg_iter_1_bleached_masked
SyNOnly_CC_4_reg_iter_1_normal_masked
SyNOnly_CC_2_reg_iter_0_normal_masked
SyNOnly_CC_2_reg_iter_0_bleached_masked
TVMSQ_bleached_masked
TVMSQ_normal_masked
SyNOnly_CC_6_reg_iter_1_normal_masked
SyNOnly_CC_6_reg_iter_1_bleached_masked
SyNOnly_CC_4_reg_iter_0_normal_masked
SyNOnly_MI_32_reg_iter_1_normal_masked
SyNOnly_CC_4_reg_iter_0_bleached_masked
SyNOnly_CC_6_reg_iter_0_normal_masked
SyNOnly_MI_32_reg_iter_0_normal_masked
SyNOnly_MI_32_reg_iter_1_bleached_masked
SyNOnly_CC_6_reg_iter_0_bleached_masked
SyNOnly_MI_32_reg_iter_0_bleached_masked
TVMSQC_bleached_masked
TVMSQC_normal_masked

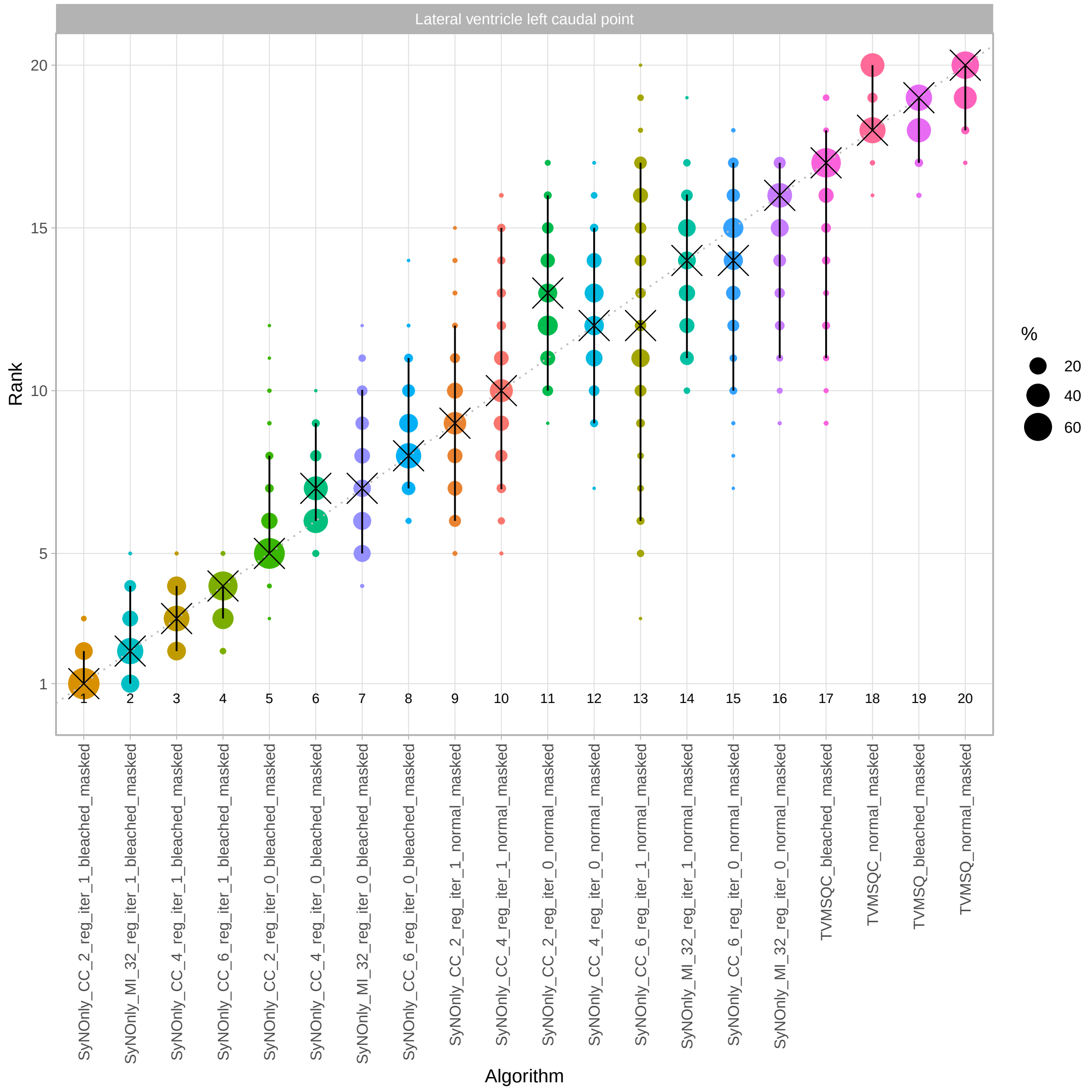

Lateral ventricle left caudal point
Rank
Algorithm
%
20
40
60
SyNOnly_CC_2_reg_iter_1_bleached_masked
SyNOnly_MI_32_reg_iter_1_bleached_masked
SyNOnly_CC_4_reg_iter_1_bleached_masked
SyNOnly_CC_6_reg_iter_1_bleached_masked
SyNOnly_CC_2_reg_iter_0_bleached_masked
SyNOnly_CC_4_reg_iter_0_bleached_masked
SyNOnly_MI_32_reg_iter_0_bleached_masked
SyNOnly_CC_6_reg_iter_0_bleached_masked
SyNOnly_CC_2_reg_iter_1_normal_masked
SyNOnly_CC_4_reg_iter_1_normal_masked
SyNOnly_CC_2_reg_iter_0_normal_masked
SyNOnly_CC_4_reg_iter_0_normal_masked
SyNOnly_CC_6_reg_iter_1_normal_masked
SyNOnly_MI_32_reg_iter_1_normal_masked
SyNOnly_CC_6_reg_iter_0_normal_masked
SyNOnly_MI_32_reg_iter_0_normal_masked
TVMSQC_bleached_masked
TVMSQC_normal_masked
TVMSQ_bleached_masked
TVMSQ_normal_masked

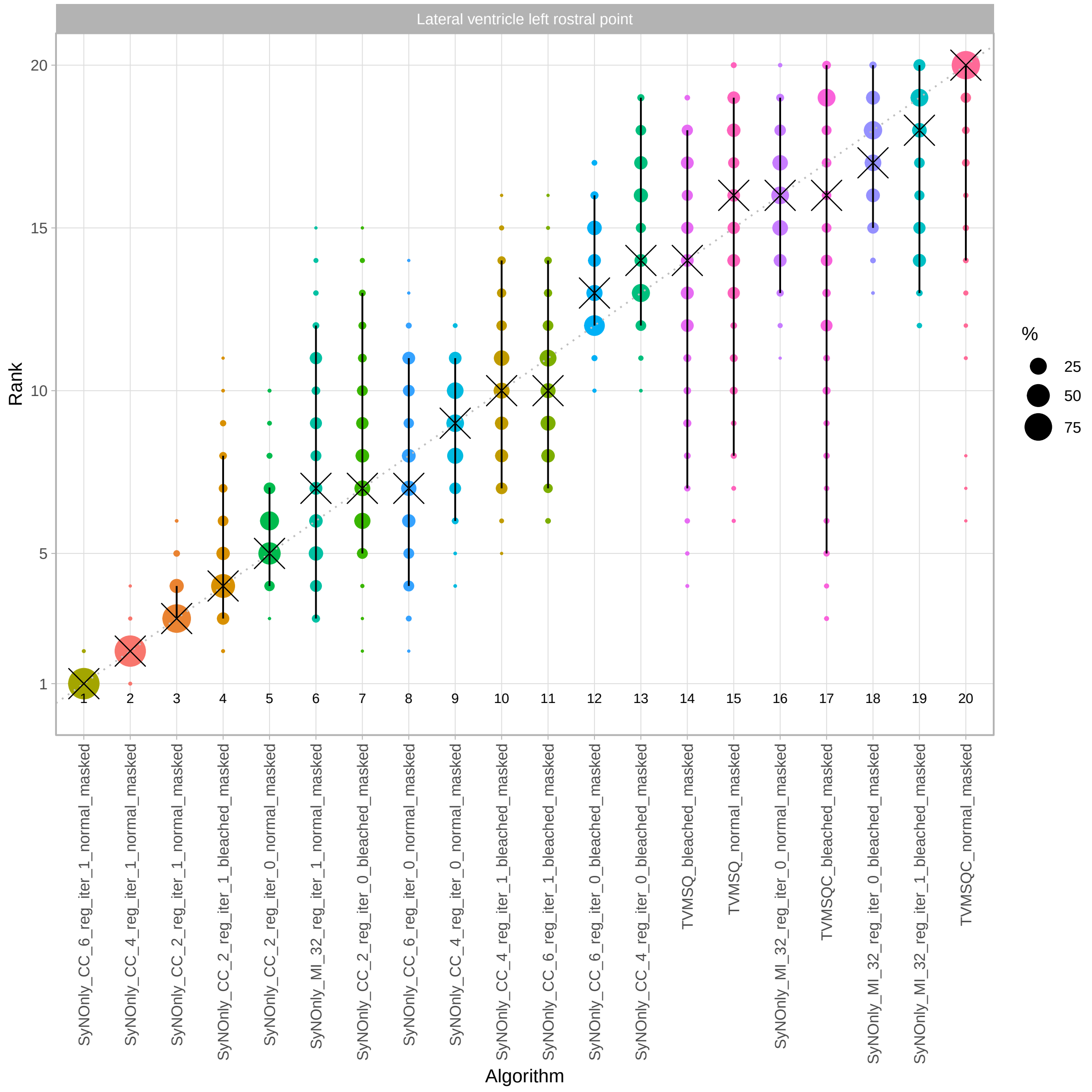

Lateral ventricle left rostral point
Rank
1
5
10
15
20
Algorithm
1
2
3
4
5
6
7
8
9
10
11
12
13
14
15
16
17
18
19
20
SyNOnly_CC_6_reg_iter_1_normal_masked
SyNOnly_CC_4_reg_iter_1_normal_masked
SyNOnly_CC_2_reg_iter_1_normal_masked
SyNOnly_CC_2_reg_iter_1_bleached_masked
SyNOnly_CC_2_reg_iter_0_normal_masked
SyNOnly_MI_32_reg_iter_1_normal_masked
SyNOnly_CC_2_reg_iter_0_bleached_masked
SyNOnly_CC_6_reg_iter_0_normal_masked
SyNOnly_CC_4_reg_iter_0_normal_masked
SyNOnly_CC_4_reg_iter_1_bleached_masked
SyNOnly_CC_6_reg_iter_1_bleached_masked
SyNOnly_CC_6_reg_iter_0_bleached_masked
SyNOnly_CC_4_reg_iter_0_bleached_masked
TVMSQ_bleached_masked
TVMSQ_normal_masked
SyNOnly_MI_32_reg_iter_0_normal_masked
TVMSQC_bleached_masked
SyNOnly_MI_32_reg_iter_0_bleached_masked
SyNOnly_MI_32_reg_iter_1_bleached_masked
TVMSQC_normal_masked
%
25
50
75

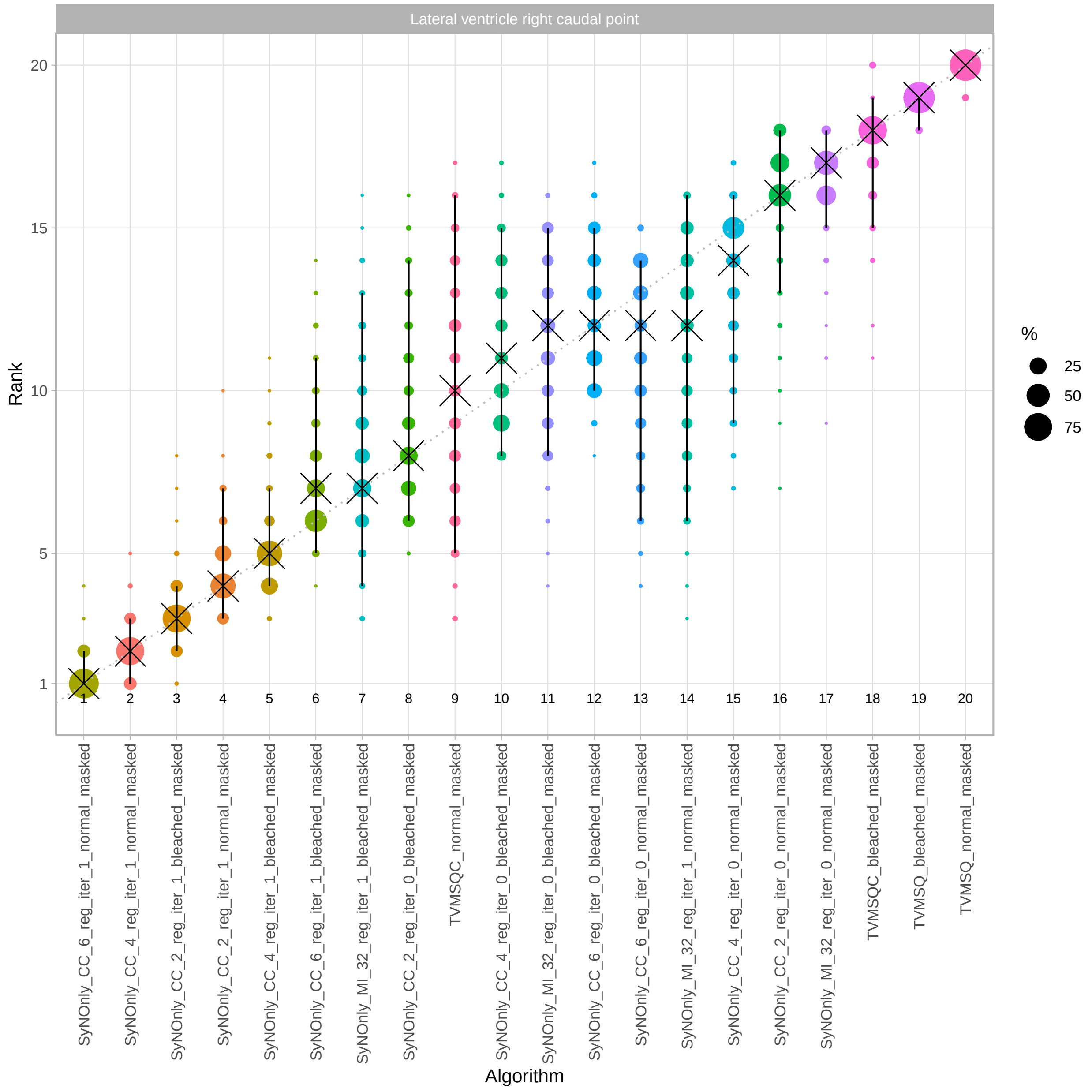

Lateral ventricle right caudal point
Rank
20
15
10
5
1
1
2
3
4
5
6
7
8
9
10
11
12
13
14
15
16
17
18
19
20
%
25
50
75
SyNOnly_CC_6_reg_iter_1_normal_masked
SyNOnly_CC_4_reg_iter_1_normal_masked
SyNOnly_CC_2_reg_iter_1_bleached_masked
SyNOnly_CC_2_reg_iter_1_normal_masked
SyNOnly_CC_4_reg_iter_1_bleached_masked
SyNOnly_CC_6_reg_iter_1_bleached_masked
SyNOnly_MI_32_reg_iter_1_bleached_masked
SyNOnly_CC_2_reg_iter_0_bleached_masked
TVMSQC_normal_masked
SyNOnly_CC_4_reg_iter_0_bleached_masked
SyNOnly_MI_32_reg_iter_0_bleached_masked
SyNOnly_CC_6_reg_iter_0_bleached_masked
SyNOnly_CC_6_reg_iter_0_normal_masked
SyNOnly_MI_32_reg_iter_1_normal_masked
SyNOnly_CC_4_reg_iter_0_normal_masked
SyNOnly_CC_2_reg_iter_0_normal_masked
SyNOnly_MI_32_reg_iter_0_normal_masked
TVMSQC_bleached_masked
TVMSQ_bleached_masked
TVMSQ_normal_masked
Algorithm

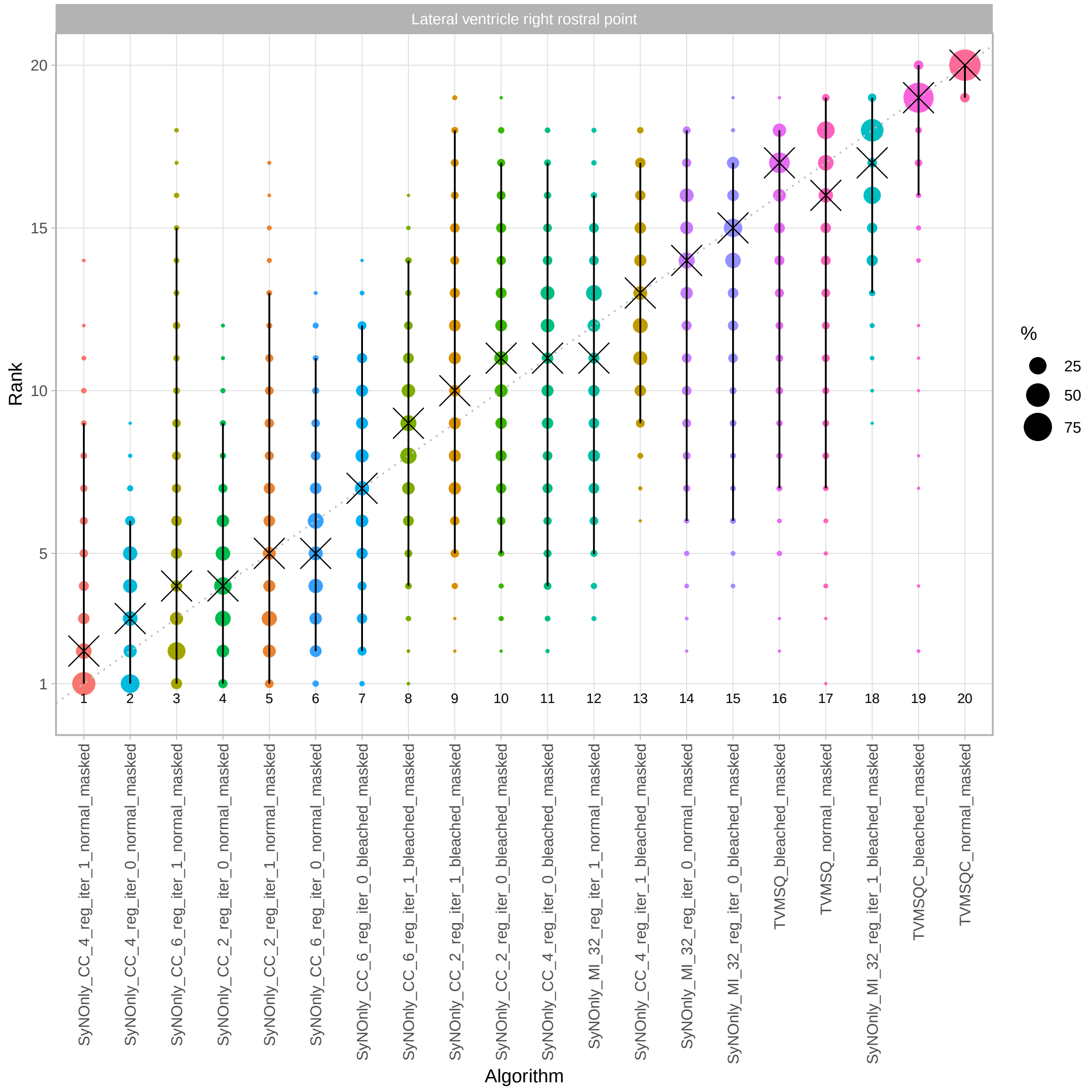
Lateral ventricle right rostral point
Rank
Algorithm
%
25
50
75
SyNOnly_CC_4_reg_iter_1_normal_masked
SyNOnly_CC_4_reg_iter_0_normal_masked
SyNOnly_CC_6_reg_iter_1_normal_masked
SyNOnly_CC_2_reg_iter_0_normal_masked
SyNOnly_CC_2_reg_iter_1_normal_masked
SyNOnly_CC_6_reg_iter_0_normal_masked
SyNOnly_CC_6_reg_iter_0_bleached_masked
SyNOnly_CC_6_reg_iter_1_bleached_masked
SyNOnly_CC_2_reg_iter_1_bleached_masked
SyNOnly_CC_2_reg_iter_0_bleached_masked
SyNOnly_CC_4_reg_iter_0_bleached_masked
SyNOnly_MI_32_reg_iter_1_normal_masked
SyNOnly_CC_4_reg_iter_1_bleached_masked
SyNOnly_MI_32_reg_iter_0_normal_masked
SyNOnly_MI_32_reg_iter_0_bleached_masked
TVMSQ_bleached_masked
TVMSQ_normal_masked
SyNOnly_MI_32_reg_iter_1_bleached_masked
TVMSQC_bleached_masked
TVMSQC_normal_masked

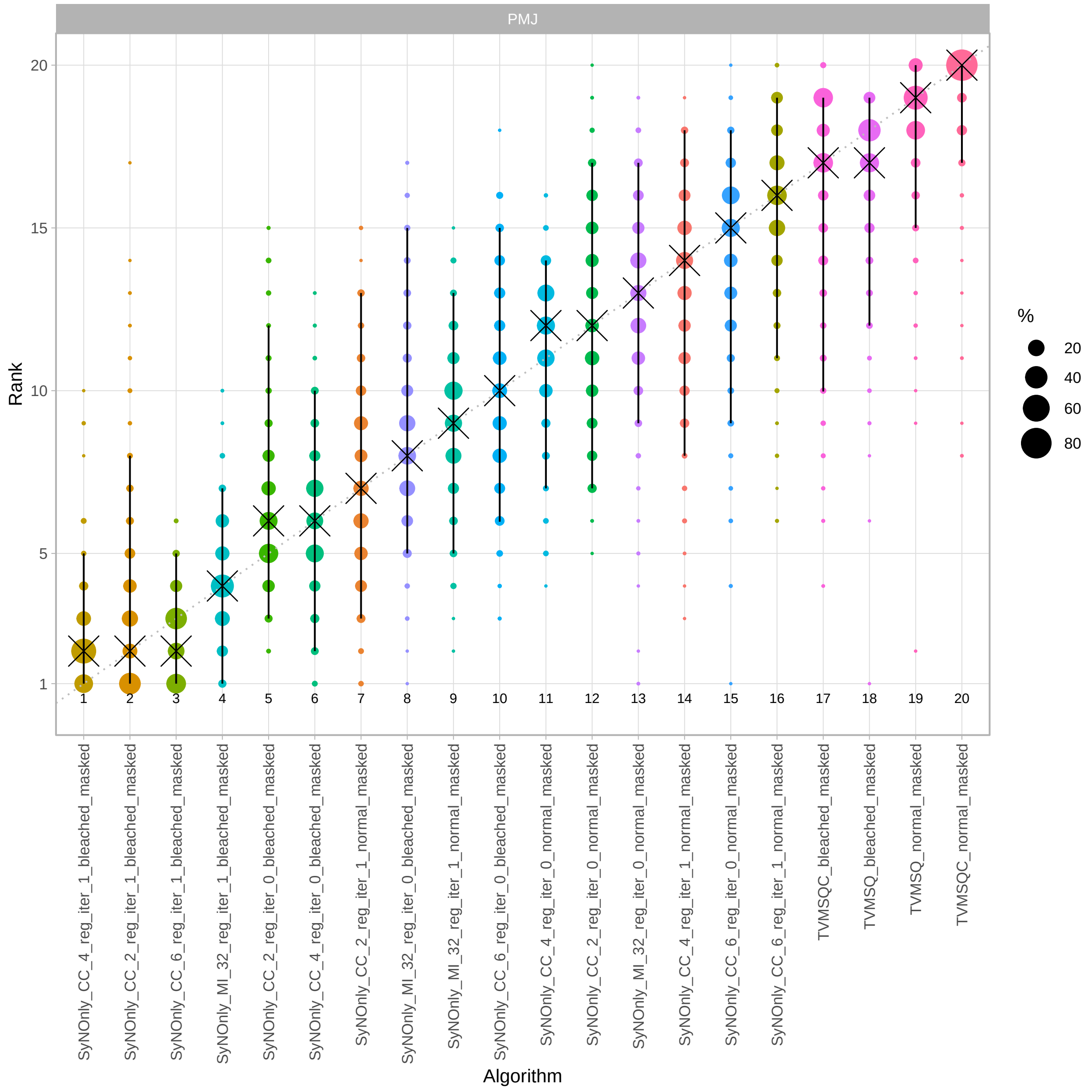

PMJ
Rank
20
15
10
5
1
1
2
3
4
5
6
7
8
9
10
11
12
13
14
15
16
17
18
19
20
%
20
40
60
80
SyNOnly_CC_4_reg_iter_1_bleached_masked
SyNOnly_CC_2_reg_iter_1_bleached_masked
SyNOnly_CC_6_reg_iter_1_bleached_masked
SyNOnly_MI_32_reg_iter_1_bleached_masked
SyNOnly_CC_2_reg_iter_0_bleached_masked
SyNOnly_CC_4_reg_iter_0_bleached_masked
SyNOnly_CC_2_reg_iter_1_normal_masked
SyNOnly_MI_32_reg_iter_0_bleached_masked
SyNOnly_MI_32_reg_iter_1_normal_masked
SyNOnly_CC_6_reg_iter_0_bleached_masked
SyNOnly_CC_4_reg_iter_0_normal_masked
SyNOnly_CC_2_reg_iter_0_normal_masked
SyNOnly_MI_32_reg_iter_0_normal_masked
SyNOnly_CC_4_reg_iter_1_normal_masked
SyNOnly_CC_6_reg_iter_0_normal_masked
SyNOnly_CC_6_reg_iter_1_normal_masked
TVMSQC_bleached_masked
TVMSQ_bleached_masked
TVMSQ_normal_masked
TVMSQC_normal_masked
Algorithm

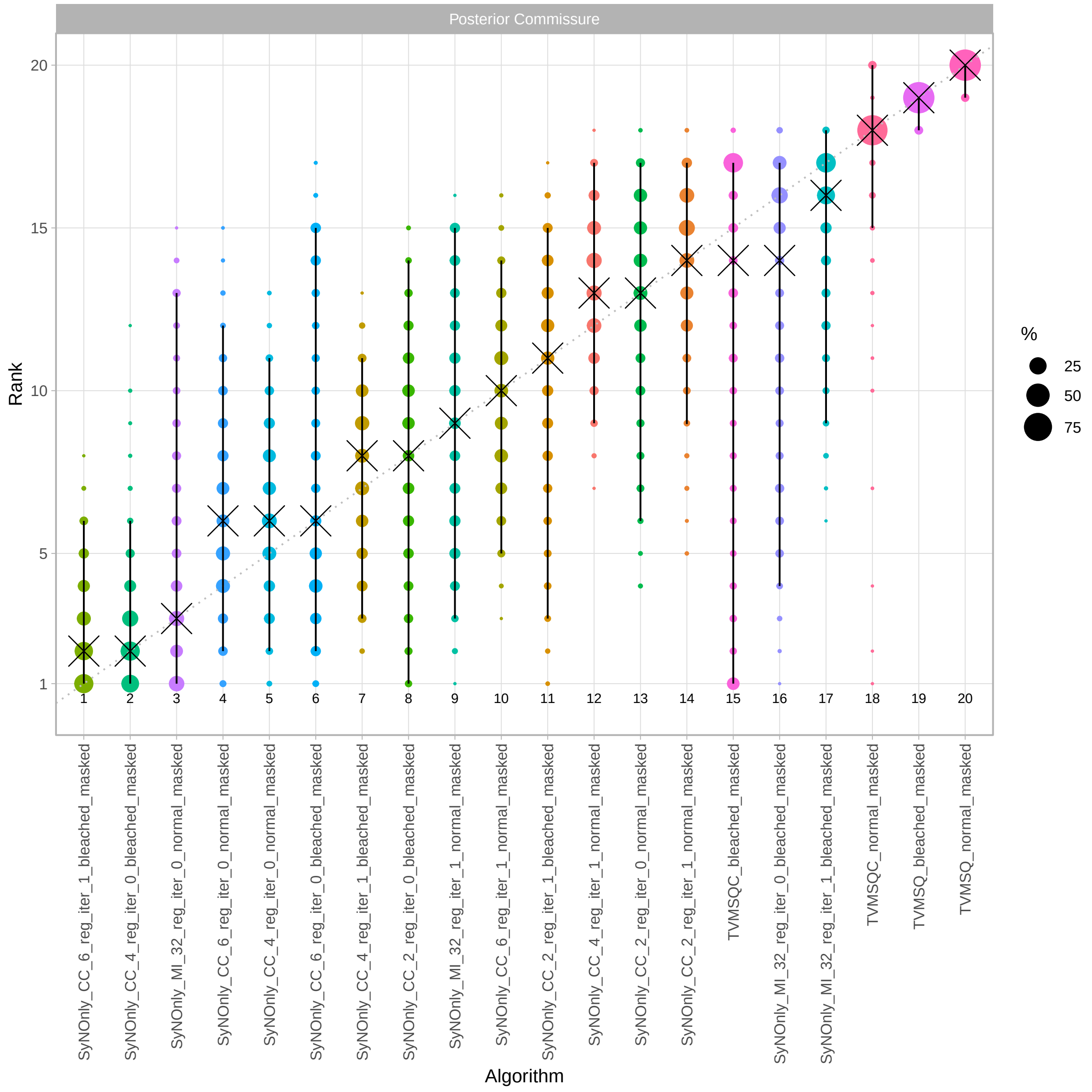
Posterior Commissure
Rank
Algorithm
%
25
50
75
SyNOnly_CC_6_reg_iter_1_bleached_masked
SyNOnly_CC_4_reg_iter_0_bleached_masked
SyNOnly_MI_32_reg_iter_0_normal_masked
SyNOnly_CC_6_reg_iter_0_normal_masked
SyNOnly_CC_4_reg_iter_0_normal_masked
SyNOnly_CC_6_reg_iter_0_bleached_masked
SyNOnly_CC_4_reg_iter_1_bleached_masked
SyNOnly_CC_2_reg_iter_0_bleached_masked
SyNOnly_MI_32_reg_iter_1_normal_masked
SyNOnly_CC_6_reg_iter_1_normal_masked
SyNOnly_CC_2_reg_iter_1_bleached_masked
SyNOnly_CC_4_reg_iter_1_normal_masked
SyNOnly_CC_2_reg_iter_0_normal_masked
SyNOnly_CC_2_reg_iter_1_normal_masked
TVMSQC_bleached_masked
SyNOnly_MI_32_reg_iter_0_bleached_masked
SyNOnly_MI_32_reg_iter_1_bleached_masked
TVMSQC_normal_masked
TVMSQ_bleached_masked
TVMSQ_normal_masked

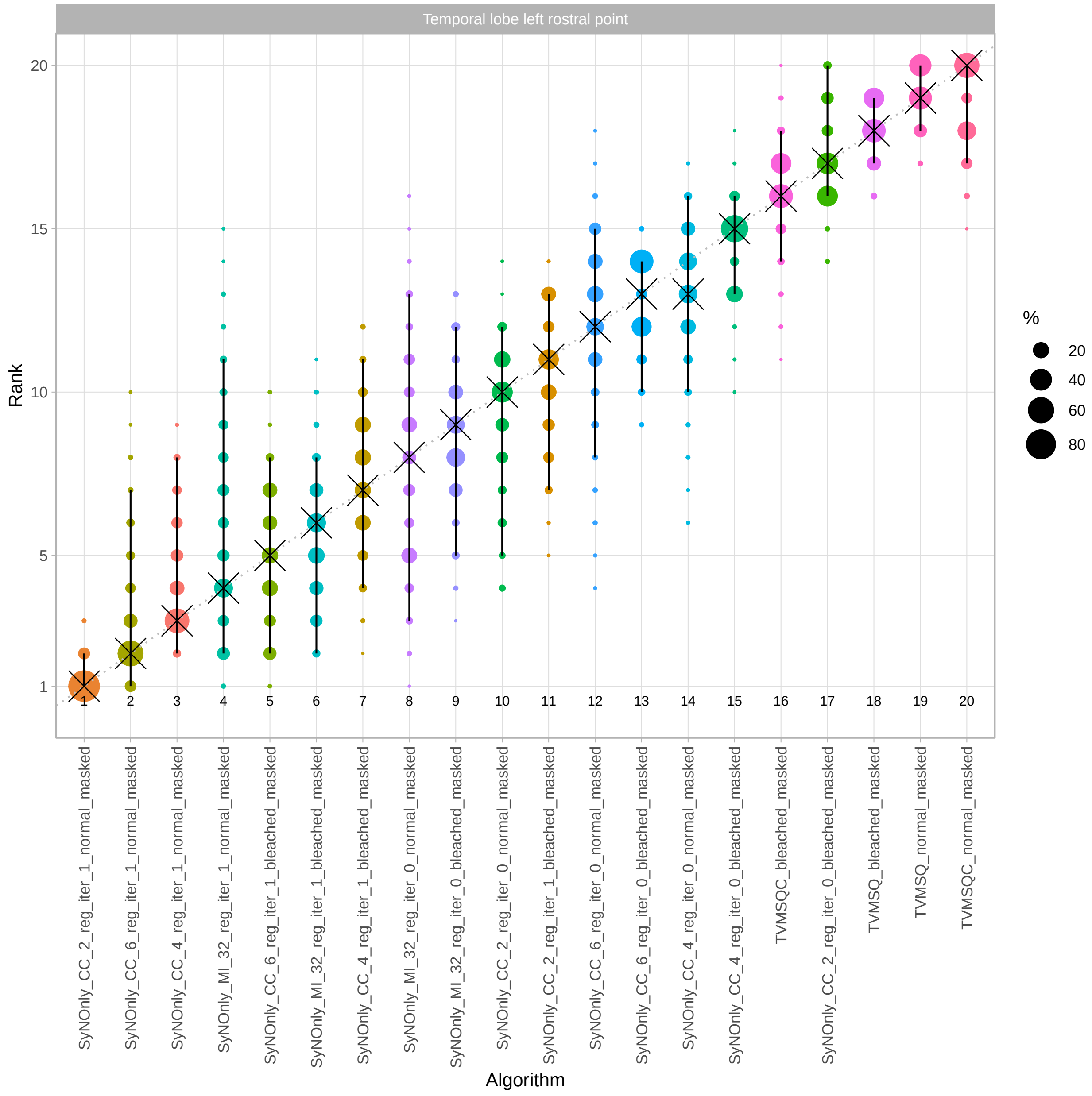
Temporal lobe left rostral point
Rank
20
15
10
5
1
1 2 3 4 5 6 7 8 9 10 11 12 13 14 15 16 17 18 19 20
%
20
40
60
80
SyNOnly_CC_2_reg_iter_1_normal_masked
SyNOnly_CC_6_reg_iter_1_normal_masked
SyNOnly_CC_4_reg_iter_1_normal_masked
SyNOnly_MI_32_reg_iter_1_normal_masked
SyNOnly_CC_6_reg_iter_1_bleached_masked
SyNOnly_MI_32_reg_iter_1_bleached_masked
SyNOnly_CC_4_reg_iter_1_bleached_masked
SyNOnly_MI_32_reg_iter_0_normal_masked
SyNOnly_MI_32_reg_iter_0_bleached_masked
SyNOnly_CC_2_reg_iter_0_normal_masked
SyNOnly_CC_2_reg_iter_1_bleached_masked
SyNOnly_CC_6_reg_iter_0_normal_masked
SyNOnly_CC_6_reg_iter_0_bleached_masked
SyNOnly_CC_4_reg_iter_0_normal_masked
SyNOnly_CC_4_reg_iter_0_bleached_masked
TVMSQC_bleached_masked
SyNOnly_CC_2_reg_iter_0_bleached_masked
TVMSQ_bleached_masked
TVMSQ_normal_masked
TVMSQC_normal_masked
Algorithm

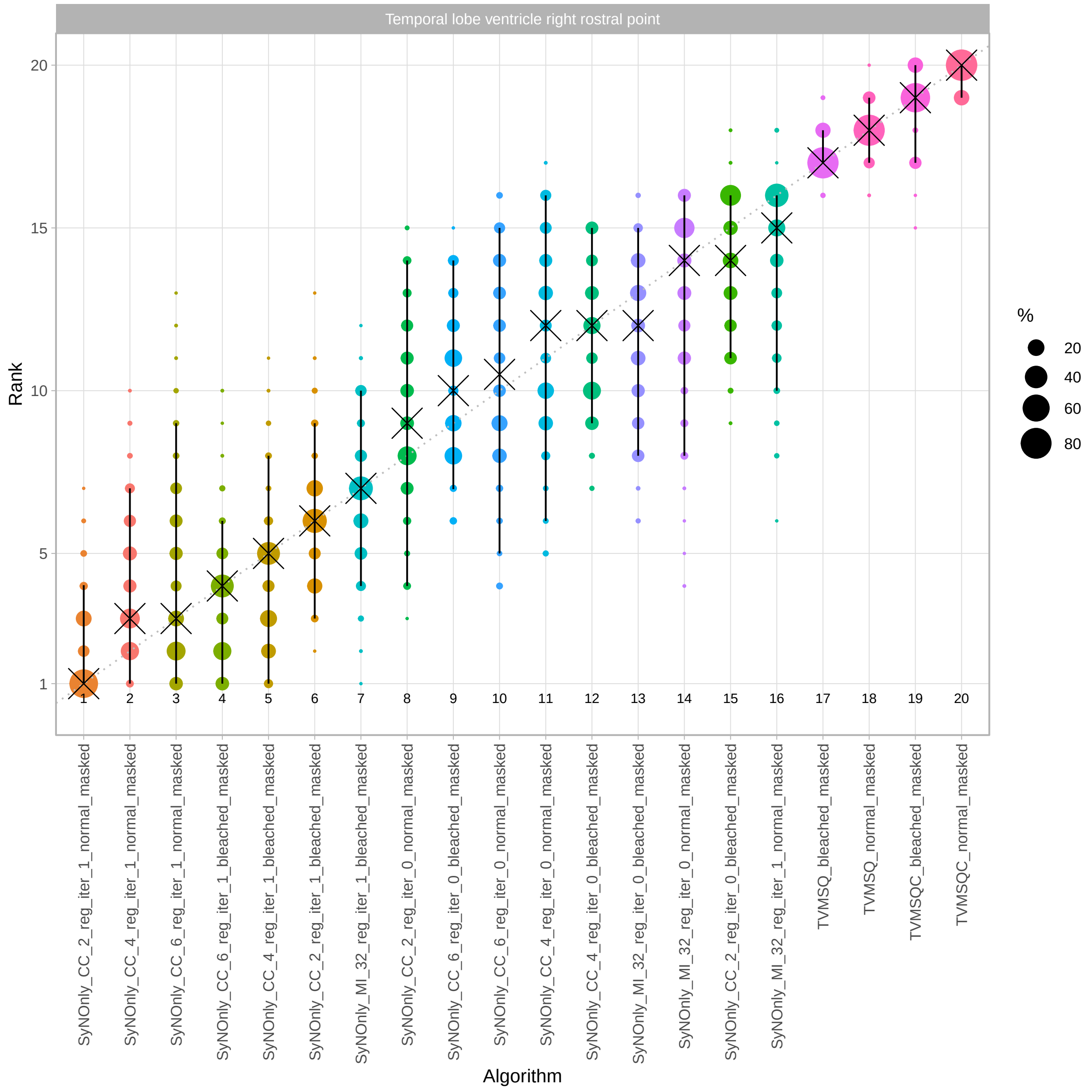

Temporal lobe ventricle right rostral point
Rank
Algorithm
%
20
40
60
80
SyNOnly_CC_2_reg_iter_1_normal_masked
SyNOnly_CC_4_reg_iter_1_normal_masked
SyNOnly_CC_6_reg_iter_1_normal_masked
SyNOnly_CC_6_reg_iter_1_bleached_masked
SyNOnly_CC_4_reg_iter_1_bleached_masked
SyNOnly_CC_2_reg_iter_1_bleached_masked
SyNOnly_MI_32_reg_iter_1_bleached_masked
SyNOnly_CC_2_reg_iter_0_normal_masked
SyNOnly_CC_6_reg_iter_0_bleached_masked
SyNOnly_CC_6_reg_iter_0_normal_masked
SyNOnly_CC_4_reg_iter_0_normal_masked
SyNOnly_CC_4_reg_iter_0_bleached_masked
SyNOnly_MI_32_reg_iter_0_bleached_masked
SyNOnly_MI_32_reg_iter_0_normal_masked
SyNOnly_CC_2_reg_iter_0_bleached_masked
SyNOnly_MI_32_reg_iter_1_normal_masked
TVMSQ_bleached_masked
TVMSQ_normal_masked
TVMSQC_bleached_masked
TVMSQC_normal_masked

3.2 *Violin plot* for visualizing ranking stability based on bootstrapping

The ranking list based on the full assessment data is pairwise compared with the ranking lists based on the individual bootstrap samples (here $b = 1000$ samples). For each pair of rankings, Kendall's τ correlation is computed. Kendall's τ is a scaled index determining the correlation between the lists. It is computed by evaluating the number of pairwise concordances and discordances between ranking lists and produces values between -1 (for inverted order) and 1 (for identical order). A violin plot, which simultaneously depicts a boxplot and a density plot, is generated from the results.

Summary Kendall's tau:

Task	mean	median	q25	q75
Chiasma	0.9572842	0.9578947	0.9473684	0.9684211
Infracollicular Sulcus	0.9070737	0.9157895	0.8842105	0.9368421
4th Ventricle	0.8985789	0.9052632	0.8736842	0.9263158
Lateral ventricle left caudal point	0.8842421	0.8947368	0.8526316	0.9157895
Hemisphere left rostral point	0.8788105	0.8842105	0.8526316	0.9157895
Lateral ventricle right caudal point	0.8656737	0.8842105	0.8315789	0.9157895
Temporal lobe left rostral point	0.8713579	0.8842105	0.8421053	0.9157895
Culmen	0.8582947	0.8736842	0.8210526	0.9157895
Temporal lobe ventricle right rostral point	0.8434211	0.8631579	0.7894737	0.9052632
Hemisphere left caudal point	0.8097684	0.8421053	0.7789474	0.8842105
Hemisphere right caudal point	0.8295579	0.8421053	0.7789474	0.8947368
PMJ	0.8216632	0.8421053	0.7789474	0.8842105
Lateral ventricle left rostral point	0.8241789	0.8315789	0.7789474	0.8842105
Lateral ventricle right rostral point	0.7461368	0.7789474	0.6842105	0.8315789
Hemisphere right rostral point	0.7461684	0.7684211	0.6842105	0.8210526
Posterior Commissure	0.7118421	0.7157895	0.6526316	0.7789474

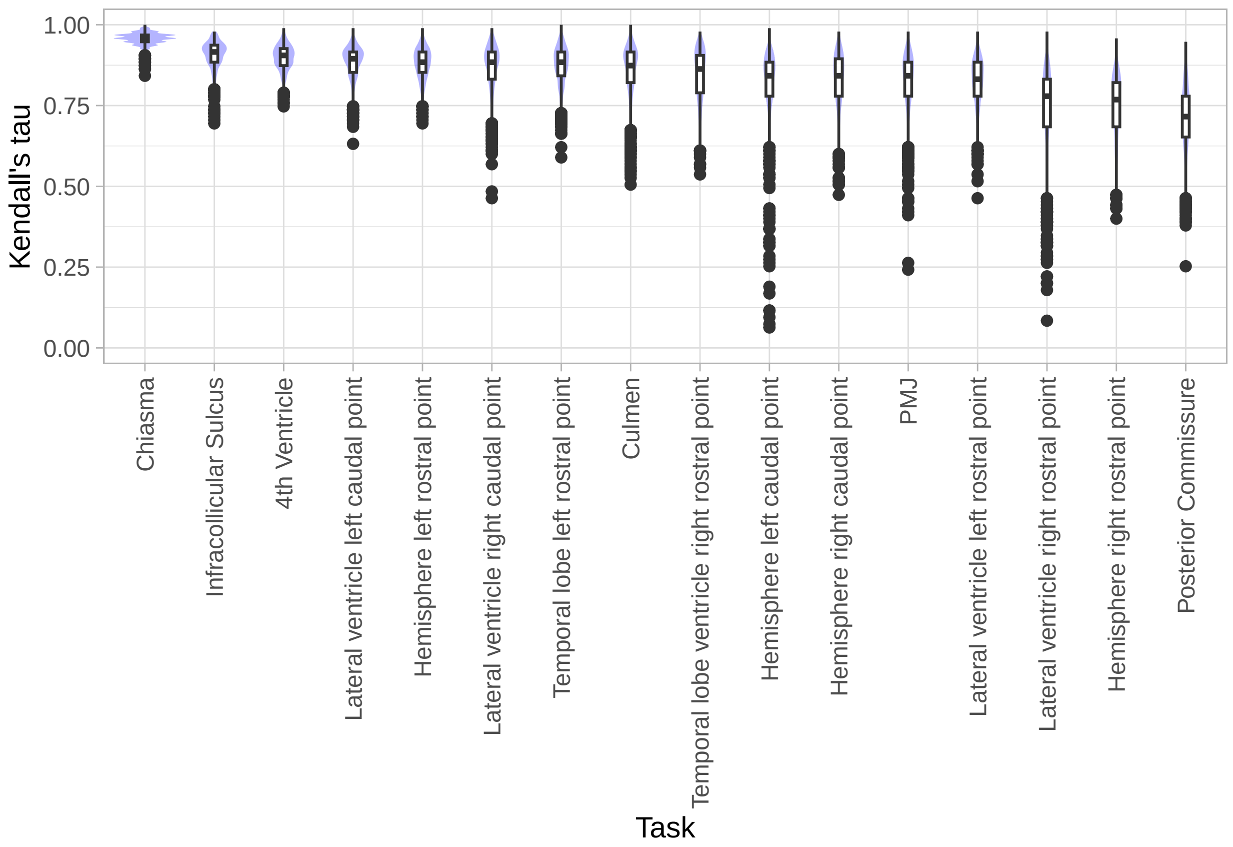

1.00
0.75
0.50
0.25
0.00
Kendall's tau
Chiasma
Infracollicular Sulcus
4th Ventricle
Lateral ventricle left caudal point
Hemisphere left rostral point
Lateral ventricle right caudal point
Temporal lobe left rostral point
Culmen
Temporal lobe ventricle right rostral point
Hemisphere left caudal point
Hemisphere right caudal point
PMJ
Lateral ventricle left rostral point
Lateral ventricle right rostral point
Hemisphere right rostral point
Posterior Commissure
Task

3.3 *Significance maps* for visualizing ranking stability based on statistical significance

Significance maps depict incidence matrices of pairwise significant test results for the one-sided Wilcoxon signed rank test at a 5% significance level with adjustment for multiple testing according to Holm. Yellow shading indicates that metric values from the algorithm on the x-axis were significantly superior to those from the algorithm on the y-axis, blue color indicates no significant difference.

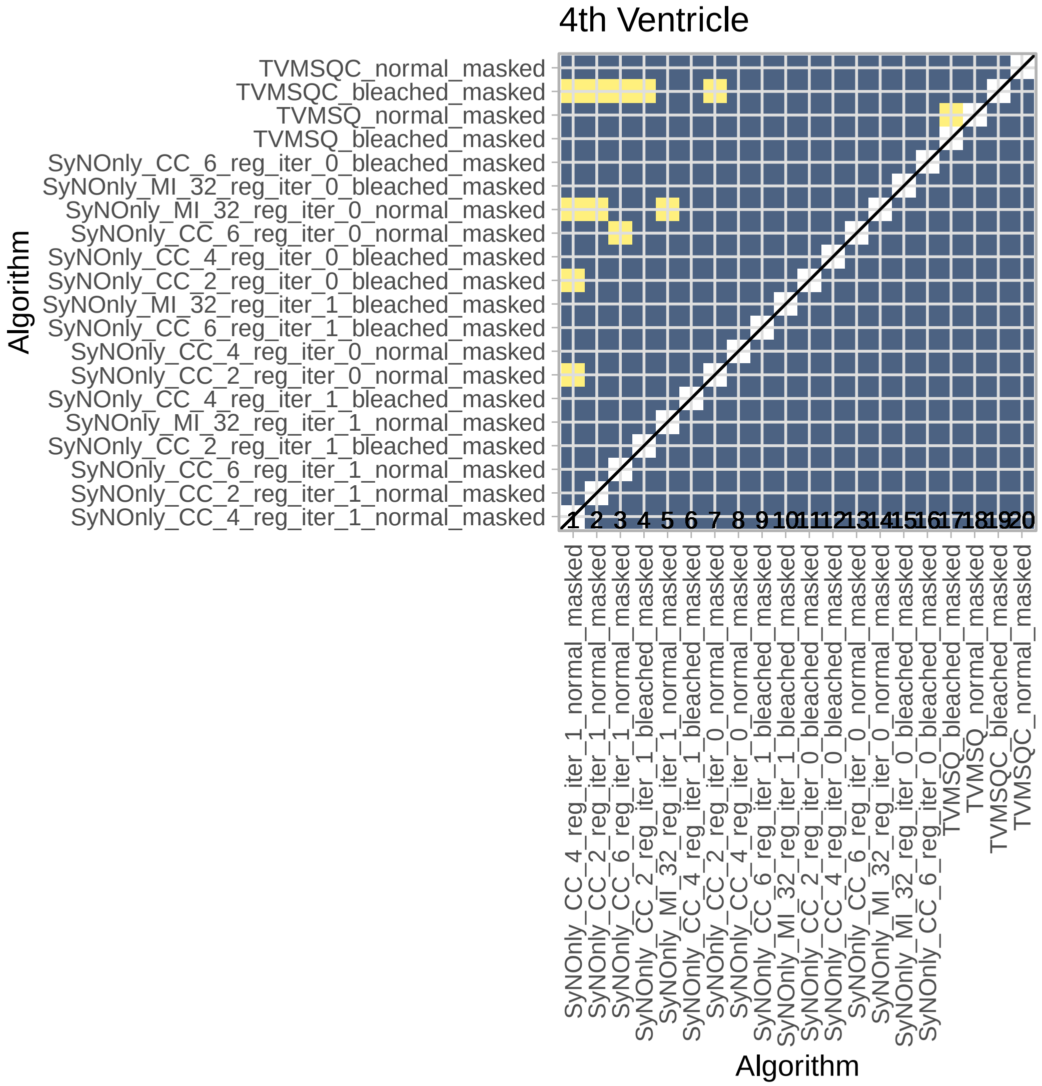

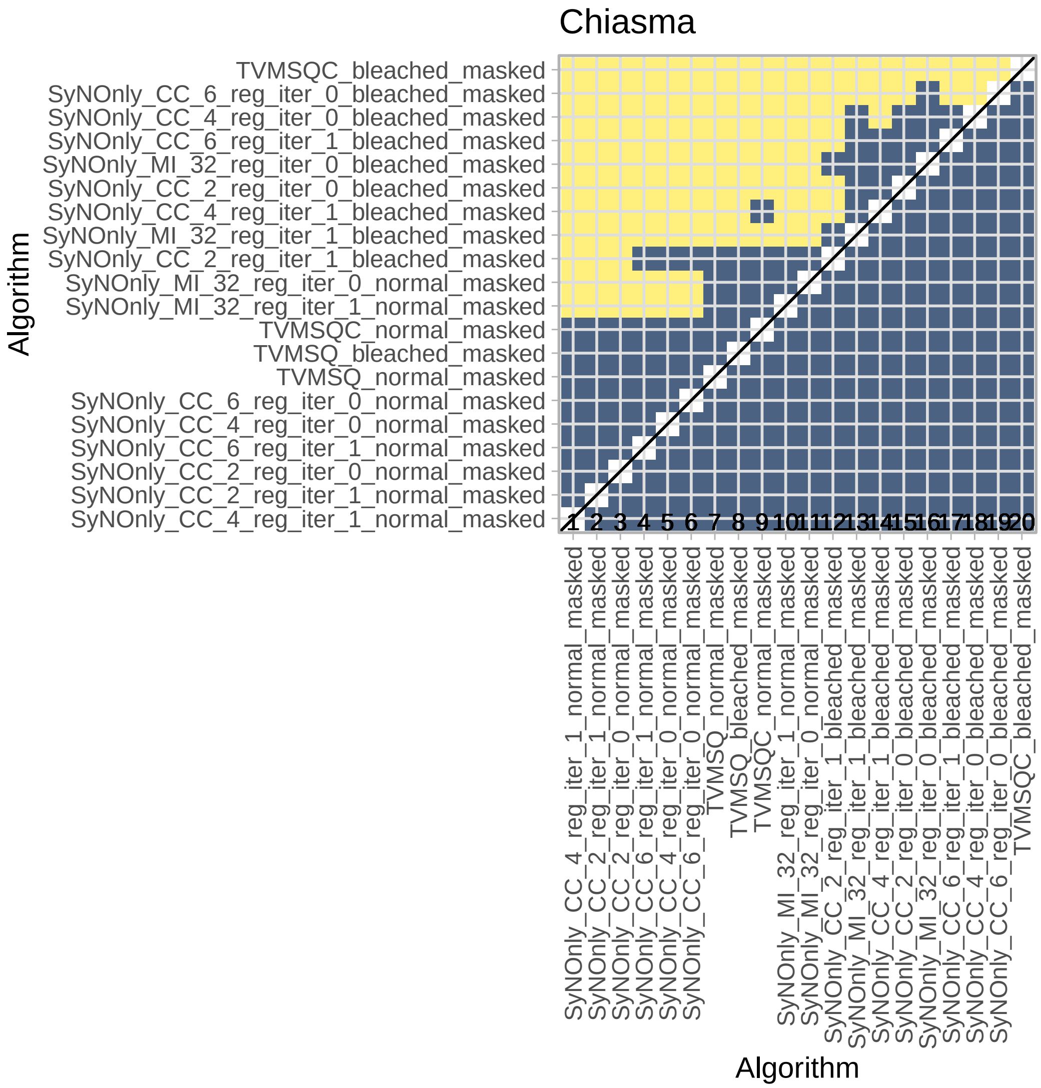

Chiasma
Algorithm
TVMSQC_bleached_masked
SyNOnly_CC_6_reg_iter_0_bleached_masked
SyNOnly_CC_4_reg_iter_0_bleached_masked
SyNOnly_CC_6_reg_iter_1_bleached_masked
SyNOnly_MI_32_reg_iter_0_bleached_masked
SyNOnly_CC_2_reg_iter_0_bleached_masked
SyNOnly_CC_4_reg_iter_1_bleached_masked
SyNOnly_MI_32_reg_iter_1_bleached_masked
SyNOnly_CC_2_reg_iter_1_bleached_masked
SyNOnly_MI_32_reg_iter_0_normal_masked
SyNOnly_MI_32_reg_iter_1_normal_masked
TVMSQC_normal_masked
TVMSQ_bleached_masked
TVMSQ_normal_masked
SyNOnly_CC_6_reg_iter_0_normal_masked
SyNOnly_CC_4_reg_iter_0_normal_masked
SyNOnly_CC_6_reg_iter_1_normal_masked
SyNOnly_CC_2_reg_iter_0_normal_masked
SyNOnly_CC_2_reg_iter_1_normal_masked
SyNOnly_CC_4_reg_iter_1_normal_masked
1 2 3 4 5 6 7 8 9 10 11 12 13 14 15 16 17 18 19 20
SyNOnly_CC_4_reg_iter_1_normal_masked
SyNOnly_CC_2_reg_iter_1_normal_masked
SyNOnly_CC_2_reg_iter_0_normal_masked
SyNOnly_CC_6_reg_iter_1_normal_masked
SyNOnly_CC_4_reg_iter_0_normal_masked
SyNOnly_CC_6_reg_iter_0_normal_masked
TVMSQ_normal_masked
TVMSQ_bleached_masked
TVMSQC_normal_masked
SyNOnly_MI_32_reg_iter_1_normal_masked
SyNOnly_MI_32_reg_iter_0_normal_masked
SyNOnly_CC_2_reg_iter_1_bleached_masked
SyNOnly_MI_32_reg_iter_1_bleached_masked
SyNOnly_CC_4_reg_iter_1_bleached_masked
SyNOnly_CC_2_reg_iter_0_bleached_masked
SyNOnly_MI_32_reg_iter_0_bleached_masked
SyNOnly_CC_6_reg_iter_1_bleached_masked
SyNOnly_CC_4_reg_iter_0_bleached_masked
SyNOnly_CC_6_reg_iter_0_bleached_masked
TVMSQC_bleached_masked
Algorithm

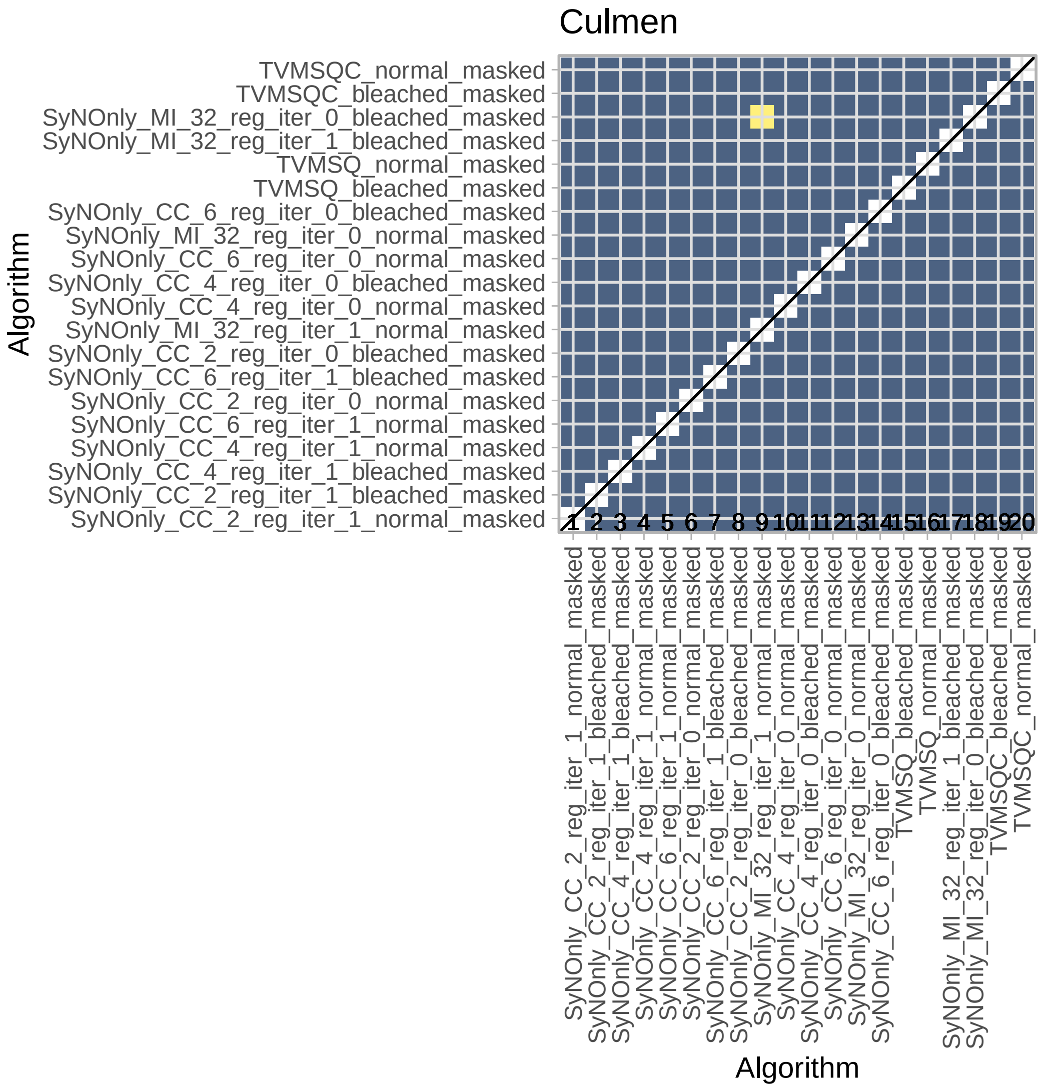
Culmen
TVMSQC_normal_masked
TVMSQC_bleached_masked
SyNOnly_MI_32_reg_iter_0_bleached_masked
SyNOnly_MI_32_reg_iter_1_bleached_masked
TVMSQ_normal_masked
TVMSQ_bleached_masked
SyNOnly_CC_6_reg_iter_0_bleached_masked
SyNOnly_MI_32_reg_iter_0_normal_masked
SyNOnly_CC_6_reg_iter_0_normal_masked
SyNOnly_CC_4_reg_iter_0_bleached_masked
SyNOnly_CC_4_reg_iter_0_normal_masked
SyNOnly_MI_32_reg_iter_1_normal_masked
SyNOnly_CC_2_reg_iter_0_bleached_masked
SyNOnly_CC_6_reg_iter_1_bleached_masked
SyNOnly_CC_2_reg_iter_0_normal_masked
SyNOnly_CC_6_reg_iter_1_normal_masked
SyNOnly_CC_4_reg_iter_1_normal_masked
SyNOnly_CC_4_reg_iter_1_bleached_masked
SyNOnly_CC_2_reg_iter_1_bleached_masked
SyNOnly_CC_2_reg_iter_1_normal_masked
Algorithm
1 2 3 4 5 6 7 8 9 10 11 12 13 14 15 16 17 18 19 20
Algorithm

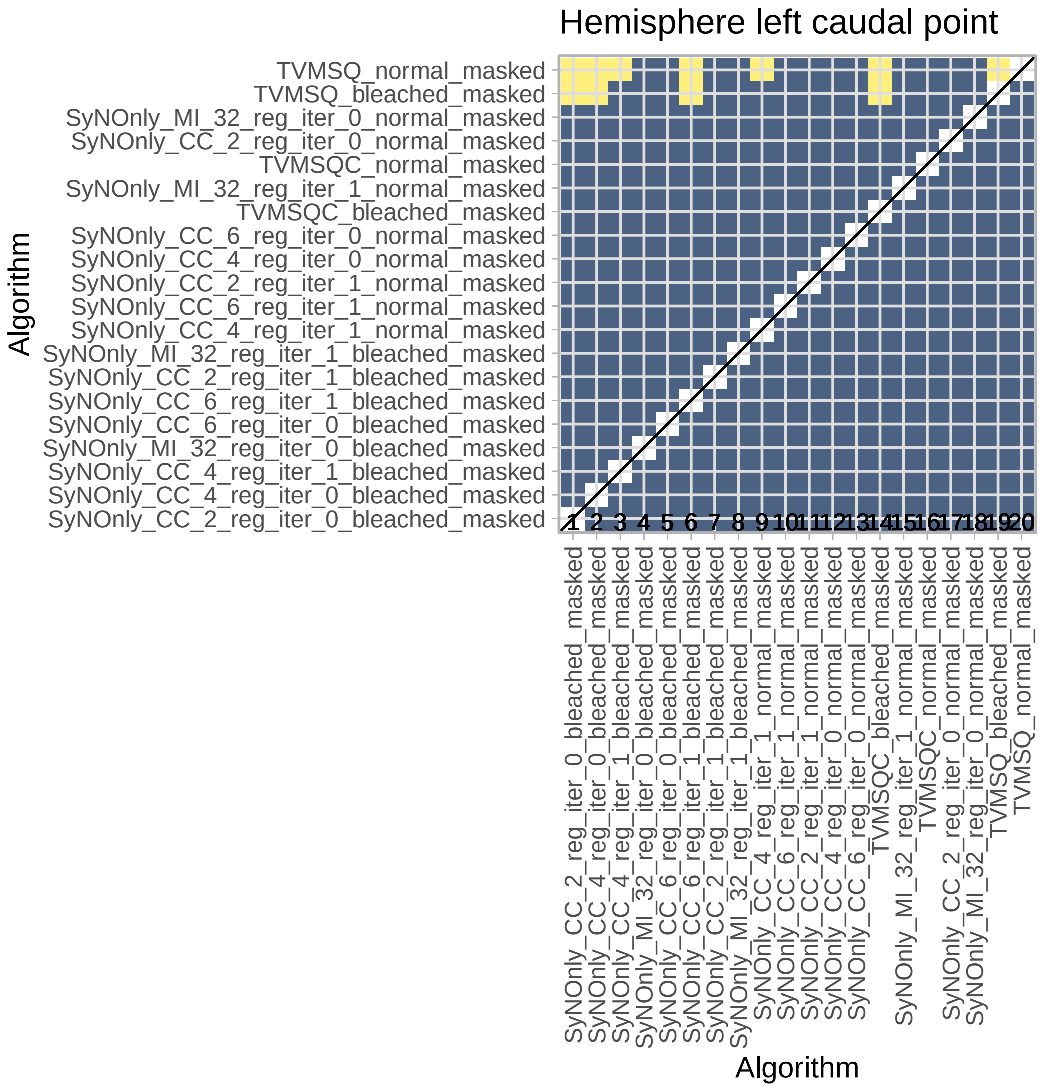

Hemisphere left caudal point
Algorithm
TVMSQ_normal_masked
TVMSQ_bleached_masked
SyNOnly_MI_32_reg_iter_0_normal_masked
SyNOnly_CC_2_reg_iter_0_normal_masked
TVMSQC_normal_masked
SyNOnly_MI_32_reg_iter_1_normal_masked
TVMSQC_bleached_masked
SyNOnly_CC_6_reg_iter_0_normal_masked
SyNOnly_CC_4_reg_iter_0_normal_masked
SyNOnly_CC_2_reg_iter_1_normal_masked
SyNOnly_CC_6_reg_iter_1_normal_masked
SyNOnly_CC_4_reg_iter_1_normal_masked
SyNOnly_MI_32_reg_iter_1_bleached_masked
SyNOnly_CC_2_reg_iter_1_bleached_masked
SyNOnly_CC_6_reg_iter_1_bleached_masked
SyNOnly_CC_6_reg_iter_0_bleached_masked
SyNOnly_MI_32_reg_iter_0_bleached_masked
SyNOnly_CC_4_reg_iter_1_bleached_masked
SyNOnly_CC_4_reg_iter_0_bleached_masked
SyNOnly_CC_2_reg_iter_0_bleached_masked
1 2 3 4 5 6 7 8 9 10 11 12 13 14 15 16 17 18 19 20
SyNOnly_CC_2_reg_iter_0_bleached_masked
SyNOnly_CC_4_reg_iter_0_bleached_masked
SyNOnly_CC_4_reg_iter_1_bleached_masked
SyNOnly_MI_32_reg_iter_0_bleached_masked
SyNOnly_CC_6_reg_iter_0_bleached_masked
SyNOnly_CC_6_reg_iter_1_bleached_masked
SyNOnly_CC_2_reg_iter_1_bleached_masked
SyNOnly_MI_32_reg_iter_1_bleached_masked
SyNOnly_CC_4_reg_iter_1_normal_masked
SyNOnly_CC_6_reg_iter_1_normal_masked
SyNOnly_CC_2_reg_iter_1_normal_masked
SyNOnly_CC_4_reg_iter_0_normal_masked
SyNOnly_CC_6_reg_iter_0_normal_masked
TVMSQC_bleached_masked
SyNOnly_MI_32_reg_iter_1_normal_masked
TVMSQC_normal_masked
SyNOnly_CC_2_reg_iter_0_normal_masked
SyNOnly_MI_32_reg_iter_0_normal_masked
TVMSQ_bleached_masked
TVMSQ_normal_masked
Algorithm

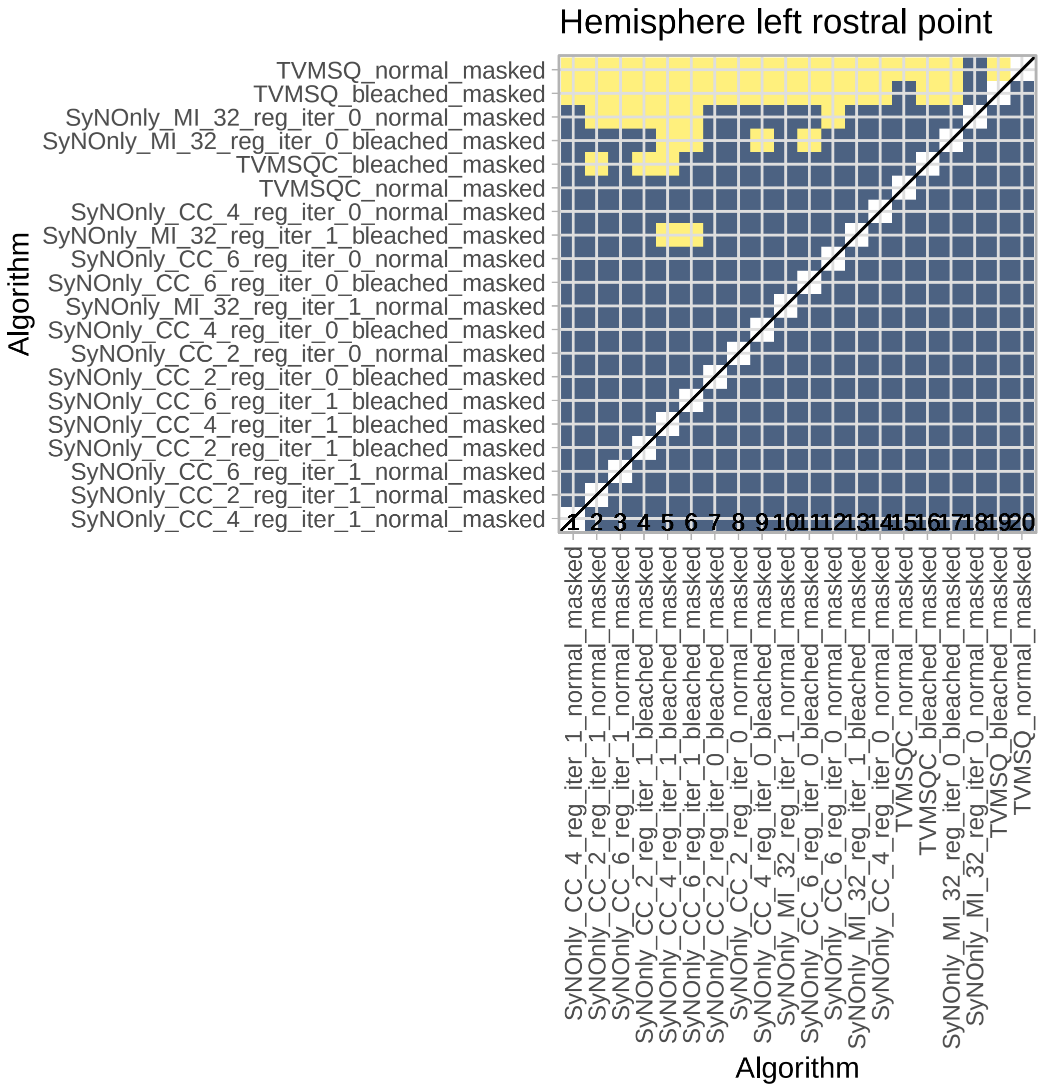
Hemisphere left rostral point
TVMSQ_normal_masked
TVMSQ_bleached_masked
SyNOnly_MI_32_reg_iter_0_normal_masked
SyNOnly_MI_32_reg_iter_0_bleached_masked
TVMSQC_bleached_masked
TVMSQC_normal_masked
SyNOnly_CC_4_reg_iter_0_normal_masked
SyNOnly_MI_32_reg_iter_1_bleached_masked
SyNOnly_CC_6_reg_iter_0_normal_masked
SyNOnly_CC_6_reg_iter_0_bleached_masked
SyNOnly_MI_32_reg_iter_1_normal_masked
SyNOnly_CC_4_reg_iter_0_bleached_masked
SyNOnly_CC_2_reg_iter_0_normal_masked
SyNOnly_CC_2_reg_iter_0_bleached_masked
SyNOnly_CC_6_reg_iter_1_bleached_masked
SyNOnly_CC_4_reg_iter_1_bleached_masked
SyNOnly_CC_2_reg_iter_1_bleached_masked
SyNOnly_CC_6_reg_iter_1_normal_masked
SyNOnly_CC_2_reg_iter_1_normal_masked
SyNOnly_CC_4_reg_iter_1_normal_masked
Algorithm
1 2 3 4 5 6 7 8 9 10 11 12 13 14 15 16 17 18 19 20
SyNOnly_CC_4_reg_iter_1_normal_masked
SyNOnly_CC_2_reg_iter_1_normal_masked
SyNOnly_CC_6_reg_iter_1_normal_masked
SyNOnly_CC_2_reg_iter_1_bleached_masked
SyNOnly_CC_4_reg_iter_1_bleached_masked
SyNOnly_CC_6_reg_iter_1_bleached_masked
SyNOnly_CC_2_reg_iter_0_bleached_masked
SyNOnly_CC_2_reg_iter_0_normal_masked
SyNOnly_CC_4_reg_iter_0_bleached_masked
SyNOnly_MI_32_reg_iter_1_normal_masked
SyNOnly_CC_6_reg_iter_0_bleached_masked
SyNOnly_CC_6_reg_iter_0_normal_masked
SyNOnly_MI_32_reg_iter_1_bleached_masked
SyNOnly_CC_4_reg_iter_0_normal_masked
TVMSQC_normal_masked
TVMSQC_bleached_masked
SyNOnly_MI_32_reg_iter_0_bleached_masked
SyNOnly_MI_32_reg_iter_0_normal_masked
TVMSQ_bleached_masked
TVMSQ_normal_masked
Algorithm

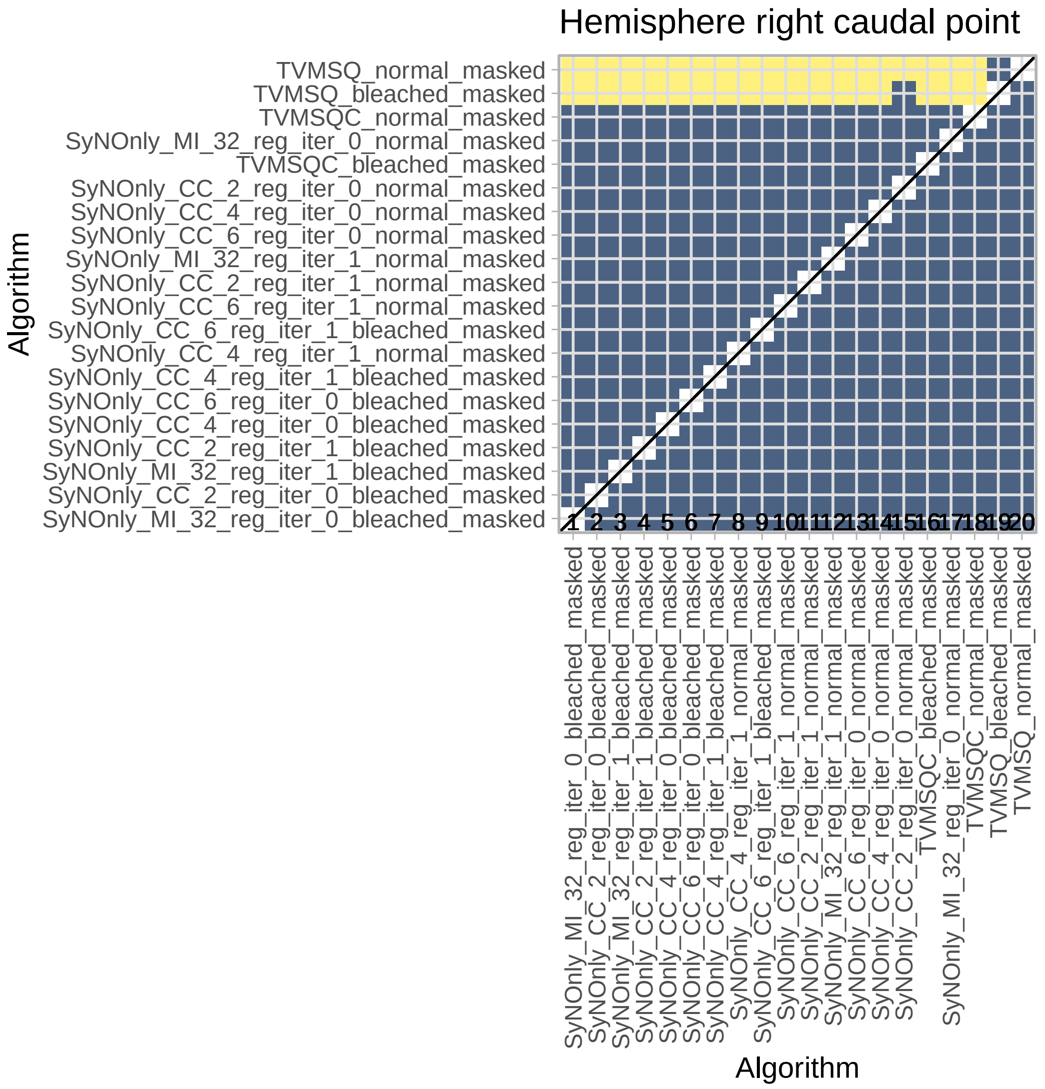
Hemisphere right caudal point
Algorithm
TVMSQ_normal_masked
TVMSQ_bleached_masked
TVMSQC_normal_masked
SyNOnly_MI_32_reg_iter_0_normal_masked
TVMSQC_bleached_masked
SyNOnly_CC_2_reg_iter_0_normal_masked
SyNOnly_CC_4_reg_iter_0_normal_masked
SyNOnly_CC_6_reg_iter_0_normal_masked
SyNOnly_MI_32_reg_iter_1_normal_masked
SyNOnly_CC_2_reg_iter_1_normal_masked
SyNOnly_CC_6_reg_iter_1_normal_masked
SyNOnly_CC_6_reg_iter_1_bleached_masked
SyNOnly_CC_4_reg_iter_1_normal_masked
SyNOnly_CC_4_reg_iter_1_bleached_masked
SyNOnly_CC_6_reg_iter_0_bleached_masked
SyNOnly_CC_4_reg_iter_0_bleached_masked
SyNOnly_CC_2_reg_iter_1_bleached_masked
SyNOnly_MI_32_reg_iter_1_bleached_masked
SyNOnly_CC_2_reg_iter_0_bleached_masked
SyNOnly_MI_32_reg_iter_0_bleached_masked
1 2 3 4 5 6 7 8 9 10 11 12 13 14 15 16 17 18 19 20
SyNOnly_MI_32_reg_iter_0_bleached_masked
SyNOnly_CC_2_reg_iter_0_bleached_masked
SyNOnly_MI_32_reg_iter_1_bleached_masked
SyNOnly_CC_2_reg_iter_1_bleached_masked
SyNOnly_CC_4_reg_iter_0_bleached_masked
SyNOnly_CC_6_reg_iter_0_bleached_masked
SyNOnly_CC_4_reg_iter_1_bleached_masked
SyNOnly_CC_4_reg_iter_1_normal_masked
SyNOnly_CC_6_reg_iter_1_bleached_masked
SyNOnly_CC_6_reg_iter_1_normal_masked
SyNOnly_CC_2_reg_iter_1_normal_masked
SyNOnly_MI_32_reg_iter_1_normal_masked
SyNOnly_CC_6_reg_iter_0_normal_masked
SyNOnly_CC_4_reg_iter_0_normal_masked
SyNOnly_CC_2_reg_iter_0_normal_masked
TVMSQC_bleached_masked
SyNOnly_MI_32_reg_iter_0_normal_masked
TVMSQC_normal_masked
TVMSQ_bleached_masked
TVMSQ_normal_masked
Algorithm

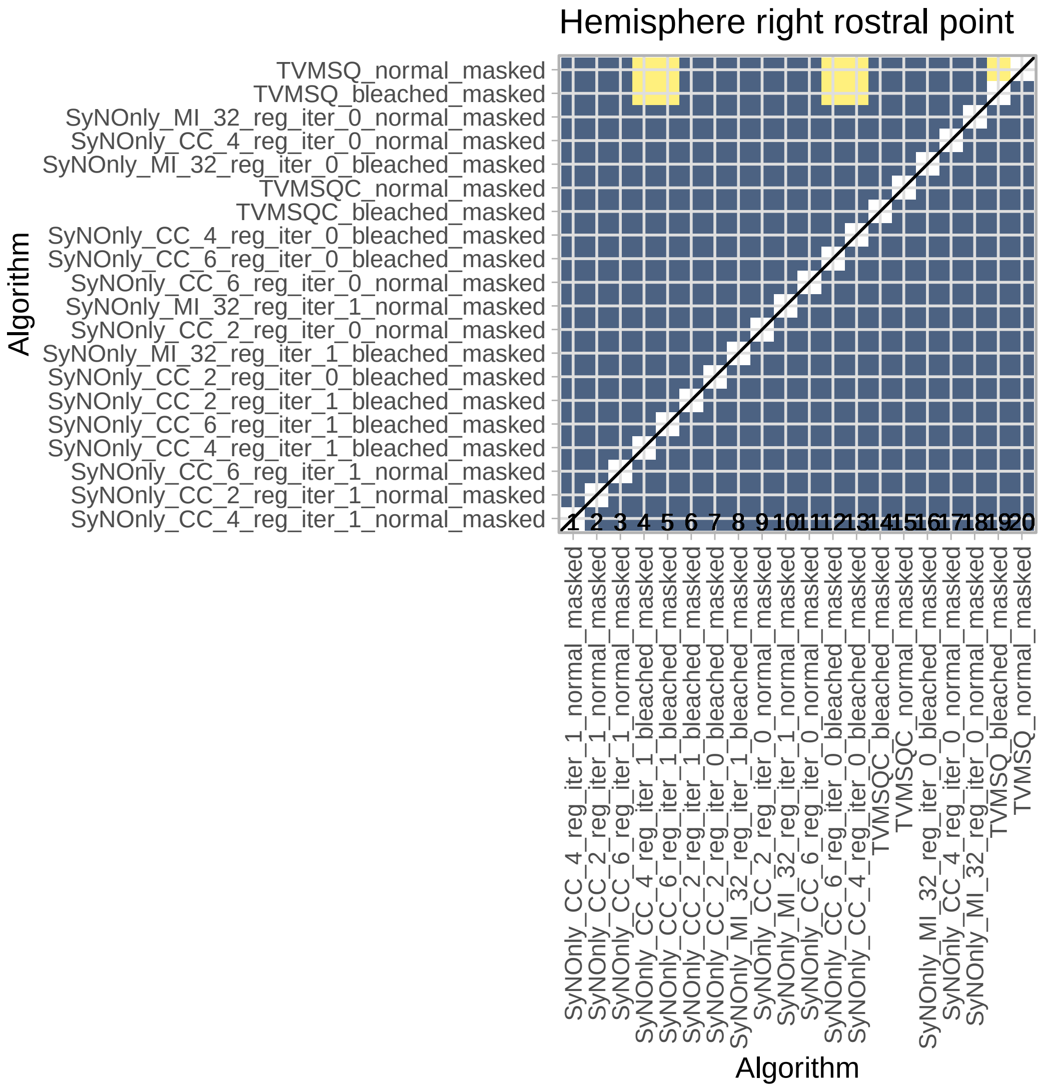
Hemisphere right rostral point
Algorithm
TVMSQ_normal_masked
TVMSQ_bleached_masked
SyNOnly_MI_32_reg_iter_0_normal_masked
SyNOnly_CC_4_reg_iter_0_normal_masked
SyNOnly_MI_32_reg_iter_0_bleached_masked
TVMSQC_normal_masked
TVMSQC_bleached_masked
SyNOnly_CC_4_reg_iter_0_bleached_masked
SyNOnly_CC_6_reg_iter_0_bleached_masked
SyNOnly_CC_6_reg_iter_0_normal_masked
SyNOnly_MI_32_reg_iter_1_normal_masked
SyNOnly_CC_2_reg_iter_0_normal_masked
SyNOnly_MI_32_reg_iter_1_bleached_masked
SyNOnly_CC_2_reg_iter_0_bleached_masked
SyNOnly_CC_2_reg_iter_1_bleached_masked
SyNOnly_CC_6_reg_iter_1_bleached_masked
SyNOnly_CC_4_reg_iter_1_bleached_masked
SyNOnly_CC_6_reg_iter_1_normal_masked
SyNOnly_CC_2_reg_iter_1_normal_masked
SyNOnly_CC_4_reg_iter_1_normal_masked
1 2 3 4 5 6 7 8 9 10 11 12 13 14 15 16 17 18 19 20
Algorithm

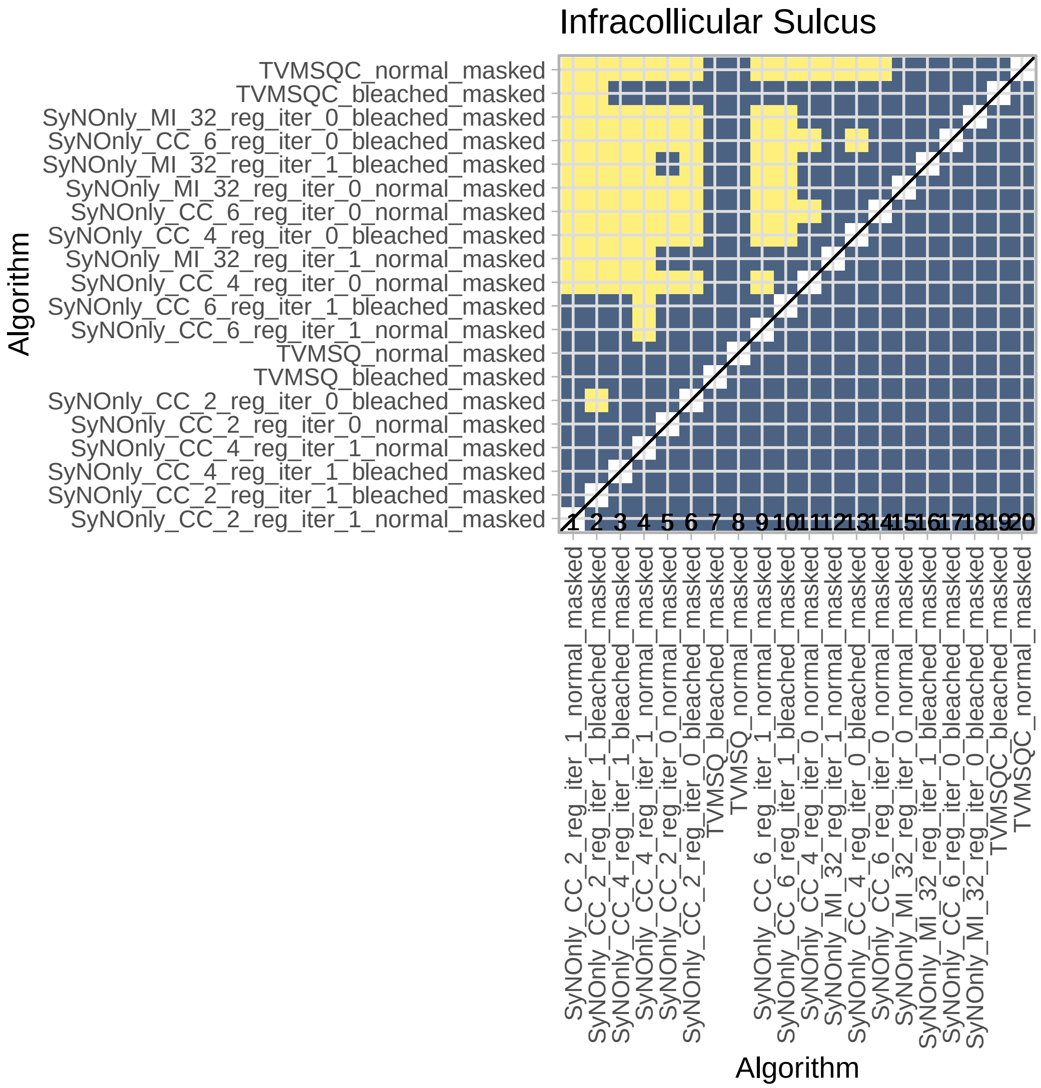

Infracollicular Sulcus
TVMSQC_normal_masked
TVMSQC_bleached_masked
SyNOnly_MI_32_reg_iter_0_bleached_masked
SyNOnly_CC_6_reg_iter_0_bleached_masked
SyNOnly_MI_32_reg_iter_1_bleached_masked
SyNOnly_MI_32_reg_iter_0_normal_masked
SyNOnly_CC_6_reg_iter_0_normal_masked
SyNOnly_CC_4_reg_iter_0_bleached_masked
SyNOnly_MI_32_reg_iter_1_normal_masked
SyNOnly_CC_4_reg_iter_0_normal_masked
SyNOnly_CC_6_reg_iter_1_bleached_masked
SyNOnly_CC_6_reg_iter_1_normal_masked
TVMSQ_normal_masked
TVMSQ_bleached_masked
SyNOnly_CC_2_reg_iter_0_bleached_masked
SyNOnly_CC_2_reg_iter_0_normal_masked
SyNOnly_CC_4_reg_iter_1_normal_masked
SyNOnly_CC_4_reg_iter_1_bleached_masked
SyNOnly_CC_2_reg_iter_1_bleached_masked
SyNOnly_CC_2_reg_iter_1_normal_masked
Algorithm
1 2 3 4 5 6 7 8 9 10 11 12 13 14 15 16 17 18 19 20
SyNOnly_CC_2_reg_iter_1_normal_masked
SyNOnly_CC_2_reg_iter_1_bleached_masked
SyNOnly_CC_4_reg_iter_1_bleached_masked
SyNOnly_CC_4_reg_iter_1_normal_masked
SyNOnly_CC_2_reg_iter_0_normal_masked
SyNOnly_CC_2_reg_iter_0_bleached_masked
TVMSQ_bleached_masked
TVMSQ_normal_masked
SyNOnly_CC_6_reg_iter_1_normal_masked
SyNOnly_CC_6_reg_iter_1_bleached_masked
SyNOnly_CC_4_reg_iter_0_normal_masked
SyNOnly_MI_32_reg_iter_1_normal_masked
SyNOnly_CC_4_reg_iter_0_bleached_masked
SyNOnly_CC_6_reg_iter_0_normal_masked
SyNOnly_MI_32_reg_iter_0_normal_masked
SyNOnly_MI_32_reg_iter_1_bleached_masked
SyNOnly_CC_6_reg_iter_0_bleached_masked
SyNOnly_MI_32_reg_iter_0_bleached_masked
TVMSQC_bleached_masked
TVMSQC_normal_masked
Algorithm

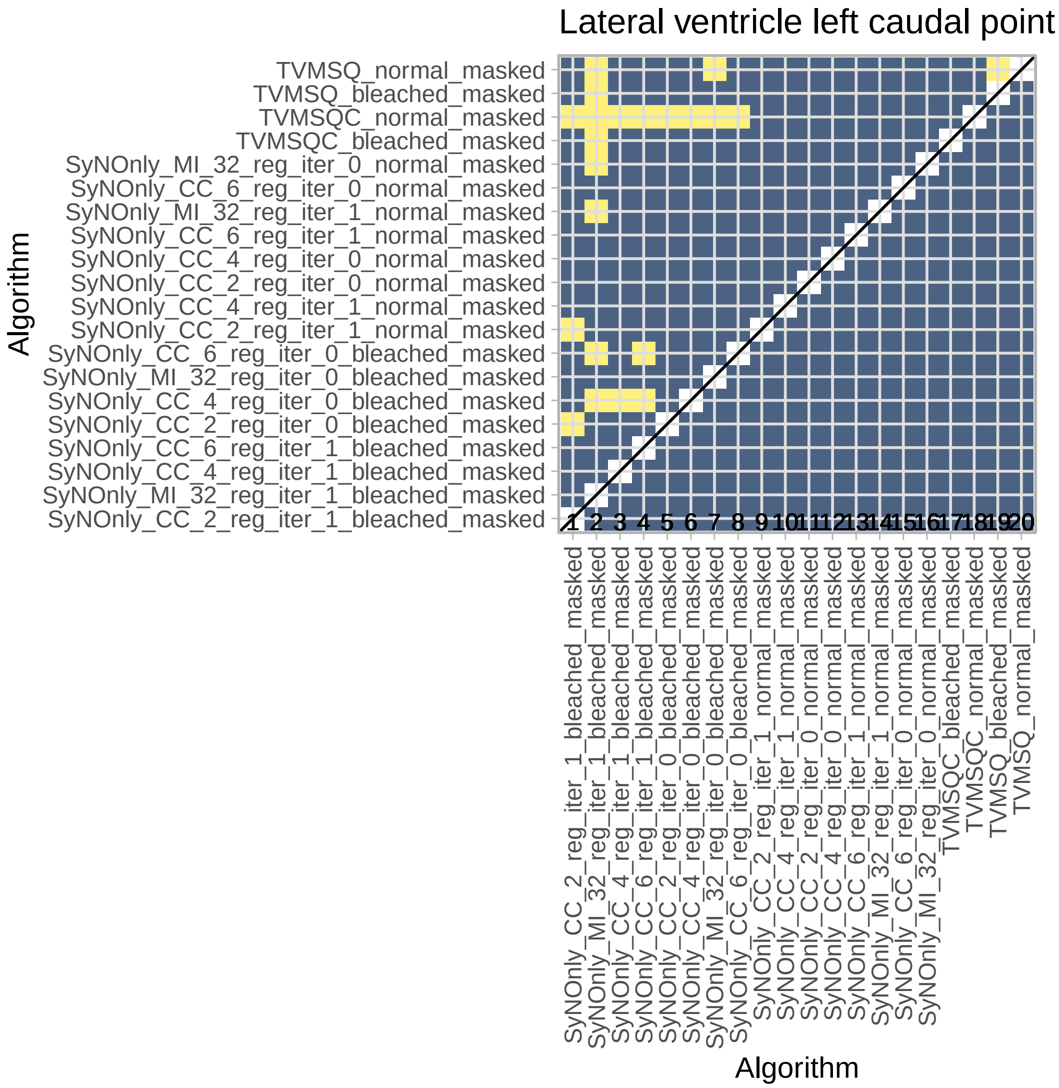
Lateral ventricle left caudal point
Algorithm
TVMSQ_normal_masked
TVMSQ_bleached_masked
TVMSQC_normal_masked
TVMSQC_bleached_masked
SyNOnly_MI_32_reg_iter_0_normal_masked
SyNOnly_CC_6_reg_iter_0_normal_masked
SyNOnly_MI_32_reg_iter_1_normal_masked
SyNOnly_CC_6_reg_iter_1_normal_masked
SyNOnly_CC_4_reg_iter_0_normal_masked
SyNOnly_CC_2_reg_iter_0_normal_masked
SyNOnly_CC_4_reg_iter_1_normal_masked
SyNOnly_CC_2_reg_iter_1_normal_masked
SyNOnly_CC_6_reg_iter_0_bleached_masked
SyNOnly_MI_32_reg_iter_0_bleached_masked
SyNOnly_CC_4_reg_iter_0_bleached_masked
SyNOnly_CC_2_reg_iter_0_bleached_masked
SyNOnly_CC_6_reg_iter_1_bleached_masked
SyNOnly_CC_4_reg_iter_1_bleached_masked
SyNOnly_MI_32_reg_iter_1_bleached_masked
SyNOnly_CC_2_reg_iter_1_bleached_masked
1 2 3 4 5 6 7 8 9 10 11 12 13 14 15 16 17 18 19 20
Algorithm

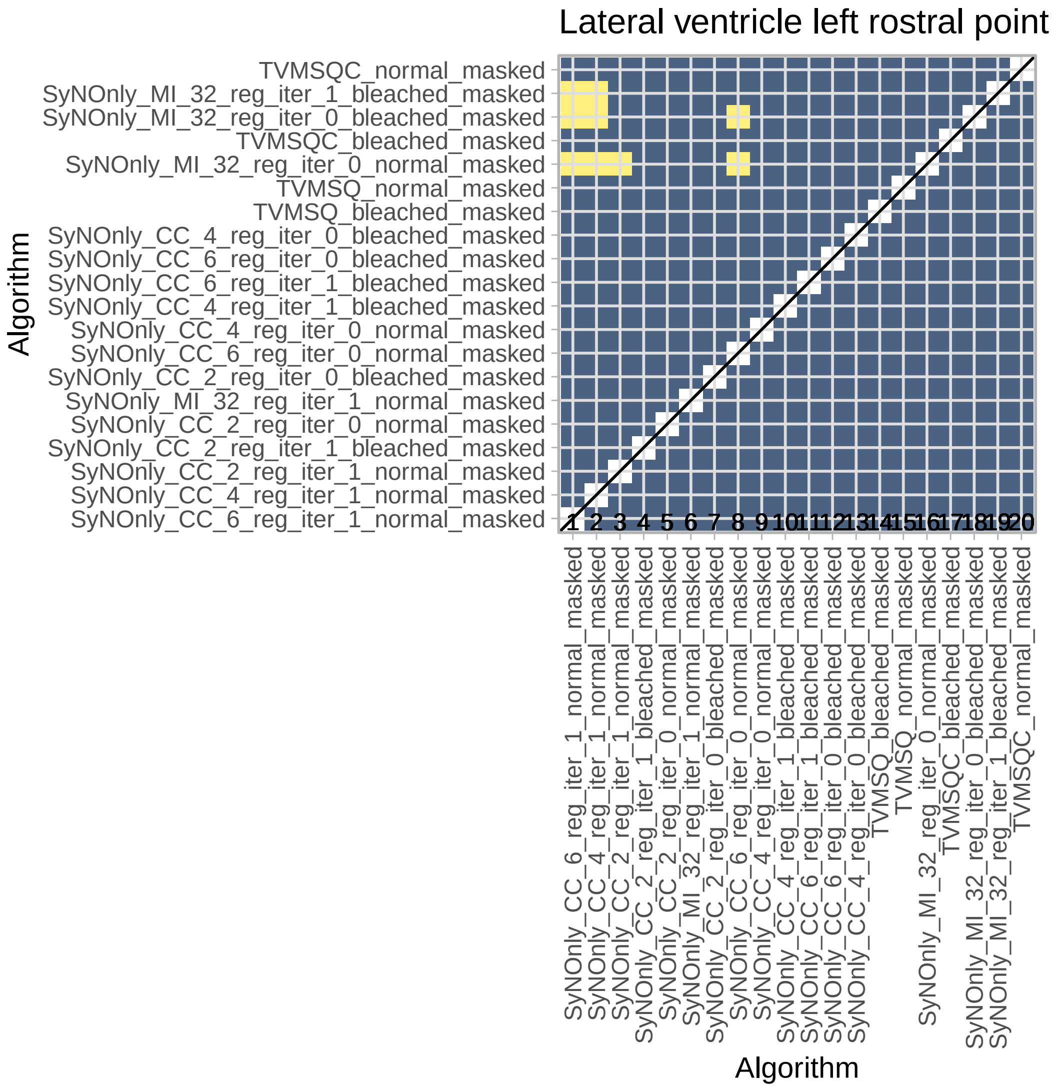

Lateral ventricle left rostral point
Algorithm
TVMSQC_normal_masked
SyNOnly_MI_32_reg_iter_1_bleached_masked
SyNOnly_MI_32_reg_iter_0_bleached_masked
TVMSQC_bleached_masked
SyNOnly_MI_32_reg_iter_0_normal_masked
TVMSQ_normal_masked
TVMSQ_bleached_masked
SyNOnly_CC_4_reg_iter_0_bleached_masked
SyNOnly_CC_6_reg_iter_0_bleached_masked
SyNOnly_CC_6_reg_iter_1_bleached_masked
SyNOnly_CC_4_reg_iter_1_bleached_masked
SyNOnly_CC_4_reg_iter_0_normal_masked
SyNOnly_CC_6_reg_iter_0_normal_masked
SyNOnly_CC_2_reg_iter_0_bleached_masked
SyNOnly_MI_32_reg_iter_1_normal_masked
SyNOnly_CC_2_reg_iter_0_normal_masked
SyNOnly_CC_2_reg_iter_1_bleached_masked
SyNOnly_CC_2_reg_iter_1_normal_masked
SyNOnly_CC_4_reg_iter_1_normal_masked
SyNOnly_CC_6_reg_iter_1_normal_masked
1 2 3 4 5 6 7 8 9 10 11 12 13 14 15 16 17 18 19 20
SyNOnly_CC_6_reg_iter_1_normal_masked
SyNOnly_CC_4_reg_iter_1_normal_masked
SyNOnly_CC_2_reg_iter_1_normal_masked
SyNOnly_CC_2_reg_iter_1_bleached_masked
SyNOnly_CC_2_reg_iter_0_normal_masked
SyNOnly_MI_32_reg_iter_1_normal_masked
SyNOnly_CC_2_reg_iter_0_bleached_masked
SyNOnly_CC_6_reg_iter_0_normal_masked
SyNOnly_CC_4_reg_iter_0_normal_masked
SyNOnly_CC_4_reg_iter_1_bleached_masked
SyNOnly_CC_6_reg_iter_1_bleached_masked
SyNOnly_CC_6_reg_iter_0_bleached_masked
SyNOnly_CC_4_reg_iter_0_bleached_masked
TVMSQ_bleached_masked
TVMSQ_normal_masked
SyNOnly_MI_32_reg_iter_0_normal_masked
TVMSQC_bleached_masked
SyNOnly_MI_32_reg_iter_0_bleached_masked
SyNOnly_MI_32_reg_iter_1_bleached_masked
TVMSQC_normal_masked
Algorithm

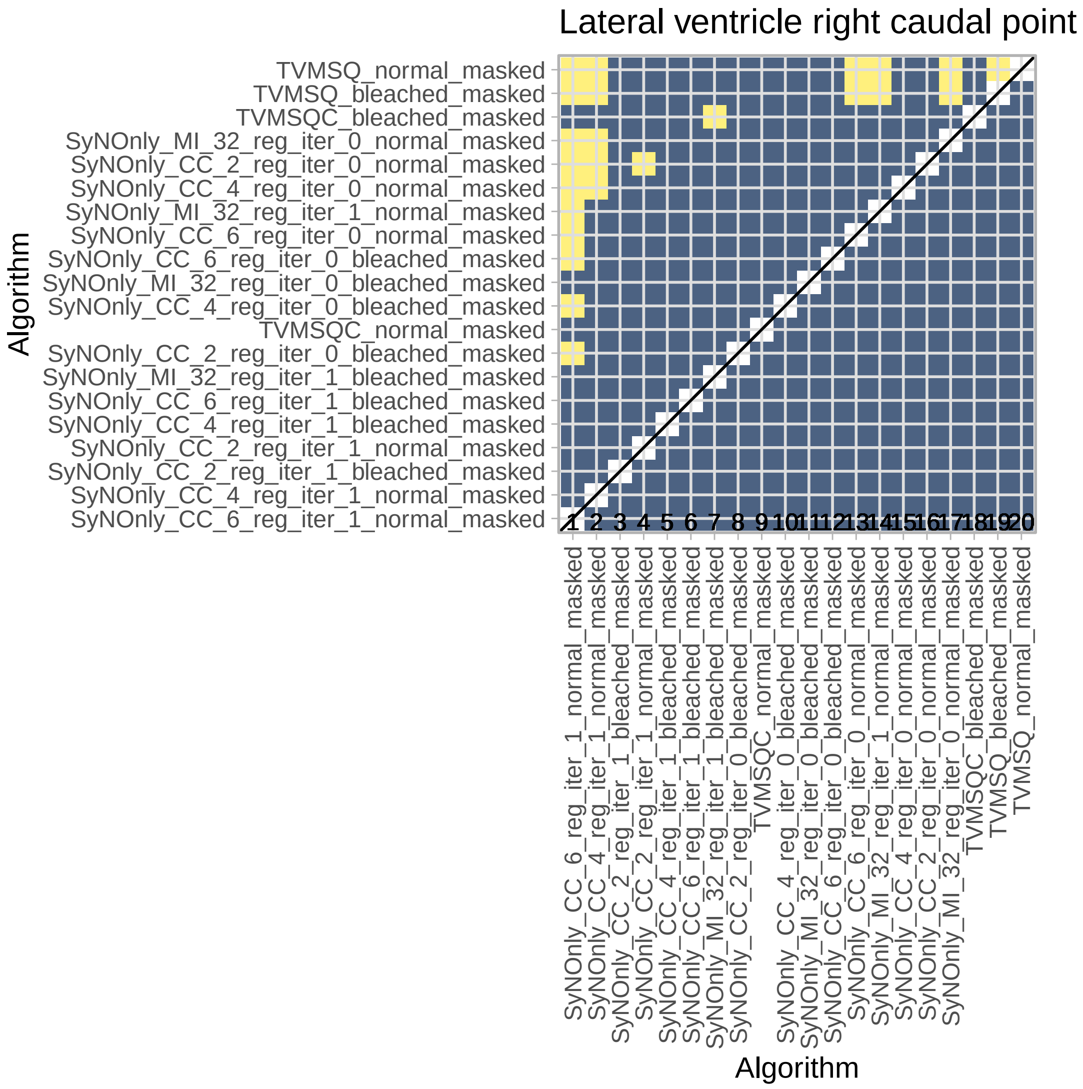

Lateral ventricle right caudal point
Algorithm
TVMSQ_normal_masked
TVMSQ_bleached_masked
TVMSQC_bleached_masked
SyNOnly_MI_32_reg_iter_0_normal_masked
SyNOnly_CC_2_reg_iter_0_normal_masked
SyNOnly_CC_4_reg_iter_0_normal_masked
SyNOnly_MI_32_reg_iter_1_normal_masked
SyNOnly_CC_6_reg_iter_0_normal_masked
SyNOnly_CC_6_reg_iter_0_bleached_masked
SyNOnly_MI_32_reg_iter_0_bleached_masked
SyNOnly_CC_4_reg_iter_0_bleached_masked
TVMSQC_normal_masked
SyNOnly_CC_2_reg_iter_0_bleached_masked
SyNOnly_MI_32_reg_iter_1_bleached_masked
SyNOnly_CC_6_reg_iter_1_bleached_masked
SyNOnly_CC_4_reg_iter_1_bleached_masked
SyNOnly_CC_2_reg_iter_1_normal_masked
SyNOnly_CC_2_reg_iter_1_bleached_masked
SyNOnly_CC_4_reg_iter_1_normal_masked
SyNOnly_CC_6_reg_iter_1_normal_masked
1 2 3 4 5 6 7 8 9 10 11 12 13 14 15 16 17 18 19 20
SyNOnly_CC_6_reg_iter_1_normal_masked
SyNOnly_CC_4_reg_iter_1_normal_masked
SyNOnly_CC_2_reg_iter_1_bleached_masked
SyNOnly_CC_2_reg_iter_1_normal_masked
SyNOnly_CC_4_reg_iter_1_bleached_masked
SyNOnly_CC_6_reg_iter_1_bleached_masked
SyNOnly_MI_32_reg_iter_1_bleached_masked
SyNOnly_CC_2_reg_iter_0_bleached_masked
TVMSQC_normal_masked
SyNOnly_CC_4_reg_iter_0_bleached_masked
SyNOnly_MI_32_reg_iter_0_bleached_masked
SyNOnly_CC_6_reg_iter_0_bleached_masked
SyNOnly_CC_6_reg_iter_0_normal_masked
SyNOnly_MI_32_reg_iter_1_normal_masked
SyNOnly_CC_4_reg_iter_0_normal_masked
SyNOnly_CC_2_reg_iter_0_normal_masked
SyNOnly_MI_32_reg_iter_0_normal_masked
TVMSQC_bleached_masked
TVMSQ_bleached_masked
TVMSQ_normal_masked
Algorithm

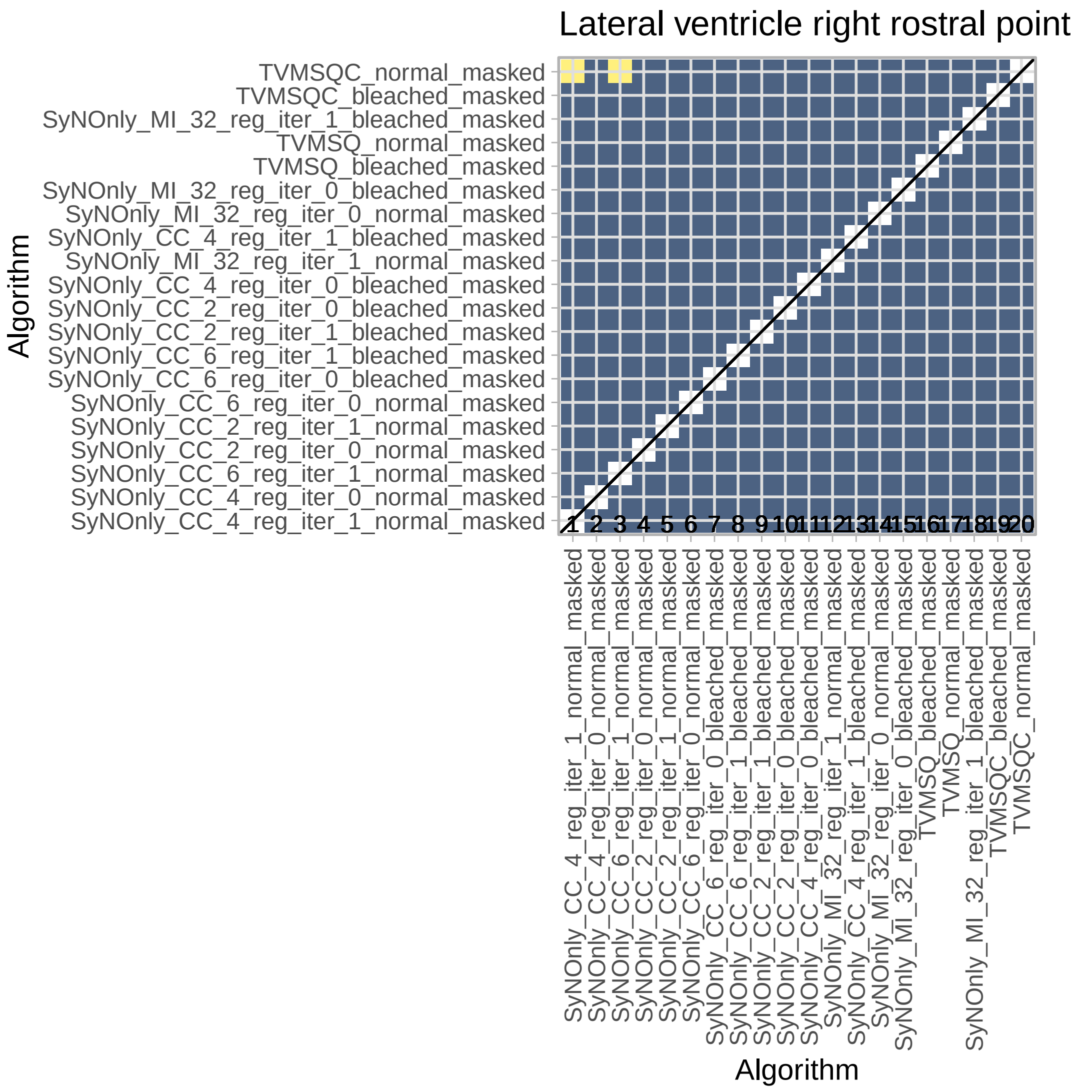
Lateral ventricle right rostral point
Algorithm
TVMSQC_normal_masked
TVMSQC_bleached_masked
SyNOnly_MI_32_reg_iter_1_bleached_masked
TVMSQ_normal_masked
TVMSQ_bleached_masked
SyNOnly_MI_32_reg_iter_0_bleached_masked
SyNOnly_MI_32_reg_iter_0_normal_masked
SyNOnly_CC_4_reg_iter_1_bleached_masked
SyNOnly_MI_32_reg_iter_1_normal_masked
SyNOnly_CC_4_reg_iter_0_bleached_masked
SyNOnly_CC_2_reg_iter_0_bleached_masked
SyNOnly_CC_2_reg_iter_1_bleached_masked
SyNOnly_CC_6_reg_iter_1_bleached_masked
SyNOnly_CC_6_reg_iter_0_bleached_masked
SyNOnly_CC_6_reg_iter_0_normal_masked
SyNOnly_CC_2_reg_iter_1_normal_masked
SyNOnly_CC_2_reg_iter_0_normal_masked
SyNOnly_CC_6_reg_iter_1_normal_masked
SyNOnly_CC_4_reg_iter_0_normal_masked
SyNOnly_CC_4_reg_iter_1_normal_masked
1 2 3 4 5 6 7 8 9 10 11 12 13 14 15 16 17 18 19 20
SyNOnly_CC_4_reg_iter_1_normal_masked
SyNOnly_CC_4_reg_iter_0_normal_masked
SyNOnly_CC_6_reg_iter_1_normal_masked
SyNOnly_CC_2_reg_iter_0_normal_masked
SyNOnly_CC_2_reg_iter_1_normal_masked
SyNOnly_CC_6_reg_iter_0_normal_masked
SyNOnly_CC_6_reg_iter_0_bleached_masked
SyNOnly_CC_6_reg_iter_1_bleached_masked
SyNOnly_CC_2_reg_iter_1_bleached_masked
SyNOnly_CC_2_reg_iter_0_bleached_masked
SyNOnly_CC_4_reg_iter_0_bleached_masked
SyNOnly_MI_32_reg_iter_1_normal_masked
SyNOnly_CC_4_reg_iter_1_bleached_masked
SyNOnly_MI_32_reg_iter_0_normal_masked
SyNOnly_MI_32_reg_iter_0_bleached_masked
TVMSQ_bleached_masked
TVMSQ_normal_masked
SyNOnly_MI_32_reg_iter_1_bleached_masked
TVMSQC_bleached_masked
TVMSQC_normal_masked
Algorithm

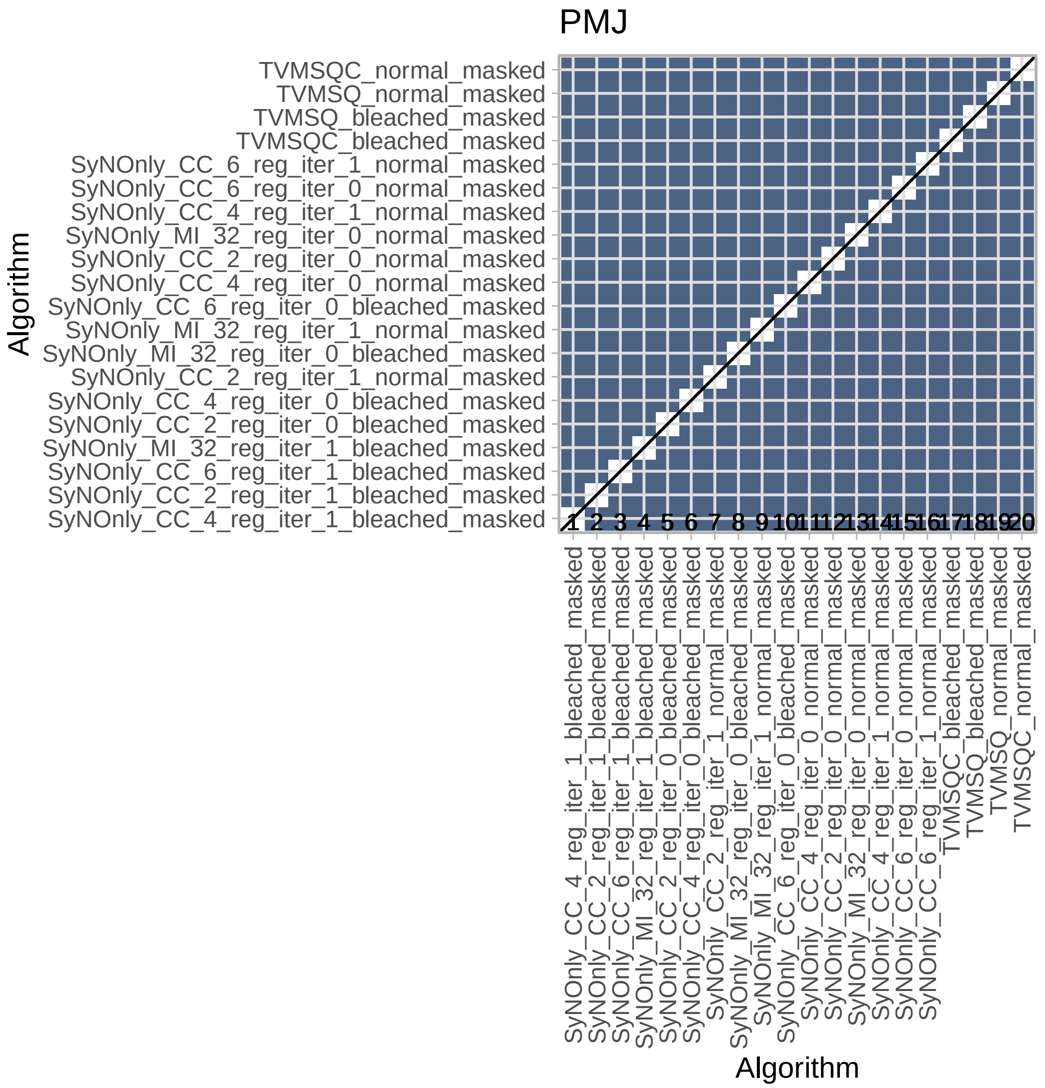
PMJ
Algorithm
TVMSQC_normal_masked
TVMSQ_normal_masked
TVMSQ_bleached_masked
TVMSQC_bleached_masked
SyNOnly_CC_6_reg_iter_1_normal_masked
SyNOnly_CC_6_reg_iter_0_normal_masked
SyNOnly_CC_4_reg_iter_1_normal_masked
SyNOnly_MI_32_reg_iter_0_normal_masked
SyNOnly_CC_2_reg_iter_0_normal_masked
SyNOnly_CC_4_reg_iter_0_normal_masked
SyNOnly_CC_6_reg_iter_0_bleached_masked
SyNOnly_MI_32_reg_iter_1_normal_masked
SyNOnly_MI_32_reg_iter_0_bleached_masked
SyNOnly_CC_2_reg_iter_1_normal_masked
SyNOnly_CC_4_reg_iter_0_bleached_masked
SyNOnly_CC_2_reg_iter_0_bleached_masked
SyNOnly_MI_32_reg_iter_1_bleached_masked
SyNOnly_CC_6_reg_iter_1_bleached_masked
SyNOnly_CC_2_reg_iter_1_bleached_masked
SyNOnly_CC_4_reg_iter_1_bleached_masked
1 2 3 4 5 6 7 8 9 10 11 12 13 14 15 16 17 18 19 20
SyNOnly_CC_4_reg_iter_1_bleached_masked
SyNOnly_CC_2_reg_iter_1_bleached_masked
SyNOnly_CC_6_reg_iter_1_bleached_masked
SyNOnly_MI_32_reg_iter_1_bleached_masked
SyNOnly_CC_2_reg_iter_0_bleached_masked
SyNOnly_CC_4_reg_iter_0_bleached_masked
SyNOnly_CC_2_reg_iter_1_normal_masked
SyNOnly_MI_32_reg_iter_0_bleached_masked
SyNOnly_MI_32_reg_iter_1_normal_masked
SyNOnly_CC_6_reg_iter_0_bleached_masked
SyNOnly_CC_4_reg_iter_0_normal_masked
SyNOnly_CC_2_reg_iter_0_normal_masked
SyNOnly_MI_32_reg_iter_0_normal_masked
SyNOnly_CC_4_reg_iter_1_normal_masked
SyNOnly_CC_6_reg_iter_0_normal_masked
SyNOnly_CC_6_reg_iter_1_normal_masked
TVMSQC_bleached_masked
TVMSQ_bleached_masked
TVMSQ_normal_masked
TVMSQC_normal_masked
Algorithm

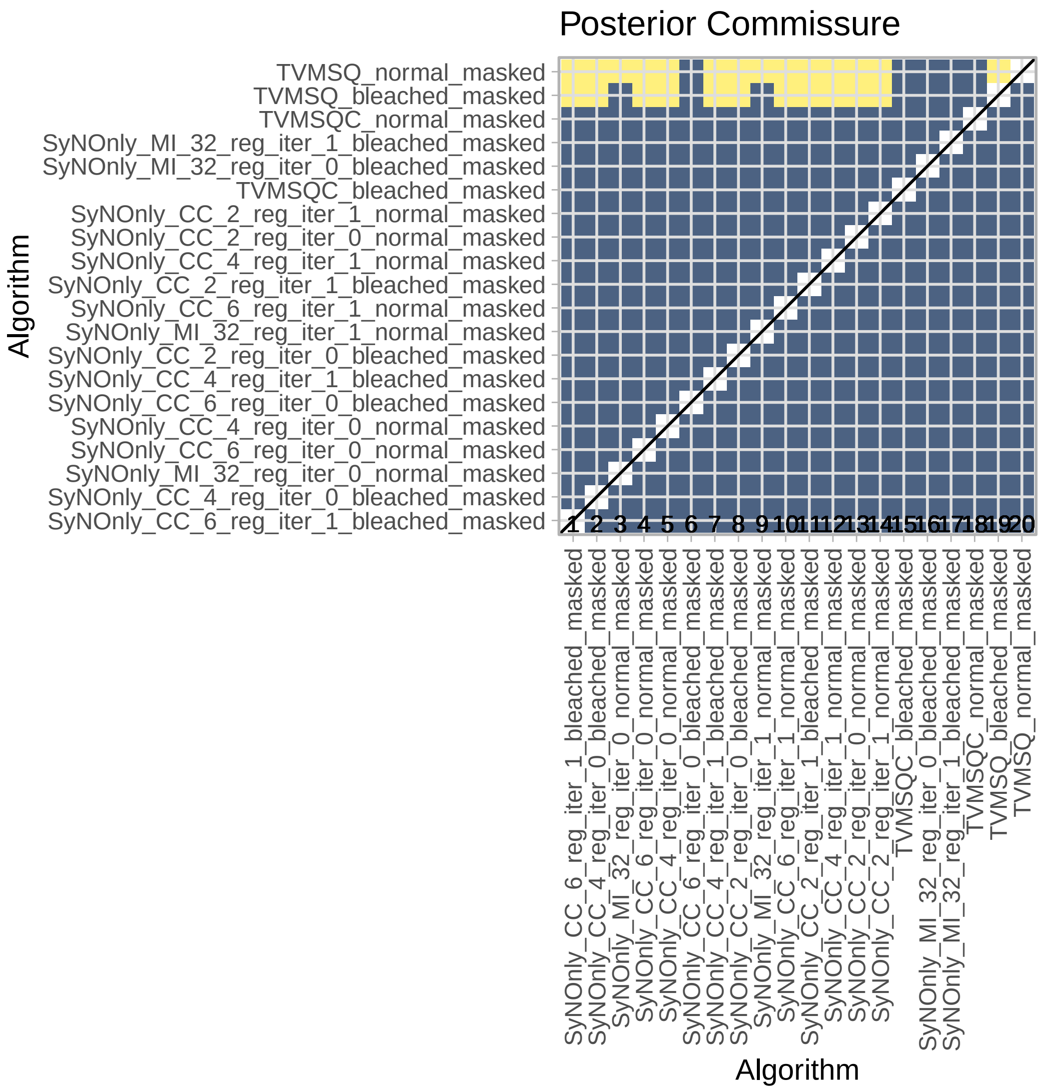
Posterior Commissure
TVMSQ_normal_masked
TVMSQ_bleached_masked
TVMSQC_normal_masked
SyNOnly_MI_32_reg_iter_1_bleached_masked
SyNOnly_MI_32_reg_iter_0_bleached_masked
TVMSQC_bleached_masked
SyNOnly_CC_2_reg_iter_1_normal_masked
SyNOnly_CC_2_reg_iter_0_normal_masked
SyNOnly_CC_4_reg_iter_1_normal_masked
SyNOnly_CC_2_reg_iter_1_bleached_masked
SyNOnly_CC_6_reg_iter_1_normal_masked
SyNOnly_MI_32_reg_iter_1_normal_masked
SyNOnly_CC_2_reg_iter_0_bleached_masked
SyNOnly_CC_4_reg_iter_1_bleached_masked
SyNOnly_CC_6_reg_iter_0_bleached_masked
SyNOnly_CC_4_reg_iter_0_normal_masked
SyNOnly_CC_6_reg_iter_0_normal_masked
SyNOnly_MI_32_reg_iter_0_normal_masked
SyNOnly_CC_4_reg_iter_0_bleached_masked
SyNOnly_CC_6_reg_iter_1_bleached_masked
Algorithm
1 2 3 4 5 6 7 8 9 10 11 12 13 14 15 16 17 18 19 20
SyNOnly_CC_6_reg_iter_1_bleached_masked
SyNOnly_CC_4_reg_iter_0_bleached_masked
SyNOnly_MI_32_reg_iter_0_normal_masked
SyNOnly_CC_6_reg_iter_0_normal_masked
SyNOnly_CC_4_reg_iter_0_normal_masked
SyNOnly_CC_6_reg_iter_0_bleached_masked
SyNOnly_CC_4_reg_iter_1_bleached_masked
SyNOnly_CC_2_reg_iter_0_bleached_masked
SyNOnly_MI_32_reg_iter_1_normal_masked
SyNOnly_CC_6_reg_iter_1_normal_masked
SyNOnly_CC_2_reg_iter_1_bleached_masked
SyNOnly_CC_4_reg_iter_1_normal_masked
SyNOnly_CC_2_reg_iter_0_normal_masked
SyNOnly_CC_2_reg_iter_1_normal_masked
TVMSQC_bleached_masked
SyNOnly_MI_32_reg_iter_0_bleached_masked
SyNOnly_MI_32_reg_iter_1_bleached_masked
TVMSQC_normal_masked
TVMSQ_bleached_masked
TVMSQ_normal_masked
Algorithm

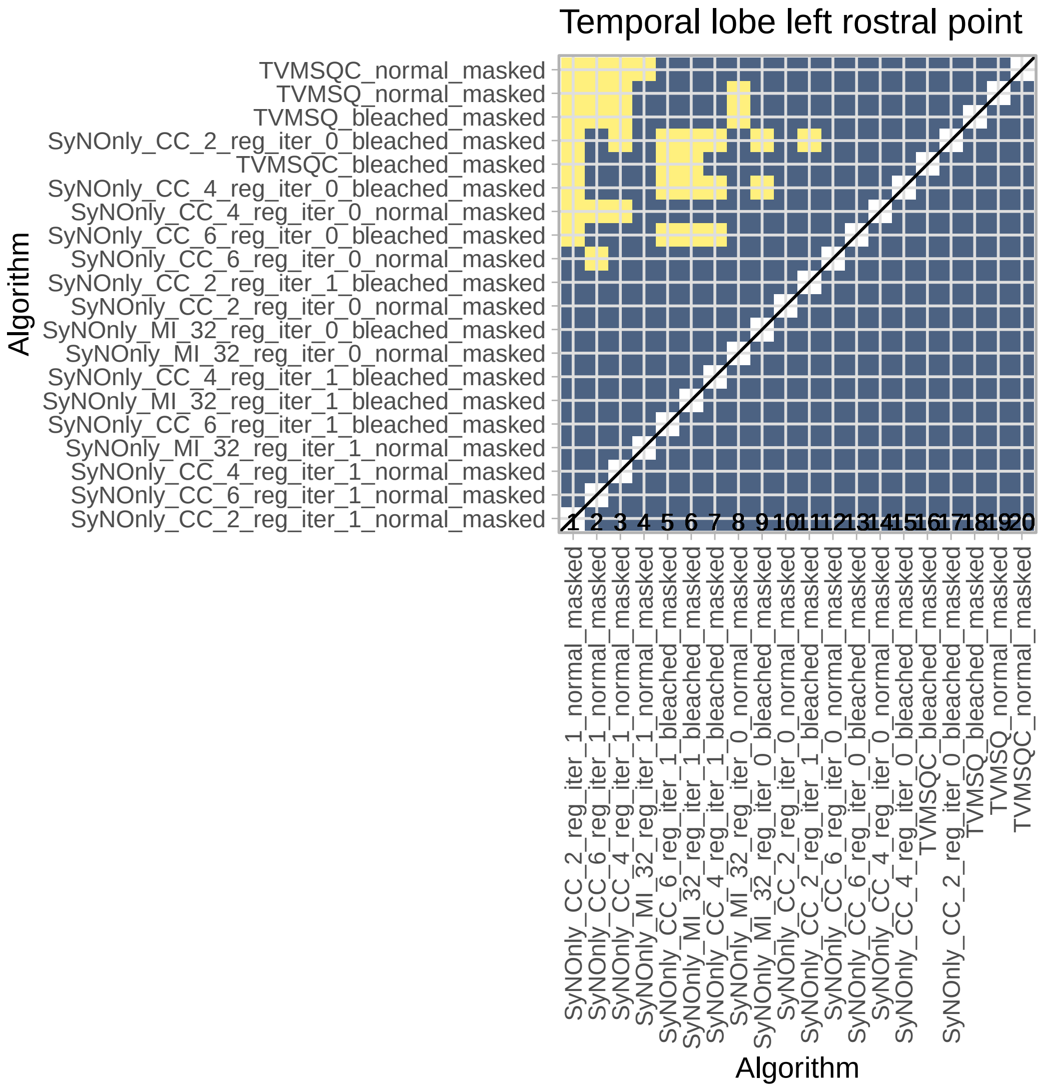
Temporal lobe left rostral point
Algorithm
TVMSQC_normal_masked
TVMSQ_normal_masked
TVMSQ_bleached_masked
SyNOnly_CC_2_reg_iter_0_bleached_masked
TVMSQC_bleached_masked
SyNOnly_CC_4_reg_iter_0_bleached_masked
SyNOnly_CC_4_reg_iter_0_normal_masked
SyNOnly_CC_6_reg_iter_0_bleached_masked
SyNOnly_CC_6_reg_iter_0_normal_masked
SyNOnly_CC_2_reg_iter_1_bleached_masked
SyNOnly_CC_2_reg_iter_0_normal_masked
SyNOnly_MI_32_reg_iter_0_bleached_masked
SyNOnly_MI_32_reg_iter_0_normal_masked
SyNOnly_CC_4_reg_iter_1_bleached_masked
SyNOnly_MI_32_reg_iter_1_bleached_masked
SyNOnly_CC_6_reg_iter_1_bleached_masked
SyNOnly_MI_32_reg_iter_1_normal_masked
SyNOnly_CC_4_reg_iter_1_normal_masked
SyNOnly_CC_6_reg_iter_1_normal_masked
SyNOnly_CC_2_reg_iter_1_normal_masked
1 2 3 4 5 6 7 8 9 10 11 12 13 14 15 16 17 18 19 20
SyNOnly_CC_2_reg_iter_1_normal_masked
SyNOnly_CC_6_reg_iter_1_normal_masked
SyNOnly_CC_4_reg_iter_1_normal_masked
SyNOnly_MI_32_reg_iter_1_normal_masked
SyNOnly_CC_6_reg_iter_1_bleached_masked
SyNOnly_MI_32_reg_iter_1_bleached_masked
SyNOnly_CC_4_reg_iter_1_bleached_masked
SyNOnly_MI_32_reg_iter_0_normal_masked
SyNOnly_MI_32_reg_iter_0_bleached_masked
SyNOnly_CC_2_reg_iter_0_normal_masked
SyNOnly_CC_2_reg_iter_1_bleached_masked
SyNOnly_CC_6_reg_iter_0_normal_masked
SyNOnly_CC_6_reg_iter_0_bleached_masked
SyNOnly_CC_4_reg_iter_0_normal_masked
SyNOnly_CC_4_reg_iter_0_bleached_masked
TVMSQC_bleached_masked
SyNOnly_CC_2_reg_iter_0_bleached_masked
TVMSQ_bleached_masked
TVMSQ_normal_masked
TVMSQC_normal_masked
Algorithm

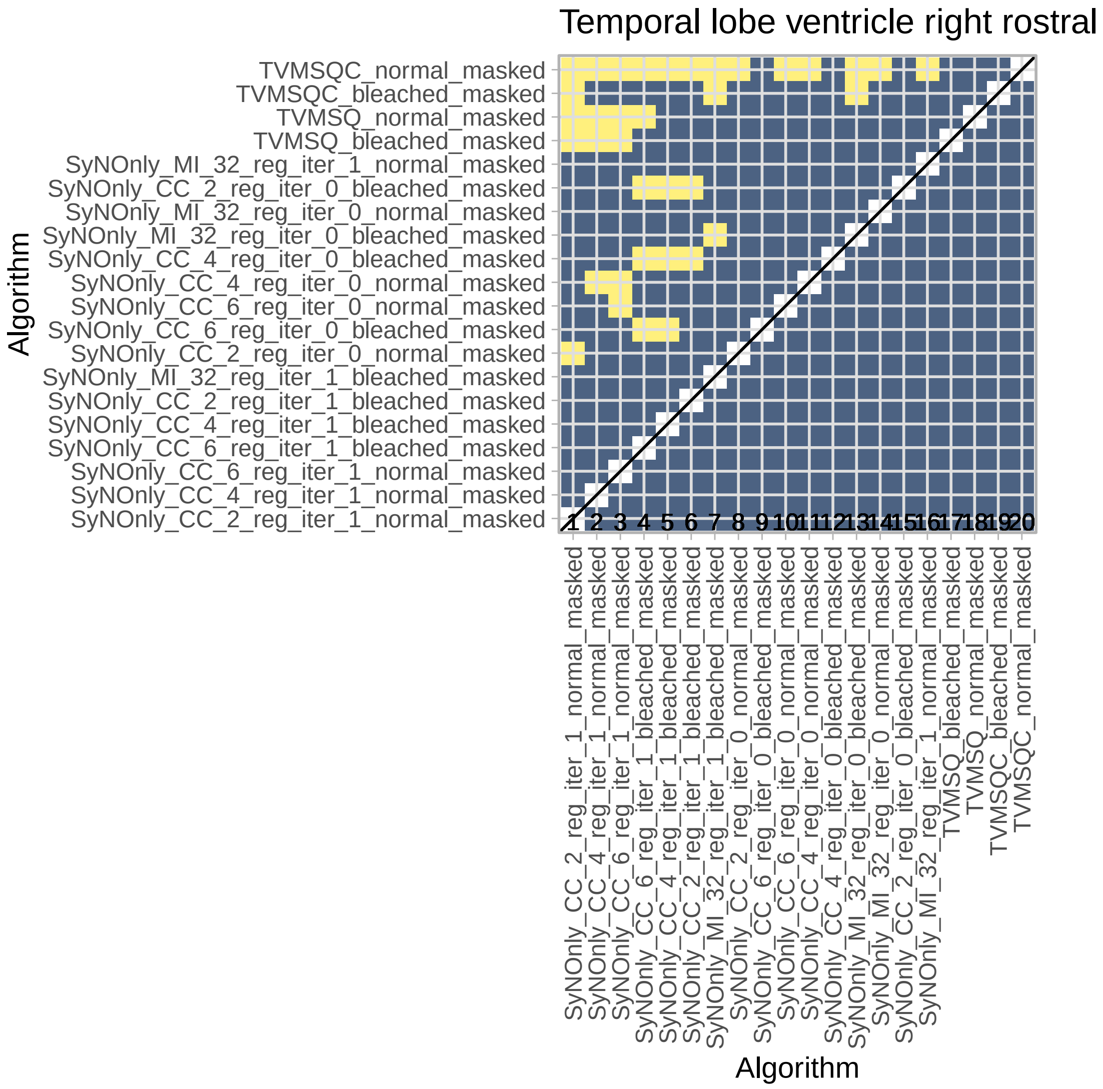
Temporal lobe ventricle right rostral
Algorithm
TVMSQC_normal_masked
TVMSQC_bleached_masked
TVMSQ_normal_masked
TVMSQ_bleached_masked
SyNOnly_MI_32_reg_iter_1_normal_masked
SyNOnly_CC_2_reg_iter_0_bleached_masked
SyNOnly_MI_32_reg_iter_0_normal_masked
SyNOnly_MI_32_reg_iter_0_bleached_masked
SyNOnly_CC_4_reg_iter_0_bleached_masked
SyNOnly_CC_4_reg_iter_0_normal_masked
SyNOnly_CC_6_reg_iter_0_normal_masked
SyNOnly_CC_6_reg_iter_0_bleached_masked
SyNOnly_CC_2_reg_iter_0_normal_masked
SyNOnly_MI_32_reg_iter_1_bleached_masked
SyNOnly_CC_2_reg_iter_1_bleached_masked
SyNOnly_CC_4_reg_iter_1_bleached_masked
SyNOnly_CC_6_reg_iter_1_bleached_masked
SyNOnly_CC_6_reg_iter_1_normal_masked
SyNOnly_CC_4_reg_iter_1_normal_masked
SyNOnly_CC_2_reg_iter_1_normal_masked
1 2 3 4 5 6 7 8 9 10 11 12 13 14 15 16 17 18 19 20
Algorithm

3.4 Ranking robustness to ranking methods

Line plots for visualizing ranking robustness across different ranking methods. Each algorithm is represented by one colored line. For each ranking method encoded on the x-axis, the height of the line represents the corresponding rank. Horizontal lines indicate identical ranks for all methods.

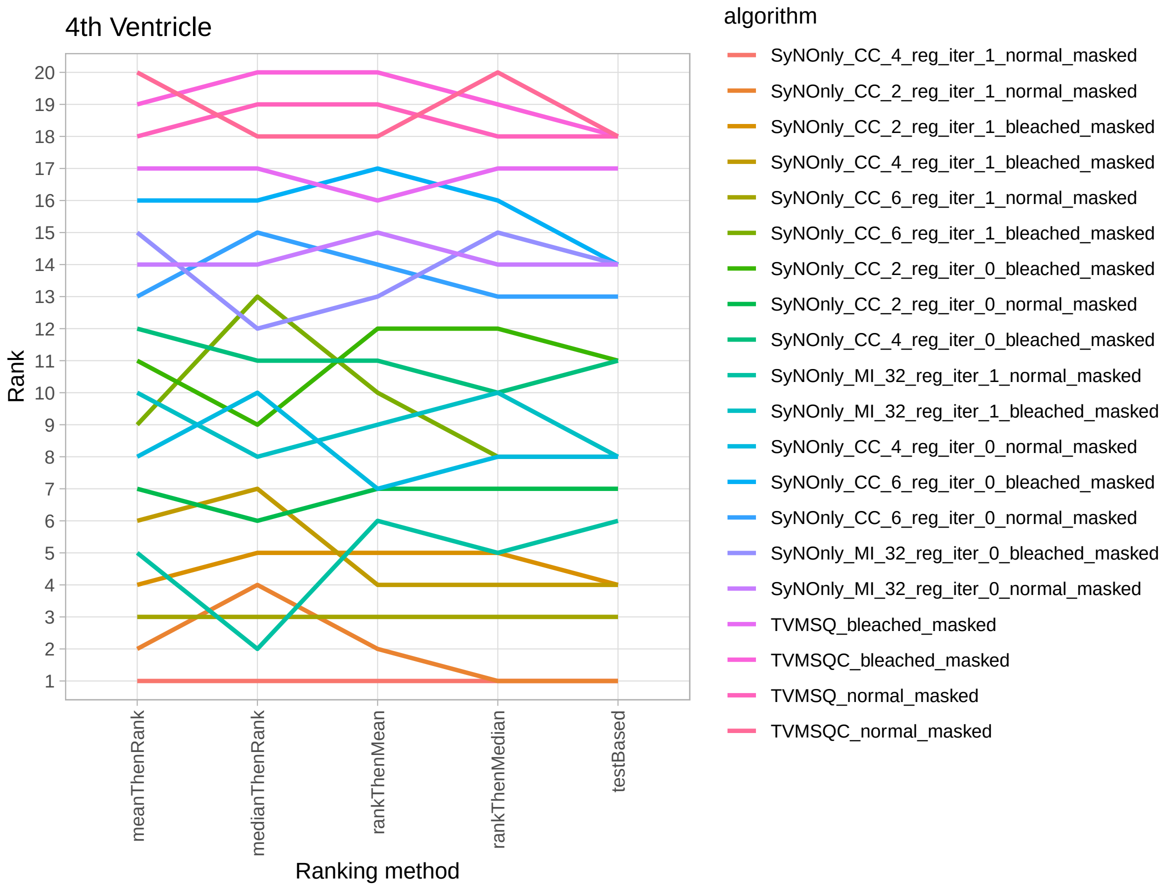

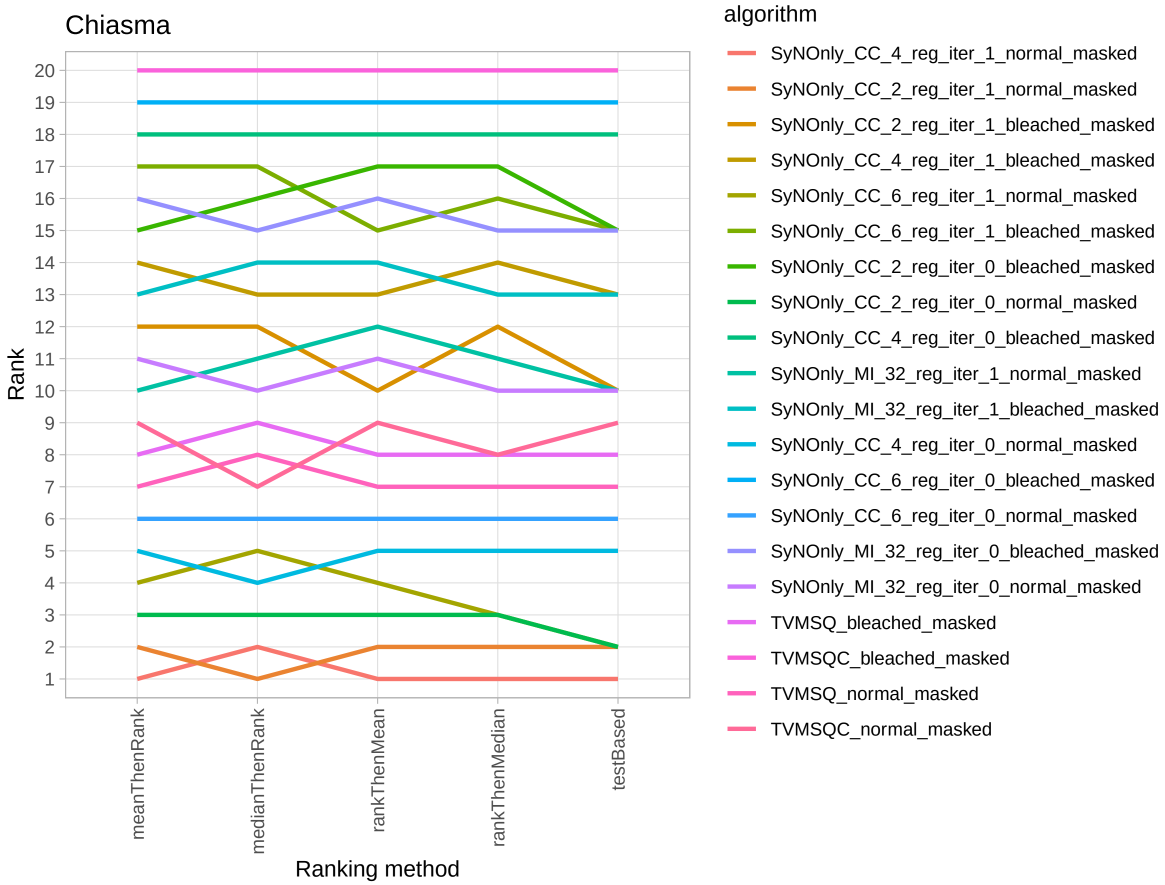
Chiasma
algorithm
SyNOnly_CC_4_reg_iter_1_normal_masked
SyNOnly_CC_2_reg_iter_1_normal_masked
SyNOnly_CC_2_reg_iter_1_bleached_masked
SyNOnly_CC_4_reg_iter_1_bleached_masked
SyNOnly_CC_6_reg_iter_1_normal_masked
SyNOnly_CC_6_reg_iter_1_bleached_masked
SyNOnly_CC_2_reg_iter_0_bleached_masked
SyNOnly_CC_2_reg_iter_0_normal_masked
SyNOnly_CC_4_reg_iter_0_bleached_masked
SyNOnly_MI_32_reg_iter_1_normal_masked
SyNOnly_MI_32_reg_iter_1_bleached_masked
SyNOnly_CC_4_reg_iter_0_normal_masked
SyNOnly_CC_6_reg_iter_0_bleached_masked
SyNOnly_CC_6_reg_iter_0_normal_masked
SyNOnly_MI_32_reg_iter_0_bleached_masked
SyNOnly_MI_32_reg_iter_0_normal_masked
TVMSQ_bleached_masked
TVMSQC_bleached_masked
TVMSQ_normal_masked
TVMSQC_normal_masked
Rank
meanThenRank
medianThenRank
rankThenMean
rankThenMedian
testBased
Ranking method

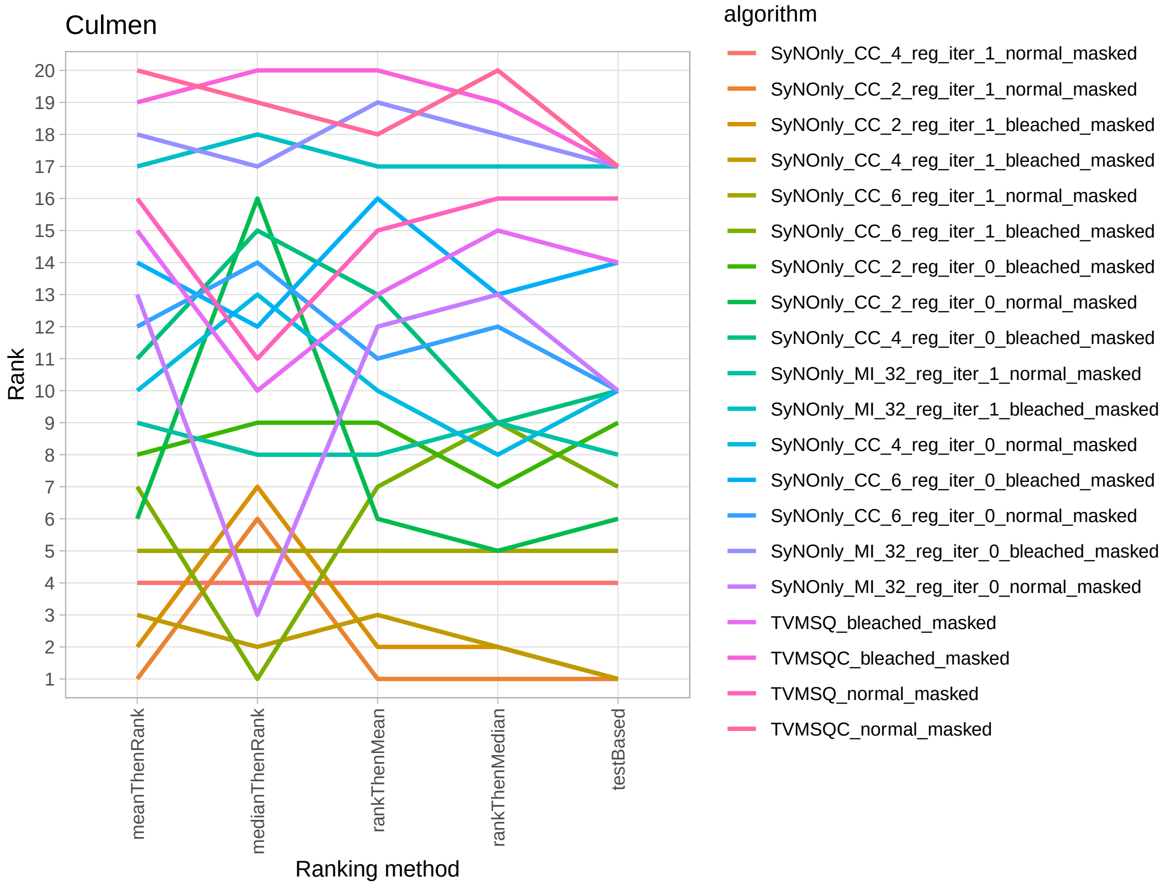

Culmen
Rank
20
19
18
17
16
15
14
13
12
11
10
9
8
7
6
5
4
3
2
1
meanThenRank
medianThenRank
rankThenMean
rankThenMedian
testBased
Ranking method
algorithm
SyNOnly_CC_4_reg_iter_1_normal_masked
SyNOnly_CC_2_reg_iter_1_normal_masked
SyNOnly_CC_2_reg_iter_1_bleached_masked
SyNOnly_CC_4_reg_iter_1_bleached_masked
SyNOnly_CC_6_reg_iter_1_normal_masked
SyNOnly_CC_6_reg_iter_1_bleached_masked
SyNOnly_CC_2_reg_iter_0_bleached_masked
SyNOnly_CC_2_reg_iter_0_normal_masked
SyNOnly_CC_4_reg_iter_0_bleached_masked
SyNOnly_MI_32_reg_iter_1_normal_masked
SyNOnly_MI_32_reg_iter_1_bleached_masked
SyNOnly_CC_4_reg_iter_0_normal_masked
SyNOnly_CC_6_reg_iter_0_bleached_masked
SyNOnly_CC_6_reg_iter_0_normal_masked
SyNOnly_MI_32_reg_iter_0_bleached_masked
SyNOnly_MI_32_reg_iter_0_normal_masked
TVMSQ_bleached_masked
TVMSQC_bleached_masked
TVMSQ_normal_masked
TVMSQC_normal_masked

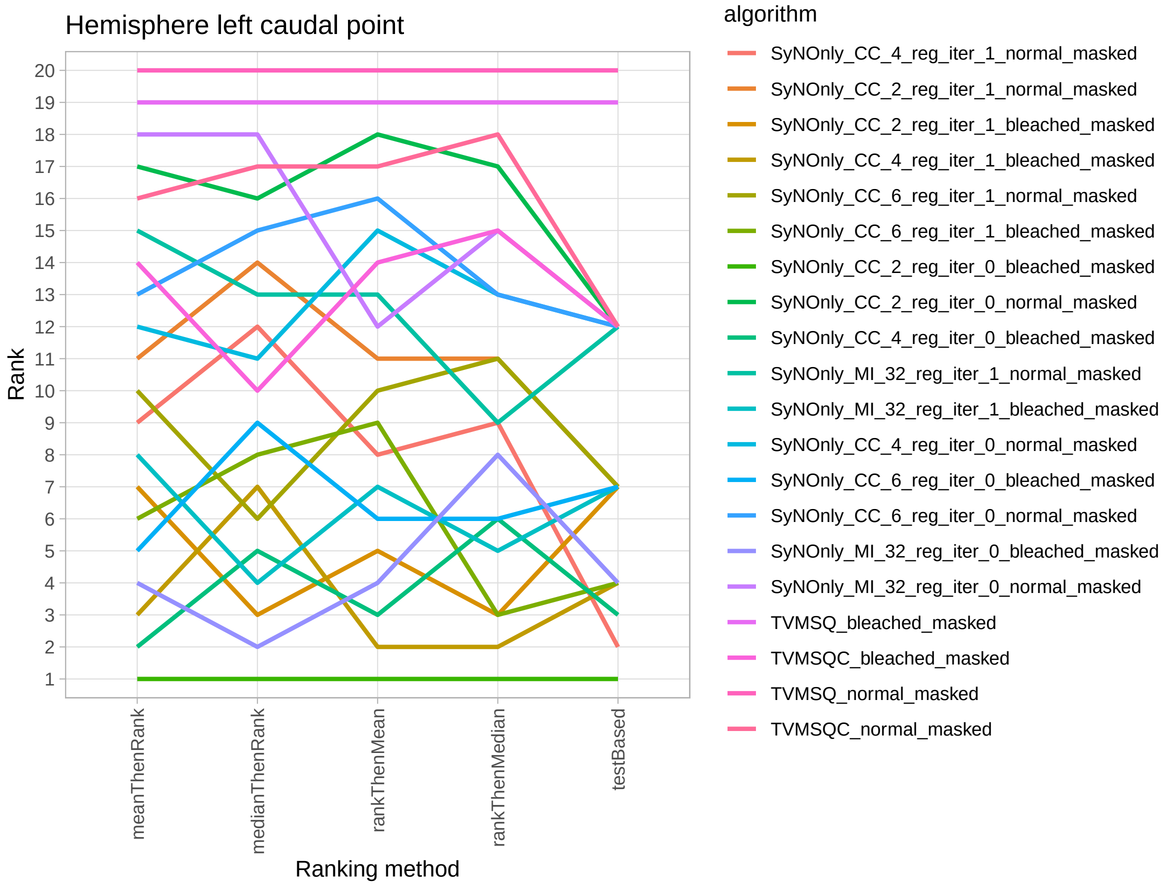
Hemisphere left caudal point
Rank
20
19
18
17
16
15
14
13
12
11
10
9
8
7
6
5
4
3
2
1
meanThenRank
medianThenRank
rankThenMean
rankThenMedian
testBased
Ranking method
algorithm
SyNOnly_CC_4_reg_iter_1_normal_masked
SyNOnly_CC_2_reg_iter_1_normal_masked
SyNOnly_CC_2_reg_iter_1_bleached_masked
SyNOnly_CC_4_reg_iter_1_bleached_masked
SyNOnly_CC_6_reg_iter_1_normal_masked
SyNOnly_CC_6_reg_iter_1_bleached_masked
SyNOnly_CC_2_reg_iter_0_bleached_masked
SyNOnly_CC_2_reg_iter_0_normal_masked
SyNOnly_CC_4_reg_iter_0_bleached_masked
SyNOnly_MI_32_reg_iter_1_normal_masked
SyNOnly_MI_32_reg_iter_1_bleached_masked
SyNOnly_CC_4_reg_iter_0_normal_masked
SyNOnly_CC_6_reg_iter_0_bleached_masked
SyNOnly_CC_6_reg_iter_0_normal_masked
SyNOnly_MI_32_reg_iter_0_bleached_masked
SyNOnly_MI_32_reg_iter_0_normal_masked
TVMSQ_bleached_masked
TVMSQC_bleached_masked
TVMSQ_normal_masked
TVMSQC_normal_masked

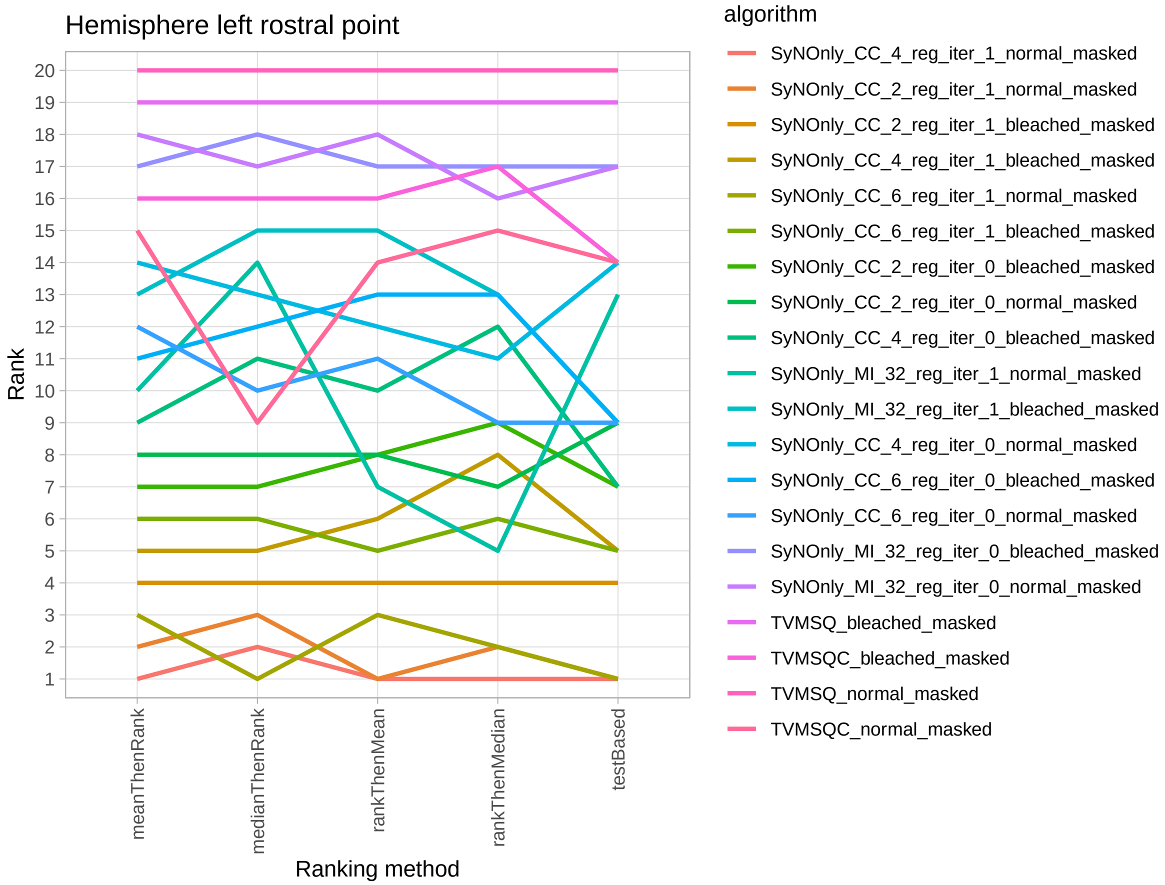

Hemisphere left rostral point
Rank
20
19
18
17
16
15
14
13
12
11
10
9
8
7
6
5
4
3
2
1
meanThenRank
medianThenRank
rankThenMean
rankThenMedian
testBased
Ranking method
algorithm
SyNOnly_CC_4_reg_iter_1_normal_masked
SyNOnly_CC_2_reg_iter_1_normal_masked
SyNOnly_CC_2_reg_iter_1_bleached_masked
SyNOnly_CC_4_reg_iter_1_bleached_masked
SyNOnly_CC_6_reg_iter_1_normal_masked
SyNOnly_CC_6_reg_iter_1_bleached_masked
SyNOnly_CC_2_reg_iter_0_bleached_masked
SyNOnly_CC_2_reg_iter_0_normal_masked
SyNOnly_CC_4_reg_iter_0_bleached_masked
SyNOnly_MI_32_reg_iter_1_normal_masked
SyNOnly_MI_32_reg_iter_1_bleached_masked
SyNOnly_CC_4_reg_iter_0_normal_masked
SyNOnly_CC_6_reg_iter_0_bleached_masked
SyNOnly_CC_6_reg_iter_0_normal_masked
SyNOnly_MI_32_reg_iter_0_bleached_masked
SyNOnly_MI_32_reg_iter_0_normal_masked
TVMSQ_bleached_masked
TVMSQC_bleached_masked
TVMSQ_normal_masked
TVMSQC_normal_masked

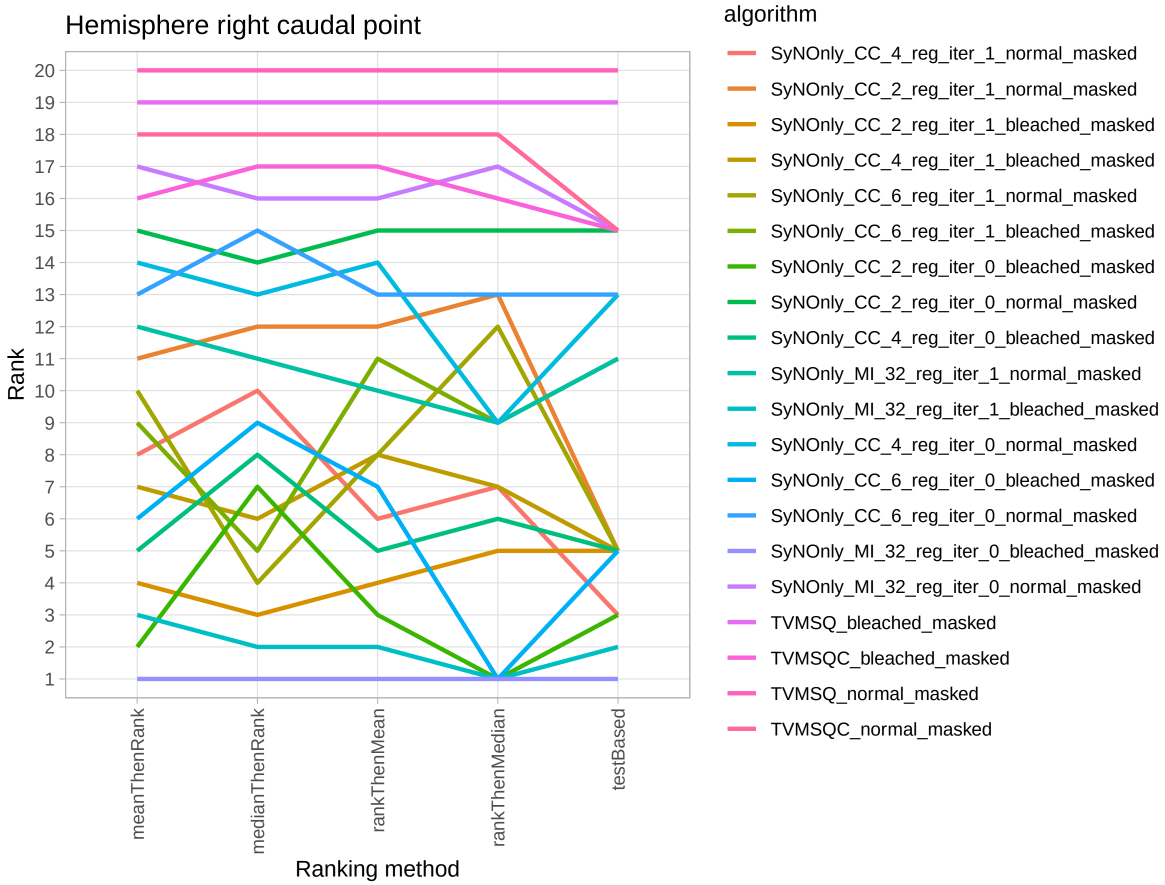

Hemisphere right caudal point
Rank
20
19
18
17
16
15
14
13
12
11
10
9
8
7
6
5
4
3
2
1
meanThenRank
medianThenRank
rankThenMean
rankThenMedian
testBased
Ranking method
algorithm
SyNOnly_CC_4_reg_iter_1_normal_masked
SyNOnly_CC_2_reg_iter_1_normal_masked
SyNOnly_CC_2_reg_iter_1_bleached_masked
SyNOnly_CC_4_reg_iter_1_bleached_masked
SyNOnly_CC_6_reg_iter_1_normal_masked
SyNOnly_CC_6_reg_iter_1_bleached_masked
SyNOnly_CC_2_reg_iter_0_bleached_masked
SyNOnly_CC_2_reg_iter_0_normal_masked
SyNOnly_CC_4_reg_iter_0_bleached_masked
SyNOnly_MI_32_reg_iter_1_normal_masked
SyNOnly_MI_32_reg_iter_1_bleached_masked
SyNOnly_CC_4_reg_iter_0_normal_masked
SyNOnly_CC_6_reg_iter_0_bleached_masked
SyNOnly_CC_6_reg_iter_0_normal_masked
SyNOnly_MI_32_reg_iter_0_bleached_masked
SyNOnly_MI_32_reg_iter_0_normal_masked
TVMSQ_bleached_masked
TVMSQC_bleached_masked
TVMSQ_normal_masked
TVMSQC_normal_masked

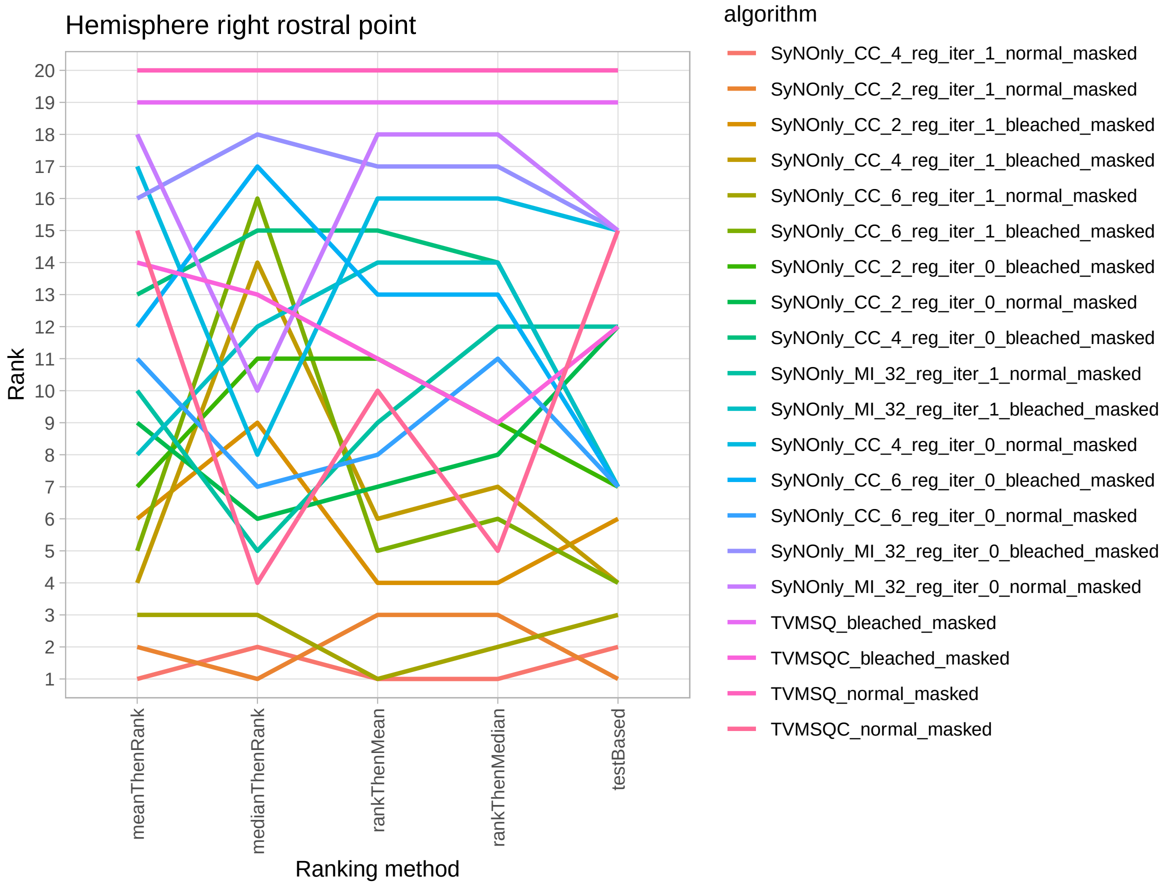
Hemisphere right rostral point
Rank
Ranking method
meanThenRank
medianThenRank
rankThenMean
rankThenMedian
testBased
algorithm
SyNOnly_CC_4_reg_iter_1_normal_masked
SyNOnly_CC_2_reg_iter_1_normal_masked
SyNOnly_CC_2_reg_iter_1_bleached_masked
SyNOnly_CC_4_reg_iter_1_bleached_masked
SyNOnly_CC_6_reg_iter_1_normal_masked
SyNOnly_CC_6_reg_iter_1_bleached_masked
SyNOnly_CC_2_reg_iter_0_bleached_masked
SyNOnly_CC_2_reg_iter_0_normal_masked
SyNOnly_CC_4_reg_iter_0_bleached_masked
SyNOnly_MI_32_reg_iter_1_normal_masked
SyNOnly_MI_32_reg_iter_1_bleached_masked
SyNOnly_CC_4_reg_iter_0_normal_masked
SyNOnly_CC_6_reg_iter_0_bleached_masked
SyNOnly_CC_6_reg_iter_0_normal_masked
SyNOnly_MI_32_reg_iter_0_bleached_masked
SyNOnly_MI_32_reg_iter_0_normal_masked
TVMSQ_bleached_masked
TVMSQC_bleached_masked
TVMSQ_normal_masked
TVMSQC_normal_masked

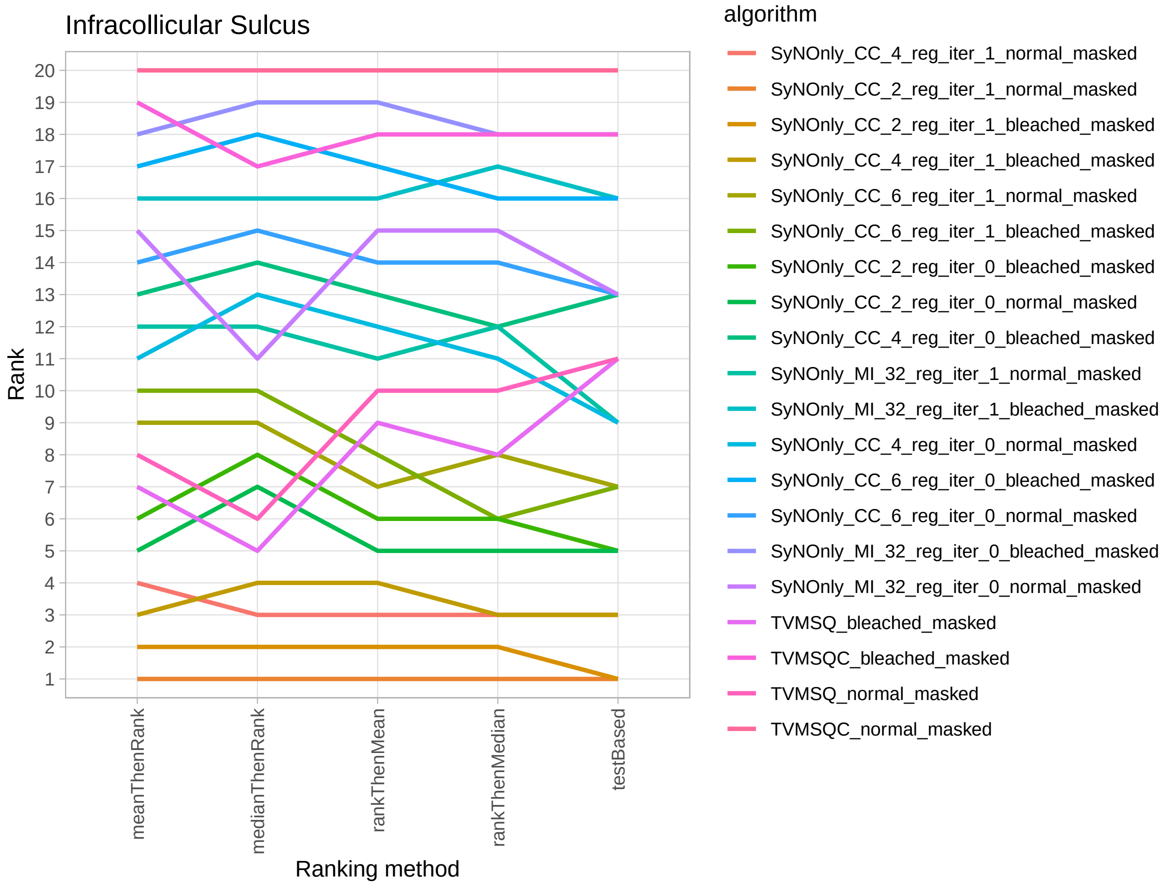
Infracollicular Sulcus
Rank
20
19
18
17
16
15
14
13
12
11
10
9
8
7
6
5
4
3
2
1
meanThenRank
medianThenRank
rankThenMean
rankThenMedian
testBased
Ranking method
algorithm
SyNOnly_CC_4_reg_iter_1_normal_masked
SyNOnly_CC_2_reg_iter_1_normal_masked
SyNOnly_CC_2_reg_iter_1_bleached_masked
SyNOnly_CC_4_reg_iter_1_bleached_masked
SyNOnly_CC_6_reg_iter_1_normal_masked
SyNOnly_CC_6_reg_iter_1_bleached_masked
SyNOnly_CC_2_reg_iter_0_bleached_masked
SyNOnly_CC_2_reg_iter_0_normal_masked
SyNOnly_CC_4_reg_iter_0_bleached_masked
SyNOnly_MI_32_reg_iter_1_normal_masked
SyNOnly_MI_32_reg_iter_1_bleached_masked
SyNOnly_CC_4_reg_iter_0_normal_masked
SyNOnly_CC_6_reg_iter_0_bleached_masked
SyNOnly_CC_6_reg_iter_0_normal_masked
SyNOnly_MI_32_reg_iter_0_bleached_masked
SyNOnly_MI_32_reg_iter_0_normal_masked
TVMSQ_bleached_masked
TVMSQC_bleached_masked
TVMSQ_normal_masked
TVMSQC_normal_masked

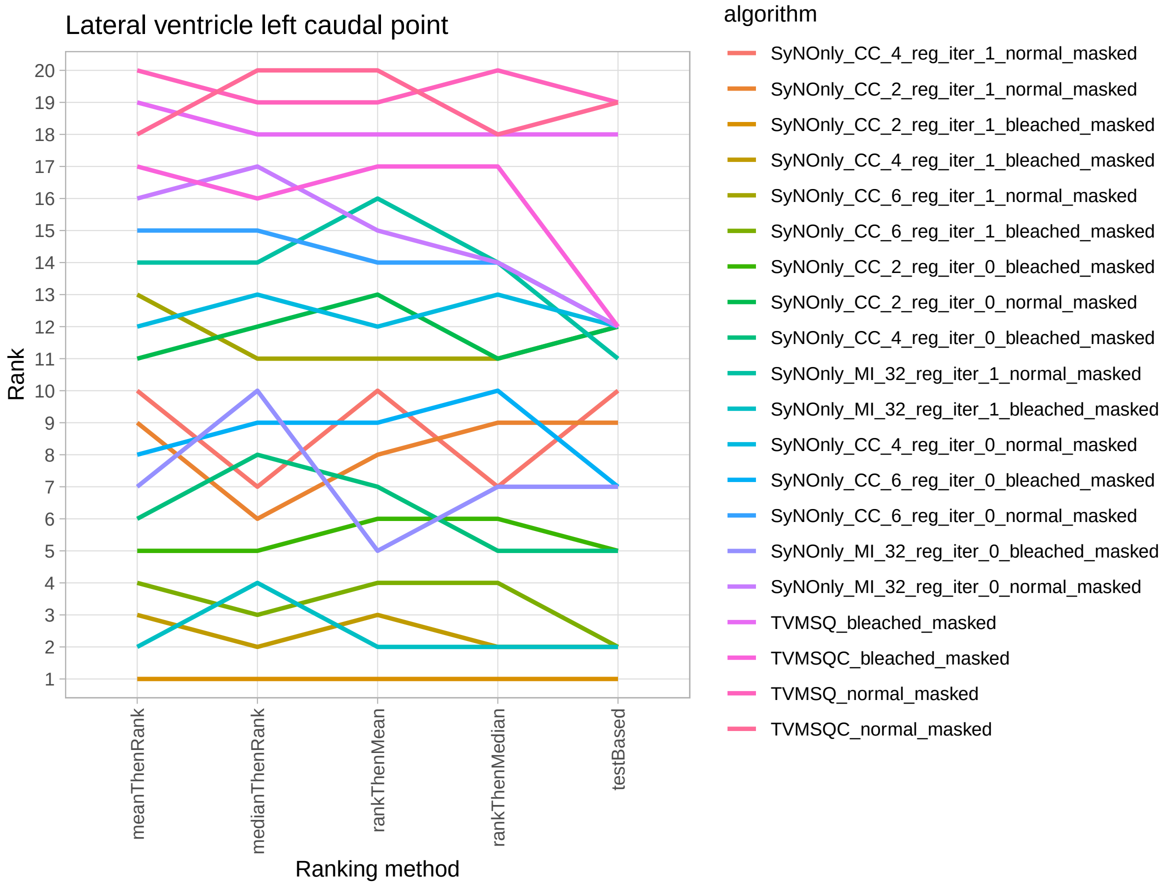

Lateral ventricle left caudal point
Rank
20
19
18
17
16
15
14
13
12
11
10
9
8
7
6
5
4
3
2
1
meanThenRank
medianThenRank
rankThenMean
rankThenMedian
testBased
Ranking method
algorithm
SyNOnly_CC_4_reg_iter_1_normal_masked
SyNOnly_CC_2_reg_iter_1_normal_masked
SyNOnly_CC_2_reg_iter_1_bleached_masked
SyNOnly_CC_4_reg_iter_1_bleached_masked
SyNOnly_CC_6_reg_iter_1_normal_masked
SyNOnly_CC_6_reg_iter_1_bleached_masked
SyNOnly_CC_2_reg_iter_0_bleached_masked
SyNOnly_CC_2_reg_iter_0_normal_masked
SyNOnly_CC_4_reg_iter_0_bleached_masked
SyNOnly_MI_32_reg_iter_1_normal_masked
SyNOnly_MI_32_reg_iter_1_bleached_masked
SyNOnly_CC_4_reg_iter_0_normal_masked
SyNOnly_CC_6_reg_iter_0_bleached_masked
SyNOnly_CC_6_reg_iter_0_normal_masked
SyNOnly_MI_32_reg_iter_0_bleached_masked
SyNOnly_MI_32_reg_iter_0_normal_masked
TVMSQ_bleached_masked
TVMSQC_bleached_masked
TVMSQ_normal_masked
TVMSQC_normal_masked

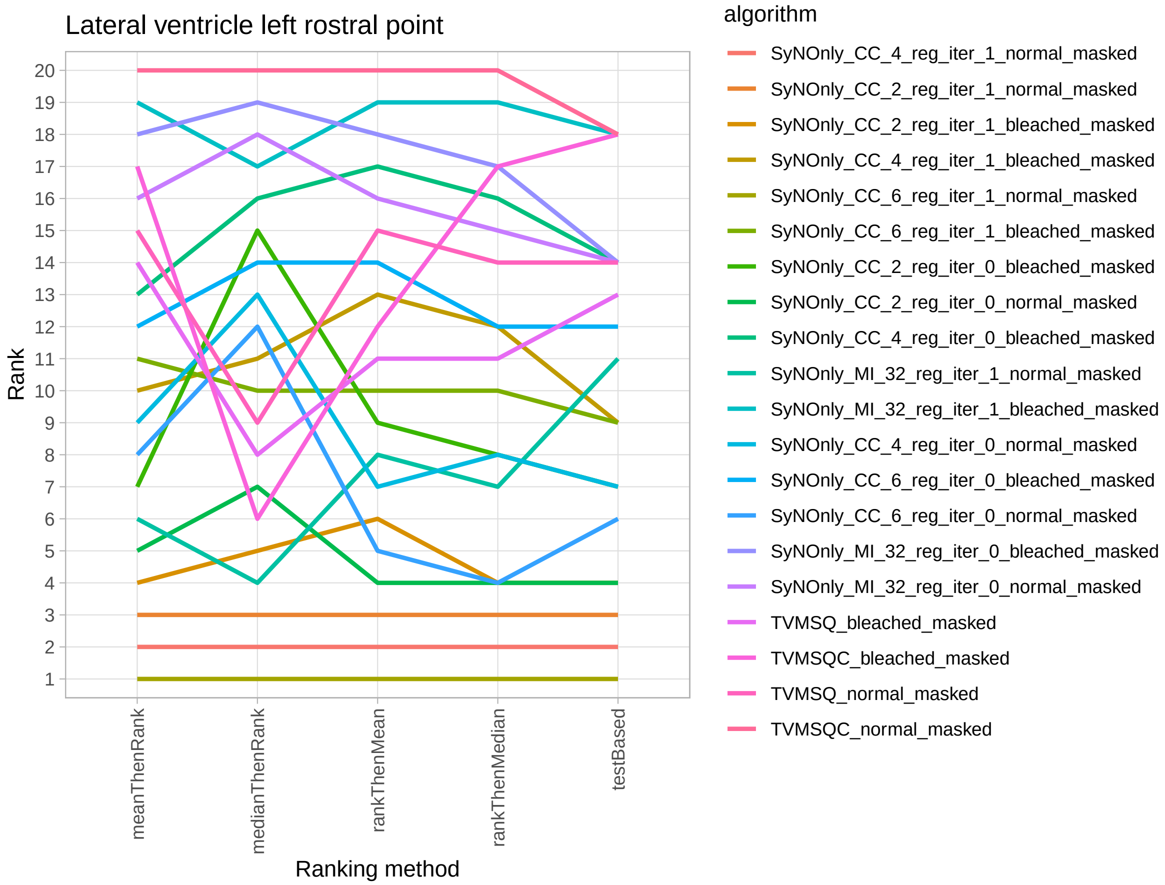
Lateral ventricle left rostral point
Rank
20
19
18
17
16
15
14
13
12
11
10
9
8
7
6
5
4
3
2
1
meanThenRank
medianThenRank
rankThenMean
rankThenMedian
testBased
Ranking method
algorithm
SyNOnly_CC_4_reg_iter_1_normal_masked
SyNOnly_CC_2_reg_iter_1_normal_masked
SyNOnly_CC_2_reg_iter_1_bleached_masked
SyNOnly_CC_4_reg_iter_1_bleached_masked
SyNOnly_CC_6_reg_iter_1_normal_masked
SyNOnly_CC_6_reg_iter_1_bleached_masked
SyNOnly_CC_2_reg_iter_0_bleached_masked
SyNOnly_CC_2_reg_iter_0_normal_masked
SyNOnly_CC_4_reg_iter_0_bleached_masked
SyNOnly_MI_32_reg_iter_1_normal_masked
SyNOnly_MI_32_reg_iter_1_bleached_masked
SyNOnly_CC_4_reg_iter_0_normal_masked
SyNOnly_CC_6_reg_iter_0_bleached_masked
SyNOnly_CC_6_reg_iter_0_normal_masked
SyNOnly_MI_32_reg_iter_0_bleached_masked
SyNOnly_MI_32_reg_iter_0_normal_masked
TVMSQ_bleached_masked
TVMSQC_bleached_masked
TVMSQ_normal_masked
TVMSQC_normal_masked

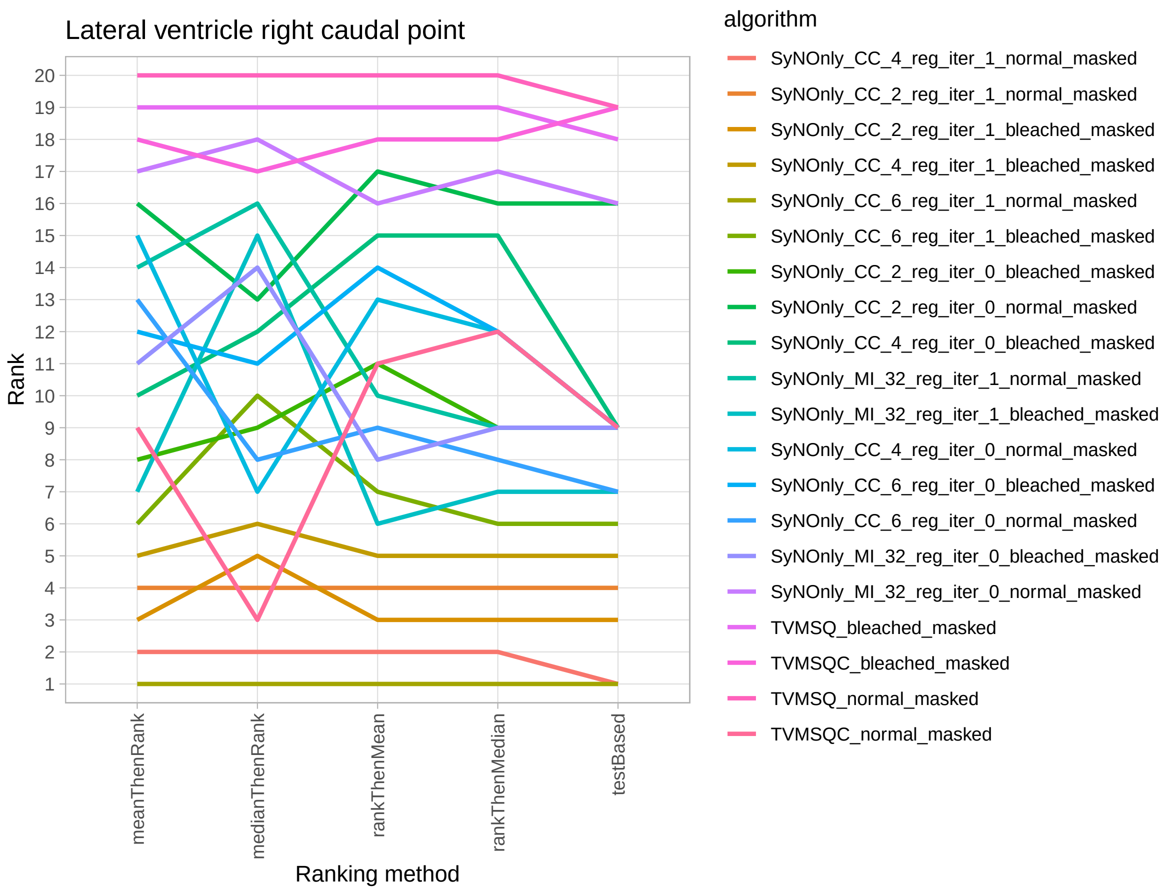

Lateral ventricle right caudal point
Rank
20
19
18
17
16
15
14
13
12
11
10
9
8
7
6
5
4
3
2
1
meanThenRank
medianThenRank
rankThenMean
rankThenMedian
testBased
Ranking method
algorithm
SyNOnly_CC_4_reg_iter_1_normal_masked
SyNOnly_CC_2_reg_iter_1_normal_masked
SyNOnly_CC_2_reg_iter_1_bleached_masked
SyNOnly_CC_4_reg_iter_1_bleached_masked
SyNOnly_CC_6_reg_iter_1_normal_masked
SyNOnly_CC_6_reg_iter_1_bleached_masked
SyNOnly_CC_2_reg_iter_0_bleached_masked
SyNOnly_CC_2_reg_iter_0_normal_masked
SyNOnly_CC_4_reg_iter_0_bleached_masked
SyNOnly_MI_32_reg_iter_1_normal_masked
SyNOnly_MI_32_reg_iter_1_bleached_masked
SyNOnly_CC_4_reg_iter_0_normal_masked
SyNOnly_CC_6_reg_iter_0_bleached_masked
SyNOnly_CC_6_reg_iter_0_normal_masked
SyNOnly_MI_32_reg_iter_0_bleached_masked
SyNOnly_MI_32_reg_iter_0_normal_masked
TVMSQ_bleached_masked
TVMSQC_bleached_masked
TVMSQ_normal_masked
TVMSQC_normal_masked

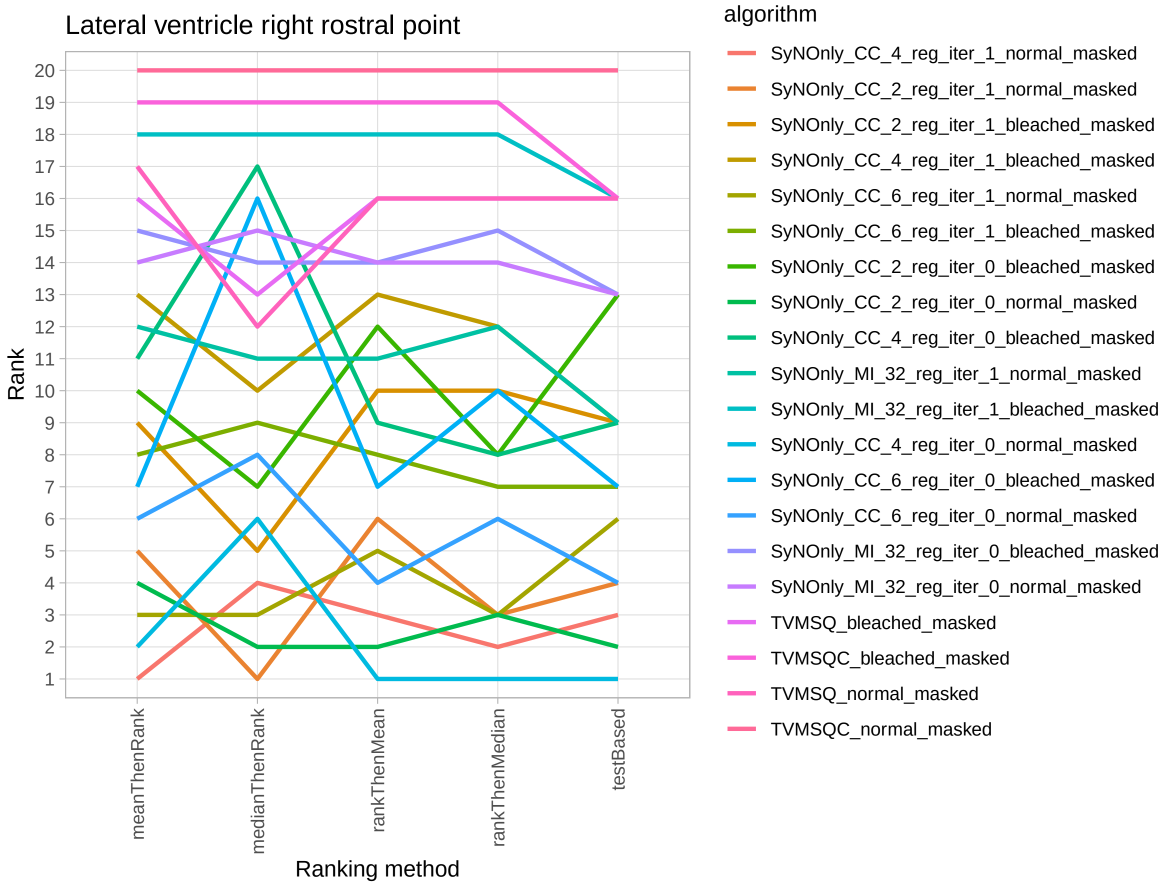
Lateral ventricle right rostral point
Rank
20
19
18
17
16
15
14
13
12
11
10
9
8
7
6
5
4
3
2
1
meanThenRank
medianThenRank
rankThenMean
rankThenMedian
testBased
Ranking method
algorithm
SyNOnly_CC_4_reg_iter_1_normal_masked
SyNOnly_CC_2_reg_iter_1_normal_masked
SyNOnly_CC_2_reg_iter_1_bleached_masked
SyNOnly_CC_4_reg_iter_1_bleached_masked
SyNOnly_CC_6_reg_iter_1_normal_masked
SyNOnly_CC_6_reg_iter_1_bleached_masked
SyNOnly_CC_2_reg_iter_0_bleached_masked
SyNOnly_CC_2_reg_iter_0_normal_masked
SyNOnly_CC_4_reg_iter_0_bleached_masked
SyNOnly_MI_32_reg_iter_1_normal_masked
SyNOnly_MI_32_reg_iter_1_bleached_masked
SyNOnly_CC_4_reg_iter_0_normal_masked
SyNOnly_CC_6_reg_iter_0_bleached_masked
SyNOnly_CC_6_reg_iter_0_normal_masked
SyNOnly_MI_32_reg_iter_0_bleached_masked
SyNOnly_MI_32_reg_iter_0_normal_masked
TVMSQ_bleached_masked
TVMSQC_bleached_masked
TVMSQ_normal_masked
TVMSQC_normal_masked

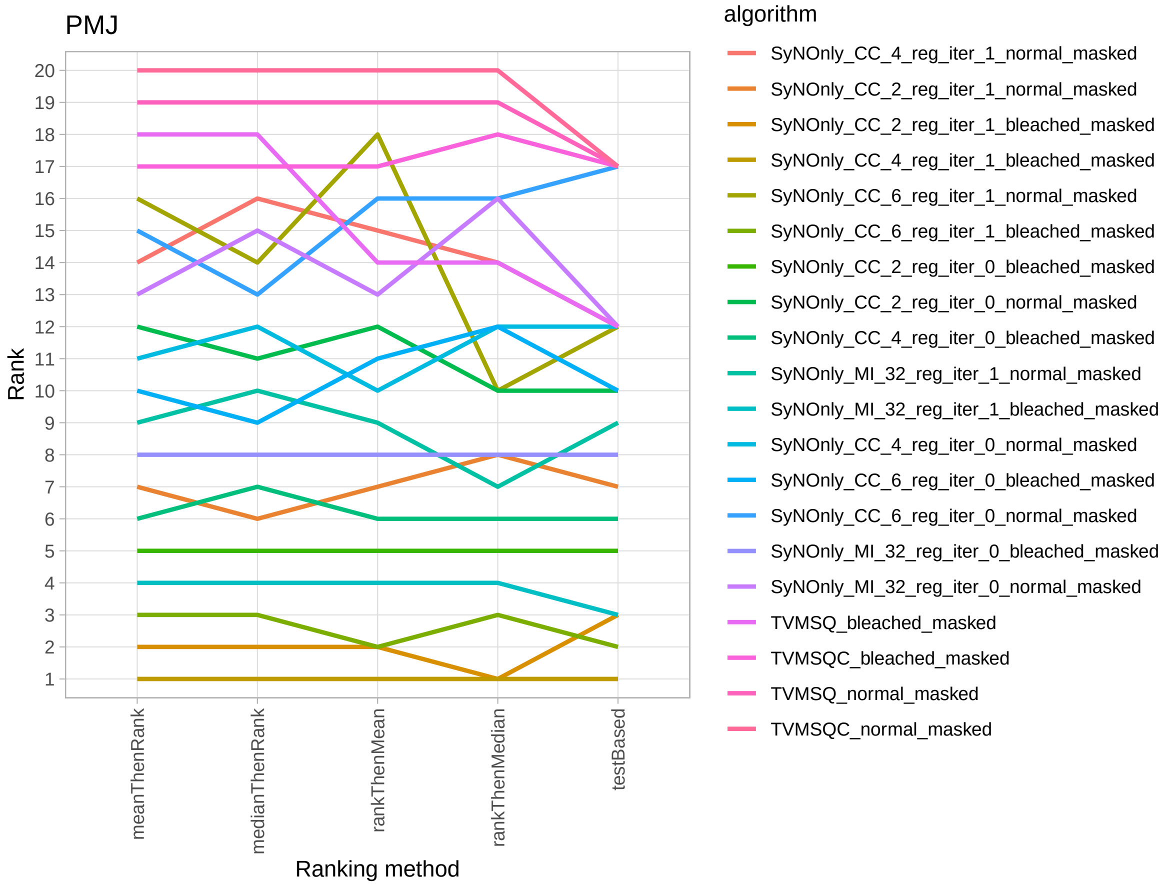

PMJ
algorithm
SyNOnly_CC_4_reg_iter_1_normal_masked
SyNOnly_CC_2_reg_iter_1_normal_masked
SyNOnly_CC_2_reg_iter_1_bleached_masked
SyNOnly_CC_4_reg_iter_1_bleached_masked
SyNOnly_CC_6_reg_iter_1_normal_masked
SyNOnly_CC_6_reg_iter_1_bleached_masked
SyNOnly_CC_2_reg_iter_0_bleached_masked
SyNOnly_CC_2_reg_iter_0_normal_masked
SyNOnly_CC_4_reg_iter_0_bleached_masked
SyNOnly_MI_32_reg_iter_1_normal_masked
SyNOnly_MI_32_reg_iter_1_bleached_masked
SyNOnly_CC_4_reg_iter_0_normal_masked
SyNOnly_CC_6_reg_iter_0_bleached_masked
SyNOnly_CC_6_reg_iter_0_normal_masked
SyNOnly_MI_32_reg_iter_0_bleached_masked
SyNOnly_MI_32_reg_iter_0_normal_masked
TVMSQ_bleached_masked
TVMSQC_bleached_masked
TVMSQ_normal_masked
TVMSQC_normal_masked
Rank
20
19
18
17
16
15
14
13
12
11
10
9
8
7
6
5
4
3
2
1
meanThenRank
medianThenRank
rankThenMean
rankThenMedian
testBased
Ranking method

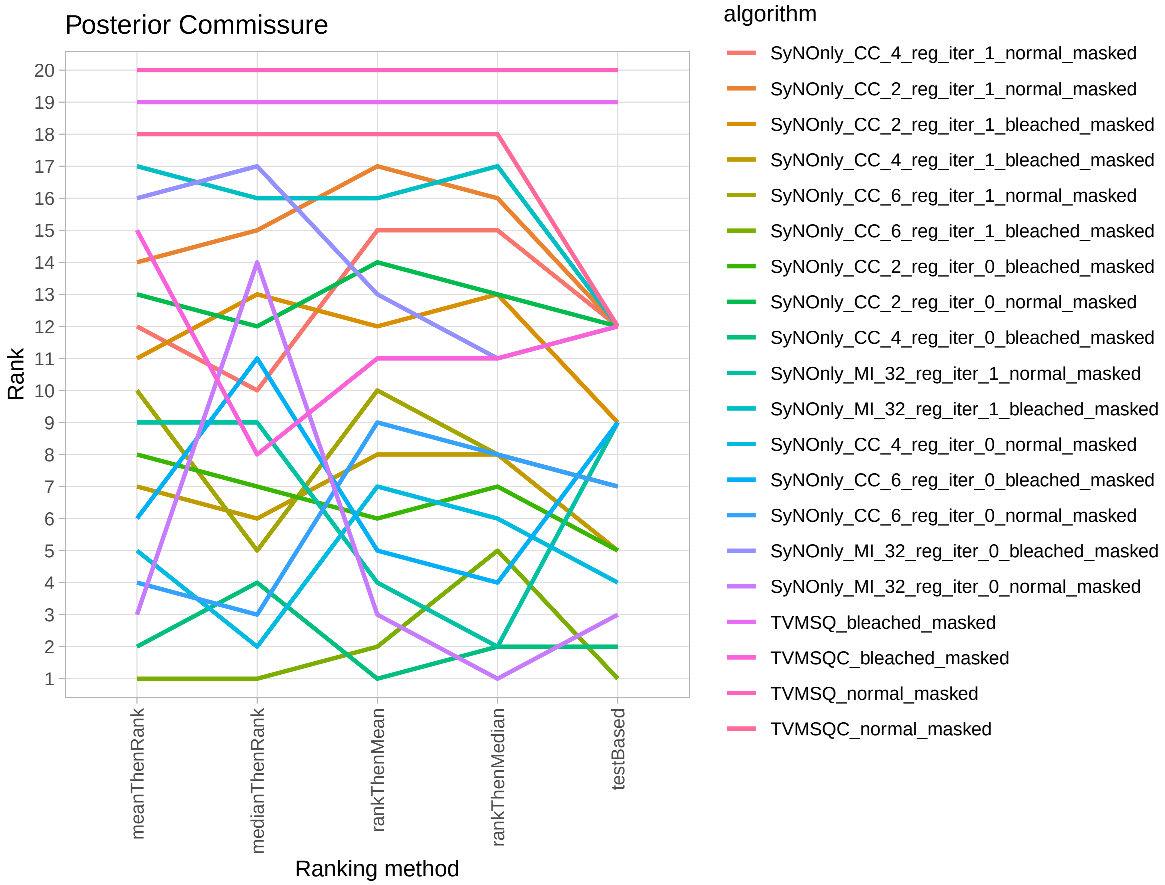

Posterior Commissure
Rank
20
19
18
17
16
15
14
13
12
11
10
9
8
7
6
5
4
3
2
1
meanThenRank
medianThenRank
rankThenMean
rankThenMedian
testBased
Ranking method
algorithm
SyNOnly_CC_4_reg_iter_1_normal_masked
SyNOnly_CC_2_reg_iter_1_normal_masked
SyNOnly_CC_2_reg_iter_1_bleached_masked
SyNOnly_CC_4_reg_iter_1_bleached_masked
SyNOnly_CC_6_reg_iter_1_normal_masked
SyNOnly_CC_6_reg_iter_1_bleached_masked
SyNOnly_CC_2_reg_iter_0_bleached_masked
SyNOnly_CC_2_reg_iter_0_normal_masked
SyNOnly_CC_4_reg_iter_0_bleached_masked
SyNOnly_MI_32_reg_iter_1_normal_masked
SyNOnly_MI_32_reg_iter_1_bleached_masked
SyNOnly_CC_4_reg_iter_0_normal_masked
SyNOnly_CC_6_reg_iter_0_bleached_masked
SyNOnly_CC_6_reg_iter_0_normal_masked
SyNOnly_MI_32_reg_iter_0_bleached_masked
SyNOnly_MI_32_reg_iter_0_normal_masked
TVMSQ_bleached_masked
TVMSQC_bleached_masked
TVMSQ_normal_masked
TVMSQC_normal_masked

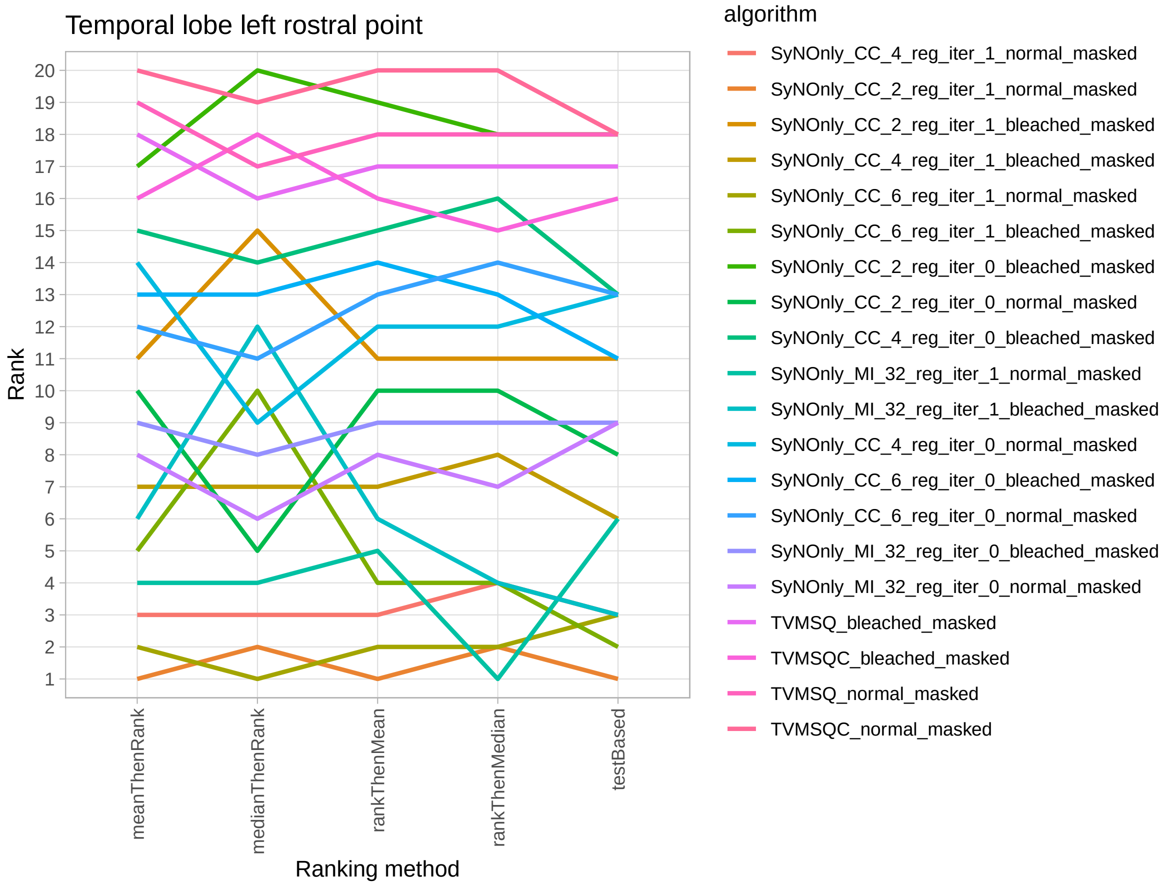
Temporal lobe left rostral point
Rank
Ranking method
meanThenRank
medianThenRank
rankThenMean
rankThenMedian
testBased
algorithm
SyNOnly_CC_4_reg_iter_1_normal_masked
SyNOnly_CC_2_reg_iter_1_normal_masked
SyNOnly_CC_2_reg_iter_1_bleached_masked
SyNOnly_CC_4_reg_iter_1_bleached_masked
SyNOnly_CC_6_reg_iter_1_normal_masked
SyNOnly_CC_6_reg_iter_1_bleached_masked
SyNOnly_CC_2_reg_iter_0_bleached_masked
SyNOnly_CC_2_reg_iter_0_normal_masked
SyNOnly_CC_4_reg_iter_0_bleached_masked
SyNOnly_MI_32_reg_iter_1_normal_masked
SyNOnly_MI_32_reg_iter_1_bleached_masked
SyNOnly_CC_4_reg_iter_0_normal_masked
SyNOnly_CC_6_reg_iter_0_bleached_masked
SyNOnly_CC_6_reg_iter_0_normal_masked
SyNOnly_MI_32_reg_iter_0_bleached_masked
SyNOnly_MI_32_reg_iter_0_normal_masked
TVMSQ_bleached_masked
TVMSQC_bleached_masked
TVMSQ_normal_masked
TVMSQC_normal_masked

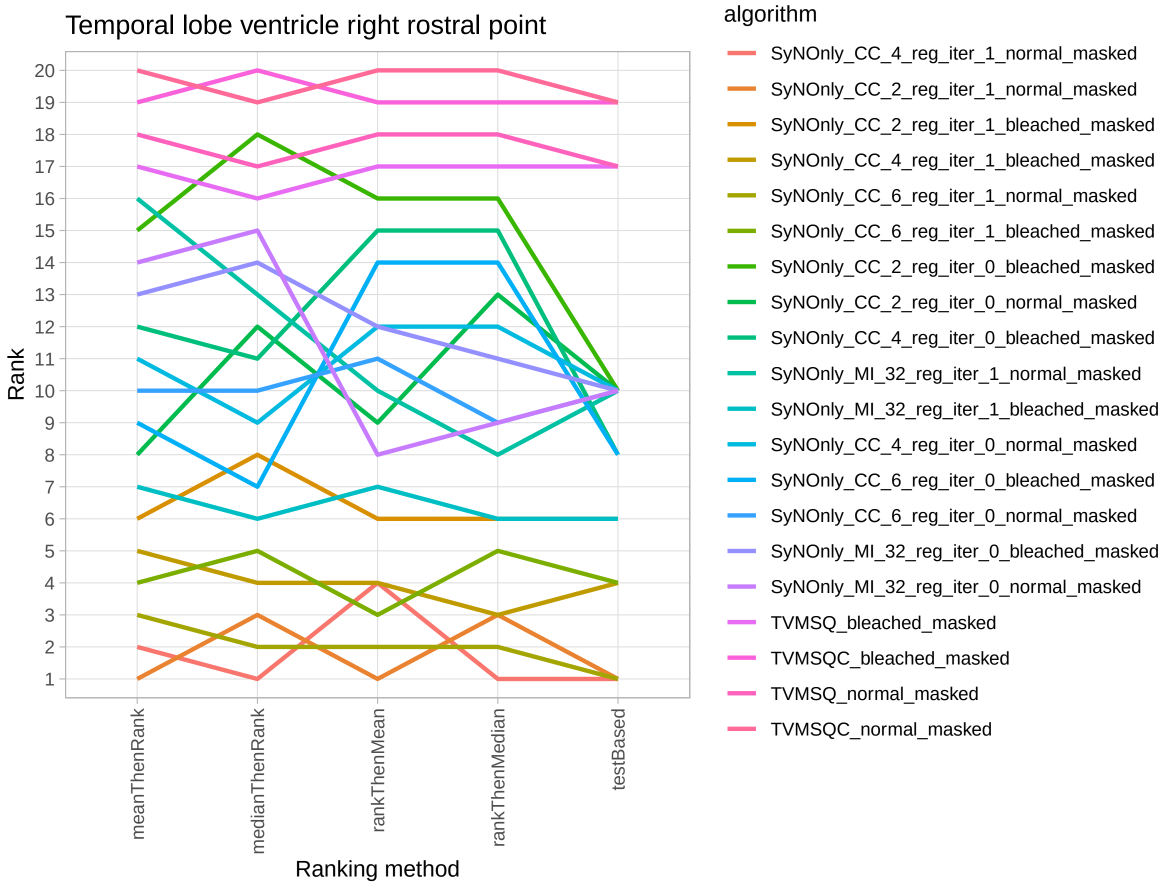
Temporal lobe ventricle right rostral point
Rank
20
19
18
17
16
15
14
13
12
11
10
9
8
7
6
5
4
3
2
1
meanThenRank
medianThenRank
rankThenMean
rankThenMedian
testBased
Ranking method
algorithm
SyNOnly_CC_4_reg_iter_1_normal_masked
SyNOnly_CC_2_reg_iter_1_normal_masked
SyNOnly_CC_2_reg_iter_1_bleached_masked
SyNOnly_CC_4_reg_iter_1_bleached_masked
SyNOnly_CC_6_reg_iter_1_normal_masked
SyNOnly_CC_6_reg_iter_1_bleached_masked
SyNOnly_CC_2_reg_iter_0_bleached_masked
SyNOnly_CC_2_reg_iter_0_normal_masked
SyNOnly_CC_4_reg_iter_0_bleached_masked
SyNOnly_MI_32_reg_iter_1_normal_masked
SyNOnly_MI_32_reg_iter_1_bleached_masked
SyNOnly_CC_4_reg_iter_0_normal_masked
SyNOnly_CC_6_reg_iter_0_bleached_masked
SyNOnly_CC_6_reg_iter_0_normal_masked
SyNOnly_MI_32_reg_iter_0_bleached_masked
SyNOnly_MI_32_reg_iter_0_normal_masked
TVMSQ_bleached_masked
TVMSQC_bleached_masked
TVMSQ_normal_masked
TVMSQC_normal_masked

4 Visualization of cross-task insights

The algorithms are ordered according to consensus ranking.

4.1 Characterization of algorithms

4.1.1 Ranking stability: Variability of achieved rankings across tasks

Algorithms are color-coded, and the area of each blob at position $(A_i, \text{rank } j)$ is proportional to the relative frequency A_i achieved rank j across multiple tasks. The median rank for each algorithm is indicated by a black cross. This way, the distribution of ranks across tasks can be intuitively visualized.

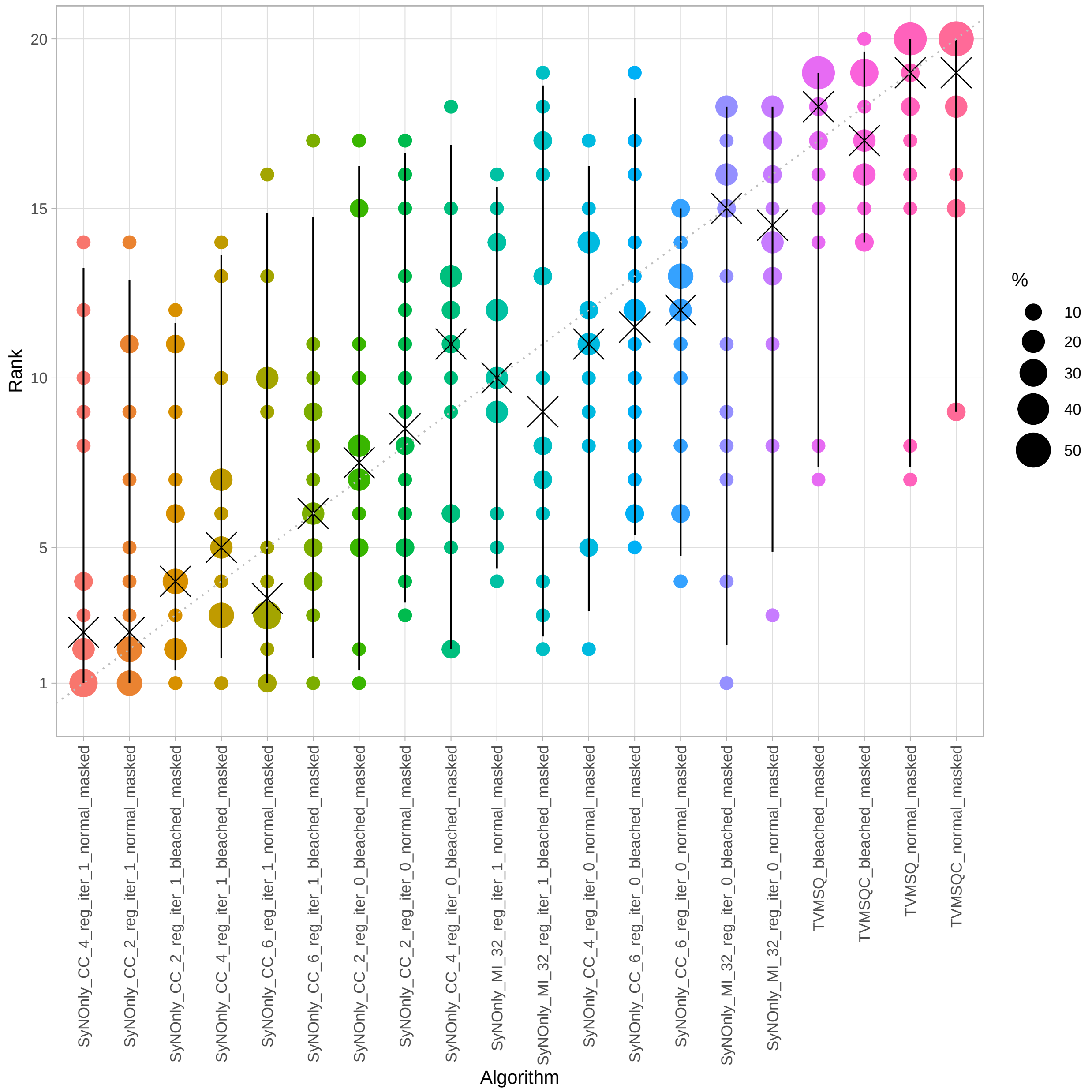

4.1.2 Ranking stability: Ranking variability via bootstrap approach

A blob plot of bootstrap results over the different tasks separated by algorithm allows another perspective on the assessment data. This gives deeper insights into the characteristics of tasks and the ranking uncertainty of the algorithms in each task.

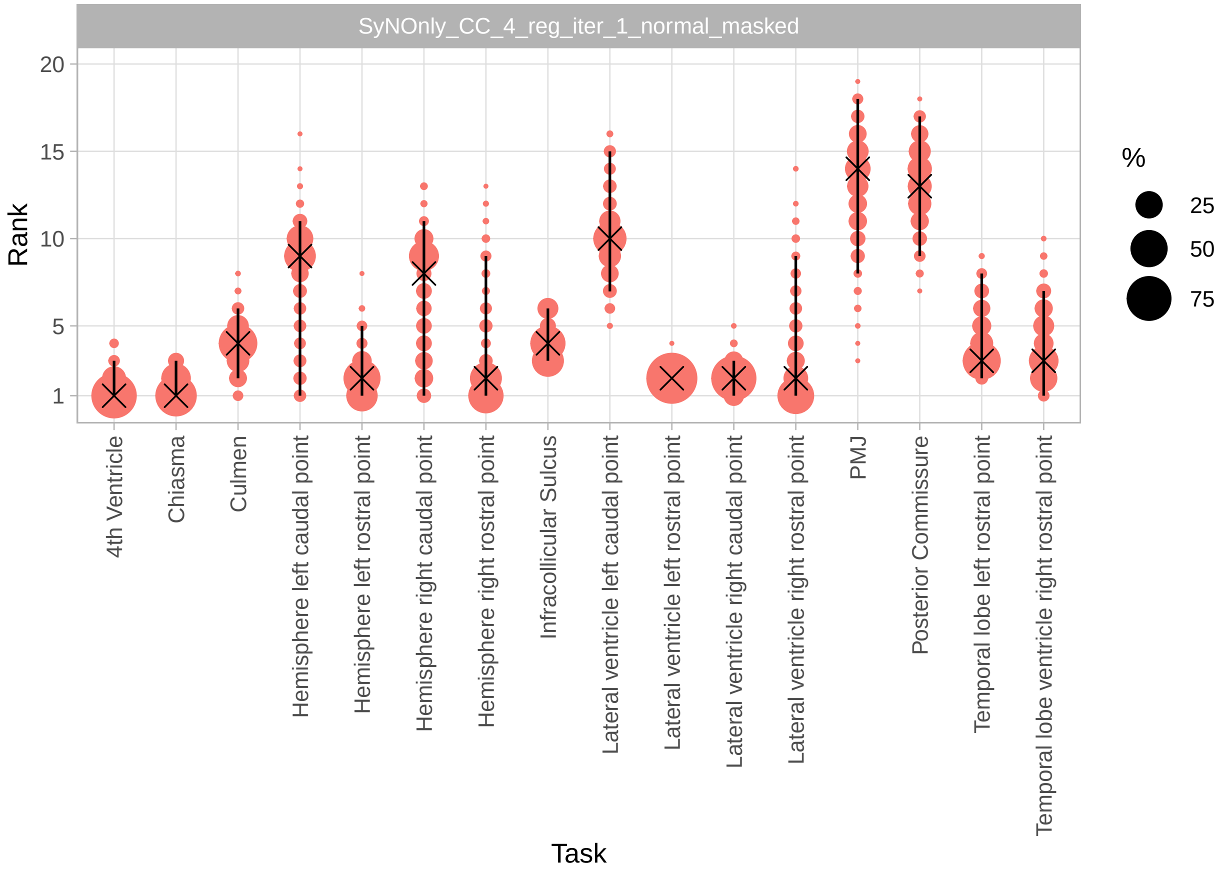

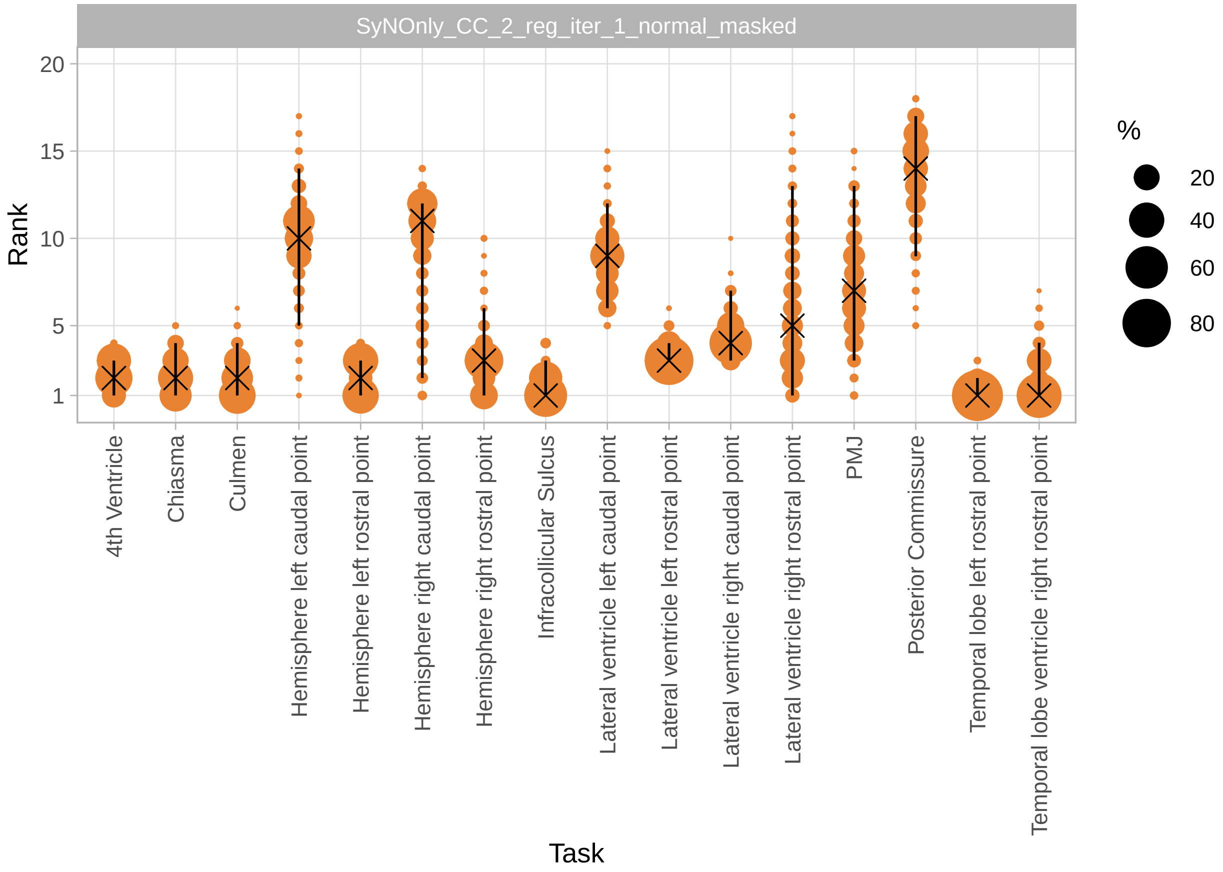
SyNOnly_CC_2_reg_iter_1_normal_masked
Rank
20
15
10
5
1
4th Ventricle
Chiasma
Culmen
Hemisphere left caudal point
Hemisphere left rostral point
Hemisphere right caudal point
Hemisphere right rostral point
Infracollicular Sulcus
Lateral ventricle left caudal point
Lateral ventricle left rostral point
Lateral ventricle right caudal point
Lateral ventricle right rostral point
PMJ
Posterior Commissure
Temporal lobe left rostral point
Temporal lobe ventricle right rostral point
Task
%
20
40
60
80

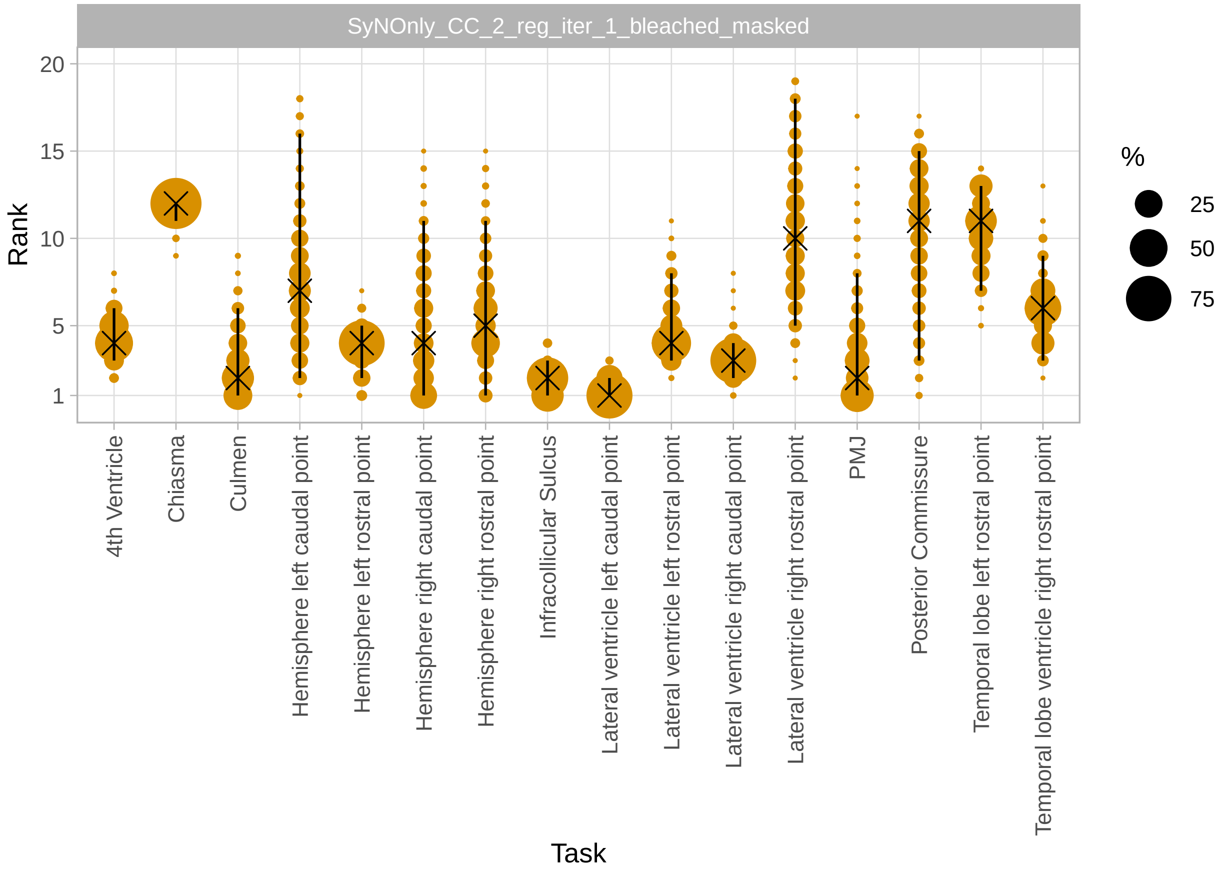
SyNOnly_CC_2_reg_iter_1_bleached_masked
Rank
20
15
10
5
1
%
25
50
75
4th Ventricle
Chiasma
Culmen
Hemisphere left caudal point
Hemisphere left rostral point
Hemisphere right caudal point
Hemisphere right rostral point
Infracollicular Sulcus
Lateral ventricle left caudal point
Lateral ventricle left rostral point
Lateral ventricle right caudal point
Lateral ventricle right rostral point
PMJ
Posterior Commissure
Temporal lobe left rostral point
Temporal lobe ventricle right rostral point
Task

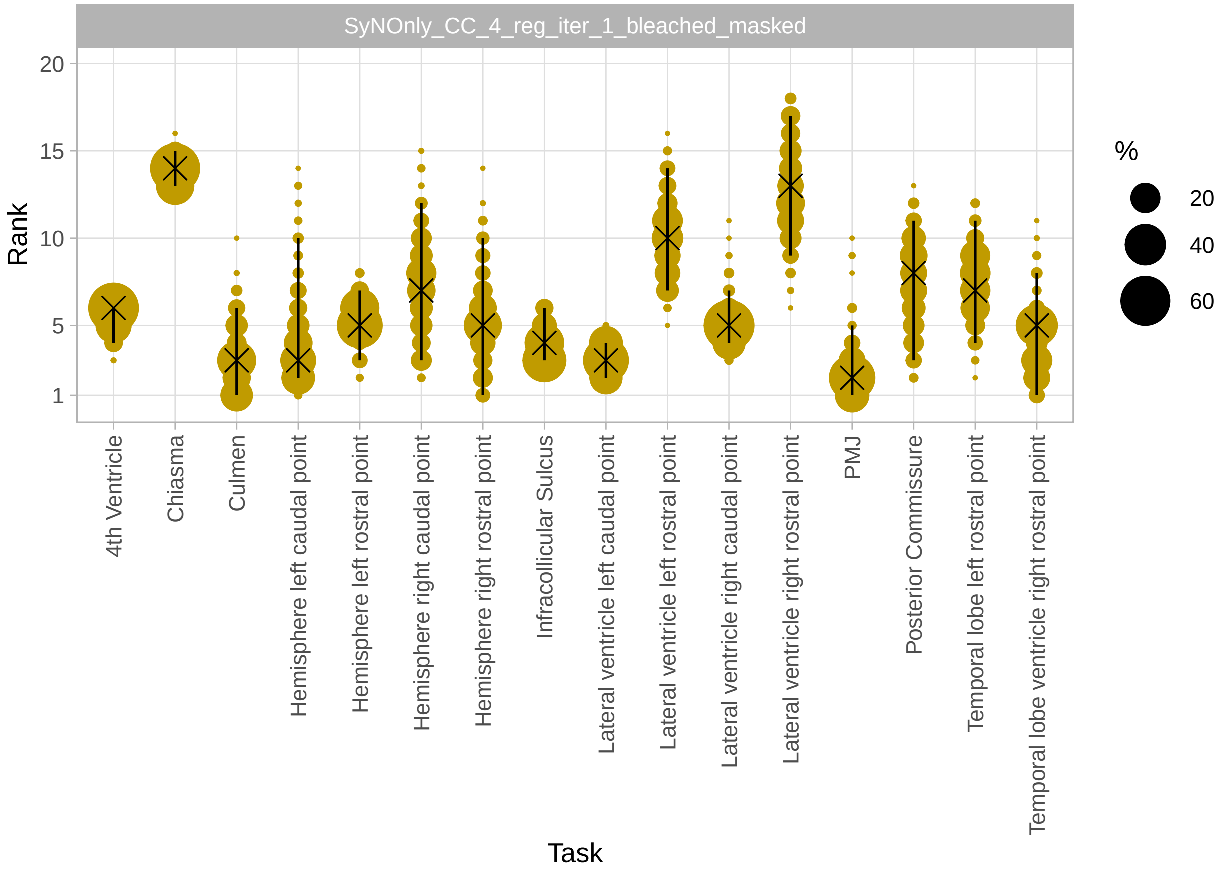
SyNOnly_CC_4_reg_iter_1_bleached_masked
Rank
20
15
10
5
1
%
20
40
60
4th Ventricle
Chiasma
Culmen
Hemisphere left caudal point
Hemisphere left rostral point
Hemisphere right caudal point
Hemisphere right rostral point
Infracollicular Sulcus
Lateral ventricle left caudal point
Lateral ventricle left rostral point
Lateral ventricle right caudal point
Lateral ventricle right rostral point
PMJ
Posterior Commissure
Temporal lobe left rostral point
Temporal lobe ventricle right rostral point
Task

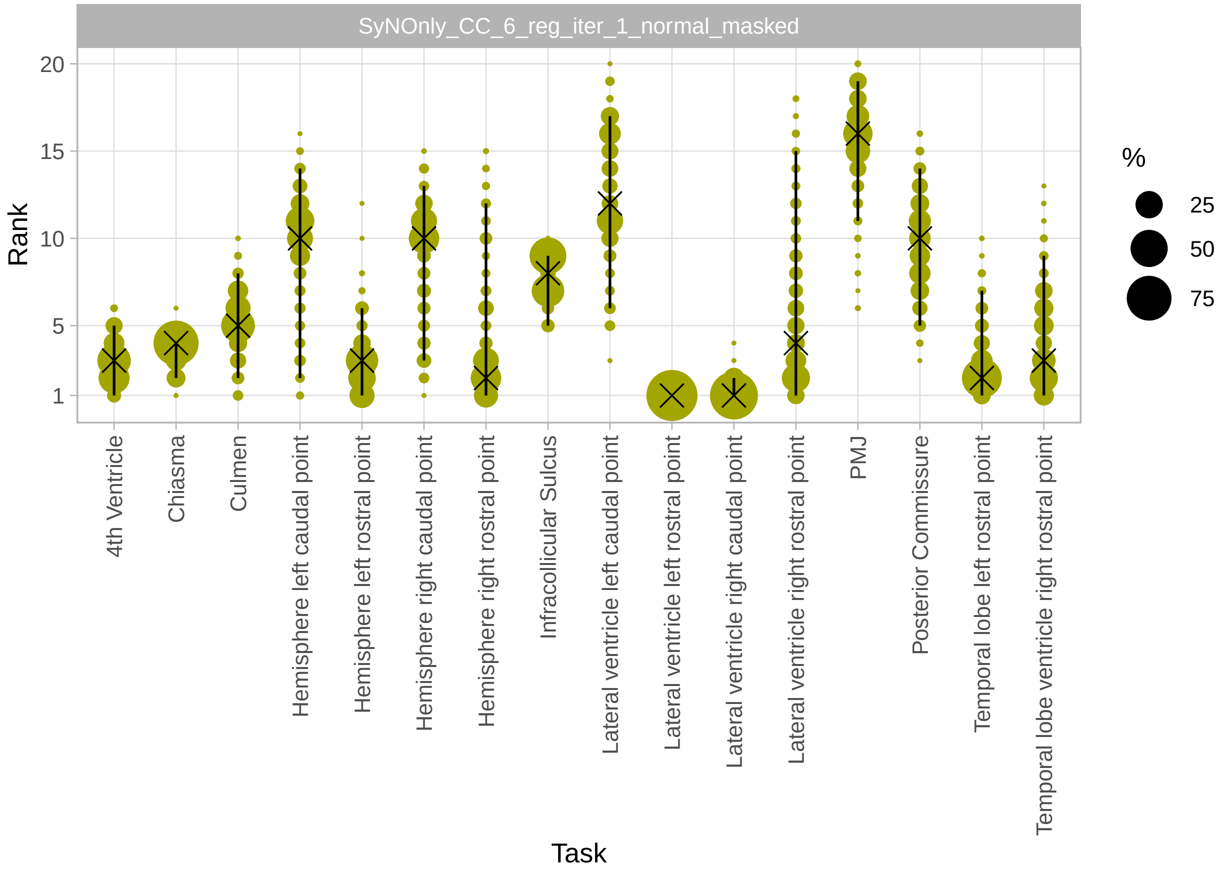
SyNOnly_CC_6_reg_iter_1_normal_masked
Rank
20
15
10
5
1
4th Ventricle
Chiasma
Culmen
Hemisphere left caudal point
Hemisphere left rostral point
Hemisphere right caudal point
Hemisphere right rostral point
Infracollicular Sulcus
Lateral ventricle left caudal point
Lateral ventricle left rostral point
Lateral ventricle right caudal point
Lateral ventricle right rostral point
PMJ
Posterior Commissure
Temporal lobe left rostral point
Temporal lobe ventricle right rostral point
Task
%
25
50
75

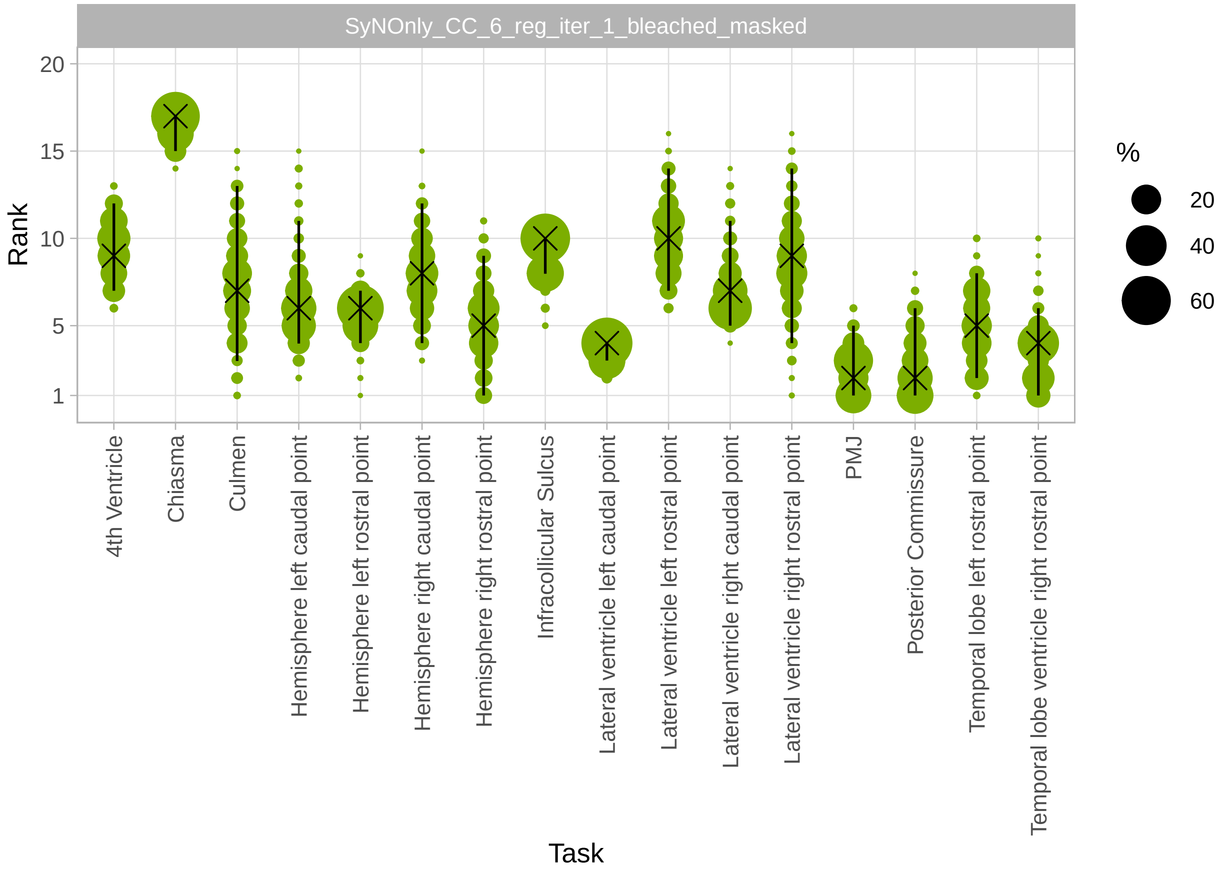

SyNOnly_CC_6_reg_iter_1_bleached_masked
Rank
20
15
10
5
1
4th Ventricle
Chiasma
Culmen
Hemisphere left caudal point
Hemisphere left rostral point
Hemisphere right caudal point
Hemisphere right rostral point
Infracollicular Sulcus
Lateral ventricle left caudal point
Lateral ventricle left rostral point
Lateral ventricle right caudal point
Lateral ventricle right rostral point
PMJ
Posterior Commissure
Temporal lobe left rostral point
Temporal lobe ventricle right rostral point
Task
%
20
40
60

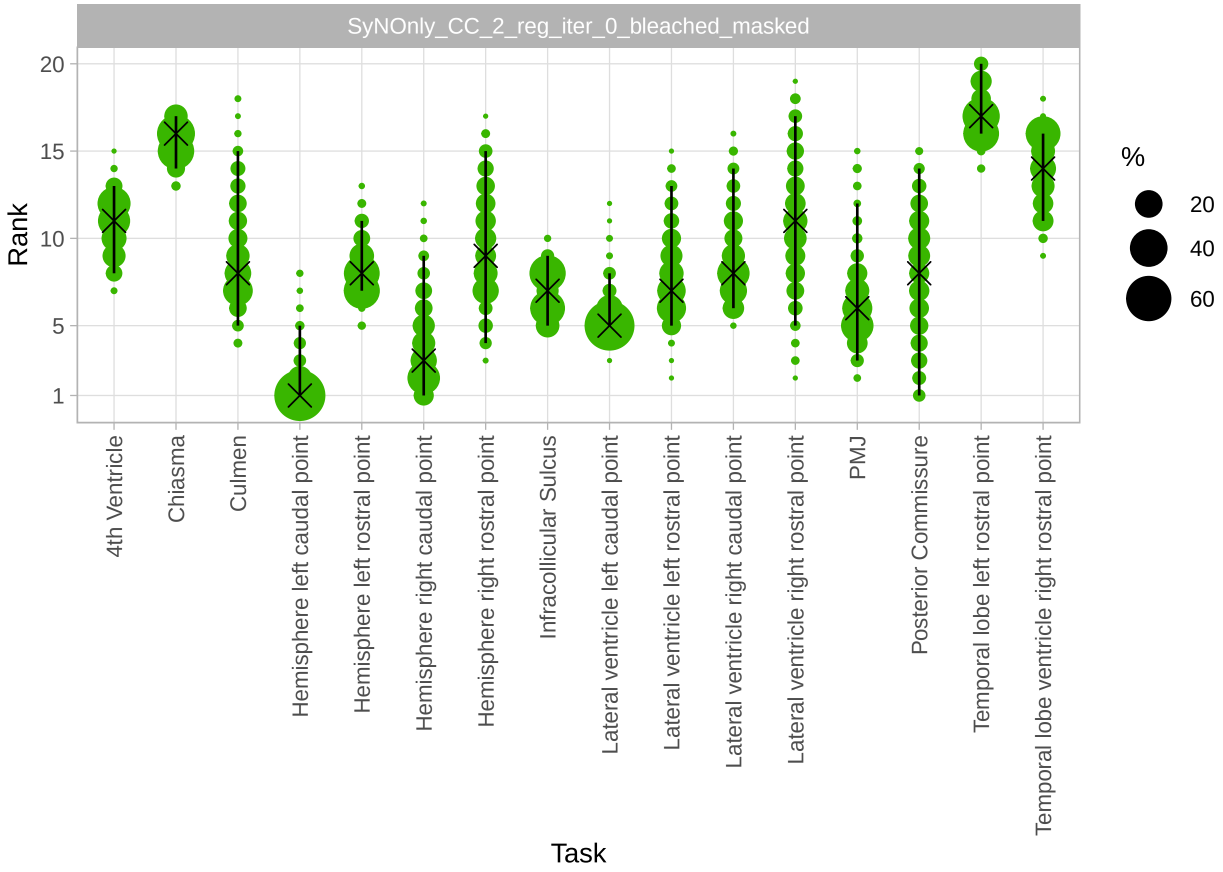

SyNOnly_CC_2_reg_iter_0_bleached_masked
Rank
20
15
10
5
1
4th Ventricle
Chiasma
Culmen
Hemisphere left caudal point
Hemisphere left rostral point
Hemisphere right caudal point
Hemisphere right rostral point
Infracollicular Sulcus
Lateral ventricle left caudal point
Lateral ventricle left rostral point
Lateral ventricle right caudal point
Lateral ventricle right rostral point
PMJ
Posterior Commissure
Temporal lobe left rostral point
Temporal lobe ventricle right rostral point
Task
%
20
40
60

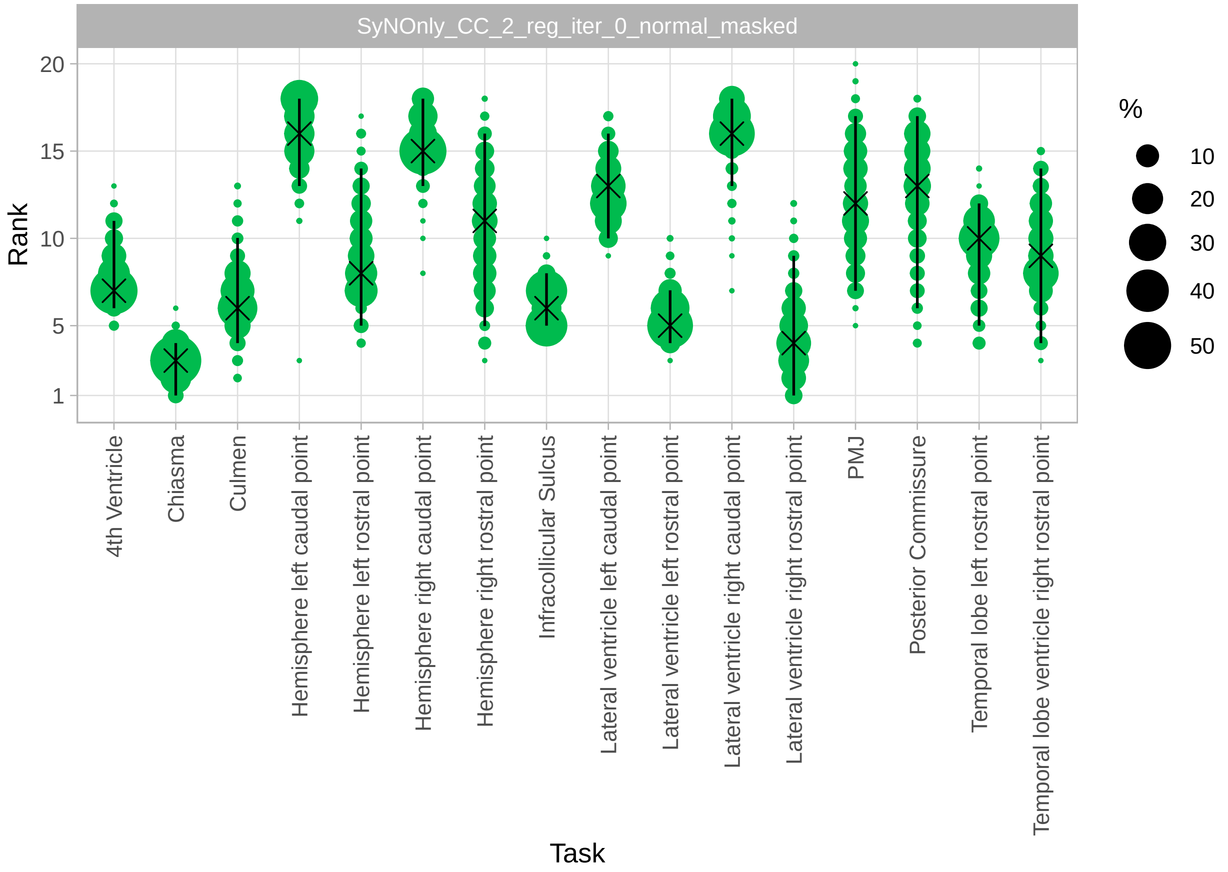

SyNOnly_CC_2_reg_iter_0_normal_masked
Rank
20
15
10
5
1
4th Ventricle
Chiasma
Culmen
Hemisphere left caudal point
Hemisphere left rostral point
Hemisphere right caudal point
Hemisphere right rostral point
Infracollicular Sulcus
Lateral ventricle left caudal point
Lateral ventricle left rostral point
Lateral ventricle right caudal point
Lateral ventricle right rostral point
PMJ
Posterior Commissure
Temporal lobe left rostral point
Temporal lobe ventricle right rostral point
Task
%
10
20
30
40
50

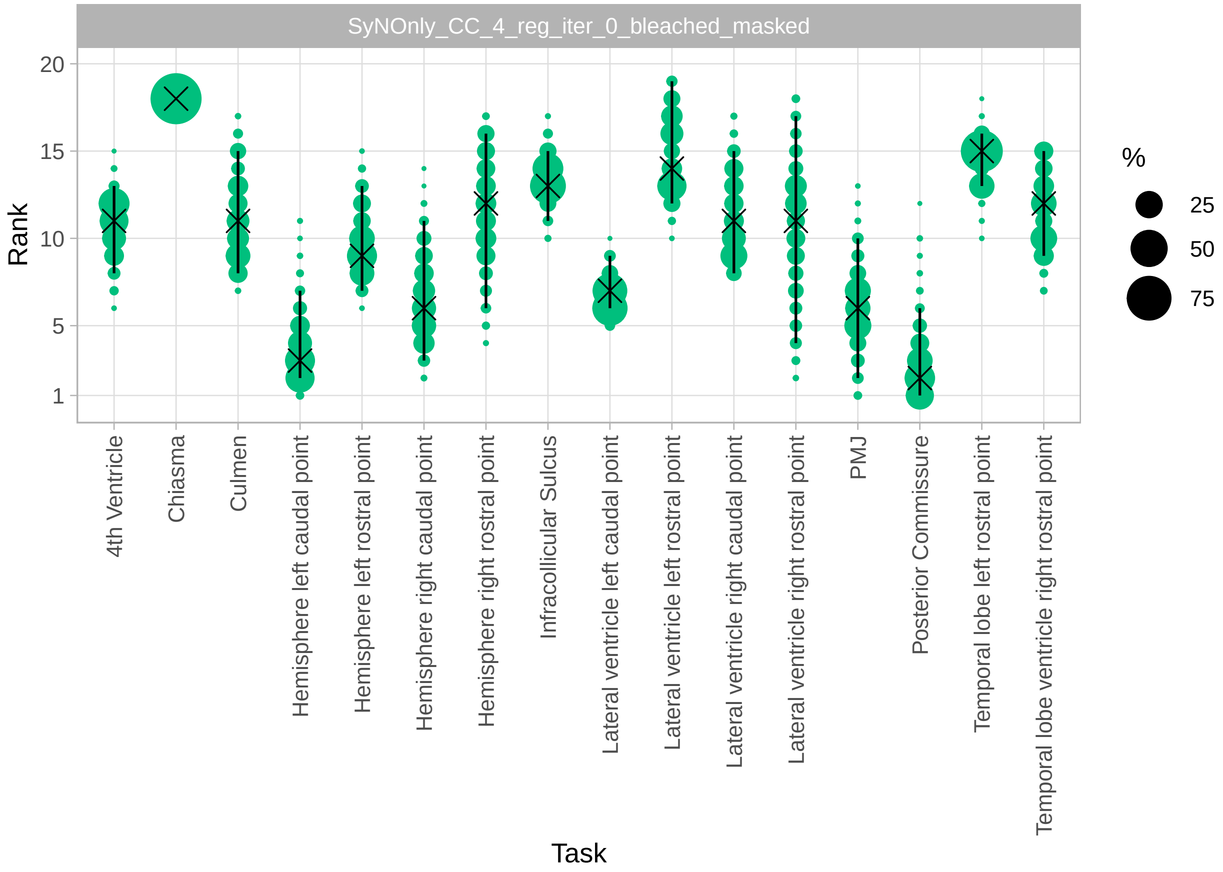
SyNOnly_CC_4_reg_iter_0_bleached_masked
Rank
20
15
10
5
1
4th Ventricle
Chiasma
Culmen
Hemisphere left caudal point
Hemisphere left rostral point
Hemisphere right caudal point
Hemisphere right rostral point
Infracollicular Sulcus
Lateral ventricle left caudal point
Lateral ventricle left rostral point
Lateral ventricle right caudal point
Lateral ventricle right rostral point
PMJ
Posterior Commissure
Temporal lobe left rostral point
Temporal lobe ventricle right rostral point
Task
%
25
50
75

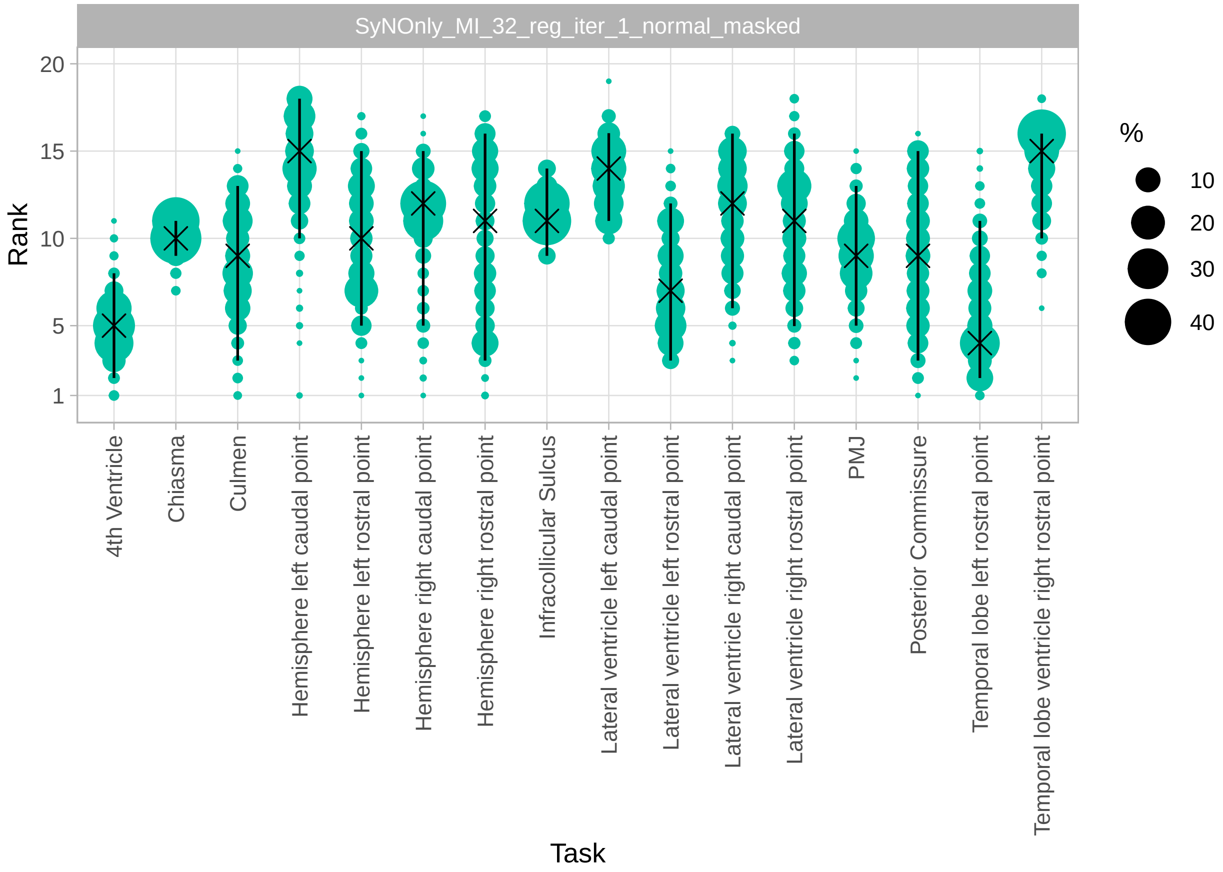
SyNOnly_MI_32_reg_iter_1_normal_masked
Rank
20
15
10
5
1
%
10
20
30
40
4th Ventricle
Chiasma
Culmen
Hemisphere left caudal point
Hemisphere left rostral point
Hemisphere right caudal point
Hemisphere right rostral point
Infracollicular Sulcus
Lateral ventricle left caudal point
Lateral ventricle left rostral point
Lateral ventricle right caudal point
Lateral ventricle right rostral point
PMJ
Posterior Commissure
Temporal lobe left rostral point
Temporal lobe ventricle right rostral point
Task

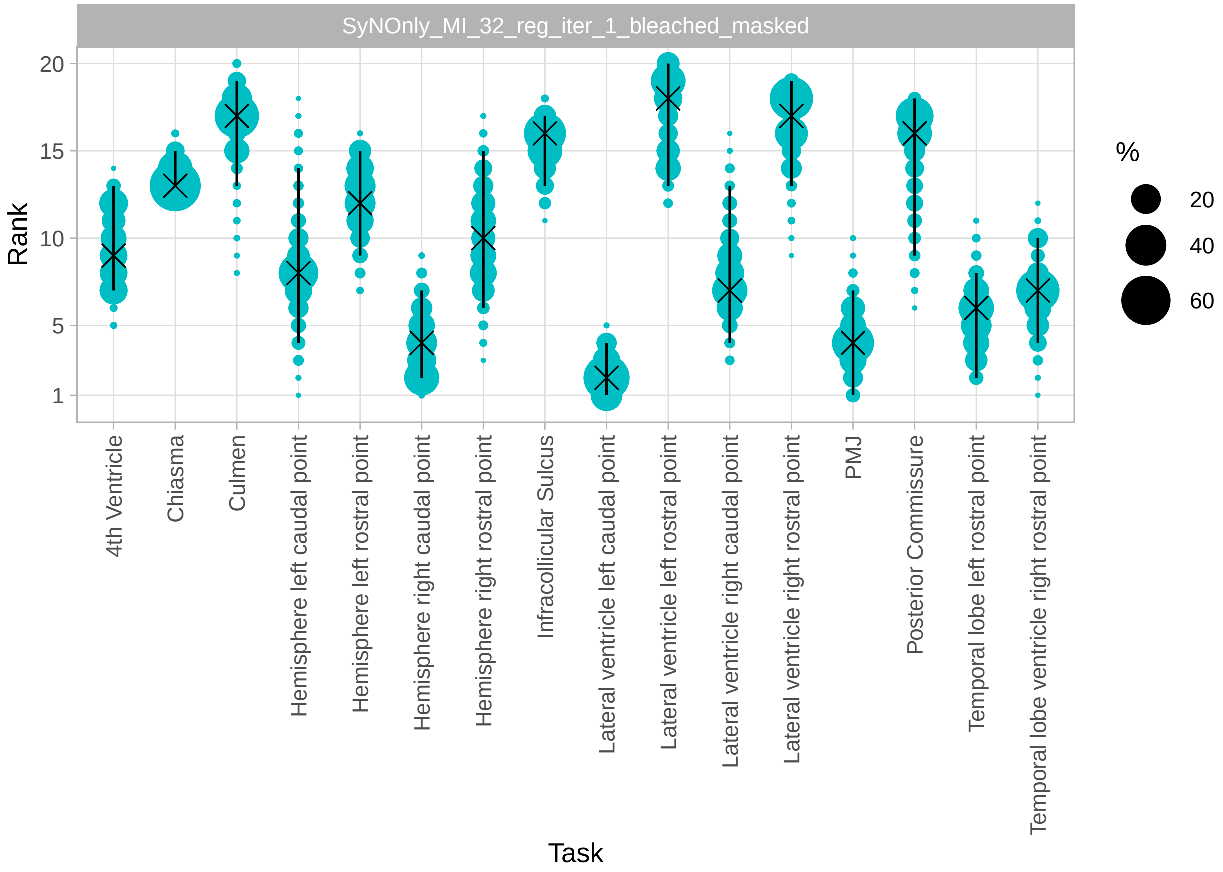
SyNOnly_MI_32_reg_iter_1_bleached_masked
Rank
20
15
10
5
1
%
20
40
60
4th Ventricle
Chiasma
Culmen
Hemisphere left caudal point
Hemisphere left rostral point
Hemisphere right caudal point
Hemisphere right rostral point
Infracollicular Sulcus
Lateral ventricle left caudal point
Lateral ventricle left rostral point
Lateral ventricle right caudal point
Lateral ventricle right rostral point
PMJ
Posterior Commissure
Temporal lobe left rostral point
Temporal lobe ventricle right rostral point
Task

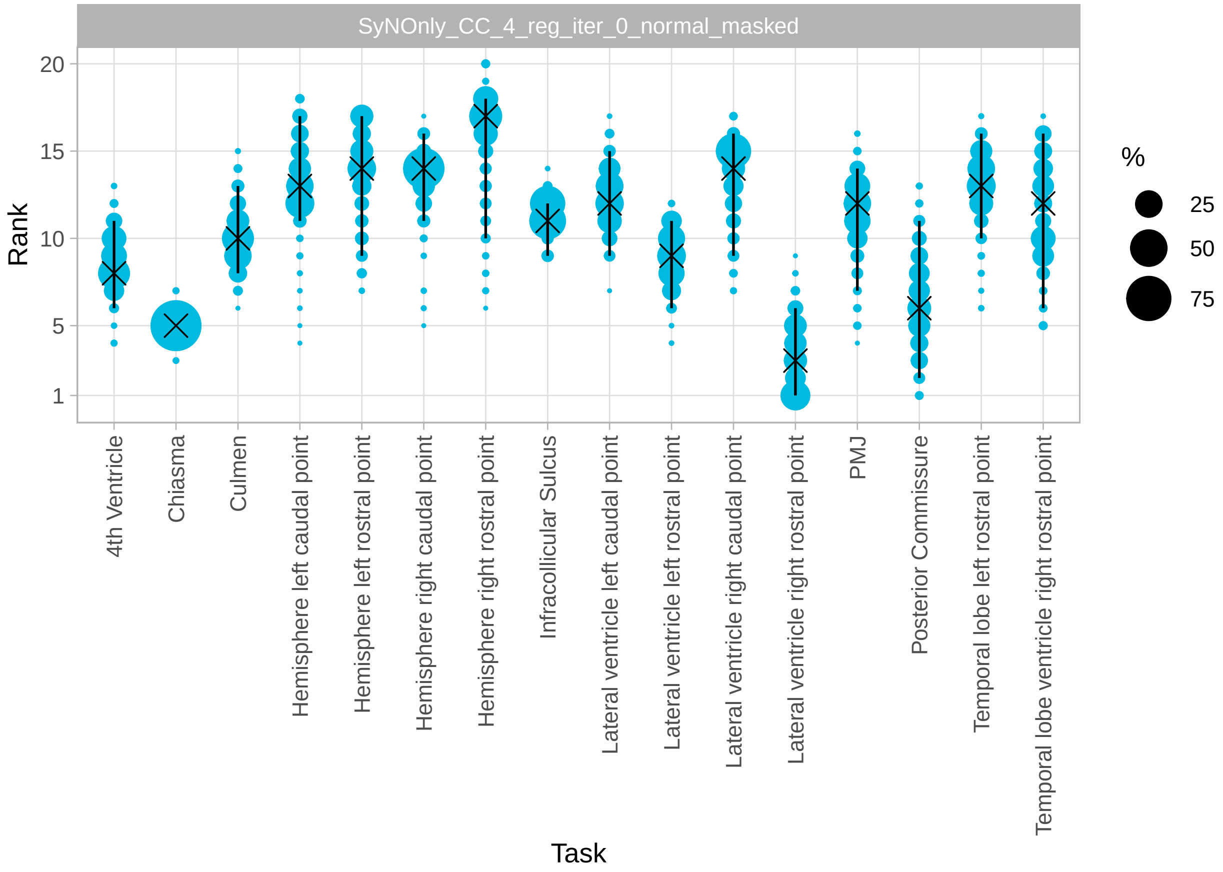
SyNOnly_CC_4_reg_iter_0_normal_masked
Rank
20
15
10
5
1
4th Ventricle
Chiasma
Culmen
Hemisphere left caudal point
Hemisphere left rostral point
Hemisphere right caudal point
Hemisphere right rostral point
Infracollicular Sulcus
Lateral ventricle left caudal point
Lateral ventricle left rostral point
Lateral ventricle right caudal point
Lateral ventricle right rostral point
PMJ
Posterior Commissure
Temporal lobe left rostral point
Temporal lobe ventricle right rostral point
Task
%
25
50
75

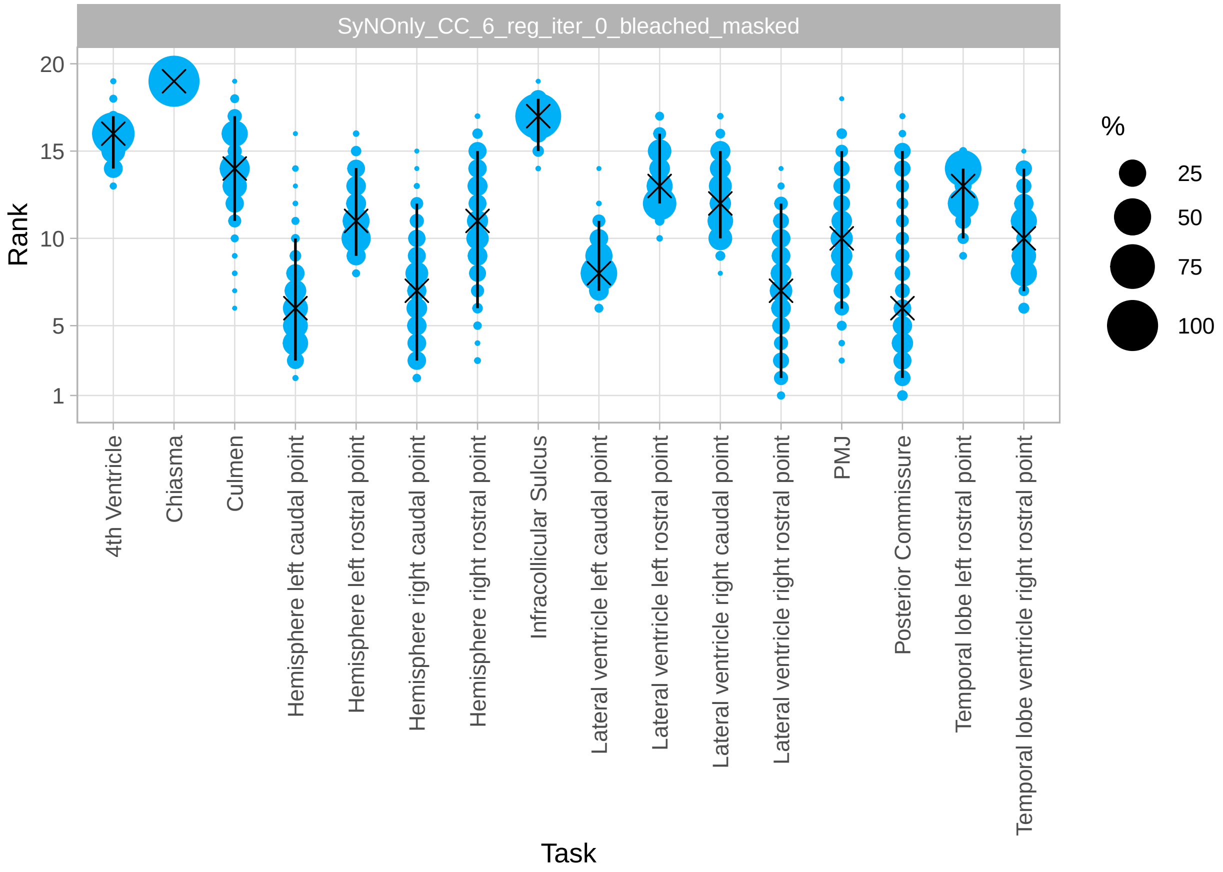

SyNOnly_CC_6_reg_iter_0_bleached_masked
Rank
20
15
10
5
1
%
25
50
75
100
4th Ventricle
Chiasma
Culmen
Hemisphere left caudal point
Hemisphere left rostral point
Hemisphere right caudal point
Hemisphere right rostral point
Infracollicular Sulcus
Lateral ventricle left caudal point
Lateral ventricle left rostral point
Lateral ventricle right caudal point
Lateral ventricle right rostral point
PMJ
Posterior Commissure
Temporal lobe left rostral point
Temporal lobe ventricle right rostral point
Task

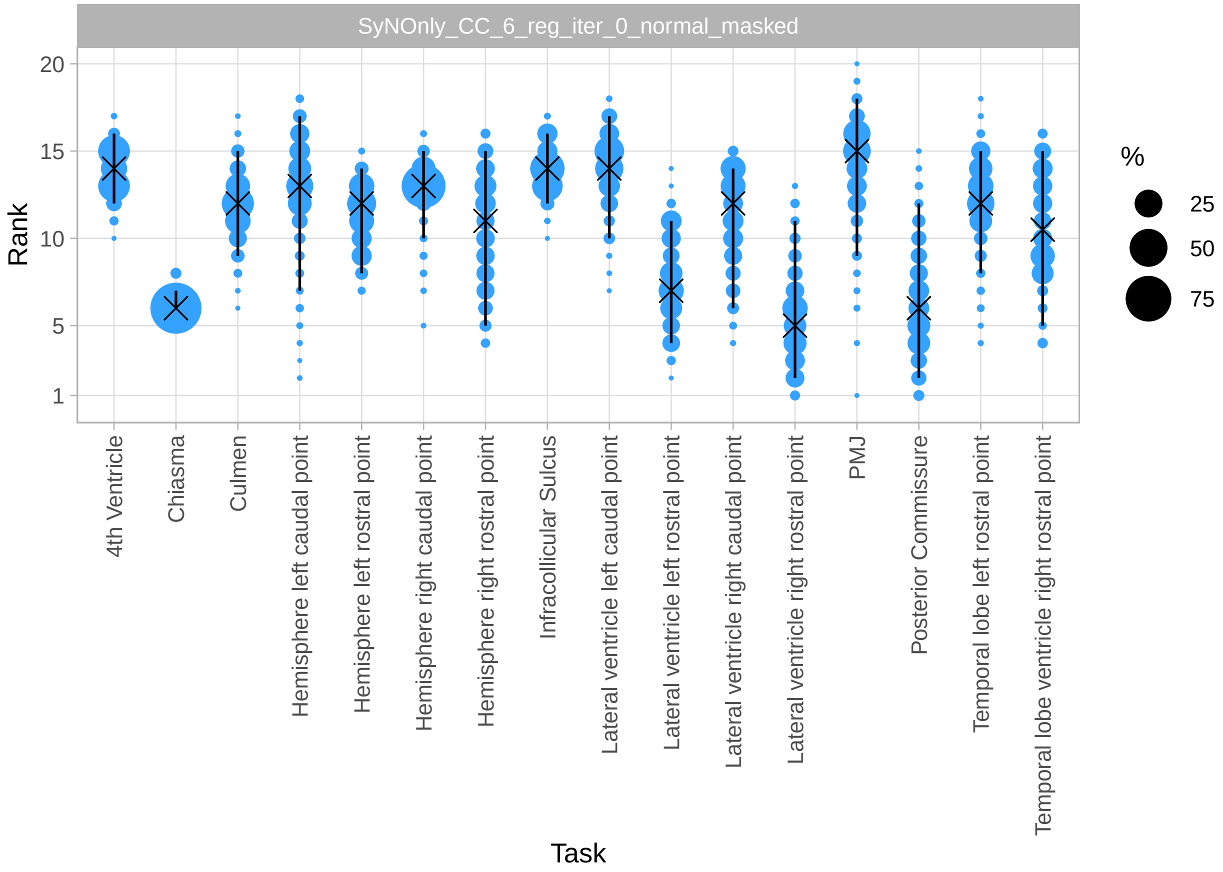
SyNOnly_CC_6_reg_iter_0_normal_masked
Rank
20
15
10
5
1
4th Ventricle
Chiasma
Culmen
Hemisphere left caudal point
Hemisphere left rostral point
Hemisphere right caudal point
Hemisphere right rostral point
Infracollicular Sulcus
Lateral ventricle left caudal point
Lateral ventricle left rostral point
Lateral ventricle right caudal point
Lateral ventricle right rostral point
PMJ
Posterior Commissure
Temporal lobe left rostral point
Temporal lobe ventricle right rostral point
Task
%
25
50
75

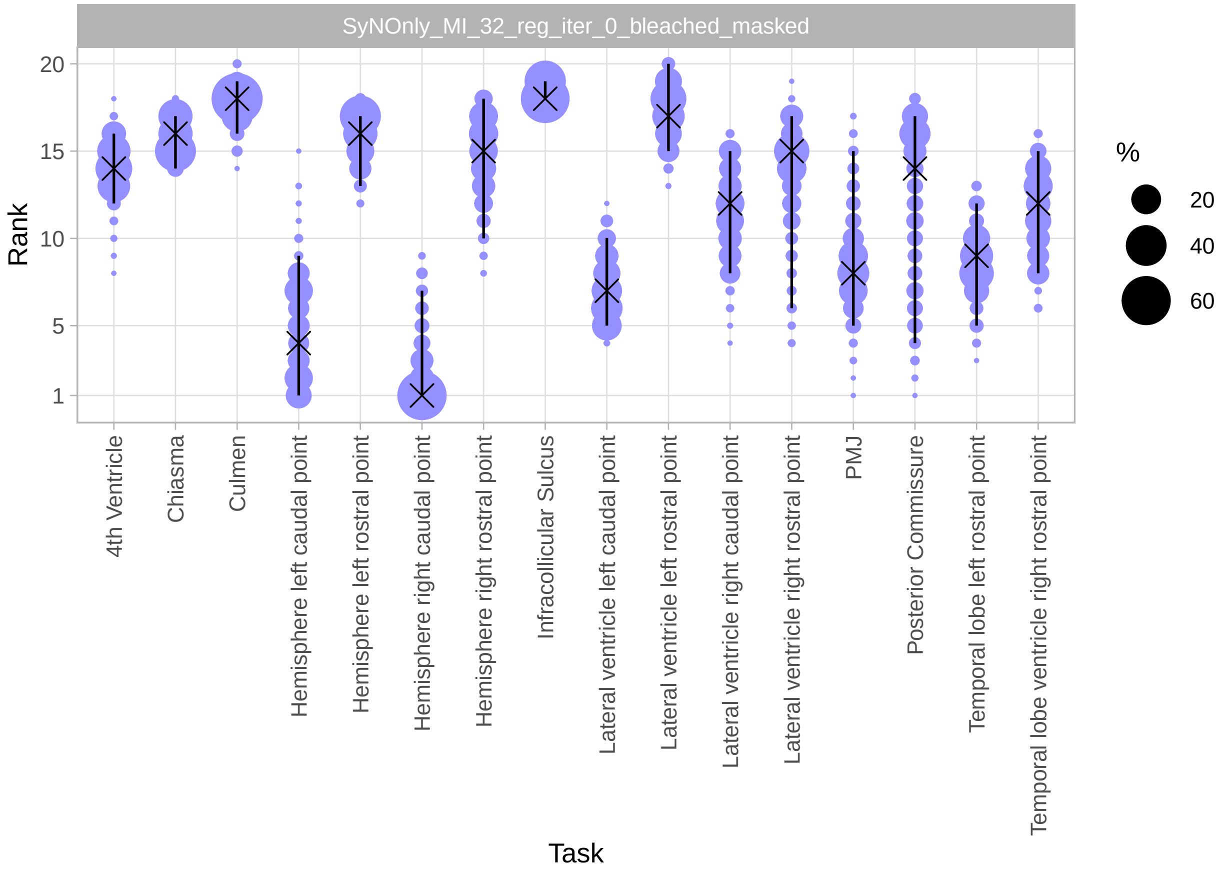

SyNOnly_MI_32_reg_iter_0_bleached_masked
Rank
20
15
10
5
1
4th Ventricle
Chiasma
Culmen
Hemisphere left caudal point
Hemisphere left rostral point
Hemisphere right caudal point
Hemisphere right rostral point
Infracollicular Sulcus
Lateral ventricle left caudal point
Lateral ventricle left rostral point
Lateral ventricle right caudal point
Lateral ventricle right rostral point
PMJ
Posterior Commissure
Temporal lobe left rostral point
Temporal lobe ventricle right rostral point
Task
%
20
40
60

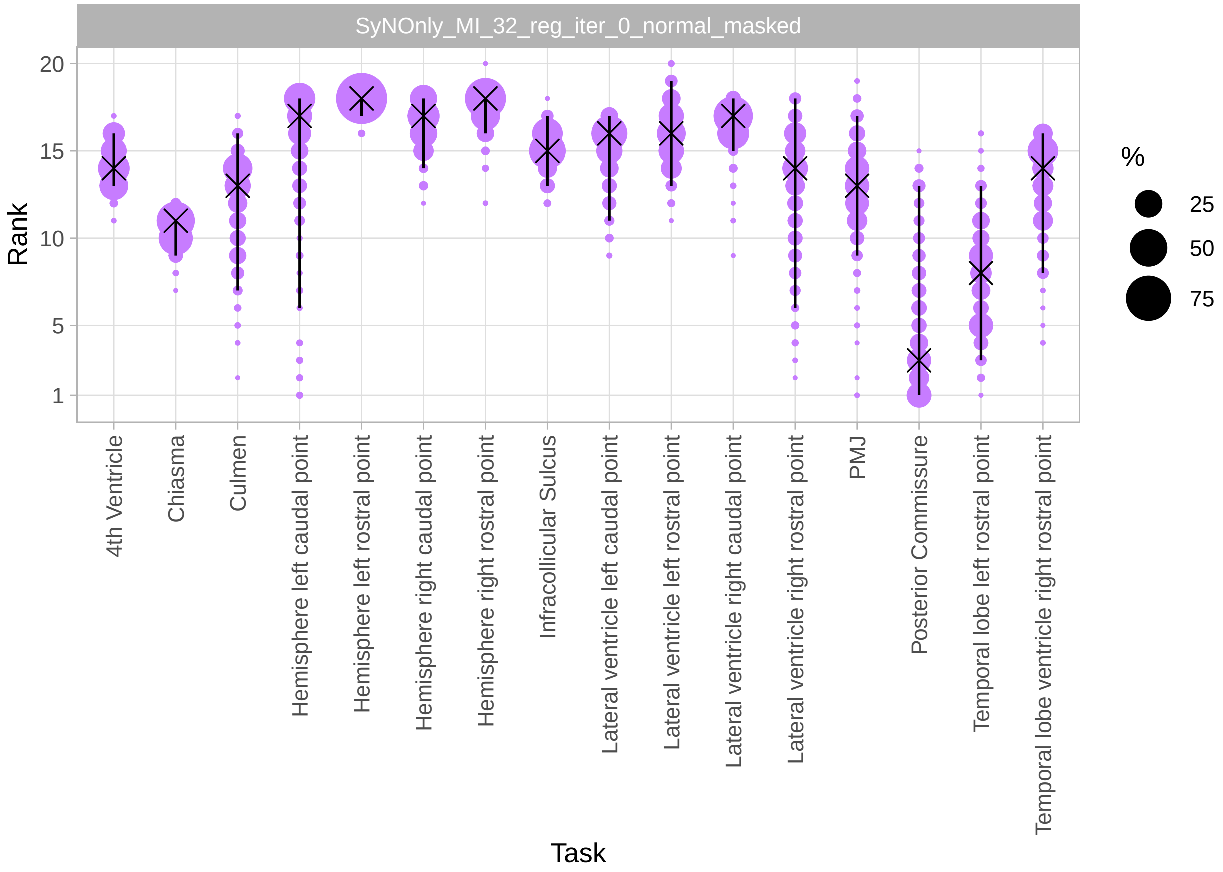
SyNOnly_MI_32_reg_iter_0_normal_masked
Rank
20
15
10
5
1
4th Ventricle
Chiasma
Culmen
Hemisphere left caudal point
Hemisphere left rostral point
Hemisphere right caudal point
Hemisphere right rostral point
Infracollicular Sulcus
Lateral ventricle left caudal point
Lateral ventricle left rostral point
Lateral ventricle right caudal point
Lateral ventricle right rostral point
PMJ
Posterior Commissure
Temporal lobe left rostral point
Temporal lobe ventricle right rostral point
Task
%
25
50
75

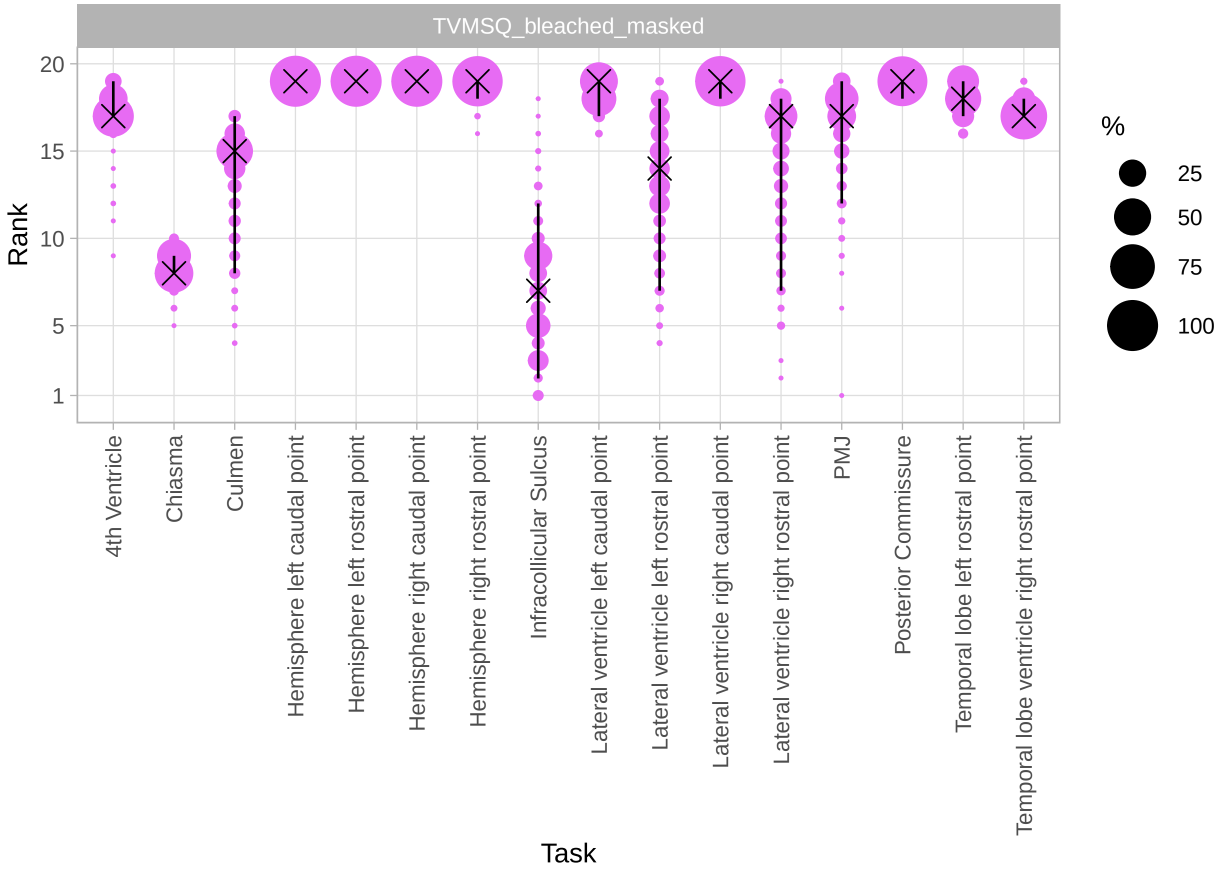

TVMSQ_bleached_masked
Rank
20
15
10
5
1
4th Ventricle
Chiasma
Culmen
Hemisphere left caudal point
Hemisphere left rostral point
Hemisphere right caudal point
Hemisphere right rostral point
Infracollicular Sulcus
Lateral ventricle left caudal point
Lateral ventricle left rostral point
Lateral ventricle right caudal point
Lateral ventricle right rostral point
PMJ
Posterior Commissure
Temporal lobe left rostral point
Temporal lobe ventricle right rostral point
Task
%
25
50
75
100

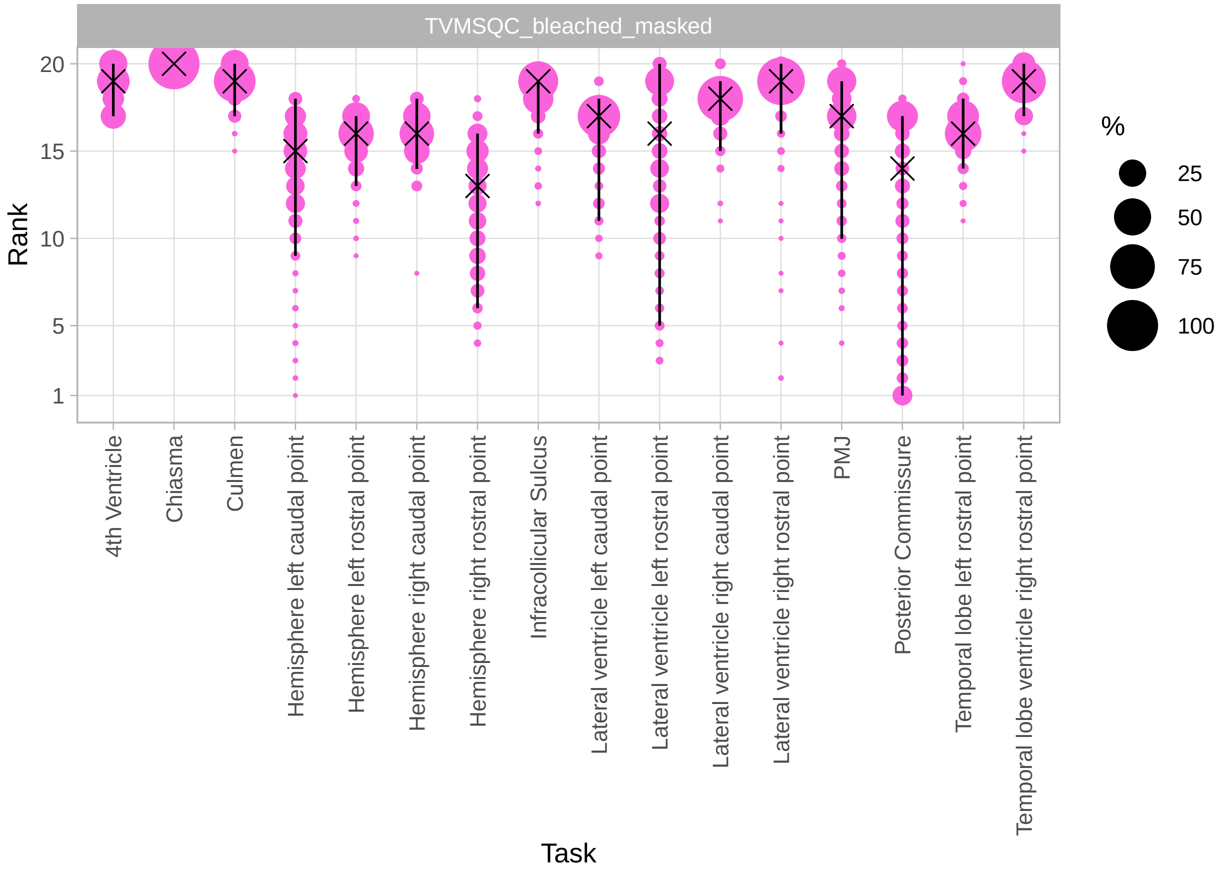

TVMSQC_bleached_masked
Rank
20
15
10
5
1
4th Ventricle
Chiasma
Culmen
Hemisphere left caudal point
Hemisphere left rostral point
Hemisphere right caudal point
Hemisphere right rostral point
Infracollicular Sulcus
Lateral ventricle left caudal point
Lateral ventricle left rostral point
Lateral ventricle right caudal point
Lateral ventricle right rostral point
PMJ
Posterior Commissure
Temporal lobe left rostral point
Temporal lobe ventricle right rostral point
Task
%
25
50
75
100

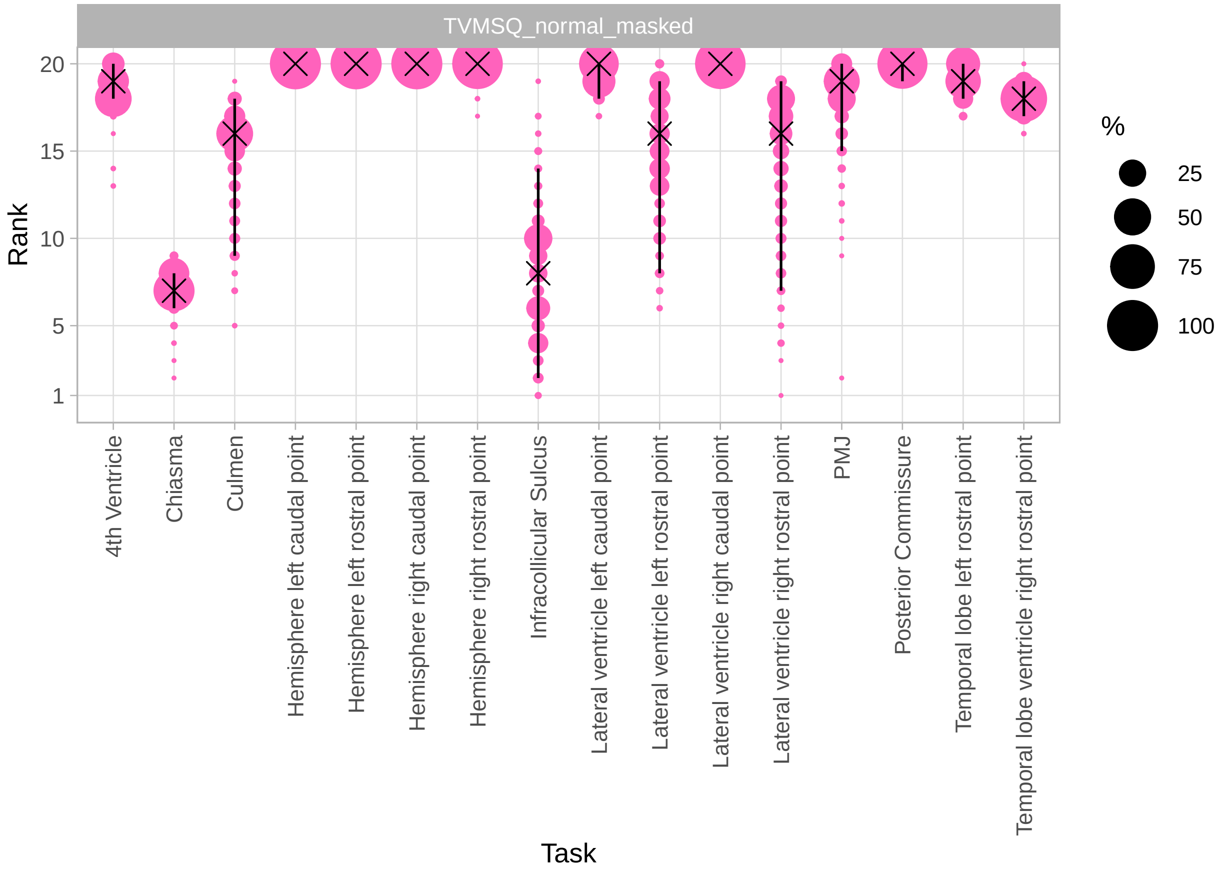
TVMSQ_normal_masked
Rank
20
15
10
5
1
%
25
50
75
100
4th Ventricle
Chiasma
Culmen
Hemisphere left caudal point
Hemisphere left rostral point
Hemisphere right caudal point
Hemisphere right rostral point
Infracollicular Sulcus
Lateral ventricle left caudal point
Lateral ventricle left rostral point
Lateral ventricle right caudal point
Lateral ventricle right rostral point
PMJ
Posterior Commissure
Temporal lobe left rostral point
Temporal lobe ventricle right rostral point
Task

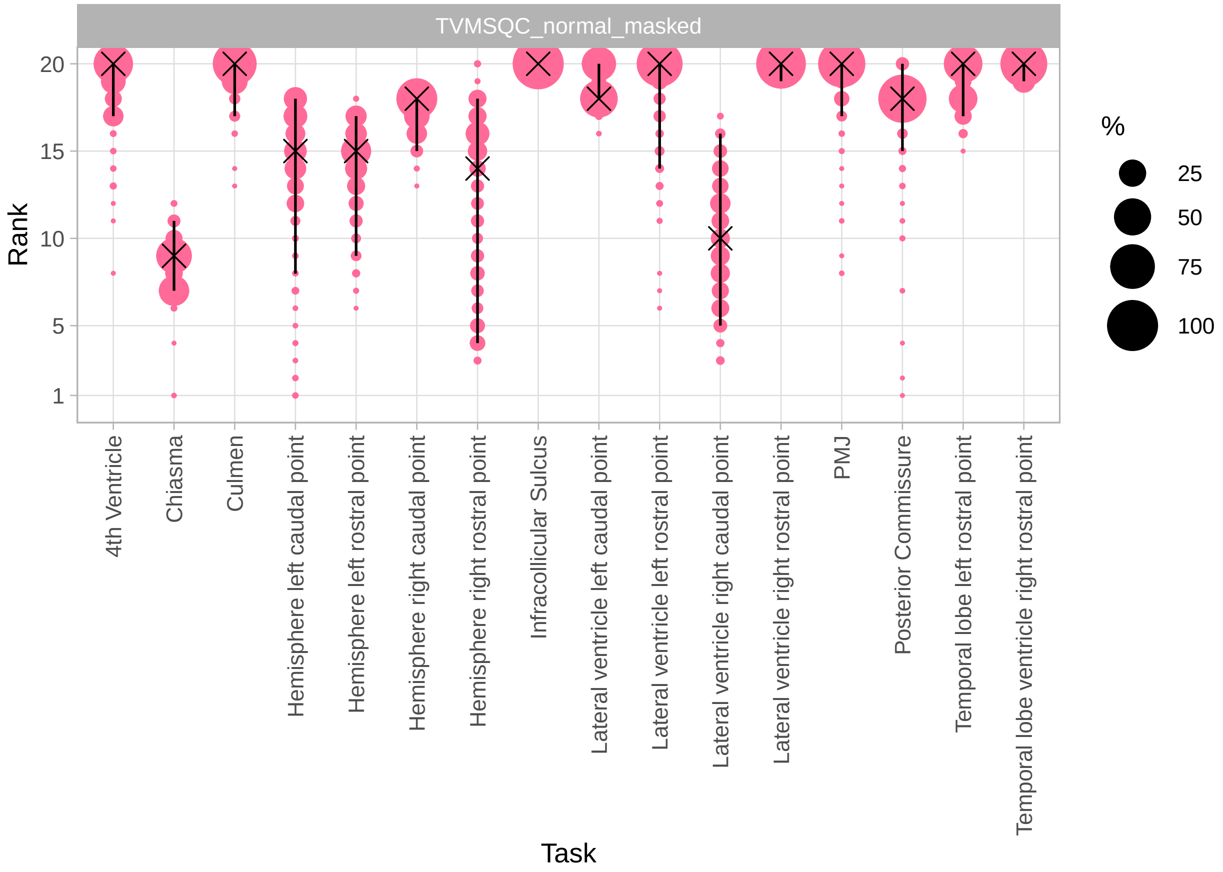
TVMSQC_normal_masked
Rank
20
15
10
5
1
Task
4th Ventricle
Chiasma
Culmen
Hemisphere left caudal point
Hemisphere left rostral point
Hemisphere right caudal point
Hemisphere right rostral point
Infracollicular Sulcus
Lateral ventricle left caudal point
Lateral ventricle left rostral point
Lateral ventricle right caudal point
Lateral ventricle right rostral point
PMJ
Posterior Commissure
Temporal lobe left rostral point
Temporal lobe ventricle right rostral point
%
25
50
75
100

An alternative representation is provided by a stacked frequency plot of the observed ranks, separated by algorithm. Observed ranks across bootstrap samples are displayed with coloring according to the task. For algorithms that achieve the same rank in different tasks for the full assessment data set, vertical lines are on top of each other. Vertical lines allow to compare the achieved rank of each algorithm over different tasks.

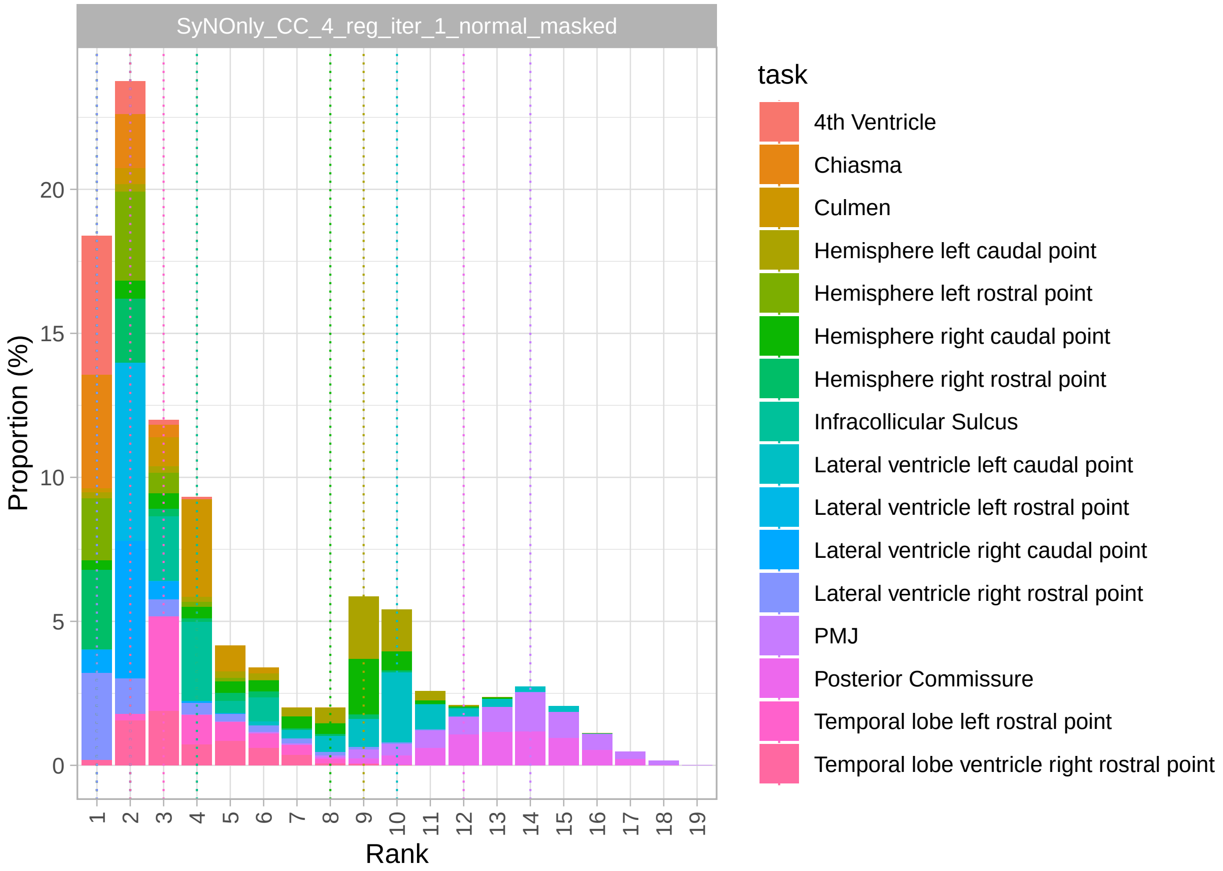

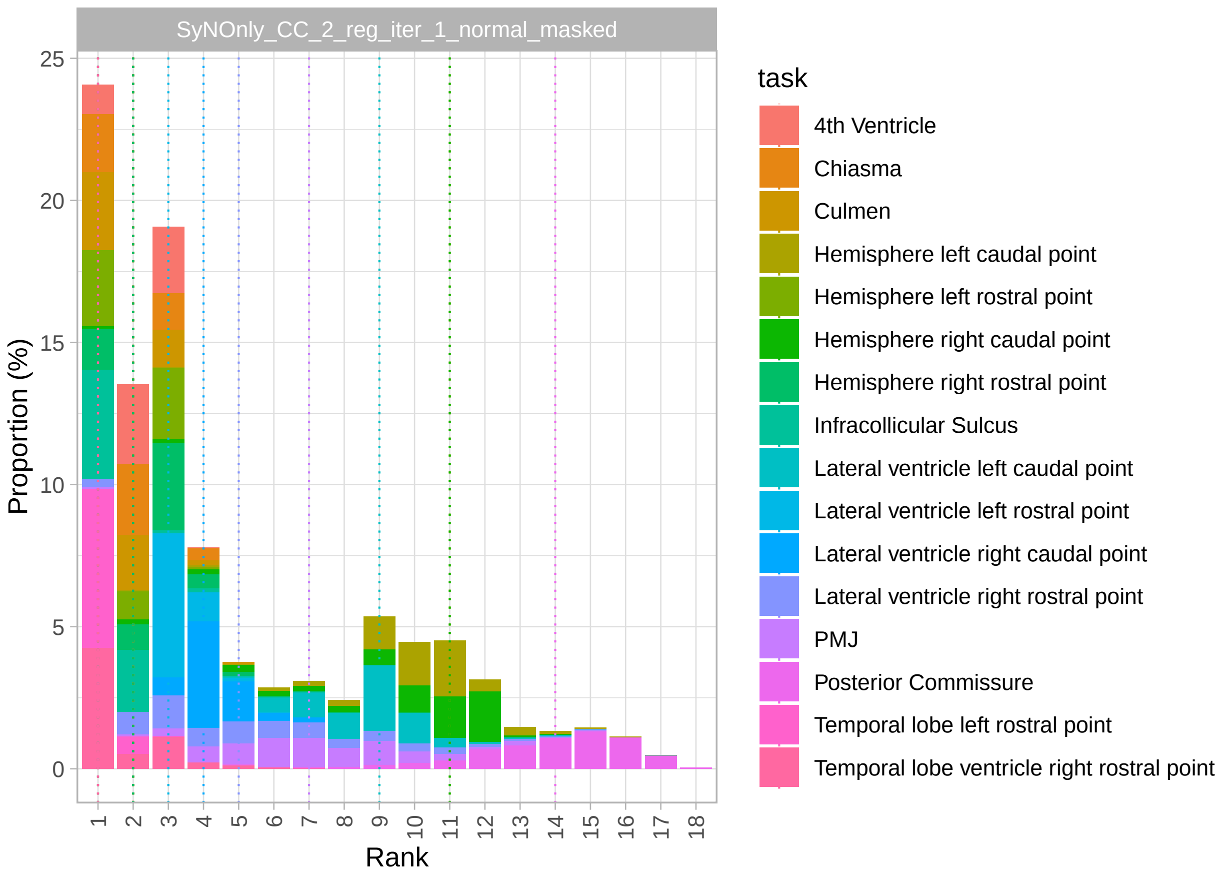

SyNOnly_CC_2_reg_iter_1_normal_masked
Proportion (%)
0
5
10
15
20
25
Rank
1
2
3
4
5
6
7
8
9
10
11
12
13
14
15
16
17
18
task
4th Ventricle
Chiasma
Culmen
Hemisphere left caudal point
Hemisphere left rostral point
Hemisphere right caudal point
Hemisphere right rostral point
Infracollicular Sulcus
Lateral ventricle left caudal point
Lateral ventricle left rostral point
Lateral ventricle right caudal point
Lateral ventricle right rostral point
PMJ
Posterior Commissure
Temporal lobe left rostral point
Temporal lobe ventricle right rostral point

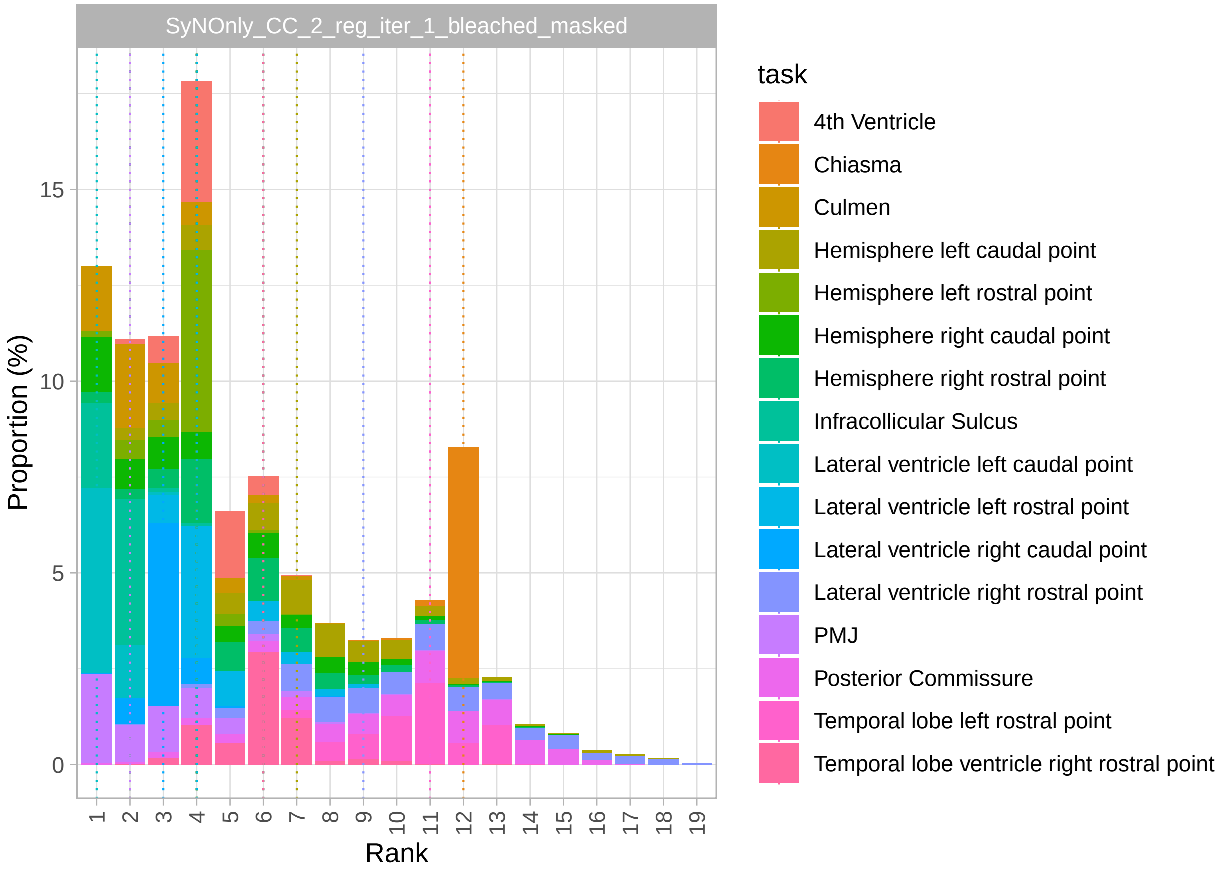

SyNOnly_CC_2_reg_iter_1_bleached_masked
Proportion (%)
0
5
10
15
Rank
1
2
3
4
5
6
7
8
9
10
11
12
13
14
15
16
17
18
19
task
4th Ventricle
Chiasma
Culmen
Hemisphere left caudal point
Hemisphere left rostral point
Hemisphere right caudal point
Hemisphere right rostral point
Infracollicular Sulcus
Lateral ventricle left caudal point
Lateral ventricle left rostral point
Lateral ventricle right caudal point
Lateral ventricle right rostral point
PMJ
Posterior Commissure
Temporal lobe left rostral point
Temporal lobe ventricle right rostral point

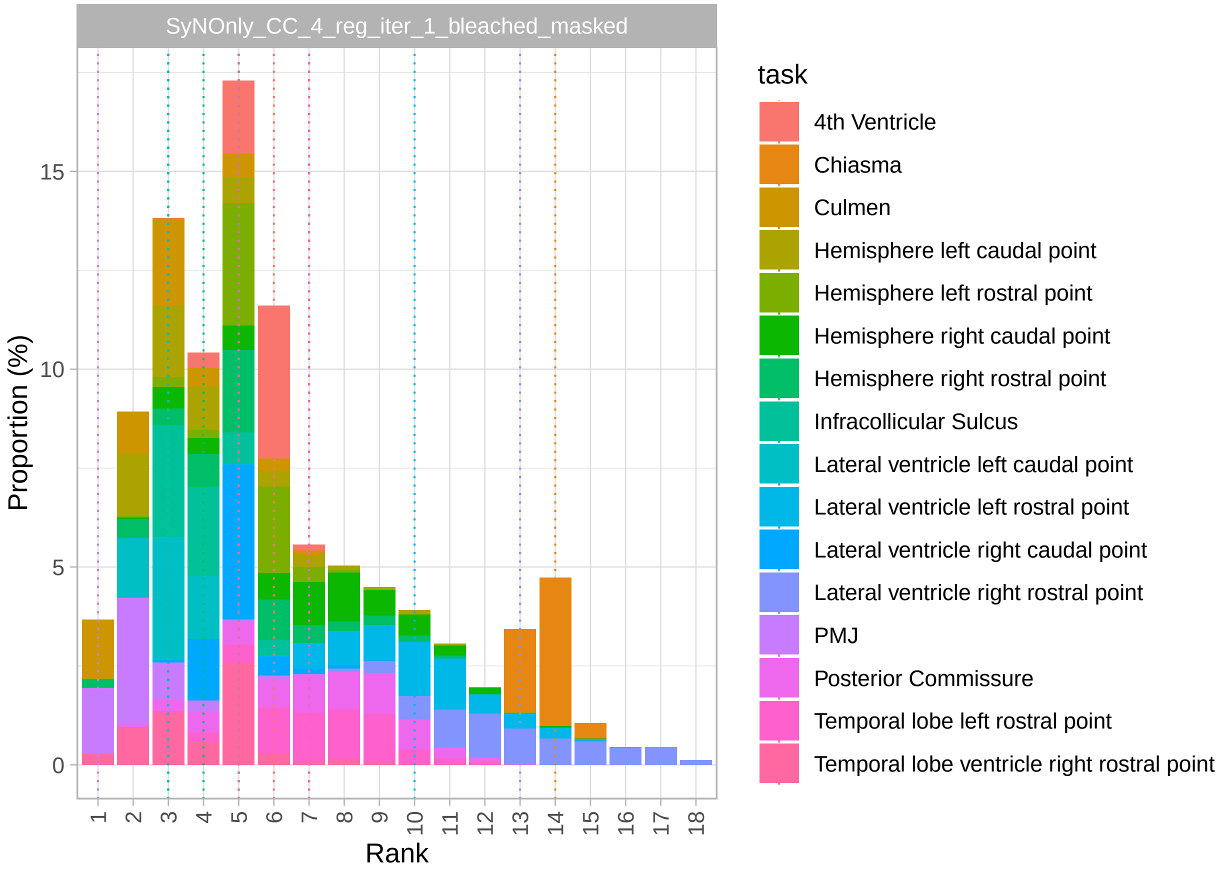

SyNOnly_CC_4_reg_iter_1_bleached_masked
Proportion (%)
0
5
10
15
1
2
3
4
5
6
7
8
9
10
11
12
13
14
15
16
17
18
Rank
task
4th Ventricle
Chiasma
Culmen
Hemisphere left caudal point
Hemisphere left rostral point
Hemisphere right caudal point
Hemisphere right rostral point
Infracollicular Sulcus
Lateral ventricle left caudal point
Lateral ventricle left rostral point
Lateral ventricle right caudal point
Lateral ventricle right rostral point
PMJ
Posterior Commissure
Temporal lobe left rostral point
Temporal lobe ventricle right rostral point

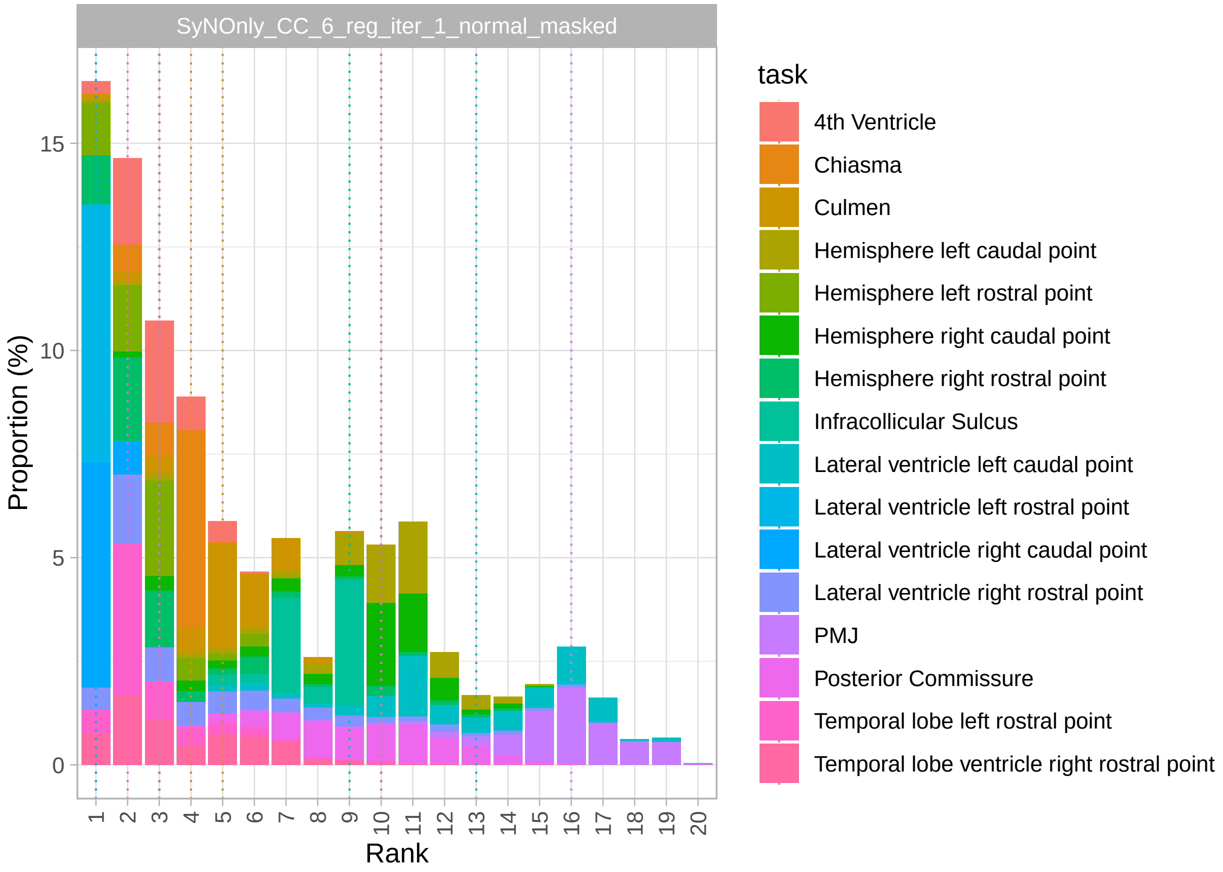

SyNOnly_CC_6_reg_iter_1_normal_masked
Proportion (%)
0
5
10
15
1
2
3
4
5
6
7
8
9
10
11
12
13
14
15
16
17
18
19
20
Rank
task
4th Ventricle
Chiasma
Culmen
Hemisphere left caudal point
Hemisphere left rostral point
Hemisphere right caudal point
Hemisphere right rostral point
Infracollicular Sulcus
Lateral ventricle left caudal point
Lateral ventricle left rostral point
Lateral ventricle right caudal point
Lateral ventricle right rostral point
PMJ
Posterior Commissure
Temporal lobe left rostral point
Temporal lobe ventricle right rostral point

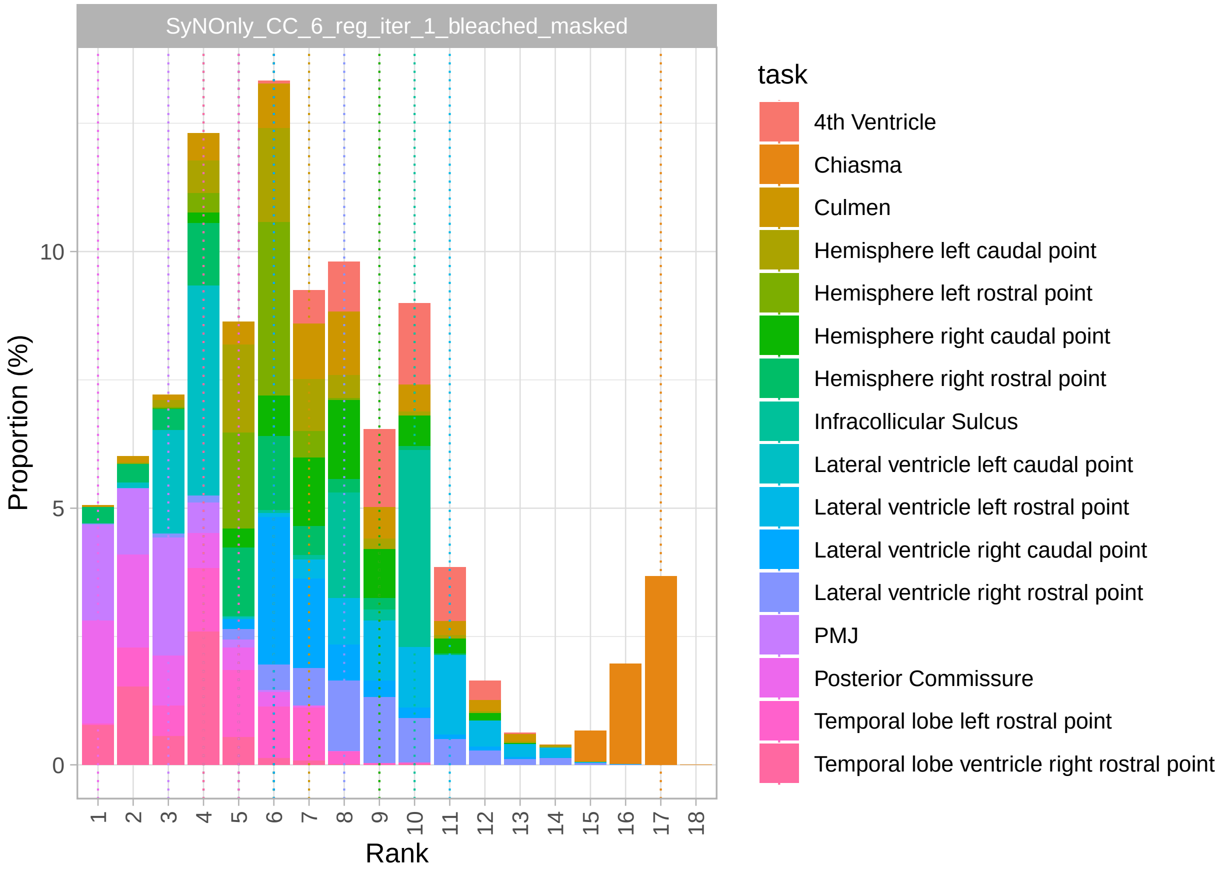
SyNOnly_CC_6_reg_iter_1_bleached_masked
Proportion (%)
0
5
10
1
2
3
4
5
6
7
8
9
10
11
12
13
14
15
16
17
18
Rank
task
4th Ventricle
Chiasma
Culmen
Hemisphere left caudal point
Hemisphere left rostral point
Hemisphere right caudal point
Hemisphere right rostral point
Infracollicular Sulcus
Lateral ventricle left caudal point
Lateral ventricle left rostral point
Lateral ventricle right caudal point
Lateral ventricle right rostral point
PMJ
Posterior Commissure
Temporal lobe left rostral point
Temporal lobe ventricle right rostral point

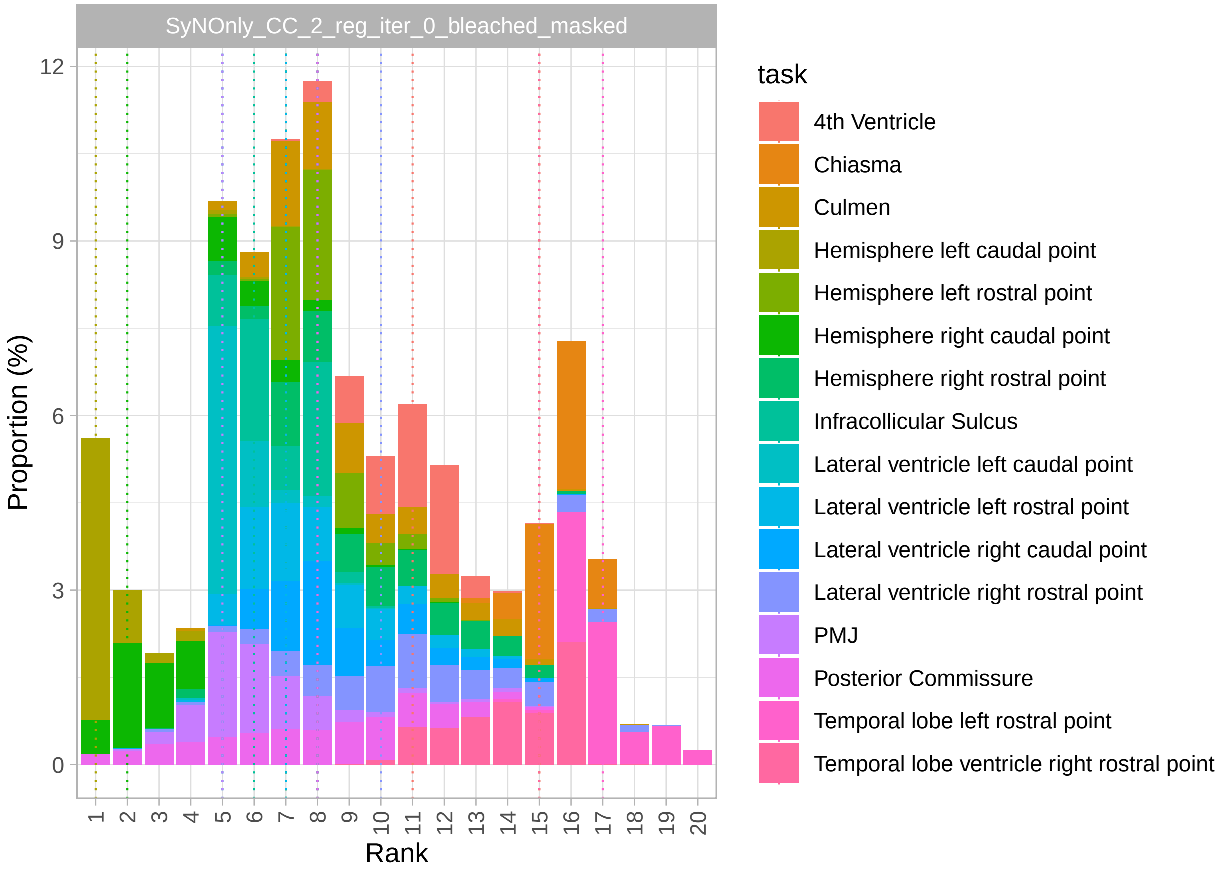

SyNOnly_CC_2_reg_iter_0_bleached_masked
Proportion (%)
0
3
6
9
12
1
2
3
4
5
6
7
8
9
10
11
12
13
14
15
16
17
18
19
20
Rank
task
4th Ventricle
Chiasma
Culmen
Hemisphere left caudal point
Hemisphere left rostral point
Hemisphere right caudal point
Hemisphere right rostral point
Infracollicular Sulcus
Lateral ventricle left caudal point
Lateral ventricle left rostral point
Lateral ventricle right caudal point
Lateral ventricle right rostral point
PMJ
Posterior Commissure
Temporal lobe left rostral point
Temporal lobe ventricle right rostral point

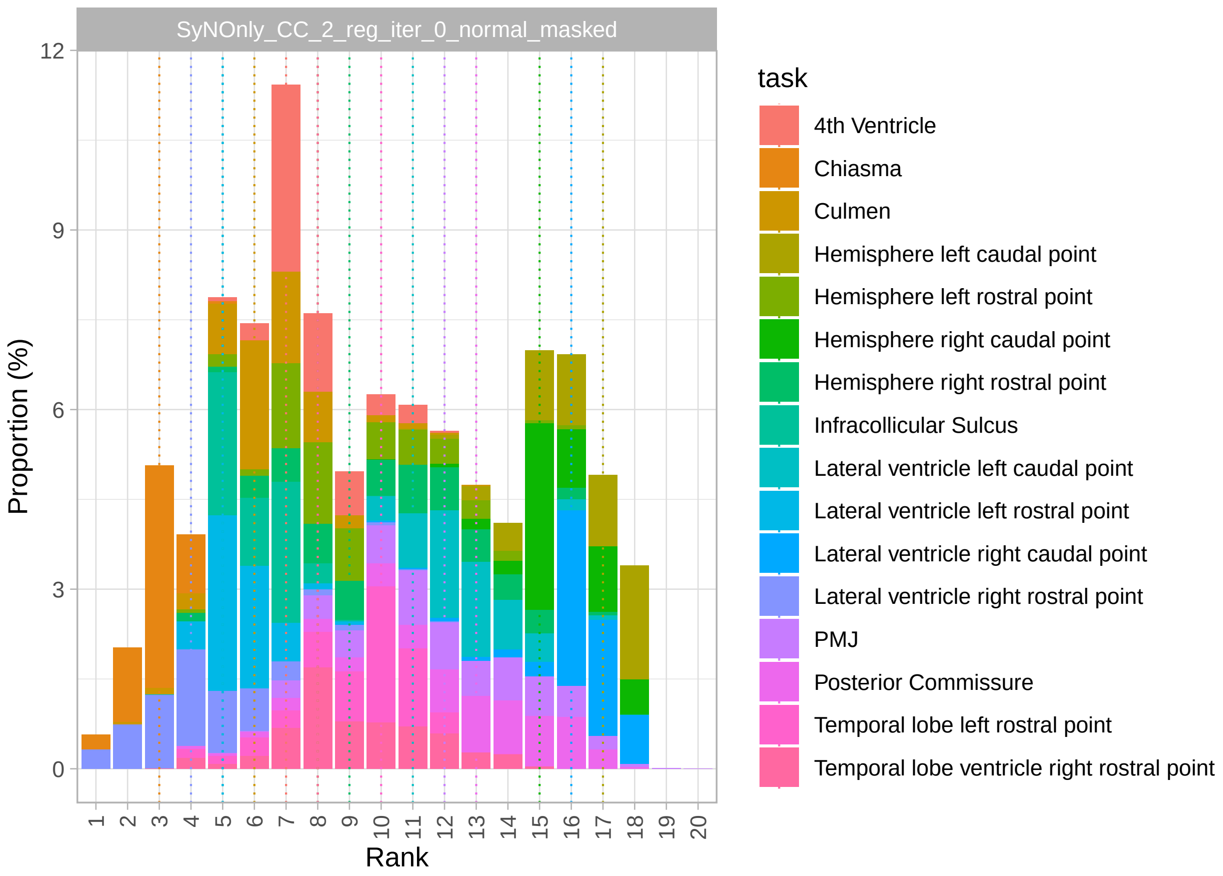
SyNOnly_CC_2_reg_iter_0_normal_masked
Proportion (%)
0
3
6
9
12
Rank
1
2
3
4
5
6
7
8
9
10
11
12
13
14
15
16
17
18
19
20
task
4th Ventricle
Chiasma
Culmen
Hemisphere left caudal point
Hemisphere left rostral point
Hemisphere right caudal point
Hemisphere right rostral point
Infracollicular Sulcus
Lateral ventricle left caudal point
Lateral ventricle left rostral point
Lateral ventricle right caudal point
Lateral ventricle right rostral point
PMJ
Posterior Commissure
Temporal lobe left rostral point
Temporal lobe ventricle right rostral point

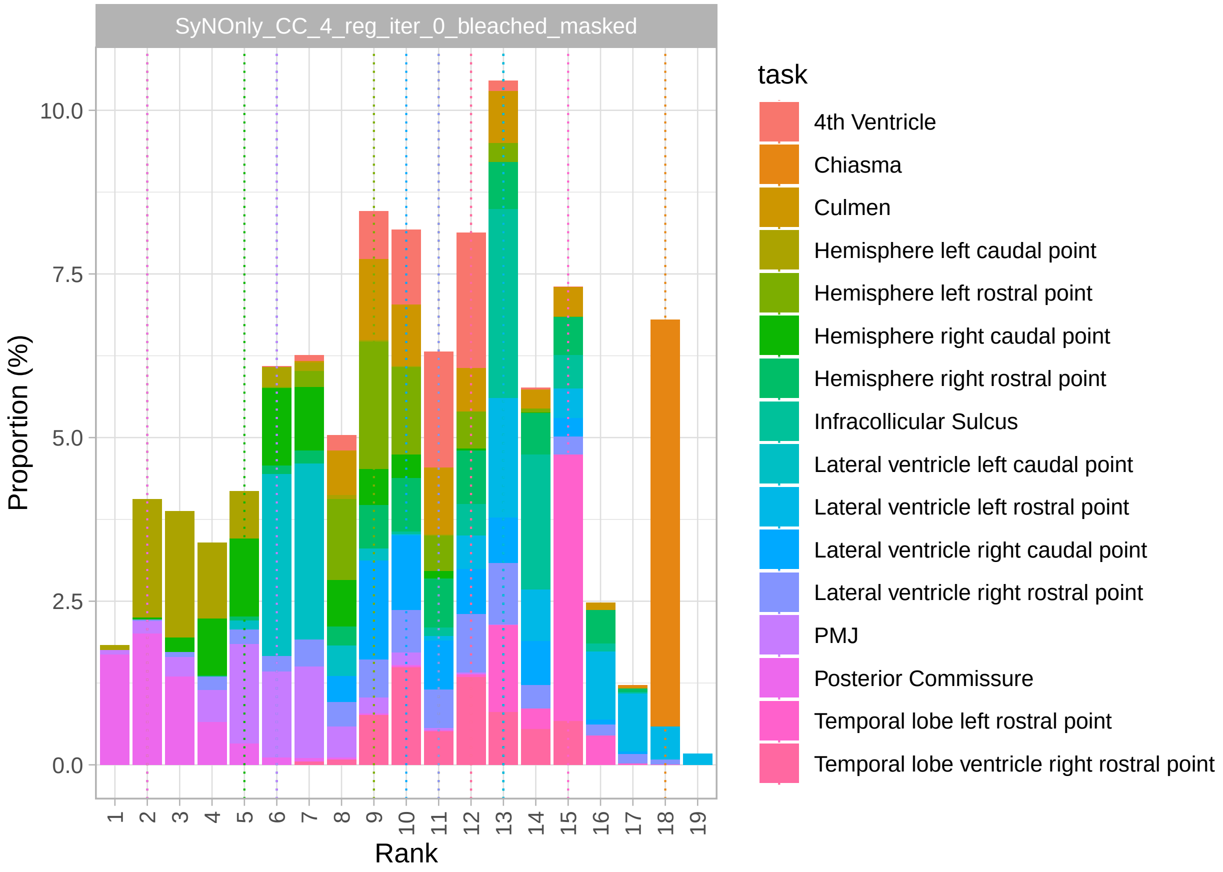

SyNOnly_CC_4_reg_iter_0_bleached_masked
Proportion (%)
0.0
2.5
5.0
7.5
10.0
1
2
3
4
5
6
7
8
9
10
11
12
13
14
15
16
17
18
19
Rank
task
4th Ventricle
Chiasma
Culmen
Hemisphere left caudal point
Hemisphere left rostral point
Hemisphere right caudal point
Hemisphere right rostral point
Infracollicular Sulcus
Lateral ventricle left caudal point
Lateral ventricle left rostral point
Lateral ventricle right caudal point
Lateral ventricle right rostral point
PMJ
Posterior Commissure
Temporal lobe left rostral point
Temporal lobe ventricle right rostral point

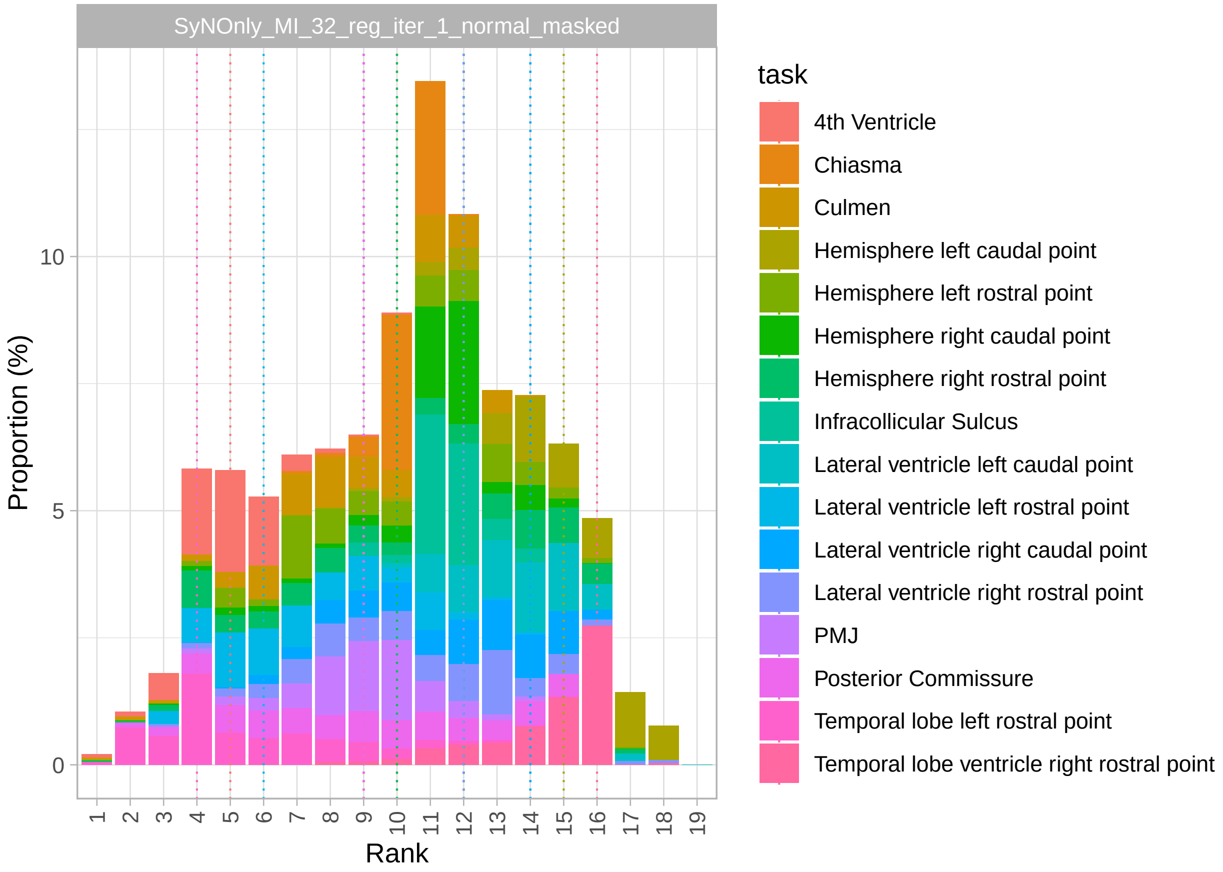
SyNOnly_MI_32_reg_iter_1_normal_masked
Proportion (%)
0
5
10
1
2
3
4
5
6
7
8
9
10
11
12
13
14
15
16
17
18
19
Rank
task
4th Ventricle
Chiasma
Culmen
Hemisphere left caudal point
Hemisphere left rostral point
Hemisphere right caudal point
Hemisphere right rostral point
Infracollicular Sulcus
Lateral ventricle left caudal point
Lateral ventricle left rostral point
Lateral ventricle right caudal point
Lateral ventricle right rostral point
PMJ
Posterior Commissure
Temporal lobe left rostral point
Temporal lobe ventricle right rostral point

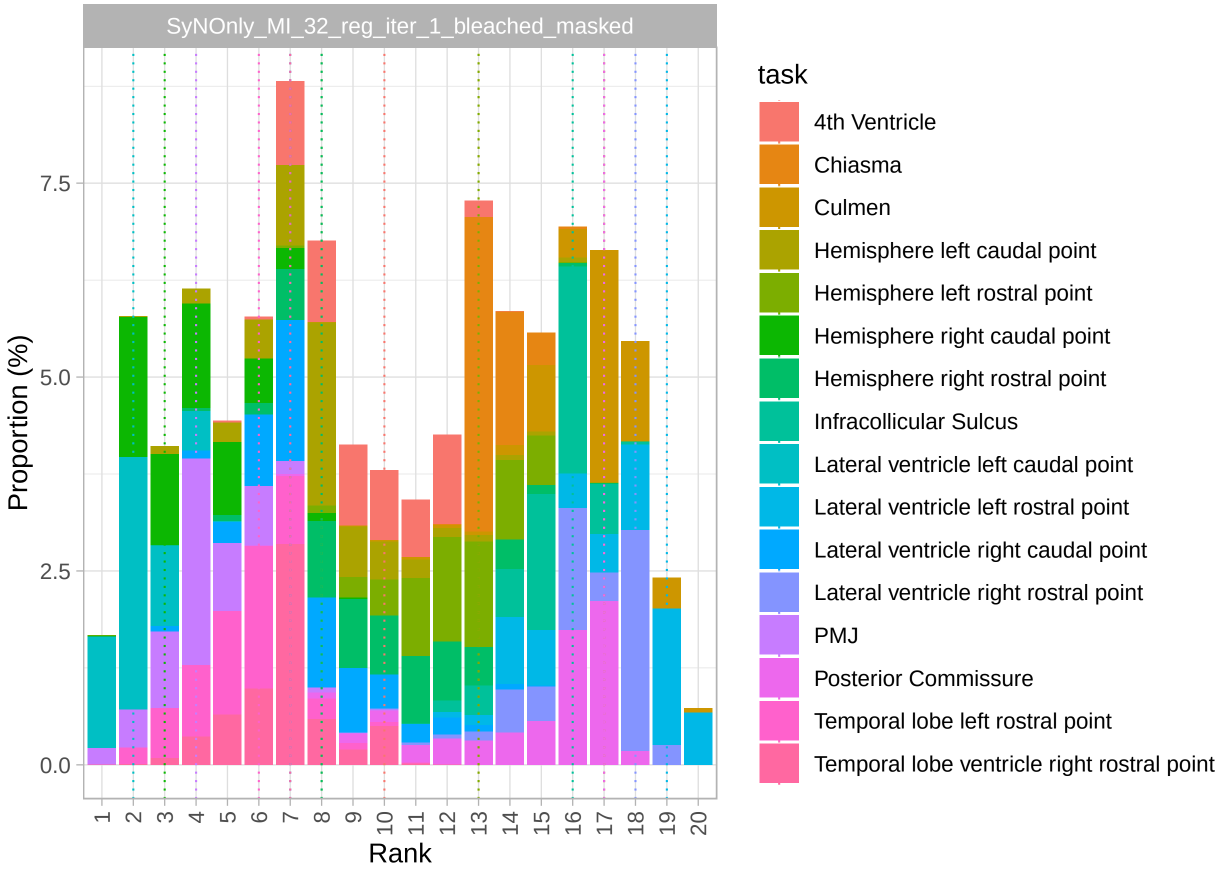
SyNOnly_MI_32_reg_iter_1_bleached_masked
Proportion (%)
0.0
2.5
5.0
7.5
Rank
1
2
3
4
5
6
7
8
9
10
11
12
13
14
15
16
17
18
19
20
task
4th Ventricle
Chiasma
Culmen
Hemisphere left caudal point
Hemisphere left rostral point
Hemisphere right caudal point
Hemisphere right rostral point
Infracollicular Sulcus
Lateral ventricle left caudal point
Lateral ventricle left rostral point
Lateral ventricle right caudal point
Lateral ventricle right rostral point
PMJ
Posterior Commissure
Temporal lobe left rostral point
Temporal lobe ventricle right rostral point

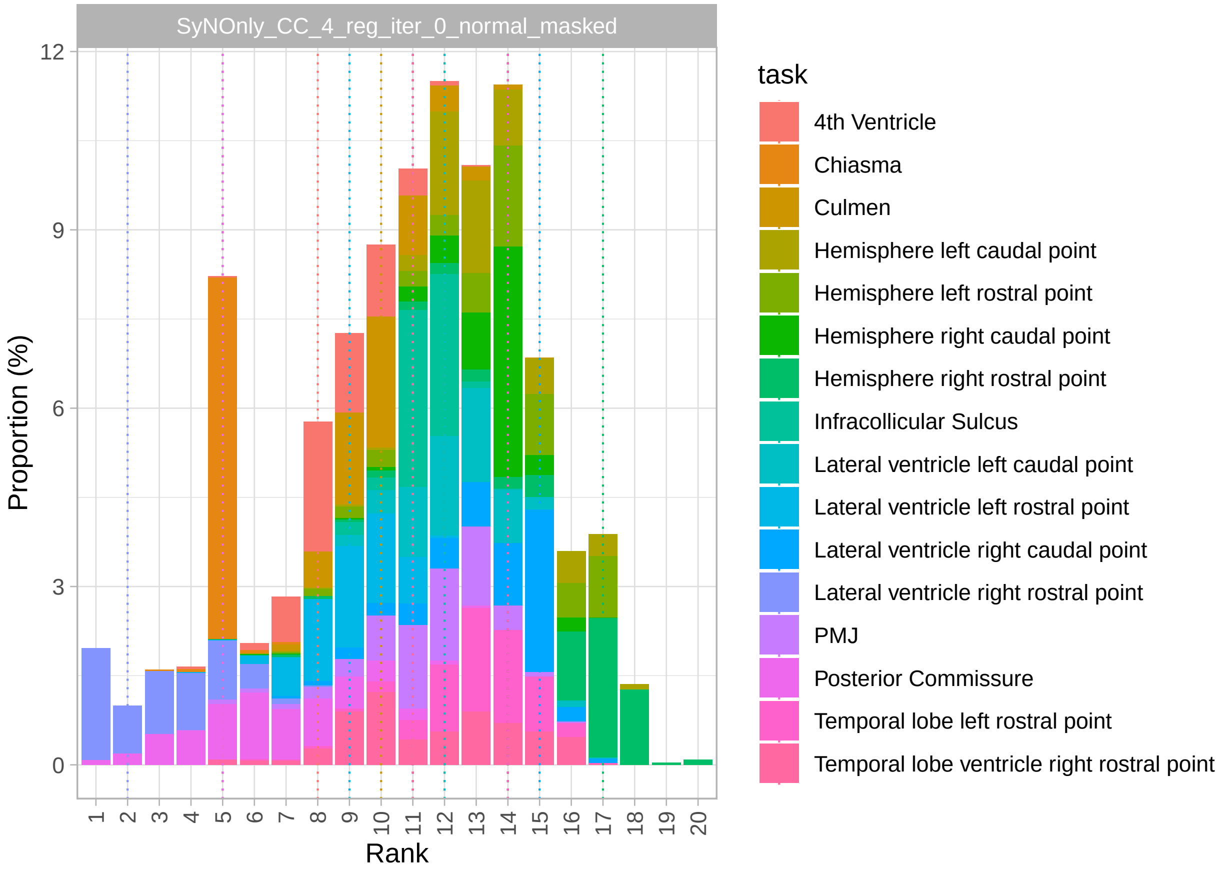

SyNOnly_CC_4_reg_iter_0_normal_masked
Proportion (%)
12
9
6
3
0
1
2
3
4
5
6
7
8
9
10
11
12
13
14
15
16
17
18
19
20
Rank
task
4th Ventricle
Chiasma
Culmen
Hemisphere left caudal point
Hemisphere left rostral point
Hemisphere right caudal point
Hemisphere right rostral point
Infracollicular Sulcus
Lateral ventricle left caudal point
Lateral ventricle left rostral point
Lateral ventricle right caudal point
Lateral ventricle right rostral point
PMJ
Posterior Commissure
Temporal lobe left rostral point
Temporal lobe ventricle right rostral point

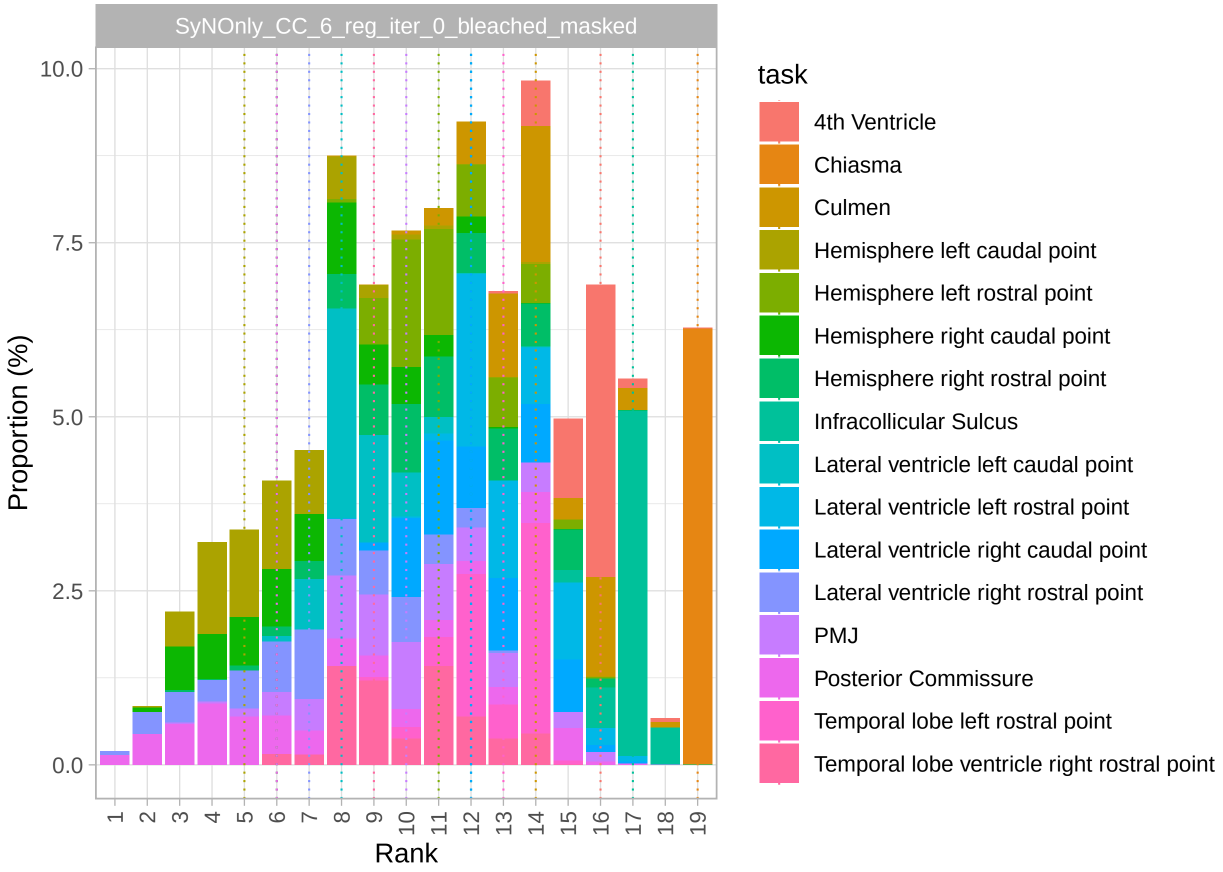

SyNOnly_CC_6_reg_iter_0_bleached_masked
Proportion (%)
10.0
7.5
5.0
2.5
0.0
Rank
1
2
3
4
5
6
7
8
9
10
11
12
13
14
15
16
17
18
19
task
4th Ventricle
Chiasma
Culmen
Hemisphere left caudal point
Hemisphere left rostral point
Hemisphere right caudal point
Hemisphere right rostral point
Infracollicular Sulcus
Lateral ventricle left caudal point
Lateral ventricle left rostral point
Lateral ventricle right caudal point
Lateral ventricle right rostral point
PMJ
Posterior Commissure
Temporal lobe left rostral point
Temporal lobe ventricle right rostral point

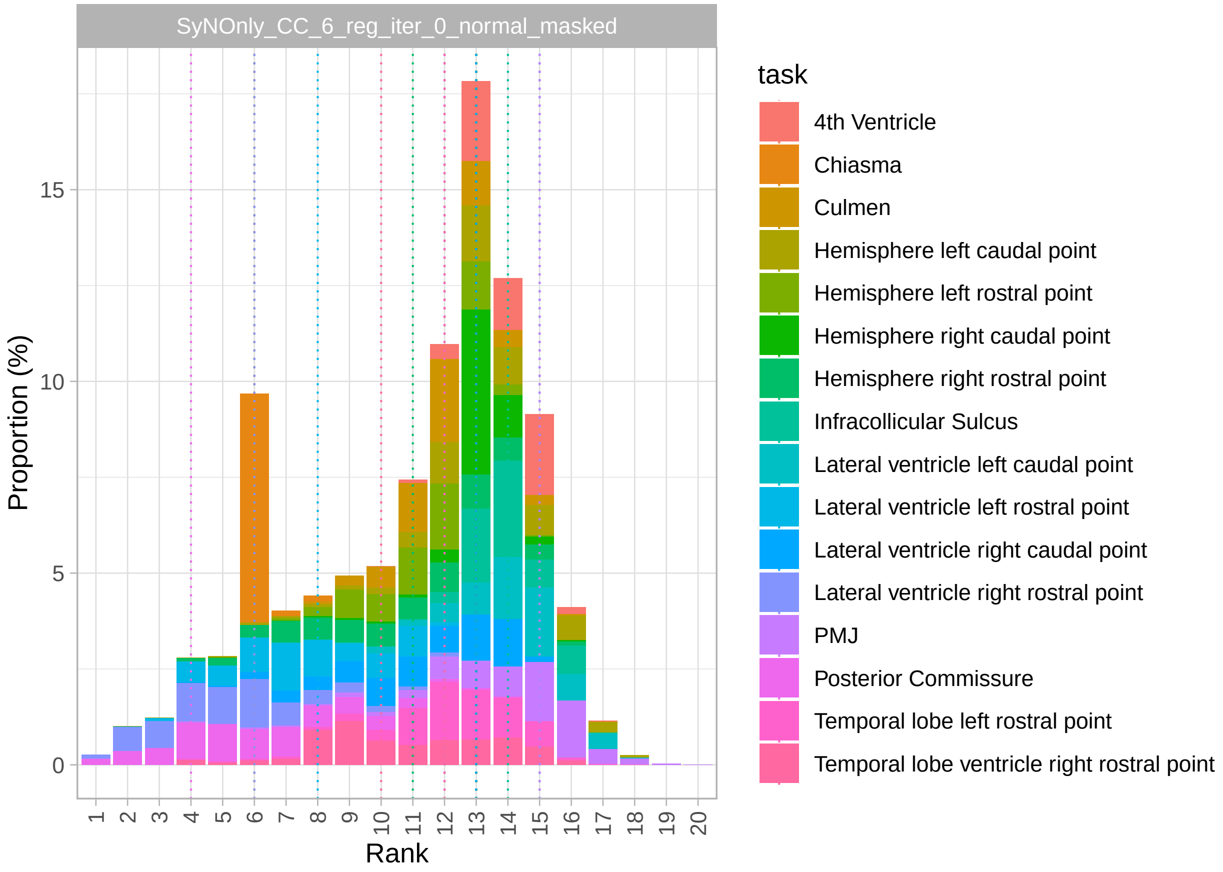

SyNOnly_CC_6_reg_iter_0_normal_masked
Proportion (%)
0
5
10
15
1
2
3
4
5
6
7
8
9
10
11
12
13
14
15
16
17
18
19
20
Rank
task
4th Ventricle
Chiasma
Culmen
Hemisphere left caudal point
Hemisphere left rostral point
Hemisphere right caudal point
Hemisphere right rostral point
Infracollicular Sulcus
Lateral ventricle left caudal point
Lateral ventricle left rostral point
Lateral ventricle right caudal point
Lateral ventricle right rostral point
PMJ
Posterior Commissure
Temporal lobe left rostral point
Temporal lobe ventricle right rostral point

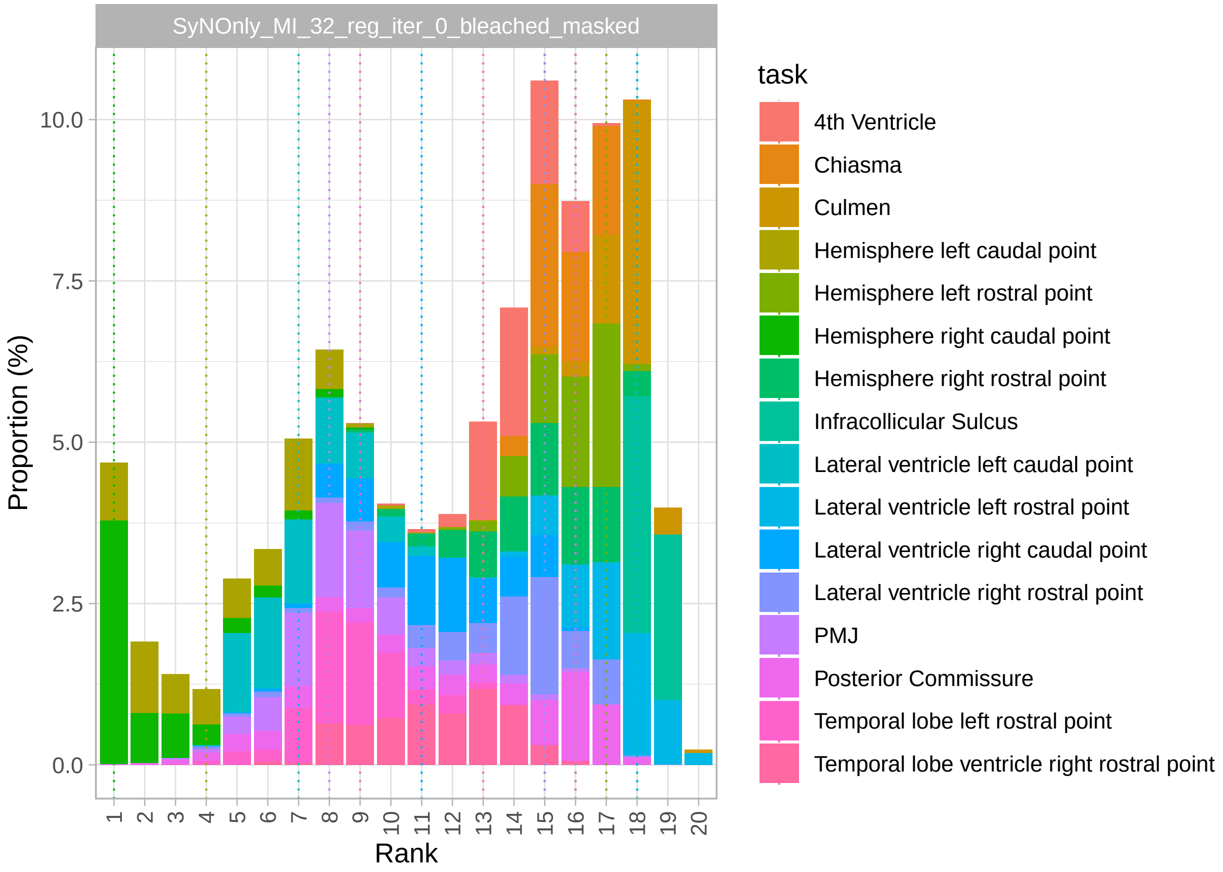

SyNOnly_MI_32_reg_iter_0_bleached_masked
Proportion (%)
0.0
2.5
5.0
7.5
10.0
1
2
3
4
5
6
7
8
9
10
11
12
13
14
15
16
17
18
19
20
Rank
task
4th Ventricle
Chiasma
Culmen
Hemisphere left caudal point
Hemisphere left rostral point
Hemisphere right caudal point
Hemisphere right rostral point
Infracollicular Sulcus
Lateral ventricle left caudal point
Lateral ventricle left rostral point
Lateral ventricle right caudal point
Lateral ventricle right rostral point
PMJ
Posterior Commissure
Temporal lobe left rostral point
Temporal lobe ventricle right rostral point

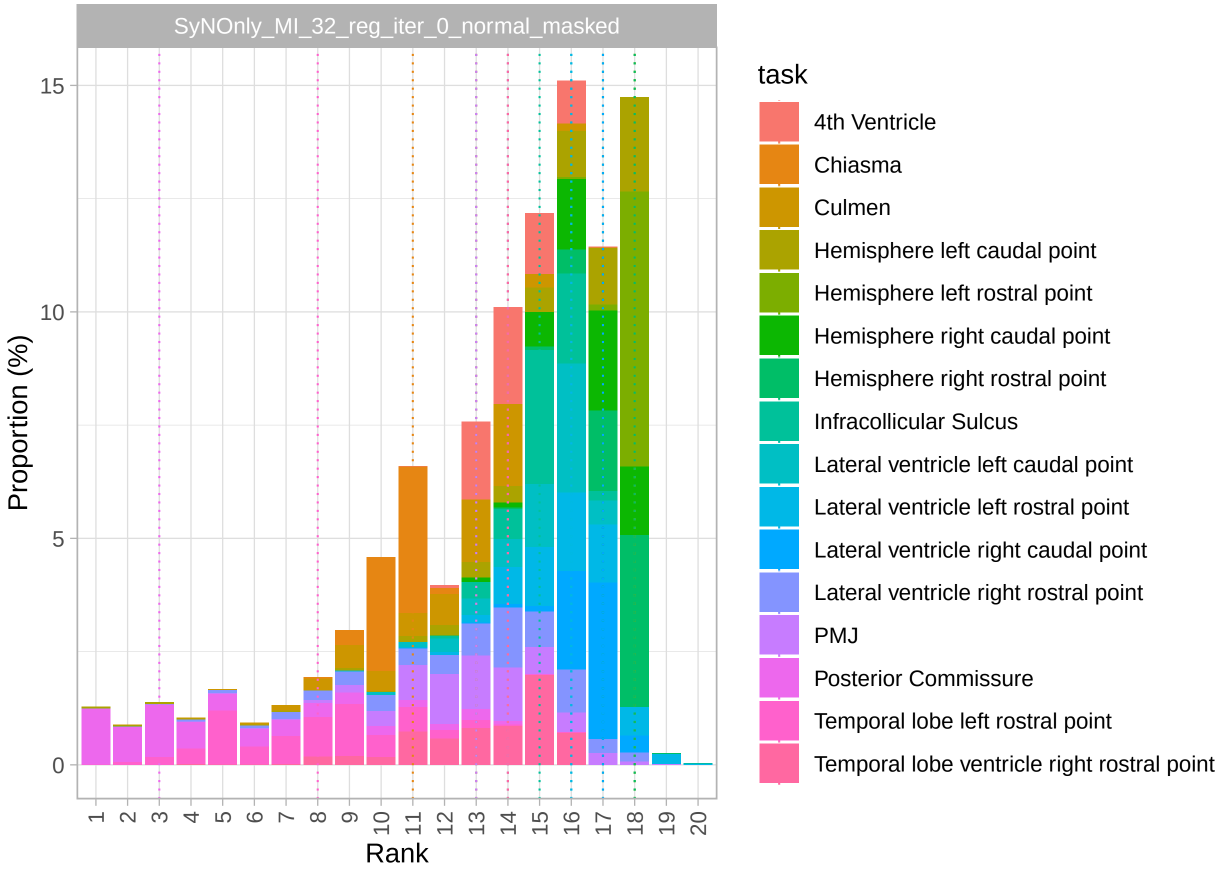
SyNOnly_MI_32_reg_iter_0_normal_masked
Proportion (%)
0
5
10
15
1
2
3
4
5
6
7
8
9
10
11
12
13
14
15
16
17
18
19
20
Rank
task
4th Ventricle
Chiasma
Culmen
Hemisphere left caudal point
Hemisphere left rostral point
Hemisphere right caudal point
Hemisphere right rostral point
Infracollicular Sulcus
Lateral ventricle left caudal point
Lateral ventricle left rostral point
Lateral ventricle right caudal point
Lateral ventricle right rostral point
PMJ
Posterior Commissure
Temporal lobe left rostral point
Temporal lobe ventricle right rostral point

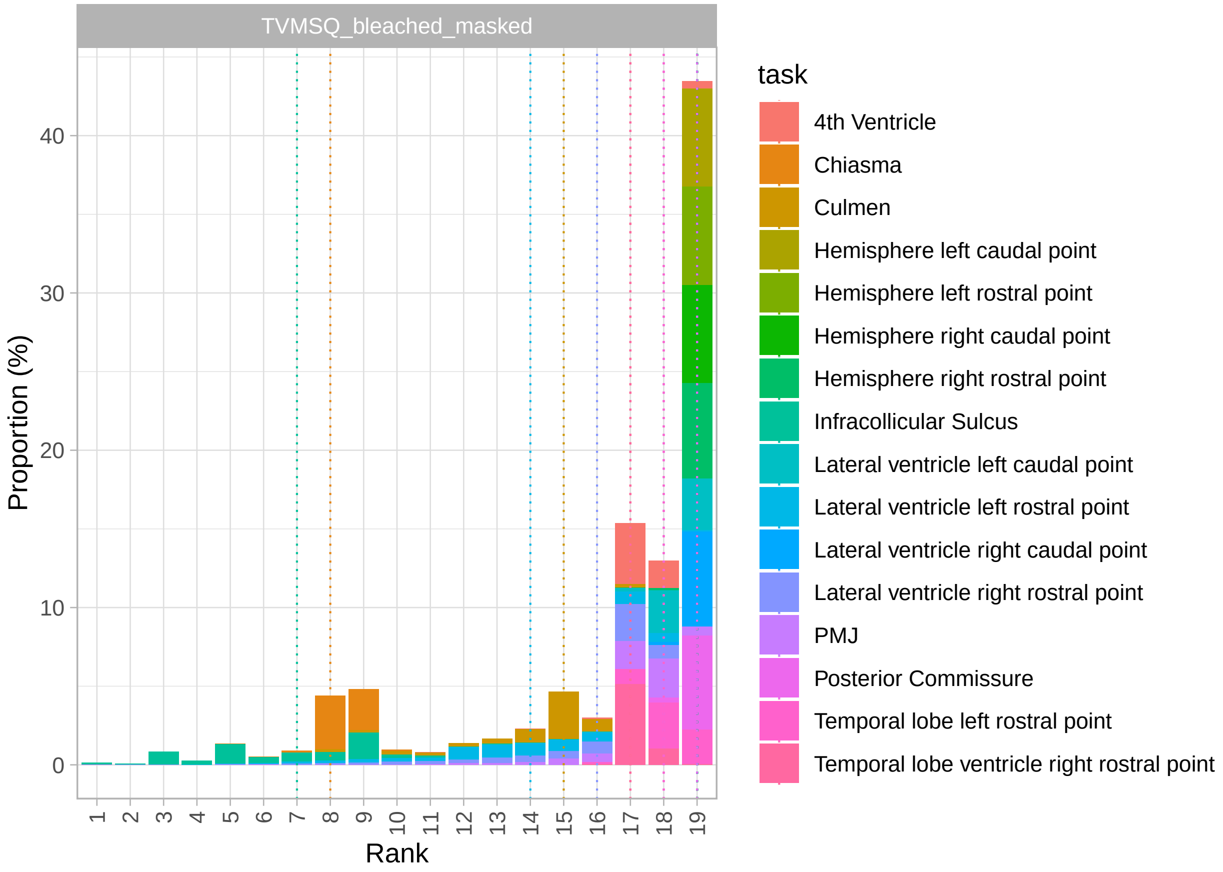

TVMSQ_bleached_masked
Proportion (%)
0
10
20
30
40
Rank
1
2
3
4
5
6
7
8
9
10
11
12
13
14
15
16
17
18
19
task
4th Ventricle
Chiasma
Culmen
Hemisphere left caudal point
Hemisphere left rostral point
Hemisphere right caudal point
Hemisphere right rostral point
Infracollicular Sulcus
Lateral ventricle left caudal point
Lateral ventricle left rostral point
Lateral ventricle right caudal point
Lateral ventricle right rostral point
PMJ
Posterior Commissure
Temporal lobe left rostral point
Temporal lobe ventricle right rostral point

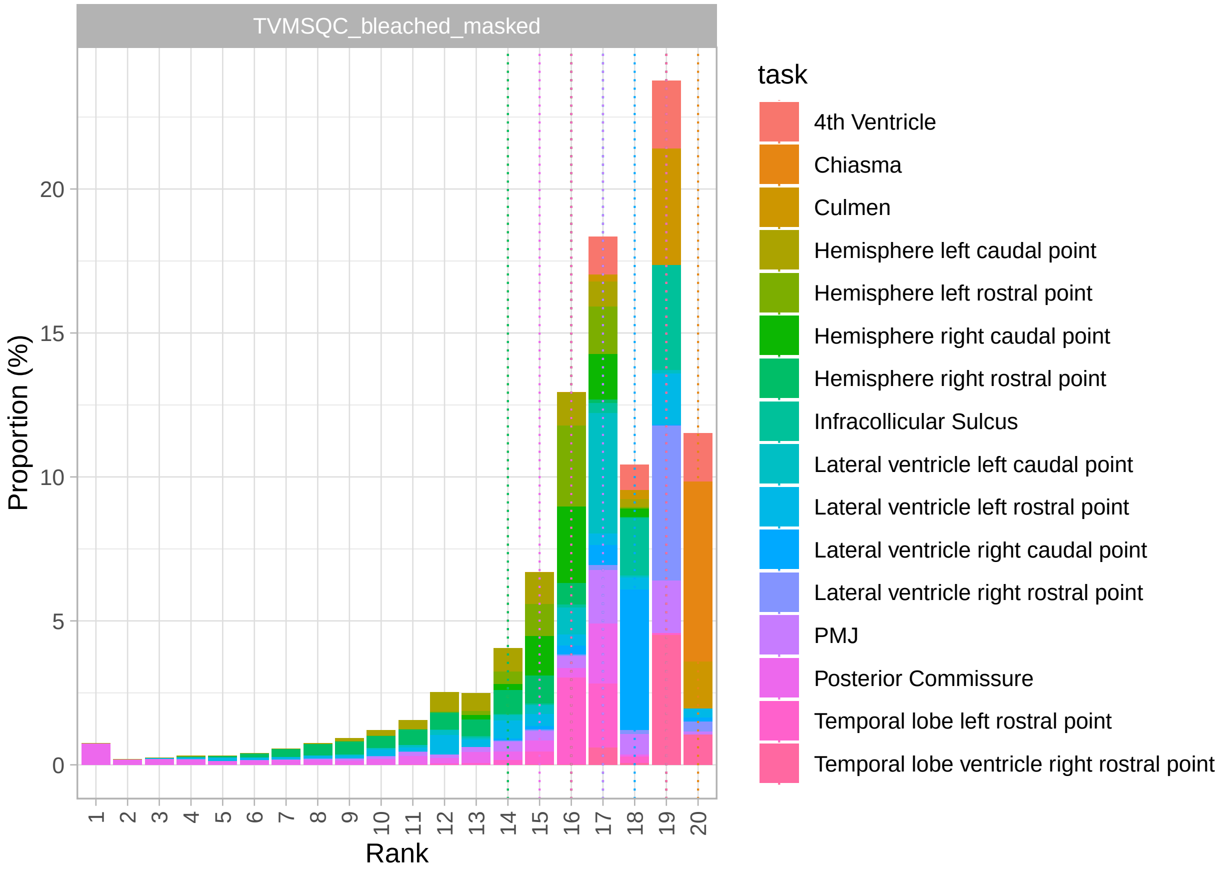
TVMSQC_bleached_masked
Proportion (%)
0
5
10
15
20
Rank
1
2
3
4
5
6
7
8
9
10
11
12
13
14
15
16
17
18
19
20
task
4th Ventricle
Chiasma
Culmen
Hemisphere left caudal point
Hemisphere left rostral point
Hemisphere right caudal point
Hemisphere right rostral point
Infracollicular Sulcus
Lateral ventricle left caudal point
Lateral ventricle left rostral point
Lateral ventricle right caudal point
Lateral ventricle right rostral point
PMJ
Posterior Commissure
Temporal lobe left rostral point
Temporal lobe ventricle right rostral point

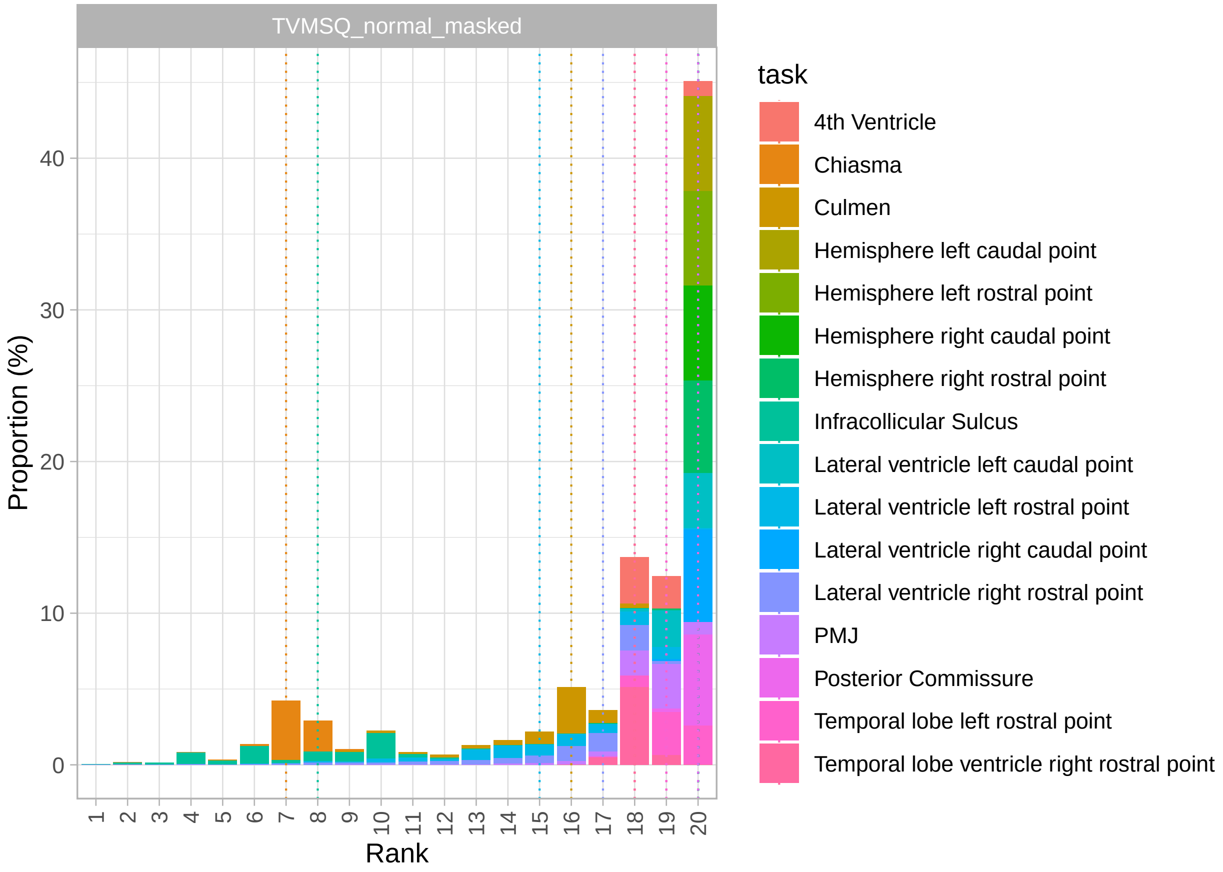
TVMSQ_normal_masked
Proportion (%)
Rank
task
4th Ventricle
Chiasma
Culmen
Hemisphere left caudal point
Hemisphere left rostral point
Hemisphere right caudal point
Hemisphere right rostral point
Infracollicular Sulcus
Lateral ventricle left caudal point
Lateral ventricle left rostral point
Lateral ventricle right caudal point
Lateral ventricle right rostral point
PMJ
Posterior Commissure
Temporal lobe left rostral point
Temporal lobe ventricle right rostral point

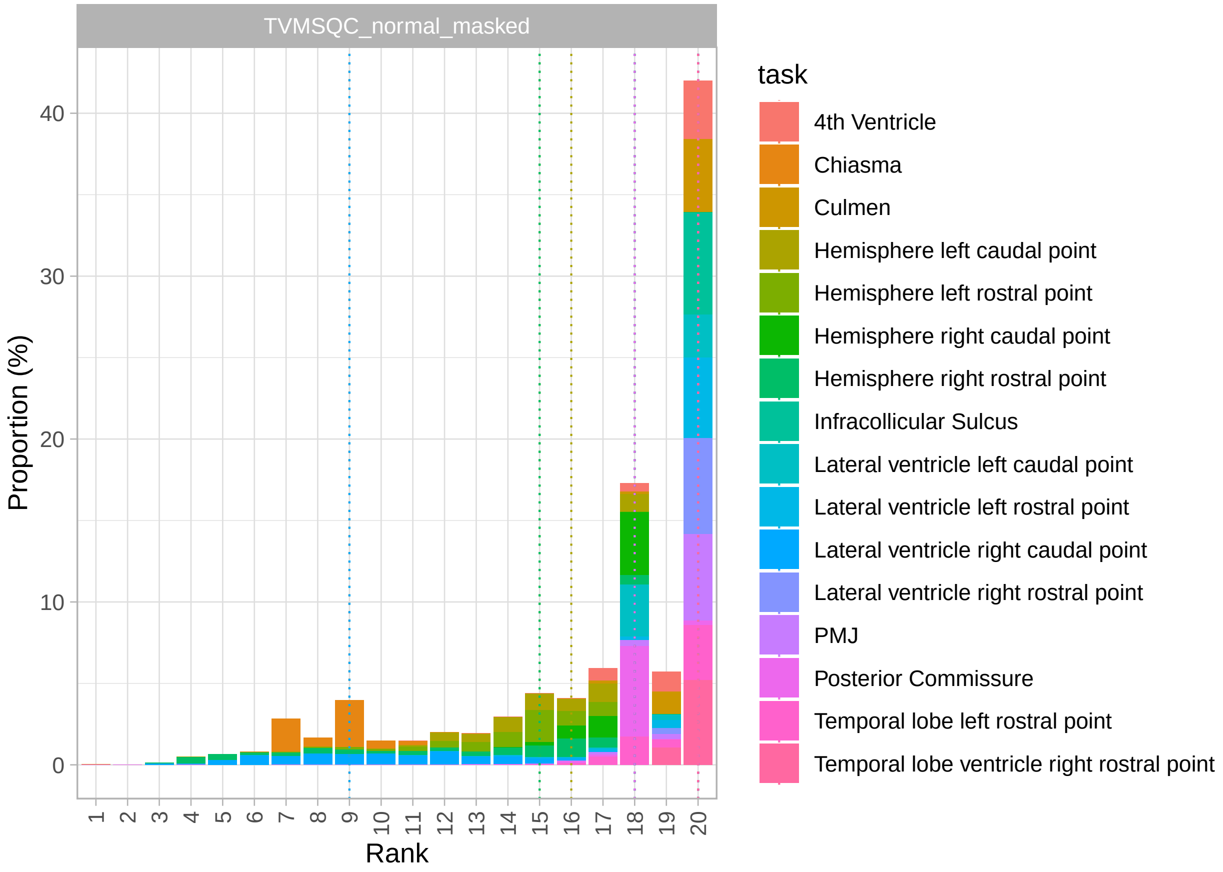
TVMSQC_normal_masked
Proportion (%)
0
10
20
30
40
1
2
3
4
5
6
7
8
9
10
11
12
13
14
15
16
17
18
19
20
Rank
task
4th Ventricle
Chiasma
Culmen
Hemisphere left caudal point
Hemisphere left rostral point
Hemisphere right caudal point
Hemisphere right rostral point
Infracollicular Sulcus
Lateral ventricle left caudal point
Lateral ventricle left rostral point
Lateral ventricle right caudal point
Lateral ventricle right rostral point
PMJ
Posterior Commissure
Temporal lobe left rostral point
Temporal lobe ventricle right rostral point

4.2 Characterization of tasks

4.2.1 Visualizing bootstrap results

To investigate which tasks separate algorithms well (i.e., lead to a stable ranking), a blob plot is recommended.

Bootstrap results can be shown in a blob plot showing one plot for each task. In this view, the spread of the blobs for each algorithm can be compared across tasks. Deviations from the diagonal indicate deviations from the consensus ranking (over tasks). Specifically, if rank distribution of an algorithm is consistently below the diagonal, the algorithm performed better in this task than on average across tasks, while if the rank distribution of an algorithm is consistently above the diagonal, the algorithm performed worse in this task than on average across tasks. At the bottom of each panel, ranks for each algorithm in the tasks are provided.

Same as in Section 3.1 but now ordered according to consensus.

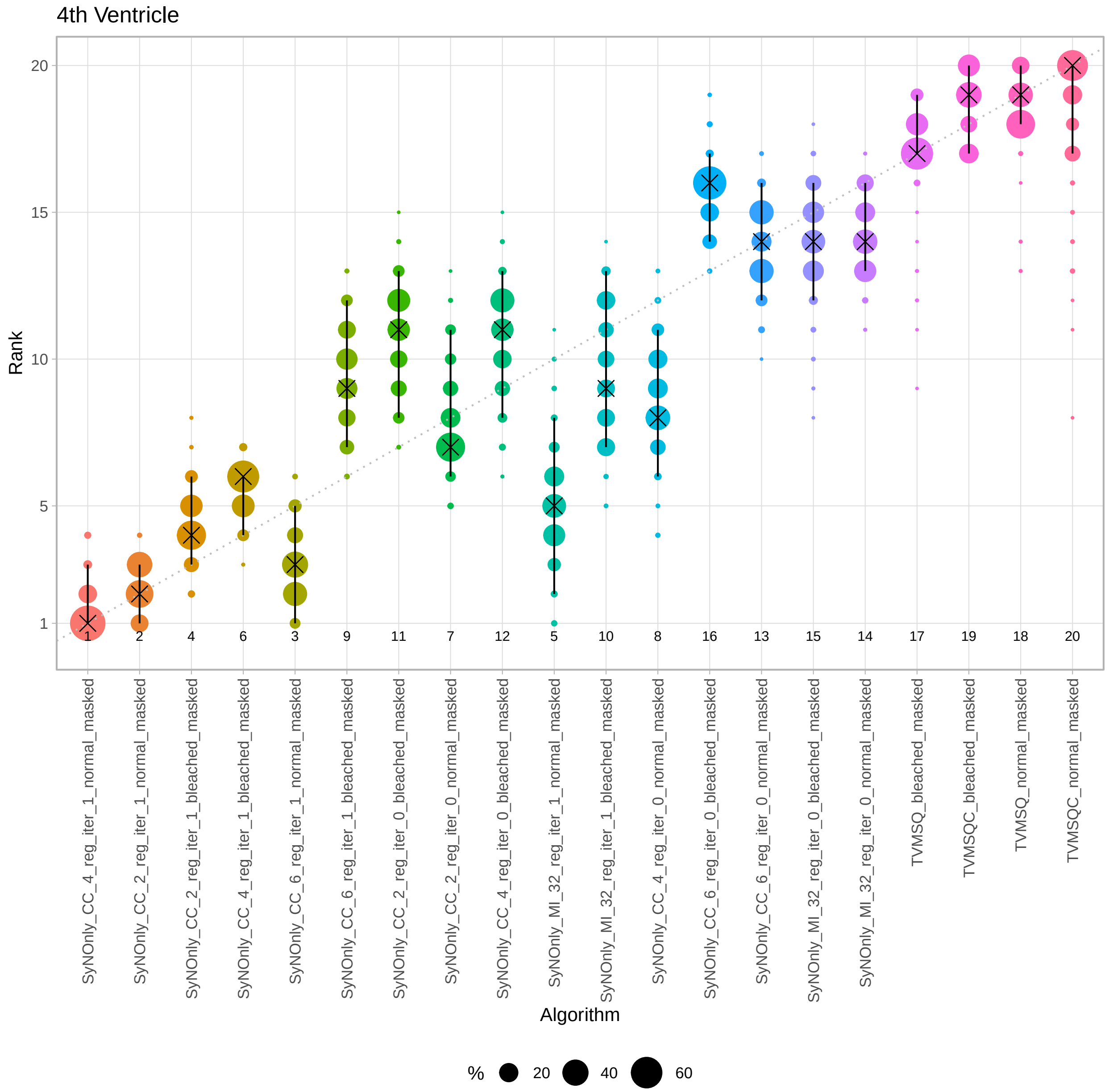

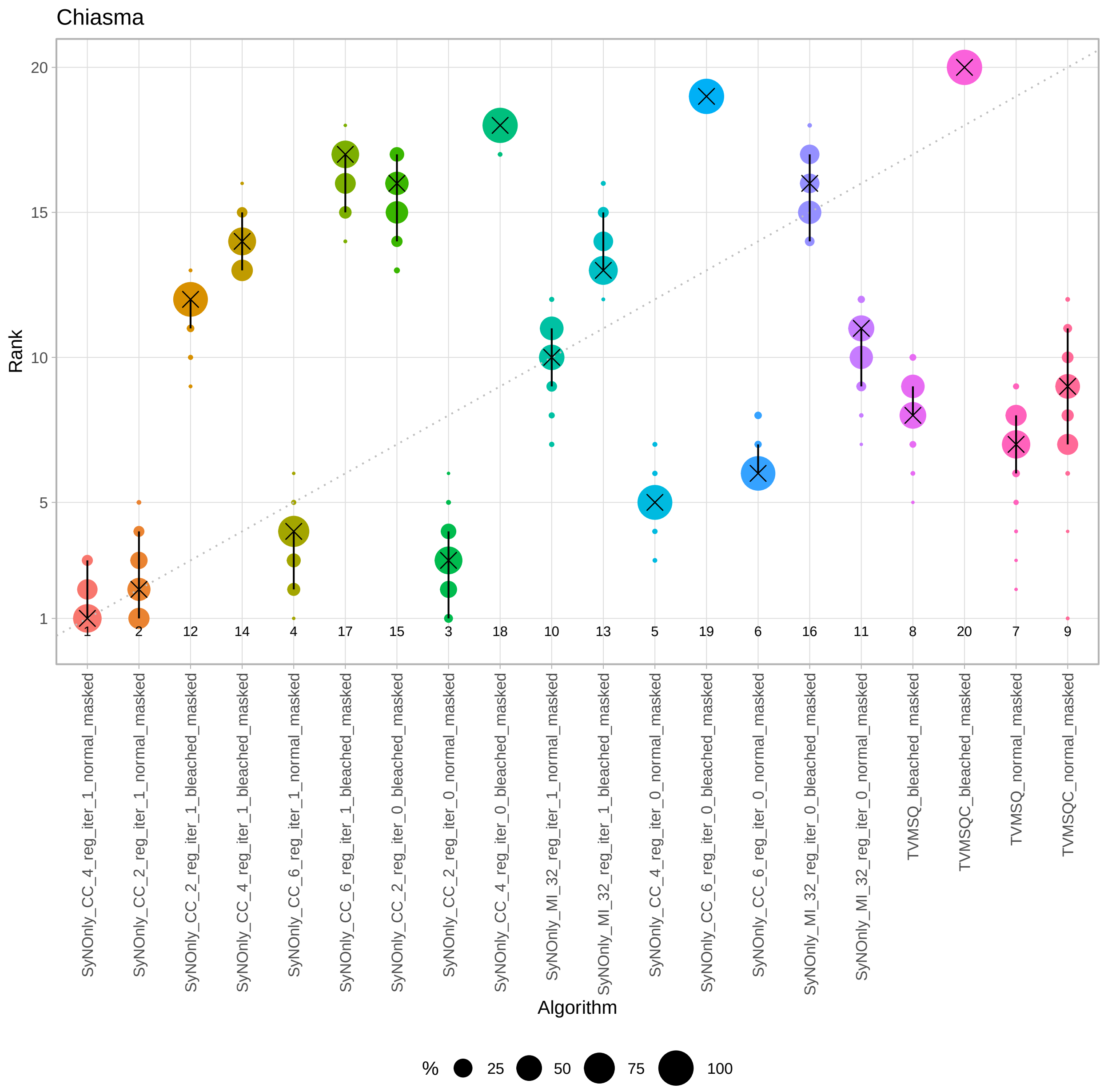
Chiasma
Rank
Algorithm
SyNOnly_CC_4_reg_iter_1_normal_masked
SyNOnly_CC_2_reg_iter_1_normal_masked
SyNOnly_CC_2_reg_iter_1_bleached_masked
SyNOnly_CC_4_reg_iter_1_bleached_masked
SyNOnly_CC_6_reg_iter_1_normal_masked
SyNOnly_CC_6_reg_iter_1_bleached_masked
SyNOnly_CC_2_reg_iter_0_bleached_masked
SyNOnly_CC_2_reg_iter_0_normal_masked
SyNOnly_CC_4_reg_iter_0_bleached_masked
SyNOnly_MI_32_reg_iter_1_normal_masked
SyNOnly_MI_32_reg_iter_1_bleached_masked
SyNOnly_CC_4_reg_iter_0_normal_masked
SyNOnly_CC_6_reg_iter_0_bleached_masked
SyNOnly_CC_6_reg_iter_0_normal_masked
SyNOnly_MI_32_reg_iter_0_bleached_masked
SyNOnly_MI_32_reg_iter_0_normal_masked
TVMSQ_bleached_masked
TVMSQC_bleached_masked
TVMSQ_normal_masked
TVMSQC_normal_masked
% 25 50 75 100

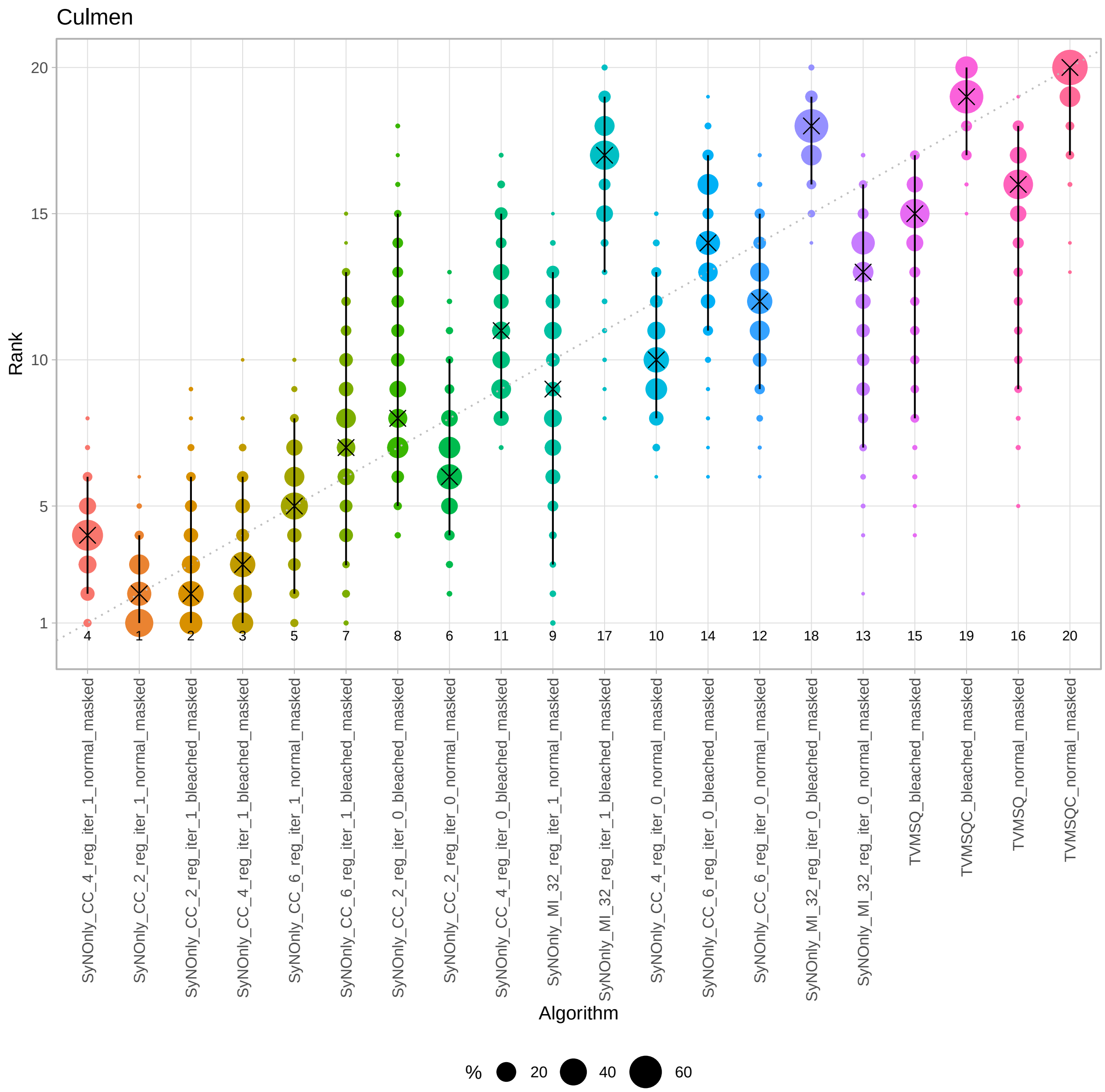

Culmen
Rank
Algorithm
%
20
40
60
4
1
2
3
5
7
8
6
11
9
17
10
14
12
18
13
15
19
16
20
SyNOnly_CC_4_reg_iter_1_normal_masked
SyNOnly_CC_2_reg_iter_1_normal_masked
SyNOnly_CC_2_reg_iter_1_bleached_masked
SyNOnly_CC_4_reg_iter_1_bleached_masked
SyNOnly_CC_6_reg_iter_1_normal_masked
SyNOnly_CC_6_reg_iter_1_bleached_masked
SyNOnly_CC_2_reg_iter_0_bleached_masked
SyNOnly_CC_2_reg_iter_0_normal_masked
SyNOnly_CC_4_reg_iter_0_bleached_masked
SyNOnly_MI_32_reg_iter_1_normal_masked
SyNOnly_MI_32_reg_iter_1_bleached_masked
SyNOnly_CC_4_reg_iter_0_normal_masked
SyNOnly_CC_6_reg_iter_0_bleached_masked
SyNOnly_CC_6_reg_iter_0_normal_masked
SyNOnly_MI_32_reg_iter_0_bleached_masked
SyNOnly_MI_32_reg_iter_0_normal_masked
TVMSQ_bleached_masked
TVMSQC_bleached_masked
TVMSQ_normal_masked
TVMSQC_normal_masked

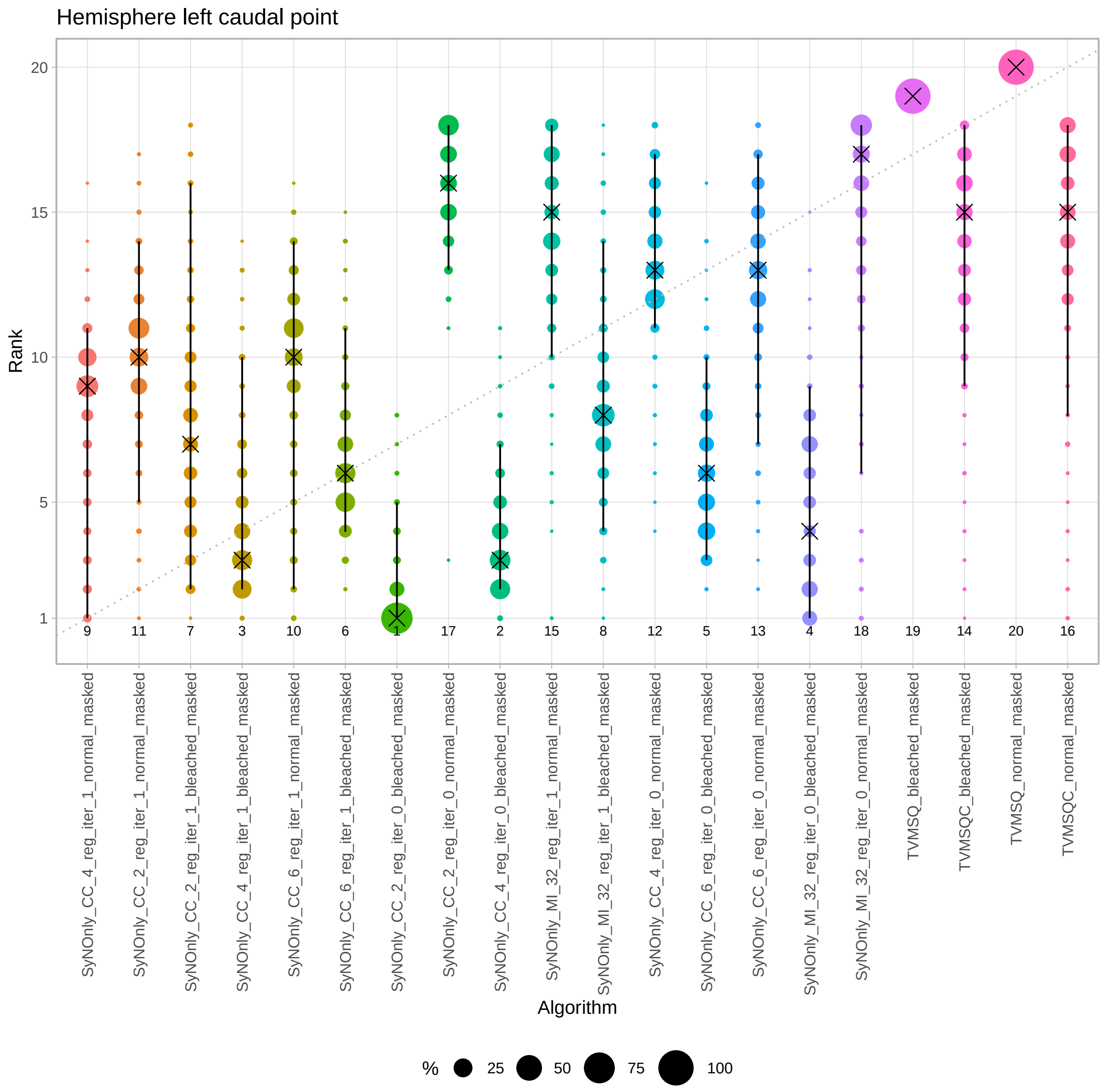

Hemisphere left caudal point
Rank
20
15
10
5
1
9
11
7
3
10
6
1
17
2
15
8
12
5
13
4
18
19
14
20
16
SyNOnly_CC_4_reg_iter_1_normal_masked
SyNOnly_CC_2_reg_iter_1_normal_masked
SyNOnly_CC_2_reg_iter_1_bleached_masked
SyNOnly_CC_4_reg_iter_1_bleached_masked
SyNOnly_CC_6_reg_iter_1_normal_masked
SyNOnly_CC_6_reg_iter_1_bleached_masked
SyNOnly_CC_2_reg_iter_0_bleached_masked
SyNOnly_CC_2_reg_iter_0_normal_masked
SyNOnly_CC_4_reg_iter_0_bleached_masked
SyNOnly_MI_32_reg_iter_1_normal_masked
SyNOnly_MI_32_reg_iter_1_bleached_masked
SyNOnly_CC_4_reg_iter_0_normal_masked
SyNOnly_CC_6_reg_iter_0_bleached_masked
SyNOnly_CC_6_reg_iter_0_normal_masked
SyNOnly_MI_32_reg_iter_0_bleached_masked
SyNOnly_MI_32_reg_iter_0_normal_masked
TVMSQ_bleached_masked
TVMSQC_bleached_masked
TVMSQ_normal_masked
TVMSQC_normal_masked
Algorithm
%
25
50
75
100

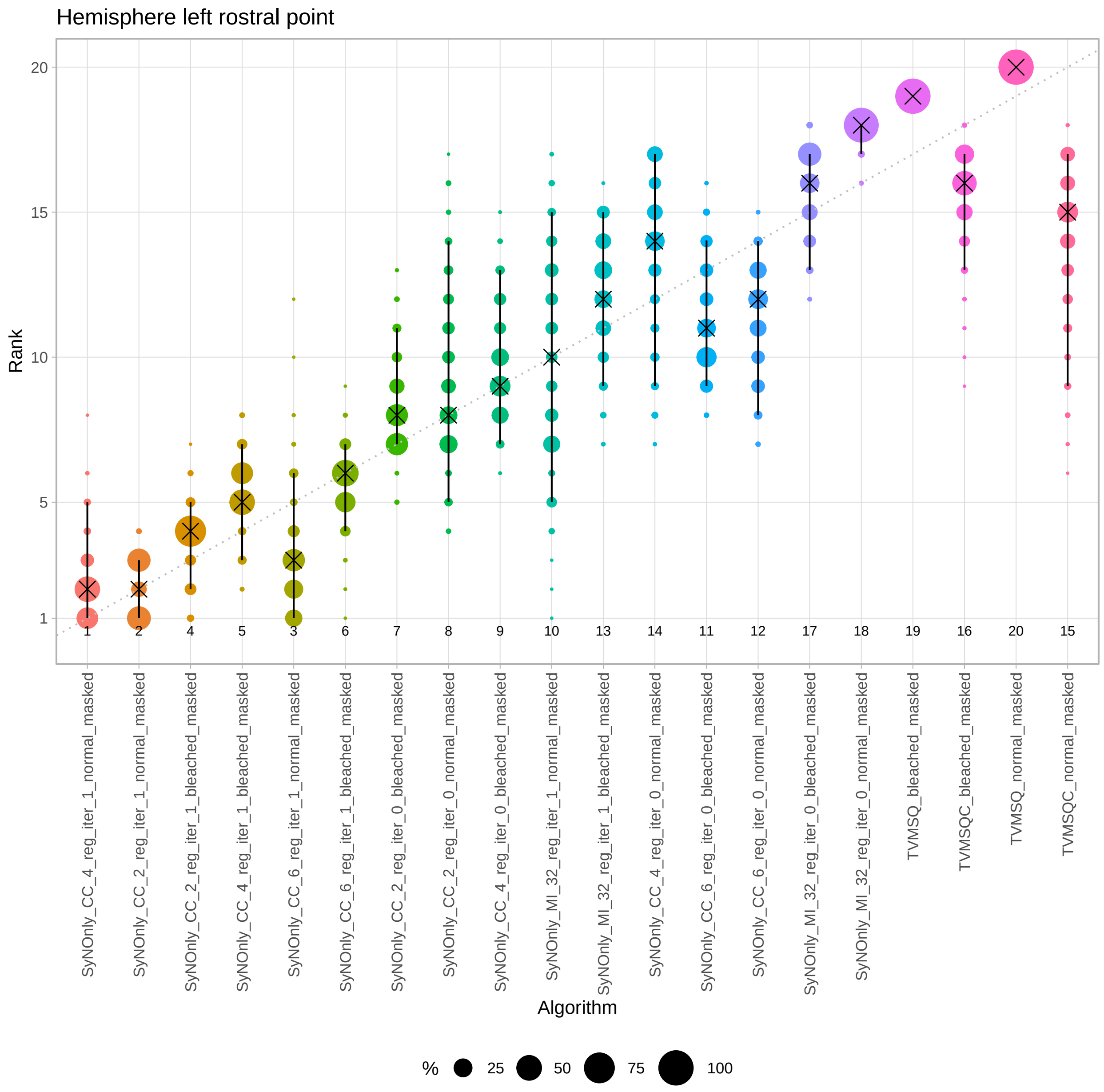

Hemisphere left rostral point
Rank
20
15
10
5
1
1
2
4
5
3
6
7
8
9
10
13
14
11
12
17
18
19
16
20
15
SyNOnly_CC_4_reg_iter_1_normal_masked
SyNOnly_CC_2_reg_iter_1_normal_masked
SyNOnly_CC_2_reg_iter_1_bleached_masked
SyNOnly_CC_4_reg_iter_1_bleached_masked
SyNOnly_CC_6_reg_iter_1_normal_masked
SyNOnly_CC_6_reg_iter_1_bleached_masked
SyNOnly_CC_2_reg_iter_0_bleached_masked
SyNOnly_CC_2_reg_iter_0_normal_masked
SyNOnly_CC_4_reg_iter_0_bleached_masked
SyNOnly_MI_32_reg_iter_1_normal_masked
SyNOnly_MI_32_reg_iter_1_bleached_masked
SyNOnly_CC_4_reg_iter_0_normal_masked
SyNOnly_CC_6_reg_iter_0_bleached_masked
SyNOnly_CC_6_reg_iter_0_normal_masked
SyNOnly_MI_32_reg_iter_0_bleached_masked
SyNOnly_MI_32_reg_iter_0_normal_masked
TVMSQ_bleached_masked
TVMSQC_bleached_masked
TVMSQ_normal_masked
TVMSQC_normal_masked
Algorithm
%
25
50
75
100

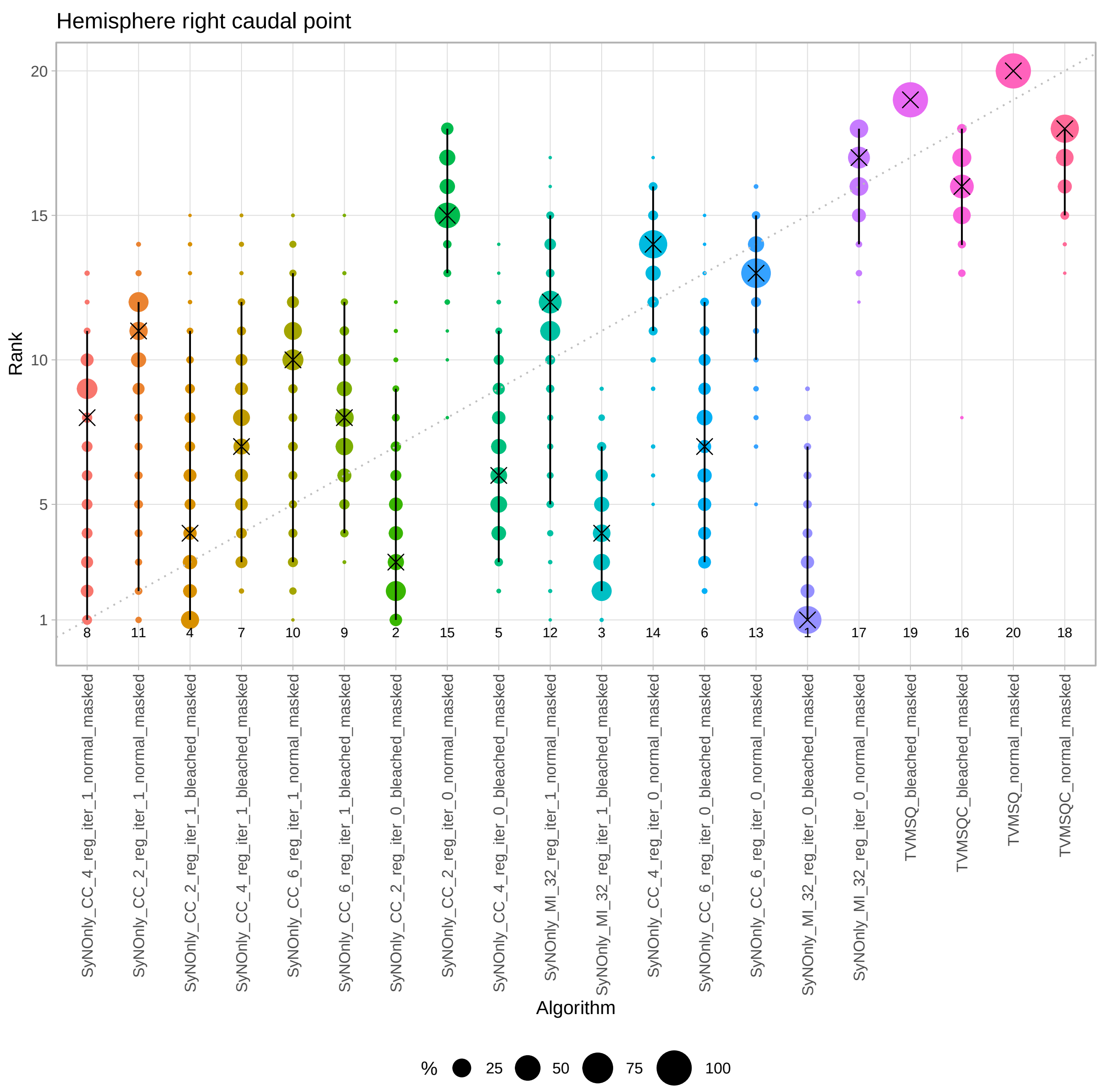
Hemisphere right caudal point
Rank
20
15
10
5
1
8
11
4
7
10
9
2
15
5
12
3
14
6
13
1
17
19
16
20
18
SyNOnly_CC_4_reg_iter_1_normal_masked
SyNOnly_CC_2_reg_iter_1_normal_masked
SyNOnly_CC_2_reg_iter_1_bleached_masked
SyNOnly_CC_4_reg_iter_1_bleached_masked
SyNOnly_CC_6_reg_iter_1_normal_masked
SyNOnly_CC_6_reg_iter_1_bleached_masked
SyNOnly_CC_2_reg_iter_0_bleached_masked
SyNOnly_CC_2_reg_iter_0_normal_masked
SyNOnly_CC_4_reg_iter_0_bleached_masked
SyNOnly_MI_32_reg_iter_1_normal_masked
SyNOnly_MI_32_reg_iter_1_bleached_masked
SyNOnly_CC_4_reg_iter_0_normal_masked
SyNOnly_CC_6_reg_iter_0_bleached_masked
SyNOnly_CC_6_reg_iter_0_normal_masked
SyNOnly_MI_32_reg_iter_0_bleached_masked
SyNOnly_MI_32_reg_iter_0_normal_masked
TVMSQ_bleached_masked
TVMSQC_bleached_masked
TVMSQ_normal_masked
TVMSQC_normal_masked
Algorithm
%
25
50
75
100

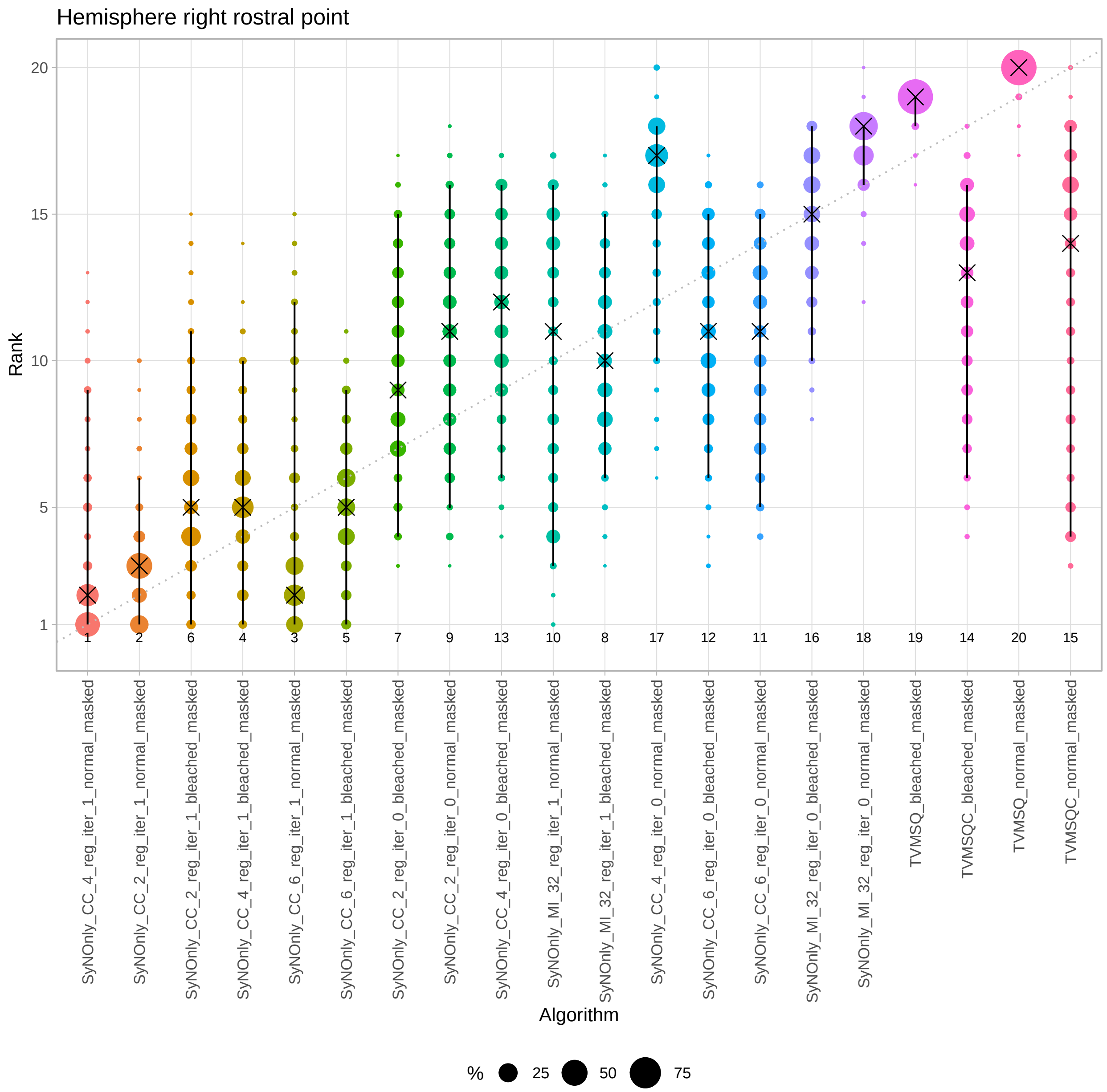

Hemisphere right rostral point
Rank
20
15
10
5
1
1
2
6
4
3
5
7
9
13
10
8
17
12
11
16
18
19
14
20
15
SyNOnly_CC_4_reg_iter_1_normal_masked
SyNOnly_CC_2_reg_iter_1_normal_masked
SyNOnly_CC_2_reg_iter_1_bleached_masked
SyNOnly_CC_4_reg_iter_1_bleached_masked
SyNOnly_CC_6_reg_iter_1_normal_masked
SyNOnly_CC_6_reg_iter_1_bleached_masked
SyNOnly_CC_2_reg_iter_0_bleached_masked
SyNOnly_CC_2_reg_iter_0_normal_masked
SyNOnly_CC_4_reg_iter_0_bleached_masked
SyNOnly_MI_32_reg_iter_1_normal_masked
SyNOnly_MI_32_reg_iter_1_bleached_masked
SyNOnly_CC_4_reg_iter_0_normal_masked
SyNOnly_CC_6_reg_iter_0_bleached_masked
SyNOnly_CC_6_reg_iter_0_normal_masked
SyNOnly_MI_32_reg_iter_0_bleached_masked
SyNOnly_MI_32_reg_iter_0_normal_masked
TVMSQ_bleached_masked
TVMSQC_bleached_masked
TVMSQ_normal_masked
TVMSQC_normal_masked
Algorithm
% 25 50 75

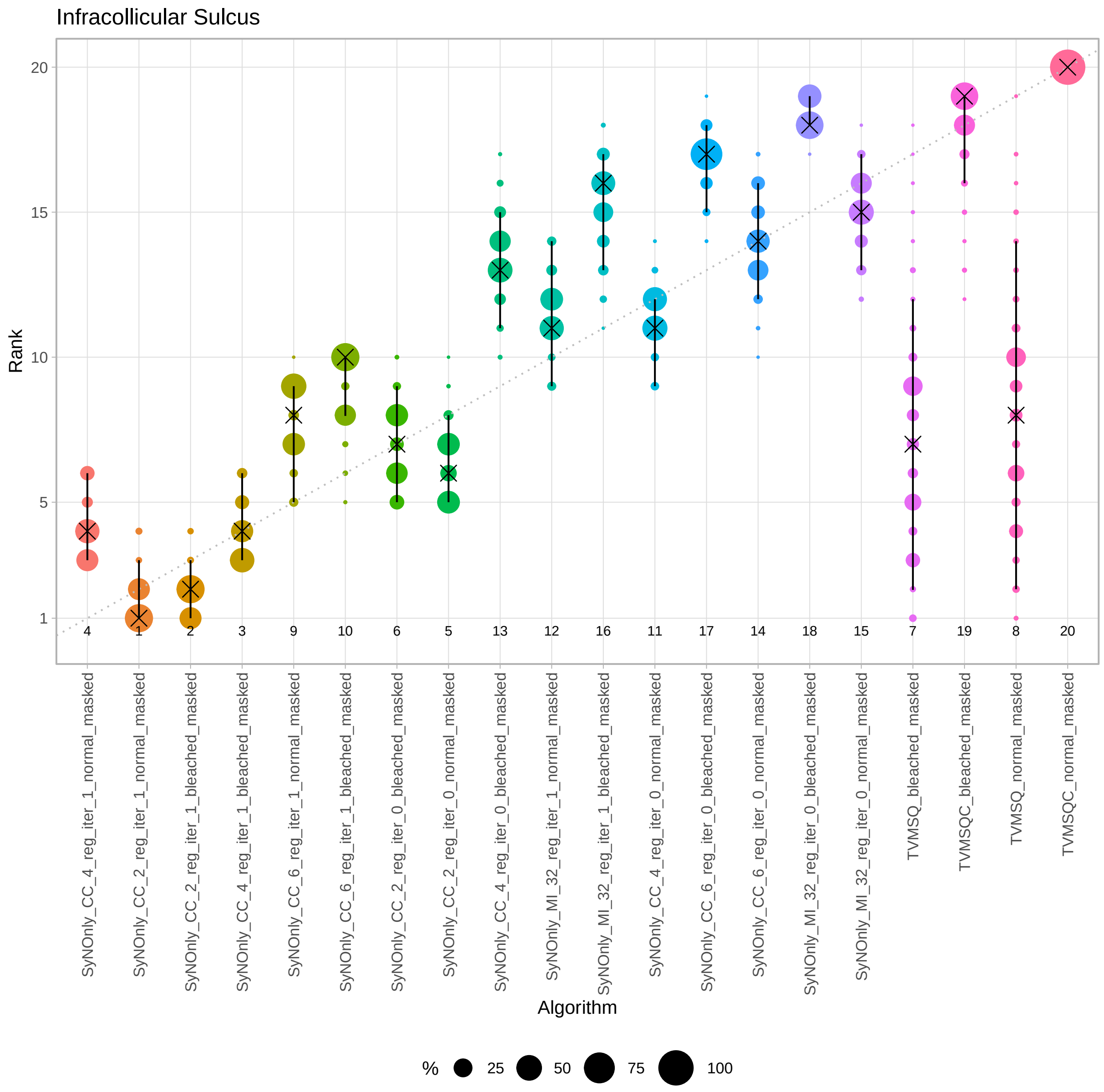
Infracollicular Sulcus
Rank
20
15
10
5
1
4
1
2
3
9
10
6
5
13
12
16
11
17
14
18
15
7
19
8
20
SyNOnly_CC_4_reg_iter_1_normal_masked
SyNOnly_CC_2_reg_iter_1_normal_masked
SyNOnly_CC_2_reg_iter_1_bleached_masked
SyNOnly_CC_4_reg_iter_1_bleached_masked
SyNOnly_CC_6_reg_iter_1_normal_masked
SyNOnly_CC_6_reg_iter_1_bleached_masked
SyNOnly_CC_2_reg_iter_0_bleached_masked
SyNOnly_CC_2_reg_iter_0_normal_masked
SyNOnly_CC_4_reg_iter_0_bleached_masked
SyNOnly_MI_32_reg_iter_1_normal_masked
SyNOnly_MI_32_reg_iter_1_bleached_masked
SyNOnly_CC_4_reg_iter_0_normal_masked
SyNOnly_CC_6_reg_iter_0_bleached_masked
SyNOnly_CC_6_reg_iter_0_normal_masked
SyNOnly_MI_32_reg_iter_0_bleached_masked
SyNOnly_MI_32_reg_iter_0_normal_masked
TVMSQ_bleached_masked
TVMSQC_bleached_masked
TVMSQ_normal_masked
TVMSQC_normal_masked
Algorithm
%
25
50
75
100

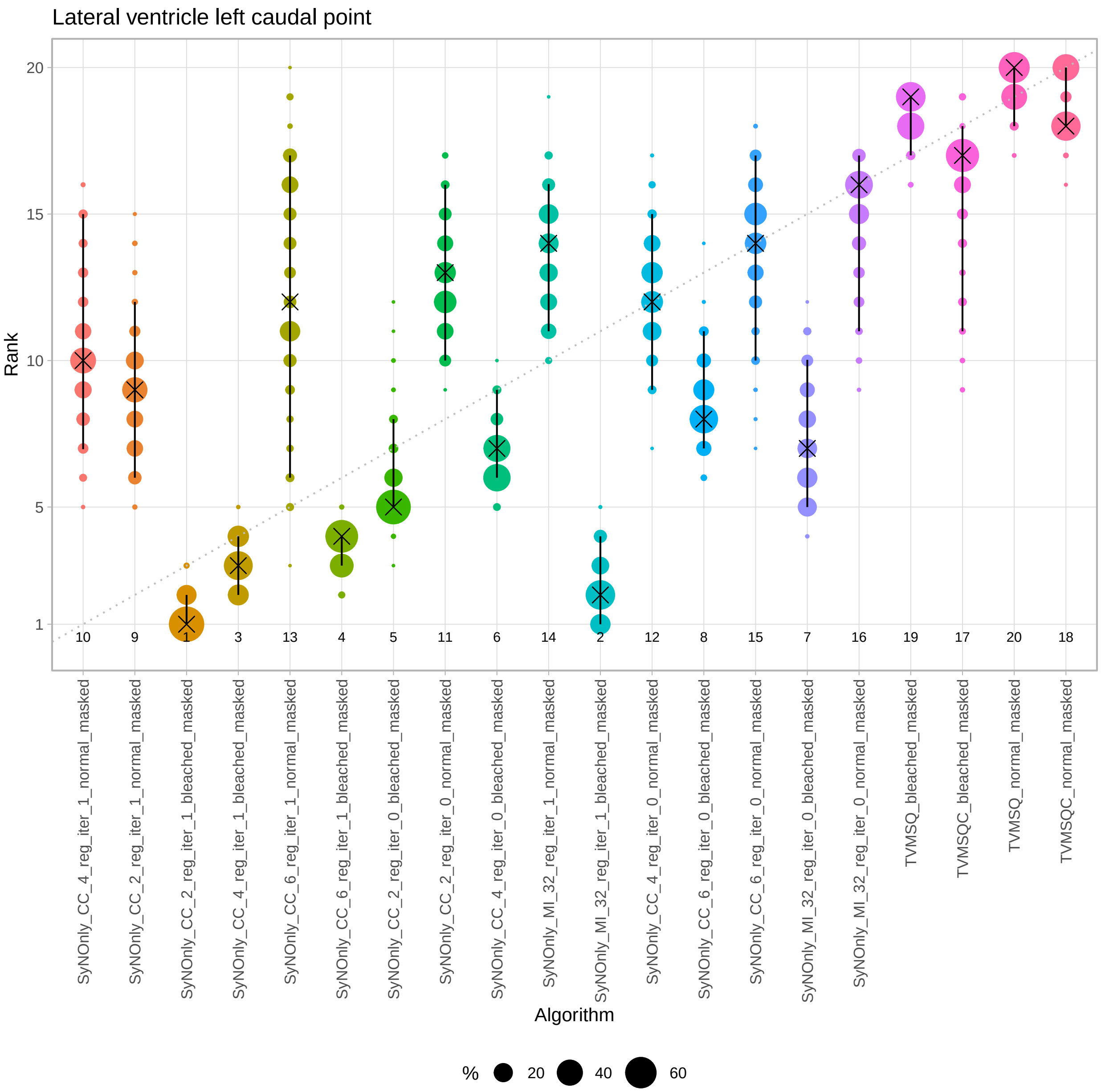
Lateral ventricle left caudal point
Rank
20
15
10
5
1
10 9 1 3 13 4 5 11 6 14 2 12 8 15 7 16 19 17 20 18
SyNOnly_CC_4_reg_iter_1_normal_masked
SyNOnly_CC_2_reg_iter_1_normal_masked
SyNOnly_CC_2_reg_iter_1_bleached_masked
SyNOnly_CC_4_reg_iter_1_bleached_masked
SyNOnly_CC_6_reg_iter_1_normal_masked
SyNOnly_CC_6_reg_iter_1_bleached_masked
SyNOnly_CC_2_reg_iter_0_bleached_masked
SyNOnly_CC_2_reg_iter_0_normal_masked
SyNOnly_CC_4_reg_iter_0_bleached_masked
SyNOnly_MI_32_reg_iter_1_normal_masked
SyNOnly_MI_32_reg_iter_1_bleached_masked
SyNOnly_CC_4_reg_iter_0_normal_masked
SyNOnly_CC_6_reg_iter_0_bleached_masked
SyNOnly_CC_6_reg_iter_0_normal_masked
SyNOnly_MI_32_reg_iter_0_bleached_masked
SyNOnly_MI_32_reg_iter_0_normal_masked
TVMSQ_bleached_masked
TVMSQC_bleached_masked
TVMSQ_normal_masked
TVMSQC_normal_masked
Algorithm
% 20 40 60

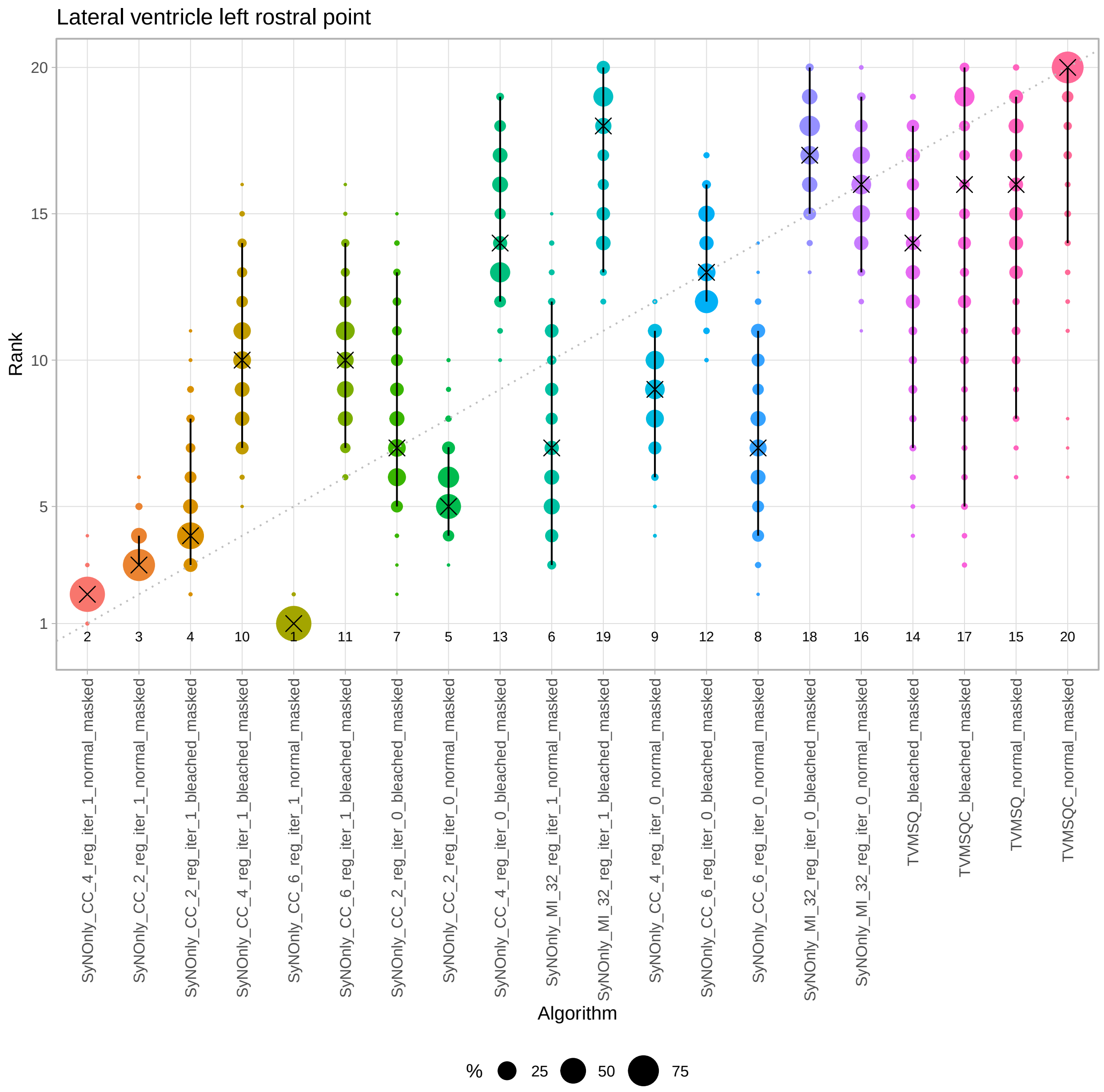

Lateral ventricle left rostral point
Rank
20
15
10
5
1
2
3
4
10
1
11
7
5
13
6
19
9
12
8
18
16
14
17
15
20
SyNOnly_CC_4_reg_iter_1_normal_masked
SyNOnly_CC_2_reg_iter_1_normal_masked
SyNOnly_CC_2_reg_iter_1_bleached_masked
SyNOnly_CC_4_reg_iter_1_bleached_masked
SyNOnly_CC_6_reg_iter_1_normal_masked
SyNOnly_CC_6_reg_iter_1_bleached_masked
SyNOnly_CC_2_reg_iter_0_bleached_masked
SyNOnly_CC_2_reg_iter_0_normal_masked
SyNOnly_CC_4_reg_iter_0_bleached_masked
SyNOnly_MI_32_reg_iter_1_normal_masked
SyNOnly_MI_32_reg_iter_1_bleached_masked
SyNOnly_CC_4_reg_iter_0_normal_masked
SyNOnly_CC_6_reg_iter_0_bleached_masked
SyNOnly_CC_6_reg_iter_0_normal_masked
SyNOnly_MI_32_reg_iter_0_bleached_masked
SyNOnly_MI_32_reg_iter_0_normal_masked
TVMSQ_bleached_masked
TVMSQC_bleached_masked
TVMSQ_normal_masked
TVMSQC_normal_masked
Algorithm
%
25
50
75

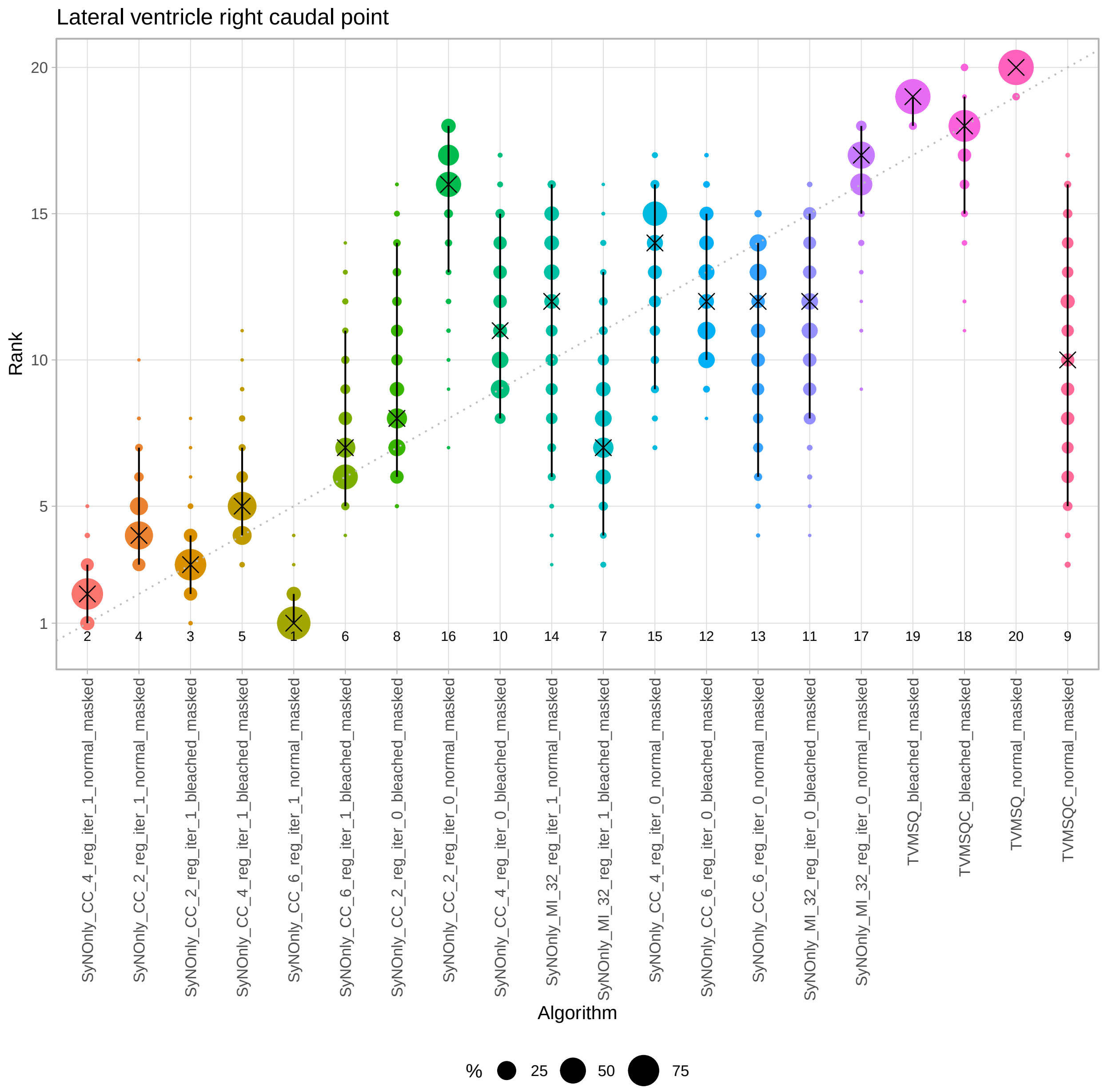
Lateral ventricle right caudal point
Rank
20
15
10
5
1
2 4 3 5 1 6 8 16 10 14 7 15 12 13 11 17 19 18 20 9
SyNOnly_CC_4_reg_iter_1_normal_masked
SyNOnly_CC_2_reg_iter_1_normal_masked
SyNOnly_CC_2_reg_iter_1_bleached_masked
SyNOnly_CC_4_reg_iter_1_bleached_masked
SyNOnly_CC_6_reg_iter_1_normal_masked
SyNOnly_CC_6_reg_iter_1_bleached_masked
SyNOnly_CC_2_reg_iter_0_bleached_masked
SyNOnly_CC_2_reg_iter_0_normal_masked
SyNOnly_CC_4_reg_iter_0_bleached_masked
SyNOnly_MI_32_reg_iter_1_normal_masked
SyNOnly_MI_32_reg_iter_1_bleached_masked
SyNOnly_CC_4_reg_iter_0_normal_masked
SyNOnly_CC_6_reg_iter_0_bleached_masked
SyNOnly_CC_6_reg_iter_0_normal_masked
SyNOnly_MI_32_reg_iter_0_bleached_masked
SyNOnly_MI_32_reg_iter_0_normal_masked
TVMSQ_bleached_masked
TVMSQC_bleached_masked
TVMSQ_normal_masked
TVMSQC_normal_masked
Algorithm
% 25 50 75

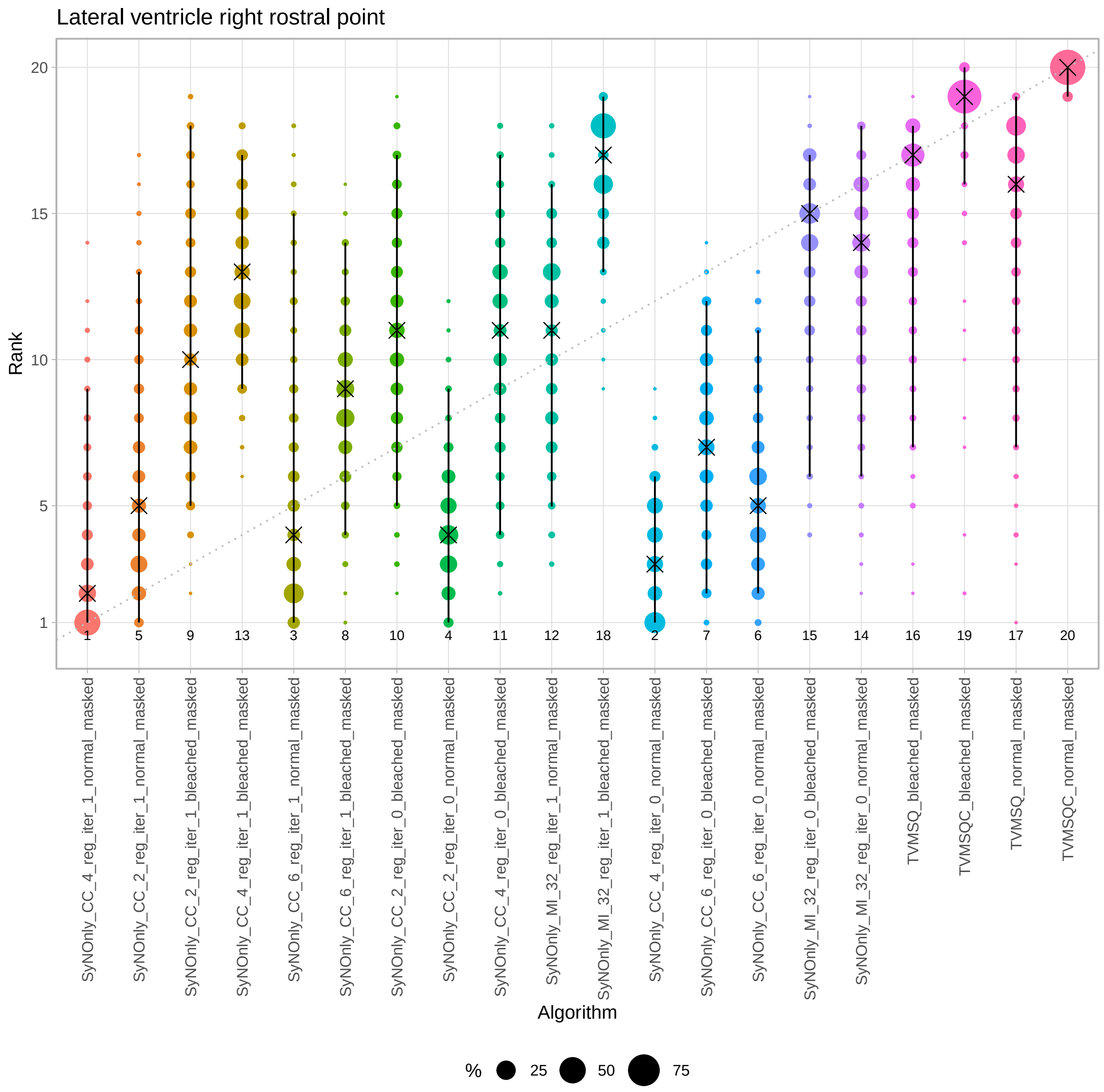
Lateral ventricle right rostral point
Rank
Algorithm
1
5
10
15
20
1 5 9 13 3 8 10 4 11 12 18 2 7 6 15 14 16 19 17 20
SyNOnly_CC_4_reg_iter_1_normal_masked
SyNOnly_CC_2_reg_iter_1_normal_masked
SyNOnly_CC_2_reg_iter_1_bleached_masked
SyNOnly_CC_4_reg_iter_1_bleached_masked
SyNOnly_CC_6_reg_iter_1_normal_masked
SyNOnly_CC_6_reg_iter_1_bleached_masked
SyNOnly_CC_2_reg_iter_0_bleached_masked
SyNOnly_CC_2_reg_iter_0_normal_masked
SyNOnly_CC_4_reg_iter_0_bleached_masked
SyNOnly_MI_32_reg_iter_1_normal_masked
SyNOnly_MI_32_reg_iter_1_bleached_masked
SyNOnly_CC_4_reg_iter_0_normal_masked
SyNOnly_CC_6_reg_iter_0_bleached_masked
SyNOnly_CC_6_reg_iter_0_normal_masked
SyNOnly_MI_32_reg_iter_0_bleached_masked
SyNOnly_MI_32_reg_iter_0_normal_masked
TVMSQ_bleached_masked
TVMSQC_bleached_masked
TVMSQ_normal_masked
TVMSQC_normal_masked
% 25 50 75

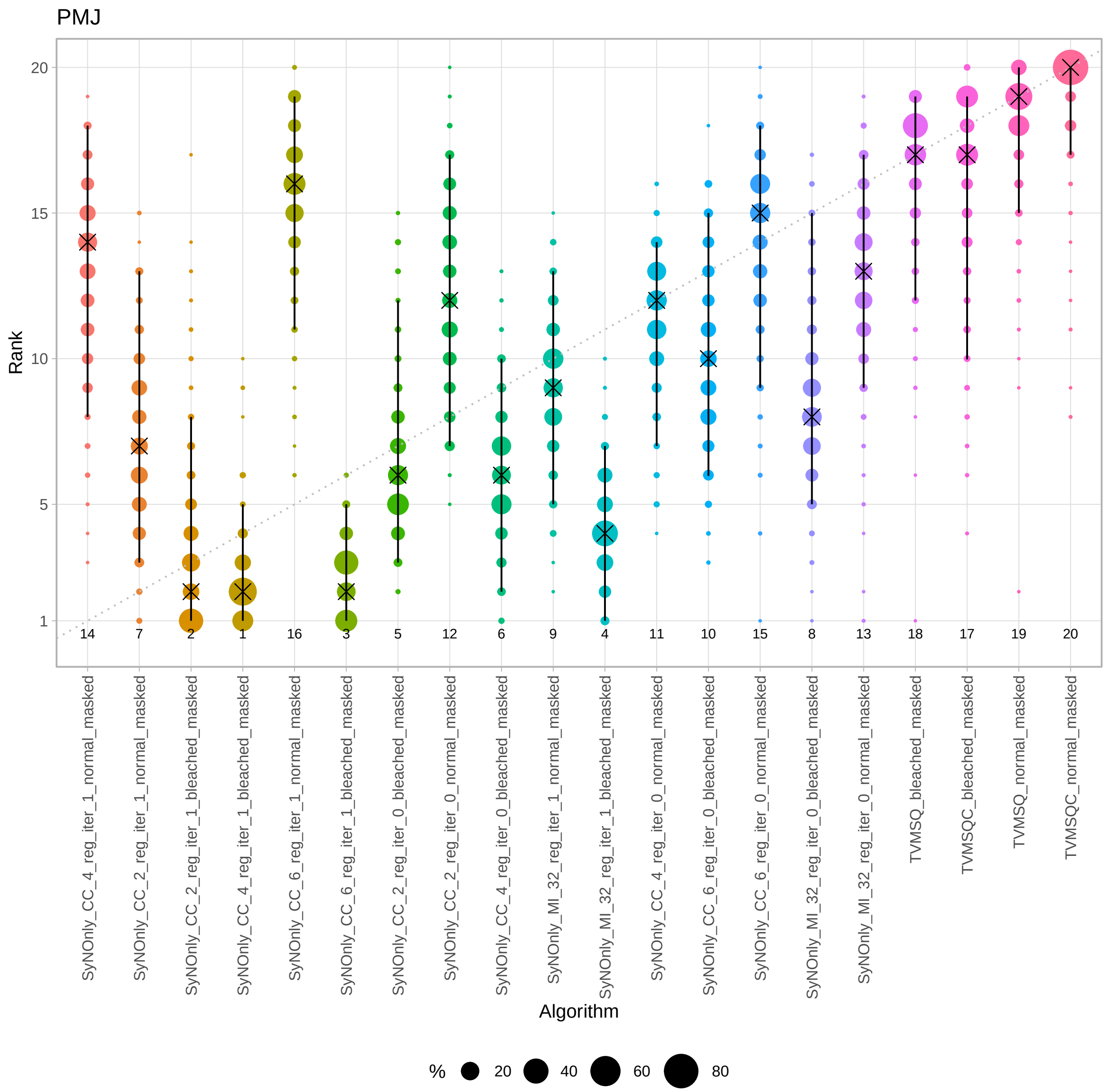

PMJ
Rank
20
15
10
5
1
14
7
2
1
16
3
5
12
6
9
4
11
10
15
8
13
18
17
19
20
SyNOnly_CC_4_reg_iter_1_normal_masked
SyNOnly_CC_2_reg_iter_1_normal_masked
SyNOnly_CC_2_reg_iter_1_bleached_masked
SyNOnly_CC_4_reg_iter_1_bleached_masked
SyNOnly_CC_6_reg_iter_1_normal_masked
SyNOnly_CC_6_reg_iter_1_bleached_masked
SyNOnly_CC_2_reg_iter_0_bleached_masked
SyNOnly_CC_2_reg_iter_0_normal_masked
SyNOnly_CC_4_reg_iter_0_bleached_masked
SyNOnly_MI_32_reg_iter_1_normal_masked
SyNOnly_MI_32_reg_iter_1_bleached_masked
SyNOnly_CC_4_reg_iter_0_normal_masked
SyNOnly_CC_6_reg_iter_0_bleached_masked
SyNOnly_CC_6_reg_iter_0_normal_masked
SyNOnly_MI_32_reg_iter_0_bleached_masked
SyNOnly_MI_32_reg_iter_0_normal_masked
TVMSQ_bleached_masked
TVMSQC_bleached_masked
TVMSQ_normal_masked
TVMSQC_normal_masked
Algorithm
%
20
40
60
80

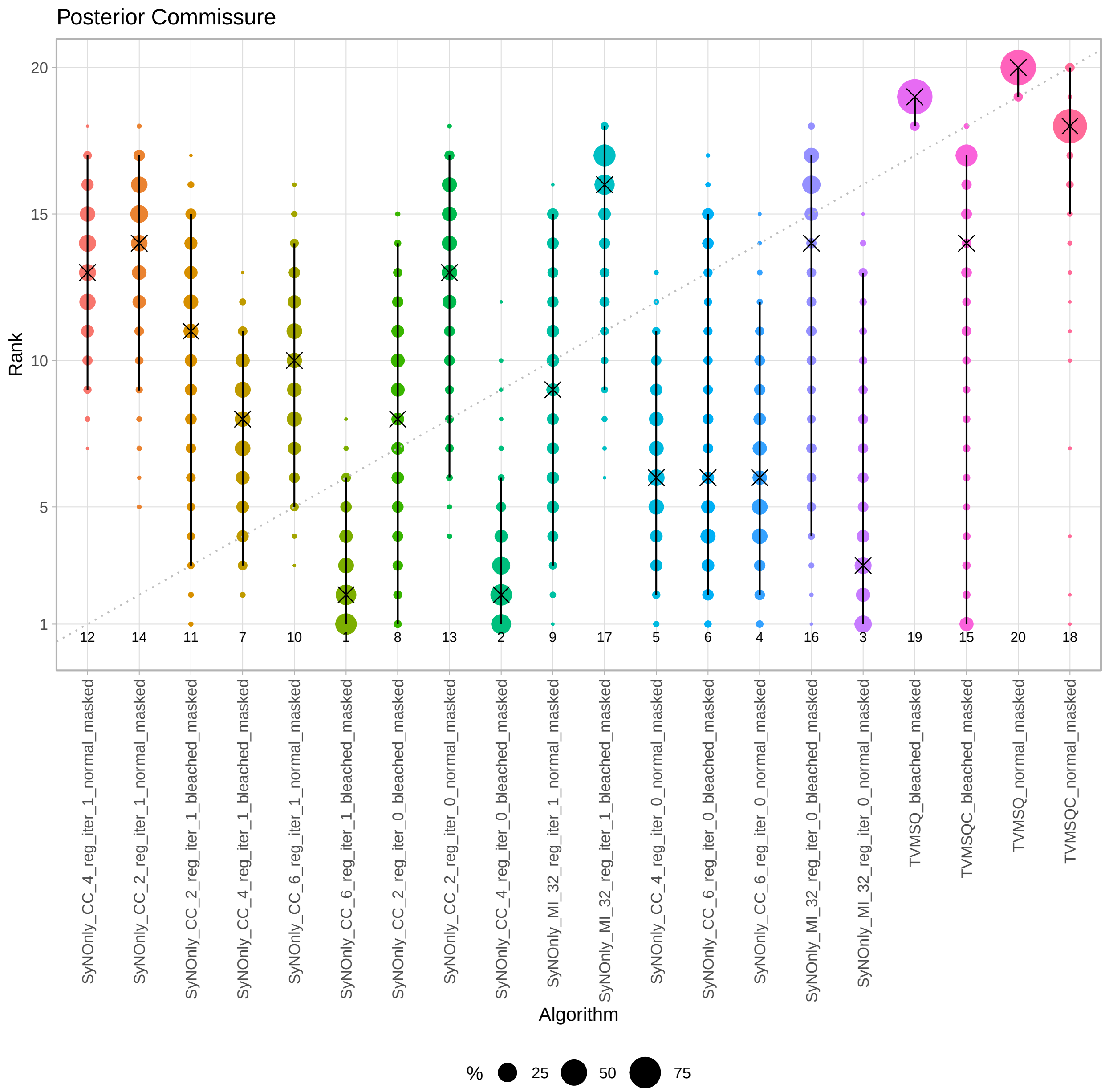
Posterior Commissure
Rank
20
15
10
5
1
12
14
11
7
10
1
8
13
2
9
17
5
6
4
16
3
19
15
20
18
SyNOnly_CC_4_reg_iter_1_normal_masked
SyNOnly_CC_2_reg_iter_1_normal_masked
SyNOnly_CC_2_reg_iter_1_bleached_masked
SyNOnly_CC_4_reg_iter_1_bleached_masked
SyNOnly_CC_6_reg_iter_1_normal_masked
SyNOnly_CC_6_reg_iter_1_bleached_masked
SyNOnly_CC_2_reg_iter_0_bleached_masked
SyNOnly_CC_2_reg_iter_0_normal_masked
SyNOnly_CC_4_reg_iter_0_bleached_masked
SyNOnly_MI_32_reg_iter_1_normal_masked
SyNOnly_MI_32_reg_iter_1_bleached_masked
SyNOnly_CC_4_reg_iter_0_normal_masked
SyNOnly_CC_6_reg_iter_0_bleached_masked
SyNOnly_CC_6_reg_iter_0_normal_masked
SyNOnly_MI_32_reg_iter_0_bleached_masked
SyNOnly_MI_32_reg_iter_0_normal_masked
TVMSQ_bleached_masked
TVMSQC_bleached_masked
TVMSQ_normal_masked
TVMSQC_normal_masked
Algorithm
%
25
50
75

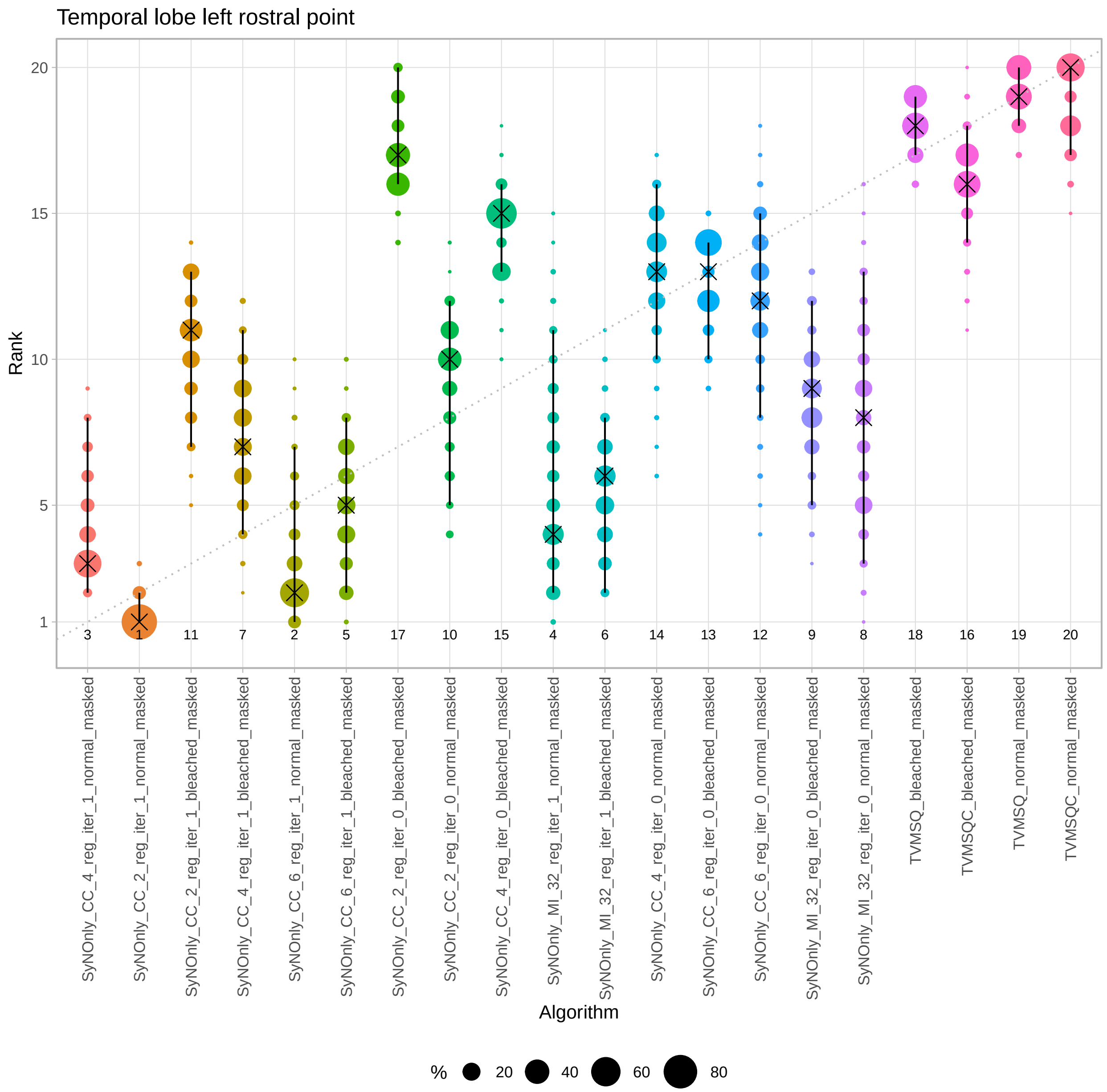

Temporal lobe left rostral point
Rank
20
15
10
5
1
3
1
11
7
2
5
17
10
15
4
6
14
13
12
9
8
18
16
19
20
SyNOnly_CC_4_reg_iter_1_normal_masked
SyNOnly_CC_2_reg_iter_1_normal_masked
SyNOnly_CC_2_reg_iter_1_bleached_masked
SyNOnly_CC_4_reg_iter_1_bleached_masked
SyNOnly_CC_6_reg_iter_1_normal_masked
SyNOnly_CC_6_reg_iter_1_bleached_masked
SyNOnly_CC_2_reg_iter_0_bleached_masked
SyNOnly_CC_2_reg_iter_0_normal_masked
SyNOnly_CC_4_reg_iter_0_bleached_masked
SyNOnly_MI_32_reg_iter_1_normal_masked
SyNOnly_MI_32_reg_iter_1_bleached_masked
SyNOnly_CC_4_reg_iter_0_normal_masked
SyNOnly_CC_6_reg_iter_0_bleached_masked
SyNOnly_CC_6_reg_iter_0_normal_masked
SyNOnly_MI_32_reg_iter_0_bleached_masked
SyNOnly_MI_32_reg_iter_0_normal_masked
TVMSQ_bleached_masked
TVMSQC_bleached_masked
TVMSQ_normal_masked
TVMSQC_normal_masked
Algorithm
%
20
40
60
80

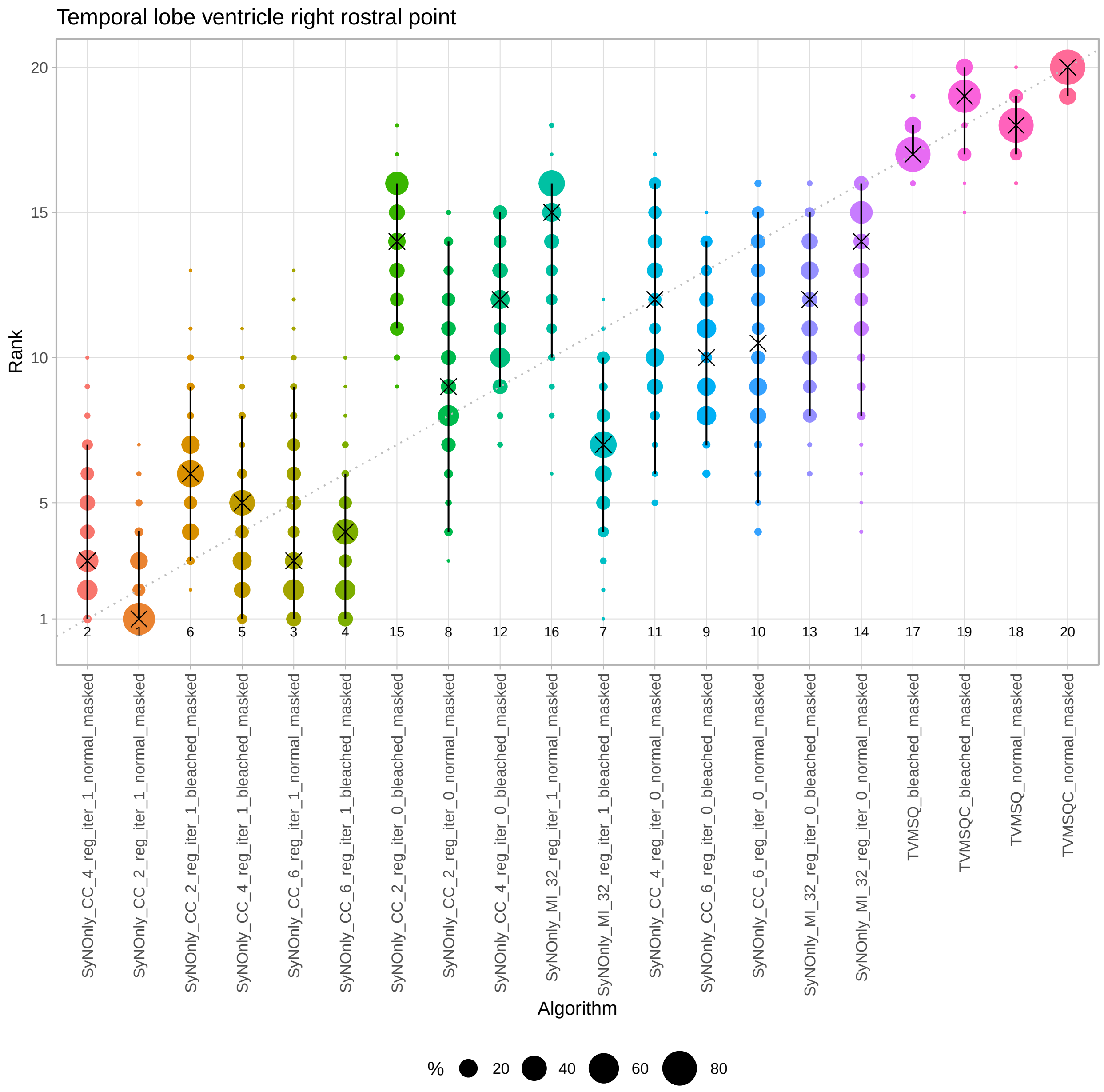

4.2.2 Cluster Analysis

Dendrogram from hierarchical cluster analysis and *network-type graphs* for assessing the similarity of tasks based on challenge rankings.

A dendrogram is a visualization approach based on hierarchical clustering. It depicts clusters according to a chosen distance measure (here: Spearman's footrule) as well as a chosen agglomeration method (here: complete and average agglomeration).

Cluster Dendrogram (complete agglomeration)

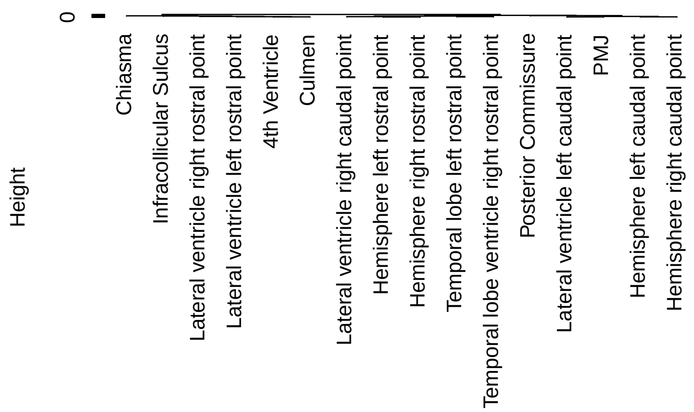

Cluster Dendrogram (average agglomeration)

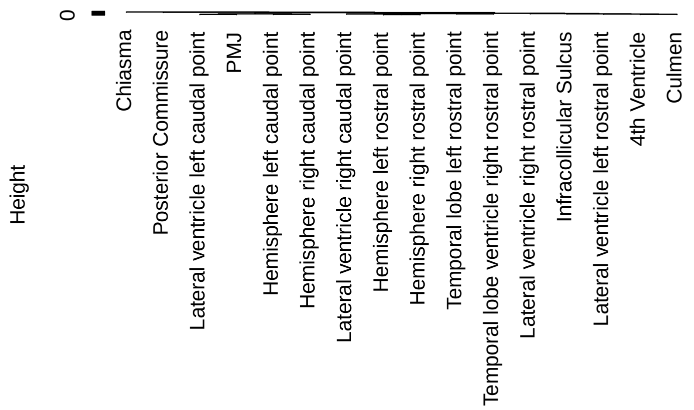

5 References

Wiesenfarth, M., Reinke, A., Landman, B.A., Eisenmann, M., Aguilera Saiz, L., Cardoso, M.J., Maier-Hein, L. and Kopp-Schneider, A. Methods and open-source toolkit for analyzing and visualizing challenge results. *Sci Rep* **11**, 2369 (2021). https://doi.org/10.1038/s41598-021-82017-6

M. J. A. Eugster, T. Hothorn, and F. Leisch, “Exploratory and inferential analysis of benchmark experiments,” Institut fuer Statistik, Ludwig-Maximilians-Universitaet Muenchen, Germany, Technical Report 30, 2008. [Online]. Available: http://epub.ub.uni-muenchen.de/4134/.
