# Supplementary material for: Characterization of dynamic patterns of human fetal to neonatal brain asymmetry with deformation-based morphometry
Source: Front Neurosci. 2023 Dec 6;17:1252850. doi: 10.3389/fnins.2023.1252850 (PMC10734644; doi:10.3389/fnins.2023.1252850)
Supplement: Supplementary file 1 [file Data_Sheet_1.ZIP › ChallengeR/Framework B to mid-age template/fetal2template_nonlinear/ranking_Fetal2Template_nonlinear_normal_and_bleached_voldiff.pdf]

Benchmarking report for Fetal to template registration nonlinear normal and bleached combined

created by challengeR v1.0.4

31 März, 2023

This document presents a systematic report on the benchmark study "Fetal to template registration nonlinear normal and bleached combined". Input data comprises raw metric values for all algorithms and cases. Generated plots are:

- Visualization of assessment data: Dot- and boxplots, podium plots and ranking heatmaps
- Visualization of ranking stability: Blob plots, violin plots and significance maps, line plots
- Visualization of cross-task insights: Blob plots, stacked frequency plots, dendrograms

Details can be found in Wiesenfarth et al. (2021).

1 Rankings

Algorithms within a task are ranked according to the following ranking scheme:

aggregate using function ("mean") then rank

Ranking for each task:

Brainstem : The analysis is based on 20 algorithms and 20 cases. 0 missing cases have been found in the data set.

	value_mean	rank
SyNOnly_CC_6_reg_iter_0_bleached_masked	0.1366925	1
SyNOnly_CC_4_reg_iter_0_bleached_masked	0.1404025	2
SyNOnly_CC_2_reg_iter_0_bleached_masked	0.1570167	3
SyNOnly_CC_6_reg_iter_1_bleached_masked	0.1700438	4
SyNOnly_CC_4_reg_iter_1_bleached_masked	0.1839926	5
SyNOnly_MI_32_reg_iter_0_bleached_masked	0.1879714	6
SyNOnly_CC_6_reg_iter_1_normal_masked	0.2034066	7
SyNOnly_MI_32_reg_iter_1_bleached_masked	0.2070512	8
SyNOnly_CC_2_reg_iter_1_bleached_masked	0.2091098	9
SyNOnly_CC_4_reg_iter_0_normal_masked	0.2102274	10
SyNOnly_CC_6_reg_iter_0_normal_masked	0.2118865	11
SyNOnly_CC_4_reg_iter_1_normal_masked	0.2149512	12
SyNOnly_CC_2_reg_iter_0_normal_masked	0.2163069	13
SyNOnly_CC_2_reg_iter_1_normal_masked	0.2307205	14
TVMSQC_bleached_masked	0.2586335	15
SyNOnly_MI_32_reg_iter_1_normal_masked	0.2664644	16
SyNOnly_MI_32_reg_iter_0_normal_masked	0.2866426	17
TVMSQC_normal_masked	0.3150396	18
TVMSQ_bleached_masked	0.3808165	19
TVMSQ_normal_masked	0.3851217	20

Cerebellum : The analysis is based on 20 algorithms and 20 cases. 0 missing cases have been found in the data set.

	value_mean	rank
SyNOnly_MI_32_reg_iter_1_bleached_masked	0.1916663	1
SyNOnly_MI_32_reg_iter_0_bleached_masked	0.1962952	2
SyNOnly_MI_32_reg_iter_0_normal_masked	0.2137709	3
SyNOnly_MI_32_reg_iter_1_normal_masked	0.2315804	4
TVMSQC_bleached_masked	0.2559604	5
SyNOnly_CC_6_reg_iter_1_bleached_masked	0.2598678	6
SyNOnly_CC_6_reg_iter_0_bleached_masked	0.2628392	7
SyNOnly_CC_4_reg_iter_1_bleached_masked	0.2704835	8
SyNOnly_CC_4_reg_iter_0_bleached_masked	0.2719923	9
SyNOnly_CC_2_reg_iter_1_normal_masked	0.2786101	10
SyNOnly_CC_2_reg_iter_0_bleached_masked	0.2808403	11
SyNOnly_CC_2_reg_iter_0_normal_masked	0.2852445	12
SyNOnly_CC_2_reg_iter_1_bleached_masked	0.2872621	13
SyNOnly_CC_4_reg_iter_1_normal_masked	0.2958634	14
SyNOnly_CC_6_reg_iter_1_normal_masked	0.2965716	15
TVMSQ_bleached_masked	0.2966520	16
TVMSQ_normal_masked	0.2966630	17
SyNOnly_CC_4_reg_iter_0_normal_masked	0.2982808	18
SyNOnly_CC_6_reg_iter_0_normal_masked	0.3011696	19
TVMSQC_normal_masked	0.3172544	20

Cortical grey matter : The analysis is based on 20 algorithms and 20 cases. 0 missing cases have been found in the data set.

	value_mean	rank
SyNOnly_CC_2_reg_iter_1_bleached_masked	0.2077415	1
SyNOnly_CC_2_reg_iter_1_normal_masked	0.2399944	2
SyNOnly_CC_4_reg_iter_1_bleached_masked	0.2597151	3
SyNOnly_CC_2_reg_iter_0_bleached_masked	0.2720678	4
SyNOnly_CC_2_reg_iter_0_normal_masked	0.2724414	5
SyNOnly_MI_32_reg_iter_1_bleached_masked	0.2784195	6
SyNOnly_CC_4_reg_iter_1_normal_masked	0.2809152	7
SyNOnly_CC_6_reg_iter_1_normal_masked	0.2936499	8
SyNOnly_MI_32_reg_iter_0_normal_masked	0.2953617	9
SyNOnly_MI_32_reg_iter_0_bleached_masked	0.2975884	10
SyNOnly_CC_6_reg_iter_1_bleached_masked	0.2977444	11
SyNOnly_MI_32_reg_iter_1_normal_masked	0.2978878	12
TVMSQ_normal_masked	0.3031790	13
TVMSQ_bleached_masked	0.3048507	14
SyNOnly_CC_4_reg_iter_0_bleached_masked	0.3179783	15
TVMSQC_normal_masked	0.3292536	16
SyNOnly_CC_6_reg_iter_0_bleached_masked	0.3354584	17
TVMSQC_bleached_masked	0.3415453	18
SyNOnly_CC_4_reg_iter_0_normal_masked	0.3531774	19
SyNOnly_CC_6_reg_iter_0_normal_masked	0.3698489	20

CSF and ventricles : The analysis is based on 20 algorithms and 20 cases. 0 missing cases have been found in

the data set.

	value_mean	rank
SyNOnly_CC_2_reg_iter_1_bleached_masked	0.3428113	1
SyNOnly_CC_4_reg_iter_1_bleached_masked	0.3999925	2
SyNOnly_CC_6_reg_iter_1_bleached_masked	0.4416183	3
SyNOnly_CC_2_reg_iter_0_bleached_masked	0.4735326	4
SyNOnly_MI_32_reg_iter_1_bleached_masked	0.4903918	5
SyNOnly_CC_2_reg_iter_1_normal_masked	0.5108680	6
SyNOnly_CC_4_reg_iter_0_bleached_masked	0.5296610	7
SyNOnly_CC_6_reg_iter_0_normal_masked	0.5438412	8
SyNOnly_CC_4_reg_iter_0_normal_masked	0.5460144	9
SyNOnly_CC_6_reg_iter_0_bleached_masked	0.5667857	10
SyNOnly_CC_4_reg_iter_1_normal_masked	0.5778893	11
SyNOnly_CC_2_reg_iter_0_normal_masked	0.5852621	12
SyNOnly_CC_6_reg_iter_1_normal_masked	0.5942107	13
SyNOnly_MI_32_reg_iter_0_bleached_masked	0.5987745	14
SyNOnly_MI_32_reg_iter_1_normal_masked	0.6539739	15
TVMSQC_bleached_masked	0.7107885	16
TVMSQC_normal_masked	0.7482376	17
SyNOnly_MI_32_reg_iter_0_normal_masked	0.7531086	18
TVMSQ_normal_masked	0.7753455	19
TVMSQ_bleached_masked	0.7763527	20

Deep grey matter : The analysis is based on 20 algorithms and 20 cases. 0 missing cases have been found in the data set.

	value_mean	rank
SyNOnly_CC_2_reg_iter_1_bleached_masked	0.2257437	1
SyNOnly_CC_4_reg_iter_1_bleached_masked	0.2289197	2
SyNOnly_CC_2_reg_iter_1_normal_masked	0.2308003	3
SyNOnly_CC_2_reg_iter_0_bleached_masked	0.2327224	4
SyNOnly_CC_6_reg_iter_1_bleached_masked	0.2344403	5
SyNOnly_CC_2_reg_iter_0_normal_masked	0.2376182	6
SyNOnly_CC_4_reg_iter_0_bleached_masked	0.2464854	7
SyNOnly_CC_6_reg_iter_0_bleached_masked	0.2543293	8
SyNOnly_CC_4_reg_iter_0_normal_masked	0.2573313	9
SyNOnly_CC_4_reg_iter_1_normal_masked	0.2576636	10
TVMSQ_bleached_masked	0.2592128	11
TVMSQ_normal_masked	0.2596007	12
SyNOnly_CC_6_reg_iter_0_normal_masked	0.2727735	13
SyNOnly_MI_32_reg_iter_1_bleached_masked	0.2890215	14
SyNOnly_CC_6_reg_iter_1_normal_masked	0.2916276	15
SyNOnly_MI_32_reg_iter_1_normal_masked	0.2981583	16
TVMSQC_normal_masked	0.3034739	17
SyNOnly_MI_32_reg_iter_0_normal_masked	0.3055568	18
TVMSQC_bleached_masked	0.3102114	19
SyNOnly_MI_32_reg_iter_0_bleached_masked	0.3132470	20

White matter : The analysis is based on 20 algorithms and 20 cases. 0 missing cases have been found in the data set.

	value_mean	rank
SyNOnly_CC_4_reg_iter_0_normal_masked	0.0417861	1
SyNOnly_CC_6_reg_iter_0_normal_masked	0.0423986	2
SyNOnly_CC_2_reg_iter_1_normal_masked	0.0601853	3
SyNOnly_CC_2_reg_iter_1_bleached_masked	0.0602923	4
TVMSQC_bleached_masked	0.0666784	5
SyNOnly_CC_2_reg_iter_0_bleached_masked	0.0711632	6
SyNOnly_CC_4_reg_iter_1_normal_masked	0.0741033	7
SyNOnly_MI_32_reg_iter_1_normal_masked	0.0790035	8
TVMSQC_normal_masked	0.0823148	9
SyNOnly_CC_6_reg_iter_1_normal_masked	0.0830406	10
SyNOnly_CC_2_reg_iter_0_normal_masked	0.0834586	11
TVMSQ_bleached_masked	0.0862282	12
SyNOnly_CC_4_reg_iter_1_bleached_masked	0.0877852	13
TVMSQ_normal_masked	0.0878040	14
SyNOnly_CC_4_reg_iter_0_bleached_masked	0.1000412	15
SyNOnly_CC_6_reg_iter_0_bleached_masked	0.1080584	16
SyNOnly_CC_6_reg_iter_1_bleached_masked	0.1082895	17
SyNOnly_MI_32_reg_iter_0_normal_masked	0.1083434	18
SyNOnly_MI_32_reg_iter_0_bleached_masked	0.1099772	19
SyNOnly_MI_32_reg_iter_1_bleached_masked	0.1108549	20

Consensus ranking across tasks according to chosen method “euclidean”:

	value	rank
SyNOnly_CC_2_reg_iter_1_bleached_masked	4.833	1
SyNOnly_CC_2_reg_iter_0_bleached_masked	5.333	2
SyNOnly_CC_4_reg_iter_1_bleached_masked	5.500	3
SyNOnly_CC_2_reg_iter_1_normal_masked	6.333	4
SyNOnly_CC_6_reg_iter_1_bleached_masked	7.667	5
SyNOnly_MI_32_reg_iter_1_bleached_masked	9.000	6
SyNOnly_CC_4_reg_iter_0_bleached_masked	9.167	7
SyNOnly_CC_2_reg_iter_0_normal_masked	9.833	8
SyNOnly_CC_6_reg_iter_0_bleached_masked	9.833	8
SyNOnly_CC_4_reg_iter_1_normal_masked	10.167	10
SyNOnly_CC_4_reg_iter_0_normal_masked	11.000	11
SyNOnly_CC_6_reg_iter_1_normal_masked	11.333	12
SyNOnly_MI_32_reg_iter_0_bleached_masked	11.833	13
SyNOnly_MI_32_reg_iter_1_normal_masked	11.833	13
SyNOnly_CC_6_reg_iter_0_normal_masked	12.167	15
TVMSQC_bleached_masked	13.000	16
SyNOnly_MI_32_reg_iter_0_normal_masked	13.833	17
TVMSQ_bleached_masked	15.333	18
TVMSQ_normal_masked	15.833	19
TVMSQC_normal_masked	16.167	20

2 Visualization of raw assessment data

The algorithms are ordered according to the computed ranks for each task.

2.1 Dot- and boxplot

Dot- and boxplots for visualizing raw assessment data separately for each algorithm. Boxplots representing descriptive statistics over all cases (median, quartiles and outliers) are combined with horizontally jittered dots representing individual cases.

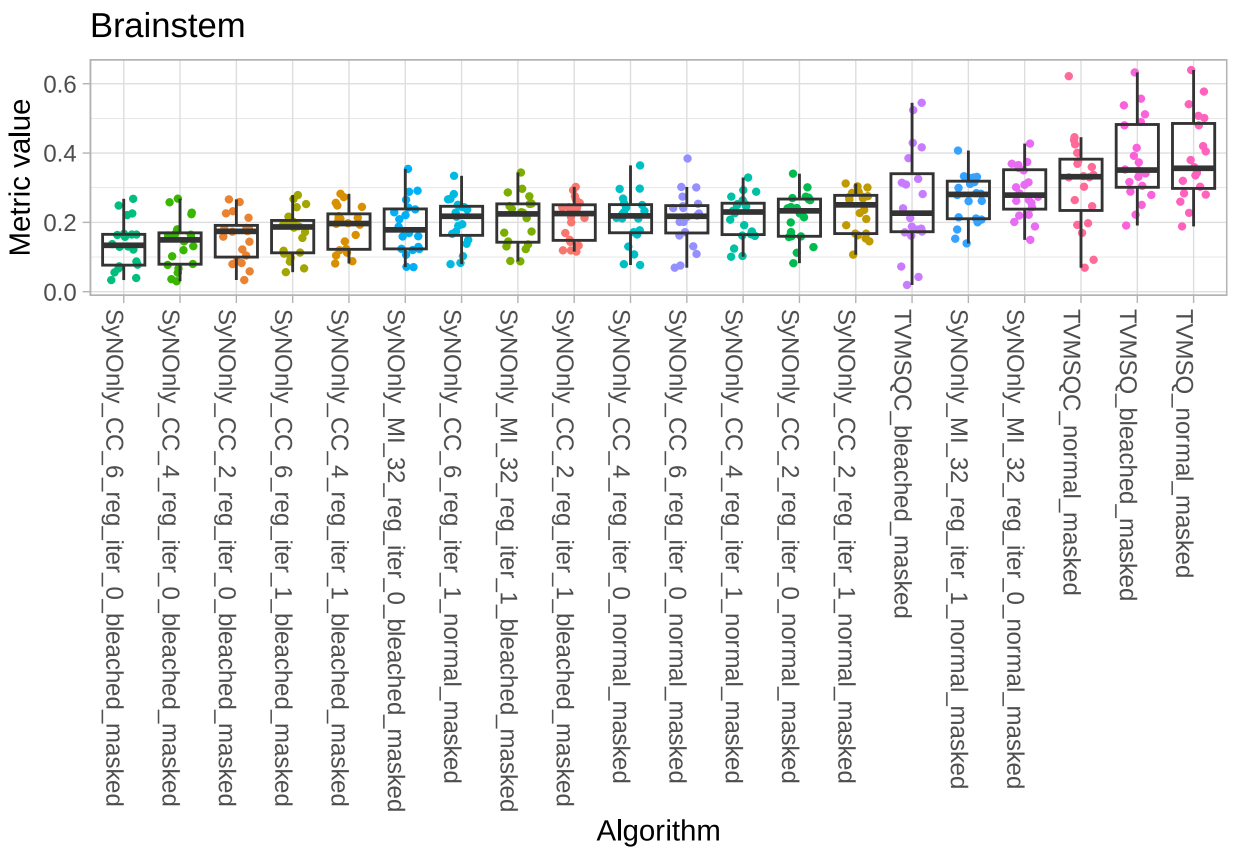

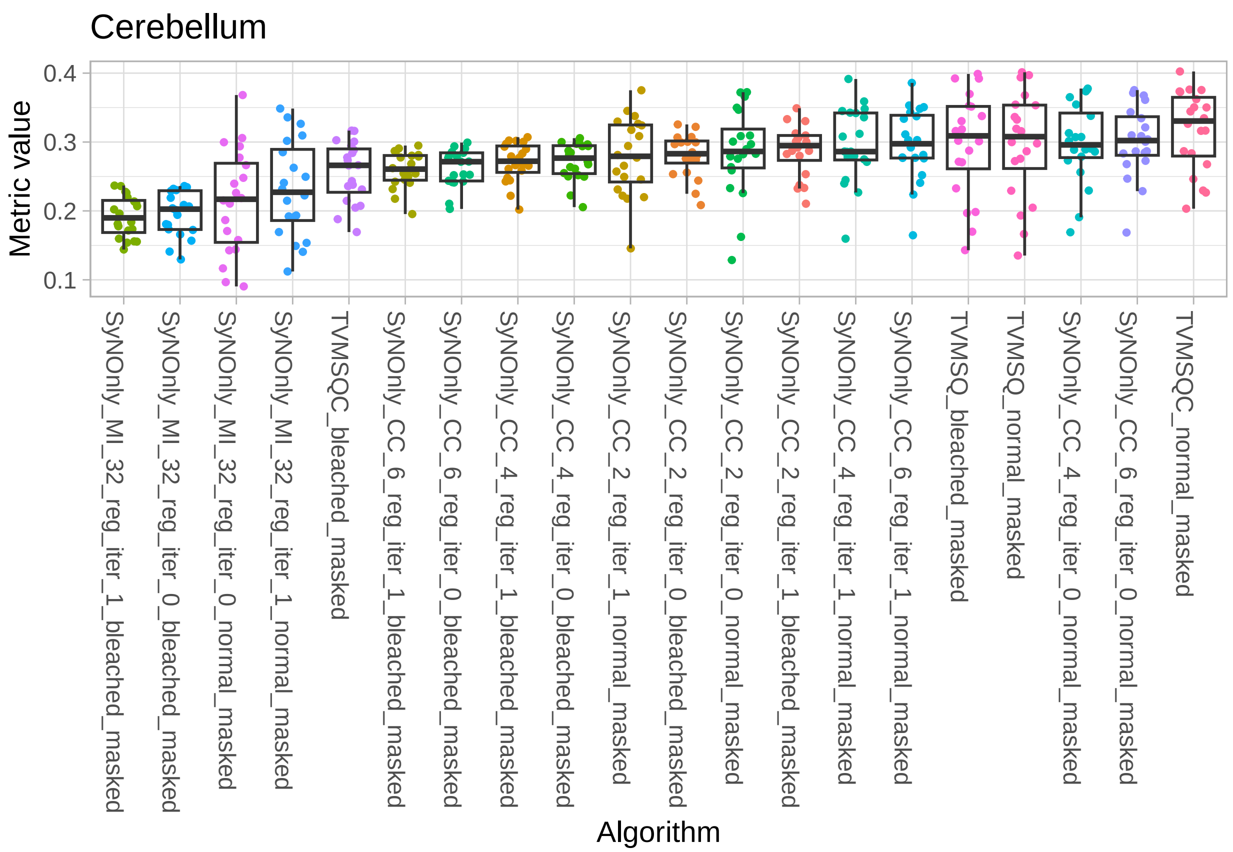
Cerebellum
Metric value
0.1
0.2
0.3
0.4
Algorithm
SyNOnly_MI_32_reg_iter_1_bleached_masked
SyNOnly_MI_32_reg_iter_0_bleached_masked
SyNOnly_MI_32_reg_iter_0_normal_masked
SyNOnly_MI_32_reg_iter_1_normal_masked
TVMSQC_bleached_masked
SyNOnly_CC_6_reg_iter_1_bleached_masked
SyNOnly_CC_6_reg_iter_0_bleached_masked
SyNOnly_CC_4_reg_iter_1_bleached_masked
SyNOnly_CC_4_reg_iter_0_bleached_masked
SyNOnly_CC_2_reg_iter_1_normal_masked
SyNOnly_CC_2_reg_iter_0_bleached_masked
SyNOnly_CC_2_reg_iter_0_normal_masked
SyNOnly_CC_2_reg_iter_1_bleached_masked
SyNOnly_CC_4_reg_iter_1_normal_masked
SyNOnly_CC_6_reg_iter_1_normal_masked
TVMSQ_bleached_masked
TVMSQ_normal_masked
SyNOnly_CC_4_reg_iter_0_normal_masked
SyNOnly_CC_6_reg_iter_0_normal_masked
TVMSQC_normal_masked

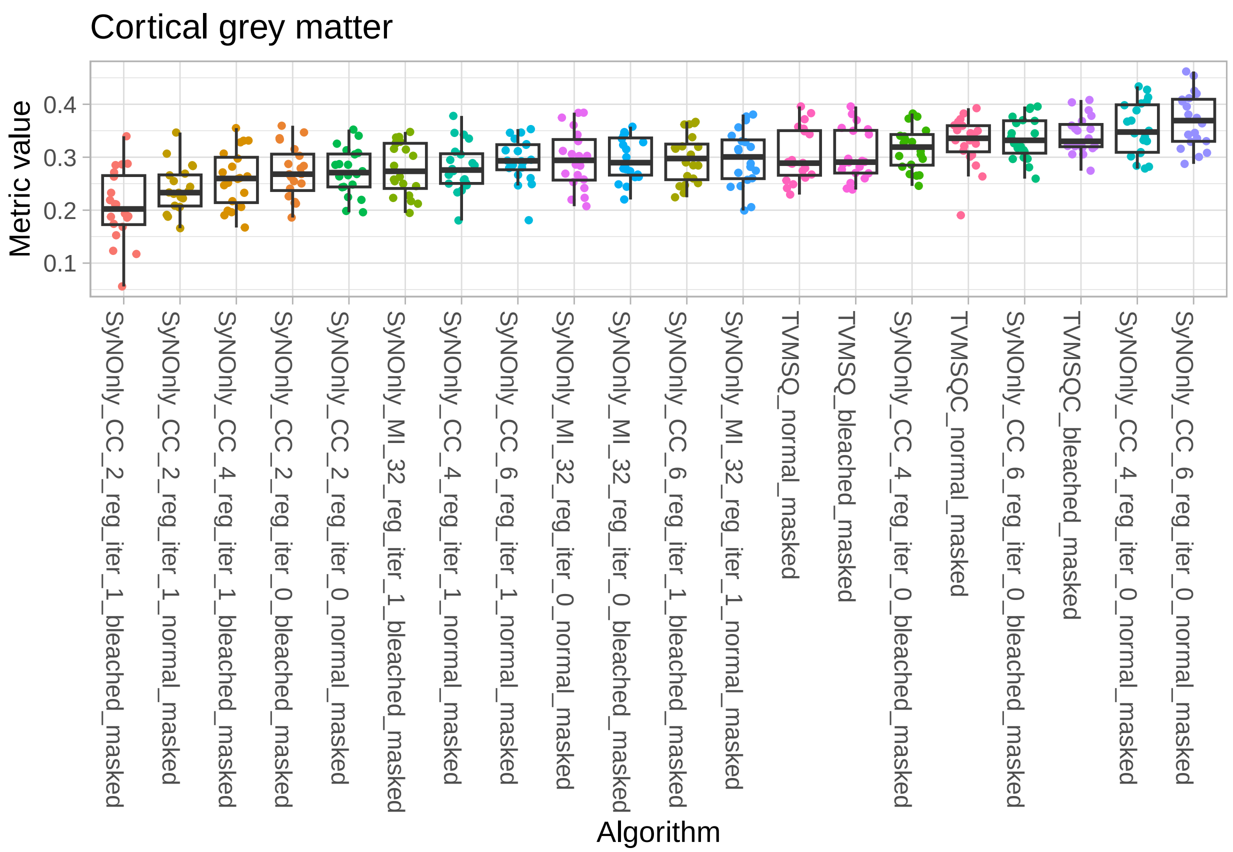
Cortical grey matter
Metric value
0.4
0.3
0.2
0.1
SyNOnly_CC_2_reg_iter_1_bleached_masked
SyNOnly_CC_2_reg_iter_1_normal_masked
SyNOnly_CC_4_reg_iter_1_bleached_masked
SyNOnly_CC_2_reg_iter_0_bleached_masked
SyNOnly_CC_2_reg_iter_0_normal_masked
SyNOnly_MI_32_reg_iter_1_bleached_masked
SyNOnly_CC_4_reg_iter_1_normal_masked
SyNOnly_CC_6_reg_iter_1_normal_masked
SyNOnly_MI_32_reg_iter_0_normal_masked
SyNOnly_MI_32_reg_iter_0_bleached_masked
SyNOnly_CC_6_reg_iter_1_bleached_masked
SyNOnly_MI_32_reg_iter_1_normal_masked
TVMSQ_normal_masked
TVMSQ_bleached_masked
SyNOnly_CC_4_reg_iter_0_bleached_masked
TVMSQC_normal_masked
SyNOnly_CC_6_reg_iter_0_bleached_masked
TVMSQC_bleached_masked
SyNOnly_CC_4_reg_iter_0_normal_masked
SyNOnly_CC_6_reg_iter_0_normal_masked
Algorithm

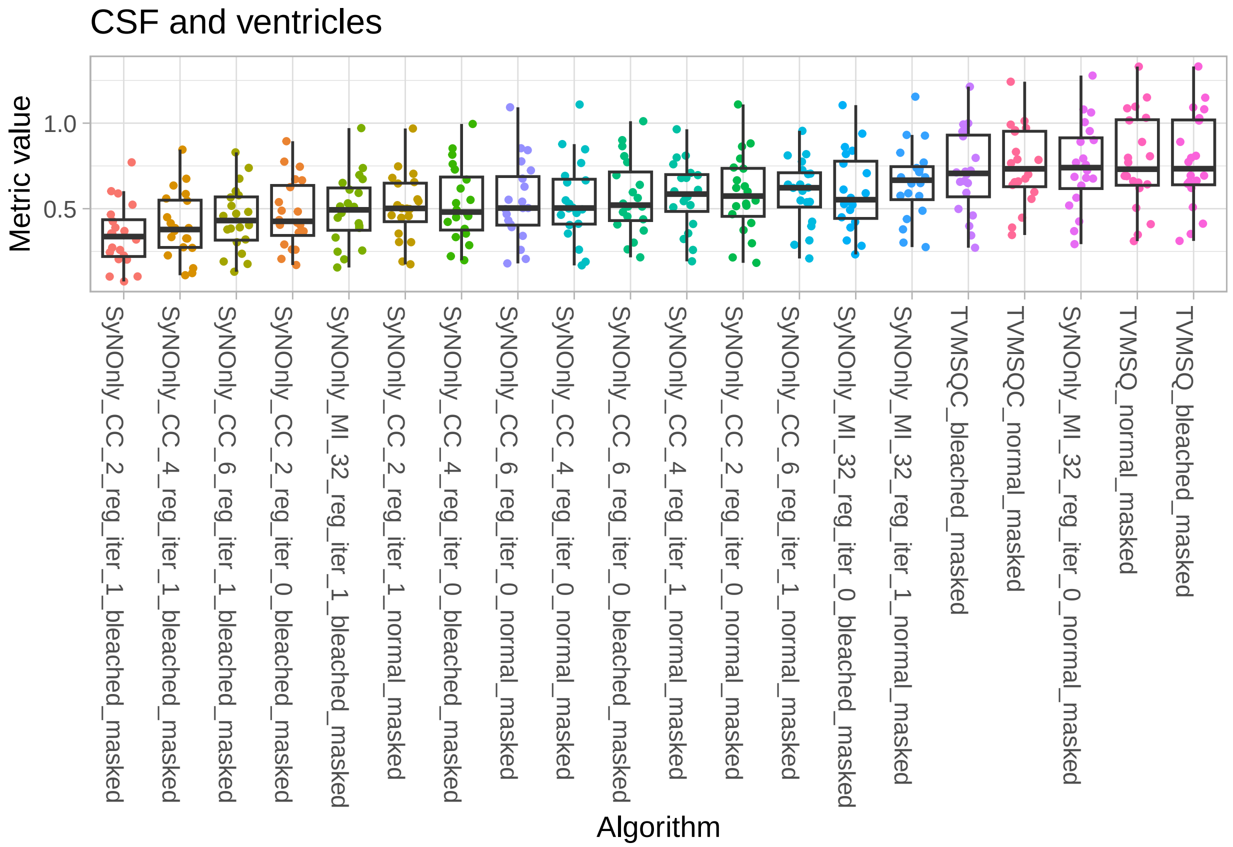
CSF and ventricles
Metric value
1.0
0.5
SyNOnly_CC_2_reg_iter_1_bleached_masked
SyNOnly_CC_4_reg_iter_1_bleached_masked
SyNOnly_CC_6_reg_iter_1_bleached_masked
SyNOnly_CC_2_reg_iter_0_bleached_masked
SyNOnly_MI_32_reg_iter_1_bleached_masked
SyNOnly_CC_2_reg_iter_1_normal_masked
SyNOnly_CC_4_reg_iter_0_bleached_masked
SyNOnly_CC_6_reg_iter_0_normal_masked
SyNOnly_CC_4_reg_iter_0_normal_masked
SyNOnly_CC_6_reg_iter_0_bleached_masked
SyNOnly_CC_4_reg_iter_1_normal_masked
SyNOnly_CC_2_reg_iter_0_normal_masked
SyNOnly_CC_6_reg_iter_1_normal_masked
SyNOnly_MI_32_reg_iter_0_bleached_masked
SyNOnly_MI_32_reg_iter_1_normal_masked
TVMSQC_bleached_masked
TVMSQC_normal_masked
SyNOnly_MI_32_reg_iter_0_normal_masked
TVMSQ_normal_masked
TVMSQ_bleached_masked
Algorithm

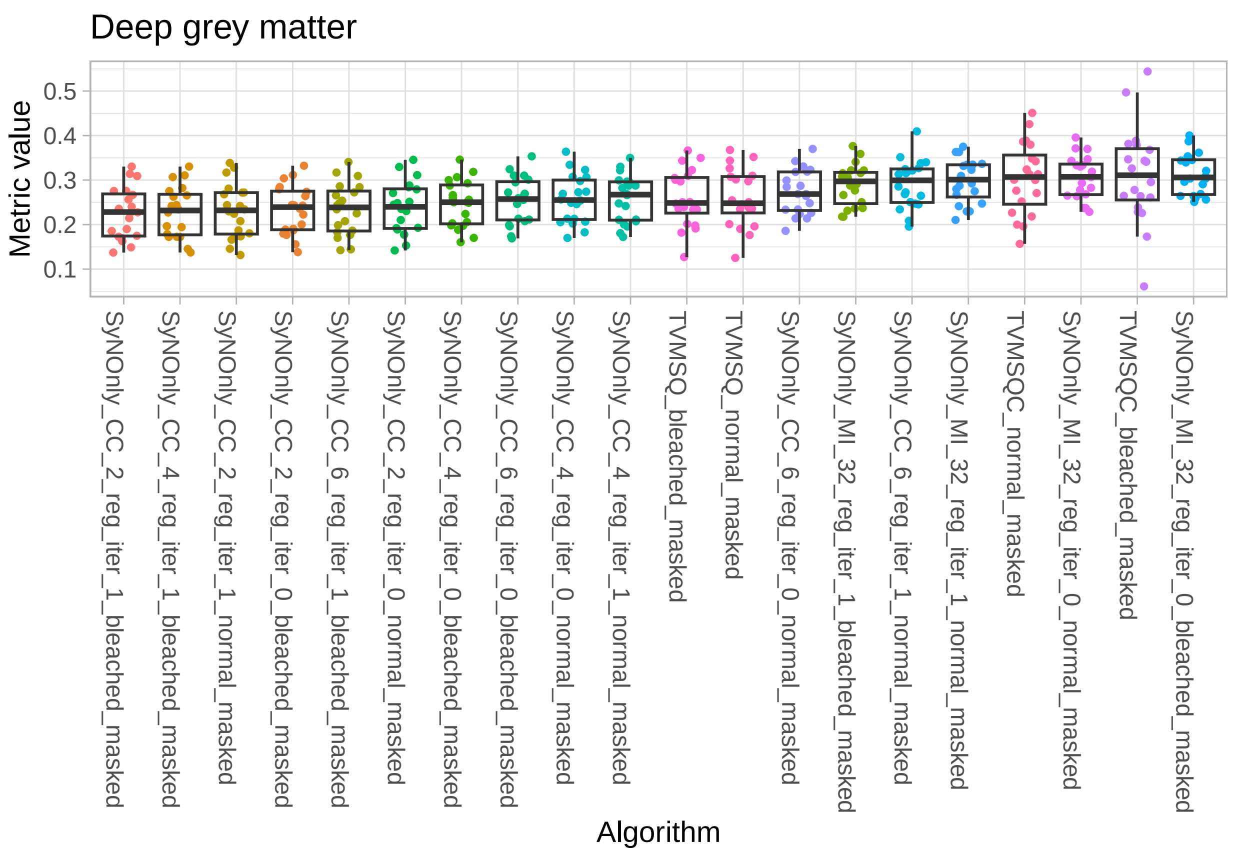
Deep grey matter
Metric value
0.5
0.4
0.3
0.2
0.1
SyNOnly_CC_2_reg_iter_1_bleached_masked
SyNOnly_CC_4_reg_iter_1_bleached_masked
SyNOnly_CC_2_reg_iter_1_normal_masked
SyNOnly_CC_2_reg_iter_0_bleached_masked
SyNOnly_CC_6_reg_iter_1_bleached_masked
SyNOnly_CC_2_reg_iter_0_normal_masked
SyNOnly_CC_4_reg_iter_0_bleached_masked
SyNOnly_CC_6_reg_iter_0_bleached_masked
SyNOnly_CC_4_reg_iter_0_normal_masked
SyNOnly_CC_4_reg_iter_1_normal_masked
TVMSQ_bleached_masked
TVMSQ_normal_masked
SyNOnly_CC_6_reg_iter_0_normal_masked
SyNOnly_MI_32_reg_iter_1_bleached_masked
SyNOnly_CC_6_reg_iter_1_normal_masked
SyNOnly_MI_32_reg_iter_1_normal_masked
TVMSQC_normal_masked
SyNOnly_MI_32_reg_iter_0_normal_masked
TVMSQC_bleached_masked
SyNOnly_MI_32_reg_iter_0_bleached_masked
Algorithm

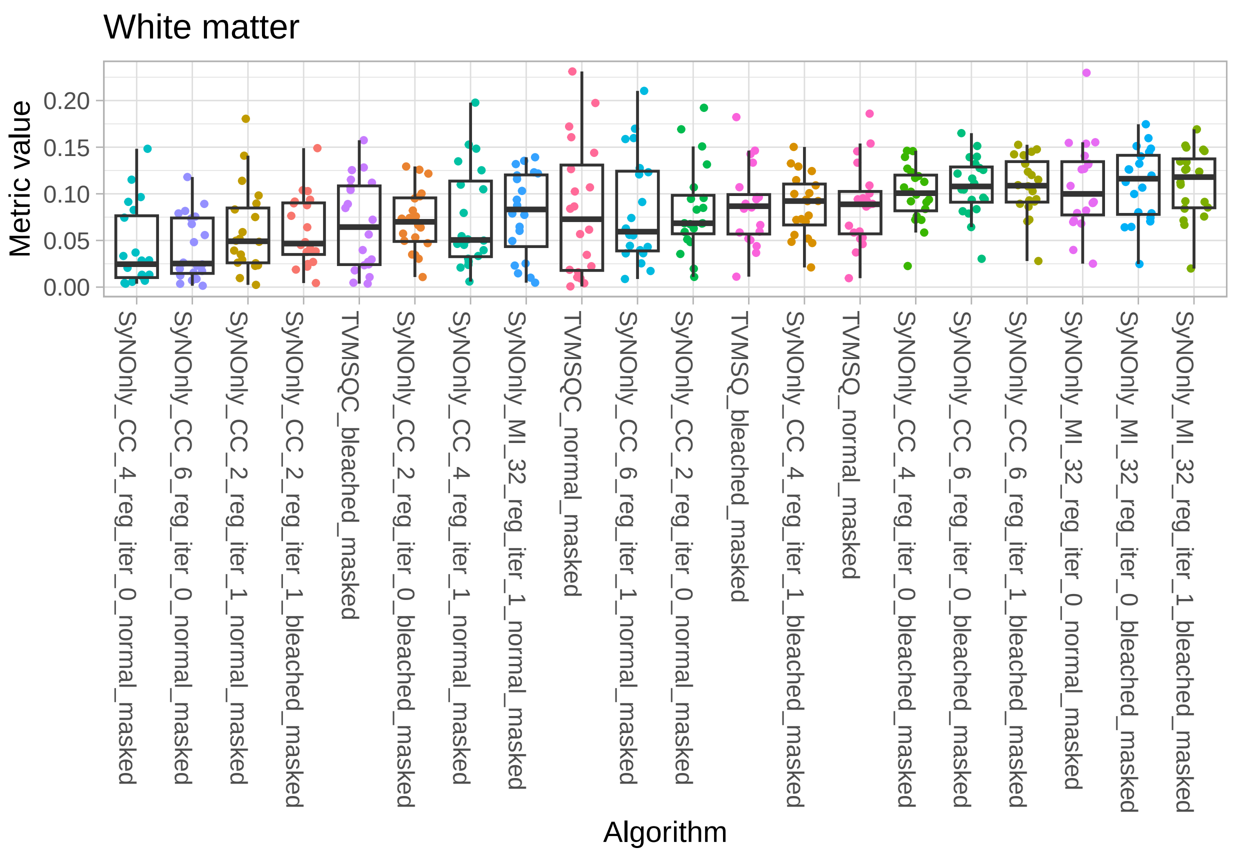
White matter
Metric value
0.20
0.15
0.10
0.05
0.00
SyNOnly_CC_4_reg_iter_0_normal_masked
SyNOnly_CC_6_reg_iter_0_normal_masked
SyNOnly_CC_2_reg_iter_1_normal_masked
SyNOnly_CC_2_reg_iter_1_bleached_masked
TVMSQC_bleached_masked
SyNOnly_CC_2_reg_iter_0_bleached_masked
SyNOnly_CC_4_reg_iter_1_normal_masked
SyNOnly_MI_32_reg_iter_1_normal_masked
TVMSQC_normal_masked
SyNOnly_CC_6_reg_iter_1_normal_masked
SyNOnly_CC_2_reg_iter_0_normal_masked
TVMSQ_bleached_masked
SyNOnly_CC_4_reg_iter_1_bleached_masked
TVMSQ_normal_masked
SyNOnly_CC_4_reg_iter_0_bleached_masked
SyNOnly_CC_6_reg_iter_0_bleached_masked
SyNOnly_CC_6_reg_iter_1_bleached_masked
SyNOnly_MI_32_reg_iter_0_normal_masked
SyNOnly_MI_32_reg_iter_0_bleached_masked
SyNOnly_MI_32_reg_iter_1_bleached_masked
Algorithm

2.2 Podium plot

Podium plots (see also Eugster et al., 2008) for visualizing raw assessment data. Upper part (spaghetti plot): Participating algorithms are color-coded, and each colored dot in the plot represents a metric value achieved with the respective algorithm. The actual metric value is encoded by the y-axis. Each podium (here: p=20) represents one possible rank, ordered from best (1) to last (here: 20). The assignment of metric values (i.e. colored dots) to one of the podiums is based on the rank that the respective algorithm achieved on the corresponding case. Note that the plot part above each podium place is further subdivided into p "columns", where each column represents one participating algorithm (here: $p = 20$). Dots corresponding to identical cases are connected by a line, leading to the shown spaghetti structure. Lower part: Bar charts represent the relative frequency for each algorithm to achieve the rank encoded by the podium place.

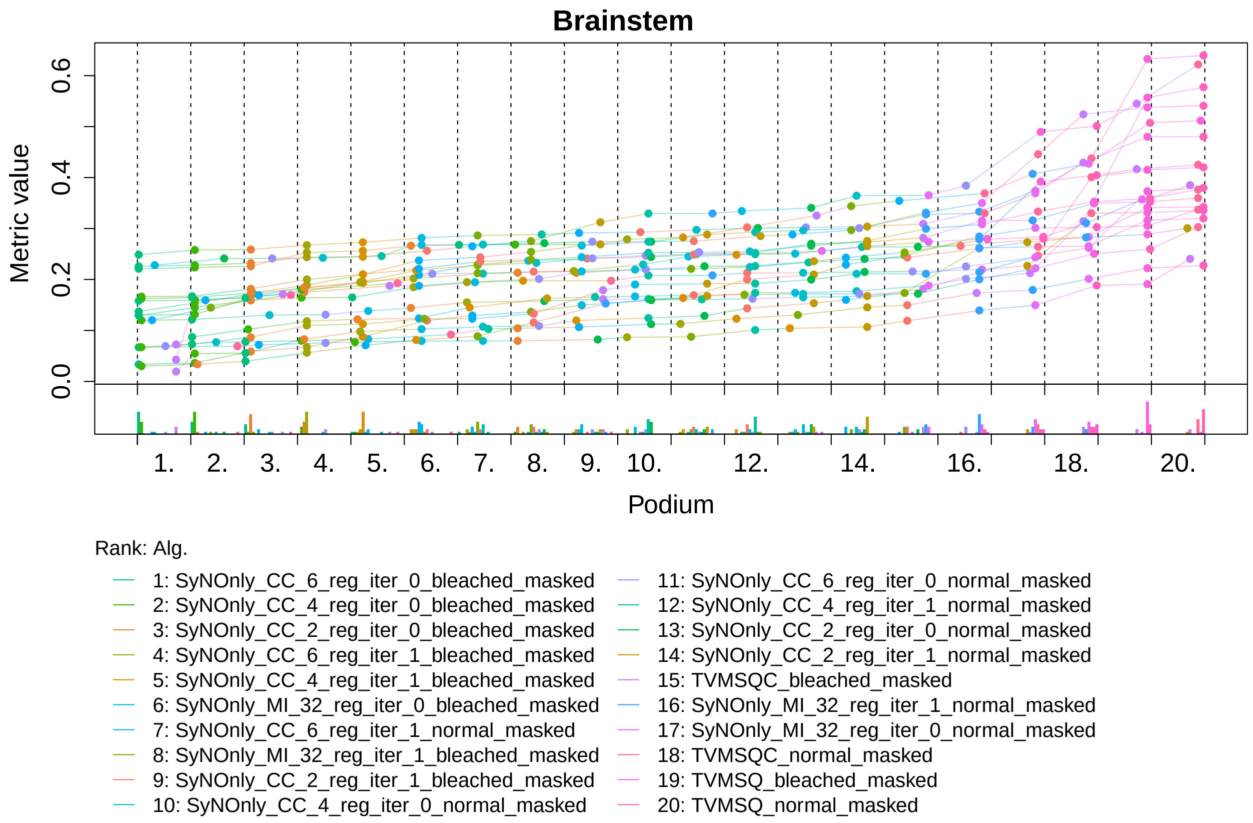

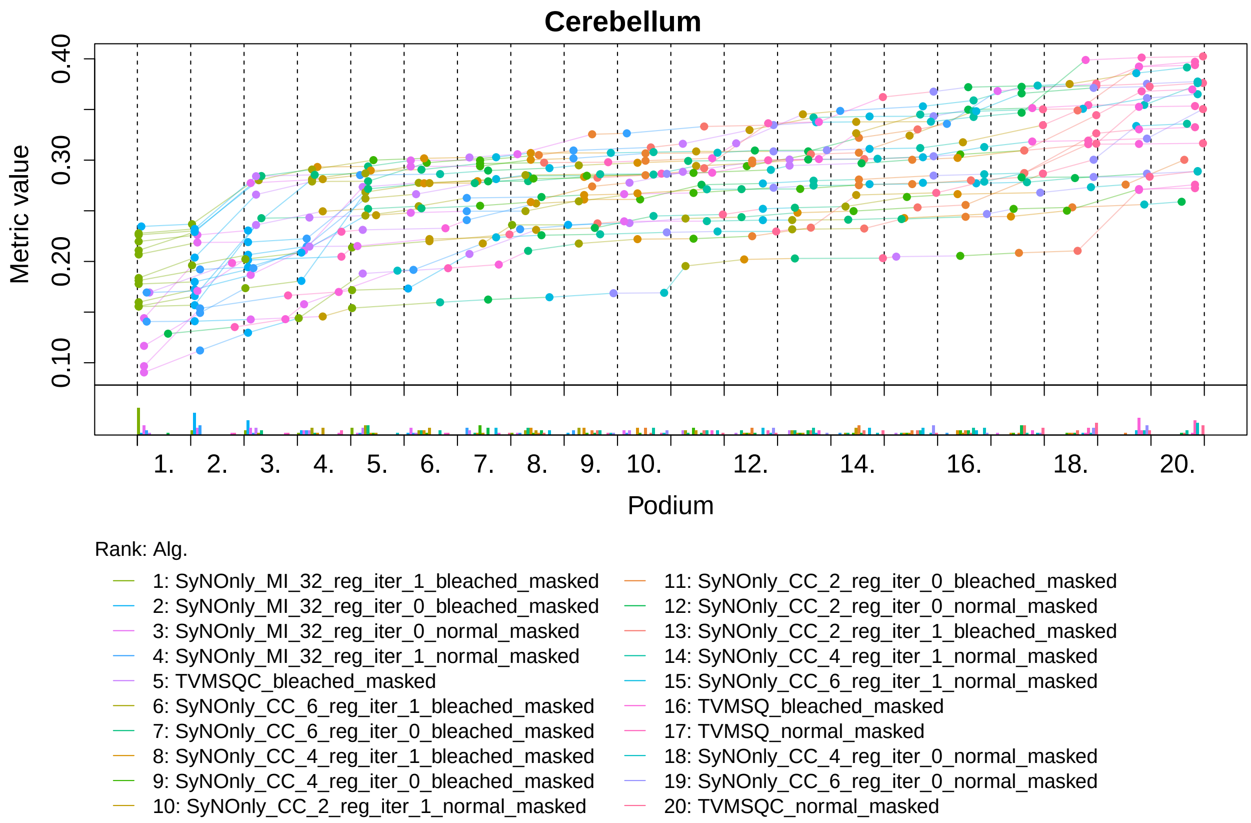
Cerebellum
Metric value
0.10
0.20
0.30
0.40
1.
2.
3.
4.
5.
6.
7.
8.
9.
10.
12.
14.
16.
18.
20.
Podium
Rank: Alg.
1: SyNOnly_MI_32_reg_iter_1_bleached_masked
2: SyNOnly_MI_32_reg_iter_0_bleached_masked
3: SyNOnly_MI_32_reg_iter_0_normal_masked
4: SyNOnly_MI_32_reg_iter_1_normal_masked
5: TVMSQC_bleached_masked
6: SyNOnly_CC_6_reg_iter_1_bleached_masked
7: SyNOnly_CC_6_reg_iter_0_bleached_masked
8: SyNOnly_CC_4_reg_iter_1_bleached_masked
9: SyNOnly_CC_4_reg_iter_0_bleached_masked
10: SyNOnly_CC_2_reg_iter_1_normal_masked
11: SyNOnly_CC_2_reg_iter_0_bleached_masked
12: SyNOnly_CC_2_reg_iter_0_normal_masked
13: SyNOnly_CC_2_reg_iter_1_bleached_masked
14: SyNOnly_CC_4_reg_iter_1_normal_masked
15: SyNOnly_CC_6_reg_iter_1_normal_masked
16: TVMSQ_bleached_masked
17: TVMSQ_normal_masked
18: SyNOnly_CC_4_reg_iter_0_normal_masked
19: SyNOnly_CC_6_reg_iter_0_normal_masked
20: TVMSQC_normal_masked

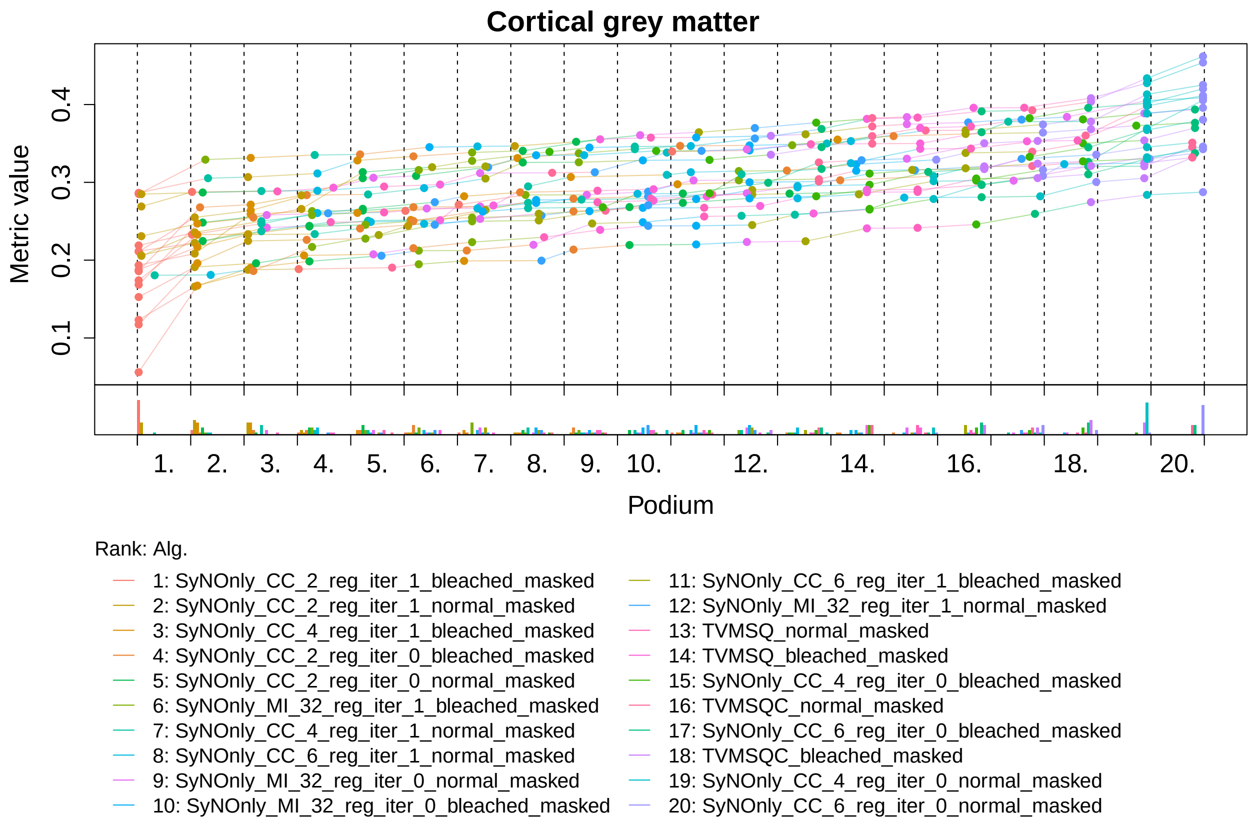
Cortical grey matter
Metric value
0.1
0.2
0.3
0.4
1.
2.
3.
4.
5.
6.
7.
8.
9.
10.
12.
14.
16.
18.
20.
Podium
Rank: Alg.
1: SyNOnly_CC_2_reg_iter_1_bleached_masked
2: SyNOnly_CC_2_reg_iter_1_normal_masked
3: SyNOnly_CC_4_reg_iter_1_bleached_masked
4: SyNOnly_CC_2_reg_iter_0_bleached_masked
5: SyNOnly_CC_2_reg_iter_0_normal_masked
6: SyNOnly_MI_32_reg_iter_1_bleached_masked
7: SyNOnly_CC_4_reg_iter_1_normal_masked
8: SyNOnly_CC_6_reg_iter_1_normal_masked
9: SyNOnly_MI_32_reg_iter_0_normal_masked
10: SyNOnly_MI_32_reg_iter_0_bleached_masked
11: SyNOnly_CC_6_reg_iter_1_bleached_masked
12: SyNOnly_MI_32_reg_iter_1_normal_masked
13: TVMSQ_normal_masked
14: TVMSQ_bleached_masked
15: SyNOnly_CC_4_reg_iter_0_bleached_masked
16: TVMSQC_normal_masked
17: SyNOnly_CC_6_reg_iter_0_bleached_masked
18: TVMSQC_bleached_masked
19: SyNOnly_CC_4_reg_iter_0_normal_masked
20: SyNOnly_CC_6_reg_iter_0_normal_masked

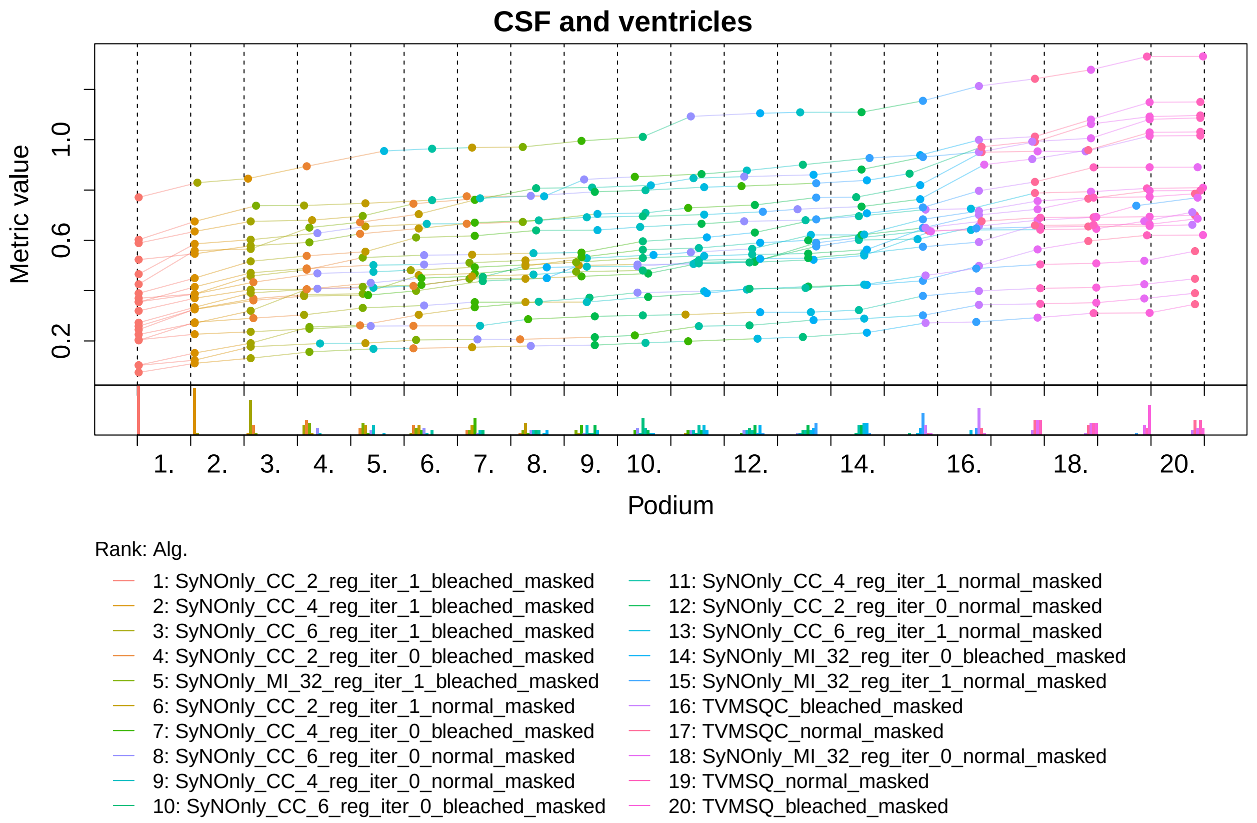
CSF and ventricles
Metric value
0.2
0.6
1.0
1.
2.
3.
4.
5.
6.
7.
8.
9.
10.
12.
14.
16.
18.
20.
Podium
Rank: Alg.
1: SyNOnly_CC_2_reg_iter_1_bleached_masked
2: SyNOnly_CC_4_reg_iter_1_bleached_masked
3: SyNOnly_CC_6_reg_iter_1_bleached_masked
4: SyNOnly_CC_2_reg_iter_0_bleached_masked
5: SyNOnly_MI_32_reg_iter_1_bleached_masked
6: SyNOnly_CC_2_reg_iter_1_normal_masked
7: SyNOnly_CC_4_reg_iter_0_bleached_masked
8: SyNOnly_CC_6_reg_iter_0_normal_masked
9: SyNOnly_CC_4_reg_iter_0_normal_masked
10: SyNOnly_CC_6_reg_iter_0_bleached_masked
11: SyNOnly_CC_4_reg_iter_1_normal_masked
12: SyNOnly_CC_2_reg_iter_0_normal_masked
13: SyNOnly_CC_6_reg_iter_1_normal_masked
14: SyNOnly_MI_32_reg_iter_0_bleached_masked
15: SyNOnly_MI_32_reg_iter_1_normal_masked
16: TVMSQC_bleached_masked
17: TVMSQC_normal_masked
18: SyNOnly_MI_32_reg_iter_0_normal_masked
19: TVMSQ_normal_masked
20: TVMSQ_bleached_masked

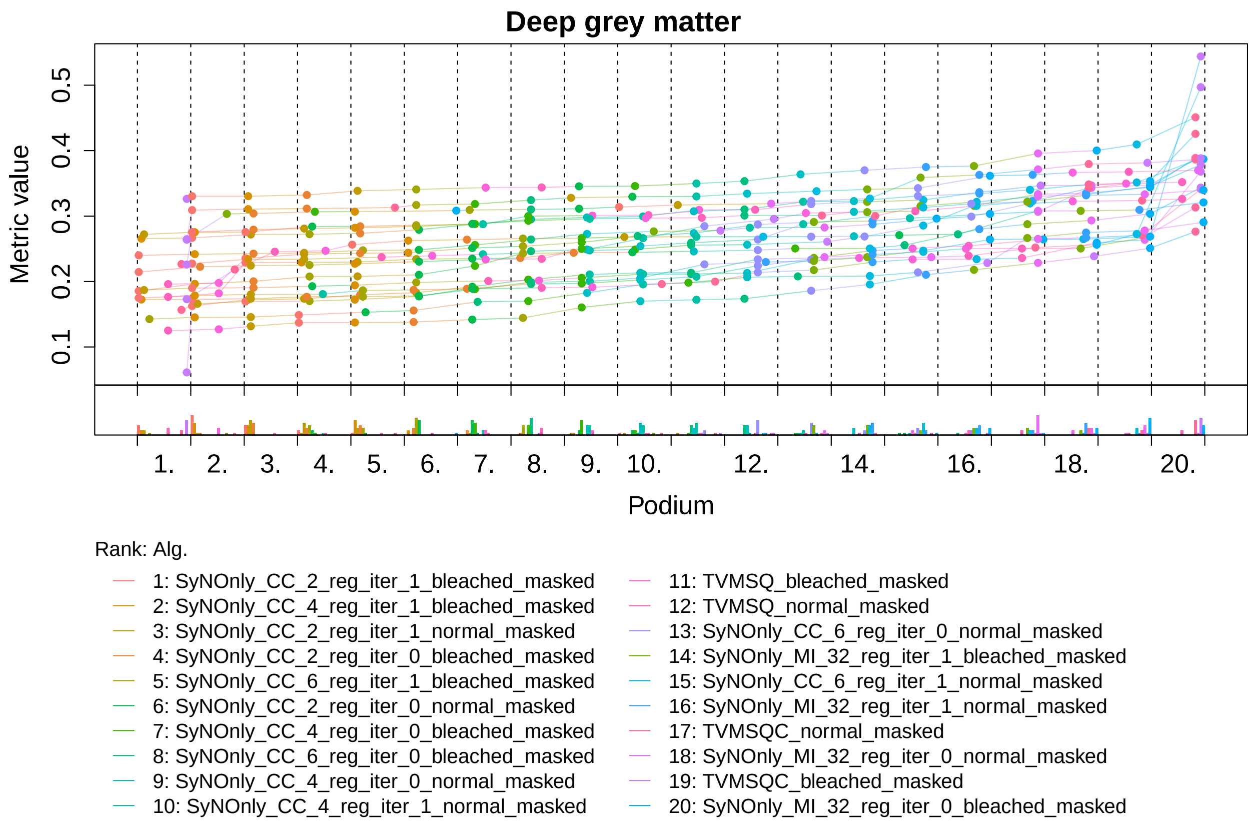
Deep grey matter
Metric value
0.1
0.2
0.3
0.4
0.5
1.
2.
3.
4.
5.
6.
7.
8.
9.
10.
12.
14.
16.
18.
20.
Podium
Rank: Alg.
1: SyNOnly_CC_2_reg_iter_1_bleached_masked
2: SyNOnly_CC_4_reg_iter_1_bleached_masked
3: SyNOnly_CC_2_reg_iter_1_normal_masked
4: SyNOnly_CC_2_reg_iter_0_bleached_masked
5: SyNOnly_CC_6_reg_iter_1_bleached_masked
6: SyNOnly_CC_2_reg_iter_0_normal_masked
7: SyNOnly_CC_4_reg_iter_0_bleached_masked
8: SyNOnly_CC_6_reg_iter_0_bleached_masked
9: SyNOnly_CC_4_reg_iter_0_normal_masked
10: SyNOnly_CC_4_reg_iter_1_normal_masked
11: TVMSQ_bleached_masked
12: TVMSQ_normal_masked
13: SyNOnly_CC_6_reg_iter_0_normal_masked
14: SyNOnly_MI_32_reg_iter_1_bleached_masked
15: SyNOnly_CC_6_reg_iter_1_normal_masked
16: SyNOnly_MI_32_reg_iter_1_normal_masked
17: TVMSQC_normal_masked
18: SyNOnly_MI_32_reg_iter_0_normal_masked
19: TVMSQC_bleached_masked
20: SyNOnly_MI_32_reg_iter_0_bleached_masked

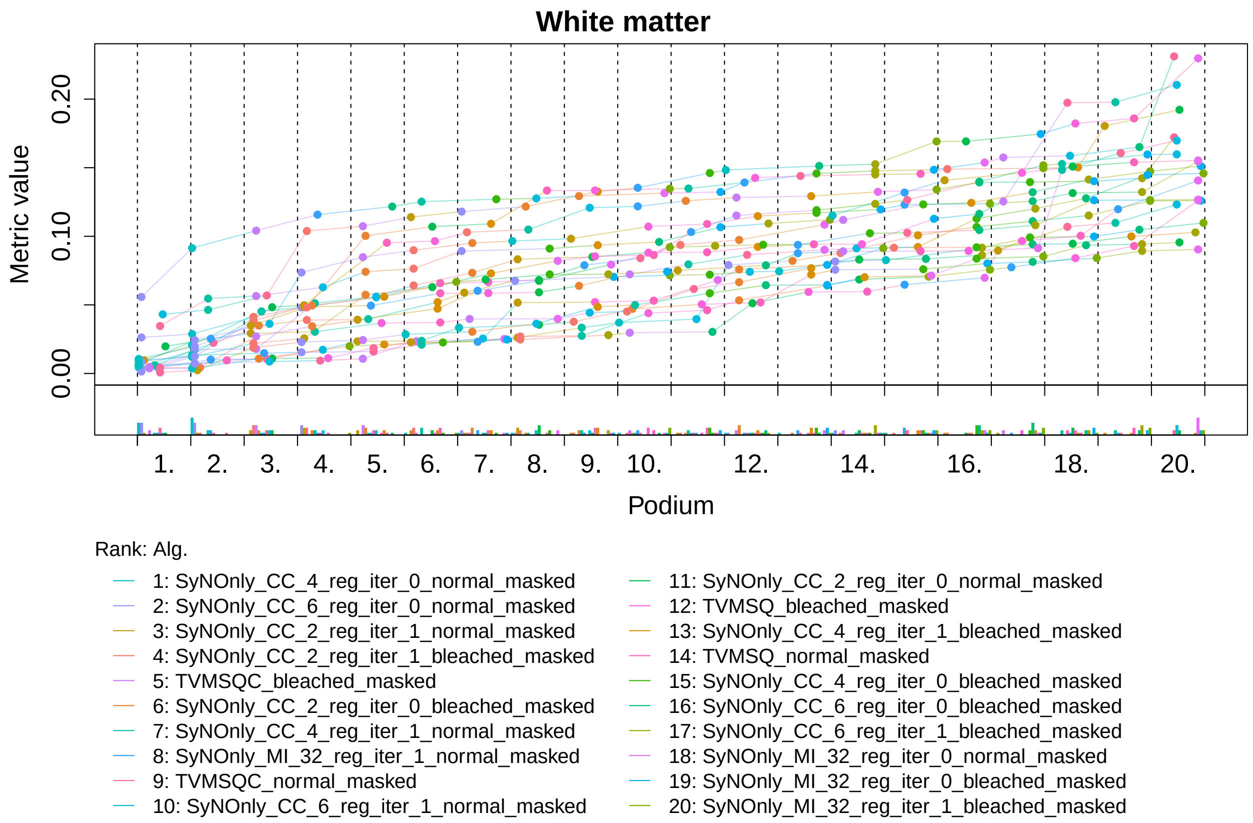
White matter
Metric value
0.00
0.10
0.20
Podium
1.
2.
3.
4.
5.
6.
7.
8.
9.
10.
12.
14.
16.
18.
20.
Rank: Alg.
1: SyNOnly_CC_4_reg_iter_0_normal_masked
2: SyNOnly_CC_6_reg_iter_0_normal_masked
3: SyNOnly_CC_2_reg_iter_1_normal_masked
4: SyNOnly_CC_2_reg_iter_1_bleached_masked
5: TVMSQC_bleached_masked
6: SyNOnly_CC_2_reg_iter_0_bleached_masked
7: SyNOnly_CC_4_reg_iter_1_normal_masked
8: SyNOnly_MI_32_reg_iter_1_normal_masked
9: TVMSQC_normal_masked
10: SyNOnly_CC_6_reg_iter_1_normal_masked
11: SyNOnly_CC_2_reg_iter_0_normal_masked
12: TVMSQ_bleached_masked
13: SyNOnly_CC_4_reg_iter_1_bleached_masked
14: TVMSQ_normal_masked
15: SyNOnly_CC_4_reg_iter_0_bleached_masked
16: SyNOnly_CC_6_reg_iter_0_bleached_masked
17: SyNOnly_CC_6_reg_iter_1_bleached_masked
18: SyNOnly_MI_32_reg_iter_0_normal_masked
19: SyNOnly_MI_32_reg_iter_0_bleached_masked
20: SyNOnly_MI_32_reg_iter_1_bleached_masked

2.3 Ranking heatmap

Ranking heatmaps for visualizing raw assessment data. Each cell (i, A_j) shows the absolute frequency of cases in which algorithm A_j achieved rank i.

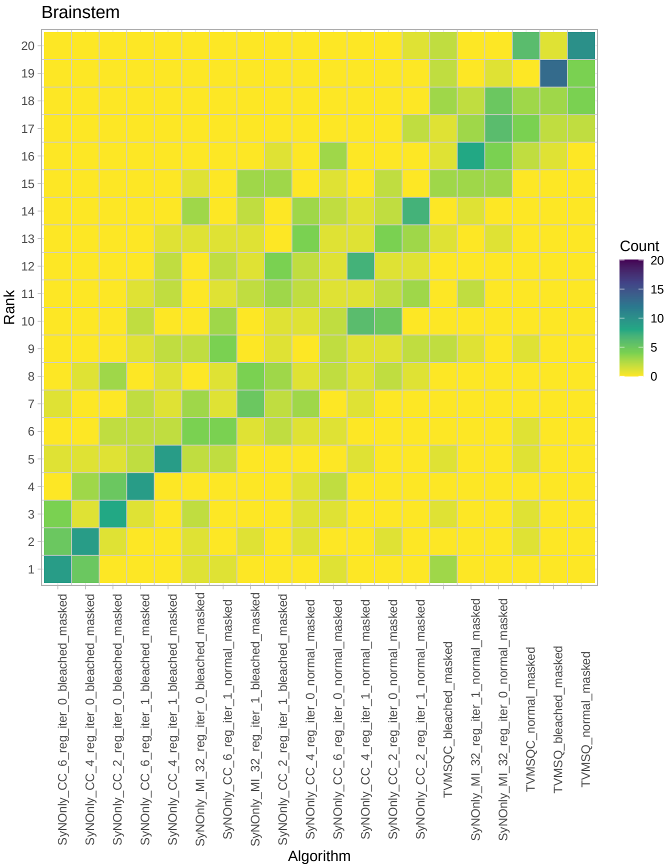

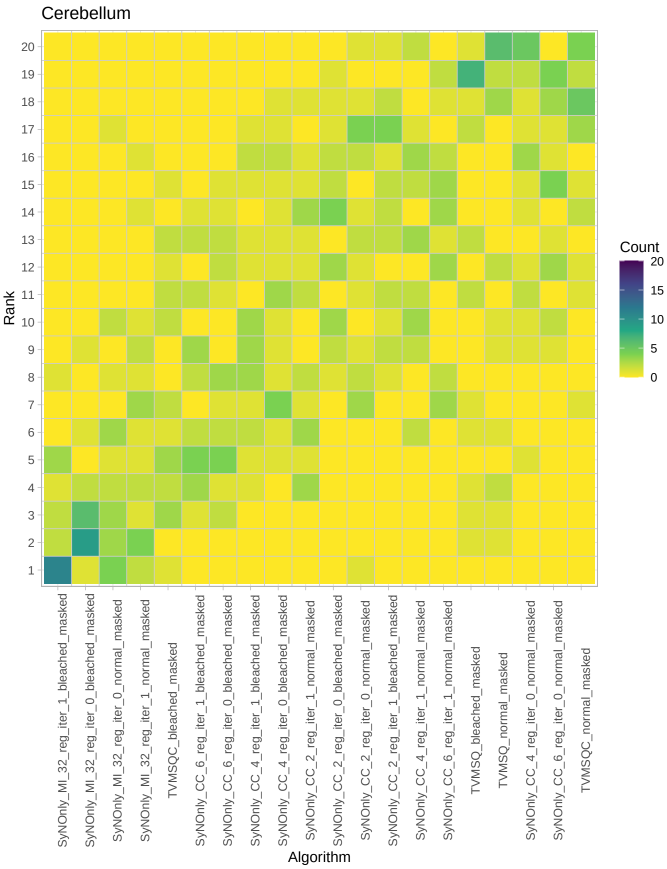
Cerebellum
Rank
20
19
18
17
16
15
14
13
12
11
10
9
8
7
6
5
4
3
2
1
SyNOnly_MI_32_reg_iter_1_bleached_masked
SyNOnly_MI_32_reg_iter_0_bleached_masked
SyNOnly_MI_32_reg_iter_0_normal_masked
SyNOnly_MI_32_reg_iter_1_normal_masked
TVMSQC_bleached_masked
SyNOnly_CC_6_reg_iter_1_bleached_masked
SyNOnly_CC_6_reg_iter_0_bleached_masked
SyNOnly_CC_4_reg_iter_1_bleached_masked
SyNOnly_CC_4_reg_iter_0_bleached_masked
SyNOnly_CC_2_reg_iter_1_normal_masked
SyNOnly_CC_2_reg_iter_0_bleached_masked
SyNOnly_CC_2_reg_iter_0_normal_masked
SyNOnly_CC_2_reg_iter_1_bleached_masked
SyNOnly_CC_4_reg_iter_1_normal_masked
SyNOnly_CC_6_reg_iter_1_normal_masked
TVMSQ_bleached_masked
TVMSQ_normal_masked
SyNOnly_CC_4_reg_iter_0_normal_masked
SyNOnly_CC_6_reg_iter_0_normal_masked
TVMSQC_normal_masked
Algorithm
Count
20
15
10
5
0

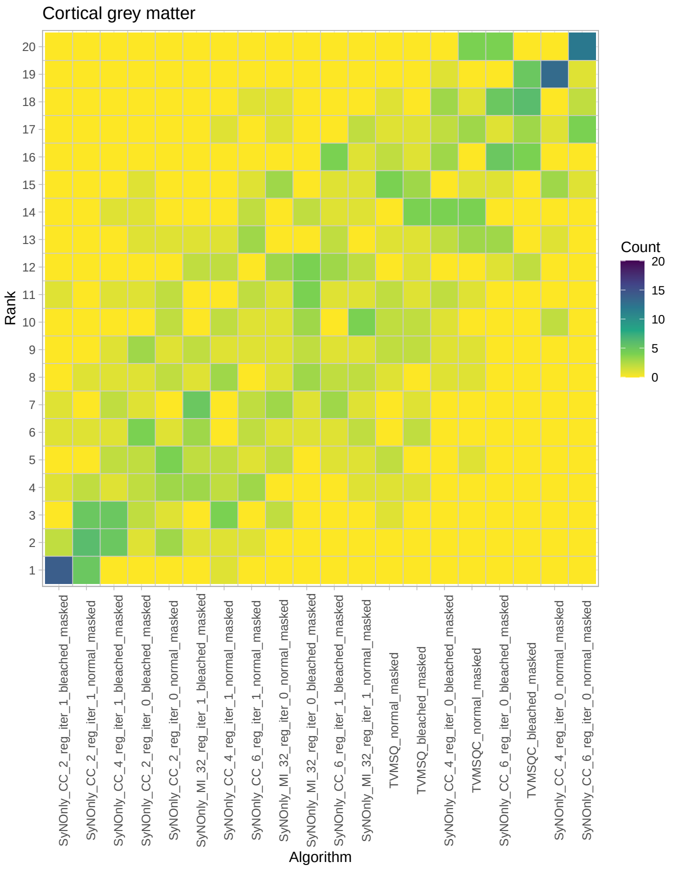
Cortical grey matter
Rank
Algorithm
Count
20
15
10
5
0
SyNOnly_CC_2_reg_iter_1_bleached_masked
SyNOnly_CC_2_reg_iter_1_normal_masked
SyNOnly_CC_4_reg_iter_1_bleached_masked
SyNOnly_CC_2_reg_iter_0_bleached_masked
SyNOnly_CC_2_reg_iter_0_normal_masked
SyNOnly_MI_32_reg_iter_1_bleached_masked
SyNOnly_CC_4_reg_iter_1_normal_masked
SyNOnly_CC_6_reg_iter_1_normal_masked
SyNOnly_MI_32_reg_iter_0_normal_masked
SyNOnly_MI_32_reg_iter_0_bleached_masked
SyNOnly_CC_6_reg_iter_1_bleached_masked
SyNOnly_MI_32_reg_iter_1_normal_masked
TVMSQ_normal_masked
TVMSQ_bleached_masked
SyNOnly_CC_4_reg_iter_0_bleached_masked
TVMSQC_normal_masked
SyNOnly_CC_6_reg_iter_0_bleached_masked
TVMSQC_bleached_masked
SyNOnly_CC_4_reg_iter_0_normal_masked
SyNOnly_CC_6_reg_iter_0_normal_masked

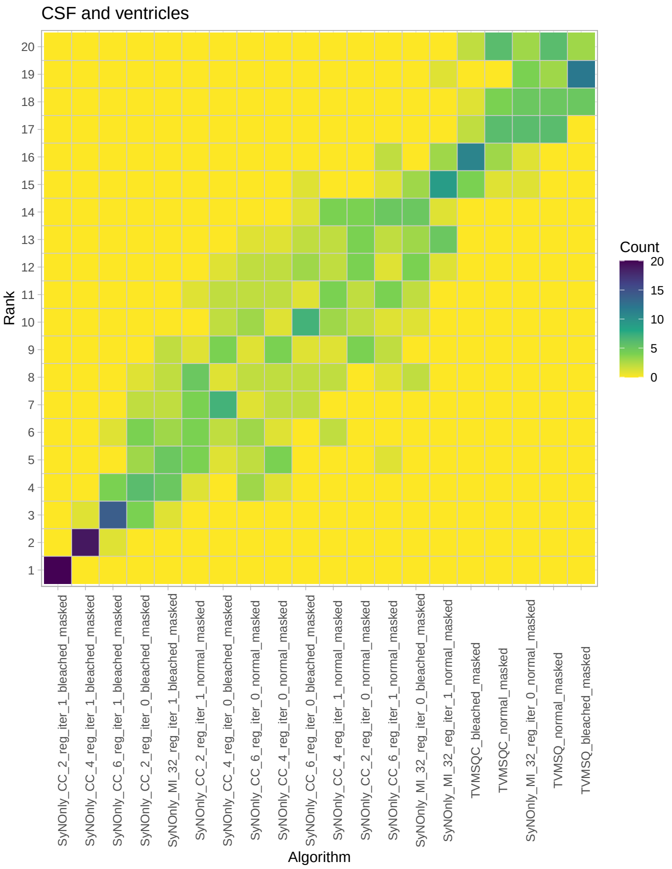
CSF and ventricles
Rank
Algorithm
Count
20
15
10
5
0
SyNOnly_CC_2_reg_iter_1_bleached_masked
SyNOnly_CC_4_reg_iter_1_bleached_masked
SyNOnly_CC_6_reg_iter_1_bleached_masked
SyNOnly_CC_2_reg_iter_0_bleached_masked
SyNOnly_MI_32_reg_iter_1_bleached_masked
SyNOnly_CC_2_reg_iter_1_normal_masked
SyNOnly_CC_4_reg_iter_0_bleached_masked
SyNOnly_CC_6_reg_iter_0_normal_masked
SyNOnly_CC_4_reg_iter_0_normal_masked
SyNOnly_CC_6_reg_iter_0_bleached_masked
SyNOnly_CC_4_reg_iter_1_normal_masked
SyNOnly_CC_2_reg_iter_0_normal_masked
SyNOnly_CC_6_reg_iter_1_normal_masked
SyNOnly_MI_32_reg_iter_0_bleached_masked
SyNOnly_MI_32_reg_iter_1_normal_masked
TVMSQC_bleached_masked
TVMSQC_normal_masked
SyNOnly_MI_32_reg_iter_0_normal_masked
TVMSQ_normal_masked
TVMSQ_bleached_masked

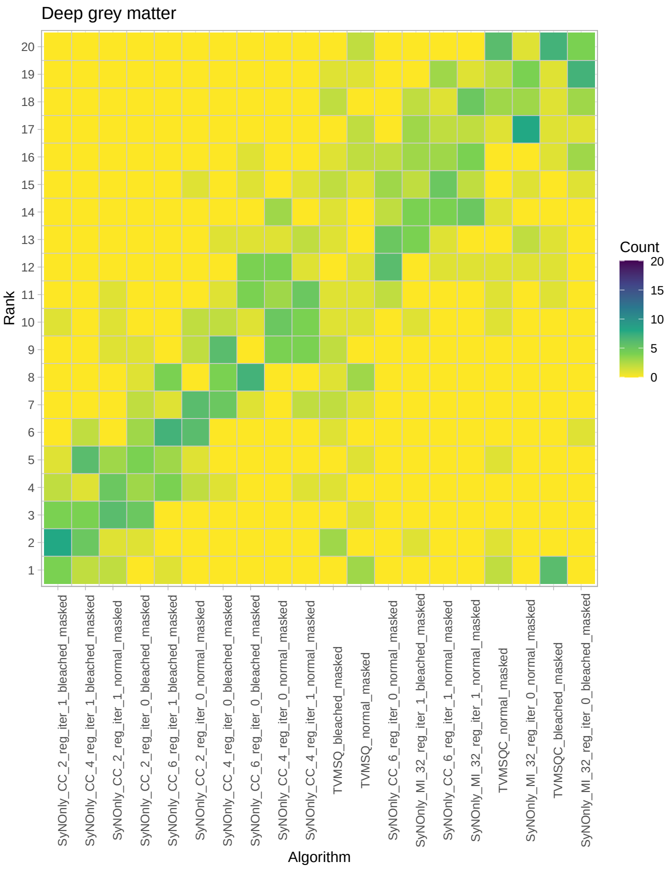
Deep grey matter
Rank
Algorithm
Count
20
15
10
5
0
SyNOnly_CC_2_reg_iter_1_bleached_masked
SyNOnly_CC_4_reg_iter_1_bleached_masked
SyNOnly_CC_2_reg_iter_1_normal_masked
SyNOnly_CC_2_reg_iter_0_bleached_masked
SyNOnly_CC_6_reg_iter_1_bleached_masked
SyNOnly_CC_2_reg_iter_0_normal_masked
SyNOnly_CC_4_reg_iter_0_bleached_masked
SyNOnly_CC_6_reg_iter_0_bleached_masked
SyNOnly_CC_4_reg_iter_0_normal_masked
SyNOnly_CC_4_reg_iter_1_normal_masked
TVMSQ_bleached_masked
TVMSQ_normal_masked
SyNOnly_CC_6_reg_iter_0_normal_masked
SyNOnly_MI_32_reg_iter_1_bleached_masked
SyNOnly_CC_6_reg_iter_1_normal_masked
SyNOnly_MI_32_reg_iter_1_normal_masked
TVMSQC_normal_masked
SyNOnly_MI_32_reg_iter_0_normal_masked
TVMSQC_bleached_masked
SyNOnly_MI_32_reg_iter_0_bleached_masked

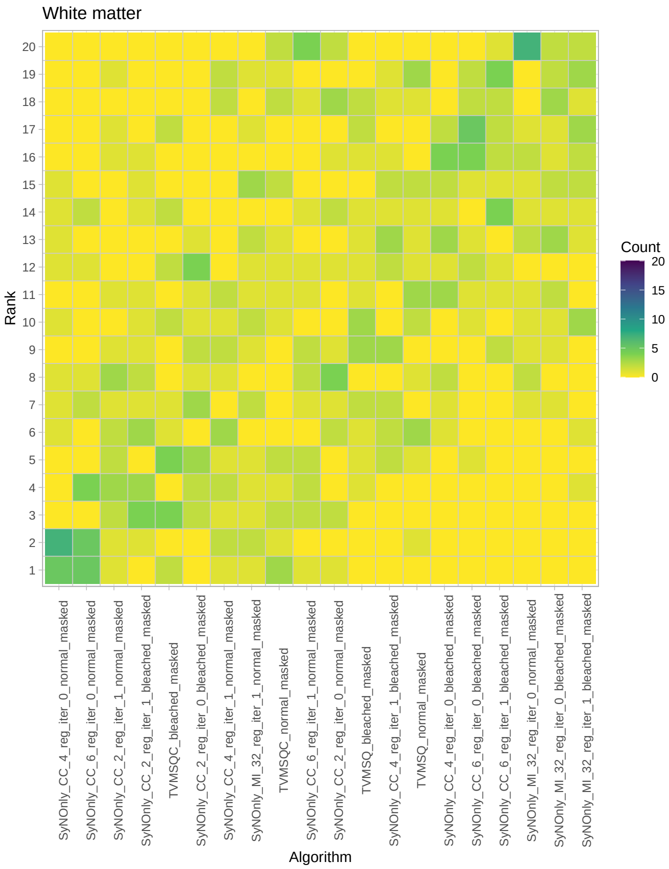
White matter
Rank
Algorithm
Count
20
15
10
5
0
SyNOnly_CC_4_reg_iter_0_normal_masked
SyNOnly_CC_6_reg_iter_0_normal_masked
SyNOnly_CC_2_reg_iter_1_normal_masked
SyNOnly_CC_2_reg_iter_1_bleached_masked
TVMSQC_bleached_masked
SyNOnly_CC_2_reg_iter_0_bleached_masked
SyNOnly_CC_4_reg_iter_1_normal_masked
SyNOnly_MI_32_reg_iter_1_normal_masked
TVMSQC_normal_masked
SyNOnly_CC_6_reg_iter_1_normal_masked
SyNOnly_CC_2_reg_iter_0_normal_masked
TVMSQ_bleached_masked
SyNOnly_CC_4_reg_iter_1_bleached_masked
TVMSQ_normal_masked
SyNOnly_CC_4_reg_iter_0_bleached_masked
SyNOnly_CC_6_reg_iter_0_bleached_masked
SyNOnly_CC_6_reg_iter_1_bleached_masked
SyNOnly_MI_32_reg_iter_0_normal_masked
SyNOnly_MI_32_reg_iter_0_bleached_masked
SyNOnly_MI_32_reg_iter_1_bleached_masked

3 Visualization of ranking stability

3.1 *Blob plot* for visualizing ranking stability based on bootstrap sampling

Algorithms are color-coded, and the area of each blob at position $(A_i, \text{rank } j)$ is proportional to the relative frequency A_i achieved rank j across $b = 1000$ bootstrap samples. The median rank for each algorithm is indicated by a black cross. 95% bootstrap intervals across bootstrap samples are indicated by black lines.

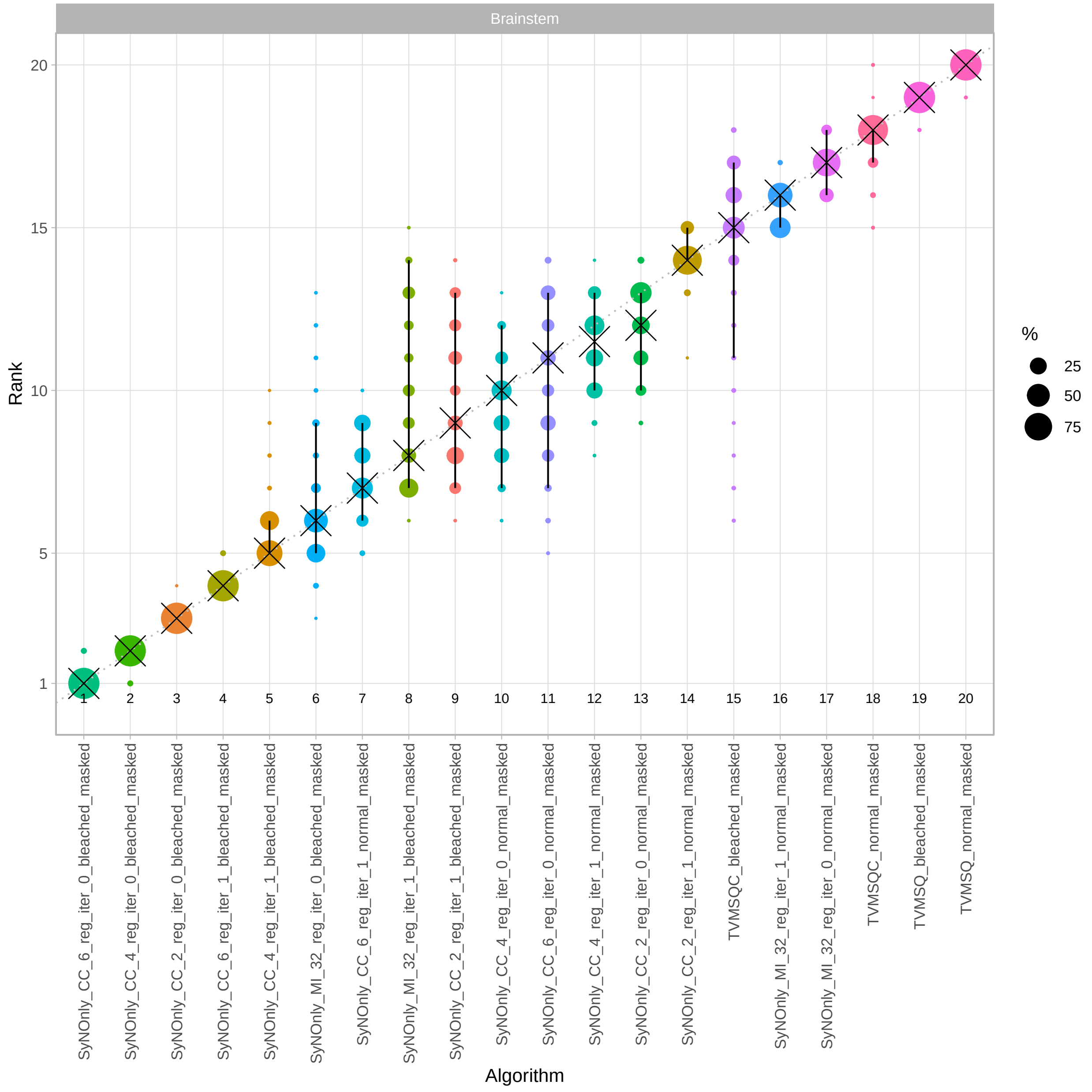

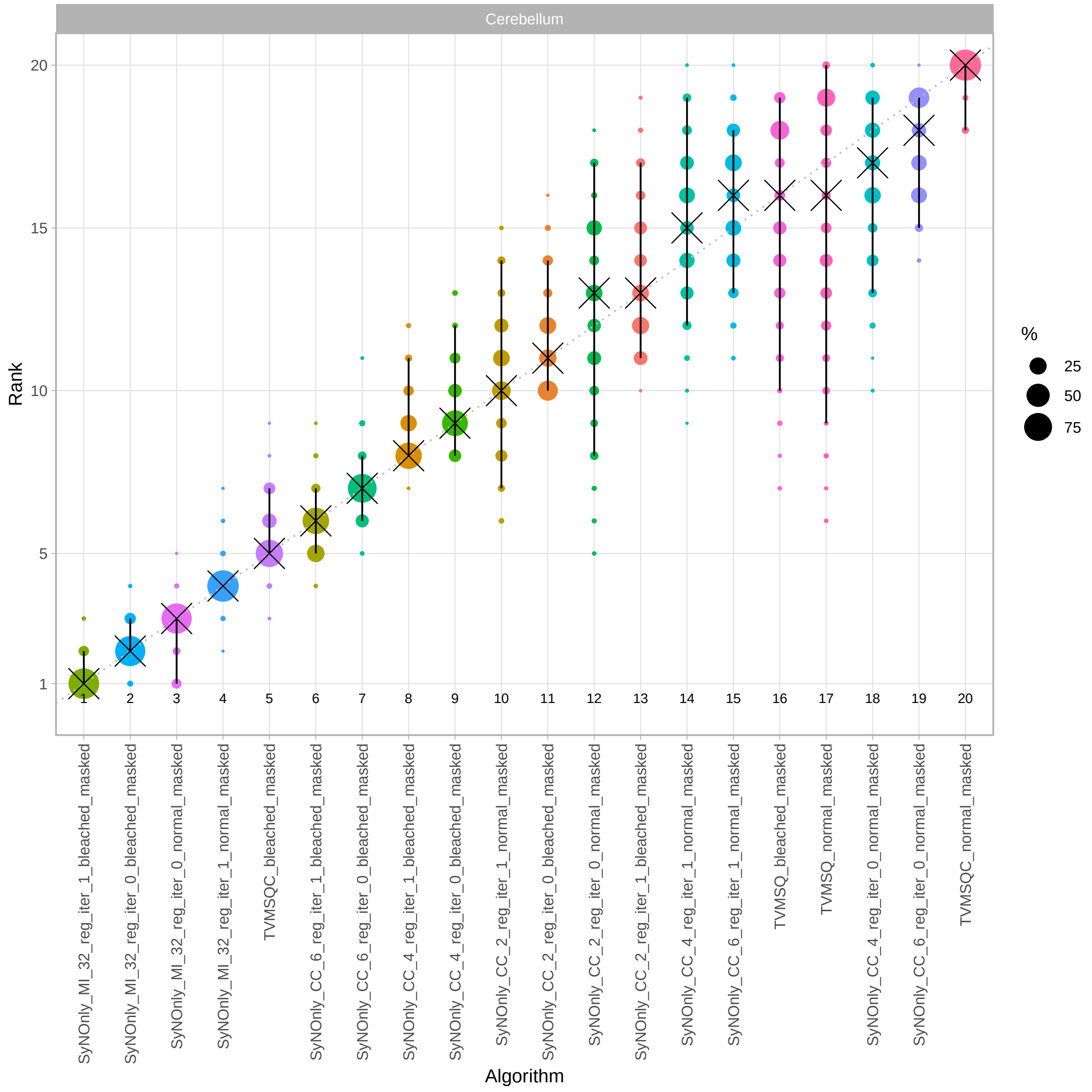
Cerebellum
Rank
%
25
50
75
Algorithm
SyNOnly_MI_32_reg_iter_1_bleached_masked
SyNOnly_MI_32_reg_iter_0_bleached_masked
SyNOnly_MI_32_reg_iter_0_normal_masked
SyNOnly_MI_32_reg_iter_1_normal_masked
TVMSQC_bleached_masked
SyNOnly_CC_6_reg_iter_1_bleached_masked
SyNOnly_CC_6_reg_iter_0_bleached_masked
SyNOnly_CC_4_reg_iter_1_bleached_masked
SyNOnly_CC_4_reg_iter_0_bleached_masked
SyNOnly_CC_2_reg_iter_1_normal_masked
SyNOnly_CC_2_reg_iter_0_bleached_masked
SyNOnly_CC_2_reg_iter_0_normal_masked
SyNOnly_CC_2_reg_iter_1_bleached_masked
SyNOnly_CC_4_reg_iter_1_normal_masked
SyNOnly_CC_6_reg_iter_1_normal_masked
TVMSQ_bleached_masked
TVMSQ_normal_masked
SyNOnly_CC_4_reg_iter_0_normal_masked
SyNOnly_CC_6_reg_iter_0_normal_masked
TVMSQC_normal_masked

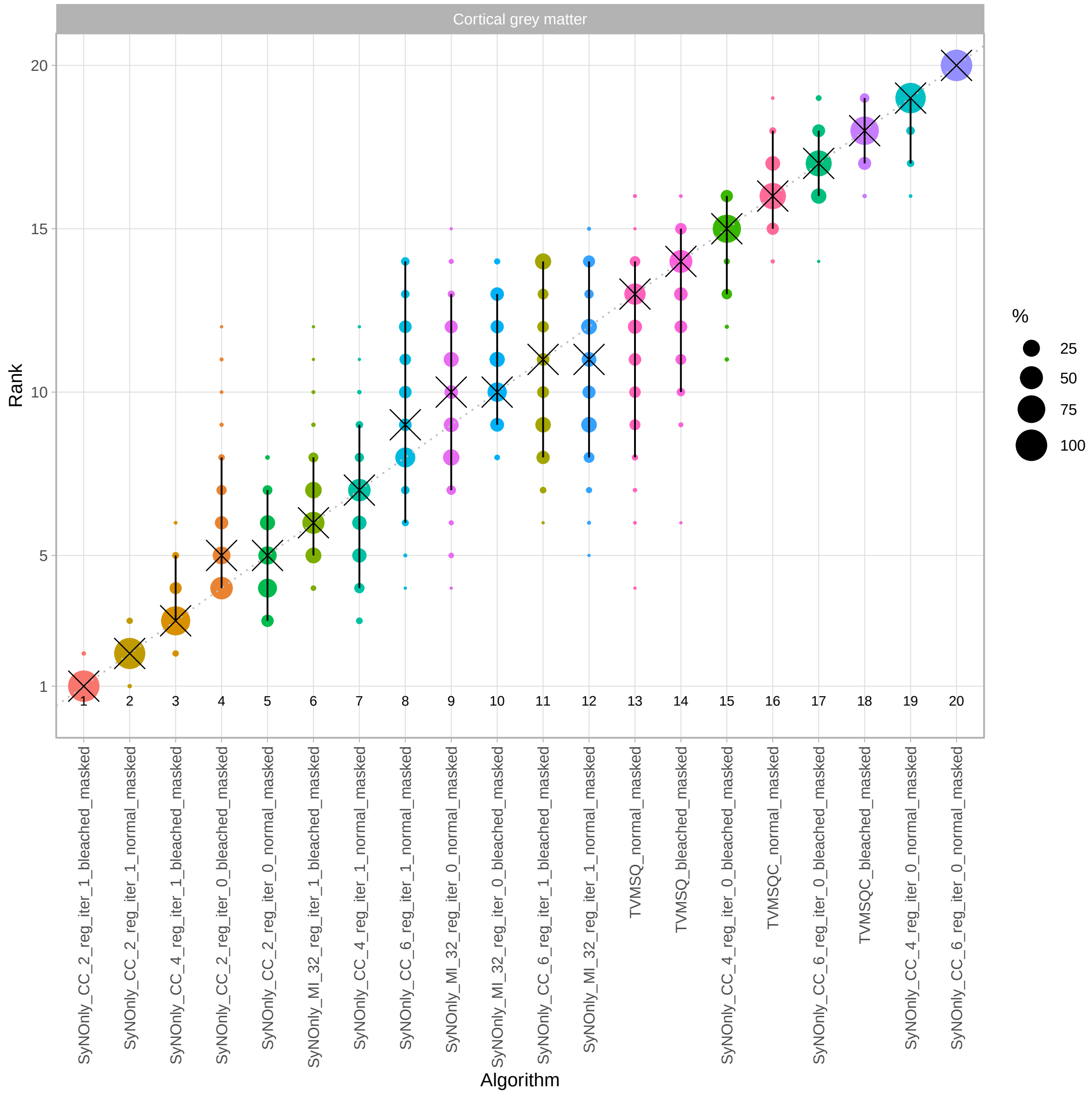
Cortical grey matter
Rank
Algorithm
%
25
50
75
100
SyNOnly_CC_2_reg_iter_1_bleached_masked
SyNOnly_CC_2_reg_iter_1_normal_masked
SyNOnly_CC_4_reg_iter_1_bleached_masked
SyNOnly_CC_2_reg_iter_0_bleached_masked
SyNOnly_CC_2_reg_iter_0_normal_masked
SyNOnly_MI_32_reg_iter_1_bleached_masked
SyNOnly_CC_4_reg_iter_1_normal_masked
SyNOnly_CC_6_reg_iter_1_normal_masked
SyNOnly_MI_32_reg_iter_0_normal_masked
SyNOnly_MI_32_reg_iter_0_bleached_masked
SyNOnly_CC_6_reg_iter_1_bleached_masked
SyNOnly_MI_32_reg_iter_1_normal_masked
TVMSQ_normal_masked
TVMSQ_bleached_masked
SyNOnly_CC_4_reg_iter_0_bleached_masked
TVMSQC_normal_masked
SyNOnly_CC_6_reg_iter_0_bleached_masked
TVMSQC_bleached_masked
SyNOnly_CC_4_reg_iter_0_normal_masked
SyNOnly_CC_6_reg_iter_0_normal_masked

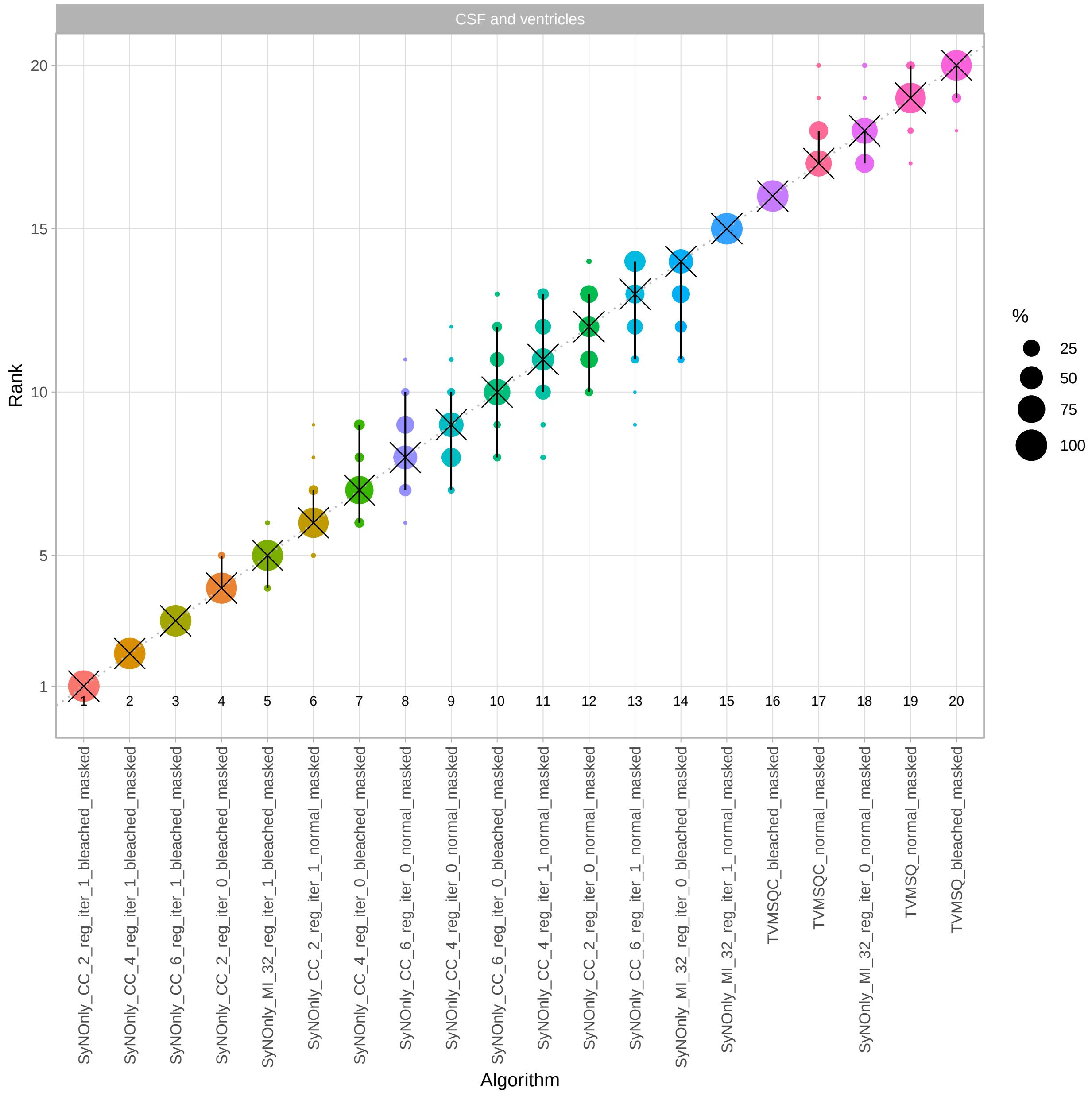
CSF and ventricles
Rank
Algorithm
%
25
50
75
100
SyNOnly_CC_2_reg_iter_1_bleached_masked
SyNOnly_CC_4_reg_iter_1_bleached_masked
SyNOnly_CC_6_reg_iter_1_bleached_masked
SyNOnly_CC_2_reg_iter_0_bleached_masked
SyNOnly_MI_32_reg_iter_1_bleached_masked
SyNOnly_CC_2_reg_iter_1_normal_masked
SyNOnly_CC_4_reg_iter_0_bleached_masked
SyNOnly_CC_6_reg_iter_0_normal_masked
SyNOnly_CC_4_reg_iter_0_normal_masked
SyNOnly_CC_6_reg_iter_0_bleached_masked
SyNOnly_CC_4_reg_iter_1_normal_masked
SyNOnly_CC_2_reg_iter_0_normal_masked
SyNOnly_CC_6_reg_iter_1_normal_masked
SyNOnly_MI_32_reg_iter_0_bleached_masked
SyNOnly_MI_32_reg_iter_1_normal_masked
TVMSQC_bleached_masked
TVMSQC_normal_masked
SyNOnly_MI_32_reg_iter_0_normal_masked
TVMSQ_normal_masked
TVMSQ_bleached_masked

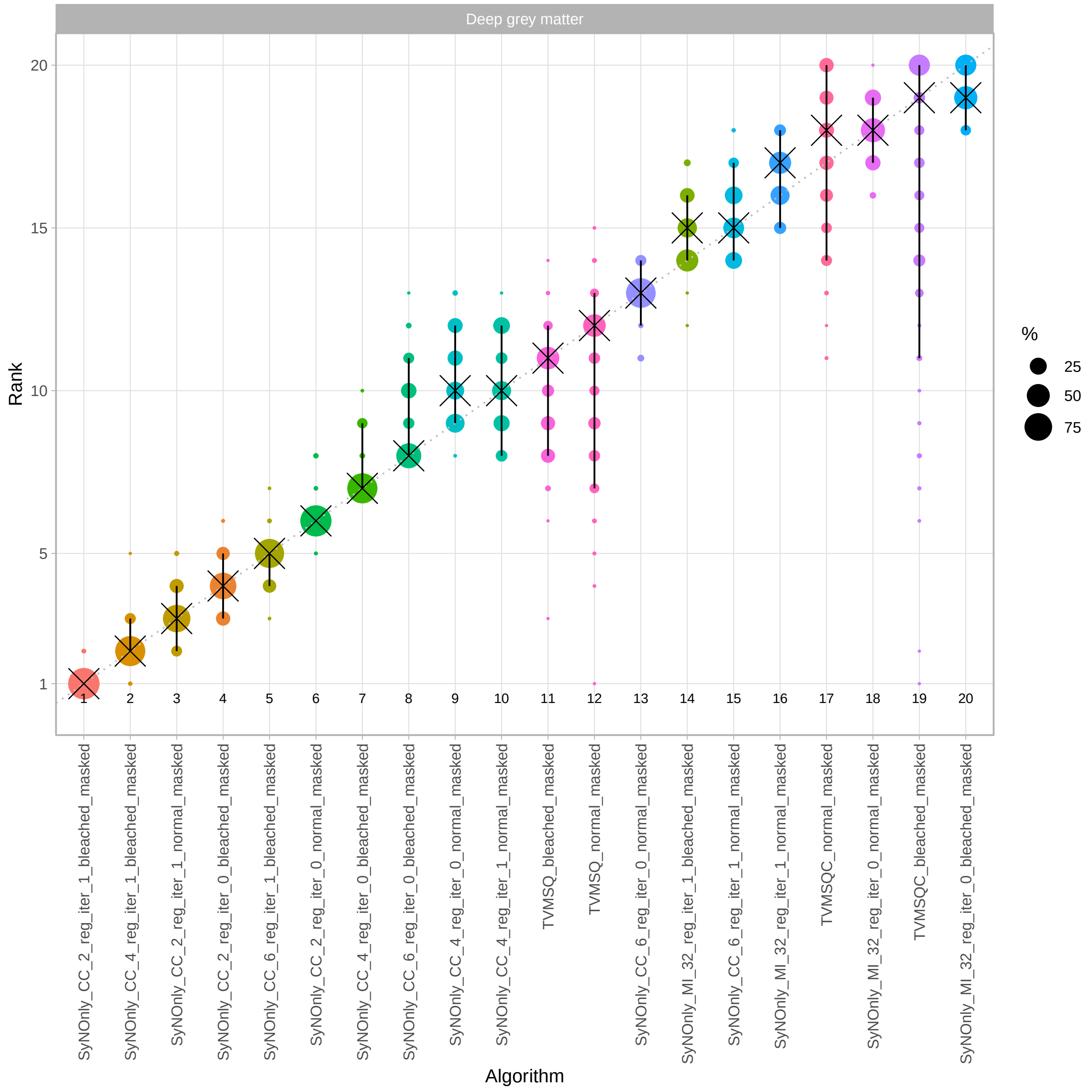

Deep grey matter
Rank
Algorithm
%
25
50
75
SyNOnly_CC_2_reg_iter_1_bleached_masked
SyNOnly_CC_4_reg_iter_1_bleached_masked
SyNOnly_CC_2_reg_iter_1_normal_masked
SyNOnly_CC_2_reg_iter_0_bleached_masked
SyNOnly_CC_6_reg_iter_1_bleached_masked
SyNOnly_CC_2_reg_iter_0_normal_masked
SyNOnly_CC_4_reg_iter_0_bleached_masked
SyNOnly_CC_6_reg_iter_0_bleached_masked
SyNOnly_CC_4_reg_iter_0_normal_masked
SyNOnly_CC_4_reg_iter_1_normal_masked
TVMSQ_bleached_masked
TVMSQ_normal_masked
SyNOnly_CC_6_reg_iter_0_normal_masked
SyNOnly_MI_32_reg_iter_1_bleached_masked
SyNOnly_CC_6_reg_iter_1_normal_masked
SyNOnly_MI_32_reg_iter_1_normal_masked
TVMSQC_normal_masked
SyNOnly_MI_32_reg_iter_0_normal_masked
TVMSQC_bleached_masked
SyNOnly_MI_32_reg_iter_0_bleached_masked

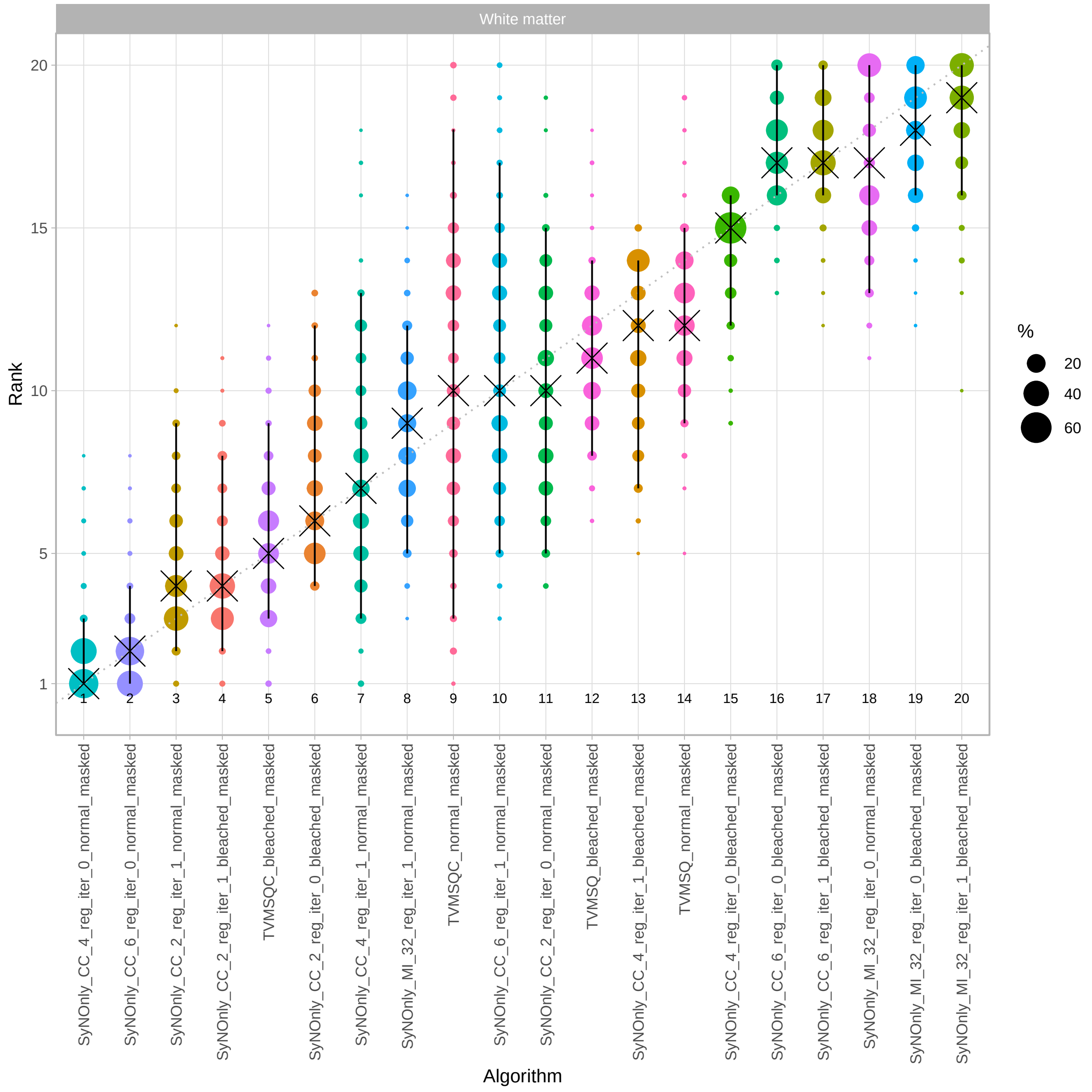

White matter
Rank
20
15
10
5
1
1 2 3 4 5 6 7 8 9 10 11 12 13 14 15 16 17 18 19 20
%
20
40
60
SyNOnly_CC_4_reg_iter_0_normal_masked
SyNOnly_CC_6_reg_iter_0_normal_masked
SyNOnly_CC_2_reg_iter_1_normal_masked
SyNOnly_CC_2_reg_iter_1_bleached_masked
TVMSQC_bleached_masked
SyNOnly_CC_2_reg_iter_0_bleached_masked
SyNOnly_CC_4_reg_iter_1_normal_masked
SyNOnly_MI_32_reg_iter_1_normal_masked
TVMSQC_normal_masked
SyNOnly_CC_6_reg_iter_1_normal_masked
SyNOnly_CC_2_reg_iter_0_normal_masked
TVMSQ_bleached_masked
SyNOnly_CC_4_reg_iter_1_bleached_masked
TVMSQ_normal_masked
SyNOnly_CC_4_reg_iter_0_bleached_masked
SyNOnly_CC_6_reg_iter_0_bleached_masked
SyNOnly_CC_6_reg_iter_1_bleached_masked
SyNOnly_MI_32_reg_iter_0_normal_masked
SyNOnly_MI_32_reg_iter_0_bleached_masked
SyNOnly_MI_32_reg_iter_1_bleached_masked
Algorithm

3.2 *Violin plot* for visualizing ranking stability based on bootstrapping

The ranking list based on the full assessment data is pairwise compared with the ranking lists based on the individual bootstrap samples (here $b = 1000$ samples). For each pair of rankings, Kendall's τ correlation is computed. Kendall's τ is a scaled index determining the correlation between the lists. It is computed by evaluating the number of pairwise concordances and discordances between ranking lists and produces values between -1 (for inverted order) and 1 (for identical order). A violin plot, which simultaneously depicts a boxplot and a density plot, is generated from the results.

Summary Kendall's tau:

Task	mean	median	q25	q75
CSF and ventricles	0.9713053	0.9684211	0.9578947	0.9894737
Brainstem	0.9315684	0.9368421	0.9157895	0.9578947
Deep grey matter	0.9151263	0.9263158	0.8842105	0.9473684
Cortical grey matter	0.9064842	0.9157895	0.8842105	0.9368421
Cerebellum	0.8831789	0.8947368	0.8526316	0.9157895
White matter	0.8105684	0.8210526	0.7684211	0.8631579

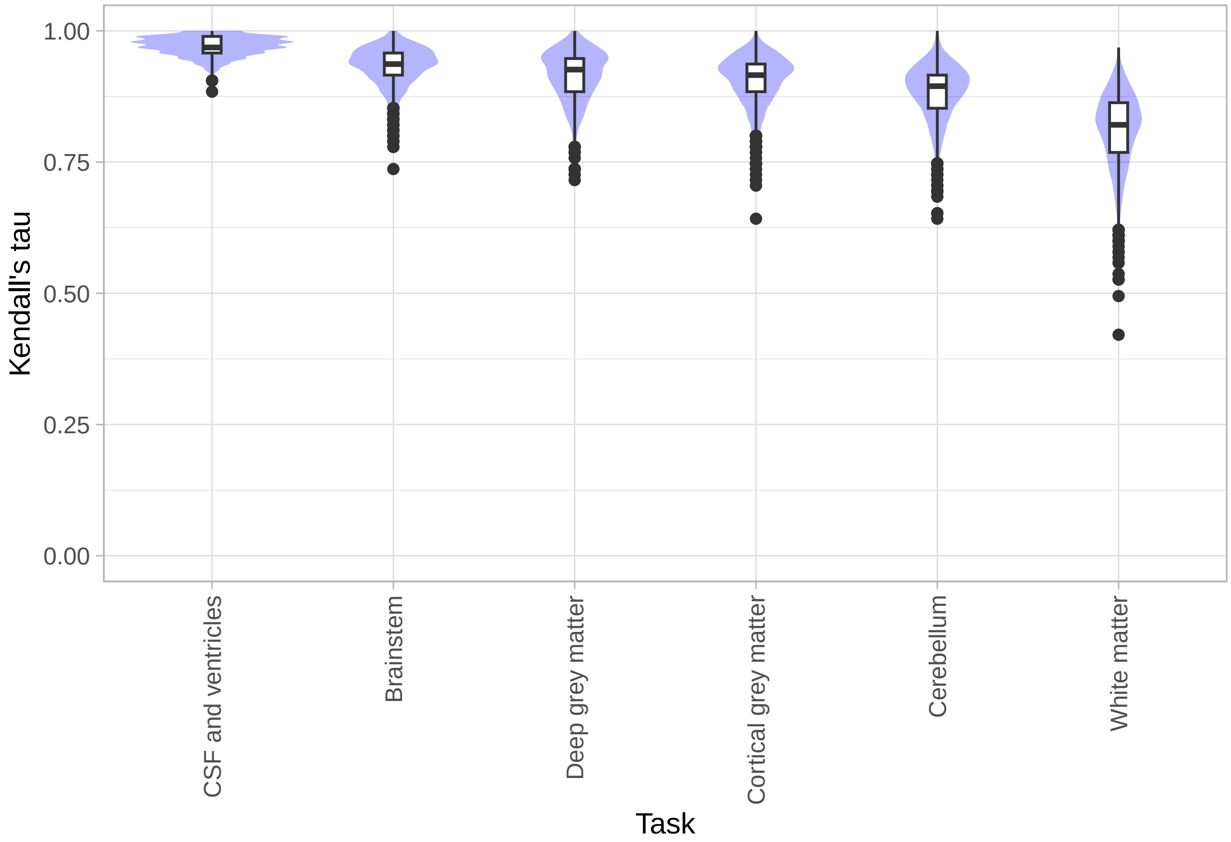

3.3 ***Significance maps*** for visualizing ranking stability based on statistical significance

Significance maps depict incidence matrices of pairwise significant test results for the one-sided Wilcoxon signed rank test at a 5% significance level with adjustment for multiple testing according to Holm. Yellow shading indicates that metric values from the algorithm on the x-axis were significantly superior to those from the algorithm on the y-axis, blue color indicates no significant difference.

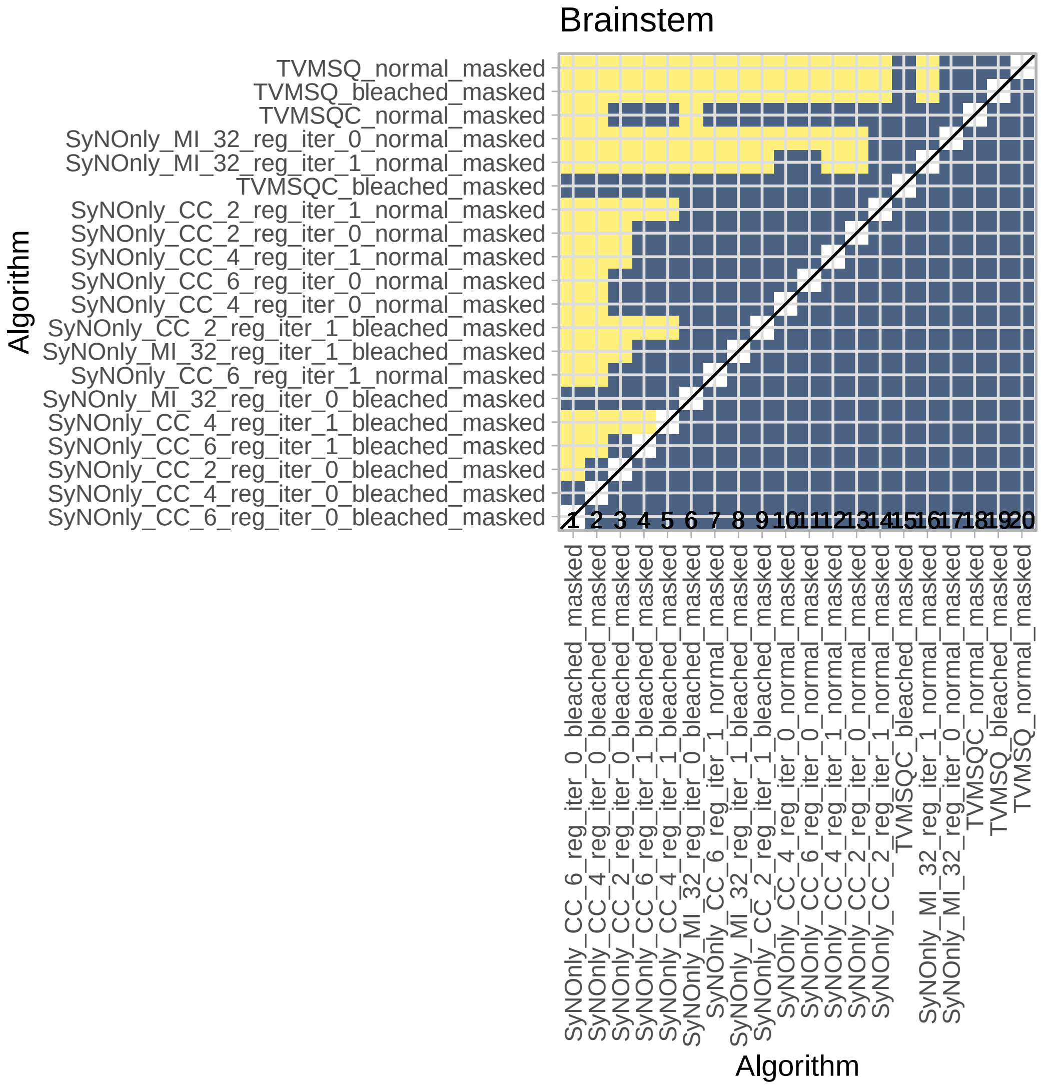

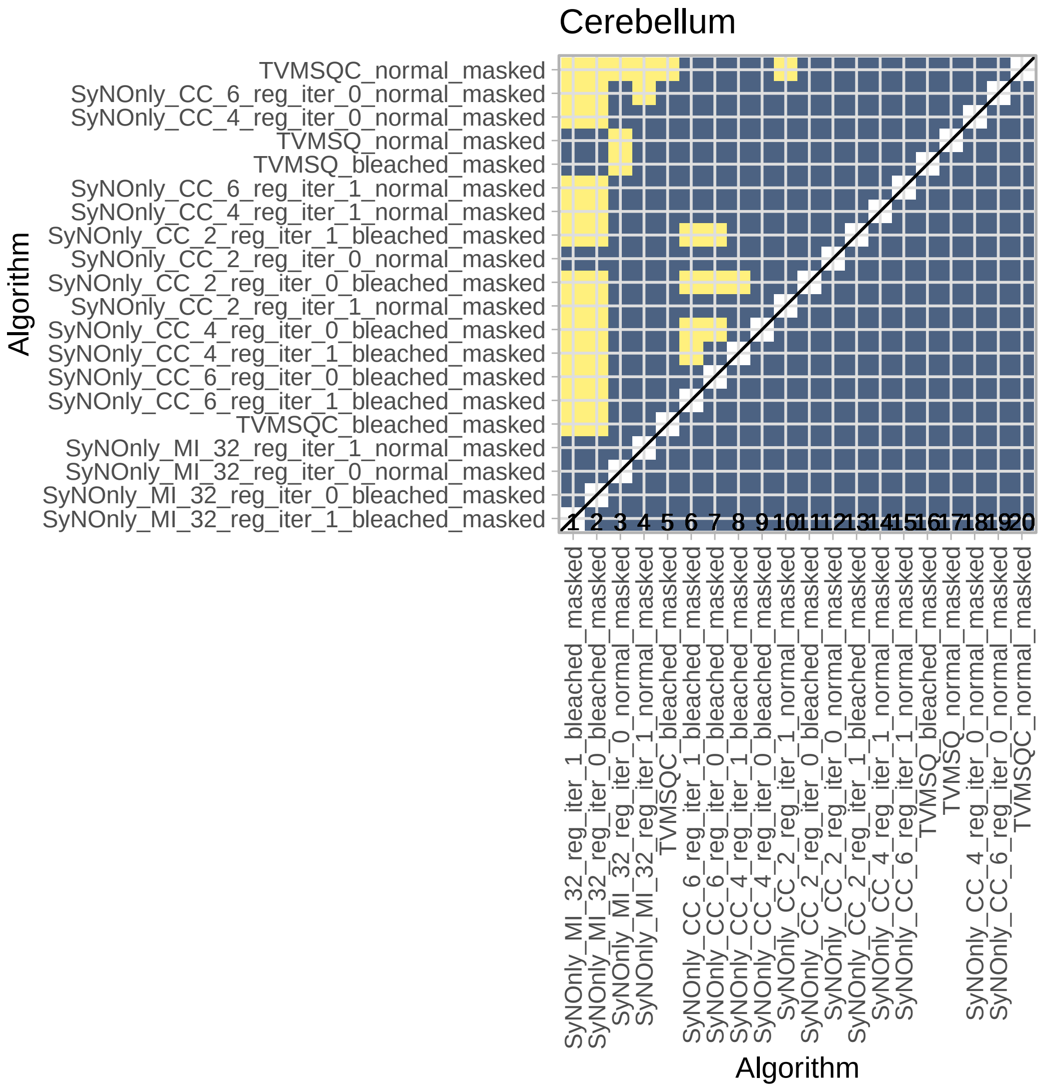
Cerebellum
Algorithm
TVMSQC_normal_masked
SyNOnly_CC_6_reg_iter_0_normal_masked
SyNOnly_CC_4_reg_iter_0_normal_masked
TVMSQ_normal_masked
TVMSQ_bleached_masked
SyNOnly_CC_6_reg_iter_1_normal_masked
SyNOnly_CC_4_reg_iter_1_normal_masked
SyNOnly_CC_2_reg_iter_1_bleached_masked
SyNOnly_CC_2_reg_iter_0_normal_masked
SyNOnly_CC_2_reg_iter_0_bleached_masked
SyNOnly_CC_2_reg_iter_1_normal_masked
SyNOnly_CC_4_reg_iter_0_bleached_masked
SyNOnly_CC_4_reg_iter_1_bleached_masked
SyNOnly_CC_6_reg_iter_0_bleached_masked
SyNOnly_CC_6_reg_iter_1_bleached_masked
TVMSQC_bleached_masked
SyNOnly_MI_32_reg_iter_1_normal_masked
SyNOnly_MI_32_reg_iter_0_normal_masked
SyNOnly_MI_32_reg_iter_0_bleached_masked
SyNOnly_MI_32_reg_iter_1_bleached_masked
1 2 3 4 5 6 7 8 9 10 11 12 13 14 15 16 17 18 19 20
SyNOnly_MI_32_reg_iter_1_bleached_masked
SyNOnly_MI_32_reg_iter_0_bleached_masked
SyNOnly_MI_32_reg_iter_0_normal_masked
SyNOnly_MI_32_reg_iter_1_normal_masked
TVMSQC_bleached_masked
SyNOnly_CC_6_reg_iter_1_bleached_masked
SyNOnly_CC_6_reg_iter_0_bleached_masked
SyNOnly_CC_4_reg_iter_1_bleached_masked
SyNOnly_CC_4_reg_iter_0_bleached_masked
SyNOnly_CC_2_reg_iter_1_normal_masked
SyNOnly_CC_2_reg_iter_0_bleached_masked
SyNOnly_CC_2_reg_iter_0_normal_masked
SyNOnly_CC_2_reg_iter_1_bleached_masked
SyNOnly_CC_4_reg_iter_1_normal_masked
SyNOnly_CC_6_reg_iter_1_normal_masked
TVMSQ_bleached_masked
TVMSQ_normal_masked
SyNOnly_CC_4_reg_iter_0_normal_masked
SyNOnly_CC_6_reg_iter_0_normal_masked
TVMSQC_normal_masked
Algorithm

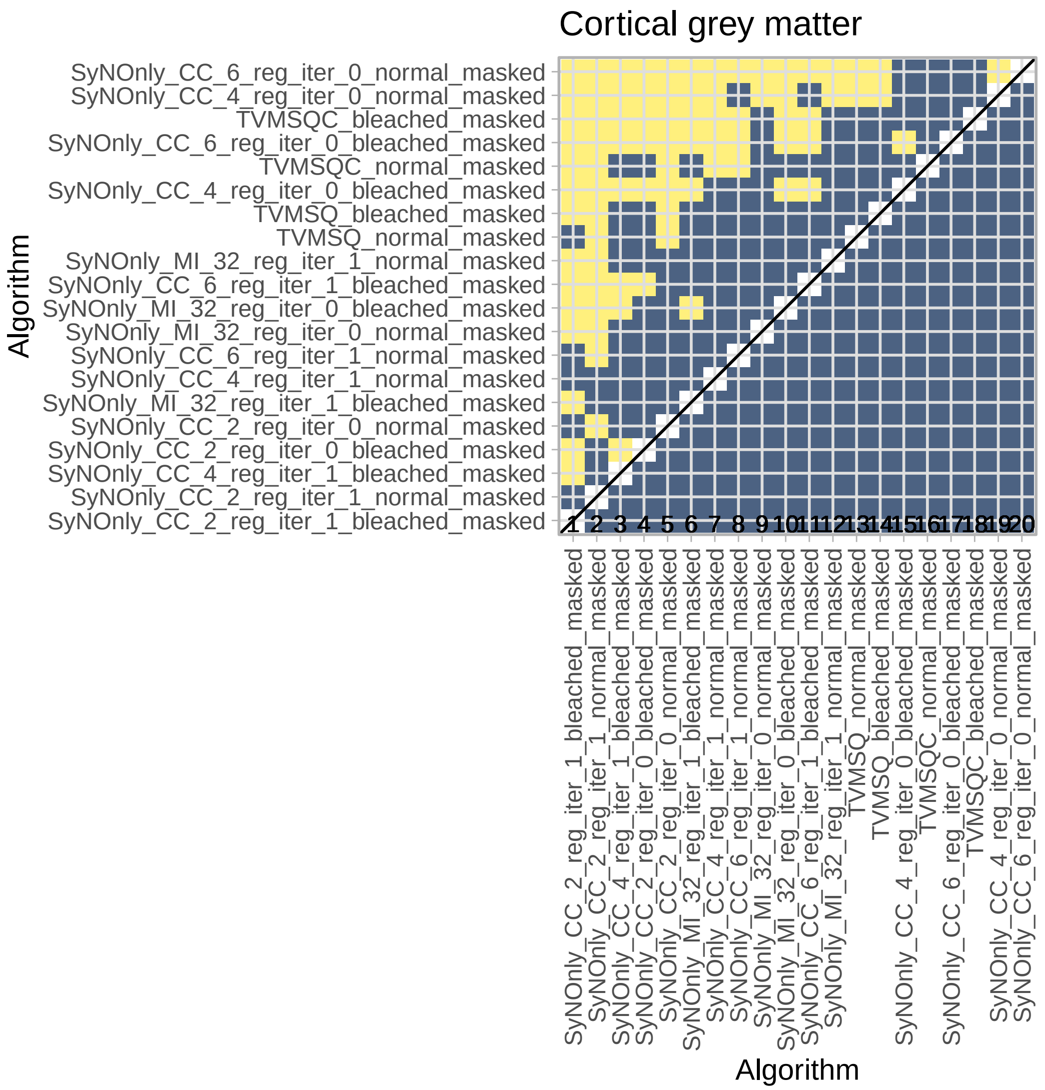
Cortical grey matter
Algorithm
SyNOnly_CC_6_reg_iter_0_normal_masked
SyNOnly_CC_4_reg_iter_0_normal_masked
TVMSQC_bleached_masked
SyNOnly_CC_6_reg_iter_0_bleached_masked
TVMSQC_normal_masked
SyNOnly_CC_4_reg_iter_0_bleached_masked
TVMSQ_bleached_masked
TVMSQ_normal_masked
SyNOnly_MI_32_reg_iter_1_normal_masked
SyNOnly_CC_6_reg_iter_1_bleached_masked
SyNOnly_MI_32_reg_iter_0_bleached_masked
SyNOnly_MI_32_reg_iter_0_normal_masked
SyNOnly_CC_6_reg_iter_1_normal_masked
SyNOnly_CC_4_reg_iter_1_normal_masked
SyNOnly_MI_32_reg_iter_1_bleached_masked
SyNOnly_CC_2_reg_iter_0_normal_masked
SyNOnly_CC_2_reg_iter_0_bleached_masked
SyNOnly_CC_4_reg_iter_1_bleached_masked
SyNOnly_CC_2_reg_iter_1_normal_masked
SyNOnly_CC_2_reg_iter_1_bleached_masked
1 2 3 4 5 6 7 8 9 10 11 12 13 14 15 16 17 18 19 20
Algorithm

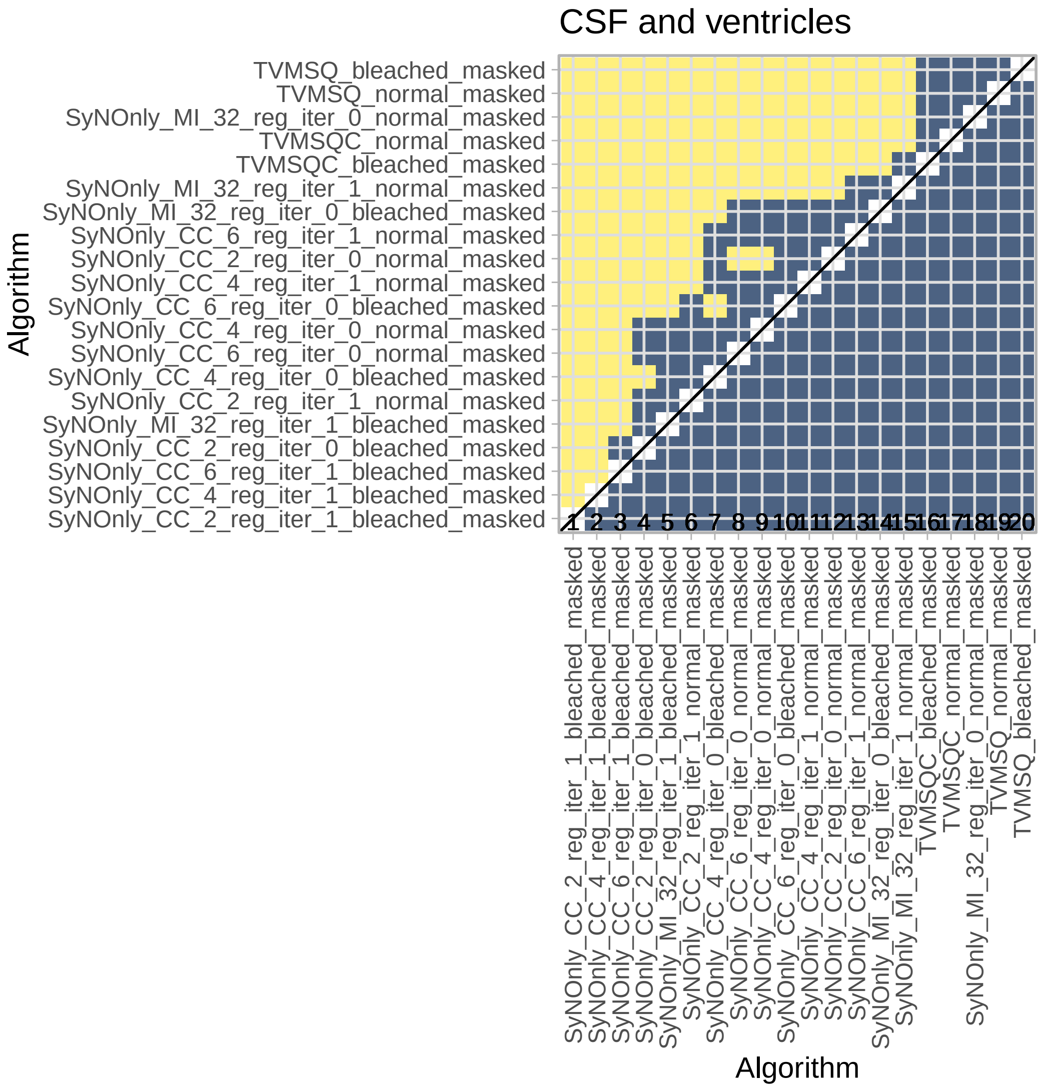

CSF and ventricles
Algorithm
TVMSQ_bleached_masked
TVMSQ_normal_masked
SyNOnly_MI_32_reg_iter_0_normal_masked
TVMSQC_normal_masked
TVMSQC_bleached_masked
SyNOnly_MI_32_reg_iter_1_normal_masked
SyNOnly_MI_32_reg_iter_0_bleached_masked
SyNOnly_CC_6_reg_iter_1_normal_masked
SyNOnly_CC_2_reg_iter_0_normal_masked
SyNOnly_CC_4_reg_iter_1_normal_masked
SyNOnly_CC_6_reg_iter_0_bleached_masked
SyNOnly_CC_4_reg_iter_0_normal_masked
SyNOnly_CC_6_reg_iter_0_normal_masked
SyNOnly_CC_4_reg_iter_0_bleached_masked
SyNOnly_CC_2_reg_iter_1_normal_masked
SyNOnly_MI_32_reg_iter_1_bleached_masked
SyNOnly_CC_2_reg_iter_0_bleached_masked
SyNOnly_CC_6_reg_iter_1_bleached_masked
SyNOnly_CC_4_reg_iter_1_bleached_masked
SyNOnly_CC_2_reg_iter_1_bleached_masked
1 2 3 4 5 6 7 8 9 10 11 12 13 14 15 16 17 18 19 20
SyNOnly_CC_2_reg_iter_1_bleached_masked
SyNOnly_CC_4_reg_iter_1_bleached_masked
SyNOnly_CC_6_reg_iter_1_bleached_masked
SyNOnly_CC_2_reg_iter_0_bleached_masked
SyNOnly_MI_32_reg_iter_1_bleached_masked
SyNOnly_CC_2_reg_iter_1_normal_masked
SyNOnly_CC_4_reg_iter_0_bleached_masked
SyNOnly_CC_6_reg_iter_0_normal_masked
SyNOnly_CC_4_reg_iter_0_normal_masked
SyNOnly_CC_6_reg_iter_0_bleached_masked
SyNOnly_CC_4_reg_iter_1_normal_masked
SyNOnly_CC_2_reg_iter_0_normal_masked
SyNOnly_CC_6_reg_iter_1_normal_masked
SyNOnly_MI_32_reg_iter_0_bleached_masked
SyNOnly_MI_32_reg_iter_1_normal_masked
TVMSQC_bleached_masked
TVMSQC_normal_masked
SyNOnly_MI_32_reg_iter_0_normal_masked
TVMSQ_normal_masked
TVMSQ_bleached_masked
Algorithm

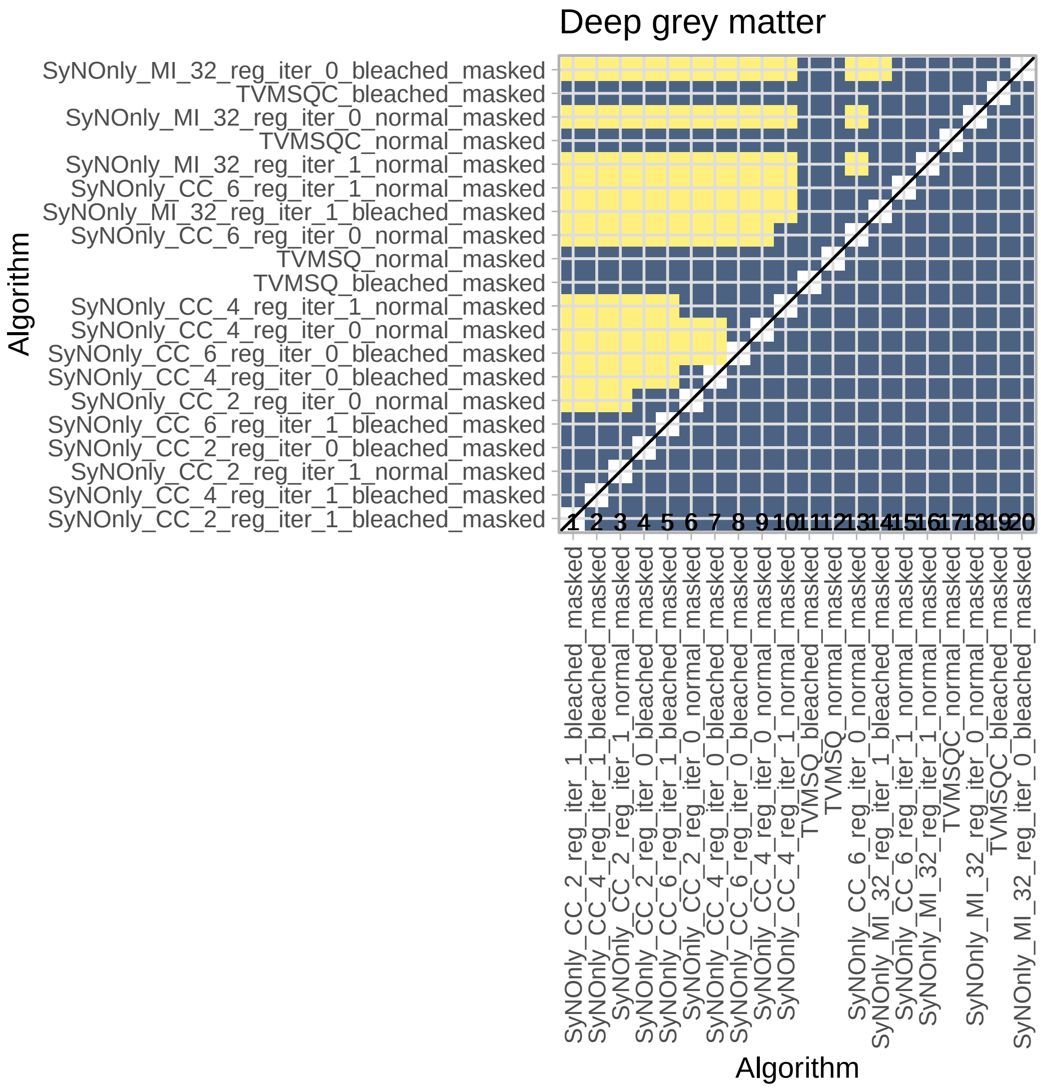
Deep grey matter
SyNOnly_MI_32_reg_iter_0_bleached_masked
TVMSQC_bleached_masked
SyNOnly_MI_32_reg_iter_0_normal_masked
TVMSQC_normal_masked
SyNOnly_MI_32_reg_iter_1_normal_masked
SyNOnly_CC_6_reg_iter_1_normal_masked
SyNOnly_MI_32_reg_iter_1_bleached_masked
SyNOnly_CC_6_reg_iter_0_normal_masked
TVMSQ_normal_masked
TVMSQ_bleached_masked
SyNOnly_CC_4_reg_iter_1_normal_masked
SyNOnly_CC_4_reg_iter_0_normal_masked
SyNOnly_CC_6_reg_iter_0_bleached_masked
SyNOnly_CC_4_reg_iter_0_bleached_masked
SyNOnly_CC_2_reg_iter_0_normal_masked
SyNOnly_CC_6_reg_iter_1_bleached_masked
SyNOnly_CC_2_reg_iter_0_bleached_masked
SyNOnly_CC_2_reg_iter_1_normal_masked
SyNOnly_CC_4_reg_iter_1_bleached_masked
SyNOnly_CC_2_reg_iter_1_bleached_masked
Algorithm
1 2 3 4 5 6 7 8 9 10 11 12 13 14 15 16 17 18 19 20
SyNOnly_CC_2_reg_iter_1_bleached_masked
SyNOnly_CC_4_reg_iter_1_bleached_masked
SyNOnly_CC_2_reg_iter_1_normal_masked
SyNOnly_CC_2_reg_iter_0_bleached_masked
SyNOnly_CC_6_reg_iter_1_bleached_masked
SyNOnly_CC_2_reg_iter_0_normal_masked
SyNOnly_CC_4_reg_iter_0_bleached_masked
SyNOnly_CC_6_reg_iter_0_bleached_masked
SyNOnly_CC_4_reg_iter_0_normal_masked
SyNOnly_CC_4_reg_iter_1_normal_masked
TVMSQ_bleached_masked
TVMSQ_normal_masked
SyNOnly_CC_6_reg_iter_0_normal_masked
SyNOnly_MI_32_reg_iter_1_bleached_masked
SyNOnly_CC_6_reg_iter_1_normal_masked
SyNOnly_MI_32_reg_iter_1_normal_masked
TVMSQC_normal_masked
SyNOnly_MI_32_reg_iter_0_normal_masked
TVMSQC_bleached_masked
SyNOnly_MI_32_reg_iter_0_bleached_masked
Algorithm

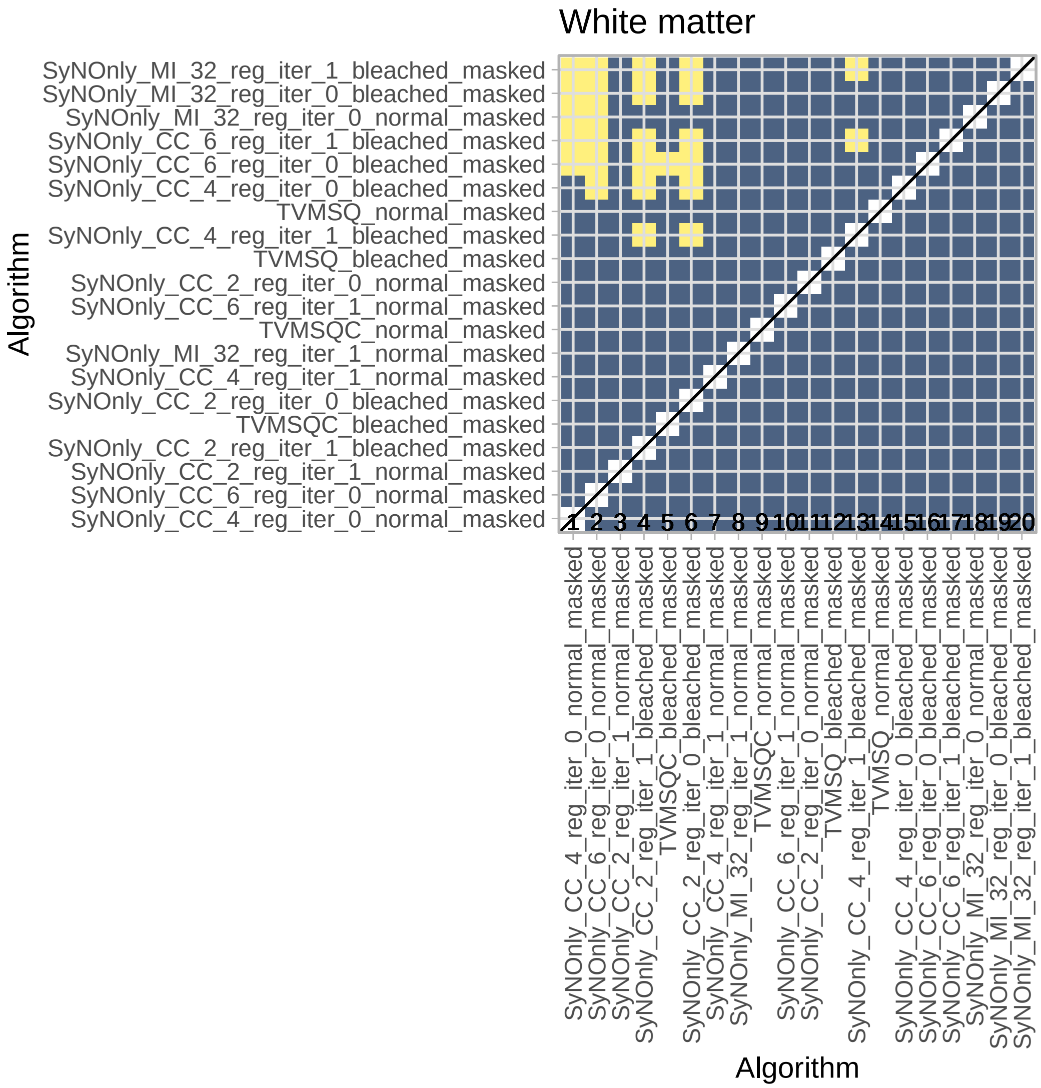
White matter
Algorithm
SyNOnly_MI_32_reg_iter_1_bleached_masked
SyNOnly_MI_32_reg_iter_0_bleached_masked
SyNOnly_MI_32_reg_iter_0_normal_masked
SyNOnly_CC_6_reg_iter_1_bleached_masked
SyNOnly_CC_6_reg_iter_0_bleached_masked
SyNOnly_CC_4_reg_iter_0_bleached_masked
TVMSQ_normal_masked
SyNOnly_CC_4_reg_iter_1_bleached_masked
TVMSQ_bleached_masked
SyNOnly_CC_2_reg_iter_0_normal_masked
SyNOnly_CC_6_reg_iter_1_normal_masked
TVMSQC_normal_masked
SyNOnly_MI_32_reg_iter_1_normal_masked
SyNOnly_CC_4_reg_iter_1_normal_masked
SyNOnly_CC_2_reg_iter_0_bleached_masked
TVMSQC_bleached_masked
SyNOnly_CC_2_reg_iter_1_bleached_masked
SyNOnly_CC_2_reg_iter_1_normal_masked
SyNOnly_CC_6_reg_iter_0_normal_masked
SyNOnly_CC_4_reg_iter_0_normal_masked
1 2 3 4 5 6 7 8 9 10 11 12 13 14 15 16 17 18 19 20
SyNOnly_CC_4_reg_iter_0_normal_masked
SyNOnly_CC_6_reg_iter_0_normal_masked
SyNOnly_CC_2_reg_iter_1_normal_masked
SyNOnly_CC_2_reg_iter_1_bleached_masked
TVMSQC_bleached_masked
SyNOnly_CC_2_reg_iter_0_bleached_masked
SyNOnly_CC_4_reg_iter_1_normal_masked
SyNOnly_MI_32_reg_iter_1_normal_masked
TVMSQC_normal_masked
SyNOnly_CC_6_reg_iter_1_normal_masked
SyNOnly_CC_2_reg_iter_0_normal_masked
TVMSQ_bleached_masked
SyNOnly_CC_4_reg_iter_1_bleached_masked
TVMSQ_normal_masked
SyNOnly_CC_4_reg_iter_0_bleached_masked
SyNOnly_CC_6_reg_iter_0_bleached_masked
SyNOnly_CC_6_reg_iter_1_bleached_masked
SyNOnly_MI_32_reg_iter_0_normal_masked
SyNOnly_MI_32_reg_iter_0_bleached_masked
SyNOnly_MI_32_reg_iter_1_bleached_masked
Algorithm

3.4 Ranking robustness to ranking methods

Line plots for visualizing ranking robustness across different ranking methods. Each algorithm is represented by one colored line. For each ranking method encoded on the x-axis, the height of the line represents the corresponding rank. Horizontal lines indicate identical ranks for all methods.

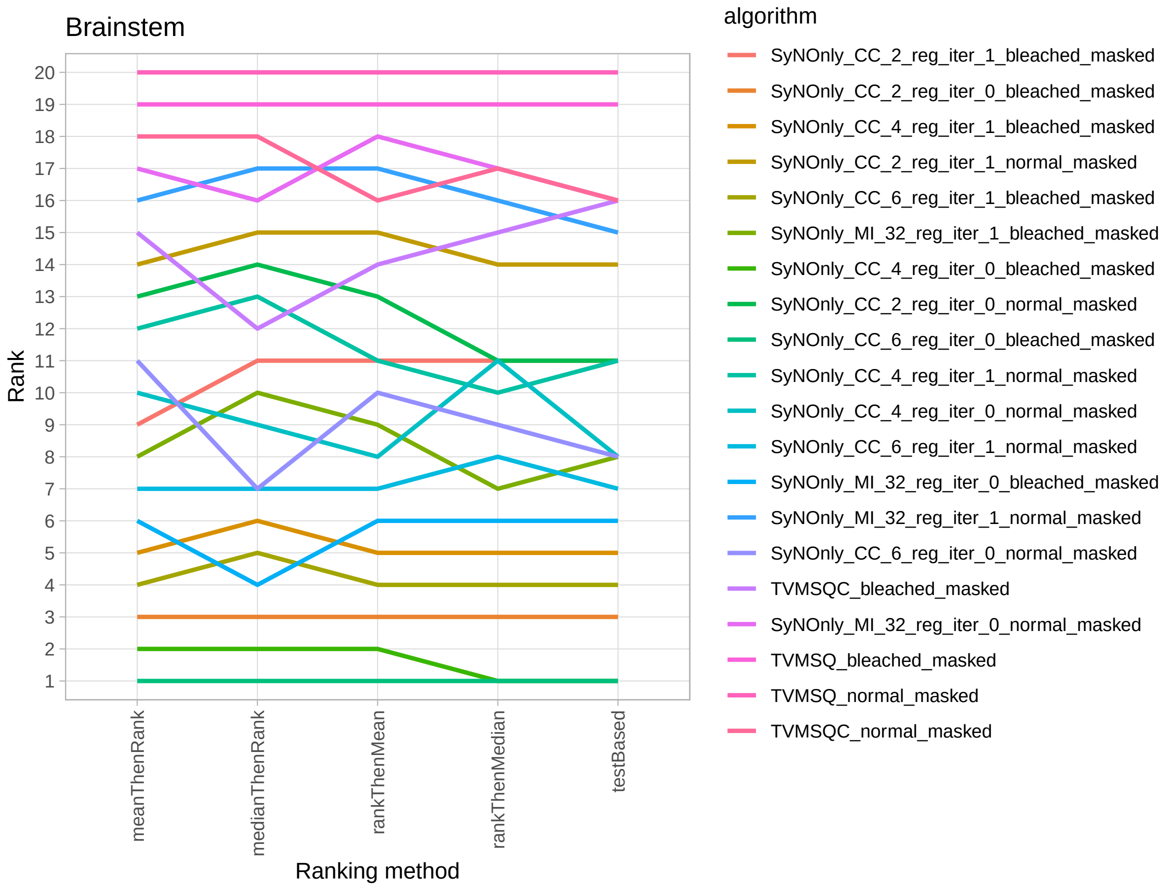

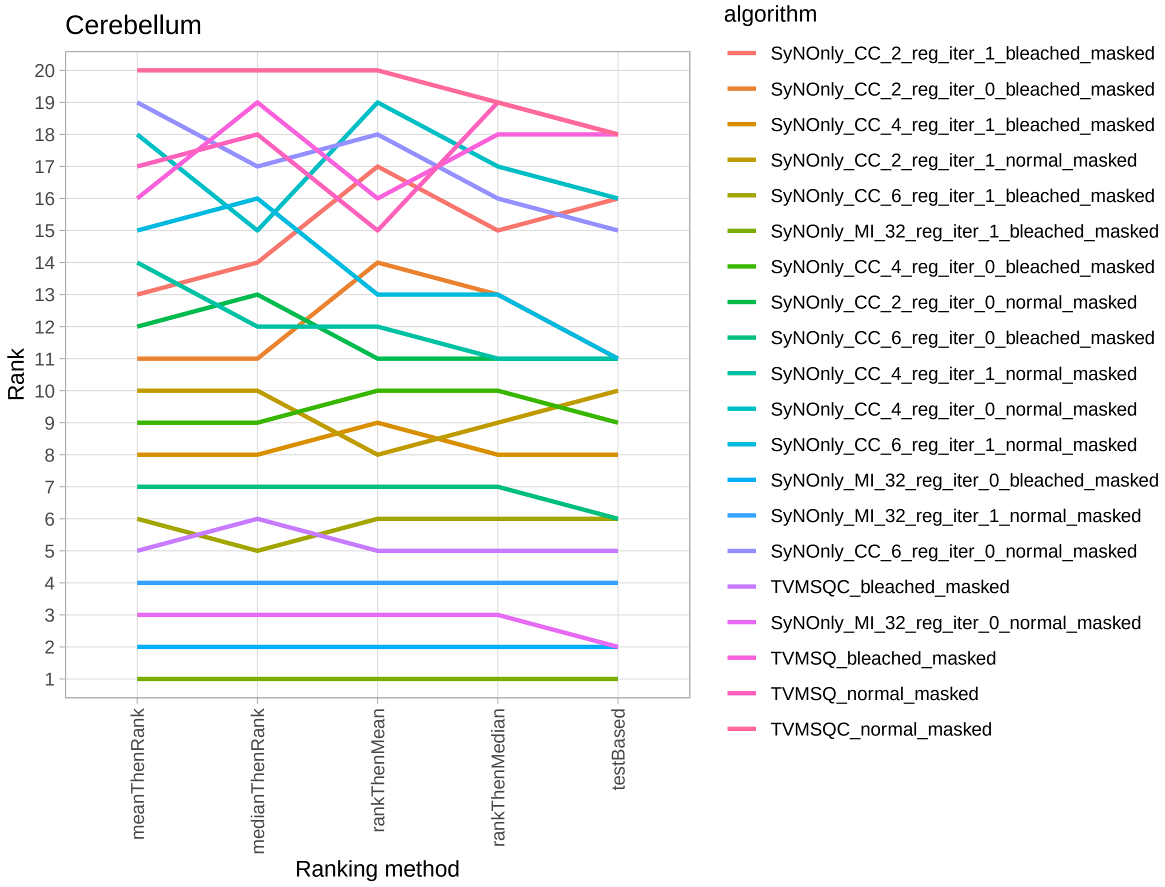
Cerebellum
Rank
Ranking method
meanThenRank
medianThenRank
rankThenMean
rankThenMedian
testBased
algorithm
SyNOnly_CC_2_reg_iter_1_bleached_masked
SyNOnly_CC_2_reg_iter_0_bleached_masked
SyNOnly_CC_4_reg_iter_1_bleached_masked
SyNOnly_CC_2_reg_iter_1_normal_masked
SyNOnly_CC_6_reg_iter_1_bleached_masked
SyNOnly_MI_32_reg_iter_1_bleached_masked
SyNOnly_CC_4_reg_iter_0_bleached_masked
SyNOnly_CC_2_reg_iter_0_normal_masked
SyNOnly_CC_6_reg_iter_0_bleached_masked
SyNOnly_CC_4_reg_iter_1_normal_masked
SyNOnly_CC_4_reg_iter_0_normal_masked
SyNOnly_CC_6_reg_iter_1_normal_masked
SyNOnly_MI_32_reg_iter_0_bleached_masked
SyNOnly_MI_32_reg_iter_1_normal_masked
SyNOnly_CC_6_reg_iter_0_normal_masked
TVMSQC_bleached_masked
SyNOnly_MI_32_reg_iter_0_normal_masked
TVMSQ_bleached_masked
TVMSQ_normal_masked
TVMSQC_normal_masked

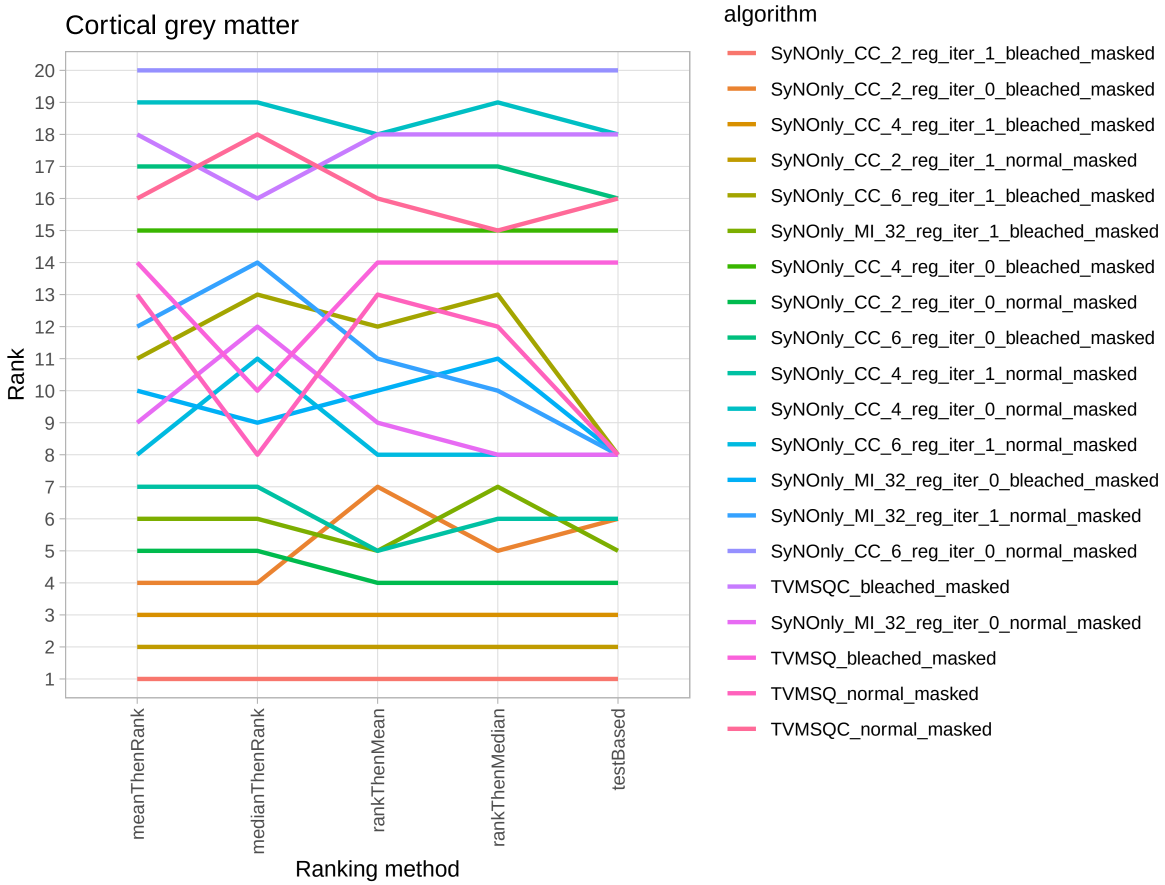
Cortical grey matter
Rank
Ranking method
meanThenRank
medianThenRank
rankThenMean
rankThenMedian
testBased
algorithm
SyNOnly_CC_2_reg_iter_1_bleached_masked
SyNOnly_CC_2_reg_iter_0_bleached_masked
SyNOnly_CC_4_reg_iter_1_bleached_masked
SyNOnly_CC_2_reg_iter_1_normal_masked
SyNOnly_CC_6_reg_iter_1_bleached_masked
SyNOnly_MI_32_reg_iter_1_bleached_masked
SyNOnly_CC_4_reg_iter_0_bleached_masked
SyNOnly_CC_2_reg_iter_0_normal_masked
SyNOnly_CC_6_reg_iter_0_bleached_masked
SyNOnly_CC_4_reg_iter_1_normal_masked
SyNOnly_CC_4_reg_iter_0_normal_masked
SyNOnly_CC_6_reg_iter_1_normal_masked
SyNOnly_MI_32_reg_iter_0_bleached_masked
SyNOnly_MI_32_reg_iter_1_normal_masked
SyNOnly_CC_6_reg_iter_0_normal_masked
TVMSQC_bleached_masked
SyNOnly_MI_32_reg_iter_0_normal_masked
TVMSQ_bleached_masked
TVMSQ_normal_masked
TVMSQC_normal_masked

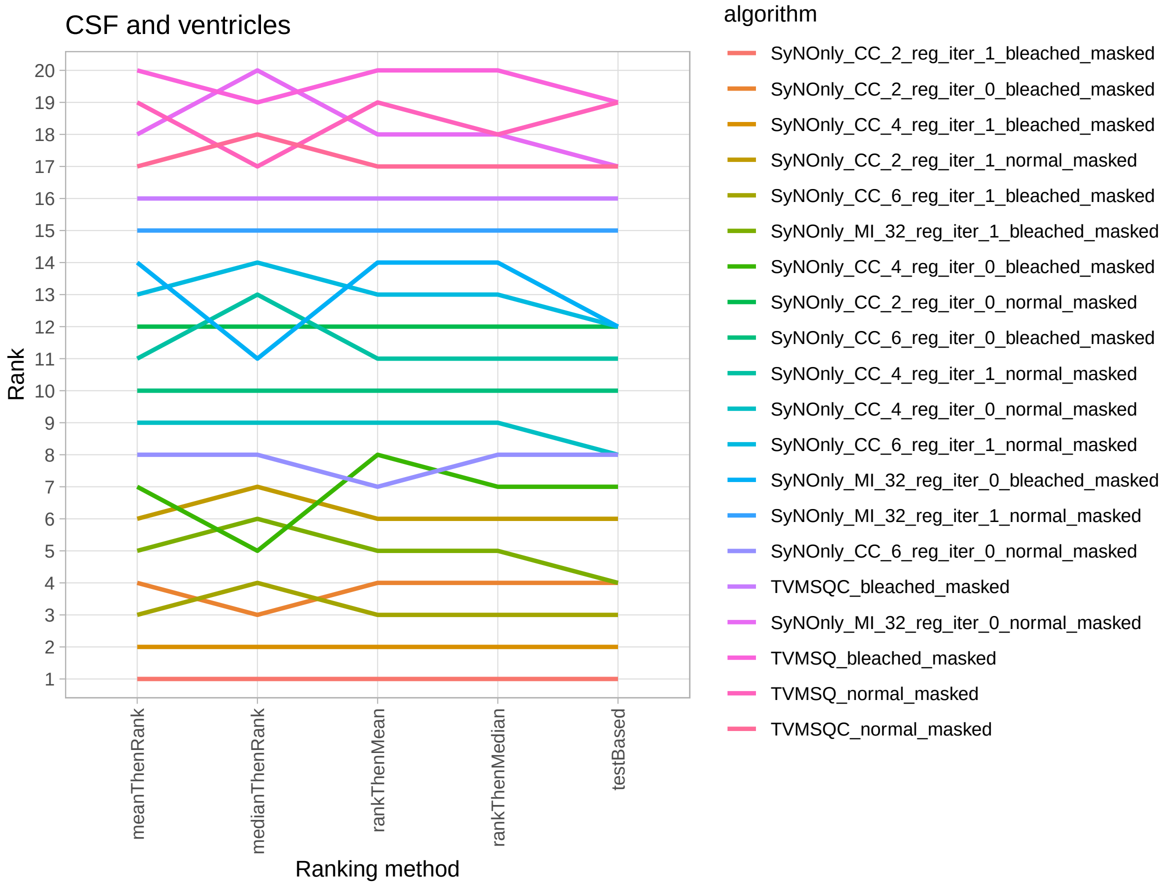
CSF and ventricles
Rank
Ranking method
meanThenRank
medianThenRank
rankThenMean
rankThenMedian
testBased
algorithm
SyNOnly_CC_2_reg_iter_1_bleached_masked
SyNOnly_CC_2_reg_iter_0_bleached_masked
SyNOnly_CC_4_reg_iter_1_bleached_masked
SyNOnly_CC_2_reg_iter_1_normal_masked
SyNOnly_CC_6_reg_iter_1_bleached_masked
SyNOnly_MI_32_reg_iter_1_bleached_masked
SyNOnly_CC_4_reg_iter_0_bleached_masked
SyNOnly_CC_2_reg_iter_0_normal_masked
SyNOnly_CC_6_reg_iter_0_bleached_masked
SyNOnly_CC_4_reg_iter_1_normal_masked
SyNOnly_CC_4_reg_iter_0_normal_masked
SyNOnly_CC_6_reg_iter_1_normal_masked
SyNOnly_MI_32_reg_iter_0_bleached_masked
SyNOnly_MI_32_reg_iter_1_normal_masked
SyNOnly_CC_6_reg_iter_0_normal_masked
TVMSQC_bleached_masked
SyNOnly_MI_32_reg_iter_0_normal_masked
TVMSQ_bleached_masked
TVMSQ_normal_masked
TVMSQC_normal_masked

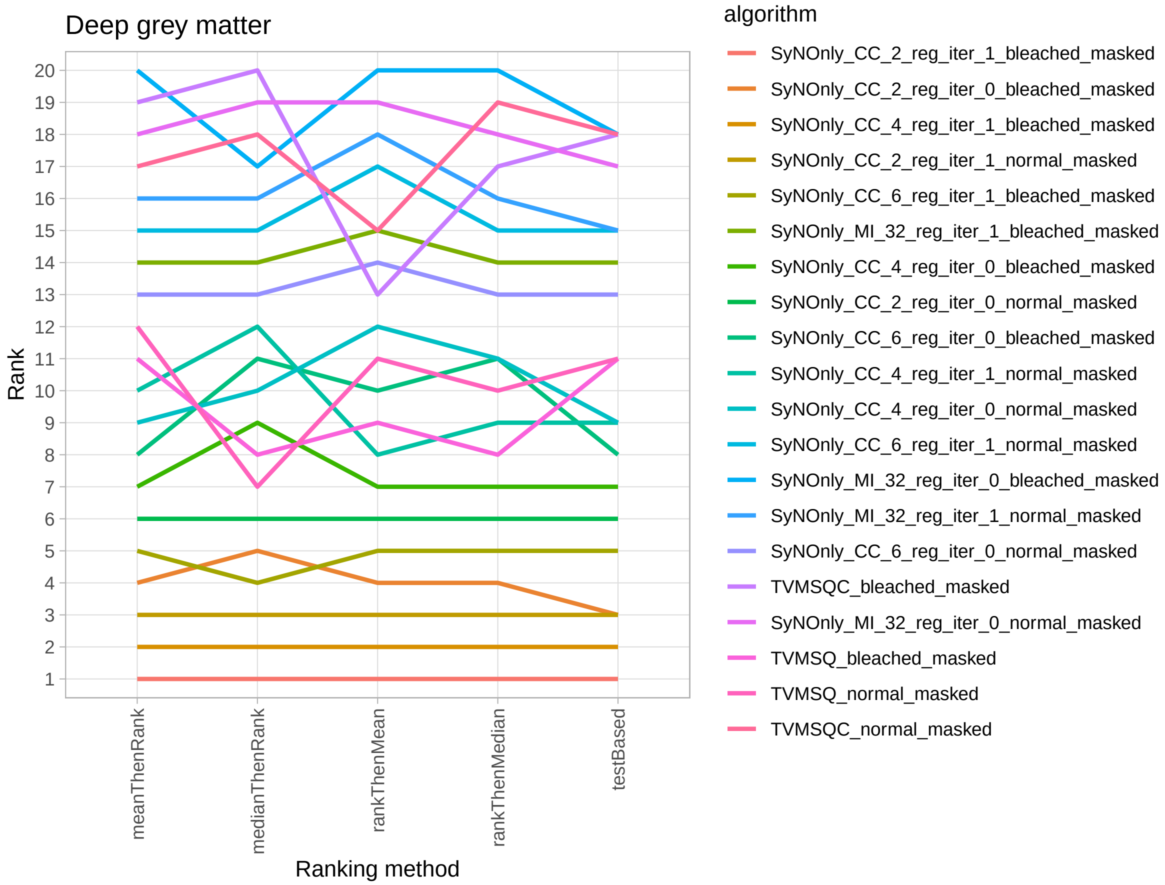
Deep grey matter
Rank
Ranking method
meanThenRank
medianThenRank
rankThenMean
rankThenMedian
testBased
algorithm
SyNOnly_CC_2_reg_iter_1_bleached_masked
SyNOnly_CC_2_reg_iter_0_bleached_masked
SyNOnly_CC_4_reg_iter_1_bleached_masked
SyNOnly_CC_2_reg_iter_1_normal_masked
SyNOnly_CC_6_reg_iter_1_bleached_masked
SyNOnly_MI_32_reg_iter_1_bleached_masked
SyNOnly_CC_4_reg_iter_0_bleached_masked
SyNOnly_CC_2_reg_iter_0_normal_masked
SyNOnly_CC_6_reg_iter_0_bleached_masked
SyNOnly_CC_4_reg_iter_1_normal_masked
SyNOnly_CC_4_reg_iter_0_normal_masked
SyNOnly_CC_6_reg_iter_1_normal_masked
SyNOnly_MI_32_reg_iter_0_bleached_masked
SyNOnly_MI_32_reg_iter_1_normal_masked
SyNOnly_CC_6_reg_iter_0_normal_masked
TVMSQC_bleached_masked
SyNOnly_MI_32_reg_iter_0_normal_masked
TVMSQ_bleached_masked
TVMSQ_normal_masked
TVMSQC_normal_masked

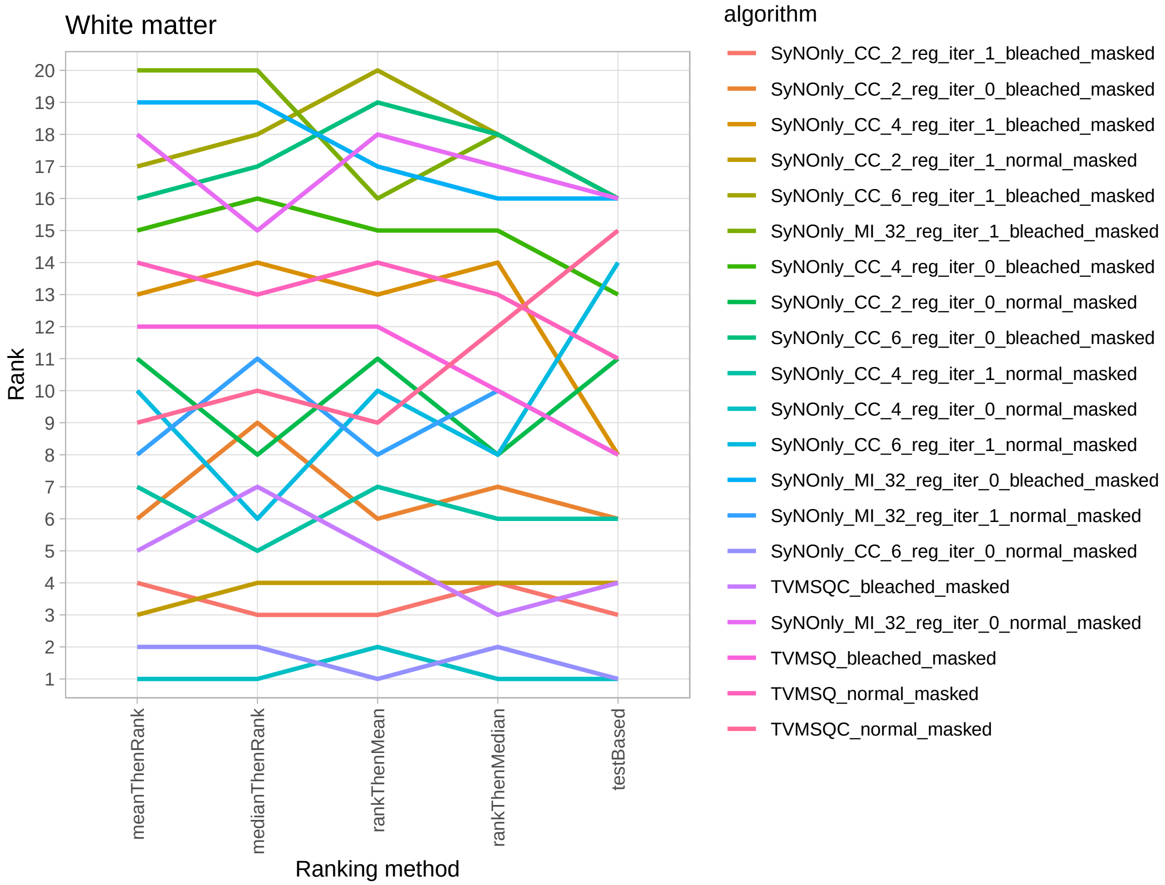
White matter
Rank
Ranking method
meanThenRank
medianThenRank
rankThenMean
rankThenMedian
testBased
algorithm
SyNOnly_CC_2_reg_iter_1_bleached_masked
SyNOnly_CC_2_reg_iter_0_bleached_masked
SyNOnly_CC_4_reg_iter_1_bleached_masked
SyNOnly_CC_2_reg_iter_1_normal_masked
SyNOnly_CC_6_reg_iter_1_bleached_masked
SyNOnly_MI_32_reg_iter_1_bleached_masked
SyNOnly_CC_4_reg_iter_0_bleached_masked
SyNOnly_CC_2_reg_iter_0_normal_masked
SyNOnly_CC_6_reg_iter_0_bleached_masked
SyNOnly_CC_4_reg_iter_1_normal_masked
SyNOnly_CC_4_reg_iter_0_normal_masked
SyNOnly_CC_6_reg_iter_1_normal_masked
SyNOnly_MI_32_reg_iter_0_bleached_masked
SyNOnly_MI_32_reg_iter_1_normal_masked
SyNOnly_CC_6_reg_iter_0_normal_masked
TVMSQC_bleached_masked
SyNOnly_MI_32_reg_iter_0_normal_masked
TVMSQ_bleached_masked
TVMSQ_normal_masked
TVMSQC_normal_masked

4 Visualization of cross-task insights

The algorithms are ordered according to consensus ranking.

4.1 Characterization of algorithms

4.1.1 Ranking stability: Variability of achieved rankings across tasks

Algorithms are color-coded, and the area of each blob at position $(A_i, \text{rank } j)$ is proportional to the relative frequency A_i achieved rank j across multiple tasks. The median rank for each algorithm is indicated by a black cross. This way, the distribution of ranks across tasks can be intuitively visualized.

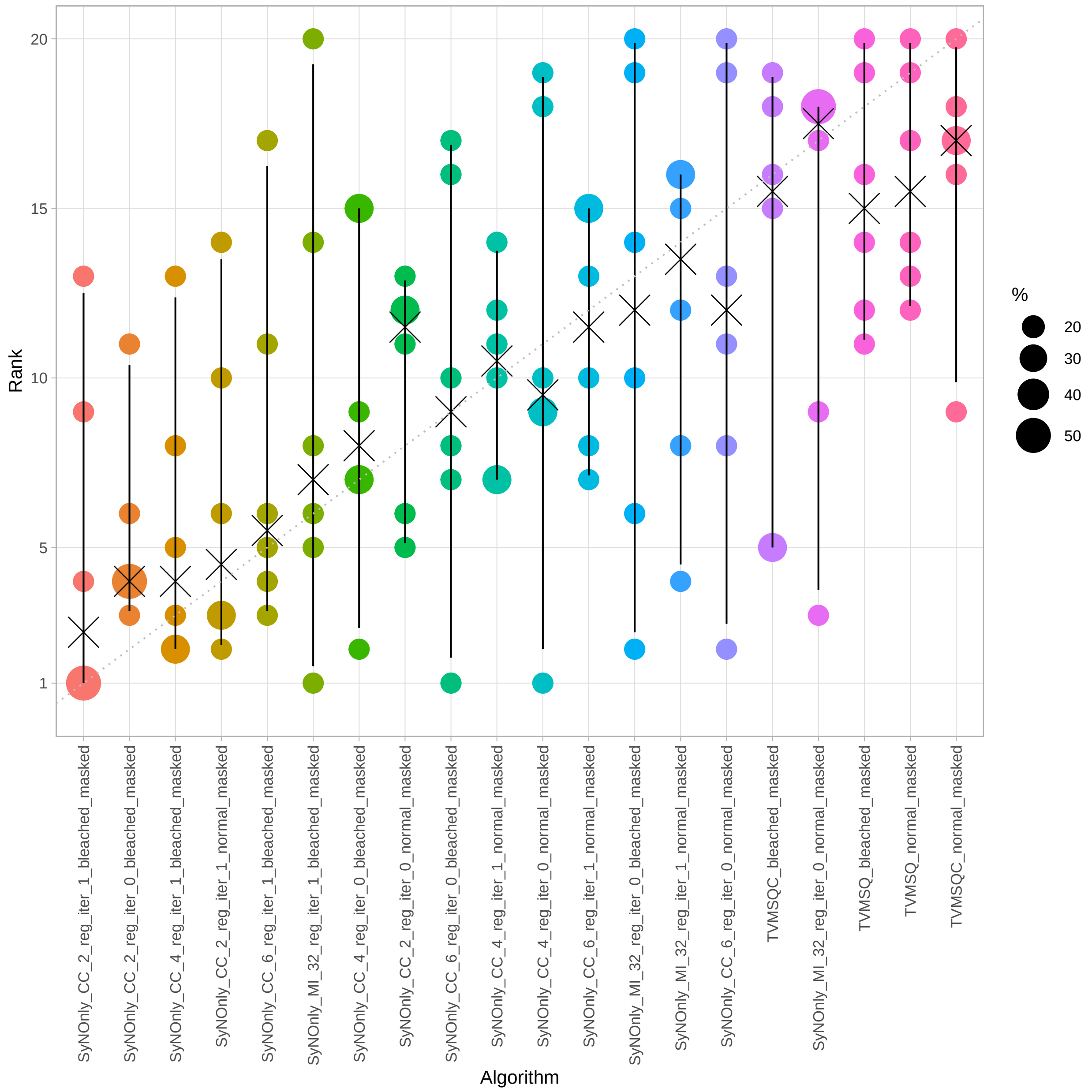

4.1.2 Ranking stability: Ranking variability via bootstrap approach

A blob plot of bootstrap results over the different tasks separated by algorithm allows another perspective on the assessment data. This gives deeper insights into the characteristics of tasks and the ranking uncertainty of the algorithms in each task.

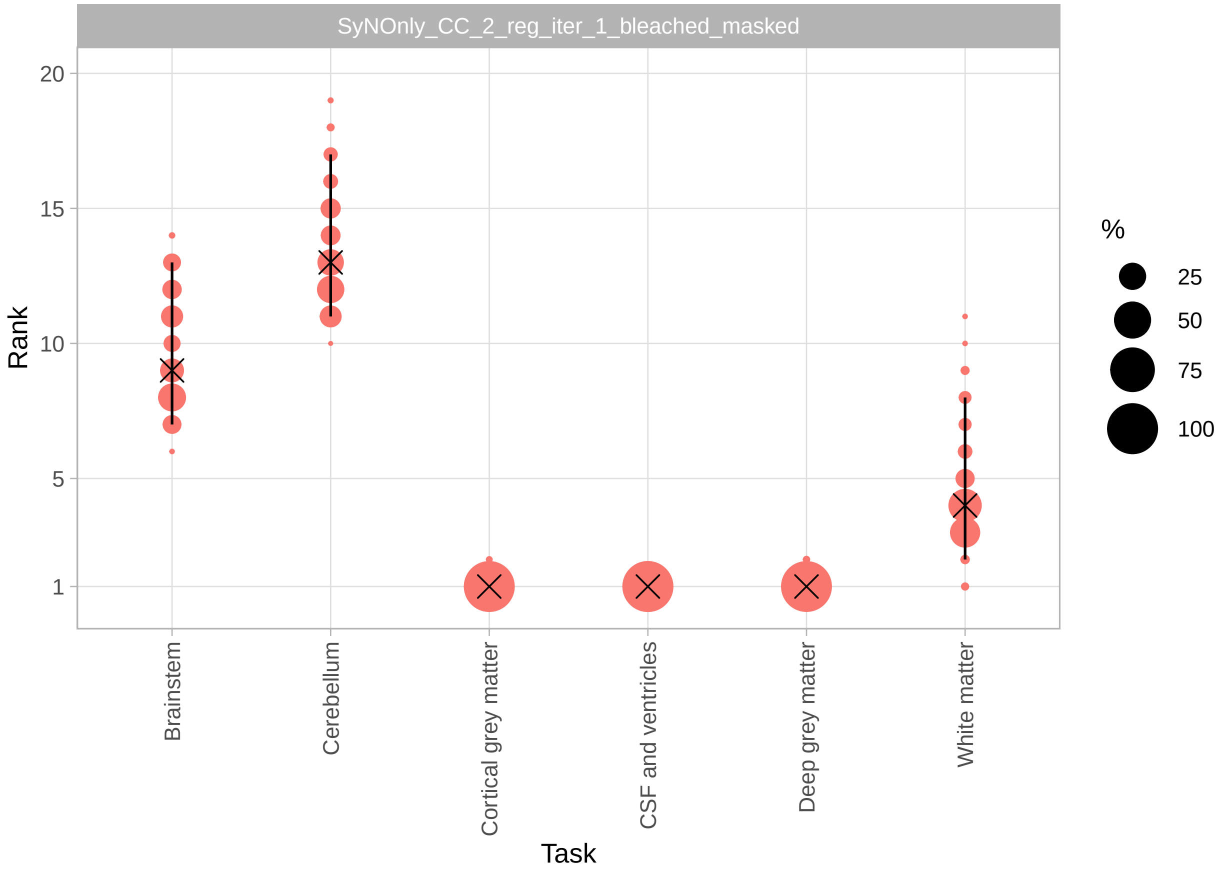

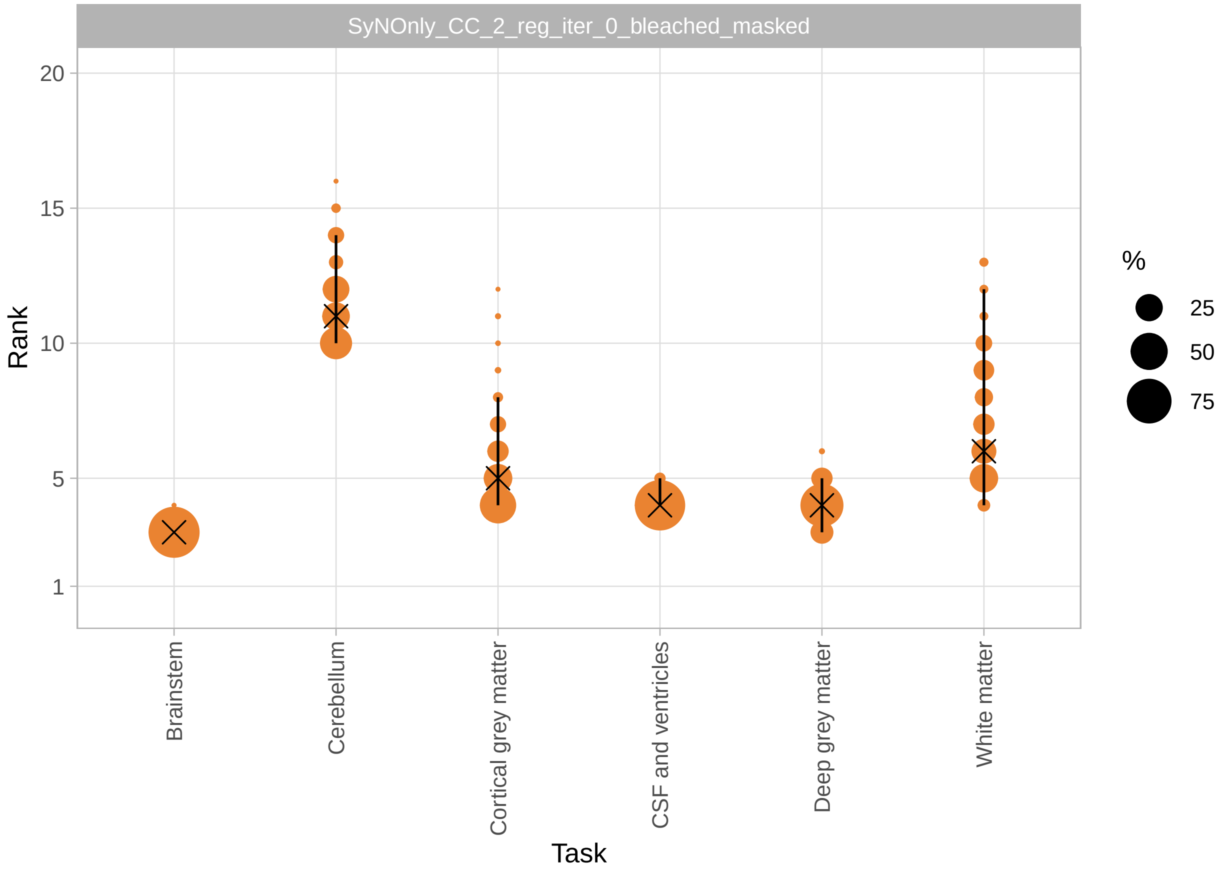
SyNOnly_CC_2_reg_iter_0_bleached_masked
Rank
20
15
10
5
1
Brainstem
Cerebellum
Cortical grey matter
CSF and ventricles
Deep grey matter
White matter
Task
%
25
50
75

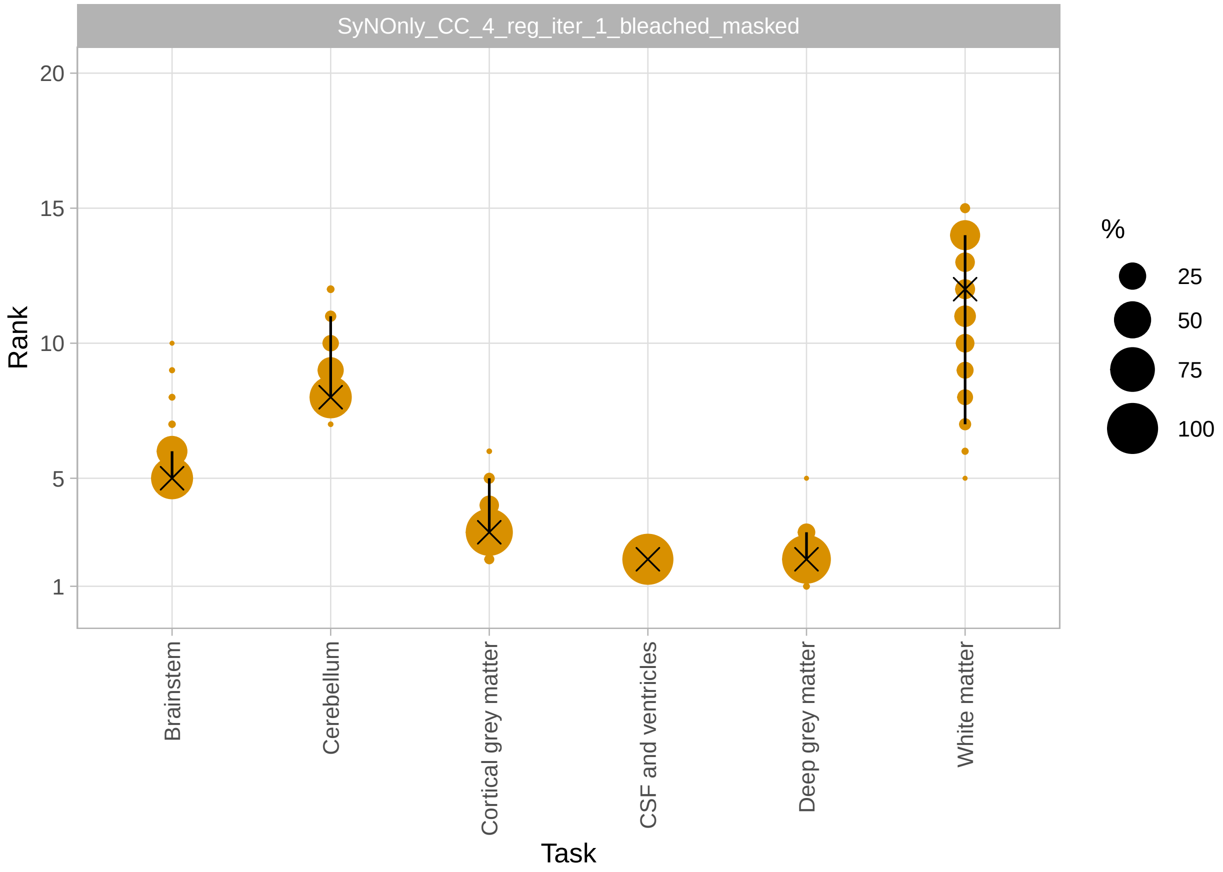
SyNOnly_CC_4_reg_iter_1_bleached_masked
Rank
20
15
10
5
1
Brainstem
Cerebellum
Cortical grey matter
CSF and ventricles
Deep grey matter
White matter
Task
%
25
50
75
100

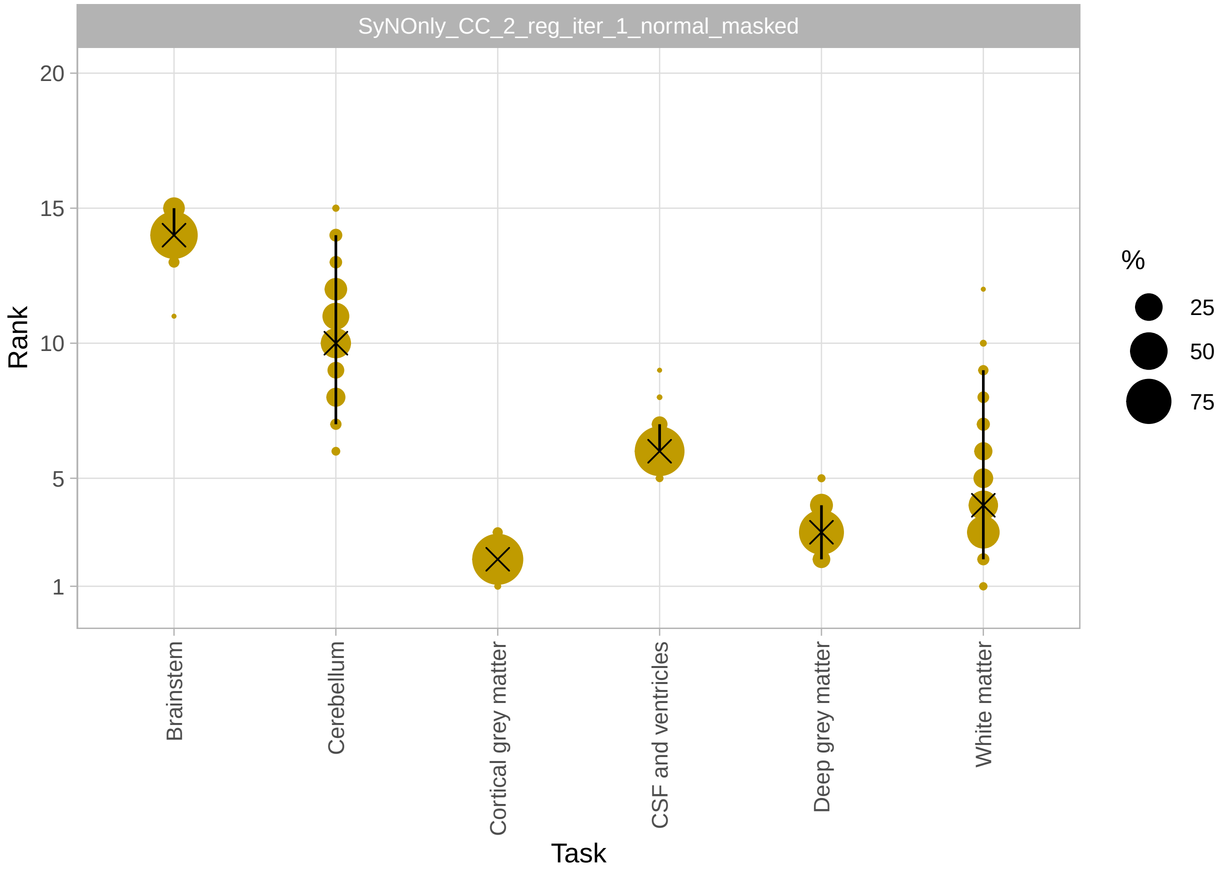
SyNOnly_CC_2_reg_iter_1_normal_masked
Rank
20
15
10
5
1
Brainstem
Cerebellum
Cortical grey matter
CSF and ventricles
Deep grey matter
White matter
Task
%
25
50
75

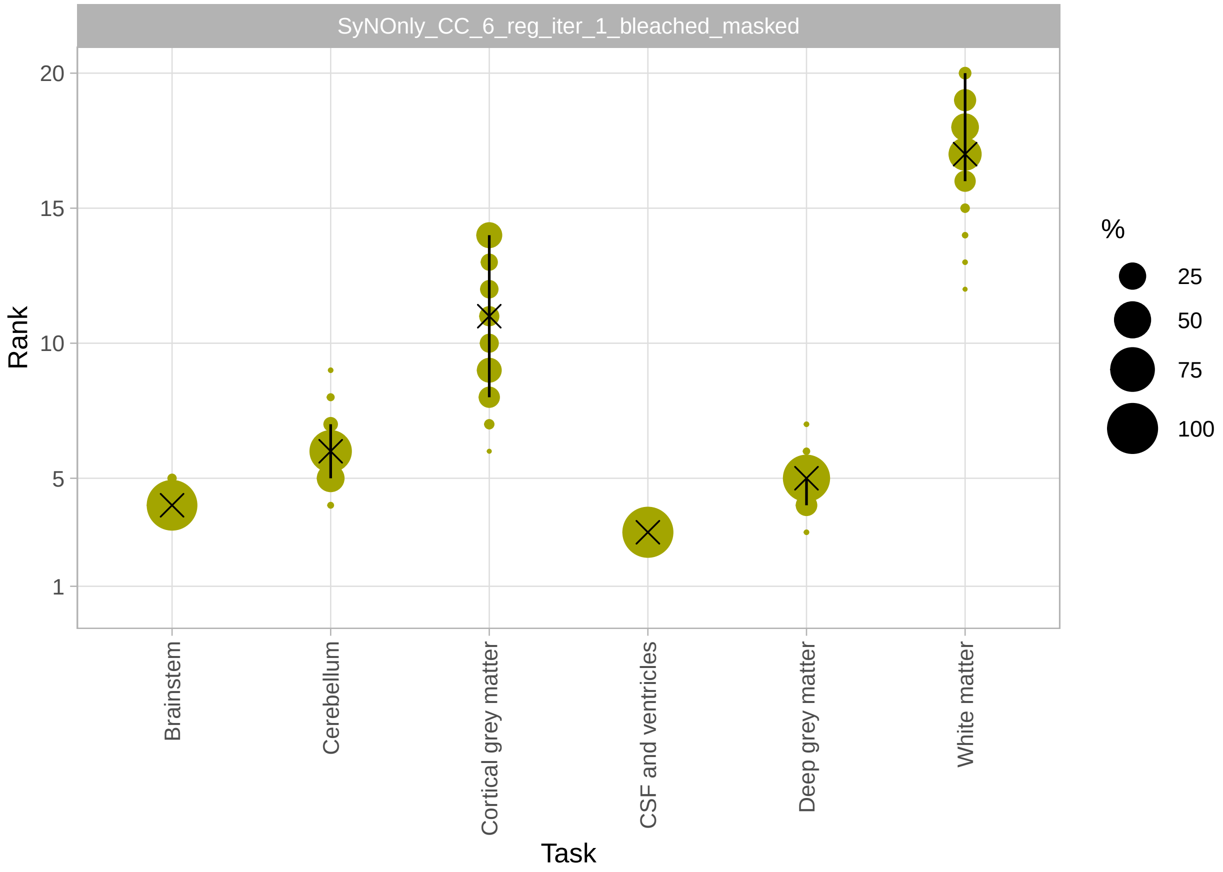
SyNOnly_CC_6_reg_iter_1_bleached_masked
Rank
20
15
10
5
1
Brainstem
Cerebellum
Cortical grey matter
CSF and ventricles
Deep grey matter
White matter
Task
%
25
50
75
100

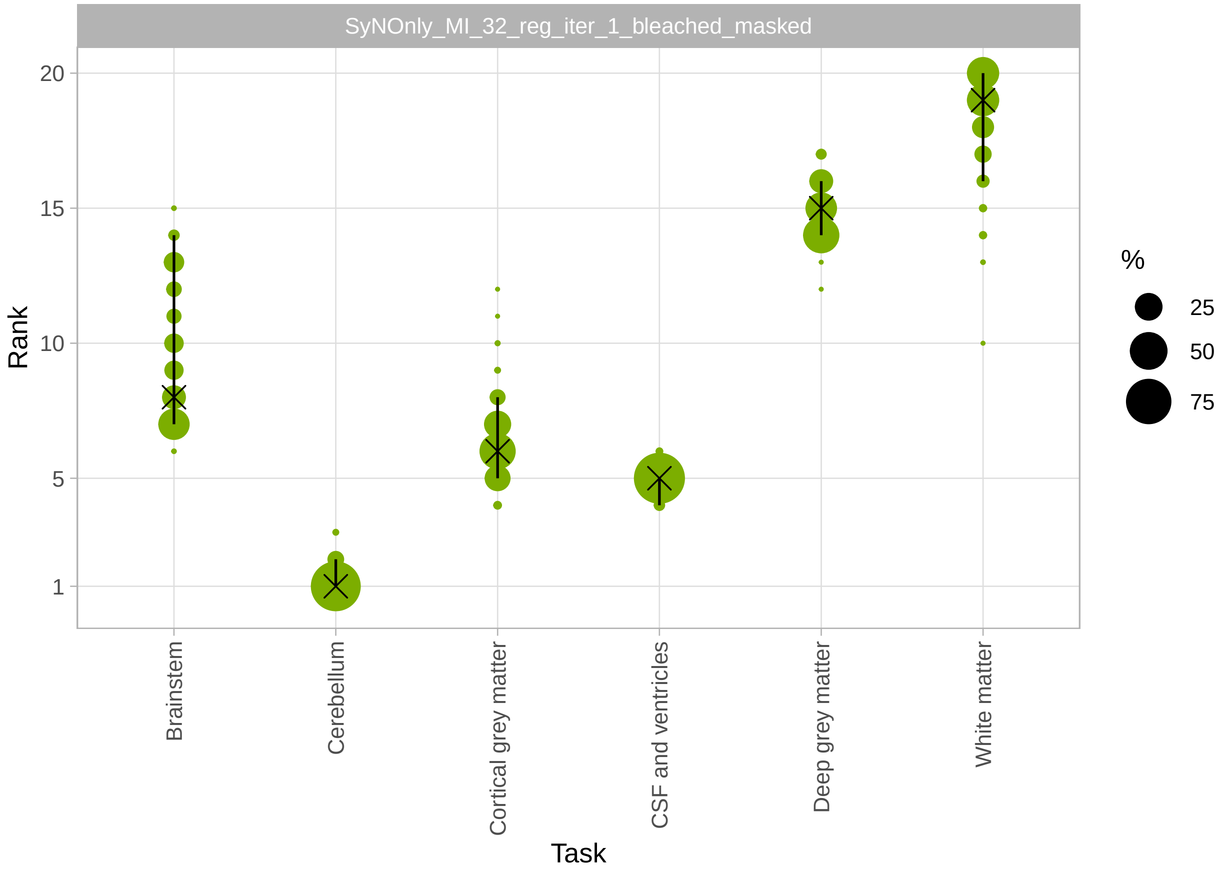

SyNOnly_MI_32_reg_iter_1_bleached_masked
Rank
20
15
10
5
1
Brainstem
Cerebellum
Cortical grey matter
CSF and ventricles
Deep grey matter
White matter
Task
%
25
50
75

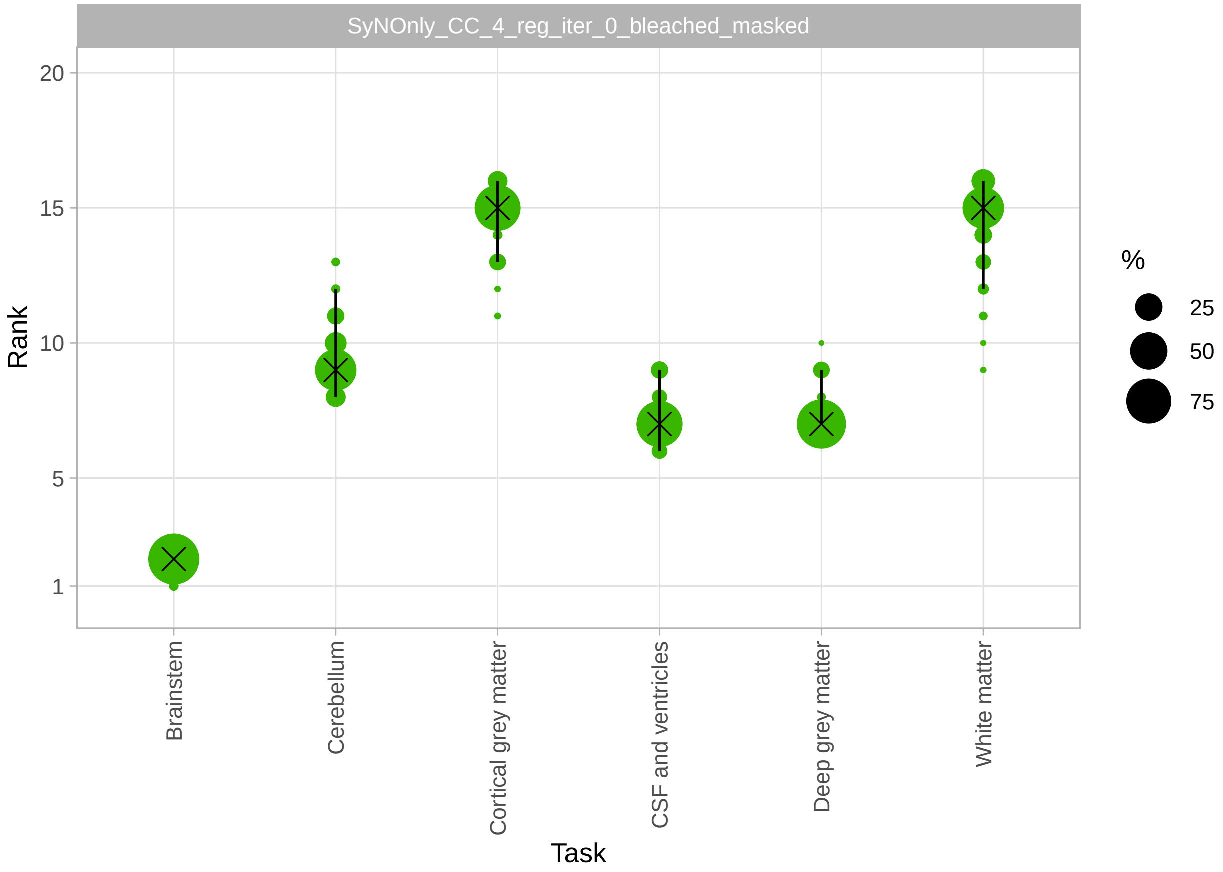
SyNOnly_CC_4_reg_iter_0_bleached_masked
Rank
20
15
10
5
1
%
25
50
75
Brainstem
Cerebellum
Cortical grey matter
CSF and ventricles
Deep grey matter
White matter
Task

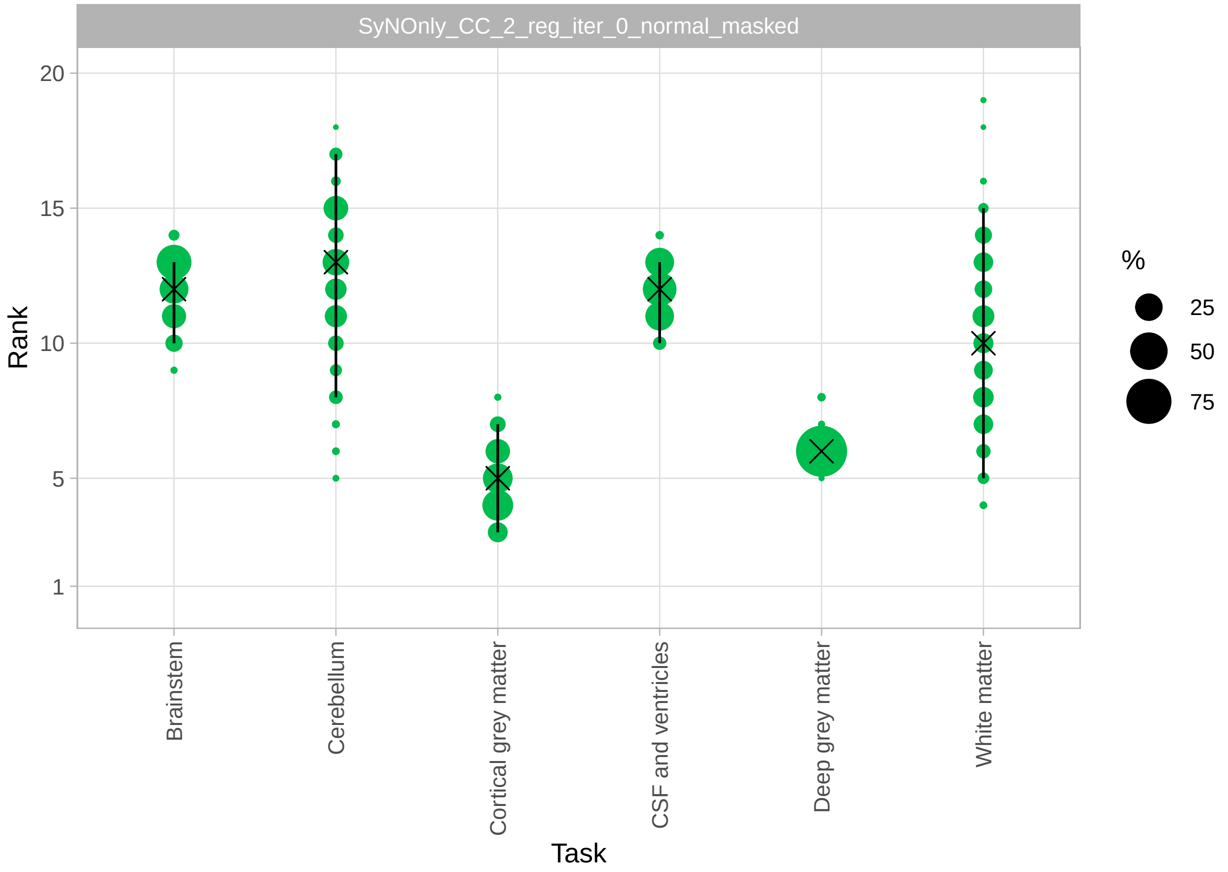
SyNOnly_CC_2_reg_iter_0_normal_masked
Rank
20
15
10
5
1
Brainstem
Cerebellum
Cortical grey matter
CSF and ventricles
Deep grey matter
White matter
Task
%
25
50
75

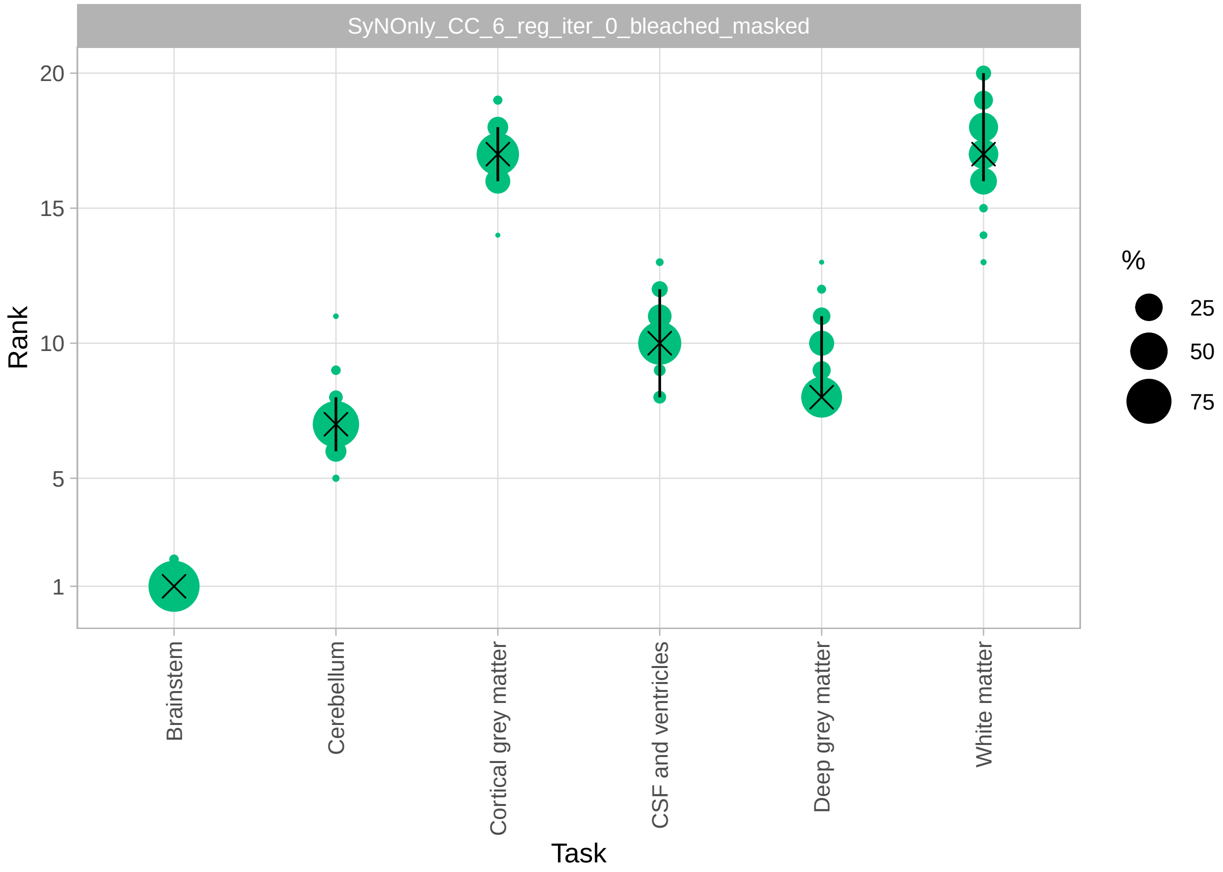
SyNOnly_CC_6_reg_iter_0_bleached_masked
Rank
20
15
10
5
1
Brainstem
Cerebellum
Cortical grey matter
CSF and ventricles
Deep grey matter
White matter
Task
%
25
50
75

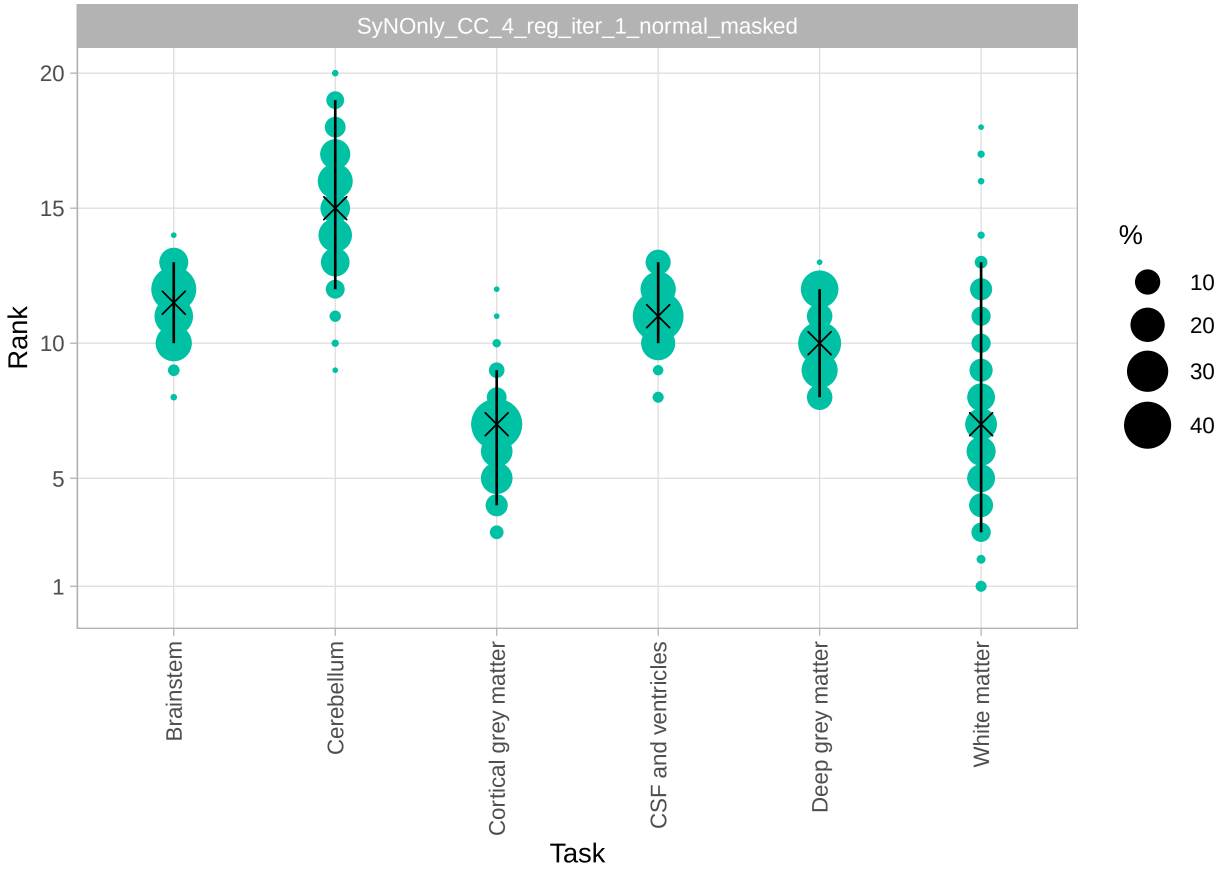
SyNOnly_CC_4_reg_iter_1_normal_masked
Rank
20
15
10
5
1
Brainstem
Cerebellum
Cortical grey matter
CSF and ventricles
Deep grey matter
White matter
Task
%
10
20
30
40

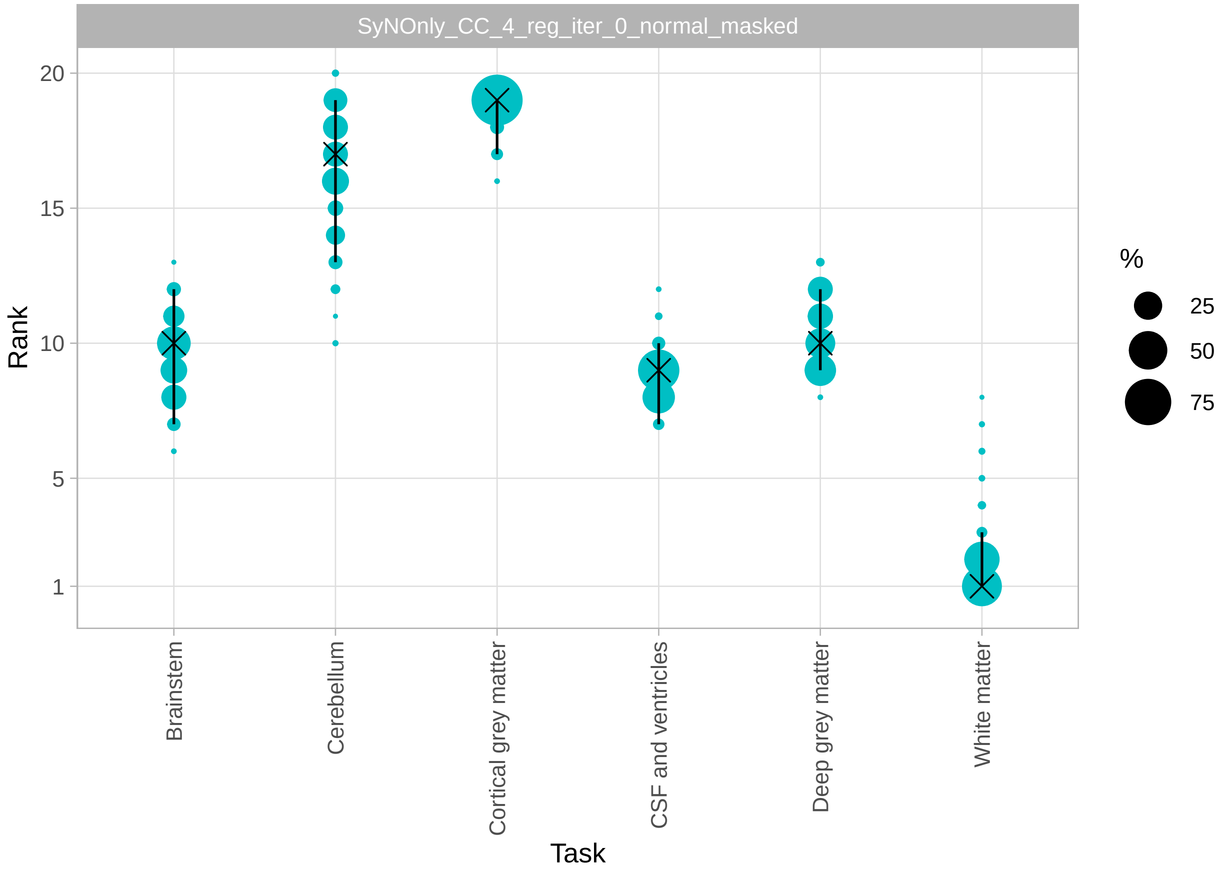

SyNOnly_CC_4_reg_iter_0_normal_masked
Rank
Task
20
15
10
5
1
Brainstem
Cerebellum
Cortical grey matter
CSF and ventricles
Deep grey matter
White matter
%
25
50
75

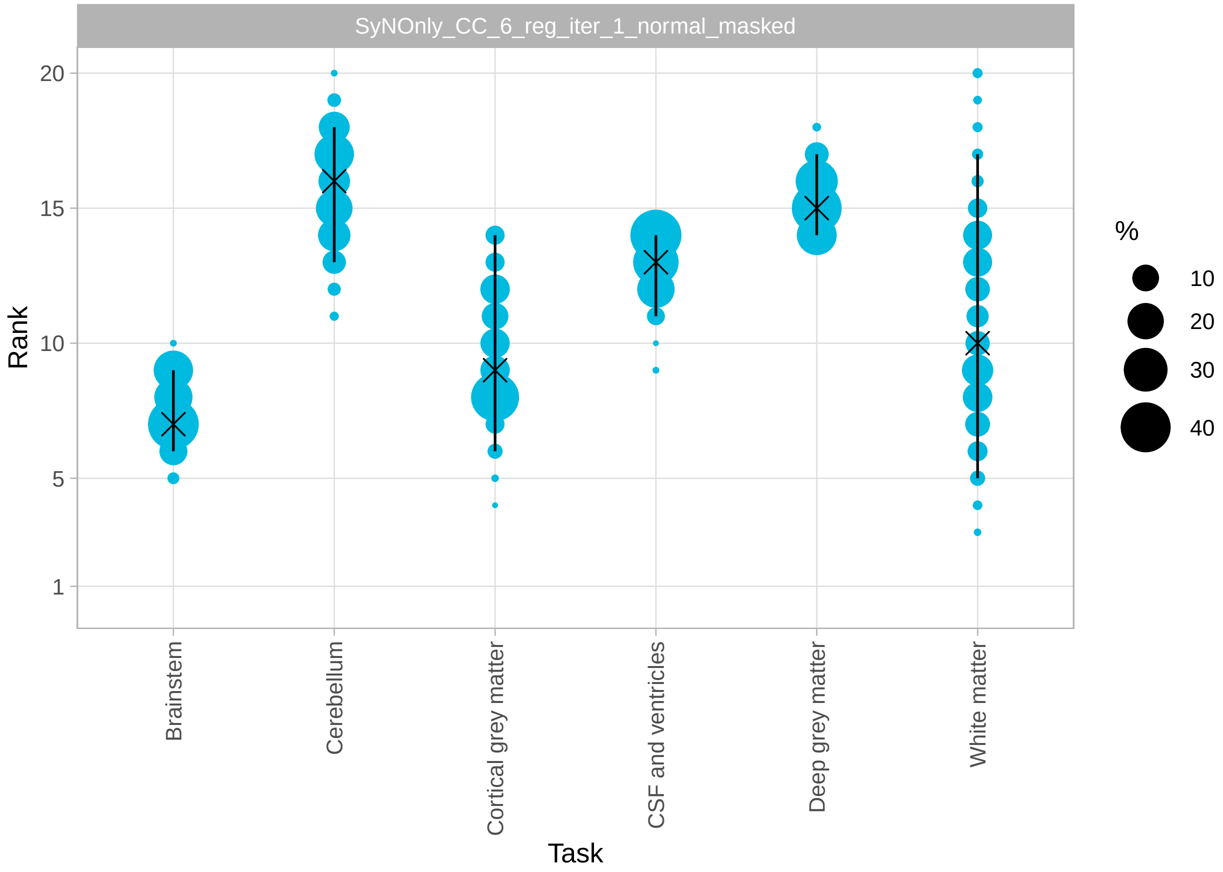

SyNOnly_CC_6_reg_iter_1_normal_masked
Rank
20
15
10
5
1
Brainstem
Cerebellum
Cortical grey matter
CSF and ventricles
Deep grey matter
White matter
Task
%
10
20
30
40

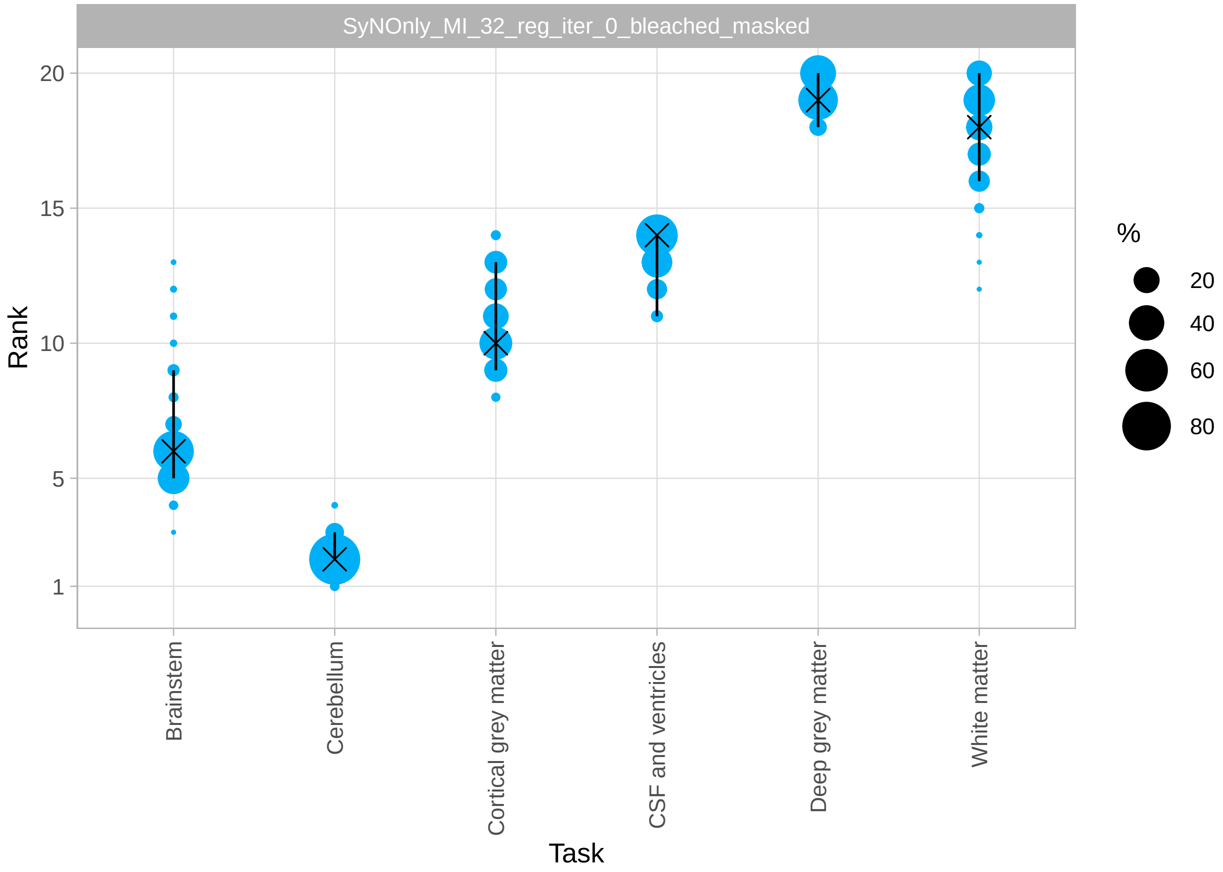
SyNOnly_MI_32_reg_iter_0_bleached_masked
Rank
20
15
10
5
1
Brainstem
Cerebellum
Cortical grey matter
CSF and ventricles
Deep grey matter
White matter
Task
%
20
40
60
80

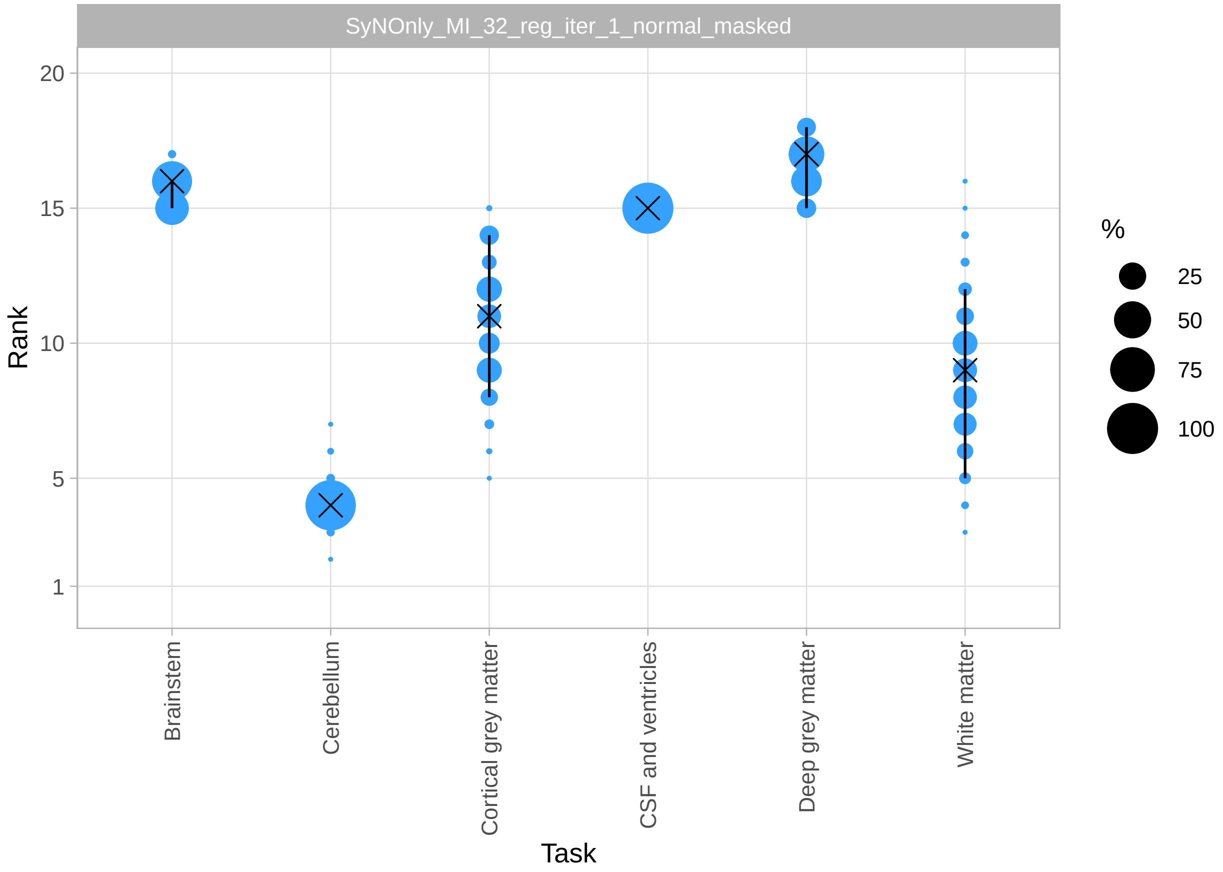

SyNOnly_MI_32_reg_iter_1_normal_masked
Rank
20
15
10
5
1
Brainstem
Cerebellum
Cortical grey matter
CSF and ventricles
Deep grey matter
White matter
Task
%
25
50
75
100

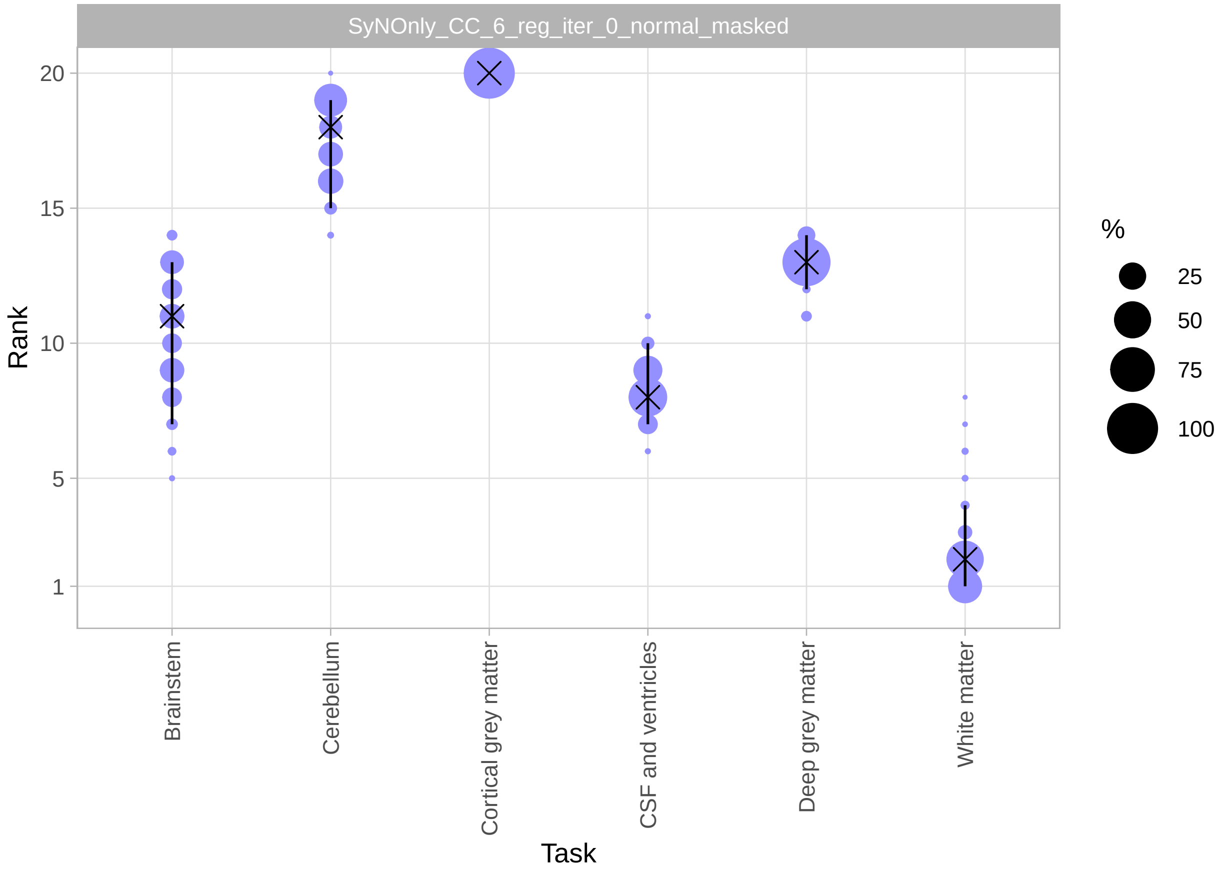
SyNOnly_CC_6_reg_iter_0_normal_masked
Rank
20
15
10
5
1
Brainstem
Cerebellum
Cortical grey matter
CSF and ventricles
Deep grey matter
White matter
Task
%
25
50
75
100

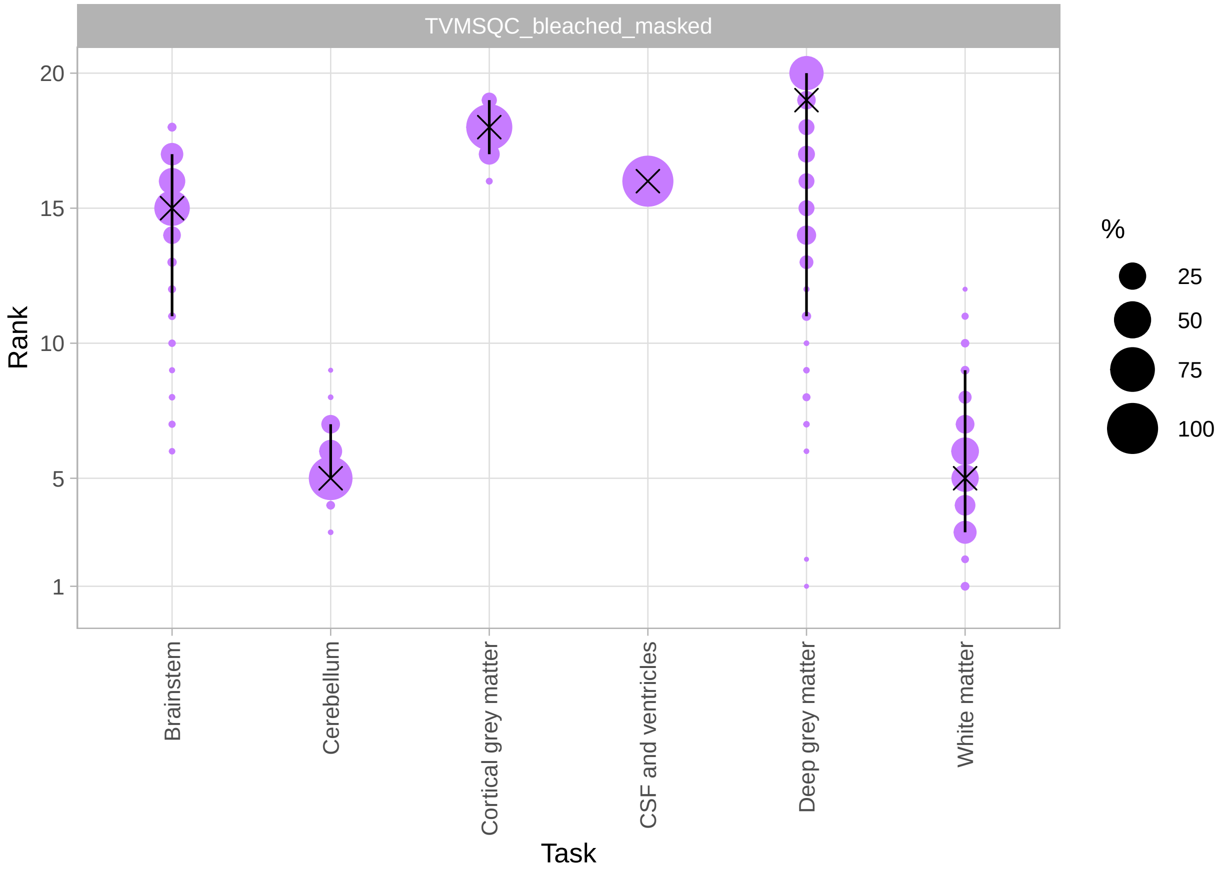
TVMSQC_bleached_masked
Rank
20
15
10
5
1
Brainstem
Cerebellum
Cortical grey matter
CSF and ventricles
Deep grey matter
White matter
Task
%
25
50
75
100

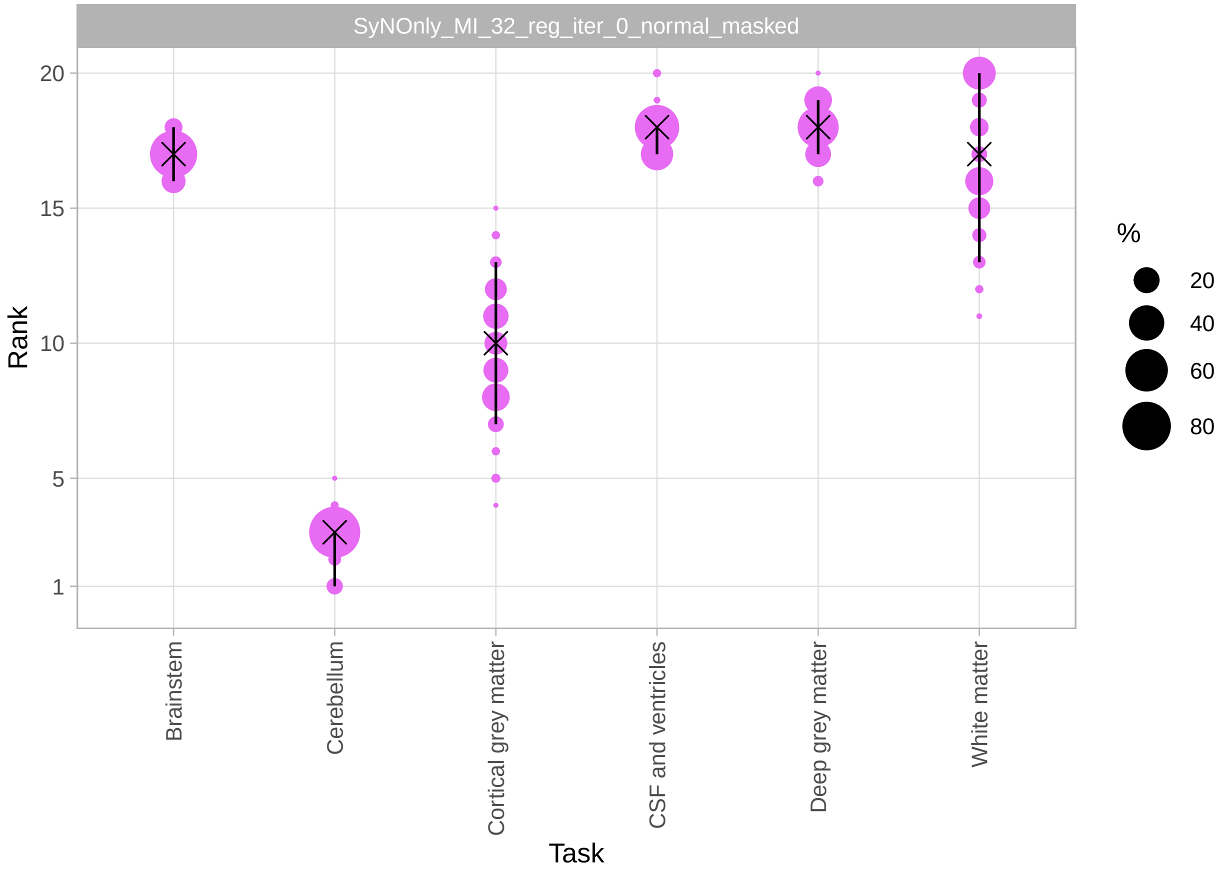
SyNOnly_MI_32_reg_iter_0_normal_masked
Rank
20
15
10
5
1
Brainstem
Cerebellum
Cortical grey matter
CSF and ventricles
Deep grey matter
White matter
Task
%
20
40
60
80

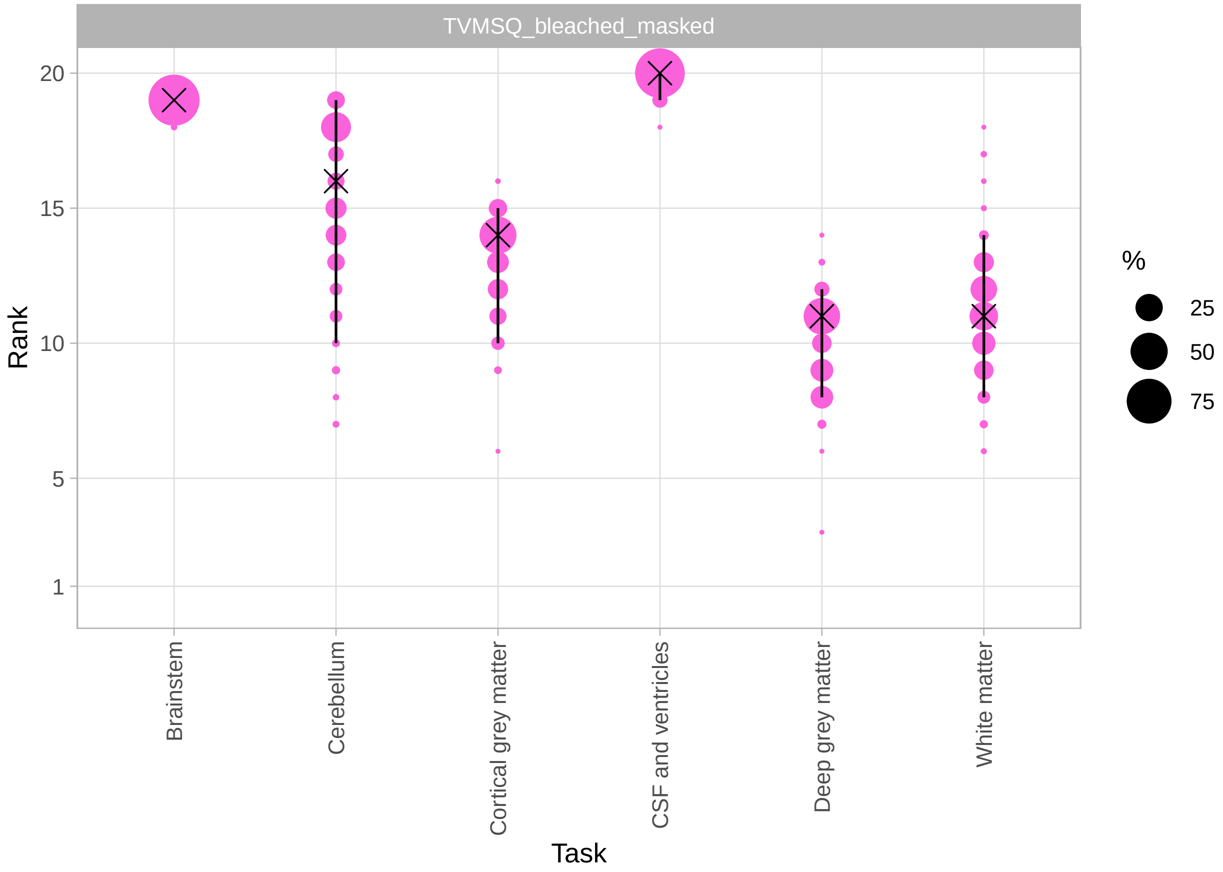
TVMSQ_bleached_masked
Rank
20
15
10
5
1
Brainstem
Cerebellum
Cortical grey matter
CSF and ventricles
Deep grey matter
White matter
Task
%
25
50
75

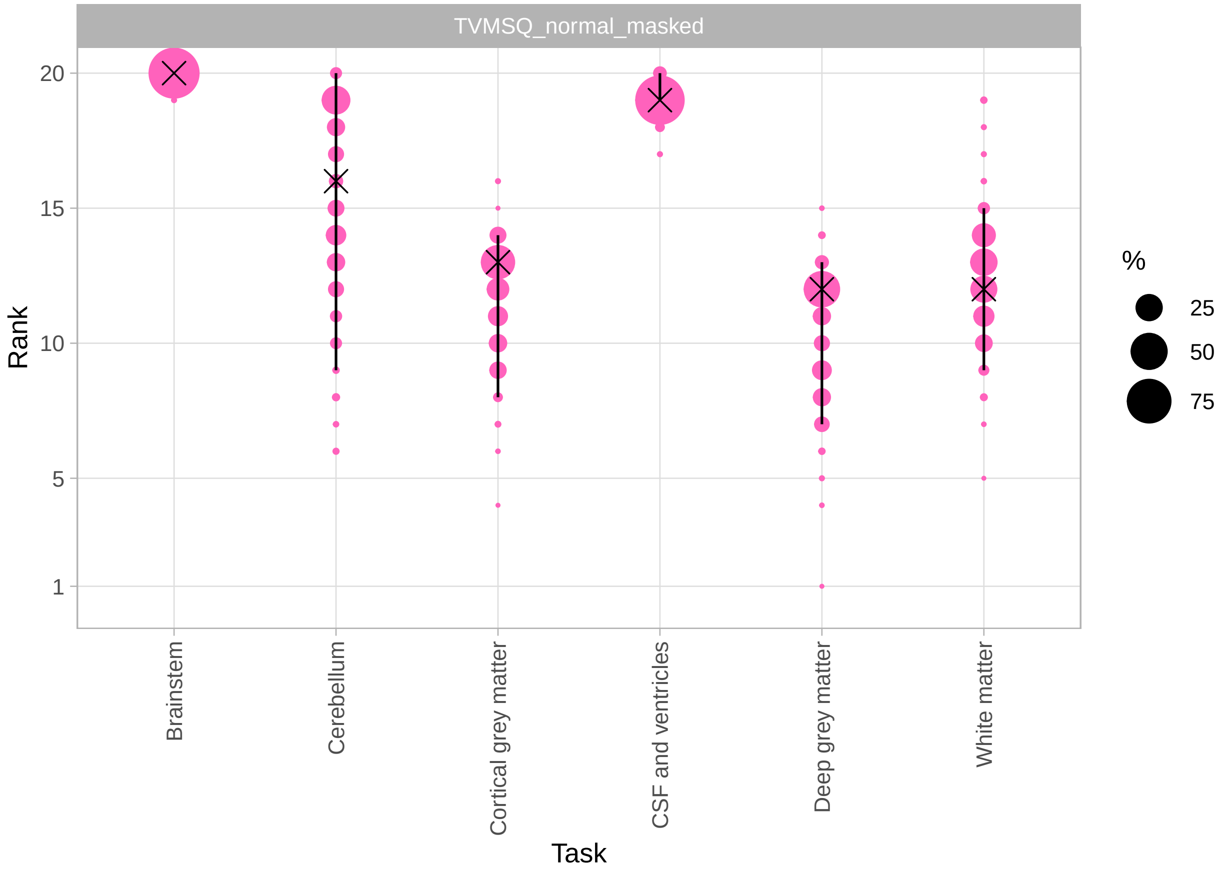
TVMSQ_normal_masked
Rank
20
15
10
5
1
Brainstem
Cerebellum
Cortical grey matter
CSF and ventricles
Deep grey matter
White matter
Task
%
25
50
75

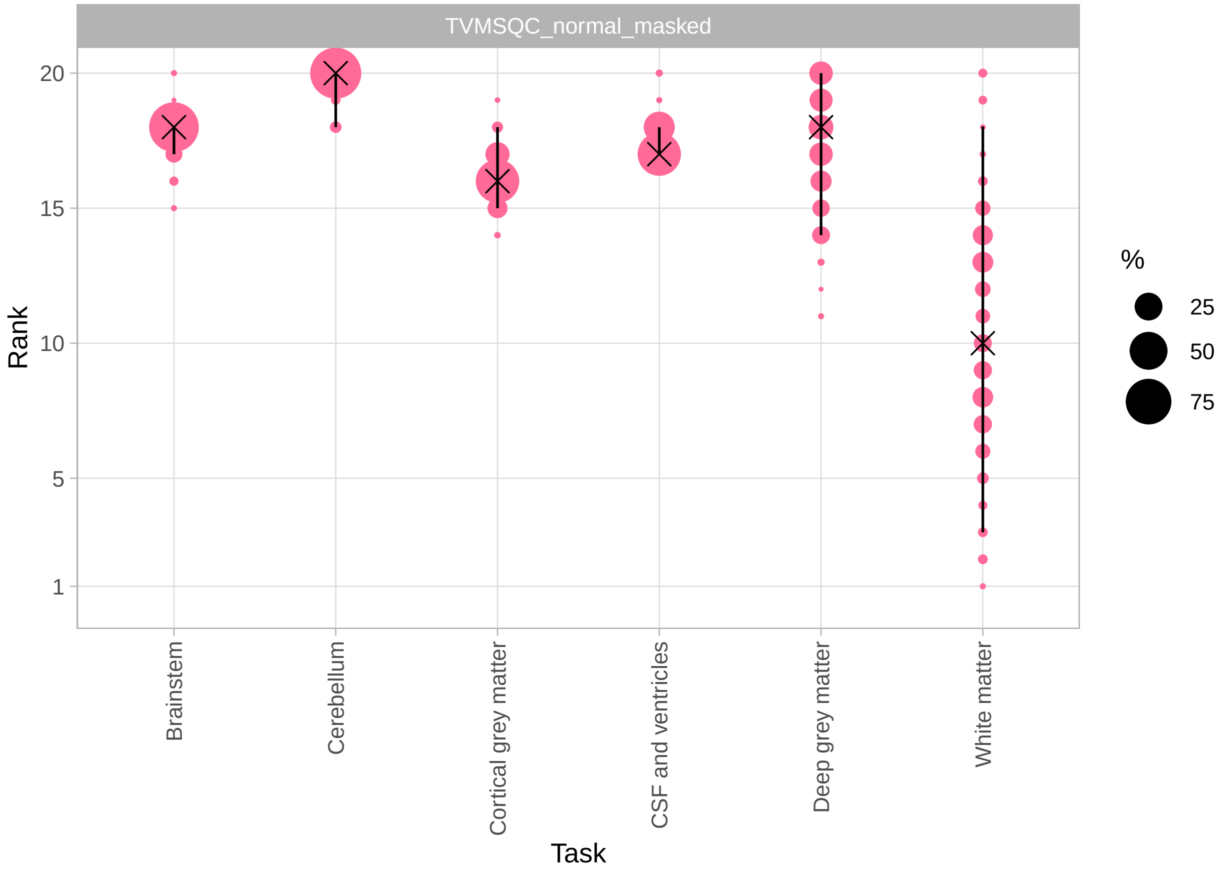
TVMSQC_normal_masked
Rank
20
15
10
5
1
Brainstem
Cerebellum
Cortical grey matter
CSF and ventricles
Deep grey matter
White matter
Task
%
25
50
75

An alternative representation is provided by a stacked frequency plot of the observed ranks, separated by algorithm. Observed ranks across bootstrap samples are displayed with coloring according to the task. For algorithms that achieve the same rank in different tasks for the full assessment data set, vertical lines are on top of each other. Vertical lines allow to compare the achieved rank of each algorithm over different tasks.

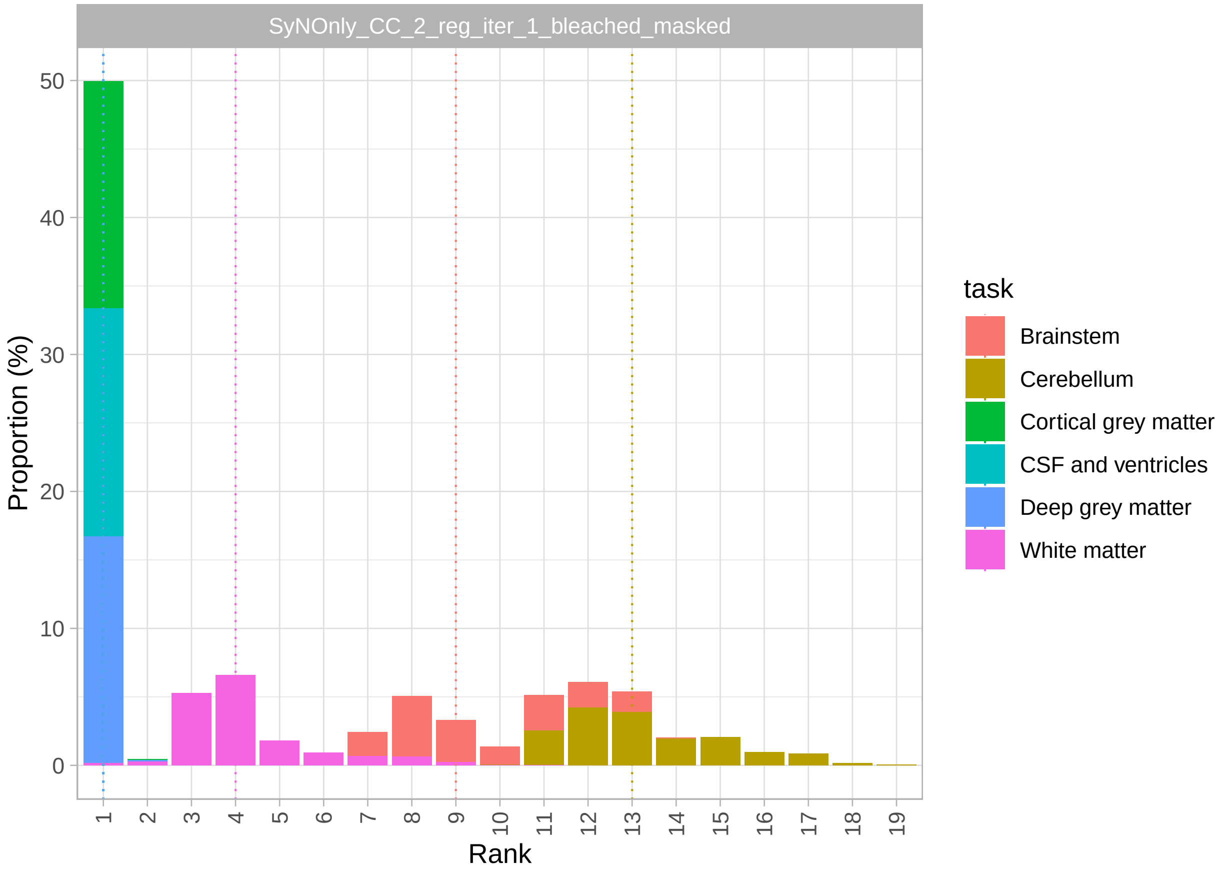

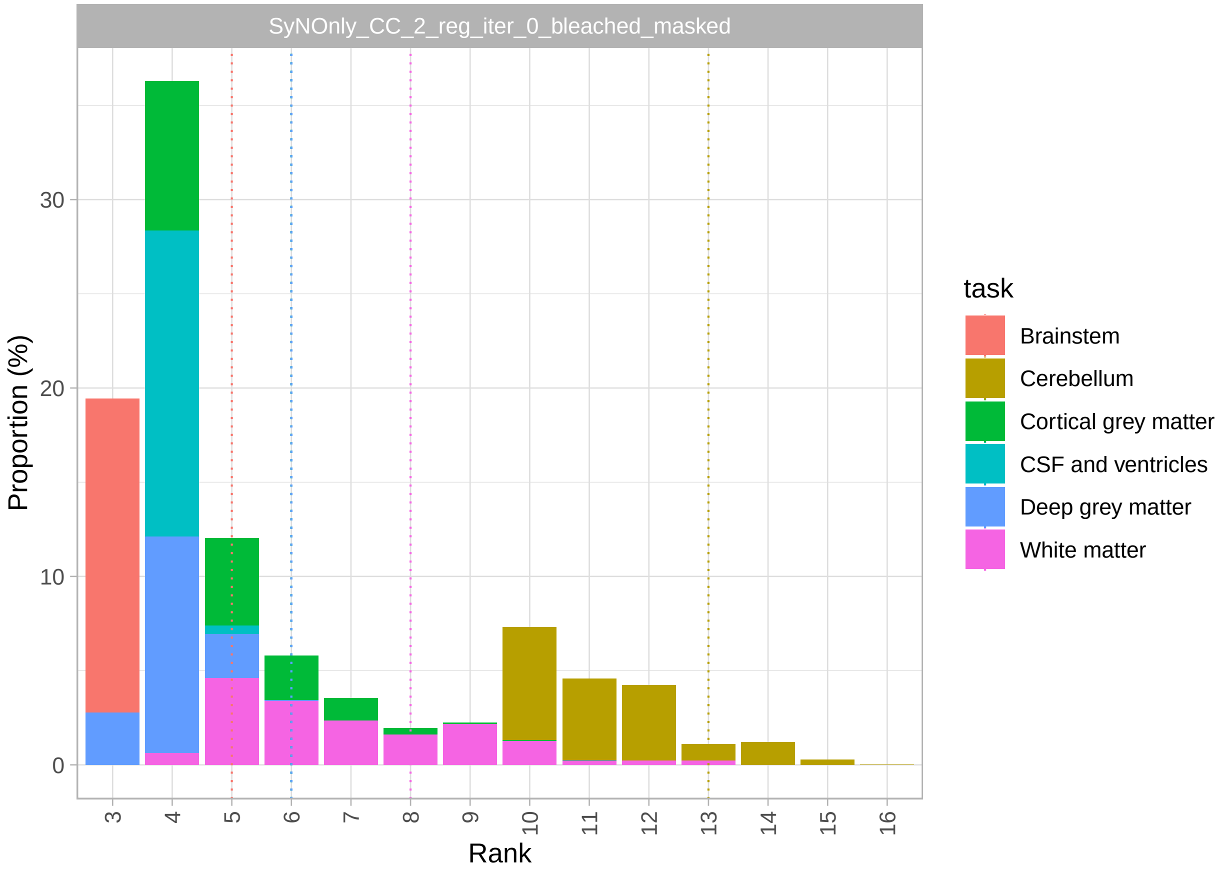
SyNOnly_CC_2_reg_iter_0_bleached_masked
Proportion (%)
30
20
10
0
3
4
5
6
7
8
9
10
11
12
13
14
15
16
Rank
task
Brainstem
Cerebellum
Cortical grey matter
CSF and ventricles
Deep grey matter
White matter

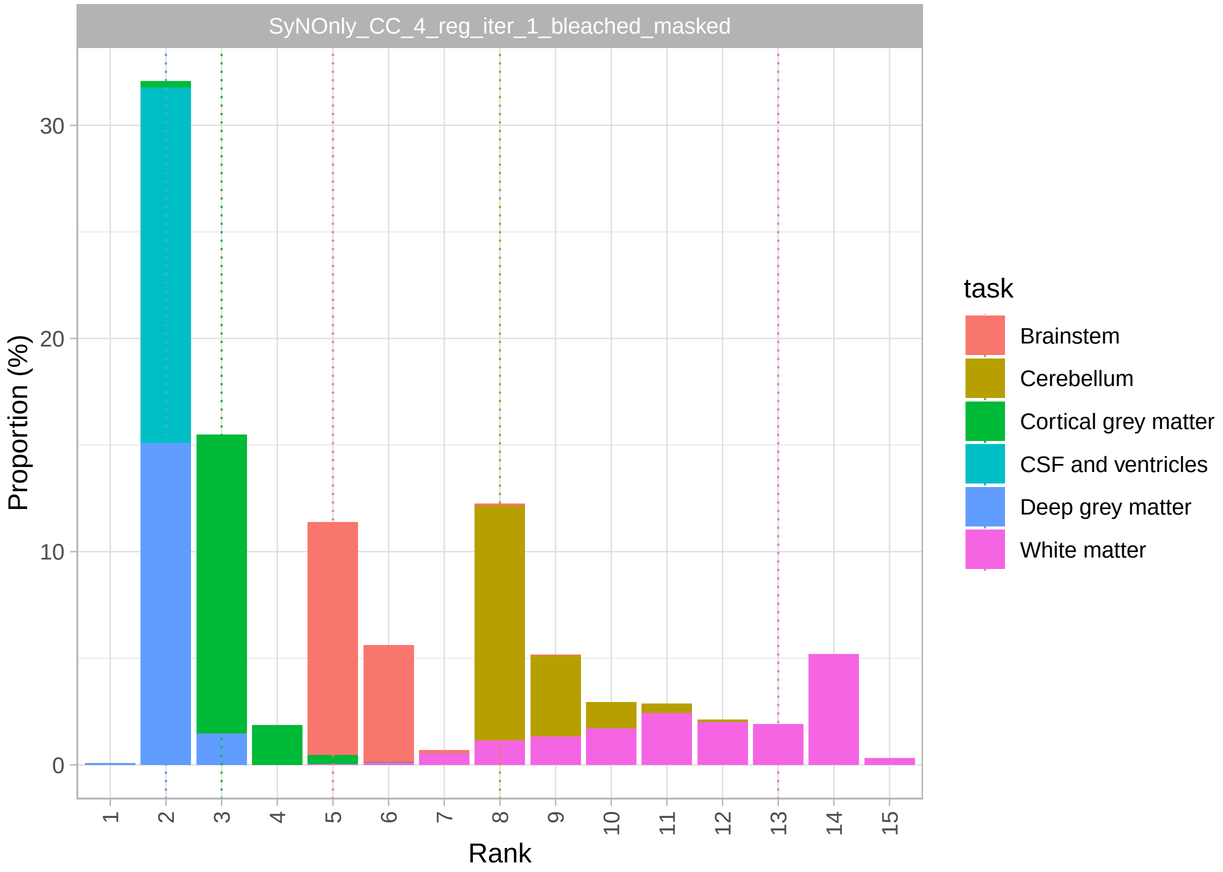
SyNOnly_CC_4_reg_iter_1_bleached_masked
Proportion (%)
30
20
10
0
1
2
3
4
5
6
7
8
9
10
11
12
13
14
15
Rank
task
Brainstem
Cerebellum
Cortical grey matter
CSF and ventricles
Deep grey matter
White matter

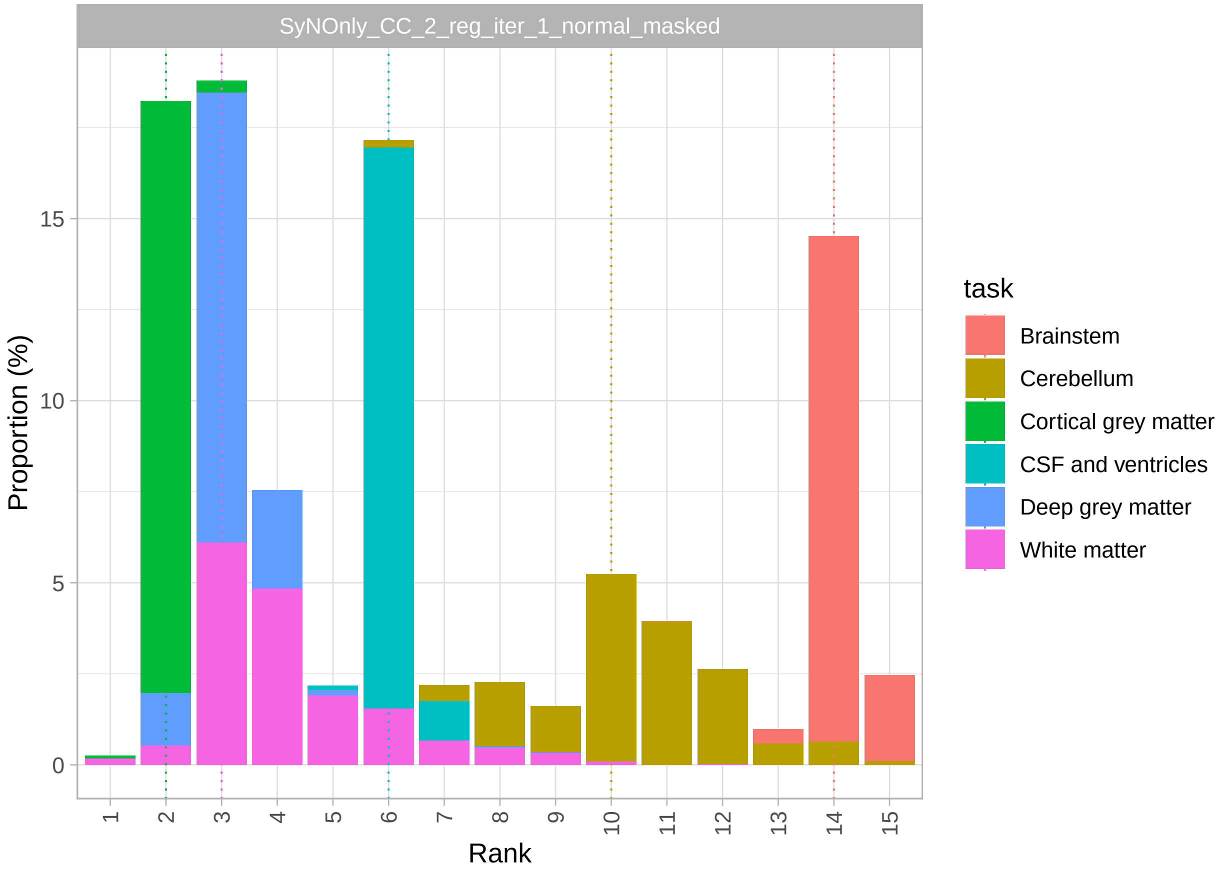
SyNOnly_CC_2_reg_iter_1_normal_masked
Proportion (%)
0
5
10
15
1
2
3
4
5
6
7
8
9
10
11
12
13
14
15
Rank
task
Brainstem
Cerebellum
Cortical grey matter
CSF and ventricles
Deep grey matter
White matter

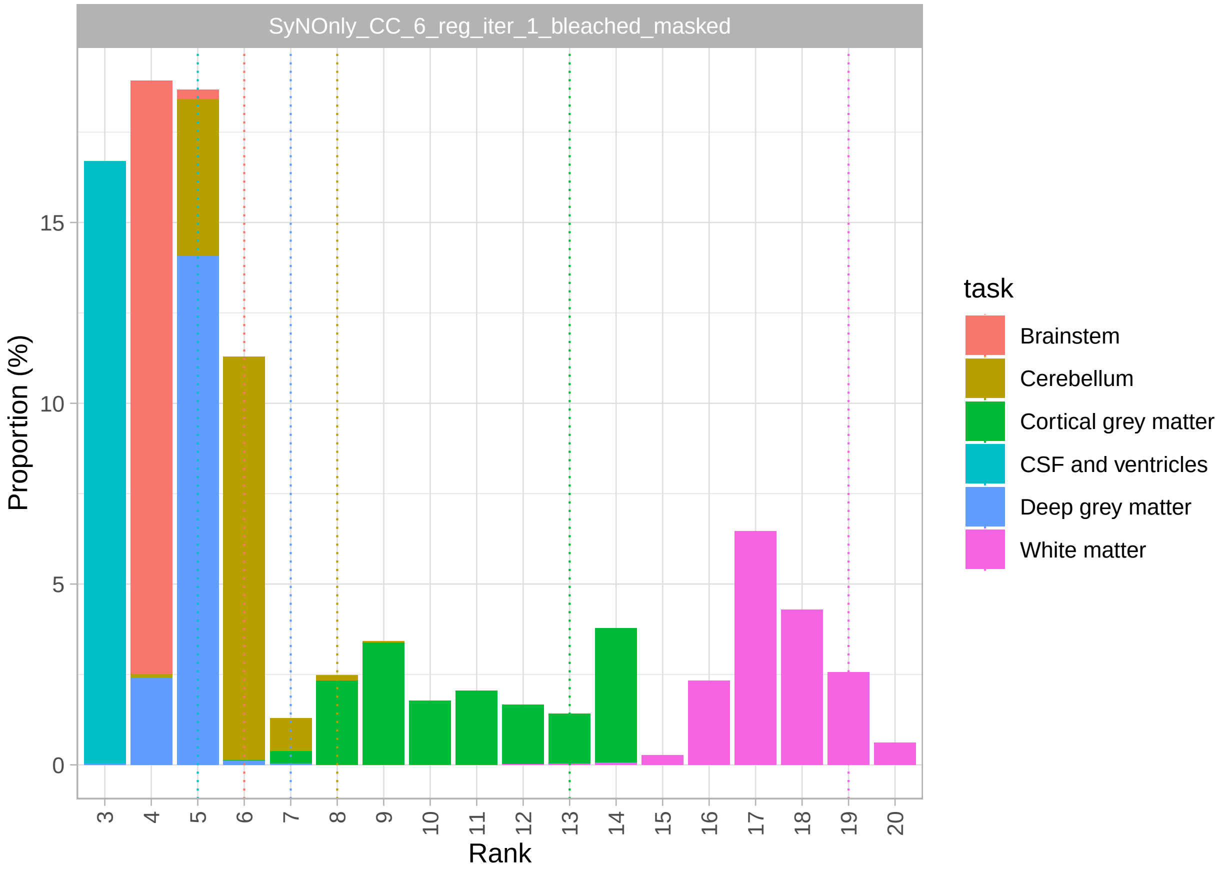
SyNOnly_CC_6_reg_iter_1_bleached_masked
Proportion (%)
15
10
5
0
3
4
5
6
7
8
9
10
11
12
13
14
15
16
17
18
19
20
Rank
task
Brainstem
Cerebellum
Cortical grey matter
CSF and ventricles
Deep grey matter
White matter

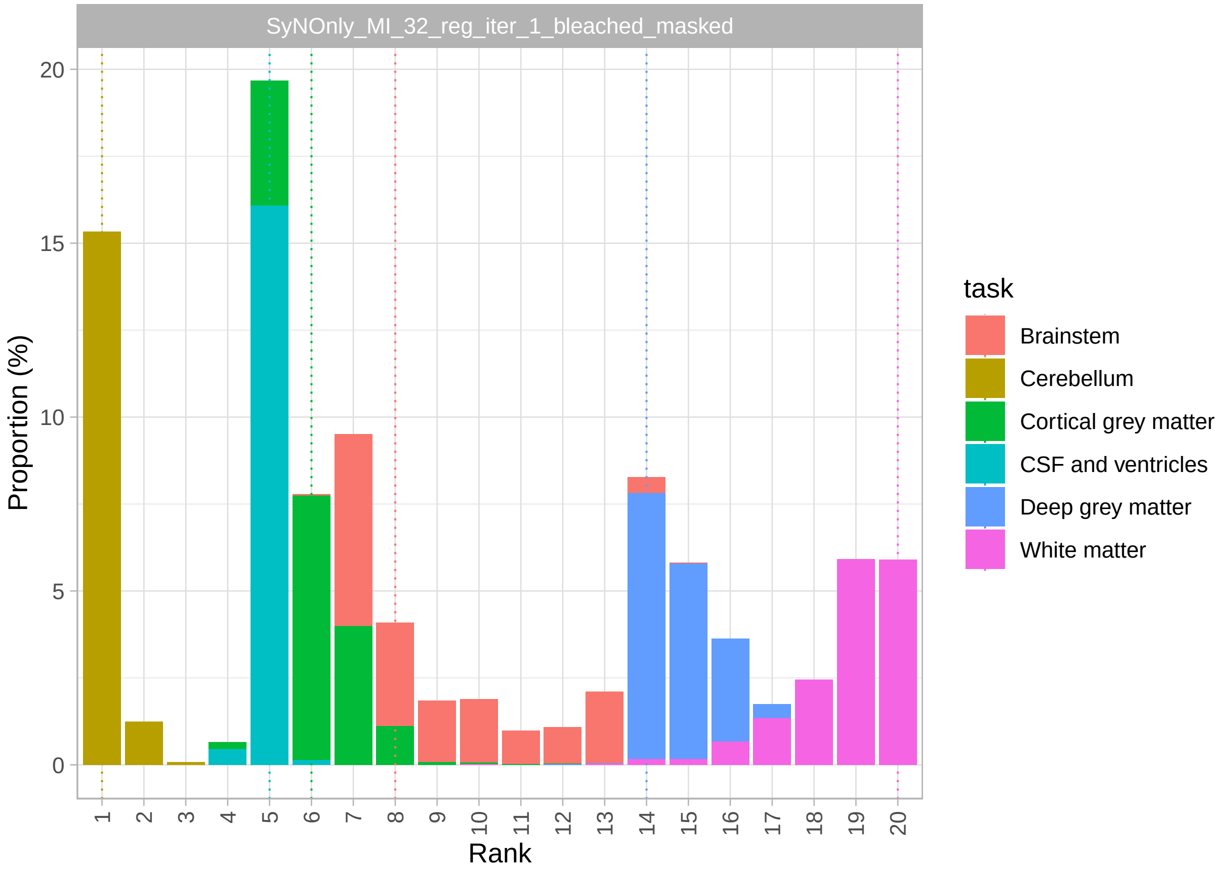
SyNOnly_MI_32_reg_iter_1_bleached_masked
Proportion (%)
Rank
task
Brainstem
Cerebellum
Cortical grey matter
CSF and ventricles
Deep grey matter
White matter

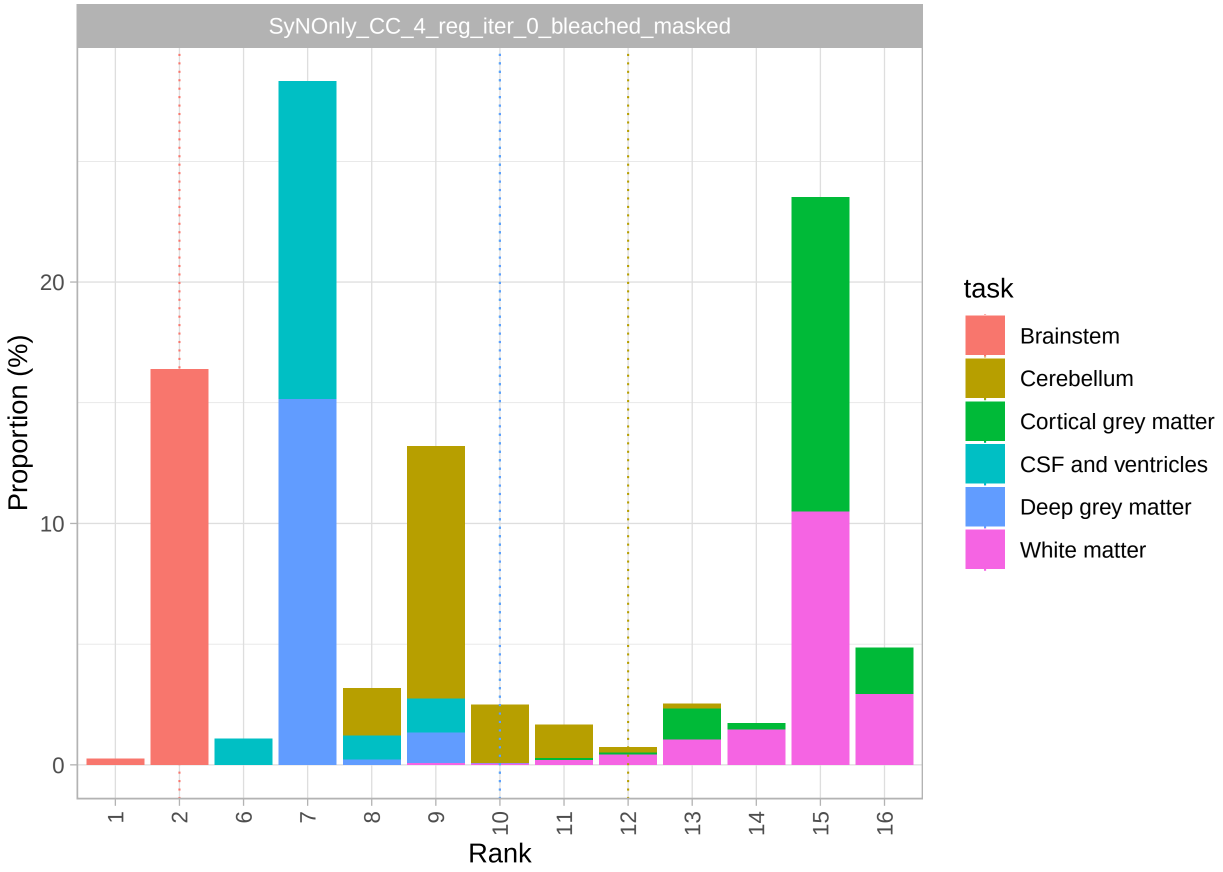
SyNOnly_CC_4_reg_iter_0_bleached_masked
Proportion (%)
20
10
0
1
2
6
7
8
9
10
11
12
13
14
15
16
Rank
task
Brainstem
Cerebellum
Cortical grey matter
CSF and ventricles
Deep grey matter
White matter

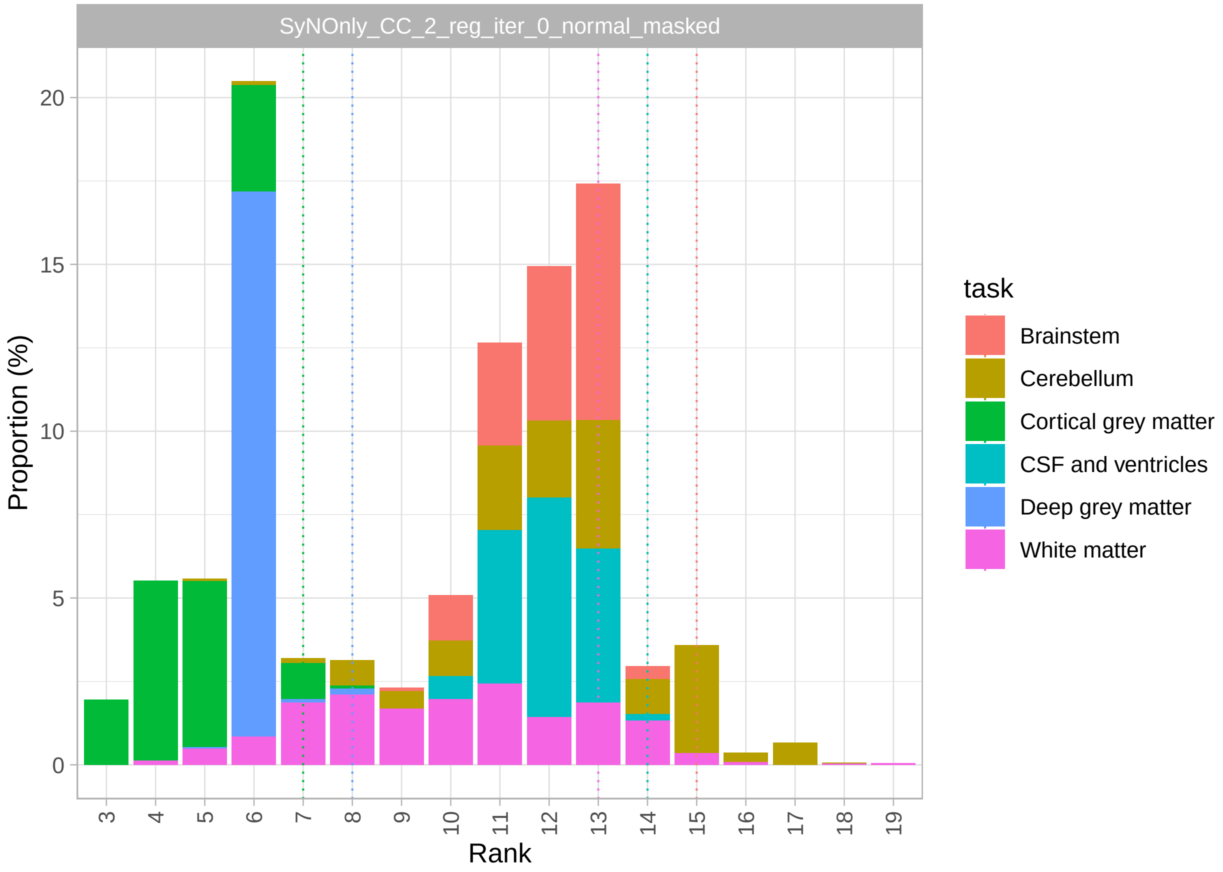

SyNOnly_CC_2_reg_iter_0_normal_masked
Proportion (%)
Rank
task
Brainstem
Cerebellum
Cortical grey matter
CSF and ventricles
Deep grey matter
White matter

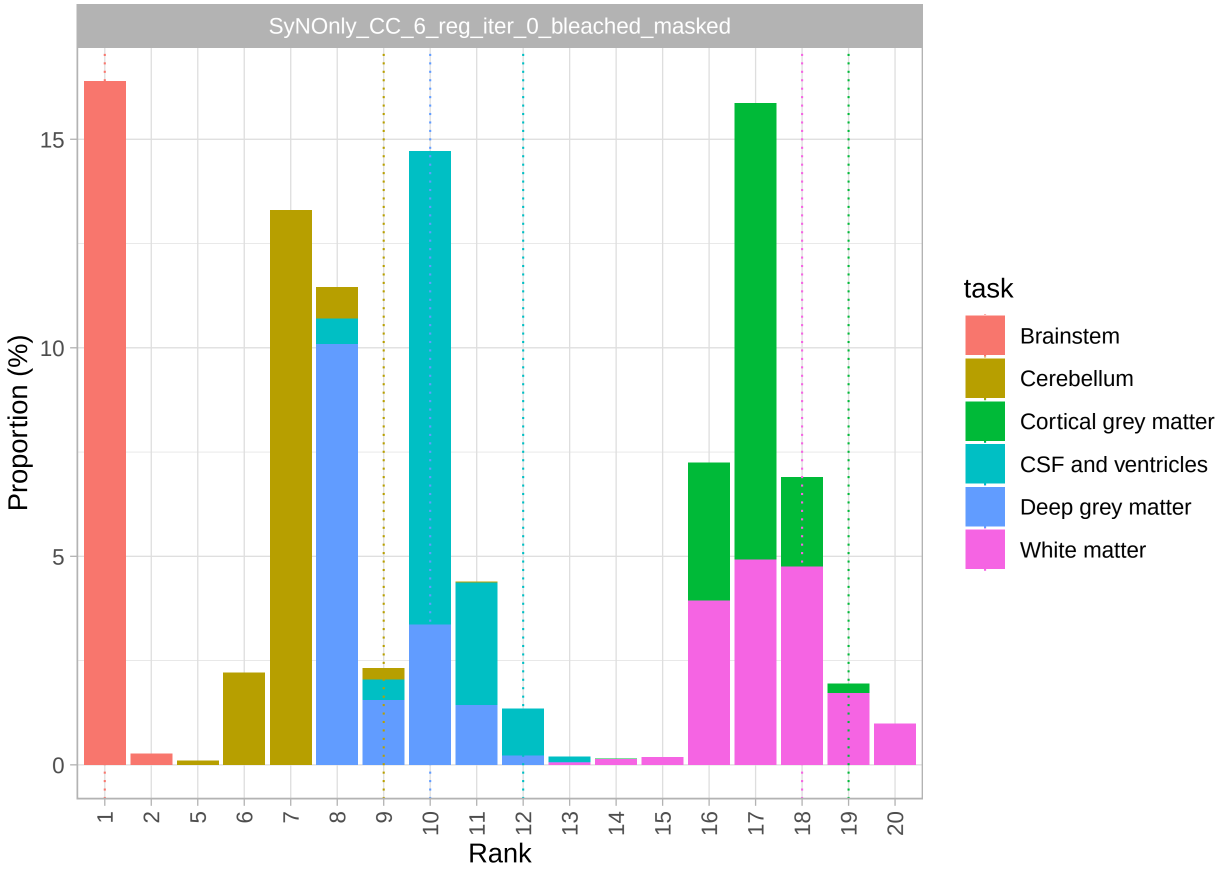
SyNOnly_CC_6_reg_iter_0_bleached_masked
Proportion (%)
15
10
5
0
1
2
5
6
7
8
9
10
11
12
13
14
15
16
17
18
19
20
Rank
task
Brainstem
Cerebellum
Cortical grey matter
CSF and ventricles
Deep grey matter
White matter

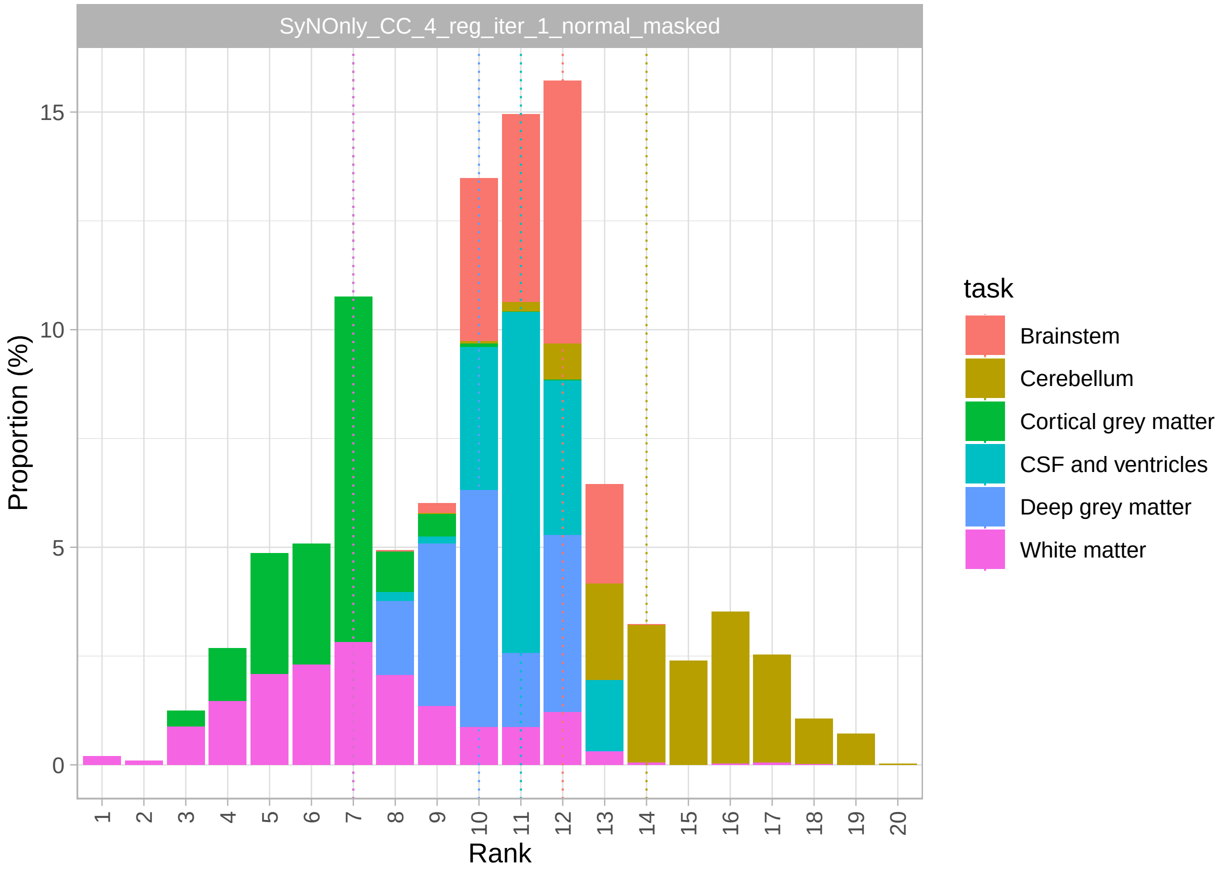

SyNOnly_CC_4_reg_iter_1_normal_masked
Proportion (%)
0
5
10
15
1
2
3
4
5
6
7
8
9
10
11
12
13
14
15
16
17
18
19
20
Rank
task
Brainstem
Cerebellum
Cortical grey matter
CSF and ventricles
Deep grey matter
White matter

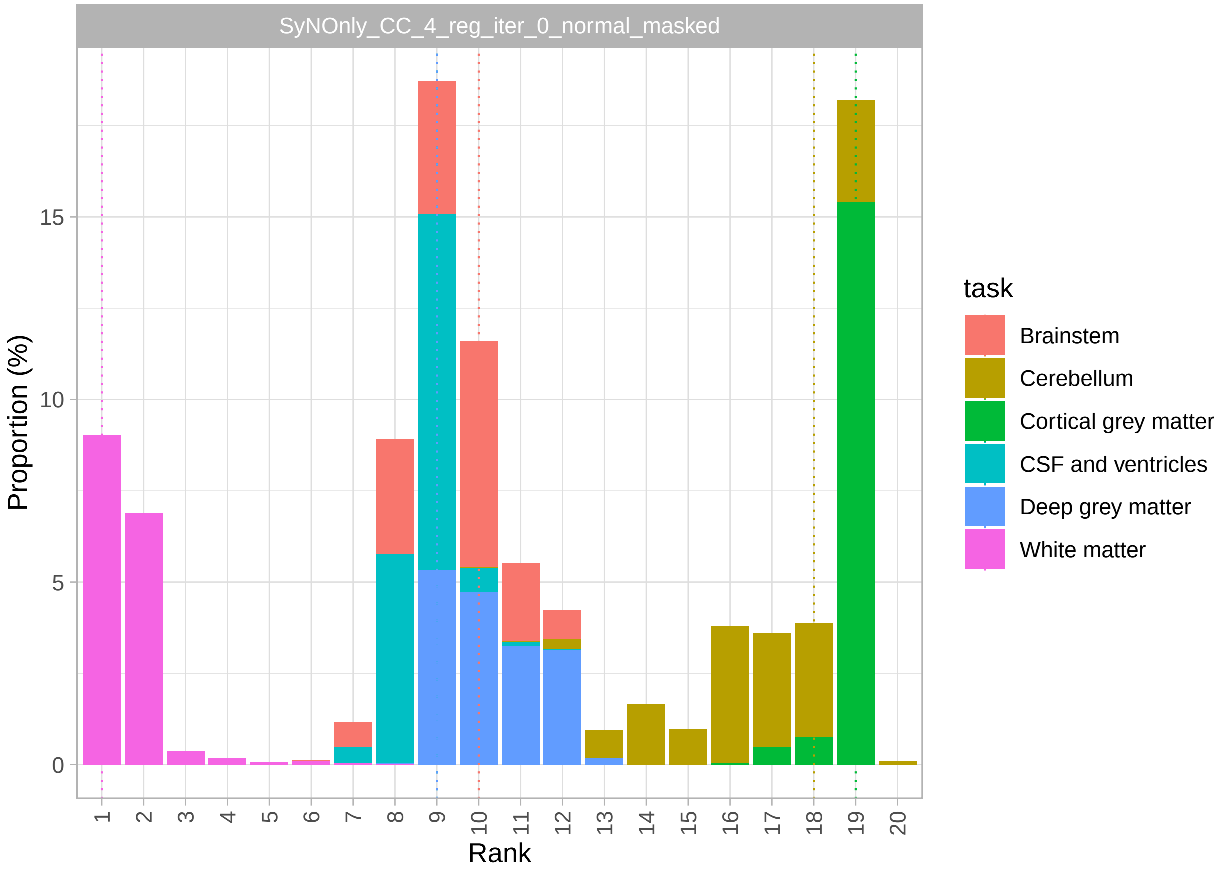

SyNOnly_CC_4_reg_iter_0_normal_masked
Proportion (%)
0
5
10
15
1
2
3
4
5
6
7
8
9
10
11
12
13
14
15
16
17
18
19
20
Rank
task
Brainstem
Cerebellum
Cortical grey matter
CSF and ventricles
Deep grey matter
White matter

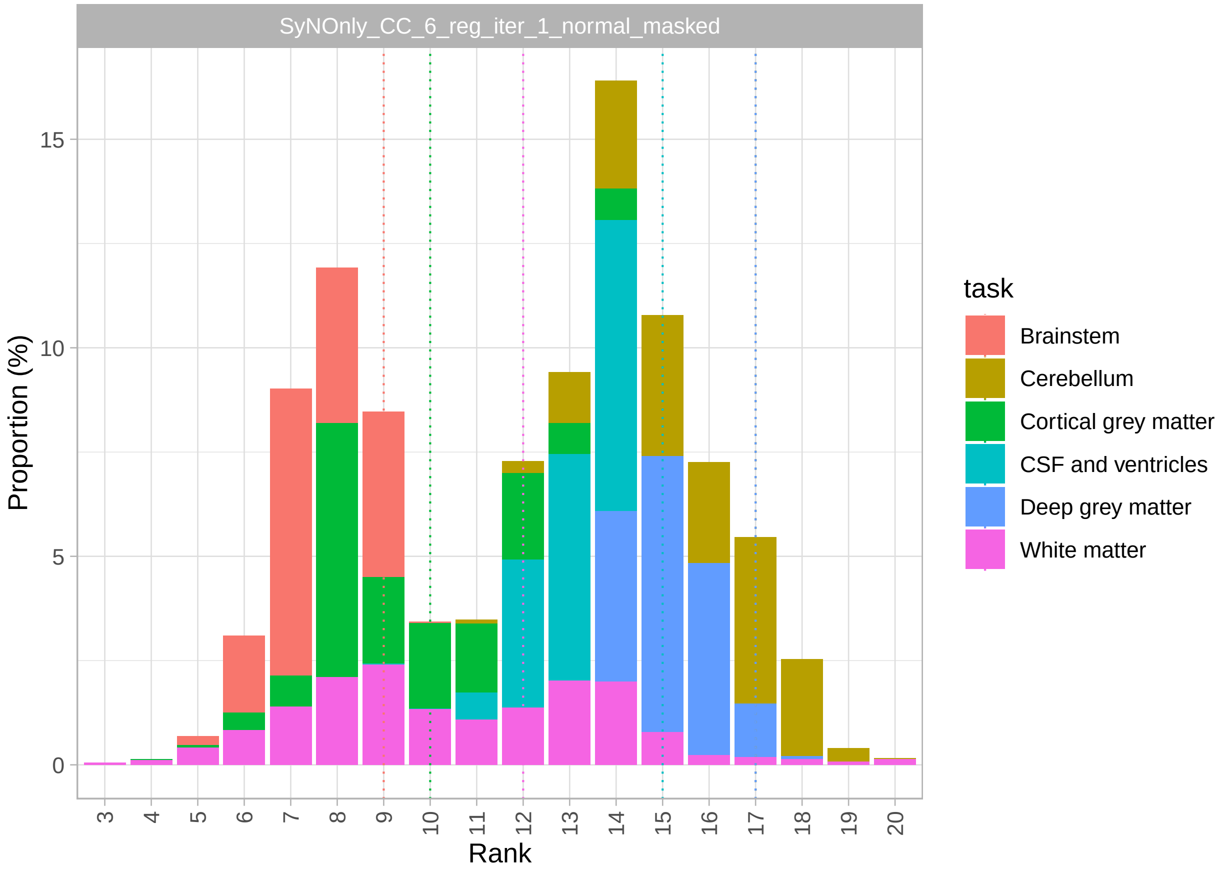
SyNOnly_CC_6_reg_iter_1_normal_masked
Proportion (%)
0
5
10
15
3
4
5
6
7
8
9
10
11
12
13
14
15
16
17
18
19
20
Rank
task
Brainstem
Cerebellum
Cortical grey matter
CSF and ventricles
Deep grey matter
White matter

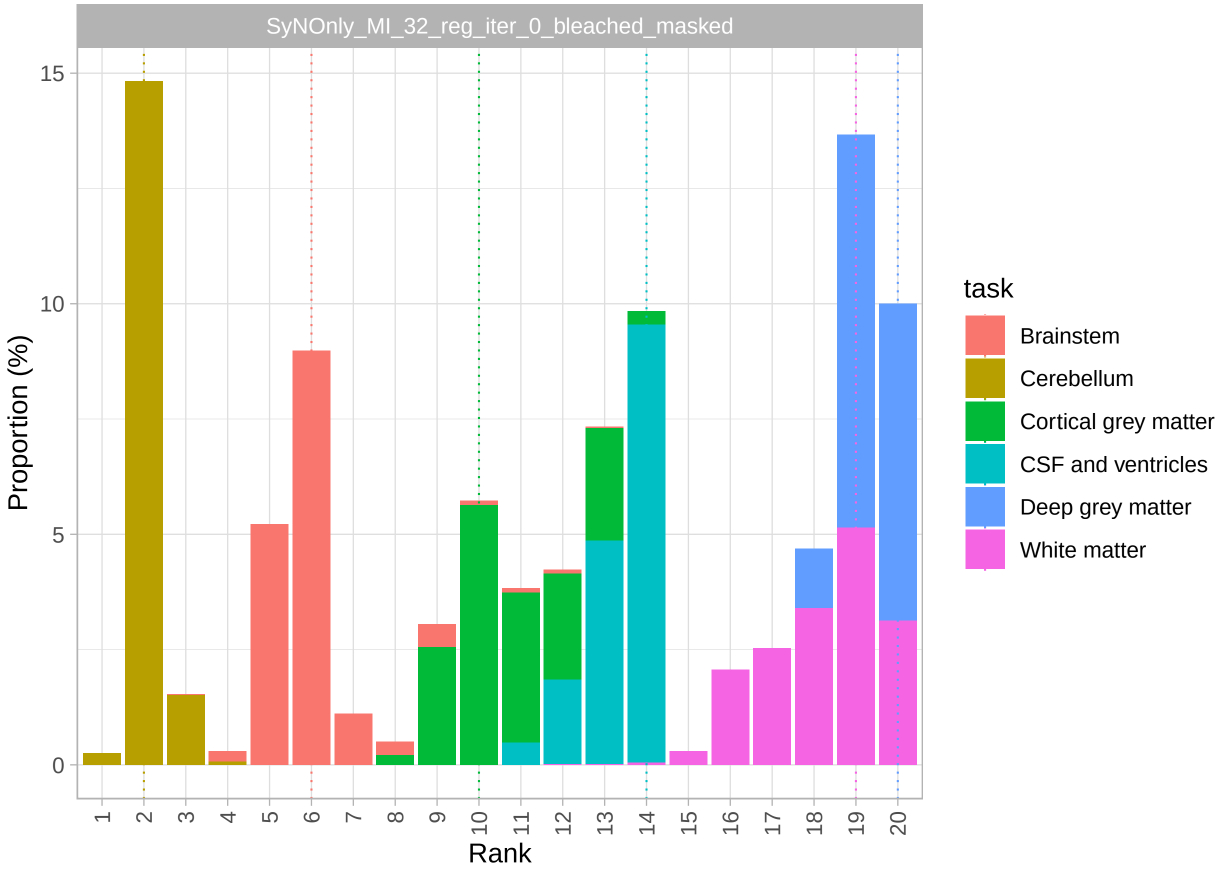
SyNOnly_MI_32_reg_iter_0_bleached_masked
Proportion (%)
15
10
5
0
1
2
3
4
5
6
7
8
9
10
11
12
13
14
15
16
17
18
19
20
Rank
task
Brainstem
Cerebellum
Cortical grey matter
CSF and ventricles
Deep grey matter
White matter

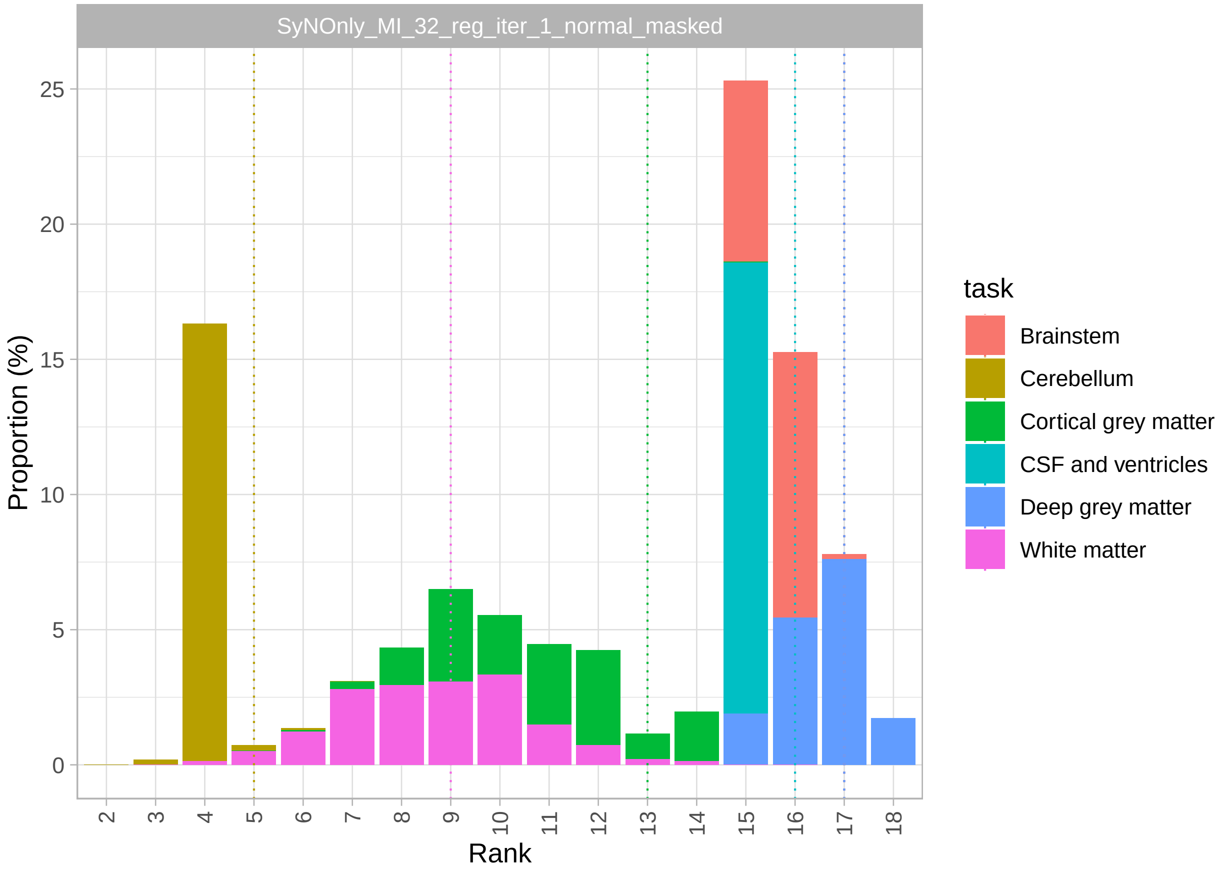
SyNOnly_MI_32_reg_iter_1_normal_masked
Proportion (%)
0
5
10
15
20
25
2
3
4
5
6
7
8
9
10
11
12
13
14
15
16
17
18
Rank
task
Brainstem
Cerebellum
Cortical grey matter
CSF and ventricles
Deep grey matter
White matter

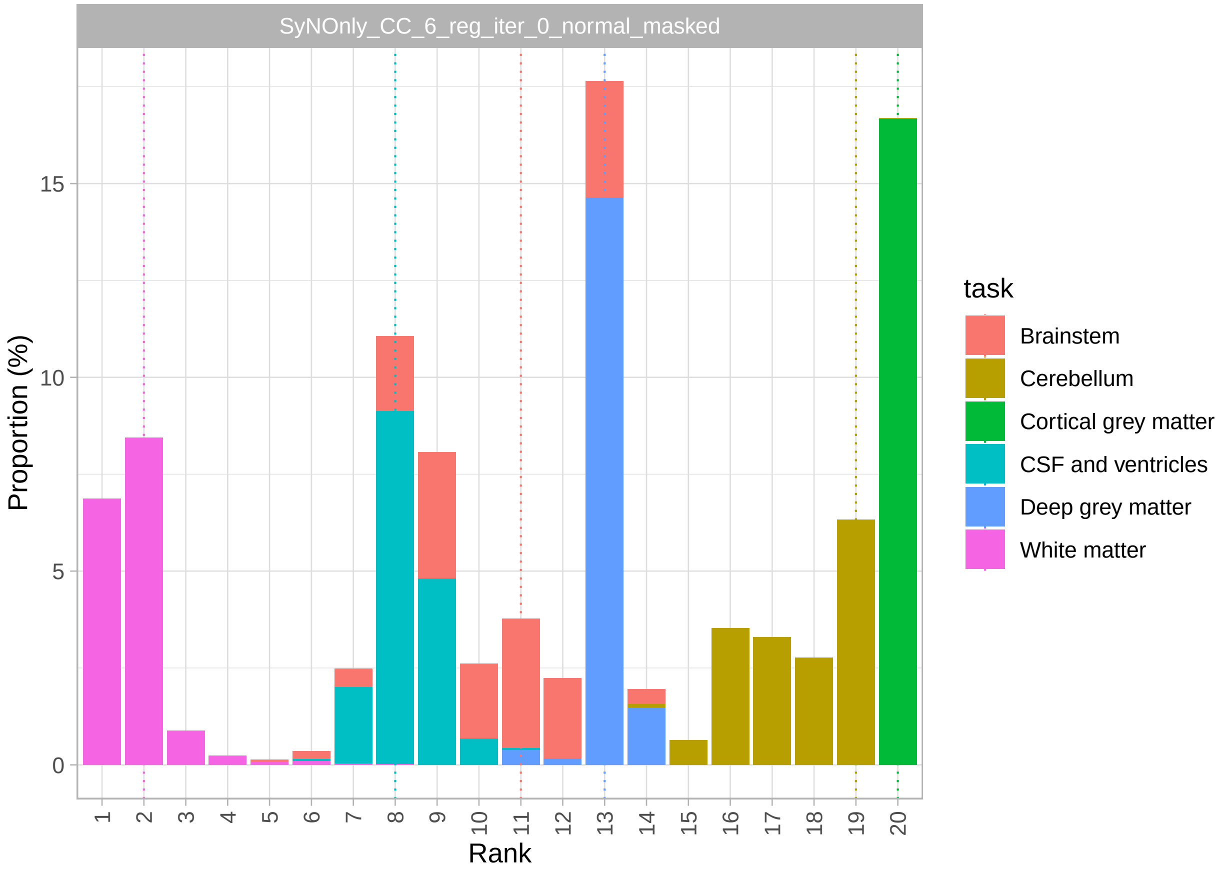

SyNOnly_CC_6_reg_iter_0_normal_masked
Proportion (%)
15
10
5
0
1
2
3
4
5
6
7
8
9
10
11
12
13
14
15
16
17
18
19
20
Rank
task
Brainstem
Cerebellum
Cortical grey matter
CSF and ventricles
Deep grey matter
White matter

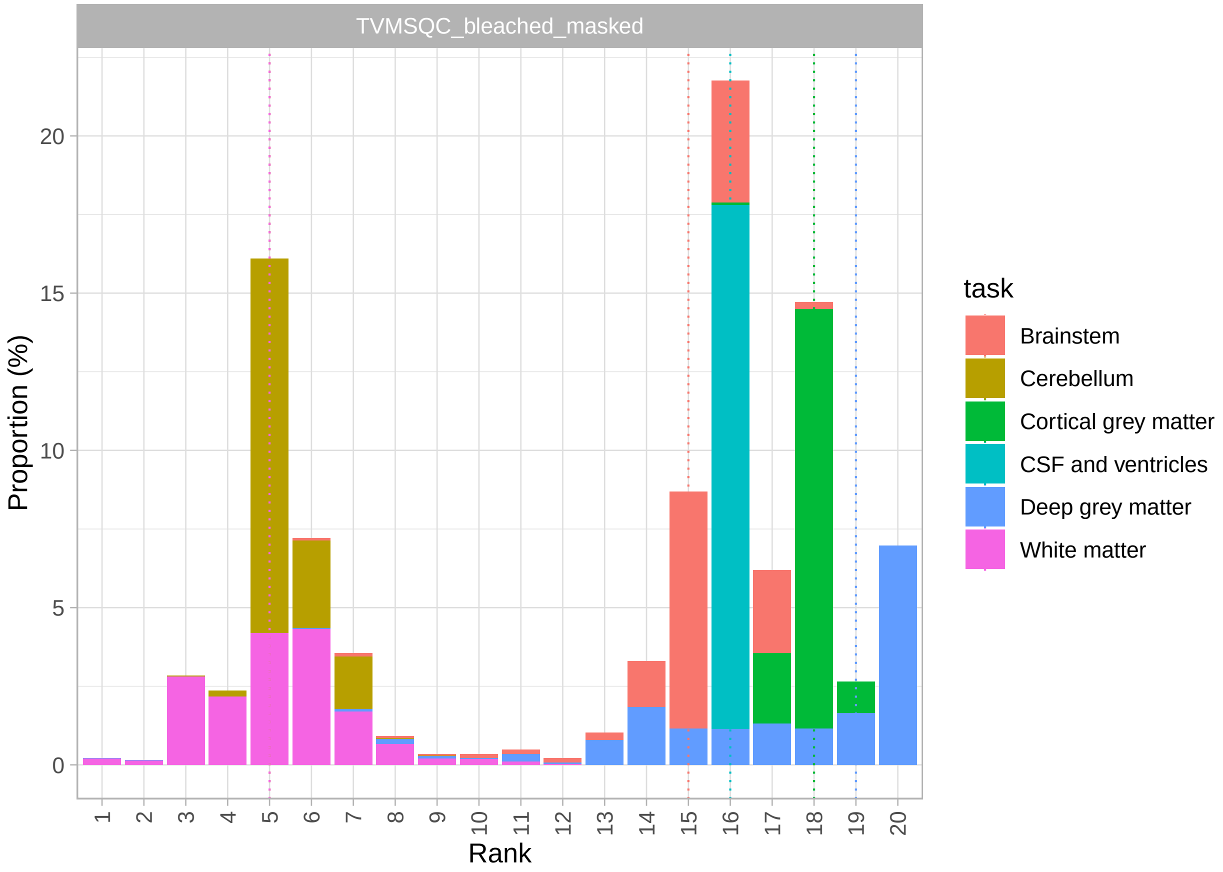
TVMSQC_bleached_masked
Proportion (%)
Rank
task
Brainstem
Cerebellum
Cortical grey matter
CSF and ventricles
Deep grey matter
White matter

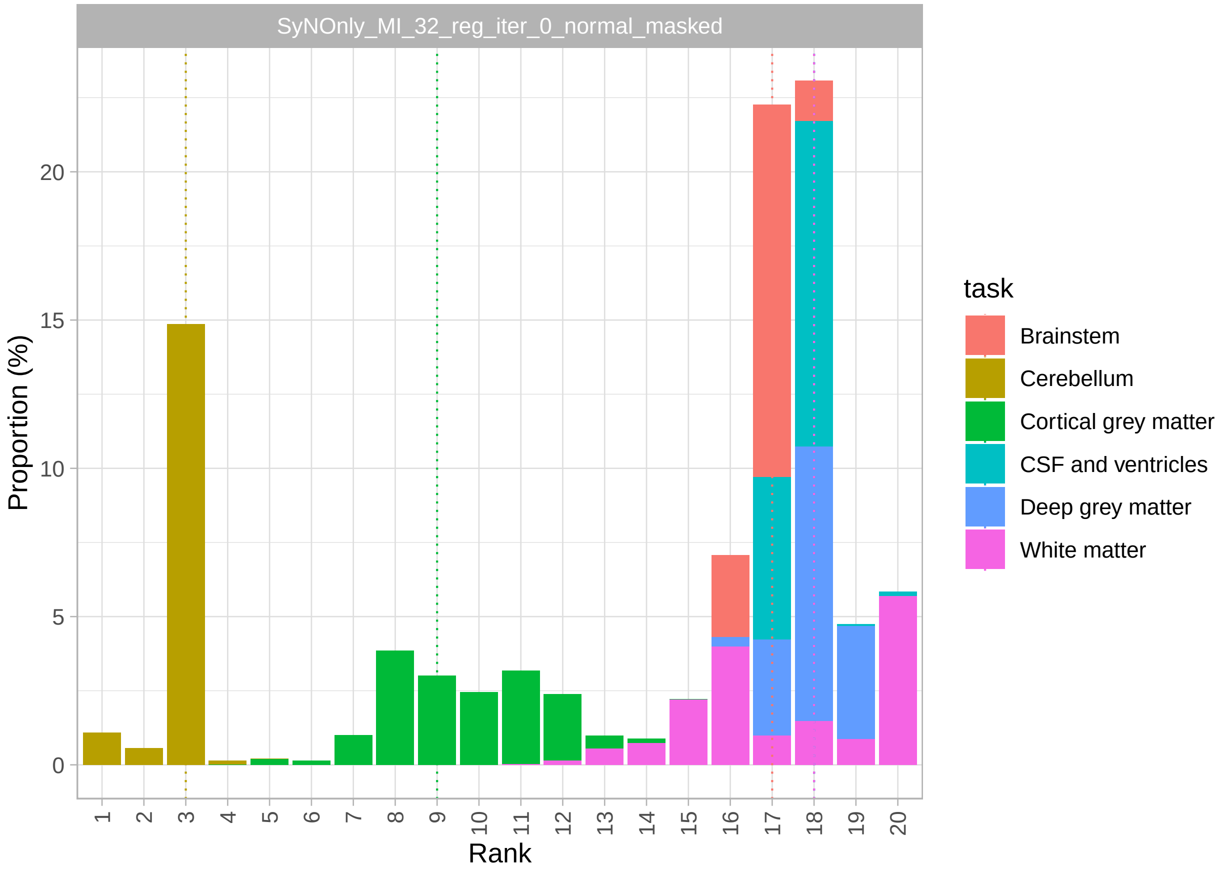

SyNOnly_MI_32_reg_iter_0_normal_masked
Proportion (%)
0
5
10
15
20
1
2
3
4
5
6
7
8
9
10
11
12
13
14
15
16
17
18
19
20
Rank
task
Brainstem
Cerebellum
Cortical grey matter
CSF and ventricles
Deep grey matter
White matter

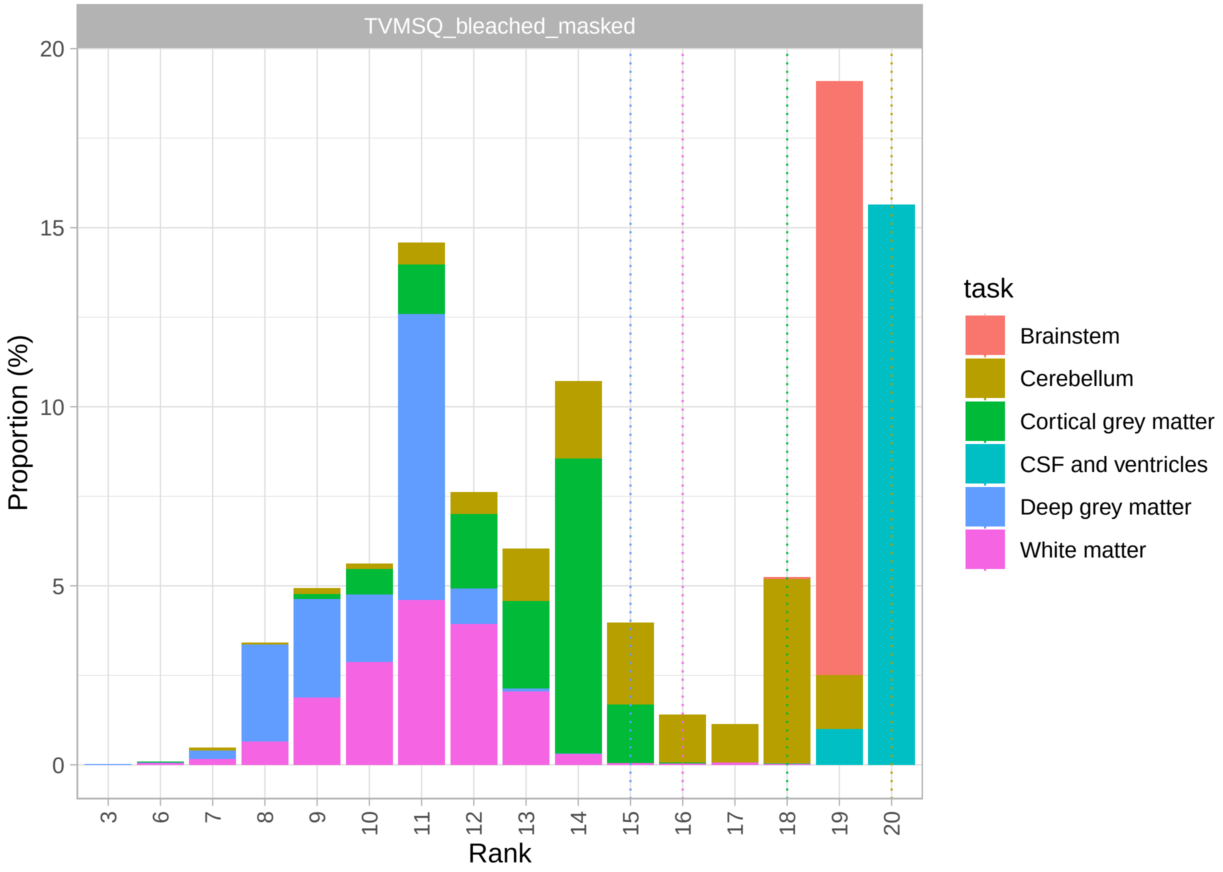
TVMSQ_bleached_masked
Proportion (%)
20
15
10
5
0
3
6
7
8
9
10
11
12
13
14
15
16
17
18
19
20
Rank
task
Brainstem
Cerebellum
Cortical grey matter
CSF and ventricles
Deep grey matter
White matter

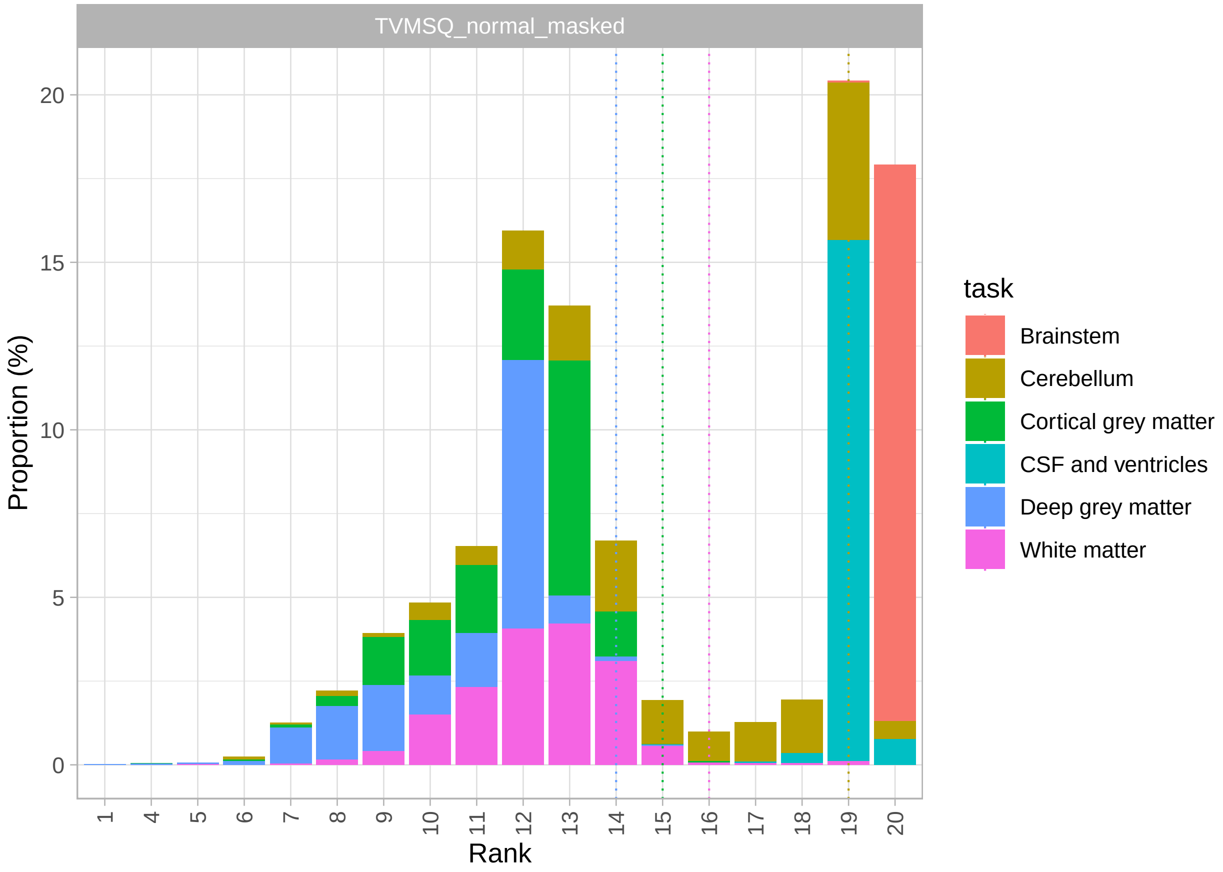

TVMSQ_normal_masked
Proportion (%)
20
15
10
5
0
1
4
5
6
7
8
9
10
11
12
13
14
15
16
17
18
19
20
Rank
task
Brainstem
Cerebellum
Cortical grey matter
CSF and ventricles
Deep grey matter
White matter

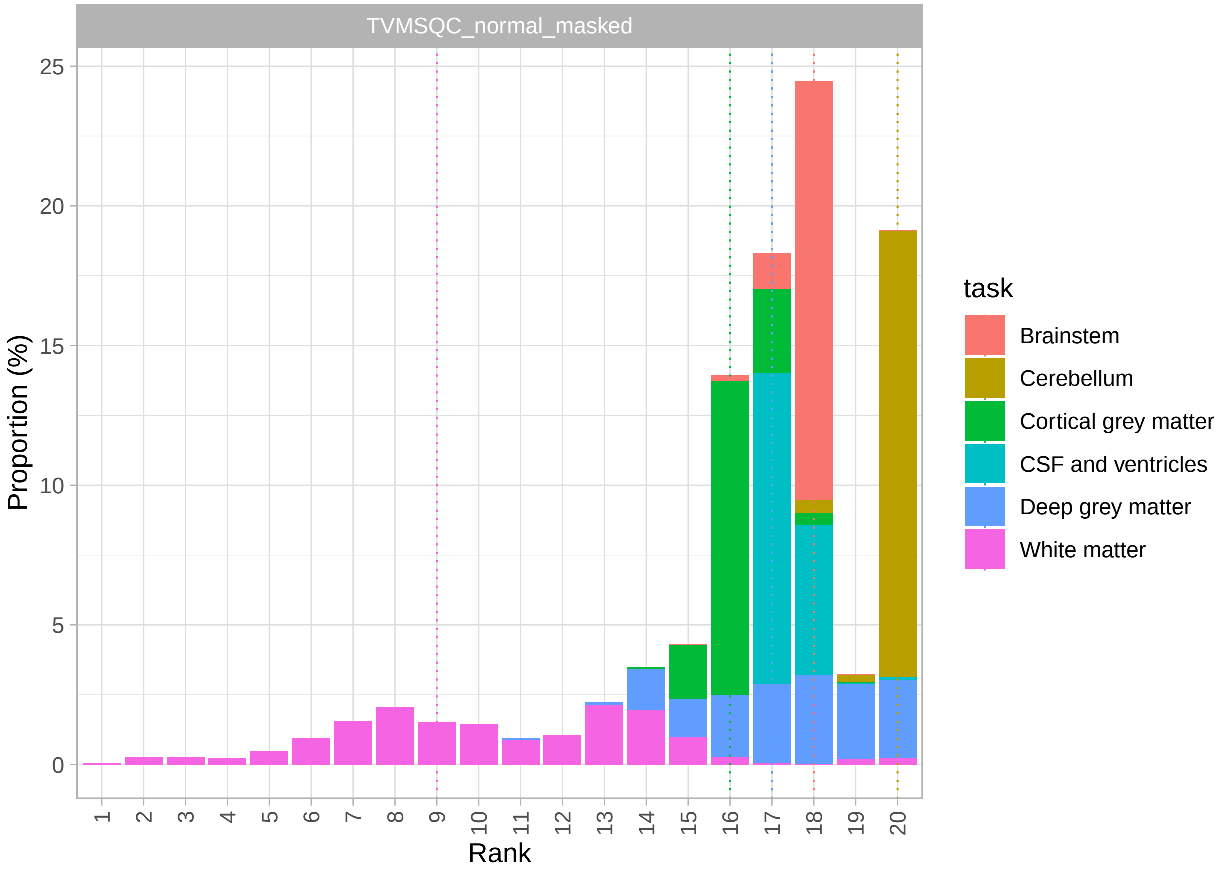
TVMSQC_normal_masked
Proportion (%)
Rank
task
Brainstem
Cerebellum
Cortical grey matter
CSF and ventricles
Deep grey matter
White matter

4.2 Characterization of tasks

4.2.1 Visualizing bootstrap results

To investigate which tasks separate algorithms well (i.e., lead to a stable ranking), a blob plot is recommended.

Bootstrap results can be shown in a blob plot showing one plot for each task. In this view, the spread of the blobs for each algorithm can be compared across tasks. Deviations from the diagonal indicate deviations from the consensus ranking (over tasks). Specifically, if rank distribution of an algorithm is consistently below the diagonal, the algorithm performed better in this task than on average across tasks, while if the rank distribution of an algorithm is consistently above the diagonal, the algorithm performed worse in this task than on average across tasks. At the bottom of each panel, ranks for each algorithm in the tasks are provided.

Same as in Section 3.1 but now ordered according to consensus.

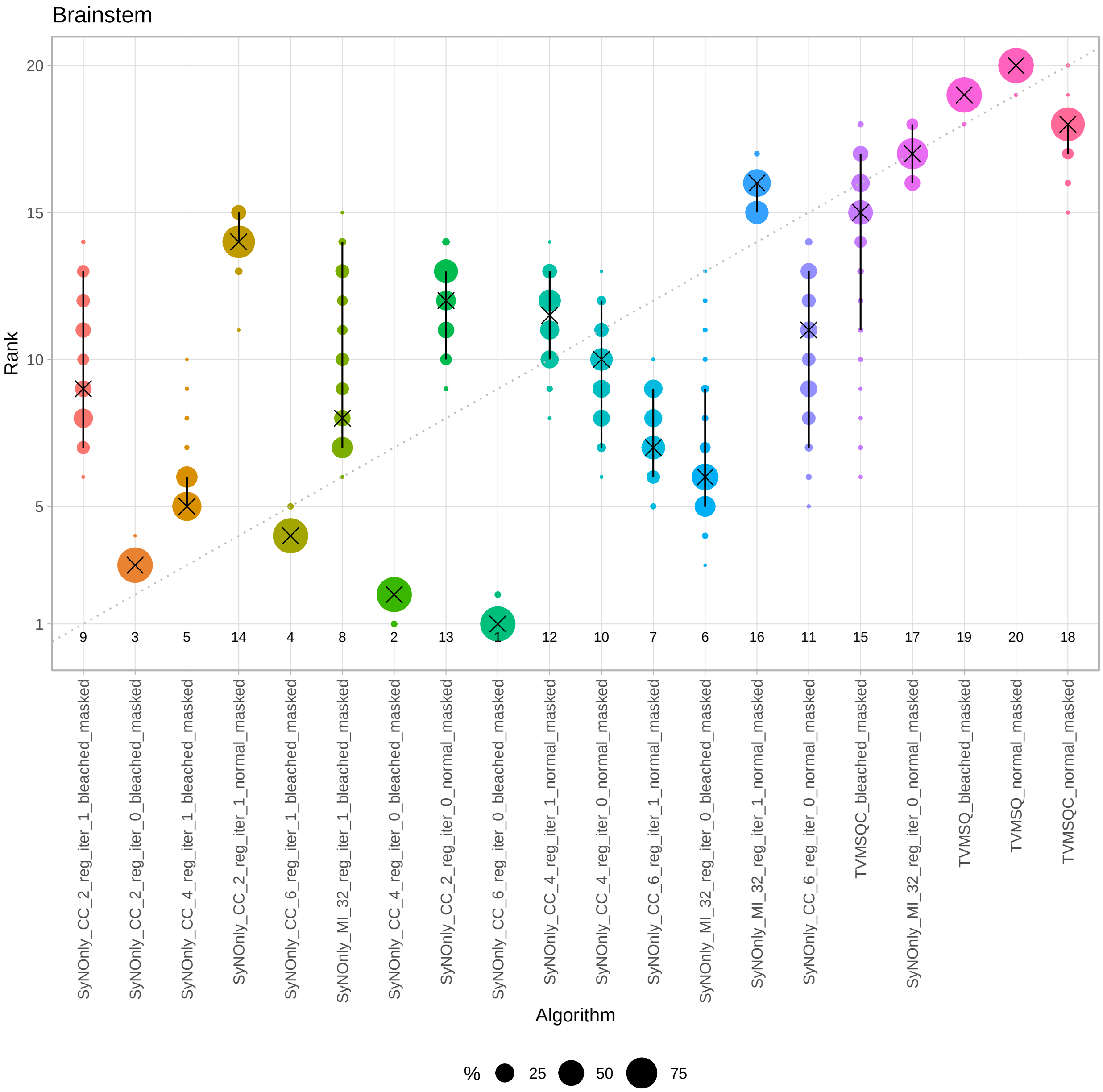

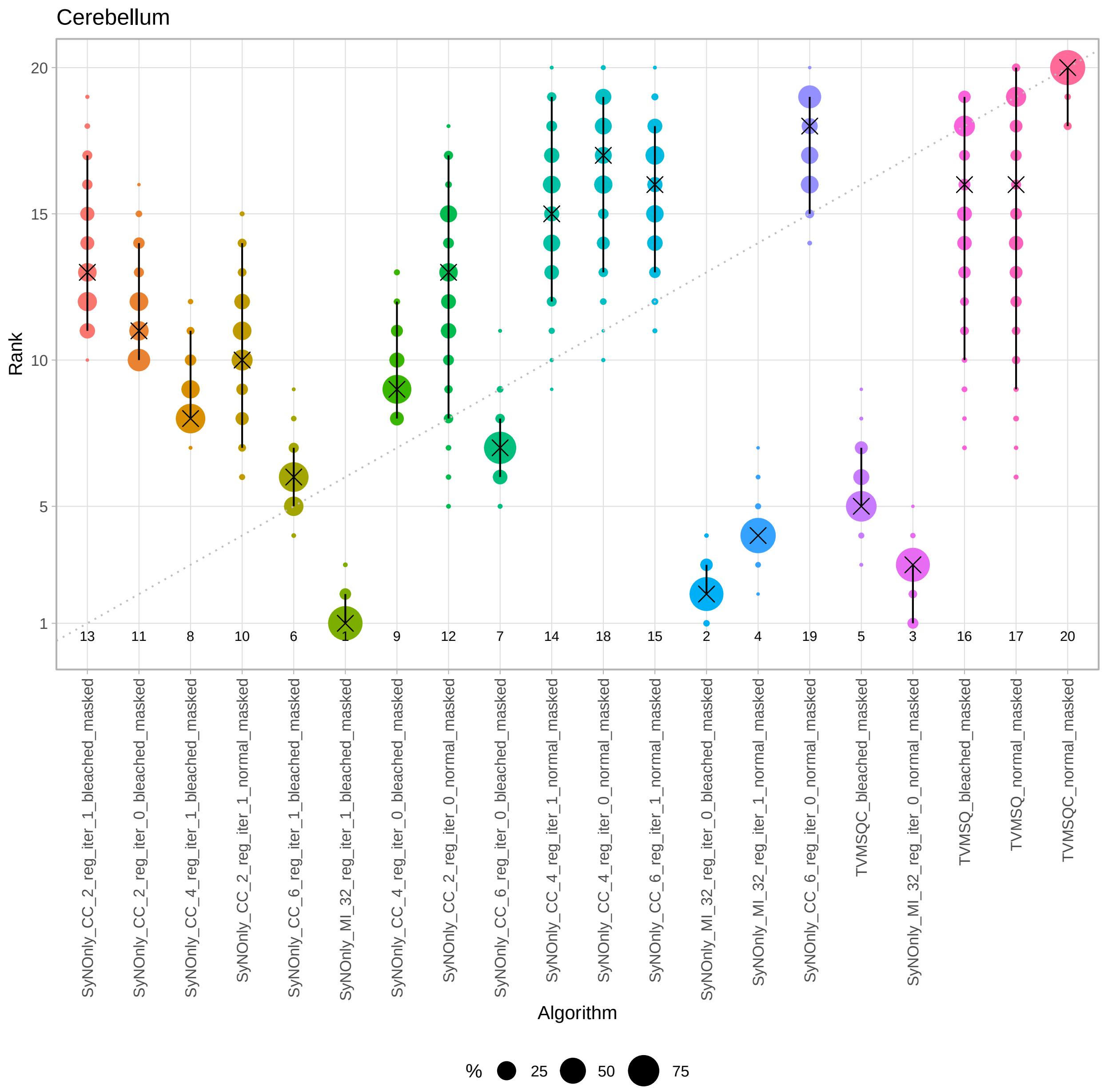
Cerebellum
Rank
20
15
10
5
1
13 11 8 10 6 1 9 12 7 14 18 15 2 4 19 5 3 16 17 20
SyNOnly_CC_2_reg_iter_1_bleached_masked
SyNOnly_CC_2_reg_iter_0_bleached_masked
SyNOnly_CC_4_reg_iter_1_bleached_masked
SyNOnly_CC_2_reg_iter_1_normal_masked
SyNOnly_CC_6_reg_iter_1_bleached_masked
SyNOnly_MI_32_reg_iter_1_bleached_masked
SyNOnly_CC_4_reg_iter_0_bleached_masked
SyNOnly_CC_2_reg_iter_0_normal_masked
SyNOnly_CC_6_reg_iter_0_bleached_masked
SyNOnly_CC_4_reg_iter_1_normal_masked
SyNOnly_CC_4_reg_iter_0_normal_masked
SyNOnly_CC_6_reg_iter_1_normal_masked
SyNOnly_MI_32_reg_iter_0_bleached_masked
SyNOnly_MI_32_reg_iter_1_normal_masked
SyNOnly_CC_6_reg_iter_0_normal_masked
TVMSQC_bleached_masked
SyNOnly_MI_32_reg_iter_0_normal_masked
TVMSQ_bleached_masked
TVMSQ_normal_masked
TVMSQC_normal_masked
Algorithm
% 25 50 75

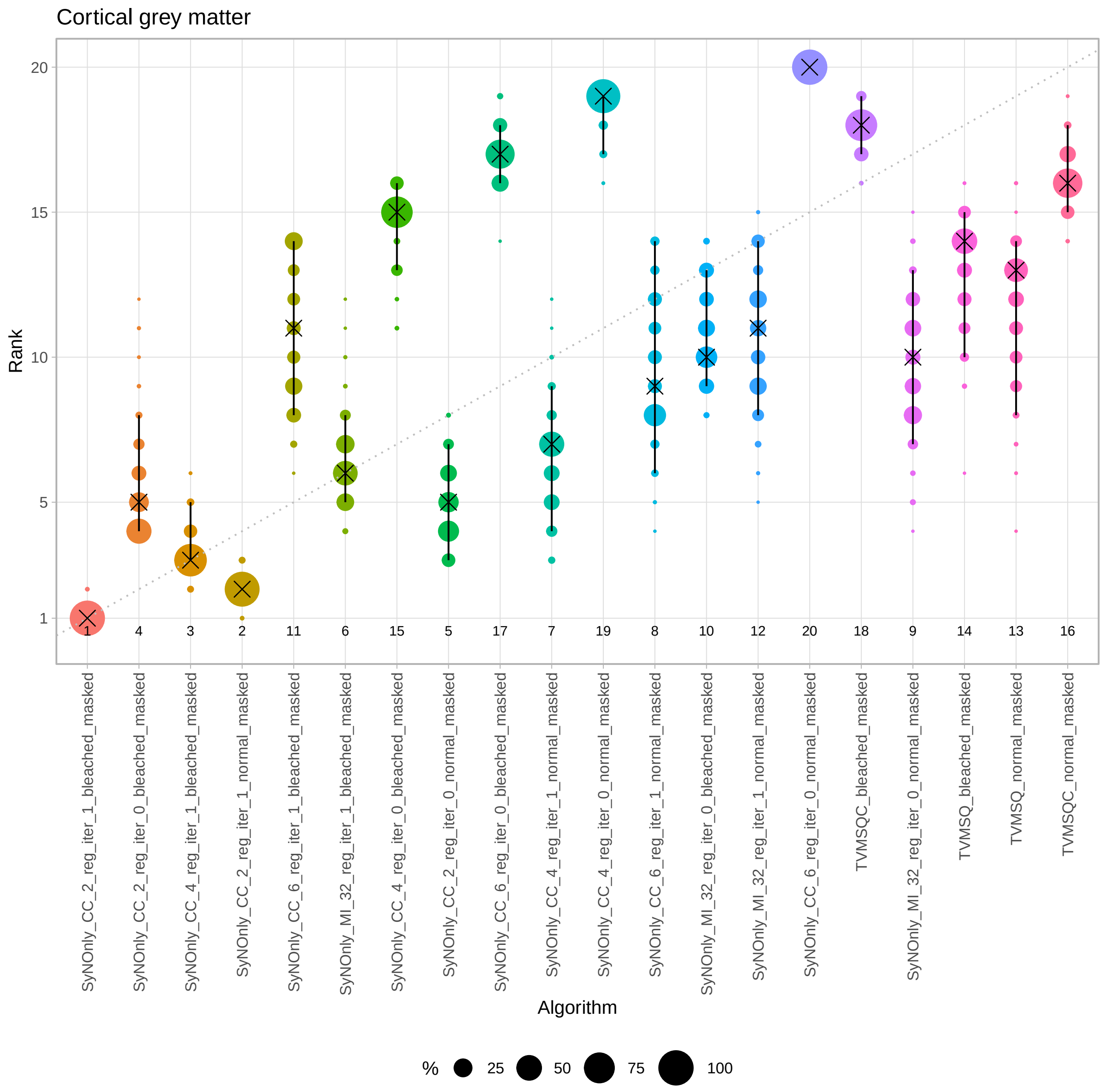

Cortical grey matter
Rank
20
15
10
5
1
1
4
3
2
11
6
15
5
17
7
19
8
10
12
20
18
9
14
13
16
SyNOnly_CC_2_reg_iter_1_bleached_masked
SyNOnly_CC_2_reg_iter_0_bleached_masked
SyNOnly_CC_4_reg_iter_1_bleached_masked
SyNOnly_CC_2_reg_iter_1_normal_masked
SyNOnly_CC_6_reg_iter_1_bleached_masked
SyNOnly_MI_32_reg_iter_1_bleached_masked
SyNOnly_CC_4_reg_iter_0_bleached_masked
SyNOnly_CC_2_reg_iter_0_normal_masked
SyNOnly_CC_6_reg_iter_0_bleached_masked
SyNOnly_CC_4_reg_iter_1_normal_masked
SyNOnly_CC_4_reg_iter_0_normal_masked
SyNOnly_CC_6_reg_iter_1_normal_masked
SyNOnly_MI_32_reg_iter_0_bleached_masked
SyNOnly_MI_32_reg_iter_1_normal_masked
SyNOnly_CC_6_reg_iter_0_normal_masked
TVMSQC_bleached_masked
SyNOnly_MI_32_reg_iter_0_normal_masked
TVMSQ_bleached_masked
TVMSQ_normal_masked
TVMSQC_normal_masked
Algorithm
%
25
50
75
100

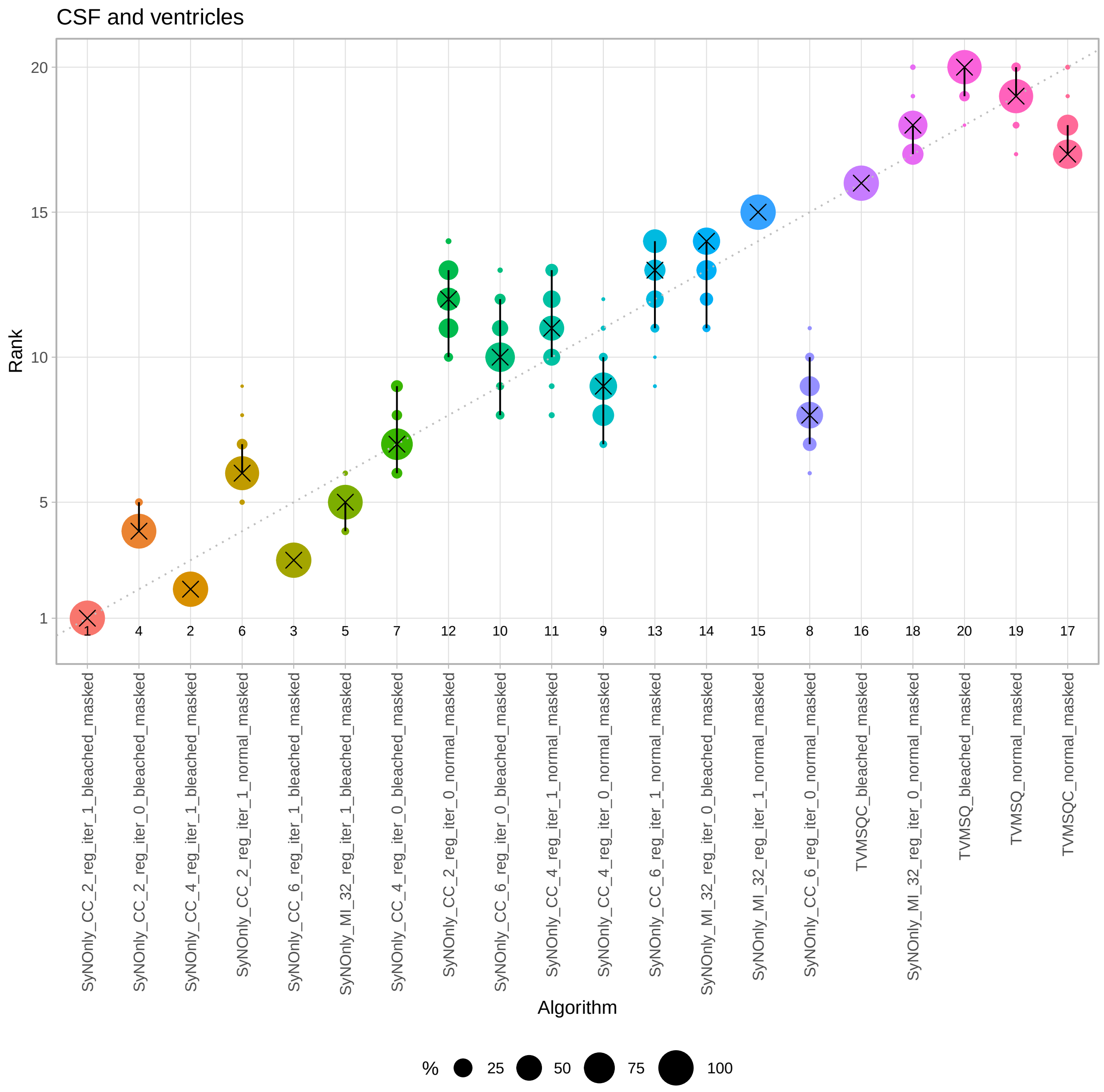
CSF and ventricles
Rank
Algorithm
1 4 2 6 3 5 7 12 10 11 9 13 14 15 8 16 18 20 19 17
SyNOnly_CC_2_reg_iter_1_bleached_masked
SyNOnly_CC_2_reg_iter_0_bleached_masked
SyNOnly_CC_4_reg_iter_1_bleached_masked
SyNOnly_CC_2_reg_iter_1_normal_masked
SyNOnly_CC_6_reg_iter_1_bleached_masked
SyNOnly_MI_32_reg_iter_1_bleached_masked
SyNOnly_CC_4_reg_iter_0_bleached_masked
SyNOnly_CC_2_reg_iter_0_normal_masked
SyNOnly_CC_6_reg_iter_0_bleached_masked
SyNOnly_CC_4_reg_iter_1_normal_masked
SyNOnly_CC_4_reg_iter_0_normal_masked
SyNOnly_CC_6_reg_iter_1_normal_masked
SyNOnly_MI_32_reg_iter_0_bleached_masked
SyNOnly_MI_32_reg_iter_1_normal_masked
SyNOnly_CC_6_reg_iter_0_normal_masked
TVMSQC_bleached_masked
SyNOnly_MI_32_reg_iter_0_normal_masked
TVMSQ_bleached_masked
TVMSQ_normal_masked
TVMSQC_normal_masked
% 25 50 75 100

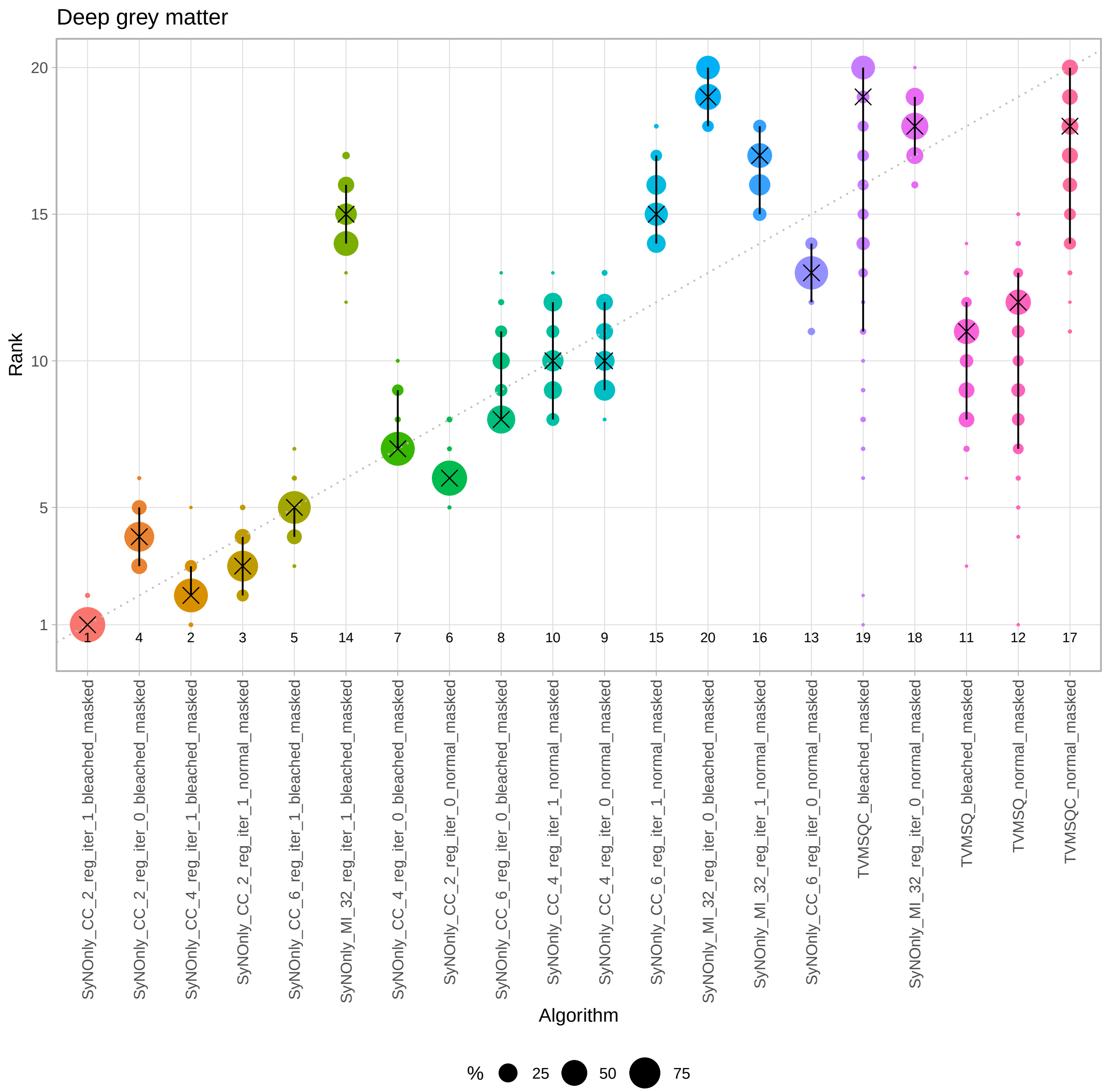

Deep grey matter
Rank
20
15
10
5
1
1
4
2
3
5
14
7
6
8
10
9
15
20
16
13
19
18
11
12
17
SyNOnly_CC_2_reg_iter_1_bleached_masked
SyNOnly_CC_2_reg_iter_0_bleached_masked
SyNOnly_CC_4_reg_iter_1_bleached_masked
SyNOnly_CC_2_reg_iter_1_normal_masked
SyNOnly_CC_6_reg_iter_1_bleached_masked
SyNOnly_MI_32_reg_iter_1_bleached_masked
SyNOnly_CC_4_reg_iter_0_bleached_masked
SyNOnly_CC_2_reg_iter_0_normal_masked
SyNOnly_CC_6_reg_iter_0_bleached_masked
SyNOnly_CC_4_reg_iter_1_normal_masked
SyNOnly_CC_4_reg_iter_0_normal_masked
SyNOnly_CC_6_reg_iter_1_normal_masked
SyNOnly_MI_32_reg_iter_0_bleached_masked
SyNOnly_MI_32_reg_iter_1_normal_masked
SyNOnly_CC_6_reg_iter_0_normal_masked
TVMSQC_bleached_masked
SyNOnly_MI_32_reg_iter_0_normal_masked
TVMSQ_bleached_masked
TVMSQ_normal_masked
TVMSQC_normal_masked
Algorithm
% 25 50 75

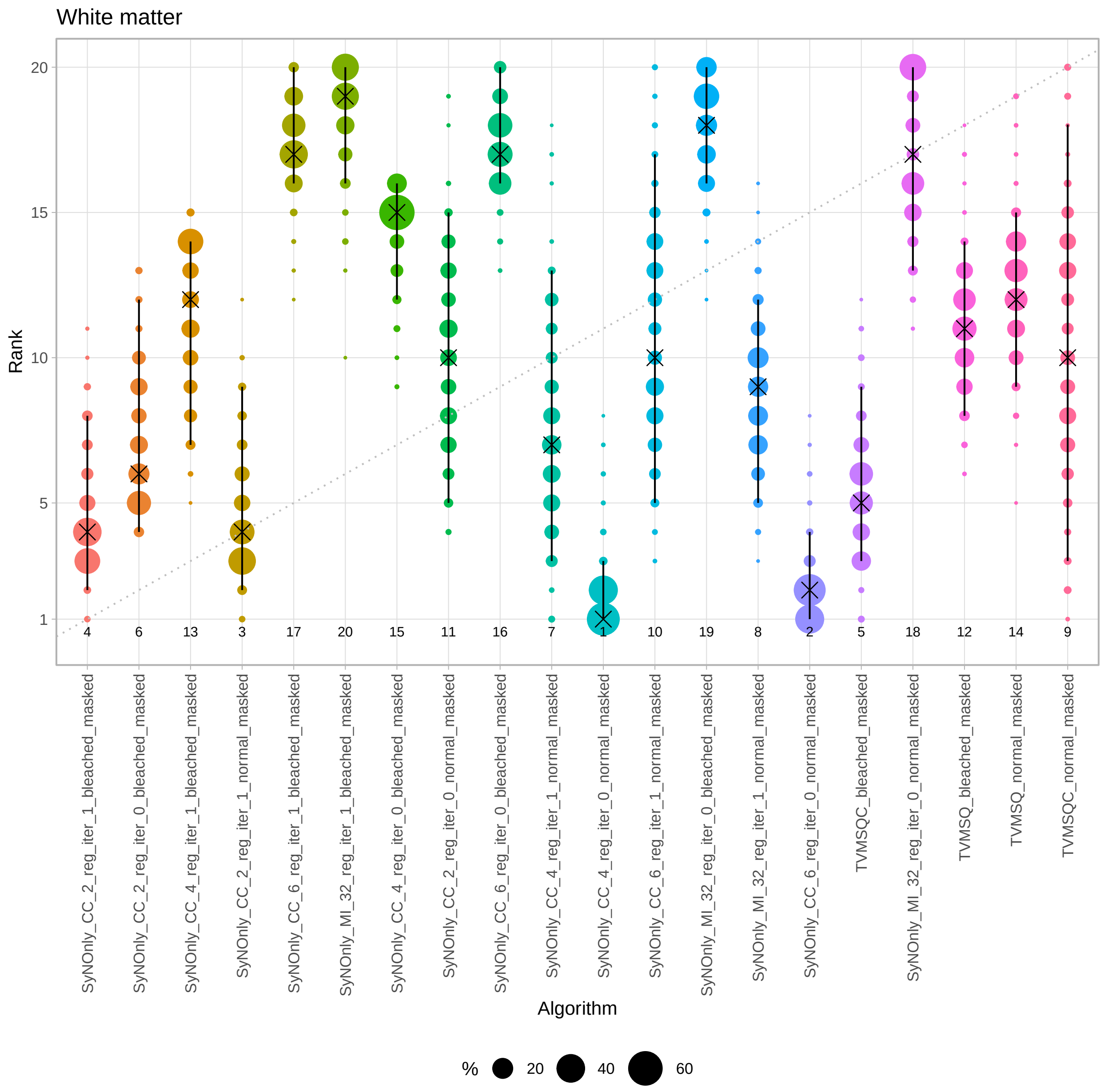

4.2.2 Cluster Analysis

Dendrogram from hierarchical cluster analysis and *network-type graphs* for assessing the similarity of tasks based on challenge rankings.

A dendrogram is a visualization approach based on hierarchical clustering. It depicts clusters according to a chosen distance measure (here: Spearman's footrule) as well as a chosen agglomeration method (here: complete and average agglomeration).

Cluster Dendrogram (complete agglomeration)

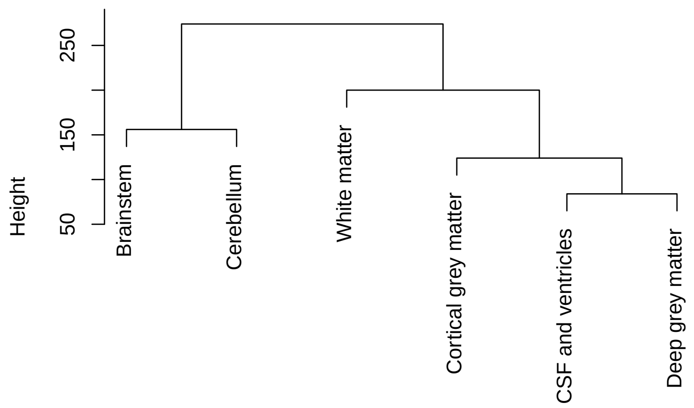

Cluster Dendrogram (average agglomeration)

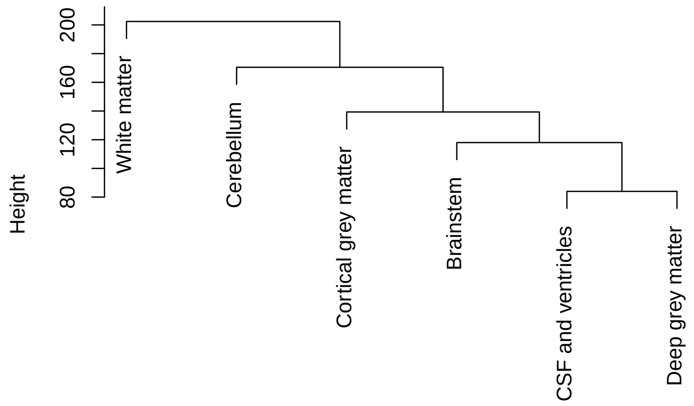

5 References

Wiesenfarth, M., Reinke, A., Landman, B.A., Eisenmann, M., Aguilera Saiz, L., Cardoso, M.J., Maier-Hein, L. and Kopp-Schneider, A. Methods and open-source toolkit for analyzing and visualizing challenge results. *Sci Rep* **11**, 2369 (2021). https://doi.org/10.1038/s41598-021-82017-6

M. J. A. Eugster, T. Hothorn, and F. Leisch, "Exploratory and inferential analysis of benchmark experiments," Institut fuer Statistik, Ludwig-Maximilians-Universitaet Muenchen, Germany, Technical Report 30, 2008. [Online]. Available: http://epub.ub.uni-muenchen.de/4134/.
