# Supplementary material for: Characterization of dynamic patterns of human fetal to neonatal brain asymmetry with deformation-based morphometry
Source: Front Neurosci. 2023 Dec 6;17:1252850. doi: 10.3389/fnins.2023.1252850 (PMC10734644; doi:10.3389/fnins.2023.1252850)
Supplement: Supplementary file 1 [file Data_Sheet_1.ZIP › ChallengeR/Framework B to mid-age template/neonatal2tempalte_linear/Neonatal2MidTemplate_linear_landmarks.pdf]

Benchmarking report for Neonatal to Midtempalte Registration Linear

created by challengeR v1.0.4

29 März, 2023

This document presents a systematic report on the benchmark study "Neonatal to Midtempalte Registration Linear". Input data comprises raw metric values for all algorithms and cases. Generated plots are:

- Visualization of assessment data: Dot- and boxplots, podium plots and ranking heatmaps
- Visualization of ranking stability: Blob plots, violin plots and significance maps, line plots
- Visualization of cross-task insights: Blob plots, stacked frequency plots, dendrograms

Details can be found in Wiesenfarth et al. (2021).

1 Rankings

Algorithms within a task are ranked according to the following ranking scheme:

aggregate using function ("mean") then rank

Ranking for each task:

4th Ventricle : The analysis is based on 17 algorithms and 20 cases. 0 missing cases have been found in the data set.

	value_mean	rank
Affine_eroded_masked	1.259509	1
TRSAA_eroded_masked	1.283071	2
terminal_MI_nomask	1.307835	3
terminal_MI_masked	1.348512	4
terminal_CC_nomask	1.355594	5
terminal_CC_masked	1.356039	6
Affine_masked	1.362579	7
TRSAA_masked	1.386661	8
terminal_CC_eroded_masked	1.389473	9
terminal_MI_eroded_masked	1.433100	10
Similarity_masked	1.557264	11
Similarity_nomask	1.561335	12
Similarity_eroded_masked	1.634710	13
Affine_nomask	1.758667	14
flirt_na	1.760502	15
TRSAA_nomask	1.785346	16
antsAI_masked	2.011277	17

Chiasma : The analysis is based on 17 algorithms and 20 cases. 0 missing cases have been found in the data set.

	value_mean	rank
Affine_eroded_masked	1.032719	1
TRSAA_eroded_masked	1.049966	2
terminal_MI_masked	1.090202	3
TRSAA_masked	1.123455	4
terminal_CC_eroded_masked	1.126702	5
terminal_CC_masked	1.132857	6
terminal_CC_nomask	1.146479	7
terminal_MI_nomask	1.151693	8
TRSAA_nomask	1.169962	9
terminal_MI_eroded_masked	1.170871	10
Affine_nomask	1.221261	11
Affine_masked	1.242152	12
flirt_na	1.442365	13
Similarity_nomask	1.651297	14
Similarity_eroded_masked	1.693840	15
Similarity_masked	1.724497	16
antsAI_masked	2.623162	17

Culmen : The analysis is based on 17 algorithms and 20 cases. 0 missing cases have been found in the data set.

	value_mean	rank
TRSAA_eroded_masked	1.593687	1
Affine_eroded_masked	1.613054	2
terminal_MI_masked	1.711821	3
terminal_MI_eroded_masked	1.735645	4
terminal_MI_nomask	1.749505	5
terminal_CC_eroded_masked	1.766456	6
terminal_CC_masked	1.800653	7
Affine_masked	1.901531	8
TRSAA_masked	1.911276	9
terminal_CC_nomask	1.930771	10
TRSAA_nomask	2.150612	11
Affine_nomask	2.152244	12
Similarity_eroded_masked	2.309475	13
Similarity_masked	2.391279	14
flirt_na	2.395021	15
Similarity_nomask	2.397877	16
antsAI_masked	2.917496	17

Hemisphere left caudal point : The analysis is based on 17 algorithms and 20 cases. 0 missing cases have been found in the data set.

	value_mean	rank
Affine_masked	2.915596	1
TRSAA_eroded_masked	2.929417	2
Affine_eroded_masked	2.960643	3
TRSAA_masked	2.990980	4
Similarity_nomask	3.286226	5
terminal_MI_nomask	3.309152	6

	value_mean	rank
terminal_MI_masked	3.343111	7
terminal_CC_masked	3.406201	8
terminal_MI_eroded_masked	3.470365	9
terminal_CC_eroded_masked	3.492172	10
TRSAA_nomask	3.583793	11
Affine_nomask	3.617691	12
terminal_CC_nomask	3.666775	13
antsAI_masked	3.760823	14
flirt_na	3.882347	15
Similarity_eroded_masked	4.288836	16
Similarity_masked	4.346921	17

Hemisphere left rostral point : The analysis is based on 17 algorithms and 20 cases. 0 missing cases have been found in the data set.

	value_mean	rank
Affine_masked	3.784574	1
TRSAA_masked	3.889136	2
Similarity_nomask	3.891857	3
antsAI_masked	4.209576	4
terminal_CC_nomask	4.459982	5
Affine_nomask	4.564502	6
terminal_CC_masked	4.583861	7
Similarity_masked	4.590841	8
TRSAA_nomask	4.610537	9
terminal_MI_nomask	4.635705	10
flirt_na	4.636939	11
terminal_MI_masked	4.678213	12
Affine_eroded_masked	4.766789	13
Similarity_eroded_masked	4.882547	14
terminal_CC_eroded_masked	5.073913	15
TRSAA_eroded_masked	5.135892	16
terminal_MI_eroded_masked	5.203307	17

Hemisphere right caudal point : The analysis is based on 17 algorithms and 20 cases. 0 missing cases have been found in the data set.

	value_mean	rank
Similarity_nomask	3.387910	1
Affine_masked	3.976000	2
Affine_eroded_masked	4.018945	3
TRSAA_eroded_masked	4.094896	4
TRSAA_masked	4.141454	5
antsAI_masked	4.182749	6
terminal_MI_nomask	4.340359	7
terminal_CC_nomask	4.384588	8
terminal_CC_masked	4.421650	9
terminal_MI_masked	4.427261	10
flirt_na	4.455442	11
Similarity_masked	4.621007	12

	value_mean	rank
terminal_CC_eroded_masked	4.631151	13
terminal_MI_eroded_masked	4.661906	14
Affine_nomask	4.675376	15
TRSAA_nomask	4.687773	16
Similarity_eroded_masked	4.790385	17

Hemisphere right rostral point : The analysis is based on 17 algorithms and 20 cases. 0 missing cases have been found in the data set.

	value_mean	rank
Affine_masked	4.380252	1
TRSAA_masked	4.532407	2
Similarity_nomask	4.577400	3
antsAI_masked	4.862605	4
Similarity_masked	5.113428	5
terminal_CC_nomask	5.178824	6
flirt_na	5.195386	7
terminal_MI_nomask	5.242078	8
TRSAA_nomask	5.267960	9
Affine_nomask	5.281126	10
terminal_MI_masked	5.312106	11
Affine_eroded_masked	5.321441	12
terminal_CC_masked	5.378409	13
Similarity_eroded_masked	5.533089	14
TRSAA_eroded_masked	5.674947	15
terminal_CC_eroded_masked	5.725501	16
terminal_MI_eroded_masked	5.746052	17

Infracollicular Sulcus : The analysis is based on 17 algorithms and 20 cases. 0 missing cases have been found in the data set.

	value_mean	rank
TRSAA_eroded_masked	0.9743556	1
terminal_MI_eroded_masked	1.0159773	2
Affine_eroded_masked	1.0173744	3
terminal_MI_masked	1.0307411	4
terminal_MI_nomask	1.0522055	5
terminal_CC_eroded_masked	1.0667636	6
terminal_CC_masked	1.1188841	7
Affine_masked	1.1370504	8
terminal_CC_nomask	1.1615859	9
TRSAA_masked	1.1650404	10
Affine_nomask	1.2790208	11
TRSAA_nomask	1.2874115	12
Similarity_eroded_masked	1.3025112	13
Similarity_masked	1.3066202	14
Similarity_nomask	1.3943681	15
flirt_na	1.4990979	16
antsAI_masked	2.1441279	17

Lateral ventricle left caudal point : The analysis is based on 17 algorithms and 20 cases. 0 missing cases have been found in the data set.

	value_mean	rank
TRSAA_eroded_masked	5.374917	1
Affine_eroded_masked	5.382700	2
TRSAA_nomask	5.421994	3
TRSAA_masked	5.457447	4
Affine_nomask	5.459999	5
terminal_MI_eroded_masked	5.466247	6
terminal_MI_masked	5.491629	7
terminal_CC_eroded_masked	5.524920	8
Affine_masked	5.566477	9
terminal_MI_nomask	5.599908	10
flirt_na	5.753427	11
terminal_CC_masked	5.815482	12
terminal_CC_nomask	6.203734	13
Similarity_nomask	6.598516	14
Similarity_eroded_masked	7.026054	15
Similarity_masked	7.088527	16
antsAI_masked	7.215909	17

Lateral ventricle left rostral point : The analysis is based on 17 algorithms and 20 cases. 0 missing cases have been found in the data set.

	value_mean	rank
flirt_na	1.499085	1
Affine_nomask	1.521864	2
TRSAA_nomask	1.523349	3
terminal_MI_masked	1.611258	4
Affine_masked	1.641749	5
TRSAA_masked	1.661949	6
terminal_MI_nomask	1.689620	7
terminal_MI_eroded_masked	1.735549	8
terminal_CC_eroded_masked	1.740930	9
terminal_CC_masked	1.779668	10
Similarity_nomask	1.967703	11
terminal_CC_nomask	2.001811	12
Affine_eroded_masked	2.177417	13
TRSAA_eroded_masked	2.215018	14
antsAI_masked	2.537153	15
Similarity_masked	2.880330	16
Similarity_eroded_masked	2.985764	17

Lateral ventricle right caudal point : The analysis is based on 17 algorithms and 20 cases. 0 missing cases have been found in the data set.

	value_mean	rank
Affine_eroded_masked	4.210769	1
TRSAA_eroded_masked	4.250220	2
terminal_MI_masked	4.339848	3

	value_mean	rank
TRSAA_masked	4.354082	4
Affine_masked	4.391795	5
terminal_MI_eroded_masked	4.399923	6
terminal_CC_eroded_masked	4.450454	7
terminal_MI_nomask	4.466936	8
Affine_nomask	4.508945	9
TRSAA_nomask	4.545749	10
terminal_CC_masked	4.606968	11
flirt_na	4.678046	12
terminal_CC_nomask	4.798637	13
Similarity_nomask	5.112113	14
Similarity_eroded_masked	5.457120	15
Similarity_masked	5.490776	16
antsAI_masked	5.685570	17

Lateral ventricle right rostral point : The analysis is based on 17 algorithms and 20 cases. 0 missing cases have been found in the data set.

	value_mean	rank
TRSAA_masked	1.676501	1
Affine_masked	1.684603	2
terminal_MI_masked	1.717603	3
terminal_MI_nomask	1.778382	4
terminal_CC_eroded_masked	1.792849	5
terminal_CC_masked	1.800407	6
terminal_MI_eroded_masked	1.854796	7
terminal_CC_nomask	1.874189	8
Affine_eroded_masked	1.896281	9
Affine_nomask	1.918985	10
TRSAA_nomask	1.927859	11
flirt_na	1.972319	12
TRSAA_eroded_masked	2.021783	13
Similarity_nomask	2.344026	14
Similarity_masked	2.748897	15
Similarity_eroded_masked	2.842379	16
antsAI_masked	3.358622	17

PMJ : The analysis is based on 17 algorithms and 20 cases. 0 missing cases have been found in the data set.

	value_mean	rank
terminal_MI_eroded_masked	0.6313966	1
TRSAA_eroded_masked	0.6318485	2
terminal_MI_masked	0.6342800	3
terminal_CC_eroded_masked	0.6363934	4
terminal_MI_nomask	0.6491479	5
Affine_eroded_masked	0.6577463	6
terminal_CC_masked	0.6722661	7
Similarity_eroded_masked	0.6918396	8
terminal_CC_nomask	0.7321668	9
TRSAA_masked	0.8200932	10

	value_mean	rank
Similarity_masked	0.8392013	11
Affine_masked	0.9050964	12
TRSAA_nomask	1.0072272	13
Affine_nomask	1.0417756	14
Similarity_nomask	1.1835630	15
flirt_na	1.3237040	16
antsAI_masked	2.1371140	17

Posterior Commissure : The analysis is based on 17 algorithms and 20 cases. 0 missing cases have been found in the data set.

	value_mean	rank
terminal_CC_nomask	1.102514	1
terminal_CC_masked	1.131453	2
Similarity_masked	1.156954	3
Similarity_eroded_masked	1.177218	4
terminal_MI_nomask	1.205144	5
terminal_MI_eroded_masked	1.210595	6
terminal_CC_eroded_masked	1.218416	7
terminal_MI_masked	1.221414	8
TRSAA_eroded_masked	1.349797	9
Affine_eroded_masked	1.367624	10
Affine_masked	1.384728	11
TRSAA_masked	1.416198	12
Similarity_nomask	1.488226	13
TRSAA_nomask	1.576726	14
Affine_nomask	1.585095	15
flirt_na	1.765276	16
antsAI_masked	1.939865	17

Temporal lobe left rostral point : The analysis is based on 17 algorithms and 20 cases. 0 missing cases have been found in the data set.

	value_mean	rank
Affine_masked	3.108947	1
TRSAA_masked	3.296178	2
flirt_na	3.569547	3
terminal_CC_nomask	3.587065	4
Affine_eroded_masked	3.608982	5
Similarity_masked	3.819303	6
TRSAA_nomask	3.834489	7
Affine_nomask	3.881372	8
terminal_CC_masked	3.895624	9
TRSAA_eroded_masked	3.906082	10
terminal_MI_nomask	3.947899	11
Similarity_eroded_masked	3.962360	12
terminal_MI_masked	4.043743	13
terminal_CC_eroded_masked	4.194540	14
terminal_MI_eroded_masked	4.309493	15
Similarity_nomask	4.672395	16

	value_mean	rank
antsAI_masked	5.647661	17

Temporal lobe ventricle right rostral point : The analysis is based on 17 algorithms and 20 cases. 0 missing cases have been found in the data set.

	value_mean	rank
Affine_masked	2.716106	1
TRSAA_masked	2.876611	2
Affine_eroded_masked	3.242908	3
flirt_na	3.360172	4
Affine_nomask	3.373299	5
TRSAA_nomask	3.391671	6
terminal_CC_nomask	3.422419	7
TRSAA_eroded_masked	3.576757	8
terminal_CC_masked	3.629807	9
terminal_MI_nomask	3.672689	10
terminal_MI_masked	3.716518	11
terminal_CC_eroded_masked	3.882029	12
terminal_MI_eroded_masked	3.944752	13
Similarity_masked	4.090676	14
Similarity_eroded_masked	4.379839	15
Similarity_nomask	5.151946	16
antsAI_masked	6.066581	17

Consensus ranking across tasks according to chosen method "euclidean":

	value	rank
TRSAA_masked	5.312	1
Affine_masked	5.375	2
Affine_eroded_masked	5.438	3
TRSAA_eroded_masked	6.375	4
terminal_MI_masked	6.625	5
terminal_MI_nomask	7.000	6
terminal_CC_masked	8.062	7
terminal_CC_nomask	8.125	8
terminal_MI_eroded_masked	9.062	9
terminal_CC_eroded_masked	9.125	10
Affine_nomask	9.938	11
TRSAA_nomask	10.000	12
flirt_na	11.125	13
Similarity_nomask	11.375	14
Similarity_masked	12.125	15
Similarity_eroded_masked	13.562	16
antsAI_masked	14.375	17

2 Visualization of raw assessment data

The algorithms are ordered according to the computed ranks for each task.

2.1 Dot- and boxplot

Dot- and boxplots for visualizing raw assessment data separately for each algorithm. Boxplots representing descriptive statistics over all cases (median, quartiles and outliers) are combined with horizontally jittered dots representing individual cases.

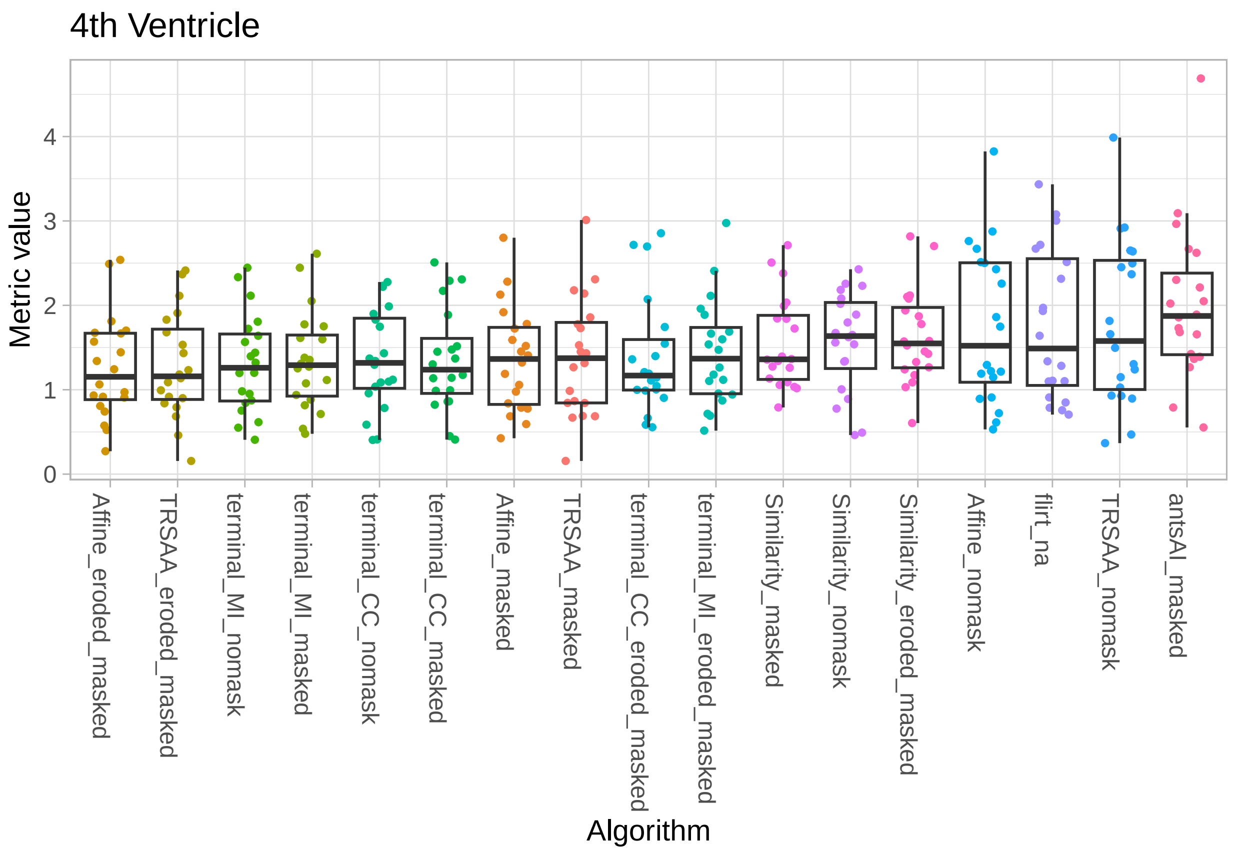

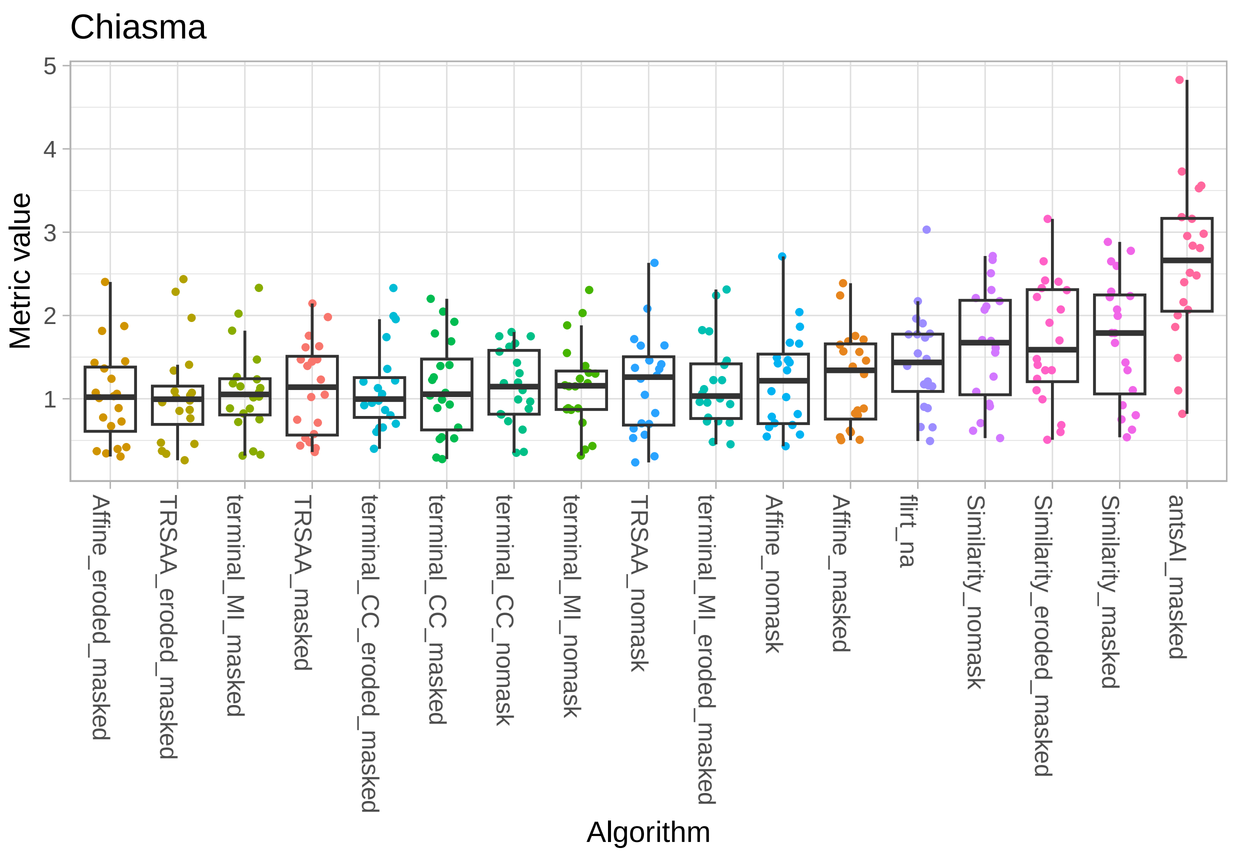
Chiasma
Metric value
5
4
3
2
1
Affine_eroded_masked
TRSAA_eroded_masked
terminal_MI_masked
TRSAA_masked
terminal_CC_eroded_masked
terminal_CC_masked
terminal_CC_nomask
terminal_MI_nomask
TRSAA_nomask
terminal_MI_eroded_masked
Affine_nomask
Affine_masked
flirt_na
Similarity_nomask
Similarity_eroded_masked
Similarity_masked
antsAI_masked
Algorithm

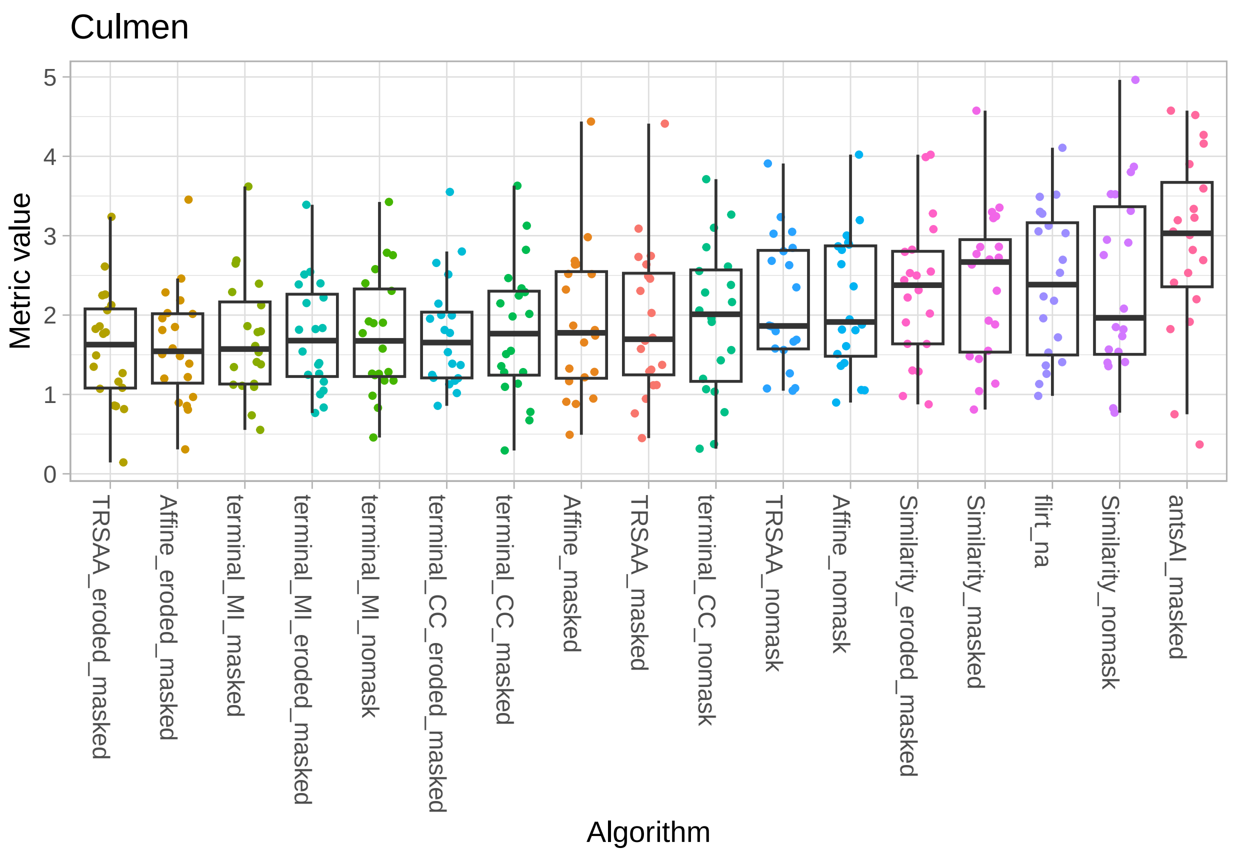
Culmen
Metric value
0
1
2
3
4
5
TRSAA_eroded_masked
Affine_eroded_masked
terminal_MI_masked
terminal_MI_eroded_masked
terminal_MI_nomask
terminal_CC_eroded_masked
terminal_CC_masked
Affine_masked
TRSAA_masked
terminal_CC_nomask
TRSAA_nomask
Affine_nomask
Similarity_eroded_masked
Similarity_masked
flirt_na
Similarity_nomask
antsAI_masked
Algorithm

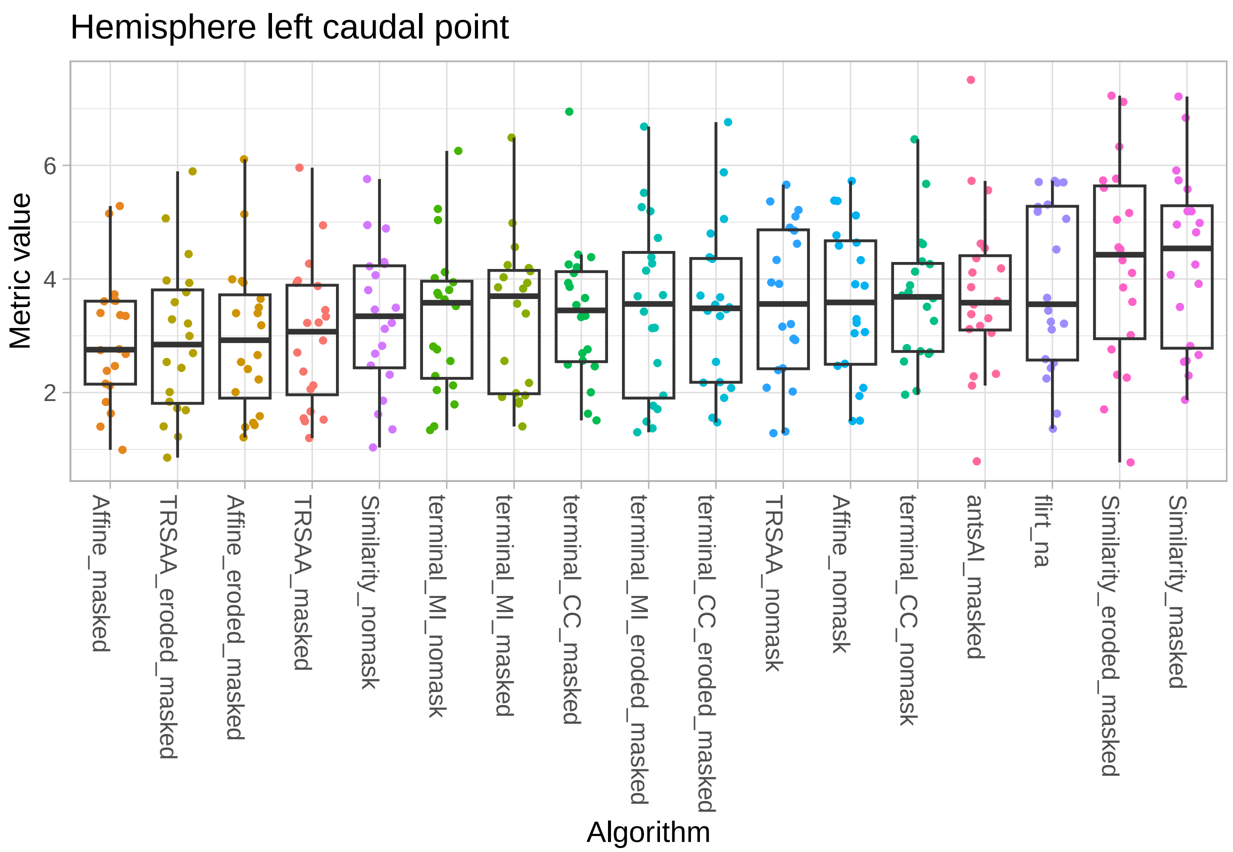
Hemisphere left caudal point
Metric value
6
4
2
Affine_masked
TRSAA_eroded_masked
Affine_eroded_masked
TRSAA_masked
Similarity_nomask
terminal_MI_nomask
terminal_MI_masked
terminal_CC_masked
terminal_MI_eroded_masked
terminal_CC_eroded_masked
TRSAA_nomask
Affine_nomask
terminal_CC_nomask
antsAI_masked
flirt_na
Similarity_eroded_masked
Similarity_masked
Algorithm

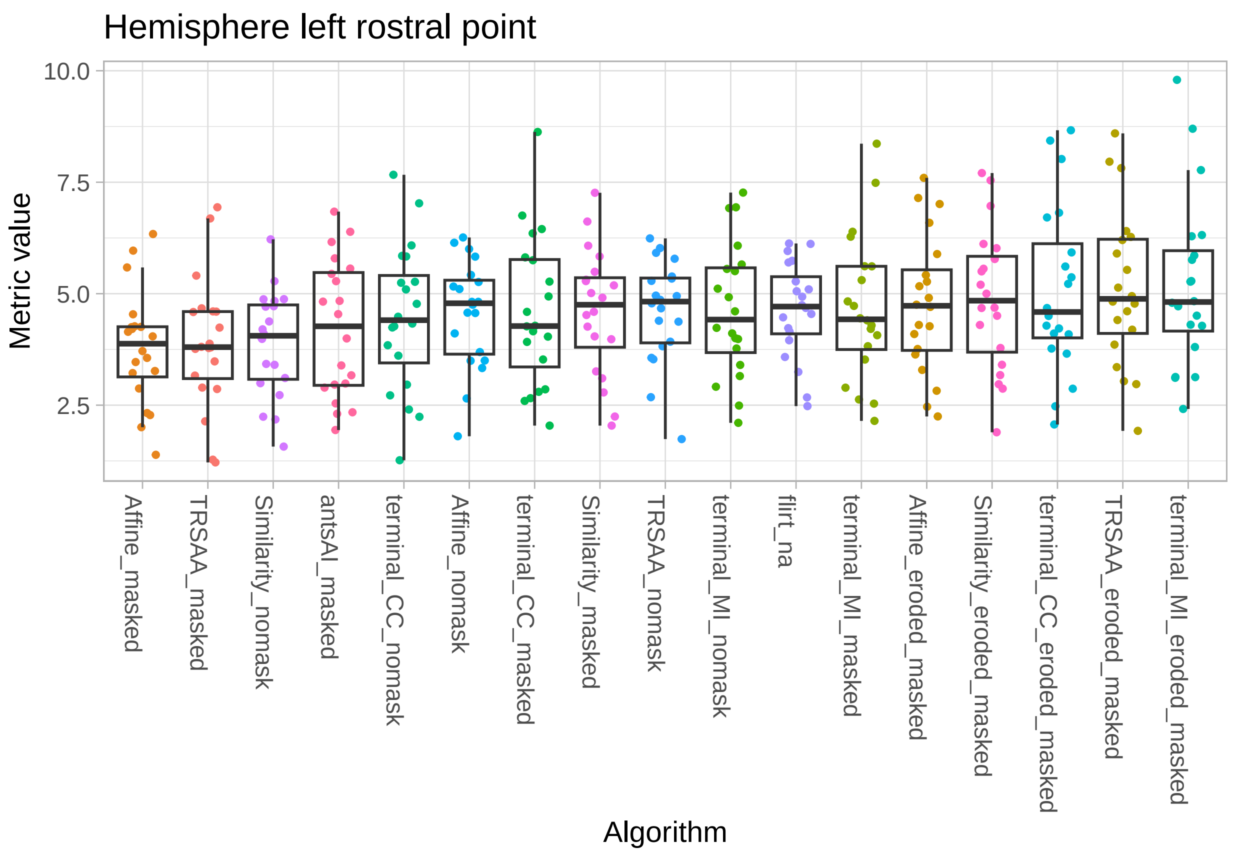

Hemisphere left rostral point
Metric value
10.0
7.5
5.0
2.5
Affine_masked
TRSAA_masked
Similarity_nomask
antsAI_masked
terminal_CC_nomask
Affine_nomask
terminal_CC_masked
Similarity_masked
TRSAA_nomask
terminal_MI_nomask
flirt_na
terminal_MI_masked
Affine_eroded_masked
Similarity_eroded_masked
terminal_CC_eroded_masked
TRSAA_eroded_masked
terminal_MI_eroded_masked
Algorithm

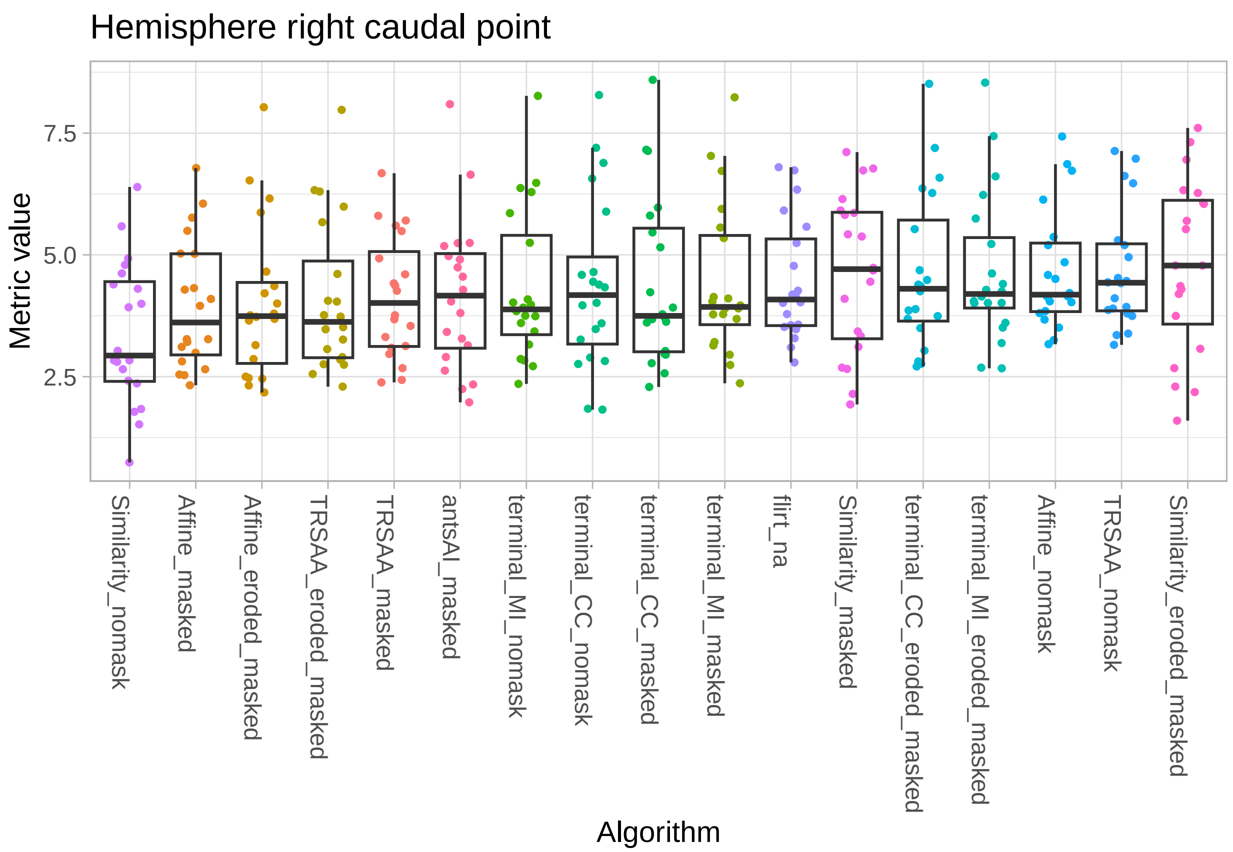
Hemisphere right caudal point
Metric value
7.5
5.0
2.5
Similarity_nomask
Affine_masked
Affine_eroded_masked
TRSAA_eroded_masked
TRSAA_masked
antsAI_masked
terminal_MI_nomask
terminal_CC_nomask
terminal_CC_masked
terminal_MI_masked
flirt_na
Similarity_masked
terminal_CC_eroded_masked
terminal_MI_eroded_masked
Affine_nomask
TRSAA_nomask
Similarity_eroded_masked
Algorithm

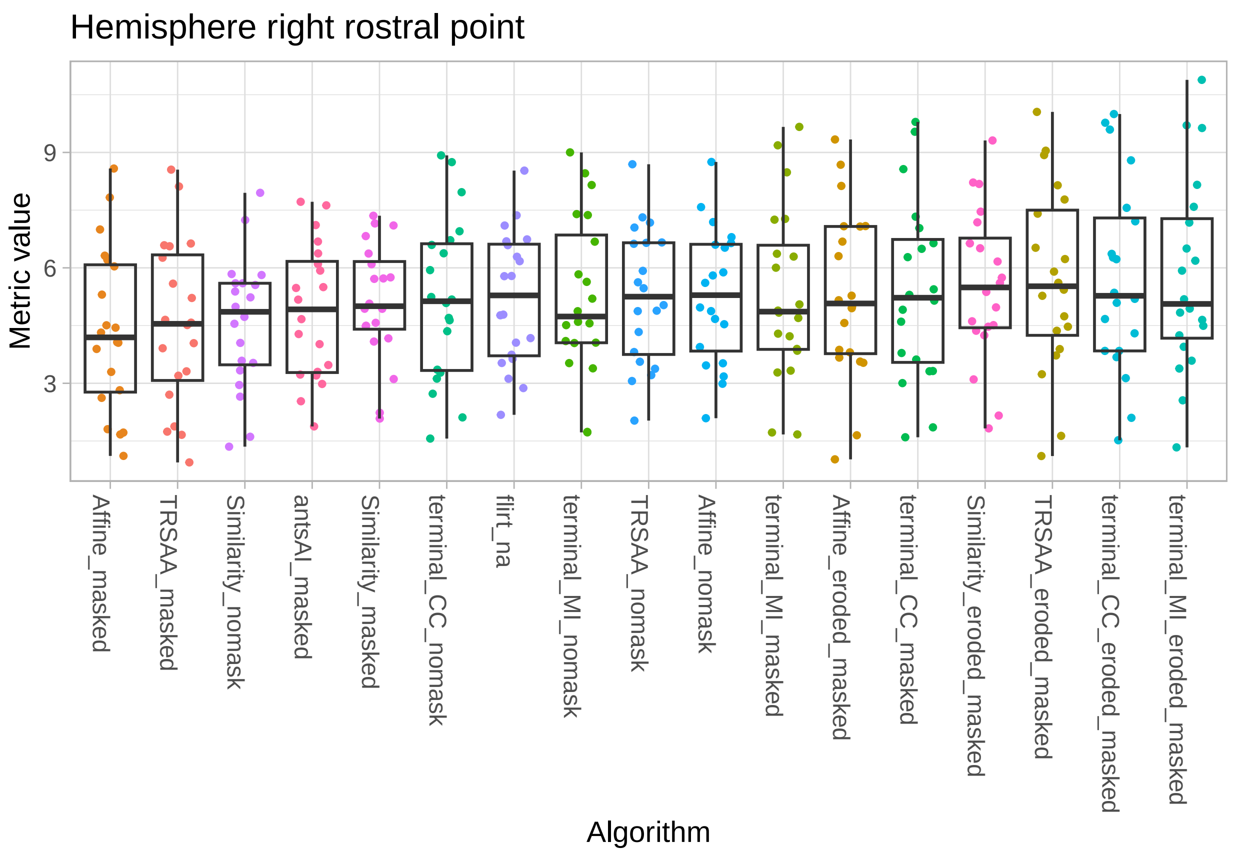

Hemisphere right rostral point
Metric value
9
6
3
Affine_masked
TRSAA_masked
Similarity_nomask
antsAI_masked
Similarity_masked
terminal_CC_nomask
flirt_na
terminal_MI_nomask
TRSAA_nomask
Affine_nomask
terminal_MI_masked
Affine_eroded_masked
terminal_CC_masked
Similarity_eroded_masked
TRSAA_eroded_masked
terminal_CC_eroded_masked
terminal_MI_eroded_masked
Algorithm

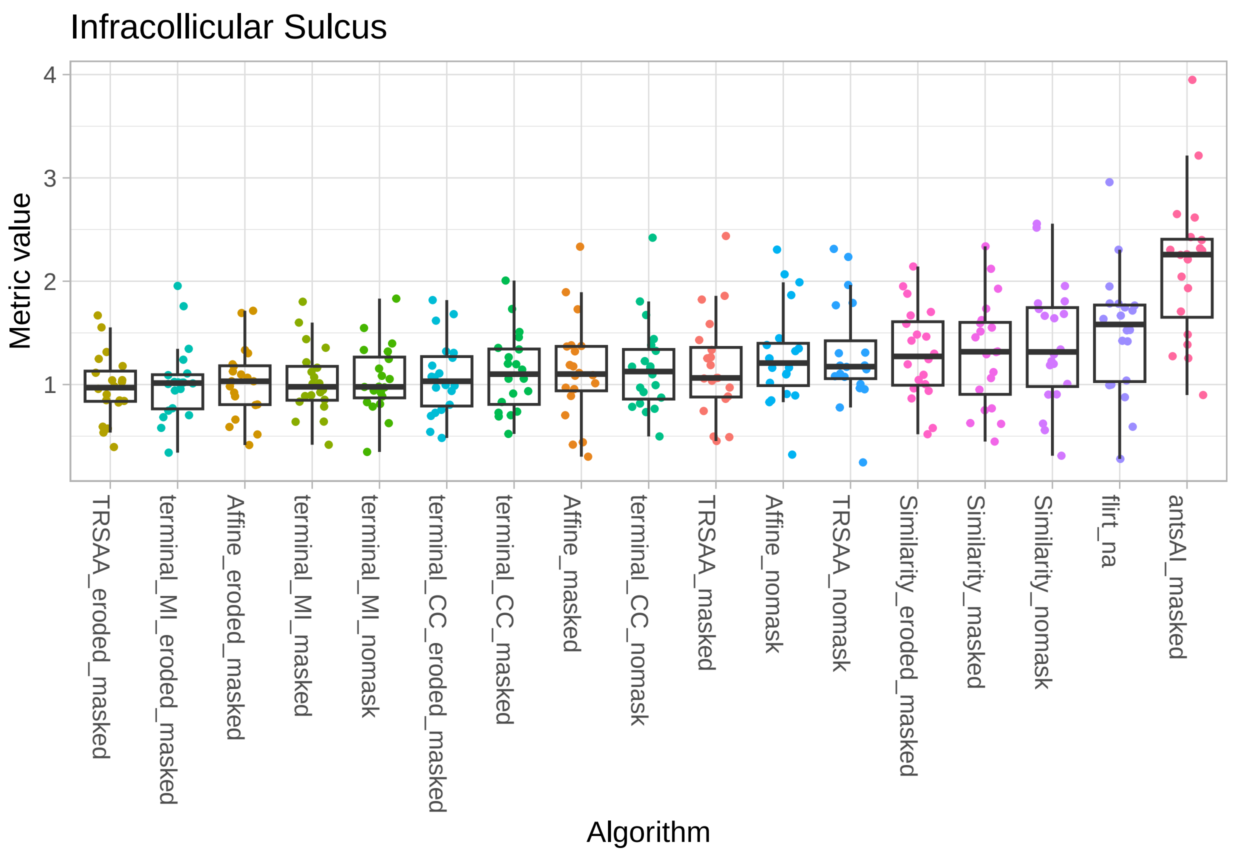
Infracollicular Sulcus
Metric value
4
3
2
1
TRSAA_eroded_masked
terminal_MI_eroded_masked
Affine_eroded_masked
terminal_MI_masked
terminal_MI_nomask
terminal_CC_eroded_masked
terminal_CC_masked
Affine_masked
terminal_CC_nomask
TRSAA_masked
Affine_nomask
TRSAA_nomask
Similarity_eroded_masked
Similarity_masked
Similarity_nomask
flirt_na
antsAI_masked
Algorithm

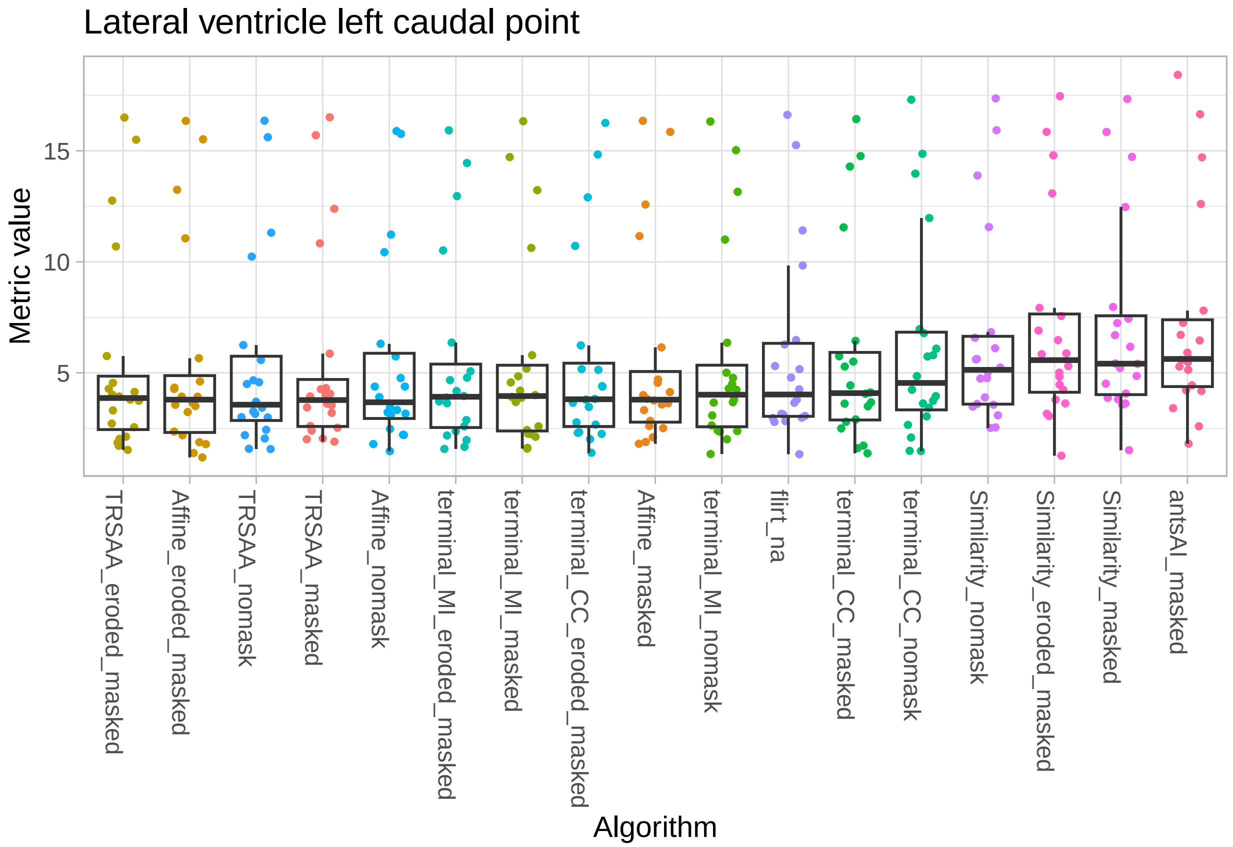

Lateral ventricle left caudal point
Metric value
15
10
5
TRSAA_eroded_masked
Affine_eroded_masked
TRSAA_nomask
TRSAA_masked
Affine_nomask
terminal_MI_eroded_masked
terminal_MI_masked
terminal_CC_eroded_masked
Affine_masked
terminal_MI_nomask
flirt_na
terminal_CC_masked
terminal_CC_nomask
Similarity_nomask
Similarity_eroded_masked
Similarity_masked
antsAI_masked
Algorithm

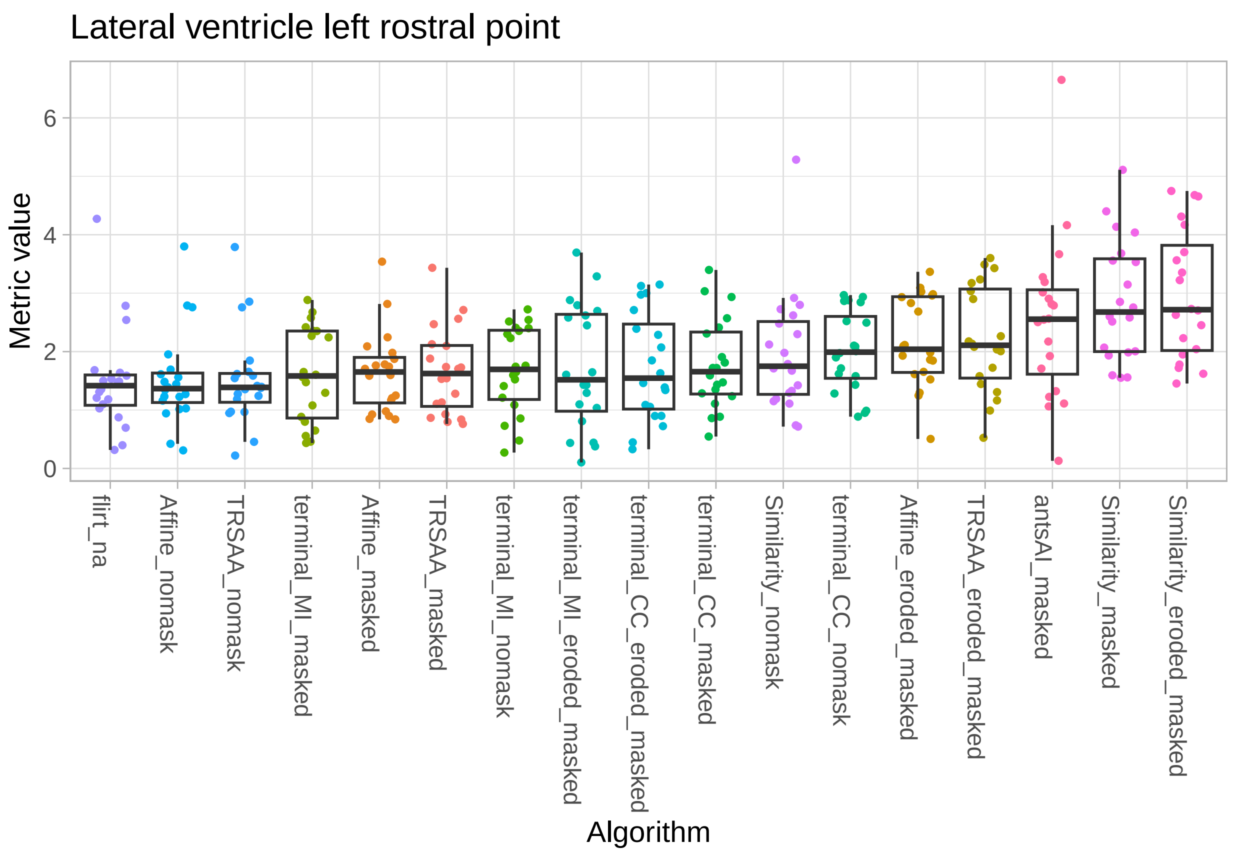
Lateral ventricle left rostral point
Metric value
0
2
4
6
flirt_na
Affine_nomask
TRSAA_nomask
terminal_MI_masked
Affine_masked
TRSAA_masked
terminal_MI_nomask
terminal_MI_eroded_masked
terminal_CC_eroded_masked
terminal_CC_masked
Similarity_nomask
terminal_CC_nomask
Affine_eroded_masked
TRSAA_eroded_masked
antsAI_masked
Similarity_masked
Similarity_eroded_masked
Algorithm

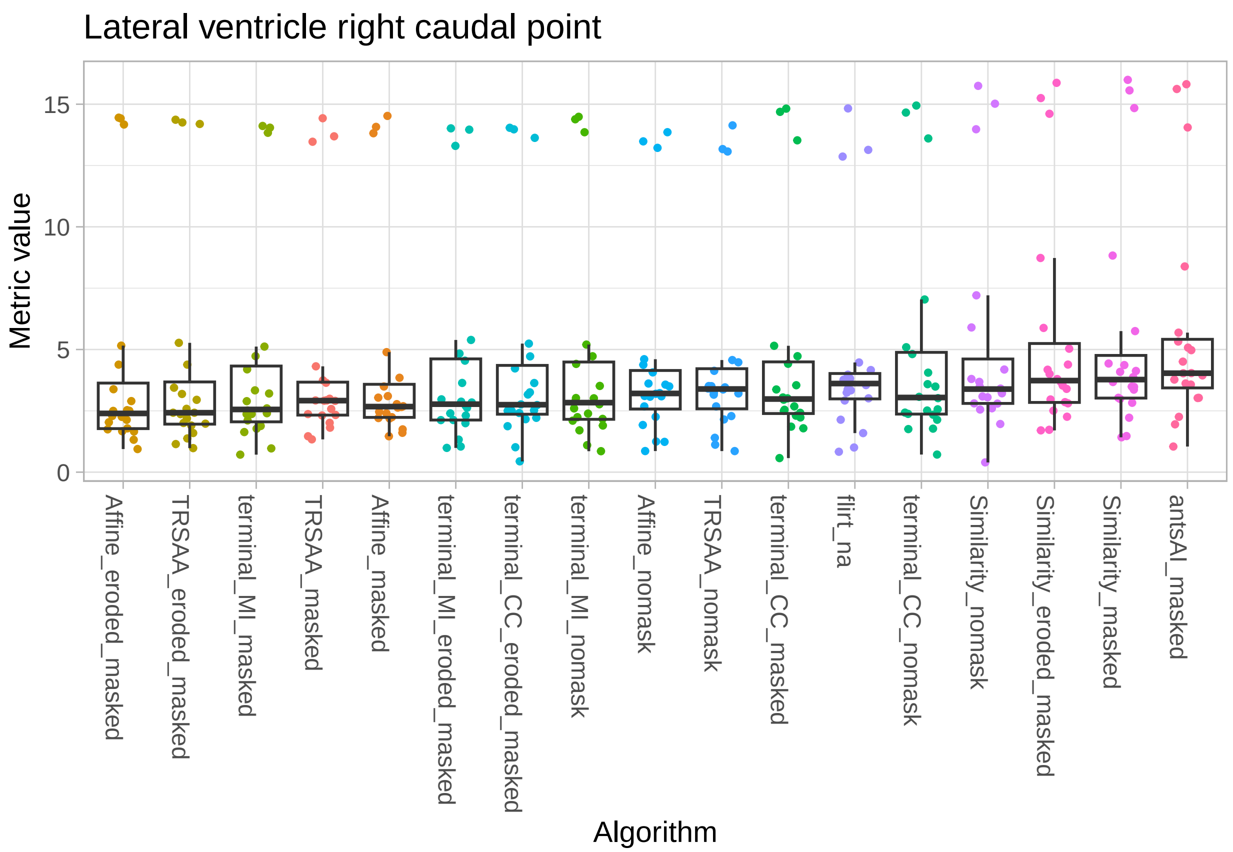

Lateral ventricle right caudal point
Metric value
0
5
10
15
Affine_eroded_masked
TRSAA_eroded_masked
terminal_MI_masked
TRSAA_masked
Affine_masked
terminal_MI_eroded_masked
terminal_CC_eroded_masked
terminal_MI_nomask
Affine_nomask
TRSAA_nomask
terminal_CC_masked
flirt_na
terminal_CC_nomask
Similarity_nomask
Similarity_eroded_masked
Similarity_masked
antsAI_masked
Algorithm

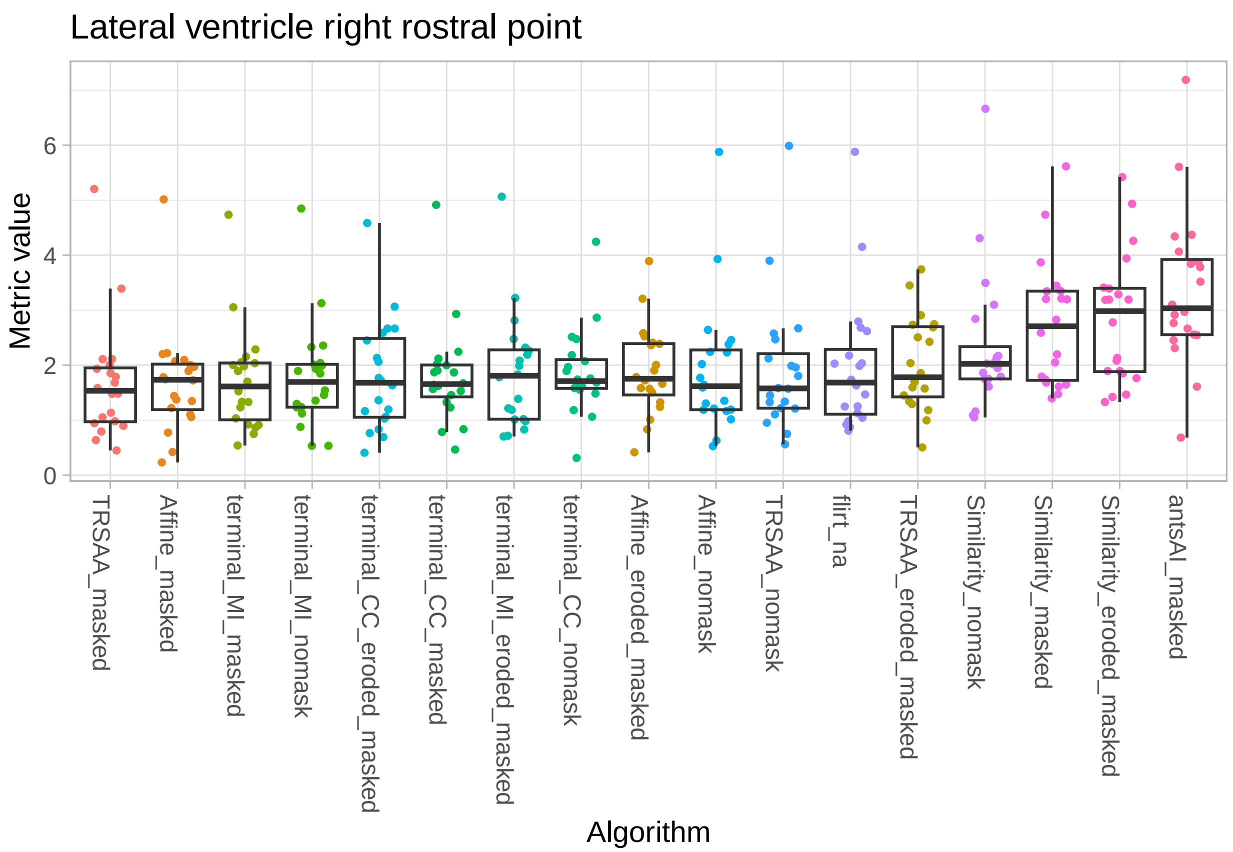

Lateral ventricle right rostral point
Metric value
0
2
4
6
TRSAA_masked
Affine_masked
terminal_MI_masked
terminal_MI_nomask
terminal_CC_eroded_masked
terminal_CC_masked
terminal_MI_eroded_masked
terminal_CC_nomask
Affine_eroded_masked
Affine_nomask
TRSAA_nomask
flirt_na
TRSAA_eroded_masked
Similarity_nomask
Similarity_masked
Similarity_eroded_masked
antsAI_masked
Algorithm

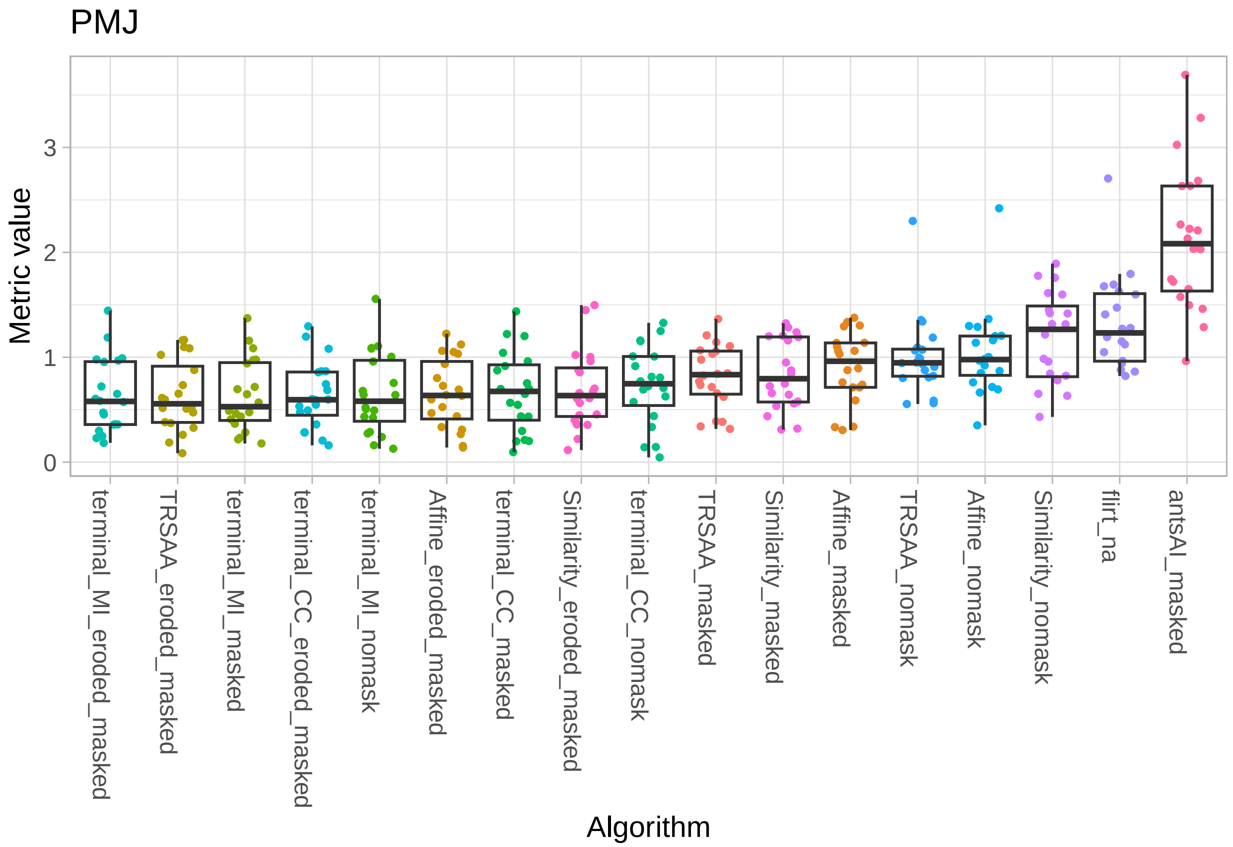
PMJ
Metric value
0
1
2
3
terminal_MI_eroded_masked
TRSAA_eroded_masked
terminal_MI_masked
terminal_CC_eroded_masked
terminal_MI_nomask
Affine_eroded_masked
terminal_CC_masked
Similarity_eroded_masked
terminal_CC_nomask
TRSAA_masked
Similarity_masked
Affine_masked
TRSAA_nomask
Affine_nomask
Similarity_nomask
flirt_na
antsAI_masked
Algorithm

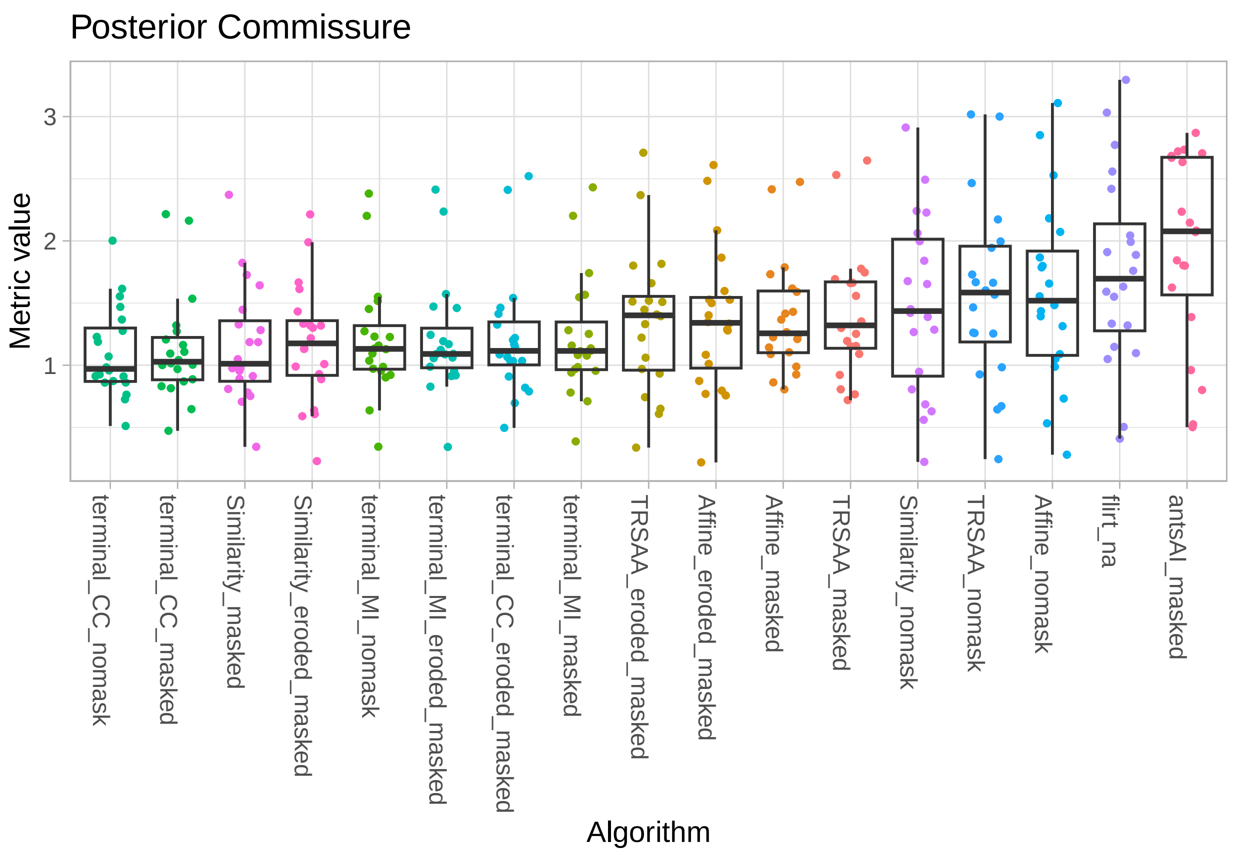

Posterior Commissure
Metric value
3
2
1
terminal_CC_nomask
terminal_CC_masked
Similarity_masked
Similarity_eroded_masked
terminal_MI_nomask
terminal_MI_eroded_masked
terminal_CC_eroded_masked
terminal_MI_masked
TRSAA_eroded_masked
Affine_eroded_masked
Affine_masked
TRSAA_masked
Similarity_nomask
TRSAA_nomask
Affine_nomask
flirt_na
antsAI_masked
Algorithm

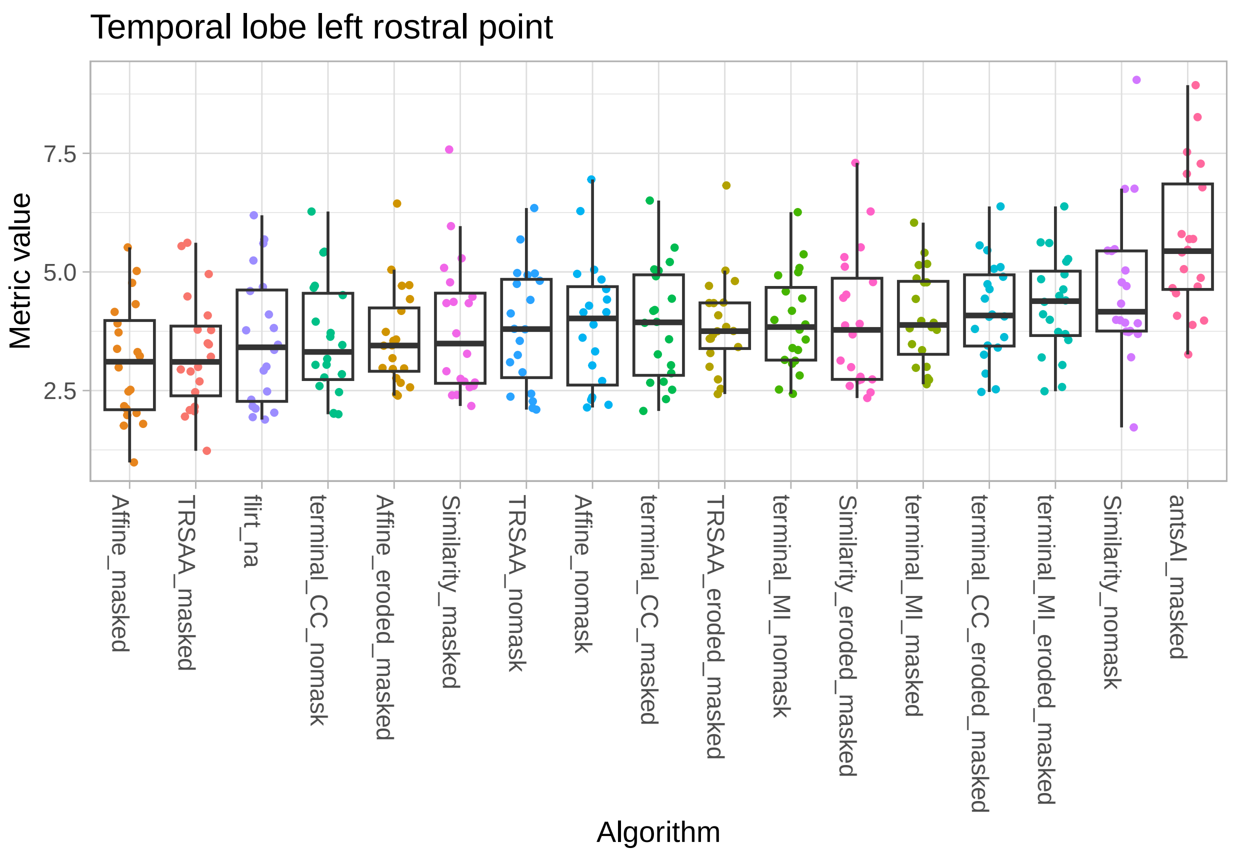

Temporal lobe left rostral point
Metric value
7.5
5.0
2.5
Affine_masked
TRSAA_masked
flirt_na
terminal_CC_nomask
Affine_eroded_masked
Similarity_masked
TRSAA_nomask
Affine_nomask
terminal_CC_masked
TRSAA_eroded_masked
terminal_MI_nomask
Similarity_eroded_masked
terminal_MI_masked
terminal_CC_eroded_masked
terminal_MI_eroded_masked
Similarity_nomask
antsAI_masked
Algorithm

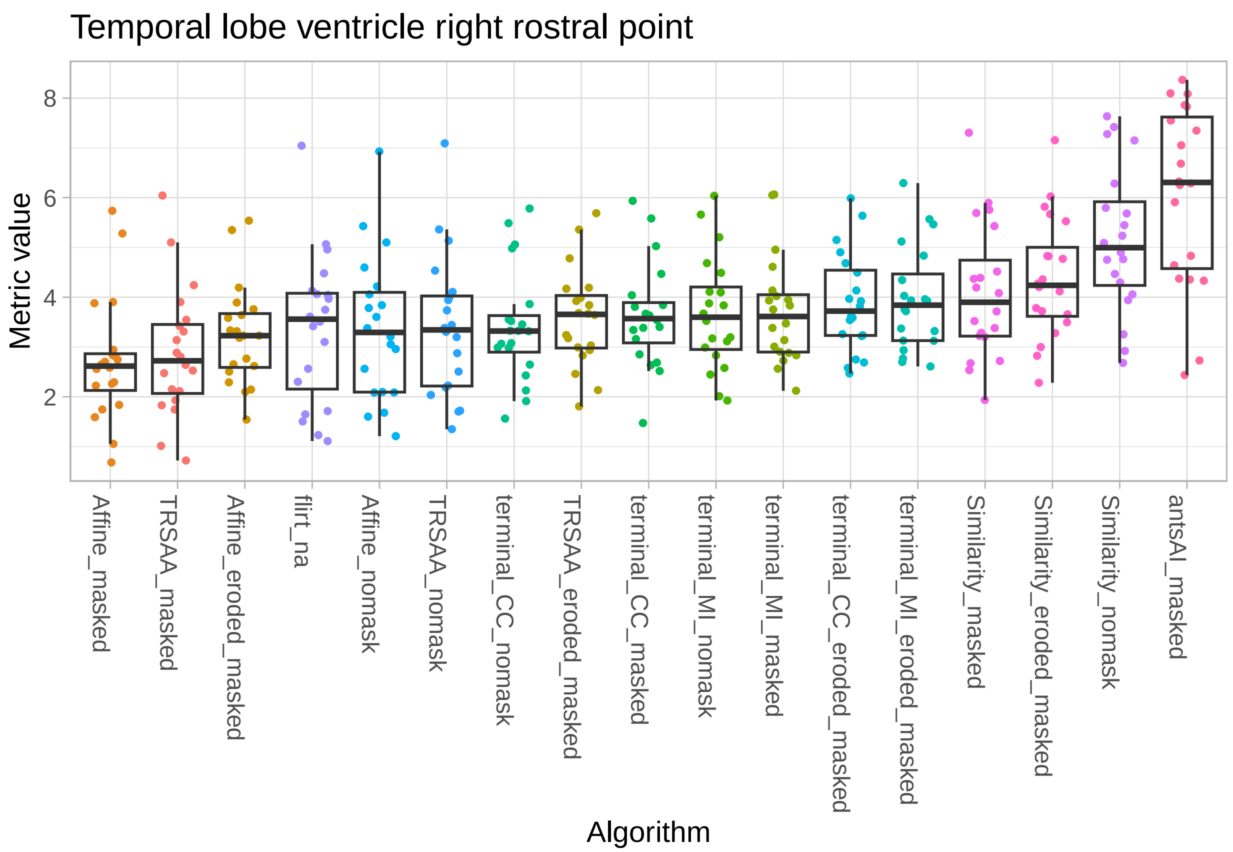
Temporal lobe ventricle right rostral point
Metric value
8
6
4
2
Affine_masked
TRSAA_masked
Affine_eroded_masked
flirt_na
Affine_nomask
TRSAA_nomask
terminal_CC_nomask
TRSAA_eroded_masked
terminal_CC_masked
terminal_MI_nomask
terminal_MI_masked
terminal_CC_eroded_masked
terminal_MI_eroded_masked
Similarity_masked
Similarity_eroded_masked
Similarity_nomask
antsAI_masked
Algorithm

2.2 Podium plot

Podium plots (see also Eugster et al., 2008) for visualizing raw assessment data. Upper part (spaghetti plot): Participating algorithms are color-coded, and each colored dot in the plot represents a metric value achieved with the respective algorithm. The actual metric value is encoded by the y-axis. Each podium (here: p=17) represents one possible rank, ordered from best (1) to last (here: 17). The assignment of metric values (i.e. colored dots) to one of the podiums is based on the rank that the respective algorithm achieved on the corresponding case. Note that the plot part above each podium place is further subdivided into p "columns", where each column represents one participating algorithm (here: $p = 17$). Dots corresponding to identical cases are connected by a line, leading to the shown spaghetti structure. Lower part: Bar charts represent the relative frequency for each algorithm to achieve the rank encoded by the podium place.

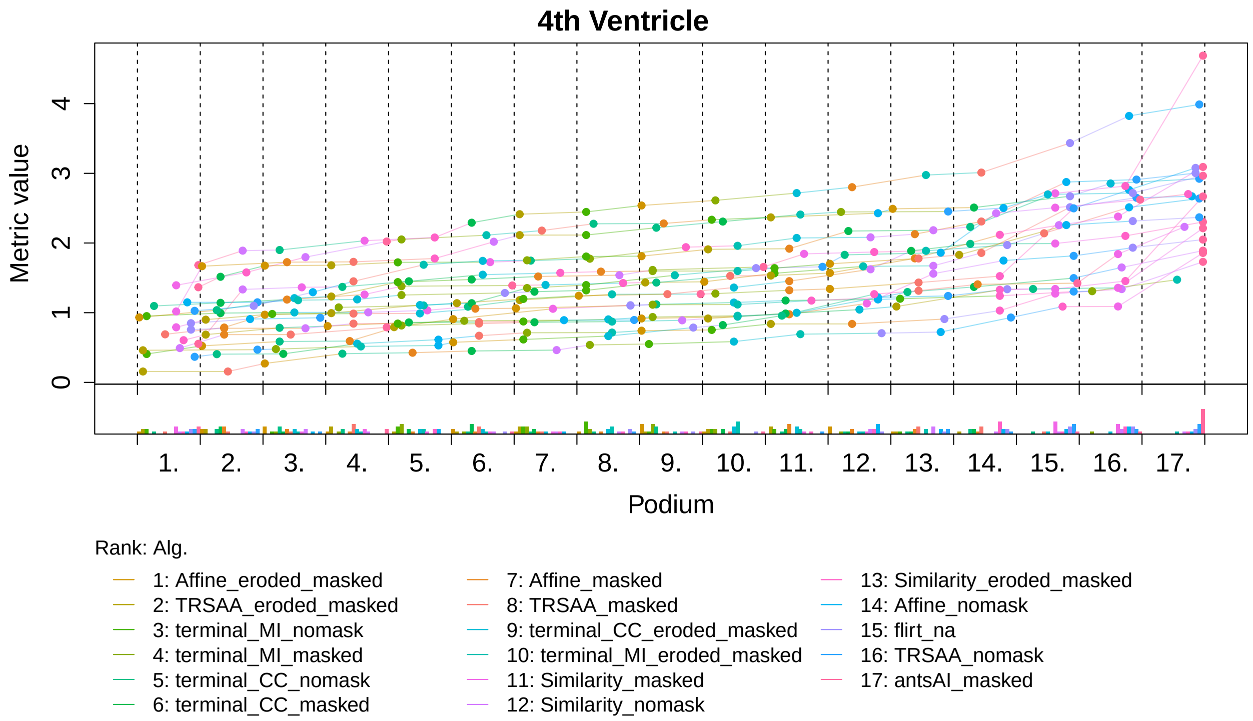

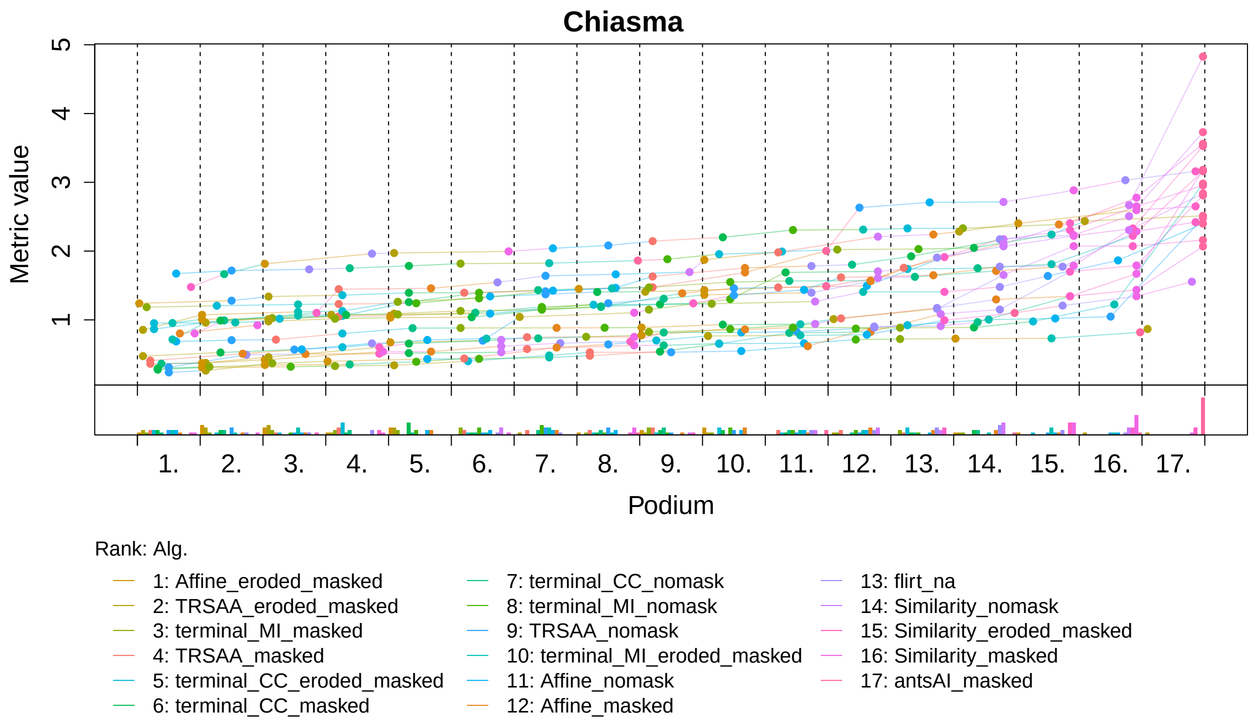
Chiasma
Metric value
1
2
3
4
5
Podium
1. 2. 3. 4. 5. 6. 7. 8. 9. 10. 11. 12. 13. 14. 15. 16. 17.
Rank: Alg.
1: Affine_eroded_masked
2: TRSAA_eroded_masked
3: terminal_MI_masked
4: TRSAA_masked
5: terminal_CC_eroded_masked
6: terminal_CC_masked
7: terminal_CC_nomask
8: terminal_MI_nomask
9: TRSAA_nomask
10: terminal_MI_eroded_masked
11: Affine_nomask
12: Affine_masked
13: flirt_na
14: Similarity_nomask
15: Similarity_eroded_masked
16: Similarity_masked
17: antsAI_masked

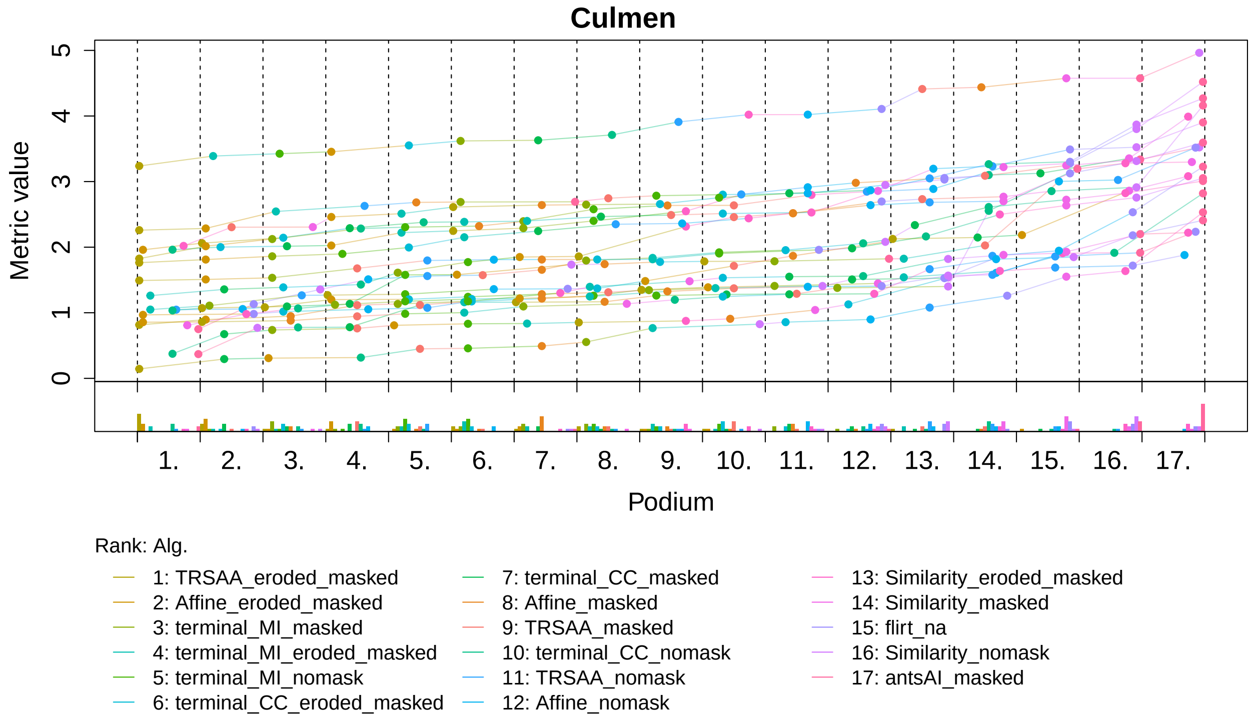
Culmen
Metric value
0
1
2
3
4
5
Podium
1. 2. 3. 4. 5. 6. 7. 8. 9. 10. 11. 12. 13. 14. 15. 16. 17.
Rank: Alg.
1: TRSAA_eroded_masked
2: Affine_eroded_masked
3: terminal_MI_masked
4: terminal_MI_eroded_masked
5: terminal_MI_nomask
6: terminal_CC_eroded_masked
7: terminal_CC_masked
8: Affine_masked
9: TRSAA_masked
10: terminal_CC_nomask
11: TRSAA_nomask
12: Affine_nomask
13: Similarity_eroded_masked
14: Similarity_masked
15: flirt_na
16: Similarity_nomask
17: antsAI_masked

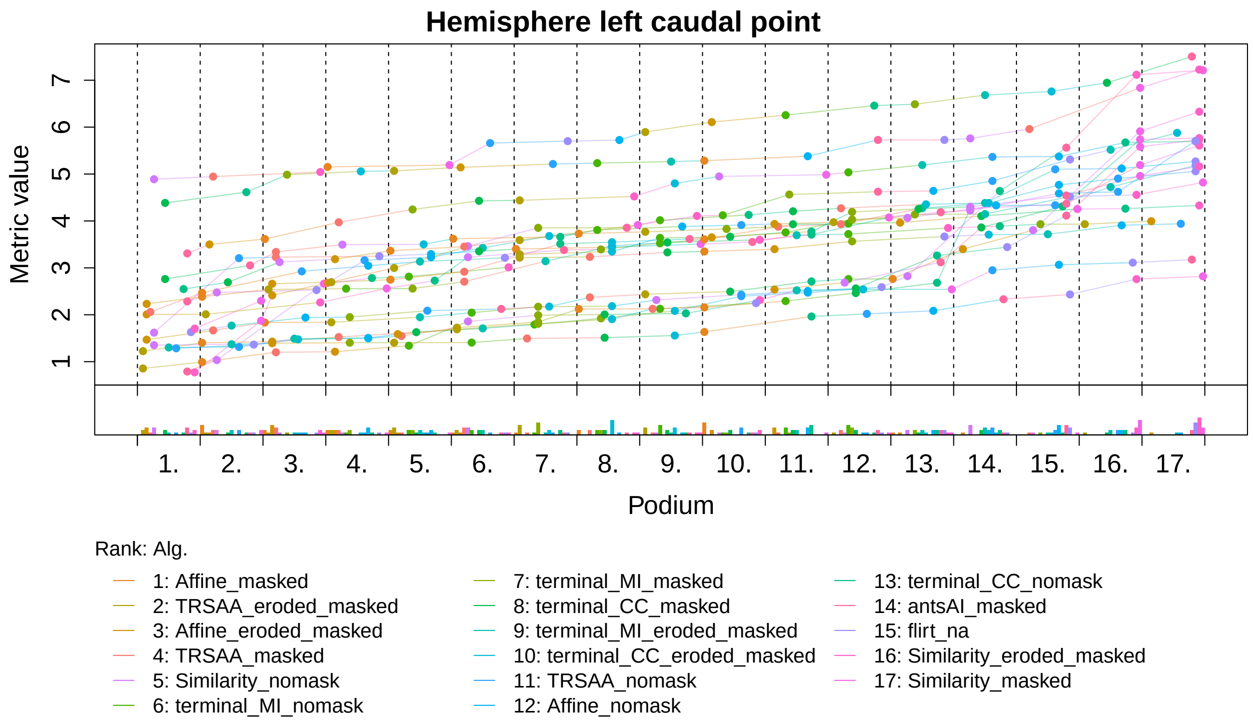
Hemisphere left caudal point
Metric value
1
2
3
4
5
6
7
1.
2.
3.
4.
5.
6.
7.
8.
9.
10.
11.
12.
13.
14.
15.
16.
17.
Podium
Rank: Alg.
1: Affine_masked
2: TRSAA_eroded_masked
3: Affine_eroded_masked
4: TRSAA_masked
5: Similarity_nomask
6: terminal_MI_nomask
7: terminal_MI_masked
8: terminal_CC_masked
9: terminal_MI_eroded_masked
10: terminal_CC_eroded_masked
11: TRSAA_nomask
12: Affine_nomask
13: terminal_CC_nomask
14: antsAI_masked
15: flirt_na
16: Similarity_eroded_masked
17: Similarity_masked

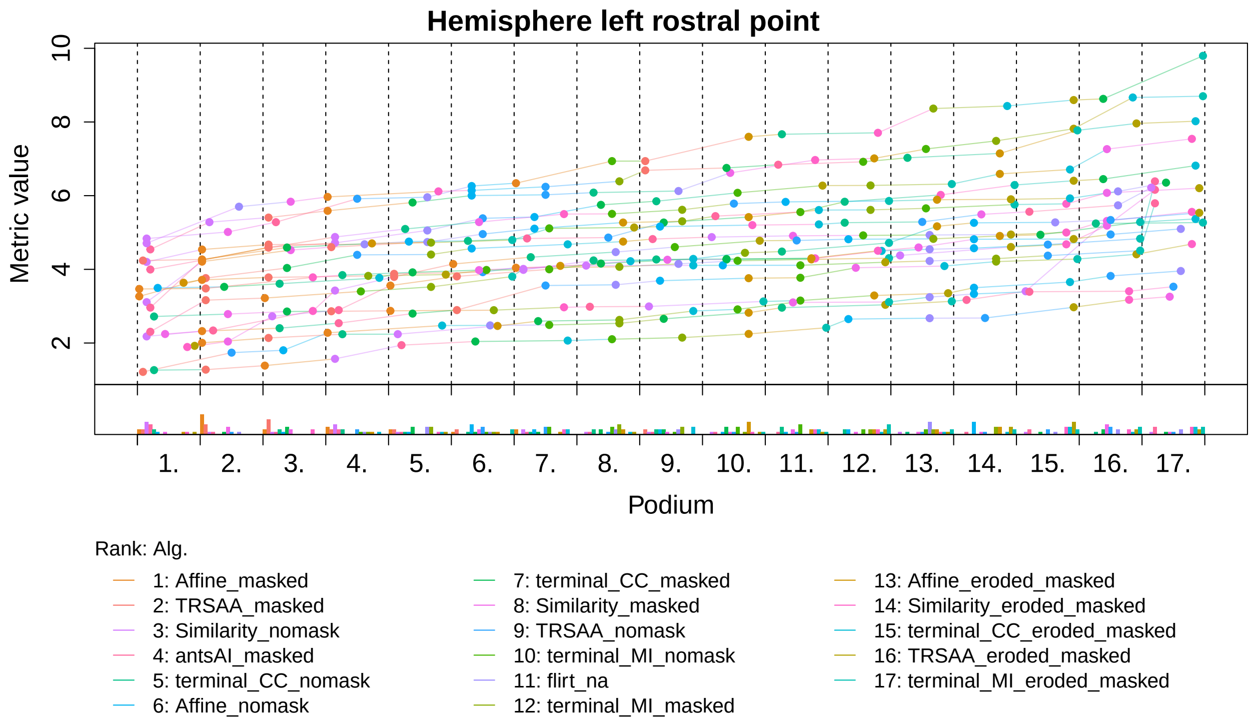
Hemisphere left rostral point
Metric value
2
4
6
8
10
1.
2.
3.
4.
5.
6.
7.
8.
9.
10.
11.
12.
13.
14.
15.
16.
17.
Podium
Rank: Alg.
1: Affine_masked
2: TRSAA_masked
3: Similarity_nomask
4: antsAI_masked
5: terminal_CC_nomask
6: Affine_nomask
7: terminal_CC_masked
8: Similarity_masked
9: TRSAA_nomask
10: terminal_MI_nomask
11: flirt_na
12: terminal_MI_masked
13: Affine_eroded_masked
14: Similarity_eroded_masked
15: terminal_CC_eroded_masked
16: TRSAA_eroded_masked
17: terminal_MI_eroded_masked

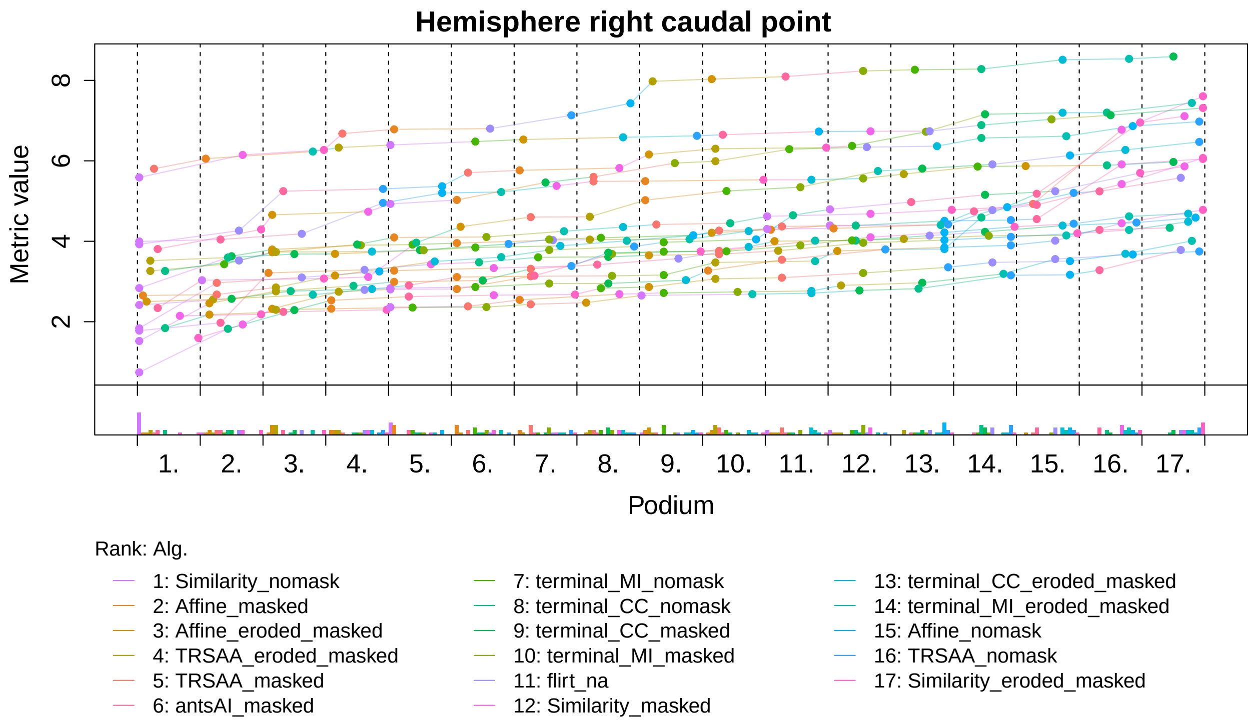

Hemisphere right caudal point
Metric value
2
4
6
8
1.
2.
3.
4.
5.
6.
7.
8.
9.
10.
11.
12.
13.
14.
15.
16.
17.
Podium
Rank: Alg.
1: Similarity_nomask
2: Affine_masked
3: Affine_eroded_masked
4: TRSAA_eroded_masked
5: TRSAA_masked
6: antsAI_masked
7: terminal_MI_nomask
8: terminal_CC_nomask
9: terminal_CC_masked
10: terminal_MI_masked
11: flirt_na
12: Similarity_masked
13: terminal_CC_eroded_masked
14: terminal_MI_eroded_masked
15: Affine_nomask
16: TRSAA_nomask
17: Similarity_eroded_masked

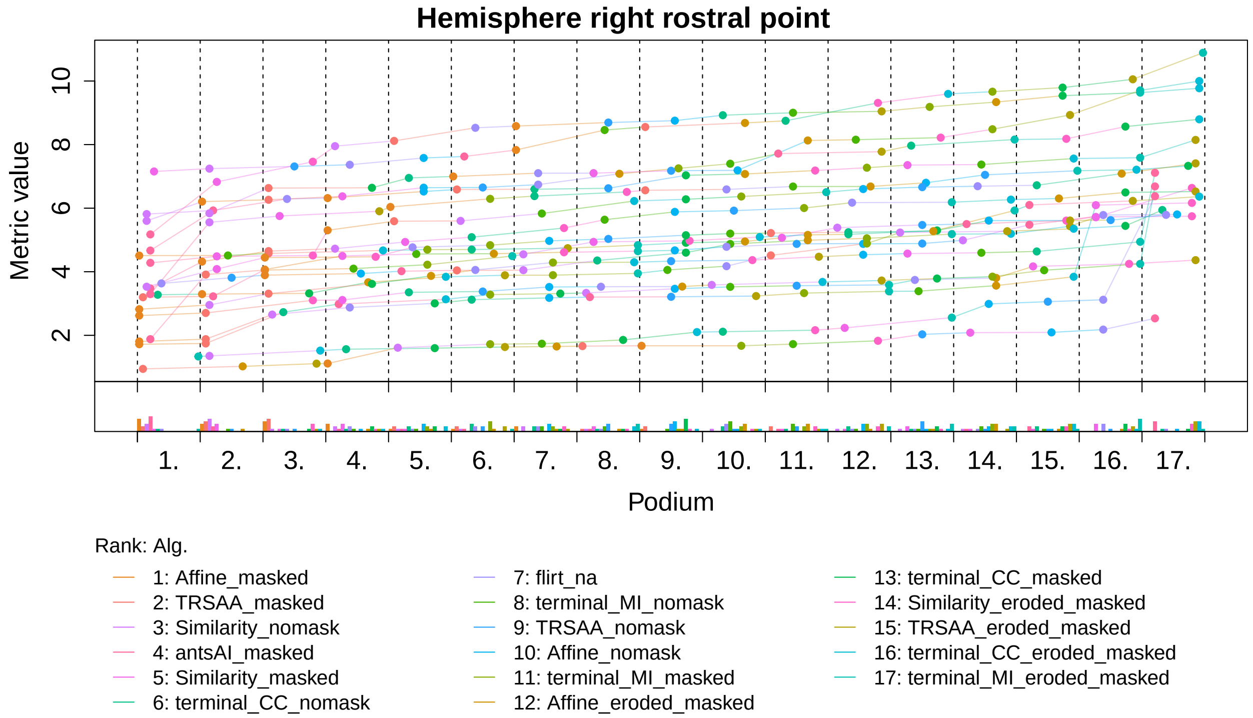

Hemisphere right rostral point
Metric value
2
4
6
8
10
1.
2.
3.
4.
5.
6.
7.
8.
9.
10.
11.
12.
13.
14.
15.
16.
17.
Podium
Rank: Alg.
1: Affine_masked
2: TRSAA_masked
3: Similarity_nomask
4: antsAI_masked
5: Similarity_masked
6: terminal_CC_nomask
7: flirt_na
8: terminal_MI_nomask
9: TRSAA_nomask
10: Affine_nomask
11: terminal_MI_masked
12: Affine_eroded_masked
13: terminal_CC_masked
14: Similarity_eroded_masked
15: TRSAA_eroded_masked
16: terminal_CC_eroded_masked
17: terminal_MI_eroded_masked

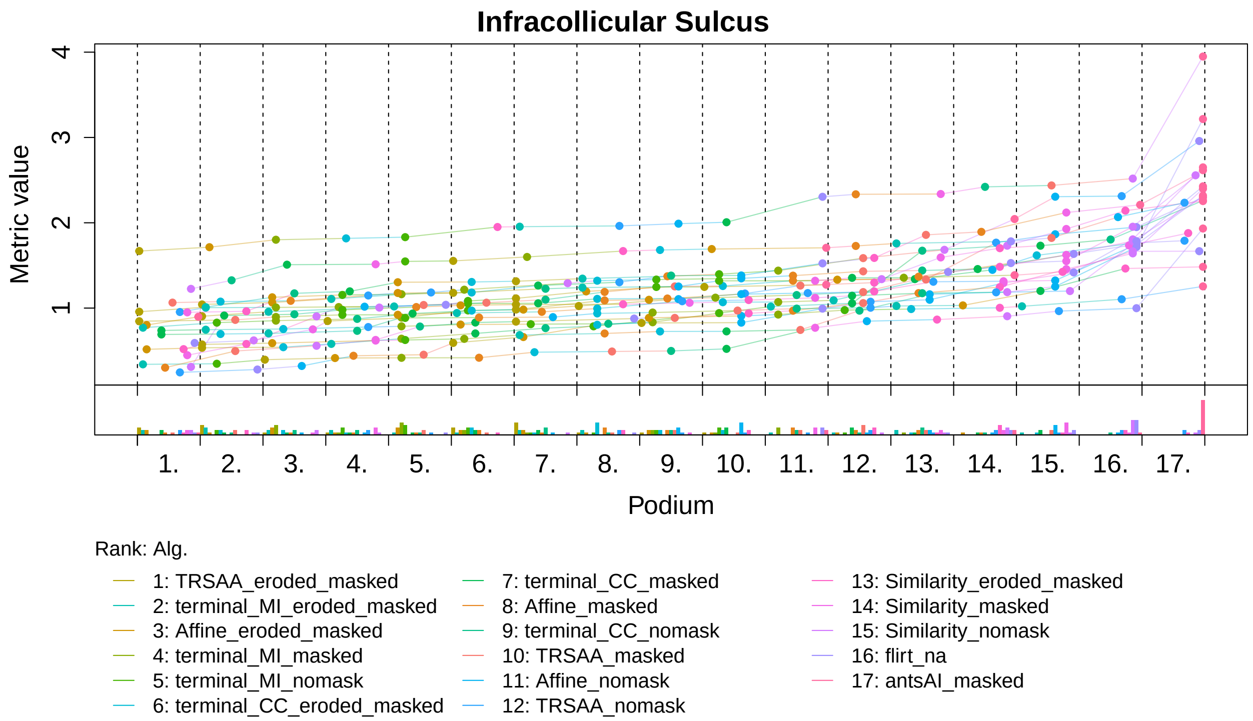
Infracollicular Sulcus
Metric value
Podium
1. 2. 3. 4. 5. 6. 7. 8. 9. 10. 11. 12. 13. 14. 15. 16. 17.
Rank: Alg.
1: TRSAA_eroded_masked
2: terminal_MI_eroded_masked
3: Affine_eroded_masked
4: terminal_MI_masked
5: terminal_MI_nomask
6: terminal_CC_eroded_masked
7: terminal_CC_masked
8: Affine_masked
9: terminal_CC_nomask
10: TRSAA_masked
11: Affine_nomask
12: TRSAA_nomask
13: Similarity_eroded_masked
14: Similarity_masked
15: Similarity_nomask
16: flirt_na
17: antsAI_masked

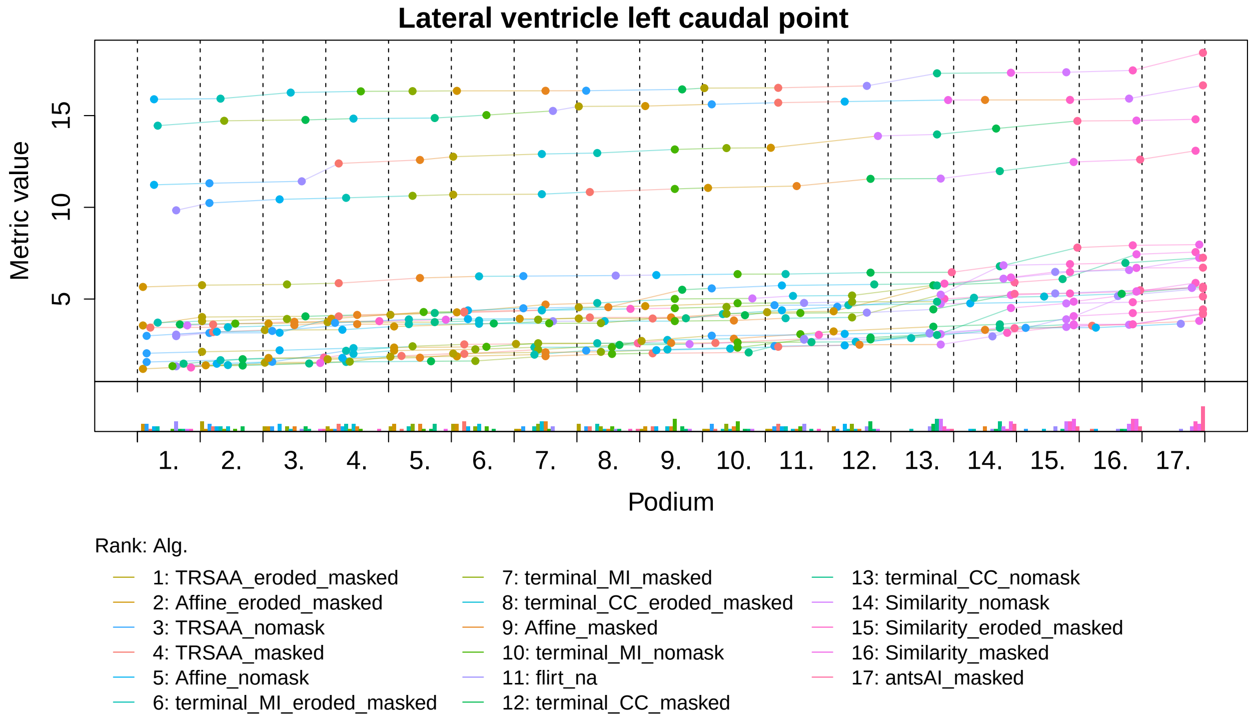
Lateral ventricle left caudal point
Metric value
Podium
1. 2. 3. 4. 5. 6. 7. 8. 9. 10. 11. 12. 13. 14. 15. 16. 17.
Rank: Alg.
1: TRSAA_eroded_masked
2: Affine_eroded_masked
3: TRSAA_nomask
4: TRSAA_masked
5: Affine_nomask
6: terminal_MI_eroded_masked
7: terminal_MI_masked
8: terminal_CC_eroded_masked
9: Affine_masked
10: terminal_MI_nomask
11: flirt_na
12: terminal_CC_masked
13: terminal_CC_nomask
14: Similarity_nomask
15: Similarity_eroded_masked
16: Similarity_masked
17: antsAI_masked

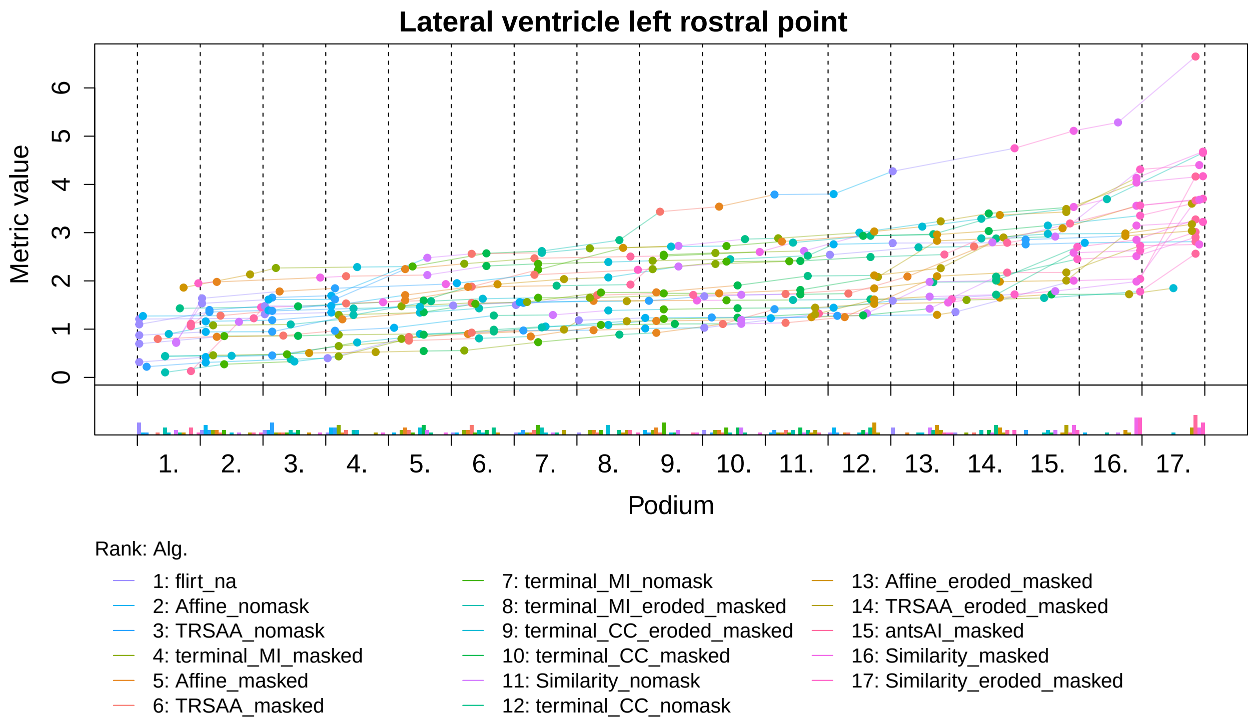
Lateral ventricle left rostral point
Metric value
0
1
2
3
4
5
6
1.
2.
3.
4.
5.
6.
7.
8.
9.
10.
11.
12.
13.
14.
15.
16.
17.
Podium
Rank: Alg.
1: flirt_na
2: Affine_nomask
3: TRSAA_nomask
4: terminal_MI_masked
5: Affine_masked
6: TRSAA_masked
7: terminal_MI_nomask
8: terminal_MI_eroded_masked
9: terminal_CC_eroded_masked
10: terminal_CC_masked
11: Similarity_nomask
12: terminal_CC_nomask
13: Affine_eroded_masked
14: TRSAA_eroded_masked
15: antsAI_masked
16: Similarity_masked
17: Similarity_eroded_masked

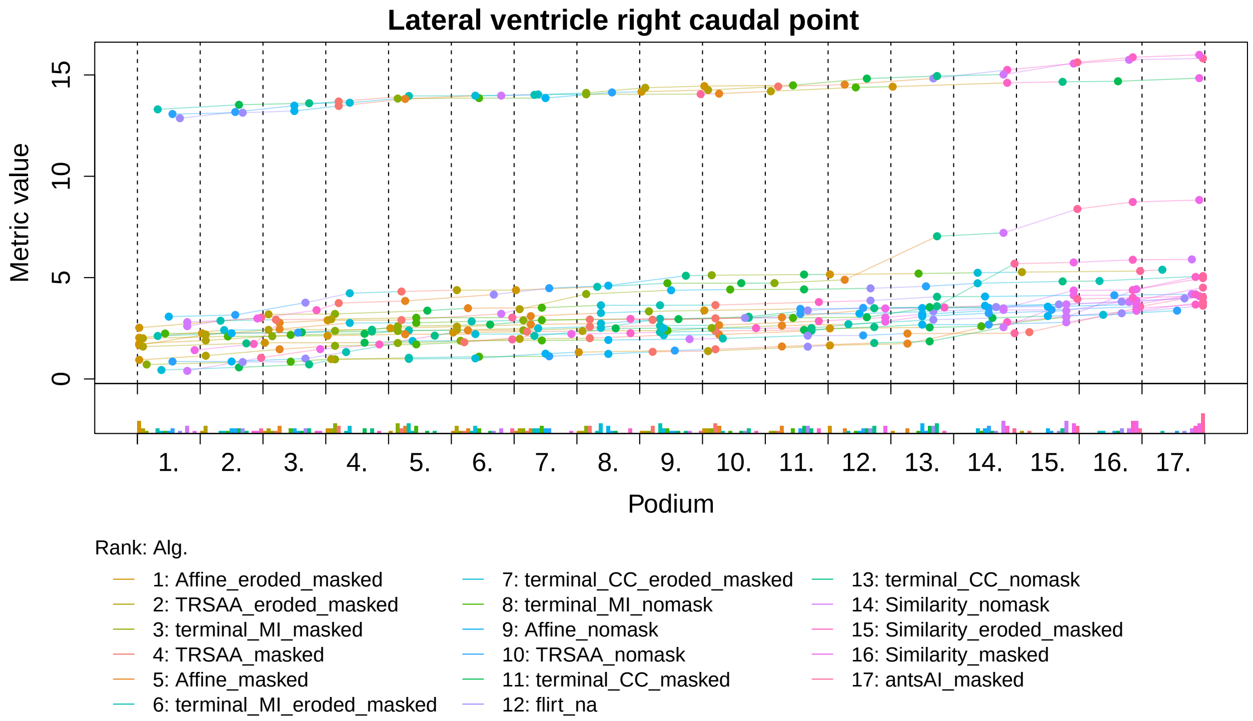
Lateral ventricle right caudal point
Metric value
0
5
10
15
1.
2.
3.
4.
5.
6.
7.
8.
9.
10.
11.
12.
13.
14.
15.
16.
17.
Podium
Rank: Alg.
1: Affine_eroded_masked
2: TRSAA_eroded_masked
3: terminal_MI_masked
4: TRSAA_masked
5: Affine_masked
6: terminal_MI_eroded_masked
7: terminal_CC_eroded_masked
8: terminal_MI_nomask
9: Affine_nomask
10: TRSAA_nomask
11: terminal_CC_masked
12: flirt_na
13: terminal_CC_nomask
14: Similarity_nomask
15: Similarity_eroded_masked
16: Similarity_masked
17: antsAI_masked

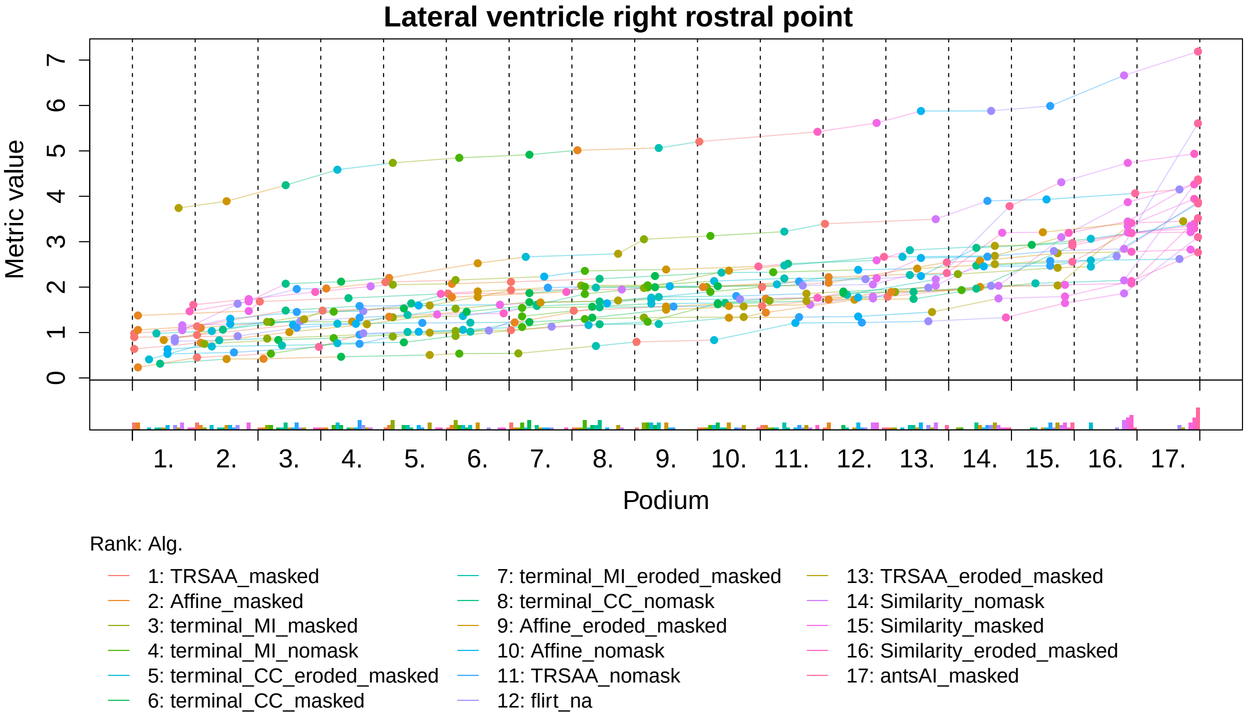
Lateral ventricle right rostral point
Metric value
0 1 2 3 4 5 6 7
1. 2. 3. 4. 5. 6. 7. 8. 9. 10. 11. 12. 13. 14. 15. 16. 17.
Podium
Rank: Alg.
1: TRSAA_masked
2: Affine_masked
3: terminal_MI_masked
4: terminal_MI_nomask
5: terminal_CC_eroded_masked
6: terminal_CC_masked
7: terminal_MI_eroded_masked
8: terminal_CC_nomask
9: Affine_eroded_masked
10: Affine_nomask
11: TRSAA_nomask
12: flirt_na
13: TRSAA_eroded_masked
14: Similarity_nomask
15: Similarity_masked
16: Similarity_eroded_masked
17: antsAI_masked

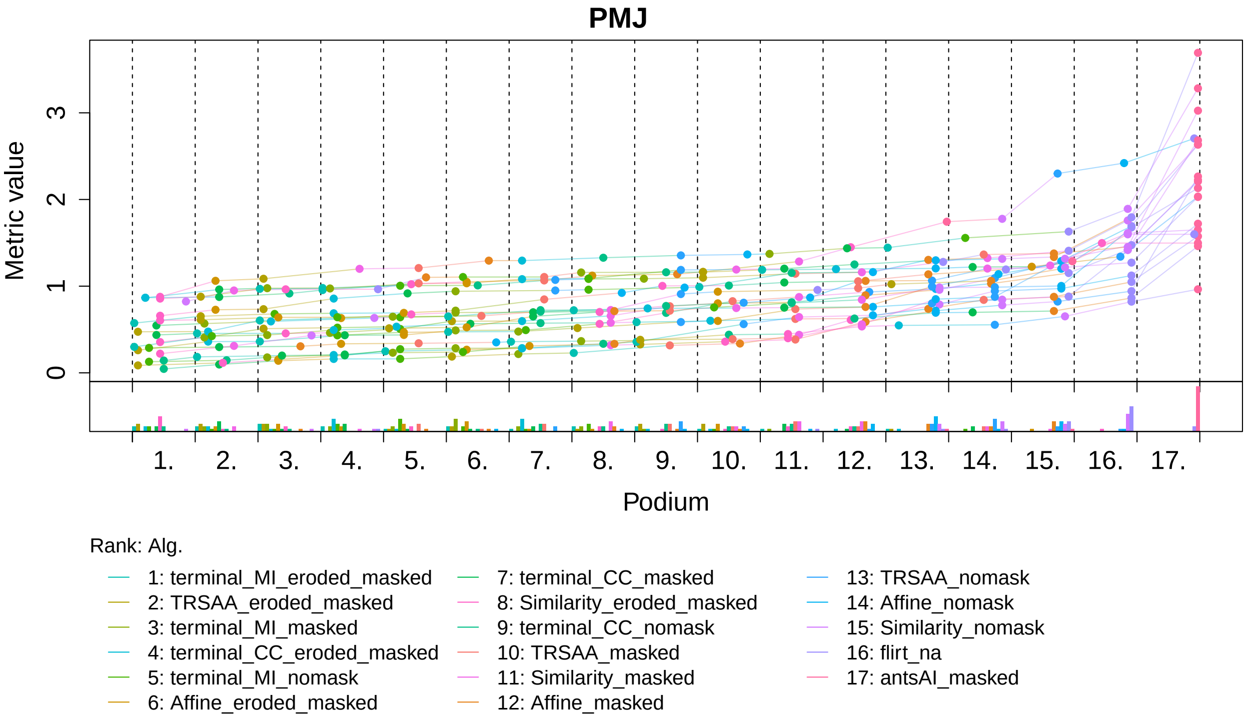
PMJ
Metric value
0 1 2 3
1. 2. 3. 4. 5. 6. 7. 8. 9. 10. 11. 12. 13. 14. 15. 16. 17.
Podium
Rank: Alg.
1: terminal_MI_eroded_masked
2: TRSAA_eroded_masked
3: terminal_MI_masked
4: terminal_CC_eroded_masked
5: terminal_MI_nomask
6: Affine_eroded_masked
7: terminal_CC_masked
8: Similarity_eroded_masked
9: terminal_CC_nomask
10: TRSAA_masked
11: Similarity_masked
12: Affine_masked
13: TRSAA_nomask
14: Affine_nomask
15: Similarity_nomask
16: flirt_na
17: antsAI_masked

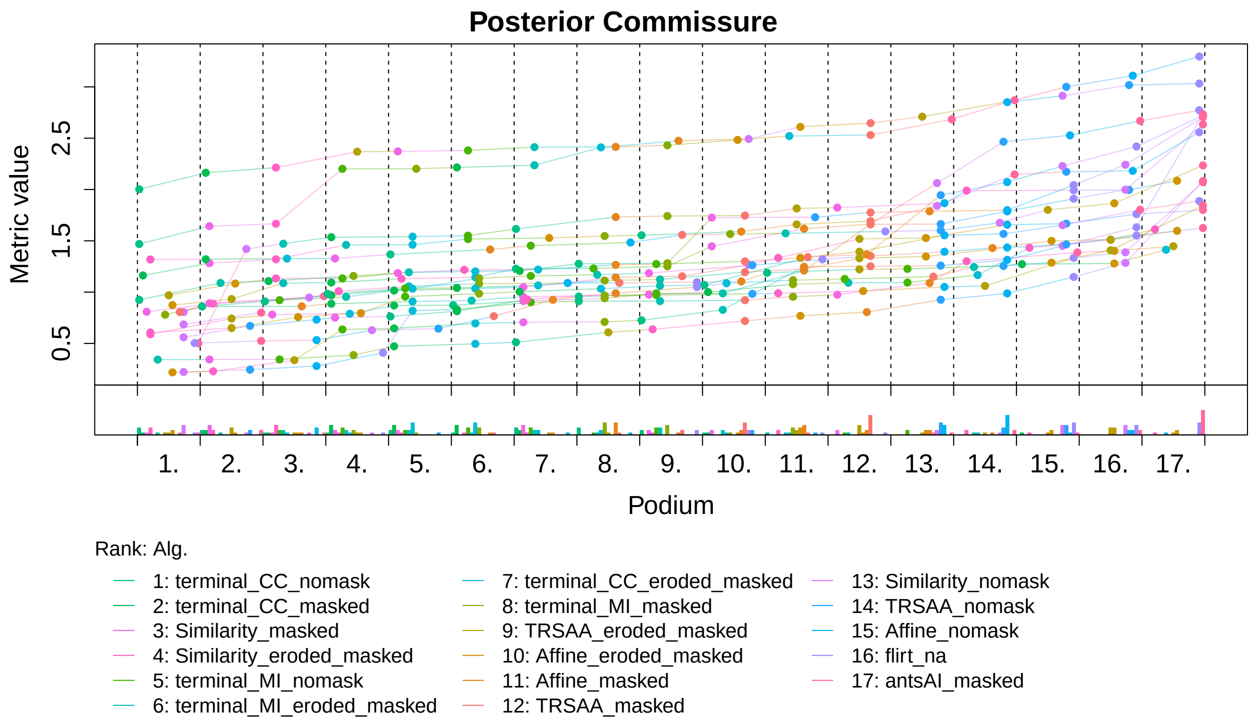
Posterior Commissure
Metric value
0.5
1.5
2.5
Podium
1. 2. 3. 4. 5. 6. 7. 8. 9. 10. 11. 12. 13. 14. 15. 16. 17.
Rank: Alg.
1: terminal_CC_nomask
2: terminal_CC_masked
3: Similarity_masked
4: Similarity_eroded_masked
5: terminal_MI_nomask
6: terminal_MI_eroded_masked
7: terminal_CC_eroded_masked
8: terminal_MI_masked
9: TRSAA_eroded_masked
10: Affine_eroded_masked
11: Affine_masked
12: TRSAA_masked
13: Similarity_nomask
14: TRSAA_nomask
15: Affine_nomask
16: flirt_na
17: antsAI_masked

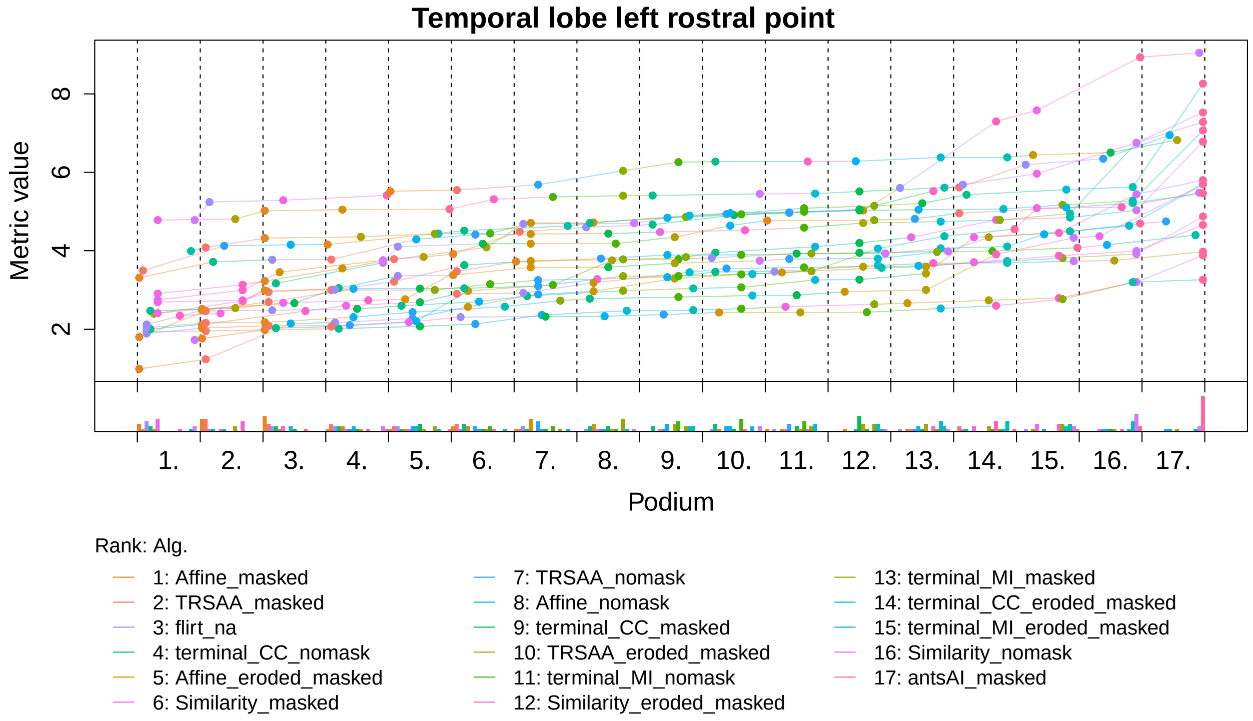
Temporal lobe left rostral point
Metric value
2
4
6
8
Podium
1. 2. 3. 4. 5. 6. 7. 8. 9. 10. 11. 12. 13. 14. 15. 16. 17.
Rank: Alg.
1: Affine_masked
2: TRSAA_masked
3: flirt_na
4: terminal_CC_nomask
5: Affine_eroded_masked
6: Similarity_masked
7: TRSAA_nomask
8: Affine_nomask
9: terminal_CC_masked
10: TRSAA_eroded_masked
11: terminal_MI_nomask
12: Similarity_eroded_masked
13: terminal_MI_masked
14: terminal_CC_eroded_masked
15: terminal_MI_eroded_masked
16: Similarity_nomask
17: antsAI_masked

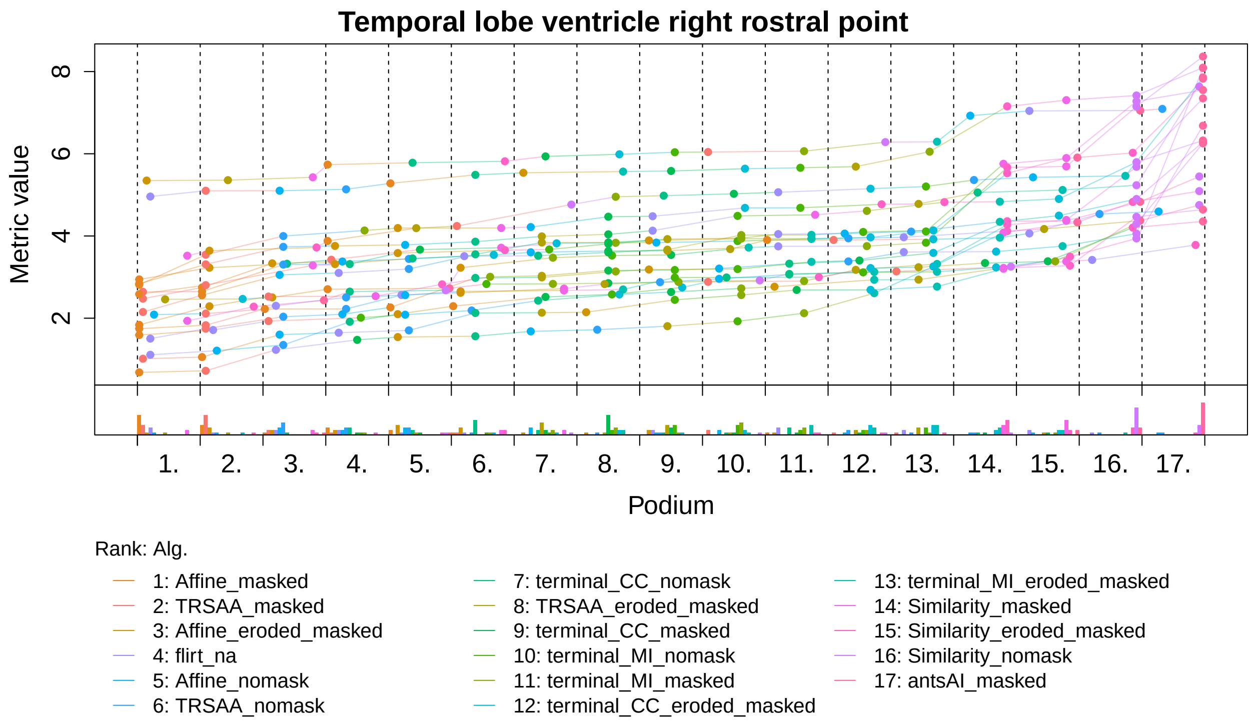
Temporal lobe ventricle right rostral point
Metric value
2
4
6
8
1.
2.
3.
4.
5.
6.
7.
8.
9.
10.
11.
12.
13.
14.
15.
16.
17.
Podium
Rank: Alg.
1: Affine_masked
2: TRSAA_masked
3: Affine_eroded_masked
4: flirt_na
5: Affine_nomask
6: TRSAA_nomask
7: terminal_CC_nomask
8: TRSAA_eroded_masked
9: terminal_CC_masked
10: terminal_MI_nomask
11: terminal_MI_masked
12: terminal_CC_eroded_masked
13: terminal_MI_eroded_masked
14: Similarity_masked
15: Similarity_eroded_masked
16: Similarity_nomask
17: antsAI_masked

2.3 Ranking heatmap

Ranking heatmaps for visualizing raw assessment data. Each cell (i, A_j) shows the absolute frequency of cases in which algorithm A_j achieved rank i.

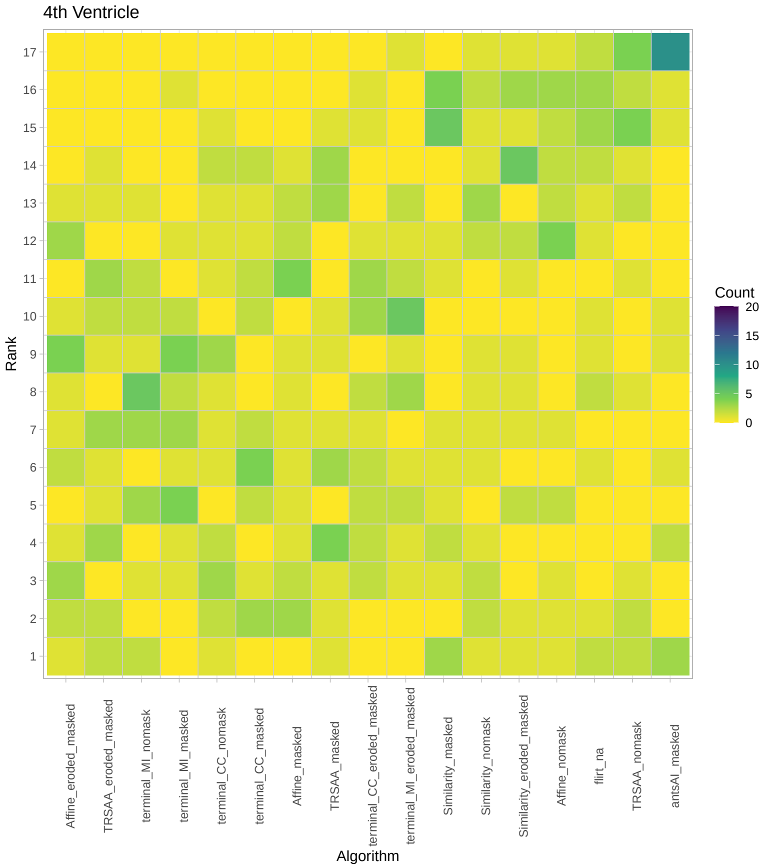

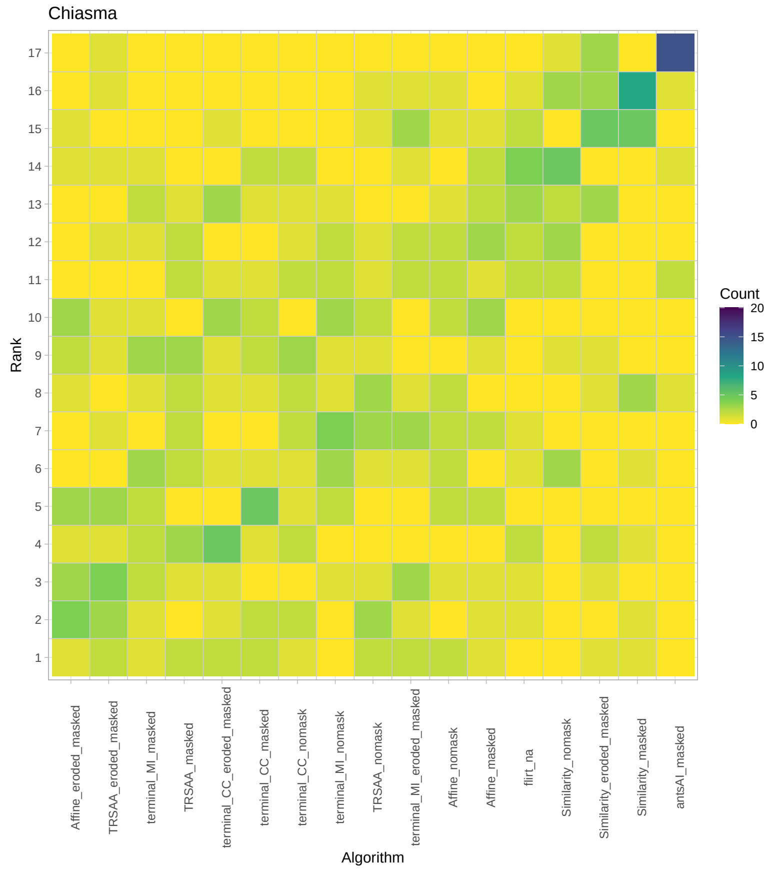
Chiasma
Rank
17
16
15
14
13
12
11
10
9
8
7
6
5
4
3
2
1
Affine_eroded_masked
TRSAA_eroded_masked
terminal_MI_masked
TRSAA_masked
terminal_CC_eroded_masked
terminal_CC_masked
terminal_CC_nomask
terminal_MI_nomask
TRSAA_nomask
terminal_MI_eroded_masked
Affine_nomask
Affine_masked
flirt_na
Similarity_nomask
Similarity_eroded_masked
Similarity_masked
antsAI_masked
Algorithm
Count
20
15
10
5
0

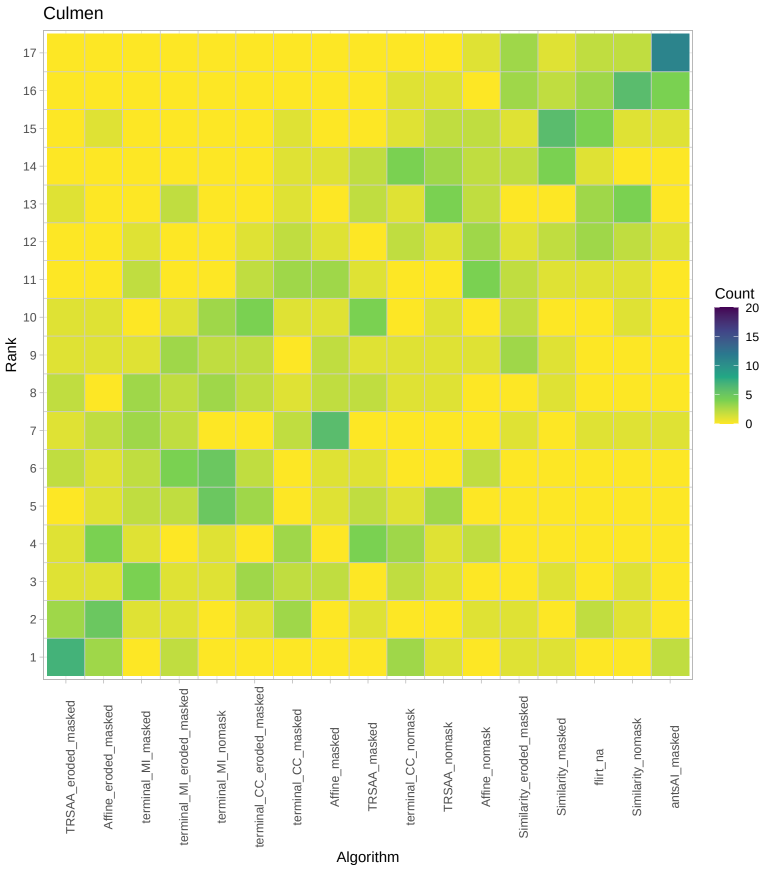
Culmen
Rank
Algorithm
Count
20
15
10
5
0
17
16
15
14
13
12
11
10
9
8
7
6
5
4
3
2
1
TRSAA_eroded_masked
Affine_eroded_masked
terminal_MI_masked
terminal_MI_eroded_masked
terminal_MI_nomask
terminal_CC_eroded_masked
terminal_CC_masked
Affine_masked
TRSAA_masked
terminal_CC_nomask
TRSAA_nomask
Affine_nomask
Similarity_eroded_masked
Similarity_masked
flirt_na
Similarity_nomask
antsAI_masked

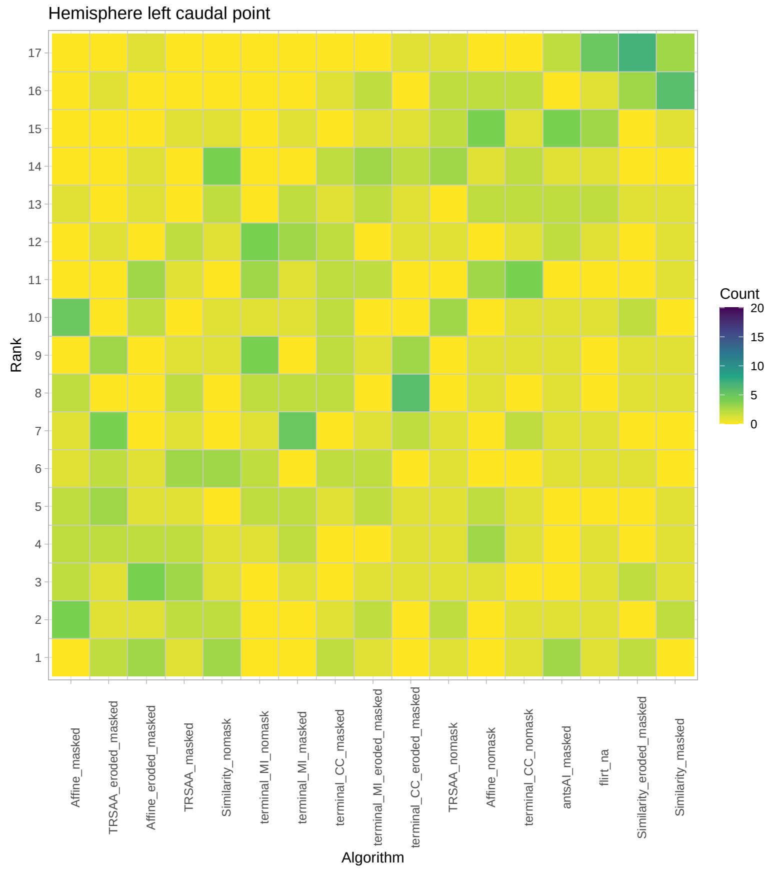

Hemisphere left caudal point
Rank
17
16
15
14
13
12
11
10
9
8
7
6
5
4
3
2
1
Count
20
15
10
5
0
Affine_masked
TRSAA_eroded_masked
Affine_eroded_masked
TRSAA_masked
Similarity_nomask
terminal_MI_nomask
terminal_MI_masked
terminal_CC_masked
terminal_MI_eroded_masked
terminal_CC_eroded_masked
TRSAA_nomask
Affine_nomask
terminal_CC_nomask
antsAI_masked
flirt_na
Similarity_eroded_masked
Similarity_masked
Algorithm

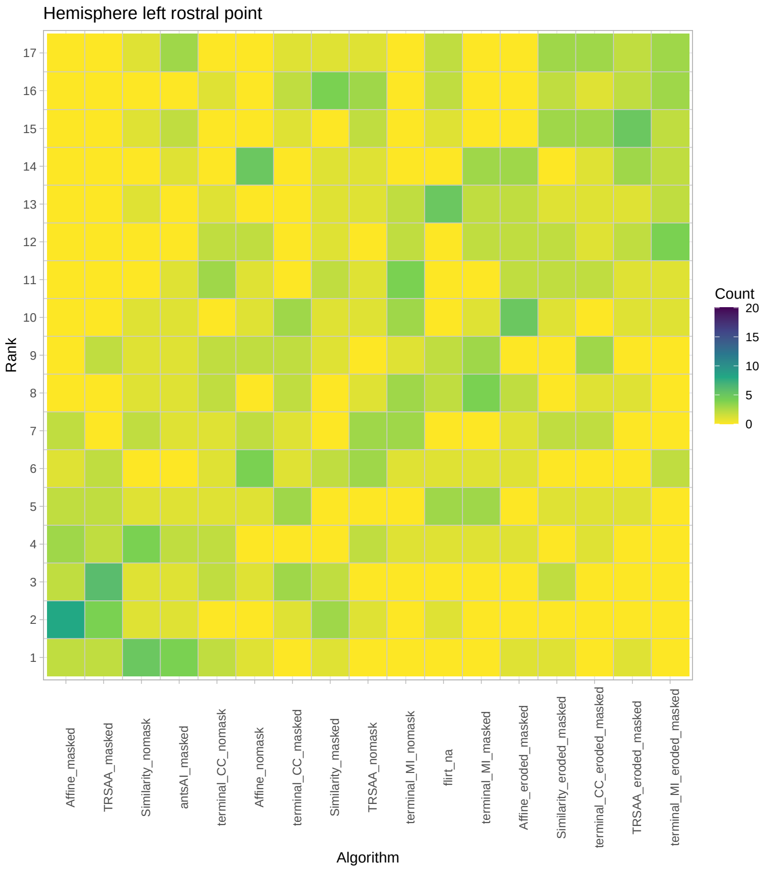
Hemisphere left rostral point
Rank
17
16
15
14
13
12
11
10
9
8
7
6
5
4
3
2
1
Count
20
15
10
5
0
Affine_masked
TRSAA_masked
Similarity_nomask
antsAI_masked
terminal_CC_nomask
Affine_nomask
terminal_CC_masked
Similarity_masked
TRSAA_nomask
terminal_MI_nomask
flirt_na
terminal_MI_masked
Affine_eroded_masked
Similarity_eroded_masked
terminal_CC_eroded_masked
TRSAA_eroded_masked
terminal_MI_eroded_masked
Algorithm

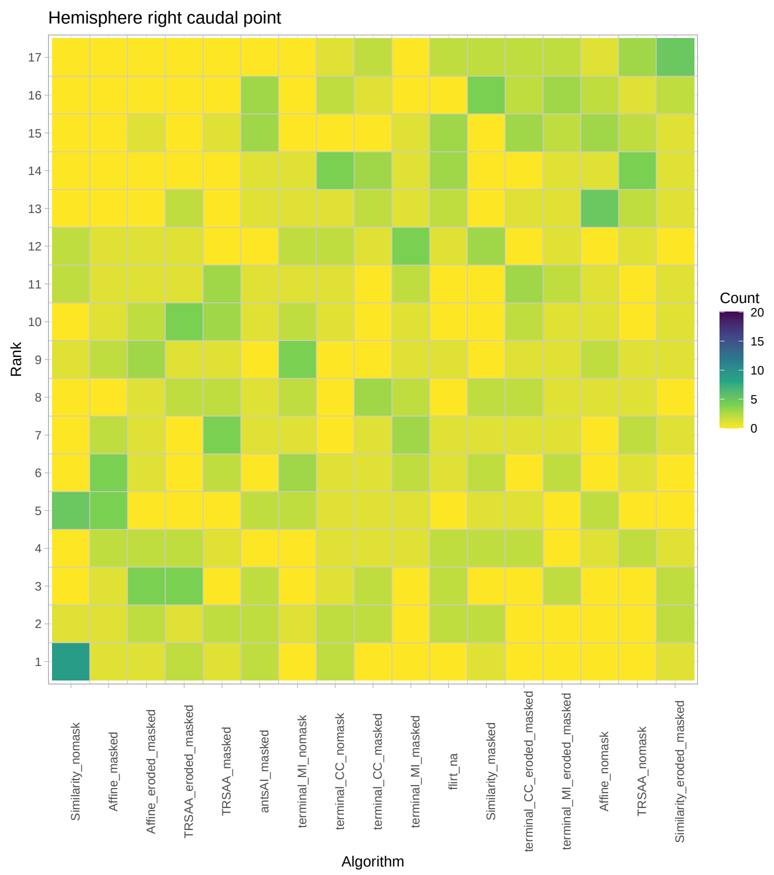
Hemisphere right caudal point
Rank
17
16
15
14
13
12
11
10
9
8
7
6
5
4
3
2
1
Count
20
15
10
5
0
Similarity_nomask
Affine_masked
Affine_eroded_masked
TRSAA_eroded_masked
TRSAA_masked
antsAI_masked
terminal_MI_nomask
terminal_CC_nomask
terminal_CC_masked
terminal_MI_masked
flirt_na
Similarity_masked
terminal_CC_eroded_masked
terminal_MI_eroded_masked
Affine_nomask
TRSAA_nomask
Similarity_eroded_masked
Algorithm

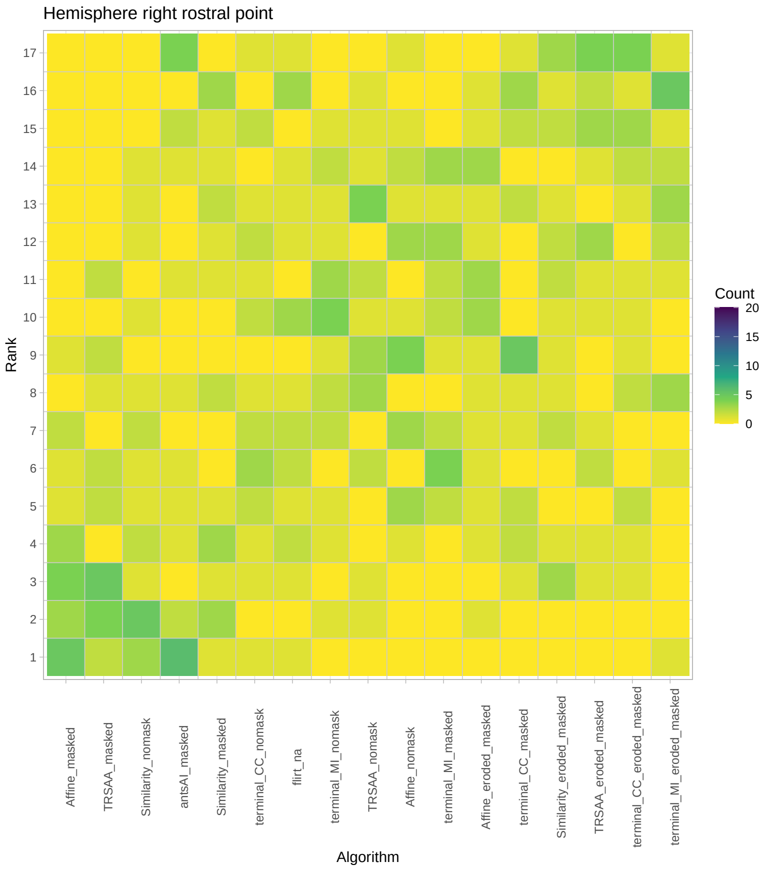
Hemisphere right rostral point
Rank
17
16
15
14
13
12
11
10
9
8
7
6
5
4
3
2
1
Algorithm
Affine_masked
TRSAA_masked
Similarity_nomask
antsAI_masked
Similarity_masked
terminal_CC_nomask
flirt_na
terminal_MI_nomask
TRSAA_nomask
Affine_nomask
terminal_MI_masked
Affine_eroded_masked
terminal_CC_masked
Similarity_eroded_masked
TRSAA_eroded_masked
terminal_CC_eroded_masked
terminal_MI_eroded_masked
Count
20
15
10
5
0

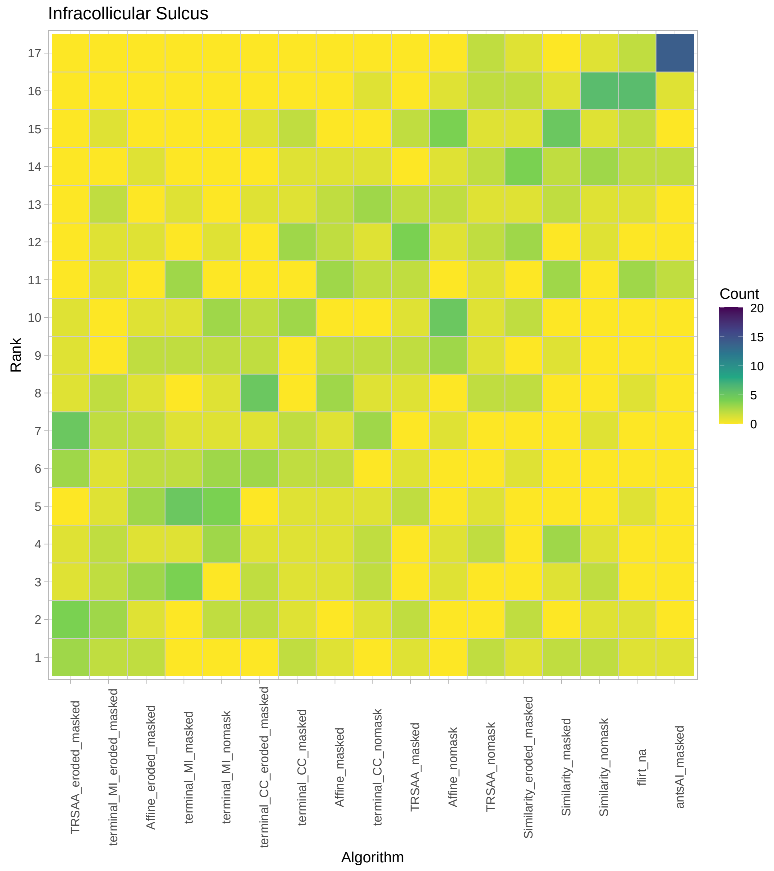
Infracollicular Sulcus
Rank
17
16
15
14
13
12
11
10
9
8
7
6
5
4
3
2
1
Count
20
15
10
5
0
TRSAA_eroded_masked
terminal_MI_eroded_masked
Affine_eroded_masked
terminal_MI_masked
terminal_MI_nomask
terminal_CC_eroded_masked
terminal_CC_masked
Affine_masked
terminal_CC_nomask
TRSAA_masked
Affine_nomask
TRSAA_nomask
Similarity_eroded_masked
Similarity_masked
Similarity_nomask
flirt_na
antsAI_masked
Algorithm

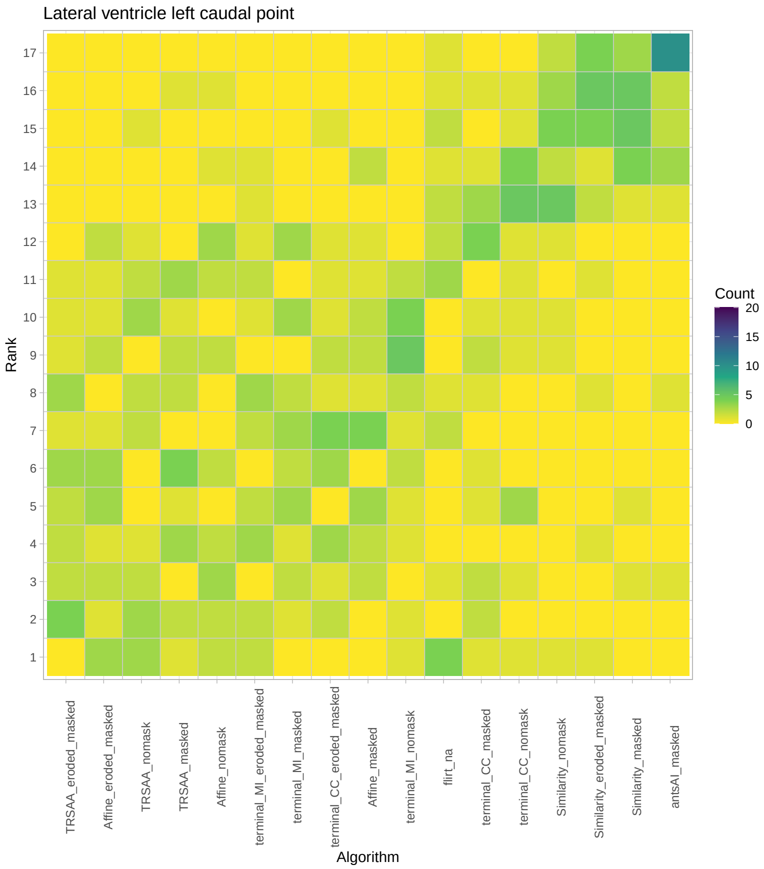
Lateral ventricle left caudal point
Rank
17
16
15
14
13
12
11
10
9
8
7
6
5
4
3
2
1
Count
20
15
10
5
0
TRSAA_eroded_masked
Affine_eroded_masked
TRSAA_nomask
TRSAA_masked
Affine_nomask
terminal_MI_eroded_masked
terminal_MI_masked
terminal_CC_eroded_masked
Affine_masked
terminal_MI_nomask
flirt_na
terminal_CC_masked
terminal_CC_nomask
Similarity_nomask
Similarity_eroded_masked
Similarity_masked
antsAI_masked
Algorithm

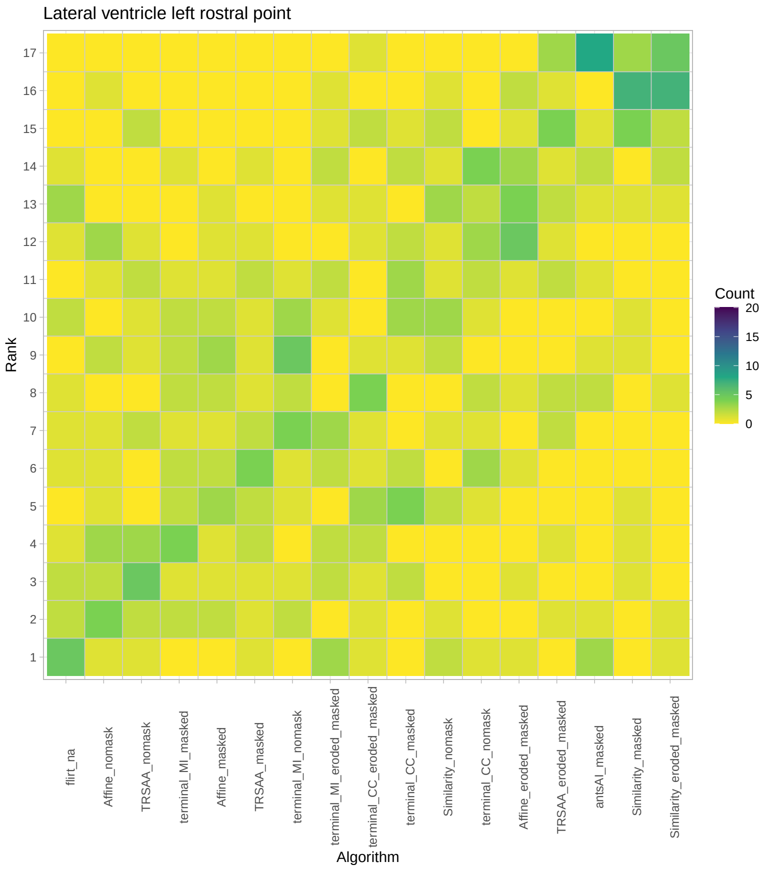

Lateral ventricle left rostral point
Rank
17
16
15
14
13
12
11
10
9
8
7
6
5
4
3
2
1
flirt_na
Affine_nomask
TRSAA_nomask
terminal_MI_masked
Affine_masked
TRSAA_masked
terminal_MI_nomask
terminal_MI_eroded_masked
terminal_CC_eroded_masked
terminal_CC_masked
Similarity_nomask
terminal_CC_nomask
Affine_eroded_masked
TRSAA_eroded_masked
antsAI_masked
Similarity_masked
Similarity_eroded_masked
Algorithm
Count
20
15
10
5
0

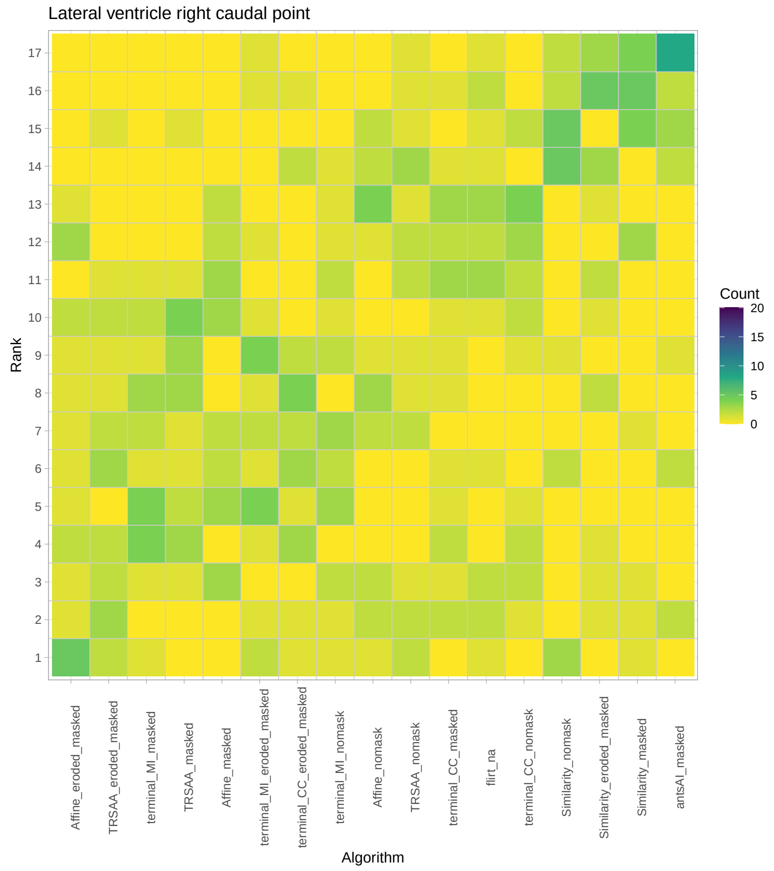
Lateral ventricle right caudal point
Rank
Algorithm
Count
20
15
10
5
0
17
16
15
14
13
12
11
10
9
8
7
6
5
4
3
2
1
Affine_eroded_masked
TRSAA_eroded_masked
terminal_MI_masked
TRSAA_masked
Affine_masked
terminal_MI_eroded_masked
terminal_CC_eroded_masked
terminal_MI_nomask
Affine_nomask
TRSAA_nomask
terminal_CC_masked
flirt_na
terminal_CC_nomask
Similarity_nomask
Similarity_eroded_masked
Similarity_masked
antsAI_masked

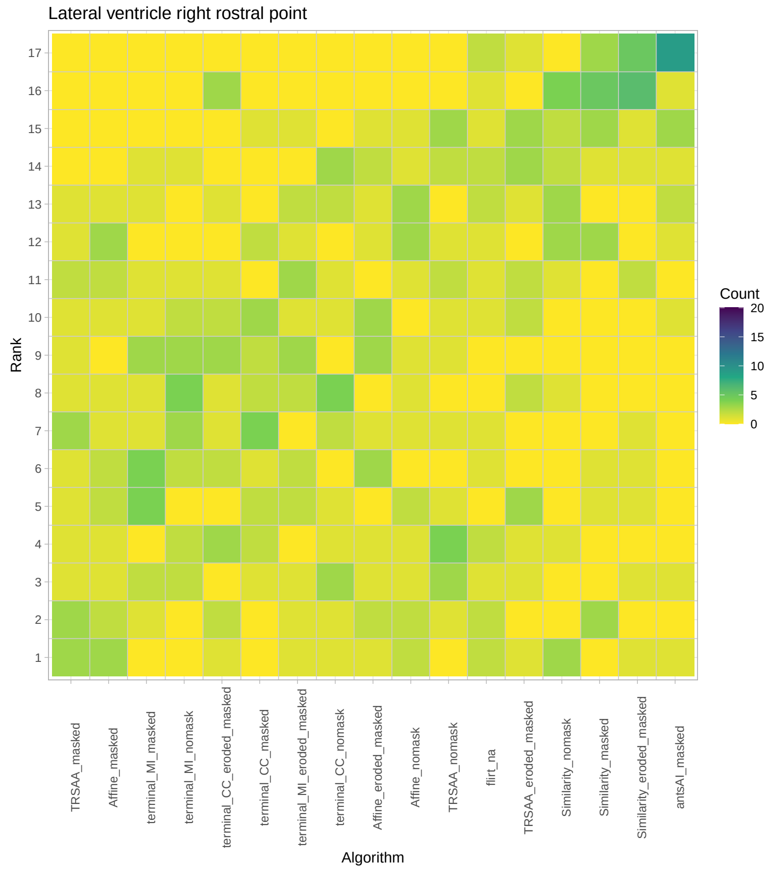
Lateral ventricle right rostral point
Rank
17
16
15
14
13
12
11
10
9
8
7
6
5
4
3
2
1
TRSAA_masked
Affine_masked
terminal_MI_masked
terminal_MI_nomask
terminal_CC_eroded_masked
terminal_CC_masked
terminal_MI_eroded_masked
terminal_CC_nomask
Affine_eroded_masked
Affine_nomask
TRSAA_nomask
flirt_na
TRSAA_eroded_masked
Similarity_nomask
Similarity_masked
Similarity_eroded_masked
antsAI_masked
Algorithm
Count
20
15
10
5
0

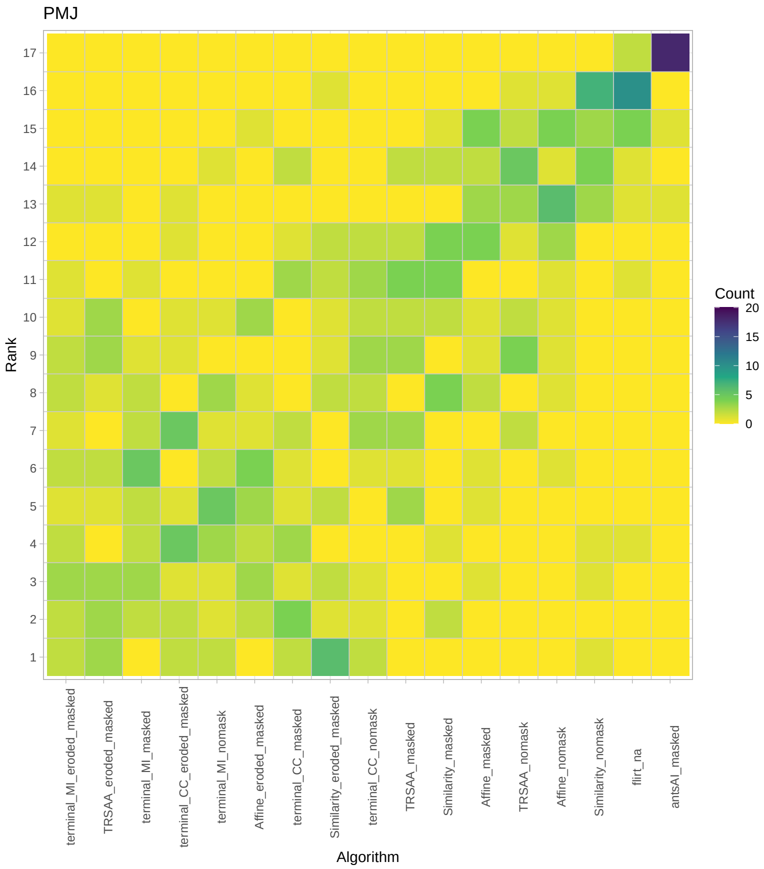
PMJ
Rank
17
16
15
14
13
12
11
10
9
8
7
6
5
4
3
2
1
terminal_MI_eroded_masked
TRSAA_eroded_masked
terminal_MI_masked
terminal_CC_eroded_masked
terminal_MI_nomask
Affine_eroded_masked
terminal_CC_masked
Similarity_eroded_masked
terminal_CC_nomask
TRSAA_masked
Similarity_masked
Affine_masked
TRSAA_nomask
Affine_nomask
Similarity_nomask
flirt_na
antsAI_masked
Algorithm
Count
20
15
10
5
0

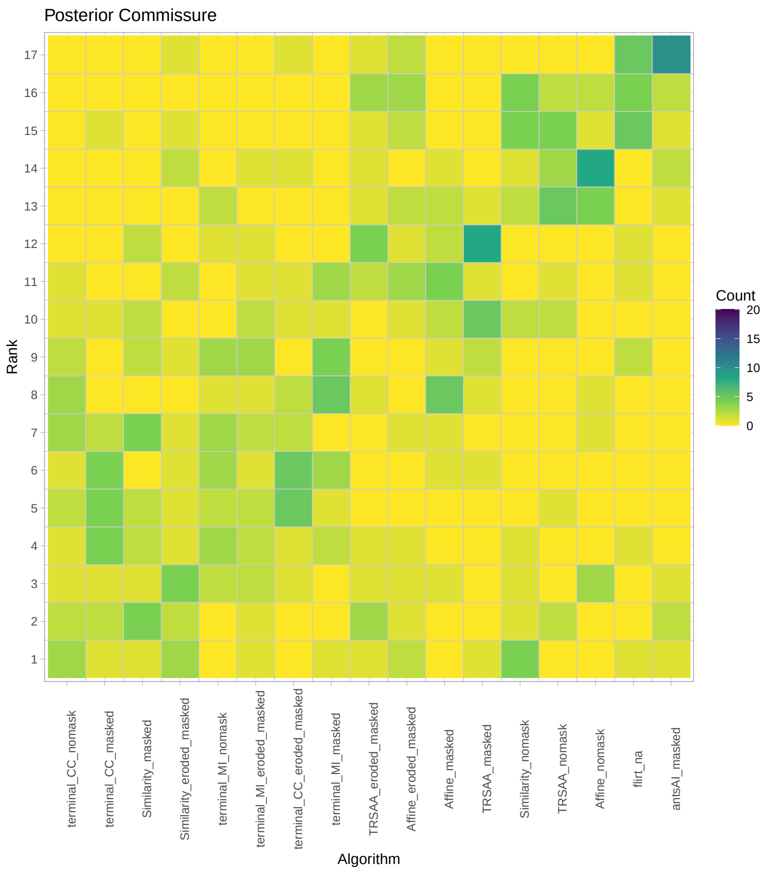
Posterior Commissure
Rank
17
16
15
14
13
12
11
10
9
8
7
6
5
4
3
2
1
Count
20
15
10
5
0
terminal_CC_nomask
terminal_CC_masked
Similarity_masked
Similarity_eroded_masked
terminal_MI_nomask
terminal_MI_eroded_masked
terminal_CC_eroded_masked
terminal_MI_masked
TRSAA_eroded_masked
Affine_eroded_masked
Affine_masked
TRSAA_masked
Similarity_nomask
TRSAA_nomask
Affine_nomask
flirt_na
antsAI_masked
Algorithm

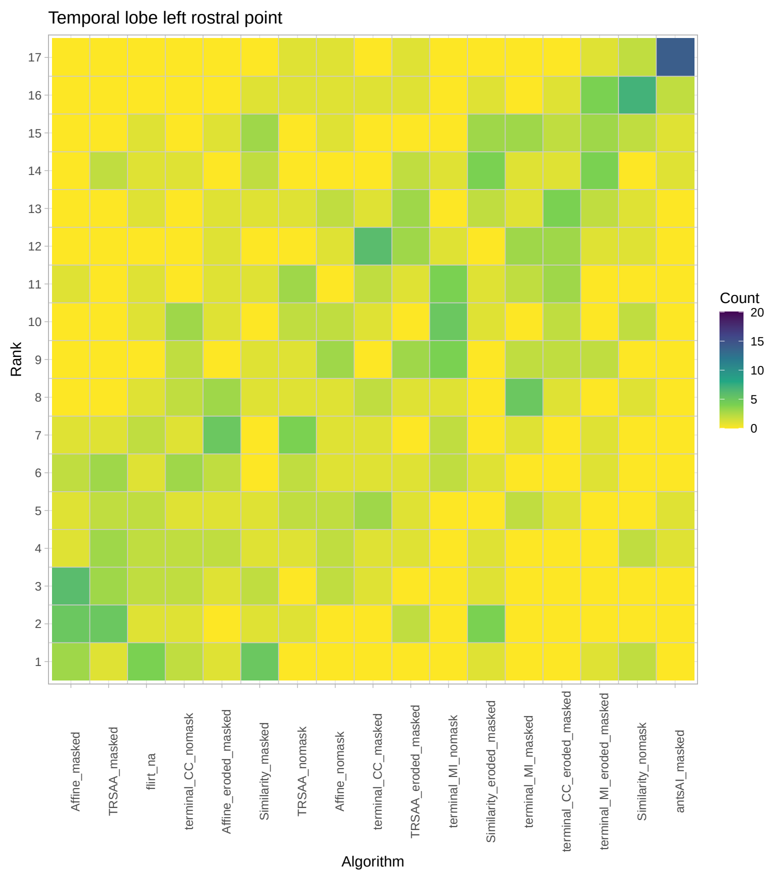
Temporal lobe left rostral point
Rank
17
16
15
14
13
12
11
10
9
8
7
6
5
4
3
2
1
Count
20
15
10
5
0
Affine_masked
TRSAA_masked
flirt_na
terminal_CC_nomask
Affine_eroded_masked
Similarity_masked
TRSAA_nomask
Affine_nomask
terminal_CC_masked
TRSAA_eroded_masked
terminal_MI_nomask
Similarity_eroded_masked
terminal_MI_masked
terminal_CC_eroded_masked
terminal_MI_eroded_masked
Similarity_nomask
antsAI_masked
Algorithm

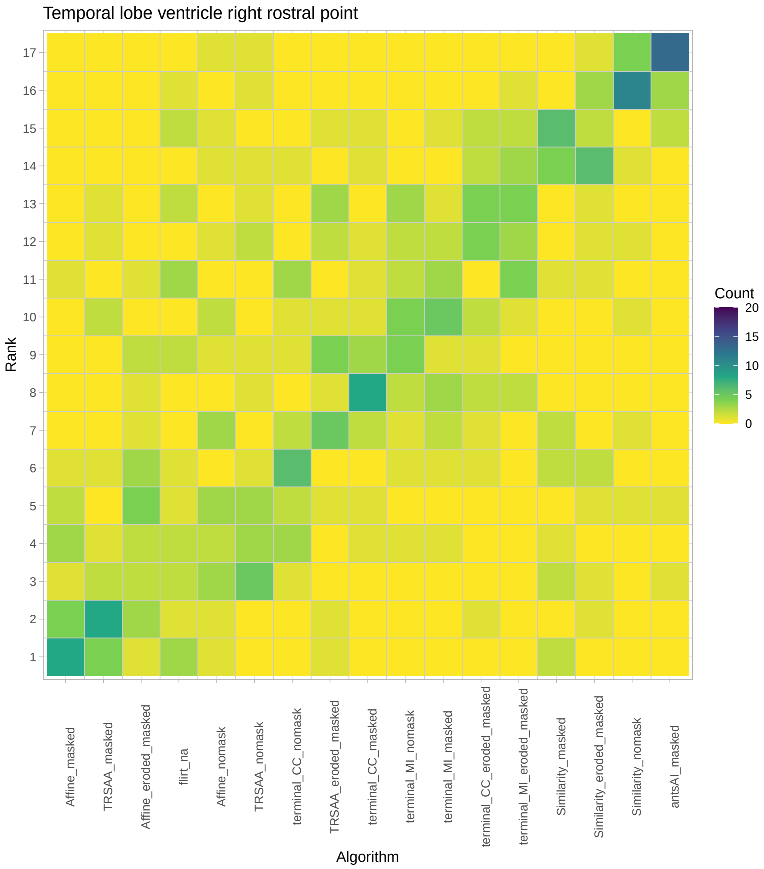
Temporal lobe ventricle right rostral point
Rank
17
16
15
14
13
12
11
10
9
8
7
6
5
4
3
2
1
Affine_masked
TRSAA_masked
Affine_eroded_masked
flirt_na
Affine_nomask
TRSAA_nomask
terminal_CC_nomask
TRSAA_eroded_masked
terminal_CC_masked
terminal_MI_nomask
terminal_MI_masked
terminal_CC_eroded_masked
terminal_MI_eroded_masked
Similarity_masked
Similarity_eroded_masked
Similarity_nomask
antsAI_masked
Algorithm
Count
20
15
10
5
0

3 Visualization of ranking stability

3.1 *Blob plot* for visualizing ranking stability based on bootstrap sampling

Algorithms are color-coded, and the area of each blob at position $(A_i, \text{rank } j)$ is proportional to the relative frequency A_i achieved rank j across $b = 1000$ bootstrap samples. The median rank for each algorithm is indicated by a black cross. 95% bootstrap intervals across bootstrap samples are indicated by black lines.

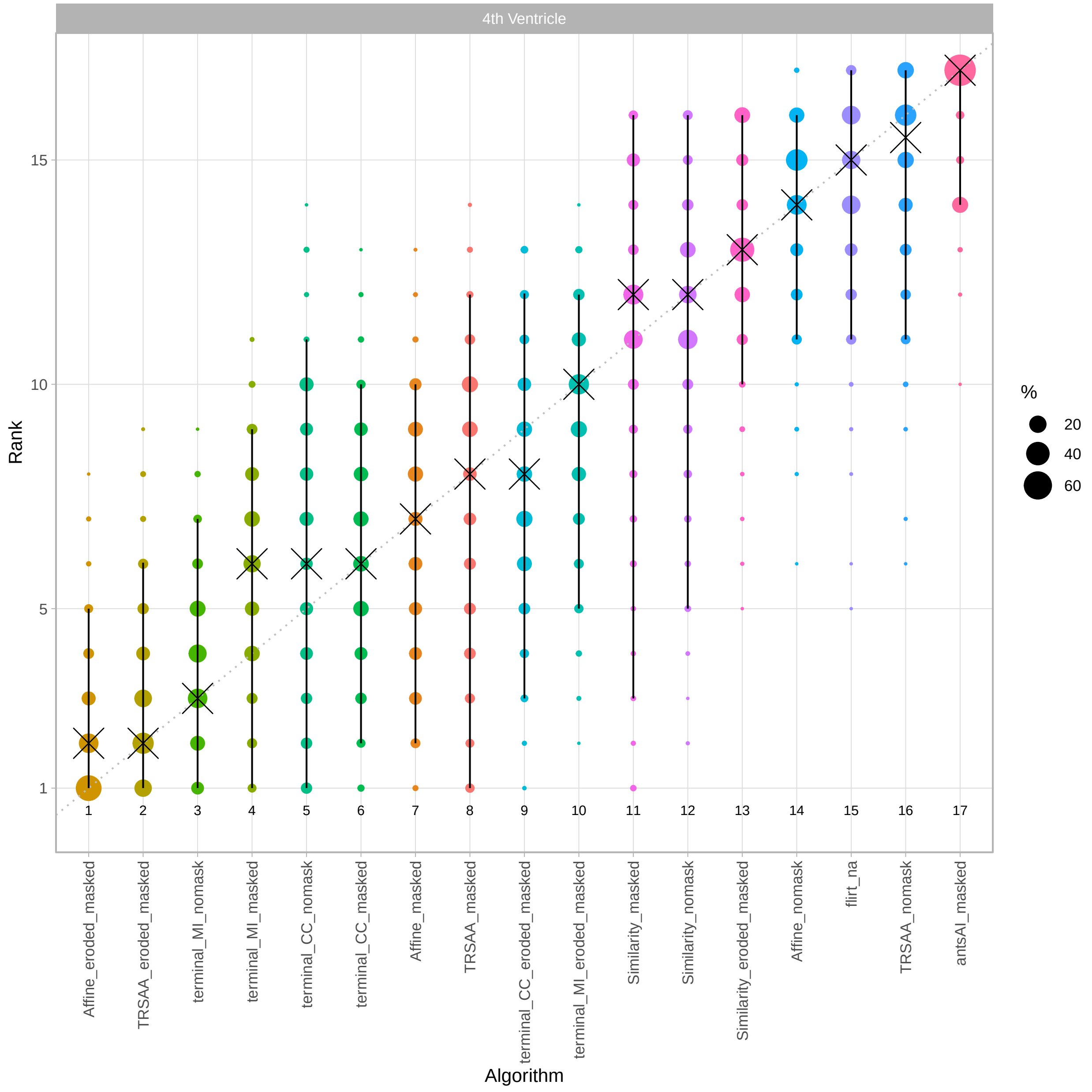

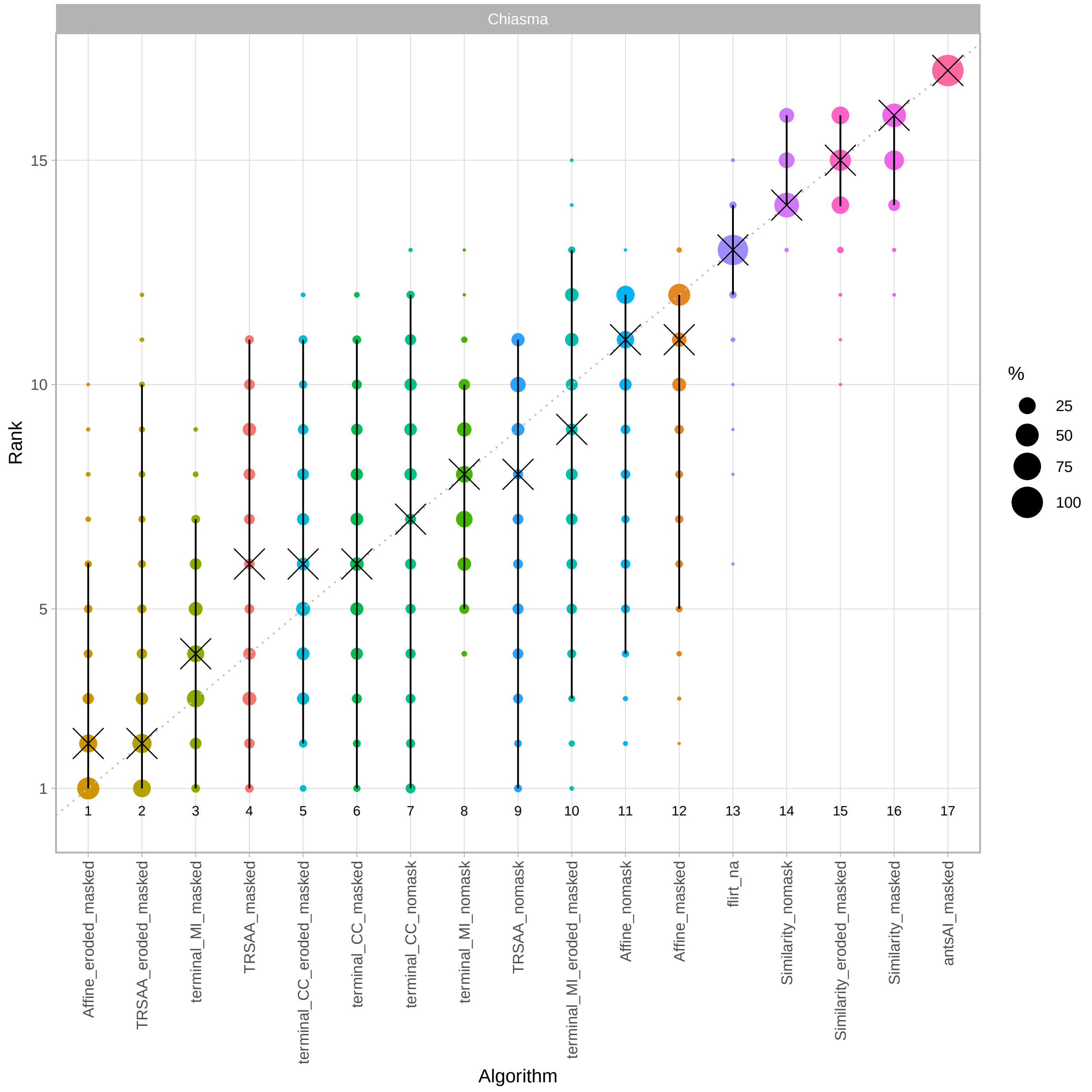
Chiasma
Rank
Algorithm
1
5
10
15
1 2 3 4 5 6 7 8 9 10 11 12 13 14 15 16 17
Affine_eroded_masked
TRSAA_eroded_masked
terminal_MI_masked
TRSAA_masked
terminal_CC_eroded_masked
terminal_CC_masked
terminal_CC_nomask
terminal_MI_nomask
TRSAA_nomask
terminal_MI_eroded_masked
Affine_nomask
Affine_masked
flirt_na
Similarity_nomask
Similarity_eroded_masked
Similarity_masked
antsAI_masked
%
25
50
75
100

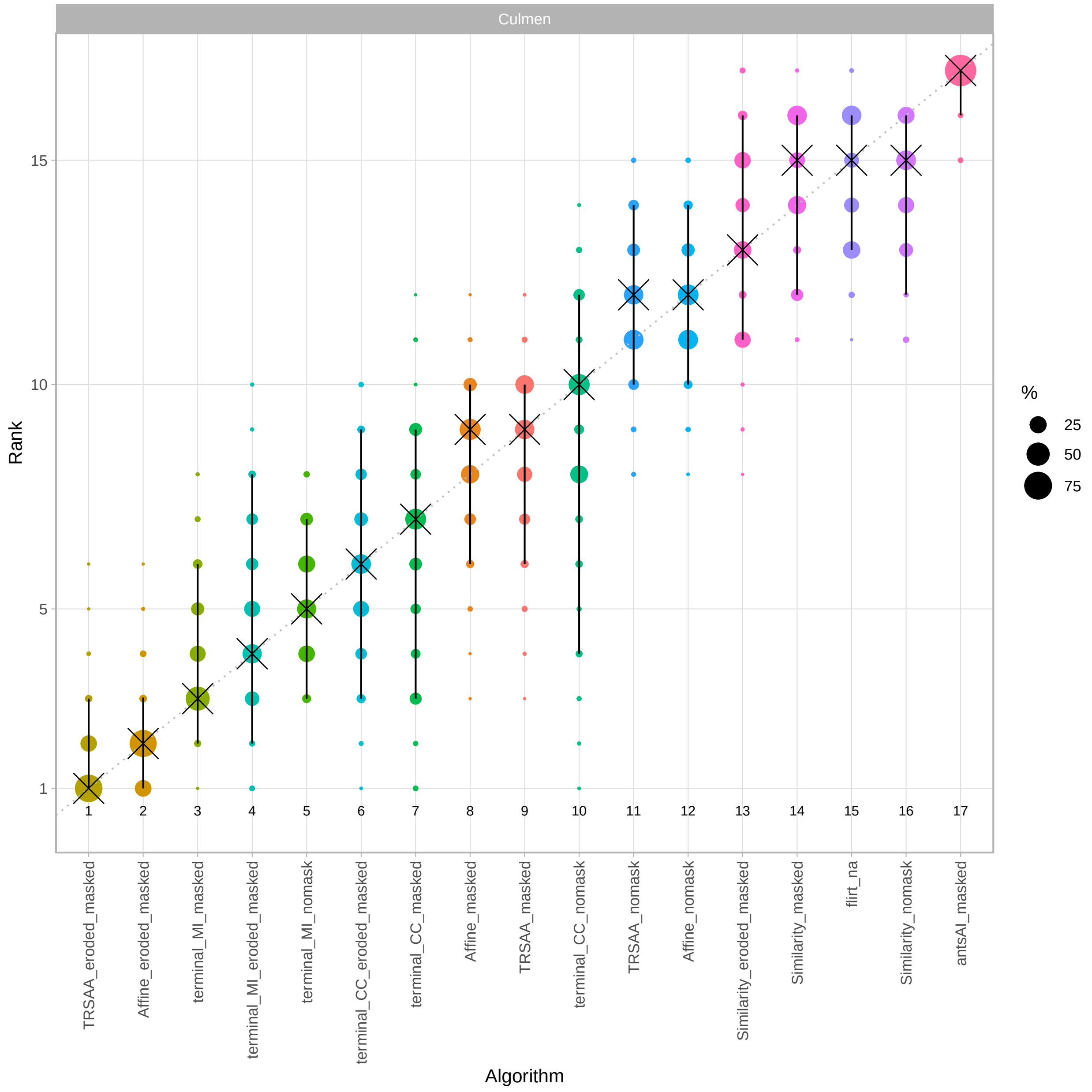

Culmen
Rank
Algorithm
%
25
50
75
1
5
10
15
1
2
3
4
5
6
7
8
9
10
11
12
13
14
15
16
17
TRSAA_eroded_masked
Affine_eroded_masked
terminal_MI_masked
terminal_MI_eroded_masked
terminal_MI_nomask
terminal_CC_eroded_masked
terminal_CC_masked
Affine_masked
TRSAA_masked
terminal_CC_nomask
TRSAA_nomask
Affine_nomask
Similarity_eroded_masked
Similarity_masked
flirt_na
Similarity_nomask
antsAI_masked

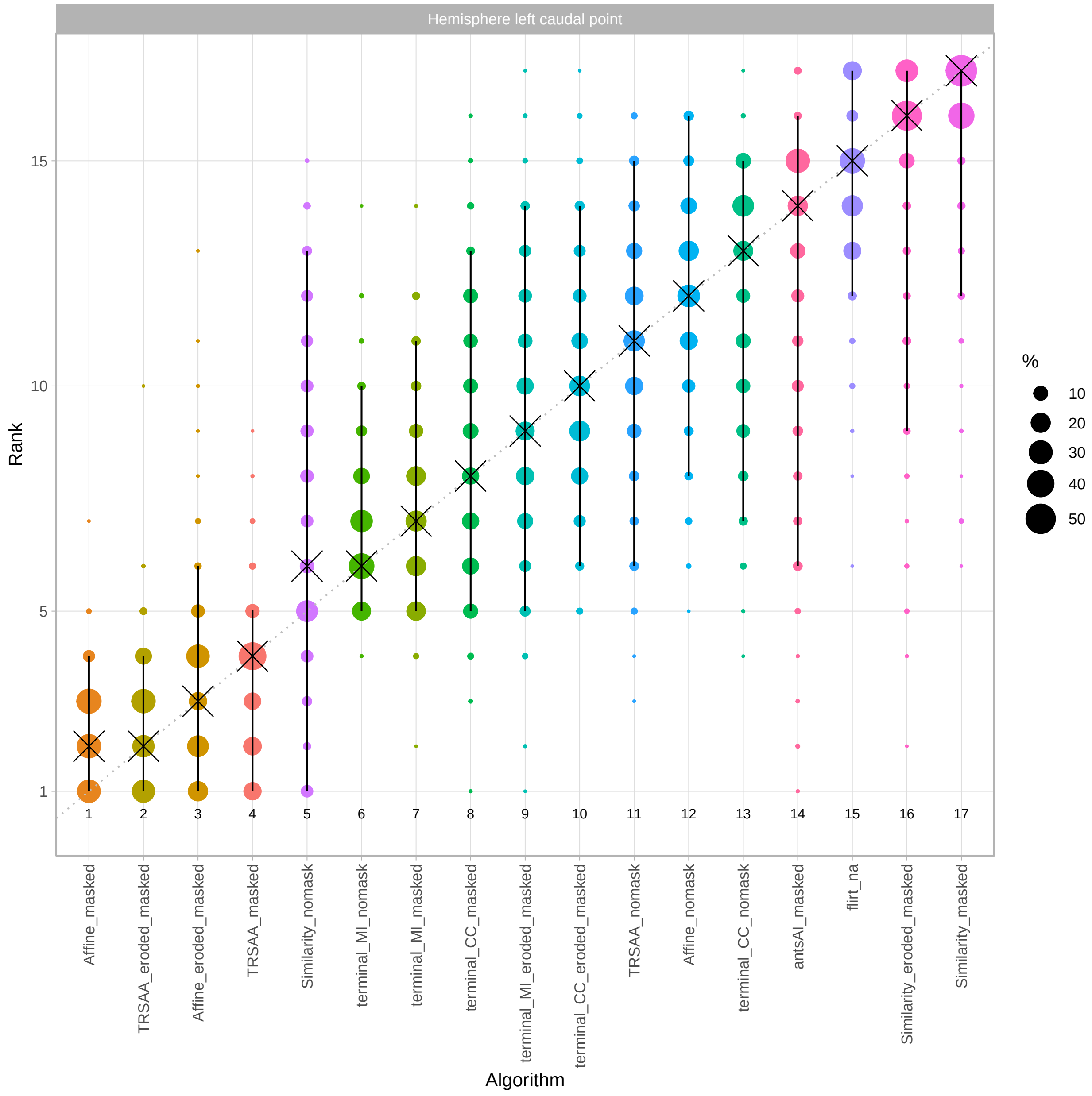

Hemisphere left caudal point
Rank
15
10
5
1
1 2 3 4 5 6 7 8 9 10 11 12 13 14 15 16 17
Affine_masked
TRSAA_eroded_masked
Affine_eroded_masked
TRSAA_masked
Similarity_nomask
terminal_MI_nomask
terminal_MI_masked
terminal_CC_masked
terminal_MI_eroded_masked
terminal_CC_eroded_masked
TRSAA_nomask
Affine_nomask
terminal_CC_nomask
antsAI_masked
flirt_na
Similarity_eroded_masked
Similarity_masked
Algorithm
%
10
20
30
40
50

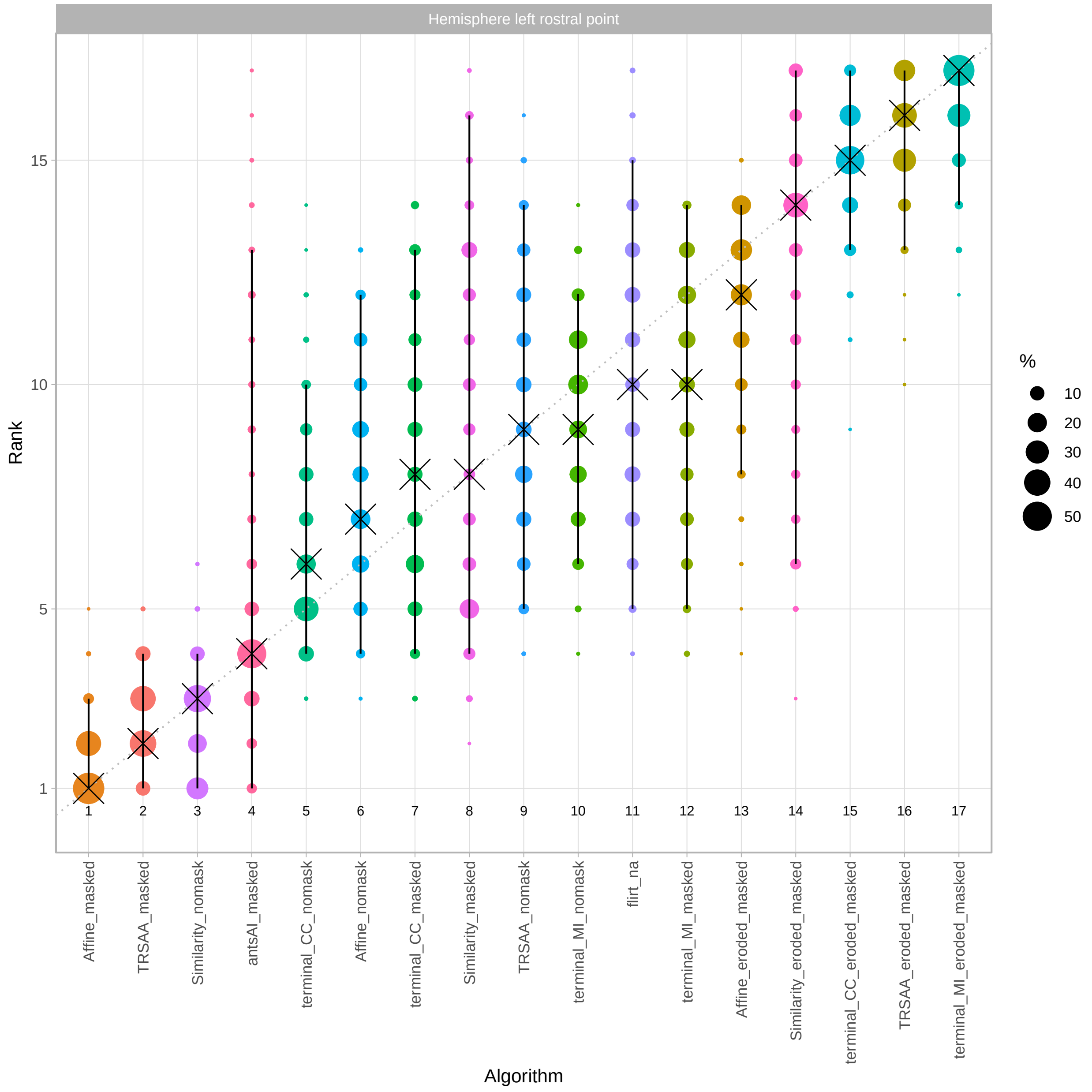

Hemisphere left rostral point
Rank
Algorithm
%
10
20
30
40
50
1
5
10
15
1 2 3 4 5 6 7 8 9 10 11 12 13 14 15 16 17
Affine_masked
TRSAA_masked
Similarity_nomask
antsAI_masked
terminal_CC_nomask
Affine_nomask
terminal_CC_masked
Similarity_masked
TRSAA_nomask
terminal_MI_nomask
flirt_na
terminal_MI_masked
Affine_eroded_masked
Similarity_eroded_masked
terminal_CC_eroded_masked
TRSAA_eroded_masked
terminal_MI_eroded_masked

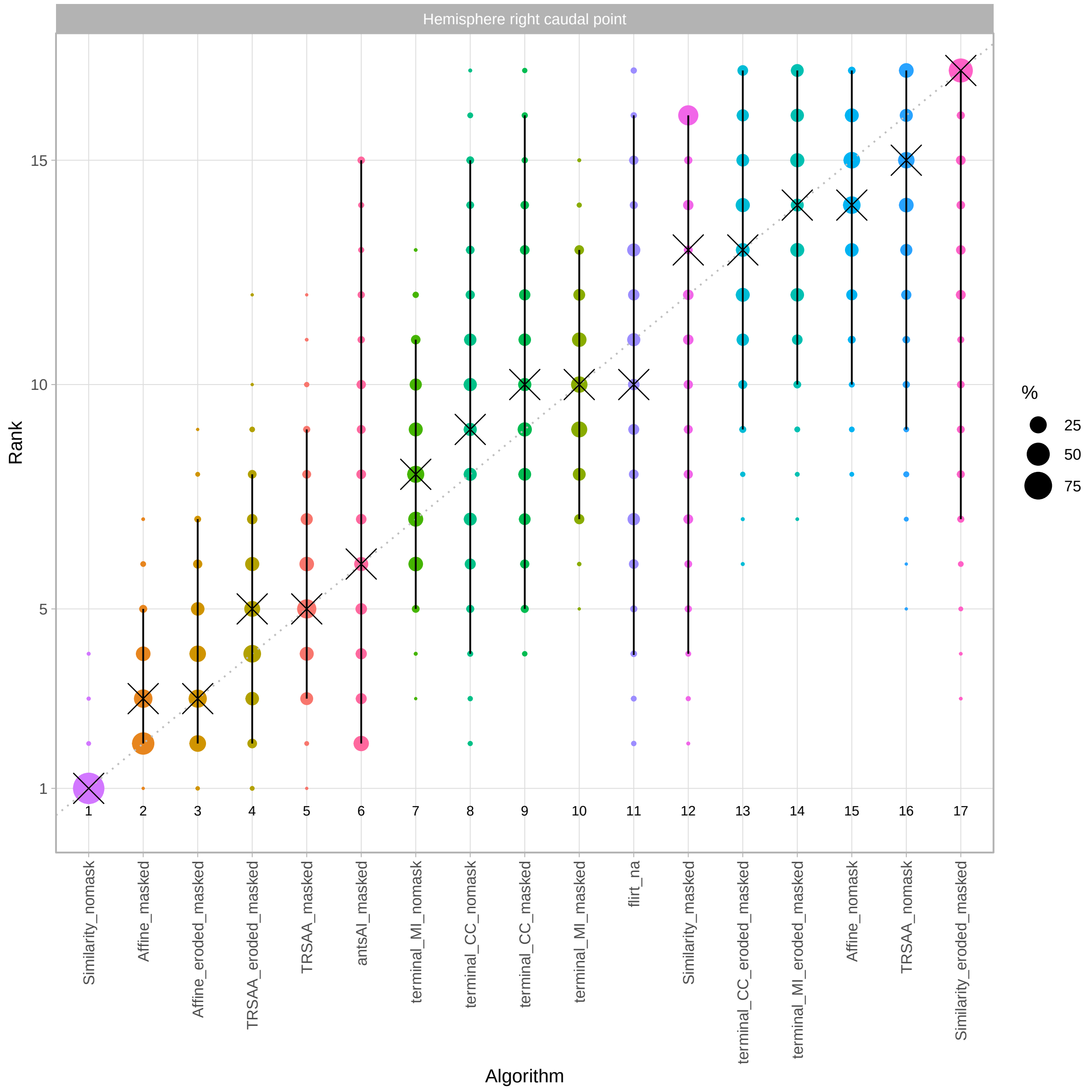

Hemisphere right caudal point
Rank
15
10
5
1
1
2
3
4
5
6
7
8
9
10
11
12
13
14
15
16
17
Similarity_nomask
Affine_masked
Affine_eroded_masked
TRSAA_eroded_masked
TRSAA_masked
antsAI_masked
terminal_MI_nomask
terminal_CC_nomask
terminal_CC_masked
terminal_MI_masked
flirt_na
Similarity_masked
terminal_CC_eroded_masked
terminal_MI_eroded_masked
Affine_nomask
TRSAA_nomask
Similarity_eroded_masked
Algorithm
%
25
50
75

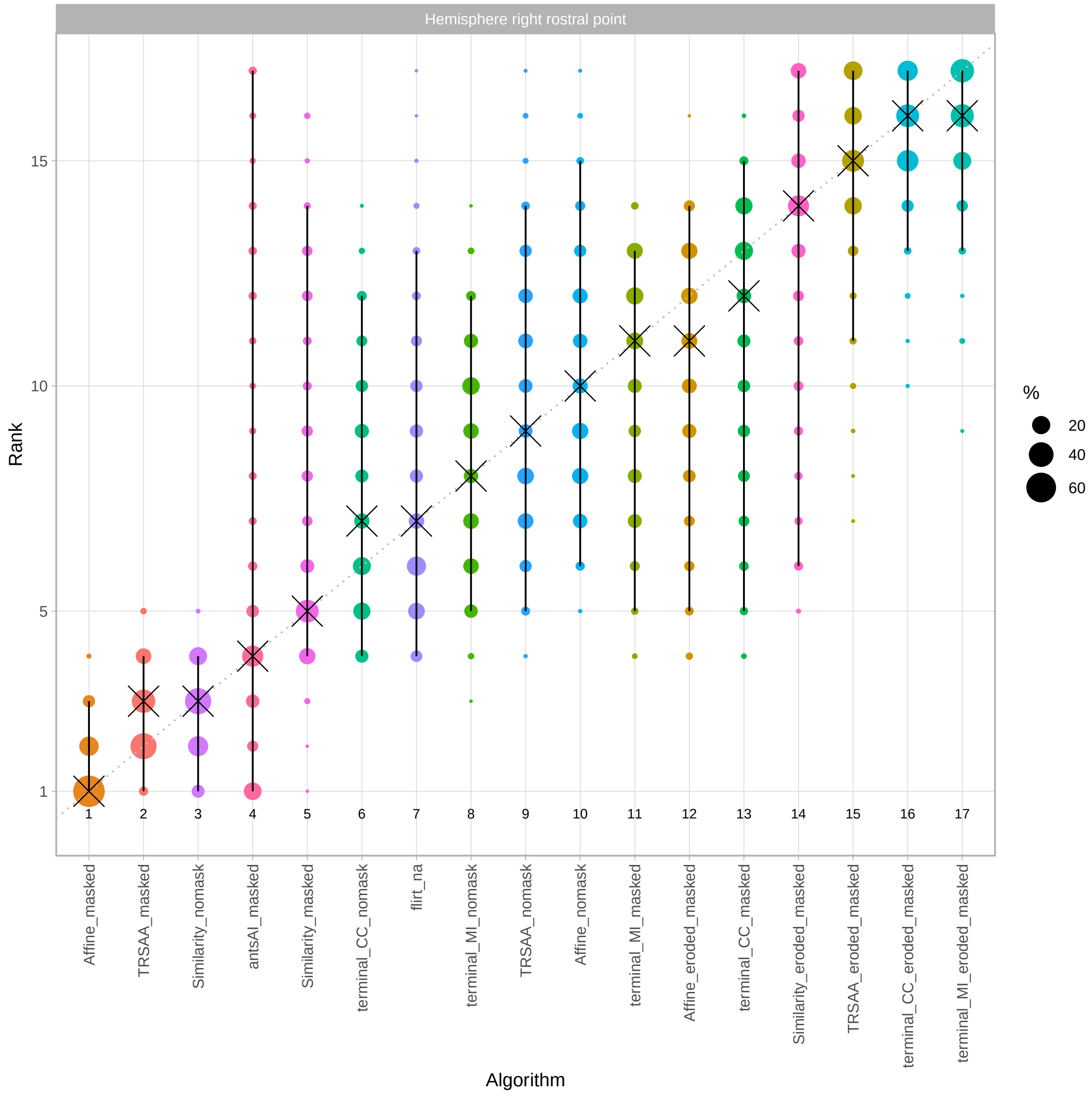
Hemisphere right rostral point
Rank
Algorithm
%
20
40
60
1
5
10
15
1
2
3
4
5
6
7
8
9
10
11
12
13
14
15
16
17
Affine_masked
TRSAA_masked
Similarity_nomask
antsAI_masked
Similarity_masked
terminal_CC_nomask
flirt_na
terminal_MI_nomask
TRSAA_nomask
Affine_nomask
terminal_MI_masked
Affine_eroded_masked
terminal_CC_masked
Similarity_eroded_masked
TRSAA_eroded_masked
terminal_CC_eroded_masked
terminal_MI_eroded_masked

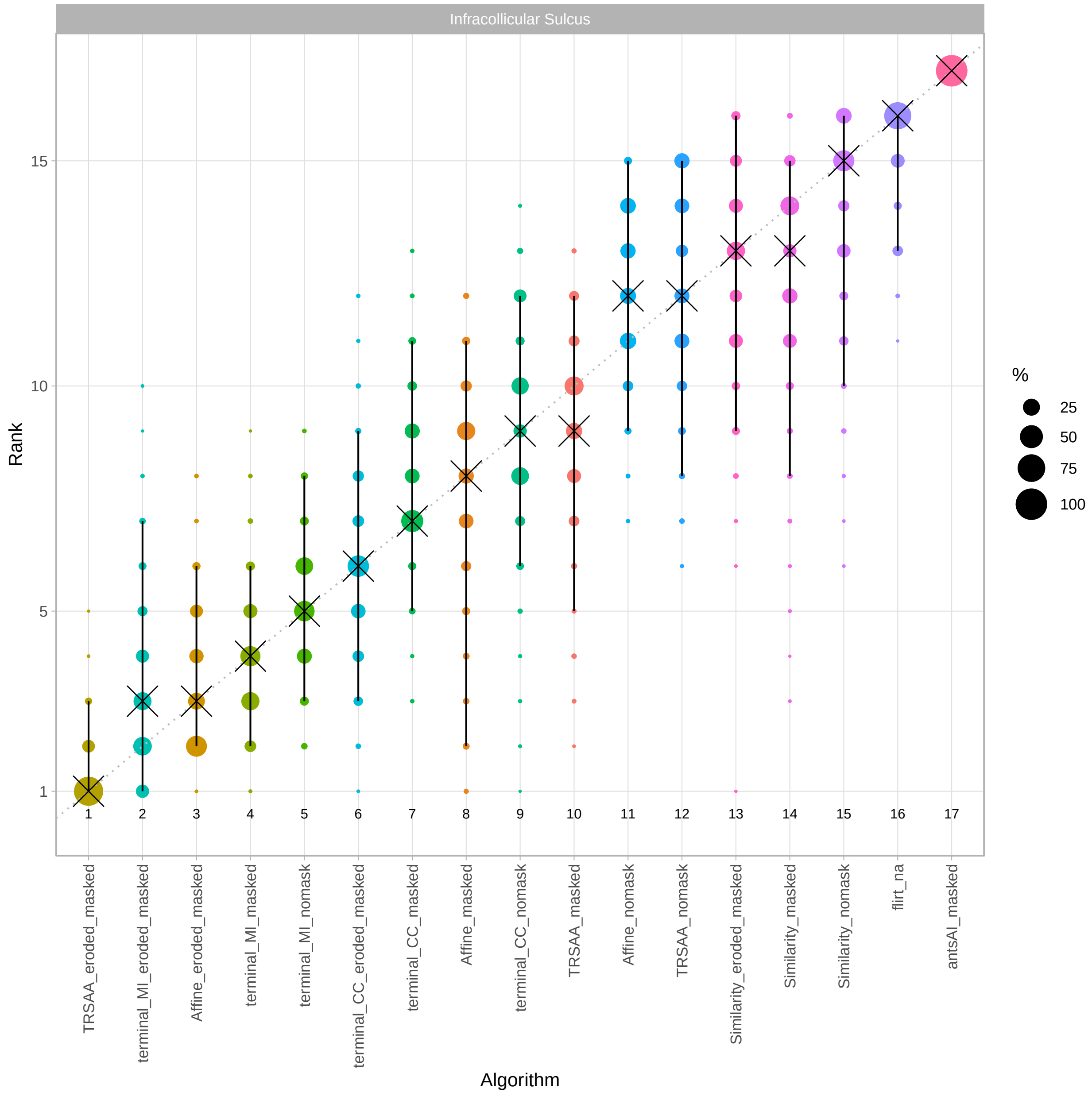
Infracollicular Sulcus
Rank
15
10
5
1
1
2
3
4
5
6
7
8
9
10
11
12
13
14
15
16
17
TRSAA_eroded_masked
terminal_MI_eroded_masked
Affine_eroded_masked
terminal_MI_masked
terminal_MI_nomask
terminal_CC_eroded_masked
terminal_CC_masked
Affine_masked
terminal_CC_nomask
TRSAA_masked
Affine_nomask
TRSAA_nomask
Similarity_eroded_masked
Similarity_masked
Similarity_nomask
flirt_na
antsAI_masked
Algorithm
%
25
50
75
100

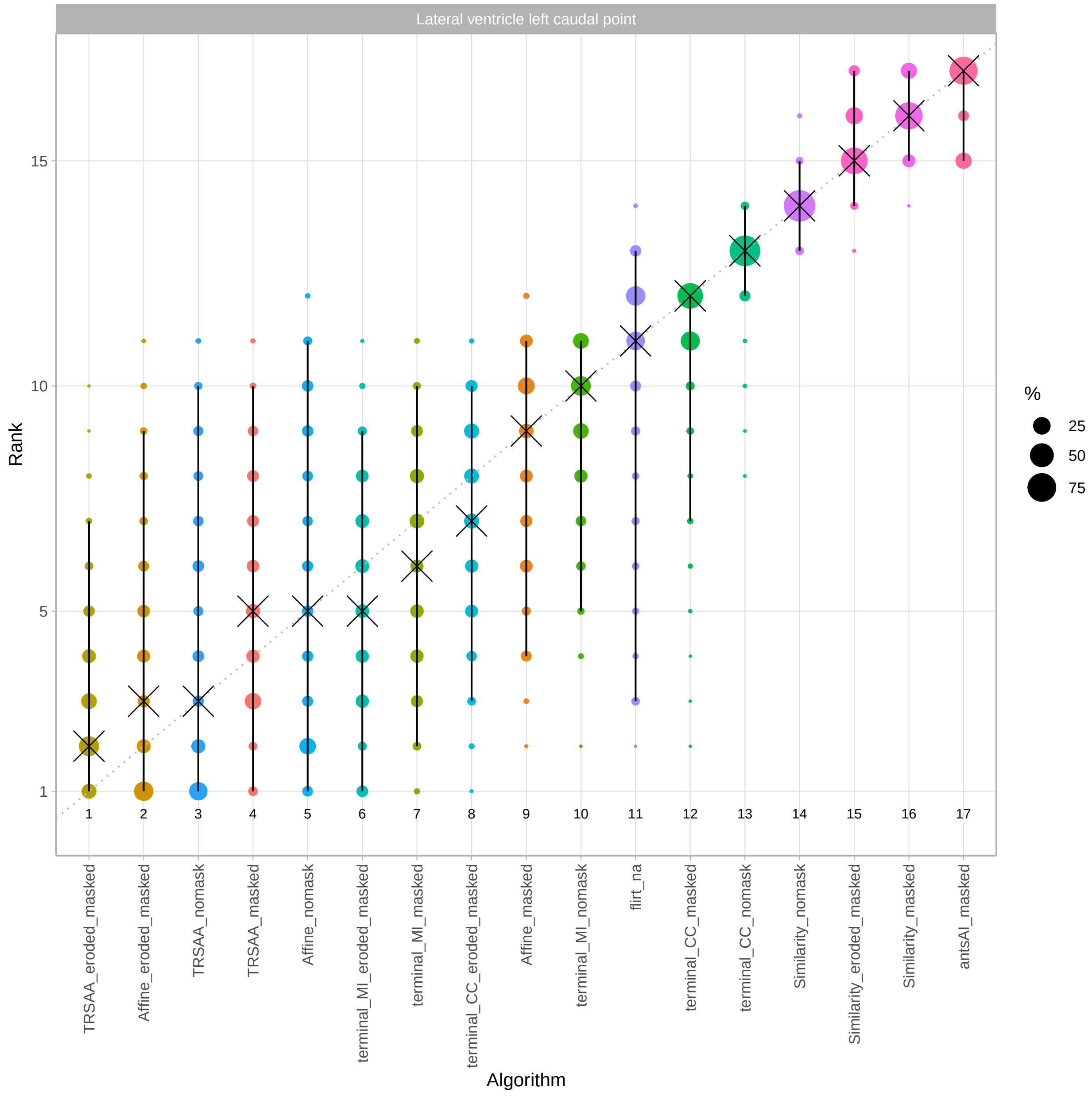

Lateral ventricle left caudal point
Rank
1
5
10
15
1
2
3
4
5
6
7
8
9
10
11
12
13
14
15
16
17
TRSAA_eroded_masked
Affine_eroded_masked
TRSAA_nomask
TRSAA_masked
Affine_nomask
terminal_MI_eroded_masked
terminal_MI_masked
terminal_CC_eroded_masked
Affine_masked
terminal_MI_nomask
flirt_na
terminal_CC_masked
terminal_CC_nomask
Similarity_nomask
Similarity_eroded_masked
Similarity_masked
antsAI_masked
Algorithm
%
25
50
75

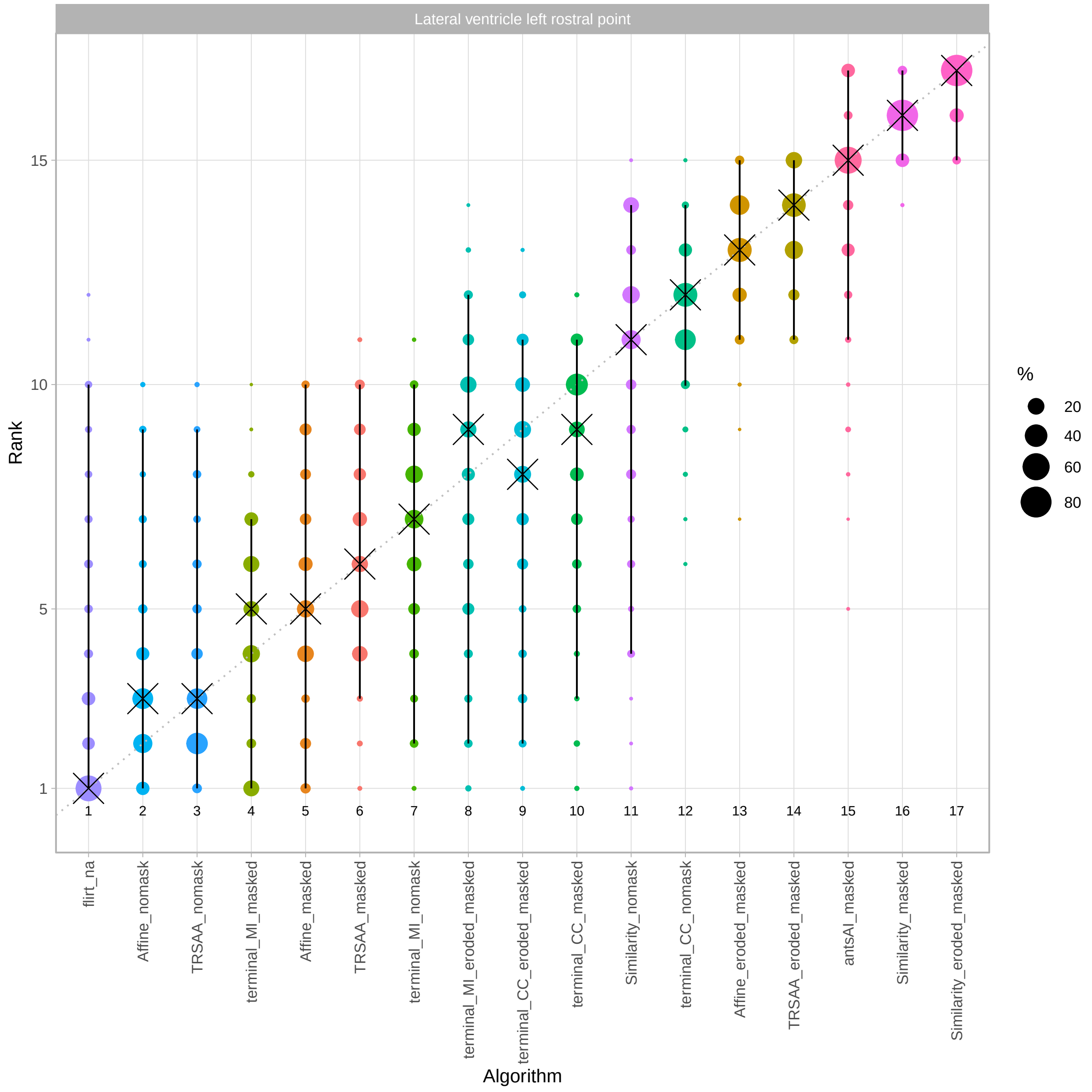
Lateral ventricle left rostral point
Rank
Algorithm
%
20
40
60
80
flirt_na
Affine_nomask
TRSAA_nomask
terminal_MI_masked
Affine_masked
TRSAA_masked
terminal_MI_nomask
terminal_MI_eroded_masked
terminal_CC_eroded_masked
terminal_CC_masked
Similarity_nomask
terminal_CC_nomask
Affine_eroded_masked
TRSAA_eroded_masked
antsAI_masked
Similarity_masked
Similarity_eroded_masked

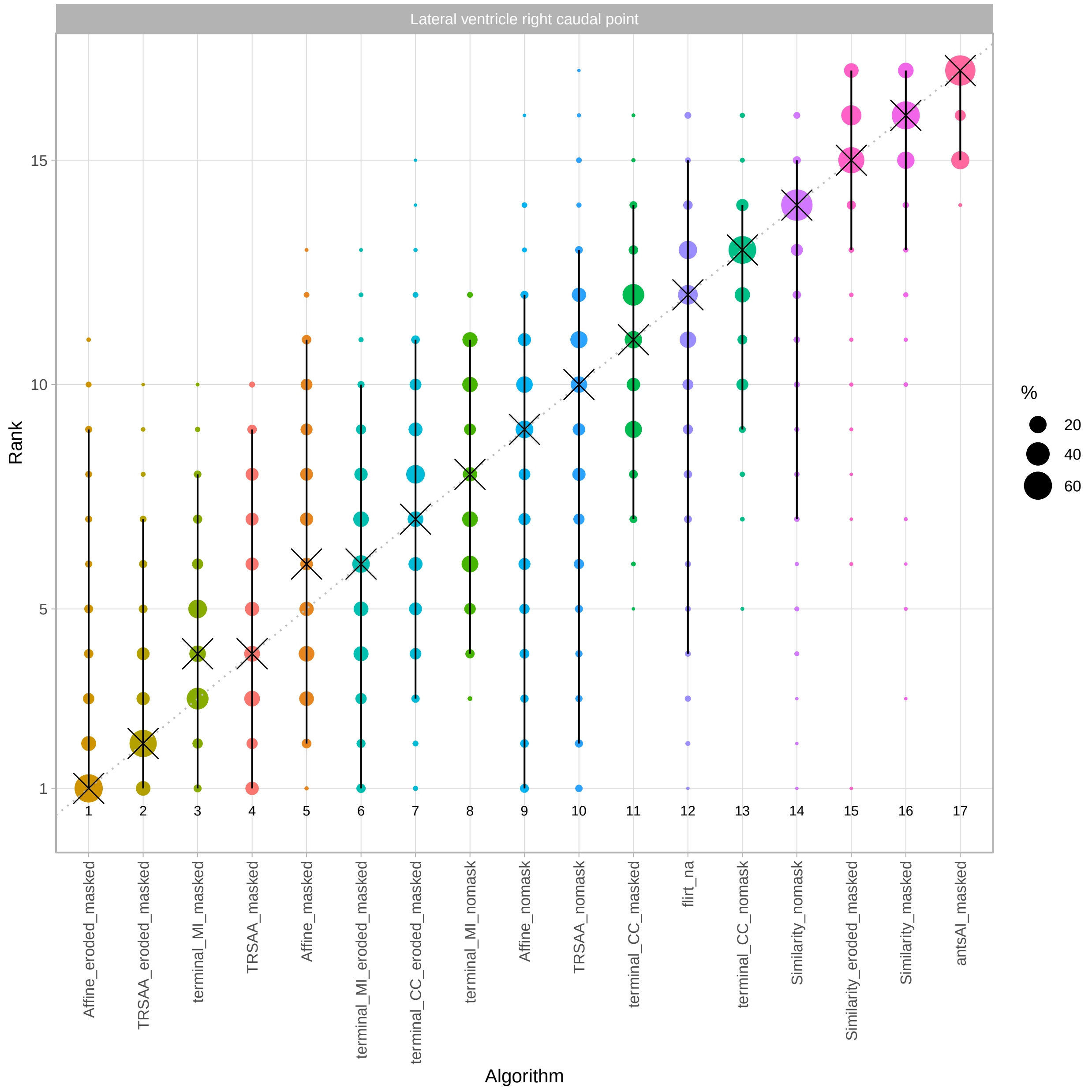

Lateral ventricle right caudal point
Rank
15
10
5
1
1
2
3
4
5
6
7
8
9
10
11
12
13
14
15
16
17
Affine_eroded_masked
TRSAA_eroded_masked
terminal_MI_masked
TRSAA_masked
Affine_masked
terminal_MI_eroded_masked
terminal_CC_eroded_masked
terminal_MI_nomask
Affine_nomask
TRSAA_nomask
terminal_CC_masked
flirt_na
terminal_CC_nomask
Similarity_nomask
Similarity_eroded_masked
Similarity_masked
antsAI_masked
Algorithm
%
20
40
60

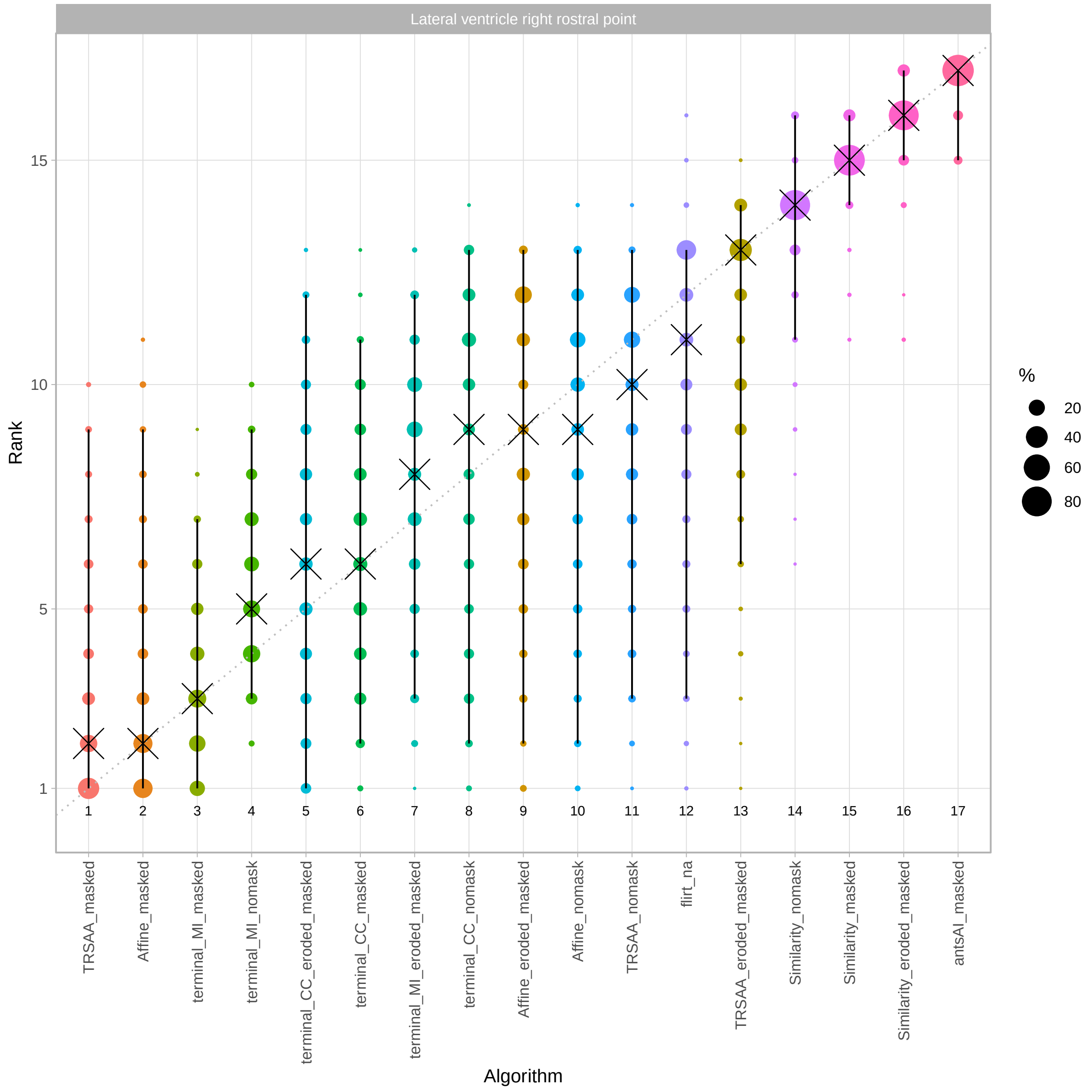

Lateral ventricle right rostral point
Rank
Algorithm
%
20
40
60
80
TRSAA_masked
Affine_masked
terminal_MI_masked
terminal_MI_nomask
terminal_CC_eroded_masked
terminal_CC_masked
terminal_MI_eroded_masked
terminal_CC_nomask
Affine_eroded_masked
Affine_nomask
TRSAA_nomask
flirt_na
TRSAA_eroded_masked
Similarity_nomask
Similarity_masked
Similarity_eroded_masked
antsAI_masked

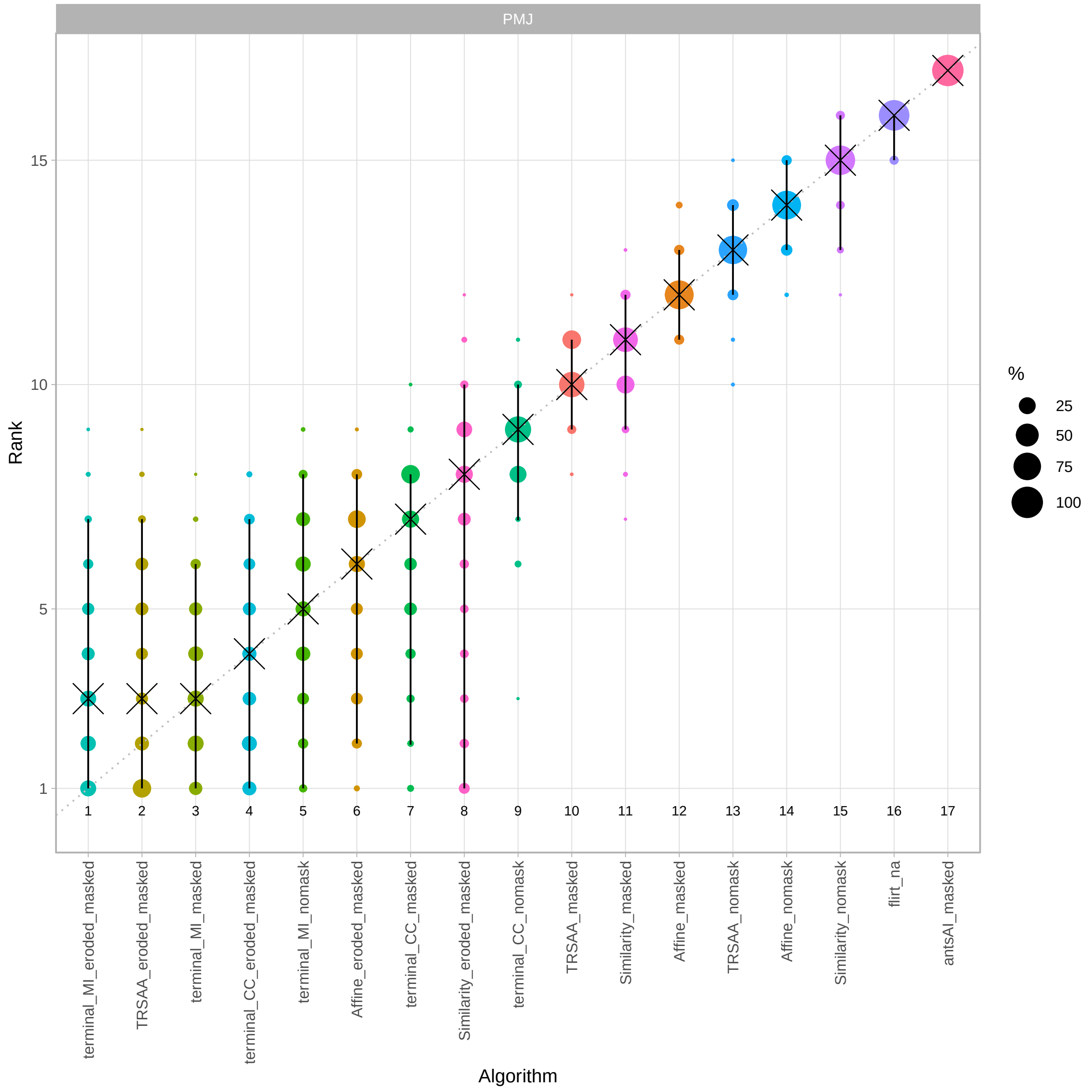
PMJ
Rank
15
10
5
1
1
2
3
4
5
6
7
8
9
10
11
12
13
14
15
16
17
%
25
50
75
100
terminal_MI_eroded_masked
TRSAA_eroded_masked
terminal_MI_masked
terminal_CC_eroded_masked
terminal_MI_nomask
Affine_eroded_masked
terminal_CC_masked
Similarity_eroded_masked
terminal_CC_nomask
TRSAA_masked
Similarity_masked
Affine_masked
TRSAA_nomask
Affine_nomask
Similarity_nomask
flirt_na
antsAI_masked
Algorithm

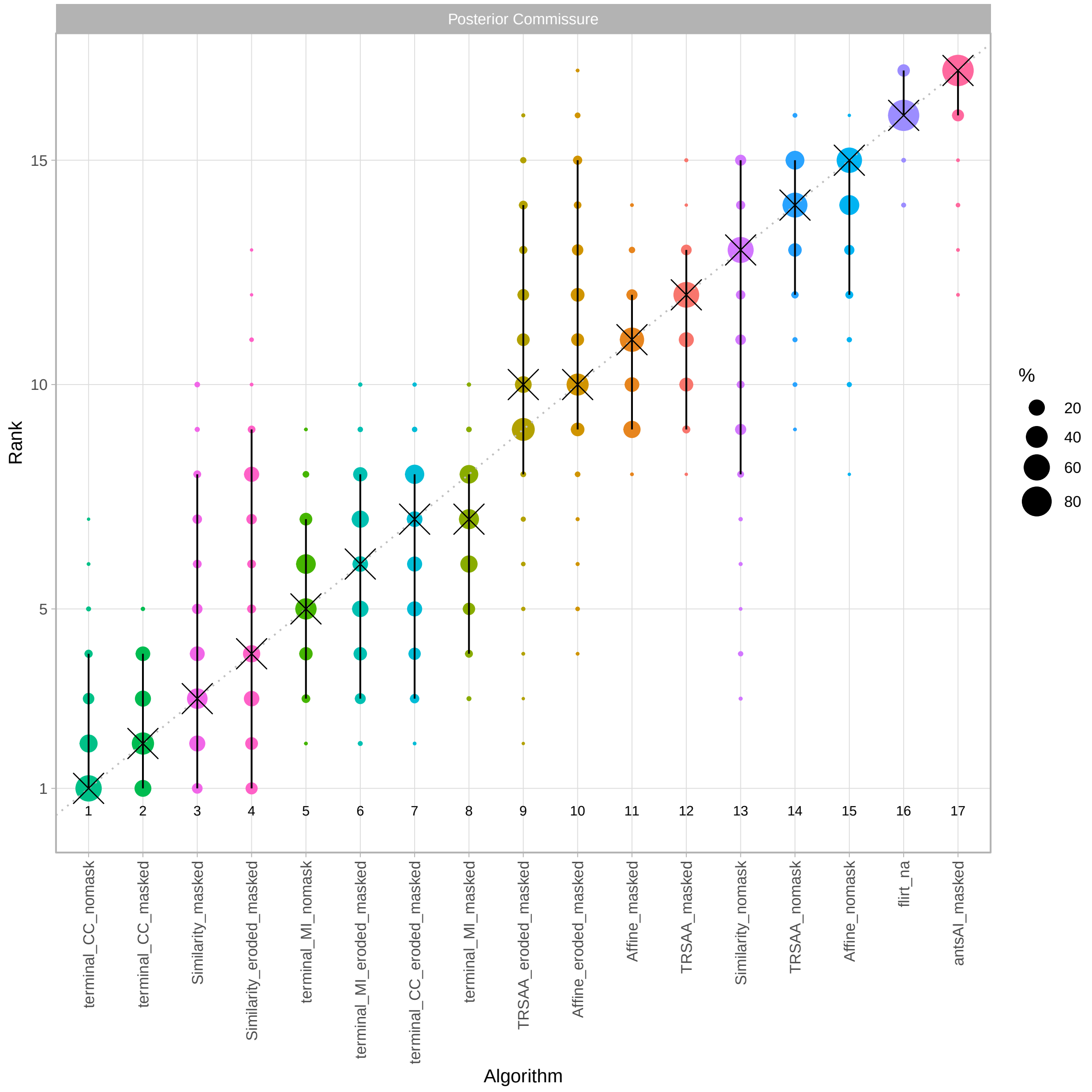
Posterior Commissure
Rank
15
10
5
1
1
2
3
4
5
6
7
8
9
10
11
12
13
14
15
16
17
%
20
40
60
80
terminal_CC_nomask
terminal_CC_masked
Similarity_masked
Similarity_eroded_masked
terminal_MI_nomask
terminal_MI_eroded_masked
terminal_CC_eroded_masked
terminal_MI_masked
TRSAA_eroded_masked
Affine_eroded_masked
Affine_masked
TRSAA_masked
Similarity_nomask
TRSAA_nomask
Affine_nomask
flirt_na
antsAI_masked
Algorithm

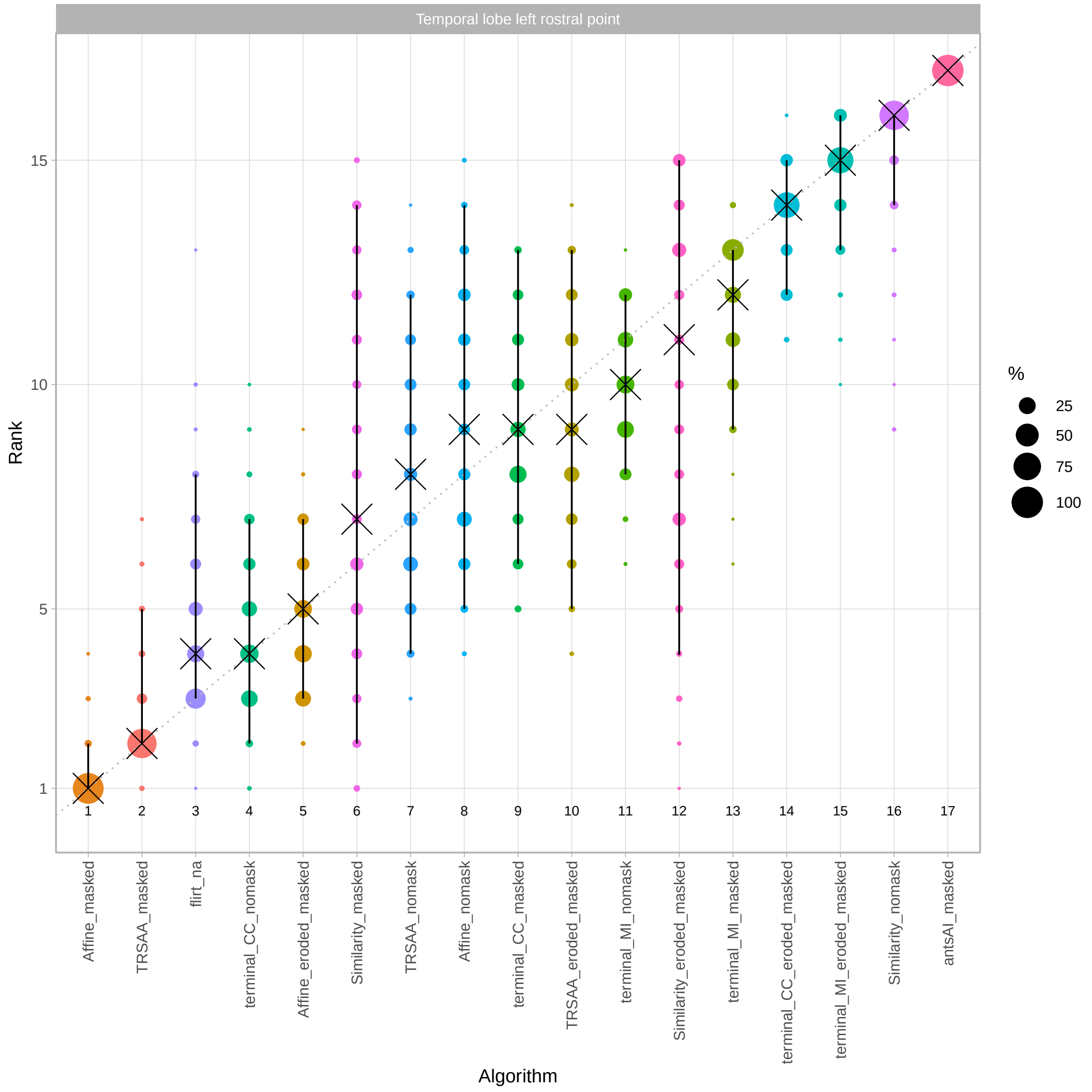

Temporal lobe left rostral point
Rank
Algorithm
%
25
50
75
100
1
5
10
15
1
2
3
4
5
6
7
8
9
10
11
12
13
14
15
16
17
Affine_masked
TRSAA_masked
flirt_na
terminal_CC_nomask
Affine_eroded_masked
Similarity_masked
TRSAA_nomask
Affine_nomask
terminal_CC_masked
TRSAA_eroded_masked
terminal_MI_nomask
Similarity_eroded_masked
terminal_MI_masked
terminal_CC_eroded_masked
terminal_MI_eroded_masked
Similarity_nomask
antsAI_masked

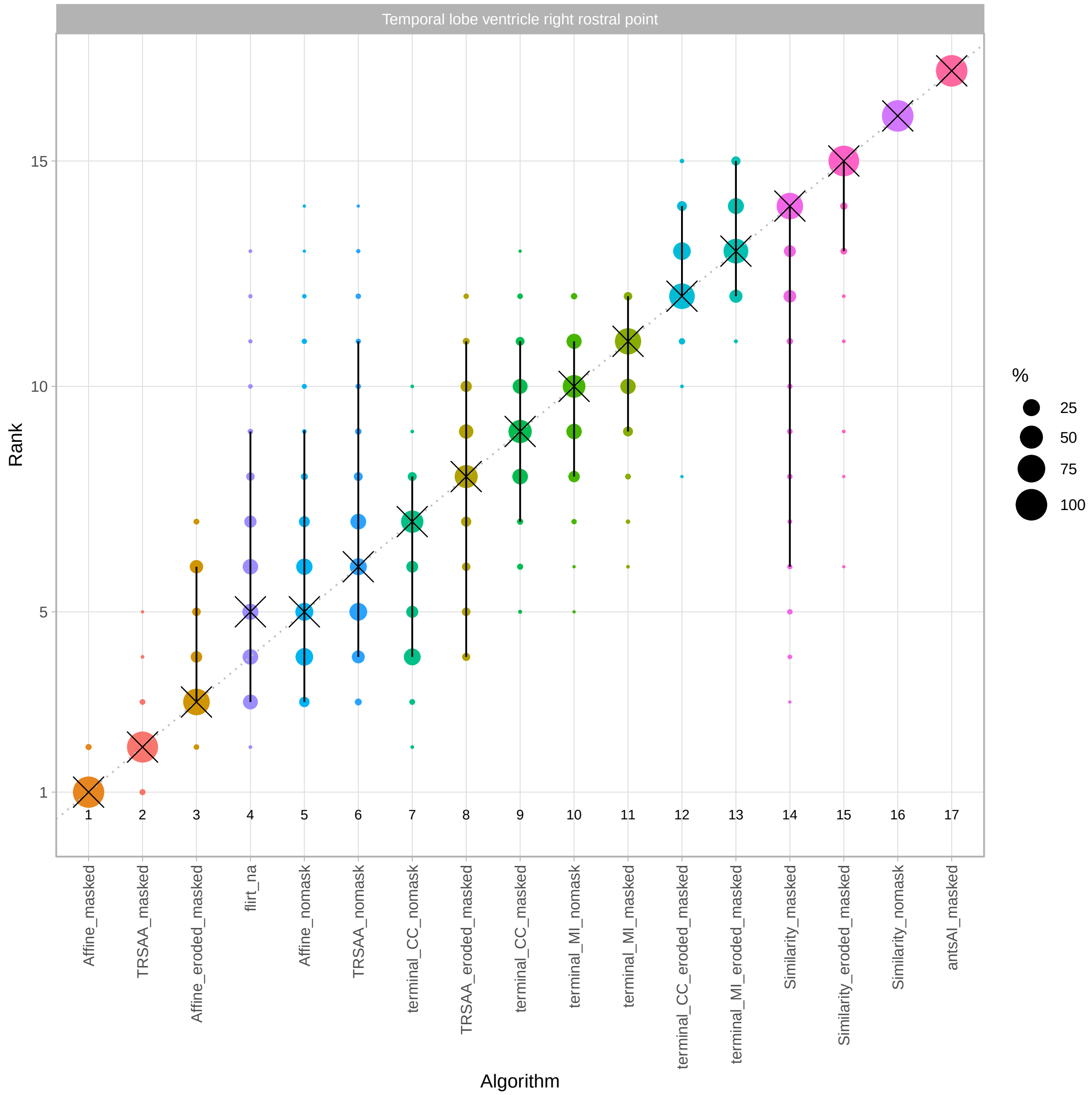

Temporal lobe ventricle right rostral point
Rank
15
10
5
1
1
2
3
4
5
6
7
8
9
10
11
12
13
14
15
16
17
Affine_masked
TRSAA_masked
Affine_eroded_masked
flirt_na
Affine_nomask
TRSAA_nomask
terminal_CC_nomask
TRSAA_eroded_masked
terminal_CC_masked
terminal_MI_nomask
terminal_MI_masked
terminal_CC_eroded_masked
terminal_MI_eroded_masked
Similarity_masked
Similarity_eroded_masked
Similarity_nomask
antsAI_masked
Algorithm
%
25
50
75
100

3.2 *Violin plot* for visualizing ranking stability based on bootstrapping

The ranking list based on the full assessment data is pairwise compared with the ranking lists based on the individual bootstrap samples (here $b = 1000$ samples). For each pair of rankings, Kendall's τ correlation is computed. Kendall's τ is a scaled index determining the correlation between the lists. It is computed by evaluating the number of pairwise concordances and discordances between ranking lists and produces values between -1 (for inverted order) and 1 (for identical order). A violin plot, which simultaneously depicts a boxplot and a density plot, is generated from the results.

Summary Kendall's tau:

Task	mean	median	q25	q75
Temporal lobe ventricle right rostral point	0.9132941	0.9264706	0.8970588	0.9558824
Culmen	0.8655588	0.8823529	0.8235294	0.9117647
Posterior Commissure	0.8620441	0.8823529	0.8235294	0.9117647
PMJ	0.8656618	0.8676471	0.8235294	0.9117647
Infracollicular Sulcus	0.8462794	0.8529412	0.8088235	0.8970588
Lateral ventricle left rostral point	0.8070735	0.8382353	0.7647059	0.8970588
Lateral ventricle right caudal point	0.7952647	0.8235294	0.7352941	0.8823529
Temporal lobe left rostral point	0.8131471	0.8235294	0.7647059	0.8676471
Hemisphere left caudal point	0.7788971	0.7941176	0.7205882	0.8529412
Lateral ventricle left caudal point	0.7885294	0.7941176	0.7352941	0.8529412
Chiasma	0.7687059	0.7794118	0.7205882	0.8382353
Hemisphere left rostral point	0.7644265	0.7794118	0.7058824	0.8235294
4th Ventricle	0.7520294	0.7647059	0.6911765	0.8235294
Lateral ventricle right rostral point	0.7525147	0.7647059	0.6911765	0.8382353
Hemisphere right caudal point	0.7288971	0.7500000	0.6617647	0.8088235
Hemisphere right rostral point	0.7353088	0.7500000	0.6764706	0.8088235

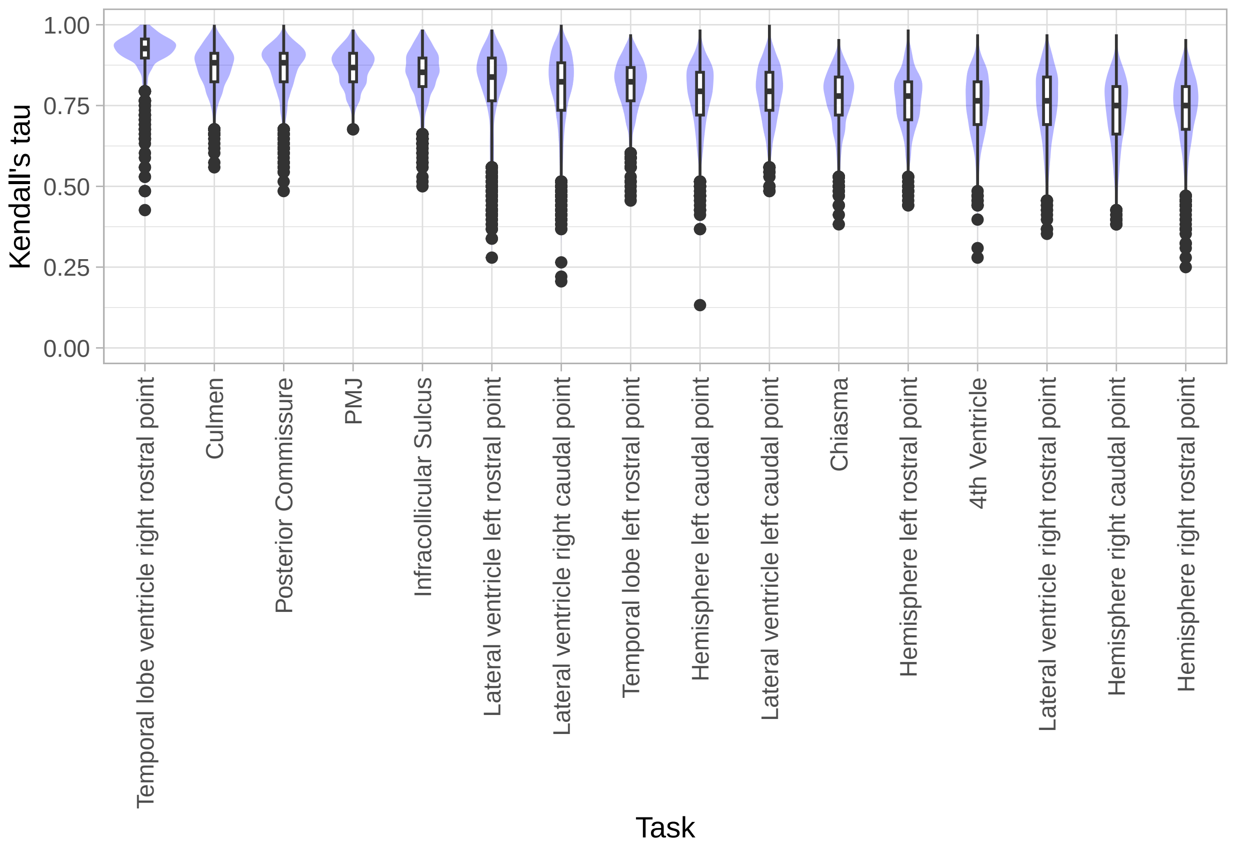
Kendall's tau
1.00
0.75
0.50
0.25
0.00
Temporal lobe ventricle right rostral point
Culmen
Posterior Commissure
PMJ
Infracollicular Sulcus
Lateral ventricle left rostral point
Lateral ventricle right caudal point
Temporal lobe left rostral point
Hemisphere left caudal point
Lateral ventricle left caudal point
Chiasma
Hemisphere left rostral point
4th Ventricle
Lateral ventricle right rostral point
Hemisphere right caudal point
Hemisphere right rostral point
Task

3.3 *Significance maps* for visualizing ranking stability based on statistical significance

Significance maps depict incidence matrices of pairwise significant test results for the one-sided Wilcoxon signed rank test at a 5% significance level with adjustment for multiple testing according to Holm. Yellow shading indicates that metric values from the algorithm on the x-axis were significantly superior to those from the algorithm on the y-axis, blue color indicates no significant difference.

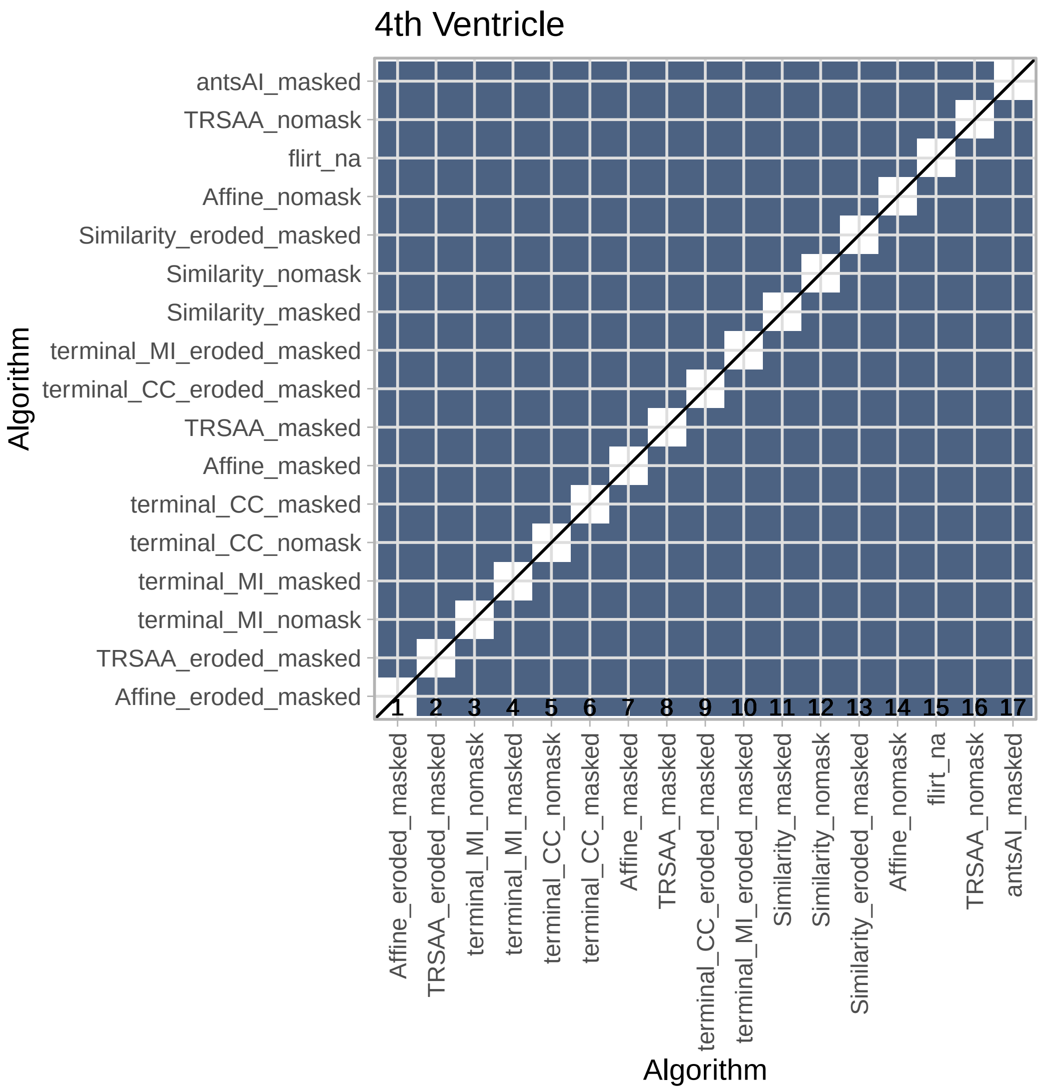

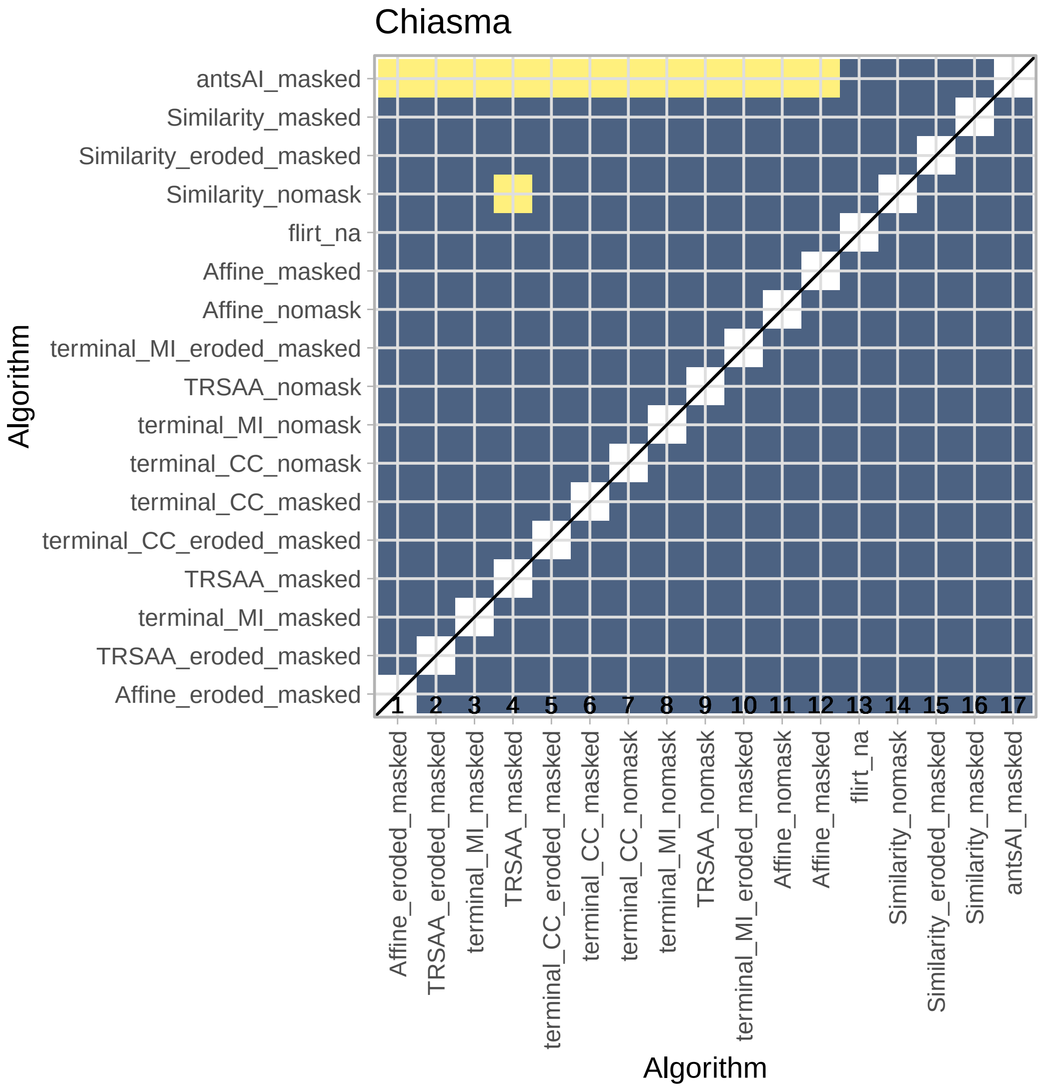
Chiasma
antsAI_masked
Similarity_masked
Similarity_eroded_masked
Similarity_nomask
flirt_na
Affine_masked
Affine_nomask
terminal_MI_eroded_masked
TRSAA_nomask
terminal_MI_nomask
terminal_CC_nomask
terminal_CC_masked
terminal_CC_eroded_masked
TRSAA_masked
terminal_MI_masked
TRSAA_eroded_masked
Affine_eroded_masked
Algorithm
1 2 3 4 5 6 7 8 9 10 11 12 13 14 15 16 17
Algorithm

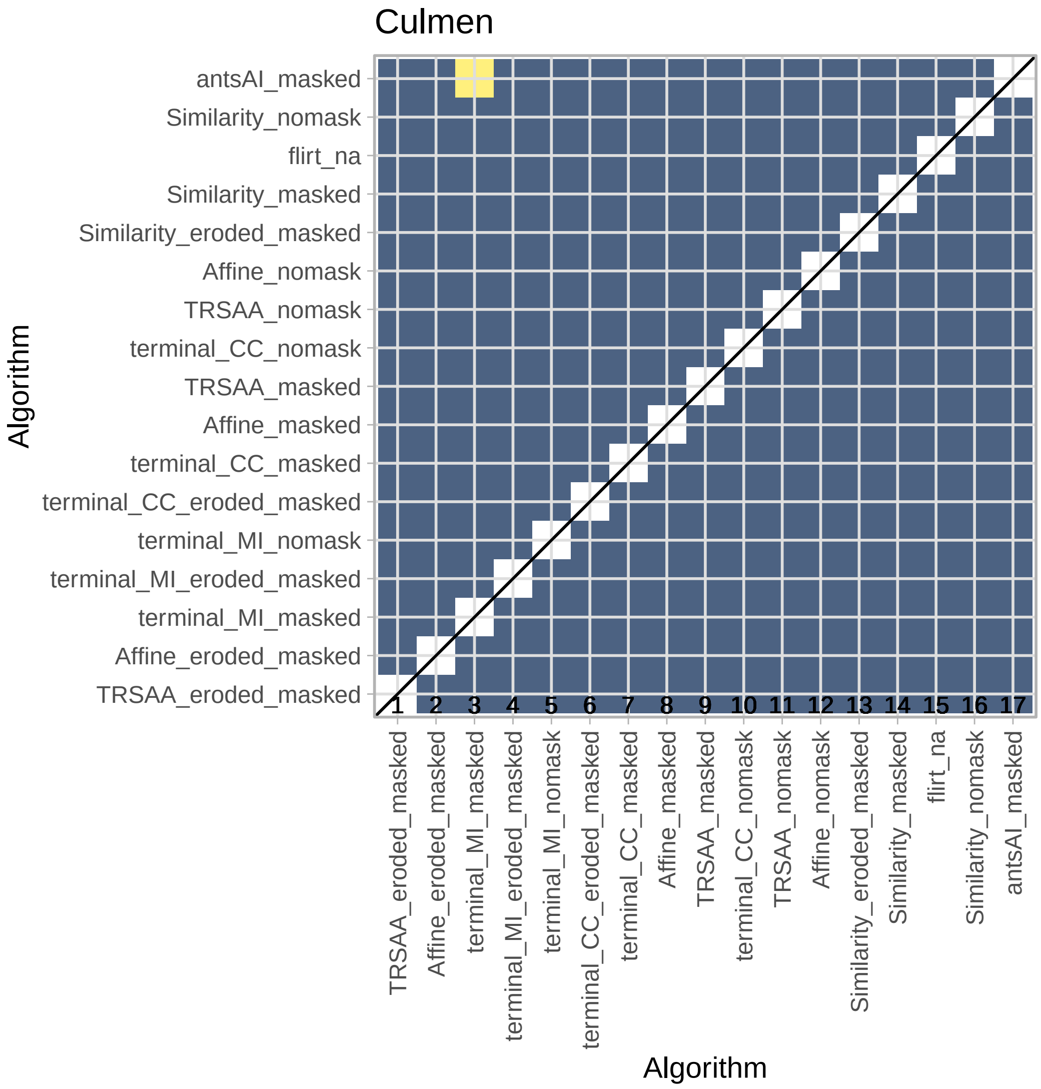
Culmen
Algorithm
antsAI_masked
Similarity_nomask
flirt_na
Similarity_masked
Similarity_eroded_masked
Affine_nomask
TRSAA_nomask
terminal_CC_nomask
TRSAA_masked
Affine_masked
terminal_CC_masked
terminal_CC_eroded_masked
terminal_MI_nomask
terminal_MI_eroded_masked
terminal_MI_masked
Affine_eroded_masked
TRSAA_eroded_masked
1 2 3 4 5 6 7 8 9 10 11 12 13 14 15 16 17
TRSAA_eroded_masked
Affine_eroded_masked
terminal_MI_masked
terminal_MI_eroded_masked
terminal_MI_nomask
terminal_CC_eroded_masked
terminal_CC_masked
Affine_masked
TRSAA_masked
terminal_CC_nomask
TRSAA_nomask
Affine_nomask
Similarity_eroded_masked
Similarity_masked
flirt_na
Similarity_nomask
antsAI_masked
Algorithm

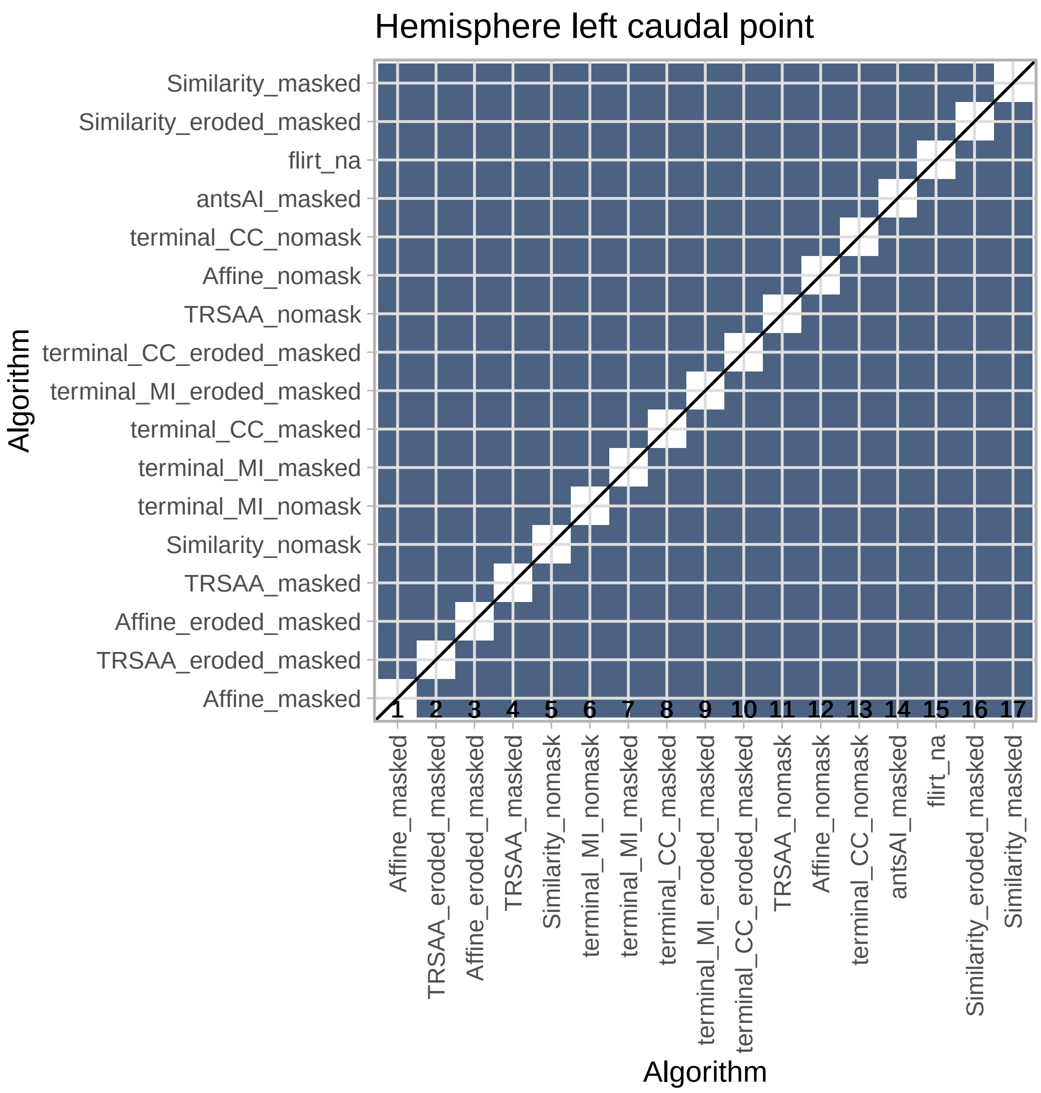
Hemisphere left caudal point
Algorithm
Similarity_masked
Similarity_eroded_masked
flirt_na
antsAI_masked
terminal_CC_nomask
Affine_nomask
TRSAA_nomask
terminal_CC_eroded_masked
terminal_MI_eroded_masked
terminal_CC_masked
terminal_MI_masked
terminal_MI_nomask
Similarity_nomask
TRSAA_masked
Affine_eroded_masked
TRSAA_eroded_masked
Affine_masked
1 2 3 4 5 6 7 8 9 10 11 12 13 14 15 16 17
Affine_masked
TRSAA_eroded_masked
Affine_eroded_masked
TRSAA_masked
Similarity_nomask
terminal_MI_nomask
terminal_MI_masked
terminal_CC_masked
terminal_MI_eroded_masked
terminal_CC_eroded_masked
TRSAA_nomask
Affine_nomask
terminal_CC_nomask
antsAI_masked
flirt_na
Similarity_eroded_masked
Similarity_masked
Algorithm

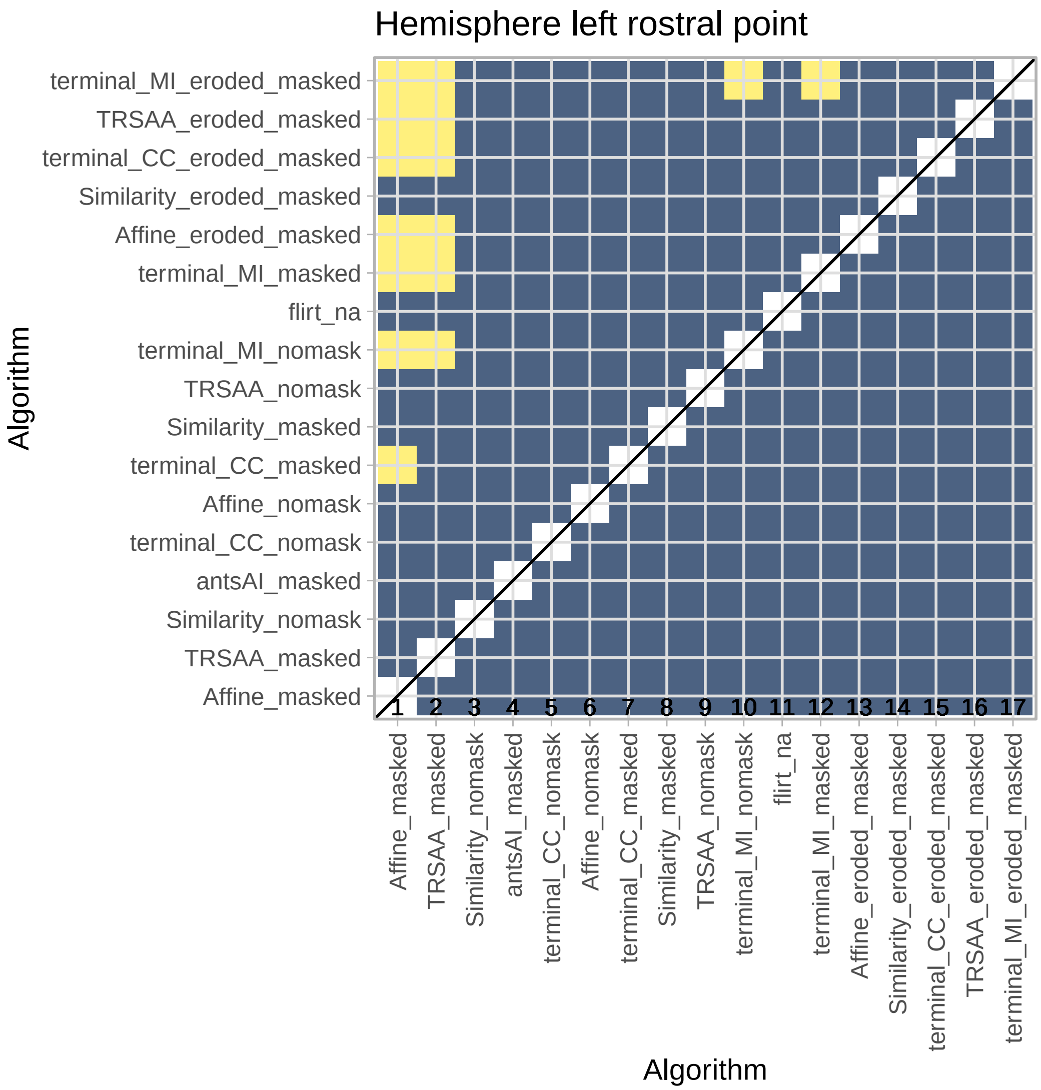
Hemisphere left rostral point
Algorithm
terminal_MI_eroded_masked
TRSAA_eroded_masked
terminal_CC_eroded_masked
Similarity_eroded_masked
Affine_eroded_masked
terminal_MI_masked
flirt_na
terminal_MI_nomask
TRSAA_nomask
Similarity_masked
terminal_CC_masked
Affine_nomask
terminal_CC_nomask
antsAI_masked
Similarity_nomask
TRSAA_masked
Affine_masked
1 2 3 4 5 6 7 8 9 10 11 12 13 14 15 16 17
Affine_masked
TRSAA_masked
Similarity_nomask
antsAI_masked
terminal_CC_nomask
Affine_nomask
terminal_CC_masked
Similarity_masked
TRSAA_nomask
terminal_MI_nomask
flirt_na
terminal_MI_masked
Affine_eroded_masked
Similarity_eroded_masked
terminal_CC_eroded_masked
TRSAA_eroded_masked
terminal_MI_eroded_masked
Algorithm

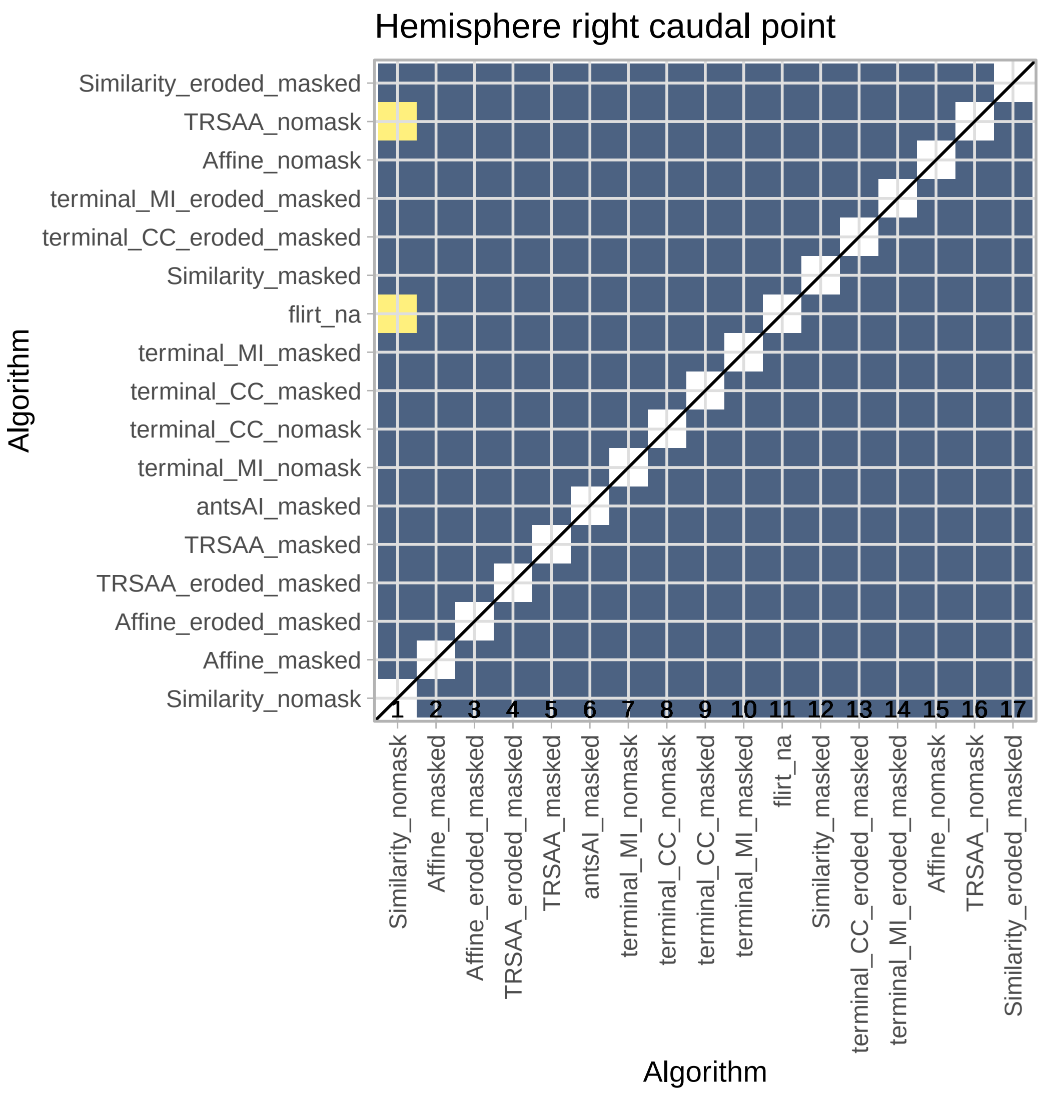
Hemisphere right caudal point
Algorithm
Similarity_eroded_masked
TRSAA_nomask
Affine_nomask
terminal_MI_eroded_masked
terminal_CC_eroded_masked
Similarity_masked
flirt_na
terminal_MI_masked
terminal_CC_masked
terminal_CC_nomask
terminal_MI_nomask
antsAI_masked
TRSAA_masked
TRSAA_eroded_masked
Affine_eroded_masked
Affine_masked
Similarity_nomask
1 2 3 4 5 6 7 8 9 10 11 12 13 14 15 16 17
Similarity_nomask
Affine_masked
Affine_eroded_masked
TRSAA_eroded_masked
TRSAA_masked
antsAI_masked
terminal_MI_nomask
terminal_CC_nomask
terminal_CC_masked
terminal_MI_masked
flirt_na
Similarity_masked
terminal_CC_eroded_masked
terminal_MI_eroded_masked
Affine_nomask
TRSAA_nomask
Similarity_eroded_masked
Algorithm

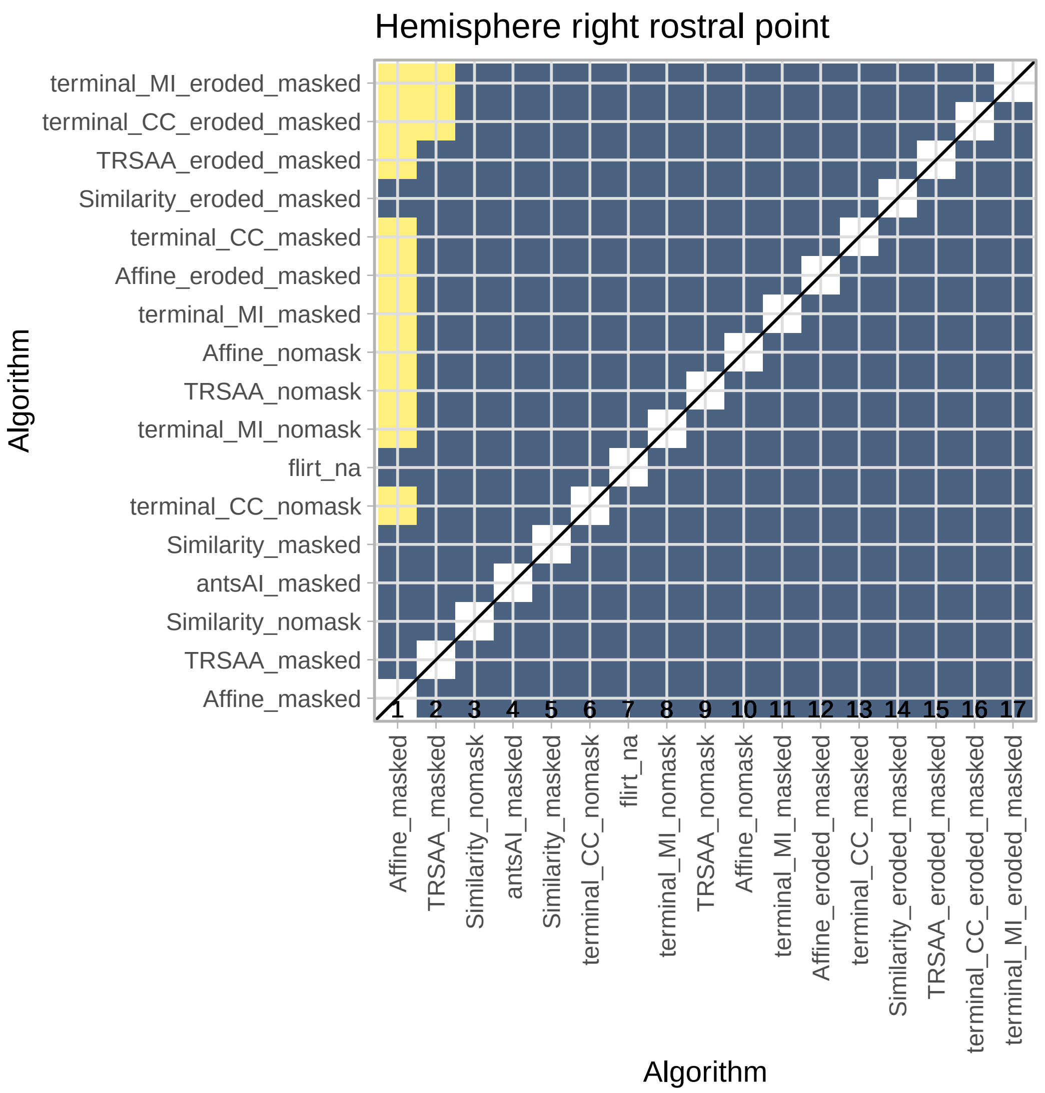
Hemisphere right rostral point
Algorithm
terminal_MI_eroded_masked
terminal_CC_eroded_masked
TRSAA_eroded_masked
Similarity_eroded_masked
terminal_CC_masked
Affine_eroded_masked
terminal_MI_masked
Affine_nomask
TRSAA_nomask
terminal_MI_nomask
flirt_na
terminal_CC_nomask
Similarity_masked
antsAI_masked
Similarity_nomask
TRSAA_masked
Affine_masked
1 2 3 4 5 6 7 8 9 10 11 12 13 14 15 16 17
Affine_masked
TRSAA_masked
Similarity_nomask
antsAI_masked
Similarity_masked
terminal_CC_nomask
flirt_na
terminal_MI_nomask
TRSAA_nomask
Affine_nomask
terminal_MI_masked
Affine_eroded_masked
terminal_CC_masked
Similarity_eroded_masked
TRSAA_eroded_masked
terminal_CC_eroded_masked
terminal_MI_eroded_masked
Algorithm

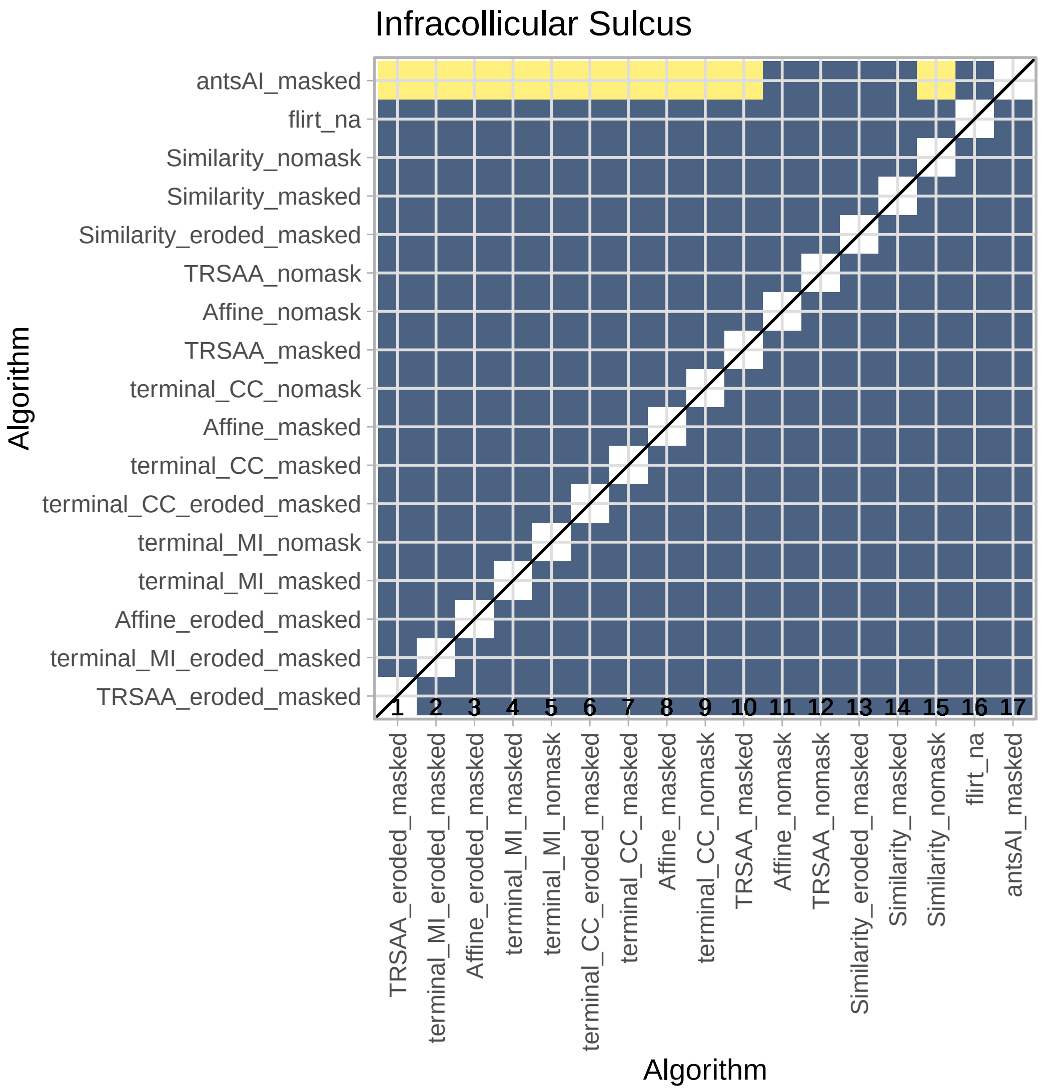
Infracollicular Sulcus
antsAI_masked
flirt_na
Similarity_nomask
Similarity_masked
Similarity_eroded_masked
TRSAA_nomask
Affine_nomask
TRSAA_masked
terminal_CC_nomask
Affine_masked
terminal_CC_masked
terminal_CC_eroded_masked
terminal_MI_nomask
terminal_MI_masked
Affine_eroded_masked
terminal_MI_eroded_masked
TRSAA_eroded_masked
Algorithm
1 2 3 4 5 6 7 8 9 10 11 12 13 14 15 16 17
TRSAA_eroded_masked
terminal_MI_eroded_masked
Affine_eroded_masked
terminal_MI_masked
terminal_MI_nomask
terminal_CC_eroded_masked
terminal_CC_masked
Affine_masked
terminal_CC_nomask
TRSAA_masked
Affine_nomask
TRSAA_nomask
Similarity_eroded_masked
Similarity_masked
Similarity_nomask
flirt_na
antsAI_masked
Algorithm

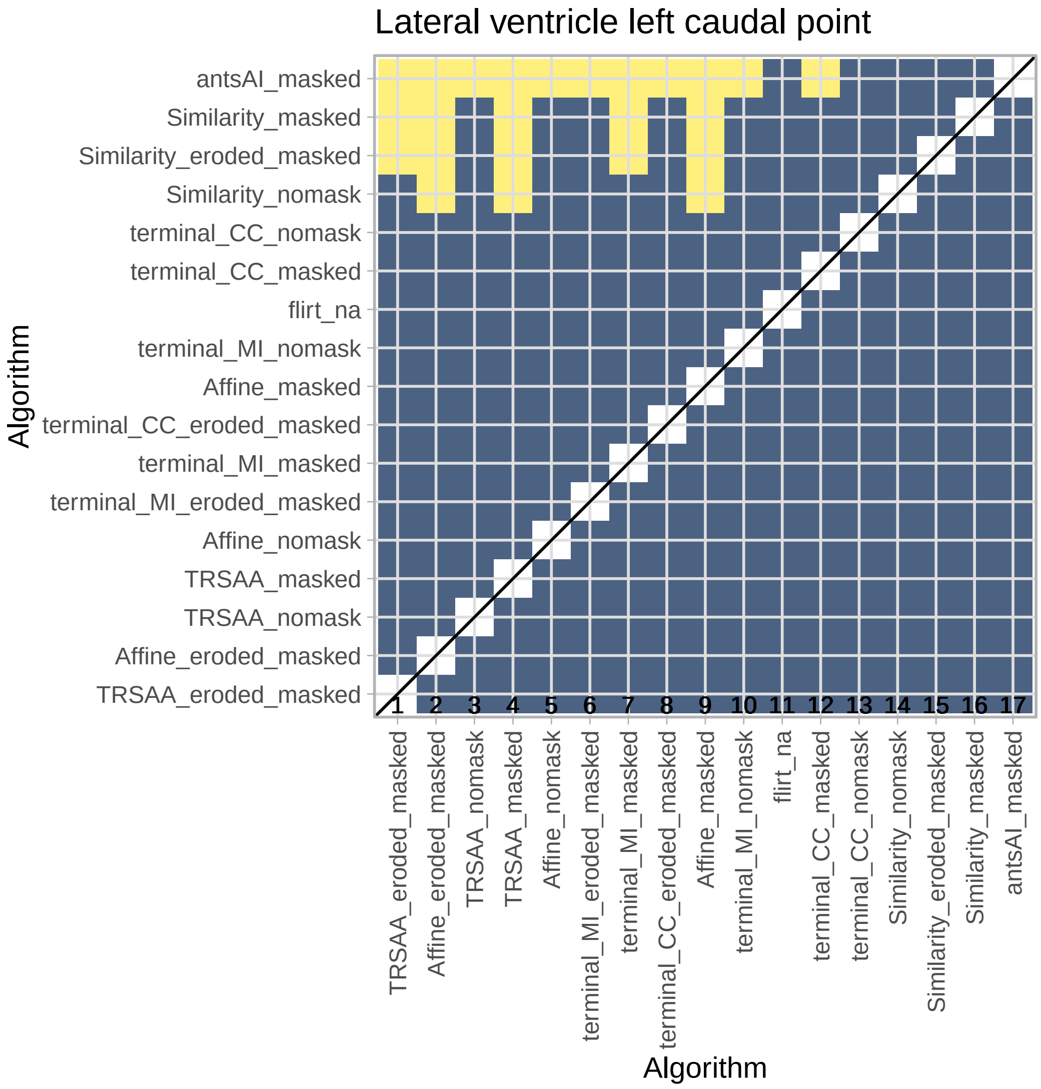

Lateral ventricle left caudal point
Algorithm
antsAI_masked
Similarity_masked
Similarity_eroded_masked
Similarity_nomask
terminal_CC_nomask
terminal_CC_masked
flirt_na
terminal_MI_nomask
Affine_masked
terminal_CC_eroded_masked
terminal_MI_masked
terminal_MI_eroded_masked
Affine_nomask
TRSAA_masked
TRSAA_nomask
Affine_eroded_masked
TRSAA_eroded_masked
1 2 3 4 5 6 7 8 9 10 11 12 13 14 15 16 17
TRSAA_eroded_masked
Affine_eroded_masked
TRSAA_nomask
TRSAA_masked
Affine_nomask
terminal_MI_eroded_masked
terminal_MI_masked
terminal_CC_eroded_masked
Affine_masked
terminal_MI_nomask
flirt_na
terminal_CC_masked
terminal_CC_nomask
Similarity_nomask
Similarity_eroded_masked
Similarity_masked
antsAI_masked
Algorithm

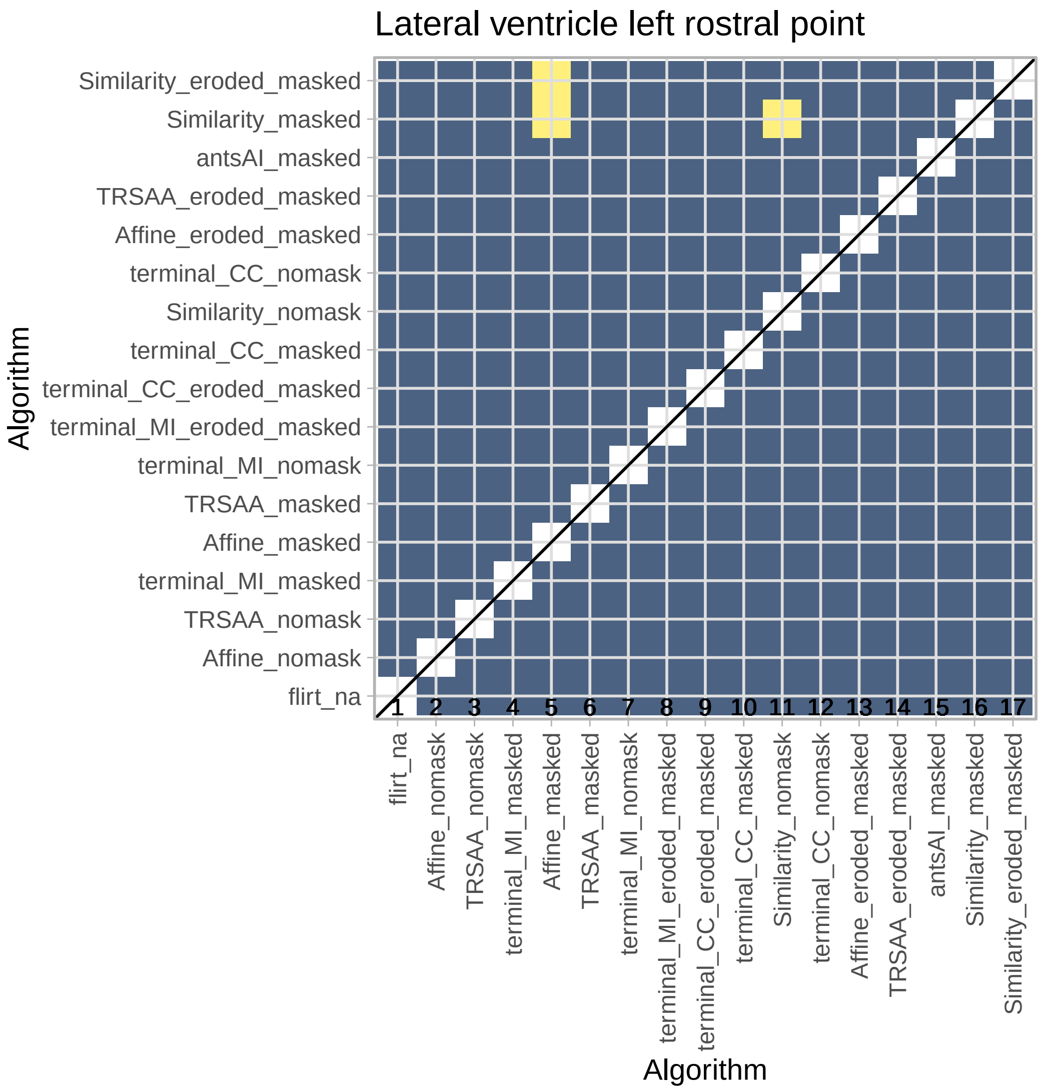
Lateral ventricle left rostral point
Algorithm
Similarity_eroded_masked
Similarity_masked
antsAI_masked
TRSAA_eroded_masked
Affine_eroded_masked
terminal_CC_nomask
Similarity_nomask
terminal_CC_masked
terminal_CC_eroded_masked
terminal_MI_eroded_masked
terminal_MI_nomask
TRSAA_masked
Affine_masked
terminal_MI_masked
TRSAA_nomask
Affine_nomask
flirt_na
1 2 3 4 5 6 7 8 9 10 11 12 13 14 15 16 17
flirt_na
Affine_nomask
TRSAA_nomask
terminal_MI_masked
Affine_masked
TRSAA_masked
terminal_MI_nomask
terminal_MI_eroded_masked
terminal_CC_eroded_masked
terminal_CC_masked
Similarity_nomask
terminal_CC_nomask
Affine_eroded_masked
TRSAA_eroded_masked
antsAI_masked
Similarity_masked
Similarity_eroded_masked
Algorithm

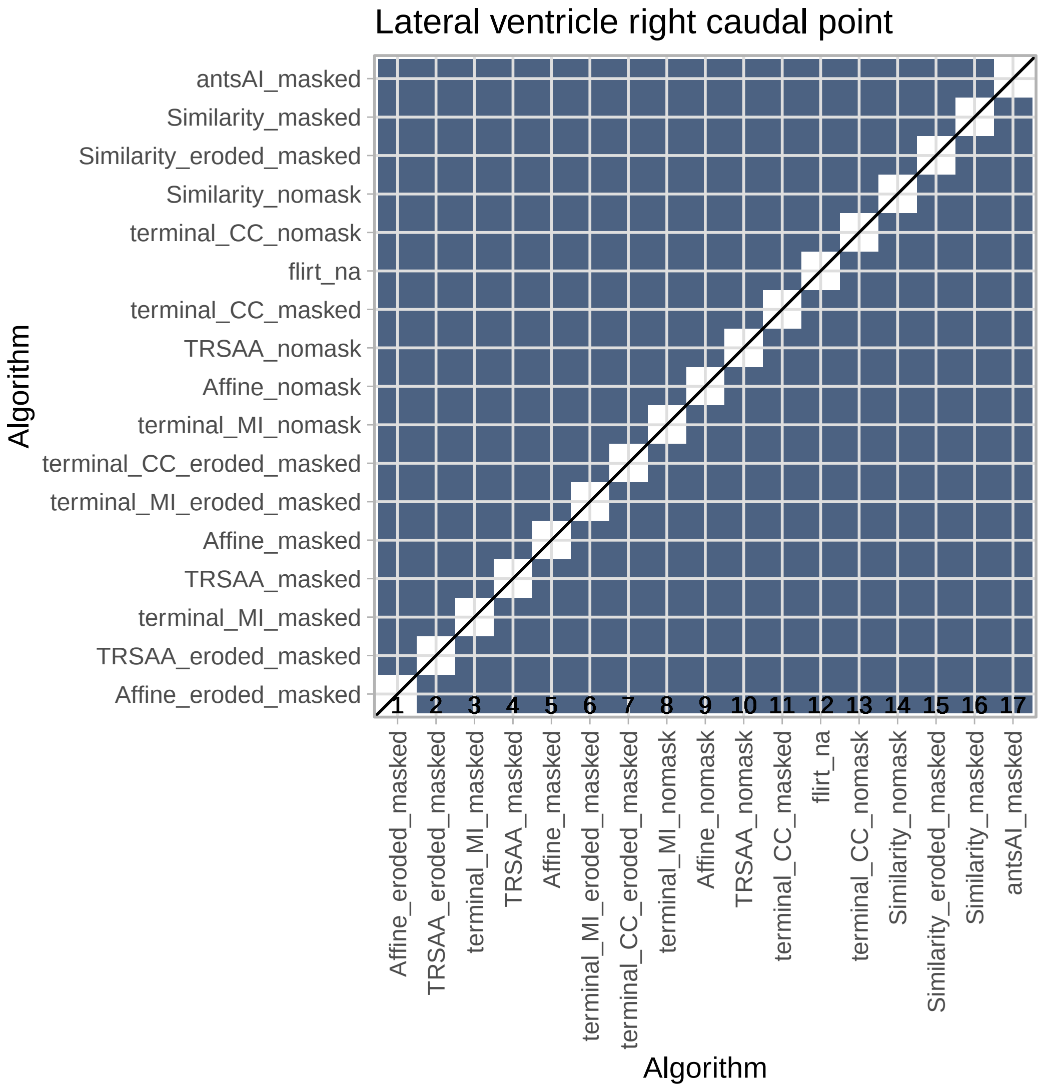
Lateral ventricle right caudal point
Algorithm
antsAI_masked
Similarity_masked
Similarity_eroded_masked
Similarity_nomask
terminal_CC_nomask
flirt_na
terminal_CC_masked
TRSAA_nomask
Affine_nomask
terminal_MI_nomask
terminal_CC_eroded_masked
terminal_MI_eroded_masked
Affine_masked
TRSAA_masked
terminal_MI_masked
TRSAA_eroded_masked
Affine_eroded_masked
1 2 3 4 5 6 7 8 9 10 11 12 13 14 15 16 17
Algorithm

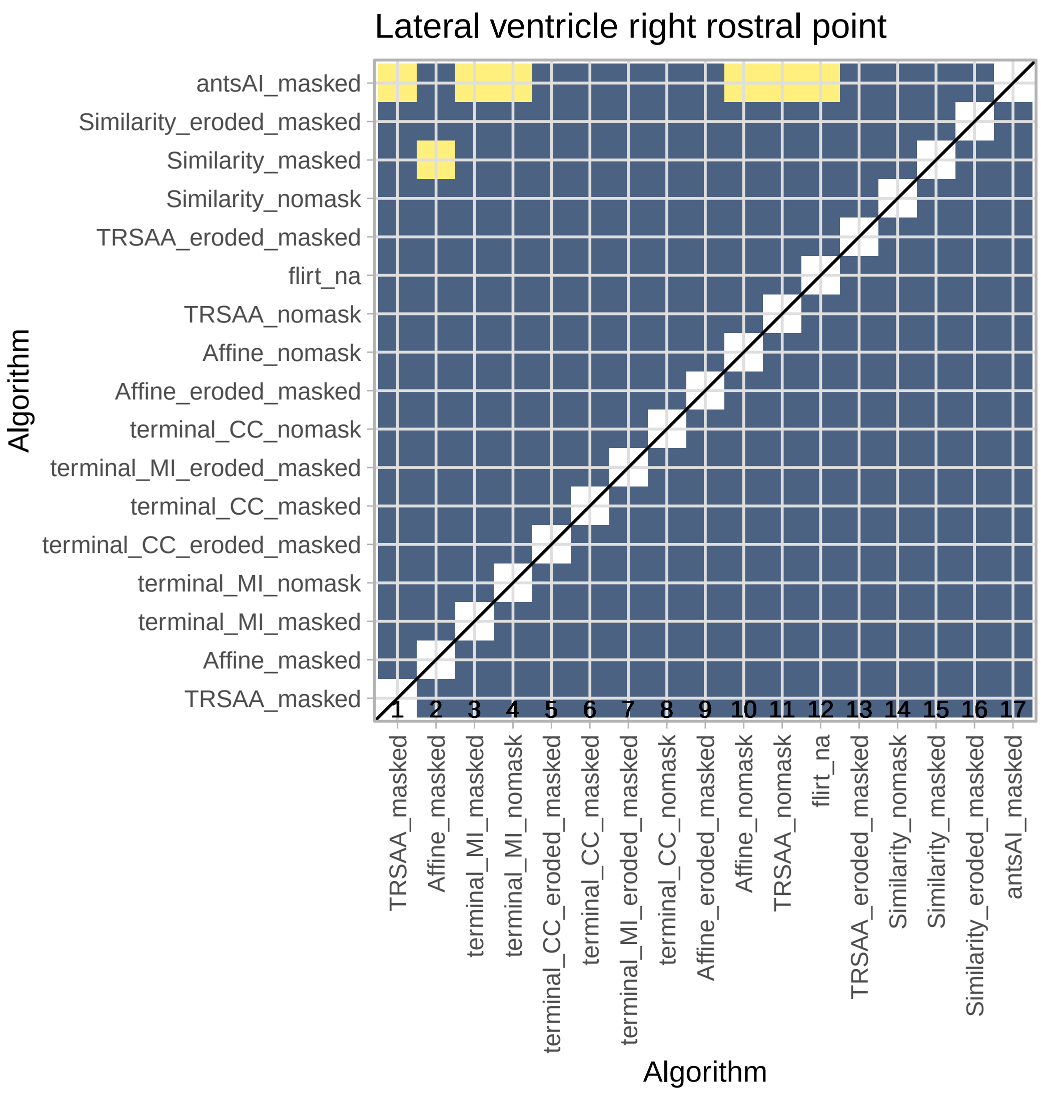

Lateral ventricle right rostral point
Algorithm
antsAI_masked
Similarity_eroded_masked
Similarity_masked
Similarity_nomask
TRSAA_eroded_masked
flirt_na
TRSAA_nomask
Affine_nomask
Affine_eroded_masked
terminal_CC_nomask
terminal_MI_eroded_masked
terminal_CC_masked
terminal_CC_eroded_masked
terminal_MI_nomask
terminal_MI_masked
Affine_masked
TRSAA_masked
1 2 3 4 5 6 7 8 9 10 11 12 13 14 15 16 17
TRSAA_masked
Affine_masked
terminal_MI_masked
terminal_MI_nomask
terminal_CC_eroded_masked
terminal_CC_masked
terminal_MI_eroded_masked
terminal_CC_nomask
Affine_eroded_masked
Affine_nomask
TRSAA_nomask
flirt_na
TRSAA_eroded_masked
Similarity_nomask
Similarity_masked
Similarity_eroded_masked
antsAI_masked
Algorithm

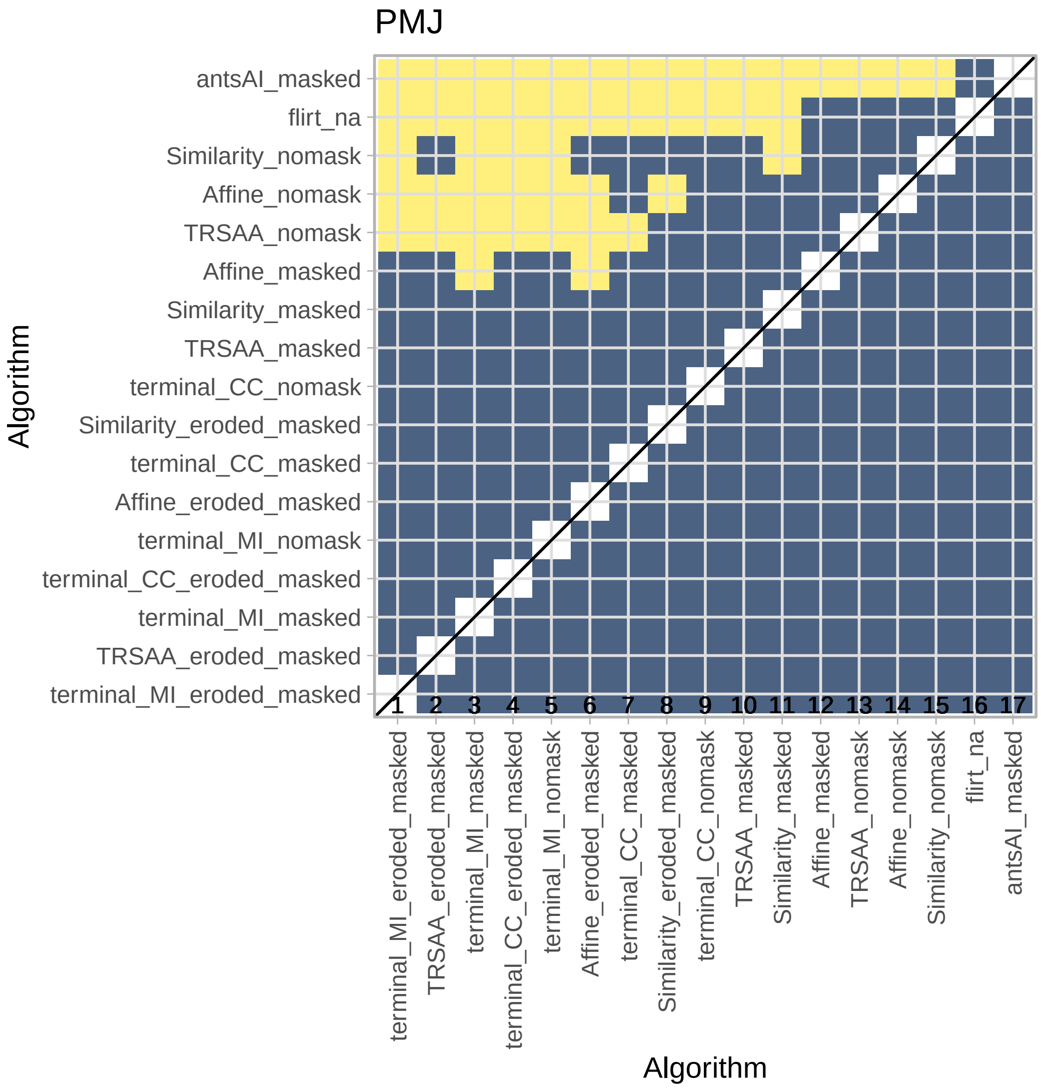
PMJ
antsAI_masked
flirt_na
Similarity_nomask
Affine_nomask
TRSAA_nomask
Affine_masked
Similarity_masked
TRSAA_masked
terminal_CC_nomask
Similarity_eroded_masked
terminal_CC_masked
Affine_eroded_masked
terminal_MI_nomask
terminal_CC_eroded_masked
terminal_MI_masked
TRSAA_eroded_masked
terminal_MI_eroded_masked
1 2 3 4 5 6 7 8 9 10 11 12 13 14 15 16 17
Algorithm
Algorithm

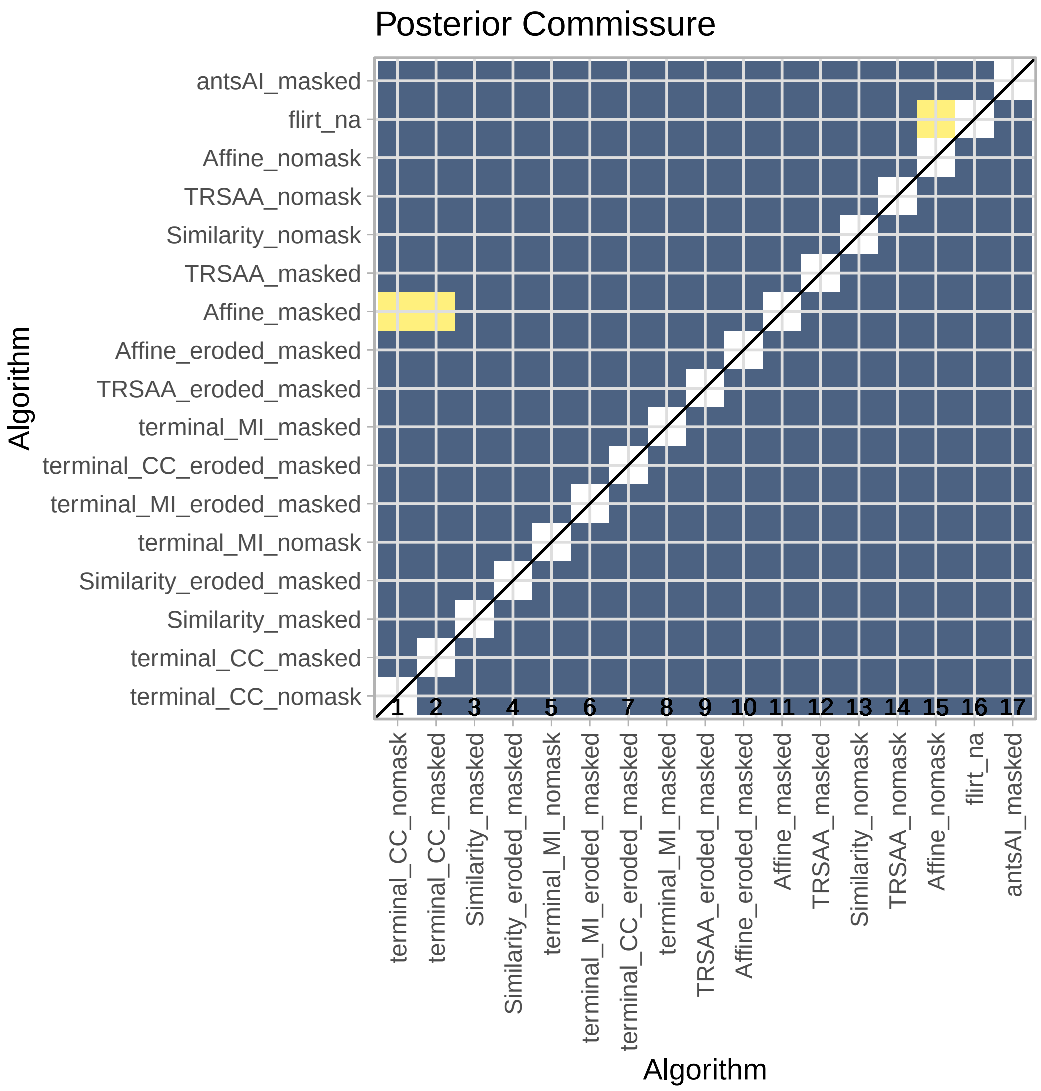

Posterior Commissure
Algorithm
antsAI_masked
flirt_na
Affine_nomask
TRSAA_nomask
Similarity_nomask
TRSAA_masked
Affine_masked
Affine_eroded_masked
TRSAA_eroded_masked
terminal_MI_masked
terminal_CC_eroded_masked
terminal_MI_eroded_masked
terminal_MI_nomask
Similarity_eroded_masked
Similarity_masked
terminal_CC_masked
terminal_CC_nomask
1 2 3 4 5 6 7 8 9 10 11 12 13 14 15 16 17
Algorithm

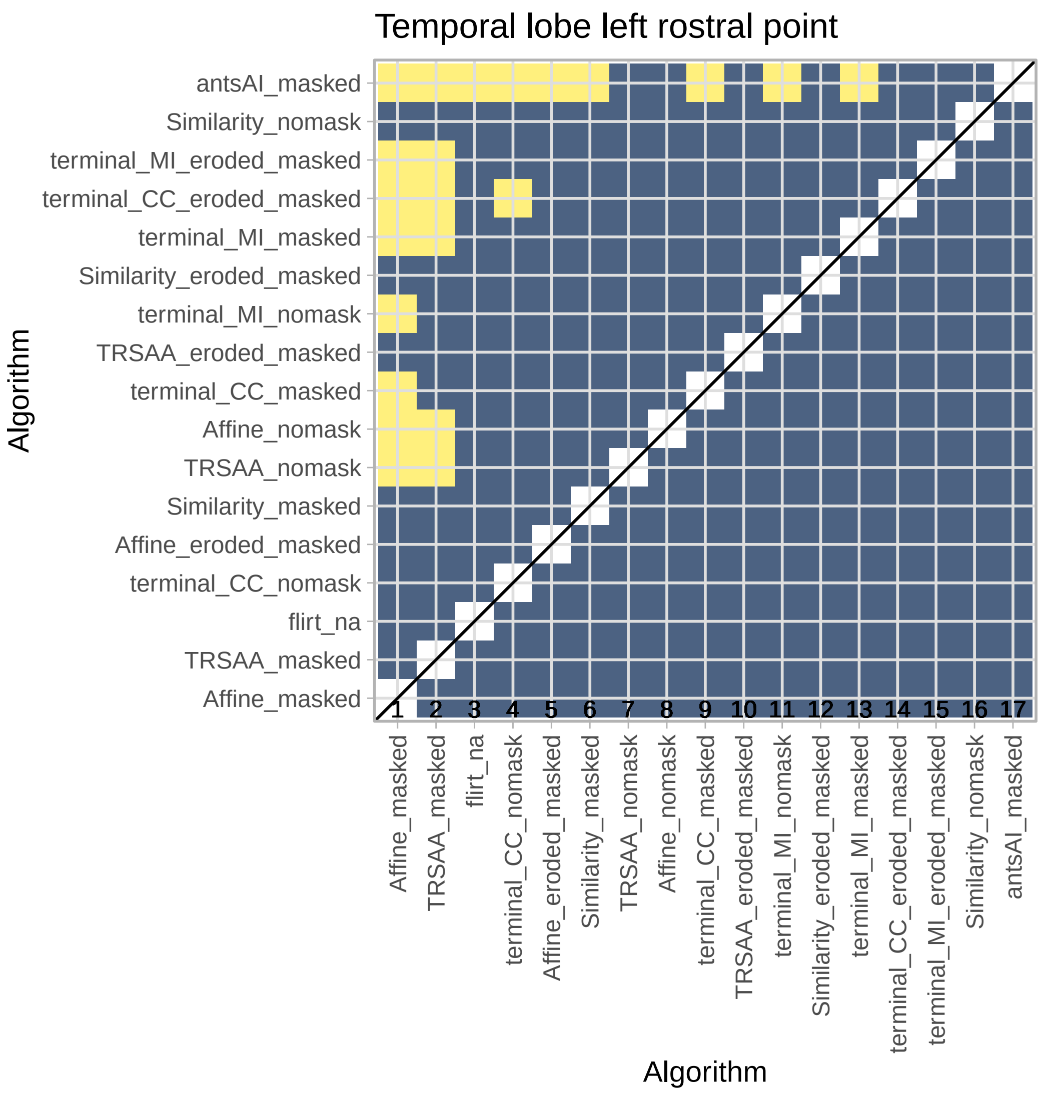

Temporal lobe left rostral point
Algorithm
antsAI_masked
Similarity_nomask
terminal_MI_eroded_masked
terminal_CC_eroded_masked
terminal_MI_masked
Similarity_eroded_masked
terminal_MI_nomask
TRSAA_eroded_masked
terminal_CC_masked
Affine_nomask
TRSAA_nomask
Similarity_masked
Affine_eroded_masked
terminal_CC_nomask
flirt_na
TRSAA_masked
Affine_masked
1 2 3 4 5 6 7 8 9 10 11 12 13 14 15 16 17
Affine_masked
TRSAA_masked
flirt_na
terminal_CC_nomask
Affine_eroded_masked
Similarity_masked
TRSAA_nomask
Affine_nomask
terminal_CC_masked
TRSAA_eroded_masked
terminal_MI_nomask
Similarity_eroded_masked
terminal_MI_masked
terminal_CC_eroded_masked
terminal_MI_eroded_masked
Similarity_nomask
antsAI_masked
Algorithm

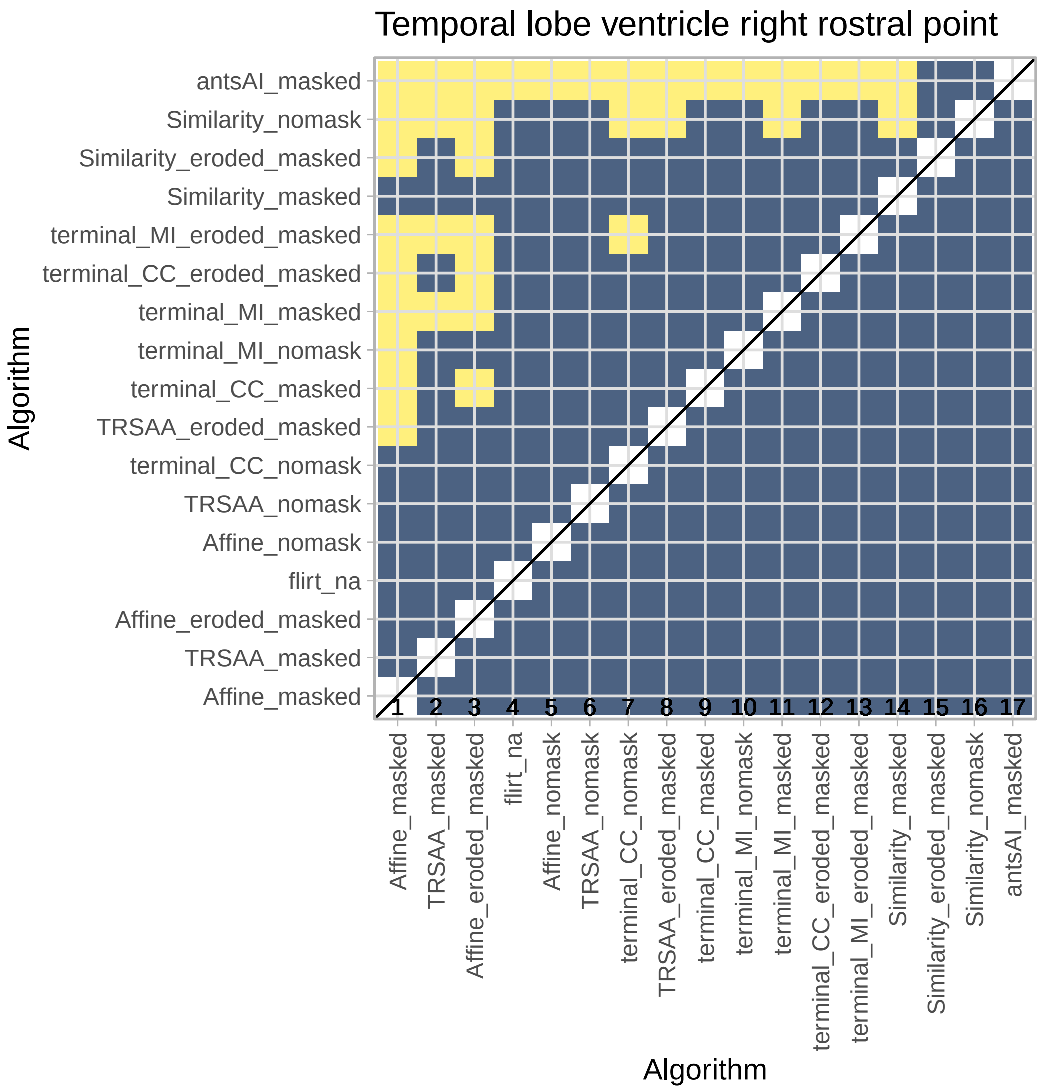
Temporal lobe ventricle right rostral point
antsAI_masked
Similarity_nomask
Similarity_eroded_masked
Similarity_masked
terminal_MI_eroded_masked
terminal_CC_eroded_masked
terminal_MI_masked
terminal_MI_nomask
terminal_CC_masked
TRSAA_eroded_masked
terminal_CC_nomask
TRSAA_nomask
Affine_nomask
flirt_na
Affine_eroded_masked
TRSAA_masked
Affine_masked
1 2 3 4 5 6 7 8 9 10 11 12 13 14 15 16 17
Affine_masked
TRSAA_masked
Affine_eroded_masked
flirt_na
Affine_nomask
TRSAA_nomask
terminal_CC_nomask
TRSAA_eroded_masked
terminal_CC_masked
terminal_MI_nomask
terminal_MI_masked
terminal_CC_eroded_masked
terminal_MI_eroded_masked
Similarity_masked
Similarity_eroded_masked
Similarity_nomask
antsAI_masked
Algorithm
Algorithm

3.4 Ranking robustness to ranking methods

Line plots for visualizing ranking robustness across different ranking methods. Each algorithm is represented by one colored line. For each ranking method encoded on the x-axis, the height of the line represents the corresponding rank. Horizontal lines indicate identical ranks for all methods.

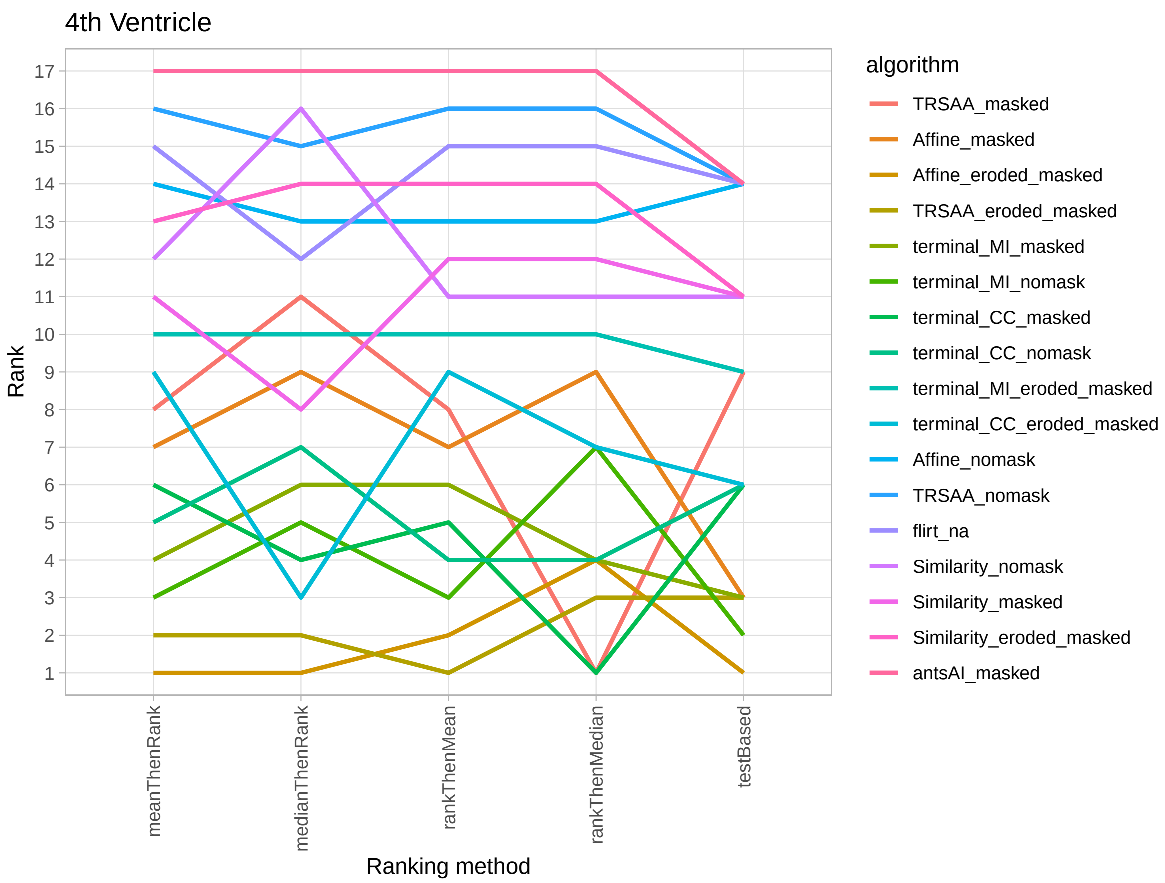

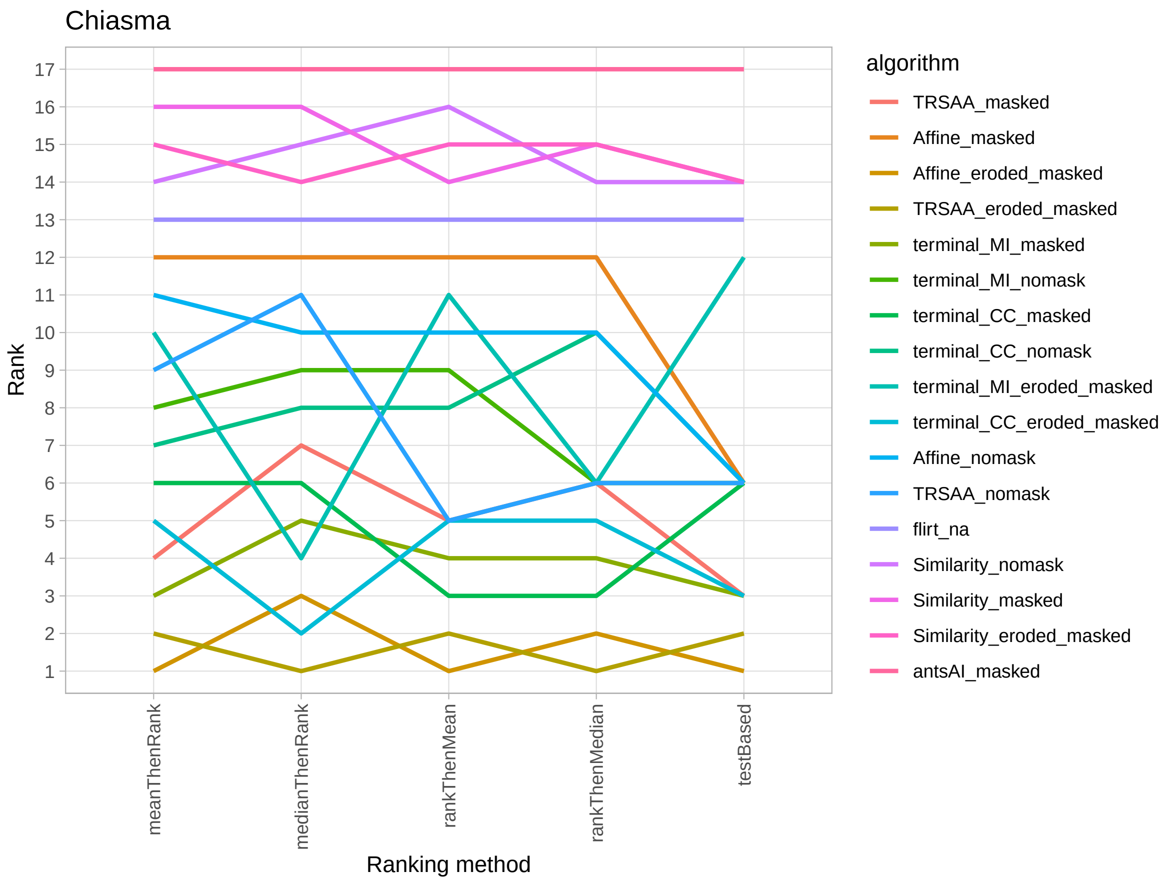

Chiasma
Rank
17
16
15
14
13
12
11
10
9
8
7
6
5
4
3
2
1
meanThenRank
medianThenRank
rankThenMean
rankThenMedian
testBased
Ranking method
algorithm
TRSAA_masked
Affine_masked
Affine_eroded_masked
TRSAA_eroded_masked
terminal_MI_masked
terminal_MI_nomask
terminal_CC_masked
terminal_CC_nomask
terminal_MI_eroded_masked
terminal_CC_eroded_masked
Affine_nomask
TRSAA_nomask
flirt_na
Similarity_nomask
Similarity_masked
Similarity_eroded_masked
antsAI_masked

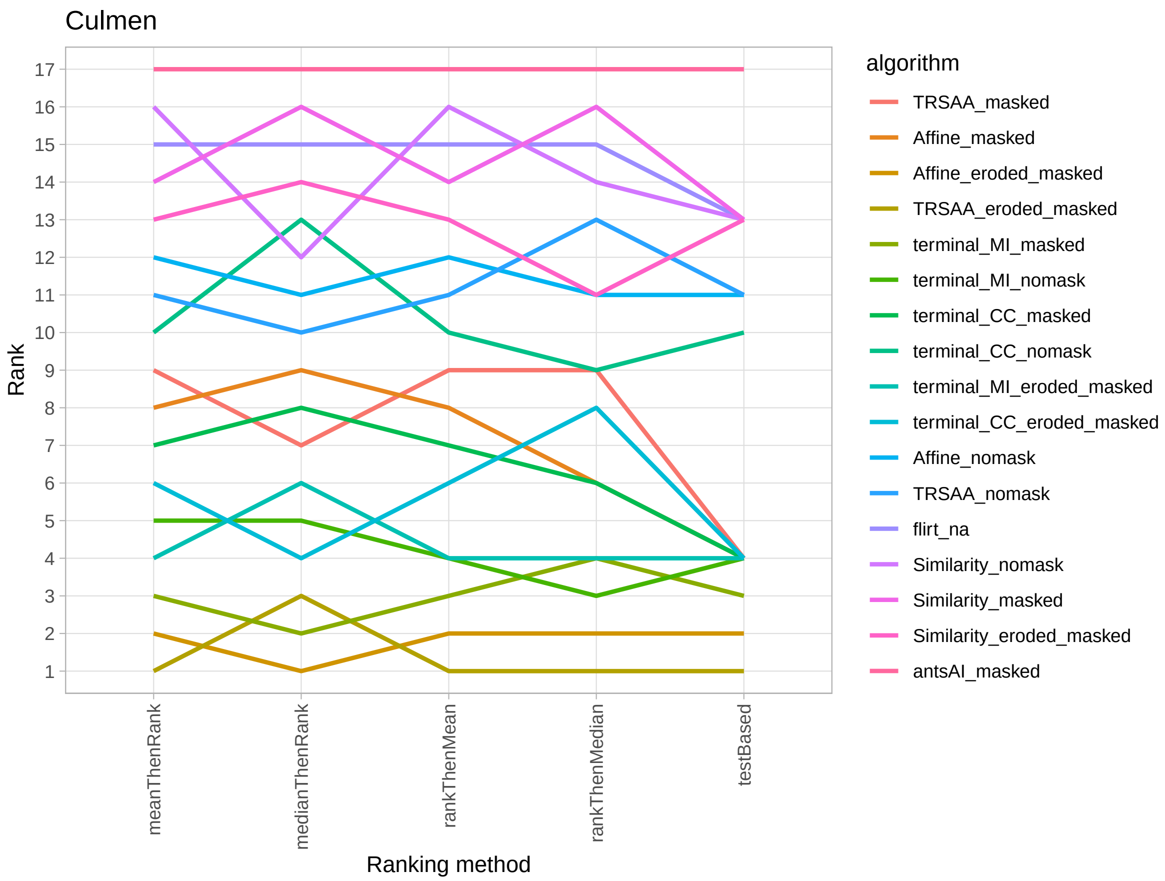
Culmen
Rank
17
16
15
14
13
12
11
10
9
8
7
6
5
4
3
2
1
meanThenRank
medianThenRank
rankThenMean
rankThenMedian
testBased
Ranking method
algorithm
TRSAA_masked
Affine_masked
Affine_eroded_masked
TRSAA_eroded_masked
terminal_MI_masked
terminal_MI_nomask
terminal_CC_masked
terminal_CC_nomask
terminal_MI_eroded_masked
terminal_CC_eroded_masked
Affine_nomask
TRSAA_nomask
flirt_na
Similarity_nomask
Similarity_masked
Similarity_eroded_masked
antsAI_masked

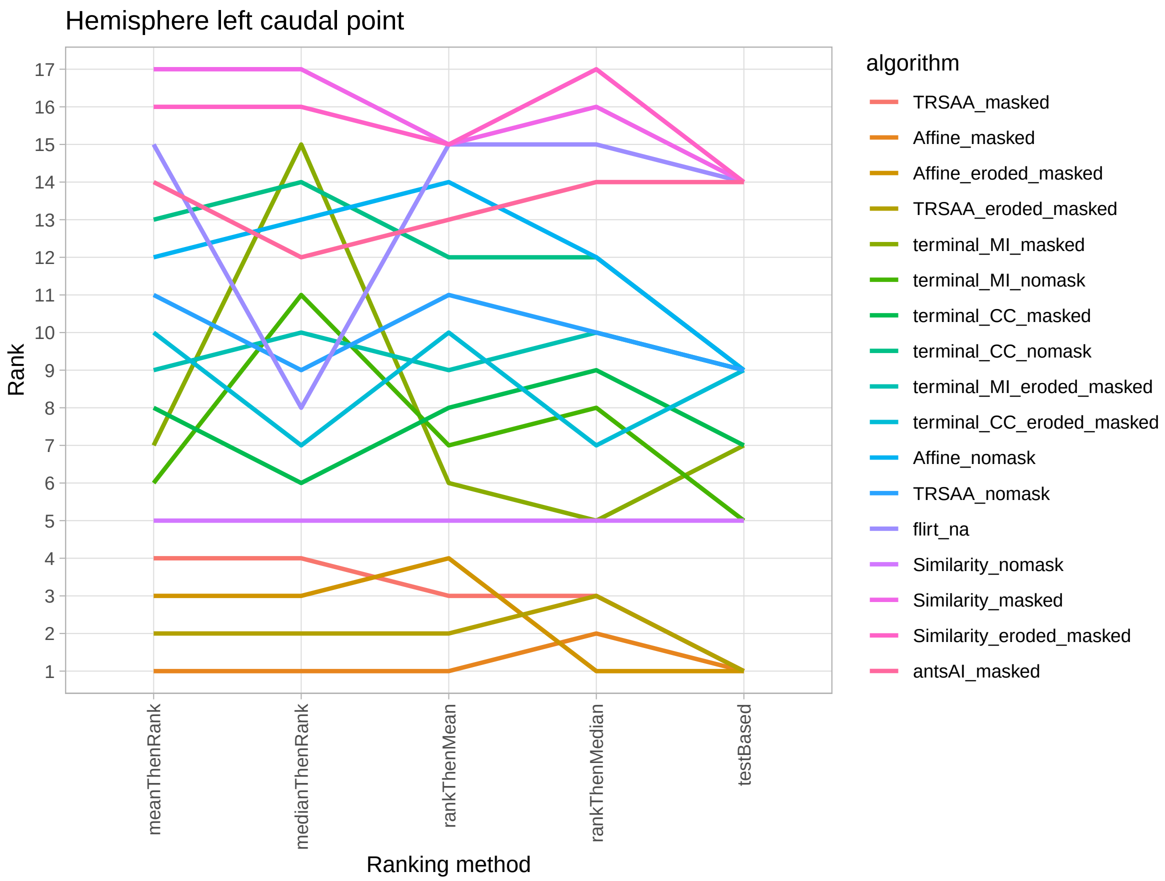

Hemisphere left caudal point
Rank
17
16
15
14
13
12
11
10
9
8
7
6
5
4
3
2
1
meanThenRank
medianThenRank
rankThenMean
rankThenMedian
testBased
Ranking method
algorithm
TRSAA_masked
Affine_masked
Affine_eroded_masked
TRSAA_eroded_masked
terminal_MI_masked
terminal_MI_nomask
terminal_CC_masked
terminal_CC_nomask
terminal_MI_eroded_masked
terminal_CC_eroded_masked
Affine_nomask
TRSAA_nomask
flirt_na
Similarity_nomask
Similarity_masked
Similarity_eroded_masked
antsAI_masked

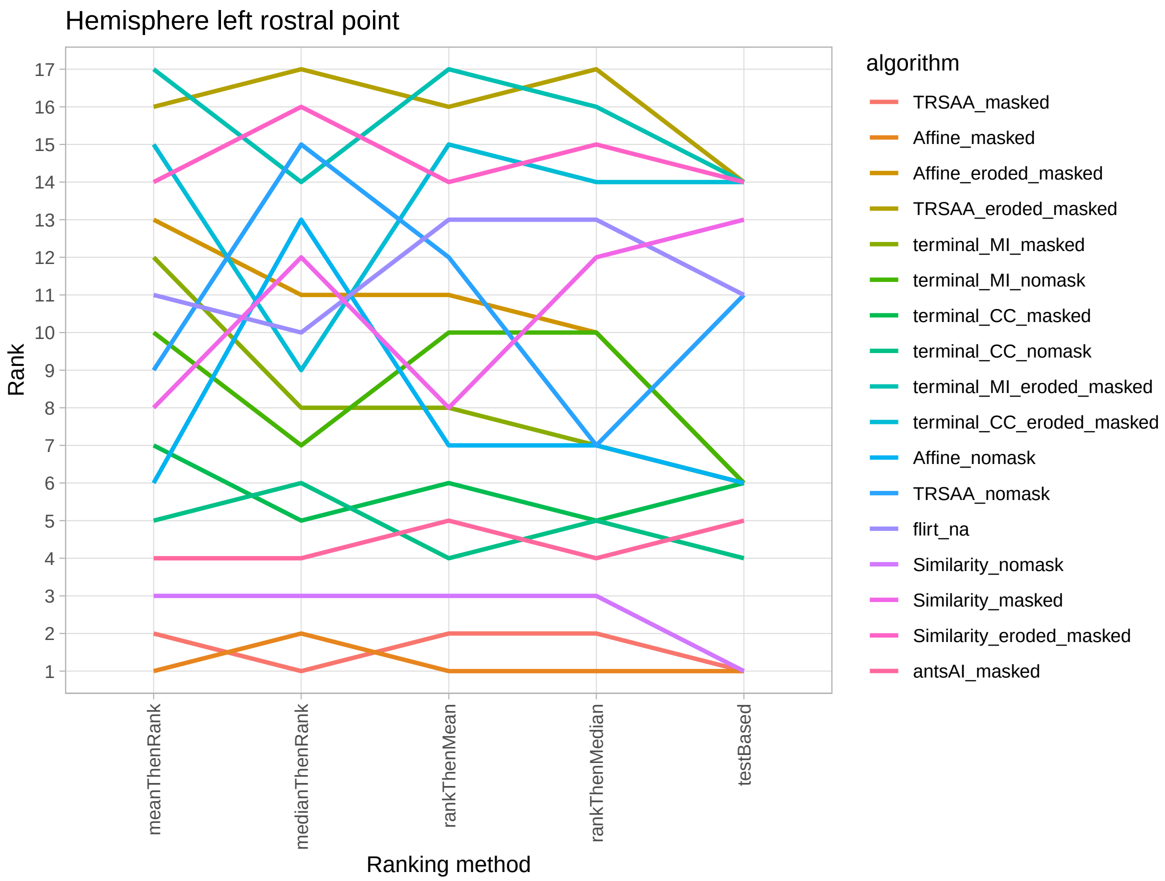

Hemisphere left rostral point
Rank
17
16
15
14
13
12
11
10
9
8
7
6
5
4
3
2
1
meanThenRank
medianThenRank
rankThenMean
rankThenMedian
testBased
Ranking method
algorithm
TRSAA_masked
Affine_masked
Affine_eroded_masked
TRSAA_eroded_masked
terminal_MI_masked
terminal_MI_nomask
terminal_CC_masked
terminal_CC_nomask
terminal_MI_eroded_masked
terminal_CC_eroded_masked
Affine_nomask
TRSAA_nomask
flirt_na
Similarity_nomask
Similarity_masked
Similarity_eroded_masked
antsAI_masked

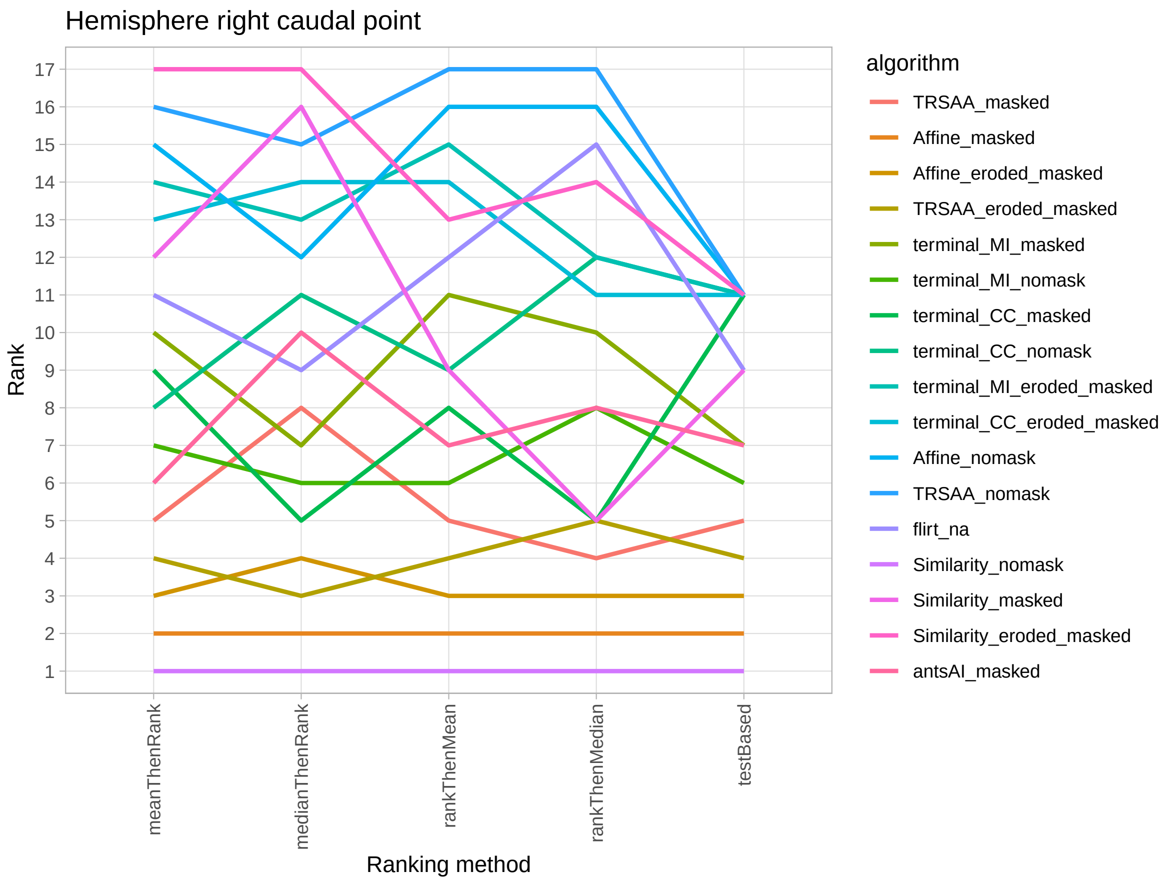

Hemisphere right caudal point
Rank
17
16
15
14
13
12
11
10
9
8
7
6
5
4
3
2
1
meanThenRank
medianThenRank
rankThenMean
rankThenMedian
testBased
Ranking method
algorithm
TRSAA_masked
Affine_masked
Affine_eroded_masked
TRSAA_eroded_masked
terminal_MI_masked
terminal_MI_nomask
terminal_CC_masked
terminal_CC_nomask
terminal_MI_eroded_masked
terminal_CC_eroded_masked
Affine_nomask
TRSAA_nomask
flirt_na
Similarity_nomask
Similarity_masked
Similarity_eroded_masked
antsAI_masked

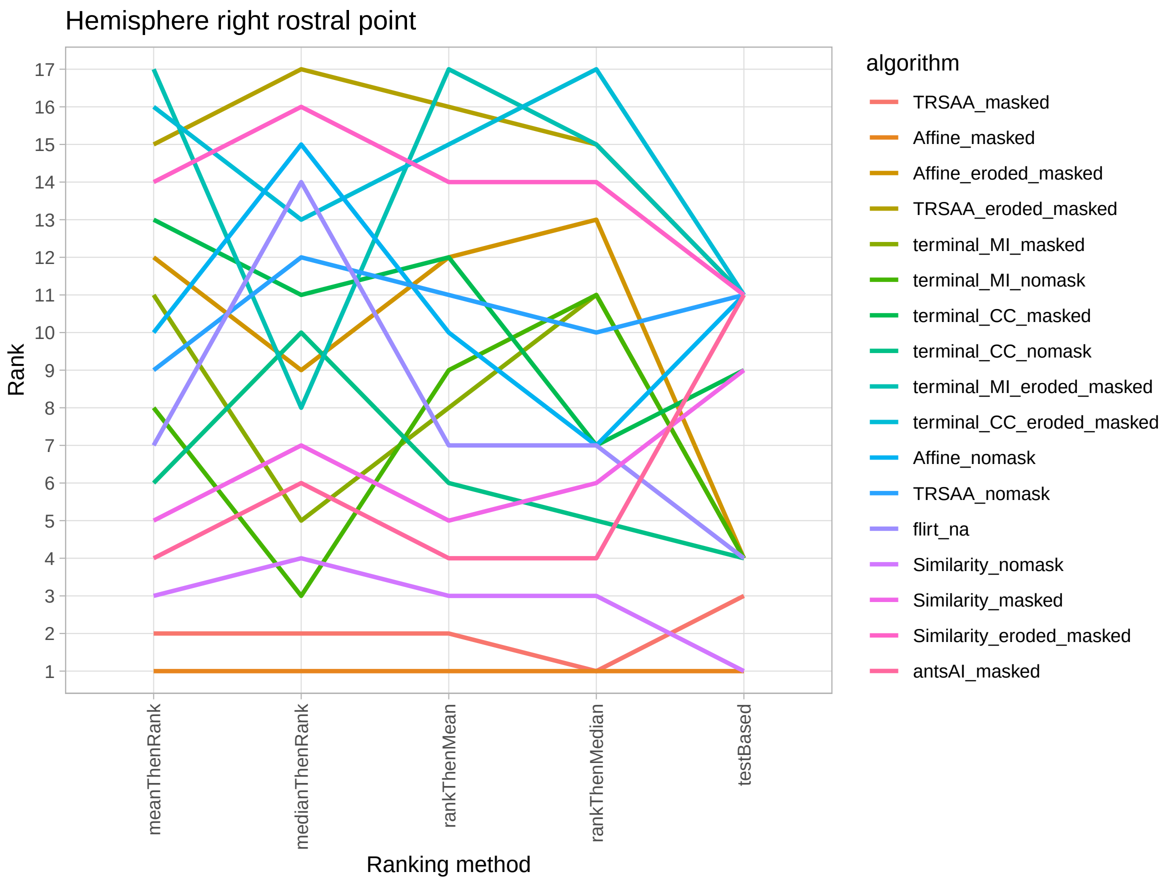
Hemisphere right rostral point
Rank
17
16
15
14
13
12
11
10
9
8
7
6
5
4
3
2
1
meanThenRank
medianThenRank
rankThenMean
rankThenMedian
testBased
Ranking method
algorithm
TRSAA_masked
Affine_masked
Affine_eroded_masked
TRSAA_eroded_masked
terminal_MI_masked
terminal_MI_nomask
terminal_CC_masked
terminal_CC_nomask
terminal_MI_eroded_masked
terminal_CC_eroded_masked
Affine_nomask
TRSAA_nomask
flirt_na
Similarity_nomask
Similarity_masked
Similarity_eroded_masked
antsAI_masked

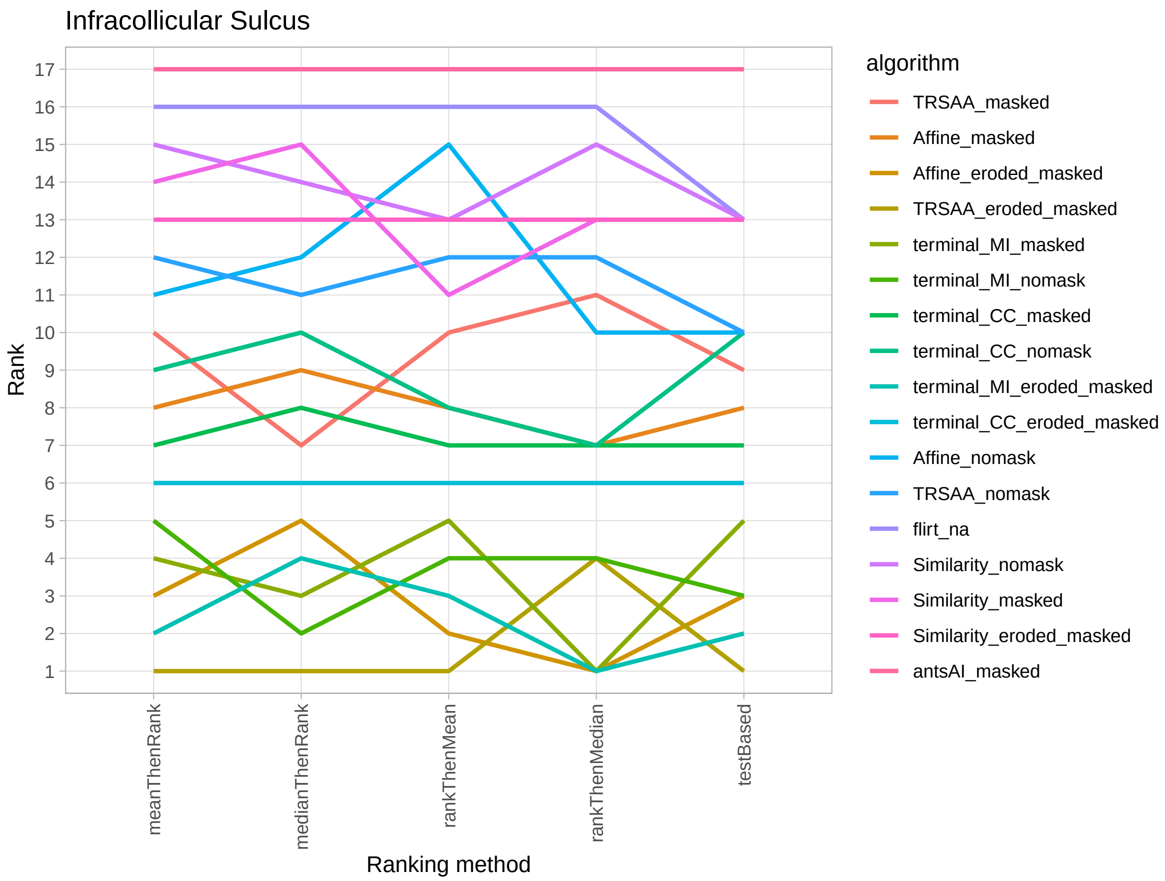

Infracollicular Sulcus
Rank
17
16
15
14
13
12
11
10
9
8
7
6
5
4
3
2
1
meanThenRank
medianThenRank
rankThenMean
rankThenMedian
testBased
Ranking method
algorithm
TRSAA_masked
Affine_masked
Affine_eroded_masked
TRSAA_eroded_masked
terminal_MI_masked
terminal_MI_nomask
terminal_CC_masked
terminal_CC_nomask
terminal_MI_eroded_masked
terminal_CC_eroded_masked
Affine_nomask
TRSAA_nomask
flirt_na
Similarity_nomask
Similarity_masked
Similarity_eroded_masked
antsAI_masked

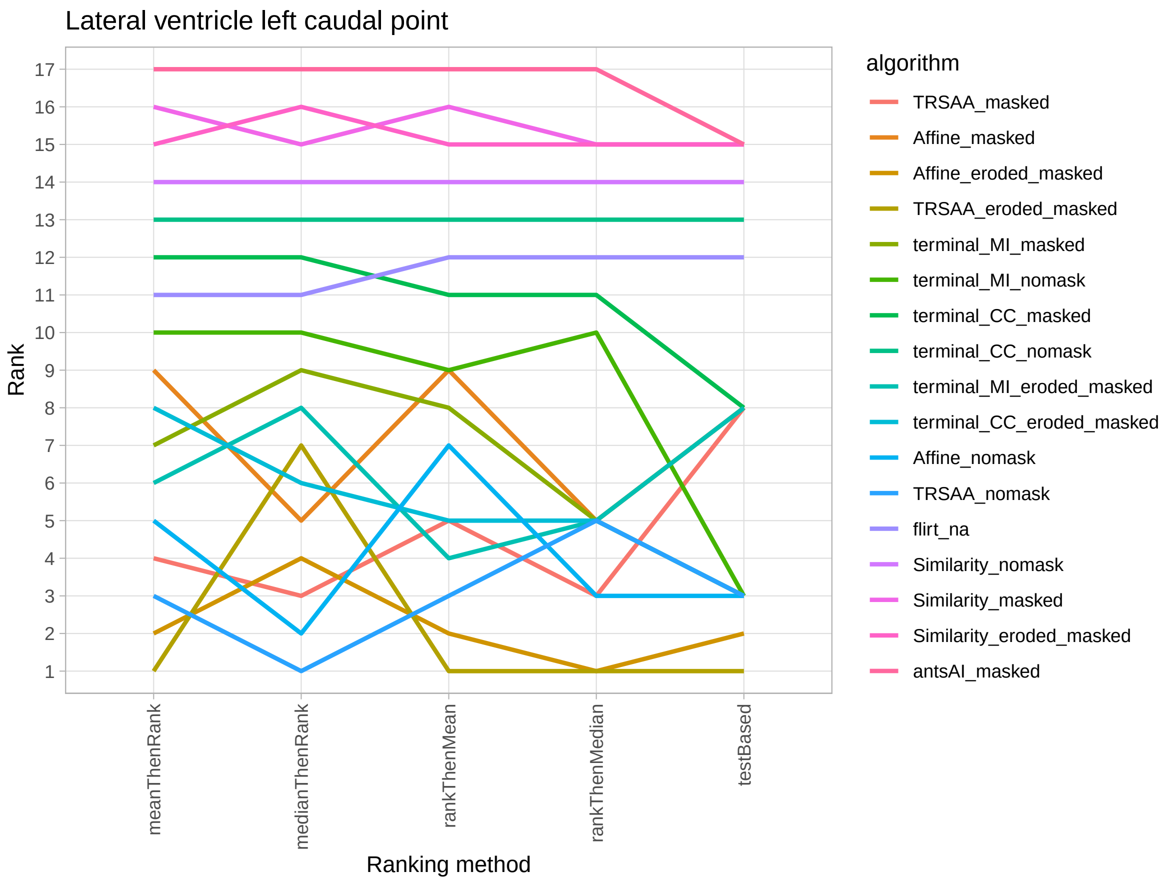

Lateral ventricle left caudal point
Rank
17
16
15
14
13
12
11
10
9
8
7
6
5
4
3
2
1
meanThenRank
medianThenRank
rankThenMean
rankThenMedian
testBased
Ranking method
algorithm
TRSAA_masked
Affine_masked
Affine_eroded_masked
TRSAA_eroded_masked
terminal_MI_masked
terminal_MI_nomask
terminal_CC_masked
terminal_CC_nomask
terminal_MI_eroded_masked
terminal_CC_eroded_masked
Affine_nomask
TRSAA_nomask
flirt_na
Similarity_nomask
Similarity_masked
Similarity_eroded_masked
antsAI_masked

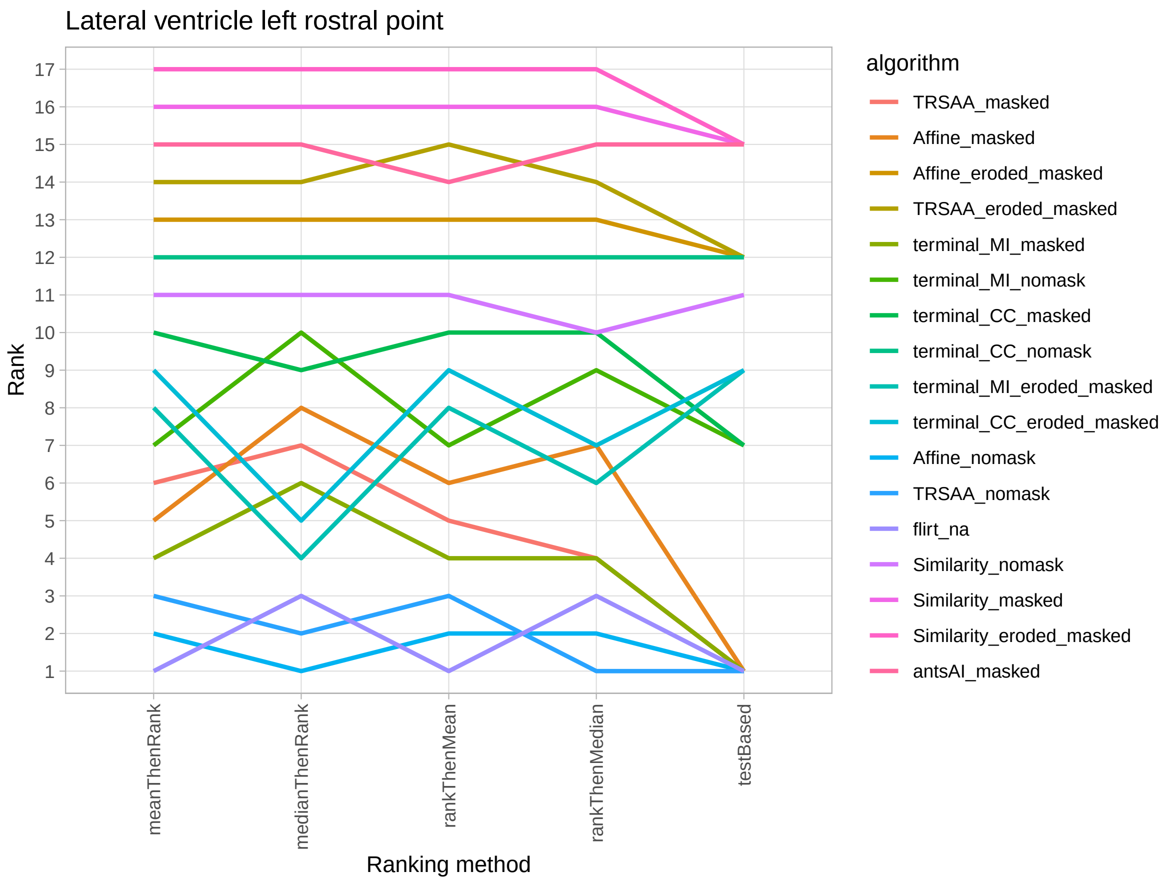
Lateral ventricle left rostral point
Rank
17
16
15
14
13
12
11
10
9
8
7
6
5
4
3
2
1
meanThenRank
medianThenRank
rankThenMean
rankThenMedian
testBased
Ranking method
algorithm
TRSAA_masked
Affine_masked
Affine_eroded_masked
TRSAA_eroded_masked
terminal_MI_masked
terminal_MI_nomask
terminal_CC_masked
terminal_CC_nomask
terminal_MI_eroded_masked
terminal_CC_eroded_masked
Affine_nomask
TRSAA_nomask
flirt_na
Similarity_nomask
Similarity_masked
Similarity_eroded_masked
antsAI_masked

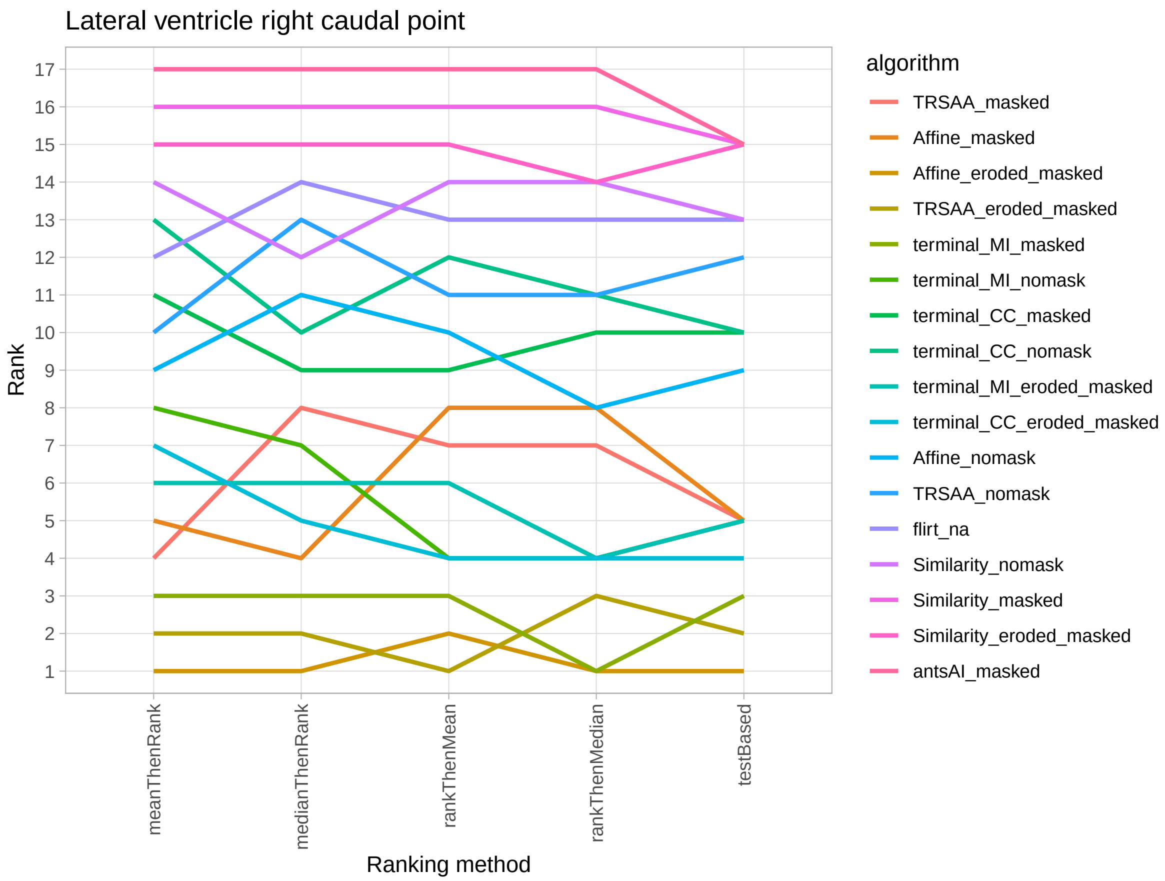
Lateral ventricle right caudal point
Rank
17
16
15
14
13
12
11
10
9
8
7
6
5
4
3
2
1
meanThenRank
medianThenRank
rankThenMean
rankThenMedian
testBased
Ranking method
algorithm
TRSAA_masked
Affine_masked
Affine_eroded_masked
TRSAA_eroded_masked
terminal_MI_masked
terminal_MI_nomask
terminal_CC_masked
terminal_CC_nomask
terminal_MI_eroded_masked
terminal_CC_eroded_masked
Affine_nomask
TRSAA_nomask
flirt_na
Similarity_nomask
Similarity_masked
Similarity_eroded_masked
antsAI_masked

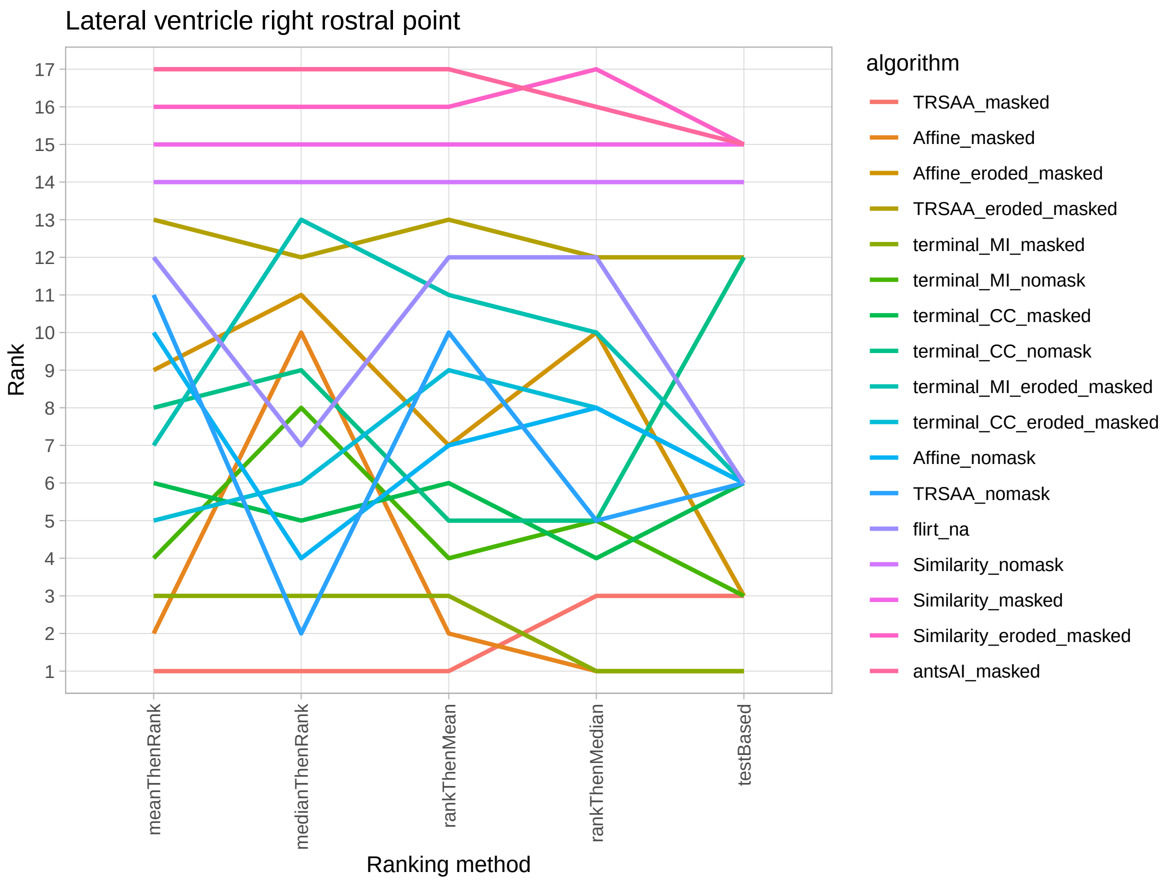
Lateral ventricle right rostral point
Rank
17
16
15
14
13
12
11
10
9
8
7
6
5
4
3
2
1
meanThenRank
medianThenRank
rankThenMean
rankThenMedian
testBased
Ranking method
algorithm
TRSAA_masked
Affine_masked
Affine_eroded_masked
TRSAA_eroded_masked
terminal_MI_masked
terminal_MI_nomask
terminal_CC_masked
terminal_CC_nomask
terminal_MI_eroded_masked
terminal_CC_eroded_masked
Affine_nomask
TRSAA_nomask
flirt_na
Similarity_nomask
Similarity_masked
Similarity_eroded_masked
antsAI_masked

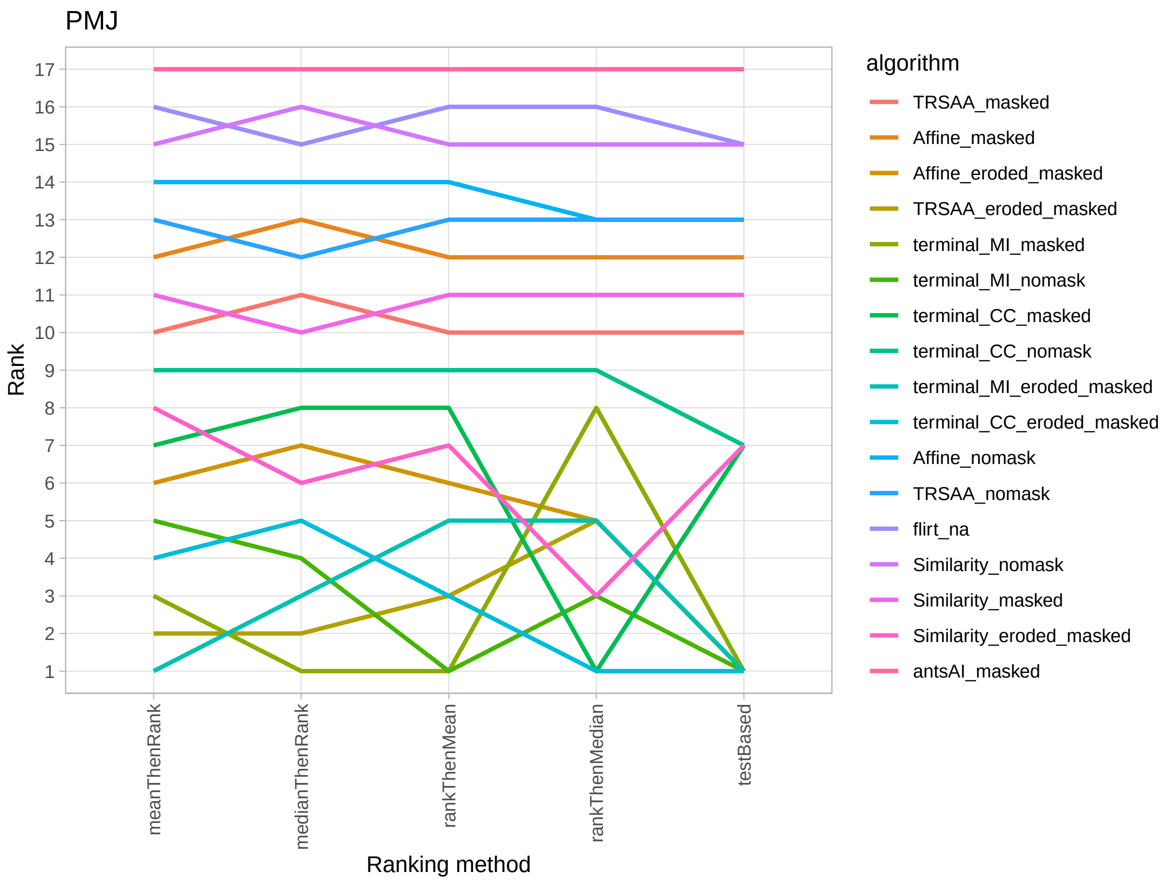
PMJ
Rank
17
16
15
14
13
12
11
10
9
8
7
6
5
4
3
2
1
meanThenRank
medianThenRank
rankThenMean
rankThenMedian
testBased
Ranking method
algorithm
TRSAA_masked
Affine_masked
Affine_eroded_masked
TRSAA_eroded_masked
terminal_MI_masked
terminal_MI_nomask
terminal_CC_masked
terminal_CC_nomask
terminal_MI_eroded_masked
terminal_CC_eroded_masked
Affine_nomask
TRSAA_nomask
flirt_na
Similarity_nomask
Similarity_masked
Similarity_eroded_masked
antsAI_masked

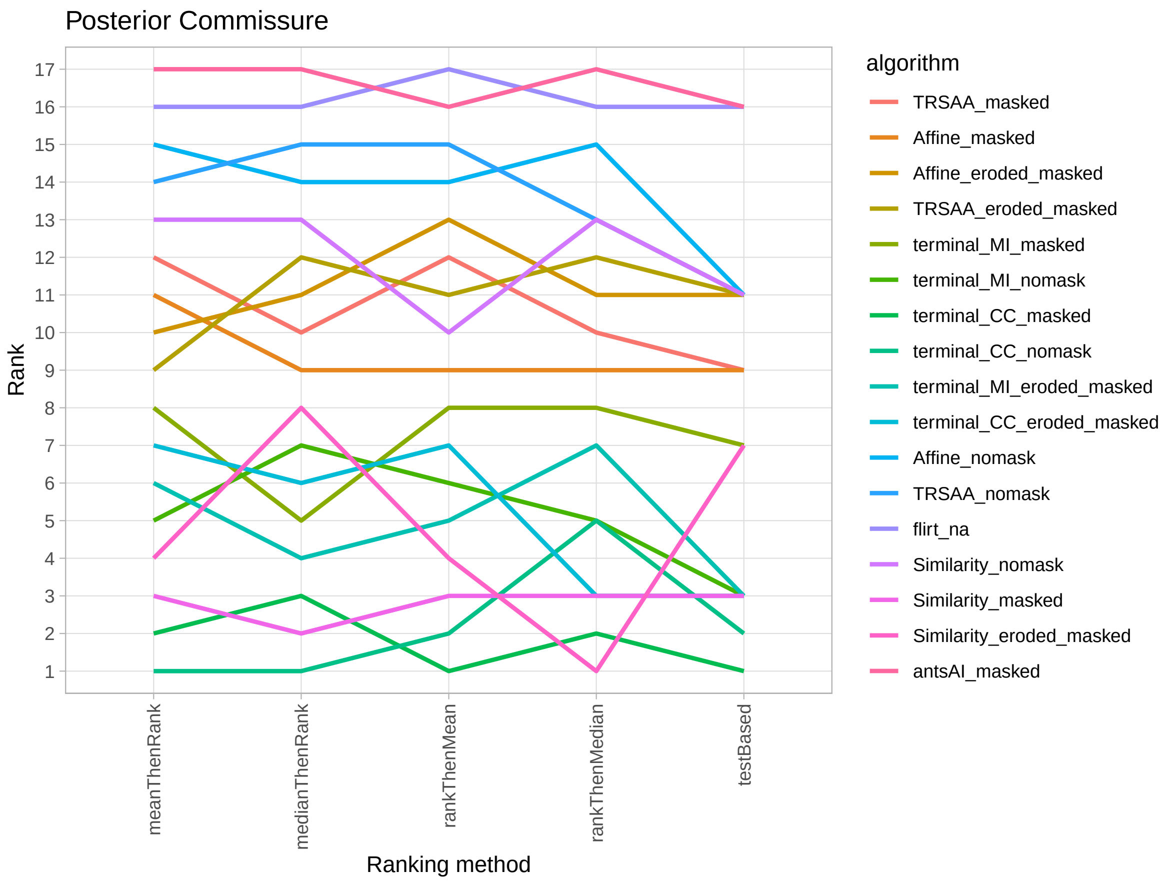
Posterior Commissure
Rank
Ranking method
meanThenRank
medianThenRank
rankThenMean
rankThenMedian
testBased
algorithm
TRSAA_masked
Affine_masked
Affine_eroded_masked
TRSAA_eroded_masked
terminal_MI_masked
terminal_MI_nomask
terminal_CC_masked
terminal_CC_nomask
terminal_MI_eroded_masked
terminal_CC_eroded_masked
Affine_nomask
TRSAA_nomask
flirt_na
Similarity_nomask
Similarity_masked
Similarity_eroded_masked
antsAI_masked

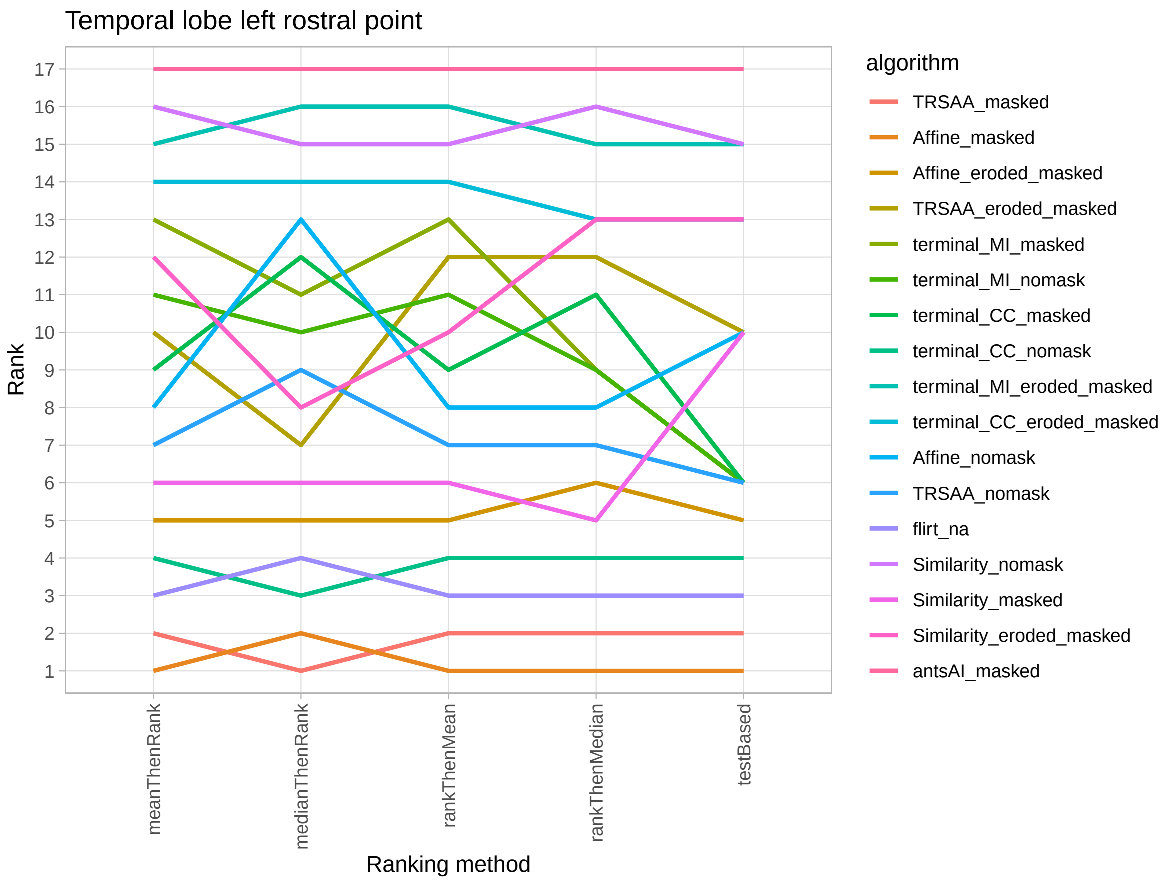
Temporal lobe left rostral point
Rank
17
16
15
14
13
12
11
10
9
8
7
6
5
4
3
2
1
meanThenRank
medianThenRank
rankThenMean
rankThenMedian
testBased
Ranking method
algorithm
TRSAA_masked
Affine_masked
Affine_eroded_masked
TRSAA_eroded_masked
terminal_MI_masked
terminal_MI_nomask
terminal_CC_masked
terminal_CC_nomask
terminal_MI_eroded_masked
terminal_CC_eroded_masked
Affine_nomask
TRSAA_nomask
flirt_na
Similarity_nomask
Similarity_masked
Similarity_eroded_masked
antsAI_masked

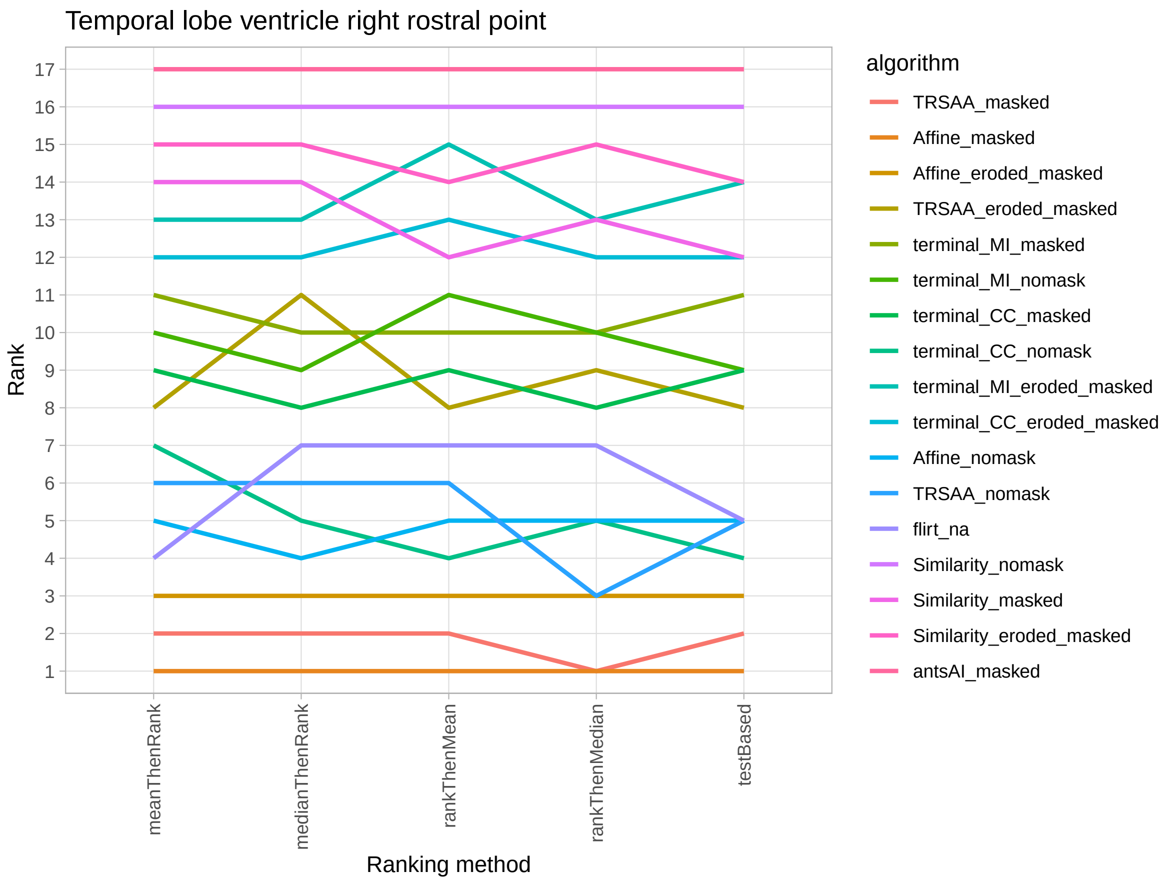
Temporal lobe ventricle right rostral point
Rank
17
16
15
14
13
12
11
10
9
8
7
6
5
4
3
2
1
meanThenRank
medianThenRank
rankThenMean
rankThenMedian
testBased
Ranking method
algorithm
TRSAA_masked
Affine_masked
Affine_eroded_masked
TRSAA_eroded_masked
terminal_MI_masked
terminal_MI_nomask
terminal_CC_masked
terminal_CC_nomask
terminal_MI_eroded_masked
terminal_CC_eroded_masked
Affine_nomask
TRSAA_nomask
flirt_na
Similarity_nomask
Similarity_masked
Similarity_eroded_masked
antsAI_masked

4 Visualization of cross-task insights

The algorithms are ordered according to consensus ranking.

4.1 Characterization of algorithms

4.1.1 Ranking stability: Variability of achieved rankings across tasks

Algorithms are color-coded, and the area of each blob at position $(A_i, \text{rank } j)$ is proportional to the relative frequency A_i achieved rank j across multiple tasks. The median rank for each algorithm is indicated by a black cross. This way, the distribution of ranks across tasks can be intuitively visualized.

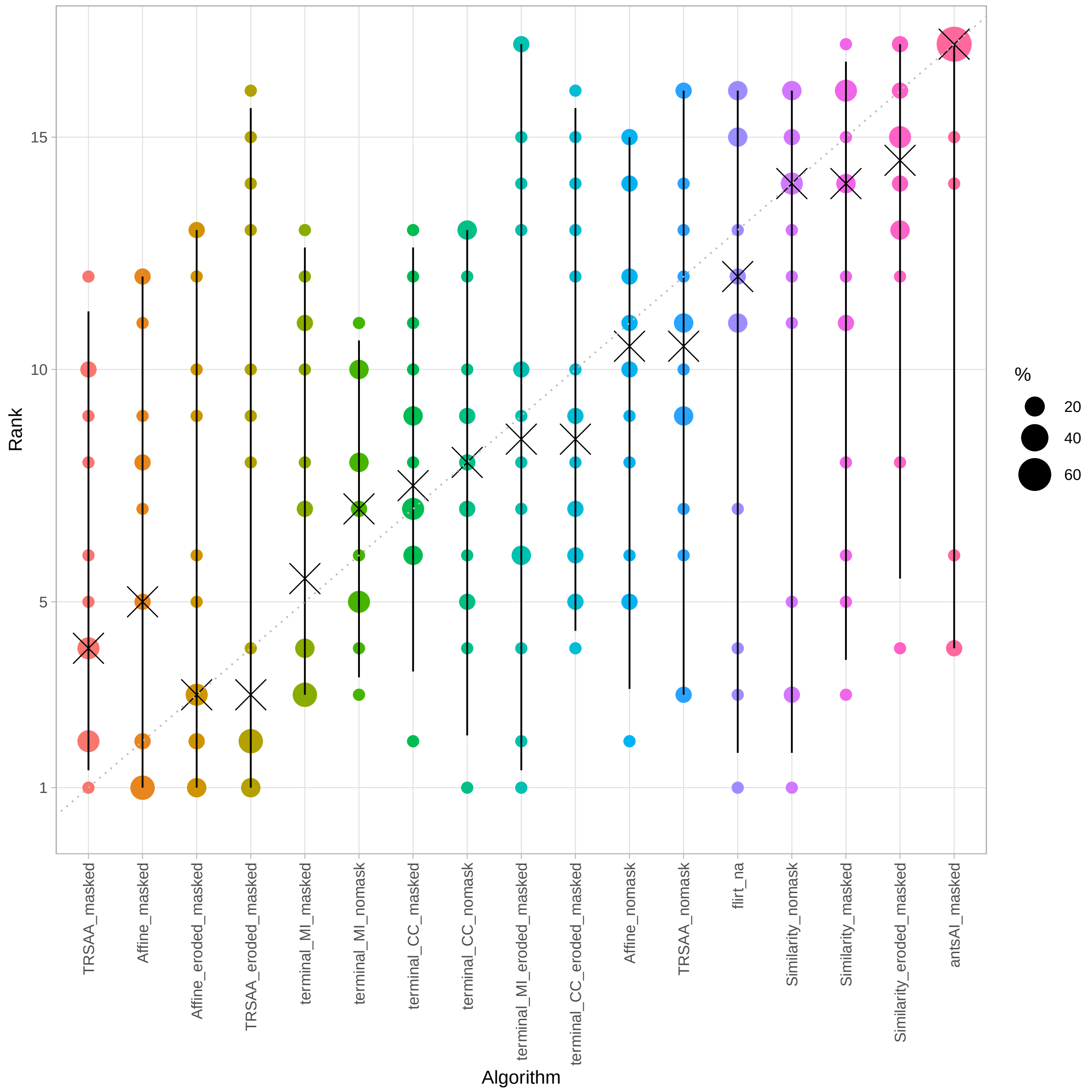

4.1.2 Ranking stability: Ranking variability via bootstrap approach

A blob plot of bootstrap results over the different tasks separated by algorithm allows another perspective on the assessment data. This gives deeper insights into the characteristics of tasks and the ranking uncertainty of the algorithms in each task.

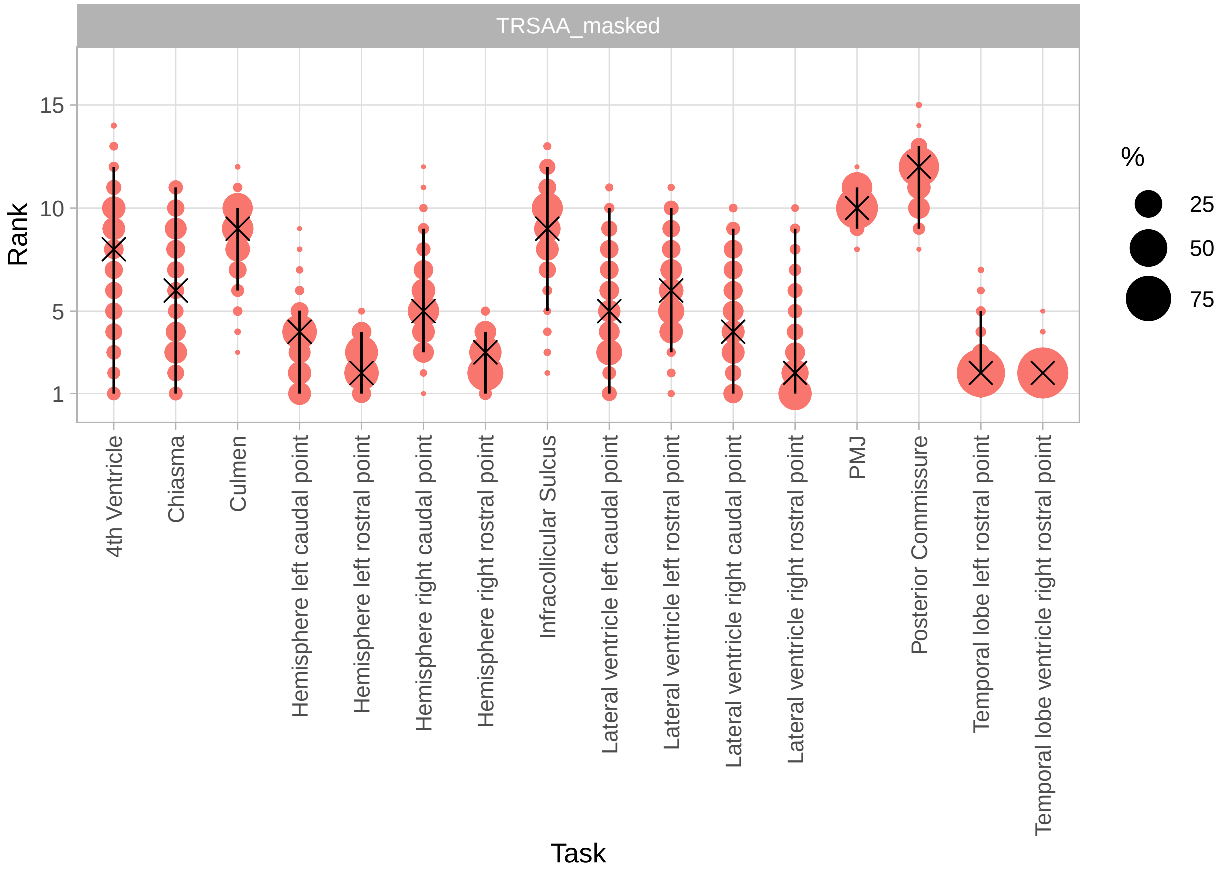

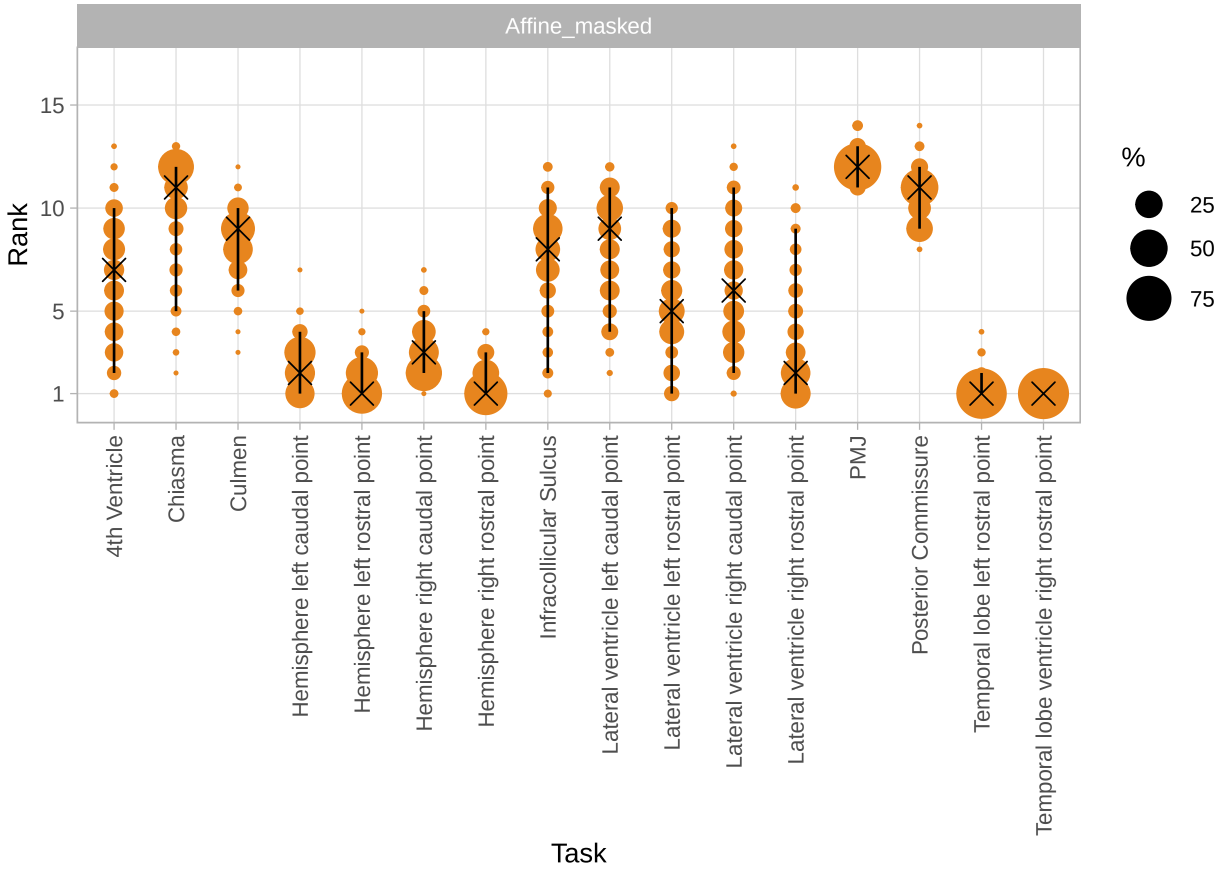

Affine_masked
Rank
15
10
5
1
%
25
50
75
4th Ventricle
Chiasma
Culmen
Hemisphere left caudal point
Hemisphere left rostral point
Hemisphere right caudal point
Hemisphere right rostral point
Infracollicular Sulcus
Lateral ventricle left caudal point
Lateral ventricle left rostral point
Lateral ventricle right caudal point
Lateral ventricle right rostral point
PMJ
Posterior Commissure
Temporal lobe left rostral point
Temporal lobe ventricle right rostral point
Task

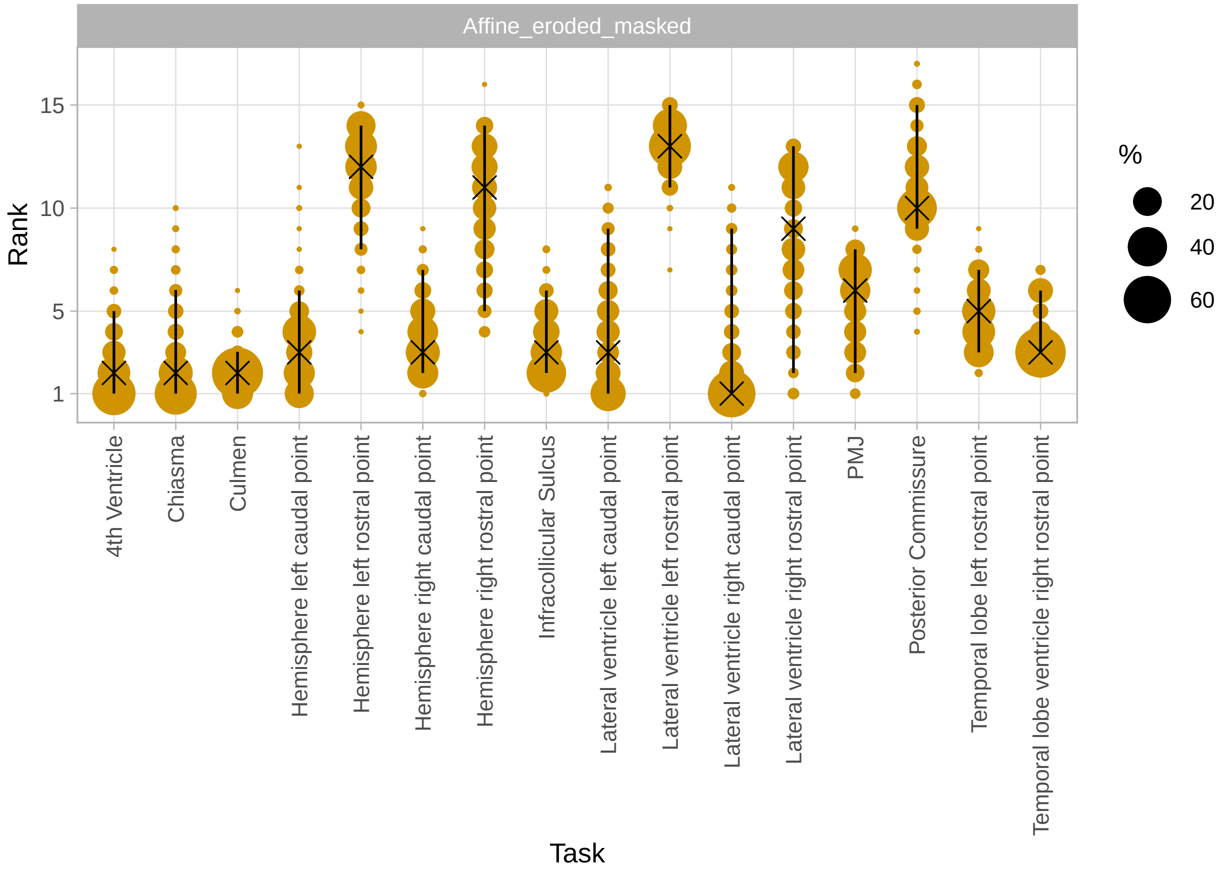

Affine_eroded_masked
Rank
15
10
5
1
%
20
40
60
4th Ventricle
Chiasma
Culmen
Hemisphere left caudal point
Hemisphere left rostral point
Hemisphere right caudal point
Hemisphere right rostral point
Infracollicular Sulcus
Lateral ventricle left caudal point
Lateral ventricle left rostral point
Lateral ventricle right caudal point
Lateral ventricle right rostral point
PMJ
Posterior Commissure
Temporal lobe left rostral point
Temporal lobe ventricle right rostral point
Task

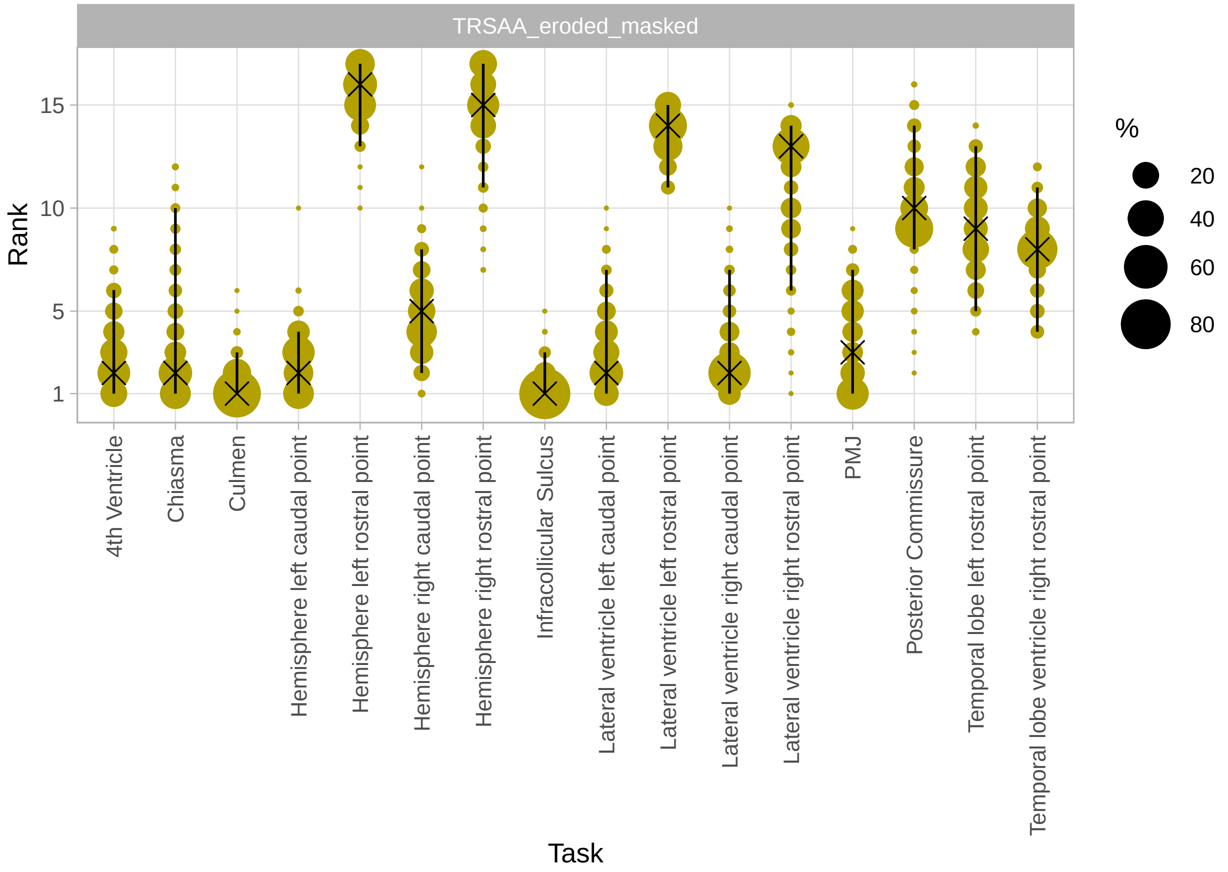
TRSAA_eroded_masked
Rank
15
10
5
1
4th Ventricle
Chiasma
Culmen
Hemisphere left caudal point
Hemisphere left rostral point
Hemisphere right caudal point
Hemisphere right rostral point
Infracollicular Sulcus
Lateral ventricle left caudal point
Lateral ventricle left rostral point
Lateral ventricle right caudal point
Lateral ventricle right rostral point
PMJ
Posterior Commissure
Temporal lobe left rostral point
Temporal lobe ventricle right rostral point
Task
%
20
40
60
80

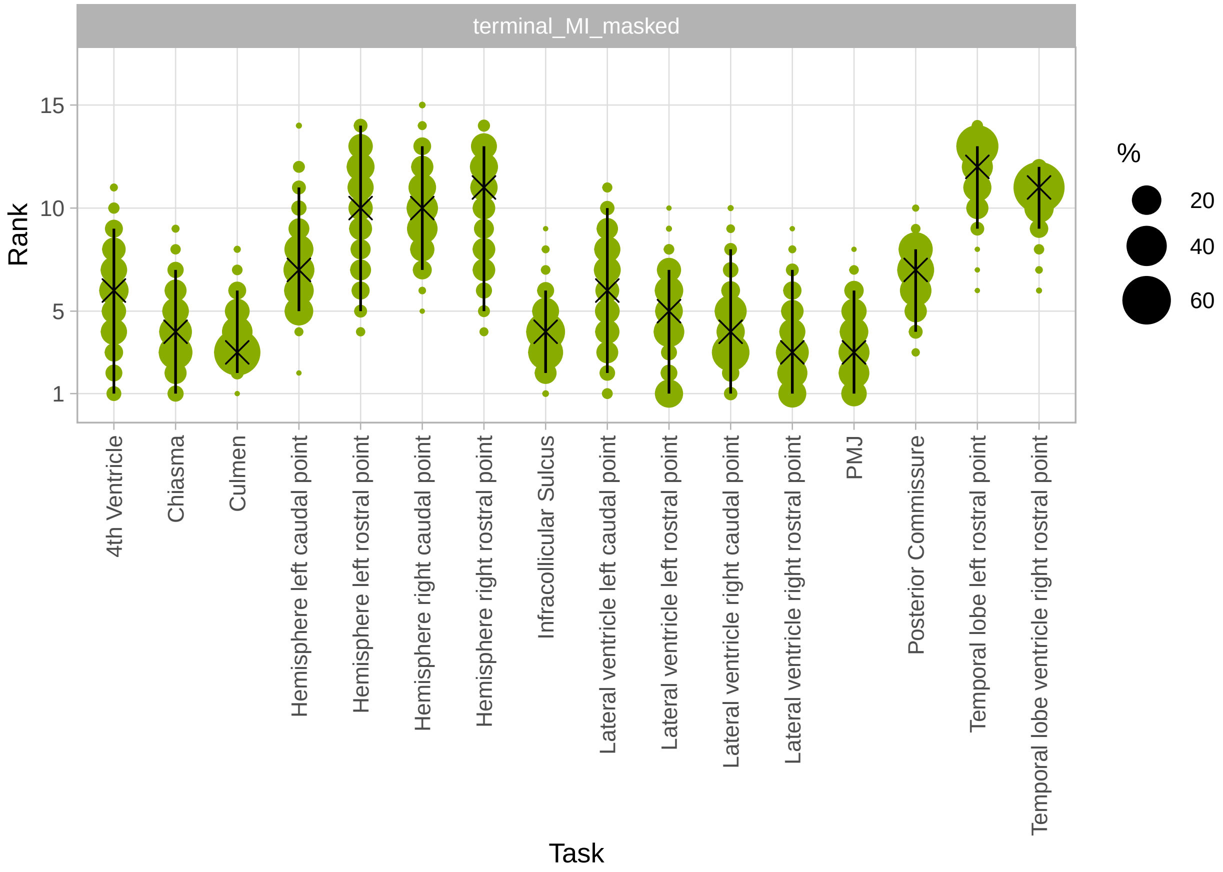
terminal_MI_masked
Rank
15
10
5
1
%
20
40
60
4th Ventricle
Chiasma
Culmen
Hemisphere left caudal point
Hemisphere left rostral point
Hemisphere right caudal point
Hemisphere right rostral point
Infracollicular Sulcus
Lateral ventricle left caudal point
Lateral ventricle left rostral point
Lateral ventricle right caudal point
Lateral ventricle right rostral point
PMJ
Posterior Commissure
Temporal lobe left rostral point
Temporal lobe ventricle right rostral point
Task

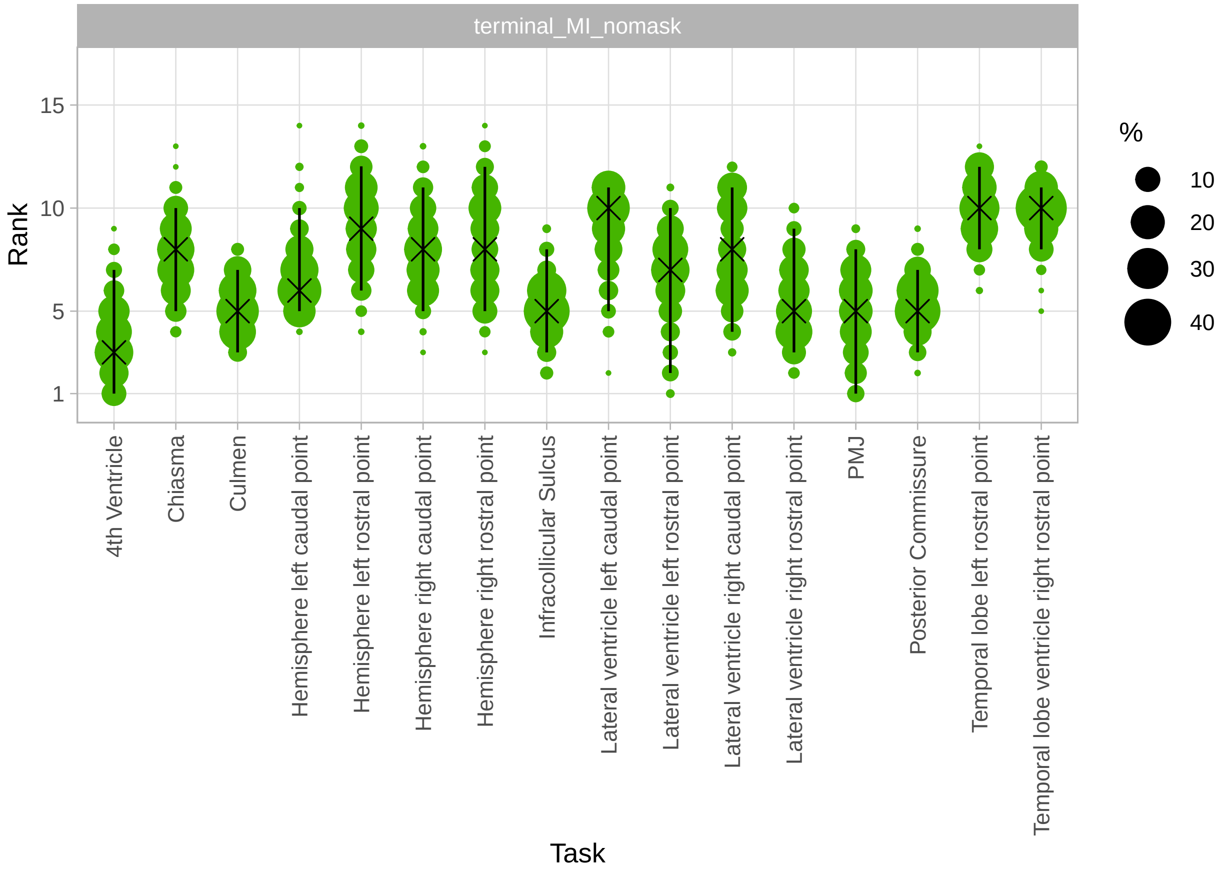
terminal_MI_nomask
Rank
15
10
5
1
%
10
20
30
40
4th Ventricle
Chiasma
Culmen
Hemisphere left caudal point
Hemisphere left rostral point
Hemisphere right caudal point
Hemisphere right rostral point
Infracollicular Sulcus
Lateral ventricle left caudal point
Lateral ventricle left rostral point
Lateral ventricle right caudal point
Lateral ventricle right rostral point
PMJ
Posterior Commissure
Temporal lobe left rostral point
Temporal lobe ventricle right rostral point
Task

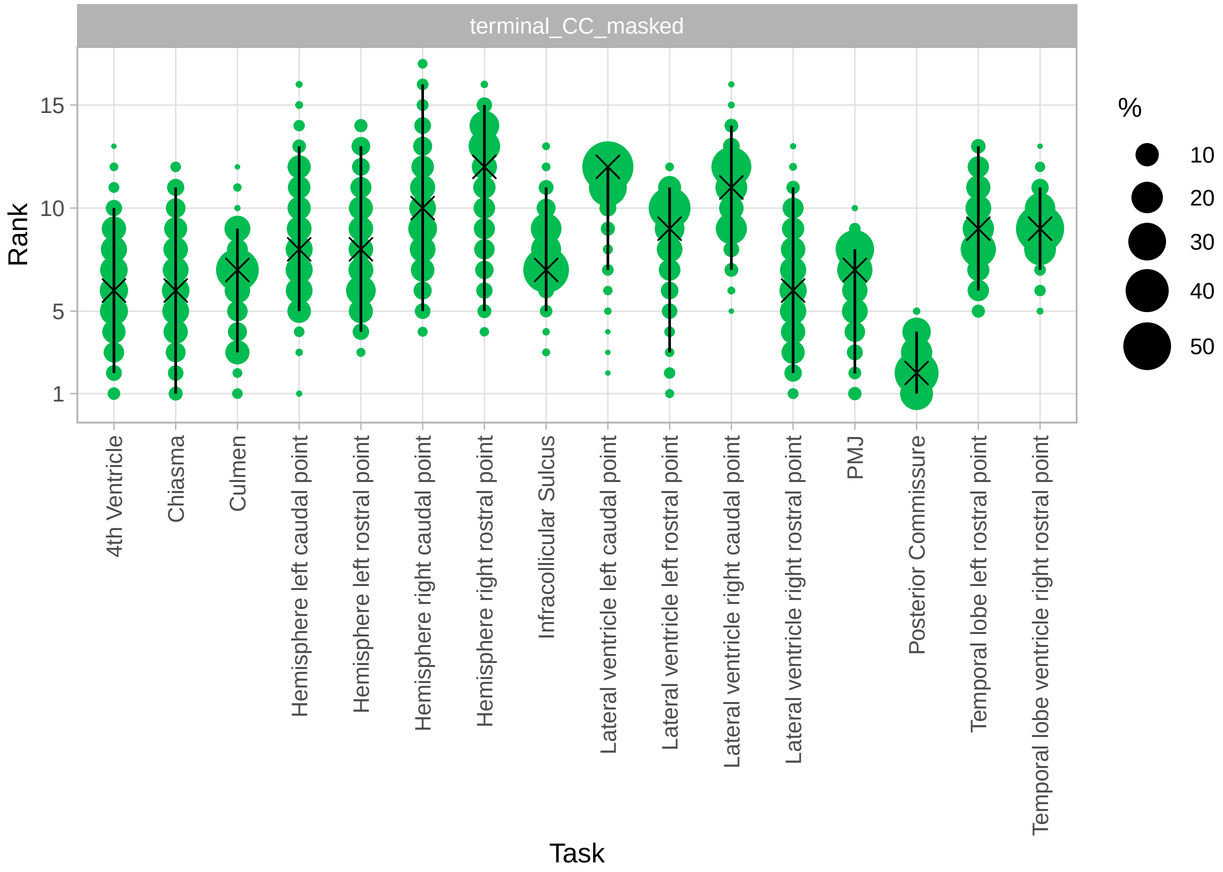
terminal_CC_masked
Rank
15
10
5
1
%
10
20
30
40
50
4th Ventricle
Chiasma
Culmen
Hemisphere left caudal point
Hemisphere left rostral point
Hemisphere right caudal point
Hemisphere right rostral point
Infracollicular Sulcus
Lateral ventricle left caudal point
Lateral ventricle left rostral point
Lateral ventricle right caudal point
Lateral ventricle right rostral point
PMJ
Posterior Commissure
Temporal lobe left rostral point
Temporal lobe ventricle right rostral point
Task

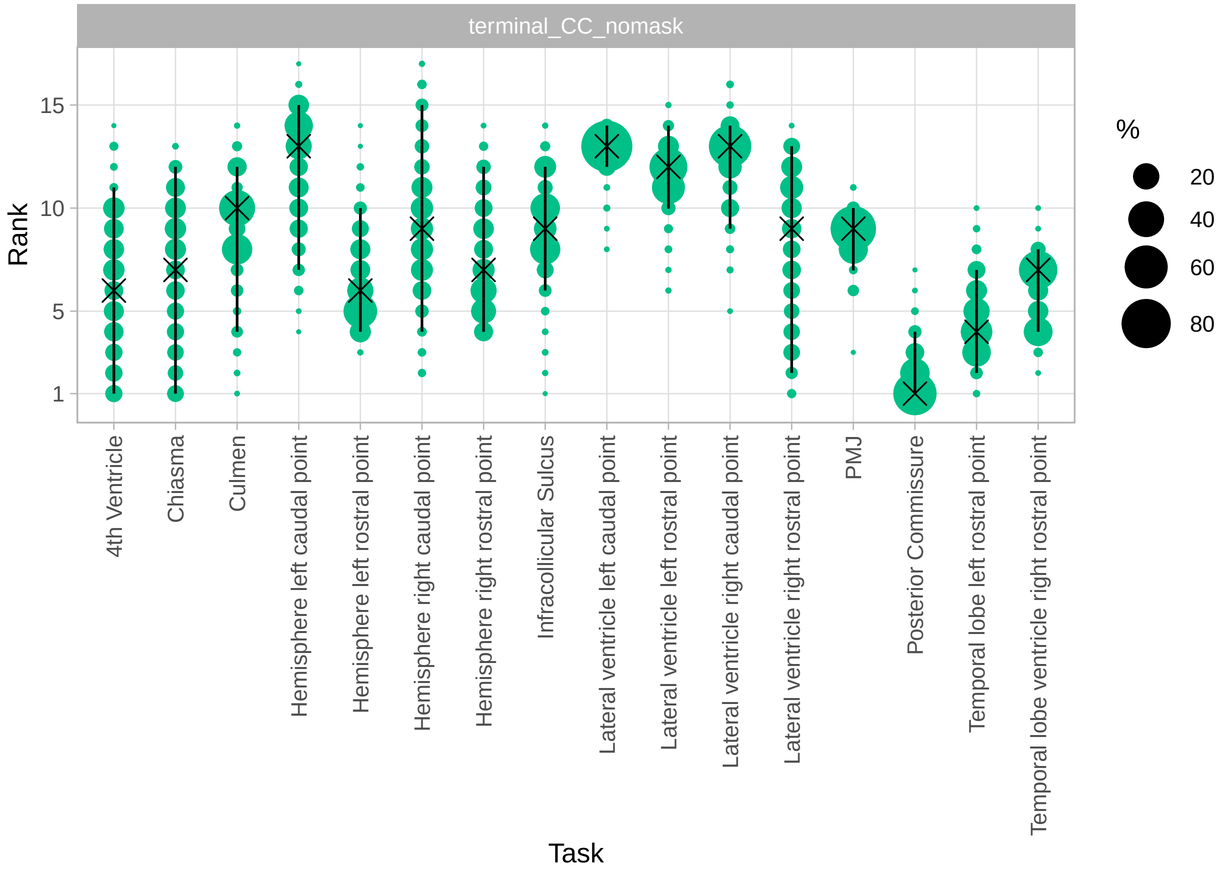
terminal_CC_nomask
Rank
15
10
5
1
4th Ventricle
Chiasma
Culmen
Hemisphere left caudal point
Hemisphere left rostral point
Hemisphere right caudal point
Hemisphere right rostral point
Infracollicular Sulcus
Lateral ventricle left caudal point
Lateral ventricle left rostral point
Lateral ventricle right caudal point
Lateral ventricle right rostral point
PMJ
Posterior Commissure
Temporal lobe left rostral point
Temporal lobe ventricle right rostral point
Task
%
20
40
60
80

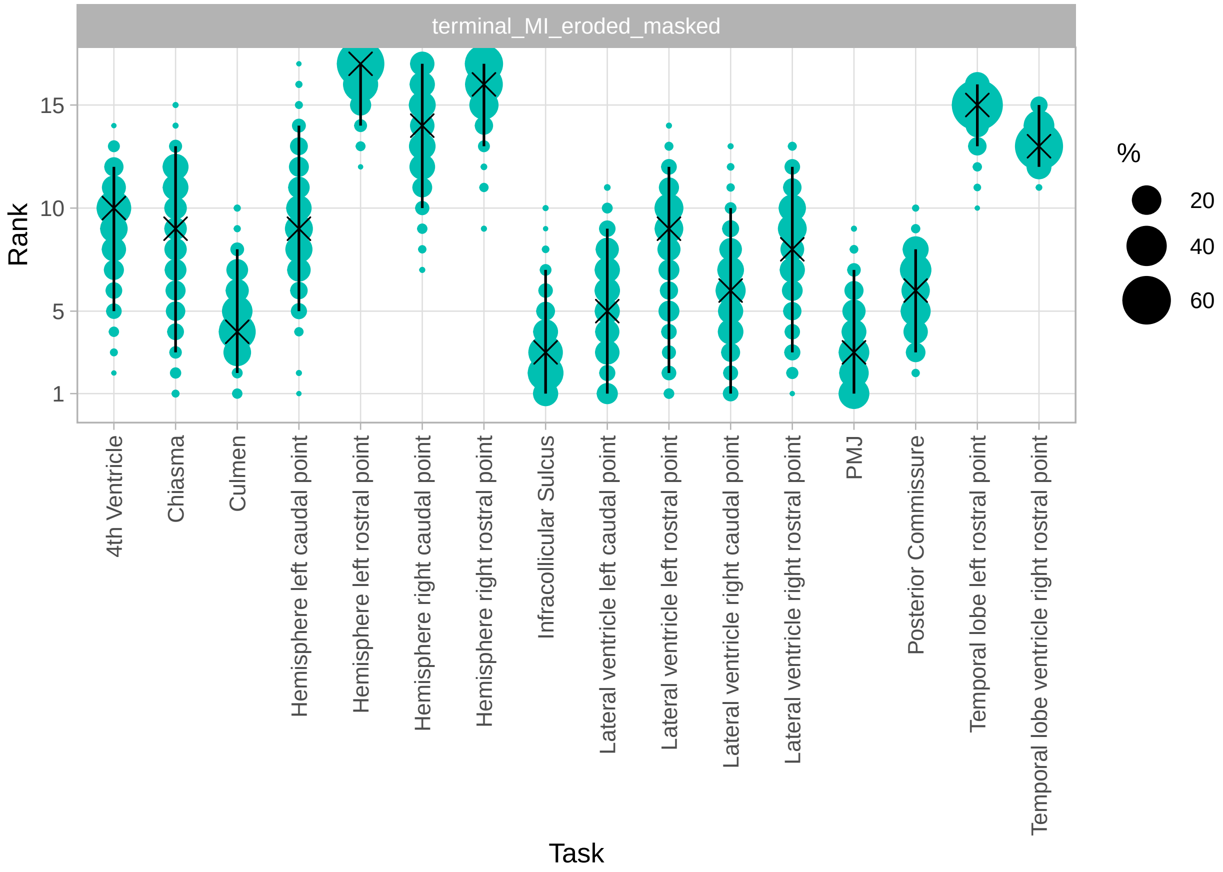
terminal_MI_eroded_masked
Rank
15
10
5
1
%
20
40
60
4th Ventricle
Chiasma
Culmen
Hemisphere left caudal point
Hemisphere left rostral point
Hemisphere right caudal point
Hemisphere right rostral point
Infracollicular Sulcus
Lateral ventricle left caudal point
Lateral ventricle left rostral point
Lateral ventricle right caudal point
Lateral ventricle right rostral point
PMJ
Posterior Commissure
Temporal lobe left rostral point
Temporal lobe ventricle right rostral point
Task

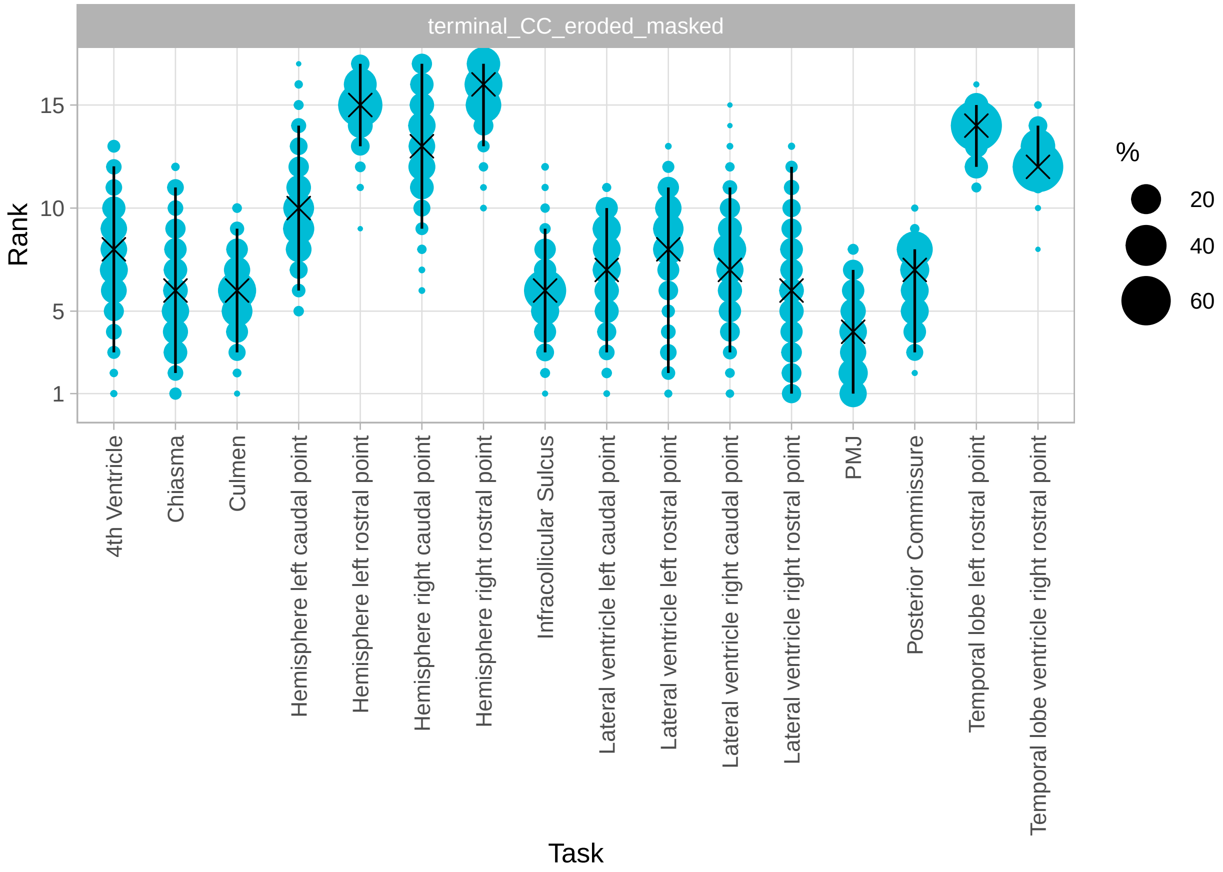

terminal_CC_eroded_masked
Rank
15
10
5
1
%
20
40
60
4th Ventricle
Chiasma
Culmen
Hemisphere left caudal point
Hemisphere left rostral point
Hemisphere right caudal point
Hemisphere right rostral point
Infracollicular Sulcus
Lateral ventricle left caudal point
Lateral ventricle left rostral point
Lateral ventricle right caudal point
Lateral ventricle right rostral point
PMJ
Posterior Commissure
Temporal lobe left rostral point
Temporal lobe ventricle right rostral point
Task

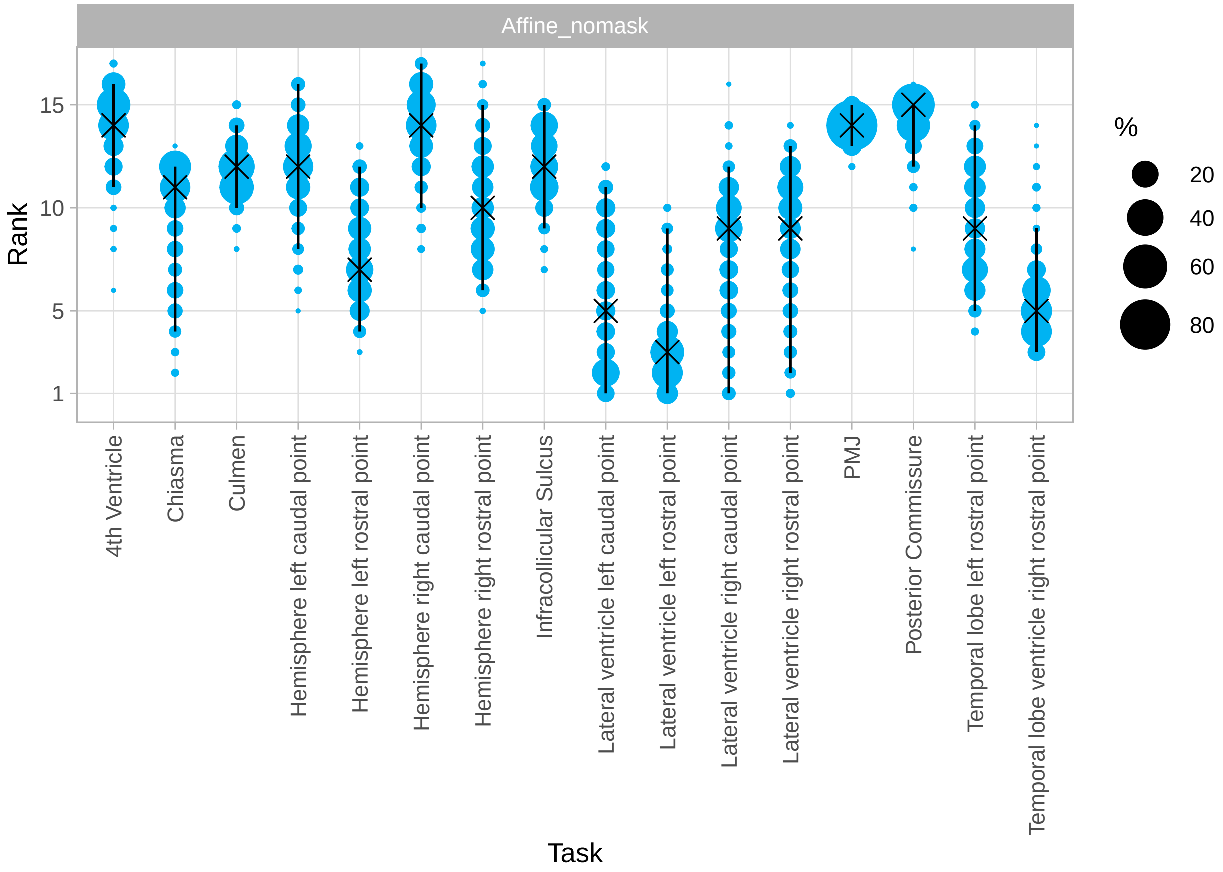

Affine_nomask
Rank
15
10
5
1
4th Ventricle
Chiasma
Culmen
Hemisphere left caudal point
Hemisphere left rostral point
Hemisphere right caudal point
Hemisphere right rostral point
Infracollicular Sulcus
Lateral ventricle left caudal point
Lateral ventricle left rostral point
Lateral ventricle right caudal point
Lateral ventricle right rostral point
PMJ
Posterior Commissure
Temporal lobe left rostral point
Temporal lobe ventricle right rostral point
Task
%
20
40
60
80

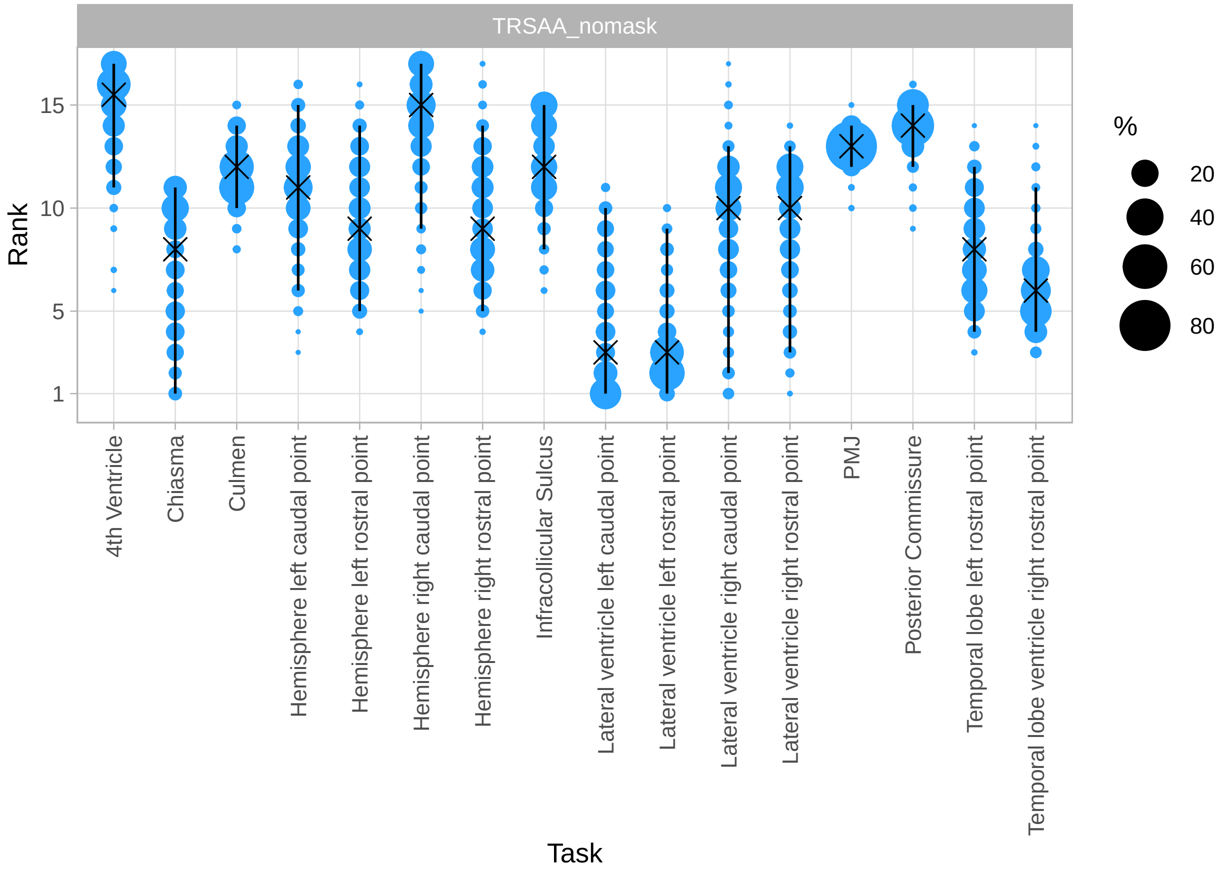
TRSAA_nomask
Rank
15
10
5
1
4th Ventricle
Chiasma
Culmen
Hemisphere left caudal point
Hemisphere left rostral point
Hemisphere right caudal point
Hemisphere right rostral point
Infracollicular Sulcus
Lateral ventricle left caudal point
Lateral ventricle left rostral point
Lateral ventricle right caudal point
Lateral ventricle right rostral point
PMJ
Posterior Commissure
Temporal lobe left rostral point
Temporal lobe ventricle right rostral point
Task
%
20
40
60
80

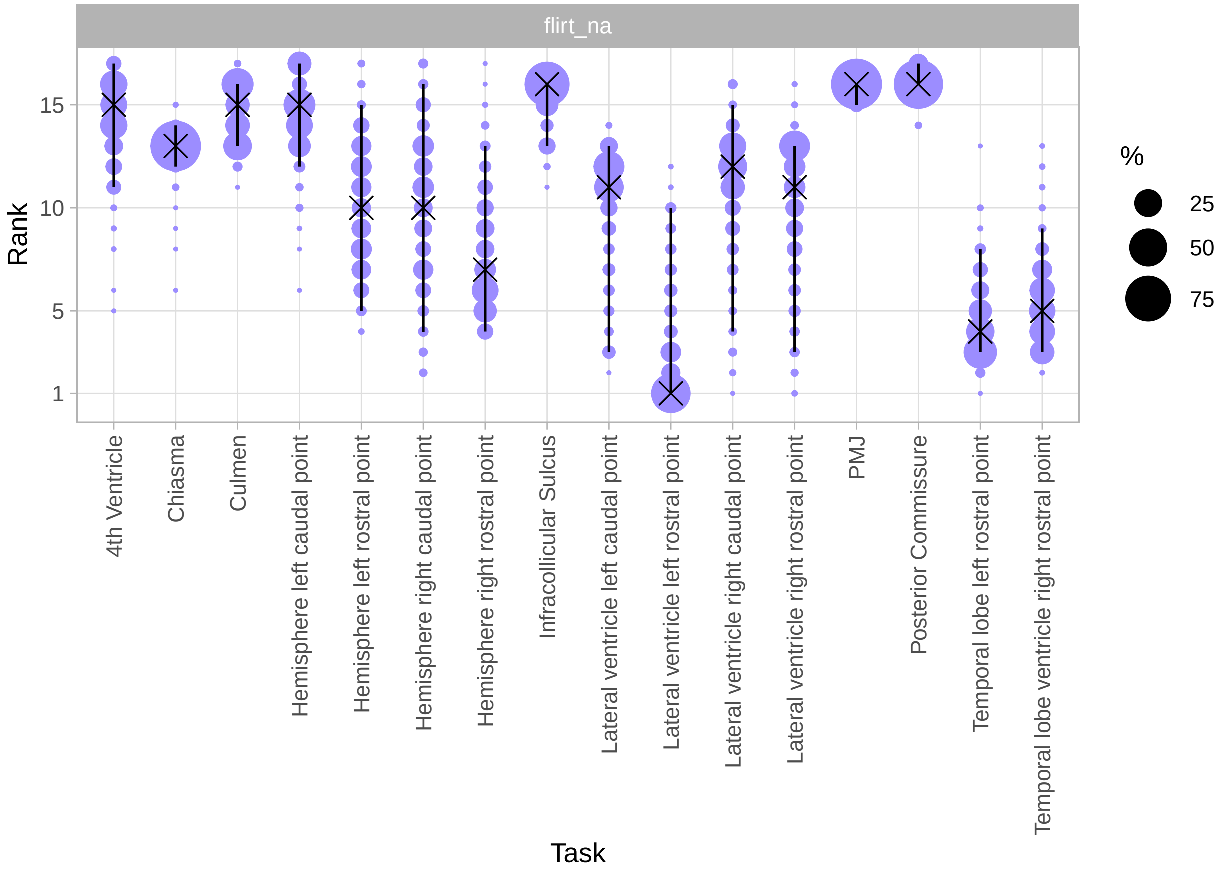
flirt_na
Rank
15
10
5
1
4th Ventricle
Chiasma
Culmen
Hemisphere left caudal point
Hemisphere left rostral point
Hemisphere right caudal point
Hemisphere right rostral point
Infracollicular Sulcus
Lateral ventricle left caudal point
Lateral ventricle left rostral point
Lateral ventricle right caudal point
Lateral ventricle right rostral point
PMJ
Posterior Commissure
Temporal lobe left rostral point
Temporal lobe ventricle right rostral point
Task
%
25
50
75

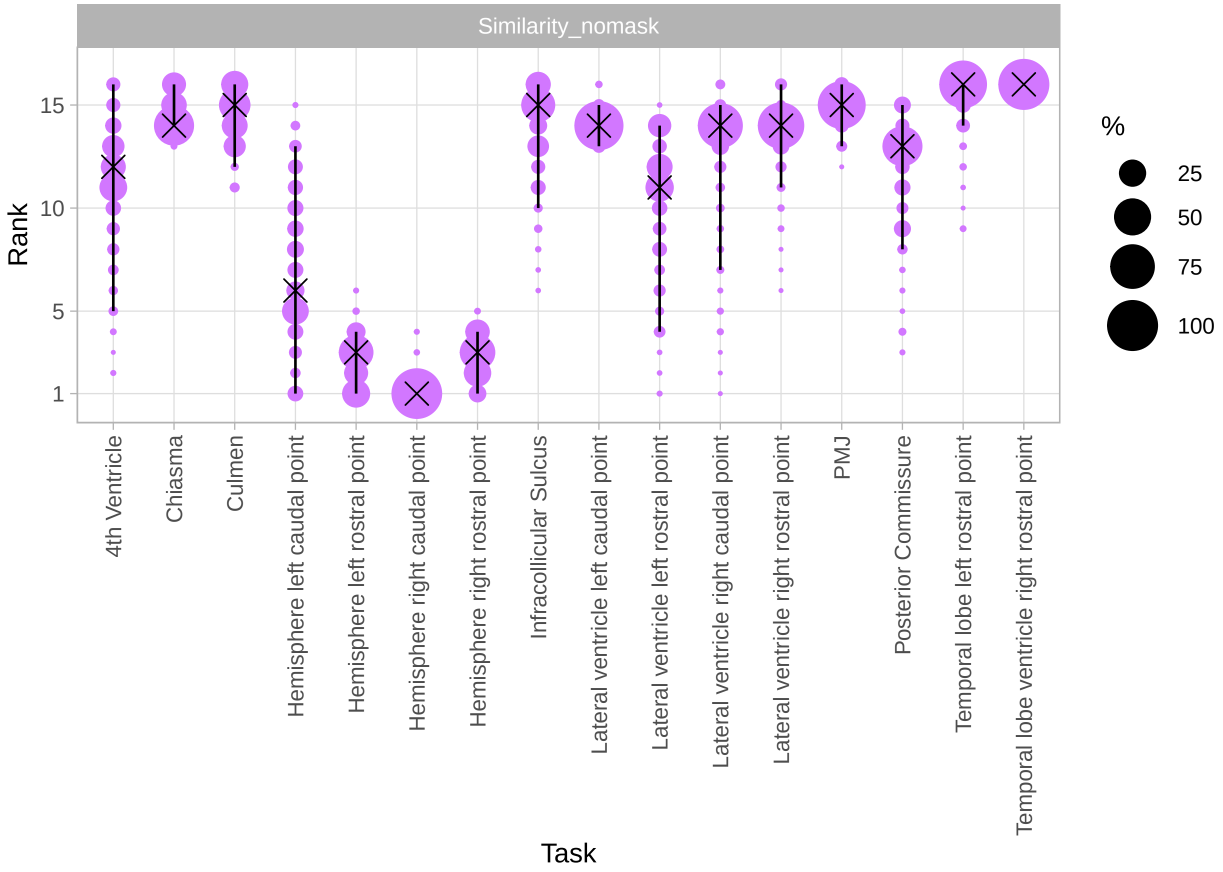

Similarity_nomask
Rank
15
10
5
1
4th Ventricle
Chiasma
Culmen
Hemisphere left caudal point
Hemisphere left rostral point
Hemisphere right caudal point
Hemisphere right rostral point
Infracollicular Sulcus
Lateral ventricle left caudal point
Lateral ventricle left rostral point
Lateral ventricle right caudal point
Lateral ventricle right rostral point
PMJ
Posterior Commissure
Temporal lobe left rostral point
Temporal lobe ventricle right rostral point
Task
%
25
50
75
100

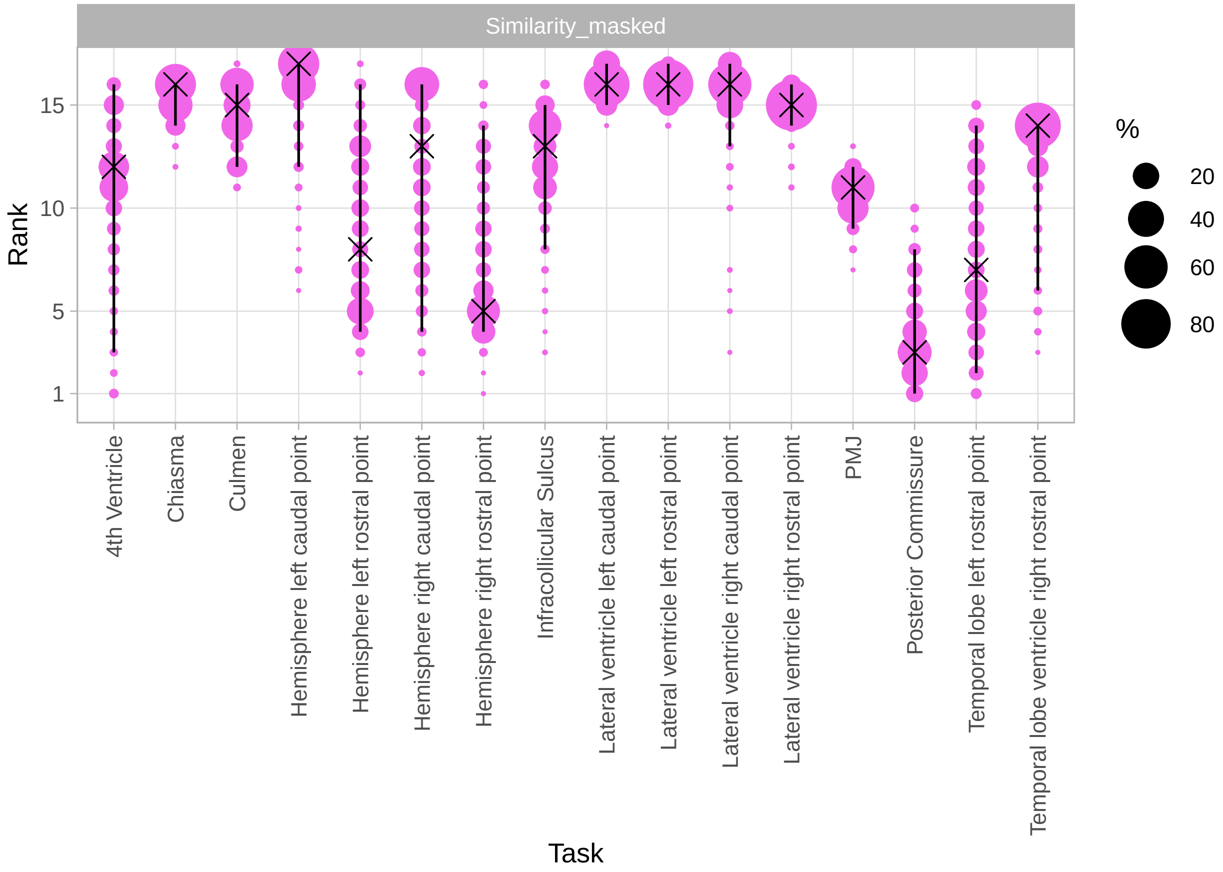

Similarity_masked
Rank
15
10
5
1
4th Ventricle
Chiasma
Culmen
Hemisphere left caudal point
Hemisphere left rostral point
Hemisphere right caudal point
Hemisphere right rostral point
Infracollicular Sulcus
Lateral ventricle left caudal point
Lateral ventricle left rostral point
Lateral ventricle right caudal point
Lateral ventricle right rostral point
PMJ
Posterior Commissure
Temporal lobe left rostral point
Temporal lobe ventricle right rostral point
Task
%
20
40
60
80

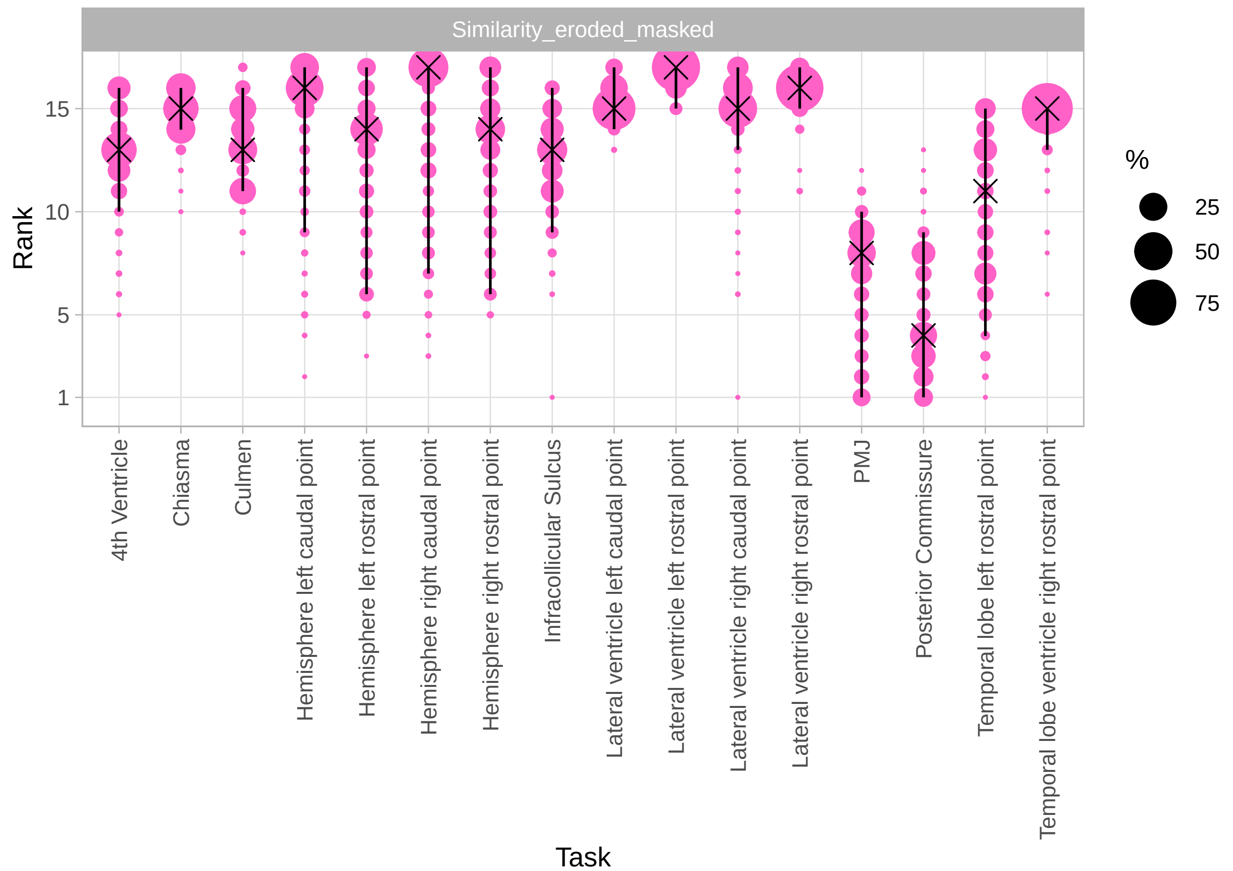

Similarity_eroded_masked
Rank
15
10
5
1
%
25
50
75
Task
4th Ventricle
Chiasma
Culmen
Hemisphere left caudal point
Hemisphere left rostral point
Hemisphere right caudal point
Hemisphere right rostral point
Infracollicular Sulcus
Lateral ventricle left caudal point
Lateral ventricle left rostral point
Lateral ventricle right caudal point
Lateral ventricle right rostral point
PMJ
Posterior Commissure
Temporal lobe left rostral point
Temporal lobe ventricle right rostral point

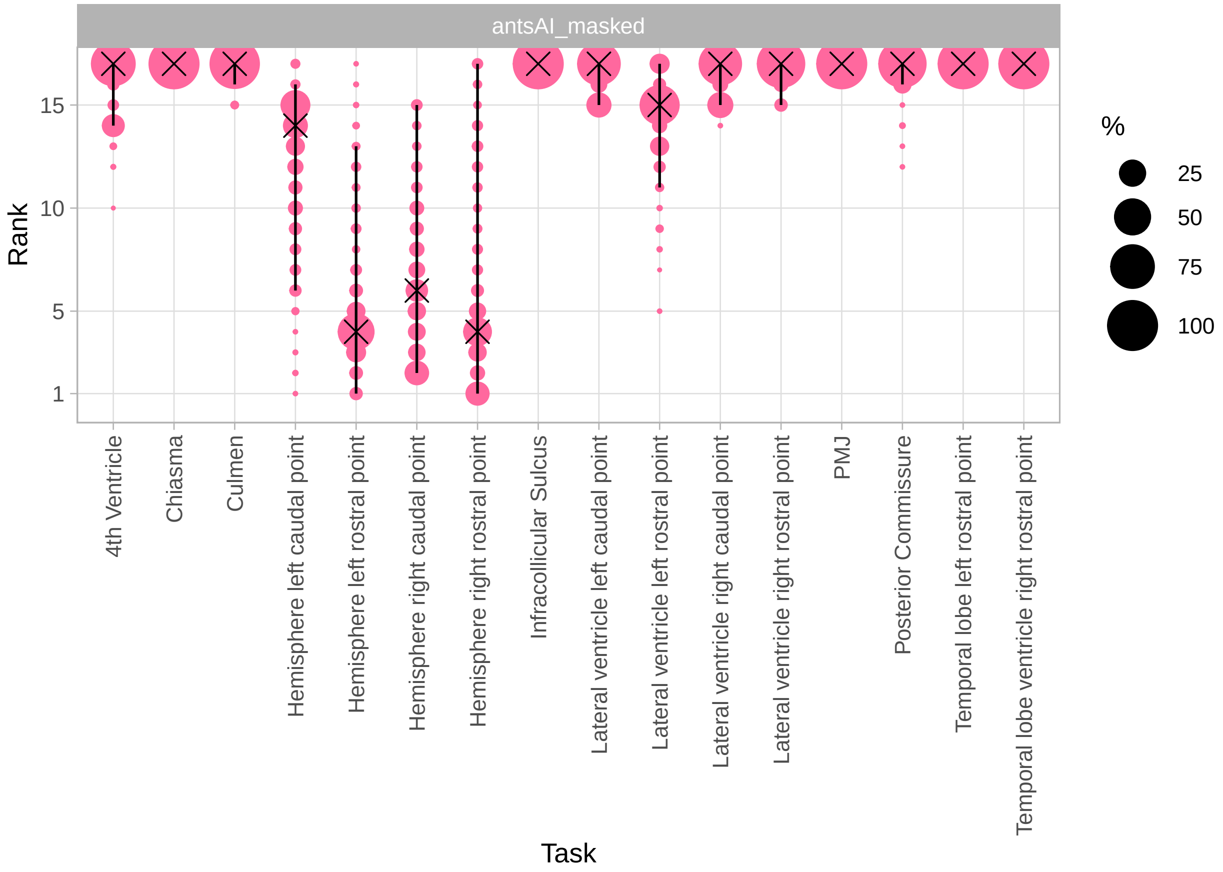
antsAI_masked
Rank
15
10
5
1
4th Ventricle
Chiasma
Culmen
Hemisphere left caudal point
Hemisphere left rostral point
Hemisphere right caudal point
Hemisphere right rostral point
Infracollicular Sulcus
Lateral ventricle left caudal point
Lateral ventricle left rostral point
Lateral ventricle right caudal point
Lateral ventricle right rostral point
PMJ
Posterior Commissure
Temporal lobe left rostral point
Temporal lobe ventricle right rostral point
Task
%
25
50
75
100

An alternative representation is provided by a stacked frequency plot of the observed ranks, separated by algorithm. Observed ranks across bootstrap samples are displayed with coloring according to the task. For algorithms that achieve the same rank in different tasks for the full assessment data set, vertical lines are on top of each other. Vertical lines allow to compare the achieved rank of each algorithm over different tasks.

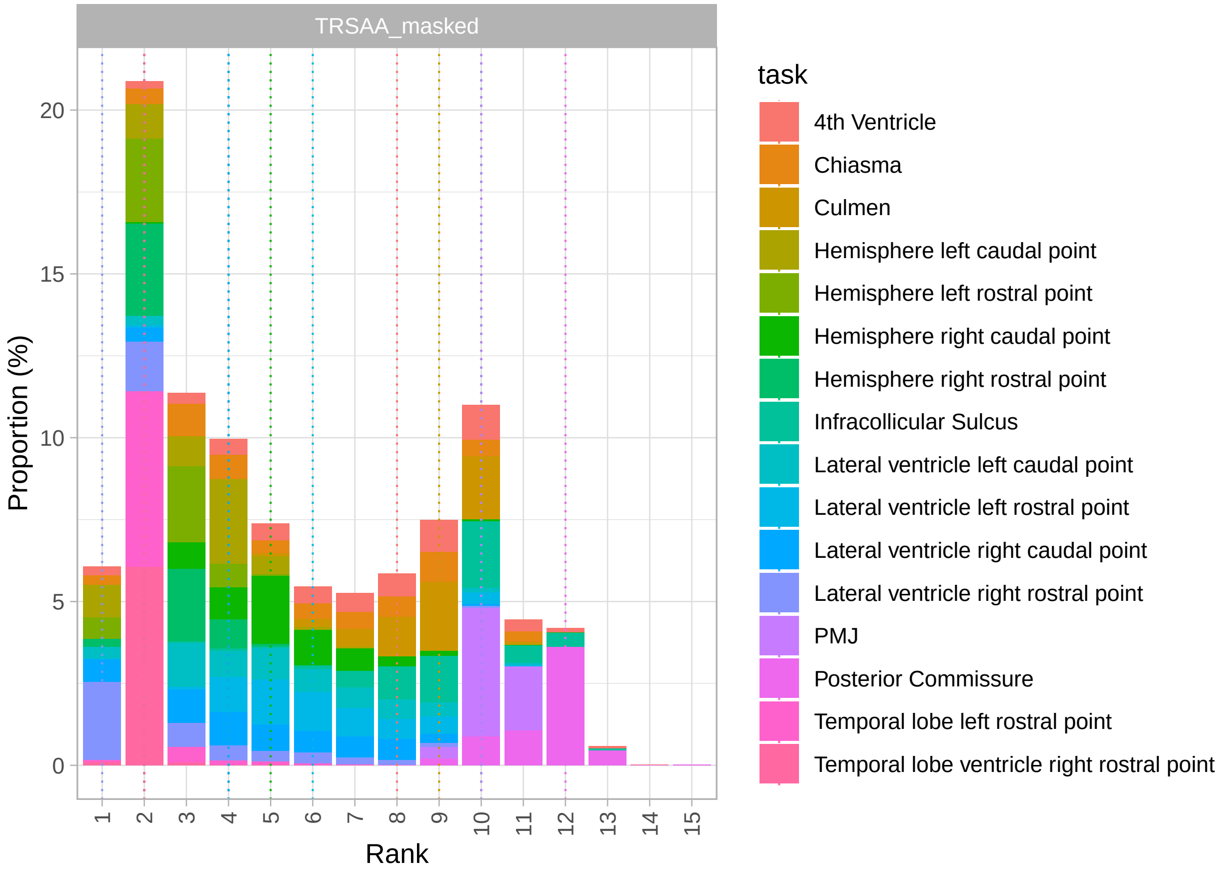

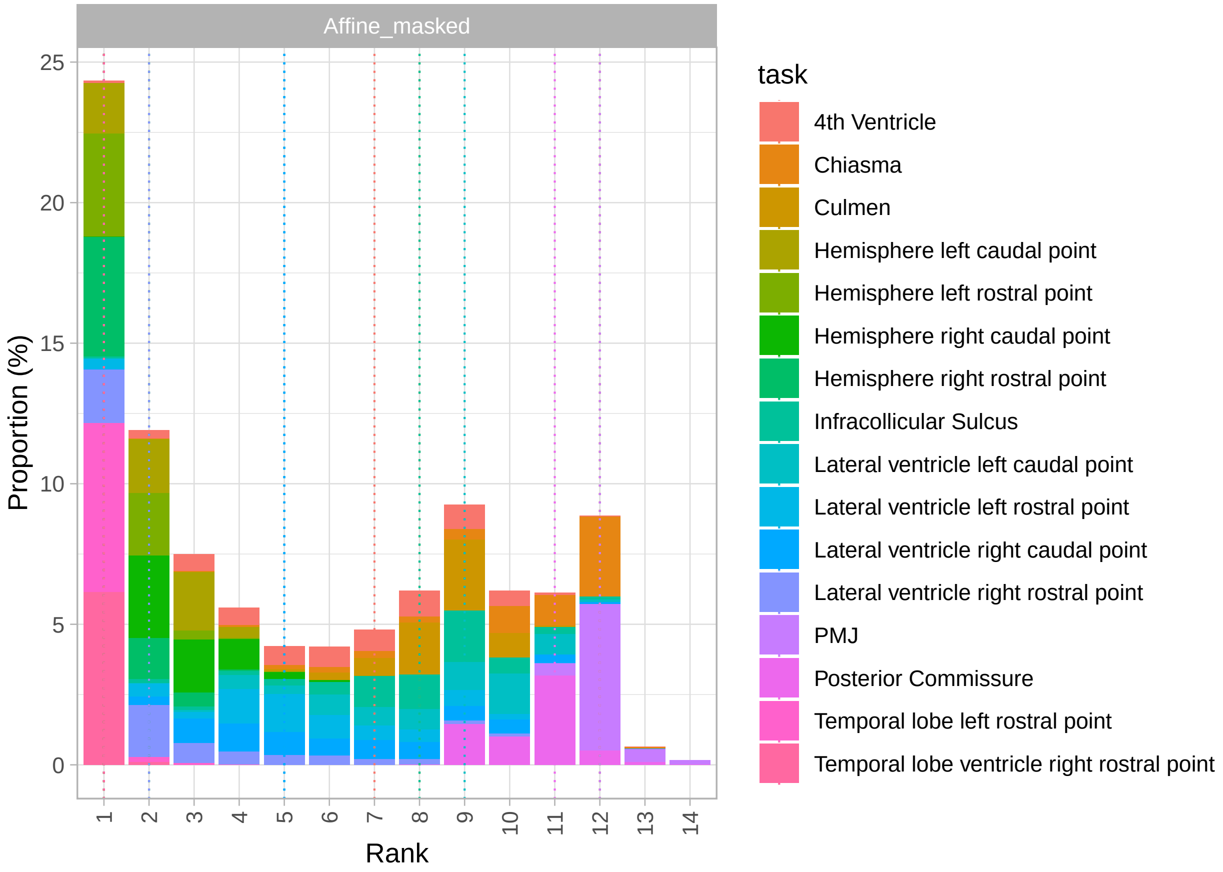

Affine_masked
Proportion (%)
0
5
10
15
20
25
Rank
1
2
3
4
5
6
7
8
9
10
11
12
13
14
task
4th Ventricle
Chiasma
Culmen
Hemisphere left caudal point
Hemisphere left rostral point
Hemisphere right caudal point
Hemisphere right rostral point
Infracollicular Sulcus
Lateral ventricle left caudal point
Lateral ventricle left rostral point
Lateral ventricle right caudal point
Lateral ventricle right rostral point
PMJ
Posterior Commissure
Temporal lobe left rostral point
Temporal lobe ventricle right rostral point

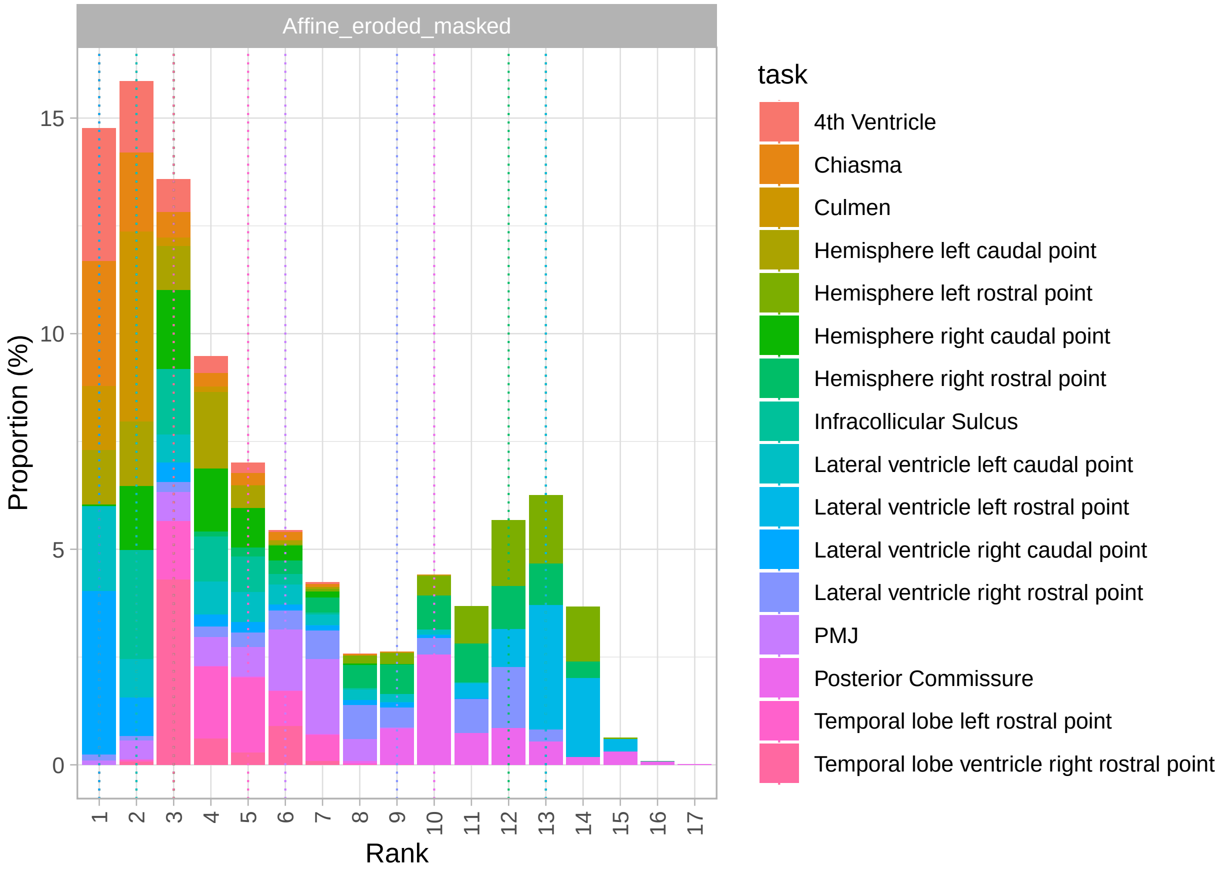
Affine_eroded_masked
Proportion (%)
0
5
10
15
Rank
1
2
3
4
5
6
7
8
9
10
11
12
13
14
15
16
17
task
4th Ventricle
Chiasma
Culmen
Hemisphere left caudal point
Hemisphere left rostral point
Hemisphere right caudal point
Hemisphere right rostral point
Infracollicular Sulcus
Lateral ventricle left caudal point
Lateral ventricle left rostral point
Lateral ventricle right caudal point
Lateral ventricle right rostral point
PMJ
Posterior Commissure
Temporal lobe left rostral point
Temporal lobe ventricle right rostral point

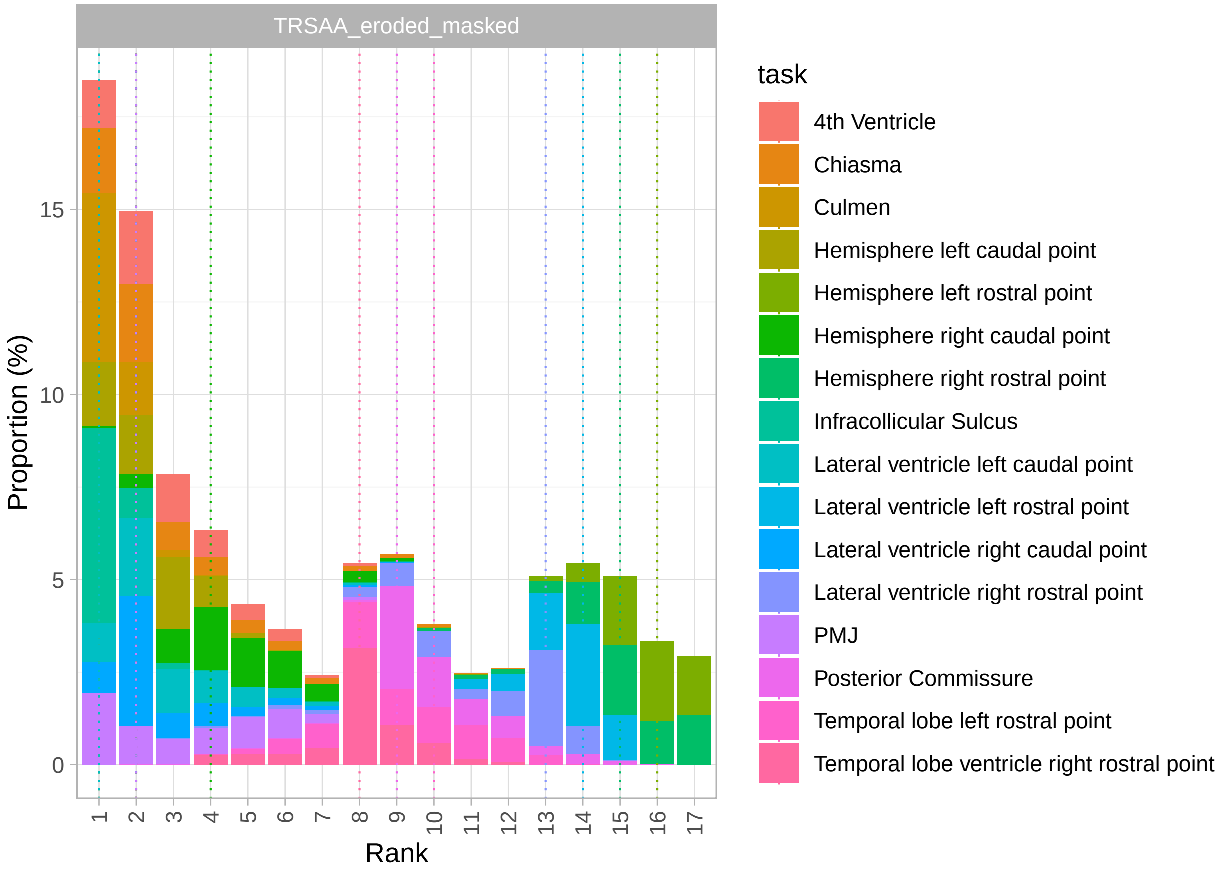
TRSAA_eroded_masked
Proportion (%)
0
5
10
15
1
2
3
4
5
6
7
8
9
10
11
12
13
14
15
16
17
Rank
task
4th Ventricle
Chiasma
Culmen
Hemisphere left caudal point
Hemisphere left rostral point
Hemisphere right caudal point
Hemisphere right rostral point
Infracollicular Sulcus
Lateral ventricle left caudal point
Lateral ventricle left rostral point
Lateral ventricle right caudal point
Lateral ventricle right rostral point
PMJ
Posterior Commissure
Temporal lobe left rostral point
Temporal lobe ventricle right rostral point

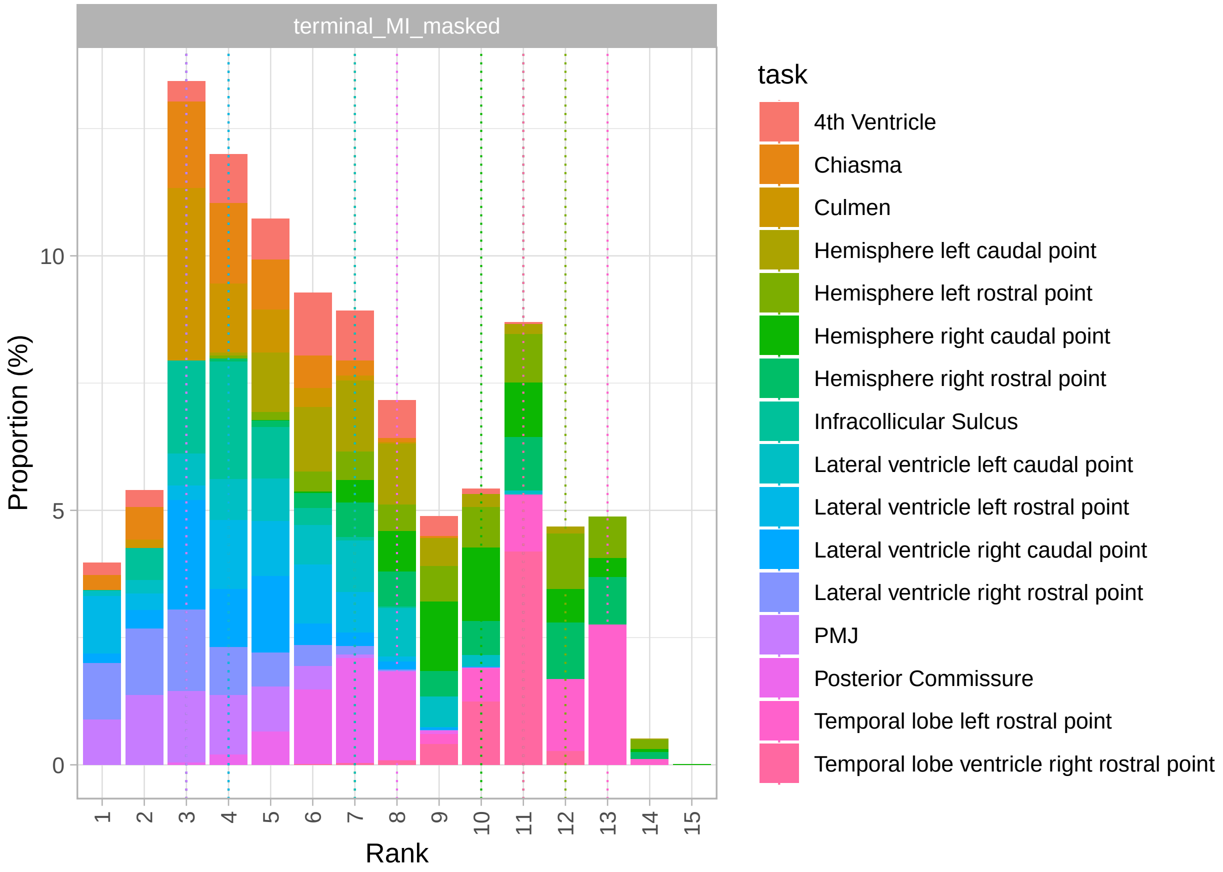

terminal_MI_masked
Proportion (%)
0
5
10
1
2
3
4
5
6
7
8
9
10
11
12
13
14
15
Rank
task
4th Ventricle
Chiasma
Culmen
Hemisphere left caudal point
Hemisphere left rostral point
Hemisphere right caudal point
Hemisphere right rostral point
Infracollicular Sulcus
Lateral ventricle left caudal point
Lateral ventricle left rostral point
Lateral ventricle right caudal point
Lateral ventricle right rostral point
PMJ
Posterior Commissure
Temporal lobe left rostral point
Temporal lobe ventricle right rostral point

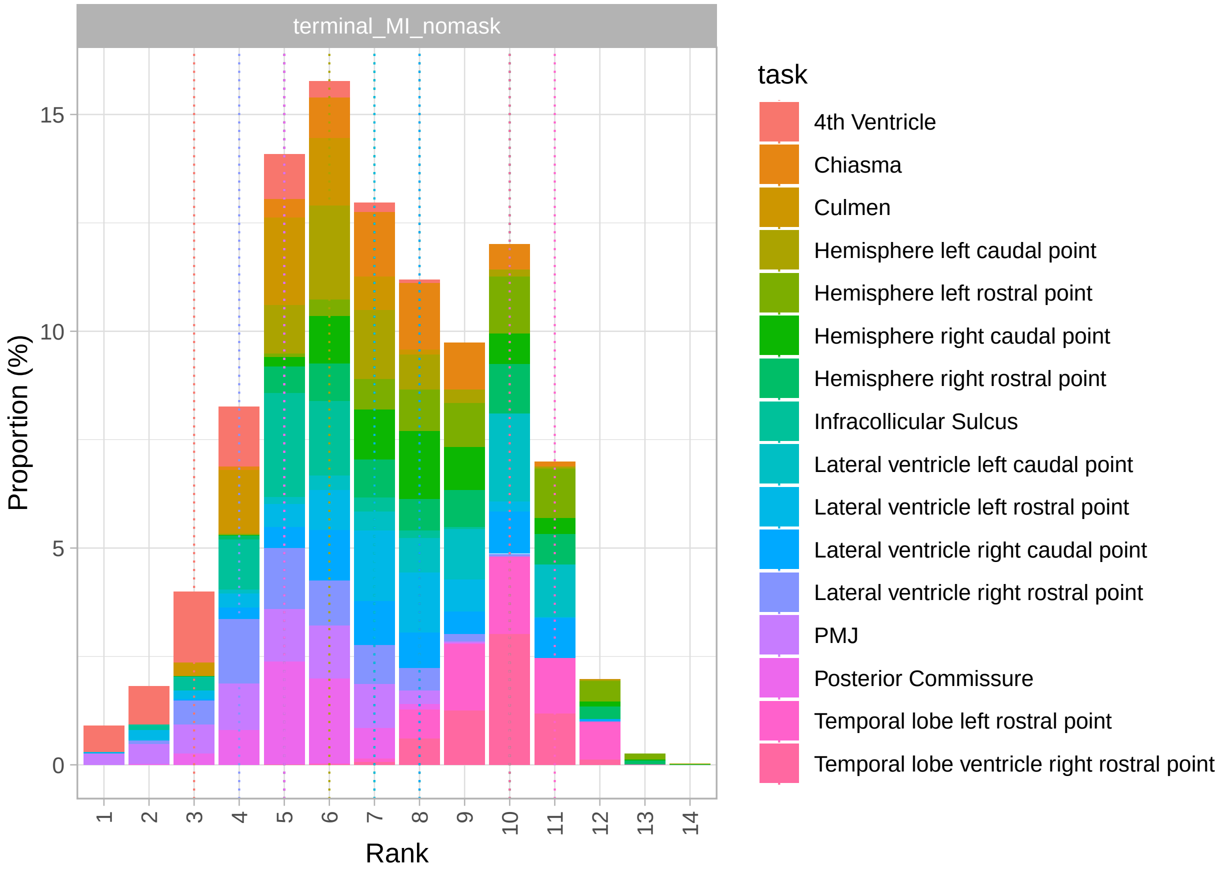

terminal_MI_nomask
Proportion (%)
15
10
5
0
1
2
3
4
5
6
7
8
9
10
11
12
13
14
Rank
task
4th Ventricle
Chiasma
Culmen
Hemisphere left caudal point
Hemisphere left rostral point
Hemisphere right caudal point
Hemisphere right rostral point
Infracollicular Sulcus
Lateral ventricle left caudal point
Lateral ventricle left rostral point
Lateral ventricle right caudal point
Lateral ventricle right rostral point
PMJ
Posterior Commissure
Temporal lobe left rostral point
Temporal lobe ventricle right rostral point

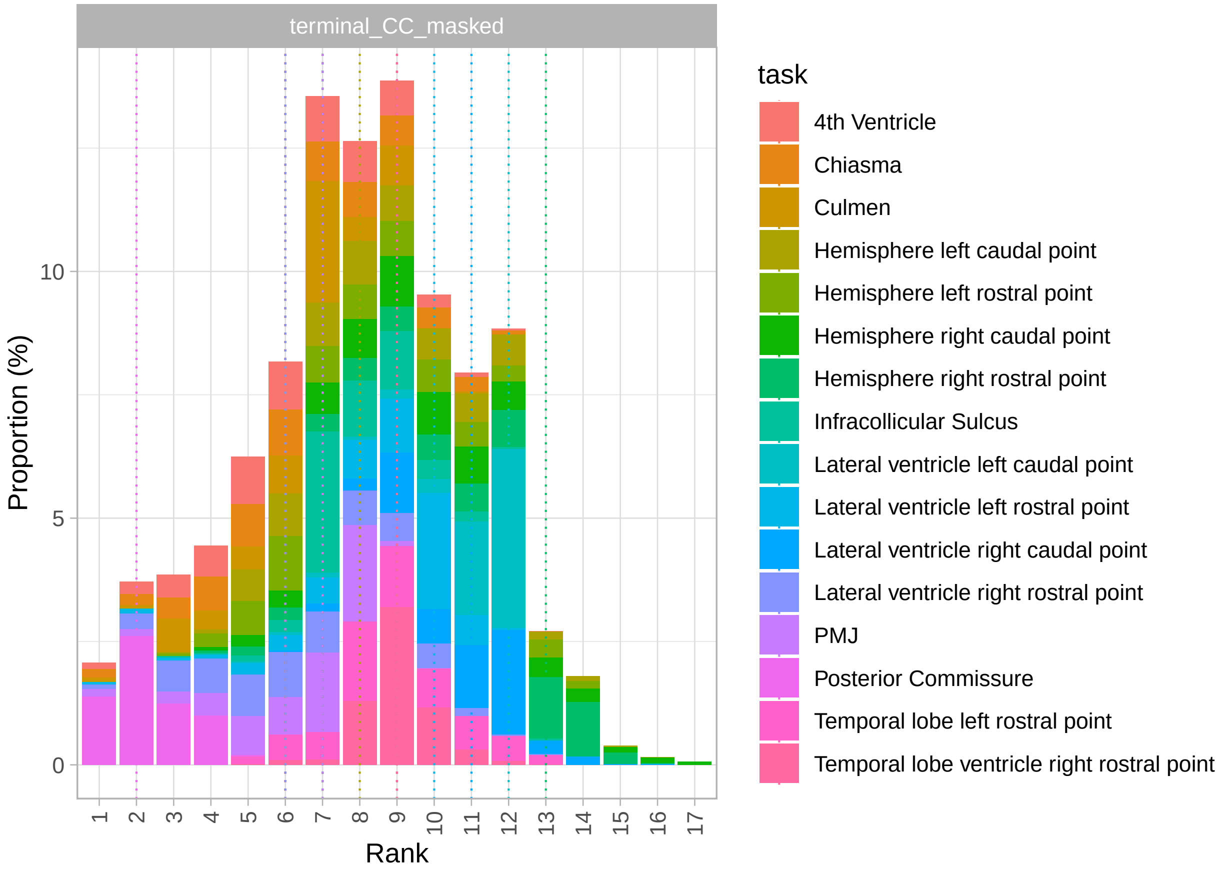
terminal_CC_masked
Proportion (%)
0
5
10
Rank
1
2
3
4
5
6
7
8
9
10
11
12
13
14
15
16
17
task
4th Ventricle
Chiasma
Culmen
Hemisphere left caudal point
Hemisphere left rostral point
Hemisphere right caudal point
Hemisphere right rostral point
Infracollicular Sulcus
Lateral ventricle left caudal point
Lateral ventricle left rostral point
Lateral ventricle right caudal point
Lateral ventricle right rostral point
PMJ
Posterior Commissure
Temporal lobe left rostral point
Temporal lobe ventricle right rostral point

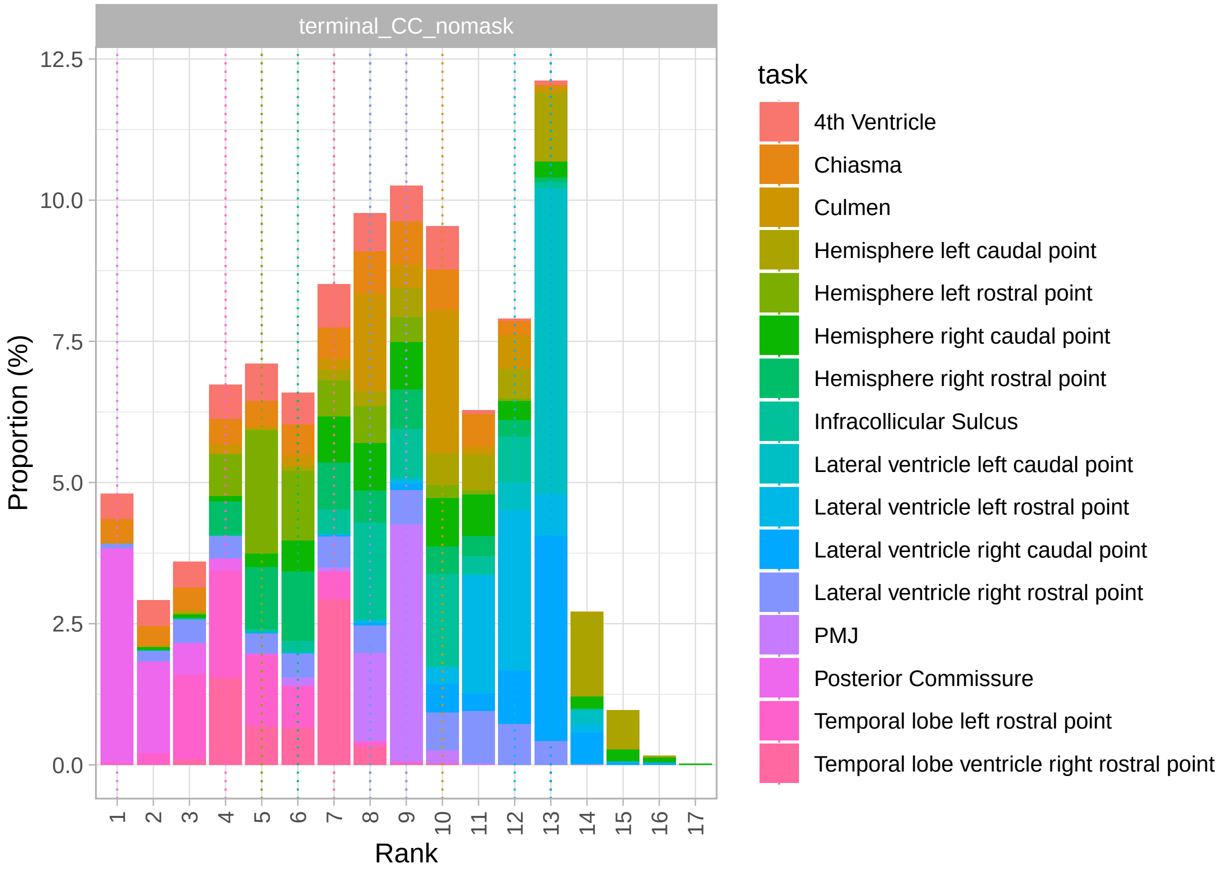
terminal_CC_nomask
Proportion (%)
12.5
10.0
7.5
5.0
2.5
0.0
1
2
3
4
5
6
7
8
9
10
11
12
13
14
15
16
17
Rank
task
4th Ventricle
Chiasma
Culmen
Hemisphere left caudal point
Hemisphere left rostral point
Hemisphere right caudal point
Hemisphere right rostral point
Infracollicular Sulcus
Lateral ventricle left caudal point
Lateral ventricle left rostral point
Lateral ventricle right caudal point
Lateral ventricle right rostral point
PMJ
Posterior Commissure
Temporal lobe left rostral point
Temporal lobe ventricle right rostral point

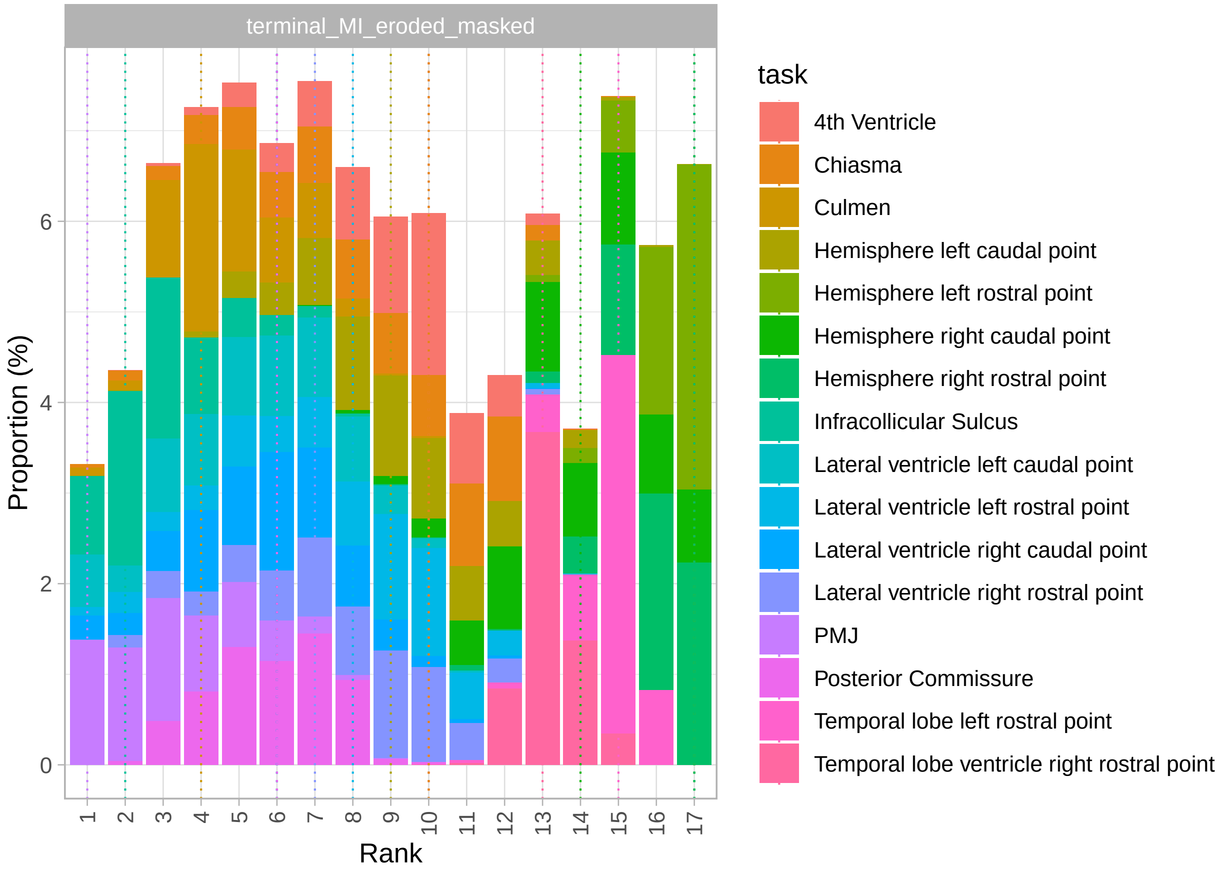
terminal_MI_eroded_masked
Proportion (%)
0
2
4
6
1
2
3
4
5
6
7
8
9
10
11
12
13
14
15
16
17
Rank
task
4th Ventricle
Chiasma
Culmen
Hemisphere left caudal point
Hemisphere left rostral point
Hemisphere right caudal point
Hemisphere right rostral point
Infracollicular Sulcus
Lateral ventricle left caudal point
Lateral ventricle left rostral point
Lateral ventricle right caudal point
Lateral ventricle right rostral point
PMJ
Posterior Commissure
Temporal lobe left rostral point
Temporal lobe ventricle right rostral point

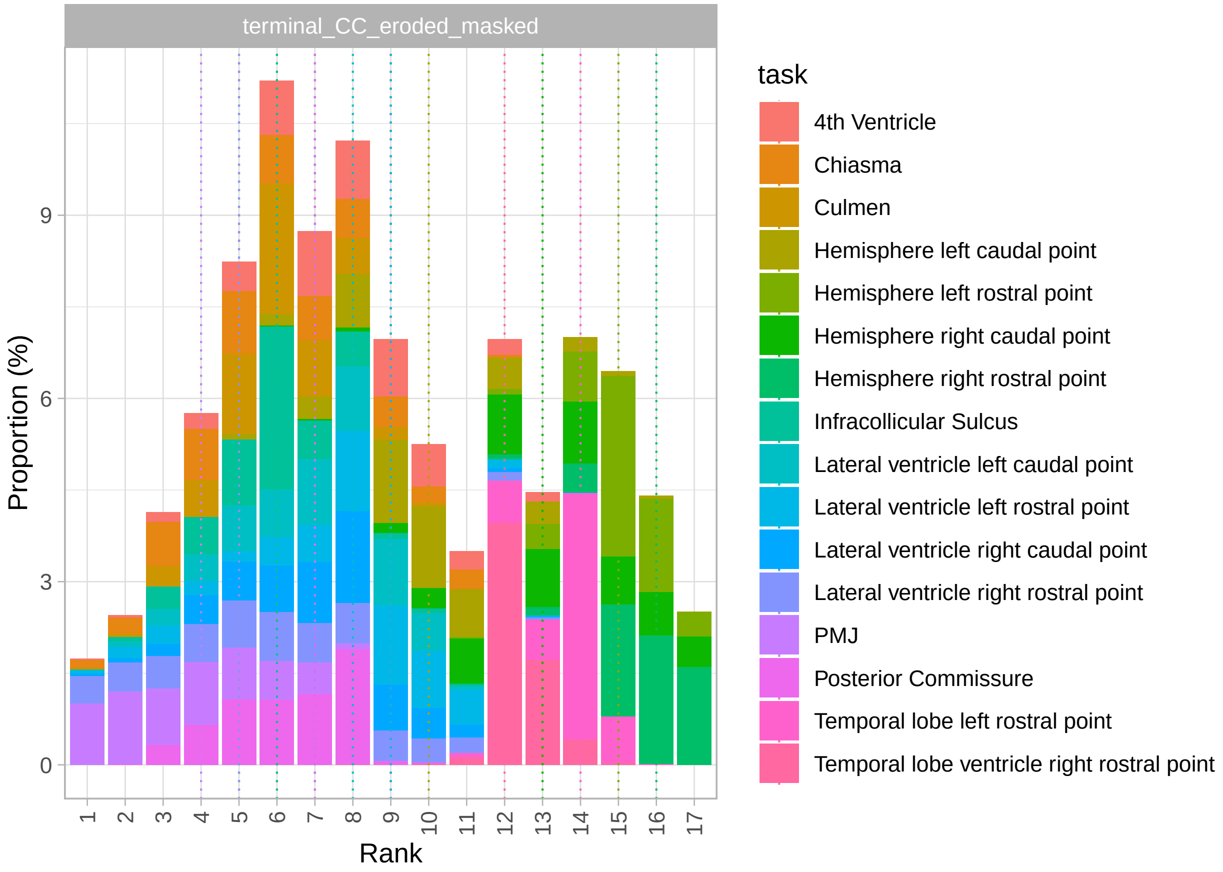
terminal_CC_eroded_masked
Proportion (%)
0
3
6
9
1
2
3
4
5
6
7
8
9
10
11
12
13
14
15
16
17
Rank
task
4th Ventricle
Chiasma
Culmen
Hemisphere left caudal point
Hemisphere left rostral point
Hemisphere right caudal point
Hemisphere right rostral point
Infracollicular Sulcus
Lateral ventricle left caudal point
Lateral ventricle left rostral point
Lateral ventricle right caudal point
Lateral ventricle right rostral point
PMJ
Posterior Commissure
Temporal lobe left rostral point
Temporal lobe ventricle right rostral point

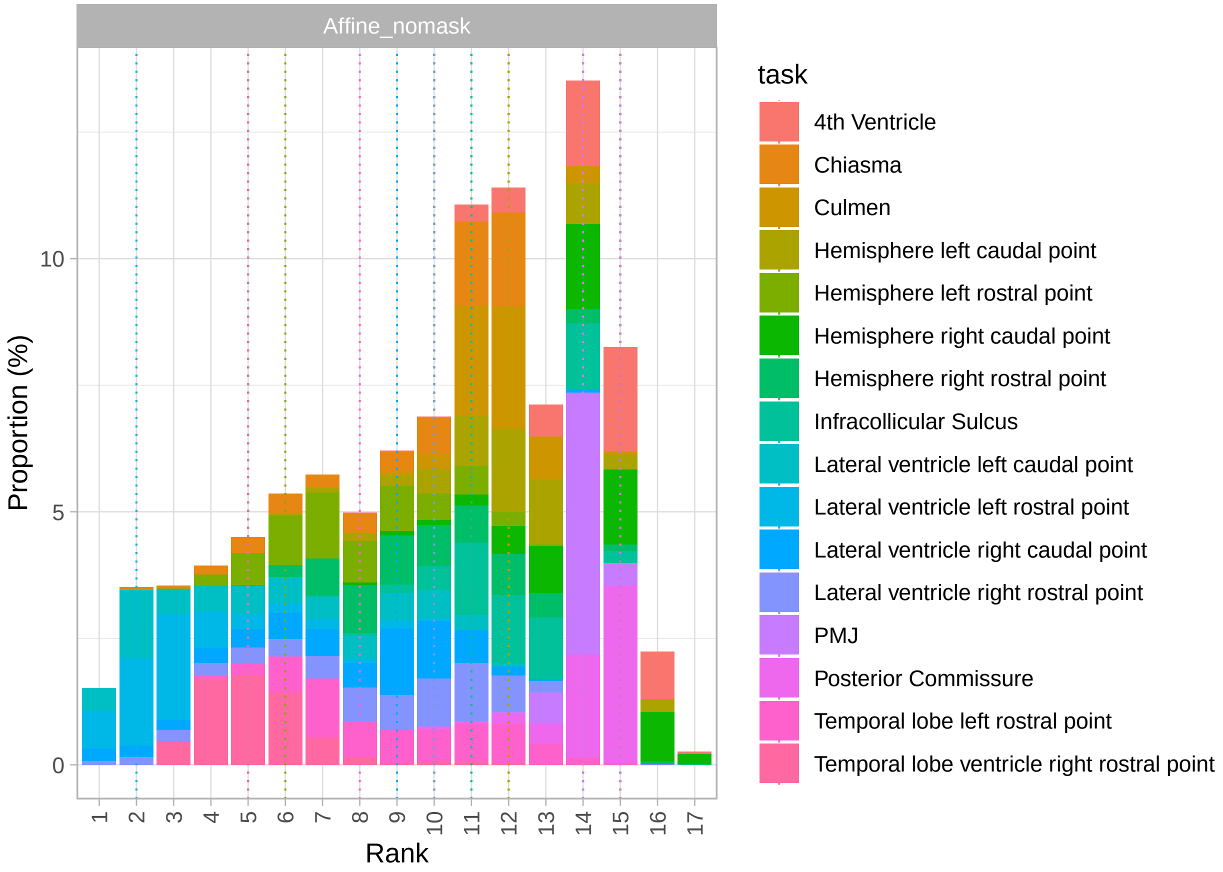
Affine_nomask
Proportion (%)
0
5
10
Rank
1
2
3
4
5
6
7
8
9
10
11
12
13
14
15
16
17
task
4th Ventricle
Chiasma
Culmen
Hemisphere left caudal point
Hemisphere left rostral point
Hemisphere right caudal point
Hemisphere right rostral point
Infracollicular Sulcus
Lateral ventricle left caudal point
Lateral ventricle left rostral point
Lateral ventricle right caudal point
Lateral ventricle right rostral point
PMJ
Posterior Commissure
Temporal lobe left rostral point
Temporal lobe ventricle right rostral point

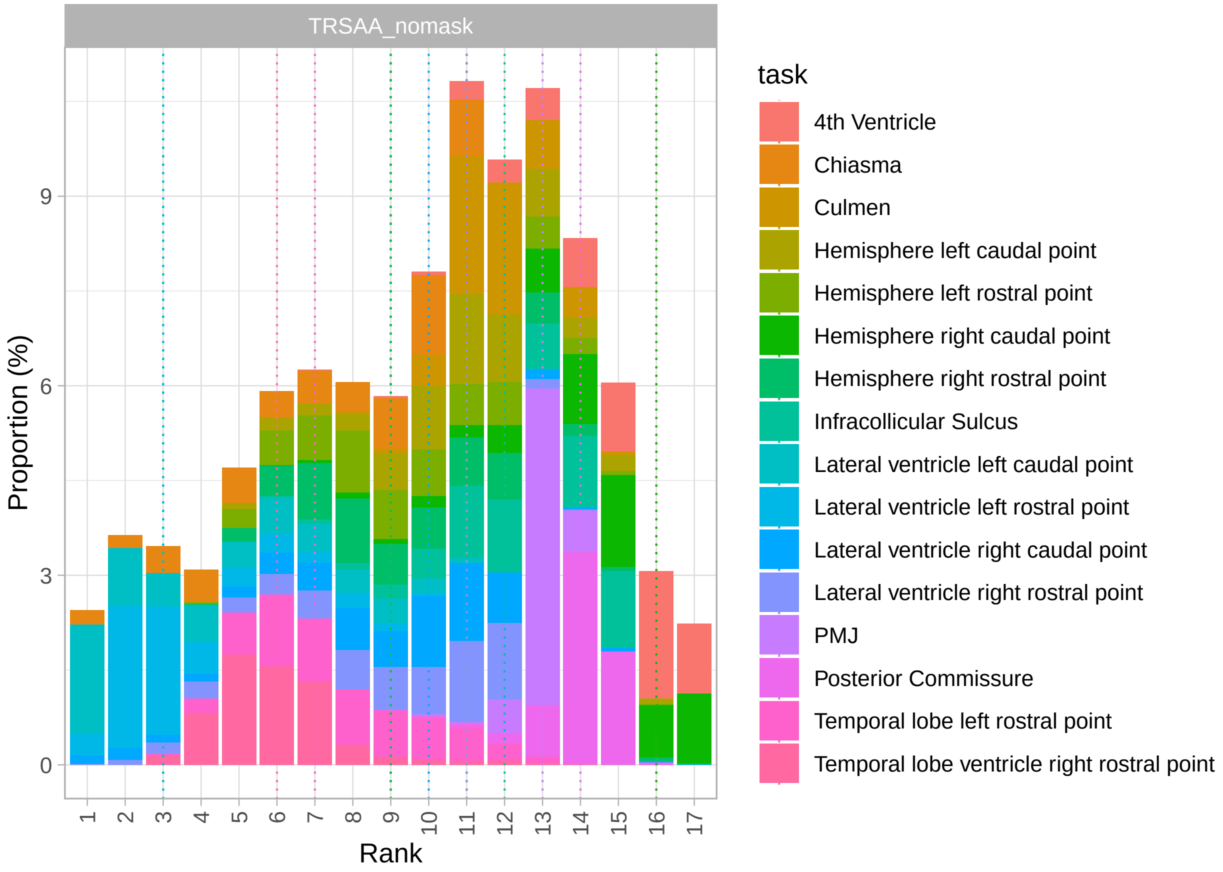
TRSAA_nomask
Proportion (%)
0
3
6
9
1
2
3
4
5
6
7
8
9
10
11
12
13
14
15
16
17
Rank
task
4th Ventricle
Chiasma
Culmen
Hemisphere left caudal point
Hemisphere left rostral point
Hemisphere right caudal point
Hemisphere right rostral point
Infracollicular Sulcus
Lateral ventricle left caudal point
Lateral ventricle left rostral point
Lateral ventricle right caudal point
Lateral ventricle right rostral point
PMJ
Posterior Commissure
Temporal lobe left rostral point
Temporal lobe ventricle right rostral point

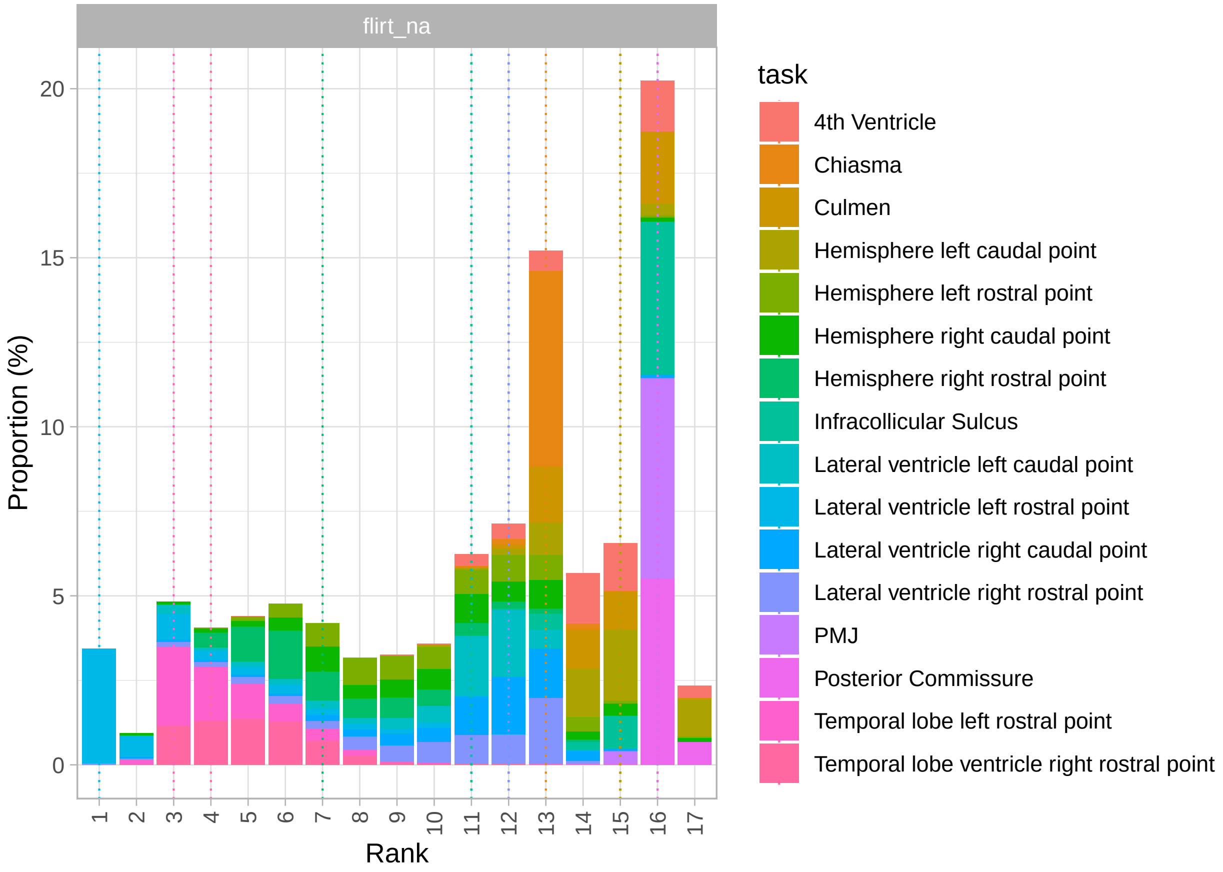
flirt_na
Proportion (%)
0
5
10
15
20
1
2
3
4
5
6
7
8
9
10
11
12
13
14
15
16
17
Rank
task
4th Ventricle
Chiasma
Culmen
Hemisphere left caudal point
Hemisphere left rostral point
Hemisphere right caudal point
Hemisphere right rostral point
Infracollicular Sulcus
Lateral ventricle left caudal point
Lateral ventricle left rostral point
Lateral ventricle right caudal point
Lateral ventricle right rostral point
PMJ
Posterior Commissure
Temporal lobe left rostral point
Temporal lobe ventricle right rostral point

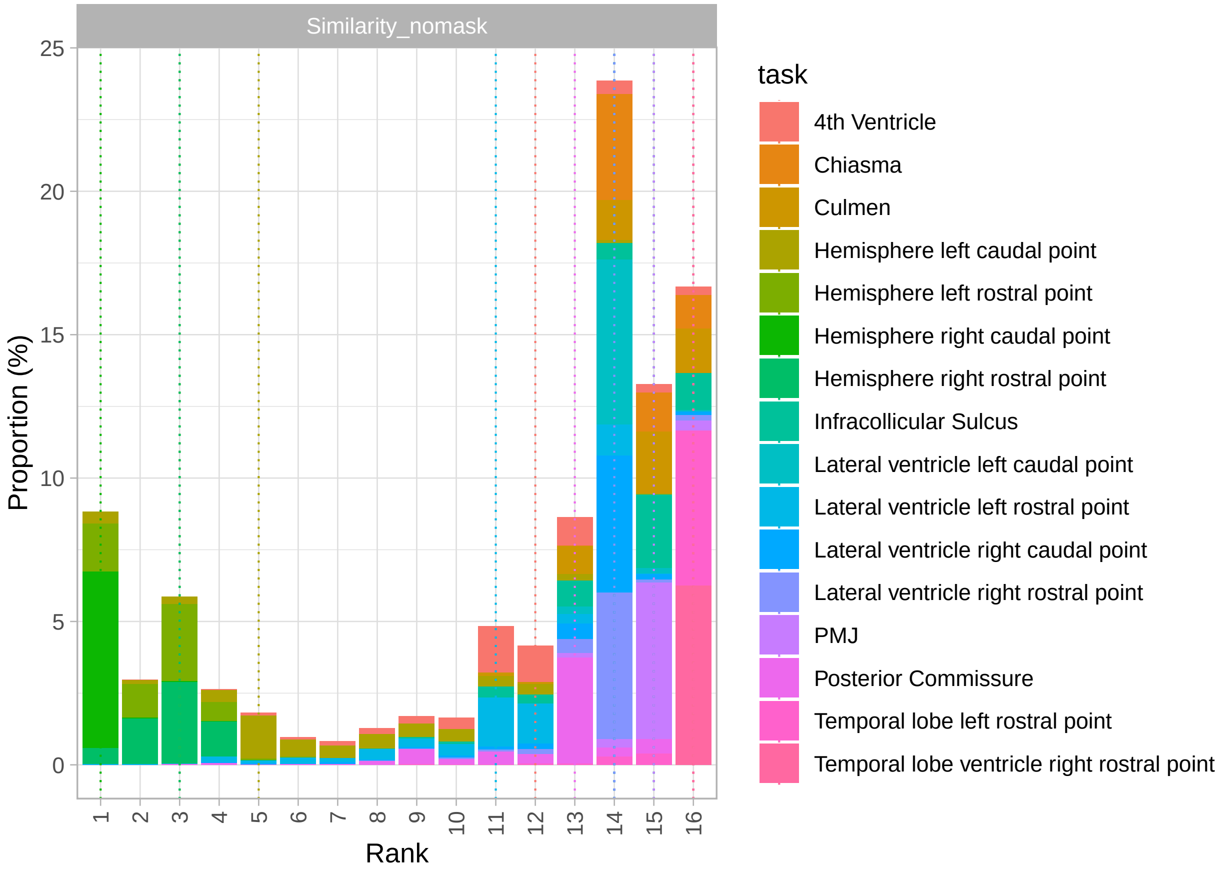
Similarity_nomask
Proportion (%)
25
20
15
10
5
0
1
2
3
4
5
6
7
8
9
10
11
12
13
14
15
16
Rank
task
4th Ventricle
Chiasma
Culmen
Hemisphere left caudal point
Hemisphere left rostral point
Hemisphere right caudal point
Hemisphere right rostral point
Infracollicular Sulcus
Lateral ventricle left caudal point
Lateral ventricle left rostral point
Lateral ventricle right caudal point
Lateral ventricle right rostral point
PMJ
Posterior Commissure
Temporal lobe left rostral point
Temporal lobe ventricle right rostral point

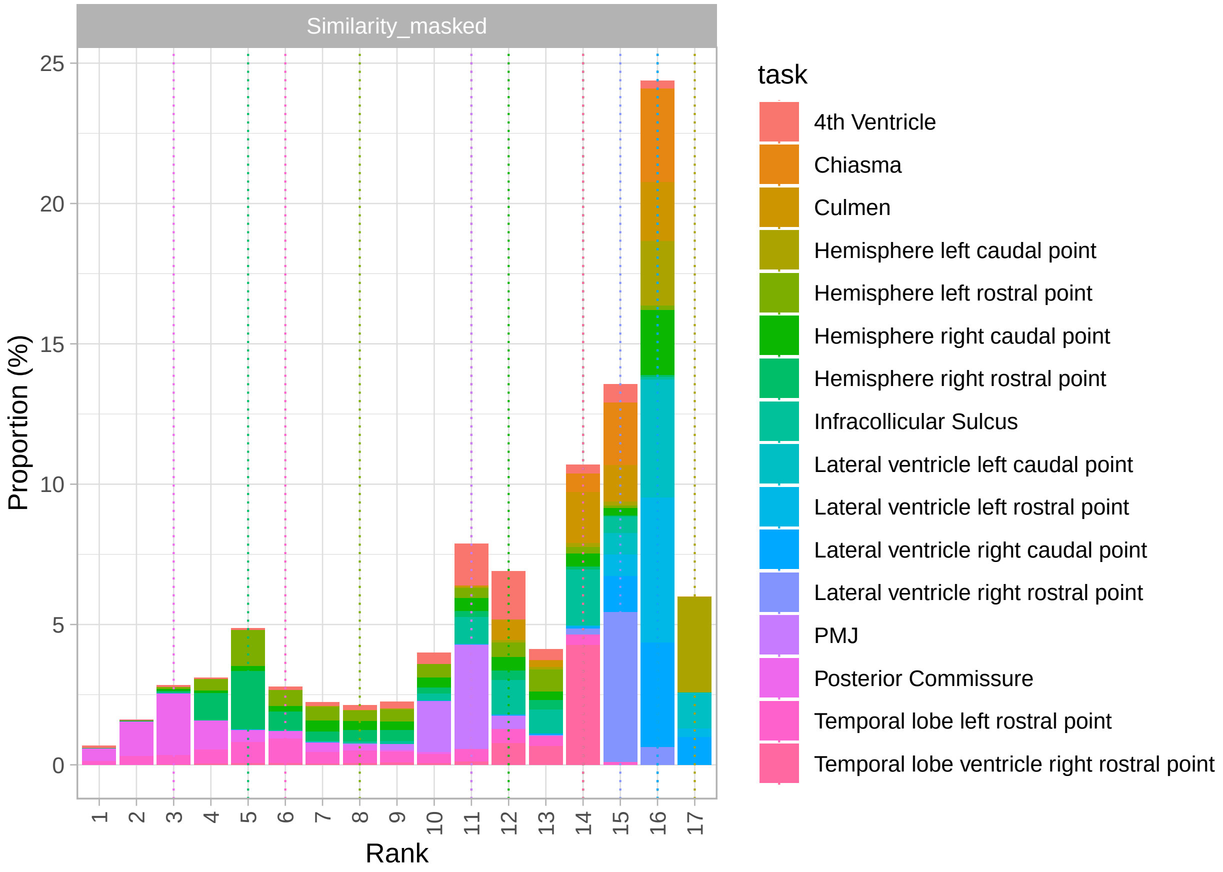

Similarity_masked
Proportion (%)
0
5
10
15
20
25
1
2
3
4
5
6
7
8
9
10
11
12
13
14
15
16
17
Rank
task
4th Ventricle
Chiasma
Culmen
Hemisphere left caudal point
Hemisphere left rostral point
Hemisphere right caudal point
Hemisphere right rostral point
Infracollicular Sulcus
Lateral ventricle left caudal point
Lateral ventricle left rostral point
Lateral ventricle right caudal point
Lateral ventricle right rostral point
PMJ
Posterior Commissure
Temporal lobe left rostral point
Temporal lobe ventricle right rostral point

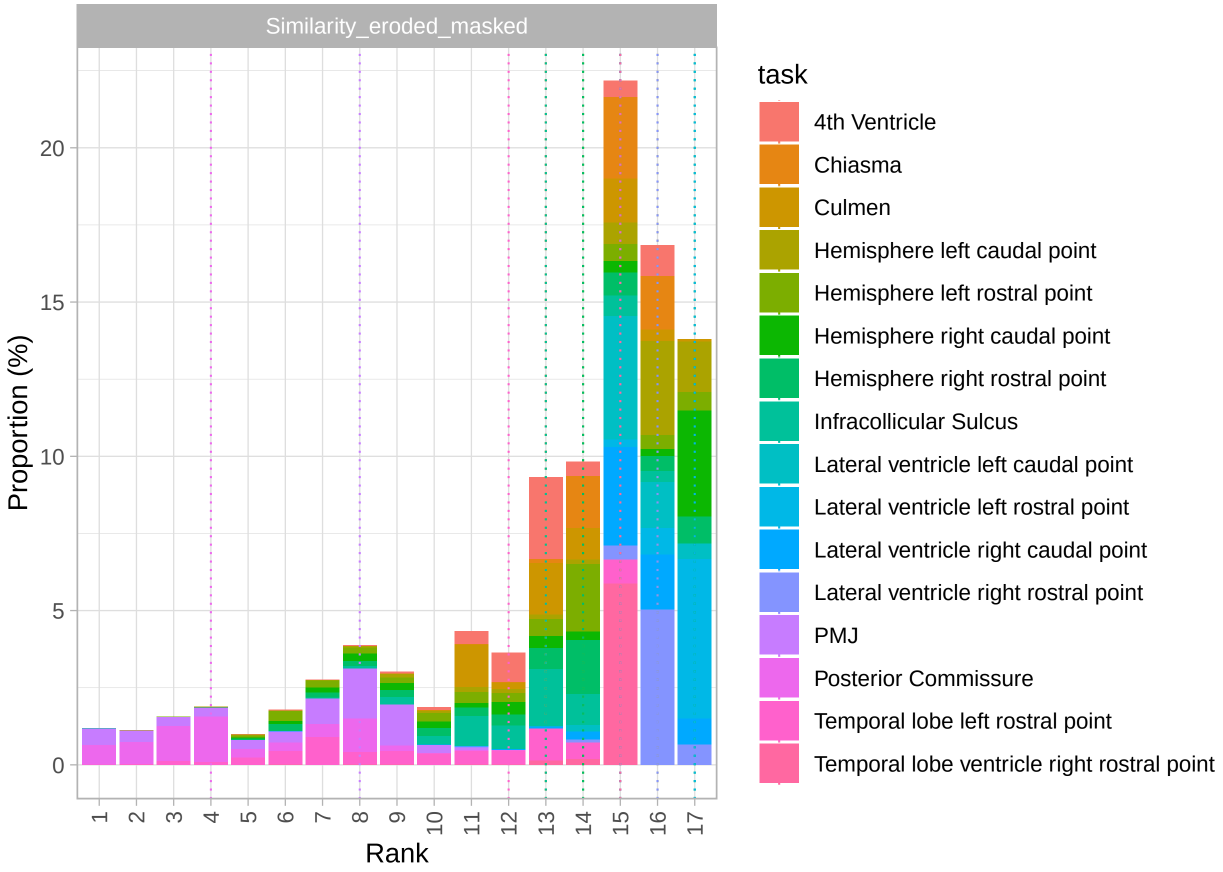

Similarity_eroded_masked
Proportion (%)
0
5
10
15
20
Rank
1
2
3
4
5
6
7
8
9
10
11
12
13
14
15
16
17
task
4th Ventricle
Chiasma
Culmen
Hemisphere left caudal point
Hemisphere left rostral point
Hemisphere right caudal point
Hemisphere right rostral point
Infracollicular Sulcus
Lateral ventricle left caudal point
Lateral ventricle left rostral point
Lateral ventricle right caudal point
Lateral ventricle right rostral point
PMJ
Posterior Commissure
Temporal lobe left rostral point
Temporal lobe ventricle right rostral point

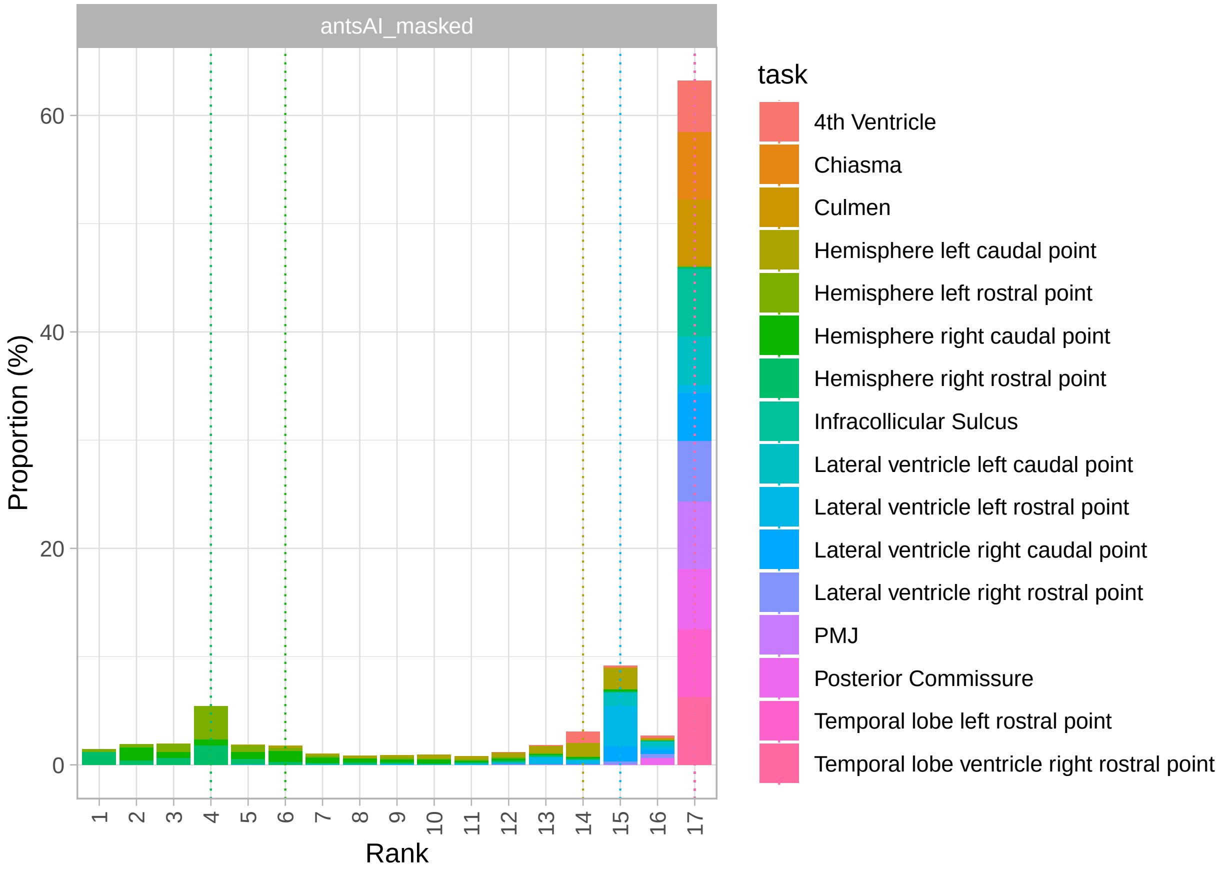
antsAI_masked
Proportion (%)
0
20
40
60
1
2
3
4
5
6
7
8
9
10
11
12
13
14
15
16
17
Rank
task
4th Ventricle
Chiasma
Culmen
Hemisphere left caudal point
Hemisphere left rostral point
Hemisphere right caudal point
Hemisphere right rostral point
Infracollicular Sulcus
Lateral ventricle left caudal point
Lateral ventricle left rostral point
Lateral ventricle right caudal point
Lateral ventricle right rostral point
PMJ
Posterior Commissure
Temporal lobe left rostral point
Temporal lobe ventricle right rostral point

4.2 Characterization of tasks

4.2.1 Visualizing bootstrap results

To investigate which tasks separate algorithms well (i.e., lead to a stable ranking), a blob plot is recommended.

Bootstrap results can be shown in a blob plot showing one plot for each task. In this view, the spread of the blobs for each algorithm can be compared across tasks. Deviations from the diagonal indicate deviations from the consensus ranking (over tasks). Specifically, if rank distribution of an algorithm is consistently below the diagonal, the algorithm performed better in this task than on average across tasks, while if the rank distribution of an algorithm is consistently above the diagonal, the algorithm performed worse in this task than on average across tasks. At the bottom of each panel, ranks for each algorithm in the tasks are provided.

Same as in Section 3.1 but now ordered according to consensus.

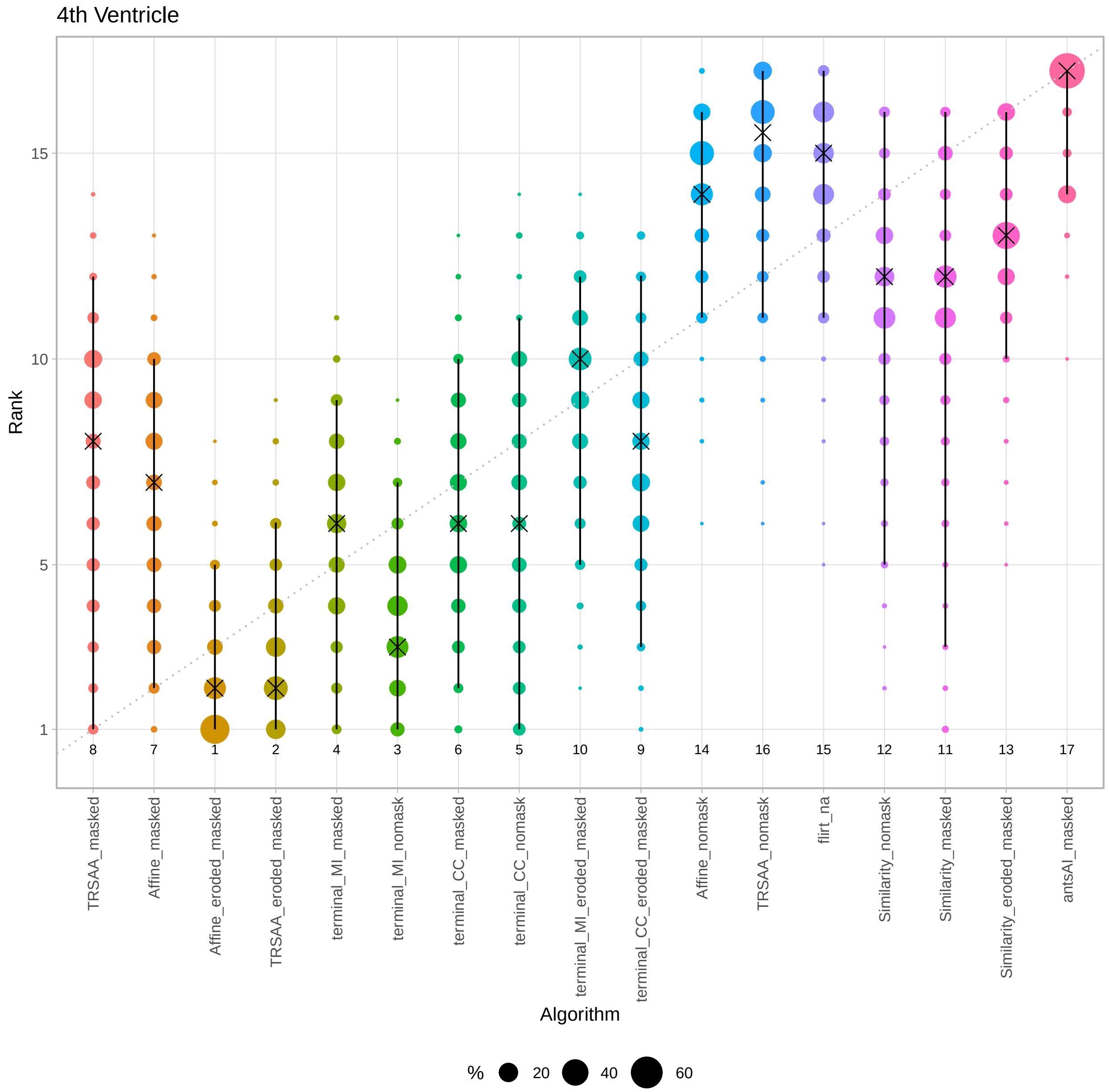

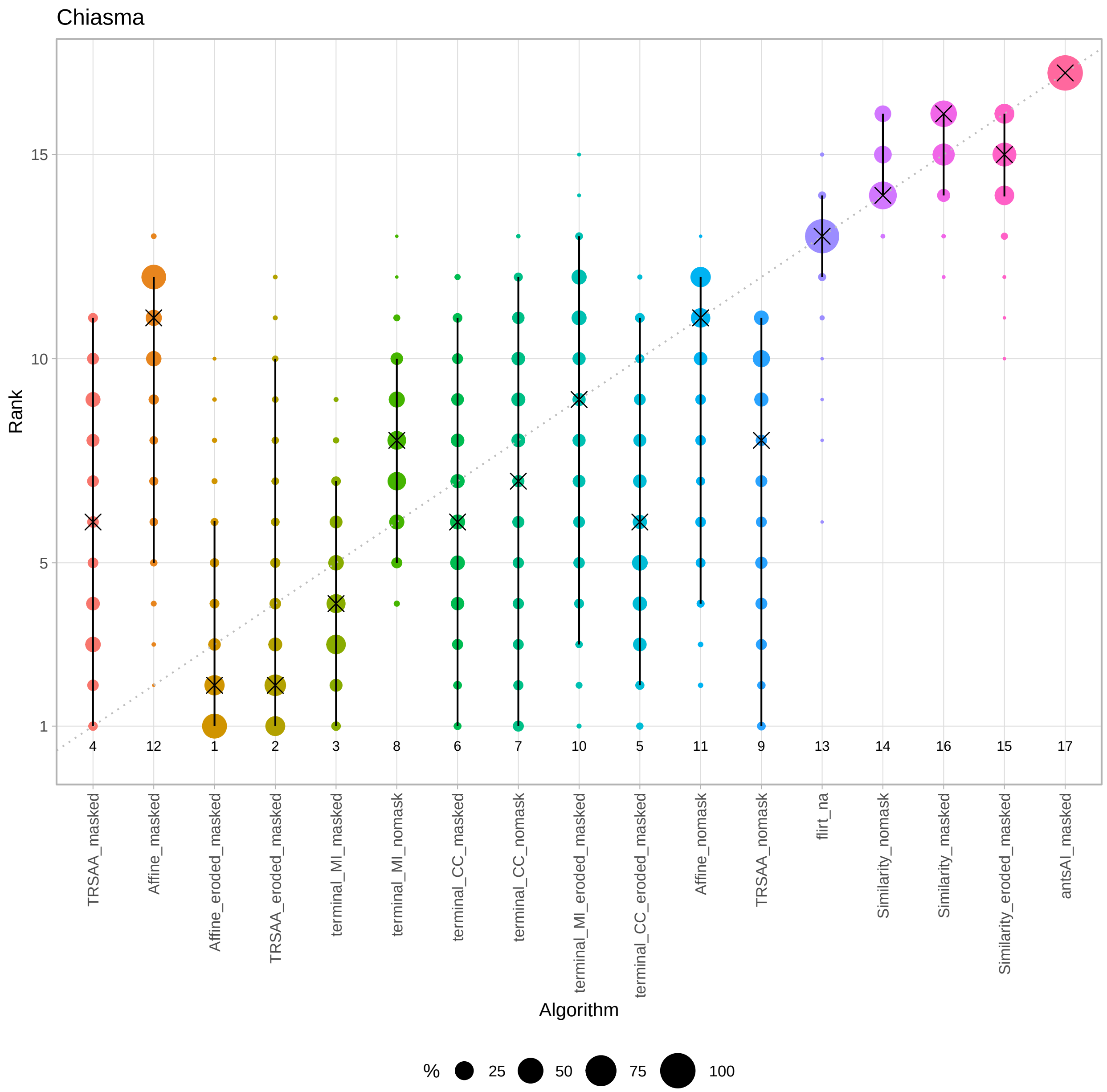
Chiasma
Rank
15
10
5
1
4
12
1
2
3
8
6
7
10
5
11
9
13
14
16
15
17
TRSAA_masked
Affine_masked
Affine_eroded_masked
TRSAA_eroded_masked
terminal_MI_masked
terminal_MI_nomask
terminal_CC_masked
terminal_CC_nomask
terminal_MI_eroded_masked
terminal_CC_eroded_masked
Affine_nomask
TRSAA_nomask
flirt_na
Similarity_nomask
Similarity_masked
Similarity_eroded_masked
antsAI_masked
Algorithm
%
25
50
75
100

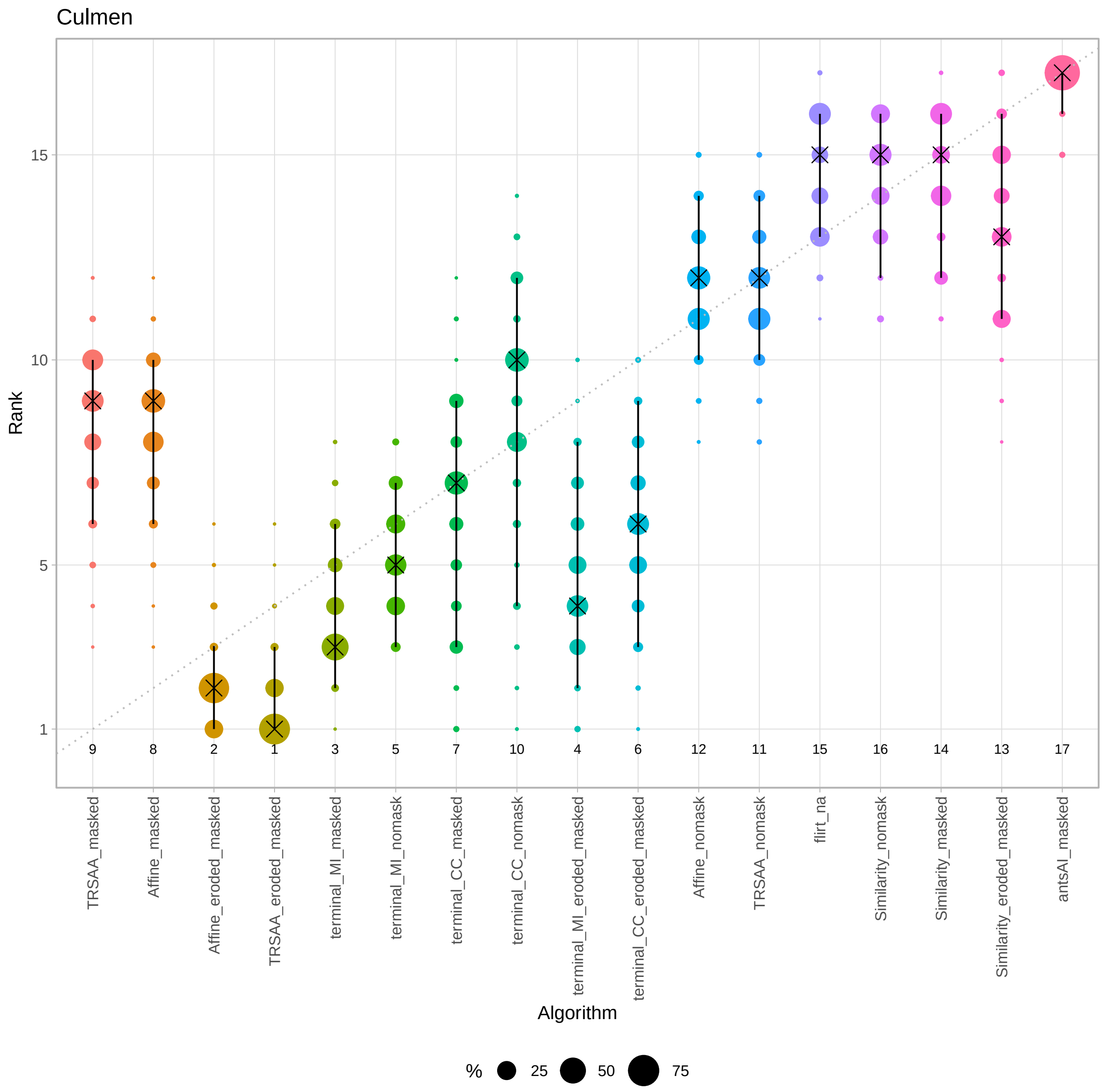
Culmen
Rank
15
10
5
1
9
8
2
1
3
5
7
10
4
6
12
11
15
16
14
13
17
TRSAA_masked
Affine_masked
Affine_eroded_masked
TRSAA_eroded_masked
terminal_MI_masked
terminal_MI_nomask
terminal_CC_masked
terminal_CC_nomask
terminal_MI_eroded_masked
terminal_CC_eroded_masked
Affine_nomask
TRSAA_nomask
flirt_na
Similarity_nomask
Similarity_masked
Similarity_eroded_masked
antsAI_masked
Algorithm
%
25
50
75

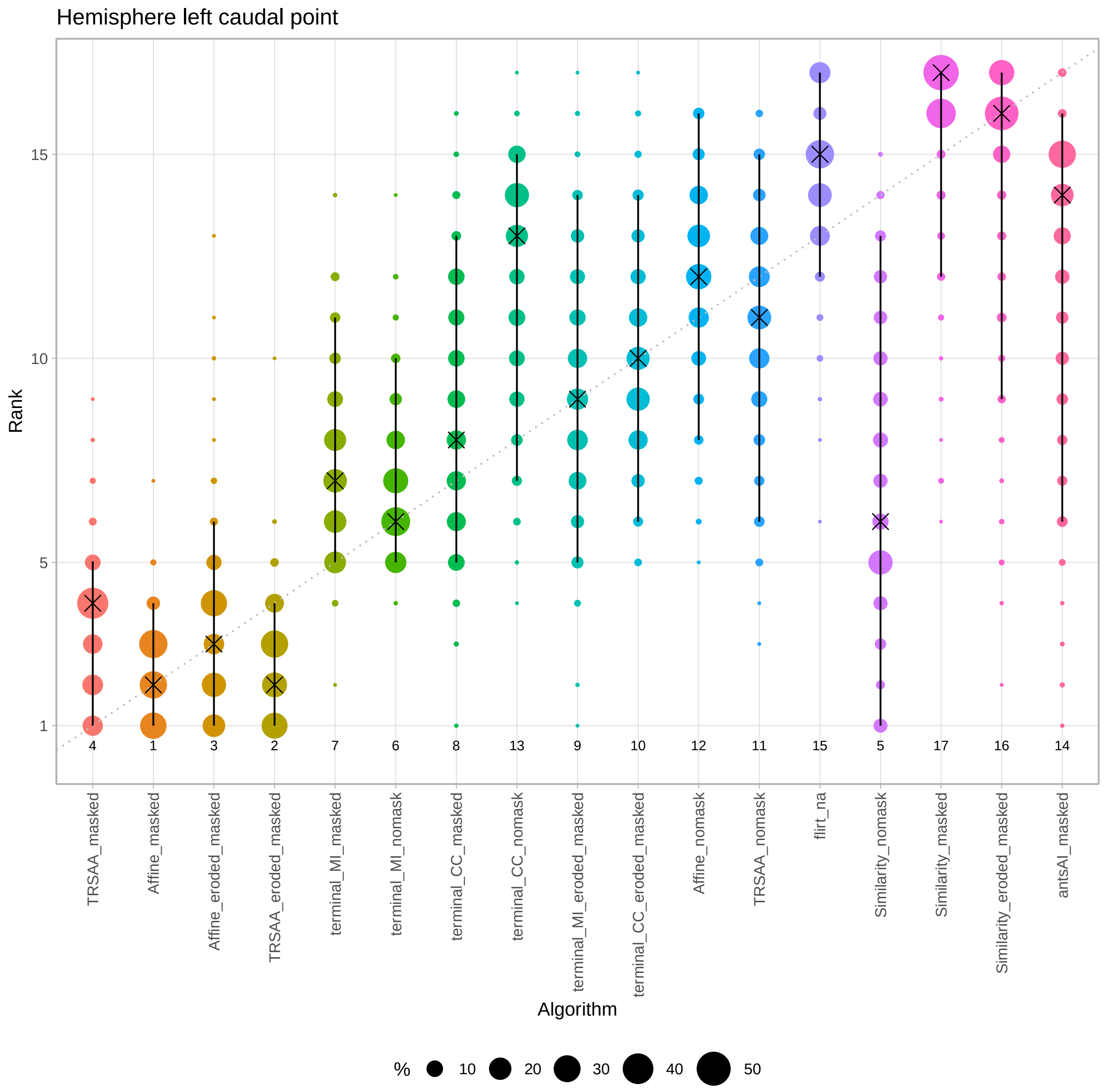
Hemisphere left caudal point
Rank
15
10
5
1
4
1
3
2
7
6
8
13
9
10
12
11
15
5
17
16
14
TRSAA_masked
Affine_masked
Affine_eroded_masked
TRSAA_eroded_masked
terminal_MI_masked
terminal_MI_nomask
terminal_CC_masked
terminal_CC_nomask
terminal_MI_eroded_masked
terminal_CC_eroded_masked
Affine_nomask
TRSAA_nomask
flirt_na
Similarity_nomask
Similarity_masked
Similarity_eroded_masked
antsAI_masked
Algorithm
%
10
20
30
40
50

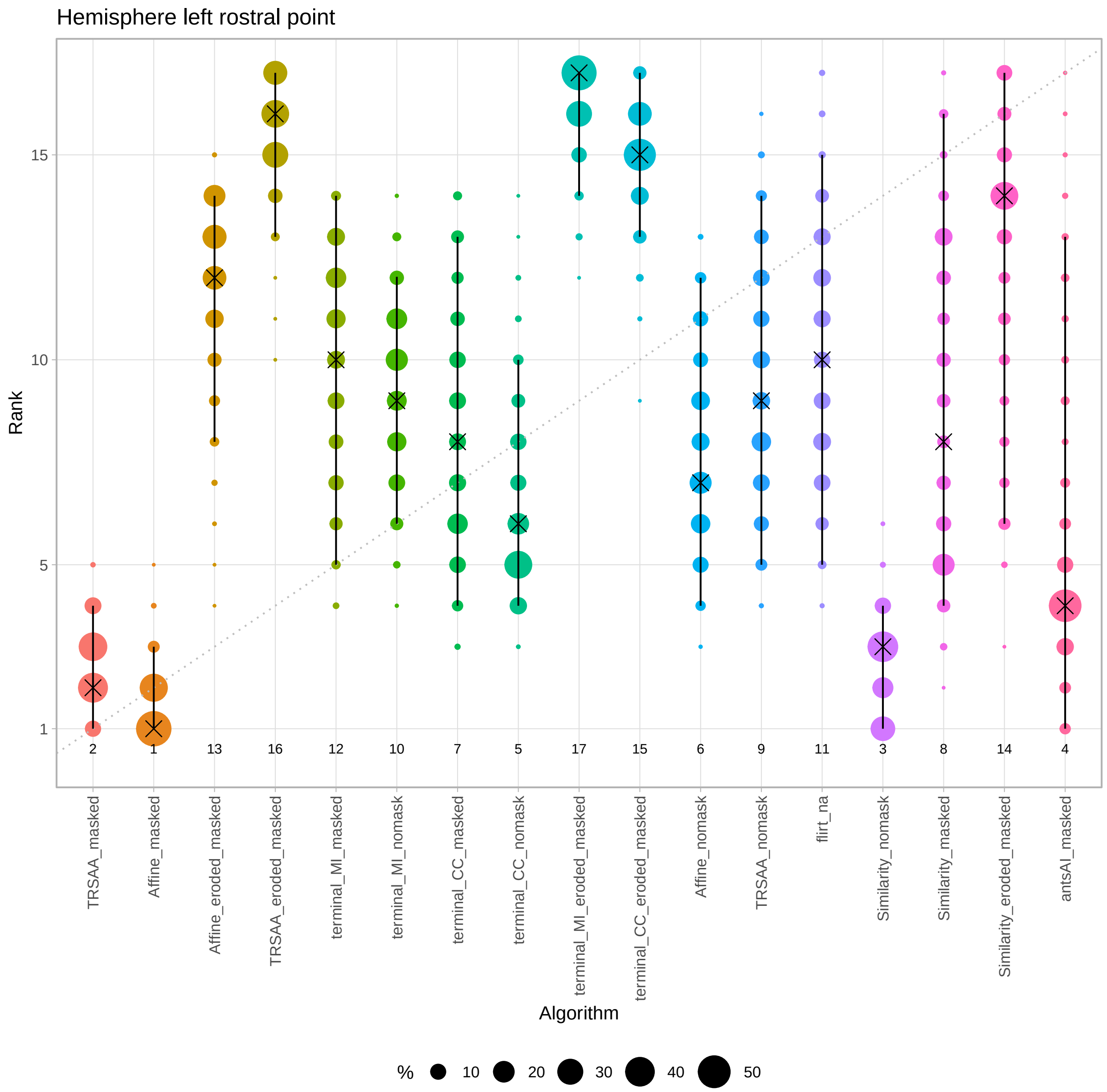

Hemisphere left rostral point
Rank
15
10
5
1
2
1
13
16
12
10
7
5
17
15
6
9
11
3
8
14
4
TRSAA_masked
Affine_masked
Affine_eroded_masked
TRSAA_eroded_masked
terminal_MI_masked
terminal_MI_nomask
terminal_CC_masked
terminal_CC_nomask
terminal_MI_eroded_masked
terminal_CC_eroded_masked
Affine_nomask
TRSAA_nomask
flirt_na
Similarity_nomask
Similarity_masked
Similarity_eroded_masked
antsAI_masked
Algorithm
%
10
20
30
40
50

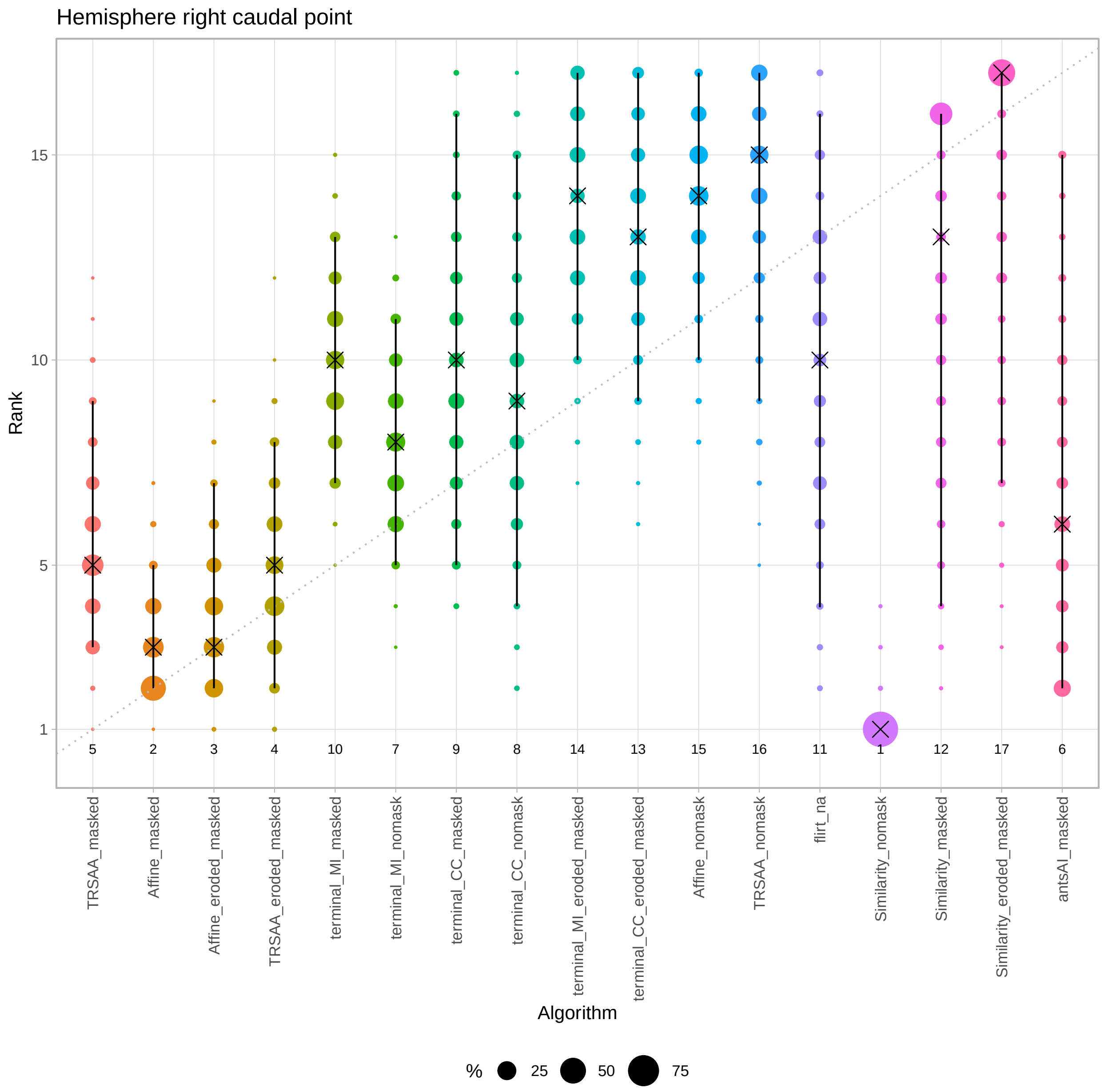

Hemisphere right caudal point
Rank
15
10
5
1
5
2
3
4
10
7
9
8
14
13
15
16
11
1
12
17
6
TRSAA_masked
Affine_masked
Affine_eroded_masked
TRSAA_eroded_masked
terminal_MI_masked
terminal_MI_nomask
terminal_CC_masked
terminal_CC_nomask
terminal_MI_eroded_masked
terminal_CC_eroded_masked
Affine_nomask
TRSAA_nomask
flirt_na
Similarity_nomask
Similarity_masked
Similarity_eroded_masked
antsAI_masked
Algorithm
%
25
50
75

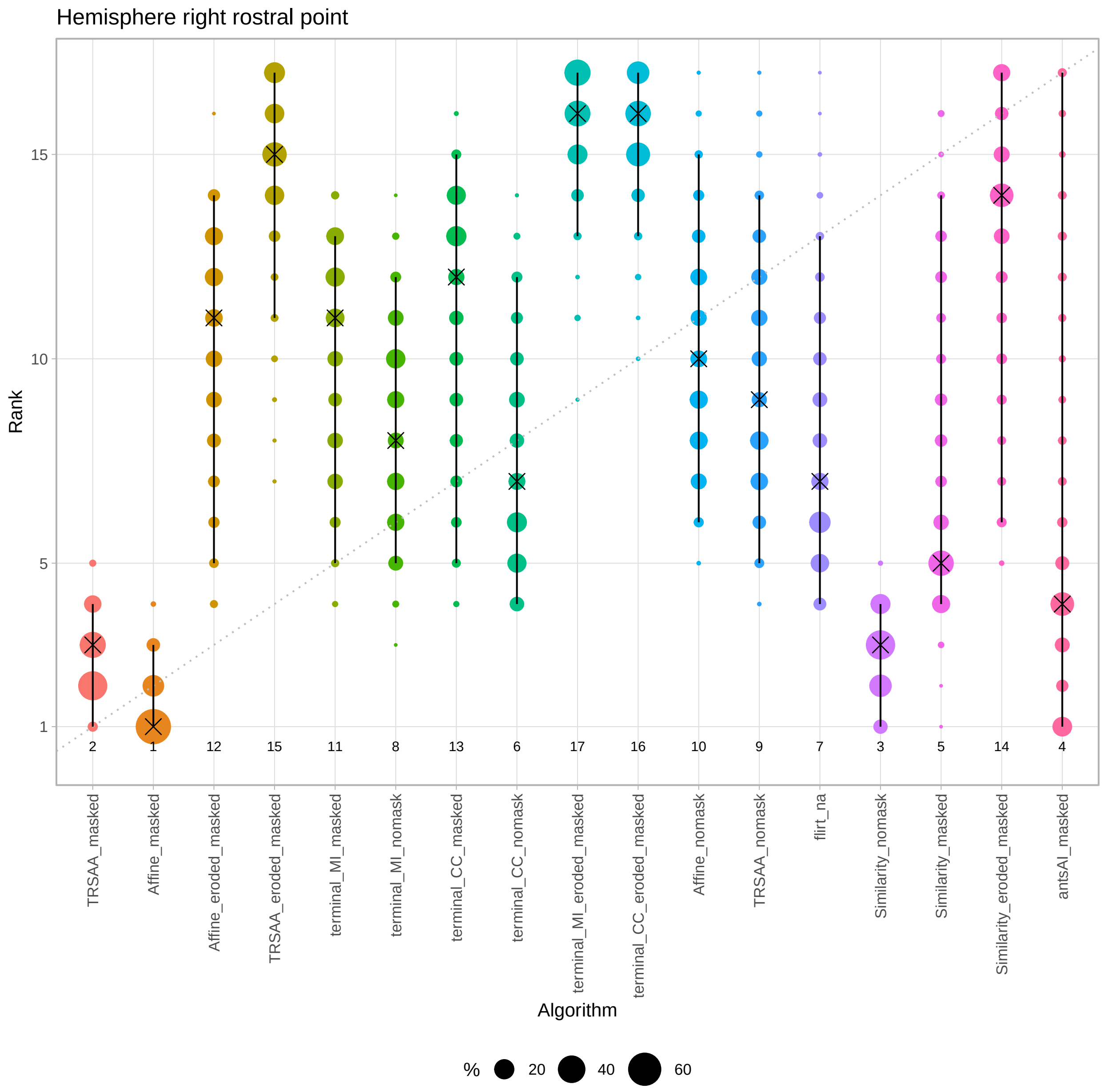
Hemisphere right rostral point
Rank
1
5
10
15
2
1
12
15
11
8
13
6
17
16
10
9
7
3
5
14
4
TRSAA_masked
Affine_masked
Affine_eroded_masked
TRSAA_eroded_masked
terminal_MI_masked
terminal_MI_nomask
terminal_CC_masked
terminal_CC_nomask
terminal_MI_eroded_masked
terminal_CC_eroded_masked
Affine_nomask
TRSAA_nomask
flirt_na
Similarity_nomask
Similarity_masked
Similarity_eroded_masked
antsAI_masked
Algorithm
%
20
40
60

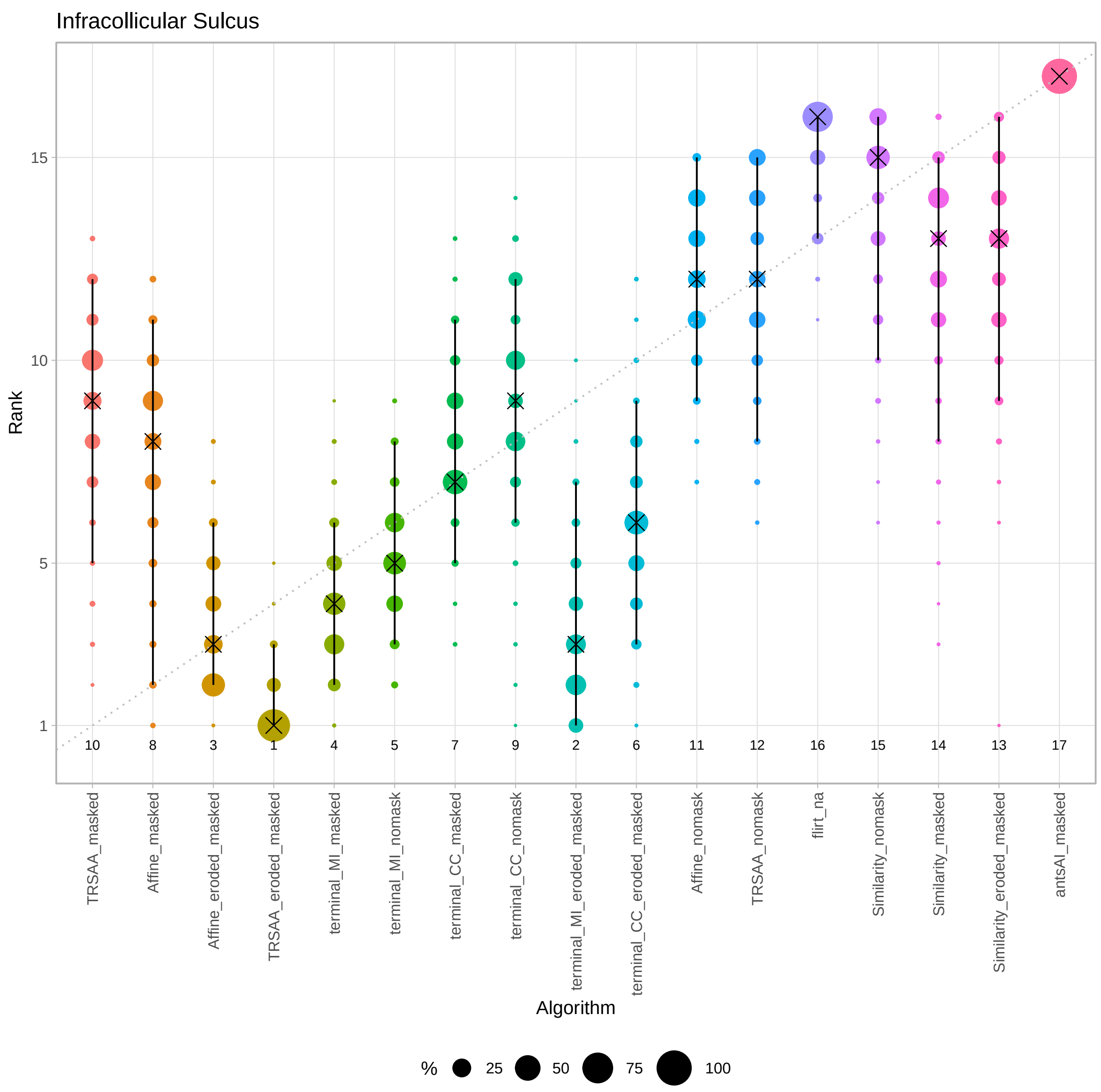

Infracollicular Sulcus
Rank
15
10
5
1
10
8
3
1
4
5
7
9
2
6
11
12
16
15
14
13
17
TRSAA_masked
Affine_masked
Affine_eroded_masked
TRSAA_eroded_masked
terminal_MI_masked
terminal_MI_nomask
terminal_CC_masked
terminal_CC_nomask
terminal_MI_eroded_masked
terminal_CC_eroded_masked
Affine_nomask
TRSAA_nomask
flirt_na
Similarity_nomask
Similarity_masked
Similarity_eroded_masked
antsAI_masked
Algorithm
%
25
50
75
100

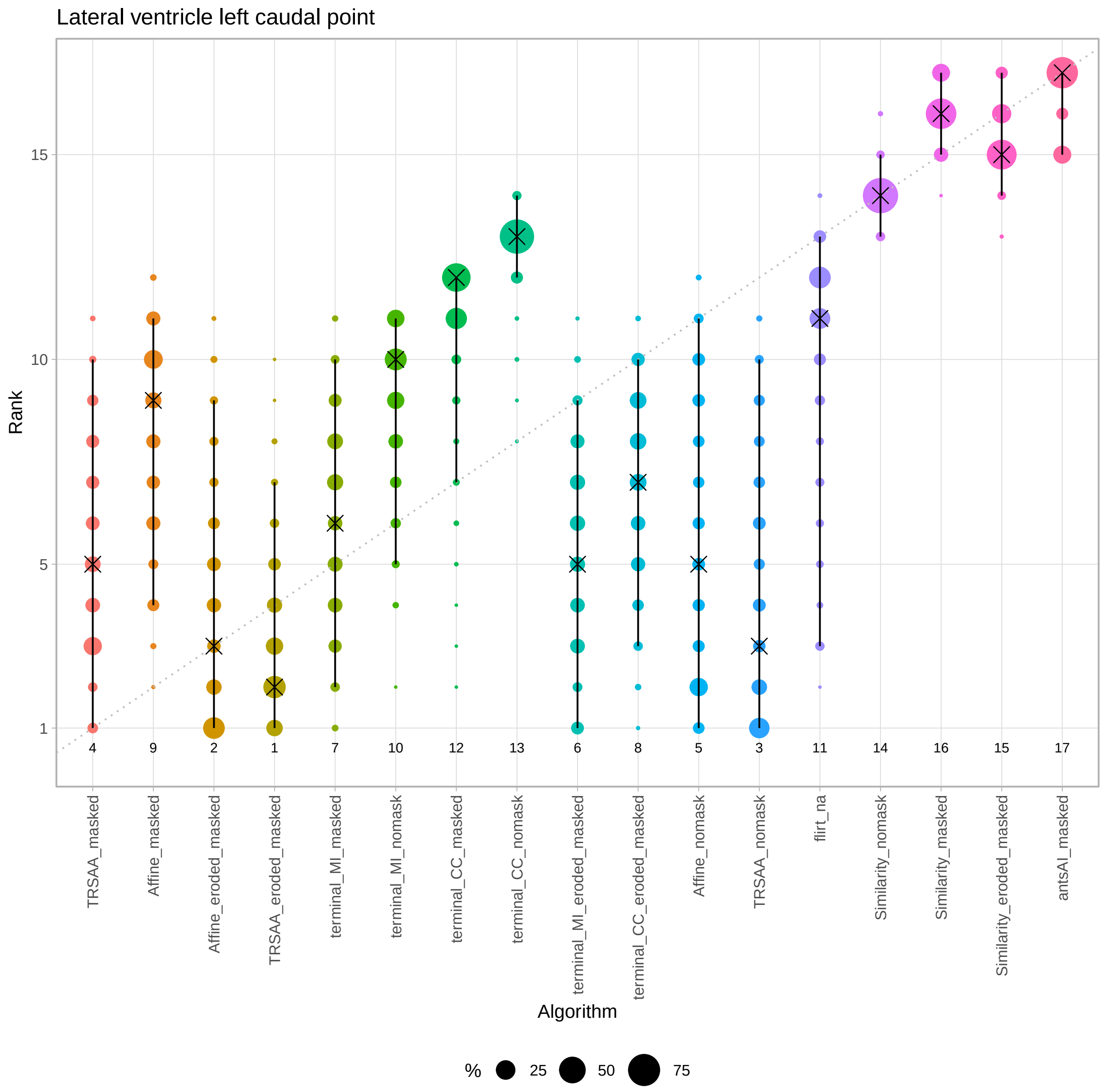
Lateral ventricle left caudal point
Rank
15
10
5
1
4
9
2
1
7
10
12
13
6
8
5
3
11
14
16
15
17
TRSAA_masked
Affine_masked
Affine_eroded_masked
TRSAA_eroded_masked
terminal_MI_masked
terminal_MI_nomask
terminal_CC_masked
terminal_CC_nomask
terminal_MI_eroded_masked
terminal_CC_eroded_masked
Affine_nomask
TRSAA_nomask
flirt_na
Similarity_nomask
Similarity_masked
Similarity_eroded_masked
antsAI_masked
Algorithm
%
25
50
75

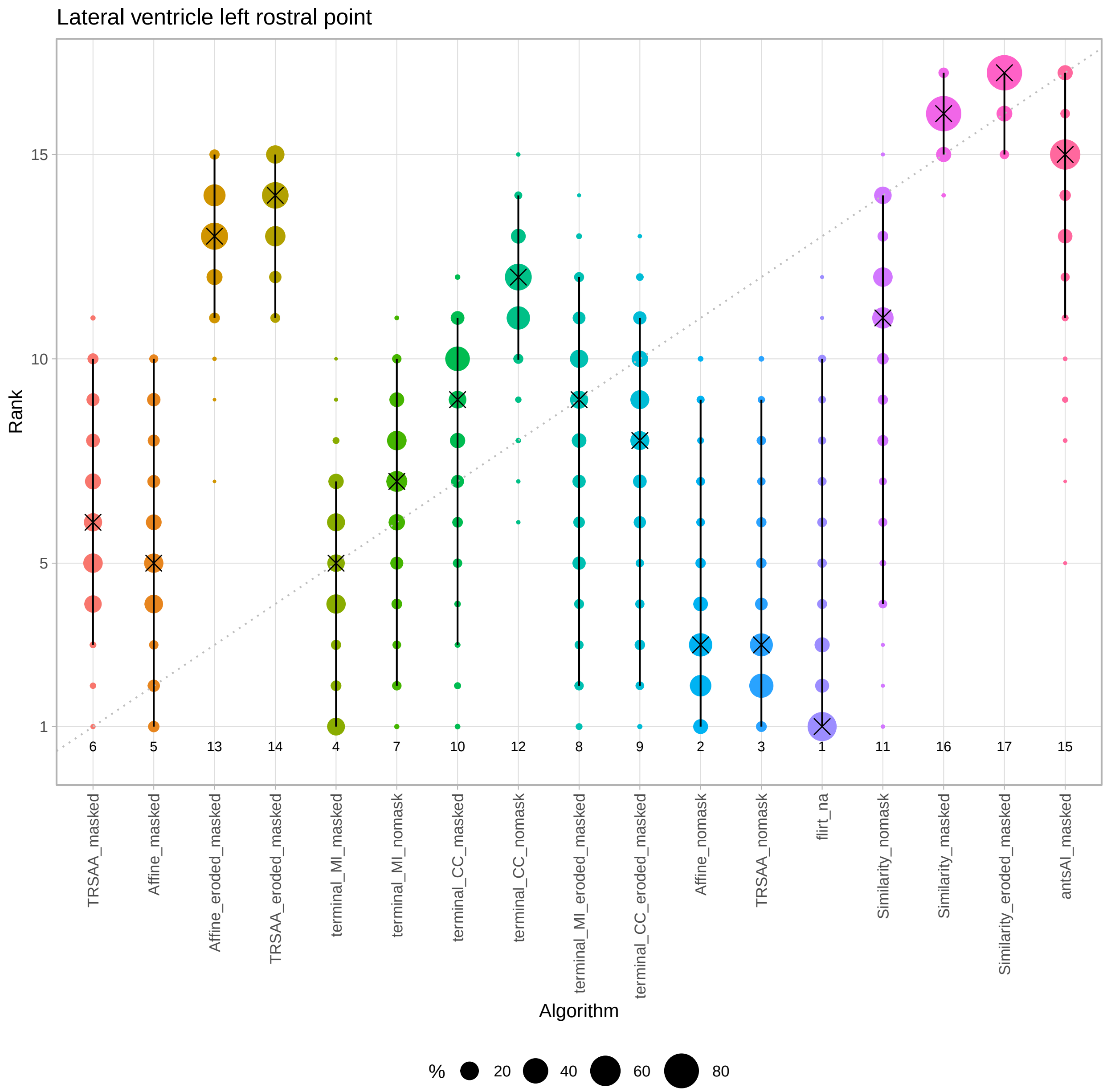

Lateral ventricle left rostral point
Rank
15
10
5
1
6
5
13
14
4
7
10
12
8
9
2
3
1
11
16
17
15
TRSAA_masked
Affine_masked
Affine_eroded_masked
TRSAA_eroded_masked
terminal_MI_masked
terminal_MI_nomask
terminal_CC_masked
terminal_CC_nomask
terminal_MI_eroded_masked
terminal_CC_eroded_masked
Affine_nomask
TRSAA_nomask
flirt_na
Similarity_nomask
Similarity_masked
Similarity_eroded_masked
antsAI_masked
Algorithm
%
20
40
60
80

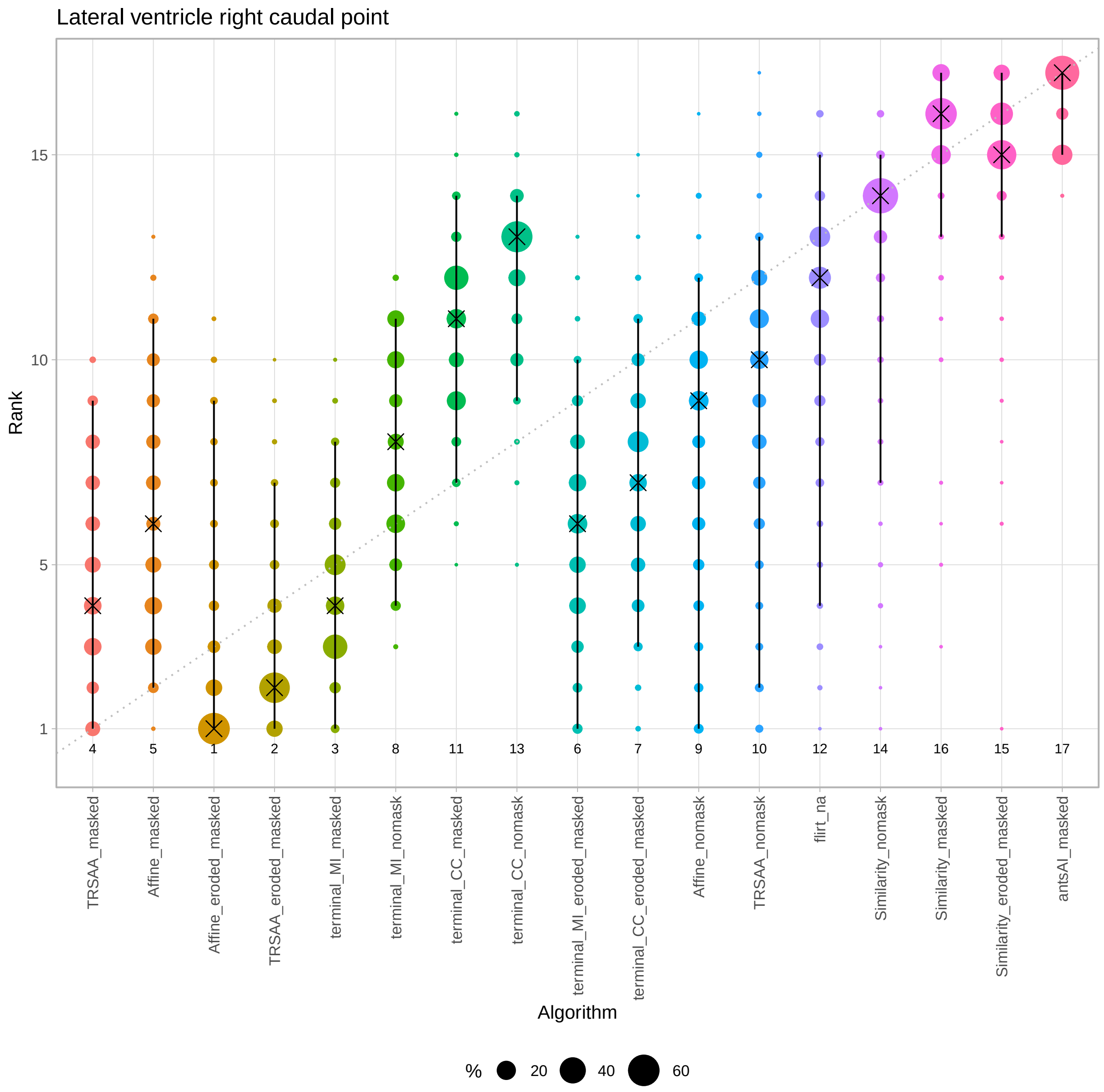
Lateral ventricle right caudal point
Rank
15
10
5
1
4
5
1
2
3
8
11
13
6
7
9
10
12
14
16
15
17
TRSAA_masked
Affine_masked
Affine_eroded_masked
TRSAA_eroded_masked
terminal_MI_masked
terminal_MI_nomask
terminal_CC_masked
terminal_CC_nomask
terminal_MI_eroded_masked
terminal_CC_eroded_masked
Affine_nomask
TRSAA_nomask
flirt_na
Similarity_nomask
Similarity_masked
Similarity_eroded_masked
antsAI_masked
Algorithm
%
20
40
60

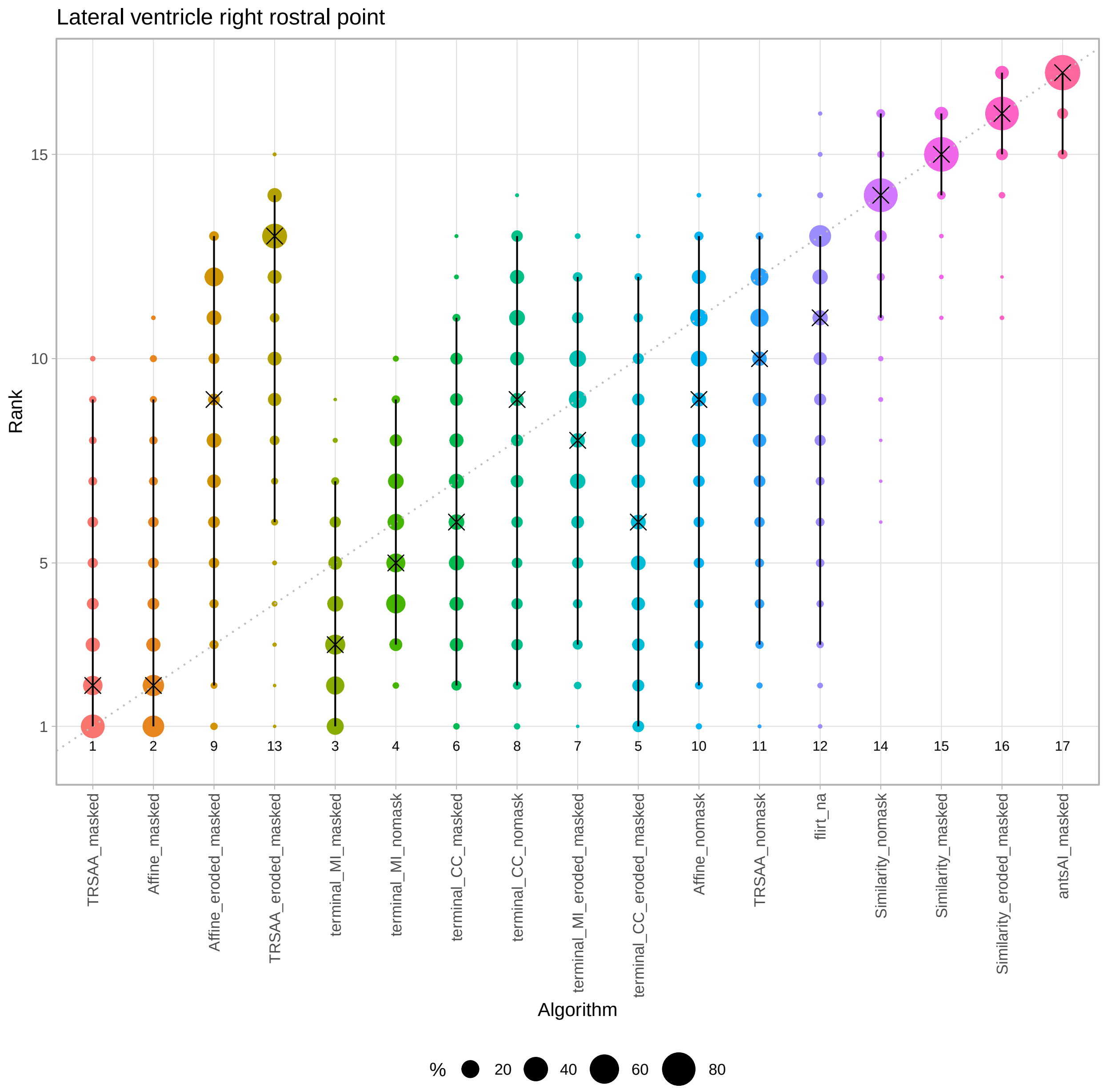
Lateral ventricle right rostral point
Rank
15
10
5
1
1
2
9
13
3
4
6
8
7
5
10
11
12
14
15
16
17
TRSAA_masked
Affine_masked
Affine_eroded_masked
TRSAA_eroded_masked
terminal_MI_masked
terminal_MI_nomask
terminal_CC_masked
terminal_CC_nomask
terminal_MI_eroded_masked
terminal_CC_eroded_masked
Affine_nomask
TRSAA_nomask
flirt_na
Similarity_nomask
Similarity_masked
Similarity_eroded_masked
antsAI_masked
Algorithm
%
20
40
60
80

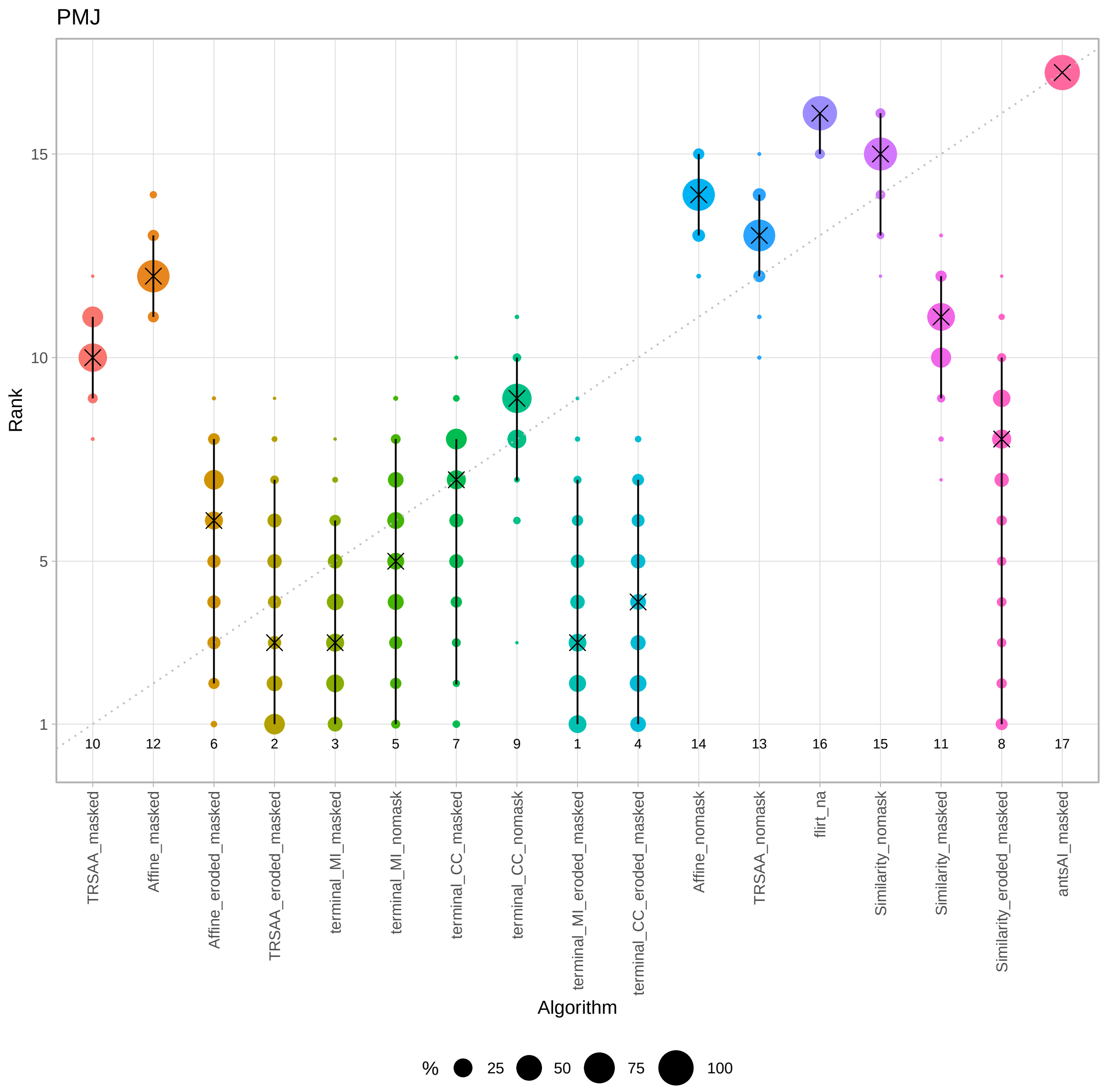

PMJ
Rank
15
10
5
1
10
12
6
2
3
5
7
9
1
4
14
13
16
15
11
8
17
TRSAA_masked
Affine_masked
Affine_eroded_masked
TRSAA_eroded_masked
terminal_MI_masked
terminal_MI_nomask
terminal_CC_masked
terminal_CC_nomask
terminal_MI_eroded_masked
terminal_CC_eroded_masked
Affine_nomask
TRSAA_nomask
flirt_na
Similarity_nomask
Similarity_masked
Similarity_eroded_masked
antsAI_masked
Algorithm
%
25
50
75
100

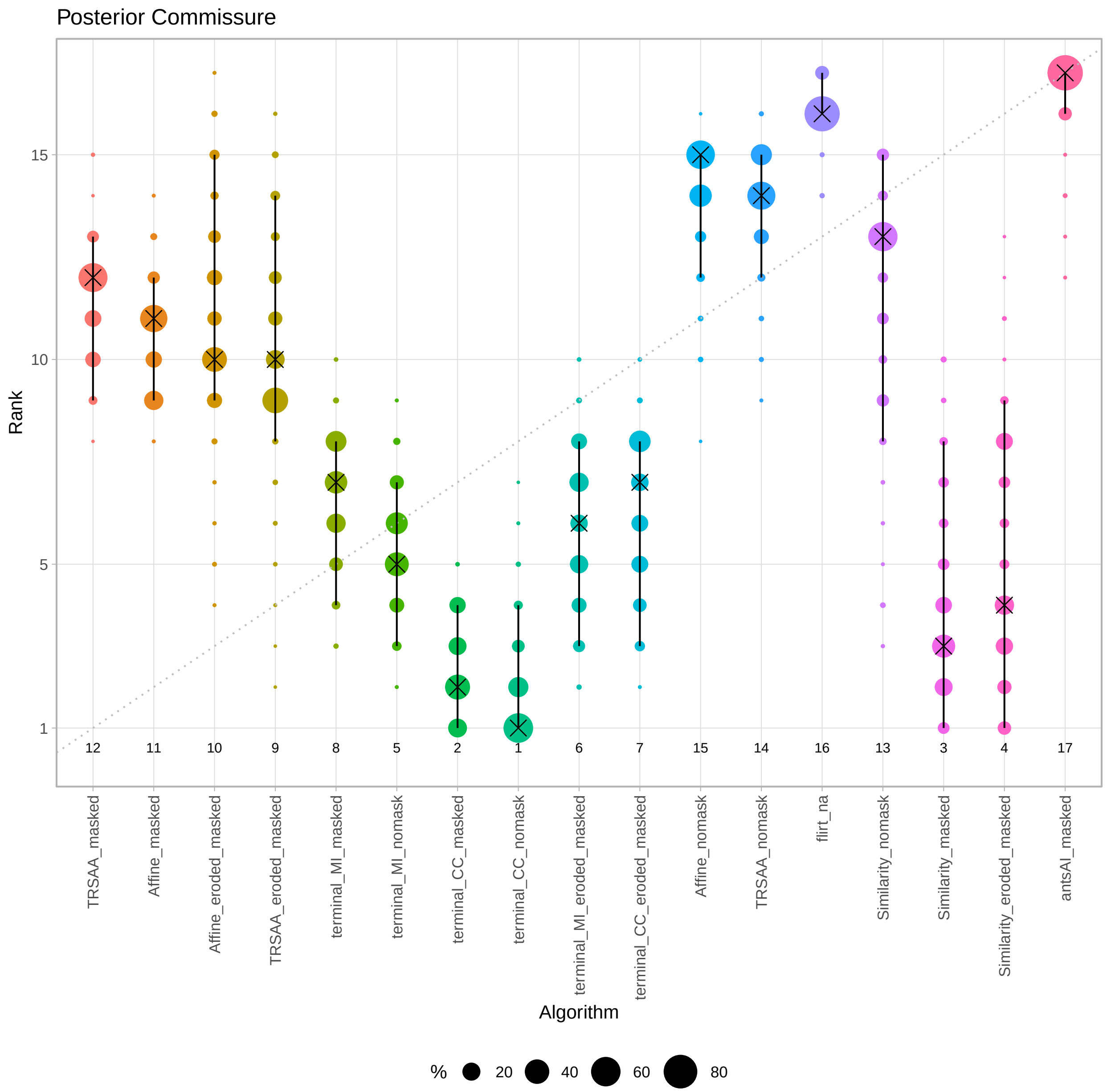

Posterior Commissure
Rank
15
10
5
1
12
11
10
9
8
5
2
1
6
7
15
14
16
13
3
4
17
TRSAA_masked
Affine_masked
Affine_eroded_masked
TRSAA_eroded_masked
terminal_MI_masked
terminal_MI_nomask
terminal_CC_masked
terminal_CC_nomask
terminal_MI_eroded_masked
terminal_CC_eroded_masked
Affine_nomask
TRSAA_nomask
flirt_na
Similarity_nomask
Similarity_masked
Similarity_eroded_masked
antsAI_masked
Algorithm
%
20
40
60
80

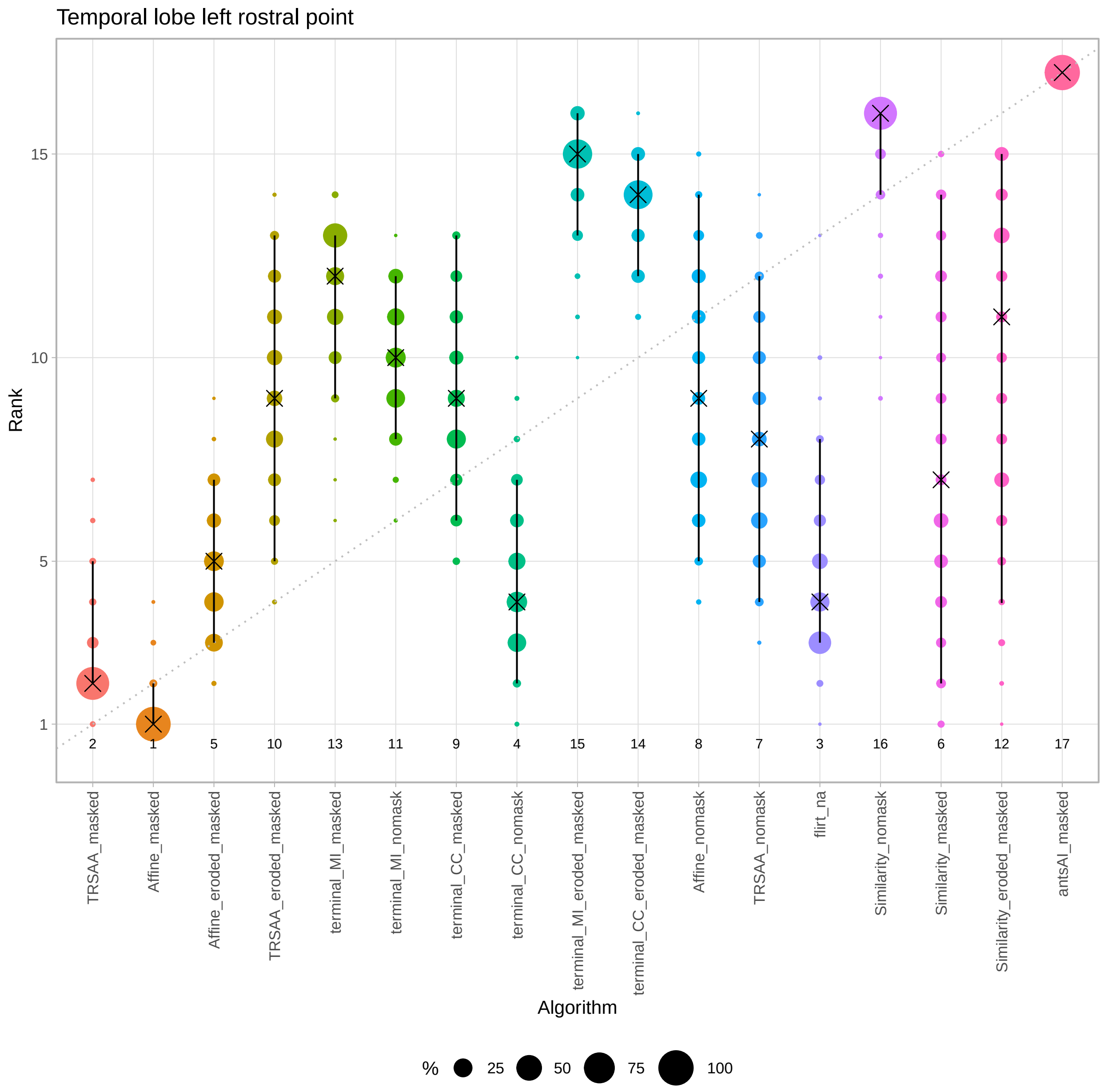

Temporal lobe left rostral point
Rank
15
10
5
1
2
1
5
10
13
11
9
4
15
14
8
7
3
16
6
12
17
TRSAA_masked
Affine_masked
Affine_eroded_masked
TRSAA_eroded_masked
terminal_MI_masked
terminal_MI_nomask
terminal_CC_masked
terminal_CC_nomask
terminal_MI_eroded_masked
terminal_CC_eroded_masked
Affine_nomask
TRSAA_nomask
flirt_na
Similarity_nomask
Similarity_masked
Similarity_eroded_masked
antsAI_masked
Algorithm
%
25
50
75
100

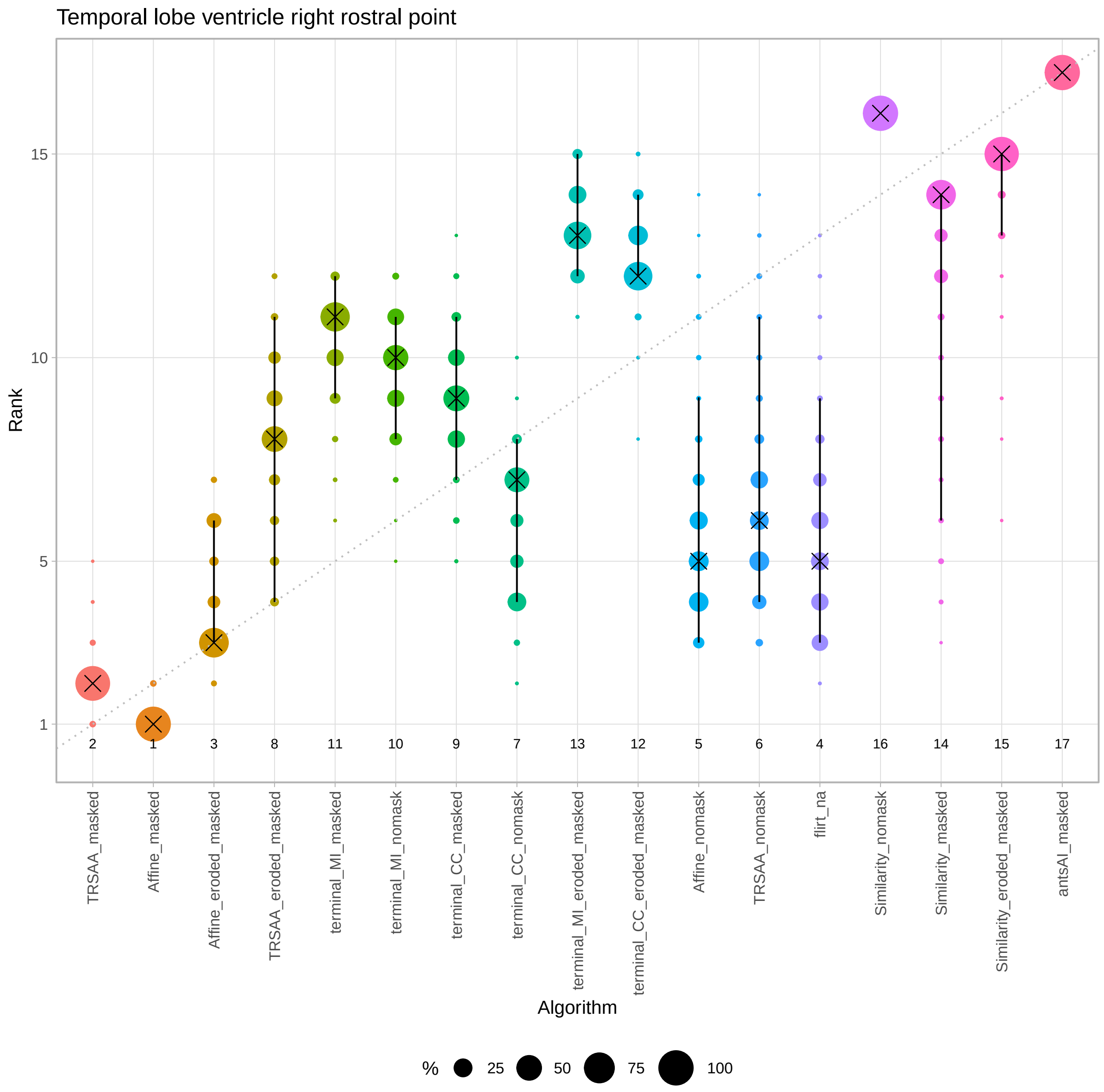

4.2.2 Cluster Analysis

Dendrogram from hierarchical cluster analysis and *network-type graphs* for assessing the similarity of tasks based on challenge rankings.

A dendrogram is a visualization approach based on hierarchical clustering. It depicts clusters according to a chosen distance measure (here: Spearman's footrule) as well as a chosen agglomeration method (here: complete and average agglomeration).

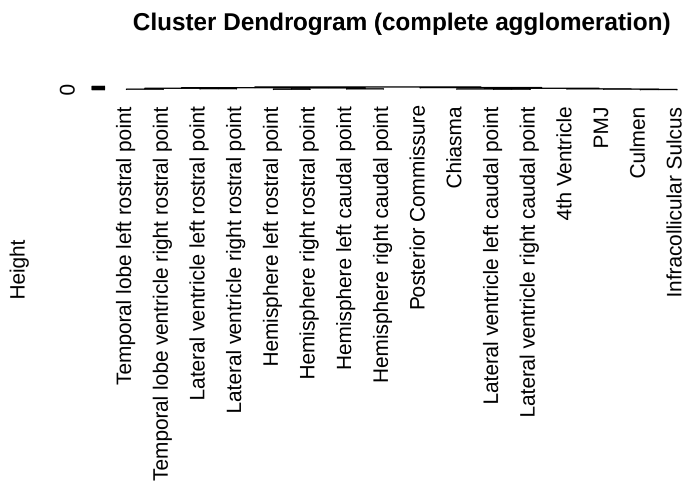
Cluster Dendrogram (complete agglomeration)
Height
0
Temporal lobe left rostral point
Temporal lobe ventricle right rostral point
Lateral ventricle left rostral point
Lateral ventricle right rostral point
Hemisphere left rostral point
Hemisphere right rostral point
Hemisphere left caudal point
Hemisphere right caudal point
Posterior Commissure
Chiasma
Lateral ventricle left caudal point
Lateral ventricle right caudal point
4th Ventricle
PMJ
Culmen
Infracollicular Sulcus

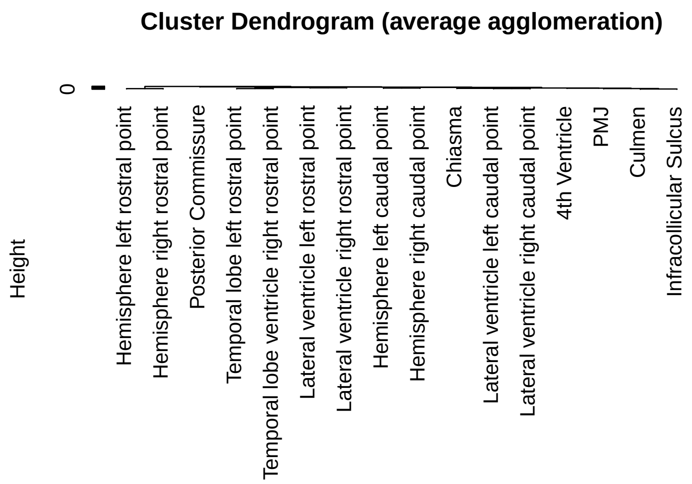
Cluster Dendrogram (average agglomeration)
Height
0
Hemisphere left rostral point
Hemisphere right rostral point
Posterior Commissure
Temporal lobe left rostral point
Temporal lobe ventricle right rostral point
Lateral ventricle left rostral point
Lateral ventricle right rostral point
Hemisphere left caudal point
Hemisphere right caudal point
Chiasma
Lateral ventricle left caudal point
Lateral ventricle right caudal point
4th Ventricle
PMJ
Culmen
Infracollicular Sulcus

5 References

Wiesenfarth, M., Reinke, A., Landman, B.A., Eisenmann, M., Aguilera Saiz, L., Cardoso, M.J., Maier-Hein, L. and Kopp-Schneider, A. Methods and open-source toolkit for analyzing and visualizing challenge results. *Sci Rep* **11**, 2369 (2021). https://doi.org/10.1038/s41598-021-82017-6

M. J. A. Eugster, T. Hothorn, and F. Leisch, "Exploratory and inferential analysis of benchmark experiments," Institut fuer Statistik, Ludwig-Maximilians-Universitaet Muenchen, Germany, Technical Report 30, 2008. [Online]. Available: http://epub.ub.uni-muenchen.de/4134/.
