# Supplementary material for: Characterization of dynamic patterns of human fetal to neonatal brain asymmetry with deformation-based morphometry
Source: Front Neurosci. 2023 Dec 6;17:1252850. doi: 10.3389/fnins.2023.1252850 (PMC10734644; doi:10.3389/fnins.2023.1252850)
Supplement: Supplementary file 1 [file Data_Sheet_1.ZIP › ChallengeR/Framework B to mid-age template/neonatal2tempalte_linear/Neonatal2MidTemplate_linear_voldiff.pdf]

Benchmarking report for Neonatal to Midtempalte Registration Linear

created by challengeR v1.0.4

29 März, 2023

This document presents a systematic report on the benchmark study "Neonatal to Midtempalte Registration Linear". Input data comprises raw metric values for all algorithms and cases. Generated plots are:

- Visualization of assessment data: Dot- and boxplots, podium plots and ranking heatmaps
- Visualization of ranking stability: Blob plots, violin plots and significance maps, line plots
- Visualization of cross-task insights: Blob plots, stacked frequency plots, dendrograms

Details can be found in Wiesenfarth et al. (2021).

1 Rankings

Algorithms within a task are ranked according to the following ranking scheme:

aggregate using function ("mean") then rank

Ranking for each task:

Brainstem : The analysis is based on 17 algorithms and 20 cases. 0 missing cases have been found in the data set.

	value_mean	rank
Affine_masked	0.0560066	1
Affine_eroded_masked	0.0573546	2
TRSAA_masked	0.0587065	3
Similarity_masked	0.0594093	4
Similarity_eroded_masked	0.0651778	5
terminal_CC_nomask	0.0676703	6
TRSAA_eroded_masked	0.0734388	7
terminal_CC_masked	0.0873339	8
terminal_MI_nomask	0.1056802	9
terminal_MI_masked	0.1127775	10
terminal_CC_eroded_masked	0.1153890	11
Affine_nomask	0.1299255	12
TRSAA_nomask	0.1317459	13
terminal_MI_eroded_masked	0.1325678	14
Similarity_nomask	0.1783086	15
antsAI_masked	0.2435671	16
flirt_NA	0.8410144	17

Cerebellum : The analysis is based on 17 algorithms and 20 cases. 0 missing cases have been found in the data set.

	value_mean	rank
Affine_masked	0.1858084	1
TRSAA_masked	0.2007643	2
Similarity_masked	0.2011993	3
Similarity_eroded_masked	0.2078954	4
Affine_eroded_masked	0.2087841	5
terminal_CC_nomask	0.2290297	6
TRSAA_eroded_masked	0.2392247	7
terminal_CC_masked	0.2552963	8
terminal_MI_nomask	0.2780396	9
terminal_MI_masked	0.2885716	10
flirt_NA	0.2902863	11
terminal_CC_eroded_masked	0.2902863	11
Affine_nomask	0.3050837	13
TRSAA_nomask	0.3078700	14
terminal_MI_eroded_masked	0.3109604	15
Similarity_nomask	0.3619141	16
antsAI_masked	0.4441090	17

Cortical grey matter : The analysis is based on 17 algorithms and 20 cases. 0 missing cases have been found in the data set.

	value_mean	rank
Affine_masked	0.1130243	1
Similarity_masked	0.1283141	2
TRSAA_masked	0.1306991	3
Similarity_eroded_masked	0.1339549	4
Affine_eroded_masked	0.1368969	5
terminal_CC_nomask	0.1542633	6
TRSAA_eroded_masked	0.1667259	7
terminal_CC_masked	0.1792415	8
terminal_MI_nomask	0.2042628	9
terminal_MI_masked	0.2140106	10
terminal_CC_eroded_masked	0.2151636	11
flirt_NA	0.2156964	12
Affine_nomask	0.2296542	13
TRSAA_nomask	0.2322221	14
terminal_MI_eroded_masked	0.2350234	15
Similarity_nomask	0.2835611	16
antsAI_masked	0.3607075	17

CSF and ventricles : The analysis is based on 17 algorithms and 20 cases. 0 missing cases have been found in the data set.

	value_mean	rank
antsAI_masked	0.2366328	1
Similarity_nomask	0.2477928	2
TRSAA_nomask	0.2746417	3
Affine_nomask	0.2761131	4
terminal_MI_eroded_masked	0.2789841	5
flirt_NA	0.2841053	6

	value_mean	rank
terminal_CC_eroded_masked	0.2875640	7
terminal_MI_masked	0.2886596	8
terminal_MI_nomask	0.2929262	9
terminal_CC_masked	0.3071100	10
TRSAA_eroded_masked	0.3120650	11
terminal_CC_nomask	0.3200545	12
Affine_eroded_masked	0.3279570	13
TRSAA_masked	0.3316990	14
Similarity_eroded_masked	0.3334090	15
Similarity_masked	0.3348166	16
Affine_masked	0.3423216	17

Deep grey matter : The analysis is based on 17 algorithms and 20 cases. 0 missing cases have been found in the data set.

	value_mean	rank
terminal_CC_nomask	0.0259390	1
TRSAA_masked	0.0307756	2
Affine_eroded_masked	0.0344350	3
Similarity_eroded_masked	0.0348345	4
Affine_masked	0.0362095	5
Similarity_masked	0.0388925	6
terminal_CC_masked	0.0430928	7
TRSAA_eroded_masked	0.0492435	8
terminal_MI_nomask	0.0661924	9
terminal_MI_masked	0.0743960	10
terminal_CC_eroded_masked	0.0752514	11
flirt_NA	0.0762553	12
Affine_nomask	0.0887794	13
TRSAA_nomask	0.0910930	14
terminal_MI_eroded_masked	0.0926492	15
Similarity_nomask	0.1374812	16
antsAI_masked	0.2020246	17

White matter : The analysis is based on 17 algorithms and 20 cases. 0 missing cases have been found in the data set.

	value_mean	rank
TRSAA_eroded_masked	0.0338609	1
terminal_CC_masked	0.0370338	2
terminal_MI_nomask	0.0391586	3
terminal_MI_masked	0.0409563	4
terminal_CC_eroded_masked	0.0446200	5
Similarity_eroded_masked	0.0462086	6
terminal_CC_nomask	0.0472999	7
Affine_eroded_masked	0.0483514	8
flirt_NA	0.0489288	9
TRSAA_masked	0.0490870	10
Similarity_masked	0.0517110	11
terminal_MI_eroded_masked	0.0517459	12

	value_mean	rank
Affine_nomask	0.0556313	13
TRSAA_nomask	0.0573846	14
Affine_masked	0.0619026	15
Similarity_nomask	0.0882617	16
antsAI_masked	0.1493308	17

Consensus ranking across tasks according to chosen method "euclidean":

	value	rank
TRSAA_masked	5.667	1
Affine_eroded_masked	6.000	2
Similarity_eroded_masked	6.333	3
terminal_CC_nomask	6.333	3
Affine_masked	6.667	5
TRSAA_eroded_masked	6.833	6
Similarity_masked	7.000	7
terminal_CC_masked	7.167	8
terminal_MI_nomask	8.000	9
terminal_MI_masked	8.667	10
terminal_CC_eroded_masked	9.417	11
flirt_NA	11.250	12
Affine_nomask	11.333	13
TRSAA_nomask	12.000	14
terminal_MI_eroded_masked	12.667	15
Similarity_nomask	13.500	16
antsAI_masked	14.167	17

2 Visualization of raw assessment data

The algorithms are ordered according to the computed ranks for each task.

2.1 Dot- and boxplot

Dot- and boxplots for visualizing raw assessment data separately for each algorithm. Boxplots representing descriptive statistics over all cases (median, quartiles and outliers) are combined with horizontally jittered dots representing individual cases.

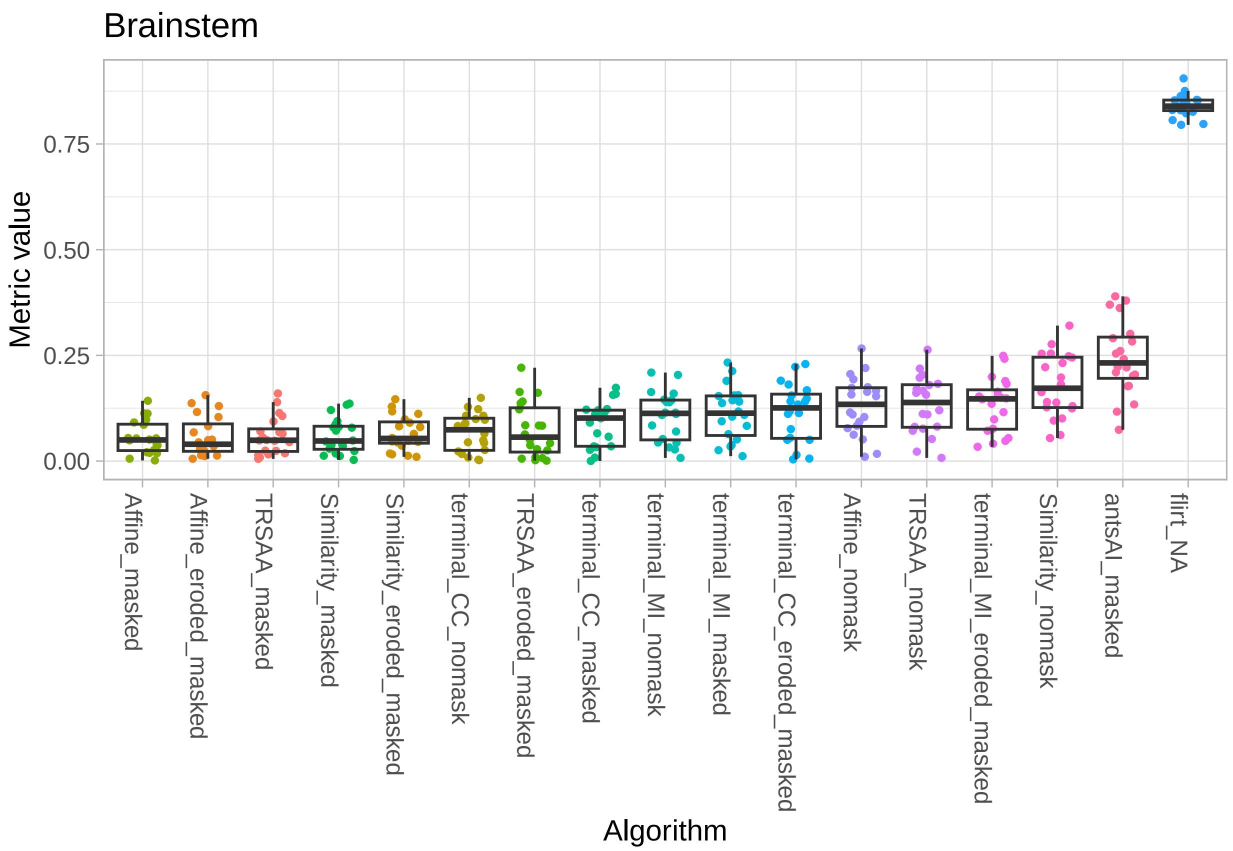

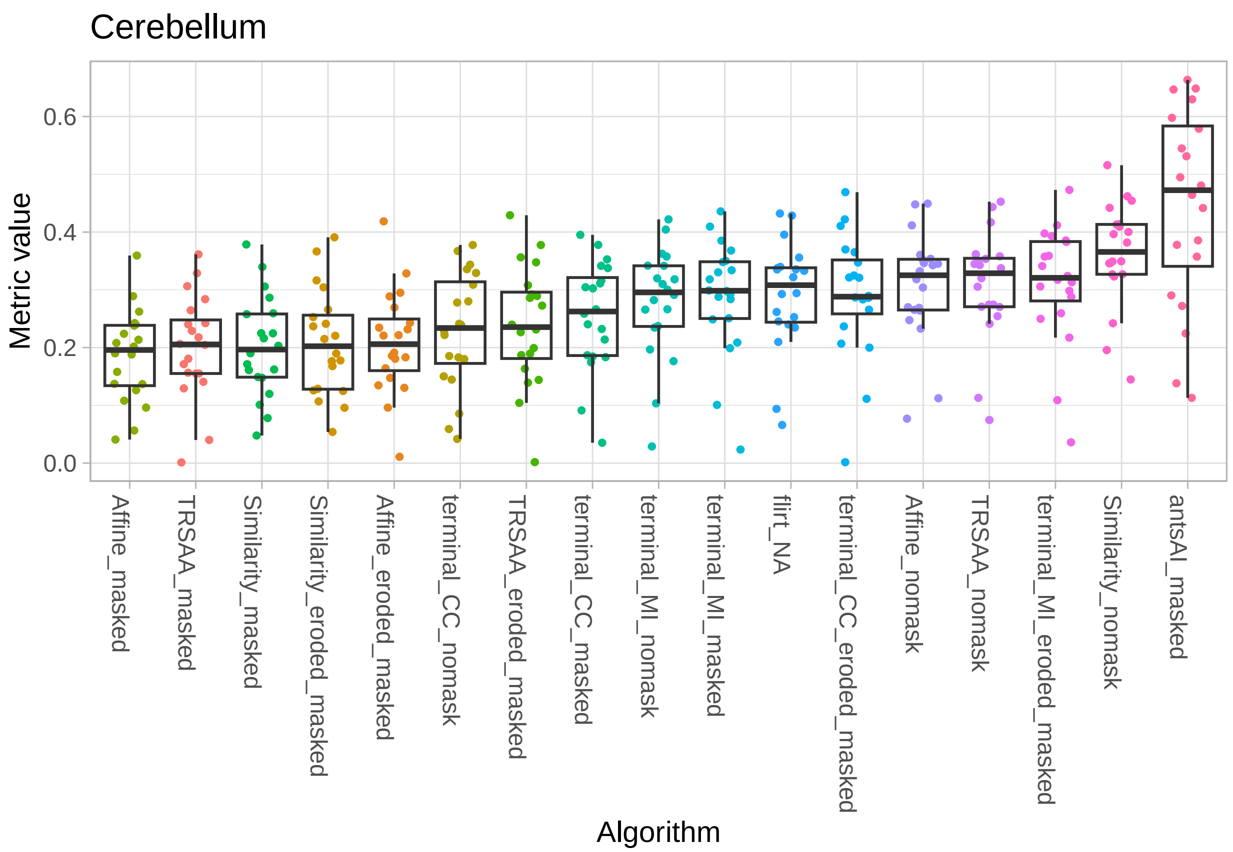

Cerebellum
Metric value
0.0
0.2
0.4
0.6
Affine_masked
TRSAA_masked
Similarity_masked
Similarity_eroded_masked
Affine_eroded_masked
terminal_CC_nomask
TRSAA_eroded_masked
terminal_CC_masked
terminal_MI_nomask
terminal_MI_masked
flirt_NA
terminal_CC_eroded_masked
Affine_nomask
TRSAA_nomask
terminal_MI_eroded_masked
Similarity_nomask
antsAI_masked
Algorithm

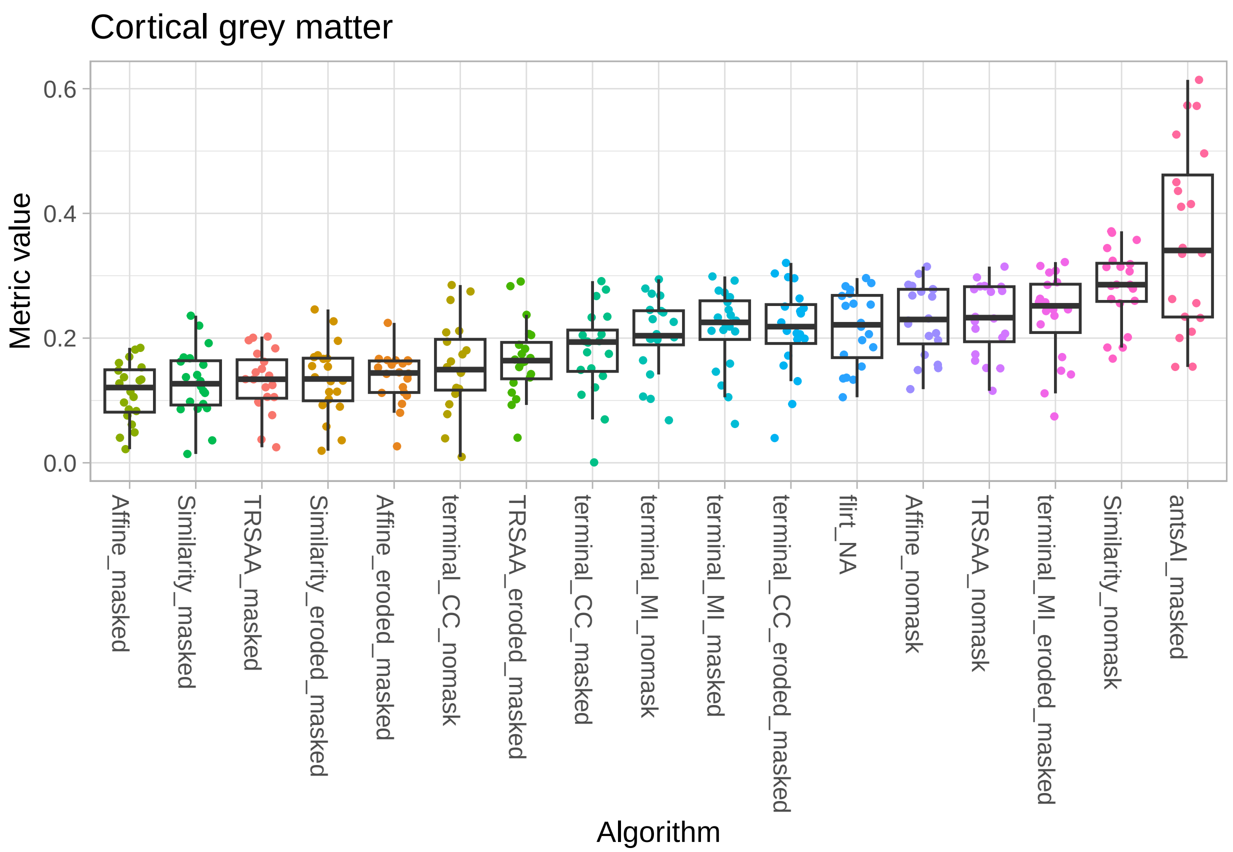
Cortical grey matter
Metric value
0.6
0.4
0.2
0.0
Affine_masked
Similarity_masked
TRSAA_masked
Similarity_eroded_masked
Affine_eroded_masked
terminal_CC_nomask
TRSAA_eroded_masked
terminal_CC_masked
terminal_MI_nomask
terminal_MI_masked
terminal_CC_eroded_masked
flirt_NA
Affine_nomask
TRSAA_nomask
terminal_MI_eroded_masked
Similarity_nomask
antsAI_masked
Algorithm

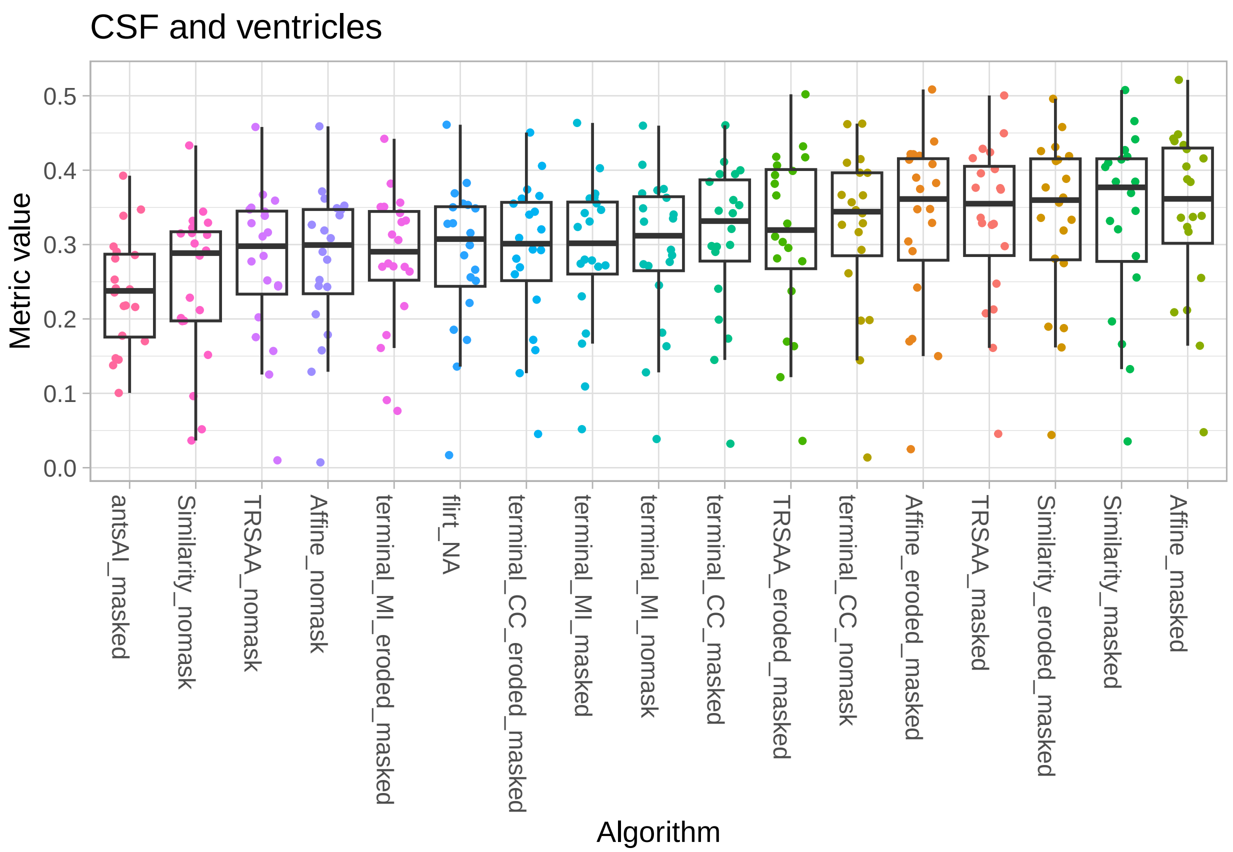
CSF and ventricles
Metric value
0.0
0.1
0.2
0.3
0.4
0.5
antsAI_masked
Similarity_nomask
TRSAA_nomask
Affine_nomask
terminal_MI_eroded_masked
flirt_NA
terminal_CC_eroded_masked
terminal_MI_masked
terminal_MI_nomask
terminal_CC_masked
TRSAA_eroded_masked
terminal_CC_nomask
Affine_eroded_masked
TRSAA_masked
Similarity_eroded_masked
Similarity_masked
Affine_masked
Algorithm

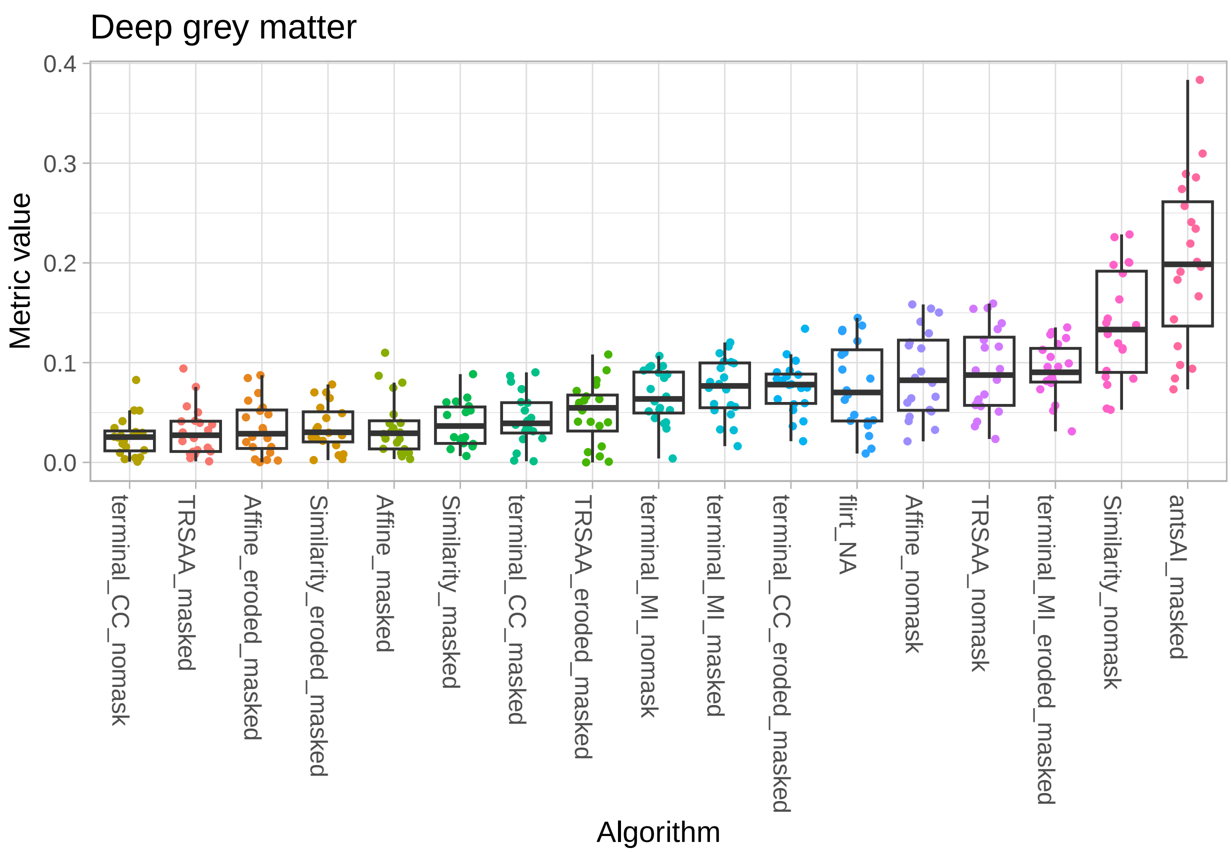

Deep grey matter
0.4
0.3
0.2
0.1
0.0
Metric value
terminal_CC_nomask
TRSAA_masked
Affine_eroded_masked
Similarity_eroded_masked
Affine_masked
Similarity_masked
terminal_CC_masked
TRSAA_eroded_masked
terminal_MI_nomask
terminal_MI_masked
terminal_CC_eroded_masked
flirt_NA
Affine_nomask
TRSAA_nomask
terminal_MI_eroded_masked
Similarity_nomask
antsAI_masked
Algorithm

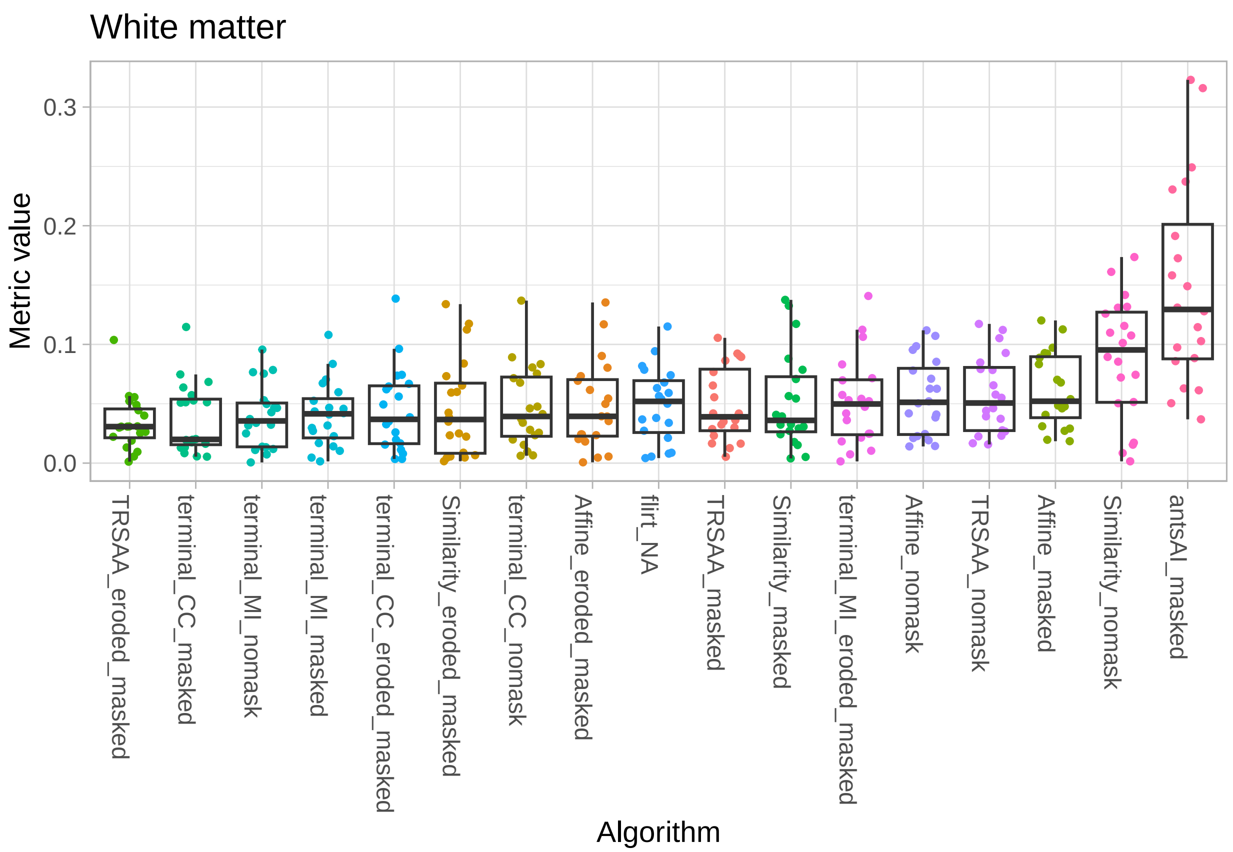

White matter
Metric value
0.3
0.2
0.1
0.0
TRSAA_eroded_masked
terminal_CC_masked
terminal_MI_nomask
terminal_MI_masked
terminal_CC_eroded_masked
Similarity_eroded_masked
terminal_CC_nomask
Affine_eroded_masked
flirt_NA
TRSAA_masked
Similarity_masked
terminal_MI_eroded_masked
Affine_nomask
TRSAA_nomask
Affine_masked
Similarity_nomask
antsAI_masked
Algorithm

2.2 Podium plot

Podium plots (see also Eugster et al., 2008) for visualizing raw assessment data. Upper part (spaghetti plot): Participating algorithms are color-coded, and each colored dot in the plot represents a metric value achieved with the respective algorithm. The actual metric value is encoded by the y-axis. Each podium (here: p=17) represents one possible rank, ordered from best (1) to last (here: 17). The assignment of metric values (i.e. colored dots) to one of the podiums is based on the rank that the respective algorithm achieved on the corresponding case. Note that the plot part above each podium place is further subdivided into p "columns", where each column represents one participating algorithm (here: $p = 17$). Dots corresponding to identical cases are connected by a line, leading to the shown spaghetti structure. Lower part: Bar charts represent the relative frequency for each algorithm to achieve the rank encoded by the podium place.

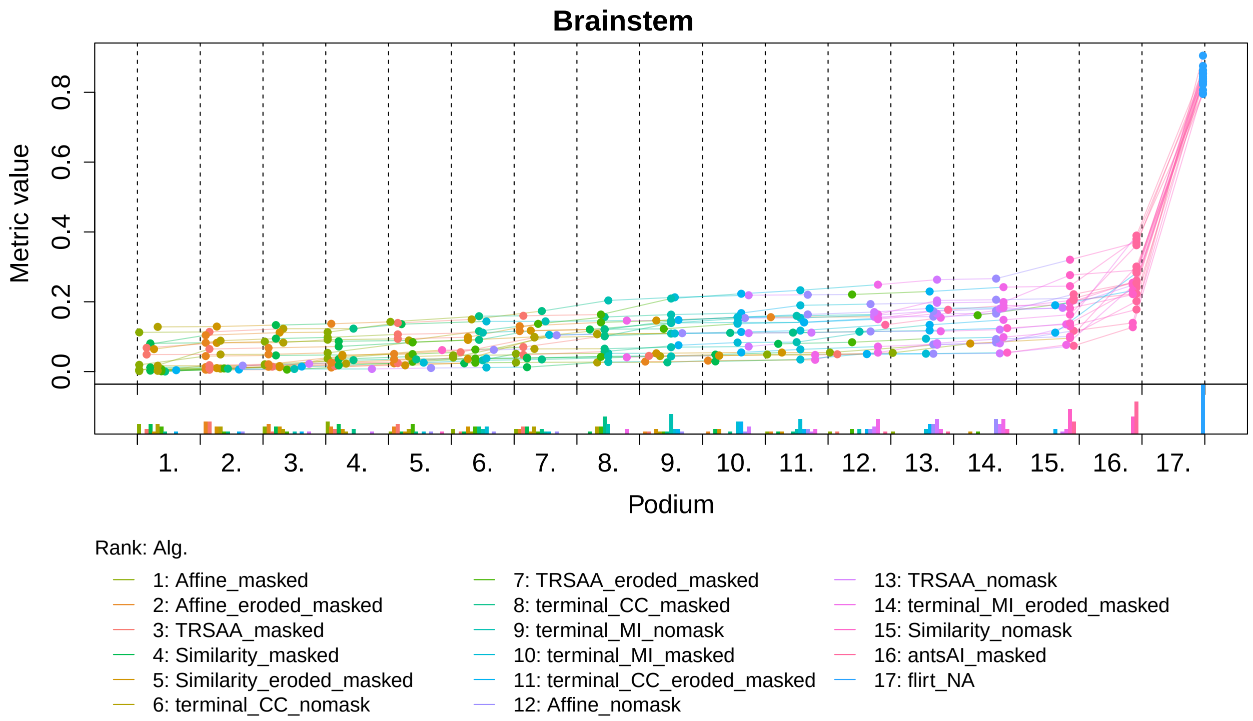

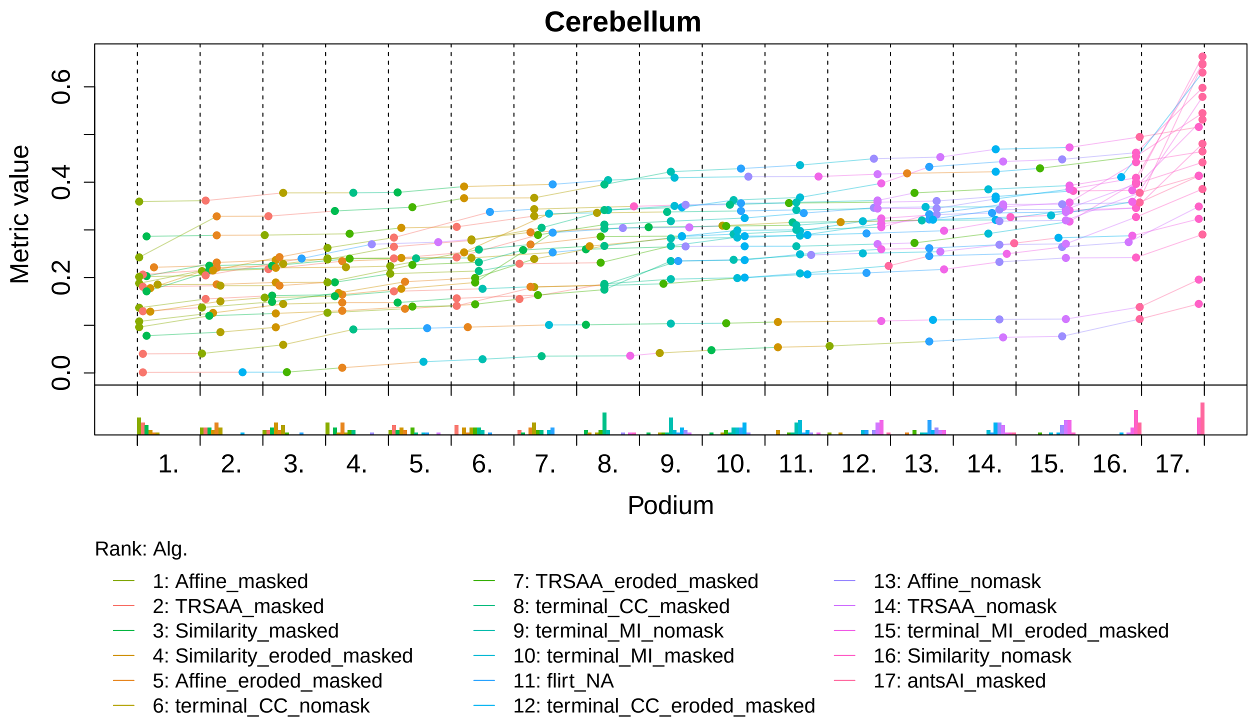
Cerebellum
Metric value
0.0
0.2
0.4
0.6
1.
2.
3.
4.
5.
6.
7.
8.
9.
10.
11.
12.
13.
14.
15.
16.
17.
Podium
Rank: Alg.
1: Affine_masked
2: TRSAA_masked
3: Similarity_masked
4: Similarity_eroded_masked
5: Affine_eroded_masked
6: terminal_CC_nomask
7: TRSAA_eroded_masked
8: terminal_CC_masked
9: terminal_MI_nomask
10: terminal_MI_masked
11: flirt_NA
12: terminal_CC_eroded_masked
13: Affine_nomask
14: TRSAA_nomask
15: terminal_MI_eroded_masked
16: Similarity_nomask
17: antsAI_masked

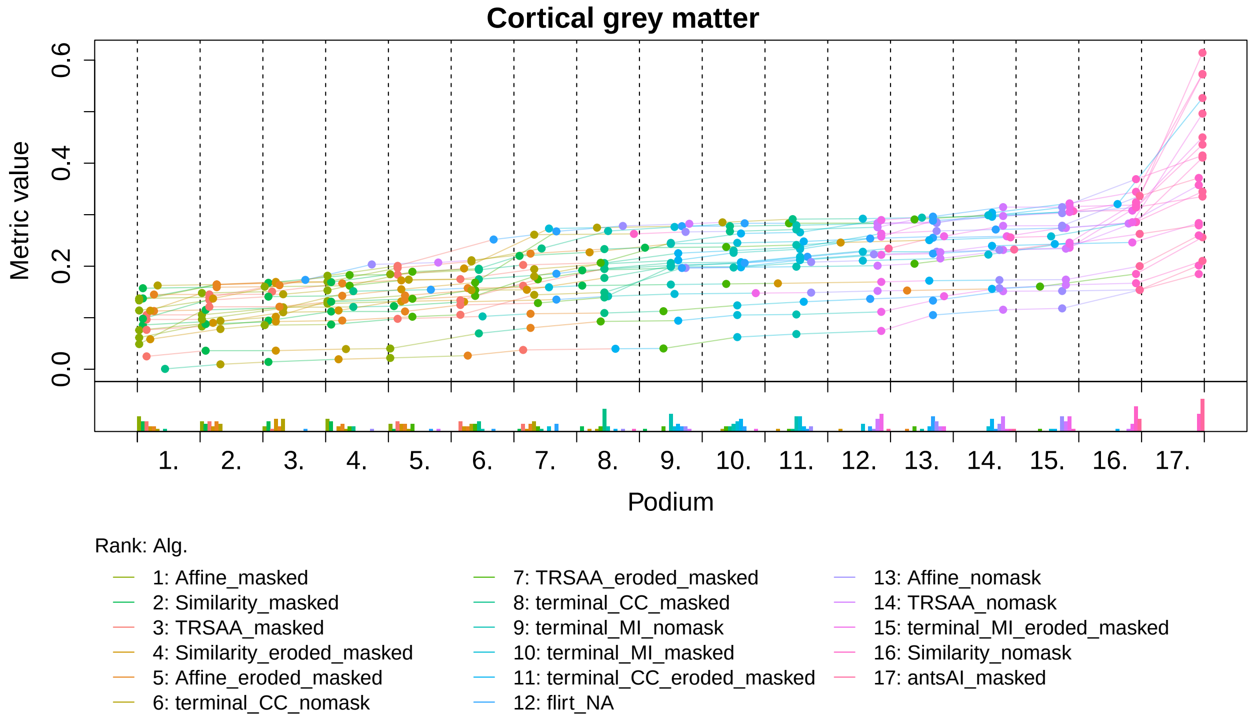
Cortical grey matter
Metric value
0.0
0.2
0.4
0.6
1.
2.
3.
4.
5.
6.
7.
8.
9.
10.
11.
12.
13.
14.
15.
16.
17.
Podium
Rank: Alg.
1: Affine_masked
2: Similarity_masked
3: TRSAA_masked
4: Similarity_eroded_masked
5: Affine_eroded_masked
6: terminal_CC_nomask
7: TRSAA_eroded_masked
8: terminal_CC_masked
9: terminal_MI_nomask
10: terminal_MI_masked
11: terminal_CC_eroded_masked
12: flirt_NA
13: Affine_nomask
14: TRSAA_nomask
15: terminal_MI_eroded_masked
16: Similarity_nomask
17: antsAI_masked

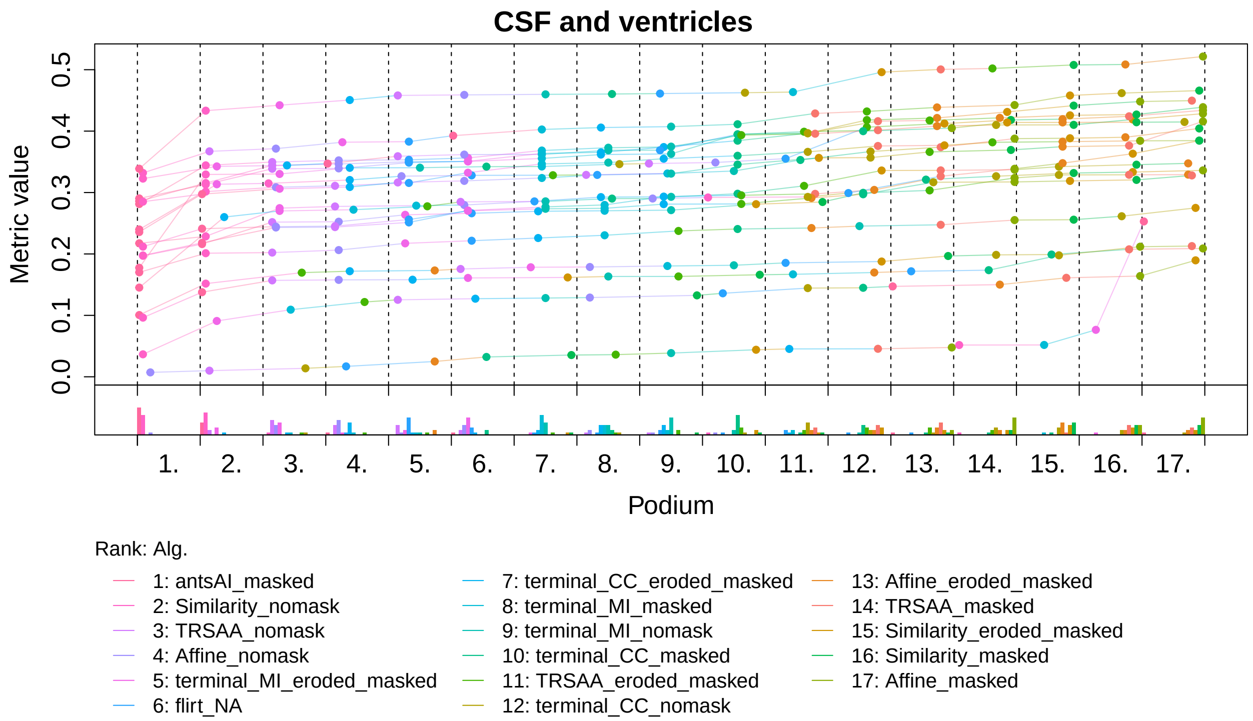
CSF and ventricles
Metric value
0.0
0.1
0.2
0.3
0.4
0.5
1.
2.
3.
4.
5.
6.
7.
8.
9.
10.
11.
12.
13.
14.
15.
16.
17.
Podium
Rank: Alg.
1: antsAI_masked
2: Similarity_nomask
3: TRSAA_nomask
4: Affine_nomask
5: terminal_MI_eroded_masked
6: flirt_NA
7: terminal_CC_eroded_masked
8: terminal_MI_masked
9: terminal_MI_nomask
10: terminal_CC_masked
11: TRSAA_eroded_masked
12: terminal_CC_nomask
13: Affine_eroded_masked
14: TRSAA_masked
15: Similarity_eroded_masked
16: Similarity_masked
17: Affine_masked

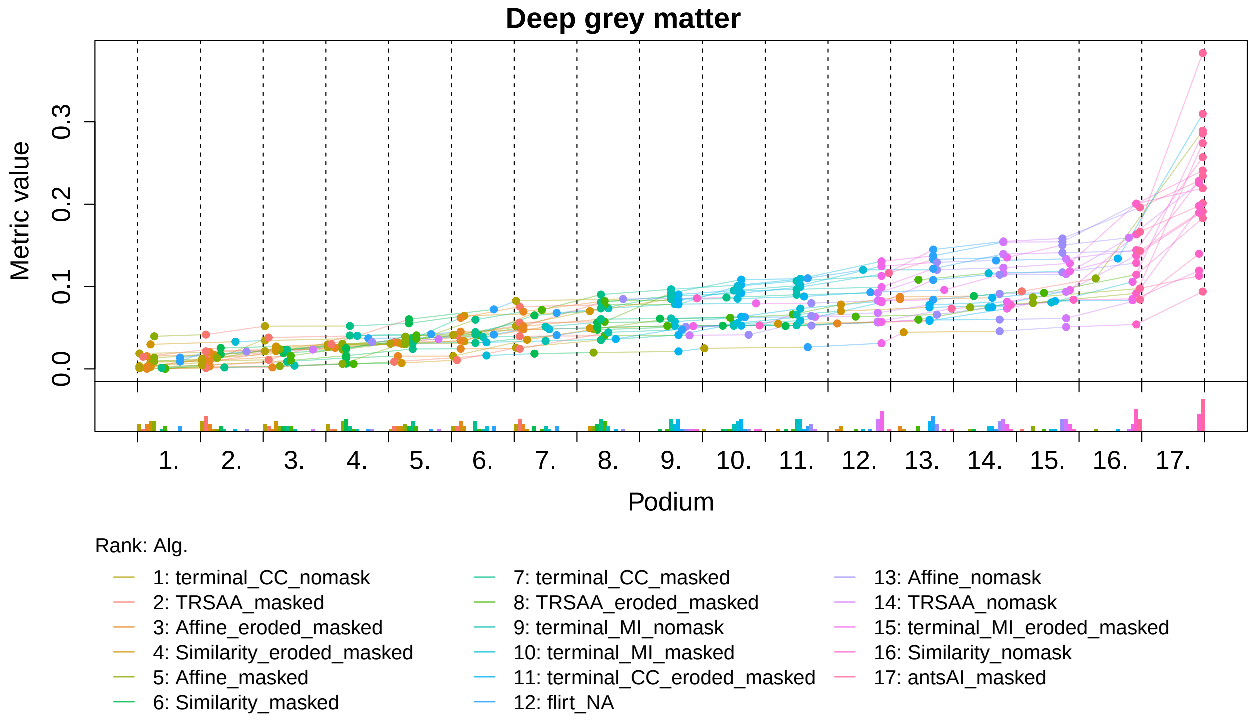
Deep grey matter
Metric value
0.0
0.1
0.2
0.3
1.
2.
3.
4.
5.
6.
7.
8.
9.
10.
11.
12.
13.
14.
15.
16.
17.
Podium
Rank: Alg.
1: terminal_CC_nomask
2: TRSAA_masked
3: Affine_eroded_masked
4: Similarity_eroded_masked
5: Affine_masked
6: Similarity_masked
7: terminal_CC_masked
8: TRSAA_eroded_masked
9: terminal_MI_nomask
10: terminal_MI_masked
11: terminal_CC_eroded_masked
12: flirt_NA
13: Affine_nomask
14: TRSAA_nomask
15: terminal_MI_eroded_masked
16: Similarity_nomask
17: antsAI_masked

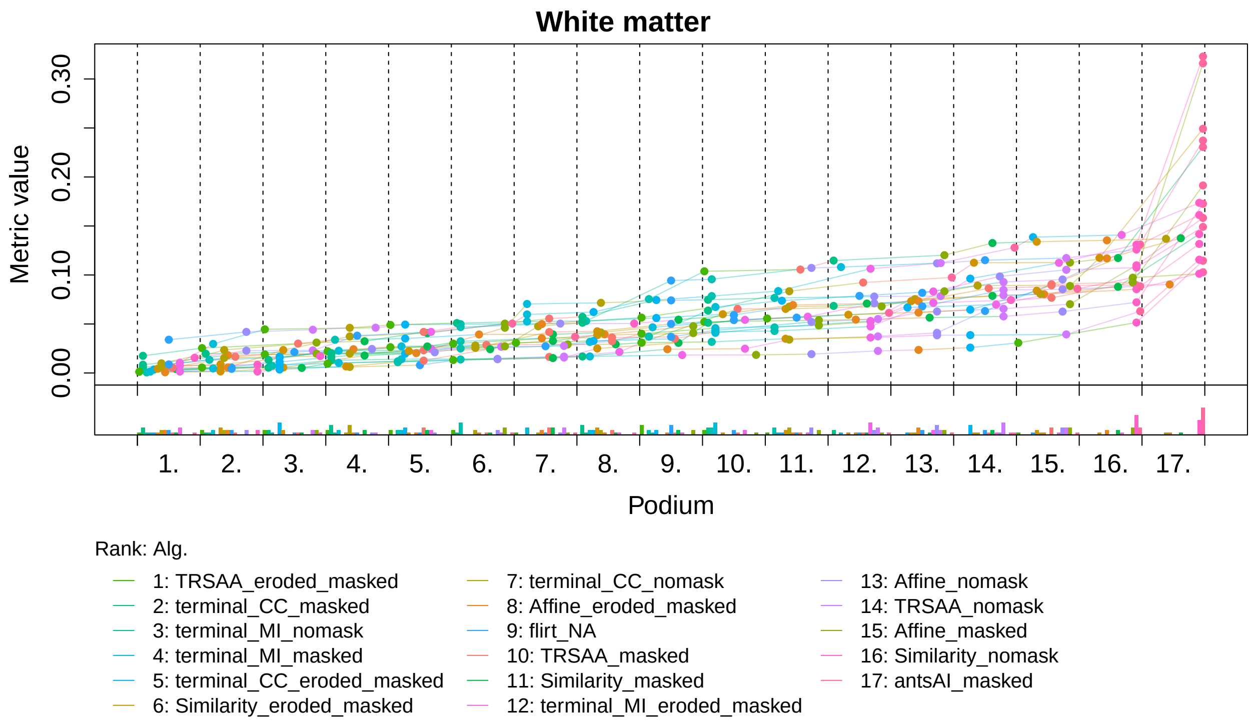
White matter
Metric value
0.00
0.10
0.20
0.30
1.
2.
3.
4.
5.
6.
7.
8.
9.
10.
11.
12.
13.
14.
15.
16.
17.
Podium
Rank: Alg.
1: TRSAA_eroded_masked
2: terminal_CC_masked
3: terminal_MI_nomask
4: terminal_MI_masked
5: terminal_CC_eroded_masked
6: Similarity_eroded_masked
7: terminal_CC_nomask
8: Affine_eroded_masked
9: flirt_NA
10: TRSAA_masked
11: Similarity_masked
12: terminal_MI_eroded_masked
13: Affine_nomask
14: TRSAA_nomask
15: Affine_masked
16: Similarity_nomask
17: antsAI_masked

2.3 Ranking heatmap

Ranking heatmaps for visualizing raw assessment data. Each cell (i, A_j) shows the absolute frequency of cases in which algorithm A_j achieved rank i.

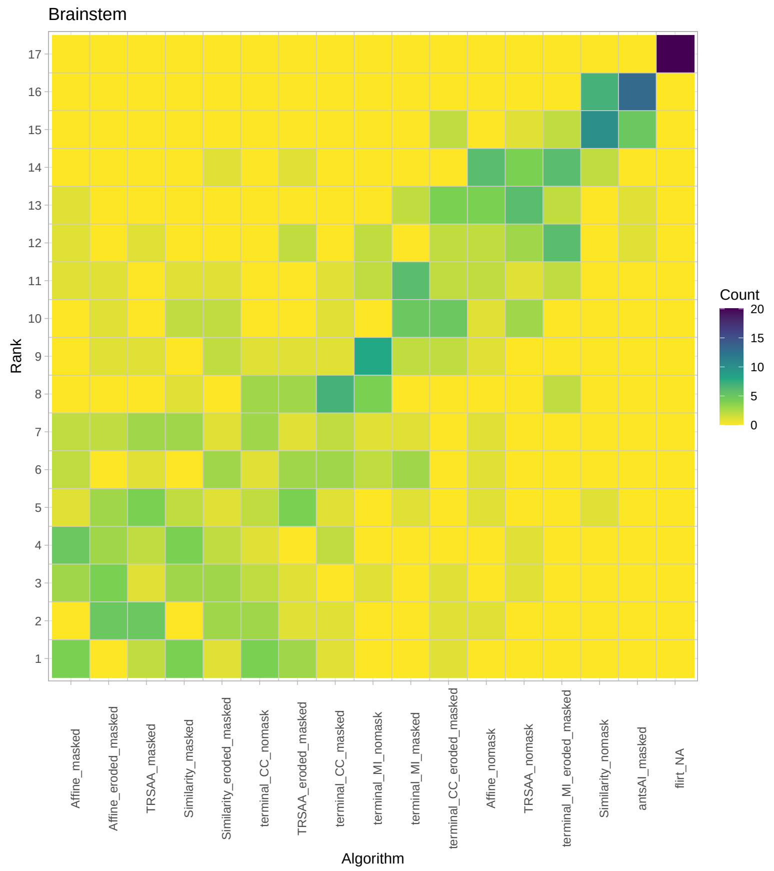

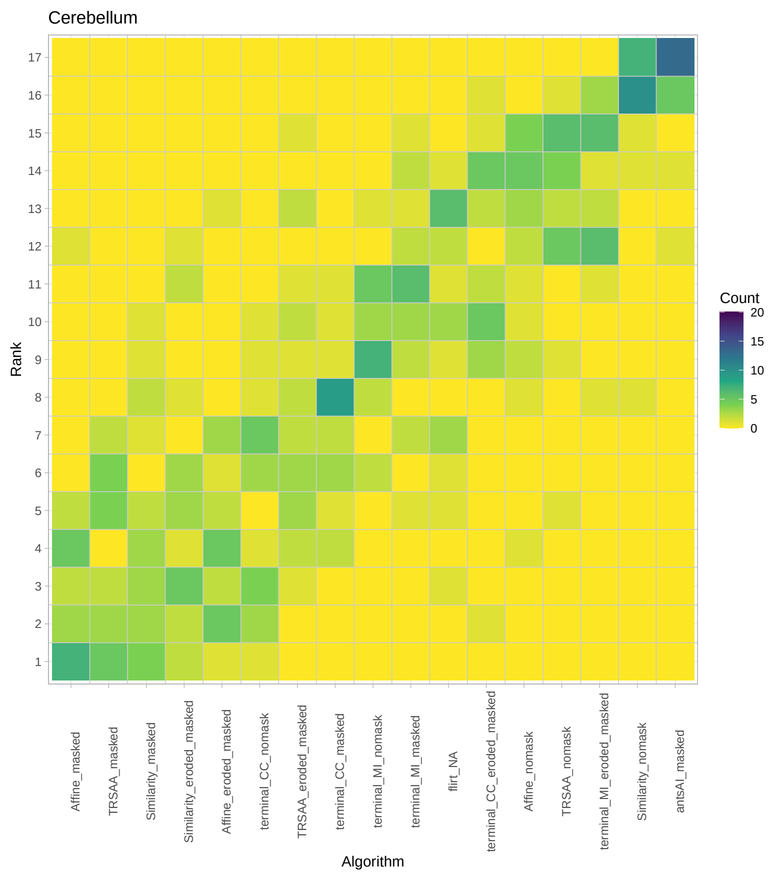

Cerebellum
Rank
17
16
15
14
13
12
11
10
9
8
7
6
5
4
3
2
1
Count
20
15
10
5
0
Affine_masked
TRSAA_masked
Similarity_masked
Similarity_eroded_masked
Affine_eroded_masked
terminal_CC_nomask
TRSAA_eroded_masked
terminal_CC_masked
terminal_MI_nomask
terminal_MI_masked
flirt_NA
terminal_CC_eroded_masked
Affine_nomask
TRSAA_nomask
terminal_MI_eroded_masked
Similarity_nomask
antsAI_masked
Algorithm

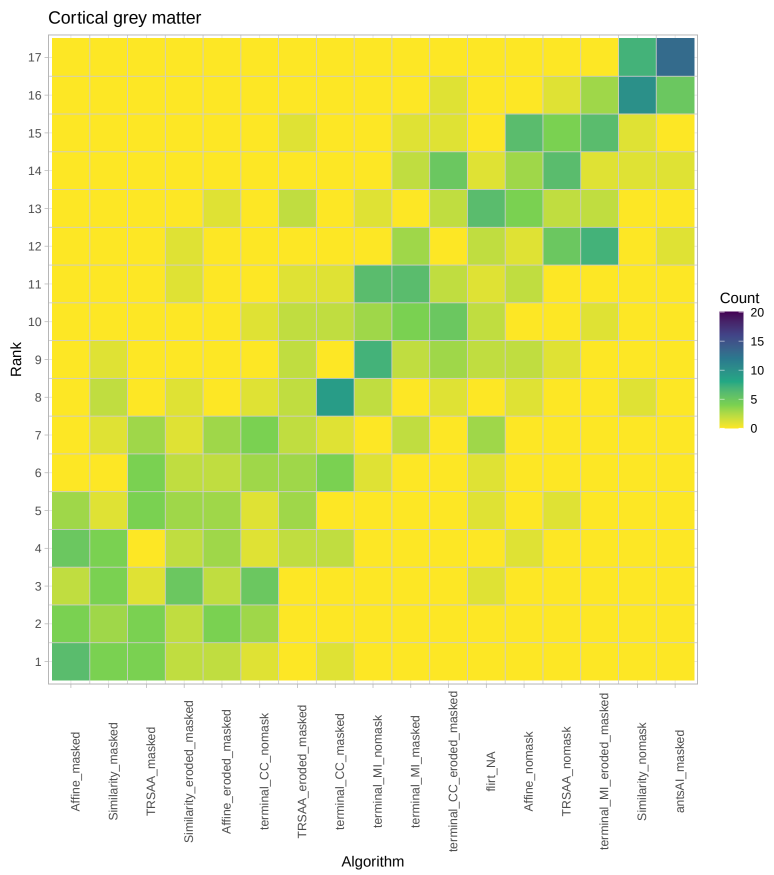
Cortical grey matter
Rank
17
16
15
14
13
12
11
10
9
8
7
6
5
4
3
2
1
Affine_masked
Similarity_masked
TRSAA_masked
Similarity_eroded_masked
Affine_eroded_masked
terminal_CC_nomask
TRSAA_eroded_masked
terminal_CC_masked
terminal_MI_nomask
terminal_MI_masked
terminal_CC_eroded_masked
flirt_NA
Affine_nomask
TRSAA_nomask
terminal_MI_eroded_masked
Similarity_nomask
antsAI_masked
Algorithm
Count
20
15
10
5
0

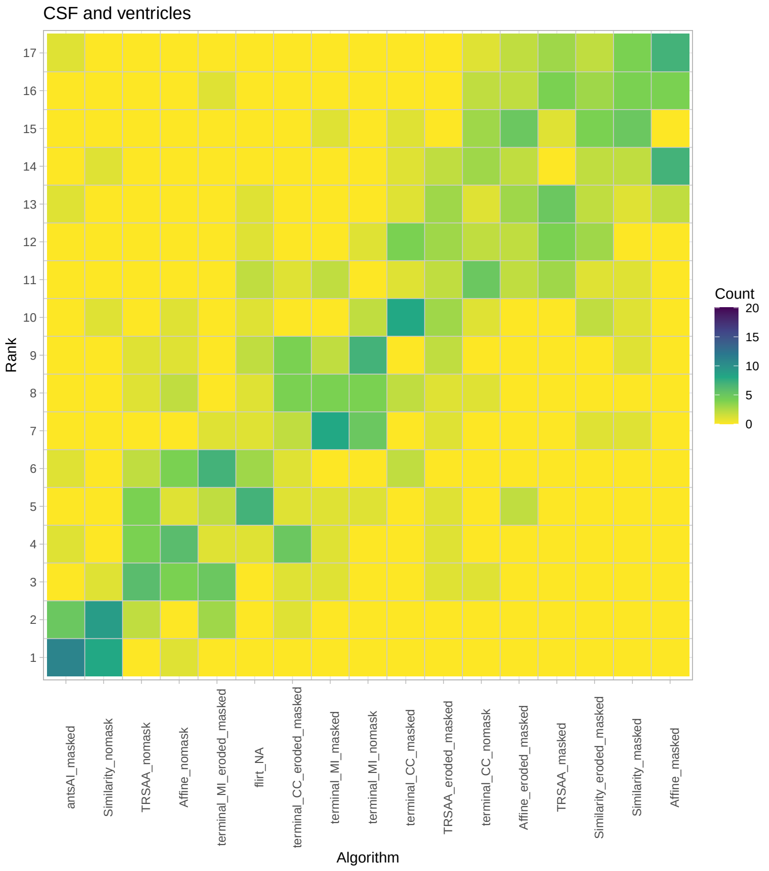

CSF and ventricles
Rank
17
16
15
14
13
12
11
10
9
8
7
6
5
4
3
2
1
Count
20
15
10
5
0
antsAI_masked
Similarity_nomask
TRSAA_nomask
Affine_nomask
terminal_MI_eroded_masked
flirt_NA
terminal_CC_eroded_masked
terminal_MI_masked
terminal_MI_nomask
terminal_CC_masked
TRSAA_eroded_masked
terminal_CC_nomask
Affine_eroded_masked
TRSAA_masked
Similarity_eroded_masked
Similarity_masked
Affine_masked
Algorithm

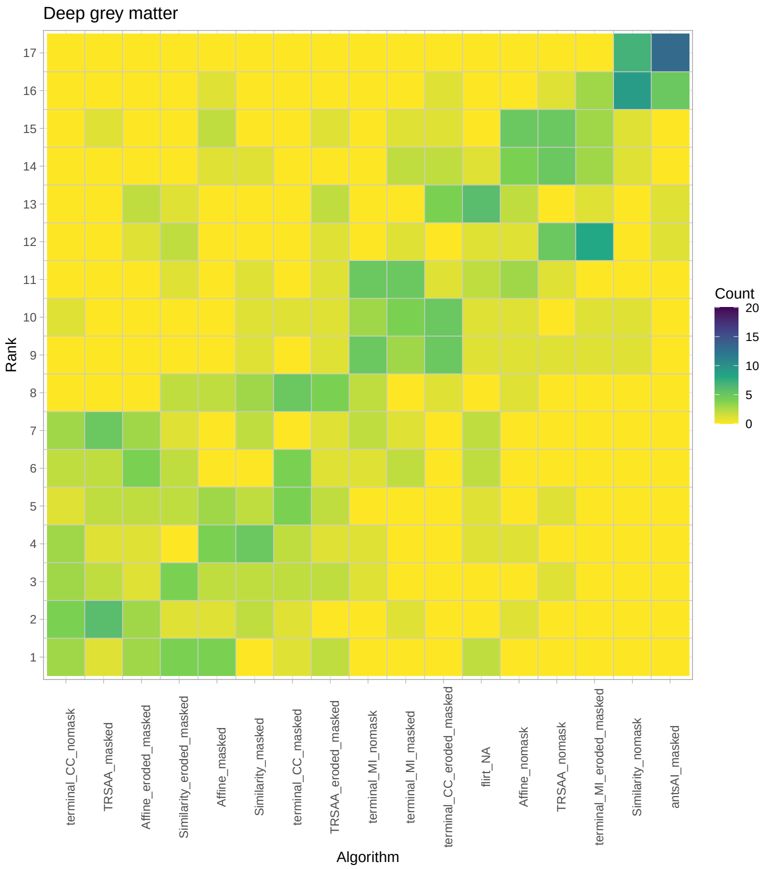

Deep grey matter
Rank
17
16
15
14
13
12
11
10
9
8
7
6
5
4
3
2
1
Count
20
15
10
5
0
terminal_CC_nomask
TRSAA_masked
Affine_eroded_masked
Similarity_eroded_masked
Affine_masked
Similarity_masked
terminal_CC_masked
TRSAA_eroded_masked
terminal_MI_nomask
terminal_MI_masked
terminal_CC_eroded_masked
flirt_NA
Affine_nomask
TRSAA_nomask
terminal_MI_eroded_masked
Similarity_nomask
antsAI_masked
Algorithm

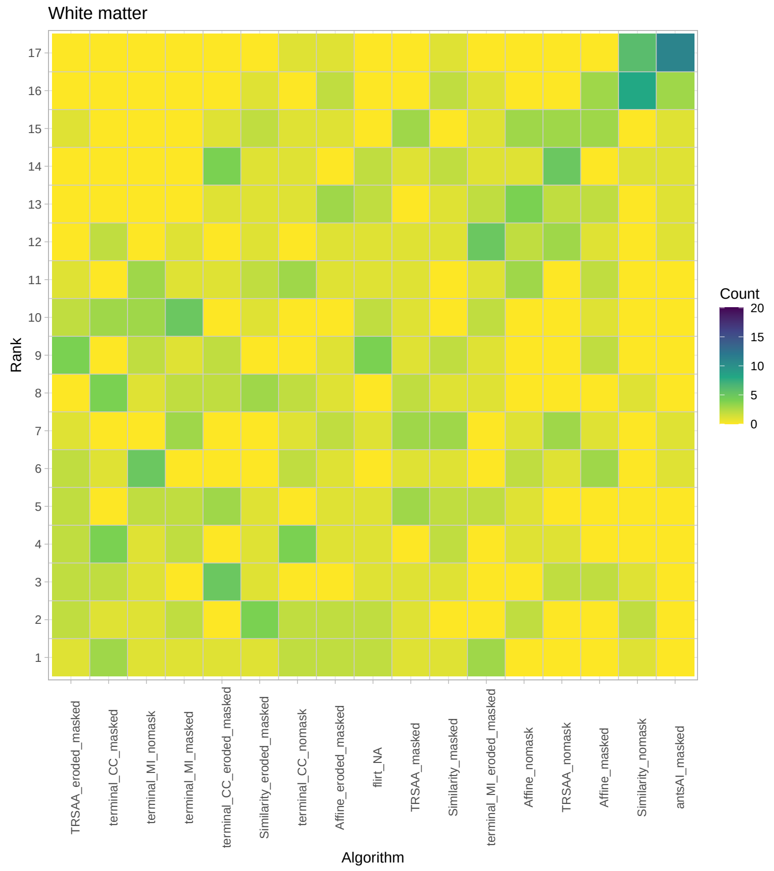
White matter
Rank
Algorithm
Count
20
15
10
5
0
17
16
15
14
13
12
11
10
9
8
7
6
5
4
3
2
1
TRSAA_eroded_masked
terminal_CC_masked
terminal_MI_nomask
terminal_MI_masked
terminal_CC_eroded_masked
Similarity_eroded_masked
terminal_CC_nomask
Affine_eroded_masked
flirt_NA
TRSAA_masked
Similarity_masked
terminal_MI_eroded_masked
Affine_nomask
TRSAA_nomask
Affine_masked
Similarity_nomask
antsAI_masked

3 Visualization of ranking stability

3.1 *Blob plot* for visualizing ranking stability based on bootstrap sampling

Algorithms are color-coded, and the area of each blob at position $(A_i, \text{rank } j)$ is proportional to the relative frequency A_i achieved rank j across $b = 1000$ bootstrap samples. The median rank for each algorithm is indicated by a black cross. 95% bootstrap intervals across bootstrap samples are indicated by black lines.

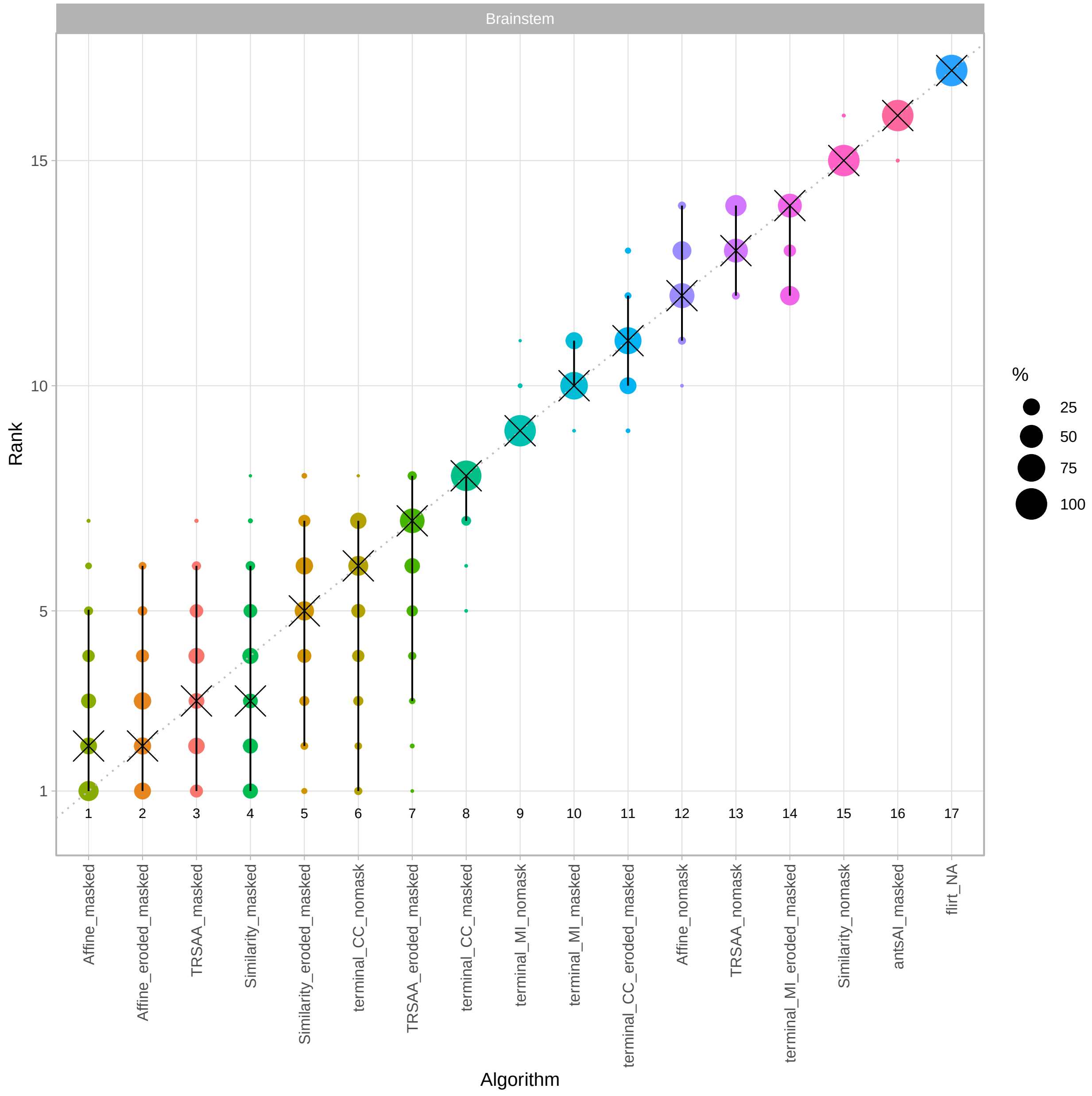

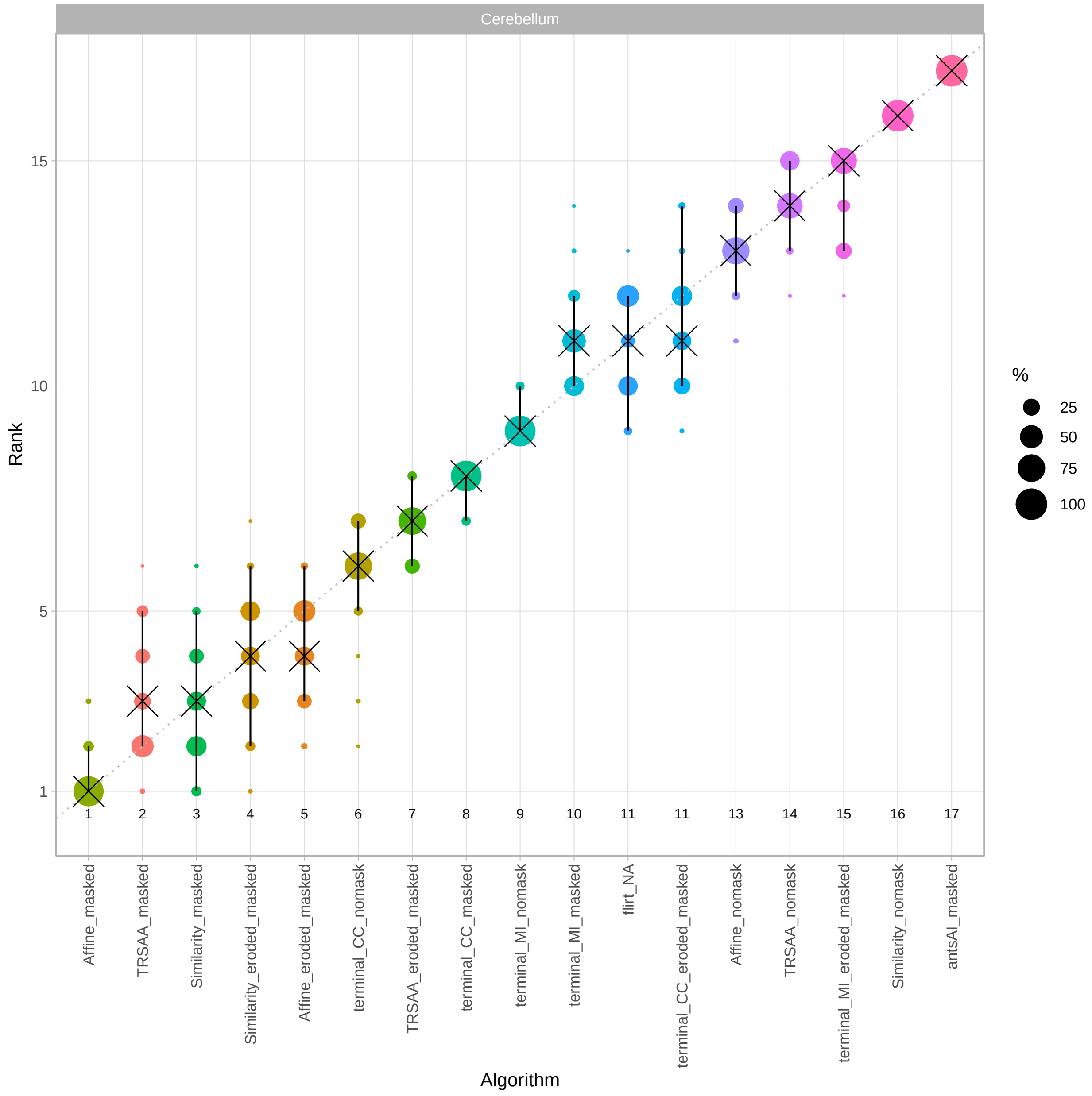
Cerebellum
Rank
15
10
5
1
1
2
3
4
5
6
7
8
9
10
11
11
13
14
15
16
17
Affine_masked
TRSAA_masked
Similarity_masked
Similarity_eroded_masked
Affine_eroded_masked
terminal_CC_nomask
TRSAA_eroded_masked
terminal_CC_masked
terminal_MI_nomask
terminal_MI_masked
flirt_NA
terminal_CC_eroded_masked
Affine_nomask
TRSAA_nomask
terminal_MI_eroded_masked
Similarity_nomask
antsAI_masked
Algorithm
%
25
50
75
100

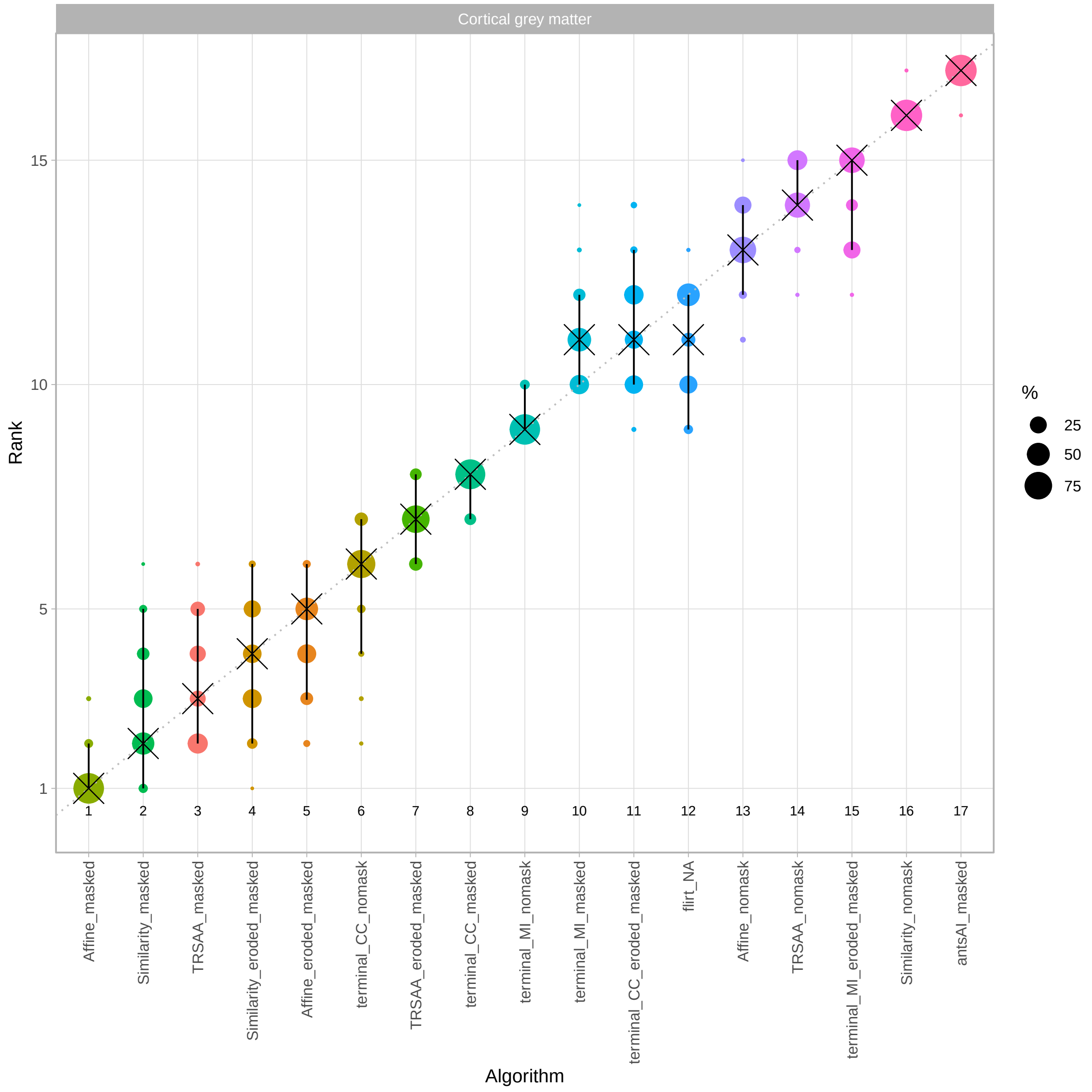
Cortical grey matter
Rank
15
10
5
1
1
2
3
4
5
6
7
8
9
10
11
12
13
14
15
16
17
%
25
50
75
Affine_masked
Similarity_masked
TRSAA_masked
Similarity_eroded_masked
Affine_eroded_masked
terminal_CC_nomask
TRSAA_eroded_masked
terminal_CC_masked
terminal_MI_nomask
terminal_MI_masked
terminal_CC_eroded_masked
flirt_NA
Affine_nomask
TRSAA_nomask
terminal_MI_eroded_masked
Similarity_nomask
antsAI_masked
Algorithm

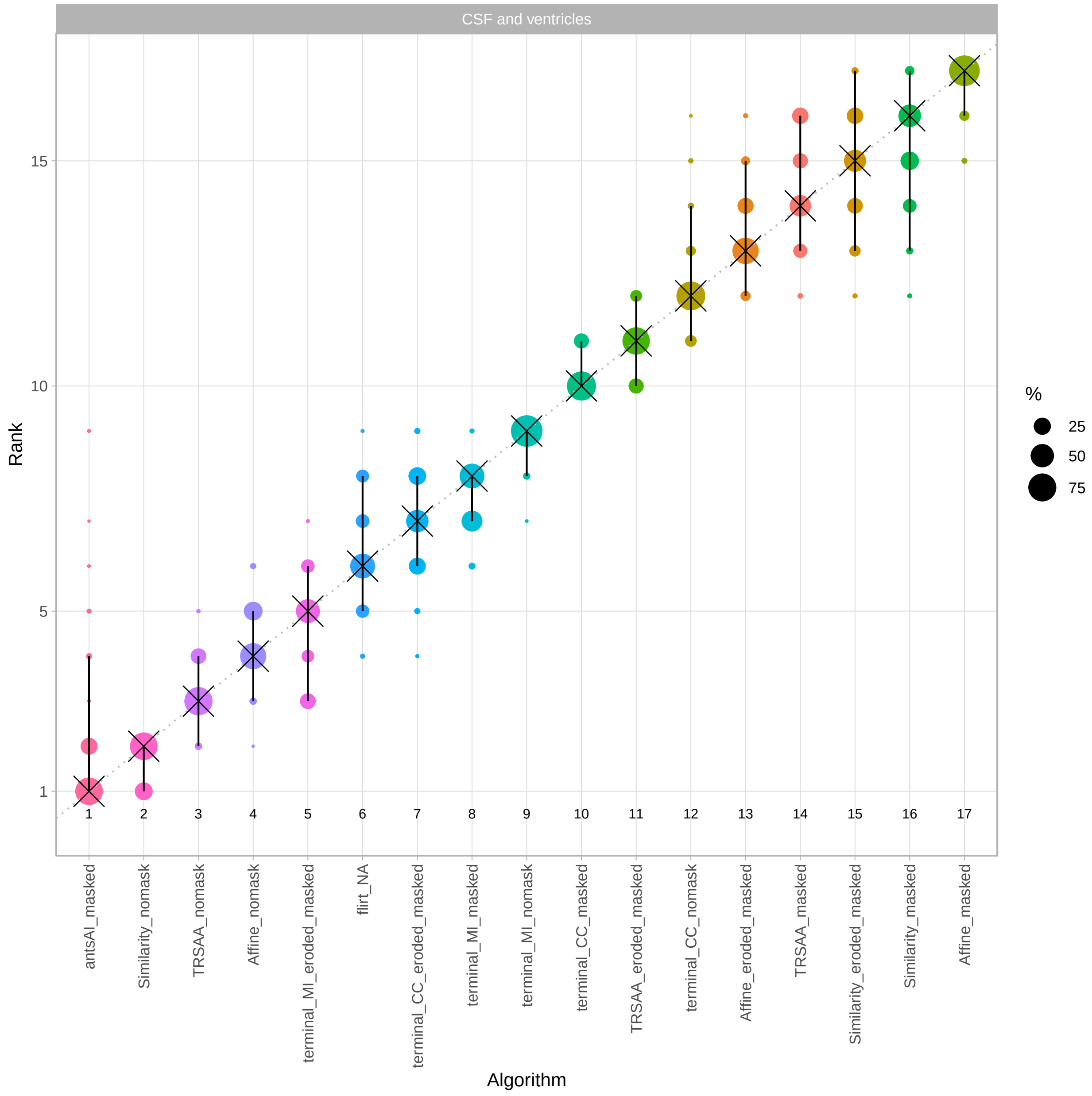

CSF and ventricles
Rank
15
10
5
1
1
2
3
4
5
6
7
8
9
10
11
12
13
14
15
16
17
antsAI_masked
Similarity_nomask
TRSAA_nomask
Affine_nomask
terminal_MI_eroded_masked
flirt_NA
terminal_CC_eroded_masked
terminal_MI_masked
terminal_MI_nomask
terminal_CC_masked
TRSAA_eroded_masked
terminal_CC_nomask
Affine_eroded_masked
TRSAA_masked
Similarity_eroded_masked
Similarity_masked
Affine_masked
Algorithm
%
25
50
75

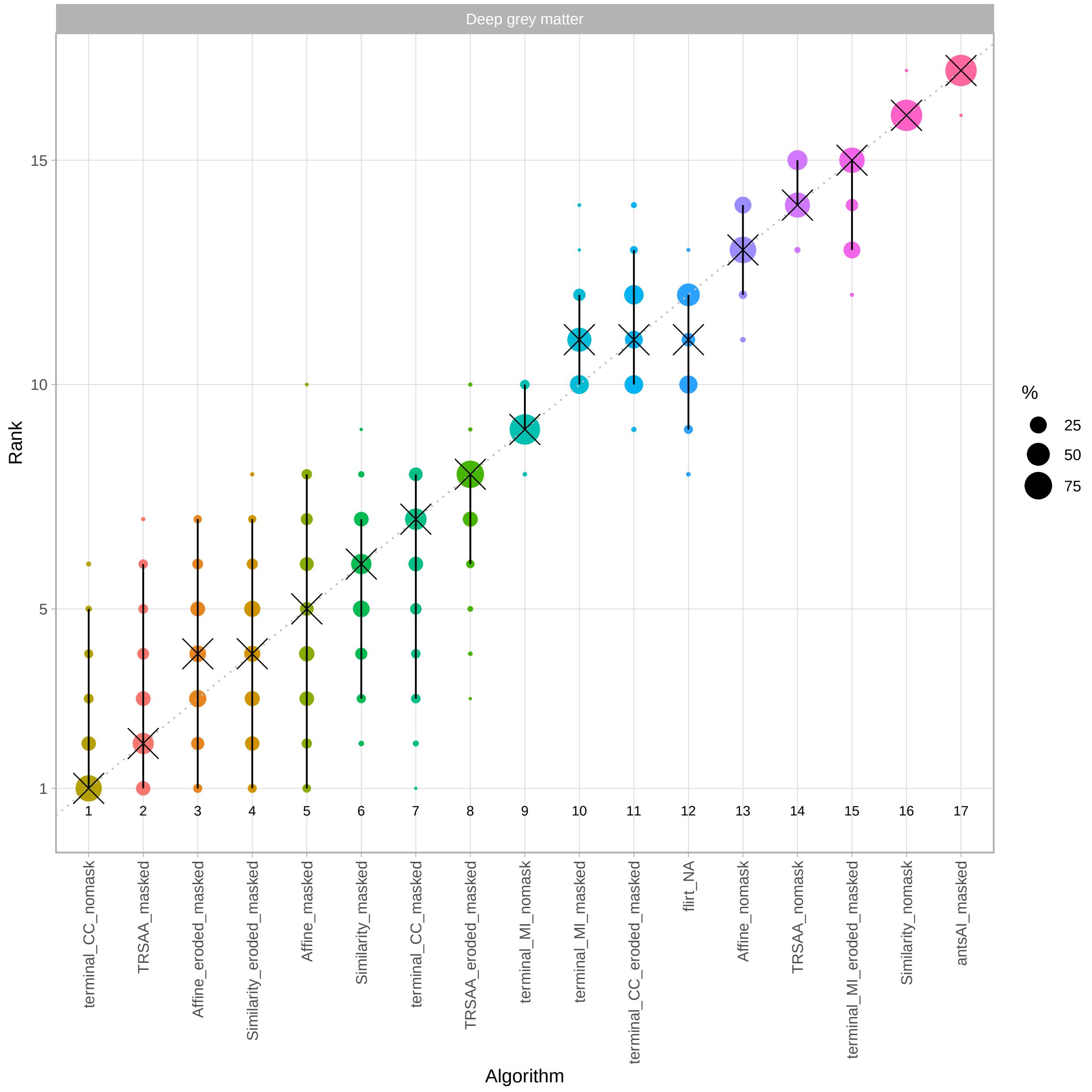
Deep grey matter
Rank
Algorithm
%
25
50
75
1
5
10
15
1
2
3
4
5
6
7
8
9
10
11
12
13
14
15
16
17
terminal_CC_nomask
TRSAA_masked
Affine_eroded_masked
Similarity_eroded_masked
Affine_masked
Similarity_masked
terminal_CC_masked
TRSAA_eroded_masked
terminal_MI_nomask
terminal_MI_masked
terminal_CC_eroded_masked
flirt_NA
Affine_nomask
TRSAA_nomask
terminal_MI_eroded_masked
Similarity_nomask
antsAI_masked

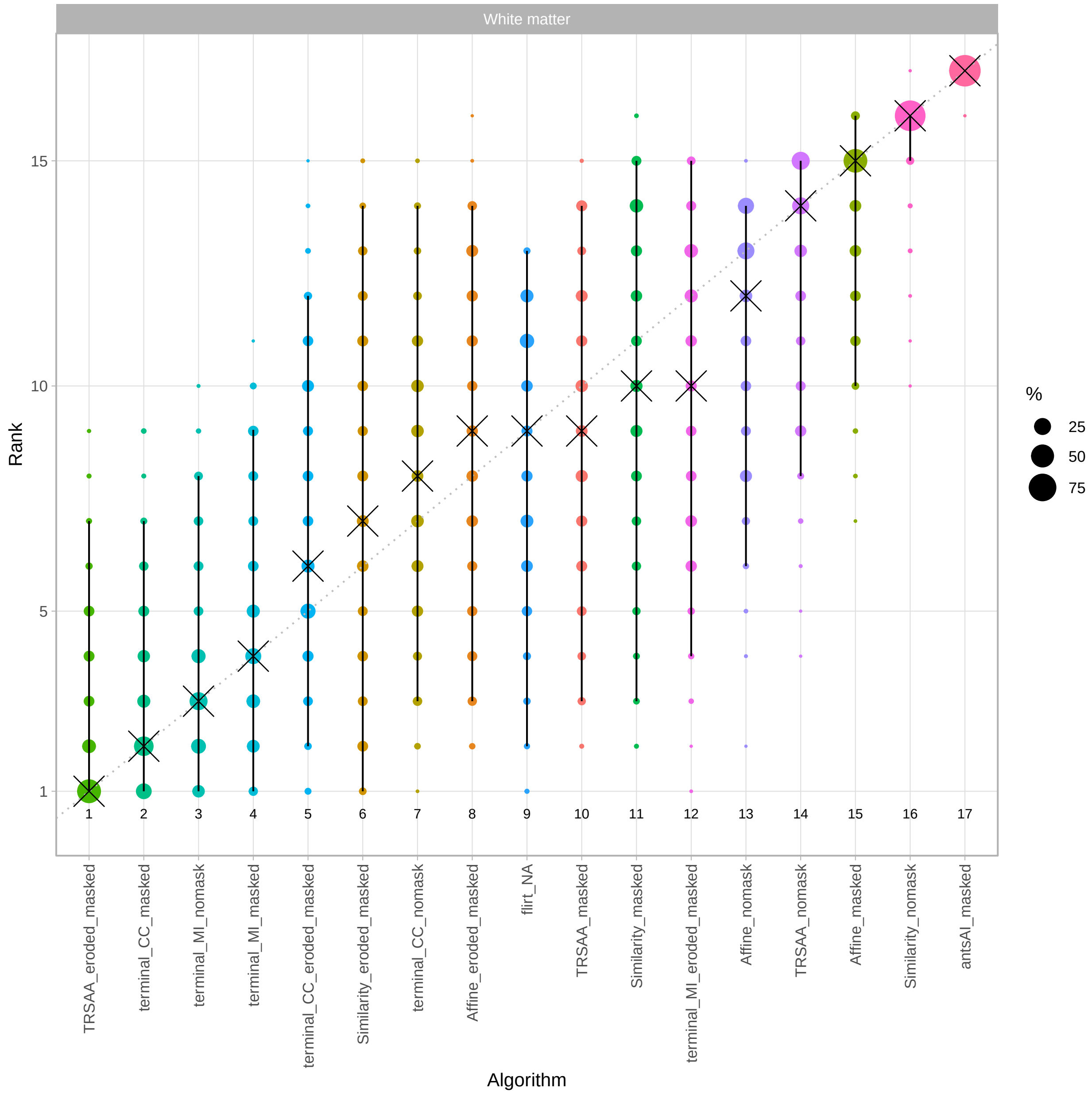
White matter
Rank
15
10
5
1
1 2 3 4 5 6 7 8 9 10 11 12 13 14 15 16 17
TRSAA_eroded_masked
terminal_CC_masked
terminal_MI_nomask
terminal_MI_masked
terminal_CC_eroded_masked
Similarity_eroded_masked
terminal_CC_nomask
Affine_eroded_masked
flirt_NA
TRSAA_masked
Similarity_masked
terminal_MI_eroded_masked
Affine_nomask
TRSAA_nomask
Affine_masked
Similarity_nomask
antsAI_masked
Algorithm
%
25
50
75

3.2 *Violin plot* for visualizing ranking stability based on bootstrapping

The ranking list based on the full assessment data is pairwise compared with the ranking lists based on the individual bootstrap samples (here $b = 1000$ samples). For each pair of rankings, Kendall's τ correlation is computed. Kendall's τ is a scaled index determining the correlation between the lists. It is computed by evaluating the number of pairwise concordances and discordances between ranking lists and produces values between -1 (for inverted order) and 1 (for identical order). A violin plot, which simultaneously depicts a boxplot and a density plot, is generated from the results.

Summary Kendall's tau:

Task	mean	median	q25	q75
CSF and ventricles	0.9461029	0.9558824	0.9264706	0.9705882
Cerebellum	0.9439621	0.9520360	0.9225155	0.9667963
Cortical grey matter	0.9402169	0.9411765	0.9264706	0.9558824
Brainstem	0.9152339	0.9264706	0.8823529	0.9558824
Deep grey matter	0.9021136	0.9117647	0.8823529	0.9264706
White matter	0.7072750	0.7205882	0.6323529	0.7941176

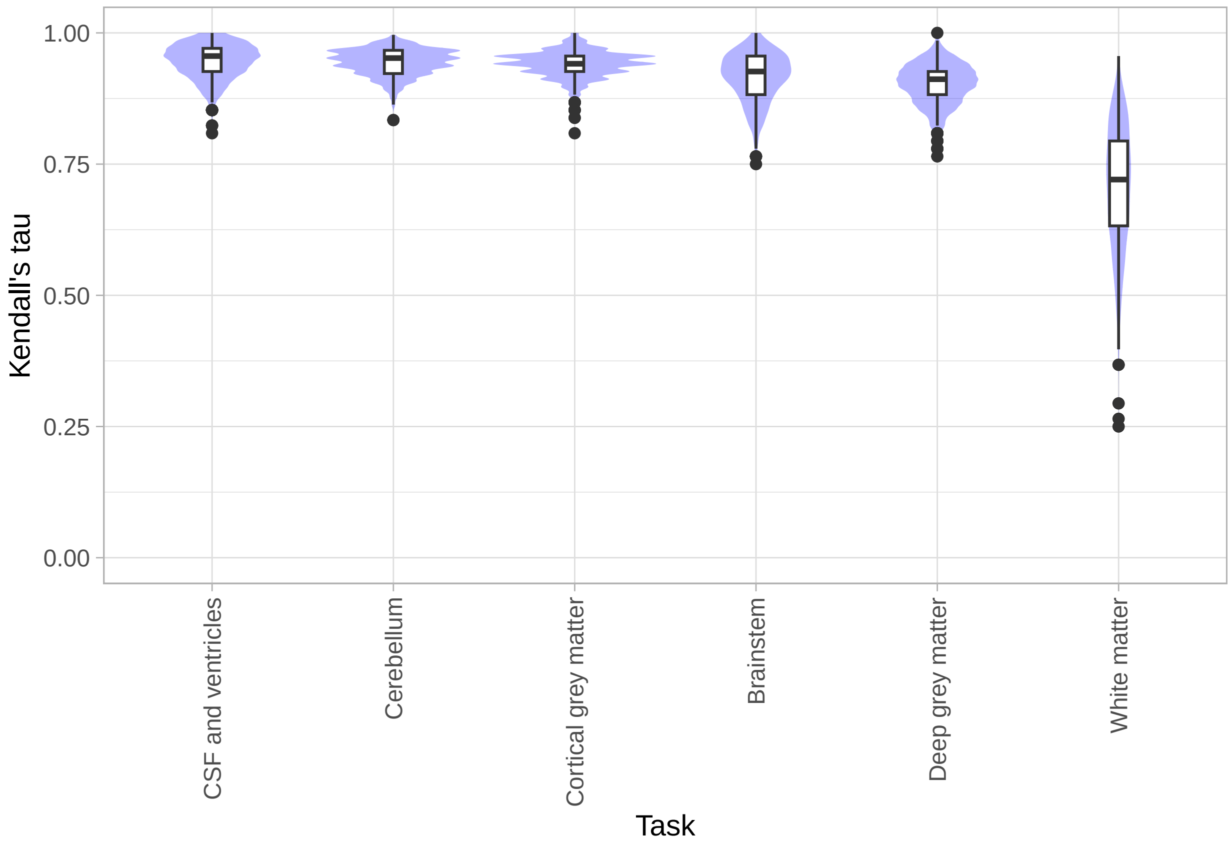

3.3 *Significance maps* for visualizing ranking stability based on statistical significance

Significance maps depict incidence matrices of pairwise significant test results for the one-sided Wilcoxon signed rank test at a 5% significance level with adjustment for multiple testing according to Holm. Yellow shading indicates that metric values from the algorithm on the x-axis were significantly superior to those from the algorithm on the y-axis, blue color indicates no significant difference.

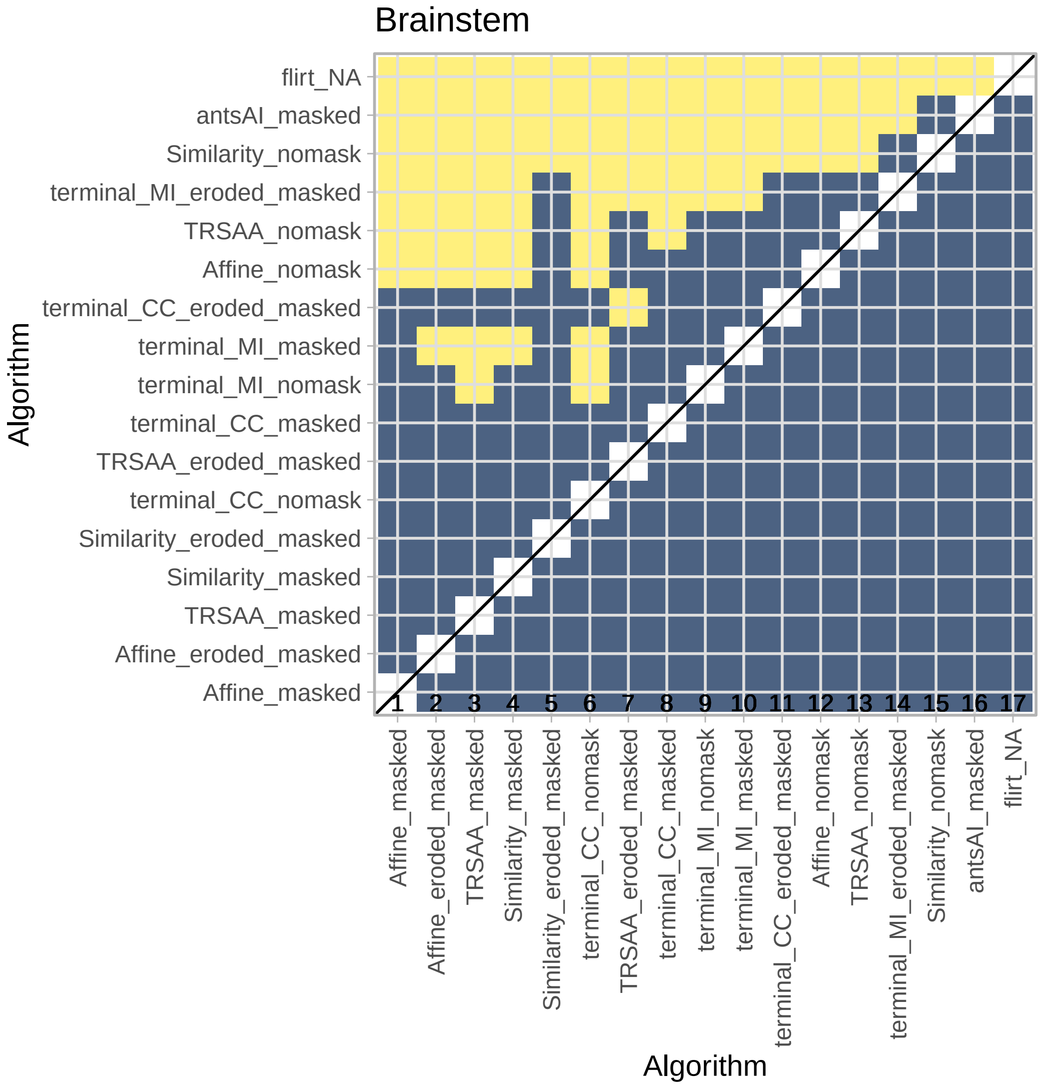

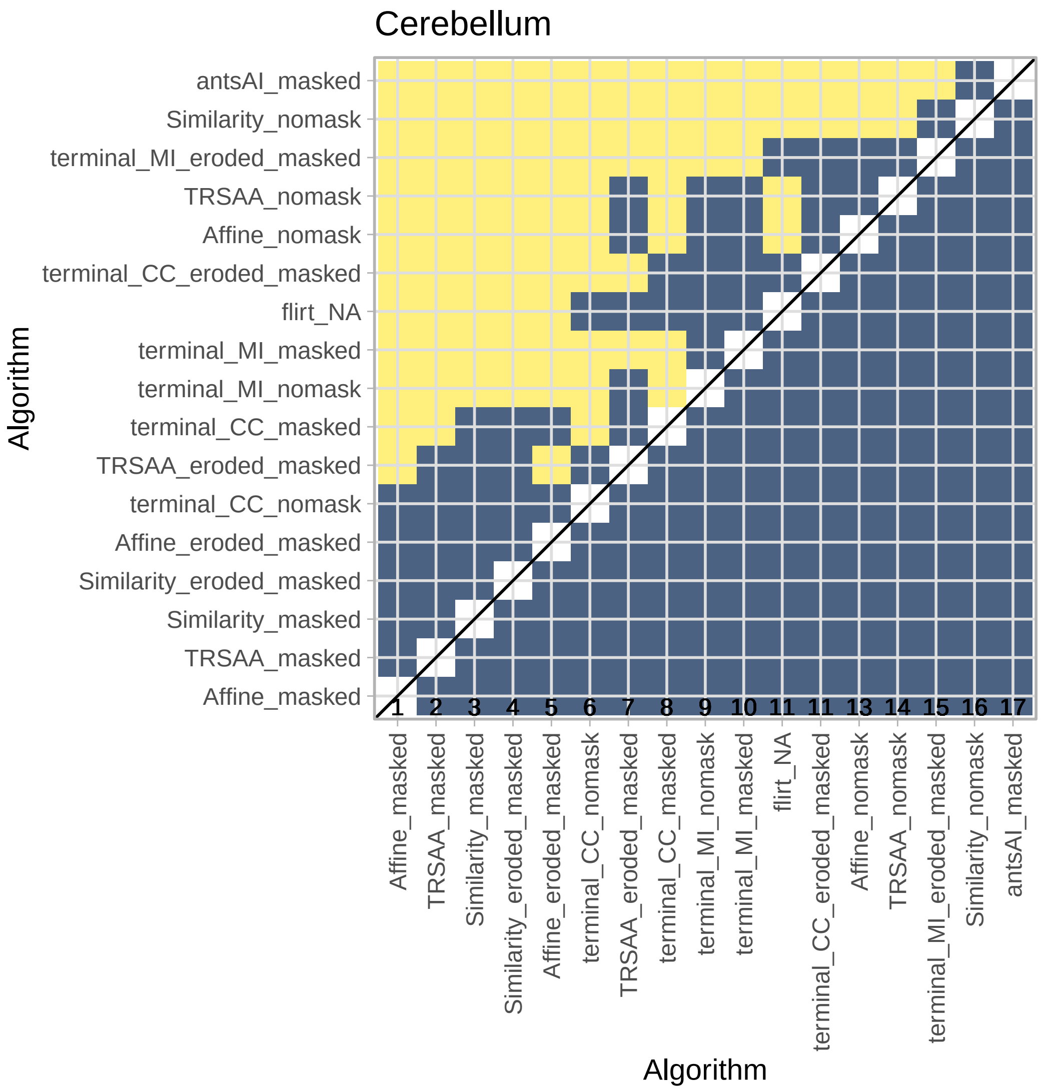
Cerebellum
Algorithm
antsAI_masked
Similarity_nomask
terminal_MI_eroded_masked
TRSAA_nomask
Affine_nomask
terminal_CC_eroded_masked
flirt_NA
terminal_MI_masked
terminal_MI_nomask
terminal_CC_masked
TRSAA_eroded_masked
terminal_CC_nomask
Affine_eroded_masked
Similarity_eroded_masked
Similarity_masked
TRSAA_masked
Affine_masked
1 2 3 4 5 6 7 8 9 10 11 11 13 14 15 16 17
Affine_masked
TRSAA_masked
Similarity_masked
Similarity_eroded_masked
Affine_eroded_masked
terminal_CC_nomask
TRSAA_eroded_masked
terminal_CC_masked
terminal_MI_nomask
terminal_MI_masked
flirt_NA
terminal_CC_eroded_masked
Affine_nomask
TRSAA_nomask
terminal_MI_eroded_masked
Similarity_nomask
antsAI_masked
Algorithm

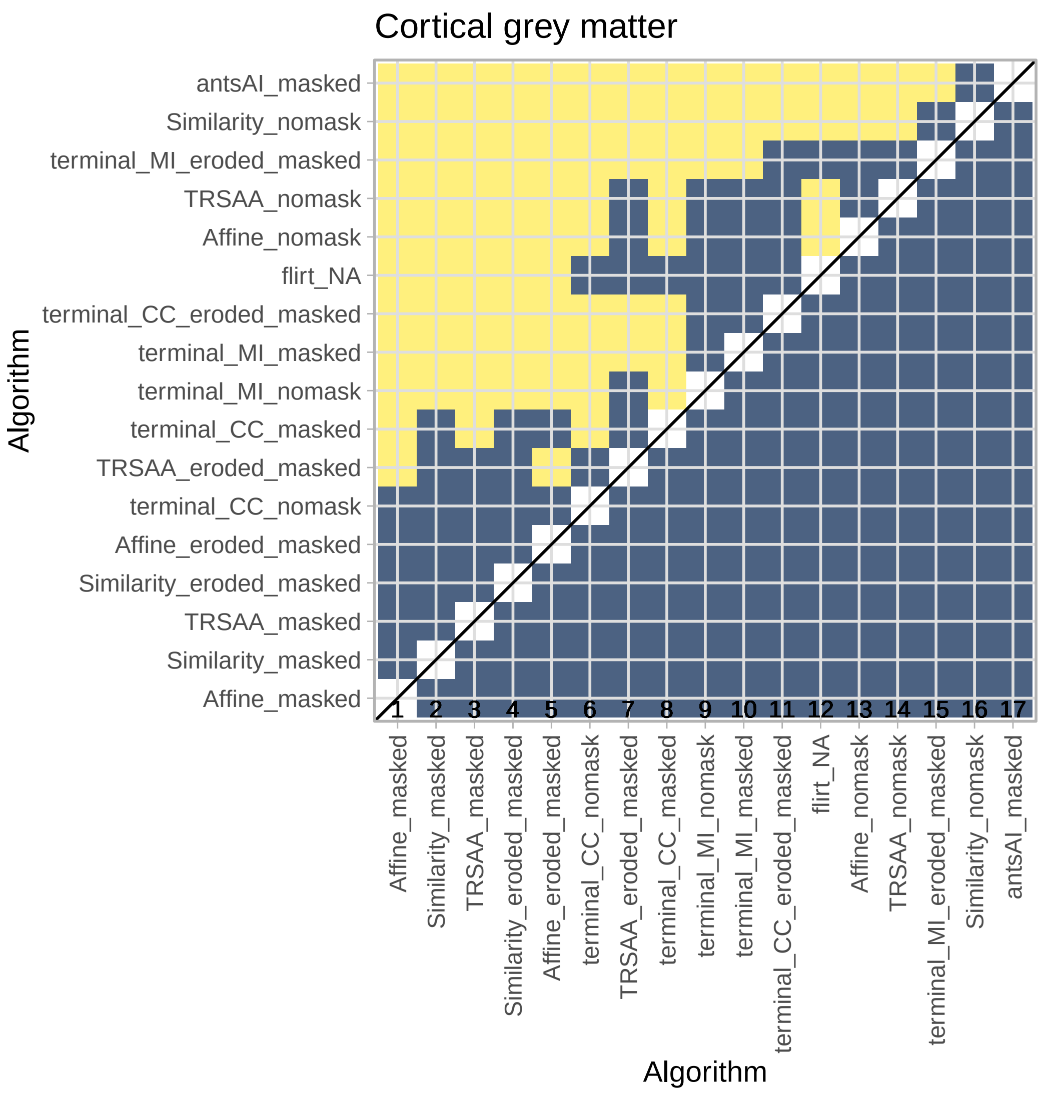
Cortical grey matter
Algorithm
antsAI_masked
Similarity_nomask
terminal_MI_eroded_masked
TRSAA_nomask
Affine_nomask
flirt_NA
terminal_CC_eroded_masked
terminal_MI_masked
terminal_MI_nomask
terminal_CC_masked
TRSAA_eroded_masked
terminal_CC_nomask
Affine_eroded_masked
Similarity_eroded_masked
TRSAA_masked
Similarity_masked
Affine_masked
1 2 3 4 5 6 7 8 9 10 11 12 13 14 15 16 17
Affine_masked
Similarity_masked
TRSAA_masked
Similarity_eroded_masked
Affine_eroded_masked
terminal_CC_nomask
TRSAA_eroded_masked
terminal_CC_masked
terminal_MI_nomask
terminal_MI_masked
terminal_CC_eroded_masked
flirt_NA
Affine_nomask
TRSAA_nomask
terminal_MI_eroded_masked
Similarity_nomask
antsAI_masked
Algorithm

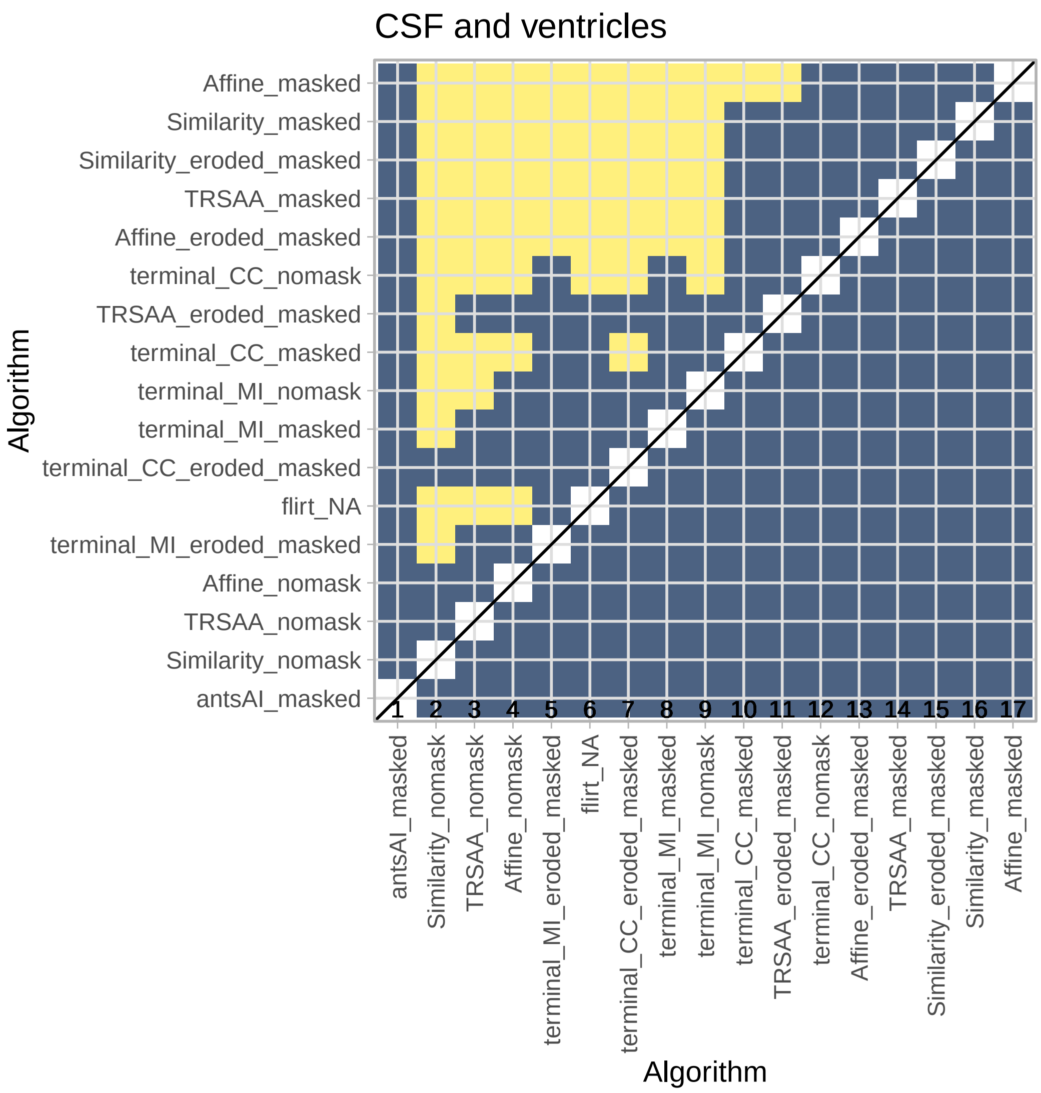
CSF and ventricles
Algorithm
Affine_masked
Similarity_masked
Similarity_eroded_masked
TRSAA_masked
Affine_eroded_masked
terminal_CC_nomask
TRSAA_eroded_masked
terminal_CC_masked
terminal_MI_nomask
terminal_MI_masked
terminal_CC_eroded_masked
flirt_NA
terminal_MI_eroded_masked
Affine_nomask
TRSAA_nomask
Similarity_nomask
antsAI_masked
1 2 3 4 5 6 7 8 9 10 11 12 13 14 15 16 17
antsAI_masked
Similarity_nomask
TRSAA_nomask
Affine_nomask
terminal_MI_eroded_masked
flirt_NA
terminal_CC_eroded_masked
terminal_MI_masked
terminal_MI_nomask
terminal_CC_masked
TRSAA_eroded_masked
terminal_CC_nomask
Affine_eroded_masked
TRSAA_masked
Similarity_eroded_masked
Similarity_masked
Affine_masked
Algorithm

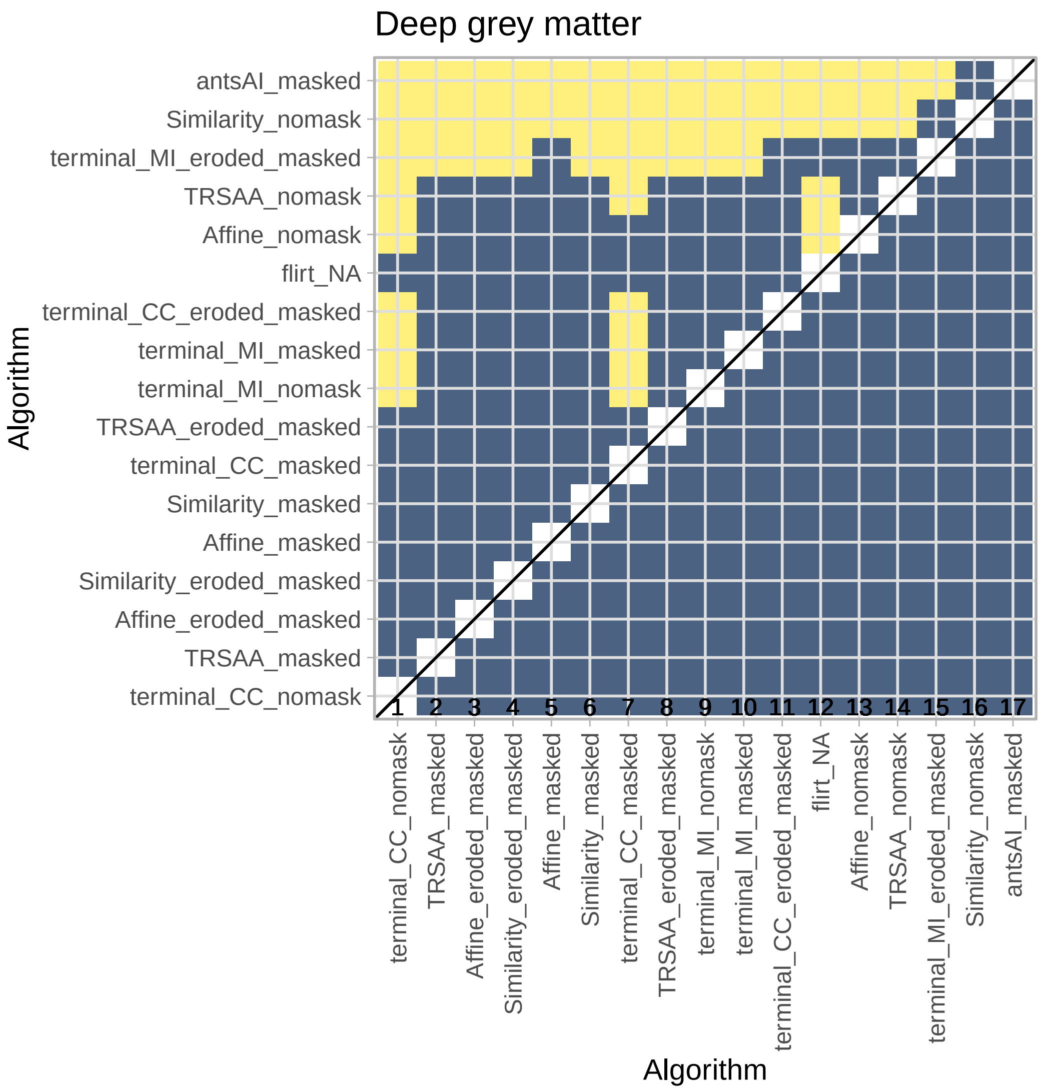
Deep grey matter
antsAI_masked
Similarity_nomask
terminal_MI_eroded_masked
TRSAA_nomask
Affine_nomask
flirt_NA
terminal_CC_eroded_masked
terminal_MI_masked
terminal_MI_nomask
TRSAA_eroded_masked
terminal_CC_masked
Similarity_masked
Affine_masked
Similarity_eroded_masked
Affine_eroded_masked
TRSAA_masked
terminal_CC_nomask
Algorithm
1 2 3 4 5 6 7 8 9 10 11 12 13 14 15 16 17
terminal_CC_nomask
TRSAA_masked
Affine_eroded_masked
Similarity_eroded_masked
Affine_masked
Similarity_masked
terminal_CC_masked
TRSAA_eroded_masked
terminal_MI_nomask
terminal_MI_masked
terminal_CC_eroded_masked
flirt_NA
Affine_nomask
TRSAA_nomask
terminal_MI_eroded_masked
Similarity_nomask
antsAI_masked
Algorithm

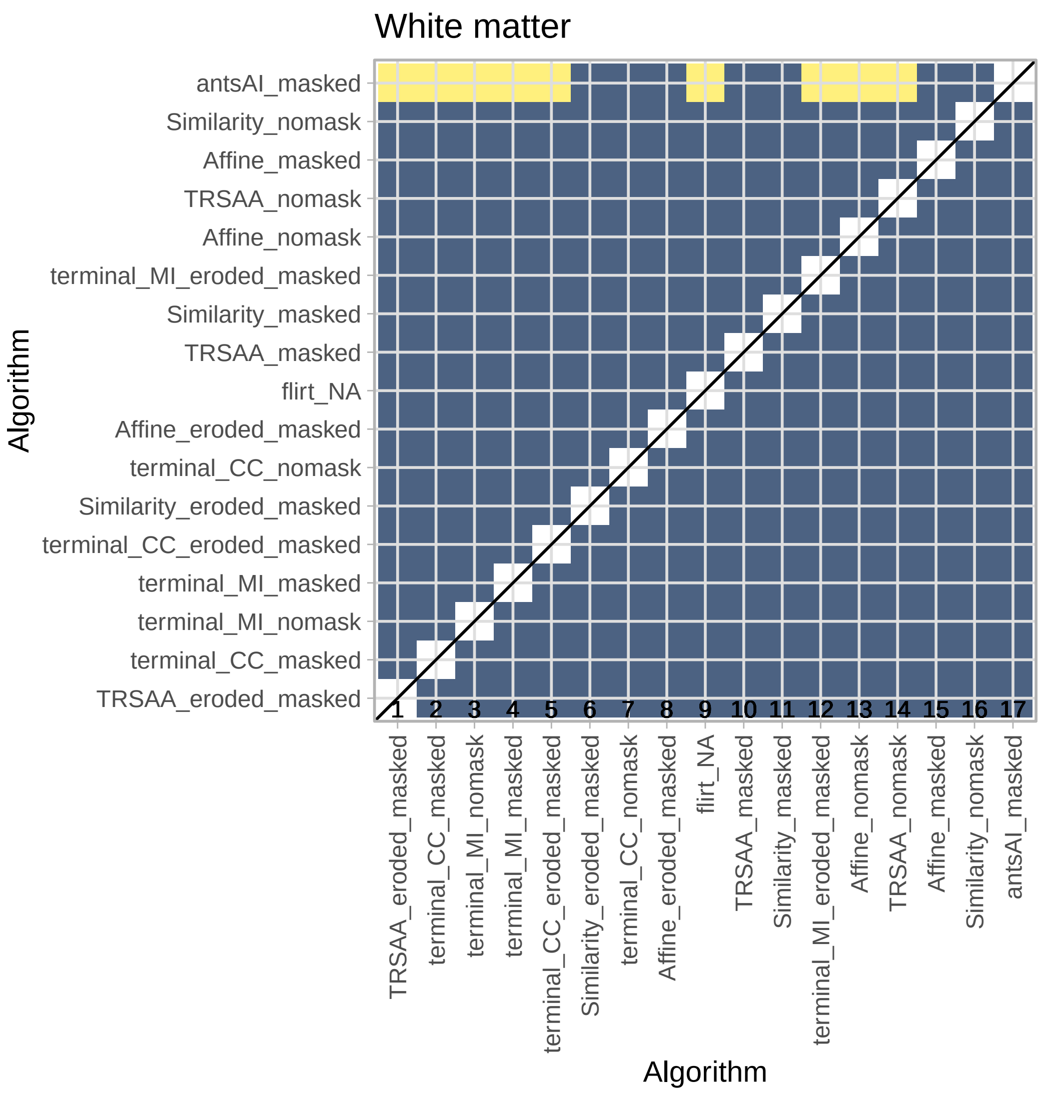
White matter
Algorithm
antsAI_masked
Similarity_nomask
Affine_masked
TRSAA_nomask
Affine_nomask
terminal_MI_eroded_masked
Similarity_masked
TRSAA_masked
flirt_NA
Affine_eroded_masked
terminal_CC_nomask
Similarity_eroded_masked
terminal_CC_eroded_masked
terminal_MI_masked
terminal_MI_nomask
terminal_CC_masked
TRSAA_eroded_masked
1 2 3 4 5 6 7 8 9 10 11 12 13 14 15 16 17
TRSAA_eroded_masked
terminal_CC_masked
terminal_MI_nomask
terminal_MI_masked
terminal_CC_eroded_masked
Similarity_eroded_masked
terminal_CC_nomask
Affine_eroded_masked
flirt_NA
TRSAA_masked
Similarity_masked
terminal_MI_eroded_masked
Affine_nomask
TRSAA_nomask
Affine_masked
Similarity_nomask
antsAI_masked
Algorithm

3.4 Ranking robustness to ranking methods

Line plots for visualizing ranking robustness across different ranking methods. Each algorithm is represented by one colored line. For each ranking method encoded on the x-axis, the height of the line represents the corresponding rank. Horizontal lines indicate identical ranks for all methods.

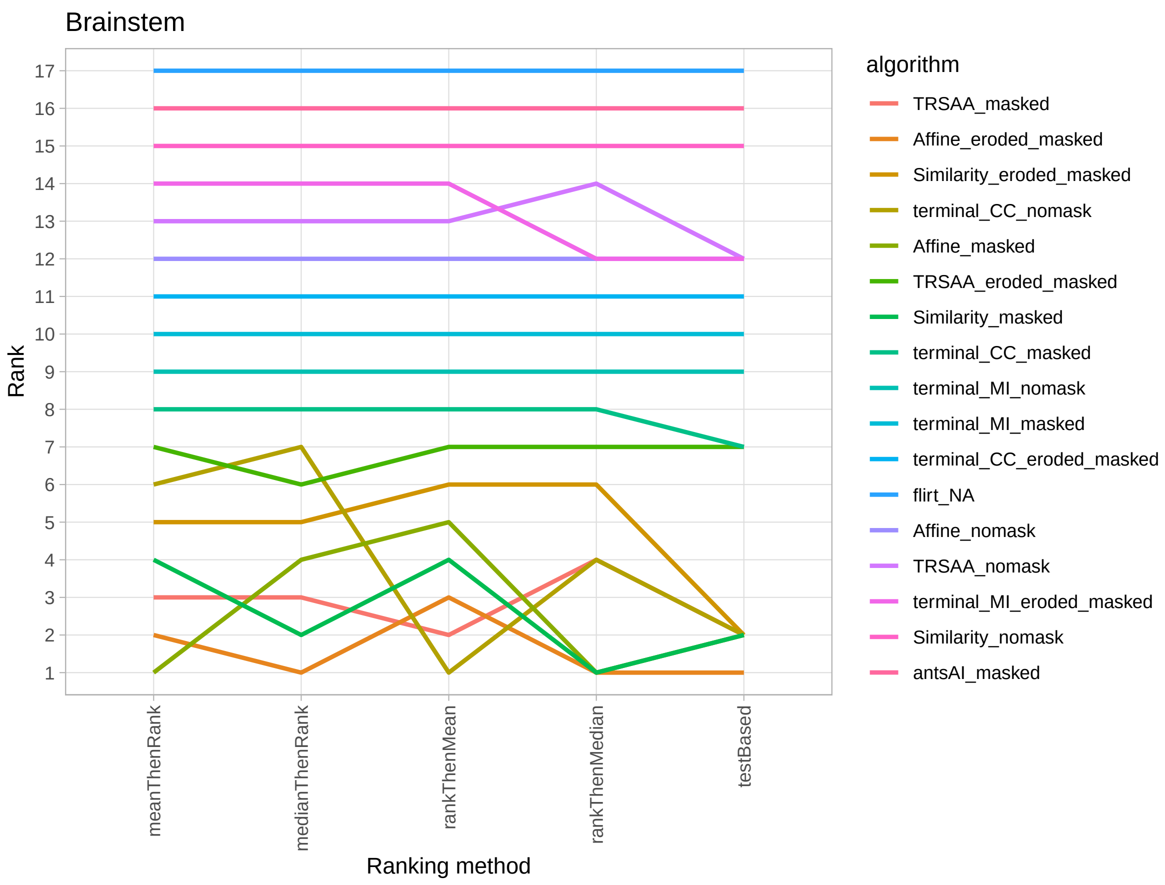

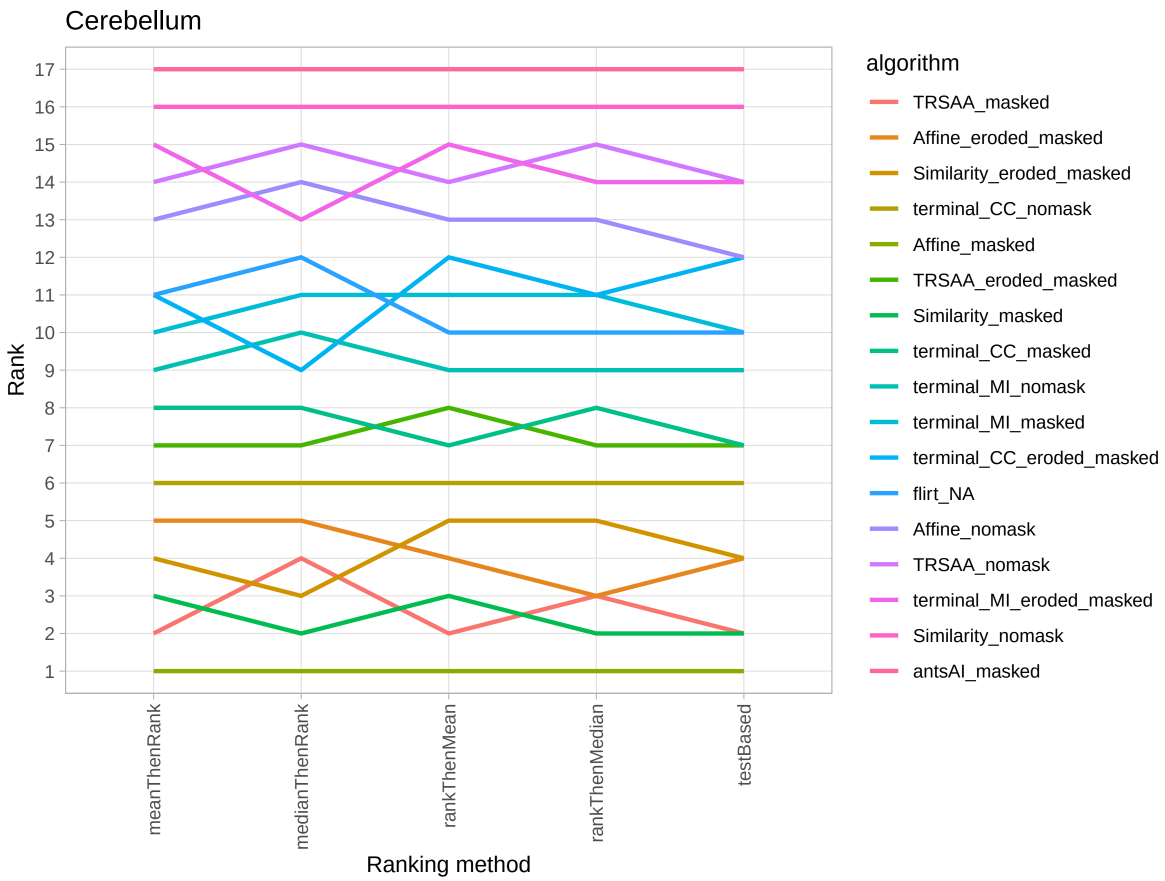
Cerebellum
Rank
17
16
15
14
13
12
11
10
9
8
7
6
5
4
3
2
1
meanThenRank
medianThenRank
rankThenMean
rankThenMedian
testBased
Ranking method
algorithm
TRSAA_masked
Affine_eroded_masked
Similarity_eroded_masked
terminal_CC_nomask
Affine_masked
TRSAA_eroded_masked
Similarity_masked
terminal_CC_masked
terminal_MI_nomask
terminal_MI_masked
terminal_CC_eroded_masked
flirt_NA
Affine_nomask
TRSAA_nomask
terminal_MI_eroded_masked
Similarity_nomask
antsAI_masked

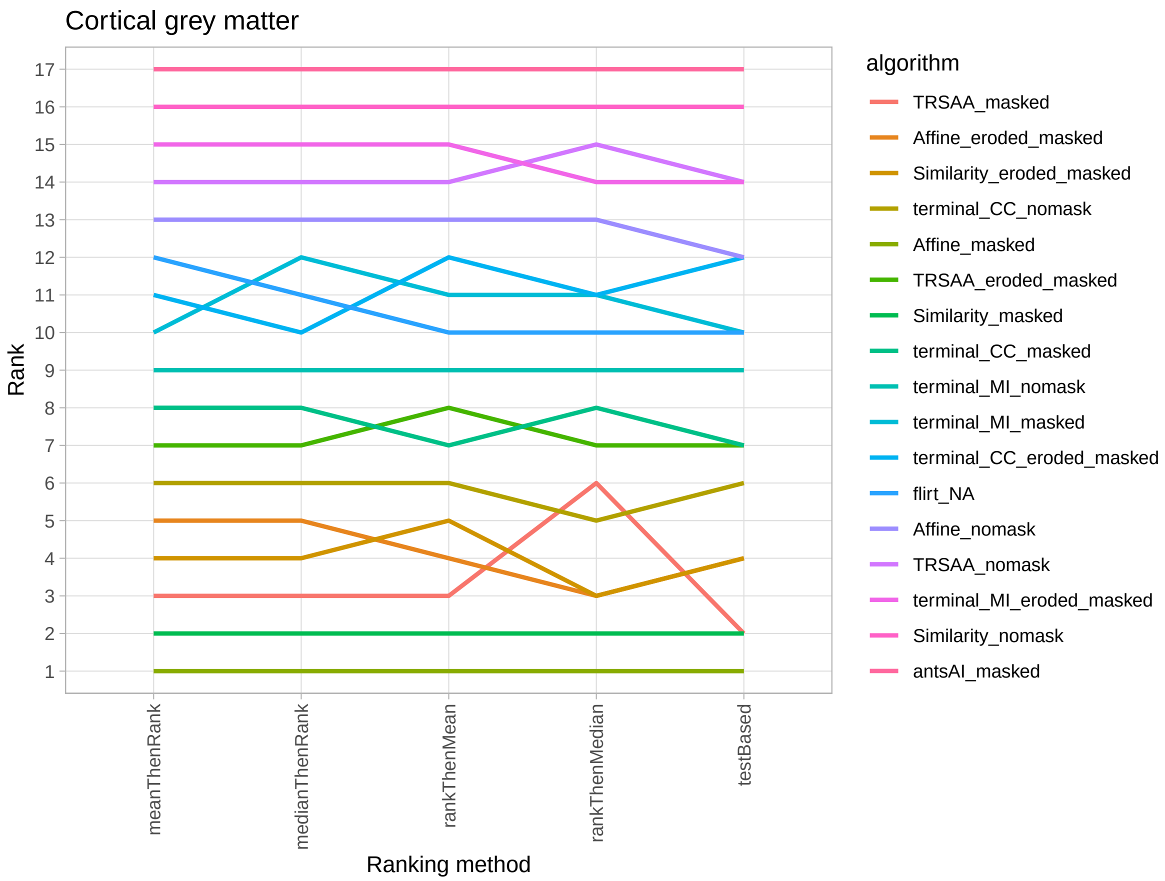

Cortical grey matter
Rank
17
16
15
14
13
12
11
10
9
8
7
6
5
4
3
2
1
meanThenRank
medianThenRank
rankThenMean
rankThenMedian
testBased
Ranking method
algorithm
TRSAA_masked
Affine_eroded_masked
Similarity_eroded_masked
terminal_CC_nomask
Affine_masked
TRSAA_eroded_masked
Similarity_masked
terminal_CC_masked
terminal_MI_nomask
terminal_MI_masked
terminal_CC_eroded_masked
flirt_NA
Affine_nomask
TRSAA_nomask
terminal_MI_eroded_masked
Similarity_nomask
antsAI_masked

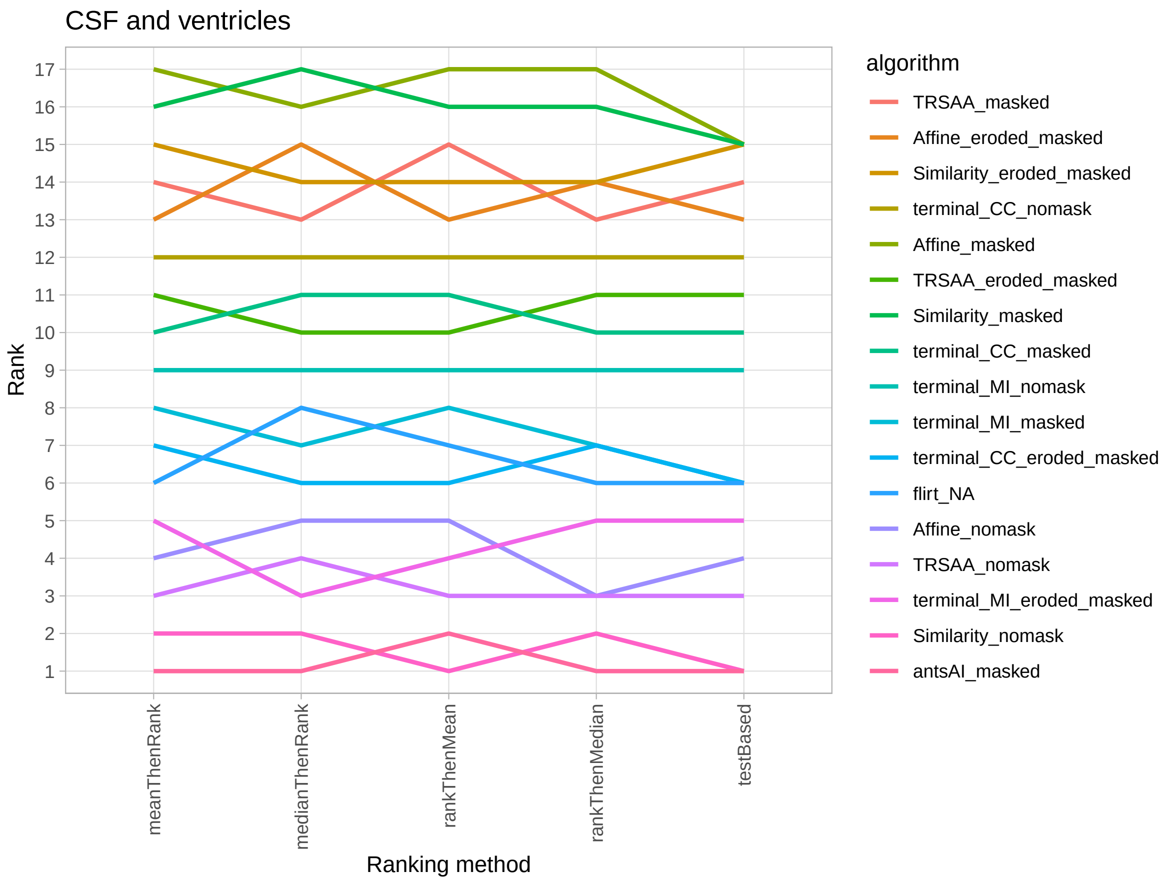
CSF and ventricles
Rank
17
16
15
14
13
12
11
10
9
8
7
6
5
4
3
2
1
meanThenRank
medianThenRank
rankThenMean
rankThenMedian
testBased
Ranking method
algorithm
TRSAA_masked
Affine_eroded_masked
Similarity_eroded_masked
terminal_CC_nomask
Affine_masked
TRSAA_eroded_masked
Similarity_masked
terminal_CC_masked
terminal_MI_nomask
terminal_MI_masked
terminal_CC_eroded_masked
flirt_NA
Affine_nomask
TRSAA_nomask
terminal_MI_eroded_masked
Similarity_nomask
antsAI_masked

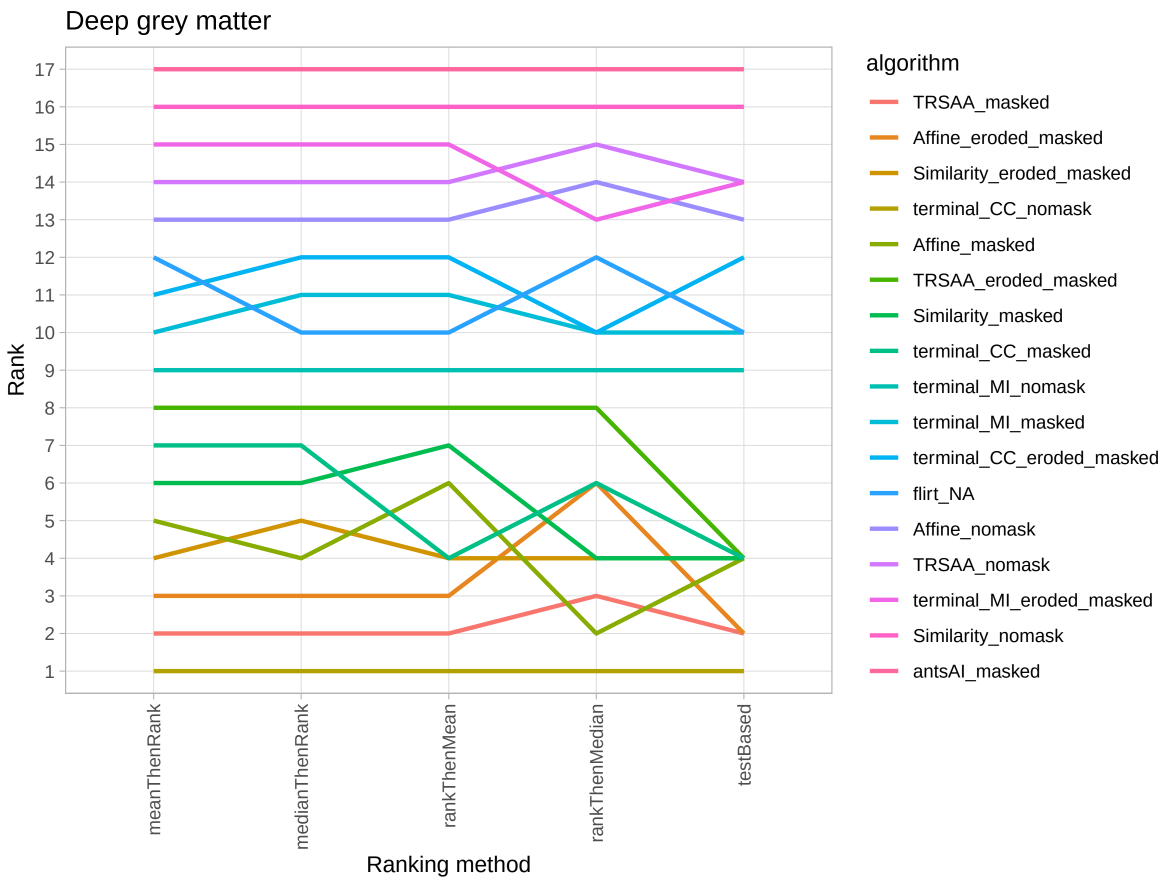

Deep grey matter
Rank
17
16
15
14
13
12
11
10
9
8
7
6
5
4
3
2
1
meanThenRank
medianThenRank
rankThenMean
rankThenMedian
testBased
Ranking method
algorithm
TRSAA_masked
Affine_eroded_masked
Similarity_eroded_masked
terminal_CC_nomask
Affine_masked
TRSAA_eroded_masked
Similarity_masked
terminal_CC_masked
terminal_MI_nomask
terminal_MI_masked
terminal_CC_eroded_masked
flirt_NA
Affine_nomask
TRSAA_nomask
terminal_MI_eroded_masked
Similarity_nomask
antsAI_masked

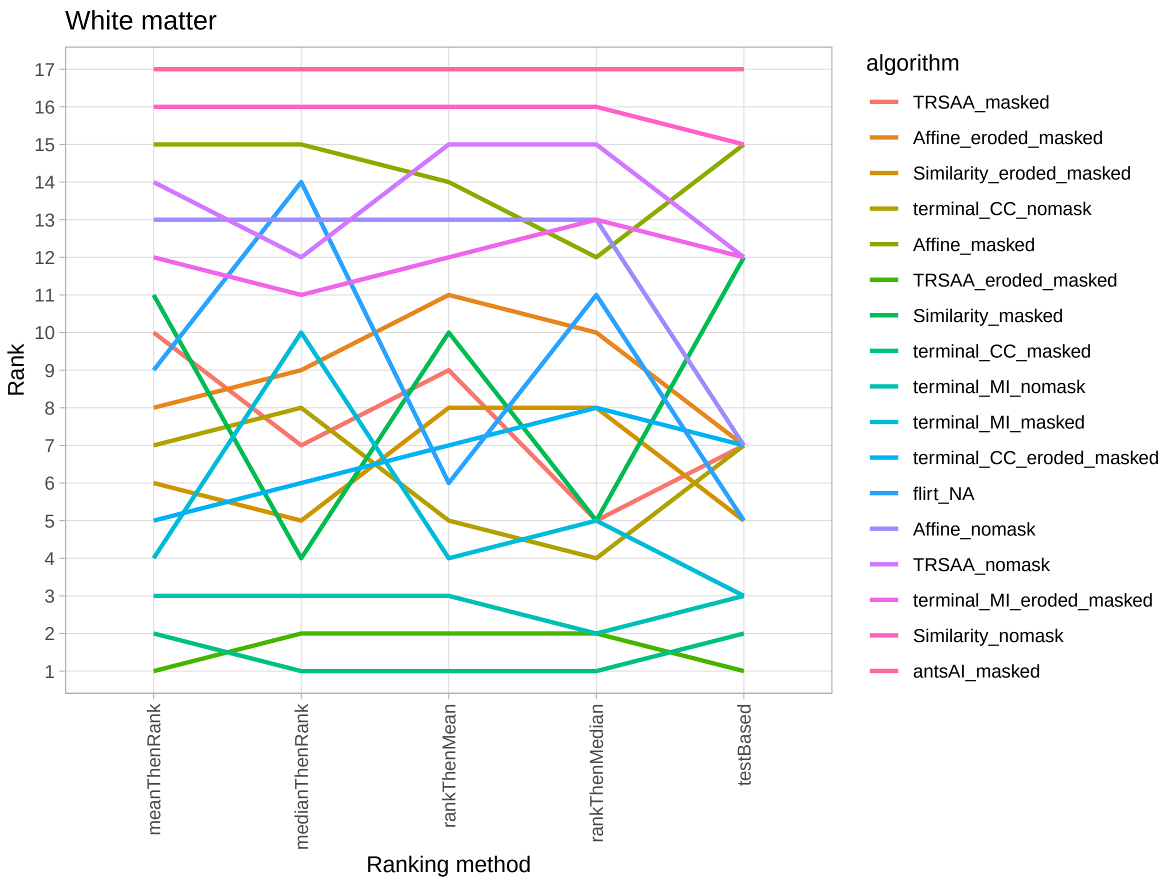
White matter
Rank
17
16
15
14
13
12
11
10
9
8
7
6
5
4
3
2
1
meanThenRank
medianThenRank
rankThenMean
rankThenMedian
testBased
Ranking method
algorithm
TRSAA_masked
Affine_eroded_masked
Similarity_eroded_masked
terminal_CC_nomask
Affine_masked
TRSAA_eroded_masked
Similarity_masked
terminal_CC_masked
terminal_MI_nomask
terminal_MI_masked
terminal_CC_eroded_masked
flirt_NA
Affine_nomask
TRSAA_nomask
terminal_MI_eroded_masked
Similarity_nomask
antsAI_masked

4 Visualization of cross-task insights

The algorithms are ordered according to consensus ranking.

4.1 Characterization of algorithms

4.1.1 Ranking stability: Variability of achieved rankings across tasks

Algorithms are color-coded, and the area of each blob at position $(A_i, \text{rank } j)$ is proportional to the relative frequency A_i achieved rank j across multiple tasks. The median rank for each algorithm is indicated by a black cross. This way, the distribution of ranks across tasks can be intuitively visualized.

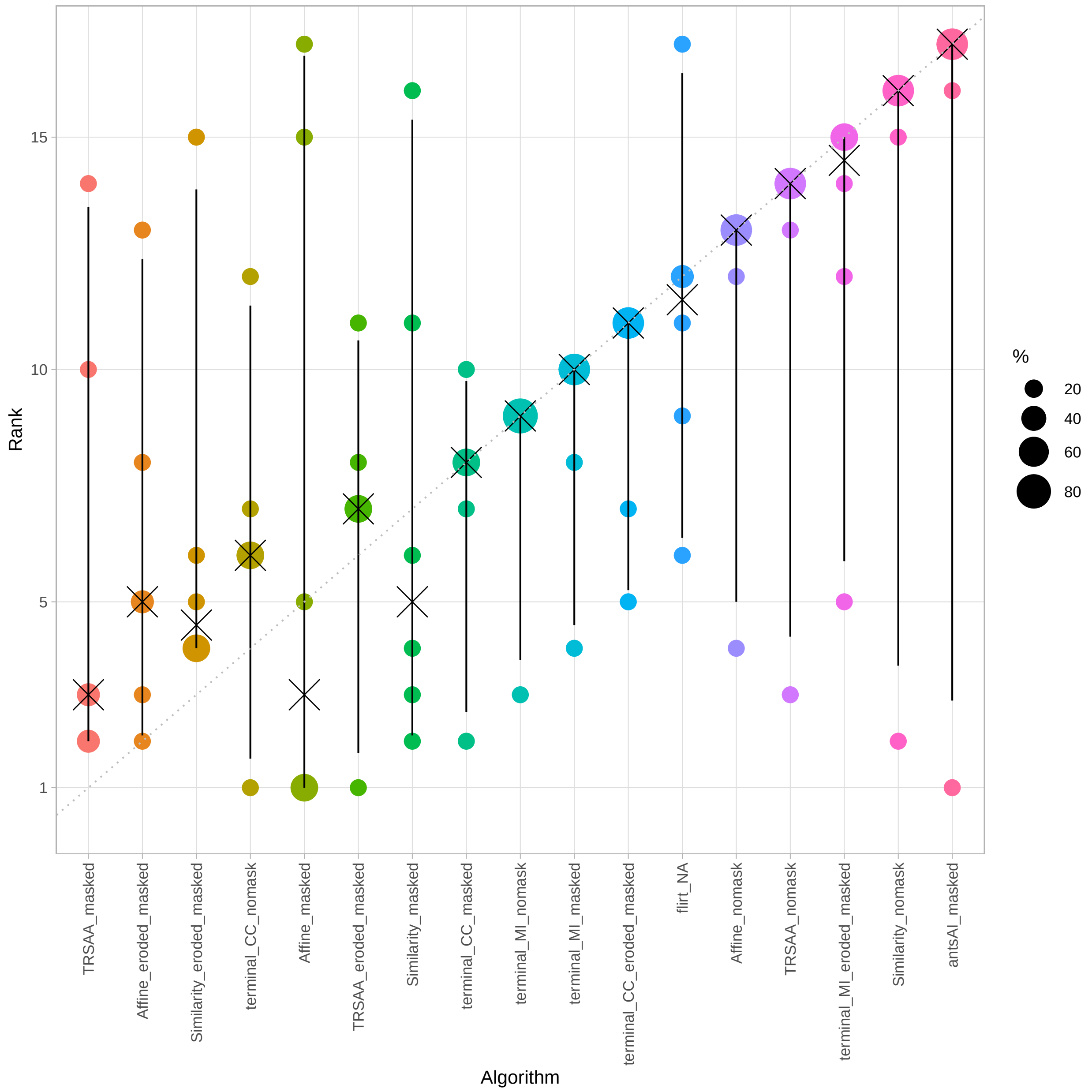

4.1.2 Ranking stability: Ranking variability via bootstrap approach

A blob plot of bootstrap results over the different tasks separated by algorithm allows another perspective on the assessment data. This gives deeper insights into the characteristics of tasks and the ranking uncertainty of the algorithms in each task.

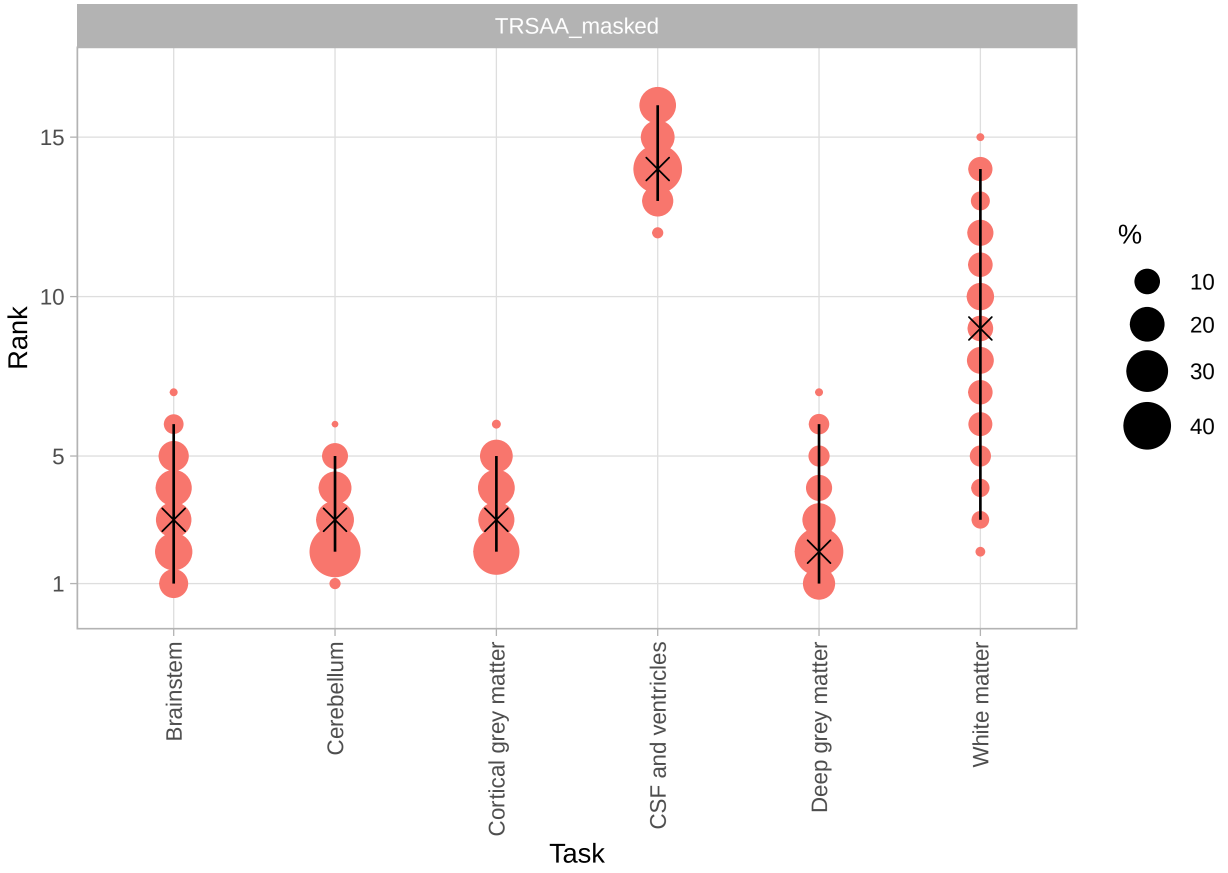

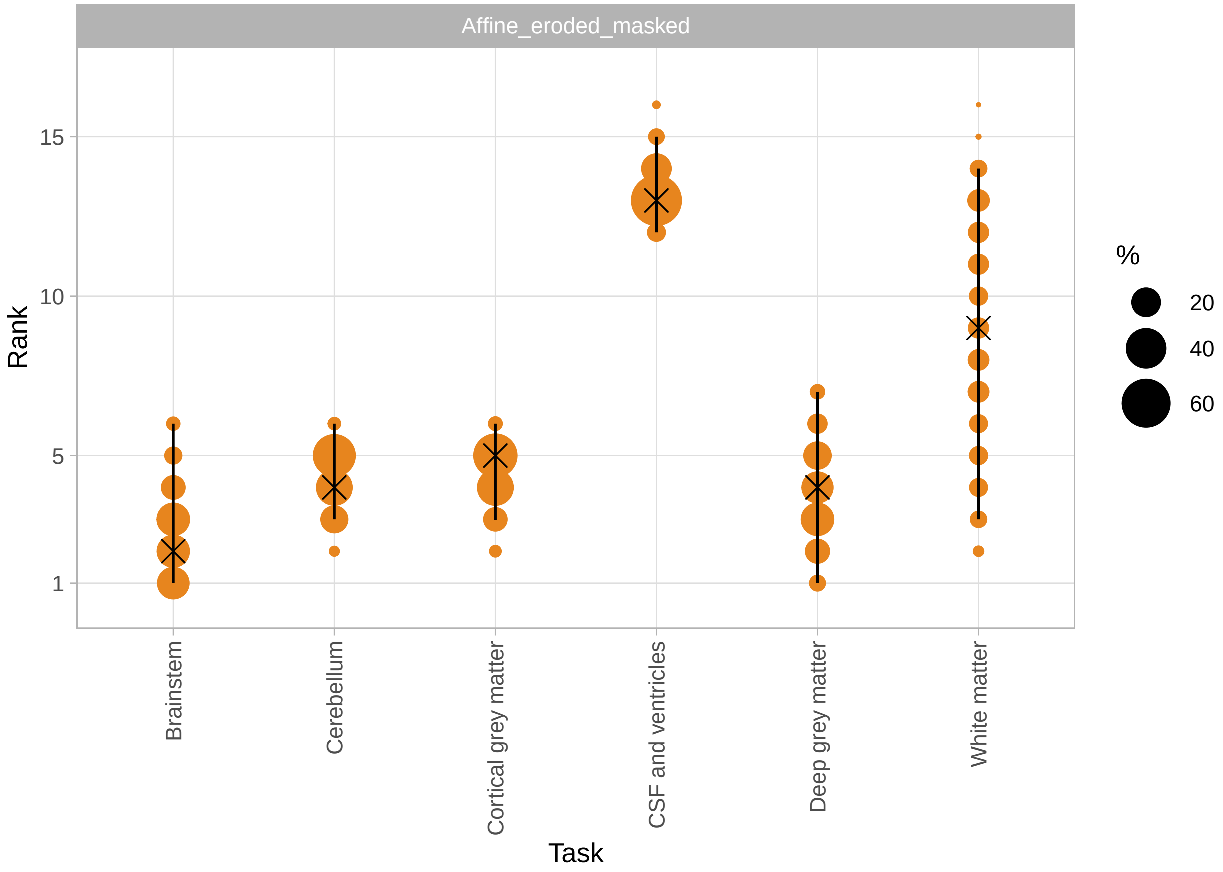
Affine_eroded_masked
Rank
15
10
5
1
Brainstem
Cerebellum
Cortical grey matter
CSF and ventricles
Deep grey matter
White matter
Task
%
20
40
60

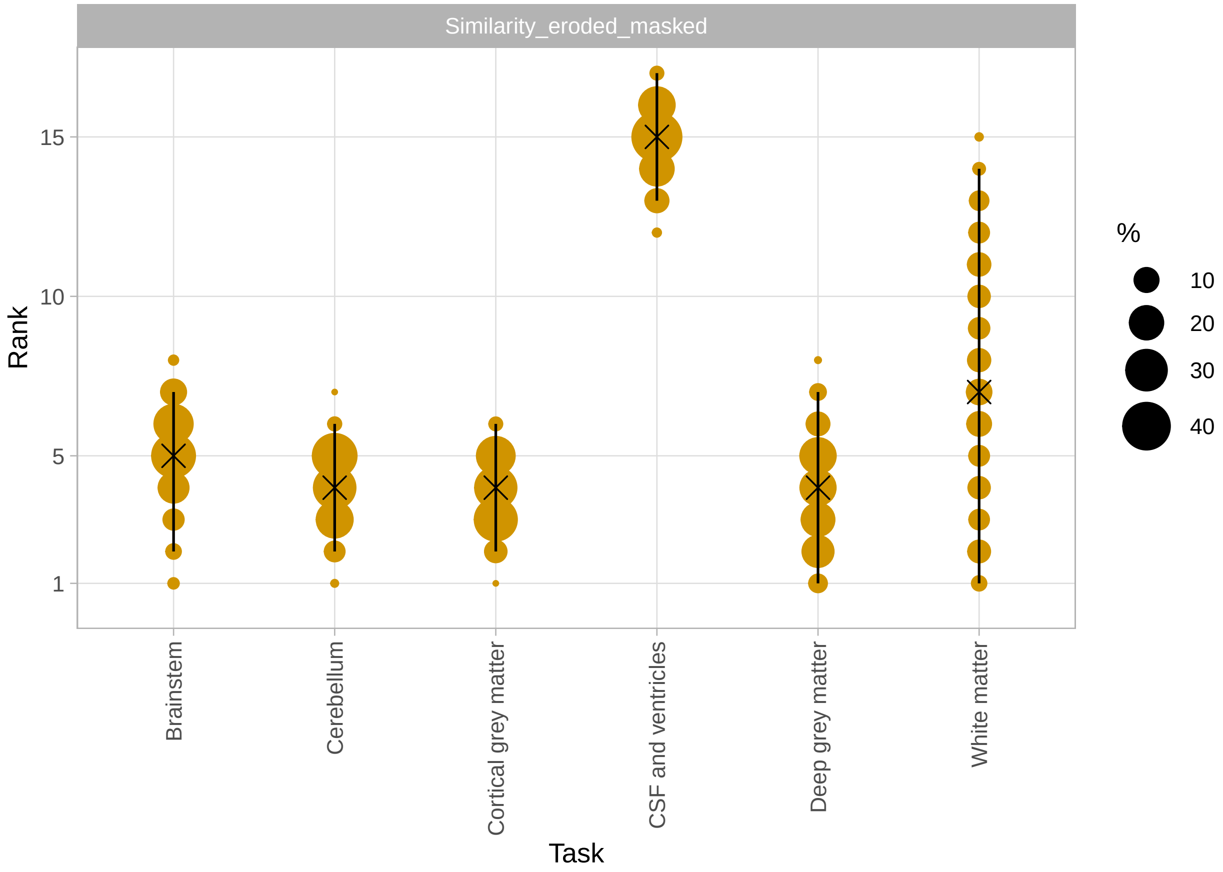

Similarity_eroded_masked
Rank
15
10
5
1
Brainstem
Cerebellum
Cortical grey matter
CSF and ventricles
Deep grey matter
White matter
Task
%
10
20
30
40

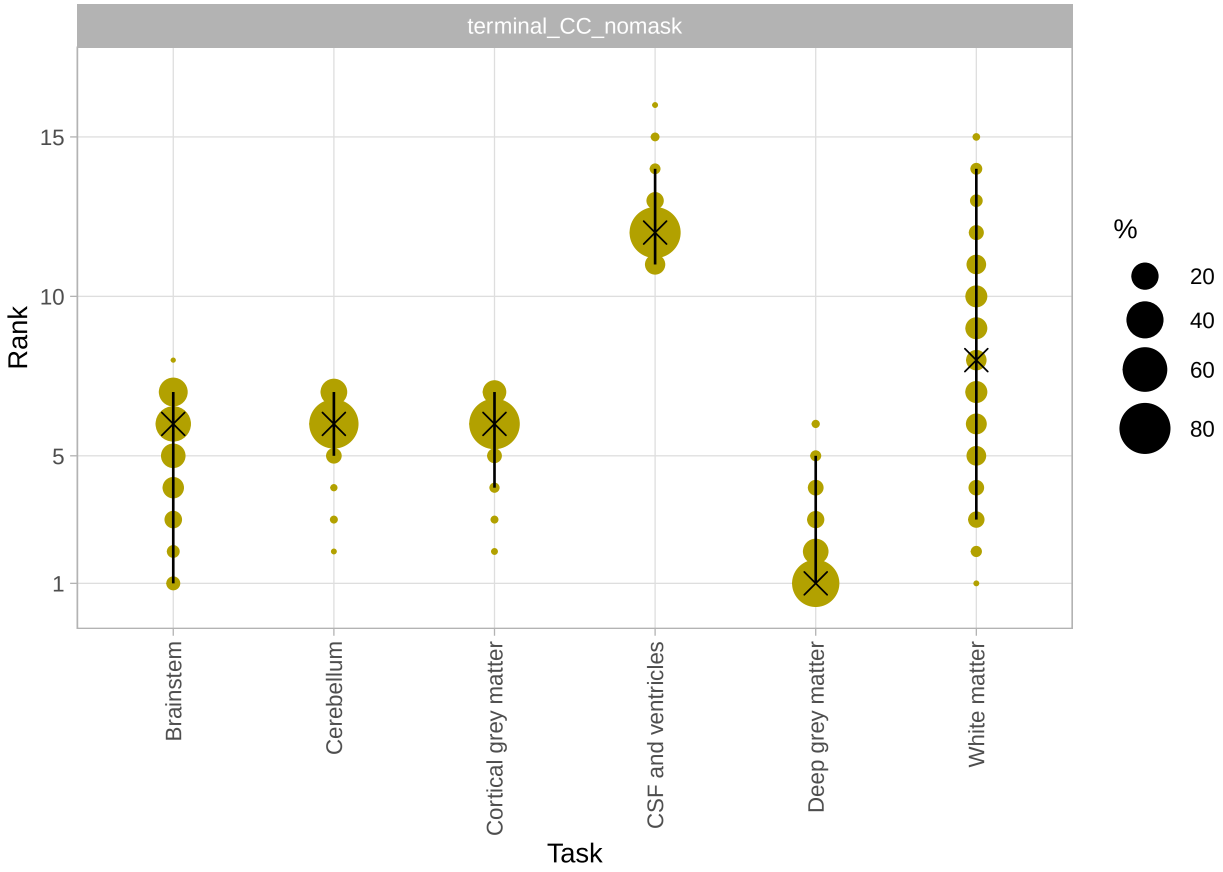

terminal_CC_nomask
Rank
15
10
5
1
Brainstem
Cerebellum
Cortical grey matter
CSF and ventricles
Deep grey matter
White matter
Task
%
20
40
60
80

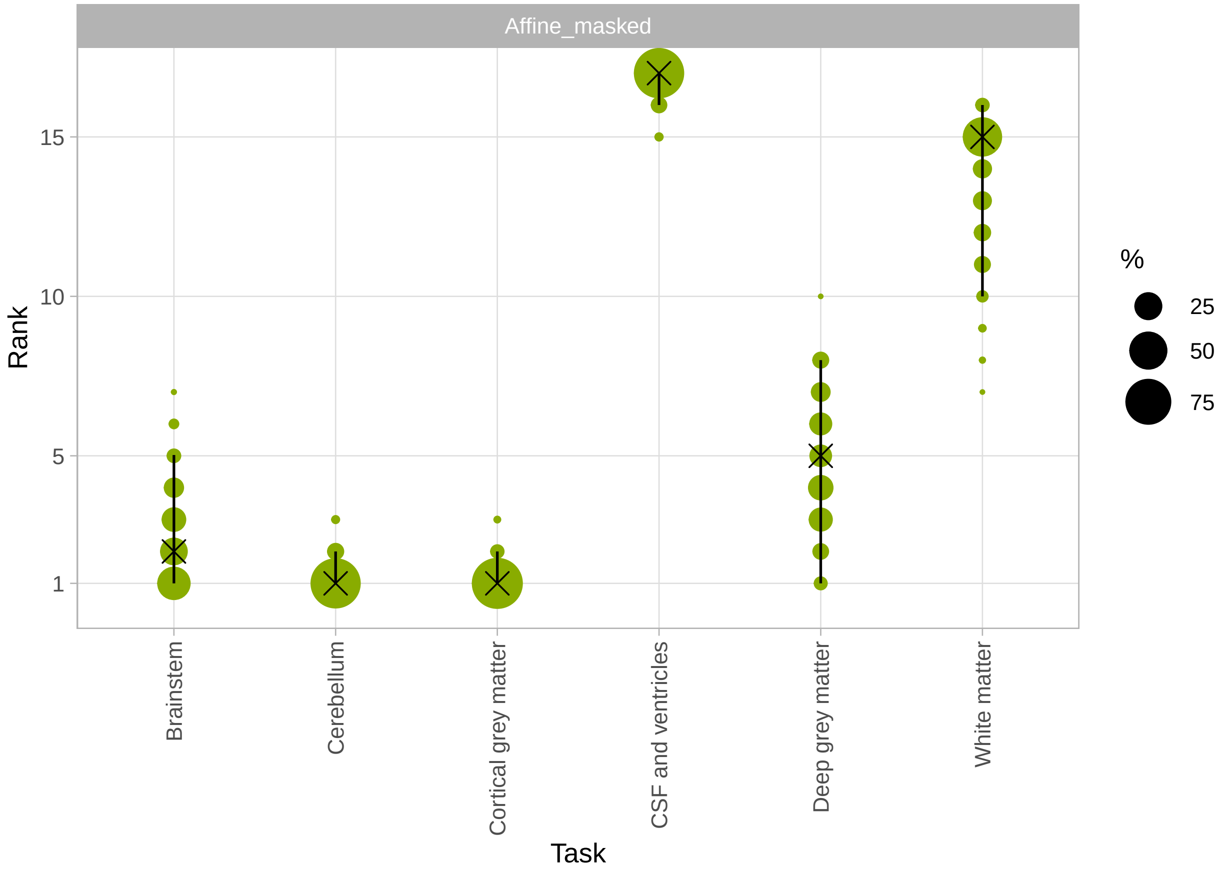
Affine_masked
Rank
15
10
5
1
Brainstem
Cerebellum
Cortical grey matter
CSF and ventricles
Deep grey matter
White matter
Task
%
25
50
75

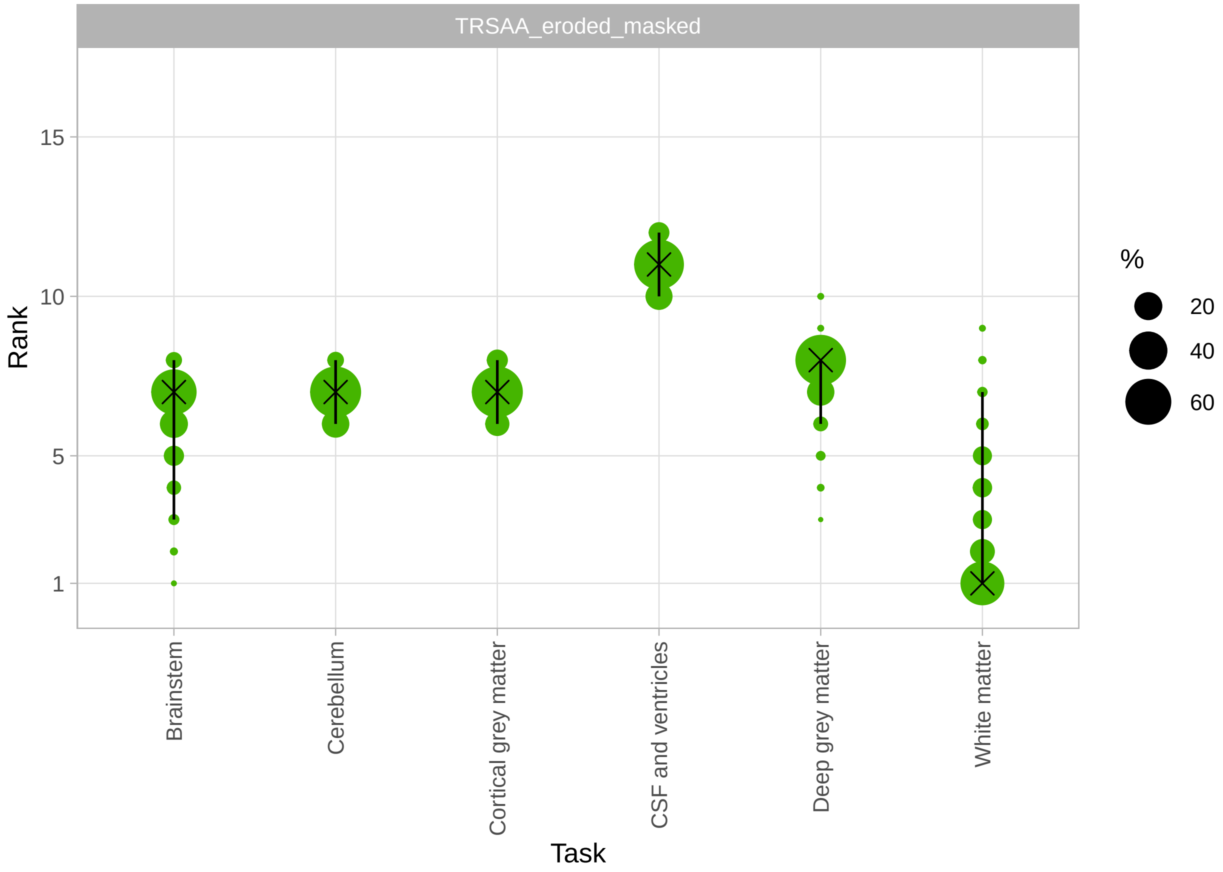

TRSAA_eroded_masked
Rank
15
10
5
1
Brainstem
Cerebellum
Cortical grey matter
CSF and ventricles
Deep grey matter
White matter
Task
%
20
40
60

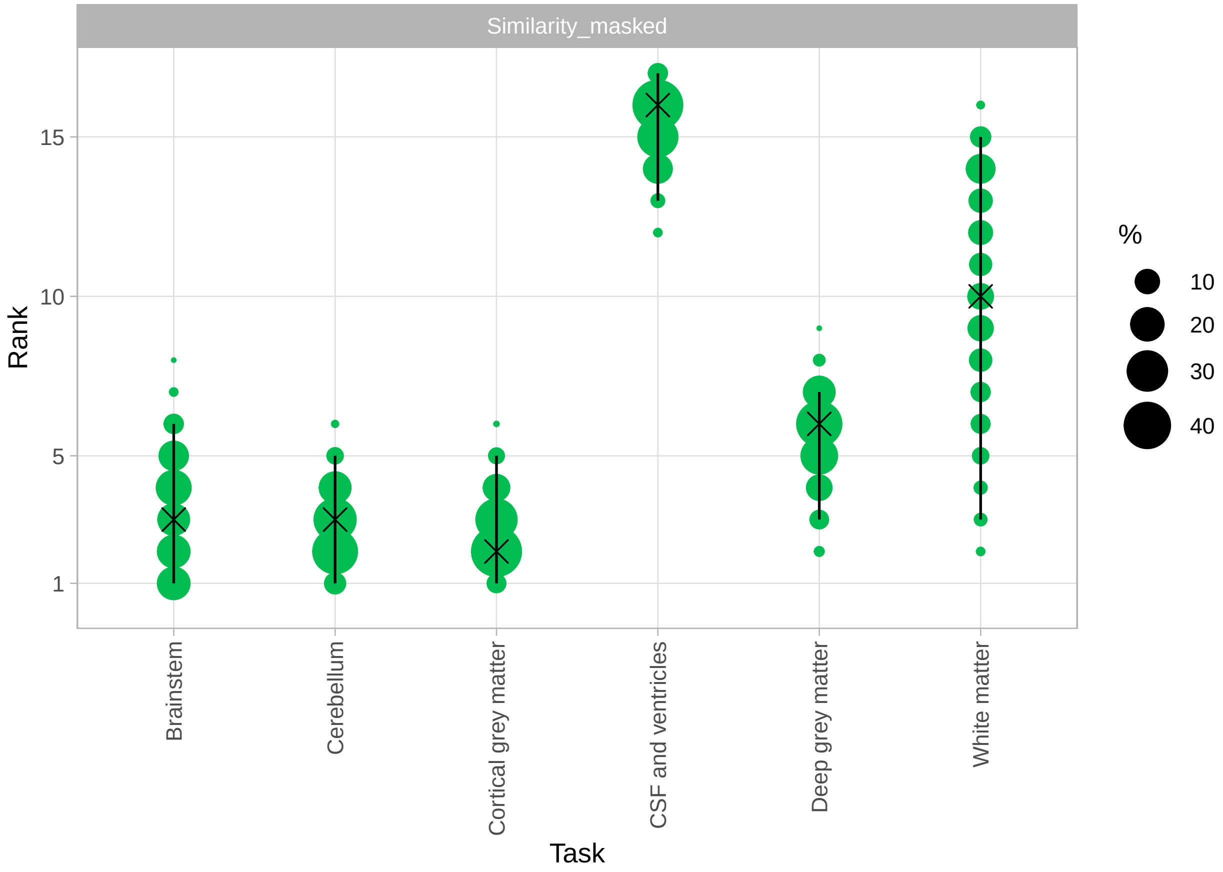

Similarity_masked
Rank
15
10
5
1
Brainstem
Cerebellum
Cortical grey matter
CSF and ventricles
Deep grey matter
White matter
Task
%
10
20
30
40

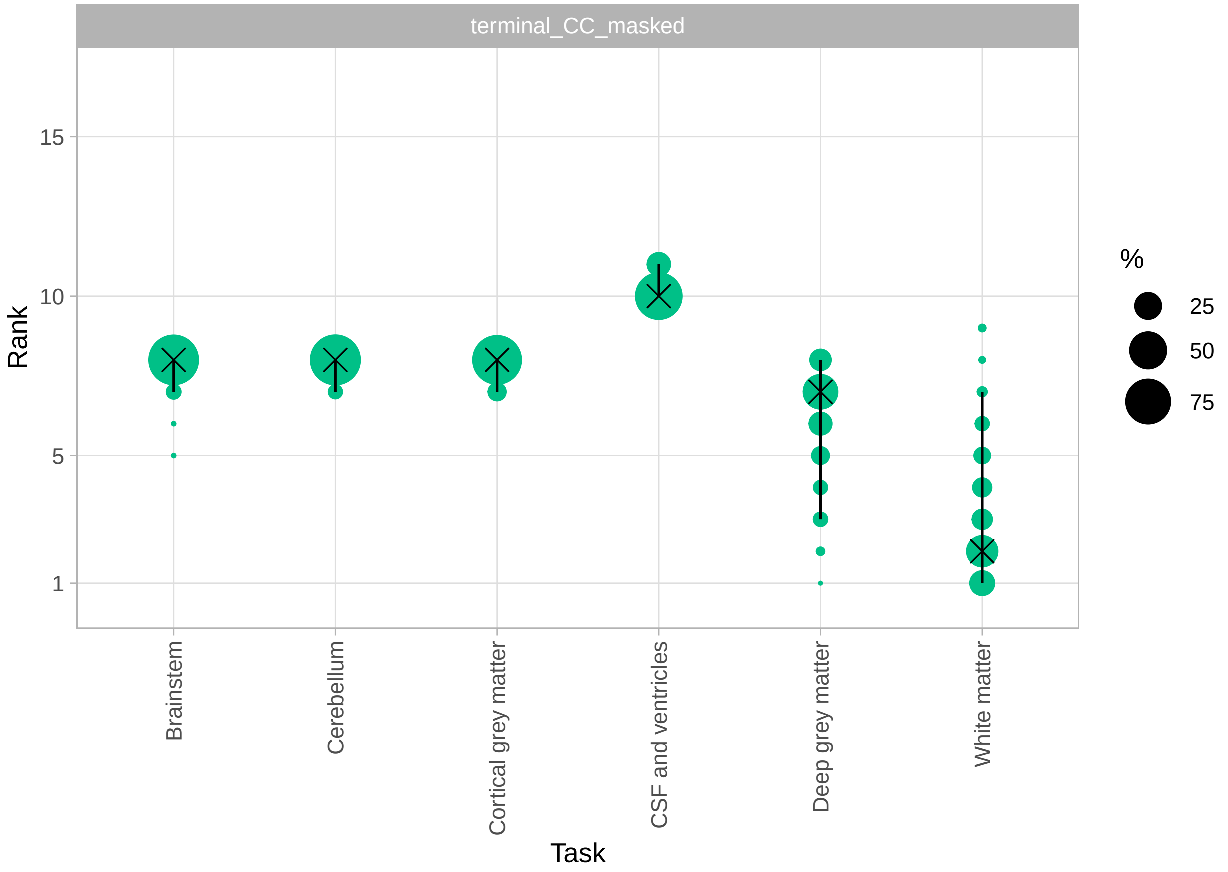
terminal_CC_masked
Rank
15
10
5
1
%
25
50
75
Brainstem
Cerebellum
Cortical grey matter
CSF and ventricles
Deep grey matter
White matter
Task

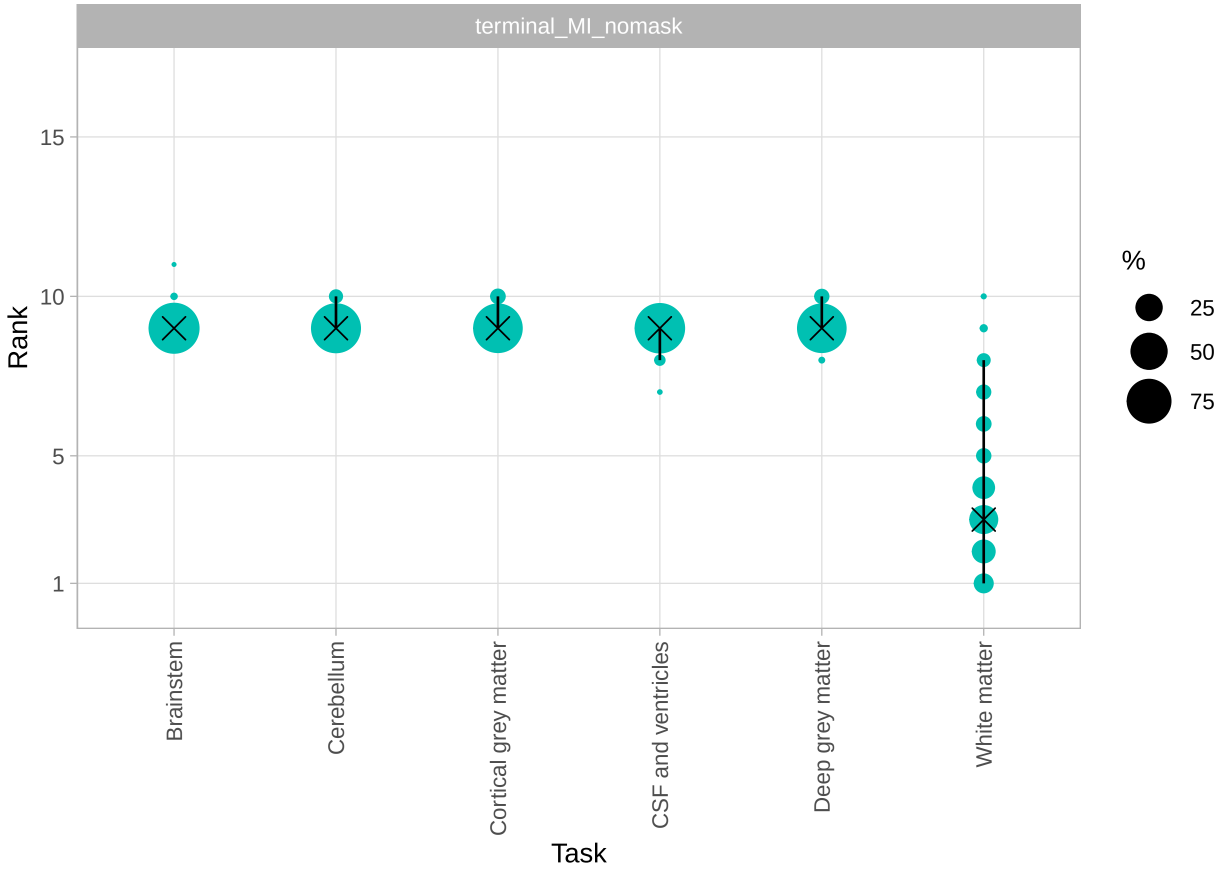
terminal_MI_nomask
Rank
15
10
5
1
Brainstem
Cerebellum
Cortical grey matter
CSF and ventricles
Deep grey matter
White matter
Task
%
25
50
75

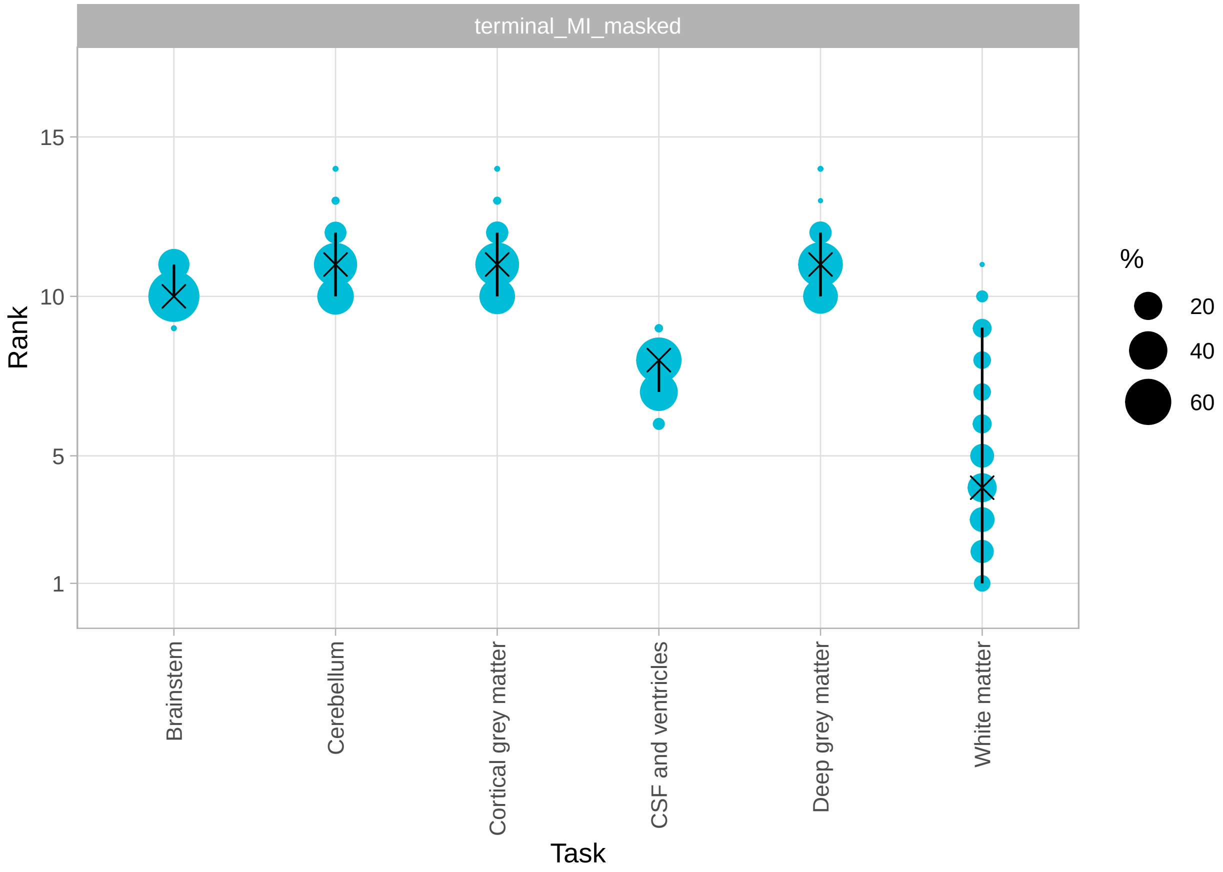
terminal_MI_masked
Rank
15
10
5
1
Brainstem
Cerebellum
Cortical grey matter
CSF and ventricles
Deep grey matter
White matter
Task
%
20
40
60

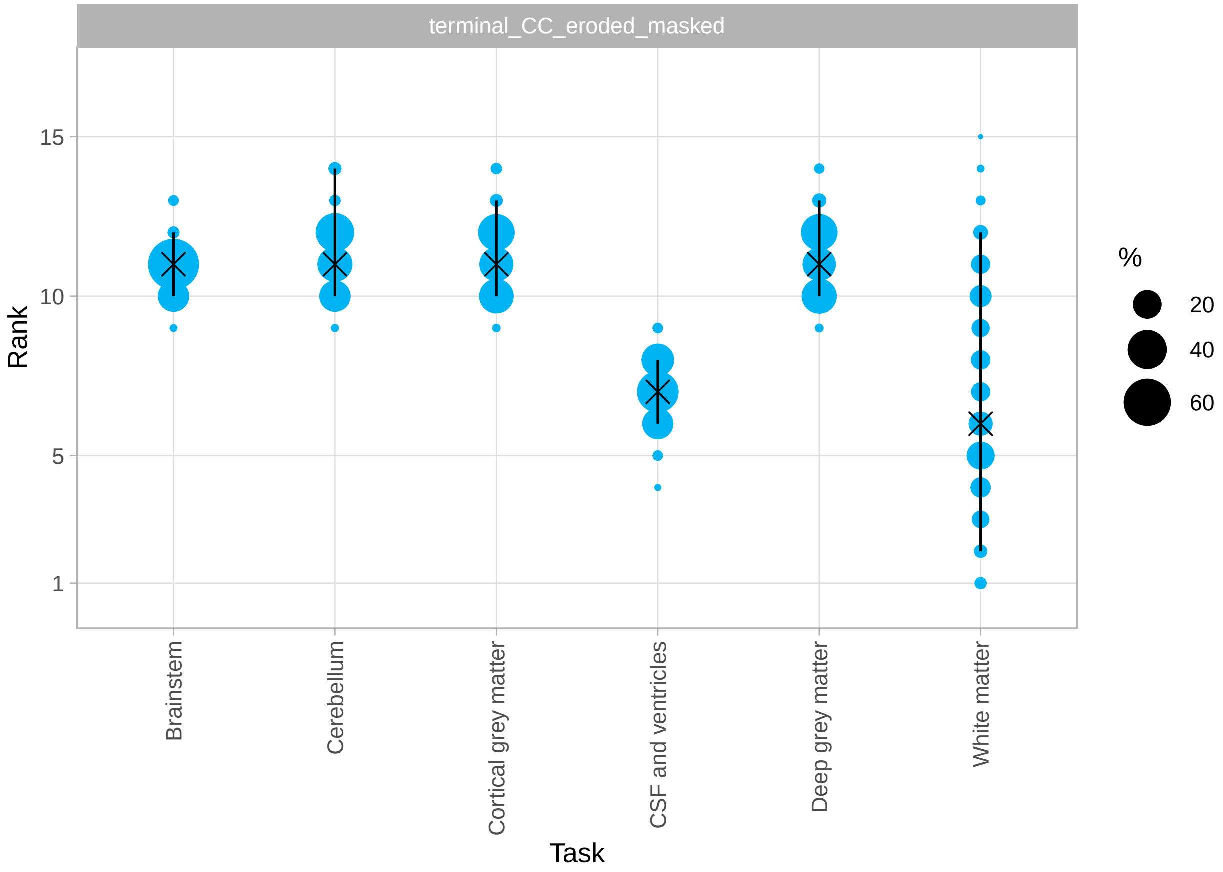

terminal_CC_eroded_masked
Rank
15
10
5
1
Brainstem
Cerebellum
Cortical grey matter
CSF and ventricles
Deep grey matter
White matter
Task
%
20
40
60

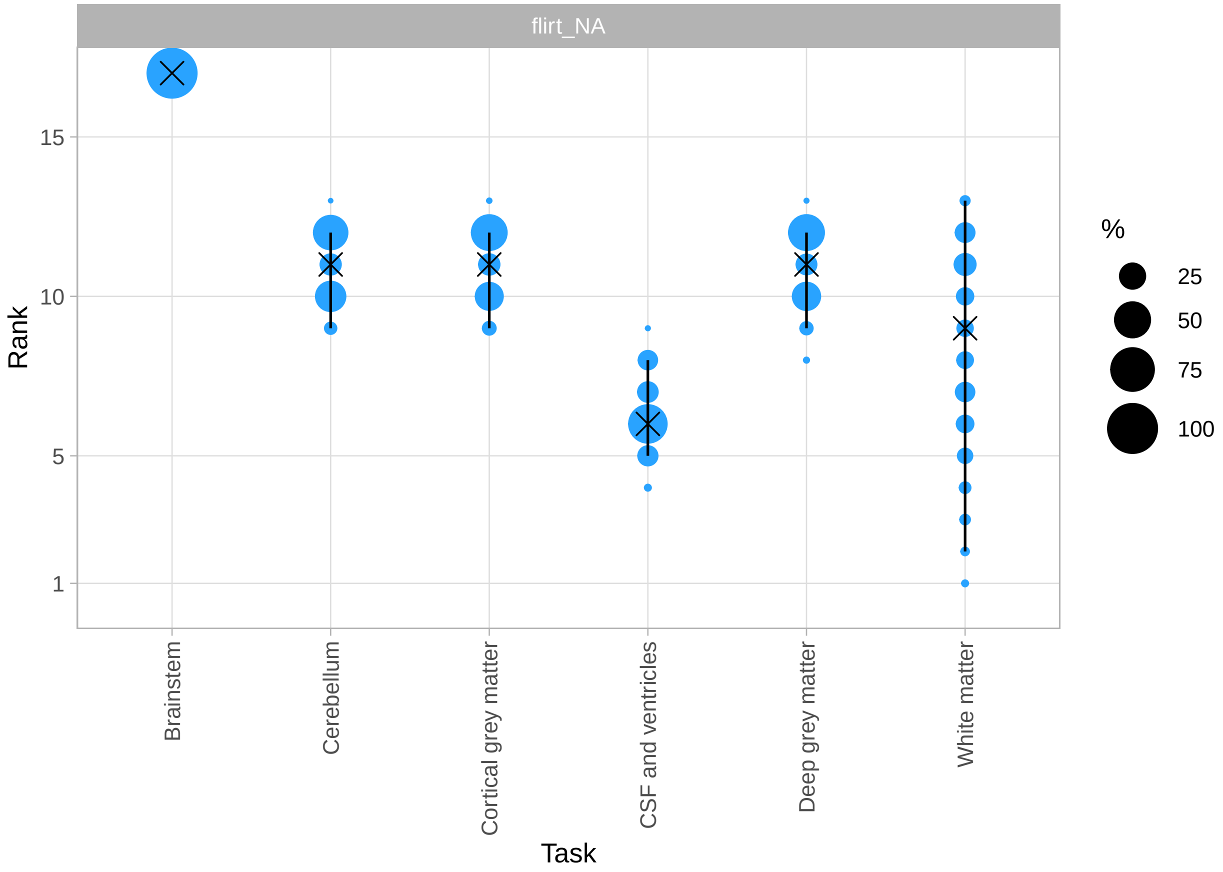

flirt_NA
Rank
15
10
5
1
Brainstem
Cerebellum
Cortical grey matter
CSF and ventricles
Deep grey matter
White matter
Task
%
25
50
75
100

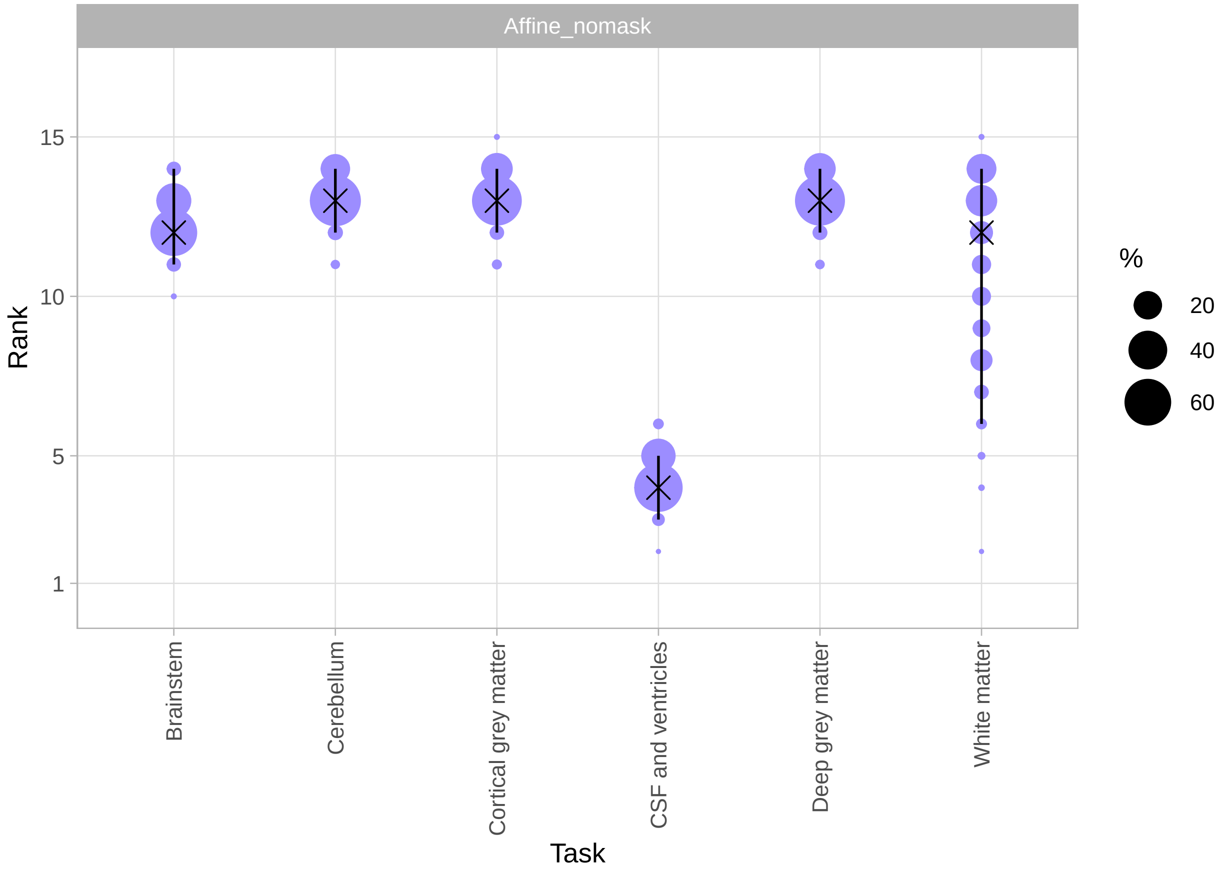
Affine_nomask
Rank
15
10
5
1
Brainstem
Cerebellum
Cortical grey matter
CSF and ventricles
Deep grey matter
White matter
Task
%
20
40
60

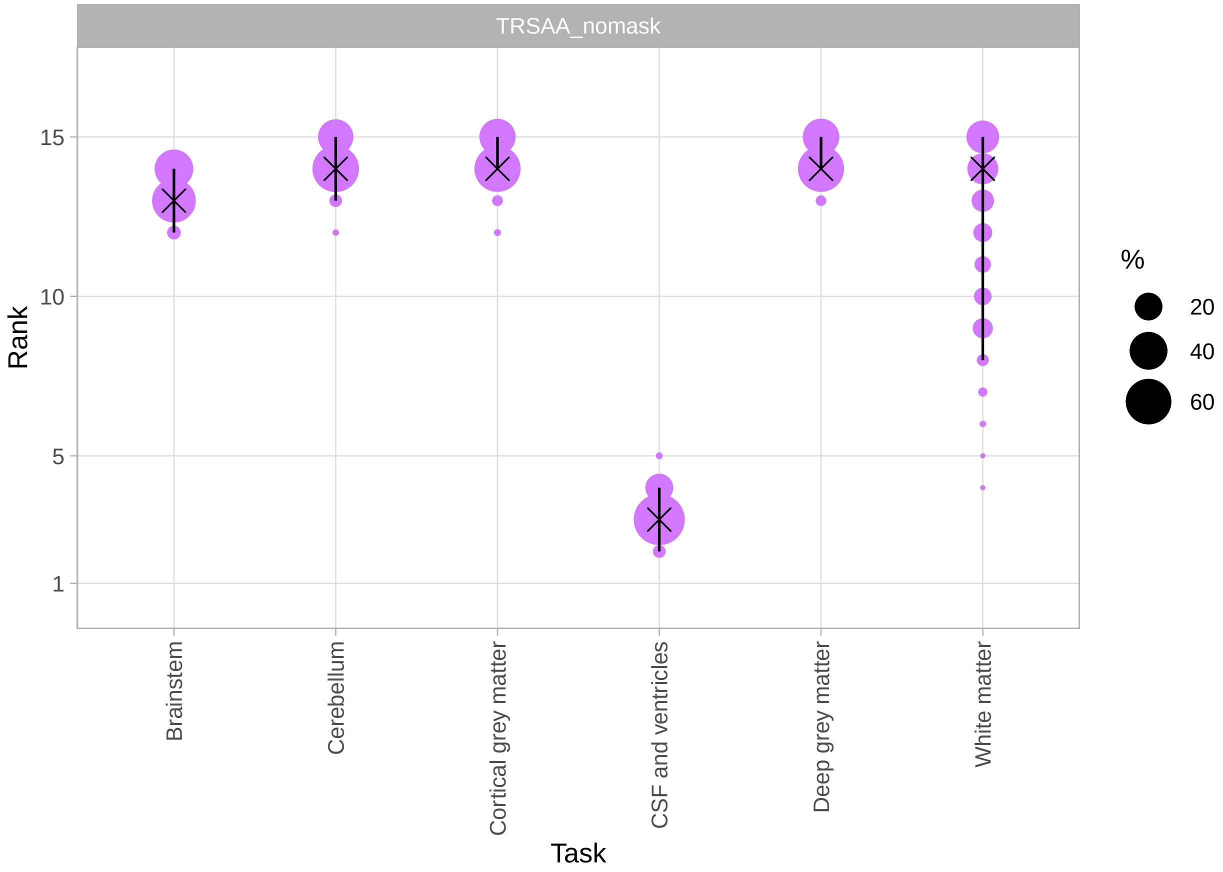
TRSAA_nomask
Rank
15
10
5
1
Brainstem
Cerebellum
Cortical grey matter
CSF and ventricles
Deep grey matter
White matter
Task
%
20
40
60

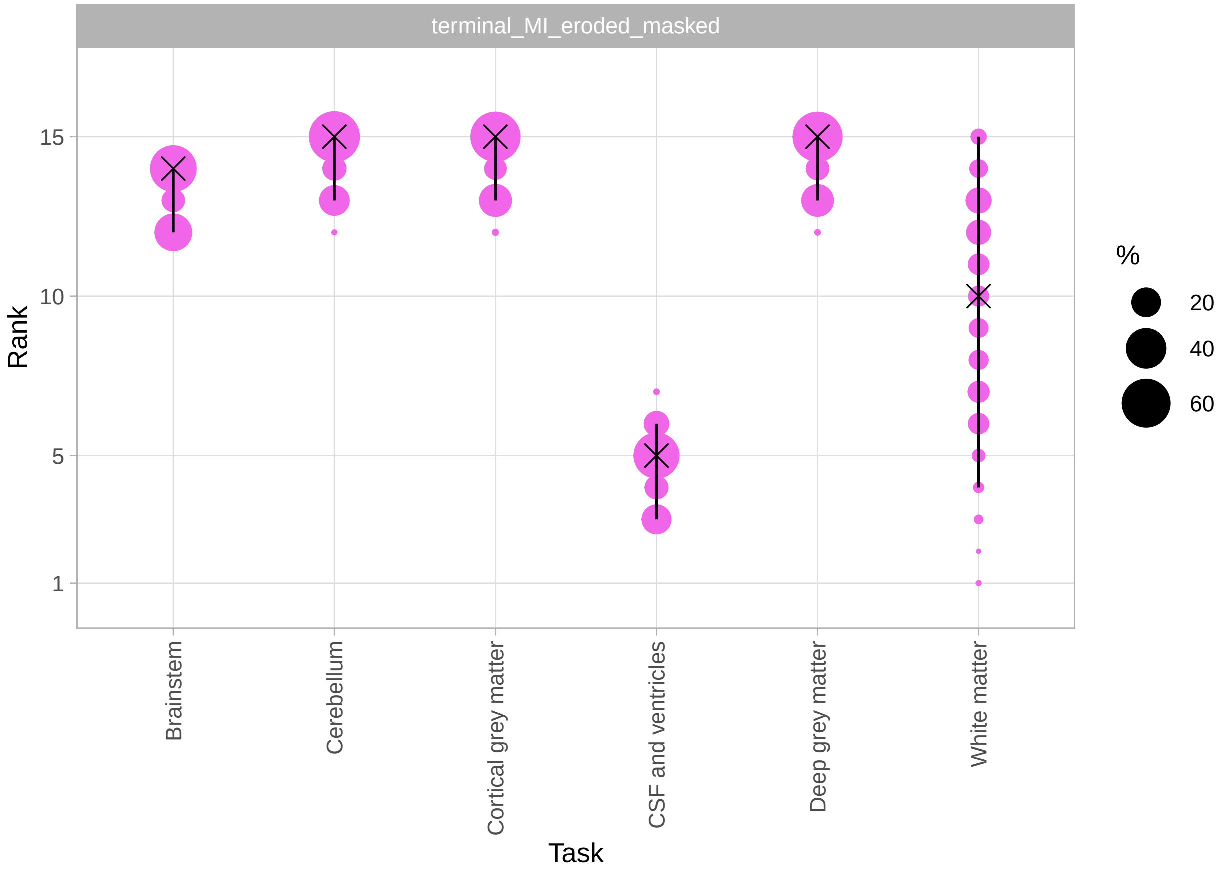

terminal_MI_eroded_masked
Rank
15
10
5
1
Brainstem
Cerebellum
Cortical grey matter
CSF and ventricles
Deep grey matter
White matter
Task
%
20
40
60

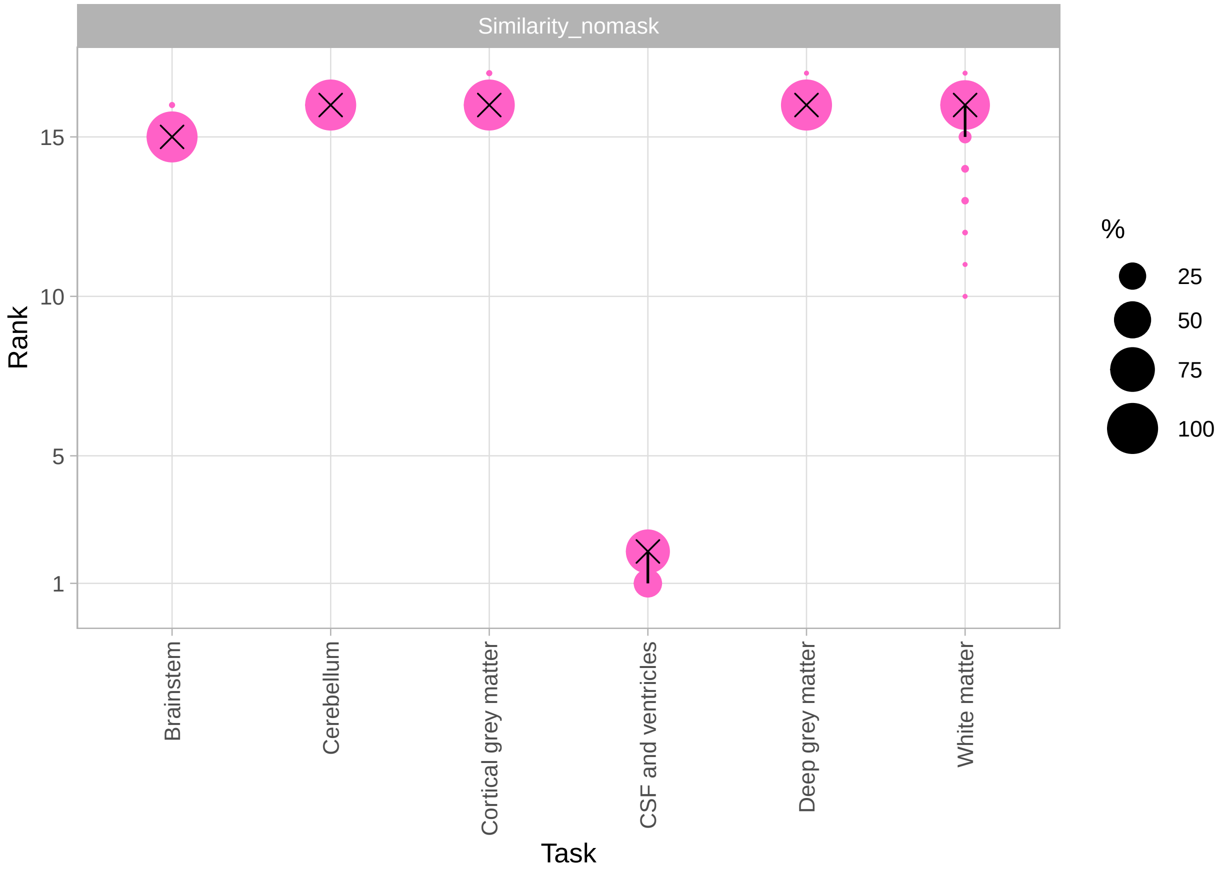
Similarity_nomask
Rank
15
10
5
1
Brainstem
Cerebellum
Cortical grey matter
CSF and ventricles
Deep grey matter
White matter
Task
%
25
50
75
100

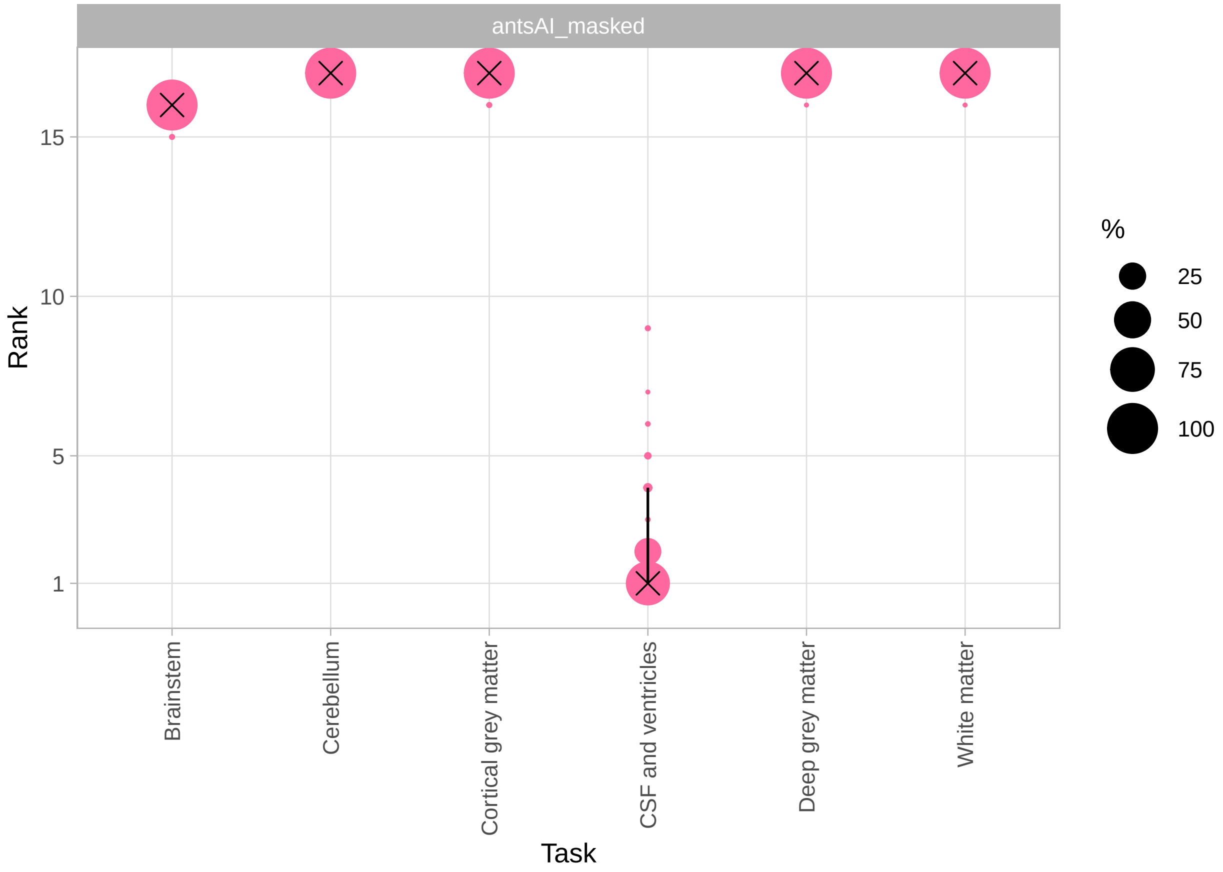

antsAI_masked
Rank
15
10
5
1
%
25
50
75
100
Brainstem
Cerebellum
Cortical grey matter
CSF and ventricles
Deep grey matter
White matter
Task

An alternative representation is provided by a stacked frequency plot of the observed ranks, separated by algorithm. Observed ranks across bootstrap samples are displayed with coloring according to the task. For algorithms that achieve the same rank in different tasks for the full assessment data set, vertical lines are on top of each other. Vertical lines allow to compare the achieved rank of each algorithm over different tasks.

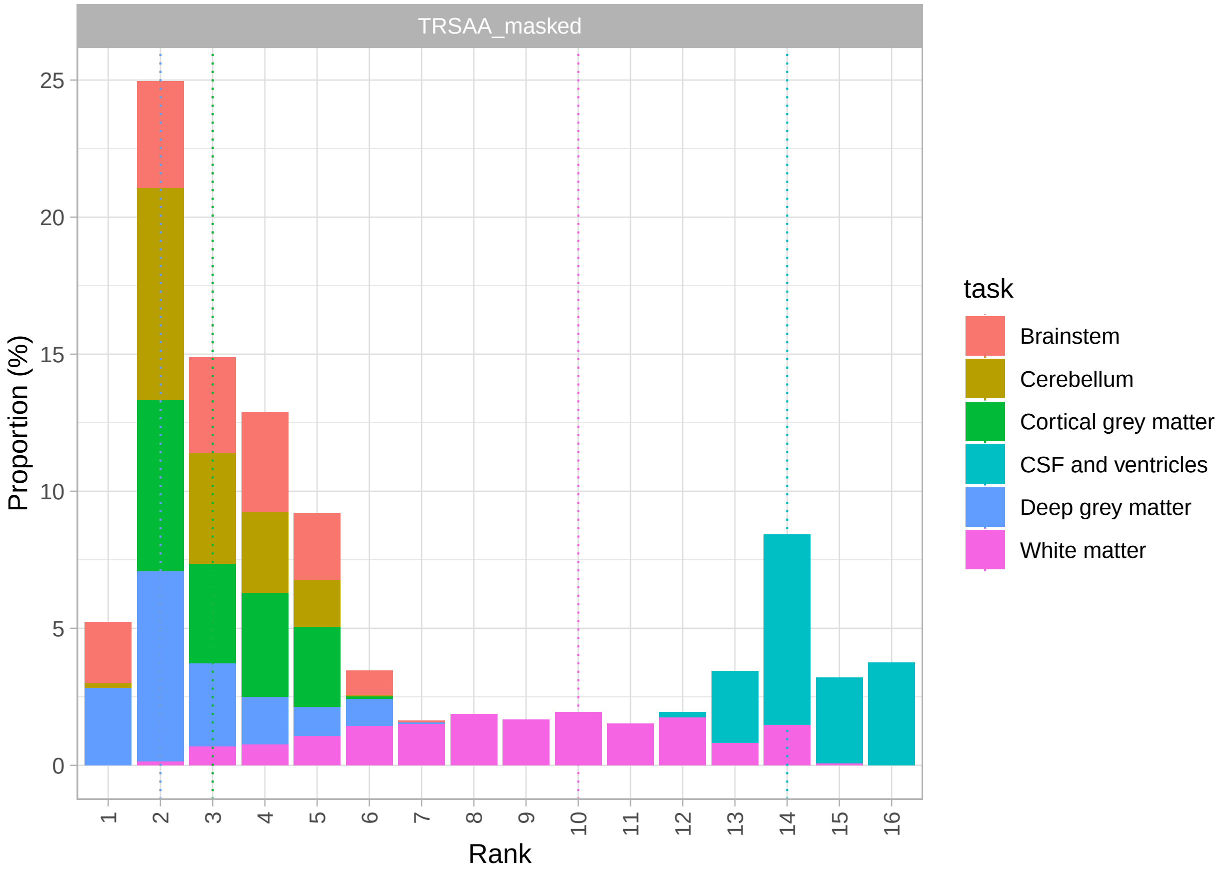

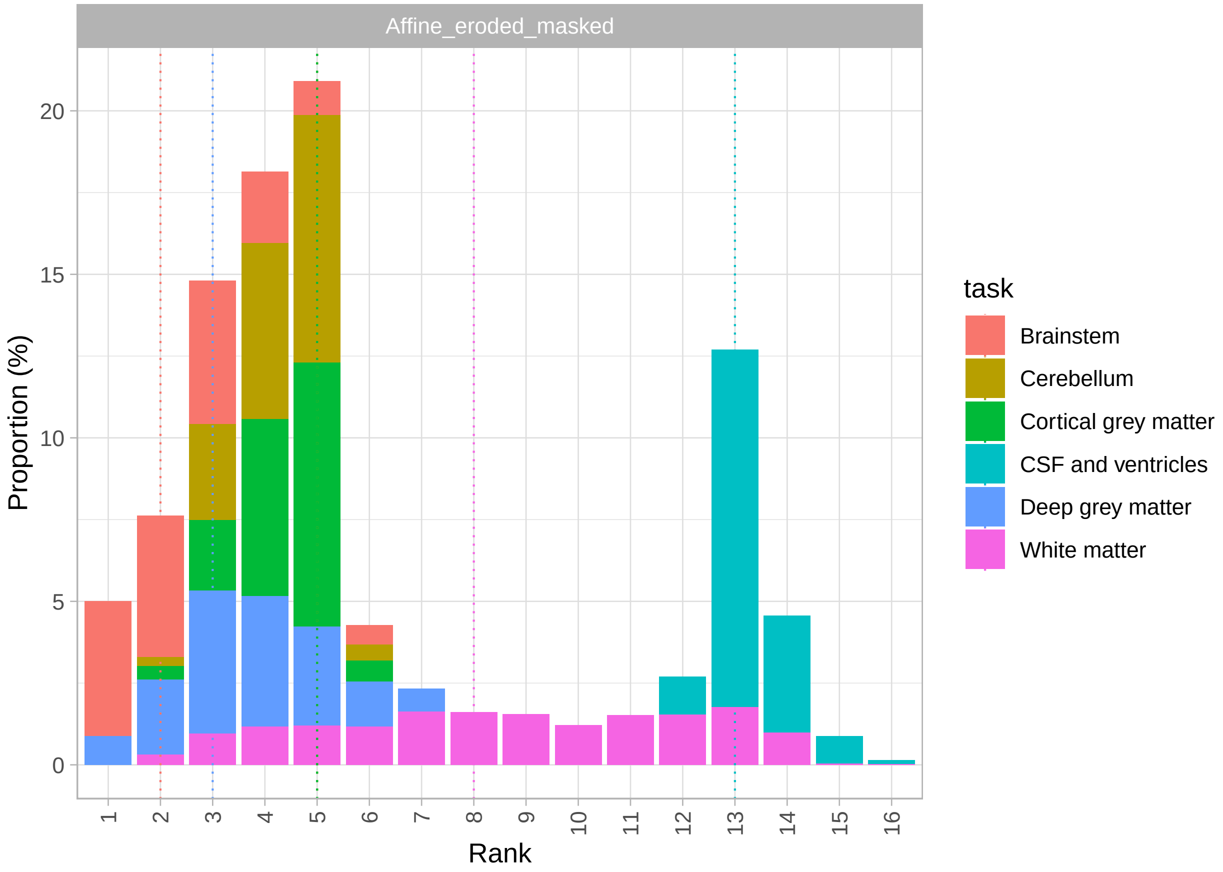

Affine_eroded_masked
Proportion (%)
20
15
10
5
0
1
2
3
4
5
6
7
8
9
10
11
12
13
14
15
16
Rank
task
Brainstem
Cerebellum
Cortical grey matter
CSF and ventricles
Deep grey matter
White matter

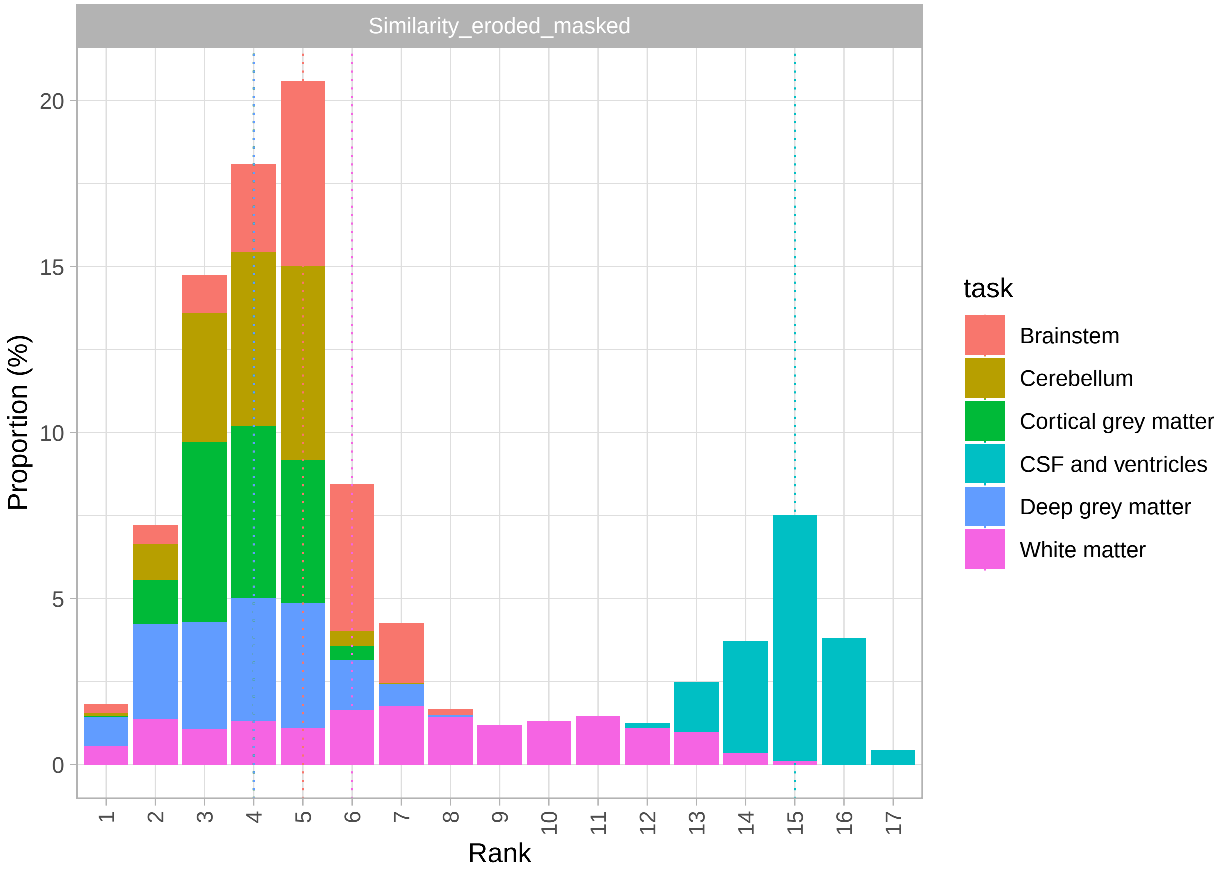

Similarity_eroded_masked
Proportion (%)
20
15
10
5
0
1
2
3
4
5
6
7
8
9
10
11
12
13
14
15
16
17
Rank
task
Brainstem
Cerebellum
Cortical grey matter
CSF and ventricles
Deep grey matter
White matter

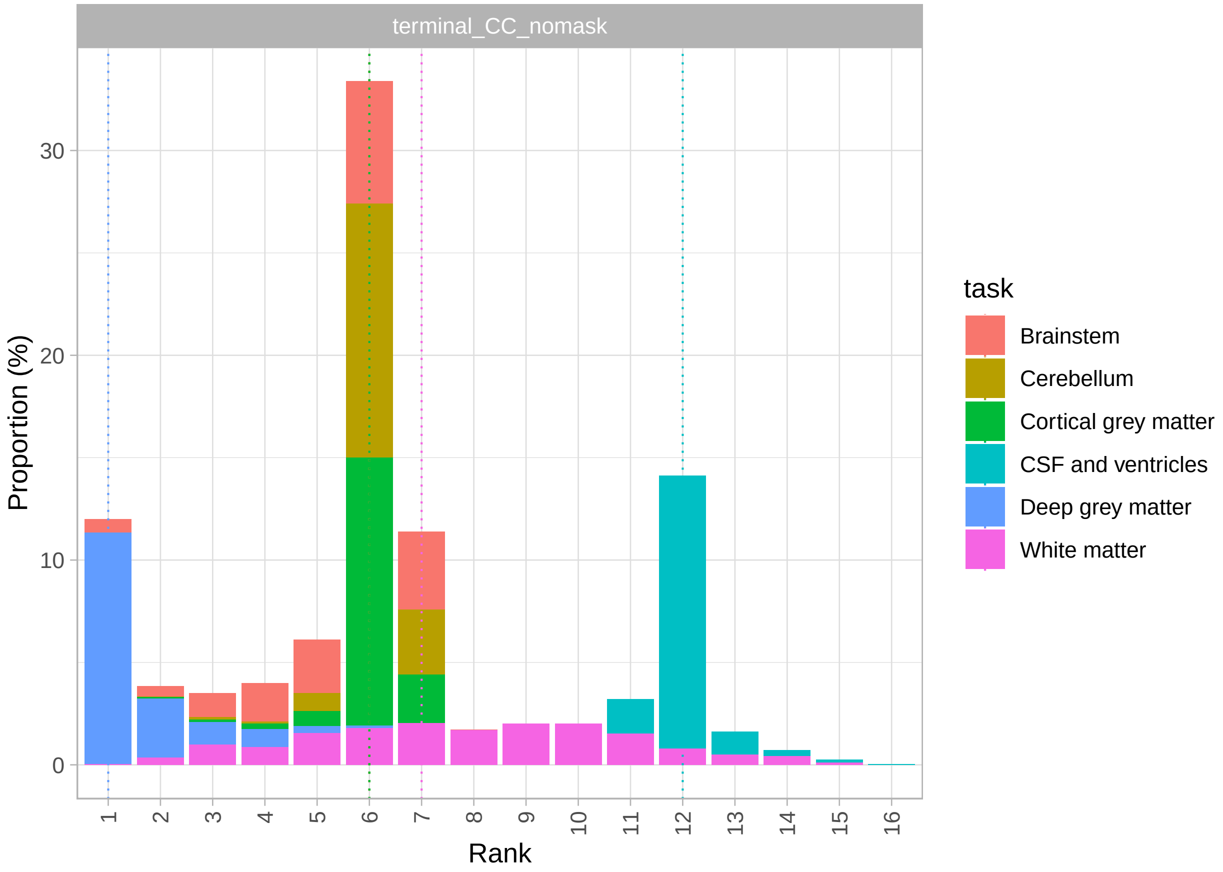

terminal_CC_nomask
Proportion (%)
30
20
10
0
1
2
3
4
5
6
7
8
9
10
11
12
13
14
15
16
Rank
task
Brainstem
Cerebellum
Cortical grey matter
CSF and ventricles
Deep grey matter
White matter

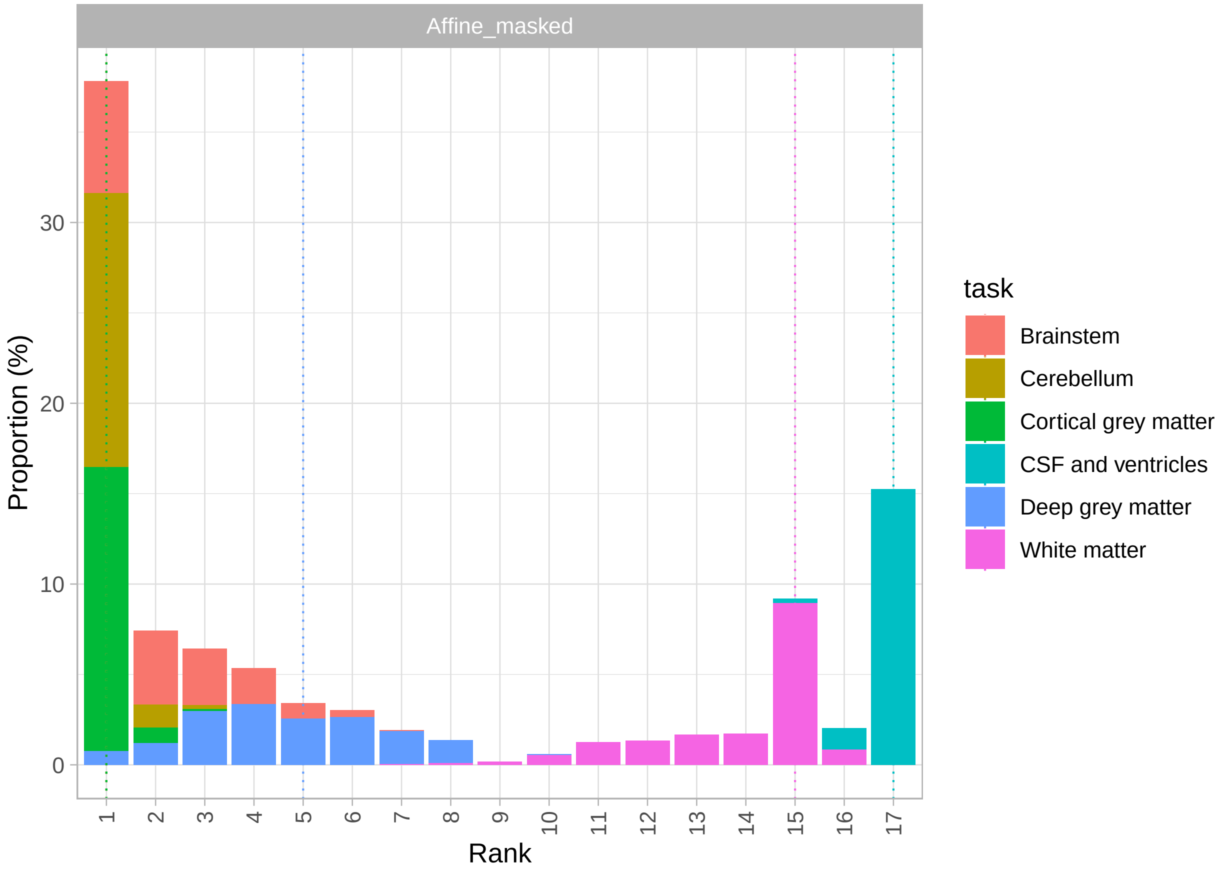
Affine_masked
Proportion (%)
0
10
20
30
Rank
1
2
3
4
5
6
7
8
9
10
11
12
13
14
15
16
17
task
Brainstem
Cerebellum
Cortical grey matter
CSF and ventricles
Deep grey matter
White matter

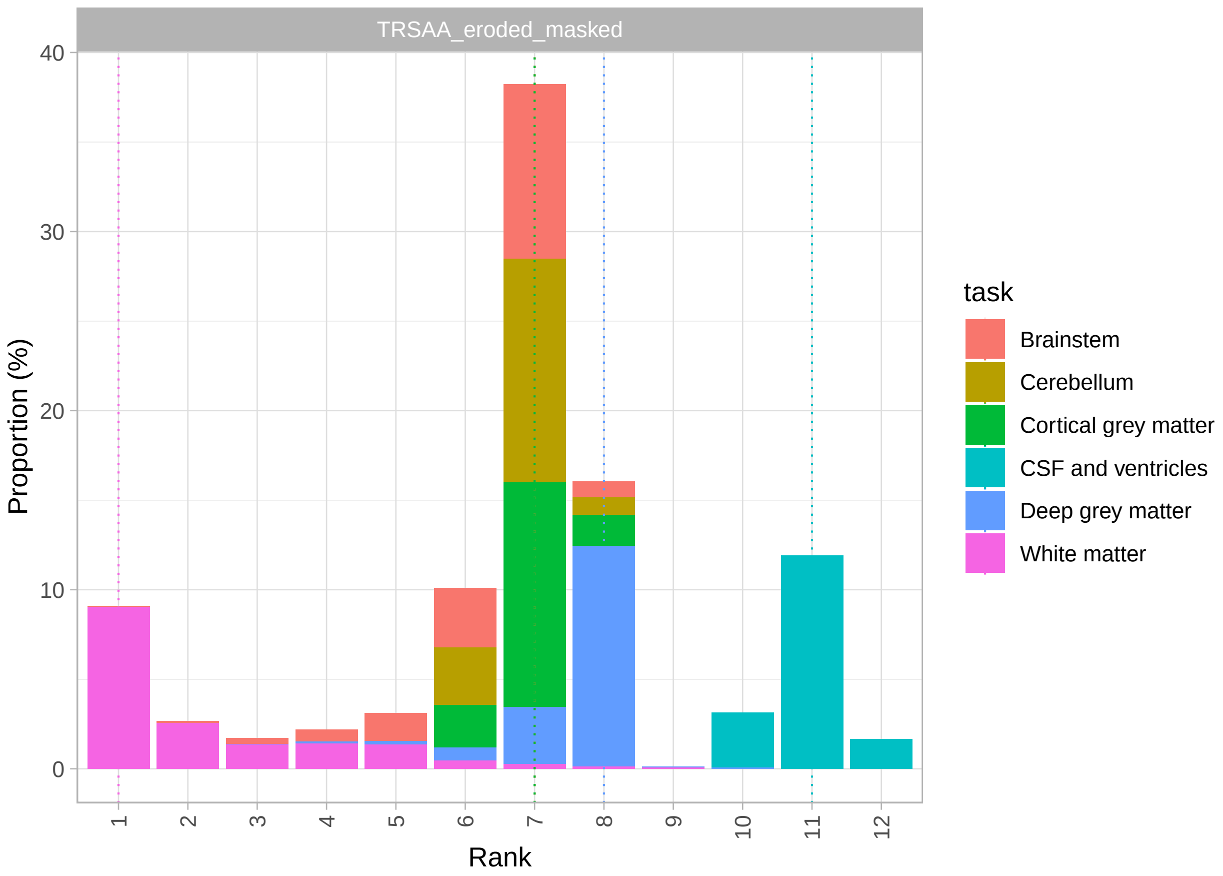

TRSAA_eroded_masked
40
30
20
10
0
Proportion (%)
1
2
3
4
5
6
7
8
9
10
11
12
Rank
task
Brainstem
Cerebellum
Cortical grey matter
CSF and ventricles
Deep grey matter
White matter

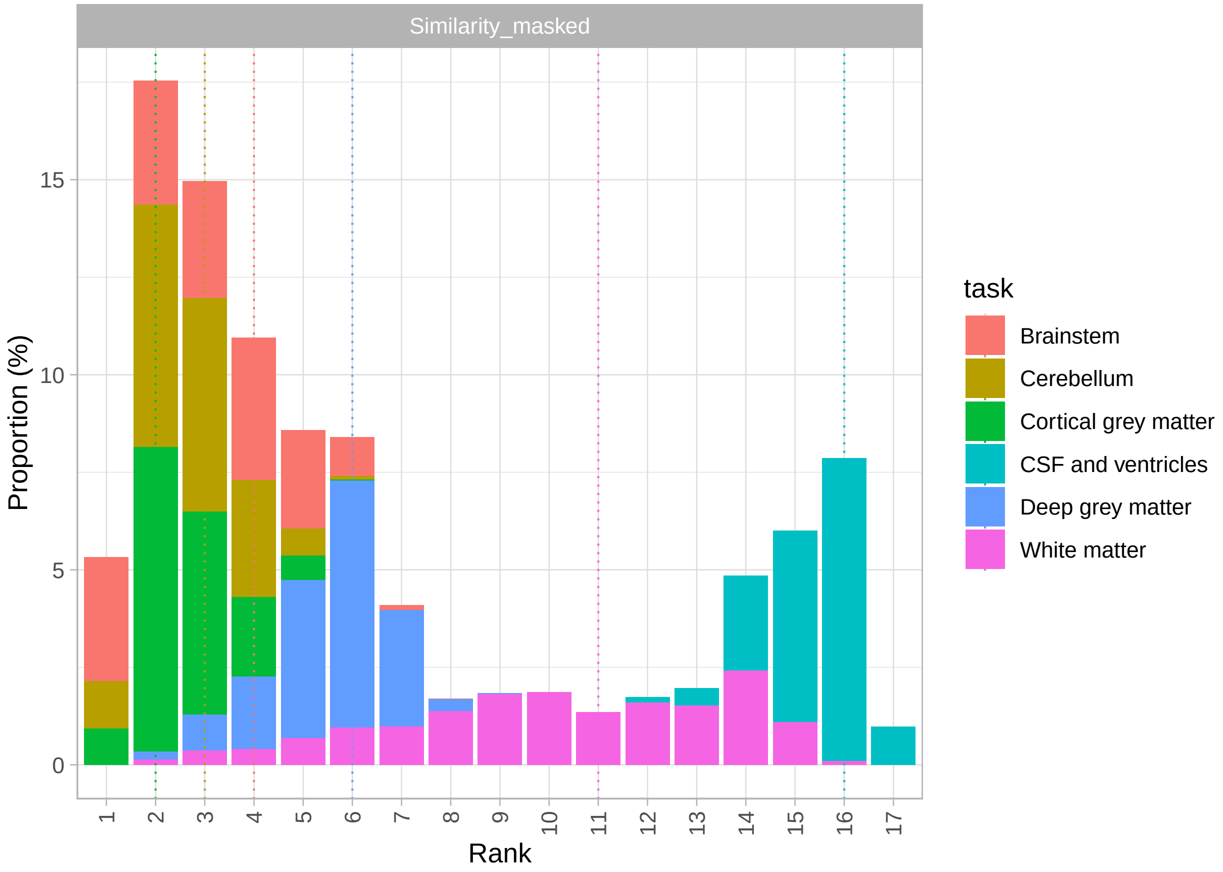

Similarity_masked
Proportion (%)
15
10
5
0
1
2
3
4
5
6
7
8
9
10
11
12
13
14
15
16
17
Rank
task
Brainstem
Cerebellum
Cortical grey matter
CSF and ventricles
Deep grey matter
White matter

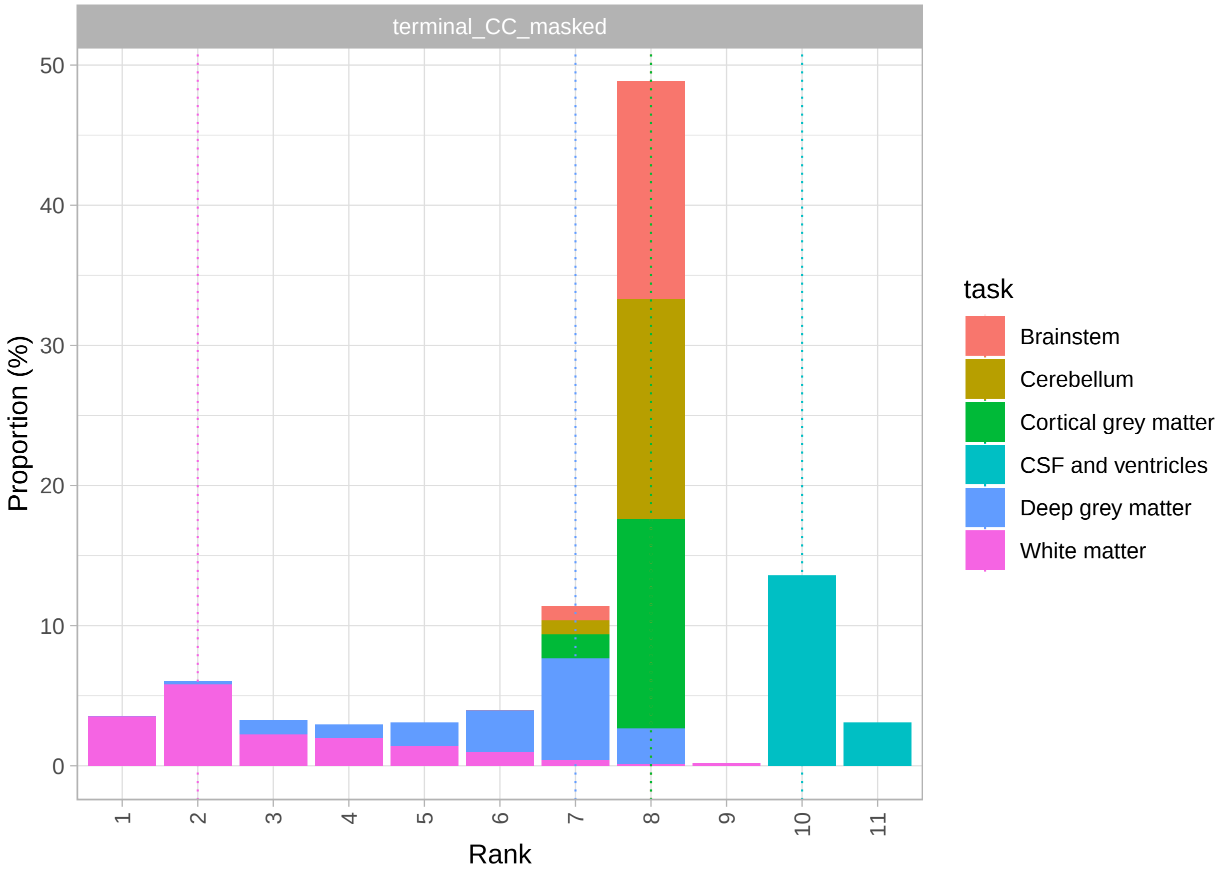
terminal_CC_masked
Proportion (%)
50
40
30
20
10
0
1
2
3
4
5
6
7
8
9
10
11
Rank
task
Brainstem
Cerebellum
Cortical grey matter
CSF and ventricles
Deep grey matter
White matter

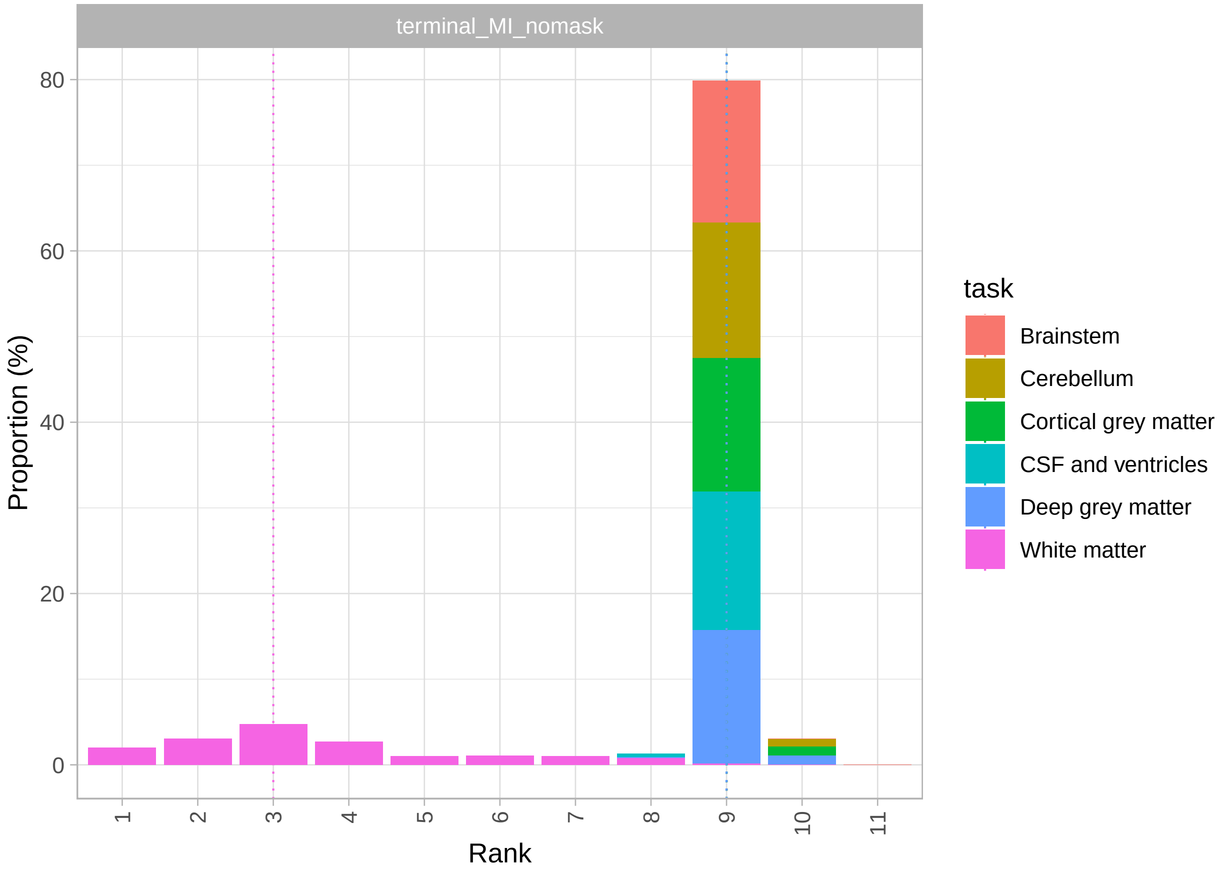
terminal_MI_nomask
Proportion (%)
80
60
40
20
0
1
2
3
4
5
6
7
8
9
10
11
Rank
task
Brainstem
Cerebellum
Cortical grey matter
CSF and ventricles
Deep grey matter
White matter

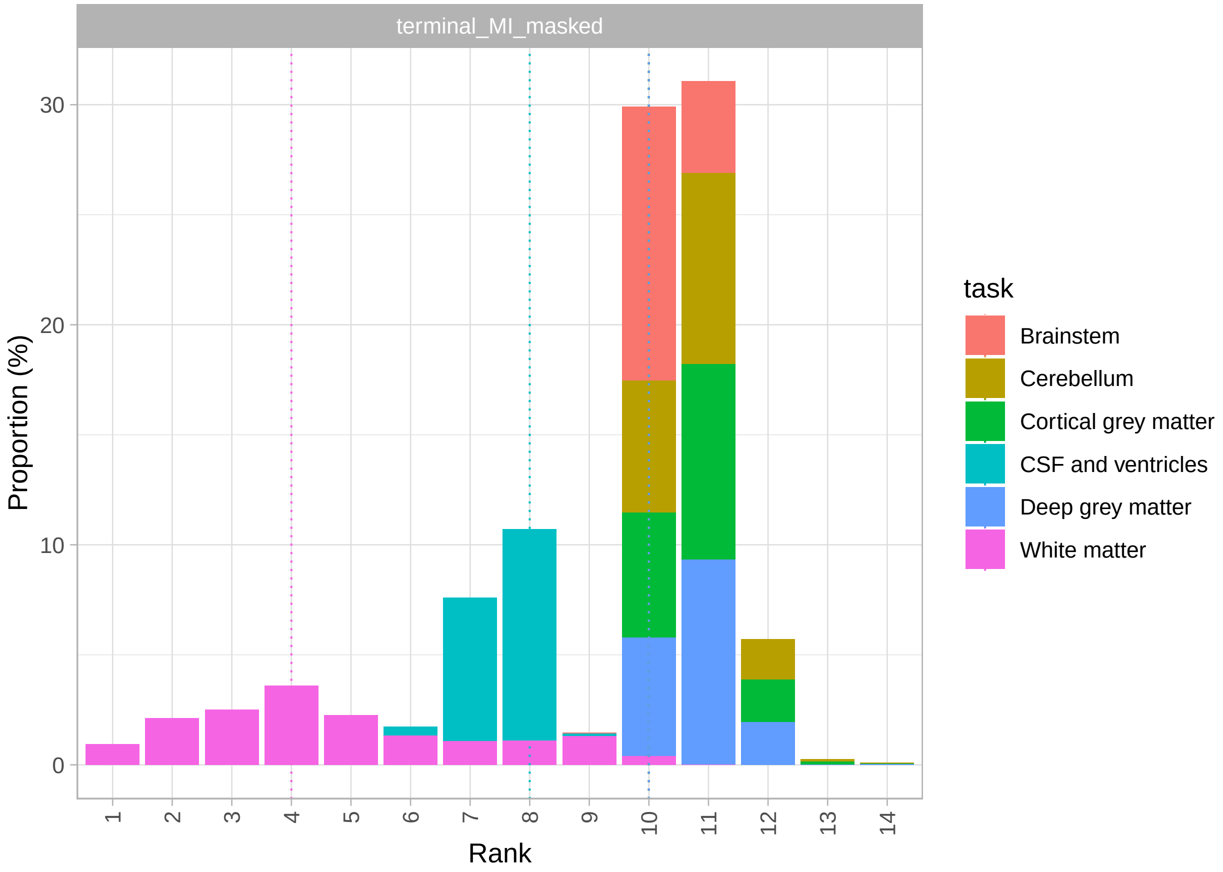
terminal_MI_masked
Proportion (%)
30
20
10
0
1
2
3
4
5
6
7
8
9
10
11
12
13
14
Rank
task
Brainstem
Cerebellum
Cortical grey matter
CSF and ventricles
Deep grey matter
White matter

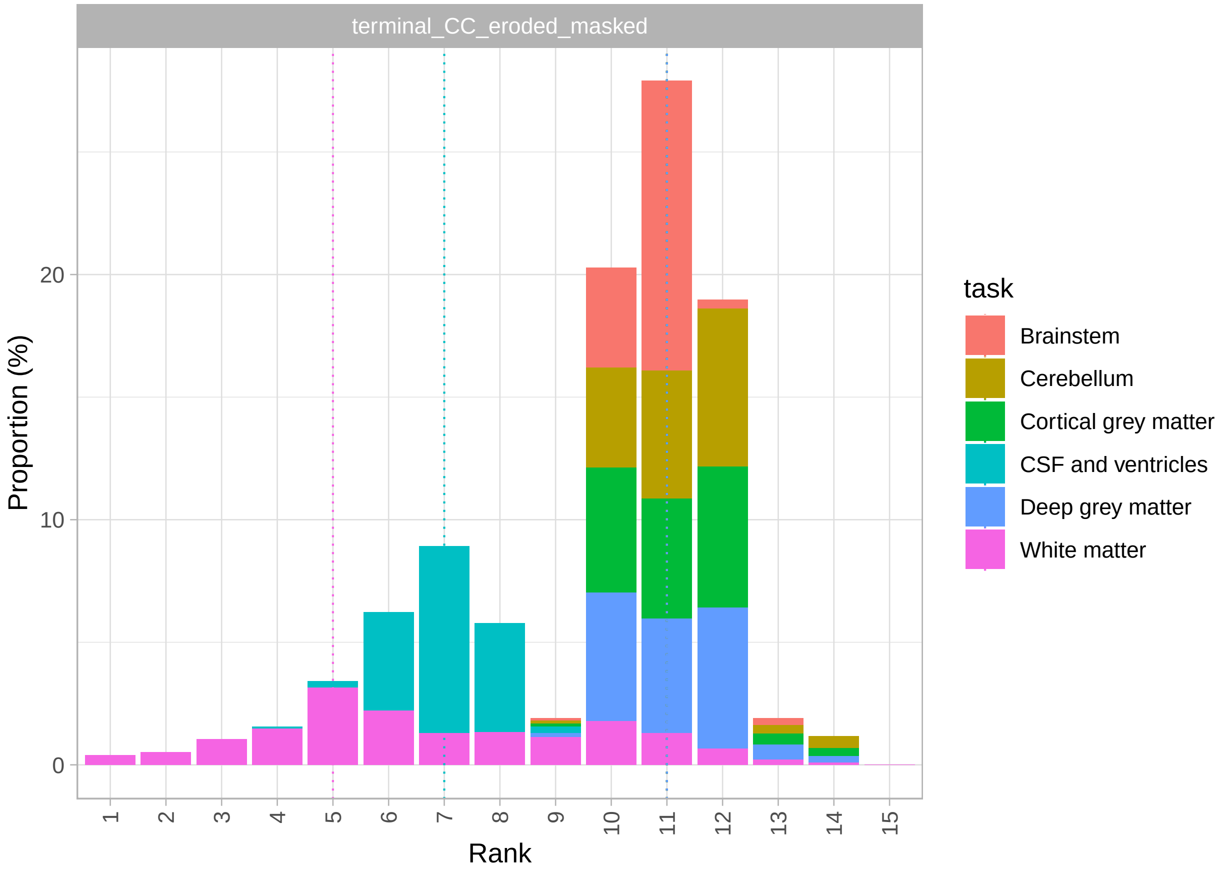
terminal_CC_eroded_masked
Proportion (%)
0
10
20
1
2
3
4
5
6
7
8
9
10
11
12
13
14
15
Rank
task
Brainstem
Cerebellum
Cortical grey matter
CSF and ventricles
Deep grey matter
White matter

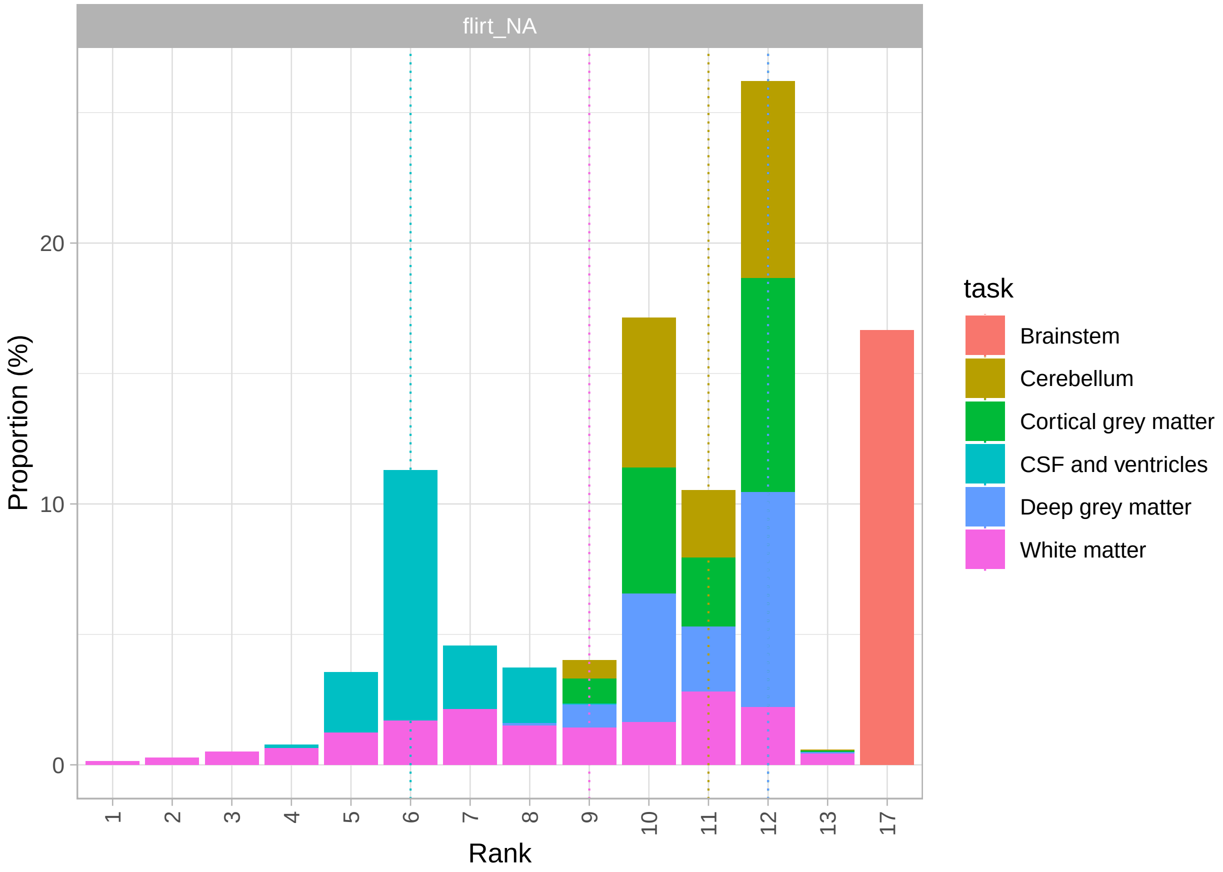
flirt_NA
Proportion (%)
0
10
20
Rank
1
2
3
4
5
6
7
8
9
10
11
12
13
17
task
Brainstem
Cerebellum
Cortical grey matter
CSF and ventricles
Deep grey matter
White matter

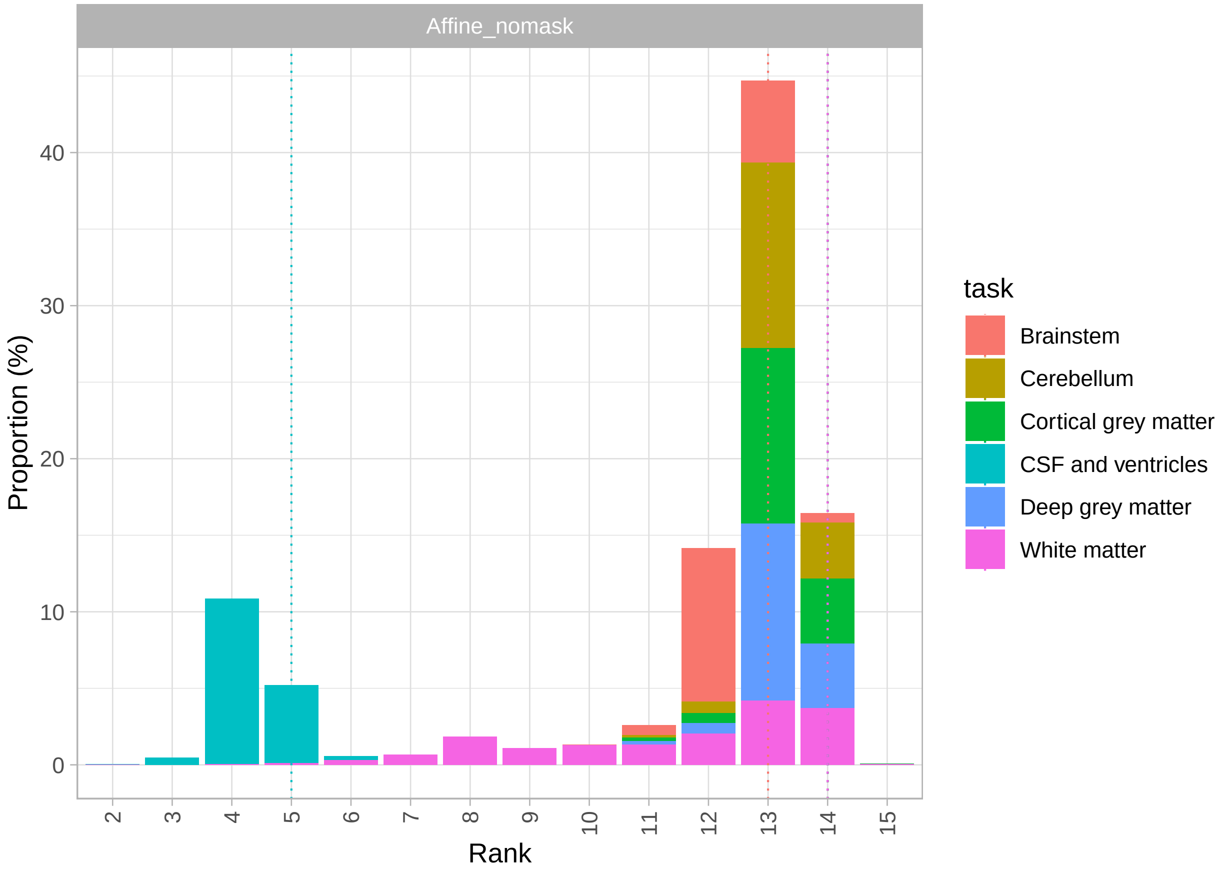
Affine_nomask
Proportion (%)
40
30
20
10
0
2
3
4
5
6
7
8
9
10
11
12
13
14
15
Rank
task
Brainstem
Cerebellum
Cortical grey matter
CSF and ventricles
Deep grey matter
White matter

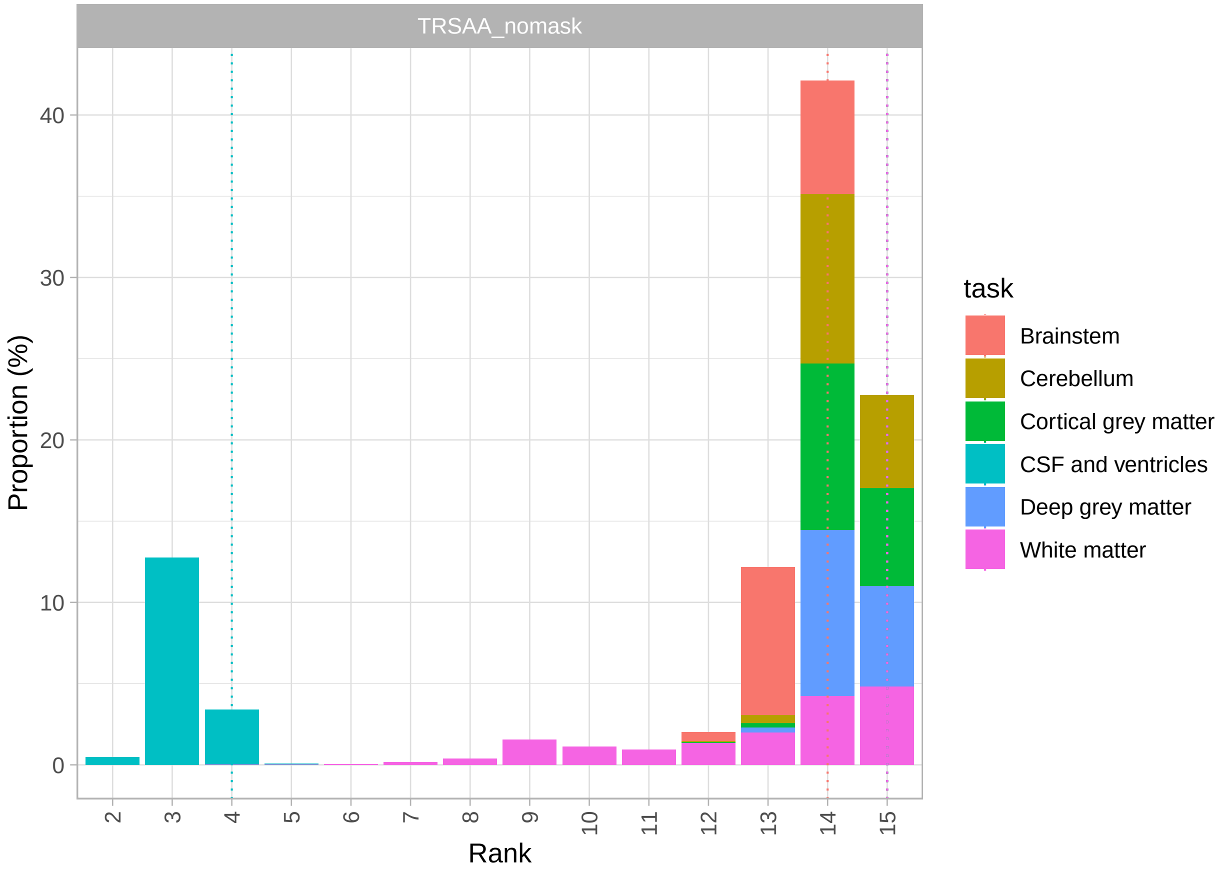

TRSAA_nomask
Proportion (%)
0
10
20
30
40
2
3
4
5
6
7
8
9
10
11
12
13
14
15
Rank
task
Brainstem
Cerebellum
Cortical grey matter
CSF and ventricles
Deep grey matter
White matter

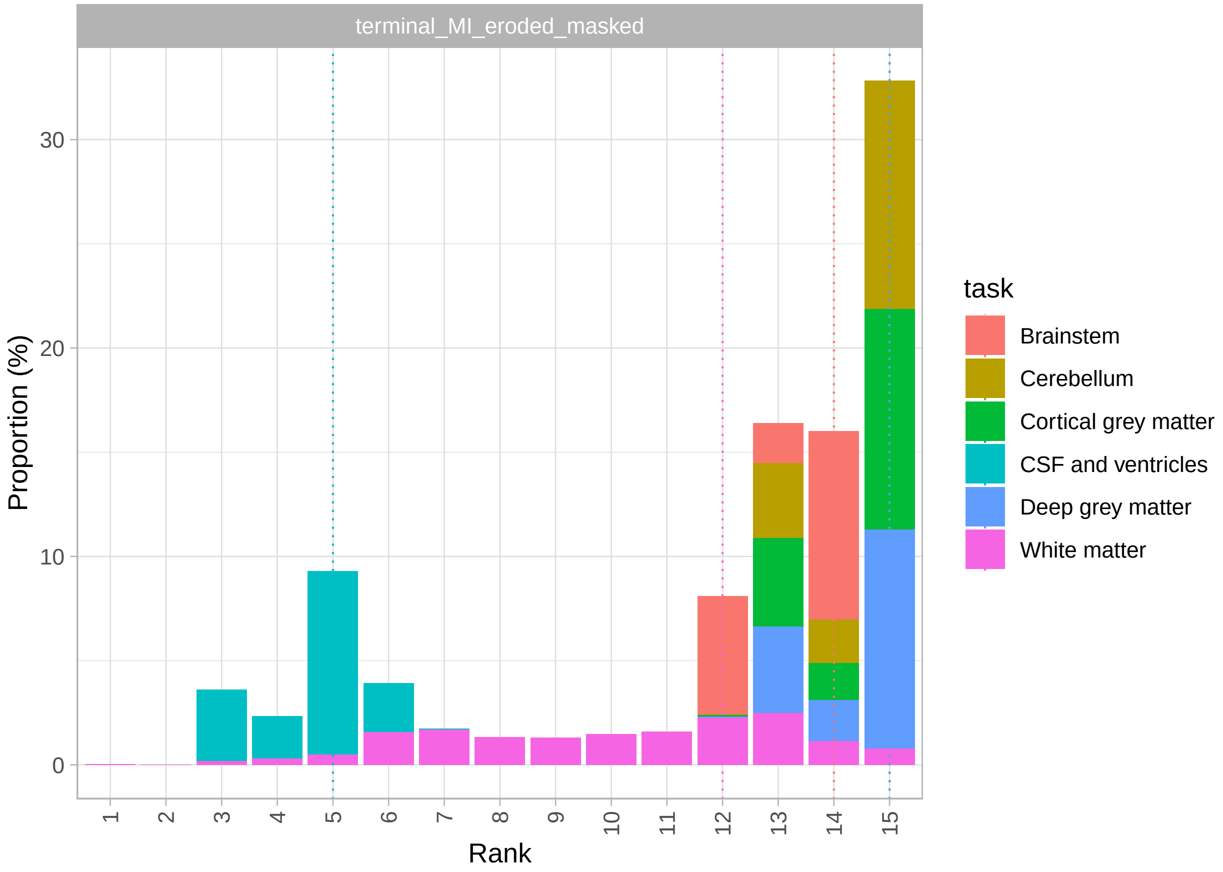

terminal_MI_eroded_masked
Proportion (%)
0
10
20
30
1
2
3
4
5
6
7
8
9
10
11
12
13
14
15
Rank
task
Brainstem
Cerebellum
Cortical grey matter
CSF and ventricles
Deep grey matter
White matter

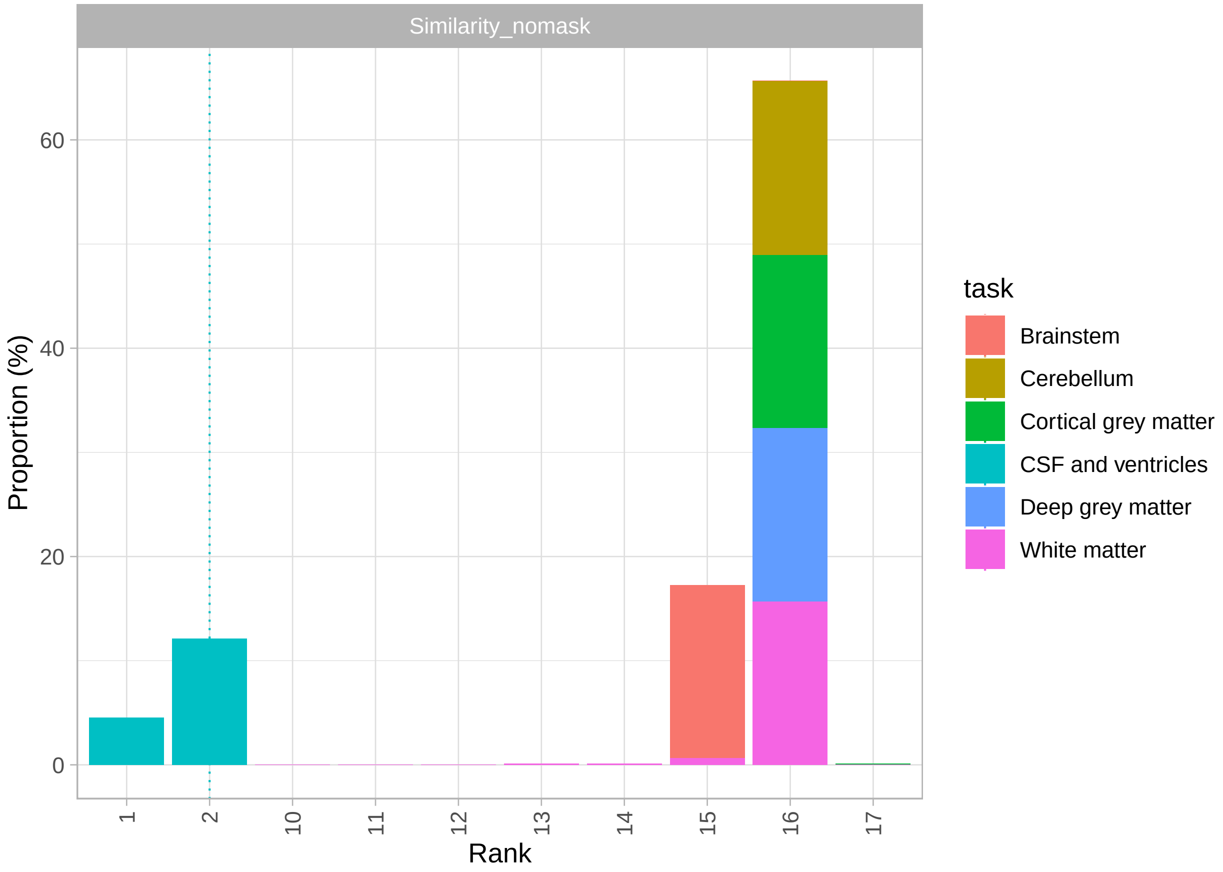
Similarity_nomask
Proportion (%)
60
40
20
0
1
2
10
11
12
13
14
15
16
17
Rank
task
Brainstem
Cerebellum
Cortical grey matter
CSF and ventricles
Deep grey matter
White matter

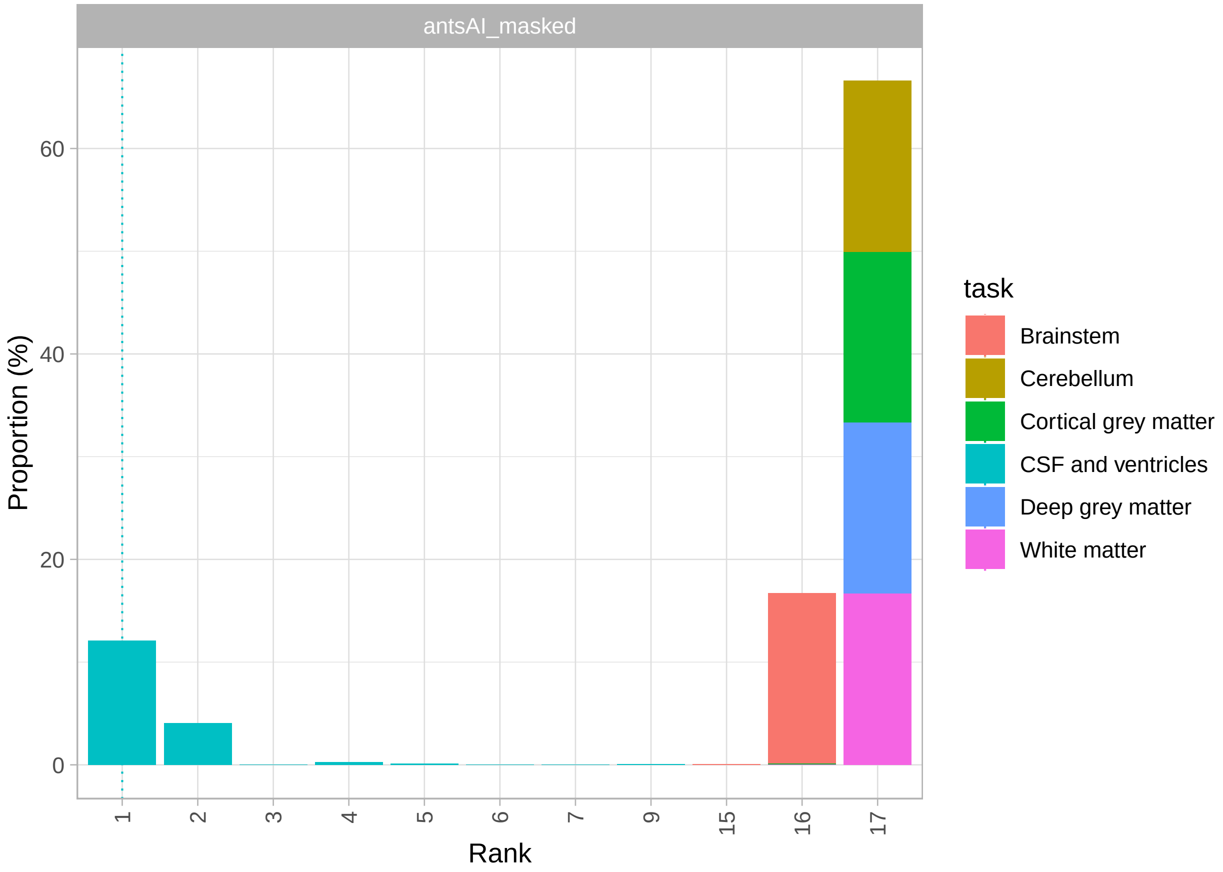
antsAI_masked
Proportion (%)
60
40
20
0
1
2
3
4
5
6
7
9
15
16
17
Rank
task
Brainstem
Cerebellum
Cortical grey matter
CSF and ventricles
Deep grey matter
White matter

4.2 Characterization of tasks

4.2.1 Visualizing bootstrap results

To investigate which tasks separate algorithms well (i.e., lead to a stable ranking), a blob plot is recommended.

Bootstrap results can be shown in a blob plot showing one plot for each task. In this view, the spread of the blobs for each algorithm can be compared across tasks. Deviations from the diagonal indicate deviations from the consensus ranking (over tasks). Specifically, if rank distribution of an algorithm is consistently below the diagonal, the algorithm performed better in this task than on average across tasks, while if the rank distribution of an algorithm is consistently above the diagonal, the algorithm performed worse in this task than on average across tasks. At the bottom of each panel, ranks for each algorithm in the tasks are provided.

Same as in Section 3.1 but now ordered according to consensus.

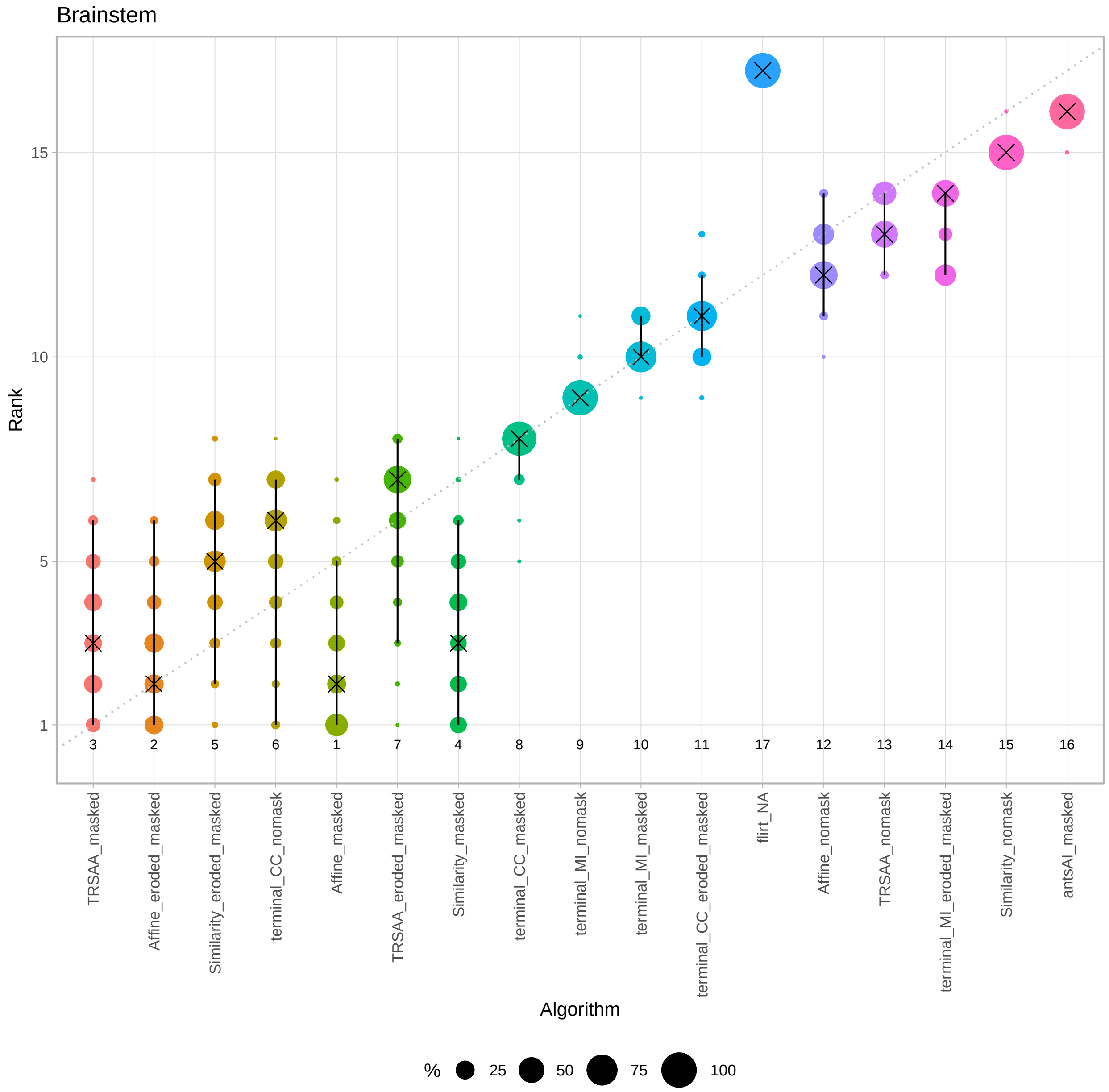

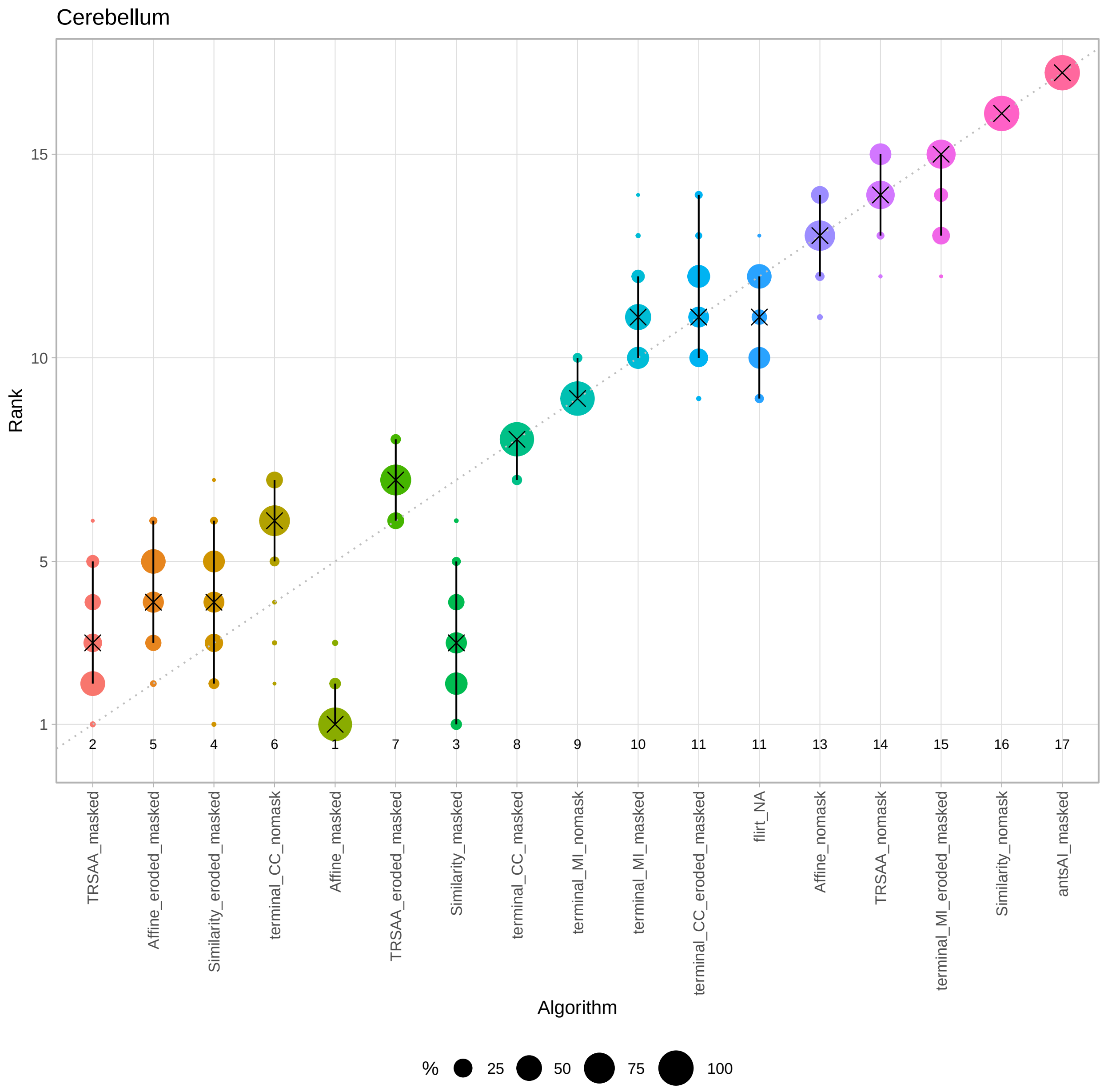

Cerebellum
Rank
15
10
5
1
2
5
4
6
1
7
3
8
9
10
11
11
13
14
15
16
17
TRSAA_masked
Affine_eroded_masked
Similarity_eroded_masked
terminal_CC_nomask
Affine_masked
TRSAA_eroded_masked
Similarity_masked
terminal_CC_masked
terminal_MI_nomask
terminal_MI_masked
terminal_CC_eroded_masked
flirt_NA
Affine_nomask
TRSAA_nomask
terminal_MI_eroded_masked
Similarity_nomask
antsAI_masked
Algorithm
%
25
50
75
100

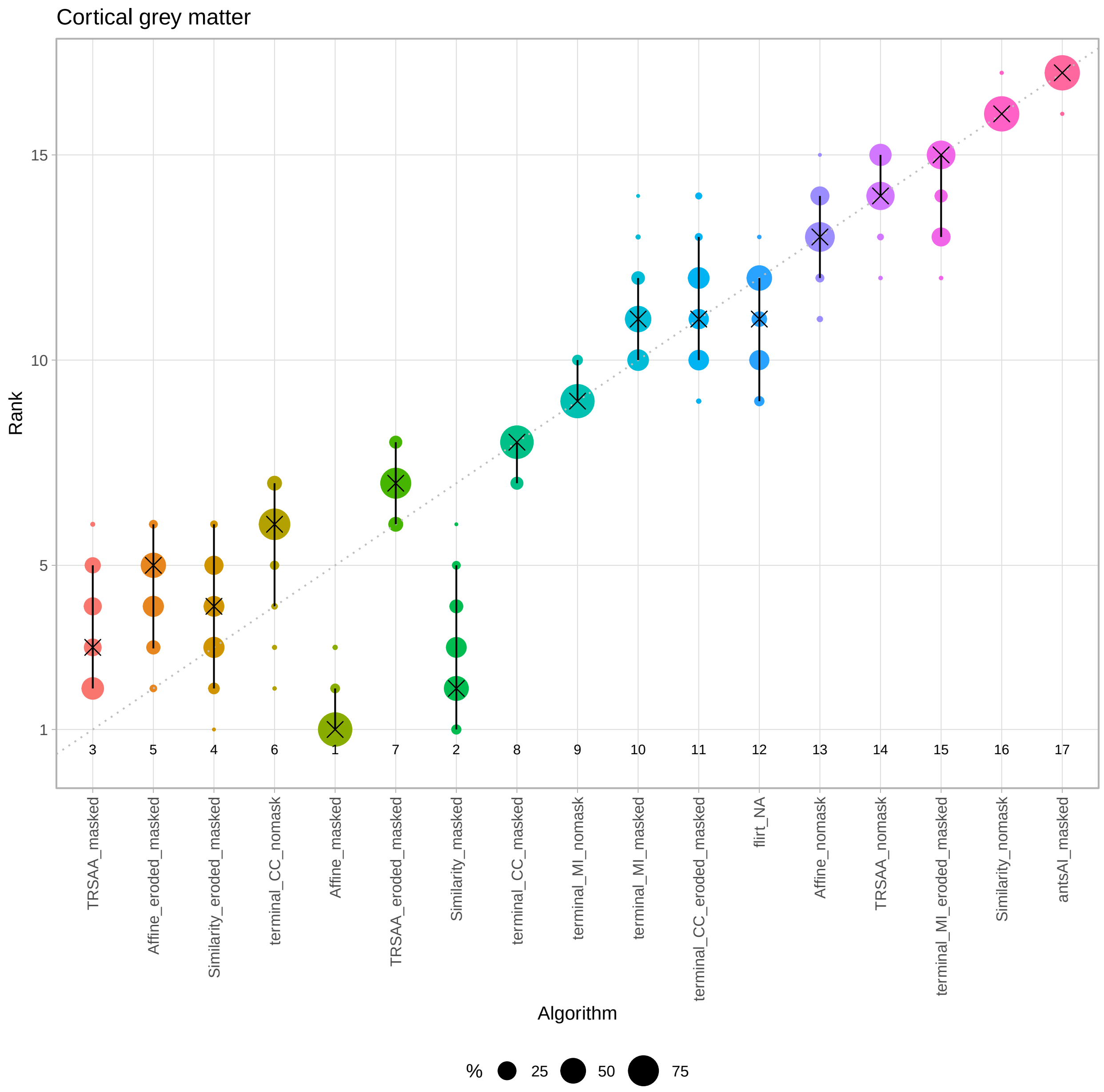

Cortical grey matter
Rank
15
10
5
1
3
5
4
6
1
7
2
8
9
10
11
12
13
14
15
16
17
TRSAA_masked
Affine_eroded_masked
Similarity_eroded_masked
terminal_CC_nomask
Affine_masked
TRSAA_eroded_masked
Similarity_masked
terminal_CC_masked
terminal_MI_nomask
terminal_MI_masked
terminal_CC_eroded_masked
flirt_NA
Affine_nomask
TRSAA_nomask
terminal_MI_eroded_masked
Similarity_nomask
antsAI_masked
Algorithm
% 25 50 75

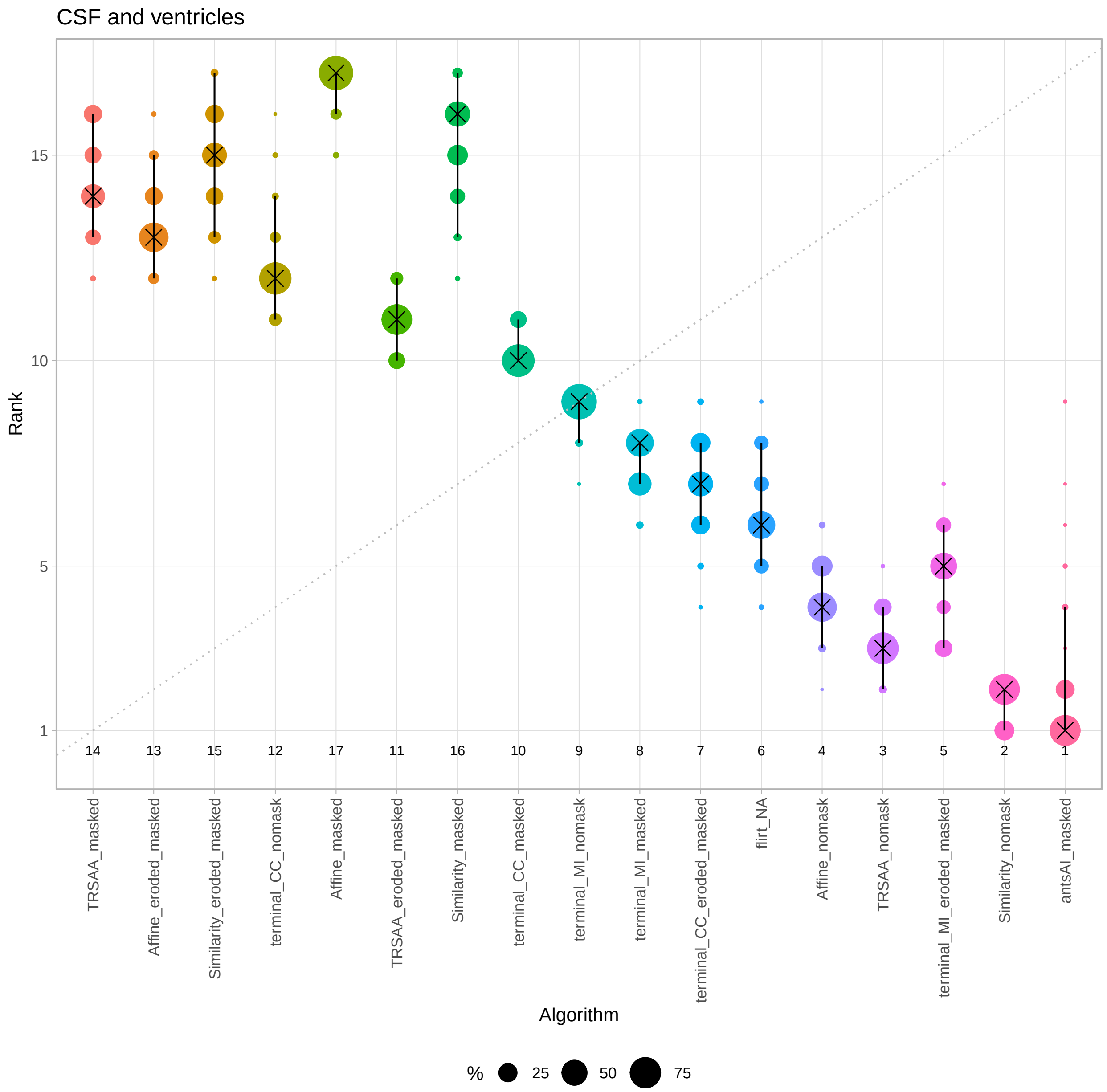
CSF and ventricles
Rank
15
10
5
1
14
13
15
12
17
11
16
10
9
8
7
6
4
3
5
2
1
TRSAA_masked
Affine_eroded_masked
Similarity_eroded_masked
terminal_CC_nomask
Affine_masked
TRSAA_eroded_masked
Similarity_masked
terminal_CC_masked
terminal_MI_nomask
terminal_MI_masked
terminal_CC_eroded_masked
flirt_NA
Affine_nomask
TRSAA_nomask
terminal_MI_eroded_masked
Similarity_nomask
antsAI_masked
Algorithm
%
25
50
75

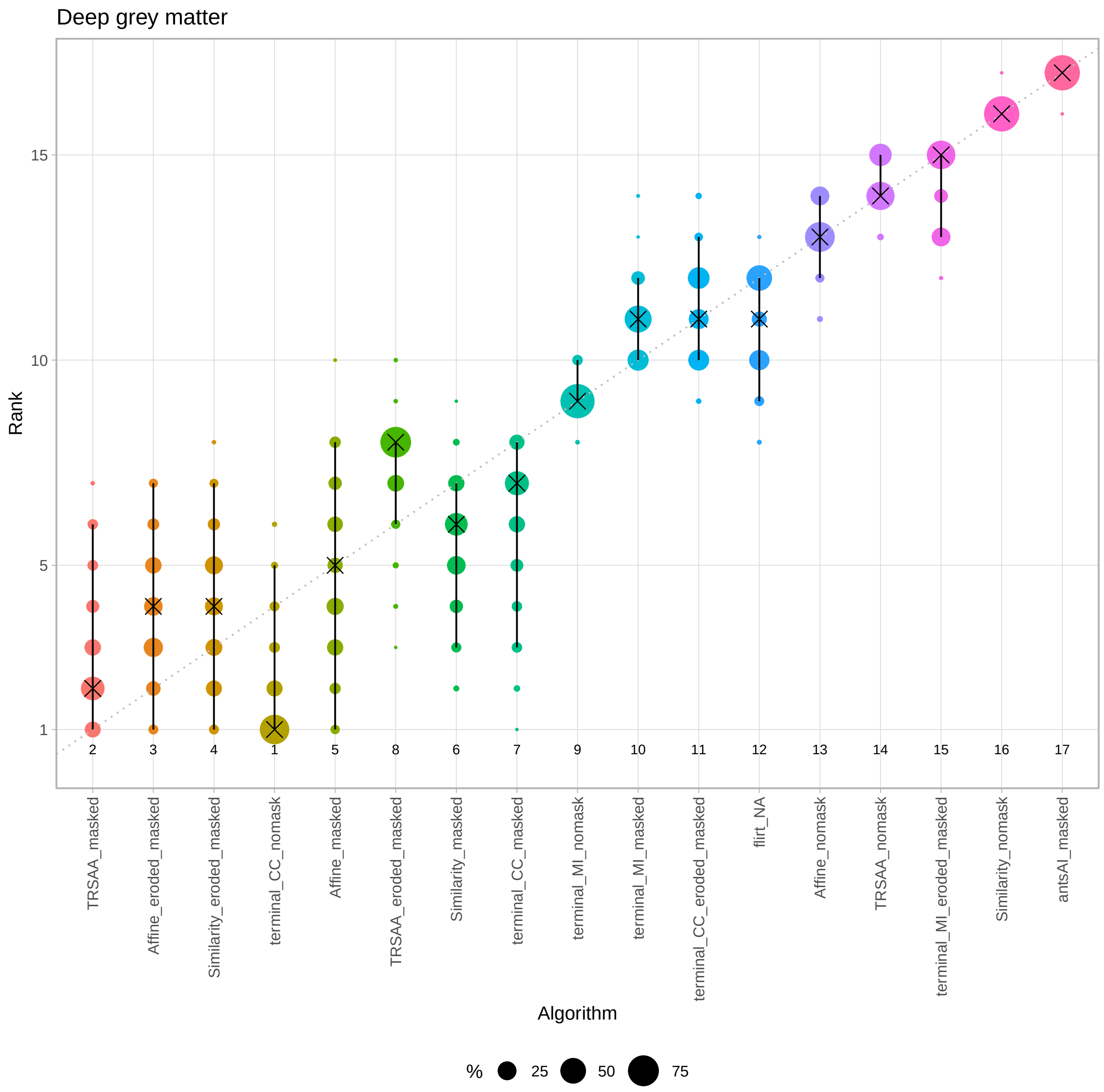
Deep grey matter
Rank
15
10
5
1
2
3
4
1
5
8
6
7
9
10
11
12
13
14
15
16
17
TRSAA_masked
Affine_eroded_masked
Similarity_eroded_masked
terminal_CC_nomask
Affine_masked
TRSAA_eroded_masked
Similarity_masked
terminal_CC_masked
terminal_MI_nomask
terminal_MI_masked
terminal_CC_eroded_masked
flirt_NA
Affine_nomask
TRSAA_nomask
terminal_MI_eroded_masked
Similarity_nomask
antsAI_masked
Algorithm
%
25
50
75

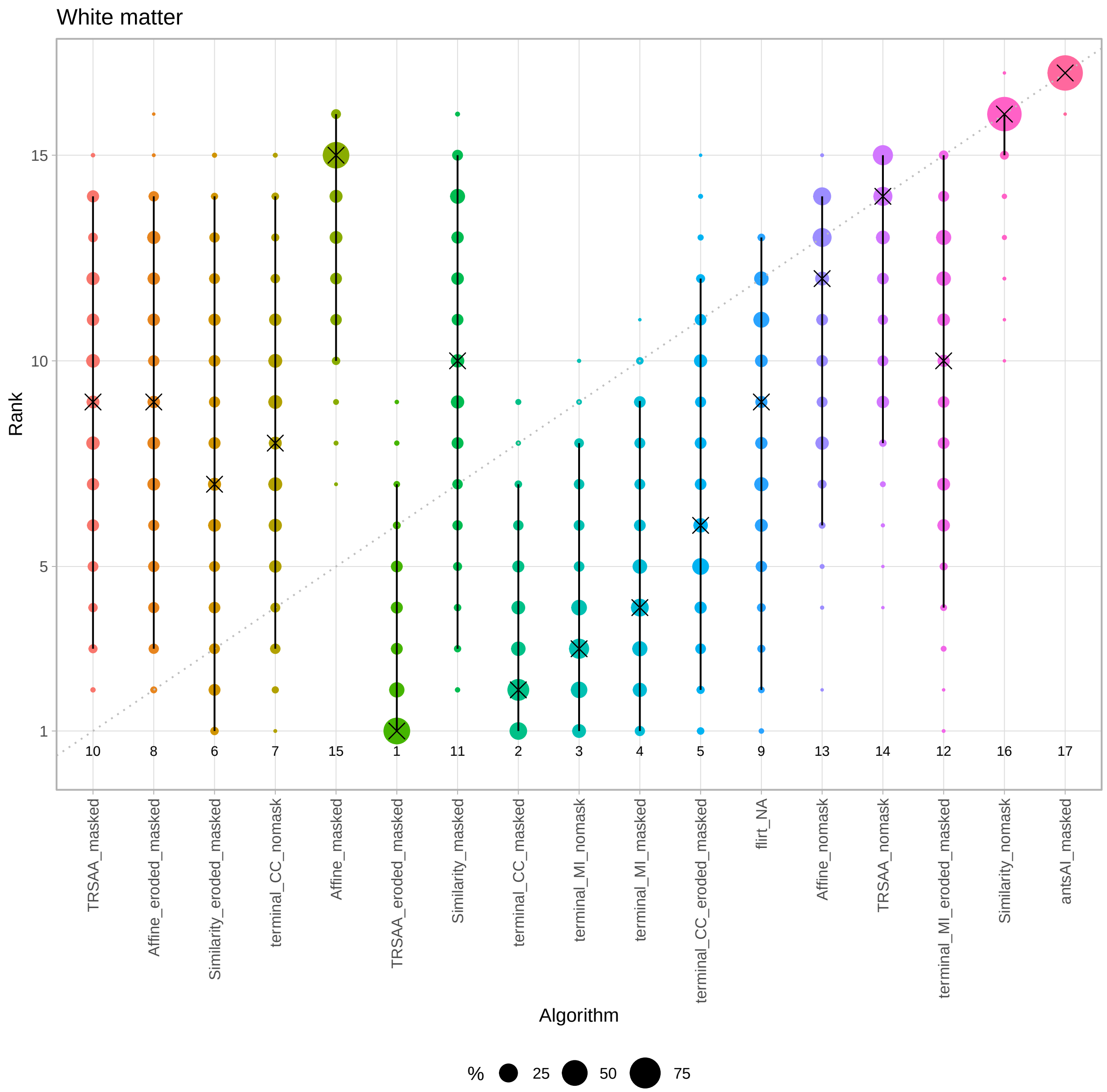

4.2.2 Cluster Analysis

Dendrogram from hierarchical cluster analysis and *network-type graphs* for assessing the similarity of tasks based on challenge rankings.

A dendrogram is a visualization approach based on hierarchical clustering. It depicts clusters according to a chosen distance measure (here: Spearman's footrule) as well as a chosen agglomeration method (here: complete and average agglomeration).

Cluster Dendrogram (complete agglomeration)

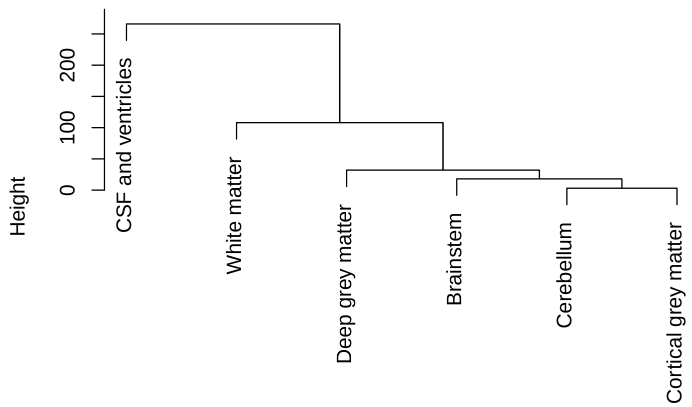

Cluster Dendrogram (average agglomeration)

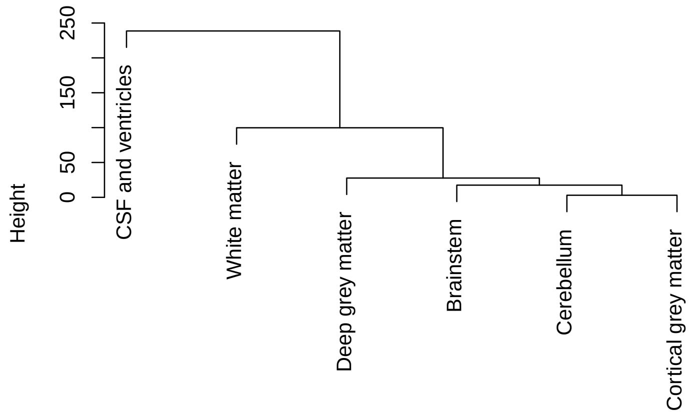

5 References

Wiesenfarth, M., Reinke, A., Landman, B.A., Eisenmann, M., Aguilera Saiz, L., Cardoso, M.J., Maier-Hein, L. and Kopp-Schneider, A. Methods and open-source toolkit for analyzing and visualizing challenge results. *Sci Rep* **11**, 2369 (2021). https://doi.org/10.1038/s41598-021-82017-6

M. J. A. Eugster, T. Hothorn, and F. Leisch, "Exploratory and inferential analysis of benchmark experiments," Institut fuer Statistik, Ludwig-Maximilians-Universitaet Muenchen, Germany, Technical Report 30, 2008. [Online]. Available: http://epub.ub.uni-muenchen.de/4134/.
