# Supplementary material for: Characterization of dynamic patterns of human fetal to neonatal brain asymmetry with deformation-based morphometry
Source: Front Neurosci. 2023 Dec 6;17:1252850. doi: 10.3389/fnins.2023.1252850 (PMC10734644; doi:10.3389/fnins.2023.1252850)
Supplement: Supplementary file 1 [file Data_Sheet_1.ZIP › ChallengeR/Framework B to mid-age template/neonatal2template_nonlinear/ranking_Neonatal2MidTemplate_nonlinear_normal_landmarks.pdf]

Benchmarking report for Neonatal to Template registration nonlinear normal and bleached combined

created by challengeR v1.0.4

31 März, 2023

This document presents a systematic report on the benchmark study "Neonatal to Template registration nonlinear normal and bleached combined". Input data comprises raw metric values for all algorithms and cases. Generated plots are:

- Visualization of assessment data: Dot- and boxplots, podium plots and ranking heatmaps
- Visualization of ranking stability: Blob plots, violin plots and significance maps, line plots
- Visualization of cross-task insights: Blob plots, stacked frequency plots, dendrograms

Details can be found in Wiesenfarth et al. (2021).

1 Rankings

Algorithms within a task are ranked according to the following ranking scheme:

aggregate using function ("mean") then rank

Ranking for each task:

4th Ventricle : The analysis is based on 10 algorithms and 20 cases. 0 missing cases have been found in the data set.

	value_mean	rank
SyNOnly_CC_2_reg_iter_1_normal_masked	0.8102403	1
SyNOnly_MI_32_reg_iter_1_normal_masked	0.8490277	2
SyNOnly_CC_4_reg_iter_1_normal_masked	0.8935448	3
SyNOnly_CC_2_reg_iter_0_normal_masked	0.9449776	4
SyNOnly_CC_6_reg_iter_1_normal_masked	0.9568431	5
SyNOnly_MI_32_reg_iter_0_normal_masked	1.0018494	6
SyNOnly_CC_4_reg_iter_0_normal_masked	1.0122273	7
SyNOnly_CC_6_reg_iter_0_normal_masked	1.0950800	8
TVMSQC_normal_masked	1.2102637	9
TVMSQ_normal_masked	1.3511604	10

Chiasma : The analysis is based on 10 algorithms and 20 cases. 0 missing cases have been found in the data set.

	value_mean	rank
SyNOnly_CC_2_reg_iter_1_normal_masked	0.8217610	1
SyNOnly_CC_4_reg_iter_1_normal_masked	0.8323557	2
SyNOnly_CC_2_reg_iter_0_normal_masked	0.8951749	3
SyNOnly_CC_6_reg_iter_1_normal_masked	0.9186495	4

	value_mean	rank
SyNOnly_MI_32_reg_iter_1_normal_masked	0.9990657	5
SyNOnly_CC_4_reg_iter_0_normal_masked	1.0427457	6
TVMSQ_normal_masked	1.0795571	7
SyNOnly_MI_32_reg_iter_0_normal_masked	1.0834125	8
SyNOnly_CC_6_reg_iter_0_normal_masked	1.1398533	9
TVMSQC_normal_masked	1.6150729	10

Culmen : The analysis is based on 10 algorithms and 20 cases. 0 missing cases have been found in the data set.

	value_mean	rank
SyNOnly_CC_2_reg_iter_1_normal_masked	1.196743	1
SyNOnly_MI_32_reg_iter_1_normal_masked	1.321600	2
SyNOnly_CC_4_reg_iter_1_normal_masked	1.399911	3
SyNOnly_CC_6_reg_iter_1_normal_masked	1.484932	4
SyNOnly_MI_32_reg_iter_0_normal_masked	1.590406	5
SyNOnly_CC_2_reg_iter_0_normal_masked	1.597700	6
SyNOnly_CC_6_reg_iter_0_normal_masked	1.700494	7
SyNOnly_CC_4_reg_iter_0_normal_masked	1.704348	8
TVMSQ_normal_masked	1.870811	9
TVMSQC_normal_masked	2.555781	10

Hemisphere left caudal point : The analysis is based on 10 algorithms and 20 cases. 0 missing cases have been found in the data set.

	value_mean	rank
SyNOnly_MI_32_reg_iter_1_normal_masked	2.458457	1
SyNOnly_CC_4_reg_iter_1_normal_masked	2.514853	2
SyNOnly_CC_6_reg_iter_1_normal_masked	2.530459	3
SyNOnly_CC_2_reg_iter_1_normal_masked	2.538105	4
SyNOnly_MI_32_reg_iter_0_normal_masked	2.556785	5
SyNOnly_CC_2_reg_iter_0_normal_masked	2.631058	6
SyNOnly_CC_4_reg_iter_0_normal_masked	2.651578	7
SyNOnly_CC_6_reg_iter_0_normal_masked	2.664413	8
TVMSQC_normal_masked	2.769062	9
TVMSQ_normal_masked	2.970517	10

Hemisphere left rostral point : The analysis is based on 10 algorithms and 20 cases. 0 missing cases have been found in the data set.

	value_mean	rank
SyNOnly_CC_2_reg_iter_1_normal_masked	3.053120	1
SyNOnly_CC_4_reg_iter_1_normal_masked	3.357722	2
SyNOnly_CC_6_reg_iter_1_normal_masked	3.458621	3
SyNOnly_CC_2_reg_iter_0_normal_masked	3.516286	4
SyNOnly_MI_32_reg_iter_1_normal_masked	3.657426	5
SyNOnly_CC_4_reg_iter_0_normal_masked	3.822831	6
SyNOnly_CC_6_reg_iter_0_normal_masked	3.827894	7

	value_mean	rank
TVMSQ_normal_masked	3.892267	8
SyNOnly_MI_32_reg_iter_0_normal_masked	3.899618	9
TVMSQC_normal_masked	4.259099	10

Hemisphere right caudal point : The analysis is based on 10 algorithms and 20 cases. 0 missing cases have been found in the data set.

	value_mean	rank
SyNOnly_CC_2_reg_iter_1_normal_masked	3.227733	1
SyNOnly_CC_6_reg_iter_1_normal_masked	3.231346	2
SyNOnly_CC_4_reg_iter_1_normal_masked	3.236120	3
SyNOnly_CC_2_reg_iter_0_normal_masked	3.287478	4
SyNOnly_MI_32_reg_iter_1_normal_masked	3.319825	5
SyNOnly_CC_6_reg_iter_0_normal_masked	3.344959	6
SyNOnly_CC_4_reg_iter_0_normal_masked	3.355279	7
SyNOnly_MI_32_reg_iter_0_normal_masked	3.470115	8
TVMSQC_normal_masked	3.854957	9
TVMSQ_normal_masked	4.120709	10

Hemisphere right rostral point : The analysis is based on 10 algorithms and 20 cases. 0 missing cases have been found in the data set.

	value_mean	rank
SyNOnly_CC_2_reg_iter_1_normal_masked	4.134985	1
SyNOnly_CC_4_reg_iter_1_normal_masked	4.396170	2
SyNOnly_CC_6_reg_iter_1_normal_masked	4.535013	3
TVMSQ_normal_masked	4.540337	4
SyNOnly_CC_2_reg_iter_0_normal_masked	4.541963	5
SyNOnly_MI_32_reg_iter_1_normal_masked	4.716314	6
SyNOnly_MI_32_reg_iter_0_normal_masked	4.769514	7
SyNOnly_CC_6_reg_iter_0_normal_masked	4.840852	8
SyNOnly_CC_4_reg_iter_0_normal_masked	4.858187	9
TVMSQC_normal_masked	5.038807	10

Infracollicular Sulcus : The analysis is based on 10 algorithms and 20 cases. 0 missing cases have been found in the data set.

	value_mean	rank
SyNOnly_CC_6_reg_iter_1_normal_masked	0.8124378	1
SyNOnly_CC_4_reg_iter_1_normal_masked	0.8262647	2
SyNOnly_CC_4_reg_iter_0_normal_masked	0.8333134	3
SyNOnly_CC_6_reg_iter_0_normal_masked	0.8366234	4
SyNOnly_MI_32_reg_iter_0_normal_masked	0.8372633	5
SyNOnly_CC_2_reg_iter_0_normal_masked	0.8403204	6
SyNOnly_MI_32_reg_iter_1_normal_masked	0.8448096	7
SyNOnly_CC_2_reg_iter_1_normal_masked	0.8693383	8
TVMSQC_normal_masked	1.0747175	9
TVMSQ_normal_masked	1.0912875	10

Lateral ventricle left caudal point : The analysis is based on 10 algorithms and 20 cases. 0 missing cases have been found in the data set.

	value_mean	rank
SyNOnly_CC_2_reg_iter_1_normal_masked	4.049994	1
SyNOnly_CC_6_reg_iter_1_normal_masked	4.151478	2
SyNOnly_CC_4_reg_iter_1_normal_masked	4.165355	3
SyNOnly_MI_32_reg_iter_1_normal_masked	4.170682	4
SyNOnly_CC_2_reg_iter_0_normal_masked	4.456710	5
SyNOnly_CC_4_reg_iter_0_normal_masked	4.610450	6
SyNOnly_CC_6_reg_iter_0_normal_masked	4.663878	7
SyNOnly_MI_32_reg_iter_0_normal_masked	4.809570	8
TVMSQC_normal_masked	4.824403	9
TVMSQ_normal_masked	5.442504	10

Lateral ventricle left rostral point : The analysis is based on 10 algorithms and 20 cases. 0 missing cases have been found in the data set.

	value_mean	rank
SyNOnly_CC_4_reg_iter_1_normal_masked	1.366216	1
SyNOnly_CC_2_reg_iter_1_normal_masked	1.371069	2
SyNOnly_CC_6_reg_iter_0_normal_masked	1.375694	3
SyNOnly_CC_6_reg_iter_1_normal_masked	1.385428	4
SyNOnly_CC_4_reg_iter_0_normal_masked	1.403740	5
SyNOnly_CC_2_reg_iter_0_normal_masked	1.441672	6
SyNOnly_MI_32_reg_iter_0_normal_masked	1.442838	7
SyNOnly_MI_32_reg_iter_1_normal_masked	1.475612	8
TVMSQ_normal_masked	1.634798	9
TVMSQC_normal_masked	1.948252	10

Lateral ventricle right caudal point : The analysis is based on 10 algorithms and 20 cases. 0 missing cases have been found in the data set.

	value_mean	rank
SyNOnly_MI_32_reg_iter_1_normal_masked	3.132465	1
SyNOnly_CC_6_reg_iter_1_normal_masked	3.204107	2
SyNOnly_CC_4_reg_iter_1_normal_masked	3.232584	3
SyNOnly_CC_2_reg_iter_1_normal_masked	3.292653	4
SyNOnly_CC_6_reg_iter_0_normal_masked	3.336737	5
SyNOnly_CC_2_reg_iter_0_normal_masked	3.354689	6
SyNOnly_CC_4_reg_iter_0_normal_masked	3.389203	7
SyNOnly_MI_32_reg_iter_0_normal_masked	3.418340	8
TVMSQC_normal_masked	3.735130	9
TVMSQ_normal_masked	4.245810	10

Lateral ventricle right rostral point : The analysis is based on 10 algorithms and 20 cases. 0 missing cases have been found in the data set.

	value_mean	rank
SyNOnly_CC_4_reg_iter_1_normal_masked	1.382802	1
SyNOnly_CC_6_reg_iter_1_normal_masked	1.393954	2
SyNOnly_CC_6_reg_iter_0_normal_masked	1.404390	3
SyNOnly_CC_4_reg_iter_0_normal_masked	1.419406	4
SyNOnly_CC_2_reg_iter_1_normal_masked	1.430081	5
SyNOnly_MI_32_reg_iter_1_normal_masked	1.452837	6
SyNOnly_CC_2_reg_iter_0_normal_masked	1.471803	7
SyNOnly_MI_32_reg_iter_0_normal_masked	1.485860	8
TVMSQ_normal_masked	1.661867	9
TVMSQC_normal_masked	2.536153	10

PMJ : The analysis is based on 10 algorithms and 20 cases. 0 missing cases have been found in the data set.

	value_mean	rank
SyNOnly_CC_4_reg_iter_1_normal_masked	0.6533394	1
SyNOnly_CC_2_reg_iter_1_normal_masked	0.6713383	2
SyNOnly_CC_6_reg_iter_1_normal_masked	0.6760550	3
SyNOnly_CC_2_reg_iter_0_normal_masked	0.6773725	4
SyNOnly_CC_4_reg_iter_0_normal_masked	0.6852035	5
SyNOnly_CC_6_reg_iter_0_normal_masked	0.6924697	6
TVMSQ_normal_masked	0.7689277	7
SyNOnly_MI_32_reg_iter_1_normal_masked	0.8560544	8
SyNOnly_MI_32_reg_iter_0_normal_masked	0.9516294	9
TVMSQC_normal_masked	0.9691643	10

Posterior Commissure : The analysis is based on 10 algorithms and 20 cases. 0 missing cases have been found in the data set.

	value_mean	rank
SyNOnly_MI_32_reg_iter_1_normal_masked	0.7391086	1
SyNOnly_CC_6_reg_iter_1_normal_masked	0.7779325	2
SyNOnly_CC_4_reg_iter_1_normal_masked	0.8031050	3
SyNOnly_CC_2_reg_iter_1_normal_masked	0.8481364	4
SyNOnly_CC_2_reg_iter_0_normal_masked	0.8680397	5
SyNOnly_CC_6_reg_iter_0_normal_masked	0.8753852	6
SyNOnly_CC_4_reg_iter_0_normal_masked	0.8927115	7
SyNOnly_MI_32_reg_iter_0_normal_masked	0.9139115	8
TVMSQC_normal_masked	1.2074563	9
TVMSQ_normal_masked	1.3851023	10

Temporal lobe left rostral point : The analysis is based on 10 algorithms and 20 cases. 0 missing cases have been found in the data set.

	value_mean	rank
SyNOnly_CC_2_reg_iter_1_normal_masked	1.892863	1
SyNOnly_CC_4_reg_iter_1_normal_masked	1.983392	2
SyNOnly_CC_2_reg_iter_0_normal_masked	2.100561	3
SyNOnly_CC_6_reg_iter_1_normal_masked	2.119758	4

	value_mean	rank
SyNOnly_CC_4_reg_iter_0_normal_masked	2.188321	5
SyNOnly_MI_32_reg_iter_1_normal_masked	2.195789	6
SyNOnly_CC_6_reg_iter_0_normal_masked	2.234581	7
SyNOnly_MI_32_reg_iter_0_normal_masked	2.432132	8
TVMSQC_normal_masked	2.947953	9
TVMSQ_normal_masked	3.270861	10

Temporal lobe ventricle right rostral point : The analysis is based on 10 algorithms and 20 cases. 0 missing cases have been found in the data set.

	value_mean	rank
SyNOnly_CC_2_reg_iter_1_normal_masked	2.080639	1
SyNOnly_CC_4_reg_iter_1_normal_masked	2.108753	2
SyNOnly_CC_2_reg_iter_0_normal_masked	2.108884	3
SyNOnly_CC_6_reg_iter_1_normal_masked	2.153300	4
SyNOnly_CC_4_reg_iter_0_normal_masked	2.217602	5
SyNOnly_CC_6_reg_iter_0_normal_masked	2.255523	6
SyNOnly_MI_32_reg_iter_1_normal_masked	2.324299	7
SyNOnly_MI_32_reg_iter_0_normal_masked	2.368357	8
TVMSQC_normal_masked	2.655745	9
TVMSQ_normal_masked	2.858510	10

Consensus ranking across tasks according to chosen method “euclidean”:

	value	rank
SyNOnly_CC_4_reg_iter_1_normal_masked	2.188	1
SyNOnly_CC_2_reg_iter_1_normal_masked	2.375	2
SyNOnly_CC_6_reg_iter_1_normal_masked	3.000	3
SyNOnly_MI_32_reg_iter_1_normal_masked	4.625	4
SyNOnly_CC_2_reg_iter_0_normal_masked	4.812	5
SyNOnly_CC_4_reg_iter_0_normal_masked	6.062	6
SyNOnly_CC_6_reg_iter_0_normal_masked	6.250	7
SyNOnly_MI_32_reg_iter_0_normal_masked	7.312	8
TVMSQ_normal_masked	8.938	9
TVMSQC_normal_masked	9.438	10

2 Visualization of raw assessment data

The algorithms are ordered according to the computed ranks for each task.

2.1 Dot- and boxplot

Dot- and boxplots for visualizing raw assessment data separately for each algorithm. Boxplots representing descriptive statistics over all cases (median, quartiles and outliers) are combined with horizontally jittered dots representing individual cases.

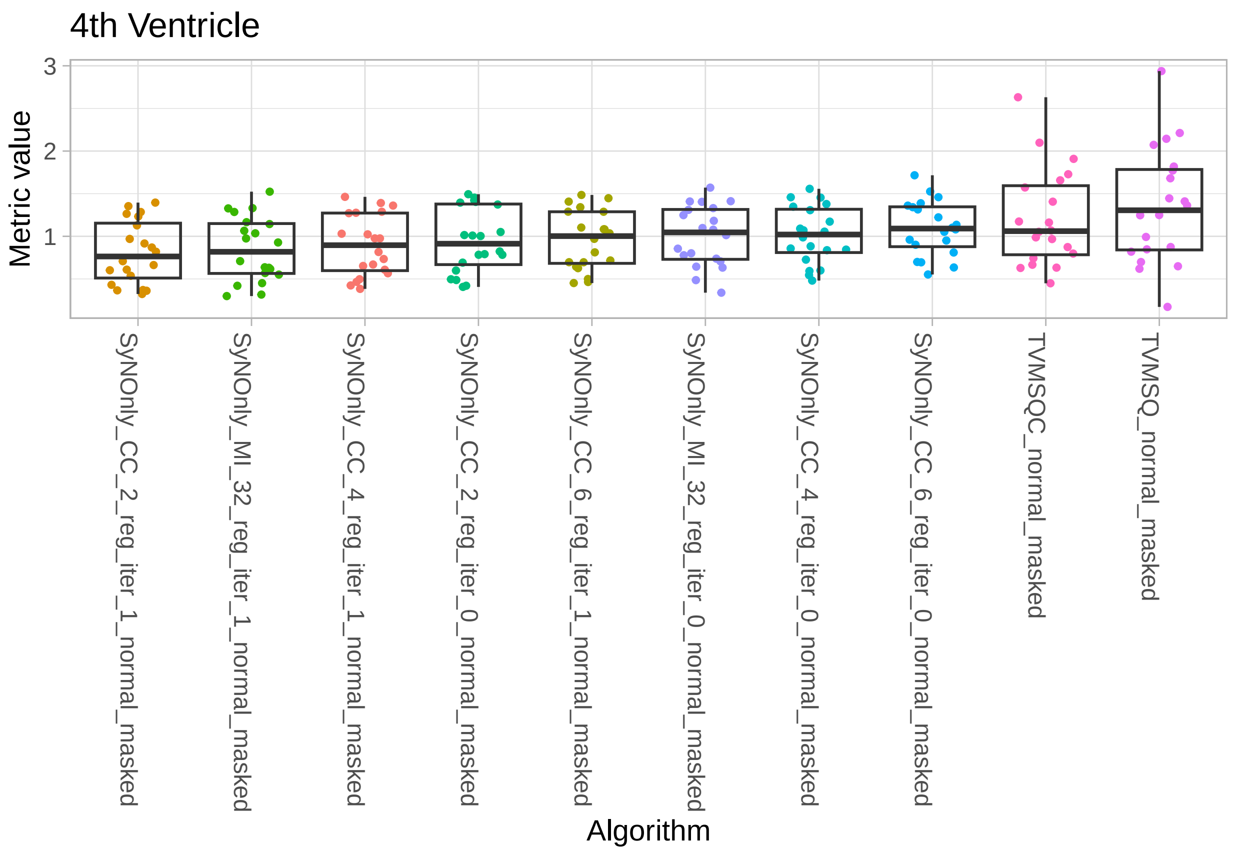

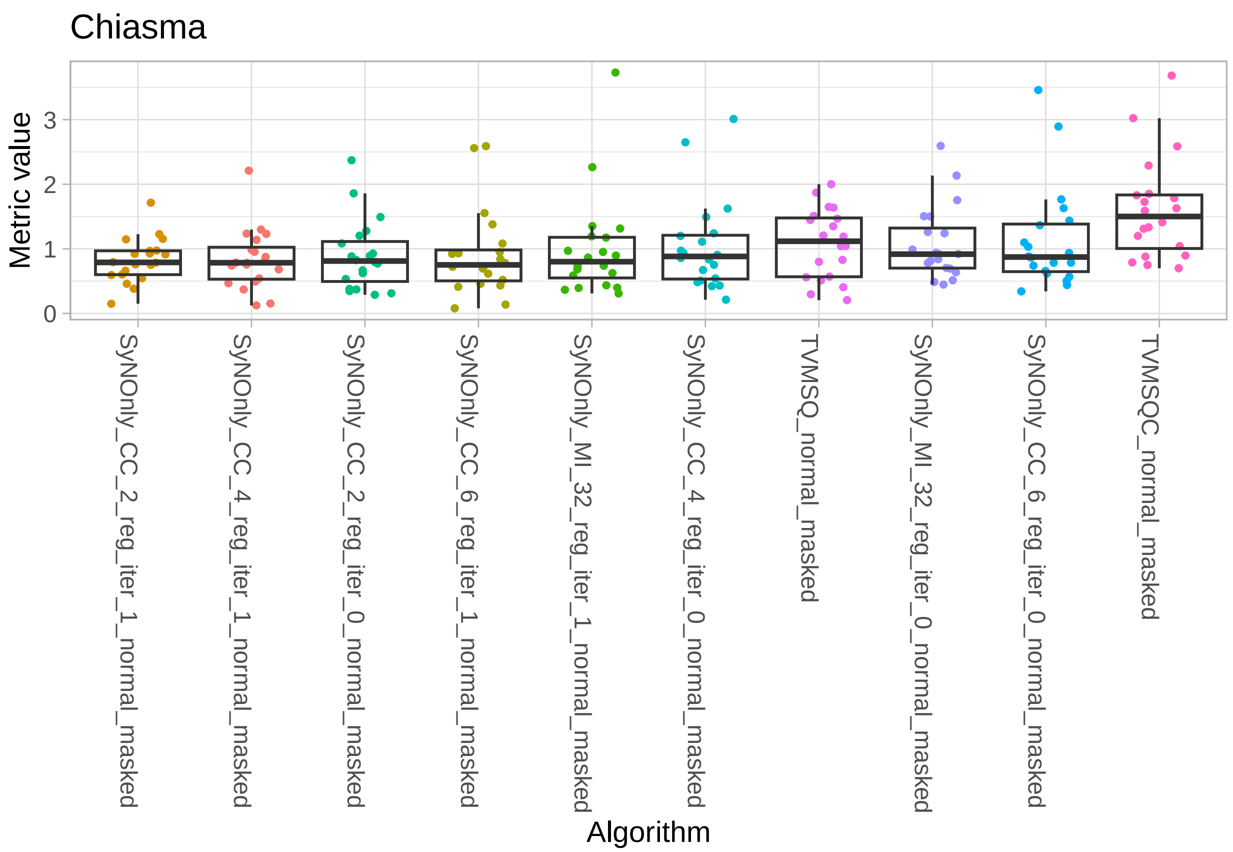
Chiasma
Metric value
0
1
2
3
SyNOnly_CC_2_reg_iter_1_normal_masked
SyNOnly_CC_4_reg_iter_1_normal_masked
SyNOnly_CC_2_reg_iter_0_normal_masked
SyNOnly_CC_6_reg_iter_1_normal_masked
SyNOnly_MI_32_reg_iter_1_normal_masked
SyNOnly_CC_4_reg_iter_0_normal_masked
TVMSQ_normal_masked
SyNOnly_MI_32_reg_iter_0_normal_masked
SyNOnly_CC_6_reg_iter_0_normal_masked
TVMSQC_normal_masked
Algorithm

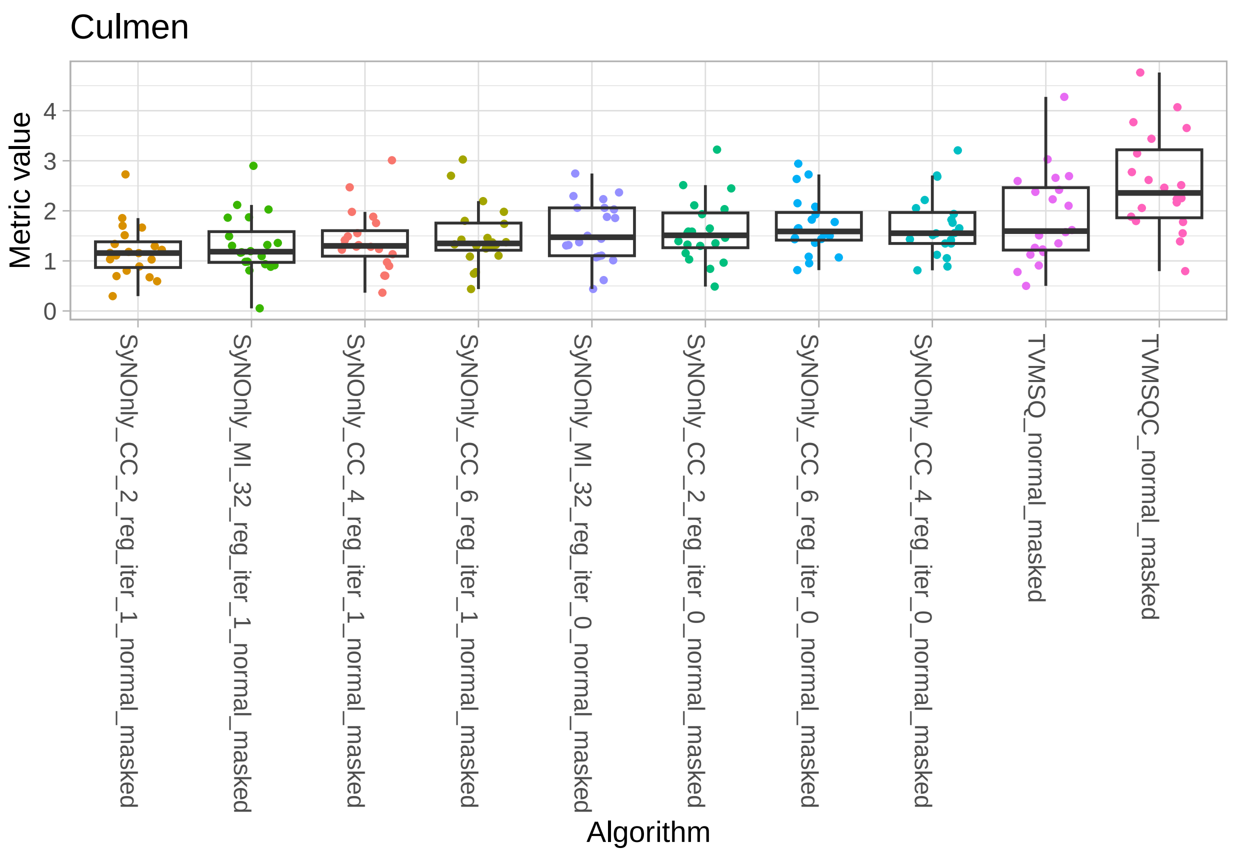

Culmen
Metric value
0
1
2
3
4
SyNOnly_CC_2_reg_iter_1_normal_masked
SyNOnly_MI_32_reg_iter_1_normal_masked
SyNOnly_CC_4_reg_iter_1_normal_masked
SyNOnly_CC_6_reg_iter_1_normal_masked
SyNOnly_MI_32_reg_iter_0_normal_masked
SyNOnly_CC_2_reg_iter_0_normal_masked
SyNOnly_CC_6_reg_iter_0_normal_masked
SyNOnly_CC_4_reg_iter_0_normal_masked
TVMSQ_normal_masked
TVMSQC_normal_masked
Algorithm

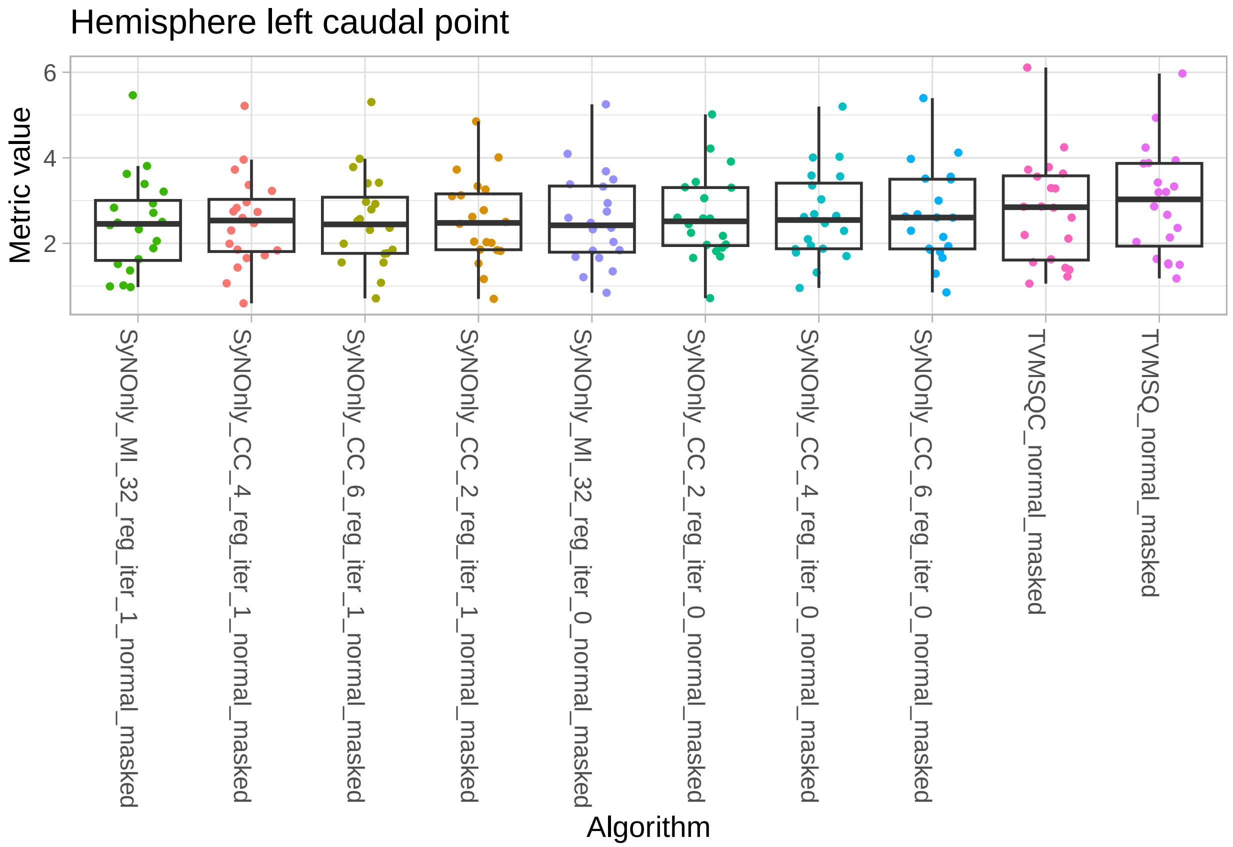

Hemisphere left caudal point
Metric value
6
4
2
SyNOnly_MI_32_reg_iter_1_normal_masked
SyNOnly_CC_4_reg_iter_1_normal_masked
SyNOnly_CC_6_reg_iter_1_normal_masked
SyNOnly_CC_2_reg_iter_1_normal_masked
SyNOnly_MI_32_reg_iter_0_normal_masked
SyNOnly_CC_2_reg_iter_0_normal_masked
SyNOnly_CC_4_reg_iter_0_normal_masked
SyNOnly_CC_6_reg_iter_0_normal_masked
TVMSQC_normal_masked
TVMSQ_normal_masked
Algorithm

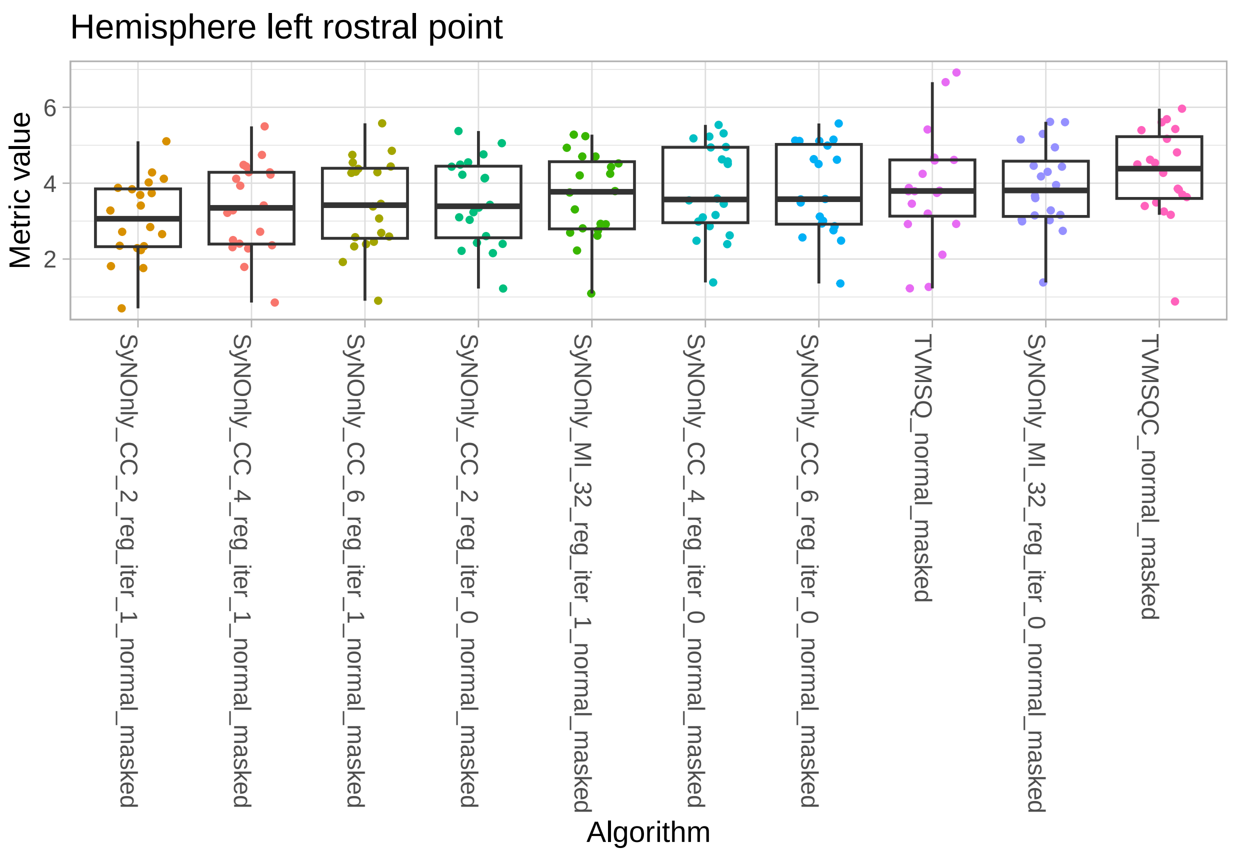
Hemisphere left rostral point
Metric value
6
4
2
SyNOnly_CC_2_reg_iter_1_normal_masked
SyNOnly_CC_4_reg_iter_1_normal_masked
SyNOnly_CC_6_reg_iter_1_normal_masked
SyNOnly_CC_2_reg_iter_0_normal_masked
SyNOnly_MI_32_reg_iter_1_normal_masked
SyNOnly_CC_4_reg_iter_0_normal_masked
SyNOnly_CC_6_reg_iter_0_normal_masked
TVMSQ_normal_masked
SyNOnly_MI_32_reg_iter_0_normal_masked
TVMSQC_normal_masked
Algorithm

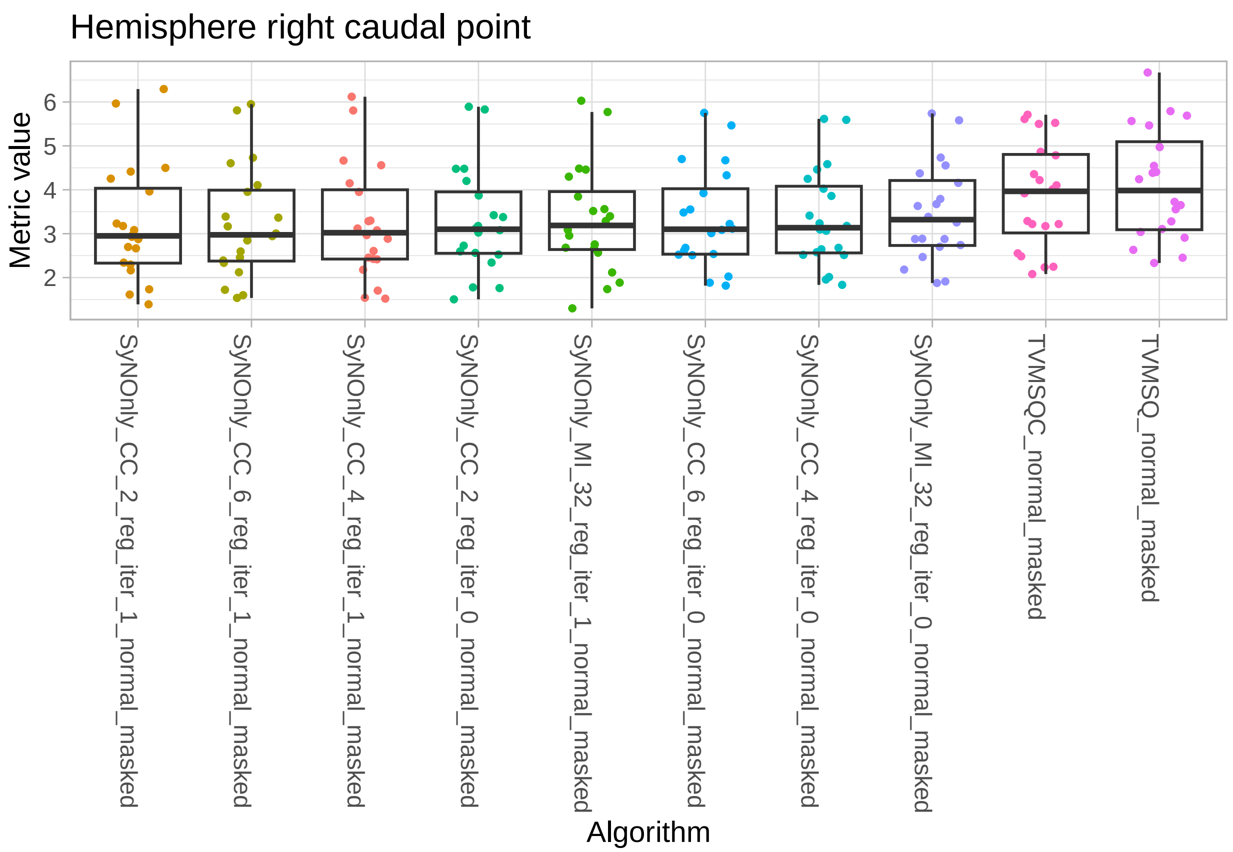
Hemisphere right caudal point
Metric value
6
5
4
3
2
SyNOnly_CC_2_reg_iter_1_normal_masked
SyNOnly_CC_6_reg_iter_1_normal_masked
SyNOnly_CC_4_reg_iter_1_normal_masked
SyNOnly_CC_2_reg_iter_0_normal_masked
SyNOnly_MI_32_reg_iter_1_normal_masked
SyNOnly_CC_6_reg_iter_0_normal_masked
SyNOnly_CC_4_reg_iter_0_normal_masked
SyNOnly_MI_32_reg_iter_0_normal_masked
TVMSQC_normal_masked
TVMSQ_normal_masked
Algorithm

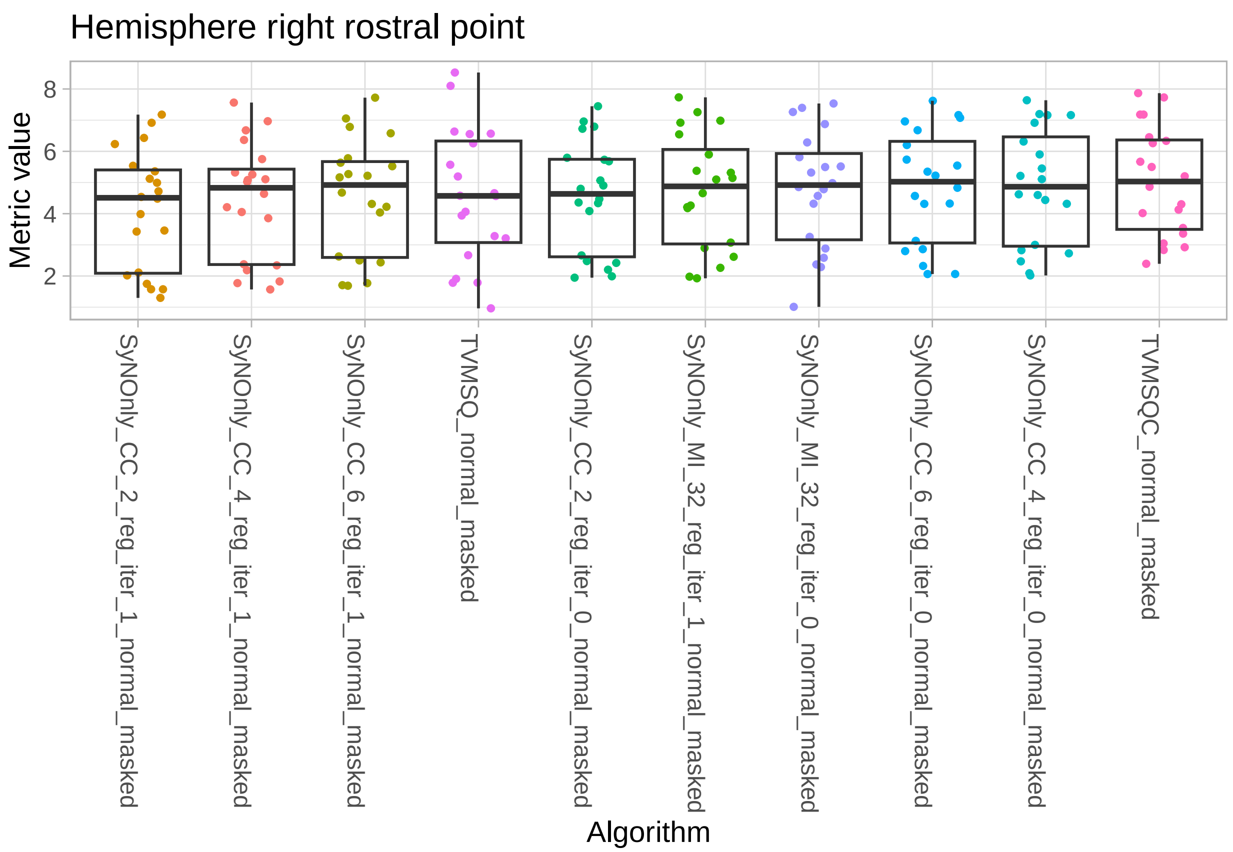

Hemisphere right rostral point
Metric value
8
6
4
2
SyNOnly_CC_2_reg_iter_1_normal_masked
SyNOnly_CC_4_reg_iter_1_normal_masked
SyNOnly_CC_6_reg_iter_1_normal_masked
TVMSQ_normal_masked
SyNOnly_CC_2_reg_iter_0_normal_masked
SyNOnly_MI_32_reg_iter_1_normal_masked
SyNOnly_MI_32_reg_iter_0_normal_masked
SyNOnly_CC_6_reg_iter_0_normal_masked
SyNOnly_CC_4_reg_iter_0_normal_masked
TVMSQC_normal_masked
Algorithm

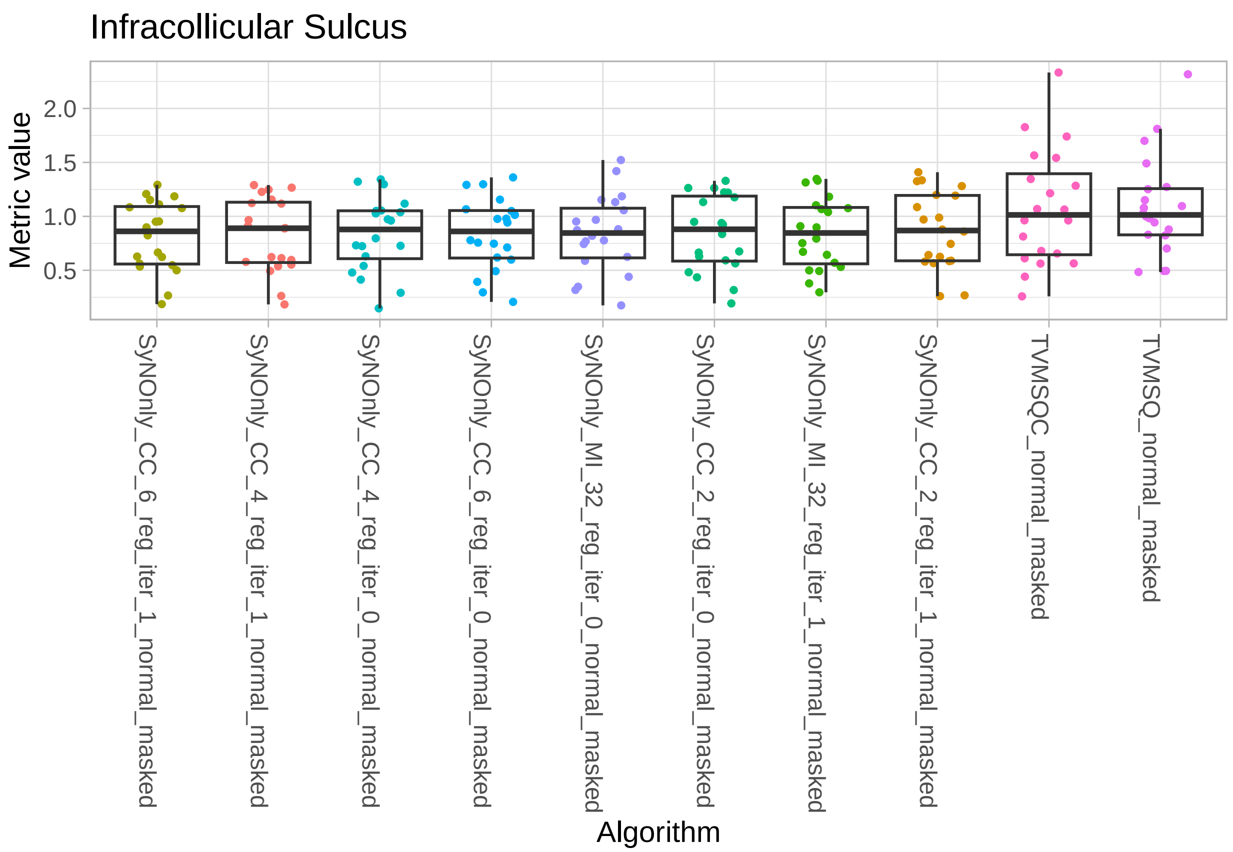
Infracollicular Sulcus
Metric value
2.0
1.5
1.0
0.5
SyNOnly_CC_6_reg_iter_1_normal_masked
SyNOnly_CC_4_reg_iter_1_normal_masked
SyNOnly_CC_4_reg_iter_0_normal_masked
SyNOnly_CC_6_reg_iter_0_normal_masked
SyNOnly_MI_32_reg_iter_0_normal_masked
SyNOnly_CC_2_reg_iter_0_normal_masked
SyNOnly_MI_32_reg_iter_1_normal_masked
SyNOnly_CC_2_reg_iter_1_normal_masked
TVMSQC_normal_masked
TVMSQ_normal_masked
Algorithm

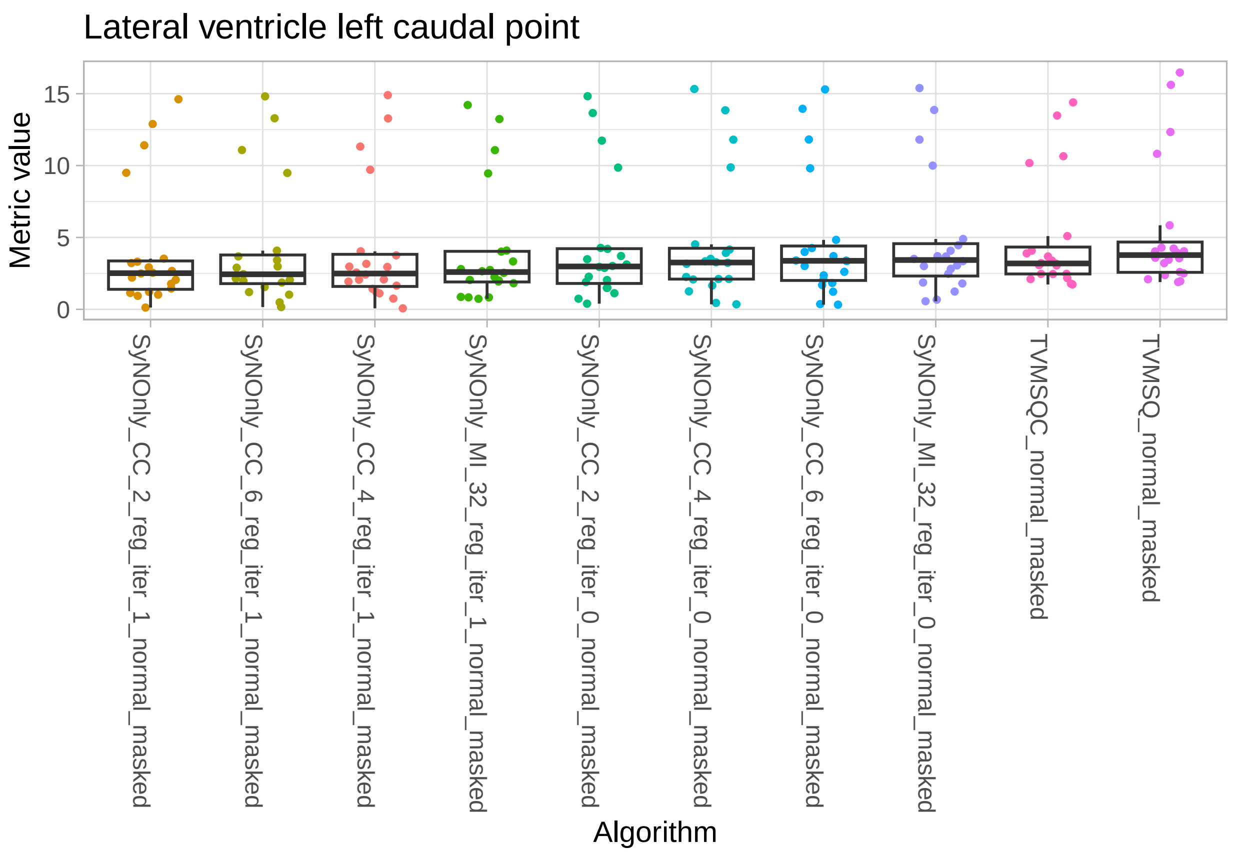
Lateral ventricle left caudal point
Metric value
15
10
5
0
SyNOnly_CC_2_reg_iter_1_normal_masked
SyNOnly_CC_6_reg_iter_1_normal_masked
SyNOnly_CC_4_reg_iter_1_normal_masked
SyNOnly_MI_32_reg_iter_1_normal_masked
SyNOnly_CC_2_reg_iter_0_normal_masked
SyNOnly_CC_4_reg_iter_0_normal_masked
SyNOnly_CC_6_reg_iter_0_normal_masked
SyNOnly_MI_32_reg_iter_0_normal_masked
TVMSQC_normal_masked
TVMSQ_normal_masked
Algorithm

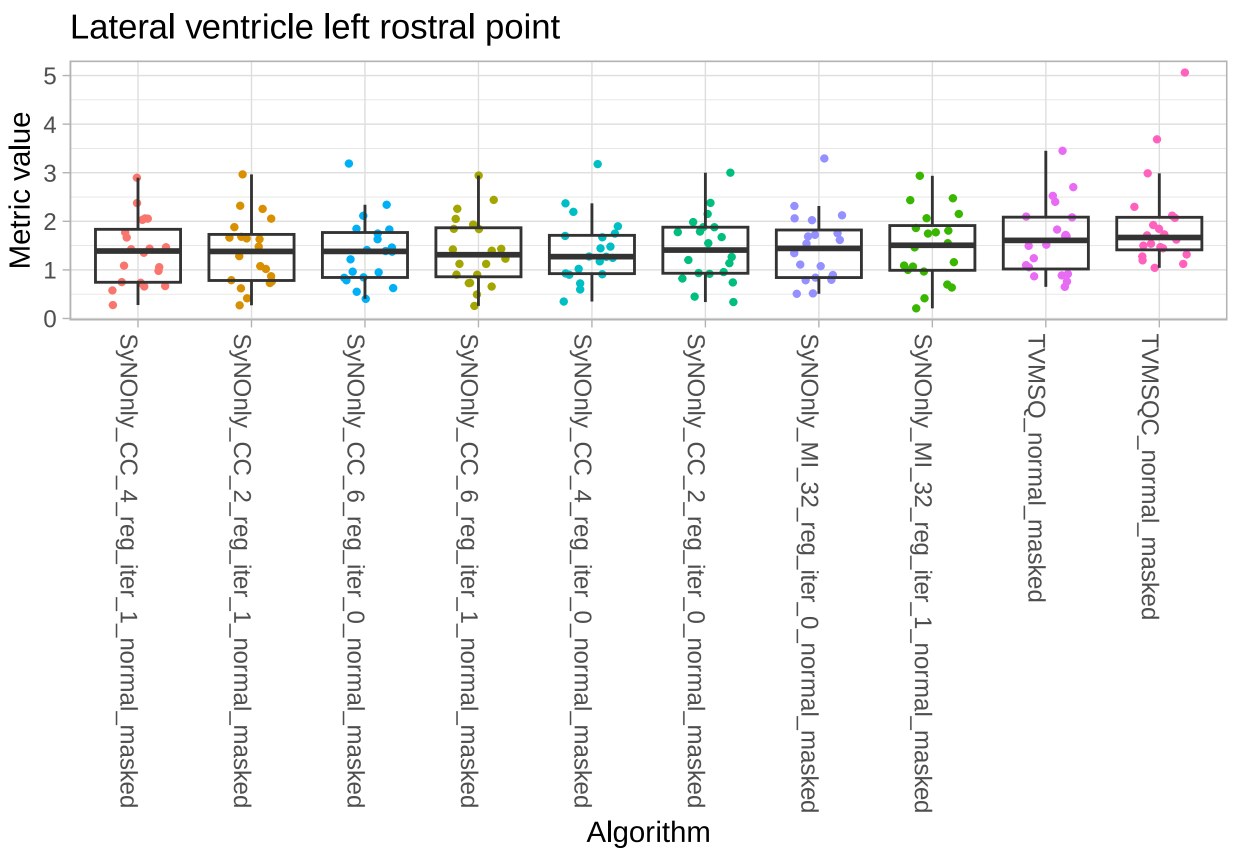

Lateral ventricle left rostral point
Metric value
0
1
2
3
4
5
SyNOnly_CC_4_reg_iter_1_normal_masked
SyNOnly_CC_2_reg_iter_1_normal_masked
SyNOnly_CC_6_reg_iter_0_normal_masked
SyNOnly_CC_6_reg_iter_1_normal_masked
SyNOnly_CC_4_reg_iter_0_normal_masked
SyNOnly_CC_2_reg_iter_0_normal_masked
SyNOnly_MI_32_reg_iter_0_normal_masked
SyNOnly_MI_32_reg_iter_1_normal_masked
TVMSQ_normal_masked
TVMSQC_normal_masked
Algorithm

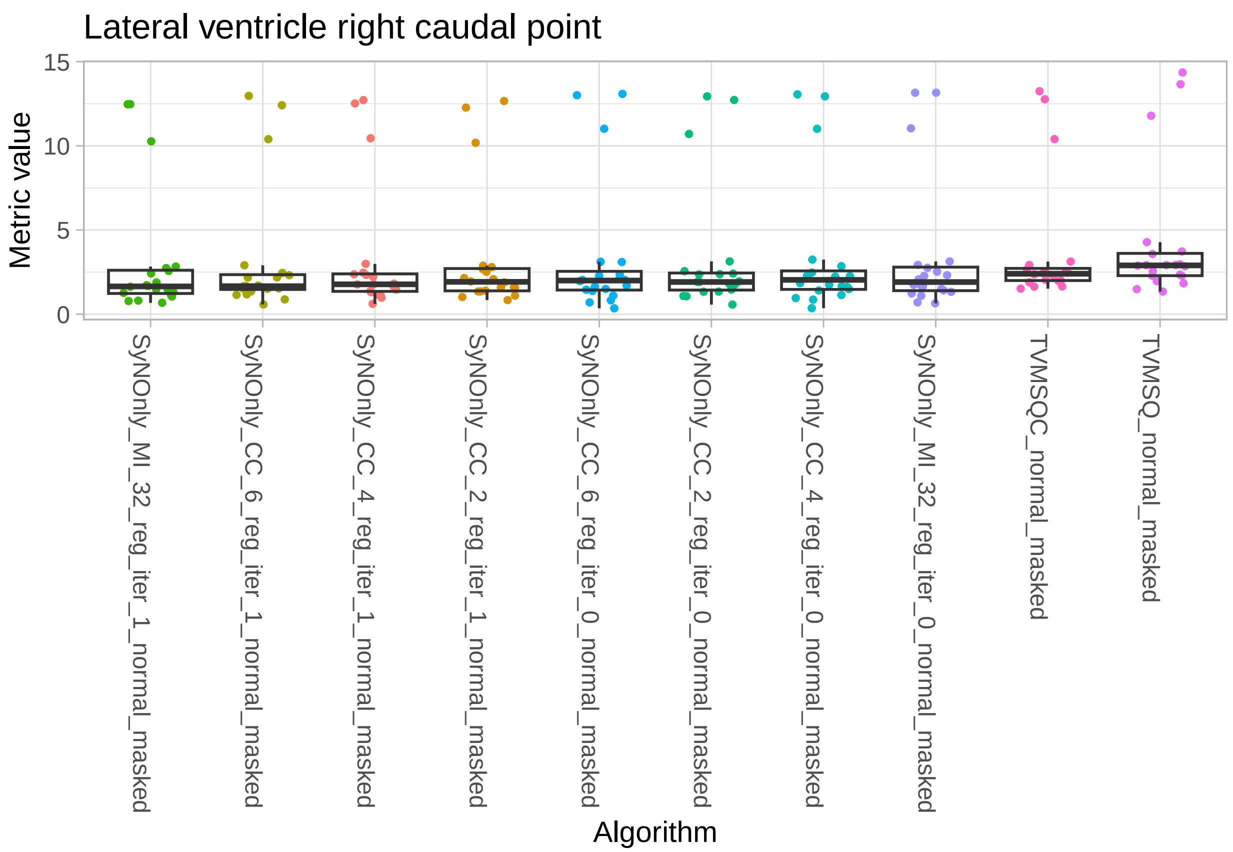

Lateral ventricle right caudal point
Metric value
15
10
5
0
SyNOnly_MI_32_reg_iter_1_normal_masked
SyNOnly_CC_6_reg_iter_1_normal_masked
SyNOnly_CC_4_reg_iter_1_normal_masked
SyNOnly_CC_2_reg_iter_1_normal_masked
SyNOnly_CC_6_reg_iter_0_normal_masked
SyNOnly_CC_2_reg_iter_0_normal_masked
SyNOnly_CC_4_reg_iter_0_normal_masked
SyNOnly_MI_32_reg_iter_0_normal_masked
TVMSQC_normal_masked
TVMSQ_normal_masked
Algorithm

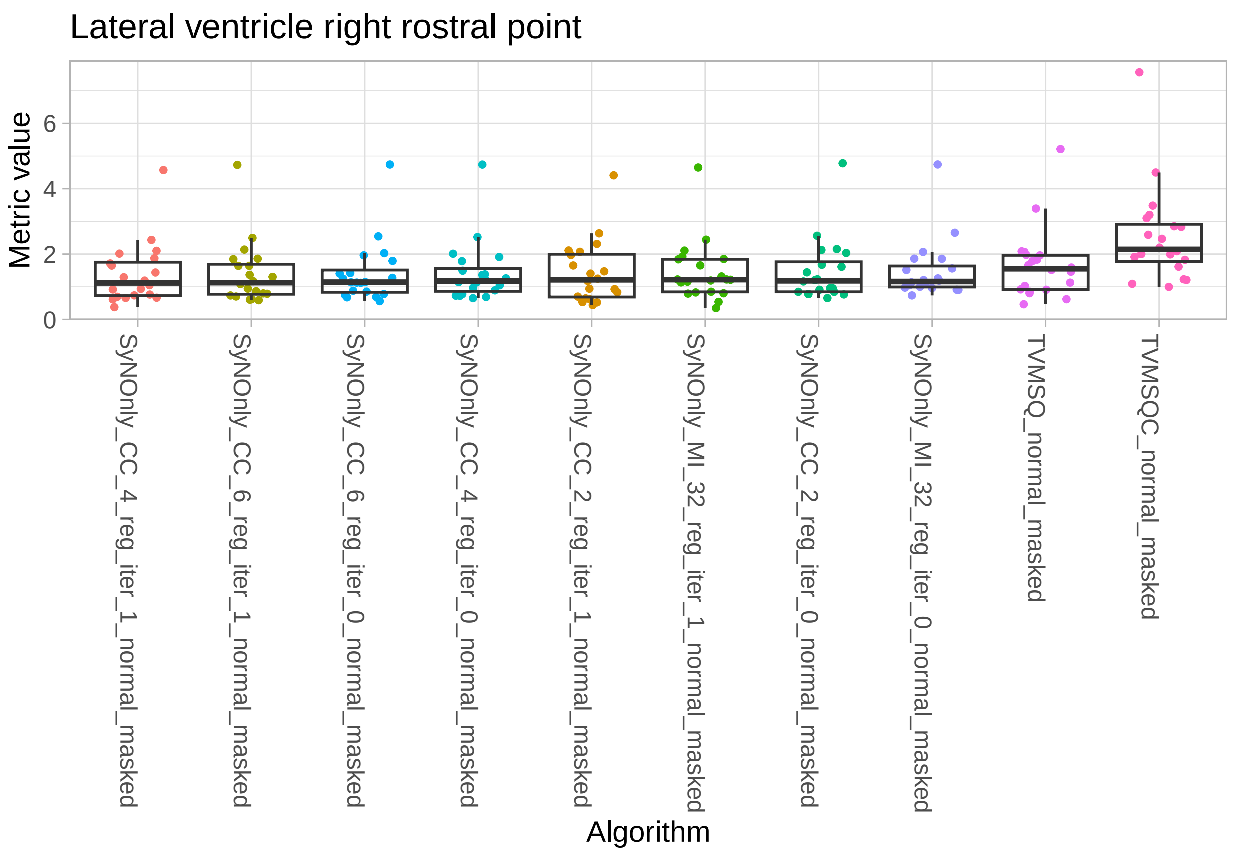
Lateral ventricle right rostral point
Metric value
0
2
4
6
SyNOnly_CC_4_reg_iter_1_normal_masked
SyNOnly_CC_6_reg_iter_1_normal_masked
SyNOnly_CC_6_reg_iter_0_normal_masked
SyNOnly_CC_4_reg_iter_0_normal_masked
SyNOnly_CC_2_reg_iter_1_normal_masked
SyNOnly_MI_32_reg_iter_1_normal_masked
SyNOnly_CC_2_reg_iter_0_normal_masked
SyNOnly_MI_32_reg_iter_0_normal_masked
TVMSQ_normal_masked
TVMSQC_normal_masked
Algorithm

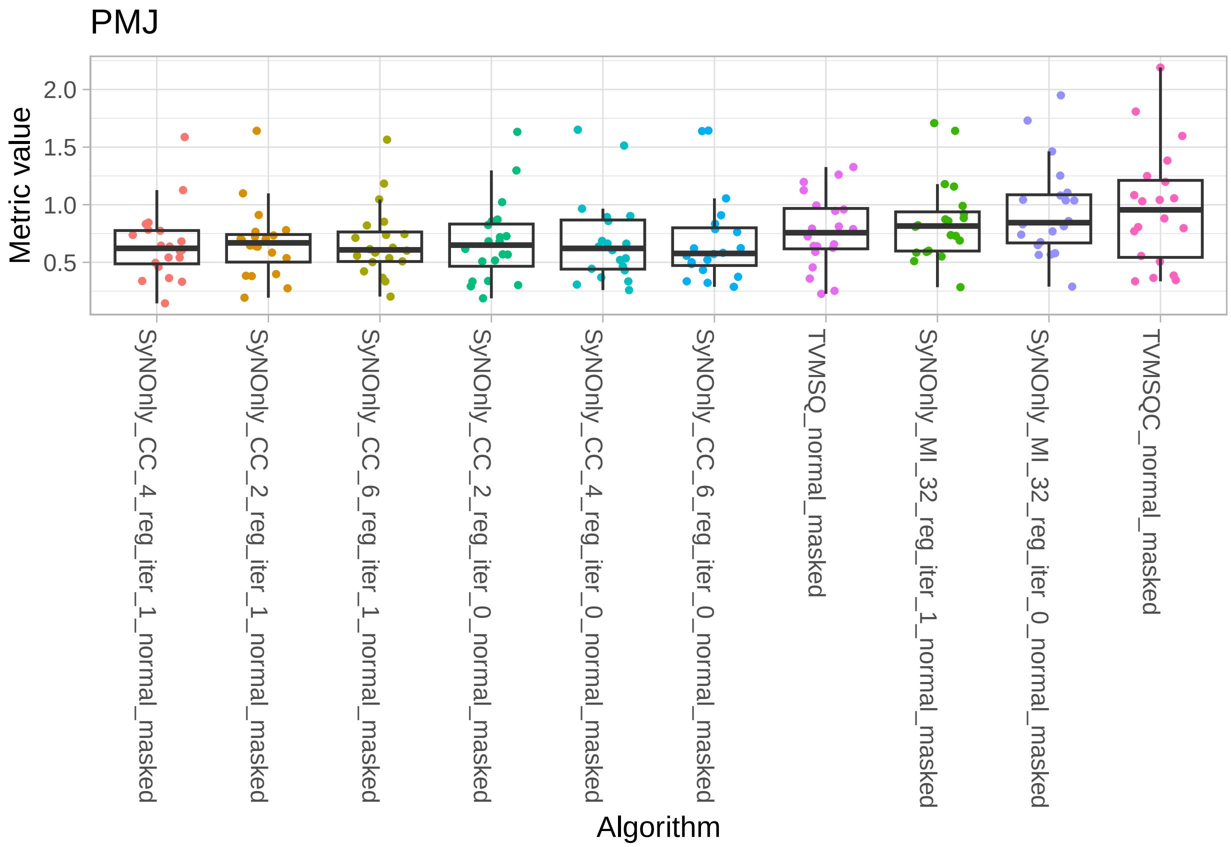
PMJ
Metric value
2.0
1.5
1.0
0.5
SyNOnly_CC_4_reg_iter_1_normal_masked
SyNOnly_CC_2_reg_iter_1_normal_masked
SyNOnly_CC_6_reg_iter_1_normal_masked
SyNOnly_CC_2_reg_iter_0_normal_masked
SyNOnly_CC_4_reg_iter_0_normal_masked
SyNOnly_CC_6_reg_iter_0_normal_masked
TVMSQ_normal_masked
SyNOnly_MI_32_reg_iter_1_normal_masked
SyNOnly_MI_32_reg_iter_0_normal_masked
TVMSQC_normal_masked
Algorithm

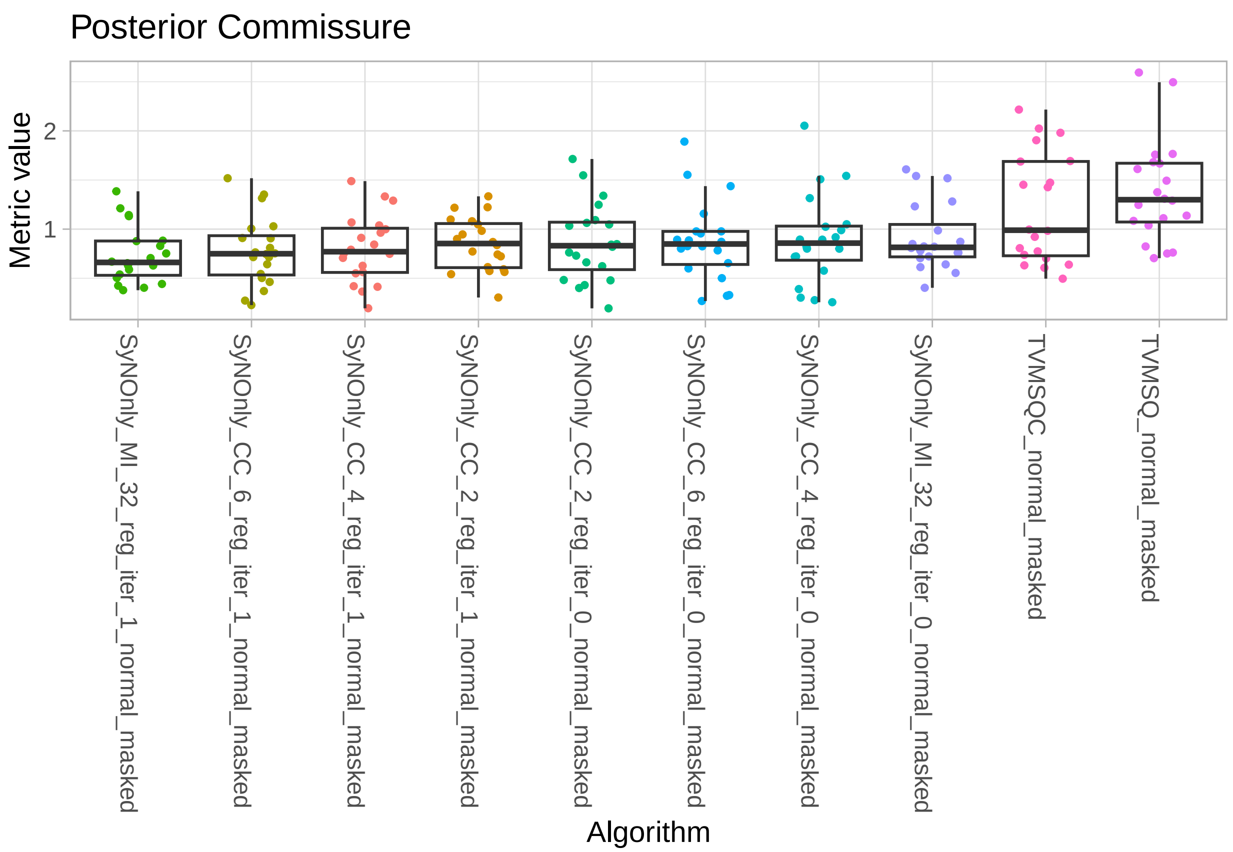

Posterior Commissure
Metric value
2
1
SyNOnly_MI_32_reg_iter_1_normal_masked
SyNOnly_CC_6_reg_iter_1_normal_masked
SyNOnly_CC_4_reg_iter_1_normal_masked
SyNOnly_CC_2_reg_iter_1_normal_masked
SyNOnly_CC_2_reg_iter_0_normal_masked
SyNOnly_CC_6_reg_iter_0_normal_masked
SyNOnly_CC_4_reg_iter_0_normal_masked
SyNOnly_MI_32_reg_iter_0_normal_masked
TVMSQC_normal_masked
TVMSQ_normal_masked
Algorithm

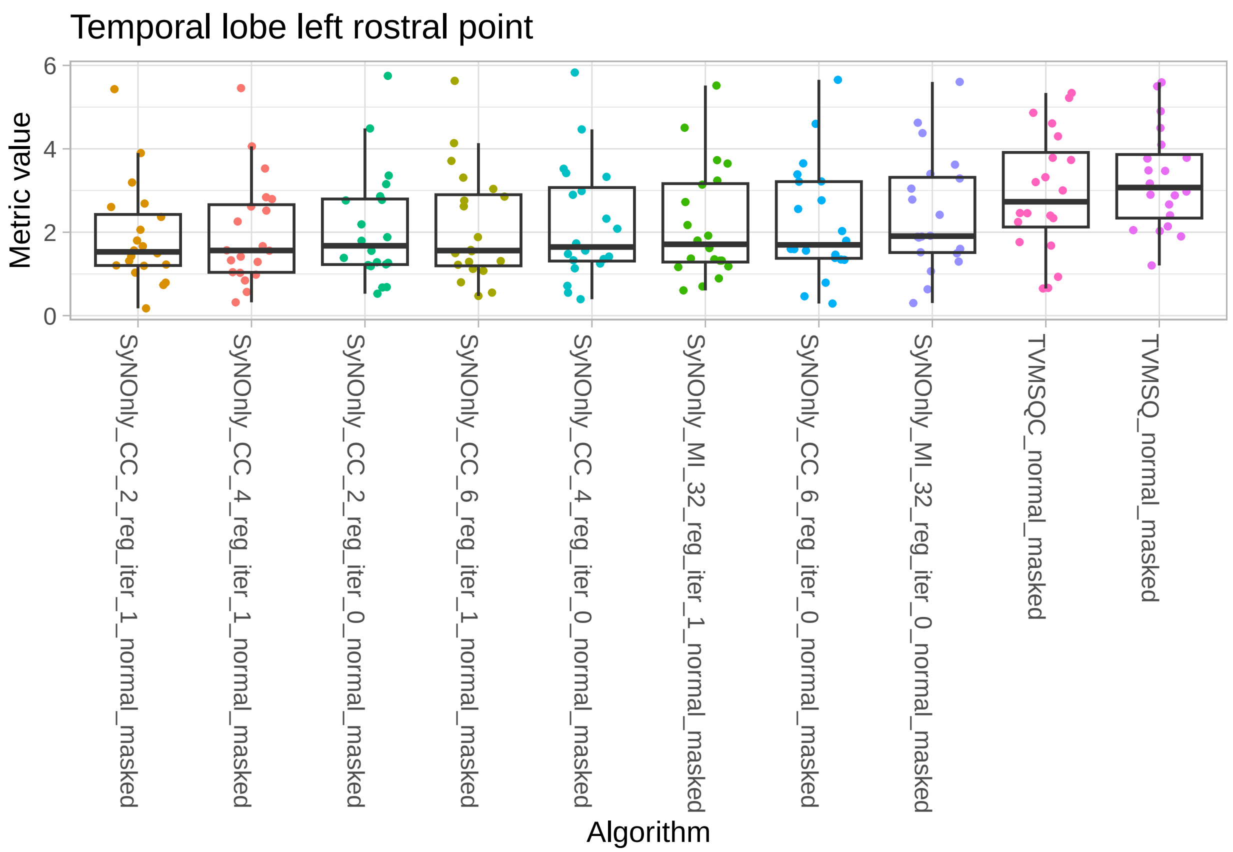

Temporal lobe left rostral point
Metric value
0
2
4
6
SyNOnly_CC_2_reg_iter_1_normal_masked
SyNOnly_CC_4_reg_iter_1_normal_masked
SyNOnly_CC_2_reg_iter_0_normal_masked
SyNOnly_CC_6_reg_iter_1_normal_masked
SyNOnly_CC_4_reg_iter_0_normal_masked
SyNOnly_MI_32_reg_iter_1_normal_masked
SyNOnly_CC_6_reg_iter_0_normal_masked
SyNOnly_MI_32_reg_iter_0_normal_masked
TVMSQC_normal_masked
TVMSQ_normal_masked
Algorithm

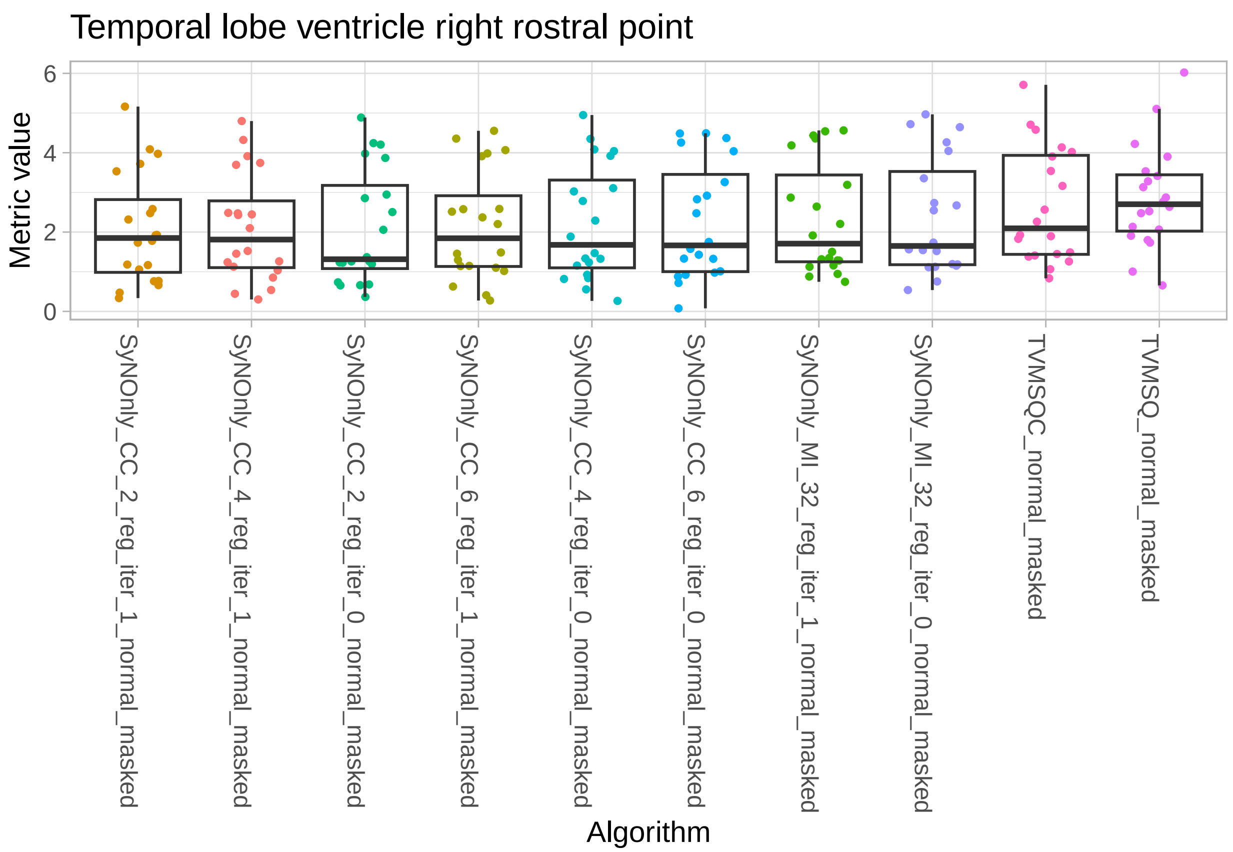

Temporal lobe ventricle right rostral point
Metric value
6
4
2
0
SyNOnly_CC_2_reg_iter_1_normal_masked
SyNOnly_CC_4_reg_iter_1_normal_masked
SyNOnly_CC_2_reg_iter_0_normal_masked
SyNOnly_CC_6_reg_iter_1_normal_masked
SyNOnly_CC_4_reg_iter_0_normal_masked
SyNOnly_CC_6_reg_iter_0_normal_masked
SyNOnly_MI_32_reg_iter_1_normal_masked
SyNOnly_MI_32_reg_iter_0_normal_masked
TVMSQC_normal_masked
TVMSQ_normal_masked
Algorithm

2.2 Podium plot

Podium plots (see also Eugster et al., 2008) for visualizing raw assessment data. Upper part (spaghetti plot): Participating algorithms are color-coded, and each colored dot in the plot represents a metric value achieved with the respective algorithm. The actual metric value is encoded by the y-axis. Each podium (here: p=10) represents one possible rank, ordered from best (1) to last (here: 10). The assignment of metric values (i.e. colored dots) to one of the podiums is based on the rank that the respective algorithm achieved on the corresponding case. Note that the plot part above each podium place is further subdivided into p "columns", where each column represents one participating algorithm (here: $p = 10$). Dots corresponding to identical cases are connected by a line, leading to the shown spaghetti structure. Lower part: Bar charts represent the relative frequency for each algorithm to achieve the rank encoded by the podium place.

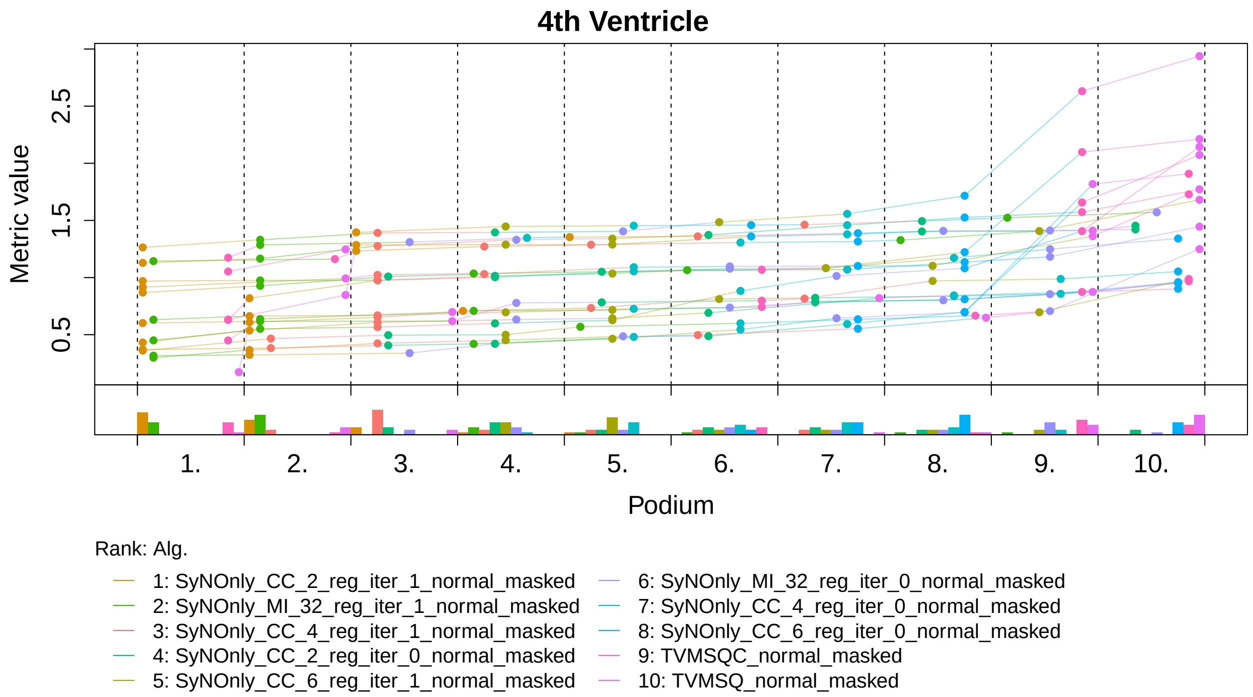

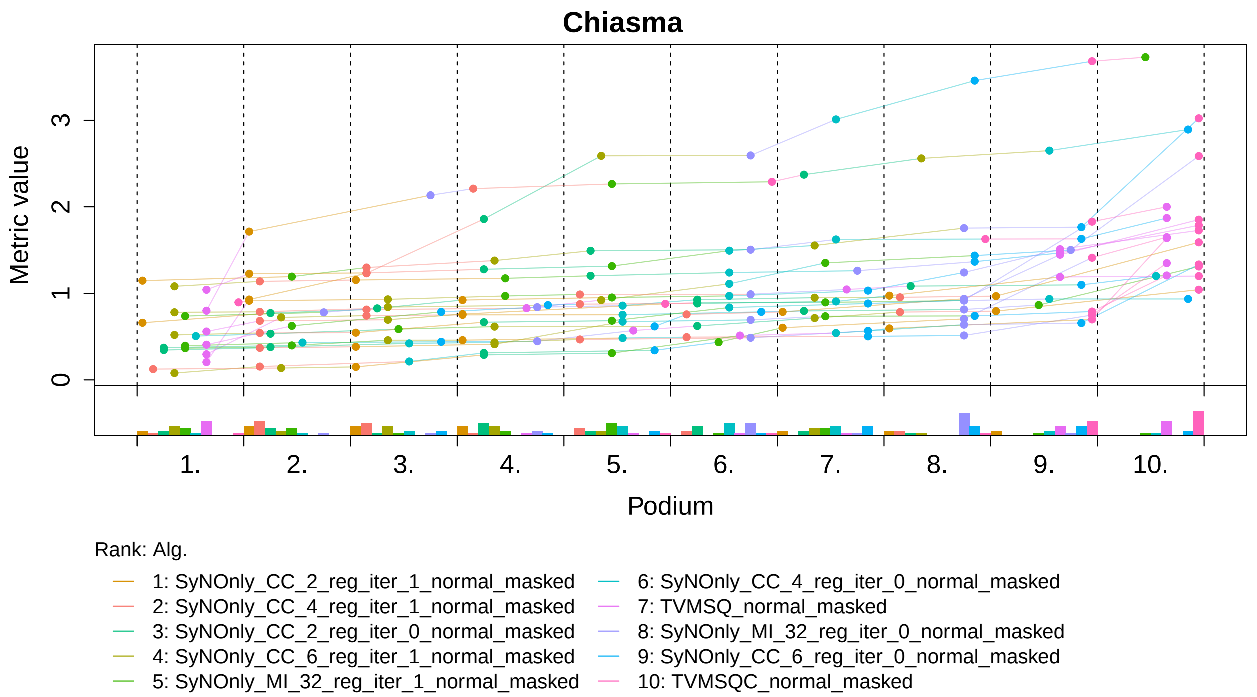

Chiasma
Metric value
Podium
Rank: Alg.
1: SyNOnly_CC_2_reg_iter_1_normal_masked
2: SyNOnly_CC_4_reg_iter_1_normal_masked
3: SyNOnly_CC_2_reg_iter_0_normal_masked
4: SyNOnly_CC_6_reg_iter_1_normal_masked
5: SyNOnly_MI_32_reg_iter_1_normal_masked
6: SyNOnly_CC_4_reg_iter_0_normal_masked
7: TVMSQ_normal_masked
8: SyNOnly_MI_32_reg_iter_0_normal_masked
9: SyNOnly_CC_6_reg_iter_0_normal_masked
10: TVMSQC_normal_masked

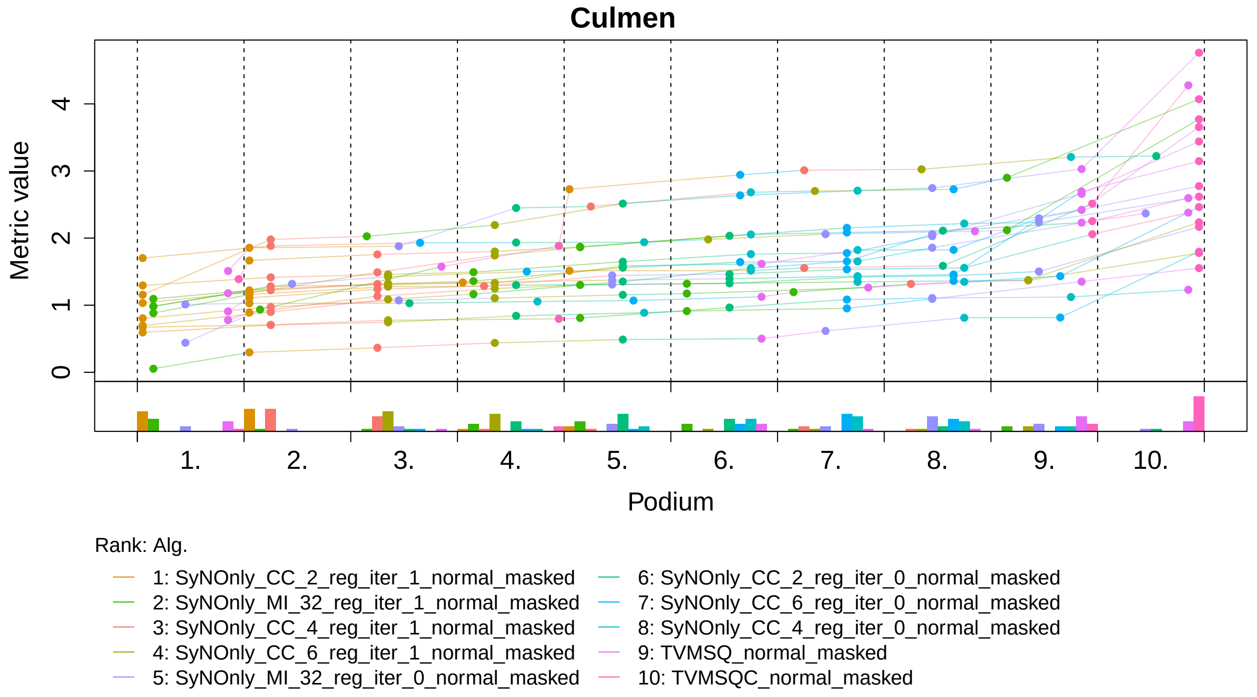

Culmen
Metric value
Podium
Rank: Alg.
1: SyNOnly_CC_2_reg_iter_1_normal_masked
2: SyNOnly_MI_32_reg_iter_1_normal_masked
3: SyNOnly_CC_4_reg_iter_1_normal_masked
4: SyNOnly_CC_6_reg_iter_1_normal_masked
5: SyNOnly_MI_32_reg_iter_0_normal_masked
6: SyNOnly_CC_2_reg_iter_0_normal_masked
7: SyNOnly_CC_6_reg_iter_0_normal_masked
8: SyNOnly_CC_4_reg_iter_0_normal_masked
9: TVMSQ_normal_masked
10: TVMSQC_normal_masked

Hemisphere left caudal point

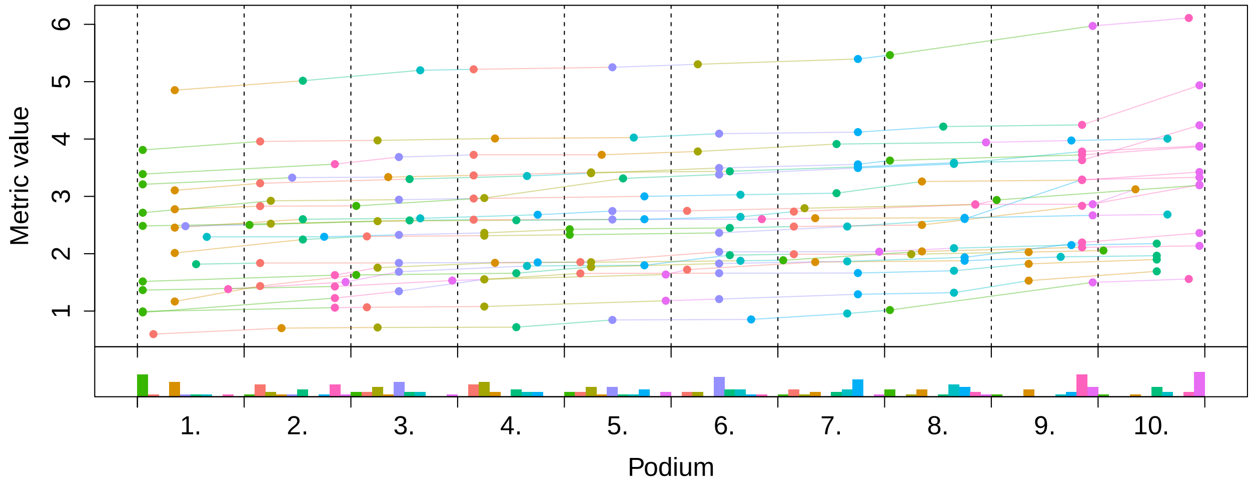

Rank: Alg.

- 1: SyNOnly_MI_32_reg_iter_1_normal_masked
- 2: SyNOnly_CC_4_reg_iter_1_normal_masked
- 3: SyNOnly_CC_6_reg_iter_1_normal_masked
- 4: SyNOnly_CC_2_reg_iter_1_normal_masked
- 5: SyNOnly_MI_32_reg_iter_0_normal_masked
- 6: SyNOnly_CC_2_reg_iter_0_normal_masked
- 7: SyNOnly_CC_4_reg_iter_0_normal_masked
- 8: SyNOnly_CC_6_reg_iter_0_normal_masked
- 9: TVMSQC_normal_masked
- 10: TVMSQ_normal_masked

Hemisphere left rostral point

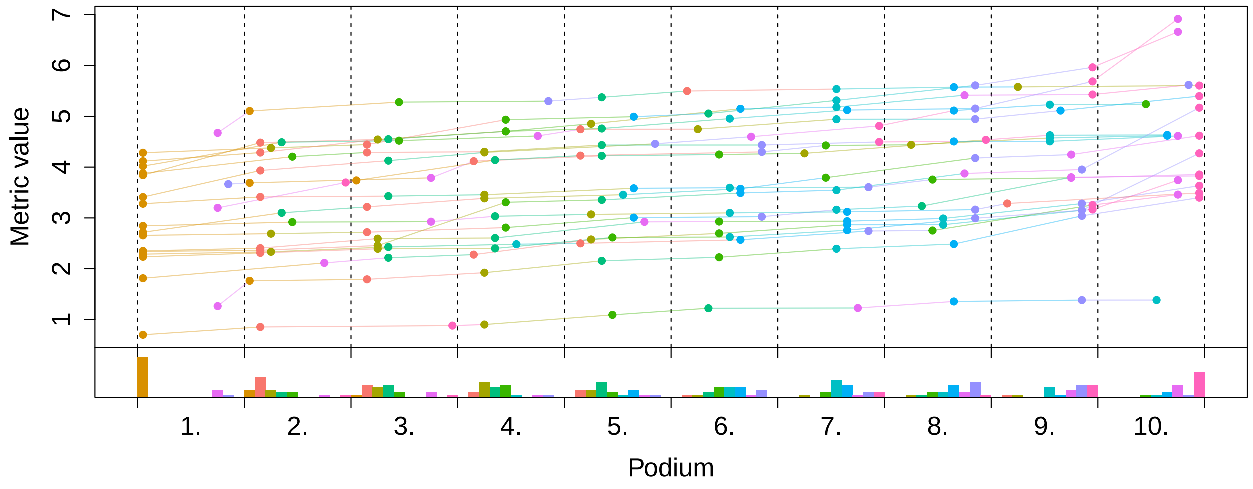

Rank: Alg.

- 1: SyNOnly_CC_2_reg_iter_1_normal_masked
- 2: SyNOnly_CC_4_reg_iter_1_normal_masked
- 3: SyNOnly_CC_6_reg_iter_1_normal_masked
- 4: SyNOnly_CC_2_reg_iter_0_normal_masked
- 5: SyNOnly_MI_32_reg_iter_1_normal_masked
- 6: SyNOnly_CC_4_reg_iter_0_normal_masked
- 7: SyNOnly_CC_6_reg_iter_0_normal_masked
- 8: TVMSQ_normal_masked
- 9: SyNOnly_MI_32_reg_iter_0_normal_masked
- 10: TVMSQC_normal_masked

Hemisphere right caudal point

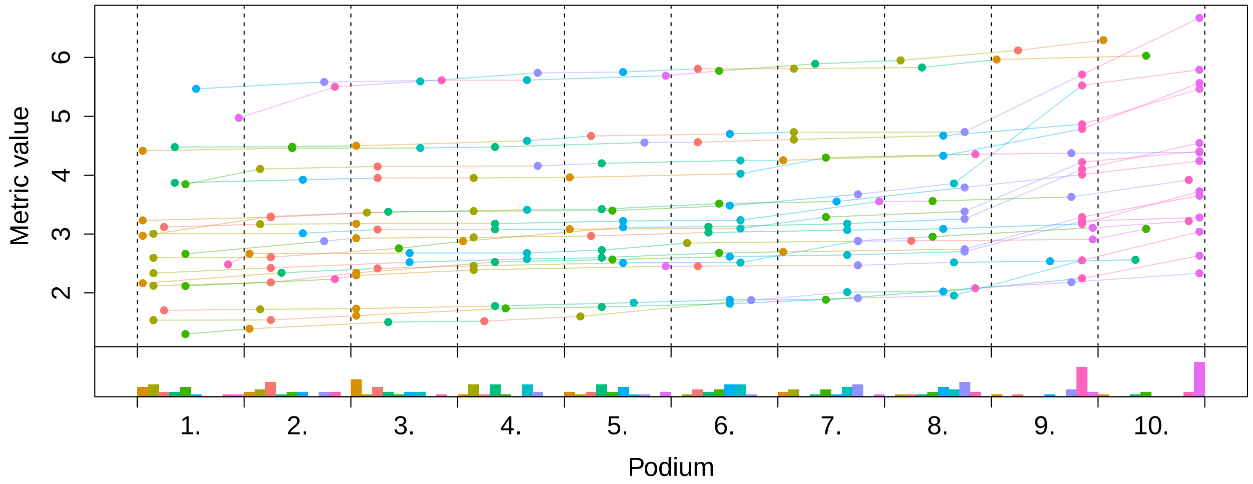

Rank: Alg.

- 1: SyNOnly_CC_2_reg_iter_1_normal_masked
- 2: SyNOnly_CC_6_reg_iter_1_normal_masked
- 3: SyNOnly_CC_4_reg_iter_1_normal_masked
- 4: SyNOnly_CC_2_reg_iter_0_normal_masked
- 5: SyNOnly_MI_32_reg_iter_1_normal_masked
- 6: SyNOnly_CC_6_reg_iter_0_normal_masked
- 7: SyNOnly_CC_4_reg_iter_0_normal_masked
- 8: SyNOnly_MI_32_reg_iter_0_normal_masked
- 9: TVMSQC_normal_masked
- 10: TVMSQ_normal_masked

Hemisphere right rostral point

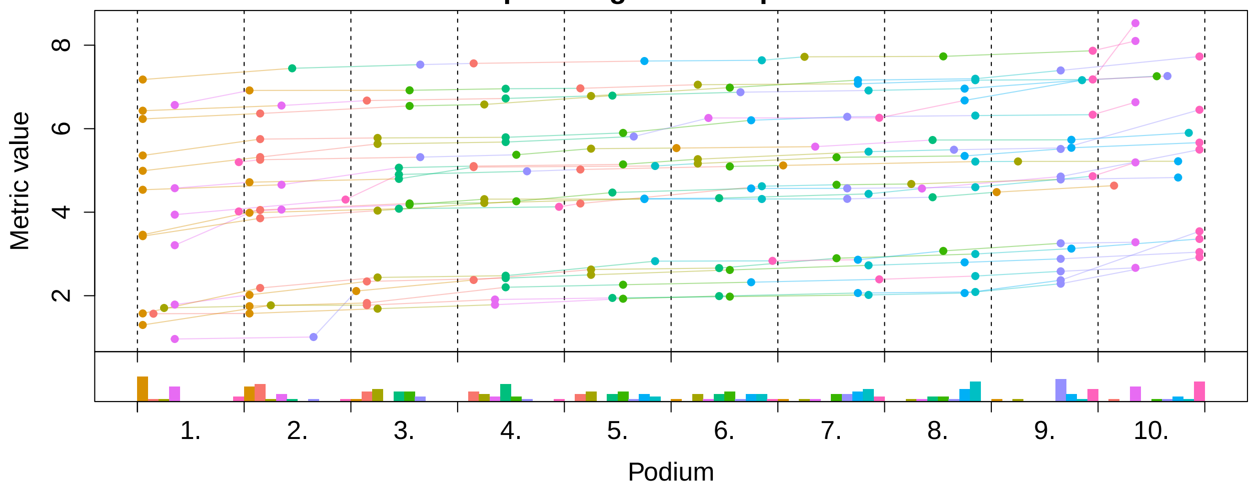

Rank: Alg.

- 1: SyNOnly_CC_2_reg_iter_1_normal_masked
- 2: SyNOnly_CC_4_reg_iter_1_normal_masked
- 3: SyNOnly_CC_6_reg_iter_1_normal_masked
- 4: TVMSQ_normal_masked
- 5: SyNOnly_CC_2_reg_iter_0_normal_masked
- 6: SyNOnly_MI_32_reg_iter_1_normal_masked
- 7: SyNOnly_MI_32_reg_iter_0_normal_masked
- 8: SyNOnly_CC_6_reg_iter_0_normal_masked
- 9: SyNOnly_CC_4_reg_iter_0_normal_masked
- 10: TVMSQC_normal_masked

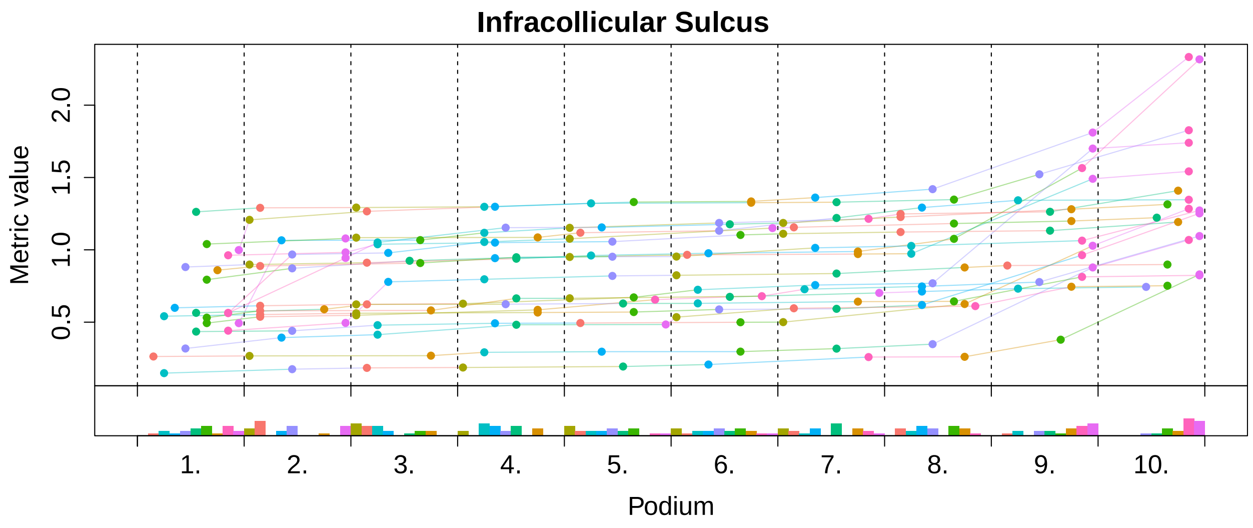

Rank: Alg.

1: SyNOnly_CC_6_reg_iter_1_normal_masked
2: SyNOnly_CC_4_reg_iter_1_normal_masked
3: SyNOnly_CC_4_reg_iter_0_normal_masked
4: SyNOnly_CC_6_reg_iter_0_normal_masked
5: SyNOnly_MI_32_reg_iter_0_normal_masked
6: SyNOnly_CC_2_reg_iter_0_normal_masked
7: SyNOnly_MI_32_reg_iter_1_normal_masked
8: SyNOnly_CC_2_reg_iter_1_normal_masked
9: TVMSQC_normal_masked
10: TVMSQ_normal_masked

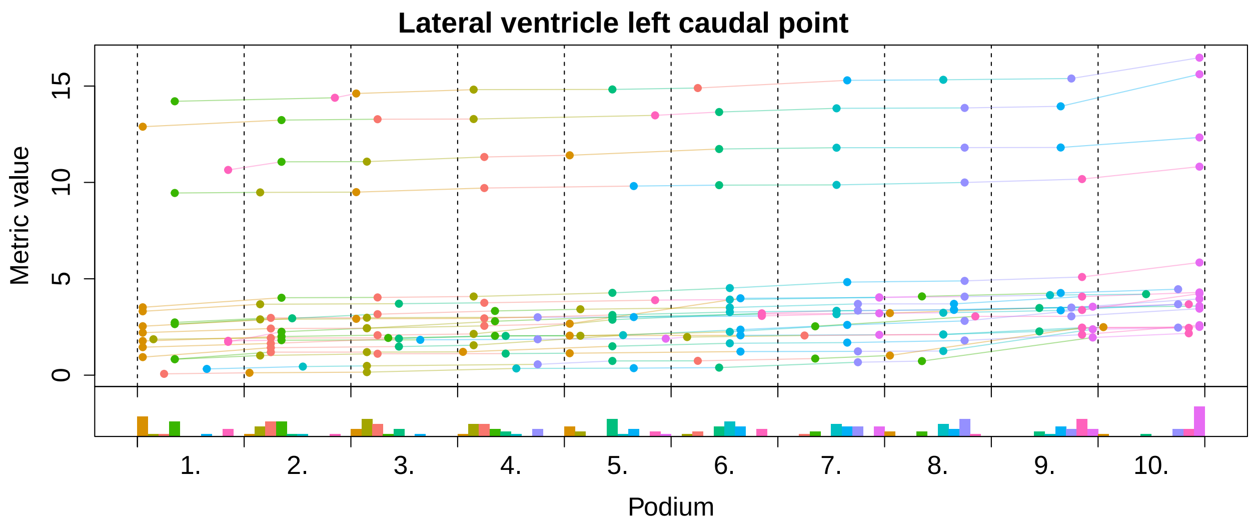

Rank: Alg.

1: SyNOnly_CC_2_reg_iter_1_normal_masked
2: SyNOnly_CC_6_reg_iter_1_normal_masked
3: SyNOnly_CC_4_reg_iter_1_normal_masked
4: SyNOnly_MI_32_reg_iter_1_normal_masked
5: SyNOnly_CC_2_reg_iter_0_normal_masked
6: SyNOnly_CC_4_reg_iter_0_normal_masked
7: SyNOnly_CC_6_reg_iter_0_normal_masked
8: SyNOnly_MI_32_reg_iter_0_normal_masked
9: TVMSQC_normal_masked
10: TVMSQ_normal_masked

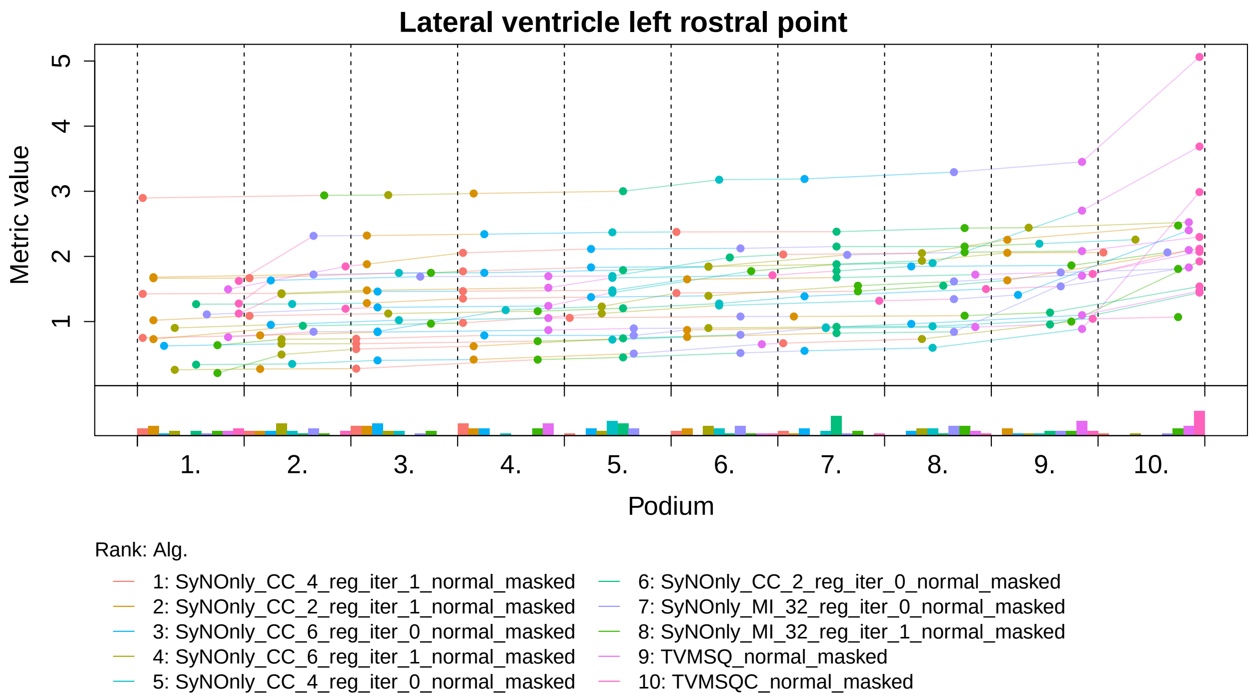

Lateral ventricle left rostral point
Metric value
Podium
1.
2.
3.
4.
5.
6.
7.
8.
9.
10.
Rank: Alg.
1: SyNOnly_CC_4_reg_iter_1_normal_masked
2: SyNOnly_CC_2_reg_iter_1_normal_masked
3: SyNOnly_CC_6_reg_iter_0_normal_masked
4: SyNOnly_CC_6_reg_iter_1_normal_masked
5: SyNOnly_CC_4_reg_iter_0_normal_masked
6: SyNOnly_CC_2_reg_iter_0_normal_masked
7: SyNOnly_MI_32_reg_iter_0_normal_masked
8: SyNOnly_MI_32_reg_iter_1_normal_masked
9: TVMSQ_normal_masked
10: TVMSQC_normal_masked

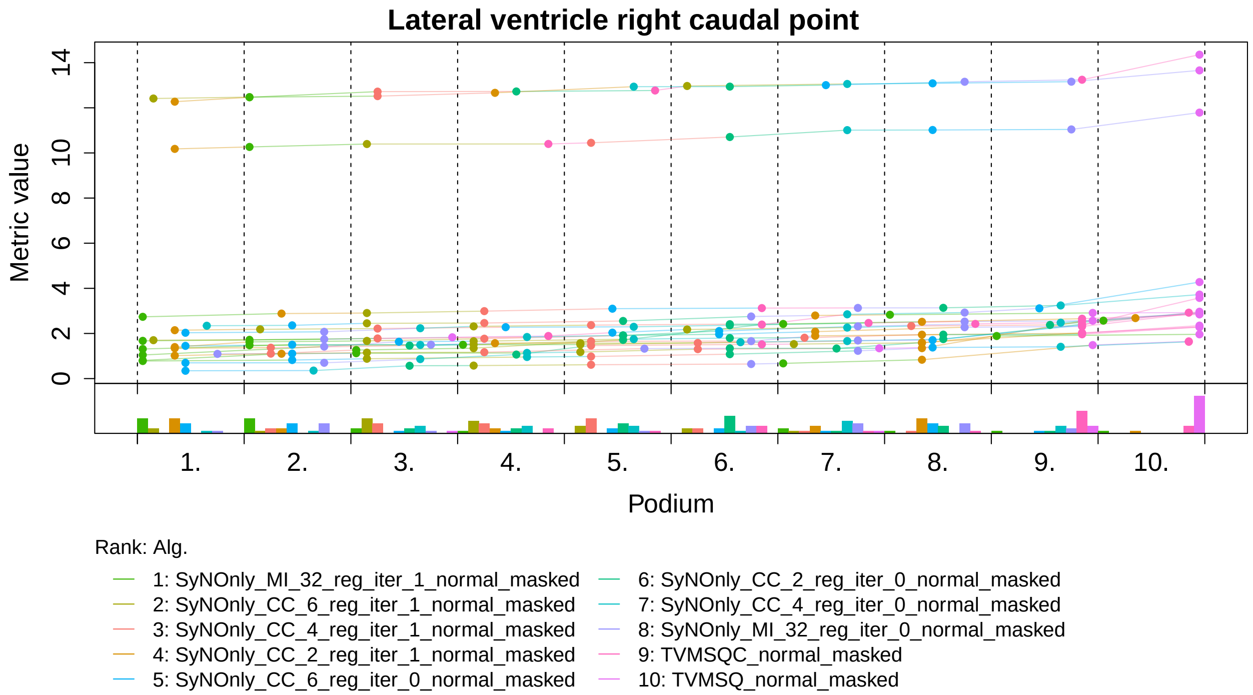

Lateral ventricle right caudal point
Metric value
Podium
1.
2.
3.
4.
5.
6.
7.
8.
9.
10.
Rank: Alg.
1: SyNOnly_MI_32_reg_iter_1_normal_masked
2: SyNOnly_CC_6_reg_iter_1_normal_masked
3: SyNOnly_CC_4_reg_iter_1_normal_masked
4: SyNOnly_CC_2_reg_iter_1_normal_masked
5: SyNOnly_CC_6_reg_iter_0_normal_masked
6: SyNOnly_CC_2_reg_iter_0_normal_masked
7: SyNOnly_CC_4_reg_iter_0_normal_masked
8: SyNOnly_MI_32_reg_iter_0_normal_masked
9: TVMSQC_normal_masked
10: TVMSQ_normal_masked

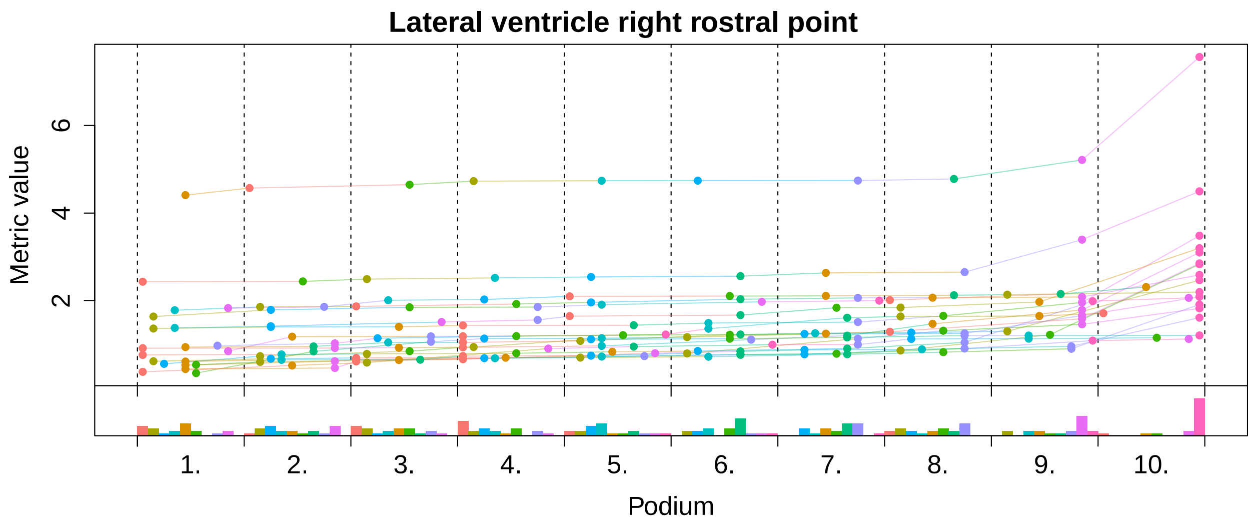

Rank: Alg.

1: SyNOnly_CC_4_reg_iter_1_normal_masked
2: SyNOnly_CC_6_reg_iter_1_normal_masked
3: SyNOnly_CC_6_reg_iter_0_normal_masked
4: SyNOnly_CC_4_reg_iter_0_normal_masked
5: SyNOnly_CC_2_reg_iter_1_normal_masked
6: SyNOnly_MI_32_reg_iter_1_normal_masked
7: SyNOnly_CC_2_reg_iter_0_normal_masked
8: SyNOnly_MI_32_reg_iter_0_normal_masked
9: TVMSQ_normal_masked
10: TVMSQC_normal_masked

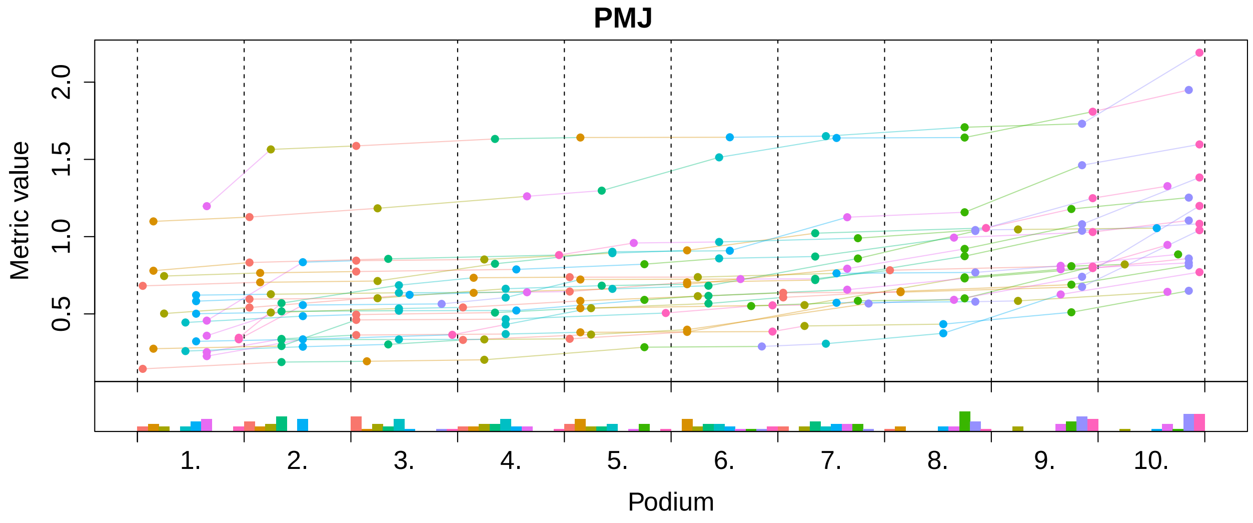

Rank: Alg.

1: SyNOnly_CC_4_reg_iter_1_normal_masked
2: SyNOnly_CC_2_reg_iter_1_normal_masked
3: SyNOnly_CC_6_reg_iter_1_normal_masked
4: SyNOnly_CC_2_reg_iter_0_normal_masked
5: SyNOnly_CC_4_reg_iter_0_normal_masked
6: SyNOnly_CC_6_reg_iter_0_normal_masked
7: TVMSQ_normal_masked
8: SyNOnly_MI_32_reg_iter_1_normal_masked
9: SyNOnly_MI_32_reg_iter_0_normal_masked
10: TVMSQC_normal_masked

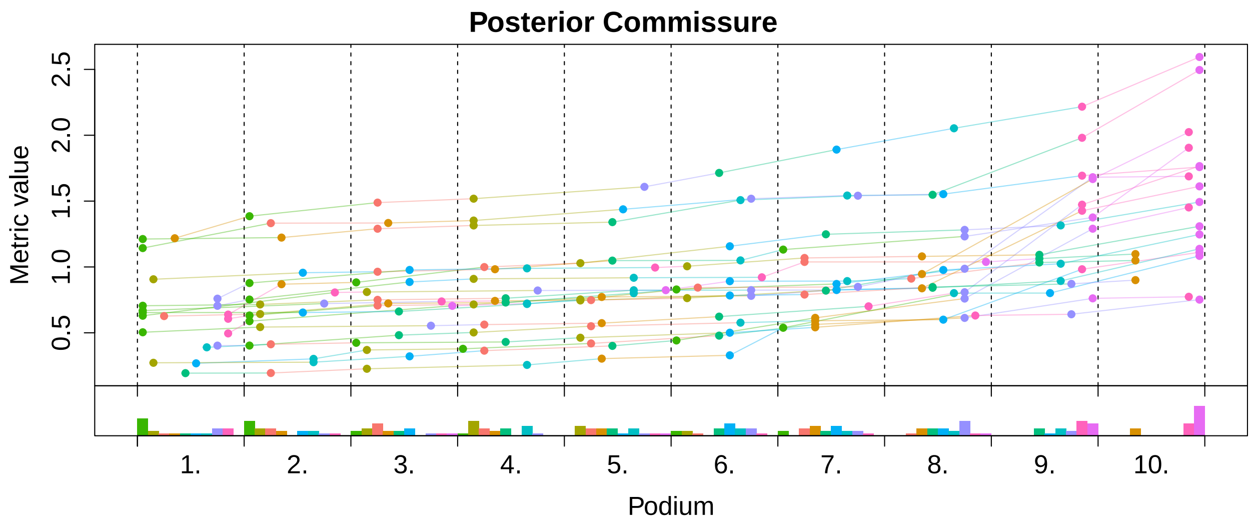

Rank: Alg.

1: SyNOnly_MI_32_reg_iter_1_normal_masked
2: SyNOnly_CC_6_reg_iter_1_normal_masked
3: SyNOnly_CC_4_reg_iter_1_normal_masked
4: SyNOnly_CC_2_reg_iter_1_normal_masked
5: SyNOnly_CC_2_reg_iter_0_normal_masked
6: SyNOnly_CC_6_reg_iter_0_normal_masked
7: SyNOnly_CC_4_reg_iter_0_normal_masked
8: SyNOnly_MI_32_reg_iter_0_normal_masked
9: TVMSQC_normal_masked
10: TVMSQ_normal_masked

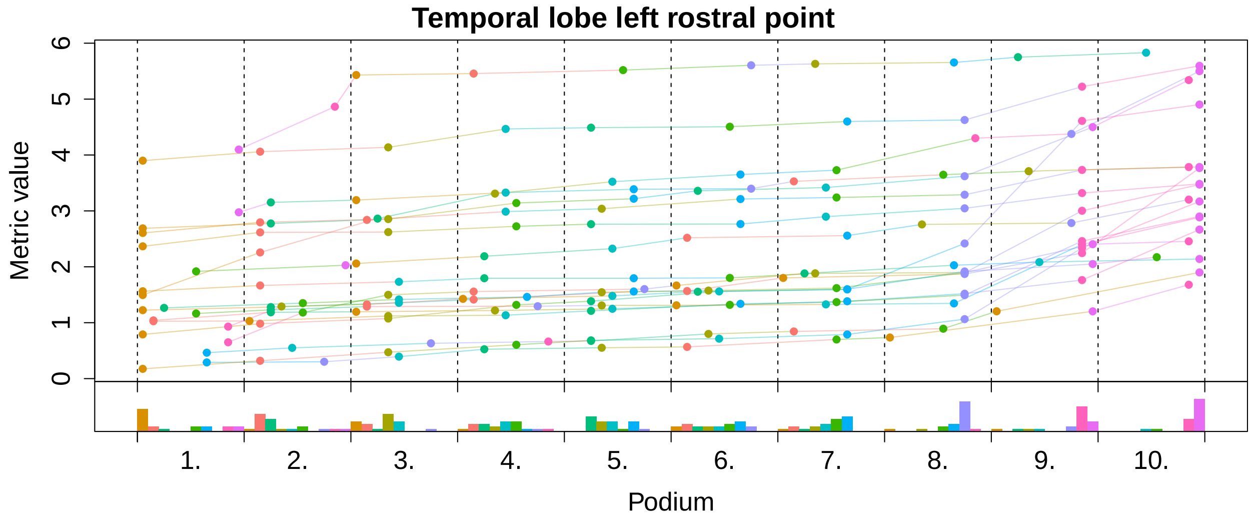

Rank: Alg.

1: SyNOnly_CC_2_reg_iter_1_normal_masked
2: SyNOnly_CC_4_reg_iter_1_normal_masked
3: SyNOnly_CC_2_reg_iter_0_normal_masked
4: SyNOnly_CC_6_reg_iter_1_normal_masked
5: SyNOnly_CC_4_reg_iter_0_normal_masked
6: SyNOnly_MI_32_reg_iter_1_normal_masked
7: SyNOnly_CC_6_reg_iter_0_normal_masked
8: SyNOnly_MI_32_reg_iter_0_normal_masked
9: TVMSQC_normal_masked
10: TVMSQ_normal_masked

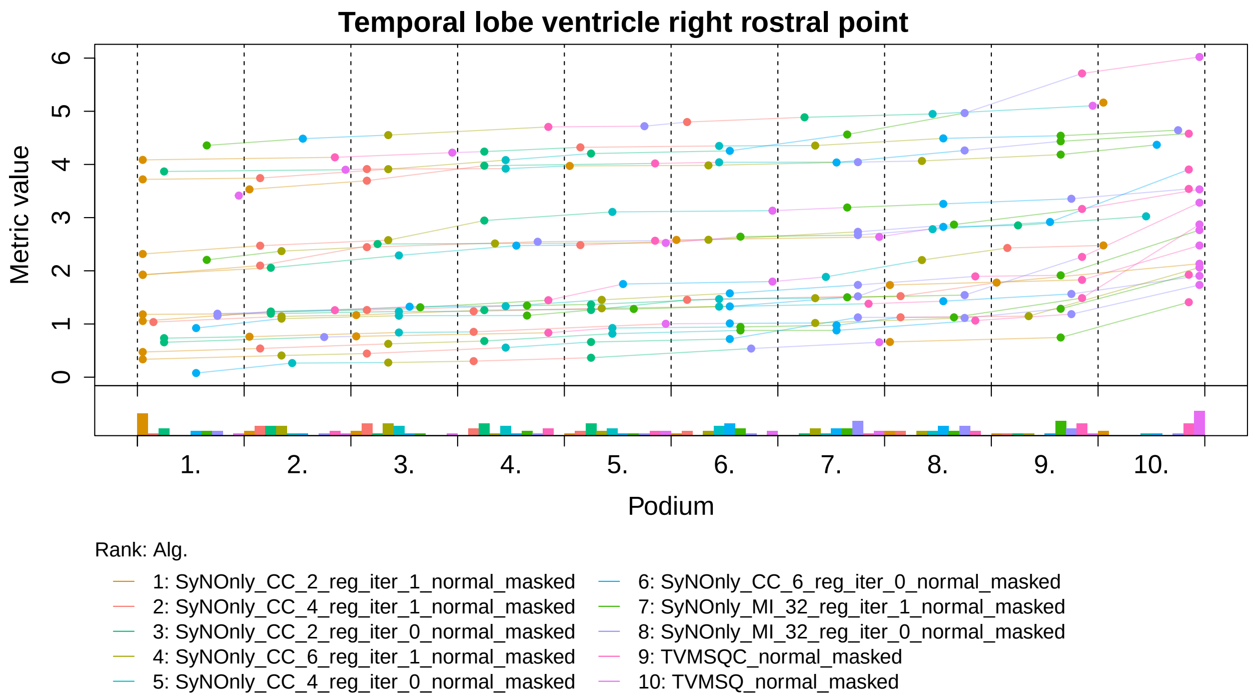

Temporal lobe ventricle right rostral point
Metric value
0
1
2
3
4
5
6
1.
2.
3.
4.
5.
6.
7.
8.
9.
10.
Podium
Rank: Alg.
1: SyNOnly_CC_2_reg_iter_1_normal_masked
2: SyNOnly_CC_4_reg_iter_1_normal_masked
3: SyNOnly_CC_2_reg_iter_0_normal_masked
4: SyNOnly_CC_6_reg_iter_1_normal_masked
5: SyNOnly_CC_4_reg_iter_0_normal_masked
6: SyNOnly_CC_6_reg_iter_0_normal_masked
7: SyNOnly_MI_32_reg_iter_1_normal_masked
8: SyNOnly_MI_32_reg_iter_0_normal_masked
9: TVMSQC_normal_masked
10: TVMSQ_normal_masked

2.3 Ranking heatmap

Ranking heatmaps for visualizing raw assessment data. Each cell (i, A_j) shows the absolute frequency of cases in which algorithm A_j achieved rank i.

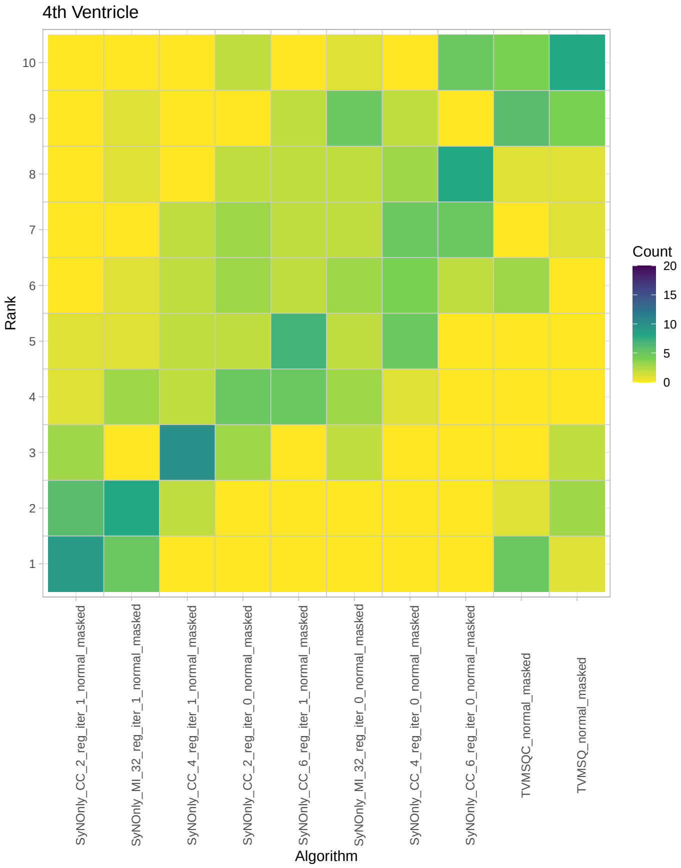

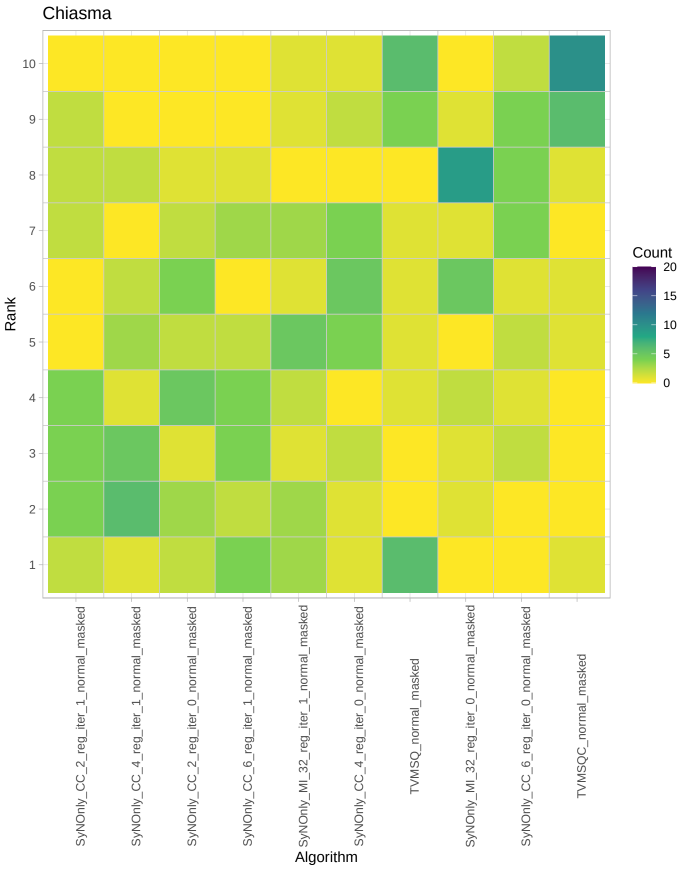
Chiasma
Rank
10
9
8
7
6
5
4
3
2
1
Count
20
15
10
5
0
SyNOnly_CC_2_reg_iter_1_normal_masked
SyNOnly_CC_4_reg_iter_1_normal_masked
SyNOnly_CC_2_reg_iter_0_normal_masked
SyNOnly_CC_6_reg_iter_1_normal_masked
SyNOnly_MI_32_reg_iter_1_normal_masked
SyNOnly_CC_4_reg_iter_0_normal_masked
TVMSQ_normal_masked
SyNOnly_MI_32_reg_iter_0_normal_masked
SyNOnly_CC_6_reg_iter_0_normal_masked
TVMSQC_normal_masked
Algorithm

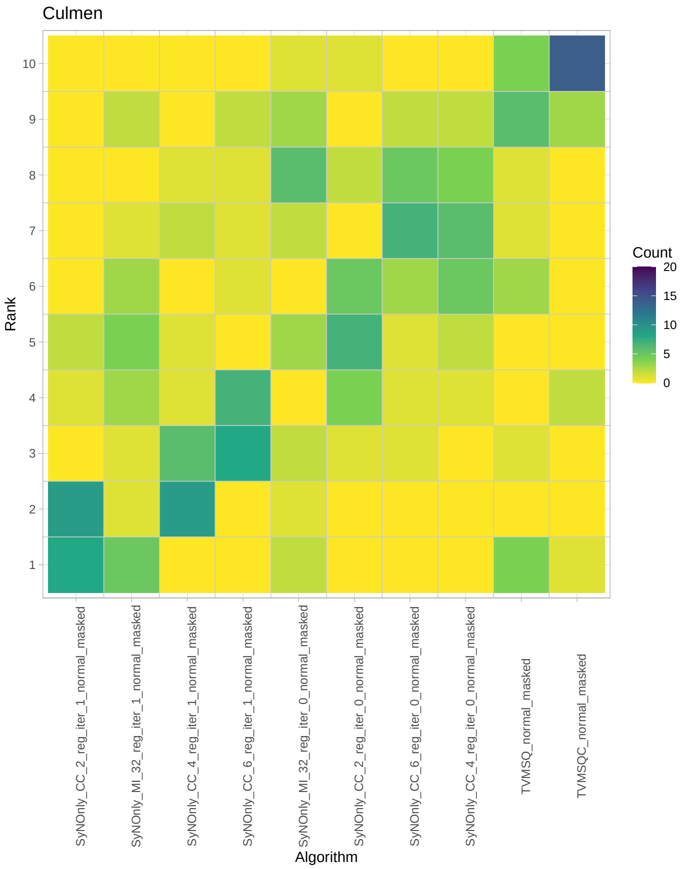

Culmen
Rank
10
9
8
7
6
5
4
3
2
1
Count
20
15
10
5
0
SyNOnly_CC_2_reg_iter_1_normal_masked
SyNOnly_MI_32_reg_iter_1_normal_masked
SyNOnly_CC_4_reg_iter_1_normal_masked
SyNOnly_CC_6_reg_iter_1_normal_masked
SyNOnly_MI_32_reg_iter_0_normal_masked
SyNOnly_CC_2_reg_iter_0_normal_masked
SyNOnly_CC_6_reg_iter_0_normal_masked
SyNOnly_CC_4_reg_iter_0_normal_masked
TVMSQ_normal_masked
TVMSQC_normal_masked
Algorithm

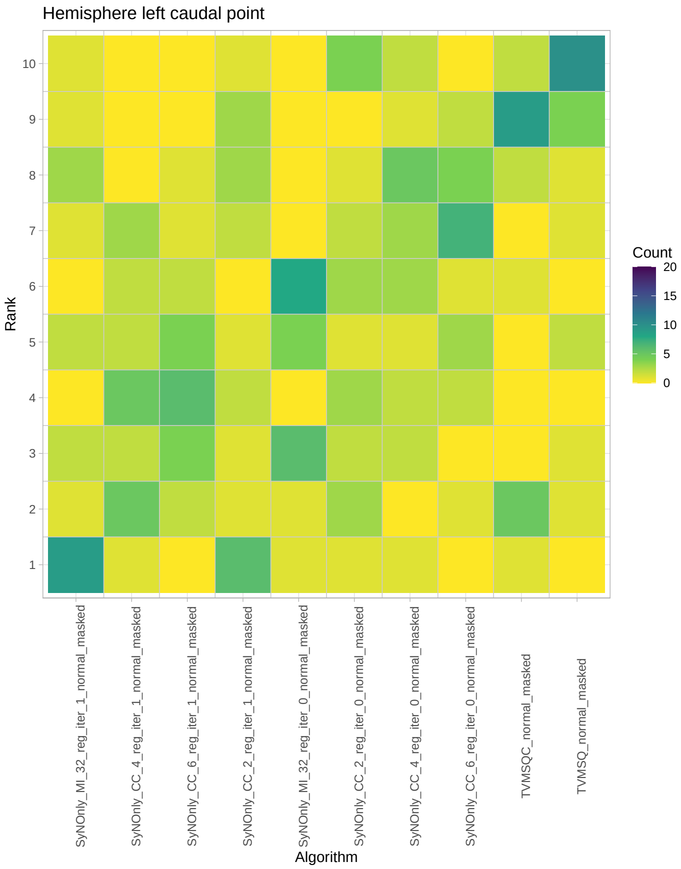
Hemisphere left caudal point
Rank
10
9
8
7
6
5
4
3
2
1
Count
20
15
10
5
0
SyNOnly_MI_32_reg_iter_1_normal_masked
SyNOnly_CC_4_reg_iter_1_normal_masked
SyNOnly_CC_6_reg_iter_1_normal_masked
SyNOnly_CC_2_reg_iter_1_normal_masked
SyNOnly_MI_32_reg_iter_0_normal_masked
SyNOnly_CC_2_reg_iter_0_normal_masked
SyNOnly_CC_4_reg_iter_0_normal_masked
SyNOnly_CC_6_reg_iter_0_normal_masked
TVMSQC_normal_masked
TVMSQ_normal_masked
Algorithm

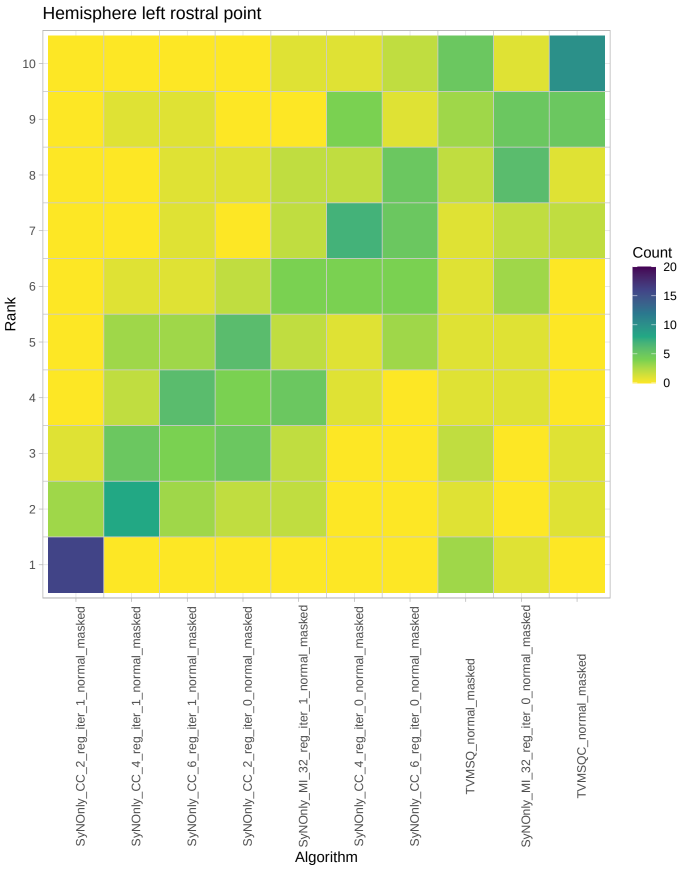

Hemisphere left rostral point
Rank
10
9
8
7
6
5
4
3
2
1
Count
20
15
10
5
0
SyNOnly_CC_2_reg_iter_1_normal_masked
SyNOnly_CC_4_reg_iter_1_normal_masked
SyNOnly_CC_6_reg_iter_1_normal_masked
SyNOnly_CC_2_reg_iter_0_normal_masked
SyNOnly_MI_32_reg_iter_1_normal_masked
SyNOnly_CC_4_reg_iter_0_normal_masked
SyNOnly_CC_6_reg_iter_0_normal_masked
TVMSQ_normal_masked
SyNOnly_MI_32_reg_iter_0_normal_masked
TVMSQC_normal_masked
Algorithm

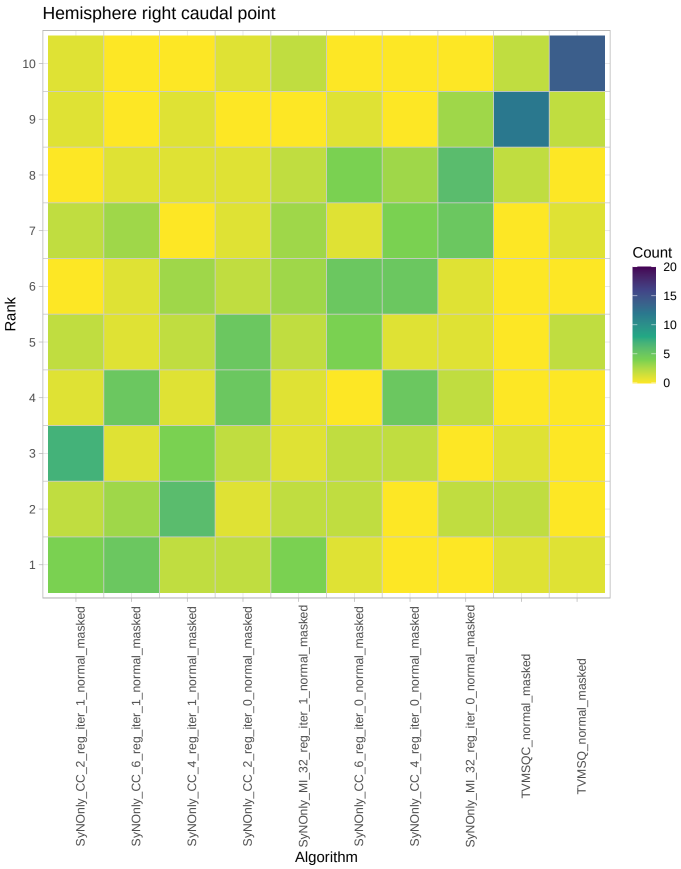

Hemisphere right caudal point
Rank
10
9
8
7
6
5
4
3
2
1
Count
20
15
10
5
0
SyNOnly_CC_2_reg_iter_1_normal_masked
SyNOnly_CC_6_reg_iter_1_normal_masked
SyNOnly_CC_4_reg_iter_1_normal_masked
SyNOnly_CC_2_reg_iter_0_normal_masked
SyNOnly_MI_32_reg_iter_1_normal_masked
SyNOnly_CC_6_reg_iter_0_normal_masked
SyNOnly_CC_4_reg_iter_0_normal_masked
SyNOnly_MI_32_reg_iter_0_normal_masked
TVMSQC_normal_masked
TVMSQ_normal_masked
Algorithm

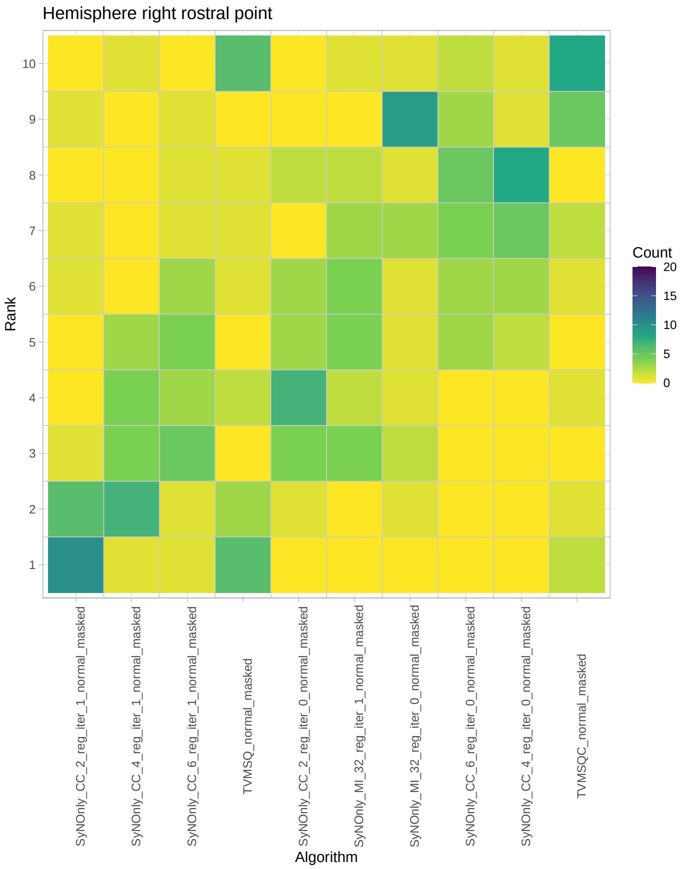
Hemisphere right rostral point
Rank
10
9
8
7
6
5
4
3
2
1
Count
20
15
10
5
0
SyNOnly_CC_2_reg_iter_1_normal_masked
SyNOnly_CC_4_reg_iter_1_normal_masked
SyNOnly_CC_6_reg_iter_1_normal_masked
TVMSQ_normal_masked
SyNOnly_CC_2_reg_iter_0_normal_masked
SyNOnly_MI_32_reg_iter_1_normal_masked
SyNOnly_MI_32_reg_iter_0_normal_masked
SyNOnly_CC_6_reg_iter_0_normal_masked
SyNOnly_CC_4_reg_iter_0_normal_masked
TVMSQC_normal_masked
Algorithm

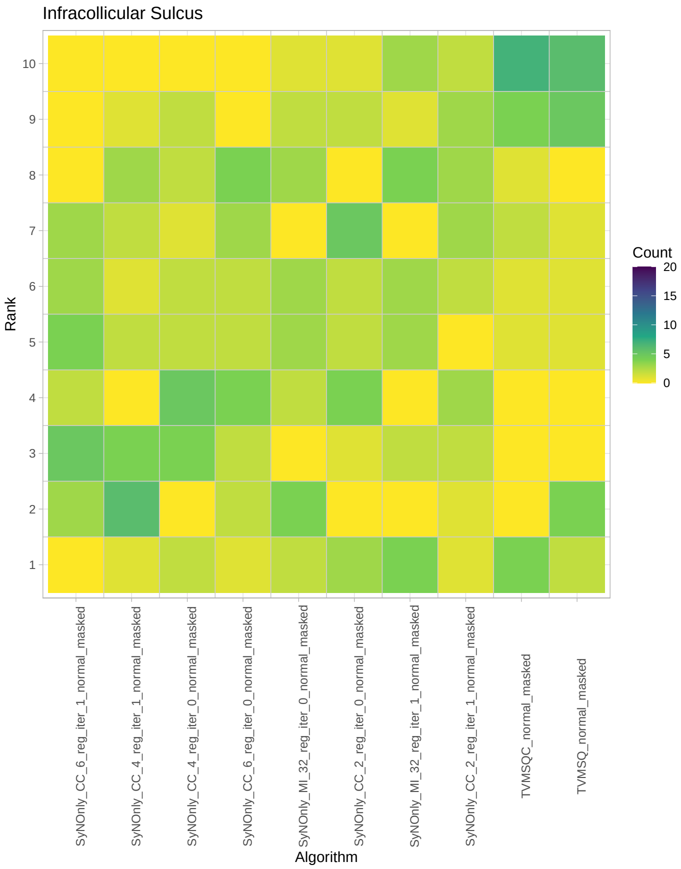
Infracollicular Sulcus
Rank
10
9
8
7
6
5
4
3
2
1
Count
20
15
10
5
0
SyNOnly_CC_6_reg_iter_1_normal_masked
SyNOnly_CC_4_reg_iter_1_normal_masked
SyNOnly_CC_4_reg_iter_0_normal_masked
SyNOnly_CC_6_reg_iter_0_normal_masked
SyNOnly_MI_32_reg_iter_0_normal_masked
SyNOnly_CC_2_reg_iter_0_normal_masked
SyNOnly_MI_32_reg_iter_1_normal_masked
SyNOnly_CC_2_reg_iter_1_normal_masked
TVMSQC_normal_masked
TVMSQ_normal_masked
Algorithm

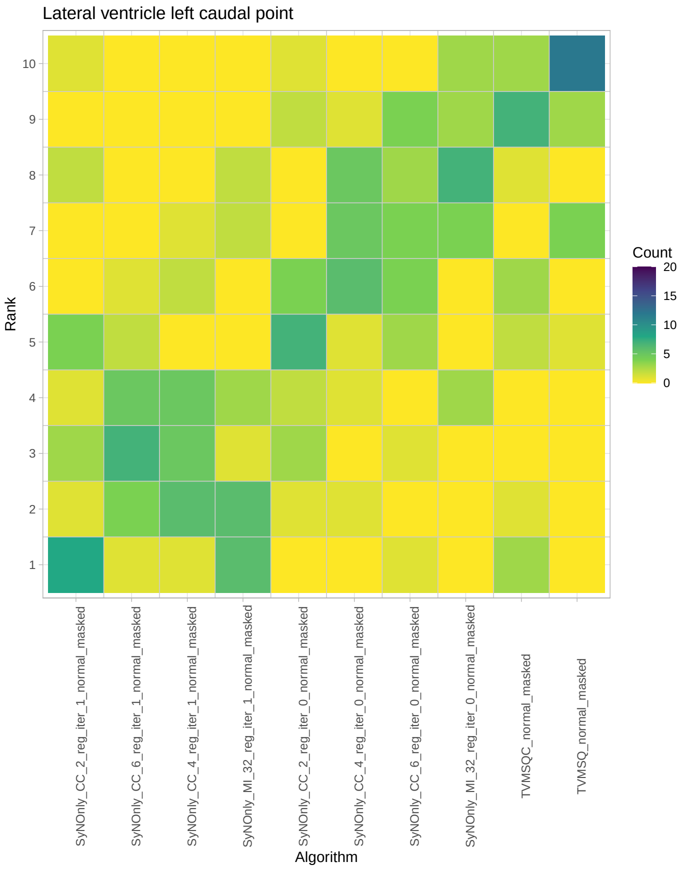
Lateral ventricle left caudal point
Rank
10
9
8
7
6
5
4
3
2
1
Count
20
15
10
5
0
SyNOnly_CC_2_reg_iter_1_normal_masked
SyNOnly_CC_6_reg_iter_1_normal_masked
SyNOnly_CC_4_reg_iter_1_normal_masked
SyNOnly_MI_32_reg_iter_1_normal_masked
SyNOnly_CC_2_reg_iter_0_normal_masked
SyNOnly_CC_4_reg_iter_0_normal_masked
SyNOnly_CC_6_reg_iter_0_normal_masked
SyNOnly_MI_32_reg_iter_0_normal_masked
TVMSQC_normal_masked
TVMSQ_normal_masked
Algorithm

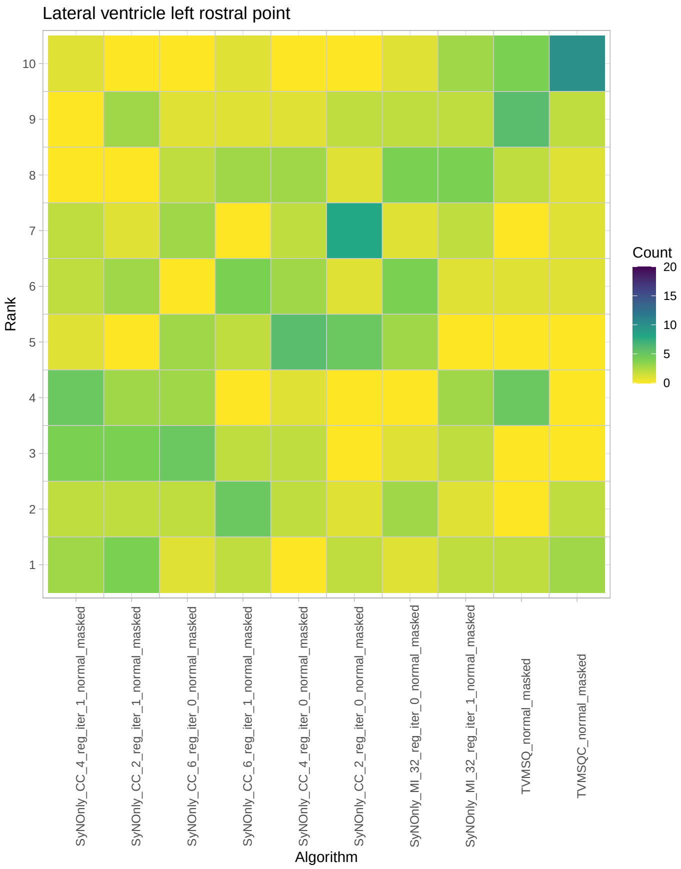
Lateral ventricle left rostral point
Rank
10
9
8
7
6
5
4
3
2
1
Count
20
15
10
5
0
SyNOnly_CC_4_reg_iter_1_normal_masked
SyNOnly_CC_2_reg_iter_1_normal_masked
SyNOnly_CC_6_reg_iter_0_normal_masked
SyNOnly_CC_6_reg_iter_1_normal_masked
SyNOnly_CC_4_reg_iter_0_normal_masked
SyNOnly_CC_2_reg_iter_0_normal_masked
SyNOnly_MI_32_reg_iter_0_normal_masked
SyNOnly_MI_32_reg_iter_1_normal_masked
TVMSQ_normal_masked
TVMSQC_normal_masked
Algorithm

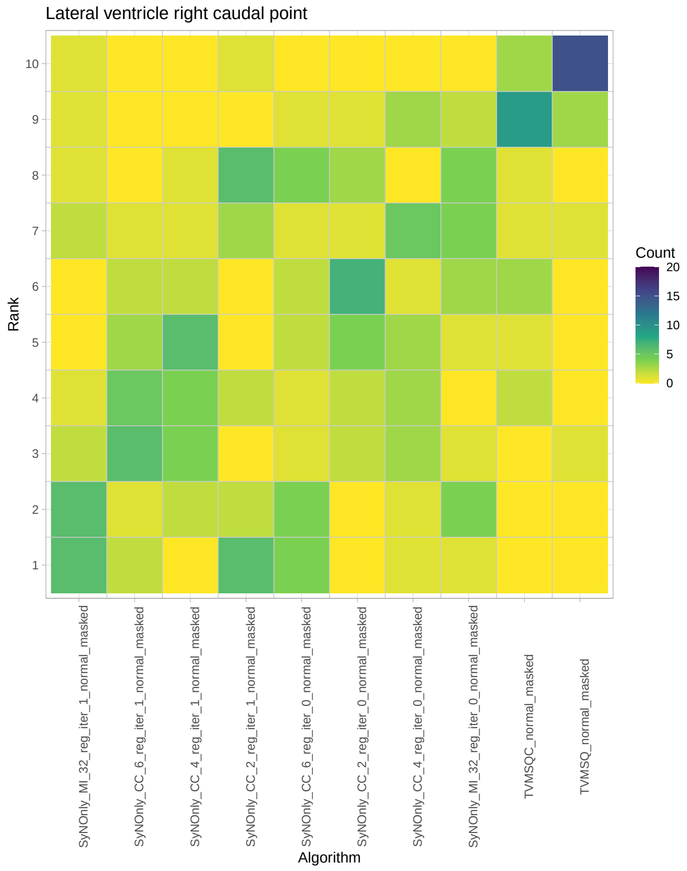
Lateral ventricle right caudal point
Rank
Algorithm
Count
20
15
10
5
0
10
9
8
7
6
5
4
3
2
1
SyNOnly_MI_32_reg_iter_1_normal_masked
SyNOnly_CC_6_reg_iter_1_normal_masked
SyNOnly_CC_4_reg_iter_1_normal_masked
SyNOnly_CC_2_reg_iter_1_normal_masked
SyNOnly_CC_6_reg_iter_0_normal_masked
SyNOnly_CC_2_reg_iter_0_normal_masked
SyNOnly_CC_4_reg_iter_0_normal_masked
SyNOnly_MI_32_reg_iter_0_normal_masked
TVMSQC_normal_masked
TVMSQ_normal_masked

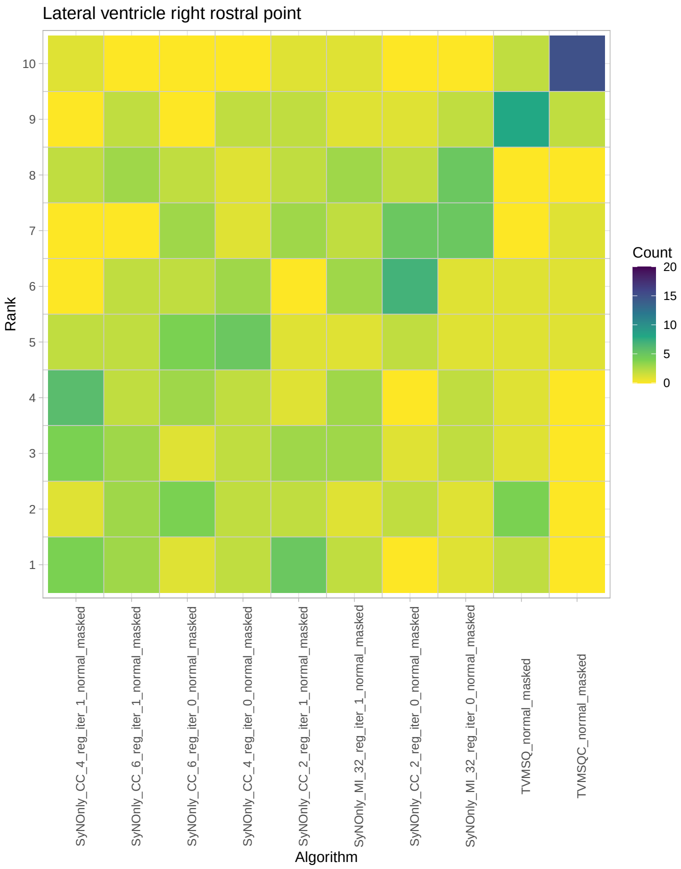

Lateral ventricle right rostral point
Rank
10
9
8
7
6
5
4
3
2
1
Count
20
15
10
5
0
SyNOnly_CC_4_reg_iter_1_normal_masked
SyNOnly_CC_6_reg_iter_1_normal_masked
SyNOnly_CC_6_reg_iter_0_normal_masked
SyNOnly_CC_4_reg_iter_0_normal_masked
SyNOnly_CC_2_reg_iter_1_normal_masked
SyNOnly_MI_32_reg_iter_1_normal_masked
SyNOnly_CC_2_reg_iter_0_normal_masked
SyNOnly_MI_32_reg_iter_0_normal_masked
TVMSQ_normal_masked
TVMSQC_normal_masked
Algorithm

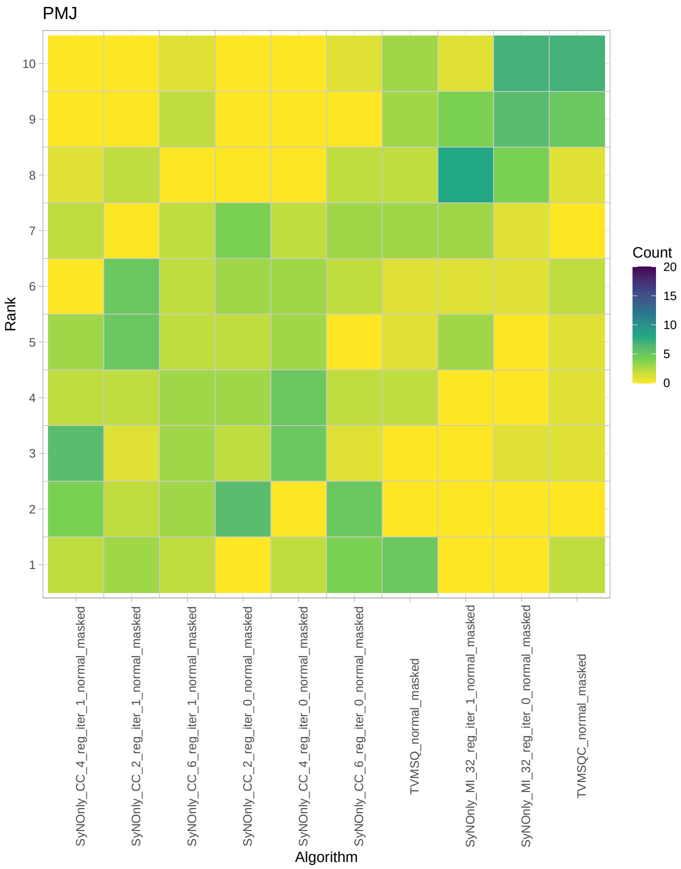
PMJ
Rank
10
9
8
7
6
5
4
3
2
1
Count
20
15
10
5
0
SyNOnly_CC_4_reg_iter_1_normal_masked
SyNOnly_CC_2_reg_iter_1_normal_masked
SyNOnly_CC_6_reg_iter_1_normal_masked
SyNOnly_CC_2_reg_iter_0_normal_masked
SyNOnly_CC_4_reg_iter_0_normal_masked
SyNOnly_CC_6_reg_iter_0_normal_masked
TVMSQ_normal_masked
SyNOnly_MI_32_reg_iter_1_normal_masked
SyNOnly_MI_32_reg_iter_0_normal_masked
TVMSQC_normal_masked
Algorithm

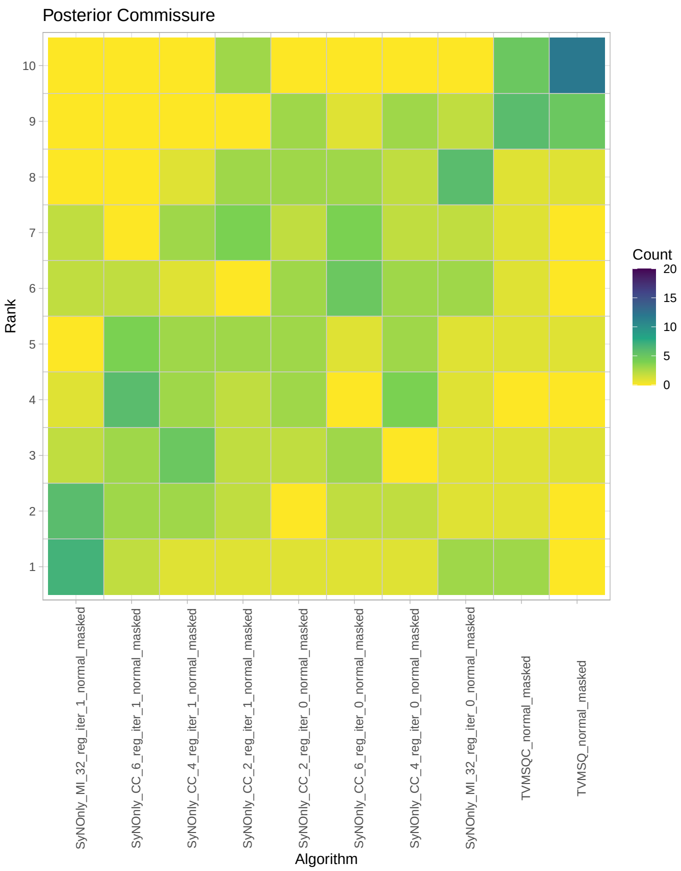
Posterior Commissure
Rank
10
9
8
7
6
5
4
3
2
1
Count
20
15
10
5
0
SyNOnly_MI_32_reg_iter_1_normal_masked
SyNOnly_CC_6_reg_iter_1_normal_masked
SyNOnly_CC_4_reg_iter_1_normal_masked
SyNOnly_CC_2_reg_iter_1_normal_masked
SyNOnly_CC_2_reg_iter_0_normal_masked
SyNOnly_CC_6_reg_iter_0_normal_masked
SyNOnly_CC_4_reg_iter_0_normal_masked
SyNOnly_MI_32_reg_iter_0_normal_masked
TVMSQC_normal_masked
TVMSQ_normal_masked
Algorithm

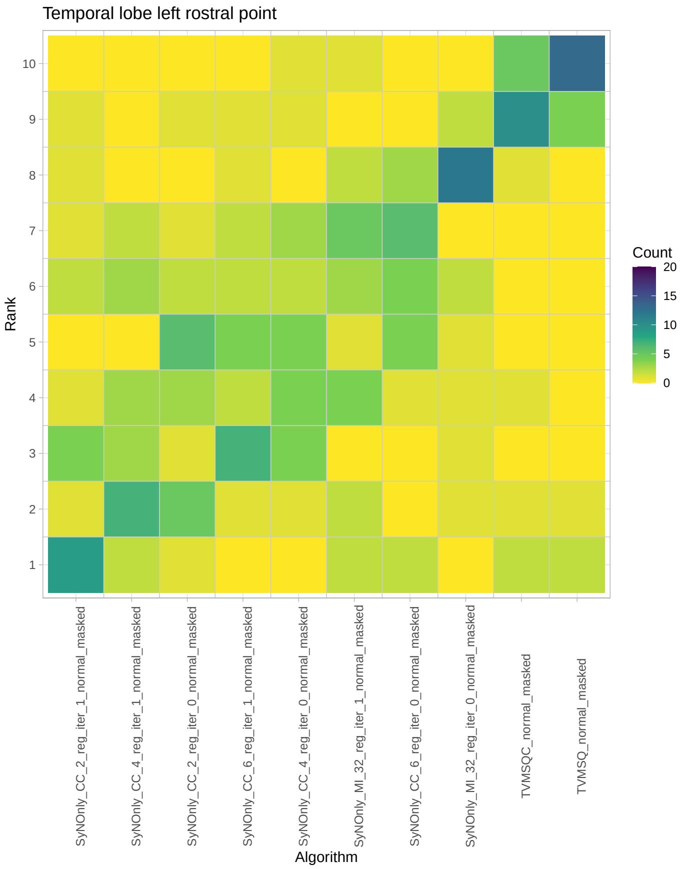

Temporal lobe left rostral point
Rank
10
9
8
7
6
5
4
3
2
1
Count
20
15
10
5
0
SyNOnly_CC_2_reg_iter_1_normal_masked
SyNOnly_CC_4_reg_iter_1_normal_masked
SyNOnly_CC_2_reg_iter_0_normal_masked
SyNOnly_CC_6_reg_iter_1_normal_masked
SyNOnly_CC_4_reg_iter_0_normal_masked
SyNOnly_MI_32_reg_iter_1_normal_masked
SyNOnly_CC_6_reg_iter_0_normal_masked
SyNOnly_MI_32_reg_iter_0_normal_masked
TVMSQC_normal_masked
TVMSQ_normal_masked
Algorithm

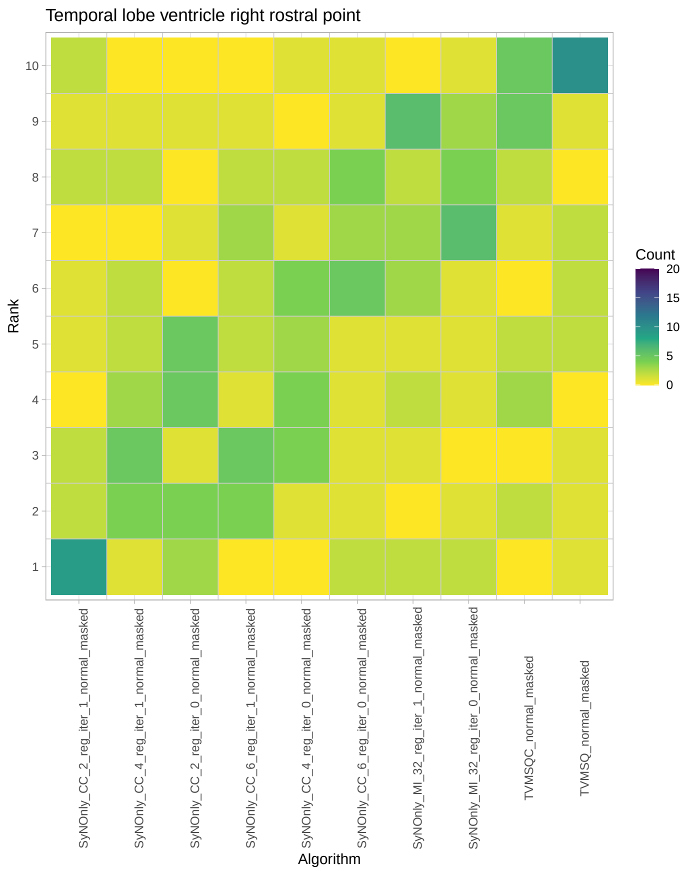
Temporal lobe ventricle right rostral point
Rank
10
9
8
7
6
5
4
3
2
1
Count
20
15
10
5
0
SyNOnly_CC_2_reg_iter_1_normal_masked
SyNOnly_CC_4_reg_iter_1_normal_masked
SyNOnly_CC_2_reg_iter_0_normal_masked
SyNOnly_CC_6_reg_iter_1_normal_masked
SyNOnly_CC_4_reg_iter_0_normal_masked
SyNOnly_CC_6_reg_iter_0_normal_masked
SyNOnly_MI_32_reg_iter_1_normal_masked
SyNOnly_MI_32_reg_iter_0_normal_masked
TVMSQC_normal_masked
TVMSQ_normal_masked
Algorithm

3 Visualization of ranking stability

3.1 *Blob plot* for visualizing ranking stability based on bootstrap sampling

Algorithms are color-coded, and the area of each blob at position $(A_i, \text{rank } j)$ is proportional to the relative frequency A_i achieved rank j across $b = 1000$ bootstrap samples. The median rank for each algorithm is indicated by a black cross. 95% bootstrap intervals across bootstrap samples are indicated by black lines.

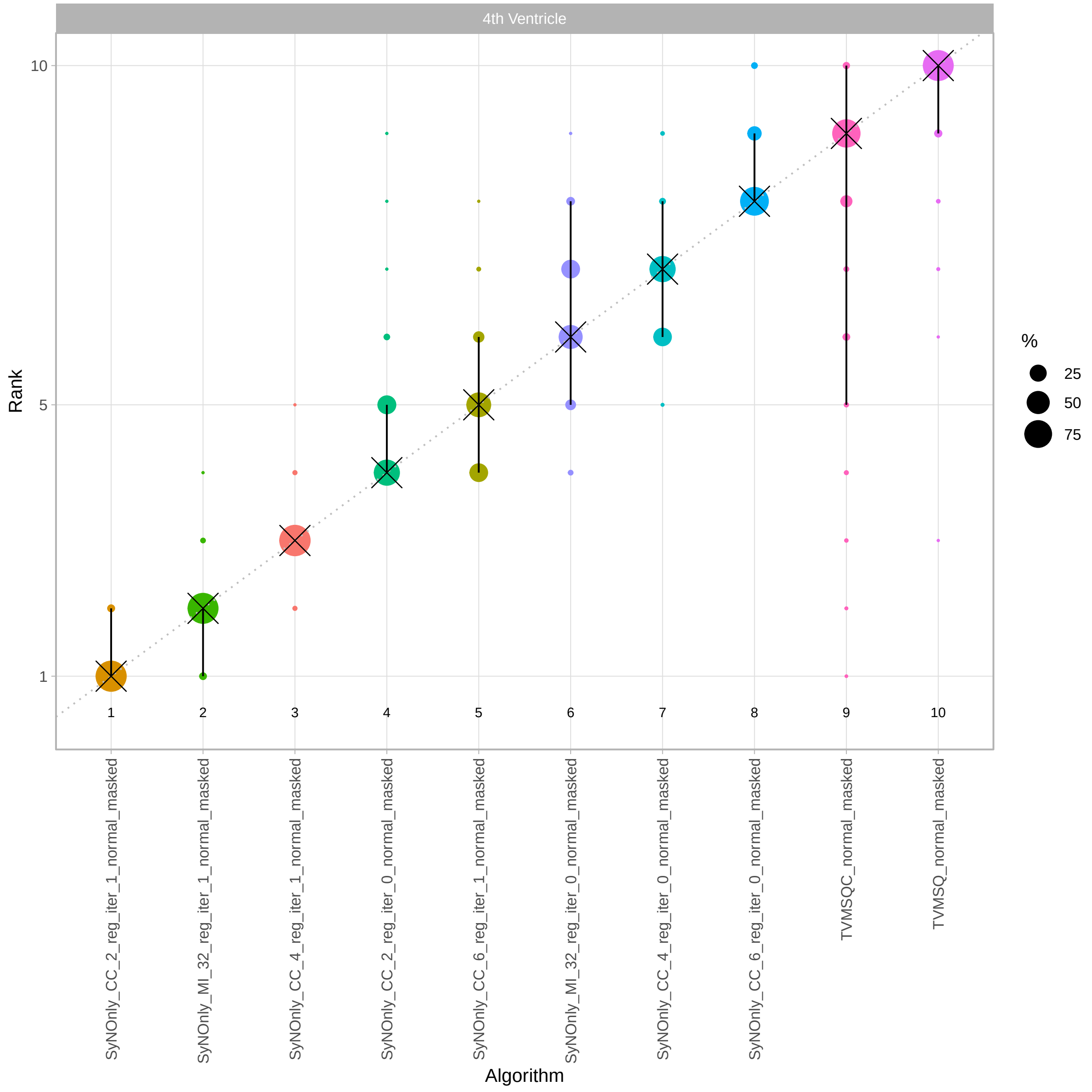

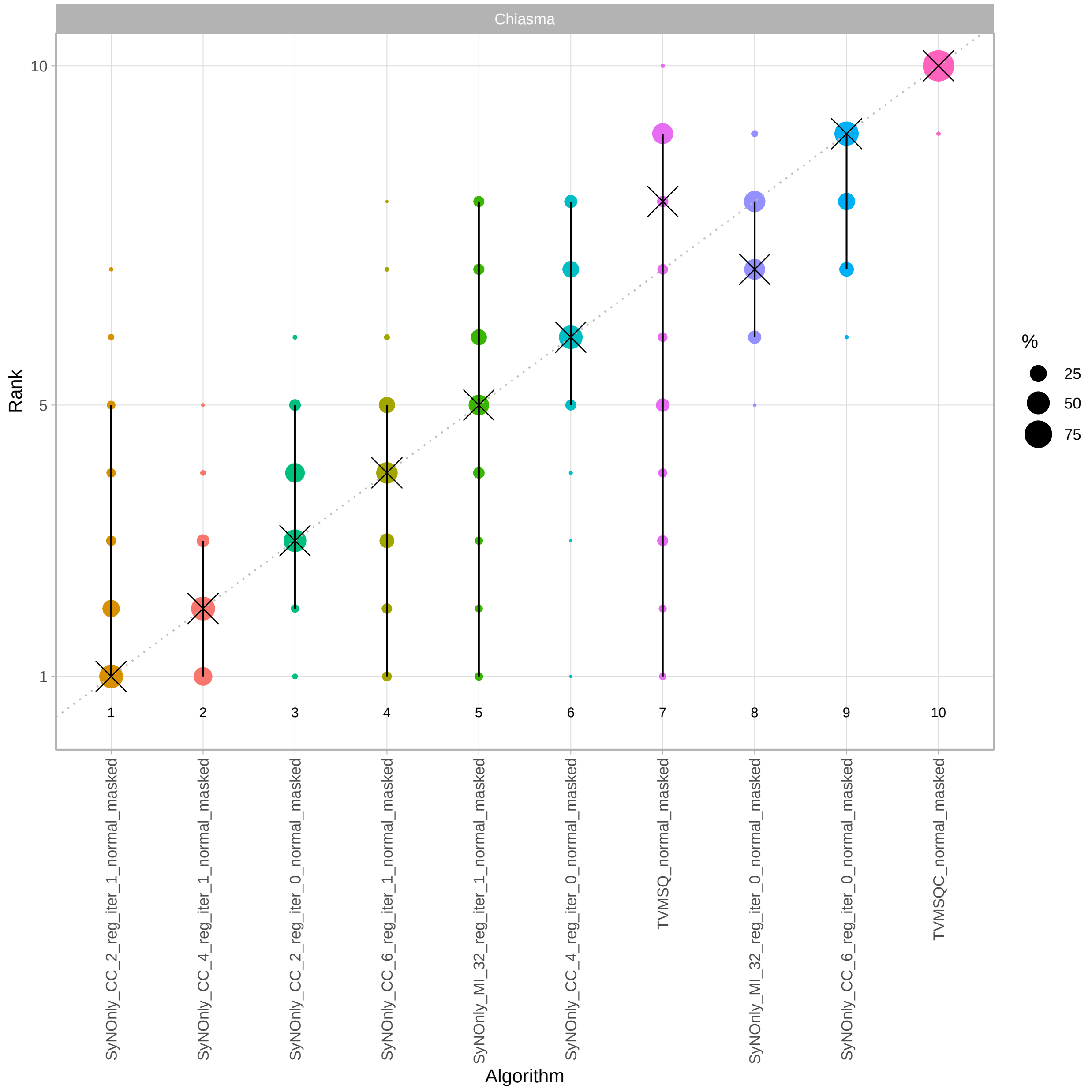

Chiasma
Rank
10
5
1
1
2
3
4
5
6
7
8
9
10
%
25
50
75
SyNOnly_CC_2_reg_iter_1_normal_masked
SyNOnly_CC_4_reg_iter_1_normal_masked
SyNOnly_CC_2_reg_iter_0_normal_masked
SyNOnly_CC_6_reg_iter_1_normal_masked
SyNOnly_MI_32_reg_iter_1_normal_masked
SyNOnly_CC_4_reg_iter_0_normal_masked
TVMSQ_normal_masked
SyNOnly_MI_32_reg_iter_0_normal_masked
SyNOnly_CC_6_reg_iter_0_normal_masked
TVMSQC_normal_masked
Algorithm

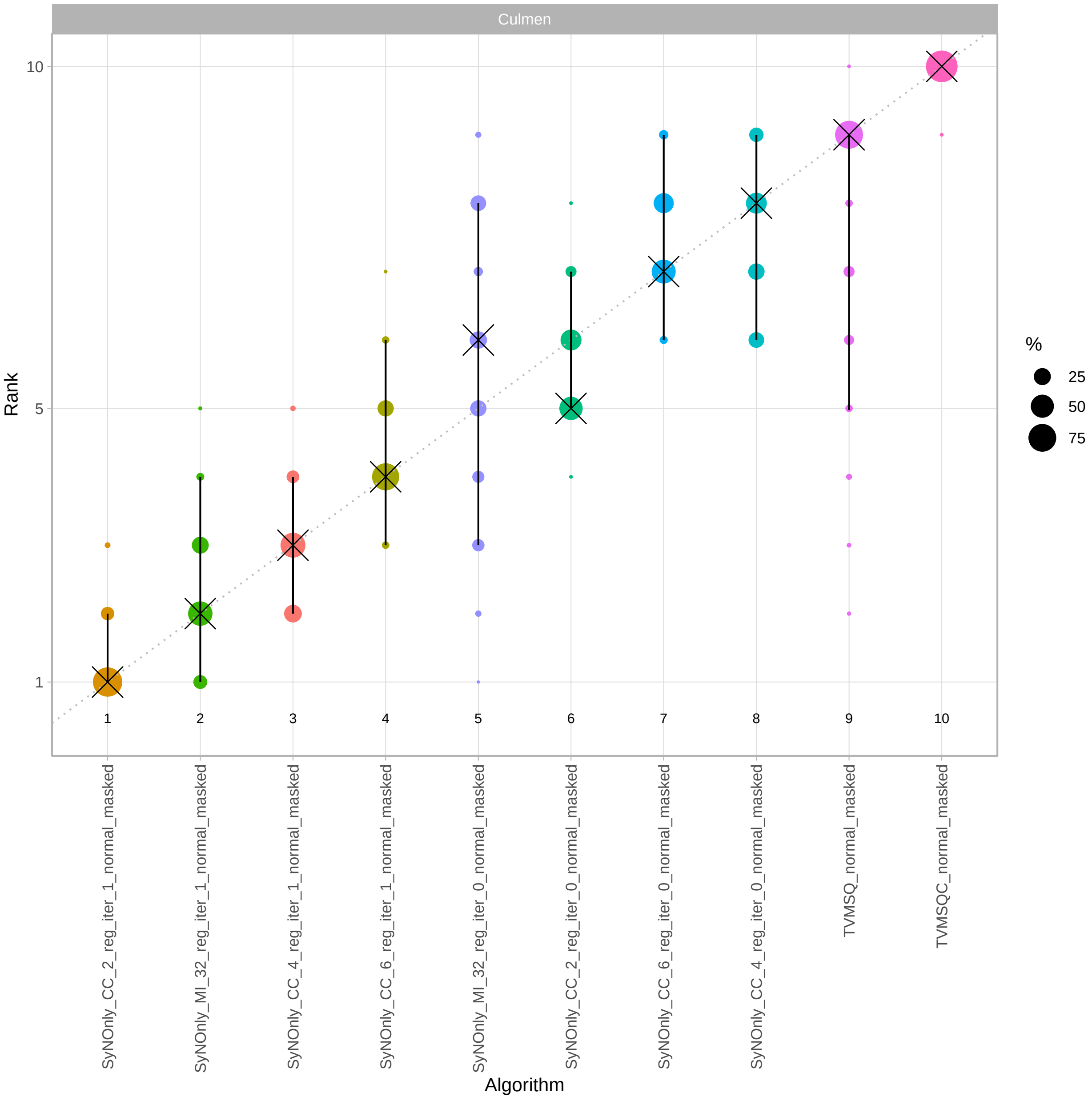
Culmen
Rank
10
5
1
1
2
3
4
5
6
7
8
9
10
%
25
50
75
SyNOnly_CC_2_reg_iter_1_normal_masked
SyNOnly_MI_32_reg_iter_1_normal_masked
SyNOnly_CC_4_reg_iter_1_normal_masked
SyNOnly_CC_6_reg_iter_1_normal_masked
SyNOnly_MI_32_reg_iter_0_normal_masked
SyNOnly_CC_2_reg_iter_0_normal_masked
SyNOnly_CC_6_reg_iter_0_normal_masked
SyNOnly_CC_4_reg_iter_0_normal_masked
TVMSQ_normal_masked
TVMSQC_normal_masked
Algorithm

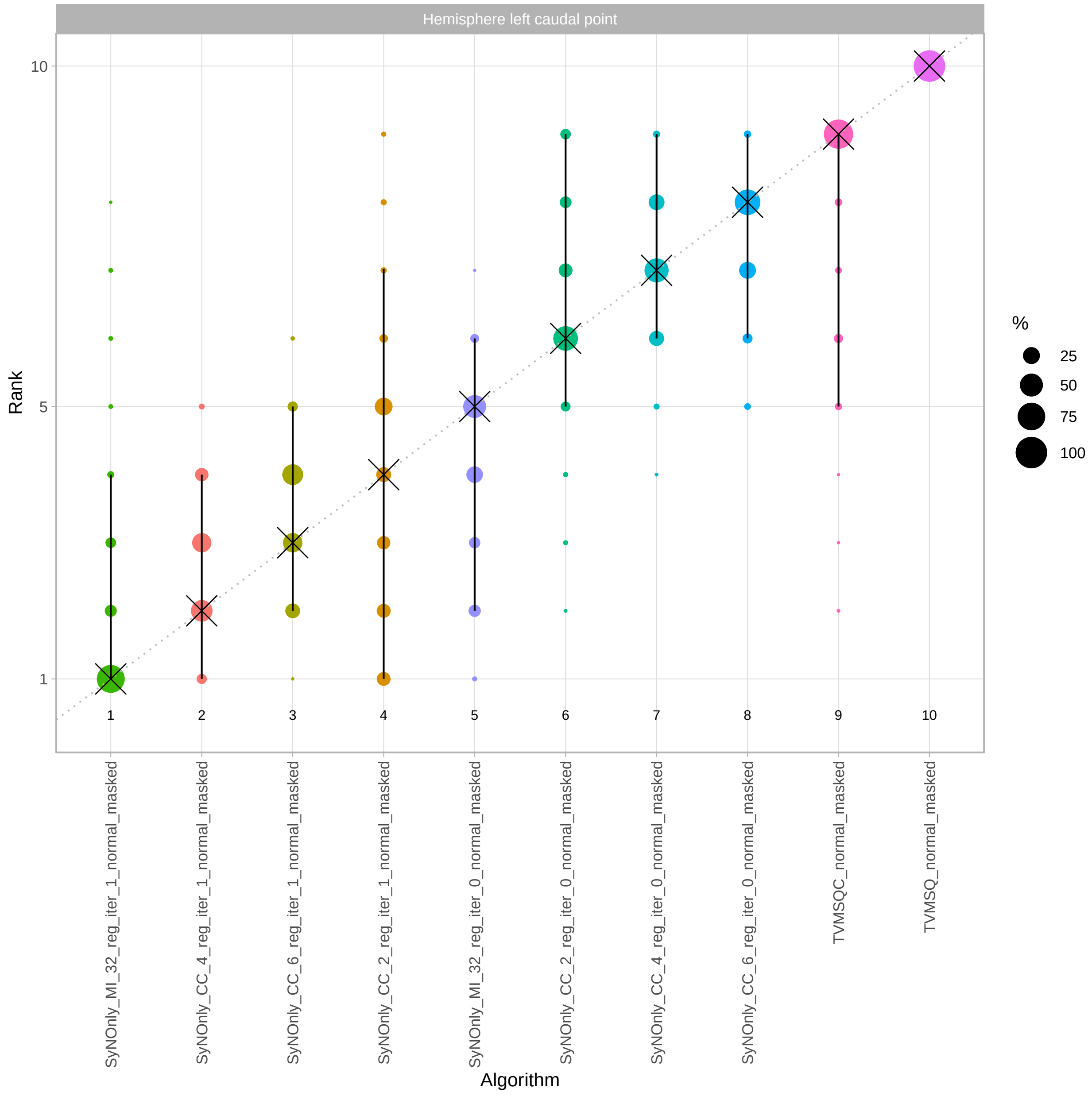
Hemisphere left caudal point
Rank
10
5
1
1
2
3
4
5
6
7
8
9
10
%
25
50
75
100
SyNOnly_MI_32_reg_iter_1_normal_masked
SyNOnly_CC_4_reg_iter_1_normal_masked
SyNOnly_CC_6_reg_iter_1_normal_masked
SyNOnly_CC_2_reg_iter_1_normal_masked
SyNOnly_MI_32_reg_iter_0_normal_masked
SyNOnly_CC_2_reg_iter_0_normal_masked
SyNOnly_CC_4_reg_iter_0_normal_masked
SyNOnly_CC_6_reg_iter_0_normal_masked
TVMSQC_normal_masked
TVMSQ_normal_masked
Algorithm

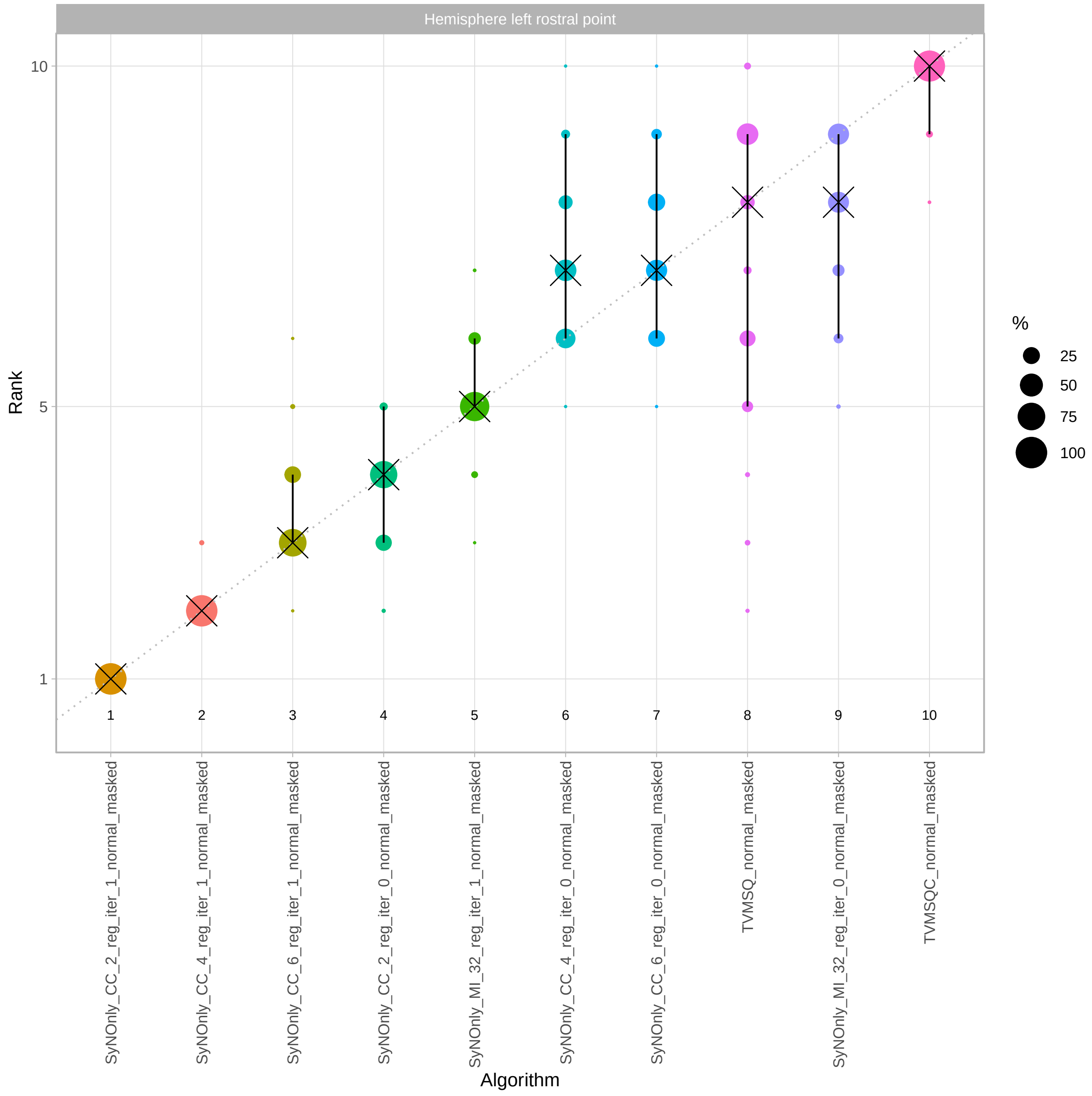
Hemisphere left rostral point
Rank
10
5
1
1
2
3
4
5
6
7
8
9
10
%
25
50
75
100
SyNOnly_CC_2_reg_iter_1_normal_masked
SyNOnly_CC_4_reg_iter_1_normal_masked
SyNOnly_CC_6_reg_iter_1_normal_masked
SyNOnly_CC_2_reg_iter_0_normal_masked
SyNOnly_MI_32_reg_iter_1_normal_masked
SyNOnly_CC_4_reg_iter_0_normal_masked
SyNOnly_CC_6_reg_iter_0_normal_masked
TVMSQ_normal_masked
SyNOnly_MI_32_reg_iter_0_normal_masked
TVMSQC_normal_masked
Algorithm

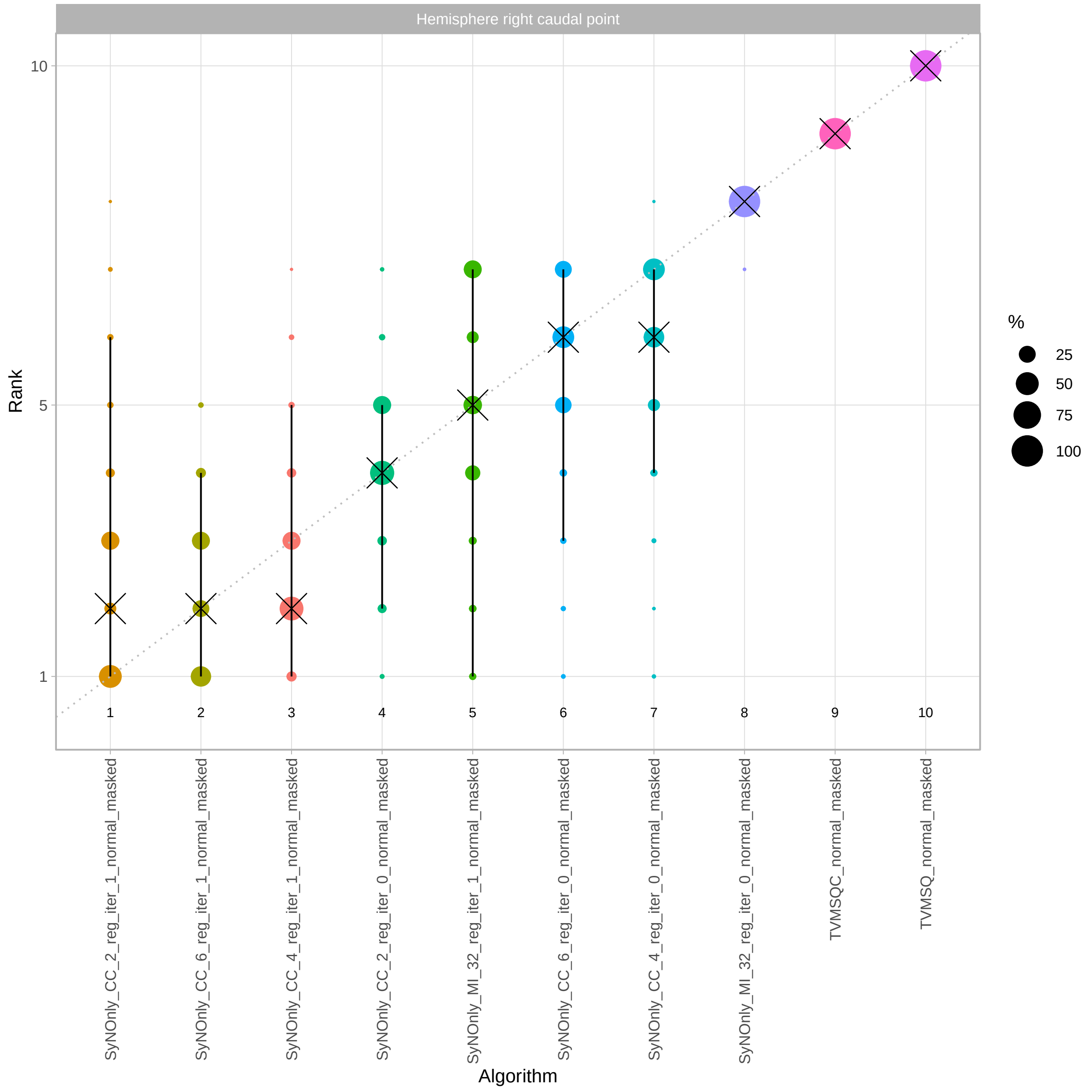
Hemisphere right caudal point
Rank
10
5
1
1
2
3
4
5
6
7
8
9
10
%
25
50
75
100
SyNOnly_CC_2_reg_iter_1_normal_masked
SyNOnly_CC_6_reg_iter_1_normal_masked
SyNOnly_CC_4_reg_iter_1_normal_masked
SyNOnly_CC_2_reg_iter_0_normal_masked
SyNOnly_MI_32_reg_iter_1_normal_masked
SyNOnly_CC_6_reg_iter_0_normal_masked
SyNOnly_CC_4_reg_iter_0_normal_masked
SyNOnly_MI_32_reg_iter_0_normal_masked
TVMSQC_normal_masked
TVMSQ_normal_masked
Algorithm

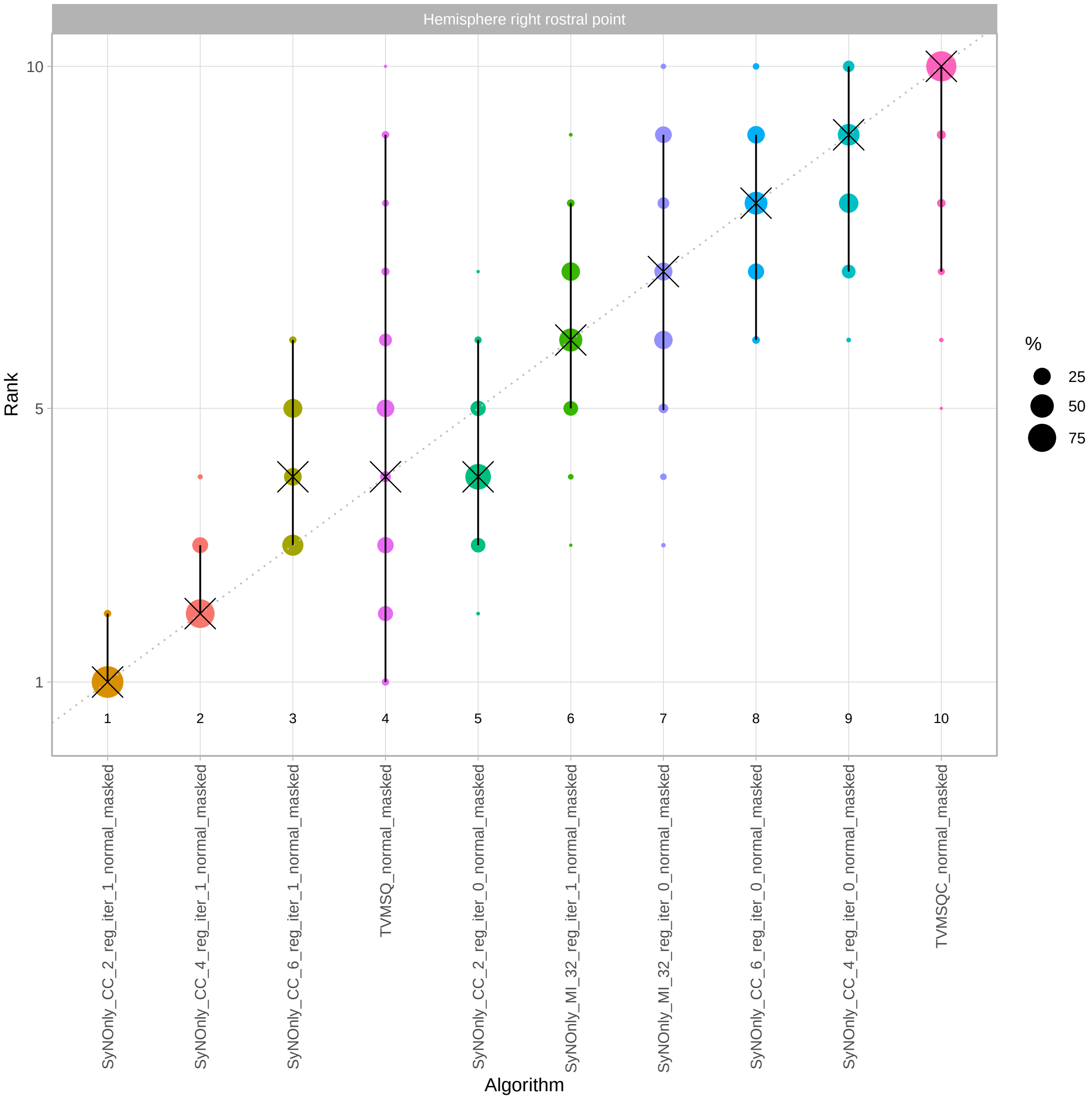

Hemisphere right rostral point
Rank
10
5
1
1
2
3
4
5
6
7
8
9
10
%
25
50
75
SyNOnly_CC_2_reg_iter_1_normal_masked
SyNOnly_CC_4_reg_iter_1_normal_masked
SyNOnly_CC_6_reg_iter_1_normal_masked
TVMSQ_normal_masked
SyNOnly_CC_2_reg_iter_0_normal_masked
SyNOnly_MI_32_reg_iter_1_normal_masked
SyNOnly_MI_32_reg_iter_0_normal_masked
SyNOnly_CC_6_reg_iter_0_normal_masked
SyNOnly_CC_4_reg_iter_0_normal_masked
TVMSQC_normal_masked
Algorithm

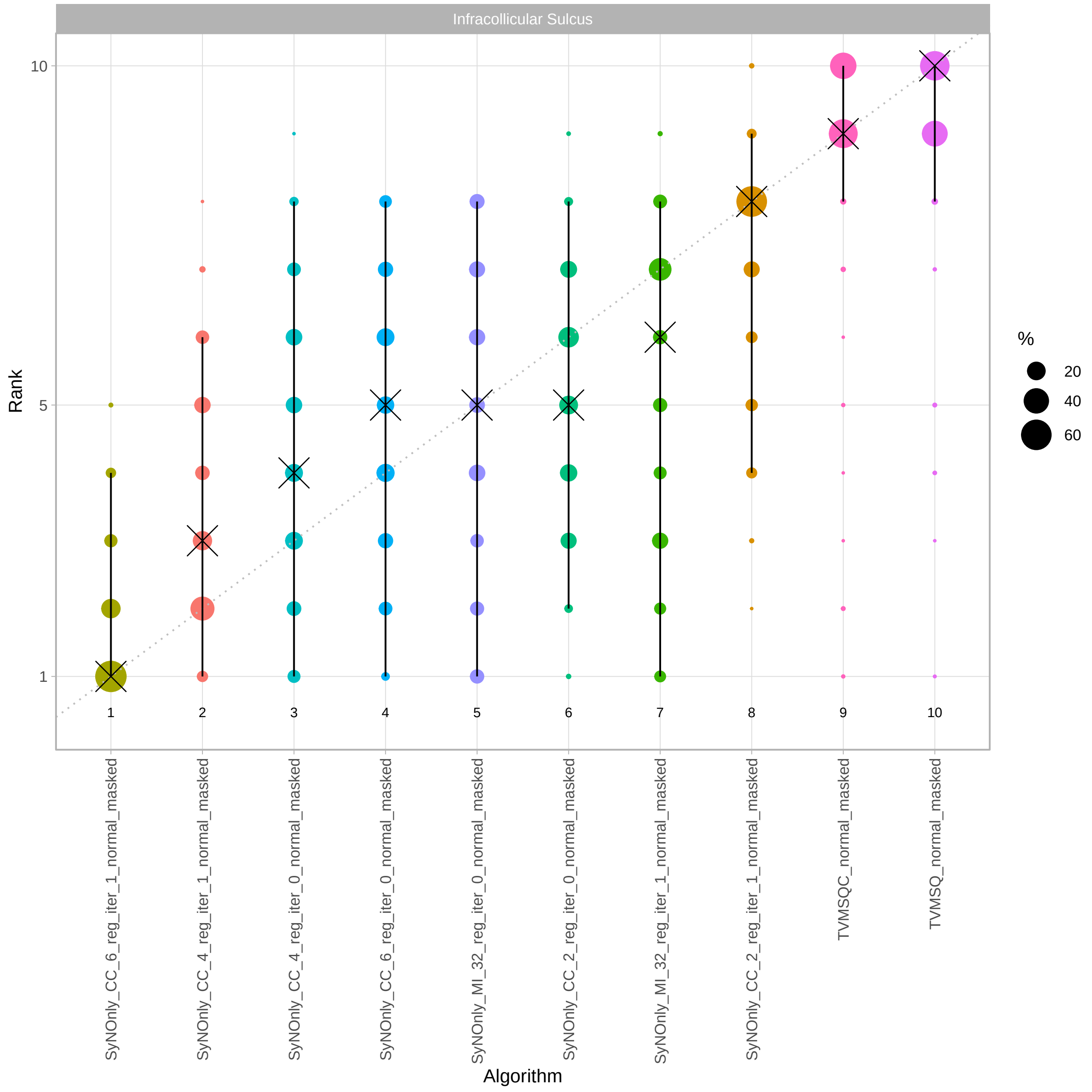

Infracollicular Sulcus
Rank
10
5
1
1
2
3
4
5
6
7
8
9
10
%
20
40
60
SyNOnly_CC_6_reg_iter_1_normal_masked
SyNOnly_CC_4_reg_iter_1_normal_masked
SyNOnly_CC_4_reg_iter_0_normal_masked
SyNOnly_CC_6_reg_iter_0_normal_masked
SyNOnly_MI_32_reg_iter_0_normal_masked
SyNOnly_CC_2_reg_iter_0_normal_masked
SyNOnly_MI_32_reg_iter_1_normal_masked
SyNOnly_CC_2_reg_iter_1_normal_masked
TVMSQC_normal_masked
TVMSQ_normal_masked
Algorithm

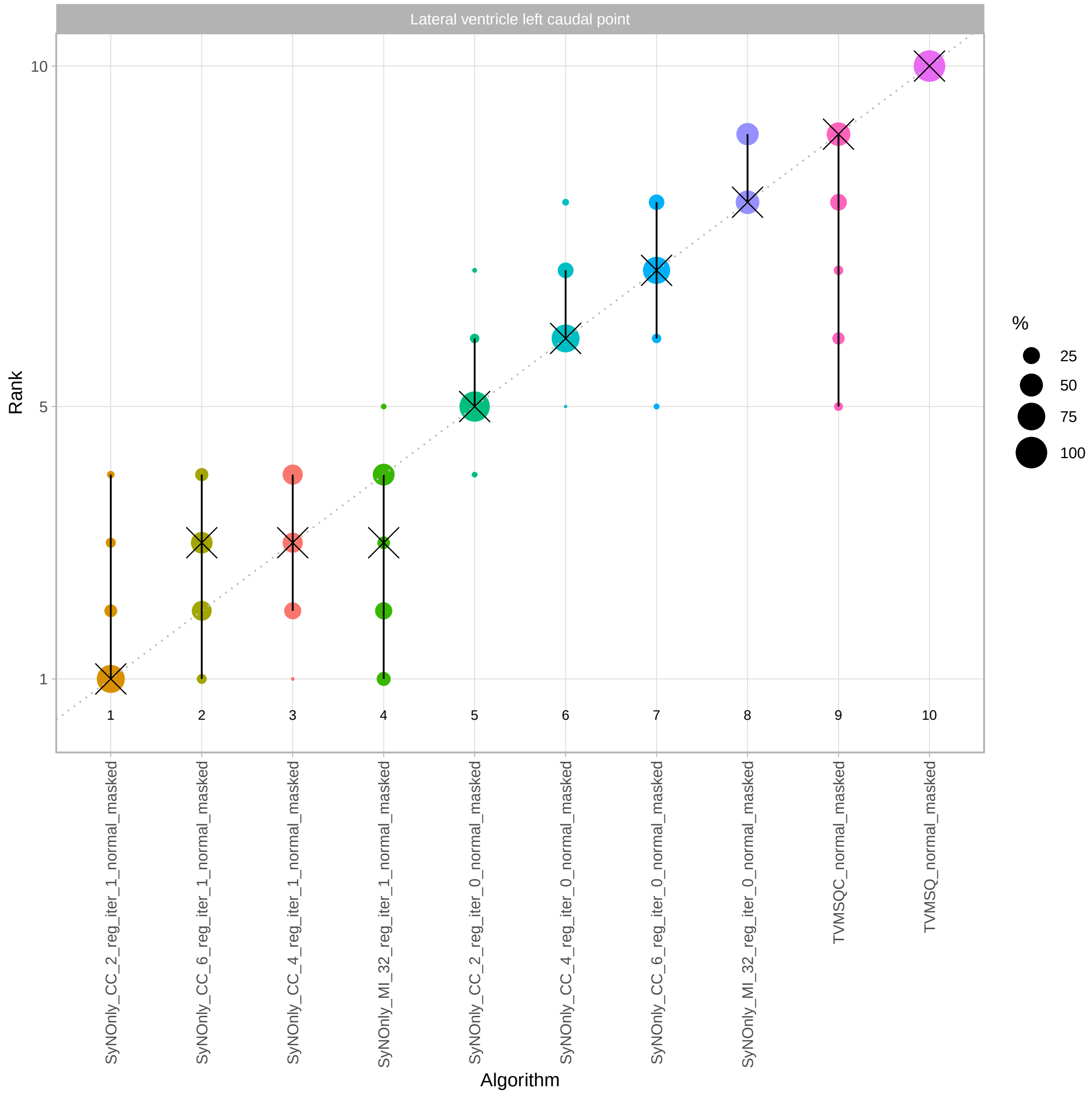

Lateral ventricle left caudal point
Rank
10
5
1
1
2
3
4
5
6
7
8
9
10
%
25
50
75
100
SyNOnly_CC_2_reg_iter_1_normal_masked
SyNOnly_CC_6_reg_iter_1_normal_masked
SyNOnly_CC_4_reg_iter_1_normal_masked
SyNOnly_MI_32_reg_iter_1_normal_masked
SyNOnly_CC_2_reg_iter_0_normal_masked
SyNOnly_CC_4_reg_iter_0_normal_masked
SyNOnly_CC_6_reg_iter_0_normal_masked
SyNOnly_MI_32_reg_iter_0_normal_masked
TVMSQC_normal_masked
TVMSQ_normal_masked
Algorithm

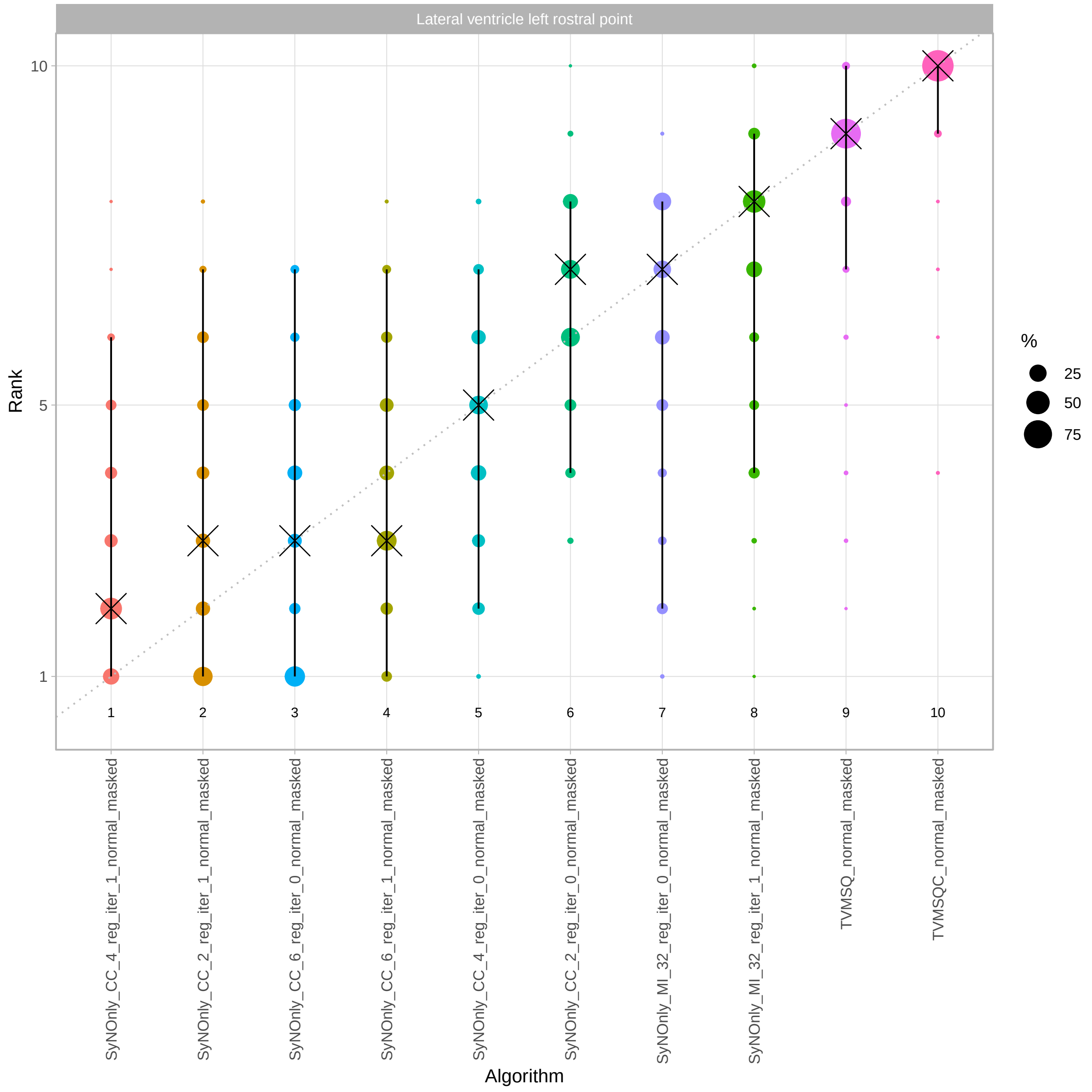

Lateral ventricle left rostral point
Rank
10
5
1
1
2
3
4
5
6
7
8
9
10
SyNOnly_CC_4_reg_iter_1_normal_masked
SyNOnly_CC_2_reg_iter_1_normal_masked
SyNOnly_CC_6_reg_iter_0_normal_masked
SyNOnly_CC_6_reg_iter_1_normal_masked
SyNOnly_CC_4_reg_iter_0_normal_masked
SyNOnly_CC_2_reg_iter_0_normal_masked
SyNOnly_MI_32_reg_iter_0_normal_masked
SyNOnly_MI_32_reg_iter_1_normal_masked
TVMSQ_normal_masked
TVMSQC_normal_masked
Algorithm
%
25
50
75

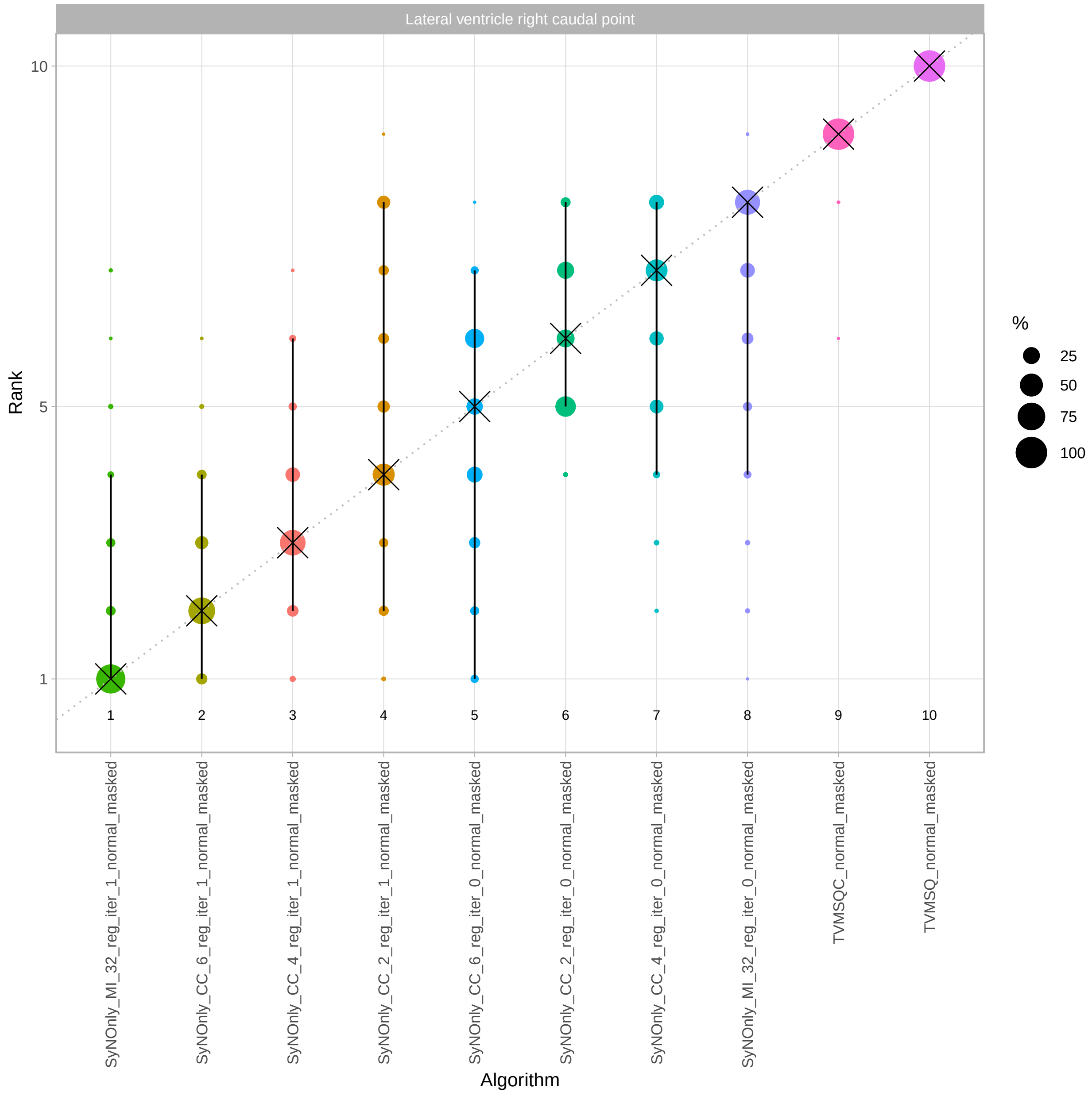

Lateral ventricle right caudal point
Rank
10
5
1
1
2
3
4
5
6
7
8
9
10
%
25
50
75
100
SyNOnly_MI_32_reg_iter_1_normal_masked
SyNOnly_CC_6_reg_iter_1_normal_masked
SyNOnly_CC_4_reg_iter_1_normal_masked
SyNOnly_CC_2_reg_iter_1_normal_masked
SyNOnly_CC_6_reg_iter_0_normal_masked
SyNOnly_CC_2_reg_iter_0_normal_masked
SyNOnly_CC_4_reg_iter_0_normal_masked
SyNOnly_MI_32_reg_iter_0_normal_masked
TVMSQC_normal_masked
TVMSQ_normal_masked
Algorithm

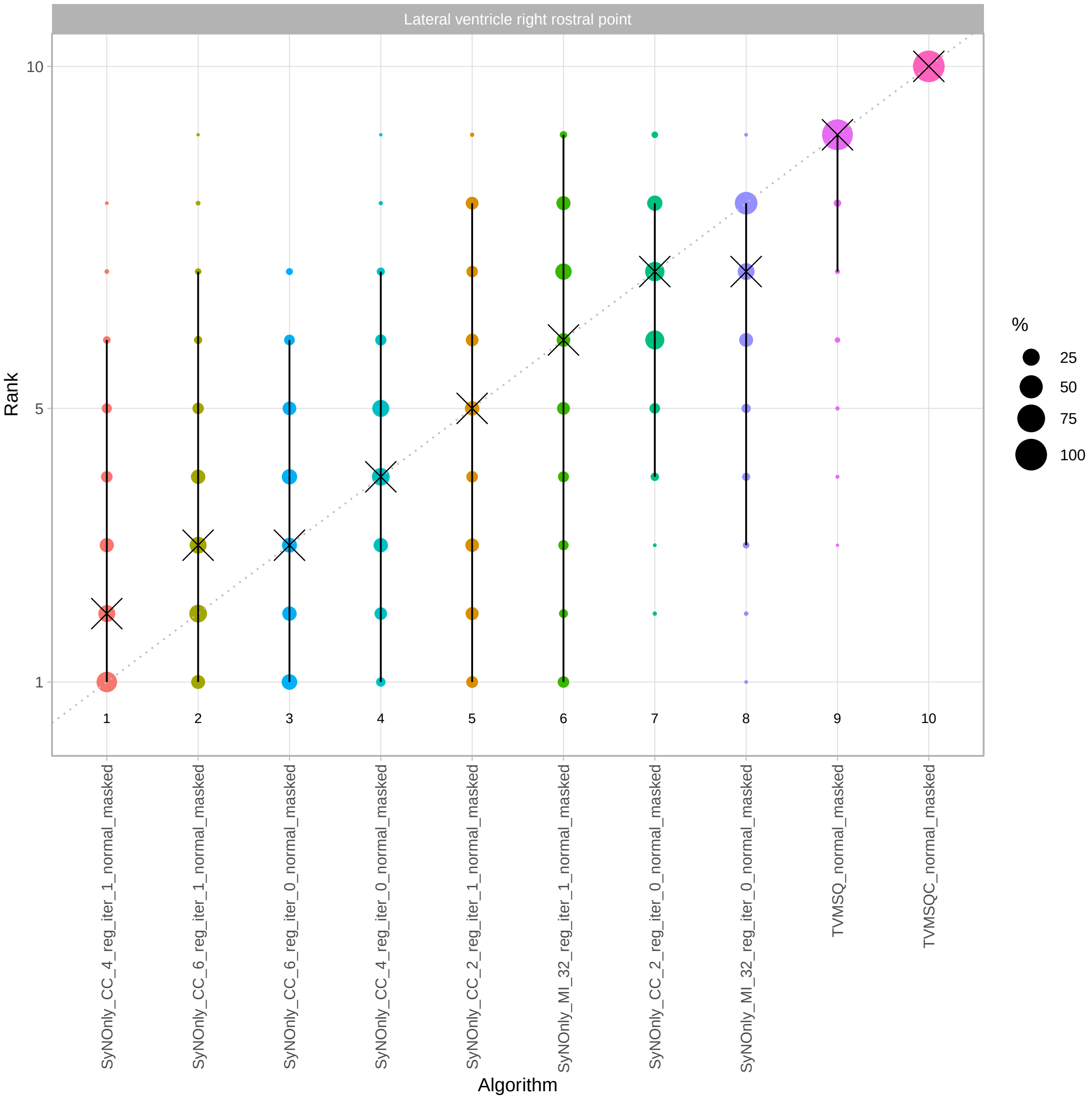

Lateral ventricle right rostral point
Rank
10
5
1
1
2
3
4
5
6
7
8
9
10
%
25
50
75
100
SyNOnly_CC_4_reg_iter_1_normal_masked
SyNOnly_CC_6_reg_iter_1_normal_masked
SyNOnly_CC_6_reg_iter_0_normal_masked
SyNOnly_CC_4_reg_iter_0_normal_masked
SyNOnly_CC_2_reg_iter_1_normal_masked
SyNOnly_MI_32_reg_iter_1_normal_masked
SyNOnly_CC_2_reg_iter_0_normal_masked
SyNOnly_MI_32_reg_iter_0_normal_masked
TVMSQ_normal_masked
TVMSQC_normal_masked
Algorithm

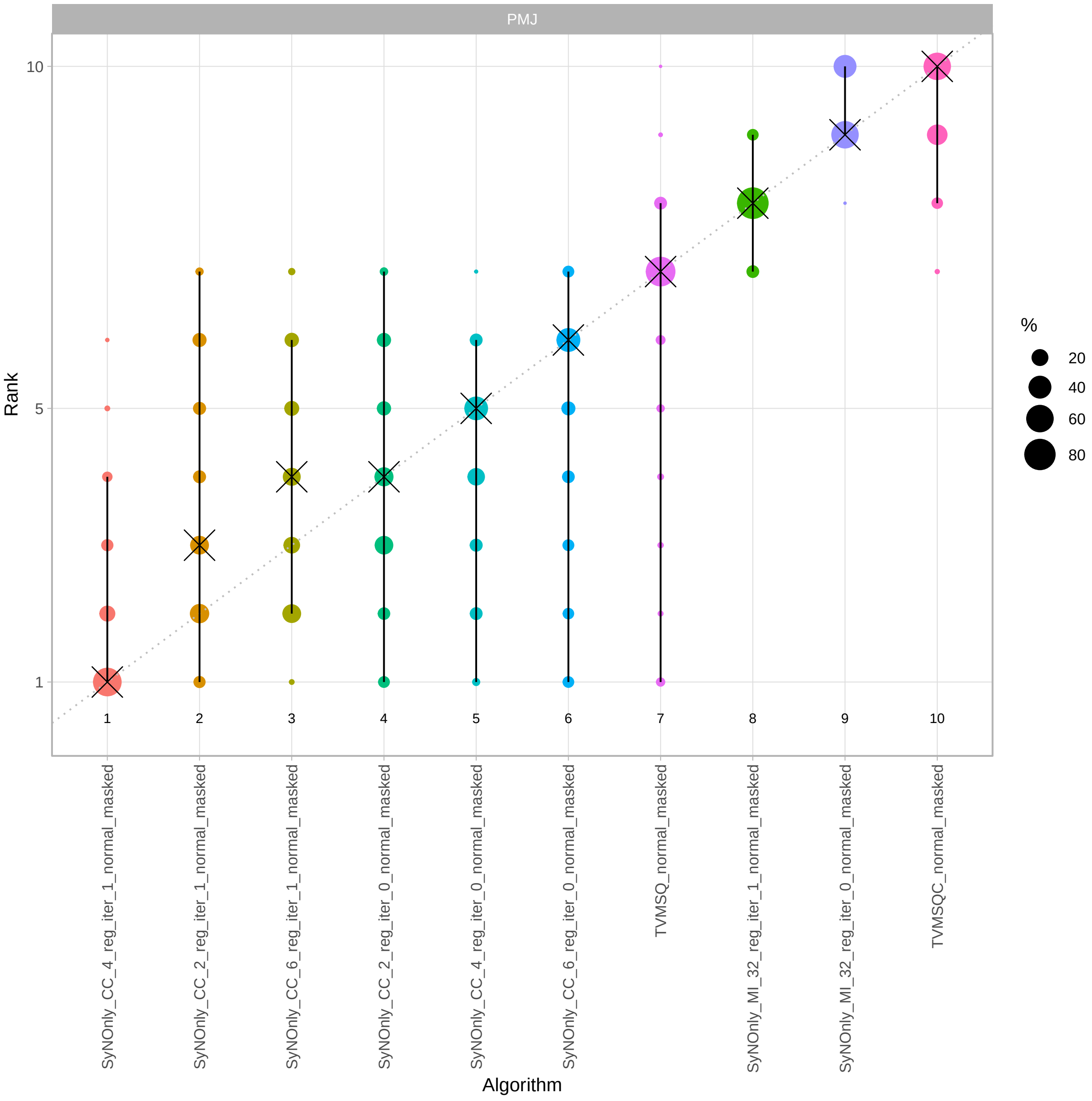
PMJ
Rank
Algorithm
%
20
40
60
80
SyNOnly_CC_4_reg_iter_1_normal_masked
SyNOnly_CC_2_reg_iter_1_normal_masked
SyNOnly_CC_6_reg_iter_1_normal_masked
SyNOnly_CC_2_reg_iter_0_normal_masked
SyNOnly_CC_4_reg_iter_0_normal_masked
SyNOnly_CC_6_reg_iter_0_normal_masked
TVMSQ_normal_masked
SyNOnly_MI_32_reg_iter_1_normal_masked
SyNOnly_MI_32_reg_iter_0_normal_masked
TVMSQC_normal_masked

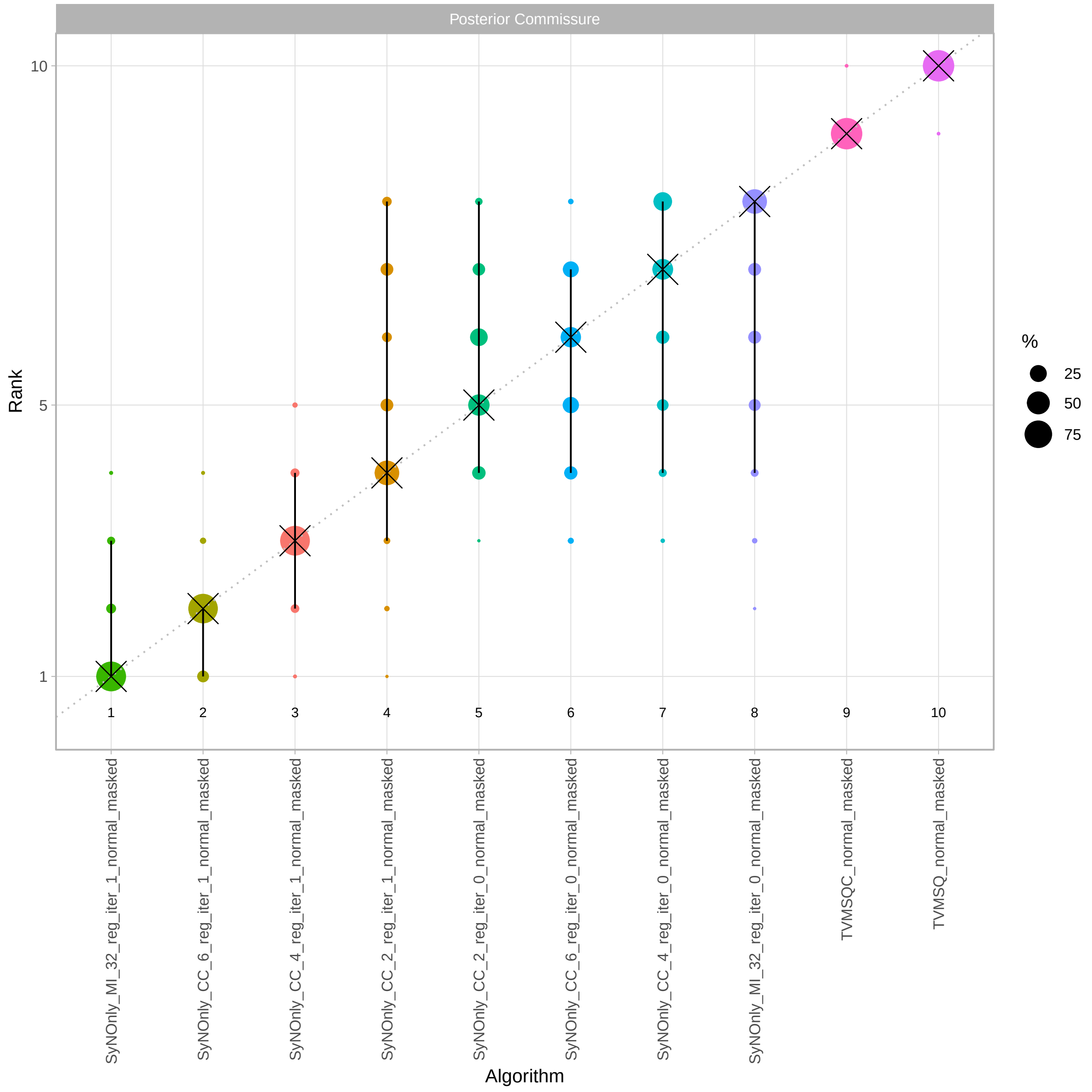
Posterior Commissure
Rank
10
5
1
1
2
3
4
5
6
7
8
9
10
%
25
50
75
SyNOnly_MI_32_reg_iter_1_normal_masked
SyNOnly_CC_6_reg_iter_1_normal_masked
SyNOnly_CC_4_reg_iter_1_normal_masked
SyNOnly_CC_2_reg_iter_1_normal_masked
SyNOnly_CC_2_reg_iter_0_normal_masked
SyNOnly_CC_6_reg_iter_0_normal_masked
SyNOnly_CC_4_reg_iter_0_normal_masked
SyNOnly_MI_32_reg_iter_0_normal_masked
TVMSQC_normal_masked
TVMSQ_normal_masked
Algorithm

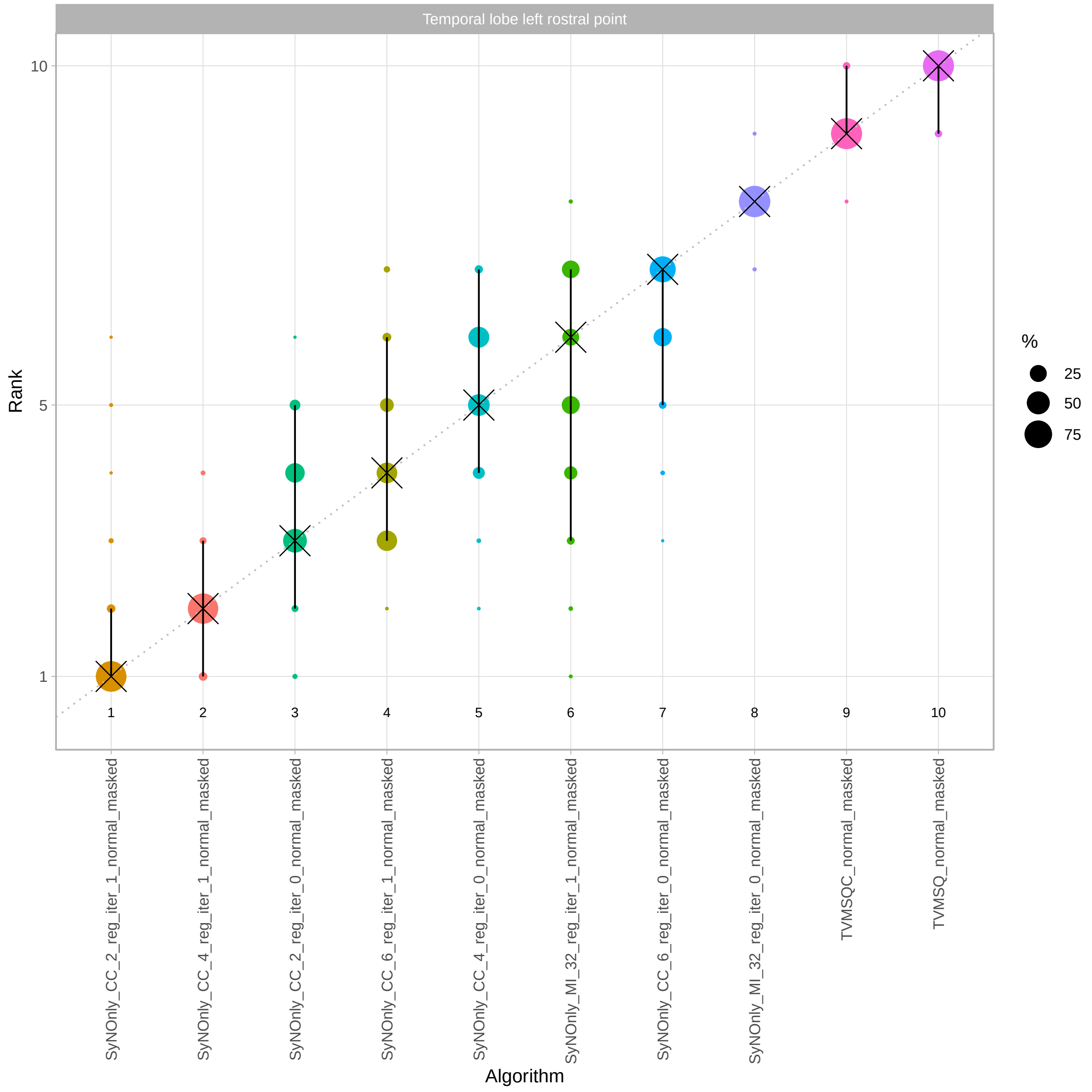
Temporal lobe left rostral point
Rank
10
5
1
1
2
3
4
5
6
7
8
9
10
SyNOnly_CC_2_reg_iter_1_normal_masked
SyNOnly_CC_4_reg_iter_1_normal_masked
SyNOnly_CC_2_reg_iter_0_normal_masked
SyNOnly_CC_6_reg_iter_1_normal_masked
SyNOnly_CC_4_reg_iter_0_normal_masked
SyNOnly_MI_32_reg_iter_1_normal_masked
SyNOnly_CC_6_reg_iter_0_normal_masked
SyNOnly_MI_32_reg_iter_0_normal_masked
TVMSQC_normal_masked
TVMSQ_normal_masked
Algorithm
%
25
50
75

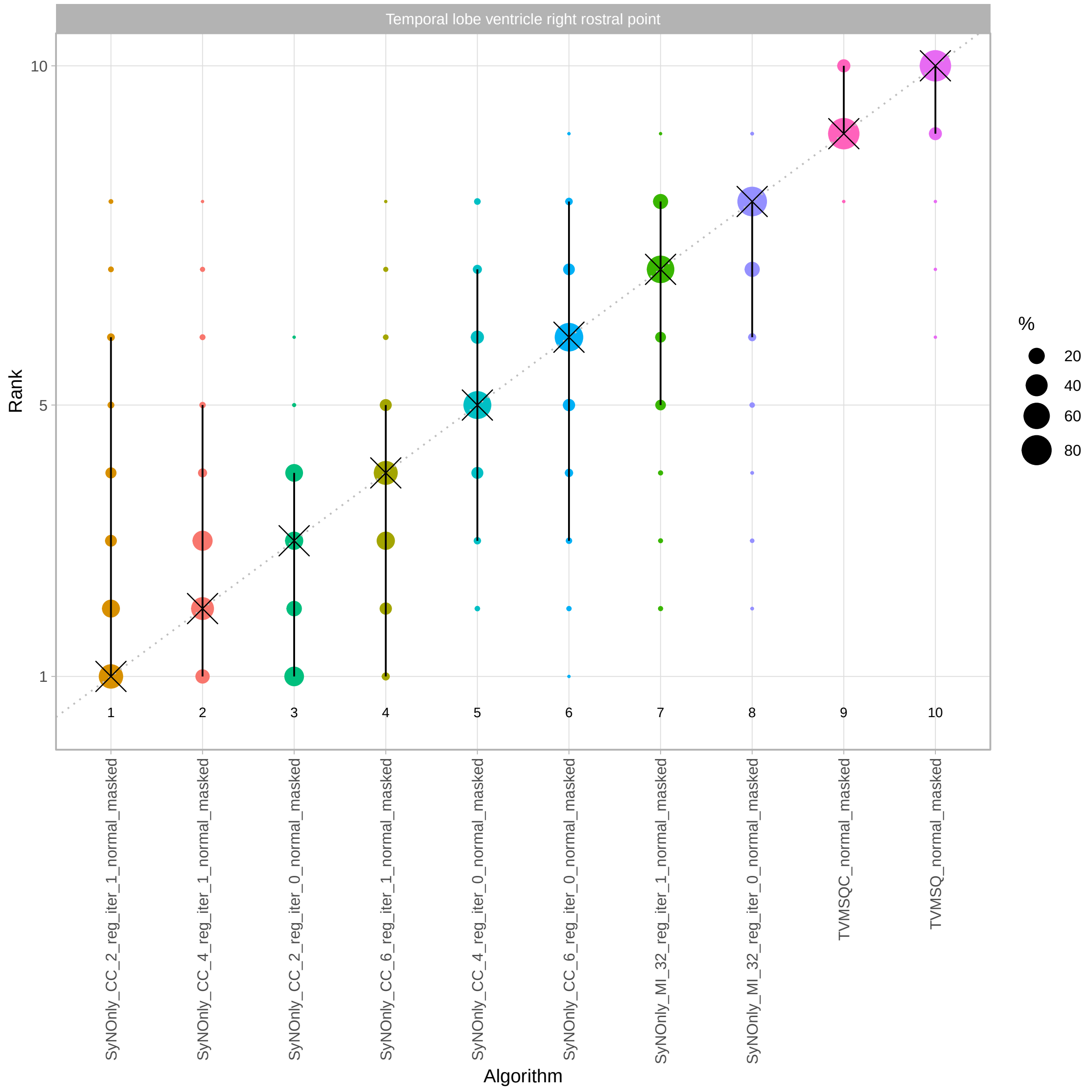

Temporal lobe ventricle right rostral point
Rank
10
5
1
1
2
3
4
5
6
7
8
9
10
%
20
40
60
80
SyNOnly_CC_2_reg_iter_1_normal_masked
SyNOnly_CC_4_reg_iter_1_normal_masked
SyNOnly_CC_2_reg_iter_0_normal_masked
SyNOnly_CC_6_reg_iter_1_normal_masked
SyNOnly_CC_4_reg_iter_0_normal_masked
SyNOnly_CC_6_reg_iter_0_normal_masked
SyNOnly_MI_32_reg_iter_1_normal_masked
SyNOnly_MI_32_reg_iter_0_normal_masked
TVMSQC_normal_masked
TVMSQ_normal_masked
Algorithm

3.2 *Violin plot* for visualizing ranking stability based on bootstrapping

The ranking list based on the full assessment data is pairwise compared with the ranking lists based on the individual bootstrap samples (here $b = 1000$ samples). For each pair of rankings, Kendall's τ correlation is computed. Kendall's τ is a scaled index determining the correlation between the lists. It is computed by evaluating the number of pairwise concordances and discordances between ranking lists and produces values between -1 (for inverted order) and 1 (for identical order). A violin plot, which simultaneously depicts a boxplot and a density plot, is generated from the results.

Summary Kendall's tau:

Task	mean	median	q25	q75
4th Ventricle	0.9426667	0.9555556	0.9111111	1.0000000
Hemisphere left rostral point	0.8929778	0.9111111	0.8666667	0.9555556
Lateral ventricle left caudal point	0.8810222	0.9111111	0.8222222	0.9555556
Lateral ventricle right caudal point	0.8488000	0.9111111	0.7777778	0.9555556
Posterior Commissure	0.8829778	0.9111111	0.8222222	0.9555556
Temporal lobe left rostral point	0.9197333	0.9111111	0.9111111	0.9555556
Temporal lobe ventricle right rostral point	0.8638667	0.9111111	0.8222222	0.9555556
Culmen	0.8676889	0.8666667	0.8222222	0.9555556
Hemisphere left caudal point	0.8388889	0.8666667	0.7777778	0.9111111
Hemisphere right caudal point	0.8530222	0.8666667	0.8222222	0.9111111
Chiasma	0.7947556	0.8222222	0.7333333	0.8666667
Hemisphere right rostral point	0.8350222	0.8222222	0.7777778	0.9111111
PMJ	0.7648889	0.8222222	0.6444444	0.9111111
Lateral ventricle left rostral point	0.7204444	0.7333333	0.6444444	0.8222222
Lateral ventricle right rostral point	0.7101778	0.7333333	0.6000000	0.8222222
Infracollicular Sulcus	0.6614667	0.6888889	0.5555556	0.7777778

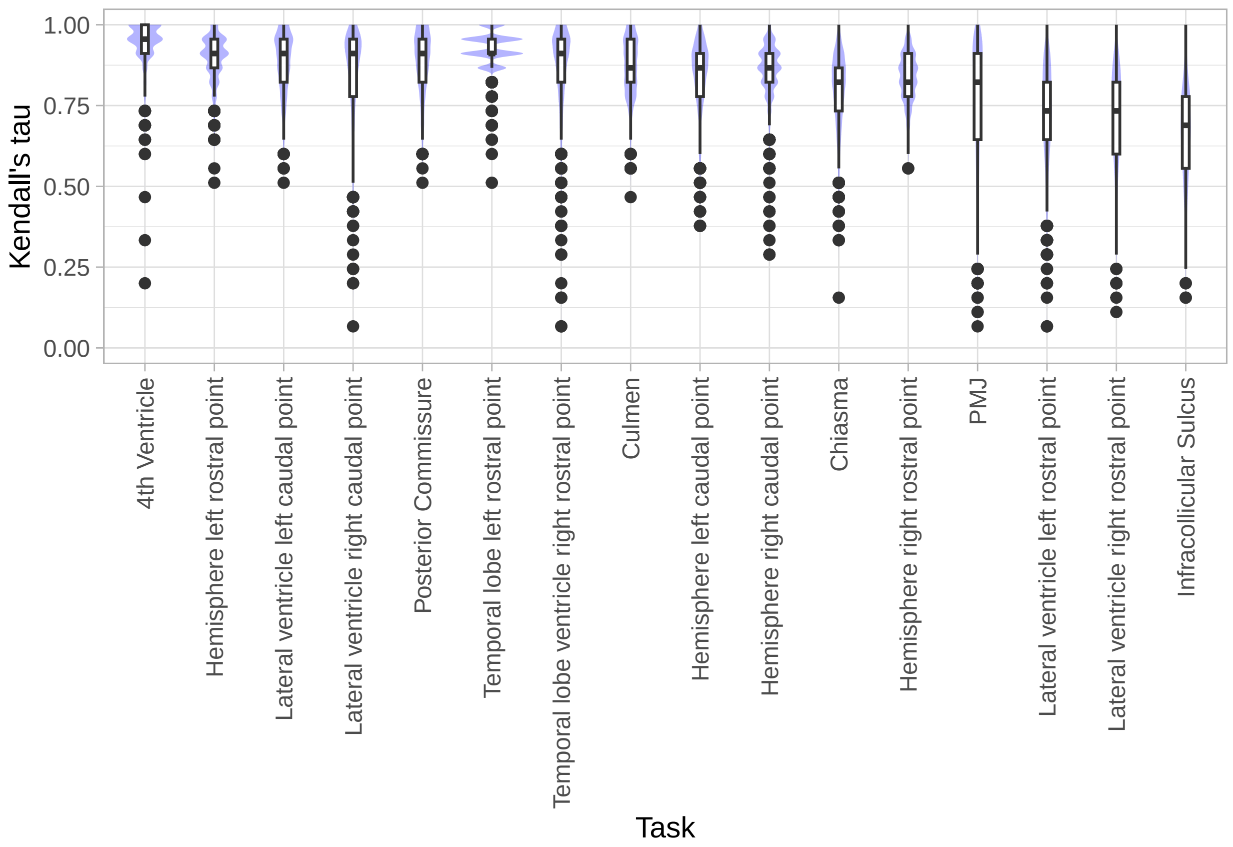

Kendall's tau
1.00
0.75
0.50
0.25
0.00
4th Ventricle
Hemisphere left rostral point
Lateral ventricle left caudal point
Lateral ventricle right caudal point
Posterior Commissure
Temporal lobe left rostral point
Temporal lobe ventricle right rostral point
Culmen
Hemisphere left caudal point
Hemisphere right caudal point
Chiasma
Hemisphere right rostral point
PMJ
Lateral ventricle left rostral point
Lateral ventricle right rostral point
Infracollicular Sulcus
Task

3.3 *Significance maps* for visualizing ranking stability based on statistical significance

Significance maps depict incidence matrices of pairwise significant test results for the one-sided Wilcoxon signed rank test at a 5% significance level with adjustment for multiple testing according to Holm. Yellow shading indicates that metric values from the algorithm on the x-axis were significantly superior to those from the algorithm on the y-axis, blue color indicates no significant difference.

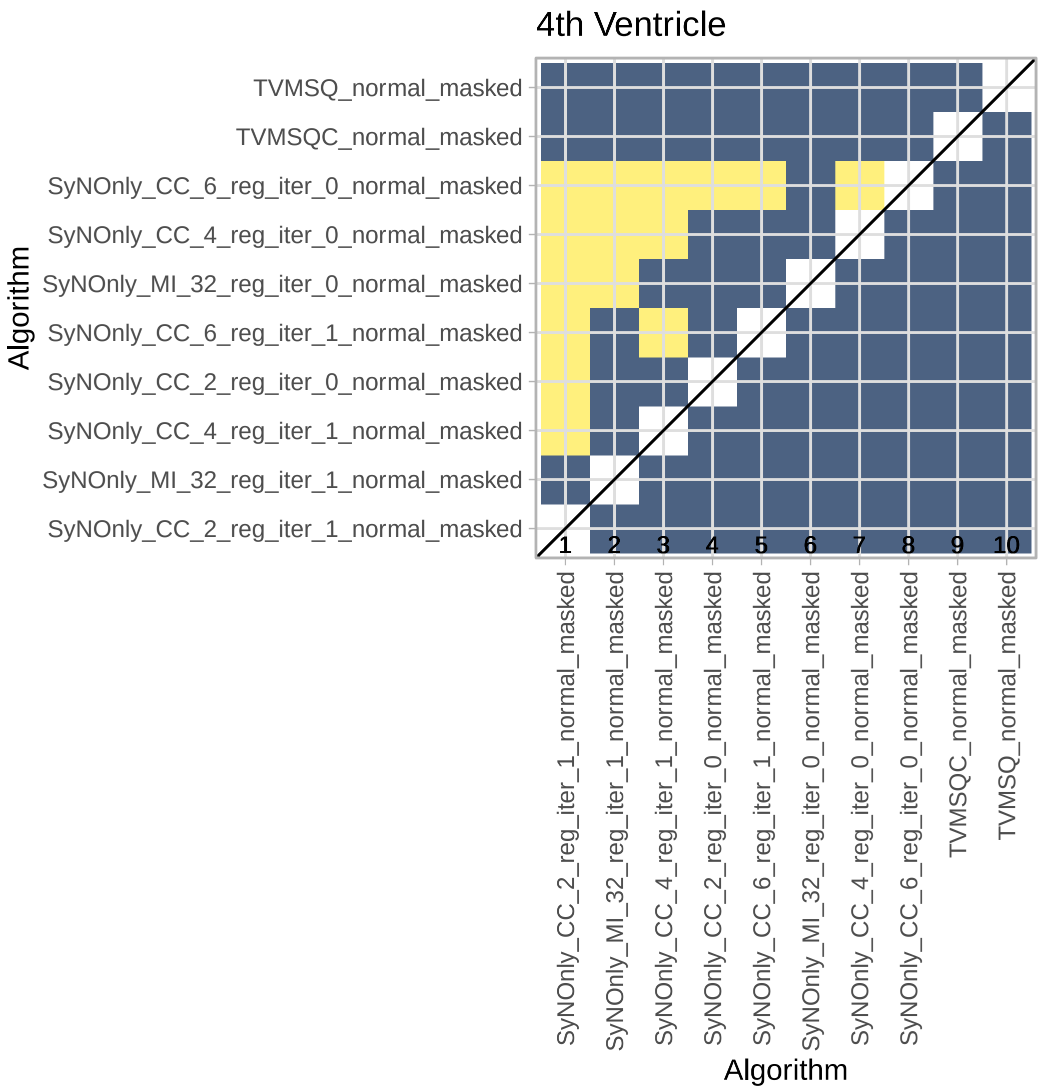

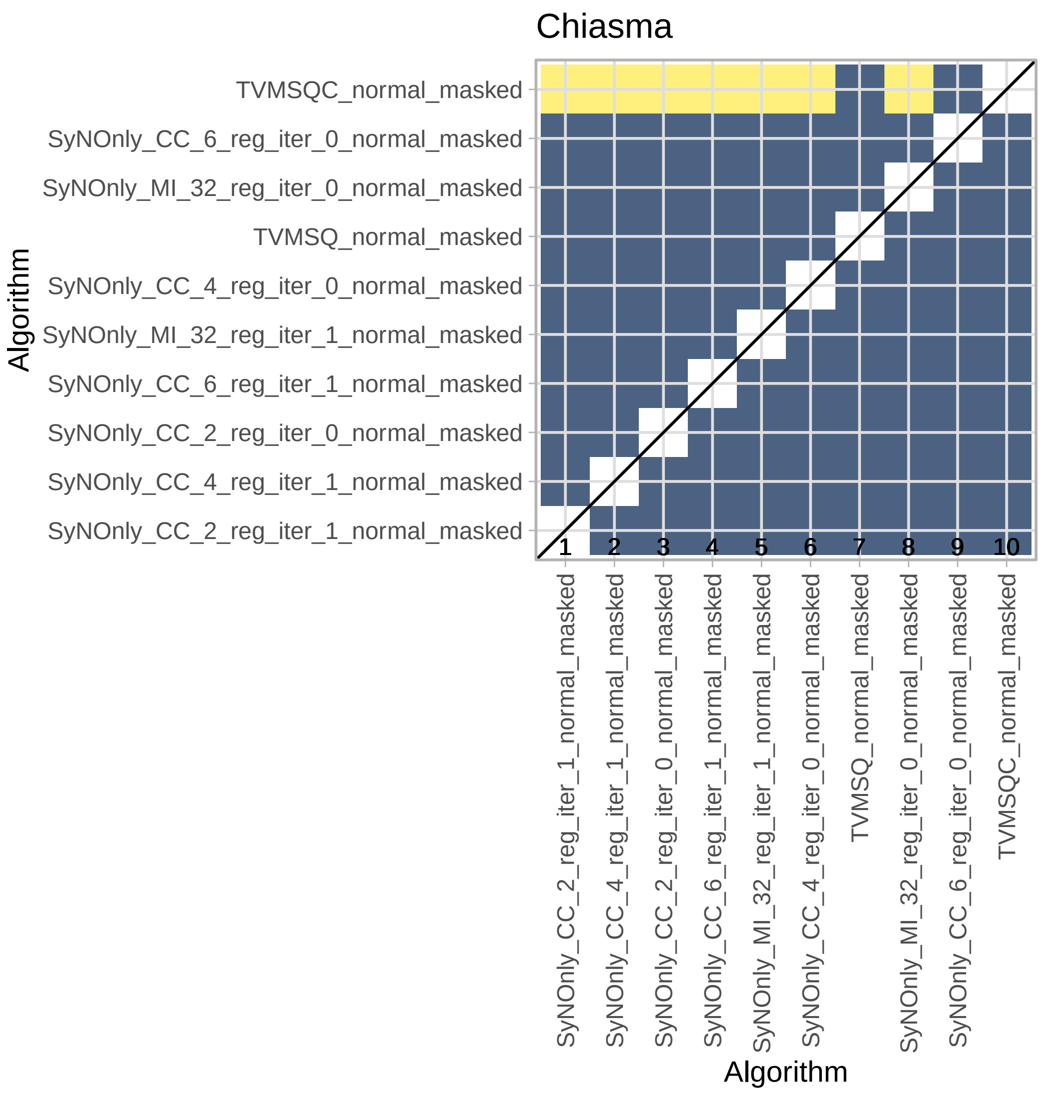
Chiasma
Algorithm
TVMSQC_normal_masked
SyNOnly_CC_6_reg_iter_0_normal_masked
SyNOnly_MI_32_reg_iter_0_normal_masked
TVMSQ_normal_masked
SyNOnly_CC_4_reg_iter_0_normal_masked
SyNOnly_MI_32_reg_iter_1_normal_masked
SyNOnly_CC_6_reg_iter_1_normal_masked
SyNOnly_CC_2_reg_iter_0_normal_masked
SyNOnly_CC_4_reg_iter_1_normal_masked
SyNOnly_CC_2_reg_iter_1_normal_masked
1
2
3
4
5
6
7
8
9
10
SyNOnly_CC_2_reg_iter_1_normal_masked
SyNOnly_CC_4_reg_iter_1_normal_masked
SyNOnly_CC_2_reg_iter_0_normal_masked
SyNOnly_CC_6_reg_iter_1_normal_masked
SyNOnly_MI_32_reg_iter_1_normal_masked
SyNOnly_CC_4_reg_iter_0_normal_masked
TVMSQ_normal_masked
SyNOnly_MI_32_reg_iter_0_normal_masked
SyNOnly_CC_6_reg_iter_0_normal_masked
TVMSQC_normal_masked
Algorithm

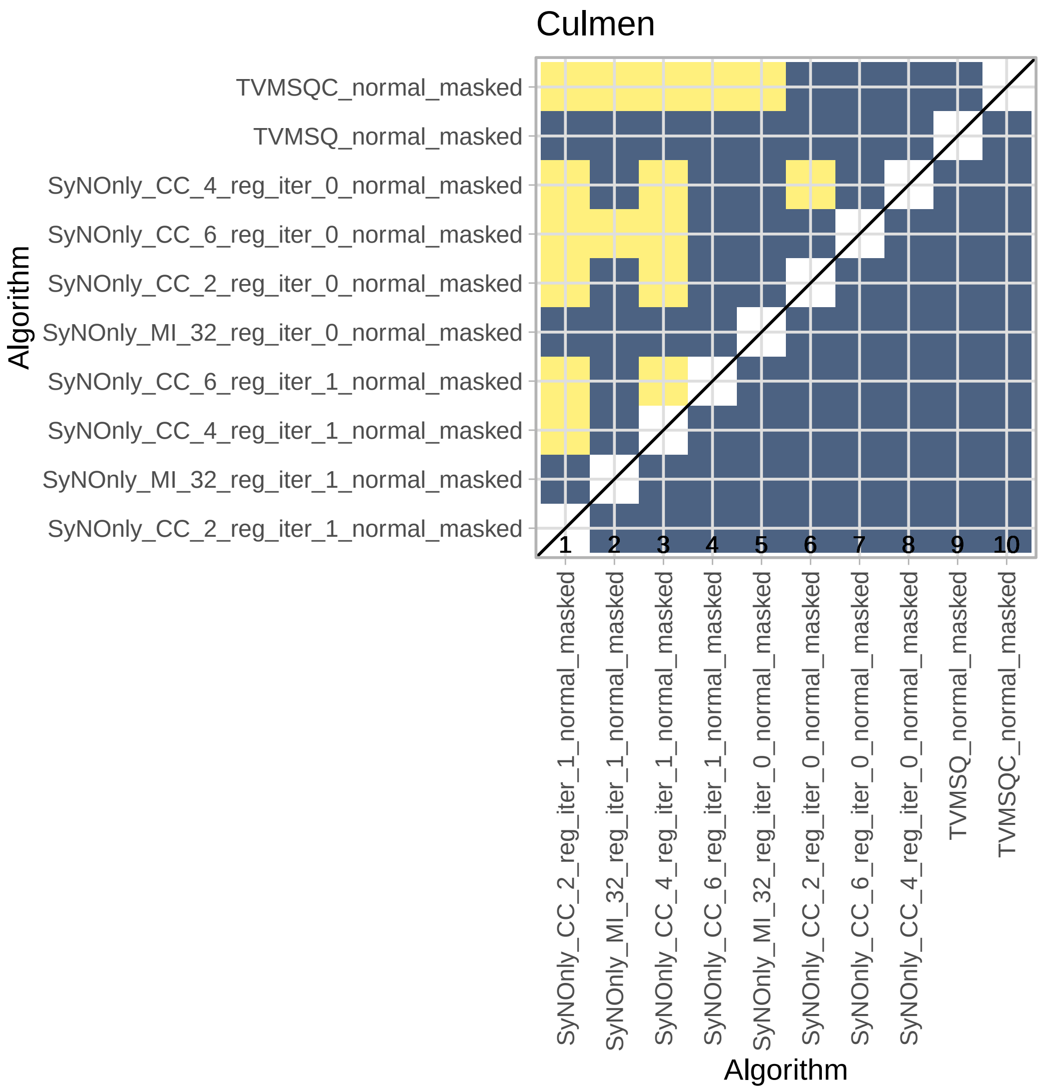

Culmen
TVMSQC_normal_masked
TVMSQ_normal_masked
SyNOnly_CC_4_reg_iter_0_normal_masked
SyNOnly_CC_6_reg_iter_0_normal_masked
SyNOnly_CC_2_reg_iter_0_normal_masked
SyNOnly_MI_32_reg_iter_0_normal_masked
SyNOnly_CC_6_reg_iter_1_normal_masked
SyNOnly_CC_4_reg_iter_1_normal_masked
SyNOnly_MI_32_reg_iter_1_normal_masked
SyNOnly_CC_2_reg_iter_1_normal_masked
Algorithm
1
2
3
4
5
6
7
8
9
10
SyNOnly_CC_2_reg_iter_1_normal_masked
SyNOnly_MI_32_reg_iter_1_normal_masked
SyNOnly_CC_4_reg_iter_1_normal_masked
SyNOnly_CC_6_reg_iter_1_normal_masked
SyNOnly_MI_32_reg_iter_0_normal_masked
SyNOnly_CC_2_reg_iter_0_normal_masked
SyNOnly_CC_6_reg_iter_0_normal_masked
SyNOnly_CC_4_reg_iter_0_normal_masked
TVMSQ_normal_masked
TVMSQC_normal_masked
Algorithm

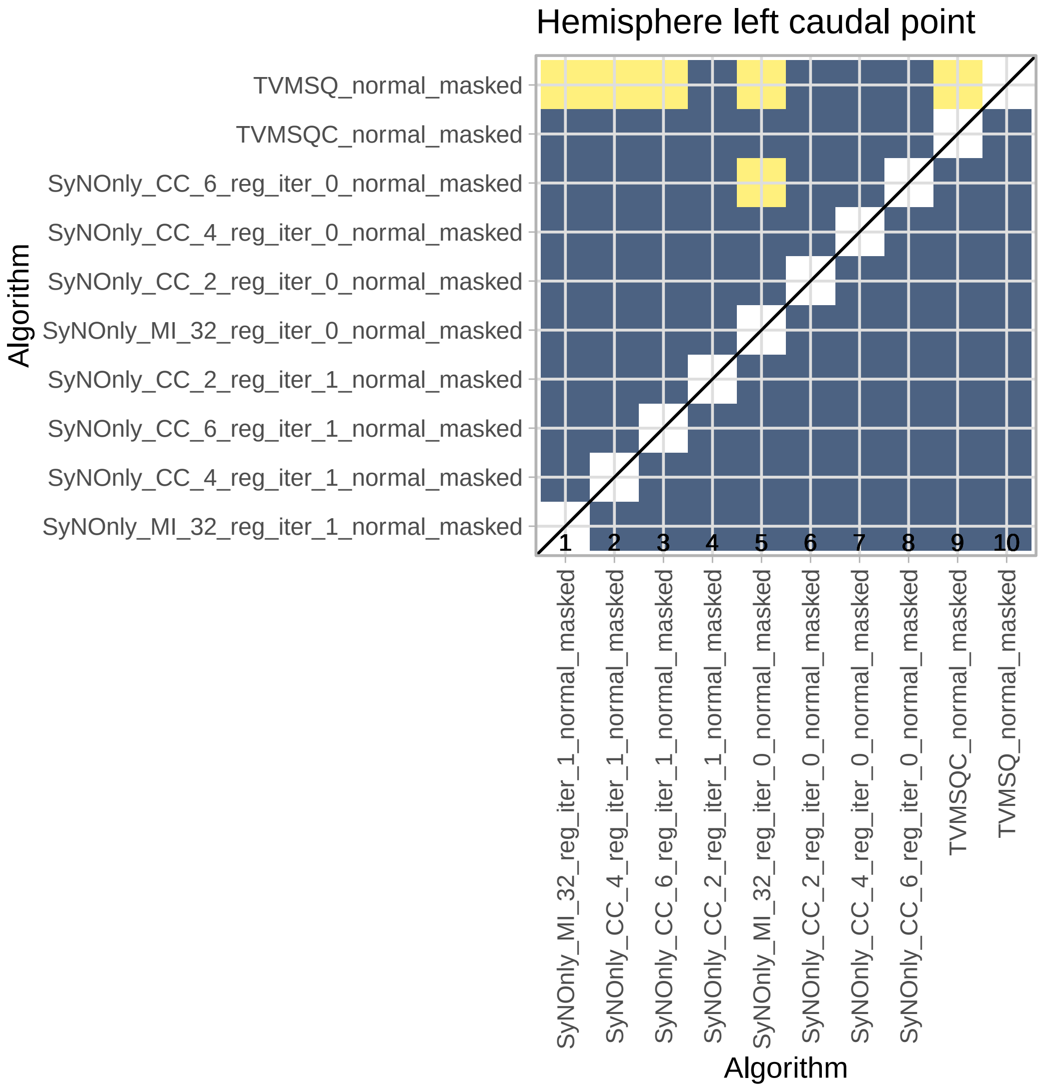

Hemisphere left caudal point
TVMSQ_normal_masked
TVMSQC_normal_masked
SyNOnly_CC_6_reg_iter_0_normal_masked
SyNOnly_CC_4_reg_iter_0_normal_masked
SyNOnly_CC_2_reg_iter_0_normal_masked
SyNOnly_MI_32_reg_iter_0_normal_masked
SyNOnly_CC_2_reg_iter_1_normal_masked
SyNOnly_CC_6_reg_iter_1_normal_masked
SyNOnly_CC_4_reg_iter_1_normal_masked
SyNOnly_MI_32_reg_iter_1_normal_masked
Algorithm
1
2
3
4
5
6
7
8
9
10
SyNOnly_MI_32_reg_iter_1_normal_masked
SyNOnly_CC_4_reg_iter_1_normal_masked
SyNOnly_CC_6_reg_iter_1_normal_masked
SyNOnly_CC_2_reg_iter_1_normal_masked
SyNOnly_MI_32_reg_iter_0_normal_masked
SyNOnly_CC_2_reg_iter_0_normal_masked
SyNOnly_CC_4_reg_iter_0_normal_masked
SyNOnly_CC_6_reg_iter_0_normal_masked
TVMSQC_normal_masked
TVMSQ_normal_masked
Algorithm

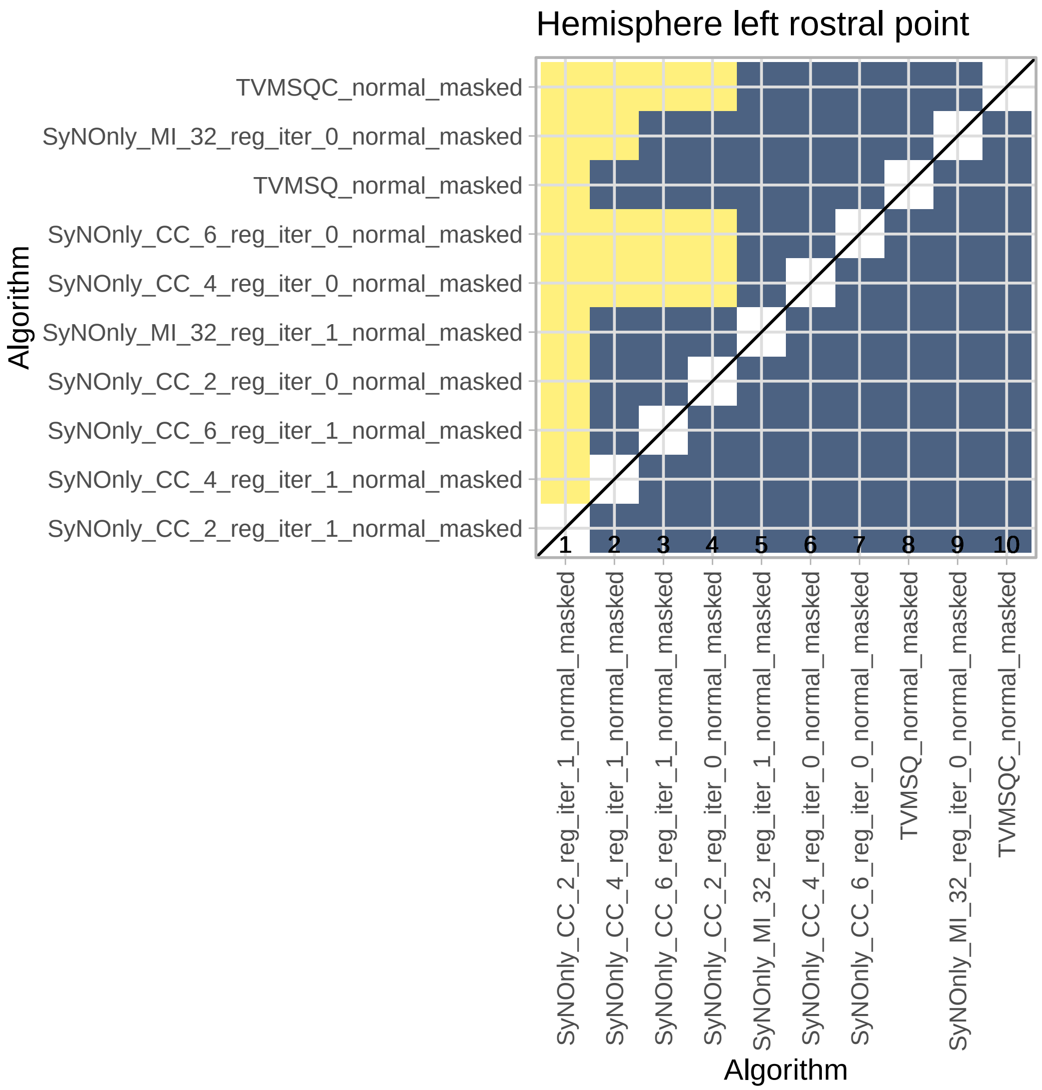

Hemisphere left rostral point
TVMSQC_normal_masked
SyNOnly_MI_32_reg_iter_0_normal_masked
TVMSQ_normal_masked
SyNOnly_CC_6_reg_iter_0_normal_masked
SyNOnly_CC_4_reg_iter_0_normal_masked
SyNOnly_MI_32_reg_iter_1_normal_masked
SyNOnly_CC_2_reg_iter_0_normal_masked
SyNOnly_CC_6_reg_iter_1_normal_masked
SyNOnly_CC_4_reg_iter_1_normal_masked
SyNOnly_CC_2_reg_iter_1_normal_masked
Algorithm
1
2
3
4
5
6
7
8
9
10
SyNOnly_CC_2_reg_iter_1_normal_masked
SyNOnly_CC_4_reg_iter_1_normal_masked
SyNOnly_CC_6_reg_iter_1_normal_masked
SyNOnly_CC_2_reg_iter_0_normal_masked
SyNOnly_MI_32_reg_iter_1_normal_masked
SyNOnly_CC_4_reg_iter_0_normal_masked
SyNOnly_CC_6_reg_iter_0_normal_masked
TVMSQ_normal_masked
SyNOnly_MI_32_reg_iter_0_normal_masked
TVMSQC_normal_masked
Algorithm

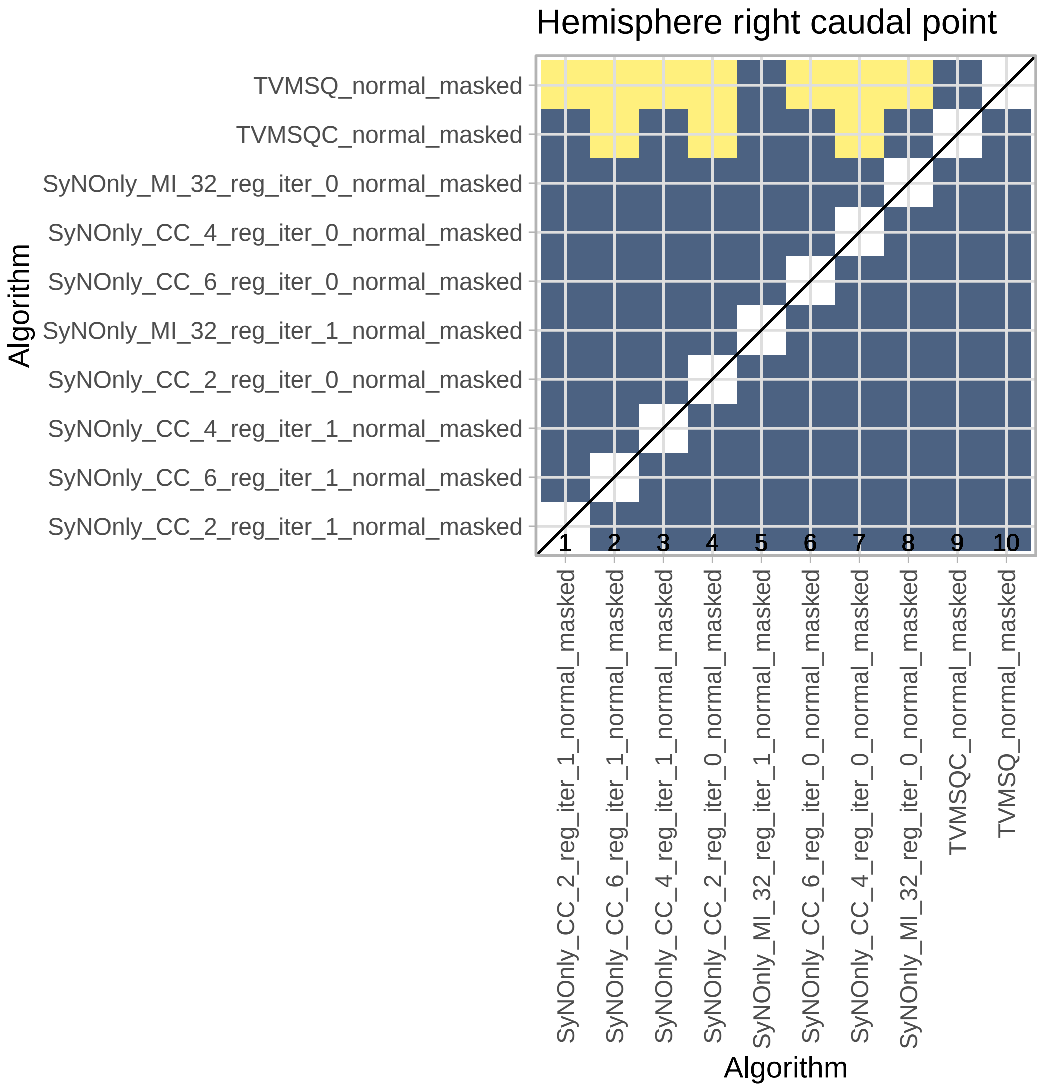

Hemisphere right caudal point
Algorithm
TVMSQ_normal_masked
TVMSQC_normal_masked
SyNOnly_MI_32_reg_iter_0_normal_masked
SyNOnly_CC_4_reg_iter_0_normal_masked
SyNOnly_CC_6_reg_iter_0_normal_masked
SyNOnly_MI_32_reg_iter_1_normal_masked
SyNOnly_CC_2_reg_iter_0_normal_masked
SyNOnly_CC_4_reg_iter_1_normal_masked
SyNOnly_CC_6_reg_iter_1_normal_masked
SyNOnly_CC_2_reg_iter_1_normal_masked
1
2
3
4
5
6
7
8
9
10
SyNOnly_CC_2_reg_iter_1_normal_masked
SyNOnly_CC_6_reg_iter_1_normal_masked
SyNOnly_CC_4_reg_iter_1_normal_masked
SyNOnly_CC_2_reg_iter_0_normal_masked
SyNOnly_MI_32_reg_iter_1_normal_masked
SyNOnly_CC_6_reg_iter_0_normal_masked
SyNOnly_CC_4_reg_iter_0_normal_masked
SyNOnly_MI_32_reg_iter_0_normal_masked
TVMSQC_normal_masked
TVMSQ_normal_masked
Algorithm

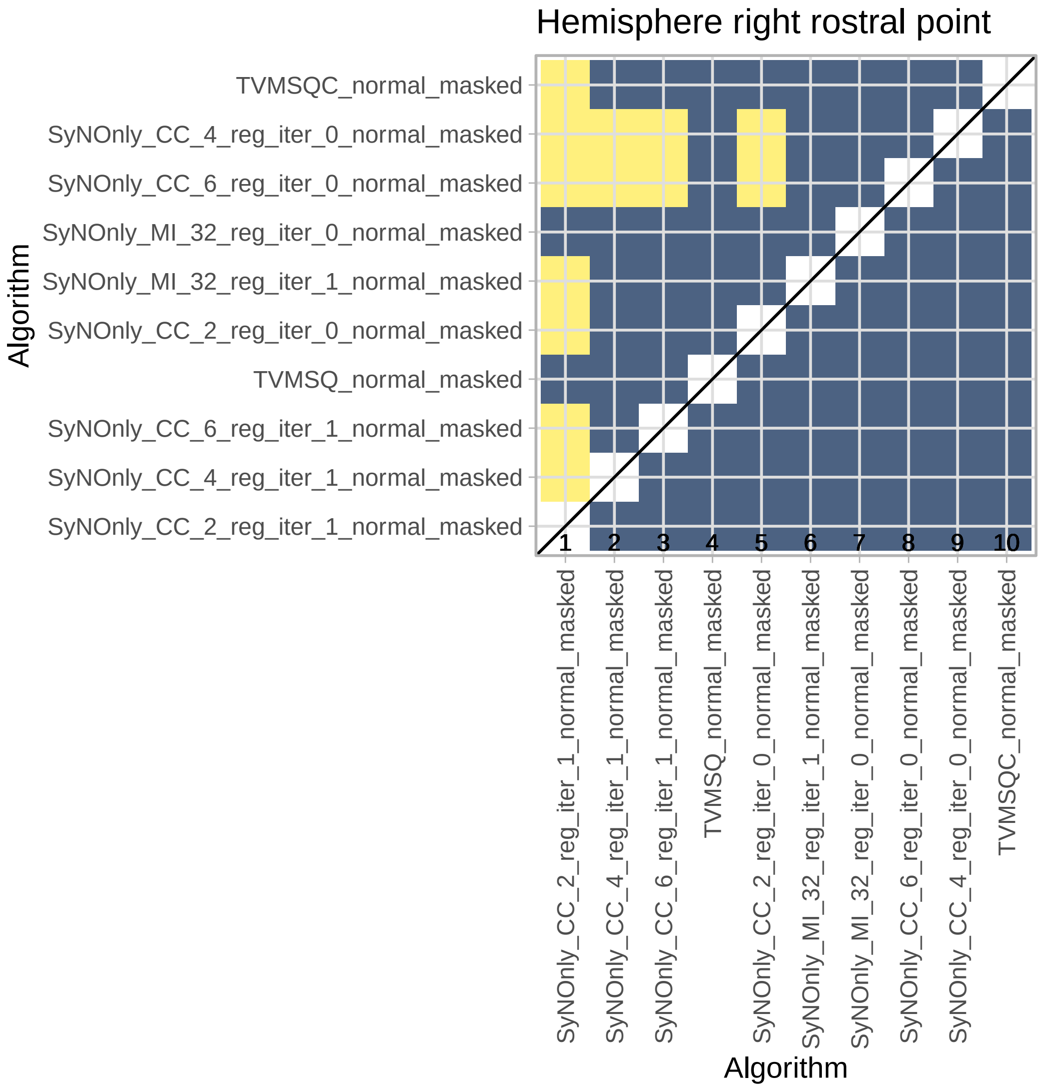
Hemisphere right rostral point
Algorithm
TVMSQC_normal_masked
SyNOnly_CC_4_reg_iter_0_normal_masked
SyNOnly_CC_6_reg_iter_0_normal_masked
SyNOnly_MI_32_reg_iter_0_normal_masked
SyNOnly_MI_32_reg_iter_1_normal_masked
SyNOnly_CC_2_reg_iter_0_normal_masked
TVMSQ_normal_masked
SyNOnly_CC_6_reg_iter_1_normal_masked
SyNOnly_CC_4_reg_iter_1_normal_masked
SyNOnly_CC_2_reg_iter_1_normal_masked
1
2
3
4
5
6
7
8
9
10
SyNOnly_CC_2_reg_iter_1_normal_masked
SyNOnly_CC_4_reg_iter_1_normal_masked
SyNOnly_CC_6_reg_iter_1_normal_masked
TVMSQ_normal_masked
SyNOnly_CC_2_reg_iter_0_normal_masked
SyNOnly_MI_32_reg_iter_1_normal_masked
SyNOnly_MI_32_reg_iter_0_normal_masked
SyNOnly_CC_6_reg_iter_0_normal_masked
SyNOnly_CC_4_reg_iter_0_normal_masked
TVMSQC_normal_masked
Algorithm

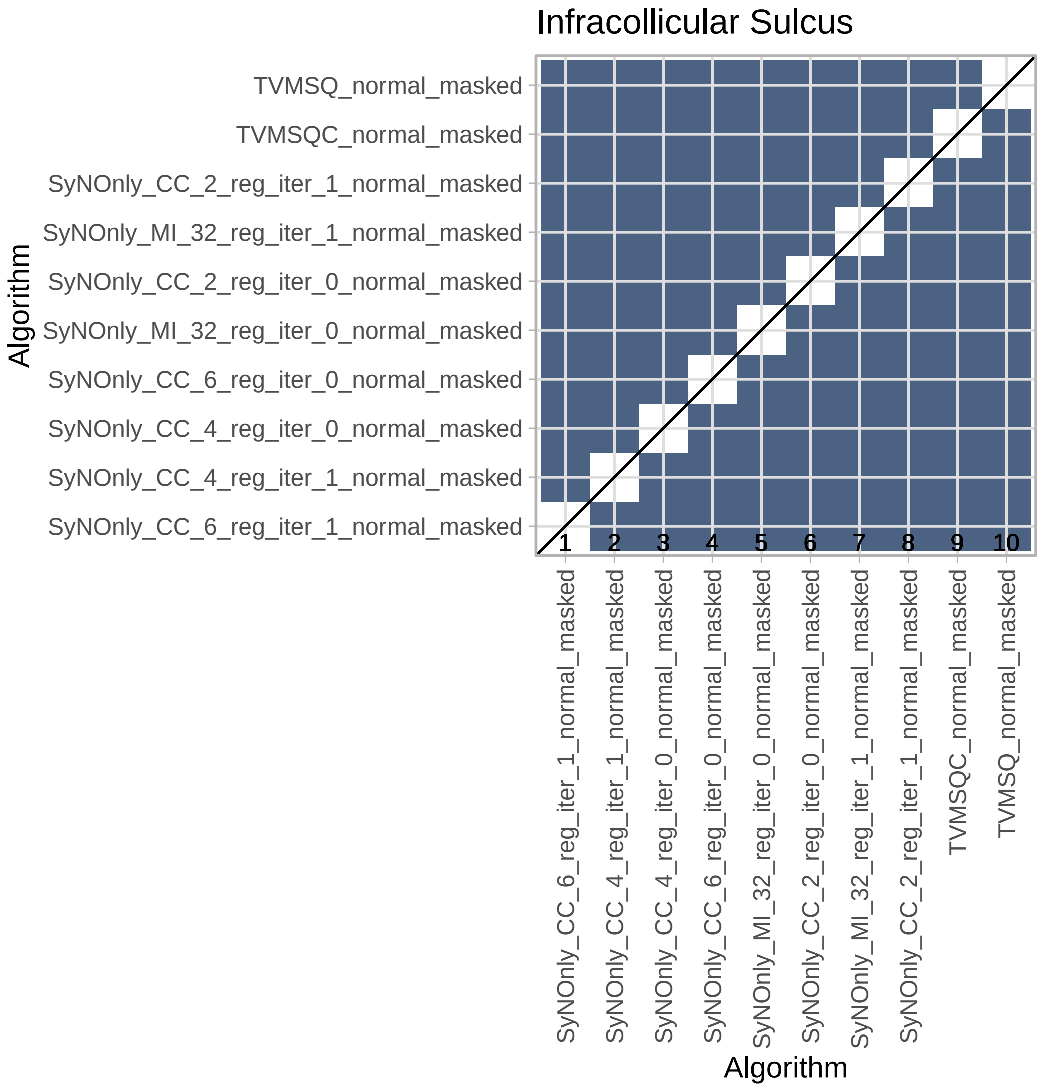
Infracollicular Sulcus
TVMSQ_normal_masked
TVMSQC_normal_masked
SyNOnly_CC_2_reg_iter_1_normal_masked
SyNOnly_MI_32_reg_iter_1_normal_masked
SyNOnly_CC_2_reg_iter_0_normal_masked
SyNOnly_MI_32_reg_iter_0_normal_masked
SyNOnly_CC_6_reg_iter_0_normal_masked
SyNOnly_CC_4_reg_iter_0_normal_masked
SyNOnly_CC_4_reg_iter_1_normal_masked
SyNOnly_CC_6_reg_iter_1_normal_masked
Algorithm
1 2 3 4 5 6 7 8 9 10
SyNOnly_CC_6_reg_iter_1_normal_masked
SyNOnly_CC_4_reg_iter_1_normal_masked
SyNOnly_CC_4_reg_iter_0_normal_masked
SyNOnly_CC_6_reg_iter_0_normal_masked
SyNOnly_MI_32_reg_iter_0_normal_masked
SyNOnly_CC_2_reg_iter_0_normal_masked
SyNOnly_MI_32_reg_iter_1_normal_masked
SyNOnly_CC_2_reg_iter_1_normal_masked
TVMSQC_normal_masked
TVMSQ_normal_masked
Algorithm

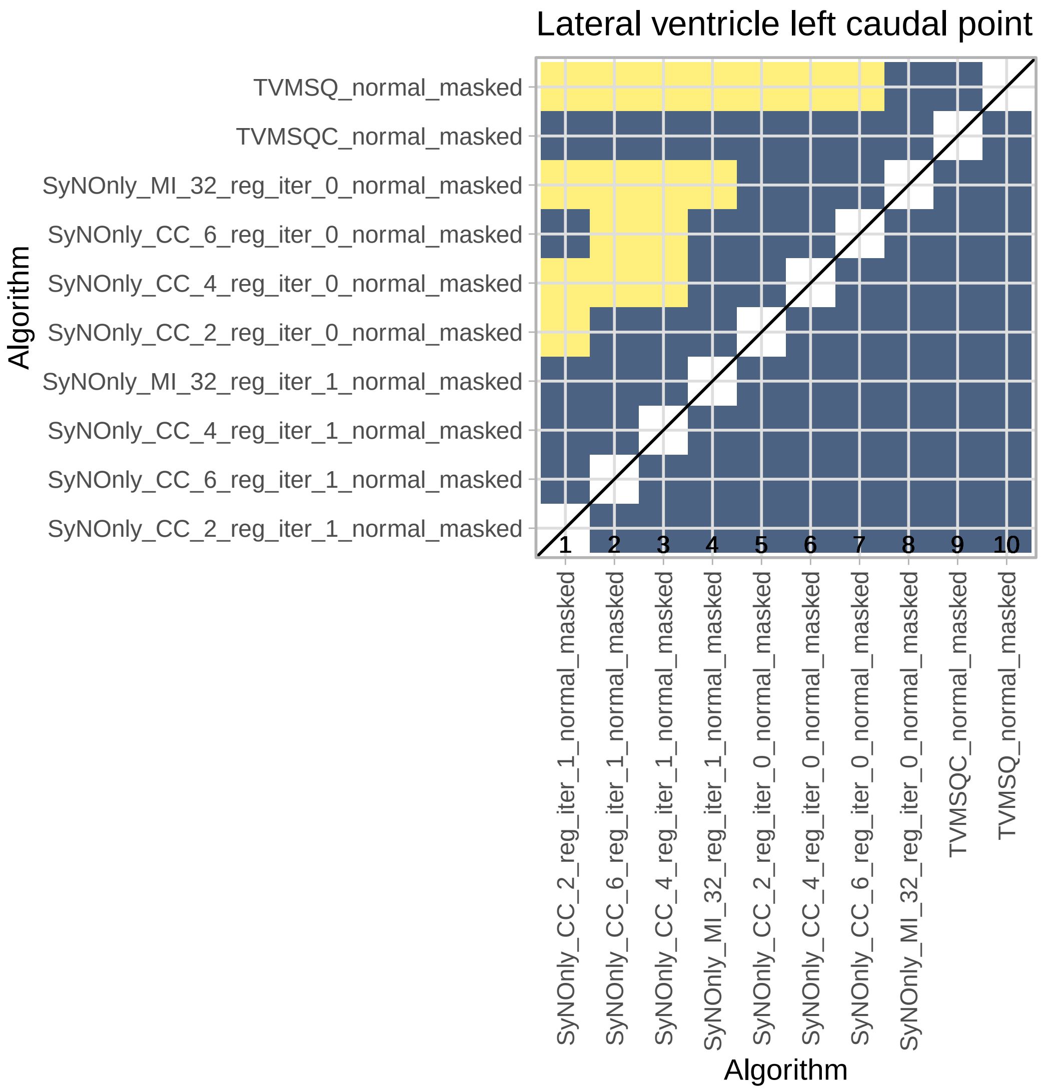
Lateral ventricle left caudal point
Algorithm
TVMSQ_normal_masked
TVMSQC_normal_masked
SyNOnly_MI_32_reg_iter_0_normal_masked
SyNOnly_CC_6_reg_iter_0_normal_masked
SyNOnly_CC_4_reg_iter_0_normal_masked
SyNOnly_CC_2_reg_iter_0_normal_masked
SyNOnly_MI_32_reg_iter_1_normal_masked
SyNOnly_CC_4_reg_iter_1_normal_masked
SyNOnly_CC_6_reg_iter_1_normal_masked
SyNOnly_CC_2_reg_iter_1_normal_masked
1
2
3
4
5
6
7
8
9
10
SyNOnly_CC_2_reg_iter_1_normal_masked
SyNOnly_CC_6_reg_iter_1_normal_masked
SyNOnly_CC_4_reg_iter_1_normal_masked
SyNOnly_MI_32_reg_iter_1_normal_masked
SyNOnly_CC_2_reg_iter_0_normal_masked
SyNOnly_CC_4_reg_iter_0_normal_masked
SyNOnly_CC_6_reg_iter_0_normal_masked
SyNOnly_MI_32_reg_iter_0_normal_masked
TVMSQC_normal_masked
TVMSQ_normal_masked
Algorithm

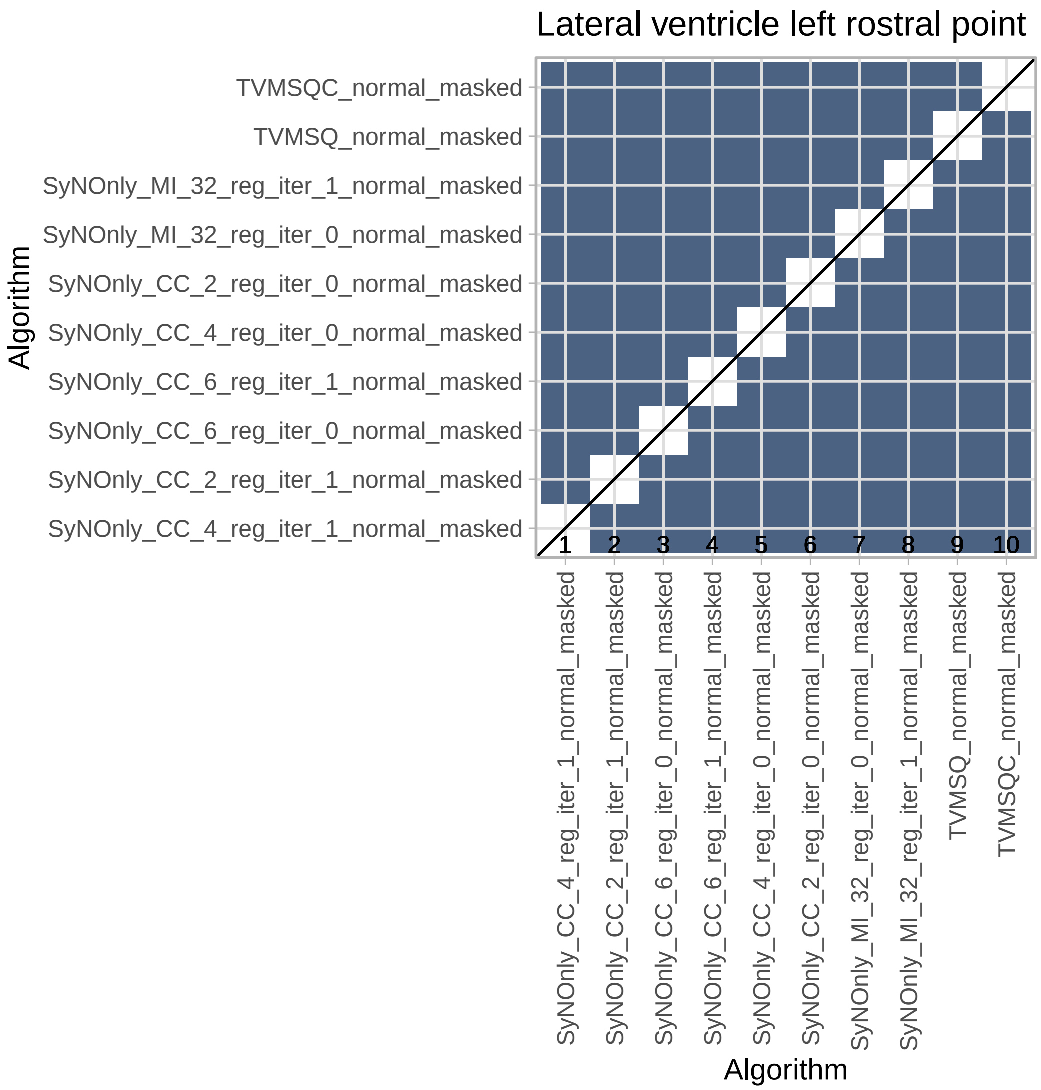

Lateral ventricle left rostral point
TVMSQC_normal_masked
TVMSQ_normal_masked
SyNOnly_MI_32_reg_iter_1_normal_masked
SyNOnly_MI_32_reg_iter_0_normal_masked
SyNOnly_CC_2_reg_iter_0_normal_masked
SyNOnly_CC_4_reg_iter_0_normal_masked
SyNOnly_CC_6_reg_iter_1_normal_masked
SyNOnly_CC_6_reg_iter_0_normal_masked
SyNOnly_CC_2_reg_iter_1_normal_masked
SyNOnly_CC_4_reg_iter_1_normal_masked
Algorithm
1
2
3
4
5
6
7
8
9
10
SyNOnly_CC_4_reg_iter_1_normal_masked
SyNOnly_CC_2_reg_iter_1_normal_masked
SyNOnly_CC_6_reg_iter_0_normal_masked
SyNOnly_CC_6_reg_iter_1_normal_masked
SyNOnly_CC_4_reg_iter_0_normal_masked
SyNOnly_CC_2_reg_iter_0_normal_masked
SyNOnly_MI_32_reg_iter_0_normal_masked
SyNOnly_MI_32_reg_iter_1_normal_masked
TVMSQ_normal_masked
TVMSQC_normal_masked
Algorithm

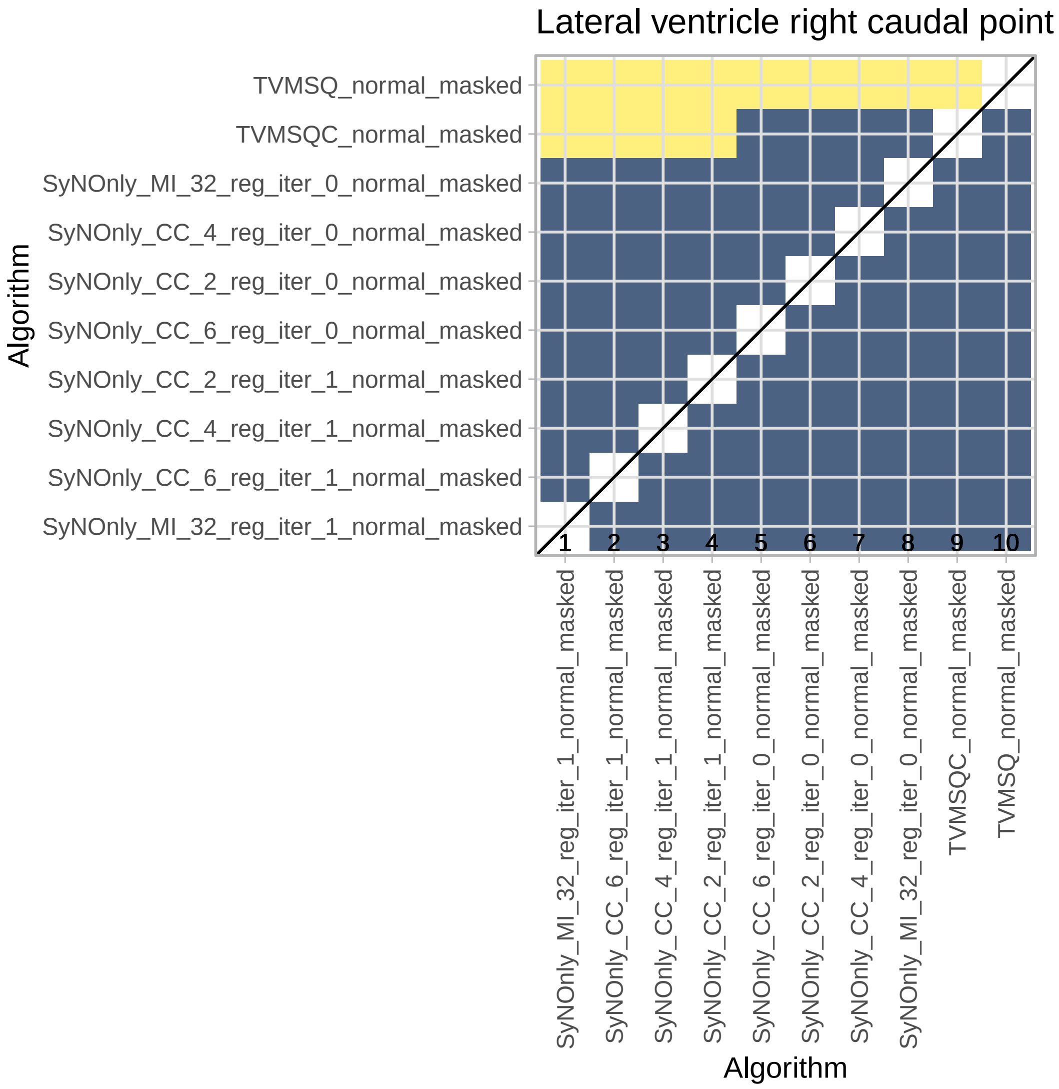

Lateral ventricle right caudal point
TVMSQ_normal_masked
TVMSQC_normal_masked
SyNOnly_MI_32_reg_iter_0_normal_masked
SyNOnly_CC_4_reg_iter_0_normal_masked
SyNOnly_CC_2_reg_iter_0_normal_masked
SyNOnly_CC_6_reg_iter_0_normal_masked
SyNOnly_CC_2_reg_iter_1_normal_masked
SyNOnly_CC_4_reg_iter_1_normal_masked
SyNOnly_CC_6_reg_iter_1_normal_masked
SyNOnly_MI_32_reg_iter_1_normal_masked
Algorithm
1
2
3
4
5
6
7
8
9
10
SyNOnly_MI_32_reg_iter_1_normal_masked
SyNOnly_CC_6_reg_iter_1_normal_masked
SyNOnly_CC_4_reg_iter_1_normal_masked
SyNOnly_CC_2_reg_iter_1_normal_masked
SyNOnly_CC_6_reg_iter_0_normal_masked
SyNOnly_CC_2_reg_iter_0_normal_masked
SyNOnly_CC_4_reg_iter_0_normal_masked
SyNOnly_MI_32_reg_iter_0_normal_masked
TVMSQC_normal_masked
TVMSQ_normal_masked
Algorithm

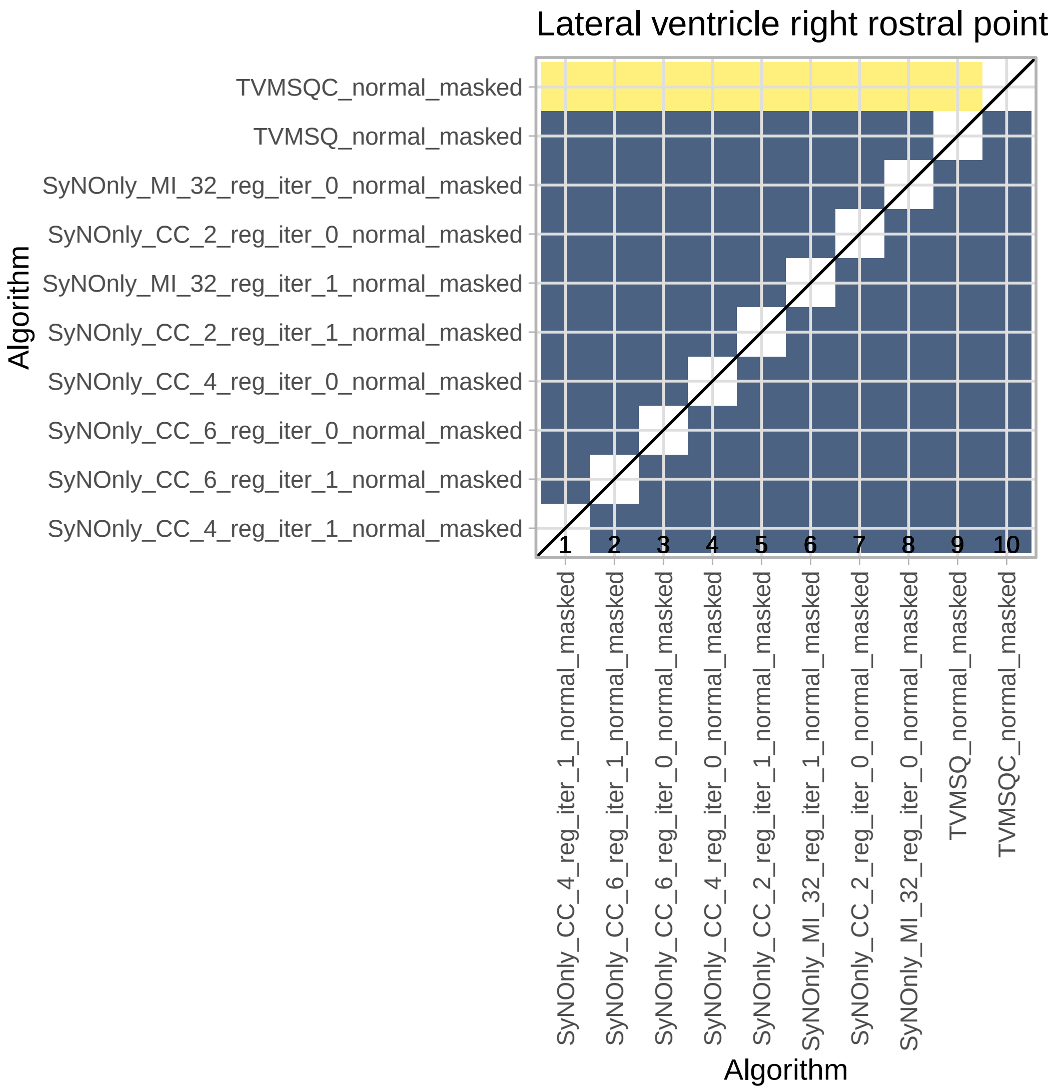
Lateral ventricle right rostral point
Algorithm
TVMSQC_normal_masked
TVMSQ_normal_masked
SyNOnly_MI_32_reg_iter_0_normal_masked
SyNOnly_CC_2_reg_iter_0_normal_masked
SyNOnly_MI_32_reg_iter_1_normal_masked
SyNOnly_CC_2_reg_iter_1_normal_masked
SyNOnly_CC_4_reg_iter_0_normal_masked
SyNOnly_CC_6_reg_iter_0_normal_masked
SyNOnly_CC_6_reg_iter_1_normal_masked
SyNOnly_CC_4_reg_iter_1_normal_masked
1
2
3
4
5
6
7
8
9
10
SyNOnly_CC_4_reg_iter_1_normal_masked
SyNOnly_CC_6_reg_iter_1_normal_masked
SyNOnly_CC_6_reg_iter_0_normal_masked
SyNOnly_CC_4_reg_iter_0_normal_masked
SyNOnly_CC_2_reg_iter_1_normal_masked
SyNOnly_MI_32_reg_iter_1_normal_masked
SyNOnly_CC_2_reg_iter_0_normal_masked
SyNOnly_MI_32_reg_iter_0_normal_masked
TVMSQ_normal_masked
TVMSQC_normal_masked
Algorithm

PMJ

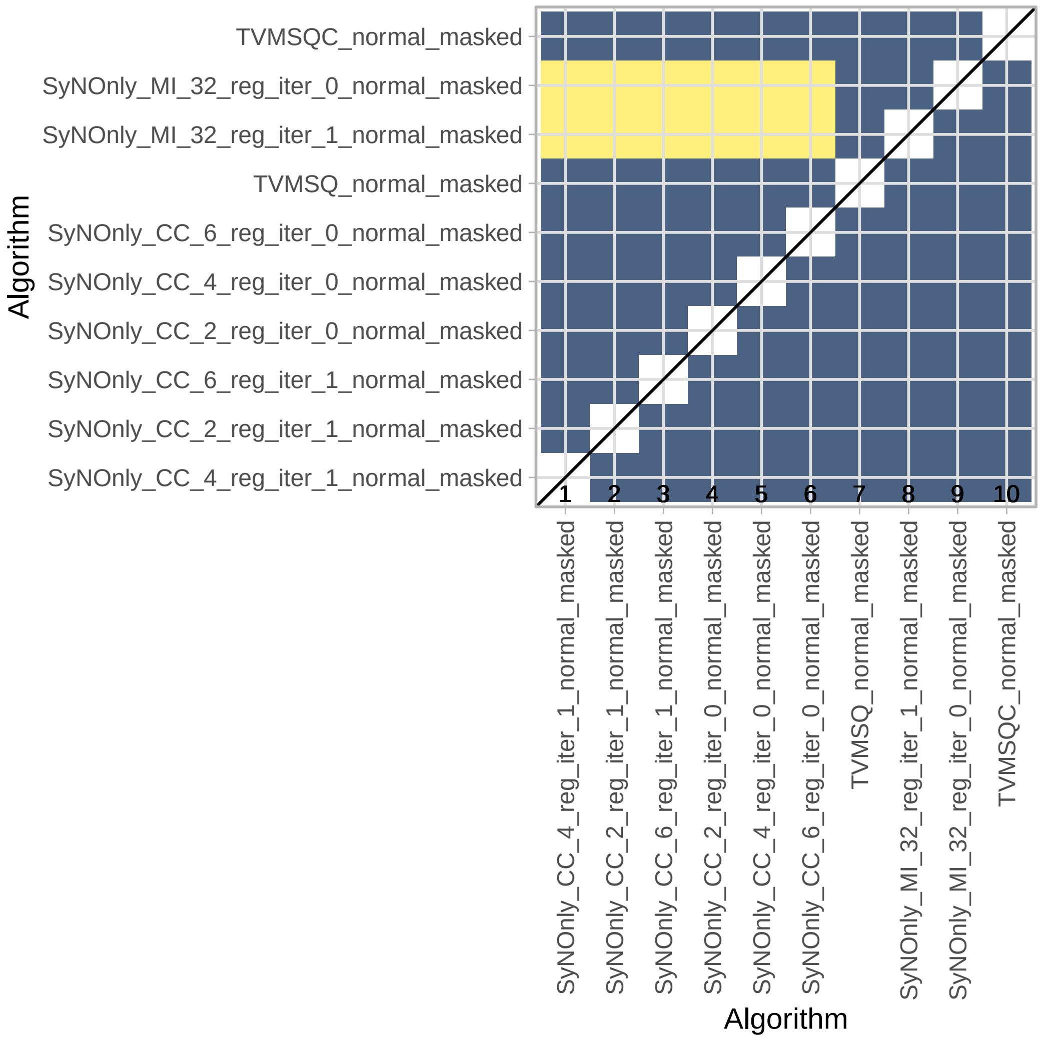

TVMSQC_normal_masked
SyNOnly_MI_32_reg_iter_0_normal_masked
SyNOnly_MI_32_reg_iter_1_normal_masked
TVMSQ_normal_masked
SyNOnly_CC_6_reg_iter_0_normal_masked
SyNOnly_CC_4_reg_iter_0_normal_masked
SyNOnly_CC_2_reg_iter_0_normal_masked
SyNOnly_CC_6_reg_iter_1_normal_masked
SyNOnly_CC_2_reg_iter_1_normal_masked
SyNOnly_CC_4_reg_iter_1_normal_masked
Algorithm
1
2
3
4
5
6
7
8
9
10
SyNOnly_CC_4_reg_iter_1_normal_masked
SyNOnly_CC_2_reg_iter_1_normal_masked
SyNOnly_CC_6_reg_iter_1_normal_masked
SyNOnly_CC_2_reg_iter_0_normal_masked
SyNOnly_CC_4_reg_iter_0_normal_masked
SyNOnly_CC_6_reg_iter_0_normal_masked
TVMSQ_normal_masked
SyNOnly_MI_32_reg_iter_1_normal_masked
SyNOnly_MI_32_reg_iter_0_normal_masked
TVMSQC_normal_masked
Algorithm

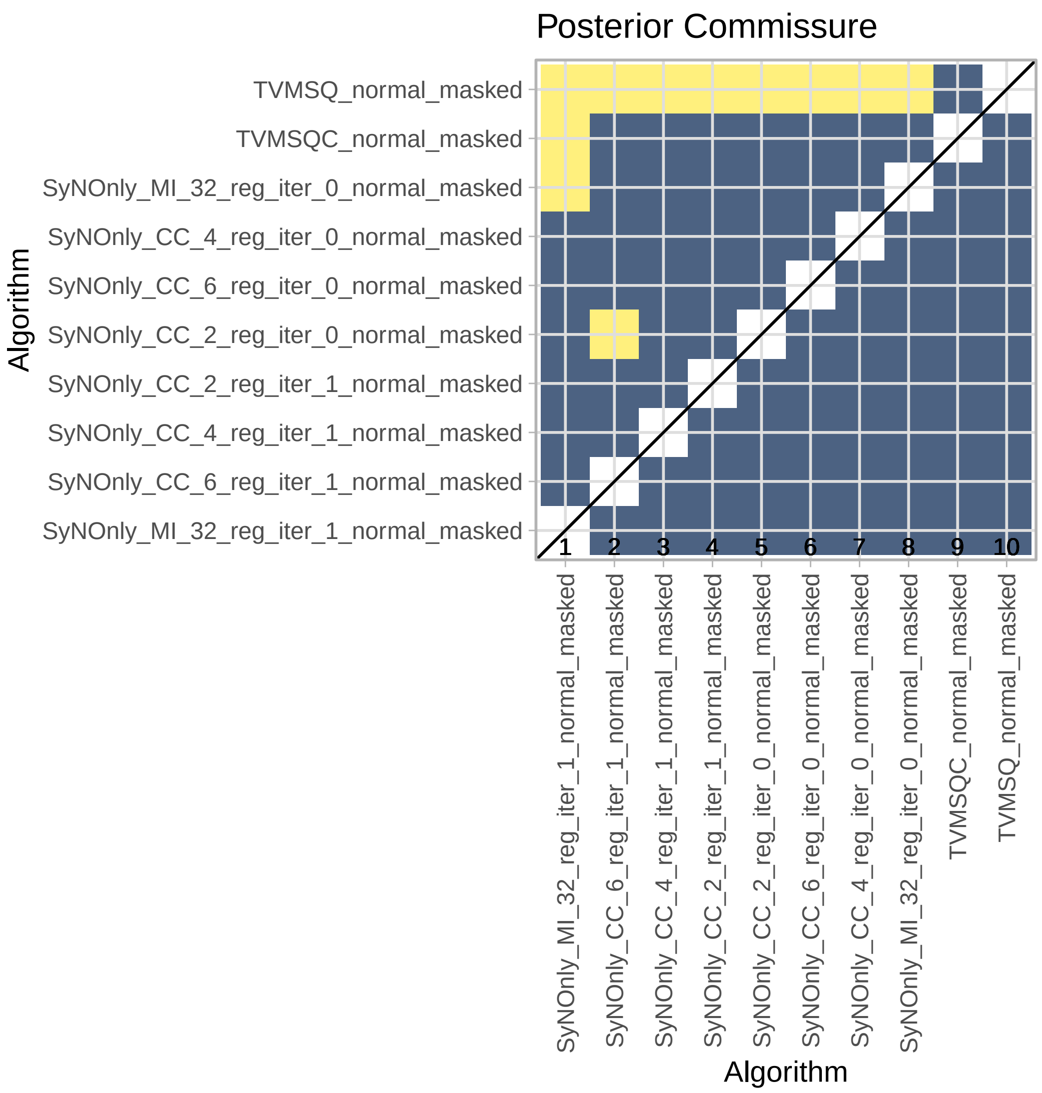
Posterior Commissure
Algorithm
TVMSQ_normal_masked
TVMSQC_normal_masked
SyNOnly_MI_32_reg_iter_0_normal_masked
SyNOnly_CC_4_reg_iter_0_normal_masked
SyNOnly_CC_6_reg_iter_0_normal_masked
SyNOnly_CC_2_reg_iter_0_normal_masked
SyNOnly_CC_2_reg_iter_1_normal_masked
SyNOnly_CC_4_reg_iter_1_normal_masked
SyNOnly_CC_6_reg_iter_1_normal_masked
SyNOnly_MI_32_reg_iter_1_normal_masked
1
2
3
4
5
6
7
8
9
10
SyNOnly_MI_32_reg_iter_1_normal_masked
SyNOnly_CC_6_reg_iter_1_normal_masked
SyNOnly_CC_4_reg_iter_1_normal_masked
SyNOnly_CC_2_reg_iter_1_normal_masked
SyNOnly_CC_2_reg_iter_0_normal_masked
SyNOnly_CC_6_reg_iter_0_normal_masked
SyNOnly_CC_4_reg_iter_0_normal_masked
SyNOnly_MI_32_reg_iter_0_normal_masked
TVMSQC_normal_masked
TVMSQ_normal_masked
Algorithm

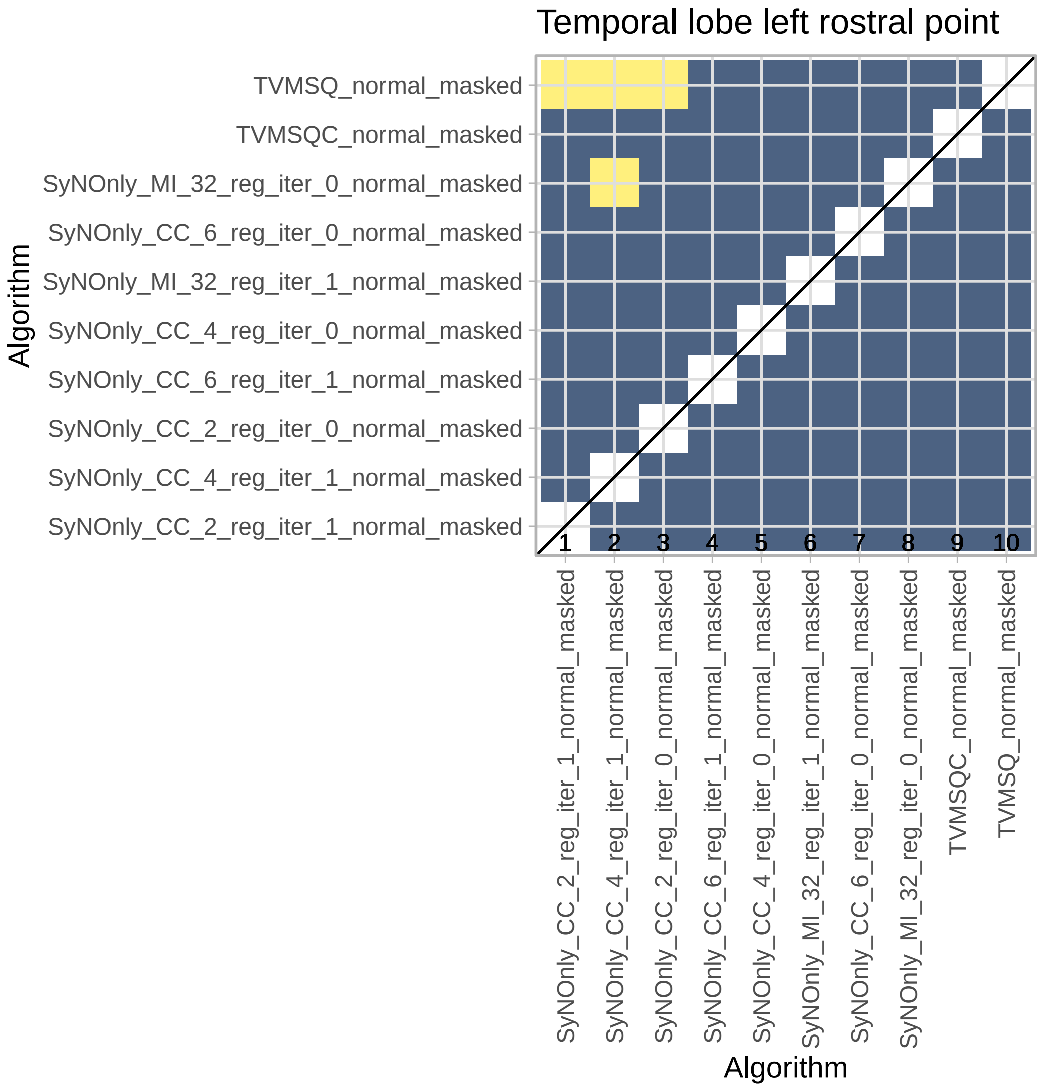
Temporal lobe left rostral point
Algorithm
TVMSQ_normal_masked
TVMSQC_normal_masked
SyNOnly_MI_32_reg_iter_0_normal_masked
SyNOnly_CC_6_reg_iter_0_normal_masked
SyNOnly_MI_32_reg_iter_1_normal_masked
SyNOnly_CC_4_reg_iter_0_normal_masked
SyNOnly_CC_6_reg_iter_1_normal_masked
SyNOnly_CC_2_reg_iter_0_normal_masked
SyNOnly_CC_4_reg_iter_1_normal_masked
SyNOnly_CC_2_reg_iter_1_normal_masked
1
2
3
4
5
6
7
8
9
10
SyNOnly_CC_2_reg_iter_1_normal_masked
SyNOnly_CC_4_reg_iter_1_normal_masked
SyNOnly_CC_2_reg_iter_0_normal_masked
SyNOnly_CC_6_reg_iter_1_normal_masked
SyNOnly_CC_4_reg_iter_0_normal_masked
SyNOnly_MI_32_reg_iter_1_normal_masked
SyNOnly_CC_6_reg_iter_0_normal_masked
SyNOnly_MI_32_reg_iter_0_normal_masked
TVMSQC_normal_masked
TVMSQ_normal_masked
Algorithm

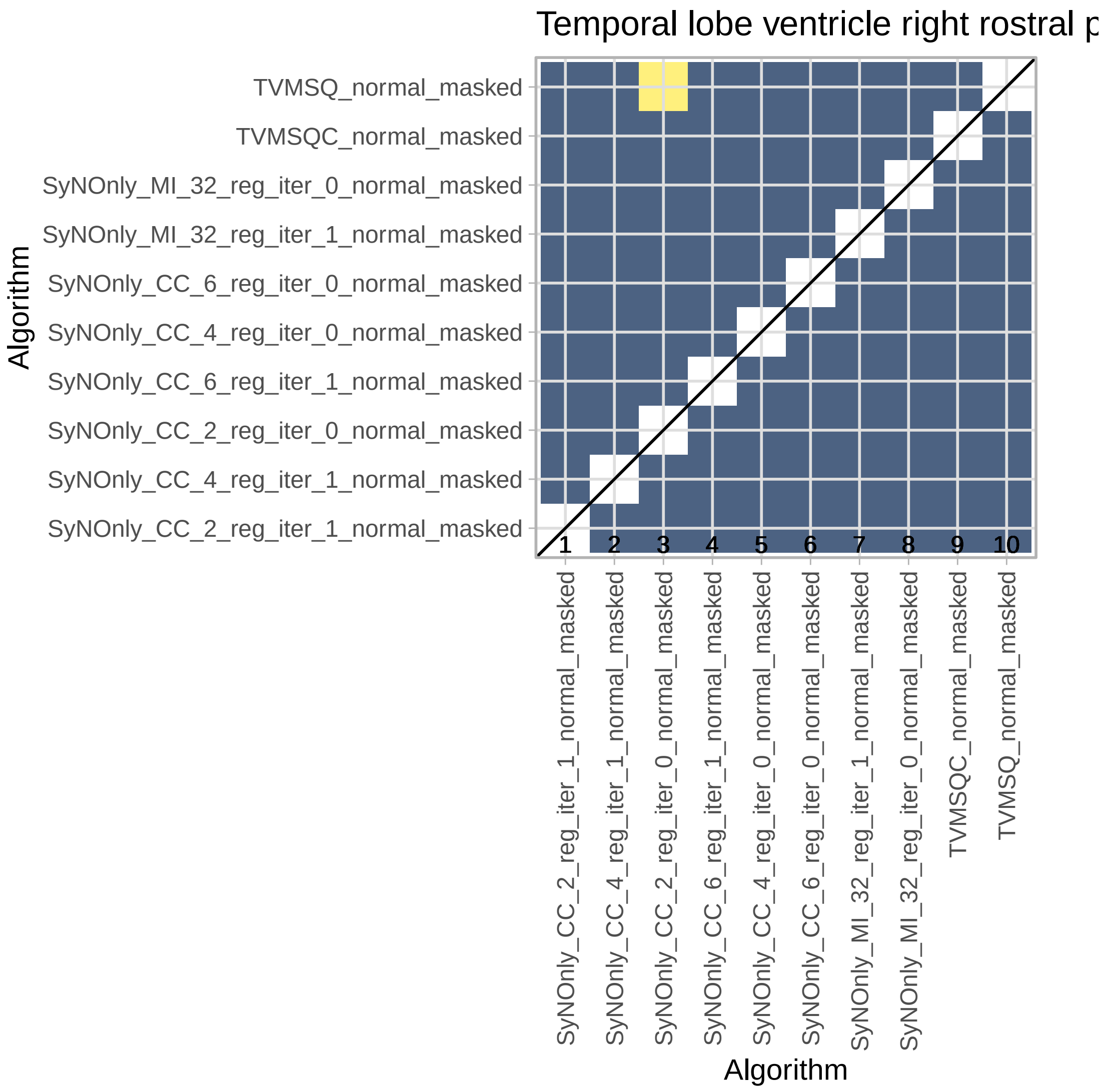
Temporal lobe ventricle right rostral p
TVMSQ_normal_masked
TVMSQC_normal_masked
SyNOnly_MI_32_reg_iter_0_normal_masked
SyNOnly_MI_32_reg_iter_1_normal_masked
SyNOnly_CC_6_reg_iter_0_normal_masked
SyNOnly_CC_4_reg_iter_0_normal_masked
SyNOnly_CC_6_reg_iter_1_normal_masked
SyNOnly_CC_2_reg_iter_0_normal_masked
SyNOnly_CC_4_reg_iter_1_normal_masked
SyNOnly_CC_2_reg_iter_1_normal_masked
Algorithm
1
2
3
4
5
6
7
8
9
10
SyNOnly_CC_2_reg_iter_1_normal_masked
SyNOnly_CC_4_reg_iter_1_normal_masked
SyNOnly_CC_2_reg_iter_0_normal_masked
SyNOnly_CC_6_reg_iter_1_normal_masked
SyNOnly_CC_4_reg_iter_0_normal_masked
SyNOnly_CC_6_reg_iter_0_normal_masked
SyNOnly_MI_32_reg_iter_1_normal_masked
SyNOnly_MI_32_reg_iter_0_normal_masked
TVMSQC_normal_masked
TVMSQ_normal_masked
Algorithm

3.4 Ranking robustness to ranking methods

Line plots for visualizing ranking robustness across different ranking methods. Each algorithm is represented by one colored line. For each ranking method encoded on the x-axis, the height of the line represents the corresponding rank. Horizontal lines indicate identical ranks for all methods.

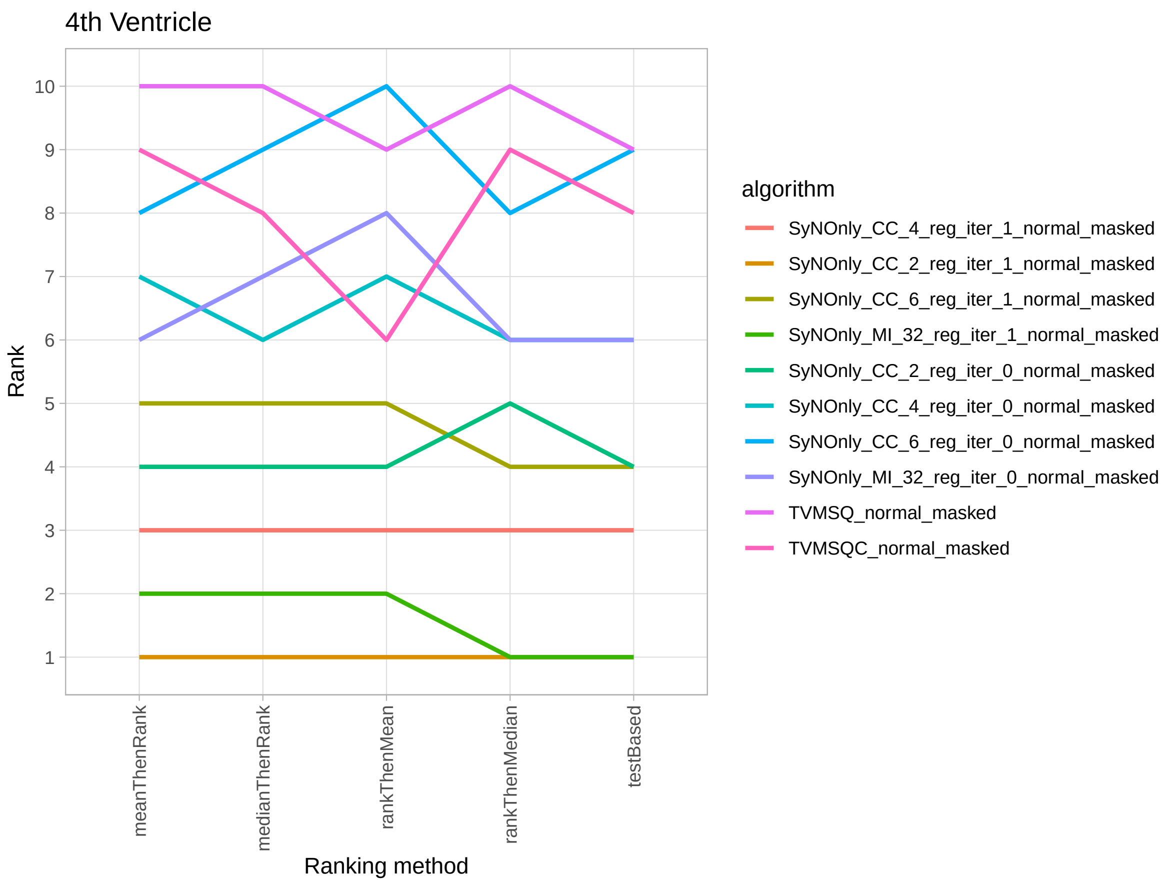

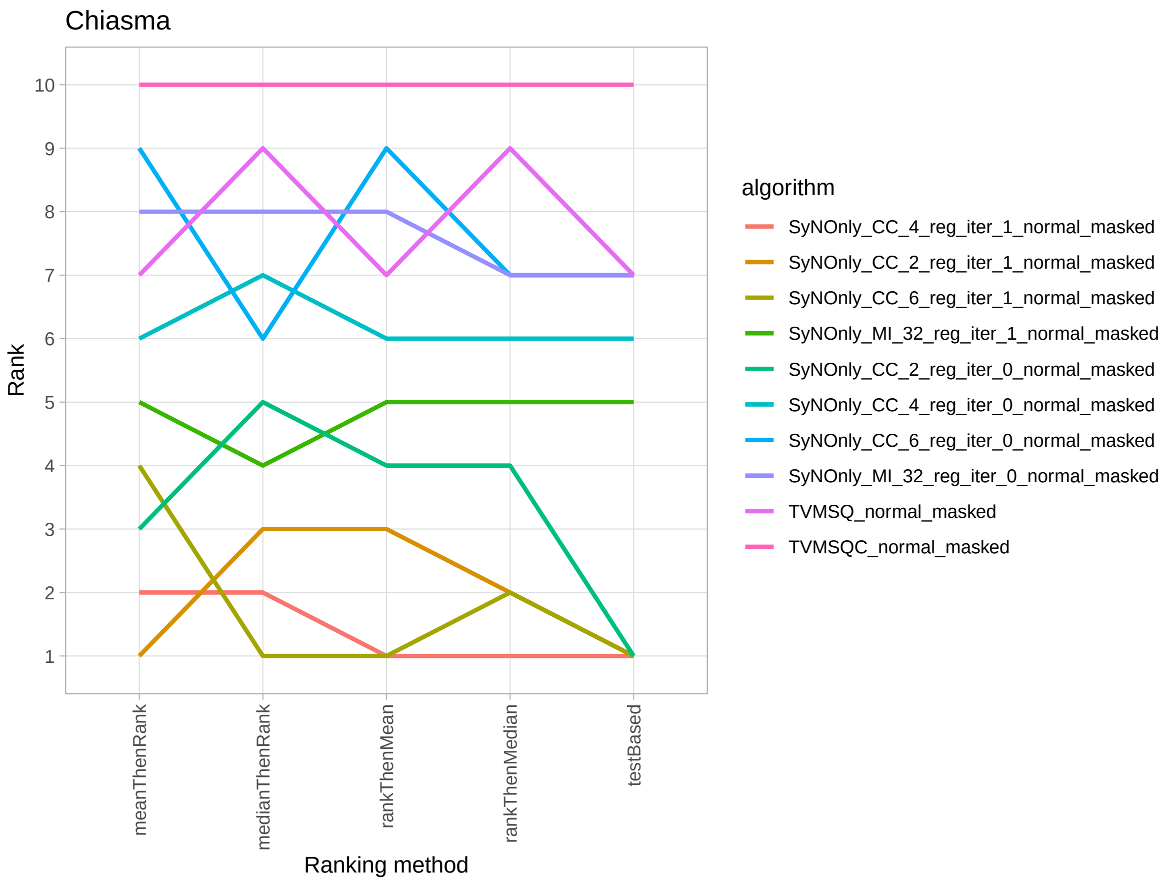

Chiasma
10
9
8
7
6
5
4
3
2
1
Rank
meanThenRank
medianThenRank
rankThenMean
rankThenMedian
testBased
Ranking method
algorithm
SyNOnly_CC_4_reg_iter_1_normal_masked
SyNOnly_CC_2_reg_iter_1_normal_masked
SyNOnly_CC_6_reg_iter_1_normal_masked
SyNOnly_MI_32_reg_iter_1_normal_masked
SyNOnly_CC_2_reg_iter_0_normal_masked
SyNOnly_CC_4_reg_iter_0_normal_masked
SyNOnly_CC_6_reg_iter_0_normal_masked
SyNOnly_MI_32_reg_iter_0_normal_masked
TVMSQ_normal_masked
TVMSQC_normal_masked

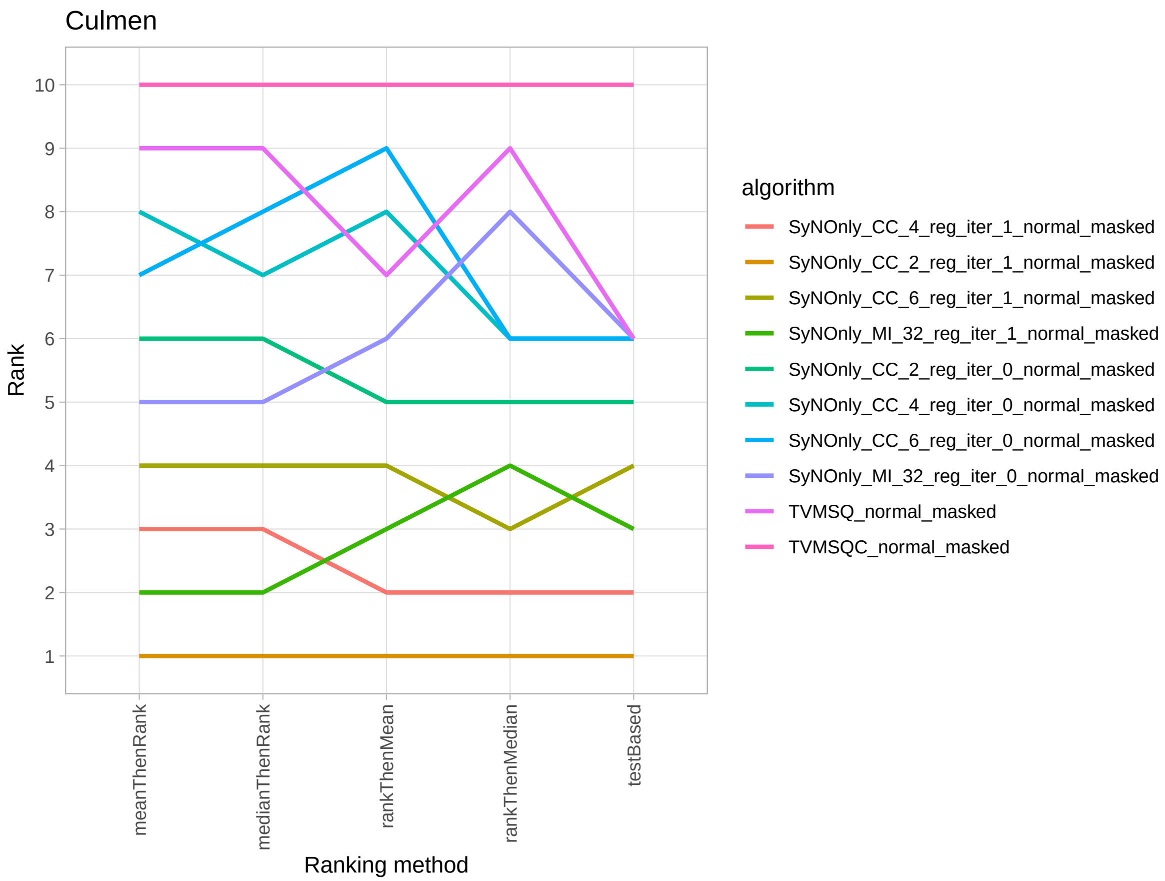
Culmen
Rank
10
9
8
7
6
5
4
3
2
1
meanThenRank
medianThenRank
rankThenMean
rankThenMedian
testBased
Ranking method
algorithm
SyNOnly_CC_4_reg_iter_1_normal_masked
SyNOnly_CC_2_reg_iter_1_normal_masked
SyNOnly_CC_6_reg_iter_1_normal_masked
SyNOnly_MI_32_reg_iter_1_normal_masked
SyNOnly_CC_2_reg_iter_0_normal_masked
SyNOnly_CC_4_reg_iter_0_normal_masked
SyNOnly_CC_6_reg_iter_0_normal_masked
SyNOnly_MI_32_reg_iter_0_normal_masked
TVMSQ_normal_masked
TVMSQC_normal_masked

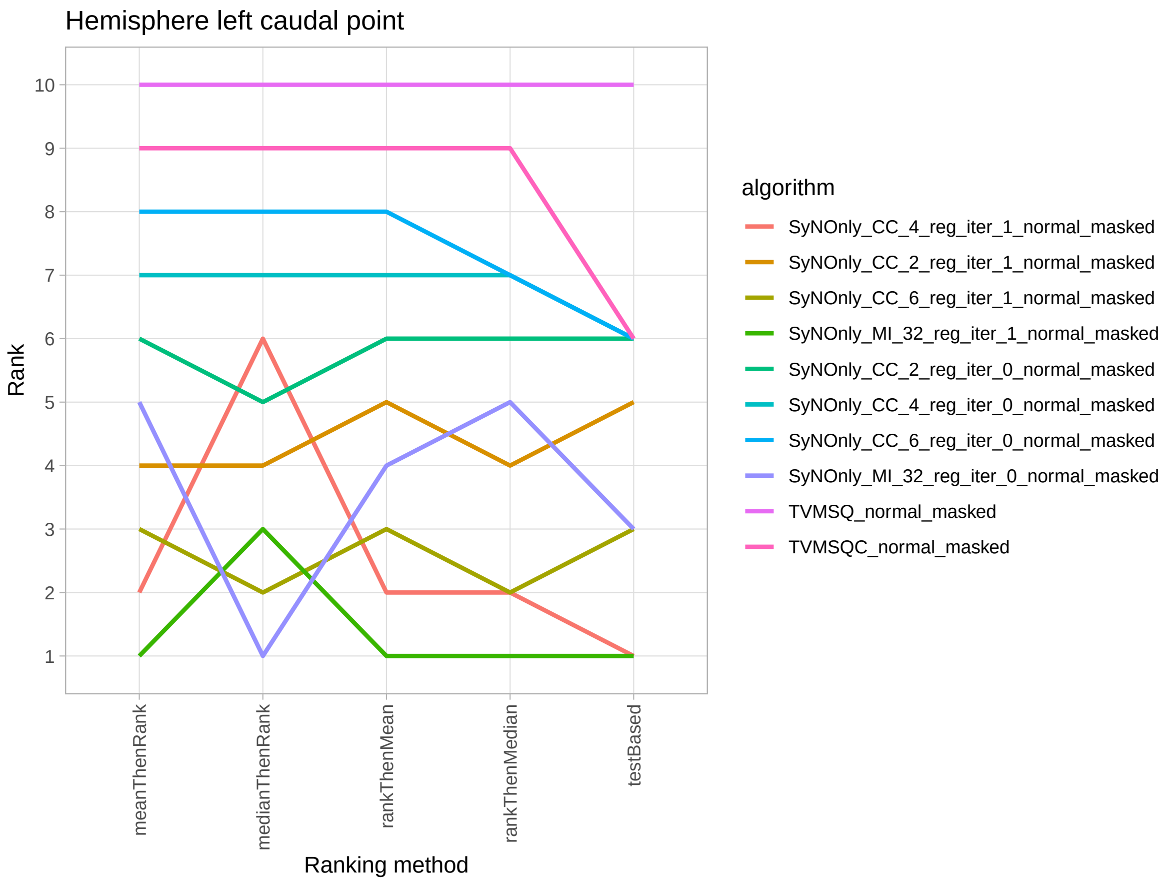

Hemisphere left caudal point
Rank
10
9
8
7
6
5
4
3
2
1
meanThenRank
medianThenRank
rankThenMean
rankThenMedian
testBased
Ranking method
algorithm
SyNOnly_CC_4_reg_iter_1_normal_masked
SyNOnly_CC_2_reg_iter_1_normal_masked
SyNOnly_CC_6_reg_iter_1_normal_masked
SyNOnly_MI_32_reg_iter_1_normal_masked
SyNOnly_CC_2_reg_iter_0_normal_masked
SyNOnly_CC_4_reg_iter_0_normal_masked
SyNOnly_CC_6_reg_iter_0_normal_masked
SyNOnly_MI_32_reg_iter_0_normal_masked
TVMSQ_normal_masked
TVMSQC_normal_masked

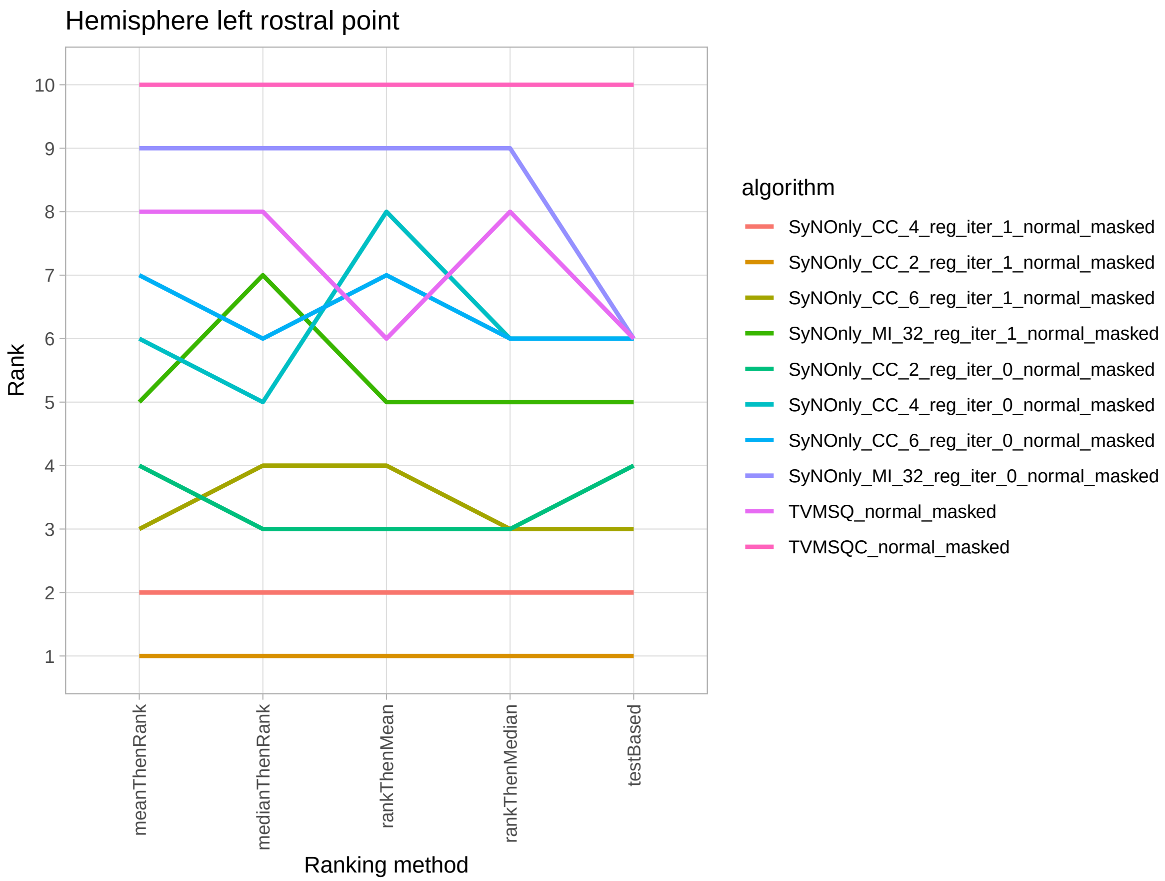

Hemisphere left rostral point
Rank
10
9
8
7
6
5
4
3
2
1
meanThenRank
medianThenRank
rankThenMean
rankThenMedian
testBased
Ranking method
algorithm
SyNOnly_CC_4_reg_iter_1_normal_masked
SyNOnly_CC_2_reg_iter_1_normal_masked
SyNOnly_CC_6_reg_iter_1_normal_masked
SyNOnly_MI_32_reg_iter_1_normal_masked
SyNOnly_CC_2_reg_iter_0_normal_masked
SyNOnly_CC_4_reg_iter_0_normal_masked
SyNOnly_CC_6_reg_iter_0_normal_masked
SyNOnly_MI_32_reg_iter_0_normal_masked
TVMSQ_normal_masked
TVMSQC_normal_masked

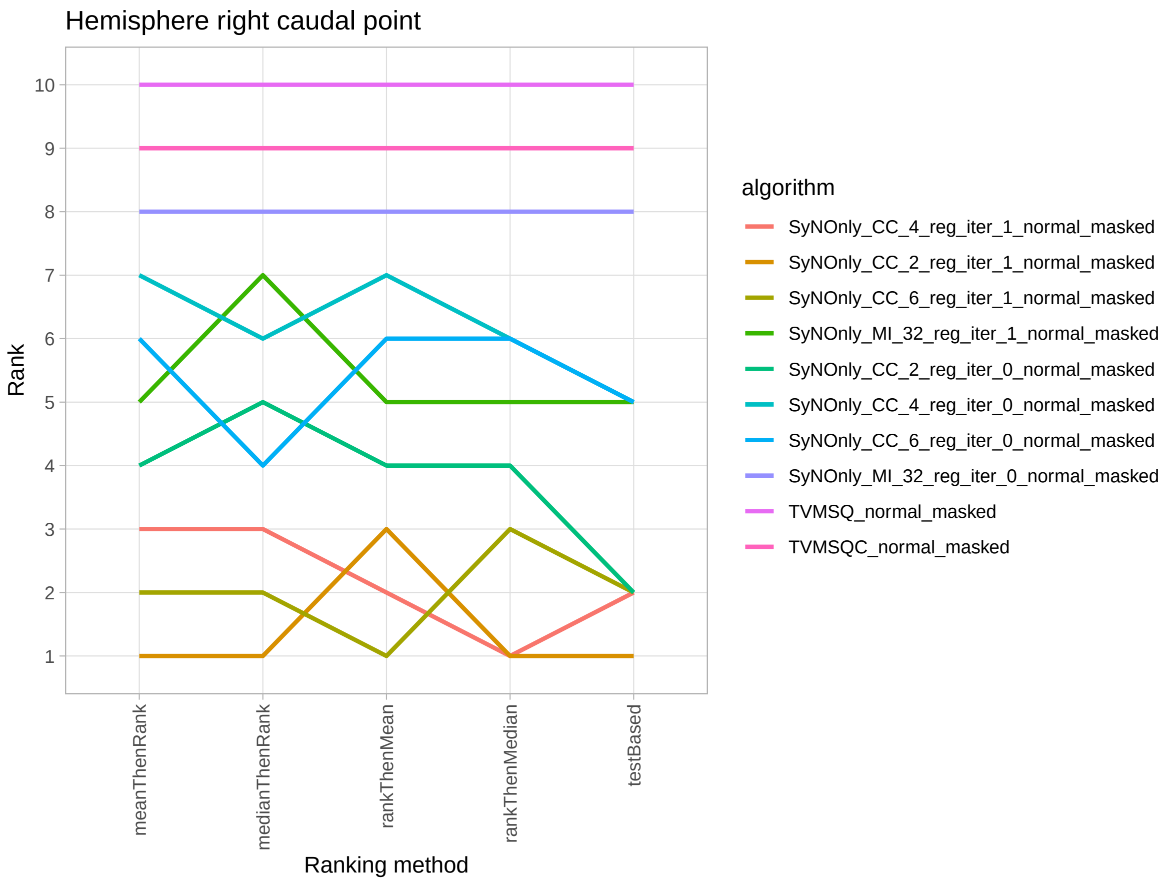
Hemisphere right caudal point
Rank
10
9
8
7
6
5
4
3
2
1
meanThenRank
medianThenRank
rankThenMean
rankThenMedian
testBased
Ranking method
algorithm
SyNOnly_CC_4_reg_iter_1_normal_masked
SyNOnly_CC_2_reg_iter_1_normal_masked
SyNOnly_CC_6_reg_iter_1_normal_masked
SyNOnly_MI_32_reg_iter_1_normal_masked
SyNOnly_CC_2_reg_iter_0_normal_masked
SyNOnly_CC_4_reg_iter_0_normal_masked
SyNOnly_CC_6_reg_iter_0_normal_masked
SyNOnly_MI_32_reg_iter_0_normal_masked
TVMSQ_normal_masked
TVMSQC_normal_masked

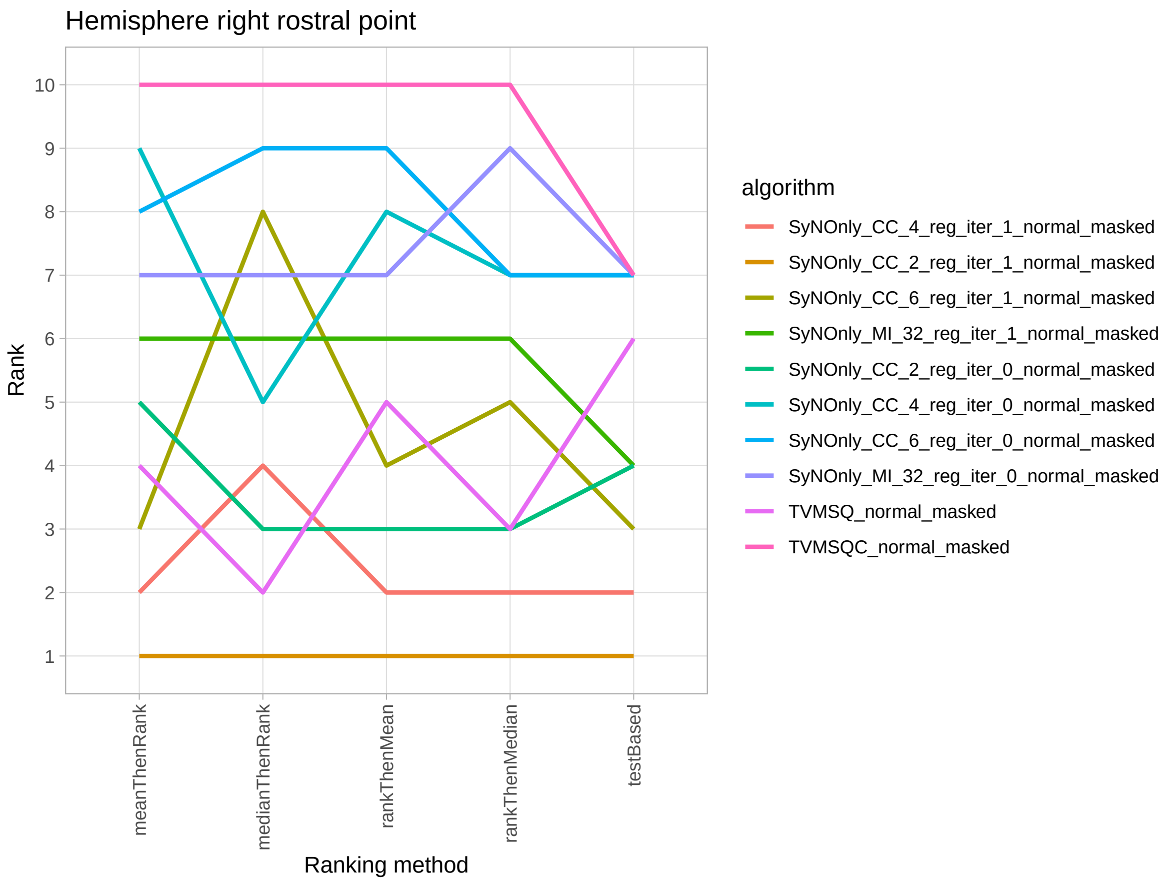

Hemisphere right rostral point
Rank
10
9
8
7
6
5
4
3
2
1
meanThenRank
medianThenRank
rankThenMean
rankThenMedian
testBased
Ranking method
algorithm
SyNOnly_CC_4_reg_iter_1_normal_masked
SyNOnly_CC_2_reg_iter_1_normal_masked
SyNOnly_CC_6_reg_iter_1_normal_masked
SyNOnly_MI_32_reg_iter_1_normal_masked
SyNOnly_CC_2_reg_iter_0_normal_masked
SyNOnly_CC_4_reg_iter_0_normal_masked
SyNOnly_CC_6_reg_iter_0_normal_masked
SyNOnly_MI_32_reg_iter_0_normal_masked
TVMSQ_normal_masked
TVMSQC_normal_masked

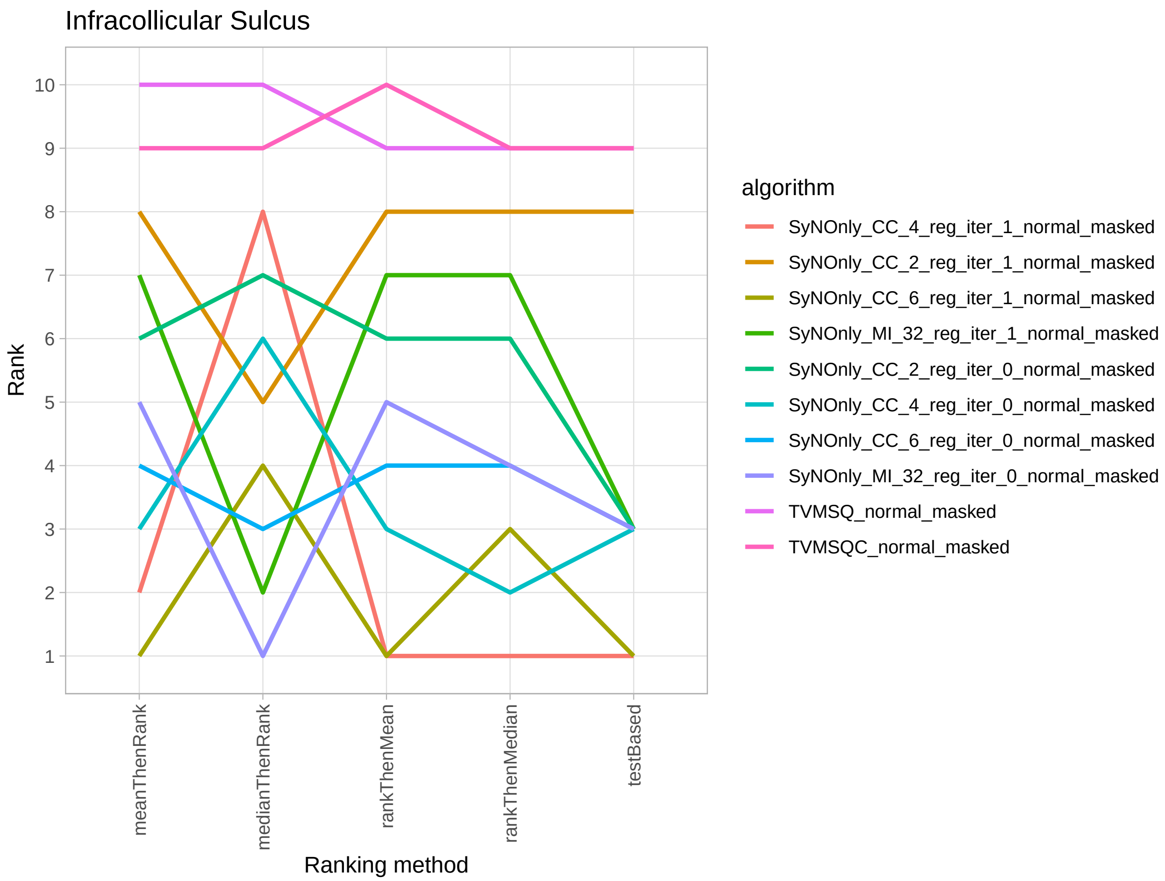

Infracollicular Sulcus
Rank
10
9
8
7
6
5
4
3
2
1
meanThenRank
medianThenRank
rankThenMean
rankThenMedian
testBased
Ranking method
algorithm
SyNOnly_CC_4_reg_iter_1_normal_masked
SyNOnly_CC_2_reg_iter_1_normal_masked
SyNOnly_CC_6_reg_iter_1_normal_masked
SyNOnly_MI_32_reg_iter_1_normal_masked
SyNOnly_CC_2_reg_iter_0_normal_masked
SyNOnly_CC_4_reg_iter_0_normal_masked
SyNOnly_CC_6_reg_iter_0_normal_masked
SyNOnly_MI_32_reg_iter_0_normal_masked
TVMSQ_normal_masked
TVMSQC_normal_masked

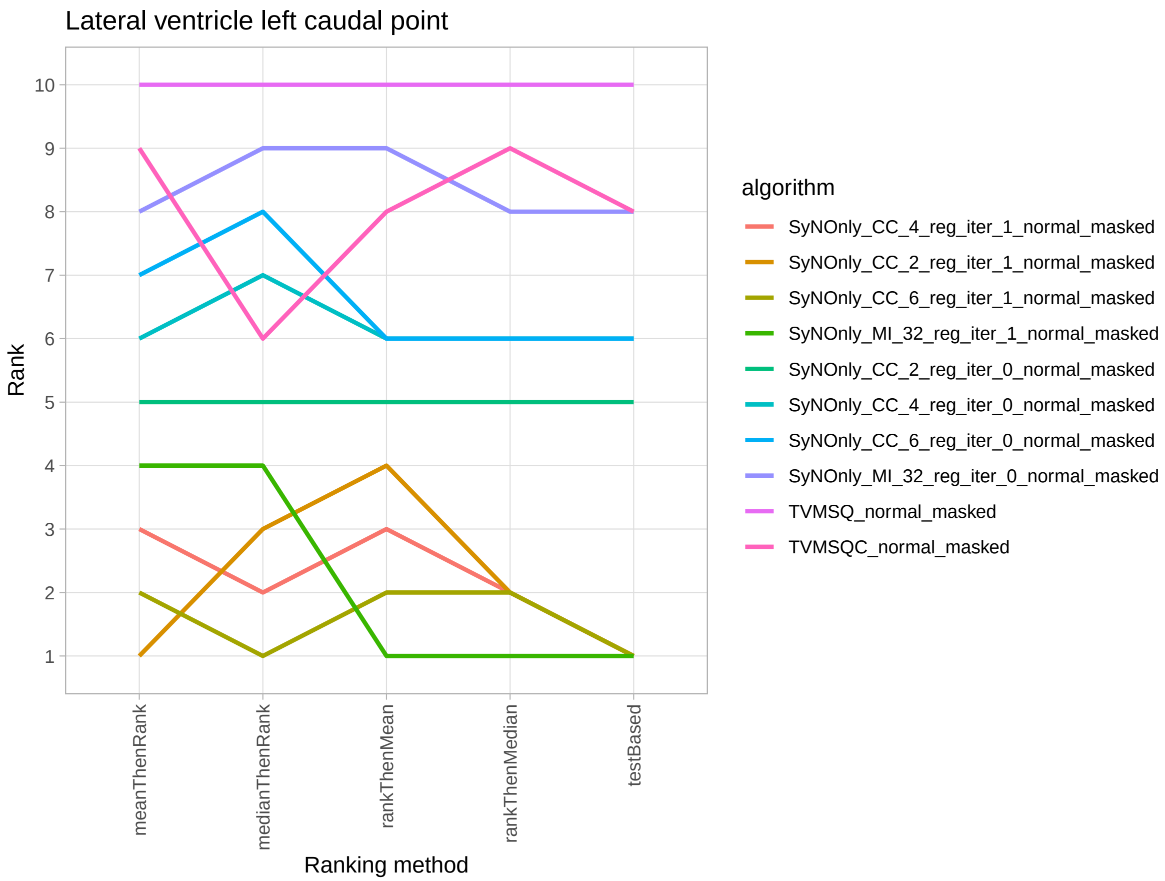
Lateral ventricle left caudal point
Rank
10
9
8
7
6
5
4
3
2
1
meanThenRank
medianThenRank
rankThenMean
rankThenMedian
testBased
Ranking method
algorithm
SyNOnly_CC_4_reg_iter_1_normal_masked
SyNOnly_CC_2_reg_iter_1_normal_masked
SyNOnly_CC_6_reg_iter_1_normal_masked
SyNOnly_MI_32_reg_iter_1_normal_masked
SyNOnly_CC_2_reg_iter_0_normal_masked
SyNOnly_CC_4_reg_iter_0_normal_masked
SyNOnly_CC_6_reg_iter_0_normal_masked
SyNOnly_MI_32_reg_iter_0_normal_masked
TVMSQ_normal_masked
TVMSQC_normal_masked

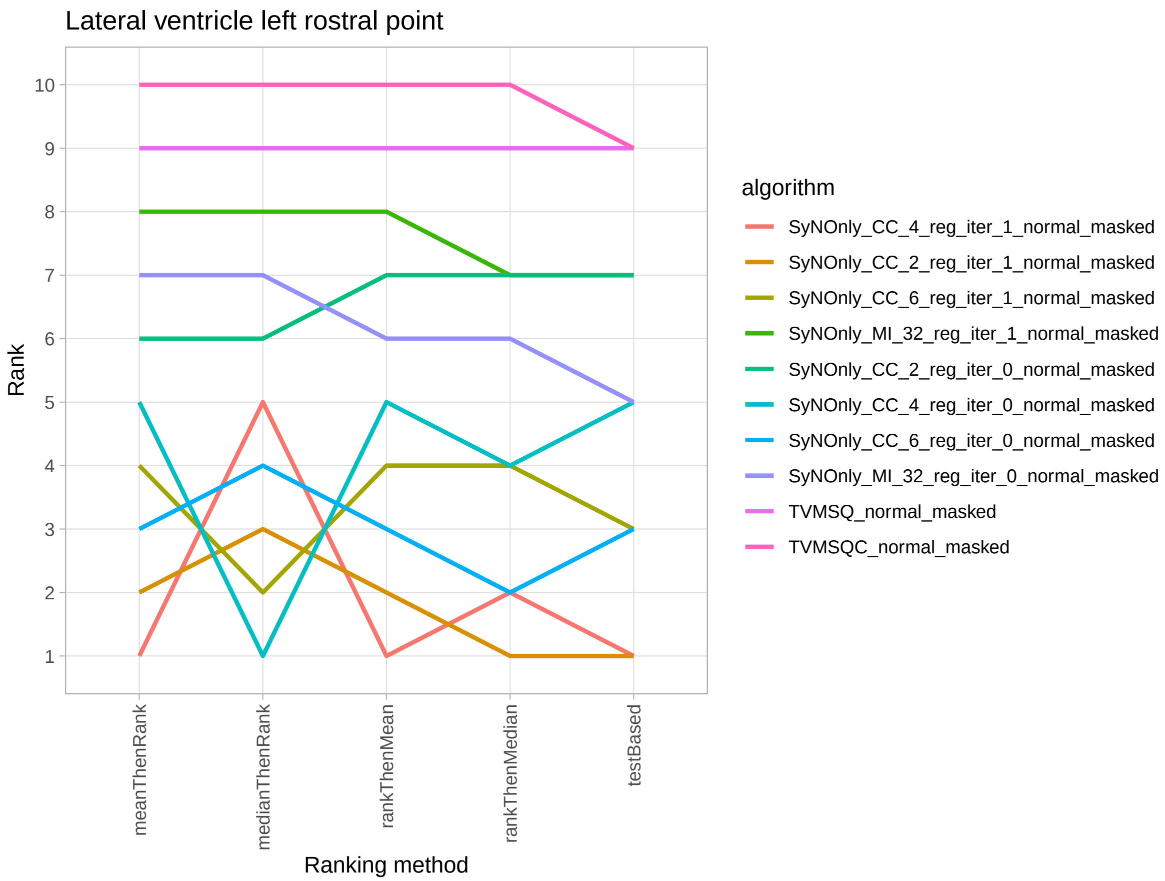
Lateral ventricle left rostral point
Rank
10
9
8
7
6
5
4
3
2
1
meanThenRank
medianThenRank
rankThenMean
rankThenMedian
testBased
Ranking method
algorithm
SyNOnly_CC_4_reg_iter_1_normal_masked
SyNOnly_CC_2_reg_iter_1_normal_masked
SyNOnly_CC_6_reg_iter_1_normal_masked
SyNOnly_MI_32_reg_iter_1_normal_masked
SyNOnly_CC_2_reg_iter_0_normal_masked
SyNOnly_CC_4_reg_iter_0_normal_masked
SyNOnly_CC_6_reg_iter_0_normal_masked
SyNOnly_MI_32_reg_iter_0_normal_masked
TVMSQ_normal_masked
TVMSQC_normal_masked

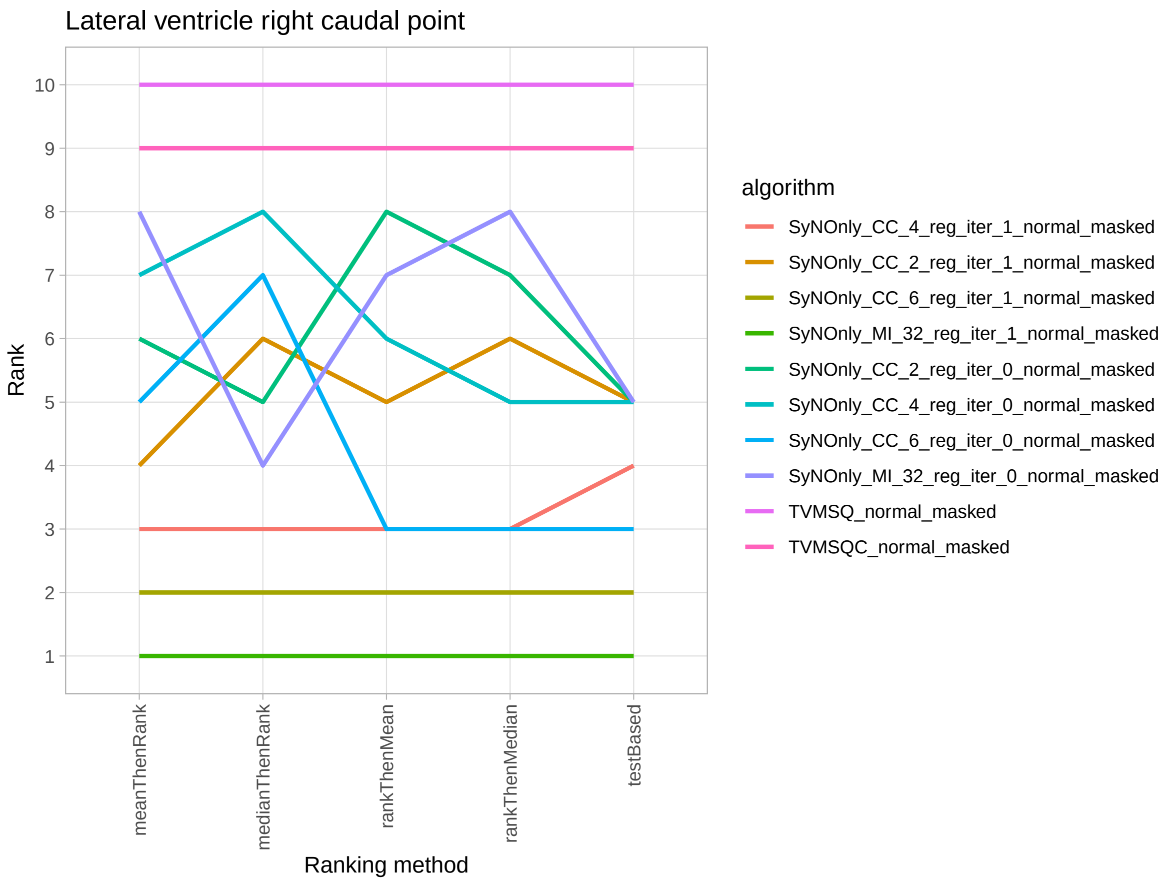
Lateral ventricle right caudal point
Rank
10
9
8
7
6
5
4
3
2
1
meanThenRank
medianThenRank
rankThenMean
rankThenMedian
testBased
Ranking method
algorithm
SyNOnly_CC_4_reg_iter_1_normal_masked
SyNOnly_CC_2_reg_iter_1_normal_masked
SyNOnly_CC_6_reg_iter_1_normal_masked
SyNOnly_MI_32_reg_iter_1_normal_masked
SyNOnly_CC_2_reg_iter_0_normal_masked
SyNOnly_CC_4_reg_iter_0_normal_masked
SyNOnly_CC_6_reg_iter_0_normal_masked
SyNOnly_MI_32_reg_iter_0_normal_masked
TVMSQ_normal_masked
TVMSQC_normal_masked

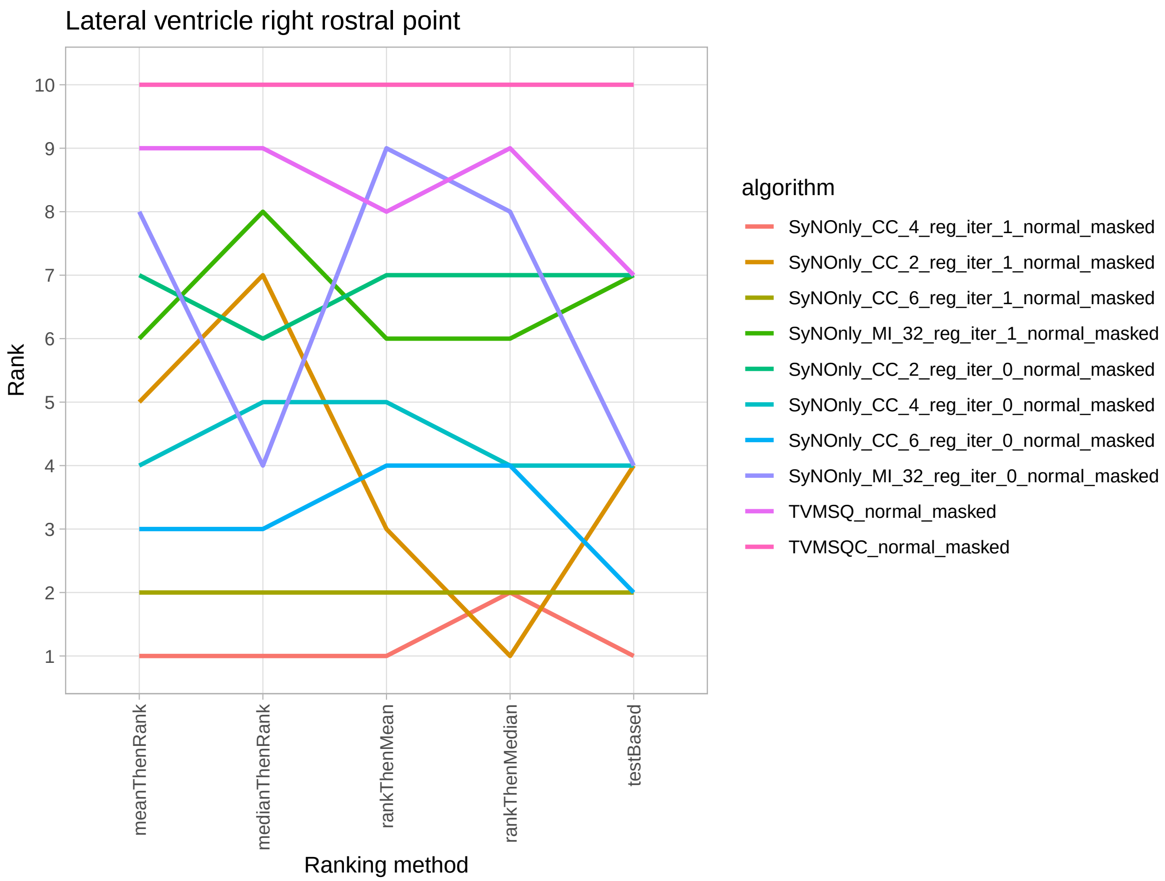
Lateral ventricle right rostral point
Rank
10
9
8
7
6
5
4
3
2
1
meanThenRank
medianThenRank
rankThenMean
rankThenMedian
testBased
Ranking method
algorithm
SyNOnly_CC_4_reg_iter_1_normal_masked
SyNOnly_CC_2_reg_iter_1_normal_masked
SyNOnly_CC_6_reg_iter_1_normal_masked
SyNOnly_MI_32_reg_iter_1_normal_masked
SyNOnly_CC_2_reg_iter_0_normal_masked
SyNOnly_CC_4_reg_iter_0_normal_masked
SyNOnly_CC_6_reg_iter_0_normal_masked
SyNOnly_MI_32_reg_iter_0_normal_masked
TVMSQ_normal_masked
TVMSQC_normal_masked

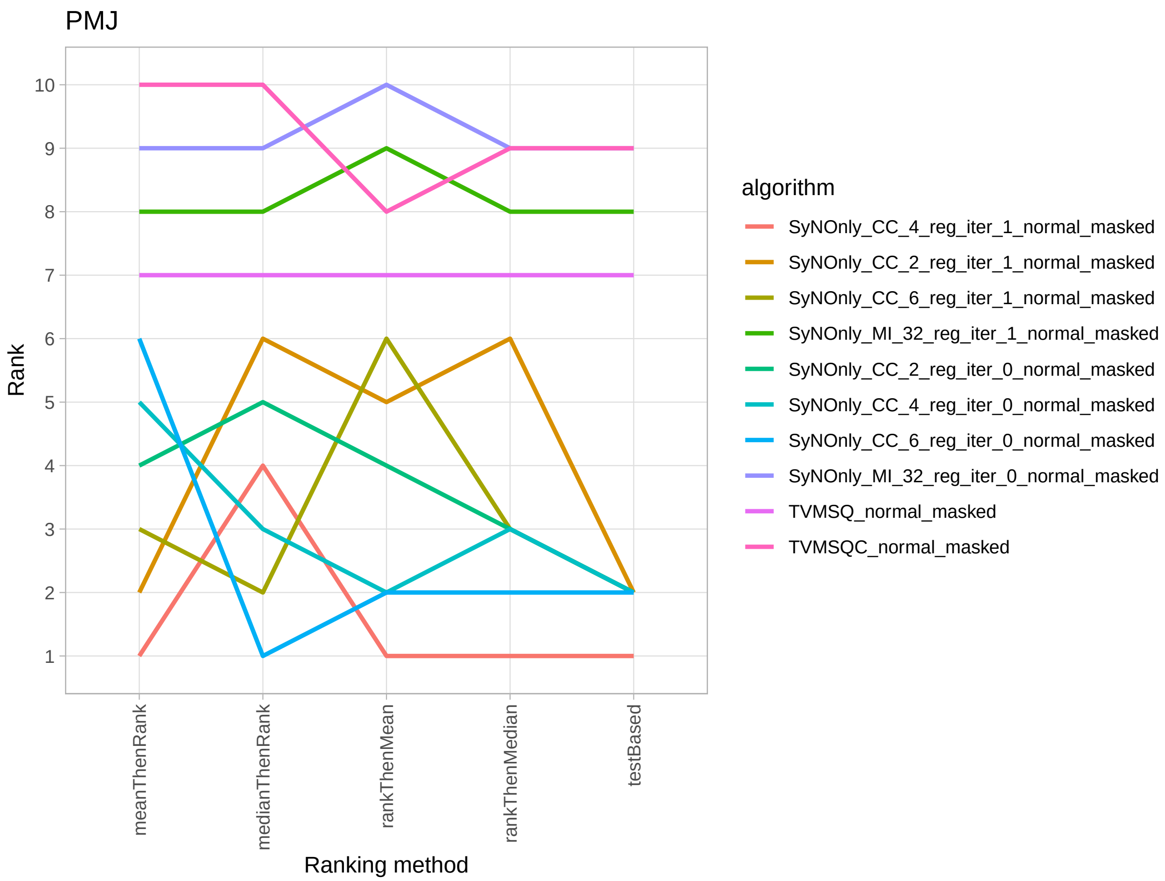
PMJ
Rank
10
9
8
7
6
5
4
3
2
1
meanThenRank
medianThenRank
rankThenMean
rankThenMedian
testBased
Ranking method
algorithm
SyNOnly_CC_4_reg_iter_1_normal_masked
SyNOnly_CC_2_reg_iter_1_normal_masked
SyNOnly_CC_6_reg_iter_1_normal_masked
SyNOnly_MI_32_reg_iter_1_normal_masked
SyNOnly_CC_2_reg_iter_0_normal_masked
SyNOnly_CC_4_reg_iter_0_normal_masked
SyNOnly_CC_6_reg_iter_0_normal_masked
SyNOnly_MI_32_reg_iter_0_normal_masked
TVMSQ_normal_masked
TVMSQC_normal_masked

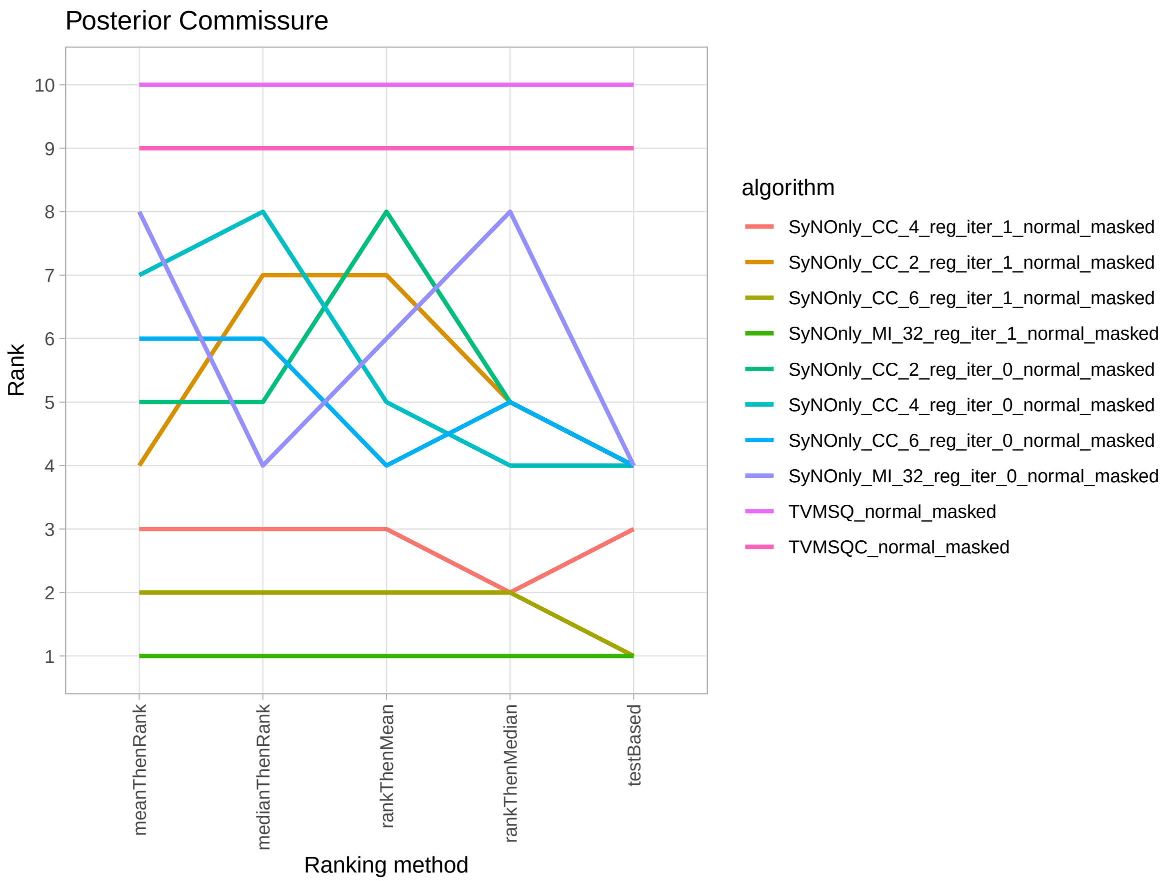
Posterior Commissure
Rank
Ranking method
meanThenRank
medianThenRank
rankThenMean
rankThenMedian
testBased
algorithm
SyNOnly_CC_4_reg_iter_1_normal_masked
SyNOnly_CC_2_reg_iter_1_normal_masked
SyNOnly_CC_6_reg_iter_1_normal_masked
SyNOnly_MI_32_reg_iter_1_normal_masked
SyNOnly_CC_2_reg_iter_0_normal_masked
SyNOnly_CC_4_reg_iter_0_normal_masked
SyNOnly_CC_6_reg_iter_0_normal_masked
SyNOnly_MI_32_reg_iter_0_normal_masked
TVMSQ_normal_masked
TVMSQC_normal_masked

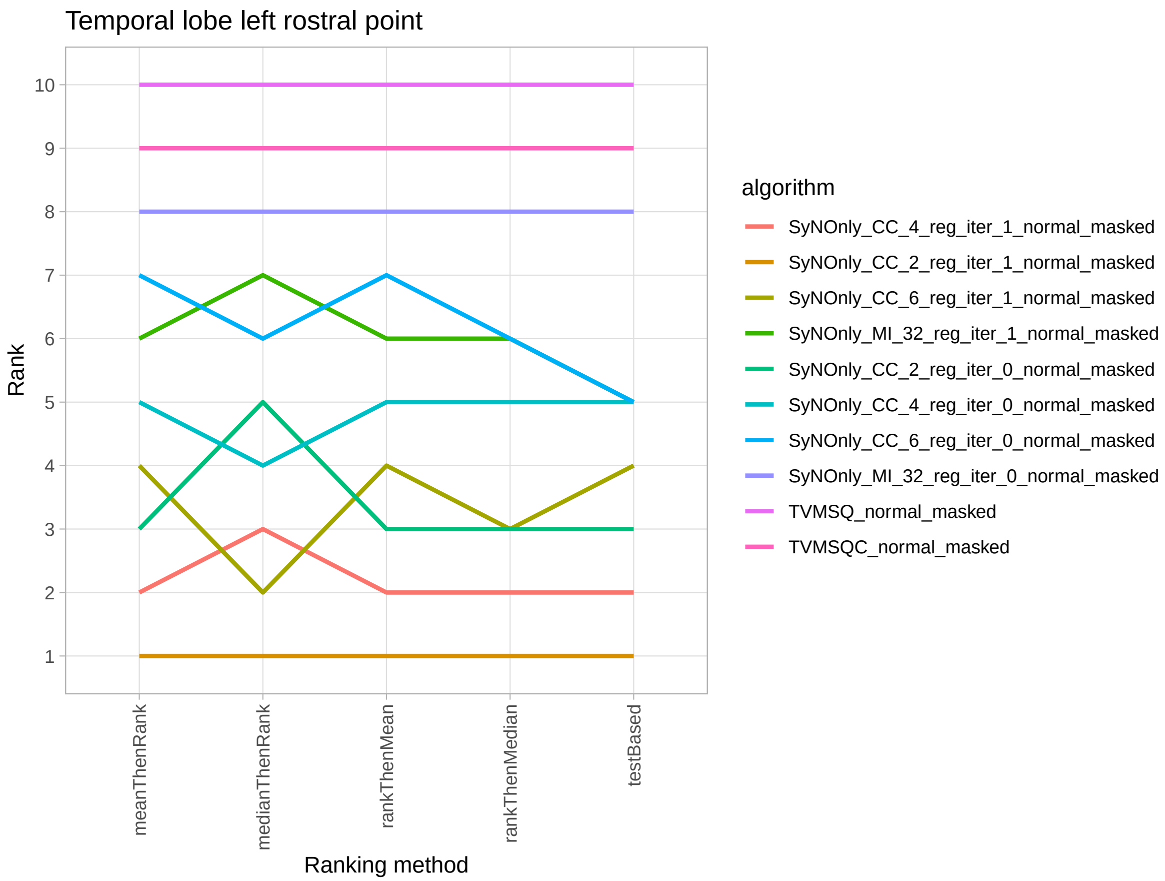
Temporal lobe left rostral point
Rank
10
9
8
7
6
5
4
3
2
1
meanThenRank
medianThenRank
rankThenMean
rankThenMedian
testBased
Ranking method
algorithm
SyNOnly_CC_4_reg_iter_1_normal_masked
SyNOnly_CC_2_reg_iter_1_normal_masked
SyNOnly_CC_6_reg_iter_1_normal_masked
SyNOnly_MI_32_reg_iter_1_normal_masked
SyNOnly_CC_2_reg_iter_0_normal_masked
SyNOnly_CC_4_reg_iter_0_normal_masked
SyNOnly_CC_6_reg_iter_0_normal_masked
SyNOnly_MI_32_reg_iter_0_normal_masked
TVMSQ_normal_masked
TVMSQC_normal_masked

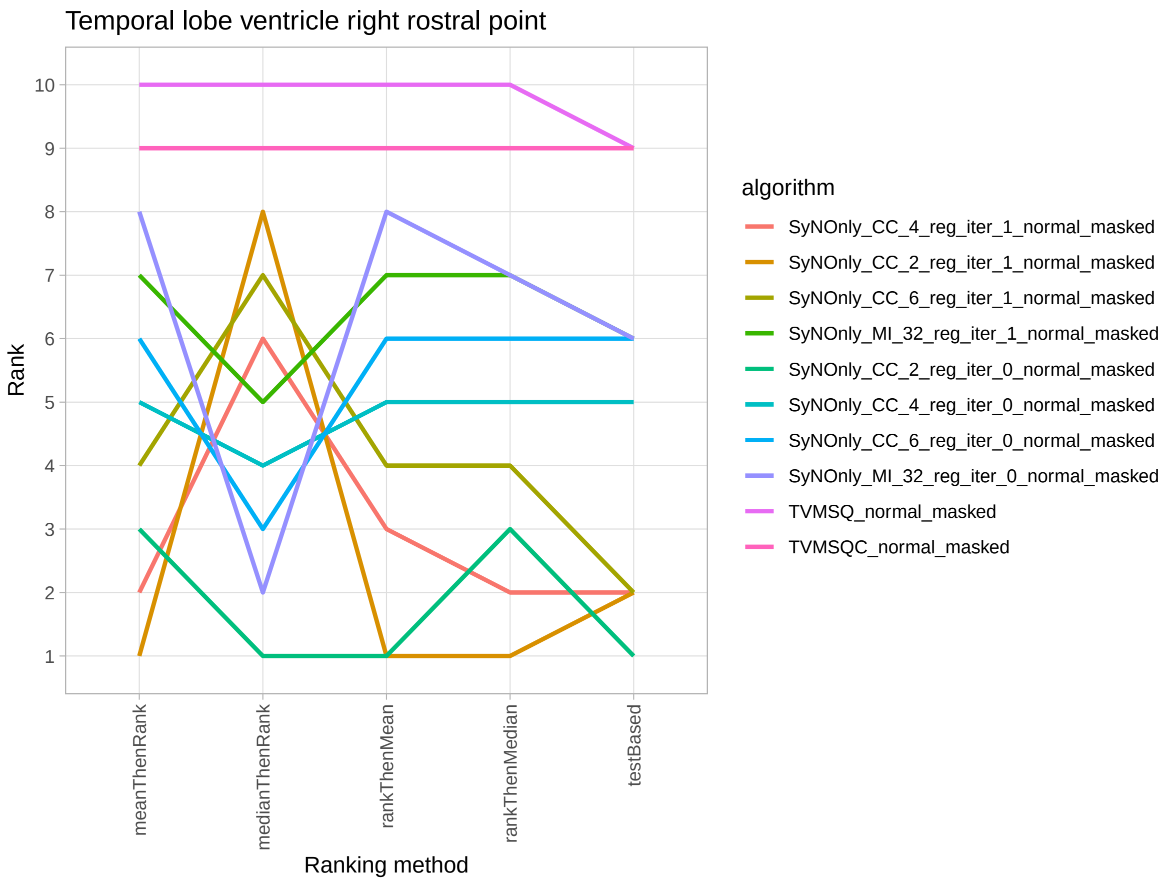

Temporal lobe ventricle right rostral point
Rank
10
9
8
7
6
5
4
3
2
1
meanThenRank
medianThenRank
rankThenMean
rankThenMedian
testBased
Ranking method
algorithm
SyNOnly_CC_4_reg_iter_1_normal_masked
SyNOnly_CC_2_reg_iter_1_normal_masked
SyNOnly_CC_6_reg_iter_1_normal_masked
SyNOnly_MI_32_reg_iter_1_normal_masked
SyNOnly_CC_2_reg_iter_0_normal_masked
SyNOnly_CC_4_reg_iter_0_normal_masked
SyNOnly_CC_6_reg_iter_0_normal_masked
SyNOnly_MI_32_reg_iter_0_normal_masked
TVMSQ_normal_masked
TVMSQC_normal_masked

4 Visualization of cross-task insights

The algorithms are ordered according to consensus ranking.

4.1 Characterization of algorithms

4.1.1 Ranking stability: Variability of achieved rankings across tasks

Algorithms are color-coded, and the area of each blob at position $(A_i, \text{rank } j)$ is proportional to the relative frequency A_i achieved rank j across multiple tasks. The median rank for each algorithm is indicated by a black cross. This way, the distribution of ranks across tasks can be intuitively visualized.

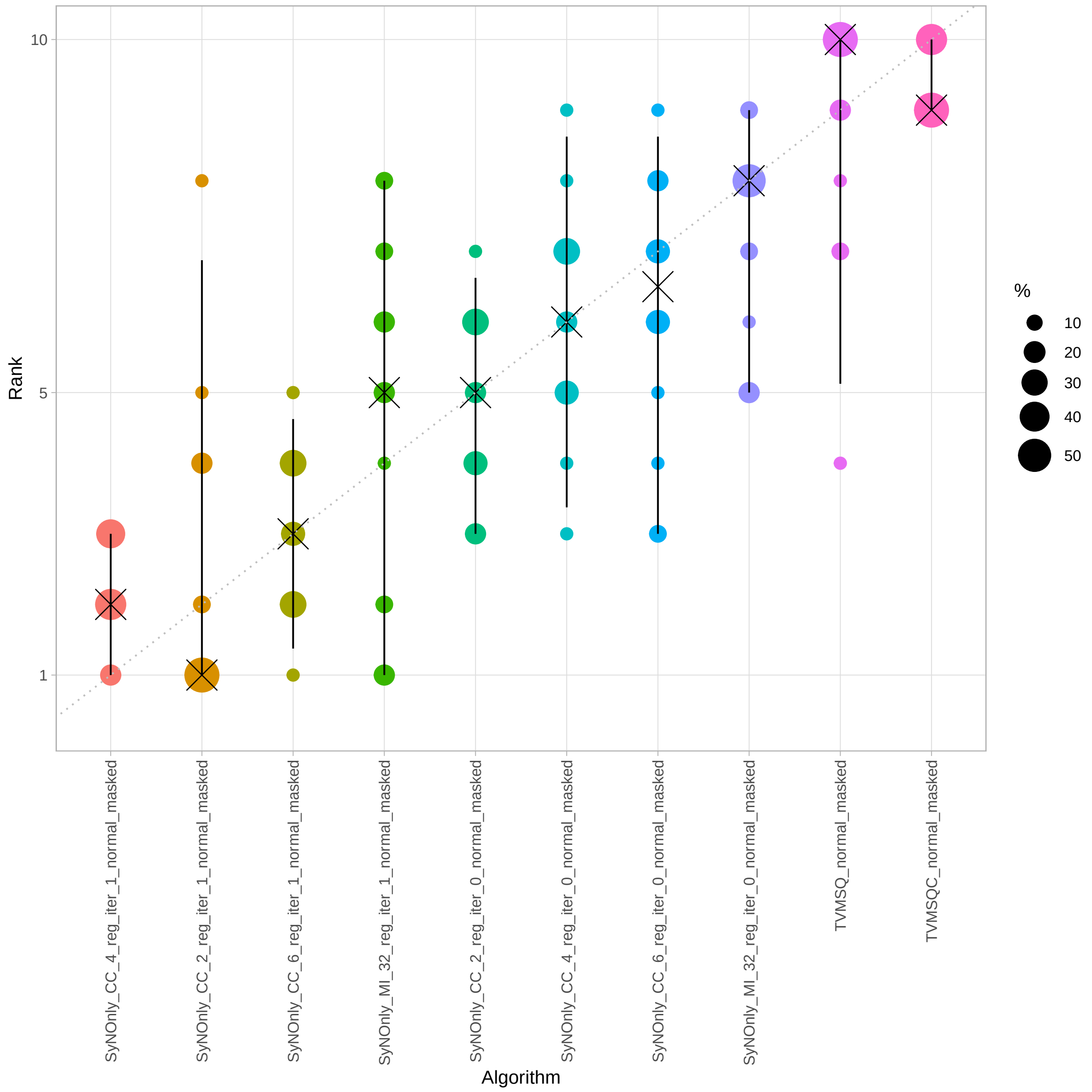

4.1.2 Ranking stability: Ranking variability via bootstrap approach

A blob plot of bootstrap results over the different tasks separated by algorithm allows another perspective on the assessment data. This gives deeper insights into the characteristics of tasks and the ranking uncertainty of the algorithms in each task.

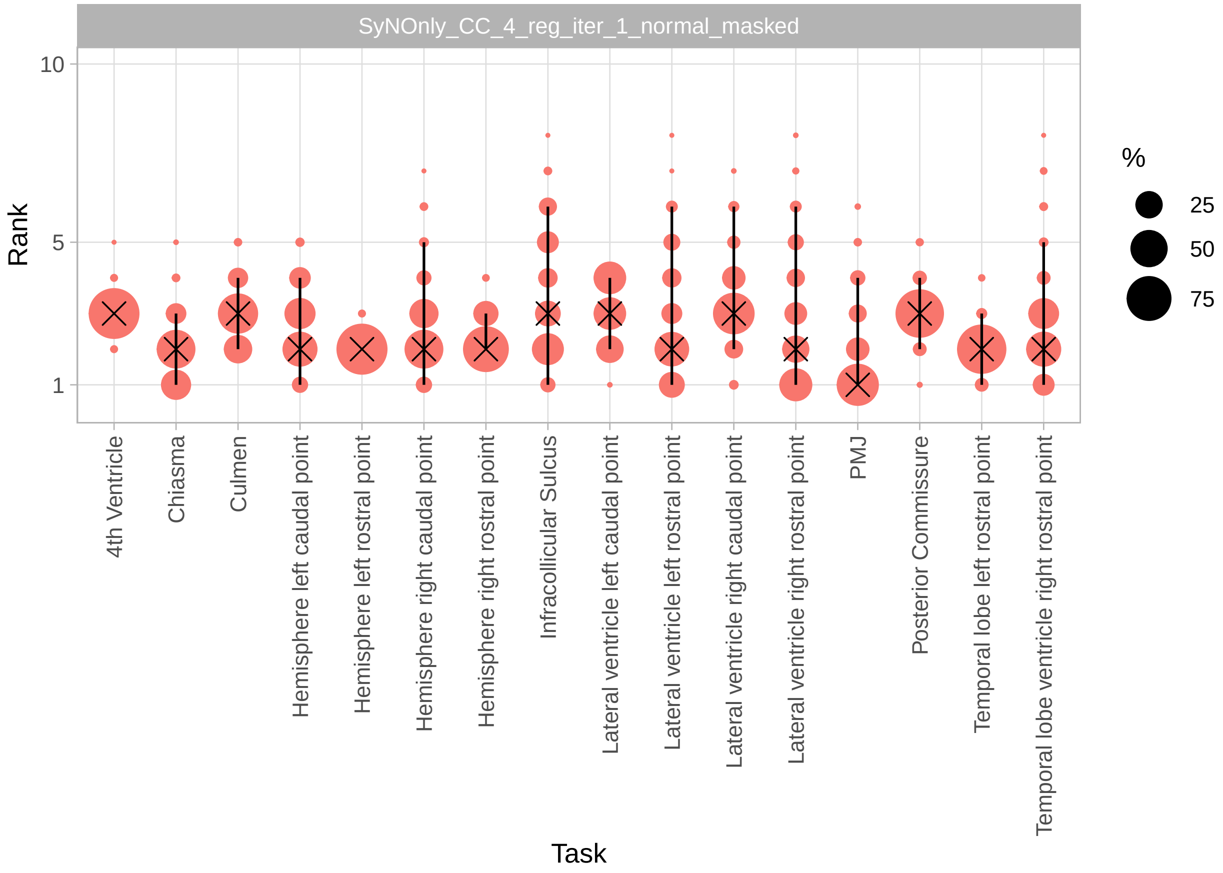

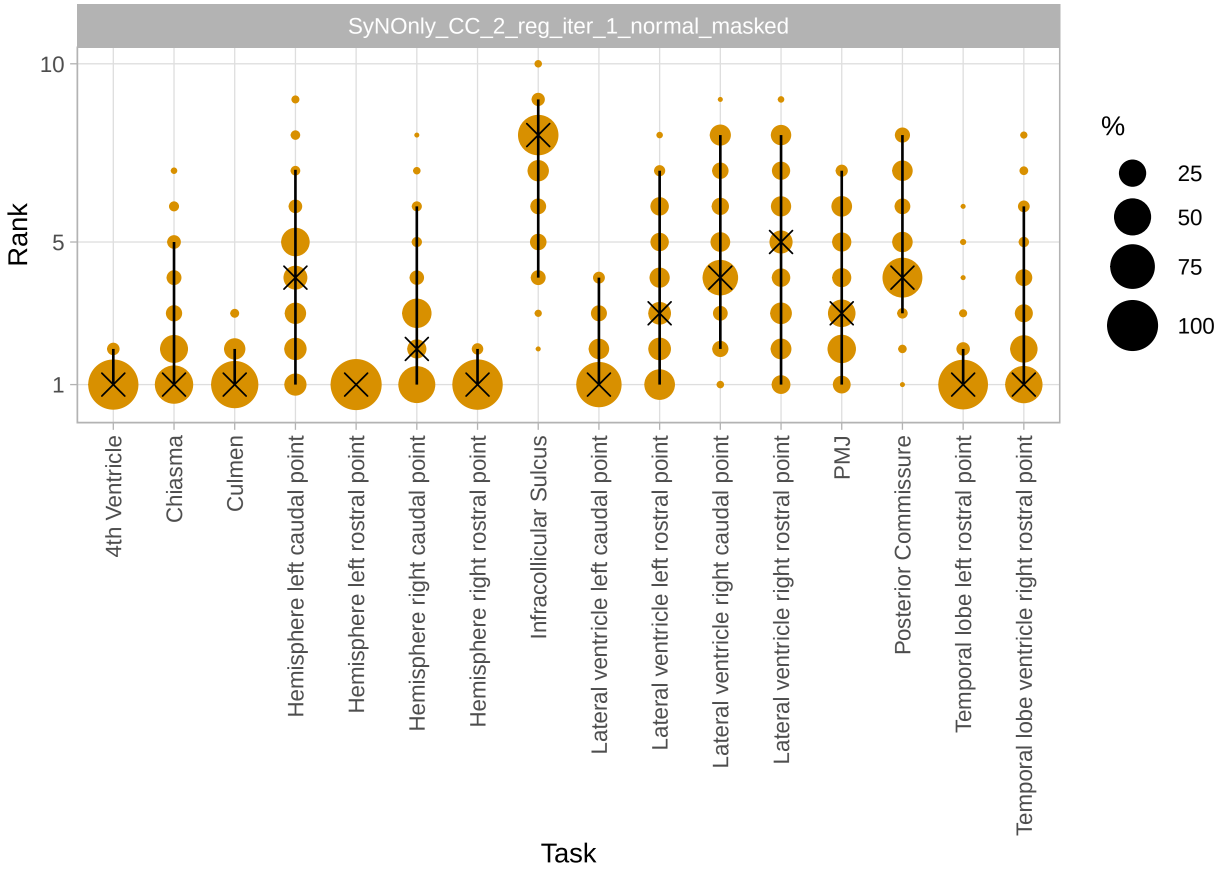
SyNOnly_CC_2_reg_iter_1_normal_masked
Rank
10
5
1
%
25
50
75
100
4th Ventricle
Chiasma
Culmen
Hemisphere left caudal point
Hemisphere left rostral point
Hemisphere right caudal point
Hemisphere right rostral point
Infracollicular Sulcus
Lateral ventricle left caudal point
Lateral ventricle left rostral point
Lateral ventricle right caudal point
Lateral ventricle right rostral point
PMJ
Posterior Commissure
Temporal lobe left rostral point
Temporal lobe ventricle right rostral point
Task

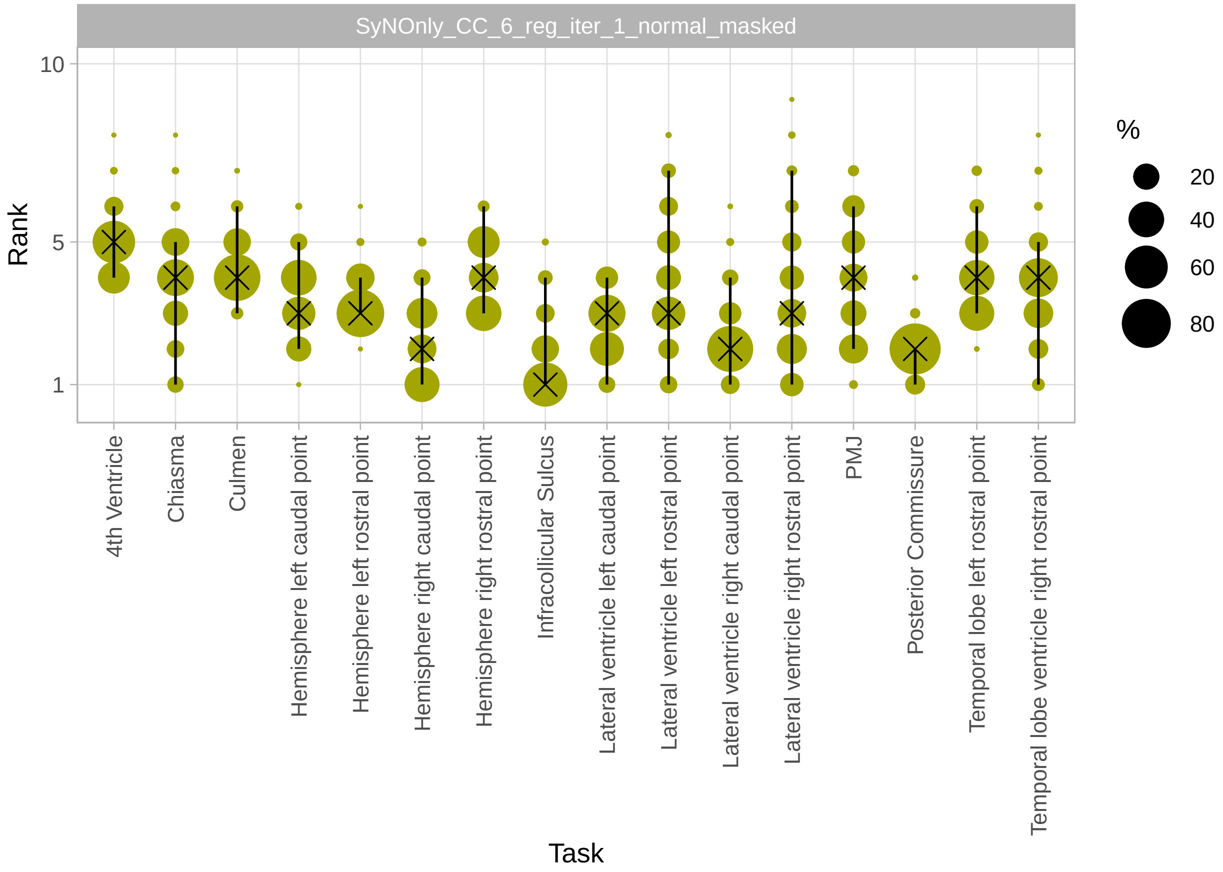

SyNOnly_CC_6_reg_iter_1_normal_masked
Rank
10
5
1
Task
4th Ventricle
Chiasma
Culmen
Hemisphere left caudal point
Hemisphere left rostral point
Hemisphere right caudal point
Hemisphere right rostral point
Infracollicular Sulcus
Lateral ventricle left caudal point
Lateral ventricle left rostral point
Lateral ventricle right caudal point
Lateral ventricle right rostral point
PMJ
Posterior Commissure
Temporal lobe left rostral point
Temporal lobe ventricle right rostral point
%
20
40
60
80

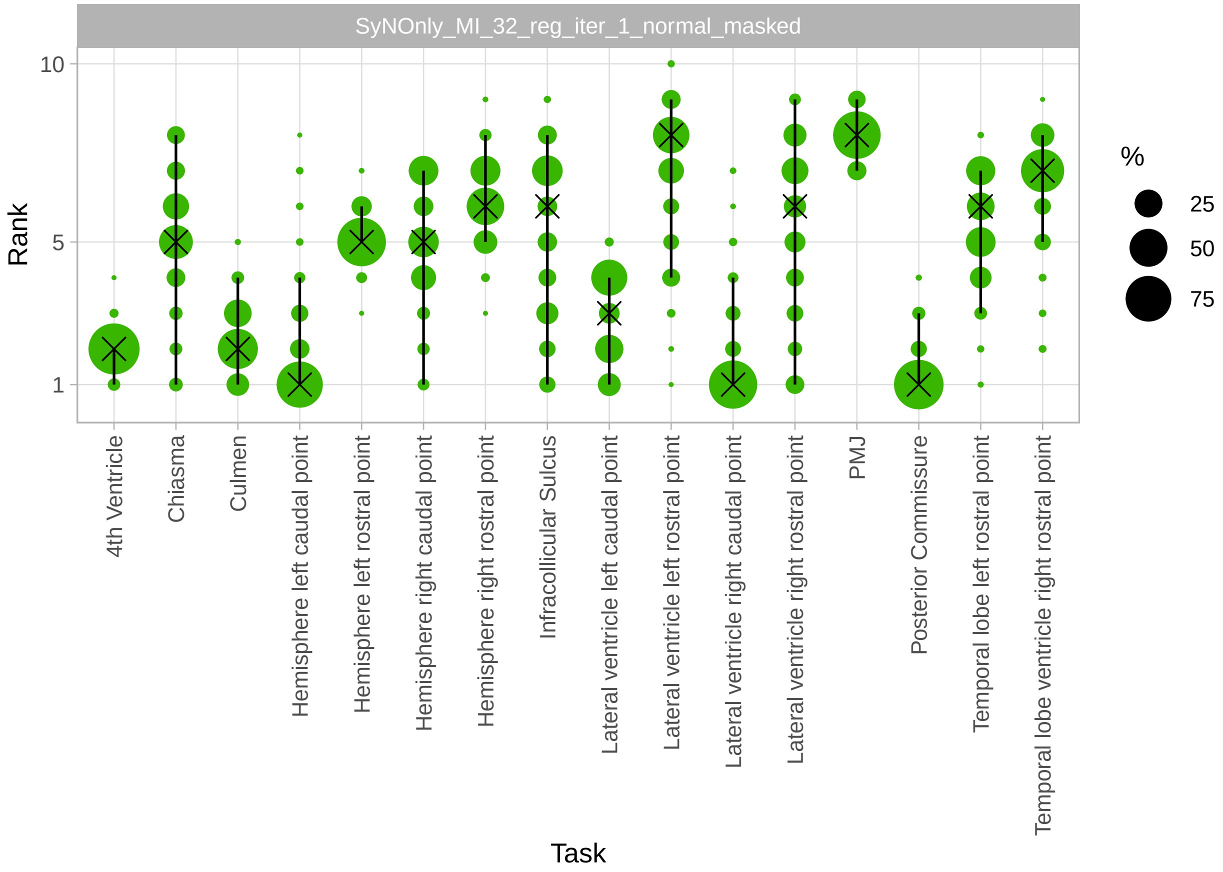

SyNOnly_MI_32_reg_iter_1_normal_masked
Rank
10
5
1
4th Ventricle
Chiasma
Culmen
Hemisphere left caudal point
Hemisphere left rostral point
Hemisphere right caudal point
Hemisphere right rostral point
Infracollicular Sulcus
Lateral ventricle left caudal point
Lateral ventricle left rostral point
Lateral ventricle right caudal point
Lateral ventricle right rostral point
PMJ
Posterior Commissure
Temporal lobe left rostral point
Temporal lobe ventricle right rostral point
Task
%
25
50
75

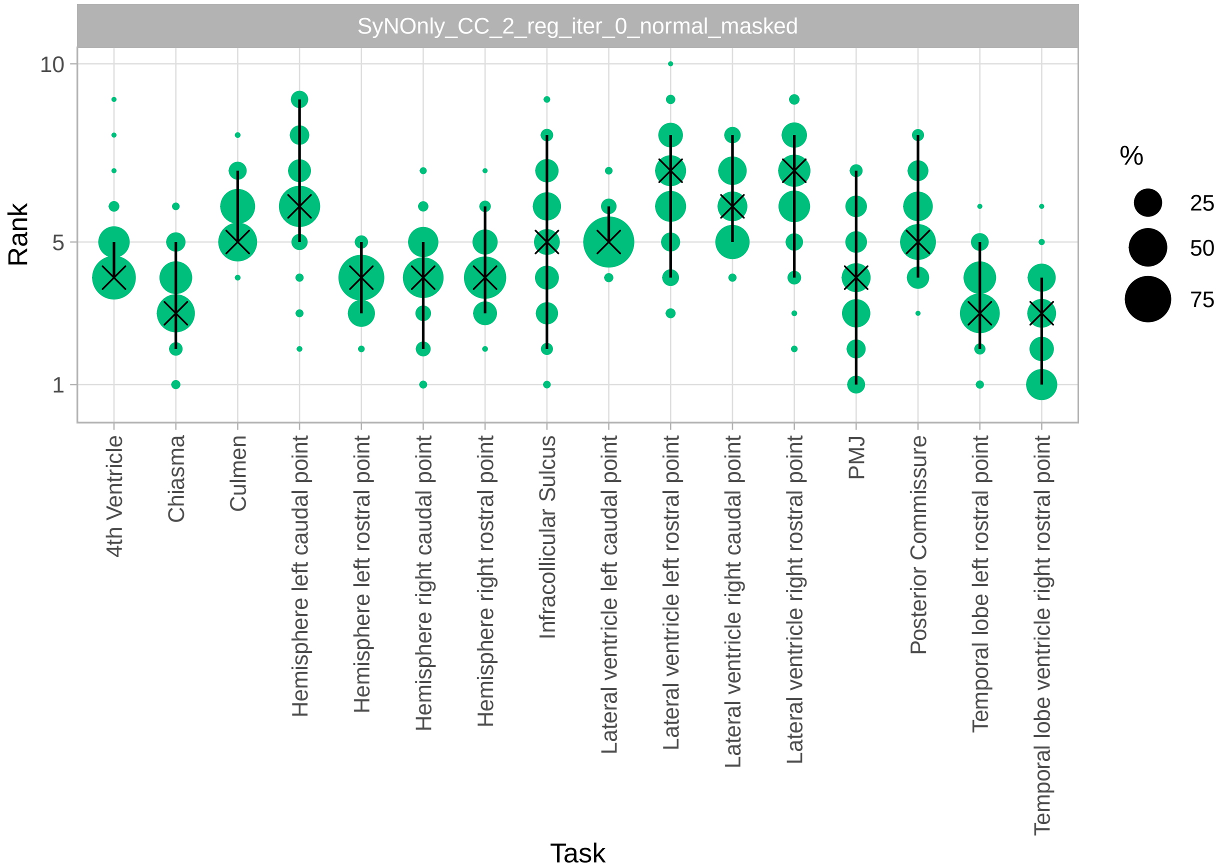
SyNOnly_CC_2_reg_iter_0_normal_masked
Rank
10
5
1
4th Ventricle
Chiasma
Culmen
Hemisphere left caudal point
Hemisphere left rostral point
Hemisphere right caudal point
Hemisphere right rostral point
Infracollicular Sulcus
Lateral ventricle left caudal point
Lateral ventricle left rostral point
Lateral ventricle right caudal point
Lateral ventricle right rostral point
PMJ
Posterior Commissure
Temporal lobe left rostral point
Temporal lobe ventricle right rostral point
Task
%
25
50
75

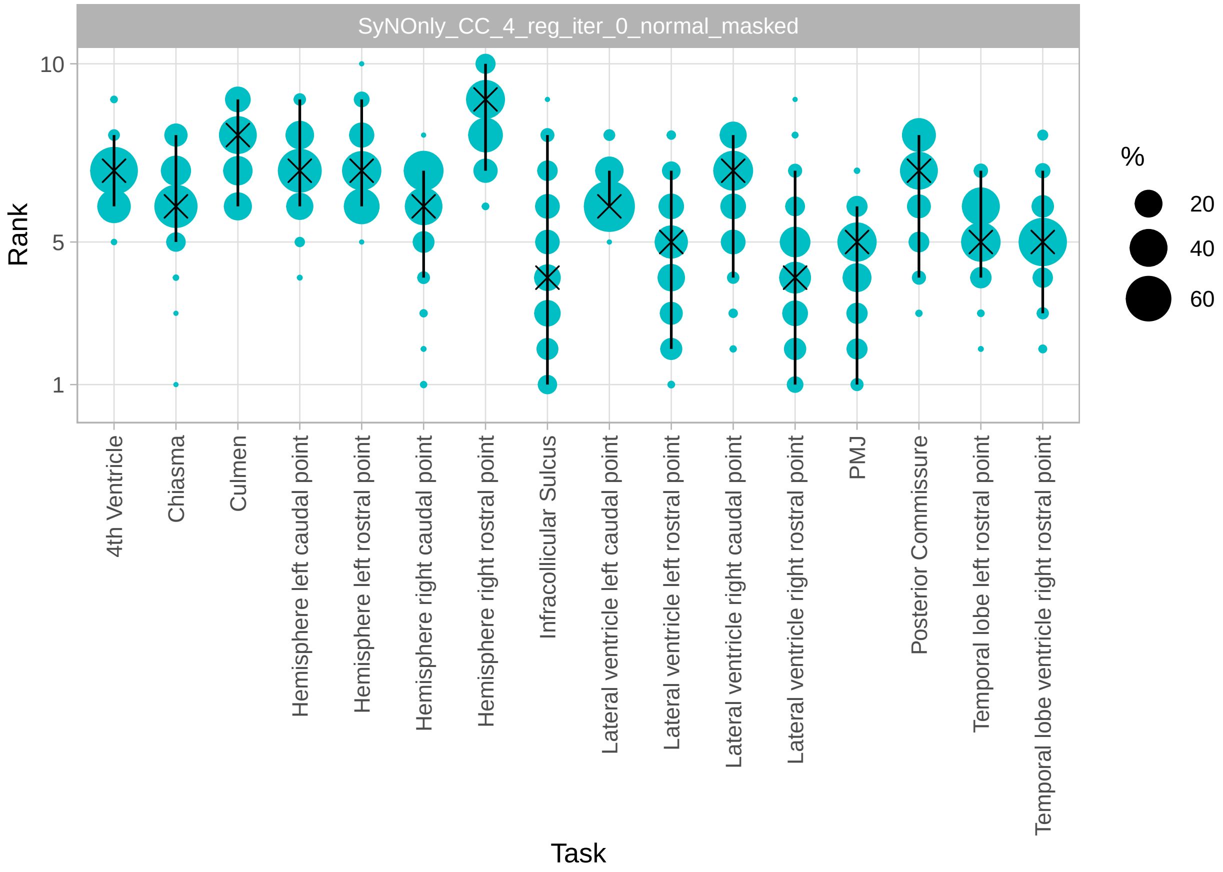
SyNOnly_CC_4_reg_iter_0_normal_masked
Rank
10
5
1
%
20
40
60
4th Ventricle
Chiasma
Culmen
Hemisphere left caudal point
Hemisphere left rostral point
Hemisphere right caudal point
Hemisphere right rostral point
Infracollicular Sulcus
Lateral ventricle left caudal point
Lateral ventricle left rostral point
Lateral ventricle right caudal point
Lateral ventricle right rostral point
PMJ
Posterior Commissure
Temporal lobe left rostral point
Temporal lobe ventricle right rostral point
Task

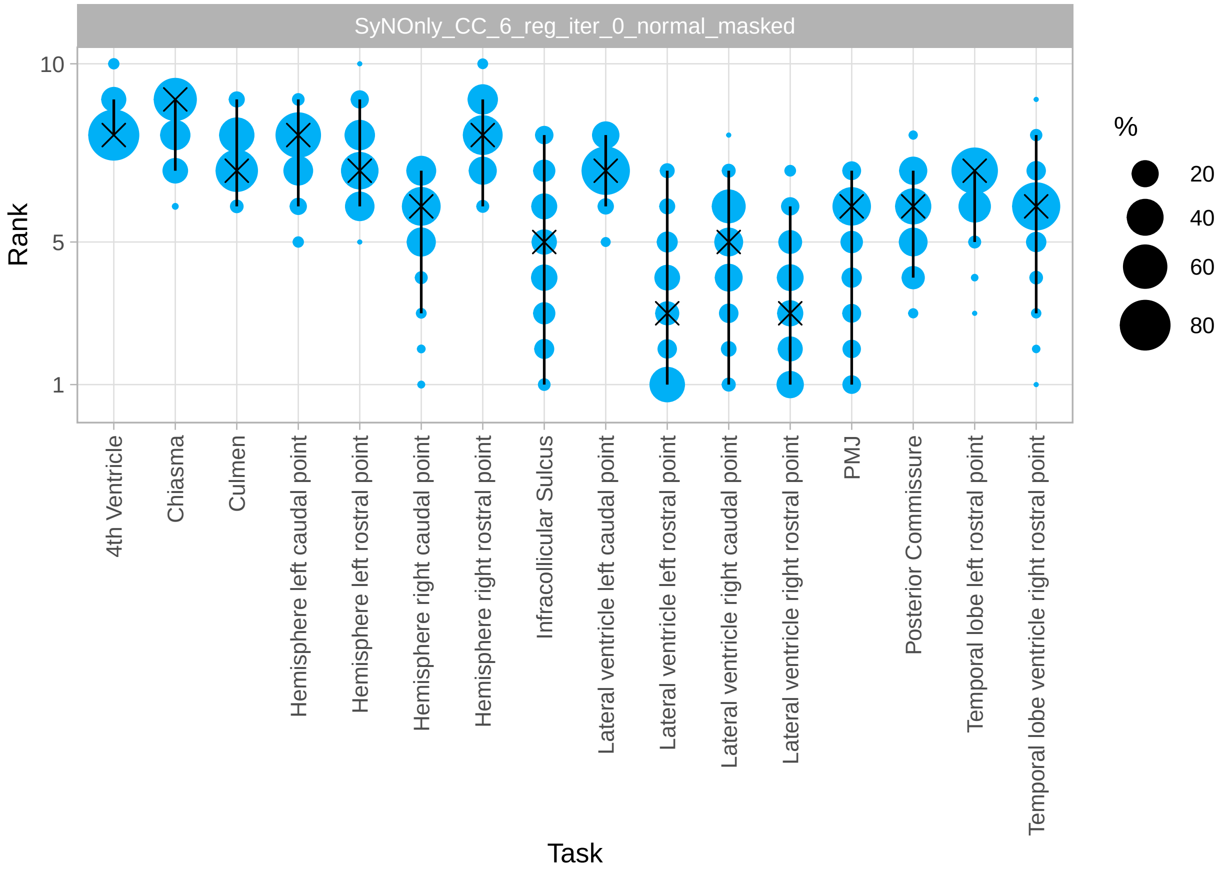
SyNOnly_CC_6_reg_iter_0_normal_masked
Rank
10
5
1
Task
4th Ventricle
Chiasma
Culmen
Hemisphere left caudal point
Hemisphere left rostral point
Hemisphere right caudal point
Hemisphere right rostral point
Infracollicular Sulcus
Lateral ventricle left caudal point
Lateral ventricle left rostral point
Lateral ventricle right caudal point
Lateral ventricle right rostral point
PMJ
Posterior Commissure
Temporal lobe left rostral point
Temporal lobe ventricle right rostral point
%
20
40
60
80

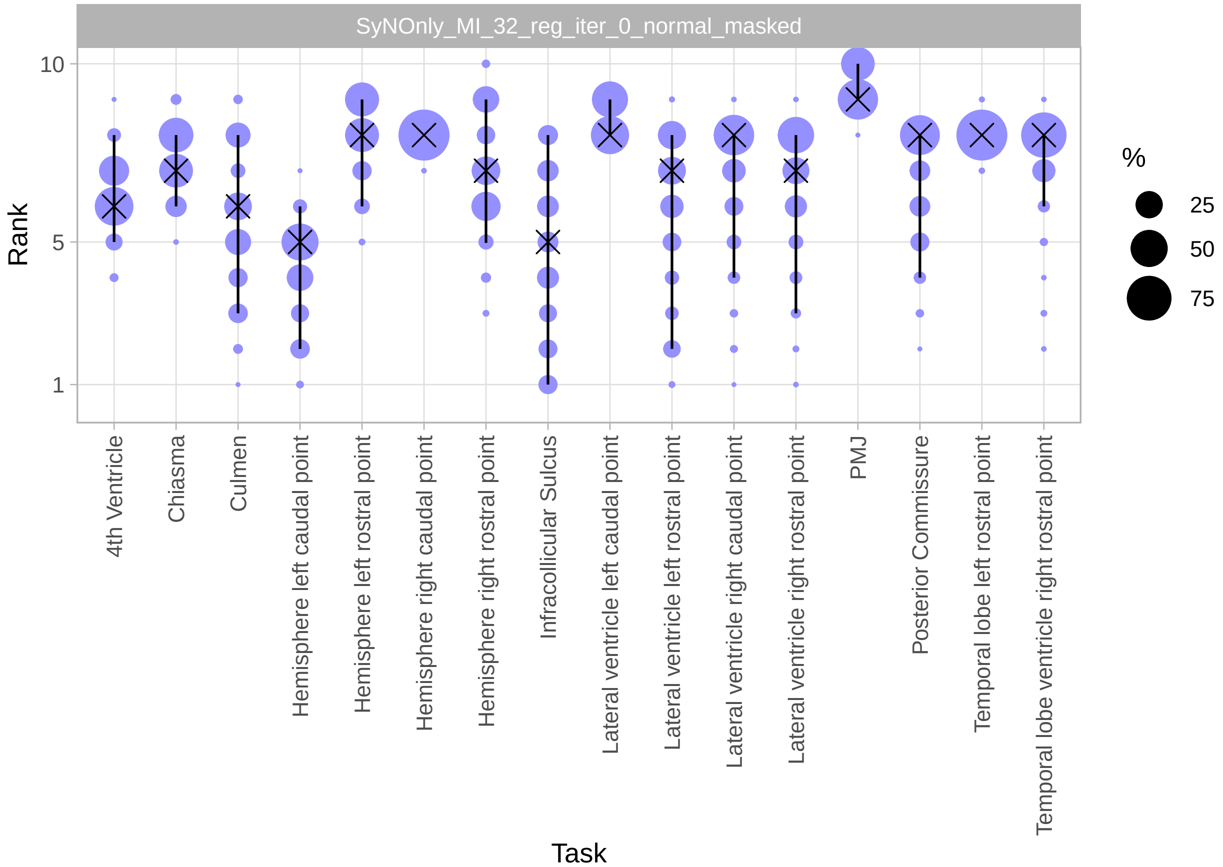
SyNOnly_MI_32_reg_iter_0_normal_masked
Rank
10
5
1
Task
4th Ventricle
Chiasma
Culmen
Hemisphere left caudal point
Hemisphere left rostral point
Hemisphere right caudal point
Hemisphere right rostral point
Infracollicular Sulcus
Lateral ventricle left caudal point
Lateral ventricle left rostral point
Lateral ventricle right caudal point
Lateral ventricle right rostral point
PMJ
Posterior Commissure
Temporal lobe left rostral point
Temporal lobe ventricle right rostral point
%
25
50
75

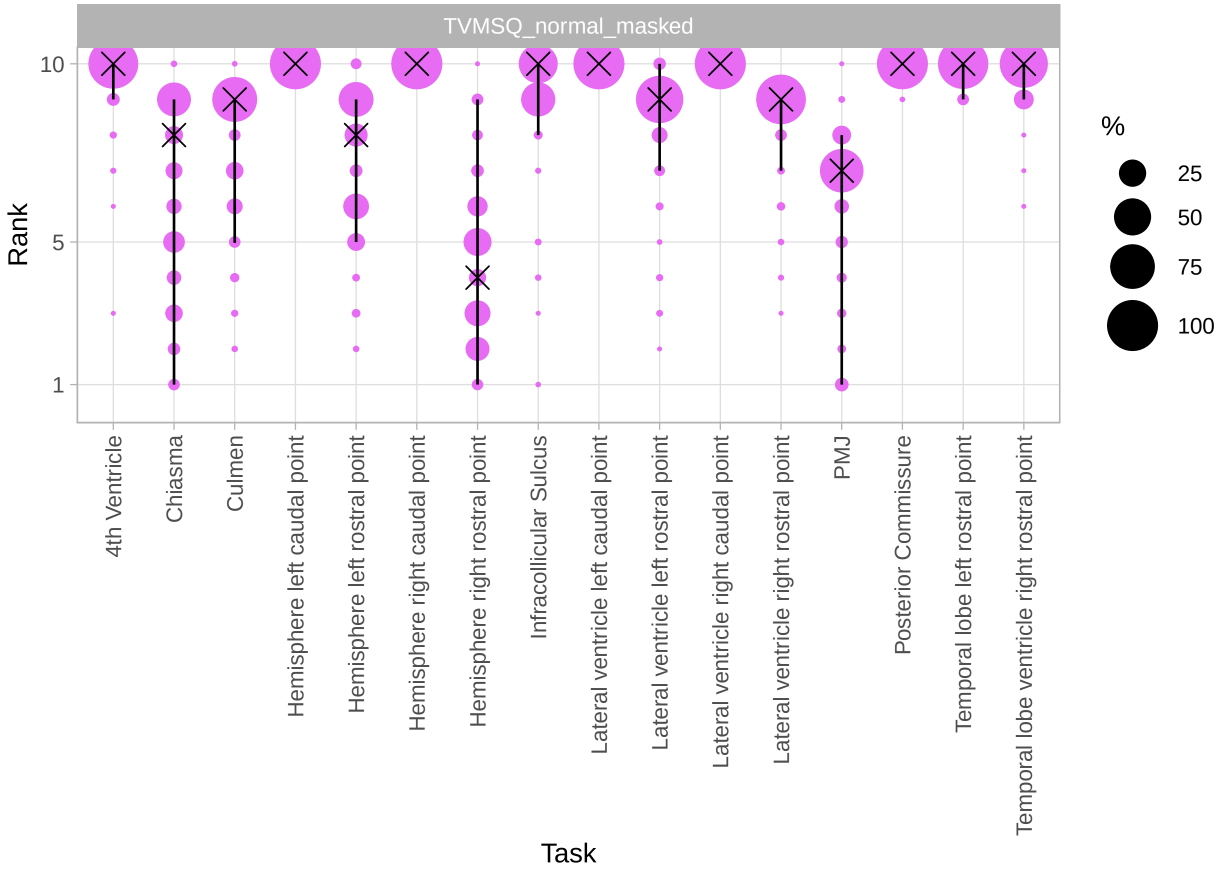
TVMSQ_normal_masked
Rank
10
5
1
%
25
50
75
100
4th Ventricle
Chiasma
Culmen
Hemisphere left caudal point
Hemisphere left rostral point
Hemisphere right caudal point
Hemisphere right rostral point
Infracollicular Sulcus
Lateral ventricle left caudal point
Lateral ventricle left rostral point
Lateral ventricle right caudal point
Lateral ventricle right rostral point
PMJ
Posterior Commissure
Temporal lobe left rostral point
Temporal lobe ventricle right rostral point
Task

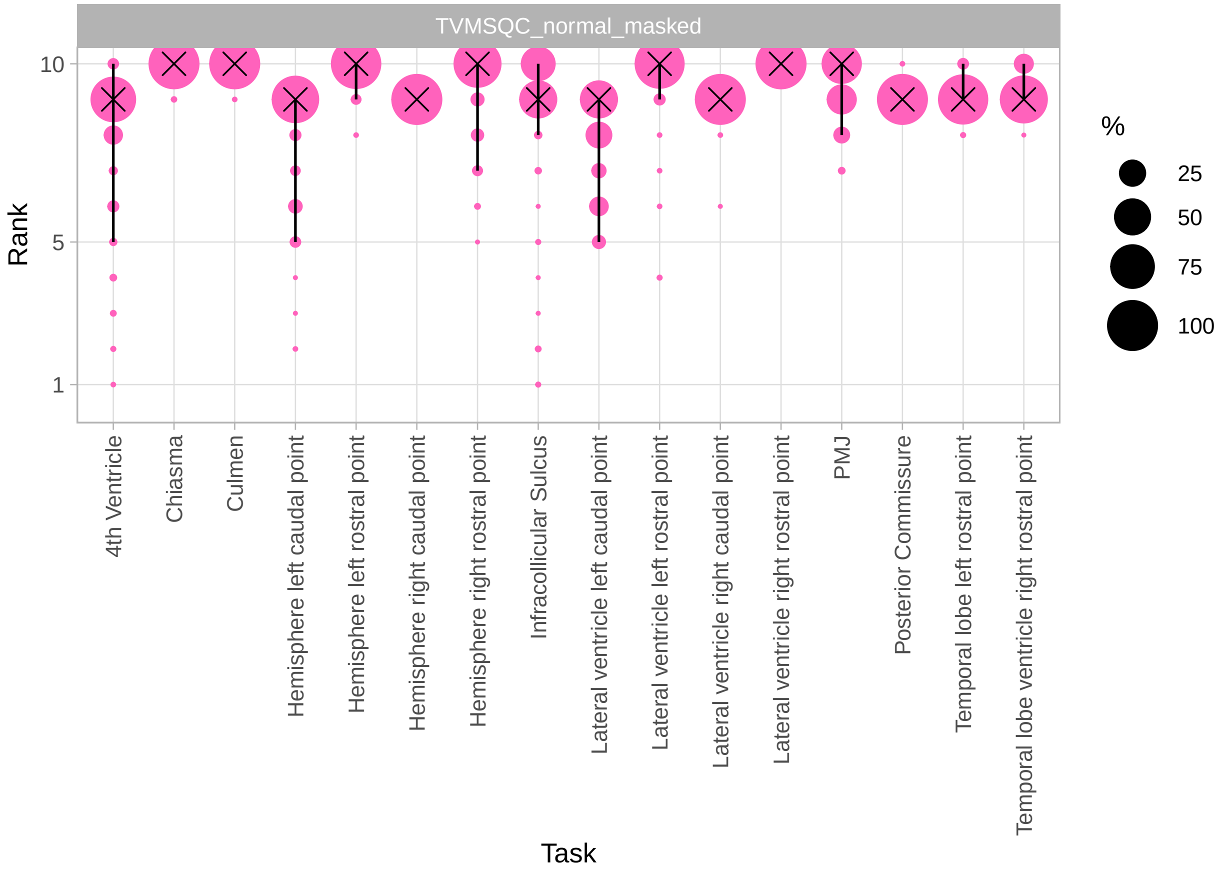

TVMSQC_normal_masked
Rank
10
5
1
Task
4th Ventricle
Chiasma
Culmen
Hemisphere left caudal point
Hemisphere left rostral point
Hemisphere right caudal point
Hemisphere right rostral point
Infracollicular Sulcus
Lateral ventricle left caudal point
Lateral ventricle left rostral point
Lateral ventricle right caudal point
Lateral ventricle right rostral point
PMJ
Posterior Commissure
Temporal lobe left rostral point
Temporal lobe ventricle right rostral point
%
25
50
75
100

An alternative representation is provided by a stacked frequency plot of the observed ranks, separated by algorithm. Observed ranks across bootstrap samples are displayed with coloring according to the task. For algorithms that achieve the same rank in different tasks for the full assessment data set, vertical lines are on top of each other. Vertical lines allow to compare the achieved rank of each algorithm over different tasks.

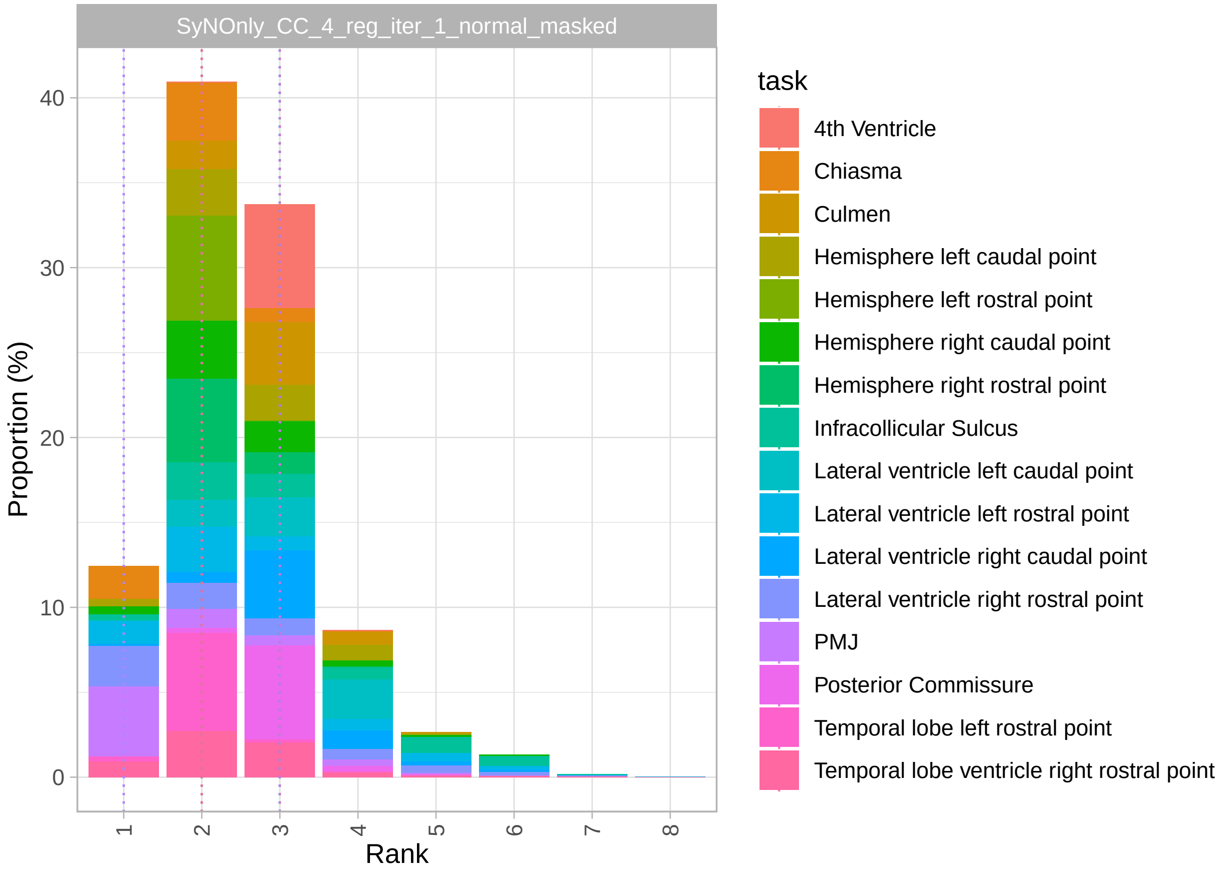

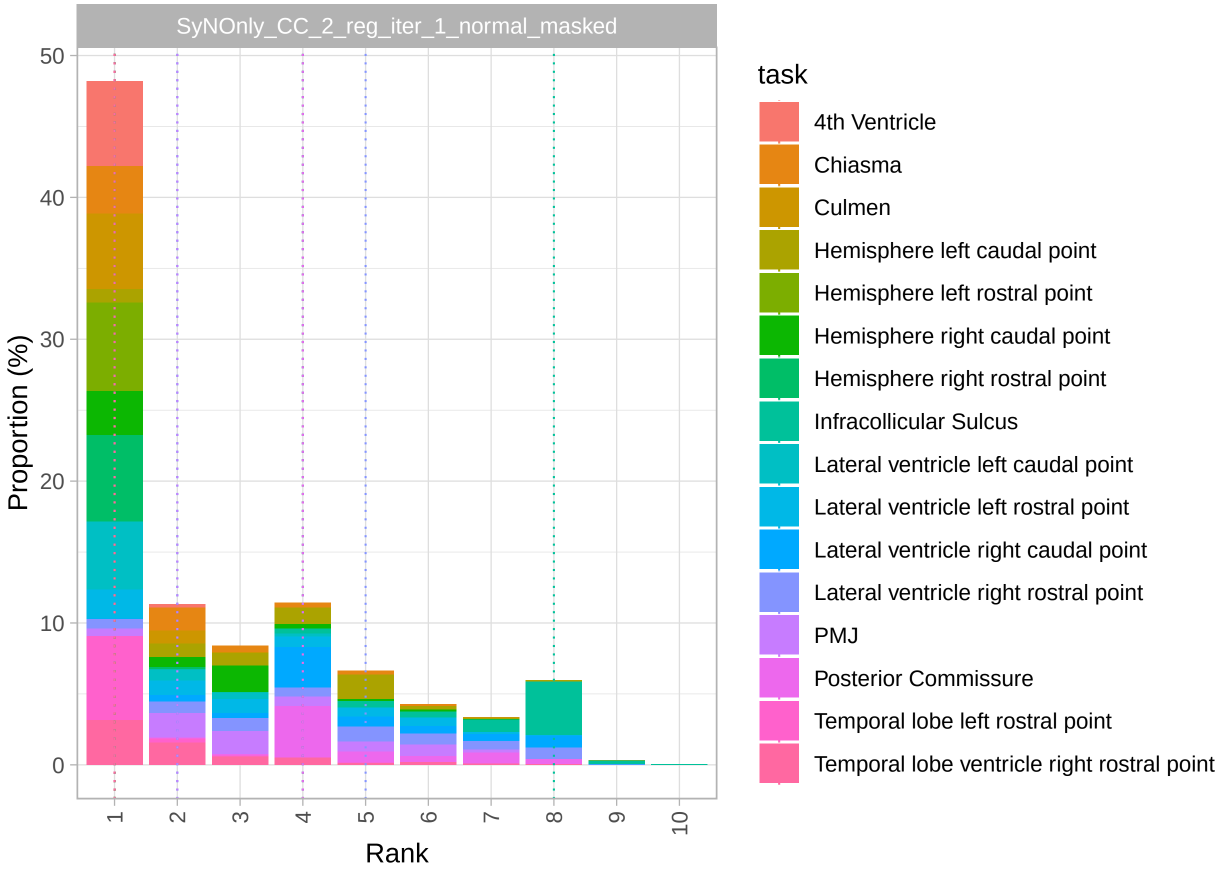

SyNOnly_CC_2_reg_iter_1_normal_masked
Proportion (%)
50
40
30
20
10
0
1
2
3
4
5
6
7
8
9
10
Rank
task
4th Ventricle
Chiasma
Culmen
Hemisphere left caudal point
Hemisphere left rostral point
Hemisphere right caudal point
Hemisphere right rostral point
Infracollicular Sulcus
Lateral ventricle left caudal point
Lateral ventricle left rostral point
Lateral ventricle right caudal point
Lateral ventricle right rostral point
PMJ
Posterior Commissure
Temporal lobe left rostral point
Temporal lobe ventricle right rostral point

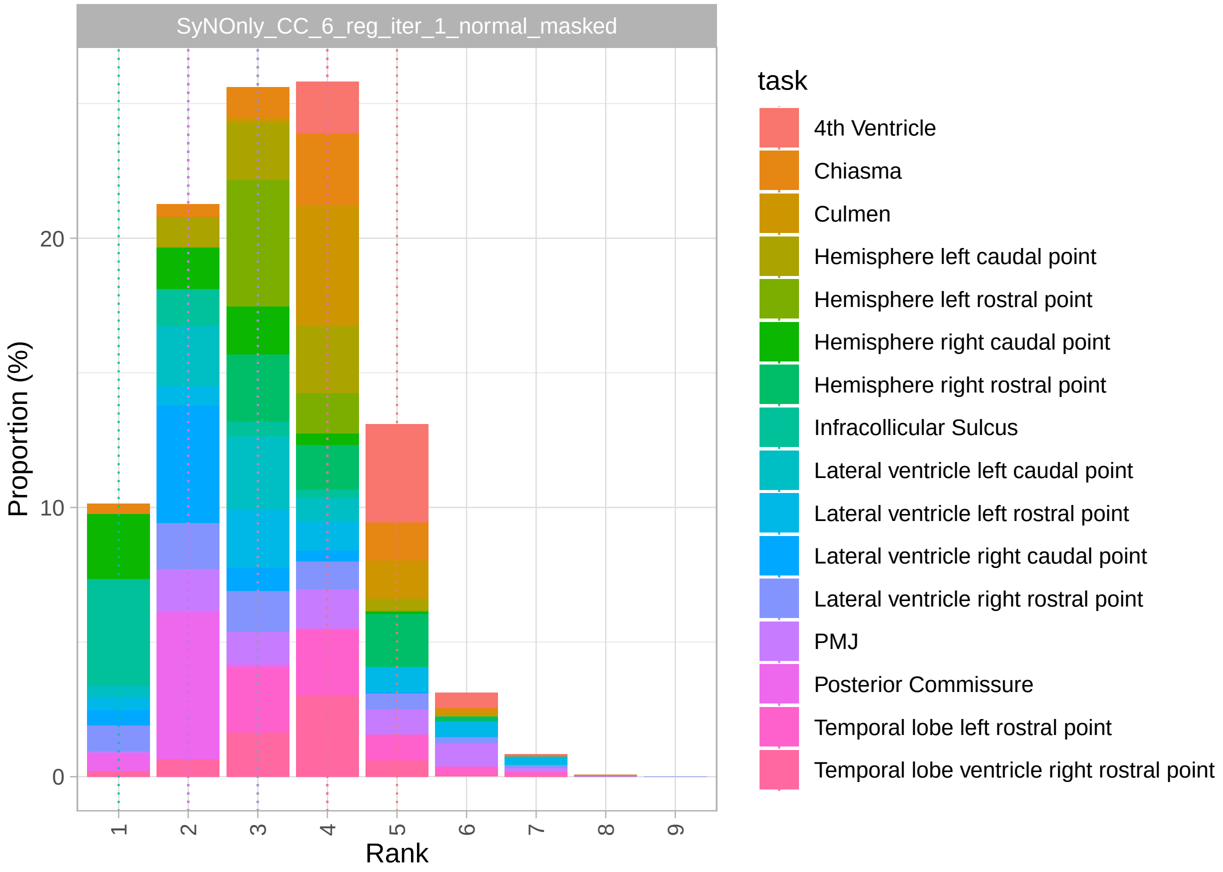

SyNOnly_CC_6_reg_iter_1_normal_masked
Proportion (%)
20
10
0
1
2
3
4
5
6
7
8
9
Rank
task
4th Ventricle
Chiasma
Culmen
Hemisphere left caudal point
Hemisphere left rostral point
Hemisphere right caudal point
Hemisphere right rostral point
Infracollicular Sulcus
Lateral ventricle left caudal point
Lateral ventricle left rostral point
Lateral ventricle right caudal point
Lateral ventricle right rostral point
PMJ
Posterior Commissure
Temporal lobe left rostral point
Temporal lobe ventricle right rostral point

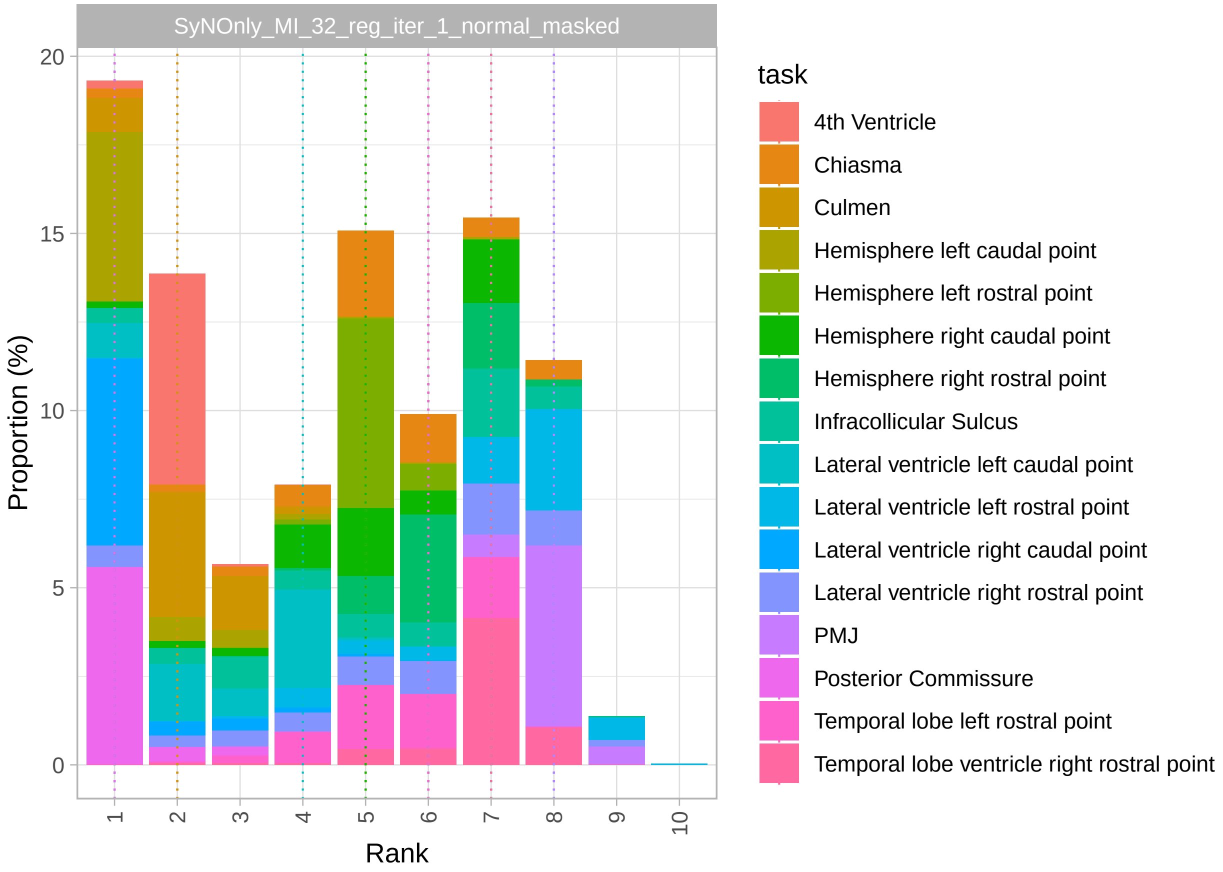

SyNOnly_MI_32_reg_iter_1_normal_masked
Proportion (%)
20
15
10
5
0
1
2
3
4
5
6
7
8
9
10
Rank
task
4th Ventricle
Chiasma
Culmen
Hemisphere left caudal point
Hemisphere left rostral point
Hemisphere right caudal point
Hemisphere right rostral point
Infracollicular Sulcus
Lateral ventricle left caudal point
Lateral ventricle left rostral point
Lateral ventricle right caudal point
Lateral ventricle right rostral point
PMJ
Posterior Commissure
Temporal lobe left rostral point
Temporal lobe ventricle right rostral point

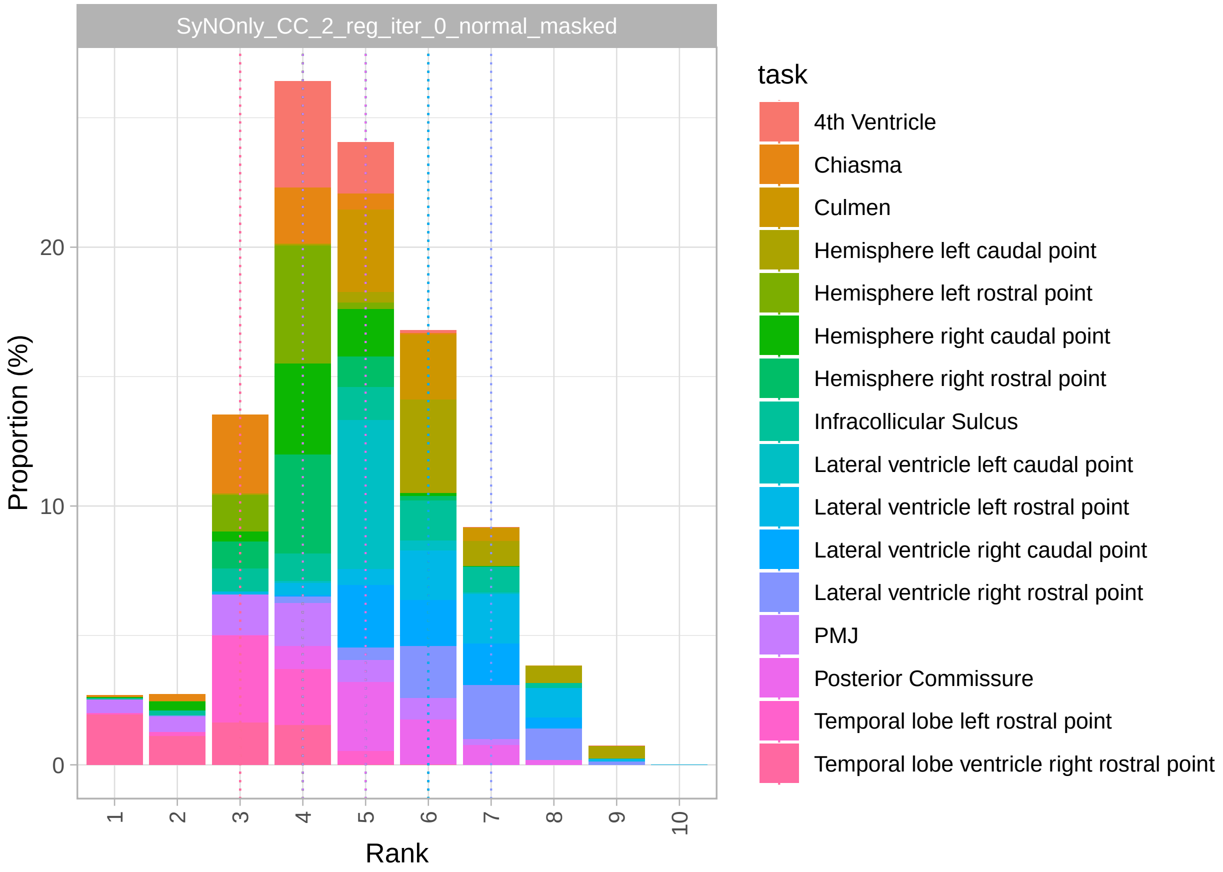

SyNOnly_CC_2_reg_iter_0_normal_masked
Proportion (%)
20
10
0
1
2
3
4
5
6
7
8
9
10
Rank
task
4th Ventricle
Chiasma
Culmen
Hemisphere left caudal point
Hemisphere left rostral point
Hemisphere right caudal point
Hemisphere right rostral point
Infracollicular Sulcus
Lateral ventricle left caudal point
Lateral ventricle left rostral point
Lateral ventricle right caudal point
Lateral ventricle right rostral point
PMJ
Posterior Commissure
Temporal lobe left rostral point
Temporal lobe ventricle right rostral point

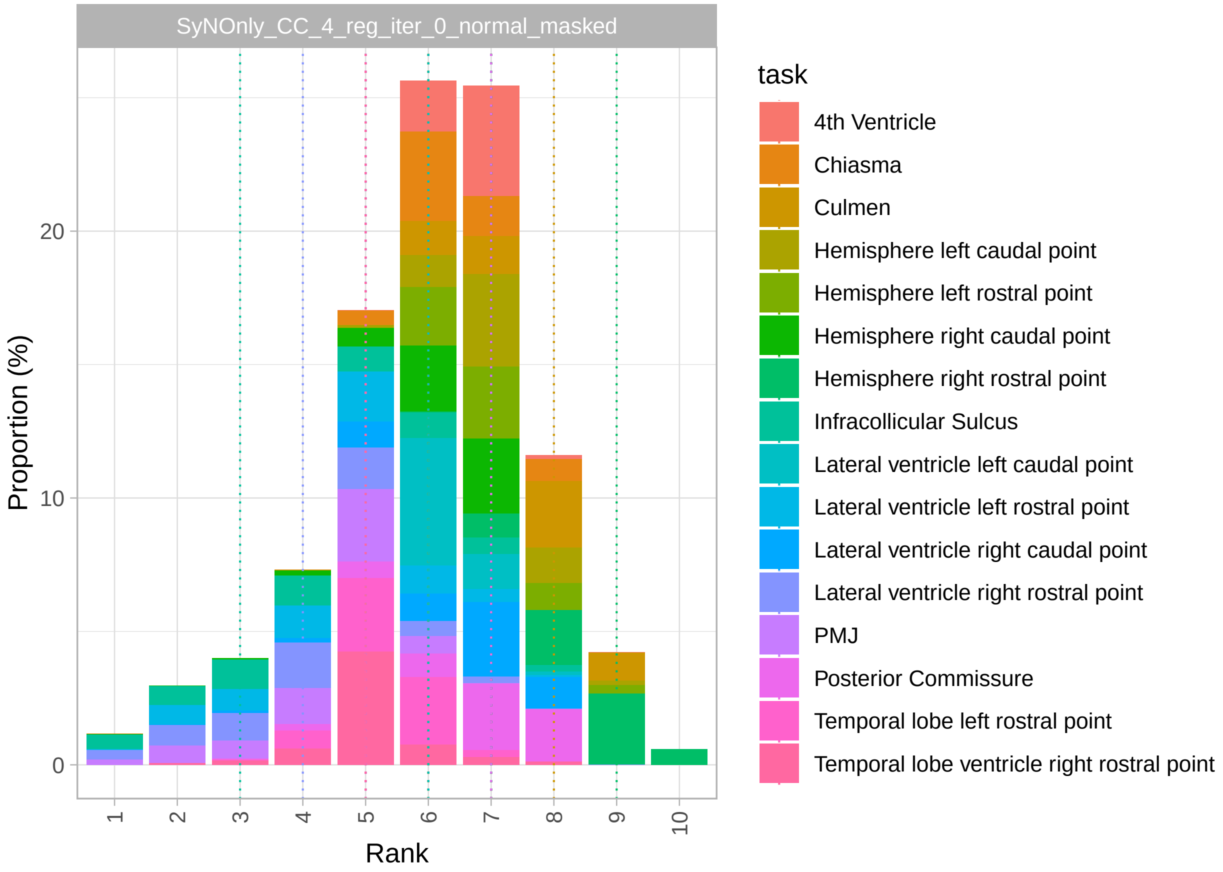
SyNOnly_CC_4_reg_iter_0_normal_masked
Proportion (%)
20
10
0
1
2
3
4
5
6
7
8
9
10
Rank
task
4th Ventricle
Chiasma
Culmen
Hemisphere left caudal point
Hemisphere left rostral point
Hemisphere right caudal point
Hemisphere right rostral point
Infracollicular Sulcus
Lateral ventricle left caudal point
Lateral ventricle left rostral point
Lateral ventricle right caudal point
Lateral ventricle right rostral point
PMJ
Posterior Commissure
Temporal lobe left rostral point
Temporal lobe ventricle right rostral point

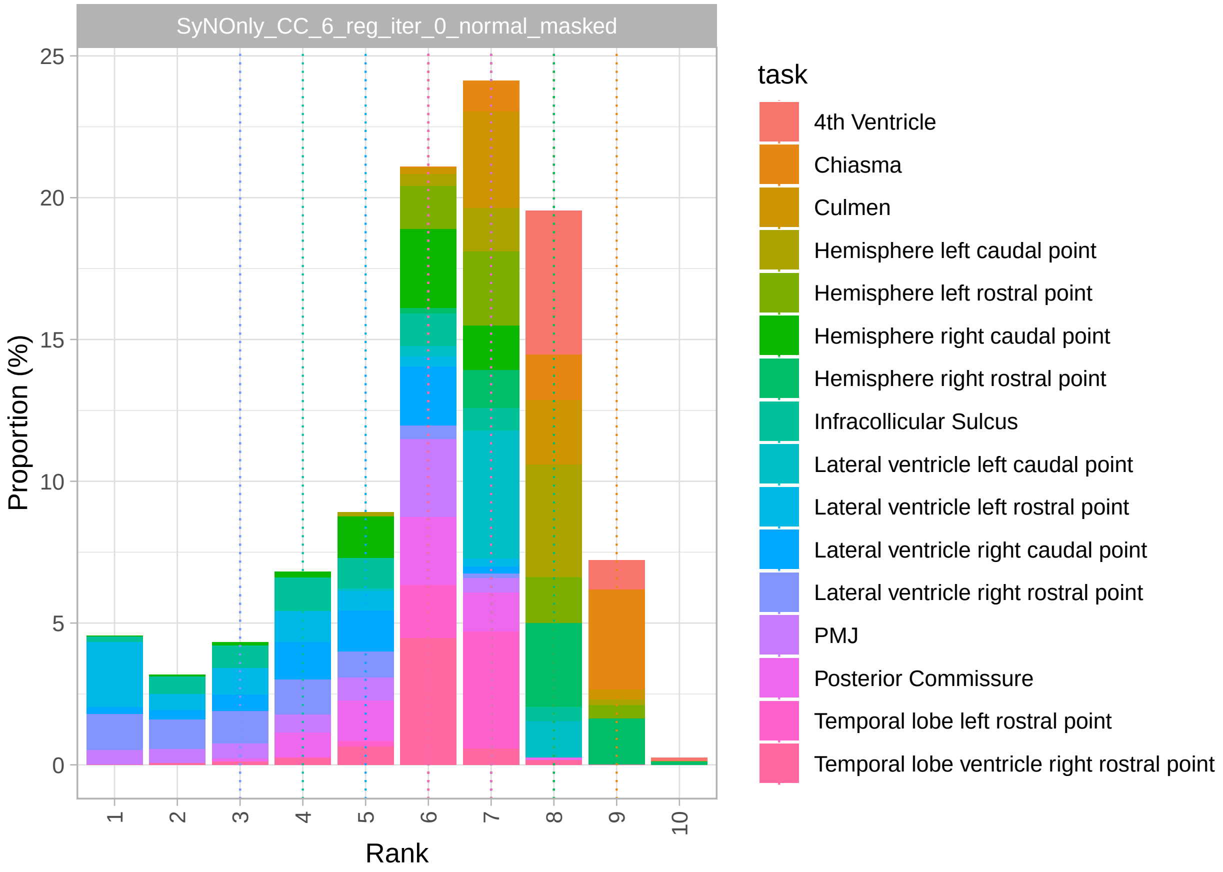

SyNOnly_CC_6_reg_iter_0_normal_masked
Proportion (%)
Rank
task
4th Ventricle
Chiasma
Culmen
Hemisphere left caudal point
Hemisphere left rostral point
Hemisphere right caudal point
Hemisphere right rostral point
Infracollicular Sulcus
Lateral ventricle left caudal point
Lateral ventricle left rostral point
Lateral ventricle right caudal point
Lateral ventricle right rostral point
PMJ
Posterior Commissure
Temporal lobe left rostral point
Temporal lobe ventricle right rostral point

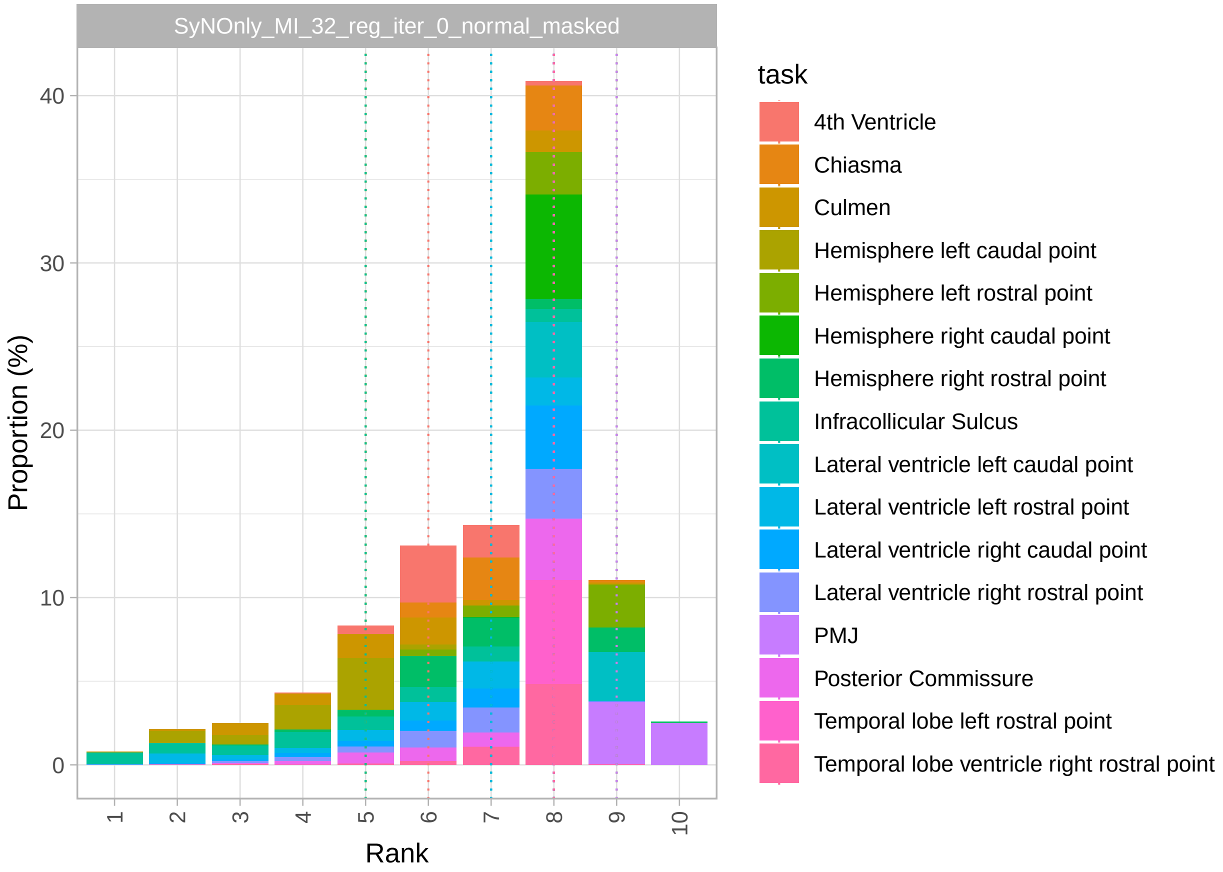
SyNOnly_MI_32_reg_iter_0_normal_masked
Proportion (%)
0
10
20
30
40
1
2
3
4
5
6
7
8
9
10
Rank
task
4th Ventricle
Chiasma
Culmen
Hemisphere left caudal point
Hemisphere left rostral point
Hemisphere right caudal point
Hemisphere right rostral point
Infracollicular Sulcus
Lateral ventricle left caudal point
Lateral ventricle left rostral point
Lateral ventricle right caudal point
Lateral ventricle right rostral point
PMJ
Posterior Commissure
Temporal lobe left rostral point
Temporal lobe ventricle right rostral point

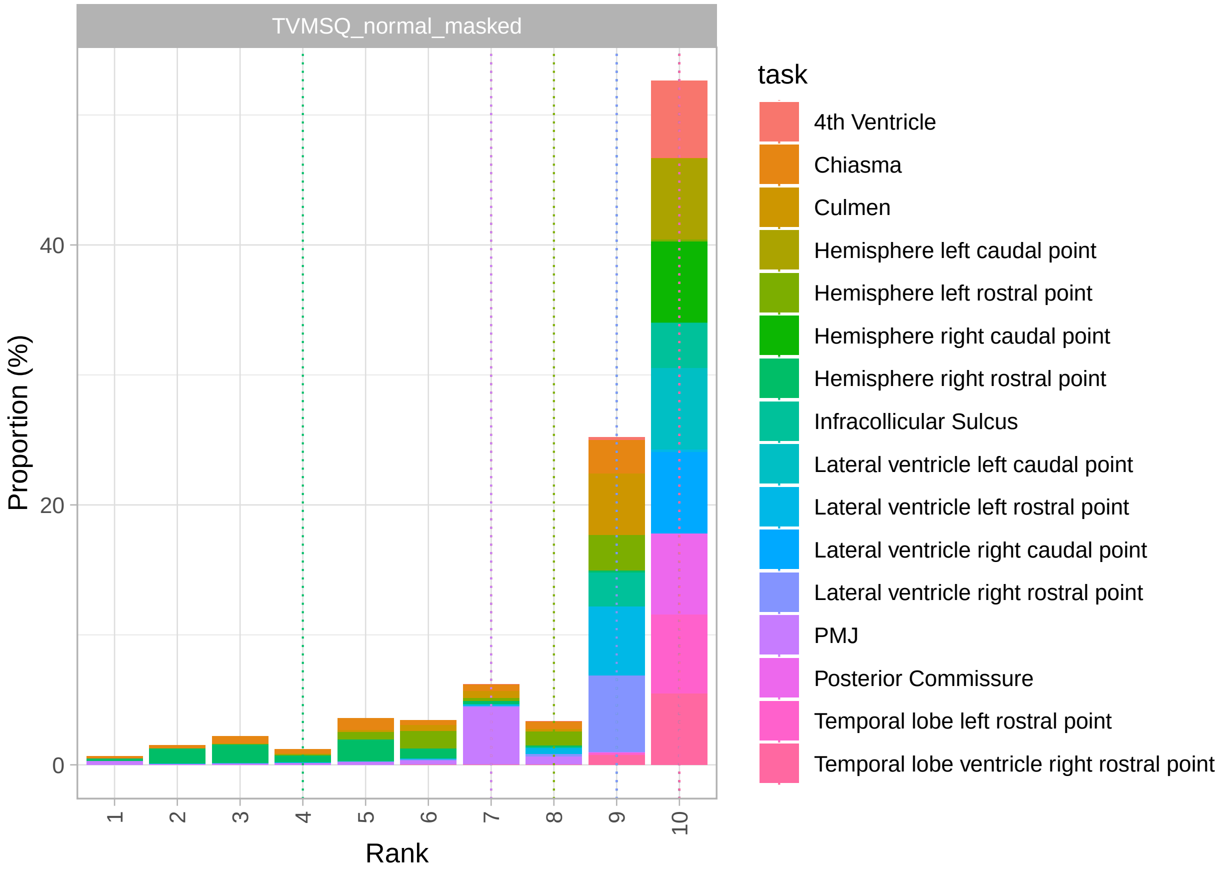

TVMSQ_normal_masked
Proportion (%)
40
20
0
1
2
3
4
5
6
7
8
9
10
Rank
task
4th Ventricle
Chiasma
Culmen
Hemisphere left caudal point
Hemisphere left rostral point
Hemisphere right caudal point
Hemisphere right rostral point
Infracollicular Sulcus
Lateral ventricle left caudal point
Lateral ventricle left rostral point
Lateral ventricle right caudal point
Lateral ventricle right rostral point
PMJ
Posterior Commissure
Temporal lobe left rostral point
Temporal lobe ventricle right rostral point

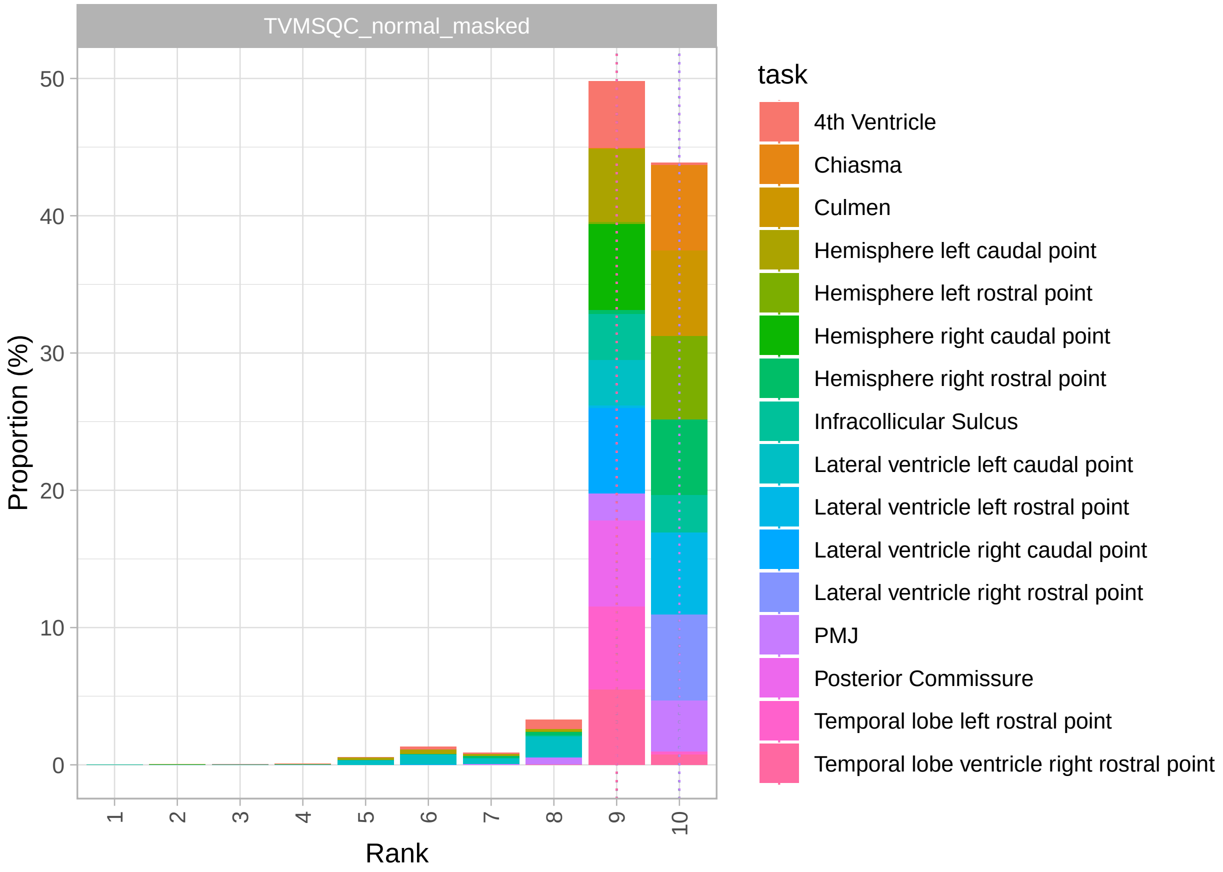
TVMSQC_normal_masked
Proportion (%)
50
40
30
20
10
0
1
2
3
4
5
6
7
8
9
10
Rank
task
4th Ventricle
Chiasma
Culmen
Hemisphere left caudal point
Hemisphere left rostral point
Hemisphere right caudal point
Hemisphere right rostral point
Infracollicular Sulcus
Lateral ventricle left caudal point
Lateral ventricle left rostral point
Lateral ventricle right caudal point
Lateral ventricle right rostral point
PMJ
Posterior Commissure
Temporal lobe left rostral point
Temporal lobe ventricle right rostral point

4.2 Characterization of tasks

4.2.1 Visualizing bootstrap results

To investigate which tasks separate algorithms well (i.e., lead to a stable ranking), a blob plot is recommended.

Bootstrap results can be shown in a blob plot showing one plot for each task. In this view, the spread of the blobs for each algorithm can be compared across tasks. Deviations from the diagonal indicate deviations from the consensus ranking (over tasks). Specifically, if rank distribution of an algorithm is consistently below the diagonal, the algorithm performed better in this task than on average across tasks, while if the rank distribution of an algorithm is consistently above the diagonal, the algorithm performed worse in this task than on average across tasks. At the bottom of each panel, ranks for each algorithm in the tasks are provided.

Same as in Section 3.1 but now ordered according to consensus.

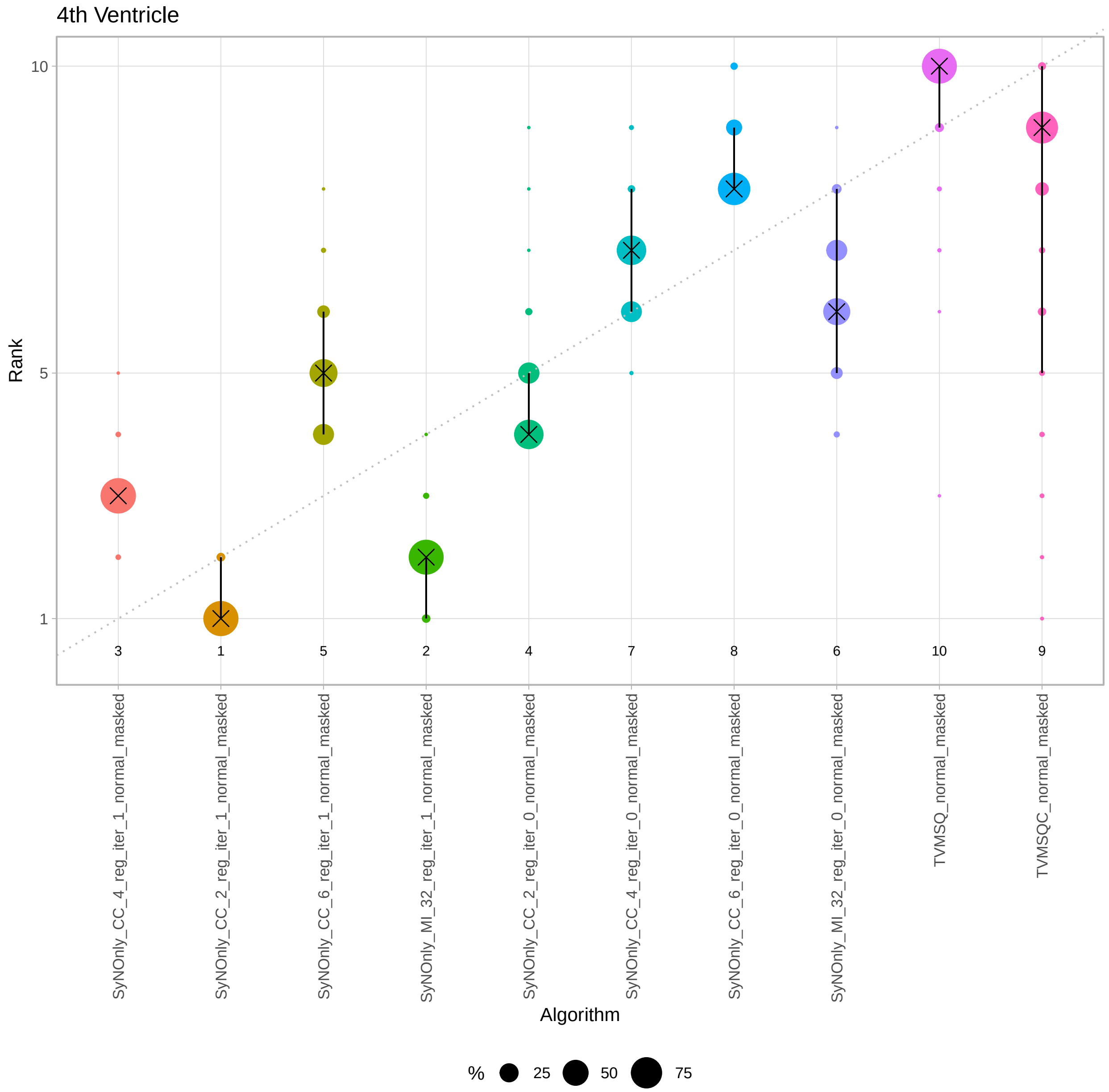

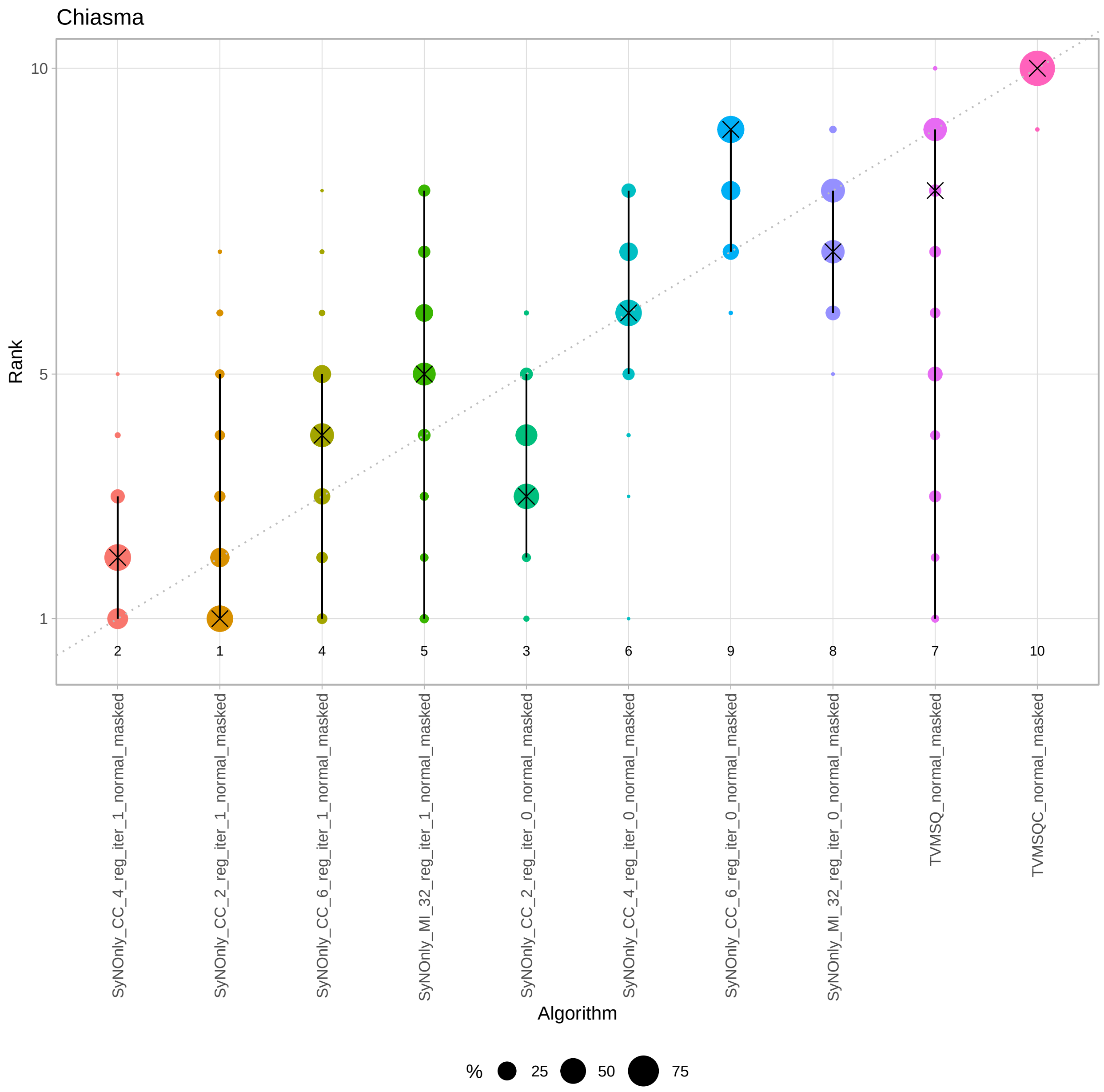

Chiasma
Rank
10
5
1
2
1
4
5
3
6
9
8
7
10
SyNOnly_CC_4_reg_iter_1_normal_masked
SyNOnly_CC_2_reg_iter_1_normal_masked
SyNOnly_CC_6_reg_iter_1_normal_masked
SyNOnly_MI_32_reg_iter_1_normal_masked
SyNOnly_CC_2_reg_iter_0_normal_masked
SyNOnly_CC_4_reg_iter_0_normal_masked
SyNOnly_CC_6_reg_iter_0_normal_masked
SyNOnly_MI_32_reg_iter_0_normal_masked
TVMSQ_normal_masked
TVMSQC_normal_masked
Algorithm
%
25
50
75

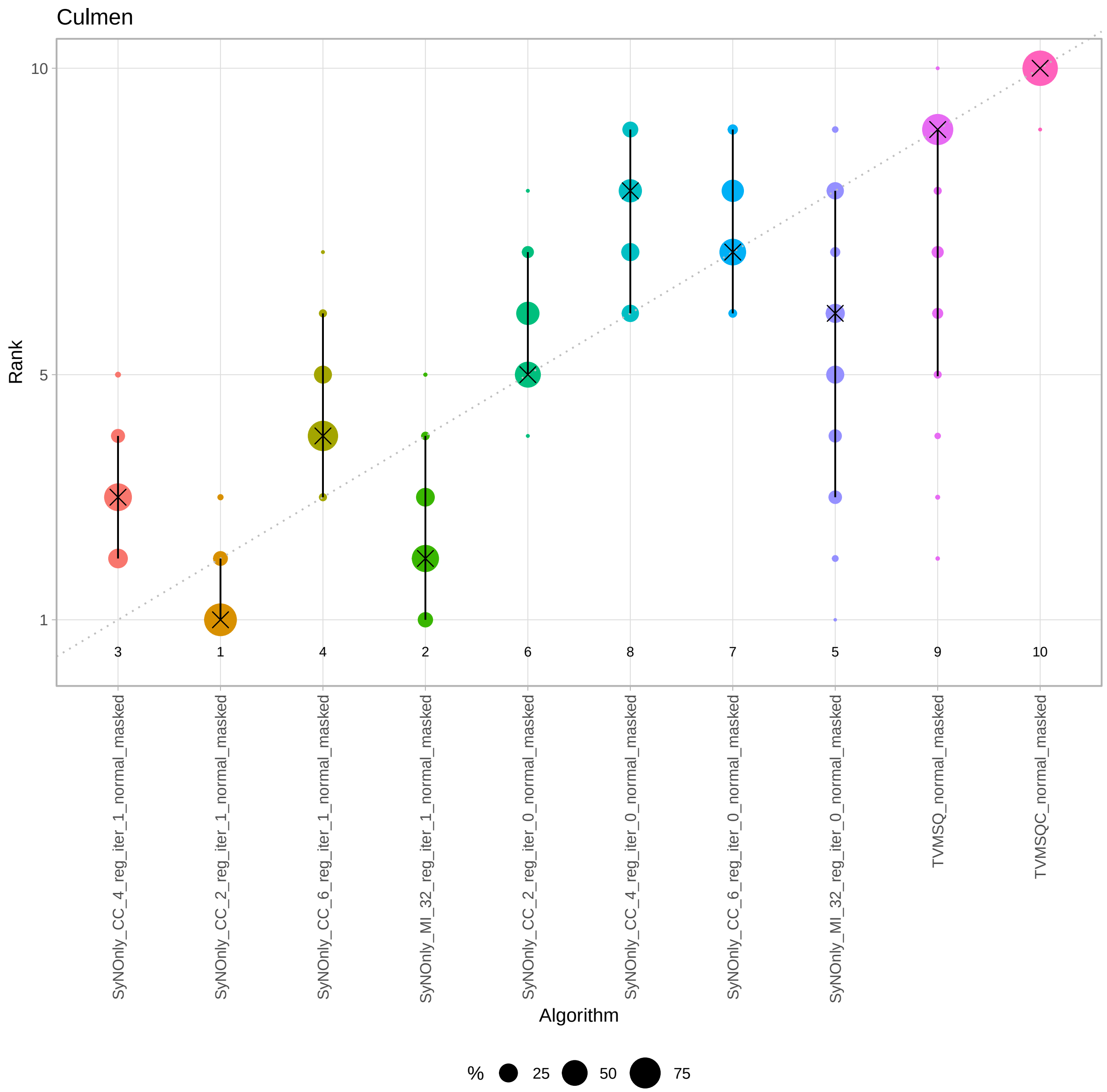
Culmen
Rank
10
5
1
3
1
4
2
6
8
7
5
9
10
SyNOnly_CC_4_reg_iter_1_normal_masked
SyNOnly_CC_2_reg_iter_1_normal_masked
SyNOnly_CC_6_reg_iter_1_normal_masked
SyNOnly_MI_32_reg_iter_1_normal_masked
SyNOnly_CC_2_reg_iter_0_normal_masked
SyNOnly_CC_4_reg_iter_0_normal_masked
SyNOnly_CC_6_reg_iter_0_normal_masked
SyNOnly_MI_32_reg_iter_0_normal_masked
TVMSQ_normal_masked
TVMSQC_normal_masked
Algorithm
% 25 50 75

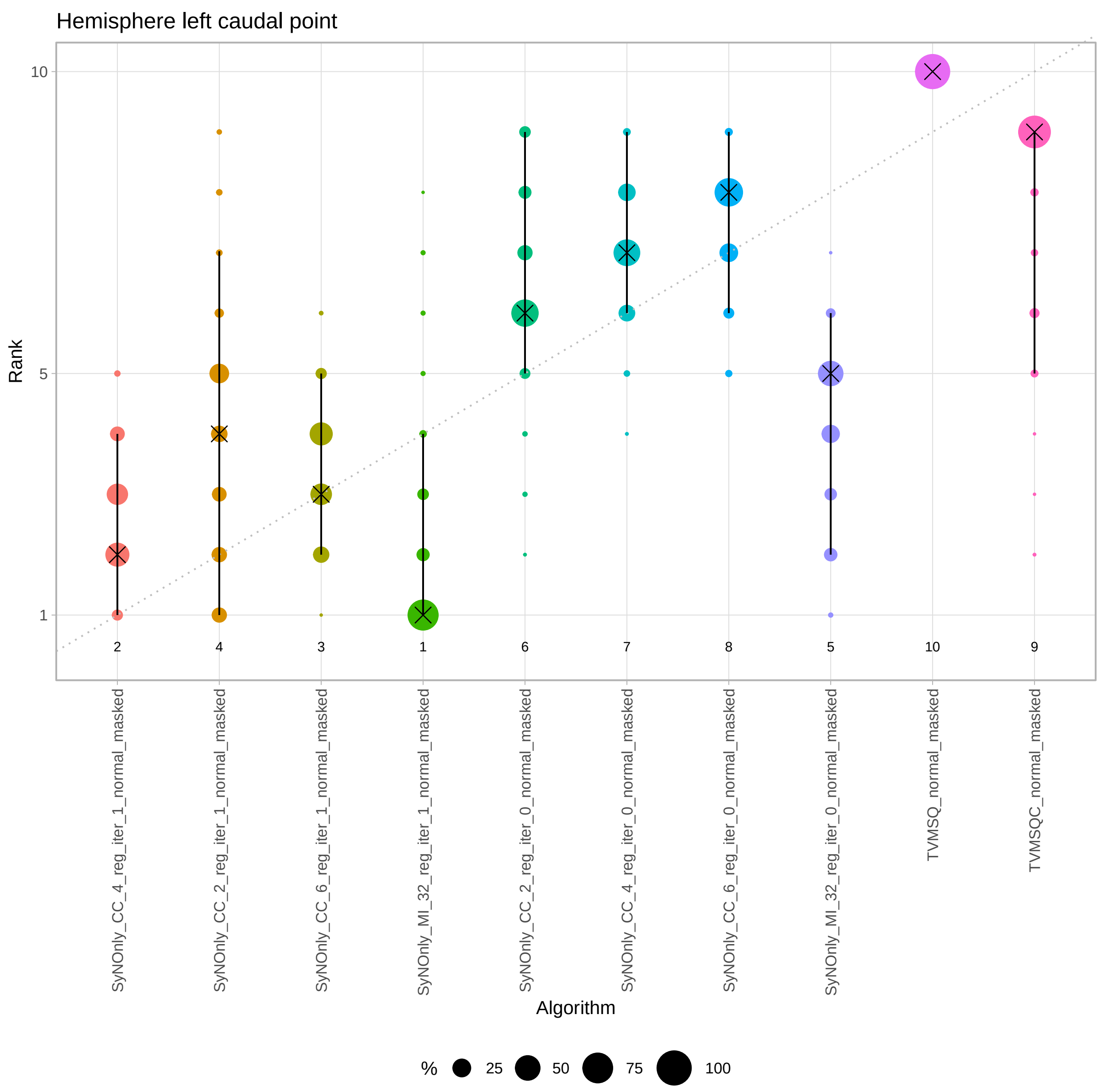

Hemisphere left caudal point
Rank
10
5
1
2
4
3
1
6
7
8
5
10
9
SyNOnly_CC_4_reg_iter_1_normal_masked
SyNOnly_CC_2_reg_iter_1_normal_masked
SyNOnly_CC_6_reg_iter_1_normal_masked
SyNOnly_MI_32_reg_iter_1_normal_masked
SyNOnly_CC_2_reg_iter_0_normal_masked
SyNOnly_CC_4_reg_iter_0_normal_masked
SyNOnly_CC_6_reg_iter_0_normal_masked
SyNOnly_MI_32_reg_iter_0_normal_masked
TVMSQ_normal_masked
TVMSQC_normal_masked
Algorithm
% 25 50 75 100

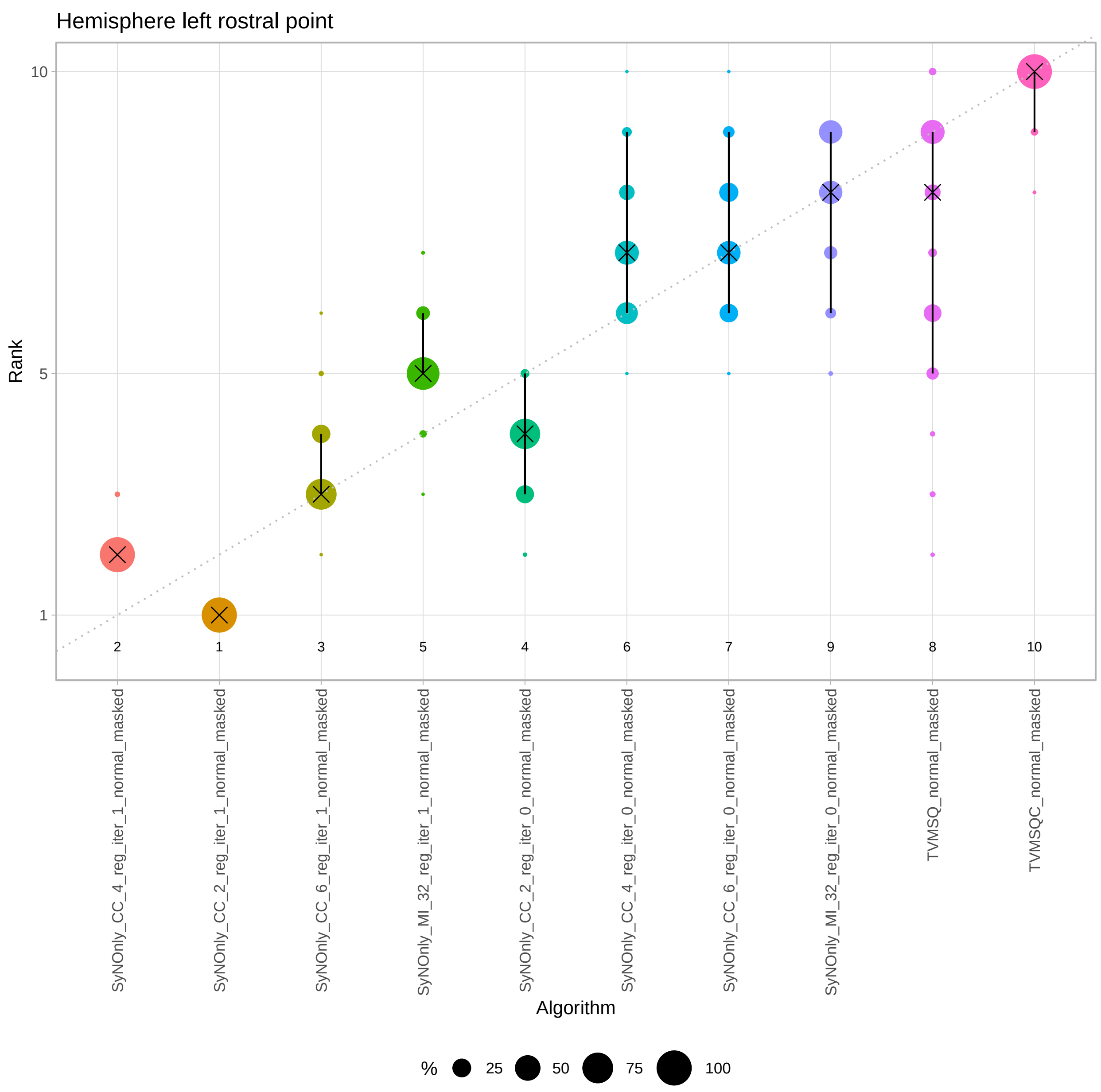

Hemisphere left rostral point
Rank
10
5
1
2
1
3
5
4
6
7
9
8
10
SyNOnly_CC_4_reg_iter_1_normal_masked
SyNOnly_CC_2_reg_iter_1_normal_masked
SyNOnly_CC_6_reg_iter_1_normal_masked
SyNOnly_MI_32_reg_iter_1_normal_masked
SyNOnly_CC_2_reg_iter_0_normal_masked
SyNOnly_CC_4_reg_iter_0_normal_masked
SyNOnly_CC_6_reg_iter_0_normal_masked
SyNOnly_MI_32_reg_iter_0_normal_masked
TVMSQ_normal_masked
TVMSQC_normal_masked
Algorithm
%
25
50
75
100

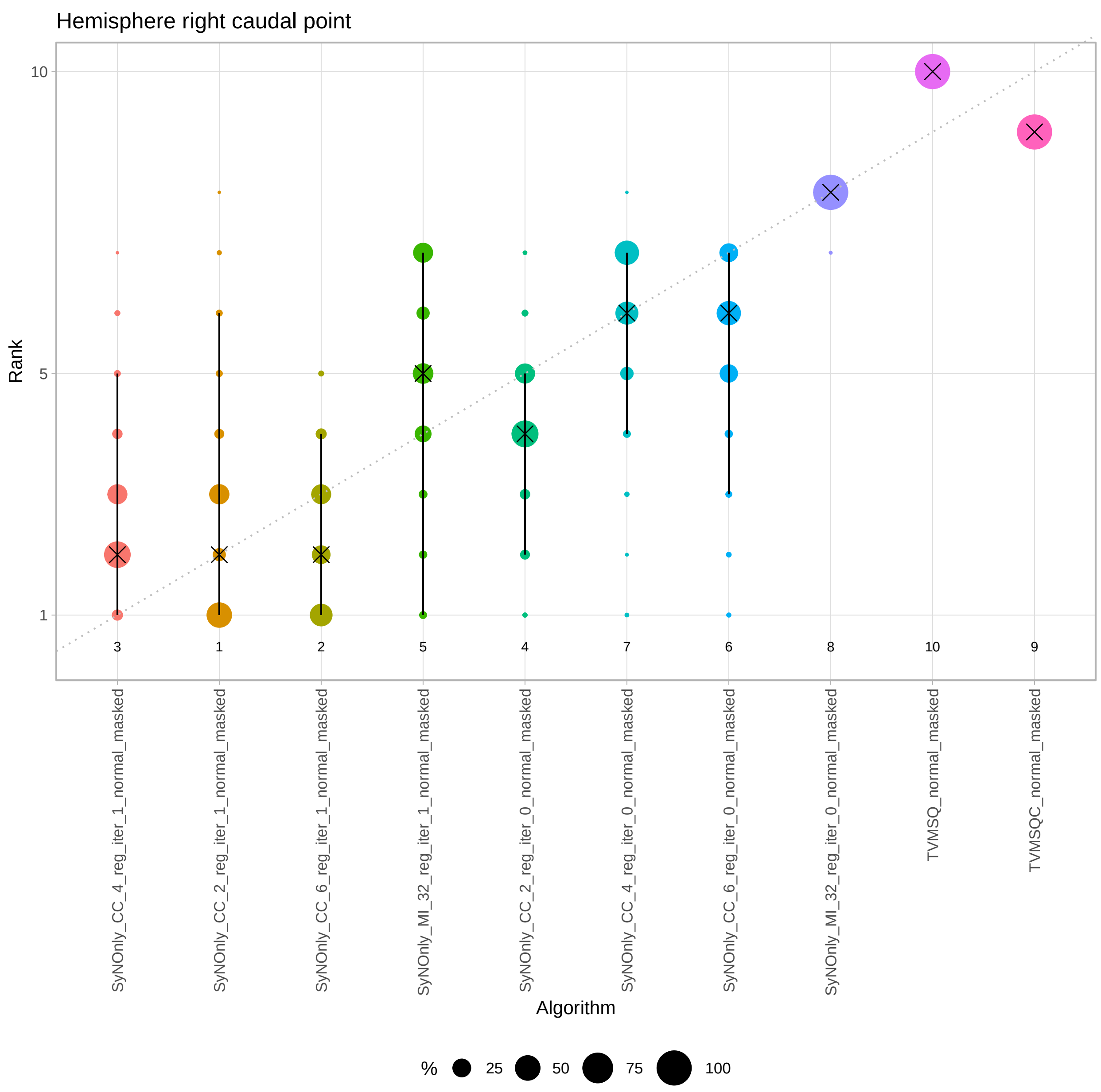
Hemisphere right caudal point
Rank
10
5
1
3
1
2
5
4
7
6
8
10
9
SyNOnly_CC_4_reg_iter_1_normal_masked
SyNOnly_CC_2_reg_iter_1_normal_masked
SyNOnly_CC_6_reg_iter_1_normal_masked
SyNOnly_MI_32_reg_iter_1_normal_masked
SyNOnly_CC_2_reg_iter_0_normal_masked
SyNOnly_CC_4_reg_iter_0_normal_masked
SyNOnly_CC_6_reg_iter_0_normal_masked
SyNOnly_MI_32_reg_iter_0_normal_masked
TVMSQ_normal_masked
TVMSQC_normal_masked
Algorithm
%
25
50
75
100

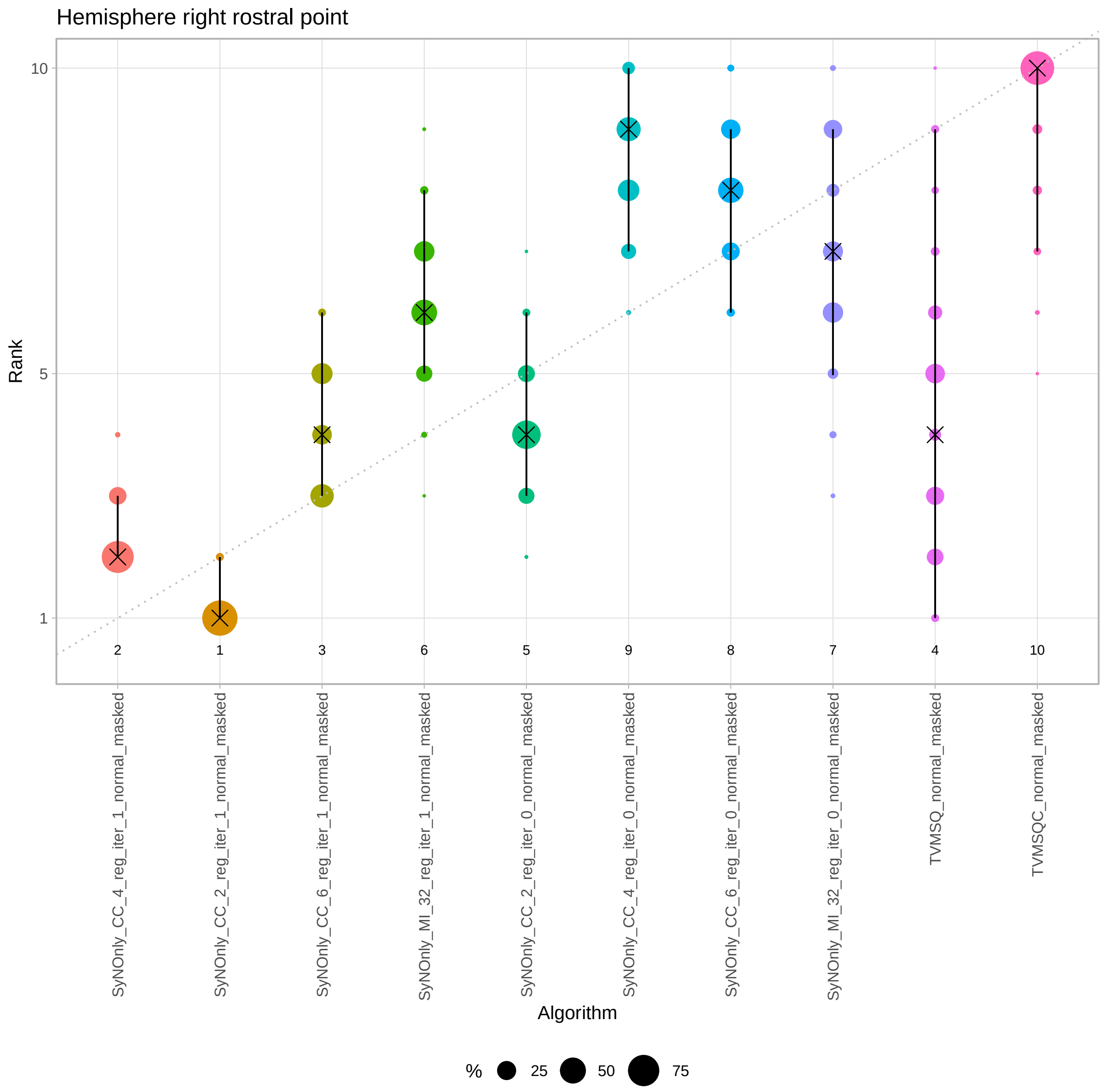
Hemisphere right rostral point
Rank
10
5
1
2
1
3
6
5
9
8
7
4
10
SyNOnly_CC_4_reg_iter_1_normal_masked
SyNOnly_CC_2_reg_iter_1_normal_masked
SyNOnly_CC_6_reg_iter_1_normal_masked
SyNOnly_MI_32_reg_iter_1_normal_masked
SyNOnly_CC_2_reg_iter_0_normal_masked
SyNOnly_CC_4_reg_iter_0_normal_masked
SyNOnly_CC_6_reg_iter_0_normal_masked
SyNOnly_MI_32_reg_iter_0_normal_masked
TVMSQ_normal_masked
TVMSQC_normal_masked
Algorithm
%
25
50
75

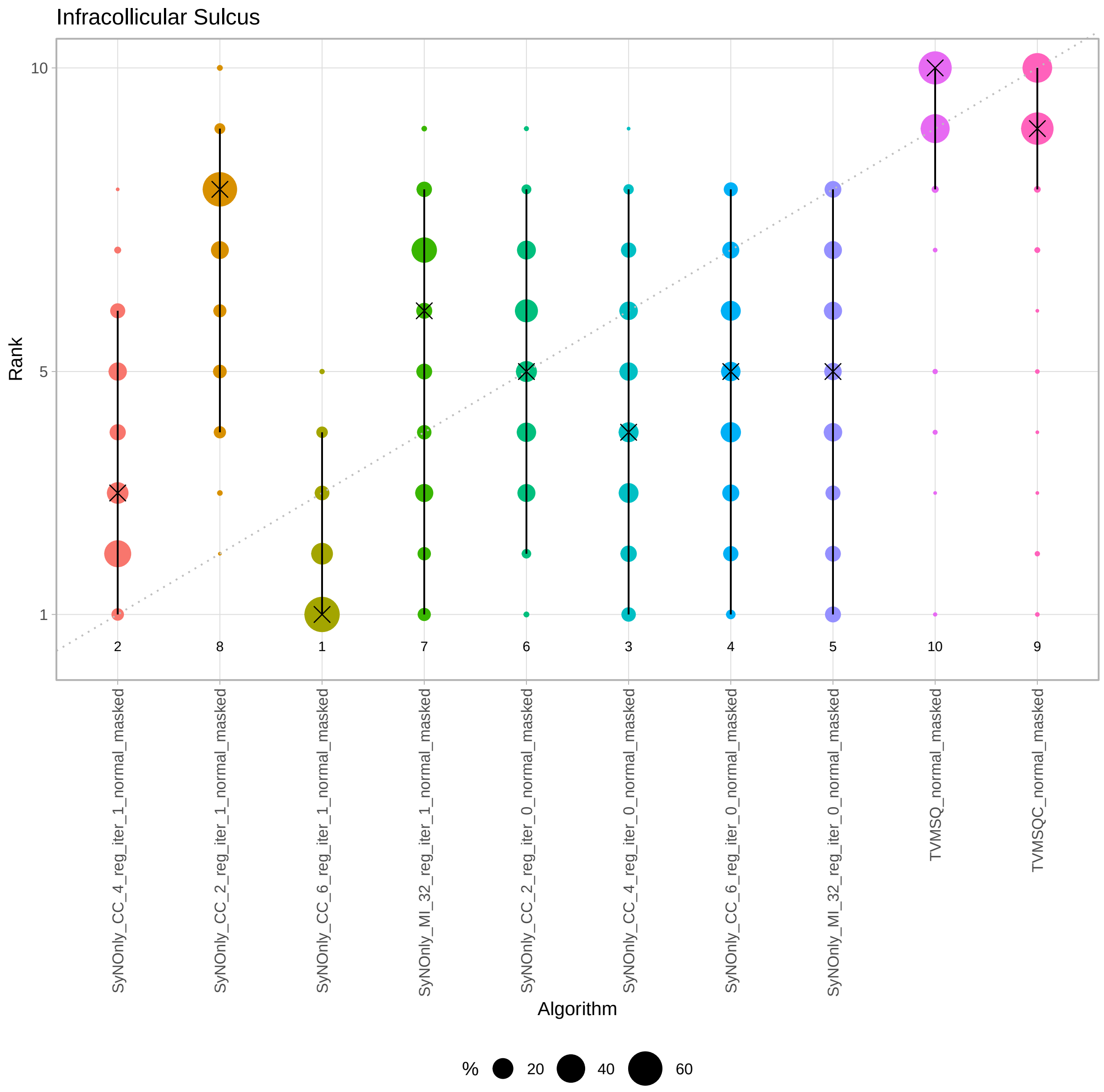

Infracollicular Sulcus
Rank
10
5
1
2
8
1
7
6
3
4
5
10
9
SyNOnly_CC_4_reg_iter_1_normal_masked
SyNOnly_CC_2_reg_iter_1_normal_masked
SyNOnly_CC_6_reg_iter_1_normal_masked
SyNOnly_MI_32_reg_iter_1_normal_masked
SyNOnly_CC_2_reg_iter_0_normal_masked
SyNOnly_CC_4_reg_iter_0_normal_masked
SyNOnly_CC_6_reg_iter_0_normal_masked
SyNOnly_MI_32_reg_iter_0_normal_masked
TVMSQ_normal_masked
TVMSQC_normal_masked
Algorithm
%
20
40
60

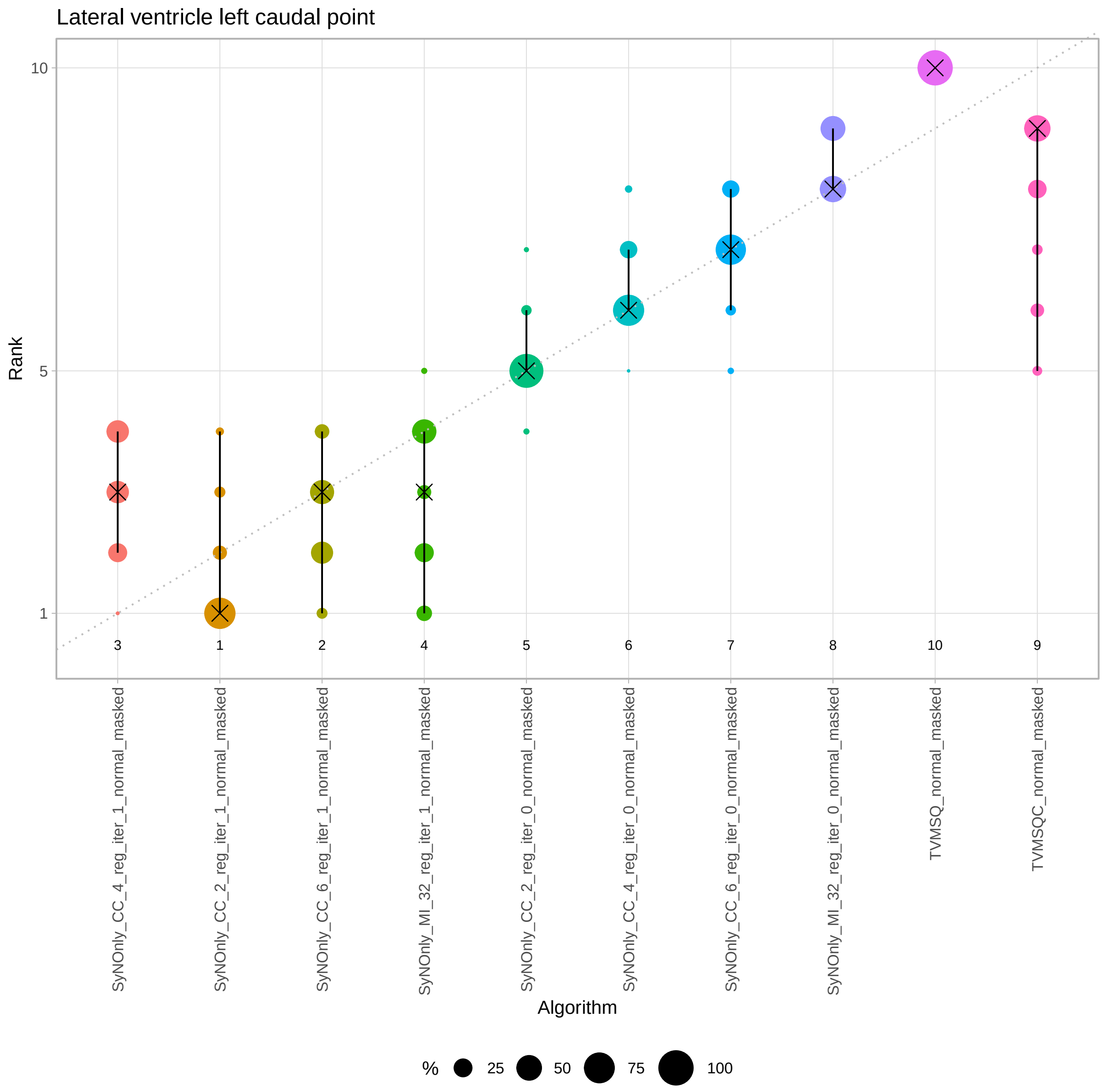

Lateral ventricle left caudal point
Rank
10
5
1
3
1
2
4
5
6
7
8
10
9
SyNOnly_CC_4_reg_iter_1_normal_masked
SyNOnly_CC_2_reg_iter_1_normal_masked
SyNOnly_CC_6_reg_iter_1_normal_masked
SyNOnly_MI_32_reg_iter_1_normal_masked
SyNOnly_CC_2_reg_iter_0_normal_masked
SyNOnly_CC_4_reg_iter_0_normal_masked
SyNOnly_CC_6_reg_iter_0_normal_masked
SyNOnly_MI_32_reg_iter_0_normal_masked
TVMSQ_normal_masked
TVMSQC_normal_masked
Algorithm
% 25 50 75 100

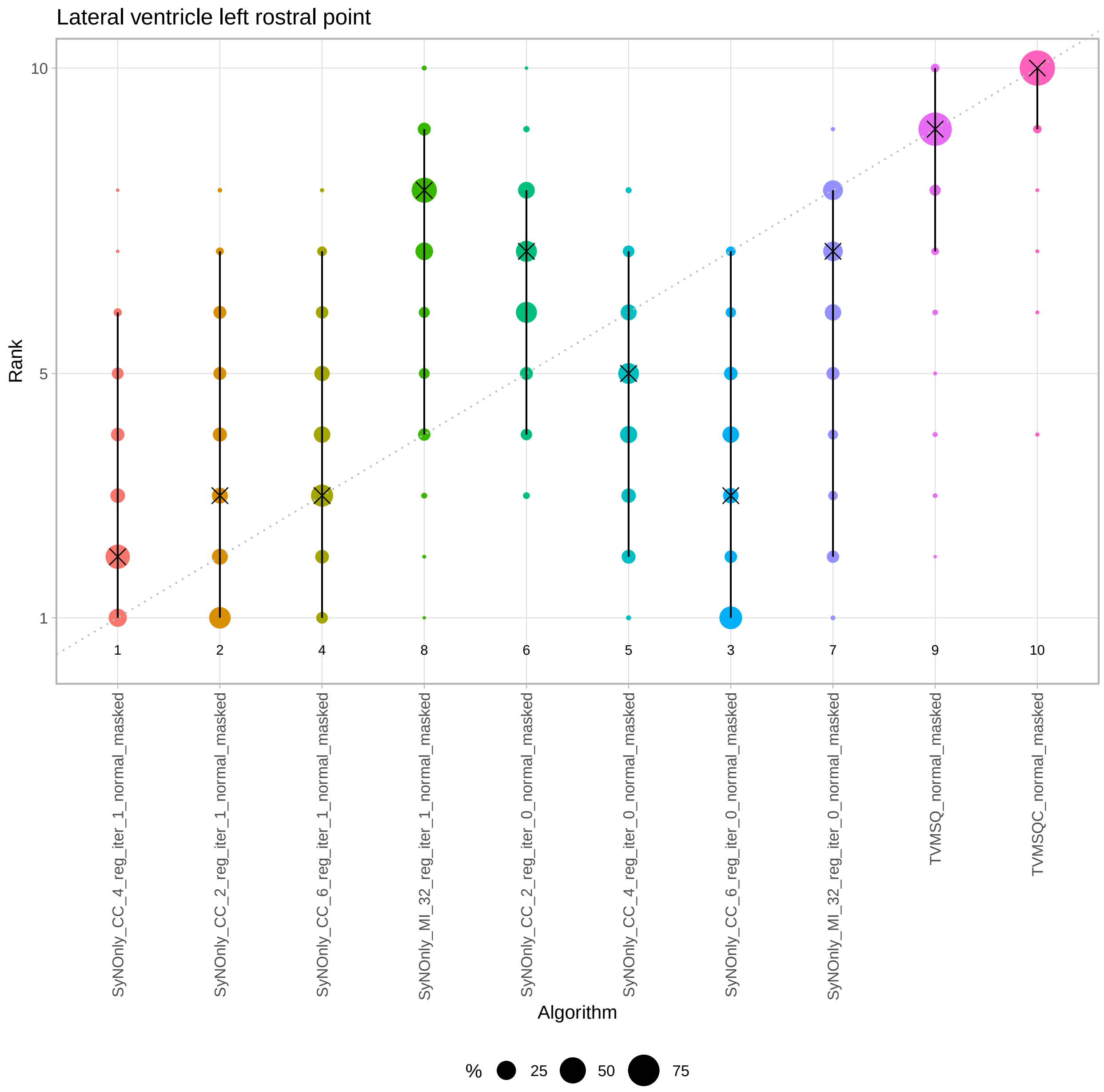

Lateral ventricle left rostral point
Rank
10
5
1
1
2
4
8
6
5
3
7
9
10
SyNOnly_CC_4_reg_iter_1_normal_masked
SyNOnly_CC_2_reg_iter_1_normal_masked
SyNOnly_CC_6_reg_iter_1_normal_masked
SyNOnly_MI_32_reg_iter_1_normal_masked
SyNOnly_CC_2_reg_iter_0_normal_masked
SyNOnly_CC_4_reg_iter_0_normal_masked
SyNOnly_CC_6_reg_iter_0_normal_masked
SyNOnly_MI_32_reg_iter_0_normal_masked
TVMSQ_normal_masked
TVMSQC_normal_masked
Algorithm
%
25
50
75

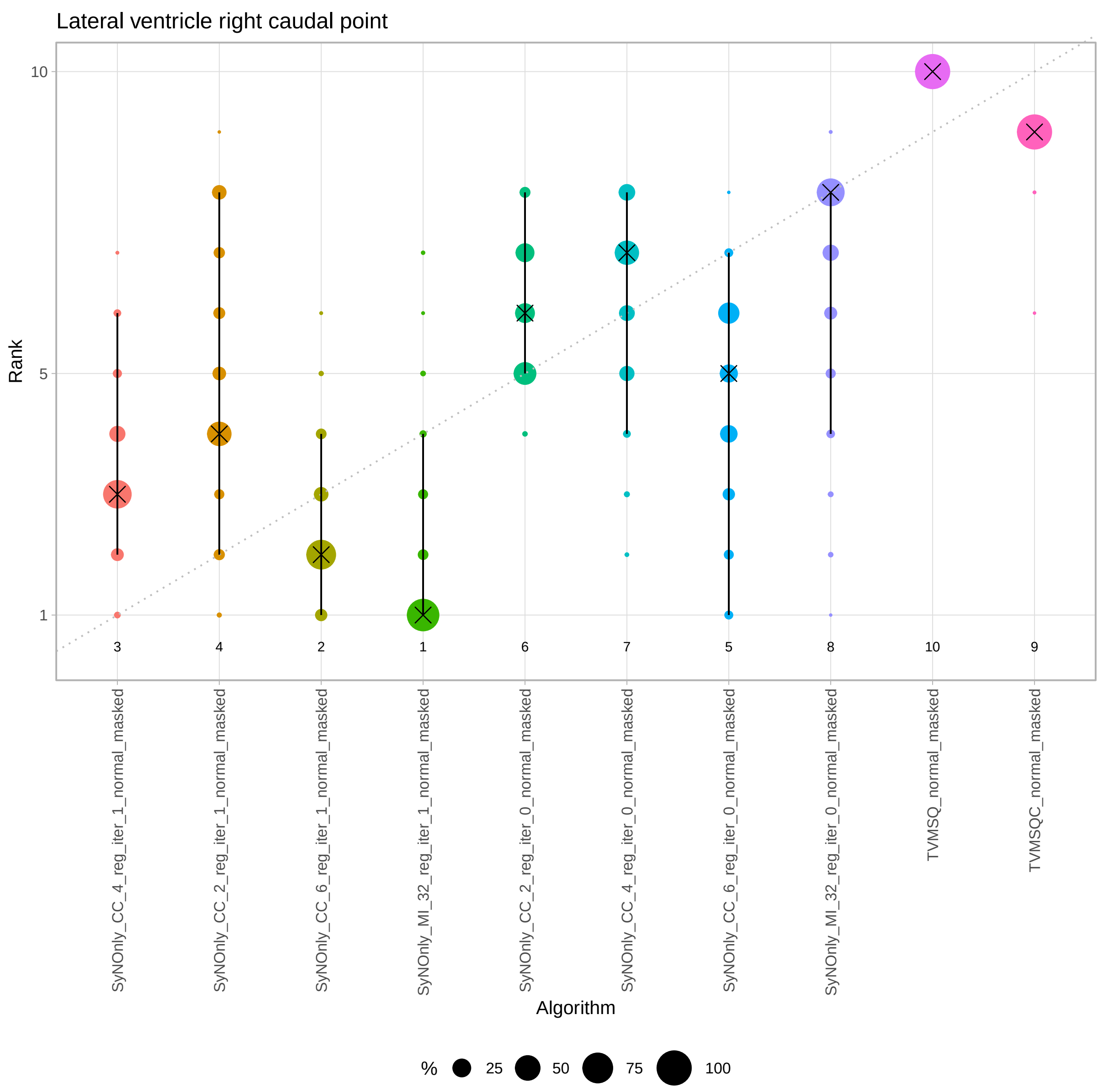

Lateral ventricle right caudal point
Rank
10
5
1
3
4
2
1
6
7
5
8
10
9
SyNOnly_CC_4_reg_iter_1_normal_masked
SyNOnly_CC_2_reg_iter_1_normal_masked
SyNOnly_CC_6_reg_iter_1_normal_masked
SyNOnly_MI_32_reg_iter_1_normal_masked
SyNOnly_CC_2_reg_iter_0_normal_masked
SyNOnly_CC_4_reg_iter_0_normal_masked
SyNOnly_CC_6_reg_iter_0_normal_masked
SyNOnly_MI_32_reg_iter_0_normal_masked
TVMSQ_normal_masked
TVMSQC_normal_masked
Algorithm
%
25
50
75
100

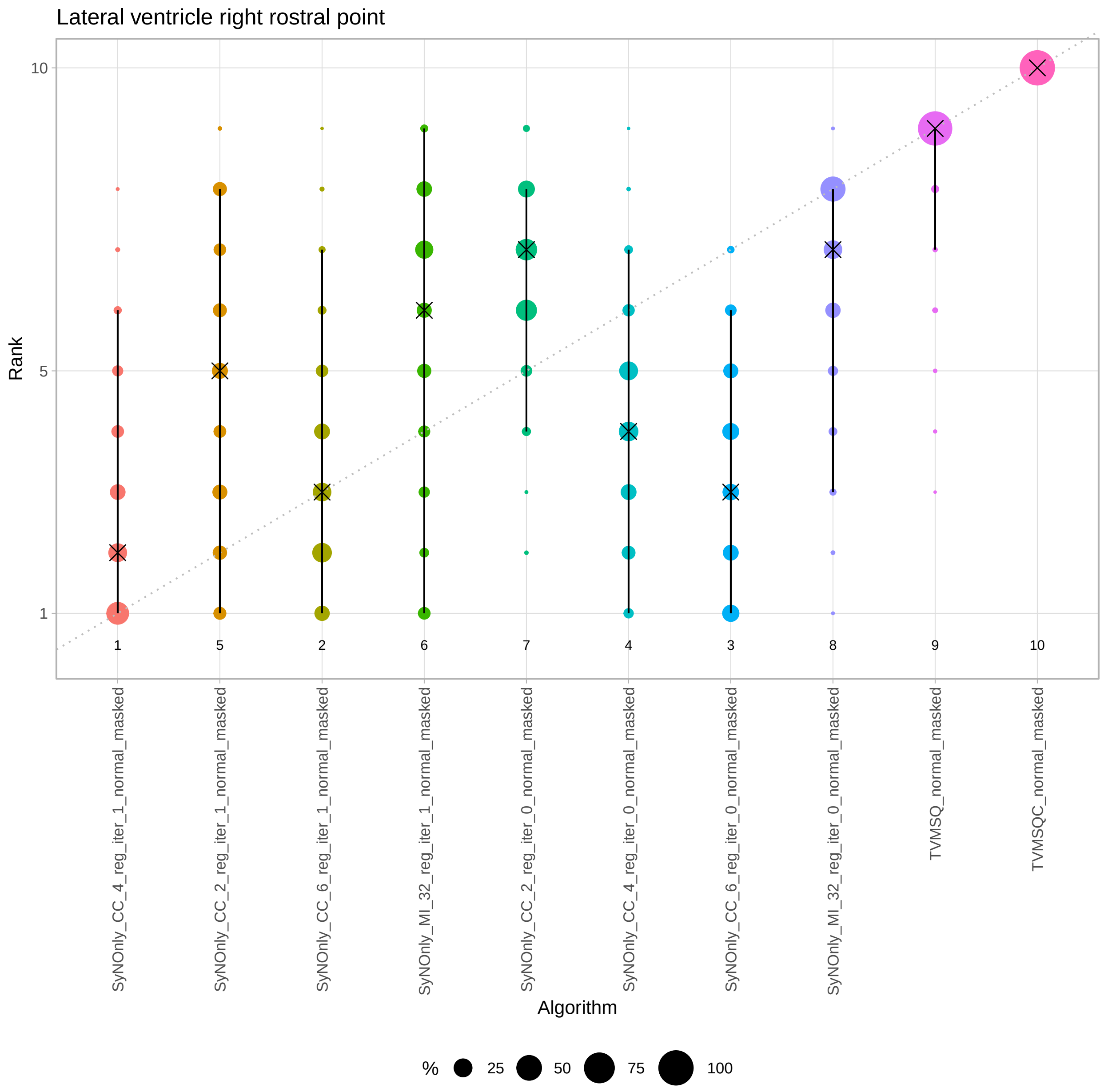
Lateral ventricle right rostral point
Rank
10
5
1
1
5
2
6
7
4
3
8
9
10
SyNOnly_CC_4_reg_iter_1_normal_masked
SyNOnly_CC_2_reg_iter_1_normal_masked
SyNOnly_CC_6_reg_iter_1_normal_masked
SyNOnly_MI_32_reg_iter_1_normal_masked
SyNOnly_CC_2_reg_iter_0_normal_masked
SyNOnly_CC_4_reg_iter_0_normal_masked
SyNOnly_CC_6_reg_iter_0_normal_masked
SyNOnly_MI_32_reg_iter_0_normal_masked
TVMSQ_normal_masked
TVMSQC_normal_masked
Algorithm
%
25
50
75
100

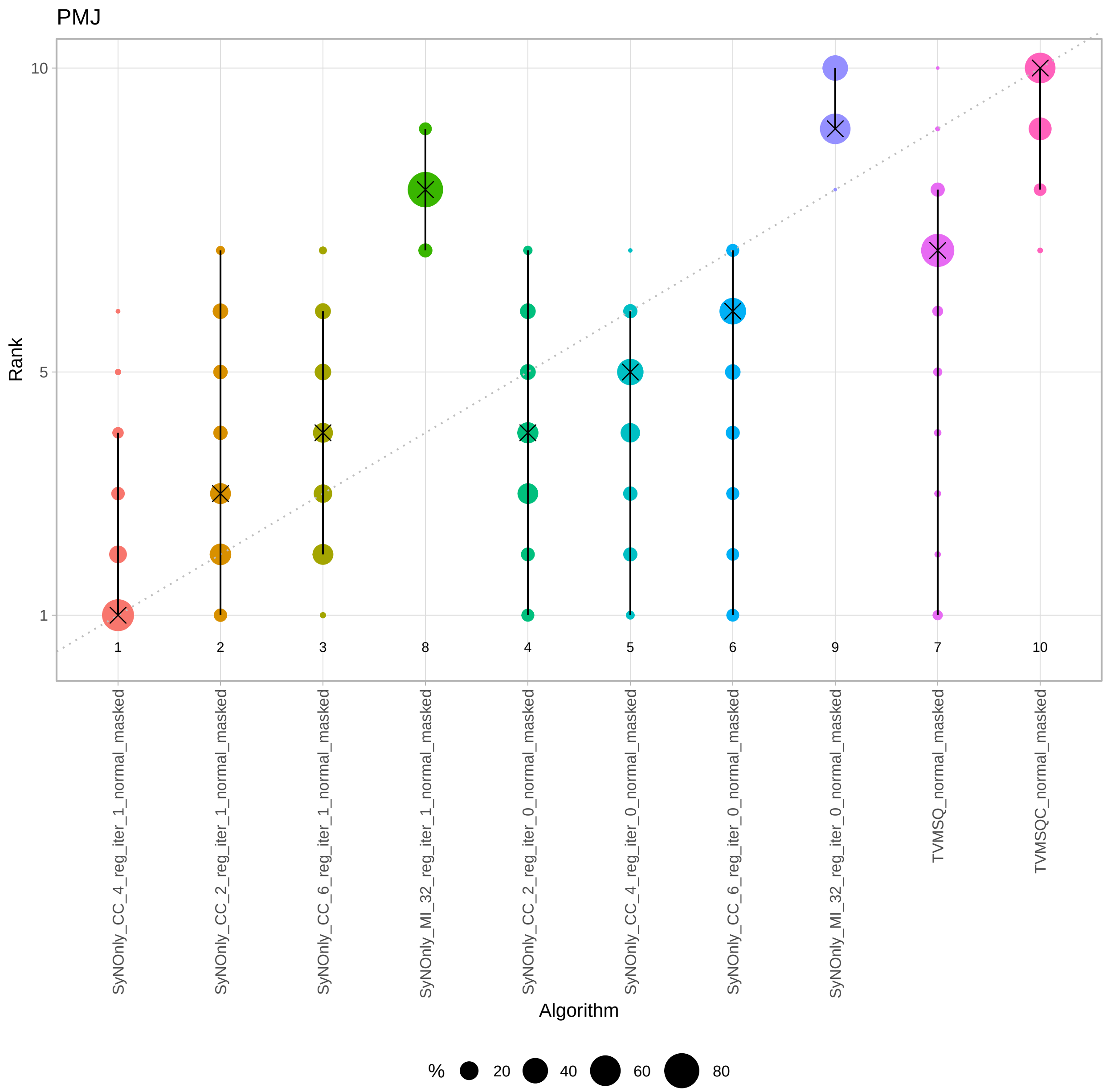
PMJ
Rank
10
5
1
1
2
3
8
4
5
6
9
7
10
SyNOnly_CC_4_reg_iter_1_normal_masked
SyNOnly_CC_2_reg_iter_1_normal_masked
SyNOnly_CC_6_reg_iter_1_normal_masked
SyNOnly_MI_32_reg_iter_1_normal_masked
SyNOnly_CC_2_reg_iter_0_normal_masked
SyNOnly_CC_4_reg_iter_0_normal_masked
SyNOnly_CC_6_reg_iter_0_normal_masked
SyNOnly_MI_32_reg_iter_0_normal_masked
TVMSQ_normal_masked
TVMSQC_normal_masked
Algorithm
%
20
40
60
80

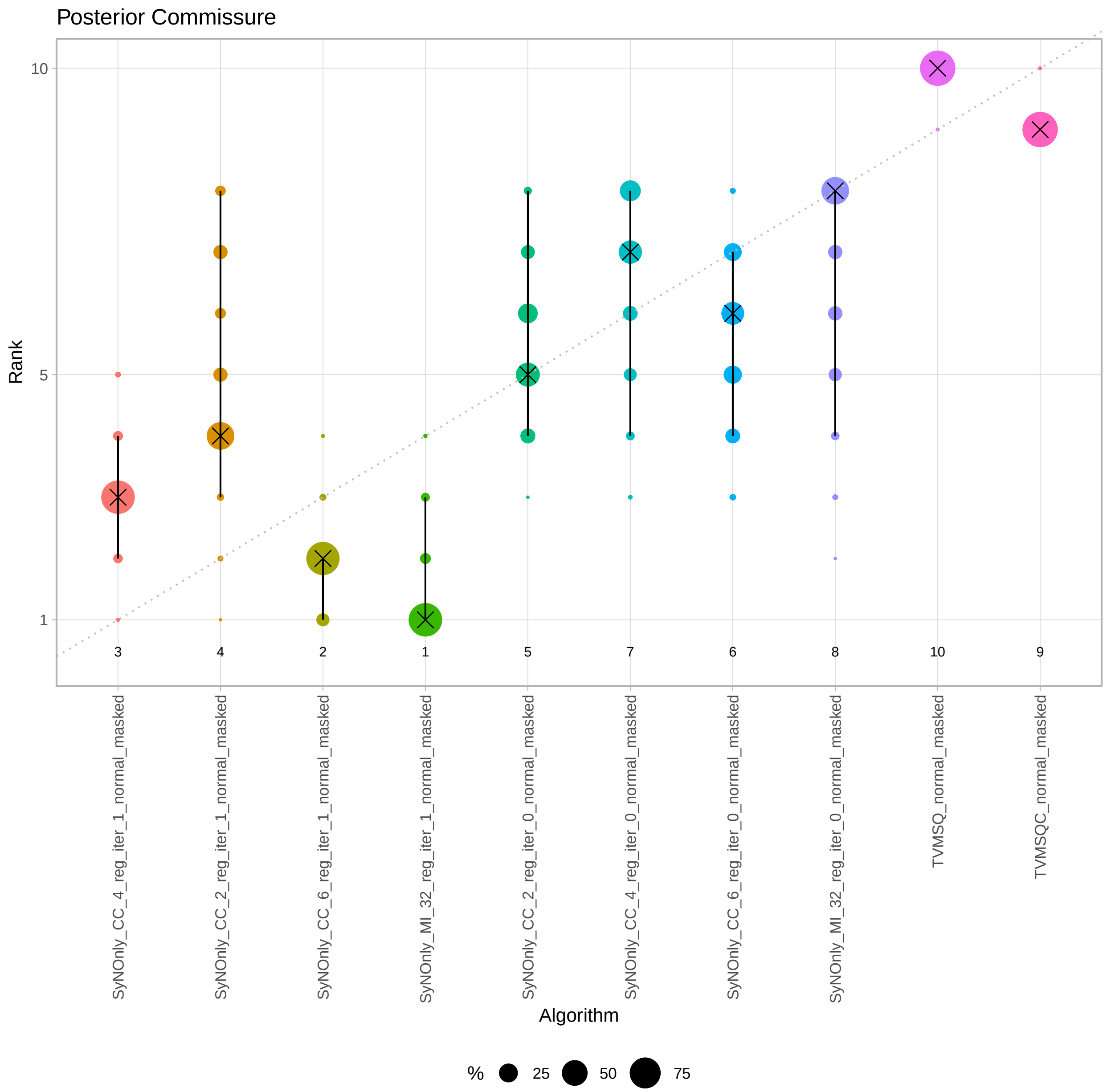
Posterior Commissure
Rank
10
5
1
3
4
2
1
5
7
6
8
10
9
SyNOnly_CC_4_reg_iter_1_normal_masked
SyNOnly_CC_2_reg_iter_1_normal_masked
SyNOnly_CC_6_reg_iter_1_normal_masked
SyNOnly_MI_32_reg_iter_1_normal_masked
SyNOnly_CC_2_reg_iter_0_normal_masked
SyNOnly_CC_4_reg_iter_0_normal_masked
SyNOnly_CC_6_reg_iter_0_normal_masked
SyNOnly_MI_32_reg_iter_0_normal_masked
TVMSQ_normal_masked
TVMSQC_normal_masked
Algorithm
%
25
50
75

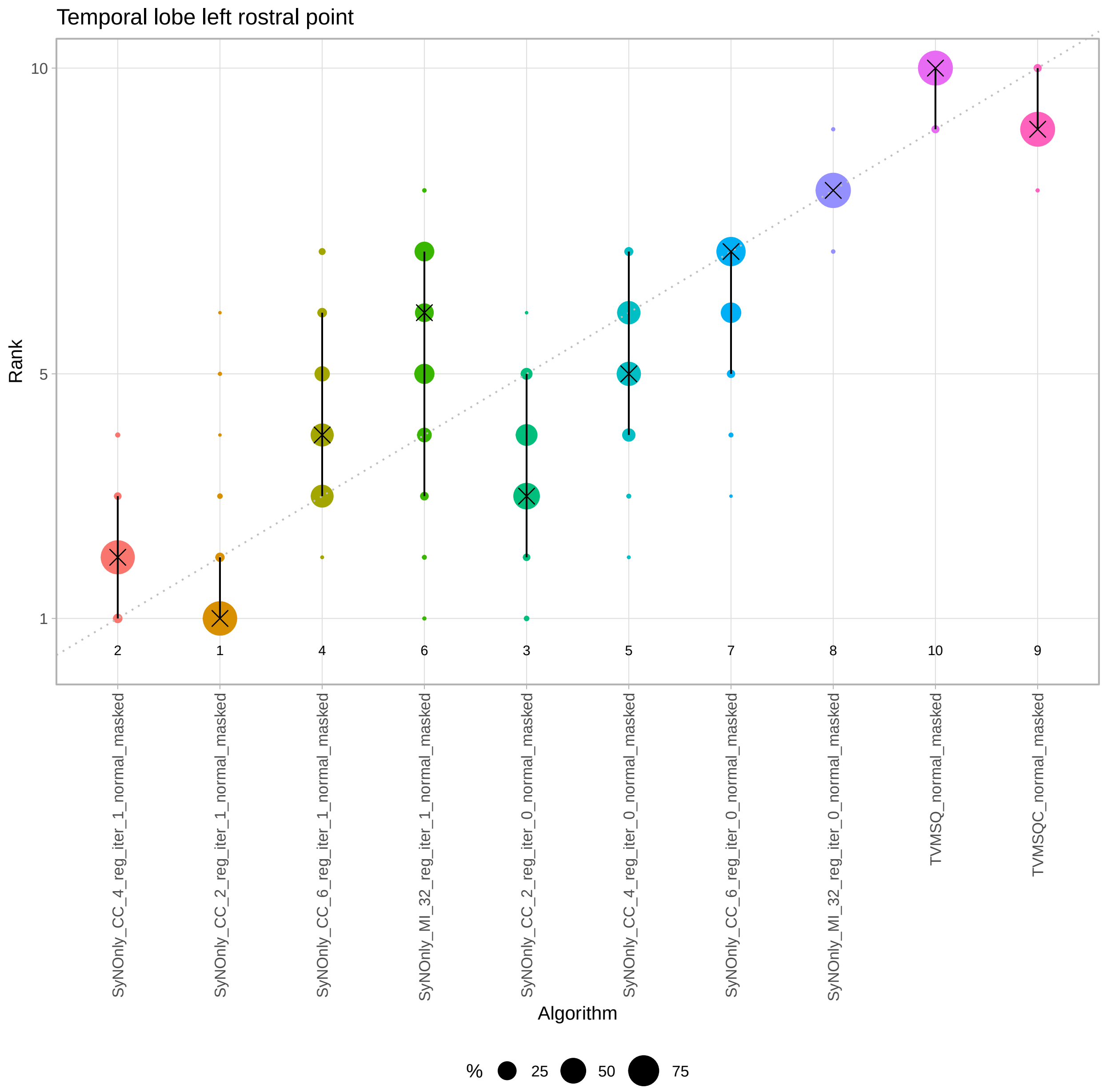

Temporal lobe left rostral point
Rank
10
5
1
2
1
4
6
3
5
7
8
10
9
SyNOnly_CC_4_reg_iter_1_normal_masked
SyNOnly_CC_2_reg_iter_1_normal_masked
SyNOnly_CC_6_reg_iter_1_normal_masked
SyNOnly_MI_32_reg_iter_1_normal_masked
SyNOnly_CC_2_reg_iter_0_normal_masked
SyNOnly_CC_4_reg_iter_0_normal_masked
SyNOnly_CC_6_reg_iter_0_normal_masked
SyNOnly_MI_32_reg_iter_0_normal_masked
TVMSQ_normal_masked
TVMSQC_normal_masked
Algorithm
%
25
50
75

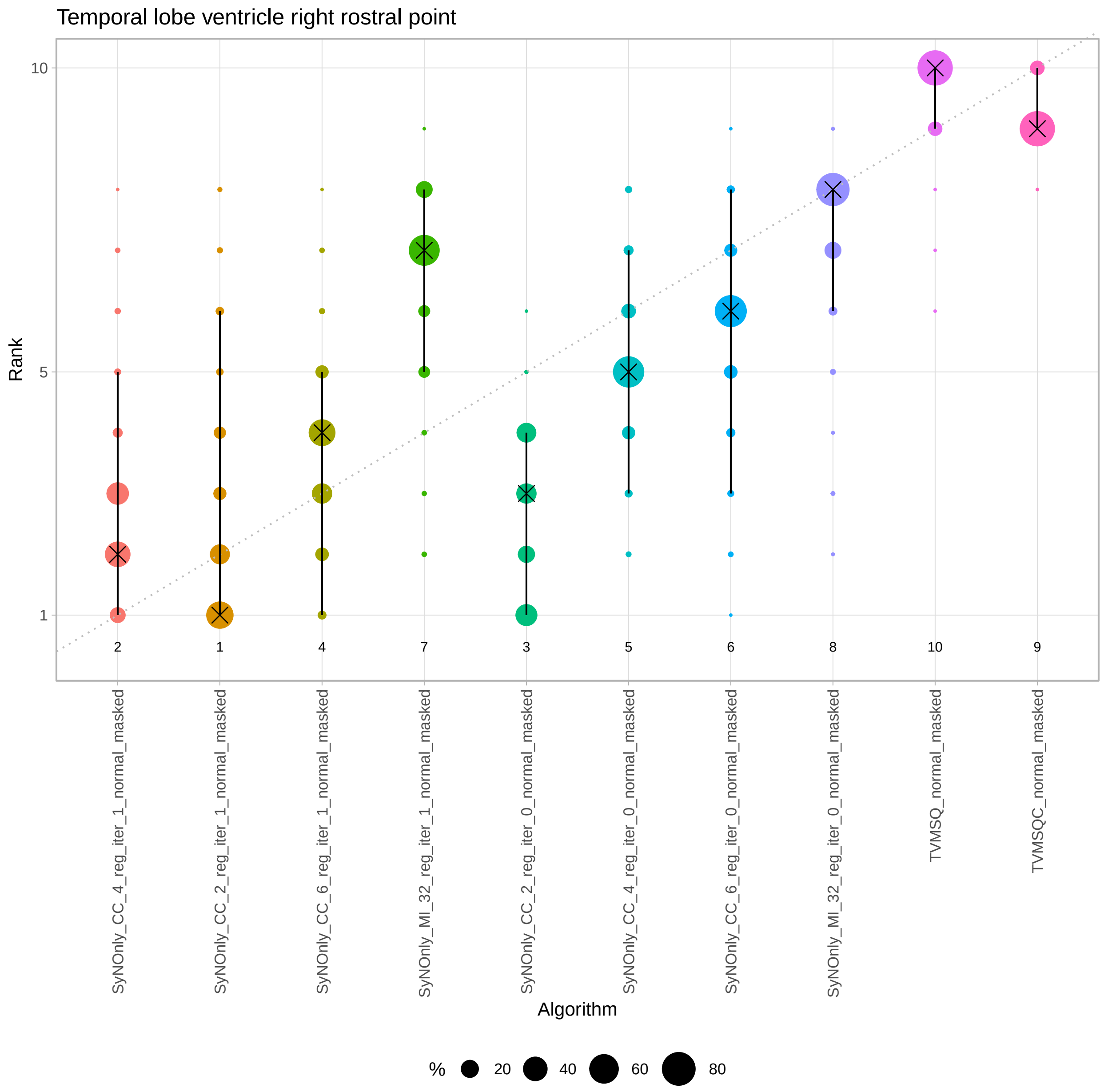

4.2.2 Cluster Analysis

Dendrogram from hierarchical cluster analysis and *network-type graphs* for assessing the similarity of tasks based on challenge rankings.

A dendrogram is a visualization approach based on hierarchical clustering. It depicts clusters according to a chosen distance measure (here: Spearman's footrule) as well as a chosen agglomeration method (here: complete and average agglomeration).

Cluster Dendrogram (complete agglomeration)

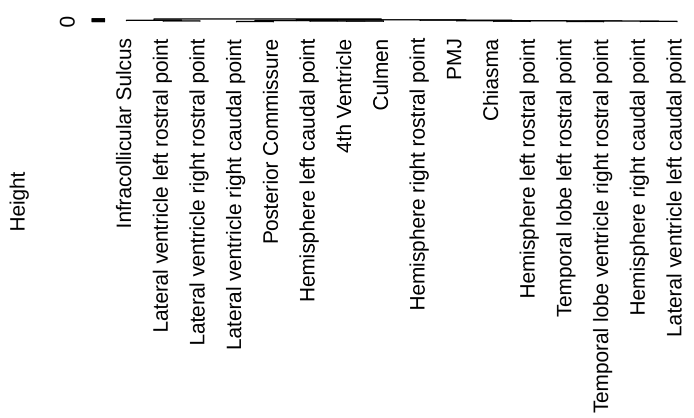

Cluster Dendrogram (average agglomeration)

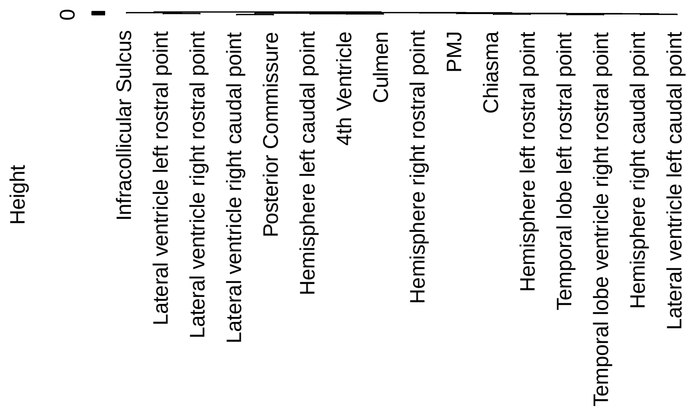

5 References

Wiesenfarth, M., Reinke, A., Landman, B.A., Eisenmann, M., Aguilera Saiz, L., Cardoso, M.J., Maier-Hein, L. and Kopp-Schneider, A. Methods and open-source toolkit for analyzing and visualizing challenge results. *Sci Rep* **11**, 2369 (2021). https://doi.org/10.1038/s41598-021-82017-6

M. J. A. Eugster, T. Hothorn, and F. Leisch, "Exploratory and inferential analysis of benchmark experiments," Institut fuer Statistik, Ludwig-Maximilians-Universitaet Muenchen, Germany, Technical Report 30, 2008. [Online]. Available: http://epub.ub.uni-muenchen.de/4134/.
